# Regulation of piglet T-cell immune responses by thioredoxin peroxidase from *Cysticercus cellulosae* excretory-secretory antigens

**DOI:** 10.3389/fmicb.2022.1019810

**Published:** 2022-11-18

**Authors:** Wei He, Xiaoqing Sun, Bo Luo, Meichen Liu, Lizhu Li, Xianmin Fan, Jingming Ye, Biying Zhou

**Affiliations:** Department of Parasitology, Zunyi Medical University, Zunyi, China

**Keywords:** *Cysticercus cellulosae*, excretory-secretory antigens, label-free quantification proteomics, thioredoxin peroxidase, T-cell immune response

## Abstract

*Taenia solium* (*T. solium*) cysticercosis is a serious threat to human health and animal husbandry. During parasitization, *Cysticercus cellulosae* (*C. cellulosae*) can excrete and secrete antigens that modulate the host’s T-cell immune responses. However, the composition of *C. cellulosae* excretory-secretory antigens (ESAs) is complex. This study sought to identify the key molecules in *C. cellulosae* ESAs involved in regulating T-cell immune responses. Thus, we screened for thioredoxin peroxidase (TPx), with the highest differential expression, as the key target by label-free quantification proteomics of *C. cellulosae* and its ESAs. In addition, we verified whether TPx protein mainly exists in *C. cellulosae* ESAs. The TPx recombinant protein was prepared by eukaryotic expression, and ESAs were used as the experimental group to further investigate the effect of TPx protein on the immune response of piglet T cells *in vitro*. TPx protein induced an increase in CD4^+^ T cells in piglet peripheral blood mononuclear cells (PBMCs), while CD8^+^ T cells did not change significantly. This resulted in an imbalance in the CD4^+^/CD8^+^ T-cell ratio and an increase in CD4^+^CD25^+^Foxp3^+^ Treg cells in the PBMCs. In addition, TPx protein initiated T helper 2 (Th2)-type immune responses by secreting IL-4 and IL-10 and suppressed Th1/Th17-type immune responses. The results showed that ESAs were involved in regulating piglet T-cell immune responses cells. This suggests that TPx protein found in ESAs plays an essential role to help the parasite evade host immune attack. Moreover, this lays a foundation for the subsequent exploration of the mechanism through which TPx protein regulates signaling molecules to influence T-cell differentiation.

## Introduction

*Taenia solium* (*T. solium*) cysticercosis is a zoonotic parasitic disease caused by the larvae of *T. solium*. *Cysticercus cellulosae* (*C. cellulosae*) can parasitize humans and pigs, posing a serious threat to human health and animal husbandry (Mendlovic et al., 2021). The disease has a global distribution, mainly in developing countries in Latin America, Africa, and Asia (Dixon et al., 2020). In humans, *C. cellulosae* can parasitize the subcutaneous muscles, eye, heart, brain, and other tissues. Among them, neurocysticercosis (NCC) is the most serious condition, which can cause epileptic seizures, increased intracranial pressure, neuropsychiatric disorders, and other central nervous system symptoms. It is often confused with other intracerebral diseases and has a high mortality and disability rate (Prodjinotho et al., 2020; Hamamoto-Filho et al., 2021; Himwaze et al., 2022). Over 50,000 people die of NCC every year worldwide, and it is recognized by WHO as one of the 20 neglected tropical diseases in the world (Engels and Zhou, 2020; WHO, 2022).

Dendritic cells (DCs) have the most efficient antigen-presenting ability. When a parasite infects a host as an antigen, DCs can process, synthesize, and present the antigen to naive CD4^+^ T cells, triggering naive CD4^+^ T-cell activation and inducing helper T cell (Th) and regulatory T-cell (Treg) differentiation (Hilligan and Ronchese, 2020). In the early stage of a parasitic infection, the levels of host CD4^+^ and CD8^+^ T cells are increased, and Th1 or Th17 immune response is the mainstay, which exerts anti-parasitic effects by secreting interferon-γ (IFN-γ), tumor necrosis factor-α (TNF-α), or interleukin-17 (IL-17). As the infection persists, the Th1/Th2 balance is disrupted, leading to a dominant Th2-type immune response in the host. Moreover, there is an increase in the percentage of Treg cells, which exert immunosuppressive effects by secreting IL-10 and transforming growth factor-β (TGF-β), helping the parasite to evade host immune attack (Anuradha et al., 2015).

Parasites can excrete and secrete certain products within their host. These excretory-secretory antigens (ESAs) can directly regulate the host’s T-cell immune responses, which is beneficial for the parasite to escape the host’s immune attack and survive in the host (White and Artavanis-Tsakonas, 2012; Sun et al., 2019). For instance, *Toxocara canis* ESAs can stimulate the expression of Forkhead box P3 (Foxp3) in host CD4^+^ and CD8^+^ T lymphocytes (Junginger et al., 2017). *Haemonchus contortus* ESAs stimulate goat peripheral blood mononuclear cells (PBMCs) to secrete IL-10, and the IL-10 secretion increases in a concentration-dependent manner with ESAs (Gadahi et al., 2016). ESAs from *Trichinella spiralis* muscle larvae induce an increase in the number of CD4^+^ T cells in the PBMCs of patients and triggered a mixture of Th1/Th2-type immune responses (Della et al., 2017). It is suggested that parasite ESAs can induce Th2-type immune responses, the differentiation of host Treg cells, and the secretion of a large amount of IL-10, which play a negative role in immune regulation and help the parasite to evade the host’s immune attack. *Cysticercus cellulosae* ESAs can be used as a specific antigen to diagnose *T. solium* cysticercosis and as a new drug target (Arora and Prasad, 2020). Our previous study revealed that *C. cellulosae* ESAs disrupted the CD4^+^/CD8^+^ T-cell ratio, altered the T-cell immune function, triggered the host to produce Treg cells, induced Th2-type immune responses, and upregulated the expression of IL-10, thereby exerting an immune evasion effect (Fan et al., 2021).

Due to their complex composition, in the present study, we further explored the protein molecules in *C. cellulosae* ESAs involved in regulating T-cell immune responses. Based on previous label-free quantification (LFQ) proteomics analysis of *C. cellulosae* and its ESAs, we aimed to screen for thioredoxin peroxidase (TPx) protein and verify its existence in *C. cellulosae* ESAs. Further, we sought to explore the effect of thioredoxin peroxidase (TPx) protein on piglet T-cell immune responses *in vitro*.

## Materials and methods

### Animals and preparation of *Cysticercus cellulosae* ESAs

Healthy piglets were confirmed to be pathogen-free and to have been raised under standard conditions. The Animal Care and Use Committee of Zunyi Medical University approved the experiments. *Cysticercus cellulosae* ESAs were prepared in the previous stage. Briefly, *C. cellulosae* were collected from the muscle tissue of cysticercosis-infected pigs, washed with physiological saline and sterile phosphate-buffered saline, and incubated in complete medium and 5% CO_2_ at 37°C for 72 h. The culture supernatant was collected aseptically and transferred into 3 kDa ultrafiltration tubes for concentration. Then, the concentrated liquid of *C. cellulosae* ESAs was stored at −80°C until use (Fan et al., 2021).

### Screening for TPx protein

The differential proteins of *C. cellulosae* ESAs were identified based on previous LFQ proteomics analysis. The ratio of the mean relative quantification values of each protein in multiple replicate samples was taken as the fold change (FC). The calculation formula was: FC_*A*/*B*, *k*_ = *Mean* (*R_ik_*, *i*∈*A*) / *Mean* (*R*_ik_, *i*∈*B*). In this formula, *A* represents *C. cellulosae*, *B* represents *C. cellulosae* ESAs, *R* represents the relative quantitative value of the protein, *i* represents the number of samples, and *k* represents the protein.

The relative quantitative value of each protein in the samples of the experimental and control groups was compared using *t-test*, and the corresponding *p value* was determined. Proteins with no quantitative information and poor reproducibility were removed, and proteins with *p value* < 0.05 were selected. When the *p* value was <0.05, FC > 1.5 was used as the threshold for significant upregulation, and FC < 1/1.5 was used as the threshold for significant downregulation. Therefore, the differential proteins of *C. cellulosae* ESAs were screened in the region with FC < 1/1.5. FC_A/B_ indicates the proportion of the change in protein expression of A relative to B. The lower the ratio, the higher the expression of ESAs. Then, the differential proteins of *C. cellulosae* ESAs were analyzed by gene ontology (GO) and biological function annotation to screen for TPx protein in *C. cellulosae* ESAs.

### Validation of TPx protein

*Cysticercus cellulosae* and its ESAs were subjected to equimolar enzymatic digestion, and the volume was adjusted to uniformity with the lysing solution. Then, 20% trifluoroacetic acid (Thermo Fisher, Wyman Street, Waltham, MA, USA) was slowly added, mixed by vortex, and precipitated at 4°C for 2 h. The solution was centrifuged at 4,500 rpm for 5 min, the supernatant was discarded, and the precipitate was washed with pre-chilled acetone 2–3 times. After drying the precipitate, 200 mM tetraethylammonium bromide was added, the precipitate was ultrasonically dispersed, trypsin (Promega, Madison, Wisconsin, USA) was added at a ratio of 1:50 (protease: protein, m/m), and the mixture was incubated overnight for enzymolysis. Dithiothreitol (Sigma, St. Louis, MI, USA) was added to a final concentration of 5 mM at 56°C for 30 min. Then, iodoacetamide (Sigma, USA) was added to a final concentration of 11 mM, and the mixture was incubated at room temperature for 15 min in the dark.

The peptides were dissolved using liquid chromatography mobile phase A [aqueous solution containing 0.1% formic acid (Fluka, Selzer, Germany) and 2% acetonitrile (Thermo Fisher, USA)] and separated using the EASY-nLC 1,000 ultra-high performance liquid chromatography system. Afterward, the peptides were injected into an NSI ion source for ionization and then analyzed using Q ExactiveTM Plus mass spectrometer. The ion source voltage was set to 2.1 kV, and the peptide precursor ions and their secondary fragments were detected and analyzed using high-resolution Orbitrap.

### Recombinant protein preparation

The TPx gene sequence of *T. solium* was obtained from the NCBI database (GenBank: AHZ89374.1) for the full gene synthesis of TPx. The TPx target gene was digested with *EcoR1* and *BamHI* (Takara Biotech, Beijing, China) and then connected to the eukaryotic expression vector pcDNA3.4 (Zhongding Biotech, Nanjing, China) to construct the recombinant plasmid pcDNA3.4-TPx. The incubated plasmids were added to HEK293 cells (Zhongding Biotech, China), cultured in suspensions at 37°C for 6 days, centrifuged at 5000 rpm for 15 min, and the supernatant of the cell secretion medium was collected. The cells were re-suspended in PBS, disrupted by ultrasonic waves, and lysed, and the cell lysis supernatant and cell lysis pellet were collected. The cell secretion medium supernatant was placed in a Nickel (Ni) column (Lanxiao Technology Materials, Xi’an, China), incubated at 4°C in a rotary incubator for 3–4 h, and then slowly added to a purified empty column. The column was washed with washing buffer (20 mM imidazole, 50 mM Tris, 300 mM NaCl, pH 8.0) at a flow rate of 1 ml/min until the OD_280_ value of the effluent reached the baseline, and the effluent and washing liquids were collected. The target protein was eluted with elution buffer (200 mM imidazole, 50 mM Tris, 300 mM NaCl, pH 8.0) at a flow rate of 1 ml/min, and the elution solution was collected. Then, the recombinant TPx protein was purified and identified by western blotting as previously described (Li and Zhou, 2022).

### Peripheral blood mononuclear cells isolation and culture

The protocol for piglet PBMC isolation was slightly modified according to a previous procedure (Islam et al., 2016). Briefly, under sterile conditions, 10 ml of heparinized anticoagulant blood was extracted from the jugular vein of healthy piglets, and the fresh blood was mixed with the sample diluent in a 1:1 ratio according to the porcine peripheral blood lymphocyte separation medium kit (TBD Sciences, Tianjin, China). Then, the same amount of diluted blood was slowly added to the separation liquid surface. The solution was centrifuged at 500 rpm by density gradient centrifugation for 30 min, and the PBMCs layer was obtained. PBMCs were collected and washed three times with cleaning solution. Erythrocyte lysis buffer (Solarbio Sciences, Beijing, China) was used to eliminate erythrocyte contamination. PBMCs were re-suspended in RPMI 1640 medium containing 10% fetal bovine serum (Solarbio Sciences, China) and antibiotics (100 U/ml penicillin, 100 μg/ml streptomycin; Solarbio Sciences, China).

To investigate the effect of TPx on the differentiation of T-cell subsets induced by phytohemagglutinin (PHA; Solarbio Sciences, China), PBMCs were labeled with carboxyfluorescein diacetate succinimidyl ester (CFSE) (Thermo Fisher, USA), and then TPx (50 μg/ml) was added. Under standard conditions, PHA (2.5 μg/ml) was added after 3 h of culture. PBMCs were cultured in 6-well plates at 37°C and in 5% CO_2_, with 1 × 10^6^ cells/well. ESAs (50 μg/ml), ConA (10 μg/ml) (Sigma, USA), and RPMI 1640 medium were added as control groups, respectively. After 48 h of culture, CD4^+^ and CD8^+^ T cells were stained and analyzed by flow cytometry.

To further analyze whether TPx can induce the production of Treg cells in PBMCs, PBMCs were cultured in 6-well plate with 1 × 10^6^ cells/well, and TPx (50 μg/ml) was added. ESAs (50 μg/ml), LPS (2 μg/ml; Sigma, USA), and 1,640 medium were added as control groups, respectively. Under standard conditions, the cells were collected after culturing in 5% CO_2_ at 37°C for 48 h. The expressions of CD4, CD25, and Foxp3 were detected by flow cytometry.

### T lymphocyte differentiation

After healthy piglets were anesthetized, 20 ml of bone marrow was extracted by puncturing the posterior superior iliac spine, and DCs derived from the bone marrow premonomer cells were isolated under sterile conditions. The cells were grown under standard conditions, and immature DCs were induced. To determine the effect of TPx on the differentiation of T-cell subsets, CD4^+^ T cells were isolated from the spleen of healthy piglets using anti-CD4 positive selection magnetic beads (Miltenyi Biotec, Bergisch Gladbach, Germany), according to the manufacturer’s instructions, and approximately 90% of CD4^+^ T cells were harvested using a flow sorter. The concentration of CD4^+^ T cells was adjusted to 1 × 10^6^ cells/ml with RPMI 1640 complete medium, and that of immature DCs was adjusted to 1 × 10^5^ cells/ml, giving a ratio of CD4^+^ T cells to DCs of 10:1. The cells were stimulated with TPx (50 μg/ml) for the experimental group and ESAs (50 μg/ml), LPS (10 μg/ml) (Sigma, USA), and RPMI 1640 medium for the control groups. The cells were co-cultured at 37°C for 24 h, centrifuged, and then added to RPMI 1640 complete medium containing 10 ng/ml porcine rIL-2 (Abcam, Cambridge, UK). The culture was continued at 37°C for 24, 48, and 72 h. The supernatant was collected by centrifugation, and cytokines including IL-4, IL-5, IL-10, IL-17, and IFN-γ were detected using an ELISA kit.

### Flow cytometry and antibodies

For cell surface staining, cells were washed three times in pre-chilled staining buffer and then re-suspended in the staining buffer. According to the manufacturer’s instructions, cells were incubated with the appropriate amount of fluorescent antibodies for 30 min at 4°C in the dark. Cells were washed twice with staining buffer and then tested by flow cytometry. The T-cell epifluorescent antibodies were as follows: CD4a: PE-Cy7 (BD Pharmingen, Franklin Lakes, NJ, USA), CD8a: AF647 (BD Pharmingen, USA), and CD25: Alexa Fluor 647 (Thermo Fisher, USA). It is necessary to break the membrane to detect intracellular antigens. After incubating cells with surface fluorescent antibodies at 4°C for 30 min in the dark, the washed cells were re-suspended in the staining buffer. According to the manufacturer’s instructions, cells were incubated with transcription factor buffer at 4°C for 50 min in the dark, and then incubated with the intracellular fluorescent antibody Foxp3: FITC (Thermo Fisher, USA) at 4°C for 45 min in the dark. All data were analyzed by Flowjo software (TreeStar, Ashland, OR, USA).

### ELISA for cytokine secretion

The culture supernatants were collected, and the levels of cytokines were determined using porcine IFN-γ, IL-4, IL-5, IL-10, and IL-17 ELISA kits (Cloud-Clone Corp, Wuhan, China), according to the manufacturer’s instructions.

### Statistical analysis

Statistical analysis was conducted using IBM SPSS Statistics software version 26.0 (IBM, Inc.). After normal analysis and the homogeneity of variance test, one-way analysis of variance (one-way ANOVA) was used for comparison between multiple groups. Pairwise comparison between groups was performed by Least-Significant Difference-t (LSD) test. All experiments were run in triplicate. Graphing was done with GraphPad Prism 8.0.2. In all figures, NS stands for not significant; *p ≤ 0.05; **p ≤ 0.01; and ***p ≤ 0.001.

## Results

### Screening and validation of TPx protein

**Figure 1:**
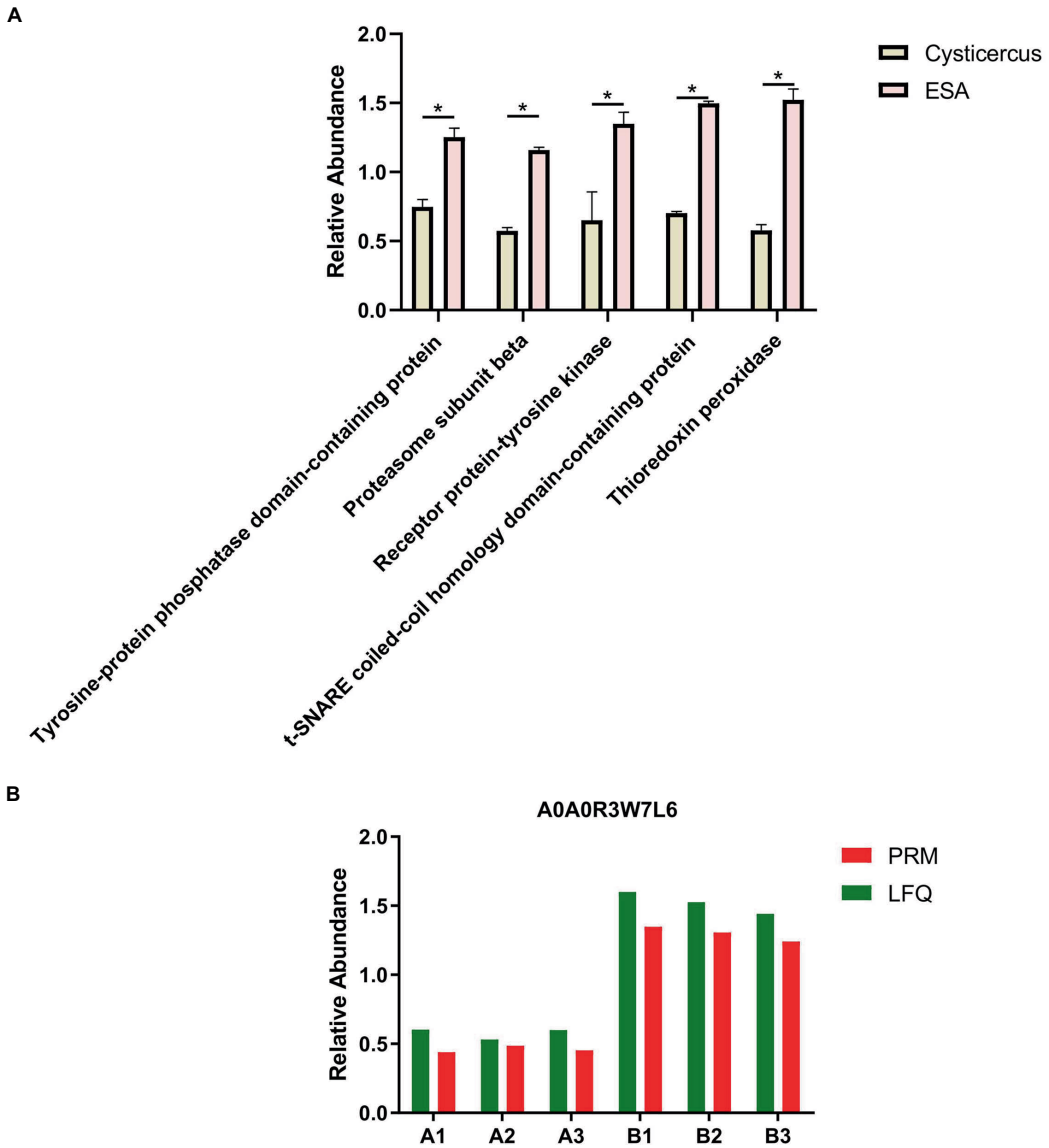
Screening and validation of TPx protein. **(A)** Statistical plots of the differential expression of five proteins, among which the TPx protein had the largest differential expression; **(B)** The change in the trend of TPx protein expression between the label-free quantification and parallel reaction monitoring results was consistent. *p < 0.05.

The differential proteins of *C. cellulosae* ESAs were analyzed by GO analysis, and 43 of them were found to be involved in the immune response process. Subsequently, the biological functions of the differential proteins were annotated, and it was discovered a total of five proteins including tyrosine-protein phosphatase domain-containing protein, proteasome subunit beta, receptor protein-tyrosine kinase, t-SNARE Coiled-coil homology domain-containing protein, and TPx played a role in regulating T-cell immune responses. TPx protein (A0A0R3W7L6) with the greatest differential expression was selected as the key research target (Table 1; Figure 1A). The screened TPx protein was verified by parallel reaction monitoring (PRM) technology. In addition, the differential expression of TPx protein showed consistent trends in LFQ and PRM (Figure 1B), indicating that TPx protein is highly expressed in *C. cellulosae* ESAs.

**Table 1:** Proteins with the function of regulating T-cell immune responses.

Protein accession	Protein description	*A*_1_	*A*_2_	*A*_3_	*B*_1_	*B*_2_	*B*_3_	FC_*A*/*B*_	*p* Value
A0A0R3WDB8	Tyrosine-protein phosphatase domain-containing protein	0.7054	0.7299	0.8065	1.3211	1.1908	1.2463	0.5965	0.00049870
A0A0R3VT49	Proteasome subunit beta	0.6016	0.5615	0.5614	1.1370	1.1783	1.1601	0.4962	0.00001026
A0A158R9E3	Receptor protein-tyrosine kinase	0.5956	0.8783	0.4806	1.4320	1.2638	1.3498	0.4831	0.01361241
A0A158R8G6	t-SNARE coiled-coil homology domain-containing protein	0.6901	0.7125	0.7038	1.4868	1.4914	1.5154	0.4687	0.00000027
A0A0R3W7L6	Thioredoxin peroxidase	0.6023	0.5306	0.5999	1.5996	1.5259	1.4416	0.3794	0.00004629

Expression levels of differential proteins that regulate T-cell function. The differential expression of TPx protein is the largest, and *p* < 0.05.

### *Cysticercus cellulosae* ESAs and TPx induced CD4^+^ and CD8^+^ T lymphocyte responses in PBMCs

**Figure 2:**
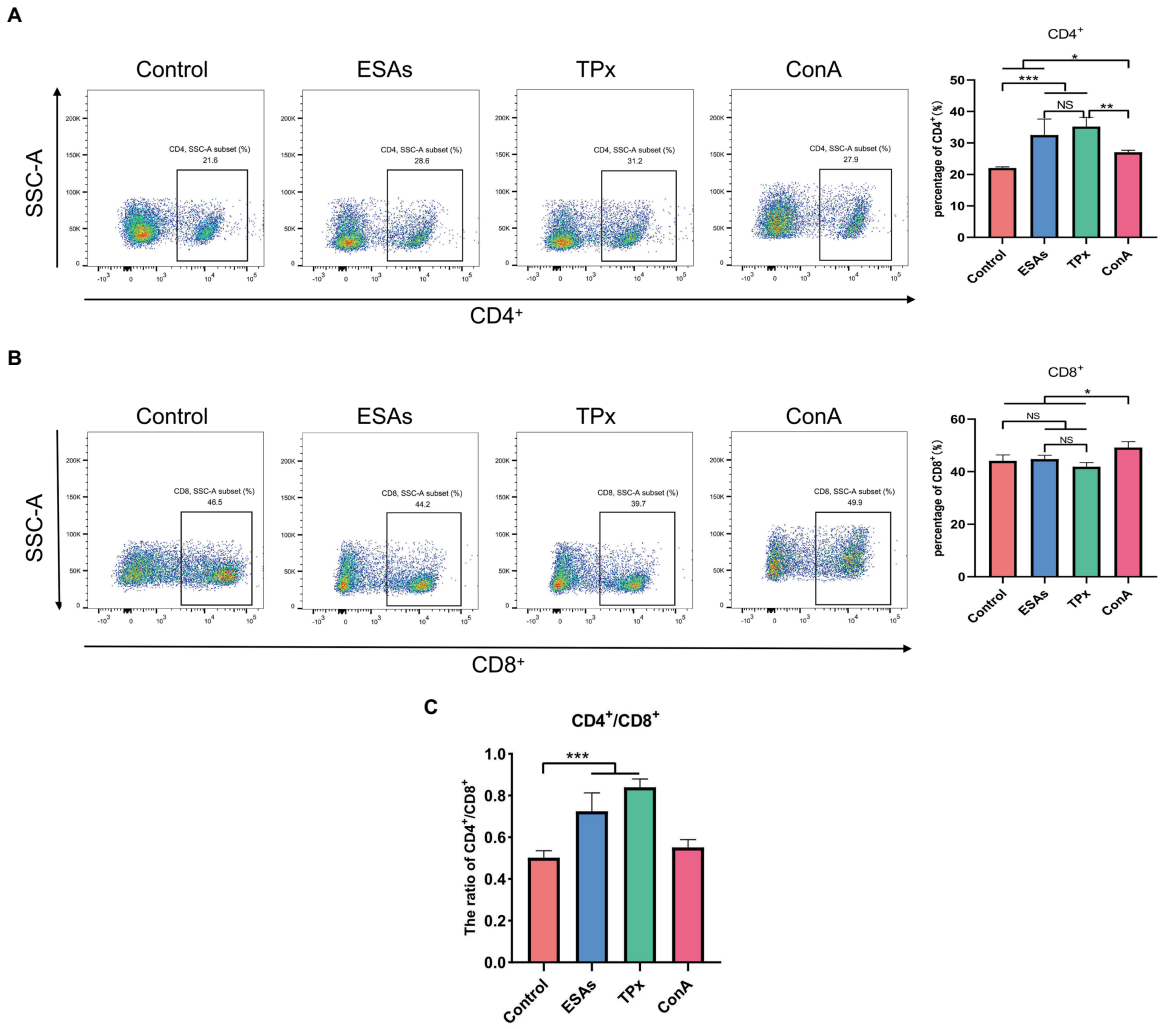
T-cell immune response assay. **(A)** Both *Cysticercus cellulosae* excretory-secretory antigens (ESAs) and TPx induced a significant increase in the number of CD4^+^ T lymphocytes. **(B)** Neither *C. cellulosae* ESAs nor TPx induced an increase in CD8^+^ T lymphocytes with statistical significance. **(C)** Both *C. cellulosae* ESAs and TPx induced a significant increase in the ratio of CD4^+^/CD8^+^ T cells. All data were represented by means ± SD, NS stands for not significant, *p < 0.05, **p < 0.01, ***p < 0.001.

*Cysticercus cellulosae* ESAs and TPx were cultured with piglet PBMCs for 48 h, respectively, and the expression of CD4^+^ and CD8^+^ T-cell subsets was detected by flow cytometry. Compared with the normal group, both ESAs and TPx induced a significant increase in the number of CD4^+^ T cells (Figure 2A), but there was no significant increase or decrease in CD8^+^ T cells (Figure 2B). The CD4^+^/CD8^+^ T-cell ratio in the control, ESAs, and TPx groups was approximately 0.5004, 0.7263, and 0.8409, respectively **(**Figure 2C**)**. Compared with the ConA group, *C. cellulosae* ESAs and TPx significantly increased the number of CD4^+^ T cells but decreased the number of CD8^+^ T cells **(**Figures 2A,B**)**. Compared with the ESAs group, TPx did not induce a statistically significant increase in the number of CD4^+^ and CD8^+^ T cells (Figures 2A,B). Therefore, both *C. cellulosae* ESAs and TPx could induce the increase in the CD4^+^/CD8^+^ T-cell ratio in piglet PBMCs, leading to T cells imbalance and immune dysfunction.

### *Cysticercus cellulosae* ESAs and TPx induced an increase in the number of CD4^+^CD25^+^Foxp3^+^ Tregs in PBMCs

**Figure 3:**
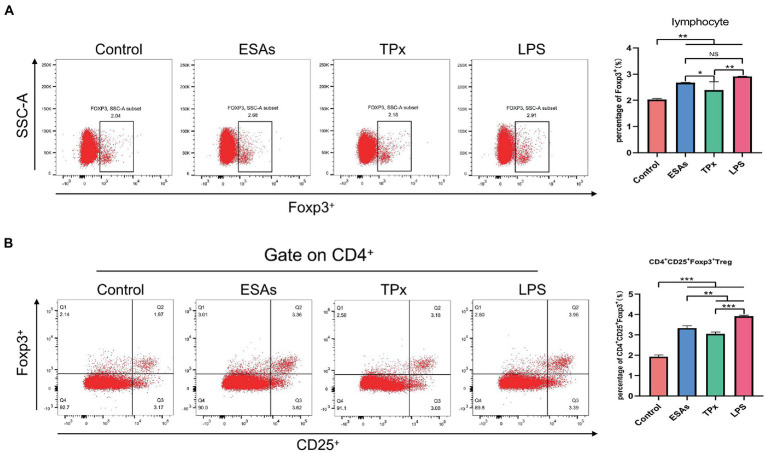
Tregs induction assay. **(A)** *C. cellulosae* ESAs and TPx significantly induced the expression of Foxp3^+^ lymphocytes, both of which were statistically significant. **(B)** *C. cellulosae* ESAs and TPx caused an increase in the number of CD4^+^CD25^+^Foxp3^+^ Tregs in peripheral blood mononuclear cells, and the difference was statistically significant. All data were expressed as the mean ± SD, NS stands for not significant, *p < 0.05, **p < 0.001, ***p < 0.001.

*Cysticercus cellulosae* ESAs and TPx were cultured with piglet PBMCs for 48 h, respectively, and the expression of Tregs was detected by flow cytometry. Compared with the control group, both *C. cellulosae* ESAs and TPx increased the expression of Foxp3 by lymphocytes in PBMCs **(**Figure 3A**)**. Tricolor flow cytometry showed that *C. cellulosae* ESAs and TPx induced an increase in the number of CD4^+^CD25^+^Foxp3^+^ Tregs in PBMCs **(**Figure 3B**)**. Compared with the ESAs and LPS groups, TPx induced a significant decrease in the number of Foxp3^+^ lymphocytes and CD4^+^CD25^+^Foxp3^+^ Treg cells in PBMCs (Figures 3A,B).

### *Cysticercus cellulosae* ESAs and TPx induced T helper subpopulation differentiation

**Figure 4:**
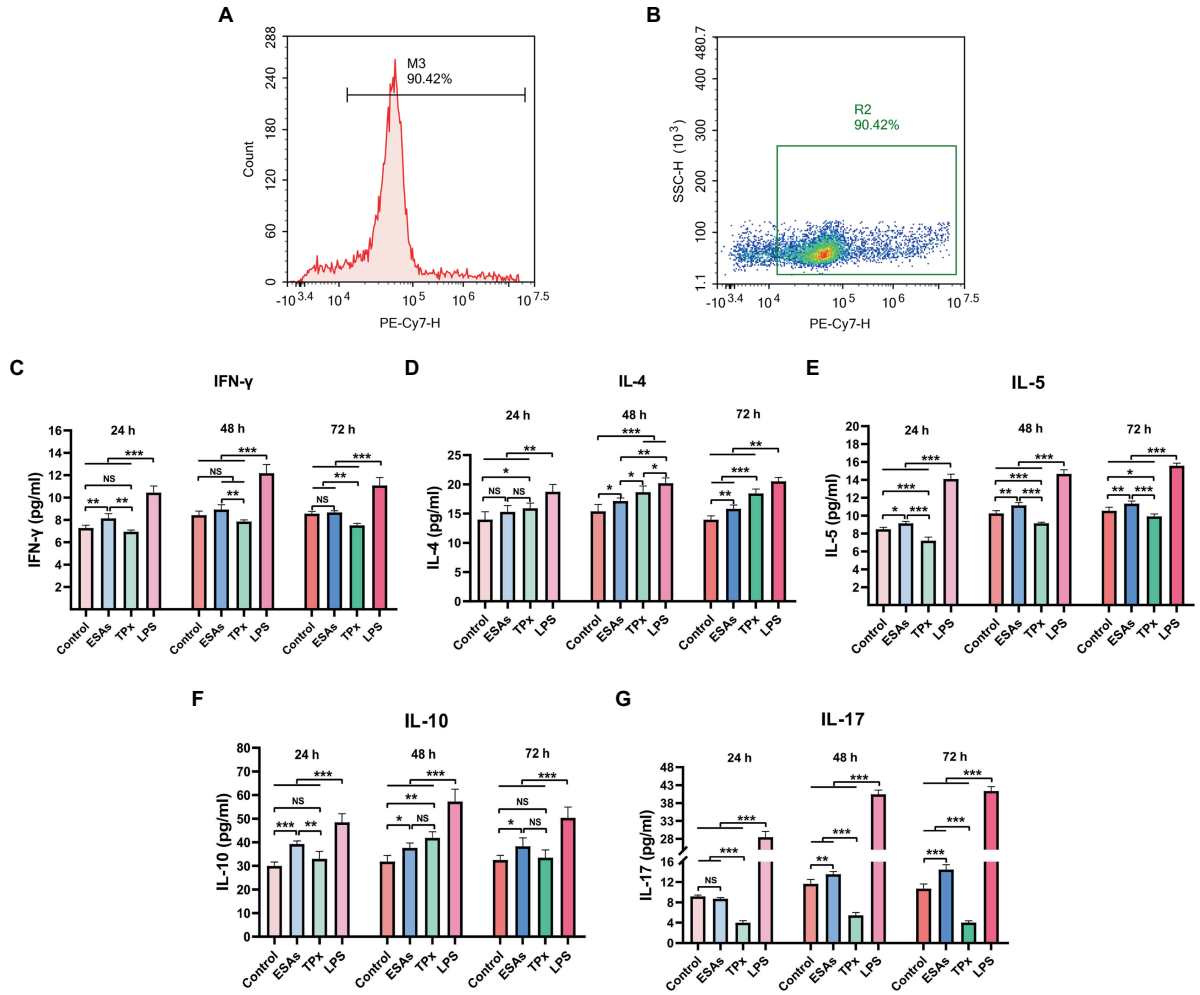
Detection of cytokines in the Th subsets. **(A,B)** The purity of naive CD4^+^ T cells was detected by flow cytometry. **(C)** Th1: *C. cellulosae* ESAs significantly promoted the secretion of interferon-γ (IFN-γ) at 24 h, but the secretion of IFN-γ was not remarkable with the extension of the culture time. TPx did not induce the secretion of FN-γ at 24 and 48 h but inhibited the secretion of IFN-γ at 72 h. **(D,E)** Th2: The secretion of IL-4 by *C. cellulosae* ESAs was not obvious at 24 h. With the prolongation of the culture time, the level of IL-4 secretion increased significantly, and the secretion of IL-5 was promoted at 24, 48, and 72 h. TPx promoted the secretion of IL-4 but inhibited secretion of IL-5 at 24, 48, and 72 h. **(F)** Th2/Treg: *C. cellulosae* ESAs promoted the secretion of IL-10 at 24, 48, and 72 h. However, the level of secreted IL-10 induced by TPx did not increase significantly at 24 h, but significantly increased at 48 h. **(G)** Th17: *C. cellulosae* ESAs did not induce a significant increase in IL-17 level at 24 h. With the prolongation of culture time, the secretion of IL-17 was significantly increased, while TPx inhibited the secretion of IL-17 at 24, 48, and 72 h. All data were represented by means ± SD; NS stands for not significant, *p < 0.05, **p < 0.01, ***p < 0.001.

To investigate the effect of *C. cellulosae* ESAs and TPx on the differentiation of Th subpopulations, secretion levels of IFN-γ, IL-4, IL-5, IL-10, and IL-17 were examined *via* ELISA at different time periods. Naive CD4^+^ T cells with >90% purity were obtained from the spleen of piglets by magnetic bead sorting (Figures 4A,B). DC-CD4^+^ T cells exposed to *C. cellulosae* ESAs and TPx produced different levels of cytokines (Figures 4C–G). *Cysticercus cellulosae* ESAs induced DC-CD4^+^ T cells to secrete significantly high IFN-γ, IL-5, and IL-10 levels in the early stage (24 h), but there was no significant increase in IL-4 secretion at this stage. In the middle and late stages (48 and 72 h), it mainly promoted the secretion of IL-4, IL-5, IL-10, and IL-17. TPx induced DC-CD4^+^ T cells to secrete significantly high IL-4 levels in the early stage (24 h), while there was a remarkable increase in IFN-γ and IL-10 secretion. Moreover, it mainly promoted the secretion of IL-4 and IL-10 in the middle stage (48 h), inhibited the secretion of IFN-γ in the late stage (72 h), and inhibited the secretion of IL-5 and IL-17 at all stages. Therefore, both *C. cellulosae* ESAs and TPx could induce Th subsets to secrete different cytokines at different time periods, which played an important regulatory role in the immune response of Th cells.

## Discussion

*Taenia solium* cysticercosis is a major public health problem and a great hindrance to pig breeding in endemic areas. The development of a protective vaccine is the most effective and economical means of treating *C. cellulosae* (Gulelat et al., 2022; Zhang et al., 2022). However, the complex immune pathogenesis of *C. cellulosae* has not been fully clarified, and this is one of the important bottlenecks restricting the development of effective vaccines against it (Rosales-Mendoza et al., 2018; Kaur et al., 2021). The reason may be that *C. cellulosae* as a parasite in the host can directly excrete and secrete mixed products. The composition of *C. cellulosae* ESAs is complex, and it is regularly updated and replaced during metabolism. Thus, the interaction mechanism between *C. cellulosae* ESAs and host immune cells is not clear (Harnett, 2014). Due to the fact that the key protein molecules in *C. cellulosae* ESAs involved in regulating T-cell immune responses have not been elucidated, this study sort to screen for TPx protein and verify its existence in *C. cellulosae* ESAs, based on previous LFQ proteomic analysis of *C. cellulosae* ESAs. The eukaryotic protein expression was used to obtain recombinant TPx protein, which further verified the effect of TPx protein in *C. cellulosae* ESAs on piglet T-cell immune responses *in vitro*.

Proteomics techniques have been widely studied and applied in the field of tapeworm research, and targeted proteomics has become a powerful tool for protein quantification, of which PRM is the mainstream method for targeted proteomics data acquisition (Shi et al., 2016; Jeferson et al., 2017; José et al., 2017). The advantage of PRM is that it is extremely specific, provides higher selectivity in complex biological samples, and more effectively distinguishes the product ion signal of the target protein or target peptide from co-eluting interference, thereby improving the quality of data analysis (Peterson et al., 2012; Sobsey et al., 2020). Given the complexity of *C. cellulosae* and its ESA components, this experiment was performed to validate the TPx protein values using PRM technology, and the results showed that the trend of TPx protein expression was consistent between LFQ and PRM. This suggests that TPx protein is expressed in both *C. cellulosae* and its ESAs, and that the expression level of TPx protein is significantly higher in *C. cellulosae* ESAs than in *C. cellulosae*.

Under normal circumstances, CD4^+^ and CD8^+^ T cells regulate each other to maintain the balance of immune function (Khan et al., 2019). In this study, both *C. cellulosae* ESAs and TPx could significantly increase the number of CD4^+^ T lymphocytes, but the number of CD8^+^ T lymphocytes did not change significantly, causing an increase in the CD4^+^/CD8^+^ ratio and immune function imbalance. It is suggested that *C. cellulosae* ESAs and TPx play a potential regulatory role in T lymphocyte homeostasis, which can disrupt the immune function of T lymphocytes. The result was similar to some of the previous studies on *Schistosoma japonicum* TPx-3, which induced an increase in the proportion of CD4^+^ T cells in mice peripheral blood (Han et al., 2017). However, this result is contrary to a previous study on the regulation of T lymphocytes by *C. cellulosae* ESAs (Fan et al., 2021), indicating that the CD4^+^/CD8^+^ ratio is in dynamic change, which may be related to the time and degree of infection (Mülayim et al., 2022). In this study, although TPx could induce immune function disorder in piglet T lymphocytes, it could not dynamically reflect the changes in the number of CD4^+^ and CD8^+^ T lymphocytes. Therefore, we need to further optimize the experiment by setting different time periods and concentration gradients, which can more objectively reflect the dynamic changes of TPx and the CD4^+^/CD8^+^ ratio.

As an important immunosuppressive cell in the immune system, Treg cells account for 5–10% of the total CD4^+^ T cells and highly express CD25 (IL-2 receptor alpha chain, IL-2Rα) on their surface. Competitive binding of IL-2 with IL-2Rα can activate the signal molecule STAT5 downstream of IL-2R, further maintain the activity of Treg cells, and inhibit the activation of effector T cells (Malek, 2003; Hayes et al., 2020; Huang et al., 2022). Foxp3 is a highly specific nuclear transcription factor in Treg cells, which plays an immunosuppressive role by secreting IL-10 and TGF-β (Georgiev et al., 2019). It was found in the peripheral blood and cerebrospinal fluid of NCC patients that the number of CD4^+^CD25^high^Foxp3^+^ Treg cells was significantly increased, accompanied by a decrease in active CD4^+^ and CD8^+^ T cells, suggesting that Treg cells play an immunosuppressive role and are beneficial to the survival of cerebral *C. cellulosae* (Adalid-Peralta et al., 2012). In this study, we found that both *C. cellulosae* ESAs and TPx induced a significant increase in CD4^+^CD25^+^Foxp3^+^ Treg cells in piglet PBMCs. This was similar to a previous study on *Fasciola gigantica* TPx, which could induce the production of Treg cells in goat PBMCs (Tian et al., 2020). The result suggests that *C. cellulosae* ESAs can induce an increase in Treg cells in piglet PBMCs, which may be one of the main roles played by TPx protein in the ESAs.

When stimulated by antigens, immature DCs recognize, uptake, and process antigens, and gradually mature in the process of migration. Mature DCs present antigens to naive CD4^+^ T cells through the major histocompatibility complex-II (MHC-II), thereby enabling CD4^+^ T cells to be activated and to differentiate into different cell subsets that secrete various cytokines, resulting in different types of adaptive immune responses, such as Th1, Th2, Th17, and Tregs (Letendre et al., 2018; Corripio-Miyar et al., 2022). Under normal circumstances, IFN-γ secreted by Th1 and IL-4 secreted by Th2 antagonize each other to maintain immune balance in the body. Once infected, the pathogen disrupts the Th1/Th2 immune balance and drifts the immune response toward Th2 (Butcher and Zhu, 2021) to escape the host’s immune attack, and the same is true between Th17 and Tregs (Zhang et al., 2021). In addition, the disease will be aggravated if the body is in an excessive inflammatory state for a long period. Currently, IL-10 plays an important role as a negative feedback immunosuppressive factor, which can weaken the antigen-presentation ability of antigen-presenting cells (APC) and play an immunosuppressive role by inhibiting the activation of T cells and the secretion of pro-inflammatory factors (Nakamae et al., 2019; Steen et al., 2020). This study found that when DCs were co-cultured with naive CD4^+^ T cells, *C. cellulosae* ESAs initiated a mixed Th1/Th2 immune response by secreting IFN-γ, IL-5, and IL-10 in the early culture, and mainly secreted IL-4, IL-5, and IL-10 in the mid to late stage, initiating a predominantly Th2-type immune response. Meanwhile, TPx could induce DC-CD4^+^ T cells to secrete IL-4 but not IFN-γ and IL-10 during early culture, mainly initiating the Th2 immune response. Moreover, they secreted higher IL-4 and IL-10 levels in in the middle stages and inhibited the secretion of IFN-γ and IL-17 at 72 h, indicating that the TPx stimulation could induce a Th2 immune response and inhibit the Th1/Th17 immune response in piglets. This result is similar to the results of previous research on *Fasciola hepatica* TPx and *Trichinella spiralis* TPx, both of which could secrete high levels of IL-4, induce Th2 immune responses, and inhibit Th1 immune responses (Donnelly et al., 2008; Jin et al., 2020).

In conclusion, both ESAs and TPx of *C. cellulosae* could induce an increase in the number of CD4^+^CD25^+^Foxp3^+^ Treg cells and trigger a Th2-type immune response in piglets in the middle and late stages. ESAs induced Treg cell differentiation and initiated a Th2-type immune response, which may be one of the key roles played by the TPx protein component, as Th2-type immune response is associated with susceptibility of *C. cellulosae* to long-term parasitism in the host (Mendlovic et al., 2015). This study revealed that TPx protein in *C. cellulosae* ESAs could regulate the host T-cell immune response. Subsequently, we will investigate the signaling pathway molecules responsible for the effect of TPx protein on host T-cell differentiation, laying a foundation for the discovery of new potential targets and the development of immunomodulatory interventions for the control of *T. solium* cysticercosis.

## Data availability statement

The data presented in the study are deposited in the 4TU.ResearchData repository, under the following https://doi.org/10.4121/21401148.v1.

## Ethics statement

The studies involving human participants were reviewed and approved by Ethics Committee of Zunyi Medical University. The patients/participants provided their written informed consent to participate in this study. The animal study was reviewed and approved by Ethics Committee of Zunyi Medical University. Written informed consent was obtained from the individual(s) for the publication of any potentially identifiable images or data included in this article.

## Author contributions

WH, XS, LL, and JY conducted the experiments. WH, LL, XF, and BZ designed the experiments. WH analyzed the data. XS, LL, BL, ML, XF, JY, and BZ assisted with the experiments. WH wrote the manuscript. BZ revised the manuscript. All authors contributed to the article and approved the submitted version.

## Funding

This work was supported by the National Natural Science Foundation of China (No. 81960378), Science and Technology Planed Project, Zunyi City, Guizhou Province ([2021]278), Special Project of Cultivating New Academic Seedlings and Innovative Exploration of Zunyi Medical University ([2020]-004), and Project of Zunyi Medical University Graduate Education Innovation (ZYK70).

## Conflict of interest

The authors declare that the research was conducted in the absence of any commercial or financial relationships that could be construed as a potential conflict of interest.

## Publisher’s note

All claims expressed in this article are solely those of the authors and do not necessarily represent those of their affiliated organizations, or those of the publisher, the editors and the reviewers. Any product that may be evaluated in this article, or claim that may be made by its manufacturer, is not guaranteed or endorsed by the publisher.

## References

[ref1] Adalid-Peralta L. Fleury A. García-Ibarra T. M. Hernández M. Parkhouse M. Crispín J. C. (2012). Human neurocysticercosis: in vivo expansion of peripheral regulatory T cells and their recruitment in the central nervous system. J. Parasitol. 98, 142–148. doi: 10.1645/GE-2839.1, PMID: 21955298

[ref2] Anuradha R. Munisankar S. Dolla C. Kumaran P. Nutman T. B. Babu S. (2015). Parasite antigen-specific regulation of Th1, Th2, and Th17 responses in *Strongyloides stercoralis* infection. J. Immunol. 195, 2241–2250. doi: 10.4049/jimmunol.1500745, PMID: 26202988 PMC4546867

[ref3] Arora N. Prasad A. (2020). *Taenia solium* proteins: a beautiful kaleidoscope of pro and anti-inflammatory antigens. Expert Rev. Proteomics 17, 609–622. doi: 10.1080/14789450.2020.1829486, PMID: 32985289

[ref5] Butcher M. J. Zhu J. (2021). Recent advances in understanding the Th1/Th2 effector choice. Fac. Rev. 10:30. doi: 10.12703/r/10-30 33817699 PMC8009194

[ref6] Corripio-Miyar Y. Hayward A. Lemon H. Sweeny A. R. Bal X. Kenyon F. (2022). Functionally distinct T-helper cell phenotypes predict resistance to different types of parasites in a wild mammal. Sci. Rep. 12:3197. doi: 10.1038/s41598-022-07149-9, PMID: 35210503 PMC8873199

[ref7] Della B. C. Benagiano M. De Gennaro M. Gomez-Morales M. A. Ludovisi A. D'Elios S. (2017). T-cell clones in human trichinellosis: evidence for a mixed Th1/Th2 response. Parasite Immunol. 39:e12412. doi: 10.1111/pim.12412, PMID: 28106258

[ref8] Dixon M. A. Winskill P. Harrison W. E. Whittaker C. Schmidt V. Sarti E. (2020). Force-of-infection of *Taenia solium* porcine cysticercosis: a modelling analysis to assess global incidence and prevalence trends. Sci. Rep. 10:17637. doi: 10.1038/s41598-020-74007-x, PMID: 33077748 PMC7572398

[ref9] Donnelly S. Stack C. M. O’Neill S. M. Sayed A. A. Williams D. L. Dalton J. P. (2008). Helminth 2-Cys peroxiredoxin drives Th2 responses through a mechanism involving alternatively activated macrophages. FASEB J. 22, 4022–4032. doi: 10.1096/fj.08-106278, PMID: 18708590 PMC3980656

[ref10] Engels D. Zhou X. N. (2020). Neglected tropical diseases: an effective global response to local poverty-related disease priorities. Infect. Dis. Poverty 9:10. doi: 10.1186/s40249-020-0630-9, PMID: 31987053 PMC6986060

[ref11] Fan X. M. Zhang Y. Ouyang R. H. Luo B. Li L. Z. He W. (2021). *Cysticercus cellulosae* regulates T-cell responses and interacts with the host immune system by excreting and secreting antigens. Front. Cell. Infect. Microbiol. 11:728222. doi: 10.3389/fcimb.2021.728222, PMID: 34540719 PMC8447960

[ref12] Gadahi J. A. Yongqian B. Ehsan M. Zhang Z. C. Wang S. Yan R. F. (2016). *Haemonchus contortus* excretory and secretory proteins (HcESPs) suppress functions of goat PBMCs in vitro. Oncotarget 7, 35670–35679. doi: 10.18632/oncotarget.9589, PMID: 27229536 PMC5094953

[ref13] Georgiev P. Charbonnier L. M. Chatila T. A. (2019). Regulatory T cells: the many faces of Foxp3. J. Clin. Immunol. 39, 623–640. doi: 10.1007/s10875-019-00684-7, PMID: 31478130 PMC6754763

[ref14] Gulelat Y. Eguale T. Kebede N. Aleme H. Fèvre E. M. Cook E. A. J. (2022). Epidemiology of porcine cysticercosis in eastern and southern Africa: systematic review and meta-analysis. Public Health 10:836177. doi: 10.3389/fpubh.2022.836177, PMID: 35372187 PMC8966092

[ref15] Hamamoto-Filho P. T. Fragoso G. Sciutto E. Fleury A. (2021). Inflammation in neurocysticercosis: clinical relevance and impact on treatment decisions. Expert Rev. Anti-Infect. Ther. 19, 1503–1518. doi: 10.1080/14787210.2021.1912592, PMID: 33794119

[ref16] Han Y. H. Zhao B. Zhang M. Hong Y. Han H. X. Cao X. D. (2017). Biochemical properties and vaccine effect of recombinant TPx-3 from *Schistosoma japonicum*. Parasitol. Res. 116, 1361–1372. doi: 10.1007/s00436-017-5415-0, PMID: 28285327

[ref17] Harnett W. (2014). Secretory products of helminth parasites as immunomodulators. Mol. Biochem. Parasitol. 195, 130–136. doi: 10.1016/j.molbiopara.2014.03.007 24704440

[ref18] Hayes E. T. Hagan C. E. Khoryati L. Gavin M. A. Campbell D. J. (2020). Regulatory T cells maintain selective access to IL-2 and immune homeostasis despite substantially reduced CD25 function. J. Immunol. 205, 2667–2678. doi: 10.4049/jimmunol.1901520, PMID: 33055282 PMC7657993

[ref19] Hilligan K. L. Ronchese F. (2020). Antigen presentation by dendritic cells and their instruction of CD4+T helper cell responses. Cell. Mol. Immunol. 17, 587–599. doi: 10.1038/s41423-020-0465-0, PMID: 32433540 PMC7264306

[ref20] Himwaze C. Mucheleng'anga L. A. Telendiy V. Hamukale A. Tembo J. Kapata N. (2022). Cardiac cysticercosis and neurocysticercosis in sudden and unexpected community deaths in Lusaka, Zambia: a descriptive medico-legal post-mortem examination study. Int. J. Infect. Dis. 115, 195–200. doi: 10.1016/j.ijid.2021.11.042, PMID: 34896266

[ref21] Huang K. Ren H. Y. Lin B. Y. Liu Y. Y. Guo Q. F. (2022). Protective effects of Wuwei Xiaodu drink against chronic osteomyelitis through Foxp3+CD25+CD4+Treg cells via the IL-2/STAT5 signaling pathway. Chin. J. Nat. Med. 20, 185–193. doi: 10.1016/S1875-5364(22)60146-8, PMID: 35369962

[ref22] Islam M. A. Große-Brinkhaus C. Pröll M. J. Uddin M. J. Rony S. A. Tesfaye D. (2016). Deciphering transcriptome profiles of peripheral blood mononuclear cells in response to PRRSV vaccination in pigs. BMC Genomics 17:641. doi: 10.1186/s12864-016-2849-1, PMID: 27528396 PMC4986384

[ref23] Jeferson C. D. L. Karina M. M. Tatiana N. B. C. Gabriela P. P. Hercules M. John R. B. (2017). Comparative proteomics of the larval and adult stages of the model cestode parasite *Mesocestoides corti*. J. Proteome 175, 127–135. doi: 10.1016/j.jprot.2017.12.022, PMID: 29317356 PMC10486185

[ref24] Jin Q. W. Zhang N. Z. Li W. H. Qin H. T. Liu Y. J. Ohiolei J. A. (2020). *Trichinella spiralis* thioredoxin peroxidase 2 regulates protective Th2 immune response in mice by directly inducing alternatively activated macrophages. Front. Immunol. 11:2015. doi: 10.3389/fimmu.2020.02015, PMID: 33072069 PMC7544948

[ref25] José N. P. Marta I. Joao A. P. Ricardo C. C. Jeanette F. B. Beatriz H. T. (2017). Quantitative multiplexed proteomics of *Taenia solium* cysts obtained from the skeletal muscle and central nervous system of pigs. PLoS Negl. Trop. Dis. 11:e0005962. doi: 10.1371/journal.pntd.0005962, PMID: 28945737 PMC5634658

[ref26] Junginger J. Raue K. Wolf K. Janecek E. Stein V. M. Tipold A. (2017). Zoonotic intestinal helminths interact with the canine immune system by modulating T cell responses and preventing dendritic cell maturation. Sci. Rep. 7:10310. doi: 10.1038/s41598-017-10677-4, PMID: 28871165 PMC5583179

[ref27] Kaur R. Arora N. Rawat S. S. Keshri A. K. Sharma S. R. Mishra A. (2021). Vaccine for a neglected tropical disease *Taenia solium* cysticercosis: fight for eradication against all odds. Expert Rev. Vaccines 20, 1447–1458. doi: 10.1080/14760584.2021.1967750, PMID: 34379534

[ref28] Khan I. A. Hwang S. Moretto M. (2019). *Toxoplasma gondii*: CD8 T cells cry for CD4 help. Front. Cell. Infect. Microbiol. 9:136. doi: 10.3389/fcimb.2019.00136, PMID: 31119107 PMC6504686

[ref29] Letendre C. Auger J. P. Lemire P. Galbas T. Gottschalk M. Thibodeau J. (2018). *Streptococcus suis* serotype 2 infection impairs interleukin-12 production and the MHC-II-restricted antigen presentation capacity of dendritic cells. Front. Immunol. 9:1199. doi: 10.3389/fimmu.2018.01199, PMID: 29899744 PMC5988873

[ref30] Li L. Z. Zhou B. Y. (2022). Eukaryotic expression and antigen epitope prediction of the LRRC15 protein in excretory secretory antigens of *Taenia solium* cysticercus. Chin. J. Schistosomiasis Control 34, 286–291. doi: 10.16250/j.32.1374.2021227, PMID: 35896492

[ref31] Malek T. R. (2003). The main function of IL-2 is to promote the development of T regulatory cells. J. Leukoc. Biol. 74, 961–965. doi: 10.1189/jlb.0603272, PMID: 12960253

[ref32] Mendlovic F. Cruz-Rivera M. Ávila G. Vaughan G. Flisser A. (2015). Correction: cytokine, antibody and proliferative cellular responses elicited by *Taenia solium* calreticulin upon experimental infection in hamsters. PLoS One 10:e0141177. doi: 10.1371/journal.pone.0141177, PMID: 26469943 PMC4607426

[ref33] Mendlovic F. Fleury A. Flisser A. (2021). Zoonotic Taenia infections with focus on cysticercosis due to *Taenia solium* in swine and humans. Res. Vet. Sci. 134, 69–77. doi: 10.1016/j.rvsc.2020.11.015, PMID: 33321377

[ref01] Mülayim S. Dalkılıç S. Akbulut H. H. Aksoy A. Kaplan M. (2022). Investigation of the relationship between lymphocyte subsets and intestinal parasites. Acta Trop. 225:106221. doi: 10.1016/j.actatropica.2021.106221 34757042

[ref34] Nakamae S. Kimura D. Miyakoda M. Sukhbaatar O. Inoue S. I. Yui K. (2019). Role of IL-10 in inhibiting protective immune responses against infection with heterologous plasmodium parasites. Parasitol. Int. 70, 5–15. doi: 10.1016/j.parint.2019.01.003, PMID: 30639137

[ref35] Peterson A. C. Russell J. D. Bailey D. J. Westphall M. S. Coon J. J. (2012). Parallel reaction monitoring for high resolution and high mass accuracy quantitative, targeted proteomics. Mol. Cell. Proteomics 11, 1475–1488. doi: 10.1074/mcp.O112.020131, PMID: 22865924 PMC3494192

[ref36] Prodjinotho U. F. Lema J. Lacorcia M. Schmidt V. Vejzagic N. Sikasunge C. (2020). Host immune responses during *Taenia solium* neurocysticercosis infection and treatment. PLoS Negl. Trop. Dis. 14:e0008005. doi: 10.1371/journal.pntd.0008005, PMID: 32298263 PMC7162612

[ref37] Rosales-Mendoza S. Monreal-Escalante E. González-Ortega O. Hernández M. Fragoso G. Garate T. (2018). Transplastomic plants yield a multicomponent vaccine against cysticercosis. J. Biotechnol. 266, 124–132. doi: 10.1016/j.jbiotec.2017.12.012, PMID: 29253519

[ref38] Shi T. Song E. Nie S. Rodland K. D. Liu T. Qian W. J. (2016). Advances in targeted proteomics and applications to biomedical research. Proteomics 16, 2160–2182. doi: 10.1002/pmic.201500449, PMID: 27302376 PMC5051956

[ref39] Sobsey C. A. Ibrahim S. Richard V. R. Gaspar V. Mitsa G. Lacasse V. (2020). Targeted and untargeted proteomics approaches in biomarker development. Proteomics 20:e1900029. doi: 10.1002/pmic.201900029, PMID: 31729135

[ref40] Steen E. H. Wang X. Balaji S. Butte M. J. Bollyky P. L. Keswani S. G. (2020). The role of the anti-inflammatory cytokine interleukin-10 in tissue fibrosis. Adv. Wound Care (New Rochelle) 9, 184–198. doi: 10.1089/wound.2019.1032, PMID: 32117582 PMC7047112

[ref41] Sun X. M. Guo K. Hao C. Y. Zhan B. Huang J. J. Zhu X. (2019). *Trichinella spiralis* excretory-secretory products stimulate host regulatory T cell differentiation through activating dendritic cells. Cells 8:1404. doi: 10.3390/cells8111404, PMID: 31703440 PMC6912532

[ref42] Tian A. L. Tian X. Chen D. Lu M. Calderón-Mantilla G. Yuan X. D. (2020). Modulation of the functions of goat peripheral blood mononuclear cells by *Fasciola gigantica* thioredoxin peroxidase in vitro. Pathogens 9:758. doi: 10.3390/pathogens9090758, PMID: 32957426 PMC7559183

[ref43] White R. R. Artavanis-Tsakonas K. (2012). How helminths use excretory secretory fractions to modulate dendritic cells. Virulence 3, 668–677. doi: 10.4161/viru.22832, PMID: 23221477 PMC3545949

[ref44] World Health Organization. (2022). Taeniasis/cysticercosis. Available at: https://www.who.int/news-room/fact-sheets/detail/taeniasis-cysticercosis

[ref45] Zhang S. Gang X. Yang S. Cui M. Sun L. Li Z. (2021). The alterations in and the role of the Th17/Treg balance in metabolic diseases. Front. Immunol. 12:678355. doi: 10.3389/fimmu.2021.678355, PMID: 34322117 PMC8311559

[ref46] Zhang Y. Luo B. Liu M. C. OuYang R. H. Fan X. M. Zhou B. Y. (2022). Analysis of immune response in BALB/c mice immunized with recombinant plasmids pMZ-X3-Ts14-3-3.3 and pMZ-X3-sp-Ts14-3-3.3 of *Taenia solium*. Acta Trop. 232:106517. doi: 10.1016/j.actatropica.2022.106517, PMID: 35595093

