# Supplementary material for: Regulation of piglet T-cell immune responses by thioredoxin peroxidase from Cysticercus cellulosae excretory-secretory antigens
Source: Front Microbiol. 2022 Nov 18;13:1019810. doi: 10.3389/fmicb.2022.1019810 (PMC9718028; doi:10.3389/fmicb.2022.1019810)
Supplement: Supplementary file 1 [file Data_Sheet_1.PDF]

1 Supplementary Material

After the recombinant plasmid pcDNA3.4-TPx was digested by *EcoR*I and *BamH*I, the pcDNA3.4 vector fragment of 6011 bp and the TPx target gene fragment of 678 bp were obtained by 1% agarose gel electrophoresis, which were consistent with the expected results (**Supplementary Figure 1A**). The sequencing results were compared with the expected TPx gene sequence, and the matching degree was 100%, indicating that the recombinant plasmid pcDNA3.4-TPx was successfully constructed. The recombinant plasmid pcDNA3.4-TPx was transfected into HEK293 cells and cultured for 6 d. By SDS-PAGE analysis, it was found that there was a target protein band with molecular weight of approximately 26 KDa on the supernatant of the cell secretory medium (**Supplementary Figure 1B**). The target protein in the supernatant of the cell secretory culture was further purified, and SDS-PAGE analysis showed that the molecular weight was approximately 26 KDa (**Supplementary Figure 1C**). The purified TPx recombinant protein was identified by Western blot. Using Anti-His-labeled antibody as the primary antibody, it was found that the purified TPx recombinant protein could be recognized and purified (**Supplementary Figure 1D**). The results showed that TPx protein was successfully expressed using the eukaryotic expression technique.

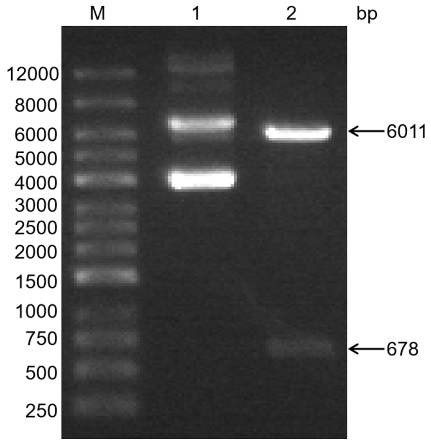

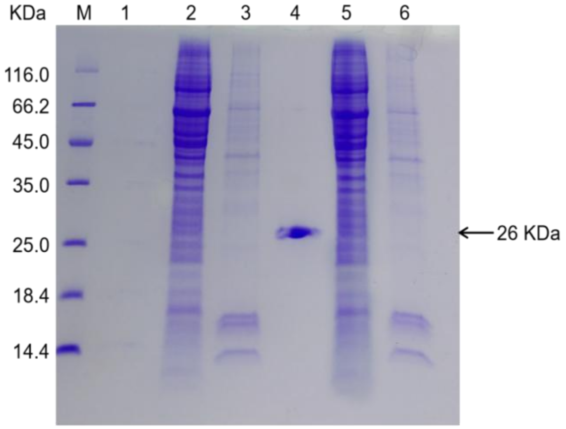

A **B**

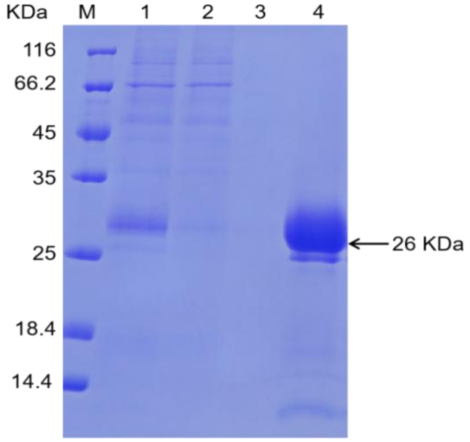

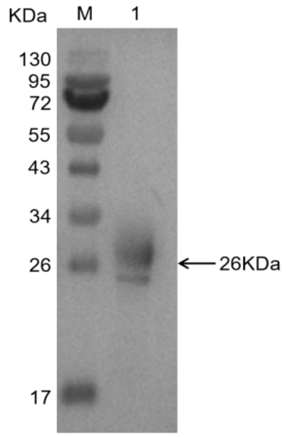

C **D**

Figure 1 Eukaryotic expression experiment of TPx protein. (A) Identification of recombinant plasmid pcDNA3.4-TPx by 1% agarose gel electrophoresis. Lane M: DNA markers; Lane 1: plasmid before endonuclease digestion; Lane 2: restriction endonuclease digested plasmid. (B) SDS-PAGE analysis of the expression of recombinant TPx. Lane M: Protein molecular weight marker; Lane 1: cell secretory medium supernatant (negative); Lane 2: cell lysate supernatant (negative); Lane 3: cell lysate precipitation (negative); Lane 4: cell secretory medium supernatant (TPx); Lane 5: cell lysate supernatant (TPx); Lane 6: cell lysate precipitation (TPx). (C) Purification of recombinant TPx by SDS-PAGE. Lane M: protein molecular weight marker; Lane 1: supernatant of cell secretory medium; Lane 2: effluent; Lane 3: washing liquid; Lane 4: eluate. (D) Identification of recombinant TPx by Western blotting. Lane M: protein molecular weight marker; Lane 1: recombinant TPx reaction with His-tag antibody (1: 1000).
