# Supplementary material for: Regulation of piglet T-cell immune responses by thioredoxin peroxidase from Cysticercus cellulosae excretory-secretory antigens
Source: Front Microbiol. 2022 Nov 18;13:1019810. doi: 10.3389/fmicb.2022.1019810 (PMC9718028; doi:10.3389/fmicb.2022.1019810)
Supplement: Supplementary file 2 [file Data_Sheet_2.ZIP › 1. Screening and Validation of TPx Protein/SPSS statistical analysis/3. Receptor protein-tyrosine kinase/3.3 (SPSS data export) Receptor protein-tyrosine kinase.doc]

Explore

Notes	
Output Created	10-SEP-2022 19:13:17	
Comments		
Input	Data	E:\×ÀÃæ\Raw Data\1. Screening and Validation of TPx Protein\SPSS statistical analysis\2. Proteasome subunit beta\2.1 Proteasome subunit beta.sav	
	Active Dataset	DataSet2	
	Filter	<none>	
	Weight	<none>	
	Split File	<none>	
	N of Rows in Working Data File	6	
Missing Value Handling	Definition of Missing	User-defined missing values for dependent variables are treated as missing.	
	Cases Used	Statistics are based on cases with no missing values for any dependent variable or factor used.	
Syntax	EXAMINE VARIABLES=Numerical value BY variable
  /PLOT BOXPLOT STEMLEAF NPPLOT
  /COMPARE GROUPS
  /STATISTICS DESCRIPTIVES
  /CINTERVAL 95
  /MISSING LISTWISE
  /NOTOTAL.	
Resources	Processor Time	00:00:00.55	
	Elapsed Time	00:00:00.44	


Case Processing Summary	
	variable	Cases	
		Valid	Missing	Total	
		N	Percent	N	Percent	N	Percent	
Numerical value	Cysticercus	3	100.0%	0	0.0%	3	100.0%	
	ESAa	3	100.0%	0	0.0%	3	100.0%	


Descriptives	
	variable	Statistic	Std. Error	
Numerical value	Cysticercus	Mean	.651500	.1181594	
		95% Confidence Interval for Mean	Lower Bound	.143101		
			Upper Bound	1.159899		
		5% Trimmed Mean	.		
		Median	.595600		
		Variance	.042		
		Std. Deviation	.2046581		
		Minimum	.4806		
		Maximum	.8783		
		Range	.3977		
		Interquartile Range	.		
		Skewness	1.137	1.225	
		Kurtosis	.	.	
	ESAa	Mean	1.348533	.0485593	
		95% Confidence Interval for Mean	Lower Bound	1.139600		
			Upper Bound	1.557467		
		5% Trimmed Mean	.		
		Median	1.349800		
		Variance	.007		
		Std. Deviation	.0841072		
		Minimum	1.2638		
		Maximum	1.4320		
		Range	.1682		
		Interquartile Range	.		
		Skewness	-.068	1.225	
		Kurtosis	.	.	


Tests of Normality	
	variable	Kolmogorov-Smirnova	Shapiro-Wilk	
		Statistic	df	Sig.	Statistic	df	Sig.	
Numerical value	Cysticercus	.274	3	.	.944	3	.544	
	ESAa	.176	3	.	1.000	3	.975	

a. Lilliefors Significance Correction	


Normal Q-Q Plots


Detrended Normal Q-Q Plots


Oneway

Notes	
Output Created	10-SEP-2022 19:13:31	
Comments		
Input	Data	E:\×ÀÃæ\Raw Data\1. Screening and Validation of TPx Protein\SPSS statistical analysis\2. Proteasome subunit beta\2.1 Proteasome subunit beta.sav	
	Active Dataset	DataSet2	
	Filter	<none>	
	Weight	<none>	
	Split File	<none>	
	N of Rows in Working Data File	6	
Missing Value Handling	Definition of Missing	User-defined missing values are treated as missing.	
	Cases Used	Statistics for each analysis are based on cases with no missing data for any variable in the analysis.	
Syntax	ONEWAY Numerical value BY variable
  /POLYNOMIAL=1
  /STATISTICS DESCRIPTIVES HOMOGENEITY
  /MISSING ANALYSIS
  /POSTHOC=LSD ALPHA(0.05).	
Resources	Processor Time	00:00:00.02	
	Elapsed Time	00:00:00.01	


Warnings	
Post hoc tests are not performed for Numerical value because there are fewer than three groups.	


Descriptives	
Numerical value  	
	N	Mean	Std. Deviation	Std. Error	95% Confidence Interval for Mean			
					Lower Bound	Upper Bound			
Cysticercus	3	.651500	.2046581	.1181594	.143101	1.159899			
ESAa	3	1.348533	.0841072	.0485593	1.139600	1.557467			
Total	6	1.000017	.4066205	.1660021	.573295	1.426739			


Test of Homogeneity of Variances	
	Levene Statistic	df1	df2	Sig.	
Numerical value	Based on Mean	2.724	1	4	.174	
	Based on Median	.778	1	4	.428	
	Based on Median and with adjusted df	.778	1	2.461	.455	
	Based on trimmed mean	2.541	1	4	.186	


ANOVA	
Numerical value  	
	Sum of Squares	df	Mean Square	F		
Between Groups	(Combined)	.729	1	.729	29.771		
	Linear Term	Contrast	.729	1	.729	29.771		
Within Groups	.098	4	.024			
Total	.827	5				
