# Supplementary material for: Regulation of piglet T-cell immune responses by thioredoxin peroxidase from Cysticercus cellulosae excretory-secretory antigens
Source: Front Microbiol. 2022 Nov 18;13:1019810. doi: 10.3389/fmicb.2022.1019810 (PMC9718028; doi:10.3389/fmicb.2022.1019810)
Supplement: Supplementary file 2 [file Data_Sheet_2.ZIP › 1. Screening and Validation of TPx Protein/SPSS statistical analysis/4. t-SNARE coiled-coil homology domain-containing protein/4.3 (SPSS data export) t-SNARE coiled-coil homology domain-containing protein.doc]

Explore

Notes	
Output Created	10-SEP-2022 19:22:06	
Comments		
Input	Data	E:\×ÀÃæ\Raw Data\1. Screening and Validation of TPx Protein\SPSS statistical analysis\3. Receptor protein-tyrosine kinase\3.1 3. Receptor protein-tyrosine kinase.sav	
	Active Dataset	DataSet2	
	Filter	<none>	
	Weight	<none>	
	Split File	<none>	
	N of Rows in Working Data File	6	
Missing Value Handling	Definition of Missing	User-defined missing values for dependent variables are treated as missing.	
	Cases Used	Statistics are based on cases with no missing values for any dependent variable or factor used.	
Syntax	EXAMINE VARIABLES=Numerical value BY variable
  /PLOT BOXPLOT STEMLEAF NPPLOT
  /COMPARE GROUPS
  /STATISTICS DESCRIPTIVES
  /CINTERVAL 95
  /MISSING LISTWISE
  /NOTOTAL.	
Resources	Processor Time	00:00:00.58	
	Elapsed Time	00:00:00.44	


Case Processing Summary	
	variable	Cases	
		Valid	Missing	Total	
		N	Percent	N	Percent	N	Percent	
Numerical value	Cysticercus	3	100.0%	0	0.0%	3	100.0%	
	ESAa	3	100.0%	0	0.0%	3	100.0%	


Descriptives	
	variable	Statistic	Std. Error	
Numerical value	Cysticercus	Mean	.702133	.0065198	
		95% Confidence Interval for Mean	Lower Bound	.674081		
			Upper Bound	.730186		
		5% Trimmed Mean	.		
		Median	.703800		
		Variance	.000		
		Std. Deviation	.0112926		
		Minimum	.6901		
		Maximum	.7125		
		Range	.0224		
		Interquartile Range	.		
		Skewness	-.650	1.225	
		Kurtosis	.	.	
	ESAa	Mean	1.497867	.0088667	
		95% Confidence Interval for Mean	Lower Bound	1.459716		
			Upper Bound	1.536017		
		5% Trimmed Mean	.		
		Median	1.491400		
		Variance	.000		
		Std. Deviation	.0153575		
		Minimum	1.4868		
		Maximum	1.5154		
		Range	.0286		
		Interquartile Range	.		
		Skewness	1.559	1.225	
		Kurtosis	.	.	


Tests of Normality	
	variable	Kolmogorov-Smirnova	Shapiro-Wilk	
		Statistic	df	Sig.	Statistic	df	Sig.	
Numerical value	Cysticercus	.225	3	.	.984	3	.755	
	ESAa	.330	3	.	.867	3	.287	

a. Lilliefors Significance Correction	


Normal Q-Q Plots


Detrended Normal Q-Q Plots


Oneway

Notes	
Output Created	10-SEP-2022 19:22:48	
Comments		
Input	Data	E:\×ÀÃæ\Raw Data\1. Screening and Validation of TPx Protein\SPSS statistical analysis\3. Receptor protein-tyrosine kinase\3.1 3. Receptor protein-tyrosine kinase.sav	
	Active Dataset	DataSet2	
	Filter	<none>	
	Weight	<none>	
	Split File	<none>	
	N of Rows in Working Data File	6	
Missing Value Handling	Definition of Missing	User-defined missing values are treated as missing.	
	Cases Used	Statistics for each analysis are based on cases with no missing data for any variable in the analysis.	
Syntax	ONEWAY Numerical value BY variable
  /POLYNOMIAL=1
  /STATISTICS DESCRIPTIVES HOMOGENEITY
  /MISSING ANALYSIS
  /POSTHOC=LSD ALPHA(0.05).	
Resources	Processor Time	00:00:00.00	
	Elapsed Time	00:00:00.00	


Warnings	
Post hoc tests are not performed for Numerical value because there are fewer than three groups.	


Descriptives	
Numerical value 	
	N	Mean	Std. Deviation	Std. Error	95% Confidence Interval for Mean			
					Lower Bound	Upper Bound			
Cysticercus	3	.702133	.0112926	.0065198	.674081	.730186			
ESAa	3	1.497867	.0153575	.0088667	1.459716	1.536017			
Total	6	1.100000	.4360078	.1779994	.642438	1.557562			


Test of Homogeneity of Variances	
	Levene Statistic	df1	df2	Sig.	
Numerical value	Based on Mean	.652	1	4	.465	
	Based on Median	.061	1	4	.817	
	Based on Median and with adjusted df	.061	1	3.089	.821	
	Based on trimmed mean	.573	1	4	.491	


ANOVA	
Numerical value  	
	Sum of Squares	df	Mean Square	F		
Between Groups	(Combined)	.950	1	.950	5227.564		
	Linear Term	Contrast	.950	1	.950	5227.564		
Within Groups	.001	4	.000			
Total	.951	5				
