# Supplementary material for: Regulation of piglet T-cell immune responses by thioredoxin peroxidase from Cysticercus cellulosae excretory-secretory antigens
Source: Front Microbiol. 2022 Nov 18;13:1019810. doi: 10.3389/fmicb.2022.1019810 (PMC9718028; doi:10.3389/fmicb.2022.1019810)
Supplement: Supplementary file 2 [file Data_Sheet_2.ZIP › 1. Screening and Validation of TPx Protein/SPSS statistical analysis/5. Thioredoxin peroxidase/5.3 (SPSS data export) Thioredoxin peroxidase.doc]

Explore
Notes	
Output Created	10-SEP-2022 20:38:53	
Comments		
Input	Data	E:\×ÀÃæ\Raw Data\1. Screening and Validation of TPx Protein\SPSS statistical analysis\4. t-SNARE coiled-coil homology domain-containing protein\4.1 t-SNARE coiled-coil homology domain-containing protein.sav	
	Active Dataset	DataSet2	
	Filter	<none>	
	Weight	<none>	
	Split File	<none>	
	N of Rows in Working Data File	6	
Missing Value Handling	Definition of Missing	User-defined missing values for dependent variables are treated as missing.	
	Cases Used	Statistics are based on cases with no missing values for any dependent variable or factor used.	
Syntax	EXAMINE VARIABLES=Numerical value BY variable
  /PLOT BOXPLOT STEMLEAF NPPLOT
  /COMPARE GROUPS
  /STATISTICS DESCRIPTIVES
  /CINTERVAL 95
  /MISSING LISTWISE
  /NOTOTAL.	
Resources	Processor Time	00:00:00.80	
	Elapsed Time	00:00:00.49	


Case Processing Summary	
	variable	Cases	
		Valid	Missing	Total	
		N	Percent	N	Percent	N	Percent	
Numerical value	Cysticercus	3	100.0%	0	0.0%	3	100.0%	
	ESAa	3	100.0%	0	0.0%	3	100.0%	


Descriptives	
	variable	Statistic	Std. Error	
Numerical value	Cysticercus	Mean	.577629	.0235218	
		95% Confidence Interval for Mean	Lower Bound	.476423		
			Upper Bound	.678835		
		5% Trimmed Mean	.		
		Median	.599976		
		Variance	.002		
		Std. Deviation	.0407409		
		Minimum	.5306		
		Maximum	.6023		
		Range	.0717		
		Interquartile Range	.		
		Skewness	-1.726	1.225	
		Kurtosis	.	.	
	ESAa	Mean	1.522371	.0456486	
		95% Confidence Interval for Mean	Lower Bound	1.325961		
			Upper Bound	1.718781		
		5% Trimmed Mean	.		
		Median	1.525888		
		Variance	.006		
		Std. Deviation	.0790657		
		Minimum	1.4416		
		Maximum	1.5996		
		Range	.1580		
		Interquartile Range	.		
		Skewness	-.200	1.225	
		Kurtosis	.	.	


Tests of Normality	
	variable	Kolmogorov-Smirnova	Shapiro-Wilk	
		Statistic	df	Sig.	Statistic	df	Sig.	
Numerical value	Cysticercus	.375	3	.	.774	3	.055	
	ESAa	.184	3	.	.999	3	.926	

a. Lilliefors Significance Correction	


Normal Q-Q Plots


Detrended Normal Q-Q Plots


Oneway
Notes	
Output Created	10-SEP-2022 20:39:13	
Comments		
Input	Data	E:\×ÀÃæ\Raw Data\1. Screening and Validation of TPx Protein\SPSS statistical analysis\4. t-SNARE coiled-coil homology domain-containing protein\4.1 t-SNARE coiled-coil homology domain-containing protein.sav	
	Active Dataset	DataSet2	
	Filter	<none>	
	Weight	<none>	
	Split File	<none>	
	N of Rows in Working Data File	6	
Missing Value Handling	Definition of Missing	User-defined missing values are treated as missing.	
	Cases Used	Statistics for each analysis are based on cases with no missing data for any variable in the analysis.	
Syntax	ONEWAY Numerical value BY variable
  /POLYNOMIAL=1
  /STATISTICS DESCRIPTIVES HOMOGENEITY
  /MISSING ANALYSIS
  /POSTHOC=LSD ALPHA(0.05).	
Resources	Processor Time	00:00:00.00	
	Elapsed Time	00:00:00.01	


Warnings	
Post hoc tests are not performed for Numerical value because there are fewer than three groups.	


Descriptives	
Numerical value	
	N	Mean	Std. Deviation	Std. Error	95% Confidence Interval for Mean			
					Lower Bound	Upper Bound			
Cysticercus	3	.577629	.0407409	.0235218	.476423	.678835			
ESAa	3	1.522371	.0790657	.0456486	1.325961	1.718781			
Total	6	1.050000	.5205049	.2124952	.503764	1.596236			


Test of Homogeneity of Variances	
	Levene Statistic	df1	df2	Sig.	
Numerical value	Based on Mean	.727	1	4	.442	
	Based on Median	.678	1	4	.456	
	Based on Median and with adjusted df	.678	1	3.910	.457	
	Based on trimmed mean	.732	1	4	.441	


ANOVA	
Numerical value  	
	Sum of Squares	df	Mean Square	F		
Between Groups	(Combined)	1.339	1	1.339	338.458		
	Linear Term	Contrast	1.339	1	1.339	338.458		
Within Groups	.016	4	.004			
Total	1.355	5				
