# Supplementary material for: Regulation of piglet T-cell immune responses by thioredoxin peroxidase from Cysticercus cellulosae excretory-secretory antigens
Source: Front Microbiol. 2022 Nov 18;13:1019810. doi: 10.3389/fmicb.2022.1019810 (PMC9718028; doi:10.3389/fmicb.2022.1019810)
Supplement: Supplementary file 2 [file Data_Sheet_2.ZIP › 2. C. Cellulosae ESAs and TPx Induced CD4+ and CD8+ T-Lymphocyte Responses in PBMCs/2. SPSS statistical analysis/2.1 SPSS statistical analysis--CD4+/2.1.3 (SPSS data export) SPSS statistical analysis--CD4+.doc]

Explore


Notes	
Output Created	11-SEP-2022 16:33:56	
Comments		
Input	Active Dataset	DataSet0	
	Filter	<none>	
	Weight	<none>	
	Split File	<none>	
	N of Rows in Working Data File	26	
Missing Value Handling	Definition of Missing	User-defined missing values for dependent variables are treated as missing.	
	Cases Used	Statistics are based on cases with no missing values for any dependent variable or factor used.	
Syntax	EXAMINE VARIABLES=Numerical BY variable
  /PLOT BOXPLOT NPPLOT
  /COMPARE GROUPS
  /STATISTICS DESCRIPTIVES
  /CINTERVAL 95
  /MISSING LISTWISE
  /NOTOTAL.	
Resources	Processor Time	00:00:02.45	
	Elapsed Time	00:00:01.66	


variable


Case Processing Summary	
	variable	Cases	
		Valid	Missing	Total	
		N	Percent	N	Percent	N	Percent	
Numerical	Control	4	100.0%	0	0.0%	4	100.0%	
	ESAs	4	100.0%	0	0.0%	4	100.0%	
	TPx	4	100.0%	0	0.0%	4	100.0%	
	ConA	4	100.0%	0	0.0%	4	100.0%	


Descriptives	
	variable	Statistic	Std. Error	
Numerical	Control	Mean	22.066667	.1699673	
		95% Confidence Interval for Mean	Lower Bound	21.525755		
			Upper Bound	22.607579		
		5% Trimmed Mean	22.074074		
		Median	22.133333		
		Variance	.116		
		Std. Deviation	.3399346		
		Minimum	21.6000		
		Maximum	22.4000		
		Range	.8000		
		Interquartile Range	.6333		
		Skewness	-1.056	1.014	
		Kurtosis	1.500	2.619	
	ESAs	Mean	32.533333	2.5378250	
		95% Confidence Interval for Mean	Lower Bound	24.456842		
			Upper Bound	40.609825		
		5% Trimmed Mean	32.353704		
		Median	30.916667		
		Variance	25.762		
		Std. Deviation	5.0756499		
		Minimum	28.6000		
		Maximum	39.7000		
		Range	11.1000		
		Interquartile Range	9.1333		
		Skewness	1.394	1.014	
		Kurtosis	1.500	2.619	
	TPx	Mean	35.233333	1.4504788	
		95% Confidence Interval for Mean	Lower Bound	30.617262		
			Upper Bound	39.849404		
		5% Trimmed Mean	35.309259		
		Median	35.916667		
		Variance	8.416		
		Std. Deviation	2.9009577		
		Minimum	31.2000		
		Maximum	37.9000		
		Range	6.7000		
		Interquartile Range	5.3667		
		Skewness	-1.204	1.014	
		Kurtosis	1.500	2.619	
	ConA	Mean	27.066667	.3009245	
		95% Confidence Interval for Mean	Lower Bound	26.108991		
			Upper Bound	28.024343		
		5% Trimmed Mean	27.051852		
		Median	26.933333		
		Variance	.362		
		Std. Deviation	.6018490		
		Minimum	26.5000		
		Maximum	27.9000		
		Range	1.4000		
		Interquartile Range	1.1167		
		Skewness	1.155	1.014	
		Kurtosis	1.500	2.619	


Tests of Normality	
	variable	Kolmogorov-Smirnova	Shapiro-Wilk	
		Statistic	df	Sig.	Statistic	df	Sig.	
Numerical	Control	.250	4	.	.944	4	.677	
	ESAs	.250	4	.	.863	4	.271	
	TPx	.250	4	.	.925	4	.564	
	ConA	.250	4	.	.932	4	.608	

a. Lilliefors Significance Correction	


Numerical


Normal Q-Q Plots


Þ÷sî]`fp.`~`~`~`~`~`~`~`~`~ÌÌÌÌÌÌÀX÷Ø<x0gûoØhs:´~ýúÔÖÖ¾ýöÛ7]áØ¹+ðü+V¼úê«ãè3gÎÔÕÕãå8qâ¾ûî+--Ä4²ÉYæàæçÚ¹sç^¼x±P4â¹ç»+½÷æYÛ--r×(¼òÊ+·Ú9ãîÆ¦ÿnccc¡GÆüæ`_à¡*8sæLä=o½õV*º~ýzCcwwwÎUÝê"#þô+WvîÜZMùE^¾|9LDdÓÁ0?7?×ÆãñðûÐ¡C£ñ_lÝº5*644$Ïììì¼ûî»ï¿ÿþtËûï¿¿jÕªÒÒÒõë×òÉ''NX±bEYYÙ2/V=ÿüóUUUQY3üô¬Ñ$àÑGíµ×ÀÁCcSSSÎ×x«dÿé ¡%lä-uÎKÙh|%²ì³ç1 ®®®åË§'oú	Ç[½zuº`þüùqù¸æwåÊ.X° Ld?þøãÊÊÊL	Ïq¶.))	¿~øáOsUKï»ï¾pO?Ü¸qcÚF<óÈ/-í.]Êlâ.]ó5Þê"#þôåË£k~Ë-»¥ÎÉ/pã^p,ÅüÆPwwwl3gÎäÏ=ûãÿxÄ3¾Ð0?wÒüÂÄ#G2¯exâ0½yóæË7áahÌ|æ=®_¿]µZ|òÉT*uòäÉèaXsxØÙÙ¦dDËFfï½÷Âôùóç3g&¥¥¥9Û3½ÍEFû_úÛ­vNÎ­÷cl,æ7ÂÃcÇéè<òHþÜ£¹[·n^Îððp´àìÙ³Ç:æà_ ÔÃt8Oh_°`Aþøã£ôQx8þüÌ5¤ç¦[®^½ù°¿¿ÿÓ_M3ÏîÁX»vmté¦æWVVóälÇ"¶ÊRWW×ÕÕ³ÓÆÒ99·jÜ%²±ßX>IhÉæ9_Â­ù¸óæÎýK,ÊlÏ¾fý¡·`9×ÿaPÏ<îQUUÕ:³õn¤×0EFëvÚX:g4ßcl,æ7²/|°´ÑrÏ³ýãópçÍ/ðï|'<Lß%jÎÓ#®NÆ±HFþË/î®òþûï3DW¹¢¬444<ýôÓ/_~íµ×BãÖ­[sßX·ùÝjç¤÷clAùÍ=;LÜ£k~éË#tÌÀ_à¡J¨?jnÞ¼ùÊ~øáèSb·o~Ñu¯îîîà(¯¾úêX$àøñãQÍ1xÃÉ'£ëyGÉùou[2¿üõäàF¬äVõ"Äëå_ûÉ'DºùE[¬'ô^^µjÕXrþâÎ;ÃÇÓ/wè2¿/¦?Ýµ$÷Z°`Az¨ìíß¦M2W;þüð;s|kÎm~ì±Ç²«÷ÜsOyKÜùåïôGÜy½zKx½eeeã3¿èV7æÝ=¶÷¥^ºÐ0?b~¨Ùþ³ýlëÖ­e7hhhísý·*ýýýamaAyæ CaÖSO=uS	8pàÀ+JJJ-ZôÆo|ùË_N½ýEnÉüòwÎ#GüFw¼Á-õê-EvåÊÇ<Zs]]ÝéÓ§Çg~ÎÎÎµk×~/¤­­mì¹wuu­^½:Zð^H·;tÌþáááÇ,²[ÀüóóóóóóP89r$>ðÀØºÁø3eÇHÿO>y÷ÝwNX´hÑ®]»§OópÇ¨ªªòì'JÍ@ó·ôçÏ¿~ýúo¾V»iÓ¦q¬êÌ3uuuÌó0Ó[>&Úüþù°CMIO2?ÌÀdU¦ìÚµkîÜ¹³gÏÞ»wï§ùË_^»vmºñäÉQcºeóæÍ¡%´ôÑGuuu¥¥¥«W¯>vìXÎõdþÝü¼ñÆ±X¬¶¶öÒ¥KÙÎtõêÕÆÆÆ°lØì;wÞÒÅËU«Võå§7?6©ªªêÈ#Ù=ÝcyVåùTù¦¿ùÍoé¶¶¶`Zaâ[ßúVæs:;;ûûû3ÚÏ?¦D-aúîyï½÷ÂD.s=:ÿ"Áç¾óïx<½ÙO=õTOx÷ÝwÃÄóÏ??BÅFØm&A4Gs¯ü½ñòË/wwwàÙ4âæ_7!æ`Ì/HL¾A¶~e_K<f×®]Þ¸¦¦_íµhÖ~¸gÏµk×Æëá=ù6iîÜ¹ÙË.X° Úìë×¯Åýåç1¿ü½qíÚµh:¬a4óK¿Òü«ò&ÀüLùejÐhNI0­ªªªÙ³g_¾|9ü®¬¬fÚ_õÕðüzè'?ùI¶åüÓcYd´Mya/²Æ1^ó[¶lYh¿zõj~)Ìoxù§ÇÑ±ÀüL¸ù-0óÒTúâYAyáÂ¬6¤Ë¬²²²ððúÆh~ùIoÒÝwß½ìüùó£eÇñò÷ìÙ÷Ýw³g¥7Æh~ãèX`~&Öü¢®¯½öZTÆé¥n*(ýýýÑ­ð;=ü"sçÎUÑüò/üì»ßýnxâ'²üñÇÃôo¾ÙÓÓþ¬á¹råJð°¥K~ôÑGÁ£K?üð#szîÜ¹éÁ"#^é8:5¿T*kööíÛó9Ù<ùäaîöíÛÓ-G­¬¬&´sçÎ1_þEºººÂÜûî»/=Ä$ó9O?ýtØæ²²²M6üñÇ·ÔaÁÃâA^îÝ»7úßXz#súÈ#a¢bx¥ãëX`~`~ÌÌÌÌÌoüüÝßýÝDÁÁÁ_þòú¡°ø÷ÿw©bj·zSHãàêÕ«R¹æ÷§ú§AþDû·;sæ~&O>ùäïÿþïõCañ¯ÿú¯?þñõÔóãÔóãÔóãóÓRc~ÌC@jÌRc~ÌC@jÌRc~ÌCHM?0?pHù1?!50?p©ùCHÌBj`~B?HÌBj`~Róãóc~Róãóc~Róãóc~Rc~àóc~Rc~àóc~Rc~àóûON>½zõêÒÒÒU«Ve'Íü8ÔÀüÀ!¤â1¿¥K~ðÁaâí·ß^¶lY¶ù½÷Þ1:ÿôOÿtòäIý 5L4¿øÅ/~øÃêÂâç?ÿy8Åè©l~TTTdß+¯¼òCÎñãÇ¿ÿýïë©AjÔfÅ`~gÎijjRíU7T¡n(5ýP´ÕÞ«W¯6442?!50?p©éb6¿/ÆãñpTÍÅü8ÔÀüÀ!¤â1¿ãÇoØ°¡¿¿?çÇ!¤æ!5ùÅb±»2`~Bj`~àRÓEk~ùa~Bj`~àRó8ÔÀü8¤æ!5æÇü8¤æÇ! 5æÇü8¤æÇ! 5æÇü8¤æÇ! 5æÇü8¤ÆüÀ! 5æÇü8¤ÆüÀ! 5æÇü8¤ÆüÀ! 5æÇü8ÔÀüÀ! 5æÇü8ÔÀüÀ!¤æ!50?p©ùqHÌBjÌùqHÌC@jÌùqHÌC@jÌùqHÌC@jÌùqHùC@jÌùqHùC@jÌùqHùC@jÌùq©ùC@jÌùq©ùCHÌBj`~àRóã8ÔóãÔóãÔóãÔóãóÔóãóÔóãóÔóãRóÔ9ÔÀüÀ!¤æ!50?p©ùqHÌCp©1?æÇ! 50?©1?æÇ! 50?©1?æÇ! 5æÇü8¤ÆüÔ8¤ÆüÔ8¤ÆüÔ8¤Æü8Ô¤ÆüÀ!¤æ!50?Á!¤æ!50?©ùq!5æÇü8¤æÇ! 5æÇü8¤æÇ! 5æÇü8¤ÆüÔóãóÔóãóÔóãRóÔóãRóÔ8ÔÀüÀ!¤æÇ!8ÔÀüÀ!¤æÇ! 50?Á!¤ÆüÔÀü8¤ÆüÔÀü8¤ÆüÔóãóc~Rc~àóc~Rc~àÈ¦¯¯OjÌùq©ùù9]]]>øà¬Y³. 5æÇü8ÔÀüÀü¡¡¡ööö»~Ekk«ÔóãRóó+*zzzZZZb±Ø]ÿêêj©1?æÇ!¤ææW$3xý¬Y³F8ß¼yóöööJù1?!50?0¿Â&L¶µµ­Y³æ®,jkk_ýõ¡¡!©1?æÇ!¤ææWØ$;vÌ7oðÍ5kÛ¶mc<¡Kù1?!50?0¿iM8Y·Ë.ìVUUíÛ·o´a¼Rc~ÌCHÌÌ¯`H&û÷ï_·n]vawýúõííí©TJjÌùq©Iùù6Db÷îÝ9»Çóc~Rc~`~M*êêêÚ¶m[yyùçÅbûöíÇ7©1?æÇ! 5ææWØD#v«««³»ëÖ­_aWjÌùq©ùùM/zsv·oß~êÔ)©1?æÇ! 5ææWØDÝºººì|/'è©1?æÇ! 5ææWØ$ÉÖÖÖÝ wª°+5æÇü8ÔÀüÀ!¦ÞÞÞx<=z#´öÉÙ¤Æü8ÄJ¥:::rv«««[[[É¤Ôóãóó+l¦¶°+5æÇü8ÔÀüÀ!&ð~ÎYØ7o^sssoo¯ÔßDÑßßÅ8ÄD3­»RæwòäÉåË7óãRó8úúúÂY5å,ìvuuM~aWj3Ñü6mÚÔÛÛÇü^õÕ¿ÇèuþÁ~ ¤&ü*5©(¯¿þúæÍKJJFß9s¶nÝzèÐ!©l~ÿ¹£ß_üÅ_|ÑùÙÏ~vâÄ	ý 5LBjá¤üÇÿhÍØáÂÜsÏ=9oÅüíoûç?ÿ¹Ôb6?Õ^uC©Aµêã(ìæü¾µúúúéSØÚL¬ö2?!50?p;Ex666ÃË±ÛÒÒH$¤ÆüÔÀü_aJ¥Þyç#vkjjÚÚÚ¦|Ä®ÔóãRó¸]Z[[/^]Ømll,Äs(óæ7ÌCHÌ"'ÝÝÝMMMÙæ«¬¬,Â.óc~ÌCHÌ"ÉdrÿþýëÖ­Ëþ0_uuuûàà Ôóãó+lzsØÝ²eKggçt±ËüóãRó]]]7nÌ9b7(/ 50?©ù1¿ÂvÁÁÁ¶¶¶+WfØ]³fÍÁo:bwhhèõ×_onnþú×¿^(o`æÇüfC£DKKKÎÂî¶mÛÆøVâø»¿û»ÿcÉÿøý/ÿþ¿øÅÏ|æ3ßþö·¥ÆüÔÀüß´ J=ztûöíååå#/íÞ½;=Æ¾¶?þã?þßù=»÷ìý½á§ùkÍAþN:%5æÇü8¤æÇü¦¨°[]]]Ø]¿~û8Fo,Z´èüãHû¢/~ñ_ÿú×¥ÆüÔÀüßÔÐ××·÷îìÂnyyy<¿KtsçÌM_ð~~ÿ÷ÿk_ûÔóãóTR©TGGÇ¶mÛ²GìVVVîÛ·/ámþï½7¨^¦ù­X±¢­­MjÌùqHÌùMÑ­sv×¬YóÎ;ïÝ?têÔ©ùóçùÛ³Ï§vÜwï+W¬þ÷yf~Ìùq©ù¡¢··7gÞ-c±«ò÷¿÷,øÃ?üÃÞFjÌùqHÌùÝQa·®®.û"ßâÅ÷íÛwK#v¥æÇ!8ÔÀü0"LY,Ëv¾ ï¼óNq|ßóc~ÌC@jÌ3Ú!Â»%gawÞ¼yÍÍÍgÏóæw×Í())a~Rc~à©Tª½½½¶¶6ûY]]ÝÚÚZ´c~3×üJnFii)óãóèëëËYØ5kVWWÂ.ó+ór¦¹CDÝìÛòEÝD"!æW$æwåÊ'xùqHùa:ÄÐÐÐhÝ¶¶6]æWØæÅJKKÎC@jÌ3Ü!¢Ânö÷­E]§-æWæ·jÕªìÿi*++'úVàÌCHÌÓÇ!Â)©±±1ga·¥¥EaùùwvUUUÂ÷Ýï~7L4551?©1?·CDÝ]æ7SÌ/z zaâüùó×¯_³gÏf~Rc~(VH$---9»ÎPÌ¯hÍïî»ïoô'Oöööç.pW©1?¥C³O½Â.ó¡æ·sçÎôxÌ ¶¶ùqHù¡h"Oa7òÂ¬ðËüßü/¼ðÂüùóÃÄéÓ§ÃD°Àµk×Nô¦3?!50?LC$;v(ì2?æ70?!50?L´CtvvnÙ²%û;vc±X8õõõéIæÇüÔÛ!ÚÚÚòvßóÑæ·lÙ²èÞ.îäÌ! 5ævgvÎ9ÙÝmÛ¶	ù1¿O.]iiíåóC¡8D*êìì¬««Ë±[YYùâ/*ì2?æ÷É;F8W]¿~27ùq©ùáö"L8p ev×­[×ÑÑ1Ñ_IæW`æþ»Ç$kóãRóÃm:DSSSÎ»¡=dêÃ|Ìùå dö§zêêÕ«ÌC@jÌÓ¡¡¡/|áQÍ*Å·¶¶ÝP/1?æEe_$7ÂC@jÌÓ vÑ·Ì ¾¾>è |ÌùÝ%KáÁ! 5æ§¦3ÝÝÝÛ¶mËùkñxüìÙ³ºù1¿1¯âa§äMg~Bj`~ÈO*joo_³fMöE¾êêê¯íkÉdR/1?æwk,X°À©1?ý0­¸páBKKKÎ»7n<zôè/ùKÁüßx8~üxØvîÜ9ÉßTÍü8ÔÀüÍÙ³g·oß]Ø3gÎ;ræÇüno£`Ô&¨°[[[>ª©©Ù¿ÿÂ.`~ÌoRc~úúúÂ!»°;kÖ¬úúú£Græ;i~SóãRóá³@cccÎï[Û±cG"àÌwÞüÂ¿YK,éééa~Rc~òv<8ï[ãÌùÒÒÒ°³Mþ¦3?!50¿F"hiiÉùkõõõ·tRàÌùcÇ½nß¾W®Ì»0?!50¿ÃhÝ`Áóv9óÃ4?c9¤ÆüßvkjjrvÃ¬qßPC0?æ7Níåóc~w<ÝÆÆÆÛ?þsæÇüæÇ!¤æWcÎÂn8ò÷õõqææ!50¿Â&L¶µµå,ìÖÖÖÞNaC0?ÜaóÞ´iSEEEØ?gÏýðÃOÂPæÇ!¤æW$æææ+ìræ;i~×®]Ë9Âc¢¿Æùq©ù4©Tª««+ga7ÝÁÂ.`~¸æ·|ùò°nÞ¼ùêÕ«áá+Wzè¡ÐrÏ=÷0?©1?dL&[[[«««G+ì)äÚ45¿²²²°¯fî¥ÃÃÃ¡%´3?©1?dÒÛÛ³°[^^Ç'³9óc~ã¤¤¤$ì´ÁöÒ-CCC¡Å]]8¤Æüvëêê²/òEÝd2É! µÂ0¿¨Ú»aÃ¨Ú~éÐ²zõjæÇ! 5æ7ÃÉSØ"ØÑÑ1	]Áüp'Í/¨^Î/_f~Rc~3ÞÞÞx<^^^³°æÅwîÜ¹fÍ+W655pæ'µ©7¿OoïøáçÎ[RR~oØ°!´Lô¦3?!50¿iH*êèèÈYØ­®®nmmca·¯¯ïsûÜºÖ=~ûî»ï³ýìíË!ó+0ß´"(]82Çb±;RØòÉ'ï]wïÞ?Ùþ	ò×ÔÔÄ!~`~àRóJÂËÏYØ7o^ssóX»Ù¬Y³æñÇÏ4¿¦ÿÝTSSÃ!~ó»ëf0?©1¿"æNvs²nÝºÿÿ_æ®Z¹C0?ý05æW2:ÌC@jÌ¯¸éëëËYØ5kVWW×íØýÆ7¾±bÅ=»÷¤ÍoåÊ»wïæÌO?LùÆ3Ï<íÿo¿ý6óãó+2¢Ânö÷­EÝD"q§þÐÐÐÐç?ÿùe5Û¶åË¯^½zppC0?ý0]Ì/1öìè¼7vf~Rc~Nð°öööÚÚÚÝ¶¶¶¸s*:pàÀW¾ò|ð¥^ºÚÇ!ó»]¶nÝÞï½ÉÙtæÇ!¤æ7	DÝìï[»]©ù½ûî»ÑQ`óæÍ¹éÌCHÌoB	ÇØÆÆÆÝ;XØåZaßµk×î¹çh0Ç±cÇ&yÓßDvkjj²»¡q»Rîæ÷òË/GGydJ6ùq©ùÝñ·ßhÝÆÆÆ";är©1¿[ýü8¤b1¿®®®àvsæÌ)ÊÂ.îùÜÒÒRæÇ! 5æ7»+W®Ìþï½¶¶6ÌOàó0?!50¿qÓÓÓÓÜÜ»Bj`~RÃL4¿T*uøðáõë×gØÅb­­­Rc~ÌC@j(lóK&AìrØ­­­:X ·åãRóßÑÓÓóì³Ïæ±»ûööÃBjÌùqHÅi~á ¹qãÆìÂîâÅÃ!t``Cx÷Jù1?©¡°ÍohhèÀÕÕÕÙÝººº£GÎ´Â.îù¹Ô0ÝÌ¯»»»©©©²²rÄyÎ9ñx<Ìó'·îËi~îçÇ! 5æ79¤Gìf«ªªöïß_ß·Æ! µ©4¿4ï¿ÿ~8¸<úè£W¯^Ãïz(´8qùqHùM(/¾øââÅsvñ­9Ô05æ2?52<<ý£yké.°´´tÅÇc~Bj`~iº»»wìØýkåååñxüìÙ³ráRÃ_t¬	¶7Âünÿs~o¾ùfxåWyälóûÁ~0Ñ¹páÂ| ¤æ_þå_N:59+L>|ø_üböÝ%K|ë[ßúçþgóçÏ>Z?H­øpó[°`A8âK»víZxþäæÍCKh¿ý«×¯_T2eßþýûÿ_N0ã¿ú«¿ÒRCq¤ö½ïïþà²Go>ÿùÏ¿ðÂ]]]²°¯In~'NÈ9Âã'?ùÉm®9sHöxÕ^uC©aTÃÊãñxyyyÎÂîD n(5L^µ7pþüù¥KVTTÌ=õêÕ.]ºýÕfÖËÊÊfù¥R©öööÚÚÚì­«««[[[ØåRÃÔß±`Áèãáwvíùq©¡XÍ¯¯¯/âb±Xö÷­Õ××wuu¹3Ðüßxã0~7440?!5½ùÃZ8ôeÞ7o^sss"ÐÛBjúaêÍïÜ¹sË-=vT]°`Á¡Cnµ'O¬ªªëpáéÓ§ÕüF+ìÖÔÔ´µµ)ìrHmº_t'çÌol¦_yå	ÝtæÇ!¤"0¿¨°;oÞ¼]G9©M;ó«ªª©sçÎ¥ÍïôéÓazîÜ¹ÌC@jÌo4òv[ZZv9¤6MÍ/:TEù]¿~Ý÷örHùå$*ìÖÔÔä,ìY¾oC@jÓÚü¢;9G×ùùïÚµ+LgßùqHm&_"hiiÉYØmllt@ãZaßñãÇsÞÉù>`~Rc~Þ(ìÖ××+ìrH­Ì/:ê­]»6Û[QQ±lÙ²I81?!5LsóK&mmm9»µµµ»R+Tó¦­ùÿ~v9¤VæØæÒ¥KË-[¼x1óãÚL3¿®®®ÝX,ZºC@jÅf~ÃÃÃÆörHmFßøÃöööðïh]ß·Æ! µÂ6¿¸Lî¾ûnæÇ! µ¢§§§çþè>óÏ8Çãñ;û¾àRÚßGTrô·wd´ïÝwße~R+bÂ!¨®®n´Â®ï[ãZQ_ày]Øe~Bj>8p`åÊÙµuëÖ=ztr»CCCâàRÃdßTÁü8Ô0É$x<>gÎì»7o>öìälFooï¾ðßøß¨¬¬üìg?ÛÙÙ)!5Lù/[¶löìÙéùóçóßd~R+R©ÔÑ£G|ðÁÝûöýô§?´ÏóáûÒ¾´g÷½²÷«_ýê¯ÿú¯wuuCHd~-1¼7: £!óãZAL&Ã¡&ç¶7>|8*ìÞÒ÷öÞ&Ï=÷Üºuëó¥þçÖÿ¹víZaq©aÌ¯¬¬,»»»Ó-!Ð2wîÇ! µ%ÓvìØ+æòòòíÛ·÷ôôd>y2ÍïË_þòW¿úÕLó?"ãRÃ$_4¼÷úõëÿm½Y7ùc~Rþ¤R©7f_ä«ªª:xð`Î»i~ñxüÁÍfjß§vüÖoýì8Ô0Iæ·páÂpL|úé§£/ ¼víÚÞ½££$óãZ¡Ð××÷â/W¶ómÙ²åèÑ£y¾cw2Í/lÉoÿöoÿ§ÿOÚü~oíï=öØcäRÃ$_8Kå¼óéÓ§Ô¦?gÏÇãÙ£7æÌ³ûö±ØLóÿ´ó7sã6Ö××/ûÝsÏ=îÈ!¤É3¿ÀÅW¬XQQQQRR2öìåËÞtæÇ!¤Û!J½óÎ;ë×¯ÏþÇµººúõ×_»NM²ùzã&Ò_ÿú×wìØ¶3ÏÅHp©aBÌoJ`~Bj.ÊÊÊÝÎÎÎ[½óä8ÔÀüÀ!¤ÙÝ`ÍÍÍã¾3óãÚÌ2¿sçÎE7sÆó.X°àÐ¡CÌC@jÓ¡¡¡öööÚÚÚì|/>pàÀm~óãÚ2¿÷ß?Ì/~åWÔ¦D"ÑÒÒ[¾Y³f¯°Ëü8¤6ÓÍ/º	Â¹sçÒæwúôiwræÚÔ2Za7Xà³Ï>ÛÛÛÿóãÚ2¿è`úiÆÝ¯_¿¦KKKÔ&d2ÙÖÖVSS]Øííí1ùqHmß¢»÷Eæ7<<¼k×®èÌÔ&<ÝÆÆÆ	=?©Í ó;~üxÎ;9ðÁÌC@j@8Ô××ç,ìE__ßDoóãÚ2¿è¨·víÚhloEEÅ²eËÂ?ß½éÌCHm§°[[[;A]æÇ! 5æ750?!µKøß²¹¹9ga7O¾1?©1?æÇ! µ;L*êêêÊYØÅbSØe~Rc~þâ¿X²dIYYY8þVTT¬X±¢¿¿ùqHíNL&[[[«««G+ìÞÛò1?©1¿þÏ9Â£§§ùqHí6éííÍYØ-//Â.óãÚL7¿èNÎÑ×]½zõG	-.d~R©Tª£££®®.û¿Ê¨°L&§ÏÖ2?©Í óÅ¥0¾«3óãÚ-§°D0èàÔvÔfºùE×üÓ-×®]sÍC@j·JOOO<///ÏYØ½³ß·ÆüÀ!¤ÆüÆIô9¿ |ááåË×¯_ïs~R#©Tª³³3çÝêêêÖÖÖiUØe~Réæw×Í ²/óãR+tÒíß¿?å,ìÿ*§aaùqHm¦_ÉÍ(--e~RËäÔ©SMMM9Gì>ûì³Ó¹°Ëü8¤6ÓÍoª`~BjÇÐÐÐÑ£G×­[oñâÅû÷ï(Ä×Åü8¤6Ìo´6_¼xùqH-ýºÂ>³°[__üøñB)ì2?©Ítóî^xaDãöíÛÝÕC@j Dá=bwÞ¼y---=ùCHwØüáø.]º¾õÖ[Ña>ÞÇüÔ¡¡¡öööÚÚÚì|ÕÕÕîý^0?©Í óûôÆ¾è¾páÂhâ¡èMg~ÌOjÓö%¼øâÙ£7[¶l9|øpÂ"KùqHmf_àÄéûÁ'aÓóÚt#ØOccã9sF_hinn.Â.óãóûôÉ'ïÑ÷yôQæÇ!0CRloo_¹reöE¾Ðøúë¯SaùqHm¦_iii8¾Ï;÷Ã?ü4ãs~eeeÌC ¸SK$Ï>ûlvawÖ¬Y=bùqHùåZÅ]wíÙ³gDc<7¶C Sß¶mÛrØ.xáÂóãÚ2¿ÑîçÌC ÈRK&mmm9»kÖ¬	³¾°Ëü8¤6ÓÍoª`~ÌOjI"hii­°;ÃwFæÇ! µâ7¿pÄÏ¬çæÈü84µT*ÕÕÕU__oóUUUíÛ·/¡ÔÔóã(ìÔ¢ÂnuuuvawÝºuííí3³°Ëü8¤Æü@Q¥ÖÛÛÛÜÜ³°Ç)óãóc~ZTØ­««Ë¾ÈÅÂ×××' æÇ! 5æÇü8;µd2ÙÚÚ³°D°££cÜùqHù1?bNm´Ânyy¹Â.óãóc~ÅZ*êèèÈYØ­®®nmmM&²`~Rc~ÿÍüòÃü¦aj»ÌBjÌo<ÜÒÒRæÇü0RjÇ³¿o-*ìöööê|æÇ! 5æ7a~ÌOjcAaùCHÌC øSëëë»I,ËYØíêêRØe~B?Hù1?15ôôô>|øÔ©SCCCR»ÍDÝìï[7o^ss³Â.óóc~bÊ¬¯¯¯ªªº÷Þ,Yò¹ÏîÂR©Tª½½½¶¶6ga·­­MaùCHù1?1ÅlÙ²eÍ5vïÙû'ÃÏ6|iùòå3¶9¾ÔF+ìÎ5+XµÂ.óÌ?ç³;#í~-Z4cÏ£·ZØsv[ZZùCHÌCLº»»×,ÏÔ¾ðsïº;::¤¡¡¡Ñ»555»ÌBj`~b:âß­¦H$ZZZ²¿o-*ìÚ)8ÔÀüß´æ?>ç·zäçüfìß<©)ì2?p©Iù¡àÍopppãÆÆöZTØ­©©ÉYØ³føp8ÔÀüPHæÑÝÝÝÑÑqêÔ© R¦óv½ÿ8ÔÀüP¨æÌÔÂ»¾¾>ga7¼óûúútó_aL&ÿìÏþìsû·¶¶VaùCHMjÌÌ¯H$ÍÍÍ»ÌRc~Ìù-©Tª««+ga7)ì2?pHù1?æW$ÉÖÖÖêêêìÂî+ÚÛÛßóÔóc~OOOOÎÂnyyy<ÿþ÷¿/5æ©ÍPóëïïÅbÌù§NÚ²eËhÝèûÖ¤ÆüÀ! µj~'O|y8/2?æWÐíß¿?ga·®®®££#³°+5æ©ÍPóÛ´iSoooóûó?ÿóÿÑùÑ~ô×ý×úay÷ÝwôÑÏ|æ3#ï×~í×¾ò¯|ïßZqpúôi©IR&°ùýç&n~0:?ýéOÿæoþF?L	o½õÖºuë²/òUUUíÚµëÜ¹sR+²íoÿöoõCaÑÝÝâÄ	ý µâ£ÍOµWµwº188xðàÁÅç,ì>|ø¦#v¥¦ÚuCHmUÓ§IæÇüðè;ÊËË³¿o­¹¹9ÌóÔßù1¿iK*:zôè>/µ¶¶F#v¥ÆüÀ! 5æÇü_Ó××·ÿþªªªÝ ã»³Ô8¤6£Ío4*Â9>gßoÎ9;vìèééóÔóc~M*zçwêêê²/òUWW·µµJùC@jÌù1¿Â&L÷[Î»õõõc±+5æ©1?æÇü¦;á=ÇçÌ³°H$¤æÇ! 5æÇü_a344ÔÖÖóVÌ+W®<pàÀ)ìJùC@jÌùq©$H´´´TVV¾Y³fÕ××OÂ[NjÌRc~ÌCL8áíÔØØ=bwÞ¼y»wïîëëÔóc~M2lkk«©©É.ì®Y³&ÌºÕ[1KùC@jÌù1¿iGTØ7o^va·±±1¼»îà]©1?pHù1?1ëêêª¯¯ÏYØï«I+ìJùC@jÌùq"*ìVWWgvkkkÛÛÛ¤æÇ! 5æÇü_aH$svãñø´:gKùC@jÌùqñ§°Å¦¼°+5æ©1?æÇ!îÉd²µµ5ga·®®®££cJFoHùC@jÌùq;IoooÎÂnyyùt+ìJùC@jÌùqñJ¥:::êêê²/òEÝI¾-Ô8¤Æü¸ónaù1?pHù1?1VÂ¹6ç,ìöööJÌBj`~Ì¯°ÉSØ­®®nmm-Â.óc~àóc~bTöïß¿xñâÝ®®®Â*ì2?æ©1?æÇ!rÐÓÓ³ûöìÂî¼yó´°Ëü8¤Æüø/R©TggçúõësvÛÚÚº°Ëü8¤Æüø¢ÂneeevóÔóéÑÝÝ½cÇìÂî9sÏ=+50?p©ù¡°"J>|xÍ5Ùùjjj^|ñÅâ+ì2?æ©1?æ7ã"lç¾ûªªª²oýúõAµ°Ëü8¤Æüßrîîî#vçÌ³cÇb±Ëü8¤ÆüßuT*õÎ;ïä,ìÆýû÷ÏÂ.óc~àóc~Åì/¾øbÎ»>øàñãÇgTaù1?pHù1¿ât³gÏnÙ²%»°,pß¾DB^ÌùC@jÌù¶C¤R©×_Ýºu9Go´··Jù1?pHù1¿Âv¾¾¾g6¾Y³fÅãq§IæÇüÀ! 5æÇüÞ!R©TWWWÎÂnt+æÑ0?æ©1?æWØL&[[[«««³»+W®<xðàL±Ëü8¤Æü_8ÄçÍ]ØÝ¶m[É]æÇüÀ! 5æÇüÞ!ºººÛÃË±ÛÒÒPÌùC@jÌù¶C<x°¦¦&»°»nÝº¡¡!ýÏü8¤Æü_a;DOOÏîÝ»çÌ]Ømjj'?]æÇüÀ! 5æÇüÛ!ÏuvvÖ××gv«ªªöíÛ700 ÃóÔó+lH&X¼xqva·¶¶öðáÃ»ÌÌC@jÌù¼C$¦¦¦ì»ååånÅÌüÀü8¤Æü_18D*:zôèÆsØmmmíëëÓ·ÌÌC@jÌù¶C¼øâ9oÅ¼fÍÃ½ÁüÀü8¤Æü_Á;ÄÙ³gãñxöE¾òòòÆÆÆ0Wg2?0?©1?æWðÑÙÙ¹eË#vCû¾5ææÇ! 5æÇüÞ!Â¬ÑFì®_¿¾££cppP2?0?©1?æWÑÞÞ³°ÛÝÝ­ßÔó+*LG,Û¿x²c~`~Rc~Ì¯8bÇQa×]ææÇ! 5æÇüÜ!óóãúAjÌùqHÌC@jÌùqHÌC@jÌùqHùé©1?æÇ! 5æ©1?æÇ! 5æ©1?æÇ!¤¦8¤Æü8ÔÀüÀ!¤æ!50?¡¤æ!50?©ùqHù1?©ùqHù1?©ùqHù1?©1?pHù1?©1?pHù1?©1?pHù1?!50?pHù1?!50?p©ùCHÌBj`~RóÔÀü8¤ÆüÔÀü8¤ÆüÔÀü8¤ÆüÔ8¤ÆüÔ8¤ÆüÔ8¤Æü8¤Æü8ÔPæwúôéÕ«W®Zµ*;iæÇ!¤æ!5ù-]ºô>o¿ýö²eË²ÍïøñãÿFçâÅ?úÑôÔ0Ñôõõ_×ÅÇ,5©%l~TTTdßK/½ÔÑyï½÷=ª¤©!gjù©¤V|ùÁojjRíU7T¡n(5ýP´ÕÞ«W¯6442?!50?p©é¢2¿»~EôðâÅñx<U³Éü8ÔÀüÀ!¤Â6¿L?¾aÃþþþs8ÔP<æÅîÊùq©ùCHM?­ùåùq©ùCHÌBj`~àRóã8ÔóãÔóãÔóãÔóãóÔóãóÔóãóÔóãRóÔóãRó8ÔÀüÀ!¤b2¿o|ã0:~øá#GôÔ0ÑüÃ?üÃ÷¾÷=ýPX8zô¨~ZñL&ÓüÎ;·wïÞ?À¯È_½ËeOÕ^0?0?0?0?0?0?|zúôéÕ«W®Zµ*CóþþþX,ýäO>ùä®BKXdáÂañ+V;vLNÃÔ2#+))É#¦$µ9FdïYöµéZö,ûÚôOÍyùÍ8.]úÁ·ß~Ù²eaâäÉË/Ïy:tèP<ÏlihhxóÍ7ÃÄ+¯¼òÈ#èÏiZT)gÔ²[òìYöµéZö,ûÚôOÍyùÍh***ÂïM6õööæt°9r$³¥²²òúõëabxx8ç'LyjçÏàFËSZì=Ë¾6ýSËe_þ©9¯1¿Ë3gþ+ÑþmÚ°aCiiéÚµkö³09éZÄÃ?<ZÂÔr¶dïYöµéZö,ûÚôOÍyùÍP®^½ÚÐÐ0888xqÕªUa"úÜXDYY©õööÞwßyrÄ¦ÝsÏ²¯MÿÔòÌ²¯MóÔ×ßÌ"¼Ýãñø'|òß½Ù£ýaÁÃÃÃÞ¸*¦uæôLíùçùåóä©J-g9÷,ûÚôO-ÿ,ûÚ´MÍyùÍ,?¾aÃþþþRíM$Ñ.o¼ñF¿ÃÿRús¦¸ÿþû?üðÃ<9bJR-Çmú§=Ë¾6ýSs^c~3X,ó¦#"zxæÌåË®_¿>ì$ÞRZUUURR²páÂÓ§OëÏiZôlôåì1%©åÌ1ÈÞ³ìkÓ?µìYöµéóóóó`~`~`~`~`~`~`~`~`~`~ÌÌÌÌÌÌÌÀá­·Þºÿþû+nðÀ¼ûî»ÿíøu9ÚæÚÚX,^ÚàààöÐRZZºpáÂë×¯ßê:ù(<öîÝW/¼ðB1ß¾ûBãÁG´8p ´?÷ÜsãX'0?Æüãà4¥¥¥¯¾újê¯½öZx?üðÃ¢1¿óçÏÆU«Vh_¾|yhO$ÌóPü<úè£Ái¾õ­oe6¾ôÒK¡qûöíÞsìØ±`NAW¯^¦ÓO¾|ùrSSÓÜ¹sÃ¬ÊÊÊ§~:³¨ÚÕÕì*ÌËvvvp©Ðr÷Ýwßÿýï½÷^xØØØ8bÃ==Ñ¬°IÑ¬÷ß4KÛ°aCh?útºå>-uuuéç¾ªª*¬ª¢¢bëÖ­/^Ì6¿ìõhÉ³©L%-ÖòÑGe6~üñÇ¡1eÍÎ9Íè¡FÌzê©§¢YÝÝÝ%%%9Fs~øáë×¯w,++K¥RanøÌiöìÙÑÇïò¬'LäÜ¼ìWúöÛogêlZ.ß|óÍ´öXÉ<p«ægS0?b¢ÂncÖ]wË4GyäêÂDx¸yóæhn$:;Fî±E³¢aúäÉÑJ2×¹gÏàvQ±õñÇ-G	Óáw~ì±Çnº`áaxBæ¶å|EÁ&çÏ^ox~-º9<<=aáÂaÁð>ýUu¸¢¢âVÍ/Ï¦`~0õæÈi~éöÈl"ax1<Î=zux¸dÉ'x"Ûµk×Ò+	Ïq-½Tôðã?N?9ØRºà~é®®®®'XfxxéÒ¥Ìmí3yÏ<óLõíoûÓ_U´~úéÌ'æ|tíÚµÑ%É[5¿<ùÀ]èºzõjfãàà`h³ò¸NÚ#ùKNúÃyÙEØ.y/0]YYYVV644V~÷Ýw§çÞt=£yØÎ;Ij¿ÃtwwwzîÉ'Ãä¬Ýüòl*æSLôY·_~9³1º×ÉéÏö÷÷g_ÊúðÃ÷íÛÕ^ÓÊ]KSoêgO=õTTä¿ÒíyÖ]cûäO¢.]Ê?÷¾ûîs£ÙaÍó³Þÿý+W®ä7¿´F½gS0?b¢¥¥¥¯¼òJtWW_µ¬¬,4ÆæÍåE¥Øô8Üès~ÑÇãz3? àÓO?<)u¾¯JN?ÛFýøñãéö<ë>ØÎopp0zfóíµ×ÒWãÂKÎä±»»;üÐ	£_äÁÃÓqnÝº5snMÀü`êîr<çþ¿_7Ä(=®¦U¤yüñÇ3µ2Cå1¿Oo|ÙFh¯¬¬Ì,çYOØÌK«íÅ^»v­¢¢"z	#jÜ6mÊüóçÏ¿£»d®3º$&òÔôÜ<ùÀ´ ØÉ<Pvûï¿?`;ÂüÞÿýè¶y+V¬8qâDzîààà®]»ªªª"[zægÒs;;;×®]láÂmmm#Ö½%aU¡ýÉ'Ñ>ÚzacÂ&E7Ìs?¿4MMM#nïÑßßßÐÐz`îÜ¹áU$ôj2×yåÊ ¶Q_ÕÕÕ>zÄ_Ì³©óóóóóóóÀ­óÿ©sl¬	IEND®B`


	®m§Cwp&<eeeÚ×u8©þ#C~DÈH~ÒÆS===Ê=¯½öZ$¹ÿ¾e£ßï7¼ªñ^$æKìÞ½[¶ÆfL~êwîÜÓCCC)ñ#K;ò#¢ÑkN§üìØ±xÁ|òImm­zª´®®.Æ|æéÓ§çÎ»jÕ*mKGGÇ%KL&ÓêÕ«oÝºuöìÙEåää¬]»6z²jÿþý¥¥¥êiMùÚYñðè£Êöèç^¥ÃËÆÃïq¼ÑiÁ¢l9®Á2Ô¡]p,?2ýõçñtæÌÊ§É'ËOyÔ»ö	K.Õ°¾víÚèDüh*å700PVVVRR"'ôÁ~ú©Ùl¶|fÌ£uVVü¿iÓ¦a£gKW®ñÚ555b>sÍ50oÞ<Ù~ûöíèÙ¸`ÁÃïq¼ùÒwîÜQs~ãÄðÇò#üÆòòûýê'«ÕÓÓøç®Î½xñbÌP3±:!?"JùÉ'OFÏE??ñÄrzýúõwFò¡lþÌ½÷Þ¿_ÍZ©-[·nD"ÝÝÝêC¹fùðôéÓrZ¡.«^ÍvêÔ)9íÚµè³â!Àd2n¾ì$/ïu~ÚÛxÇðVMøcùE~cùÕÕÕÉrZæ-[ÿÜÕ¹µµµêÛRÌÏÏðM±ü$yPÓò8³½¤¤DNúé§êÃ7nÈÅÅÅÑ× «m¹÷nôÃ?mýè.2 ª««ÕÒ¨òËÉÉ1üF·Oà"ÑÚËYl6Û3gm,cx«&|Á±üÈÆ"¿±üÔ«!£-îÑ4üÆûC'"äGDS/?yì7ÍóçÏÃÑÛõsfú½¯3ñBg<ØEUZZªëÔó.%Ú5å"ñÆdÔAËàÄóèÄ.8ÙXä7~â3Fiñ~î	nÿ~èDühêå'½ñÆò¡¶Ú¨§cf§dãXøÃªÕU:::DfcAåR¬ÔÕÕíØ±ãÎ;GµµµòËE&,¿ñÖ/8YË_~~¾ysñX~îjÎOVn?t"B~D4-ò6nÜ¨½¨_mQo#]¿~ýÀH6mR¯¼üÔ¼ßï£¼ôÒKcA@WWzÎQÜÐÝÝ­æóT'O4üÇqÉ/ñà¨)æJÆ;ªãú)x½øâ2°·nÝR¯ºüÔ­+ËõÈèÉé%Kåç®¾âîÝ»årº¼¼|Â?t"B~D4]ò»yó¦öê.µ%Æ¼Þ«¤¤D«ìdä·nÝºè«-..ÿ£ßßjxì1ý³Ë/Oðmë"ã_âÁÑdYy£:®YÌ÷31ù©¥G~þ½½Ï?ÿüd~èDühZä'©gB£·_½zµ¶¶6g¤ºººx¯ë/,úúúäÚä:.;wîÉYÛ¶mhQVVÖ¼yó^yåzH»èä/2.ù%'OþL&Zñ.¦qê¸~d?þ¸ºfÍváÂÉO:útuuµ|#gì?÷3gÎ,]ºT]ðÀÚö	ÿÐùý®¡¡¡ÇLéaú.BDDÈùò#""""äGDDDDÈ!?""""B~DDDDüùò#"""B~DDDDüùò#""""äGDDDDÈR¼'OZ,É´fÍÞ4±ÏËeÇ~¢SÛ·mÛf6edëêê>ùäíRHD¶Ë'çääÈi~ùQjTZZ*éëëyÁ$üôÛN§lïììÓgÏÓ,ÐÎmoo×¤xêÔ)~ùQìP¦P3|ó¦[~999²``ÀðRõõõr®úÿñÇç·¥û¢õ³gÏÂÂÂüüüûöÅ|ÚC=T]]­mìîîVµ-ë×¯-²ýÆ6-//Ïd2-]ºTÍé¯'úë&¾È+¯¼b±X¬VëíÛ·õb»÷®ÃáËÊÍÞ½÷¸&/ãÉ¯¼¼/P¾³îß¿?wî÷îÝÿÕöK.­ZµJ¶È-ïåÓO?å%#þÔé§~ZN<xöÙg£?çôéÓÑ¢Éök×®Éé`0(§e^¾|¹zTÎeee×ý¥_D<÷ÆoÈ	§Ó©¿ÙÛ¶mÓò	Çû÷ï6z_¹Cýç<yRÛ²fÍ>ø@»zòW+§kkkå´lÑ°800à÷ûåÄÊ+ùí""äGDÉ+?Å"§FÒóK?¦¸gÏáÉB9äÈuÖ¥KöîÝ[]]-³²²¯'Fc/¢nRaa¡þ²%%%êfß¿_NÀ&öíÇÔÓÓ#Õü'ß¬Ú¾cÇíCåÑ;wÊi¹mÊ¾rV8æW%µüÔûUµíòab´JKKóóóïÜ¹#ÿÍfálé¥äó7nÜøÁD_6æz¢?ËEâÝ$abô¤Rã¸æüOgg§ÚïTó±|	ùGhÓóæÍÓÇ;w®ºBãÇóÛEDÈW~eeeÑs~ÚäY8p@ÎZ»v­ö4ëðço¸?Òåø"ÚMZé/[\¬.;Éo?^êKË-ÓLcºtéÒðÈj/§NRïÖ¦'%£ünê[õ4îóÏ??*úúúÔL¡ü¯½ýB	òÊ+rUc_âìÝ»÷Í7ßO<ñþ²?þ¸~õÕW?úè#íµß%KdGGÇðçk¸<öØcÚµ´´h)£¤ÔkµZåÐðÖ­[r¢²²ß."B~D¼òD"b¬üGåjëÖ­jmPÉl6îÞ½òK|3gÎÈ¹+W®ÔÞb³êò;ä6çää¬[·nª÷°ÃáÛU\¼mÛ6ù*²Ñ¢Eò9W¯^Õ®!È¢¯®®N½=yõêÕÑCDüùò#"""B~DDDDüùòÆ~ñ_~=xüñÿ÷óKÓíÛ·Õ(ºH$òÉ'0úB¡ÐÝ»wîÝ»á;Øx]»vãééû·û·7o2úþå_þå¿þë¿ßÔôãÿXðÉ¿O?ÿùÏ'v¤ôî£>úõ¯Í8ÄôÿñgÎaô½ÿþû<bé?~ùË_2úÎ=+ÊabúÍo~óÁ0úN>ýÿùÈù!?äüòC~Èù!?äüòC~Èù!?äüòC~Èù!?äüòC~üòC~Èù!?äü!?äüòC~Èù!?äGÈù!?äüòC~ÈùòC~Èù!?äüòC~Èù!?äüòC~Èù!?äÇ8 ?äüòC~Èù!?äüòC~Èù!?äüòC~Èù!?äüòC~Èù!?äüòC~Èù!?äGÈù!?äüòC~ÈùòC~Èù!?äò»páÂÒ¥KM&Ó%K.^¼üòC~Èù!?äüÒV~,8wîxýõ×+++õò;uêÔNä×××w¾Ø¥K~õ«_11ýö·¿íèè`ô?>01]»víÿñï¾ûî7®^½úÞï1úÞzë­Û·oÏðMaùE§ß¡C~Áýìg?û%é:sæÌ;ï¼Ã8Äô_üBþT`ô½ýöÛ]]]CLÂ>yÄbôÉ°Èà01	åoKÆA_ww÷Ñt_OOOCCÏöòl/Ïöòl/Ïöòl/Ïöòl/Ïö¦í³½ª»wïÖÕÕ"?äüòC~Èù!¿tìNç­[·ôg!?äüòC~Èù!¿ô_WW×ÚµkûúúÏE~Èù!?äüòC~é#?Å2'*äüòC~Èù!?ä¶òKòC~Èù!?äüòC~Èù!?äüòC~Èù!?äüù!?äüòC~Èù!?B~Èù!?äüòC~ÈòC~Èù!?äüò#äüòC~Èù!?äüù!?äüòC~Èù!?äüòC~Èù!?äüòC~Èù!?äüòC~Èù!?äüòC~Èù!?äGÈù!?äüòC~ÈùòC~Èù!?äüòC~üòC~Èù!?äü!?äüòC~Èù!?äGÈù!?äüòC~Èù!?äüòC~Èù!?äüòC~Èù!?äüòC~Èù!?äüòC~Èù!?äüòC~Èù!?äü!?äüòC~Èù!?äGÈù!?äüòC~ÈùòC~Èù!?äüòC~üòC~Èù!?äüòùõööz<H$üòC~üòC~i+?Ù¹mÞ¼977wÎ9çÏG~Èù!?B~Èù!¿t_¿Ûí®ªªÓéD~Èù!?B~Èù!¿ô<ú×××gggÏùb²eóæÍÈù!?äGÈù!?äòò3äSº`0l#üòC~Èù!?äüÆ×ùóçëëëôæ«©©ñz½É92Èù!?äüòC~ÈoLÃáÖÖÖeËéÁg6wíÚå÷û|dòC~Èù!?äüßèr]QQÞ|Á$ù!?äüòC~Èù¯p8ìõzkjjôïÞÈÍÍÝ¾òOò!?äüòC~Èù!¿QMMM¥¥¥úI¾ªª*Ç¢ÃüòC~Èù!?äü>+¨I>=ørssëëë>_JòC~Èù!?äüòÍÍÍf³Yo¾Å»ÝîP(#üòC~Èù!?ä¹òD"²/²ÛíñÖaîêêJwo ?äüòC~Èù!?ãB¡Ûí®¨¨0¹¥¥¥¿¿?ýFù!?äüòC~È/³ä'ÖC?É'mØ°¡½½=§ëÈ ?äüòC~Èùeüd âlÍb±¸ë×¯§ýÈ ?äüòC~Èù¥¹ü|>ÓéÌÍÍÕÏf³y½Þtz%òC~Èù!?äü_&ÊOFÆãñNò¹@ i#üòC~Èù!?äGuuu[³Z­­­­iüJ>äüòC~Èù!¿H<'ªÛNò566fà$òC~Èù!?äü_º%¤ØÅäKÝ­!?äüòC~ÈßgÃa¯×k³ÙôàûÒ¾ät:Sý`kÈù!?äüò#ä÷»­¹ÃI¾ªªªç.ÃòC~Èù!?äüR¾H$âõz¶ëp8ÔñÛüòC~Èù!?äü¥P($³E?ÉWQQáv»£Çù!?äüòC~Èù¥^HDö|²ÅápÈ¹úuòC~Èù!?äü_*ÜnwEEáÁÖä1W>!ÞeòC~Èù!?äü_j$¦ÃpÏn·Nò!?äüòC~Èù!¿T*<xÐð`kÅårÁ1^òC~Èù!?äüòKÞýdSSS¼uÛÚÚFäC~Èù!?äüòC~IZYl§b×l6×××OxfäüòC~Èù!?äD;FËe¸DË+=:888ëG~Èù!?äüòC~³y$X¢¥««kJ¾òC~Èù!?äüòµzÝn·á$_UUUKKKÿ~9äüòC~Èù!?ä7ù|>ÃQPP ä«¯¯ÇÍñ¾ù!?äüòC~Èù%W¡P¨¥¥eÅúI¾ÅOù$òC~Èù!?äüò¬Ã¬^É7|Èù!?äüòC~Èoæáñxâ­ÃÜÜÜ|ýúõ»1Èù!?äüòC~ÈoZë0ÛíööööäC~Èù!?äüòC~ÓZÙf³ÅäB³5,Èù!?äüòC~Èoj.ËpoÃ'NùI>äüòC~Èù!?ä7ç¼^¯á:ÌåååMMM3ùJ>äüòC~Èù!?ä7-B!y3Ùf³	gù!?äüòC~ÈùM*ñÜO'ùW HÎaA~Èù!?äüòC~c-¹Ýîý$ÕjmmmÃÉ<,Èù!?äüòC~Èoôâ­Ãët:>_JòC~Èù!?äüòÛí6Y6z<$?:0òC~Èù!?äüò=Ïçt:sss¶¢kÈù!?äüòC~ÈïJ|°5yDÅuòC~Èù!?äüßÔÈÏçól-;;Ûn·Ë3ÙhA~Èù!?äüò£ñÉ/·¶¶Z­Vý$Z¢%¦Í° ?äüòC~Èùe¨üá$¶s/ÑüòC~Èù!?äG£ÈO<'ª«©©1ä&í:ÌÈù!?äüòC~Èo¬ò»ví2Íé±DòC~Èù!?äüÅÊ/´··oÞ¼Y¿DKj­ÃüòC~Èù!?äüâÊïúõë.Ëb±Nòµ´´¤ý$òC~Èù!?äü_ú÷Â/|÷»ß-((Ð/Ñ²yóæ'N¤Á-Èù!?äüòC~Ýàà Ûí®¨¨ÐOò655õööfòø ?äüòC~Èù¥Cê`kúI>ióæÍíííé·DòC~Èù!?äü_f%;xð áÁÖ¾ò¯455É®ßäüòC~Èù!¿ÔÎï÷Ç[yÙ²eO>ùäíÛ·ùA~Èù!?äüòKáÂáðÑ£GÅvzðåææîÚµKMòMà¸½Èù!?äüòC~È/Y»ÒÒR½ùV¬Xáñx¢ß½üòÞúúú,òC~Èù!?ä7µÃá¶¶6«Õª_AAÃáðûýúK!?äü¦±îîîÊù!?äüòª@ss³á:Ì+V¬8|øpÛ!?äü¦±uëÖÉý3ü^~ùåÊàÚÛÛåöOôÅþáþAþ$`bºtéÒ©S§ï¼óÎùóçzzz:::Òìòù|Ï?ÿüòåËõàûÒ¾d³ÙþöoÿVî)¯äí·ßëá7$&ù;¡³³qÐ÷óÿüòåË3üESX~ÝÄøò;vìØNäwýúõôÅÞï½÷ßqé×¿þµ<b1úäï¿ßÏ8ÄôÏÿüÏï¾ûnÚ|;ÝÝÝ[·nýò¿¬7ßýÑíß¿_ÇxUò§ÂÇÌoHL~ø¡àqÐ'r_»vm¿h:Ëgy¶gy¶gy¶7^HD~íívvv¶þ`k6l8úôx¶Æ³½<ÛË³½Èù!?äü_râlM6ÊYýýý»fäüòC~Èù!?ä,ÉãÃá0äír/ï$òC~Èù!?äüòK®DcÇð`kE/B¡Ð|!äüß¬üòC~Èùù|>Ã­eggÛíöÉOò!?äüòC~Èù!¿Y.·¶¶®Ãl±XW0¯üòC~Èù!?äüf®@  °ÓOòI6ÍëõNí$òC~ÈïóKVVVòC~Èù!?ä7%çDub;ýÃ(P,("ü_æÊ/k´L&òC~Èù!?ä7ÉB¡ìð¶fµZ[[[ÃáðÝäü2W~³òC~Èù!¿4ZÙp¢¢¢ÆÆÆäC~Èù©'xù!?äüòoB«xë0[­VÇ3öÊùÉ#ì_þå_îÞ½»µµuÔN"¿Åb1L¼Îù!?äüßdòù|N§3777æE¶Èv9wÖG&Cä·uëÖßû½ßûvÍ·ívû/_ø¨'È/³ä·dÉýef³yppù!?äüòµë0ËÆÙäË@ùµµµýÁüÁvühßûÔ¿¯Wý?øòC~#wÎ¾¾¾ÒÒR9!àóÍ7åDCCòC~Èù!?ä xë0çææ:$ÜÉgü¾÷½ïmX¿AcüÛ¾m»üòûü*FB=9qíÚ5áÈÏÏG~Èù!?äüô%X¹¢¢Âív'­®2A~µµµ¿hùímÚ[PPüßgÍ;Wî«ÝÝÝ@@N<õÔSê«º ?äüòI âlÍápLùÁÖßzúé§-]-?»Ýþo|ù!¿ÏÚ½·ö~w`!?äüòC~Ã#|ñÖa¶X,²?B)12 ¿ÁÁÁ|ðßüæ®»ö6íøá¿½ùeü¤Ë.È	Q`uuõtßtäüòC~É/¿`08[C~«¿¿ÿ?øÁïÿþïù¾ñoú®jäqòòC~Èù%­üÔÁÖìv»á:Ì3v°5ä73!?äüòC~È/CåT[C~ÈùMü*++ÕÚ.¬äüòC~)?u°5ÃI¾äYù!?ä7ò[°`A´ö´xo/òC~ÈùeüB¡P¼­%Û:ÌÈù!¿) OîÞ===3¬äüòC~³+?Ù	;ý$Z¢%-wÑÈù!¿a³Ù,÷ó'òC~Èù!¿YÐÇívlM­Ã*K´ ?äü&ÒÅåÞ¾mÛ¶»wï"?äüòKcùùý~§Ói¸³ÝnOþuòC~SóyóæéÿòãÈù!?äòë=zÔjµÆ[¢%fÈÈ ?äüçÏÏ;<òC~È/-åâ­ÃÒK´ ?äü&t#ùýþ¾éÈù!?äü¦©ßþö·Ï>ûìêÕ«õà+**jllLÑuòC~S ¿ÞáüòC~éüßÔÔ4wîI¾£Gf¸òC~Ã]]]²GØ½÷Ïù#?äüòÚ¨ÃuäÅ!?äüF®"N¼Ãù!?äü¿þþþgyÆpæÕ«W·¶¶Ê'ðüòû²âÄ;<òC~È/iD"mmm555¹¹¹úI¾-[¶x<~=òC~IòC~Èù!¿	ÔÛÛ+ûÏòòrý$Õjðõ÷÷Oà¸½Èù1"?Å2þü	òC~Èù!¿q¥¶¦äËÎÎ®¯¯÷ù|Úg"?äü_ÜL&ì8fþ¦#?äüòK"Ç³xñbý$üéÞÜÜ¬?ØòC~ÈùÅ­³³Sv²ïI ?äüòKüÐ7lØ »®ø`kÈù!?äÿ*xo/òC~Èù%SBVUUé÷ÌcYù!?äüâÆòC~È/Iòù|N§SÿJ>©¦¦FÆ¸ð*òC~Èù%]Èù!?äüTê|äC~Èù!?äüòC~ÉN`WTTd¸³×ëSòC~È/QCCCëÖ­ËËËM~~þ¦Mf@$Èù!?ä±òÃ¢:Í¦(P,8É¶òC~È/n÷îÝ3|ÇtÆù!?äü2P~Á`ÐårNò©u§Ä%Èù!?ä·ÊgýúõwïÞ6nÜ([/_üòC~ÈoJD"ò´Ûíú%ZrssNgô:ÌÈù!?ä7òËÉÉ]Oô¢PCCC²E¶#?äüòd¡PÈívWTTè'ùªªª¦jù!?äüÆZVVìDÚp8,[XÕù!?äü&:Øá$lÖ òC~ÈùÅM=Û»víZõl¯ü/§eËÒ¥KòC~Èù·K´TTT¸ÝîòC~ÈùÅM¨gøÙq ?äüòñÖaÎÎÎv8	¶üòC~3'¿á·÷nÚ´©°°0++Kþ_»v­lîüòC~é!¿p8ÜÚÚjµZõB[,Ù×B¡¾IÈù!?ät!?äü_ªË/.Ñm·Ûgrù!?äüòC~ÈùMKâ¹xë0[,±`0ÝaA~Èù!?Ý%G+++ù!?äü_tBºòòrý>S (­I>äüò¥¬ø!?äüòéüùó<ò~¢¢"ËjXòC~Èo¬íÜ¹SíÎ^ýuäüòËpùB¡ææfÅbx°µÖÖÖé>Ð%òC~ÈùMü.^¼¯³^Øù!?äü2P~~¿ßét®ÃÜØØl|Èù!?ä7¾jkkÕNíÔ©S3sÓòC~È/	å',hii1<ØÚ+ÜnwoooòòC~ÈùÅíøñãj§¶~ýú¼éÈù!?äTòóù|»víÒ/Ñët:/_¾BÃüòC~Ý»woùòåêÍ3|ÓòC~È/äÛÚÚâ­Ãìv»ûûûSnXòC~È/¶_|QíÚ¶lÙ2+7ù!?äüfW~~¿¿¾¾Þð`k²ýÄI²DòC~ÈùMüXÏù!?äòloo¯©©Ñ¿Ãl6»ë×¯§ú° ?äü_lY£e2òC~È/ä·oßï`kI»DòC~ÈùMüf=äüòùE"¶¶6Íf8É×ÜÜ<ë[C~Èù!?äü!¿ÉÊ¯··Wv5Â;ýZ/^|ôèÑÁÁÁ´äüòC~Èù!¿L_$9qâÍf3|÷Óé_³ôäüòC~Èù!¿ô_(áÁÖ***<|B&òC~Èù!?äü_ÚÊ/ÈøØívÃ­9Ù§î-Èù!?äüòC~ôÙ~ù/þâ/¶¦Þ½!|Èù!?ä7Ú%YÏù!?äÊÉÄápÈÊpÙ_§Í-Èù!?ä7ò^ºÏP~¬çü_úÉOöbÙzòÉ'St2L¶ÝnwUUáÁÖWú-ÑüòC~S ?­Ùc>úè£wïÞåÿ7Ê¹ ?äüÒI~(..¶Ûíßw|ßúMë<pùòåÏçt:õo×l6×ëÍ¨Wò!?äüßS+]Eï1dKii)òC~È/mäç÷û¿üå/?ñØ÷ä>õïÛ5ß~ðÁÿÇiÇc8ÉWTTô§ú§ÝÝÝü ?äüß¯b$Ñ^üxòC~é$?ÁÓÿZõ¿4öÉ¿½MÅÉ¼±ÏçkllÔlM²Z­ê`k8n/òC~òËhùÈn´®®îÞ½Ã#Ç2_¿~½líÈù!¿tß·¾õ­hùÉ¿âââ$xNT'¶3äíòC~Èùûn`øéþ%@~ÈùÍ¤üü~¿°éG;~¤±oó¦ÍÉöl¯.Á$àUÿüòC~ÈoÜ]»vmÁyyyYYYùùùK.½ûötßtäüßLÊOÚ±cÇW¿úÕïïÖøo§  àüùóÉðÝÃa¯×k³ÙôàËÍÍu:>/ÞeòC~Èù¥FÈù!¿tøðáoë[ò^mmm2¼±7ºÃI¾ªª*ÃI>äüòC~Èù!?äJE"¯×à`kcß9 ?äüòwW®¬¬ÌÏÏWïç-))9vìòC~ÈùMy¡PHîûE?ÉWQQáv»Çû¨üòC~Èo|©£Ø¦N:tù!?äü¦¤H$"·ßpO¶89wbë0#?äüò_¥¥¥²ó½rå&¿.ÈéÂÂBäüòdê`k[ýÀ$"üòC~ÈoW1:¡ä'á¸½Èù!¿Iï`kÙÙÙv»Â|Èù!?äü&ZÉYÍóüöìÙ£þG~Èù!¿ñà`k²Wq¹`p¿òC~Èù!¿ñÕÕÕe¸ó¹sçòC~Èoì%XÙf³y½Þ)äC~Èù!?ä7ÙnÝºU]]­ÞÛWYY9µ#?äüÒX~	Öaºè­!?äüòùÍJÈù!¿T_u­Vkkk« pºoòC~Èù!¿q^ÅçoìÐºûveeeyy9òC~ÈùéK°D(°±±qZ'ùòC~ÈùM±üxo/òC~ÈO_u­VëX¶üòC~ÈoväW^^>'asçÎE~Èù!?Ü[áÁÖN§Ïç­üòC~ÈoLÝ¸q#k$íèÑ	û?üòËpù%X¢E6ÎÊ$òC~Èù!¿I%Îî'vòC~)'¿xë0ËÃ<w^äüòC~©òC~È/	å788ØÚÚjµZõ|n·;Ù5òC~Èù¯¡¡¡ÊÊÊüü|mKqqñÓO?üòË(ùùýþíÛ·ëhÉÎÎv8Su°5äüòC~³,¿yóæÅ¼½WíîòC~i/¿|Eî§¡P(Gù!?äüßøÊÉÉ]¼ü¹¯m¹xñ¢l),,D~Èù¥±üâMò©­%í$òC~Èù!¿I¥ÞÞCý"HYVVf2-ZÔÙÙüòKùçN8a8É'ÜµkWôßÈù!?äüÒM~3ÙãïØ±CgéÞ½ûöí-¥¥¥¼æºººW_UN:thË-Èù!¿Ù_(r¹×Å­!?äüòeùõôô®ä|áÂI^³ÙlV²²X,zù=ýôÓ?#¢éï©§ª®®6L1wsÙ²zõê^x!""J¦]~üÅ¼hÑ¢¼¼¼¬¬¬üüüNÉßÐÑËêdÎ9?æü¦Î¯··WîhK´>|8&?ócÎ9?æüóK¢_)üò1ùÉ';Ãu»ººRâÝÈù!?äüRI~%%%CCCÃ#ÏöÊiäüßtË¯¿¿ÿàÁ/6äKþ%ZòC~ÈùÍü®¢sV³t¢´cÇMþjÇ+¯¼"'äÿºº:äüßôÉÏçómß¾]?Ém·ÛO86|Èù!?äü&UGGö ¡ä§N:th×ÜÝÝ]ZZ*×YVV¦¿òC~Èoòòlkk3Ål6§å$òC~Èù!¿I¥x¸rå&?Q+9#?ääòMMMë0Ûív¯×+(ÌA~Èù!?ä7Î«i8jõfáZîù!?älòÃñ¶f6wíÚ%Q#üòC~Èo|¨ÕûüöìÙ£ÙüòKù½üòË|Ë-;zôhº¾ù!?äüßTÊ¯««Ëp%çsçÎ!?äüf½p8ìõz×¬Y£¿æææ:ÎË/gòø ?äüòw·nÝª®®VïíÍËË«¬¬ÓÓòC~»¡Ëå2ä«ªªòx<<!?äüòKòC~E"¯×k·Û³³³×aÎð;òC~Èù!?äü_:ä~a±Xô|>øàøC±òC~ÈùMA|òÉüùósrrä&//oÑ¢EÈù!¿)9sÆpO¶89÷ßÿýß'pÜ^äüù!?ä<¾Ãcº×@~ÈùB!·Û]QQ¡¿Z,èuÇÜ^äüòC~ÈÏ8µs]]Z÷õîÝ»[¶l-eeeÈù!¿iJ~ùá$ÝnäÅ,ÑüòC~ÈùOáJÎÑ3rZ[Õù!?ä7ÉCÛí®ªª2äs¹ÞVüòC~ÈùOáßÐÐ¶åÞ½Ìù!?ä7µù|>§Ó«7Ífóz½×aF~Èù!?äü§ðu~uuu>µ¿X½z5¯óC~ÈoJÇÇc8ÉWTTär¹ÀX®ù!?äüòÂg4MOû"?äÞòóù|ñ¶fµZ[[[ÃáðØ¯ù!?äüòùeÉdB~Èù1µsMMá$Xp|Èù!?äüß´Èo¶B~)'¿¿û»¿³ÙlUUUßýîwý~?òIH×ÜÜ¬^8«ääÁÖòC~Èù!¿©_¼E§güRK~7o~pþßw|¿ñÏ¿óíï?ùi»6lÍétú|¾É	äüòC~Èoxª^çwàÀõõõ¬êü´ÚÛÛ¿úÕ¯îùö=¹OýÛ¼ió>áòëíímii)//×OòUUUMrù!?äüòùðäª¤¤äöíÛòák¯½¦º¦éåÈ/å÷äO~çÛßÑØ§þ«Õ¿3P~ò«»aÃý-ê`kS2ÉüòC~ÈùMüGføÔãVYY:±qãÆé¾éÈ/µäWó¿k<`´´´Ä;ØÛíÖ¶üòC~Èù%¯üÔA;|øðÜtäÇ³½)$¿óçÏ744èÍgx°5äüòC~È/yå·uëVõ¦½-ñÑGE~È/ºÌ|G8öx<Ë-Ó¯¼¼¼¹¹9ÞÁÖòC~Èù!¿$Éd±ÂÂÂK.G½Î/''ù!¿èþú¯ÿÚjµ.X° ¶¶öòåËÓôUD~r3víÚe¸óÚÛÛ§ù!?äüòùÉ#ÙÞ½c6:NÞÛüf(¿p8ÜÖÖ&ÀÕ/ÑRZZ*¾òC~Èù!¿_¼õünÝºü_æÈO¾®Ëå2ä[¶lYkkët¼ù!?äüòiùÍVÈù%üÂá°:Ø~O¸k×®lù!?äüòK.ùÉ[ôó¹?D~È/ýä'¤ØlmÙ²eGMªäüòC~Èù!?ä7îÂápkk«ÕjÕÏl6744tuu%á° ?äüòC~Èù!¿qã½ÏãñÌî+ùòC~Èù!?äüßdD"^¯×f³éÁ'lhh8þü/ÑüòC~Èù!?äGS,¿P($¿fEo¾ªª*ÇBÈù!?äüòC~ÈÏ H$"H²Ûíú·ëæææ:TüÅC~Èù!?äüòC~_Hvèn·»¢¢B?É'å¬ÔÝã#?äüòC~_âòK!ùù|>§Óókm·ÛÅLÉÿJ>äüòC~Èoå5Z&	ù!¿$ìÁ=OUUþOÅâr¹Á`zòC~Èù!?ä7Ì1<_ÆÊ/Á-6Íëõ¦ú$òC~Èù!?äü_¦Ë/Á:Ì¢@±`lù!?äüòC~ÈùM Á`ÐårNò	SkäüòC~Èù!?2_â%ZN§ÏçËaA~Èù!?äüòKgù%^9¥hA~Èù!?äüò£Ïä×ÖÖæp8ô|²%E×aF~Èù!?äüòC~_H­Ãüµ¯Ípù	B;8Èù!?äüòC~iRâuÓoäüòC~Èù!¿+Á:ÌEEEé´3òC~Èù!?äü_æÊÏçóÅ[ùÿøî¹p8Ìù!?äüòC~È/K¼ó®]»ü~ÿÄÛü!?äüòC~ÉR­-^¼X8888¨>ù!?äüòC~Èù¥dápØëõÚl6=øâ­ÃüòC~Èù!?äüR¬[[¶lY­!?äüòC~Èù!¿Ô(x½Þ[»|ùrâk@~Èù!?äüòC~É^â­µ´´ôööåzòC~Èù!?äü_DD$ñ&ùGWW×¸®ù!?äüòC~Èù%]¡PÈívWTTè'ùÊËË<Øßß?«E~Èù!?äüòC~IÔåËëëëõ|²eóæÍíííYù!?äüòC~ÈùÍ~ýýýmmmë0»ÝîP(4ù¯üòC~Èù!?äüf³Ë/744NòÉ#LÕ×B~Èù!?äüòC~³Z¢¥¦¦Æð`kMMMÁ`pÊ¿(òC~Èù!?äüòÑÀÎl6ëÍgµZ[[['óJ>äüòC~Èù!?ä7ûE"¶¶6Í¦÷FQQQcc£pºoòC~Èù!?äüòvo%äKp°5äüòC~Èù!?äòÓ^Éï`k>oæüòC~Èù!?äü¦²P(ôÌ3Ïlm&'ùòC~Èù!?äüßt%Ø¼ys¼­Íúù!?äüòC~ÈùM65Ég±Xô|n·;IöÈù!?äüòC~ÈoR¿¸<òáÁÖxb×aF~Èù!?äüòC~³ ¿ë×¯·´´¾Ïb±ÈøOÉÁÖòC~Èù!?äüßlÊOÖápNòÙíödäC~Èù!?äüòC~ãNvgn·»ªªÊpÏårMÇÁÖòC~Èù!?äüßÊÏçó9ÎÜÜùl6×ëMæI>äüòC~Èù!?ä7záp¸µµÕjµêÁWTTär¹fà`kÈù!?äüòC~Èozå'¤kllÞlM8((LÑaA~Èù!?äüòC~¿KlÍf³NòSqù!?äüòC~Èù¡P($#f¸³Õjõx<ó7óøyyy,øÉO~üòC~òC~Èù¥ü"<ÞÛívÃ­9NÏ'&òà¾ïøþ¾'÷ýÐùÃ¯ík;vì@~Èù!?B~Èù!¿ÔZ¢¥¢¢B?ÉWUU%ÔÓv^áp¸   áÿ4ûÔ¿íøQ~~~J¬áüòC~Èù!?äÑòSë0ëh-²]?h@ ª²Jcú·bÅööväüò#äüòKFùÉÎÈãñ®Ãáv»ãí­Áà¼yóbä·dÉùE~Èù!?B~Èù!¿ä_¼%Z²³³ÇX¶f±X6nÜ¨±ïOêÿ¤°°°··ù!?äüù!?äü¢ãÇ¿úê«ë0äd|B¡Ð¯Êï÷ýë_øáå¿ò¯¤®òC~Èù!?äüÒª@ ÐÔÔ¯ä³ÛícäÓ'Lliiihhxþùç¯_¿ºüòC~Èù!?äçÚÛÛyäý-Åår¥è»qòC~Èù!?äüßÿÔÛÛÛÒÒRZZªb×f³y½Þ	Lò!?äGÈù!?äü_rÕÞÞ¾yófý-EEEuuuW¯^å~üòC~Èù!?äÚõ÷÷·´´,^¼X?É·lÙ²ÖÖVÙõLà¸½ÈùòC~Èù!?äDùý~§Ó©ä+((íçÏ×ØE~Èù!?äüòC~)Y8>xð á$_EEEKKËàà`ÌEòC~Èù!?äü_uùòåÆÆFý»7²³³m6[ïù!?äüòC~Èù¥FmmmË-3äægF]ù!?äüòC~Èù%~¿ûöíú­Iv»]88Æ%ZòC~Èù!?äüÒJ~%=ä[[[¶VZZêr¹À¸®ù!?äüòC~È/ä×ÝÝ½páBQ<ùýô§?ýM*$;Ð?ÿó?àôæ«®®~î¹ç>þøã	ÈïÆ¿¡/ÖÓÓséÒ%Æ!¦`0øöÛo3úäÈ_ýêWCLW¯^÷Ýwï¼óüÎ8ÄôáÊ#ã ï­·Þú×ý×þ¢),¿uëÖÉ,ü9òA÷þûïÿä'?Y¹reVVVø~øáÖÖÖÉÏ~ö³Ë/@_¬««Kþ(gô¿§NbôÉù¹sçÞï½ÆAü%^21	û:;;ííí.]á/ÚÏöþî&Æ_?ÛõØµZ­'x¶gy¶gy¶gy¶gÓçÙÞTd³Ùørssyä®®®)¼räüòC~Èù!¿Ô67ò;qâÄâÅÝnwÿ_9òC~Èù!?äü_jËÏÐ©+¿ÈHÓtåÈù!?äüòC~È/SB~Èù!?äüòK7ùÅù!?äüòC~Èù!?äüòC~Èù!?äüòC~üòC~Èù!?äü!?äüòC~Èù!?äGÈù!?äüòC~ÈùòC~Èù!?äüòC~üòC~Èù!?äüòC~Èù!?äüòC~Èù!?äüòC~Èù!?äüòC~Èù!?äüò#äüòC~Èù!?äüù!?äüòC~Èù!?B~Èù!?äüòC~ÈòC~Èù!?äüò#äüòC~Èù!?äüòC~Èù!?äüòC~Èù!?äüòC~Èù!?äüòC~Èù!?äüòC~Èù!?äüòC~ÈòC~Èù!?äüò#äüòC~Èù!?äüù!?äüòC~Èù!?B~Èù!?äüòC~Èù!?äüòC~Èù!?äüòC~Èù!?äüòC~Èù!?äüòC~Èù!?äüòC~Èù!?äü¸_!?äüòC~Èù!?äGÈù!?äüòC~ÈùòC~Èù!?äüòC~üòC~Èù!?äü!?äüòC~Èù!?äüòC~Èù!?äüòC~Èù!?äüòC~Èù!?äüòC~Èù!?äüòC~Èù!?äüòC~üòC~Èù!?äü!?äüòC~Èù!?äGÈù!?äüòC~ÈùòC~Èù!?äüòC~Èù!?äüòC~Èù!?äÇ8 ?äüòC~Èù!?äüòC~Èù!?äüòC~Èù!?äüòC~Èù!?äüòC~Èù!?äGÈù!?äüòC~ÈùòC~Èù!?äüòC~üòC~Èù!?äüòC~Èù!?äüòC~Èù!?äüòC~Èù!?äüù!?äüòC~Èù!?äüòC~Èù!?äüòC~Èù!?äüòC~Èù!?äüòC~È/3å÷WõW^¯÷z÷òË/_»ví:±ÎÎNùq)¼öÚk>ùÊçó11øá?ýéOo¾ùæ+WÞï½·ÞzqÐ÷÷ÿ÷Á`p¿hâ?NRX~rßÛ·oßèó?#:X"""¢	ù!?""""B~DDDDüùò#""""äG±]¸paéÒ¥&iÉ%/^-]]].--êîîÎÌa¹råÊòåË£A§¬¬LméììdXÔ°èèMGGÇ9smXÂá°ÓéÌÉÉ)//]#£;^Ã;Þx#lû^äJ-X°àÜ¹srâõ×_¯¬¬f³ùÆrBþ·X,9,²9yò¤Áuuu¯¾úª8tèÐ-[5,úßFFDd×ÉòÓËþýûöÙû÷ïtæÏÏÈh#Ã×ð^Ã7ÞÈ$Û¾ù¥jyyyò¿ìÕ!GåÿLÞ5«N>-Q©ý²:¢ñÐÐP&ïcEÿûÃÈh#óôÓO¿ðÂ,?ý°x>úè#D?2ìxï5ìxãL²íÙÍ¥d===Ã#³ëòk$¿^ò¿lÌä?°eä/*ùÐd2igEÎðaÑÿþ02jdnÜ¸Q]]-Z.?ýýèÅ_àæ>`d´aÇkx¯aÇodmßüR¯»wïÖÕÕÊéåË«?ÊeO´råÊ9¥mÌÉÉaXÔ°è526m:öìïvÌùñ~täÈ9qõêUy$cd´aÇkx¯aÇodmßËn.Åºyó¦Óé¼uëc¦AöÎCCCÃ#O:D£'ÃEÿûCjdæ|1E»±1v¼÷v¼ñF&Ùö½ìãR©®®®µk×öõõi[äÏÍ+W®È	¿ß/fæ°,X°@Âd|äÃáxåWäü/c1,jXô¿?è]6Ã¢ËÖ­[ßxãá7·Z­VFFv¼÷v¼ñF&Ùö½È/²X,1I|òÉ'j¹ù_Ngæ°xqÑ¢E2kÖ¬QQuwwfeeÉÎaQÃ¢ÿýadbþgX¢e```Ó¦M²¥ºº:02ÚÈ°ã5¼×°ã72É¶ïE~DDDDCRùò#""""äGDDDDÈ!?""""B~DDDDüù!?""""B~DDDDüùò#""""äGDDDDÈ!?"J^íµU«Vå´fÍãÇaÿ5RÊìmn­Åbompp0f»l1Leee÷ïßïu!?"J½öíÛ7G×ÒI~ÍÍÍ²ñðáÃ1Û<(Ûzê©	ò#¢ëâÅbÉôÒK/EF:rä|(/]º6ò»víldIÌöÊö`0üùQú÷è£iöÙèÏ?ÿ¼l¬¯¯vOgg§ÈIP¸téR9­ò;wå,³Ù¼cÇè'UÏ9#º³ä²§O±l;wîªU«N:%:ÖÞÞøzÔYrÔYñ¶víZÙ~áÂmË¹sçdÍfÓ¶ìß¿¿´´T®*//¯¶¶öæÍzùé¯?fKJDÈh67o¨åÆÑ?ýôSÙh±X¢eSOO:wãÆ1gmÛ¶Må÷û³²²/¥>TçnÚ´éþýûbÇH$"çÊÿ"§üü|õò»×#'oþ;ýõ×£9«áòÕW_ÕØs%kÖ¬¯üÜT"B~DD³zb×`5g8,Z6[¶l¹;×¯_¯ÎUÐQvTSw"6uV]],ÓÝÝÝêJ¢¯sïÞ½b;õdëã?.[N<)§å9ýØcz=¢FùP>!ú¶~G¢Éââbù~ûúúäCù_n¹pshhHBYY¾áÏÎËË¯üÜT"B~DD³/?ÉP~Úv%&éæÍò¡I¸téRùpþüùO<ñíÞ½ÚÈçÄÌ¢iR~úé§Ú'´'|å9æÌQ¯G)Þ¾;ú¶ÅMÞÎ;å¬^xaøóg´wìØý	¢@x´ººZMIW~	n*!?"¢YNMtÝ½7zãàà l³XGsa PøÓ £½8Oÿ$l¥¢×RÓf³9'''ËÏ;W;wÔëç°®¢*§å9í÷ûµs»»»å>k<vù%¸©Düf9õZ·_|1z£Zë$æÚkûúúôSY.]jnnVÏ½jdTrÚÓ©£úlÛ¶mêI^ù¿¡¡AÛàzÔÛ­[·Ô·oßNü>Ü+WÊ¹j!kôYê¾rVGGÇÀÀ@bùi*U£¡à¦ò#"åÔ;$L&Ó¡CÔª./½ôRNNþÝë×¯åÃaõT¬ö>:?õò¸@ ýAÁ;vÔ»nµuU¦Þl«¾zWW¶=Áõ¨ö©×ùªÏL ¿#Gh³qò-G¥^òè÷ûå«È Ä²¦XY>MÄY[[nJDÈhöS«Ç´ÿþÿÙ¤`¤Ö'ÕÞT¡õøãG³2ºcÇ%ßðÈÁ6d»Ùl~"8ÁõÈÍ¾aÚûjã³÷îÝËËËSßBÌsÜëÖ­þÅÅÅò¿ZØ%ú:Õ¤rªvnJDÈ()¬Y³&g¤U«V©7ØÆÈ¯££C-·hÑ¢³gÏjçîÙ³§´´TiiçÎápX;÷ôéÓÕÕÕ²²²2Çsú["W%Û·nÝ³=ÞõHrcä&©¬ç§ÕÐÐ³¼ª¯¯¯®®NF °°P¾`0¨­PB[5V6íÂ1_1ÁM%"äGDDDDÈ!?""""B~DDDDüùò#""""äGDDDDÈùò#""""äGDDDDÈ!?""""B~DDDD4þþ? «Ö5IEND®B`


^ù!?B~ü_÷ôÓOWo¬VìSÿþïêÿ[WWü¦Ïç_Ý¨ØµX,n·;¦ý/òC~üòC~:­¼¼Üù]g¸ü¾cÿÕjE~M~]ív»ö-ÙÙÙCÏ»Qò#äGÈùÍØç÷ùý~Ëõ-¥¥¥i6zù!?B~ü±kù;¿©åõzNgÔko¤ñèäüùòC~Æîå_ÎÉÉ©¬¬¬ø?í¸Y>zù!?B~üáóù|mmmòÌùüâ/%ùµ4Í³yôòC~üù!?Jsù1zù!?B~ü¥¹üÁ ÛífôòC~üù!?Jgùõ÷÷×××G½a·ÛgóèäüùòC~&òÏêl6öÀ®ÙlßIÏÇ*@~È!?äGÆßïoll,--ÕØµZ­­­­ìäC~È!?äG¬tÃ­=EÓéôz½,säüùòÍf3ò£äËOÞ°X,Ú|ååå.ÑÈùò#ä7uvv.Z´HÞh%S~ñGo$ÿ·ùò#äüfüÖ®]ëõzãÈï¯ÿú¯?Mn~øaWW×§¤ã~üãÎ¦pÇÞÞÞ¿ú«¿Z¹reFFFù¾ò¯|÷»ßõx<,ÞiéÒ¥KôËAÏÉÿr?øàMÖ¹sçX:ïÝwßýäOüM,¿ÿy±å×ÚÚz#¹*>ûì³¤ãæ?ûÙÏ&u+W®üà?øýßÿýð	ÿøÿø7Þøå/ÉÆd;øñÇ³ôÜçÞÞÞÎrÐsýýýï½÷ËAçÎÿÎòãh/=âÑÞ¾¾>§ÓÇv9ÚKíåh/q´75ÎS!?9ùAËe±X´§åã»Èò#äb"?.ùÉ­û÷ï:zcóæÍÞ@~ü!?äü/¿±±±¶¶¶ªª*íN¾¢¢¢úúú	ÿ0ò#äGÈ/!?JD~Bº#GD½öÆSO=uúôé@ ÀBC~ü!?äü-?ùõ¨®®ÖîäËËË«««ëííeY!?B~ÈòC~Æ®»»»©©)êN>ÙÒÒÂN>äGÈùòC~ÈÏð©S´|ùË__vv¶½166ÆRB~ü!?äülmmz³Ù¼gÏÏÇRB~ü!?äüÝÀÀ@½ðN`×jµ9s|Èò#äü±Ïy<õë×k¯½ý'ò'§Of)!?B~ÈòC~ÆÎï÷766F½a±X;&_0©«·ò#äüù!?ä§»dÉ;ììì	¯½ü!?äGÈù!?CÝn·ÅbÑîä+**jllìïï¸òC~ü!?äü®¾¾^]5z#Îµ7ò#äüù!?ägÆÆÆZ[[m6ö-¢@§Ó©ÝÉü!?äGÈù!?åóùdF=EKyy¹Ëåòûý<òC~ü!?äüô¬G»Ý®ÝÉ'sdþd×2òC~ü!?äütßïw¹ååÚ|f³YÖïÔ®½ü!?äGÈù!?åõzN§vôFff¦Ífóx<ríäüù!?B~Èù¥>5zÃjµjwòëëë'½ü!?B~Èù!?½gôÅbq»ÝÁ`pº¾òC~ü!?äüRºÀnÔS´dgg;YÓþMò#äüù!?äÔâWfÊM	¢ù!?B~üòC~ú-ÎvôòC~üù!?äüRxîÌ3QGoÍæé½ü!?B~Èù!¿T¦FoE½ÀnkkëLïäC~Èò#äü_2FÔko¨ìz½Þ<+äüù!?B~ÈùM[@ ¹¹yÙ²eÚ|K,Iü»Èùò#äüòÓo^¯w×®]Q¯½Q[[ÛÑÑä»ÈùòC~üòæÄsmmmV«U`·  `Ï=©:°ü!?äGÈù!¿ik`` ±±1êµ7ªªªÜnw ÐÛsF~Èò#äüßäº|ùòæÍ£¯®®Nt¥Ûgü!?äGÈù!¿---K,Ñîä+))e~ãÆÿÈùòC~üò ¯×»gÏ¨£7ª««åwW£7ò#äGÈù!?ä7õÔèÍ¦½·k×.ýïäC~Èò#äüß9r$êèeË577§ö´|Èùò#äüòúúúv'Ì±Ûíþéò#äüù!?ä÷Àï÷»¨£7ÌfsCCÃÀÀ@¼Ìò#äüù!¿Y-?yÚN§S;zCïÌ3F½ü!?B~Èù!¿è©ÑE>Qà®]»tuíäüùòC~ÈùM¥8×ÞX²dËåJ×òC~ü!?ä7[äçñxìv^^^ø²³³kkkåù§ýËù!?B~ÈòKsù577ÇºöAOÑü!?B~Èù!¿ßÓéÔîä¬Vk[[[:Þ@~È!?äüf£üÄs§O^³fö´|óæÍ«¯¯OËÑÈùò#äüßìß7äg/((Ðîä³X,.köØE~Èò#äüÒS~ê-ÕÕÕÚ|ÙÙÙv»=ùKùòC~üù!¿iN]`·´´4êµ7d9ø|>^]ÈùòC~ü±å×××çt:µ;ùÔµ7<Ïl½ü!?B~Èù¥üÁ Ûí¶Z­QGoìß¿ÖÞ@~È!?äüÒJ~­­­EEEÚ|Ë-knnå£7ò#äGÈù!¿´_¿vôFoo/¯äüùòC~È/Ýä'­Y³FÞhhh`'òC~üù!?äÎò»|ùò,¼öòC~üù!?ä7åGÈùò#äüò#äüùòC~ÈùòC~ü!?äü!?B~ÈòC~È!?äGÈù!?äGÈù!?äGÈù!?äGÈùò#äüùòC~üù!?B~ü!?äü!?äGÈòC~Èò#äGÈù!?äGÈùò#äüò#äüù!?B~Èù!?B~ü!?äü!?B~ÈòC~Èòc9 ?B~Èù!?B~È!?äGÈò#äGÈùò#äüù!?äüù!?B~üòC~ü!?B~Èù!?B~È!?äü!?äGÈùòC~Èùò#äüù!?äüùòC~üòC~ü!?B~I¬«««¢¢Âd2-]º´§§ùòC~üù!¿´ßÂ/]º$§N*++ÓÊ¯££ãWÉ­»»ÛçóýtÜÕ«W½^/ËAÏ]¿~½··å çîÞ½ÛÙÙÉrÐs@@äÇrÐy"¿û÷ï'ùX~áåäähåwôèÑóÉ­­­íÜ¹sçIÇ½ûî»¬#'+HVËAÏ½÷Þ²¹c9è¼ýèG,Ö¶t_www]]G£½í%öG9Ú¶GU###555@ùòC~üù!¿´ßß¤>½û¶Ãá-äGÈùòC~ü-¿ð:::V¯^=88õVäGÈùòC~üÒG~f³yNXÈò#äGÈù¥­üâüù!?B~ÈòC~Èù!?B~ÈòC~È!?äGÈù!?äGÈò#äüò#äüùòC~üù!?B~ü!?B~È!?äGÈò#äüò#äüùòC~ÈùòC~üù!?äüù!?B~ÈòC~È!?äGÈ/ä÷Ãþ°µµõFrûÑ~ô³ýìé¸öövù/ËAÏuuuÉÖå ç>ýôÓ¿û»¿c9è¹k×®<yå óþæoþ¦¿¿?ÉßÔï÷§§ü®^½zðàÁ¿ """¢ßÿèöÄÍò#""""äGDDDDÈ!?""""B~ºèêÕ«Ë/7L/îìì9===ÅÅÅjN;Hë¨«««¢¢Bæ,]ºTÖHëHuáÂ9sØétAÃURRÒÑÑÁ"Òá:õ²hÑ"í+R^øÆMol`;A²Î=+.]*(('OÊÄñãÇ·lÙÂ"Òá:Z¸p¡LËÄ©S§ÊÊÊXD:ÒØØùév:tè¥^^,X°E¤Ãu$oÞ¼ùàáÍf3H'ElÜôÆ¶¹vþüù¥KªWlebttW>×Qx999,®£_|ñW^A~º]GâÏ?ÿ¢çu$"¿û¶LÈGt®"6nzcÛÜð/«ðÔ©Sò©ÉdÝ>MúYG¡º»»ëêêX>:oÞ¬¬¬­!òÓó¶îÕW_ÿ;	)>ùä×QOO¬ ùT>Êæå£´7½±mn¢]¸p¡°°P&222B3³²²X2:ª@ ÀÑá:Ú¸qãÅ½B~:ÞÖ½öÚk2ñÅ_È;KFëhùòåj¿¬pÅ,=¤Ý¸éls'¢º¼ÞFGG<ÜmîÒÏ:zððØÃá¸sçËDëhÎoÇbÑç¶NW;*H»8¥Ã´7½±î-ðêÕ«]½zµLØíö×_]&äcMMHë¨££C&Y8º]Gá[I>×Ñ;Þ|óÍZ,×Ñ+Ô¾¾¾åË³ôF@5¡76°Í ÅËÿ¥V­Z¥v uvveddËËE¤Ãud6Ù¤óuüô¿7nÜ(s*++½^/HëèÚµkê</òQ¦YDúÞØÀ6hÖE@DDDüùò#""""äGDDDDÈ!?""""B~DDDDü!?""""B~DDDDüùò#""""äGDDDDÈ¥Mo¼ñÆÊ+s¶jÕªwÞyç·¶_3ÌÖ6Ú³5Íò£ù2Çd2Oö1¯ÎÑtøðát_CCÌlnnìØ1ÿÂ/Lá1¬1Éd:qâÄØÃ^í5ùTf^¹r%mäwýúu¹téÒù-ùýýýÈ¥[·nÓ¼ôÒKá3=*3kkkÃÝÓÞÞ.rVTTÈtèïÝ»WWW/7ìÞ½;ü ªÇã]ÉMrßóçÏGXJæÌ;wåÊçÎOívÄkkkÿ8ê&yJê¦.ÄRÚêÕ«e~WWWhÎ¥KdÕjÍ9tèPQQ<TNNÎ¦Mnß¾­öñ#æÄyªDüRÙO<!j¹yóføÌ[·nÉL³Ù.º»»Õ­6l¸içÎê¦¾¾¾¨÷Rª[7nÜ8>>.vÌÊÊ[å£È)77Wýù]Ç¨OOû:u*³!<y2Ä¾YµjÕdåç©ò#"JqêÀnmÖ9â°pÙlÙ²eäa2!®[·NÝª £ì¨vÝØÔM555jg¡Lwvvª	ÌíÔÁÖmÛ¶É³gÏÊ´|éçnÂÇ5Ê§òáÏ-êO$?¾ü¼ò©|g.ÜU_P\,wáÁoçääLV~q*!?"¢ÔËO*¿Ð|%&éöíÛò©IZQQ!.X°`ûöí"¶û÷ïD¾&b/Zè^êÓ[·n¾X´:à+eÚãñLø8¢LùôîÝ»áÏ-Ößä=ÿüórÓ+¯¼òà7G´wïÞþ¢@x´²²Rí¬üâ<U"B~DD)Níè	d¦ÜÇ:!z½^¿tB§=a©ðs©ÈtAAAVVV0;wnèÖ	'Ã"ºzõªBªLËGîëëÝÚÙÙ)O êQãÄåç©ò#"JqêoÝ^õÕðê<B888¨ÝuåÊuì5DFµC.t8uBíÜ¹Säuuu¡ùqGíc»sçúôîÝ»ñÇá®X±BnU'²°ß¤ÆùÊM./¿JÕÒÝç©ò#"JqjÉd:~ü¸:«Ë'²²²´£1Ö­['Ê	êPlh®ú;?õçq^¯7üwïÞ-NR£nCçUê35ØV÷Ðü8£þ°Oý_ P_G~¯½öZhoüÈá7©?yìëëï"!ü5ÅÊòe"ÎM6ßç©ò#"Jê,Ç:tè·_S0MUÚ¶m[8+Ãûí·ãÈïÁÃmÈüðÁqGFø«õÃÞ¿?''GýÇ¸×®]þ-æÏ/Õ]ÂSí¥º5ÎS%"äGD¤D'«V­ÊzØÊ+ÕÛù]¸pA6oñâÅ/^ÝöíÛWTT¤´ôüóÏÁÐ­çÏ¯¬¬»ÇÔ>y(¿cÇù±G'#OIh0ÎùüBÕÕÕEÞE588XSS#K ??_~þþþÐjÂsxxXh«Õjíêêøq*!?""""B~DDDDüùò#""""äGDDDDÈ!?""""B~DDDDÈ!?""""B~DDDDüùò#"""¢É÷ÿ8Ò6«¦yIEND®B`


X8gÎ)++ëÃ¯âÄEåççÞí²ô)ðà÷ÚñãÇ_½z5[2â¥^bçÎîí¡n2ª÷îØÓçÅX\ø¹Ùþ)?P~À0*¿`åÊYgÎºç?øA<ïêêáÛ0³©©)í]=ìMºýè[·nmÛ¶-Ì	56håÝðæÍaº³³3+²áÐòü^[UU¾:t¨§÷à_ýêWkÖ¬6VTT´´´t»æ±cÇ&LðÔSO%æ¼ûî»óæÍËÏÏ_¼xñµk×N81gÎ¥K&V½üòËEEEÑfÍð#õÏ<óL¼í5xýõ×ÃÌ6¤ýö&©?:ÄbäC-nC©?¨o7ìÍSzÃÔKùÅb±Ù³g«+gùÏâÇ?~bõ¥Kúü¤ÊèÏò»uëVqqñäÉÃDêðÇÜ"áÝÞ­óòòÂ×U«VÝK·µtÑ¢Eá=>ñíÓO?(n×dIæ2eJÿÉ'$ÏáfÎ1#íïø°7éö£oÞ¼ùÍ9ó¡Næëóóõ¦üzó555EÏlÂ3g2?ïÑ¥çÎëö#¢ëôíIÐå&9<üüÜsÏéåËß¼/LoÃÌäkîØ±£««+µælÚ´)<y2ú6ÜsøöØ±ca:DFtÛho¶£GéK.%_ÔSäçç§|ÛG¼IOûù%Øvá¤T¾ao²Þ_o ðíñãÇÃttÍuëÖe~Þ£K×¬Yý:ÑÇ×ç'P~@?_ÞÔÃtxî6òäÉaúã?¾½råJøvÒ¤IÉ÷¸41çöíÛÉß¶µµÝûífÓäw÷P!B@,0Cz`ù¤ýEÒÎïÃMk/Ü6$KYYY,K»Ðz³pÒ>ª>ß°7OYoÊ¯7OP´7d²0'óó|Ã´¿ÂÃ>éòú¿üÂaaá´iÓîÞ½<?uÌ,u§·Piï3ó·!YºmñÌÐ.¢¢¢h[gjÞuÄ=ôæ&=-.´Þ,z´o7ìÍSÖòëÍ:ðÙ­ÒzzÞ3<þ><éòú¿üþðáÛÄ9P¢Ñût·Ñ©0³7ùÛÙ³gGgWy÷ÝwCõ&¢Q®è+[¶l¹yóæo¼f®Y³&mùõæ&.¿]8	¾ao²nY±oå7nÜ¸0ÝíàâÞ<ïÑ_bX1Yt@ùR~ÁÊ+;õGs¢ÃH/_~ë¾U«VE=zùEã^MMM¡QöíÛ×hll¶9n8yòd49räHÚßñaoòPåyáDK²ã¾nwò°Kõ¡²(¼^íµ°`¯]»íu×òUhåp?aééyóæõæy~â¶mÛÂ?¦§NÚç'P~À@ßÕ«WwEsZZZºíï5yòäÄ¡²R~Ë-K¾ÛI&¯ÉÇ·¦Ì_ûÚ×R·B>þøã~ÍºÉC_æ8!s·³1÷a©>ÔSÖí÷-((è[ùE§B|àa.©sRíõÕWåI0 åD[BçøákÖ¬)¸¯¢¢¢§ýú6,ÚÚÚÂ½ûáòÂ/=ÿüóÚÚÚ9sæäååM2å­·Þúâ¿8ÑoòPåyá9r$Ä_~~~tÆ»nj©>ÔSvëÖ­g6ºç²²²Ó§O÷­ücÇ-0,·ðÔÕÕõþyÅbóçÏnøÊ+¯$æ÷ùIÀéììüÚ×¾ÕÃÀÝå üP~(?Êåò@ù üP~Êåò@ù üP~@6;räHIII~~þ%KEv_ß®ÓÛöR[[Û¦M&LÂ)S¶oßÞÑÑÑ·»ÇãáNÂ+((Ó^]ò¢¢¢P*¡~¿TCùÈqß¥KºººÞ~ûíp·Ë-ëÛ½544ú­£GzuÊfkþ<Ì×_ûå_÷sèÐ¡~y´ë×¯÷öÙg½ºå»®J®¨íÛ·?~Ü¸q;wîìvµ/~ñ.LÌ<yòd431gùòåaNåÊ²²²±cÇæççÏ?ÿøñãiï'ùçf¾É[o½URRRZZúÉ'¤ßíÛ·+++ÃmÃÃÞ¶mÛC^Î7/ÜÏÝ»wÓ^ai466TTTtäÈh~WWW´ÉøÎ;áë¤I¼ÀåÇø¦¿õ­oéºººPZaâÛßþvòu;ÖÖÖ|Ûhaþ¥KÂtKKKsÂôã?mëÅÅÅiï'ùGg¾Iè¹þða¢ªª*õa?ÿüóa:wÞ	/¿ür·¨íV·É¢ÝòÒ.ÌKãµ×^kjj¡ÿ¢ù'NßüÓkÖ¬	Óa üaZ~!bÂtç©ù:%ÑöíÛïÝÓo¼ñFtÑûï¿¿cÇyyyiï§[e¾IôÆzÛÉ'G»««+LL:µ÷¿~òË¼4îÜ¹MæoÙ²%ÓQ§¾ðÂ^`òiù%gPrÓô4`J«¨¨hÜ¸q7oÞ_C$ùûöí×_¹rå||Ûn÷ümonÒÓC<°Uc/ÇüfÎæß¾;söô£»usøÑaQÜ»¿:LO2ÅP~À0-¿âââäQ®ÄàYÃ)^yåpÑÒ¥KYðm×½,¿Ì7I<¤	&¤ÞvÒ¤IÑmûðëïØ±#ÜöwÞI½¨7K#1ÖnÞÿ¯1@ùÃ±ü¢]ßxãh3î«¯¾úÀòkkkÆÆÂ×ÄáQ3]¸p!ÜU/Ë/óMBýèG?Ï=÷möÙ0ýöÛo_¼x1±¯a/Ýºu+$Ý3®Ú1zjU/Fb:ºò=÷®Ê^åÇCc»o×®]i¯jÓ¦MÑ©LsÇ¿mÛ¶^_æÄb±pé¢E$_§££cË-á1,[¶ìã?~¨%î3´c¸y×;wîöáëÍÒHLÏ3'L|øá«5779óæÍóÊå üP~(?ßûÅ/~qùòeOáàÇã¿úÕ¯,"áÅúilä¦ßüæ7Á¿ÿû¿ß¸qÃrP~ýé¯þê¯BüyßÝ»wÿöoÿÖr òî»ïþçþ§å@ðOÿôO³pãþõ_ÿÕòS~ÊåòCù¡üòCù¡üP~(?å§üP~(?ÊOù)?ÊåòCù¡üP~(?ÊåòCù)?åòCù¡üP~ÊOù¡üP~(?òS~ÊÊå§üòCù¡üP~ÊOù)?åòCù¡üP~ÊOù¡üP~(?òS~(?Êå§üÊåòCù¡üP~(?YV~§O?~~~þ¼yóÎ;§üÊåòcÄß3N:&80sæÌÔò;zôèMÝµk×~ö³YD;výúuËàäÉ¿þõ¯-óçÏ755YC"Ë/ÙØ±cSËoïÞ½ÿÈ ûÅ/~ñüÄr ^á%a9ÿüüç?·b±ØßýÝßYCb$ß3g6lØ`k¯­½ØÚ­½ØÚËÝÚ¹ûvEEEGGòS~(?Ê W¯^­ªªºvíZêEÊOù¡üP~(?FNù566.]º´­­-í¥ÊOù¡üP~(?FNùJ¢üÊåòcÄ_fÊOù¡üP~(?ÊåòCù¡üP~(?ÊOù)?ÊåòS~ÊåòCù¡üòS~ üP~(?å§üÊåò³òS~(?Êå§üÊåòCù)?åòCù¡üP~ÊOù¡üP~(?ÊåòCù¡üP~(?ÊOù)?ÊåòS~ÊåòCù¡üòS~ üP~(?å§üÊåòCù)?åòCù¡üP~ÊOù¡üP~(?òS~(?Êå§üÊåòCù¡üP~(?ÊåòCù¡üòCù¡üP~Qù]¼x±££Ã¢S~ÊOù¡üP~Øòï)|úé§G]WWgÑ)?å§üP~(?F`ù]¼xqëÖ­EEE£~kñâÅòS~Êåòcä_ò ß¨ßæ|ÙÒS~ÊOù¡üP~dù¥ò%¯¿þú7,:å§üÊåG_æA¾Õ«W;v,BS~ÊOù¡üP~dqùeä>zmmíõë×-+å§üÊåGv¿M|÷»ßê©§zäkhh0È§üòCù¡üÈnÍÍÍ=òÍ5«¶¶Ö|ÊOù)?Ê¬_Ø¿iiijð%ùâñ¸¥üòCù¡üÈbÍÍÍ7n8qbjóÍ1£¶¶¶µµÕRR~ÊOù¡üP~d÷AYYYÚA¾+VìÛ·ï¿ü¥¥üòCù¡üÈbÍÍÍÕÕÕiùfÍUSSíÉ×ÏíEù)?åòCù1ü=ò3¦²²²Û;²òS~ÊOù¡üP~dÌ|ííí©·R~ÊOù)?Ê¬ÇëëëËËËSÏÉvOù)?å§üP~(?²O4ÈWRRv¯®®.í òS~ÊOù¡üP~dÌ|UUUgÏíý½)?å§üÊåÇpÔ/|ÊOù)?åòCù1|ÅãñX,Ö_|ÊOù)?åòCù1µ¶¶÷ÐþäS~ÊOù)?Êá¥±±qùòS~ÊåòcXhooß½÷Ô©SSùJKKûeOù)?å§üP~(?R<ohhX»ví1cºßÄ7nÜØ|ÊOù)?åòCù14._¾[;úôAäS~ÊOù)?ÊÁÖØØ¸~ýúöä´÷Må§üòCù¡ü(×¯_ß½wÚA¾¹sçîÙ³çÆùxòS~Êåò£ÿÅb±+V¤îÉ7zôèªªªÆÆÆx<>øJù)?å§üP~(?úMkkëîÝ»çÎ:È7uêÔ=ö+áÃS~ÊOù)?ÊGÇßï½µk×¦Ý/Ìµô?Nå§üòCù¡üè»ë×¯ïÙ³'íoL>½¦¦f ×U~ÊOù)?P~(¿Åzä[½zõPíÉ§üòS~(?òë77nÜØ³gOQQQÚO×­­­V|ÊOù)?åòCùÑáÝíé§N¸neee¸tò)?å§üÊåÇChnn®®®N;ÈWRR²÷î¡=ù)?å§ü@ù¡üúa¥Z___VV|A¾áp¸®òS~ÊOùòCùõ]KKKuuõÄÓ/»ùòS~Êåò#x<^___^^z¸n³bÅ¬äS~ÊOù)?Êßòe8'_öò)?å§üÊåÇÉ<ÈWYYÅÿáºÊOù)?åÊåIkkkx·J;ÈfFÌ òS~ÊOù¡üP~9*Çb±ùÂü7È§üòS~(?_ÎÉÙA>å§üòCù¡ürA>å§üòCù¡üF¾Ì|ÕÕÕ---9øbP~ÊOù)?Êoäù*++ä«¯¯Ï©A>å§üòCù¡üF ÖÖÖéÓ§äS~ÊOù)?Êod2È§üòS~ üùåaoâÄùòS~Êåò	Â»LÚA¾ ¬¬¬¾¾>Û?]Wù)?åòCùëå×ÞÞ^SS3kÖ¬ù=ÝÊOù)?åòCùewùE|cÆ1È§üòCù¡üågOù)¿á¢­­­¤¤Dù)?Êo =¶ªªÊ òS~ÃÂÉ'gÏþüòCù¡üúQ]]]ÚA¾è|ùòË-Êoß¾gtaå~ôèQËÈO~òð°ÂÂÿØù|óÍ7/_þ©Oª[ðååå~ë[ß:ú´§òÑýýßÿcc£å0$²¸üþû!ö ?þñ¯1è®rìØ1ËHx1üæ7¿±N8ñË_þrx>¶>úè»ßýnÚsòýþïÿþæÍÏ9ãìGçÎûà,!1ËÏÖ^[±µ[3ëiO>¼ak¯­½Êåòc_´'_ii©O×U~(?Ê[~gÏÝ¸qãÄò)?ÊåÇÈ,¿ýû÷äS~dùõDù)?Ê/hjjÚ¼ysO|±XÌ òCù¡üP~dwùÝ¸q£¶¶vÁiùÂDkk«'Eù¡üP~(?²»üÞï½ªªªÇ,íoäS~(?Ê¬/¿°ò©­­;wnÚO×ÝºukSSgAù¡üP~(?²»üÂÚ~Ã©Í®ëÓu(>|xÛ¶mßøÆ7b±òS~Êåòcxß7êêêxâÔà¸qãÆóçÏ[ì½ù3ùÌ²?]öô<ýéOúÏÿüÏt¸òS~ÊOù¡üP~½õÞï­^½:õ7Å<x°££Ãï½¿þë¿þÜç>·ýëÛw~cgø·õ­¡8 üòCù¡ü²òËp¸îc=¶yófçäë/|áÿçÿe_ôïË+¿üÕ¯~Uù)?åòCù1åwöìÙv=®»ÿ~|böìÙÏý¿Ï%ßÿ®üßO?ý´òS~ÊåòcðÊ/úàéÓ§§=|ý¥²²rÉ%Éå·èÉEßøÆ7òS~(?Q~±Xlýúõ©¼íÉçpÝþÕÜÜøôÓOoÛºmë[¿°ø%%%z¦kå§üòCù¡üîµ··÷´'A¾uñâÅeËl|XÎ_ùÊW®_¿> ?Nù)?å§üP~ätù5veeeOë¾ùæ!-ÃCù)?å§üP~äbù«©©5kVÚÃu«ªª-:åòS~ÊåGvßÙ³gCØ¥ä+--u¸®ò#ËÊoÔäåå)?åò#×Ê¯½½½®®.í _´'A>åGV_Þäçç+?åò#w|ÿûßÿêW¿aÏáºÊ,.¿!§üÊáÀ ÊOùÝ»uëÖsÏ=§üÊììÙ³!ìÒ¯´´4ä Ãu#³üJJJòóóíç§üP~äh/´]jðM0!´`(BKIù)¿[~óæÍKýã/,,è¶òCù1È2òíØ±Ã=Êoä_AAAøokk+**!ø~ô£6(?åòcÈ0È7fÌªªªh¯Ûò#ûÊ/úã!õÂÄ¥KºººÂÄ¸qãòCùÕä5kV·=ùÊ/'ÊoÂ	apòäÉ°/½ôR4á¬.ÊåGöþïß¿ÿ|Ý(?_Nß¶mÛÇstÛçCù)?Ù¥¥¥¥÷|Êåå¼òÊ+&M§O¡.Ð]ù)?ý¥££ãðáÃeee£Gîý òCùåhù	å§üP~<ºæææ]»võ4ÈWSSÓûsò)?òS~ÊåÇ0ýC>xð`O|XÁ*?_®ßÌ3£s»8³òCù1Ìµ´´lÝºµ¤¤$uoîÜ¹5È§üP~¹X~3fÌH®½Çö*?ÃÊ±cÇJKKSùÂh/?Êý+?_N_¼°â8sæLWW×`>tå§üP~ôÆÅÃ³°°0í ßîÝ»oÜ¸Ñ/?Hù¡ür¢ü¢µÉ gòS~(?2ÇãxqOë666>â òCùåbù;w.¬GþùÛ·o+?åòcÈ577WWW§ÝoÁµµµÞOù¡üß2eJêúÅÊåÇ ÿm644,^¼8uíÉ×ï|Êåå7mÚ4Gx(?C¨©©éÅ_LN¾'xbÏ=4È§üP~¹X~ÑÊ%¬wù¡+?åò#­^½ºÏÉ§üP~Êï&Oìåòc0µ¶¶îÙ³gúôéi?x£¶¶¶¿×U~(?å×]cccX×lÛ¶-òS~(?N<Åbk×®Í0È7 ò)?ò»7ªðP~(?úKkkkXéÄo(?ò8y=pòCùñ¢A¾òòòùúp]åòS~ÃòS~(¿,Ã _´'ßõë×ÛcV~(¿(¿°b6mÚÅòCùñâñxÚA¾'nÜ¸ñìÙ³ÃöÁ+?_N_~~~X%þCW~Êå7´´´ôôÁ¥¥¥uuuÃaO>åòS~÷?VL»víºuëÖ`ÛEù)?ßa/útÝá<È§üP~¹X~íU~(?Ë¡2òÍ5++ùÊ/ËÏ±½Êåg9ôÞHäS~(¿¿¡¢üÊ/»¼A>åòS~ÊOù¡üèþ'aoð?]Wù¡üß£êììlÙØ±cÃlÜ¸q«V­C=òCùsÍÍÍÕÕÕ'NL;È7L>xCù¡üßÃ¹sçNÚ#<úc|òCùÛ¿úúú²²²ÔãäS~(¿¿Ù³gÕÙòåËoß¾¾½uëÖÊ+ÃÇ)?_NÉµA>åòËÅò+((+µäìììsÂ|å§üP~9òGÑÓ ßèÑ£Gð òCùåbùåååµ[¨½ä`ã¬.Êå7âEë¦ä>zMMMkkkî,åòËò¶ö.]º4ÚÚ¾é0gþüùÊOù¡üF¤çäùb±Xòåòcä_H½´GxÜ¼ySù)?_fíííÛ¶m[°`ÁÜ¹s7lØ0üwËpN¾äS~(¿¿÷ï]µjÕøñãóòòÂ×¥K9ýÐò#ÛË/DÒg?ûÙ'âÙûþèþhÎ9Ã3þò)?òbÊOùíå·iÓ¦'xrç7v&þòõÕWÕ/ò4¬mÒòá¢äS~(?å§üÊ¯·,Xðì³Ï&ßºuëÊËËÃïÇc±XO|a¾A>åòË­òõ yyyÊOù¡ü2xâ'þoÕÿM.¿¯¬ùÊ¿üå¡ýò)?òK#¯gÊOù¡üzãßüæ9sv¼¸#Q~ö¹ïïCòäS~(?å×/¼ðB´®<pàòS~(¿Ì¯¥Ïþó³fÎZ[±6ü9sæüÉ~]eä«®®niiñD+?òëîÜ¹sãÆëÊ²²²ä;+?åòëIè¼ÚÚÚ/ùË+V¬øÎw¾ÓÑÑ1h<ä«¬¬ìi¯¾¾Þ òCù)¿ôÖ¬Y­1=:8]ù)?F@ùÖÖÖéÓ§äS~(?å÷ÐÞyçh¹|ùòÁ|èÊOù¡üVXiäS~(?å×GwîÜyüñÇ£9?>È]ù)?_/uttÔÕÕÍ;7uoâÄùÊOù=Øk¯½­7×­[7$]ù)?_fÑo¬X±"ä]jóKÃëÙ¨üP~Ê¯·t>?åÃµüZ[[wïÞvO¾¢¢¢êêêææfÏòCù)¿÷ ùùùÊOù¡üÙ±cÇÊËËÇº'ßêÕ«ìÉ§üP~Ê/+)?åòK¸~ýzX'ô4È·uëÖË/²Êåò#Ë/744TVV>öØc=òÙOù¡üP~(?²»üZ[[÷ìÙã7Êåòc$_ccãÚµkº®òCù¡üP~Øò»~ýzuuuQQA>åòCù¡üå^o«W¯N¸nyyùáÃò)?^~Îç§ü`@Ë¯¥¥e÷îÝS§NM]½îÚµ«µµÕòW~(?©üOÝ¶üÏOù¡üú Çb±öäsN>åòchÊ/y½ÖÈÏ<óÌíÛ·Ã·áëÊ+Ã'N(?åòë½êêê´ëFçä»~ýº®üP~qùõròÁ;;;£5õ#Þó¹sçóóóçÌsüøqå§üåºnyyyê _°bÅçäS~(?QùEkçPÝÊïÑ÷ó«¨¨xûí·ÃÄÞ½×­[Z~¡?:t7oÞoöýìgÿñÿÑ·Û~ôÑGññÏ|&5øÂÌ¯ýë~ø¡%EN:uùòeËàÿù/^¼h9/¿É'Õt¨´;wîoÃ|yæ?úhbWWW%%%©å·gÏ¿gÐ?~¼¡¡Ár ^á%ñP7ÅbßùÎw|òÉÔ½Ã0?®cÙf£Gzâüì>ËaHxù8q"í|ðÁ#Þsò1"©ÇØÚkk/Y·µ·µµ5üåöôÁá"ëÚÚ­½÷­½Á¥KfÌ1vìØðÿõqãÆÍ?¿_þø·(?åG_t¸nÚ=ù|ðòCùå7@&Oí>¾¦n;V~Êá_~­­­555Ó§O÷ÁÊåòÊÊÊ·Þz+L¯ÊOù-åò?áùêëëò)?ÙZ~.9sæ¸qã¢í³'O>tèÐ£ßíÉ'Â>Zù)?ùäS~Êo_t&çäOl¦÷îÝ; ]ù)?OùeäÊÊÊò)?#¤üÂýÂò;út?~¼òS~xï¼óÎîÝ»ÓòM8±ººº¹¹ÙRR~(?FNùE«øh"*¿®®.Û«üñÂ`eeå§>õ©ù|ðòCù1Ë/:s4ÎÊ¯³³sûöíÑ>=ÊOù1ò´··×ÔÔÌ5Ë Êåå×ØØöLÎ§NR~ÊäüùóUUU!ïò¡üP~¹[~Áµk×.Û;vìØ3gÂáÊOù18nÜ¸Q[[;wîÜ´|_úÒ.`)¡üP~9T~CBù)?Úùóç7oÞüØc¥6_iiéþýûÃá¡>½åò#ëË/q`GBøË9sæÔ©Sò#Ý¸q#T]O|7nLÞOù¡üP~¹^~íU~d£ð7µ~ýú´|,9ØÞÞÞí&ÊåòËò:uê¨&L üÙòÖÕÕ¥äÛ¼yóùóçº­òCù¡ür¢ü®w_âÓ;ìçwòc;öl»´ë.X°àÍ7ßLäS~(?_._Bè¼Þ°«üßqãÆaò$<x°´´45øCöþ|ÊåòË­ò*ÊOùex<¾cÇÇlòäÉ&LØ¸qãPíÅÃO>t±_ÎÉ§üP~(¿Ü*¿ÎÎÎ3g7.1gÒ¤IßúÖ·ò#áÏþìÏþàþ`ãoÜùß<öì¯~õ«ùÚÛÛkkkxâÔà¸uëÖ>ðòCù¡ür«ü¦LÒíðÞèíd×®]ÊOù´´´L8qÛÖm!û¢Û¿¾=ÌO9kjjzñÅÓîÉ¯££ãQî_ù¡üP~¹U~á-$¼»$æ;w.Ì?~¼òS~È¾èßO>yøðáû¡ñx¼¾¾~Å£GNÝ¯?]Wù¡üP~¹U~Ñá½]]]¿s¿)'ùS~Ê/g½÷Þsþ9ÝÊoîÜ¹ñãBÒmÞ¼9ä]ê _yyyÈÁGäS~(?_N_qqqxGÙ²eK´cø;wvîÜí<¤üÑ¶¶bm"ûÖ­[÷¿÷ýoø)û÷ïïépÝ­[·&Ì+?ÊOùõÑ3gÒÉùôéÓÊOùyï½÷&NøäO~éK_q²¯ü7nÜvO¾²²²èqÄÊåòË­ò®^½:gÎ±cÇæåå7nöìÙaÎ@?tå§ü²Kkkë=6lØðê«¯^¾|¹_ùJJJª««ûå§(?ÊOùÊOùå¬³gÏ¦ä=zt´'ß`,Pù¡üP~ÊOù)?ú_]]]A¾ÁTÊåòË¹ò»páBt2çèxÞÉ':tHù)?úKO|ÞªOQ~(?_Î_Xï'Þ¢ò¦÷îÝ«ü"Ã _¨À~<'òCù¡ü_oEzáÂDù>Úâ|ûéºÊåò£Ê/z7ºtöæ®®®0¯ü%+ùÊåÓå7yòäèìQùuvvnß¾=Úå)?z)Ã _ôéºÃgOù¡üP~9]~iÏä|êÔ)å§üÈ,ä5kVÚA¾ÐÃpOù¡üP~9]~Áµk×.Û;vìØ3gÂÙ%òËjgÏ­ªª3fLÖò)?Ê/×ËoH(?åFÀ òCù¡üòS~<ÀäS~(?_®_xv§MVPPÞÃÆ;gÎ¶¶6å§ü¸×A¾PYý*?Ê/·Ê/¥=ÂãâÅÊOùå²ÌëQ8~MåòCùåVùEgr®¨¨èèèßÞ¾ÝºuaNqq±òS~9(ó9ùFÀ òCù¡ürºü¢·´äÓ³:+?å;rdOù¡üP~9]~Ñ_gggbÎ;wù)¿Z û÷ïO;È7fÌªªª6È§üP~(¿.¿h?¿|áÛ7o.^¼Ø~~Ê/477÷4È7kÖ¬:È§üP~(¿.¿Q2@òÂ%Ë|ÊåòËéòËüü|å§üF|ÊåòËõò*ÊOùæ¯a¯²²2_ÊåòË­òëé¤ÍW¯^U~Ê/Û577WWW÷4ÈWSS|ÊåòËéòo¯¼òJ·ë×¯wVåÕ¿cYYA>åòCù)¿ß/¼N<9úÿÁ~½AÐîÊOù¨ÖÖV|ÊåòS~¬_¿>zk,..&V®Ð]ù)¿þý¥<¸zõê1cÆt¾Ñ£GäS~(?òû'NH¼S¾þúëðÐòë/_¯¥ÔA¾éÓ§×ÔÔ´¶¶zÒÊå§üþÇ¦M¢wÊèó<gyFù)¿á,×××?ýôÓ£GN;ÈÅ?åòCù)¿ÿÞ,ÇÿþûïßKÚÏ¯  @ù)¿a¨µµ5¼xSùæÎkOù¡üP~Ê/ã]µcÇn3«ªªÛ«üx<ÞÐÐPVV:È®öìY|ÊåòS~ÐÓùü®]»¦üßpÐÚÚº÷î´|S§N/¤7nxZÊå§ü5å§üèØ±c=íÉ·~ýúÆÆF|ÊåòS~½»å¨QÉÛs3«üß`ºqãÆ=Ç%[°`Amm­A>åòCù)?åGÖ_xI§ï±Ç[¿~½òCù¡üò#ëËïúõë»ví:ujÚsòÙOù¡üP~(?FBù×À+RùÂÕ«WKíÉ§üP~(?Ù]~íííá0úôöä»~ýºgJù¡üP~(?²¸üâñx,«¬¬L»'_UUA>åòCù¡üÈúòkmm­©©I;È7kÖ¬ÚÚÚOòCù¡üÀòËLù)¿Gäëé7¼ÊåÇ`_Þäçç+?å×gùêêêÚÛÛ=ÊåòcÊoÈ)¿Y~Ñ _yyyÚA¾ªªª³gÏzÊåò#»Ë¯µµ5<§%%%ùÊåòS~ÃB<+÷^z©©©©¿î0Ã =ùÊåòS~C£µµõsûÜ)S.éOúÿøå³12òÕÔÔäS~(?ÊOùü¯ÒÿµãÅ;¿±3|â'ÊËËöNò)?Êå§ü»óçÏö³²/ú·ýëÛ'MÔûOËÈ0È7út|ÊåòCù)¿á¢¡¡áó>È¾èßÌ3x°m<¯¯¯O;ÈæTVVÆb1¼¡üP~(?òFú0æ×ÒÒR]]a¯µµÕU~(?ÊOùGóæÎë¶ßþé¦^Í òCù¡üòS~Y/õØÞn~áÛð¼¤ýà|ÊåòCù)¿,Çÿñÿñ¥^:þ|bÜ.L444¬]»vÌ1©|åååùÊåòS~Y)ù3<._¾¼÷î©S§¦òçÈ òCù¡üP~Ê/ëË/­^½:í|ùÊå§üò	ÚÛÛÿò/ÿrÒ¤Iiùª««[ZZ,%åòCù)?å§ü²Ûï½WYY9qâÄÔæ[±bÅáÃò)?òCù)?åÝÚÛÛkjjæÎ|EEEáY¸|ù²¥¤ü,ÊOù)¿ìÖØØ¸~ýúÔÃu?üÃ?<|øðÝ»w-%Êå§üúM[[[IIòL×¯_ß½wÚA¾©S§VWWôÑGcAù¡üP~Ê¯<yröìÙ!8ßàÅbUUUi×]½zuCCC´'_òY]@ù¡üP~Ê¯,[¶¬¹¹9CùÿûßÿgÙ'¾ùÍo¦ýàâââ6=z4ùúçÏï6^á%a9ÿ=ø³þáþá¿øå0$²¸üþû!ö õõõyÇ_»vm^^^jó~ïßû·û·Ô[ýú×¿>vì¥Gä§?ýiKKå@ðóÿüÂAø?À¹sç,!1ËÏÖÞ¾iooß³gOh»Ô»EEE[·nmjjÊps[±µ[±µ×ÖÞ~ë¼òaUVV¦=¬¬lÿþý¼åòCù¡üß¶ òDÑ9ùfÍ|'NÜºuëCOù¡üP~(?å§ü£|¥¥¥û÷ïïÃ9ùÊåòS~MùeÐÚÚaoãÆÍÍÍ¾såòCù¡üòzñx<UVV¦ºòÕÕÕµ··?âOQ~(?ÊOù)¿¡ò¥='_aaáÖ­[/^¼Ø_?Kù¡üP~(?å§ü@æA¾ÔÕÕE¼ÑÊåòS~ÊoPeä×Í|N>åòCù¡üòî2òÕ××÷áp]åòCù¡üòF2ïÉ·yóæG9ù¡üP~(?å§ü^4ÈW^^voñâÅo¾ùfo>xCù¡üP~(?ßðÕÚÚ~ÇÔà>]wÐùÊåòS~Êoe8'ßáÃzO>åòCù¡üò</¾øb·=ùª««jOù¡üP~(?å§üPSSS4æW^^^__?Èò)?Êå§üß ª««kiiLù¡üP~(?å§üròCù¡üP~ÊOù)?ÊOù¡üòS~(?Êå§üÊåòCù)?åòCù¡üP~ÊOù¡üP~(?ÊåòCù¡üP~(?ÊåòCù¡üP~ÊOù¡üP~(?òS~ÊOù¡üP~(?å§üÊåÊOù)?åòCù¡üP~ÊOù¡üP~(?òS~(?Êå§üÊåòCù¡üP~(?ÊåòCù¡üòCù¡üP~(?å§üP~(?ÊOù)?å§üP~(?òS~Êåòå§üòCù¡üP~(?å§üP~(?ÊOù)?ÊåòS~ÊåòCù¡üP~(?ÊåòCù¡üP~ÊOù¡üP~(?òS~(?Êå§üò³P~(?òS~ÊåòCù)?å§üÊåòCù)?åòCù¡üP~ÊOù¡üP~(?òS~(?ÊåòCù¡üP~(?ÊåòS~ÊåòCù¡üòCù¡üP~(?å§ü(?ÊOù)?åòCù¡üòS~ÊåòCù¡üòCù¡üP~(?å§üP~(?ÊOù)?ÊåòCù¡üP~(?ÊåòCù)?åòCù¡üP~ÊOù¡üP~(?òS~ÊÊå§üòCù¡üP~òS~ÊåòCù¡üòCù¡üP~(?å§üP~(?ÊOù)?ÊåòCù¡üP~(?ÊåòCù)?åòCù¡üP~ÊOù¡üP~(?òS~ÊÊå§üòCù¡üP~(?å§üP~(?ÊOù)?ÊåGîßéÓ§çÏ?oÞ¼sçÎ)?åòCù¡ü±å7cÆS§NÌ93µüÿ?]GGG(?ËH(¿ð°Â×¯^½j9üË¿üË/ùKËaHdqù%;vljù½úê«Çt?ýéOÿæoþÆr âÅ@BCCCX?X?ùÉO=j9P~gÎÙ°a­½¶öbk/¶öbk/#vkoäöíÛÊOù¡üP~(?FTùú­èÛ«W¯VUU]»v-õÊOù¡üP~(?²»ü566.]º´­­-í¥ÊOù¡üP~(?FNùJ¢üÊåòcÄ_fÊOù¡üP~(?ÊåòCù¡üP~(?ÊOù)?ÊåòS~ÊåòCù¡üòS~ üP~(?å§üÊåò³òS~(?Êå§üÊåòCù)?åòCù¡üP~ÊOù¡üP~(?ÊåòCù1ÊïßüfýeÝG´ÿ~ËÈÛo¿ÖïÁüãóçÏ[Ç?qâå0$ÚÛÛGfù]¸paçÎÀoeÞ":Ê°'­½(?Êåò@ù1àN>=þüüüüyóæ;wîÞýî¨ªª*((:ujcccò¯]»6*¥7â_ÉOw^^^òÃÃçÌsüøqK/_Ö¹öbo³gÏþüO<iÍ üÈ&3fÌ8uêT8pàÀÌ3ÃÄË/¿üío»««+ümO6-ùÊQh¡åÎ!áí·ßÞ»woò03LùëÖ­³ôrùÅ`Ík/ÂÂÂ+W®ðµ¤¤ÄAù­Æ¾ÿ¨]¼x1íÂÊýÈ#Tî¼".]Z²dI·+UøïAèììì¶ê'×^Ö¹öb6mÚÕ«WÃDøÚmÀAù5Î9³aÃ0ÿÚk¯?ïð÷üÁtûßÒ¥KÃ.áZh#þÅYµjUÓí:áev|1X3äÚáÜ¹sá=bÔ¨Qák·×5ò#;Ü¾»¢¢¢££#Lçåå½ñÆa"¬ÁÃz<íõÃÿóæÍg¹øCÐÜÜ¼hÑ¢Ô«%ïéUPP`¹åòÁ!×^?þx´i($`·5ò#uUUÕµk×¢o'OÜÿ±ùÎÃ½ûû¾öÚk©×¯ÎÎÎ÷·é$¿fÈÁ5C®½2ìY3(?»ÆÆÆ¥K¶µµ%ælÚ´é?üa¸páBiiiògÌÑÒÒ­Â­,½ÿbzê©÷ß?õÊo½õV_+**,½ 1X3äÚaÑ¢Eá"L455=þøãÖÊlRRRÒít·nÝZµjU´ËNssó¿î_tæÌèHþÅG»÷2²_÷îáDûkÿÏÚäþE'O,**ÊËË+..>ú´¥Ë/k{1üêW¿Áñð5L[3(?Êåò@ù(?Êåò@ù üP~(?ò@ù üP~ýá?øÁSO=5ö¾%K¼óÎ;¿³þº/kÖ¶émIIIøÕ:::ºÍsòóó»ººö>vîÜ9*Å+¯¼2Êo×®]aæë¯¿Þm~mmmÿÒK/õá>eÎ;&??ß¾ñûÞxãðmùþûïò»téR9oÞ¼nógÏæ·´´(?@ù#ß3Ï<æÛßþvòÌW_5Ì~r÷?~<SÂùóçéÄoÞ¼¹aÃñãÇ·lÙ¼Q5ºÛ;v¬[K9&Lxê©§=¾­¬¬ìöÀ2ßOtQxHÑEï¾ûnO¶téÒ0ÿôéÓ9§NsÊÊÊs^~ùå¢¢¢pWcÇ]³fÍÕ«WSË/õþ»ÍÉðPå0¦LªåÊ+É3?þøã0³¤¤$¹lº9sæLtéÊ+»]ôüóÏG555ååå¥½UômtéªU«ºººB;ÄãñpiøÊiÜ¸qÑîwî'L¤x©¿és6o¿ýv"ûºÝÉ%K¶ü2<T@ù±hÃnuÖ¨Q¡ÃËfÝºu·ïáÛåËGF¡µc4t-º¨¢¢",Ó'Oî$ù>wìØÚ.ÚØúì³Ï9G	ÓákþÚ×¾öÀû	Õ¾WH~li£P&M¿o[[[ø6|<äfgggtâââpÃð+ÜûíÖá±cÇ>lùex¨òúòÒ_b~T6Q0W¯^ßf¾?~øvÚ´iÏ=÷¶;wî$î$Û(ZâVÑ·üqâÊ¡|Ã×0Åx?¡2Ã·|òIòcëi¼^x!Ýï~÷Þo·hoÙ²%ù¡Cù]¸pa4$ù°åá¡Ê`E]·oßNÙÑÑf2´N¢£øKNbç¼Ô°ÝZ*ùaº°°°  àîÝ»áÎ'L¸ô÷ÓSusáÂ(RÃtø¦<y2<´[_~* üX´¯Ûk¯½<3:×I·#<û¶µµ¥e½ÿþû»ví¶½&1KlN`=ÿüóÑFÞðuÃùî'c»víZôí'|ù8ÜEK£Ù`M¾(:Î7î»ïÞºu+sù%ª4ZK3<T@ù±èüüü½÷FguÙ·o_AAAêÑË/s÷îÝhSlâ8Üh?¿h÷¸æææä£Ü²eKè¤è¨ÛÄyUÒöYt°môÓó3ÜO´c_´_GGGtÍå÷Æo$FãÂ¯|Q´ËcSSSø)a!ôT~QkVWÅ¹fÍäK3<T@ù½è,ÇÝ¼üòËÿ³þº/£Ätb;iâ g69+:t(CùÝ»ÿaa~aaaòà÷FòKWÛÓ/çÎ±cÇF¿B·mÜË-Kþ&M_£»$ßg4$ujâÒP~ÃB¨%KÜ÷ÔSOEØv+¿wß7:mÞ9sN8¸´££cûöíEEEQ-½ðÂwïÞMìØ± +..®««ëv©$ÜU¿iÓ¦nóº <ð¢f8_ÂºÞ%ÒÖÖVQQÀøñãÃoÑÒÒ8CMòÞºu+¤m´¬ÊÊÊN>Ýí'fx¨ò@ù üP~(?Êåò@ù(?Êåò@ù üxxÿ?OÄé¾2´IEND®B`


Detrended Normal Q-Q Plots
