# Supplementary material for: Regulation of piglet T-cell immune responses by thioredoxin peroxidase from Cysticercus cellulosae excretory-secretory antigens
Source: Front Microbiol. 2022 Nov 18;13:1019810. doi: 10.3389/fmicb.2022.1019810 (PMC9718028; doi:10.3389/fmicb.2022.1019810)
Supplement: Supplementary file 2 [file Data_Sheet_2.ZIP › 2. C. Cellulosae ESAs and TPx Induced CD4+ and CD8+ T-Lymphocyte Responses in PBMCs/2. SPSS statistical analysis/2.2 SPSS statistical analysis--CD8+/2.2.3 (SPSS data export) SPSS statistical analysis--CD8+.doc]

Explore

Notes	
Output Created	11-SEP-2022 17:11:20	
Comments		
Input	Data	E:\×ÀÃæ\Raw Data\2. C. Cellulosae ESAs and TPx Induced CD4+ and CD8+ T-Lymphocyte Responses in PBMCs\2. SPSS statistical analysis\2.1 SPSS statistical analysis--CD4+\2.1.1 SPSS statistical analysis--CD4+.sav	
	Active Dataset	DataSet0	
	Filter	<none>	
	Weight	<none>	
	Split File	<none>	
	N of Rows in Working Data File	26	
Missing Value Handling	Definition of Missing	User-defined missing values for dependent variables are treated as missing.	
	Cases Used	Statistics are based on cases with no missing values for any dependent variable or factor used.	
Syntax	EXAMINE VARIABLES=Numerical BY variable
  /PLOT BOXPLOT NPPLOT
  /COMPARE GROUPS
  /STATISTICS DESCRIPTIVES
  /CINTERVAL 95
  /MISSING LISTWISE
  /NOTOTAL.	
Resources	Processor Time	00:00:01.45	
	Elapsed Time	00:00:00.99	

variable

Case Processing Summary	
	variable	Cases	
		Valid	Missing	Total	
		N	Percent	N	Percent	N	Percent	
Numerical	Control	4	100.0%	0	0.0%	4	100.0%	
	ESAs	4	100.0%	0	0.0%	4	100.0%	
	TPx	4	100.0%	0	0.0%	4	100.0%	
	ConA	4	100.0%	0	0.0%	4	100.0%	


Descriptives	
	variable	Statistic	Std. Error	
Numerical	Control	Mean	44.100000	1.1496376	
		95% Confidence Interval for Mean	Lower Bound	40.441340		
			Upper Bound	47.758660		
		5% Trimmed Mean	44.138889		
		Median	44.450000		
		Variance	5.287		
		Std. Deviation	2.2992752		
		Minimum	41.0000		
		Maximum	46.5000		
		Range	5.5000		
		Interquartile Range	4.3000		
		Skewness	-.857	1.014	
		Kurtosis	1.500	2.619	
	ESAs	Mean	44.766667	.7419943	
		95% Confidence Interval for Mean	Lower Bound	42.405310		
			Upper Bound	47.128024		
		5% Trimmed Mean	44.735185		
		Median	44.483333		
		Variance	2.202		
		Std. Deviation	1.4839886		
		Minimum	43.3000		
		Maximum	46.8000		
		Range	3.5000		
		Interquartile Range	2.7667		
		Skewness	1.034	1.014	
		Kurtosis	1.500	2.619	
	TPx	Mean	41.900000	.7778175	
		95% Confidence Interval for Mean	Lower Bound	39.424638		
			Upper Bound	44.375362		
		5% Trimmed Mean	41.961111		
		Median	42.450000		
		Variance	2.420		
		Std. Deviation	1.5556349		
		Minimum	39.7000		
		Maximum	43.0000		
		Range	3.3000		
		Interquartile Range	2.7500		
		Skewness	-1.414	1.014	
		Kurtosis	1.500	2.619	
	ConA	Mean	49.200000	1.1098048	
		95% Confidence Interval for Mean	Lower Bound	45.668106		
			Upper Bound	52.731894		
		5% Trimmed Mean	49.238889		
		Median	49.550000		
		Variance	4.927		
		Std. Deviation	2.2196096		
		Minimum	46.2000		
		Maximum	51.5000		
		Range	5.3000		
		Interquartile Range	4.1500		
		Skewness	-.883	1.014	
		Kurtosis	1.500	2.619	


Tests of Normality	
	variable	Kolmogorov-Smirnova	Shapiro-Wilk	
		Statistic	df	Sig.	Statistic	df	Sig.	
Numerical	Control	.250	4	.	.957	4	.762	
	ESAs	.250	4	.	.946	4	.690	
	TPx	.260	4	.	.827	4	.161	
	ConA	.250	4	.	.956	4	.754	

a. Lilliefors Significance Correction	


Numerical

Normal Q-Q Plots


Detrended Normal Q-Q Plots


ONEWAY Numerical BY variable
  /POLYNOMIAL=1
  /STATISTICS DESCRIPTIVES HOMOGENEITY
  /MISSING ANALYSIS
  /POSTHOC=LSD ALPHA(0.05).


Oneway


Notes	
Output Created	11-SEP-2022 17:11:41	
Comments		
Input	Data	E:\×ÀÃæ\Raw Data\2. C. Cellulosae ESAs and TPx Induced CD4+ and CD8+ T-Lymphocyte Responses in PBMCs\2. SPSS statistical analysis\2.1 SPSS statistical analysis--CD4+\2.1.1 SPSS statistical analysis--CD4+.sav	
	Active Dataset	DataSet0	
	Filter	<none>	
	Weight	<none>	
	Split File	<none>	
	N of Rows in Working Data File	26	
Missing Value Handling	Definition of Missing	User-defined missing values are treated as missing.	
	Cases Used	Statistics for each analysis are based on cases with no missing data for any variable in the analysis.	
Syntax	ONEWAY Numerical BY variable
  /POLYNOMIAL=1
  /STATISTICS DESCRIPTIVES HOMOGENEITY
  /MISSING ANALYSIS
  /POSTHOC=LSD ALPHA(0.05).	
Resources	Processor Time	00:00:00.05	
	Elapsed Time	00:00:00.02	


Descriptives	
Numerical  	
	N	Mean	Std. Deviation	Std. Error	95% Confidence Interval for Mean			
					Lower Bound	Upper Bound			
Control	4	44.100000	2.2992752	1.1496376	40.441340	47.758660			
ESAs	4	44.766667	1.4839886	.7419943	42.405310	47.128024			
TPx	4	41.900000	1.5556349	.7778175	39.424638	44.375362			
ConA	4	49.200000	2.2196096	1.1098048	45.668106	52.731894			
Total	16	44.991667	3.2348450	.8087113	43.267939	46.715394			


Test of Homogeneity of Variances	
	Levene Statistic	df1	df2	Sig.	
Numerical	Based on Mean	.210	3	12	.887	
	Based on Median	.183	3	12	.906	
	Based on Median and with adjusted df	.183	3	10.717	.906	
	Based on trimmed mean	.210	3	12	.888	


ANOVA	
Numerical  	
	Sum of Squares	df	Mean Square	F		
Between Groups	(Combined)	112.457	3	37.486	10.107		
	Linear Term	Contrast	30.918	1	30.918	8.336		
		Deviation	81.539	2	40.770	10.992		
Within Groups	44.507	12	3.709			
Total	156.963	15				


Post Hoc Tests


Multiple Comparisons	
Dependent Variable:   Numerical  	
LSD  	
(I) variable	(J) variable	Mean Difference (I-J)	Std. Error	Sig.	95% Confidence Interval	
					Lower Bound	Upper Bound	
Control	ESAs	-.6666667	1.3617799	.633	-3.633730	2.300397	
	TPx	2.2000000	1.3617799	.132	-.767063	5.167063	
	ConA	-5.1000000*	1.3617799	.003	-8.067063	-2.132937	
ESAs	Control	.6666667	1.3617799	.633	-2.300397	3.633730	
	TPx	2.8666667	1.3617799	.057	-.100397	5.833730	
	ConA	-4.4333333*	1.3617799	.007	-7.400397	-1.466270	
TPx	Control	-2.2000000	1.3617799	.132	-5.167063	.767063	
	ESAs	-2.8666667	1.3617799	.057	-5.833730	.100397	
	ConA	-7.3000000*	1.3617799	.000	-10.267063	-4.332937	
ConA	Control	5.1000000*	1.3617799	.003	2.132937	8.067063	
	ESAs	4.4333333*	1.3617799	.007	1.466270	7.400397	
	TPx	7.3000000*	1.3617799	.000	4.332937	10.267063	

*. The mean difference is significant at the 0.05 level.	
