# Supplementary material for: Regulation of piglet T-cell immune responses by thioredoxin peroxidase from Cysticercus cellulosae excretory-secretory antigens
Source: Front Microbiol. 2022 Nov 18;13:1019810. doi: 10.3389/fmicb.2022.1019810 (PMC9718028; doi:10.3389/fmicb.2022.1019810)
Supplement: Supplementary file 2 [file Data_Sheet_2.ZIP › 2. C. Cellulosae ESAs and TPx Induced CD4+ and CD8+ T-Lymphocyte Responses in PBMCs/2. SPSS statistical analysis/2.3 SPSS statistical analysis--CD4 and CD8/2.3.3 (SPSS data export) SPSS statistical analysis--CD4 and CD8.doc]

EXAMINE VARIABLES=figure BY variable
  /PLOT BOXPLOT NPPLOT
  /COMPARE GROUPS
  /STATISTICS DESCRIPTIVES
  /CINTERVAL 95
  /MISSING LISTWISE
  /NOTOTAL.


Explore


Notes	
Output Created	06-OCT-2022 17:14:05	
Comments		
Input	Active Dataset	DataSet0	
	Filter	<none>	
	Weight	<none>	
	Split File	<none>	
	N of Rows in Working Data File	17	
Missing Value Handling	Definition of Missing	User-defined missing values for dependent variables are treated as missing.	
	Cases Used	Statistics are based on cases with no missing values for any dependent variable or factor used.	
Syntax	EXAMINE VARIABLES=figure BY variable
  /PLOT BOXPLOT NPPLOT
  /COMPARE GROUPS
  /STATISTICS DESCRIPTIVES
  /CINTERVAL 95
  /MISSING LISTWISE
  /NOTOTAL.	
Resources	Processor Time	00:00:02.45	
	Elapsed Time	00:00:01.54	


[DataSet0] 


variable


Case Processing Summary	
	variable	Cases	
		Valid	Missing	Total	
		N	Percent	N	Percent	N	Percent	
figure	Controll	4	100.0%	0	0.0%	4	100.0%	
	ESAs	4	100.0%	0	0.0%	4	100.0%	
	TPx	4	100.0%	0	0.0%	4	100.0%	
	ConA	4	100.0%	0	0.0%	4	100.0%	


Descriptives	
	variable	Statistic	Std. Error	
figure	Controll	Mean	.50169281	.016870207	
		95% Confidence Interval for Mean	Lower Bound	.44800428		
			Upper Bound	.55538134		
		5% Trimmed Mean	.50127770		
		Median	.49795682		
		Variance	.001		
		Std. Deviation	.033740414		
		Minimum	.464516		
		Maximum	.546341		
		Range	.081825		
		Interquartile Range	.062580		
		Skewness	.649	1.014	
		Kurtosis	1.638	2.619	
	ESAs	Mean	.72460645	.043987756	
		95% Confidence Interval for Mean	Lower Bound	.58461778		
			Upper Bound	.86459512		
		5% Trimmed Mean	.72129613		
		Median	.69481356		
		Variance	.008		
		Std. Deviation	.087975512		
		Minimum	.660508		
		Maximum	.848291		
		Range	.187783		
		Interquartile Range	.156796		
		Skewness	1.365	1.014	
		Kurtosis	1.269	2.619	
	TPx	Mean	.83983584	.019929680	
		95% Confidence Interval for Mean	Lower Bound	.77641070		
			Upper Bound	.90326098		
		5% Trimmed Mean	.84052373		
		Median	.84602690		
		Variance	.002		
		Std. Deviation	.039859360		
		Minimum	.785894		
		Maximum	.881395		
		Range	.095501		
		Interquartile Range	.074194		
		Skewness	-.881	1.014	
		Kurtosis	1.642	2.619	
	ConA	Mean	.55137052	.018557990	
		95% Confidence Interval for Mean	Lower Bound	.49231071		
			Upper Bound	.61043033		
		5% Trimmed Mean	.55017366		
		Median	.54059881		
		Variance	.001		
		Std. Deviation	.037115981		
		Minimum	.520388		
		Maximum	.603896		
		Range	.083508		
		Interquartile Range	.067399		
		Skewness	1.392	1.014	
		Kurtosis	1.787	2.619	


Tests of Normality	
	variable	Kolmogorov-Smirnova	Shapiro-Wilk	
		Statistic	df	Sig.	Statistic	df	Sig.	
figure	Controll	.266	4	.	.953	4	.733	
	ESAs	.258	4	.	.840	4	.196	
	TPx	.261	4	.	.951	4	.725	
	ConA	.263	4	.	.888	4	.372	

a. Lilliefors Significance Correction	


figure


Normal Q-Q Plots


i·OøìÚÃ¯X±¢ì^Ë/÷ÝwïC¿É¤¤Âç/pÿþý#Ø9çÏ¯©©ÁêÔ©Ç¬¸¸8üíîaÀU>)Iä')Çä7mÚ´«W¯æü^yåI)íÜ¹3Ã½ë&iïÞ½ÃÝ9#Þ¿·¾¾>ÃUä'ü$X¡õë×çüÎ?Qõßû^<¿÷n¸>/]ºö®õ­[·^|ñÅ°%kÜäÝðæÍárÿ¯$òta444?><Y~úÓ>ùäÑRi]]],ðÇ>úã?ØrâÄÅ¯X±âÚµk§NZ¸paIIÉªU«ß_|õÕW+**¢µËðW$®ÌLO=õTØ¼öÚ·o_ØØØØökîMRÿêÅ°%<Èaío¦þE#¸aêU>'bÁa?¯^½:ìÌäk»·Áæjkk÷¾ð0ÊpçD~òA~·nÝª¬¬5kV¸jO>ù¤¼¼<á3­¨¨(ü¹aÃOÓ­>öØc7Jìð+W®Ì,¿Ù³gí×¯_OÞd6Î;7í×8Üø«oÞ¼½ç7oÞ¼aíÌòÙ3ËïÃ?L¾6|uÃ_ò/]ºèüùó2ùIÊyùGI~,ÙÏ>ûl¸¼víÚ÷ÂacògîØ±ãîÝ»Ñ»VÑ­[·ÆãñÓ§OG?~<nýÈÚ±cÇÂå+W®$_5üÓnO¾íÞd°óK¼':ÜöQø®Jþ0-««»ûv@üúõë+¿ä9û	[N<.GsÜ¼y³§D~òA~¡ð=ßãl5kV¸üÉ'D~üñÇáÃ3g&ßCâÚÄä»»»?ýù²i²?úûûü8-[½çt_ù¤ýBÒnÁMµn;eÊ¶¶¶´;m(;'í£ñ3È/úáÅk×®%ßç°ä<ÇèÞ[<e$ò'ò8+//øáûúú·§¾gúCotC¡É2¬xf`JTEEE´<Ê»KÜÛPnV?a(;g0ìöçPlùÚÔ98Ç§D~òD~¡ïÿûáÃÄ9P¢Î¼;6Å?`Atv'NE~ÑÑ	Vêêê¶oß~óæÍ7ß|3l|òÉ'ÓÊo(7±ü»søø_Âvúwø)SÂKä')oåZ¿~âçú£-Ñ¿k×®½u¯èÉ¶nÝúàòÞ÷ºtéRàÈþýû"¿öööhÙ1`ñôéÓÑûyQGIû5÷&Ã_æíÉÞ¸áîÕ¡ìÏè'ótïÜ¹`·fÍT¾þúëaoõfÞáÑCPv]¸¼xñbOü$åü®^½ø¯hK,ð#_³fÍJ*û òKÐ$jæÌáÏäã[Ó>æ§~:u!òGÉðeë&Ã_æ8ëò³1`¯e^¸p!ùüñäkìÌòÎöIä')OäVB·ÿä'?yòÉ'KîUWWz<ÇÈä×ÝÝî-Üg0ÐóÏ?0®zî¹çîË¯7ÞxcáÂEEE³gÏ~ûí·xâÄ1Â~aÉ/óÎ9räHÀ_qqqâyÉk¯q÷=zôá_f¸Ãh=qürøðgþºsçÎÝ÷MÖãÇ/[¶,Ü[ø*Z[[=_$ò¤¯¿¿ÿé§È8v7Éþ¢Ååèèìx<	~ÁþH"?IÊ·¢óÐ7¿ùMFùIR¾ÕÛÛûâ/Î=;ZÒ^yå»EùI$ü$IÈO$Iä'I$ò$IùI$ü$ID~$I"?I$$IÈO$Iä'ID~$I"?I$¤BëÈ#UUUÅÅÅ+W®ï¬ìsrÛ!ÖÝÝ½uëÖéÓ§0öì^z©··wü÷À(~EÈOÒWQQÀôÇP~ysïuåÊ»wï¾óÎ;án×¬Y3»:þ|MMùI"?IYüªÝàkù½úê«á~><!ü$¤ñvU²9^zé¥iÓ¦M2eçÎ>í'X¶lYbãéÓ§£-k×®[Âö?þ¸¦¦¦¬¬¬¸¸xÉ%'OL?Éoæ¼ýöÛUUUË/¿~ýzªnß¾]__nö/¾8¬7//^î§¯¯/íµöFxHGIÝ©,Ã]$ò4®ø.ãß[[[´Âo~óÉsüøñîîîäÛ¢íW®c±X¸¶Ë<òÈ±cÇÂpUØXYYö~ÿêÌ7	ûþ÷¿.444¤>ìç.ðÞï¯¾úêÐmry+óÞxýõ×/]º.ÿÆ¸ÄWù®ÈOùIùÄËý÷JåWêic^zé¥Oï½§.¿ùæÑU.±cÇ²eËÂÆ¢¢¢´÷3À:o=¤iÓ¦¥ÞvÖ¬YÑÃ¾÷n¸0gÎ¡ùäyoÜ¹s'ºîa0Æ%¾ÒÌwE~ÈOÒÈ/A&¹ ­)S¦Ü¼y3üY^^d¶ïß¿?|þúõëüãgÀMòC¹É`)01ù½HC|ÏoÞ¼yaûíÛ·3£0³ðîË¸áÞ$¤1_eeeò[S7Ï2 äµ×^W­Zµ*±Ì*))	Þ½×åù&4úôÔÛÎ93ºí¾ü;vÛ¾÷Þ©WeoQ~Ã½+I"?Ic.¿è@×7ß|3ZÆýÖ·¾u_twwGoh?_DÐ¹|ùr¸«!Ê/óMÏ~ðÏ>ûlêmyæpùwÞùè£?k8ÄnÝº6wîÜ?þ8Ø1zëqÃCÜÉ§M8XdÀW:Ü»$ò4æòÇãÁXSîÕÒÒösRÛºuk¸vË--G-//zñÅ(¿Ì7ikk×>öØcCL?§··wûöíá1¬Y³æO>Ö÷ìnð¸sçÎègø²7/9r$<èØ¾ÒáÞ$$IÈO$ü$ID~$I"?I$$IÈotúû¿ÿûÎÎN#ÌPooï¿ÿû¿Û¹Õý×Z.Nm¸§©iÝ¾ÛÔW~üÇðgúÏÿüÏóçÏÛ¦¦±îÚµkÿðÿ`?äVÿñÿñáÚ¦F~äÇ25CÈÔÈüB¦&òcùCÈÔÈÏ~`ùCÈÔÈO!S#?òcù!djäG~ajöù!djäG~aj"?1©üÄ¦&òCÈ!ìSù!LMäÇ25CÈÔÈüB¦&òcùCÈÔD~!S#?òcù!djäG~!S#?1LüÈ!djä'©ßÏ:wîÜ%K/^:iòcSù!LMù#¿¹sç9s&÷ÝwçÍ*¿cÇÝÔàýë¿þëéÓ§íSÓX÷Óþô>°r«ù	ßbìSË¿rX~É¥ÊoïÞ½hðÚÛÛÿú¯ÿÚ~05LÍÔ§|ßùóç­öZ745YíuCS³òvµ7êöíÛuuu½½½äÇ¦&òCýÏò»zõjCCCxUM½üÂÔD~bSSþÈ¯½½ÕªUÝÝÝi¯%?05ÂÔ?ò«ªªù1©üÄ¦f?ä­ü2G~aj"?1©üÄ¦&òCÈ!dj"?1©ù1LMäÇ25ò#?©üB¦F~äÇ25CÈÔÈüB¦F~bùCÈÔÈO!S#?òcù!djäG~aj"?1LüÈ!LMä'05ÂÔD~bSù1LMä'05ò#?©üB¦F~äÇ25CÈÔÈüB¦&òcùCÈÔÈO!S#?òcù!djäG~!S#?1LüÈ!LMä'©ù1©üÄ¦&òCÈOaj"?©üÄ¦F~äÇ25CÈÔÈüB¦&òcùCÈÔD~!S#?òcù!djäG~!S#?1LüÈ!djä'Ð¸N­§§üÈ!dj"?P>O-8p`ùòå555äG~!Sù1òsj]]]UUU~ÞGD~äÇ25C(¯¦§'Oô½üòËäG~!Sù1òajñxüÐ¡C555R?þ¾ûêGýÈ!dj"?P~N-nÀÂnÔäÉkkkSäÇ25C(ß¦-ì0ß3c±XÁîCòcÈ!'SË°°[]]ÝÚÚZhçp!?©üD~y8µÌ»mmmv ù1LMäÇÊí©eXØmjj*ä]òcÈOä'S³°K~bSùüòjt»wï¼³°K~bSùüò¶>ø`íÚµvÉOaj"?_ÞfaüÈ!LMä'òËÿ[ØZØ%?1©üD~ùPGGGÚ#v§MÖÔÔ®µÈOaj"?_nya÷ÿðÛÛÛí%òCÈOäÛea×ÔÈüÂÔD~"¿Ü®£££©©iÆiØM^Ø55ò#?05È/'Çãmmm-ìîÞ½;õ]S#?òcSùür¬ÙÔÈüÂÔD~"¿)óÂîOÅljäG~aj"?_¶aawþüùC?³©ù1©üD~Ù[æÝá~75ò#?05È/Åb-ì677ìwìùCÈOäEE»µµµ'O`¾ªªªÝ»wW9S#?òcùür»ÖÖÖ´§b|ÿý÷üwìùCÈO1Á¶°[ZZ¶_¼xÑÔÈüB¦F~"¿/|#N»°»hÑ¢=öñ]S#?òcSùü²·ÞÞÞÖÖÖùóçÝÂ®©ù1©üÄùå§N:|åååÛ¶mKþ»¦F~äÇ25òùådñxüèÑ£vMüÈ!djä'òËízzzìÒ±[SSsèÐ¡1ZØ55ò#?05büºxñbcccêÂniiiCCÃü75ò#?05b;~üxÚß±[]]½÷îQ?b×ÔÈüÂÔD~b	xýÙ³gÏ9s²aa×ÔÈüÂÔD~b1éÒ¥K[¶lI]Ø1cFSSÓX±kjäG~aj"?1Ä±»zõêÔ#v'|a×ÔÈüÂÔD~bQ©ÙµkWUUUÚÝ¶¶¶	_Ø55ò#?05âA»xñâ-[JKK³|a×ÔÈüÂÔD~bÇ<¸bÅ´Gì¶¶¶fÛÂ®©ù1©üÄÃ®³³3|Ç,//¾É'×ÖÖfíÂ.ùù1©üÄÃ(|¬¯¯O=zcÆÍÍÍ±XÌÔÈüB¦&ò#¿Ü®¯¯ïÀË/O]Ø?~®,ìùCÈO©®®®ðÍqÆ©»õõõyðMüÈüÂÔD~bOÃ¿À|ZØ%?ò#?05âÇãvÃU¦&òcÈürÛÑÂnê©ófaüT~ÝÝÝá5ù1©üÄQ²°K~(¿Ó§O/X° ük&?05Üvsú]ò#¿µfÍòÛ¿ÿ?hðøÃÚ¦¦±.ü_¨©ÚØuìØ±ßû½ß5kÖðÔ·BÏµ¡ÃòûÙC þç~M÷üäÔ©Sö©i¦¾!Ù¹Õ?ÿó?ÿÑÊòyüøñ¯|å+©»=ôÐ¶mÛÎ?ojJ-ågµ×º¡©Éj¯òoÝ0:t¨¦¦¦v­öâj/ù1©üTP¤ìÝÚÚZßõÈüÈ!LMä§|0DtÄniii±K~äG~aj"?!2,ìVWWòÂ.ù®üüÂÔD~Ê]CÜ¸qcÏ=sæÌI»°ÛÖÖPhLäG~äÇ¦&òSn"ÃÂnSS]ò#?òcSù)çÑ××wðàA»äG~äÇ25òS>¢··7í»¡Õ«W[Ø%?ò#?05òÁÍÍÍåååÀWZZÚÔÔtñâÅÂÙáÑ[á«þú×¿>âçùù1©ühkkÛ¸qcêóÍ3§¥¥%° övooïÒ¥Kíá_ûò_þÂ¾ðK¿ôKßþö·ÉüÈ!djä§Ü_ Nkkë¢ERvôÑæÂî¶mÛ/^¼ãå;ÿ`gø¯ékMgÏ%?ò#?05S#?å¤ü:::fÌzM6ø¿Ù³gÿÏ­ÿ3b_ôß¾ð¯ýëäG~äÇ¦fjä§_<ßjkkð¯¼¼¼¥¥åÆöö´©ÓoøEÿùË_þÚ×¾F~äG~aj¦F~ÊùõôôìÙ³'í»K.=pà@__ýõßûozÉò[¸pakk+ùù1©ù)Ûå§ÍÍÍ©»¥¥¥õõõ¾7¥vöìÙÏ|æ3k×®ÝñòmÏmì×[´pQoo/ùù1©ù)åwüøñÕ«W§.ìÎ3'|W²°+W®,++5kÖïþîïì´ÕäG~äÇ¦&òÓË/îÀóçÏO]Ø]±bEÁ±+^ù1LMäÇC2DGGÇ¶mÛ¦Nº°ÛÐÐ0óüÈüB¦F~Ê.CÄãñ¶¶¶´¿c·¢¢"|êêê²É¯°ä7é~CÈÔÈO¹eÝ»wWWW§ý»Áq	"¿|_Ñý*..&?©rÅ±X,í©ðwìùecäÇ¦&òÓ"ZØM*æêêêÝ»w;büÈïþÝºuëÙg%?©²Öííí­­­ivkjj:ä]ò#¿ôUUUû9?©r¢X,öÕ¯~uÚ´iiØíèè°ÈümñâÅ©ÿ·T^^>Ö?K~aj"?«»UUUáÛÊÈN/,ò+,ùçLwwwEEE¸À÷ü $?©²¡@:»ä§Ñ_ôÌ	õÂ+W®Ü½72eù1Lü4±vÄnIIÉÚµküÈoØM>=<N>ÝÑÑ.¼òÊ+Ñguaùiß#2,ìoUA~ä7^|ñÅÄñÉO­åËCÈÔÈOã°öwì&/ì2ùßÈíµ×fÎ.;w.lÙX?tòcSù)¹X,ÖÜÜöTÌfÄäG~9ù1©üya7í»A~äG~!SùåRH»°»|ùòpU#vüÈoäÍ7/:·393Lü4uvv¾ðÂ©»'ON]ØeòÓhÊoîÜ¹ÉÚKäØ^©F½ööö7¦]ØíêêbòÓØÊ/ /<åÂ÷ª»wïçC'?05_áÔÛÛÛÚÚºtéÒ,ì2ùi4åW^^xãÌ>òcSùH---iv×­[wöìY ?«üÂÂ3ð¹ç»û6ù1Lü4ZvÄnPà/¼DÈä§	_höìÙ©o¿;Â!djä§±»hÑ¢Ôï,ÕÕÕáªÞÞ^ ?Mü~øaGx0Lüì/½ðÂ©æÛ´iSûp!ÈO£/¿è9yéÒ¥q~èäÇ¦&òËÚÚÚêëëSvËËË[ZZ:::B¦-ò5k#<B¦F~öÃêííë­·ª««SßäôÑG[[[oÜ¸Á2µì_x¾øâäÇ25òÓPêììljj:ujê»6m:~üø(.ì2ùi4å7iáÁ25òSjá¥Ýºuivü] ?¹üÉ!S#?%êééyã7Ò±»téÒûöóÂCùåXäÇ¦&òËnÜ¸^±ÓyÓ¦MµëüÈoUUU=üðÃôù1Lü®¯¯Oý»ÑÂîX±Ëä§1_qqqxÿC'?05_vyùòåivÇô] ?¹üN<Ì---·nÝÏs»CÈ/ÛêêêJ»°Z½zõè!ÈO#?Çö2LüÈ/ì´¿cwÛ¶m±X!djy"?Çö2LüV~ñx|°Ýùóç«Æù] ?¹ü&*òcSùM`ÑÂnUUUê»õõõÙÿúÌäG~äÇ25ßýË°°ÛÜÜ»A~eùõ÷÷¯Y³¦¬¬,<ó§L²aÃq8ÔüÂÔD~ãYæÝÖÖÖ©å¿üîÜ¹ö±þÙòcSùOvkkksô¥!LüFØÂóíÚµ·oßÞºukýúõaË#<B~!S#¿.?vü4ò+))	¯É§hêïï[Âvòcùåbá%ýÐ¡C555ù±°Ëä§Ñ_QQQx-ÚKléëë[Õ!djäsÒåßÂ.CFS~ÑjïªU«¢ÕÞðg¸¶,Y²üB¦F~¹R´°ú;vó`a!LM£)¿@½´GxÜ¼yüB¦V òkiiY½zõÜ¹sxâÜziÊ°°[]]»ajMùzïðÞ6L6­¨¨(ü¹jÕª°e¬:ù1©)KÚ²eKEEÅæÍ¾Öôå/ù¡:xð`ö?ìÌ»mmmÙó;vB¦]òÈ!LMÙÐÅ§OþûÛçìþûê¯Î1#kkÙ§vòoa!LMäÇ25N­­­+~cEÑÕ¿VéÒ¥l¨NÅß»ajùMº_EEEäÇ2µ¼ï­·ÞúÇcüæü?s>úè£ìyOÅ÷»ajùù1L­pÅbÓ¦Mö<`ß¦ºMûÜç²ÄRáu²¾¾>/OÅÌ¦¦qß`=ÿüóÑËÊ»ï¾K~!S+^íµ©/éK¿]ÿÛ+V¬>zûÄ>¤¾¾¾ÁvçÌSh»ajùaL2%¼¬ÔÔÔ$ØüB¦ß]»vmÿþý«W¯ÞºuëÄ®óF»A¢©æ«­­ÿý÷pa!LM£/¿'|2ze9vìØø<tòcSSöÈ/Îä<ØÂnyyù¶mÛ:::L!LM£ ¿÷Þ/zqY»víx>tòcSùúóÝùóç§¾É·hÑ¢=öôööCFA~wîÜyäG¢9N<9ÎüÂÔTàòÅbÍÍÍ©»'O^·n]a±Ë¦¦±ßë¯¿½ÄlÞ¼yB:ù1©©`å^ýjkkÓ±ûÂ/tvvCFY~ÎçÇ25³üzzzZ[[Ó.ìVWW¿õÖ[ØeSÓXÉ¯è~CÈÔÈoTÅbMMMiv7nÜØÞÞna!LMc+¿	üÂÔTòla·¼¼¼¥¥¥OÅÌ¦&òCòD~ØôÑGÃUÞäcSù!LM9/¿§b^·nÝÑ£G!LMä'05å¼üÂ444¤.ìVTT8b!djäG~ajÊùÅãñC­^½:õM¾pU__Ì25ò#?05å¶üºººvíÚUQQ1|¥¥¥ÙðáB¦F~?¿¥óù1LM#ßÅÓ.ìVUU7§åcZÖÉ/ùÔiåç|~!S#¿Åãñ®X±"õ5sùòåØeZöÊ/Ñ'ÂkÖSO=uûöíðaøsýúõaË©S§È!djäÕÙÙ^µÊËËSOÅla!dj¹$¿è,ùÿSûûû£ÑðÃt+++.òäIòcSS.Ê/ØØ8ØÂnWWÈ2µ_ô´7@~þs~uuuï¼óN¸°wïÞÍ7§Êï?üa¯¯³³óÌ3ö©i¬û·û·³gÏØxãÆ·Þzëóÿ|êÂî¯ÿú¯ÿÉüIOO]7]¹råÜ¹sö©å_c.¿Y³f² ´;wîÃ_¹víÚ°%lðwïÞ½Q2üÏqªüöìÙóÿiðÿê¯þÊ~05óÔ>üÕ¯~uêÔ©©Ç½éK_ÿ+ky®ÉÔÆ®1ß©S§Òáñãÿøï9ùÔãE¬öZ745eÛjïÙ³g3±ka×º¡L-VCW®;wnYYYø?Ú)S¦,Y²äúõë~·ÉëÅ%%%äÇ¦¦ì,µ´´,Z´È»!S+ùQ³fÍ~|0üºvL~ajÊó577?ôÐC©GìÖ××bù£ðºùöÛoáÏºº:òcSSö^ÂkTêÂî3,ì2L-ÏåwùòåyóæM2%Z5kÖáÃünO>]QQî³²²òÜ¹säÇ¦¦	¯¯¯ïÀóçÏla×ïØeZË/:sòol.ïÝ»wL:ù1©i<ëêê/;3fÌH]ØÝ°aÃþýûí"©ü¢ß5~ùòåüÎ;.O6üB¦Ýwaw¿·W!SËUùE¯ÑH~wïÞõB¦ëE»Ë/¿ïÂ.ù1L­äÉ9z/È¯¿¿ÿ¥^Î_E~!SËÅ2,ì¦=büB¦V@òkooO&ç3gÎCÈÔr«»ÍÍÍ±X,í­È!dj$¿èUoÙ²eÑ±½eeeóæÍìõüB¦eXØ?þØ%?©ü&$òcSÓ7Ü]òcZ¡Ë/q`G¢ë×¯Ï7oÎ9äÇ2µ¬-X-íïØÍ¼°K~!S#¿òëïïwl/CÈÔ²³x<þ »äÇ2µß9s&elúôéäÇ2µì)ZØ­ªªõß±K~!SËùüñÇE÷JüöäûÞï=òcZ64»äÇ2µ_¢à¼±^Ø%?05 x<þþûï§]Ø7oÞw¾óQùÈ!dj$¿üÂÔa7îÚµ+õR&Oü¿ù.üÌg>S^^þ+¿ò+Ç'?©ß0êïïÿ÷<eÊÄ3g~ãß ?©/^lhh:ujÚÝ.ðñ_Üñò°ó+_ùÊC=ÔÖÖF~!S#¿¡6öì÷F¯³---äÇ2µñ):t(íÂî¢EZ[[£ÝW^yåÑGæKü÷ßüïË-#?©ßP+))	¯­.]Jl	S	[¦MF~!SënÜ¸±k×®êêêTó­X±âèÑ£O~â'¾ò¯$Ë/üWVVF~!S#¿¡Þ÷îÝ_¸ßüCÈÔF·ð?MMM©»'Onllüè£RoÒÐÐ°níºdömnÛç>÷9òcùµÊÊÊðR»ûöèô§wîÜÙ¹sgØRQQA~!Sõ¢Ý´»o¼ñFØÝöèÑ£¿üË¿üûÛ?!¿Ï/ûüÓO?M~!S#¿¡¾K¥=ó¹sçÈ!dj£Ø°v+üoêg?ûÙÕ_[[;ïÿ÷È#<ø¹]È!dj$¿ÐÕ«W.eÊ-cýÐÉ!L­pÅb©»¥¥¥Ãº·ðÒñõ¯Û¶mo½õÖ~Wù1L­Ðå7!CZÞÇ=ºnÝºÔ_¿Q]]^FëTÌäÇ25ò#?©MXtiOÅª­­ÿý÷²°K~bSÓÈïòåËÑÉ£ãygÍuøðaòcÚêèèØ¶mÛ3ROÅüÂ/$@üÄ¦¦	ß'/Íü¢Ë÷î%?©±èÝ7¦.ìVTTìÚµëÆYûàÉ!dj$¿ð¢^/_¾ß¹sçÉ!djC¬§§gÏ=UUU©»>úhàà¨A~bSÓèÈ/zþ4éìÍwïÞÉ!djêèèhhh(--¾©S§Ö××gçÂ.ù1L­Ðå7kÖ¬èìüúûû_zé¥p9ü<ù1L-µ§b®®®ÞµkW±K~!S#¿4µ··§=ó3gÈ!djÉÒíÞ½;í©£#v³aüB¦Vèò^õ-[Û[VV6oÞ¼X,6ÖüÈÏÔr¨¦¦¦´GìíiÇ.ù!djY*¿	üÈÏÔ²¿x<ÞÖÖ6ØÂîîÝ»snaüB¦F~äÇ2µeXØÌªS1CÈÔÈoýô§?øáKJJÂkzYYÙÂ»»»É!TSlawd¿cüÄ2µì_ø÷´GxõîùZVU »äÇ2µB_t&çºººÞÞÞðáíÛ·7oÞ¶TVVC¨¦ya÷Ð¡Cù´°K~!S+tùE¯ïÉ¯ìárâ¬ÎäÇÊã©Åb±B[Ø%?©ºü¢÷üúûû[îÜ¹ã=?PO-ZØ­­­Mý»y¿°K~!S+tùE?çWWWÀ>¼yóæ+üC(/§H×ÚÚZÈ»äÇ2µBß¤û5FË¾äG~¦6YØ%?©ßÿ©è~C(w§hiv«ªªÂÓ°ÐvÉ!dj.¿üÈÏÔÆ´¾¾¾ûö-Z´ÈÂ.ù1Lüþo´ùêÕ«äÇÊÅ©Åb±_~9:x+uaqÈ!dj-¿ðýàµ×^°qË-ÎêÂÊ¹©çÔºuë,ìCÈÔÈoÐðÂ7Y³f]¿~=|ø½ï/úV1F?ÞG~ägj£^ Ý`»+V¬°°K~!S#¿_hË-Ñ7ÊÊÊèÂúõëÇú¡ùÚ×ÙÙÙÜÜº°;yòd»äÇ25ò´S§N%¾gìÛ·o:ù©=HíííivçÌÓÒÒÅÌüB¦F~éÛºukô=#ñÎÁSO=E~ä§,ZoookkëÒ¥KSvÃÆXØ%?©_¦Ã÷iÓ¦]¸páÓ¤ó+))!?òSöL­³³³¥¥%õTÌ'OÞ´iÓXÿÒòCÈÔòD~á;Ç;llhhpl/ù)K¦ÖÖÖ¶qãÆ´Gì¾ñÆ]]]v;ù1LüÚ`çó/äG~À©õöö¾õÖ[ivkjj8>Á'?©_nD~ägj±;uêÔÔS1oÙ²åâÅö3ù!LüyËI×s3H~ä§ñZû`»á)sãÆüÄ¦F~äÇÊí©Åãñ._¾<í©=Ú××gßÂÔD~¡ÜZ,ìTÌ[¶l9ö¬]J~bSù1r~jáßÚÝ_~¹³³ÓÎ$?1©üB¹=µ¾¾¾Ì?ß©ÉOaj"?_ÞN­««+ü³O*æuëÖ§óÂÔD~¡Ú`»AáàTÌä'05ü2G~ä§Z´°öÝ°1]òCÆI~E÷«¸¸üÈO#ZÝúúzÿòÉOajoùMxäG~yÙ#GV¯^va·¹¹9ÙEä'05È/·Ë°°;þ|»ä'©ùß Ý¸q£­­íøñãa'dùCµ°K~bùù¼ï~÷»Ó§Oÿü²Ï/]º´¬¬ìÏþìÏ²óq+444¤.ìN:ÕÂ.ù!djäG~äwÿ?þÙÏ~öÙÿñìÎ?Øþkü½ÆÏ|æ3Yõ]9gXØýÎw¾súôiÿzÉO!S#?ò#¿û÷[¿õ[7lØý÷ÅU_lllÌÇ-ìVUUeXØõÓä'©ùßP?~Ó×å÷Ûõ¿]SS3±j°ÝÔ#vÉüÄ25ò#?òjYõ_æÝÖÖÖS#?1LüÈüFXü_ÝÚÚÚÿtÉüÄ25ò#?òFßýîw§NÛ;úô7ß|s<ÿö¿cw(Gìù!djäG~ä7¼nÜ¸qü^]]]ãó7f>sÚ]S#?1LüÈür¬X,ÖÜÜz*æÐNÅljä'©ù_66ØÂnyyù¶mÛ:::LüÄ25ò#?òËí¢Ýùóç§¾É·téÒÖÖÖÞÞ^S#?1LüÈür»Áv'O¼iÓ¦ð½?ù!djäG~äÛlµµµiv[ZZFñwìù!djäG~11õôô´¶¶¦]Ø]±bÅüM>S#?1Lü~VwwwUUù1ÄøÅÒ±»nÝº¶¶6Sù1LüF³Ó§O/X° |£%?·âñxP]ÚÝ^x¡³³ÓÔD~!S#¿ÑoÍ5ä÷§ú§ÿK÷£ýèoþæoì!öÁlß¾ýWõWSßä7oÞk¯½vîÜ9SSÚÂ¿S35ZÃòûÙC êÔàýÓ?ýÓßþíßÚ÷-ì¥ßùßyè¡¯¨¨hýúõãüÏÌÔrô¹öw÷wöCnuéÒ¥S§NÙ¦å³ü¬öZ7|À¢ÝÔ7ùªªªÂ?°qûÍo¦fµWÖejºÚøÖK~1võõõµ¶¶>úè£©æ:4êGìù!djä7TCVá_NyyùðÖ××Ê©MüÄ25ò#?àÎ=ÛÐÐzÄnuuõîÝ»oÜ¸aj"?©_öF~1âñø-Zº°[SS3±»¦F~bùCN±Xl×®]©»¦&òcûÁÔÈüÈ/ç;öì¦MRv£#vzzLMä'05ò#?òËí¢#v.]+»¦F~bùCä+mii©ªªJ»°[ßÉüÄ25ò#?H_û¦MðrqaüÈO!S#?òcû×××wðàÁ´§bÎ¡]ò#?1LüÈ!2Å;b7¾ûù!djäG~ñi÷ºuërô]ò#?1LüÈ!î_ ]kkkuuuêÂîòåË8»»äG~bùCü¬X,ÖÜÜ<cÆà<yr~,ìù!djäG~ñvkkk[Øíêê25ÂÔD~ÊmCôõõ8p`þüù²°K~ä'©ù¢!2,ìnÜ¸± ¾§ù!djäG~yk0ÄúúúÔÝòòòÝ»wç÷Â.ùB¦F~äW°°K~ä'©ùå¿![Ø---Ý¸qciÁüÈO!S#?òËC¶°±­ù!djäG~¹m»K.-ä]ò#?1LüÈ/ÑÕÕÆ4Ø»gÏe>ò#?1LüÈ/ç1ØÂnEEEKKË7LüÈO!S#?òËmCD»Ë/la···×üÄ25ò#¿Ü6Ä`»¥¥¥yü;vÉOäÇ25ò#¿B4Dêû|ÍÍÍØ%?òCÈÔÈüòÍGM^Ø=xð ]ò#?1LüÈ/?Ç«««ëëë/]ºdÏù!djäG~yn§h!?òCÈÔÈüB¦F~bùCÈÔÈO!S#?òcSù!djäG~aj"?1©üÄ¦&òCÈ!dj"?1©üB¦&òcùCÈÔD~!S#?òcÈ!djäG~!S#?1LüÈ!djä'©ù1LüÄ25ò#?05B¦F~äÇ¦&òCÈOaj"?1©üB¦&òCÈ!dj"?©ù1LMäÇ25ò#?©üB¦F~äÇ25òCÈÔÈüB¦F~bùCÈÔÈO!S#?òcSù!djäG~aj"?1©üÄ¦&òCÈ!dj"?1©ù1LMäÇ25ò#?©üB¦F~äÇ25CÈÔÈüB¦F~bùCÈÔÈO!S#?òcù!djäG~aj"?1LüÈ!LMä'05ÂÔD~bSù1LMä'05ò#?©üB¦F~äÇ25CÈÔÈüB¦&òcùCÈÔÈO!S#?òcù!djäG~!S#?1LüÈ!LMä'©ù1©üÄ¦&òCÈOaj"?©üÄ¦F~äÇ25CÈÔÈüB¦&òcùCÈÔD~!S#?òcù!djä7ÜÎ;·dÉâââÅ§NüÂÔD~bSSþÈoîÜ¹gÎ	Þ÷Ýyóæ¥Ê¯½½ýkð®^½ú£ýÈ~05u]]]ÁëöCnõÉ'©åe9,¿äÊÊÊRå÷­oë¸ïØ±cGµLM¦¦´SûË¿üKûÁÔò¯|_~cc£Õ^ë¦&«½²nhjöCÞ®öFÝ¾»®®®··üÂÔD~bS³òJ~~^ôáÕ«WÂ«jêgCÈOajÊmù%×ÞÞ¾jÕªîîî´×CÈOajÊùUUUMJüÂÔD~bS³òV~#?05ÂÔD~bSù!LMäÇ25ÂÔÈüB¦&òcùCÈÔD~!S#?òcÈ!djäG~!S#?1LüÈ!djä'©ù1LüÄ25ò#?05B¦F~äÇ¦&òCÈOaj"?1©)ä÷GôGêÔà]¸páÈ#ö©i¬ûÇüÇ¿ø¿°r«£GÚ¦õôôä§ü._¾¼sçÎ?$IÒÏË¼":ÉÛ$IV%ID~$I"?I$$IÈO$Iä'I$òÓ§~øaeeeqqñÂO<ú	'N4égó½víÚ¤¤rsMøÔGVTTvØ©åå¹ýS;wîÜ%KÂ'/^¼8úýkÙ?5ß×È¯àª««çwÂ½÷nÞ¼yÀµñx<¼%^°>ÜÐÐ0ô+¦(Ü$|~Ú9jb§6gçZöOmîÜ¹gÎ	Þ÷Ýyóæy®åÄÔ|_#¿«¼¼üîÝ»áBUUÕk¿ño|ûÛßN"<=92ô+¦uåÊ+W6GMìÔòÌòþ©%WVVæ¹Sóü®âââ´Cüñ²eËÂ að´«V­¶ÿä'?É|seÉÔ¢6lØpþüùÁæ¨ÚPYkÙ?µDá¹ÖØØè¹Sóü®è¿¢JJJXáÔ©SÞûA±·ºzõêâÅ3ß3µÇ,õ~sÔÄNm(Ï,ÏµìZÔíÛ·ëêêz=×rhj¾¯_5kÖ¬þþþOï½­.ÿÂP±7n®ìÚ«¯¾úúë¯§½+¯kÙ0µ¡<³<×²j®]»æ¹CSóü«úúú·ß~;ÿOM?Ý¤ÕÞX,½º­Zµj7×ÄN-ôøã_¸p!ñaê5±SÊ3Ës-û§ÖÞÞ.tww®åÐÔ|_#¿ëôéÓEEEçÎKECòçÏ_°`Aqqñ+Âd°+«¦ýlôËÍQ;µ¡<³<×²jUUUÞq÷þ©ù¾F~$I"?I$$IùI$ü$ID~$I"?I$$IÈO$Iä'I$ò$IùI$ü$IÈO$Iä'I$ò$IùI$ü$ID~$I"?IÓ÷¾÷½Ç¼ì^+W®|ï½÷~áõë^9ójîÑVUU/­··wÀö°¥¸¸¸²²òîÝ»Ã½OI"?I¹×Î;'¥ôÚk¯åüZZZÂÆûöØþÆoí¯¼òÊîSÈORõáÓïß¿?~¯7ß|3|6^¸p!oäwåÊ°qñâÅ¶/X° lÅbä'ü$åO=õT0Í7¿ùÍäßúÖ·ÂÆ-[¶$»çäÉANK,	|óæÍÆÆÆiÓ¦«ÊËË·oß¼¨ÚÖÖt®·=~üøK-Ó§OüñÇ;>¬¯¯ðÀ=ù~¢«ÂC®:qâÄ`J[µjUØ~îÜ¹Ä3gÎ-555-¯¾újEEE¸«²²²'|òêÕ«©òK½ÿ[2<TIä'IÙìÙ³Z>þøãä|òIØXUU,?>ºvýúõ®zî¹ç¢«.]ºTTTöVÑÑµ6l¸÷n°cIII<×?¦Lýø]û	Ò>¼Ô¯ôÝwßMælï¼óNîdåÊÃ_*ü$ivÓ¼fM,Í7ß¾W¸>vmtmÈÑ[wAlÑUuuuÑáòéÓ§£;I¾Ï;vÛE­Ï<óLØräÈp9ü.?ýôÓ÷½ ÆðaøäÇö+9sføz»»»ÃáÏðÈ7ûûû£O¨¬¬7_Â§?_.++®ü2<TIä'I/¿PZù%¶G²Àºzõjø0)úpÉ%áÃ~øÙgb»sçNâNÂçx-q«èÃO>ù$ñÉAKßðg¸ÜÖÖvßû	Ê^¿~=ù±ö3yÏ?ÿ|¸êÛßþö§?_ÑÞ¾ò'ù.[¶,zKr¸òËðP%$MpÑ]·oßNÞØÛÛ6«2X'áÂ	è$~8/uv¥Ï¥.ôõõ;>zâÚûÞÏ`ÐåË#¤ËáÏpùÒ¥KkO>@ÚUã¡Ë/ÃCD~4ÁE?ëöúë¯'oÎu2àÄÏvww§¾uáÂhí5AÆè¹ÄrêöÜsÏE¼áÏÆÆÆÄö÷½ÇvíÚµèÃë×¯g>÷±Ç×F'²	`M¾*:Î7âÄ[·ne_B¥ÑÞHá¡J"?Ià¢#$÷îÝÕeÿþý%%%©Gc¬]»6(§¯¯/ZMý_ôãqÉ? ApûöíÁIÑQ·óª¤õYt°mô····'¶g¸èû¢óëíí>3üÞ|óÍÄ»qáKN¾*úÇK.¿%ìÁäY3X9|ZçO>|m*ü$iâÎr< W_õÿ¾~Ý+Qârb4qPE¢gy&É>|8ü>½÷Ë6Âöòòòäà÷FòKW;ØçÎ²²²èK°Æ½fÍä¿bæÌáÏèÄ.É÷½%(rjâÚUùIRVt²råÊ=þøãÑ¶äwâÄè´y.<uêTâÚÞÞÞ^z©¢¢"ÒÒóÏ?ß××¸öøñãË- «¬¬lmmp©$ÜUØ¾uëÖÛ»Px0á!E'Ìp>¿DNïÕÝÝ]WWöÀ´iÓÂWÅg¨I¾Ï[·nÚFûª¦¦æÜ¹sþÆUùI$ü$ID~$I"?I$$IÈO$Iä'I$ò$IùI$ü$IÈO$Iä'I$ò$IùI$ü$ID~$I"?I$¿ÿïþ;^qIEND®B`


r&Wî!	Õ/Ú)jÕk~aÆçÂÊKò|,w!ßòÃ©Í²¼dÉY>sæLVVr2gÏeë""äGDúÕjU¿æxñ,^|ñE9«ªª*°õáï¦GLN5Cù¿Jà!	­¯»xñbåºóüö§K¹ky²©¦+W®<ú´ÎÎNe^pàåI""äGDzÐMÙc«ìÆõÕWÉ£;wî(¯Ê×ÀôE×®]¡üÂ_eÿþý?üáeaëÖ­Á×þùçeùÄ÷ÎS~¥¥¥²¾»»ûáï>Ãå¹çQSSSà2JzËËËeAh866&l]DüH¿òóûýb¬ô©É#¥íÛ·+qX#T2Í÷î¡üÂ_ÅétÊ¹«V­L1Ñ|êò®]»ä1L¦µk×ÎjRítåFjkkåñÅïØ±CîEÖËe>ÿüóÀ-É¢Ïn·+Ó+**Ô!"B~DDDDüù!?""""B~DDDDüù-L?úÑcy<p»ÝlOÑèßÿýßÿë¿þqx¿ùÍonÞ¼É8D£7npÈµhäñxFGGhô³ýìþç7>>þ«_ýùE½¿ÿû¿üÅøèØÄ£ÑåËÿó?ÿqxwîÜùñÌ8D£÷ßÿ×¿þ5ã¿?úè#Æ!ýÛ¿ýr¼D8©õöyÈù!?äGÈù!?äüòC~ÈòC~üòC~ÈòC~ü!?äGÈù!?B~ÈòC~üòC~ÈòC~üòC~ÈòC~üòC~ÈùòC~ÈòC~ÈùòC~ÈòC~ÈùòC~ÈòC~Èù!?B~Èù!?äüòC~üò#äüòC~üò#äüù!?äüò#äüù!?äGÈù!?ä7mË-3¥¥¥ê³ä¤Õj³Éü!?äüù!¿8_~~þ¥KdáÔ©Sê³ìvû'dáÈ#555Áòëìì¼ÃFGGÏ?¢PooïÏþsÆ!âýâ¿¸xñ"ãÞ÷]1ãñä7è~È8D£®®.ùqx|òü¹ã;cù©KKKS4Í² £Øl¶`ù?aòT¬ùE!ùK´§§qxï½÷<×3ÑHÞÿÆ!â	©»»»hÔÑÑ!f3ïwÞq:1¾ÓDßåËëêêÔkFcÈeöö²·ØÛËÞ^ööÙÛÇò»wïÝn÷z½ê!°l2ò#äü!?ä÷òu8cccõ999Ê6*_eù!?B~ÈùòC~ñ-¿ªª*ùý|VmmíñãÇeA¾Úíväüù!?äGÈùÅ·ül6Û"U¿ÄS½½½Å`0X­Ö¾¾>äüù!?äGÈùÅ·üæòC~üò#äüòC~üò#äüòC~ÈòC~üòC~ÈòC~üòC~ÈòC~ü!?äüù!?äü!?äüù!?äü!?äüù!?äüòC~ÈùòC~Èù!?äGÈù!?B~Èù!?äGÈù!?B~Èù!?äGÈù!?B~Èù!?äüù!?äGÈù!?äüù!?äGÈù!?äüù!?äGÈùòC~ÈòC~ÈùòC~ÈòC~ÈùòC~ÈòC~Èù!?Æù!?B~Èù!?äüù!?äGÈù!?äüù!?äGÈù!?äüù!?äGÈù!?äü!?äüù!?äü!?äüù!?äü!?äüù!?B~ÈùòC~Èù!?B~ÈùòC~Èù!?B~ÈùòC~Èù!?äGÈù!?äüòC~ÈòC~üòC~ÈòC~üòC~ÈòC~¤Gù"?äüòC~È_ËÏår=ñÄ©©©n·ù!?äüù!?äüS~ÃÃÃ7oNIIY4øù!?äüù!?äüM~^¯W~eggg/Re6æiù!?B~ÈùòC~ý~kk«ÍfS/55U~<Xäüù!?äGÈ/Ùå×ÞÞ§6_JJJ]]ÝÈÈH,òC~üò#ä¼òëïï¯¬¬ûÉÒü!?äüù%µüä¼¾¾>55Um¾®®®Xäüù!?äGÈ/¹äçóù322Ôæ³Ùlöûý=°ÈùòC~È_²ÈOÌ×ÜÜ¬Æ!z½ÞdXäüù!?äGÈ/)ä×ÕÕ¥Æ!%ä4äüù!?äü_òÊÏívWTThÌ·~ýúDÆü!?äüòKFù;ÀÑ8/_ît:s`ò#äü!¿Çã_¾iåØ±c	?ù!?B~Èù!?ä,òØµ´´hÀ&ßÅI2ù!?B~Èù!?äòs:Ir4äüòC~ü_òÊ/äÑ8ª««].ãüæõËÉf³¯SojÈùòC~Èb#¿Ó8ÊËËcüù% üza'9sF6»é®ü!?äü(âò9#77·½½=§q ¿µvíÚ¡¡¡òö;w.üþéþéæt:;::Þ¡(ÔÙÙÙÝÝÍ8D<Õ·ÞzqF2°çÏg"èäí·ßf¢ü_dá1víÚ¥1ß¾ð-[¶ðë/L]SÅøNãX~¿¡ä_UUe4ËÊÊ5-òïü~ñ=&O?þñoÞ¼É8D¼>øqF===·oßf"Ûí`¢èä×¿þõtç<yòOþäOÔæKMMÝ½÷/ùK.|~úiï41åhtt´´´½½ìí%öö²·½½ñ½½!§q$çÑ8ØÛ«ùI&	ù!?B~ÈùQåçv»CC,Èp!¿_~~¾l§^ó«ªªB~ÈòC~ùy<ùl6[[[Ó8ßÈO9)(**2?äüù!?äGóßtGã8|ø0Gã@~ºù!?B~ÈùÑ¬äwúôiÍÑ8RSSëëë9òC~Èù!?B~È/qêïï/--eòC~Èù!?B~È/9£¢¢iÈù!?äüù!¿ÄiºiLã@~Èù!?äGÈù%H»Ci¦q¤¦¦~÷»ßeòC~Èù!?B~È/qjkkÓLãHIIÙ¹sçÉ'grÜ^B~Èù!?äGÈùÅAòk1øhµµµÊãNwB~Èù!?äGÈùÅS!ÀV^^®ÆüòC~ÈùòC~ñ<yÖ××gdd¨Í|4äüòC~Èò×|>ü*Ô/;;ûå_³/üòC~ÈùòC~ñßïommÍÍÍÕLÝßgºk!?äüòC~ü_%¿ûV®yKÃáP¦q	ù!?äüò#äüâ¦ÁÁÁõë×kÌWYY9ÃsäüòC~Èòi©©©iN§sæ7üòC~ÈùòC~ºÎï÷¿üòËÁÓ8ZZZf6äüòC~ÈòÓ¯ùZ[[5Gãxä4äüòC~Èò³úûûÀ6iÈù!?äü!?ä7¹Í7ó4Ó8çyËÈù!?äü!?ä§<Ï=4æí4äüòC~ÈòÓuÊ4ììlµù,Kkkël§q ?äüòC~ü~;út§q ?äüòC~üOÍÑ8æ?ù!?äüò#äüôÕàà`¦q ?äüòC~ü^òx<ÑÆüòC~ÈùòC~Ïçkjjö4äüòC~Èò[àÚÚÚl6ælÑÆüòC~ÈùòC~Óé9#öOzÈù!?äü!?ä­×¯_¿è÷ö4äüòC~Èòiããã;wîÌÈÈP¯°°°½½òC~Èù!?äGÈùE2¯×ÛÔÔd6Õæ---±ÆüòC~ÈùòC~±¨½½=77Wó¾ÆÆÆññq=<<äüòC~Èò@ýýýË/×¼¥oóæÍõ>äüòC~Èò|.kýúõOf^¹r¥XPoù!?äüò#äüæØÈÈÈ=RSSÕæËÍÍíèèXð·ô!?äüòC~ü_dØ:tH3u×l6777½^Ý>läüòC~Èò]§OÖLãHMMÝ³gO,ÆüòC~ÈòC~ÑM~éhÆ!UWW»Ýî¸[äüòC~ÈòtCCC"<ùÊËËãëçù!?äüò#äüÂ522RWW§º[XXØÚÚªÏiÈù!?äü!?ä7ë¼^¯ü¢ÉÎÎVONêáhÈù!?äüù!?ä±Z[[Æ±sçNýOã@~Èù!?äüù!¿æt:§qÔÕÕÇûØ"?äüòC~üßo¬¬¬ÔoýúõW¯^M±E~Èo×|TùòC~ÈâZ~òüãp84Ó8òòò:::ilòtGe4!?äü(Nåçñx§qäææ¶´´èùhÈùEK~zù!?B~ÈùÍ9¿ßßÚÚj±X4SwåKâù!¿È411±uëVäGÈù!?/ùÉ¯ÍÔÝmÛ¶ÅõÔ]äü",?Íf4y8òC~üâW~!§qÄÑØòüJKK§wÍæ¼$.¿Áë¬V«p³¸¸8ùòC~ÈoæOã(//ñ¯äüâC~&I~Bä·òßøCå#æyË½½½EEErSÁgÙíö'NÈÂ#Gjjjò#äüßR¦qdddh¦qÄãØòüYêÉÂ7&''e!==·¼víÚ¡¡¡ò3Ír/² [jðòc|òäÉÏcØ§~ÚÕÕõ9E!!õO~òÆ!â]½zõwÞa¢Qww÷'|Â8D<ù;ð½÷ÞÔ­öÙgÿøÿ(¿MÔæËÌÌüÖ·¾%?É6¶2 lcïÒ¥K|ðAï4êòËÊÊÞÞ^Ei/¼ð²©Ou	)?õßÈïÔ©S?aò#0ý¢POOüÏ8D¼?þXTÍ8D£óçÏË_CÄ?/^¼:vìØ%K4ïM¯­­½råJr­ÈOÄÀ6ñúúú1¾Ó¨ËoïÞ½ùwHDO~êé#&½½ìí%öö²·½½3)ä41_2Lã`o/Fjnï/¾¸xñbYÛÊ°¬¬¬,b1ürrrmT¾Ê2òC~üòÓ8òäR~ò÷ÙñãÇeA¾Úíväüù!?ä7]!§qäåå%Û4äüâR~ÊÉÞÞ^Åb0¬Vk__òC~üònº£q:tó!?ä7÷Ïvá	ù!?B~:ÓéÌËËK¶£q ?äuùåçç«µ(RsòC~ÈùÑ¬ä×ßßÏ4äü¢%?AüDÉ¯gåöôòC~ü_rÊiÈùE]~ÊÇ`êÈùòC~I(?¯×Ë4äüb!¿ùéÚ±cÇ½÷!?äGÈ/ÆòØ;v,77iÈùÅB~æÐáAÈù!?Æ!6ògûÍ4¦q ?ä-ù-]ºÈù!?B~1Ëåª­­Õ¼¥iÈùE]~Êüêç»B~ÈòK`ùiÈùÅH~999Ìð@~Èùòüä¹½±±1;;[m>ÍÖÜÜÌ[úòüzzzäoïÞ½>ùòC~ü¢Ôw¿ûÝÇLm¾Cy½^gVµ··¦¥¥ýÑýÑþýûå×7òC~³¹ib!?äü("uuuOã¨««apf[KKËüÁ<óÌ3ûë÷oýæÖÂÂõë×#?ä7ÓÄB~ÈùÑ<h^Yxúé§9äóù222ê¾QwàÛûþvß¿øÅW^yù!¿8ù!?B~È/ÌWWW§ùdæ/ùËÎ1Ø§ü[³fÍ7¿ùMäüfÍf[ºtéàà òcG~Èßüóù|ÁGã°X,ÍÍÍ?ùÉOùäv»,Y¢ßÿù¶mÛòiF£Q~&uõ]!?äGÈùÅc~¿_x§Ts4Ç3ãöÒ#³Ùl6l°oë7·¦¥¥=zù!¿&âÌÆÆÆ	|¶òC~ü_ÜÕÕÕ§Æ±mÛ¶À3òH.Kl½|ùò§zêñÇ³²²þå_þÈo67ÁÜ^äü!¿ùY¤ººZóKdóæÍiÈ/Ry<Ã×ÕÕýÃ?ür äüfsòC~üæütïÜ¹Ss4+Wöôô_ùE/äüâ;äüù!?çñxêëë5Ó8l6ÛéÓ§§;òC~Èù!?äGÈùÅY»AÚ|f³ùðáÃáüòÓüdsY»vmZZüô¦§§oÜ¸qa§z ?äGÈùé³ÂÂBÍ4;w?òºÈù!?]Èïþýû!gx,àa|ò#äüô<-WVVÃívÏðòC~º_QQüô®[·îÞ½rrbbbÃ²fÅÈòC~$¶«­­ÕoåÊýýý³ºäü.äg2ägXýzd¬G~üòKæ<OCCfên^^^[[Ûnù!?ä§ùùIVo1>OÖð©.üòKÚü~ÿ¡C²³³§qx½Þ¹Ý&òC~ÈOòSööVUU)å«,ËeË!?B~Èù%ammm£q¤¦¦Ê3³ÇãÏÍ"?äüt!?¡^ÈwïÞE~üòKªBNãp83ÆüòÓ»üNMïÝ¸qcff¦Á`¯UUU²f¿+äüù!¿r(0òC~ÈO/òÓ[ÈùòC~1ËãñlÛ¶-xÓéì!?äüòC~üßrliiîlÈù!¿xß¢Ge0!?äüµÖÖÖhLã@~ÈùéT~éC~üòKàoP`+**4G`Ô4äüNå7]»wïVN:üù!?äHM7cpp0÷üòÓüÒÓÓgÝò#äü"[Ì¦q ?äüâC~6mR:;;ü»B~ÈòT!§qX,ÖÖÖhLã@~Èùé]~gÏUÖ­[§ïù!?B~È/"Å~òC~ÈO¿ò»ÿþ+ÉºròC~üß<[¨iÈù!?Êïõ×_Wjjjô6ÐÈùòC~sNlW]]½PÓ8òC~:çüò£¦q ?äüt*?Ã£2ÈòC~qßïojjÒÃ4äüNå§çò#äüf^GGGaa¡æl5ù!?äüòC~ÈùE¥ÊÊÊàiúü¡C~Èù!?äüù!¿¹444¤ÏiÈù!?äüòC~È/bïÜ¹S3£¤¤D'Ó8òC~Èù!?äü_òûý6ÍjóÉÉææfýLã@~Èù!?äüòC~ó5ß±cÇÆÑÐÐ ·iÈù!?ÊÏóC~ÈùQÏéth¢7oÞ<<<_üò[Hù©?º/¤üø<?B~Èù-ln·»¶¶Vóä^Þßßüò[Hùêîî§göÞ½rR¾nØ°AÖxùòC~ÈoAòx<£qtuuÅÅ[úòC~úòfaõSl=Ê¿Ïó¬V«Ñh,..Ö¸jllLýtü!?ä§¤Ãf³i¦qÈÊø5òC~ÈOGòSVÔ["¿ù¿ÏÏn·8qB9RSS£>ëÌ3cº+üÎ?ïaòKÔétz)]ºtixxqxòK´··qF===ò×éÜõéÓ§.]ªÆ±cÇÛ·o'ÀÀ^¿~``,uuuMLL0ï³Ï>s¹¾Ó¨Ë/''G÷ïßrëÖ­5²~þ¯&NNN*¿_Õg	ûÎ;F~ò×í»1ìwÞyë­·Þ¥(ôöÛoã'«tvv2ÑHä9!Æw*!OãxüñÇÅ	3°òT Ol`Ñ¨££ãÂCÄû·©b|§QßÅCÎðÿkòê9"ù"ùùùUUU²²¬¬,øuTöö²·ØÛ<CNã¨¬¬t¹6°ìíeo/u±·WºqãP,--Í`0¤§§/[¶ìöíÛó¿YõþbÉò2£££¥¥¥ÈùòKBùM7£½½=!ù!?ä§ùE©eC¯aö£ù!?B~-¿Ó8,Kkkk¼Oã@~Èù%¯üjkk?.òÕn·«ÏÊÏÏw»Ý§^ó«ªªB~È_òÈ¯½½]s4úúúG¾³ùòC~ßµk×ÒÓÓý³999gÎÿÍöööÊ°rV«µ¯¯ï·xê3EEEF£±¢¢Bðü!¿d_¿<éiÞÒçp8ägù!?ä§ù)ä¬>b²|äÈú®ò#ä`ò¬¬¬Æ§Gã@~ÈùÅ±ü,<]»v- ¿¾¾>YÎÌÌD~üòg²ý;µù/_ãg9äüùýî&~wü&''9n/!?äüæÇãg³ììlµùl6[sssOã@~Èùé]~Ê'9+¯óüdÓÙ·oòôüù!?ä7v­­­Ê@B@yrKøiÈù!?½Ë¯§§'ä'9_ºt	ùòC~Èo¶ÉÓæh)))Û¶móx<I>°Èù!?]ÈO+++Sæö¦¥¥(¹üù!?ä7óBNã¨®®^ØgTäüùé=äüùÅü§q$á4äüÞåØèöíÛ¹¹¹ÈòC~áS¦qhÀðGã@~Èù%üdëan/!?äüÂfoéC~ÈùéN~¹¹¹Âüù!?ä2§Ó©9[JJJòù!?äò»yó¦aªÀÑ;Ô	ûÎ=üù!?ä§iº£qÈzù!?ä§_ùç-à]äüùÅüBNãÈËËs:òC~È/nä§Ãò#ä§+ùÆÝÒÒÂ4äü_ÉO¶ôôôÀÅ¿ôÒKÈòC~!§q¤¦¦2ù!?ä¯ò[²dfz¯òÔÖØØüù!¿d_ð4ÉápðÉÌÈù!¿8Édç2ËX300 k233!?äòëéé	91G~ÈùÅ½üé½¿w»Aòüù!¿dhxxxíÚµÁÓ8ÚÚÚäü_"ÈÏjµÊóÚ®]»|>¼ÿþÏ G~ü_òÄ4äü()ä'¿C~s__ò#äü"[WW×·¿ýí½÷¶··ëê;vL§ùdæ¦q ?äüM~ÒèèhqqqZZÁ`HOO/**5ø]!?äG'?Ï÷äOZ­Ö¯ýÕ×þê¿²X,Ï>û¬^Ks:+W®Ôüék·ÛÆüñy~ÈòcüÒ¾´ïo÷øöù'6íßûÞ>¤«W¯Oã(//ÿçþç·òC~ÈùòC~¡[¹rå_?û×ûÿkÃÿäÁÈ6¹sçÎài­­­~¿&Çí%äü_ËïÚµkÊ9+óysrrÎ9üù!¿¶|ùò­ßÜªß3Ï<S]]ãáóù§q;v,°ëù!?äG,¿îîîÀÓ"?eùÈ#ÈòTãñÇÕòûJÙW^xáX>öööÜÜÑ8êëë5Ó8òC~ÈòSJtíÚµüúúúø$gB~È/²ÍæÊÊÊ½öÊ¿5kÖüñÿñøøxlî½££#xÇúõë/üò£DòøPõéÍ²l4!?äÁYëÖ­ËÎÎÎÊÊúêW¿É³2JÁÓ8ª««¯^½:ÝUòC~ÈòËÉÉQ>½Ol:ûöíeÍüù!¿øM6<ÃªÆqúôéð&üò£D_OOOÈOr¾téò#äüâ1¯×+O#OfùÑ8òC~ÈòÆÆÆÊÊÊ¹½iiiû¦Èùò[»Ó§OÛl6ùäYeæGã@~ÈùQËOo!?äGÈoutth¦î*Ó8fû§,òC~Èò#äüôËåzâ'4æ[¹reiÈù!?J^ù]¿~éÒ¥&I+ÓÒÒåò#äüôl][¶lÑcùòåííís>(0òC~ÈY~N§3äÁÁAäGÈùé6¯×[__|4_~yÎæC~ÈùQâËOù$g»Ý.O£ròÞ½555²Æjµ"?B~ÈO	ì>¬ÆÒØØ8óiÈù!?JRù)Oê?e9ð©ÎÈòÓUmmmyyy[¶là' ?äü(å§¼æ§Þbîß¿Ïk~üÞ§à£q,_¾¼¿¿?²wüò£Dò>?»Ý.àwïÞ­¨¨à~ü~r»ÝµµµóÌgòC~ÈT~Uìwû"?äGÈOÉãñ444h¦îÍæ¦¦&Ï¥;E~ÈùQ"ËÏð¨F#ò#äübßï?tèælB@`D¦q ?äü(Iå§Ãò£$_Èi7oÍ%òC~ÈòîCGGG!?äãBNã¨¨¨ÛÑ8òC~ünbÑ¢_|Q³rË-|ª!?äËBNã(,,lmmÆ4äü%©üDxòôsûöm9yòäIå	7öoïC~ÈS~gÛ¶m!§qÄØ|Èù!?J|ù=zOyªµZ­ÊÂð»B~ÈD~a¦q(GZòC~àò.^¼xÚmnn^Øïù!?Jùµ¶¶FûhÈù!?B~!Ú¾»ò«ÏCzöÙg!?ä¥äÄxuÕÕÕ:ùòC~Èò3òyåÊª÷ùL&äGÈùE¶Ó8òòòÚÛÛõó òC~Èò§Ýýû÷kV:æöòC~,ä4ÍvìØ±Æüò£$ßtç7666Ï[°Z­F£±¸¸Xãª0g!?äG	&¿Ó8RSS£w6äü!¿Xg·ÛO8!G©©©áYÈùQ"É/ä4=öèö1#?äü(å'O¾êý¹áOÎ-³Ù<99)²9Úl¶¥ÈïðáÃ?aòDÿÖ[oý¢PWW×»ï¾Ë8D¼[=?Bù).--Õï/ÿò/;¦ó±íìì|ï½÷ØÆ"ü-@a¢QGGÇÅçt:Ï?ã;cù©?Zó¹ÐaÎRä÷öÛoOÄ°ÿøÿÿÝ	B|ðÛíf"Þ7ä^í£>úêW¿ª1(P~9ÅÅØÊß*·nÝbx?ýéO/_¾Ì8D#ù;ðW¿úãñ×Çã;cù©oA3S8ÌYìíeo/ÅïÞÞé¦q´µµémÙÛËÞ^booå£lòUgxòC~òØ577k¦qÈÉ¦¦¦<òC~È_ìäW[[üøqY¯v»g!?äGq'¿Ó§Ok¦q¤¤¤ìÜ¹3NÿëòC~üæRoo¯ÅbÛ±Z­[î,äü(îäçr¹6oÞ¬yK_yyùàà`ü-òC~È_LC~Èô/¿ááa1æ-yyyñõ>äü%üÂüù!¿y<ù!ÍÈÈP?cX,ÖÖÖx7òC~ÈV~Güa+È_ËÏçó577ò4GãYá`Â-òC~ÈP~zù!?Ò¡üººº5Ó8ÛíN°±E~ÈùòC~üW~òóXYY©yC¬ëiÈù!?B~Èòû½äÿ.xên^^ÓéLà±E~ÈùòC~üK~ò¿V__ÀÓ8òC~ü!?ä÷Û£qhÌ`Ó8òC~ü!¿d_kkkðÑ8GRý'"?äüù!?B~	.¿«W¯®2y¦q ?äüù!?B~É(?·Û|4ÿ"?äGÈù!?äü(òóx<uuuó%É4äü!?äGÈ/Yäçóù²³³vòC~Èò#äòkii±ÙlI>ù!?äGÈùòKpùÉOVEEÓ8òC~üòC~,¿áááõë×kÌÌÓ8òC~ü!¿ßøøøÎ;5ÌäÓ8òC~ü!¿Ànºi^¯1D~ÈùòC~üD~­­­¹¹¹Ý»Lã@~ÈùòC~È/Îäçñxÿæoþæ¥^JÚ©	aäòhO<ñD?$òC~ÈòC~ñ$¿¡¡¡/~ñ_ùÊWzê©¿Xý===È/08ë×¯×|2saa¡ÓédSD~ÈùòC~È/þä÷å/yí×Öøöåß_?û×=öX~ø°F~ãããóÙl¶Ó§Oû|>¶Cäü!?äüâO~###ø¸¿~@~ò¯¨¨(	_öÈO`wèÐ!ÍÔ]9¹gÏÆüò#äü_ËÏår¨Ù'ÿþôOÿ´½½=9å'ßx^^Ó8òC~Èù!?äòóz½ßÚþ­ûöýí¾Ç,	çytttäççkÌ÷ôÓO3ù!?äGÈù!¿tðàAÍ¶õ[vïY¾lù3Ï<TC:44´eËÍ[úV®ùòC~üòK4ùI¯¾újvvvNNNVVVMMMòÌ`ß¶mÆ|¹¹¹ù!?äÇ8 ?äüS~Jr³ÉÃ¯×ÛÔÔd±X4Ó88òC~üòC~I!¿ä©­­Íf³©Íòo|OéC~ÈòC~Èù%Nb»ÊÊJÍ4ÚÚÚááá·òC~üòC~qÐÐÐÐæÍ5æ+//Lã@~ÈùòC~Èù!¿¸/ä4¼¼¼öövõûòC~üòC~È/óx<f³Ym>9ÙÔÔ<ù!?äGÈù!?äüâ2¿ßßÒÒ­ÆæhÈù!?B~Èù!?ä9Îà#°mÞ¼Ùív¹òC~ÈòC~ÈùÅSýýýÁSw+**f²ý#?äüù!?äü_|4<<ìp8§q´¶¶Îðã©òC~üòC~ÈOïy<ÙÂ322ÔæËÎÎ>tèÐ¬Iüò#äüòC~úmºiÛ¶mÎöÖòC~üòC~ÈO§µµµOã¨­­?ù!?äGÈù!?äüâ©Ó8ÊËËç¹#?äüù!?äür»ÝóÆüò#äüòC~zÏãñ444hÌ7iÈù!?B~Èù!?ä§ß¦Æ!Ã4äü!?äüòÓiÆüò#äüòC~º+ä4Y½-ù!?äGÈù!?äübÝtÓ8ÚÚÚ¢z¿Èù!?B~Èù!?ä»b0ù!?äGÈùÅü¬V«Ñh,..Ö¸jllLýËù!¿øJ`'¼Á4äü!?ä7ò³Ûí'N#GÔÔÔ¨Ï:sæÃáîÈùé¹XNã@~ÈùòC~q#?³Ù<99)²9Úl6õYÂ¾sçÎß~ð_Ä°þô§òcóB===|òIb|/]]]ög¦1¬ñæªôé§¾ûî»l`ÑèüùóCÄ»zõªüUÏ8D£ÎÎÎë×¯3ïòåË1¾Ó8Ñh¹,åççWUUÉÊ²²²`Mü¾ÿýï_aòg¨üR¿FQèÂýýýñþ]¼ÿþû_ÿú×M&Ú|K,ùÎw¾³Pi``Àét²E£îîn1ãñä7¨ü)È8D#üÍ8D¼ÞÞ^ùs%ÆwÇò3eùò2£££¥¥¥ìíeo¯nóx<öìÉÈÈÐLãhiiÁ4öö²·½½ÄÞ^ööêZ~êI999Ê(_eyº«£ù!?=$°;vìÅbYÀiÈù!?B~ÈO×òSW[[üøqY¯v»]V~~¾òvøÑÑÑªª*äüôl%%%·ômÞ¼9ÆÓ8òC~ü_ÜÈ¯··×b±«ÕÚ××÷ÛG<õr h ¨¨Èh4VTTþòÓO.K6Kùª««õÆ,äü!?ä !?ä· 8ÔÔTµùrssÛÛÛuøhòC~üòC~Èo.y½Þ¦¦&Í'3gdd:tHÎÒçcF~ÈùòC~Èù!¿Ùå÷û5Ó8Ä|õõõ###zXäü!?äüòE!§qÔÖÖêÜ|Èù!?B~Èù!?ä7/%%Em¾+W:ÎxXäü!?äüòD###Áæ+,,ìêê¯E~ÈùòC~Èù!¿ióx<ÁGãhjjòù|q7°Èù!?B~Èù!?äºÓ§OÍfÍÑ8öìÙ££q ?äüù!?äü_dr:+W®ÔóÑ8òC~üòC~Èo¾ÉVTYY©1ßO<ár¹``òC~üòC~Èïÿ%÷¸~ýzùrss»ººü~b,òC~ÈòC~È/ÙårÙlnnnß·ô!?äüù!?äüßïåóùdËÑ-%%¥¾¾>ÁÌüò#äüòKjùµ¶¶æååcxx8Qù!?äGÈù!?ätòM¥¢¢"xÇÕ«W`òC~üòC~I$?·Û][[«1_IIIÜù!?äGÈù!?äü¦MÆ¡9ÅbimmM©»Èù!?B~Èù!¿dÀîÐ¡Ci©©©²Í$ä4äü!?äü_Ê¯­­M3#%%ÅápÄûÑ8òC~üòC~Èïÿ×ßß|4Y388´üò#äüòK4ù;Í[úòòòNg,òC~ÈòC~È/qäçñxdKÐ#Ù¦q ?äüù!?äü »Í49Ó8òC~üòC~,?§ÓrG,þüòC~Èò£èÊoppiÈù!?B~Èù!¿_Èi%%%1Þ!?äüù!?¢üÆüò#äüòK|ù	ìw<¦q ?äüù!?äüY~òÿ[RRÂ4äü!?äü_"ËÏívWWWÏa.Ã.`äü!?äü_Èo|||Û¶msÆÑÓÓc±Xrssóòò²³³Ï=Ë¨"?äüù!?äüt*¿¦¦&Í'3äf2cxxø±ÇÛôô¦ß> ÿþ÷ÿý/|9¿Èù!?B~Èù!?=öÒK/éK_Æáõzgrõ½÷>þøãûO=õÔ×¾ö5ù!?äGÈù!?ä§£úûûËËËç9£ººúëµ_WËoÛßlJ2¼Èù!?B~Èù!?]$"©¬¬Ô¼¥oýúõs8Ç¶mÛ¾öW_SË¯¦¦¦¢¢AF~ÈùòC~Èù-pÃÃÃµµµóÎù?T®U÷:ßÚþ-«Õú½ï¡F~ÈùòC~Èù-X§¾¾^s4³Ù¼sçÎ_üâó¹åÖÖÖÌÌeË­øÊ¹ýï|ç;6òC~ÈòC~Èoaòûý---£q¤¦¦644grÜÞ°Òétvuu0àÈù!?B~Èù!¿I4VXX¨ÆQWW ZDäGÈù!?B~Èù!¿¬¿¿?øhË/w¹!?äüù!?äü_'>G^^^[[[ð'3#?äüù!?äü_õz5Ó8,Ë±cÇ¦;òC~ÈòC~ÈùÅ_§OÎËËÓ¼¥¯¾¾>üÑ8òC~üòC~È/r:%%%·ômÞ¼Ùív?òºÈù!?B~Èù!?ä>ýôÓóUWW_½zu·üò#äüòC~zÏãñìÜ¹355Um>ÍÖÓÓ3«ÛA~ÈùòC~Èù!?ýæóù^~ùe³Ù¬6_vvvSSSø·ô!?äüù!?äü_<uõêÕÜÜù222äÄãñÌíòC~üòC~ÈO§«_ís8ó<`òC~ÈòC~É"?ùåd³Ù×X­V£ÑX\L.ä·°;vLÌWQQÿäü!?äüB~½½½EEEbà³ìvû'dáÈ#555Áòëééù?1LåßyçÿCSý÷ÿwGG|È­øá£££jÄ»téãÞï½»wï2ï¿üå+Wh$òûÍo~Ã8D<aßàà`ï4å·víÚ¡¡¡ò3Í² £¿((òõÕW»bØÛo¿-Öé¢($ÛÙÙÉ8°ÑÆ×F+ÃË8D<y*xë­·hô¯ÿú¯BÂl´ñ½·÷ÿ=ÄPò3!ÙÛx±·½½ìí%öö²·½½I±·7üC`Ùd2!?äGÈù!?B~È/þä^~999Ê6*_eù!?B~ÈùòC~ñ'¿^Y[[üøqY¯v»ù!?B~ÈùòC~	(?ådoo¯Åb1V«µ¯¯ù!?B~ÈùòC~ ¿9ü!?äüù!?äü!?äüù!?äüò#äü!?äüò#äü!?äüò#äü!?äGÈù!?B~Èù!?äGÈù!?B~Èù!?äGÈù!?B~Èù!?äüòC~üòC~ÈùòC~ÈòC~ÈùòC~ÈòC~ÈùòC~ÈòK¤þîïþ®­­m8Éÿë©S§);wîÊ+CÄûøãÛÛÛhôüà³Ï>c"Þ~øöÛo3ÑèûßÿþÏþsÆ!âÉßï¾ûnïÔãñ$ü®]»vàÀ¿'"""J²Âïö+±DDDDIò#"""B~DDDDüùò#""""äGDDDDÈOwX­V£ÑX\¬9bÏçs8&)77·§§'üi>;66¶H£7ç±U£Á``£ÞÀ²ÑFjlåI ¨¨H9«··6zËF©±ýüóÏËÊÊä·Ø¦M&&&ô°Ñòß9ìvû'dáÈ#555ê³<øÊ+¯LNNÊÏÏÒ¥KÃ_æ3°gÎ2hóÛ@r96zËF©±5Í7oÞùj³ÙØh£7°l´Û+VôõõÉÂàààöíÛõ°Ñ"¿Y$?BYxðàò³Hä.ÿ©3¼0Íg`åÉèÜ¹sÚüÇVéÆkÖ¬a£êÀ²ÑFjlåÏ¿ÑÑQY¯Êl´QX6ÚH­Ñh,geeéa£E~³Hýÿ§^VN¾þúëiiiò3£P<Ìi>_UU%ëËÊÊôvìø[¥7^¾|y¦¹,m¤Æv``@-Z$_áe£ÒÀ²ÑFjlKKK¯]»&'OTÎZðùÍ"å-;J&IsÖo¼ñðwôÃ_æ3°äoSùbôæ<¶ÒÐÐÐªU«fxaóÀ²ÑFjlW¬X¡ì©(#ÌF¥e£ÔØ~ôÑGBê×^M¾êa£E~³(''çÁ§^¡eÍYÂ¹0Íg`ÕñD?±8õ6Ê×_¦9,m¤Æ6øÅ6Ú(,m®_¿^TT¤ùÍ¢ÚÚÚãÇË|µÛíê³¶oßþæoÊÂµk×ÊËËÃ_æ3°ùùùn·[ùK´ªªÑóØJ«W¯¾råÊ/LsX6ÚHíªU«g.kÅl´ÑX6ÚH­äÀÀÀäääk¯½öâ/êa£E~³¨··×b±«ÕªLÕy8õ	òubbbãÆÊ["¦»0Í`/_¾¬|ú@EEòdÛØ*Ê+o4saÿÀ²ÑFjl¯_¿..¯²ÌF½e£ÔØöôô,Y²Dêêêü~¿6ZäGDDD,!?""""äGDDDDÈ!?""""B~DDDDüùò#""""äGDDDüùò#""""äGDDDDÈ!?""""B~DDDDü(1;yòäêÕ«Ó¦Z³fÍÙ³gï¹lª¸yæõhm6|k^¯W³^ÖF«Õ:999ÛÛ$"B~D8p`QP/¾øb"É¯±±QV677kÖ>|XÖ¿ðÂs¸M""äGDqÖÀÀÆh4=zÔ?Õo¼!'eå+WF~7nÜ¥¥¥õEEE²Þív#?"B~Dø=ûì³bW^yE½òÕW_[¶lQ»çÂ"'Aá²eËd9pá»wïÖÕÕeffÊYf³y×®]êªN§St%gÉu»ºº45YYY«W¯îììµµµÖÑÑþv³ä!)guwwO§´ªª*Yß××XséÒ%YSYYXsðàAÅ"7¶iÓ¦ÑÑÑ`ùß¾fMJDÈh![²d¨åæÍê·nÝ6M-M/_VÎÝ°aæ¬;v(g¹ÁòZÊIåÜ7NNNM&ßïså«È)==]yû]Û/ø;=uê³8q"À>Í¬Y³f¶òóPù-pÊÝÏ_ÃÔ²©©©¹7,ÈÉuëÖ)ç*ÐQì¨¼t'bSÎ²ÛíÊ²ÜÛÛ«Üú6÷ïß/¶Sv¶>ÿüó²æÜ¹s²,_eù¹çäíå¤ýØB~G¢ÉÅË÷çÎ9)_å7<x jµÊå[xø»½Ãiii³_JDÈháå'_`½"LÒèè¨3)'-[&'.]ºuëVÛýû÷7"Ñ¼¸ròÖ­[;|å«,;ÎGÞ(SNÞ¾[ýØ¦OÞîÝ»å¬×^íáïöhïÚµKQ ÈO<ZVV¦¼$9[ùy¨Dü8å®÷î©Wz½^Y)g±NÀCCCþÐ	¼9/x'¬ÆRêÏRe³Ùl2|>ÜxVVVàÜGÞÎtÓtíÚ5©²,_eÙårÎííír¯ñÌåæ¡ò#"Zà÷º½þúëêÊghfxÞxçÎà²®ÒØØ¨ìQyA.°;õ>Û±c²W¾ÖÕÕÖ¹å5¶±±1åäíÛ·ÃÏÃ]µj«|U2ÏWÎêîî/¿JÑæ¡ò#"ZàF£ñÈ#Ê§º=zÔd2ÏÆX·n(Ççó)»bóp÷ù)oR¿APà®]»ÄIÊ¬ÛÀçªô2ÙV¹÷Àú0·£¼±Oy×ëU.F~o¼ñFàÕ8ùÕg)oyt¹/2ÓÉO±¦XY.&âÜ´iúÜ0ÑÂ§|Ê±¦þÿç²©ûI*=ÿüójVª;sæLù=:Ø¬7ÍêÁanGúæÕN÷ÍÞ¿?--Mù4û¸×®]«¾ÅËWå]Ô·©¼$HqjàÜ0.¬Y³Æ4ÕêÕ«	¶ùuww+W\|ñâÅÀ¹^¯wß¾EÑÒîÝ»>_àÜ®®®²²2ÕjmiiÑÜfð#õÛ·o×¬îv$y0òóy~êêê4ï¢tçÎ»Ý.#)ßÛí|Bú6'&&¶ÊXUVVöõõiî1ÌC%"äGDDDDÈ!?""""B~DDDDüùò#""""äGDDDüùò#""""äGDDDDÈ!?""""B~DDDD4³þ/×Aìr©Æ·IEND®B`


`` Zðwøðá~:tHçÚft:qâÄLgÖÃãù¹Eç,ðGDàfl,iÓ¦MQ¿îîn¥Õ_~ÙívONNÊv»]óªfz=<<¼ÿ~Y#êþÔ=z$ËÓ<üÑ´aµZåë©å£>Úºu«3­¨¨èëëóùÎ5kÖxÖ´¶¶._¾Ü`0¬]»öÁ7nÜ(,,LJJ*--õ~ñÈ#ÙÙÙjS~ç¬@lÚ±c¬÷N<)++++5Ç^ÄÿGeÜÈÏþ?hô?Ëçä?¢  @Æyýúõ2Þçº¶@ÿR[[üâò_)gñx!D#øÎÉÉÉÊÊÜ¿ßh4zC¾ÓGi			òuóæÍµ¦MW¯^-¾ñxäçóëÖ­ÓÇß¢EdýÃ½W.eåÒ¥K5Ç^ÄçG?zôH½ó7£ÁÑÇßì.¨¿ïså·)þ¼ÿív»:é©»»ø#¢XÀ,tÉû0o<óÌ3²^þèI² 'e¥÷w<xprrR½w¥ÖìÚµËívwtt¨rÍr²¥¥Eê²êãkW¯^å»wïzAs½÷e¼H ÏüyÞéàhÞªY_Ðç,ï6Y®¨¨ÇoÚ´i¦øóþë5×¯_eõÿ¸ûv2Dàb¼´Ë²¼Ìû¬ÏÊÊåû÷ï«÷îÝ.ô¾Ï¹5âïCCC??õ&ÈÄÄàOÌQ\¬ÞyIII¿æúY|rÙÔÔÔ¶¶6ÍAÎàhÞªY_Pê<ð¾ÎáÏûÿQ]w²ø#¢ØÁøÌh4.Y²d||Ü½ÿ;gþÕMG'>'>S:RQegg«yjáyhâsmÓ¹®ô¿a:¤³» ÎxNwúçúÿ?úìøø#¢ØÁôÊ+¯ÈIÏQÔJå3÷¨dåtH¡²  @ír¥µµUp6ü©·'Õ^W***öîÝûèÑ£Ó§OËÊ­[·jâo:5þf:8fÁ ßùóðN¬¯?à©©©²ÀÅ2þ¤M6y>æ¯Ö¨mËËË¤>X¶k×®àñ§Þý²Ûí"S§NMíííjþQ¼ØÑÑ¡ÞÕS]ºtIówéEf?ýÁQ#ézÏÌtT§3êSzÝ±±1±Ýüixüøqm¢ÀWÀÕM+Ë÷ËÐÉòòåËyÈ?"5üx>ì¥Öôõõù|ü+++Ë³ñl0øóèDµpáBùê½Å«æm~ê©§üg$W®ókÎè"3ÂþàxvÈì³£æYêtÆóÖ­[ÞW¸fÍïs!))Ijÿ¼ø#¢ØÁ¤¦D½×ðÁ[·nMzREEÿæ³ÃßÐÐ0hß¾â!9k÷îÝS¬¾¾¾°°0!!aÑ¢EgÏýÚ×¾æÙj8øÌúséÒ%ñÁ`ðì9Ï»ê4÷Ê+K,_S®PÍ¤¶hO?ý´úq%%%]]]S¾ÕÚÒÒR\,×&¿ECC"ðGDMLL<õÔSJswÈOÍ2«íµÝn·B|AA÷"DD1úÌOÇcdüÅ`.kÿþý-Rs»²pøðaÀ?""""ðÇ?""""DDDDþüø#""""ðGDDDDàÀ?""""DDDDþÀQð]ºtÉd2uëÖûyíI³ûé7À?ï³RRR***¸·ø#¢¨/;;[#²q»Ýñ?kó^SWW'ËÛ·oçÞBDà¢ÿÉ%¤ÛÍ'þÅ²Â½ÀÅü¼¡sàÀôôôÔÔÔCù|Û×¾öµââbÏÊµÒ³¦¼¼Èú÷îÃ+®_¿®y=Þ?Wÿ"gÏ5Lf³ùáÃþ8µX,rY¹Ùû÷ïÅ[ÓÇß| nÿää¤ÜT9)+¹/ø#¢hòZ~î¹çd¹¡¡A°%ÇóþÏ½	dýÝ»we¹¯¯Oe,¯òêÕ«² gÉÊÍëñþÑúÒ½òÊ+²`µZýoöîÝ»eY¾áâÅ²päÈÇ>Ï7üðddyçÎê¬9yúôéãÇËÂáÃ¹ø#¢¨ÄÉdå'ùÌÿ5eÄ<~ò¡":ëÖ­[,..VÛLh^¹ô/¢nRzzºÿe³²²ÔÍ¨ÉÂâÅï5êÈù«³ä-]ºT~hjjj^^ä^DDà¢Á³,r2T­ììl1Ð£Gä«ÑhT:uê|ÿ¦MÞï=ïËú÷ÉéÐMy¿½§àªwþ4áÔ7xO?"2üåääx¿óçyMGBG³JKK=ó­RRR|Ò4ñ§ÏMÊÈÈð¿ìÂÕeC2SþÊÃÃÃYYYkÖ¬ò>xð?"JüÞÔÔ­Ïá¦ÐÐÐz¿P¾z¶ÆP¼sç4ñ§¾úê«²ðÌ3Ïø_öé§å^z©··×ó¹Ã¹ÃßÞ½å¬[·n©M^<C$"Deøs»ÝÂ¬Ô'ÕÔÔLGBÒ®]»¼·®b4ÓÓÓ÷ïß?Müé_¤­­MÎ]½zµgïïq¹2¹ÍIII6l¸ÿþÜáOx*ë×®]«N®Y³FNÚívîHDþüø#""""ðGDDDþüøÿnÞ¼ÙßßÿÿñÿÁ1TòÉ'³Ø%jrrÒáp0!L¸~ñ_0¡jhhèç?ÿ9ãªÆÆÆîÝ»Ç8°?üü¬ïïâ¿¸[Ü¹sç§?ý)PõàÁú§bBÕÿ÷_½zqaü½Â>øàý×eBHúwÞaBØ+Wþçþü?ðþÀø#ðþÀøþÀø#ðþÀøþÀø#ðþÀøþÀø#ðþÀøàü?ðþü?ðþÀ?ðþü?ðþÀ?ðþü?ðþÀ?ðþÀ?ðþÀø#ðþÀ?ðþÀø#ðþÀ?ðþÀø#ðþÀøàü?ðþÀøàü?ðþÀø#ðþÀøþÀø#ðþÀøþÀø#ðþÀ_â¯««kÅaùòå===àÀøþÀøeü-]ºTÝÏ;ç¿ÖÖÖÑèïöíÛÿò/ÿ2J!J$ÝÙÙÉ8ªÁãÂÞ~ûíû÷ï3¡êý÷ß?¡Põ³ýìæÍCüýçþghãÏ»ü?~ü­èï?øÁµk×Þ¢õÆo¼þúëC¨joo¿|ù2ãÂÓ×¯_gBUë>Ê]qaóòøëîî®¬¬dÚöeÚi_bÚi_¦cyÚW5::ZQQár¹À?ðþü?ðãø°Z­òrîø#ðþÀ?ðþbííí¥¥¥CCCç?àüøà/¦ðg2xþü?ðGàü¿XÆ~àÀøþÀøàü?ðþÀ?ðþÀø#ðþÀ?ðþÀø#ðþÀ?ðþÀø#ðþÀø#ðþÀøþÀø#ðþÀøþÀø#ðþÀøþÀøàü?ðþÀøàü?ðÇÃü?ðGàü?ðþü?ðGàü?ðþü?ðGàü?ðþü?ðþü?ðþÀ?ðþü?ðþÀ?ðþü?ðþÀ?ðþÀ?ðþÀø#ðþÀ?ðþÀø#ðþÀ?ðþÀø#ðþÀ?ðþÀø#ðþÀøàü?ðþü?ðþÀ?ðþü?ðþÀ?ðþü?ðþÀ?ðþÀ?ðþÀø#ðþÀ?ðþÀø#ðþÀ?ðþÀø#ðþÀøàü?ðþðþÀ?ðþÀø#ðþÀ?ðþÀø#ðþÀ?ðþÀø#ðþÀøàü?ðþü?ðþÀ?ðþü?ðþÀ?ðþü?ðþÀ?ðþÀ?ðþÀø#ðþÀ?ðþÀø#ðþÀ?ðþÀø#ðþÀ?ðþÀøàü?ðGàü?ðþü?ðGàü?ðþü?ðGàü?ðþü?ðþÀøàü?àü?ðGàü?àü?ðGàü?àü?ðGàü?ðþÀøàü?ðþÀ?ðþÀø#ðþÀ?ðþÀø#ðþÀ?ðþÀø#ðþÀø#ðþÀøþÀø#ðþÀøþÀø#ðþÀøþÀø_###àü?ðGàü?qüÙíögvñâÅë×¯àü?àüQlâOní3g|>-11Ñét?ðþÀ?ðþ(vðçv»¶lÙ¶À¯çü?ðþü?ðG±?ÃQ]]m2üÍ¸mÛ¶æææññqðþÀø#ðþÀE1þFFFÌfó­rsskkkåGæ?ðþÀøàü¿iåv»ÛÚÚ,Krr²¿ù233m6Ýnð!àü?ðþÀøS¤?½[VV&¢´é]ðþÀøþÀøYúÓ»ùùùµµµ¶1/øàü?àüÍ8yA×ÞÞ'vðþÀøàü?ð÷ËNgmmm~~~ éÝ¦¦¦hÞàü?ðGàü?íÄs¢:±/f¦wÁøàüøàÏ7y~¶Ùl±7½þÀøàÀø¿LgzW2ÍÑ>½þÀøàÀøwüéOïL¦êêjÃÛCþÀøàü?ðûøÓÞMNN¶X,mmmn·;ü?ðþÀøà/fñ§¿£>Y/çÊ÷ÄÕ?ðþÀøàüÅþôÃ'Ó»àü?ðþü¿ØÇÎqØâmzü?ðþÀ?ð³øò8lq8½þÀøàÀø5üéOïÆêúÀøàüøq¿¾¾¾@Ó»1s6ðþÀø#ðþÀ_¼ã/®ÃþÀøþÀø_üÅáqØÀ_¤444d2À?ðþüç%é[ßúæqØÞaz,((;ø#ðþÀ¿¹kÊã°Ék.Ó»à/mØ°ÁápèàïÕW_½ý½óÎ;ï¾ûî]Qï¿ÿþ[o½Å8ª¾¾>yæbBØo¼ñÏÿüÏC¨êêêêîîffÝ?þã?îØ±ã3ù¿ù6nÜØØØøoÿöoÔ¬»|ùòOúÓ0ÿÐ(Æß/ob`ü=ö'ÑßÛo¿ýÃþð'¢äeàúõëC¨²Ûí¯½öãÂ®]»&ï1¡êæÍÃLçÉ¿üË¿üüç?¯¹£¾ßýÝßý«¿ú«ÎÎN*øäïçðÿÐXÆÓ¾Ä´/Ó¾Lû2íË´ïNÅhzwß¾¯¼ò£Ä´/øàüøÑÝÏþóºº:Í-9¼Ã6»cûøàü?à/RÌW__¿víZÍcrÍfã°?ðþÀøàü¿¨¬³³Ójµ¦¥¥ù/;;»ººÚáphbü¿ÈüøàÀOýýýµµµË-ÓÞÝ¹sg»ÛítqðþÀøàü?ð766hnÉQTTT__?888åõ?ðþÀøàü¿®···ººÚd2ùÏh4VUUÙíöé_øàü?ðþÀøÄÔEEE¡=øàü?ðþÀø Äs-[¶hn½[[[ÌqØ4ñçr¹ÚÛÛ[ZZúûû¹Ë?ðþÀø#ðþÂQgg§ÍfËÎÎÖßQ_?ÅÍÍÍÅÅÅ¿÷¿ö×ý×ÜëÀøàüøsÕÈÈHccãÚµk5Ã¦¦w½wÔZüÙíö~cç7çüÛ³ÉdrÇàü?ðGàü2·ÛÝÒÒb±X4wÔ·xñâ¾¾¾ÿüÙl¶%?õoËæ-²;øàü?à/49êêj£ÑhzWG¡Å_YYÙ[þÜ¶oÙ>ÿùÏsÇàü?ðGàüÕÈÈHCCÙlÖÞþúB?ÍVú¿K½ñ÷§[ÿwþÀøàüø³Ìív·µµY,Í­wg±£¾ÐâOó3/^äþÀøàÀøYjzWsçÌÁì¨/´øüéÖ¾EEE«V­ÊÈÈøîw¿Ë½ü?ðþÀ?ð7Ýô§wóóóÜQ_Èñ÷øÓýü	bæbðþÀ?ðþ(ñ§?½i³Ù"á¹#|?ðþÀøàüÓé¬­­ÍÍÍ¨é]ðþfuÉ©JHHàü?ñ?ñ¨Nl'ÂÀé]ðþfSÂTðþÀø#ðoø§ ÍÉÓ»àüEeàÀø9øSÓ»ùùù³a%%%5½þÀ_è~ægÀøàÀ_lãOz×d2UWW;hOðþ¦Ü¹ùàüøüéLïªã°µµµÍÝqØÀøOü-_¾§ä.ü?ðþüÅþG:Ó»f³¹¡¡add$JÇü¿i$w÷¡¡¡ììlYó½úê«²PYY	þÀøþbjGiii10½þÀ_pWñ$YíÉÂÝ»w'''e!55ü?ðþüE;þzöYÍõEïô.øA!y:¿xdáðáÃj]½?ðþüE/þWcccIIæÑ>½þÀ_Píß¿ß³yÏü?ðþüEþÔqØ¬V«æô®Ñh¬ªªºûv¬'øÓíèÑ£.®®.YÏõMþÀø£âOç8lRYY¼GþúÀøþæ%ðGàü?.ëüùó[¶lÑ9Ûàà`'øàü?ðþÀ_ÌâÏn·WUUFÍéÝ?øó¿¼¼<µÃvòþÀø#ðáøSÓ»/4½ÛÔÔ4×»ªà/ºñ·téRoðybk_ðþÀ¿ÈÁÚ#Ðônnnnï?ðTâ<yØtwwONNó¦?àüÑtðg·Û÷ìÙ£y6YiµZ;;;cfGàüêÓaø#ðþÀéãÏår9s¦¨¨Hszwýúõq;½þÀ_PõôôÈ£h÷îÝ£££àü?ðGào~ñ§¦w-77÷ùçïïïgèÀøªEù?ÀØàü?ðGà/øs8ÕÕÕ&Éÿ%I h³ÙÞà/4ø[²d	|?ðþüÍþnÝºÕÐÐ`65§w-[V[[ët:+ðþB?õè²Ûía¾éàÀøñÞýã?þcÿ©õíÙ³Gx«ü¿Ðã/++>ÀøþÂÎônbb¢ÚQ_Ìü¿ùÄ_»<ÞöïßæGø#ðþÀ_22¢3½«ÃÆô.øáÀß±ÁøàÀ_HµÕjMNNö­IOO·X,<êÁø+þÄàü?Á¤Ã¯3½ûþûïÏúØ¾þÀ_þü?ðêÄvÇaóÞÅ±	ü¿`3LK,éííàü?Á$^Íè8lrÿ£ü?ð7ø3ò°ÿMþÀøt¦w%³ÙÜÐÐ022¢yYðþÀß<àïúõëòà¬©©ç_À?ðþ¢:ýé]ÉT]]íp8ô¯ü?ð7øck_ðþÀ¿%¤4½l±XÚÚÚ¦¹sfðþÀß<à­Áøþ¦þúô§wÁø¿ùüøà/*RÇa³X,;êæô.øàü?àüEz:Çaéô.øà/²ð711±aÃy<§¦¦nÞ¼9[~?àüEfÓ9ÛL§wÁø¿±±1Í÷êüøà/¢ÒÞ´£>ðþÀ_ôá¯  @Õååå£££rrxxxÓ¦M²fåÊàü?ðGñ?§Ó)ÏÉÓ»ã°ÍÑ;àü¿yÀ_RR<¼½?´111!kd=øàüQãoFÇa£Àøó¿yø¼d»zàüQ¬âoÇaàüÅþÔ´oii©ö¯²,kV¬XþÀøKøÓ9Ûï?ðþ"¢=Í>=zþÀøøÏýýßÿQQÑoüÆoÚQ___ß¼Ü6ðþÀß<àïñ~7oÞ _KKKeÍtðGàü¿¹NMï¦¥¥ÍÝúÀøQ¿y	üøàoîhzW?ò-Z$ß	7ü?ðþÀøàüÍ2·Û-/TEó­>1Ùl®ü¿=5×ñ?ðþ"¦*!!ü?ðþ(*ð×ÛÛ[WW·xñbÍ-9zÛ·oóúÎ!õ////Bhàü¿ðá/!pàü?ðGQ¿æææ@Çaõ.â¿èaü«ÚWÊ´/ø£¸Ã_ öíÛ§5Î;þÀøø»û¶ÕjÕ9ÃáPßév»¿¼æËê?_A~BÆü?ð7øëéé¿å£¤¤ÄÏàü?ðG?u6£Ñ¨ùV_YY¼Pùï¨oppð÷ÿ÷ë·~+??_çwîÜér¹Àø£xÇßÖ­[ÕsGØ^'À?ðþ¦x®±±Ql§ùVß²eËêêê<oõJxûöííàüÍ'þ.^¼¨>ÊËËÃyÓÁ?ðþ¦¬³³sÏ=ÙÙÙþæ3UUUòD:¿;êàüEþÆÆÆV®¶í¸~ýzo:ø#ðþÀ_ Ng]]]nn®æôî-[4§wÁ?ð§×ñãÇÕÈöíÛçå¦?àüùäv»Åvþæ[¼xqMMMooo,'øà/|øc?àü?üÙíö@[r¨­w£zü?ð7ÿøK*ÁþÀø4§øêúúúeËiþ^TT$çÄðx?ðþÂ¿yüøq?·ÛÝÖÖf±X4·ÞÍÍÍ­®®±é]ðþÀøþÀ_<âÏápìL&æqØÊÊÊcrzü?ðþÀ?ðGøihht¶U«VÕ××Æáx?ðþÀøàüÅþô§w333+++§Ü93ø#ðþÀøþ"SNï655EÎ1ÖÀø#ðþÀø4üéOïæçç×ÖÖ:Nü?ð7øc?àü?!þä	Mgz×f³qÇàüÍ3þ¼wé§?öóþÀø£)ñçt:kkkóóóu¦wcã8làü¿¨Ç§ÖÖVyÚ±cÇèè¨¯6m57nÜàü?ÒÄxNT'¶Ó<Ó»àü¿Æ:÷n¥&&&dMvvv×ÜÓÓc0¯_¿þü¿èïþîï¾ùÍofff2½þÀøVü©ç,þÿÌ_EEÅK/½$'NØ¾;ø#ðþ¢7é]Él67662½þÀøüeeeÉ3@mllLNºòòrY#ë¼f£Ñ899©4i2üñ×ÐÐp+úóÍ7oÜ¸qBÔøÃk×®1¡êÝwßg.ÆaÖuuu;vìþà4?-ÏñqñâEjÖµ··¿õÖ[C¨êììlmmeBØåËÃÿCçÍ?dßï½ ¯ÙÿÍGòéþ~ô£ÉX9)DõööÞ¼yqU?ûÙÏ^í5Æa]½zõßøÆg>óÿ§Ç¤¤¤?ù?9þ|?d===òRÇ8ª?üP<Í80ùûùþýûaþ¡s?éîÝ»K.MII?mSSSW¬XñðáÃà¯ÖâX+ö%¦öüôwÔ'ë¿ýíoüñÇÓ¾Lû2íÅÓ¾sWVVú(¡|õDþÀ_ä¤6ÉT]]­Ã6»cûøà/.ð'O£gÏùZQQþü¿Lç8lAyzïü?ðþbÂ¼¼¼ÔÔT5QuáÂà¯V"³³³å:srrºººÀ?ð9M9½+çÊ÷h>³?ðþÀ_tãOíäÙûnjùÄszÓÁ?ðþô§w§³£>ðþÀøzüeggËSÆ¿®®.YNOOàü¿©¯¯OgzwýúõÍÍÍÞÓ»àü?zÛ;yVÛü?ðMç8lÓ¿BðþÀøzü©<«wûP[·?ðþÀ_ô&w$Í¦y¶´´´Yü?ðþ¢íííwëø#ðþæ"ýã°455inÉþÀø£xÁz¹-..V[û¦¤¤äååõõõÍõMþÀ_rzWsúûûÿAàü?ðøÀ?ð¦Þmooáàü¿¨Çg;O>ÌËË[¼x1øàüElSNïÚQøàÀ/þ&&&ØÚü?ð©õmÙ²EszWþj­®®Éô.øàbò¹@·ðþÀøúúúþyÍç®´´4ÅÚé]ðþÀÅþîÝ»ð$Ï±=¼ù]¼xü?ðþæ=µ%GIIæ[EEEs4½þÀø£Ç±:í+Ôë^ðGàüÍ"»Ý^YYè8lòá¿Uàü?ðõø¯À?ð§Y½æeeeMMMãããóuó¼ñ×ÞÞ^WW×ØØ8§2þÀ_ñ711êY³páÂçü?ðþÂxNdmÉ¡Ãæt:çýv*ü¹/ùË-úêW¿ºzõêßüÍß¼ví÷7ðþÀ_tàO¼|6øUOµ555àü?ðä¼ªªJsGjz7¢î_ÿú×V|öà¡ï_ßñõ´´´#Àøó¿¤¤$yõþèLOO¬IOOàü¿¹ËétÖ××çææFæô®>þ>ûÙÏVí«RòSÿVþ¯çÏç.þÀøü©~'''ízývþþÀø!ÉívËé¶mÛ"|zWü±ÑhôüûÊW¾"·»øà/ð#O¸÷îUa:tHÖdgg?ðþÀ_³ÛíUUU&Éß|ÉÉÉV«5*þëÕ;ß´~Ó¹_Èmnnæ.þÀøüuwwkîä¹««ü?ðþopp°¾¾Þl6k¾Õ'ëÃ¼£¾àO~#ùËYùïÀ·|eíW¾ô¥/Eà$5øþ´(,,LIIIHHHMM-((5sÓÁ¿ØÆÛínjjÚ¶mæúL&SuuµÃáº!õìê¥±±Ñø¤¯~õ«ò¢Ëýü?ð5øÀ¿XÅN`­9½k±XÚÚÚQ:¤>;y×ZÞðàü?ðGà/ñ722ÒÐÐ`65?IuÓ»ÓÄ?ðþ¢ÂµgµoVVÖÀøào:¹Ýî¶¶6ÅKÓ»àü?Yüµ¶¶z¦þÔò'ÀøàO'5½«¹õnbbb´Oï?ðþ(fñ§>#ñà¯««<?ðþ¥?½ù;êàüQ¼ãO=_?öÚ±óää¤,ðþÀøó¤?½ÇaàüøÓ.++KíÕOáobbâÀê:àü?ð÷øÉqØjkk£î8làü?Úµ··kÎÝÌõ½üøpüçDub»(=øàÀÞËmqq±ÚÚ7%%%//¯¯¯o®o:ø#ð±øÿÍÏÓ»àü?zÌ~þÀø±?5½¯9PRRWÓ»àü?àü¿ØÄþônìí¨ü?ðGàïñG´dÉ¤¤$y¢OII),,àüÅ<þt¦wcà8làü?ÚÉ»æOoo/øà/&ñ'/gÎ	4½3ÇaàüøÓNíä¹¢¢ÂårÉÉÑÑÑíÛ·ËðþÀ_,áÏívwvvZ,¦wÁø×øSÏûÞ3;²ìÙá3øà/ð×××÷üóÏké]ðþÀÅþÔ;5ccc¼óþÀ_är¹þáþáK_úÓ»àü?¿Jæ¯¢¢BÌ''=z´víZ>óþÀ_Tg·Û+++ÓÒÒüÍg4å,ü?ðGñ¿S5Gó¿àÀ_Ès:uuuÇa6nÜØØØÈ[àü?wü%LÁ`àüEr.KThGûÜçêëëûûû(ðþÀ¿ùüø¾ÎÎÎªª*Íõ¥¥¥Y­ÖýèG/_f Àøþ~U ý9?ðþ"3u¶eËÚÃs¶ÙÛÀøËø£Gú¬Ü¹s'»zà/Òr»Ý¢ºmÛ¶iNïæççËÃJú½/þÀøþ|äÉËFVVÖÃåäË/¿¬^Hæè£~àÀß,ºûvuuµæúÔÖ»Màü?ðGàO£;wªWµ°iÓ¦¹¾éàÀß444ÍfÍéÝµk×9sF'Pàü?ðGàO»7nx^QN<þüÊív·µµY,äädóåææÖÔÔ8ÎéøàüøÓh×®]êEEíCÚ±cøà/ü9@Ó»Á7¶··Ïè8làü?ðGàÏ7Á ¯+ééé·nÝzìõ¿¤¤$ðþÀ_xÒÞ-**s¶äàüø³½<è³Òjµ²µ/øsþônvvvUUÃáæG?ðþÀ?ßíçO^ÁøsÎônbbbYYgGAþÀøþ"%ðGq?ýéÝüüüÚÚÚinÉþÀø#ðþfxÉ¼'võO?ðþL~)«Õª9½i³Ùæè·àü?àü¿ð¥Ã?×Ó»àü?àü?ð7oçDub»@Çaùô.øàÀøàüÍCrãm6[fff§wÁøþÀøà/|éLïJf³¹¡¡add$ü7ü?ðþü?ðþBþô®Édª®®rGàü?àü?ð7ÿ	éMï&''[,¶¶¶ü?ðGàüÍ9þôàüù§¿£¾yÞàüøS0Uü?ð§Ò?[$Lï?ðþü¿HüQTàOç8l5½þÀø#ðþÀø³o:Ça¨é]ðþÀ?ðþÀøqúÓ»ó²£>ðþÀ?ðþâ---ßùÎwöïßßÜÜþæ.§Ó)÷IÍéÝðü?ðGàü¿xÇPcãÆ¿ó;¿³þÿ¬ÿ£?ú£ÏîsåååÑèHÆ_äü?ðGàü¿xÇßsÏ=÷/|áÀ·úÎ!ù'_üâkjjÀ_H´ã°?ðþü?ðïø[µjÕ×w|]ÉOýûÆÎo,_¶üÎqØ¢zzü?ðGàü¿¨Ç_QQÑ3ÿïoüÉIQøEÓ9[___ì'øàüøQ?«Õúµ_ñÆßÚ/¯ý³?û3ð7£t¦w£bGàü?àüÅþN§Ñh,))Ù_µÿÀ·þïRLL¾;5ø	4½Çaàüøà/Þñ'õ÷÷ù222JKK#ób¿ñññh<øàÀøà/5þúúúí¨/¦wÁøþÀøq¿úúz³Ù¬¹%GüLï?ðþü?ðþbn·[î?[rÆªªª¸ÞàüøàüÅ&þÔúMïnÜ¸±©©)§wÁøþÀø1¿õë×kNï®ZµêäÉñ<½þÀø#ðþÀøüuvvêl½[UUó»¿àüøàüÅ>þôÃ¶mÛ¶ææf¦wÁøþÀ_d544d2ÀM¿û÷ïÿûßß²eæônQQQ½¸àüøøYPP /Øà¦Ýn·ÙlýìgýÍ¶sçÎÎÎNÞêàüøÛþ¾ÿýï·E¯¿þzKKKÍ¶W_õ[ßúÖ%KüÍ¿oß>á5ë._¾Ì °×^­µµqà)42;§ÜEhbüýò&Æßo¼ñèïÇ?þ±÷4Ã>ùä!ÝöíÛ5·äøíßþíêêê¾¾>*È3ãÂnÜ¸ñàÁÆ!TÝ¹s§··qUC»råÊØØXh,ãißøL¬,°ÓÜQ_bbbEEÅßþíß3PLû2íË´/1íË´oàÏó*þÈ'µ£>³Ù¼@«¢¢"9wpppÖÇö%ðþÀ?ð)ñÛínkkÓÙQÍf»û¶çûÁøàÀøà/*ÓÞ-++ÓÜQøàüøÑ¿@¿XMz7??¿¶¶VgGàü?ðGàü?ðÉh éÝÌÌLÍ6Õ?ðþÀ?ðþÀ_D§¶²²²¦¦¦éo½þÀøþÀøxNT'¶Ó<ÛÓ»àü?àü?ð	Îl6[fff0Ó»àü?àü?ðÑéLïJf³¹±±1ø3?ðþÀ?ðþÀß|¦?½k2ª««G¨~øàüøàüÍO:Ó»ÉÉÉ¥­­ÍGàü?àü?ðMéï¨OÖË¹ò=sôÓÁøàÀøà/My¶ÐNï?ðþü?ðþÀßü¤s¶¹Þàüøàü¿°6åqØætzü?ðGàü?ðþÂþô®ÑhÜ³gÝnÛ[àü?ðÇ8?ðþÀßÔ××hz711qýúõgÎq¹#ÁøàÀøà/¨ôwÔ·lÙ²ºººþþþ¹µàü?ðGàü?ð7ËôwÔ·sçNù/¯é]ðþÀ?ðþÀøMSíüùóÁü?ðGàü?ðþæ³)§wkjj"gzü?ðGàü?ðþfþôneeeggg¤Mï?ðþü?ðþÀßÌÒÞMLLvmcc£<D×x?ðþÀ?ðþÀo2tEsz×d2ÕÔÔ£t<ÁøàÀøàï©õåææú/--Íjµó8làü?àü?ð7'766ÚÃl6×ÕÕÿ8làü?àü?ðÊÜnwee¥Ñhô7_fffUUUggg=ÌÀøàÀøq?§ÓyòäIÍéÝÄÄÄ²²²¦¦¦ÝQøàÀøàüM+·Ûþüù-[¶hNïæçç×ÖÖFïàü?àü?ð÷Ëd(öìÙ£¹£>Yi³ÙâÇCàü?ðGàü¿Åßàà`CCÃªU«âpzü?ðGàü?ð/øÏÉIl·Ó»àü?àü¿ØÇÃá¨®®t6«ÕÀøàÀøQ?y4<yRszWí¨¯¡¡!fvÔþÀø#ðþÀøSü·´´Þ5LÕÕÕøàüøà/ºñ§¦w5wÎ,´X,1p6ðþÀ?ðþÀ_¼ãOÍl63½þÀø#ðþÀøeüWl6æLï?ðþü?ðþbN§sß¾_øÂÞàüøàüÅ,þÄsÍÍÍeeeÇa[¶lYmm-Ó»àü?àü¿¨ÇÝn¯ªªÒÜCVÊYò¼ÕþÀø#ðþÀønüÉÃ©¾¾¾¨¨Èß|			ø(wx;øàÀøà/ñ×ÞÞ¾eËÍéÝU«V544¼ùæ³>¶/?ðþÀøàüEDr/^¬9½ûì³ÏPÔôî,íKàü?àü¿Èår566ø¿Õ¼~ýúææfô?ðþÀ?ðþÀ_ôáOÀaµZÓÒÒ4wÎ¨yAðþÀø#ðþÀøü9ÎÚÚÚüü|óeggÛl6»Ý®àü?ðGàü?ðøÿQÿé]Y³eË9klàü?àü¿èÀßÈÈ÷ÙêëëNç®ü?ðþü?ðþ¢Ree¥ÑhæØ»àü?ðGàü?ð5øÇËåæÀøàÀøà/jð|àü?ðGàü?ðþü?ðGàü?ðþü?ðGàü?ðþü?ðþü?ðþÀ?ðþü?ðþÀ?ðþü?ðþÀ?ðþÀøàü?ðþÀøþÀøàÀøþÀøàÀøþÀøàÀøàü?ðþÀø#ðþÀøþÀø#ðþÀøþÀø#ðþÀøþÀøþÀøàÀøþÀøàÀøþÀøàÀøþÀøàÀøàÀøàüøàÀøàüøàÀøàüøàü?ðþÀøþÀøàÀøþÀøàÀøþÀøàÀøàü?ðþÀøàü?àü?ðGàü?àü?ðGàü?à/2êêêZ±bÁ`X¾|yOOø#ðþÀ?ðþbK.UwÁsçÎåååùãï7Þþ~üãøác¢úûûåÏÆ!TòÉ'¯½öãÂnÜ¸ñïÿþïC¨úÉO~ÒÛÛË8*§ÓyóæMÆ!	þþë¿þ+Ì?4ñç]JJ?þþæoþæèïõ×_ÿÁ~ð¨ÖÖÖ«W¯2!L¹&¾víãªZÄ8*¹sòÚ._¾þøëîî®¬¬dÚöeÚi_bÚi_¦cyÚW5::ZQQár¹À?ðþü?ðkø[ðiêäÀÀÕjsÿïþÀø#ðþÀ_ÔãÏ»öööÒÒÒ¡¡!ÍsÁ?ðþü?ðSø3L¼þÀø#ðþÀ_,ãO?ðGàü?àü?ðþÀøàÀøàüøàÀøàüøàÀøàüøàüøàü?àüøàü?àüøàü?àü?ðþÀøàü?ðþÀøãáþÀø#ðþÀøþÀø#ðþÀøþÀø#ðþÀ_¼áï»ßýnSSSô×ÖÖ&/ý¢Þ÷ÝË/3¡Jþ29sæãÂ.`·ÛPõæo¾ýöÛC¨zÿý÷/^¼È8°Ó§OüñÇaþ¡###±¿;wî:tèDDDDäþÔèÞ%"""Àø#""""ðGDDDDàÀ?""""±Á`(,,¼~ýú¯ýx kÚÛÛÔ7wtt0z3RÿÔùfÅxvuu­X±BÖ,_¾Èè9ªÖÖVy`èÏññq«Õ´xñb9Ñ~HyUf<?øàââb¹CnÝºuxx8B^x®	G/½ô,8qbûöíß#ß çÊÑh¼wï,ÈWÉÄèÍtHýp:ãÏxN<.]ªþtîÜ¹¼¼<F/ÈñÜn·xüd<9rìØ±ÉÉI!Ë%K½àW¥`ÆsåÊò³,ôööîÚµ+B^x®	GòÈg"YÐ|äÜ½wÝºujY­dA¾òÌ5!õÀ)Çf4Þ¥¤¤0zÁçsÏ=÷â/¿gaa¡¼Ê2h!R^OÁàYÎÈÈ$kÂ÷ÿ½÷²§Í7www«åyAùêYIÓRÿrüiFãéINVVV2zAç½÷åÅüêñ~üøq9)Lyï½÷½àW¥`ÆsùòåwîÜ_~Y	/I<×#õa>URRÏ¹cõêÕ+W®T¶ÊãÍ=MsHýPüi¦ã©­¨¨p¹^ã)ûÝ¸qãñO3t!y¼>úñ§µbôR^Où$//OÜüâ/ª©HxIâ¹&eeeMLL<~ò¯,ûäÈù;u:@ÐtÔõÇf:ÌþX­Ö0tÁç_ÑþñÎShhW¥ _åUôQAAA¼$ñD,ËÙ³geA¾VTTø»fÍ[·nyNÊßUê-b»Ý.o1z3RÿÔéx¶··1n!Ï_=#¿Pç®]»^yåYõf³Ñ~HyUf<.]ÚÓÓ399ùâ/=z4B^x®	GÙÙÙ			999j«ï'ú¤¤$õÙOÏòè¿®ä«,3z3RÿÔüfõxL&Þ©áx¿ÐçðððæÍeMqq±Ãá`ôR^OùkyÑ¢EòB_YYév»#ä%ç"""¢8ü?""""DDDDþüø#""""ðGDDDDàÀ?""""DDDDþÀ?""""DDDDþüø#""""ðGDñÞË/¿¼fÍ'­[·îâÅ¿öüõ¤¨y¶Õºµ&I~5Ëå³^ÖÉÉÉ^'ø#¢¨ìÐ¡Cü:zôh,á¯¦¦FV<yÒg½¬?|øð,®üQôÕÓÓ#¬1§Nr?éôéÓrRVÞºu+fðw÷î]Y¹|ùrõ²¾¯¯üø#¢¸hÇÂcÇy¯|ádåÎ;½ésýúuÁ¸pÅ²ìùæGUVV¦§§ËYF£qïÞ½Þ³«mmm,9K.ÛÒÒâÃ)Y±fÍ«W¯ÊIÅâsÃ®¢=ê,¹Iê¬ÖÖÖ@P+--õ]]]5ï¼ó¬)))ñ¬9räHvv¶ÊÖ­[üñçý>ktn*?"¢ynÑ¢E÷îy¯¼ÿ¾¬4LÞ¸ñ©»»[»iÓ&³vïÞ­Î²Ûí			R'Õ¹7o>&%%¹Ýn9W¾RSSÕGñt®G4oÿozîÜ9oÑz|ùÒK/yäçs%ëÖ­)þtn*?"¢ùOÍðj<g-X óÆÍöíÛG$r²¼¼«¬£ø¨ÞÀ´©³***Ô[²ÜÑÑ¡®Äû:<(¼S³®O?ý´¬¹té,ËWY~ê©§¦¼£oð¾m¿ráÂòûÉIù*·911¡¾!''G.(¿ÂãO§SRRf?JDà("ð'iâÏ³^áFIÂ&urÅrrÉ%Ï<ó mllÌs%ò=>ï¥y.¥NÞ¿ßóÍ&ÏÌ¯|å¶¶¶)¯G )'>|èÛ>oß¾rÖ/¾øøÓ©í½÷z@Pð'$-..VoLÎ:7ÀÑü§Þîõ^ér¹d¥¥ÃòÇ:êùÏÆúpÊ+²l4ÆÇÇåÊ322<çNy=(æÓ;wSeY¾Ê²Ýn÷ÛÑÑ!7@súxúøÓ¹©Dþæ?õ¹·ãÇ¯T;@ñÙàÃó¹À¡¡!ÿ7´nÝºUSS£&a=jToËyæU§$ÚîÝ»Õl¯|­¬¬ô¬×¹õNÛÔÉêo»zõj9WíÝFÌêÚòWÎjmmÖÇ¦j4<çêÜT"DDóÚ`Â`08qBíêåÔ©SIIIþgtÆÇÇÕ¬gË?õQ9ÃáýaAeÁ½÷Ôv¸­hMm~«~z»g½Îõ¨ù©Ïü¹õ:ø;ú´ç=9ù½ÏR´ÛíòSdáOqS¸,ß&èÜºu«÷¹:7ÀQD¤vìÓ#G~õüõ$e#Ï²gÂÔ³§§~Ú[Þ]¸pACÖFïaëáÃ<[ÚúeÇÆÆRRRÔ¯à3Ù½aÃï±páBùªööâêIOªsun*?"¢HI²nÝº¤'­Y³Fmrë¿ÖÖVµ;½ÂÂÂ7nxÎu¹ÈÎÎV`Ú·oßøø¸çÜââb1YNNNCCÏuúß¹*Y¿k×.õ®G#7IíPg?*++öù¢ª¨¨HOOß¢¯¯Ï³ÛïëÝª±*))éêêòù:7À?""""DDDDþüø#""""ðGDDDDàÀ?""""DDDDàÀ?""""DDDDþüQ¸úÿÇ-ôxfY IEND®B`


õÛßþ¶ÿ¨áÙßdZø<8/^ÿÿóÖ¨Nqx/_¾¼hÑ"ù1å=éþ#åËmÛ¶)ß®ªªª··÷µ^½zµ¼¼M~ÖÖV^/DàH¿øâµ»þSö2+Çkû|>ñÅÅÅ<CüÅaÊ¿ ^yåFÀQæñxöïß¿páBeß®,¼ôÒK?""""DDDDà! """DDDDþüø#""""ðGDDDDàÀ?""""DDDDþü?"¢tñâEÉd0V­ZíÙd3»ÎTn;ÅFFFvîÜ-°páÂx<ÝÏç;*Ë<»üî2Pô±¢üóOvóæÍ3gÎÈÝ®Y³ff÷vùòå¤_wåÊ]DþH[¹û-o®öáÃå~Î??'vëÖ­roÊßÛ¶mãÙEDàôH«@H8p +++##ãÐ¡CAWûö·¿]^^î_ÙÝÝ­¬ô¯Y»v­¬õ·oß®ªªJOO7K.íèèy?ß7òM^õUÉTYYyÿþ5þÆÆÆ,ÜVöþýû§õfii©Ü×ëyiÑèììd4/^¼¨¬Pö?zôHþÎÉÉá	FDàtê?eùå_åÖÖVÁ,¼òÊ+×¹zõêÈÈHàmEi²þæÍ²ìv»eYÖÈò²eËr¬ÌÏÏy?ß:òMto¼ñ,X­VõÃÞµk,Ë. rmpS¦èÈ£qüøq§Ó)B@eWW|)å7Ê²¬á	FDàô?q,O¦ú5EEx<ù!,:uJ¹èúõë,//ÉÉÉ!ï'do¢<¤¬¬,õmóòò=11!Sÿñ#à/òh<zôHYPÖïÙ³GùRªîÝ»'?"Ò/þ%Èp	¶FcFFÆäïÜÜ¬?yò¤ýúõ~øaàmî'ðË©Ü$ÜC)~¼§ÀqüÊú±±±È.÷­è,ßZâñähY^¸p!O0"D¤_üåçç~Öåÿ-ÂÑGV¯^íßß*¥¦¦ÊMoâHÙÙÙêÛæää(·ÁðàA¹íÔMe4üË~³uýúucDþH§øS=uê²?÷G?úÑñ722¢|B&ûÆPØ400 w5EüE¾í§?ý©,ìØ±CÛmÛ¶Éò3gýó§ØÃEu/¾û¶ðQùrÃSÿ²råcÇùïY®b""ðGDºÃÏçfeLÖØØò:êvîÜ©ßÄ¿æòåË¹¹¹YYYû÷ï"þ"ßÄápÈ¥Ë/÷qxÇ³gÏyÌ©©©kÖ¬¹sçÎ´F@îSø(7¿:¤ÌçÊhøKJJdáÆþ«¹YSZZÊsÀ?""""DDDDþÀ?""""óÓ?üÃ?Üºu+ÊßôóÏ?ÿÏÿüO7uçÎÿú¯ÿb4êöíÛÊGHnÞ¼éõzr»ÝÿýßÿÍ8hÔ'|ò?ÿó?FüñÇàoÎúá(þò7ýðÃÿõ_ÿ§²FðÁ÷îÝc4ê½÷Þû÷ÿwÆA£þþïÿÿj×ßþíßò_íºzõ*¶Ö®Ë/ÿïÿþ/øþÀø#ðþÀøàü?ðGàü?ðþÀ?ðþÀøàü?àüøàü?àüøàü?àüøàü?ðGàü?ðþÀøàÀøþÀøàÀøþÀ?ðGàü?àÀøþÀøàÀøþÀøàÀøþÀøàüøþÀøàüøàÀøCüõöö.]ºÔ`0öõõ?ðGàü?à/ñ·xñby»³gÏªñ×ÞÞ>ÝD'|òÉiSOOÏgÆ8hügIþëÂ8hTggççÎ8hÃáø·û7ÆA£Þ~ûíÿøÿ`4Jð'ÿ3ò7aü®ÆßñãÇßnòß#Ù½KÚ$ ùÿ=ã QW®µë­·Þêèè`4JÞ>ÿîïþqÐnxå/F]ºt)úß4ðwíÚµíÛ·³ÛÝ¾Än_vû²ÛØíËnßxÞí«466V[[ëñxÀø#ðþÀ?ðçø»÷®Õj	ðþü?ðGàüÅþ:;;W¯^=22òRðþü?ðGàüÅþL&SR@àüøàÀøgüEü?àüøàü?àüøàü?ðþÀø#ðþÀøàÀøàü?ðþÀ?ðþü?ðþÀ?ðþü?àÀøþÀ?ðþü?ðþÀ?ðþü?ðþÀ?ðþü?ðþÀø#ðþü?ðþÀø#ðþÀ?ðþÀø#ðþÀ?ðþÀø#ðþÀ?ðþÀøþÀ?ðþÀøþÀø#ðþÀøþÀø#ðþü?àüøþÀø#ðþü?àüøàü?àüøàü?ðGàüøàü?ðGàü?àü?ðGàü?àü?ðGàü?àü?ðþü?JüïÛ·ottü?ðþÀ?ðGñ¿þþþuëÖ¥¥¥%%%	Àøàüøøóù|mmmeeeIL&YþÀøàÀøñ?ÙÊµ´´Íæ¤ß,--ÍjµÎË_ðþü?ðGàüÍ»ÆÆÆ±OÖÈú[·nÍ×àÀøþÀßv»­Vkfffû;6_Çy?ðGàü?àoTWW§¤¤±¯¢¢BÈ5/3üÀø#ðþÀ?ð7Çy½Þ¶¶¶%K$©zá¢oðþü?ðGàüiÒððð±cÇFcù233wïÞív»uøÁø#ðþÀ?ð7ívÂ;å	æbøþÀø#ðþæ&y¯¯©©QOì[²dÉéÓ§½^¯Îü?àüøOÎçóÙl¶¢¢"õÄ¾êêêÎÎNÏþÀ?ðþü¿Y5::*oñ&)È|)))V«ÕétÆÖð?ðGàü?à/tn·»®®N¢f ¼éÇâð?ðGàü?à/¸pû*++m6[¬ìáàÀøþÀ_¤"<õ^± ÞÎØþÀ?ðGàü¿nb_ZZÕjÕçûÀø#ðþü?ð7í"OìÓóûÀø#ðþü?ð7ÂMì+**õàüøàÀøûU0±ü?àüø_Lìkjj2Íq?±ü?àüøKhü%ÚÄ>ðþü?ðGà/Añ'oÍ%Ñ&ö?ðGàü?¿pû¤ªªª¸ØþÀ?ðþü%þ!'ö¹^ðþü?ðGà/Nðçv»wïÞ­Ø'kmbøþÀø#ðÏøëììpÆ>¯×Ë¨?ðGàü?ñ?»Ý¾dÉõÄ¾êêêDØþÀ?ðþüÅþ¼^ïéÓ§Ô'jÞºu+ìàÀøþâÃÃÃòÄ>Y³oß>çàÀøþâýýýV«5333Gåxðþü?ðþÀ_àOÖTUU©'ö%æÁøàüøq¿ÑÑÑcÇ©oJJÊ¦MdÍX?ðGàüøñ?·Û]__¯>c_ZZZ]]ûÀøàüøqRÿªU«Ôgì+((÷Y6àü?ðþü¿8©³³sÝºujöUTT0±ü?ðþÀ?ð'êÄv!ÏØ'7VØþÀøàÀø=j2ØñýïÿÖ­[øàü?à/N¶®V«5---Á¦¦¦7ß|sº¿ÛÀø#ðþÀøºËçóÙíögì[¹r¥bß´~·/?ðGàüøºktt´µµUÆ>é^èéé	¼2øàü?ðGàüÅjCCCòþ¨>c¬Ù½·ÛíVßü?ðþÀø#ðþbrjµZÕ§n1Í­­­~/øàü?ðGàüÅL>ÏápØ'+ívûOÝþÀøàüø1P¸)))eêoàü?ðþÀ?ð§ë"Lì«««9±ü?ðþÀø#ðþbr;rbÉd·ÅûÀøàü?à/f0±¯¨¨h¿ü?ðþÀø#ðþô×ëÛ<cXpNÞÁøàü?àoþ7±/--Íjµº¹úFàü?ðþÀ?ð7	ìBNìÎxbøàü?ðGàüé«ûÌf³Ífóz½Z|_ðþÀøàÀø6ûÎ;nbp6Çs?ðþÀøþÀ^òx<---ê5[­VÙ*Fá1?ðþÀøþÀæ9Îººº'jnhhîÁøàüøàO§ÉWuuµúx³ÙÜÔÔ4çÇs?ðþÀøþÀß<äóùìvEEzb_YYÙ,OÔþÀøàÀøzÉëõ¶¶¶<£¦¦Fëã9Àøàü?à/J¹ÝîûöL¦'jÔÃàü?ðþü¿ÙÖßßo±XÔûrssôóPÁøàü?àoæ;Q³¬x<½=`ðþÀøàÀøvÊÕÇs¤¤¤lÝºµ¿¿_·ü?ðþÀø#ðþ¦Ñ­[·êëëC±OÖË¥:^ðþÀøàÀøRN§Ójµª'ö;v,úgìà/ð722b2Àø#ðþÀ®Rÿ¦MÔì«¬¬<wîÜüºü¿Æ_wwwqq±¼ÂáïoþæoîE·¤M¢ù7n0õî»ï~òÉ'FN>ûì3ÆA£ÚÛÛoÝº¥GrçÎÖÖÖ¥Kª'öÕÔÔ¼ùæ±8¼W®âi¦Q.]úüóÏ£üMckÖ¬q¹ðwòäÉ¢Ãáèêêú´éwÞÿ1õ³ýìÿñºzõêï½Ç8hè¤··w~Cggç;¾ò¯±/==Ó¦MÂ¾ØÞ·Þzëý÷ßçi¦Q/_þ7íÝ¾_<Äðøc·/»Ý¾ìöe·¯Ö¹Ýîºº:õñ&éèÑ£º:c»Ùíó»Áø#ðþhñ'ï2555êK,9wî×ëáàü?ðþü%4þ|>Íf«¬¬T¨¹ººº§§'¶çàü?ðþü¿Ð655Íæ¿×årÅåð?ðþÀøþ&öÉÛM¬±ü¿8Á_¸Àø#ðþÀßì·Å¢ØWTTd³Ùâl/øàü?ðGà/Añab_UUUôß_Àøü%=©äädðGàü?Òþsbøs¿ä'e0À?ðþH?ø7±OÖÄýÄ>ðþæóøþÀøb'öÅÍûÀøOü=|øpÇàÀø4øóù|v»½ªªâO¶-ýýý	ûÑiâÏd2æüøàt?&öEÃÃÃÏ=÷^^qQqzzz]]]"þªÿGëñxÀ?ðþ(Êøs»ÝLìþä^xýÜçÖ<ôCßÛó½EíÞ½!ü¥¦¦ÊjddÄh4Ê<~úÓÊÂöíÛÁ?ðþ(jø7±OÂÄ>-ð×ÖÖöõ¯]Øçÿ³ã»;233ùð/þñ§¼´dA´'7oÞðGàü?Ò'ö9,¢þ«¾Y?ù7<<Ì¨Æ9þ²³³åÖÝÝír¹dá¥^R8Õ?ðþHSüØÂÄ¾(à¯­­mÉ%òÛ½k·¨mÇ?þöïßï?¼#ðµWYY	þü?ðGZà/òÄ¾¡¡!-øóx<_ýêWkÖÔ(ò;XP,øÝï~!üIGÉÉÉÞÞ^YkýÐÁø#ðþ²¡°Z­LìÓþ$ËõÌ3Ï|ñ×¿ñoÆçDÁß¼þÀ?ð8ø7±¯¢¢B.bWã¼àïñäKÙz·µµõ÷÷ó¯þÀ?ðþh¶îÙ³çÙg9±opp!_üQâ¯°°P9á'y&ðþÀÍUá&ö¥¥¥ÕÕÕÉ¥øó¿ÅÏGûøàf¼57±¯µµ5?ð7ÏøçÉòÚµkÑ|èàüøqV3öýîïþ®ÍfcJøºÀ_nn®¼,£,?ðþü¿xjtt´µµ5äû,ËáÃÇÆÆ%ðþô¿¾¾>yîÚµ+Ê¯Lðþü¿8hhhH¶ç!'öíÞ½[Ø7­ßíKàüi?iáÂêè9àÀøù%rb_AAAcccàÁøúÂß¢E8àÀøàoù|>Ãrb_YYÙ¹sçÔûÀøúÂòu:Q~èàüø±U555mmmáçàüéyyyðAàü?ð!yî=ztÆgìàüéòÞ¿øþÀþØY­ÖÌÌÌ ö	&ö?ðþbIaâàü%r===555êã9*++§u¢fðþÀ¾ð&ø ðþÀ_æóùN>]QQ¡Ø·iÓ&yÏà>ÁøúÂß|þÀ?ð§«<OSSúx444ÌæWñ?ðþô?É´hÑ¢ÁÁAðGàü¿ÄÌåríÛ·O=±O (ý¯âàüéA^áÑèàüøó^OOÅb	9±o/øàO_øëèè×yccãÃ£yÂðþü¿yÌápðØ§±oÎ7Îàü?á£	ü?Jüy½ÞõÄ¾´´4«Õ:àü¿ÁGûøøçO½ÑhTÏ!äÙOìàüÅþæ+ðþü¿èÔßßoµZÓÒÒÔÇsØl¶(áü?ðþÀø#ðþ¢Ä'j®ªªr8su<øà/öð7>>¾fÍôôtÙ"dddlØ°!G~?ðGàüi×ëµÙleeeê5[­VËåÇþÀøÓþäò­w?ðGàüÍyÃÃÃ²u5L!'öMñWñ?ðGq¿ââbÙ.¬]»vllL¾|øðáúõëeÍ²eËÀ?ðþb%·Û]WWòDÍ­­­ÏþÀø1ü¥¦¦ÊÖ!pæÇøø¸¬õàÀøúO¶¥á&öÙíö¨MìàüÅþe!àó¯ñz½²S½øàOÏêl6[QQzbÅbþü?ð3øSvû®^½ZÙí+Ë²¬Yºt)ø#ðþÀ7±¯®®N£5?ðþâ¢½|<xðüøàOW)ûyA[l Ö'jàüÅþOð»aÃ¬¬¬äädùõêÕQx?ðGàüM½pû*++m6&ö?ðþb	óøþÀßS&öðÔûÄºØþÀøàüø3/ÜÄ¾´´4«ÕªÃàü?½ã/éI%''?àüE¿ØþÀøÓ;þÃþü?ð7/ØWTT¤ÿàü?½ã/{÷îU¶5gÏþÀøBñ1±ü?ðøëëëËÈÈPNxÎgðGàü?mjj2Íq0±ü?ðcøÛ¸q£²Å¹råJt:øþñ7±ü?ð3ø»pá²ÅY»vm4:øþ²é³X,ñ7±ü?ðøâ²eËc;:::¢üÐÁø#ðPø7±OlûÀøzÇßñãÇÎæÍçå¡?ðGà/Að§Lì+((9±ÏårÅýð?ðþt?ÎóGàüÖøÚ¾nnnÐvÁñ7±ü?ð§wü%?)Áþü?ð7³ëêê233CNìóz½	5¼àü?]àoÞàÀ_OÞ«««ÕÇsÄýÄ>ðþÀøàÀ_áÏëõ¶¶¶ªÏØ'Ü½·ÓéLäáàü?ðþüÅþd¦Ø§±O.exÁøàü?ñ?§Ó¹uëVõÞ%K477ÇåûÀøàüø	?QÝnyÆ¾êêê¶¶6ØþÀøàüøüy½ÞææfõûRRR¬VkOìàüéçÀø£©ãoxx¸±±Ñh4m*eÍ¾ûäRÆü?ð§wüÒ/$þ8Ï?ð7ã|>Ëå ðçt:­VkÈ_ÅÛÜÜìñxxr?ðþbþÚÛÛe+¶eË±±1ùRþ^¿~½¬éêêþÀßºtéÒoÿöo<S`6³³³Ï?ø¶ÊjUUUÈ3ö9&ö?ðþbÊé	·bãããÊ¾ðGàüÍà(++ë·üñ¡?²õOäKÙzÆþEEEê_Å»uëÖDøU¼àüQãOÙ¨	øðÇ?ào=ÿüók×®Uä§üùÎw¾óø1¿[·nÕ××/X° òäÎØþÀøüåååÉ¦­¶¶Vyez<ÙpËYþü¿éVTTT÷ÿêñ'_~ík_ÓùÃniiÙ²ez¯ÙlnnnåþÀøüuuu<àCþü¿éö­oë/âï6þQUU>­rÆ>yxêm`YYûÀø£¸=ÏßÍ7/^±téÒû÷ïkýÐÁø£¸Ä¼ùË_ÞñÝþý~úé.èíqûU¼V«µ¿¿§øô<?ào*ýå_þeffæïÿÞ²ß[&ñ¡«§ü*^õÄ¾ììì·ÛÍü?àÀø^òP¯N¦«$ä)òf³yß¾lÀø£ÂßÀÀ@aaaFFro^^^ÎËþÀÅ1þtÏçs8!'öÉJ»Ý.WÖïö%ðþÀ_lãO9Ésà¯tSO8þü¿.ÂÄ>Å¸	àüQáOùU~üõööÊrVVø#ðþb´pûdM]]zbøàÊQYPð711Áïö%ðþb÷yrbÉdÍN¸3ö?ðþ(ð§äYù´Oð7>>~àÀeC	þü¿X)ÂÄ¾¢¢"Íùàü?J üuvv<É³¼Ó?àOÿy½^±zbr<Ç·3àü?J üIò]^^®í^XX3]?ðGàoØfµZ].×Ôïü?ðG¿y	ü?3N`rb@0ÂÄ>ðþÀ¿É»øõqþîß¿_XXXPPþü?]abÙl¶Ùl^¯wf÷þÀø£Æßøø8Gûøzc_"ÂÈÇs?ðþü=.((HXvv6ø#ðþæ½¡¡¡ææfõ&+%%ÅjµÊ³nN¾øàâ·oßNÌÿ»=ù]¸püøóXsøàâþzZïáàÀß´êïïß´if³¹©©iÇs?ðþüÍàüøJ9c_ee¥z"JYYÙOÔþÀø#ð7¥ÆÇÇ322ükrrr^~ùeðGàüE-ÇÓÒÒb2Ôì«©©ýñàü?ÿ×ÂøU6¸àÀøÓ:ËuôèQõF£l%¢pÂyðþÀ%þRSSe;ët:ýkúúúdMVVø#ðþ´«§§'äÄ¾%K´¶¶z<h>ðþÀ%þ~'&&~ã~U'ÿAÈüü|ÁPRRÒÑÑþÀ¿Çgì÷ª'j®®®í¬ÖxÁøèøÉ6wÏ=ÊñåõyèÐ!eË,ï¹¶¶öÌ3²pâÄÍ7?ðG	?åxpgìÇÇþÀø£Âßµk×Bä¹··w÷«| 8>>n2ÔøóÍ7?nÂÍþþþÏHÞ÷Ý>úqÐ¨ðòºûÞ÷¾'ÿ¥ÚÎäääüéþiww÷¼?ÂwÞyGôÉÓL£~ö³üñÇF]¹råÓO?e4Jðçv»£üM5Çt÷îÝôôôäääââbY3û»< úT¿×^m ºuvvjHD'ò	ÆA£G___Ì=ì/nØ°A[X~~þ÷¿ÿý>ø@'³½½]ÊÓL£Þ~ûí_üâF½õÖ[N§qÐ¨K.EÿF8kP6ýìöe·/%Ôn_y¯[·N<ÇÊ+ÛÚÚæeb»ÙíËn_b·¯¶ååå+»eü?Jüy½^ÙVVTT¨'å]JoìàüQ"âo``@9Ï³òY@íüùó³¿[Åòê«¯Êü][[þÀÅ7þFGGÔ'j^°`Aý­[·ôüàÁø@økoo÷o£ü)Ë'Nå=wwwF¹Ïüü|õá#àüQÜàÏív×ÕÕ©OÔ¯t-~/øþÀßÌS¿ðãO ÆI	ü¿©$¯âõÄ¾²²2»Ý®Ï=¼àü?Jtü)[êÇ'veõñ¹àÀøSÕÙl¶ÊÊÊ'jîìì!ö?ðþ(áð§ÕOÁßøøødYf>ðGàü)ûÌfsùÒÒÒ¬VkÔ~/øþÀßÌÿ£<É³¼Ó?àÏ_¸òÅXØþÀø#ð÷«ä»¼¼Ú7==½°°0ÿàbòRµX,êEEE6-æöð?ðþüÍOàüÎñab_UUUô_¿àüøàü?ð§I'ö¹ø^ðþÀ%þ>ýôÓE)¿|3==½¤¤dddüøK@üØ'kâ`bøàÀß9|?¿Èû¼^o|/øàÊIkkk=|966¶yófYþüÅ=þ|>Ýn¯ªªJàü?w1Yàñz²ì?á3ø#ð¯øKÀàü?_¤|ò7>>î_#/Q>ù#ðÇøs»Ý	8±ü?ðGàï9µµµÊ+óÁ+W®dÎ¿¸Ä_¸f³9&ö?ðþüMÞÅÒhÿ/øMüÉË-ÜÄ>ù`|¨ü?ðGàoJ%?)ÁþüÅ(þ¼^oKKzb_JJJNìàüøÏÀøÓ[B«þþþùýý¹s¿áááeR¯zbßÐÐÏXðþÀ%(þÂÏùîÝ»àgÓ¦M999ÅEÅ_úÒÖ¬Y£Ã¦?ùØ½wff&ûÀøþBÝERÒ#GVnÝºS½Pâà¯¦¦¦liÙþûýàÐï¨¨¨øæ7¿øëéé	9±O~@»ÝÎÄ>ðþÀ¿/äÉC^^Þýû÷åË×_]y·ÐhªøzkppÐh4¬?(òSþÈòÓO?Ýßß+øóz½6­¨¨HÆ¾^xAo?øþÀß<ãïñäç|Ê[E~~¾²°~ýz­:øúyaÿþòß÷ËOùóoØívýãoxx¸±±Ñd2©'ö5440±ü?ðGà/l]]]þ·(<tðþtRÿ³Ï>¿EõôôèN§³¾¾>äÄ¾æææÄ<Q3øàÀßTÛ¹s§ò¶á?0pË-à>ïgyþùçýòûÎw¾óÕ¯~Uo~òã/Üû*++Ï;ÇÄ>ðþÀ¿'d0äm#++ëúõëæü¥¦¦?JüI.K´·dÉU«VýÎïüÎÓO?ít:õö åõÒÒÒ"RÍ¾uëÖ1±ü?ðGàoÊwtðàÁ V«£)qð÷xòl/mmmÇ³ÛízûÌoxxøèÑ£_þòC±ÏívóÜàü1ào;ÏÖïâàüÑØÕÕÕ©'öL¦ÖÖV&ö?ðGàüÅRàüQäÕ±nÝºõÄ>»Ýîñx"ðþü¿iÞ2))pÇnä/Á¿èî¢À?ø?p8øþÀøà/ÅÔûöíÛçv»§û»	ü?ðGàü?ð§Óq³Z­ê=¼F£ñØ±c~ð?ðþü?ðþÀ_çóùGÈ3öÉÊ¶¶6¯×xðþÀ?ðþÀøÉf³Y=±Ïb±]?ðþü?ðþÀ_ab_CCCä3ö?ðþü?ðþÀ_Ìär¹BNì3ÍMMMS9cøàÀø-þ"þüÍ¾û*++m6ÛÔ/øàÀøþÁ`þf5553xò?ðþü¿ÇüðþtX¸iiiV«uÆ¿ü?ðGàü?ðþôU¸AyÂÏòWñ?ðþü?ðþÀ^'sÈf³Ùf³±ü?ðGàü?ðþb²p¿W9Q³Ãáúñàü?àü?ð§ßFGGCNìKII±Z­.kÎ¿#øàÀøàüÍCÊÄ¾´´´û4ú¾àü?àü?ðÕ"Lìkmmåñàü?àü?ð§|>_v»'ö?ðþü?ðþÀß¼¥Lì3Lê%ÊO`ðþÀ?ðþÀøÓ*·Û]WW§>CÖÈú¨ü?ðGàü?ðþô<--úDÍ&©±±Qëàü?àü?ð|>Ýny<GEEÍfÎÄ>ðþÀ?ðþÀøÓ¶ÑÑÑÖÖV³Ùòxè?KÁøþÀøàOB¨9--m÷îÝZ¨ü?ðGàü?ðþæç¡Z­VõÄ>£ÑxìØ1ü?ðGàü?ðþ¦Ïçs8!'öÙív¯×«ÛþÀø#ðþÀøSMTg³ÙBNì[·n]OOÏ¼ÏþÀø#ðþÀøsr¢æpûæåàü?àü?ð7÷	ì¬V« Oý«x=:44[ÃþÀø#ðþÀø¡§VMMúx+Wêáàü?àü?ð7êÄv!ogggL/øàÀøàüýªááá¦¦&ÉëûÀøþÀøà/RN§³®®.33SÆ>yÅÜÄ>ðþÀ?ðþÀø<Ö­[§Øg6m6Çã³áàüøàü%"þeee!¯ÃáÑã9ÀøþÀøàï7=zô¨úDÍiiiV«Uo¿ü?àü?ðþfÛíÞ¾»úDÍ²FHÂD^ðþÀ?ðþÀ_üã¯§§§ºº:ÜÄ>=ÿ*^ðþü?ðþÀøÞæ¬ªªJÍ¾øØþÀø#ðþÀøK,üy<õabøàÀøà/Qðçv»ëëëÕgìS&öÅÓûÀøþÀø	?§Ó¹iÓ&&ö?ðþü?ðþâ>O^TvÆ>ðþÀ?ðþÀ_báott´µµUÆ>&ö?ðþü?ðþân·»¡¡!ÜûØþÀø#ðþÀøüÉ¥V«5äÄ¾ÖÖÖ9Q3øàÀøà/ÎñçóùìvUUûÀøþÀøq?·Û=88èõz#Oì+3bàü?àü¿î¯ÿú¯¿ò¯|ík_vÑ³á·~ë·ØþÀ?ðþÀøÏnÝº%¶ûæªo.)ê©§ØþÀ?ðþÀøÛ|>ßÆ³³³ØþÀ?ðþÀøçÂMìê©§óóó"ðþü?ðþÀ_<444$ÿê3ö¥¦¦VVVîøîÍ7¯àÀøàüÅváÎØ÷ÔSO=÷Üsûöî;ôCßÛó½¿ú«¿b¸Àø#ðþÀø1Ïçs8áÎØg·Û9ò¥/©¬¬¬¢¢BþìÏþAàÀøàüÅ^^¯×f³©'öIÅÆ>Yp»ÝòRÆü?àü?ðcØVWW×ßßxå©ün_àüøàüé1ËrbÑhljjy¢fðþÀ?ðþÀø±"Lì+++kmmõx<ánþÀø#ðþÀø1S555mmmO<Q3øàÀøàüÅ@£££SØþÀø#ðþÀø±ÛíÞ©Ùg4BNìàüøàü¿ØKßb±¨çxâÄ>ðþÀ?ðþÀø|>Ýn¯®®VOìáügìYàü?àü%:þFFFL&øÓC£££ÇSÏYWWçr¹fÿ-ÀøþÀøKhüuww/Àßüæv»ëëëÕûsõÀøþÀøKhü­Y³ÆårEÀßk¯½öËèöî»ï¾ÿþû¿L~òlØ°!999EEEGíëëÛo×ÑÑ!þû%iÃáøùÏÎ8hT?ã Q¢>úqÐ¨+W®üÓ?ýã Q¿èÓÆß¯bxü½ñÆîèÖÕÕuýúuw¼÷É'477¯X±B=±oÝºuÚ»Øú¿ø´Il-ÛwÆA£ÞyçÁÁAÆA£7nÜ`4ê­·ÞÍ>ã Q.]úçþç(ÓxÆ»ç<¯×úôé²²²Y±Ý¾ìöe·/±ÛÝ¾Änß©RO	üE¹áááúúúÜÜÜ öÉê9ØþÀø#ðþÀøÁ¦9NÅòOülàüøàÀøüÊ+Õ'j®ªª±&ûÀøþÀøOüÍ8¯×ÛÒÒ¢>c_ZZÕj3ö?ðþü?àO555©'ö-X°@ÖO÷Wñ?ðGàü?àOïµ´´±ïôéÓÑ9ü?ðGàü?ðþ¢×ëU~WÇºuëäýàü?àü?ðÕìv»ÖgìàüøþÀ?ðþü?ðþÀ?ðþü?ðþÀø#ðþü?ðþÀø#ðþÀ?ðþÀø#ðþÀ?ðþÀø#ðþÀ?ðþÀøþÀ?ðþÀøþÀø#ðþÀøþÀø#ðþü?àüøþÀø#ðþü?àüøàü?àüøàü?ðGàüøàü?ðGàü?àü?ðGàü?àü?ðGàü?àü?ðþü?àü?ðþü?ðGàü?ðþü?ðGàüñTàÀøþÀ?ðþü?àÀøþÀøàÀøþÀøàü?ðþü?ðþÀø#ðþÀ?ðþÀø#ðþÀ?ðþÀø#ðþÀ?ðþÀøàü?àü?ðþü?ðÇ8?ðþÀø#ðþÀ?ðþÀø#ðþÀ?ðGàü?ðþÀ?ðGàü?àü?ðGàü?àü?ðGàü?àü?ðþü?ðþÀøàüøàÀøàüøàÀøàüøàÀøàü?àü?ðþÀøþÀø#ðþÀøþÀø#ðþü?àüøþÀø#ðþü?àüøàü?àüøàü?ðGàüøàü?ðGàü?àü?ðGàü?àü?ðGàü?àü?ðþü?àü?ðþü?ðGàü?ðþü?ðGàüøþÀø#ðþü?ðGàüøóþüÏÿÜn·ßno¿ýöûï¿´éÒ¥K?ÿùÏºpáÂGÄ8hÔo¾ùË_þqÐ¨üä'ü1ã Q¯½öÛíf4êÔ©Sÿò/ÿåo:::ø8tèÐ( È»Fø<(qDDDDàÀ?""""DDDDþüé¥¾¾¾üü|ÁPRRÒÑÑxÑ½÷|eýðööö.]ºT®*7dôævxÚÛÛ¿¤¹^¯×kµZSSS:;;½¹^ÒââbåÊÝÝÝÞlWý­MÓáÕÃ[ýàjkkÏ9#'NØ¼ysàEçÏÂ)^f?¼/~ï½÷dáìÙ³ÞÜ¯äóùdþ´ÞÃ¿òÊ+²¹_´h£7·ÃûömY¿M&£7áU?WykÓtxõðÖÆF?8Ù¦È?,mSdësñâÅ)^f?¼¥§§3zs>¼/¿üòücð§Åð2h¯¼Þ½Wäol=ËáU?WykÓtxõðÖÆF?8ÁrYÑúêÕ«eeyyù7"_f?¼þ®]»¶ûvFon÷öíÛ²,,ð§ÑÆáøñã²e|øáÞÜo__­<uåoÙ>0z³^õs·6MWomlôKNNö/§¦¦¼ü_³´´tW¦¯ÒØØXmm­Çãaôævx7lØÐÕÕõÅVüi³q8uê,V,ÞÜï²eËOSDË/gôf3¼êç*om¯ÞÚØè7>>þxòÃ[Yw5å_wW¦¯²­·Z­÷îÝcèæ|x~3FoÎ7|v¢ÝðòÑÔ¯ú¹Ê[¦Ã«·6¶øÁY,W_UäoQyàE/v»ÝÊ?ÛêÕ«#_f?¼²022Â¸i1¼ÿ·@~ïÎ;ßxãY¨¬¬dôævx/_.+N§sÙ²eÞlWý­MÓáÕÃ[ýàº»»Fcrrr~~~oooà»ãµk×¬RkòÊ4WÃk2øhJ»áïÃ7lØ LSs¹ÞÜï§~*æ5ò·,3z³^õs·6MWomlô(ðGDDDþüø#""""ðGDDDDàÀ?""""DDDDþüø#"""DDDDþüø#""""ðGDDDDàÀ%z¯¿þú+Ò'[µjÕ~cû5YÌlmC=ZÉ$?Çã	Z/kC~~þÄÄÄtïüQLvèÐ¡$UG'ü566ÊÊ õÍÍÍ²þ¥^Á?"½úúú5áäÉ¾ÉN:%_ÊÊë×¯ÇþnÞ¼)+KKKÖËz·ÛþüQB´eËaÍ+¯¼¸òG?ú¬Üºuk :::OâÂ¥KÊ²ÿÊ<Ø¾VV»gÏÀ½«C%Ém¯^½Ä)Y½bÅ+W®È%è]¾|9òý(ÉCR.jooµÕ«WËúÞÞ^ÿ÷ÞOÖTUUù×>|Øh4Ê]¥§§oÜ¸ñîÝ»jü©ï?hMJDàh[¸p¡ÀåöíÛ+ïÜ¹#+M&S nºvíréúõë.ÚµkrÓéLNNy+åKåÒ6LLLSSS>222©xîGB><õOzöìÙ@ÑúyæÌ¿üîdÕªUÓÅ_JDàhþSöðØf%%	Åq³yóæ±ÉdA¾v­r©bÊx6å¢ÚÚZå#CYîîîVî$ð><(¼SöºnÛ¶MÖxQåoY~ñÅx?GùR®øØBþDÊùyGFFäKù[¹s||B~~¾ÜP~Ç¿ÞM>]üEx¨Dþt?)$þüëÜ(fîÞ½+_/.]*_.Z´hÇ¶GùïD®ôYÿVÊwîÜñ_YÀäßó+Ë²Ãáxâý4åËû÷ï>¶póóöîÝ+ýøÇ?~üë]Ûöì	¼@Pð'$-//W>.þ"<T"DDóòq×ØØXàJÇ#+å¢ÜñÓÐår)þó[Ç?QO½76S'XåÜÜÜÔÔT¯×+wí¿ô÷bA(Neù[N§ÿÒîîny!wO*?"¢ùO÷vüøñÀÊ	PøðÏQ uýúõÆÆFe'¬_ÊÇrþýªO$Ú®]»½½ò÷öíÛýë#ÜòIÛ½÷/ïß¿ùÈÜåËË¥ÊÙmÄ¬)GþÊEííí>??LÑð_á¡ø#"ÿ&Ã'S½<y255UpÆÚµk:^¯WÙ'ë?2Wó§Ls¹îÙ³G¨¤ë?ÙJH¢)ß*ß½³³Ó¿>Âý(ü9G¹fü:uÊÿüÈ)ÓN§|pøS¸)«	:7nÜxiJDàH)'@êðáÃÿ·ýL±Ù¿ÃÔ¿mÛ¶Ê2°óçÏGÀßãÉ_Å!ësss÷G¸yÌ¤m¸öÑ£GéééÊ´³Í5ß"''GþVÎöxÊþªú/ðPüé%ÊªU«R'[±brÈmþÚÛÛÓétuuù/õx<0öîÝëõzý^½zµ¼¼ßÚÚtêG"w%ëwîÜ´>ÜýHò`ä!)' p?Û·o:çÒÈÈHmm­@VVün·ÛÚÀû|øð¡èV«ªªªÞÞÞ ïá¡ø#""""ðGDDDDàÀ?""""DDDDþüø#""""ðGDDDDàüø#""""ðGDDDDàÀ?""""VÿAÊ­ÉâEIEND®B`


Detrended Normal Q-Q Plots


ØÚçãó²?Ï=ÏÝýóùÃ=÷«Þ$IÒ=S!$I?I$Á$IàO$Ið'I$ø$IüI$	þ$I$I?I$Á$IàO$	þ$I$I?I$Á¤ÿÿcW*ª©©Y¶lYWW×Wïïï_¹råLÞÔ»¸b2ÿÙg½[W43÷ôèÑ£?üpÍxË/?|øðm78õU---û÷ï¿Á¹ãÇÆ3gâAN§ãÚ¶P°hÖ÷$øæ6þòÛ¶mÛOdå¿ÚÚÚ«W¯Îü=úè£Å;k÷îÝ%¶6­U&<ìÛ·oºsÇÃÉdr×»~ýúàO?IÔ7nÜxöÙgkjjbf__ß½¿hÍ5sýýýV¿öµ¯Ýºu+&âÛyþüù	75ÝU;vì9¡®Ã_²âõë×c:ÍNq$øtçÎxúé§^t9uêÔâÅÓéô%KN<Y§ÜF¸Àüùózè¡ëæ.|îÜ¹+W&G$7lØ044¥­­­ÉÝÝÝ7u²ÍÞvÅ°qãÆøzôèÑÉÆäÍ7ßvmr;;;.9q¥qÕq~øák×®9s¦¥¥¥ººº½½=ÿUÆ=ö444$1ã*r&»ÁûÜçb~þAØ(°37mÚ4áþî*ÅW^9q#§58Åî`ÅÉo¹ÄDó§>õ©Ìü¥mm²ýXú&	þ¤JÃ_x%f666&ß?>JåÿÞíïï/ñË8¹pGGGus_ÕùK×­[ÿUq¥oÒmW,¾ï7nÜÈd2õõõ1Q<&o½õV]]]þvâJË¿¿Å×»lÙ²äÔ¤pIN~bEiü-0æ¿óÎ;Å;«¹¹yÂý;ÝU®úúõëÉ+-Öàü;[±4þòÆ½.þ¦ø¸Râ/,K¦;;;ãÛîííÍ'ZÁêÉ·»víºuëVòZÎm×]³fMüÖË'/7¥ñ;8¾Õokå_WÍ^qÂû~ìØ±üWÂò/¿yóæ^½zõõñb"¾ÝßdÎ-[ÆÆÆl9¾=yòdþLÞ¾vâÄ¾|ùrþ¢Énp:ìäÖý«Lö¿Ü+£Ó	oÕ¯8áã­x¿ããq5]üMñq+	þ¤Å_îH_ò±übN_Æo½õVnÎm×Í½^L¥RÉ·óæÍËÉªà(^Í^q²û1~ÍÌ¯¯¯Ï¿GW®o,X0ÙýMæ?ò¿Me'w0ÿd³ÙÀ_£­­-ÿ¾À_NäIý®?¤±næÊ+O:5á Mep&¼Uw¼b	ü%k×®åosZøâãVüI¿ÀJÌ¬««ìÕ É|Ê)ñJR	âL÷·õlv²ùqãþ655æÏ/~å¬øpÅ÷w*ß4¸ïIÉqêbá:·©¬2±ðSÉHzg+ÏþpâãVüI¿äð¼6áÉ¥­3­uK¿óÎ;ïä/-±ÙÒ+¸GosÌL|VðU§> ùß.^¼8ùÈîîîÀÙTð¼<|êJggç¶mÛ®_¿~ðàÁ¹víÚ	ñ7UîÓ¼â|å/Ç»ä¿7%¼ÄLüI¿ømJ~7ç>ê%y7U¸!~w&'Ï.Y²$Y¼)~x¼	X·´µ$oá'Û)x×-½biè¬Y³&÷6ÿdNr.ðêÕ«olmË-É«_çÏ»°ÿþ©àïôéÓÉñÇ¸Ë½½½É«zIÇpÿNwiá¯ôà<6òî¨Ne<wéÅÞ	Û­Zµª÷îÑ |Kx$ø*ÅmÝº5wâógsoÿÏènò2añ/ÑëS¨(áQþ¹¥%6[zÅÒÐ¹zõjîÍ^ÉÁÁÁ·Õ××çÞPøAðÓIÒrï,¡6ï¬¥KØÅÓZeZø+=8ü¦;ªSÏ×_==ôPþÒAÈc>Ù5xI?©¢ð¿W¬XQüç¿N<ÙÖÖßèÈÍ?vìXÌ	låÊÝ×½­Ç·¶¶d½í¥¡ÍÿÆo¬]»¶z¼ÎÎÎâÓ;îCCC±µØf0hûöíá¡¹Kìgw|áÂúô§?;kø¯2-üÇFAÓÕ)ïñãÇânÆ#é¹3ãÛGy$¹º+WöõõÝö¥Ö0Ið'I³V6Ý°aCþ+µ?UÊ¿ä%Þä|í±±±ñ/öàOT%ïù+èÉ'42üI*°ááá;v,09¶>ú¨aàO$Ið'I$ø$IüI$	þ$I$I?I$Á$IüI$	þ$I$I?I$Á$IàO$Ið'©Ì;vìXccc:^±bÅL?awgÊºSlhhhË-óçÏAX¸páÎ;g~îâ=4iaÐØØØ=¿p^óx/_¾uëVWWWlvÕªUw°©þþþ+WÂ$øTÞÏåm4þöìÙÛ9zôè¬$üI?I³@«|vìÜ¹³¶¶vÞ¼y»wï.¸Ø§?ýé¶¶¶ÜÌÞÞÞdfnÎêÕ«cNÌ¿råÊÊ+kjjÒétkkkOOÏÛÉ¿ÞÒ«:t¨±±qùòåï¼óN1nÞ¼¹~ýúX7nö;¦õæ%Kb;£££.-1§OÔÐÐpìØ±â,±?Ið'i¦ýL?ñÄ1àÀÀVL<ùäù9yòäÐÐPþº¡´ùòåéÓK.=qâDLÄ¢Éd&ÜNþU^%HwäÈØ¸qcñÍÞºukLÇ^xáØ³gOÆ_Xs2r½÷?>&I.wOKoþ$Á¤ÙÁ_8&¦³ã¬øµ2;wî|ü­>xð`²èõ×_ßµkW[[[ÌL¥Rn§;¥WInRmmmñºõõõÉÍ¾uëVL<ðÀS¿û%ðWz4FFFéØÂdËÝÓÒ?Ið'ivð/¡ÉX_`«¡¡aÞ¼y×¯_¯uuu¿ÿþ¸ü5kÎ;WÂ7ùßNeÉnRH1ÿå½S|åoÑ¢E1ÿæÍ¥]Xy·Üt7%Ið'i&ðÉdò_ Ê½VÂ%?þx,jooÏoª««ãÛ[ãM¥WÉÝ¤ùóç¯»`ÁdÝ;¸û»víu_xáâES)âoº$ø4øKN=xð`r<÷©§º­KµâkîlÄ:.MM¥W	¢=÷Üs1±yóæâuyäîêêºxñbîSìÆA±æææ+W® ;::¦8ùÓµµµ¹sGîét7%Ið'i&ð766Ì7Þc=6áeÛ²eK,ýüç?süøñºººÀÐ;¦¿Ò«:u*.[¶,wÆIþe·mÛ·¹ººzÕªUo½õÖ´F ¶|ÕÃ¯¡ÀÝ»w'ïçÊhäO;v,n@òYÙ÷tº$ø$IüI$Á$IàO$Ið'I$ø$Iü÷w÷ýï&¯ñ_þå_þã?þÃ#¯ÜºvíÚ7C¹;åßþíßC¹Ob3üÌ©©ôÿù.]2eØ?ÿó?ÿà?¿réÿðÃ3ÌMÈ(ÃÎ?.7åVìØ5Æ¡Q>ÃÏJ·nÝúÛ¿ý[ãP>úßÿýßáþ?øüÁüÁàOð?ø?øüÁàOðð?øü	þàþàOð?øüÁü	þàOð?ø?Áü	þàOðð?Áü	þàþàOð'ø?ÁüÁü	þàOð'ø?ø?Áü	þàþàþàOð?ø?øü	þàOðð?øü	þàþàOð?ÁüÝ»øÈd2étº¥¥¥§§'ÑèèèÆ«««xàøüÁàþs¾ÎÎÎ®®®Ø·oßºuëòíÙ³çÉ'ú¦¦¦büuwwßÁ¾ño¼ýöÛ7UfûÛßþÞ÷¾gÊ­Ø)±kC¹ObñTfÊ­÷Þ/ðgÊ°k×®Íä5Þø«««ÞÅD6mllÌ_ÔÒÒrñâÅÉVüíÝ»÷3Ø'âAðY/¿üò+¯¼bÊ­Ø)±kC¹ObñTfÊ°_|Ñ aòW_u&¯ñÀ_:p:ù6xWSSÓÔÔtîÜ9å°¯Ã¾rØ×a_9ì;çK¥R¹éêêêE7Þx£­­þð'ø?Áß¯¾¾>Í¾?~Ø7¦å¦^?Áü	þàOð7'[¿~ý¡Cb"¾vvvæ/Ú²eË#GbâÂË/?Áü	þàOð7çëíímhhH¥RL¦¯¯ï÷­ª*yêèèH§Ómmm.]?Áü	þàOðwï?øüÁàþàþ?øüÁüÁàOð?ø?øüÁàOðð?øü	þàþàOð?ø?øü	þàOðð?ø?øüÁüÁàþ?ø?øüÁàþð?øüÁàþàOð?øüÁüÁàOð?ø?øü	þàOðð?øü	þàþàOð?ÁüÁü	þàOðð?Áü	þàþàOð'ø?ÁüÁü	þàOð'ø?ø?Áü	þàþàþàOð?øüÁü	þàOð?ø?øü	þàOðð?Áü	þàþàOð?ÁüÁü	þàOð'ø?ø?Áü	þàþàOð'ø?ÁüÁü	þð'ø?ø?Áü	þT¶ø»xñâßüÍß¼öÚk£££vüÁüÁàþT±ø[½zõ>ô¡¥K677ô£õðð'ø?U,þ¶oßþñÿöñ¿»c÷ïï¿ú«¿zÿý÷Û)ðð?øSâoþüù_ØòD~É¿%K<ÿüóvüÁüÁàþTiøï½÷êëëóåÿ~é~éOþäOìø?ø?Áü©ÒðøÃþíßúí|ü-^¼Ø+ðð?øSeâïK_úÒ>ø;['Ø·ëïúä'?ÙØØè=ðð?øSeâ/Ú¶mÛOýÔOý÷æÿ^__¿téÒÁÁAþàþàOðªXü½?þæ¿oë[o¿ýöØØÝðð'ø?U8þðð'ø?Áàþàþð'ø?ø?Áàþðð'ø?ø?ÁüÁü	þàOð'ø?ø?Áü	þàOðð'ø?Áü	þàþð'ø?ÁüÁü	þð'ø?ø?Áàþðð'ø?Áàþàþð'øüÁüÁàþðð'øüÁàþàþ?øüÁüÁàþ?ø?øüÁàþàþàþð'ø?ÁüÁàþð'ø?ø?Áàþs¢L&N§[ZZzzz/ÐÝÝ]UU?øüÁà¯êìììêêûö­[·®`éØØXkkëdøûë¿þëÁ¾þõ¯ïßûWY¯½öÚ¹sçC¹;%vq(·âI,ÊC¹uåÊãÇ2ìW^yóÍ7gòï	üÕÕÕÅÿxb"Í666,â'~úéÉðwðàÁs3Xwwwÿ9Y¯¾újoo¯q(·b§Ä®1åV<ÅSq(·¾óïþCvòäÉoë[3y÷þÒéôÓQüO¨­­-hè°¯öuØWû:ì++©T*7]]]¿¨££ãÌ3ÿuWáOð?øüUÆ°Ö××g³Ù÷ÇûÆôÝÃþð'ø?ÁßoýúõøÚÙÙ9ñ]õÊàþð'ø«aííímhhH¥RL¦¯¯oBíÁàþð'øó!Ïð'ø?Áü	þàþàOð'ø?ÁüÁü	þð'ø?ø?Áü	þðð'ø?ÁüÁüÁü	þàOðð?Áü	þàþàOð?ÁüÁü	þàOð'ø?ø?Áü	þàOðð'ø?Áü	þàþð'ø?ÁüÁü	þð'ø?ø?Áàþðð'ø?Áàþàþð'øüÁüÁàþðð'øüÁàþàþðð'ø?ø?Áü	þðð'ø?Áüø?øüÁàþð?øüÁàþàþ?øüÁüÁàOð?ø?øüÁàOðð?øü	þàþàOð?ø?øü	þàOðð?Áü	þàþàOð?ÁüÁü	þàOððð?øüÁàï®á¯êv¥R)ø?øüÁàþ*©ÛN§áþàOð?øsØþàþð'ø¿ÊÂ_<5lÞ¼þàþð'ø¿Ä_ccc:ö?ø?Áü	þTùø[²dIñÙuuuÃÃÃðð'ø?ÁüUþª««CCCC1æî¹çbbÓ¦Mðð'ø?ÁüUþúb"´/_Ç_LÌ7þàþð'ø¿JÃßüùóz½½½.]G4ðQ/ð?øü©ñ·cÇÜéùoû[¾|9üÁü	þàOðx¶ïã?¾`Áèëë`[[ÛØð'ø?Áü	þî¡àOð?øüÁüÁàOð¿É[´hQò/>äþàOð?U8þóÁËÙ¾ð?øü©ñÎöõ÷÷ÇÃnÎíøüÁàþÓ«®®.ð7å?øüÁàoÚþ¶nÝzóæMø?øüÁàO¿háÂUE9áþàOð?U þðð'ø?Áîü%ì£OÐð'ø?Áü	þ¦W½>àþð'øÓ½¿¸K¿;vÂüÁàþªpüUM>àþð'øSe~Èó9áþàOð?UæG½ÌÝàOð?øüM¯ÆÆÆ¦¦¦/ÎâÝÈd2étº¥¥¥§§'Q___kkk,Z²dIþð'ø?Áß*Ë¯ vvvvuuÅÄ¾ûÖ­[¿¨¹¹ùìÙ³1qøðáEÁàþð'øû@õôôþì±x:­|©««K®:Í666Nv±büýÕ_ýÕÿÁ^yå×_ýÿ¨Ìúæ7¿ùÚk¯r+vJìãPnÅX<rëÂÇ7eØË/¿|îÜ¹¼Æ8þÊálßüK&;Ñ¤¿¿Ó¦MÅø;räÈàvêÔ©Ø+*³z¿ýíor+vJìãPnÅX<rëþéÆ¡ëîîþîw¿;×øÇ_9íÍêêêâÜ¼y³³³sxxØa_9ìë°¯öuØWûÎùêëë³ÙlrØ7¦^½zuãÆ×®]+^þð'ø?ÁßÜkýúõøÚÙÙY0âíííCCC®?øüÁàoÚe³ÙU«VÕÔÔTUUÍ7¯££cÏüèíímhhH¥RL¦¯¯ï÷müäÆÆÆüw"Âàþð'øû@LxÂÇøS¿ð'ø?Áü	þ¦×âÅz«W¯¾yófò¤°fÍ³téRø?øüÁàþ*ÕÕÕA½±±±Ül6s&<ëþàþð'ø¿¹¿T*ÔKN¶M93ùQ/ð'ø?ÁüÁüÍþÃ¾íííÉaßøÓ1§µµþàþð'ø¿JÃ_hoÂ>®_¿ð?øüÁ_~ÔËÈÈHGGGmmm*¯ííí1gNìøüÁàþ÷Pð'ø?Áü	þàþàOð'ø?Á_Á·+JÁüÁàþðW!øKMüÁü	þàOð§JÃßdmß¾=ÁßáÃáþàOð?ø«XüÌ7/Ø·råÊüÏ|?ø?Áü	þà¯Òð·víÚä¿'NÌ¡ý?øüÁàoz½ðÂ	ûV¯^=çöü	þàOð¿©622²téÒäÜ¹¸?àOð?øüM©½÷&/ø­[·nîîøüÁàþS[ÓçüÁü	þàOð§©ÛN§áþàOð?øóçÝàþàOð?ø?øü	þàOðð?øü	þàþàOð?ÁüÁü	þàOð?ø?Áü	þàOðw×ð·hÑ¢êêjóð'ø?Á*ÍÍÍùàó9ð?øü©ñÎöõ÷÷ÇÃnÎíøüÁàþÓ«®®.ð7å?øüÁàoÚþ¶nÝzóæMø?øüÁàO¿háÂUE9áþàOð?U þðð'ø?Áîü%ì£OÐð'ø?Áü	þ¦W½>àþð'øÓ½¿¸K¿;vÂüÁß=Ø»ï¾;66ð'øÓ½¿ªIrÂüÁ_Å÷Õ¯~õþûï¯««?þg>óP üÁàO¿Ô$9áþà¯âå÷ºüú_ßýû»wþÞÎåXþO|þàOð§ÊÇßþwÜý÷ßÈ/÷ïgögO:ð'øüÁüÁ_¥õÞïÕÕÕåË/þ­X±â+_ùüÁàO¿l6»jÕªªªªyóæuttÌáOðwgÍ?ÿw·ÿn>þ-[öùð?U8þFFF&<ácNü¿;nõêÕ¿ð¿ßolüøààà üÁàO¿Åõâ×@ò·ãIaÍ51géÒ¥ððWÁ½÷ÞË/ÿØÇ>¶bÅ_üÅ_¼ï¾û^zé¥»ð?ø+GüUWWõò?å+ÍÆðß©S§¾ò¯8pàí·ß¾ë?øüÁ_9â/Jõ|¹9£££1ÇG½Àü	þàOð§=ìÛÞÞö¯1sZ[[áþàOð?ø«4üö&<áãúõëðð'ø?ÁüUàG½tttÔÖÖ¦R©øÚÞÞsæÄþ?Áü	þàOðw?øüÁàoz566655]¼xþàþð'øSåã/NWUÍÕWáOð?øüM¯Àßc=Oså¯ºÁàþð'ø»ÓMLR*?ø?Áü	þà¯?äyÂ|È3üÁàþªü555µ´´Ïéý?øüÁàoJ%Ò÷ýñÃ¾sâ/ü	þàOð¿;Ç_ò'?ãð?øü©ÂñÉdªJæø?Áü	þT9øëïï?~òú_B='|Àü	þàOð§Å_®¹â<øüÁàþþ¶/ü	þàOð¿Ùk`` É¤ÓéÒîlü	þàOð¿r©³³³««+&öíÛ·nÝºÒîlN~;vìhhhx`«[¸pá*³âÆ¡Ü»Æ8[ñ$6ÃÏbõõõ¡»ÿþûgøWÿÃ_]]]ò³Ùl<ï^tgsòÛ¾$IRõÇßo¼qñîHÁy$ÅîlüI$øûGÇ?ç¥µµõÍ7ßüà[ËÿtÀêêêÒîlN~_úÒvïÞýÕ,®îÏÿüÏ¿ª2ëþè¾üå/r+vJìãPnÅØ?sj*uuuñ_4eØ®]»80×øÇ_°/'Ít:ýðÃÞñÖêëë³Ùlr6¦K/º³9Nø>ð!'|8áCNø¸/^S`uuõªU«î`;ë×¯?tèPLÄ×ÎÎÎÒîlü	þàOð¿»öÈ;qâDMMMÕþy·ÞÞÞX7ÉôõõýðV½.^tgsàOð?øüÝßù¯üÕÖÖnÚ´©ü÷ü	þàOð¿éo¾uëÖ]¹re®ìøüÁàþÓÜÄøyúÔ§._¾<çöü	þàOð¿éuW>áþ?øüÁßÜÀßþð'ø?Áß´»páÂ¢EæÍá[__ôèQø?øüÁàO¿îîîÜ		þéûöÁüÁàþðWiøkhhê]¸p!¿¾¾¾äÓ^àþàOð?ø«À³aÎá/É)Àðð'ø?ÁüUþêëëzÉ«¿l6»sçÎnll?ø?Áü	þà¯Òðw©j¢Î=ð?øüÁ_ííÚµ¶¶¶älßEÎý?øüÁàïþð'ø?ÁüÁü	þð'ø¨+W®´¶¶ÖÖÖ¦Æ¹rÌþð'ø?Áß4zäGª&ióæÍðð'ø?Á*GM·ÿþááádæÈÈÈ±cÇù'O?ø?Áü	þà¯BðÉdBx,^üÁ7óð'ø?Á*ÕÕÕ!¼âEÙl6ÅàþàOð?ø«üåþªÛdK?õð?øüÁ_à¯ïàþàOð?ÁüÁü	þàOðs¥?ø?Áü	þT9øKÝ®t:ð?øüÁ?ïð?øüÁüÁàOð?ø?øüÁàOðð?øü	þàþàOð?øüÁü	þàOð?ø?Áü	þàOðð?Áü	þàþàOð'ø?ÁüÁü	þàOð'ø?ø?Áü	þðð'ø?ÁüÁü	þð'ø?ø?ÁüÁü	þàþàOð?ÁüÁü	þàOð?ø?Áü	þàOðð'ø?Áü	þàþàOð'ø?ÁüÁü	þð'ø?ø?Áü	þðð'ø?Áàþàþð'ø?ø?Áàþðð'øüÁàþàþð'øüÁüÁàþððð'ø?Áü	þàþð'ø?ÁüÁüøüÁàþàþ?øüÁüÁàþ?ø?øüÁàOðð?øüÁüÁàOð?ø?øü	þàOððWªL&N§[ZZzzzòõõõµ¶¶Æ¢%KÄÅàOð?øüÍù:;;»ººbbß¾ëÖ­Ë_ÔÜÜ|öìÙ8|øð¢EàOð?øüÍùêêêâAÙl¶±±q²ÕÔÔãï©§:9?~ü¥^:©2+1O8aÊ­Ø)±kC¹ObñTfÊ°_|Ñ a3ÿ«ÿÀ_:p:¿þþþM6ã/<þg°3gÎý_Yÿ÷?88hÊ­Ø)±kC¹ObñTfÊ­üàñ%ãP½úê«7nÜÉk¼'ðJ¥rÓÕÕÕÅ¸yófggçðð°Ã¾rØ×a_9ìë°¯öUý¨®¯¯Ïf³Éaß.¸äÕ«W7nÜxíÚµâÀàþð'ø­_¿þÐ¡C1_;;;F¼½½hhhÂáOð?øüÍ½zR©T&éëëûáQ°±±±*/øüÁàþ÷nð'ø?Áü	þàþàOð'ø?ÁüÁü	þð'ø?ø?Áü	þðð'ø?Áàþàþð'ø?ø?Áàþðð'ø?ø?ÁüÁü	þàOð'ø?ø?Áü	þàOðð'ø?Áü	þà¯²ðwòäÉ_þå_nnn¯Ç÷ð'ø?ÁüU,þþìÏþì#ùÈ¯uüÚoÿÖoÿ¯5ÿkÁÏ?ÿ¼6ø?Áü	þà¯ñ÷î»ïÞwßsóîßßüûõ¿þ¡httÔÏüÁàþðWiø;uêÔÿhû9ù%ÿ>þñ_¼xÑÏüÁàþðWøkmm-Àß>ð?øüÁ_âïÝwß­©©qØþàOðððwOàïýñ>~æg~&ÿçÎüÁàþðWøÿGõòà~òôQ/ð?øüÁ_ãOð?ø?ø?øüÁàþð?øüÁàþàþCâïOÿôOþç¾¦¦æcûØ®]»Æ?ø?øüUlºï¾ûÖþÚÚ]ÿ×æßÜüs~î3ùa?ÁüÁü	þ*³~ô£ýìgsa¹ó÷vÞÿýñ4jdàOðð¿Jkxxø#ùHÁ_¯Y±bÅ3Ï<cpàOðð¿Jktt´¶¶vçïíÌÇßÿ?öY?ø?øüU`K.mÿdN~_Øòyóæù»Õð'ø?ø?Á_Å>cþôOÿô²eË>ûÙÏþÊ¯üÊ?üá?þã?6,ð'ø?ø?Á_e;¥»»û_üâÊ+7lØpòäIc?ø?øüU2þ|È3ü	þàþàOð?ÁüÁü	þàOð?ø?Áü	þàOðð'ø?Áü	þàþàOð'ø?ÁüÁü	þð'ø?ø?Áü	þðð'ø?Áàþàþð'ø?ø?Áàþðð'øüÁàþàþð'øüÁüÁàþððð'ø?Áü	þàþð'ø?ÁüÁüÁü	þàOðð?Áü	þàþàOð?ÁüÁü	þàOð'ø?ø?Áü	þàþàOð'ø?ÁüÁü	þð'ø?ø?Áü	þðð'ø?Áàþàþð'ø?ÁüÁàþð'ø?øüÁàþðð'øüÁàþàþðð'øÝ2L:niiééé)¾@wwwUUü	þàOð¿J¨³³³««+&öíÛ·nÝº¥ccc­­­áïë_ÿúÈöÍo~óÚµk#*³¾óïüã?þ£q(·b§Ä®1åV<ÅSq(·Æ¡ëééÉk¼'ðWWWÿãl6ÛØØX°ô'xúé§'Ãß¿üå¯Ï`ñùÊ+¯|]eÖK/½ôòË/r+vJìãPnÅX<2ìÅ_4eØÌÿê¿'ðN§'®ÒÖÖ4tØWû:ì+öÃ¾¿T*®®®Î_ÔÑÑqæÌÿº«ð'ø?Áü	þæîPVý¨®¯¯Ïf³ïöé	/»0ü	þàOð¿¹Ýúõë:ñµ³³s2,Ï?Áü	þàOð7÷êíímhhH¥RL¦¯¯oBíÁàþð'øó!Ïð'ø?Áü	þàþàOð'ø?ÁüÁü	þð'ø?ø?Áü	þðð'ø?ÁüÁüÁü	þàOðð?Áü	þàþàOð?ÁüÁü	þàOð'ø?ø?Áü	þàOðð'ø?Áü	þàþð'ø?Áß½ÕüÁ<ÿüóßÁâêþáþáû*³º»»Ï=kÊ­Ø)±kC¹Ob3üÌ©©tùòå¿ø¿0eØ#G¾ûÝïÎä5¾÷Þð7i.½÷J$UJ¥_¯òb¬$IÒ½üI$Á$IàO$Ið'I$ø$IüI$	þ*§L&N§[ZZzzz/ÐÝÝ]UõÃÝ¸qcuuõ<púôi£W&û%öÅâÅ÷öö½ÙÚ5×®]«Êk*ûQ³²_úúúZ[[ãÂK,^ìâ'7Ã~õ_ýw¿ÎÎÎ®®®Ø·oßºuëÅSdîçpÏ=O>ùä­[·b÷755½2Ù/uuuW®øÚØØhôfk×=z4"§¾5[û¥¹¹ùìÙ³1qøðáE½2Ù/ÅOn*ý2ë¿ú=î~áØ£1ÍfÝðÄO<ýôÓ¹Ãø/ÂÅZ¹íøi¼zõjLÄW(Å]ÏÇú~Ôlíüjjj^ùì'7Ã~õ_ýw¿t:=áôûã/#µµµÅã#÷sØ»wo<W0Î;gôÊd¿ÄNoãk¿Ñ­]ÓÜÜÜÞÞ3c½ñÆ¥/¬YÜ/¹âçeÓ¦MF¯LöKñÊäylvõ4ÜýR©Tnººº:QGGÇ3gþkÜôs>xð`LÄ"F¯LöËÒ¥Kÿ-[fôfk×äºzõê%K¦xaÍü~Iºyófggçðð°Ñ+ýRüä¦2yÝ_ýw¿úúúl6ûþøk¿1ýcÃýã%ö2Fî//É®É/y>â5Ãû%ùÅ¶qãÆk×®ºòÙ/ÅOn*ç±Ùýã¡p÷[¿~ý¡Cb"¾Æ'÷ýnÙ²åÈ#1qáÂåË½2Ù/Ë-=çÏ_ºt©Ñ­]ÓÜÜ<88À¢½½ûQ3¿_N>CCCÆ­¬öKñÊa¿Ìú¯~»_oooCCC*Êd2þàå¾½qãFGGGòVK.½2Ù/o¾ùf/öK|i£7[»¦¿¿?ùÌ~89gÂkÖ÷Kcc£WÊp¿À_yîYÿÕïÑ It$Ið'I$ø$IüI$	þ$I$I?I$Á$IàO$Ið'I$ø$IüI$Á$IàO$Ið'I$ø$IüI$	þ$Itû¾öµ¯=ôÐC5ã­X±â^ø±ç¸ñæÌ3òD·¶±±1îÚððpÁüN§3Ì­[·¦»MIð'Is²Ý»wWõøãWþì±ùì³ÏÌægbþ£>zÛ4÷Ö¤Óéýû÷wðàÁø6f¾þúë¿Ë/ÇÌ%KÌ_¼xqÌ?Ið'éèsûæÉ'ÌùÔSOÅÌÏþóùôééé	<[[[c:wáë×¯oÚ´©¶¶6ÕÕÕmÛ¶-ÿèê©S§X±(Ö=yòd§bÎüùózè¡'NÄ·ë×¯/¸aÇ/½dQÜ¤dQww÷dPkooù¹9gÏ9+W®ÌÍÙ³gOCCClª¦¦fíÚµW¯^-Æ_ñöæ¸©àOf¹?ó­·Þù¸)¨¿¿?YºfÍE[·nM?>JM¸Vòm²´££ãÖ­[ÁÇêêê±±±X_OóæÍKÞWb;11áÍ+¾§ÎmÎ]]]9ùldÅÓÅ_*	þ$iöKðNð¼VUËÇÍºuënñíêÕ«¥u>&/àÚEÉK1ÝÛÛl$»víÞ%G]yäsìØ±¯1½aÃÛn'àßÆòoÛ÷(@¹`Á¸¿CCCñm|[âÌf³É2L¬wáý&®©©.þJÜTIð'Ie¿hBüåæ'¸IÌ]½z5¾6%ß¶¶¶Æ·MMM7o´ä6)x--·Vòí[o½»p)wä7¾Æô©S§n»f|ûÎ;ïäß¶ÉÞ·ûöXôôÓO¿ÿ£CÛÛ¶mË¿@@0ð$mkkK^.þJÜTIð'I³_òr×Í7ógÇÌXT;9^ºt)ñ_Î:¹7ê-àTþ¬Ät]]]uuõèèhl|þüù¹¥·ÝÎd+èÂSc:¾ÆôùóçsKãLxøxêø+qS%Á$Í~ÉûÞöîÝ?3ù>rï*~Aëõ×_ì±Ç°95&/Ëå«Þh[·nMöÆ×M6åæØNòJÛµk×oßyçÒgæ.[¶,&nfÍ_ùº»»oÜ¸Q9&£[Zâ¦J?Iý&Òéô¾ûzÙ¿uuuñÉ«W¯è&Çdsgæ&ïùKÞ*wéÒ¥ü7&Ü¶m[P)97÷a+-9ý6¹öÓ§OçæØNò&¿ä=ÃÃÃÉ%KàïàÁ¹×äâ.ç/JÞþxþüù¸Éðp3¸t®]»6i*	þ$©,J>¹ =öüÿç¸ñå¦sLsçXäzäGòeßÑ£GKàïýñ?ÅóëêêòØNÜü;Óv²;;22RSSÜÝ«V­Ê¿Ä×äÓ^ò·¼0+¡jni*	þ$© ¬X±¢z¼z(9å¶ÝÝÝÉÇéµ´´9s&·txxxçÎ	¶oß>::[zòäÉ¶¶¶0Y&9pà@Á6oIl*æoÙ²¥`þdÛâÆÄMJ>°ÄçüåÚ´iSÁg¾$uvvÆÔÖÖÆ½ÌlMþ6oÜ¸ºMÆjåÊ×Xâ¦J?I$Á$IàO$Ið'I$ø$IüI$	þ$I$I?I$ø$IüI$	þ$I$I?I$Á$IàO$Ið'I¤»Ñÿ8±0z;DSIEND®B`


;ØnýúõiøÒK/Ñ@ðÍ?àyv0Ið'þ&·sçÎÌ:¯Í¼ü?ó¦»ÑaÂÉ¿Dól_NAE²¯ÄÌ,Ís³ù7Ìë×¯g^ìÍÈyùWeeeæ÷¿N¢.]ydý<õÔS¿Y+W®Ìó-Õ&³Â_þÁÉÙ7²í¨Îd<Ï9kÖ¬É^3kÌ§ûyv0Ið'þÂ/ÅuëÖMþó_Rá7ú«¯¾ßÑÑæle¿ËÝ·½£¢·Ç7ÞÐÐ0ùíú¦»Ù;n:Ñ)Ñìù/^Ü¼ysb¢T*5ùò»Ãßàà`¸µpA»wïÊ;¿À^~ùåðèÂ_¶lÙ¡C>ûÙÏf®¾÷Mf¿ü³oä4«Qáð=ztùòåáaÎ¤g®hnÝº5úrMMM§O¾ã¡Ö<;$ø¤V:~ê©§²ÔÞM~Ñ!ÞèzíÑÑÑñµµµö	þ$IXô¿^|ñE##Á$©nkk[¶lYtn7L<ûì³E?I$Á$IàO$Ið'I$ø$IüI$	þ$I$Ið'I$ø$IüI$	þ$I$I?I$Á¤Å[GGGuuu<_·nÝ|?MtwëÌdÛßì¢ùÃÃÃ;wî¬¨¨#S^^J¥Þ~ûíÌV£££a~X9Hi$ø´hªªªÄ,üMßÚÚæwuué'OéÌÒ£Gf°xìØ1$ø´xPæHQó|÷î7þD?444åVO>ùdXÜºu«½HüIZLòËÐ=ÊËËËÊÊöíÛ³Úg?ûÙÆÆÆÌÌhffÎÂ0ÿêÕ«MMM¥¥¥ñx¼¡¡!:~6ùv²¿nþM:T]]½zõê7nLFÛ­[·ZZZÂ¶án·µµÍêætøøáÃüÚÚÚðpr-Y²$ÜÏÛ·oK.æ9sfÍ5aN¸'á±vÍ&	þ$-PÿEÓø¦_õÕ­0ñâ/f¯ÓÙÙ988½mPZùòå0=00¦Ã0½råÊèdhXf&É)o'ûKçß$îµ×^­­­ïöÎ;ÃtXáõ×_Ï?ÿüøT/æËsqò:9ëÖ­;wîè,pnÞ¼yss2^êïï«V­²wI?IÕÕÕa:=ÑdM>¢qÏ=ãÃôÁ£EgÎÙ»wocccÅ¦¼åß$ºKååå·­¬¬îöØØX»»Sooo iáÁFówíÚù4"éîÝ»Ãt¸oÃ¢»$ø´Ðñ]Á>Í/¤­ªªª²²²7oA`aþÂú7n<wî¶9·ýéL6î.)fºà8«#yÆ§««+:ÀiÈáK<>qÆ9L/[¶,L···/Y²$ºÁ0¯¿þº½KüIZÐøK&ÙGþ2Ðòé^3ç[ÇvµÄØD3Ä_þM2w)èjò¶K.¶½Ç?]Ñ÷0LglÓ3gÆ'ÞæØ±cÑÂ(þÞ¢S·ÑùÜ¯ýëwÒàà`t¼0|Ì!òüùóá¦f¿üìÝ»÷;ßùNØ¾ûäm·nÝ¦>|áÂÌëïõõõaþñãÇÇöÆ.O=õTfþäOþ$³f¥¾«W¯Aï¼óNX±b½KüIZÐøÌ*è¹ç»£¢vìØ½ïIfNÐREEEyyy[[Ûñ'N¥«V­ÊóÌ»ví÷9H¬_¿~VÙNwj8ÜHKKK¸?±XléÒ¥;wî_%Ì¯««ëx1s.]sùR©TtÁòÚµk³×$ø$IüI$	þ$IàO$Ið'I$ø$Iü-è~ðre>¿âûï¿ÿÎ;ïØÃ¹[·n]¿~Ý8sÿýßÿ=«7Qáõ?ÿó?ÿñÿa¹Û·o_½zþ@_ûÚ×ÿæó+ïô~ô#;1÷ÿùÑjPÑþøÿøÆ¡û¯ÿú¯Ó§ObîæÍÿðÿð'øü	þð?ÁàOðð'øü	þð?ÁàOðð'øü	þð?ÁàOðð'øü	þð?ÁàOðð'øü	þð?ÁàOðððððð?ÁàOðð'øü	þð?ÁàOðð'øü	þð?ÁàOðð'øü	þð?ÁàOðð'øü	þð?ÁàOðð'øü	þð?ÁàOð?ÁàOðð'øü	þT=ö7Þèïï?øü	þ*ä.ðK=ºêÑzèÓþôï½ð'øü	þTçUTT<±þÏìÿö~uï'>ñÕ«WÂü	þ?Zo¼ñÆ/ÿò/GòþíùÊ_ø_¿àþ?Á­_~¹éÓMÙøÿV¬Xàþ?Á­ÎÎÎüãÙòz÷Óeeeï½÷üÁàOð'øS¡5::ZWW÷©5ÚûÕ½üj©mmmwÁü	þ?dï¾ûî£>úÁ~ðG)++òÉ'áþ?ÁgÏÞä%þàOð'øü©?øü	þ?ø?ÁàOð'ø?øü	þ?ø?ÁàOð'ø?øü	þ?ø?ÁàOð'ø?øü	þ?ø?ÁàOð'ø?øü	þ?ø?ÁàOð'ø»úúúÉd<¯««ëêêÊyJ-É*ÿÊð'øü	þ©TêðáÃabÿþý[¶lÉ^ÔÞÞÞÚÚ:ÃáOð'øü	þþ***ÆÆÆÂD:®®®Î^ä×ÑÑ1Ã#üýñÿñ÷æ±¿þë¿þ«¿ú«ï©³È> û:;;=:Ï_tã/O9ª©©inn3/^¼åÝÝÝéyìßÿýßÏ=VwåÊ¾¾>ãPÌ]»ví­·Þ2ÅÜOúÓS§NbîÝwßíééç/ºñÅ2ÓDbÊu®_¿^__ÇöÓ¾rÚWNûÊiß¿ÊÊÊH¯ácnµzùW?ÁàOð'ø[èøkii9tèPS©Tö¢ñ#ÍÍÍùW?ÁàOð'ø[øëéé©ªªÅbÉd2óÃ½±Kooommm<_»vmðßt+ÃàOð'øü-üÍað'øü	þð?ÁàOðð'øü	þð?ÁàOðð'øü	þðððððð'øü	þð?ÁàOðð'øü	þð?ÁàOðð'øü	þð?ÁàOðð'øü	þð?ÁàOðð'øü	þð?ÁàOðð'øü	þð'øü	þð?ÁàOðð'øü	þð?ÁàOðð'øü	þð?ÁàOðð'øü	þð?ÁàOðð'øü	þð?ÁàOðð'øü	þð'øü	þð?ÁàOðð'øü	þð?ÁàOðð'øü	þð?ÁàOðð'øü	þð?ÁàOðð'øü	þð?ÁàOðð'øü	þð'øü	þð?ÁàOðð'øü	þð?ÁàOðð'øü	þð?ÁàOðð'øü	þð?ÁàOðð'øü	þð?ÁàOðð'øü	þðððððð'øü	þð?ÁàOðð'øü	þð?ÁàOðð'øü	þð?ÁàOðð'øü	þð?ÁàOðð'øü	þð?ÁàOðð'øü	þð'øü	þð7]Éd2×ÕÕuuue/?KaQX-z-Éþ?Áàoá/J>|8Lìß¿Ë-ÙjjjN:&9²bÅ0ÑÞÞÞÚÚ:ÝMü½ùæ7æ±ÿøÇáþ¸üä'a/5ÅÜ¿üË¿ôôôbî_ÿõ_O<i¹û·ûû¿ÿûyþ¢ccca"NWWWO·Ziiiøä×ÑÑögöÖ<¾Ó'NxKEÜ÷¿ÿý¿ù¿1Å­üøqãPÌýà?øÞ÷¾g¹ð?ÀÎÎÎyþ¢ñx|Êéìz·mÛlnn«566^¼xÑi_9í+§å´¯ö]døÅbéD"1y[·n¥R©áááì×¯_¯¯¯?ÁàOð'ø[dø«¬¬L§Óã§ÃtÎÒ¼ÖÖÖðÜ:yÃÉR?ÁàOð'ø[èøkii9tèPS©Tö¢îîîæææÁÁÁÌaü	þ?Áß"Ã_OOOUUU,K&èm««sÞØ¥···¶¶6¯]»6øþ?Áàoáo?ÁàOð'ø?øü	þ¿ny§²¯Õ?ÁàOð'ø[ÜøÝ©éÞþ?Áàoñáoq?ÁàOðwñ744´ûvø³ÓÃq?ãÆþÕÕÕñxÜkþàOð'øü©ðñW__?ùj¿º?ÁàOðWøK$AUUUa"ï;ßùNØ¶müÙéáÏ8Àq?ã¿ÌßØÚ/_eeeðg§?ãÆþüþ,Y¨×ÓÓséÒ¥0ñì³ÏFÞêþàþàþàþà¯ñ×ÖÖ¹¼#ûe«W¯?;=üø3ðgà¯Ððzá.]&Â.&Ôã?ÁàOð'ø÷&Ïð'øü	þð?ÁàOð7+V¬ÞðÅ<ÃàOð'øSã¯¦¦&|ððððWøÎìëíí[°C?ÁàOð77ø«¨¨ø[Èò?ÁàOð'ø3üõõõüíÜ¹óÖ­[ð?ÁàO¿Ð²eËJ&åø?ø?ø?ø¿ÄßòåË]ð?ÁàOÅ¿ýýýyèáOð'øü	þæ.ø?ÁàOð§bÁ_wwwÀ_[[ÛÈÈüÁàOð'øSã¯dððððWoò<e.ø?ø?ø?ø?ø+Ì·zYøÁàOð'øüÍþª««/_~áÂø?ÁàOð§ÂÇ_</)YèGßüæ7yäßú­ßZào¸?Áà¯ð×ÕÕð÷ÜsÏ-Ø7|)Hü>ñÄ¿´ü>ßòùßýòï>þÇþçþÄ~ÒàOð'øüÝGü¹Ú÷AáïÛßþöG>ò=_Ù³ïÑ¿Ïmú4øü	þ÷®öPøægÿÌãùEÿ.]úþûïûa?ÁàOðw¿ð·(*HüýþïÿþgûL¶üö~uï%Ký°ÁàOð'ø¿BÃ_xDúÐ~o×ïeð÷øgÿÕ_ýU?ið'øü	þî/þÒéôúõëKKKKJJÊÊÊ6mÚ´Ð®ü(Ô«wíÚõáø7~ã7>ßòù_[ýk.]ò?Áàï>âïöíÛS^ð± þÔo¿ÕËo¼ñÛ¿ýÛ=öØÞ½¯âÇþ?ÁßýÅ_mmm ÞnÝº>Ú¸qc³råJø³ÓÃq?ãÆþD"Pott43'N9a>üÙéáÏ8Àq?ã¿X,¨À322æx«ø?ø?ø?ø¿=íÛÜÜöÃtÓÐÐvzø3ðgàÏ8À_¡á/hoÊ>Â£?;=üø3ðgà¯Ðð7>qÁï¦MÊËËc±XøØÜÜæ,¨Ç	?ÁàOð7îMáOð'øü	þàþ?Áà/gË;ÅàÏNÆþüø+üÅ¦þàOð'øüi¼HNûîÞ½;Âß#GàÏNÆþüø+Xüõõõö555e¿ç3ü	þ?Á_¡áoóæÍÑ¿cÇ-À¡?ÁàOð'øü½þúëû6lØ°`þ?Áàï^ñwûöí+WF×vtuu-ä¡?ÁàOð'ø»'ü½ôÒKÑ¿-[¶,ü¡?ÁàOð'ø÷>ð'øü	þ3*v§âñ8üÙéáÏ8Àq?ã¿Åü	þ?ÁüÁàOð'øüÁü	þ?ÁüÁàOð'øüÁü	þ?ÁüÁüÁüÁüÁü*þV¬XH$¼Ïü	þ?>þjjj²Á7ïó×××L&ÃíÔÕÕåüù¸Éò¬?ÁàOð7gøÎìëííÛ;J¥>&öïßóGä&/Ê³r¨­­­ªªêáy¬ºº:`ôaqaøð?l¹~ô£öû È[¶lÙ<$tßñWQQð7çòn9ºÙt:~æ_gåÐîÝ»K$I û¿¾¾¾ðevîÜyëÖ­¹Å_öãÈåYþ$IüÍþBË-üïýì[H$ùåY9´oß¾ßùßùÚ<öôÓOoß¾ýk*âÚÚÚ¾ô¥/bnÏ=_üâC1÷Õ¯~uëÖ­Æ¡ægZ[[çùÞwü-_¾<|sxÁGeee:Îäéüò¬ì¹àC.ø>ä9Ã_Ä¾þþþ9¿s---ác*Ê¿(ÏÊð'øü	þs¿ÊÊÊûsÁGOOOUUU,K&èLöäES®?ÁàOð7Çøëîî kkkY°C?ÁàOð7>§ïÇð'øü	þ±iº÷>àOð'øü	þþEð'øü	þð?ÁàOð7ûÒéôúõëKKKKJJÊÊÊ6mÚt?.þ?ÁàOð'øðø»ûö|,¨áOð'øü	þæµµµz6lþ¶ïÐÐÐÆÃ+WÂþüø3ðWhøK$z£££9ét:ÌüváOð'øü	þ=þb±X ^ôwu£FFFÂoõððððW°§£Ó¾ácsàÏNÆþüø+4üíMyÁGx´ðg§?ãÆþüþÆ'.øÝ´iSyyy,Ãõ8áOð'øü	þÆ½É3ü	þ?Áßìª®®^¾|ùàþ?Áñx¼¤d¡A?ÁàOð'øüuuuü=÷ÜsCCCí¯ºÁàOð'øüÍ1þJ¦)Áþüø3ðWhøM7y?ø?ø?ø?ø+ü-_¾¼®®nxxxQ=ü	þ?Áß=á/ú¾ã§Ô^øü	þ¿¹Ç_ô'£?ãð'øü	þTàøK&%ysÁüÁüÁüÁüÁ_áà¯··wÉ%Ññ¿z.ø?ÁàOð§Å_¦æ<øü	þ¿û¿Eü	þ?ÁüÁàOð'øüÁü	þ?ÁüÁàOð'øü_¼xþàOð'øü©Xð½ÏKCCÃÛo¿ð'øü	þTàøìË¼±s<_»víÀÀüÁàOð'øS!¿æïâÅµµµ&õë×Ãþüø3ðWø;vìXii©?ï?ÁàO¿þþþì#åååÛ¶m?;=üø3ðgà¯Ððm¾ÒÒÒ-[¶÷,´¡?ÁàOð'ø««ãñøc=vùòå;ôð'øü	þs¿ü/ð'øü	þs¿Eü	þ?Áßáïüùó+V¬(++®ð­¬¬loo?ø?ø?ø?ø¿ÄßñãÇ3|Dø¦÷ïßvzø3ðgàÏ8À_¡á¯ªª*Pïüùóü=z·ø³ÓÃq?ãÆþÑq¾ñýßñ·z.?;=üø3ðgà¯ÐðWYY¨íøK§Óöì	ÓÕÕÕðg§?ãÆþüþº»»K¦êÔ©Sðg§?ãÆþüþ¢'¸ÆÆÆèjßÒÒÒ+V,¨Ç	?ÁàOð7îþàOð'øü	þàþ?Áàoâ444Ç&uuuí/ü	þ?ÁßàoëÖ­%Ó´ûvø?ø?ø?ø?ø+üµ··GÎ;pàÀððp4óöíÛÑüÎÎNø³ÓÃq?ãÆþÉd2ïàÁEðÍûüÁüÁüÁüÁüþDÞíÛ·'/J§ÓaQXþìôðgàÏ8Àq¿Á_æ¯ºM·4úSoð'øü	þ¿<¼?øü	þ?øüu.?ÁàoQâ/ðð~øÃ~ä#ùÅ_üÅ_QZZúÍo~þ¿E¿ØÇãðg§?ãºråÊ?øÁÍÛ¼ïáß¶/nè¡¾ýíoÃàOð·ð·¸?Áß¬­­í×ý×#ùEÿ>ßòùøü	þàþØO<´¿ðïç~îçàOð'ø?øü`_þòüñlùmûâ¶~ô£ð'øüÁü	þ°ðÓ÷¡hû¶GòÛó=¬xäé§?ÁàþàOðWýéþé>ðµZ»nÝºzè7ó7á_àOð'ø]Éd2×ÕÕuuue/?KaQX-zÍ~£øü-´þâ/þâå_îîî-?ÁßìJ¥Rû÷ïß²eKö¢S§N#G¬X±"L´···¶¶NwS'OüßyìÊ+ÿ«"îÚµkÆ¡ûéOðg¹7nübî½÷Þøç/ºñWQQ166&Òétuuõt«A~yð÷GôGGç·7ß|ó¨;ûì²hþ÷E¿ì·î-£·mÛlnn«566^¼xÑi_9í+§å´¯ö]døËþqDbò·nÝJ¥RÃÃÃÙ3¯_¿^__?ÁàOð·ðÅFeee:8í¦sÖÈkmmÏ­od²áOð'øü	þÆø¿Cð1Je/êîînnnÌÌ©©©PÁàOð'øü-2üõôôTUUÅb±d2ùáVWWç¼±Kooommm<_»vmðü	þ?Áß"Ãß?ÁàOðð'øü	þð?ÁàOðð'øü	þð?ÁàOðððððð?ÁàOðð'øü	þð?ÁàOðð'øü	þð?ÁàOðð'øü	þð?ÁàOðð'øü	þð?ÁàOðð'øü	þð?ÁàOð?ÁàOðð'øü	þð?ÁàOðð'øü	þð?ÁàOðð'øü	þð?ÁàOðð'øü	þð?ÁàOðð'øü	þð?ÁàOð?ÁàOðð'øü	þð?ÁàOðð'øü	þð?ÁàOðð'øü	þð?ÁàOðð'øü	þð?ÁàOðð'øü	þð?ÁàOð?ÁàOðð'øü	þð?ÁàOðð'øü	þð?ÁàOðð'øü	þð?ÁàOð¿³gÏ¾ñÆáãèè¨?øü	þðW°ø÷Ýw]õèÇ>ö±ð±ººº®®.@ÄO ü	þ?ø+Lü­òSk>µ÷«÷=³/ükjjþsüþ?Áü þÎ=û±,#¿èß²eËü?ÁàOð¿£G>ºêÑlùüÄ'¿ûÝïú!?ÁàOð¿³gÏ&É#ÕÕÕ~	ÁàOð'ø¿Ä_èüäêGW·=ÝØ·ç+V¯^ý+¿ò+###~áOð'øüÁ_âoxxøsûÜÒ¥Kyä|àë×¯ï½÷üÂàOð'ø¿ÂÄ_T_øÅóî»ïúÙ?ÁàOð?ÁàOð'ø?øü	þ?ø?ÁàOð'ø?øü	þ?ø?ÁàOð'ø?øü	þ?øü	þ?ø?ÁàOð'ø¿¬úúúÉd<¯««ëêêÊyJ-É*ÿÊð'øü	þ©TêðáÃabÿþý[¶lÉ^ÔÞÞÞÚÚ:Ã#üM®ÎcA~áUEø/q(æÎ?ÿýïß8s?þñ»»»C1÷üäïþîïæù.büUTTt:]]]½(È¯££c+Gøûó?ÿóÍcA~áý#q§Nêêê2ÅÜøÃ¿ýÛ¿5ÅÜ[o½uâÄ	ãPÌõöö?~|¿è"Æ_<r:TSSÓÜÜf666^¼x1ÿÊNûÊi_9í+§å´ï"À_,ËL')×¹~ýzýW?ÁàOð'ø[øË¾£²²2NOÉÓÓmQ/ÿÊð'øü	þãüÈ_KKË¡CÂDøJ¥²ÕÔÔOùknnÎ¿2ü	þ?Áß"À_OOOUUU,K&è `ooommm<_»vmðßt+ÃàOð'øü-üÍað'øü	þð?ÁàOðð'øü	þð?ÁàOðð'øü	þðððððð'øü	þð?ÁàOðð'øü	þð?ÁàOðð'øü	þð?ÁàOðð'øü	þð?ÁàOðð'øü	þð?ÁàOðð'øü	þð'øü	þð?ÁàOðð'øü	þð?ÁàOðð'øü	þð?ÁàOðð'øü	þð?ÁàOðð'øü	þð?ÁàOðð'øü	þð'øü	þð?ÁàOðð'øü	þð?ÁàOðð'øü	þð?ÁàOðð'øü	þð?ÁàOðð'øü	þð?ÁàOðð'øü	þð'øü	þð7ßýÁüÁw¿ûÝ+óXøiïìì¼¢".üÖ?vìq(æþéþéÍ7ß4ÅÜ¹sçÞxããPÌýó?ÿó_þå_Îóÿý÷áoüüùóûöíû$IR¡ÿlgã±$IÅüI$Á$IàO$Ið'I$ø$IüI$	þeÉd2×ÕÕuuue/immM$?üpww÷øÄýdeô~ÈþvÇb±ü+«HöÏÅ¶çÿÚÚÚhQOOçûÀBxðÔsO¥R©Ãýû÷oÙ²%ÑóÏ?ÿâ/ïúòåËÃöööÀAV<û@¦°BX:ÃUØûçbÛ***®^½:>ñ7?«««=ØÂóüÝSá;x&ÒétôÍáÂì9á;ÝÑÑaÐgº|ùòºuëf¸²~ð<Plû@øÏÿõë×ÃDøð<`xàÏðwOÅãñ)§£O_zé¥ÒÒÒð>wîSSSÓÜÜæ766^¼xÑèü>µiÓ¦ÞÞÞ®¬ß<Û>Ð××~ÑnàyÀ>ðÀàï^ÄH$r<x0Lïkøîf/ö¯¯¯7z¿.]º´jÕª®¬bØ<Û>°råÊè,P@´'x°<ðçø»§*++ÓéôøÄaÞ0³(ÏÿíüÀÃ>0>ñÒÏ^zi+«öÏÅ¶L> äyÀ>ðÀàïjii9tèPS©Tö¢;v¼öÚkaâüùó«W¯8Ì;00I¿¹¹Ùèü>Z³fÍ3gf¸²að<PlûÀªU«Âo0Ñßß¿råJÏöð<÷TOOOUUU,K&§Oþ¿c:qÙöÐÐÐ¦M¢3ú.]sz£½×®]½öS½Dÿ¥^geÕ>ày Øö·ß~;ü¾ßñð1L°,çø$I*¢àO$	þ$I$I?I$Á$IàO$Ið'I$ø$IüI$	þ$I$I?I$ø$IüI$	þ$I$I?I$Á$IàO¦î[ßúÖ5kJ'Z·nÝë¯¿þÿ=¯M´h§º·ÕÕÕá¡çÌsâñx2ímJüIZíÛ·¯dR/¼ðB!áï¹ç3_yåù/¿ürÿì³ÏÞÅmJüIZ|õõõÖÄãñNtðàÁðiyæÌÁßåËÃÌúúúùµµµaþÀÀüI?IEÑ¾ðÀ_|1æ×¿þõ0óÉ'Ì¦OWWWÀSpaCCCÎ¬|óæÍmÛ¶E»víÊ>»zâÄ¬°(lÛÙÙÃ©0gÉ%kÖ¬9vìXø´¥¥%ç=z4ÿíDÂ]?~|:¨577ù§OÎÌ9uêTÓÔÔóüóÏWUU*--Ý¼yóõë×'ãoòíçÌÉsW%Á$=à-[àrõêÕì×®]3«««³qSooo´tãÆ9vîÜ-êïïÅbSn-Ý´iÓØØXàc"KÃÇ§²²²è¥xyn'LLy÷&?Ò#Gd6ãËÃgäs#ëÖ­-þòÜUIð'I¾èïÏe%%bÙ¸Ù²eË­ÂDøtÃÑÒÈ:£xmÑ¢T*2Ó===ÑdßæÞ½ï¢³®[·ns:::Âtø¦zê©;ÞNcø4¬ß¦|DK.wpp0|>ÄN§£ÉdØ0<ñ&.---þòÜUIð'I¡)ñá&2SèúõëáÓÀ¦èÓðéòåË·oßÐvûöíÌur¥e¶>½víZfå¦Ìßð1L8qâ· >½qãFöîõy»wï¾ñoÿìÔö®]»²Wø$mllLÎyîª$ø¤_t¸ëÖ­[Ù3ÃÌ°(w24¼téRä¿u2/Ô|66SÙo°¦+**ÄÈÈH¸ñ%KdÞñv¦£XNçÏ¦ÃÇ0ÝßßYÚÓÓîÀ§g¿<wUüIÒ/zÝÛK/½=3z>2¯|@ëÌ3Ï=÷6£Æè°¼ê¶sçÎèloø¸mÛ¶Ìü<·içw¢OoÜ¸ÿÊÜU«V¥Ñ»Û³f/®ü?>44F£Yç®J?IzðELÄãñýû÷GoõràÀD"1ùâ6èDçd3WæF¯ù^*wéÒ¥ìFÜµkW RtnæÍV¦$ZtùmôÕ»»»3óóÜNô"¿è5ÃÃÃÑyðwðàÁÌ1¹ð³E/ìïï_%Âtø¸¸VèÜ¼ysöÒ<wUüIÒ(zäþùÿ÷¼6Qd£ÌtæiæL[·nÍevíííyð7>ñ§8Âüì3Âyn'Üì;¹ÒvºûöíÒÒÒè!äì^¿~öXºtiø½ÛKömF&3ETÍ,ÍsW%Á$-PÖ­[hÍ5Ñ%·9ø;~üxôvzuuu'OÌ,Þ³gOUUU¦Ý»wdvvv666%ÉW_5ç6'ßpSaþ;ræOw;¡pgÂ]Þ0ÏûüeÚ¶m[Î¾D¦R©0åååáQdÞ¶&û6n£±jjj:útÎWÌsW%Á$IàO$Ið'I$ø$IüI$	þ$I$I?I$Á$IàO$	þ$I$I?I$Á$IàO$Ið'I$ø$IÒìÊxCÈ×IEND®B`


µµµ8A6###sE.-wÎ7¿ùÍ0ÝÚÚjkfÿKI5c7o&ñX¦+++m-àOR9È/:>úhUUÕ¼yó¶oßw¶ûï¿¿©©);³»»;³fÍ0'Ìïïï_µjUÐR<ollìêê÷rr¯·ø*û÷ïO¥RË/ÿðÃqvåÊ¶¶¶°n¸Ù[·nÆ[Çß3gb±XtûGGGÃM'ÃLÛ$ø4üM?õÔSaú¹çØO?ýtîy:::ò>÷èæ?>L;w.L9azéÒ¥G	aQL&Ç½Ü«.¾J Ý/¼&Ö¯__x³7mÚ¦Ã^~ùå0±sçÎ«|oøÂã¦¿üå/GvìØNîÛ·o÷îÝaâñÇ·I?I³©T*LgÆ*Xá;jôÑ«coF$<yrÛ¶mMMMÑ1ã^N¹¯Ý¤ªªªÂukjj¢ &n½õÖëÜ9Ñ-	WäwùòåhQ¸¢E+7o^8i+f%þâñxv:LÙ(*`«¶¶6èÒ¥Káguuu$¡½÷ó¯]»öwÞÉ]7ïrrONfnRðYîÛKõÎß¸=óÌ3ÑrwKüIeøK&¹ïüeßB+"¡'|2,jnnÎîo%prt¬Iâ¯ø*Ù4þüÂu,X­[A¸æ]¾|ùrMMM:^±bE ïÅmEàOÒ¬Ä_Ð[´ë6ÚûÌ3ÏÑûágöh§N5Iü_eÛ¶m/¾øbØ°aCáº<òH>pàÀéÓ§³;üøð·yóæ°èäÉÑ!/Ù!JüIeø	Ì7Ö;&#¡ÐÆs>|¸ºººªªjëÖ­Ä_ñU=.[¶,ÄIîyÈÂmN$«W¯~ÿý÷?>üùwßwtrÅád__IüI$	þ$I$I?I$ø$IüI$	þÊ¤üàï½÷ÞL^ãÏþóÿüÏÿ´å°ÁÁÁi|®¹¡öÿñ6Ôwþüùÿú¯ÿ2%ìÊ+|ðq(m?ûÙÏnªu®ãïë_ÿzðßs3û§<Uþå_þåÄÆ¡´;vìßÿýßC	»xñâÛo¿mJÛ÷¿ÿýãPÂ.ðÿðÆ¡´½ñÆá×?ø?ÁüÁàþàþàOðð'ø?ø?øüÁü	þàþàþððððð'ø?øüÁüÁü	þàþðð?ø?ÁüÁüÁüÁüÁü	þàþàþð?ø?ø?ÁüÁàþàþàOððððð?ø?ø?ÁüÁàþàþàOðð'ø?ø?øüÁü	þàþæ.þzÉd<ohhèêê*<CgggEEüÁàþàOðåP:>pà@Ø³gOkkkÞÒÆÆÆðOU|ðÁ®óçÏ?~Ü8¶ðT^C	ûÙÏ~öÖ[oÒöúë¯ÿó?ÿ³q(açÎûáhJ[WWWøõo&¯qNà¯ººztt4Ld2T*·ô©§Úµk×DøÛ½÷÷g°#Gàû*]¯½öÚ«¯¾jl¨6Ô9Ø+¯¼ügJØÑ£Gm¨e°¡Î	üÅãñq§CýýýMMMvûÚí+»íöµÛWvûÚí[&øÅbÙéD"»¨¥¥%¼ýò®Âü	þàþðWÃZSSÉd®íöÓ¿q3ø?ÁüÁàþfmmmû÷ïág:ÿ®zçþð?ø+aíîî®­­ÅbÉd²§§gÁü	þàþðçKáþð?ø?ø?ø?ø?øüÁüÁü	þàþðð?ø?ÁüÁüÁàþàþàþàþàþðð?ø?ÁüÁüÁàþàOððð'ø?øüÁüÁüÁàþàOððð'ø?øüÁüÁü	þàþðð?ø?øüÁüÁü	þàþðð?ø?ÁüÁüÁàþàOððð?ø?ø?ÁüÁàþàþàOðð'ø?ø?øüÁü	þàþàþàOðð'ø?ø?øüÁü	þàþàþð?ø?ø?ÁüÁüø?ø?øüÁü	þàþàþð?ø?ø?ÁüÁàþàþàþàþàþàþð?ø?ø?ÁüÁàþàþàOðð'ø?ø?ø?ø¿Y¿öööûï»ÿ±ÇûàüÁüÁü	þà¯lñ·yóæO~ò¬yàKm_ºkÙ]ÕÕÕgÏ5>ððð'ø¿2Ä_x¸å[¾ºù«ÛÛý»÷ó÷ÞqÇÆþàþàþðWøâ'>Ïç³òÿ¶mÛüùóüÁüÁü	þà¯Üð÷ØcÝ·ú¾ÕÕÕFþàþàþðWøûÎw¾³páÂm_ÛßÒ_øÌg>c|àþàþàOðe¿ûî»¯¾¾þ¡úÿ÷÷~þÞ[n¹%¼0ø?ø?øüÁ_â/þÆ7¾±|ùòE­[·îG?úÁ?ø?ø?Áü3þðð?ø?ÁüÁüÁàþàOððð'ø?øüÁüÁüÁàþàOððð'ø?øüÁüÁü	þàþðð?ø?øüÁüÁü	þàþðð?ø?ÁüÁüÁàþàOððð?ø?ø?ÁüÁàþàþàOðð'ø?ø?øüÁü	þàþàþàÏ8ÀüÁàþàþàOðð'ø?ø?øüÁü	þàþàþððððð'ø¿¹¿>úè¡úÄ'>1þü?ú£?êëë3Jððð'ø¿òÄ_ßïýÞïÝu×]¹å/·mÛ½¿7(ðôéÓþn|½½½Éd2744tuuå.êééill,YÎð'ø?øÓ$ñ÷ÄOÜÞxûöÇ¶gÿö³ýÂ¾` àïÆN§8&öìÙÓÚÚ»hÑ¢EÇ¬¯¯/ÄßßýÝßýbíµ×~òüB¥ëÿñß|óMãPòõìÙ³Æ¡:uÊZò¾÷½ïýô§?5%ìÇ?þñ~ðìÉæææ/~ñ¹øÛð7|úÓ6PSÝPß÷Ý¼Æ9¿êêêÑÑÑ0ÉdR©ÔDg«¬¬,Äß¾ûÞÁ:;;O8ñJ×[o½uôèQãPÚ¾ûÝïÚPK¾¡Ro¨o¿ý¶q(aÇÏÝP×®]ûÀrñ÷g_þ³5¥:::fxCøÇããNç^ÉÚÛÛíöµÛWvûÚík·¯&¹Û÷ðáÃüä'¿ºù«ü¶mÛm·ÝöäO(»o|±X,;H$ÏÒéôàà üÁàþàOÄ_hóæÍ·ÜrË=»ç¾ûîûýÏüþç>÷¹7¾L&sul·o.Ü×¯_yW?øüÁü©þ¢W®'xâ+_ùÊßþíß%ø»)jkkÛ¿?ÓétÞ777»"üÁàþàOÅñ'ø»ëîî®­­ÅbÉd²§§çW÷­â÷.JUäð'ø?øüÁßÜþàOðð'ø?ø?ø?ø?ø?ÁüÁüÁàþàOððð'ø?øüÁüÁü	þàþððð'ø?ø?øüÁü	þàþàþð?ø?ø?ÁüÁàþàþàþð?ø?ø?ÁüÁàþàþàOðð'ø?ø?øüÁüÁàþàþàOðð'ø?ø?øüÁü	þàþàþð?ø?ø?øüÁüÁü	þàþðð?ø?ÁüÁüÁàþàOððð?ø?ÁüÁüÁàþàOððð'ø?øüÁüÁü	þàþàþàþàþàOðð'ø?ø?øüÁü	þàþàþð?ø?ø?ø?ø?ø?øüÁü	þàþàþð?ø?ø?ÁüÁàþàþàþàþàþðð?ø?Áü×*ÁüÁü	þàþà¯Lð»VñxþàþàOððvûÂüÁüÁàþà¯¼ð|³aÃø?ø?ÁüÁü!þR©T<÷?ø?øüÁü©üñ·dÉÂ£=ª««áþàþððWnøK$Aµµµa"ïÅ_íííðð?ø?ø+7üEoõ ½0qþüùÑÑÑ01oÞ<ø?ø?ÁüÁüþæÏ¨×ÝÝöìÙ0ñøãG¾êþàþð*CümÝº5xGîÇþ/_ðð'ø?ø¿2<Ú÷É'`Aèéé	MMM³âñ?øüÁü	þàoð'ø?øüÁüÁüÁüÁüÁàoâêëë£/|ñ%Ïðð'ø?øSãoÑ¢E¹àËæh_ø?øSyàïG?úÑw¾óðsddþàOðw58/°/¼úÎºÇþàOðE|ymmí]ËîZ¸paCCÃ|ð§¹¿êêê¿Ù(?ø?ÁüïOþäOn¿ýöm_Û¶ý±íáßªU«ÿn÷ÿàþàïÆà¯··7àoÓ¦M³ñ üÁàþ&ê£>Z°`ÁÖ¿ÜÉ/ú·páÂçÀüÁßÁ_(<Täø?øÓ¬Æ___ßâÿµ8W~áßÿûÎoûÛðÓø«««sÀüÁü©üð÷ÑGýÎïüNÞ;©O¥¼óëøØ~AüÁàþôËÏü5þÆgþn»í¶ááaø?ÍiüÕÔÔ8àþàþTøËíçwFGû¾÷Þ7ÏÍ?ø¿¿pþ¶nÝzóü.ð'ø¿Ï_¸ý¾çþc1Aø?ø?þnÚàþàïÆà/6Aø?ø?ÁüÁÊó«^foð?ø?ÁüM­T*UWWwúôéÜ ÞÞÞd2Çººº/Þø?ÁüÁàþ¦_pUEEÉÞAL§Óöìimm-¾hzsrôÑGo¿ýöU3X¸º»ï¾J×+î¸ãã`Cµ¡ÎÁm¨¥íÿðm¨ÇúÙÏ~v&¯ñcÇ_WWWÀß;._¾|ý_øR]]]H&I¥RÅMoNn[¶l©$I*£>vüöhßÜÃDò)½9ð'Iàoúöhß&â¦7'·mÛ¶Ýwßëg°pu>øàz®/~ñkÖ¬16TêlõêÕ=ôq(aú§úÀÒvï½÷Îðú±ã¯´ÕÔÔd2h/m.¾hzsðá9àÃø>ðq³ÔÖÖ¶ÿþ0~¦Óéâ¦7þàOðð'ø¿ë*É¬^½º²²²¢¢bÞ¼y---Ó>ò£»»»¶¶6%É_ÝÊ±½×¦7þàOðð'ø¿é7444îgÅú?øüÁü	þàoj-^¼8PoÍ5ÑÿÀ µk×9K.?ø?øüÁüÁ_¹á/Hêdçd20§ðÐZø?ø?øüÁüÍzüÅb±@½èÚ¨ááá0gz_õ?ø?ÁüÁßM¿h·osssô?0üÓaNcc#üÁüÁàþàþÊá?Þ¸|t	þàþàOððeøU/CCC---UUU±X,ülnnsfÅãð'ø?øüÁßþàOðð'ø?ø?ø?ø?ø?Á_Þ×*ÁüÁü	þàþà¯Lð8ø?ø?ÁüÁÊµeË?ø?øüÁüÁ_Ùâ¯··wÞ¼y«V­ÊýÎgø?ø?ÁüÁüþÖ­[½áwäÈYôxÀü	þàþð7µ^~ùåkÖ¬uüÁàþàOðmhhhéÒ¥Ñ±]]]³ññ?øüÁü	þàoRíÞ½;zÃ¯µµuö>ð?ø?ÁüMnMßóð?ø?ÍüÅ®U<?ø?øüÁüÁ?ïðð?ø?ø?ÁüÁàþàþàOðð'ø?ø?øüÁüÁüÁüÁüÁàþàOððð'ø?øüÁ_ÉðW__H$|ÏüÁü	þàþTþø[´hQ.ø|ÏüÁü	þàþTÎøÎì¯¾£££³îñ?øüÁü	þàojUWWüÍFùÁü	þàþð7åzþ6mÚ4ÿÂü	þàþð7å.>àþàOðð§2Ä_]]>àþàOðð§¹¿³ññ?øüÁü	þàojÕÔÔ8àþàþð+øw)àoëÖ­ÃÃÃðð?ø?9þ*&Èðð'ø?øSy~Éó¸9àþàþð*Ï¯z½Áü	þàþððððð'ø¸L&³zõêÊÊÊyóæµ´´Ìáþð?øZCCCãð1+þ?øüÁü	þàoj-^¼8PoÍ5ÑÿÀ µk×9K.?ø?øüÁüÁ_¹á/Hêdçd20'Ì?ø?øüÁüÁ_~ÕK ^_vÎððpã«^àþàOðð§²ÝíÛÜÜý?ÃtÓØØðð'ø?ø¿rÃ_ø7î.]?ø?øüÁüÁ_~ÕËÐÐPKKKUUU,?ÃYñxÀü	þàþð7?øüÁü	þàoj¥R©ºººÓ§OÃüÁü	þàþTþøÇã³õDø?ÁüÁàþ¦VWWWÀß;ifË_u?øüÁü	þàoº1A±XþàþàOððeø%ÏãæKáþàOðð§òÁ_]]]CCÃààà¬~<àþð?øTÑô½:¶ÛwVìá?øüÁü	þàoúøþ¤oôg|áþàþð*sü%É¢9àþàþð*üÛùóçGïÿEÔsÀüÁü	þàþT¶øË6[ð'ø?øüÁ¿íð'ø?øüÁßl«··7LÆãñ®®®ÜE===aÑ%KÂÙàþð?øõ¥Óé=ö´¶¶æ.Z´hÑñãÇÃÄÁëëëáþð?øõUWWGV8É¤R©ÎVYYY¿¿ù¿ykëèèþK¥ëÍ7ßüîw¿kJÛ«¯¾jC-ùÚÙÙil¨7yÁÓ6ÔcCíîîÉküØñwæÌ¿Ü#N&:úäÄíííø;|øðÀöúë¯?~@¥ë§?ýiøOeJ¾¡þüç?7%ììÙ³6Ô÷Úk¯õ÷÷öüäøñãÆ¡´=zt7ÔÑ÷¼466¾ûî»7¹ß)H$ÏpåÊt:]øÇèìöµÛWvûÚík·¯ìöµÛwjöe¿Ø9ß÷ÝçÎþëÂtMMM&vûéÂMyýúõá·ðBàþð?øNgÎY¼xqdDbõêÕ3v7ÚÚÚöïß&ÂÏt:7âÍÍÍ½ð'ø?øüÁßô=räHeeåÿy·îîîÚÚÚpÉd²§§çW÷mìMÁT*ûGçàþð?ø+A¹ïüUUU]qð'ø?øüÁßÔÊ5_eeekkkÿly<àþð?øâEçqÏ=÷?~Ö=ð?ø?ÁüM­ø/ð'ø?ø?ãð7ÓøÕÁü	þàþð7åN:U__?oÞ¼èß^z	þàþàOðð§2Ä_gggöÑô=àþàþððWnø«­­Ô;uêT===Ñ·½ÀüÁü	þàþà¯ö¾<9¿ÑÑÑè`ø?ø?ÁüÁüþjjjõ¢wûþ2Ì£>¦S©üÁüÁàþàþÊá.U×ñãÇáþàþððWGû'µ¦¦¦èhßÊÊÊúúúsçÎÍÇþàOðð'ø¿9üÁàþàOððððð¿ñêïïoll¬ªª&fË>_ø?ÁüÁàþ¦Ð#<R1A6l?ø?øüÁü©|ð÷ÒK/EÎÛ»wïàà`4shhèÐ¡CÑüø?ø?ÁüÁü	þÉdÞ¾ûEðÍ÷üÁüÁàþàOå¿D"744T¸(ÉEáðð?ø?ø+üeÿªÛDK£?õðð'ø?ø¿2Á_ÞÁüÁü	þàþððð'ø?øµø+üÁüÁàþàOå¿ØµÇãðð?ø?øóçÝàþàþàOððð'ø?øüÁüÁü	þàþðð?ø?ø?ø?ø?øüÁü	þàþàþð?ø?ø?ÁüÁàþàþàþàþàþðð?ø?ÁüÁüÁàþàOððð'ø?øüÁüÁüÁàþàþàOðð'ø?ø?øüÁü	þàþàþð?ø?ø?øüÁü	þàþàþð?ø?ø?ÁüÁàþàþàOðð?ø?ø?ÁüÁàþàþàOðð'ø?ø?øüÁü	þàþàþàOððð'ø?øüÁüÁü	þàþðð?ø?ÁüÁüÁüø?øüÁüÁü	þàþðð?ø?ÁüÁüÁàþàþàþàþàþð?ø?ø?ÁüÁàþàþàOðð'ø?ø?ø?ø?ø?ø?ÁüÁàþàþàOðð'ø?ø¿bõöö&Éx<ÞÐÐÐÕÕUxÎÎÎø?ÁüÁàþÊ¡t:àÀ0±gÏÖÖÖ¼¥###ð?ø?Áü	þª««GGGÃD&I¥RyKzê©]»vM¿gy¦c;|øð«¯¾Ú¡ÒuäÈ0ªÆÁjCµ¡ª$ê+¯¼bfû:'ðÇÇõ÷÷755N¿àñÿÁ;600ðß*]á÷ÔþðÆ¡´½ùæôq(a|ðÁ'CiOàÿöoÿfJØ/~ñ·ß~Û8¶×_ýòåË3ys±X,;H$rµ´´oýò®Úík·¯ìöµÛ×n_Ùík·ïìÊ_¦kjj2L´Û7L¶ìáþð?øÝµµµíß¿?Létz",Î?øüÁü	þàoöÕÝÝ][[ÅÉdOOÏ¸Ú?øüÁü	þàÏ<Ãü	þàþððððð'ø?ø?øüÁü	þàþàþð?ø?ø?ÁüÁüÁüÁüÁü	þàþàþð?ø?ø?ÁüÁàþàþàOðð'ø?ø?ø?ÁüÁàþàþàOðð'ø?ø?øüÁü	þàþàþðð'ø?ø?øüÁü	þàþàþð?ø?ø?ÁüÁàþàþàþðð?ø?ÁüÁüÁàþàOððð'ø?øüÁüÁüÁàþàOððð'ø?øüÁüÁü	þàþðð?ø?ø3ððð'ø?øüÁüÁü	þàþðð?ø?ÁüÁüÁüÁüÁüÁüºÁÁÁ°utt¼÷Þð'ø?øüÁü3þ=ZUUuÛm·ÝyçóæÍÛºuëÈÈü	þàþðe¿³gÏþvåo·~¡uûcÛÃ¿¯nþê§?ýéíÛ·ÃàþàOððWøûæ7¿y×]wEòþýùú?ÿÝßý]øüÁü	þàþÊ_ùÊWþøÿ8áßoýÖoÁàþàOððWøöÙgï¸ã©íK-?ÁüÁàþà¯ñ^ª?ñO¬¾wõ¶¯mòkÿ?íáäÁáOðð'ø?ø+CüúúúR©Ô§>õ©?ø?¨¬¬üë¿þë¹ð ÂüÁü	þàþæ(þ¢Î;8888GDø?ø?ÁüÁßÆßþàþàOððð'ø?øüÁüÁü	þàþðð?ø?ÁüÁüÁü	þàþðð?ø?ÁüÁüÁàþàOððð'ø?ø?ø?ÁüÁàþàþàOðð'ø?ø?øüÁü	þàþàþððððð'ø?øüÁüÁü	þàþðð?ø?ÁüÁüÁüÁüÁü	þàþàþð?ø?ø?ÁüÁàþàþàOðð'ø?ø?ø?ÁüÁüÁàþàOððð'ø?øüÁüÁü	þàþðs½½½Éd2744tuuå.^¿~"¸õÖ[ÃèÃü	þàþð7ëK§ÓöìimmÍ]´sçÎ§~ztt4]]]!þ^íµ¡ìÍ7ß¯C*]ýýýo½õq(má55¨Ú8°÷Þ¯§§Ç8¶×_ý_ÿõ_C	;þ|øuÚ8¶®®®¼Æ9¿êêêÀ»0ÉdR©Tî¢Ó§OO´bÀß7¾ñ×f°W^yåßûÞk*]G16TªU6TjÔÀ_<w::¹÷îÊÊÊºººwÞyÇn_»e·¯Ý¾vûÊn_»g±X,;H$òíÛ·/L9s¦©©	þàOðð'ø¿YYÅ¯Ó555LæêØnß0¶ÜyoÂü	þàþð7+kkkÛ¿?Óétî¢7¾ðÂaâÔ©SË/?øüÁü	þàoÖ×ÝÝ][[ÅÉdOOÏ¯îÛØAZ---ñx¼©©éìÙ³ð?ø?ÁüÍÝàþð?ø?ø?ø?ø?øüÁüÁü	þàþðð?ø?ÁüÁüÁàþàOððð?ø?ø?ÁüÁàþàþàOðð'ø?ø?øüÁü	þàþàþàOðð'ø?ø?øüÁü	þàþàþð?økýÕ_ýÕ·¿ýí÷f°pu?þñßSé:yòä¡CCiñÅO:eJXxAýû¿ÿãPÚ^xáÓ§O~EyåWCÉ7Ôú§Ék,þÛû_xyÛ¾û×%IÊ¥â5+¼+I4w?I$ø$IüI$	þ$I$I?I$Á_ùÔÛÛL&ãñxCCCWW×owN±XìêØ·~/^¼8:sww·ÑÆ¨a3kzCÚÓÓÓØØæ,Y²$¬hôJ2ªQá	ÁÐdH×¯_H$n½õÖ°ÔèdT½N]ç9s¦©©)lëÖ­þÄ×òSúÒéôÂÄ=Z[[Ç=O8CX&ª««ûûûÃDøJ¥Þ4Fµp'óhJCºhÑ¢ãÇÖ××½jhdd$¨þJ5¤;wî|úé§GGGWêêê^IFÕëÔuéÒ¥KÃïÏaâôéÓ7n¼^¤<ã¾ðÿ$<õL&3îÿóçÏ¯2OO.:öç=UMoTÇð¦:¤¹UVV½RêSO=µk×.ø+Õ644×WVÚQõ:uCÇ³ÓóçÏ¿^¤<ã¾Ü9w:[KKË'¢éÞÞÞðRúÃÏìLMiTÇð¦:¤ÙÂÉööv£WQíïïojj¯ðWÂÿû»wï'QÞyç£WQõ:uCºdÉS§Nç>ZtÃ_¤<ã¾èÃ|QD"oéÙ³g-[=¹téÒè÷Ôð¿+w¾&?ªcXü!Ð44êÊ+étzppÐèdTÃïÇ»:öi`CWªÿûûöí»úëOY½ª×©ëÒðH ó®]»¢='7üEÊ3Né«©©Éd2WÇÞÎÓyKwîÜ~1Ìïä¨añ@ÓÒ«c|Ö¯_ñâECWªQ­øÍ^IþïF-ù¨zºþþ¨wßwñâÅ7Ã§Ò×ÖÖ¶ÿþ0~¦Óé¼¥+V¬8yòdödø-*z7¸¯¯/üveô¦1ªcXü!Ð4ô7Þhnn0n%Õÿy"&¿éÆ_xá0æ/_¾ÜèdT½N]ç.Z´¨··wttt×®]O>ùäÍð"å§ôuww×ÖÖÆb±d2àûäH$¢yfÿÂïRág6zÓÕÂ1÷Ìº!M¥RÞ£*ù¨Â_ÉôòåË---aNSSÓÙ³g^IFÕëÔuiøåyáÂáÕ¿½½ddäfxò#I4?I$ø$IüI$	þ$I$I?I$Á$IàO$Ið'I$ø$IüI$	þ$IàO$Ið'I$ø$IüI$	þ$I$I?IºvÏ?ÿü+*ÇZ¹råË/¿üÏqcÍgäñnm*wmpp0o~ÇÉäèèèT/SüIÒ¬lûöí=ùäå¿;vÏ>ûlÞüoë[aþã?>Ë4ûêíí¬Çã÷îkß¾ádyòäÉ²ÁßùóçÃÌ%KäÍ_¼xqîÜ9øæD>ø``ÍÓO?;óg	3¿üå/çÒ§««+à)¸°±±1LgÏ|éÒ¥öööªªª°¨ººzóæÍ¹W=u;::ò8æÌ?ÅG	'ÛÚÚònØáÃ_N´(Ü¤hQggçDPknnózz²s?æ¬Zµ*;gçÎµµµá¢*++×­[wáÂBü^~Þ"7UüIÒnáÂ.ýýý¹3ßÿý03Jåâ&¯'NDK×®]·hÓ¦MÑ¢¾¾¾X,6îZÑÉhiKKËèèhàc"	KÃÏ§yóæEÅ+r9abÜWxO<+Ú¬/8_Þ¬rªø+rS%Á$Ýø¢=¼ã<¯UTåâ¦µµõÊXa"fM´4²NÄÇè¼¶hQ:Þ2ÓÝÝÝÑä^æ¶mÛï¢½®<òHsèÐ¡0~é~øàN3äÞ¶qïQåÂý'ÃÏpË83Ltd2Vwáê¯wWVVNEnª$ø¤¡qñá&2SèÂád`St²±±1¬««Û°aC@ÛÐÐPöBÂyòÞKË®|ÿý÷³g`Êîù?ÃôÑ£G¯y9áä~Û&ú|Þ-[Â¢]»v]ýõ®íÍ7ç!@0à/´©©)zcrªø+rS%Á$Ýø¢·»®;spp0Ìp'KÃ³gÏFþËZ'ûA½Â½±yÊý0]]]H$ÃÏ??»ô3Åò:uêTäÔ0~é¾¾¾ìÒîîîpÆÝ<yü¹©àOn|ÑçÞvïÞ;3ú¼>²(|CëäÉ;vìvÂfÕ½-Ý¯zM¢mÚ´)ÚÛ~¶··gç¹è¶/F'?üðÃâGæ.[¶,,¾Ý&5wQtäoXÔÙÙyùòåâøËÂ4ìÒ"7UüIÒ/:`"ïÙ³'úª½÷&Â3Ö¬Y 3<<íÍæ/ú¨ÜÙ³gs?,YpóæÍJÑq¸Ù/[hÑá·Ñµ¿ñÆÙùE.'ú_ô¿ÁÁÁèEð·oß¾ìrá.ç.>þØ××®%ÂDø¸¸ÎÐ¹nÝºÜ¥Enª$ø¤¢èóÚ¹sçÿ<ÇÙ(;Ýa=Æ"Û#<+ËÜ^zé¥"ø»:ö§8ÂüêêêÜ=ÂE.'ÜÜ=Òv¢;;44TYYÝ¼Ý«W¯Î½Ñ·½ä^fôÆd¶ªÙ¥Enª$ø¤¥+W&ÆZ±bEtÈmþ:;;£¯Ókhh8vìXvéààà£>Z[[iË-ÃÃÃÙ¥MMMÁdÉdò¹çË»ÌÂ[.*Ìß¸qcÞü.'nL¸IÑù¿líííyßù500N§ÃTUUqîÜ¹ì×Öä^æåËn£±ZµjUOOOÞ5¹©àO$Ið'I$ø$IüI$	þ$I$I?I$Á$IàO$	þ$I$I?I$Á$IàO$Ið'I$ø$IüI$©ý0ÃUÁ-IEND®B`


e^·µµY7áx©.ÑÑQøUiåoÒW,½ï7oÞÌf³õõõ1Q:&ï¾ûn]]]ávâEJ+¼¿¥×»téÒäÔ¤pI^~EbEyüÍ7/æ¿ÿþû¥;kÁcîßÉ®RtÕ7nÜHÞù[¸pá¤§üàßÝåñ×ßß_¸4îÝdñ7ÁÇ­$øªÉü`Y2ÝÞÞ¿vwwÇtOOO!ÑVO~Ý¹sçíÛ·÷rî¸î5kâU?.¼ÝNJÆkpü«ß-Ö*¼®2-¿â÷ýØ±cï^~Ó¦M1½zõê£ÅDü3Ç»¿ÉÍ7$7,ÙrüzòäÉÂ;||íÄ1åÊÂEãÝàt:=ÞÉ¯ûWï3ùwF';8cÞª»^qÌÇ[é~ÇÇãj²øàãVüIU¿ü¾ä#bÅ2/Æï¾ûn~Î×Í¿^L¥RÉ¯³fÍ*|Ëªè(^Í_q¼û1^ææ×××Þ£«W¯Æ¯sçÎïþ&sÂ¿&Ç²;XxKr¹/ÌÑÒÒRxßËà//òñ¤þW)ÒX7såÊ§NsÐ&28cÞª»^±þÄõë×·9)üMðq+	þ¤*Ä_`%fÖÕÕ÷nÐxLI~åy'©q&ûjo~Üå¸¿óçÏ._úÎYéàJïïD~hú,sßãÔ¥Â+"u~Ye"c5æ&28ãôîV,3ýá4ÁÇ­$øª(<á#y/mÌ+Ë[gRë#çý÷ß/³åW,s9¿æ¿%ø¬è=ª"O|@]´hQò+]]]³à/y2ùÖööö­[·Þ¸qãàÁ1síÚµcâo"«Ü5þ&;8ùîzÅøÎ_wÉÿÞð20Ið'UþâÕ.¼6ç¿ê%ù4U¸!^;g/^,J>?8Ú/¢eÖ-/§D-ÉG¸bãÉv>à5æfË¯X:kÖ¬ÉÌ?¼zõê£%[Û¼yóGÇ_òî×ùóçã.ìß¿"øýõ×ãqzzwõ;6æþì*Â_ùÁ)zl6ÙQÈx&Ò½?44¶[µjU)÷ìÙ£@øð20Ið'UþJÛ²eKþ2¥çÏæ?þÿÒÝämÂÒÑ2ëS¨(áQáù¥e6[~ÅòÐ¹víZþÃ^ÉË/ü«¾¾>ÿÂ¿¼NæÎÿdý<ñÄ¥;kÉ%evñ¤VþÊNÑc£°ÉêDÆóìÙ³|ôÑGBþóñ®±ÌLüIU¿xQbEé?ÿuòäÉT¼¢8p ?ÿØ±c1'°Uø-wrJ¾/6ÞÜÜuãmö+NrH´pþÅ×®]­½½½ôô»ÃßÀÀ@l-¶Ú¶m[x(oîòáâÞÅ7oÞ¡Cì±üYÃIá¯üà=6Ô¨Npx?>þü¸±ÁäHzþæøuÃÉÕ­²··÷oµyI?I¶r¹ÜO<QøNíObÊ/y79_dd$Aü¢E<B$ø$UaÉgþzöÙg¤*lpppûöíóæÍKíÆÄO>iX$ø$IüI$	þ$I$I?I$Á$IàO$Ið'I$I?I$Á$IàO$Ið'I$ø$IüI¡;v¬±±1N¯X±bªÈF»»ËLdÝ	600°yóæ9sæÄ Ì7oÇw·©ØHÜ°L&Ó]àORÅÕÐÐX	M=V*á¼£]¹råöíÛ±ÙU«VÝÝÖ?^óãN8áÑ%	þ$UÞ³É½m*oÞ½ºÙ»wïí=zôÜÚ/|á±µäç<º$Á¤J¤U!¤vìØ1öìY³fíÚµ«èb=öXKKK~fOOO23?gõêÕ1'æ_½zuåÊµµµétº¹¹¹»»Ìí^oùU:ÔØØ¸lÙ²÷ß¿·nÝZ·n]¬7ûöízsñâÅ±ááá1×_=nRCCÃ±cÇù·oßNÅÏ¹sçzI?Iê¿dúgé¶bâÙg-¼ÌÉ'×¥Åü+W®ÄôåËc:æÄô%K±(ff³Ù1·SxÕåW	Ò9r$&Ö¯__z³·lÙÓq^z)&vïÞ]äÚ"à|DoÌ)?öì9þ|Lù§O_CÀ1½víÚ9`àORåâ/Ó¹ÑJVúZ¢¢;v|8ú&YL<x0YtöìÙ;w¶´´ÄÌT*5æv@V~ä&Í=»tÝúúúäfß¾;&~øáßý2ø+?CCCÉtl!¿uëÖäMÊN¨ºmÛ60Ið'©rñW(¡BÖ÷¶Y`«¡¡aÖ¬Y7nÜuuuá¤¿ÿþ¸ü5kÎ;W¸nÑvÈ*ãÝ¤báÛ	'øÎßÂcþ­[·Ê»p¼«.¢sÅ£G¢czÞ¼y`àORåâ/Í¾×­ÌÙO?ýt,jmmÍo2Lüz´	â¯ü*ù4gÎÒuçÎ¬wçÎ±îK/½Tºh"£Îµ¨³gÏzI?I¿äÔ×&Çsî¹;âo`` y,~æÏÆHØtáÂØÔñW~ Ú/¾6m*]wÃ1ÝÙÙùöÛoç?w8ÁnÞ¼ª[°`ÁÕ«WÉmmmütrá?ø?Èo9._bI?I¿`Ö¬Ñzê©1/SÚæÍï7ÉÏ9~üx]]ÝìÙ³·oß>Aü_åÔ©S±téÒ¥ù3N/388¸uëÖ¸ÍLfÕªUï¾ûî¤F ¶|ÕÃ¯¡À]»v%çÈhä§bââÅù]ºt)æ,^¼ØcLüI$	þ$I$I?I$ø$IüI$	þfpùùÃþp*¯ñïÿþïÿùÿÙ#¯Òº~ýúÍ7C¥;åþáC¥ObSüÌ©ô/ÿò/.]2ØßýÝßýèG?¿Jé«_ýjøo¹	ØùóçÃåÆ¡Ò»Æ8T Ê§øSéöíÛña*°×_ýþéàþ?øüÁüÁàOð?ø?øüÁàOðð?øü	þàþàOð?øüÁü	þàOð?ø?Áü	þàOðð?Áü	þàþàOð'ø?ÁüÁü	þàOð'ø?ø?Áü	þàþàþàOð?ø?øü	þàOðð?øü	þàþàOð?ÁüÝ¿øëïïÏf³étº©©©»»»pÑðððúõë3ÌÃ?£?øüÁàoÆ×ÞÞÞÙÙ÷îíèè(÷îg6þ$bèçÏ_¿®®®[SØw¾ó÷Þï*¬ïï?øÁC¥;%vq¨´âI,ÊC¥õÁþCÖÝÝýúõ©¼ÆûuuuÁ»ÈårÞ~ûíñVüíÙ³ç;SØ'âAðUX¯¼òÊ«¯¾j*­Ø)±kC¥ObñTf*°_~Ù T`ò×^m*¯ñ¾À_:s:ù5xW[[;þüsçÎ9ì+öÃ¾ûÊaß_*ÊOg2¢E/¶´´Ààþð'øñÕ××çr¹GûÆtÑ¢ütÑð'ø?Áü	þfdëÖ­;tèPLÄÏöööÂE7o>räHLpaÙ²eð'ø?Áü	þf|===©T*ÍöööþÛ«©I¤ÚÚÚÒétKKË¥KàOð?øÈ³âöíÛ÷Ö[o[ø«ªàOð?ø+jëÖ­>øàgF/éK###FþàþàOðªBü½ðÂ?÷s?÷å­_Þõ;»â¿ßÚö[=ôÐ3Ï<cáþàþð§*Äßg>óÇ<_ò_üúÈ#aø?ø?Áü©ñ·hÑ¢MÿsS!þ~ó¿ùÉO~ÒÃüÁü	þàOU¿Ç¼õÿm-Äßûåÿ¶víZ#ðð'ø?U!þ.]º4gÎ5kÖìüí!¿_kûµùñðð'ø?U'þ¢7ß|ó~áæöó?ÿóo¼ñá?ø?øüÁªI£Xø?ø?ÁüÁß?ÁüÁü	þð'ø?ø?Áü	þðð'ø?Áàþàþð'ø?ø?Áàþðð'ø?ø?ÁüÁü	þàOð'ø?ø?Áü	þàOðð'ø?Áü	þàþð'ø?ÁüÁü	þð'ø?ø?Áàþðð'ø?Áàþàþð'øüÁüÁàþðð'øüÁàþàþ?øüÁüÁàþ?ø?øüÁàþàþàþð'ø?ÁüÁàþð'ø?ø?ø?Áü	þàþàOð'ø?ÁüÁü	þàOð'ø?ø?Áü	þðð'ø?ÁüÁü	þð'ø?ø?Áàþðð'ø?Áàþàþð'øüÁüÁàþð'ø?øüÁàþð?øüÁàþàþ?øüÁüÁàþàþðð'ø?Áàþàþð'ø?ø?Áàþðð'øüÁàþàþð'øüÁüÁàþ?ø?øüÁàþð?øüÁàþàOð?øüÁüÁàOð?ø?øü	þàOðð?øü	þàþàOð?ÁüÁü	þàOðð?Áü	þàþàOðÆþðÅõ÷÷g³Ùt:ÝÔÔÔÝÝ]z®®®øüÁàþÕPgggLìÝ»·£££héÈÈHssóxøûó?ÿóÿ3ûÛßþÁ~ðTa½ñÆçÎ3VìØ5Æ¡Ò'±x*3ÖÕ«W?n*°W_õwÞÊk¼/ðWWWÿÇ¹±±±hé3Ï<óüóÏ¿ÂºººúúúÎ©Âzíµ×zzzC¥;%vq¨´âI,ÊC¥õÖ[oþCvòäÉ7ß|s*¯ñ¾À_:s:ÿjii	:ì+öÃ¾ûÊaß*Á_*ÊOg2ÂEmmm§Oþ×»?øüÁà¯:µ¾¾>Ë8zØ7¦ÿÃ=üÁàþð'øñ­[·îÐ¡C1?ÛÛÛÇ¾«ÞùüÁàþÕ1¬===©T*Íööö©=øüÁàþ¾äþð'ø?ÁüÁü	þð'ø?ø?Áàþðð'ø?Áàþàþð'ø?ø?ø?Áü	þàþàOð'ø?ÁüÁü	þàOð'ø?ø?Áü	þðð'ø?Áü	þàþð'ø?ÁüÁàþð'ø?ø?Áàþðð'øüÁàþàþð'øüÁüÁàþ?ø?øüÁàþàþ?øüÁüÁàþàþðð'ø?Áàþàþð'ø?ãð?øüÁàþàOð?øüÁüÁàOð?ø?øü	þàOðð?øü	þàþàOð?ÁüÁü	þàOðð?Áü	þàþàOð'ø?ÁüÁü	þàOð'ø?ø?Áü	þàþàþàOð?øüÝ3üÕÜ©T*ð?øüÁ_à/u§Òé4üÁü	þàOðûÂüÁàþðW]ø§M6ÁüÁàþðWøkllL§Ó>óð'ø?Áª/.=Û£®®nppþàþð'ø¿jÃ_&	í444ÄDïÅ_7ÂüÁàþðWmøKÞêÐ^Lr%11kÖ,ø?øüÁàþªsæÌ	êõôôt)&|òÉdÂW½Àü	þàOð§*ÄßöíÛó§w~ìoÙ²eðð'ø?ÁüUáÙ¾O?ýôÜ¹sc¢··7&---3bÀàþð'ø»?Áü	þàOðð?Áü	þÆoáÂÉ¾øgø?Áü	þTåø[°`A!øò9ÛþàOð?U!þÂyÁ¾¾¾¾xØÍ¸ý?øüÁàorÕÕÕþf¢üàOð?øüMºþþþÀß-[nÝºð?øü©ÊñÍ7¯¦$'|Àü	þàOð§*Äßüùóðð'ø?Áîü%ì¡OÐð'ø?Áü	þ&W½>àþð'øÓý¿¸K¿íÛ·ÃüÁàþªrüÕ>àþð'øSu~Éó9áþàOð?UçW½ÌÜàOð?øüM®ÆÆÆùóç¿ýöÛÓx7úûû³Ùl:njjêîî.ÛÛÛÜÜ/^?Áü	þàOð÷WÕÔLó;ííí1±wïÞÂE,8sæLL>|xáÂð'ø?Áü	þ>RÝÝÝ¿§z*¦ë_êêê«Îårã]¬¶¶¶ú§ú¿§°W_õìÙ³ÿ[Öw¿ûÝ7ÞxÃ8TZ±Sb×J+Äâ©Ì8TZ.~ü¸q¨À^yåsçÎMå5þÄñW	gûÞ&7n,Åß#G.Oa§N½rYVOOÏ÷¾÷=ãPiÅN]c*­x§2ãPiýíßþmàÏ8T`]]]ßÿþ÷§òâø«³¡ÉdJ/pëÖ­öööÁÁAå°¯Ã¾rØ×a_9ì;ã«¯¯ÏårÉaß.ZzíÚµõë×_¿~½tEøüÁàþ3¯uëÖ:t(&âgÑ·¶¶¹"ü	þàOð¿IËåV­ZU[[[SS3kÖ¬¶¶¶)>ó£§§§¡¡!Je³ÙÞÞÞ»o£ç 766~þð'ø?ÁßGjhhhÌ>fÄ?õ?øüÁàor-Z´(¨·zõê[·n%OkÖ¬9K,?ø?Áü	þà¯ÚðÉdz###ù9¹æyÖ-üÁü	þàOð3©T*¨l4<<s¦ò«^àOð?ø?ø"ü%[[[Ã¾ñ3¦cNss3üÁü	þàOðÕ¿ÐÞ'|Ü¸qþàþð'ø¿*üª¡¡¡¶¶¶Ù³g§R©øÙÚÚsfÄþ?Áü	þàOðw?øüÁàþàþ?øü­y§R©üÁü	þàOðU¿ÔøÁüÁàþª6ü×¶mÛü>|þàþð'ø¿ªÅ_ÿ¬Y³+W®,üÎgø?øüÁàþªk×®MÞð;qâÄÚð'ø?Áü	þ&×K/½°oõêÕ3nÀàþð'øhCCCK,IÎíèîîûþð'ø?ÁßÚ³gOò_GGÇÌÝð'ø?Áü	þ&¶¦ïù?øüÁàO÷þRw*NÃüÁàþðçw?ø?Áü	þàþàOð'ø?ÁüÁü	þàOð'ø?ø?Áü	þðð'ø?Áü	þàþð'ø?Áß=ÃßÂ3ïù?øüÁàOÕ¿Ï÷üÁü	þàOð§jÆ_8/Ø×××»·?àOð?øüM®ºººÀßLü	þàOð¿I×ßßøÛ²eË­[·àþàOð?U9þ¢yóæÕäø?Áü	þTø?¾>àþð'øÓý¿3ô	þð'ø?Áßäª¯¯wÂüÁàþº_ðw)ð·ûöááaø?øüÁàOU¿qrÂüÁàþªÎ/y3'|Àü	þàOð§êüªü	þàOð?ø?øü	þàOð7~¹ÕªUµµµ555³fÍjkk)'ÿÂàþð'øð1#Nþ?Áü	þàOð7¹-ZÔ[½zuòoûÆÂ5kbÎ%KàþàOð?ø«6üe2 ÞÈÈH~N.91þàþð'ø¿*üª ^/?gxx8æøªø?Áü	þTµ[[[Ã¾ñ3¦cNss3üÁü	þàOðÕ¿ÐÞ'|Ü¸qþàþð'ø¿*üª¡¡¡¶¶¶Ù³g§R©øÙÚÚsfÄþ?Áü	þàOðw?øüÁàor566Î?ÿí·ß?ø?Áü	þTýøK§Ó553õDøüÁàþ«»»;ð÷ÔSOÅÓÁLùWÝàOð?øüÝí&Æ)JÁüÁàþðW_ò<f¾äþàOð?UþæÏßÔÔ4888£÷ü	þàOð¿	ü¾öGxáOð?øüÝ=þÒ7ùg|áþàOð?U9þ²ÙlMÙð1þÞzë­o~ó^ÕàOð?ø1øëëë3gNòþ_B='|L¤ÎÎÎO|âÿiÁú/KÿËÇ?þñ_ú¥_úÇüGð'ø?ÁüU:þòÍçUþÂyûØÇ>·ês»~gWü·ó·w>òÈ#Ë-ñ·?øüÁßÌÀßnñ÷Ío~óSúT"¿ä¿ÿßààï½çoþð'ø¿ûýýýÙl6N755uww_tws¦/¼ðÂÏ¬(Ä_ü·páÂ7ß|Óßü	þàOð÷#þÚÛÛ;;;cbïÞ½åÝÝÂ¶oßÞÐÐððTU__ÿÓ?ýÓszà~ê§~ê¡zXÓZüBcc£q¨´b§Ä®1Ö¼yó¦òSz1ØÏþìÏÆ_ÍT^ãÃ_]]]òçr¹xÞ/¿èîæ¶mÛ¶I¤*ê'¿/ÞCü;RtIé¢»$	þ>ÂãÑïyinn~çw>úÖ¿0É_twsûÝßýÝ]»vý¯),®îÿøÿðÿð«_ýê¾ûþ*£ßû½ßûÚ×¾f*­Ø)±kC¥ObSüÌ©ÔÙÙùå/Ù8T`;wî<pàÀT^ãOÁ¾¼4ÓéôòåË/_¾|×[«¯¯ÏårÉQÚ.¿èîæLã	ú>ð!'|8áCNøpÂGuðqñâÅEåÉdV­ZuÛY·nÝ¡Cb"~¶··_twsàOð?øüÝ³GÞ'jkkkîöwëééihhu³Ùlooï¿ÝÊÑ£×¥înü	þàOð¿ðÚYøÎßìÙ³7nÜXùûþð'ø?Áßä*4_mmmGGÇÕ«WgÊþ?Áü	þàOð7ÉMçñÙÏ~öÊ+3nÀàþð'øä^àOð'ø?ÁüÍüÍèàOð?øüMº.,pÖ¬YÉ¾õõõG?ø?Áü	þTøëêêÊðà/Þ»w/üÁü	þàOðÕ¿ ÞòøëííM¾íþàþð'ø¿*<Û7ùæ<þâñð?øüÁ_µá¯¾¾>¨¼ÛøËår;vìéÆÆFø?øüÁàþªqjÆêÌ3ðð'ø?ÁüUáÙ¾×¯_oiiIÎö­­­]¸páåËgÄþ?Áü	þàOðw?øüÁàþàþ?øüÕÕ«WgÏ-&fÊ1_øüÁàþhÃ5ã´iÓ&ø?øüÁàOÕ¿£G&ÎÛ¿ÿàà`2shhèØ±cÉü'OÂüÁàþðW%øËf³!¼.JþÁ7ßóð'ø?ÁªL&744Tº(ËÅ¢¸üÁü	þàOðU¿ü¿ê6ÞÒäz?ø?Áü	þà¯JðWwðð'ø?Áàþàþð'ø¿¿òÁüÁàþªü¥îT:?ø?Áü	þàÏ?ïð?øüÁüÁàOð?ø?øüÁàOðð?øü	þàþàOð?øüÁü	þàOð?ø?Áü	þàOðð?Áü	þàþàOð'ø?ÁüÁü	þàOð'ø?ø?Áü	þðð'ø?ÁüÁü	þð'ø?ø?ÁüÁü	þàþàOð?ÁüÁü	þàOð?ø?Áü	þàOðð'ø?Áü	þàþàOð'ø?ÁüÁü	þð'ø?ø?Áü	þðð'ø?Áàþàþð'ø?ø?Áàþðð'øüÁàþàþð'øüÁüÁàþððð'ø?Áü	þàþð'ø?ÁüÁüøüÁàþàþ?øüÁüÁàþ?ø?øüÁàOðð?øüÁüÁàOð?ø?øü	þàOððW®þþþl6N§º»»õöö677Ç¢ÅÇÅàOð?øüÍøÚÛÛ;;;cbïÞ½,XpæÌ8|øðÂáOð?øüÍøêêêâA¹±±q¼ÕÖÖâï¹ç;9?~ü[ßúÖIUXñyâÄ	ãPiÅN]c*­x§2ãP½üòË¡úþûétzÌéÂúúú6nÜX¿ðøÿÂN>=00ðUaýÕ_ýÕåËC¥;%vq¨´âI,ÊC¥õ£ý(þgÉ8T`¯½öÚÍ7§òïü¥R©üt&)½À­[·ÚÛÛöÃ¾ûÊa_å°ï¬æÇÅt.KûÆtÑ%¯]»¶~ýúë×¯nþð'ø?ÁßÌkÝºuøÙÞÞ^4â­­­c®?øüÁàoæÕÓÓÓÐÐJ¥²Ùlooï¿Ý·Ñ7k?Áü	þàOðwÿ?øüÁàþàþ?øüÁüÁàOð?ø?øüÁàOðð?øü	þàþàOð?ø?øü	þàOðð?ø?øüÁüÁàþ?ø?øüÁàþð?øüÁàþàOð?øüÁüÁàOð?ø?øü	þàOðð?øü	þàþàOð?ÁüÁü	þàOðð?Áü	þàþàOð'ø?ÁüÁü	þàOð'ø?ø?Áßhï½÷Þ¦MÿââOúÓ;wî?Áü	þàþTøù566>²ä6üÆúßøÅ_üÅ¦¦¦>øþð'ø?øûIêÀûöíë­·àoêÛ¼yó~ä?ïú]ùÿ-ZôÜsÏÁàþð÷¾@Æ<°ü¿.ÿÌg>óà~éK_¿©ìÓþôñ×ÑÑñ¹Ïþð'ø?ø»÷÷ëãÿø¦ÿ¹)1Çomû­zèþèào*äG~cýoâï××þú¯þê¯Âàþð÷¸/~ñûÜçÙñøã/_¾þ¦²¯|å+MMM;g²bâáþú×¿?øüÁüÝãB~¡½BüýæóøüMeÃÃÃK,ùÔÂOý÷öÿþëk=ä·jÕªtðþàOðð3¦íÛ·/ÿ¯Ëñ÷+¿ò+¿üË¿SÜàààïÿþï?öØckÖ¬ùú×¿>£åð'ø?ø¿Êí?üá>¸ö×Ö&òû_ø<ðÀzyð%Ïð'ø?ÁüÁß¤ÜsçÎøÿyøüäÇ>ö±_|qÝxø?Áü	þàþ&ÝÈÈÈÛ£Ï¬[ð'ø?ÁüÁßüÁàþðð'øüÁàþàþðð'ø?ø?Áü	þðð'ø?ÁüÁü	þð'ø?ø?Áàþðð'ø?Áàþàþð'øüÁüÁàþð'ø?øüÁàþð?øüÁàþàþ?øüÁüÁàOð?ø?øüÁàOðð?øü	þàþàOð?ø?øü	þàOðð?ø3ð'ø?ø?Áü	þðð'ø?Áü	þàþð'ø?ÁüÁàþð'ø?ø?Áàþðð'øüÁàþàþð'øüÁüÁàþ?ø?øüÁàþàþ?øüÁüM¦þþþl6N§º»»K/ÐÕÕUSS?øüÁà¯jooïìì½÷vtt-innßþö·¦°ï~÷»×¯_RõÖ[oýÍßüq¨´b§Ä®1V<ÅSq¨´Æ¡ëîîÊk¼/ðWWWÿÇ¹±±±hé3Ï<óüóÏ¿¯íkßÂâ/óÕW_ý¶*¬oë[¯¼òq¨´b§Ä®1V<ÅSq¨À^~ùePMýKÿ¿t:=ættõêÕ ¡Ã¾rØ×a_9ìë°¯ö­ü¥R©üt&)ÖÖvúôé½«ð'ø?Áü	þfîPÖü¸®¯¯Ïåröé1/¿0ü	þàOð¿Ýºuë:ñ³½½<,Î?Áü	þàOð7óêééihhH¥RÙl¶··wLíÁàþð'øó%Ïð'ø?Áü	þàþàOð'ø?ÁüÁü	þð'ø?ø?Áü	þðð'ø?ÁüÁüÁü	þàOðð?Áü	þàþàOð?ÁüÁü	þàOð'ø?ø?Áü	þàOðð'ø?Áü	þàþð'ø?ÁßýÕW¾ò?û³?ûáW÷×ý×?TÕÕÕuæÌãPiÅN]c*­xâgNM¤+W®üÉüq¨À9òýï*¯ñ>¿q»páÂ®]»¾*IT-o¾Æ±$I÷Oð'I$I?I$Á$IàO$Ið'I$ø«úûû³Ùl:njjêîî.ýúõbNooosssñâÅ±¢Ñ«ýÔÕÕUø«*a×¯_¿>É<üðÃ¯¿þºÑ«ýûbÑ¢EÉzzÞ´ìÒ¿2Ö4îié÷Âvïkooïìì½÷vtt.:zôh<ç,X°àÌ31qøðá½Ù/ÑÈÈHüqÂ_¥íÝ»w?ûì³·oßçÐùóç½Ù/uuuW¯^øÙØØhô¦e¿þu¹°¦q¿LûK¿¶_<	Æ>Wô$OÇoÅÚÚZ£W9ûågyþùçá¯ÒvMü_õÛo¿mÐ*m¿ÄKÚµk×b"~BùtíÒ¿2Ö4îié÷ÂvïK§ÓcN'Øomm---/^,××·qãF£W!ûåêÕ«1ÉðWi»&¦÷ìÙO!sçÎ½Ù/ýýý±Sâï%~Æ³ÑýRú×QæÂÆý2í/ý^Øî©T*?ÉdÆ¼LüÏñâÅó¿Þºu«½½ppÐèUÈ~ikk;úô¿þÀ_í¸ðÁc"Ìò0z²_,Y¼·t©ÑýRú×1¨©ß/ÓþÒïíÞW__Ëå>ï7¦Ç»XþÁÏë×¯¿~ýº¡«ýRó3z³k/àÊÙ/ÞaªýRú×1Á¨)Þ/ÓþÒïUíÞ·nÝºCÅDüÔ.Z°`ÁåË½ÞÚÚúáè)r1100`Ü*j¿üû_ùUØ®Ù¼yó#GbâÂË-3z²_.]$&Î?¿dÉ£7-û¥ô¯£Ì5ûeÚ_ú½°ÝûzzzR©T6ííí-4D___òmË/O>ÝØØè¦Ü/ðW±»ææÍmmmÉ§Í.]ºdô*d¿¼óÎ;a¾?cÚèMË~)ýëóÂöý2í/ý^Ø$Iî£àO$	þ$I$I?I$Á$IàO$Ið'I$ø$IüI$	þ$I$I?I$ø$IüI$	þ$I$I?I$Á$IàOîÜ7¾ñG´v´+V¼ôÒKÿá9n´ó<Ö­mll»688X4?æ¤Óél6ûöíÉnSüIÒl×®]5%=ýôÓÕ¿§z*fîÛ·¯hþ/¼ó|òÉ»Ø¦$ø¤W°&Nïß¿d´Æ¯1óìÙ³U¿+W®ÄÌÅÍ_´hQÌ¿|ù2üI?I÷Eÿüç5Ï>ûláÌç.f~á_(¤Owwwà)ÜÜÓùß¸qcãÆ³gÏEuuu[·n-<ºzêÔ©V,uO<YÄ©3gÎGôÄñëºuënØñãÇËo'Y7)YÔÕÕ5ÔZ[[c~ooo~Î3gbÎÊ+ósvïÞÝÐÐª­­]»víµk×JñWºý¢9enª$ø¤inÞ¼y«W¯Î|÷Ýwcfccc!nêëëK®Y³¦hÑ-[EçÏO¥Rc®ü,mkk»ûvð1ÉÄÒøx5kVòQ¼2Û1o^é==|øp¡hó¾ìììÌË¯h#+V¬,þÊÜTIð'IÓ_rwçµ X!n:::nñëêÕ«¥u>&oàÚEíííÉ[1ÝÓÓl¤p;wîÞ%G]7lØs;Óñ3¦xâ;n'à¿ÆoÛ÷(@9wîÜ¸¿ñkü[âÌårÉ²Ùl¬wáÃ&®­­,þÊÜTIð'I¿hLüåç'¸IÌ]»v-~6%¿677Ç¯óçÏß´iS mhh(¿¸LÑiùµ_ß÷ÝüLù#¿ñ3¦O:uÇí4ã×÷ß¿ð¶÷ù¼mÛ¶Å¢çþÃÚÞºuká¿ iKKKòÆädñWæ¦J?Iþ·»nÝºU8spp0fÆ¢2ÜÉÓðÒ¥KÿòÖÉP¯ôhl§¿`%¦ëêê2Ìððpl|Î9ù¥wÜÎx+êÂSc:~ÆôùóçóKzzâyøxâø+sS%Á$MÉçÞöìÙS83ù¢>ò(CëìÙ³O=õTr6¯Æäm¹üqÕ;mË-ÉÑÞø¹qãÆüü2ÛIÞi»~ýzòëûï¿_þÌÜ¥KÆÒäÛmÂ¬3cQWW×Í7Ëã/Ód4òKËÜTIð'IÓ_rÂD:Þ»woòU/û÷ïÏd2¥'g¬^½: 3<<Í|æ/ù¨Ü¥K?,XpëÖ­A¥ä<Üü­I´äôÛäÚ_ýõüü2ÛI>ä|æopp0¹dü<x0ÿåÂEÉÇÏ?×0þnãbÎµk×.-sS%Á$UDÉ µ÷î-±Q~:À4E¾6Ê²°£GÁß£ÿGÌ¯««+<";q3oXþLÛñîìÐÐPmmmrv¯Zµªð*æÎ?o)ÜfòÆd¾ªù¥enª$ø¤J)²bÅÌh>úhrÊmþººº¯Ókjj:út~éààà;0mÛ¶mxx8¿ôäÉ---a²l6àÀ¢mÞØTÌß¼ysÑüñ¶Å|aïùË·qãÆ¢ï|Ihoo=vÜË/ç¿¶¦p7oÞÝ&cµråÊÞÞÞ¢k,sS%Á$IàO$Ið'I$ø$IüI$	þ$I$I?I$Á$IüI$	þ$I$I?I$Á$IàO$Ið'I$ø$IÒ½èÿc¨v®´IEND®B`


­­­óI¥Rk3Lg üîï½÷^ºººJ¥ÒÍ[Gicå(¿zêíí)Ë|áb¦X,9r$q^(j8£üåW§ï©åßÕ3gxàL&³÷îùùùÌf³©T*Ë%ö[«å(¿õHùÊOù(?å üòS~ÊOù(?å üòS~k¹SY5Ëòh.´@ù)?å(?å üå§ü üòòP~ üP~Êò@ùòS~(?P~ÊåÊOù4°ßüæ7¯­BR~«yø¬P~Ê`-|çáô=m~»¸²S~ð8­øîñ¥ã°@ù)?µPüÿúÍF~òA]Nñ¥ã°@ù)?å(?å üå§ü(?å§ü(?å ü(?å ü(?å ü(?å ü(?å ü(?å üå§ü ü¬½¹¹¹Õ±òå§ü 1|øá,-i»ÛÜ X,Zw§¿ûûÿÒýw_ûÆºâKÇ7`-òS~ÐHâ)æ]½5ôèc-ußµÊOùòc-|gûßÕ·üâ°@ù)?P~¬¿çÊOùÊOù)?P~ÊP~ÊOùòS~ ü,å§ü@ù)?P~(?åÊOùòCù)?P~ÊÊP~ÊÊP~ÊÊÊÊòòS~ ü üòå§üå§ü(?åÊå§ü@ù)?P~(?åÊOùòCùY ü(? ü(? ü(?(?å§ü@ù¡ü@ù)?@ù)?åÊOùÊOù)?P~ÊP~ÊOùòS~ üh4Oluw_ö2ñÕ8­øîñ¥ã°@ù)?P~¬ßÿþ÷ï¯BË-«yø¬P~Ê°Ë¹Årå§ü@ù¡üå§ü@ù¡üå§ü@ù¡üå§ü@ù¡üå§ü@ù¡ü@ùÝÎ9ÓßßN§&''cfff&Ë%35Q~ üP~ üê©³³óòåË1ó|>B¡066ÑÑÑÎ(?P~(?P~õÔÓÓsõêÕÄy,Ë1(JIÖjfAù½úê«ï_xÅîßrXò³àKÕ¨å733ÓÚÚÛ8t:]¹6×jfAùýò¿üÀ&bñÔ¶Ö¡¤ü,øR5jùmÚ´ivv6IÀÍ7Ç JU®Íd25q´íÅÑ^p´·¿3×ÕÕU*£´1®áòåòåWO7o>þ|Î;·iÓ¦Å#GÄ ÎBg(?(¿ºmâü½÷ÞàK§ÓqãÌf³©T*ËMMMÕpFùòCùò[(?(?å(?(?å(?(?åMéÆï¯Â/~ñØý¿¿:Öòòµ¼iw_öþòk÷gþâ«+¾Ôòòµ+¿î¿ùÛ|P¯z¸kuww·¬BÜÝ2å§ü@ù)¿Æðþûï¿½ãòS~ üòS~ üP~ÊÊOùòP~(?P~ÊOùÊ@ù)?P~Ê@ù)?P~Ê@ù)?P~Ê@ù)?P~Ê@ù)?P~Ê@ù)?P~(?åÊå§ü@ù(?(?å§üå ü(?å ü(?å ü(?å ü(?å ü(?å ü(?òåòS~ üÊòS~òP~ÊòP~ÊòP~ÊòP~ÊòP~ÊòP~ÊÊOùò@ù)?P~ÊåÊ@ù)?@ù(?åÊOù(?åÊOù(?åÊOù(?åÊOù(?åÊOù(?åÊå§ü@ù ü(?åòå ü üòå§üòå§üòå§üòå§üòå§üòåòS~ üP~ÊòS~ÊòS~òP~ÊòP~ÊòP~ÊòP~ÊòP~ÊòP~Ê[~-UR©TÌÌÌÌär¹t:=00011QÃåÊ@ùÝÆÆÆFGGcP(b¸822RÃå÷ë_ÿúÿKçHÝËÏZø]~.]Úºuk2îìì,Ë1(Jù|¾3Êï?üáI`iñ¹¿osËÏZø]~ÃÃÃÓÓÓÉ8NWæq­fíGí­³¹¹¹Í7W.&¿íÈd25Q~ ü_8pà¥^ªêê*J7o¥qg|ÑòûW©?k©+k ÙÊoË-¿ûÝï*Åâ#GbçB¡3Ê¾hùµÔµÐ$åWÙ¦g2äO1Ùl6Jår¹©©©Î(?ø¢å÷¯ïËí[êuR~ÍS~õ¥üàNÊÏïù(?åÊOù(?åÊOù(?åÊOù(?åÊOù(?åÊå§ü@ù ü(?åòå ü üòå§üòå§ü»üÎ?ß×××ÖÖJ¥âbWW×±cÇ(?åÐlå7>>Þò/òKÆ£££ÊòhªòËf³±>þ|¥ü¦¦¦bÜÞÞ®ü@ù)?¦*¿ä¾d_¹q:V~ ü@S_WWWl÷ù¢üJ¥ÒþýûcÏç(?åÐTåwæÌÏóÎ;ï(?P~Ê ©Ê/|ðÁÉßö¶¶¶öõõ]¼x±Aòå üÖåÊ@ù-)Ï÷ôôÌÎÎ*?P~Ê ÉË/N7ÓVXùòP~K­ð÷¾÷½ë×¯ËeåÊOù4mùµ,!ùl?åMY~ñø[Ù)ÿà·þ2?°â»ÇIùÔ­üRKðIÎÐ¬þð?¼[?ýéO[VçíåXõ)¿&£ü &iøü*:tÈ2P~Ê ®åç÷üÖKùUÿn_uùù=?f+¿jårùÓO?üñÇ£üæææ@Ó_"âïøÐÝ­ü¼üÊår_&Q~Í ¥RéðáÃQ~­­­Ê ©Êo©¿íÝ¾»òhªò[üß;2Ìã?Þ KDùÊo½P~òS~ë¾üú=¿t:ÝÕÕõÂ/(?&)¿Åÿ½#ù×mñ¾û@3ßüü|[[ÛöíÛ?ùä¸ç>úèÀÀ@ß|óÍ(¿ÎÎNåÐå×××yW**3öYÌÅdÃý_å(¿¥âùùùÊL%øæ¡ü¤ü²Ùlò¹Í~úéÍ[ÿ´7ÆÉAÞééiG§üÆÇÇ?÷o'&&ÁîÝ»@3_¸råJ___kkk*óÇÌÍ[îy(?@ù­ÊP~Ê`½_òqÍ7þÉµÊ áË/Â.ù¸ÔëÃ üÔÓÓ300|K3Q~ò[(É´´üñî`WùÊïDíEóÝ¸qCù4yùår¹ÛòMR~ÓÓÓÉ;Içùæ,¿Æ<å(¿uMùÊOù(?å üòS~ÊOù(?å üòS~ÊOùÊOù)?@ù)?å§ü`=ëîînY^åwóÆ;wîÌd2ñÃtæÌÉårétz```bb¢3Êò«§üà?(Ë===1S(ÆÆÆb0:::22RÃå_bmh;å·¬ÙÙÙêÎÎÎÁJ¥|>_Ãå÷³ýìò³o]3Z~étú¥^jmmíééy÷Ýwêkk8³ üÆÆÆfò³o]3Z~©TêÕW_ÁÊµL¦3ö£½öÖSWW×wæb¦T*%Gikk5£ü@ù)¿zÚ³gÏo¼óçÏ?ôÐC1(GAÎ(?P~Ê¯n?q~ýúõááát:=888773Ùl6Jår¹©©©Î(?P~Êo=R~ üòòS~òS~ÊP~ÊOùòCù)?P~(?åÊå§ü@ù¡ü(?òå§üòå§ü üò&Öõo²÷Þ½ÿë+;ýY¦í¾®ÜïáøÛßþÖZP~ÊÖBdß#ÿøOßùoÿ½.§Î|¯]³òS~°F²÷=lä'ÔåÔý7k×¬ü(?òåòS~ üÊòS~ üòS~ üòS~ üòS~ üòS~ üòS~ üìòåòS~ üP~ÊòCùòS~ÊòS~ÊòS~ÊòS~ÊòS~ÊòS~ÊÊOùÝýÅâ#«pîÜ9Ë@ùÙ5+?å× ?a«sâÄ	Ë@ùÙ5+?å×$]ÜrP~ÊOù)?åòS~ÊOù)?ÖPÇ½÷µÔÏ=mvÍÊOù)?ÖÈW2÷´Ô]³òS~Ê5â=?å§üP~ëßóS~Êå üòS~(?å§üòCù(?å§üò@ù)?å§üÊÏ®Yù)?åòCù)?åòCù)?åòS~(?åÀm6×ù­~»¸²S~ðÞ-#+¾»ÿÞ¦üò`í¼õÖ[¯­Blíî¹Õ<Â~h-(?åWû?müßúæÜº²SúÏ;þúß~cÅwÏÿõ×Ï;g-Üý¯óí[òk_ÉÜóÍùÇªËÉÿðP~(?å·vüÎ/¡»»»elÌòS~Ê@ù¡üòS~ üòòS~òS~ÊòCùòS~(?P~ÊOù)?P~ÊOù)?P~ÊOù)?P~ÊOù)?P~ÊOù)?P~ÊOùX~|ðAõÿrN§&&&j8£ü(¿z:vìØÎ;«gÂØØXFGGGFFj8£ü(¿zì;~üxõLggg¹A©TÊçó5YP~/¾øâwæ«_û«:_þÁoYYP­QË¯··whh(N^¸p!fb6×jfAù>úsgþ¼­½¥®¬,¨Ö¨åWqõêÕ6Ä JU&3Lgí]±¯dî©oùYYÐG«%ÖÕÕU*£´1®áò[±ûºþª÷?ïþ÷Ûö×åÔy¯ÍP~½½½/^¼yë=¿¡¡¡Å#GÄ ÎBgßùP~«ûnËôôt:~øá#þbfrr2Í¦R©755UÃå§ü@ù­GÊOùòS~(?P~ÊOù)?P~ÊOù)?P~ÊOù)?P~ÊOù)?P~ÊOù)?P~ÊOùÊOù)?@ù)¿Úüµ´ä¶>øíâÊNùÁ'z¾õ÷+¾|u+ò[#o½õÖk«é6<<¼GøðÃ­P~Ê¯~þZZb[ üòòS~òS~ÊP~ÊOùòCù)?P~ÊåÊOù¡ü@ù)?(?åòå§ü¼íVãµ×^³@ù)¿ÆpèÐ¡çWáý÷ß·@ù)?å§üòÊP~Ê@ù)?å§üòP~Ê@ù)?å§üòP~ÊP~(?@ù)?å§üòP~Ê@ù)?å§üòP~Ê@ù)?@ù¡üå§üòP~Ê@ù)?å§üòP~Ê@ù)?å§üåòòP~Ê@ù)?å§üòP~Ê@ù)?å§üòÊP~Ê@ù)?åWããã--ÿïÉårétz```bb¢3ÊP~u6??¿qãÆJù±±±ÔpfAù8qâ4 .¿ïÿû¬_ggg¹A©TÊçó5YP~/¿üò@jÔò»|ùòàà`$Z¥üÒétåÚdGGëixxøìÙ³üÿ¥üR©TåÚL&SÃå(¿º~g*fºººJ¥ÒÍ[Gicå(¿»%A±X<räHâ¼P(ÔpFùÊ¯Î©·àâääd6M¥R¹ùÆZÍ(?@ù­GÊP~Ê@ù)?å§üòP~Ê@ù)?å§üòP~ÊP~ÊOùÊOù(?å üòS~Ê¯¾?þÐC=Ð^í5åòP~(?Êåò@ù üX;³³³ßýîwÛÛÛÓétWW×=>úè£Õ<à/¾¸àÖt²´ÊmN>½ûöäG%ÎcfñC<y2îçjSþ0T_L¥R­­­7n|ùå-7»åGxã7º»»_ýõR©ã<Æ<ðÀ+WV³ÛP~¸³¿ý=ºuëÖ©©©r¹ü¨Döõ÷÷>|xÁ-wìØñÔSOÅ¾ÄRmÊÅ3.è¡:dqÙØ(?îjsssñ_üòîàÁÛ¶mS~Ê¯Zü¨|òÉ'&/]ºÍf«g¢ï½÷ÞÏ>û¬££#iD¾üø°¸ìlwµ§~ztttñ|<WÇÆÆ*O:500N§ã<ÆÕ;'Nôôô¤R©¸69ð·øÀÐÙ³g;;;4åw7_&]öqbÇ Î«×oõ_¨µµuhhèNF)¿ä'$ÎÅbõÑ½ãÇËg]íîdSò£nâÕÞ¥Knx2çr¹ÇyWW×ôôtåi¿uëÖä @Ü,ùw1ÞµkW¼àKvó·4åw7ßÏþól6úÛßlçÎ¯¿þúÍ[Gc}Ueß?o¼ñF¿ÅÞ4åwáÂ6Äà£>>øôÓOcüñÇÇK¾ÅoÓÜ»e7(?ê&^¨-xbÇNºz÷_9OûØÜ/Þ,(¿¹¹¹;|4åWßüíÿ¼#;v¬££#úoÇ±Mm²?¨Hï~=Æ±¿_p'mjjÊ¢n²òU|öìÙ¾¾¾Ê«W_uÏ=1«,Ãõ¶XvSò£n¼D[ê6Éoþ&bÜÞÞþ¹;¥Êï>ò»Kvð+6ßñ?ðÙgÍårq¯äebbbbhh¨rñÑG­>´oß¾ááá±±±wÞyÇn ¡Ë¯Z<·oß¾àHß-[öïßÿÌ3ÏXëp°ì¦åGÝô÷÷_¾|ùn÷?÷¨îß<ò»Ë¯Úüüü¡Cúúú*3»víZ»wï®{ãÆ§z*éÅ÷ÞÏboÐò[ö^÷ñÔNÞòa½íÝ ü¨ûö-õ)7râå]ßó»GS~R~IüUvÉñê¿¼víZggçâ·÷®^½zôèÑÞÞ^½YË¯P(ìØ±cïÞ½à:ÜÜù¦åÇZëîîçäù'N8p oß¾½ú<büØc­¸üîäÑßÝY~Ùlvñ»t±)ß¸qc2õ»à1sæÌ¶&wù½ùæ1ZjíÓÄ»/º)@ù±¦:ÔÛÛOæùùù¸xåÊýèG?üðgÜ`jj*vùÉ/òOOOÇxrròöOû¶¶¶K.%/øÜæNMÜåwøðáØI=zôã?NïÜ¹sÅb±²5úé§XìBb>G#¾þúëÉëþÅ	Hs_ò÷¼Éßö&çëïzÛ,»)@ùQg§Næ­­­©T*6ñR¯ò¯<csÚ°aCõóy©§ý+¯¼¹åswË>ò«×~Ù?ïÈòÉ'ÛÛÛÿÙµmÛ¶Êç;DÏÅj]|4'f*Þ¯â'-~0â1ã`||Übo¾òÕÞæóüÖÛ.àN6(?Ê@ù üP~(?Êåò@ù üÊåò@ù üP~(?ÆØÔÞ²ÊÝá(?å ü$ ü¾,'OÒ*§z*fN8pà@6M§Ó­­­O>ùäÕ«W«íÔ©S[¶lYm·¿ãÄÄÄâÚ7Æx©ò;útÜ,n_ËúÀÊËåöööL&3??ã<2«­­-æzkùS[·n­N´T*çÃÃÃ¢mÙ;.0==½¸üÎ;<þâ(?ØµkWDÕñãÇcç1Þ±cGrU.'Oñ¥KbÜÚÚZhÏ>ûl4âÅDÛ²wùäÄÅmÛ¶-.¿B¡¼;ãÉÉÉä^Ö üV.ÒªrÀ7Îc|úôéÊµ¥R).opp0y¯:Ñ®òÿ7µz ööw¼víZrñêÕ«q±½½ñÄä·+7P~+Q.;;;3Ì7ÒétGGGr¨÷æ­wÚâªùUh[.¶eïø'Ûèøº¯]|P¸Ê`öîÝäóÝ»wWæûûûcæ¹ç¿~ýú²WYö/_N.^»vm©÷üÚÚÚb¬ @ùÔÌ;ï¼ÉdâüÌ3ùt:3çÎ+Ë¯¼òÊß²wÜ¶m[4ß7ãË?.®¾åððpyæx(ÈoØ°ÁÊÀjåóùH«ÎÎÎê¸=öXõÁÖï½7ÎÏg¹ù-Ç$+ãhÄÅ2==½àhï±cÇ¬)@ù¬Öþýû#­öìÙS=yíÚµB¡ÉdÚÛÛ÷íÛwñâÅ¸ÍÞ½-¿eï8>>¾qãÆh¾³gÏ~îS§N¦R©wøða«	P~(?åò@ù üP~(?ÊåòP~(?ÊåÀÚú¿ù©ýIEND®B`


ONEWAY figure BY variable
  /POLYNOMIAL=1
  /STATISTICS DESCRIPTIVES HOMOGENEITY
  /MISSING ANALYSIS
  /POSTHOC=LSD ALPHA(0.05).


Oneway


Notes	
Output Created	06-OCT-2022 17:14:38	
Comments		
Input	Active Dataset	DataSet0	
	Filter	<none>	
	Weight	<none>	
	Split File	<none>	
	N of Rows in Working Data File	17	
Missing Value Handling	Definition of Missing	User-defined missing values are treated as missing.	
	Cases Used	Statistics for each analysis are based on cases with no missing data for any variable in the analysis.	
Syntax	ONEWAY figure BY variable
  /POLYNOMIAL=1
  /STATISTICS DESCRIPTIVES HOMOGENEITY
  /MISSING ANALYSIS
  /POSTHOC=LSD ALPHA(0.05).	
Resources	Processor Time	00:00:00.02	
	Elapsed Time	00:00:00.01	


Descriptives	
figure  	
	N	Mean	Std. Deviation	Std. Error	95% Confidence Interval for Mean			
					Lower Bound	Upper Bound			
Controll	4	.50169281	.033740414	.016870207	.44800428	.55538134			
ESAs	4	.72460645	.087975512	.043987756	.58461778	.86459512			
TPx	4	.83983584	.039859360	.019929680	.77641070	.90326098			
ConA	4	.55137052	.037115981	.018557990	.49231071	.61043033			
Total	16	.65437640	.147993970	.036998493	.57551598	.73323683			


Test of Homogeneity of Variances	
	Levene Statistic	df1	df2	Sig.	
figure	Based on Mean	1.434	3	12	.281	
	Based on Median	1.073	3	12	.397	
	Based on Median and with adjusted df	1.073	3	6.347	.425	
	Based on trimmed mean	1.427	3	12	.283	


ANOVA	
figure  	
	Sum of Squares	df	Mean Square	F		
Between Groups	(Combined)	.293	3	.098	32.983		
	Linear Term	Contrast	.014	1	.014	4.717		
		Deviation	.279	2	.140	47.116		
Within Groups	.036	12	.003			
Total	.329	15				


Post Hoc Tests


Multiple Comparisons	
Dependent Variable:   figure  	
LSD  	
(I) variable	(J) variable	Mean Difference (I-J)	Std. Error	Sig.	95% Confidence Interval	
					Lower Bound	Upper Bound	
Controll	ESAs	-.222913643*	.038478031	.000	-.30675007	-.13907722	
	TPx	-.338143031*	.038478031	.000	-.42197946	-.25430660	
	ConA	-.049677711	.038478031	.221	-.13351414	.03415872	
ESAs	Controll	.222913643*	.038478031	.000	.13907722	.30675007	
	TPx	-.115229387*	.038478031	.011	-.19906581	-.03139296	
	ConA	.173235932*	.038478031	.001	.08939950	.25707236	
TPx	Controll	.338143031*	.038478031	.000	.25430660	.42197946	
	ESAs	.115229387*	.038478031	.011	.03139296	.19906581	
	ConA	.288465320*	.038478031	.000	.20462889	.37230175	
ConA	Controll	.049677711	.038478031	.221	-.03415872	.13351414	
	ESAs	-.173235932*	.038478031	.001	-.25707236	-.08939950	
	TPx	-.288465319*	.038478031	.000	-.37230175	-.20462889	

*. The mean difference is significant at the 0.05 level.	


SAVE OUTFILE='E:\桌面\1.sav'
  /COMPRESSED.
