# Supplementary material for: Regulation of piglet T-cell immune responses by thioredoxin peroxidase from Cysticercus cellulosae excretory-secretory antigens
Source: Front Microbiol. 2022 Nov 18;13:1019810. doi: 10.3389/fmicb.2022.1019810 (PMC9718028; doi:10.3389/fmicb.2022.1019810)
Supplement: Supplementary file 2 [file Data_Sheet_2.ZIP › 2. C. Cellulosae ESAs and TPx Induced CD4+ and CD8+ T-Lymphocyte Responses in PBMCs/4. Flowjo analysis data export.pdf]

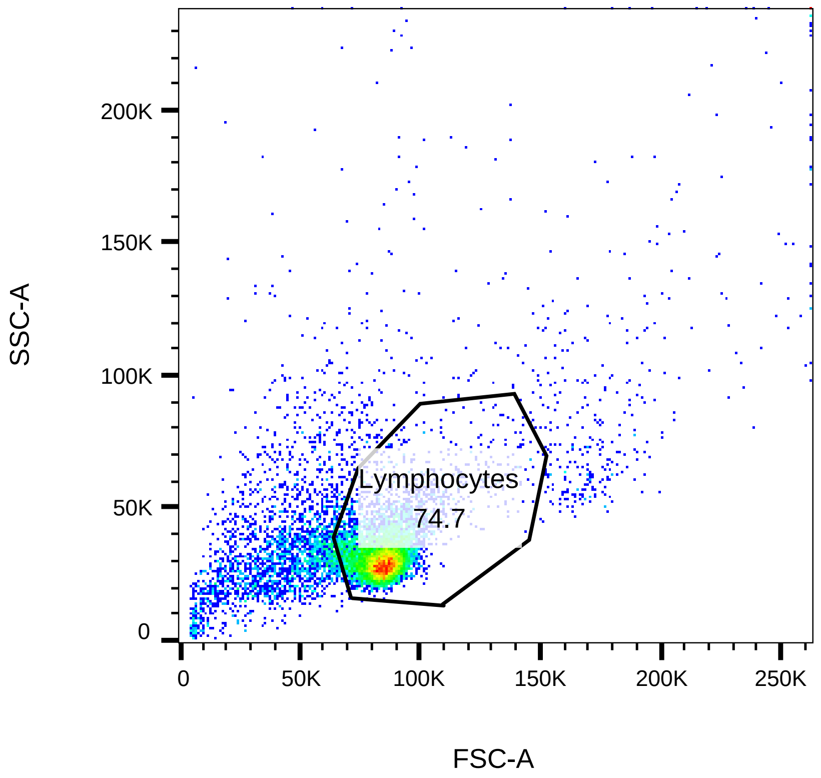

Specimen_002_ESA-1_016.fcs
Ungated
11877

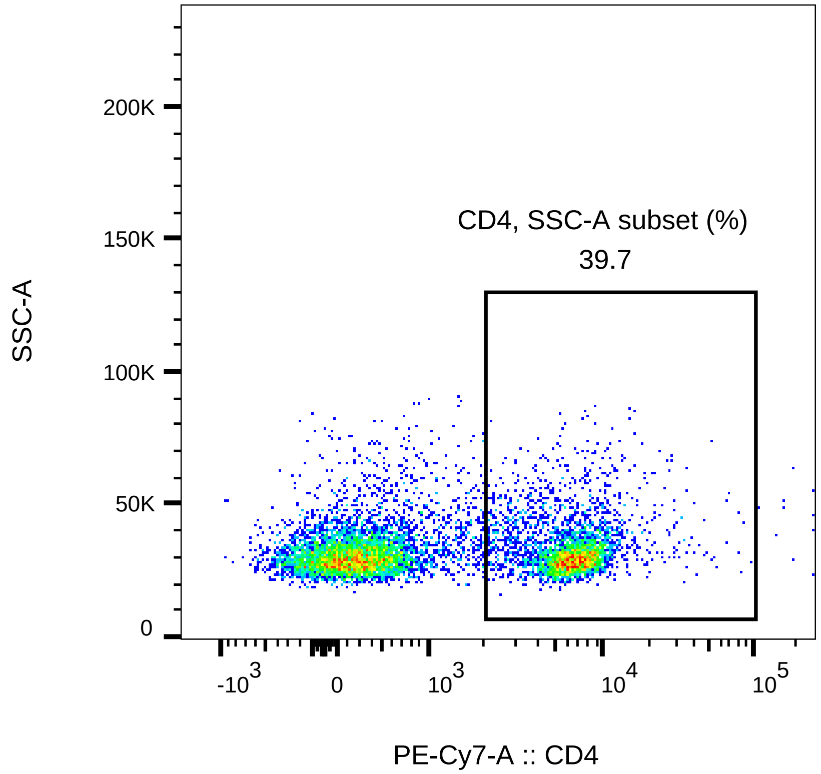

Specimen_002_ESA-1_016.fcs
Lymphocytes
8878

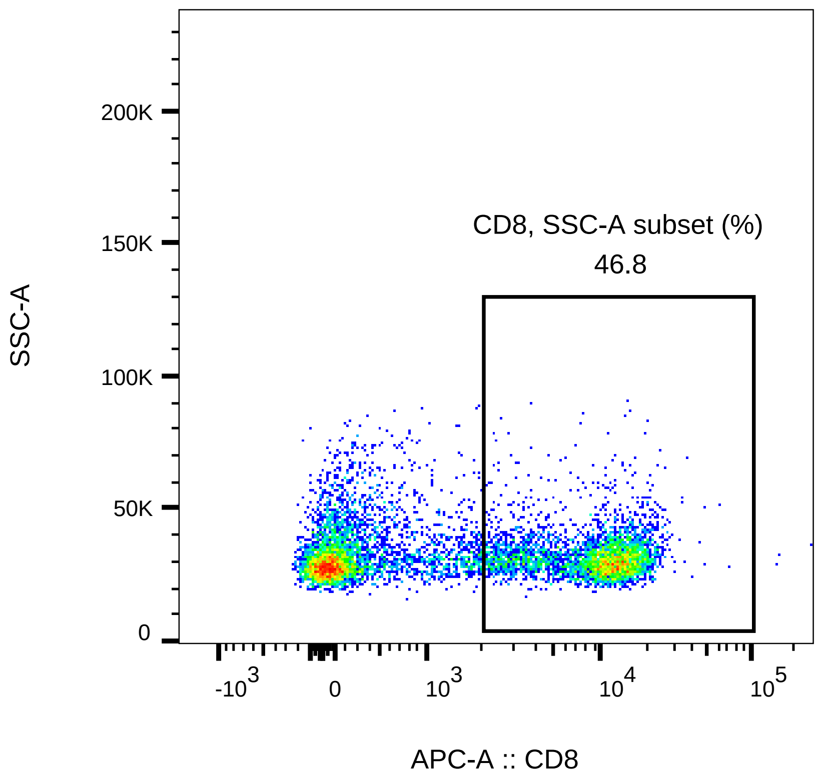

Specimen_002_ESA-1_016.fcs
Lymphocytes
8878

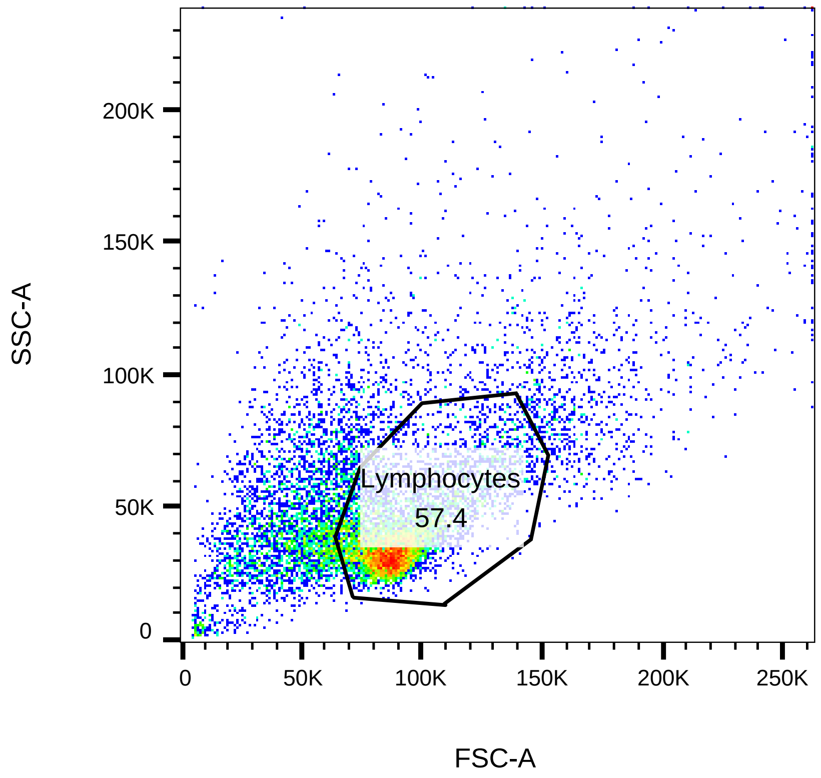

Specimen_002_ESA-2_017.fcs
Ungated
11976

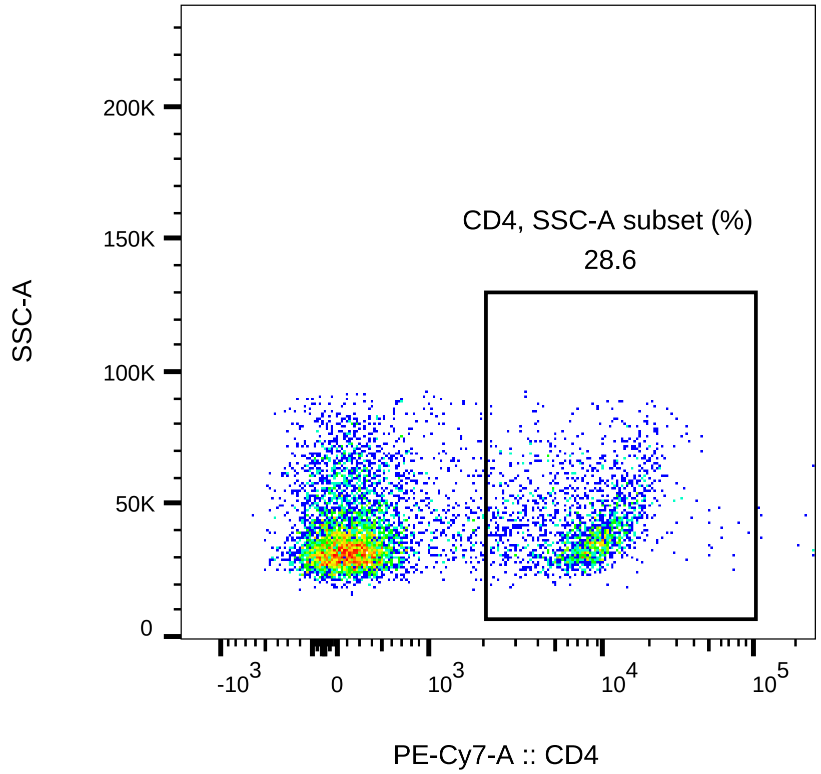

Specimen_002_ESA-2_017.fcs
Lymphocytes
6871

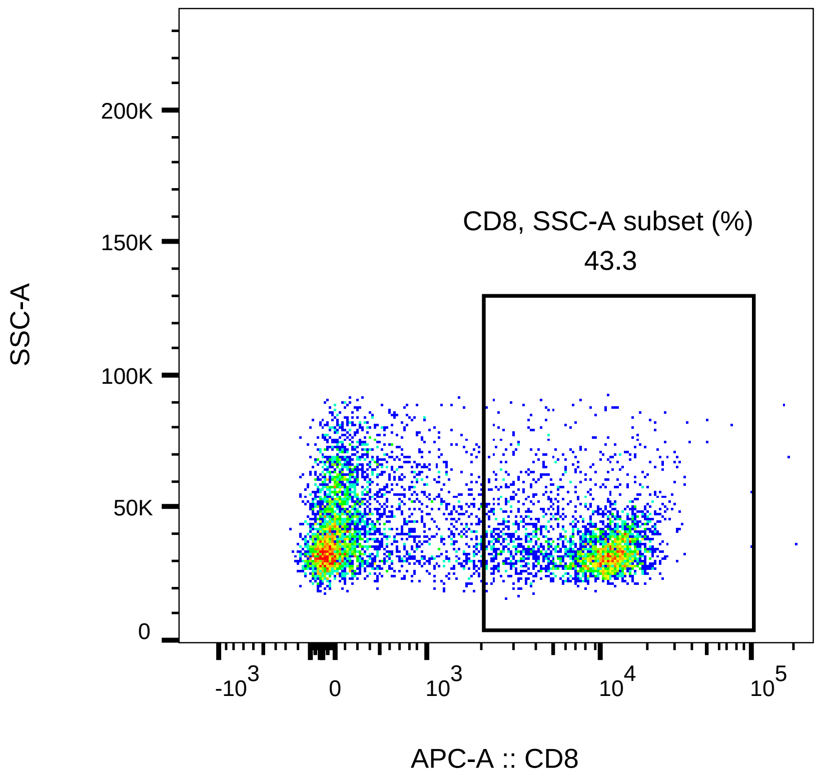

Specimen_002_ESA-2_017.fcs
Lymphocytes
6871

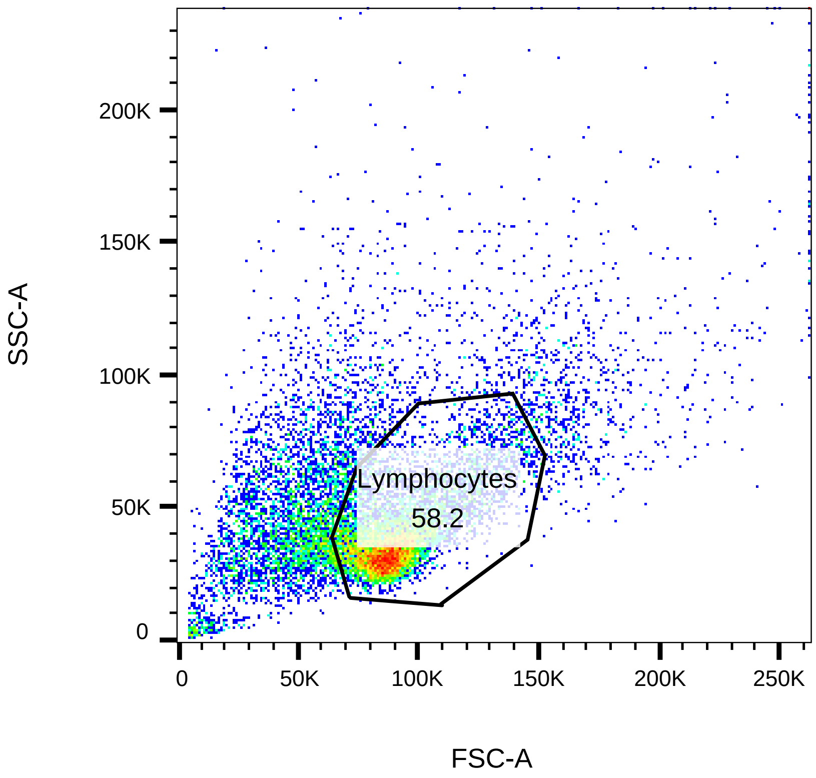

Specimen_002_ESA-3_001_018.fcs
Ungated
14097

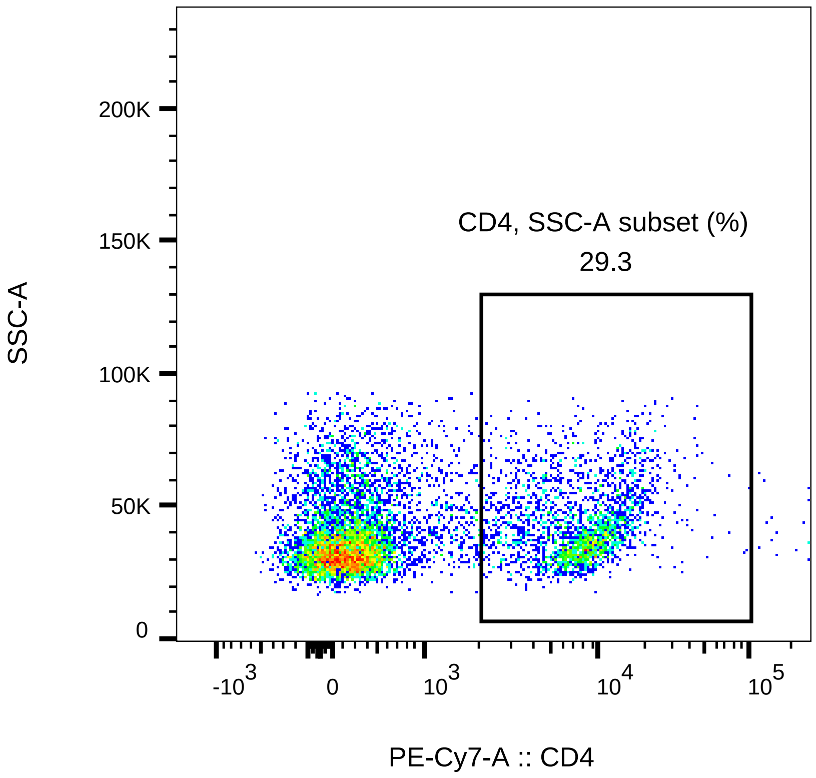

Specimen_002_ESA-3_001_018.fcs
Lymphocytes
8206

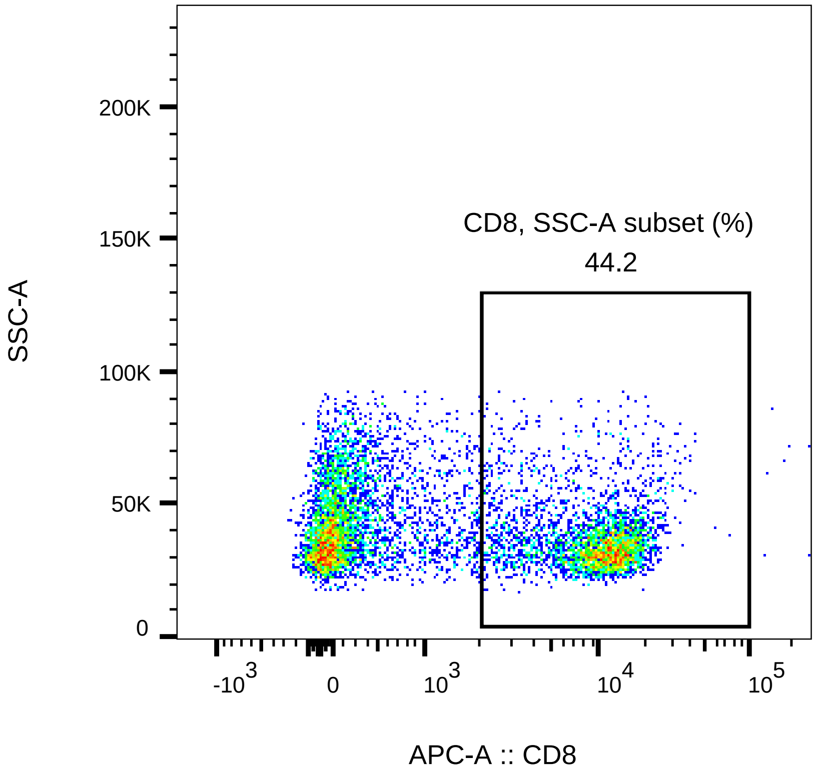

Specimen_002_ESA-3_001_018.fcs
Lymphocytes
8206

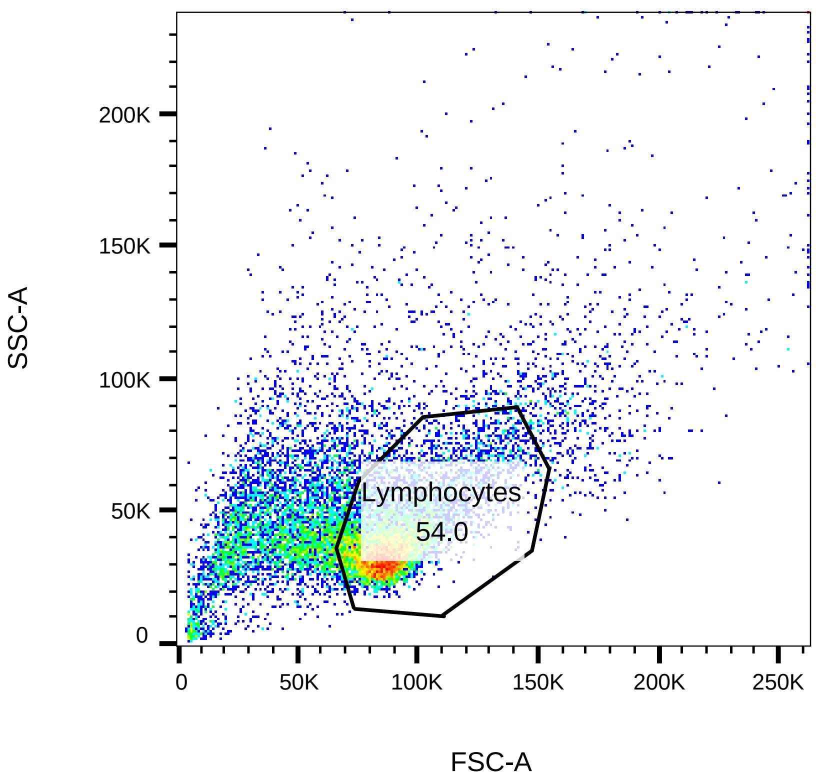

Specimen_002_TPx-1_010.fcs
Ungated
14694

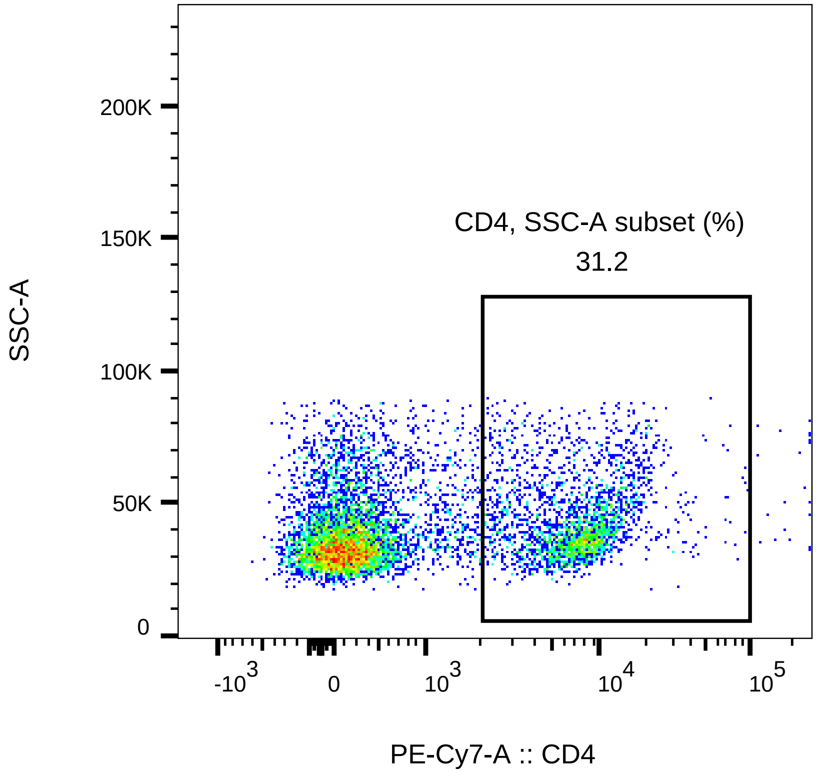

Specimen_002_TPx-1_010.fcs
Lymphocytes
7937

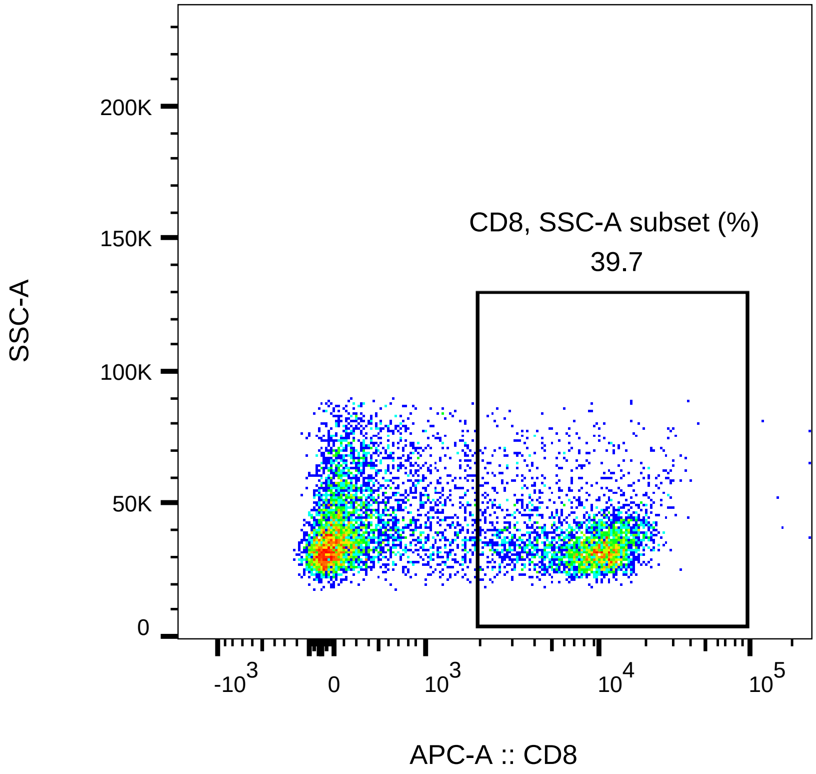

Specimen_002_TPx-1_010.fcs
Lymphocytes
7937

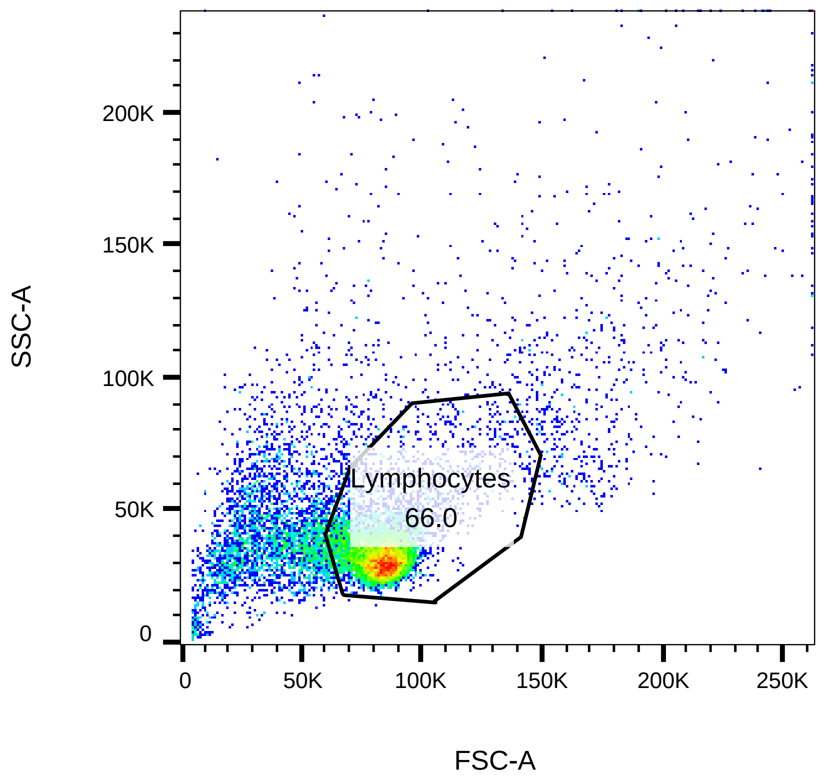

Specimen_002_TPx-2_011.fcs
Ungated
13309

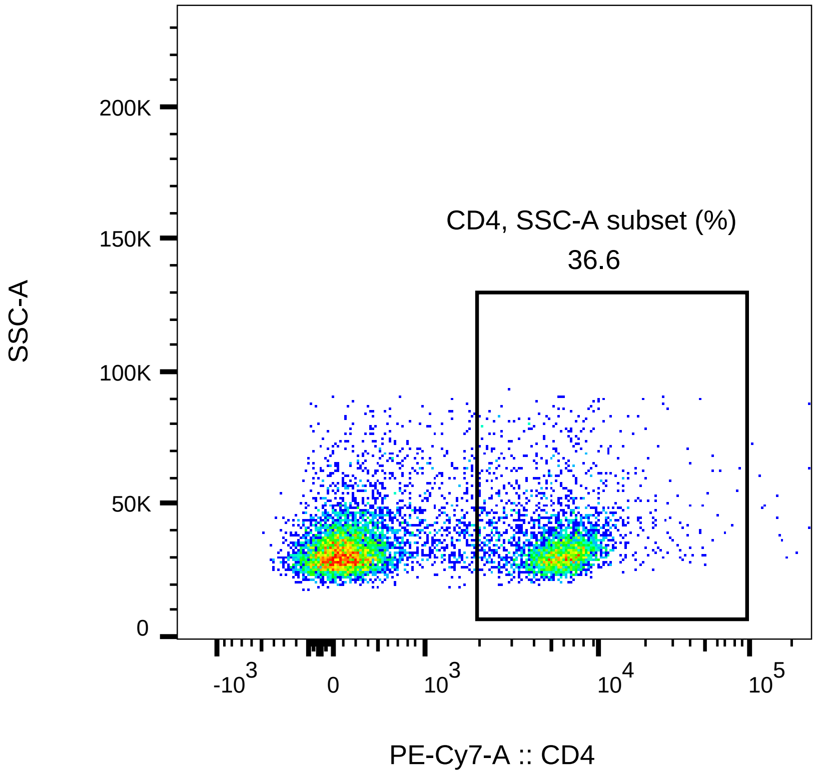

Specimen_002_TPx-2_011.fcs
Lymphocytes
8781

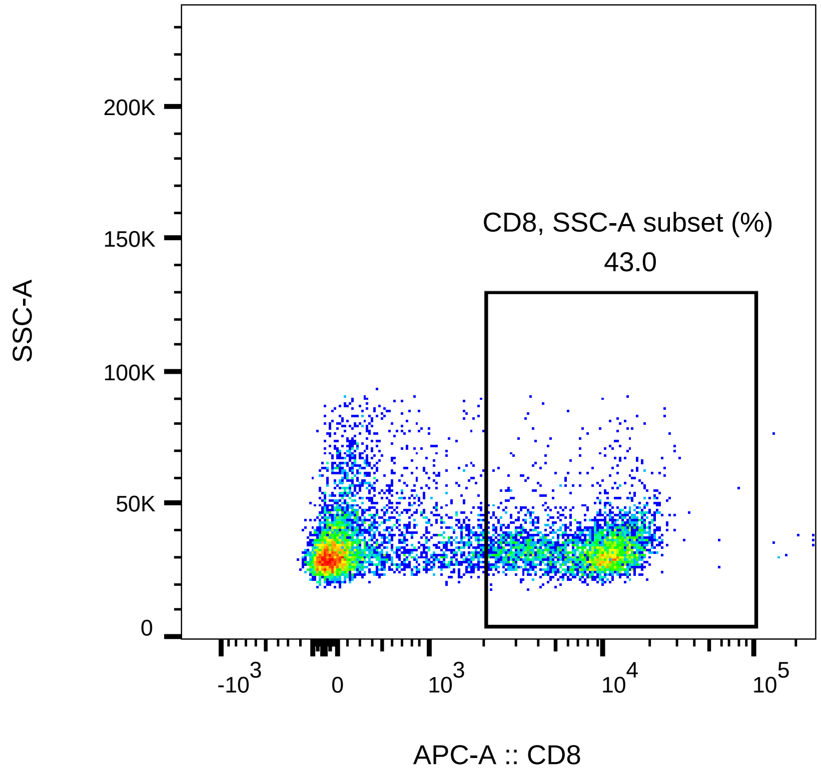

Specimen_002_TPx-2_011.fcs
Lymphocytes
8781

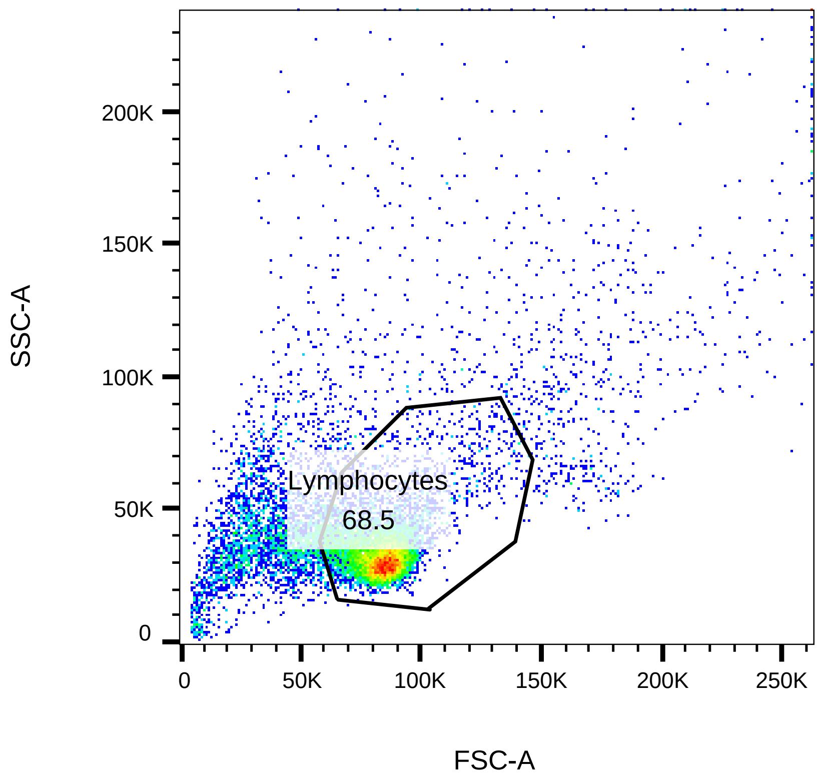

Specimen_002_TPx-3_012.fcs
Ungated
13243

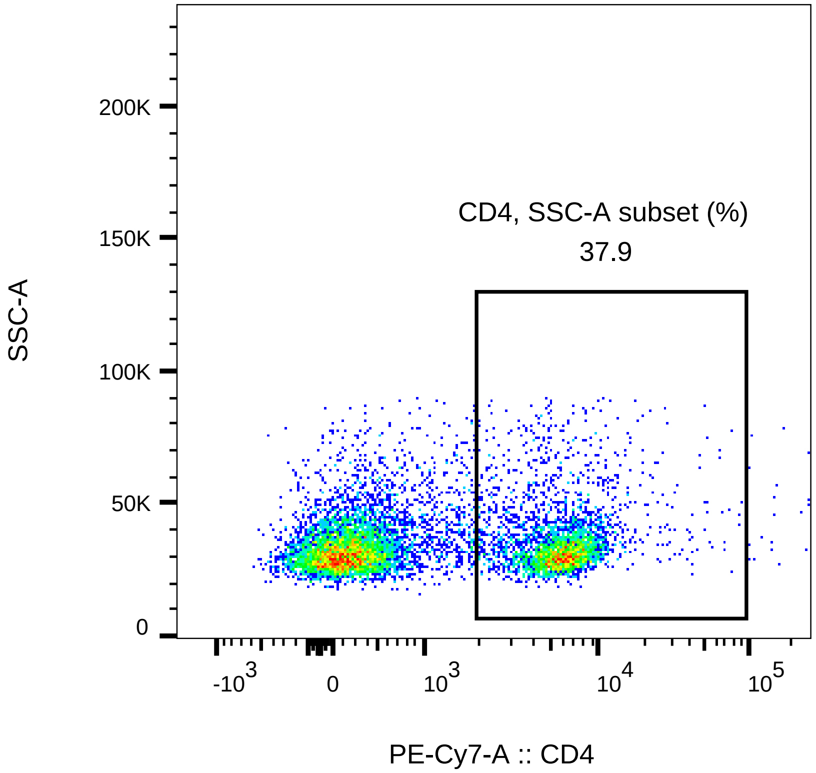

Specimen_002_TPx-3_012.fcs
Lymphocytes
9076

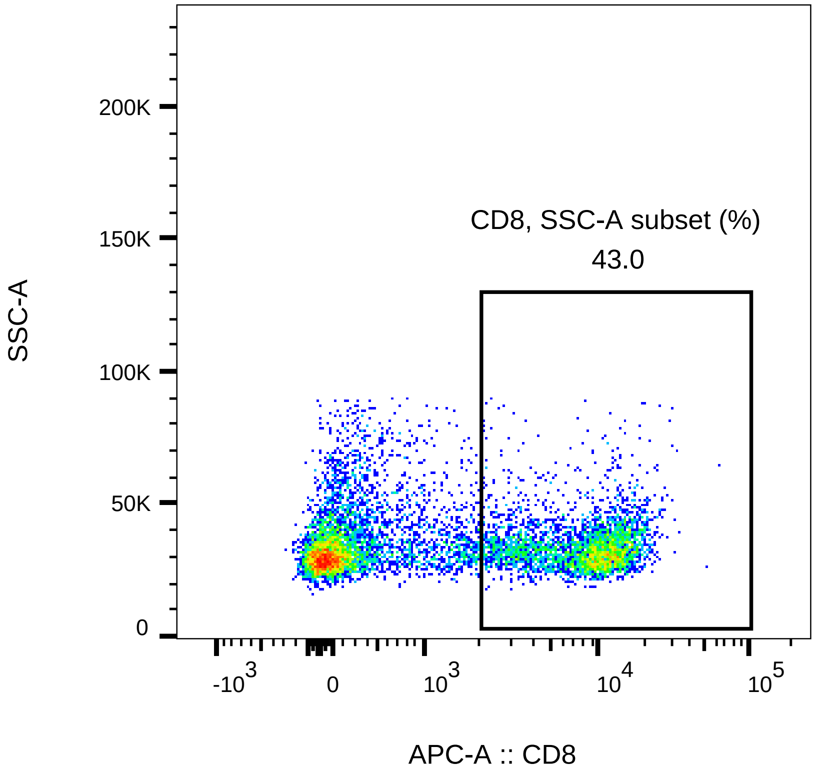

Specimen_002_TPx-3_012.fcs
Lymphocytes
9076

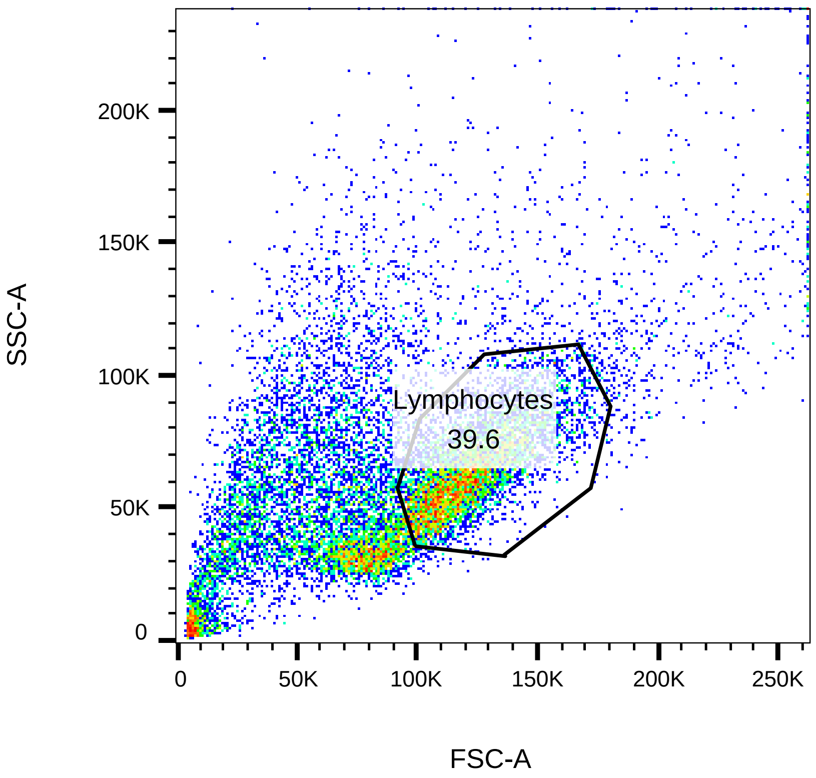

Specimen_002_conA 1_004.fcs
Ungated
16351

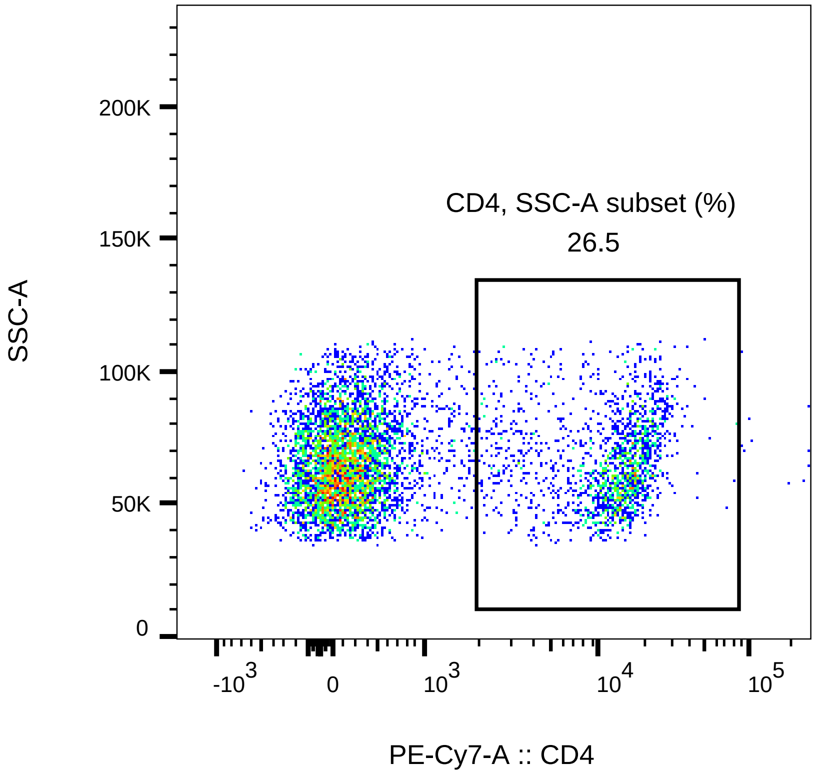

Specimen_002_conA 1_004.fcs
Lymphocytes
6475

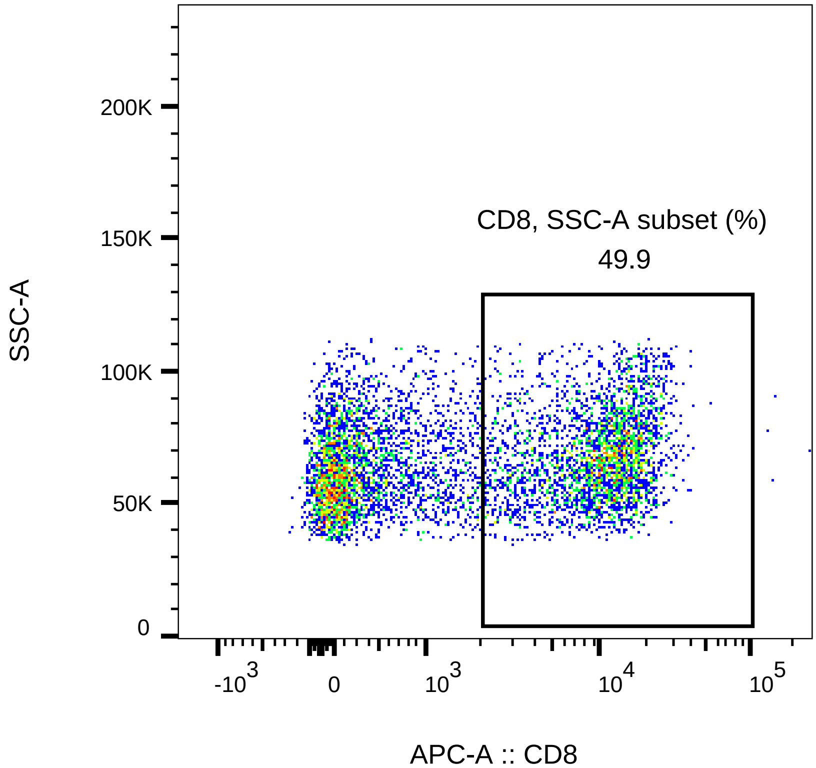

Specimen_002_conA 1_004.fcs
Lymphocytes
6475

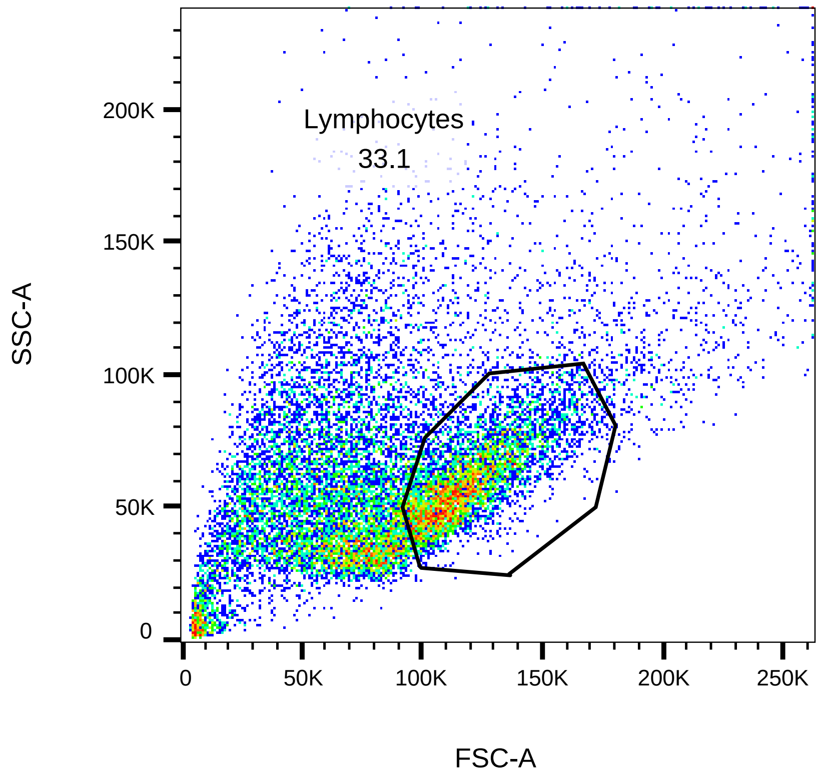

Specimen_002_conA 2_005.fcs
Ungated
17614

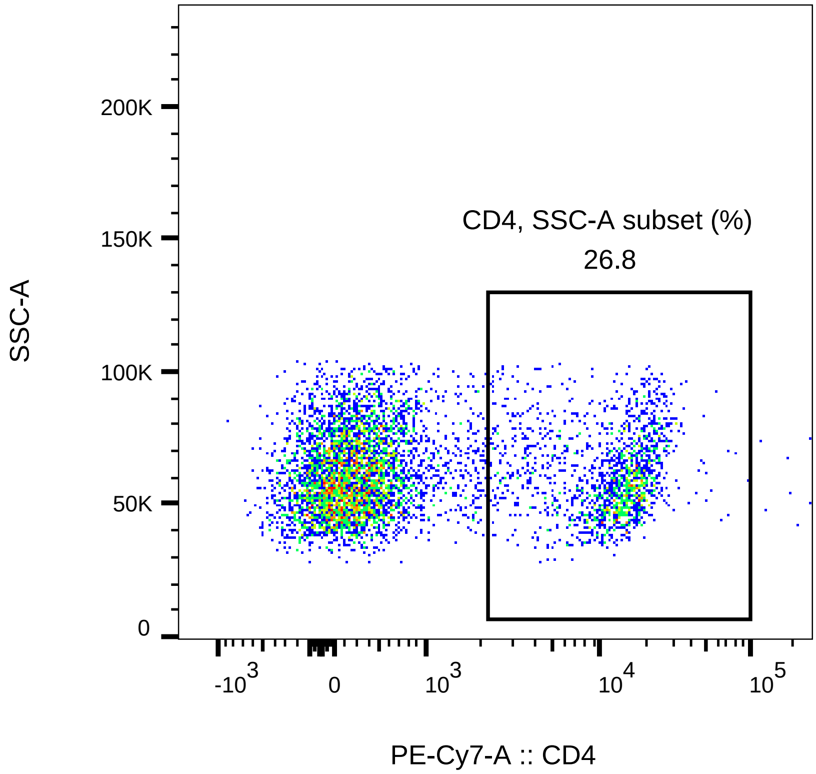

Specimen_002_conA 2_005.fcs
Lymphocytes
5835

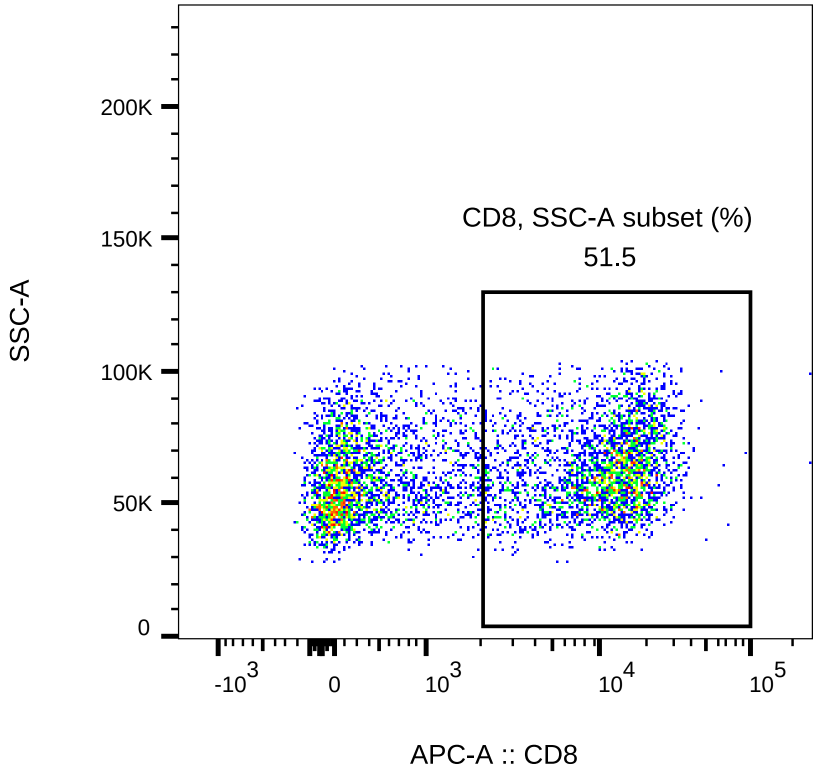

Specimen_002_conA 2_005.fcs
Lymphocytes
5835

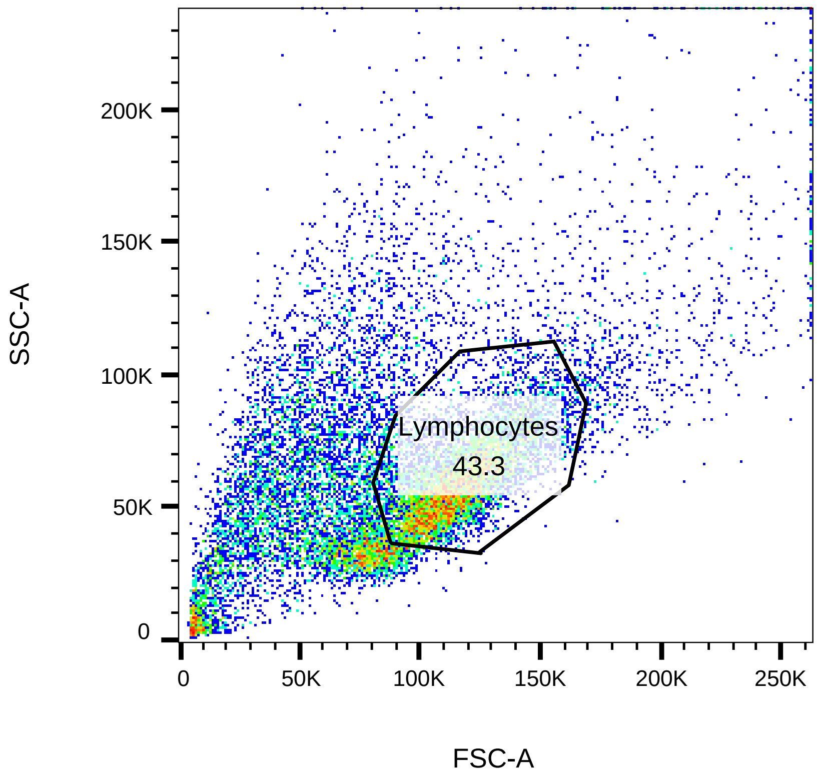

Specimen_002_conA 3_006.fcs
Ungated
16622

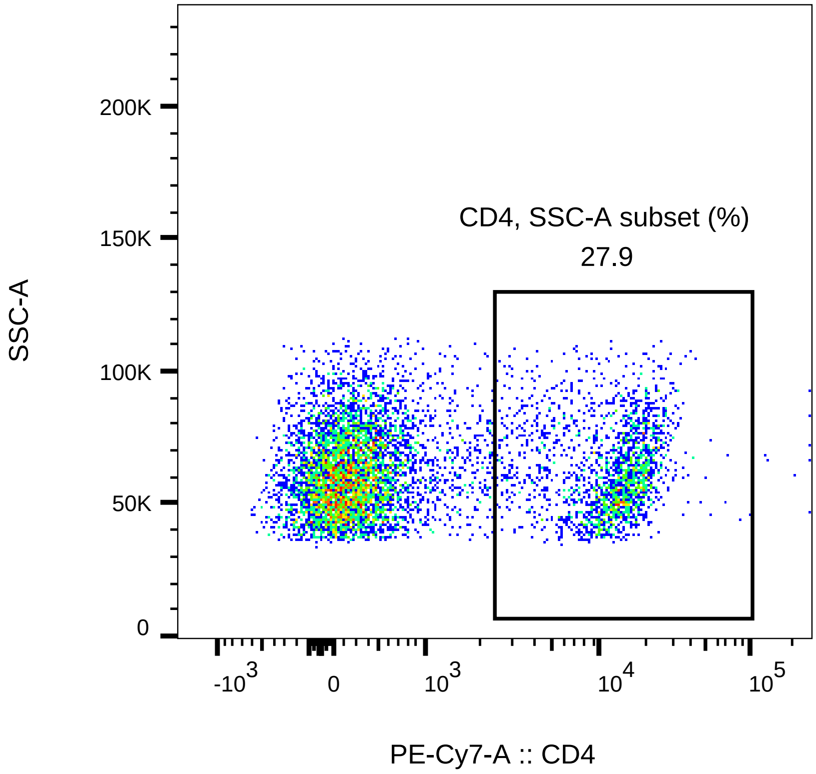

Specimen_002_conA 3_006.fcs
Lymphocytes
7199

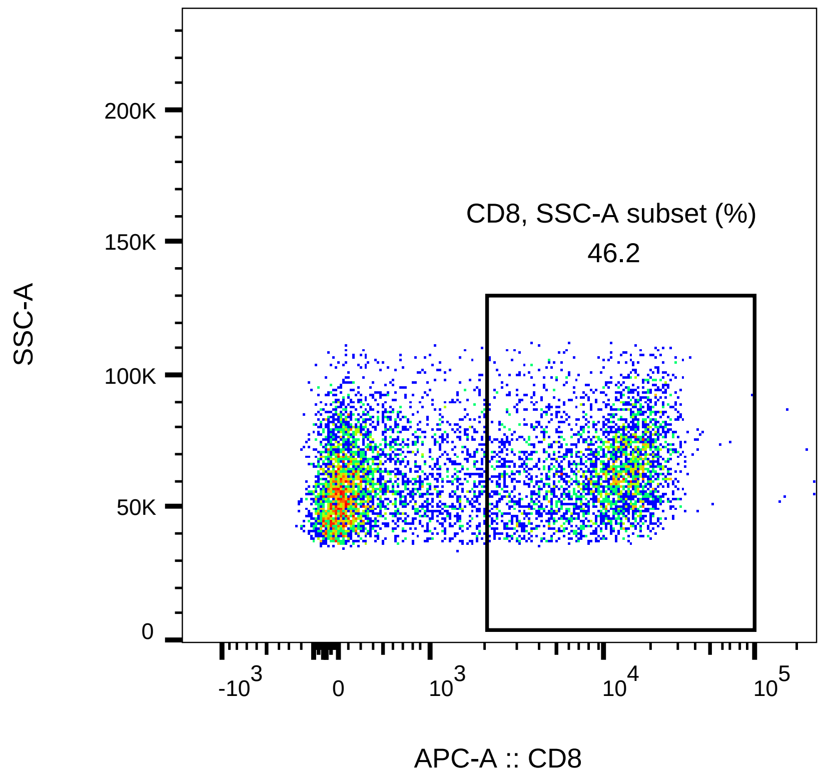

Specimen_002_conA 3_006.fcs
Lymphocytes
7199

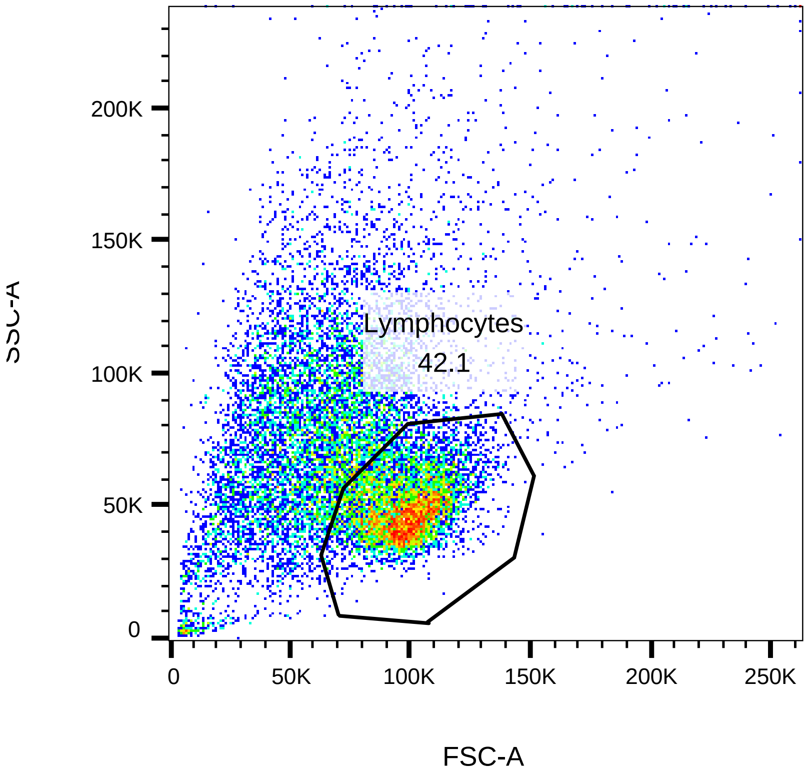

Specimen_002_1640-1_001.fcs
Ungated
16460

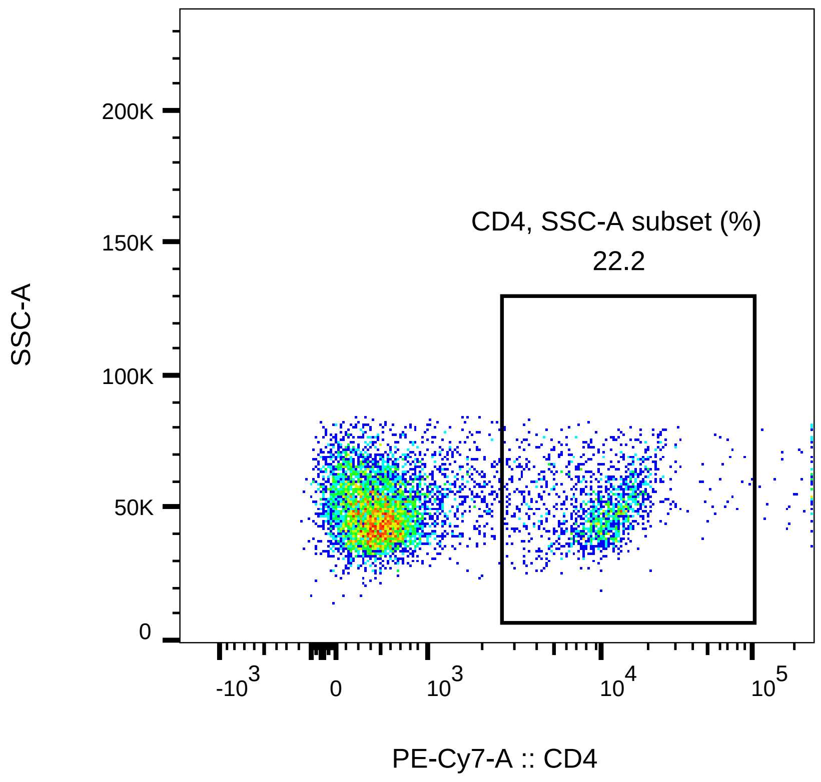

Specimen_002_1640-1_001.fcs
Lymphocytes
6922

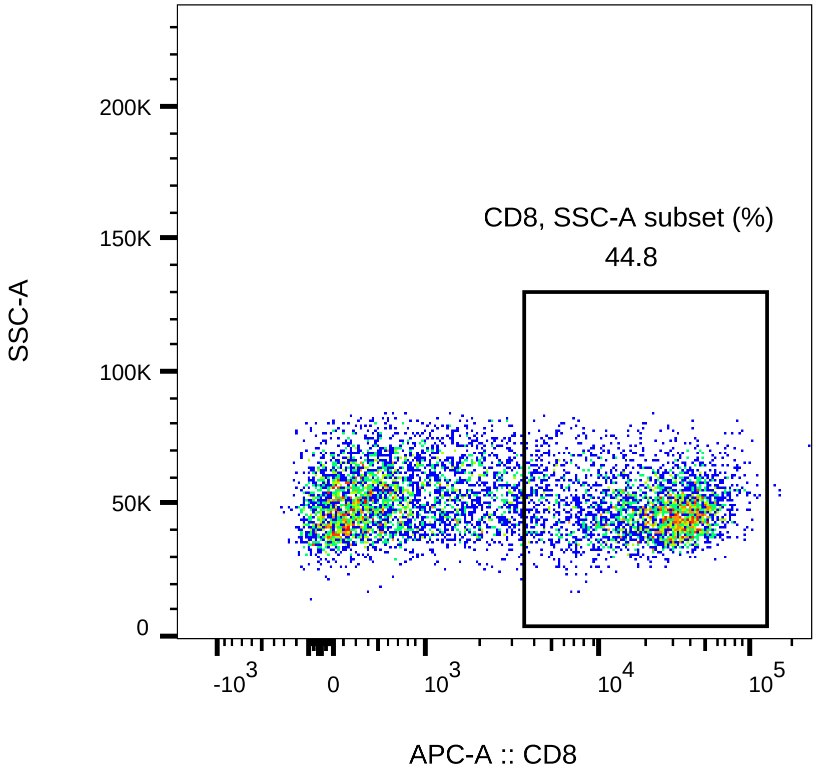

Specimen_002_1640-1_001.fcs
Lymphocytes
6922

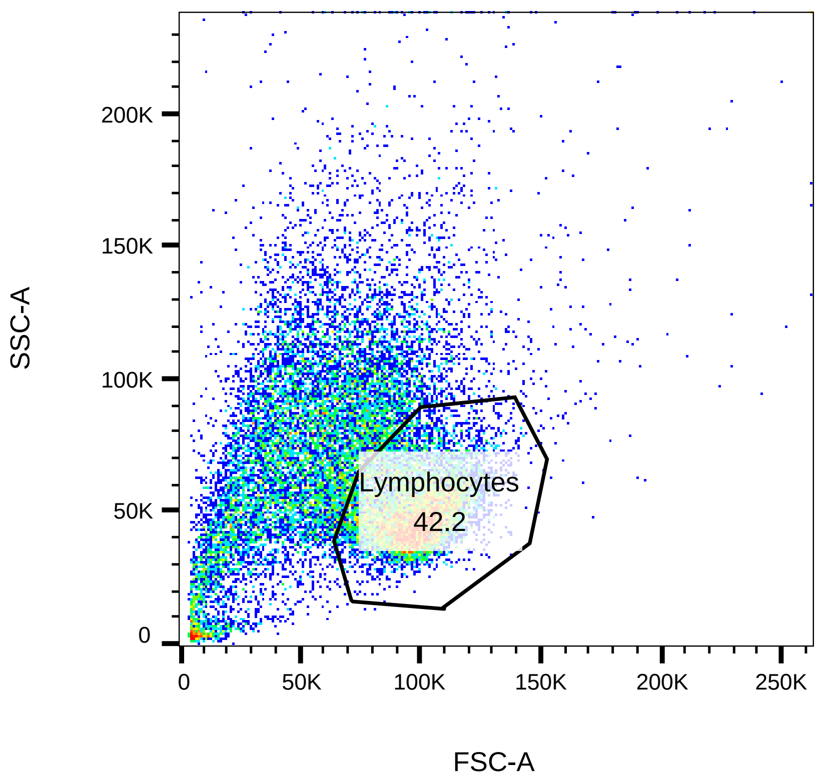

Specimen_002_1640-2_002.fcs
Ungated
21258

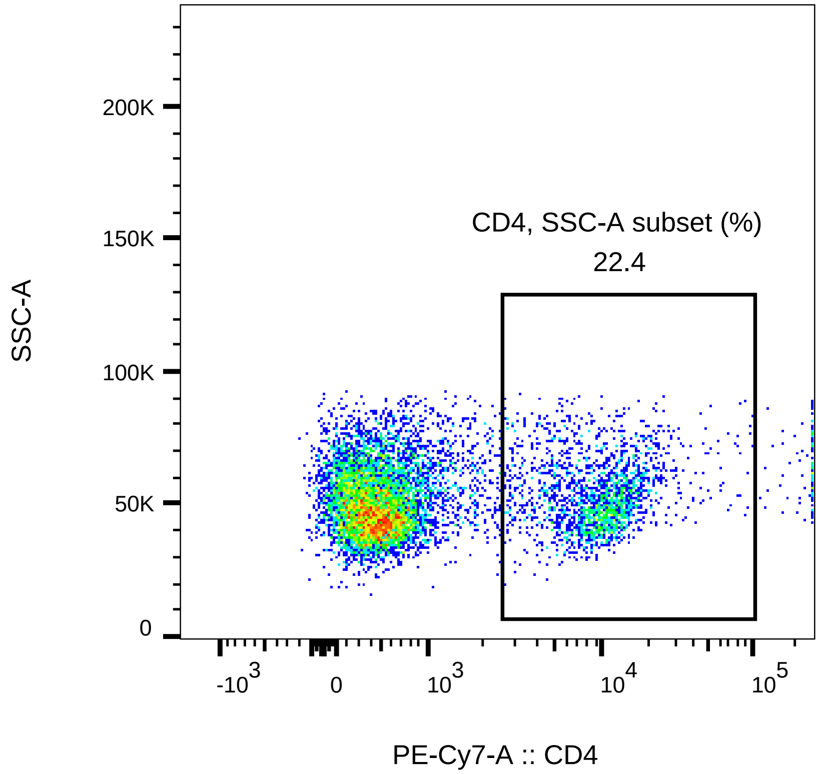

Specimen_002_1640-2_002.fcs
Lymphocytes
8971

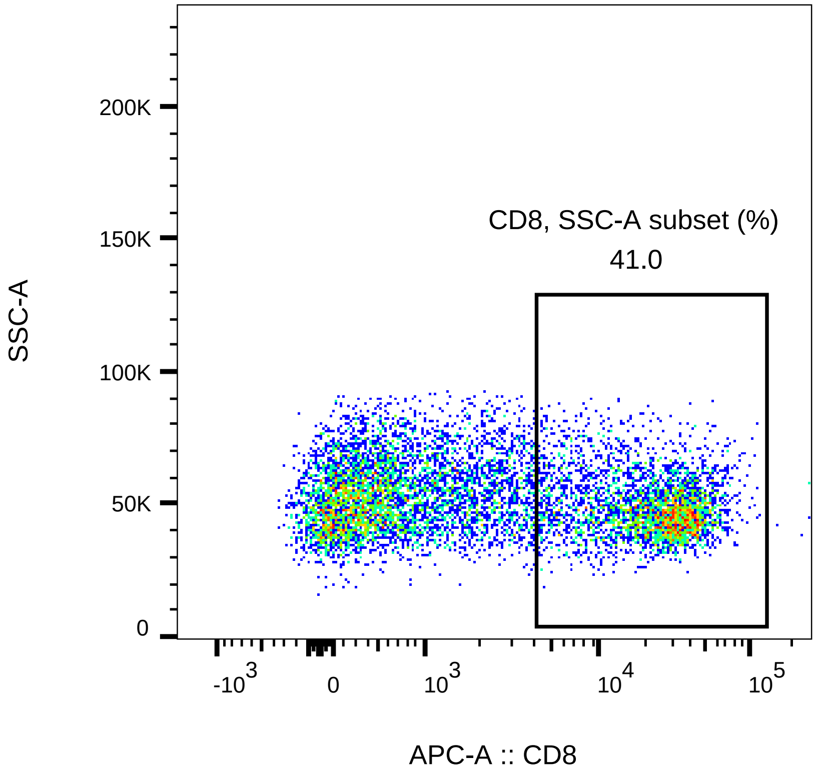

Specimen_002_1640-2_002.fcs
Lymphocytes
8971

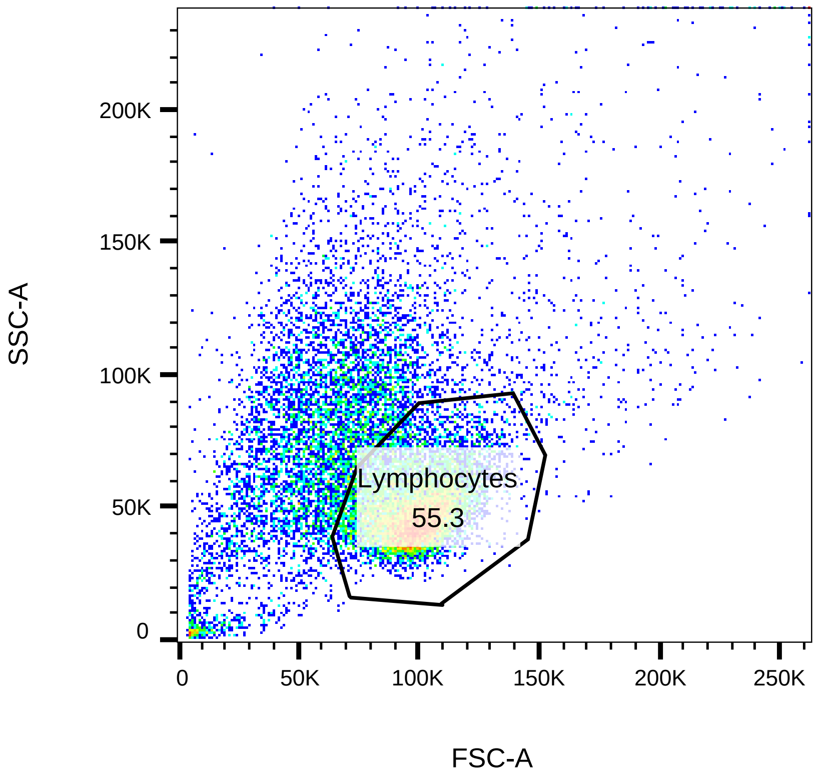

Specimen_002_1640-3_003.fcs
Ungated
17702

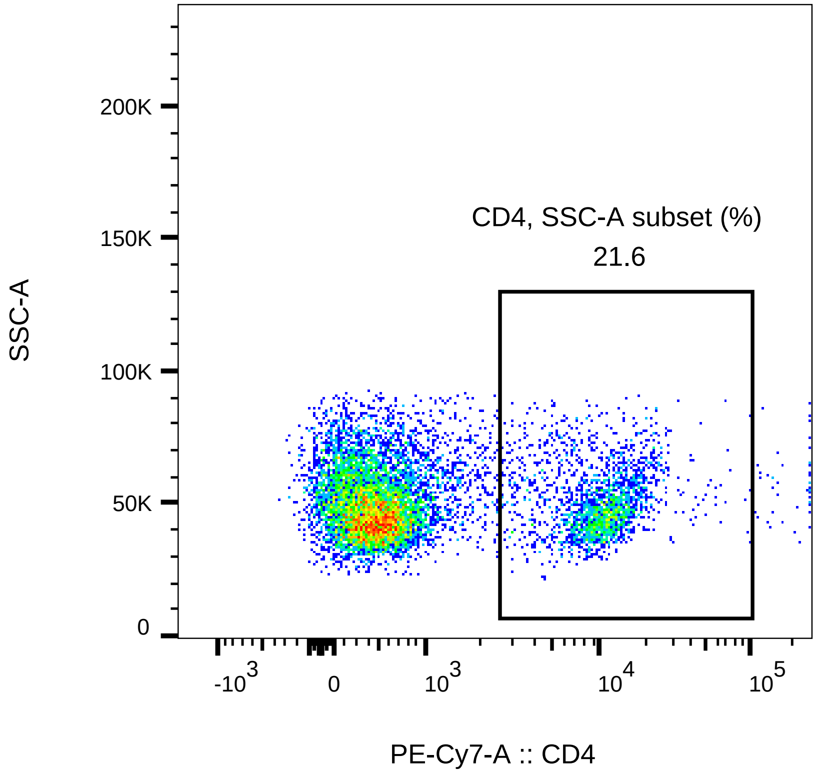

Specimen_002_1640-3_003.fcs
Lymphocytes
9786

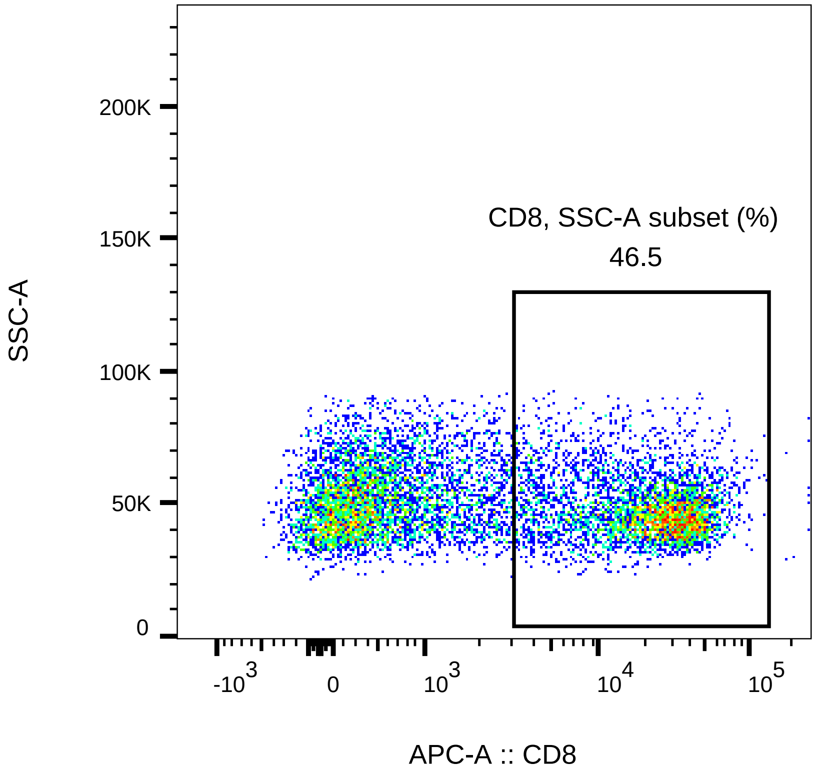

Specimen_002_1640-3_003.fcs
Lymphocytes
9786
