# Supplementary figures and images for: Regulation of piglet T-cell immune responses by thioredoxin peroxidase from Cysticercus cellulosae excretory-secretory antigens
Source: Front Microbiol. 2022 Nov 18;13:1019810. doi: 10.3389/fmicb.2022.1019810 (PMC9718028; doi:10.3389/fmicb.2022.1019810)

CD4<sup>+</sup>

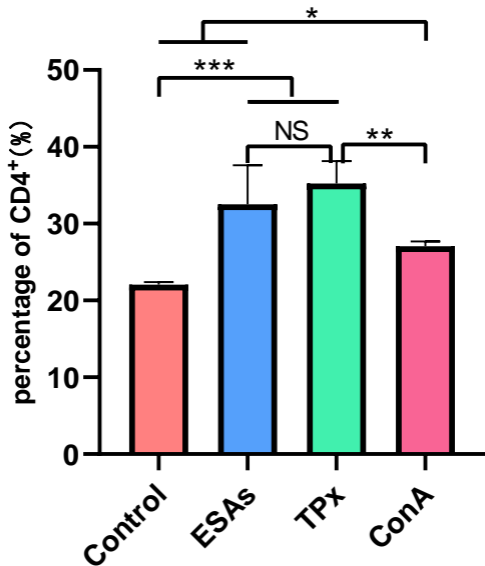

Supplement: Supplementary file 2 [file Data_Sheet_2.ZIP › 2. C. Cellulosae ESAs and TPx Induced CD4+ and CD8+ T-Lymphocyte Responses in PBMCs/3. Create statistical graphs with GraphPad software/2.1 CD4.pdf]

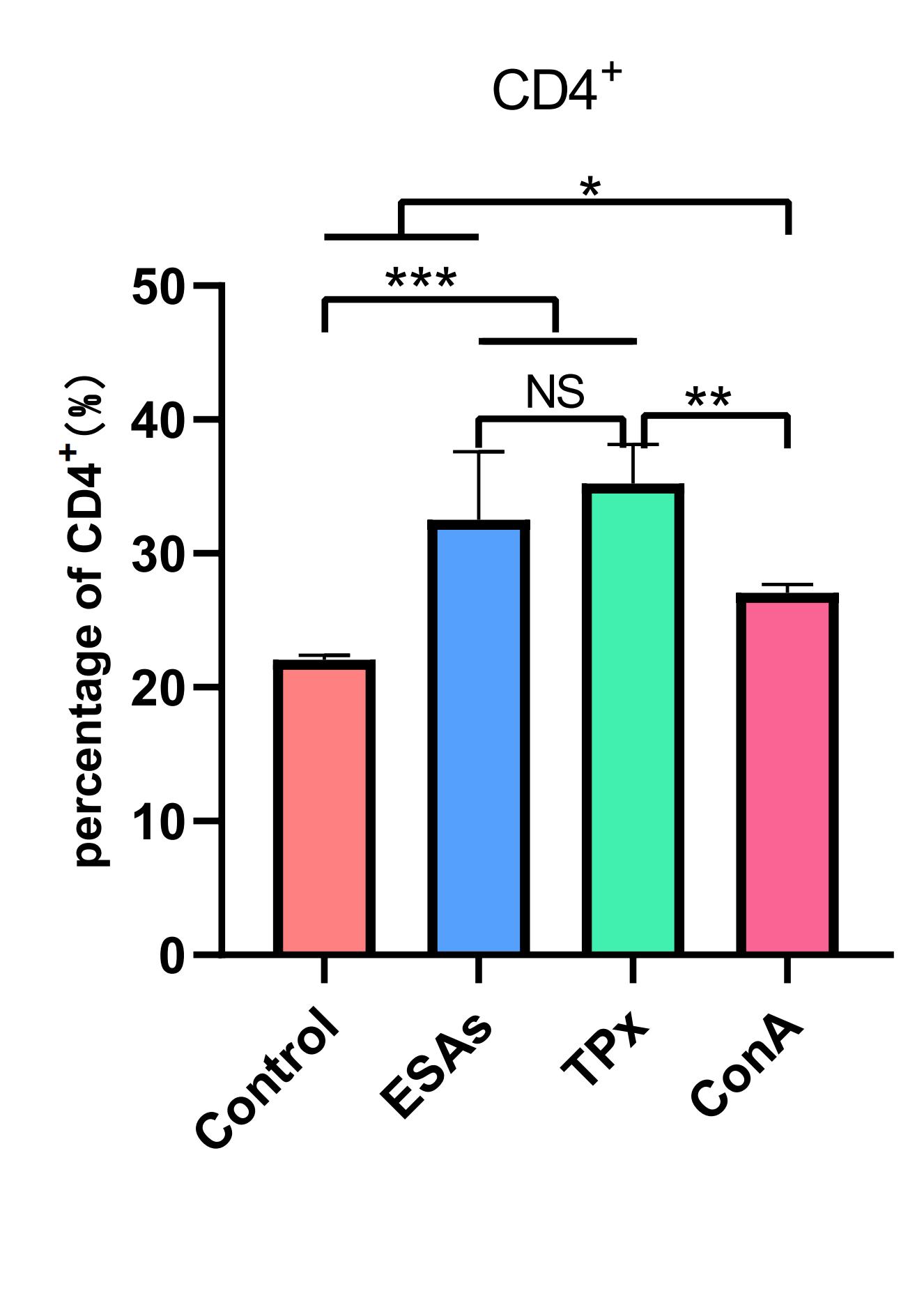

Supplement: Supplementary file 2 [file Data_Sheet_2.ZIP › 2. C. Cellulosae ESAs and TPx Induced CD4+ and CD8+ T-Lymphocyte Responses in PBMCs/3. Create statistical graphs with GraphPad software/2.2CD4_00.jpg]

CD8<sup>+</sup>

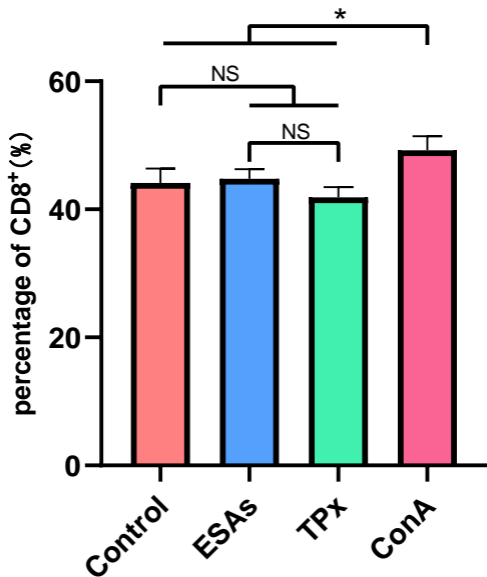

Supplement: Supplementary file 2 [file Data_Sheet_2.ZIP › 2. C. Cellulosae ESAs and TPx Induced CD4+ and CD8+ T-Lymphocyte Responses in PBMCs/3. Create statistical graphs with GraphPad software/3.1 CD8.pdf]

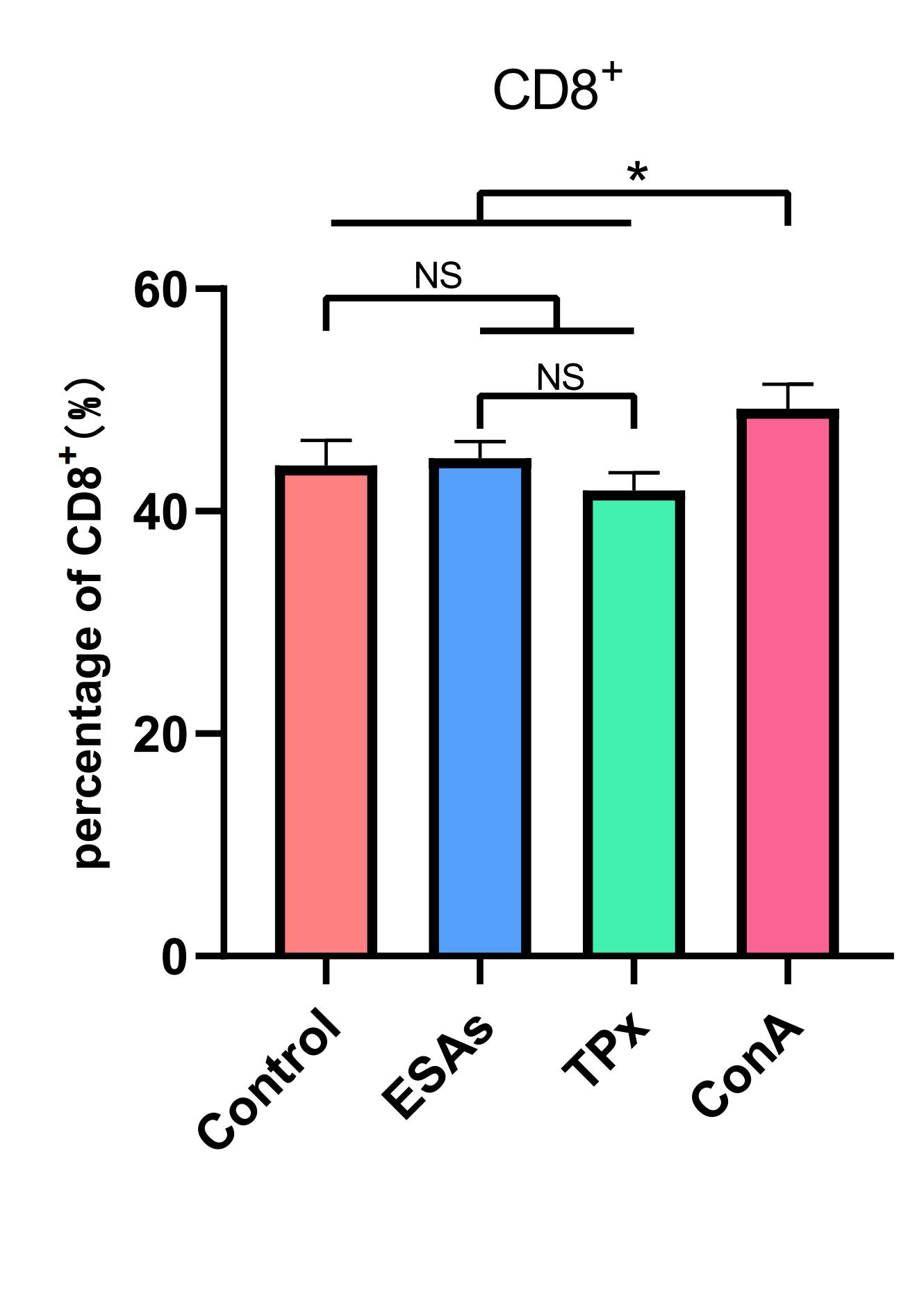

Supplement: Supplementary file 2 [file Data_Sheet_2.ZIP › 2. C. Cellulosae ESAs and TPx Induced CD4+ and CD8+ T-Lymphocyte Responses in PBMCs/3. Create statistical graphs with GraphPad software/3.2 CD8_00.jpg]

# CD4<sup>+</sup>/CD8<sup>+</sup>

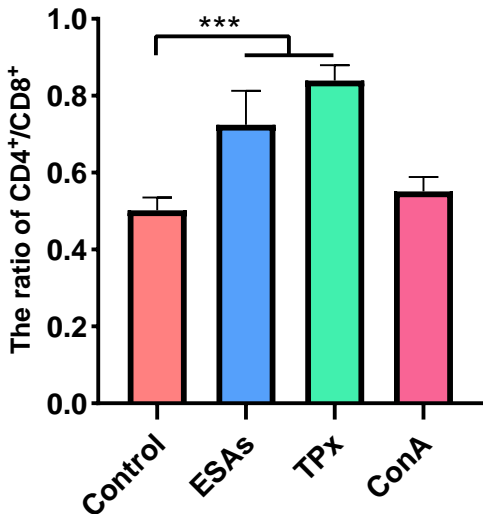

Supplement: Supplementary file 2 [file Data_Sheet_2.ZIP › 2. C. Cellulosae ESAs and TPx Induced CD4+ and CD8+ T-Lymphocyte Responses in PBMCs/3. Create statistical graphs with GraphPad software/4.1 CD4+_CD8+.pdf]

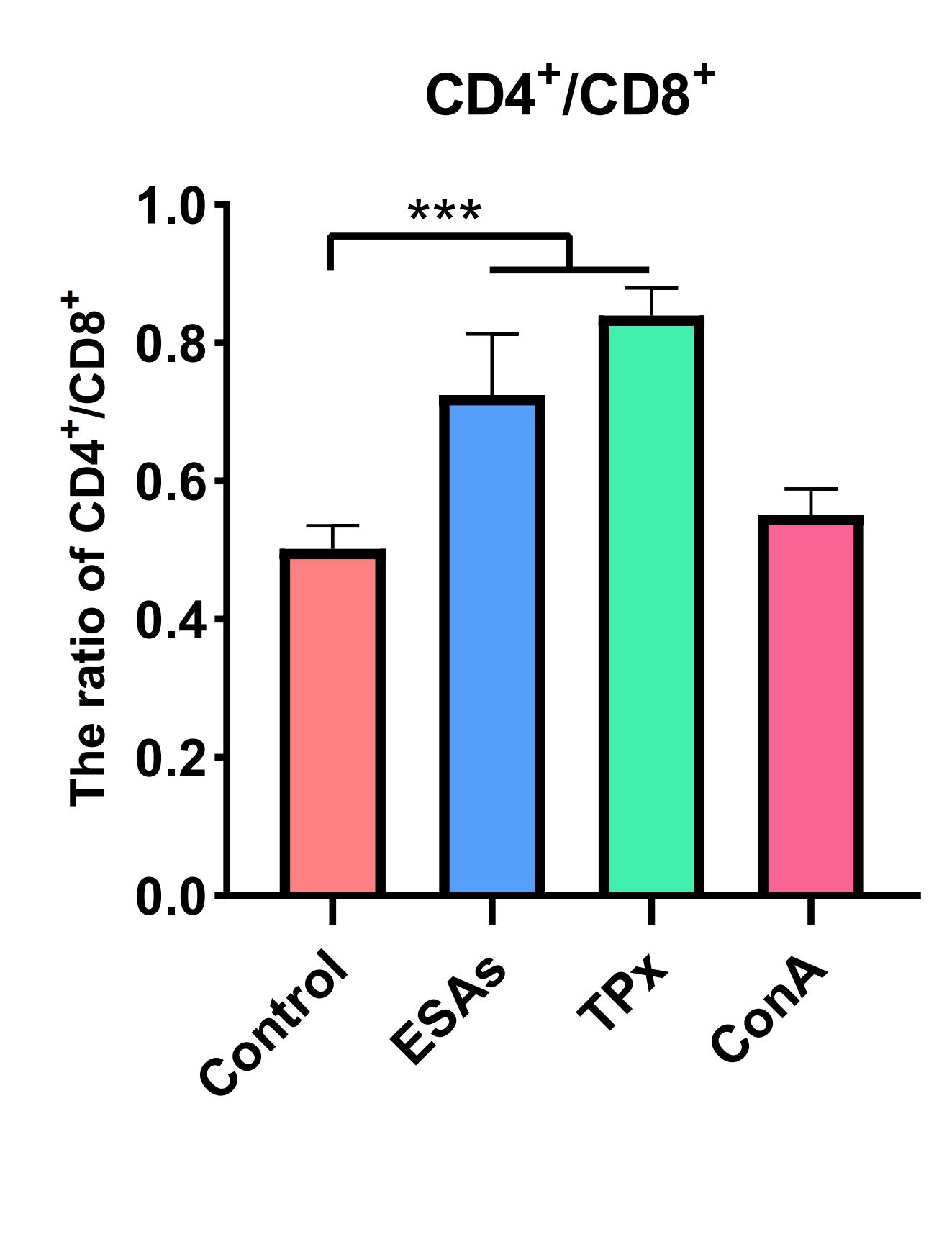

Supplement: Supplementary file 2 [file Data_Sheet_2.ZIP › 2. C. Cellulosae ESAs and TPx Induced CD4+ and CD8+ T-Lymphocyte Responses in PBMCs/3. Create statistical graphs with GraphPad software/4.2 CD4+_CD8+_00.jpg]

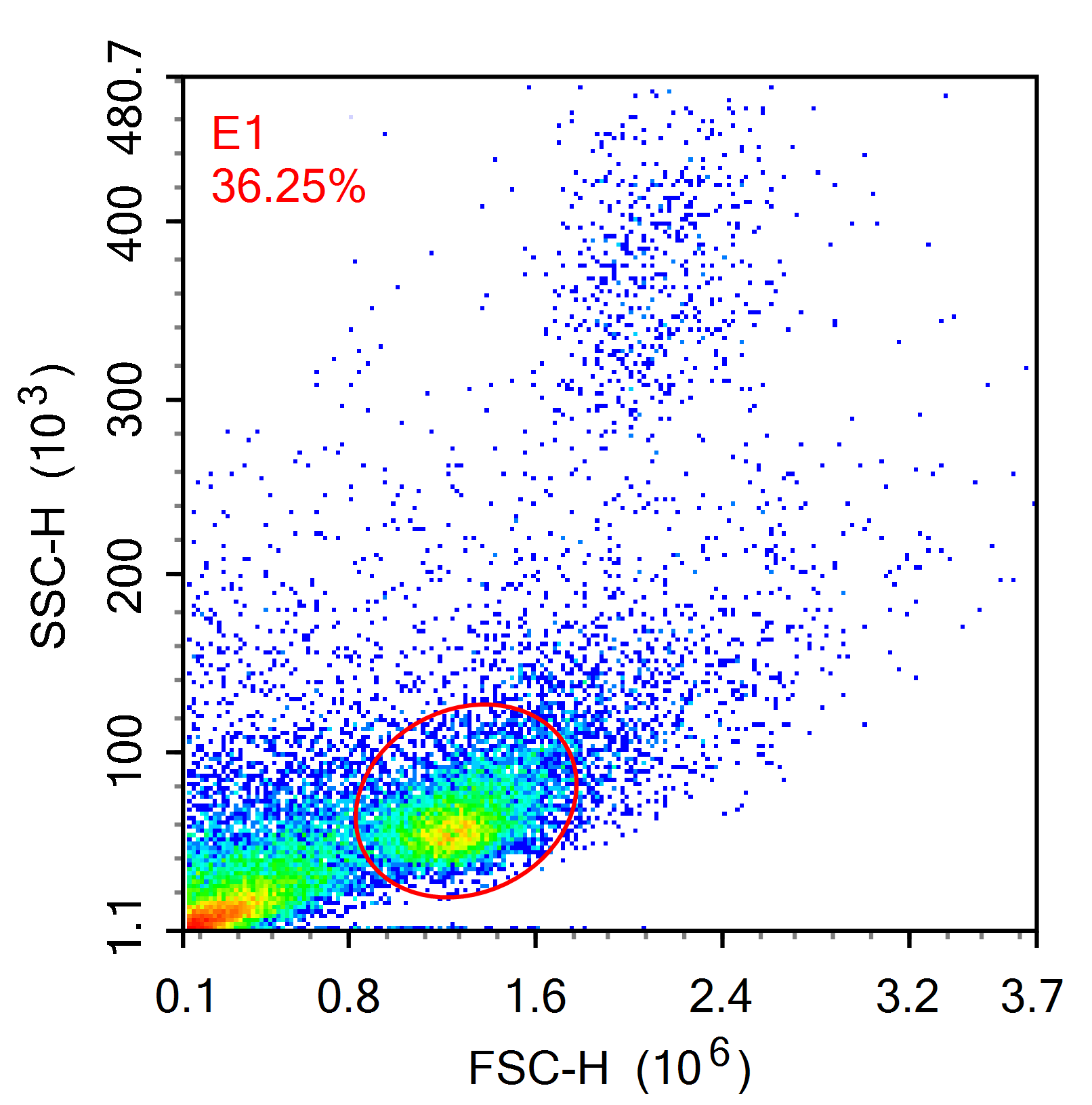

Supplement: Supplementary file 3 [file Data_Sheet_3.ZIP › 4. C. Cellulosae ESAs and TPx Induced Th Subpopulation Differentiation/1. The purity of naive CD4+ T cells was detected by flow cytometry/2. Flowjo analysis of exported images/1. Before sorting-1/figure 1.tiff]

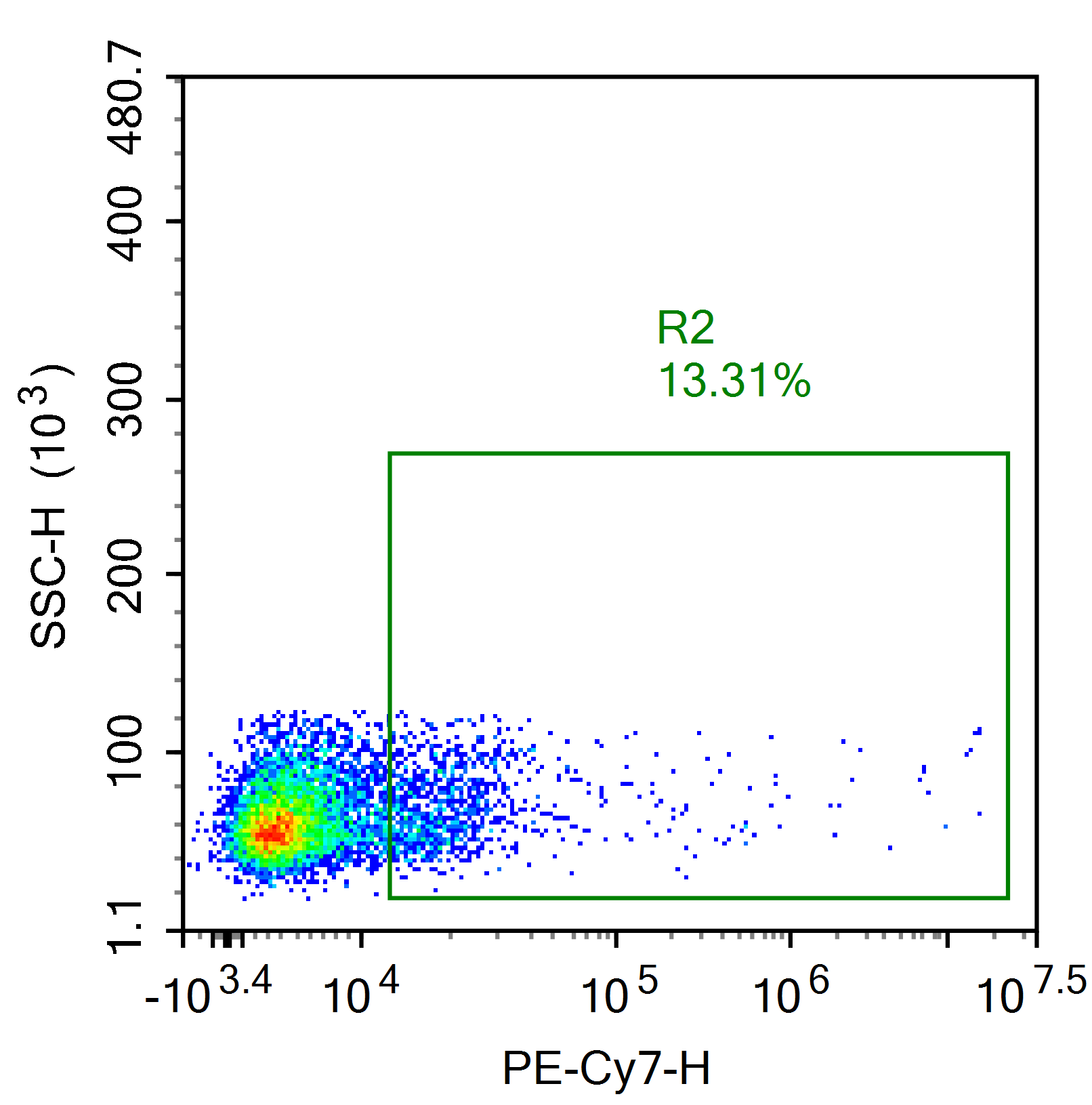

Supplement: Supplementary file 3 [file Data_Sheet_3.ZIP › 4. C. Cellulosae ESAs and TPx Induced Th Subpopulation Differentiation/1. The purity of naive CD4+ T cells was detected by flow cytometry/2. Flowjo analysis of exported images/1. Before sorting-1/figure 2.tiff]

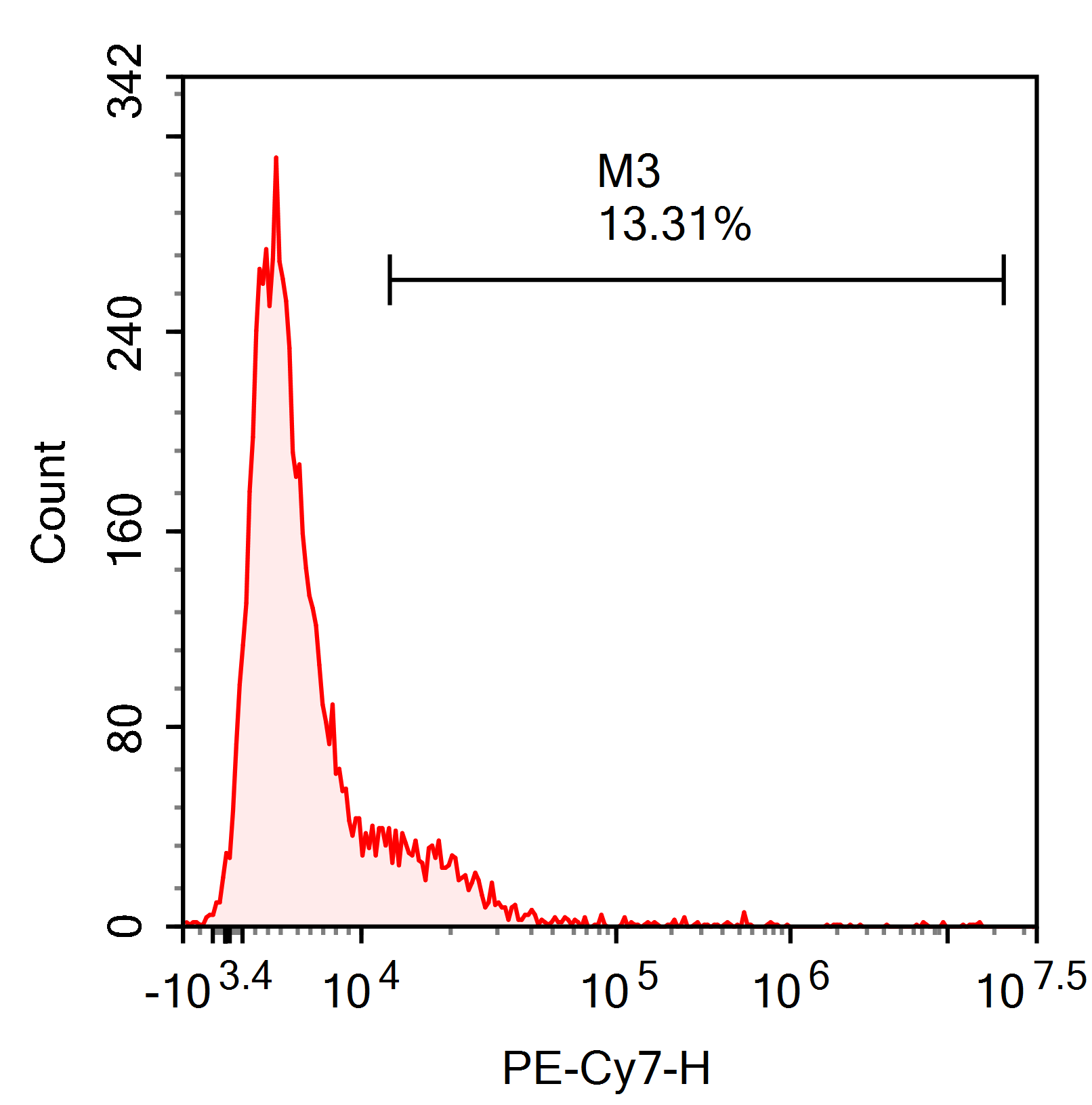

Supplement: Supplementary file 3 [file Data_Sheet_3.ZIP › 4. C. Cellulosae ESAs and TPx Induced Th Subpopulation Differentiation/1. The purity of naive CD4+ T cells was detected by flow cytometry/2. Flowjo analysis of exported images/1. Before sorting-1/figure 3.tiff]

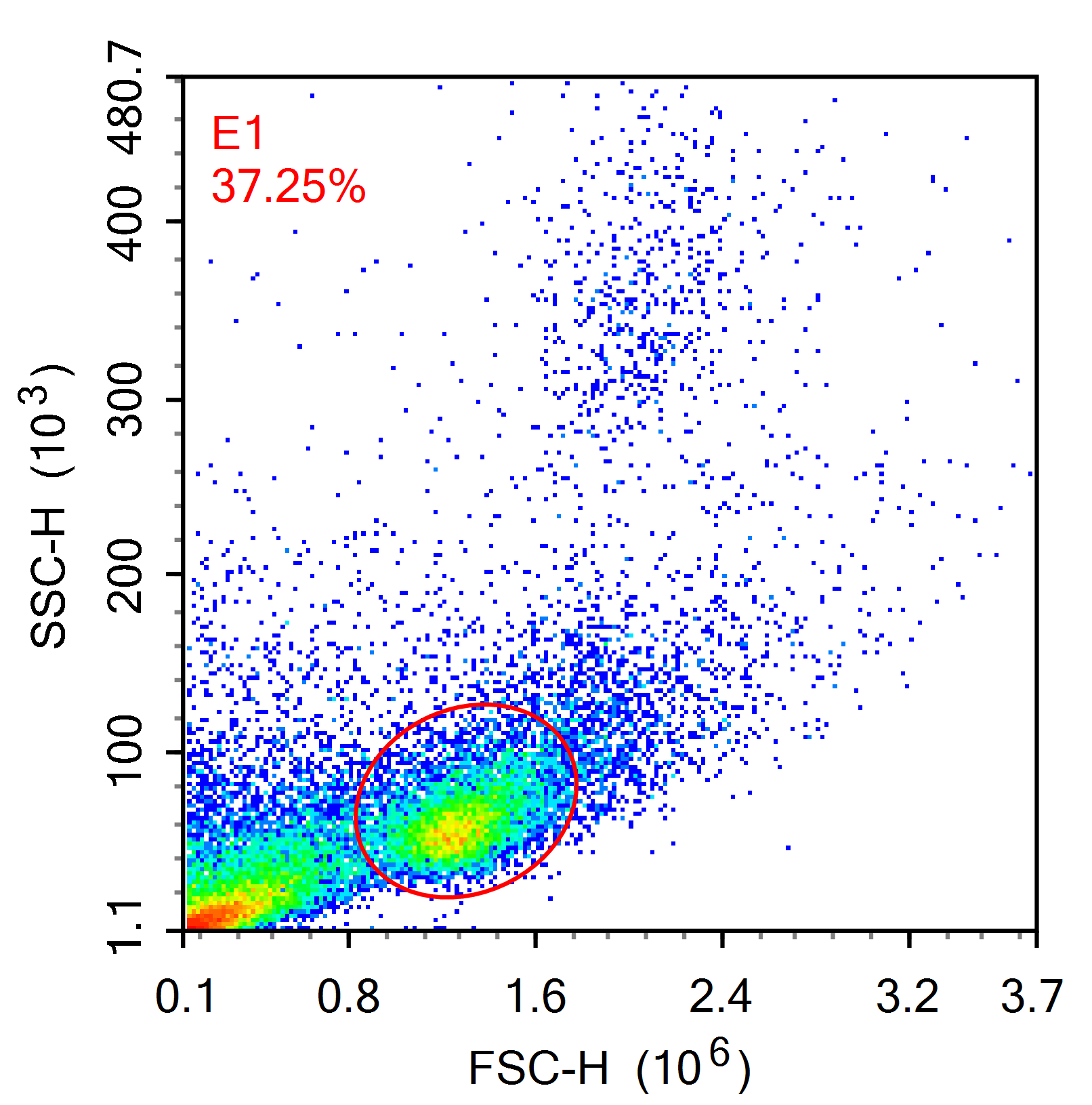

Supplement: Supplementary file 3 [file Data_Sheet_3.ZIP › 4. C. Cellulosae ESAs and TPx Induced Th Subpopulation Differentiation/1. The purity of naive CD4+ T cells was detected by flow cytometry/2. Flowjo analysis of exported images/2. Before sorting-2/figure 1.tiff]

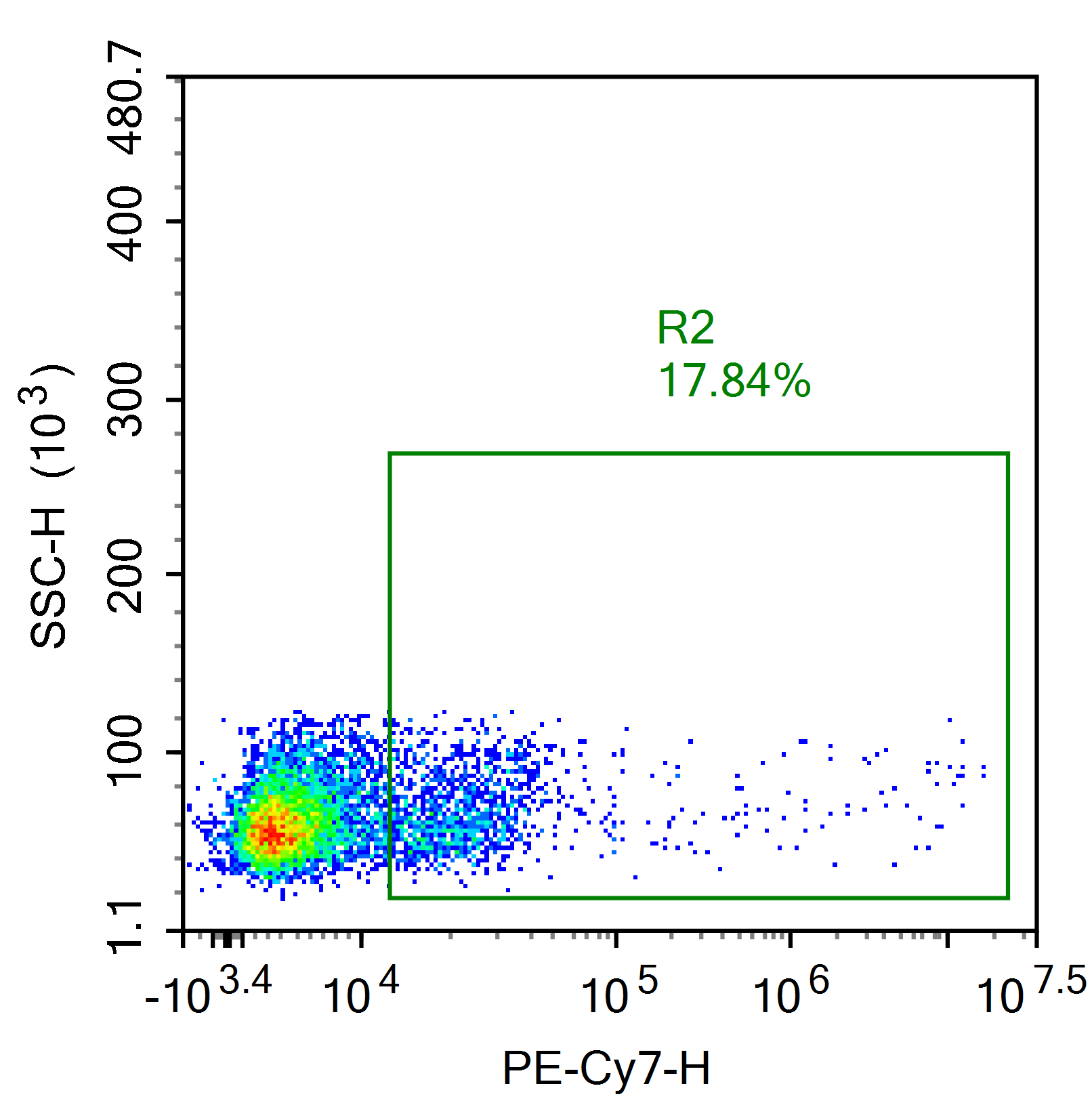

Supplement: Supplementary file 3 [file Data_Sheet_3.ZIP › 4. C. Cellulosae ESAs and TPx Induced Th Subpopulation Differentiation/1. The purity of naive CD4+ T cells was detected by flow cytometry/2. Flowjo analysis of exported images/2. Before sorting-2/figure 2.tiff]

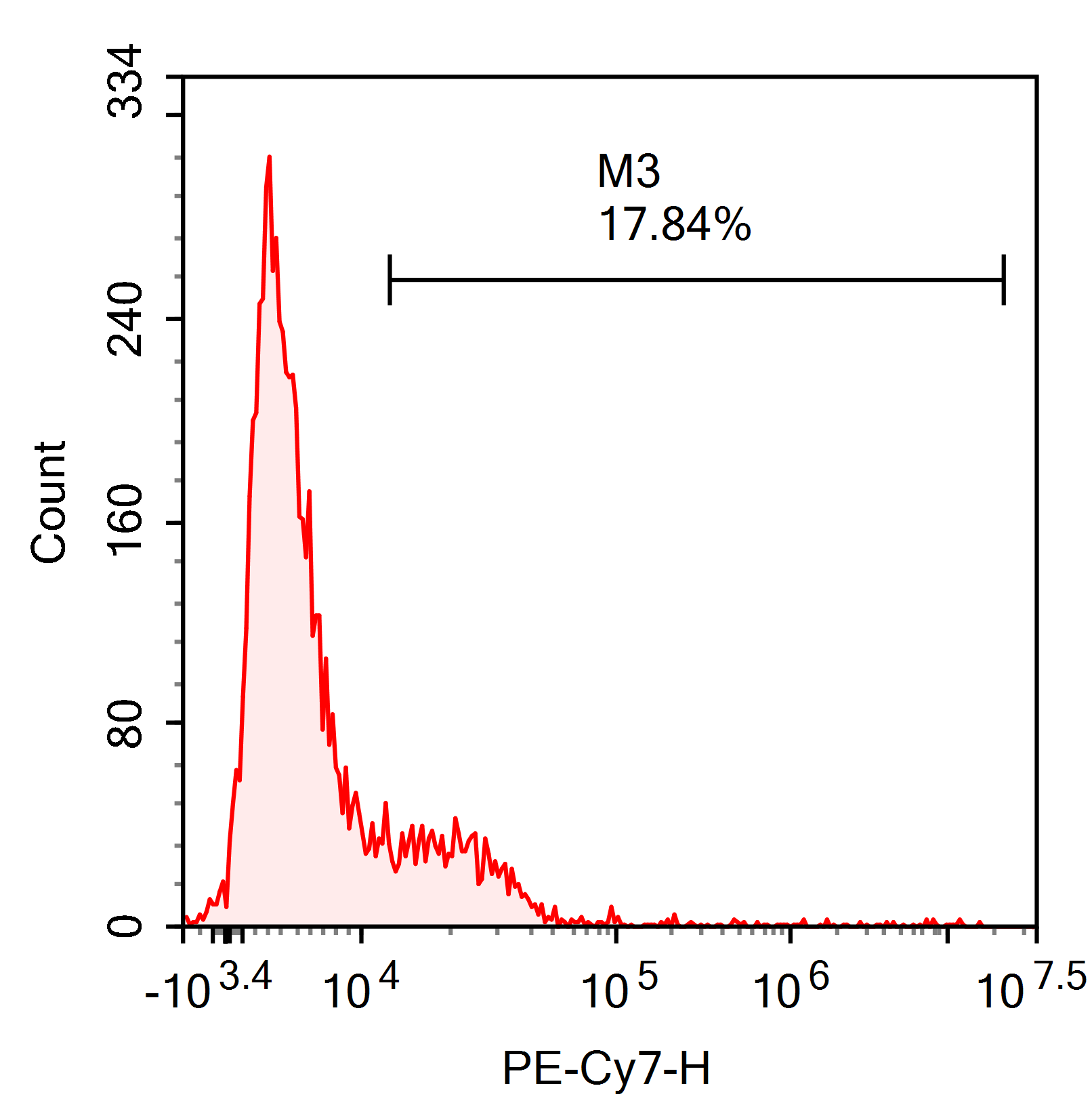

Supplement: Supplementary file 3 [file Data_Sheet_3.ZIP › 4. C. Cellulosae ESAs and TPx Induced Th Subpopulation Differentiation/1. The purity of naive CD4+ T cells was detected by flow cytometry/2. Flowjo analysis of exported images/2. Before sorting-2/figure 3.tiff]

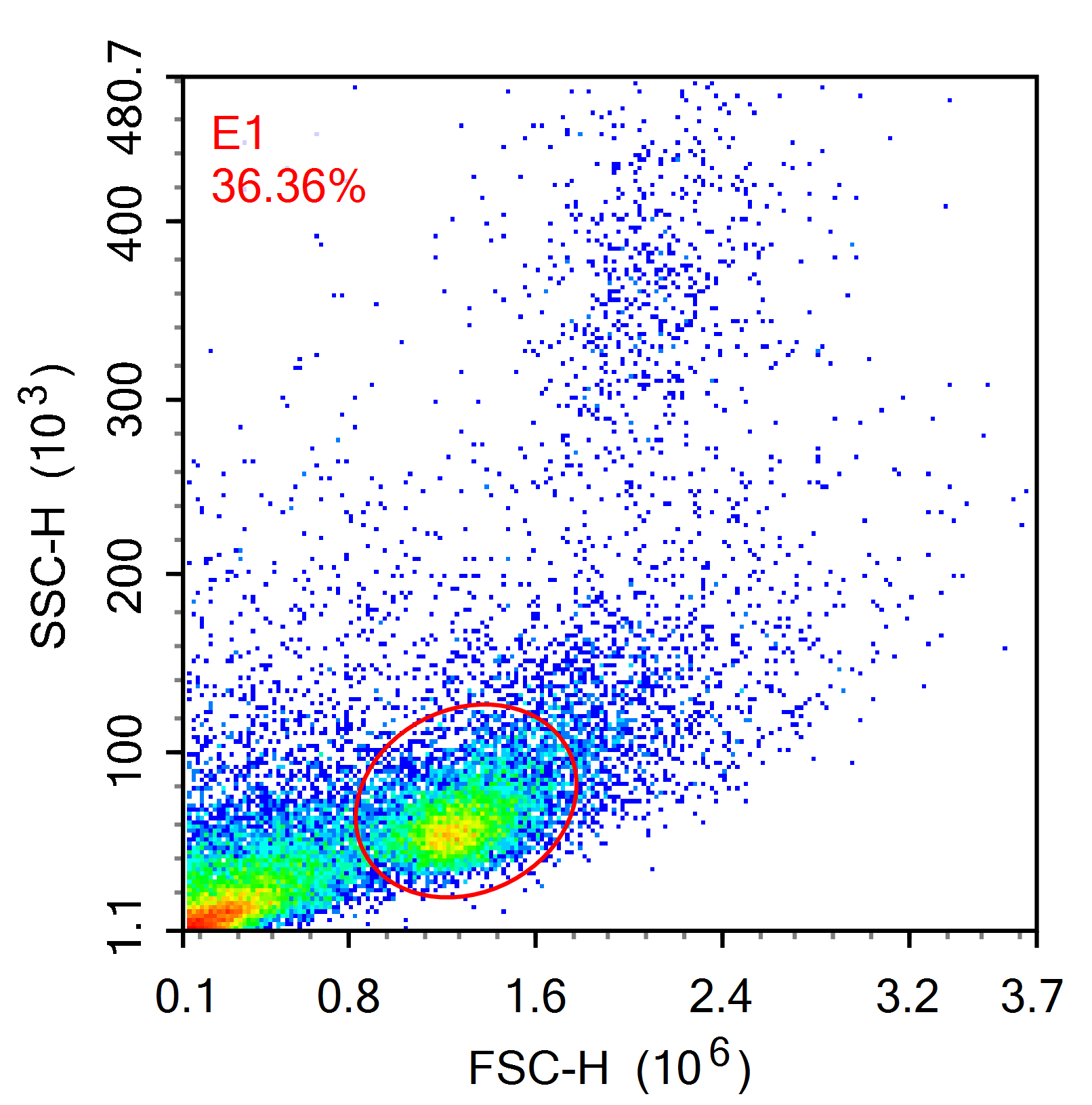

Supplement: Supplementary file 3 [file Data_Sheet_3.ZIP › 4. C. Cellulosae ESAs and TPx Induced Th Subpopulation Differentiation/1. The purity of naive CD4+ T cells was detected by flow cytometry/2. Flowjo analysis of exported images/3. Before sorting-3/figure 1.tiff]

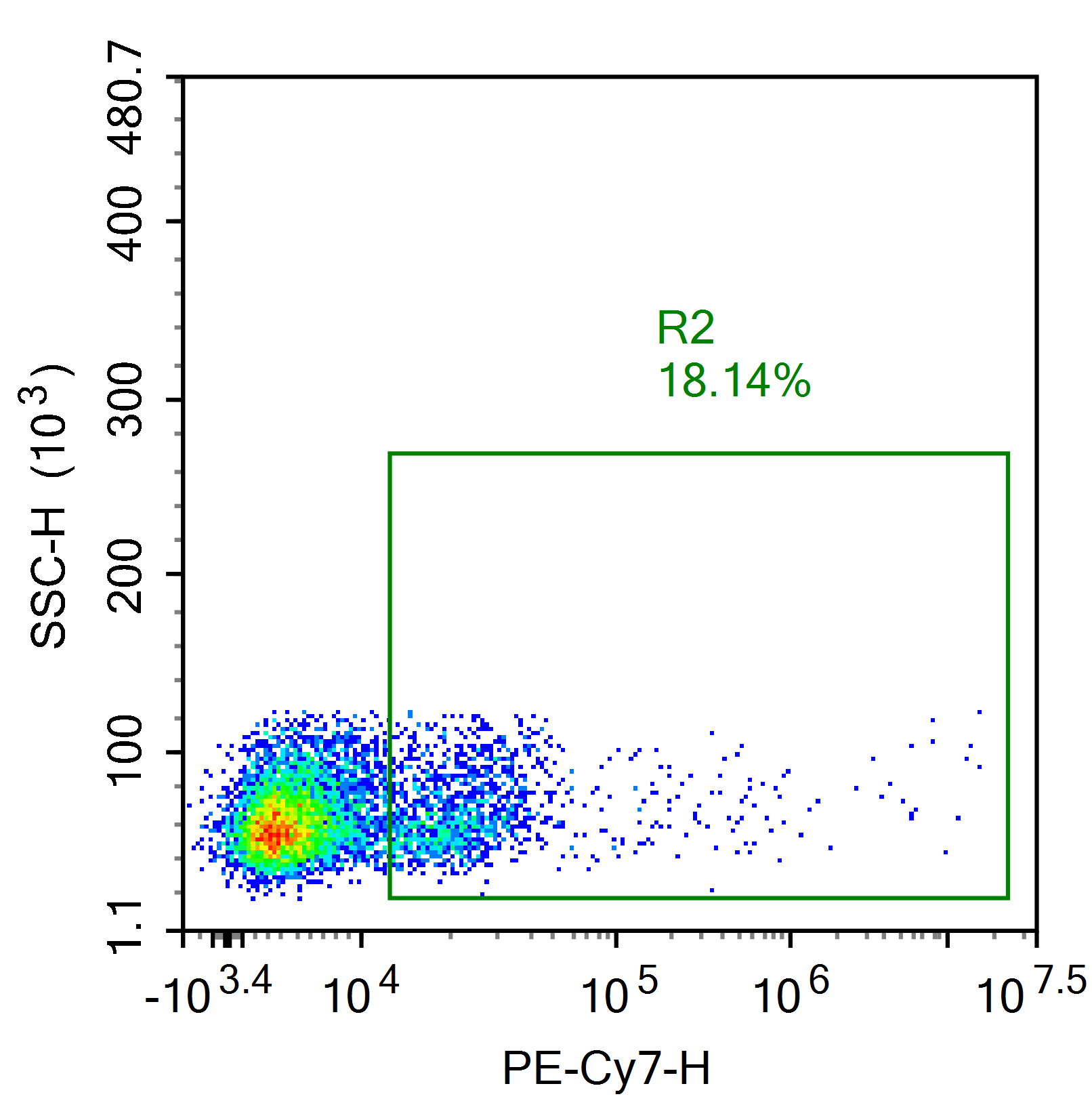

Supplement: Supplementary file 3 [file Data_Sheet_3.ZIP › 4. C. Cellulosae ESAs and TPx Induced Th Subpopulation Differentiation/1. The purity of naive CD4+ T cells was detected by flow cytometry/2. Flowjo analysis of exported images/3. Before sorting-3/figure 2.tiff]

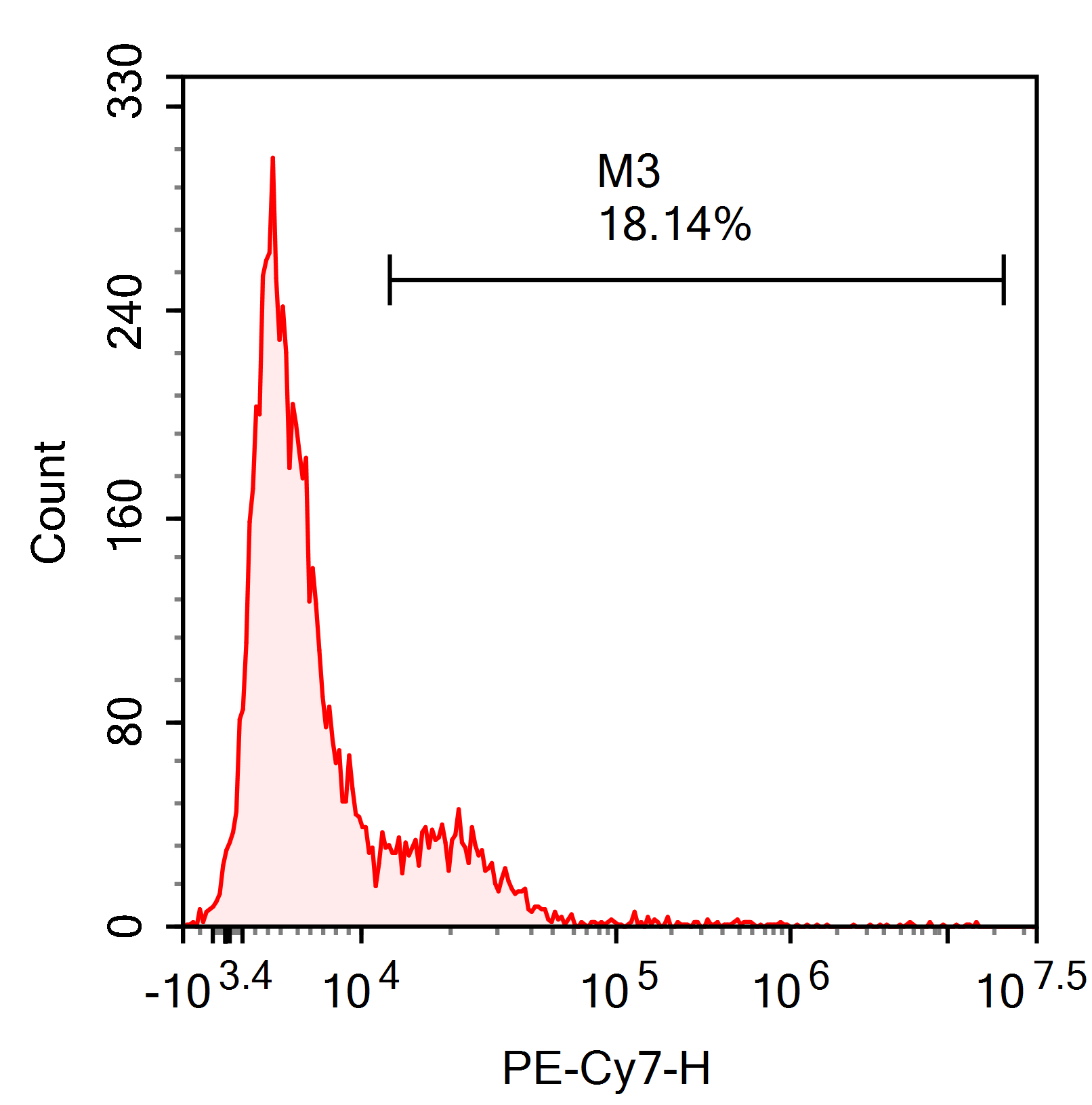

Supplement: Supplementary file 3 [file Data_Sheet_3.ZIP › 4. C. Cellulosae ESAs and TPx Induced Th Subpopulation Differentiation/1. The purity of naive CD4+ T cells was detected by flow cytometry/2. Flowjo analysis of exported images/3. Before sorting-3/figure 3.tiff]

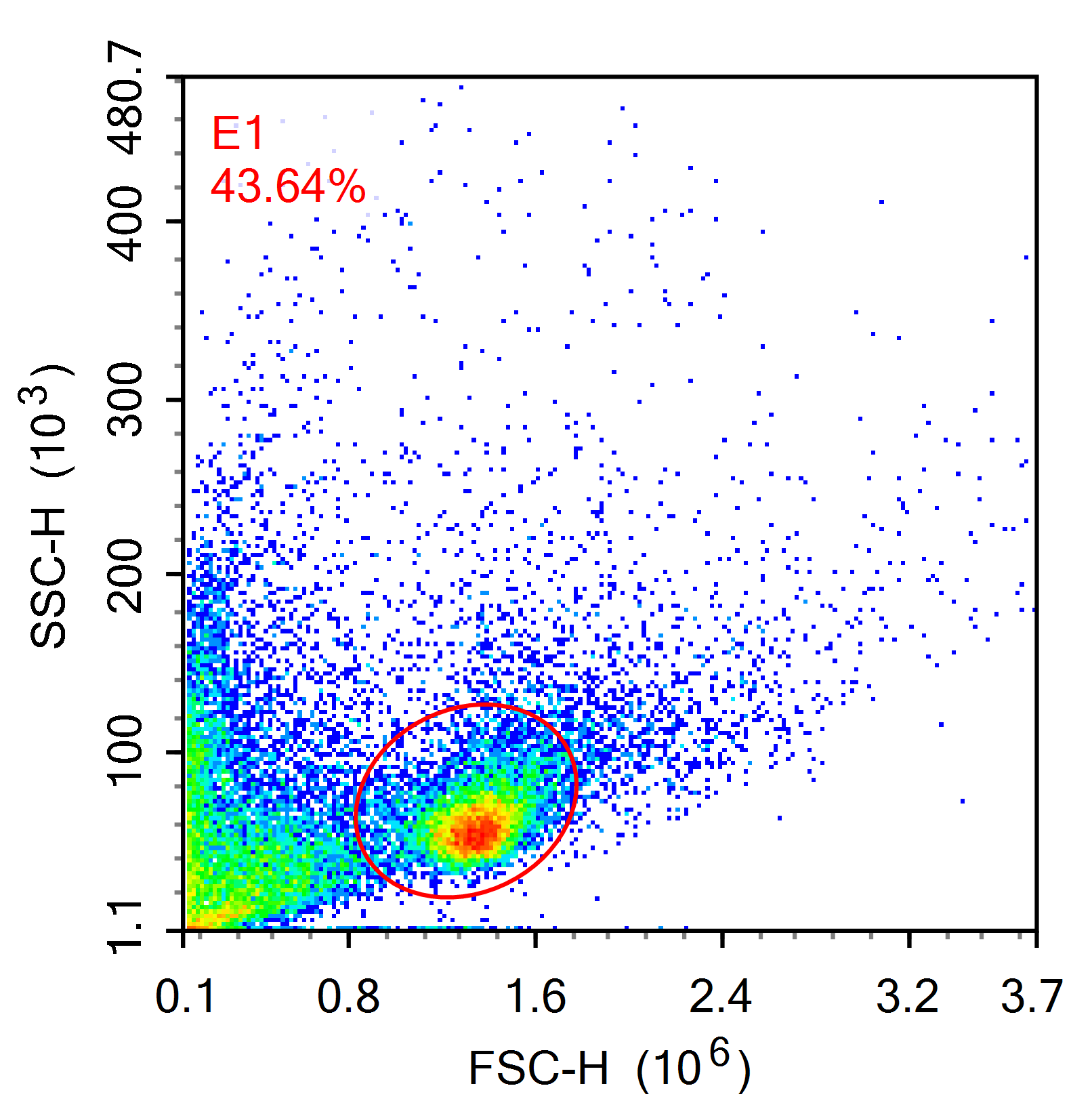

Supplement: Supplementary file 3 [file Data_Sheet_3.ZIP › 4. C. Cellulosae ESAs and TPx Induced Th Subpopulation Differentiation/1. The purity of naive CD4+ T cells was detected by flow cytometry/2. Flowjo analysis of exported images/4. After sorting-1/figure 1.tiff]

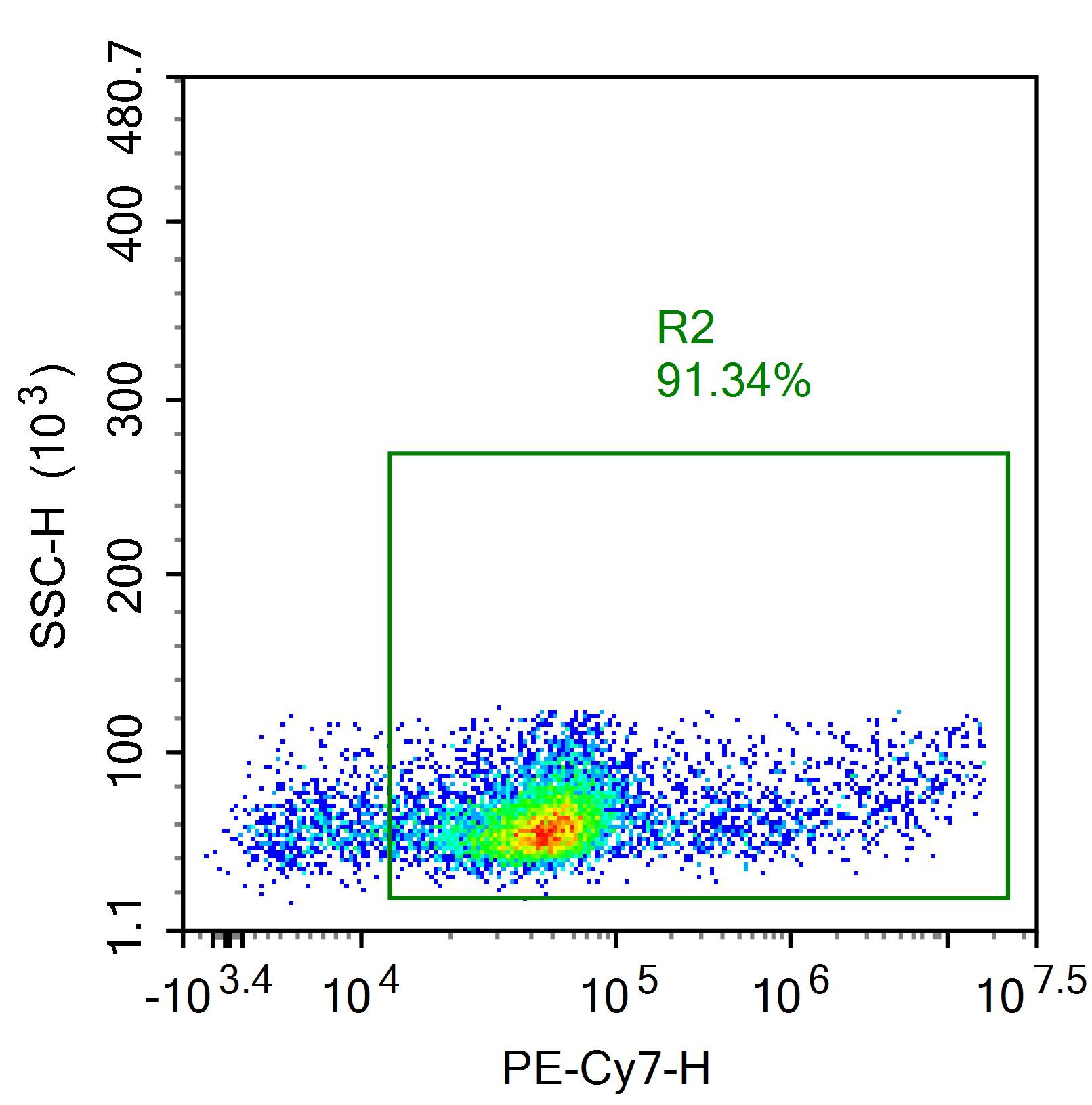

Supplement: Supplementary file 3 [file Data_Sheet_3.ZIP › 4. C. Cellulosae ESAs and TPx Induced Th Subpopulation Differentiation/1. The purity of naive CD4+ T cells was detected by flow cytometry/2. Flowjo analysis of exported images/4. After sorting-1/figure 2.tiff]

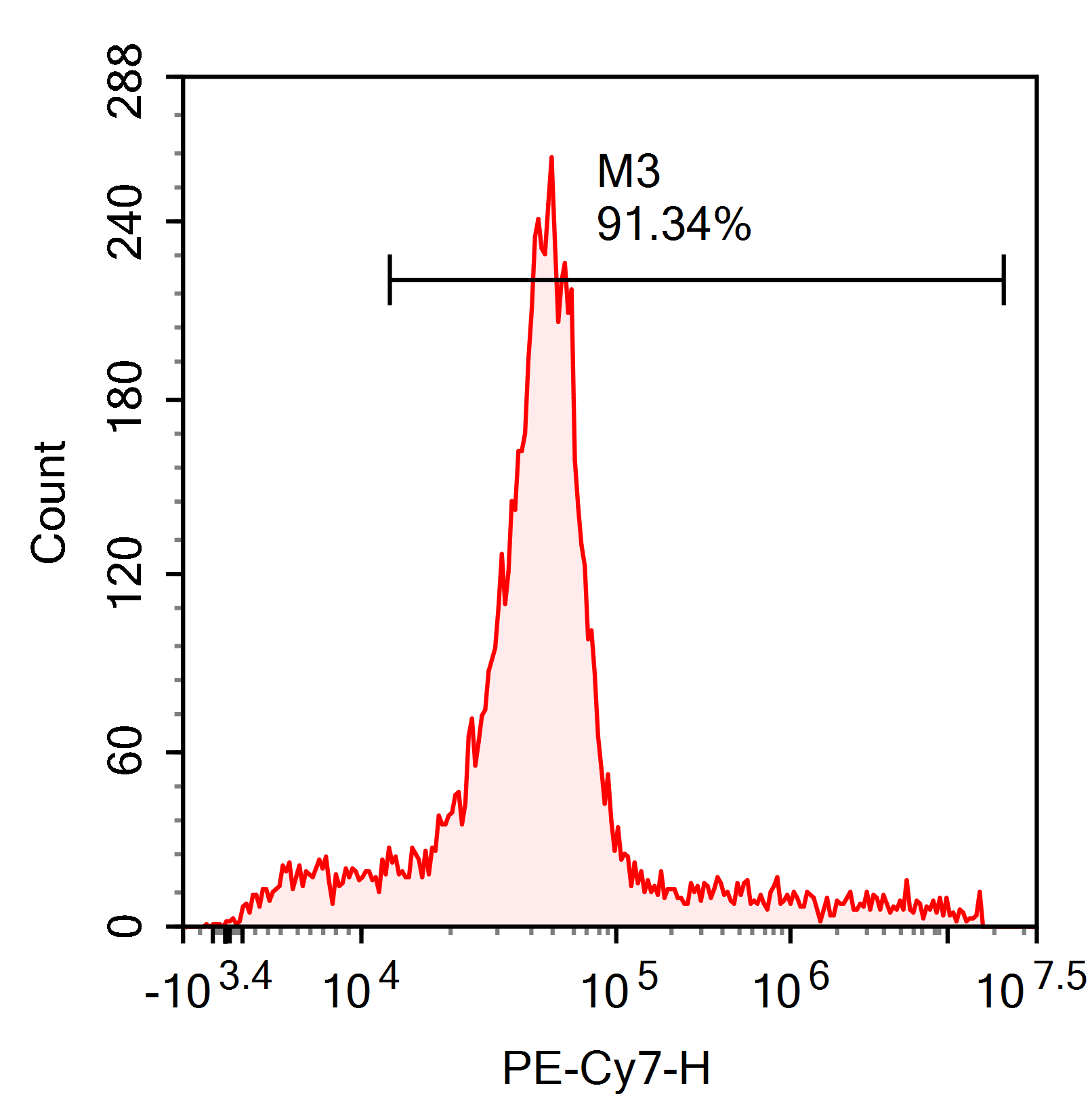

Supplement: Supplementary file 3 [file Data_Sheet_3.ZIP › 4. C. Cellulosae ESAs and TPx Induced Th Subpopulation Differentiation/1. The purity of naive CD4+ T cells was detected by flow cytometry/2. Flowjo analysis of exported images/4. After sorting-1/figure 3.tiff]

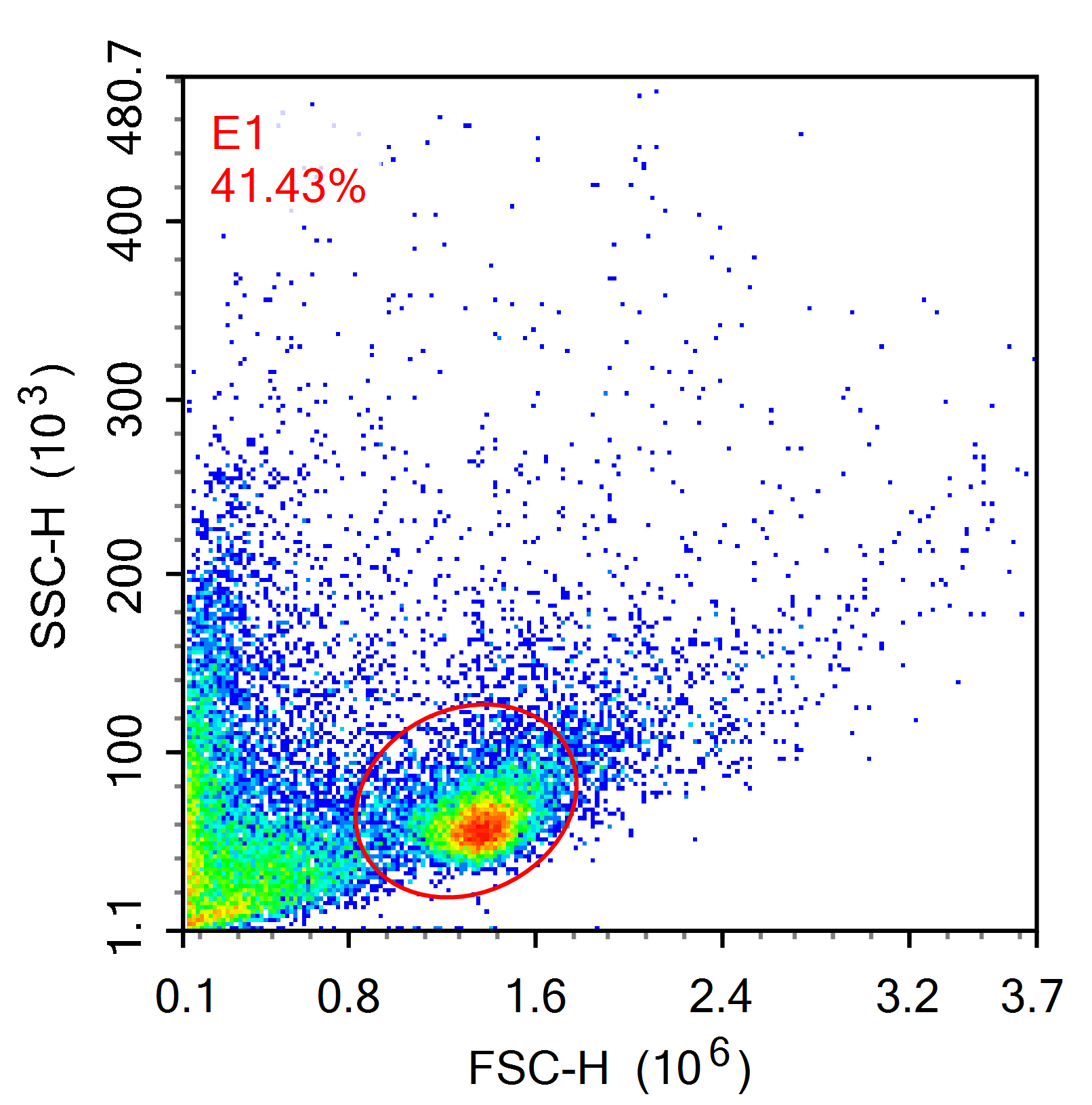

Supplement: Supplementary file 3 [file Data_Sheet_3.ZIP › 4. C. Cellulosae ESAs and TPx Induced Th Subpopulation Differentiation/1. The purity of naive CD4+ T cells was detected by flow cytometry/2. Flowjo analysis of exported images/5. After sorting-2/figure 1.tiff]

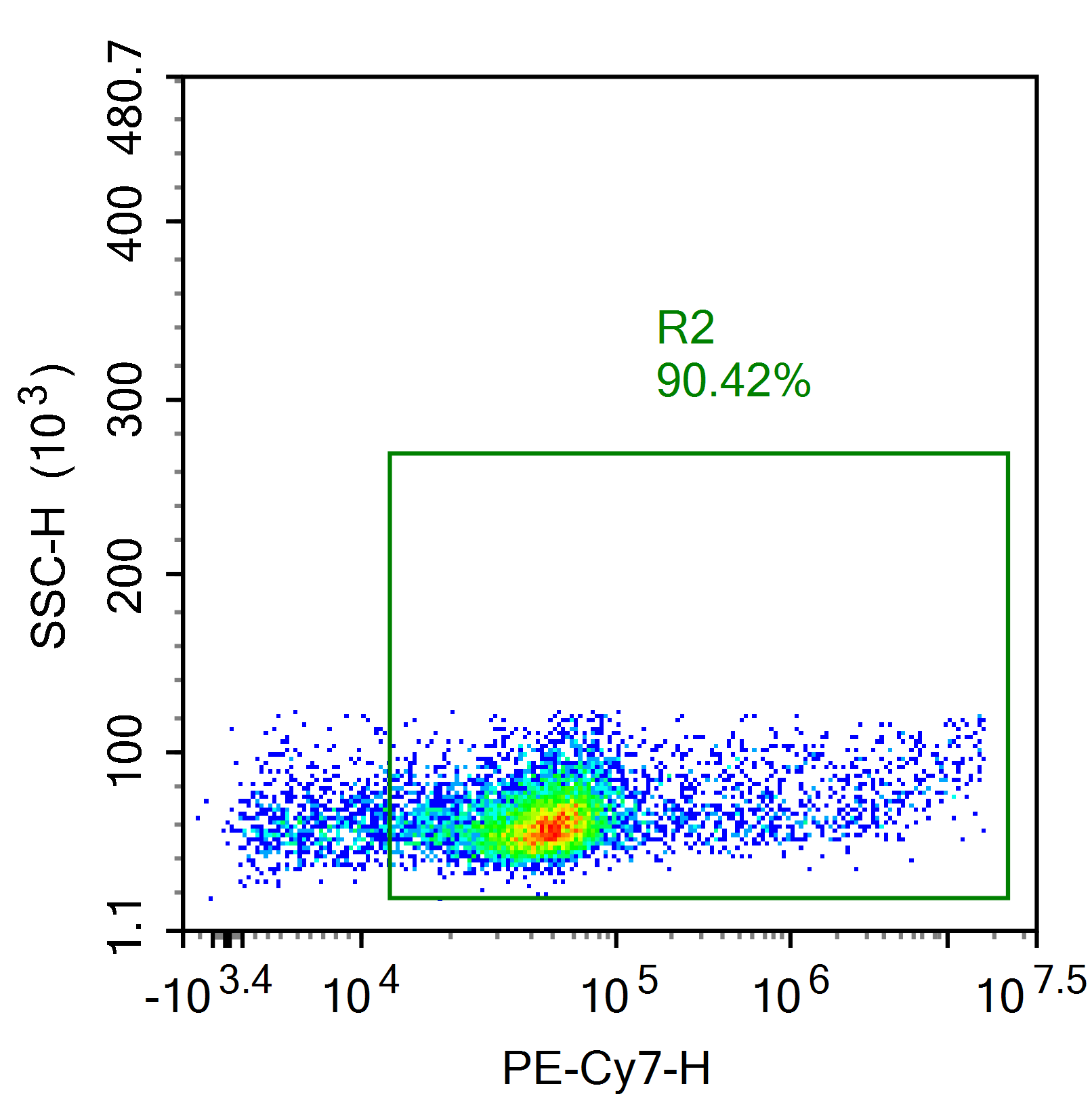

Supplement: Supplementary file 3 [file Data_Sheet_3.ZIP › 4. C. Cellulosae ESAs and TPx Induced Th Subpopulation Differentiation/1. The purity of naive CD4+ T cells was detected by flow cytometry/2. Flowjo analysis of exported images/5. After sorting-2/figure 2.tiff]

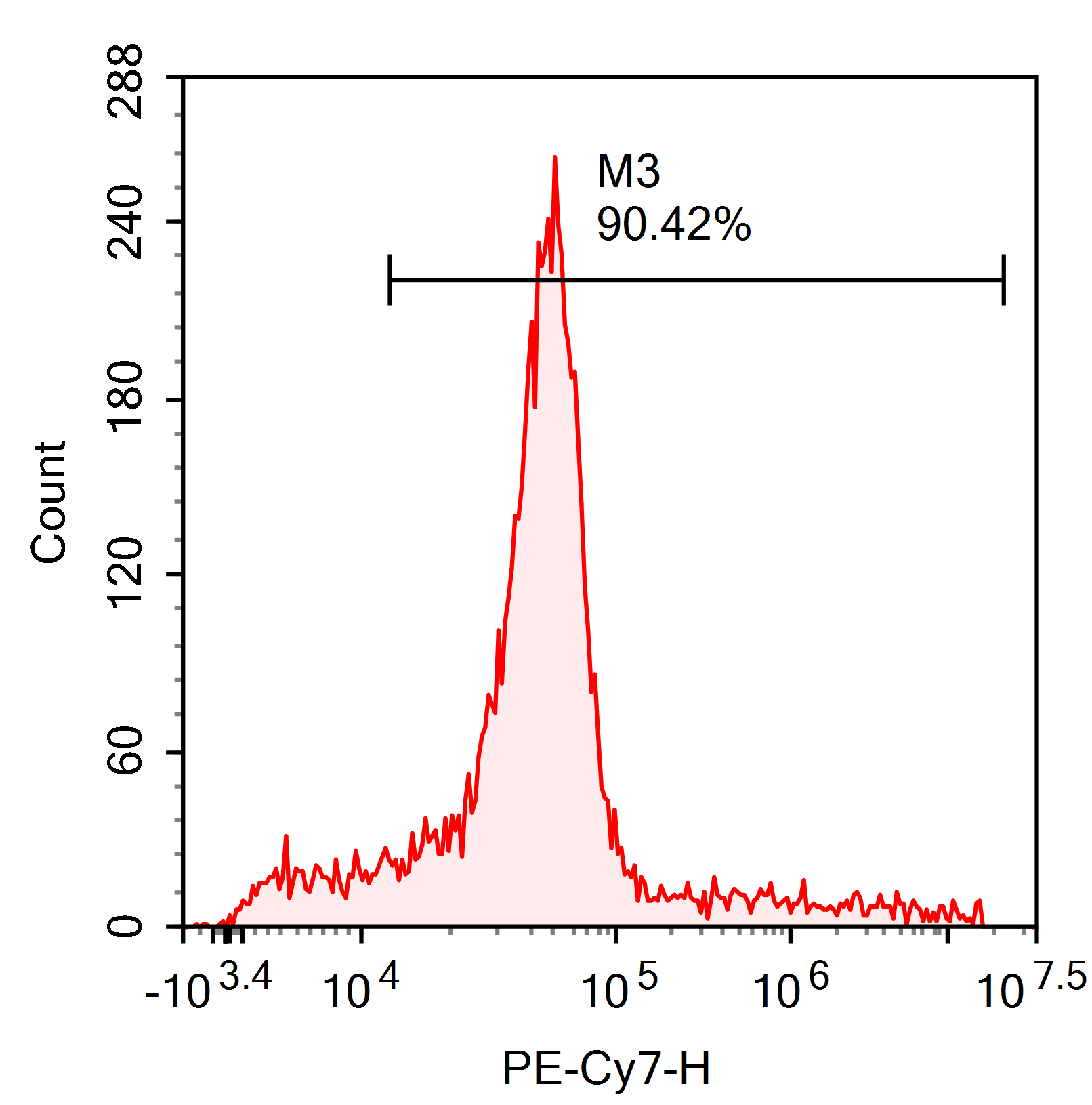

Supplement: Supplementary file 3 [file Data_Sheet_3.ZIP › 4. C. Cellulosae ESAs and TPx Induced Th Subpopulation Differentiation/1. The purity of naive CD4+ T cells was detected by flow cytometry/2. Flowjo analysis of exported images/5. After sorting-2/figure 3.tiff]

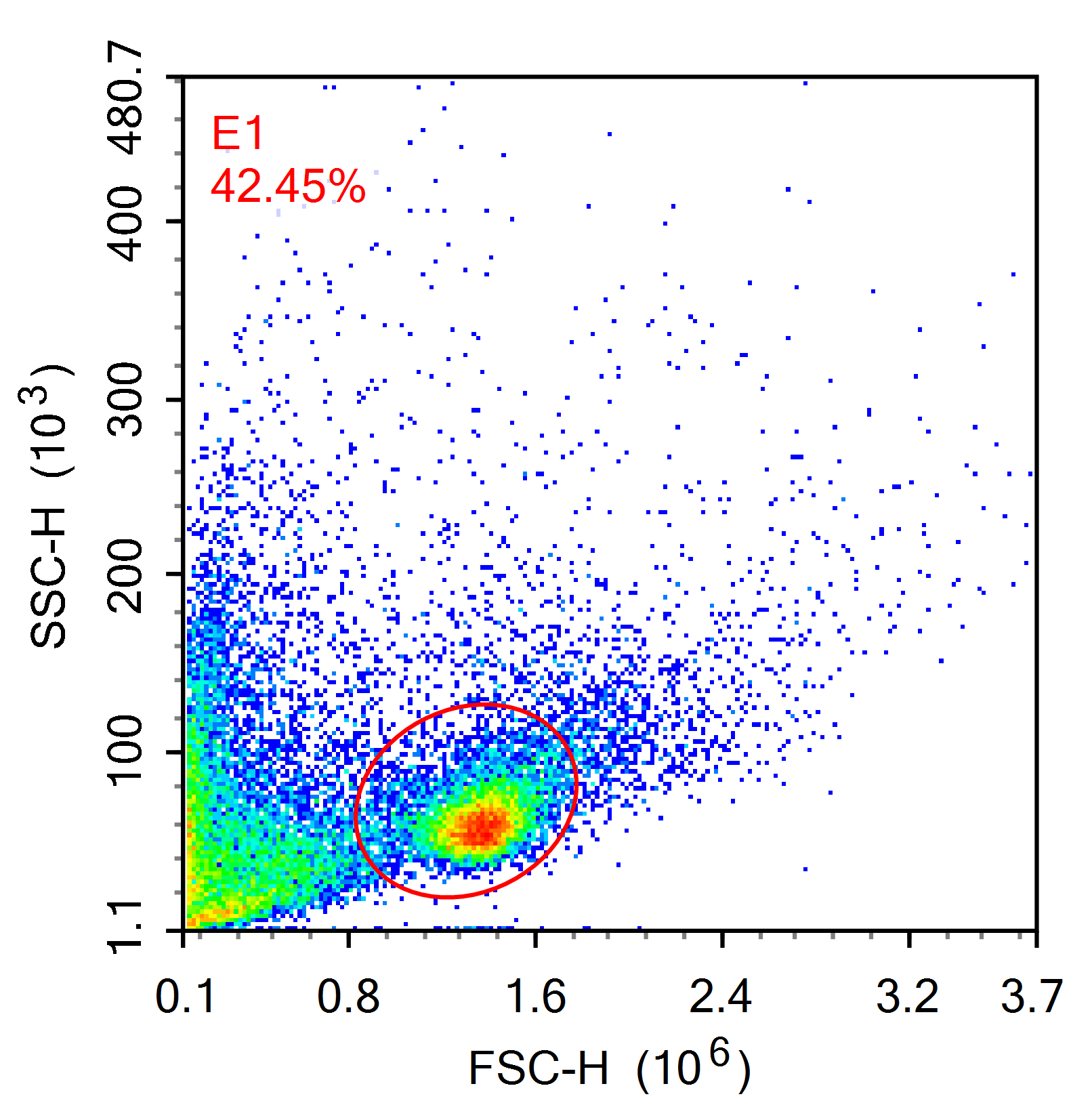

Supplement: Supplementary file 3 [file Data_Sheet_3.ZIP › 4. C. Cellulosae ESAs and TPx Induced Th Subpopulation Differentiation/1. The purity of naive CD4+ T cells was detected by flow cytometry/2. Flowjo analysis of exported images/6. After sorting-3/figure 1.tiff]

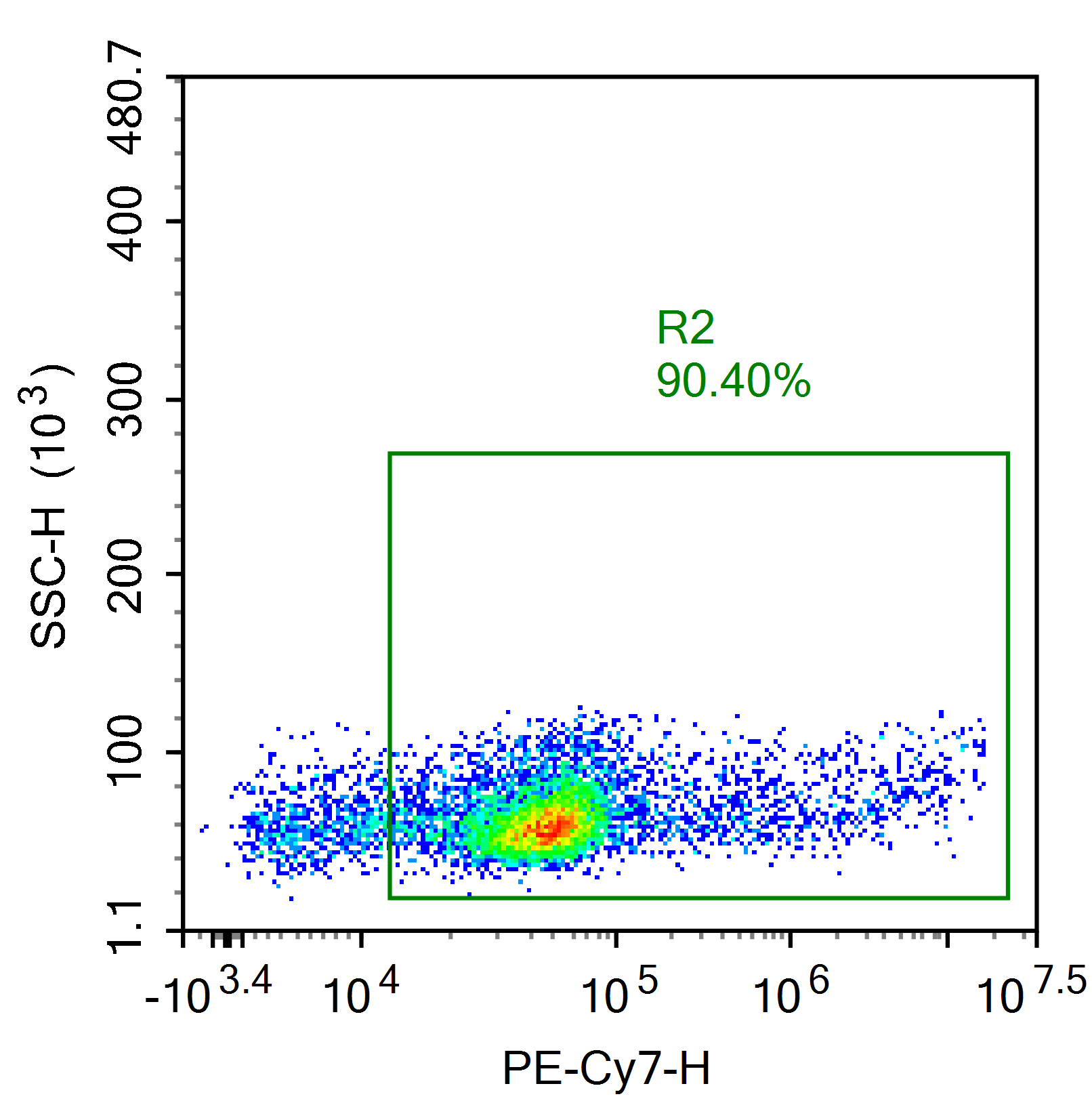

Supplement: Supplementary file 3 [file Data_Sheet_3.ZIP › 4. C. Cellulosae ESAs and TPx Induced Th Subpopulation Differentiation/1. The purity of naive CD4+ T cells was detected by flow cytometry/2. Flowjo analysis of exported images/6. After sorting-3/figure 2.tiff]

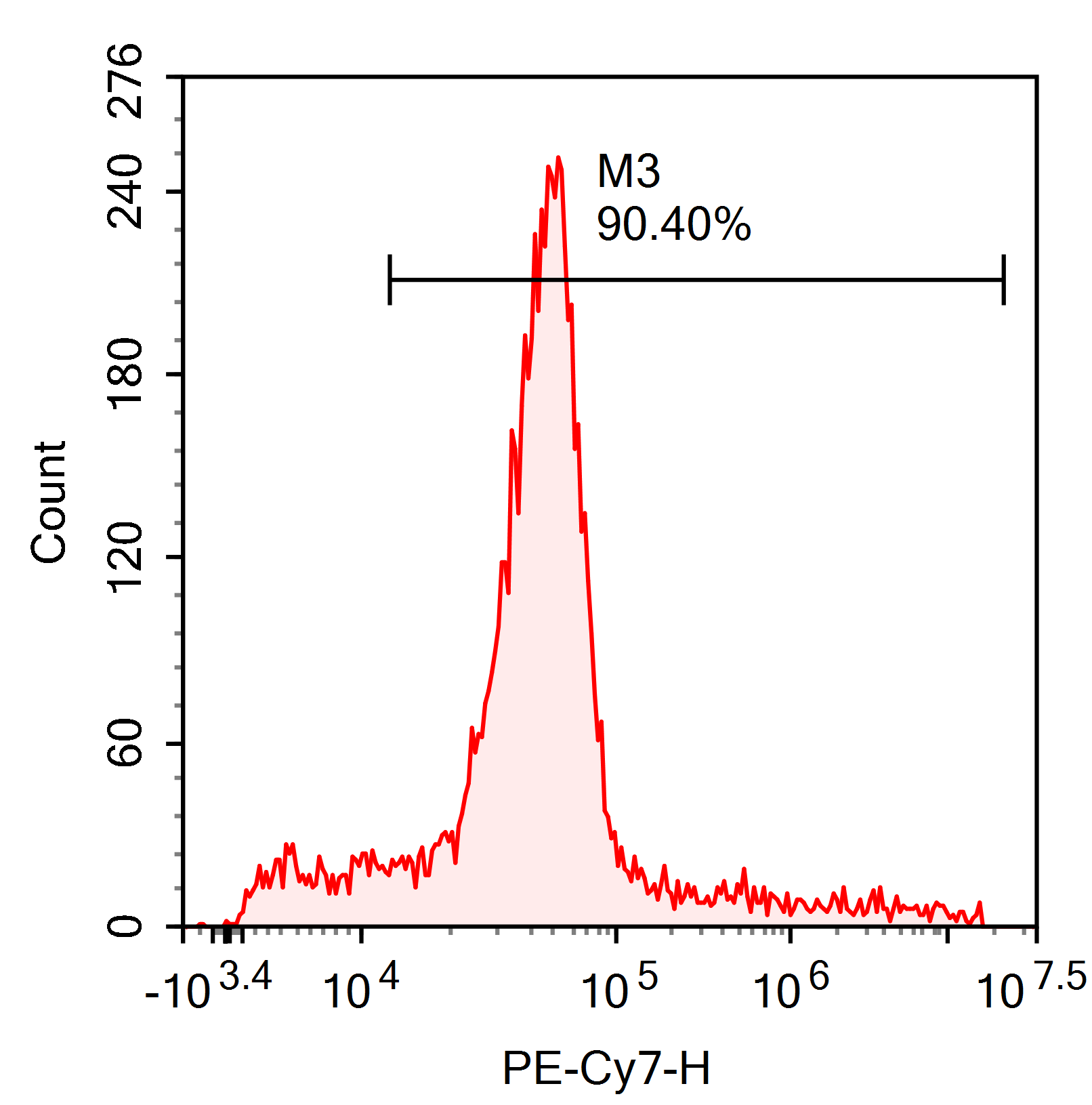

Supplement: Supplementary file 3 [file Data_Sheet_3.ZIP › 4. C. Cellulosae ESAs and TPx Induced Th Subpopulation Differentiation/1. The purity of naive CD4+ T cells was detected by flow cytometry/2. Flowjo analysis of exported images/6. After sorting-3/figure 3.tiff]

# IFN- $\gamma$

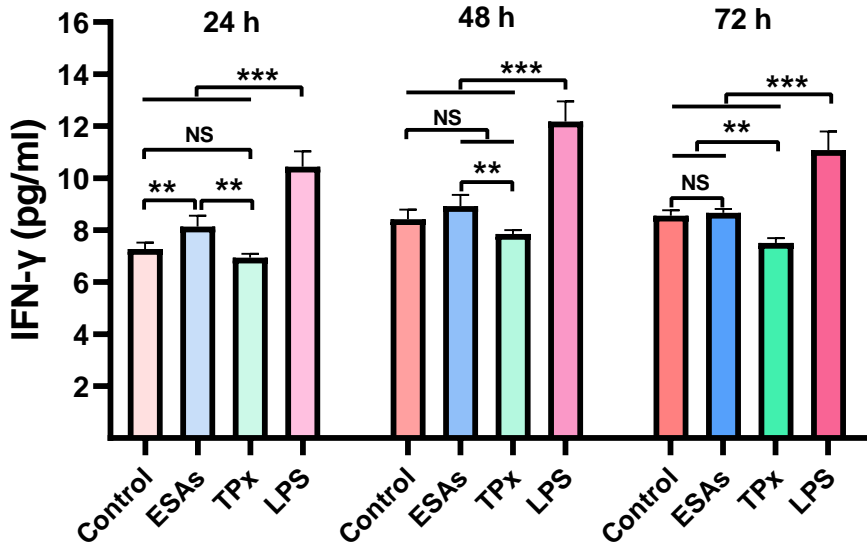

Supplement: Supplementary file 3 [file Data_Sheet_3.ZIP › 4. C. Cellulosae ESAs and TPx Induced Th Subpopulation Differentiation/4. Create statistical graphs with GraphPad software/1. IFN-γ/IFN-γ.pdf]

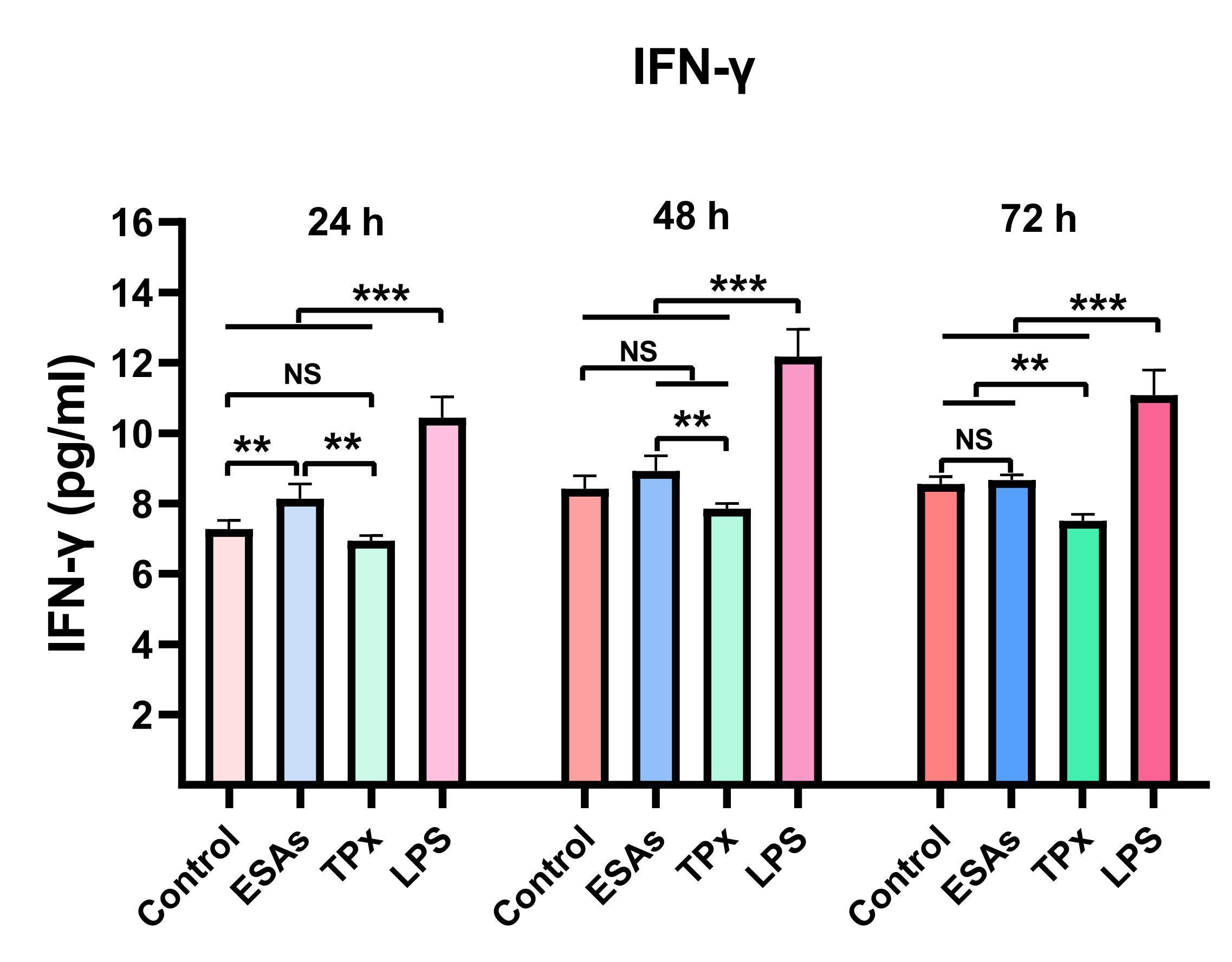

Supplement: Supplementary file 3 [file Data_Sheet_3.ZIP › 4. C. Cellulosae ESAs and TPx Induced Th Subpopulation Differentiation/4. Create statistical graphs with GraphPad software/1. IFN-γ/IFN-γ_00.jpg]

## IL-4

24 h

48 h

72 h

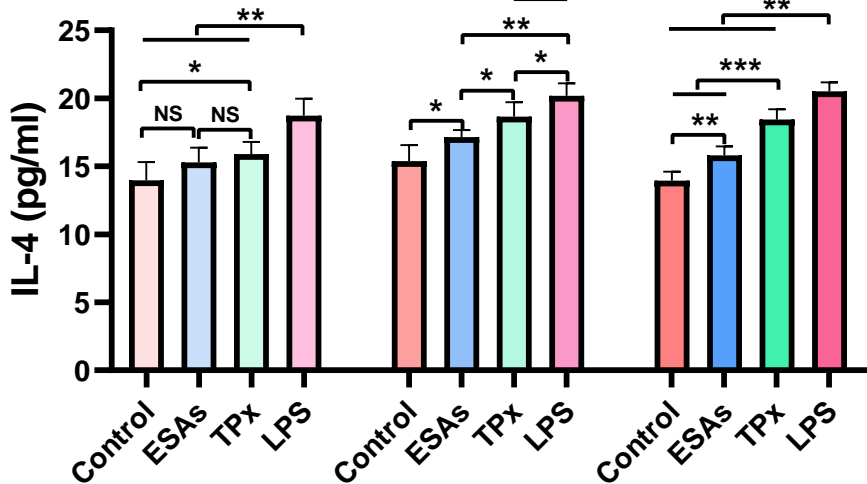

Supplement: Supplementary file 3 [file Data_Sheet_3.ZIP › 4. C. Cellulosae ESAs and TPx Induced Th Subpopulation Differentiation/4. Create statistical graphs with GraphPad software/2. IL-4/IL-4.pdf]

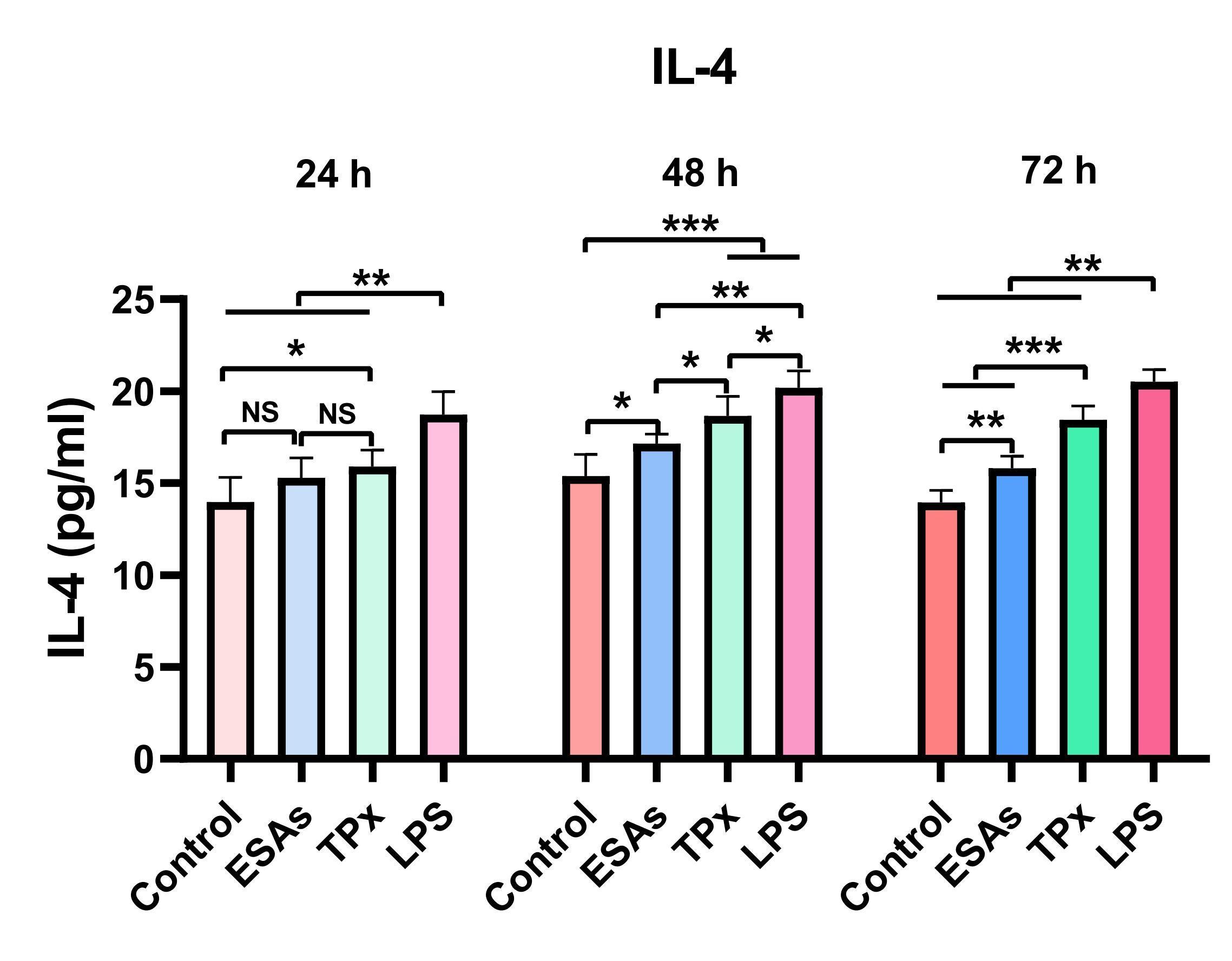

Supplement: Supplementary file 3 [file Data_Sheet_3.ZIP › 4. C. Cellulosae ESAs and TPx Induced Th Subpopulation Differentiation/4. Create statistical graphs with GraphPad software/2. IL-4/IL-4_00.jpg]

# IL-5

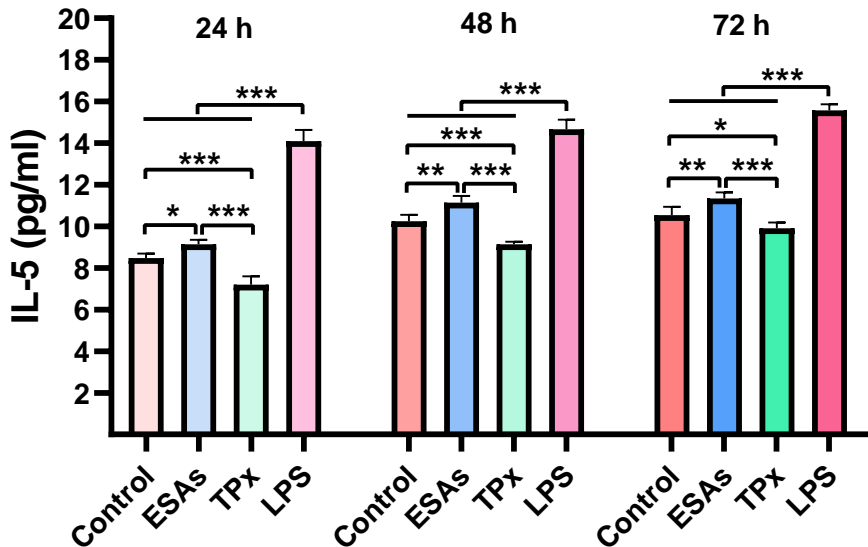

Supplement: Supplementary file 3 [file Data_Sheet_3.ZIP › 4. C. Cellulosae ESAs and TPx Induced Th Subpopulation Differentiation/4. Create statistical graphs with GraphPad software/3. IL-5/IL-5.pdf]

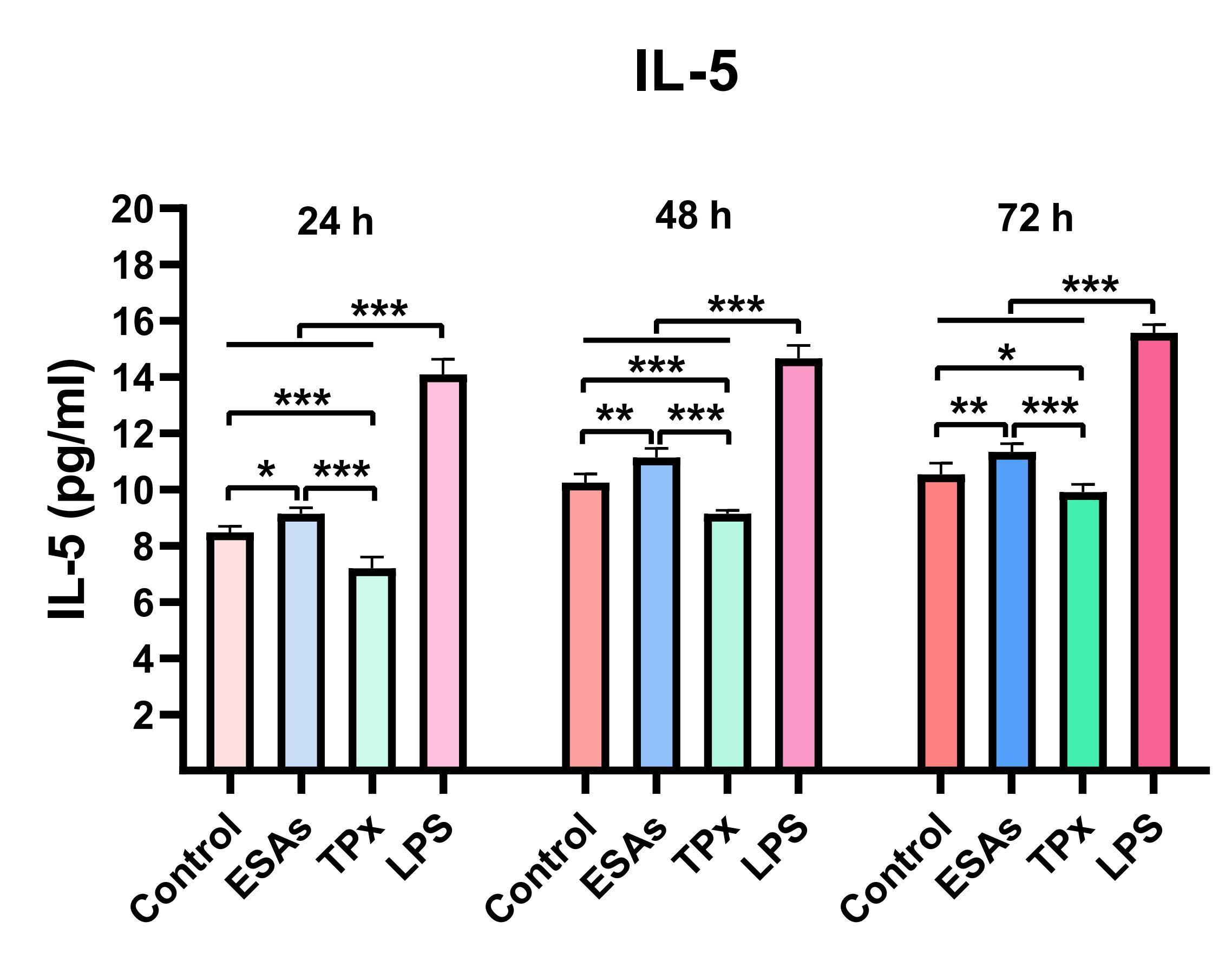

Supplement: Supplementary file 3 [file Data_Sheet_3.ZIP › 4. C. Cellulosae ESAs and TPx Induced Th Subpopulation Differentiation/4. Create statistical graphs with GraphPad software/3. IL-5/IL-5_00.jpg]

# IL-10

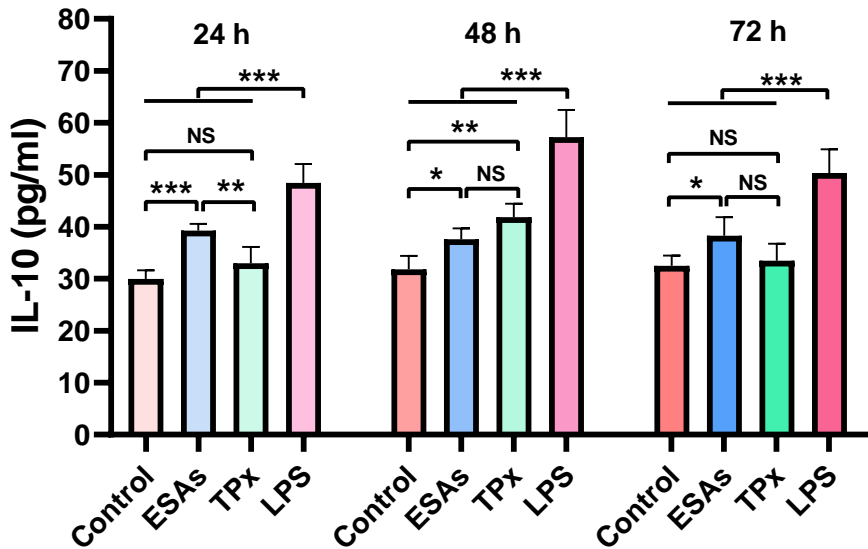

Supplement: Supplementary file 3 [file Data_Sheet_3.ZIP › 4. C. Cellulosae ESAs and TPx Induced Th Subpopulation Differentiation/4. Create statistical graphs with GraphPad software/4. IL-10/IL-10.pdf]

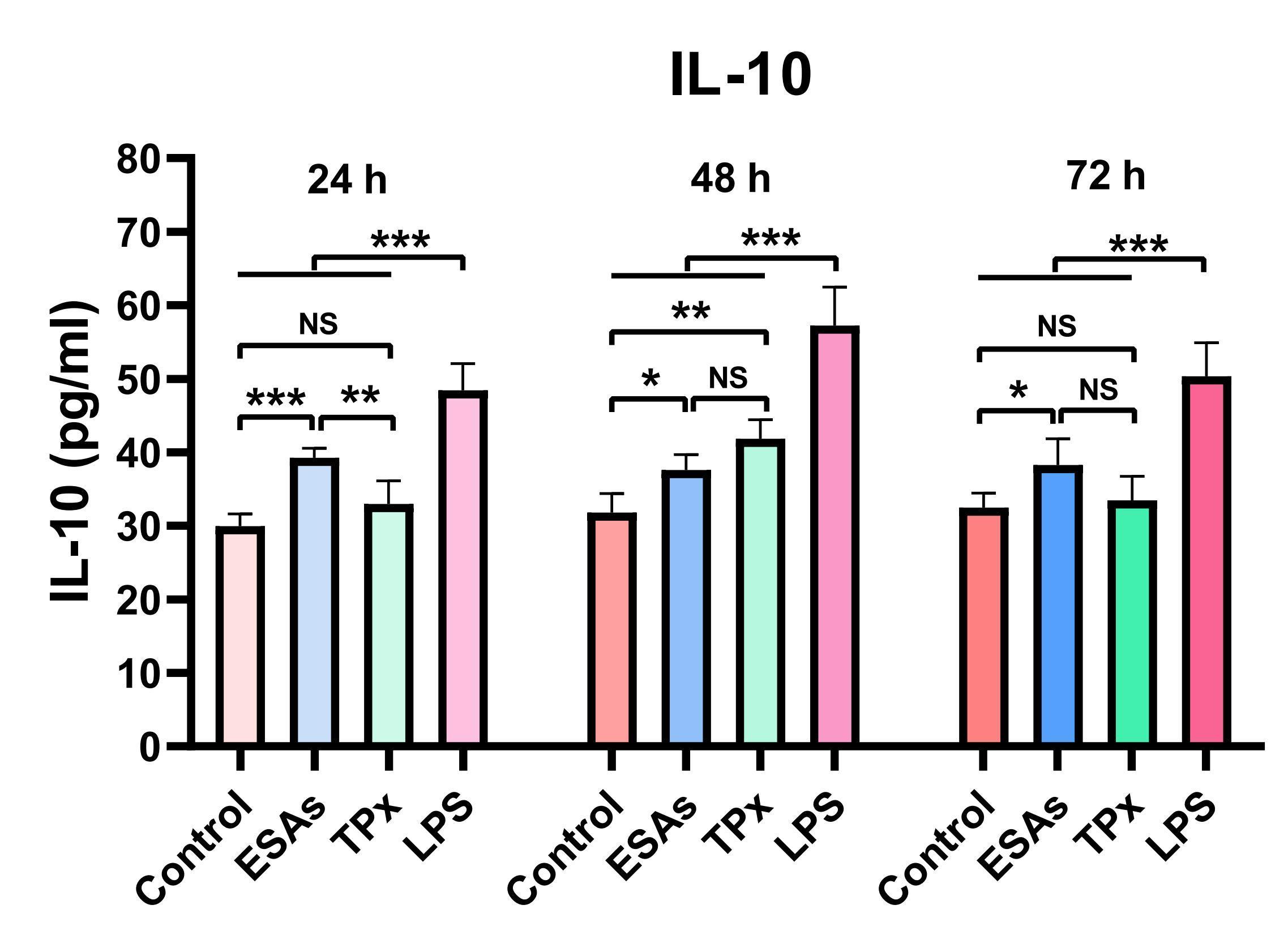

Supplement: Supplementary file 3 [file Data_Sheet_3.ZIP › 4. C. Cellulosae ESAs and TPx Induced Th Subpopulation Differentiation/4. Create statistical graphs with GraphPad software/4. IL-10/IL-10_00.jpg]

# IL-17

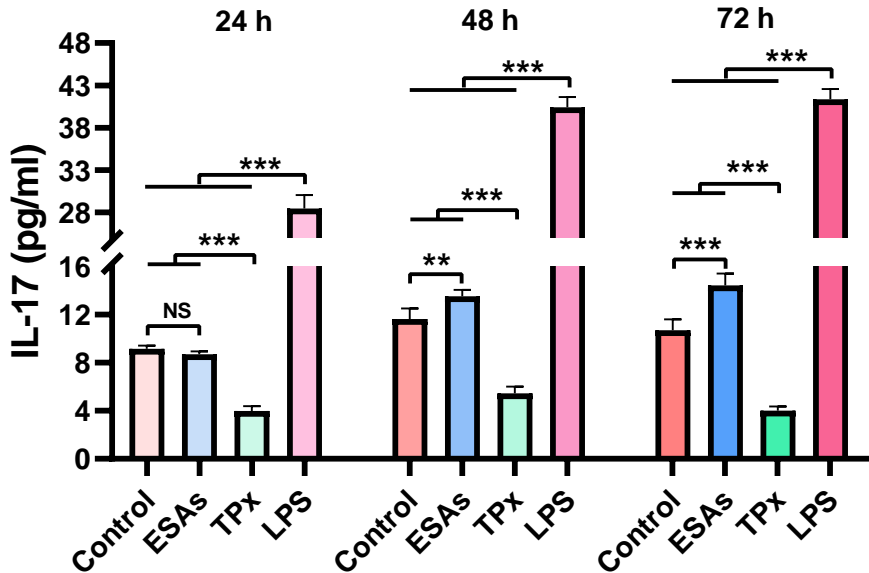

Supplement: Supplementary file 3 [file Data_Sheet_3.ZIP › 4. C. Cellulosae ESAs and TPx Induced Th Subpopulation Differentiation/4. Create statistical graphs with GraphPad software/5. IL-17/IL-17.pdf]

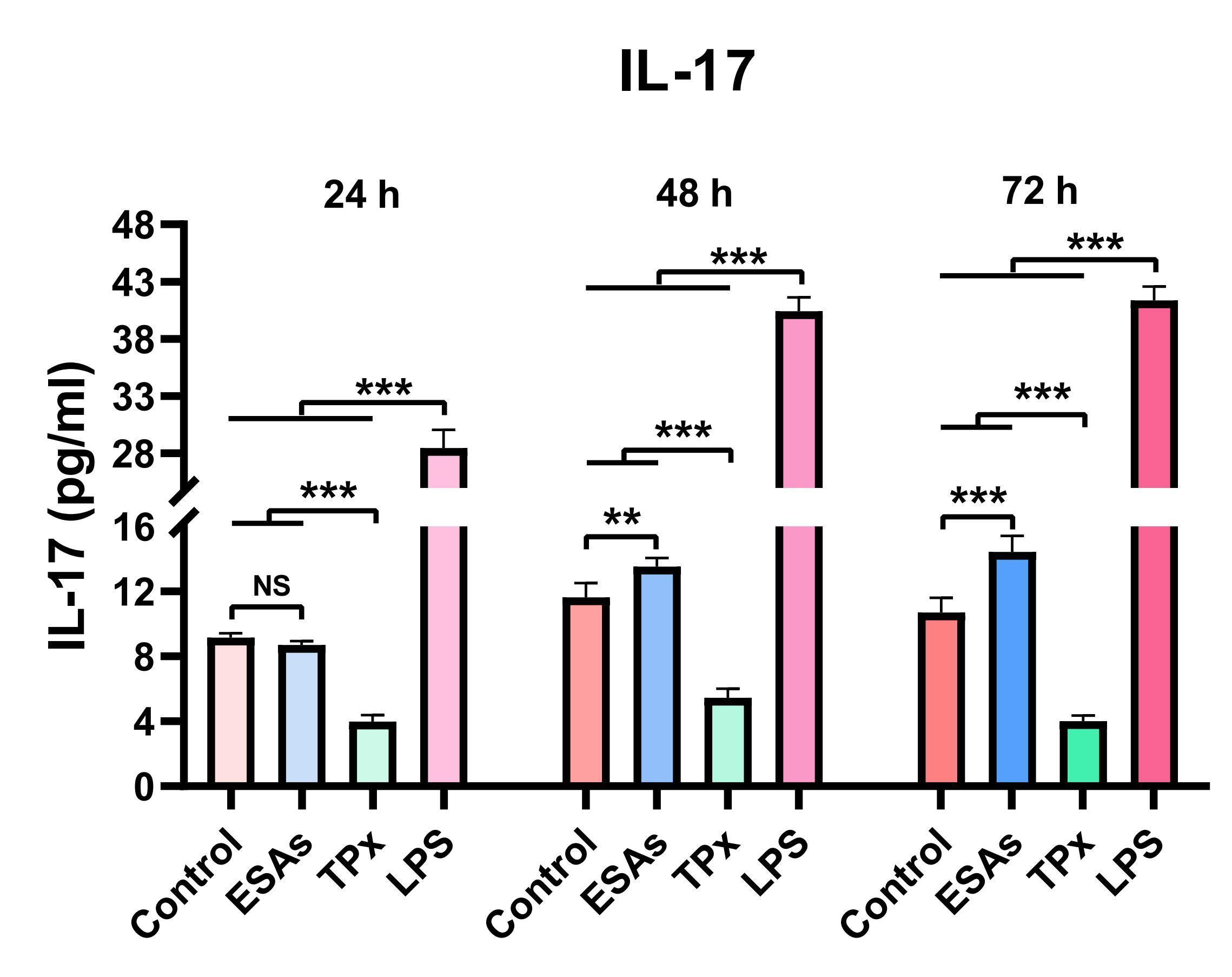

Supplement: Supplementary file 3 [file Data_Sheet_3.ZIP › 4. C. Cellulosae ESAs and TPx Induced Th Subpopulation Differentiation/4. Create statistical graphs with GraphPad software/5. IL-17/IL-17_00.jpg]

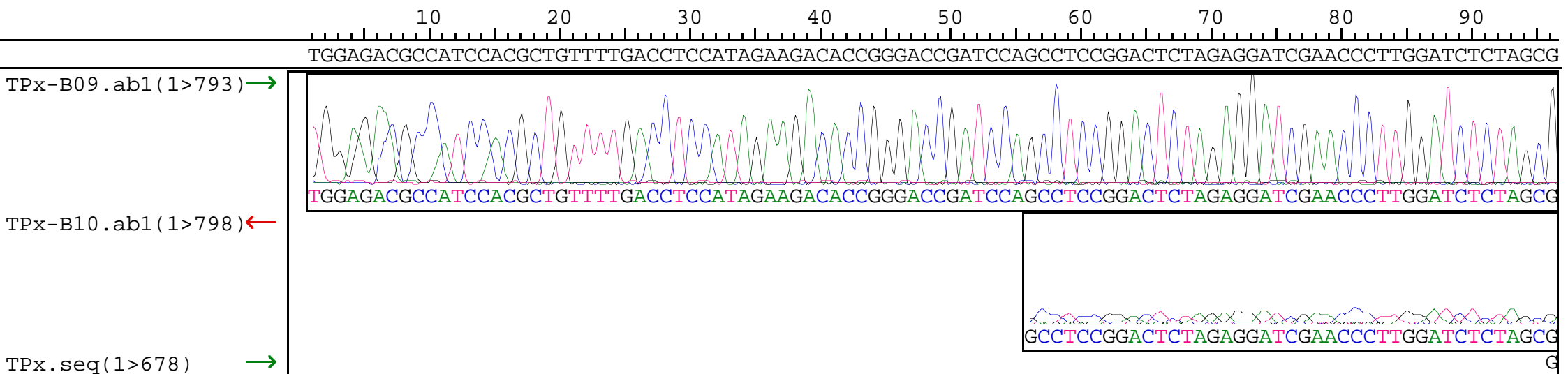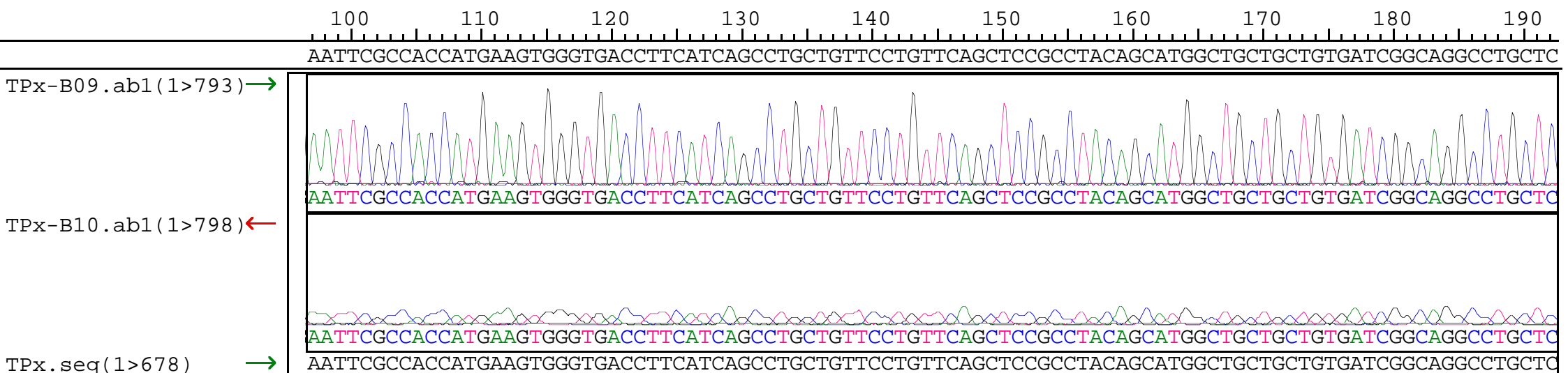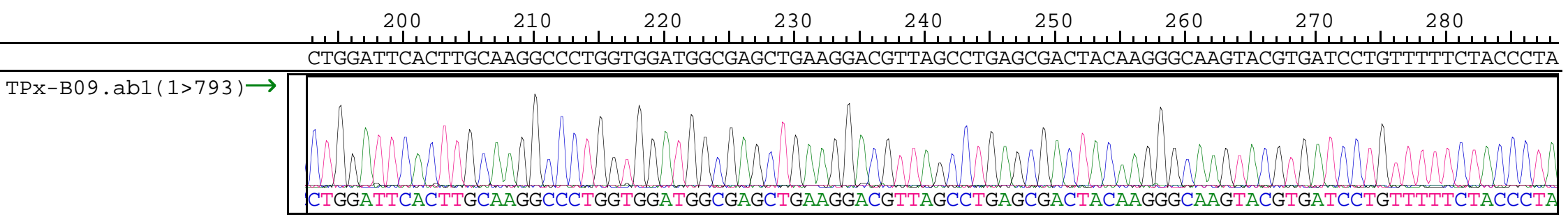

Project: Untitled.SQD Contig 1

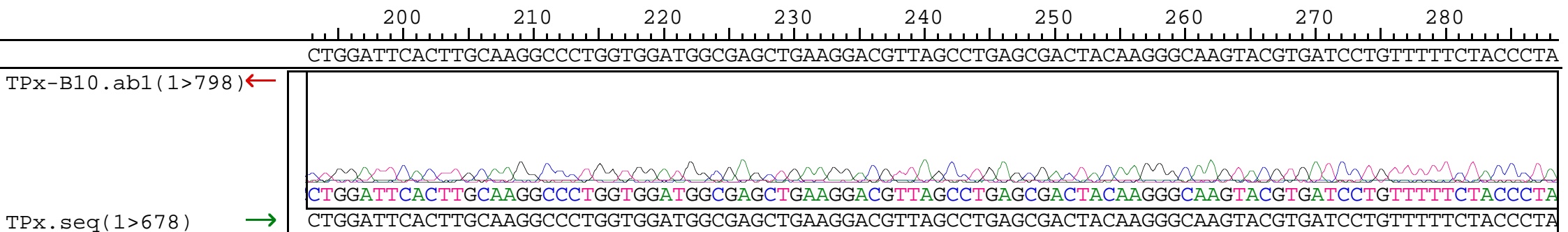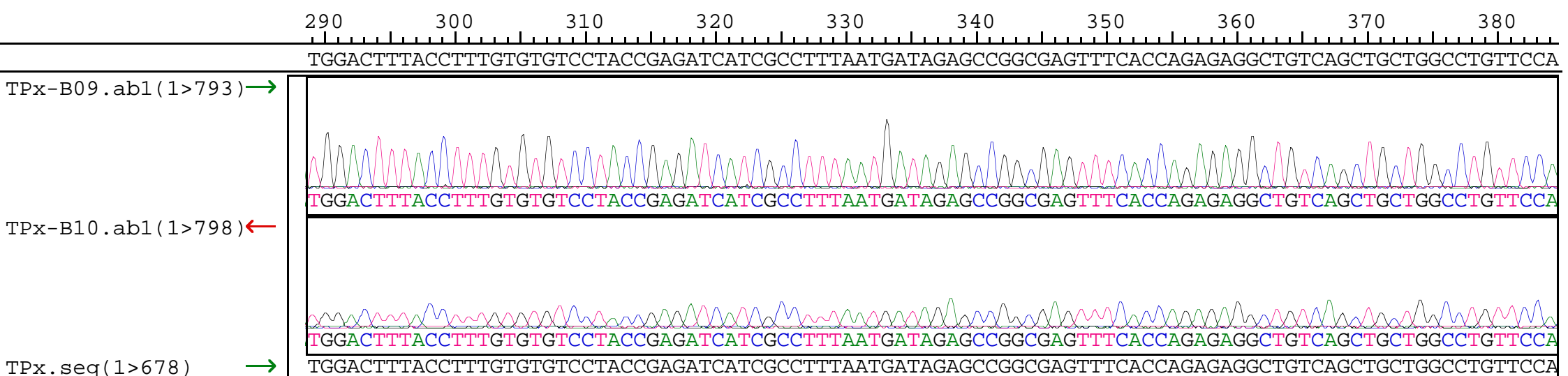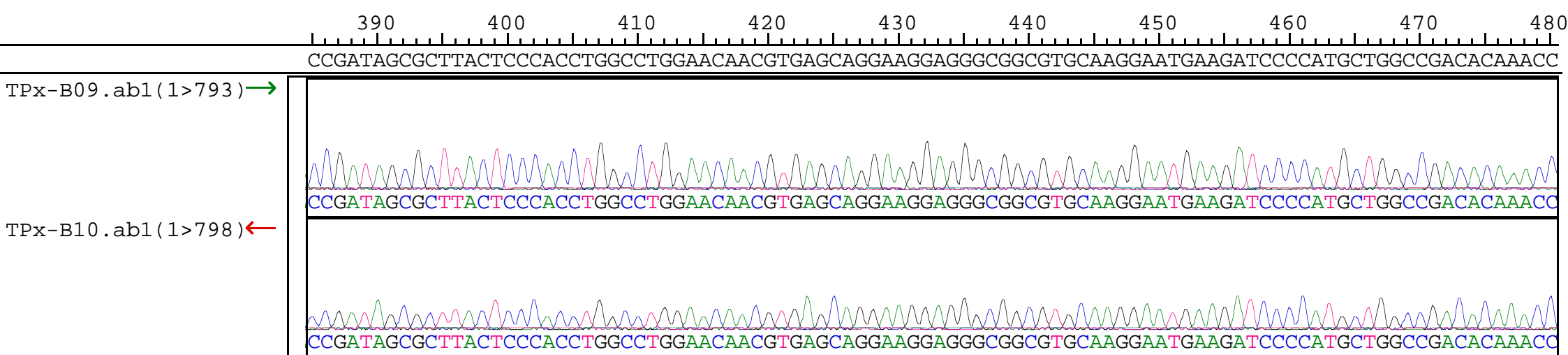

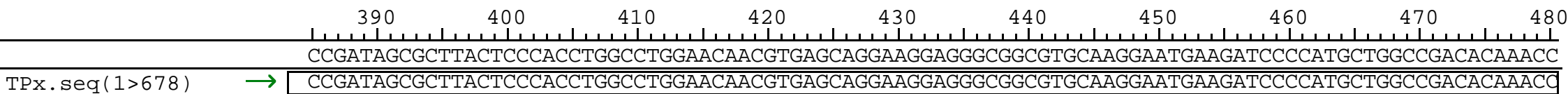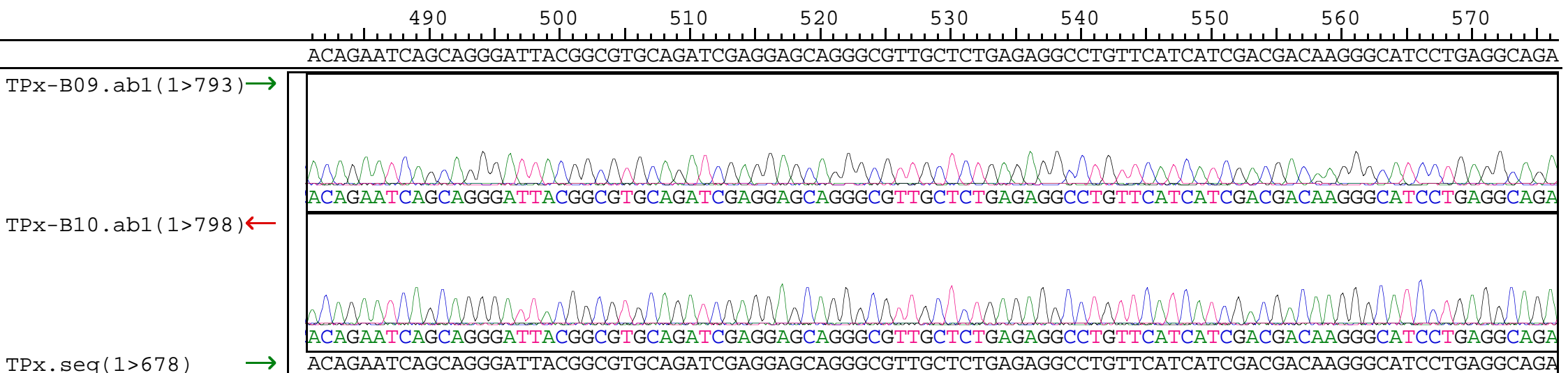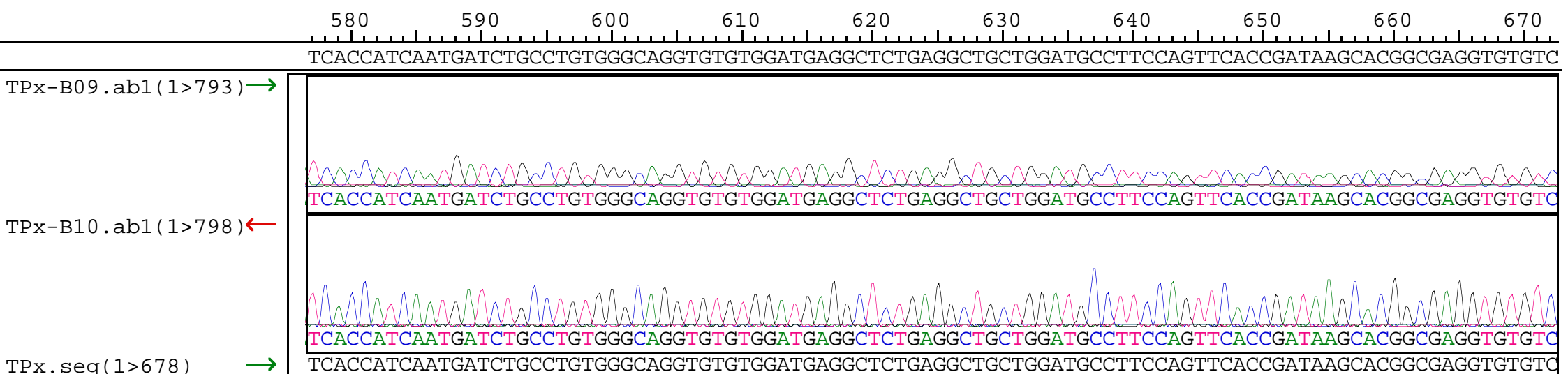

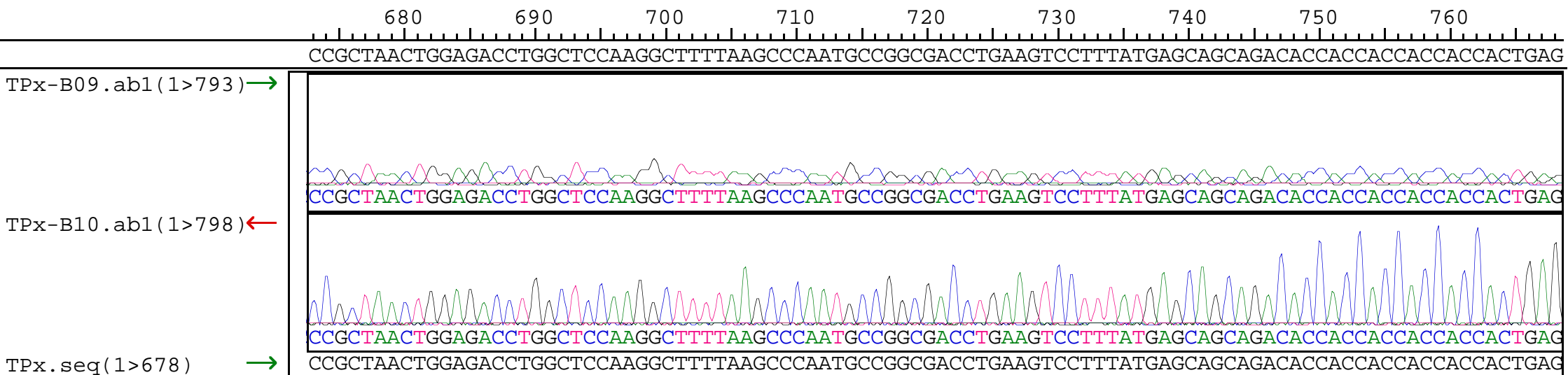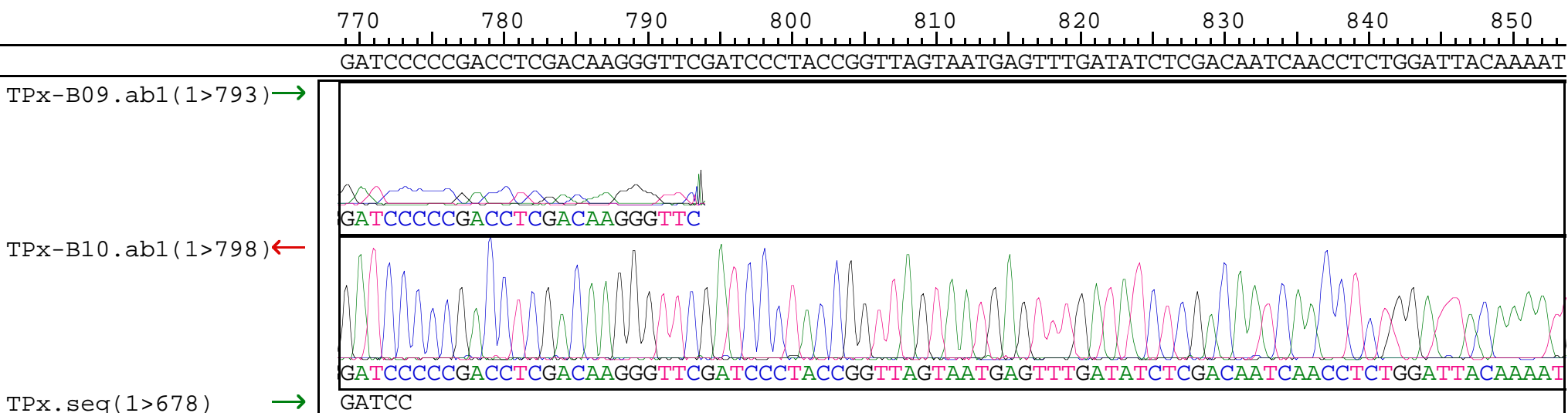

Supplement: Supplementary file 3 [file Data_Sheet_3.ZIP › 5. Supplementary Material/1. TPx gene synthesis/Sequencing file/Sequencing alignment.pdf]

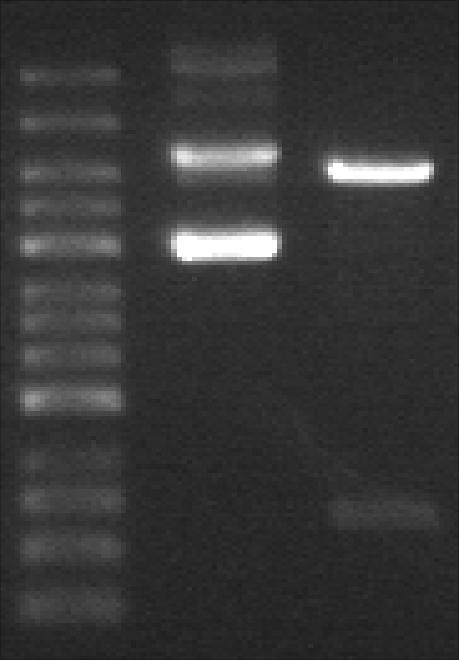

Supplement: Supplementary file 3 [file Data_Sheet_3.ZIP › 5. Supplementary Material/2. TPx recombinant protein expression, purification and identification/1. Identification of recombinant plasmids by enzymatic digestion.jpg]

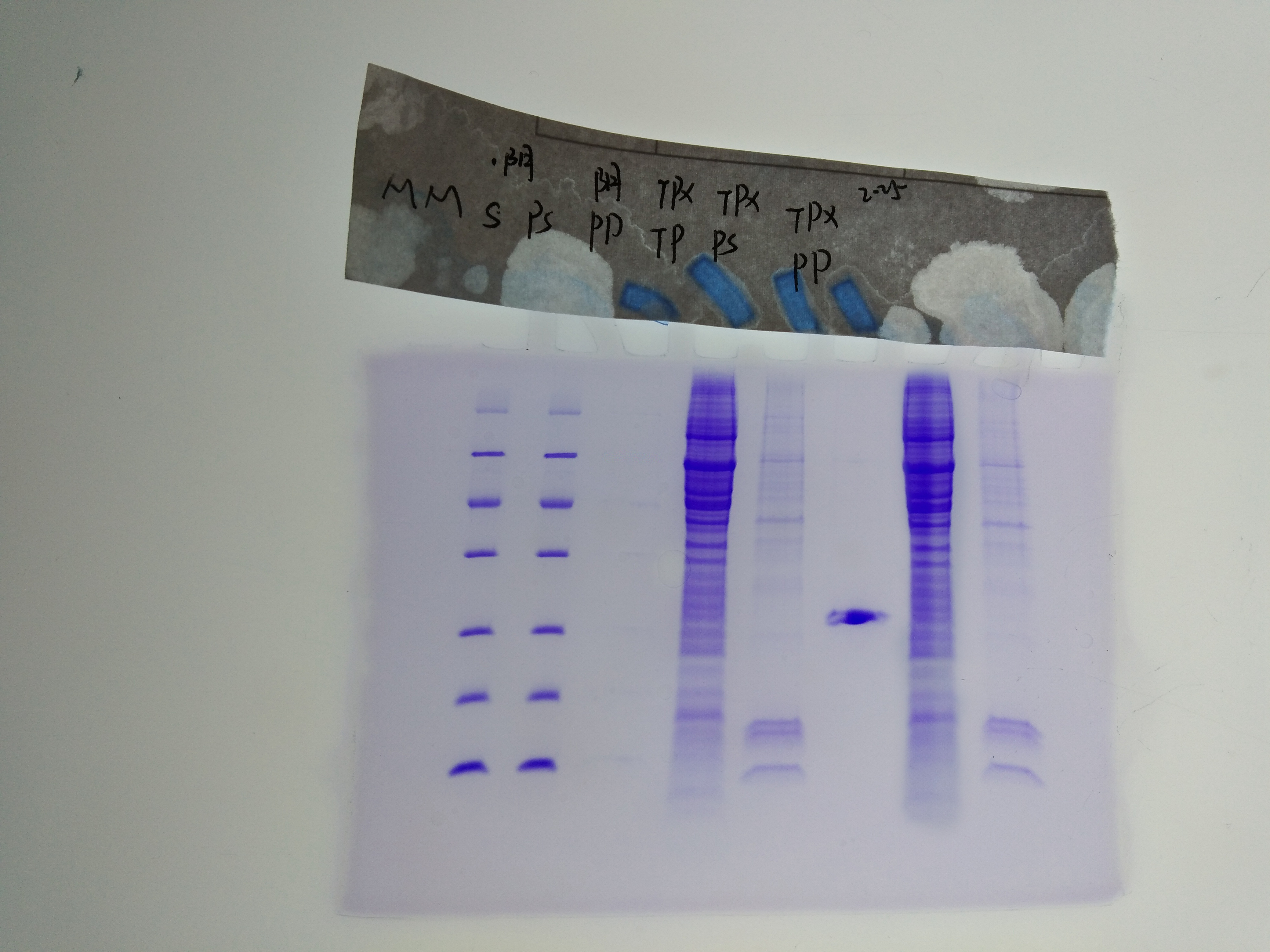

Supplement: Supplementary file 3 [file Data_Sheet_3.ZIP › 5. Supplementary Material/2. TPx recombinant protein expression, purification and identification/2. SDS-PAGE analysis of TPx recombinant protein.jpg]

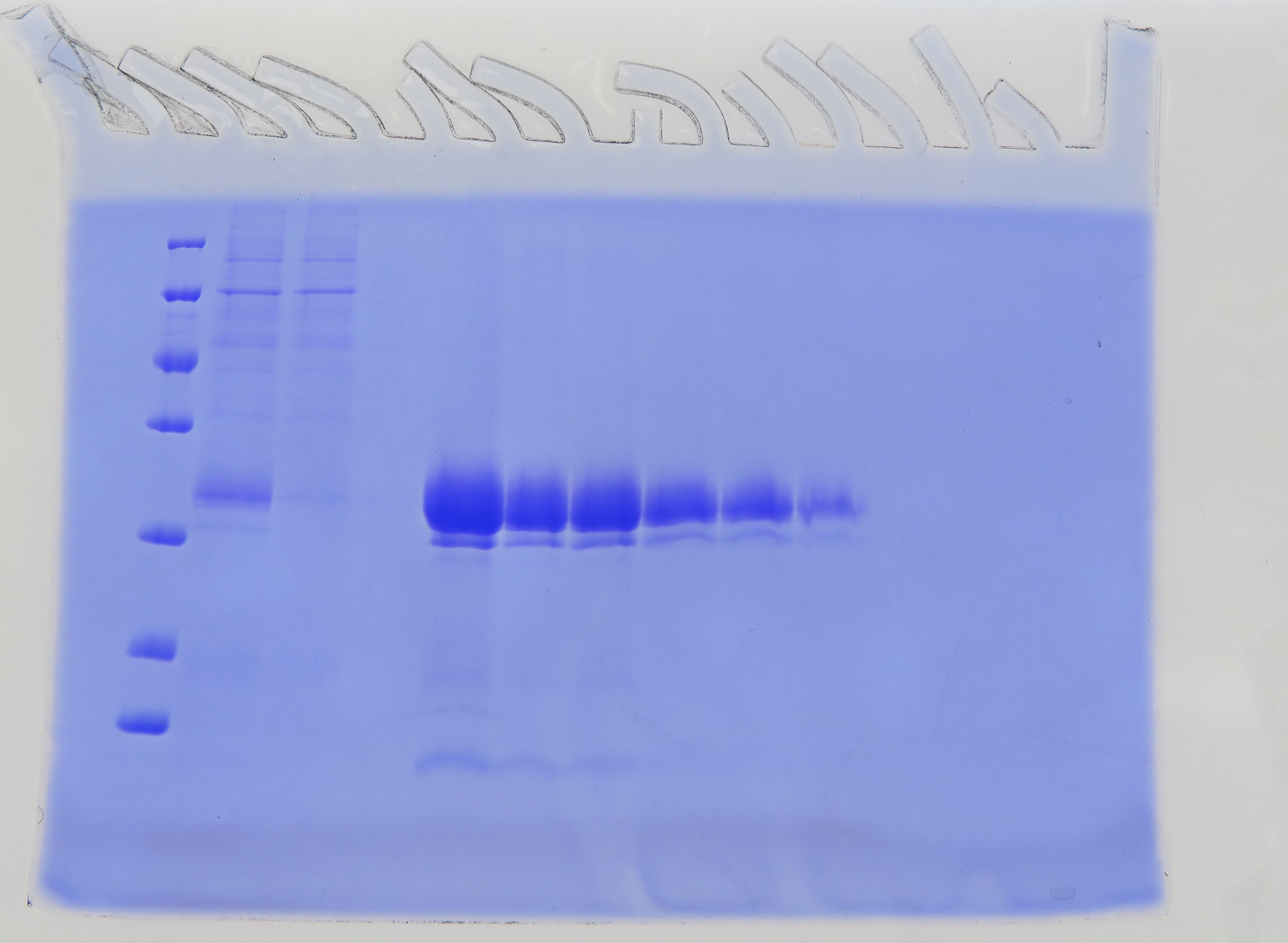

Supplement: Supplementary file 3 [file Data_Sheet_3.ZIP › 5. Supplementary Material/2. TPx recombinant protein expression, purification and identification/3. Purification of TPx recombinant protein.jpg]

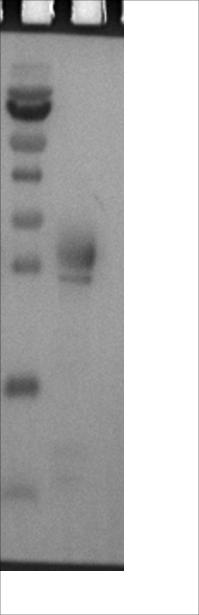

Supplement: Supplementary file 3 [file Data_Sheet_3.ZIP › 5. Supplementary Material/2. TPx recombinant protein expression, purification and identification/4. Western blot identification of TPx protein(1).jpg]

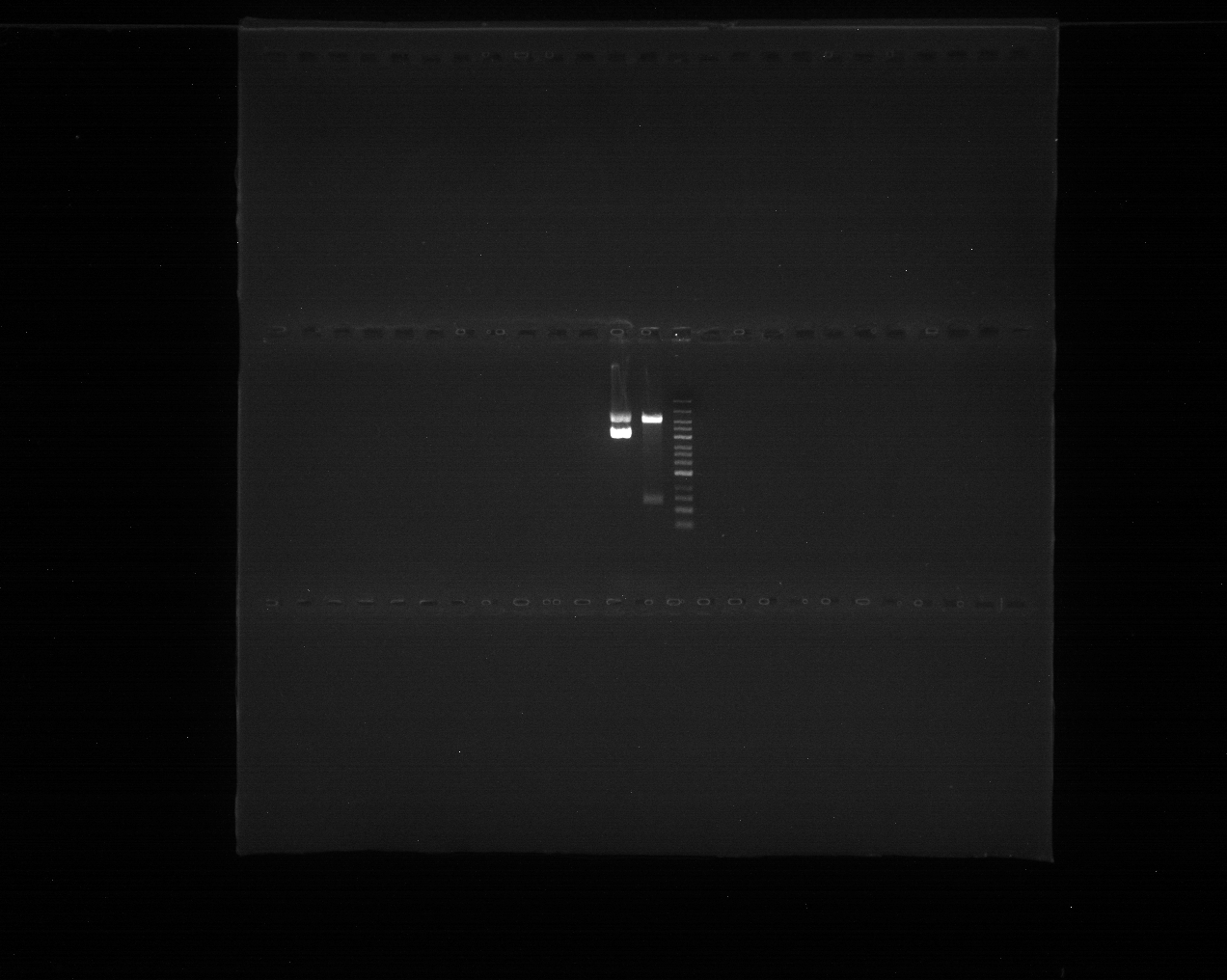

Supplement: Supplementary file 3 [file Data_Sheet_3.ZIP › 5. Supplementary Material/2. TPx recombinant protein expression, purification and identification/Figure 10 Identification of recombinant plasmids by enzymatic digestion.Tif]

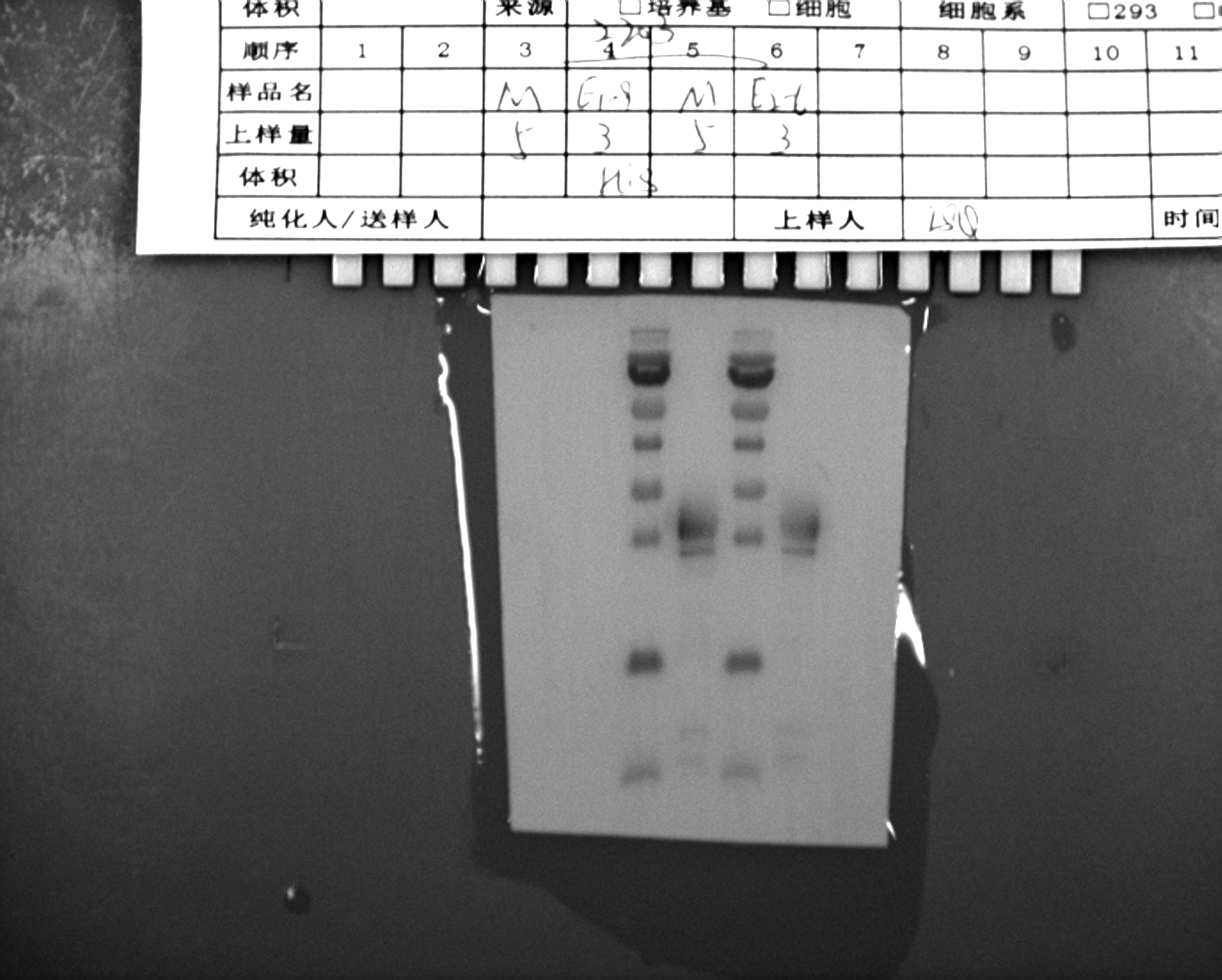

Supplement: Supplementary file 3 [file Data_Sheet_3.ZIP › 5. Supplementary Material/2. TPx recombinant protein expression, purification and identification/Western blot identification of TPx protein.Tif]

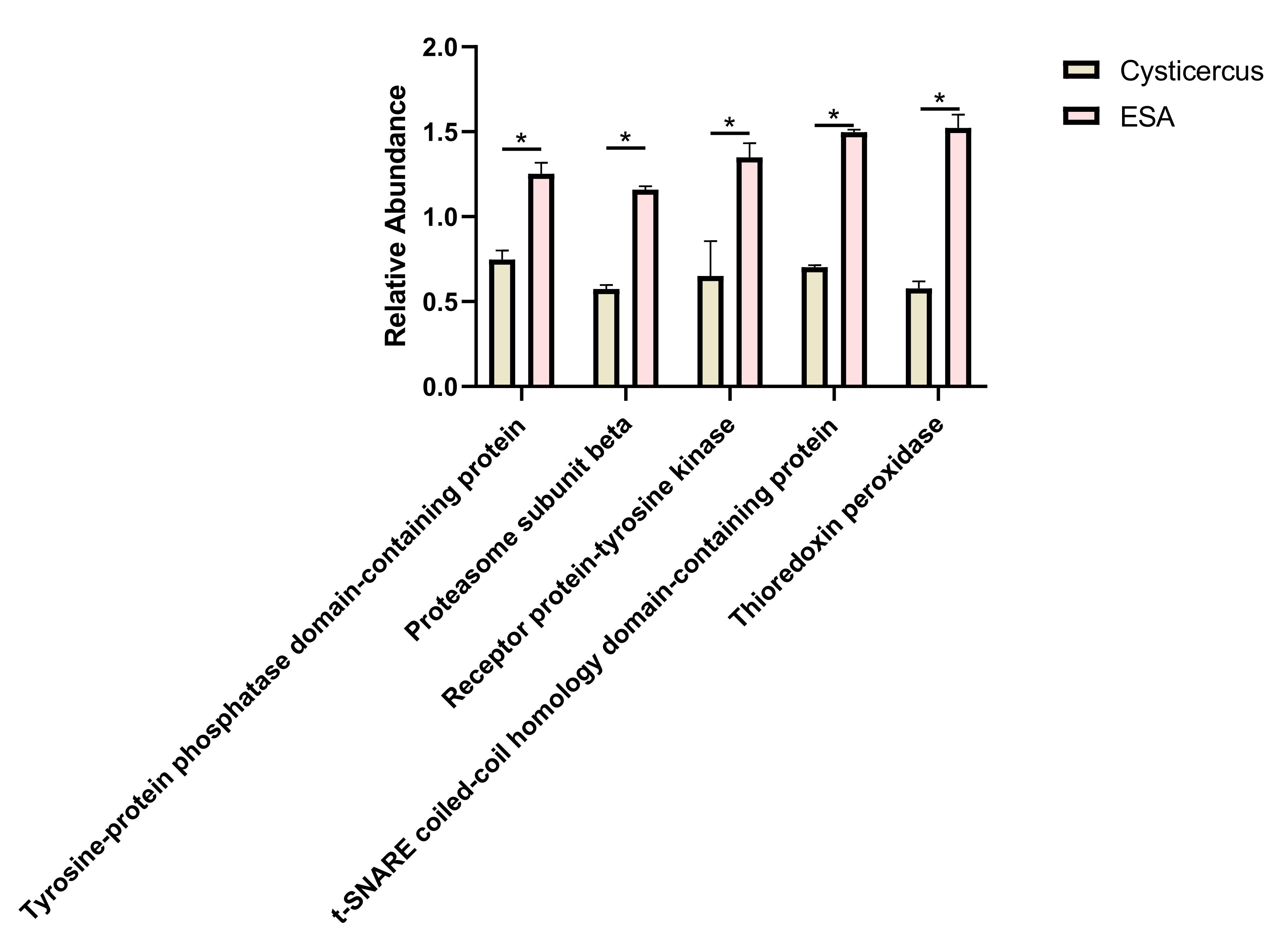

Supplement: Supplementary file 3 [file Data_Sheet_3.ZIP › 6. Image in the original article/Figure 1A.jpg]

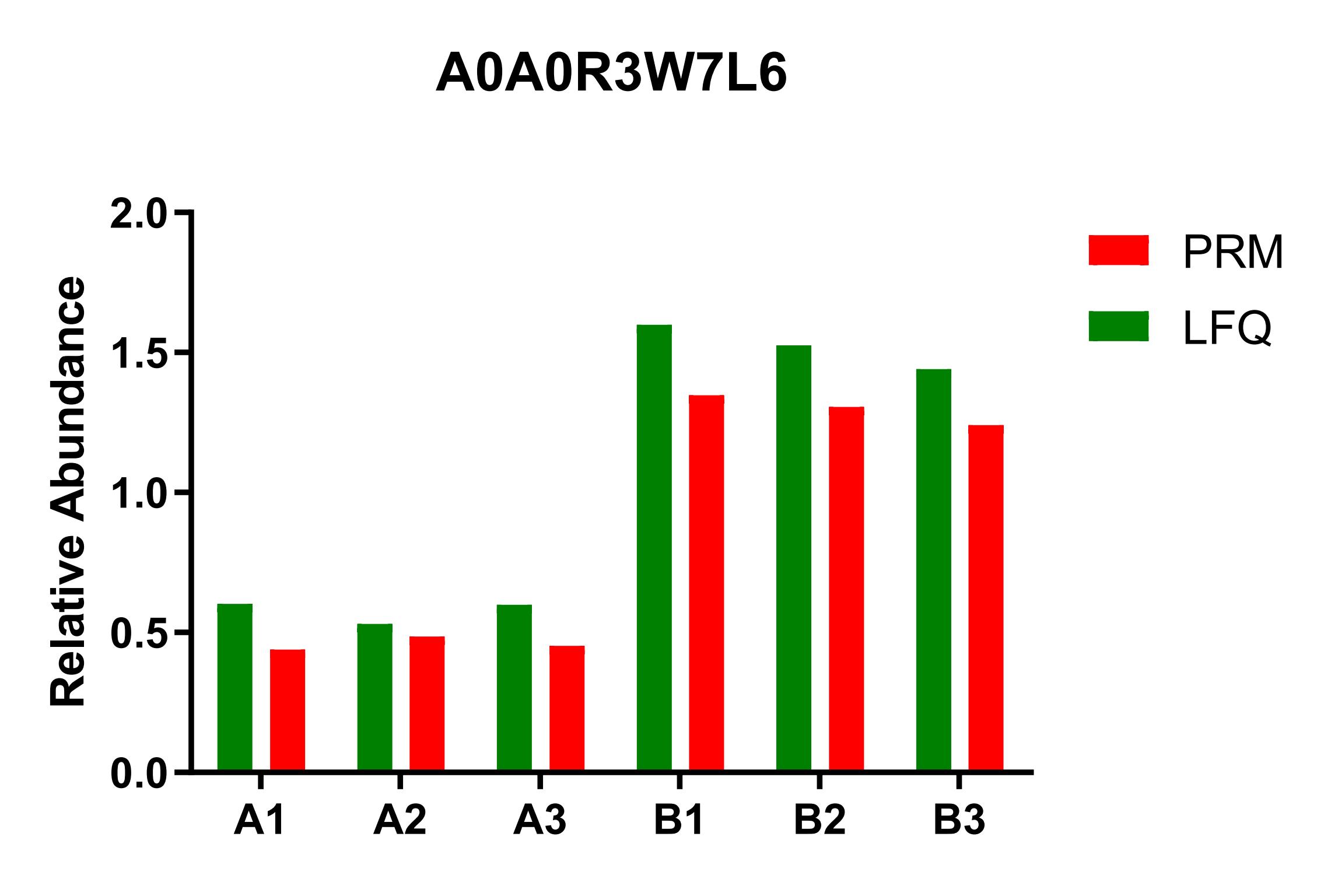

Supplement: Supplementary file 3 [file Data_Sheet_3.ZIP › 6. Image in the original article/Figure 1B.jpg]

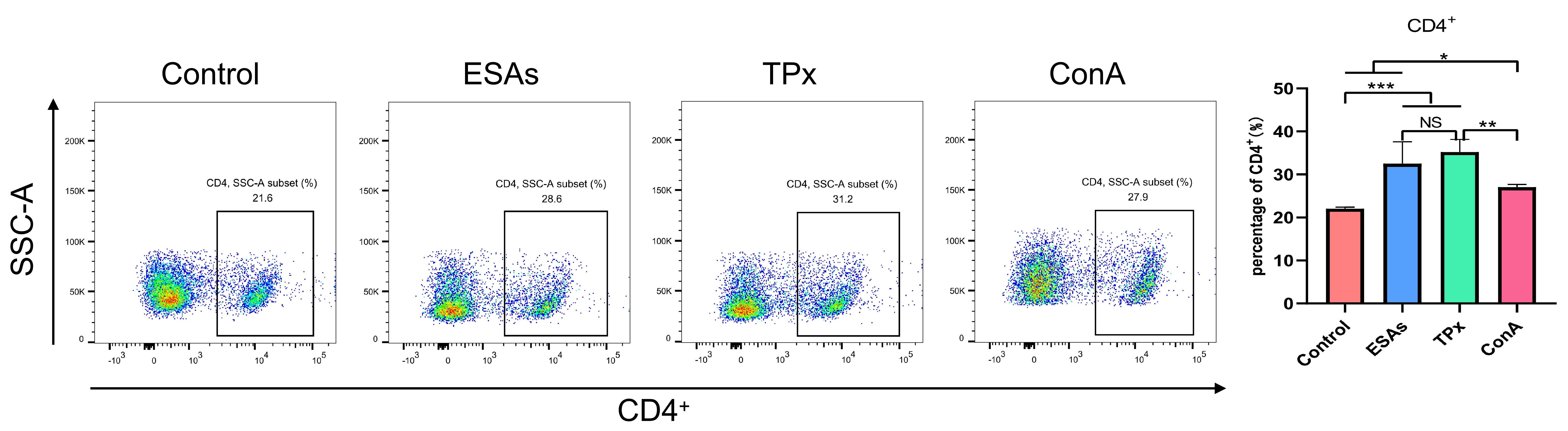

Supplement: Supplementary file 3 [file Data_Sheet_3.ZIP › 6. Image in the original article/Figure 2A.jpg]

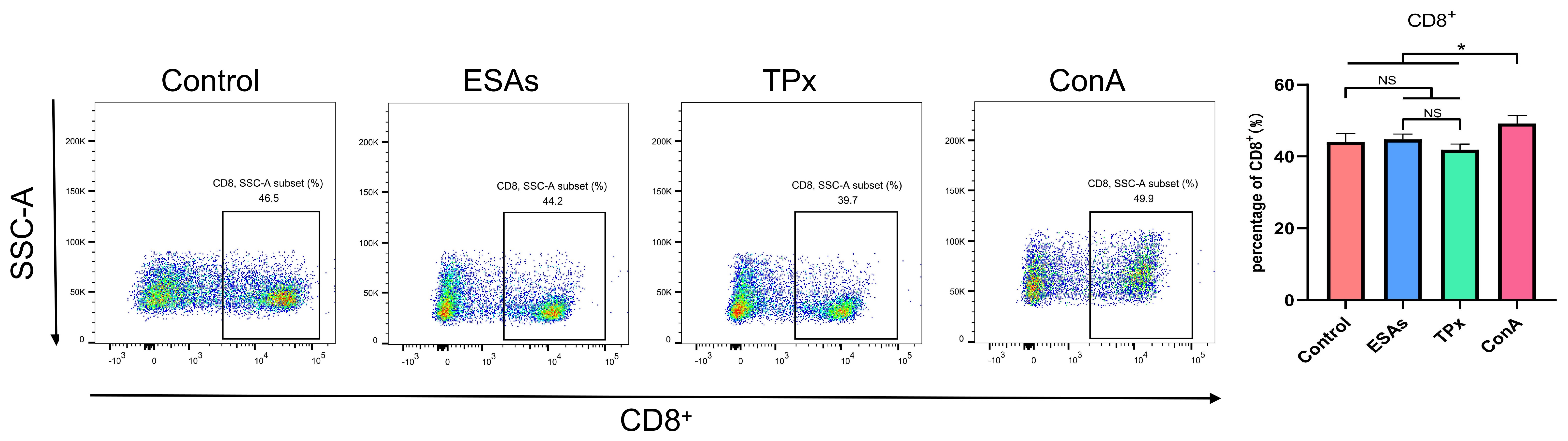

Supplement: Supplementary file 3 [file Data_Sheet_3.ZIP › 6. Image in the original article/Figure 2B.jpg]

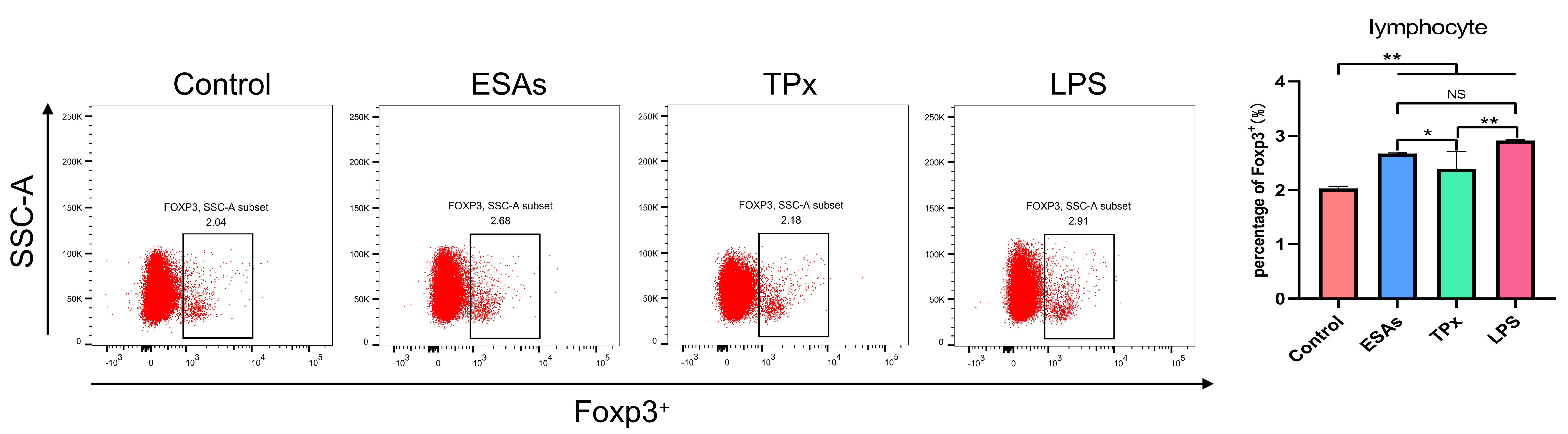

Supplement: Supplementary file 3 [file Data_Sheet_3.ZIP › 6. Image in the original article/Figure 3A.jpg]

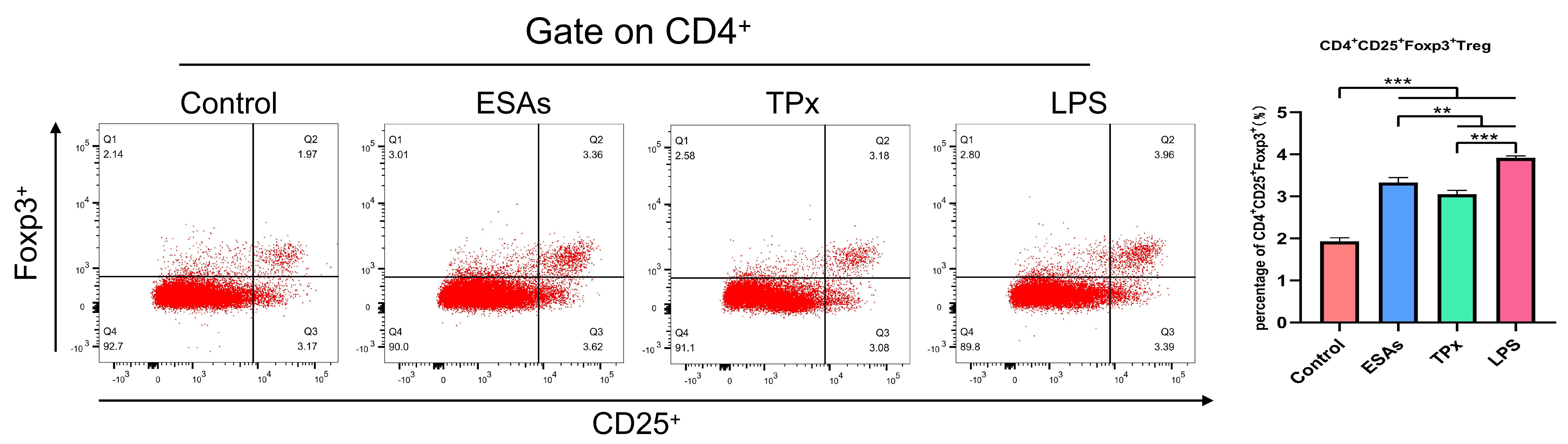

Supplement: Supplementary file 3 [file Data_Sheet_3.ZIP › 6. Image in the original article/Figure 3B.jpg]

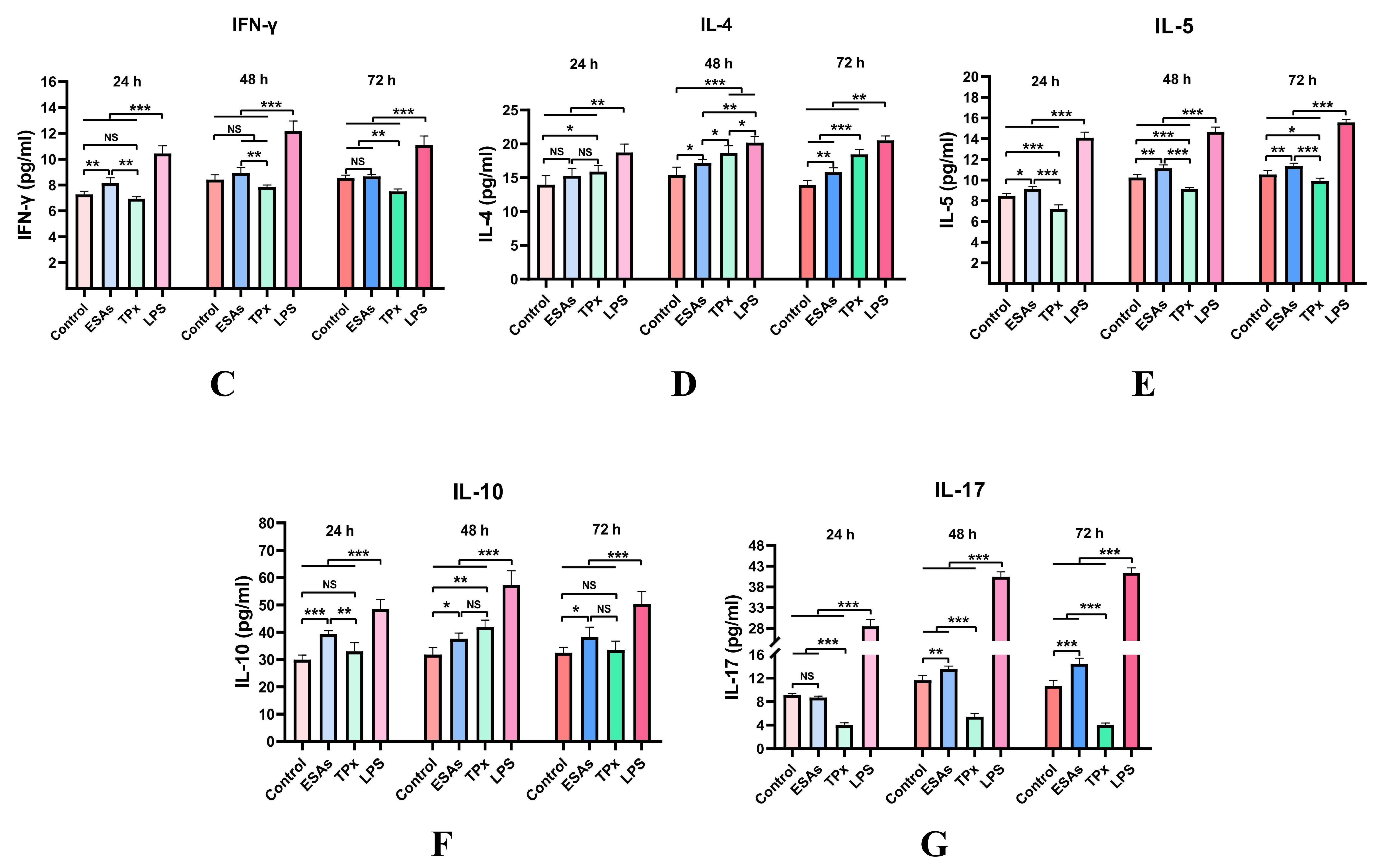

Supplement: Supplementary file 3 [file Data_Sheet_3.ZIP › 6. Image in the original article/Figure 4C-G.jpg]

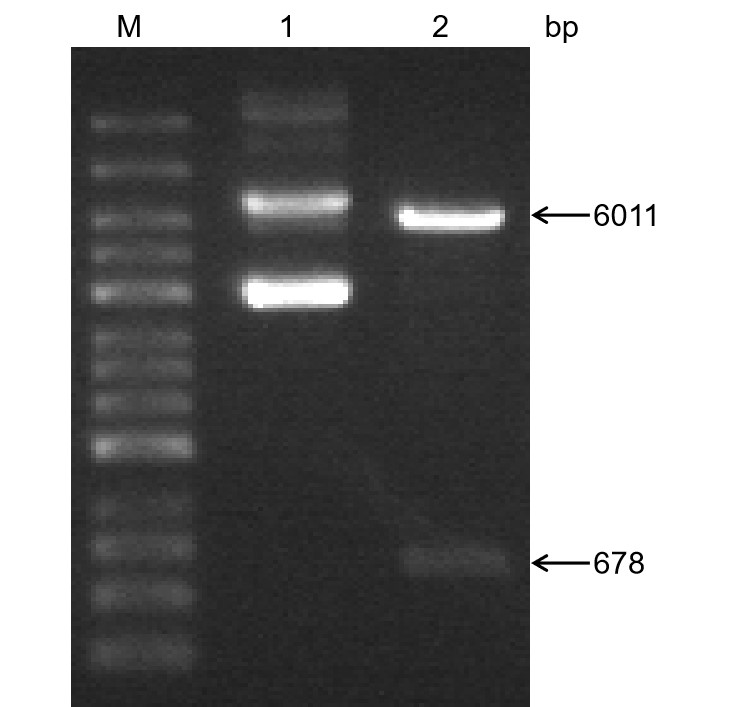

Supplement: Supplementary file 3 [file Data_Sheet_3.ZIP › 6. Image in the original article/Supplementary Material-figure 1A.jpg]

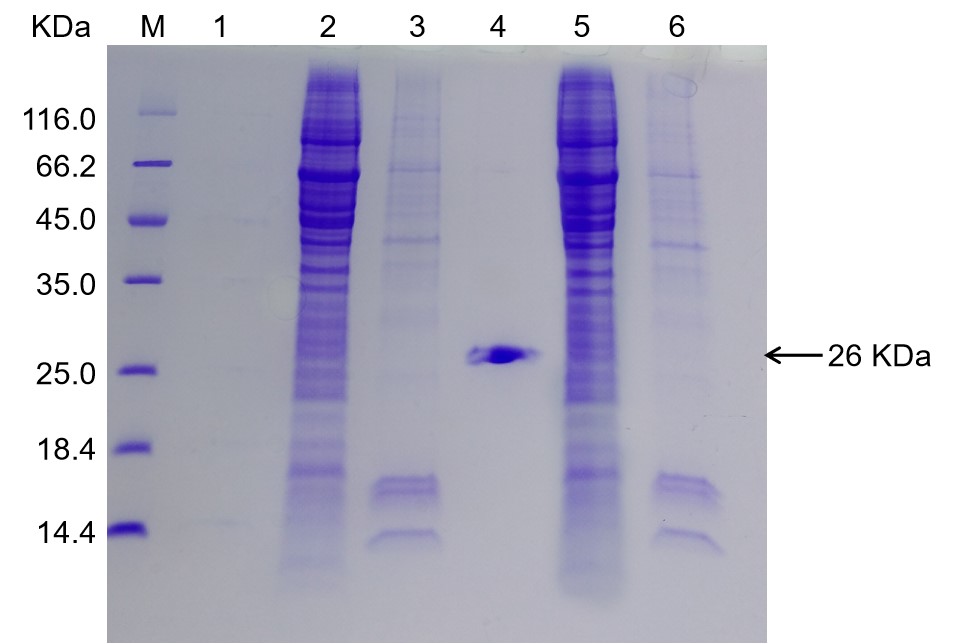

Supplement: Supplementary file 3 [file Data_Sheet_3.ZIP › 6. Image in the original article/Supplementary Material-figure 1B.jpg]

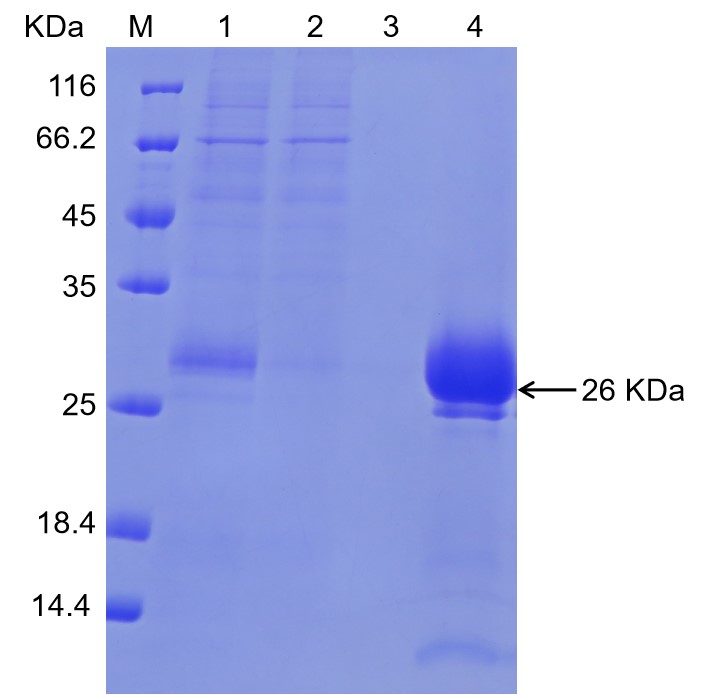

Supplement: Supplementary file 3 [file Data_Sheet_3.ZIP › 6. Image in the original article/Supplementary Material-figure 1C.jpg]

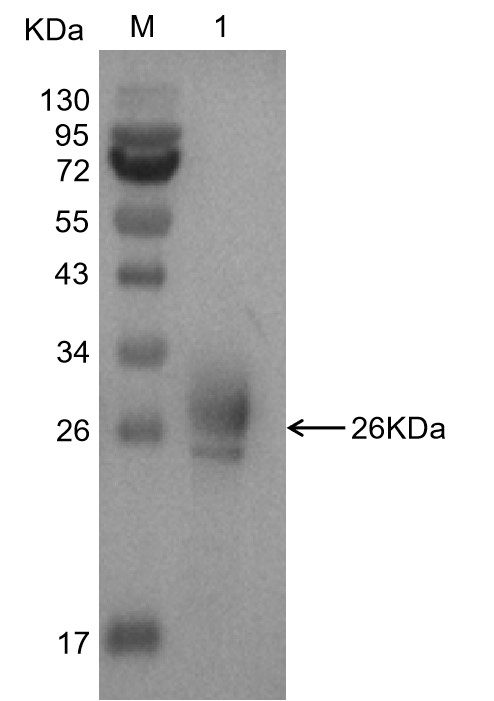

Supplement: Supplementary file 3 [file Data_Sheet_3.ZIP › 6. Image in the original article/Supplementary Material-figure 1D.jpg]

# lymphocyte

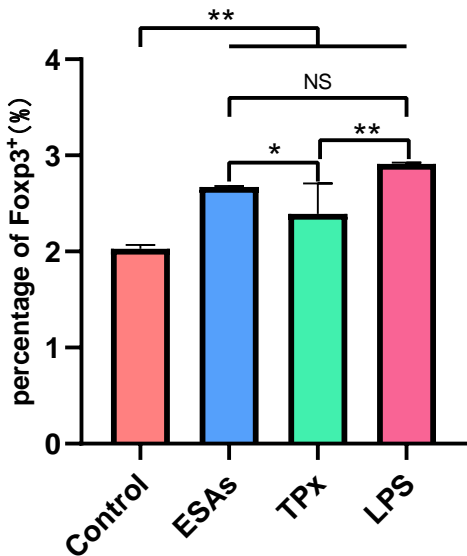

Supplement: Supplementary file 4 [file Data_Sheet_4.zip › 3. C. Cellulosae ESAs and TPx Induced the Increase in the Number of CD4+CD25+Foxp3+ Tregs in PBMCs/3. Create statistical graphs with GraphPad software/2.1 Foxp3.pdf]

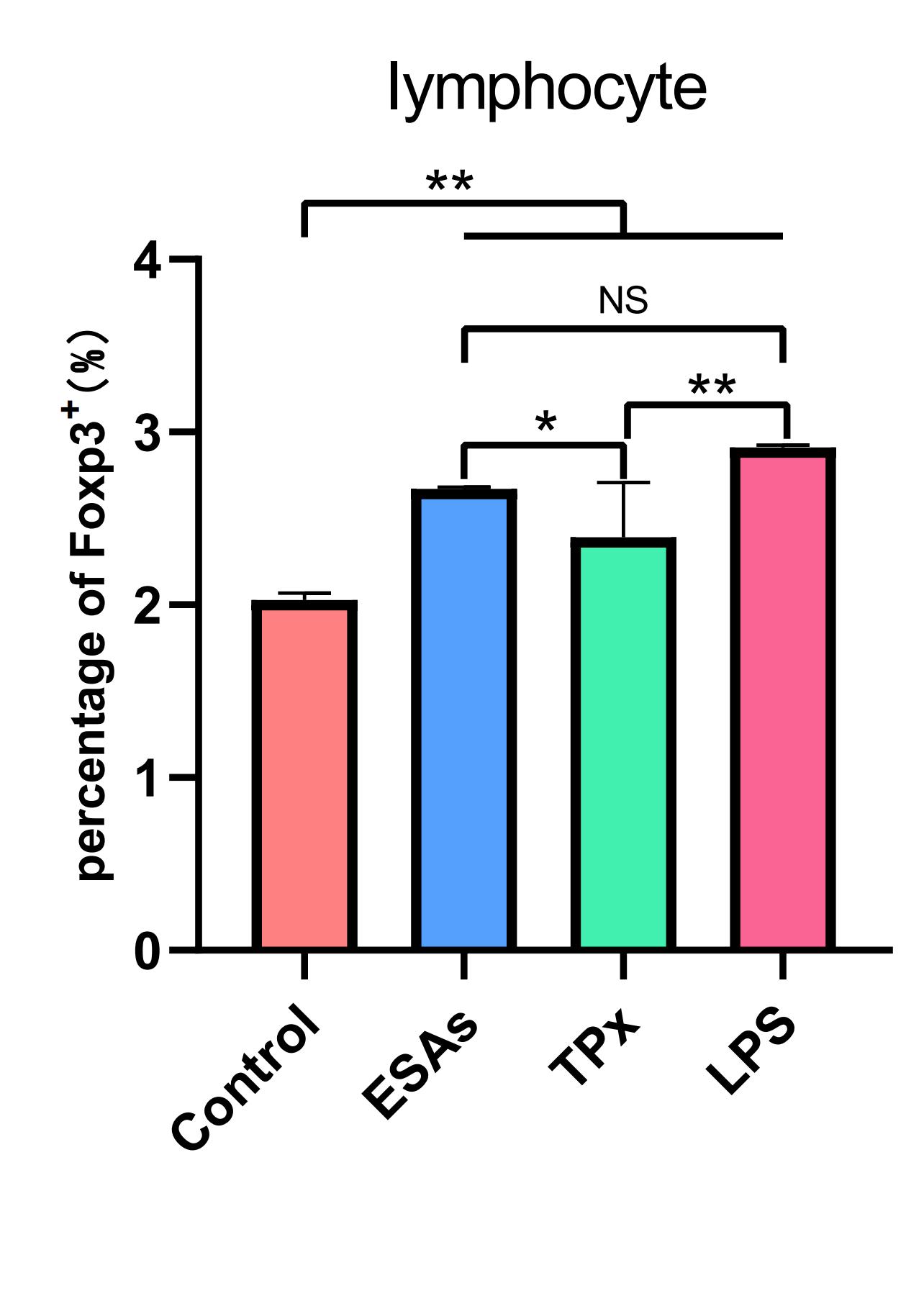

Supplement: Supplementary file 4 [file Data_Sheet_4.zip › 3. C. Cellulosae ESAs and TPx Induced the Increase in the Number of CD4+CD25+Foxp3+ Tregs in PBMCs/3. Create statistical graphs with GraphPad software/2.2 Foxp3.jpg]

# CD4<sup>+</sup>CD25<sup>+</sup>Foxp3<sup>+</sup>Treg

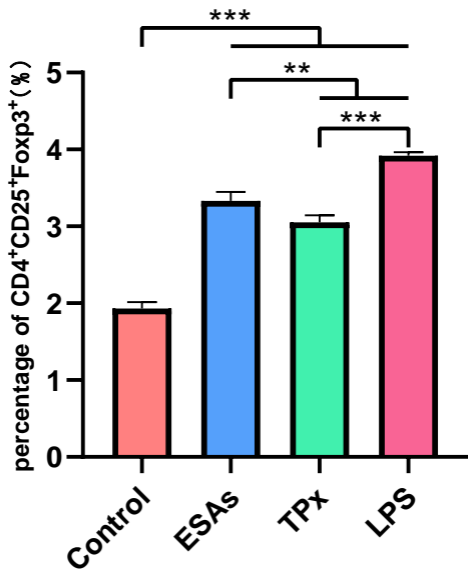

Supplement: Supplementary file 4 [file Data_Sheet_4.zip › 3. C. Cellulosae ESAs and TPx Induced the Increase in the Number of CD4+CD25+Foxp3+ Tregs in PBMCs/3. Create statistical graphs with GraphPad software/3.1 CD4+CD25+Foxp3+ Treg.pdf]

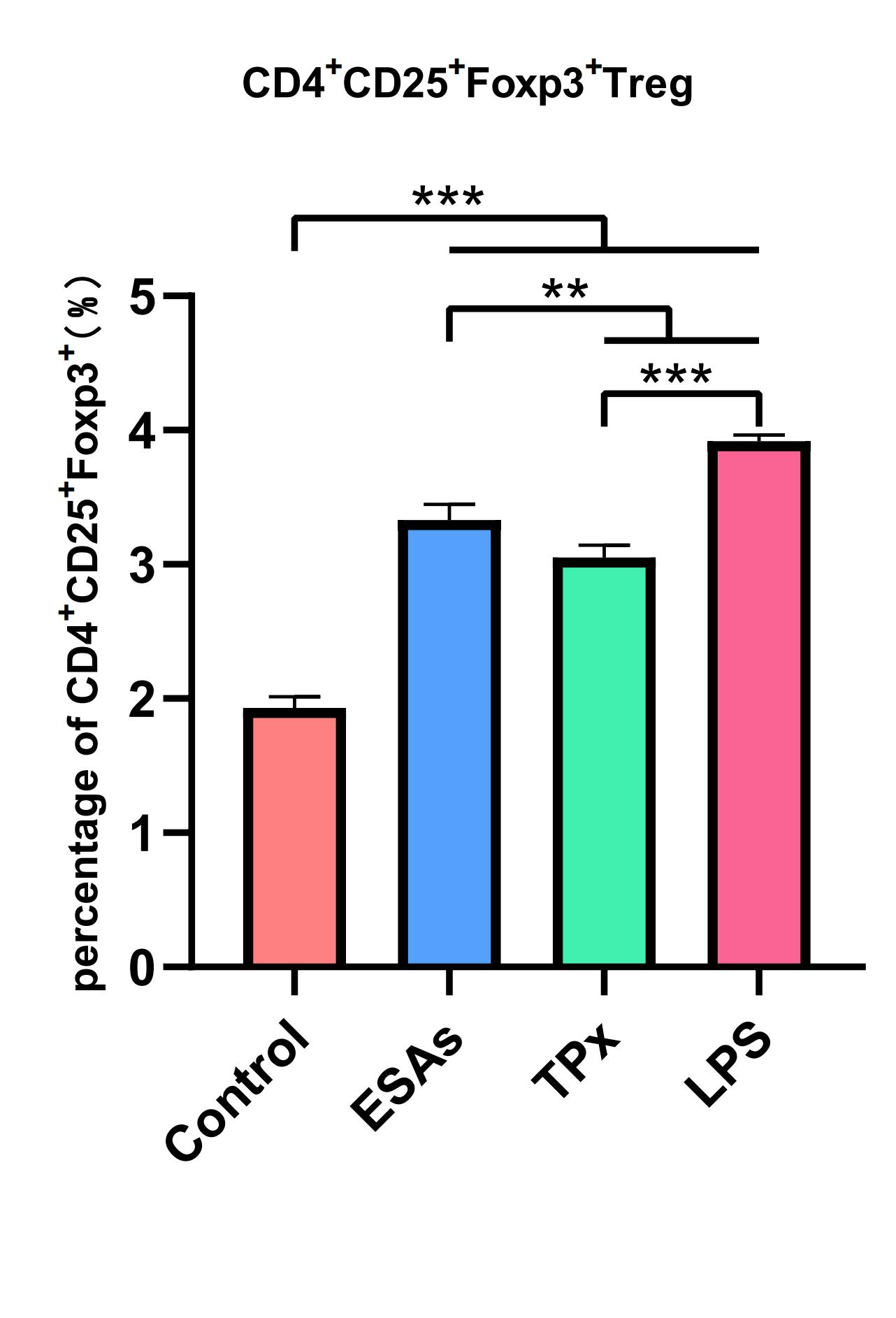

Supplement: Supplementary file 4 [file Data_Sheet_4.zip › 3. C. Cellulosae ESAs and TPx Induced the Increase in the Number of CD4+CD25+Foxp3+ Tregs in PBMCs/3. Create statistical graphs with GraphPad software/3.2 CD4+CD25+Foxp3+ Treg.jpg]
