# Supplementary material for: Regulation of piglet T-cell immune responses by thioredoxin peroxidase from Cysticercus cellulosae excretory-secretory antigens
Source: Front Microbiol. 2022 Nov 18;13:1019810. doi: 10.3389/fmicb.2022.1019810 (PMC9718028; doi:10.3389/fmicb.2022.1019810)
Supplement: Supplementary file 3 [file Data_Sheet_3.ZIP › 4. C. Cellulosae ESAs and TPx Induced Th Subpopulation Differentiation/3. SPSS statistical analysis/1. IFN-γ/1. IFN-γ--24h/1.3 (SPSS data export) SPSS statistical analysis--IFN-γ--24h.doc]

EXAMINE VARIABLES=Figures BY Variables
  /PLOT BOXPLOT NPPLOT
  /COMPARE GROUPS
  /STATISTICS DESCRIPTIVES
  /CINTERVAL 95
  /MISSING LISTWISE
  /NOTOTAL.


Explore


Notes	
Output Created	12-SEP-2022 21:54:01	
Comments		
Input	Data	E:\桌面\Raw Data\4. C. Cellulosae ESAs and TPx Induced Th Subpopulation Differentiation\3. SPSS statistical analysis\1. IFN-γ\1.  IFN-γ--24h\Untitled1.sav	
	Active Dataset	DataSet0	
	Filter	<none>	
	Weight	<none>	
	Split File	<none>	
	N of Rows in Working Data File	20	
Missing Value Handling	Definition of Missing	User-defined missing values for dependent variables are treated as missing.	
	Cases Used	Statistics are based on cases with no missing values for any dependent variable or factor used.	
Syntax	EXAMINE VARIABLES=Figures BY Variables
  /PLOT BOXPLOT NPPLOT
  /COMPARE GROUPS
  /STATISTICS DESCRIPTIVES
  /CINTERVAL 95
  /MISSING LISTWISE
  /NOTOTAL.	
Resources	Processor Time	00:00:02.47	
	Elapsed Time	00:00:01.55	


Variables


Case Processing Summary	
	Variables	Cases	
		Valid	Missing	Total	
		N	Percent	N	Percent	N	Percent	
Figures	Control	4	100.0%	0	0.0%	4	100.0%	
	ESAs	4	100.0%	0	0.0%	4	100.0%	
	TPx	4	100.0%	0	0.0%	4	100.0%	
	LPS	4	100.0%	0	0.0%	4	100.0%	


Descriptives	
	Variables	Statistic	Std. Error	
Figures	Control	Mean	7.27700	.122793	
		95% Confidence Interval for Mean	Lower Bound	6.88622		
			Upper Bound	7.66778		
		5% Trimmed Mean	7.27617		
		Median	7.26950		
		Variance	.060		
		Std. Deviation	.245586		
		Minimum	6.984		
		Maximum	7.585		
		Range	.601		
		Interquartile Range	.455		
		Skewness	.183	1.014	
		Kurtosis	1.500	2.619	
	ESAs	Mean	8.13925	.210145	
		95% Confidence Interval for Mean	Lower Bound	7.47047		
			Upper Bound	8.80803		
		5% Trimmed Mean	8.13411		
		Median	8.09300		
		Variance	.177		
		Std. Deviation	.420291		
		Minimum	7.677		
		Maximum	8.694		
		Range	1.017		
		Interquartile Range	.786		
		Skewness	.639	1.014	
		Kurtosis	1.503	2.619	
	TPx	Mean	6.93825	.075322	
		95% Confidence Interval for Mean	Lower Bound	6.69854		
			Upper Bound	7.17796		
		5% Trimmed Mean	6.93822		
		Median	6.93800		
		Variance	.023		
		Std. Deviation	.150644		
		Minimum	6.754		
		Maximum	7.123		
		Range	.369		
		Interquartile Range	.277		
		Skewness	.010	1.014	
		Kurtosis	1.500	2.619	
	LPS	Mean	10.43600	.294181	
		95% Confidence Interval for Mean	Lower Bound	9.49979		
			Upper Bound	11.37221		
		5% Trimmed Mean	10.43172		
		Median	10.39750		
		Variance	.346		
		Std. Deviation	.588361		
		Minimum	9.757		
		Maximum	11.192		
		Range	1.435		
		Interquartile Range	1.096		
		Skewness	.388	1.014	
		Kurtosis	1.500	2.619	


Tests of Normality	
	Variables	Kolmogorov-Smirnova	Shapiro-Wilk	
		Statistic	df	Sig.	Statistic	df	Sig.	
Figures	Control	.250	4	.	.954	4	.743	
	ESAs	.250	4	.	.963	4	.796	
	TPx	.251	4	.	.945	4	.683	
	LPS	.250	4	.	.961	4	.786	

a. Lilliefors Significance Correction	


Figures


Normal Q-Q Plots


õÕ0séÒ¥Yå×Ü±ünwpÝñû.¿¾ìóKÚ.¸¿÷ñ1bDv¢qj§OÞ¸qct9y´$ò_ùvóÝúÉ7òGs¢3.ífÑKûã?~÷òö577¼òÊ+_ccc´W)`ñØ±cÑþ¼¨ýû÷gýo÷&·%¿Þ'ÉÄÍÒîävGõä½3/À·££#Ànþüù.|é¥Âà÷>þÑ7ï#¦§NúcFï×liiéåÈ»$ò/ù]ºt)ù®hNkkkÚ¼ÊÊÊ§ÊÞü3fLøz~kÖeþú×¿yäñèåÇ¼­Üüzä2§óêÈïôéÓ©÷?kÖ¬ÔkÓÆ$X­wùEõh¯jj=ö'D~r@~¡èHhêü?üpéÒ¥E7«­­Í<ãÎäwõêÕpoá>|òÉ¡äôî­[·N2¥  `ìØ±¯¿þú¿üåä>§»¿ÉmÉ¯÷ÁÙ¿À_aaaò#ñR»­Q½ù80~üøðSû§'OgÏ¢G¯©©ijjºå>×CÍ1#Ü[ø¡¶oßH$Ö¯_íL|«²³³ÓK"?Iêººº¾þõ¯ßÖgÈÝÁMr®è lt²vwwwúªª*¿0ÈOò­è~i½ðÂFFùIR¾H$Ö­[7vìØènxî¹ç$ò$IùI$$IÈO$Iä'I$ò$IùI$ü$ID~$I"?I$$IÈO$ü$ID~$I"?IC­ýû÷WVVÎ3çvo;eÊûî»¯¥¥%9'L9S§N½ÖÍîìúrÛ;îêÕ«?þøèÑ£Ãà;výúõDb6âýùsI"?IC·òòò îîîÛ½í¦MÂm·lÙóâ/97nÌuùäM¼ÙùóçoÜ¸±cÇð@óçÏ¿»:yòdMMùI"?I1Ø^Ü2N>n;öìä|0Ì9uêÔÀ,dÿ	)BíÞ½eÉOùIê/ö¥:cýúõ£F1bÄÒ¾íË_þò3Òî!¹Ë0L_¹r%L9ÑU/^¬©©)...,,6mÚ#G²Þ[ê£÷~×_½²²²ºº:<P¦®_¿¾|ùòpÛ°ðëÖ­vaÎ5+Ü[îù£>êãÈL:5ÜsgggÖk¥ÆÆÆ°aöïß9Â#ÙË]ùýD~úÑô·¿ýí0½ûö`¬0ñÂ/¤~Ï¡C"á¥¶råÊpÕ«¯¾¦Ã×0]__]õÀ<x0L?>Ì¯¨¨Èzo©ÐûMçÞxã0QWW¹ðO<ñDßðæoM6ãÆÓ×®]knnÑ.ÉÌ2%`±'õ>J/½ôRôXÁö³÷~W~9%¤~_ÀJîºY&¼²¾°±±1páÂ0½xñâ5ÉkO>ýôÓOÏ1#Ì/((ÈzoiÊéý&Ñ5*ó¶eeeÑÂß¸q#Lóá;Ãô´iÓ­zÚµ^ä×û(uttDÓáz_ògïý®ürJ"?Iý(¿Tîôd´³F¾3HDUÃèªW^y%Ü*pð½÷ÞËÔOÖèËMzZ°ÀÄÔÝx÷îÝ;zôèhNiii´;°/ûü&Mæ_¿~½wö.¼Þ§ïlÀ%ü$ÝùUTT¤îvÝ"Ë/×&¿&ç97nÖGùõ~äÌeÞvÌ1ÑmÓ¯»»ûàÁuuu©;oÙÓO?¾?H1óª¾Råwg.Iä'éÈ/:¡õÕW_Þvöâ/ö"÷îMî<ã7ÒXsöìÙèý_ï7	ûþ÷¿&V®yÛÇ,LïØ±ã>ðÕÕÕaú½÷ÞûøãÃÄ¤Iú8,×®]8qâÅ&£K,éã(¥NGG£cÍi?û¸$¤ ¿îîî «7KýL¾Þ!ÒÑÑi_ó8PZZÜ³nÝº>Ê¯÷444k|ðÁä&©ßH$Ö¬Y¼¨¨hþüùÑi¼|µµµÑÉÂ³gÏþðÃû>2áQ&£cÙ6lÞÃ×QJÞ¿X¤è²Ó~ö;pI"?I$ò$IùI$ü$ID~$I"?I$ßíõüäÂü ôÑ¿ÿû¿û½sW®ôÓOC»víÚ¿þë¿8×ÑÑ1ðXÝVÿõ_ÿõÿðÆ!æuÖùÝþøÿ8àoôoÿöo?þøc¿Êqî>øÇüGãç.^¼øw÷wÆ!Îÿ>ýõ_ÿµqsÿñÿqøðaãó:ôÿùäG~"?ò#?òùùÈüD~"?ò#?òùÈOäG~äG~"?òùÈüÈüD~"?òùùÈOäG~"?ò#?òùüÈOäG~äG~"?òùüÈOä'ò#?ÈüD~"?òùùÈüD~"?ò#?òùÈOäG~äG~"?òùüÈüÈOäG~"?òùùÈOäG~"¿Ü_SSÓ´iÓ§NzêÔ)òùÈOäG~y+¿'?~<LìÚµkÒ¤Iò;xðà§[Ø;wîSÅ¸Ó§O¿ÿþûÆ!Îýüç??yò¤qsçÏÿñlâÜåË*CÌûí·¯2ÀÃòK­¸¸8S~/¿üò_lï¼óNccã_+Æ544üèG?2qîÝwß=|ø°qsáË8Ä¹üä'o½õqy8vìØ?h>ÈïäÉõõõöÊÑ^Gåh¯íu´7oöF]¿~½¶¶6HÈüD~"?òËgù]ºt©®®.+¶ÈOäG~"?òùåüçÎõêÕ¬×ÈüD~ä'òËùUVVÞùüÈOä'ò#¿¼_ïÈüD~ä'ò#?ò#?ò#?ùüÈüÈOä'ò#?ùùüD~ä'ò#?ò#?ùüD~ä'òùÈOäG~"?ùüD~ä'òùÈüÈüD~ä'òùùÈüD~"?ò#?òùÈüD~äG~ä'òùÈüÈüD~"?òùùÈOäG~"?ò#?òùÈOäG~"?ùüD~ä'òùÈüÈüD~äG~ä'ò#?ò#?ùüD~äG~ä'ò#?ÈüÈüD~ä'ò#?ùùüD~ä'ò#?ò#?ÈüD~äG~ä'òùÈüÈüD~ä'òùÈOäG~"?ùüD~ä'ò#?ò#?ùùüÈüÈOäG~"?ùùüÈOä'ò#?ò#?ùüÈOäG~äG~"?ùüÈüÈOä'ò#?ùùüD~ä'ò#?ò#?ùiÉ¯»»»½½Ý8ùùüÈOù,¿`¾×^mÜ¸quuuÆüÈüÈOäG~ÊOùuvvnÛ¶-ï¾6ìÂüÈüÈOäG~Ê+ùómÝºµ²²ò¾_lûöíüÈüÈOäG~Êù%Í7'÷ó%[±bEss³q&?ò#?òùòA~íííÁ|#GLß°aÃ/_~æÌ#L~äG~ä'ò#?åü>ùä7¤oÑ¢EajlÉüÈüD~ä§|ßåËöÙ4óEÇvÌA~äG~ä'ò#?åüÚÚÚV¯^=lØ°´ý|õõõÞÏG~äG~ä'ò#?òËùµ¶¶Þ¥oøðá+V¬WIò#?ò#?ù_>ÈïÂuuuyíùùüD~y+¿°%|yæy»«W¯f>ò#?ò#?È/OäwæÌ~8óØîÚµkÛÚÚùùÈOäò¯P-J3ßÈ#ùìç#?ò#?òùüòD~áÕpÁûùV¯^m?ùùÈOä'ò¯555i'pó=ûì³íííFüÈüÈOä'òËÞ÷Ý©S§¦oäÈ7n´üÈüÈOä'òË²îç+--uùùÈOä'uwwïÙ³'«ùþyÇvÉüÈüD~"¿<1ßÎ;'Lf¾#FlÞ¼ùÈüÈüÈüD~ùl¾ð"øæo"ò#?ò#?ò#?_>/ÚÏ×÷¿Û+ò#?ò#?È/ÇÌWYY¹mÛ¶ä±]ò#?ò#?ùüòÐ|åååÁ|D"õÉüÈüD~ä'òËóùùüÈOäWæ<yòîÝ»;;;º!ùùÈüD~9SûæÍ3ÍæìÙ³'°÷ùùüÈOäæ/a%%%æÛ½÷-ÍG~äG~ä'ò#?_oòäÉÙÏG~äG~ä'ò#?_oæÌ·e>ò#?ò#?ùübZggç-[2Í7úô;3ùùÈüD~±+HlÝºµ¼¼ü^íç#?ò#?òùÈ/vµ··?ÿüóûùjjjîÕ+ùùÈüD~o¾ðòTZZÚæ#?ò#?òùÈoðÍ÷ì³Ïöë~>ò#?ò#?ùü¹O>ùä©§ý|äG~äG~"?òù¦ùÖ®];|øð4ùùÈüD~Z[[ÛSO=5(æ#?ò#?òùÈoºpáÂÚµk¾p±®®®¥¥eÀüÈüÈOäG~"¿~,ÀnÕªUYÍ×ÚÚ:ÀC~äG~ä'ò#?_¿`W__óùùüÈOä×/µ´´¬X±"Væ#?òËgù]½zµ²²üD~ä'òøÉòåËch>ò#¿¼ß±cÇªªªÂ3üD~ä'òÈÍÈC=[óùå­üæÏßÒÒÒü^yå¿Ø~øÃnþ­bÜ»ï¾ûWõWÆ!Î=zôG?úqsï¼óÎüÁwìØ1gÎ´j)((X¸pá¾ûbµ¨ó7óÖ[où]ya55ÀÃòûïEìY~ùùñÀØ÷á~¬~éßï=ãçÞÿý'N8wîÜ¹ÆÆÆ!õ#:tè·~ë·ÒÌWTTôÈ#<y2ü/ÿò/aý®Æ¼¶µµðæ³üí£½öÊÑÞ»,¼ÔÔÔ¤¯¤¤$¼Ê´··Çv±íu´7öÈüD~ÌG~äG~ä'ò#?ß5ùùÈüD~CÅ|äG~y.¿"?ùüúXww÷Î;'Oi¾Í7_¾|9ç~"ò#?ò#?ùü²oÂ	iæ+--Ý²eË'|£?ùùÈüD~·6_yyùÖ­[sëØ.ùùÈüD~·g¾qãÆó%<XGäG~äG~"?òÓP_/æÛ¾®/üöìY·nÝ3Ï<ÓÐÐ@~äG~ä'ò#?_Ìæäù¢,XðK¿ôKóþ¼ÿoÞ¾ðo|ãá§#?ò#?òùÈ¯GóM<y÷îÝfyú§úk¿ökëÿïúÏlÿÖ>¹6(p×®]äG~äG~"?òÓ_gggVóM>=ÌÏ9óEéK_ú?þÑ¿ÿµø=òÈ#äG~äG~"?òÓ_x())I3_uuõrÔ|QUUU+eªüþ÷òÿ=oÞ<ò#?ò#?ùiÈÉ¯'óÍ=;×Íµ|ùò9sæ¤ÊïÁ/>øÌ3ÏùùüÈOCH~½oß¾y`¾¨ÒÒÒyóæ­[»ník¿4ûKmmmäG~äG~"?òÓß'|Õ|-êéCOr;ö¿ù£F?òW¿úÕ¬üÈüÈOäG~Ê7ùµ··oÞ¼9Ó|,øXE~äG~ä'ò#?åüzÚÏ÷ÐCqóùùüÈOù#¿Ë/¯^½zäÈiæ7oÞ3g¬ò#¿Áß·ª  üD~"?òëmmmõõõiæ6lX]]]kk«õB~ä7ò+¸Uä'òù__jiiÉÜÏÇ|äG~1ß G~"?òSÈ/<gïòüÈ/åwíÚµ+WÈOäG~=ÕÒÒ²|ùræ#?òË=ùUVVzÈOäG~éÌ3-J3_IIÉ³Ï>Ë|äG~qßÔ©S3Oï(--M$ä'òù_jvYÍ6éíííFüÈ/äWTT·W¯^-//|ßÿþ÷ÃD=ùüD~äÜ~2ù_>È/zö@½0qþüù7n#FÈOäG~,`>ò#¿<ßèÑ£ÃsøØ±c---aâ¹ç&|ªÈOä7Äå¶Ò555iob>ò#¿Üßºuëçs¤>·«««ÉOä'òòkll¯ÌG~äçö~ë[ß3fLhjjA3fÌèïE'?ù)ò;pàÀý÷ßÏ|ä§Ï|3ùüD~y,¿ûöÍ9ùÈOäG~"?_ÞÊ¯»»çÎûù&L°ûvæ#?òËCùM4)úlä,òùùEæÂË4_®5äG~y(¿'¦j/sE~"¿|ó®üòÂ³ýäÉ7nÜÈE'?ùiàåÇ|ä§¡.¿ÒÒÒð`öÈü4Àòkooß¼ys¦ùÆÇ|ä§!$¿S§NgþO<qýúuòùüòO~Á|a[RR¹oß¾ÌG~Zò;ö¾á!òùåºüz2ßÌ3íç#?Qù?Þ"?_É/¨"«ù&Ol?ùiHË/Ú477ð¢ÈüÔµ··?óÌ3#GL3ßìÙ³ü4ÔåWVVæÈ/?Ìu?_uuµý|ä'òûïÃvaÝºuä'òùåùfÏ=ð[ZùÅZ~÷õ3<D~"¿Ü5ßôéÓíç#?_zÈ"?_kmm]µjU¦ùjjjÞyç¾ÿÝ^ü+òùîØ|uuuÃK3ß¢E?ëóßíùi(Ê¯²²rüøñáåüD~"¿5_MMMØ¾%¿üÈOä×ca«1ðN~"?òÓÝoÑ¢EËE~ä'òë±#GmÇÆ¯]»6íB~"?òÓ/\¾|ù3g²ÞüÈOä×ó]8·Wä'òe---«W¯ÎÜÏ÷ÐCörCò#?_9·Wä'ò[ÍÍÍ?üpæ~¾ººº¾¼-üÈOä»ÈOäG~ÊìÄ,È4_`ßOÅ#?òùùÈ/Ö9s¦§ý|½Û%?òùÝ^]]]óçÏ/..[#F,Y²dNõ ?ù)i¾yóæ¥½ïÎÌG~ä'ò»EYÏðèï?ãK~"?òÓ'îÕ~>ò#?_ªªª^¿~=víÚâÅÃxüD~"¿þÛÕÔÔ¤ýøðáwi>ò#?ß-***Ô¿êÝÕÕæùä'òùÝóÂ¦oÞ¼yiûùJJJÂV±½½ý<ùÈ¯ÇÂF'h/9§³³3Ìñ©."?ß½-ëûùî­ùÈüD~·(:Ú;wîÜèhoø¦ÃiÓ¦ÈOäw¯^?ª««À|äG~"¿[¨õ°á ?rK~ßüæ7gÎyÿý÷×××·µµú"566N>ÀÌG~ä'ò»uK,5jTAAAø:wîÜ0§¿üD~äwoK$<ðÀ)S~·îwWþþÊY³fýÊ¯üÊ`á¯»»÷îÝÁ l>ò#?_L#?ùÝÛ¶lÙ2yÒä§zzÃ3¢ÕÕÕõõõòjqÿý÷ùÈüD~äG~ä§!!¿¥K>pá_ýïÕO<y aÏ=áÑ|äG~"¿ny«ÈOä§ß#<òÕ¥_MßïÖýîÔû§ÀCwwwïÛ·/óØneeåüÈOä½#?rQ~;wîüÕ_ýÕuk×%å7úô§zª¿ÍwÂ	iæsÂüÔJ%?ù¦üzêÉ'6[»ví"?rH~¡z(àï«K¿úÈ#L6Ê)DbHüÈOä××N:5bÄ°Ùª©©Iý`gòù)Wä÷ÙÍ=¿ó;¿³`Á?ù?é§Ã¬q6ùÈ¯O-]º4Úr<xp`üD~äsuvvÆÜ|äG~"¿[ôæoF[®ä¢Èür¨ööö°Õ*))¹ùÈüD~=ÖÑÑñÀD's9rdüD~äÓæ>ú=âf>ò#?_ö^zé¥hãµlÙ²AYtòù_îoß¾ñ4ùÈ¯[ú<?È/ïÌG~ä'òË^Á­*,,$?üz2_MMMx¿ùÈüD~1üD~ä+æøùÈüÈüD~ÌG~ä'ò#?ò#?ßÕÖÖÕ|-jhhÈÝüÈOäG~äG~"¿ÿWkkk]]Ý°aÃ2ÍwæÌéÈüD~äG~ä'òËóùüÈüÈOä×£ùÂÅ0³¹¹9ÖùÈ/ã>ÏOä§!#¿Ì8ëüÈOä^êG÷eÏóù)ä7ÔÌG~ä'ò»Eá7/lôÑë×¯áëâÅÃ£GÈO¹+¿ð»i¾áÃ¯Zµ*ÍG~ä'ò»E¥¥¥akúÁô]]]aNyyù]Þó©S§***§LräÈòùßÀtæÌEeoõêÕmmmCaùüz¾í¥ÉïîßçW[[»cÇ0ñòË//[¶,S~?üáÛ'þùÿ9¡÷³ýìç?ÿ¹qsçÎÿ¯ç²­Ê¾ô¥´÷®=úÿðXÎ:À=zô¨ßÕ8÷oÿöoï¼óqyo¿ývû?h¿Ë¯¬¬,lÒ:::ÂÅð.sÂü»ßxãÆòÛ²eË»[Xáá]Å¸ð¬°54qîðáÃa5Åm©þùªªª4ó9òk_ûÚ~ð¡¶<èw5Î9räÀÆ!æuÖÔ?h¿Ë/ü¿0ëï½÷Þ]Þsê9"ç8Ú+Gí½'íÛ·oæÌi[°°	ÿYëÈÑ^Gåhoo?~âÄÅÅÅ#F6mÚ+WîþnSÈüîm'N¨©©a>ò#?_,*++Þ>¾f;&?ùÝÍæ¸ºº:Í|&LØ¾û7ùÈoÐZ¾|ùë¯¿&Â×ÚÚZòùß]ÖÝÝ½gÏÌc»Á|;wîLýò#?òùõØÙ³g'M4bÄèølYYÙÞ½ïþn;V^^î³¢¢¢©©üD~äw7æ¶Âc>ò#?ß]sê_l¦_~ùå~]tòù__êììÜ¶mÛ¸qãüÈOäwäW^^¶¡gÏMÊ¯©©)L5üD~Dù%-[¶TVV2ùÈïÉ/ÚFünÜ¸áïöü4òíÚÏG~ä'ò»÷ò>É9ÚÏä×ÕÕµ~ýú0ùÙËä'òSË/:¶ëý|äG~"¿þ_cccÖOr>~ü8ùü4`òK$Á|íùüúW~¡¡3fDçöO4©µµµ¿üD~äÕÞÞ6%%%iæ«©©ihh`>ò#?ß=ß D~"?òëÅ|¿ ?ü'v$»råÊ¤IÆG~"?õüüÈüÈ/.òëêêrn¯ÈOý$?æ#?ùü2?.!­Ñ£GÈO÷P~­­­«V­b>òùß È/l»nüë©ö½ùæä'òÓ=_0_]]Ý°aÃüD~ä78òK×ßvÉOä7då×OæûäO¬ò#?_.E~"¿ü_?ïþè>ÿùÏ=zÕªU>óüÈOäwuuuM4iÄÉ9cÆùö·¿M~"?Ýü²/3[ZZîæ¡7mÚôË¿üË+åg6¬~buUUÕ#<bùün£±cÇ¦Þm¦7nÜH~"?Ýüz1ßÝ>|"(..þÇÿ °/ú·þÿ®/))¹KMÈOCK~EEEaÓÜÜÜsêÔ©0gÔ¨Qä'òSå×¯æ©I&%Ùýûâ¿¸oß>+üÈOä××¢ÓoÜ¸ñ÷ñ!ä'òSVùù¢ÚÚÚ*++~êéTùÝÿýVùÈ¯¯UTTÍô5k:;;ÃÅ69åååä'òS/òOáùýú¯ÿúoÿÖo'Ù·lÙ²ÏþóÎó%?òùÝF'OÌúIÎMMMä'òSVù÷»ß­©©IÛhô«ù¢ZZZÆó¿ñ_ùÊWª««ûìð#?òùÝv.]2eJqqqAAÁ#ªªªÂþ^tòùåbáiÈ5ðæKvùòåmÛ¶Õ××¿øâ.FÈüD~¹ùürÎ|ûù>`æùÈüÈü48æ+))	ÏâöövãC~"?òË1ù=6ú0çè|Þ²²²½÷ÈOYÍ÷¹ÏnåÊÌG~"?òËIùß¼ä=_4ýòË/Èo(oï2ßÏíçûû¿ÿû¾ÿÝ^Èüâ%¿òòò°A?ölR~MMM>ÉYä7·t3gÎìåØnÿn¯ÈOäG~q_´Yÿ,åÓoÜ¸¦ÉOäÇ|ïç#?òù_Ë¯¬¬,úô¾H~]]]ë×¯Óä'òÊæ9rdÖs8ÈüD~äÃòkllÌúIÎÇ'?ßÐ4_yyù-[z:üÈOäG~9,¿PÀÐ3¢s'M4ÎE~"¿A¬³³sÛ¶m&LÈj¾D"ÑËmÉüD~äÛòÈOä7æ«¬¬¼óùüÈüÈOäuwwoß¾=Ó|¥¥¥4ùÈüòA~çÎ?~|QQQx(..2eÊÕ«WÉOäOæÛ¹sgæ±Ý0'Ì×ÞÖ½ùüÈ/å×ÐÐõðL~"?æ#?òù_^É/ú$çÚÚÚèXÏõë×-[æTTTÈùÈüD~äWò^R_ÂtòSÉOäsµ··çWiii¦ù^íµ»1ùÈür^~Ñ>¿®®®äûüD~¹k¾4óM>ß¾wo>ò#?ùå¼ü¢÷ùÕÖÖðEÛÙ³gÈùÈüD~ä·G©ûÈ/çÌG~ä'ò#¿_Á­*,,$?_¯ºººÌG~ä'ò#¿ß`E~"¿n¾xZùüÈ/å×Ó6_ºtüD~q«­­mÍG~ä'ò#¿_xÍøÖ·¾6sÅ>ÕEä«Z[[ëêê6æ#?òù_ÎË//¼x]¹r%Þ÷¾½ôÓÛûÈOä»æ#?òù_ÎËï³ø¢hbñâÅý½èä'ò»3óafKKË`-ùÈür[~¡£G&_W¶mÛ6N~"¿;3_¸jpüÈOäG~¹-¿Ç<z]þGèÑG%?óùüÈ/ßäWXX^WFuúôéÏRÞçWTTD~"?æ#?òù_^É/¼´<ýôÓi3Ã+sE~ÌG~ä'ò#¿|_Oç×ßB"?_ô;i¾g6æ#?òù_ÎËo°"?qùßÿ´j	æOööö8/9ùÈürO~á5&õxnïÉOäÇ|äG~"?ò#?òù!óùüÈüÈOäwëóÀ|äG~"?ò#?òùÝÂ|,H;cÂ	7oÎ9óùüÈüÈOä½Ìý|Á|;wîìîîÎÝüÈOäG~äG~"¿_0_uuuþüÈOäG~äG~"¿_ØLL>=Í|Û¶mËóùüÈ/å×ä'òëAuûöí0aBæ~¾×^-oÌG~ä'ò#¿_Á­*,,$?__ÌlWYYi¾`Á<3ùÈürU~ù)×å×ÙÙ¹uëÖqãÆ¥oæÌöìÉKóùüÈüÈOCN~Á|Û¶mRûùÈüD~äG~ä§Û«µµ5À(¬÷D"£òK~uKKKÓÌW]]ÝØØ÷æ#?òùùn] Ñ×¾öµÏþó_üâ«ªªzZû±_ûÖ­[3÷óÕÔÔý|äG~"?ò#?òS_[·nÝÄ×­]·ááß²eË/òæ¿®%%%iæ=öÀÿÈüD~äG~qoôèÑðøDìþUWW¿øâ1ßåËzê©Ló-X°`¨íç#?òùùúTYYY*ûÂ¿¯|å+¿÷¿[ùóÙÏG~ä'ò#?ò#?ÝI9´Ï/oÕªUÃK3ß¢EÂoìÝÏG~ä'ò#?ò#?õµx_kkk]]ÝÈ#3í8qÂJ$?òùùúTtnoIIÉÌ3«ªªû³~ç È/2_Öý|gÎ±úÈüD~äG~ä§;Ö¾ûÚÛÛú_Vó+V¬¿-VùÈüÈüÔüzÙÏ×ÒÒbEùüD~ä§|_OûùÂÌ¬o=ùÈüÈüò¿r,Èj¾ÀAO~ä'òùòA~á­¦¦&íÀ.óùüD~ä§¼óùüD~ä§ü_Vó>ùÈüD~"¿~éêÕ«ä§_Vóßº^>PFäG~"?ßwìØ±ªªªðK~0ù1ùüÈOä78Í?¿¥¥¥ù÷»ßýÙÀö£ý¨©©égq?þñÞîàá×iæÌiæ5jÔ7¾ñðh`ïaÇ÷ÝwCÿËýáhâÜ3g<hbÞ[o½õÞïðæ°üþß=.lAï¿ÿþÅ¸@óþô§·uðT]]f¾ÏîsO>ùäÙ³gé=/l¦Cûà9bâëÛo¿mb^Ðù?ýÓ?ðæ³üíÕÝííîîÞ¹sçäÉÛu´Wö:Ú+GÍyQä§þ_d¾	&0ùüÈOä;îüüD~ä'ò#?òòËj¾ÊÊJæ#?ùüâù©òëîîÞ½wæy»AÁáZF~"?òùùå¼üê^íµÌý|ÌG~"?òùùåü¢ý|÷ß?óÈüD~äG~yÛûï¿ÿï|Ç~>òùÈüÈ/ëììÜ²eË¾ð4óM>Û¶máZCD~"?òùùåù¶nÝ:nÜ¸4óUWWïÙ³Ç~>òùÈüÈ/OÌ·eËÊÊÊLóíÛ·ùÈOäG~"?ò#¿|¨½½óæÍæ2eÊýÙòùÈüÈ/ÌW^^ù~¾ÆÆÆ¾ÿÝ^ÈüD~äG~±6ßóÏ?i¾3g8q"úò#?ùüÈüòÓ|³gÏæK?ùÈüD~äG~¹ÚåË×®];räÈ4óÍ7/üdÃA~ä'ò#?ù_þoÁ=ÝüÈOäG~"?ò#¿­­­¾¾>Ó|-:sæLï·%?òùÈüÈ/·Í·|ùò[üÈOäG~"?ò#¿Ü¨µµuÅÃÏ4_ö¾ßùÈüD~äG~ñ­­­mÕªUÃK_¸Ø÷ý|äG~"?òùùÅ½æææ~8«ùàîì>ÉüD~ä'ò#?òW­­­wæè¡ZZZîæÉüD~ä'ò#?òK'N¨««Ëº/pðîïüÈOäG~"?ò#¿Á¯¹¹yÑ¢Ei'pó^¸pá^=ùÈüD~äG~l¾¬ïç[½zõ¿üÈOäG~ÆüÈüâUXG³gÏNÛÏ7|øðµk×Þc»äG~"?òùùÅÂ|555iæ9rd0ß=<¶K~ä'ò#?ùß`ÖÐÐðÐCeîç[½zu[[[?:ùÈüD~äG~ô+>sæÌLó=ûì³ííí³äG~"?òùùõo=Û«iÀÌG~ä'ò#?ù_ÿ¶÷îéÓ§§¯¤¤dË-/_øå!?òùÈüÈ¯_~¡3íoÜ¸q÷óùüÈOäG~ä×/uwwïÜ¹3Ó|a¥$Á]<ò#?ùüÈüîM»wï0aB¦ù¶lÙ2èæ#?òùÈüÈï^öðÃ§¯´´tëÖ­ÝÝÝñYBò#?ùüÈüîMÍÍÍÑa7nÜk¯½6ïç#?òùq ?ò#¿~oíÚµ;wîÉ±]ò#?ùüÈünäG~"?òùùÈOäG~"?ò#?òùüÈOäG~äG~"?òùüÈOä'ò#?ÈüD~"?òùüÈOä'ò#?ùùüÈOä'ò#?ò#?ùüD~äG~ä'ò#?ùò#?ò#?ò#?ùüÈüÈOä'ò#?ùùüD~ä'ò#?ò#?ùüD~ä'òùÈOäG~"?ùüD~ä'òùÈüÈüD~ä'òùùÈüD~"?ò#?òùÈüD~äG~äG~ä'ò#?ùùüD~ä'ò#?ò#?ÈüD~äG~ä'ò#?ÈüD~"?òùüÈOä'ò#?ÈüD~"?òùùÈüD~"?ò#?òùÈOäG~äG~"?òùÈüÈüùüÈOäG~äG~"?ùüÈüÈOä'ò#?ùùüÈOä'ò#?ÈüD~"?òùüÈOäG~äG~"?ùüÈüÈOäG~"?ùùüÈOä'ò#?ò#?ùüÈOäG~äG~"?ùüÈüÈOä'ò#?ùùüD~ä'ò´¦MVXX8uêÔS§NÈüD~"?òË[ùM8ñøñãab×®]&MÊ_ccãl'OlkkûOÅ¸³gÏ¶´´8wþüù3gÎ8wåÊcÇ8H$üCÌòëèèàÍaù¥V\)¿_|ñÐÀvàÀRë­·¬£VPXMÆ!Î½ýöÛasgbÞ~ð`eò;yòd½£½r´×Ñ^9Ú+GíÍÛ£½Q×¯_¯­­M$ä'ò#?ÈüòJ~÷ýOÑÅK.ÕÕÕeÅùüÈOäG~"¿Ü_jsçÎ½zõjÖkÉOäG~"?òùåü*++ïKüD~ä'òù_ÞÊ¯÷ÈOäG~"?òùùùÈüD~äG~ä'òùÈüÈüD~"?òùùÈüD~"?òùüÈOä'ò#?ÈüD~"?òùüÈOäG~äG~"?òùüÈüÈOäG~"?ùùüÈOäG~"?ò#?òùüÈOäOòûæ7¿¹gÏÛ~ðþô§ã9þK`âØ8÷³ýì/þâ/C;wîÜ;CÌûó?ÿóÖÖÖ~ÐöööüßÙ³g7lØðÇ$Iúz?"z=±$IC$ò$I"?I$$IÈO$Iä'I$ò$IùruuuEEEãÆkllL½*ªª*,,2eÊ±cÇÕ ýú¦TPPzÕ©S§***¢utäÈcÏÕÔÔÔ4mÚ´°¦NV±á::|øp¸Ê@Åsõòj¥¬£X±Á3¹Ç6mÚôÂ/Ü¸q#¬°ñãÇ§^UZZzñâÅÏnþªÊÊJc5èíØ±ãå_NS[[f0Ù²e(«iâÄÇ»ví4i!á:uww _l×Q/¯VÉ:<,Àü>ÈzUxj]ºt)L¯fÞùóççÌ63<ÍÂv0LtuuÑylWSjÅÅÅF)ëèÛßþöw¾óòí:êåÕJ1YG±bgr¾ôÒKáÕ(¬¤÷Þ/õªS§Nùa;¾<yÒXnK,ÉaõeV¬VS²pU½Qá:ºxñâ3Âÿ£È/Î»^­u+6x&÷XAAÁ«¯¾&>üðÃ°áK½êþÖå>h¬±¬« õ=EEE*«)êúõëµµµDÂ@Åp±£G~vó=LF)¶»^­u+6x&÷XYYYêÿ¨ÒþÕÓUà6mÚþ³uõuuuvóhoêªT¬VÓg7ÔÕÕüñÇF)ëè¾_Ì@Åssç%)æë(Vlð4î±Çü7ÞgÏ­®®N½*=ÌÍÍÍòÆj5kÖéÓ§3ç/_¾üõ×_ákmm­çjjll;wîÕ«WQl×Q*R<×Q/¯VÉ:<ìÚµkK,	61cFKKKê¶ïÜ¹saÍ«Â×0m¬±¢¢¢èL´×§cÇTTT455¨x®¦ÊÊJûb¾È/þë(ë«bµbÅÏdI¤¡ùI$$IÈO$Iä'I$ò$IùI$ü$ID~$I"?I$$IùI$ü$ID~$I"?I$$IÈO$Iä'I$ò$IùIÊ¾÷½ïÍ5«øfsæÌyóÍ7aûu³ÙÚf[ÚÊÊÊð£%´ùaNaaaEEÅ7n÷>%ü$å^6l¸/£oë[ù$¿7Û¶mK¿uëÖ0ÿ¹ç»û$òc:u*¦°°ðW^é¾Ù«¯¾.§OÎù?>Ì:ujÚüªªª0¿µµü$¤üïÑG¦yáRg¾øâaæ+RÝsäÈ §ÂiÓ¦éä7úé§õõõ£FW®Y³&õ jCCCÐU¸*ÜöÐ¡CisF=kÖ¬Ë/O[°ô~?ÑUa¢«>ÜÒæÎæ755%ç?~<Ì©©©IÎÙ´iSyyy¸«âââ¥K^ºt)S~÷6§ED~4;6¨åâÅ©3?úè£0³²²2U6i<y2ºvñâÅiW=ñÄÑUÍÍÍYo]®]²dÉ7º»»ÃµákÓ#¢·ßõr?a"ëâeþ¤»víJål;vìH²/íNæÌs»òëeQ%$rÑÝ,Û¬ûîKÍ²eË®ß,L.® Ù1ÚuÄ]U[[í,ÓÇî$õ>~úé`»è`ëc=æìß¿?L¯aúë_ÿú-ï'¨1ßºlY¢ É1cÆ÷êÕ«ábø<p³««+úpÃð#|ö?GoW~½,ª$ò¤Á_(«üó#ÙD`]ºt)f.N6-?~üÊ+Ø:::w¾'m/ZòVÑÅ>ú(ùÍAKÉ¾áknhh¸åýeW®]¶Þ÷äO«¾óï|ö?G´×¬YúAA~Á£3fÌvIÞ®üzYTIä'I£ëúõë©3D®êÅ:I¶´´DøKB'ùæ¼Ì°iJý,0]ZZZTTÔÙÙî|ôèÑÉkoy?=9,­³gÏFHÓáknnnN^ìØ±°Y÷]~½,ª$ò¤A.z¯ÛK/½:3ú¬´3<ï¼zõjæ®¬Ó§OoÜ¸1:ö$c´C.y8õ>â'¢¼ákr~/÷ícûøã£W®ý<Ü|0MkêUÑy¾áªÃ_»v­wù%UFòÚ^UùIÒ !QXXøòË/GêòÊ+¯e±páÂ ÎÎÎèPlò<Üè~ÑÛãZZZRß ApÍ5ÁIÑY·ÉÏUÉê³èdÛèÑó¹èÑûüDô½ÈïÕW_Mî?rêUÑ[Ã£AèI~5Ã·q.]º4õÚ^UùIÒàÊqZ6múÛ¯E0JN'&OªHöØc¥²2µ½÷ö"¿Ïnþ±0¿´´4õ@p/÷#uÁçÕöôÃvttG?BÚ1îùóç§>Ä1cÂ×è]Rï3Ú%,rjòÚ^UùIR,:3gNÑÍfÍ`&¿ÃG7eÊ£G&¯M$ë×¯///´ôäOvvv&¯=tèÐ3È***¶oßvKî*ÌüñÇÓæ÷t?¡°0a¢ìåóüÕ××§¼KÔÕ«WkkkÃ5*ü­­­ÉO¨I½Ïk×®ÚFcUSSÓÔÔö½,ª$ò$IùI$ü$ID~$I"?I$$IÈO$Iä'I$ò$IùI$$IÈO$Iä'I$ò$IùI$ü$ID~$Iºýþ®¬¦¸²IEND®B`


611a±Xä¾²ñÇSSbÓ²²2y4Y/|ÿþýEÊoíÚµ²¾   ««Ëï¦éééôôtùò633SóñÂ¶ùÑ²àO-ç;ßå¦¦&1,¼ñÆ¾ÓÖÖ¦çÛä¦.È²¼åªª*uÓÔÏ;wîÈúììì æ»áï"÷ÝweÁjµnü¡CdY>à½÷Þ3gÎhPüøñàà ,¨)ÉPÇð2±ïÇzU[³yóæO>ùD»Úù»ûvYÞ³g,Ë ÛÀ³E^~f³Y§fWÐ;;;5îìÜ¹SN»u``àÄ%%%²>!!!è£ùÙ+ü]Ô¥¥¥Þ7++Kmüôô´,·d¥|¤,e<y²°añ«··WªùOY­?räö®êÑ£G³DDÈQ~ê¼Tm½¼;+Y)))òGíÐ5ê¦óçÏË½|òï#ø=ï»s¹K¨&úNÑ)5^¾|9==]­1j:pîs~aÆ­££C0ç»w[Ü,ôÑ£GOgv=Ëò5knÏ:"B~DyùeggûÎù©i³Yd±XäVí­¶^1=Óåþ.Ú	¤ï©îë·y^¯÷Úµkê²p^Ã*µ1²Í²¬QÕ¯oò#¢eºJËÔq~gÏ._¾¬AçÝwßÕÖ+G©ãÿæ"¿ðw9qâÄ÷¿ÿY8pà@à÷ïß/Ë/^VWeeii©,ËFGGe!//oò+**õíííO?½Ë+¯¼ò4ôndý·ùÑ2ÊÏëõ®Rfò½&_xùMNNª=­òÖ÷r'#£ÑvìØ±9Ê/ü]ÜºqãFíD¿k,9rD¶<11qÛ¶mêZÁVee¥:YxÓ¦M7oÞ×°îµX,²òÅfff:tH>ïÓO/jíûøÚE­¼DDÈùò#""""äGDDDDÈ!¿y÷ÑGÝ½o¡422òË_þqÐ¹ñs»Ýògtnrrò¿øã s^¯÷öíÛþýû¿ÿàEÝ_äûö·¿-øã	*ýó?ÿ³ºöéüÒpëÖ-ÆAçþó?ÿóO>atîÑ£G?þñòäÉßÿýß3úwíÚµÿùÿA~ÈùòC~ÈòC~Èù!?B~ÈòC~Èù!?B~ÈòC~Èù!?B~ÈòC~Èù!?B~ÈòC~Èù!?B~ÈùòC~Èù!?äGÈùòC~Èò#äüù!?äGÈùòC~üò#äüù!?äüò#äü!?äüòC~ü!?äüòC~ü!?äüòC~ü!?äüòC~ü!?ä·âä×ÓÓS\l0úûûòC~üò#ä·òËÍÍ½qã,t)///P~ò½yD	E!Îýd&ÆAçnÞ¼ùñÇ3:wçÎüÇdtnttô?ü!ã ï¿ÿþøøxìnËÏ·äää@ù;wîÇôã···øáÎ9ø`tNêògtNØ÷Á0:÷ÑG	AýkmmíêêÝíùõööVUU±·½½ìí%öö²·½½ÄÞÞ¸ÝÛ«¨¬¬ôx<Èù!?B~ÈùògùX­ÖÑÑÑÀòC~Èò#ä?òëììÜ²eËøøxÐ[òC~Èò#ä?ò3Í«|B~ÈùòC~È_ÜÊ/|Èù!?äGÈùòC~Èò#äüù!?äGÈùòC~üò#äüù!?B~ÈùòC~Èù!?äüòC~Èù!?B~Èù!?äüù!?B~Èù!?äüù!?B~Èù!?äüù!?B~Èù!?äüù!?äGÈù!?äü!?äGÈù!?B~Èò#äü!?äGÈùòC~Èò#äüù!?äGÈù!?äüòC~Èù1ÈùòC~Èù!?äGÈùòC~Èù!?äGÈùòC~Èù!?äGÈùòC~Èù!?äGÈù!?B~Èù!?äüù!?B~ÈùòC~ü!?äüù!?B~ÈòC~ü!?äüòC~üòcòC~Èù!?B~ÈòC~Èù!?B~ÈòC~Èù!?B~ÈòC~Èù!?B~ÈòC~Èù!?B~ÈùQÈoll¬±±ñðáÃÈù!?äGÈùòOùy½^ÉîÝ»SSSWÍt÷î]äüò#äüùÅüä×ÁéÓ§Ífóª_¯¶¶ù!?äüù!?B~ñ ?ù577®())Éb±tww#?äü!?äGÈ/¶å'¯ÿÕÕÕæËÉÉ©¯¯Î¯ù!?B~Èò£9ÉO¾)v»½¼¼<Ô$ÈÄëõFóWü!?äGÈùÑ,ò|Ít/??¿®®ÎívÇÄWü!?äGÈùQpùÍe/¶¾:äüù!?B~Èüåçt:CMòåääÄÐ$òC~ü!?äGÁóz½µµµÛ¶m[½zµødÅbq8Q~$òC~ü!?äG³¤&ù¯É§Mò¹8ø2ò#äüù!¿×ëµÛíñ:Éü!?äGÈùQ¸I>Y)ÌI>äüù!?B~ÈoåæõzG¨I>Y/Àø¯ÿú¯xýòò#äüù!¿Ëå<Ì:É·¿ÛüòC~ü!¿¨hÖI>¿#ùòC~üòC~È/öã$_Èù!?B~Èù!?ä3Í:Ég·ÛÃ®üò#äüòC~1Ëåª««ËÉÉ	:Ég³ÙNç¬üò#äüòC~Ñä³X,äC~ÈùòC~ü_´$|Èù!?B~ÈòêÄA'ù¤òòòyMò!?äüù!?B~È/s»ÝuuuùùùàËÈÈ°ÙleF~ÈùòC~Èù!¿§&ùBMòÉ-É'B~ÈùòC~Èù!¿È¤Ã$òC~Èò#äü"I¾ÒÒÒæææ¥äC~ÈùòC~ü_dr»ÝMMM¡&ùª««ûµù!?äGÈù!?äü½¾¾>«Õªÿ$òC~Èò#äütÊãñê"8Éüò#äüù!¿eoxxX`ÙI>äü!?äGÈù-ãÖÔÔ´iÓ¦Àë0«I¾¾¾¾nòC~ÈòC~Èù-Í/¬£ÑtO8èv»#¾Èù!?B~Èù!?ä·¨¯åÊ+ë×¯u$_d'ùòC~ü!?ä·Éëdmm­ð.'ùòC~ü!?ä·¨·Ûí[·n_RRÕjªI>äü!?äGÈù-¤ÁÁÁ |ùùùÑ9Éüò#äüù!¿ùmmìNò!?äüù!?B~ÈoNÛl6³Ù»|Èù!?B~Èò×ëµÛí;vì¼&_RRÅbÝßÑÈù!?B~Èù!?ä÷×ÖÖL¦ |uuu17Éüò#äüù!¿_Ëëõ¶´´TTTÄß$òC~Èò#äüþ/y­³Ùlq<Éüò#äüù­tù©ÓuËËËÁ·zõêxäC~ÈùòC~üV®üN§ÍfzM¾ºº:ËÇ#üò#äüòù©ÓuÉ§&ù|LÜ<òC~ÈòC~È/å§&ù^o%Lò!?äüù!?B~ñ/?&ù_Ô5>>.ÿA~ÈùòC~Èo	s¹k4è$¬VÔ$òºººäYüò#äüßâóz½#Ô$¬_|È/ZÚ¶müf#¿óçÏ÷Q_ü ^¿~qÐ¹?ü°³³qÐ9yªËqÐ9aßøCÆAç>þøc!ÈR=<ÔÿñgeeNòÉJ¹i	?W¬×ÚÚÚÛÛ»ÛÃòû¿M-¿¿û»¿¥ÑÑ7nüë¿þ+ã s31:744ÔÝÝÍ8èÜíÛ·åÿ9ÎÝ»w¯­­mrÿþýïï_úÒNòÉz¹U>Ñöíý÷ßÝígù±·½½ìíeo/±·½½A$Ífs:2ò#äüùÅ°üÔ|%Ô|v»#ùòC~ü!¿ØËåª««ËÉÉaù!?äGÈùò[ùÉoÃ;v0ÉüâD~¡B~Èù!?B~+Y~ccc¯¿þzÐI¾&ùòC~ü!¿x_ww·ÅbIJJ4_yy¹Ýnû2Èù!?B~È_ËÏív×ÕÕ=ÿüó¡&ùxQB~ÈùòC~üb^~UUULò!?äüòC~üâV~ßûÞ÷^ýõ^x!|F£±ººZ^ÿWìø¸úúúÃËÛ»wï"?äüù!?B~±Zww÷Þ½yæ@ómÝºÕn·<>>ùÌg^|ñÅ^z©´´TÈù!?B~Èù!?=s$Ñh¬©©d<yòì³ÏîýêÞ¯TÿDÉ?=äü!?äGÈ/s$_iiiss3×äÓêîî.üÆ>õO¸ÜÙÙüò#äüùEon·»±±qýúõàöÙgwïÞÍ$_`­­­_Üè'¿_|ñÊ+Èù!?B~È_¾tWWW§¦¦O ØÜÜüðáÃüÝÞÓéLIIùÆohì;Vs,33sxxù!?äGÈùò¢<OKKKiiià[ËÈÈ¨©©Ñ^XæûwWTò'ò;¿ó;´ï_·~=77×jµ.Õ#?äüù!?äüÛðð°ÀÎh4äë­·üNP@~áû«¿ú+ß3Ï<#ü¿ø%<ù!?äGÈù!?ä·ÀÄsMMM¥¥¥AO×­ªªu^òTÈù!?B~Èù!¿y×××W]]tO8þ|Èù!?äüòC~È/Ú±577äVUUuwwÏe×$òC~Ñ%¿U³üò#äüVNòj/??¿©©i^F~È/ºä0[ù!?äGÈùÅa&ù¬Vk__ßÂù!¿(_ÄC~Èù!?B~mi'ùòaù=~üøÀÈù!?B~È/þÙíöòòò¥äC~È/fäg6Çù!?äGÈùÅwòSo³ÙNò.rù!¿Ø_QQQÐk?Yù!?äGÈùÅJa&ùDÕÕÕK2Éü_lÈ/11Qúããã&I|ßÿþ÷e¡ªªù!?äGÈùÅtzNò!?äòS?² Ô;wîLOOËBJJòC~ÈòÅ¼^¯þ|ÈùÅüÒÓÓå'¡««K~ÊÂ©S§ÔWuA~Èò¹N§Íf3ÍúOò!?äò;vìv>ßOòC~ÈòÔ$_EEÅêÕ«#5Éü_lÈOzíµ×233e¡§§GD%%%Ë½éÈù!?äGÈoñEÏ$òC~1#¿üòC~üÜ¬|ÿG~Èù!?äüòC~ÍårÉoP|ÍÍÍB®hØNäü¢W~yyyêÚ.ù!?B~È/:óz½#'ùòùåææújOsò#äü¢¡0|ë×¯I>äübC~<ùáéííÖsÓòC~È_ÂLò%%%©Óuåc¢vûòRùFù)ÒÈù!?äGÈ/T.«®®.'''è$_ccc¤N×E~È/ä×ßß/?K@~ÈùòC~JMòY,ÀI¾ÔÔÔûöÅÖoäü¢T~Ò5kÿ_ÅÈùòC~úfOVFð|ÈùÅ¡üÖ­[ÇÈùòC~úf/))i÷îÝòk"äC~È/&å§~ÆuÞtäüò£+?·Ûj/??¿¡¡all,Öù!¿(_VVgx ?äGÈùé¼ò[,¤¤¤ÀI>Yïp8bwù!¿Ø_gg§üÈ;vLçë!!?äü­ù©I¾üüüÀI¾µk×ÊM±x$òC~1)¿U!âäüù!¿Å×ÝÝmµZ'ùV¯^½cÇxäC~È/6ä"Îð@~Èò[pccck×®Y0Luuu.+¾ù!¿(_¤B~Èù!??ùy½ÞÖÖVÅ8É·÷n¹5^'ùòùÍæuëÖ#?äüù!¿çr¹^ýuÉôtÝººº88]ù!¿xÁ`Ký7ù!?äü(äçõz¯òÕ¯~5è5ù,Ë©G~È/Jå×ÑÑ!?§O~üø±×vA~Èù!?iù¹ÝîÆÆÆPx#.O×E~È/äÇ¹½ÈùòC~óª»»;è5ùV¯^g×äC~È/åÇ¹½ÈùòC~s¤LSSÓúõëCMòÅýéºÈùÅü"òC~ÈùQ¬ÈO^¦öíÛÁ$òC~Èù!?äGÈ/>åçv»^xáÀI>³Ù,/ãLò!?äòÚ¶m[rr²ü0§¤¤ìÚµKS=òC~È¢V~N§óðáÃA'ù***äC~È/å799ôåþ3¾Èù!?äGÑ&?yåoii)--¼D|ÈùÅüäGzûöíòîãÇwîÜ)k6lØüò#ä·Bä'?µµµLò!?äÿòKLLmßé©©)Y#ëòC~üâ[~¢æææÒÒRäC~Èo¥È/!!A~ÂE¾OVYÃU]ò#äÇòçuuu¨I>»ÝÎ$òC~ñ)?µ·wË-jo¯¼eYS\üò#ägò?Ég³ÙN'#ü_<ËO¨ôy¥@~Èùò<¾öµ¯åææ¢Î=Í3dJ~Lò!?äüþ·ÉÉÉ]»v¥¥¥%$$ÈÛ-[¶ÈåÞtäüòÝ:;;ÓÓÓ¿üå/øþðå?|î¹ç¬VktnªÛíþË¿üËÏîsLò!?äü"òC~ÈùÅnýìgwîÜyòÕêß±cùÌgúúú¢j#CMòIåååLò!?äüòC~üfÏãñÆµ'4ùÉ¿M6544DÃæ©?¼ôH>Q ÍfãüßJßªÙJHH@~Èùòúý·~ë·üä÷²/466FvÃÂLò­_¿Þn·/÷%ú	ù!¿è_BèòC~üÂ÷»¿û»_þò5öãÈ7RRR#²1³Nòöw	ù!¿ø_¨=ª^/.]ºüò#ä4yòÆ7~ík_Û±Édú³?û3ý7#Ì$@°¹¹YMò-àïöòC~ñ/¿þþ~ù?«:ò×÷ÂÎÈù!?B~=|øðÕW_ÝºuëüÁ´´´èù©Õ$_~~~ÐI>± ßsù!?äüüÛ³gzÕñÒgÓòC~Èæ[__ÕjMJJ?ÉçòC~Èùýª÷ÞO½jlß¾]ÏMG~Èù!?cn·»¾¾~î|Èù!?ä¤ÉÉÉ6¨9:::tÞtäüò£Y¬ªªJMM×$òC~Èùù÷æoª×½÷FdÓòC~ÈBåv»ÃLòÍëªÑÈù!?äÇõüòC~È/ëîîØÆ |MMMÂù>&òC~Èù»Ê`0 ?äüùé¦æææç>|IIIe1/Èù!?äùòC~È¤ááaÍf6ÍS__ÿðáÃE~äüòC~Èù!?äÉ¼^okkëÖ­[W¯^í>Y£&ùäcäs!?äüòC~Èù!¿È$O¼ÚÚÚ Gò­]»¶®®Îår-ígD~Èù!?äüòC~z; ¥¥eÇA'ùvïÞíp8jù!?äüò#äü"ÓÝ»wm6ÉdÒmù!?äüò#äütÍëõ¶µµUTTä³Z­Ë4ÉüòC~÷äz~Èù!?ä·<9ÎP§ëæçç×××ß½WÏíA~Èù!¿_»_Pùq=?äüùÍ÷7½Ýn///zM¾Ý»wGêEù!?äü~U»¼*½üòËò®¼Ý¹s§¬¹~ý:òC~ÈßçÍfËÈÈzM¾Ó§O/÷|ÈùòkêÊ¾ÇLMMÉÉ´ÈGîïïÏÎÎ6Èù!?3ùävìØ¡Û|ÈùòóCÌ$Úóßâó«¬¬¼xñ¢,;wnïÞ½ò	y<?ûÙÏûù¡¡!ÆAçnß¾-ÿ'/D<ßüæ7ÓÓÓÁ÷ÙÏ~öÕW_ýùÏ=[ër¹®_¿ÎÓOçÜíííþü~ùË_Æîö/»ü²²²ä¥J699)ïÊ§Ü¾»¬õMV4Íò«¯¯ÿ>üð>3:÷Ã§º<ácwûÇ©S§^|ñÅÀ#¤eÍ¿øÅoë[ò1Q¸Ùò»§Îutt´¶¶2ú÷ü ¦·Ùå'ÿºbñd|Ï	<_½½ìíeo/c¨0§ëæääèpM>öö²·ØÛût©®çwçÎÜÜÜäädù?kJJJqqñÿ°¾ûòC~sòóz½v»=Ô5ù,Ëòýáäüù-ü©¬¬,uø ¼ÜwüòC~ÑËå© |²RnæI>äüò@ò¿á·ß~[ämee%òC~È¢_~^¯×ápäõ11ÉüòC~áÊËËKIIQûg³²²._¾¼øíêê2LòÙÙÙ===Èù!?fùÅÓ$òC~ÈùL]ÉÙ÷/¶©åsçÎ-ë¦#?äü_4|Èù!?ä2É$/pCCCüzzzd9--ù!?äGq,¿ð|6ÍétÆÇ°#?äüÏCÌ¤ü¦§§ù»½ÈùQ¼ÊOMòY,P|v»=Ö'ùòC~È/dêJÎjOä755uüøqõ_^äüÅüW]]]NNNÜOò!?äü_È:;;^ÉùÆÈù!?ù­ÀI>äüò×èèhII:·7999//Oÿø"?äüßrf/###'ùòC~È/êB~Èù!¿åK^^NòIåååv»]~+¯aG~Èù!?øôÄ­äåå­]»ù!?äG±%?·Û]WWjoe~ÓòC~È/ü¦¦¦8·ù!?-ù©I¾¤¤¤@ó¾õÖ[+jù!?äüü[»víª°¥§§#?äü(ÊåfÏh4ÖÔÔ3ìÈù!?ä÷ôÞ½	3i½Ã7aßï½üò£¨___Õj:É·~ýúø>]ù!?B~L·Ü;vòC~´Tòs»ÝMMM¹¹¹Aä«®®dòC~È/êB~Èù!¿y¥&ù®Ë|ÈùòkSSSyyy)))ÚÌÌÌï|ç;Èù!?ù©I¾ Gò%$$L&äC~Èß<Z³fßé½ê%õôéÓÈù!? üÂÉõ[¯æhÍoÿöo;ù!?B~s-11Q^Féïï5iiiÈù!?Ò_~j¯´´4|¿ñ¿Q²¾äëÖ¯|õ¤ú·yóæúúzù!?B~sMÞ;==ýkp?äü-·üúúúª««322®û×ý×Çjiìê?ù!?B~s-;;[^U9¢<yò¤¬1LÈù!?ÒA~òâc·ÛËËËÁºoß>¡ºDËK/½TVV¦±ïëÖ¯Ë¬¿½ü!¿¥_oooÐ+9÷ôô ?äühYå'ß£Ñt¯±±ÑívûÞllÌl6®às_ÜôÅôôô/2ªÈùò_###ÉÉÉ			)))²f¹7ù!?ä·bå§Mò^¢%##ãðáÃa®É'lhh¨ªªúÓ?ýS.Ýü!¿	ù!?ä·kkkÛ½wjjjà$_~~~½ß$!?äGÈù!?B~±÷+°©©)è|IIIV«µ¯¯QB~ÈNòRsVçófee]¾|ù!?äGoxx¸¦¦&èéºùùùÂA&ùò#ä§«üÚÛÛ/ÿôÓ+9;wù!?äGþ×ÚÚºuëÖÀ#ùyæÅÂ?òC~ü"#?É$¯ÅCCCüzzz¸3òC~´°Ngmmm¨I¾'Oòü!¿HÊO½"?õ¹zóôô´,äüÍ1¯×ÛÒÒ²uëÖÀ?¶&k´I¾üÝ^B~ÈßRÊ/++K]½OÉojjêøñã²l6òC~4kÃÃÃµµµA¯ÉSWWç$òC~È_å×ÙÙôJÎ7nÜ@~ÈùQ¨¼^o¨#ùdÅbq8êoøüò#äaùI£££%%%êÜÞäää¼¼<þòC~È/Fs¹¿þºÙl5É'ê¾Èù!?B~_DB~ÈùÅV^¯·­­­¢¢"ðH¾0|Èù!?B~Èù!?äK9NÍ¶°I>äü!¿¨ßíÛ·×­[(¯ãÉÉÉãããÈù!¿×ëµÛíAäõsäC~Èùò.ùÉkwÐ3<òC~+3Ë%?¡A'ùd¥Ü4÷I>äü!¿èºsee¥Çãw'&&öîÝ+k²³³òC~+*¯×+ÿI>äü!¿èzY÷5eíªÎÈù!¿ÐòMò!?äüùEüÔßÔÔ¶frr9?äüVB:Lò!?äüùEüÔq~>õ±iÓ&óC~È/¾s¹Ë=Éüò#ä]ò[5[Ë´Ûù!?ä§jÏb±Ìëo ?äGÈùÅüfË`0 ?äüb½0|ó½&òC~ü_Ë/R!?äüt(â|Èù!?B~Ñ%¿PmA~ÈùÅnn·;&ùòC~ü¢K~òkàµ×^ó[¹oß>®êü_&?Ye1]ù!?B~È/nå'Â_	YYY<wßyçõKbïC~Èù-ScccuuuùùùQ5Éüò#ä]ò:3Ã§~=dgg«;w.÷¦#?äüªîîn«Õx$_RRRÄ'ùòC~ü¢N~Òõë×µß:l:òC~Èo¹Ýîõë×Nòåçç×ÕÕÉDÛ6#?äüùE^~T¿-Ôßó^~ùeäü_Ôæp8ª««FcÐI¾hþÉB~Èùò°üüÂHKKxês_bb"òC~È/ª3É'7Eá$òC~È_tÉO~g8qÂo¥ÕjåÜ^äü¢§îîîªªªÀÓuwìØÑÖÖ=Gò!?äGÈùEµüB]Ïottù!?äñß¡&ùL&ÍfÓuò#äübI~ù!?äþY±oß¾ÀI>©¢¢ÂápÈ/Xväü!¿ÈÈO~øîÏÿ.òC~ÈOÔÞxþùçNòÕÔÔÄúùÈÈù!?B~Èù!?ä÷¿O xCzáZZZ<O;òC~ÈòC~ÈoåÊïáÃMMMb»@ðÆêêjyzÄÓ°#?äüù!?äüV¢üNgÐÓuÕ|---Ñäüù!?äüò×ëµÛíA'ùâãH>äüù!?äüò*®ªª*ðoH6mjnnÑÓuò#äüòC~ÈïW¯þ¢ºÒÒÒ9Éüò#ä-òòC~ÈoÉãWWW=¯¼¼|Lò!?äüùEüfË`0 ?äüöro·ÛÅvà[»vmmmíäC~ÈùòùE<äüâO~ò6-è_×­¨¨ÆÊ_×E~ÈòC~Èù!¿¯ï¡&ùÌf³XÐét2àÈù!?B~Èù!¿_+W®È[&ÆD~Bº |êH>&ùòC~òC~Èùù766öùî¹çä­Ùl^¿~ýÃ£Y~ê|«W¯ö(P,¸äC~ÈòC~ÈùkÃ_(ûÂÚ'_=)ÿJKKUÑ)?5É'<5É·O×E~ÈòC~ÈùÍãËî¹ç4öÉ¿ãß<966=òcù!?B~Èù!?ä·µ¶¶~~ãç5ö©yyyÑ ¿0|¥¥¥+ó|ÈùòC~Èù!¿ùQ8ççõzG¨I¾êêj&ùò#äüòC~©¸¸Øï8¿ßÿýß_îOJ~.KLò!?äGÈù!?äü¥±±±6<÷Üs/¾øb¤Îíeù!?B~Èù!?ä§ë8r¥¯¯Oçëù1Éü!?äüòó¿ûÝïä[îSL!?äGÈù!?ZöÔ$ß³Ï>t¯©©Éív3JÈùòC~Èù!¿.Ì5ù,OHäüù!?äü_Ìæ|999õõõ:PBÈù!?B~Èù!¿e,Ì$¬yé¥þæoþF³Iù!?äGÈù!?ä·ä[»vm]]ËåZØßí%äüù!?ä²ññqùíü>äÛ½·ÃáÐ&ùòC~üò[Êºººä.òC~ËÝùüîüò#äüßR¶mÛ6ùÍF~ßýîwJ?ýé~ØÝÝÍ8, þþ~Q]YYYBBødÍ¾ô¥sçÎ	¬Þ÷úL¡ÎÝ¸qCðÎõõõ	A!ã ­­­?ùÉObwûcX~ÿ·¡åg·ÛïÒÝ»ô<Gyõ£ýèàÁ¿ù¿8Ég2=*ÿ½½½òëÔ9yªwuu1:7<<ÜÑÑÁ8èÜÏ~ö³¶¶6ÆAÿÞÿý;wîÄîöÇ³üØÛËÞÞì=ÿ0¯VEEüWo§ë²·½½ìí%öö²·½½Kà<òC~KÛØØØéÓ§322NòÙl6§Ó9¯D~ÈùòC~ÈoY,üßbr8III¡&ùäåuüò#äüòC~ÑËåª««ËÉÉ	Ñh´ÙltòC~ÈòC~úü_¨ÕjuM¾%ùÃÈù!?B~Èù!?ä±WÐI>Yô|ÈùòC~üò±:;;¿úÕ¯ä³X,¾xù!?B~ÈòC~1ÙÝ»wëëë×®]8Ég6å²´|Èù!?B~Èù!?äÇ;NòUTT,Ó$òC~ÈòC~Èùé×ØØXýóÏ?I>äü!?äüòÓ£0§ëê6Éüò#äüòC~ËËåjhhzº®Ùl^ÀÞ@~Èò#äü_ÔætÝ»Ý®ÿ$òC~ÈòC~Èù-eáO×ì$òC~ÈòC~Èù-A^¯×ápX,häC~ÈùòC~Èù!¿Eæ¯ëfddDÕ$òC~ÈòC~Èù-¤0|Ryy¹ÝnW½hþòC~üòC~Èo>|ØÔÔô|j/V8üò#äüòC~!ëëë³X,©©©æ+--ë­·¢|ù!?äGÈù!?äüféáÃùùùà3555ÃÃÃ±8ìÈù!?B~Èù!?ä÷«ÂLò­_¿¾¥¥%ÚN×E~Èò#äüßü3ÉQ]]=88Ãüò#äüò[Ñò³#ùò#äüòC~ÈÏ?ÇÓÔÔj/väC~ÈòC~Èù!¿_ÕÝÝ]UU%¼z$_cc£Ûí×aG~ÈùòC~Èù­ùçÄvñ$òC~üòC~ÈoEËO>WI¾æææx:ù!?B~Èù!?ä·¢å×ÐÐà¾ÔÔÔûöõõõÅô%Zò#äüù!?äçËåÒþÒnÜÉü!?äüò[Ñòªªª>¼äC~ÈòC~Èù­tùòC~ÈòC~Èù!?B~ÈòC~Èù!?B~ÈòC~Èù!?B~ÈòC~Èù!?B~Èù!?äüòC~Èò#äü!?äGÈùòC~Èò#äüù!?äGÈùòC~üò#äüù!?äüò#äü!?äüòC~ü!?äüòC~ü!?äüòC~ü!?äüòC~üò#äüòC~Èò#äü!?äGÈùòC~Èò#äüù!?äGÈùòC~üò#äüù!?äüò#äü!?äüòC~ü!?äüòC~ü!?äüòC~ü!?äüòC~üò#äüòC~Èò#äü!?äGÈùòC~Èò#äüù!?äGÈùòC~üò#äüù!?äüò#äü!?äüòC~ü!?äüòC~ü!?äüòC~ü!?äüòC~üò#äüòC~Èò#äü!?äGÈùòC~Èò#äüù!?äGÈùòC~üò#äüù!?äüòC~Èù!?B~Èù!?äüù!?B~Èù!?äüù!?B~Èù!?äüù!?B~Èù!?äüù!?äGÈù!?äü!?äGÈù!?B~Èò#äü!?äGÈùòC~Èò#äüù!?äGÈù!?äüòC~Èù!?äüù!?ä·Tõôô¢¢¢þþ~äü!?äüùÅ­ürssoÜ¸!.]ÊËË_ggçÓÿ÷?ýÓ?Ý½qÐ¹û·»yó&ã s¿øÅ/ðàAWWã sGäÇ8èÈOØ»ÛÃòó-999P~gÏm£¶¶÷ß_¦þÃ.1:'Ou]ÿ>øàÖÖVÆAÿaÿÁ~À8è_¬<È¯···ªª½½ìíeo/±·½½ìí%ööÆíÞ^ÕÄÄDee¥ÇãA~ÈùòC~È_oÕ§©wGFF¬VëèèhàG"?äü!?äGÈ/¶åç[ggç-[ÆÇÇÞüòC~ü!¿øÙl^åòC~ÈòC~üâV~áC~Èù!?B~ÈòC~ü!?äGÈù!?B~Èò#äü!?äGÈùòC~ÈòC~Èù!?äüòC~ÈùòC~Èù!?äGÈùòC~Èù!?äGÈùòC~Èù!?äGÈùòC~Èù!?äGÈù!?B~Èù!?äüù!?B~ÈùòC~ü!¿xß·¾õ-»Ý~îÞýàzëììüÑ~Ä8èÜÇ,OxÆAç~úÓþíßþ-ã sÿñÿÑÜÜÌ8èß[o½õóÿ<v·ßívÇ§üN<ùm""""ú´ðDW1©KDDD´BB~DDDDÈ!?""""B~DDDDüùÏ÷É§ßFGGo5ýýýÙÙÙ¡°°°££Ñ[a¼)ðA®³³³  @=»ºº|o|zó×aØzzå¦¢¢"pðú;/ïö¸yçÇ5Æºxñâ¹sç|×|Ùjµú®©¬¬ùÈ½÷2hË1ì7~#hÁÆ÷îÉ¼5ÍáÞ<áuöÜÜÜ7nÈÂ¥KòòòxÂë3ì¼¼GdØãþåùÅRwîÜÙ¼y³ßJy:^½zÕï	===-SSS¡Ð´Èa¼)ðAnÝºu###² oe9üÓ'¼Ãî[rr2Ox÷>ÛãøåùÅR»víêííõ[)ÿß²eÁ`())¹yó¦¬eíVßeZÂa¼)ðA®¿¿_x±jÕ*yë7øOoð:»¬¯ªªâ	¯Ï°óòÁg¿¼#¿éÖ­[7nóò¢¢"Yð="-11¡[¦auö ·aÃááaõêì7ÈOoð:»jbb¢²²Òãñð×sØyy×ØãûåùÅLgÎyóÍ7Ãz!ÈÊÊz:³;@ºeö07ñ¼ÈÂLl>½yÂë0ìêwÕjå	¯ç°óòaïwä3®ÏÍÍu:êuyË-²`±XÞ~ûmY·òtn9=ð¦Ào-8ùßöÐÐ,ÊÍo|zó×aØ;;;åY=>>Î^Ïaçå="Ã÷/ïÈ/fÿg¨õÍ9·¼··W¾iÓ&u¼jWWÉdJHHÈÎÎîééaècØoüFÐ»û¶¼Ë`Ê[YöùÀ§7OxÝl6û]Õ'¼ÃÎËD=î_ÞÑJ	ù!?""""B~DDDDüùò#""""äGDDDDÈ!?""""äGDDDDÈ!?""""B~DDDDüùò#""""äGDñÓ;ï¼SVV<ÓæÍßï½_ý)f^mm­Ùl/Íãñø­5!;;zzz¾IDü(ö:yòäª^íµxßéÓ§eecc£ßúYêÔ©<&ò#¢«¿¿_Lc0Î?ïéÂò®¬ùÝ¹sGVù­/((õN§ùò#¢øïå_Ó¼ñÆ¾+Ï=++÷íÛçë °¸¸Xµ~ôèQUUUZZÜd49â»SÕáp®ä&¹o[[¥dMzzzYYÙµk×ä]Åâ·a­­­áGÝ$¤njoo¥´-[¶ÈúmÍ7dMyy¹¶æÌ3&I*99yÏ=###ò||¿5a6Q$[³f¨åÞ½¾+ïß¿/+Íf³¯lüêííU·îÜ¹Óï¦C©ÞK½«nÝµk×ôô´Ø111ÑëõÊ­òVä¢¿ó8²tó¿ÒK.ùrVÃåÅ5öù=ÈæÍç+¿0JDÈ(Â©»A^³V­ùÊfïÞ½3É¼»ûvu«²£º±©*++Õd¡,wuu©ñÌ'NíÔÎÖýû÷Ë«W¯Ê²¼åW^yeÖÇ5Ê»ò¾Ûô+MfffÊ×;>>.ïÊ[ÙráæÔÔúììl¹£|	O?Ý;<_ùÙT"B~DDT~Úz%&iddDÞ3©wåÝuëÖ8p@Ä699©=|ß,v/õîýû÷µ-i;|å­,;YG)ï>xðÀwÛBwôèQ¹éÏÿüÏ~ºGûÈ#¾ ùGKJJÔä|åfSùE85Ñ511á»ÒãñÈJ¹)u4ÞºuKáOvp^àNX?Kù^KEFcbbâ'OäÁÓÓÓµ[gPókhhH!Uå­,j·vuuÉÝk<wùÙT"B~DDNëöæoú®T×:ñ;ÃC;p||<p*k``àôéÓjß«FF5!§íNÕgR;yåmUU¶>Ìã¨9¶ÑÑQõîÂ»qãF¹U]ÈFÀê:ÏWnjooüøqxùi*U£¡ÝfSùE8uÁ`8wîºªËùóçÏÆØ¾»(çÉ'jW¬v®:ÎOwëÖ-ß9"NRgÝj×U	ê3u²­úìÚú0£ìSÇùy<õaäwáÂm6N¾dßÔ!òYdBÉOYS¬,&âÜ³gï­a6QäSW9öëÌ3¿zýIÁH[Ööj'Uhíß¿ß¾]¾|9üÎü±Yo4wyÙßÓÎ«õÅNNN&''«/Áo÷¶mÛ|?Eff¦¼UvñL5%©¥ªÝfSùEE¢Í7'ÎTVV¦N°õ_»ºl^aaáõë×µ[=ÏñãÇM&ÒÒÑ£G<y¢ÝÚÖÖVRR" ËÎÎnjjòÌÀ-õô[êq$ÙÙ$u¡Á0×óÓªªªò»¼j||¼²²RF --M¾§Ó©]¡Æ÷1?~,´UcU^^ÞÓÓã÷Ãl*!?""""B~DDDDüùò#""""äGDDDDÈ!?""""B~DDDDÈ!?""""B~DDDDüùò#"""¢ù÷ÿüñ?]oIEND®B`


ûö¹M×ü!?ägÎÏùíÚµKyÈÍÍU¶nÝº÷ùòm­­­¥¥¥úéZº»»ú[#?äGÈùÅü¤7n_-÷^!?B~3¯££C·Óµ ?äGÈùS~÷îU^-¿ç!=óÌ3ÈÁóù|;wîÔÝµÙl---Opè.òC~ü©äg±XäÕ"33³··÷ês~)))È1­©©ÑO×²Cwò#äüL%?yÁ8tèf¥Ûífl/!?ä	¢º*¡»ÈùòC~¦ßtóùÝ¹sg×ÜÓÓk±X5®+W¿n!?B~-566Úl6ýt-CCCxÃò#äüâI~Óé¼pá,>ºªªJVss³¼äGÈO×ë]µjfÇ-[ý¶!?äGÈùÅüäeC<7úÉ¹=99©¼0Øl6õYÂ¾Ë/Gß©S§Þaï½÷Þo¼ñ>µë×¯Íó¾ç;ßY³fÆ|_ÿú×Ï9c[xãÆyéâ§Ô°½ýöÛ­­­lÃvåÊ6aç<büMãX~ê¹ 5óBçççWTTÈÊ½¦E~o¾ùæDûùÏ.îµ®®.ù9I´-¿lÞ¼Yc>yî³u;ñ_x½^~J[__ßÀÀÛÁýÛ¿ýÑÑ¤®¿¿ÿG?úQ¿iËOÓ)..æh/q´WÝøøxuuµfè®Õjõx<±®£½í%ör´£½¿Úð yIP^dyºèQü(aåôuWN644Ä~ºäüù!?ä7×ùóçeAþw:ê³òóó?$:22RQQüùB!ÇM=<]KuuµaÍü!?äüþ«öövyëÉÍÍíìì_³üßÕÕUPP`±XÊËËÈY~ùòòò4éßûò#äüß¼B~8òõ¢¢"ù¶oß®¼)!?äGÈùÅü¢üù-hÝÝÝCó¼+--íèè¯;ü!?äòKzyX!¿'Ïçs»Ý¡»EEE^¯×Cwò#äüÌ ?#üÈ¬òóûýúéZòòò9]òC~üòC~üæh>ùÁÖO×"+@5äüù!?äüùýºP(ÔØØ¨1_rrr]]Á§kA~ÈòC~Èß,òx<v»]c>·ÛGCwò#äüò#ä÷äg¸¼¼t×ápÈË°Éäüù!?äü(qåqºYãjäGÈùòC~Èù-`§k±ÛíMMM&~tò#äüò£Ä_ÄéZ¬Vk×ü!?äü!?­ù¦®ÅCwò#äüò#ä÷ùt-Çjµuºäüù!?äüù^"L×ü!?äü%ºügºäüù!?äü(qå'¯ úéZÊÊÊÌ:]òC~üòC~ò8]K"ÝE~ÈòC~ÈE~ãããµµµ	8]òC~üòC~(ò/¿ür"O×ü!?äü_~¡P¨±±Ñf³iîVWWc>äüù!?äüÈ<ò½°fºiË-	8]òC~üòC~dZùuwwWVVêÍ×××ÇCü!?äüD~òÒ¸sçNÍÐÝ¼¼<¯×ËÆG~ÈòC~ÈL"¿ááaýt-b¾sçÎ¶<òC~üòC~dùùýþºº:Ít-©©©òãùò#äGÈùIäO8¡®E1ßøø8[ù!?B~üA~¡PÈãñäååiqìÚµkhhíü!?B~ÈL"?¯×»fÍùCwò#äGÈùyä'¶áiÌ·jÕªþòC~üù!?S%ó¯ÿú¯<¿jvÎòr¹¡»b>ÙÕAvjÈùò#äüLÕ±cÇzê)ÃQYY¿víÚxüûcsÏçÛ¹sgjjªÚ|V«µ±±÷ùò#äGÈùP~Ë-«þ_ÕÿÏaåß+ùoñanù8p@ó>°®®éZò#äGÈùV~/¿ü²Ãá³OþÕì«yúé§Í*¿`0(wY3]Ðív³C~È!?ägfùíß¿ÿ[ßüZ~ò/--Í|òBçÎ³Ùlaýýýì¼òc; ?B~ÈÏüòåçç«Ù÷ß·þ÷É¯µµÕn·kÌ·fÍþê.ò#äüù!¿_0­]»ö/÷þå¡ÚCßúÖ·¾üå/·µµF~òs¢®Eî²ÜGq ?B~ÈòK,ù=úë´þçþôÓOgdd|ãßèîîÇ¡Ïçs¹óÙl6ÇÃt-Èò#äüT~æH-¿ÑÑQ·Û­ºuüøqî"?B~ÈòC~&ßïýÐÝºººx¤ùò#äüò£õööþÝßý]ÄéZ|>ÛùòC~üò3Iç+_ùæ#C^ÏØ8Èò#äüIòòrùÊÊÊbüãAÈùòC~ÈùÑæóù*++5æ³ÛíéZ!?äGÈù!?444¤ºû;¿ó;ßùÎw0ò#äüù!?äg¡»éZdeûLþn/!?B~ÈòC~F/?~t·ººZ®å±·!?äGÈù!¿80ÇãÑüÕ]ýt-ÈùòC~üòïäQ.**Ét-ÈùòC~üâ@~ccc6M¿¾§§'77×b±êÉüL_ww·oæÓµ ?äGÈùò3ºüÚÛÛä]Óé¼pá,>ºªªù%N>O?t÷±Óµ ?äGÈùò3ºü6mÚ488Q~ÙÙÙÊkþMAß?üÃ?üßö/ÿò/òÒõi!ûðÃyæ¤¤$µùzê©oûÛ·nÝ~YùM ··mhÌäá»víÛÁ°utttww³ü2|åÊ¶aëêêêììñ7cùýú&FÅb¸ßw¿ûÝvóæM¯×;@¼êìÛ·/33Sm>9Y]]-Ï¨~ð~%Å>1½Èí`ØnÜ¸ñÃþí`Ø®^½ÊF0líííï¿ÿ~¿©9å^NIIáh¯;wîÍfnºÆÑ^öG9ÚKí5¢üÂ¯îÑå#¯Êk,#?SvéÒ%ýÐ]Ít-ÈùòC~läÇòhAýJËuþüyYÿN'ò3Yòðë§kéèèÛ"?äGÈùòKù)'ÛÛÛ­VkRRRnnngg'ò3ÓkÌÆ5æ[³fM[[Û|®ù!?B~Èß#frF~ÆiºéZ.]ºeºäüù!?¶òC~È/­©©ÑÏjµ644Ìß|ÈùòC~üò3D~¿¿¾¾>++Km¾yÀüFÈùòC~üò[´B¡Ð¹sç¬V«fºÑÑÑ'þíò#äüù!?ä·8555åååiqTWWÏaºäüù!?¶òC~ÈÏ uwwé§kõúò#äüù!?ä»úúú¶lÙ¢ÆQZZêõzcðÝò#äüù!?äõCwW­ZõD¦kA~Èòc; ?äüßïÇ"##C?]K0å-A~Èò#äüßB?®®åÀããã±¿=ÈùòC~üò[Î;·jÕ*Ít-n·!¦kA~Èòc; ?äü'Ùì¥¥¥¡»;wîWÅ½aÈùòC~üòæ+//×OØ××gü!?äGÈù!¿'Ïç«¬¬ÔoÕªU²3ÙÐ]äüù!?B~Èù-lCCCn·[3]K^^^,§kA~Èòc; ?äü¶Óµdgg8q"ð#?äGÈùòC~ÈoÖE®ENÊ6öf#?äGÈùòC~Èovæóx<v»ÝPÓµ ?äGÈù±òC~O¸ÖÖVù$Ëåóùââö#?äGÈùòC~ÈïñÉt8óÉoaäüùòC~Èù-`>ÏåriÌg·Û[[[ãî¾ ?äGÈùòC~È/r§kóy<£M×ü!?äÇv@~ÈùÍ±Óµdee?~<NÍü!?äGÈù!?m§kIMM5øt-ÈùòC~läüßìÌ7Ýt-ñ2tù!?B~ÈòC~O6TQQQüN×ü!?äÇv@~ÈùÍÈ|&®ù!?B~Èíüò¦kA~Èòc; ?äü"7::ª®%///~§kA~Èòc; ?äü´)ÓµhîÊIYLÿÜC~Èò#äüB~æ®ù!?B~ÈíüòûuMMMæ®ù!?B~ÈíüòÔÖÖVZZªÆQYYhæC~Èò#äüÌ,¿þþþ;wjqrºäüù!?¶òC~	*?ÙAëÍg·ÛÌ=tù!?B~ÈòK ùÖÖÖ¦¦¦ªÍâÄ`0Èsù!?B~Èò3üvõõõ¡»uuu3tù!?B~Èò3¹üÄ|6M?twhhçòC~ü!?ägù]ºtIc>iË-ýýý<Óò#äüù!?ÈÏëõê§k5<tù!?B~Èò3ü|>ß-[4æcè.òC~ü!?äg*ùùÜn·fºî"?äGÈù!?äüL%?¿ß___¯®Åf³y<Ìü!?äüò3üÄ|rG²²²ÔæÇ<ò#äGÈù!?3È/y<«Õª®¥¦¦f||'òC~üù!?ägù555ÙívÍ0Ëåóùx!?äGÈòC~&_ww·ÃáÐOÖÈz<Èùò#äü©ä×ÐÐ 6_QQ×ëåiü!?B~ÈùP~Á`Pù³ÊÐ]¦èC~È!?äXòé×ß¹sGýö9ä';w¡»Èùò#äüQ~ííízØIÍÍÍn·ºüÚÚÚþ_ûå/ùÎ;ïü?2j·oßf;3ùÅIäÇv0llc&¿8üØMØ'¿;ÅøÆ±ü6mÚ$»òö]¾|9ü;æao¾ù¦<÷¼dÔÞxãk×®±Û+WØ<§ùç<büMãûhïç71üòóó+**,KIIþT³þÝ^âh/G£½ÄÑ^ööhoù)..F~ü!?äGÈ/þä§´]~RJJò#äüù!?B~ñ'¿Ô¯ÌÏÏWþ¦ÅÈÈHEEò#äüù!?B~&r²«««  Àb±þ!?äGÈùò3üæò#äüù!?B~Èù!?B~ü!?äü!?B~ÈòC~Èòc; ?B~Èù!?B~È!?äGÈò#äüò#äGÈùòC~ÈùòC~üù!?äüù!?B~üòC~ü!?äÇv@~ÈùòC~ü!?äü!?B~Èú¿ù¦¦¦¡&ëÅÈ¨y½ÞÎÎN¶1U|÷»ße;6ùµV~f;³ý×=ö,ÛÁ°½÷Þßÿþ÷cüMý~ÂÉo``àðáÃKDDD`E?ì¹wb$äGDDDüùò#""""äGDDDDÈqn·;%%%//¯­­íTURRÛÊP,((°X,íííl+£=@|òIIIµcÇ	¶Õ"¼$L¿ëééÉÍÍU>üÉ">@ÒØØÍfc+óêììzµ<åÙüâ¬#G=ztrrR^·V¬Xñk.púôi¶¡ììlåo¸Éÿìø­]»Vv¦þÞ½ÙV~æt:e¥,Èúªª*6Ñ ùmV~³p°qùåççðÁ²pñâÅ+W"¿8K~åW¦(_pûöí6°¡öè3FFFdAþì´ü6^^ºt)Ûj±¸_Äë¦þÎ2¿8ðÚ´iÓàà ò3ì¤.--ùÅYòútòäIyäD7oÞÔÁ¶mÛºººØPFtzzzd½ìå >@ÅÅÅ²ðê«¯ªH1.âLýðèðúõ:ò3ö$ÉY»wïF~qVRRÒÙ³gýæcIså·®uëÖ±øè¬]»Vy·IÈcdÀH ¸råJAá+¯¼²¸¿'rÓíÁÔZJIIaCíB~qñÝ¿ßétägåääDùÝ÷È#'Od+ðÑáMã?nÝºUPPÀ¶Z¦ÛÉ÷ðáÃGSGÕ"äB~ÆFFFÜn÷;w÷ò#2öîÝûÚk¯ÉÂÀÀ@YYæÜõë×÷öö²øèÈïaÊÁÄþþþµk×²­öåçç÷ôôLNN¾òÊ+/½ôÛjQnær¹Î?/ò¿ÓédCíB~ÚÚÚ***ÆÆÆýò#2&&&¶mÛf±XJJJ5Ï·åsÐd´GçÖ­[>9Kþe¶Ñ Ù3._¾A»wïBl«EI¿Sööv«Õ«Á&C=@ÈÏàÍfSÏùüùò#""""äGDDDDÈùò#""""äGDDDDÈ!?""""B~DDDDüùò#"""B~DDDDüùò#""""äGD4§^õÕõë×§MµaÃ×_ýû²©âfÏéÖÚl6¹k@@³^ÖX,ÜÜÜÉÉÉÙ^'ò#¢øëðáÃKt½ôÒKf_½¬lhhÐ¬?uê¬ñÅçpDDÈâ¬1Åb9sæLhª³gÏÊIYÙÛÛkùÝ¾[VkÖÈzÏüùùægÄ4GU¯<vì¬ÜµkÚ=ï¾û®ÈIP¸zõjYñ½÷vïÞ)geggïß¿_PµµµUt%gÉe½^¯ÆR²féÒ¥ë×¯¿vít¹võêÕè×£%7I9ëúõëÓ)­¢¢BÖwvv×|ðÁ²Æáp×9rÄjµÊU¥¥¥íØ±cddD/?ýõkÖD¹©Dü³åËZ>ýôSõÊÏ>ûLVÚl6µl4uuu)çnÝºUsÖ¾û³úûû"^J9©»mÛ¶ÉÉI±cJJJ(såSzzºòñ»(×#oþ^¼xQÍÙ0./fæJ6lØ0[ùE¹©Dü9åÀný×%â0µlªªªîO%rróæÍÊ¹t;*oÝØ³N§òf¡,···+W¢¾ÎCí­Ï=÷¬¹|ù²,Ëÿ²üì³Ï>özDrR¾@Û"Þ#Ñä²eËäþÉIù_n¹póáÃÊäææÊå.<úÍÑá´´´ÙÊ/ÊM%"äGD´øò"Ê/¼^&iddDN«W¯+V¬Ø³gíÁá+¯Ñ¼¾rò³Ï>±h)|ÀWþåÖÖÖÇ^(SNÞ½WÛ¦ûLÞóÏ?/g½òÊ+~sDÿþýê/üÄ£%%%Ê[³_JDÈhSÞèºÿ¾ze rVë]888¨à/ðóôa5RÏ¥"ËÙÙÙ)))Á`P®|éÒ¥ás=Ó9LÓÀÀTYÿe¹¿¿?|n»ÜGg.¿(7Ñ"§|ÖíäÉêÊáÏéßÊêíí­¯¯W½É¨¼!>úXíÛ·O9È+ÿïÞ½;¼>Êõ(ï±Ý¹sG9y÷îÝèãp×­['ç*ÙXÕg)ã|å¬ë×¯OLLD_X¥ÊÖå¦ò#"ZäåôéÓÊ¬.gÎIIIÑÆØ¼y³('*bÃãpÏù)T@PàþýûÅIÊ¨Ûð¼*¦¶U¾[[[xëQ>Ø§|Î/(_E~gÏ¿'wYòÇþþ~ù.²¦bM±²|sÇês£ÜT"B~DD2Ë±¦#Gü×¾l*FáåðqÒð pÏ=÷ê£ÈïÑÔÛõÙÙÙêÁQ®GnúÇÕNwg<x¦ÜÍ1îM6©¿Å²eËäebõu*oIS>7ÊM%"äGDdD'6lHjýúõÊ[ü®_¿®LWXXxãÆð¹@à^°Z­þù`0>×ëõÈrss5×©¿%rU²~ïÞ½õÓ]$7Fn2Ñ`ùüÂíÞ½[3½ÒØØÓé-)÷Âçóg¨Q_çÄÄÐVÙV£³³Só£ÜT"B~DDDDüùò#""""äGDDDDÈ!?""""B~DDDDÈ!?""""B~DDDDüùò#""""äGDDDD3ëÿ(ÏKD¿IEND®B`


$;wîÔ^wÇ2ýþûï÷ööª=¼2³¼¼oÞ¼ùðáC(**¹ò[²duùòenmméíÛ·ó@ùÓòRWcBÇä^~O<QZåÿÐ½IýÍæ¬¬¬ûö³ü¢_ÅårÉ¹+V¬hzÏ·wï^¹ç©©©ëÖ­SñJðUWW«+**nß¾=¡Å¢Ý#eQÈO´Ùlrçe9Ì?÷îÝrx ü@ùò ü@ùòå?ùÉOîß¿ãúàÁÿøÿày10å!«5øð_üâç?ÿ9ËÊ¿þë¿þçþ'å7=¾÷½ïIüÅø~ñÅ>ä©ñ/ÿò/wîÜa9àéØh²r`9àéØ°÷wÇrréÒ%ùcò£ü@ùòåÊò£ü@ùòåÊò£ü@ùòåÊò£ü@ùòåÊò£ü@ùòåÊò£ü@ùòåÊò£ü@ùòåÊò£ü@ùòåÊòåÊòåÊòåÊ(?ÊòåÊ(?ÊòåÊ(?ÊòåÊ(?ÊòåÊ(?ÊòåÊ(?c_ggçÒ¥KM&Ó%Kº»»)?P~ ü@ùòKØò+,,¼víL9s¦¨¨H[~/^|[?ýéOïÞ½ûxüøË1,Y-ÈÊåÑ××÷ùç³ |öÙg=ñ5pùJOO×ß'~[?üáÛÚÚ~üô§.Kþ²g9@ÈjAV,üä'~ú)ËJkkkh"_WWWMMÁÞ^°·ìívo¯2<<]íóù(?P~ ü@ùòKäòëëë³Ûíº±EùòåÊ_â_[[Û5ktÏ¥ü@ùòåÊ/qÊÏb±Ì	AùòåÊ_Â_t(?P~ ü@ùQ~(?P~ ü@ùQ~(?P~ ü@ùQ~(?P~ ü@ùQ~(?P~ ü@ùQ~ ü(?P~ ü@ùQ~ ü@ùò(?Ê(?P~ ü(?Ê(?P~ ü(?Ê(?P~ ü(?Ê(?P~ ü(?Ê(?P~ ü(?Ê(?P~ ü(?Ê(?P~ ü(?Ê(?P~ ü(?P~,P~ ü@ùQ~ ü@ùòåGùQ~ ü@ùòåGùQ~ ü@ùòåGùQ~ ü@ùòåGùQ~ ü@ùòåGùQ~ ü@ùòåGùQ~ ü@ùòåGùQ~ ü@ùòåGùQ~ ü@ùòåGùò(?P~ ü(?P~ ü@ùò£ü(?P~ ü@ùò£ü(?P~ ü@ùò£ü(?P~ ü@ùò£ü(?P~ ü@ùò£ü(?P~ ü@ùò£ü(?P~ ü@ùPooommmUUåGùQ~ ü@ùòKL@ µµµ¢¢bîÜ¹sÆÜ¸qò£ü(?P~ ü@ù%þþþcÇÍæ9ÿÃá ü(?Ê(?P~	¢­­­ªª*--mÆ¤¨(?ÊòåÊ±666k/77WÞúïß¿o _ò£ü@ùòå7nÜp8Úæ³Z­çÏ÷ûýû¥(?Ê(?P~øÒsN§SÚNw#_mm­±6òQ~(?P~ ü Ããñ8p@òNw#_KKÏç3úïHùQ~ ü@ùòKvN§³²²28DKPffæ=zzzæ7¥ü(?P~ ü@ù%©þþþÝ£7dfblä£ü(?P~ ü@ù%;yØívíF¾´´4ÍûwsÊò£ü@ùòå7Í|>_KKKYYv#_AAACC×ëMì%@ùQ~ ü@ùòK|²zÔ¢eîÜ¹6ÍårdXåÊ(¿eÅ"oÜ'©åGùòåÊ/¹ÝîºººììlíF¾ÊÊÊäÙÈGùQ~ ü@ùòKXÒsRuºC´X,iA)Âd~>P~(?P~ üÇãihh(((ÐÙét&çF>ÊòåÊ_B7_Í¦ÝÈ]WWÇò£ü@ùòågx^¯·¹¹Ywæòòò¿ßÏò£ü@ùòåglòôv8Ú£7ÒÒÒìv;O~ÊòåÊáùýþòòrÝ/[knnNøq)?ÊòåÊ_~²r4DKbÙåGùQ~ ü@ù!YÊ/03åGùQ~ ü@ù!ÁËONÞU%ïò£ü(?P~ üå§Æa2DKÃLùQ~(?P~Hòóz½ÑÇafÊò£ü@ùòáËO«v»=--M»Ïáp°Z£ü(?Ê(?¾ü|>ßéÓ§ËÊÊ¢ò£ü(?P~ ü°åwãÆÆa¦ü(?Ê(?$lùù|¾«Õª=z#77÷Ø±clä£ü(?Ê(?¾üÔ8ÌºGo¸Þ ü(?Ê(?»ü¤ç"Ãl68ÐÛÛËDùQ~(?P~0vùÝ¿¿®®N;³¨¨¨hjjòù|<4åGùòå¹²²RûI¾´´4ÃÁF>Êò£ü@ùòáË¯¿¿¿±±1??_»¯¬¬¬©©ippÇò£ü(?P~ ü`ìòëèè¨­­Õ½VUUû·EP~(?P~ ü¦Y 8öì²eË´ù=ò£ü(?P~ ü`ìòëíí=pàvf±aÃóçÏK²Ø)?ÊòåÊ.?é9§ÓùÒK/iÞÈÏÏw@>ÉGùQ~(?P~0|ùy<yÓ¢EB|åGùòåÃ_!Z²³³ëëëÝn7ò£ü(?P~ ü`ìòóz½ºC´=/Ø¥ü(?Ê(?¾üäic·ÛÓÒÒ´ùù(?ÊòåÊ/?¯×ÛÜÜ\ÝÈW^^.g±òKÞò°X,(?P~HòÃáÐÑf·ÛyQ~É^~ííí%%%ò ü@ùòqËÏï÷;N«Õª;sCCù(?Êï¿¬[·NÞY£ßÉ'¿­Ï?ÿóà/~ô£µµµ± dµ +DggçÅ'Ï?¿ûví­¥¤¤üÖoýVcc£À>ýôÓøñ5pùý÷] |òÉÃØõûíÛ·^ÃrÕ¬X÷ïß¿|ùòþöoÿvõêÕÚ!Z~õWuÿþý_~ù%Ë*È'Æ?4Ë½½`o/ØÛ¸òóÿ|Û¶mºã0[­V§ÓÉ8Ììíeo/åÊOÞl6î8Ìuuu¬.(?ÊòåÊ¿ßÏR¢ü(?Ê(?[¤q333O	P~ÓòåÊ±"ÑR\|âÄO>ù¥ÊòåÊÆvÿþýúúz³Ù|sçÎµÙlê-ißÛÊò£ü@ùòC¼.«²²RôÙãñ/Lùò£ü@ùò!õ÷÷KØåççkwìJJjh¡ü@ùQ~ ü@ùÁ`äýeË-ºGoìÙ³çþýû®åçõz^íµ7ß|³··çåGù(¿$ÅÓØØ¸xñbíF¾²²²Ó§OF¿(¿þþþo|ãß,ùfeeå·*¾5oÞ¼3gÎðÜ ü(?P~(¿Ä¡hÑ~ÁîÜ¹seþø_ì	P~/W¾Ê:¬þÇþY,Q6sò£ü@ùò1ø|¾æææeËéÑÒÐÐðÌ|	V~@à¹çÛW»/X~ò¯¼¼üôéÓ<[(?ÊåÊÏ¨zzzöìÙ­m¾ªªªI? F/?¯×k6C³Oþ­Zµêøñã<g(?ÊåÊÏ`ü~ÿÙ³gËËËµÁ___?Å×uìíþùç¿mûv0û8h±XOÊòåGùò3·ÛàÀí8Ìâ¥^whí-ÉY~N§ó¹ç«ÚT%Í·óÿíxñêÕ«§eáP~åÊf$Kkk«î8ÌRµµµ===ÓøãcTYbßüæ7åW~å×~í×öíÛçóùx"Q~(?Ê_<~ü¸îF¾eË=Öï÷Oûe$gP~(?P~)yw°ÙlÚq³³³·mÛ6½ù(?P~(?P~^¯·¡¡¡¸¸Xw#ßñãÇå3(?$lùÍyÊ(?Äfål·Ûge#åd)¿g1L(?P~9~¿¿¥¥EwâââØlä£ü,å7ë(?P~ ü¼úvæ´´4ÍûwÊÉ^~CCC;wî¤ü@ùòÃ4N§Ójµj7ò444Ä~#åd,?Åb2ø(?P~!GVûòvö^3wîÜÊÊJË'CS~Hüò[²döo/³Ù<Ó?R~ ü@ù%<é9©:Ýq¥ëêêÜnwaÊ_~©©©òÈÍÍ		¾>úH&jjj(?P~ ü09jíÆ«Õêt:ãóûÄ(?$~ù©×¡LHêÉÄ½÷FGGe"##òåÊefÃç¯;Ê_~óæÍd»¼eâÈ#jQ]@ùòÃøy½ÞææfÝqËËËå¬Y?zòå÷_öíÛ<#ìJùòåg2DÝn7Ö²¥üøå'ÞxãùóçËDgg§LHÎô]§ü@ùò3´èã0ÇÃ-(¿8BùòågPn·»®®N»oîÜ¹³;3åÊò£ü@ùòjæHC´ÈZÝãñýw¤üåWTT¤Æva$gP~ ü æ¸¢òå§£°°0´ö8¶(?¨!Z´ù²³³ãpfÊß³IäÉk¸««ktt4wòåÊ/n©q#ÑÒÒÒâ÷ûò§üøåg6åãì£ü@ùòO²(ìv»AÇa¦ü@ù=[ww·¼¤wïÞ=<<Lùòå¢Ñbq)?P~ã²`ÁíK#<@ùòKQÆa6ú-(?.äP~ üí·VC´X­Ví_þòå7ÓÓÓã»Nùòå7+Ô8Ìhq¹3DåÊOGNNGxòåð¿¦ôT]¤qrÊ¶¶6yÙïÛ·/ÆèS~ ü@ùÅ¢¥  @»c×jµ&Ò8Ì(¿qÜDáÊÑ©qvÊ8Â(?òûýI83åÊ/~Q~ ü@ùM;·Û]SS£è¢Ån·3l!åÊï©ÅbY¸paoo/åÊAóçÏkÞ(..NÚ!Z(?P~:L&¬b×)?P~ ü¦.ÒF>IÀ$òå÷lW®uDýÐÐP,Çv¡ü@ùò´(ù¤÷ìÙÃ-(¿7Á±½ ü@ù§üäy[WW§ÝÈ'ÊÊÊ>)åÊ/"íåÊÏå×ÖÖVQQ¡¾´´´mÛ¶É¯À°|(¿øEùòå7÷ïß?vìî-2³±±±¿¿òåGùQ~ ü`ìòkmmÝ°aö|2gË-mmm<v(¿ÉY·n]zzº¬P2226mÚC=(?P~ üt666.[¶L»Ïb±ÈÊ|(¿)=ËuðéÞ)?P~ üÂtttØíöÌÌLí:¹²²²µµOòQ~ ü¦ª¤¤DÖ)ë×¯CCC7n9Ë/§ü@ùòÁÁÁcÇh/77·®®!Z(?P~ÓV~©©©²r	ý;rddDæÈ|Ê(¿uãÆÚÚZÝ!Z¬Vëùóçù]Êß4_JJ¬b¤ösdE#sÕ(¿âõzu?É--ûoÔåd)?µ·wÍ5jo¯ü/Ó2géÒ¥(?P~Ó¾r8wÚæ+//oiiafÊßÌ¤î?¦ü@ù!É#øÊ+¯J3ýÙýÙ¤~Aùùý~©:¹ÚmfffMMq¿8òåg°òSOôM6eee¥¤¤ÈÿkÖ¬93×)?P~¶¶¶yóæ­^½ºæÿÖüÁ¶?øßøµk×N.þf´üÜnw]]îF¾Å766òhR~ übZ~³òå©ÔÛ¸qãáCÕ¿ýßÝÿë¿þëN§3NÊOTîLee¥væ´´4»ÝÎF>ÊåGùòÃ¸ø|>³Ù|ðÀÁ`ùÉ¿ßþíßÞ³gÏ¬ÇãõÅbÑý²µææf¯×Ë#HùòuùÍyÊâßïî¹çÂÊï¥µ/÷»ßÅòuÍfÓý²5û5(?P~ÿ#%2Êâßoþæo®]»6µ¯×>ÿüó.+öåçõz#ÙÇãáñ¢ü@ùÍrùEòúë¯«Ö3g(?P~[òðÍæ²²²W^yåw7þî7¾ñ×^mr75éòkÙíö´´4íF¾ÊÊJÉP¾lòå¿å×ÝÝ¡Øòåø488xèÐ¡^zé÷~ï÷äÕê¢ÆaÖ¢%;;/[£ü@ù ü6oÞ¬V[/^Í]§ü@ù!N¿üä9f¾lòåïå÷ñÇ«ÕÖúõëcy×)?P~0Jù©!Z¬V«îF>iAK(?<¿/_®æ¸råJï:åÊñ_~jfh¡ü@ù¾üÞ÷]µòÚºuë¬ÜuÊâ¹ü:::òåÇx~(?$rùùýþ¦¦¦üü|íZ±   ¡¡|(?C_Ê³L&Ê§üdTSSÃ-åÄ,¿YGùòC<ðz½þç^RRÂ8Ì ü@ùQ~ ü°zeE¤;DÕju:lä£ü@ùQ~(?ôöèÌÌÌ=öôôô°(?ò£ü@ùÁØúûûëêê´ùO>íóùXJåÊòå²¶©ªªÊÌÌÔÑ"ó?ùäFiåÊòåcljjÒ977·¡¡A½1ÑïíåÊÏxåÇx~ ü(¿ÖÛÛk·ÛµäÑú»(?$~ùÝ§[~çÊãõzËÊÊt7òÕÖÖºÝníµ(?P~Hüòº|ù²¬_õÕááa9)ÿoÜ¸Qæzòå£èéé9pàî-ååå---QÞ ü@ù!ÊÏl6Ë1tØªõ÷ño¹»»;//Ïd2-Z´èÊ+(?L;Yw9Î6èÑâp8Æó(S~ üDå§VRaå7õÏùUWW¿ÿþû2qâÄ­[·jËïóÏ?÷ÅVGGÇ¿ÿû¿ûïg?ûÙ­[·XÆõå_¾öÚk_ÿú×µù^|ñÅïÿûã¼)Y-ÈÊE100àr¹XP>ûì3¯×ã:ãå#+J©4ùCGNÊ~½ÌùSß8::ªRÒb±hËïøñã?-y%7üèG?Ãr0âwäÈ¥Kj/==íÚµX±ÈjAV,[+W®´¶¶² ÈA1þ¡3^~W¯^Õ=ÂãæÍS¼åÐcD´Ç°·ìíÅ¸Ýîºº:ÝOò466Êæ»eöö½½H¢½½âÞ½òçrJJJFFü1ýèÑ£©ßlèþâÔÔTÊ&!¸ÊÊJí'ùÒÒÒl6¬I¦ø»(?$WùÍõñAù_»ïòåè<OCCî­ÉL9kÒù(?P~ ü¦ü-þÞïÉü_]]MùòÃ8ÉÊAV ºã0Ë|Ë5Å|(?$uùÝºu«¨¨(##CíÍÉÉ9wîÜÔo¶½½=77Wn3//¯³³òåè¼^osssqq±v#Åbú²µiGùòCÉ9ôÛÔô'fô®S~ üúÚt8ºGoX­V§Ó9½ù(?P~HÞòËÍÍuë­[·å×ÙÙ)ÓYYY(?Ì(¿ßßÒÒR^^®>©ÀºººØ<F(?$Qù©¬På7::Ê÷öòÃiõek1»3(?$Qù©Õv>)¿ýû÷«OÕP~ ü0½Ô­Y­Vmð¥¥¥ÙíöY)0Ê¨üÚÚÚtGr¾víåÊÓÅãñÈ«^þ¤Ô®m§kÊ(¿g¯õJKKÕ±½éééEEEn·¦ï:åÊ/¨q£ÑûõåÊÉ^~³òåØ¼^oôqghÊ(¿¨7ñË;=zTTTOùòÃä^hv»=--M»¯²²rÚÇa¦ü@ùòRùpl/(?LTqÕ-1ø	åÊ¾üüü9QÍ7òåñ%æXÑBùòå§ã«¯¾JüöPü1åÊQHÏE¢E*PZÐ(KòåÄ/¿ é¼Þ±KùòK0n·;Ò8ÌË->DËàà óåÊoÖÊo¶P~ üGÑRYY©;DË¶mÛ:::¢TÓé|þùçÍfó¼yóÖ®]ÛßßOùòåëò)**ÊÈÈÎ?þo¾IùòCÇã2DË±cÇ£ß¼Þ¿öµ¯½òÊ+ÞÿÝýßªøÖ/¼àóù(?P~ übZ~,;¼W­Íëëë)?P~×i¤q«ªªZ[[Ç¹ëöÅ_Ü´id_ðß¢o.jnn¦ü@ùòiù¥¦¦ÊJ¼§§'8§»»[ædeeQ~ üV!Zrssåõ;Ñq³2³8Z~¿ó;¿óÚk¯Q~ ü@ùÅ´üÔá½£££ÿëv5üQ~ üD¤qEyy¹ÓéÜ.ÚüÑ®?-¿Õ«WÿÉü	åÊ_LË///OVè÷îUnÉþðáÃêÏzÊ_ò5@KK´î-µµµ½½½S¹ý=ö,^¼8¸ÙÏñãë_ÿzGGåÊ_LË¯««Kw$çÎÎNÊ_2å0é!ZÆÏçóÉ­½°ðÊÊÊÕ«WKö½õÖ[q²(?P~H¢ò-JOOOIIÉÈÈ())93×)?P~³+D9--Ín·ß¸qcz¢ßït8G­(?$cùÍÊßléïï4DKqqñtmä3Ê(?Ê_ºqãÝn×¢eÃmmmÉùd ü@ù!¹ÊïÖ­[j0gu<oNNÎ¹sç(?P~	Ãëõ655-[¶L»/??¿¾¾~¢C´P~ ü@ùµü._¾|På§¦O8Aùò3:·ÛàÀíÑsçÎ­¬¬lmmUõ'9Ê¨ürssåmàÖ­[Áòëììd$gP~&='U'm§Ïb±ÔÕÕIò ü@ù!ËO½<½yttT¦M&åÊÏpúûûëëë%ï´;v­V«Óéç­Q~ üÄ,¿5z*¿ýû÷«(?ikkÛ²ev#_vv¶Ãá`ðBÊ(¿ÿ~·ÐÉùÚµk(¿ø×ßßìØ1Ý!ZÊËËpÊ(¿g¯õJKKÕ±½éééEEE1øåÊo:::thQã02(?P~qòå79MMM/Önä+((hhh`#åÊåGùÁðå×ÓÓSSS©¢Åf³¹Þ ü@ùò¶»wï.055UÞBÒÓÓ-Z400@ùò~¿ÿìÙ³åååÚ|E^JI>3åÊß¸Ý#<z)?P~³K^Cº©!Z(?P~ ü&L½©TWWû|>99<<¼uëVGùò~¿¿¥¥Ew#_vv6ã0S~ ü@ùMá&Æn9éà¨Î(¿X»äp8´_¶¦häËÖ(?P~ ü¦Dmó	Þ³Í_,É[N§Ójµênä¤K(?P~ üNãçüª««åé.'?~Áçü@ùÅÇã×î­33åÊß4ßg¡Ý¾¹üüÑUYY©;³Ífý«òc9òCR_Ê³L&Êßtñz½º_¶Æ8Ì(?P~3^~³òC²<ýìvZZî-ÃLùòåò4hs__åÊoê¼^osssqq±î8ÌÑBùòåÓò·7Þx#læ¶mÛÕßE¢Åjµ23åÊß,¼åää<zôHN~ðÁêi>ÞGù!áË/ú8ÌÒû¾`Ê(¿Ä)¿§c[øÔÛR^^Ø¸qãLßuÊW~n·»®®.Ò8ÌÑBùòåå'®^½|jjjÁ]§ü0å»ÝNUP~ ü@ùÅQùíÚµK½K¿þÕW_¥ü@ù=Sôq¢òåÊ/îÊÏd2É»TVVÖõë×|Î/55òåI[[Û-[´ùdã0S~ ü@ùÅoùÉÕÁÃfÚívíå§åõzËÊÊthgµÇãaÙR~ ü@ùÅoùEÏo¦ò±Ê¯§§§¶¶Öl6ëÃÌ-(?P~Æ(¿ÙBùÁå'=×ÚÚZQQ¡;Dã0S~ ü@ù£üä+tnô°üåYªÝÈ'-[ÖÒÒÂÑ(?P~åÃËåÚ°aöv333ívGG;v)?P~ ü(?ÊÆ.?ÇÓÐÐ°xñbíF¾ãÇû|>åÊåGùÁØåwãÆ»Ý®;DÕjeÊ(?ÊòáËïË/¿<öì²eËt7ò;v!Z(?P~ ü(?ÊwãÆßÿýßÏÊÊÒ6Õjmmmõûý,%Ê(?ÊòIÏIÕmØ°A»c7;;»¶¶¶··¥Dùòc9 1Ë/:ÊDÃüfê°!Z¢òc9òC"_Ê³L&ÊFäu«»OäëÖ­ãý(?$EùÍ:Ê3Êív×ÕÕéÃæt:oÞ¼9ïíåÊåGù!^©ºÊÊJíF¾´´4ÃÑÓÓ£.9ÑïíåÊåGùÇã'´ËåLßHTE»oñâÅýýý¡§ü@ùòåGù%CÍ?Å+,YùÑGú×'ÍfÓ¹ªªJêV÷ËÖ(?P~ ü@ùQ~ï/þâ/òòòþxï>tXþÇþ©@#¾íy½ÞââbíF>Å"O°èã0S~ ü@ùò£ü_aaánÿCêßÚ5kkjjô+Èãh·ÛÓÒÒ´ù*++#mä£ü@ùòåGù%ÌÌÌÐìß¶ÛjµÆÿ=÷ûý---åååÚ|ÙÙÙuuun·ü·FùòåÊòK|FÜæ'æp8$ï´Í'!(98/[£ü@ùòåGù%>ÎOÑbµZu7òIN%Ý(?P~ ü@ùQ~IáÐ¡C_ûÚ×^|ñÅÅÇç±½jfÝ!Z§þek(?P~ ü(¿dáñxZ[[å9WãùËifÍ6ÏÊ(?P~f¢¥  @»OfÊYSßÈGùòåÊòÃ,Sã0OqÊ(?P~å¿¼^ossó¤Ça¦ü@ùòåGùQ~ B¤!Z¬V«Óé¡|(?P~ ü(?ÄHôq§8DåÊ(?ÊòjéòåÊ_Ü°X,_rRã0G¢¥ªªª££cïåÊ(¿éÔÞÞ^RR"oó_²ñx<òøêÃ;C´P~ ü@ùòeëÖ­wÖ(å÷WõWÿ[òzîììü'ÌyLåqOIIÑ6ß·¾õ­¿üË¿ìîî»zu¬dåÀr¸~ýúgÆròé§Þ¼y3Æ?ÔÀå÷ßw1rù9Îû±õ÷ÿ÷?ûÙÏîcºÝºuëÐ¡C/¼ðî-;vìøño÷¹««ë/¾à±Õ¬XwïÞýáÈrrñâÅû·ñMäòcooEj·Ûµã0²²²³gÏÆföö½½`o/ØÛ;;§P~MÃ¬;DKfffìh¡ü@ùòå/-Hù%©¥Hã0KJÆÃÑ(?P~ ü(?ÊoòÔ-V«Uw#_UUUìMÊ(?P~AùYwyìØ1£lä£ü@ùòåGù!¢¶¶6ÝqÅäáÛ£7(?P~ ü@ùQ~ß¸ø|¾æææââbÝ|õõõn·;~MÊ(?P~_Rëíí­©©Ñ¢¥ªªªµµuV¾`òåÊåGùMàÑÚ»f³yÏ=R÷[S~ ü@ùò£üÛí>pàî-´òåÊå¤îÑRuuuÉD(?P~ ü(¿çñxdÉçææêÃÜÒÒÀù(?P~ ü@ùQ~É¢££Ãf³i7ò¥¥¥Ùíö$|Û£ü@ùòåGù%ÁÁÁüü|íF¾ââbÙåÊ(?ÊòHoMMMfffXðÍ;×f³îËÖ(?P~ ü@ùQ~_8¯×efYì3åÊ(?ÊÏØä·Û³gv¹sçVVVºCÙåÊ(?Êòê÷ûO>]^^®ÝÈ§hI/[£ü@ùòåGù%uùI¸8p@wòåÊååZZZ"mäs8åÊ(?ÊÏðåçñxêêê,C´P~ ü@ùò£ü³üËåÚ²eã0S~ ü@ùò£ü¶ü?®»OÑÂF>Ê(?P~áË¯££Ãn·k7òÉªª*y.2DåÊ(?ÊÏØåçóù´ùÌfsmmíýû÷y)R~ ü@ùò£ü]~===vºC´TTT0DåÊ(?ÊÏðå'=×ÒÒbµZµ;v333ívoo/¯=Ê(?P~±ËÏívGÚÈ·lÙ²ÆÆÆÁÁA^u(?P~ ü(?_ p:Þýb¡ü@ùQ~ ü@ùQ~ÓÌãñÈo­;DK~~þ±cÇä¼Ì(?P~ ü@ùQ~Æ.?ù¹6M»OÁnkk+C´P~ ü@ùò£ü_~ÒÅÅÅºC´ÔÕÕQ(?P~ ü(¿Ä)¿@ vôÓéôù|¼¢(?P~ ü@ùQ~	U~âØ±cÁ!ZnÜ¸ÁòåÊå°å×ßßßÜÜÌF>Ê(?P~_â(?P~ ü@ùQ~(?P~ ü@ùQ~(?P~ ü@ùQ~(?P~ ü@ùQ~(?P~ ü@ùQ~(?P~ ü@ùQ~ üX ü@ùò£ü@ùòåÊò£ü@ùòåÊò£ü@ùòåÊò£ü@ùòåÊò£ü@ùòåÊò£ü@ùòåÊò£ü@ùòåÊò£ü@ùòåÊò£ü@ùòåÊòåP~ ü@ùQ~ ü@ùòåGùQ~ ü@ùòåGùQ~ ü@ùòåGùQ~ ü@ùòåGùQ~ ü@ùòåGùQ~ ü@ùòåGùQ~ ü@ùòåGùQ~ ü@ùòåGùQ~ ü@ùòåGùòåGùòåGùòåÊåGùòåÊåGùòåÊåGùòåÊåGùòåÊåGùòåÊåGùòåÊåGùòåÊåGùòåÊåÊåÊåÊ(?P~åÊ(?P~åÊ(?P~åÊ(?P~åÊ(?P~åÊ(?P~åÊ(?P~åÊ(?P~åÊ(?P~3¥³³séÒ¥&iÉ%ÝÝÝ(?P~ ü@ù%lù^»vM&Î9STT¤-¿¶¶¶_ÄVWWÇãùð_üó?ÿóíÛ·Y²ZËbxxØår± HùÉ1þ¡.¿PéééÚòûí·/ÅVkkëÅ/.:å!«Y9° >ûì3úÁ~ûå'L×ÔÔ°·ìíÁÞ^°·7a÷ö*ÃÃÃÕÕÕ>òåÊ(¿*¿9¿¤NöõõÙívÝØ¢ü@ùòåÊÏØåª­­mÍ5ºçR~ ü@ùòå8åg±Xæ ü@ùòåÊ/aË/:Ê(?P~ ü(?Ê(?P~ ü(?Ê(?P~ ü(?Ê(?P~ ü(?Ê(?P~ ü(?P~(?P~ ü(?P~ ü@ùåÊ(?P~åÊ(?P~åÊ(?P~åÊ(?P~åÊ(?P~T~ú§êt:ïÇÖ~ðüÇ¼Ü¿ßÖÖöãÿå!«Y9° äoÂ¿ù¿a9@ùë¿þk·Ûãêõz³ünÝºuøðáïà¢ïÃX$AùP~ ü@ùòåÊoÝ¾»´´455uóæÍCCC¡g=|øpNU°X,ÁÝÝÝyyy&iÑ¢EW®½d³ÏÖÉödÐÃÊ'CxX3°>hùòå2ÑÛÛ»k×®Ð³Î;g·ÛYD	¯½½½¤¤$ôÅY]]ýþûïËÄ'¶nÝzá(g!	¬%íÉ ÃÊ'öÂñ°f ü"¿ÏÓóæÍ=K¶.°ÞºuëîÜ¹úê5Í£££2122ö÷³ÏÖÉödÐÎaåÀAáxX3P~-Y²äÖ­[2ñÁV (,,fÌ,--½û6Ë*±¾zC	aÏ(g!	¬%íÉi+ñ¶f ü"ºyófQQQzzú;ï¼#ÿë^¦¯¯Oe<¯çàtjjjèÅ¢$|>°àÍO(Å5å÷lwïÞ-))t./ã¤z=çää<Ûk#Ó¡rðùÀZ7V<¢_e¶Ö_DÝÝÝ£££ï¼óÎo¼vÛíVÍ¾fÍUò¼m6Ûï½'òuuuèÅ¢$|>°àÍOÝ´õ5åQ[[Û$Ékjj@èÙÕÕURRb2***äÁcY%Ïë¹½½=777%%%//Oú¼îYHÚçk	ÞìY9ðdÐ5å4Ê" ü@ùòåÊ(?P~ ü(?P~ ü@ùòåÊ(?	ã>X¹reúU«VüñÇÿký5Æ0k[½k±XäWóù|aóeÉdÊËËèmåÀx><Gã7ÞH¤ò«¯¯MMMaóeþ#G&q@ù0îîniÉtòäÉÀS§NÉIyýúõ)¿÷îÉÌ%KÍ/))ùn·ò@ùH|¯¾úª4Í[o½:óí·ßÛ¶mí+W®H9I.]ºT¦~üøqMMMVVe6÷îÝºSÕårI]ÉYrÝK.µÌ7oÞÊ+/^¼('m6[Økmm~;ê,¹Kê¬Ë/Gª´5kÖÈüÎÎÎàk×®É«ÕsôèÑÜÜ©ôôôÍ7÷õõiËOûas¢ÜUÌ¦Hµ|õÕW¡3<x 3-KhÙéêêRçnÜ¸1ì¬Ý»w«³zzzRRRt¯¥Nªs7mÚ4::*íä_Ê)##Cü.ÊíÈîÝÓþ¦gÎ	ÍÙ`ÿþûÁì»U«VM´ü¢ÜUÌ2µcWg5gtXhÙlÝºuxLÈÉõë×«sUè¨vTî¤ØÔYÕÕÕjc¡L···«	½ÍJÛ©­;vì9.iù_¦·oßþÌÛjrÐû¦ûIMÎ?_~ß9)ÿË=ÜQÈËË+Ê¯ðôÓÓÓ'Z~Qî*Êf¿ünùç«²QÁ$úúúä¤4:¹téR9¹páÂ;wJ±=yò$x#r°­hÁk©<^Xj)¸ÃWþiËõÌÛÊ=½o>÷úë¯ËYï¼óÎÓ_îÑÞ»woè¤¥ü¤GKKKÕ&É_»òY¦6tÎôù|2SÎÒ:Á.¼sç¿`è?§Ý	ÖR¡c©È´ÙlNMMõûýrãóæÍûÌÛÔaanÝº¥"U¦åîéé	ÛÞÞ.w@w¯ñøË/Ê]@ùÀ,Su÷ÝwCgª±NÂð~p``@»)ëúõëõõõjßk0Õ¹àîÔgöÙîÝ»ÕN^ù¿¦¦&8?Êí¨ml>T'=zý8Ü+VÈ¹j 	ÖÐ³Ôq¾rÖåË¢_°JÕÒå® ü`©#$L&Ó'Ô¨.'OLMMÕ±~ýz©¿ß¯vÅÃUóS»sçNèUîÝ»W:IuWE·ÏÔÁ¶ê§·µµçG¹õÁ>õ9?Ï§.¥üN:Ü'¿rèYê#===òSd!D*?ÕÒÊr1)ÎÍ7å® ü`ö©QÃ=zôÖ_cT§ûIUíØ±#4+C;w.Jù=û²o6CwG¹¹¡w,x¤_öÉ'éééêWÛÇ½nÝºÐ1þ|ù_ìzjdêÔà¹Qî*ÊâÔÉªU«RÇ¬R`V~/_VÃæ-Z´èêÕ«Ás>ßþýûsssU-½þúë~¿?xî¥KJKK%ÈòòòÃnSOä¦dþ®]»ÂæGº!wFîh0Êx~A555aÃ»(ÕÕÕ²²²²ä·p»ÝÁjBoshhHÒV-+«ÕÚÙÙö£ÜU(?P~ ü@ùòåÊ(?Ê(?P~ ü@ùòÀÄý`Ì¡ É3IEND®B`


Detrended Normal Q-Q Plots


£Ñ'NÈ¹«Smö¶+æÜM6GjLÞ|óÍ7F÷0J]ºt)çÙ7n4Üt¸«W¯¾~ýú©S§ÄÚµk³÷/îÙ³§¦¦&:¬n"³hª;üÐCùÙÇ^Pêafû¤ÏïLWÉ¿éaN¸3I÷Ée+nÅÂå³4</áýc[xï`þÒi¾C¡1UUUmÛ¶mxxØ/P~PJåb%Ì¬­­.Äb±ì÷ã¾¾¾å]¹¥¥¥Àº+7æì¥­­­Ù;«¦:®W`³WÌìCCCÉd²ºº:LäÉÕ«WÃÛyövÂ5s-ûñæßî+¢¨P!ìË¹æ5k÷Í%KÂüwÞy'ÿÉZºté¤ÏïLWÉ¹é5Ñ>¿úúúNáÁ/zÅé_övÂiùMóe¼aÃû¼uëV¿X@ùA)_4?4Y4J¥ÂÅîîî0úôéì>ËY=ºøøãGqn»nxãoùáúÑÆIÑÒð.ÕoMkeßVÍ^qÒÇ~ôèÑì`Ù×ß¼ys^¿~ýÍ	a"3§z¼Ñ-[¶Ew,Úr¸ØÕÕý£O³?~<L_¾|9ÑTw8Oõ@2ëÎr©>HÙ':ÓÁô^½bá¦Ï¾ý2Mé³éß4_ÆQ^¹r%Lg3L/X°À/P~Pzå9À,,[S ü®^½sÛu3ûÏ¢£á4ºÞ>³wVå­+°ÙÂ+NõØ£@oê9ó«««³Qx/^<ÕãæÚÈ¾Â`ö=I§Ó¡Ba455e?öåÉñ©2«diX7fssóÉ''´éÎ¤÷ªè§_~Ñ+äúõëÙ71£òæË¸±±1««åþ	122â·(?(±ò¥fVUUMõv;U£DCâx«.Ð73o.b³SÍ9<Þðþ=::=?YþÞòït.ÊÌ9âyÛsTkjj¢ÃÓùyÓÓ-LgéÕ¤WÎàLÕ£Å­8ý£½³uMóe<88Å_¦;æ(?(¥ò;pà@öÑ^´³,§:3Z·ð>wÞy'iÍ^±ÀxáÂÅÌw D3£8ËÙ;ÓÄÓìË-¾]åÄ¡Ì¦S~ÑÉèVR©Ô¶mÛnÞ¼yðàÁ0sãÆßtV)ºüf:8E¯8ýòÎ>¿LÛEÿÔ)0þ^o³gÏîÞ½;:Ä9P~0×Ë/¼·Þ3ßê½hïÑ©²Ë/Er0é[fugS,Ñç´ÂÆ£íä|kÒÍ^±pFlØ°!óAþhNtæïúõë&D[Û²eËìË/Úï500Âþýû§S~===Ñ^¥ðO>íÏ=ztÒçw¦«Ì¨üNÎk#ÛLGµò>^###!ìÖ­[ß÷îê0Dpáñ¿í_èó¼ÊæJùåË>?1ÿlÙÌçý3_ºí ÌË,°nálIµQö¥6[xÅÂqíÚµÌ'º¢9.]ÊùWuuuæC³)¿LD/^ùÔc¾yøáó¬ûï¿¿ÀS<£UfT~'çµm¦£ZDù=6û«V­Ê^3&Ì§º¯·Ì)D>ú¨_, ü 4Ê/¼®Y³&ÿ÷êêêjjjÞÎ8ôèÑ0'Vö·ÙMsÝÛfSô5xaãù_Ë7Õfo»báfÏ¿xñâÆR©TþùÅß7ÂÖÂ6Cmß¾=ÄP&¸÷ÍsÏ=]xàK,9tèÐç?ÿùÌ>§Ù¯2£ò+<89¯3Õ"Ê/8vìX]]]xÔaûÑñôÌéÌábè³èÖo»Ïuª×Ûððð;¢©!ßÃS9::ê(?E:~øágôrE¬Rr¢½ÑÉÚcccQÐ/[¶Ìå&ú_§zÊÈò Üwtt,Y²$:¤&víÚeX@ù üP~(?Êå üP~(?Êåò@ùwÞÑ£Gkkkãñø5kfºnCCCEEÅàà`fNs/_>_X»ÎtÖ-Ú7¶lÙ²hÑ¢08K,Ù±cÇðððú%þa>.@ùóWMMMP9ccc3]wÏ=aÝg63çé§svïÞ]êå"oéË/wvvZ·n]êëëknnV~òæÀïYDÆÙ³gÃº«W¯ÎÌY±bEÓßßgîäWHQÔ9rä®°òðae_vgìØ±cáÂ,Ø¹sgÎÕ>ÿùÏ755ål!³Ë0L¿óÎ;a:Ì]¹r¥¹¹¹²²2766vwwOºµì[/¼Ê¡CjkkW®n(¿nÝºÕÖÖÖw¾££#ÚÚtÕªUaka~ØòÕ«W§92Ë/[tiQêéé	w2ÂÑ£GóG8$lÊëP~ÀÑôO>¦8+L<õÔSÙ×éêê/ÛæÍÃ¢éð3L···Gî¿ÿþãÇË/ùÉdrÒ­eßÂ«áÂÄ¦MòïüÖ­[Ãt¸ÂK/½&öìÙfÞwßazhhh`` LD»$óåKÅ©Ú«ð(íÝ»7º­Ðùw2ç±Þ' ü±üB¬éôüðô===aÑúõëÃô¢¬É,=öìã?ÞÔÔæÇb±I·S9WîØÂó×­®®îüøøxÍfkéÆÆÆVSíÀTò+<J###ÑtØÂTåyì7åÅ	(?àC,¿ìÜª]rÌZ°`A¸æððptP5Ìíß¿?¬rðÜ¹sùõ3éÎ*SÝ±Ù»ñ¢j<räÈ¢E¢9UUUÑîÀéìó«¯¯óoÝºU8^áéâ@ùLù%Éì]PÑn³ÛH[[[XùH$Âñ	Ó,¿Â«dîX¹üu/^­s÷ÆÆÆ?¾iÓ¦ì·õøãëRÌ_4Qfù7àÊøÊ/:¡õàÁÑÇÎ~úééÈ#G2;Ï^xá¬9þ|ôù¿é_áUBç;ß	7oÎ_÷ÑGÓ.ð+W®ÓçÎ»~ýz¨¯¯æ°[ºté+WBMF;#[ZZ¦9JÙÓÑçèXsÎc/nÀðßØØX¨«²¿¯pDGZÃÏLùÇ«ªªÝÓÑÑ1Íò+¼ÊÉ'ÃÒ+VdN4É¾Îððð¶mÛÂ=O$ëÖ­NãÁJ¥¢W¯^ñâÅéL¸PÑ±ì;wî>Ã7QÊ>zôh¸KÑeç<öâ@ù(?Êåò@ùÝ¯½öÚÛo¿íu0§zõ?ÿó?³144ôoÿöoÆÙøÙÏ~öãÿØ80Ko½õÖý×)¿¹âë_ÿz?¯Ë9å?üáõë×³qåÊþç6ÌÆÿ÷8qÂ80K§Oþÿøå§üP~(?ÊOù)?å§üP~(?òS~ÊÊå§üòåòCù)?å§üP~ üP~ÊOù)?(?òCù¡ü@ù¡üÊåòå§üP~(?(?åòCù¡üP~ÊOù¡üP~(?òS~ÊOù¡üP~(?å§ü(?ÊOù)?åÊåòS~ÊOù¡ü@ù¡üÊªüúûûÉd<ohhèîîÎ^4::ºiÓ¦D"qß÷õôô(?åòåò+m©Tª³³3LìÛ·¯µµ5Ñ=zê©ñññuuuùåwüøñÌ%¯¿þúo¾iýèGÆÙøéOÚÕÕe¥üÇ|ë­·îä-Îò«ªªm&Òétmmmö¢.Lµb(¿¯3|ïßnïÿû'N0ÌÆk¯½öòË/féW^yõÕWïä-ÎòÇãNG÷îÝ[YYYWWwîÜ9GíÅÑ^p´GK[,ËL'E/^ljjR~ÊåÊåWÚª««ÓéôûGÃtÎ¢ÌtÎî@å§üP~ üP~¥§­­íÐ¡Ca"üL¥RÙ¶lÙòÂ/óçÏ¯Rù)?(?_ÉkMMM,K&½½½?lÿóèZZZâñxSSÓàà òS~(?P~(¿yJù)?(?òCù¡üP~Êå§üP~(?(?åòCù¡ü@ù)?ÊåòS~ÊåòCù¡üòS~ üP~(?å§ü(?ÊOù)?åÊåòS~ÊOù¡ü@ù¡üòS~(?P~(?åòCù¡ü@ù)?ÊåÊOù¡üP~(?P~ÊåòCù¡üòCù¡üP~(?å§ü(?ÊOù)?åÊåòS~ÊOùòCù¡üòS~(?P~(?å§üÊÊOù¡üP~(?P~ÊåòCùòS~(?ÊòCù¡üP~(?å§üP~(?ÊOù)?åÊåòS~ÊOùòCù¡üòS~ üP~(?å§üÊÊOùy]*?(?òCù¡üP~ üÊåòå§üP~(?(?åòCù¡üP~ÊOù¡üP~(?òS~ÊÊå§üòåòCù)?å§üP~ÊåòS~ÊOù¡ü@ù¡üÊåÊå§üP~(?(?åòCù¡ü@ù)?ÊåÊOù¡üP~(?òS~(?Êå§üòåòCù)?å§ü@ù¡üP~ÊOù)?òCù¡üòS~(?P~(?åòCùòCù)?ÊåÊOù¡üP~(?P~ÊåòCùQ¦FGGòCù¡üP~³·ß~ûÓþô¢Eªªªî½÷Þ®®.å§üP~(?eè½÷Þµ·ò­ìx¬cç;|ðÁ~ô£/¾ø¢òS~(?ÊróüóÏ/[¶,4_æÏS_üÄ'>¡üÊåò£Üüå_þåç>÷¹ìò~åW~Eù)?ÊåG¹yöÙgõïggßæ¿Øü±LùÝýýýÉd2744twwç_!üí­¨¨P~ÊåÊâ~ô£äáG¢ìÛñ¬ÿäc=¦üîT*ÕÙÙ&öíÛ×ÚÚ³tll¬±±Qù)?(?fãðáÃ+V¬X³fÍ=÷ÜóÇüÇ£££Êï.¨ªªétº¶¶6géO>ùÌ3ÏLU~ÿ÷ÿKþáþ¡··×80gÎùþ÷¿o7ÞxãøñãÆáUñµ¯í«_ýêÂä¶×ÿ~øáx'ïá¼(¿x<>étôOÿ¦¦¦ÐSß/¾ø6sÉ«¯¾ú/ÿò/ÆÙ8wîÜë¯¿nK.½òÊ+ÆYêîîþÑ~t'oq^_,ËL'ìE---§Núêh¯£½8Úörg9Úû¡¨®®N§ÓÑÑÞ0ýKð)?åòåò+mmmmág*ü¡Úç§üP~ üP~å1¬555±X,LöööNzÊOù¡ü@ù¡üæ5å§üP~ üP~ÊåòCù)?òCù¡üP~ üÊåòå§üP~(?ÊOù)?ÊåòS~ÊOùòCù¡üòS~ üP~(?å§ü(?ÊOù)?åòåòS~ÊOù¡ü@ù¡üÊåòå§üP~(?(?åòCù¡ü@ù)?ÊåòS~ÊåòCù¡üòS~ üP~(?å§ü(?ÊOù)?åÊåòS~ÊOù¡ü@ù¡üòS~(?P~(?åòCù¡ü@ù)?ÊåÊOù¡üP~(?P~ÊåòCù¡üòCù¡üP~(?å§ü(?ÊOù)?åÊåòS~ÊOùòCù¡üòS~(?P~(?åçu©üP~ üP~ÊåòCùòS~(?ÊæOùUÜN,S~(?ÊåWå»x<®üP~(?Ê¯Ê¯<(?åòåò­¡¡¡Í7+?Êåò+·ò«­­Çã>çòCù¡ü ÌËoùòåù§wTUU+?Êåò+«òK$!õnÜ¸QSS&Bðç;ß	íííÊåòCù¡üÊªü¢|a"¤^¸|ùòøøxX°`òCù¡üP~(¿²*¿EÎlpp0LìÚµ+ð­.(?ÊåWnå×ÑÑ9#û£~+W®T~(?ÊåWVå|ãßX¼xqèíí¡JâùP~ÊåÊå7_(?åòåòS~(?ÊOù¡ü&S__·orFù¡üP~PÎå·téÒìÚËpn/ÊåòCù[ùÈÍ×××7>>^rÏòS~(?P~(¿¨ªªåWÙ§üÊÊofúûûCùmÝºõÖ­[ÊåòCùA9_°dÉ<Îð@ù¡üP~(¿r+¿ºº:gx üP~(?å5ßÀÀ@)>ÊOù¡ü@ù¡üf ººÚ(?ÊæEùõôôòëèèU~(?ÊÊ¹ü*¦àÊåò+Ãor3<P~(?Ê¯¿Õ¥t)?åòåòÚÚÚººº.(?Êåe^~ñx¼¢â.ï;ìïïO&á444twwg/êíímll/_®¦üÊÊ¯x¡´BùíÞ½hhèn·K*êììûöíkmmÍ^´téÒ3gÎÃ×××+?åòöÙ/ùËÏ=÷Ü»ï¾ëT~(¿¹[~sáÜÞªªª(:ÓétmmíTW«¬¬Ì/¿¿ú«¿:ÎìØ±_~Ù80á%^H¥ro;::,XðéUþ£?ú£êêêo~óÄ9òëÈ ProjzùÍs³okªÛíëëkooÏ/¿W_õgÌ%áºvíq`6Þzë­sçÎÄ]ýñü|äÙùÄÎèOssóïüÎïxïºïïÆYzíµ×Þ÷Ý;yzùÍÙûDþnÝºJ¥íu´Gço~ó+V¬Èd_ø³ã+;Bæÿ¾ÂÑ^íGçêêêt:íÓ9K¯]»¶iÓ¦IcBù)?ßÝuàÀ?ø?È.¿ð§ªªê½÷Þó<*?ß-¿Ð[ëÖ­«¬¬¬¨¨X°`AKKË>Õ£­­íÐ¡Ca"üL¥RÙzzzÖ®]ãÆIWT~Êåwwýà?¸çv|eG&û¾Ôö¥0Ç¨üP~s´üFFF&=ÃãNþ7¾aXkjjb±X2ìííýùcø®ÚÚÚì¥üÊo®ùâ¿ø[¿õ[ÿbsÈ¾|°ººúØ±cDåò£å·lÙ²PTë×¯¿uëV¸844´aÃ0çþûïûÏòS~(¿»nlll×®]¿ù¿YYYùO|âÛßþ¶gPù¡üænù%Ðyá7WfN:s&=ÓBù¡üP~(?_	_,`sîä·º(?åÊåò»åí]»vmt´7üÓaNcc£òCù¡üP~(¿²*¿záqóæMåòCù¡üP~eU~ïOÞÛÒÒ²páÂX,~®]»6Ì)çCù)?(?ß|¡üÊÊOù¡üP~(?åòË¬y;Ùÿ®òCù¡üP~(¿.¿ØÔÊåò÷çÃÑÞíÛ·GåwøðaåòCù¡üP~åY~ýýý,Í×ÜÜýÅÎÊåòCù¡üÊªü6nÜíê;~üx	=ÊOù¡ü@ù¡üfà¥^oýúõ%÷|(?åòåòûï¿?:£»»»å§üP~ üP~··wïÞhW_kkké>ÊOù¡ü@ù¡ü¦±¦ïóCù¡üP~0OÊ/v;ñx¡üP~(?_9_yP~ÊåÊå§üP~(?òCù)?ÊåÊOù¡üP~(?P~ÊåòCù¡üòCù¡üP~(¿Ò*¿úúúD"áûüP~(?yù-]º4»ö|Êåò²-¿y¡ùúúúÆÇÇKîùP~ÊåÊå7UUU¡üJ1ûòCùòCùÍL(¿­[·ÞºuKù¡üP~(?(çò,YRÇ(?ÊåWnåWWWçÊåó¢ü¢æ(ÅçCù)?(?ßTWW;ÃåòCùÁ¼(¿P~£££ÊåòCùA9_ÅáòCù¡üP~eøMÎrÊåòCùá·º.å§üP~ üP~ÊåòCù)?ßdÒéôºuë*+++**,XÐÒÒR*§ú*?åòåòIÏð(SòCùòCùÍÀ²eËBç­_¿>ú6lØæÜÿýÊåòCù¡üÊªüDè¼±±±Ìt:æùÊåòCù¡üÊªüb±Xè¼P9£££aouAù¡üP~(¿ò<Ú»víÚèhoø¦ÃÆÆFåòCù¡üP~eU~!õ&=ÃãæÍÊåòCù¡üÊªüÞ8½·¥¥eáÂ±X,üvmSÏòS~(?P~(¿ùBù)?(?ßÔÖÖÖÕÕ]¸pAù¡üP~(?(óòÇã¥ºïPù)?(?ßtwwòÛ½÷ÐÐP©ü§mÊOù¡ü@ù¡üÚÄb±òCù¡üP~(¿²*¿Ø|3ÊåòCùIùÕÕÕ544ôó¡üÊÊïö¢ÿ®÷ý£½%q`Wù)?(?_¢ÿ®7ú/zÊåòr.¿d2YQ3<P~(?Ê¯LÊ¯¯¯oÑ¢EÑ¿¨óáòCù¡ü <Ë/£T"Où)?(?ß¼¦üÊÊOù¡üP~(?åòûõ÷÷'Éx<ÞÐÐÐÝÝ]xQqs²íÜ¹óGù:sÉÿùïØ±Ã80=öØæÍ³±÷îzÈ80K6mzâ'îä-Xù¥R©ÎÎÎ0±oß¾ÖÖÖÂmûöíåâC/¿/~åWUU5>>&ÒétmmmáEÅÍQ~ò+öñÄWº466¾ùæ³ßZöiÂ9§ç/*nN¶ûK~ý×ýcûq`6Â?óÉ¤q`6~ã7~ãî1ÌRÈ%KÜÉ[üÐË/4_&3CZ­^½úÒ¥KEo-ûûDáEÅÍq3<p8ÃgxÌöï²eË2	kÝºuEl§ºº:NGgÃtáEÅÍQ~ÊåÊå÷?~üxeeeE±ÿ[[[Û¡CÂDøJ¥/*nòS~(?P~(¿YÈÞç·páÂöööâ¨¦¦&Tc2ìííýù½ø¸bþ¢âæ(?åòåò+RvðUVV¶¶¶ß¹¥ò|(?åòåòÉ&&Nìxà._¾ÏòS~(?P~(¿ø@¾ÌEù¡üP~(?_	_IS~ÊåÊå73çÏ¯¯¯_°`At>ouuõ#GÊåòCù[ù¿3<¢ò¦÷íÛ§üP~(?Ê¯¬Ê¯¦¦&tÞùóç3å×ÛÛ±òCù¡üP~(¿²*¿Ì)¿ñññè_åòCù¡üP~eU~ÕÕÕ¡ó¢ý|¡üÒéô;Âtmm­òCù¡üP~(¿²*¿É9sFù¡üP~(?_Y_Þ§¢s+++ëëë/]ºTÏòS~(?P~(¿ùBù)?(?òCù¡üP~Êå÷ëµ±±qáÂ±	a¢¡¡¡Tõ*?åòåò®G´b7oV~(?ÊåW&åwäÈ(òöïß?<<Í9zôh4¿««Kù¡üP~(?_9_2ywðàÁüEÑÿçæûüP~(?Ê¯LÊ/H¼É_N§Ã¢påòCù¡üP~åP~ÿ´mª¥Ñÿä¦üP~(?Ê¯Ê¯@Û)?ÊåÊOù¡üP~(?_	_aÊåòCù¡üÊ¤üb·ÇÊåòCùCùå§üP~ üP~ÊåòCù)?òCù¡üP~ üÊåòå§üP~(?ÊOù)?ÊåòS~ÊOùòCù¡üòS~ üP~(?å§ü(?ÊOù)?åòåòS~ÊOù¡ü@ù¡üÊåòå§üP~(?(?åòCù¡ü@ù)?ÊåòS~ÊåòCù¡üòS~ üP~(?å§ü(?ÊOù)?åÊåòS~ÊOù¡ü@ù¡üòS~(?P~(?åòCù¡ü@ù)?ÊåÊOù¡üP~(?P~ÊåòCù¡üòCù¡üP~(?å§ü(?ÊOù)?åÊåòS~ÊOùòCù¡üòS~(?P~(?åçu©üP~ üP~ÊåòCùòS~(?ÊòCù¡üP~ üÊåòCù)?åòCù¡üP~ÊoúûûÉd<ohhèîîÎ^ÔÛÛÛØØ-_¾<ù)?(?_iK¥Rabß¾­­­Ù.]zæÌ0qøðáúúzå§üP~ üP~¥­ªªj||<L¤ÓéÚÚÚ©®VYY_~û·ÛÇ~Õ7ÌÆ«¯¾zòäIãÀlôöö¾üòËÆYêêêzýõ×ïä-ÎòÇãNgcÑÞÞ_~Gý)sIøçÑÅ³qþüùð¶m«W¯÷lãÀ,õôôüë¿þë¼ÅyQ~±X,3H$ò¯pëÖ­T*5<<ìh¯£½8Úöâhoé©ø0]]]N§££½a:ç×®]Û´iÓ¤1¡üÊÊ¯Ä´µµ:t(L©T*QOOÏÚµkoÜ¸1éÊOù¡ü@ù¡üJoXkjjb±X2ìííýùcØX[[[Eù)?(?ß<¥üÊÊOù¡üP~(?åòS~(?ÊòCù¡üP~ üÊåòCù)?åòCù¡üP~ÊOù)?P~(?òS~ÊÊå§üòåòCù)?å§üP~åáÂùÌg~õWõ×~í×R©ÔOúSO·òCù)?å§üP~eX~?ùÉOªªª;ëxlûc¿·ò÷jkkß÷]Ï¸òCù)?å§üP~åfÓ¦MkÖ¬ÙùÄÎÌO5~j×®]qåòS~ÊOù¡üÊÍ§>õ©Í±9»ü|ðÁÏîsqåòS~ÊOù¡üÊÍïþîï>òð#Ùå÷/üÉüg¡üòS~(¿r³gÏß^öÛõñ(ûÂÄ÷Ý÷w÷wqåòS~ÊOù¡üÊÍèèè<pï½÷~ö?ûÀg¸çÚÛÛÇÆÆ<ãÊå§üòCù¡Ðyßþö··mÛÖÑÑqòäIÏµòCù)?å§üP~¾ÉåòS~ÊOù¡ü@ù¡üÊåÊå§üP~(?(?åòCù¡ü@ù)?ÊåÊOù¡üP~(?òS~(?Êå§üòåòCù)?å§ü@ù¡üP~ÊOù)?òCù¡üòS~(?P~(?åòCùòCù)?ÊåÊOù¡üP~(?P~ÊåòCùòS~(?Êå§üÊåòCù)?å§ü@ù¡üP~ÊOù)?P~(?òS~ÊågP~(?å§üÊÊOù¡üP~ üP~ÊåòCùòS~(?ÊòcÂßüÍß477òüÓ?ýÓåòCù¡üò+O_øÂ>^÷ñ/µéËÿûËýÃÏ~ä#ùÁ~ üP~(?òS~åæØ±c÷Þï¯ìØùÄÎèÏZ¾ðñ¡üP~(?å§üÊÍO<ñÀgÈd_ôgñâÅÃÃÃÊåòCù)?åWnå÷Ù?ü¬òCù¡üP~ÊOù?GQ~(?P~ÊoqÊåòS~Êoùë¿þë+W.]ºtãÆo¼ñÆtVQ~(?ÊOù)¿ùBù¡üP~(?å§ü(?ÊOù)?åÊåòS~ÊOù¡ü@ù¡üòS~(?P~(?åòCùòCù)?ÊåÊOù¡üP~(?P~ÊåòCù¡üòCù¡üP~(?åWþþþd2Çº»»ó¯þöVTT(?åòåò+y©Tª³³3LìÛ·¯µµ5géØØXccãTå×ÓÓóKúúú~òfãòåËo¼ñq`6Cùféµ×^û÷ÿ÷;yó¢üªªªÆÇÇÃD:®­­ÍYúäO>óÌ3SßÓO?ÝÅìØ±ãÇf#¼^~ùeãÀ,÷»ß5ÌþMíW^¹·8/Ê/O:ôijj]èh¯£½8Úöâho9ÅbéD"½¨¥¥åÔ©SÿóPòCùòCù¨_ÓÕÕÕétúý£½azÒ«e®¬üÊÊ¯µµµ:t(L©TjªRÌ©üÊÊ¯ôµ¦¦&%ÉÞÞÞISOù)?(?ß¼¦üÊÊOù¡üP~(?åòS~(?ÊòCù¡üP~ üÊåòCù)?åòCù¡üP~ÊOù)?P~(?òS~ÊÊå§üòåòCù)?å§üP~ üP~ÊOù)?(?òCù¡üP~ üÊÉ×¾öµ_|ñmæï~÷»ÿôOÿdÞÞÞ®®.ãÀl¼ùæÆY:räÈÀÀÀ¼Å÷ÞOùMéüùó;wîü:@Y(|0³ÂnXyBù(?Êåò@ù1^Yb±Xö¢þþþd2Çº»»Å½zÃiùòåáEe¬(âU9qâDXd (îU4::ºiÓ¦D"qß÷õôô(?æ»ÎÎÎûöeÏI¥Rafó[[[Å½.]zæÌ0qøðáúúzCD¯¢`ll,üBùQô«hÏ=O=õÔøøxÈ¾ºº:åÇ¼vùòå5kÖäÌ¬ªªCÂD:®­­5J÷BÊVYYi(îUôäO>óÌ3Ê¢_E.wÏ»¯¥¥¥¯¯/gf<tfôBÊÚÛÛE¼®ÒÔÔþ-ªüÍÚÞ½Ã¿?ëêêÎ;§ü¿W¬X??ûDÂ@QÜ)rëÖ­T*5<<l (âUÞÈO:õþÄ§¸E¿©<x0Lx1üCBù1íÙ³'ü3(~uuu:~âho6P÷B®]»¶iÓ¦ë×¯%Uü2Eqojé» Ë+»lÕªUgÏÍßÖÖvèÐ¡0~¦R)Eq/¤µk×Þ¸qÃQô«(;Å½¶lÙòÂ/óçÏ¯Rù1%èLß­§O®©©ÅbÉd²··×@QÜ©¶¶ÖÞfù*R~ÌþU444ÔÒÒÇÊåò@ù(?Êåò@ù üP~(?ò@ù üP~(?Î·¾õ­U«VUNX³fÍK/½ôK¿à&Ì¯ãÉîmmmmxhÃÃÃ9óÃx<L&ÇÇÇgºM@ù;wVäùÆ7¾QNå·÷î0óùçÏÿÜsÏù»ví*bò(1ýýý¡iâñøþýûÇ&<x03Ï=[6åwùòå0sùòå9ó-[æ_ºtIùÊ(=ôPh§z*æÓO?fþÙýYv÷twwrQØØØ¦3W¾yófûÂÃ¢ªªªmÛ¶eT=yòd¨«°(¬ÛÕÕÓRaÎ¢EV­Zuüøñp±­­-ç;v¬ðv¢Eá.EN81U¥­]»6ÌïííÍÌ9sæLÓÜÜ³gÏ°©ÊÊÊ7^»v-¿üò·3§À]ÀÝ´dÉP-W®yõêÕ0³¶¶6»lrôõõEK7lØ³hëÖ­Ñ¢X,6éZÑÅhiKKËøøxhÇD"166¡,Xü®ÀvÂÄ¤w/ÿ>|8;g3qÙÙÙÉ¾¬Y³f¦åWà®Êà.ìNòK­¢"tXvÙ´¶¶Þ&ÂÅõë×GK£ÐÚ1Úu-ZJ¥¢aúôéÓÑF²·ùøã¶¶>úè£aÎÑ£GÃtø¦~øáÛn'Tc¸®ß&D¡&/^ï7ÂÅð3Üóét:ºB2+ðþ/WVVÎ´üÜU@ùÜýò&-¿Ìü¨l¢`®]».f.666uuu7oÅ622ÙH¸NÎ^´ÌZÑÅ«W¯f®j)sÀ7üÓ'O¼ívBeï¼óNöê3yÛ·oyæ÷qDÛ¶mÙWÊ/ôhSSS´Kr¦åWà®Êà.vtÝºu+æððphLFñ	ÌóòÂæ´Töw©éªªªD"1::6¾hÑ¢ÌÒÛngªËqþüù(RÃtø¦2KO>îÀ¤G§_~î* üî²è³n÷îÍ×IÎÏÞ¸q#WÖÙ³gwïÞÍ$c´C.s8õ¶¶uëÖè oøÙÞÞ_`;Ñ>¶ë×¯GßyçÂçá®X±",¾È&kö¢è<ß°èÄCCCË/S¥Ñhd¸«ò¸Ë¢3$âñø¾û¢ouÙ¿"È?cýúõ¡rFGG£C±óp£ÏùEÌþ`Û¶muù^Iû,:Ù6ºõÌüÛ>ØÎoxx8ºfò;xð`foÈÙ¢<[	0UùE­Z9çÆ³¸«ò¸û¢o9Î±gÏÿÿnBFéÌqÒÌI>úhvVf;räHòâ?Ûó«ªª²ØN¸Ùw,s^íTvdd¤²²2z9Ç¸×­[/?£/vÉÞf´K2#êÔÌÒwP~sB¨5kÖ$&¬Zµ*:Á6§üN8m^CCÃ©S§2KwìØQSSÕÒöíÛGGG3K»ººB%Éäl3ÿMù[¶lÉ?ÕvpgÂ]¾h°À÷ùe´··ç|½KäÆ©T*ÀÂÃ£¸téRæj²·944Ò6«æææÞÞÞ[,pWåò@ù üP~(?Ê@ù üP~(?Êåò@ùçÿ¤ßõIEND®B`


×1¢W¿6Ëb¼¦Q^ÆÐ2J«(Ë"MQ21!Ñ¨E%RzG=2æ¥QF3`ÔbÞÍÏÊûÍÿõv»³Û	ÎÝùó²þ÷ÿßÿïûÿÿgßÿÿþçºw$I4;ª³	$IÈO$Iä'I$ò$IùI$ü$ID~$I"?I$$IÈO$ü$ID~$I"?I$$IÈO$Iä'ÕÎOTA©Tª¡¡aÉ%äì§O^±bÅL>Ô÷pÆdü3Ï<ó^£YÓC-[¶¬a¬ûî»ïàÁwäg)>tttìÝ»wgÚÏS§NÅ0NÇw/ZBÝUÈD~R5É¯°­[·ÞUUüæÎåÊjß£>Zº³vîÜYfiSe¢çÃ=¦ºq¦½3LþûvwwD~î2nÜ¸ñÌ3Ï444ÄÈ¾¾¾Ù ¿hÍ5U!¿Ó§O'Tî¹çFFFFGGc nÆÈq5ÕYJÛ·o1¡±_2ãõë×c8ËUÔ®D~RMÉ/éÉ',:ÜrâÄöööt:½hÑ¢cÇÉ)¿d î0oÞ¼¥K7ç7ÞxcÅÉÈzèÚµkß´³³3ñøñãEu¢ÅÞqÆ¢°aÃøzèÐ¡¶Éo¾¹nÝºäf³Ù.Ý³p1ñMã[ÇX¶lÙÕ«WO:ÕÑÑQ__¿råÊÂã»wïnnnNNkÆ·ÈOèîsñç^£zÜ´iÓ¸ûwª³~ëÀb9¥3î1¹Â¦7cyùMýÏØìúÔ§bÛ?:X:uOãjlÉÀtLjllÜºuëÐÐ_,ùIÕ$¿ÀJliiIn¤R©Â×ãÓ§O_rçµk×7çxa.º~ýúÂU×+³Øò3®û72LSSSnË/ÇËyárâED+Òï»dÉäjR($Ï¾¢._¾¼¼o,Xãß~ûíÒÕÖÖ6îþê,Eß:Xó[¸pá6Nù?í'/¿þþþÂåÄÊNU~|¯Y³¦è1oÙ²Å/ü¤j_2>Lg³Ù¸ÙÓÓÃ½½½>+=¹¹cÇÑÑÑä(ÎçÎxÉû'IÉÔxÅ1û­±b®ÂïUf±ågwÝ>¬ðþ7oáÕ«W_+âfh1?üðÈÈHòÀ%ÇÍcÇ®`òn¶£GÆðÅ'MôÓéôD+÷]Î2ÑéòÇD§ºqÆTÓ±¼éo>ÂôyM^~|'"¼téRÇÞá9sæøÅ"TòËàKÞVX)#¿Ë/çÇÜqÞüñ³ä¬b¼&7ãå³ð`UÑÙº2-?ãDë1^ÔÆ755®Q¼ÀÇÍùóçO´¾ÉÐFáÍäv²$ËB]]]ë^F~yOÄôw9Ká&ycc®X±âÄãn´ÉlqÕ´g¼ügÈÕ«W¿Åä7É§qgggÜlmm¹Æ!nß¾í·D~RÉ/¤#'z¹È(ÉÍ Nê2¾êkó4;ÑøXåXßxý._zÌ¬ôMo¥ë;¡Ì¢3w¼Fµ¹¹99=]Ê»"Oç0Y&³­Æ½Ãd6ÎDÞ?Ûûî]|&øËðÈ#~±Hä'UüöÙÂ+<£hEWYN:S·ü1·ß~»pjÅ±Ìxþùçãfþ3P	ÎNxò¤ðfòé*ÇMF~ÉÉäV²ÙìÖ­[¯_¿¾oß¾¹nÝºqå7Y¦-¿©n|ÓqòòÌ1¿¼íÿêÙþeoIgÎÙµkWr9´$ò*]~ñÚJ^óê¼â2¹TvÑ¢EÉ¤äMNCcûYfÞòlJÈ¼O+,§è].¶üå±fÍüù1É¿«W¯¾1V²´~øÝË/9î500«°wïÞÉÈïäÉÉQ¥XåÞÞÞäx^ÒáÃÇÝ¿SeJò+¿qMu«NC~É;óâÉpûöíÝªU«J]øÔSOÅÆËoÿ;þ$ï×,sæ]ùI"¿Ò¯O,½Z6ÿ~ÿüî&K_2ËÌ[MA¢ÄFZæ§YlùË3âÊ+ùwt%c.Pô¯¦¦¦üßüòI?~þ]e|óÐCî¬ÅÙÅSeJò+¿qMu«NC~gÎ)Ò¥K§müæ2Ï·ü%Dù6nÜèD~RuÈ/^/_^ú×½;ÖÕÕóg6?þðáÃ1&¤Uøiv÷lJ>/ÞÙÙYú±|-ö3gDr&´püùóç×­[W?V6-½czò»víZ,-Ú¶m[`(îò¾yúé§cíbÅ,X°ÿþOúÓùcNï~)É¯üÆ)zn5¥­:ùEGimmµå'çÓó3ÇÍðYòÝW¬XÑ××wÇc®=ßyää`jð=våðð°_,ùIÒ])Ë=ôÐCSú¹iÌRu%ÇzµGFFÐ···ÂHä'Iªµ÷ùõøãÛ2ùIj­¡¡¡íÛ·/X° 9¥>ú¨Í"$IÈO$Iä'I$ò$IùI$ü$ID~$Iä'I$ò$IùI$ü$ID~$I"?I$$IÈORtøðát:½|ùò©ÎÛÑÑQWW788Ã1fÑ¢ESø-6Öôî3y§ù»µ¤düÐÐÐ-[cÍ;7Í¾ùæù¹FFFb|Ü¹¾¾>=»$¤Êª¹¹9¤ríÚµiHe÷îÝ1ïüÁäÇ<ñÄ1f×®]µ!¿Òñ6lñ===1|êÔ©nkkËO=räH^GõìD~*ìÈ»ÀÓ3gbÞeËåÇ,Y²$Æô÷÷ÏÌyùÕ××Çø7n;×>S¯7nôìD~*ÊyäGæÎ;gÎ;wÝíÓþtWWWÑòcøí·ßáLºtéÒ+Òétgggr¬tiß½ü,û÷ïoii¹ï¾ûâúìÖ­[ÝÝÝ1o<øíÛ·'0Ã¦K.¥ÅøXòåËß¥üî½÷ÞßÞÞÞÛÛ[4itttÞ¼yñ½nß¾_çÏ÷ñô$¤»¿dø±Çág6?þxá;¯°Í7Ç¤ûöÅp|áM6%/^ñ¼xñbÏd2ã.­ð%<÷üóÏÇÀJü-[b8îðÂ/ÄÀîÝ»óP»qãÆÀÀ@$$'zßD&.¼ÏáÃóc/_þÆoägINþ®^½:×­[Ã1fÜÇàY'ü$½ÿòkiiáÜX¥ð÷'OÌsgÍ5	éòSÏ9³cÇ®®®J¥Æ]Z½ÊÏ<°¹sçÎÛÔÔ<øÑÑÑoÅÈ¸gwvve§·Y:út5ï¿Xr2~ëÖ­ùP·mÛön$¤»(¿äºÔüø¸yG³æÌ÷JNhÆdÒÞ½c®àào¼Q¸¢¥ÞÌ,=°`bá!ºD7o^2¦±±198ùc~e¶[OOO`®ðìv¸9¾éõë×ß;õÃ,÷1xÖI"?Iï¿ü2Lá1¿ä°ÙÔÝÝSó_óã!FÇ¤üÊÏ`©ÒyçÏÌ[ôðFFF=?X8¥Í2QÉÇÃyªuæÌi?I"?IwQ~É§´ìÛ·/yßO<1:t(ç>?>qäÙ³g÷ÿMF~ågÙ±cÇ7¾ñØ¼ysé¼7ná;w.9»#ï»ï¾]½z5..å·hÑ¢üøñw~ú.=ôÐ;ÀMöc$òtå722º3VágòßíÛ·3­ñµðãNFsçÎÝ¾û$åW~'NÄÔ%Kä/4)úå­[·Æ#¯¯¯_µjUr	m`+Í&/[¶ìüùóSÚ,¥gc±ÝÝÝñceçÏ¿eËø¾ïüôC­ÿPëi?I"?I$ò$IùI$ü$ID~$I"?I$ßûÓ÷¾÷½·ÞzËó`ÆºpáÂ?ÿó?Û^.=e;T~ÿðÿð÷ÿ÷¶CåwñâÅú§²*¼ùùáH~5ÞW¿úÕÀ§ûuòäÉüä'¶C744ôòË/Ûßo¾ùù¶Cå/4U©Ê,t~âÄ	ò#?ùüD~äG~ä'ò#?ÈüÈüD~ä'òùùùùüÈOäG~äG~"?ùüÈüÈOä'ò#?ùùüÈüÈOäG~äG~"?òùüÈüÈOäG~"?ùüD~ä'ò#?ò#?ùüD~äG~ä'ò#?ÈüÈüD~ä'òùùùùüÈOäG~äG~"?ùüÈïÎõ÷÷g2t:ÝÑÑÑÓÓS8ixxxÃõõõ÷Þo°üÈOäG~"?_uÍf8öìY¿~á¤Ý»w?þøã£££aÖÖÖRù=zôºfªï~÷»/_¶*¼ýèGñëÏv¨üþüÏÿÜv¨üâè­·Þ²*¼¿û»¿;~üx¬È¬_cccØ.r¹á¤sçÎM4cÈ/°ø§©ÂÙ§N²*¼ØG±§lÊ/þ+F·*¿^zéW^±*¼ïïßúÖ·j`EfüÒéô¸ÃÉÍ§zª¡¡¡µµõ7Þp¶×Ù^9Ûël¯í³½Õ]*Ê×××MÚ·o_?¾««üÈOäG~"?_u×ÔÔËåÞ;ÛÃEòÃEÉüD~ä'òùU_ÝÝÝû÷ïøÍf'=üðÃÏ?ÿ|=ö¾ûî#?òùÈOäWÝõöö677§R©L&Ó××÷oëV÷¯k?lk×®M§Ó]]]äG~"?òùüfiäG~"?òùüÈOäG~"?ùùüÈOä'ò#?ò#?ùüD~äG~ä'ò#?ùüÈüÈOä'ò#?ùùüD~ä'ò#?ò#?ÈüD~äG~ä'ò#?ÈüÈüD~ä'òùùÈüD~"?òùÈOäG~äG~"?òùüÈüÈOäG~"?ùùüÈOäG~"?ò#?òùüÈOäG~äG~"?ùüÈüÈOä'ò#?ùùüÈOä'ò#?ò#?ùüD~äG~ä'ò#?ùùüÈOä'ò#?ò#?ùüD~äG~ä'ò#?ÈüÈüD~ä'ò#?ùùüD~ä'ò#?ò#?ÈüD~äG~ä'òùÈüÈüD~ä'òùùÈüD~"?òót'?ùüÈüÈOäG~"?_EtîÜ¹_|ñµ×^&?ò#?òùÈ¯6ê­^½úîY¼xq[[Û?øÁò#?ò#?ùüj°|ðÃ¿øáí_Þ¾ó·wÆ¿_ù_?þücò#?ò#?ùüj­yóæñá/&ìKþìcûú×¿N~äG~ä'ò#?_MuóæÍ¦¦¦BöÅ¿ûï¿ÿ÷ÿ÷ÉüÈüD~ä'ò«µ>ðlþÍÍòkkkñÅÉüÈüD~ä'ò«µ|òÉL&ðÝñ[;î¿ÿþ_üÅ_|Ï¯ð%?òùÈOäWmÝºõg~æg>Òö¦¦¦Å_¸pá=ÿäG~"?òùü*¥7o¾þúë?úÑFFFîÆòÉüD~ä'òùÍÈüD~ä'òùùÈüD~"?ò#?òùÈOäG~äG~"?òùÈüÈüD~"?òùùÈOäG~"?ò#?òùüÈOäG~äG~"?òùüÈüÈOäG~"?ùùüÈOäG~äG~"?òùüÈüÈOäG~"?ùùüÈOä'ò#?ò#?ùüÈOäG~äG~"?ùüÈüÈOä'ò#?ùùüÈüÈOäG~äG~"?òùüjY~ýýýL&NwttôôôÞáøñãuuuäG~"?òùüª¾l6àÀØ³gÏúõë¦tvvùüÈOä'ò«GGGc Ëµ´´Mì±Ç|òÉä÷GôGÿW3Õ±cÇ¾ÿýïÛ^ì£ØS¶CåxåWlÊïøñã¯¿þºíPá9sæèÑ£5°"³B~étzÜáèÒ¥K]]]áÂä÷Ío~ó-ÍTßùÎwmï¯ÿú¯ãÊv¨üúûûÿìÏþÌv¨ü¾ûÝï?Þv¨ð~øÃ~ûÛß®òK¥RùáúúúÂIk×®=uêÔ¿®ª³½ÎöÊÙ^gål¯í­r¹¶7ÿÃþÇÈüD~ä'òùUwÝÝÝû÷ïøÍfÇ_UÇüÈOäG~"?_ÔÛÛÛÜÜJ¥2L__ß¸Ô#?òùÈOä7«#?òùÈOäG~"?òùüÈüÈOäG~"?ùùüÈOä'ò#?ò#?ùüÈOäG~äG~"?ùüÈüÈOä'ò#?ùùüD~ä'ò#?ò#?ùüD~äG~ä'ò#?ÈüÈüD~ä'òùÈüD~"?ò#?òùÈOäG~äG~"?òùüÈüÈOäG~"?òùùÈOäG~"?ò#?òùüÈOäG~äG~"?ùüÈüÈOäG~"?ùùüÈOä'ò#?ò#?ùüÈüÈOäG~"?ùùüÈOä'ò#?ò#?ùüD~äG~ä'ò#?ùüÈüÈOä'ò#?ùùüD~ä'ò#?ò#?ÈüD~äG~ä'ò#?ÈüÈüD~ä'òù§;ùüÈOäG~äG~"?òùüÈüÈOäG~"?ùÍùÕÝ©T*E~"?òùüÈ¯äºSétüD~ä'òù_-È¯6"?òùÈOä÷n§øæÍÉOäG~"?ùÕüZZZÒé´÷ùüÈOä'ò#¿ß¢EJ/ïhllòùÈOäG~5%¿úúú Þµk×c ^3¾ñoÄÀ¦MÈOäG~"?ùÕü|1Ô/ÆÀ9sÈOäG~"?ùÕüæÍÎëííG4ð©."?òùüÈ¯Öä·ûöüõoõ»ï¾ûÈOäG~"?ùÕü¢¯íkóçÏ¾¾¾vuuUÅúùüÈOä'ò-ùüÈOä'ò#?ùüD~äG~ãµpáÂä³]|³ÈüD~"?ò«eùµµµj/kE~ä'òù_­É/æ;úôèèhÕ­?ùÈüD~"¿)ÔØØò«FöùüÈOä'òZýýý!¿-[¶ÜºuüD~ä'òù_-Ë/Z°`A]I®ðùÈOäG~µ&¿ÖÖVWxüÈOä'ò#¿Y!¿Ä|Õ¸þäG~"?òùü¦PSS+<D~ä'òùß¬_¼ü¶oß><<L~"?òùüÈ¯åW7A®ðùÈOäG~µ&¿Ô¹ÂCäG~"?ùÕüª:ò#?ùüD~S¨¥¥¥µµõÜ¹sä'ò#?Èüj étº®î>vØßßÉdâtttôôôNêëëëììI-»ùüÈOä'ò~!­ß®]»âiý~¶K6=pà@ìÙ³gýúõÚÚÚ^õÕ8xðàÂÉüD~ä'òù½ETÀµ½	:s¹ËDwkhh(ßïýÞïÕLuäÈÁÒÕ·Æ²ªâÊò«oÆºëò«k¿×Dß÷ôéÓ6m*ß+¯¼òÿ4S½üòË7oÞ´*¼üÇìéé±*¿ÁÁÁüà¶CåwêÔ©ë×¯ÛÞÐÐÐw¾óX».¿J¨ðøbénÝºÍfc§:Ûël¯íu¶WÎöÊÙÞê®©©)Ë%gc¸hê+W6lØpõêÕÒÉüD~ä'òùM­ðÖªU«êêêæÌ³víÚ¾Ô£»»ÿþý1_³Ùl5V®íÚµqg$?òùÈOä7nß¾=î3ùg|S©T&éëëû·uû¬ÂGE~ä'ò#?Èoúµ··¨V¯^ëÖ­¸Oî5kÖÄÅWþúùüÈOä'òBõõõá¼ücÆ½ÒüÈüÈOäG~"¿*_*ç%X$ÇüTò#?ùüD~3!¿älïÊ+³½ñ5cLgg'ùüÈOä'ò#¿_PoÜ+<®_¿N~"?òùüÈ¯¦ä÷ÎØå½k×®;wn*¯+W®1U±þäG~"?òùüfKäG~"?òùüÈOäG~"?ù_~Î;Uø·tÉOäG~"?òùU±üRG~"?òùüÈ¯¦ä7QÛ¶mKäwðàAòùÈOäG~µ)¿þþþ9sæùV¬XQøÁÎä'ò#?ùÙäWSò[·n]r¨ïèÑ£U´þäG~"?òùü¦Ð/¼oõêÕU·þäG~"?òùü&ÕíÛ·/^ÑÓÓSëO~ä'ò#?ÈïÎ=õÔSÉ¡¾õë×WïúùüÈOä'òÄ>ÏOäG~"?ùÍù¥îT:&?ùüD~äWò«ÈüD~ä'òùÈüD~"?ò#?òùÈOäG~äG~"?òùüÈüÈOäG~"?òùùÈOäG~"¿êßÂëëëÈüD~"?ò«qùµµµjÏçùüÈOä'ò#¿_ /ÌwúôéÑÑÑª[ò#?ùüD~S¨±±1äWì#?òùÈOä7µúûûC~[¶l¹uëùüÈOä'ò#¿Z_´`Áº!ò#?ÈüjM~­­­®ðùÈOäG~³B~ùªqýÉüD~ä'òùM¡¦¦&WxüÈOä'ò#¿Y!¿x-ùmß¾xxüD~ä'òù_-Ë¯n!ò#?ÈüjM~©	rÈüD~"?ò«5ùUuäG~"?òùüÈOäG~"?ùßxår¹U«V544ÔÕÕÍ3gíÚµÕr©/ùÈüD~"¿)tûöíq¯ð¨KÉüD~ä'òùM¡öööpÞêÕ«¿ÛOî5kÖÄÅÈüD~"?ò«)ùÕ××óFFFòcr¹ñä'ò#?ÈüjJ~©T*ÚË1>ÕEäG~"?ùÕü³½+W®LÎöÆ×1ä'ò#?ÈüjJ~A½q¯ð¸~ý:ùüÈOä'ò#¿ß;c÷®]»vîÜ¹©T*¾®2ÆTÅúùüÈOä'ò-ùiä74K~ä'ò«ùµ´´´¶¶;wüD~äWÔë¯¿¾hÑ¢ÆÆÆùóçä#6/ùÈ¯ºåN§ëêªõØ!ùîüçÍ·fÍ¿µcçoïükþçÏþìÏØÂäG~"¿*_OOOÈo×®]ñ´®?ÚF~ä§ß<ðÉO~2Ìÿ÷ßÿÛÿÌg>cùüªX~uJ¥ÈOä7å×ÞÞ¾ù77Êïÿûþðmaò#?_Ë/5A>ÉYä7Ëåwÿý÷ÿ¯îÿU(¿ÏîóÿøÇmaò#?_õÉ¯µµµ£££Ú¯×#?òÓÝßÓO?ï½÷nÿòö1Ðú­=ö-L~ä'ò«>ù%®÷±³½UqbüÈO3,¿hëÖ­÷ÜsÏý÷ßÿË¿üË?÷s?÷/|¡ðo|üÈOäW5òKþò'zÉOäG~500ðÌX¯½ömK~ä'ò«Vùe2º²¹ÂCäG~"?ùÕüN>=oÞ¼äÈ_â<WxüÈOä'ò#¿Ú_¾jAùÈüD~"¿YùÈüD~"?òùÈOäG~äwëïïÏd2étº£££§§§ü¤é)lçÎ¿þë¿þUÍT¿ök¿ö;¿ó;¶Cû(öíPùéK_úâ¿h;T~ñB/7¶C÷»¿û»ÿüçk`EªL~ÙlöÀ1°gÏõë×4½1mÛ¶­N$©Vºëò;þü(¿ÆÆÆÑÑÑÈår---å'MoùI$òî	ã±téìì|óÍ7ßýÒ/.ºd¸tÒôÆ¶ûöæææ5SýüÏÿül/öQì)Û¡òûàX¶Cå/4~õU~úÐjãWß]_/ÏÌ Õ²eË.0í¥~þsùIÓãWxÈ®ð+<äwÎ·½½=OÀ0ÖªU«¦±¦¦¦áò¦7üÈOäG~"?ßÐèèèÑ£Gê¦û×Ûº»»÷ïßñ5Í4½1äG~"?òùüÞUÇüæÎ»iÓ¦i,§···¹¹9ÔÉdúúúþíQ½]±tÒôÆùüÈOä'òfàkhhX¿~ý¥KªeýÉüD~ä'òùMecv|êSºxñbÕ­?ùÈüD~"¿©ýê©Þõ'?òùÈOä7["?òùÈOä7µÎ=»páÂ9sæ$×ó655:tüD~ä'òù_­Éïøñãù+<ù%Ãöì!?ùüD~äWSòknnç=6/¿¾¾¾ä]ÈOäG~"?ùÕüò8/¿ÑÑÑä_òùÈOäG~5%¿¦¦¦p^r/äËåyänii!?ùüD~äWSò×òºñzõÕWÉOäG~"?ùÕü¢«W¯vuu%×ö644,ðÂU±þäG~"?òùüfKäG~"?òùüÈOäG~"?ùßìÒ¥KsçÎMÕrªüÈOäG~"?ßdÛ¸qcÝmÞ¼üD~ä'òù_ÈïÐ¡C	òöîÝ¯ÉÈÛ·o>|8ìØ1òùÈOäG~µ ¿L&¼Û·o_é¤äï¹ù<?ùüD~äW#ò«¯¯ÞÝ¾»tR.IqòùÈOäG~µ ¿ümhjòÜÈOäG~"?ò#?ò«ù±ùüÈOä'ò#?ò#?ò#?òùÈ¯åW>òùÈOäG~5"¿ÔJ§Óä'ò#?ÈüjA~µùÈüD~"?òùÈOäG~äG~"?òùüÈüÈOäG~"?ùùüÈOäG~"?ò#?òùüÈOäG~äG~"?ùüÈüÈOä'ò#?ùùüÈOä'ò#?ò#?ùüD~äG~ä'ò#?ÈüD~ä'òùùÈüD~"?ò#?òùÈOäG~äG~"?òùÈüÈüD~"?òùùÈOäG~"?ò#?òùüÈOäG~äG~"?òùüÈüÈOäG~"?ùùüÈOäG~äG~"?òùüÈüÈOäG~"?ùùüÈOä'ò#?ò#?ùüÈOäG~äG~"?ùüÈüÈOä'ò#?ùùüD~ä'ò#?ò#?ùüD~äG~ä'ò#?Èü<ÝÉOäG~"?ò#?òùÈOäG~äG~"?òùüÈüÈOäG~"?ùùüÈOäG~"?ò#?òùüÈOäG~¬¿¿?É¤ÓéÂI1iÑ¢Eq7ò#?ùüD~Õ]6=pà@ìÙ³gýúõÚÚÚ^õÕ8xðàÂÉüD~ä'òùUw£££1ËåZZZ&º[CCC©üþðÿð´fª^z) n;Tx±=j;T~ñ_©Û¡ò;vìØþéÚÞk¯½V¿úfüÒéô¸ÃÅ¶Ø´iS©ü>ücÍTñß©¿ù¿±*¼·Þz+öíPù½ñÆßÿþ÷mÊïå_¾páíPáýíßþí·¿ýíXY!¿T*®¯¯/½Ã­[·²ÙìÐÐ³½ÎöÊÙ^gål¯í­ÂþÓb¸©©)Ë%gc¸èW®°aÃÕ«WKB~ä'ò#?È¯ÊêîîÞ¿Ä×l6[D+W^»vmÜÉüD~ä'òùUY½½½ÍÍÍ©T*ÉôõõýÛºlii©+üÈOäG~"?ß,üÈOäG~"?ùüÈOä'ò#?ò#?ùüD~äG~ä'ò#?ÈüÈüD~ä'ò#?ùùüD~ä'ò#?ò#?ÈüD~äG~ä'òùÈüÈüD~ä'òùùÈ¯´?þã?~à~õWu÷îÝ7oÞ$?ùùÕTÃÃÃ¯½öÚ#GÉO³Y~###¾_ø_øÌºÏþþsçnii¹páùüÈüÈ¯F:wîóGÚ>òO|âø@¼ÚÉO³S~O?ýt°oû·ïüíÉ¿_ºï>ùÉOÈüÈüjäµüC>ô?VýäEîÿóHû¾ðòÓìßºuë>³î3yöÅ¿/oûò¼yóÈOäG~äG~µÐ/¾ø±¬ðuîK[¾¯s###ä§Ù)¿Ïf?[øÿ"?ùùÕHO?ýôÿ²¢ðu.þ555ýøÇ?&?ÍBùÅOÄG~tÇoíÈÿ8¬ü¯+ãùüÈüÈ¯zñÅÿSûrÌOäÏüO|â¿±á76ÿææûï¿ÿîy¿®|"?òùù½÷¯åüà½ÏOäoxxø+_ùÊÇ?þñ~ô£<ð@Õ]ØK~ä'ò#?ò+×¿_Û»ä_¯íýìg?ëÚ^ÍfùÕ@äG~"?ò#¿rõb_|ñEç'ò#?ÈüD~"?òùùÈüD~"?ò#?òùÈOäG~äG~"?òùüÈüÈüD~"?òùùÈOäG~"?ò#?òùüÈOäG~äG~"?òùüÈüÈOäG~"?ùùüÈOä'ò#?ùùüÈüÈOäG~"?ùùüÈOä'ò#?ò#?ùüÈüÈüÈüD~"?òùùÈOäG~"?ò#?òùüÈOäG~äG~"?òùüÈüÈOäG~"?ùùüÈOä'ò#?ùùüÈüÈOäG~"?ùùüÈOä'ò#?ò#?ùüÈüÈüÈüD~"?òùùÈOäG~"?ò#?òùüÈOäG~äG~"?òùüÈüÈOäG~"?ùùüÈOä'ò#?ùùüÈüÈOäG~"?ùùüÈOä'ò#?ò#?ùüÈüÈüÈüD~"?òùùÈOäG~"?ò#?òùüÈOäG~äG~"?òùüÈüÈOäG~"?ùùüÈOä'ò#?ùüD~äG~Y&I§Ó===¥w8~üx]]ùÈüD~"¿ª/Í8p öìÙ³~ýú¢©###É/,ò/©ÂñëÏv¨ðnÞ¼ÿ²*¿¿ú«¿úÁ~`;T~§NºvííPáýä'?	ùÕÀÌù566Æ@.kii)úØc=ùäÉï'8¦êÈ#/½ôíPáÅ>=e;T~GýÖ·¾e;øÕ§÷êWßüÉÔÀÌù¥Óéq£K.uuuíu¶WÎö:Û+gålo-J¥òÃõõõÖ®]êÔ©]Uò#?ùüD~ÕúÐZ755år¹wÆÎöÆð¸wËßüÈOäG~"?_×ÝÝ½ÿþ¯Ùlv")$?òùÈOäWeõöö677§R©L&Ó××7.õÈüD~ä'òùÍêÈüD~ä'òùÈüD~"?ò#?òùÈOäG~äG~"?òùüÈüÈOäG~"?òùùÈOäG~"?ò#?òùüÈOäG~äG~"?ùüÈüÈOäG~"?ùùüÈOä'ò#?ò#?ùüD~ä'ò#?ÈüÈ¯úÊW¾òÍo~ó-ÍTÏ?ÿ|¼PÙÞùóç¿þõ¯Ûß©S§zzzlÊ/^hþâ/þÂv¨ðî¹X7oß=vçÎ_$IªÊÌíò$I=$IùI$ü$ID~$I"?I$$IÈOÕù*(JtõêÕÂ¶ÞLvòäÉöööt:ÝÑÑÑÛÛ[8©¿¿?É$zzzÆ£JØS1iÑ¢E±üLUæn*Ý)~ *sOÍ)¿t·:pàÀ=ÊO:tèÐl«÷¥ÆÆÆK.Å@|mii)ÍfcÅ@ì¦õë×;F°§ÚÚÚ^õÕ8xðàÂýLUæn*Ý)~ *sOÍ)òÓ]éâÅË/¿ã¤ø:|ø°Íõ¾ÔÚÚzåÊ¯1qtt4r¹±t*aOÖÐÐàgª2wSéNñUÉ?P5ÿ"E~º+­]»öôéÓwÔÖÖ¶råÊt:ÝÕÕuþüyÛm&ëïï+ÔÕÕÅ×¢¤h¸t*aOåñ6mò3U»©t§øªä¨"?½÷.Y²dJâÿ^-²éf²Å;w.ù=X´Sß Y__?îUÂJºuëV6ò3UÉ»©p§øªØ=5^¤ÈOï»wï~ê©§¦:É¯¿®ÌQ¦¦¦÷ÎØ©¨w*aO%/H6l¸zõª©JÞME;ÅTÅî©Ùð"E~zï[ºté3g&3©­­íÂÉ«×Ê+mº,þ_öìÙÿNêîîÞ¿Ä×l6;îUÂ:yòdüàvÍÏT%ï¦Òâª2÷Ô,y"?½÷Å7/ÿûóì§ÃM:útruý²eË·ÜjÆzóÍ7ã·^lüøÃª···¹¹9Je2¾¾¾qÇ¨öTKKKÑGNøªÀÝTºSü@Uæ%/Rä'I4["?I$ò$IùI$ü$ID~$I"?I$$IÈO$Iä'I$ò$I"?I$$IÈO$Iä'I$ò$IùI$ü$ID~$I"?I³¨çnéÒ¥c-_¾ü^ø¿àÆª_Çã=ÚXµ¡¡¡¢ñ1&Ng2ÑÑÑ©.SùIRõµsçÎº¾öµ¯ÕüvíÚ#yæ¢ñO?ýtôÑG§±LIä'IUV&NïÝ»wd¬ûöÅÍyæÌßÅcä¢EÆ···Çø.$òTûîsÓ<þøã#xâùàº§§§'ä(ìììáü¯_¿¾iÓ¦¹sçÆ¤ÆÆÆ­[·T=qâDè*&Å¼Ç+²T7oÞÒ¥K=7»»»Ø#GÊ/')tüøñ¶råÊß××óê«¯Æ+VäÇìÞ½»¹¹9ÕÐÐ°nÝº+W®Ê¯tùEcÊ<TIä'Iïg,µt©päåËcdKKK¡l:út2uÍ5E¶lÙLH¥RãÎÜL¦®]»vtt4ìX__?22SãkÈiÎ9ÉÛïÊ,'Æx¥kzðàÁBÎæqyàÀ<û²|ùò©Ê¯ÌCD~ô>ØçZ]]8¬P6ë×¯¿5VÄÍÕ«W'Sè$vLÝØIÙl69XÃ½½½ÉB¹cÇ°]r²uãÆ1æðáÃ1_cø¡ºãrBq3îPøØÆ]£Ðäüùóc¯]»7ãk<òàf.KîÉdbÆXw~zv¸¡¡aªò+óP%$½ÿòÆ_~|"LÑ+Wâf)¹ÙÙÙ7[[[7oÞb»ûv~!q¢£hù¹/_Îß9´?á_cøÄw(3n¾ýöÛm¢÷ämÛ¶-&=ùäïüôöÖ­[ïùG»ººCS_*ü$é.9ÐuëÖ­ÂCCC12&±NÞ	þòÐÉ¿9¯ô$l¥?K%ëëëcáóæÍËO½ãr&rXQgÏMÃñ5òSãÖxòò+óP%$½Ï%ïuê©§G&uRtGþ½×®]+=uæÌ]»v%ç^ódLÈåO§ÞÑg[¶lINòÆ×M6åÇYNríêÕ«ÉÍ·ß~»üu¸K,©ÉÙX'%×ùÆ¤ãÇß¸q£¼üò*M¶F~j*ü$é.¹B"NïÙ³'ùT½÷Ö××^±zõêPÎððpr*6nò>¿äíqoL ¸uëÖpRrÕmþsUÆõYr±mòÝO<_f9Éû÷ù%÷,#¿ûöåÆÅ*NJÞò800ß%6ÂDòK¬V»8×­[W8µÌCD~ôþ|ÊqQ»wïþ÷_pc%0ÊçÏæ/ªÈ·qãÆBVvèÐ¡2ògìmÄøÆÆÆÂÁe£ðå¯«heoß¾ÝÐÐ¬BÑ9îU«V~ùóçÇ×ä]Ì85?µÌCD~TN/_^?ÖÒ¥Kläwüøñäcó:::N::44ôÈ#477'ZÚ¶mÛððp~ê±cÇºººdLæÙg-Zfé#EÅø~¸hüDËâÁÄCJ>h°ÌçùåÛ´iSÑÇ»$]»v-ÍÆ;wn¬ÅòPS¸Ì7nmmµbÅ¾¾¾¢ïXæ¡J"?I$$IÈO$Iä'I$ò$IùI$ü$ID~$Iä'I$ò$IùI$ü$ID~$I"?I$$IÈO$Iä'I¤þ?]uÎÐIEND®B`


.ò,D~ª£æææpLðetttºóîÚµ+æý?ùÂç.ÆìÜ¹³ªåWfùÅcâÇpww·g$òT%¯&ïOçÏyW¬XQ³lÙ²3000;wò£_X9ëëë=$¤ªa_±fzê©yóæ544lß¾½äj_øÂ:;;KPØeÃ·nÝáLºvíÚªU«Fétº£££¯¯oÂ¥ßzùY<ØÒÒòÈ#ÄwØÝ»wzzbÞ¸ó[·nMvaM/_Kñ±äë×¯Hò»téR*JÑØØXÜù¸#=Ç$¤Ã_2üÌ3ÏÄðþýûÃX1ðì³Ï_çäÉã?Ó¶qãÆtàÀ1ÜÛÛLZºté'bàêÕ«1>ÍN¸´â;P~ðÜ#Gb`Ããïü¦Mb8®ðòË/ÇÀ®]»bäC=ÃwîÜdäà®üw±~bø«_ýj2içÎÉªØ³gOìØ±Ã³KùIªµ´´Äpþ~ãá5áÏ9Ö®]ÃëÖ­KHWzþüùmÛ¶uvv&§DL¸´]%¹cóæÍ?oSSSrçÃd1æqÍîèèË¼S<&¹o±ð`_°27½xñâ¸­­­qÑ³KùIª¥ÓéÂpÄÅÉT(Ðk'UcL2iß¾1Wpðí·ß.^BÉÒ/NeÉîXP¬x7^¢Æ£GÎ??ÓØØìü@öùMXrKT|¬ÈOR%Ê/ÍïóKv=;===1µð³0>ÉÄ±ûMQ~åg)Ü±ÀÜøy,XÌ[r÷FGGO8±aÃâ3X9wîÜijjÊårË/ß¼yÓ³KùIª%ßÒràÀäs~Ï=÷ÜTäwôèÑÂÎ³#GÆ'¼páBòù¿©È¯ü,Û¶mé¥b`ãÆãçüñÇcøÐ¡C/^LðÆÈGy$ß~ûípX´¶¶~xòÛ¼ysL:þüÙ³g?(Iä'©å7::ºj¸_ñwòß½÷#­ñ³ð¡·èøñãóæÍÛºuëåW~Ó§OÇÔeËN4)¾ÎððpØ+îy&Y³fMro/Ë%'¯X±bº'ÛN]~¡Õâ/¸Y¾|yôD~$I"?I$$IùI$ü$ID~$I"¿ªé¯ÿú¯ß÷ÝÙ¼Å+W®ø[LØøÃÿú¯ÿ²*p»ÌàO¥éÃîêÕ«¶KenÿüÏÿ´*­ýèGµ]æºü¾ùÍoþfóÏ9óÿñþ'TZßýîwïÞ½k=TZßûÞ÷þýßÿÝz¨´^ýõû·³*­x;+þ"LUHgÏý×ýWò#?ÈüD~äG~ä'òùÈüÈüD~ä'òùùÈüD~äG~äG~ä'ò#?ùüÈüD~ä'ò#?ùùüD~ä'ò#?ò#?ÈüD~äG~ä'ò#?ÈüÈüD~ä'ò#?ò#?ÈüD~äG~ä'òùÈüÈüD~ä'òùùÈüD~äG~äG~ä'ò#?ùüjJ~Ùl6N·µµõõõ¿Â©S§êêêÈOäG~"?òùU¹Ð¡C1°wïÞîîî©£££ÉïÄ·g±W_õúõë·UaÅ/ÿôOÿd=TZ¯½öíRÛåÚµkÖC¥uæÌwß×z¨´¾ûÝïþèG?Í[òkll|>ßÒÒR2õgÙ½÷dò,~hÆ¯ËßWe»TìvùÞ÷¾g=TZ¯¼òíb»¨b·Ë_:p8_L;;;ÃöÊÑ^Gåh¯£½r´·J¥RáL&S<©««+^¤þç¡ÈüD~ä7£ïg%_¥ÔÔÔÏçß»´7êþtä'ò#?ùM½·Þz«½½½±±qÁùÌgâ¢UM~===øËå&~¨öùüÈOäG~ÓéòåËóçÏ_·nÝ¶ÿ»mû×·ÿæºßüÙýÙÁÁAkü>úÕÚÜÜJ¥²ÙlÿÔ#?ùüÈoZéK_úµ_ûµ0_áßçýó¿ýÛ¿mm_µF~"?òùßd-Y²dãÿÞX,¿'~ÿOúÓÖ6ùùÈüTkòôÑG¿ÜóåbùýÎW~çá¶¶ÉüÈüD~ä§ZßóÏ?ÿÐCmý?[öÅÀ¢O-zæg¬mò#?ò#?ù©ÖämÞ¼ùãÿø£>ú«¿ú«øÄ'xâÑÑQküÈüÈOäG~ªAùE/ÜïÍ7ß´ÉüÈüD~ä§ZÈüÈüD~ä'òùùÈüD~"?ò#?òùÈüÈüD~"?òùùÈüÈüD~äG~ä'ò#?ÈüÈüD~ä'ò#?ùÈüD~ä'ò#?òùÈüD~äG~ä'òùÈüÈüD~ä'òùùÈüD~"?ò#?òùÈüÈüD~"?òùùÈü¬òùùÈüD~"?ò#?òùÈüD~äG~"?òùÈüÈüÈüD~ä'ò#?ò#?ÈüD~äG~ä'ò#?ÈüÈüD~ä'òùùÈüD~äG~ä'òùÈüÈüD~ä'òùùÈüD~"?ò#?òùÈüD~äG~"?òùÈüÈüÈüD~ä'ò#?ò#?ÈüD~äG~ä'ò#?ÈüÈüD~ä'òùùÈüD~äG~ä'òùÈüÈüD~ä'òùùÈüD~"?ò#?òùÈüD~äG~"?òùÈüÈü¬òùÈüÈüD~"?òùùÈüD~"?ò#?òùÈOäG~äG~"?òùùÈOäG~"?ò#?òùÈOäG~äG~"?òùüÈüÈOäG~"?òùùüÈOäG~"?ò#?òùüÈOäG~äG~"?ùüÈüÈOäG~"?ùùüÈOäG~äG~äG~"?òùùÈOäG~"?ò#?òùÈOäG~äG~"?òùüÈüÈOäG~"?òùùüÈOäG~"?ò#?òùüÈOäG~äG~"?ùüÈüÈOäG~"?ùùüÈOäG~äG~þ'ÈüD~äG~ä'òùÈüÈüD~ä'òùùÈüD~"?ò#?òùÈüD~äG~"?òùÈüÈüD~"?òùùÈOäG~"¿h`` Í¦Óé¶¶¶¾¾¾âIýýý1©½½=®F~"?òùÈ¯ºËår½÷vwwOZ¼xño¼nmm%?ùüÈOäWÝ566Å@>oiiìjõõõãå·oß¾·f±W^yåÍ7ß|KÖÉ'mÊÜ.ñõ`»Èv©Þ¾óï|ÿûßÍ[òK§ÓwîÜ¹ÞÞÞñòûË¿üË³ØéÓ§¯^½zSVlþðÖC¥õê«¯Ú.X__ß+W¬Jëµ×^ûÇüGëÁvòK¥RáL&3þwïÞÍårÃÃÃöÊÑ^Gåh¯£½r´·ºkjjÊçóÉÑÞ.zãÆ6L¨`òùÈüD~UVOOÏÁc ~ær¹i­^½zhhhÂÉOäG~"?òùUßjmnnN¥RÙl¶¿¿ÿÇ­î]KKK]Qä'ò#?ùüæhä'ò#?ùüÈüÈOä'ò#?ùùüÈüÈOäG~äG~"?òùüÈüÈOäG~"?ò#?òùüÈOäG~äG~"?ùüÈüÈOäG~"?ùùüÈOäG~"?ò#?ùüÈOäG~ä'ò#?ùüÈüÈOä'ò#?ùùüÈÏz ?ùùüÈOä'ò#?ò#?ùüÈüÈOä'ò#?ùUºüêT*"?ò#?ùüÈ¯äzPétüÈüD~ä'ò#¿Z_mD~"?òùÈïýO¾7ùÈüD~äWkòkiiI§Ó>çG~ä'ò#?j íííãOïhll&?ò#?ùüÈ¯¦äÉdzCCCÍÍÍ1àé¥b ··üÈüD~ä'ò#¿_²/z1põêÕ±±±hhh ?ò#?ùüÈ¯¦ä7þüp^<°Ë/ÇÀ;ßêB~ä'ò#?ùÕü¶nÝZ8£ø£~<òùùüÈOäG~5%¿èé§^°`Aô÷÷Ç@(°³³³*¶ùüÈOäG~"¿¹ùüÈOäG~"?ò#?òùüÈOä7Q­­­Éw»ø&gò#?ùüTËò[¼xq±ö9·üÈOäG~"?ò«5ùòÂ|çÎ«ºíA~"?òùÈo566üªä'ò#?ùü¦×ÀÀ@ÈoÓ¦MÕøÆI~"?òùÈoz-°nð ?òùÈüjM~-rùÈüD~òKÌ788XÛüD~ä'ò#?ß4jjjrùÈüD~òÊü¶nÝ:22B~äG~"?òù©åW7IÎð ?òùÈüjð'ÌäG~"?òù_~«KõF~"?òùÈoµ´´,Z´èâÅäG~ä'ò#?j étº®®Z÷ÈüD~ä'òF!¿;wÆ®ê¾ÛüD~ä'ò#?ßtáÜ^ò#?ùü4GäçÜ^ò#?ùü4WäWÕÈüD~ä'ò#?ò#?ÈüD~Ïç×¬YS___WW×ÐÐÐÕÕU-§zÈüD~ä'òF÷îÝðªø3¾ä'ò#?ùü¦Ñ%KÂyk×®MÞ8ãi·nÝº³téRò#?òùÈüjJ~L&7::ZÏçcL'?ò#?ùüÈ¯¦äJ¥Ây¡½Âã[]ÈüD~ä'ò#¿Ú<Ú»zõêä3~Æpéèè ?ò#?ùüÈ¯¦äïáqûömò#?òùÈüjJ~ïÝ?½·««kÞ¼y©T*~®^½:ÆTÅö ?ùüÈOä7W"?ùüÈOäG~äG~"?ùüs>¨T*E~äG~"?òù_-È/5yäG~ä'ò#?ÞG·lÙÈïðáÃäG~ä'ò#?ùÕ¦üÂ|«V­*þbgò#?òùÈüjJ~ë×¯Ovõ8q¢¶ùüÈOäG~"¿iôòË/'æ[»vmÕmòùÈüD~SêÞ½K.MNæèëë«ÆíA~"?òùÈïÁíÙ³'ÙÕ×ÝÝ]½ÛüD~ä'ò#?ßæô~äG~"?òùiÈ/õ Òé4ùùüÈOäG~µ ¿ÚüD~ä'ò#?ùùüD~ä'ò#?ò#?ùùüÈüÈOäG~"?ùùüÈOäG~äG~"?ùüÈ¯ºä×ÚÚÉd|ùÈüD~ªqù-^¼¸X¾ÏüÈOµ-¿çþù?ùÉO~ö³=uêùü4·äÈó;wnllìý/m`` ÍÛÚÚJþðøI3SÜ7¾ñíÛ·ÿù,¶mÛ¶ýû÷ÿ¹*¬Ø.ßúÖ·¬Jëë_ÿz¥m0ßÇ>ö±ööö_ù_ùô§?/]]]sp»ìÛ·Ïó³Ò·³?ýÓ?µ*­?üÃ?åíò¡Ë¯±±1ä÷°/Êår½÷üEàñf6¦¸-[¶ÔI$ÕJºüâf6mÚô,G&Ìçó---å'ÍlùI$òy.Ã3;Ã£øÓ%?ifcÈO$ß[´hQ±öÞçÅ^Ìd2å'Íl3<ägxÌ¬£GÆËÈ×6mû×·'ÿ~÷«¿û3?ó3sítgx8ÃCsúÄ|ÈjjjÊçóÉÁÙ.?ifcÈOäG~3îáþøÇ?þ¥/éßâ_üâÇ>ö±?ø?kÛüÈOsZ~Á©ºîÆ@üÌårå'ÍlùüÈïýôØcâhhhøä'?ùÇüÇsp»ùiNË/(òÛºuëÈÈÈ²S©T6íïïÿñ½¼Ðzü¤!?ùüÈOä7ÓEL¿áA~ä'ò#?ùÕà79O¿áA~ä'ò#?ùÕàßí­ÞÈOäG~"?òùùÈOäG~"¿ÊçókÖ¬©¯¯¯««khhèêêú Nõ%?ùYä'ò#¿ß½÷&<Ãã9ÕüD~äg=Èü*H~K,	ç­]»6yã§ÝºuëbÌÒ¥KÉüÈOäG~"?ò«)ùe2pÞèèhaL>1ãÿTùùüÈOäG~Õ-¿T*ÎKþBZÒÈÈHñ­.äG~"?òù_mí]½zuòÆ?c8ÆtttùÈüD~äWSò÷Ë	Ïð¸û6ùùüÈOäG~5%¿÷îÞÛÕÕ5oÞ¼T*?W¯^cªbÈüD~ä'ò+ÈüD~ä'òF----ºxñ"ùùüÈOä§_:®««Öä'ò#?ùü¦Q___ÈoçÎñ«?ÚF~"?òùÈoF¤T*E~äG~"?òù_MÉ/5I¾ÉüÈOäG~"?ò«ù-Z´¨­­mxx¸ª·ùüÈOäG~"¿ü¹Þ÷îí­»ä'ò#?ùüfXòçz?ÑK~äG~"?òù©åÍfëÊæò#?ùüÈ¯FäwîÜ¹ùóç'þç9ÃüÈOäG~"?Õ¦üUòÈOäG~"?òùÍéÈOäG~"?òùùÈOäG~"?ò#?òùùÈüÈüD~ä'òSËïÒ¥KäG~ä'ò#?æü¯téèèxçwÈüÈOäG~"?Õ²üÂ|ooN§Ó+V¬¸råùùüÈOä§ýß¥K,YR `&Y³fùùüÈOäG~5(¿¤±±±'NÔ××ûëmäG~"?òù©få788X¼ÏoÞ¼y½½½äG~ä'ò#?ùÕüÁW__ßÝÝíÚµjÙä'ò#?ùü¦³û'v|îs»zõjÕmòùÈüD~Ó¨¿ÌüD~ä'ò#?ßüD~ä'ò#?ßôºpáBkkkCCCr>oSSÓÑ£GÉüÈOäG~"?ò«5ù:uªpG"¿dxïÞ½äG~ä'ò#?ùÕüÃy.È¯¿¿?ùbò#?òùÈüjJ~É¾÷~ò|ß»ÿÎÉ	¿äG~ä'ò#?ùÕüÂyÉ~¾_>ê©§b¸¥¥üÈüD~ä'ò#¿_P¦n¢Þxãò#?òùÈüjJ~ÑÍ7;;;sëëë[[[¯RÛüD~ä'ò#?ßüD~ä'ò#?ùùüD~ä'òûé®]»ÖÑÑ1oÞ¼Ôýb ­­­ZõÈüD~ä'òj?þxÝ$mÜ¸üÈüD~ä'ò#¿ßÑ£GäíÛ·oxx8yïÞ½cÇ%ãO<I~äG~"?òù_-È/Íï80~Rò÷Ü|ùÈüD~äW#òËd2Á»÷îÏçcRüÈüD~ä'ò#¿Z_á¶M65ùKnäG~ä'ò#?ùÕüÊØüÈüD~ä'òùùÈüD~äWò+ùùüÈOäG~5"¿ÔJ§ÓäG~ä'ò#?ùÕüj#òùÈüD~äG~ä'òùÈüÈüD~äG~ä'ò#?ò#?ùüD~äG~ä'ò#?ùùüD~ä'ò#?ò#?ÈüD~äG~ä'ò#?ÈüÈüD~ä'ò#?ùÈüD~ä'ò#?òùÈüD~äG~ä'òùÈüÈüD~äg=ÈüÈüD~ä'òùùÈüD~äG~ä'òùÈüÈüD~äG~ä'ò#?ò#?ùüD~äG~ä'ò#?ùüj Ùl6N·µµõõõOêïïïèèIíííq5òùÈüD~Õ].;tèPìÝ»·»»»xÒâÅßxã8|øpkk+ùüÈOäG~"¿ê®±±qll,òù|KKËdW«¯¯/¿?û³?ûÛYìäÉó7ó·ª°¾óïØ.¹]¬JëÔ©S¶Ken·ÞzËz°]æüÒéôÃÅ;w®··w¼ü¾ýío¿;ýÕ_ýÕåËßUÛåþá¬JëôéÓ¶KenK.YÖ«¯¾j»T`?øÁfóçüR©Ta8É¿ÂÝ»ws¹Üðð°£½r´×Ñ^9Úëh¯í­¾ê~R755åóùähoÆ6l¸yóæøÈüD~ä'ò«²zzz<ñ3ËHkõêÕCCCÎH~"?òùÈ¯úVksss*Êf³ýýý?~l÷w¶´´ÔE~"?òùÈoF~"?òùÈüÈüD~"?òùùÈüÈüD~äG~ä'ò#?ÈüÈüD~ä'ò#?ò#?ÈüD~äG~ä'òùÈüÈüD~ä'òùùÈüD~ä'ò#?òùÈüD~äG~"?òùÈüÈüD~"?òùùÈü¬òùùÈüD~"?ò#?òùÈüÈüD~"?òùùÈüÈüD~äG~ä'ò#?ÈüÈüD~ä'ò#?ùÈüD~ä'ò#?òùÈüD~äG~ä'òùÈüÈüD~ä'òùùÈüD~"?ò#?òùÈüÈüD~"?òùùÈüÈüD~äG~ä'ò#?ÈüÈüD~ä'ò#?ùÈüD~ä'ò#?òùÈüD~äG~ä'òùÈüÈüD~ä'òùùÈüD~"?ò#?òùÈüÈüD~"?òùùÈü¬òùùÈüD~"?ò#?òùÈüD~äG~"?òùÈüÈüÈüD~ä'ò#?ò#?ÈüD~äG~ä'ò#?ÈüÈüD~ä'òùùÈüD~äG~ä'òùÈüÈüD~ä'òùùÈüD~"?ò#?òùÈüD~äG~"?òùÈüÈüÈüD~ä'ò#?ò#?ÈüD~äG~ä'ò#?ÈüÈüD~ä'òùùÈüD~äG~ä'òùÈüÈüD~ä'òùùÈüD~"?ò#?òùÈüD~äG~"?òùÈüÈü¬òùÈüÈüD~"?òùùÈüD~"?ò#?òùÈOäG~äG~"?òùùÈOäG~"?ò#?òùÈOäG~äG~Ô+W.^¼822B~"?òùüÈ¯få÷æoþâ/þâ§>õ©ÖÏ´Ö××:tüD~ä'òù_ÊïÝwßý_øõ¿µ~û×·Ç¿ßÛð?ÿó??ËküÈOä'ò#?ò#¿ÙhëÖ­>úhÂ¾äß¿øÅÏþóä'ò#?ÈüjM~¿ñ¿ñå/ËïßâSúùüÈOä'ò#¿ZßO<ñù_ÿ|±üº»»W¬XA~"?òùüÈ¯Öä+ðç~îçzÿWoÂ¾¯mþÚ/ýÒ/ë[ß"?ùüD~äWçö¾ôÒKóçÏÿìg?ûðÃ¿ñoTÝC ?òùÈüÈoªÇC;~üø+WªñþùüÈOäG~ä7W"?òùÈ¯jÈf³étº­­­¯¯oüN:UWWG~"?òùÈ¯êËårÉßØ»wowwwÉÔÑÑÑÉäûïYìµ×^ÿÿ­+ñ?Óz¨´Bä·oß¶*­ù¿üË¿XV|hhÈz¨´BäÿüÏÿ<·8'ä×ØØ866ù|¾¥¥¥dê3Ï<³÷îÉä÷ÜsÏÅ?þÊ+¯Te»Ø.²]lÕÆvòK§ÓG×®]ëìì:Ú+Gí£½öÊÑÞZ(J3Lñ¤®®®xúJ~"?òùÈ¯J«ûI1ÜÔÔÏçß»´7'¼ZáÊä'ò#?ùüª¸Æ@üÌårIqüHòùÈüD~Õ·ZS©T6íïïzä'ò#?ùüætä'ò#?ùüÈüÈOä'ò#?ùùüÈüÈOäG~äG~"?òùüÈüÈOäG~"?ò#?òùüÈOäG~äG~"?ùüÈüÈOäG~"?ùùüÈOäG~"?ò#?ùüÈOäG~ä'ò#?ùüÈüÈOä'ò#?_-õGôGßþö·ßÅ9òüà]UX±].^¼h=TZññ÷ÿ÷ÖC¥õÒK/]¸pÁz¨´âíìïþîï¬JëèÑ£³yå1ëò¯íÛ·S$©&*0³ÎnXI¤9ùI$$IÈO$Iä'I$ò$IùI$üª¾6d2zèÌ3?µÞJ¥RÖUl¸¸dÉt:ÝÖÖvöìYëªB¶Ë¥K:;;cÒúõëýÙ~üÅj`` Í&ÿ_úúú¬«Ù.ÑÐÐPKKµTQÛ¥¿¿¿££#þ¿´··Çÿò«ÍvíÚõì³ÏÅÛØ¢E&¼Î¡CöîÝk]UÈvill¼víZÄO¯³].]/1pñâÅ'|ÒºúHÿbËåbdÄøîîn«¨B¶KüÖ¿Á<¬Ú./~ã7bàðáÃ­­­äWÅïÁñFUæW¯^]¹r¥U9Û%ÀqãÆa]³¿]â·äÂðüùó­«ÙoÂ«øM)¤ù|ÞoJ³]Ö¬Ysùòeò«´íR}=ùÕfñvµgÏØÀa·ß~üºººÎ;gEUÎvññ?mÊÙ.ííí._|±Xµ&|±*Þ¶Kål¿»_En(&õöö_mJ¥8ðÞO>¥T25~'[¶lµTQÛeéÒ¥Én§  ­S9Û% ØÚÚ(Ü½÷Gû»òÜl²«â1e2+ªB¶ùUòv¹÷n.&¿Ú¬©©©Ì/Ä»víÚ³gµTQÛÅ>ýÿôÎ;ï,Y²Äºå&±MÏçß»´·xóé£Ý.äW±ÛåÆ6l¸yóæG=3>Ä|òÉ#GÄÀyä©Ë/?þ¼µTQÛ%~QK*.]ºÔºªí²xñâ±±±Ý»w?ýôÓÖÕ,7ÙUOOÏÁc ~ær9+ªB¶ùUæv9sæÌêÕ«>òèñ!vçÎ®®®t:ÝÙÙyùòåÿL&ùp´*g»¼óÎ;¾?cØºªí¯.ÿ2½½½£££ÖÕ,7þÅ*Ù.gÏmnnN¥RÙl69ùZ°]È¯2·KKKKñw¾$IÈO$Iä'I$ò$IùI$$IÈO$Iä'I$ò$IùI$ü$ID~$I"?I$$IÈO$ü$ID~$I"?I$$IÈO>¸^|ñÅåË×ßoåÊ/¿üòO½ÀÝ¯j^'º·---ñÐKÆÇt:ÍfÇÆÆ¦»LIä'IÕ×öíÛëÆõôÓO×üvîÜ#_xáñÏ?ÿ|ß±cÇ)ü$©ÊÓ¤ÓéûöÞïÀq1F?¾fäwõêÕÙÞÞ^2~É%1þÊ+ä'ü$Õ~_ùÊWÂ4Ï>ûlñÈç.F~õ«_-vO___È)PØÑÑÃ+ß¾»··wÞ¼y1©±±qóæÍÅUO>ºI1ïÉ'K,cæÏ¿|ùò'NÄÅ;vüøñòËI&Å]J&:uj2¥­^½:Æ÷÷÷Æ¼ñÆ1fÕªU1»víjnnEÕ××¯_¿þÆãå7~ù%cÊÜUIä'Ie.µv­xäõë×cdKKK±lJ:wîuÝºu%6mÚLL¥RÎ¦vuu3ÌèèhL!§äãweÞ½ñôðáÃÅ-àòÐ¡Cö,dåÊÓ_»*ü$é#.9°;ÁZ]]8¬X6ÝÝÝwïqqíÚµÉÔ:]w!¶dR.KvÆðÙ³g/sÛ¶ma»ä`ëã?c;Ãñ3ì±.'ÔãÅ÷mÂG`A<Þ¡¡¡¸?ã7óù|rl63ÆCxï'Gëëë§+¿2wUùIÒG/¿hBùÆ'²IÀÝ¸q#.qqÑ¢E7n±Ý»w¯°¸NÉ^´ÂÅë×¯®Z*ð1|úôé.'oÝºU|ß&ûLÞ-[bÒîÝ»ßûÉíÍ7_!òvvv&»$§+¿2wUùIÒG£ëîÝ»Å#cdL*c/_¾à¯ÂóÆ-±Tñw©Äpccc&Ï?¿0õËÌa%]¸p!AjÇÏ,L=ölÜ	O]~eîª$ò¤¸ä³nöì)|×IÉÏßuþüù;w&Ç^dLvÈ§>Ðg6mJòÆÏÞÞÞÂø2ËIö±Ý¼y3¹xëÖ­òçá.[¶,¦&_d`-çN:uçÎòò+¨4Y©eîª$ò¤¸ät:½wïÞä[]öíÛÉdÆ±víÚPÎÈÈHr(¶pnò9¿äãq/_.þ`ÁÍ7³nß«2¡Ïm[?sæLa|å$ìK>ç7<<ü8PØ¹xRòÇÁÁÁ¸X	É/±fX9®â~ñÔ2wUùIÒG_ò-Ç%íÚµëÿ¿ÀÝ/Qa¸p´pRE¡Ç¼Å=z´üÞ»ÿÇ6b|cccñà2Ë»Q|ÇçÕNö`ïÝ»W__<cÜkÖ¬)¾ÄÏä]ì,8µ0µÌ]D~TNV®¹ßòåËlKäwêÔ©äkóÚÚÚ^ýõÂÔááá§zª¹¹9ÑÒ-[FFFSO<ÙÙÙ Ëf³û÷ï/YæøñO>ùdÉøÉÅ»|Ñ`ïó+ÔÛÛ[òõ.ICCC¹ÖÀ¼yóâQr¥ð5ÅË¼sçNÐ6YW«V­êïï/¹Å2wUùI$ü$ID~$I"?I$$IÈO$Iä'I$ò$I"?I$$IÈO$Iä'I$ò$IùI$ü$ID~$I"?I$ëÿ /Ü=äÖIEND®B`


+V¬Í»ús1ÿÜsÏý¼nhvé¡C-[V;Ö%K^xáÛ.pê³>455íÝ»w+gÆÏ'O'a2·^´IºK6$òÊI~ñ6oÞ|G5v÷È¯®®îêÕ«å"¿;vßXÛ·o/±´iÍ2ÙóaÏ=Ó]93^L¦p»ä'¤;7n<÷Üsµµµadooo5È/´zõê²____DÕç~dddtt4aäÀÀÀî,ã]]]aLÐØ¬É/ñúõëa8ÏßUFùI%¿¨Ý»wín9~üxccc2lnnîîî.Sa!Ñ@¸ÂÜ¹s.]ZbÞÂÏ9³bÅè@äºuëã7ÚÒÒÍxìØ±¢»:Ùbo;cÑX¿~øyèÐ¡ÉÖÉë¯¿¾víÚèær¹/]3þx£1áFÃM;°lÙ²k×®<y²©©)JµµµÅ÷/îÚµ«¡¡!:¬n¢0i²;üùÏ>©nßéÎ2þ¦Ãp'§µr&Ü'of3_ÑÔ°]Â3$¬öz(¬ÛÒÇOâÓ85¬Éé0)NoÞ¼yhhÈ/ü¤r_ÀJÍf£D"þ~Ü××WB~Ñ×¬YSbÞÂÃs|j|gÕdÇõJ,¶ôãû72L¿N®ÞÎãË	×,"Züñ¿Ýx ÚR`_Ñ5/_^Ú7óçÏãß~ûíñkáÂnßéÎRtÓ5Ñ>¿EMkå^ù3qêòëïï/'<ØéÊoOãÕ«WÝçM6ùÅ"TNòÆEÃ¹éé	Ã§Nû¬höèâ¶mÛFGG£½8·7¼q·üpýhGc@R45¼ãaöc¹â·Ub±¥gð±>|8¾,~ý7áU«V]+aäd7óøãDw,Zr¸ØÝÝÑ§Ù=/]º4ÙN&=Â¼ïsÉ>HWØ':Ý3á½ñ¥M¿Ó|6uùMñiðòåËa8lÍ0<gÎ¿X$òÊO~|ÑÇÂâ1%äwåÊÂÛÎ[ØUï£ÑÅðößYUt´®ÄbKÏ8ÙcÞÔÆ×××ÇQxçÍ7ÙãÆmÄ/F°£¿'ù|>X!£µµ5þØKÈ¯ÀñÉþ>g¯Ò0oX+V¬8~üø+m*+gÂ5ã§.¿èríÚµøMLK~S|·´´,rÿ¸uëß*ùIe&¿ 02NOöv;Q¢8%ÞªKøfºïÍ3XìdãÃC7¼ÇÇßg6þCoãïT.eñ¼í9ªÑáéñ¼+òta	Se*ëjÂ+LeåLæÑÍ8õ£½ïÿÙ5Å§ñ"üDxäÈ¿X$òÊI~ûöíáíE+:Ër*ÐÖ¼¥÷Ù¼ýöÛñ©%[zÆwàÅ_ßpV´wªÈÄS_!ñÑ·«;v,Èl*òvLF_°Ëå6oÞ|ýúõýû÷k×®P~SeÆòîÊ)4ã§.¿©ìó+Ø.ú¯Nõ_âùuúôé;wFçAK"?én_xoÞßê½4wÊèTÙæææhRô!§¡±&|Ë,1oi6Ed>§-§èS.¶ô¥±zõêÂù£1Ñ¿«V­º1V´´ÇüýË/Úï500ÂÞ½§"¿'NDÂC>uêT´?/êðáÃnßéÎ2-ù^9EÏxÓ]«3_ôÉ¼ðd¸uëVÝÊ+Ç»ðÙg+?è0 ¸ôú¿íK ú¼æJyD~ÒÝ"¿ñÅÏO¶láóþ/ÝvË,1oi6E6hYZb±¥g,Í«W¯>Ñ¹xñbÑg¼êëë"|?ò+X$jÞ¼yO=ðÍºuëÆo¬ÅØÄÓeZò+½rñ¦»Vg ¿Ó§OÇ¿téÒøÔ¢uR8Á|²[)ñ|+BThÃ~±Hä'üÂ[àòåËÇÿu¯îîîÖÖÖ ¨ðv¾oß¾ÂøÃ1AZño³â¼·eSô5xaá---ã¿o²ÅÞvÆÒÆÇ?~íÚµ©±r¹Üøó9f&¿ÁÁÁ°´°Ì` -[¶À]Ú7_ûÚ×Â£|þüùøìg?[ØçôþgüJ¯¢çFQÓZ«3_èÈ#,:,?:^89>nÅ½½½·Ýç:ÙómhhhëÖ­ÑÎÔÀ÷°)ýbÈOîHù|~ÝºuÓú¹ÌRvEûz£µGFF"Ð766zÂHä'Iª´¢ÏùõôÓO[3ùI*­¡¡¡®®®ùóçGtÃÀ;¬ü$ID~$I"?I$$IÈO$Iä'I$ò$I"?I$$IÈO$Iä'I$ò$IùI$ü$ID~î¶>ÍfÉäòåË§;oSSSMMÍcÂpÓÜÜ<_gcÍì:Sw¿dKÞb(HÔÖÖær¹ÁÁÁhÒÐÐÐ¦MÒétXuuuaÒë¯¿î	&ü$ÝE544Ç¾LwÞ]»vyÿôOÿ´0æg	cvîÜYÙòÃÃíííÑÅõë×===aøäÉaxáÂ`ÈOÒÝôÛäàéôéÓaÞeËÆ<ðÀaLÿì;löo1X9×ÖÖFS©T¸xãÆO*Iä'éîe_[·n­««3gÎöíÛ®öÙÏ~¶µµµh	]aøí·ßÃaL4éòåË+V¬0J&---ÑÎ°ñKßzéY8Íf,Ynh¼ÃnÞ¼ÙÑÑæw¾««+ÚlºtéÒ°´0>,ùÊ+wN~÷Ýw_¸ØØØxêÔ)O-Iä'éîÅ_4üÔSOáûöc§~:~îîîÂgÚmÜ¸1LÚ¿?Ãpggg4iñâÅG.]ã3ÌKßÒ³Ï½øâa`ýúõãïü¦MÂp¸ÂK/½víÚUÐØ7Â@´Kr|3ßèèhX?aø_øB4éðáÃ._¾üÌ3]ÈOÒÝ+¿l6óc×<qâD´jÕª0¼zõêt©§OÞ¶m[kkktJÄK+2VéY¢;VWW7~ÞúúúèÎ`¾02·´´ËÏlµÝ·°ðÀ¾øáÝ¾¾¾×ÂuÂzI"?Iw©üÉda8¥ôÞØ®¯9sækEUÃhÒÞ½ÃgÎ/¡hiñSe²;(ß©ñÐ¡CsçÎÆ¤ÓéhwàÏe_zzz5ã¾%ü$ÝuòËd2ñ~Ñn³Ûr§££#L-ü,Îxkò+=KáÌwÞ¼yÑ¼EwoddäèÑ£Ñ·ÓZ-Ó_(ºááxI"?Iw©ü¢oiÙ¿ô9¿gyf*Ü9tèPaçÙ/¾X9òìÙ³Ñçÿ¦"¿Ò³lÛ¶íßüfØ¸qãøy7lØ<xîÜ¹èo¹dÉ0|æÌk×®EÝ9ù577IÇÃG	ÃëÖ­óD~îRù]Í+þ|¥åwëÖ­èHkøÿÐ[ÐO:®««ëêê¢üJÏrüøñ0õ(h¿ÎÐÐÐæÍÃ=O¥R+W®NãàËårÑÉÂË-;þü´VËø#Â%VE¸ÅpçÃz7oÞ¦MÂ]òD~$I"?I$$IùI$ü$ID~$I"¿²é»ßýîo¾9·xñâÅ|>ïWýèG?úïÿþoë¡:»téÒ´þì*lëÿ×ýõP½ñÆwÕÖ¯vùõ«_øÍ[<qâÄþçz%Tgßþö·oÞ¼i=TgßùÎwþã?þÃz¨ÎÂMü+*UU:uêßÿýßÉüD~"?ÈüÈOä'òùüÈüD~"?ÈüÈOä'òùüÈüD~"?ÈüÈOä'òùüÈüD~"?ÈüÈOä'òùüÈüD~"?ÈüÈOä'ò#?ò#?ò#?ÈOä'ò#?òùüD~"?ò#?ÈOä'ò#?òùüD~"?ò#?ÈOä'ò«(ùõ÷÷g2d2ÙÔÔÔÓÓ4<<¼~ýúT*uß÷uÈOä'òùw¹àÁa`Ï=íííñI»vízúé§GGG¹,X0^~G½>½òÊ+W®®ª,ü·ä_ÿõ_­êìÕW_µõ«¶ðôæoZÕYø?ÿo¼1·XòK§ÓÁva Ïg³Ùø¤¦¦¦sçÎM6c_Àâ÷f±Í'O~OU­_å[ÿ;ßùõP½üòË¶¾­?kUüÉäÃÑÅg¶¶¶vÁgÎq´WöÊÑ^9Ú+GË»D"QN¥REöïßÎ?ßÚÚJ~"?ÈOäWÞÕ××çóù÷Æöá¢Iá¢Ýä'òùüD~åWGGÇÂ@øËåâüñ_|1=vÉ%ä'òùüD~e¿ZD&éííýÉc«ùßG^lkÖ¬I&­­­.?ÈOä'ò«ÒÈOä'òùüÈüD~"?ÈüÈOä'òùüÈüD~"?ÈüÈOäG~äG~"?ò#?ÈOä'ò#?òùüD~"?ò#?ÈOä'ò#?òùüD~"?ò#?ÈOä'ò#?òùüD~"?ò#?ÈOä'ò#?òùüD~"?ò#?ÈOä'ò#?òùùÈüÈOä'òùüÈüD~"?ÈüÈOä'òùüÈüD~"?ÈüÈOä'òùüÈüD~"?ÈüÈOä'òùüÈüD~"?ÈüÈOä'òùüÈüD~äg=ÈüÈOä'òùüÈüD~"?ÈüÈOä'òùüÈüD~"?ÈüÈOä'òùüÈüD~"?ÈüÈOä'òùüÈüD~"?ÈüÈOä'òùüÈüÈüÈOä'ò#?òùüD~"?ò#?ÈOä'ò#?òùüD~"?ò#?ÈOä'ò#?òùüD~"?ò#?ÈOä'ò#?òùüD~"?ò#?ÈOä'ò#?òùüT9òëîî~â'ºººþú¯ÿÚÖ$?ò#?ÈO+¿Gyä#ùÈÃ~øÓú~éV­Z522bùÈOä§JßýÙÝÿý[¿²uûÛÃ¿0ð«¿ú«O>ù¤mJ~äG~"?*M~üä'»ã·#öEÿ~çó¿óO|Â6%?ò#?ÈO&¿ÆÆÆ¿·1.¿/ñK¿ò+¿bùÈOä§Jß£>Úö©¶¸üþôÃk×®µMÉüÈOä'òS¥ÉïÂsçÎ]½zõ¶ßßØ÷ÈG~á~a``À6%?ò#?ÈO&¿Ð~ðìcóÆºÿþû¿ÿýïÛ äG~ä'òù©2å54MI~äG~"?*_~"?ò#?ÈOä'ò#?òùüD~"?ò#?ò#?òùüÈüD~"?ÈüÈOä'òùüÈüD~"?È¯å×ßßÉdÉdSSSOOÏø+;v¬¦¦üD~"?È¯ìËåröìioo/:22ÒÒÒB~"?ÈOäW	¥ÓéÑÑÑ0Ïç³ÙlÑÔ§zj÷îÝÉï/ÿò/ÿaëîîþû¿ÿûPU¶~¿õPýíßþ­­_µ;vì?øõ`ëÏNU!¿d29ápèòåË­­­ÁÉï[ßúÖ³ØßýÝß]¸páMUeÇ·õ«yëÿó?ÿ³õP½òÊ+çÏ·ª³þð³yU!¿D"QN¥RñIkÖ¬9yòäÿ>TGåh¯í£½r´·ª¯¯ÏçóÑÑÞ0ü3ðg#?ÈOä'ò+ï:::8ÂÏ7ñCµÏOä'òùü*cµ644$L&ÓÛÛ;!õÈOä'òùüª:òùüD~"?ò#?ÈOä'ò#?òùüD~"?ò#?ÈOä'ò#?òùùÈüÈOä'òùüÈüD~"?ÈüÈOä'òùüÈüD~"?ÈüÈOä'òùüÈüD~"?ÈüÈOä'òùüÈüD~"?ÈüÈOä'òùüÈüD~äG~ä'ò#?òùüD~"?ò#?ÈOä'ò#?òùüD~"?ò#?ÈOä'ò#?òùüD~"?ò#?ÈOä'ò#?òùüD~"?ò#?ÈOä'ò#?òùüD~"?ò#?ùYä'ò#?òùüD~"?ò#?ÈOä'ò#?òùüD~"?ò#?ÈOä'ò#?òùüD~"?ò#?ÈOä'ò#?òùüD~"?ò#?ÈOä§ê_ÍíJ$äG~"?Èü*A~ÛL&ÉüD~"?ùUü*#òùüD~"¿÷[xoÜ¸üÈOä'òù_¥É/Í&Ió#?ÈOä§_ssóøÓ;ÒéôÐÐùÈOä'ò#¿_*Ôlhh|ßüæ7Ã@gg'ùÈOä'ò#¿_´/êK.9sæùüD~"?ò«(ùÍ;78/<°.;vD¾ÕüD~"?ùUüºººçsÄ?ê·dÉò#?ÈOäG~%¿ÐO>9oÞ¼0ÐÛÛ[[[ËbÈOä'òùUKä'òùüD~äG~"?ÈOä7Q-¾ÛÅ79ÈOä'òS%ËoáÂqírn/ùüD~"?ò«4ùäóõõõÝö ?ÈOä'òFét:È¯ÙG~"?ÈOä7½úûûü6mÚToiä'òùüD~Ókþüù5ãrùüD~"?ò«4ù-X°Àä'òùüTòÌ700PÛüD~"?ÈoÕ××;ÃüD~"?ªB~2A~]]]ÃÃÃäG~"?ÈO,¿IrùüD~"?ò«Àor0gxÈOä'ò#¿üVòüD~"?Èoe³Ù;wüÈOä'òù©ÂåL&kj>àýýýL&Ü¦¦¦ø¤ÞÞÞ0©¹¹9üD~"?ÈoæiùíÜ¹3<­?¨ïvÉåröìiooOZ¸pák¯½^xáEÈOä'òù½EÜçö¦Óéù|>ÍNvµÚÚÚñòû?ù£³Ø#GªZ³õmUíÖÿ¿ùëÁÖî¸üîsã·5Ùíöõõuvv_øøÿÅ^õÕwß÷ÿª*;qâDø¿õPûÛßÿï·ª³'O^¿~Ýz¨Î¾ûÝï¾óÎ;³yw wCñý©TjünÞ¼Ëåí£½r´WöÊÑÞò®¾¾>ÏGGÃpÑÔ«W¯®_¿þÚµkãg$?ÈOä'ò^Á[+W®¬­­­©©3gÎ5kfùTð3ËI«­­mpppÂÉOä'òùü¦Ñ­[·&<Ãc6ÿoX­D"Éôööþä±×L6ß+òùüD~"¿×ØØDµjÕªè--<¹W¯^Æ,^¼øîßä'òùüD~Ó(JçÆäóù0fÂ3-ÈüÈÏz ?È¯åH$ó¢,¢ÃÙüVòùüD~"¿Ù_t´·­­-zK?ÃpÓÒÒB~ä'òùüÈ¯¢äÞÉ&<ÃãúõëäG~"?Èü*J~ïÞ»fÍºººD"~¶µµ1e±=ÈOä'òùüª%òùüD~"?ò#?ÈOä'ò+Ìy»âKüÈOäG~äG~"¿2_bòÈüD~"?Þ«£½[¶lä÷Â/ùüD~"?ò«Lùõ÷÷Ï3'oÅñ/v&?òùüÈÏz ¿ßÚµk£]G-£íA~"?ÈOä7^zé¥È|«V­*»íA~"?ÈOä7¥nÝºµxñâèdrÜä'òùüD~·ïÙgvõµ··ïö ?ÈOä'òÂ¾ÏüD~wqï¼óÎÈÈùüD~?ù%nW2$?òùÍ~_ÿú×ï½÷Þt:=wîÜ|ð­·Þ"?ÈïýÊ¯2"?_å±ïÃé?¶î±íOlßú­Éûï¿hhüD~"?ò#?_¥uï½÷Fì+ükþµæûöÈOäG~ä'ò«¨Þ÷Ýt:g_ø÷¹Ïî_ü"ùüD~äG~"¿jddäñ»¾Üß§>õ©?ú£?"?ÈüÈOäWiîs[ºti_úâî¹çïÿûä'òùùü*­·ÞzëþûïÿXãÇ>óÏüæoþf`ßÓO?7Ü1ò#?ëü*G~-J¥R¾ÏüD~wCÃÃÃûöíûÒ¾´cÇ»aoùüÈ¯¢ä·páÂ¸ö|ùüD~"?U¬üòùúúúFGGËnÈOä'òùM£t:äWì#?ÈOä'ò^ýýýA~6m*Ç·4òùüD~"¿é5þüq9ÃüD~"?ùUü,XàòùüD~ªùEæ(ÇíA~"?ÈOä7êëëáA~"?ÈOU!¿@ ¿®®®ááaò#?ÈOä§J_Í$9ÃüD~"?ùUà79O3<ÈOä'òù_~«KùF~"?ÈOäG~ä'òùüD~ÏçW®[[[SS3gÎ5kÖË©¾ä'òùüD~ÓèÖ­[áQ§úÈOä'òùM£ÆÆÆà¼U«VEoiáÉ½zõê0fñâÅäG~"?Èü*J~©T*8odd¤0&Ï1a<ùÈOä'ò#¿_"ÎÚ+c|«ùüD~"?ò«Ì£½mmmÑ[ZøÃò#?ÈOäG~%¿ðN6á×¯_'?òùüD~äWQòoìôÞ5kÖÔÕÕ%ð³­­-)íA~"?ÈOäW-ÈOä'òùM£l6»`ÁsçÎùüD~"?U¸üÉdMM¹î;$?ÈOä'òF===A~;wîOërù£mä'òùüD~3ZÄ$%	ò#?ÈOäG~%¿Ä$ù&gòùüD~äW!ò[°`ASSÓÐÐPYoòùüD~"¿Ûý¹Þ÷ÆöÅ]òùüD~"¿ý¹ÞèOôùüD~"?U²ü2LMÉáA~"?Èü*D~sçÎöüEÎsùüD~"?U¦üòÈOä'òùüª:òùüD~"?ò#?ÈOä'ò»Ãõ÷÷g2d2ÙÔÔÔÓÓSzÒÌÆÄÛ¾ûc=öÕYìw÷wÿðÿð«ªÊÖ­[÷ðÖ­¯j+¼Ñ·ë¡:[¿~ýO<1·XfòËåröìioo/=ifcâmÙ²¥F$©Rºãò;þüÏQ~étztt4äóùl6[zÒÌÆ$I"¿0ûJ×_ýý/-~pÑ)Ãã'ÍlL¼®®®ûf±ï½wþüù÷©*O6[ßÖ­/[ÿvÇåÌW`f Õ²eË.^¼8ã¥Å¿ÿ9J4³1Îð3<ä9ÃCÎðx¿Ç|ÆZ¹råS__Ïç£³a¸ô¤!?ÈOä'òû94::zôèÑÚÚÚþõ¶ð3Ë4³1ä'òùüD~ï«ø>¿ºººÎÎÎ­  ÆL&ÓÛÛû9öqÅñf6üD~"?ÈoÅÁW[[ÛÞÞ~ùòårÙä'òùüD~ÓYÄØ=ôÐ¥KÊnÈOä'òùM£Ë¹ÈOä'òùüÊ:òùüD~"¿éuöìÙEÍ3':·¾¾þÐ¡CäG~"?Èü*M~Ç+áÉ/Þ³gùÈOä'ò#¿_CCCpÞÙ³gòëíí¾ØüÈOä'òù_EÉ¯ðçòNø%?òùüD~äWQò«¯¯Îöóùåóù­[·ál6K~ä'òùüÈ¯¢ä(S3Q¯½öùÈOä'ò#¿_èÚµk­­­Ñ¹½µµµ-ºxñbYlòùüD~"¿jüD~"?ÈüÈOä'òùü~¶Ë/·´´ÔÕÕ%ÆMMMår¨üD~"?ÈoªmØ°¡f6nÜH~ä'òùüÈ¯BäwèÐ¡y÷îFÞºuëðáÃÑøîînò#?ÈOäG~ ¿L&x·ÿþñ¢¿çæûüÈOä'òù_È/JÞÝºukü¤|>&+ùüD~"?ò«ùþhÛdS£¿äF~ä'òùù_%È¯íÈüD~"?ÈüÈüD~"?_Ê¯täG~"?Èü*D~ÛL&ÉüD~"?ùUü*#òùüD~"?ò#?ÈOä'ò#?òùüD~"?ò#?ÈOä'ò#?òùùÈüÈOä'òùüÈüD~"?ÈüÈOä'òùüÈüD~"?ÈüÈOä'òùüÈüD~"?ÈüÈOä'òùüÈüD~"?ÈüÈOä'òùüÈüD~äG~ä'ò#?òùüD~"?ò#?ÈOä'ò#?òùüD~"?ò#?ÈOä'ò#?òùüD~"?ò#?ÈOä'ò#?òùüD~"?ò#?ÈOä'ò#?òùüD~"?ò#?ùYä'ò#?òùüD~"?ò#?ÈOä'ò#?òùüD~"?ò#?ÈOä'ò#?òùüD~"?ò#?ÈOä'ò#?òùüD~"?ò#?ÈOä'ò#?òùüD~"?ò#?ò#?òùüÈüD~"?Èü¦X&I&MMM===ñI½½½---aRsss¸ùüD~"?_yËå<öìÙÓÞÞ´páÂ×^-¼ðÂ-"?ÈOä'ò+ïÒéôèèhÈçóÙlv²«ÕÖÖßÿù÷Íb/¿ürhª2[ßÖ·ª³îîîïïÖ­?;UüÉäÃñÂºèìì/¿Ã¿5?~ü7ÞxKUÙ+¯¼réÒ%ë¡:ëééùÑ~d=Tg¯¾úêÅ­êìÄÿò/ÿ2·XòK$áT*5þ7oÞÌårCCCöÊÑ^9Ú+GåhoùUóÓÂp>öá¢k^½zuýúõ×®]¿òùüD~"¿2«££ãÀa üÌårEÒjkkpFòùüD~"¿ò[­D"Éôööþä±íÌf³5±ÈOä'òùüª4òùüD~"?ò#?ÈOä'ò#?òùüD~"?ò#?ÈOä'ò#?òùùÈüÈOä'òùüÈüD~"?ÈüÈOä'òùüÈüD~"?ÈüÈOä§uîÜ¹|ðCúÐ=÷ÜËåÞzë-òùüÈüD~Øüãt:½bÅ®/wyËcÉod³ÙwÞyüD~"?ò#?_¥µ~ýúåËobáßÇ[>¾cÇòùüÈüD~ÖÇ?þñ¿·1.¿Gô3ùùüD~äG~"¿JëøÄcëËï5üÖoýùüD~äG~"¿Jk×®]küØ¶ßß±/ÜwßñA~"?ùÈ¯Ò~è¡>ò<üézð¡ï½·³³sddüD~"?ò#?_÷o|cóæÍ]]]ÇñrÈü¬ò#?òù©Z"?ò³ÈüÈOä'òùüÈüD~"?ÈüÈOä'òùüÈüD~"?ÈüÈOä'òùüÈüD~"?ÈüÈOä'òùüÈüD~"?ÈüÈOä'òùüÈüD~äG~ä'ò#?òùüD~"?ò#¿ìÇ?þñ#<rÏ=÷Ì;÷á¾xñ"ùüD~"?ò#¿lhhèþûïÿõ_ÿõ/oùòÖ¯lmûTÛ>ô¡ªÂùõ@~"?ò#¿jé©§úxËÇ·?±½ðoÙÿYöè£ÈOä'ò#?ò«´Ö®]ßÆßÛøÑ~üD~"?ù_¥Ø÷ÈGâòlÝcÍ¿ÖL~"?ÈüÈ¯Òúú×¿ýH¶ëË]ùô£Ý¶mùüD~"?ò#¿lÃþðzð¡~øùW­Z5<<L~"?ÈüÈ¯2;räÈW¾ò®®®o|ã###UõØÉü¬òùùüD~"?ùÈOä'òùùüD~"?ùÈOä'òùùüD~"?ùÈOä'òùùüD~"?ùÈOä'òùùüD~"?ùùùüD~äG~"?ÈOäG~ä'òùüD~äG~"?ÈOäG~ä'òùüD~äG~"?ÈOäG~ä'òùüD~äG~"?ÈOäG~ä'òùüD~äG~"?ÈOäG~äG~äG~"?ùÈOä'òùùüD~"?ùÈOä'òùU¡üúûû3L2ljjêéécÇÕÔÔÈOä'òù¹àÁa`Ï=íííESGFFZZZ&_ ØÿÌb¯¾újxýÿª²ðd¯ë¡:î¿~ýºõP<yrppÐz¨Îûÿíßþm6o±*äN§GGGÃ@>Ïf³ESzê©Ý»wO&¿gy¦;räÈË/¿Ü­ªÌÖ·õ­[_¶þ®*äL&']¾|¹µµ5¸ÐÑ^9Ú+Gåh¯í­Da8JÅ'­Y³æäÉÿûPÉOä'òùüÊ´ëëëóùücGÃðW+üD~"?È¯ëèè8pà@?s¹ÜdR?üD~"?È¯üVkCCC"Èd2½½½RüD~"?È¯ª#?ÈOä'ò#?òùüD~"?ò#?ÈOä'ò#?òùüD~"?ò#?ùùüÈüD~"?ÈüÈOä'òùüÈüD~"?ÈüÈOä'òùüÈüD~"?ÈüÈOä'òùüÈüD~"?È¯úã?þãoë[oÎb/¾øâøÃ7U­îÜ9ë¡:û«¿ú«ú§²ª³ðFóÿøÖCuvèÐ¡Ù¼Åwßü&íìÙ³Û·oÿª$IRETú`fÝ°$IUùI$$IÈO$Iä'I$ò$IùI$üÊ¾óçÏ·¶¶¦R©µk×ûµk×jbYWÔàà`6-ïïÏd2Éd²©©©§§'~ÍTñ[ß/ßúñÚ·õ?ð×¾_7w°Å÷öösçÎ=þøãñIZ¿~½UTy:uª±±1þbÎåröìioo_¹Ä$UüÖ÷K â·þø1^û¶þÝðÚ'¿;XøÿîÜ¹ñIa«>|Ø*ª¼V®áÂø«=N|>_ôÿ¿Tñ[ß/ßúãÇxíÛúwÃküî`ÍÍÍgÏÏ?ÿ|¡¶µµ­­­çÏ·®*¬ø«=¾é%&©â·¾_¿õ'ãµ_å[ÿíßìÌ3-ª­­Ý½wø9áu®^½h]Uðë?HS©Tüj%&©â·¾_ÕüÞïµ_Í[ÿíßlôúë¯766N6ÕË¾²_ÿõõõù|þ½±Ã:a8~µTñ[ß/j~ï÷Ú'¿ðµO~w°ö÷÷îÞ½ûÉ',tñâÅümmmÖU¿þ;::8ÂÏ¿ZIªø­ï@5¿÷íWóÖÿÀ_ûäw;qâÄüùóè;;;GFFâÏ¾¾¾ÆÆÆd2¹lÙ²°í­«~ý:uª¡¡!Hd2èÂ&¤*Ùú~	Tç¿×¾­ÿ¿öÉO$©jjH$$IÈO$Iä'I$ò$IùI$ü$ID~$I"?I$$IùI$ü$ID~$I"?I$$IÈO$Iä'I$ò$IùIªþù¥KÖµ|ùò^zég~ÁU6¿'º·Ùl6<´¡¡¡¢ñaL2Ìd2£££Ó]¦$ò¤òkûöí5ãzòÉ'+I~;wî#î¹¢ñ_ûÚ×Âø;vÌ`ÈOÊ¬þþþ`d2¹wïÞ±öïß.§O®ù]ºt)lnn.ßØØÆ_¼xü$¤Êïóÿ|0ÍÓO?ùÌ3Ï_øÂâîééé	r(lii	Ã+_¿~½³³³®®.LJ§Ó7oT=~üxÐUæíîî.²T3wîÜ¥K=z4èè(ºcG)½hR¸KÑ¤cÇM¦´¶¶¶0¾···0æµ×^cV¬XQ³k×®°¨ÚÚÚµk×^½zu¼üÆ/¿hL»*ü$élþüùA-/_¼råJÍfã²)ª¯¯/ºzõê¢I6m&$	ç.FS×¬Y3::ìJ¥FFFÂÔð3ÈiÎ9ÑÇïJ,'Lx÷Æ?Ò^x!ÎÙ.<X`_ÑB/_>]ù¸«ÈO>à¢»üR«©	Ë¦½½ýæXa \µjU45NdÇh×][4)ËE;Ãð©S§¢Ä¹mÛ¶`»è`ëÂÃáð3¯[·î¶Ë	jÃâ÷mÂG49oÞ¼ðxÃÅð3ÜóÀÍ|>]!ÉÃCxï§Gkkk§+¿wUùIÒ/¿Ðò+d)tõêÕp1)ºØÒÒ..X°`ãÆAl·nÝ*,$h/Za®èâ+WWZ*ð?ÃðñãÇo» Ìpñí·ßß·É>·eË0i÷îÝïýôöæÍãWòmmmvINW~%îª$ò¤¸hG×Í7ã#ÂÈ0©u.¼páB¿tÎ¶ÈRñïR	Ãét:JÏ;·0õ¶ËÌaE=6Bj?ÃðÀÀ@aê©S§Âð¨ñÔåWâ®J"?Iú>ëöì³ÏÆGFßuRtGá³ãwe>zçÎÑ±×£rÃ©·õÙ¦M¢¼ágggga|åDûØ®]»]|ûí·KûÀ©ÑÙ°Æ'EçùIÇ»qãFiùT­ÂÔwUùIÒD2Ü³gOô­.÷îM¥RãÏÆXµjUPÎððpt(¶pnô9¿èãq.@0àæÍ¢³nß«2¡Ï¢m£[?qâDa|åDì>ç744]³üöïß_Ør|RôÇp+a%L&¿ÈÁÊájAk×®O-qW%$ðEßr®]»þÿ/¸±"ÇI'UÚ°aCñ:TB~ïý±0>NÇXN¸ñ;V8¯v²ëÖ­ÚÚÚè!ã^¹reü&æÍ~F_ì_f´K²PäÔÂÔwUùIÒ]QÐÉòåËSc-]º4:Á¶H~Ç¾6¯©©éäÉ©CCC[·nmhh´´eËáááÂÔîîîÖÖÖ²L&³oß¾¢e¿'aQaüã?^4~²åÂ	w)ú¢ÁßçW¨³³³èë]¢s¹uuuáQx±ð5ñeÞ¸q#Ð6ZW+V¬èíí-ºÅwUùI$ü$ID~$I"?I$$IÈO$Iä'I$ò$I"?I$$IÈO$Iä'I$ò$IùI$ü$ID~$I"?I$ëÿéAu$%ÅÓKIEND®B`


allÌVhåòíîî®ÎårQõööW_rþ¢$3Ê/5ÚÚ¼h§üT,zzÂ½¤2ÏçGGGÃ`xxxpp°úÂó%Q~ ü_CØ±cÇÌÌLuùuvvËå0(JÕ¯ÞvQòù¿ÀýÓ¿ùÍoZË¦ÉÊ¯òü 2¢è¶ãÛ.J2SS~¯¿þúàþéßúÖ·¬eÓôåJ¥*ãt:]±ùÌ8Úö:ÚÛ å×ÕÕU*âÃµa}±ùÌ(?P~Ê¯AË¯P(A8ÍçóÕ¿(Éòå§ü´üÅb6M¥R¹²úó%Q~ üßJ¤ü@ù)?å(?å§üå§ü üòåòS~ üP~ÊÊOùòCù)?P~(?å÷Éo~ó_-,ß¶mÛ¹ÀïÿëPù)?h¸;BÁ:T~Ê@ù)?å§üòP~ÊP~Ê@ù)?å§üòP~Ê@ù)?å§üòP~ÊP~ÊOùÊOù(?å üòS~ÊOù4Qùõ÷÷···Á±cÇ2LEù|^ù´ZùíÚµ«­­-JËå¶*»wïV~-U~¡ó¦¦¦Î?===.]0¯üZªüâWøÂ`ëÖ­a044O¦R)åÐRå×ÞÞ:onn.~ñïòåË^óhÁòëïï¯ün_6½ù¯ú=?V+¿`Ó¦M©T*dßÜÜÆ>úhSüÿ üVå(?å ünçìÙ³ë×¯oooßÏÛÕÕuìØ1åÐjåwòäÉÊ;<âòÇÃÃÃÊ ¥Ê/ÍÎ;öl¥ü&''ª@_å+åÿ·(@K_WWWè¼øu¾P~¥RiÿþýaÜÝÝ­üZªüâ¿Ø1ßéÓ§@K_ðÑGõõõÅïíÍd2ë×¯ÿàâÿ¯üå·R(?@ùÝÝÝk×®=wîòhñò¢(~o¯òhñòå÷üóÏÏÍÍËeåÐ²å×¶ø³ý@ë_j>É ÕÊ¯©)?@ù)?å7ÿ&üÀ)¿êßí«.¿õ~çÏïëëK§Ó?þøÜÜ¢éééþ¡ÞÞÞñññ3ÊP~÷@¹zõêc=ÊoffæÜæ¦M&''ÃàÜ¹s÷î­^ÏçGGGÃ`xxxpp0áòß=â/ß5kîÉ­U¿v¸jÕªêEñ'J¥îîî35åð4@K¨CùÒåN§ïÉ­mØ°áìÙ³apäÈ#ÈÕgãqòûÅ/~ñ°ÜåW*>Ê/ÉÜ|ï½÷Ö¯_níàÁ5·Yý&84Ì8Ú8Ú§7±;wÞÛïõý÷ßïéé©éêê¡çf'Q~ò»CóÿzG:~ì±ÇîÕ·¸nÝºééér¹|ðàÁïÿûÕÂÈÈHÓ|>pFùÊ¯AMLL¬^½:Ôä=nÜ¸ñçï»íOßy±XÌf³¡5s¹þß$3ÊP~+òß§¹DQÔÕÕUs|Vù4qùÍÿëñn«@+ß7ÚÛÛwîÜyåÊp6>úè£½½½aüÆoòëììT~­P~ë×¯yrJìÚµkafûöíaòþ_åPçòéVÞuóÖ'çÅÁÿ1åÐ"åÍfãÏm¾zõêÍ[´7ã¼SSSö´Nù<yò¶ïíöìQ~­P~Á¥Kâ?­J¥Âi·ßÃ?æ¡üê_~ÍKùÊOù(¿Ê5oÍÿG¼Tù4ù°?®%µFþ0å(¿GùÁ½ýº/^´ò&Ù¡Ü±±1ë AË¯Y~OùAãt¡7)¿&AåÊ@ù)?@ù(?å(?å§üå ü ü¼üçoxÊ EÊ/µÃP~-R~­AùòP~ÊP~ÊOùÊ@ù)?@ù(?å(?å§ü@ù ü(?òåòS~pßüñüÞÝ	å÷õ¯ýnnáÌ36òS~pß]¼x1¤Ûû÷×ë+üëccc6òS~°å÷¹ìêÁT¯¯ø§òP~ÊÊOùò@ù)?P~ÊOùÊ@ù)?P~Ê@ù)?P~Ê@ù)?P~Ê@ù)?P~Ê@ù)?P~Ê@ù)?P~Ê@ù)?P~(?åÊå§ü@ù(?å(?å§ü@ù)?å§ü@ù)?å§ü@ù)?å§ü éÊï³÷àÃ[êõõ_üòe*¿¶zS~ÊOùÁ2ßg¢´òP~ÊVDùù=?å§ü@ù)?åwLLLôôôDQÔÛÛ[,«MOOçr¹xÑøøxÂåÊ@ù5¨ÎÎÎ?ü0Âiwwwõ¢|>?::ÃÃÃ	g(?å× Ö®]ùòå0§aår9J¥RIfjÊïÅ_üw`)?ûÙÏVuæêX~ë¶ügÖXßôôt&ikk§SSSÕ¢(ª'©)¿°ßþö·ÿ­Î¯ùýÏþô§6Àâ¾ü6mÚtîÜ¹87oÞ(JUÆét:á£½àh/£½jíºººâ¶§apFùòP~jóæÍgÏ3gÎlÚ´©zQ¡P	pÏçÎ(?P~Ê¯A½ÿþû!ø¢(§aüçï»íOßy±XÌf³©T*ËMNN&Q~ üßJ¤ü@ù(?å(?å§ü`¥_:ÓþÕýßõúêîÝªüòåð»ßý®­­-ûù/Üñ×gÒíëÊÝñÕÃ¿þæoÚÊOùA3ìnÚÚü¸(?åÊå§ü@ù ü(?òåòS~ üòs?å ü üòòS~òP~ÊZÿj¡ü¶mÛ¶È.^¼h(?åM²C¹;cccÖ!òS~ÊOù(?å ü ü÷ä.uwlP~ÊOùÊOù5[Zá üòòS~òS~ÊP~ÊOùÊOù)?P~ÊåÊOù¡ü@ù)¿æøÃ¿ì(|íëwüõtû?ÿ÷¯ÞñÕ¿ú/¿þõ¯mP~Êï¾»xñb[[Ûvª×Wô_ÚÇÆÆlP~Êo9ÊïsÙÕ?þ¨^_ÿðOåÊOù)?@ù)?å(?å§ü@ù)?(?å§ü(?å§ü(?å§ü(?å×å÷7û·mu¥ü@ù)¿e*¿¶zS~ üß2_Çß=øÅþýõúêZÛ§ü@ù)¿e*?¿çÊOù)?åÊOù)?åÊOù)?åÊOù)?åÊOù)?åÊOù)?@ù)¿Y~>É¸ýÃÉÝÙ¶muÊOùµàc+óg?ìo­P~ÊOùÊP~ÊOùÊP~ÊOùÊP~ÊOùÊhæò«~óW*ª^4==Ëå¢(êííO8£üp7»âº°RÊ¯btttxx¸z&ÏÉ0ó	gjÊ/äàuKØ[áÐ¤?¼uü,Ï/öïÿîw¿k+@-R~.ºukÍdggg¹R©ÔÝÝp¦¦ü<ø¿¸w.*<x|ík_[äo¾ù¦u¨îåv¶$Ñ"å700055U3EQÍ8É£½|HèÐ¡CÖ!4ævËÏÑ^XAGgff6oÞ<¾ú×þÒétÂå ü@ù5®¼ôÒKóç»ººJ¥ÒÍ[GrÃ8áò¸òóP~ËdË-ï¾ûîüùB¡022á4Ï'Q~Ê_ãJ§Óñ»4ª÷Aá´X,f³ÙT*Ëå&''Î(?;(¿ÞR¯¯öÏ=¤ü`ßý£üßóå§üòå§üòå§ü±ü¶=ûoõúzðá-ÊòX&­^ó¥ÜzÇ_ÑgW=ô÷ßñÕ?ÿÐÆÆÆlP~Ê l|,(?å üå§ü üòò¨£B¡°mañ_`[ä§N²Aù)?&y8¹;/¾ø¢uÊOù(?å üòS~òS~ÊOù(?å üòS~ÊOù(?å üòS~ÊOùÊOù(?å üòS~ÊOù(?å üòS~ÊOù(?å(?å üòS~ÊOù(?å üòS~ÊOù(?å ü üòS~ÊOù(?å üòS~ÊOù(?å üòS~òS~Ê¯®_¿þäO¦Óé5kÖLLLT/ÎårQõöö'Q~òkPøÁ~P.Cö­]»¶zQ>áááÁÁÁ35åwüøñÿÐ¾üzÏ;wÛE¡Ã T*uww'©)¿_~y %4ùEQôÒK/e2µk×¾÷Þ5jÆIfíímP©TêÕW_óçÏ÷õõÕ,ªÓétÂå(¿ÕÕÕU×¼hJ¥·äÆK2£üå× öîÝûÚk¯ÁÙ³gyäêEBadd$Âi>O8£üå× æææ¢(êëëùó÷Ýö§ï¼X,f³ÙT*Ëåâ_iL2£üå·)?@ù)?å§üòP~Ê@ù)?å§üòP~Ê@ù)?å§üå§üòP~Ê@ù)?åW_o½õÖ#<² %)?ò@ù üP~(?ÊåÇwîÜ¹'x¢££#¢®®®½÷~üñÇws?üáïäÛæ®[¿½ÆÂ*9uêÔÎ;ãûI83óoêí·ß×§Öj«ÞªÏ¦R©L&³qãÆ_~ÙzkMä^±ÐvO¸@ùQO¯½öÚ5k=Z*ÂÙpÆ«W¯¾téÒýØw(¿¦ÛÝÇ9²uëÖÉÉÉr¹ßOÂ½§§çðáÃ5Ü½÷®]»Âs	kµUïógÎ?ÿÈ#:tÈêjíò[|»'ßK ü¨yó_á;xð`¿ò³»¯÷+W®ÔL^¸p!ÍVÏ=þ<píÚµU«VÅVBùÅCZ]+ªüj¶Â½Êzzúé§çÏGîÑÑÑÊÙ'NôööFQNÃ¸zGpüøñµk×¦R©°4~Uþ¡wÞy§³³³¯¯oÉ[³E¶üÒéô¹sç¼°AÂ VoÜàäÉa£(Élß¾=É­ÑDåßIÂi¡P¨>ÖÿÖ[oy¸Ë¯²Ýï%P~ÔSx-~Ðs¹:ÃiWW×ÔÔTeG°uëÖø¸p¸Xx¿ã§zªÇÅoÍiØòýõ×Ã÷ú_ìÉ'<zôèÍ[ÇÂ¸zQå¹A¸3¼öÚk===V+ßùóç7lØüqxRwõêÕ0þäOÂS¾ù¯Ñ2åWÙîÉ÷(?ê)J-yÐváqºº*Ã üØÏß/ÔßÌÌLÂ[³Eê¸»_üíÁ±cÇV­Zöì»wïy2..øøPïÜÜû¾áÖ&''­êÖ+¿°ßyçõë×Wò_õÕ½÷Áàà`Xd¶dùÍßîIö(?ê¬ò*ÝâßüãÛî*¿;»5dw_³¯;÷ç.ËkÅ¯ðÅÆÇÇ·oß^9ûè£V¿­ohhh```ttôôéÓ~°ÙË¯ZøÞ¹sgÍ[8·lÙ²ÿþûöY­T~Kn÷%÷(?ê¬§§çÃ?ü´;ÛÕMX~Éo,¿j7nÜ8tèPxÆ_yê©§jöìÙSYzýúõ]»vÅ«V­zÿý÷­öæ-¿%¯ú>ühÇ/Ó2åw÷	u644´Ð§pU¥'v÷ð5¿ä·F_¼[¯|¨·úâ³³³ó_Þ»|ùò#GÖ­[gµ·pùåóùÝ»w?óÌ3VàJ.¿½ÊúY³fMx®?~üøâñÎ;«3/wìØqÇåüÖh¨òËf³ó_¥U·qãÆx<>>6nÍÂÌÄÄÄ=|¡)Êï7ÞÏ*Ã`ûöíÝh½ò[r/ò£!:thÝºu¡ÀÂ3³pöÒ¥K/¼ðÂ¿üåk×®Å?ÏñoéNMMq±XüÚÛÛ/¿üSsä·FCßáÃÃ#G|òÉ'ñÞüÌ3B¡ò¸þôÓOÏÿ»á)DÇaïôèÑø%ÀêwÓbå¿7~ooü>_Ç|WHù-¹@ùÑ(N8g2T**ðÀì«<~ÇÀ·aÃêG÷ÊïW^IßrÛEÂ[cw÷K¾½7ì¾üñø7õ÷÷W>'ìâÃ6`7ÌT>½%<w³p¯·¶þÉ'­ö,¿ðH_ýQ>Ï¯vÉwÑì%P~(?Ê@ù üP~(?Êåò@ù üÊåò@ù üP~÷Cwww&¹zõjÍ|¢(ËËåO·W½å./ðFÀ§ðüóÏÆzùåkæ:æ¿óï|ê½ªò@cºpáBh¬6ÔÌ÷ôôù>øà~í| üßöíÛCfMNNVfN>f¶mÛV9pà@6¢(É<þøã/_®N´'N¬ZµjË-ó£mñ+èK7nÜÆß©S§B«2ÿÉ'ìÙ³§££#,êììÜ·oßüÃÖÊà/=2ë'¨ÌìÚµ+ÌVê­í¯mÝºµ:ÑR©T8¨¶%¯Xcjjj~ù9s&¾ýùÛ¹sgÍ-<óÌ36( ütãÆx ¢ÙÙÙp6Òêèè(Jñr¹ª·ß~ûæÎd2ÕöÜsÏËåøÐpu´-yÅÁÁÁ+·A8Ûßß?¿üòù|üê`ÅøZñ¢¸?üðÃ0ÿJ···Û òXÌÐÐPÈ¦ñ/¼Æûöí«¾@¨ÀV¡ðúúúâùªíÒ¥KÙ«þõÚÅ¯·fpùòåp6äæü	5/ìU.¶qãÆpvíÚµO?ýô[o½uíÚ5P~K8ölPaNÃøÌ3¥Åb±³³³&¿ª­ú_ª.yÅ¿Ú·µEQ4éüÂ|ã¯RÇ·5å°Í7xúö·¿NCNU/ßç<yrnnnÉ«Ì,yÅø@íÍ[zÍ¯½½=+ç÷Ýwþùp±gSÊ`	¯¾újåÅ³áááêEQÅ¯ËåW^y%yù-yÅþþþÐ|×¯_/ál8#qÒíÛ·/ÜH(ÈêÏ Ï/þ=Â0N§Ó6% üpíÚµL&r½råJõ¢;vTlàÂiüù,ßWÓ°2®b®¾©©©£½ÇÅï©öÔSOÙòXÚ=j>Þ%6;;ÏçÓétGGÇÐÐÐ|PùüÅËoÉ+<yrãÆ¡ùzßyçÛÞHpâÄ¾¾¾T*Ëå>¿zõêþýû³Ùlá¸~ýºí(?ò@ù üP~(?ÊåòP~Vò@ù üP~(?ÅÉÇ±äÁ IEND®B`


ONEWAY Figures BY Variables
  /POLYNOMIAL=1
  /STATISTICS DESCRIPTIVES HOMOGENEITY
  /MISSING ANALYSIS
  /POSTHOC=LSD ALPHA(0.05).


Oneway


Notes	
Output Created	12-SEP-2022 21:54:55	
Comments		
Input	Data	E:\桌面\Raw Data\4. C. Cellulosae ESAs and TPx Induced Th Subpopulation Differentiation\3. SPSS statistical analysis\1. IFN-γ\1.  IFN-γ--24h\Untitled1.sav	
	Active Dataset	DataSet0	
	Filter	<none>	
	Weight	<none>	
	Split File	<none>	
	N of Rows in Working Data File	20	
Missing Value Handling	Definition of Missing	User-defined missing values are treated as missing.	
	Cases Used	Statistics for each analysis are based on cases with no missing data for any variable in the analysis.	
Syntax	ONEWAY Figures BY Variables
  /POLYNOMIAL=1
  /STATISTICS DESCRIPTIVES HOMOGENEITY
  /MISSING ANALYSIS
  /POSTHOC=LSD ALPHA(0.05).	
Resources	Processor Time	00:00:00.02	
	Elapsed Time	00:00:00.01	


Descriptives	
Figures  	
	N	Mean	Std. Deviation	Std. Error	95% Confidence Interval for Mean			
					Lower Bound	Upper Bound			
Control	4	7.27700	.245586	.122793	6.88622	7.66778			
ESAs	4	8.13925	.420291	.210145	7.47047	8.80803			
TPx	4	6.93825	.150644	.075322	6.69854	7.17796			
LPS	4	10.43600	.588361	.294181	9.49979	11.37221			
Total	16	8.19763	1.451590	.362897	7.42413	8.97112			


Test of Homogeneity of Variances	
	Levene Statistic	df1	df2	Sig.	
Figures	Based on Mean	.965	3	12	.441	
	Based on Median	.947	3	12	.449	
	Based on Median and with adjusted df	.947	3	7.136	.467	
	Based on trimmed mean	.964	3	12	.441	


ANOVA	
Figures  	
	Sum of Squares	df	Mean Square	F		
Between Groups	(Combined)	29.789	3	9.930	65.562		
	Linear Term	Contrast	13.698	1	13.698	90.446		
		Deviation	16.091	2	8.045	53.121		
Within Groups	1.817	12	.151			
Total	31.607	15				


Post Hoc Tests


Multiple Comparisons	
Dependent Variable:   Figures  	
LSD  	
(I) Variables	(J) Variables	Mean Difference (I-J)	Std. Error	Sig.	95% Confidence Interval	
					Lower Bound	Upper Bound	
Control	ESAs	-.862250*	.275186	.009	-1.46183	-.26267	
	TPx	.338750	.275186	.242	-.26083	.93833	
	LPS	-3.159000*	.275186	.000	-3.75858	-2.55942	
ESAs	Control	.862250*	.275186	.009	.26267	1.46183	
	TPx	1.201000*	.275186	.001	.60142	1.80058	
	LPS	-2.296750*	.275186	.000	-2.89633	-1.69717	
TPx	Control	-.338750	.275186	.242	-.93833	.26083	
	ESAs	-1.201000*	.275186	.001	-1.80058	-.60142	
	LPS	-3.497750*	.275186	.000	-4.09733	-2.89817	
LPS	Control	3.159000*	.275186	.000	2.55942	3.75858	
	ESAs	2.296750*	.275186	.000	1.69717	2.89633	
	TPx	3.497750*	.275186	.000	2.89817	4.09733	

*. The mean difference is significant at the 0.05 level.	
