# Supplementary material for: Regulation of piglet T-cell immune responses by thioredoxin peroxidase from Cysticercus cellulosae excretory-secretory antigens
Source: Front Microbiol. 2022 Nov 18;13:1019810. doi: 10.3389/fmicb.2022.1019810 (PMC9718028; doi:10.3389/fmicb.2022.1019810)
Supplement: Supplementary file 3 [file Data_Sheet_3.ZIP › 4. C. Cellulosae ESAs and TPx Induced Th Subpopulation Differentiation/3. SPSS statistical analysis/1. IFN-γ/2. IFN-γ--48h/2.3 (SPSS data export) SPSS statistical analysis--IFN-γ--48h.doc]

EXAMINE VARIABLES=Figures BY Variables
  /PLOT BOXPLOT NPPLOT
  /COMPARE GROUPS
  /STATISTICS DESCRIPTIVES
  /CINTERVAL 95
  /MISSING LISTWISE
  /NOTOTAL.


Explore


Notes	
Output Created	12-SEP-2022 22:22:06	
Comments		
Input	Data	E:\桌面\Raw Data\4. C. Cellulosae ESAs and TPx Induced Th Subpopulation Differentiation\3. SPSS statistical analysis\1. IFN-γ\2.  IFN-γ--48h\1.1 SPSS statistical analysis--IFN-γ--48h.sav	
	Active Dataset	DataSet1	
	Filter	<none>	
	Weight	<none>	
	Split File	<none>	
	N of Rows in Working Data File	20	
Missing Value Handling	Definition of Missing	User-defined missing values for dependent variables are treated as missing.	
	Cases Used	Statistics are based on cases with no missing values for any dependent variable or factor used.	
Syntax	EXAMINE VARIABLES=Figures BY Variables
  /PLOT BOXPLOT NPPLOT
  /COMPARE GROUPS
  /STATISTICS DESCRIPTIVES
  /CINTERVAL 95
  /MISSING LISTWISE
  /NOTOTAL.	
Resources	Processor Time	00:00:02.56	
	Elapsed Time	00:00:01.58	


Variables


Case Processing Summary	
	Variables	Cases	
		Valid	Missing	Total	
		N	Percent	N	Percent	N	Percent	
Figures	Control	4	100.0%	0	0.0%	4	100.0%	
	ESAs	4	100.0%	0	0.0%	4	100.0%	
	TPx	4	100.0%	0	0.0%	4	100.0%	
	LPS	4	100.0%	0	0.0%	4	100.0%	


Descriptives	
	Variables	Statistic	Std. Error	
Figures	Control	Mean	8.41650	.185806	
		95% Confidence Interval for Mean	Lower Bound	7.82518		
			Upper Bound	9.00782		
		5% Trimmed Mean	8.42422		
		Median	8.48600		
		Variance	.138		
		Std. Deviation	.371611		
		Minimum	7.908		
		Maximum	8.786		
		Range	.878		
		Interquartile Range	.693		
		Skewness	-1.018	1.014	
		Kurtosis	1.509	2.619	
	ESAs	Mean	8.92500	.217796	
		95% Confidence Interval for Mean	Lower Bound	8.23188		
			Upper Bound	9.61812		
		5% Trimmed Mean	8.92756		
		Median	8.94800		
		Variance	.190		
		Std. Deviation	.435592		
		Minimum	8.370		
		Maximum	9.434		
		Range	1.064		
		Interquartile Range	.809		
		Skewness	-.314	1.014	
		Kurtosis	1.500	2.619	
	TPx	Mean	7.84675	.076300	
		95% Confidence Interval for Mean	Lower Bound	7.60393		
			Upper Bound	8.08957		
		5% Trimmed Mean	7.84506		
		Median	7.83150		
		Variance	.023		
		Std. Deviation	.152601		
		Minimum	7.677		
		Maximum	8.047		
		Range	.370		
		Interquartile Range	.285		
		Skewness	.583	1.014	
		Kurtosis	1.494	2.619	
	LPS	Mean	12.18025	.385851	
		95% Confidence Interval for Mean	Lower Bound	10.95230		
			Upper Bound	13.40820		
		5% Trimmed Mean	12.19222		
		Median	12.28800		
		Variance	.596		
		Std. Deviation	.771702		
		Minimum	11.146		
		Maximum	12.999		
		Range	1.853		
		Interquartile Range	1.444		
		Skewness	-.794	1.014	
		Kurtosis	1.498	2.619	


Tests of Normality	
	Variables	Kolmogorov-Smirnova	Shapiro-Wilk	
		Statistic	df	Sig.	Statistic	df	Sig.	
Figures	Control	.251	4	.	.947	4	.698	
	ESAs	.250	4	.	.959	4	.774	
	TPx	.249	4	.	.964	4	.801	
	LPS	.250	4	.	.960	4	.777	

a. Lilliefors Significance Correction	


Figures


Normal Q-Q Plots


êèè¸zõ*ã G2°2¼NÉjAVêô'|²ÿþ_~Yó=Ðùùùõõõò=ÚìûäyNuvv^¹rq¦×5Ýå'¶jáqçÎy^³û1"ÞÇ°·½½ÄÞÞ°ÚÛkµZkkk5ÝPó³Äà$Ììíeo/£s>¿÷îåää$&&Êß¸IIIEEE?ÿÕºï/NHH@~È_6>>~ôèÑåËÏ5	3ó³ ?äü¢J~:®Þ>(ÿzï;F~ÈßÂ&+_ùù!?B~sÎl6¿÷ÞrBþ­ªªB~È_84>>.ªÛ1?òC~£òÈÍÍMJJRûgÓÓÓÏ??ÿ«íééÉÈÈëÌÌÌ&òC~ü05?ÀNxçm¾åË3?òC~òS39»b:üøq]ïòC~üBÝnollÔ9--­¶¶öúõën/!?äü"L~²úpÉOFr:%%ùòC~Óéloo¯¨¨Ðüh]uèzÄð¹½üòTù©õ :¡ä7==ÍçöòC~Õjmhh0L¾æg±Ùlîßüò£ÉYmçùMMMíÙ³G­!?äAÉÃ¯½½½¼¼Ü×¡2ó³ ?äü(ä×ÝÝ­9óõë×!?äÍ8?ÿC7òC~CòSk½ulobbbnn®Ç®äGÈùaòxkkkÓÜÈ§Ý¥çòC~[ò[ò#ä7çrMMÑhô6_ii©ppNEäüÅüv¸züøqnnnVVò#äüÂ*ÃÑÚÚZ\ÜÖE~ÈùQLËojjc	ù!¿°ª»»»ººÚf5?¯C7òC~üþ»¬¬¬E~KMME~üßÂ644tèÐ!ÍIe¡¬Fò¦däüE¿üãçúô÷.~üß¤&a®¬¬Ô¹ººº££c>ùòC~sòs%ÎÓÇ.òC~üfY½úl!ïùYGGGþCýËOÕ!òC~È/Jä·P!?äGÈÏ]WçÎ+++ó5	³<y¸oò³Z­ßùÎw^xá£ÑøÍo~S^x("?äü"^~SSS¹¹¹III®%K,y÷Ýw!?ägåÎ;5'aVó³`¦üFGG|¯¼òÊÞú½ûÞÙ·eË¯~õ«aüòxù-]ºÔãð^µÎ=pàò#äütêÑ£GMMMó³dddÔÕÕÝ¸q#d7FS~û÷ïé¥Ä|®ÿ~oÓïðhD~ÈùE¶üdUÛßßïZÒ××'KRRR!?äÜä"kÒïùYB¹o6òõÕW·lÙâ.?ù/11G#òC~È/²å§ïv_è=Éò#äüæÓàà`CCÉdòÑhxfýäg±X^[ÿ;ûvîØùõ¯G#òC~È/²å)kÞ]»v©×ìÉÉÉûö©.Èògããã­­­¥¥¥Þó³Hííí~ä¬¦ü:::¾ño¼µë-ü^,yñ7ÞàÑüòlùõööjÎä,&äGÈù<ÁÍf³æ^Ý¬¬¬efýä'ÉÄ¿õ[¿µöµ"ÔÜoç®X±B Ë£ù!?äÙò<xPPP/Kô¾ÈùQTÊÏn·766æååiÎÏRYY)kRýæg	®ü_ÿôOÿtçÎ§Nâáüòù-HÈùQ4ÉO0'·°¢¢Bs¯®@°©©éÑ£Gáyãùäüù!?B~ÈoVÙl6_n¤¥¥ÕÖÖ?ªòC~[òP9«ãyÓÓÓÏ?üù!??Éïº½½Ý×F>5?K¤<òC~Còëêêr­¬üÔéãÇ#?B~ÈÏ;«ÕZ[[«ù©&iaçgA~Èùò!õÉè.ùÉÃ	ù!?³x/>>^ÍÏnn ?äüùy¦VÜÏÜfoÓùòC~êãg#<ívD/òC~ÈbH~éééjö>%¿©©©=ö¨:ò#äËò÷3?Ùl¡ùòC~»òëîîÖÉùúõëÈ_ÊOÍÏâkf p0Ê&4F~ÈùQÉO­õJJJÔ±½¹¹¹![ù!?7ù©I³³³57òY,håòC~ÈbK~òC~&òs:þçgimmîO-C~ÈùòC~ü¢_~V«Õÿ$Ì7?òC~ÈßÌÝ½wÙ²e			²ºOLL,((A~ü¢U~jæòòrÍ÷øÊr97¦~Èù!?!ùÉËæÚppùò2ùù%'aF~Èù!?ä7·ÔLÎUUUC¾Ø¼y³,ÉÌÌD~ü¢C~3NÂ5ó³ ?äüùÍZû»¯ôå´kVgäGÈ/¢å'yÅâg~Hù!?äüßÜRÛü¦¦¦&''ÙæGÈ/¢å§ægñ5	sÏÏüò#ä7Cê~UUU>ùòÉ'eee¼Ï_$ÊÏ5	sÌÎÏüò#ä7CfJ§Ý¾ÈùQåg·Ûå9åk~=tù!?äGÈÏ³¸2È_xÊOÍÏ¢9	³:t#ÖægA~ÈùòÓò£ùÈOMÂìk~y~qèòC~ÈF¾&m~ðàò#änònkkÓeñâÅf³9ÆçgA~Èùò!yÁð¾êêjfu!äVòknnö5	s^^n ?äüùÍ*¼r¤§§?~üX¾<sæz-ÑéíÈùÑÏ555åææúúh]ÈüòcßÜª®®V/$êÄô¾ÈùÿnÜ¸a6ùh]äüù!¿ áqíÚ5×JKKKîòC~¤Ùøøxsssvv6­ü!?ä§ü¶oß®^WÔçyH[·nE~üB¿©¬¬ôþ¤µøøøÍ7:tC7ò#äüæ+?Á /-)))·nÝzæö>¿äGÈ/544hNÂ,Õ××Ë7ü¹½üò#ä÷¿W½÷z,´X,ÛKÈO×NçÅ×®]«9	see¥¬qùòC~ü¾óùé-$äüb6ÍÖÐÐ`45çgillôù!?äGÈùñÈùER~6ò-^¼Øb±ÈãÓ×;ùòC~üß¼ä'/6îûsýüùÍ'?ù^zé¥'aF~ÈùòC~Èù!¿°nhhHçÙÙÙÞùD555·oßåU!?äüù!?äü_8æt:;::^í5oðI¥¥¥mmms(äü!?äüò¯ÔF¾¬¬,oðÉÂúúzÍØ5#?äüù!?äü_Xäg#,©¨¨´Ísfäü!?äüò[à¬V«¯I³²²:4::üò#äüòC~Óøøxkkkii©7ø/^]-íà~ÒòC~Èò¯üüüùi>jkkÓÒÒ¼2ÅÅÅÂÁ`mäC~ÈùòC~Á_ÜLäGÈOe·Ûóòò¼Á'Î~~äü!?ä·ò[ðòÿN§xËl6kÎÏR^^ÞÞÞ»üò#äüòC~z¥>uCóÐY(gY­ÖPÞäü!?äübH~v»]òÚ¯ÓÛÈJnEE~ó³ ?äüù!?äüüõÎ;ï,Y²dåË+?øàä§ÇcÃ×¡ÙÙÙï¼yÈù!?B~ÈùÅüþú¯ÿ:33ó­]oígü÷?ç«T$ÊÏÿü,f³y¡6ò!?äüù!?äòËÉÉùá?TìSÿ½²æä7nï¼Í'ÏF~ÈùòC~È/&äìÎ>ùïûæï#¿ÀRó³dggû%<üò#äü_LÈm~AÉÏ¡Riii¸mäC~ÈùòC~È/åÇûüæÙàà`]]æ¡²0ôó³ ?äüù!?äüüõÎ;ï|õ«_ùåþÇöÎ6¹%çÎ+++ó5?KÈ&aF~Èù!?äüò[v»½££CÌç7cj#_FF¯Im6[Ä=òC~üò!ùED+?1±¯ùYâããÃg~äü!?äüò×¯µ¦¦&99ÙÛ|Ë/ollô±E~ÈùòC~ÈùÅ´üÆÇÇ½Á'Ê,r7ò!?äüù!?äüßOÂ©9	sYYÙ©S§ÂùÝÈù!?B~Èù!?ä7sv»ýÐ¡CYYY´V__ó³ ?äüù!?äüÏN§<÷|mä+..nii¾|Èù!?B~È/òä722b2ò¬ÁÁÁyyÏh4ÖÕÕõ÷÷ÇÈØ"?äüù!¿p_OOO~~¾¼H#?ä7§Ô'­­]»VóÖÔ$Ì#¦Æù!?äGÈù»üÖ­[gµZýÈïoþæo~Ú~üãËäç¤O·oßÏ5|øá[·nýÚ×¾æ¾oë[üÇüÉ'ÄæØÞºuëã?æ1¦S²Zã S×®]¿ó¿	úÓ2:õÑGÝ¹s'Ä?4å§ò#¿ööö¡ÐöÙgýÓ?ýÓéümô/ÿò/``àðáÃ%%%Þà«¬¬¼xñâ¿þë¿ÆòØÊÀÊðòÓ)Y-ÈÊqÐ)qÉ_|Á8èTww÷/~ñÆA§:;;CÿêÍòcooïíUó³Íf_n477Ûív½½ìíeo/±·½½a*?×Ë6òC~~Ï566fggkNÂ,'[ÔLÂüòC~ÈùE­ü4-üJ0×ÞÞ^QQ¡yèFqqq[[[¬ºüò#äüò6ùY­V_ó³¤¥¥ÕÖÖÊ¯|Èù!?B~È/âåç+äòSó³/>>^ÍÏÊúE~üò#äü(øò¡®­­MKKó6Édß>n ?äüù!?äü"[~?nmm---Õüh]³Ù,|a¯.òC~ÈòC~È/²±ýÞ÷¾§9?K^^pp||QB~ÈùòC~ÈùEpj~±C7%äüãüòC~kfÍùYJKKÛÚÚ8tù!?B~Èù!?äÙÙívùÝiÎÏb4ëëë­V+£ü!?äüòàÔü,0«ùYÄ%òa;òC~üòC~È/S0kÎÏ×ØØèe®ÛKÈù!?B~Èù!¿°H×ÖÖæk~ÅâýJüò#äüòC~I~ægA~ÈùòC~Èù!¿ÈH<'ªó5?KCCÃn ?äüù!?äü_¸'¿³Ùì=	óZù!?äGÈù!?äüÂ4?0L¦Í6§+D~ÈùòC~Èù!¿ðJMÂìk~ù|´.òC~ÈòC~ÈùK6­¡¡AsfùYù!?äGÈù!?äü8?0/^¼Øl6ëüò#äüòC~Õjl~äü!?äGÈùEFj~ÍIEbA^äòC~üòC~È/¤wÐÏF¾¶¶6]eüò#äüòC~º7>>î~'aF~Èù1ÈùòC~aÅÿ$ÌÍÏüò#äüù!¿pÉf³:t(;;[s#í<çgA~ÈùòC~üß'®jkk+//÷5?KÀ0#?äüäüù!¿p©¿¿¿®®.##Cs#ß¡C;?òC~Èò#äüBÝèèhkkëK/½ä¾øøøêêjÃÜÈüò#äüòC~óM0×ÑÑQYYé½WWÎÿÖò#äü!?ä·Àù:tÃh4Z,ÁÁÁ°½ñÈù!?B~Èù!?ä7s¦ÍÖO:åp8Âüwüò#äüòC~þòsè,Ü¹sgh&aF~Èù1ÈùòC~zåp8|ÍÏ"UTTx1|Ý@~ÈùòC~Èù!¿oÅbINNÖ¥¾¾Þf³Eèïù!?äGÈù!?äüþv»Ý×GëªI»»»#n#òC~ÈòC~ÈùýO®ÖÕÜ«-|ôèQt<CòC~üòC~1*?»Ý.÷Ñd2y/99Ymä²gòC~ÈòC~È/¶ä'ôioo÷??K8|ÒòC~üò#äüÏjµ644¤¥¥yOÖÖÖFýKòC~ÈòC~È/Êå'Öikk+--]¤,scÄCÈù!?B~Èù!¿¨mm­æF>£ÑX__A0#?äüäüù!?ÆÇÇ[[[5çgÖ®]ÛÑÑéó³ ?äüù!?B~±.?¹Áf³yñâÅÞàËÊÊ444ËÏäü!?äü_ÄËoÆIå%967ò!?äüù!?äü¢D~jf_ó³,_¾<&aF~Èù1ÈùòQùÙl¶_0WWWËõ°ù!?äGÈù!?äÁòqæÖÖÖhù!?äÇ8 ?äGÈ/äW__ïkæºººþþ~Èù!?B~Èù!¿(_mm­ù***ÚÛÛ¡òC~ÈòC~È/ÚäwãÆ>ÉÔÐÐk0#?äüäüùÅü¤;wÆì$ÌÈù!?Æù!?B~±%?B~ÈùòC~ü!?äüù!?äü!?äüù!?äüò#äüù!?äüò#äü!?äüò#äü!?äüòC~Èù!?B~Èù!?äüù!?äGÈù!?äüù!?äGÈùñPF~ÈòC~ü!?äüù!?B~ÈòC~üòC~ÈòC~üòC~ÈùòC~üòC~ÈùòC~ÈòC~ÈùòC~ÈòC~Èù!?äü!?äüòC~üò#äüòC~üò#äüù!?B~ÈùòC~üò#äüù!?B~ÈùòC~Èù!?B~ÈùòC~Èù!?äGÈù!?äüòC~ÈòC~üòC~ÈòC~üòC~ÈùòC~üòC~ÈùòC~ÈßÂ?Caaa__òC~üò#äü¢V~999×¯_gÏÍÍÍõ_ww÷¯C[oo¯Ýnÿ5éÈÏáp0zô«_ýJäÇ8è¬dåÀ8èÔàà àqÐ©7nüò¿dtJä799âÁòs/11Ñ[~G¹Ú::::;;/>øáü1ã G2°2¼NÉjAVNôÑG¬xu]cÅ«ëëZèh4ÈOþ®©©ao/½½ìíeo/±·½½Q»·W511QUUåp8ò#äü!?äUò[ôÔ<°X,ØB~ÈòC~ü_dËÏ½îîî5kÖhü!?äüù!¿èÉdZäòC~üò#äü¢V~þC~ÈòC~üòC~ÈòC~üòC~ÈùòC~üòC~ÈùòC~ÈòC~ÈùòC~Èò#äüù!?äGÈùòC~Èò#äüù!?äGÈù!?äüù!?äGÈù!?äü!?äüäüòC~üò#äüòC~üò#äü¢I~ögÖÞÞ>Ú>üðÃýìgC¤Ommm²~gôÈjµ9sqÐ)Y-ÈÊqÐ©O?ýô³Ï>ctêâÅwîÜatêoÿöom6[èøøxtÊo```ß¾NDDDD¿É×ÑÅÊÞ^""""B~DDDDü!?""""B~DDDDü¢£EnÅÅÅ¹ÕÝÝo0zzz«ò3ê¬«W¯2VÁÞ7oÉY2ÔUpWÕÕÕ%ë*¸cûôéSÅ%ßÆXwxy]_~ùeII<D7mÚ466¯kÈo¶>úøñãîKFãðð°M&C@~Æ°ªªJÆÈ°oÞ¼±îðæää~]N=677±îðJN§Slü>¶<|øðôô´eÙ²eUp×µù·bÅùÓZNnß¾=_×ß¬ºwïÞêÕ«=ÊJçÁrBþeX~ÆPV@²rSSS¬>¼î%&&2VAÞwß÷èÑ£È/èc[PP /¨NÃËëÚü3®Ó©©©aøºüfÕÆ=öõõÉK¦¬Ùå_ïsi6ùC÷'ûiÊðºå555Upwxx¸¤¤DVñÈO5Ã±cÇd¹¸äÎ;Up×µùWXX800 'Î9ãñâ&¯kÈoæ¬VëÊ+½¯X±Býé)OÍo ó3îïªLHH`¬;¼ªªª*ÃÁXwxåoÅk×®=þFa*èk'O>ûÍ»©«à/¯kóOþ ÉÍÍ:=zÔcJ¼®!¿;xð üé½RóÏÏ¦§§OMM=¾UVÁÞgÏ÷æX,2PAÞEÿ;Æ*¸kV¼ú/¯kAìîÝ»ùùùaøºüfnÕªU·nÝò^.©-ºýýýòw@~ÆÐl6¿÷ÞrBþ­ªªb¬;¼ÝÝÝkÖ¬aô^w2PÁÛíÛ·¿ÿþûrB¾¡´´±îðòº6ÿrrrúúú¦§§=ê«0y]C~3 Þé±6ÎËCþ*å4ØDÞc¨·§§'###...33S'EA^ÉÄF)ýùé7¶ccc7n³JJJ¬V+cÜáåumþÉßÕK.9ÔÔÔ8Î0|]C~DDDD±ò#"""B~DDDDüùò#""""äGDDDDÈ!?""""B~DDDDÈù!?""""B~DDDDüùò#""""äGDDDDÈE[gÎYµjUâóV¯^áÂ÷s=/Rîæ­5Lr×ÇrYb0233§§§çzDDÈ"¯ûö-òÊò;pà,liiñXÞÜÜ,Ë÷ïßÀu!?"°úúúÄ4áÄÎç<yR¾·nÝùÝ»wOz,ÏÏÏå6ùò#¢èoëÖ­bÃ»/<rä,¬®®vwÏÕ«WENÂ¢¢"9íúæ'OÔÔÔ¤¤¤ÈYF£q×®]î;U¯"º³ä²/_ö°,IMM]µjUgg§|i6=nXGGÿëQgÉMRguuuùRÚ5kdùÍ7]K®_¿.KÊËË]K<!W¸iÓ¦xËÏûú=ø¹©Dü²¥KZÝÞ¿_L&wÙxÔÛÛ«ÎÝ°aÇY;vìPgõ÷÷ÇÅÅi^J©ÎÝ¸qãôô´Ø1!!ÁétÊ¹ò¯È)))I½ýÎÏõÈ	ÍçOÏ=ëÎY.O>íbÇ¬^½z®òósSù-pjÇ®÷rY(sÍæÍ'''äËõë×«stÕ¦;:«ªªJm,Ó===êJÜ¯sïÞ½b;µ³õÍ7ß%.]Óò¯~ã7f¼Q£|)ßà~Û4ïhrÉ%rGFFäKùWn¹psjjJCff¦îÂ³ßìNLL«üüÜT"B~DD/?IS~®åJ6LÒäK1ú²¨¨H¾lÙ¶mÛDl®+ïñØæºúòþýû®o-¹vøÊ¿rúÊ+3^(S¾|üø±ûmóõ¼·ß~[Î:zôè³ßìÑÞµkû7E~âÑµIr®òósSù-pjC×ÄÄûBÃ!å,?Öq¹Ðjµ*ü¹ ãzs÷NXK¹Ï¥"§FcBBÂÓ§OåÊSSS]çÎx=¾æÑÀÀBªåt¿ëÜ¹g/??7Ñ§ÞëvìØ1÷j®#<ñÞuëÖ­¨¯.2ªr®Ý©3úlÇj'¯ü[SSãZîçzÔ6¶ª/?~ìÿ8Ü+WÊ¹j"«ûYê8_9«««kllÌ¿üU£á:×ÏM%"äGD´À©#$ÃñãÇÕ¬.'NHHHð>cýúõ¢§Oª]±®ãpÕûüÔÛã¬V«ûwíÚ%NRGÝºæUÑô:ØVýôîîn×r?×£ÞØ§Þççp8ÔwúßÉ'][ãä.»¥ÞòØßß/?EÁü5ÅÊòm"ÎM6¹ëç¦ò#"ZøÔ,Ç<xÐõjë´k?©ë Wo¾ù¦;+Ý;þ¼ù=þa²Üh4ºïös=r3Üoë¸Z_wvrr211QÝÜëÖ­sÿK,ÕÄ.î×©6IºRNuëç¦ò#"D'«W¯NxÞªU«Ô¶òëêêRÓævÍu®ÃáØ³gOFFÒÒÛo¿ýôéS×¹/_.))eff¶¶¶z÷-«åÛ·o÷Xîëz$¹1rÔD~æósUSSã1½jdd¤ªªJF %%EîÍfsÍPã~cccB[5Vååå7oÞôø~n*!?""""B~DDDDüùò#""""äGDDDDÈ!?""""äü!?""""B~DDDDüùQäW^^þDDDDÕ)ò-b b¡ÿÞ i·MNIEND®B`


<!"""¢ùÑùò#""""äGDDDDÈ!?""""B~DDDDüù!?""""B~DDDDüùò#""""äGD³óý<^½îþ9¿cÁ.½páBiiiòx%%%çÎð'>%$$ÈÇ/[¶ìÔ©SÓ®®.³Ù</¿½½ÕªUA>»ß-,RüÇò#¢h_ZZÚýû÷cE~G	tÏáÃCÜÚ®V'OêàL³³³µÏk±X!?"5`I6m	ùuuu)ª¾öÚk^¯wllL6ä]Ùét:uojªWñûÔCCC=¢±ÉO]ñÑ£G²=::Uÿ5Dü(¶ågµZåíñâöíÛ[¶lQJ+**zý>²¹¹9==õêÕÚ+VÒÒÒþþþöööeË%&&®]»Ö÷õÅ£Gfee©Ãò)´ùfûöí²ß÷Ø«T__/;+++u¿Æ©^%ðSeÜÉ)îkr¾Mï¡åçw©ü¿È°¯[·NÆ6ô«úþ·Jv»]nMÆAþgå"í"TIÁ´÷íÛçv»ùæ"B~D¥òÊÎÎÎÌÌ@Ü»wO~ûZD>Òh			òvóæÍOôG®ZµJp£½+ÑØç÷kÖ¬	íÅËþøîYÊÎÜÜ¯qªWñûÔÂõ_^^Þ'4à¦ÅÉË¯»»Û÷väªü|ÿ[N§zW«««Kð¦Müîó=øæ"B~D¥òK.ù¾æ]»vÉö'ò®ìôýÈC©W­ÔÝ»w½Þõ®Ü²¼ÛÜÜ,Û¢@u]õ×lW¯^í;wîø^Ì7Aw¿ïugx`H§½&:ÕÁÑ½WÓ¾b?òó»¢M¶+**FFFÄôÏ&/?ßÿV¹ÙÓÚÚ*Ûê¿uÛ¶mêïÞ½+Ûò¿)Û)))|s!?"^ùIòåºßþÌÌLÙ¾wïzW~ÀË»-ò½íRmhÃ÷Ý>ùô°©/8FGGÅ"ââbõ"ÓòKLLÔýBt÷Oã*¾¢ëbÌf³Ýn×´Éî½ö'/?õ·ýýý¾bJòóýoU·æìQÊ»K.¹Ê¯?æ;ùQ´ËOpf4åç·ÇãñÝøYà½	éto3ô»¢L¿#ÎQÍÊÊR§y§AÄïÖ&s`c2á MfpytzWüÑÞÉØ.ô¥ÿ­~KÞ¨Â&Â+W®ðÍEü(ªå'½þúëò®¶Ú©pæ÷êì B¿[PP VWiiiMF~êIµÀJEEÅ¾û=ztúôiÙ¹eË]ùMæ*ÓßTGkÚW¼ü&óf;qèñOII=~»~ýzMM:Ä¬Í&"äGDÑ+¿'ã­¯ý!¿Ú£fþnØ°ah<õ£÷îÝ3zÝËét?N:5ùµµµ©Wêõ<Õ¥Kt¿Æ©^eJò=8j$ÝãùÝÈTGuòS'ðüø±Ànýúõ.<~ü¸¾èPzüÕ=7ËÇËHÊö+|¿Lõ÷.+Äw"B~D]ò»ÿ¾ö]jOoo¯ßßxeffjSeg"?Í"ªEÉ[ßù­º÷ùùç<ò¸råÊ_æ®2%ùmAf¿Õ§1ªÓßõë×oõêÕ¾úX-´üÔ²ºS^Ô«ª¾íÜ¹o."äGD1 ?I	õÝÿÁlÙ²%q¼ÀùÓßÃåÖä6Å@û÷ïiöÍ'-[°xñâW_õË_þ²öÓÌ¯2%ùK.	þ¶$oSÕiÈOºråÊÒ¥Kå«ÛWÇÓµéÌò®øLv³ÙÜÙÙ9ák®ÍÍÍÅÅÅrkòE544hûÝn÷ÁÕ©Âwù¯ôx<|s!?"¢°4::úüóÏOi¹iæRaÕdm¯×«@_PPÀQ¼¥þÎÏ¯^z!"äGDo¹Ýî,^¼XÒ#G0,Düù!?""""B~DDDDüùò#""""äGDDDDÈ!?""""B~DDDDÈ!?""éÒ¥K&É`0¬Y³fª×]¶lÙ¶G¶eÏ+¦ð,6Þô>f2×æsk@j¿ÛíÞ³gÑhKKK«¨¨¸û¶v-¯×+ûåeG!?"®²²²D*>T=*×­««Óö¼üòË²§¦¦&>ä¸ßjµÊþÖÖVÙnooíÜÜÒ+W®hR¼zõ*."B~DeO"3ÀÓõë×åº¥¥¥ÚU«VÉîîîÈÜÉÈË/11Qöé^kÇr©z»sçN]Dü(êØç«¦¥¥¥¤¤>|ØïÃ¾üå/ûÝö¡l?xð@¶eºèîÝ»f³999Ù`0ª×ÉoÍ÷³¾Ê«¯¾j2JJJäúlddÄb±ÈuåÎ8p@½)6]½zµÜì[¾wïÞå·dÉÙ_PPÐÑÑáwÑØØXzzº|®ÇËÛEi>Þ "B~Dü©íï~÷»²ÝÐÐ Æ^zÉ÷cð|ÛµkúôiÙ·²]YY©.Z¹r¥:âyçÎÙ­k¾w ôUÄs¯¿þºlX­ÖÀ;¿gÏÙxóÍ7eãèÑ£ÔN§l¨$ý_0û~Ì¥K´=kÖ¬¹yó¦vuðwÃ²½eËÙ=º÷G!?"ùL&Ù/^ºØÖÖ¦qgÓ¦MtÚ¥×¯_?tèPqq±ìOHHÐ½5?¾ºciii×ÍÌÌTw~llL6Ä[²S>R¶Å²gzÃâWWWUóÜ²Ú¿oß>í]%Ôýû÷Ïä>!?"£üÔ¼Tm¿¼;!Y)))òn·[Ð=ê¢S§NÉµ7oÞô½¿[ów2W	vÇ¾/Ñ)5^¸p!==]í1êåÀÉ¿æbÜZ[[s¾G·ÅÍòI=zôdüÐ³l/^¼X÷>ð¨#"äGDs/¿ììlß×üÔËf2Èb±È¥Ú[m¿16Þ$åú*ÚH^wÑ¢Eêº~wÏëõ^½zUÍÉÕ^,Ò°KÝ¹Ï²­QÕ¯ë×¯Oû>!?"£üÔ*-§OVç÷òË/OA. óúë¯kû#oÝº¥þþo2òC½ñÆ²±k×®ÀëîÜ¹S¶Ï=ÛÓÓ£®ÊÎÙõ÷÷ËF^^Þå·bÅÙßÒÒòäÓ5þù'Á¸ýÓ¾DDÈÂ(?¯×+ºJÏwM¾Ðòüø±:Ò*o;Æ´´´LR~¡¯b·ÛåÒU«ViMüÖXÞ·oÜóÄÄÄõë×«)´­5Y¸´´ô>Ò°µX,rå]´hÑ=äó>ùtQkßÛ×µö "B~DDDDÈ!?""""B~DDDDüùM¹þðüqìÞÿ¾¾¾îÁCúÅ/~Á8D¸ÿùÿ¹÷.ãá~ùË_þ×ýãùXð(þå÷ï|Gð»÷ÿßþíßîß¿ÏC0ÂýÇüÇíÛ·'¿¤ýä'?a"ÜÃÿõ_ÿqp?þçþgÆ!ò]¹råÿ÷äüù!?äGÈùòC~Èò#äüòC~Èò#äüòC~Èò#äüòC~Èò#äüòC~ÈòC~üòC~ÈùòC~üò#äüù!?B~ÈùòC~ü!?äüù!?B~ÈùòC~üò#äüù!?äGÈùòC~Èù!?äGÈùòC~Èù!?äGÈùòC~Èù!?äGÈùòC~Èoúuvv+Vtww#?B~Èò#ä·òËÍÍ½víl;w.///P~W¯^³É3²ËåzDíÆN§qpnÃÁ8D¸>ú¨½½qpò+ý»ï¾Ë8D>ßàà ã Å°ü|KNNßÉ'ßÙÞyç¶¶¶÷)²Ùívùuqp­­­---Cï½÷÷Ãþð­·Þb"ßåËU<È¯«««²²£½ÄÑ^öG9ÚKíÛ£½ª·Ûüù!?B~È_<ËOldµZûûû/B~ü!?äGÈ/~ä×ÖÖ¶víZyòÒ½ùòC~ü!¿øÉdZàò#äüù!?B~q+¿Ð!?B~Èò#äü!?äGÈù!?B~Èù!?äüù!?äüò#äüù!?äüò#äüù!?äüò#äüù!?äüò#äü!?äüòC~ü!?äüù!?B~ÈòC~ü!?äGÈù!?B~Èò#äü!?äüòC~üòC~ÈùòC~Èù!?äGÈùòC~Èù!?äGÈùòC~Èù!?äGÈùòC~Èù!?äGÈù!?B~Èù!?äüù!?B~ÈùòC~ü!?äüù!?B~ÈòC~ü!?äGÈù!?B~Èù!?äüù!?äüò#äüòC~Èò#äüòC~Èò#äüòC~Èò#äüòC~ÈòC~üòC~ÈùòC~üò#äüù!?B~ÈùòC~ü!?äüù!?B~ÈòC~üòC~ÈùòC~Èò#äüòC~Èò#äüòC~Èò#äüòC~Èò#äüòC~ÈòC~üòC~ÈùòC~üò#äüù!?B~ÈùòC~ü!?äüù!?B~Èù!?äüù!?äGÈùM"ÇüòC~ü!¿xßððpYYÙÖ­[òC~Èò#äòóz½v»Ýb±$%%-O6DÈù!?äGÈùòùõõõÕÖÖæää,¨®®ù!?äüù!?B~1/?¯×ÛÔÔTVV¶páÂ@ó	¼æüò#äüùÅ¶üWuuµÉd_FFÍf»qãÆ¼(äü!?äGÈ/ÞäçñxÌfs ø.&Îù¼Èù!?B~È_|ÊÏápØl¶@óL&ÁC__ß|(äü!?äGÈ/æýõ×ÿñÿ±¤¤$|IIIÅn·½^ù!?äGÈùòáÕj5æËÏÏohh'S7ò#äüòC~qZEllêQB~Èù!?äüùÅpÚ"Ìºë³466ÎÏ©ÈùòC~Èù!¿ø©¯¯O~ôë®Ï";ÿèþèÃ?dò#äü!¿N­Ï¢»³ZEMÝÞyòC~Èò#ä©Eu×gÉÏÏ¯­­õ]ù!?äGÈù!?äüb¯ááá`ë³X­VÝ©Èù!?B~Èù!?äK©õYwºS7B¯Ïüò#äüòC~1PèõYª««].×7üò#äüòC~ÑZ%ÄÔ)Zù!?äGÈù!?äü¢15uCw¿©Èù!?B~Èò½Ôú,f³9§ÖE~ÈùòC~Èù!¿¨ÈápØl6ÝõY&ºüò#äüù!¿H­ÏSë"?äüù!?äüß¤ZWw³Ù<¥©Èù!?B~ÈòÆBZwë³ ?äüù!?B~È/zjÝNÝ@~ÈùòC~ü_Tär¹MÝ<µ.òC~Èù!?äGÈùÅ^n·»¡¡¡¨¨(ðE¾Yºüò#äüù!¿¹IT%¬ë³ ?äüù!?B~Èo.¬¯¯öÙg#?uù!?äGÈùòC~Ên·WVV¦¦¦ê¾È×ÔÔî©Èù!?B~Èòo.«¦¦&'''|F£Q.ºqãFTÝaäü!?äüòZçüùóëÖ­º!9sÆívGá=G~ÈùòC~Èù!¿ÉvãÆªª*£Ñì/ùz£ùþ#?äüù!?äüß¹ÝîúúúÒÒÒ@ð©E/^¼%ÉüæüäK~Õ@~ü!?ä7»ÉÃcÇº/òåçç;v,b0#?ä÷IòD~ü!?ä7+ÔÖÖ.Y²$|©©©V«5gZC~ÈO¿õë×»ò;uê#f÷ÝwE®l?øÁÚÚÚ÷/ÿò/ò³qpï¿ÿ~KKãá:;;¯^½¿ÿû¿ÿâ¿h¾½÷ÆôÓ£È*å÷É].¿ïÿûý1|öôôôSdëîî¾yó&ãáþýßÿ]~2N~s~ï½÷÷ñÇ¸£ê.çªªª~úé@ðÉNßµk×â`ä/_¾ü_üG Ïòãh/q´£½ÄÑ^öê6<<ÜÐÐPRR²@/³Ùl·Û=OÜ<Gãáh/ò#äüù!¿)åõzt%))Iwºüò#äüù!?ÿÄsµµµºgÝ6nÜ(?4cqêòC~Èò#äü>Éãñ455éu#??¿¾¾~`` îGùÅüüù!?B~óY~.«ºº:###|²Óf³M8Íù!?äü!?äGQ-¿	§n455ÅÓÔäü!?äGÈo>ÊÏápØl6ÝùÔ©u].×¼yäü!?äü_<ÈOMÝÈÏÏvjÝ=ëòC~Èò#äü>I[%ØÔá`ô¯Ï"÷°®®nïÞ½òVòC~ü!?ä÷k©©&)|IIIV«5V¦nÈ#ð3ùÌ¾ô¥¯|å+_üâ322N'òC~Èò#äü>YÅl6ëNÝ())ihh¡/çgÙ¶uÛá«_ÝôÕÏîsa:0üò#äüòùº!;ctê<üaÆ>õï7ó7òC~ü!¿y'?µ>K°©eee1½>PlÕWùÉO¾Ø7n ?äüù!?B~óH~òó+Ø©usrrbbêÆõöö¦¤¤|kß·4öÙ¾iKNNv»ÝÈù!?B~È_üË/Äú,¢Àø[å/ÿò/?÷¹Ïãùoû¾¾ãë/>zôh>òC~ÈòC~È/*ä§Ög	vjÝº1¥þáþ!77÷©§?~<|ù!?äGÈù!?ä7Çòëíí½>KOOüò#äüùÅ°üÔú,Á^äöÙgÏ?¯/ò!?äüòC~ÈùÍNçB¬ÏÂ|È/Vå·`¢òC~üß|H­ÏRRR¢û±¼¼\»ë³ ?ä÷%LÁ`@~ÈùòC~ñ]E/_^[[¦	ùEZ~sò#äüùÍUÃÃÃÁÖgINN¶X,ÍÍÍñ´>òC~444´k×.äü!?äO©õY-Âôw÷wßÿþ÷(äçò3L¿óC~Èò×Ô"Ì999à36Mlzçí%äKò[±bî·ANÏüù!?B~ËëõX¥´´´±±ÑwêòC~ñ/¿ÄÄDyôËSLVVløÞxãÙ¨¬¬D~ÈùòC~1ZOOO°Eeç±cÇWàµòù©oÙêÉÆ;wÆÆÆd#%%ù!?äGÈùÅVÚú,/ò%%%þÑü_üË/==]¾:::ä·Ù8räÚ`Uäüù!¿J«²²2Øú,uuuÞòC~ñ/¿hó9üNAüò#äü¢<ñÜ'tÏºa³Ùzzz&¿>òC~ñ/?éÅ_hltvvÊ(°¸¸8ÜwùòC~ü¦`N¬P^^®;uÃl6755Mã¬ÈùÍùÍIÈò#ä7zzzªªªF£îÔêêjÝ©Èù!?äü!?ä3y<`/òÉ²²2»Ý>ó³n ?ä7/ä§Öva%gäüù!¿hËétVUU©¥ÇüÊÉÉ©­­íëë­Ïü_üË/77×WZÌíE~ÈòÃMÝHJJ²X,³ò"òC~óN~<ù.êêêä]G~ü!?Ýä§ÕjMMM4_~~~CCÃððp>5òC~ñ/?õG²fò#äüùù¥N­»dÉ`ë³8pßäüâ_~ÝÝÝòMµgÏäü!?äá<OSëõE>äüæü¤Å~³1Ãù!?B~È/¬¹êêjÝ³nÈÎ®ÏüòÓoéÒ¥Ìð@~ÈòXÚ©uÁ§ÖgÞ"ÌÈù!¿ÉÝÄxN§3ÂwùòC~4ßäçp8¬VkRRR°Eçö"?äÿòËÌÌdòC~ü_øRS7òóóu_äÓú,Èù!?ýÚÚÚäïÀ~iùòC~ßòÌ	éMÝÎî"ÌÈù!¿ÉÝDáü!?ä7½ÔÔÉ4ë³ ?äüôK3<ò#äü¦ZÅl6ë¾ áõYòC~Ñò#äü(näçp8l6îú,jêFä×gA~Èùé'ßK.íééA~ÈùòC~SJ­ÏlêZ%J¦n ?äü>É`0È·häï:ò#äü(Få§¦nX,ÝõYrrr¢pêòC~ÈïZ[[åµ¦¦fhh(k» ?B~ÈbN~â9yöÖº!ªõYòC~An¹½ÈùòC~!ªSë"?äüfsò#äüÕÓÓlêF¯Ïüò®!?äGQ+¿áááúúú7¾È7ç§ÖE~Èù!?äüù!¿ÙéÆ÷î±>ËZù!?ä7ÓFGG×¯_,ßØ)))7oÀTäGÈùQôÈoxx8Ø©uãcêòC~ÈïWrÝá~ùòC~òknn¶Z­F£1Øú,ñ1uù!?ä÷Iòí½aÃywhhhÓ¦M²gåÊÈù!?B~ñ*?ùo=vìØ.|©©©6­­­m>¼Èüß¼_bb¢|û~ÊÙüò#ägògû/Íæ`ë³9sfpppÞ;òC~ñ/¿ùníi<ìaUäüùÅüN§ÍfÓ]YvÖÔÔÜ¸qaG~È/þå§ö®]»Ví·²-òC~üb]~òË|cc£ÙlÖ=µî;ìvÏüòÓO¨§;ÃãÑ£GÈù!?B~³ËåúÚ×¾[RRòòË/Oø't3Ãá¨¬¬Ôº/OÂò?ËÿòC~óN~ê¾yóæ´´´y»víZÙî»üù!¿ùS[[[zzúsÏ=·ë/výéö?]üÙÅ_ÿú×Ã$?µ>KQQQ°ùDópêòC~ÈoC~üßüé·~ë·6mÚtøÃêßªO?ýtèóMU~9ùÙ¹uëÖ¤¤$Ý©õõõóyêòC~Èù!?äGÈ/¹Ýn£ÑxèÛ4ùÉ¿ÒÒÒ'NÌüz«««­ÏRYYÉÔäüßüò#ä7ó<ÏÓO?í'¿ß[ýõõõ3ßððpCCCIIîQÝòòò/2uù!?ä÷I	ÁC~ÈùòÝ~÷w÷¹çÓØ÷­ßJIIéééüÕjÕ=ª»dÉªª*ËÅ#?äü&ÕþýûÕÓÇ¹sçòC~üf%¡ÑhjÕ×¾öµ6>ùÌßüÍß¾J üúúúZW*//'U¦n ?äü&[ww·ü*Of³ÙwagäüòC~3oppð^X·nÝÿñ?~Â×ä'³ÛíE÷¬Aá ë³ ?äü¦Ö-[ÔÈÕ«W#s×!?äG¡åwùòeyªÔ=ëFFFÍf=;òC~:½ùæêydÃ¼ëÈò#Ý<OSSÓÚµkµ?¼öÍl6Ë¥LÝ@~ÈùMçñ½råJ5£µµ5ÂwùòC~äËåª®®ÎÈÈÐ=µ®Ôäüß4;~ü¸z6Ù¶mÛÜuäGÈùJZ7Øú,Ån·3uù!?zÂz~ÈùòéÍfÓ/77÷ßüf__£üÍü&Ê`0 ?äüù#µ³îú,IIIV«UD8íóöòC~È/C~üß<L÷,K°Sëê#òC~üòC~È/&±sFFîÔäü!?äüò¥Ô"Ìeee0ËÙbäü!?äüòÔú,º0çääÔÖÖN8uù!?äGÈù!?äü¢ºë³$%%Miäü!?äüòÒB¬Ïâ7uc!?äüh6åÇz~Èùòyâ¹`S7RSS+++Nçônù!?äG³)?ß¥ûtåÇz~ÈùòºlÒÒÒÆÆF·Û=Oüò£ÙVKK<Omß¾ddDÞ·6m=íííÈù!?B~~õõõÉÓîÔÙYUUuãÆYùDÈù!?üF£<aùþÅñèè¨ìÉÊÊá-wwwgggeËµ¶¶"?B~È/vóx<MMMºë³HjÙ=µ.òC~ÈÂ"?õ´%ÚóßÌÿÎ¯¢¢âìÙ³²qòäÉmÛ¶ÊïÝwßuÇl?úÑ>úè#7E6ñGOOãáþó?ÿS~Õ·_þüã?ÿó?OOOß3Ï<óWõW2>áø¼?ÿùÏ;::xøE8·Ýng"Èï¿ÿû¿	ÿJdä)Ï_¢4ùEGÞO¹aÃÙ#ûgþjâØØ¢¤Éd_]]Ýb¶·ß~[äúlïÇ8D¸æææùöU_¾|ù[ßúVAAA øzê©?üÃ?<vì!|w@nüêÕ«<ü"«qï8Avùµ··ëÎð¸yóæoÙwHà|öG9Ú8µn~~þ'"p78ÚËÑ^öRXä'Ý¹s'777999!!!%%¥°°ðÁ3¿YßãÅÈòæBZ755uÇ³5uù!?B~s,¿0©þ|PÞ;F~ü_4äõzCLÝ())illüC~ÈùQÉÏb±¼úê«²!o+**!?äUõööVUUé®ÏµwïÞi/Âü!¿¨ß­[·òòòRRRÔñÙÌÌÌ.Ìüf;::äÙSn3;;»³³ùòC~ÑÇã¹xñbyy¹î_ò­[·®±±Q>fÎï'òC~ÈÂ"?µ³ïÛÔöÉ'Ãz×!?äánÜ¸QUU¥j]ù5U.r¹soòC~ùÉó<ëÝºuK_gg§l§¥¥!?äü(ä×××W__¿|ùò@ð-P-Â/ò!?äüHÈO=ý©%¿±±1ÎÛüÅºü¼^o[[ÕjÕº_[[+(Úûüò£°ÈO­ä¬^çù<xPù!?äG±(¿yÉÉÉ	_RRXÐápDÿWüò£°ÈO~'Ö]ÉùÚµkÈù!?!ùy<óçÏ¯[·.Øú,ÃÃÃ±2ìÈù!?ü¤þþþââb5·7999//¯··7ÜwùòC~³UOOÏ·¿ým£Ñ¾êêê¨ºü!¿9ßüù!¿688xæÌÀù¢yêòC~üæR~ÚÄ­äåå-Y²ù!?äGÑ)?§ÓlêÉd§hºü!¿èßèè(sò£(ßÀÀ@mmm°©Ån·½ÞøväüÍ¦ü,Y² déééÈù!?ùy<ù©PVV¦ÖüüüØºü!¿9ßÝ»wÆÓÎÞá°ïÍ7ßD~ÈùÑÜÊÏårUWWºa³ÙbbäüùÍ½ü´Äyá>°üù!¿)åñxKJJtHÍæ9º188ÉCÉÈù!?üæ*äGÈùæp8l6î©uM&ÓÏ"ægFczzúºuëÈùòUùæåå¥¤¤h-ZôÝï~ù!?äGßððpCCC~~¾î©uçvê°ï7¿ñç¿qøÃÿßÁ/|éK_B~È_¬ÊoñâÅ~ÓÕ³mMMòC~ÈÂ-?yØº§Öæg¾¾ãëÂ>íßg?ûY(òC~übR~òët:µ=ÝÝÝ²'--ù!?äGaxNT§û"_TMÝ6¾ìkÖ¬©««C~È_LÊOMïûµÛXäù!?äüf×ëµÛíeee±rj]¹ÃO?ýôª¾òûyæÌäüùÅ¤ü²³³å	wß¾j¢<è>,²²²òC~4[òëíí­®®6LQ5uc2å+_Y½zµÆ¾]±+555'7G~ÈùQXä×ÕÕ¥»nBgg'òC~Èf(?ù²©©I÷E>íÔºQ~ÖÏþó_(øÜÛßÿýßOOO?öl>/òC~ÈÂ"?I³lÙ²äääùòoù¹`ë³äääDÃÔ)ùµ¡¡A¾#GDLcÈù!?üæ$äGÈ/.å§ÖgÑ]9þN­ü!?äüùÍSùX9§n ?B~ÈoþÊïÖ­[j1g5733óÂÈù!?übbäGÈù!¿ÿ«¥¥EVòSÛ'OD~ÈùQ0ù©õY¬Vk©?µ.ò#äüßeeeÉ3õ­[·4ùuvv²3òC~L~òn°ùÔú,XñùòC~Èoº71ÞÕÇÆÆdÛ`0 ?äüHÛínjj*//|©ÈùòùeffªÕûüFGG<¨~wG~ÈùtùòåíÛ·ëNÝX¾|ù'ºü!¿_[[îJÎ×®]C~ÈùÍçÄsuuuÏ>ûlàóCjjêÖ­[åÙùò#äcòúûûÕÜÞäää¼¼¼üò#äµ	8,ð.Ð|Á·ÛÍ(!?äGÈ/Vå7'!?B~ÑÖÀÀÀ±cÇrrrÁVUUåt:yù!?B~ÈùòásçÏ_·n]RRRàú,f³¹®®.Ü'ò&äüòüäGéÒ¥Kå>99yÙ²eòüòûzzzl6ZÚÉ/£Ñ¨­Ï2½óöòC~ü¢Q~v»]wüH@~ÈùÅe!N­»páÂ7ÊS°ïQ]äü!¿øúu¿¢¢BýÉöÈÈÈ¶mÛdOvv6òC~È/Îr8V«5ð¨®Z¥¶¶Vwäü!¿øzÒ÷ýý^¶µUòC~qP___°³n¤¦¦îØ±C¾CLÝ@~Èùòù©×üFGG÷¼æü_¤N­k±XtO­»nÝºÉ,Âüò#ä?òSçWQQ!wy÷Ñ£G¥¥¥üòC~1Ëåª®®6L/òÉ/òÝ7¥5;òC~üâG~&*L!¿YÏãñ455ÍæÀoä¤¤¤òòò¶¶6ù©Þ,òC~È_üÈ/a¢òC~È/Ês86M÷ÔºùùùõõõÓ¾qäü!¿øßüùÍ<µ>îÔQ XPD8óÏüò#ä?ò¶hs¸YüùM;mêîú,%%%ºü!?ä7ïä'?'^|ñE¿;vì`Uäü¢0µ>î©u322ª««].×¬Räü!¿øO~fdff>xð@Þíµ×ÔO0ýyò#ä7ÔÔ²²2ÝõYd¿©ÈùòC~óN~OÆ_áS?B²³³ÕÆ¦MÂ×!¿É¤ÖgÑºa2´Së5äü!¿¸ÔÞÞ®ý8©¯¯À]G~üBúÔºÅn·8ëòC~üòÚîÝ»µ%^ÕÆöíÛòC~sRSëæçç×ÖÖÎdäüù!¿ù.?Á ?QÒÒÒ®_¿þÄçïüòC~+Ä©uEe¿_òC~üâG~òsåÐ¡C~;­V+sò@jÝ©áXù!?B~Èo¾Ë/Øz~ýýýÈù!¿ðâÔº³¸3òC~üòÍOùy<ÆÆFÝ©êE>¹4|ë³ ?äGÈùÍSùÉÏßã¹¡ßE~ÈùÍ¼§ÖUë³cfäüù!?äüòÃÃÁ¦nh0Gläüù!?äüòýBZw®ÖgA~ÈòC~Èù!¿ÙL<'oÝ©s¾>òC~üòC~ÈùÍB!N­=ë³ ?äGÈù!?äüßr¹¦n¨õY¢vêòC~üòC~ÈùMªÐ§ÖUS7¢mäüù!¿ù+¿Ð!?äü%Ý`S7Ôú,½½½ñ4ìÈù!?B~1/¿2Èù!?ß&<µ®ÝnòõYò#äüæ©üæ<äG±"?Á©jºü!?äüòC~ÔÓÓS]]½dÉè?µ.òC~üòC~ÈïWõõõ577ÛíöÁÁAä:¯×ÛØØh6­Ï§ÖE~ÈòC~ÈùÒßþíß¦¦¦þNñïÊÆo¼ütëíí=vìXNNNÌZù!?B~Èù!?ä÷577æ3Ùõ»¿pXþýõÏ-Z£)Ã$?·ÛþüùuëÖ¾È'6nÜ¯S7ò#äüò7ùýÁüAùærÅ>õï¹µÏUVV"?I~²ÊPè.Â__ßS7ò#äüò7ùåççÛ¾ióßXþÄl6Ïgù¹ÝîÆÆFÝ£º©©©bA§ÓÉóòC~ÈòC~±'?^óóMuòµÆ`S7<³ ?äüù!?ä«òãïüOÝî|¢Ày;uù!?B~Èù!¿xßñ¹½ÉÉÉÅÅÅ¿=ïæö¶µµíØ±C÷LkêÔº¼Èü!?äü_ïÉøz~òÕÜÜ<OÖós¹55º0L&¹(ÎN­ü!?äüò·&Ûínhh())Ñ=µ®ÕjGÝ¼Zù!?B~Èù!?äòSgZ3LºS7¬Ïü!?äüòmùçDuEEEºS7æÏ©uò#äüòC~q+?¯×k·Û-îÔÒÒÒ3gÎ0uù!?B~Èù!?äÛòÿý÷åÁ£T7++«ªªõYò#äüòC~±Çãijj*--MHH<µnYY<k0uù!?B~ÈùÍñ3ÉdB~4ÍfÓ=µn~~~mmm__£ü!?äüæ¸ùñüh©©ºë³ºü!?äü¢«õë×»òû§ú§Äl­­­ò¤üCßûÞ÷¾úÕ¯>õÔSæûÂ¾pàÀ÷ßQd2àmmmCëêêz÷Ýwwýúõ·ß~q|"?AÃòûä._SSÓÇ1[»Óéüf/ÃñÂ/|þó×º±÷î÷ÞïG?úQww7cánÞ¼)øc"ÜOúS7ãánß¾ýÎ;ï0ïòåË?ûÙÏ)åÇÑ^zòéú,eee.Ôº!¿!ÈÇ¨Æy£½í%ör´£½atùÑ¹`gÝÈÉÉÑºüò#äü_tßEäG¿Çãillvj]Åb·ÛµùòC~ü!?ä9`ë³LòÔºÈù!?B~ÈùÅdÈoþ400P[[?óõYòC~üòC~È/J³Ûí;vìHMM6ucªgÝ@~ÈùòC~Èù!¿¨üÿ.Y²$ðE>ÉT]]ÝÛÛ;½[F~ÈùòC~Èù!¿¨Èãñ477oÜ¸1p	§n ?äGÈù!?Æù!¿Ø¨§§§ºº:+++Øú,NÝ@~ÈòC~òC~QÛínll,--Õ]yëÖ­mmm3|ù!?äGÈùòC~sü@²Z­S7$³Ù|æÌÁÁÁp|^äü!?äüòPuuuEEEà3ßþö·].WXïòC~ÈòC~Èù=»Ý^^^h¾göÌ3³õ|ÈùòC~Èòúúúä*'''|©©©6ÍétFòþ ?äüù!?äüß,çõzÊÊÊMÝhnnÌ|Èù!?B~ÈòW===UUU!Ög	ÓÔäüù!?äÇ8 ?ä¡Ôú,Ë/_RRÒÖ­[å'Ðì®Ïü!?äGÈù!¿&³ÛíEwê@°¶¶¶¯¯/zî0òC~ÈòC~ÈùM9ËU]]m2ÁQUU5ë0#?äGÈùòC~È/¢y<¦¦&³Ù¼@¯¢¢¢3gÎ¸Ýî¨½ÿÈù!?B~Èù!?ä7qÃf³eddè¾È·wïÞp/Âü!?äGÈù!¿ð6<<ÜÐÐ¾555y<Xväü!?äüòó/ôÔµ>KTMÝ@~ÈòC~òC~SN<'ªÓ=ë(P,("Â©ÈùòC~Èq@~Èo²©©ºgÝJJJæä¬ÈùòC~üòµÔú,Á¦nØl6Ã7xäü!?äüß|ºQRR¢»>Ùl­©ÈùòC~Èq@~ÈO'ÃaµZu§nL¦êêêXù!?B~ÈòC~ASS7­ÏëS7ò#äüãüß'ë³º!ÑõYò#äüù!?ä÷«Äs2JÁN­gS7ò#äüãüæ£üæÉú,ÈùòC~üß¼ÓéÜ»w¯îú,q?uù!?B~Èù1Èo^ÈoppðÄEEEÁN­+ßq?uù!?B~Èù1È/Îå'#°uëVÝ£º&©¦¦F~âò(G~ÈùòC~übX~'NX²dI°SëÊÈÄß"ÌÈùòC~üß<×ëmnn.//×]yùòåµµµóvêòC~üòc_ÈOZWwÔÔÔ½÷Þ¸q¿äC~ÈòC~übX~çüùóÁN­+ûÝn7cäüù!?äü_ËÏápØl6ÝõY²²²Xù!?B~ÈòyùÖÕÕéZ7))õYò#äüù!¿xßððpyyy°Sëx¼"?äGÈùòC~ñ ?Éï¥¾ÔÔTÅÒÖÖÆÃù!?B~Èò7ù;vL¯¨¨h>Zù!?B~ÈòùØl6§ÓÉãù!?B~ÈòsùòC~ü!?äüù!?B~ÈòC~ü!?äGÈù!?B~Èù!?äüù!?äüò#äüòC~Èò#äüòC~Èò#äüòC~Èò#äüòC~ÈòC~üòC~ÈùòC~üò#äüù!?B~ÈùòC~ü!?äüù!?B~ÈòC~üòC~ÈùòC~Èò#äüòC~Èò#äüòC~Èò#äüòC~Èò#äüòC~ÈòC~üòC~ÈùòC~üò#äüù!?B~ÈùòC~ü!?äüù!?B~Èù!?äüù!?äGÈùòC~Èò#äüòC~Èò#äüòC~Èò#äüòC~Èò#äüòC~ÈòC~üòC~ÈùòC~üò#äüù!?B~ÈùòC~ü!?äüù!?B~Èù!?äüù!?äGÈùòC~Èò#äüòC~Èò#äü_äëìì,,,4+V¬èîîF~ü!?äGÈ/nåíÚ5Ù8wî^^ üÚÚÚ~³9ÿüç¿¤ÈöÓþôÃ?d"ÜGtóæMÆ!Âõ÷÷ËS(ãáFFFìv;ãùD~£££Ãòó-999P~/¿ürsÌöÖ[o]½zµöy¹<ãáÞ~ûmùYÈ80ìó¤Ë/3ªx_WWWee%G£½í%ör´8Ú·GU###n·ùòC~ü!¿¸ßOSï¬VkàG"?B~Èò#äÛòó­­­míÚµòä¥)ò#äüù!?B~ñ#?É´À'äGÈùòC~üâV~¡C~ü!?äGÈù!?B~ÈòC~üòC~ÈùòC~Èù!?äGÈùòC~Èù!?äGÈùòC~Èù!?äGÈùòC~Èù!?äGÈù!?B~Èù!?äüù!?B~ÈùòC~ü!?äüù!?B~ÈòC~ü!?äGÈù!?B~Èù!?äü(ä÷×ý×MMMÇlo½õÃáø"[kkk;ãáÄï¼óãá~üã_¼xqp~øá÷¾÷=Æ!ò>úg?ûã Ç§ünÝºuøðáïÑ§>"º=8ÚKDDDDÈ!?""""B~DDDDüùÅFmmmaÙ²e¾uvvÊE+V¬èîî=ýýý|bôÂ1ì,­>¸µµÑÓÈûBBøYì>(..NLLÜ²eËÐÐïEoðvÞçdØyzG~QÑh¼÷®lÈ[ÉäQnnîµk×dãÜ¹syyy²qáÂ«ÕÊ uØ¹¢¢âìÙ³²qòäÉmÛ¶1zay-mjð³ØÊ+²ÑÓÓ³÷îÐoðvÞçdØyzG~QÑÒ¥KÕyå­lû°äädy+ÙK.1haöÀA¬ÉÆèèh0¬Ðl=àïÜ¹³fÍ`ÿ4½¶úáÍ>ÃÎÓû;OïÈ/*êîîoûÈÛ®®.Ýýê×Äµk×ÊÃº¸¸ø>`ôÂ1ììû<â»MáxÀoÞ¼YÛÏ~¶Z±bÅ­[·dãµ×^ó>¼yÀG`ØyzaçéùEE+W®ìééQ?W­Zø###n·Ûwçýû÷åÁÍèoØYýÍ*11ÑßÈ»Ýÿð3ìæÍyyy¢íW^yE½Â¤øðæaçé§wä7ÇþCV«µ¿¿?ð<FÃ7ì~9::údüpl3záù£G?~<ÄÿÍ°Û·oøî	|xóÀ°óô>WÃÎÓ;òûV­Z¥^v:+W®ô½¨­­míÚµ>Ôöäææöööª§¹ÑÇ°²ÅbyõÕWeCÞÊ/è^F^Z½zõõë×yÀÏz2ÝÝÝccc¯¼òÊ/¾èQàÃ|§÷9vÞ_´üR"?ÿ¼íOF||¶¹ÉdòÞÕÕ¥VÄ(--U&O³>ìÜÑÑ­¦Q8F^ý®þÚZÅ~¶g,^¼X·²²ÒëõúàÃ|§÷9vÞ!?""""äGDDDDÈ!?""""B~DDDDüùò#""""äGDDDDÈùò#""""äGDDDDÈ!?""""B~DDDDü(~íµ×V¯^<Þ5kÞ|óÍ_þ/fmõî­Éd/Íívûí=!;;lllª·IDü(ö:|øð^|ñÅx_MMì¬¯¯÷ÛâÄ	ÙäÈiÜ&ò#¢«»»[Lc0N:åïôéÓò®ì¼~ýzÜÈïÎ;²sÅ~ûdoo/ò#"äGDñßöíÛÅ4/½ôïÎ_~YvîØ±Ã×=­­­"'Aaaa¡lküèÑ£ÊÊÊ´´4¹Èh4îÛ·Ï÷ ªÝn]ÉErÝææf?KÉôôôÕ«W_½zUÞµX,~wìÊ+¡oG]$wI]ÔÒÒLik×®ýÚk×®É³Ù¬í9zôhVVÜTrrò-[îß¿(¿ÀÛ÷Ûâ®ò#"Ë/^,j¹÷®ïÎ÷îÉNÉä+¿ºººÔ¥6mò»hÏ=ê"§Ó -õ®ºtóæÍcccbÇÄÄD¯×+Ê[SJJúó»·#ºw/ð+=wî/g5=Vcß¬Y³fªòqWùÍqêÀ®ÎsÖâ0_ÙlÛ¶md<Ùw7lØ .UÐQvT/ÝØÔEêÅBÙîèèP7âÛ©­;wî=.]my+ÛÏ?ÿü·#jwå|ïîW$h|½>wå­Üsáæèè¨úììl¹¢|	O>=:<Uù¸«Düæ^~®ü´ýJ6LÒýû÷å]1z·°°PÞ]ºté®]»Dl?ÖnD>ÆïU4íZêÝ÷îi,ZÒøÊ[Ù¶ÛíÞ(SÞðàïö7yû÷ï^yå'ÑÞ·oïE~âÑââbõäTåâ®ò#"ãÔ]###¾;Ýn·ìBXGs¡ËåRøÓ £ýq^àAX?Kù®¥"ÛF£111ÑãñÈ§§§kNx;Áæ×­[·Re[ÞÊ¶ÓéÔ.íèè; ÔxòòqWùÍqêoÝ?î»S­uâ7ÃCû[À¾uýúõuìU#£zAN;:¡ÏöìÙ£òÊÛÊÊJmÛQ¯±õ÷÷«w<xzîªU«äRµÕ÷"5ÏW.jii-?M¥j4´KCÜU"B~DDs!a0N<©Vu9uêTbbbàl6r<:«ÍÃUç§þ<Îårùþ à¾ûÄIjÖ­¶®®ÏÔd[õÙÛÚÚ´ý!nGýaú;?·Û­>2üN>­½'_²ïEêON§|`òSÖ+Ë8·lÙâi»JDÈhîS«ûuôèÑ_=§`¤mkÇIµIZ;wîôe¥o.!¿'ã'ÛýF£Ñ÷@pÛ»áÇ´yµÁ¾ØÇ'''«/Áï÷úõë?Å¢Eä­ZØÅ÷6ÕKZÊ©Ú¥!î*!?"¢¨Ht²fÍÄñV¯^­&ØúÉ¯¥¥E-·lÙ²öövíR·ÛðàÁ¬¬,¥¥ýû÷<íÒæææââbYvvvCCßmÞ¹)Ù¿÷n¿ýÁnG;#wI-4b=?­ÊÊJ¿å]T>¬¨¨HKK¯¢··W[¡Æ÷6¶j¬Ìfsgg§ßgqWùò#""""äGDDDDÈ!?""""B~DDDDüùò#"""B~DDDDüùò#""""äGDDDDÈM½ÿ î(r4IEND®B`


?ÏDDDD4;Ã" """B~DDDDüùò#""""äGDDDDÈ!?""""B~DDDDÈ!?""""B~DDDDüùÑô<uòäIÍù`É®=wîÑ£Ìfóo¾9îNü&sâÊËË¯_¼xñ©S§¦°p:;;-Ë~ü+W®¬X±Â`0Èwss¤GDÈô.¿»wïfü^|ñÅD÷<x0Å½Mê&ÉhuâÄÉ.)/ÆØ÷µÙlÈM°¤6dü:;;Ußxãh4:::*rQföööjÞÕdo2æ[íÝ»WæÆÒ&?uÃû÷ïËôÈÈ®V!?"ÊnùÙívùÿÜ¹sÉxqóæÍÍ7«M¥MMM@`ÌW^¼xqÞ¼y+W®Í¹téR]]Á`hllüòË/¯²xñâÕ«WÇ/:t¨¼¼Öo»*oyæ¿íU:yò¤ÌlnnÖü'Äo-X9ò 'µp4ÇäâÚSËoÌµ²^jkke±¯Y³FmêÑÁÄkãW«äõzåÞd9È«b7¡ÊLËU¥¥¥»wïÃ<¹éT~CCCeee2;wîÈÛy¼Eä+Ç-//Oþß¸qãC­Í+V¬ÜÄ.Bbìó«V­JíÈüø"K¹hÑ"Íq²7ó­5jÌ¯ººzR'5à¦|ÃË¯««+þ~ä¬üâWkoo¯º«³³SñÆ<æç'ò#"ÊO&Þyçø1°xìØ±C¦×­[wÿQ2!efüW8p`ttTZ©9;wîF£íííê¢Ü³xñ¢LÕmÕÞl.é[·nÅ_Ì7As~ümó&Év¤Nváh>ª)ß0ÅN~cn(bé¦¦¦éc>¸üâW«ÜÌ¹|ù²L«ÕºuëVõÅJ·oßiY2]\Ìù~å'É¹LËúùeee2çÎuQÞàåâüùóãï!vmlh#þâàààÃ¯6ÆcddD¬ ÂhhhPLãÊ¯  @óÑ?ÄJn+±X,^¯Ws¡Mdáh>ª)ßpâòSû2~ùåñßbRò_­êÞâ9êª¥KÊÅOááaYDÈô.?ÁYii©¼G"øùcf;½	é4ï3õEQæ-ã~Fµ¼¼Nä]"cîm"7I¶LÆ]hY8É<:µN|kïDlúÚÄÕ:æ7ê*¿ß¯ða[[O."äGDºôÖ[oÉÅØ1PÔL³1£S2s"H±¶¶V]åÒ¥K"³ÈOLª¬455íÞ½ûþýû§O7oÖßDn2eùMváÄò'.¿ùÅl'îO½üeÎÇ×ÝÝÝÒÒ¢61Ç>MDÈô+¿öÖíÈ¯æ¨Oþ®[·nèQê­çÎ/?5îÕÛÛ+þ8uêÔDäçóùÔ¨`±½½]ç©ÞyçÍq²7üR/µ$Ãs']ªSÚ3Oà;<<,°[»vm¢;&_t(N½üÕ#7Ë×ËéºººøSí¯é÷ûSly'"äGDúßÝ»wct©9@`Ì>^eee±Ê>übQÍ?_þÿ|«æc~î¹ç·<.[¶,Å9©LJ~©NìÌcÆ<¥:ùuwwÇßÿÊ+ã¯³LÄj©å§¨ù5ªßöíÛyr!?"ÊùIjKhüü/¾øbóæÍjjjJü<ÇÔä788(÷&÷)záC±¤öÍñãÇ/^·`Á×^íé§9=þM&%¿ÔçwÞüØ!ñâÔRü¤¶¶¶ÊO-÷¯¶§Ç>Î,Ågê»[,qÇ^¼ØÐÐ ÷&?ÛíÍÃûöíS©ÂwYH'ò#"FFFî¹ICn7ÉºÔFXõaíh4ª@_[[Ë/!?"¢íç7¦W^y%CDÈ(×Ã÷î]°`Ú¤+/¾ø"!?""""äGDDDDÈ!?""""B~DDDDüùò#""""äGDDDDÈùò#""""äGDß;ï¼c2ÃªU«&ÛÅÏ3Çï÷ÇæÈ´Ì©««ÄËÙ£¦ö5¹íT^aU^^^QQQSSÓàà ¿EDü(;*//Ç_¢ÑèdoèÐ!¹íÑ£Gcs9"sZZZ²Z~)î?~üà2½uëV~ùQ¼<º»»å¶±9+V¬9]]]éyXY¦ø-""äGDYÃ¾xÍìÛ·¯¤¤¤¸¸øàÁc¾ìé§nhhs±!Ci£®ºû¶Åb¥K^¾|YóÞâ¿ê¼öÚk&Él6Ë7JtØl6ÜVüÞ½Õ¦ØtåÊro2_îùÎ;3$¿/¾ø"//OýD£££òàå¢ÌäwéjúÇ?þ±L»Ýn1L¼òÊ+ñ_sñâÅÄÚvìØ!W>Z¦ånnnVW-[¶ìÂ2qëÖ-_QQ¡yoñ õMÄso½õLØíöÄÿüóÏË´|ÁÛo¿-2=44ÔÛÛ+jHRs¾ÉÊOx'ËG¦·mÛ¦®jiiQâØ±c2ñâ/òÛEDÈô+?É$Ó#Jæ>O®Z·nLoØ°A.vmww÷ÔG"4ïm®RßD=°ÄÛ©/&	1Ì¯é¥Ke#Èã,ø9ê±Éûê*ùÖ-Q\]-ùí""äGDúÁ`MË XÂ,|e8VUeºêÔ©Sr+áàõë×ãïaÌ½Å_ÈM=0¡Xü0Rã¹sçæÍ§æªáÀióÓLÀEßVNDüHò«¨¨óSÃfãrÇf³Éµ±ÿcódÎè£&(¿Ô7=0ÁmçÏ¯n;æáE£Ñ.ØíöøÁÂ),qÅÐÐPYYYSSÓÊ+EÃ_~ù%¿]DüH¿òSGi9ú´ÚÏïÈ#ß¹sçbgo½õVl¾rä7Ôþ_ê8pà'?ùLìØ±#ñ¶Û·oé×_ýóÏ?W[xe¦Ùléë×¯Ãd¢ººzæä·÷n¹ª»»»½½=~gD""äGDz_4]?*þ|©å7<<¬¶´Êÿ±Þ¤¶¶¶ÒÒÒ½÷NP~©oâõzåÚ+VÄ>hÿ5ápXì%¼  `íÚµêc¼¾¦¦&õaáÆÆÆÉ~Øvâò­ÆàfåÊr±··_0"B~DDDDüù!?""""B~DDDDüù¥£ýìgiþ¦wîÜùÏÿüO~oôÜÀÀÀýû÷YznhhèßþíßXznxx8ý/°4©þû¿ÿûW¿úËAçÉ:5ü¦§ýèG¿4ÓÿüçO_çþùçÿüÏÿÌrÐs·oßþ§ú'?>ùäû¯ÿú¯K.±tÞÅýë_#?äGÈù!?äGÈù!?äGÈùò#äüò#äüùòC~ÈùòC~ü!?äü!?B~ÈòC~È!?äGÈù!?äGÈò#äüò#äüùòC~üù!?B~ü!?B~ÈòC~Èò#äGÈù!?äGÈùò#äüò#äüùòC~ÈùòC~ü!?äü!?B~È_vÈ¯££céÒ¥¡®®®««ùòC~üù!¿ß¢E®^½*o¾ùfuuu¢ü.p?½ÉKáÍ7ïëîîþì³ÏXzî¿üegg'ËAÏÝºuë§?ý)ËAÏõ÷÷*X:ïý÷ßHó7ÍbùÅWTT(¿'N|Þ>øàÏ÷	é8¯×ûÑG±ôÜÇ|éÒ%öÉ;ËAÏýìg?ï½÷X:¯­­­½½=Íß4ä×ÙÙÙÜÜÌÖ^bk/[­½ÄÖ^¶öæìÖ^ÕÂá0ò#äüùòC~¹,¿»wïÚívMl!?B~Èò#ä;òóù|«W¯Ô¼ùòC~ü!¿ÜÉdò#äüùòC~9+¿Ô!?B~Èò#äüòC~Èò#äüò#äGÈùòC~Èùò#äüù!?äüù!?B~ü!?B~È!?äGÈò#äGÈùò#äüù!?äüù!?B~üòC~ü!?B~Èù!?B~Èò#äüò#äGÈùòC~Èùò#äüù!?äüùòC~üòC~ü!?B~È!?äGÈò#äGÈùòC~ÈùòC~ÈùòC~ÈùòC~üù!?äüù!?B~üòC~ü!?äGÈù!?äGÈò#äüò#äGÈùòC~Èùò#äüù!?äüù!?B~ü!?B~È!?äGÈò#äüò#äüò#äüò#äüùòC~ÈùòC~üù¥·p8DòC~Èò#äËÉÛss³Ñh<|ø0òC~ÈùòC~ür°P(tôèÑúúú9_µdÉh4ü!?B~È_î$ïïv»Ýh4ÎIèÚµkÈùò#äüùeýýýG5ÍàËÏÏß¶mÏçcÌù!?äGÈùòËî¼^¯Íf;wn¢ù[[[ÁàãäüùòC~ü2xîðáÃ555ànÛ¶­§§g¿òC~üù!?B~é.?~Ó¦Mùùùæ«¯¯w»Ý¡PhÚ¿/òC~üù!?B~éËï÷;NÉ¾ÒÒÒýû÷ËÌÜwG~È!?äGÈoÆD"Çb±ÌÑjùòå­­­yfäüùòC~ü2äÓ<>Édr¹:Èü!?B~ÈßDZ[[Åjµ¶µµ¥aù!?B~ü!¿kv8|UUU.kZÏü!?B~È_ÆBn·;Ù Ífóz½sfäüò#äüùe¾|555äC~È!?äGÈoR|a.,,´ÙléWòC~üù!?B~Ó¼Ýìw|3tfäüò#äüù¥¯`0èr¹òÙívySÖùü!?B~È_ª¢Ñ¨×ëµÙlgZ3ÍzäC~È!?äGÈoB©A¾ªªªDðFÃ¡ÿA>äüùòC~üRFÛÚÚ¬Vk²A¾ôiù!?äüù!?B~3X¿Ëåª¬¬Ôäs:é<ÓòC~ÈùòC~ü¦?µ'ÕjÕü¸®Åbñx<Y:Èü!?B~Èßoêëës:|&)ùò#äGÈùÑl_lOsO>5Èñ3­!?äü!?äGÈï±êëëkii)--MÌÜ¿ò!?äGÈò£Y'¿h4zöìYÅb/7öäC~È!?äG³W~@`6ò!?äGÈò£Ü_4õx<%|ùùù[¶lÃáÙ¶ò#äGÈùQNÉO^uwíÚ¥9ÈWUUår¹îÝ»7kWòC~üù!?ÊùÃáÔ|>/'?®ü!?B~ÈfüäevÏ=ò!?äGÈò£ÚoÍ5à+,,Ü²eËµk×äC~È!?äGÙ-?¿ßït:ËËËÍ·dÉËY#Èùò#äü(åDZ[[Ífs"øFã=Øù!?B~üe½üü~¿ÃáÞ%¯¾¾þÌ3³ðø,Èùò#äü(§äÜnw²A¾]»vÍª0#?äü!?äG¹)?ywÓäËÏÏ·Z­³äLkÈù!?äGÈùQ.ËOòÕÔÔ$òL&§ÓXÔÈù!?äGÈùQvËOÞÎìvaaa²A>>ºüòC~üe·üB¡ËåJ6È'o¬ù!?äüù!?ÊnùE£Q¯×k³Ù4ùd¾ òC~ÈùòC~Ýò.«ªª*ÙÖäC~Èù!?B~Èùe·üÔ ÕjÍÏÏO<Ó|Èù!?äGÈ_.È/Êû£ÉdJä«©©q¹P¥üòC~üùe±ü¢Ñ¨ÇãI6Èg·ÛåýüòC~üùe·üü~¿ÓéÔä3Ín·A>äMòßfäGÈùò£øþã?þãàÁEóLkA>äòkoo¯­­_bäGÈùò#äK<Ó|È/ëå·víZùýN!¿S§Ný<½øáÂÍûøãÿáþå ç®òÑG±ô°ï>`9èm¥´´´,Y²$|sçÎÝ¼yó¹sçXJzë½÷ÞûÇüÇ4Ó,ßobrùýýßÿýéMØ÷Å_|I:N~é¯_¿ÎrÐsöÙµk×XzîæÍ>å .^¼øÜsÏýÎïüN¢ù-[vüøñ¾¾>>»páB0Ló7Íeù±µØÚËÖ^bko®Ün·ÙlÖÜïßûÞéÓ§YJlíÍ©­½Èò#ä7÷ÃlO¾ÖÖÖH$2µóöòC~Èùò#ä§Ô _MMæ Óéôûý±/F~Èù!?B~È_V&og6­°°0Ñ|ÅãñD"17A~È/gå,äGÈùòËêÁ Ëåà òC~ÈùòC~ü²¯h4êõzm6[âÖRò!?äü!?äGÈ/R|UUUSäC~Èù!?B~È_väóù¬Vëãò!?äü!?äGÈO×õõõÉ»æ|&iR|¹'¿ÃïÞ½ÛívÃaäü!?äGÈ/+D"Éöä9V«ÕãñD£ÑÇùÙ.?YsçÎývã·¿óïÔÕÕýþïÿ~0D~ÈùòC~ü²©÷î=zÔd2%äÓò²Z~¼ßýÝßýçþäàªBÀU«V!?äüù!?B~Ù×ëµZ­)É÷|¹$¿³gÏÍæûäß¾?ß7oÞ¼Éîìüò#äüù¥5¿ßßÒÒ¢ùq]ér¹¦k/äwüøqÑp¼üä_YYÙ½÷òC~ü!?ÝDÎ=»fÍÄ=ùív»×ëÞA¾Ïç«¨¨Ø÷çûbìvÛ³_ÿú×sï÷ù!?B~ü_v×ÓÓ³gÏÒÒÒÄA¾åË»ô|R!Û?áaµZ|òÉßÛ!ìÛÒ´¥¬¬L$üò#äüùé¢p8ÜÚÚj±XÁWXX¸~ýú4¿%e»ü"È~ð¢¢¢RNþÚ ?äGÈòË²zzzN§æ _MMËåBéTÉù!?äGÈùò¶îÝ»çv»/_¾¹sçÚl¶k×®eðá!?äü!?äGÈoÒ577FÍA¾£GêáÈù¥[~sÆ+//ùò#äü²¥þþþãÇkiM(ý<ZäüÒ-¿¼ñ2È!?ä§ó¢Ñ¨:ÓæA,YræÌUù!¿tË/ã!?B~Èßãå­DóLk¢À]»vÉkþù!¿ÜßÐÐÐ;!?B~ÈOo©A>«Õxf©±±±µµUÿ'@~È/ò?ûùò#äüôp:|F£qÿþý===Ùò³ ?ä1ùÕÕÕ%>JKKgz¯äGÈùòHHÄãñhòÉ/×Ê×d×ü_ÆäWPP OÁÁÁòòrðýä'?ææfäGÈòË`~¿ßétjÅd2ÉU@ K4äü2&?õ	¡LÜºukttT&!?B~È/ý©A>Í3­ÅùtûÑäüô.¿yóæÉs©½½]þ´_|QMpTB~ü_7 ÃlOÞ8Á`n¬#äü2&¿½÷Æ>Ïÿ3ÍÈ!?äB¡Ûí÷ÍA>Íæõz³ù!?½ÈOzùåçÏ/2!lhhéüù!?B~ò²l¯ªªÊåråÌ òC~:_FB~üÍZù©A>Í3­æä òC~ÈùòC~4ëä'¯Ãv»]óLkAË%(ëù!¿LÊ¯ººZÛ#9ò#äüf"ñ¨.Ù XP^gÕ:B~È/cò[´hQ¼öbñÙ^B~üß´¼ð&ä3Ín·ò!?ä§ù	òäé×ÙÙ9::Îüù!?ÊaùÝ»w/Ù Ñht8³mù!?½È¯´´Tifò#äü(Wå'¯í6mJ<ÓÚ,äC~ÈO/òëêêgãóÏ?ÿàÁäGÈòZýýýÇ_²dæ¹àwíÚå÷ûY5Èùe^~Ò¨|Â!?ä7nÑhÔëõnÚ´IsO¾úúú3gÎD"VòC~zßÂù!?B~Èo²A§Ói2ÁW^^.WÉÓuüîä§¥½½½i~èÈò£lä³ÙlsçÎM4Åb9sæò!?ä§_ùñ	B~üß¸õõõ>|XsÏh4îÙ³G¾üÞåçóùäI»wïÞ4ïüù!?Êùµµµ­Y³&ñãº2gýúõ'³ØòËùÍIð äGÈoË¯¯¯O^«+++5?®ÛÒÒrïÞ=6òC~Y&¿¼$ñ	B~üf§ü"Çã±Z­|6MÞù¸.òC~Ù*¿Lüù!?Òüä	èt:Fcâ _UUUKKò!?äõò3L.Lÿgï!?äG:ú¸îúõë5÷äÛ²eÏç¯aÁ"?äò3òÜNÿCG~üe @ Ù1ùªªªò!?äkò»|ù²<Ã[ZZÒyläGÈùQ¦äFíÉ7wîÜmÛ¶]»vA>äG|¶ùò#äÝòóûýÉùêëëO<ÉAå¸üøl/!?B~9/?õq]Å¢yfÃ!/,7äG³B~ùòC~ùùý~æÇuÍfskk+ÇgA~ü!?B~Ù-?Ï'ªÛ%ä² ÍRù¬]»¶¨¨H^7nÜz ?B~ÈfèÎn·Ï;7Ñ|Åãñ0ÈühVËoxxXó3ýÒüù!?ÆB¡ÛíÖä3LN§A>äGÈïª­­×uëÖ=xð@.mØ°Aæ,[¶ùò#ä§ÿÔ _aaaâA­V«Çãáø,ÈßÿVPP /ñ¯###2Gæ#?B~üt[(r¹55|ßýîwK	ùò[^^¼Löbs"Ìá¨.üùé3yñ´ÙlÉù¼^ïÀÀÀÔÎÛKÈr_~jkïêÕ«ÕÖ^ù_¦eÎÒ¥K!?B~ú)&äSgZ/P_9Ùóöò£Y$?¡æ'<äùò£Ü_(Ú»woý%Kõ^Úh4êõzm6[âÖe¾fO>äüù¥jxxxãÆ%%%yyyòÿêÕ«eÎL?täGÈù¥¿`0øÍo~sù²åÛµbÅßû½ßéðÑÊK¥æÖjjjW²3­!?äGÈOw!?B~È/ýíÜ¹ó©åOüáÁØ?Á_ss³®¤ä³Z­ÉùÆñD~ÈòC~ÈßÃúúúíÛ·ÇË¯ùÏkjjtòðÓéL6Èçv»ò!?äGÈo¼[W^^ò#äG9&¿åËÿ©ýOãå'ëÔeöQE"ÇlÏn·Ëk×¤îù!?B~cËKò#äG¹*¿^ziñâÅöÉoÉ%û÷ïÏÔãñûýN§Óh4>æ òC~ü¦Ø/¼ ^tÞ|óMäGÈrL~Häþàjªk¶4mµµµK.ÃéÇb±$oj|Èùòt]]]ÅÅÅêÞñvF~ü(gä÷ðÑç'?þGôGë×¯?räHÙç÷ûæ Ùlò òC~ü&×æÍÕKÏÒóÐ!?ä7ÏêÄvàsù!?B~íí·ßV¯>ëÖ­KçCG~üßlH^vÒ0Èü!¿ñ^¶lú0ÇåËÓüÐ!?äÃ©A>Í3­ÍÄ òC~üÆéØ±cê5hëÖ­yèÈòËÉäuÆn·¦sù!?B~ãÝãùò#ä7çW¦ùò#ä7Nyãe0!?B~©SgZ³ÙläC~ÈÞC~ü_V].WUUU²A>¿ß©Çü!?äü!¿é©­­MóLkj¯µµ5dö"?äGÈù!?äGÈï±R|ÉöäknnÎà òC~üòC~ü¦!µ'ß-[4ùêëëÏ9þó¿!?äGÈù!?äGÈoN§ÉdJÌ«>9òC~üòC~ü&T$ñx<Éöäù­­­ÑhTÏ?òC~ünÉñüùòKøõK1È·ÿ~Ýò!?äGÈoâÝ§)?çGÈfüRò­Y³æüùó:äC~ÈßDß<yiæg<x åÿ6È+W® ?B~Ûò×]»viòUVVîß¿¿¯¯/.äüù%­´´T^ãâÿ9åååyÏ]]]añâÅ/_F~üNBn·[óø,ùùùë×¯ûìäC~ÈßïâQ¢½1òüýü^ýu8qâÄÖ­[å÷áÓÛµk×þõ_ÿ5L:îÓO?ýå/ÉrÐs7oÞ¿ë²ôÁ_¹rå»ßý®æÖ.ÒK/ýêW¿Êuå'åwUÏýû¿ÿû|ÀrÐyï¿ÿ¾ü¡æo:ãò+++<QÚðð°o¹nÝ:#ó4qttTQÒd2%ÊïèÑ£§7YÂÍIÇÉXòjÈrÐs.]Õ]ùÝwßýÁ~ðo|#|O<ñÄÊ+ÿò/ÿ2~ñ¼^ïø]Õs/_nkkc9è<YG²¦ÒüMg òw¡æ'<®_¿þ÷ÿÄÏ°µØÚËÖÞ4$¯36Ms¯²²òðáÃÁ`0÷Ö[ÙÚKlíMÕ­[·-ZTTTW\¼téÒÇ¿ÛøíÅÈòK[¡P(ÙÖDbAÏÕò!?äGÈOw©ÝåÿÄmÇÈòöÔÖò	ýýý9¿ò#ääÅ÷µ×^	ù¿©©	ùòC~3W0ÕUUU%Ïh4:yúÏuü!¿TÝ¸q£ººº¸¸Xm-++;wîÜãßmyy¹ÜgEEEGGò#äü¦=5Èì Ìf³¹µµ5Ì¶uü!¿¤©#9Ç±MM8qbF:ò#äü§`0(/#aV|~¿Ö®#äüù%­¼¼(oÜ¸_GGL ?B~¤7ùE£ÑgZ³X,rí,äC~ÈßïâQjBÉottóöò#½ÉÏï÷;NÍA>)WÍæA>äüùM4u$g5Î'òÙ·oz%E~ü(ãòD"Çb±hiÍjµÊµ9||äüùM³ü|>æ¯^½üùQåç÷ûÑhL6ÈXÈùòt¡õÙÞ¢¢¢êêê4¼"?B~ÈO³H$ÒÚÚj6òy½^ùò#äe!?B~È/ñl¯ªªÊåråäÖò#äVùÅ>Øk`` ººº²²ùò£4È/¹ÝnÍA>u¦5ùò#ä7òá³½ü(òK1È§Î´&(d#?äGÈoäWYY9'eóæÍC~üh&ä7î _ú_!?ÊqùÉkwÞ£bgïOØ÷öÛo#?B~4½òK1È·|ùrá |ÈùòùÅçÍô]äGÈoËOòÕÔÔ$;ÓÚµk×X°ÈùòKü2ò#ä7ä'O:»Ý^XXh>ù³sÕªUýýý,Räüù¥O~###ÕÕÕÅÅÅ±9óçÏÿñüùÑåbï·~ë·¬òÙmÏ~ç÷kkkÿøÿEü!¿ôÉoÁc>Þ«^[ZZ!?¬üRòë[ßzâ'þlûüáAõoßï3où!?B~é_AA¼"÷ööÆætuuÉäGÈ&(¿|¥¥¥£§§G^gª««cìSÿzê©óçÏ³Tò#ä&ù©÷þûM8Èò#äGòK1È·fÍ3gÎÃaõÁ`Ðd2Ø ^~K,ñù|,Uäüù¥I~ò½÷îH$"<(sÊËË!?J&¿P(är¹étÊªL¼íO>ùÿïcìÛºuë×¾öµ÷î±Tò#ä&ùuvvjÉ¹££ùò£ÄÎ;·víZÍA¾ÆÆFÇäKÌï÷Ï?ÿ[ßúÖw¾ó³Ù,ìcÀù!?B~it÷îÝÅååå×ÖÖÊ~èÈ_vÕßßl¯¼¼¼¥¥%Lä~ÁàÑ£G9Ò××ÇE~È_ºå!¿¬(¶µµmÚ´)??_sO®¯aA!?B~Èù!?B~Yç©æ9¾¿þõ¯»`0ÈRB~ü_öÉïÆê`Îêó¼eeeçÎC~üfg)ù·lÙò×ý×ÝÝÝ,(äGÈùe¥üä7/þdJ¿:ó'!¿Yßïw:&)q¯¦¦ÆårB¡S:o/!?B~ÈO/ò+//õ7nÄä×ÑÑÁ	ùÍ"Çã±X,à+,,´ÛíòÄÿzäüù!¿,zwôæÑÑQ6È_Î/m§Ó©þüKäs»ÝjoLÈùòC~Y,¿²²2uô>%¿ûöÉ´ÉdB~ür25ÈgµZ÷ä3cÌ òC~ü_îÈÏçóiÉùêÕ«È_ÕÛÛ»gÏá]âSÞl6'äC~ÈòËùI¡õÙÞ¢¢¢êêê	ùòËÂáðÙ³g5÷äS|~¿â÷ü!?äÝòËHÈ_R|¥¥¥|­­­êÝù!?B~Èù!?B~:*ê/_®9Èçt:'5Èü!?äSò»yóæÂä]¡¨¨hñâÅÈ_6&¿äcîÜ¹cÀoµZ=Ïùò#äürG~^¯WóòüùeK¡PÈív×ÔÔ$>M&ÓéÆwò#äü²X~êP^MMMápX.>xð`ëÖ­2§¢¢ùòÓò<²Ùl|ò§]4Þïü!?äÅòSoñï2;ª3ò#ä§ÏB¡ËåJ6È'Ï¯`08Cßù!?B~È/å§ÆüFFFbsó#ä§Ïä3¯×lOæÏÄ òC~ü_îÈOíç×ÔÔ$àS¯ìçGÈOoAËUUU8È'3åªäC~ÈòËùÍ¯ÚìüùM$5È§y¦µ´ò!?äGÈùåüòÆË`0 ?B~é/ÊÓÄd2e|ù!?B~È/wä©!?Í¢Ñ¨ÇãÑä+,,ÌÈ òC~ü_îÈ/ÙAïÞ½üù¥3¿ßït:5ùjjjW(ÒÃãD~ÈòËbùÉÊË/¿<fæ¶mÛ8ª!¿ôD<ÅbIäKÿ3ù!?B~ô0÷ó7²²²¹øÆo¨·Ú½ùòå÷ûÑhÔù òC~ü_îÈïá£>õfSQQ¡&6lØ0ÓùÑ¬_$imm5ÍÙ2Èü!?äSò®ï9yòd:ò£Y(?ùÌÆA>äüù!¿ßÎ;Õ:ôÌ3Ï ?B~ÓU8nmmÍ¢=ùò#äG9+?Á o?%%%ÝÝÝãöó+((@~ü³]»vjò¹Ýî¬äC~ÈòËùÉ;ÐÆÌ´Ûí|¶ßÃ'OT'BLäçü*fïOü!?äÅòKv<¿ò£_OOÏ¶mÛ4ùªªª²tù!?B~È/wä©åüÄs'O¬©©IÑht8"ÂYGÈùòC~Ù'?yCßú"ò#ä§Y4½víÚ¶mÛÏ´&Õ××?~<ùò#äüò£Y-¿¾¾>Q]UUU"øÊËËåÉìÙuòc9 ?B~Èù!¿ÇJ0'/ë×¯×äkllôx<H$·×òC~üòC~ãò6L|»víòûý³d!?äGÈù!?äG¹)¿h4êõz­V«æ ÅbimmÍÕ­ºÈùòC~ÈùÑl____KKæñY*++÷ïß/_0;×òC~üòC~#òD"çÏ_³fM"øòóó­V«Ûùò#äürA~©C~4Ûäç÷û÷ïß;õA>§Ó9öäC~ÈåüòÆË`0 ?òBn·Ûl6kþ	dµZÛÚÚfù òC~ü_ÖË/ã!?Ê¸üä÷ÁápÆDð-Y²ÄårÖòC~üòC~ÅòK1Èo³Ùä©Î òC~üù!¿Ù^ 8þ¼¬¸p8ò_»Ý^XXh¾ËÕßßÏZF~È!?ä7ÛF£Ï>ûì×¾öµ§zª¶¶Öh4öôôdüB¡¨Nl>Q XP~1XÅÈùò#äüè7íÝ»wÑ¢E÷ì=øÃòoÓÆMeee31ò7½òß1ÍlÏívY¹Èùò#äüèÿ4oÞ¼ïïü¾búWWWçñxô)¿`0lÏh4:ùò#äGÈùv¡P¨¬¬,òïÛßþ¶èJWòSgZ³ÙlgZ3Íò!?äGÈò£ñÓù_0ß(Él0#?äGÈò£¦¹ßLMJ~ÑhTôiµZòµ¶¶F"VòC~üù!?Dê³½F£qùòåµµµ¥¥¥ýlo p:Éù8ÓòC~ÈùòC~ÈïqSÇóóz½3··ùE"d|2GæËµò!?B~ÈòC~ÙQ2ùùý~§Ó©y¦5É$Wq¦5äGÈùòC~È/»åDZ[[iÍjµz½^Î´üù!?B~Èùe·üü~¿ÃáH6È'¿?Á`Åüù!?B~ÈùeqñlÏf³1Èüù!?B~Èùe²vGIII¢ùÊËËå=ù!?äGÈù!¿ì.¹ÝnÍ3­åççoÙ²Åçó1Èüù!?B~Y ¿ÁÁAÉü(Ùê°ÛíæºöäC~ü!¿¬_mm­¼#?/ê4ùD7n<ö,K	ùòC~ü²L~k×®õûý)ä÷7ó7¦·>ú¨££ãSÊD===õW%¿O<ñD¢ù¾ño¼ôÒKW®éO*°¸ôÜÕ«W?þøc¿r?üðCÎ_/ÀrÐyï½÷Þõë×ÓüM³X~¿yÉåçñxúÒ¨â³Ï>ë£ô&oB?üá¿ùÍo&ï·û·~úiùMøùõÅBó_üâ,4='¯|ò	ËAÏþùç/_f9è¹@ ðþûï³tè<öö¶rY~líÍí¢Ñ¨×ëÕ<ÓT__ïv»Ï7Áóö[­½lí%¶öêÂy*ä7²~M&S"øF£Ãáì¶ÈùòC~ü²F~D~³¤h4êñxò-_¾üìÙ³|ÈùòC~üòË¦úûû=ZYY©yf§Ó9ñ0#?äGÈùòËbù%ùå@Ñh´­­mýúõ|'OÃºOäüù!?B~Èùé«þþþÃkîÉWZZºk×.ÜÔîù!?B~ÈòC~ºHòiîÉ'sd¾Çãì òC~ü!?äüôUA¾ªªªi<ÓòC~ü!?äü2SêA>Íæõzåk¦ñ;"?äGÈùòC~È/Ý¥8&_MMÍ4ò!?äGÈùòC~È/3E"óçÏ¯Y³&q¯°°Ðf³ÍôZC~Èò#äüß'är:ååå|gZC~È!?äü_6¥ù5ùìv3­!?äGÈòC~È/;J1Èg6Ó6Èü!?äGÈù!¿*x<ÍA>£Ñèp8Ò<Èü!?äGÈù!¿éÏï÷ïß¿?Ù _kk« 0ãù!?B~ÈòC~S/»Ýn±]"øF£ÓéêçÑ"?äGÈùòC~Èo*õöö:á]¢ù,ÇãÑÃ òC~ü!?äü¦Þ½÷Ô _â|&Io|ÈùòC~üòJ×®]Û¶mÛÜ¹sù=Ïôiù!?B~üòC~éîÞ½Ç¯¯¯O_iié=SÙò³ ?äGÈùòC~È/éO´iÓ¦Ä­ºjO>|ù!?B~üòC~S¯¿¿?Å Î÷äC~È!?äüßD7mÚTXXlO¾p8Õ? òC~ü!?ä7Ûå].Weee"øÊËË÷ìÙ½|ÈùòC~üòû¢Ñ¨Çã±Z­òÉ|ù!?B~üòC~¨¯¯ÏétL¦ÜÛù!?B~üòC~ÿS$I1Èg±XÎ?¯ÿcò!?äGÈòC~Èoü@²oôõõÍ§òC~ü!?ä7[ä'Y,1òåö òC~ü!?ä7åwþüùòòr§ÓfáÓù!?B~ÈòEòF£9öq]äüùòC~ÈùòC~ü!?äü!?B~ÈòC~È!?äGÈù!?äGÈò#äüò#äüùòC~üù!?B~ü!?B~ÈòC~ÈòC~ÈòC~Èò#äGÈù!?äGÈùò#äüò#äüù!?B~Èù!?B~ü!?äü!?B~ÈòC~È!?äGÈù!?äGÈùò#äüùòC~üù!?B~ü!?äü!?äü!?äü!?äGÈòC~Èò#äGÈù!?äGÈùòC~üòC~üù!?B~Èù!?B~ü!?äü!?B~ÈòC~Èò#äGÈùò#äüùòC~üù!?B~Èù!?B~Èù!?B~Èù!?B~È!?äü!?äGÈòC~Èò#äüù!?äüùòC~üòC~üù!?B~Èù!?B~ü!?äü!?äGÈò#äGÈùò#äüùòC~üòC~ü!?B~Èù!?B~È!?äü!?äGÈòC~Èò#äüù!?äüùòC~üòC~üù!?B~Èù!?B~Èù!?B~Èù!?B~È!?äGÈò#äGÈùò#äüù!?äüù!?B~üòC~ü!?B~ÈoöÉ¯££céÒ¥¡®®®««ùòC~üù!¿ß¢E®^½*o¾ùfuuu¢ü|>ß¯Ó[ggg0ü5é¸7nøý~»uëVOOËAÏ´··³ôù±tÈoxx8Íß4å_QQQ¢ü9r1½µµµ]¸pá"é¸÷Þu¤ódÉjb9è¹÷ß_^îX:ïÝwße!°Ëùuvv677³µØÚËÖ^bk/±µ­½9»µWõàÁ¦¦¦p8üù!?B~ü_NÉoÎW©wïÞµÛíØB~ü!?äGÈ/»åÏç[½zõàà æµÈò#äüùåüL&Ó¸!?äGÈòËYù¥ùòC~ü!?äüòC~ü!?äü!?B~ÈòC~È!?äGÈù!?äGÈùò#äüùòC~üù!?B~ü!?B~È!?äGÈù!?äGÈùò#äüò#äüùòC~ÈùòC~ü!?äü!?B~È_.Éï¥^òx<éíÝwßýÅ/~ÑG:îòåËò'ËAÏuttÈ«!ËAÏúé§÷wÇrÐs7oÞ|ýõ×Y:ïoÿöo@¿i(ÊMùÝ¸qãàÁ?""""¢¯J½Et#±DDDD³$äGDDDüùò#""""äGDDDDÈ_¦MñWE"»Ý^PPPYYéóùXV:)®"ý¬&yúÔÖÖÅ···³¬t¸¾øây¹Û¼yóÐÐË*S¥x²tuuUTT¨«._¾Ì²Òá:M&òË^ýõ'NÄÏ9tèÐ+¯¼2::*«yáÂ,"®£_M¥¥¥·oß~øè|n:yMdYGË-ëèèxøè¼;wîdeªO¦¦&Yq2!ënëÖ­,+®#  Pþ²B~YÐ­[·V­Z5f¦p^^Y8z^Gã^EzXMòÓÝ»weBþç(®#Á7oK)S¥x²8FGGebdd¿ ô¹Ö®]ë÷û_v´qãÆÎÎÎÄÂcÇÉª½~ý:KIëhÜ«H«©««KGòj(ÿ³¦ô¹êêênÜ¸!o¼ñF¼)Í¥x²Ä¯Ö>×ÑoÈüôÅóóòòN>ýð«=`XP:©¯"¬¦eË©ásyÅdeésÉ·ÕÕÕòNöê«¯Êÿ,¨LâÉ¿kfAAËJëùeM:vìXâü²²2þÀÒù:Jéd51VO%ÕÍ7kkkYP*ÅEÞFFF>ÚÚÿöDúYGÈ/kZ¹rewwwâü;w¾õÖ[2qãÆ³ÙÌÒá:Jéd5ÉÅjKboo¯ü¹ÌÒá:Z´hQWW×èèè«¯¾úòË/³ 2U'Ífíµ×dBþojjbYép!¿¬©  @í6;fµmÜ¸QDßÐÐà÷ûYP:WÞVÓÍ7åõQJò¿L³ t¸|>ßäÚæææh4ÊÊTOµÚÛÛËËËóòò***Ô±Ioëùò#""""äGDDDDÈ!?""""äGDDDDÈ!?""""B~DDDDüùò#""""äGDDDDÈùò#""""äGDDDDÈè1zã7V®ô¨U«V½ýöÛÿçõëQYój«õhM&ühápxÌ|c0***FGG'DDÈ²¯ÎIèå_Î%ùµ´´ÈÌ'Oüøqÿâ/Ná>eY]]]bÁpêÔ©è£N>-efwwwÎÈïÖ­[2³®®nÌüÚÚZ!?"Êýyæ1Í+¯¼?óÈ#2sÛ¶mñî¹|ù²ÈIP¸téRñýû÷KJJäªÒÒÒÝ»wÇoTõz½¢+¹JnñâÅ19óæÍ[¹råä¢ÍfóÀÚÚÚRßºJºêÒ¥KÉ¶zõjßÑÑsõêUc±Xbs:T^^.wUTT´yóæ»wï&Ê/ñþÇÌIñPùe²Znß¾?óÎ;2Ód2ÅËfLêÚ6¹êùçWWõööæååiÞJ]T×nÜ¸qttTìXPPFåZù_äT\¬v¿Kq?2¡ùðÒ7ß|3³1þúë1ö¹U«VMV~)*!?"¢§6ìj¼fÍ#ÍÖ­[<J&äâºuëÔµ:ÊjèNÄ¦®jjjR2ÝÞÞ®î$þ>8 ¶S[·oß.sÞyçÿeú¹ç÷~DrQ¾ þ±iþD¢ÉùóçËÏ;88(åyäÂÍõrCù~µu¸¨¨h²òKñPùe^~¦üból¤»wïÊE1º¸téR¹¸páÂ;vØcw"_3f-v+uñÎ;±/-Å6øÊÿ2íõzÇ½Q¦lÉöÉáäªW_õáW[´wïÞÿ¢@x´¡¡AINV~)*!?"¢§º<x?3ËL¹*ub.ôûý1èÄvÎKÜ;ÆRñÇRéÒÒÒH$"w>oÞ¼ØµãÞO2éÆ©2-ÿËtoooìÚöövy['.¿QSûº;v,~¦:ÖÉOxÄöLÊêîîniiQÛ^cdTr±Í©ãúìùçWyåÿæææØü÷£ÆØ¾üòKuq`` õçpW¬X!×ªÙXã¯Ró«.]º444Z~1ª¥»6ÅC%"äGDáÔ'$Ã'ÔQ]N:UPPøiuëÖr"Úû®ÚÏOíç÷ûãwTÜ½·8Iê6vM©ÛªïîóùbóSÜÚ±OíçÕW¦ßéÓ§c£qò#Ç_¥vyìííï"!ü5ÅÊòe"ÎÍ7Ç_â¡ò#"Ê|ê(Çc:tèÐÿ¾~=JÁ(6ÛNûPE¬íÛ·Ç³2¾sçÎ¥ßÃG'Ûù¥¥¥ñSÜ<øû²vxx¸¨¨Hýc¶q¯]»6þ[Ì?_þWv¿O5$K95vmJDÈHNV­ZUð¨+WªØß¥KÔaó/^|åÊØµápxß¾åååJK/¼ðB$]ñâÅYEEÛísDîJæïÜ¹sÌüd÷#É¤4âx~±ÇÞE588ØÔÔ$K ¤¤D~@ ;BMü	mÕ²²X,c¾cJDÈ!?""""B~DDDDüùò#""""äGDDDDÈò#""""äGDDDDÈ!?""""B~DDDDühòýÂÕåö#°4IEND®B`
