# Supplementary material for: Regulation of piglet T-cell immune responses by thioredoxin peroxidase from Cysticercus cellulosae excretory-secretory antigens
Source: Front Microbiol. 2022 Nov 18;13:1019810. doi: 10.3389/fmicb.2022.1019810 (PMC9718028; doi:10.3389/fmicb.2022.1019810)
Supplement: Supplementary file 3 [file Data_Sheet_3.ZIP › 4. C. Cellulosae ESAs and TPx Induced Th Subpopulation Differentiation/3. SPSS statistical analysis/1. IFN-γ/3. IFN-γ--72h/3.3 (SPSS data export) SPSS statistical analysis--IFN-γ--72h.doc]

EXAMINE VARIABLES=Figures BY Variables
  /PLOT BOXPLOT NPPLOT
  /COMPARE GROUPS
  /STATISTICS DESCRIPTIVES
  /CINTERVAL 95
  /MISSING LISTWISE
  /NOTOTAL.


Explore


Notes	
Output Created	12-SEP-2022 22:31:32	
Comments		
Input	Data	E:\桌面\Raw Data\4. C. Cellulosae ESAs and TPx Induced Th Subpopulation Differentiation\3. SPSS statistical analysis\1. IFN-γ\3.  IFN-γ--72h\3.1 SPSS statistical analysis--IFN-γ--72h.sav	
	Active Dataset	DataSet1	
	Filter	<none>	
	Weight	<none>	
	Split File	<none>	
	N of Rows in Working Data File	20	
Missing Value Handling	Definition of Missing	User-defined missing values for dependent variables are treated as missing.	
	Cases Used	Statistics are based on cases with no missing values for any dependent variable or factor used.	
Syntax	EXAMINE VARIABLES=Figures BY Variables
  /PLOT BOXPLOT NPPLOT
  /COMPARE GROUPS
  /STATISTICS DESCRIPTIVES
  /CINTERVAL 95
  /MISSING LISTWISE
  /NOTOTAL.	
Resources	Processor Time	00:00:01.37	
	Elapsed Time	00:00:00.99	


Variables


Case Processing Summary	
	Variables	Cases	
		Valid	Missing	Total	
		N	Percent	N	Percent	N	Percent	
Figures	Control	4	100.0%	0	0.0%	4	100.0%	
	ESAs	4	100.0%	0	0.0%	4	100.0%	
	TPx	4	100.0%	0	0.0%	4	100.0%	
	LPS	4	100.0%	0	0.0%	4	100.0%	


Descriptives	
	Variables	Statistic	Std. Error	
Figures	Control	Mean	8.55525	.105183	
		95% Confidence Interval for Mean	Lower Bound	8.22051		
			Upper Bound	8.88999		
		5% Trimmed Mean	8.55267		
		Median	8.53200		
		Variance	.044		
		Std. Deviation	.210365		
		Minimum	8.324		
		Maximum	8.833		
		Range	.509		
		Interquartile Range	.393		
		Skewness	.642	1.014	
		Kurtosis	1.505	2.619	
	ESAs	Mean	8.66325	.072116	
		95% Confidence Interval for Mean	Lower Bound	8.43374		
			Upper Bound	8.89276		
		5% Trimmed Mean	8.66111		
		Median	8.64400		
		Variance	.021		
		Std. Deviation	.144232		
		Minimum	8.509		
		Maximum	8.856		
		Range	.347		
		Interquartile Range	.270		
		Skewness	.763	1.014	
		Kurtosis	1.509	2.619	
	TPx	Mean	7.50800	.088978	
		95% Confidence Interval for Mean	Lower Bound	7.22483		
			Upper Bound	7.79117		
		5% Trimmed Mean	7.51228		
		Median	7.54650		
		Variance	.032		
		Std. Deviation	.177957		
		Minimum	7.262		
		Maximum	7.677		
		Range	.415		
		Interquartile Range	.331		
		Skewness	-1.136	1.014	
		Kurtosis	1.500	2.619	
	LPS	Mean	11.08450	.356766	
		95% Confidence Interval for Mean	Lower Bound	9.94911		
			Upper Bound	12.21989		
		5% Trimmed Mean	11.09561		
		Median	11.18450		
		Variance	.509		
		Std. Deviation	.713532		
		Minimum	10.128		
		Maximum	11.841		
		Range	1.713		
		Interquartile Range	1.335		
		Skewness	-.797	1.014	
		Kurtosis	1.496	2.619	


Tests of Normality	
	Variables	Kolmogorov-Smirnova	Shapiro-Wilk	
		Statistic	df	Sig.	Statistic	df	Sig.	
Figures	Control	.250	4	.	.963	4	.795	
	ESAs	.251	4	.	.960	4	.780	
	TPx	.250	4	.	.935	4	.623	
	LPS	.250	4	.	.960	4	.777	

a. Lilliefors Significance Correction	


Figures


Normal Q-Q Plots


ÀÄ0É(?Êåò@ù üP~(?åò@ù üP~(? 7ÏçöîÝqýß±¡.=pàÀ+Ên¨®®~íµ×nzÃ¿Ê¤EEEáë/^üâ/ÞÆà8q¢¦¦æ6~ü£G._¾¼¸¸8|÷A·0iyòïå7mÚ´óçÏJù=óÌ3éÝ³ûö,·vKW*­öìÙs«sÛÃ8öìä÷­¯¯W~òrXÁÚµk¢üN8¥ê«¯¾H$ÂBø4¬ìììÌxS·zAßúòåËO>ùdXjlÔÊ/ºâ~¯_¿W¿@ù]~áãÊýìg6l6nÜ¸±»»ÐW¶µµM>ýîI®9|øð%KW¬XñÁ=ztñâÅ%%%+W®L_Ü±cGEEE´Y3|äECõÍ<Ö§nöîÝVnÚ´)ãÏx«WIÿÖ!Ãp'oip2ÎÉ¥º½+f/¿AßË¢EÂ°×ÖÖ±Í>;~iê¯5hoo·Æ!üfÃEÉ+B#b:^^¾eËþþ~O.P~@ßåËgÏ=kÖ¬°çÎoç©-¾rP¢ëÖ­û(ÓæÈåË¸I~*$¾òÞïÍÞ7sæÌ	ë/º2eX¹`Á?ã­^eÐ·YÍù-ð'ÀÝö_~'OL½ðÃÞjù¥þZ;;;£ON8ñÚµkÝçÇÌ§å::<òHX^½zõ7ðiXúO?ýôÀÀ@4k­yôÑGÄ±cÇ¢OÃ-OÛÚÚÂr¨ÀèºÑÞlo¿ývX>sæLêECõMqqqÆõ©×ýWjGºäè­NÆuÛWÌ²ß +bË7n¼zõjhúd¿üR­ávÂ#Gåè×zÿý÷G_áÙ³gÃrømå)S¦xròò·üðFÃú õ³fÍËçÎ>oðáÓ3g¦ÞBòÒäP©^¼xñ£ÿÜl×¯_­cÙ²eÑ$ÓMË¯¤¤$ãqýmµ¨ÂuCÄÔÔÔ´··g´áNÆuÛW~ùEû2~ðÁ©ßâÊ/õ×ÝZª°&ºhéÒ¥áÓyóærÿ¸víg(? ßË/ÄYyyyxÿÇã©ëÓçÌÒwzIñ6³*sÐÏ£ZQQmNÏ»dºµá¨1¹é gpêÑÛ»âð·ö§í²_þktÊè¢X,Å_²[[[=¹@ùy]~Áo¼>M%ZÅÙ Ù©°r8ýÓEEgW9|øp(³á_41`eãÆ[¶lùðÃ_zé¥°rÃËo8W¹íò»ÕÁIºí+¿ü3çl»ÐýÙÇÊ)aÍ S:uª©©)ÚÄ<P~@þßG7öÖOîÈ­ü]½zõå¢·öGôã_4ïÕÙÙúãÅ_NùuttD³J!;ÍçE:ñg¼Õ«ÜRùeh$ûot#·:ª·Q~Ñy!|¯]»ÂnÕªUé]øüóÏÁu"8ûøG÷0tsøú0ayÉ%©?f´¿f,Ë²åP~@~ßùóçtEkº»»íã5kÖ¬ä¡²§ü-9sføz|kÆûüÐC¥oy¼ûî»³ü·t[*¿ì<!ó ³1ßÆ¨ÞFù:u*õöï¹çÔKIhµìå1ã!/Ñ¬jª~ØPåD[BS×¿ÿþû6l(¹aãÆéÇsÜ^ù]¼x1ÜZ¸ÍÐ@O<ñD¡ä	A²÷ÍîÝ»/^gÎo~óÿüçsNÿ*·T~ÙçÐ¡C!þ§ÄKuK£zå´¶¶Î7/üÔáö£íéÉÃÃ§¡Ï¢ï^SSsüøñÎ¹¶µµ-[¶,ÜZø¡öíÛßßÿÔSOE©!ßÃ¯2rò×¯_è¡nér·qmÖN$QÐ/Z´ÈP~ãM´ß Ï>û¬ÀxÓßßÿäOÎ3'Ú¤yæÃ(?ò@ù üP~(?Êåò@ù(?Êh:TYYY\|ï½÷Þêu/^<iÒ¤X,Ã%KÜÂÖ·÷5Ã¹îm»xñâ£>:úô08sæÌyê©§úûûGéE|$.@ùWEEEP9DâV¯»cÇpÝ]»v%×<÷ÜsaMSSS¡_¼79sf``àW^	ßhÕªU·qS'N¨©©Q~òòàõâcDÆ©S§ÂuW¬X|ùò°æäÉ£s'G®¢¨=pàÀ°ò0RÙÚO=õÔ´iÓ¦L²ûöA_öùÏ~Ù²en!9e/Ãè¢³gÏÖÔÔ/]ºôÈ#o-õ»g¿Ê7¿ùÍÊÊÊêêêðÒéêÕ«õõõáºáÎ?ùäÑfhÓî¹'ÜZXnùÜ¹sÃ%K[Çã/Í2JáNA8tèPú§dòø0"ñ-ík_ËûöíöÙÔ¯ikk/Õ#<.zé¥Ârø7mÚ]t÷Ýw¿ýöÛaáÌ3aýìÙ³3ÞZêÈ~Ðso¼ñFXhhhH¿ó=öXX_ðæo;vsçÎË/_îììÑdºôa	±8Te¥ç>ú^¡ÿÒïä =ûMypÊÁò±¯ß^wìèè­^½:,¯]»6Êä¥§Nzúé§-[Öe¼µAý*Ñ6mZúugÍÝù°/¬_.]Òj¨	¼²_öQºvíZ´na¨òKþìÙoÊP~À_jîÕ.Ì2eJøÊþþþh£jX]ôâ/k|ï½÷Òë'ãÎUºc!S§ñ¢j<pàÀôéÓ£5åååÑtàpæü.Ö_½z5f/¼ìË·7àÊÈMùÍ=;u*6»iÔ××KëKJJÂY~Ù¯¼c!æÒ¯;sæÌèºî^"xûí·R'oêé§_J1ý¢áÒ0Ëïö@ù9(¿èÖ^z)Úíì¹çN8p 9yöÆoÊÓ§OGûÿ§ü²_%ÔØ·¾õ­°ðÈ#¤_÷áË¯¼òJWWW´7¬¬®®Ëï½÷Þ|.Ìa¹|ùrè°=6Ôd4¹nÝºaRêr´Å9ÚÖ<èg¿½P~@Ê/HºrCê9ù²Èµk×¢-­ác¦äúÖÖÖòòòÐ=O>ùä0Ë/ûUÚÛÛÃ¥Ë/Ohú5ýýý[¶l	÷¼¤¤dÕªUÑa¼!ø6nÜ,¼bÅ÷ßø#¾K¨Éh[vHÀíÛ·GûðgR:îRt¢ìA?ûí8òP~(?Êåò»5ùÙÓÓ3ÊßôÜ¹sÿò/ÿâqCn]ºtéþé¹õ¯ÿú¯gÏ5äÖÀÀÀOúSã üÆÀýÑøåoú×ý×|ðÇ¹õ³ýìïÿþï¹uñâÅ¿ú«¿2äÖ¿ýÛ¿½ýöÛÆAù)?P~(?ÊOùòCù¡üP~ÊÊåòS~(?P~(?òCùòCù¡üÊÊå§üP~(?P~(?åòCùòS~ÊOù¡üP~ üòS~(?Êå§ü@ù¡üP~(?åÊåòCù)?P~(?ÊOù¡ü@ù¡üP~ÊåÊåÇ8,¿ãÇ/]º´¸¸xÉ%'OT~(?P~(?Æmù-X°àwÞ	¯½öÚÂÓË/<ø>]?üáÃôSï¾û®q ·~þó=zÔ8[áo½õqÈg ©ÊÊÊÒËoÏ=?]ögÖÑÑñCÈ©ïÿûíííÆÜúÁ~ÐÖÖfÈ­cÇµ¶¶|6ÊïÄ6m²µ[ÁÖ^líeÜnízuãÆýýýÊåÊåÇx.¿óçÏ744d-åòåòcü_GGÇÊ+ÃWÆKÊÊñS~R(?(?ã¶ü²S~(?P~(?òCùòCù¡üÊåÊOù)?åòCùòS~ÊOù¡üP~ üòåòCù¡ü(?Êå§ü@ù¡üP~(?åòåòCù)?(?ÊOù¡ü@ù¡üP~ÊåòS~(?òCù¡ü@ù)?å§üP~(?P~ÊOù)?ÊòS~ üP~(?òåòCù¡ü(?Êå§üP~ üP~(?åòåòCù)?(?ÊOù¡üP~ÊåòS~(?(?å§üÊåÊOù)?åòCùòS~ÊÊåòS~ üP~(?òåòCù¡üÊÊå§üP~ üP~cª«««±±qýúõÊOùòCù¡üÆ­¶¶¶ÚÚÚÉ'Oº¡§§Gù)?P~(?ß¸ÒÛÛbcîÜ¹þ»mÛ¶)?åÊåòDr/©´´ô¾ûîýüP~ÊåÊåc½½½ÍÍÍóçÏæ®»îÚ¹sç¥KýgT~Êåò	]~Y&ùÂ°>¾f|ü°ÊOù¡üP~0AË/Ë$_UUÕÞ½Ã³ß òS~(?L¬ò&ùêêê2îÉ×ÐÐÞîÇëoPù)?Ê&JùEëVVVfäÛ·oß+WÆ÷oPù)?ÊÆyùeä«¯¯/èÃuòCùòCùý»ì|ÍÍÍã~Où)?ÊÆyùäS~Êåòñ_~Y&ùæÏ?'ùòCù¡ü`¼_"hiiÉ8É7þÎÉ§üÊ´üº»»³Lò¿sò)?åòåÇÄ*?|ÊOù¡ü@ù1þËÏ$òS~(?P~óòË>ÉÖäS~Êåò/¿,ëá"|ÊOù¡üP~ÊÂ.¿,çä3É§üÊã¤ü²LòUTT455äS~ÊåÊÂ.¿Nòµ´´ô÷÷û-(?åòåG_ooossóüùóÓ'ùÊËËzzz¾òS~(?P~pùE|õõõé|Á5kZ[[íÉ§üÊ]~Y&ù***¶nÝÚÙÙi´òCùò£Ë/û$_mmmkkk<7ÎÊOù¡ü@ùQÀåwåÊ¡&ù*++c±áU~ÊåÊÂ.¿ðf]___ZZÞ|+V¬hii±'òS~(?P~vùE|UUU÷ä3É§üÊã¡üÂeCCÃP|¯¿þºsò)?åòS~(?»üúúúöîÝq¯¼¼|ëÖ­ÝÝÝPù)?P~(?»üÂÛqCCÃÔ©SÓ¯¦¦fÿþý×U~ÊÊÂ.¿K.íÞ½;ãáº3fÌØ¼ysWWS~ÊÊÂ.¿M6eä«®®6É§ü(?ïÊ+öìùÌg>3Ô$Ãuòåò£àÇÒPçä«®®Þ·o_B£¤ü(?,H¼üòËwÝu×P|ámÑ()?åÊåGaëêêaWQQÞ|.4É§ü(?¯¿¿¿¥¥¥¶¶6=øJKK~ô£ÝÞßíEù)?P~(?òEww÷ã?>cÆôæ«ªªJNòÝÆßíEù)?P~(?òB<ýõ×«««'OqoÐ|ÊOù)?P~(?O,Û¶mÛM'ùQ~ÊOùòCùQ0âñøþýû«««Ú/ûáºÊOù)?P~(?@,Û¼yóP|ÍÍÍÃ9ù)?åÊåGþÇã---555'ùêëëoéÍTù)?åÊåG>ÅbsOù)?å§üP~(?òWn'ùòíW®ÊÊJåòåÇMuwwç|Où)¿ÑsìØ±EÇ«òCùòc(D¢¥¥¥®®.ã9ù>æ$òS~£gÕªU±X,Kù½øâ=º¾÷½ïçÏ_CNuttüùÿ¹q ·~øÃ>|Ø8o|è¡fÍ>É÷Ï|æË_þrxyÉíw<qâDkk«Ïg ÿq.¿oûÛ®ï¿ÿþSï½÷ÞÉ'¹þçüüÀ8KçÎÛ·oßg?ûÙôI¾/|ámmm#ô­Ï?ÿÖ[oùä³ñ ¶öbk/ØÚ;¡DòUVVÞÒÞÈ![míU~ üP~¬D"ÑÞÞ>Ô|7ýÃÊå§üP~(?@ooox¿ÃI>å§üòCù¡üYDâàÁù0É§ü_¾P~(?P~ãO__ßÎ;çÎ>ÉW]]=ú|ÊOù)?åòCùuuu¥¥¥oÆ7o«I>å§üòCù¡üÈ¾¾¾]»våí$òS~ÊOù¡üP~ä@ýÔ©SGúo(?òCù¡üMMMóçÏOä+wíÚuéÒ¥¼½ó#]~½½½aüñð±§§Ç£Eù)?(¿ï¾ûîKßoòäÉÑ$_ø<ÿ)F´üÂ#öùí×~í·~ë·/_ósâSù)?P~(?Íce<'ßÜ¹sÃÊÏ2råÇï¸ãûï»û·Gÿî¿ÿþyµ£òS~ üP~)üî»ï¾ôsò5ë×¯oooÏÿI¾Q+¿0VÿÅÉìþÝyçHÊOù¡ü@ùå¯è|÷ä+¸I¾Q+¿ÖÖÖå¿º|Pùýê¯þêÁ=¢òCùòËGaÌ×¯_q¯¡¡¡£££à&ùF­üº»»§Lò¥-_Jfß[9sfWWÇòS~(?P~y¤···±±q¨¿®;SÑ#<~ÿ÷ÿüÿñú?!ûÂÇèR~ÊåÊ//ÄãñfÜ¯´´týúõãïÐÔ>«Ë/¼ïøDHÀ?þã?.ô)Rå§ü@ù¡üÆX,ÖØØ8cÆçäkjj*Ü=ùÆ¶üP~ÊåÊ/_ÄãñI¬Y³fìÉ§üòS~(?ßD×ÝÝ=Ô$_UUUSSS__ßDå7qËoÒÍ)?P~(¿H$ZZZêêê2®ß÷<xpBí¦ü&nùÝLqq±òåò+PÑ$_ÆÃuçÏ_ÐçäS~Ê¯ )?(¿Ë>ÉW___xCù1âåwùòåGyDùòCùÞÞÞðÎq/¬MÌI>å§ü2OââbûùòCùD"ÑÞÞ>Ô$_X?Á'ùòlÉ%éÿ=*//ïïïW~ üP~yË$òS~·£¤¤$<IÂKLEEEXÁ÷­o+,lÚ´IùòCùå|ÊOù¼¸!,ÔgÎS¦LQ~ üP~ù#û$_cccww·QR~Êï&¦O3ÇÅbaágÕÊ/D|õõõCMòµ´´äS~Êo¸|òÉäñ©O§êêjåÊå7zçÏoOù³ò¾úÕ¯Î93,?~<,lÙHßuåòåI>åÇÈßP~(?P~dä1cI>åòS~(?ßxÞ2Nò555---ñxÜ£Bùò[¸patngråòMW®®ªªj/y0(?rY~,H­½$ÇöòCùh¯´´4½ùª««_~ùe|Ê)¿yáivâÄÑ¼ëÊå°ü²LòoÝºµ««Ëo_ù1åiáù6ÊÙ§üP~0ÑÊ/¼744dä»ë®»^ýuë*?F£üN<u=öØÕ«W(?_n]¹reß¾CíÉ·yóæÎÎN¿nåÇè_0gÎô'¤#<@ù¡ü>æKîP|öäS~YùÍ7Ï üP~¹Ï|Ê1.¿èÙ8úíÊåã¬ü²ïÉ·wïÞ~³Ê1.¿Y³f9ÂÊï¶E|ÕÕÕöäS~@ùutt'çO>9Êû[(?zùÕv!ï2Nòíß¿ß|Ê¼+¿ICp(?_F¡çZZZjjjÒß;¦Núà[§hQ~äiùÁ üP~Äb±­[·F'µ'ò£ Ëo¬(?Jù%'ù&O¾'ßã?nO>åGÁ_eeå¼yóFÿåòü/¿wßwÓ¦MS§NMä«ªªÚµkI>åG_qqqxþ]W~(?ÈÛòÇãûöíË¸'_iiiCCCx9õ;R~dù9r$<._¾<çvQ~(?ÈÃòëêêÚºukÆÃu«ªªBäS~vù9¶Ê/·¶¶ÖÖÖ¦ïÉgOù1®ÊÏ±½ üÈå×ÝÝ½mÛ¶|ÕÕÕ&ùã­üÆòCùÁ_"xýõ×kkkÓÿØZô7Lò)?òåGÁ_WW×¶mÛ2Ï$òcBßõë×W­ZUVVöS¦LY·nÝ(ê¡üP~0åH$ÚÏ$òcßµk×2á1ÒlQù¡ü`tÊ¯··wçÎ&ùP~Êï£E'ÿêÕ«¯^½>½|ùòÚµkÃ»ï¾[ùò£pË/H´µµÕÕÕÙå§üþKIIIxHýÓÚ×¯_kÂzåÊB,¿îîîÆÆF|(?åAQQQx-µÇÃguåGa_ø?|KKK]]ÝPòÅb1«üßD/¿hkïÊ+£­½ácXk.]ªü@ùQåoÿþý#½ë6Ê)¿zðøðÃ(¿æ^¨©©©ªªúÂ¾ÐÕÕçåzÎ$ÊOùÝ²k×®­[·nÚ´iEEEáãÊ+Ã¾ëÊåG^I$!¡þç¼ÿùÛüöæßÛüÙÏ~¶¼¼¼³³3?Ë/$]ccãPxÃ$ÊOùååò#¯<xðÓþôSÿßSÛ¿¼=úWûµáÂyU~Ñ$_MMMzðäCù)?å§üP~×¿üåÿõ¹ÿÌ¾ð/TàÔ©Só¤ü²Oò9å§ünvÍ)**R~ ü&¯|å+«ýùmbk(­±-¿Nò9'ÊOùKÑÐ(¿	(¼"òüÒ/%Ëï×WüúÆêþüèG?zðÁMò¡üÈMùå'^Y^í5åÊoBÙ²eË§>õ©5«×|±þÕ¿V]YYÙ××7Ê÷!Ë$_iiiCCI>òË'ON2%¼¸WÔ;+?P~ÇÁû·»¶¶öþàzGó[Çb±Í7gä«ªª2Éò#å·aÃèõeÔÊåÝäÛ¿uuuzðâ0Éò#Çå÷æoF/1«W¯Í»®üP~Lpï¾ûnI¾]»vµ··%9+¿k×®Ý÷ÝÑÁGå»®üP~LLñxüõ×_¿é|·ñwAù)¿!=ÿüóÑÍý÷ß?&w]ù¡ühb±ØÖ­[+**2Nò577§îÉ§üP~ä²üÏ£#H¼þúëµµµ¥¥¥é|õõõ_Ê_ÑÍ+?P~|===Y&ùvïÞåp]åò#å7æÊñ*H´¶¶®_¿~òäÉé|kÖ¬ikk_ýFÊå§üP~äµæææªªªôI¾ÊÊÊ;wÿÔÐÊåòS~(?òTÆI¾°&¬/wñxünPù¡üP~ÊåG~éëëkjj?~ú$ßÜ¹s·mÛÖÓÓs·¬üP~(?åò#_W°ºººôÃuÚÚÚÖÖÖîÉ§üP~(?åò#¯õõõíÚµ+ã|Ñ$_,ËÉ7R~(?rY~ÎçÊ[ÒÖÖöàfä[³fÍÁ?æ$òCù1åzê¾åç|~ ü.]º4Ôáº¹äS~(?F°ü>^¿xà«W¯OÃÇµk×5GU~ ü&¬D"ÑÞÞ¾fÍ©S§¦7_MMMKKKn'ùÊÑ(¿òòòð*úúuýúõ°¦¢¢âcÞòÉ'gÏ]\¼xñâ#G(?¡··7¼@UVVò$òCù1å½ÚT~?¿7¾òÊ+aaÏ=÷ßzùïßë]áeôþáú!§ºººþöoÿÖ8º+W®|ç;ßùÜç>~N¾à×ý×ÿäOþ$|Í¨ÝüÇ<vìß¹õÏÿüÏ¡üC>ñò5kVxQvíÚµðiø«W¯kÂú?800¥døßszùíÚµëÏG×w¿ûÝ9õg7Âõ­oëá=vzðÝqÇá?®ú§:ú÷ª½½=¼Cûísá8!xù=z4ãï½÷ÞÇ¼åÔcDÒ±µ[[Ñ|õõõÿðF]]ÝHïÉgk/¶ö2[3gÎ,X° ¬¬¬¨¨hÊ)K.½páÂÇ¿ÙÔíÅ%%%ÊåGèíímnnÎø7*++»»»ÇüN*?#U~#dÖ¬YÑîácú¶cåòcô|£s¸®òCù1Ë/¼¼~óßáãÆÊ±råÊ¡ÎÉ7cÆÆÆÆÑ9ù¡üÈò;úôÂ§Lm5kÖ>þÍ;v¬¢¢"ÜæìÙ³?®üP~¾h/ãÞ&ùâñx~ÞsåòcDÊ/:sê_l÷ìÙ3¢w]ù¡ü98É§üP~FùUTTÂÓ§O'ËïøñãayÚ´iÊ_ÁÉ2ÉW]]½ÿþ¼äS~(?F£ü¢Äh!*¿·_a¹råÊ¾ûäÛ¼ysþOò)?£Q~Ñ£y¾P~×¯_ê©§¢S(?P~ù/<¯ÆÁ$òCù1å×ÑÑñLÎï¼óòå·Æß$òCù1åZ¶lYtloYYÙÂGá,¦ÊåÇm?Çå$òCù1Jå7&Ê[rÓI¾ðO?¯òCù1"å<°#éÂ.;w®òåÂ37]È»|!C¿Zù¡ü¥ò»~ýºcAù¹h/´]zðÖ××þkòCùQ¨å7wîÜIYM>]ùòY&ùªªªvïÞéÒ¥q?ÊåG.ËïìÙ³E7$ÿzGªo¾ù¦òå7âñøþýû³Oò%	2ÊåG.Ë/)tÞHoØU~(?²Åb¿û»¿þÏÞ|wÞygssó¸ÜOù¡üò+ÊåG4É·téÒ¡&ù:::&Î$òCù1åwýúõN2%¹fæÌ_ûÚ×(¿ÅÚï~á~ï÷~¯¯¯oòCù1"å7gÎA÷F/¾MMMÊ_neÙoòäÉ-úbýÿ÷ÿ½lÙ2c¥üP~Hù×ÜÎÎÎä'O5Ó¦MS~ ür%566fäûÅ_üÅß_Úò¥í_Þý+++3bÊåÇ_txïÀÀÀ»Ý´ü)?P~·!·´´ÔÔÔµ'_]]ÝêßZl¾ðïñÇ¿ã;òCù1"å7öìð¼eËè/]^»vmûöíaMEEòåwÛz2Nò¥®ÛÚÚú©O*uÂïWýÊC=dÊ)¿'Nd<óñãÇ(¿ÛÐÖÖVSS3yòäa®þçùÉO~rågWÖÕÕÍ?ÿî»ïçpQ~(?F©üóçÏ/^¼¸¬¬¬¨¨hÊ)-kFú®+?ß8ÅBºõ7²®^¾ò¯<þøã/¿ür´ýåòc¤ÊoL(?ßøH$ZZZÖ¯_>É7cÆÍ7çòCù¡üÊ¯°uwwïÜ¹³¢¢"¯¦¦&ä Ù;åò#ËïôéÓÑÉ£ãygÍuàÀåÊ/£D"qðàÁ5kÖdäkllÅbÊåGßáÃ¯ÚQùEËöìQ~ üRõôôgnyyyú$_uuõþýûMò)?ù^~ÑÓ§O'ËïøñãÎäÊ/U[[[mmíPòäS~(?¦ü¢ïRÎÞ<00LðòëëëÛ¹sçwÞiOù¡ü'å7kÖ¬èìQù]¿~ý©§ËÊ&lùµ··ßwß&ùÊñV~ÏäüÎ;ï(?hå×ÛÛ»k×®çä«®®Þ·os,+?]~A¡eËEÇö-°»»¤ïºòCùå¶¶¶,|ÎÉ§üP~òÊåzzzvíÚ5wî|ÊåòS~0>Ë/H´··×××äS~(?&÷­yóæý²²²ÅWåã²üzúëº&ùÊq^~áÿýðèêêR~0nÊ/Ë$_iiiCCI>åòcB_t&ç7ö÷÷O¯^½zÿý÷5³gÏV~0ÊÏ$òå§üRnâD":7<«³ò-¿h¯®®Î$òå§üþK4çwýúõäk×®óÂ-¿ÞÞÞðüª¬¬4É§ü@ù)¿Á¢ýü6nÜ/|úá®X±Â~~Ppå¯¾¾~ôq(?yW~nf6û*?_®däknn6É§ü@ù)¿ÿPt3ÅÅÅÊò°üLò)?P~Ê¯`(?ßmË2É7þ||ÊòËöâqýùóçäUù%|aMêqú(?P~Ê/í&&MúêW¿:hå>è¬.?å×ÝÝÝØØe¯··×/BùòS~7/¼yÌ5ëÂáÓW_5z;¡ÝûÊoøLò)?ãò#Çå÷Ñ¾è½döìÙÑÂÚµkGú®+?_===»víN·iOùò#å=z4ù¾²wïÞQ¸ëÊå.H´µµÕÖÖfä«««3É§ü@ù)¿ëÑGÞZ<ðòÑ,¿ÞÞÞ]»vÍ;7¯²²2<_Lò)?P~ä üÃ[Ë´iÓN:õQÊ~~%%%ÊF¡ü:::êêêJKKÓ¯¶¶Ö$òåG.Ë/¼»<ýôÓV6448¶F´üúúúöîÝ;þüôà«¨¨OX,flq@ùãòê|~#]HÊ	[~áí¼¾¾>oòäÉµµµ/¿ür¿QEù¡üò+ÊV~.]Ú¹sgÆI¾òòòÆÆÆòCù1"åÞlR·çfÿTùÁÇ)¿wß7ã$_PSSÓÞÞÇ#ÊåòS~pù]¹re÷îÝ'ù*++»ººÊåòS~vùuvvnÞ¼yêÔ©÷äkii1ÉòCù¡ü]~W®®ªªJä1c=ùP~(?òc<ß·¿ýíM6§7_]]][[[BòCù¡ü,ôÜ®]»>ýéOg<'_hAçäCù¡üP~Ê½C®[]]ÝÒÒâo üP~äWùe§ü ]=ùÊËË·mÛÖÙÙiP~(?ò®ün¦¸¸XùAê§¡¡!ã$ßòåËwìØáo üP~äoù9åGA¸råÊ¾û:óæÍ]]]·÷wAù¡üP~Ê|e¯ººzÿþýÉsò)?Êå§üø÷¿TÛÞÞÞÖÖå>ßt/ýp]åòCù¡üßD÷Â/L>ýWýÊ]wÝ5uêÔo|ãy~ÃÃ#]È»|!:'òCù¡üP~ÊoBkkkûä'?ùÈï>²ýËÛÃ¿Mÿï¿å8ÞÕh/´ÝP|7½ÛÊåòCù)¿	ísûÜúuë£ìþýæÊßÜ´iS^ÝÉÛäS~(?ÊOùñ_ªªª6ÿÞæÔòûbýkjjòá¾eä+--mhh¸Õ¹IåòCù¡üßs~±Xl¨I¾ªÃäS~(?ÊOùñ_òj?¿x<¾ÿþNò)?Êå§üøo^xá©S§FÇöþÒ/ýÒÛ;B|ÊåòCù)?»téRÛ£ùGzOù¡üP~(?åÇØË>É×ÜÜI>åòCù¡üc&·´´ÔÔÔdä«¯¯ÑòCù¡üP~ÊÑÅ3NòÝyç»wïI>åòCù¡ü£'û$ß÷Ý×ÑÑH$FçÎ(?Êå§üÝÝÝCMòÍ;·¹¹y&Q~(?ÊOùcD¢¥¥¥®®nòäÉ÷äkkkµI>åòCù¡ü2¿rUVV*?>h/<Ò'ùæÏ¿k×®ÑäS~(?Êo°cÇ-Z´(¼=+?nCI¾°fýúõc8É§üP~(?ß`«V­ÅbYÊïßøÆß®ïÿûÇÿ[ò[xUúßù;î¸#ã$ß¶mÛÚÛÛóêÿÃüÅ_ü_¹uâÄïïÆÜúñÜÚÚjòYßÜÅ¡Ë¯¥¥¥gt·ç¿û»¿ë!/ýüç?õÕWã7~£¨¨hPð5k×®ýÌ0<yòG?úß ¹õü¤££Ã8[gÎyë­·C>Ïågk/ÞÞÞðxÈ¸'ßÜ¹swîÜyéÒ¥|¾ÿ¶öbk/¶ö2A·ö&ß°ÃÞØ:Á<ÙOù¡üP~(¿á¶ òc¾¾¾]»vÍ;w¨Ãuó|Où¡üP~(?åÇM$¶¶¶5kÖf<½½½ &ùÊåò»5ÊoBéîînjjÊ¸'ß]wÝUp|ÊåòCù)?Çã¬©©IßoÆ7o¿qðc*?Êå§ü&´îîî;w§OòUWWïÛ·ïÊ+ãæU~(?ÊOùMDDâàÁkÖ¬Éx¸nCCÃøäS~(?ÊOùMh]]]Û¶mË8ÉWUU5Î&ùÊåòS~Q"hmmh|ÊåòCù)¿%566f<ÜOò)?Êå§ü&è|555éÁWZZZ__?ú¿Våòå§üòË±îîî,|ÍÍÍgOù¡üP~(?å7>õ÷÷uN¾	;É§üP~(?òo¢=ùfÌaOù¡üP~(?å7>EëVWW§ßäÉMò)?Êå§üÆÞÞÞ¡öä?~sssø£¤üP~(?ò+`ñx¼¥¥¥®®.O¾h¯½½=H(åòCù¡ü_ëééÙ¹sçÜ¹sÓ'ù*++Lò)?Êå§ü[´'_mmmú$_°fÍp©I>åòCù¡ü_aëëëknnÎø×u+++Ã/¥§§ÇóJù¡üP~(?åWØÚÚÚÖ¯__ZZ¾'_]]=ùÊåòS~¯§§'uÆ=ùÂJò)?Êå§ü^"hoo¯¯¯Ï²'_<÷R~(?ÊOù°ÞÞÞ0¶ÏÉW^^ÞØØÅ<sÊåòS~-¼Id<'_°bÅþþ~ÏåòCùå§üØ+W«ªªÒ¯¢¢bëÖ­&ùÊòS~ãD___ú»uuu´'òCùòS~Êo¼	gO>åòå§üß(¿¶¶¶:'òCùòS~ÊoÊåÊOù)?åòCùòS~ÊÊåòS~ üP~(?òåòCù¡üÊOù¡üP~(?åòåòCù)?(?ÊOù¡ü@ù¡üP~Êåòå§üòCù¡ü@ù)?å§üP~(?P~ÊOùòCù¡üP~ÊÊåòS~ üP~(?òCùÊå§üP~ üP~(?åòåòCù)?(?ÊOù¡üP~ üòS~(?(?å§üÊåÊOù)?P~(?ÊOùòCù¡üP~ÊÊåòS~(?P~(?òCùòCù¡üÊÊå§üP~(?åòCù)?ÊòS~Êåòå§üòCù¡ü@ù)?åÊåòCù)?P~(?ÊOùòCù¡üP~ÊåÊåòS~(?P~(?òCùòCù¡üÊå§üP~(?åòCùòS~ÊOù¡üP~ üòS~(?(?å§ü@ù¡üP~(?åÊåòCù)?P~(?ÊOù¡ü@ù¡üP~ÊåÊåòS~(?P~(?òCù¡üÊå§üP~(?P~ÊOù)?ÊòÇ_ºtiqqñ%KN<©üP~ üP~Ûò[°`Á;ï¼^íµ¦_GGÇÿ]'Nèííý¿S?ýéOòr+ü75¼r+ò3ù¬Ë/UYYYzù=÷Üsm£«µµ5<âÛ §Âê­·Þ2äÖw¿ûÝðeÈ¹ï|ç;!ò;qâÄ¦MlíÅÖ^°µ[·[#W¯^Ý¸qc¿òCùòCù1®ÊoÒ>=þ|CCCÆØR~(?P~(?»üRutt¬2¼xe¼Tù¡ü@ù¡ü?åWYY9)òCùòCù1nË/;åòåòCù)?(?ÊOù¡üP~ üòS~(?(?å§üÊåÊOù)?P~(?ÊOùòCù¡üP~ÊÊåòS~(?P~(?òCùòCù¡üÊÊå§üP~(?åòc<ßþá¶´´ô®ï|ç;ó7Ó9uôèÑ#GrëÇ?þñÁ¹õóÿüå_6ùìÊ+ã³üN>½ûö?à?eß":É´'­½(?Êåò@ùE/^¼øØ±c©?~|éÒ¥á¢%K<yÒX«VäðáÃ&yn³ÇU<ohh());wnø2cENW7)Cùòòò³gÏð±²²2õ¢¼óÎ;aáµ×^[¸p¡±"W­ HÿW(?rø¸Ú±cÇ³Ï>;00ÞªçÍg¬ÈÉã*ûKÊ¯ð×ÇóçÏð1ËkeYY±"­¯ík_ÿú×9|^¼¸««ËÛÇÕ0ß%Q~ãäÉ¡êÂpøxâÄ_ÖoÚ´ÉX«Vø¯ó²eË9|?ÿüóax~ï½÷9yç]åWHî¾ûîèÉáÁ½|ùòô/¸zõêÆûûû¹zh­[·îèÑ£ÿþÌT~äîqUTTôÒK/÷ß?ü×ÂXÇÕMß%Q~&ü/9ãräüùó|ð"­Iÿ±"'«Y³fey5ÛDùð?Ó§OÎÎÎð?Ô:::V®ñâE£DnZ©	h ÈÕãêÑGã7ÂBøêêjcENWÃy)Cùýìgá¡þ>åÔ÷ãÊÊJ3ÄCKù1«Ë/¯[·.lÙ²X,f¬ÈÉã*ãE(?Êåò@ù üÊåò@ù üP~(?Ê@ù üP~¹öê«¯ÞsÏ=e7Üï½o¾ùæýº¡`^m3ÝÛÊÊÊð£õ÷÷ZÖÏ=``àVo@ùgûöíÒ|õ«_Oå×ÔÔVîÝ»wÐúÝ»wõÏ<óÌmÜ&òÌÉ'CÓ¿øâ^zé¥ðiXyêÔ©qS~gÎ	+,Y2hý¢EÂúîînå(?`üàBÓ<ûì³©+î¹°òÁLí#GrQ¸téÒ°üâ?üpÓ¦MÓ¦MoÙ²%u£j¨«pQ¸n[[Û k¦O~Ï=÷¼ýöÛáÓúúúAw¬µµ5ûíD»]tøðá¡*måÊaýñãÇkÞyç°¦¦¦&¹fÇá¦ÊÊÊ6lØpþüùôòK¿ýAk²ÜU@ù¥9sæj9ölêÊsçÎ©e3È'¢K×®];è¢Ç,º¨³³³¨¨(ãµ¢O£K×­[700Ú±¤¤$HKÃÇPNS¦Lv¿Ër;a!ãÝKÿI_íµÔMÆå+¯¼Ì¾A7rï½÷Þjùe¹«òcÑÝ¯Y&K-ûï¿ÿêa!|ºzõêèÒ(t¢v¦îB±EmÜ¸1,ËÇn$õ6~úéÐvÑÆÖ~8¬9tèPXÃòC=tÓÛ	Õ>_zß2þD¡&gÎ~Þ/OÃÇpÏCn^¿~=úÙ³g+á£ÿÜ:«åå®Ê`ìË/ÈX~ÉõQÙDÁ?>|)útéÒ¥áÓyóæ=òÈ#¡Ø®]»¼ð5fÑ×>=wîC-%7øa¹½½ý¦·*3|záÂÔû6Ô>yO<ñD¸èë_ÿúGÿ¹EË-©_*0_èÑeËES·Z~Yî* üÆX4ÑuõêÕÔýýýae¸(Kë$»0EñäÎyéaµTê¹TÂryyyIII<7>úôä¥7½¡:lÓ§OGÃÇ°ÜÙÙ¼ôØ±cádÜj<üòËrWå0Æ¢ÝþùÔÑ¹NáÜðâÅéSY§Njjj¶½&1KnN½i=öØcÑFÞðqÓ¦MÉõYn'cûà¢O/ý8ÜåËK£Ù`M½(:Î7øðáË/g/¿dF£¼4Ë]À(..Þ³gOtV_|±¤¤$ýhÕ«WÊÇãÑ¦Øäq¸Ñ~~Ñîq±X,uÁ(·lÙ:):ê6y^l÷äú,·íØíç×ßßeòé¥³qáGN½(Úå±³³3|0C_Ô¡ÃâÜ°aCê¥Yî* üÆ^tãAvìØñ_¯_7Da'MTôðÃ§feªd)¿nü±°¾¼¼<uCpÛ	w#õ%«ê½víZYYYô#ÚÆ½jÕªÔo1sæÌð1:±KêmFSIQ§&/ÍrWåBÜï½%7ÜsÏ=Ñ¶ÊïðáÃÑió/^|ôèÑä¥ýýýO=õTEEETKO<ñD<O^ÚÖÖ¶lÙ²d³gÏÞ·oß ÛL¿'á¦ÂúGtÐú¡n'w&Ü¥èDYÎç´iÓ¦A§wxqãÆa¦M~îîîäjRoóòåË!m£±ª©©9~üø ïå®Êåò@ù üP~(?Êå üP~(?ÊåòàÖýÿ8GùIEND®B`
