# Supplementary material for: Regulation of piglet T-cell immune responses by thioredoxin peroxidase from Cysticercus cellulosae excretory-secretory antigens
Source: Front Microbiol. 2022 Nov 18;13:1019810. doi: 10.3389/fmicb.2022.1019810 (PMC9718028; doi:10.3389/fmicb.2022.1019810)
Supplement: Supplementary file 3 [file Data_Sheet_3.ZIP › 4. C. Cellulosae ESAs and TPx Induced Th Subpopulation Differentiation/3. SPSS statistical analysis/2. IL-4/1. IL4--24h/1.3 (SPSS data export) SPSS statistical analysis--IL4--24h.doc]

EXAMINE VARIABLES=Figures BY Variables
  /PLOT BOXPLOT NPPLOT
  /COMPARE GROUPS
  /STATISTICS DESCRIPTIVES
  /CINTERVAL 95
  /MISSING LISTWISE
  /NOTOTAL.


Explore


Notes	
Output Created	12-SEP-2022 22:47:19	
Comments		
Input	Data	E:\桌面\Raw Data\4. C. Cellulosae ESAs and TPx Induced Th Subpopulation Differentiation\3. SPSS statistical analysis\2. IL-4\1.  IL4--24h\1.1 SPSS statistical analysis--IL4--24h.sav	
	Active Dataset	DataSet1	
	Filter	<none>	
	Weight	<none>	
	Split File	<none>	
	N of Rows in Working Data File	20	
Missing Value Handling	Definition of Missing	User-defined missing values for dependent variables are treated as missing.	
	Cases Used	Statistics are based on cases with no missing values for any dependent variable or factor used.	
Syntax	EXAMINE VARIABLES=Figures BY Variables
  /PLOT BOXPLOT NPPLOT
  /COMPARE GROUPS
  /STATISTICS DESCRIPTIVES
  /CINTERVAL 95
  /MISSING LISTWISE
  /NOTOTAL.	
Resources	Processor Time	00:00:01.11	
	Elapsed Time	00:00:00.88	


Variables


Case Processing Summary	
	Variables	Cases	
		Valid	Missing	Total	
		N	Percent	N	Percent	N	Percent	
Figures	Control	4	100.0%	0	0.0%	4	100.0%	
	ESAs	4	100.0%	0	0.0%	4	100.0%	
	TPx	4	100.0%	0	0.0%	4	100.0%	
	LPS	4	100.0%	0	0.0%	4	100.0%	


Descriptives	
	Variables	Statistic	Std. Error	
Figures	Control	Mean	13.96800	.677927	
		95% Confidence Interval for Mean	Lower Bound	11.81053		
			Upper Bound	16.12547		
		5% Trimmed Mean	13.93867		
		Median	13.70400		
		Variance	1.838		
		Std. Deviation	1.355854		
		Minimum	12.634		
		Maximum	15.830		
		Range	3.196		
		Interquartile Range	2.524		
		Skewness	1.055	1.014	
		Kurtosis	1.551	2.619	
	ESAs	Mean	15.28575	.537633	
		95% Confidence Interval for Mean	Lower Bound	13.57476		
			Upper Bound	16.99674		
		5% Trimmed Mean	15.27572		
		Median	15.19550		
		Variance	1.156		
		Std. Deviation	1.075266		
		Minimum	14.068		
		Maximum	16.684		
		Range	2.616		
		Interquartile Range	2.004		
		Skewness	.495	1.014	
		Kurtosis	1.517	2.619	
	TPx	Mean	15.89050	.455643	
		95% Confidence Interval for Mean	Lower Bound	14.44044		
			Upper Bound	17.34056		
		5% Trimmed Mean	15.90106		
		Median	15.98550		
		Variance	.830		
		Std. Deviation	.911286		
		Minimum	14.692		
		Maximum	16.899		
		Range	2.207		
		Interquartile Range	1.705		
		Skewness	-.606	1.014	
		Kurtosis	1.480	2.619	
	LPS	Mean	18.72400	.629666	
		95% Confidence Interval for Mean	Lower Bound	16.72012		
			Upper Bound	20.72788		
		5% Trimmed Mean	18.69222		
		Median	18.43800		
		Variance	1.586		
		Std. Deviation	1.259332		
		Minimum	17.548		
		Maximum	20.472		
		Range	2.924		
		Interquartile Range	2.333		
		Skewness	1.180	1.014	
		Kurtosis	1.543	2.619	


Tests of Normality	
	Variables	Kolmogorov-Smirnova	Shapiro-Wilk	
		Statistic	df	Sig.	Statistic	df	Sig.	
Figures	Control	.253	4	.	.943	4	.675	
	ESAs	.252	4	.	.961	4	.787	
	TPx	.248	4	.	.964	4	.805	
	LPS	.252	4	.	.929	4	.589	

a. Lilliefors Significance Correction	


Figures


Normal Q-Q Plots


>ßÔÔ,È²Òn·ÜÔl¯ô¥ÇÆÆ9"kDcQºâ£GdyrrRW»Å¶ü¬V«üßÜÜwïÞÝ¹s§:TZQQát:>óÚµkË-Û°aÍõë×CYYÙÃoÞ¼YXX¼iÓ¦ÀçO>m2ÔaMùþÂùfÏ=²>ðØ«ÔÐÐ +«ªªB~³½öKeÜÈYNÈçäÛ#Ë/èRÙ/2ì7o±üì öÒÀÝ*µ··ËÖddÏÊEþ+Pe$ÓrÑh<tèÇãáùNå766%Z<xð@Î-"D´¤¤$ùÇ:¹~ýzÁÿCQA¹qãÆÈ¾Y¹r¥¬²¹¹¹!¿ÇÙ^%èKkÔs~yyy³Èóg.¿ÞÞÞÀíÈ7;[ùîV»Ý®>ô×ÓÓ£>yûöíA·ùÀür!?"Ò©üdáêÕ«Ï"`ß¾²^þèq² ÊÊÀÏ<~üøÔÔzÖJ­Ù¿¿ÏçëììTÊåÃk×®É²(P]WÍfkkkå÷î^Î7!äúÀë>áUÂM¤ó?':ÛÁ	y«æ|Åü®(båññq1½ßg3_àníÈ7nÈ²Ú­»wïV¬DxÿþY½)Ëiiiür!?"Ò¯ü$y eyPZ%Ë<PÊ¼|¸|ùòÀ-ø/õ¯m~822òÙ¯crrR¬ Â())QO2M+¿äääßHÈõs¸J ¨äºÅÒÞÞrÐf28!oÕ¯8sù©¹>ü³_ànU[LÖ¨åÃÕ«WO	~³é]~3£Ñ(ß^¯7p½ö93í¤7!]ÈmFþPtÄsÚ×¨L&uxZË;?D¶6«im&Î£s»âÌöÎÄv/ÕîÖ SÞ¨Â_­­­ür!?"Òµü¤·ÞzK>ôE­T8zvJVÎ?,((PgW¹~ýºÈl&òSOLª¬TTT:tèÑ£G.;wî)¿Îòíàøóg.¿<çç·¸?òø§¥¥É vûöíÚÚZuÙÿ:h"B~D¤_ùöx¶¾"¿Z£^ù[^^>ö8õÐ¾ÿþ'zÞËn·?Î??ùutt¨gêù<ÕÕ«WC~³½Ê¬äypÔHz´Ùêä§fæ	|'&&v[¶lÑºðìÙ³2ø¢CApäñW·PÜ,/#)ËEEEß¦¯ép8"y'"äGDúßàà FZãt:æxeeeù_*û$òó[Dµ|ùrù?ðõ­!oóóÏ?¯=ò¸víÚßæ¬®2+ùEÿ	ÎÆ<Qünß¾¸ý6^4&bµÈòS§Eùõ¬j`/¼ð¿Èb@~:¸þÓO?Ý¹sgòã***´¯çüFFFdk²M1Ð/¾(ò$²oêëëV®Æo<óÌ3þçü*³_äÁ¹zõªàÏ`0øOØ¬FuòZ[[W¯^-ßµl_O÷¿Y>©¯n±Xº»»§ÎõÚµk%%%²5ù¦ýë=ÏÑ£GÕ©ÂwÙ^¯_."äGD´ MNN>ÿüó³:Ü®s©°êÅÚ>O¾  "B~DDñçÔ«¯¾ÊÈò#"·<Ï#GV®éÊÂ©S§"B~DDDDü!?""""B~DDDDüùò#""""äGDDDDÈ!?""""äGDDDDÈ%ZW¯^5ÍaãÆ³½naaá%K,Ë¢¢¢YÜa=nn3ëÎ¹ýû÷/[¶LgåÊGõx<Qº_Èï%n&I!Êñù|³½îéÓ§åºgÎñ¯yíµ×dMmmm¬ËOû¸÷îMMM]¼xQ¾Ð-[æ°©Åüùî/·oßëù×¬_¿^ÖôööFçF.je!?"Z(ö:ãèÑ£iii'Oú´gy¦¤¤$hþ§eyxxXeºèþýû%55Õ`0ß¸q#äÖ¿zä«¼ñÆf³¹´´T¾VHãããr]¹ñGQOaM7lØ [õ²åÌpddË^¯7ä¥F©££Cn¤ÂÕ«Wµ#¬Éâç-þÔòË/¿,Ëb,YxõÕW?çÚµkJxíÛ·O.ºpá,Ëÿ²¥.Z»vm[[,Ü»wOÖgggÜZà|ñÜ[o½%V«Uã8 Ëò	W®Ó§OËÊU«VÉòØØÝnõ¤6í°ÃÙ+ò(=V-ñöFï7Å'!?"Z@ù	VdyòqZxØÑÑ!ËòöíÛküÞ¾ûøñã%%%²>)))äÖù*êeddh¯¥nüÔÔ,ùd¥|¦,­Â=²ò<JjY¶N~þï=ò¦øá$"äGD(¿@î³KPÂ¬´´4ùLÇ£ªÊuÑùóçåZÂÁ;wîhõòÌä*án01ði<¥ÆæææeË©5F£Q=8çüòòòdýøøxdF^äå¹8ò#¢ù_vvvàSPêi³i!RYY)úÿ÷¯ONN5S¡ü"_ÅÃsÚë._¾7èæù|¾¶¶6«Õødá´?~_¤¨½h&£4CùÍmÀÍüÔZ/ ¦½öÚk3Hss³ÿÉ³·Þz+5ýýýjþßLäù*¢±üà²°oß>íu_xáY¾xñâ'|¢ðÊÊÒÒRY¾sçÎÃe!//oÃ266&ËÍÍ½ÿ¾hR=¹cÇRà²:â¬5ïsp""äGDó ?Ï'ºJ9ù"CdbbBiÿLþõ­­­F£QÜsäÈÊ/òUÚÛÛåÒõë×û_hø9çÐ¡CrË·lÙ¢^Æ+à«¨¨P/.++ûôÓOg>2òUDêX¶ðäÉjßLF)pùêÕ«rÔ²¾÷¹8ò#"""B~DDDDüùò#""""äGDDDDÈovýøÇ?òðàÁý×ñs£ç=zÄ8è¹±±±ÿøÿ`ôÜÄÄDôï`iVýïÿþï?ÿó?3:Oöì)ä7?ïßüEùþä'?yøð!?ÊzîO>ùùÆAÏÝ¿ÿþé='>øáûÿùë×¯3:ïÚµk?ÿùÏò#äüò#äüò#äüùòC~ÈùòC~üù!?äüù!?B~ÈòC~È!?äGÈù!?äGÈò#äüò#äGÈùòC~ÈùòC~üù!?B~ü!?B~È!?äGÈù!?äGÈùò#äüò#äüùòC~ÈùòC~üù!?äüù!?B~ÈòC~È!?äGÈ/6ä×ÝÝ]\l0z!?äGÈò[ùåææÞºuK.]º§_[[Û£è&wwïÞD:îöíÛü1ã ç~ö³õôô0zîÞ½?úÑ=744$ª`tÞ;ï¼3<<å/Ãò,55U+¿sçÎÝÞ÷ÝIÇµ··¿ÿþûûà®_¿Î8è9a<b1zîÇ?þñÛo¿Í8è¼ÖÖÖÎÎÎ(Ñx_OOOUUG£½í%öG9Ú·GUãããùòC~üù!¿xßàà Õj-äGÈùòC~üâG~6m	y)ò#äüù!?B~ñ#?³Ù¼$ äGÈùò#äüâV~C~ü!?äGÈù!?äü!?äGÈù!?äGÈò#äüò#äGÈùòC~ÈùòC~üù!?B~ü!?B~È!?äGÈò#äGÈùòC~ÈùòC~üù!?äüù!?B~üòC~ü!?äGÈù!?äGÈò#äüò#äGÈùòC~Èùò#äüù!?äüù!?B~ü!?B~È!?äGÈò#äüò#äüò#äüò#äüùòC~ÈùòC~üù!?äüù!?B~ÈòC~È!?äGÈù!?äGÈò#äüò#äGÈùòC~ÈùòC~òóz½N§ÁD~Èù!?B~ÈùÅ³ü;f2òóó>ãüòC~üÅ¡üÕjMIIYò«ÚÛÛOäüò#äü(~äçóùDxe¦Æù!?äüù!?ùy<ÆÆÆ ð-]º´²²2ú¤Èù!?B~ÈÍ¿üêêêÌfsù233kjjxmòC~Èùò#äò³ÛíÕÕÕéééAæËÉÉºÝnù!?äüùòyùÉ#ã¶mÛ.]d¾ÒÒRÍÆËxòC~È!¿N`·fÍ&ó!?äü!?B~q+¿ÑÑQy@4ÚÉ|ÕÕÕLæC~Èù!?B~üâA~¯¿þúÁµvÌüòC~üùÅOòð÷õ¯=))É|Èù!?äü!¿øÌëõìÖ­[Çd>äü!?äGÈ/ns»ÝáÎÌÇd>äüòC~È_$ªÛðÌ·bÅ=ö0ù!?äüò#äÉcÜÖ­[Ãï¿ÿû¿gõ¾½üòC~üùé.5/??_;O èàùûöòC~Èùò#ä§»<´iìÌüòC~ü!¿ÌápX­VíÝU«V;3òC~ÈùòC~üb)Ï×ÞÞn±XhZ³fMcc£Çã	w]äüò#äüùÅFn·[`£5ßÖ­[å±fÚ³1#?äü!?äGÈOïÌnµZg~f>äüò#äüùé7y	9Ïd2½òÊ+³=3òC~ÈùòC~ütÏçkjj7Ïf³EÌüòC~ü!¿ØH½åv2:3_WW×lù!?äüù!?B~ºÈétÖÔÔÌ'ëçåmvòC~Èò#ä·ÈÉSee¥v2Ùl®««s¹õòC~Èò#ä·8ù|>ÍVZZr2_KKË´giA~Èù!?B~ÈÞs»Ýòxd6µùöÙB~Èù!?B~È_ôr¹'ó¥¤¤<xÐáp,èWG~Èù!?B~È_4êëëÛ»w¯Oû6»gÎÂm@~Èù!?B~ÈßÂÖÑÑ±mÛ6í8JKKm6Û¼OæC~Èù!?B~È_´óz½­­­ëÖ­Ó¾Ãb±DÿÁù!?äüù!?B~ózGÈÉ|V«u¡'ó!?äü!?äGÈ/Ùív±Ö|f³Yfû6»Èù!?äGÈùòÓc»víÒÃd>äüò#äüù-HêlÌùùù!ßfwQ&ó!?äü!?äGÈoÇ£Ñd¾ÌÌÌêêêyy]äüò#äüù-rv»½ªª*===È|999uuu>ù!?äüù!?B~OÏçäÍ7ë|2òC~ÈùòC~üÈ|»íd¾ÊÊJ½MæC~Èù!?B~Èß:vìXÈÉ|555úÌüòC~ü!¿ÙÕÕÕn2_cc£'ó!?äü!?äGÈoFù|¾¦¦&Åò-×ä"ýOæC~Èù!?B~Èß4¹Ýîºººù¬V«ÜÇÁ>B~Èù!?B~È]~N§³¦¦Fûk²F5WÜì#äüò#äü(qå'÷ÏV«UÍæõzãl!?äü!?äG	'¿ÈùÚÛÛcz2òC~ÈùòC~ü~Ûínll7ÏápÄ÷>B~Èù!?B~ÈB~3ù!?äüù!?JEÌ»gæC~Èù!?B~Èßÿ°ùòK8ùÍfäGÈùQbÊ/òd¾ø83òC~ÈïuvvÈo8ò#äü(Ñäçr¹ä~>Á'ó!?äXòÛ²eÃá ¿óçÏÿ$º½÷ÞÂÍûàþáþqÐs7oÞ|ÿý÷='ì÷Ýwë«¿þúëåååIIIAæ3Í'Nèèè`IÿøÿøöÛo3:Oöì©(Ñß/obxùýýßÿýÃè&ìûôÓOú;wî0zîã?îêêbôÜÝ»wXQþ¢<øÛ¿ýÛ6h'óÉJ¹H>]ãïßÿýß¯]»Æ8è¼¶¶6Ëå/Ïòãh/q´£½GÌÇÑ^ör´ùòC~ÿòs¹ÎÌ'ëN'ûù!?äüù!?yùê*++ÓÓÓ93òC~ÈùòC~·òëèèØ¼y³ölÌ¥©©)ÏÌüòQÈò£×ëýõ××¬YÃd>äüò#äü(nå744òÌ|F£±¦¦f``G~Èù!?B~Èb^~N§³ººZ;OÖ>|Øãñ0ÔÈù!?äGÈùQÌËOî·nÝªÌßÐÐÀ8òC~Èò£ÏçkjjZ·nölÌ¥¥¥%Á_À!äø~ûÛß~ùååÎù!?äüù!?IùÕÖÖÆ/ààÌ|Ãáxê©§þàþàk_ûÚ7|9==½££ù!?äüù!?%ùê;¦Ìg2êêêã©úÒ¾´å«[N8©þ=·ç¹Ïþós>ðüòC~üEU~r'l±X´vsrrl6×ëe$ýÍfóñcÇýòO?ýôöC~Èù!?B~È¢!?ÇÓÚÚr2ß¶mÛ2YÝnÏËËdüûÃ?üÃäüò#äüHòs»Ý!ÏÌ¾k×®¾¾>.SSS÷ý¿~ö9|dùòås~òC~ÈùòC~´Pò³ÛíÇy6f¹7æ,-3éÌ3ÙÙÙß¬ü¦°¯ê[U_úÒ¬Vë·üòC~üÍ¿üÂMæ[³fMSSùfUKKKAAÁoüÆoüöoÿö'ä°8òC~ÈùòC~4oòëèè°ÙlùùùZómÞ¼¹½½É|òC~ÈùòC~4¹Ýî'NhÏÒrðàAÎÌüù!?B~üâ!QÕjáoÕªUuuuCCCò£ÄßéJJJB~üùÅDáÞf·´´Ôf³q`ùòû,iºò#äGÈOÏ	éBNæ~å+_þ,!?ä§_ù-zÈò£9§ÎÌg6ÌY]]Ý××7Û÷í%äG	-¿±±±ûö!?B~üôV¸É|¢@ÿùfõ¾½ü(áä'÷y~üùé¹OæC~È_Ø´/ï0ùò#ä·èEÌ'yGü!¿°%''Ë=ÈÈÈÉdß~ðY¨ªªB~üù-b'óE83òC~üÂoâq² Ô÷îMMMÉBZZò#äGÈoQÕí´o³8/BÈùòÛ²eËäÞ¤³³ÓápÈÂ©S§Ôgu!äGÈ/úÍËùò#ä¶#Gø_Ït/üùòNj2ÜñÎ|2òC~üæØK/½´|ùrYèîîQ`IIÉBßtäGÈùÑgá'ó¥¤¤X­Ö¹½Í.òC~ütò#äü¼£Ñ8·É|ÈùòC~ÈùòºººvíÚ¥Ì?/o³ü!¿Hååå©s»p&gB~ü´ÖÖÖÍ7ÏËd>äüùÍ¥ÜÜÜ@íùãµ½üùÍWn·»¾¾>''g'ó!?äGÈo.	òäÞ§§§gjj*7ùòC~¨îàÁÚ3óÆW^yÅår-ÄE~È_ØÔäâ(³ùòC~qÝnß»w¯v2ßÓO?]__ïõzîK#?äGÈ/l½½½rOtàÀññqäGÈßæóù´gæÊÊÊÚÛÛüÈùò¢V®½âüùÍ*ÇÓÐÐ ÌnµZåg;j·ù!?B~a[½z5¯ð äGÈïIRgæ3LÚÉ|r_7::åÛü!¿ðxÝnòMG~ü_ÔÕÕeµZCNæ³Ùl:ù!?B~s)++Wxò#ä7Å¢*#+å¢(LæC~Èßèè»ª#GDùoSäGÈùÅb§±±1??_f¾vuuéáF"?äGÈ/ü&ÂÄ+<ùòÌårÕÔÔhÏÌ'ke2òC~üæRRx!?B~ª¾¾¾gæ3ÍgÎq»Ýz»ÁÈùòÓ]ÈòÓyêÌ|!'óÍïÛì"?äGÈ/zò¿YW¯^ÍM!?B~ÈOç¹Ýîºº:íù.]ZYYÙ××§óÛü!¿°¹;þMG~üs:!'óL¦W^yÅápÄÄwü!¿°Ý¸qCîÔjkkÇÆÆ¢ynäGÈùé*¹yf¾×_]ùò#ä7§MðÚ^B~Àò0OVFçmvò#ä=ùñÚ^B~òs»Ý!'óY­ÖX9°ü!¿ØùòC~U¸É|êÌ|.+Ö÷òC~üòC~ü"Mækll­É|Èùòc[¶lIMM»¿´´´;vDá¥ÈòZq9ù!?B~sibb"ä+<úm|!?ä"Oæû¸ÜGÈùò[AAÜ	ËcccÛ·o5k×®E~ü(våçr¹ä~&'ó!?äGÈo.%''Ë]aàÉÉIY#ë!?Eù%Èd>äüùÍ¥¤¤$¹Cíù×x½^YÃY]ùQlÉOþmooOÉ|ÈùòKêhï¦MÔÑ^ù_eMqq1ò#äG1!¿ÄÌü!¿¹$Ôù¹ã@~ü¼¿ú«¿²X,ùùù²kt(¿pgæõN§31wòC~ü"511±cÇ¤¤$ùÓ¦M²f¡o:ò#ä§ç|>ßÖ­[oõï=·ç¹êoWÿ>÷¹ÏÙívýÈOnLuuuzzzÂNæC~È_,üùé¹+VýîÑ'Nª_ÙôM¼Iòkoo/--Õ¾CxÚÔÔ8ùò#äüò£ùéÄ_ý¯úÙ'ÿDééé(?Çc³ÙÌü!¿9]sº!¿í/þâ/þdóÊïð333E~CCCµµµÚÉ|)))ÕÕÕýü!¿iJò#äGòëù/|á;¾ãß7|yçÎQÝn·Z­ÚÉ|«V­ûÌü!¿yèÅ_Tw¬.]B~ü¹CýÖoýÖ¶òmß¬üæúõë³³³¢#?uf¾²²2íáuëÖ1ù!?B~óPoooZZ%xbgäGÈ/akiiyî¹ç6oÞ|üøñµrAåçñxêêêBNæÛ»w/ùò#ä7?íÜ¹SÝ½¶µµEç¦#?B~1ÚÉÏårÕÔÔÍfíd¾Ã«§	ù!?B~OÚ+WÔÝkyyy4o:ò#äüTv»×®]Ú³´åbtt1G~Èß<ÈobbbíÚµêÅ7nÜòMG~ü >¯µµ5äÛì®Y³æòåËLæC~Èß¼ÉïìÙ³êv÷îÝrÓ!¿×ëmhhÈÏÏ×NæÛµkWWWü!¿yçó#äGÑßÀÀÀ±cÇL&ömv>ì	!?äGÈoå4]ùò£ùüöY­Víd¾ÇÃÀ"?äGÈoå·è!?B~	"?ùM9¯¬¬¬©©Éëõ2¤ÈùòC~ÈùQlËOHg³Ù´ù¤ÊÊJÎÌüòC~Èùòù¹Ýnùù6»V«Uv1cüù!?äüùÅ¼üN§ØNd>É$¿ò.ÑC~üòC~üb^~òë¼uëVí8JKKm6ù!?äü!¿Ïç9OXYYýßqB~È_kr>?B~ôòSù´o³Y]]ít:(äGÈùéH~§î)?ÎçGÈBÖÙÙ¹ûvíd>õ6»"BùòC~º?ùÉ»ì=öËò¿Ü§Ë7o"?B~XäÉ|¼Í.ò#äüb@~F£Qî¸ï²'''ÕkñpË½½½ÙÙÙ¡°°ðÆÈ_a2@É|Èò%ù©pÑ^ü|_EEÅÅeáÜ¹s»wïÖÊï½÷ÞóD·®®®û·óûè£~ö³1:ippðÏÿüÏW¬Xd¾oë[ýýý>s¹oÞdôÜþç¾ûî»ÎçwÜnw¿èË/++KîÇEiò¡|ÉòòrY#ëüÙÄ©©)EI³Ù¬ß3g>n²?°äÞqXôÞ|óÍmÛ¶¥§§O~¯÷ìÙÓÜÜÌé¹ööö¶¶6ÆAÏÝ¸q£µµqÐy²dOEù.¸üäïÂ¯ð¸sçÎn9ð5"Ú×p´8Ú«Ï¦Ì7Û÷í%öG9Ú«££½Ò½÷rssSSSÒÒÒ|³Ç!?=§&ó	ï¦Ìü!?äÛò[ ²²²ÔôAù_ìùòÓIáÎÌ§ÞfWf>äüù!?ä¢ÊÊÊ7ÞxCäÿäGÈOoêª««333guf>äüù!¿Ø_^^^ZZ:>ÕÜÜüäíìì4L²ÍìììîînäGÈO?Ì?íùò#äübX~êLÎïØ¦Ï;· 7ùò~^¯÷ÉÏÌü!?äÃò3Lr¿ßßßï_ww·:_ò#ä7©É|Ú»sx]äüù!¿º÷WJ~SSS¼o/!¿¸ÉápX­VíÛìÊ_555CCC³Ý òC~ü_ËOÉY=Ï'ò<zô¨âüùÅn>¯½½Ýb±hÏÖ¹fÍË/O¦xäüù!¿8_GGGÈ39ßºuùòÅÔd¾íïõæÍü÷ù!?B~È/å'	JJJÔkSSSóòòf5éùòÓI.+äd¾ôôôÚíöyù*ÈùòC~±-¿E	ùòßm«Õª=K:3ßì"?äGÈùÅüü/ìð7<<·jÕ*äGÈOçù|¾¦¦¦pù¦=3òC~üùýâÍÖxm/!?çv»CNæÛ¶m[ûBù!?B~È/Vå·jÕª%[¶lò#ä§ÃNgMMMÈ3ó>|8tò#äübO~rßô8ÿ»w&ì»råò#ä§«äW¦²²R;Ïd2É/ÔèèhtnòC~ü_ìÉÏ8o¡ì"?Jùy7ïõù|6­´´TûÜ¼¬liiñz½Ñü6ò#äübX~ò£xÝnÿýßÿýÏîsË/ÿâ¿(?ió²ÙÑÑÑ3gÎQYYýß äüù!¿ßääd^^^ZZ<t½üòËÈßÌöùÏ~Óo:~ìøÉ'Ýñlzzú<ÏãñÔ××ËvÀg4<èp8ñûE~Èòaù­2èå½ê¦¶¶ùòI§NZ»v­ÏÿoóW6ýë_ÛÖDub;í8V­Z5ïgæC~È%üåA%ðùÞÞ^YüùÍ¤gyæßøF üª¿]3ÛítuuíÚµKûÙTCCC'ó!?äGÈâöòÐ255õkÛÕäùòÕjÝV¾-P~º÷O×­[7Ã«û|¾Ë/<3ÅbYÐ3ó!?äGÈN~ÙÙÙòsèÐ!õÂÄÄÄÉ'Õy"!¿ÔÚÚúÔSOçÐwû~÷è¿øÅ^ziÚ+ÕÔÔÍæ ð-]ºT4¹¸ùò#äGñ)¿grîîîF~üfØ·¾õ-£Ñ¸ù+¿úÕ¯þÎïüNyyyä'êN§Ø.%%E6fùÕp¹þfò#äübX~Òàà`aaajjjRRRZZZAA¬Yèü(ä÷Ùãgþ¾ûÝï9räòåËØ'÷#7o9¯±±Ñívëÿ;E~Èòmù-JÈâL~óx<---ëÖ­É|Èùò#äüùÍºÑÑÑW^yE5o¾Îùüù!?B~3­¿¿_ÌY½7++«¹¹ùòÂìveeeNæC~ÈÅ§üä'Ïÿ¤ä§Ï;üùÍ-ùÙÞºukLOæC~ÈÅ§üL&< õ÷÷ûå×ÝÝÍ	ùÍ!¯×ÛÐÐCgæC~È%üÔÃÒggoeÁüùÍ°õwT|LæC~ÈÅ§ü²²²ÔÙûü&''=*Ëf³ùòÉØN`733S,èt:ão!?äGÈùÅ°ü:::BÉùÖ­[È_¸|>_SSÅbÑþîÄÇd>äüùQÜ¾¶W0TRR¢^Û'*Å¨üt»pùñ1ù!?B~Äùü%´üüÜÆÓùò#äGÈùòñhùò#äGq+¿»wï®^½:99YÉRSSGFF!?Ï×ÞÞùò#äGñ)?y`ùyF~°ò</nÎÌü!?J8ù©3UTTx<ùp|||÷îÝ²&;;ùQÊ/òd>ÃÁ>B~Èòaù©GµÀ'0dÙVgäG#¿ùbúmvò#äGÈïÿRÏùMNNú×LLLð%ü"LæËÏÏ·Ùl^¯ýü!?ä'òSóü***|êþ¢¬¬y~òÒ	ìÂMæþÏ'ò#äüù-¸üL×öE~´ò7/%%É|Èùò£x_ÒtäGq#?Q]ÈÉ|f³Y~&ó,-Èùò£ßbü(ò0¯´´Ôf³%òYZò#äG	$¿p'mD~ò7oéÒ¥[·ne2òC~ü(±ä'/½ôRÐÊ½÷rVuùÖÖÖÍfíYZª««í-×ò#äGÈïðä±0++kxxX>|óÍ7Õ£ãMïC~ùÉF>¢Ëµºº:&ó!?äGÈW~=~O=.fgg«íÛ·/ôMG~´òëêêÚºu«ö¥©©É|Èùò#ä÷nÞ¼élhhÂMG~4òóx<6íé§Ö¾cÛ¶mÂAù!?B~ü~ÙþýûÕc¤z?iÏ=ÈbB~£££ò³d493ò#äüùMÁ`ÉÛ·o0Ï/99ùå')¶ÓNæÊOÁD~È!?Í&,9~üxÐJy@åµ½¤[ùÉOÎ¶mÛ´ùòóóÏ9ãñxFäüò#äºpçó[h!!?­ü¼^oCCz_é 83ò#äüùé:äG3Ûí9oéÒ¥»víêëëcÜ!?äGÈ/â5,	<ùCäG%?ËuìØ±ÌÌL&ó!?B~ÈòC~q+¿po³»fÍ&ó!?B~ÈòC~ñÐÇ_¿nÝ:­ùLæC~ü!?äüâ¡¡¡¡ÚÚÚßýÝßÕ¾ÍîáÃÌüù!?B~ÈùÅCN§³ªªJ;Ïd2ÉÏ	o³üù!?B~ÈùÅCò3òmvKKKëëëÌüù!?B~ÈùÅ|>Ïf³åççk'óù._¾Ì!?B~Èß<Ë/rÈ"uf>³Ùôó^]]m·Ûgû¾½üù!?B~Ó4]ùÑ<æt:ÅvÚÉ|¢ÀÀÉ|ÈùòC~üæ_~òK"Læ³Ùl>/ðò#äüù!?ä©É|Â;í[®Ex]äüù!?B~ÈùÍg¢«®®.¯×»Û7/%%Åjµ:ÎÈ·ù!?B~ÈòC~óÏçÛ¾ffæÚµksssW¬Xa·Ûçqû3Ìü!?äüòC~Þ#G|G9yâ¤üûÚ×¾öÔSOÍËióÂMæËÏÏ×NæC~ÈòC~Èù!¿oÙ²e¶ÿÏûÔ¿¢¢¢¦¦¦9opnùò#äüòC~Èoas»ÝYYYìôGTWW7·­Ésrræ0ù!?B~Èù!?äü¼yyÎïÉ'ó!?äGÈù!?äüßrßÌ­&»¬²²òÉ'ó!?äGÈù!?äüßøÚÞ+VôõõÍäZ!'óIe~÷#òC~ü!?äüæ3»ÝÞÔÔÔÕÕ5í«z½^occc~~~ÈÉ|cÞoòC~ü!?äü¢ÝÀÀì#íd>Yóäùò#äüòC~ÈOõõõEÌ·@oûü!?äGÈù!¿èåóù._¾É|ÈùòC~Èù!?ä·8y½ÞúúzÉ¤ÌWYY)Íü!?äGÈù!¿ÉårÉ0AæKOO¯©©K£ò#äüù!?ä7ÏÙíö½÷j'óåää444,Ü8ò#äGÈùÅ­üFFFÌf3òÓO>¯¥¥¥¬¬L;¯´´T.Zèp ?äGÈòOùuvv)r»Ýõõõ«V­ßÒ¥KwíÚåÉ|ÈùòC~ÈùÅü¶lÙâp8"Èï¯ÿú¯?nï¿ÿ~ww÷G	V[[Û7¾ñßüÍß2_FFÆ=®¢«[û£ýHþ$øtÜ­[·>øàÆAÏÉßrï½÷ã çúúúäÎqÐyo¿ýö;w¢üEcX~¿¼áå×ÔÔ4ÝDüñ@ÂÔÒÒ²ûöäää óá_8qâD¿o³Ðü§?ýéé8¹üðÃ=÷É'Ü¸qqÐsN§ówÞatèü_ÿõ_£üEãY~í]¸DÕ!ÏÌ÷ôÓO766Nû¦mí%ör´8ÚKíóTÈoQòz½6Mx§ÌgµZív»ÏçÓù·ü!?äGÈ/fäÒÈ/ÉÍfíÛìÖÔÔLûì1ò#äüùòC~1|SV«5%%%È|&©®®ntt4¶¾äüù!?B~1,¿p!¿'¯µµÕb±h'ó­Y³Æf³-úùò#äGÈù!?ä÷¤	é×­[§Ì·wï^ÃÓßòC~ü!?äü~ÑÐÐPMMMÈÉ|µµµ14ù!?B~Èù!?äüÂf·Û+++ÓÓÓµgi©¯¯Ñ»ÈùòC~üòûµdÊÊÊ.]d>YÙÚÚªÿ³´ ?äGÈòC~Èo<ÍfËÉÉ	yf¾¾¾¾xý5C~Èò#äüH~28Aæ3²ÞårÅ÷¯òC~ü!?äòëëë³Z­Ú»999:Ë5äüùòC~ÈùÍâgëÙgÕÏb±´··Çßd>äüù!?äü_ÂÉÏëõ644¬Y³F;¯²²2'ó!?äGÈù!?äüH~CCCÇ3LÚÉ|ûÉ|ÈùòC~Èù!¿ÃáØ»w¯ömvsrrêëëd2òC~üòC~È/åçóùÚÛÛC¾Í®¬lii§³1#?äGÈòC~	-?a_¢ù!?B~üòKPùIþWrdffÖÔÔ$òd>äüù!?äü_Ë¯±±QÏívóëü!?äÇ8 ?äÏòó=_$äüùòC~È/þåGÈùò#äüò#äüù!?B~Èù!?B~ü!?äü!?B~ÈòC~ÈòC~ÈòC~Èò#äGÈùò#äüùòC~üù!?B~Èù!?B~È!?äü!?äGÈòC~Èò#äGÈù!?äGÈùòC~üòC~üù!?B~Èù!?B~ü!?äü!?äü!?äü!?äGÈò#äGÈùò#äüùòC~üòC~ü!?B~Èù!?B~È!?äü!?äGÈòC~Èò#äüù!?äüùòC~üòC~üù!?B~Èù!?B~Èq@~üòC~ü!?B~È!?äGÈò#äGÈùòC~ÈùòC~üù!?äüù!?B~üòC~ü!?B~Èù!?B~Èò#äüò#äGÈùòC~Èùò#äüù!?äüù!?B~üòC~ü!?B~È!?äGÈò#äGÈùòC~ÈùòC~üù!?äüù!?B~üòC~ü!?äüòC~ü!?äGÈù!?äGÈò#äüò#äGÈùòC~ÈùòC~üù!?~!?äGÈò#äGÈùò#äüùòC~üòC~ü!?B~Èù!?B~È!?äü!?äGÈù!?äü!?äGÈùòC~Èùò#äüù!?äüùòC~üòC~ü!?B~È!?äGÈò#äGÈùò#äüùòC~üòC~ü!?B~Èù!?B~È!?ä òëîî...6EEE½½½Èò#äGÈùÅ­ürssoÝº%.]ÊËËÓÊ¯££ãçÑ­§§Çårýt¿Ãá`ôÜ½÷úúú=7<<ÜÙÙÉ8è9Ç#òctÈobb"Ê_4åXjjªV~¯½öÚµèÖÚÚÚÖÖvtÜÛo¿Í>Òy²d71zîwÞ»;ÆAçýð?dØGÚâA~===UUUí%ör´8ÚKíåhoÜíUWTTx<äGÈùò#äüâJ~K~úpppÐjµÄò#äüù!?B~±-¿À:::6mÚ422òRäGÈùòC~üâG~f³yI@Èò#äGÈùÅ­ü"üù!?B~ÈòC~Èù!?B~ÈòC~È!?äGÈù!?äGÈò#äüò#äüùòC~üù!?B~ü!?B~È!?äGÈò#äüò#äüùòC~ÈùòC~üù!?äüù!?B~ÈòC~È!?äGÈ/ä÷ùMMMÑí?üáOúÓÒq7nÜ?	=×ÝÝ-÷ûè£þîïþqÐswïÞ½xñ"ã óþæoþÆétFùºÝîø_ÿÉ'¿GDDDD¿*òÑ%<KDDD !?""""äGDDDDÈ!?""""B~DDDDüôÒÈÈÙlöØÝÝ]\l0zî£þþþµk×Ê>*,,ìììd|t¸T×¯__²!î£.	ñÑá>òz½V«599yÕªU>wSàïQRRòÓâÀ½ÜÜÜ[·nÉÂ¥Kòòò"î#ßÕ«WeAöÑhdt¸$Ï'DAÝî£ææfQ#£çtúôéW_ujjJØ·zõjH·wwª/;wùé½-[¶8pN©©©÷Ñµk×"î£_~ùûßÿ>òÓí>ö©¿ H·ûHþÊýäOxHºwïÞÆõp¹ÏÙ0zpêéé©ªªbpô¹|>_FF¬¼té£Ãtÿþý©©)ä§Û»iÓ&Á êÓO?ept¸dï=655uõêÕwîÜapôÌ;v_ïÂñññÇÃàèYç×¯_ÏÊÊbpt¸äNðæÍávéê÷hppçÎõ¹. Bs:£Û_%Ã±~ýz½Ü6vÏv¡ÜZ­Ö22:ÄR38:ÜGK~=Gç¿GÉÉÉ÷Qà_¶Ü×éùWéôéÓgÏE~±º;::6mÚ422Â°èvåææö÷÷öøØ²³=«öéù÷Èétª¿uù=Òç>Ú¿ÿ[o½õÙã28º½»Û°aÃíÛ·_¬îB³ÙÌs:ßG½½½òðÆyjùÑÜöQOOOAAü	þî£±±±;v¨¹ÁÑíÝ]rròÔÔò#""""äGDDDDÈ!?""""B~DDDDÈ!?""""B~DDDDüùò#""""äGDDDDÈò#""""äGDDDDÈÑzóÍ77lØú¸7^¹rå×î¿3÷¶¡n­ÙloÍãñ­5!;;jjj¶Û$"B~D<yr¦^z)äW[[++Ö×××ËúS§NÍaDDÈb¬ÞÞ^1Á`8þ¼ïq.eåíÛ·ãF~÷îÝEEEAëd½ÓéD~Dü(þÛ³gæÕW_Úk¯ÉÊ½÷ºçÆ"'Aaqq±,û?ùÑ£GUUUrÑh<tèPàAÕöövÑ×½víZ¥dÍ²eË6lØÐÖÖ&VVVÝ°ÖÖÖÈÛQÉMR]¿~=Ò6mÚ$ë»»»ýknÝº%k,ÍéÓ§M&l*55uçÎZùi·´&ÂM%"äGD´­RÔrÿþýÀ<f³9P6Aõôô¨K·oßtÑÔEv»=)))äµÔêÒ;vLMM>ÿÒÒÒÔô»Û7Oû^ºt)³~¼xÑÏ¾ lÜ¸q¶òpSù-rêÀnû¬%KÄa²Ù½÷øãdA>,//W*è(;ª§îDlê¢õd¡,wvvªnóøñãb;u°õ^5W¯^eù_þùi·#jåo[ÈïH4¹|ùrù~GFFäCù_n¹psrrRBvv¶¾Ï~ut855u¶òpSù-¾ü¤òó¯W²Q`åC1ú°¸¸X>zõ¾ûDlþÈç=æ¿úðÁþO-ùøÊÿ²ÜÞÞ>ívDòáððpàm7'ïÅ_¾ÿýïö«#ÚüQ ÈO<ZRR¢­ü"ÜT"B~DDz¢k||<p¥ÇãrQëø]èp8þüÐñOÎÓ²Tà¹TdÙh4&''½^Ùø²eËüN»pª¿¿_!UåY¶ÛíþK;;;å<j<sùE¸©Dü95×íìÙ³+Õ¹N^áá822¢*ëöíÛµµµêØ«ê	9ÿáÔivàÀuWþ¯ªªò¯°õÛÃÕÃÃÃ_»~ýz¹TÈFÀxz¯ýúõ±±±Èòó«TÿÒ7Ñ"§^!a0Î;§Îêrþüùäädí«1ÊËËE9^¯Wõ¿WÍóSÓãGàAÁCÔ«nýçU	é3õb[õÕ;::üë#lGMìSóü<úÌò»páÿÙ8ù/RSív»|pòSÖ+Ë§8wîÜxiJDÈhñSg9êôéÓÿwÿõ8#ÿ²ÿ8©ÿEþ^xá@VÖÜÜA~=~³Yo4GØÜÀæ]m¸ovbb"55UAÇ¸·lÙø%/_.ÿ«»nS=%éO9ÕiJDÈHN6nÜü¸6¨ØÉïúõëê´y7oÞô_êñx=j2^|ñE¯×ë¿ôÚµk%%%²ìììÆÆÆ mjolJÖïß¿?h¸íHrcä&©F8¿ªªª Ó»¨FFF***d222ä»p:þ3ÔnsllLh«ÆÊb±twwÅ7!?""""B~DDDDüùò#""""äGDDDDÈ!?""""äGDDDDÈ!?""""B~DDDDüùÑìûÿòGbÁ¥:HIEND®B`


Ò§OIùutt¨W$;;;ÕëyÊ¥KB~³½Ê¬Ê/òÎQÒ3-èFf»WçP~jdï'O$ì6nÜ¨íÂ'OÊÎ:¼ÿÕJ7ËçËå+W~j¼æ;w"¼óò[å7<<ìÑ¥Ö¸ 1^yyyþSe§üü-¢äääÈÿç·Üæ7ÞxCûÎãêÕ«#|³ºÊ¬Ê/òÎñOÈ4óöêÊ¯··7ðö×­[xiÐ>V jZÄ§¼¨WUíÞ½@ùòêÐÀõ·oßÞ¾ê´êêjíùs+¿ÑÑQ¹5¹Mi Hù'Ü7MMMeee)))K.ï½÷¾ñoø_szþ«Ìªü"ïK.Iüét:ÿxfµWçP~¢­­mÙ²eò]Ëí«÷Óý§3ËÒgê«Æ®®®g¾æzåÊ¹5ù¦ìv»½Çã9tèz1Uò]~^¯@ùÀ¼|ã7f5Ü®wÔ°êdmÏ§¾´´;ÊçäwÞaÏ ü Ñx<.]ºT½¥+o¾ù&»åÊòåÊ(?P~ ü@ùòå@ùòåâÒ¥Kz½^§Ó­_¿~¶×-++[´hÑ;wükdYÖ¬rG±isûÇVµÞãñìÛ·/77WöXVVVuuõÝ»wý×òù|²^>955U¹w üÄüü|)ÑÑÑ9Ê±cÇäº'Nð¯9~ü¸¬©¯¯OòÓ®·X,²þêÕ«²|íÚ5Y^¾|¹ÿÒ¶¶6)^¾|Ê@D#zåºþ5k×®5===ÑÙÈè_jjª¬y­]»vÉ¥êÿÝ»wsï@ù¹ì¬Ceeeedd=z4èÓ¾ñoTTTÝÿ%CY~ôè,ËuÑýû÷FczzºN§+//W¯io-ð«G¾Êï½§×ë|!mMLLÍf¹®lüÁÕKÒ¦ëÖ­[õrË<xÎò+,,õ¥¥¥AMMMeggË×zòäüãïã¹mP~æ%þÔòÛo¿-Ëv»]KÞyçÀÏ¹rå*¼@öìÎ9#Ëò¿,[­VuÑêÕ«Õ;÷îÝõ!o-p"_Ezî>Å¢ÝøûöÉ²|ÂG$ÇóÚøøx__,¨$Ãá×ÄséÒ%ÿõë×ßºuËõæï¦Mdyûöí²,kBn÷:/?½^/ËÓ´ár `GG?w¶lÙ¢Îiooï#G***dJJJÈ[j¯ÈWQ¥½n^^Úø©©)YÞò²^.-ëõzç¶[twwK¡úûOnY­¯­­õ¨õÀÏ³@ùÇòSç¥ú×ËÏÌ É¬ùLÇ£ÞÐ5ê¢Ó§OËµ$oÝºxA·øáL®nÃ$_¢SÕxñâÅììlµ&77W½8ó×ü"ì·«W¯JÌ¾»-Ý,_ôñãÇO§ßzå¥KÜîu(?_~¯ù©ÍAf³Y.õÿï_¯N6Ãò|ÿIHi¯£®´y>ïòåËê³Ú-á¨meªéííó6å`ËOÍÒræÌ5Îïøñã3É /úCç>ð¯WÙßß¯ÆÿÍ¤ü"_åÈ#~ø¡,ìÙ³GÝÝ»wËò¹sçÔ»«²Ò`0È²dÙÃe¡¸¸ø9ËoåÊ²¾½½ýés¸¼ñÆOÃOp#ëç¼@ùÇòóù|RWÓçä O<Qï´ÊÿÓHåææfee<xpåù*N§S.]»v­ÿD 9kkkeËSSS7nÜ¨N¡Øª®®V'WVVÞ¾V»Eû°Ü¬Ùl-o6''gß¾òu~9©uàíû'µó6å@ùòåÊ(¿YûÉO~288å/úàÁÿüÏÿä~1:ý!5ð_üâßþíßØPþõ_ÿõ¿þë¿(¿ã?øÄ_¿è_|ñðáCîÊÿò/ÿrçÎöNOû'öNÏOùw÷wì(W®_(?Ê(?P~ ü(?Ê(?P~ ü(?Ê(?P~ ü(?Ê(?P~ ü(?Ê(?P~ ü(?Ê(?P~ ü(?Ê(?P~ ü(?Ê(?P~ ü(?P~ ü(?P~ ü(?P~ ü@ùò£ü(?P~ ü@ùò£ü(?P~ ü@ùò£ü(?P~ ü@ùò£ü(?P~ ü@ùò£ü(?P~ ü@ùòòëêê*//×ét+W®ìéé¡ü@ùòåÊ/aËoùòå×¯_óçÏkËïòåË£ëóÏ?¿÷îcàñãNc?@ÈaAìáááÏ>ûýåÓO?ôèQ¿h_ ôôtmù:uêóèúÑ~ÔÑÑñ9ðùçN§S~³g?@ÈaAìüä'|ò	ûJ[[[ggg¿h"_ww·ÕjåÝ^ðn/x·¼ÛÞíMØwêêjÇCùòåÊ_"ßðð°Åb	[(?P~ ü@ù%NùuttlØ°att4ä¥(?P~ ü@ù%NùéõúE(?P~ ü@ùòKØòòåÊ(?ÊòåÊ(?ÊòåÊ(?ÊòåÊ(?ÊòåÊ(?ÊåÊ(?Ê(?P~åGùòåÊåGùòåÊåGùòåÊåGùòåÊåGùòåÊåGùòåÊåGùòåÊåGùòåÊåÊýÊ(?Ê(?P~ ü(?Ê(?P~ ü(?Ê(?P~ ü(?Ê(?P~ ü(?Ê(?P~ ü(?Ê(?P~ ü(?Ê(?P~ ü(?Ê(?P~ ü(?Ê(?P~ ü(?P~åÊåÊ(?P~åÊ(?P~åÊ(?P~åÊ(?P~åÊ(?, ±±1ÊòåÊtHUUUnn®Ûí¦ü(?P~ ü@ù!y<¶¶¶5kÖ,úÒ'(?Ê(?P~H(###õõõK,Yô«åGùòåÊ	ÂårY­Vmó644ðn/åÊ(?$É´xñâ æ[µjÃáðù|®KùQ~ ü@ùòC¤°34ªªªnÜ¸1¡ü(?P~ ü@ù!¦¹ÝîÆÆÆ¢¢¢ à[¼x±ÅbÕò£ü@ùòåår¹l6v0^¯ê0oåGùòåÊqCÂE;¯¤¤äù(?Ê(?P~tN§Óh4jóÉÊçÏÊòåÊ×ëu8/d0åGùòåÊ1jhhHB;OÖÈz¹ô~-ÊòåÊ´Cæ+**²Ûíáfc¦ü(?Ê(?P~ñD!Ü`¾9ÀAùQ~ ü@ùò£üb¹¤¤D;Ïl6G¡"(?Ê(?P~wn·[:A¯×kóÙl6ËÍ ü(?P~ ü@ùaIÕÕÔÔhOà(**jllÁ|åÊ(?DTÙlÖÀa0g6fÊò£ü@ùòåCBÎÆ,	h2¢åÊ(?¼x^¯·¹¹YGZZÅbÚ`>ÊòåÊæÑÈÈHCCC¸Ù£<ò£ü@ùòåy100`µZµùJJJ×ë©­¥ü(?P~ ü@ùa.N§ÉdÒ6_UUUkkk¬5åGùòåÊ³&I×ÒÒ²bÅí	åÆ±¼ñåÊ(?ÌÈØØ<×çççkOà8|øp,ÀAùQ~ ü@ùò£ü]ëêê233O¯×777KÆË7BùQ~ ü@ùòCX7nÜØ¶mv0ß5k¤ùbs0åGùòåÊ³àóù.`0´Í'!(ý´ ò£ü(?P~ ü@ù½Hn·»¹¹¹°°PÕjëïò£ü@ùòåÿ688xøðá³1744$À÷HùQ~ ü@ùòKvòx±X,iii!gc¿ÀAùQ~(?P~ üæN¸Fã"ÊÊÊÖÖÖ8ÌGùQ~ ü@ùòÃÿðz½£¨¨H;oÇ	üð¡ü(?P~ ü@ù%·Û-Ï×ÚÁ|iii555	¥ü(?P~ ü@ù%Ër0^¯gðDÌGùQ~ ü@ùòK^òìl2´3ó­X±Âápx<äÙåÊ(¿Ääóù$ìv0ÙlþS6åGùQ~ ü@ùòñÜnwcc£ö%KÔÔÔ¸¤½?P~(?P~ üTÍfÓÀTù(?Ê(?P~	NÍÆ¬Ì§fcN¼ù(?ÊåÊIW~t---!gcÉ9ò£ü@ùòåhÜn·Ýn9³ÅbáxHùQ~ ü@ùòKCCC!gc5²^.åçNùQ~ ü@ùòr9¯¨¨Èn·såGùòåÊ/ÊOaÃæs:ÀAùÍÑÑQ½^OùòåÊ/ÔlÌ%%%!óq?§üæWgggii©Üá(?P~ ü@ùÍ+·Û-O¬z½^;Ïf³%ólÌ_ôlÜ¸QY#ßéÓ§¿®Ï>ûLróà/~üãwtt° ä° öDWW×åËãk[[[·oß©yïÞ½èÇ'|òÿøQþ¢q ¿ÜÄðå÷ÃþðatÉñýöíÛ§± ä° öÄàà`¼l­<nÝºUÇêÕ«<xÀô9É¯CCCQþ¢ ¼ÛÞíïöwg+ÜlÌ&Ùy·ò£ü@ùòC"_¸ÙÓÒÒ,ù(?ÊòåÊP~áfcÎÍÍmhhãgGùQ~(?P~ûò0óÙ³g½^/?5Ê/>P~ ü@ùò ÂlÌmmm4åGùQ~ ü@ù!îËO.ÜlÌ;vìà¾JùQ~(?P~HòS³1kóeffÖÕÕñ¢ü(?Ê(?Äù¹Å¦ùÄÀAùQ~(?P~Hò§BÉ¤=Ã`0´´´ø|¾ÄÛÉ7oÞlhh¨­­µÛíò£ü(?P~ üàå'Içp8$ï´ù¶mÛ&Ï	Ù|â/þâ/rrrFã«¯¾ZZZº¢lÛí¦ü(?Ê(?$fùIè466Ùjµ&ðî½yó¦d_Íwj~ï¨ú·ö­ýã?þcÊò£ü@ùòC¢Ëå²Ùl!gcå¾^£ÑèÏ>ù·ßþ¯|å+åGùòåÄ)¿p³1;ä¯¶¶öÕo¼X~ò/==ò£ü(?P~ ü÷åçóùZZZF£¶ùªªª¤*u0_8gÏ]¾|y`ösË7+**(?ÊòåÊq n·ûÄÚÁ|åæÍÉ¹½^ïªU«V¯^ýÝ½ßìÛ¶uÛK/½ÔÑÑAùQ~(?P~ËòËþýûµùdÍfs¹¾=OMMMAAAzzzEEÓéå­¥ü(?P~ ü@ùa0ÝnO8åGùòåÊ/XGGÇæÍµÍg4u6fÊò£ü@ùòCrÇã±Ûí%%%ÚÁ|f³9úÏq ü"^óYRRR(?P~ ü@ùiOÖÔÔÔ0òÅòKyNGùòåÊ/:#333¨ùôz½<¯1òÝò[p(?P~£òs:&I;¯¤¤Äáp0òïòß³gåÊ¼üÂæS'p0òËòÓëõ:q~ ü@ùòóßÐÐP½v0_ZZÅbáBùÅkù­RûLnn®üCùòå$,¿ÁÁÁºº:)<í	æ£üâ¾üRSSåÞ<::/|~ø¡,X­VÊ(?$?ø?9³Ãáðz½ì"Ê/îËOÝ§eAROîÝ»755%(?P~Htv+V¬9Ïétrå8å-÷ìÎÎNy7ß|S-0«(?P~Hxn·[´ù/^l±X¸cP~	X~ôÏx§7(?P~HT.KÚN;/33Óf³13&ð¹½o½õVNN,tuuÉT`EEÅ|o:åÊ<û¯¨¨èí·ßþáÈ.Bß ü@ùòC4ù|>Ãa0´ùd¥yV·åGùòåãv»´ùL&Sàå¤(¿ââb5·39òåDâr¹l6[¸Ùµù(?$~ù-_¾<°öü8·(?Ä/ùJÛióEòCâD<º»»§¦¦¢¹é(?P~xá|>_KKÑhÔæÉlÌ¿ürssåñåì£ü@ùòÃåv»ív»v0ß¬fc¦üøå×ÓÓ#ûöMLLP~ ü@ù!î½¨Ù)?$~ù¥KjCâP~ üãäQn0ßÜfc¦üøå·lÙ2ÎðåÊý_ä$Ü`>»ÝîÊß/ÿb[___7òåÊ³¥fc.))	9¯¥¥e&ù(?$uùåååq(?P~ìçv»åC¯×kóÍæõBù!ñË¯££C9|¢;åÊÄÈÈH]]"h0_MMÍóQ~Hêò[gxòåuóæÍ]»veff=Céõú³1S~ ü"I	3<@ùòÃB'ÊÊJí«%%%ã9óQ~Hêò[((?P~âõz/r6æªªª(<kP~HüòÓëõË- ü@ùòÃBihhÈÏÏ¾ÌÌÌ¨=Z)?$~ùét:yhEÓ)?P~ ü ëêê´ùçc0å¤.¿«W¯Ê¬¾¾~||<s»P~ ü@ù%9y0LÚ¿Àa0Gg ü,åÇ¹½ ü@ùQ~ÑäóùZ[[W¬X¡êÙ¼y³Óém£üøåÇ¹½ ü@ùQ~Ñáñxµùöïßý¿&Eù!Ëo¡P~ ü@ù%¡¡!9ì/Y²$¨ùòóóëëë£<òåGùòåyqóæÍÍ7kó­X±âÂ2òC²ßäääÆÓÓÓå¡±uëÖ(êAùòåØÚÚÚCÈù:::æo6fÊß3îå!ÏðïßÃ(?P~ üÇãinn.))	zZY¼xñ®]»büqGù!ñË¯´´T6mÇÇÇ·lÙ"kV¯^MùòåÙ¿¿v0_nn®ÍfýoòCâ_jjª<,_u5²òåÊ3<ºZ,´´4íÙmjjµÁ|ºüRRRäÁ)µç_#QYÃ¬. ü@ù!2Ïçt:FcÈÁ|ò¬ù(?$uù©w7lØ ÞíÿeYÖS~ ü@ù!$¯×ëp8´ù¬VküîOÊ_~z!Ïðxüø1åÊ_ôýå_þ¥Ñh,))ù£?ú£XÜ8Ûí93¬±Ùlqg üøå§îè[·nÍÊÊJIIÿ7lØ kæÓ)?P~ üø|>ÉôÕe_ýµ×k¾SSõU1²£WÈÁ|EEEv»=vfc¦ü@ùÅ"Ê(¿ ­­­¿ýÛ¿èÿ:ú½£êß¶­Û¾úÕ¯.øÉáZT;³Ñhlii»Á|(?Ê(¿÷½ïïë¯|Ýê_NNÎB½&Içp8´³1KÍæèÃ)?P~s/¿EÏBùòåMú§úJÕ+ÙwäðììlÇå-ÖlllÔÀ±dÉË¨wÊ	[~)áQ~ ü@ù-9*¾ôÒKRû'þòûú+_ùå£¹RuÒvÚ8ôz½·c0åd,¿p8 äçÏ§ü@ùò²ÚÚÚßú­ßÚ¼ió·ÌßúÃïçææFí*Çd³Ù¬ÌWRRâp8i0åÊï¿õôôddd¨»;S~ ü@ùESkkëë¯¿^UUuäÈ(L"I×ÒÒr6fYù(?P~O·oß®ç/_Î¦S~ ü@ù-,·Ûm·ÛµùÒÒÒ,Kr>@(?$~ùôÑGê¡¾iÓ¦hn:åÊßB7s2æ£ü¤å'÷ïÕ«W«9®^½åM§ü@ùò>¹Û[,í`¾¢¢"ÃáõzüÎ@ù!aËïäÉêÑ¾sçÎÙtÊ(¿hr:áóÉEIråä-?æóåGù!ÊO®µµuÅÚÙ-Krp¤üåò,:òåÊ/~EÙf³%ðlÌ(¿XDùòå7OFFFöïß«Ì'-Ì'pP~ ü(?P~ ü§ünÞ¼i±X233oÍ5---æ£ü@ùQ~ ü@ù%Bù9NÁ ´]UU%Ï^ü)?P~(?Êq_~^¯×n·¯ZµJÇ;úúúøùR~ ü(?òCÜÛí¶ÙlùùùÚ8><22ÂOòåGùQ~ ü÷å'wÝí_àÐëõcccüL)?P~k2(?ÊñV~rØ4LÚ¿À¡Nàà/pP~ üÂº/dù1(?P~1BÍÆò;vÈ'íR~ üfª½½]¯½öÚÄÄ|(ÿoÙ²EÖvòåÊoa©8´³1§¥¥ÕÕÕðã£ü@ùÍê3ð÷ÅÉÉIYÿ·ÜÓÓSPP ÓéÊÊÊ®^½Jùòå7sV«U;³¬©¯¯g0åÊo®71Mj/¨ü_uuõ¹sçdáÔ©S;wîÔßgæ®7nüÇüðx~þó÷÷÷³ ä° ØÙ96¾òÊ+ÚÁ|_ûÚ×þê¯þJÙüu:ì(~ú©Ûíò÷òËËËTü¢#ÊÜ´i¬õÏÿjâÔÔJI½^¯-¿'Nü8ºäG(Ô?þñ¦± ä° ß	ïÿû¥¥¥ÚÁ|/¿üò»ï¾ËO*®^½ÚÖÖÆ~"w¹KDùÎù]»v-ä·nÝzÎ[<GD¾ïöwÁ»½ÛínllÔæ[¼x±Õje6fÞíïö¾Èò÷îÝ[¾|yzzzJJJFFFyyù£Gÿfß/NMM¥ü@ùòâr¹l6vf¾ÜÜÜ¡¡!~:(¿_~ó$//OÿµïS~ üÌå'_Ñb±hó­X±¢¹¹ùc@ùò9f³ù½÷Þù¿ººòåÊÏçó9N£Ñ¨c#+òåòëïï/..ÎÈÈPïÏæåå]¼xñùo¶³³3??_n³   ««òåd.?¯×ëp8Bæ³X,Ü)?P~Q*?5sà_lSË§N×M§ü@ù!IÊÏívËO;OÖÈzóQ~ ü¢Z~ùùùrêïï÷_WW,geeQ~ ü@ù=Ëe±XÒÒÒ¯¨¨Èn·K²ó)?P~Ñ.?uRªü¦¦¦ø»½ ü@ù=9¾L&í	F£±¥¥Á|(¿+?5³zOÊorròÐ¡C²¬òåÊ/2I:ÃQRR¢Ìg6£ÐåÊ/XGGGÈ¯_¿Nùòå7Cj0üÎ¬ÌWSSãr¹ØÏ(¿(?uÔ«¨¨Pçö¦§§Gá EùòCb0¥í´'pHÊÁ|(¿+¿AùòC¼ÄÌf³v0Á`p8æ£ü@ùÅbùùOìðôèQqqqaa!åÊ$]KKv6fI@ÉÄ`>Ê_ßää$çöòå§åv»ív»v6æ´´4ÅÂ`>Ê_ì_aaá¢²³³)?P~ üúúúp³13òåëåwÿþýiþ¿ÞH²ï£>¢ü@ùòs¹«533S;³Ãáðz½ì@Ê_tÞ|¿±KùòC<¬Ü±cGÈÙN§:cll39(?P~ñT~òåØ,?Çc·ÛCÀa±XüùçþçK,ùÍßüÍììl	DÞð¥ü@ùÅGùMNNgddø×äää¼ýöÛ(?$[ùEÌg³ÙýW9vìØïüÎïXÿõè÷Ö¨¹âåßû½ßcOR~ üâ ü.]tz¯:ÒÉáòå$)¿ÖÖÖpù^Ïóx<éééßÝû]É>ÿ?	A§ÓÉÎ¤ü@ùÅzù¥¦¦ÊÑ­¯¯Ï¿¦§§GÖdeeQ~ üðnÜ¸ñÊ+¯øOwYr0ä>9`fü[¿~ý'Ø(¿X/?u¼úÛÕLòGùòCâÓ%ï´sZíØ±Cr0ÂôzýÃGËOnêìÙ³ìUÊ_¬_AAéjkkÕÜr§?zô¨¬ÉÏÏ§ü@ù!ñÈ±NM;óâÅÍfó§tþÝßýÝ¯¿òuöÛòíÌÌLfr¦ü@ùÅAùuwwÉ¹««òåD200PSS#¿Öî~ã7~ãÛßþvà	Ï$wÜÜÜU«V½úê«ÿ»òggg;w=LùòòÃÃÃeeeééé)))¥¥¥²f¾7òå¨¹qãÆæÍµ3óØívË5«¿Û«¸Ýî¦¦&«ÕúgögC¥Aùòõò[(?DçÀr0ÑhÔ	3ÿ»½ ü@ùQ~(?Ä¯×k·ÛµùÒÒÒ¬VëÀÀ@à'S~ ü×ßß¯&sVçóæåå]¼xòåxäv»ôzPóeffÖÕÕh¯BùòC_»ÿÈ¨ÊO-:uòå8âr¹BÎÆØØØá¯«Q~ üDå§Nsëïï÷_WW39òCÉdÒÀa0.r6fÊ´üÔññiÀìÍSSS²¬Óé(?P~et#Ü	39Ê¨üòòòÔìªü&'':$Ëz½òåØäv»CÀ±k×®ÙfåÊIT~!gr¾~ý:åÊ±ÆårÙl¶%K²äU9ªÍá6)?P~H¢òSG½unozzzqqqþåÊ³üZ,³1;gæ£ü@ùò[H(?Ì$Óé4!ó½ÃåÊåÊÌëõ:í`¾Å[,øã£ü@ù!¹ÊïîÝ»Ë-KMMCjzzzYYÙèè(åÊehhHÚÁ|²FÖË¥/öËQ~ üDåçt:Cáô×(?P~ù¡ÌWTTd·Û#ÌÆLùòå7#j&çêêjÇ#NLLìÜ¹SÖP~ ü5r@7¯¥¥åyNà ü@ùò¸iGUYöÏêLùòÃ¼R³1hóÍæè%(?P~H¢òS¯ùMNNÞïyÍæÛíã^¯×æ³ÙlQ[òåd,?5Î¯ººZîîòáãÇ+++çÊóGª®¦¦FGQQQccã<æ£ü@ùò¾g§·)?P~IHõf³YÁ`xÎÙ)?P~ üf$åYt:åÊÏC®¥¥E$ Édþ¡òåä-¿BùòKn·Ûn·kgcNKK³X,ÑÌGùòå÷ßÂMÚ<<<LùòÃE9úù(?P~ ü¦obÑ¢·Þz+hå®]»Õæ&ÂlÌÃëõÆÚS~ üDå''Gä¼¼¼GÉï¿ÿ¾:FÏÓð>Ê_7ó5kæ6fÊ(¿Úµk::¨-[¶Ì÷¦S~ üFÙ-Ë|OEùòå7k×®]ó¬£°é(¿n6æ´´´9òåÊïWìÝ»W¬Õßó¯½öåÊDÙn·ÇÔ	(?P~ÿC§ÓÉÁ:++«··÷iÀ8¿ÔÔTÊ´äk2´'p¬Zµjgc¦ü@ùòÙM,ZtäÈ sAù!Ìg0´ùÌfs[[[<6åÊIW~áæóïB¢ü@ùÅ·ÛÝØØr6æýû÷ÇË`>Ê(¿DùòRu6M;¯°°°¾¾~hh(1¾MÊ¿üäØø~nä)?P~ÉF,!gc^µjÕâ÷]Ê(?Ê~IÎétjgc4L­­­	Ö|(?P~(¿däõz/r0_ÍÌGùòåGùQ~ ü"©««Óæ54òåÊòåÔúúúÌfsff¦ö'NÄ×lÌ(?P~åÊ/´í`>±fÍ¹(!óQ~ ü@ùQ~ ü×ëmnn^µjUÈÙ£ÿ¤ü@ùò^ùEFùòK$!gæ5	åÊßÓgÑét(¿Ð××'m§Ì§×ëåa<ù(?P~Hêò[p(¿ù&±ÊÊJílÌ%%%#ÙóQ~ ü@ùQ~ ü×ë°¼Óâ0I;òåÊòåÖÐÐ<æNçØØX¼|Sn·»¾¾>??_ÅbaÊ(?Ê_ÇÏÌÌ|¹âåòòrYøðÃcüÛ¶KKKÓÀ!µdòåÊò£ü0òúK/½´çÿî9ú½£òïÛoçääÄl8NÉ¤ÌWTTd·Û9òåÊò"ß+¯¼²më6êßnøC«ÕSÛ¯þÌ®vf>5Or8(?P~ ü(?ÊÏ.¿ïÔß·Ìß-^¯9¡ü@ùò£ü(?Ì¢übö5?Ërf¾%KØl¶$òåÊò£ü0òÁq~ò`	7¯±±Á|(?P~å9ßÓésÓÓÓ+**Ê¿¶çöú|>Ãa0´ùd%³1S~ ü@ùQ~^@ù=Ï¯­­Mö2Ûínll,**Òæ3LÌÆLùòåGùQ~xå·PÍf[²dIPó¥¥¥Y,óQ~ ü@ùQ~¡üäþ)m§Ì§fcf0åÊåGù!îËÏçó9N£Ñ¨ÌWTTäp8¼^/?/Ê(?ÊòC|$v0³1S~ ü@ùQ~§üä! ÌÇlÌ(?P~åÄ)?ù¢áó13åÊ_|ßèè¨^¯§ü@ù=9Ü`>fc¦ü@ùòûòëìì,--'6ÊÉ j6æfc¦ü@ùòKäòÛ¸q£<³F(¿¿þë¿þYtÉã¹««ëgÀÏ~vmÚ¼~Ï?ÿü;ßùÎW¾ò àKIIGGôïÿGrp`?@ôöö~úé§ì(|òÉ­[·¢üEã¸ü~¹áË¯¥¥e0ºþáþáç?ÿù 08ØÝÝýÅ_ÌÓwvv¾þúë¿þë¿Ô|²FÖË¥ìÿ"98° îÞ½û£ýýåòåËÿþïÿå/ÈåÇ»½H¼wå^m6µ'pèõzfcæÝ^ðn/x·7ÑÞíõ?ÏQ~H¶òDÈ8JJJÌGùòåå²)?$pùy½Þ³gÏ<CB0úwrP~ ü@ùQ~^|ù¹ÝîÂÂÂ³1ÇÚåÊßB¢ü¿å788xøðaí_àÈÍÍ;öÐÐ»òåÊò£ü÷åwóæÍcÕªUMMMKùòåGùQ~ûòÇee¥¶ùFc[['pP~ ü@ùQ~â¾ü<Ãá(**9ü@ùò£ü(?$Bù544hóÉóQ~ ü@ùQ~¤üúúúvíÚr6æ¦¦&¯×ËÞ£ü@ùò£ü(?ÄùÉ|óæÍÚæ3­­­æ£ü@ùò£ü(?Äù¹ÝîææfílÌf³ùæÍì.Ê(?ÊòCÜßÈÈÜCæ«««s¹(Ê(?ÊòCÜûøã¿ùÍofff5_QQQSSÛífQ~ ü@ùQ~âÜñªªªRRR´ùòåGùò£ü¼^oÈùÄæÍåÞHóQ~åÊòCÜs»Ý!óýÚ¯ýÚþýûÙEåÊå¸çr¹,KZZvf¾#Güô§?eòåÊòCÜÉd93Ãáðù|³ý»½ ü@ùò£ü(?ÄI:	;É;í`¾mÛ¶>»S~ ü@ùò£ü¯Ünwcc£öÌÌÌmäQ~ ü@ùò£üÍfÓÀ¡×ëånnf>Ê(?P~âÉÀÀ@MMv0ß+<OëR~ ü@ùò£üNç;´Íg4å¢ÌÌGùòåÊòCLóz½---kÖ¬	¾´´4³Ù<«'oÊ(?P~bÔØØXss³^¯j¾%KÔÕÕÍa6fÊ(?P~bÎÐÐÐáÃ333/??¿©©)Ü	(?P~ ü(?Ê/ôõõÌg0Z[[½^ïóÜ8åÊ(?Ê±ò`¼Ó6ß¶mÛ^ÔÝòåÊåvöìÙ à¬©©¹yóæüB(?P~ ü(?,ü#èY366öÂ¿åÊ(?ÊÌív§¥¥Ió­Zµê³1S~ ü@ùò£ü(¿øvâÄÖÖÖÌÆLùòåÊò£ü@ùòåÊò£ü@ùòåÊòåÊ ü@ùQ~ ü@ùòåGùQ~ ü@ùòåGùQ~ ü@ùòåGùQ~ ü@ùòåGùQ~ ü@ùòåGùQ~ ü@ùòåGùQ~ ü@ùòåGùQ~ ü@ùòåGùQ~ ü@ùòåGùòåÊòåGùòåÊåGùòåÊåGùòåÊåGùòåÊåGùòåÊåGùòåÊåGùòåÊåGùòåÊåGùòåÊåÊ(?ÊåÊ(?P~åÊ(?P~åÊ(?P~åÊ(?P~åÊ(?P~åÊ(?P~åÊ(?P~åÊ(?P~@ùòåÊòåÊûåÊ(?P~åÊ(?P~åÊ(?P~åÊ(?P~åÊ(?P~åÊ(?P~åÊ(?P~åÊ(?P~@ùòåÊòåÊ@ùQ~ ü@ùòåGùQ~ ü@ùòåGùQ~ ü@ùòåGùQ~ ü@ùòåGùQ~ ü@ùòåå×ÕÕU^^®ÓéV®ÓÓCùòåÊ_ÂßòåË¯_¿.çÏ/..Ö_GGÇ/¢«»»hhèÀ/~ñÏÿüÏ·oßf?@ÈaAì	§ÓÉ~"å'¿DùÆqùJOO×ßñãÇ¯DW[[ÛåË¯W®|2ý!98° >ýôSîðûøã£ÿE¡üäi«ÕÊ»½àÝ^ðn/x·¼Û°ïö*ÕÕÕòåÊ(¿*¿E_R[,±EùòåÊ_|_ 6¼òåÊ(¿Ä)?½^¿(åÊ(?P~	[~Q~ ü@ùòåGùQ~ ü@ùòåGùQ~ ü@ùòåGùQ~ ü@ùòåGùQ~ ü@ùòåGùò£ü@ùòåGùòåÊ ü(?P~ ü@ùò£ü(?P~ ü@ùò£ü(?P~ ü@ùò£ü(?P~ ü@ùò£ü(?P~ ü@ùòK¤òûþ÷¿ßÒÒ2]üñ?ýÓ?ÿ÷Ï~ÃØò;áßþíß² üÍßüËåòu»ÝY~ýýýGý¾ùÑE¼$(?Ê(?P~ ü@ùÅÑÑQ½^ïÿ°¿¿õêÕ:®¬¬¬³³ýÌw¥½½Ñ"JÉ~gxøðá¢ìd¾3x½^ÅZXXØÑÑÁþIòûCà!%%òuÒv¥¥¥Çq	¾K.ÉÂõë×sssÙEÉ|g>¯¼¼gzî/^'öwqìØ±wÞygjjJ²oÙ²eì")sçÎ:uòu7n¼sçNÈá+WV®.Jò;ÃÛo¿ýî»ïR~Ü$ûÔïàÎPVV600ÀáþèÞ½ë×¯-äéjf»Ió2OVV¬<þ<;'ï÷ïß¯¨¨ßì)?îË/ß°aN§»ÄíÛ·Ù9É|g»ÁÉ'ÓÓÓ-[vëÖ-vÙ ¶nÝÚÝÝMùÅñðéôè®¼¼<vN2ßä|íÚµp÷$çaxxwüÎræÌYßä7v;wî¬]»6V¶Ïïê;vN2ßý*vG%55ÌwÀxàþðtzèçÉ')¿xý._¾¼¿¿_ººº6lØÀÎáÉþ)¯ùqg>2¸§Ó¯ùqdHò;ÃÞ½?øà§ÓsAvÏëÖ­ëíí¥üâõGØÓÓSVV&¿Æ­_¿þáÃìÊòãÎ º»»KKKåÈPYY)ñÇÎIæ;ÃøøøÖ­[Õ Ï;wî°sx¦HMM¢ü@ùòåÊåÊ(?P~ ü@ùòåÊòåÊÌÉûï¿¿nÝºôië×¯ÿè£~åø5-n¶¡¶V¯×Ë·æñxÖËNWPP0555ÛÛÊ@ü9zôè"·Þz+Ê¯¾¾^V677­ojjõo¾ùæn(?q¦§§GF§Ó>Ú7íÌ3ò¡¬ìííMò»wï¬reÐúÒÒRYïr¹(?Ä÷Úk¯IÓ¼óÎ;+?.+wíÚØ=W¯^r(,//eÿ'?~üØjµfeeÉE¹¹¹µµµoª:N©+¹H®åÊ 5ÙÙÙëÖ­»|ù²|h66¬­­-òí¨dÔEíííá*mÃ²¾««Ë¿æúõë²Æh4ú×;v,??_n*==ûöíÃÃÃÚòÓÞ~Ðò´téR©û÷ï®|ðà¬Ôëõe¤»»[]ºeË öíÛ§.êëëKII	y-õ¡ºtëÖ­SSSÒ©©©>O.ÿ¥222Ôð»·#!7Oû?>0gýqyîÜ9öÝÈúõëg[~6åL½±âµhtX`ÙìÜ¹sb,È6mRªÐQí¨^ºbSUWW«e¹³³SÝHàm9rDÚN½Ùº÷nYséÒ%Yÿeù7ÞxæíH5Êò	Ûò;ÌÉÉïwttT>ÿeË%7'''Õ'Èå[xúå»Ãééé³-¿ò/?²üüëUÙ¨`ÃÃÃò¡4ú°¼¼lÙ=¤Ø<yâ¿ù WÑü×R>xðÀÿÉRKþ7|åYv:Ï¼©LùðÑ£GÛnLÞä¢wß÷éïh×ÖÖ~T ôhEEzIr¶åaSP~°ÀÔ]+=¬"´¿ïÜ¹£âÏ:þÁyÚ7aZ*p.YÎÍÍMMMõz½rãÙÙÙþKy;á:,H¿TYÿe¹¯¯Ïigg§l@Èwg^~6åLu;yòdàJ5×IÐþ±£££Ú²zëëëÕ¯þdT/ÈùßNfíÛ·O½É+ÿ[­Vÿú·£^cøð¡úðÑ£GÏÃ]»v­&²`¼Hç+µ··G.?ª½á¿4Â¦ ü`©3$t:Ý©S§Ô¬.§ONMMÕ±iÓ&©¯×«Þõ«Æù©áqwîÜ	 ¨B°¶¶V:IuëW%d©mÕWïèèð¯p;j`ççñxÔgF(¿3gÎø_o9ð"5ä±¯¯O¾ìpå§ZSZY>MsûöíFØT,<5ËqcÇýÏñk#ÿ²ÿRÿI~»wïÌÊ@/^P~O§ÿØ¬ÏÍÍ|#8ÂíÈfnÿ¼Úpßì'OÒÓÓÕ·ô÷Æ¿DNNü¯&v	¼Mõ¤êTÿ¥6å1Aêdýúõ©ÓÖ­[§N°*¿ööv5m^YYÙµk×üz<Cåçç«Z:pà×ëõ_zåÊ	²»ÝtÚ-õ÷îZîvllh0Â|~~V«5hzett´ººZö@VV|.Ë?CMàmKÚªe4»ºº¾bM@ùòåÊ(?P~ ü@ùò ü@ùòåÊ(?ÌÞÿ÷Þ/J²TIEND®B`


û÷ï_¶lYê ÝÇ| A»ÊåÇß)++Þ>¾¦;V~(?²Www÷=R'j)//­OÚU~(?²¬üjjj¾ñoðµººZù¡üÈ.]jhhÔ|³gÏÞ»woooïhý åòc¬ÊïÌ3óçÏ/..Ï:tèÎ¯¶££#ìÃuÎ5ëøñãÊåGVÅbõõõÓ¦MKj¾Å777e¢åòcâË/É9ñÛ¢åûöéMW~(?²E(°Í7O:5©ùV¬XÑÞÞ>F?Tù¡üòY9s&^~ÇËÓ§OW~ ü&¹ÖÖÖÔæ>i÷ôéÓcú£Ê1)¿hG-Då×ßßïsAùMfÍÍÍ+V¬H´»cÇOÔ¢üP~L|ùE39G¯óò»yóæöíÛ£T~ ü&íîÚµëÒ¥KãvKÊ1)¿ööö´39¿óÎ;Êßäª.4_øOoê'íîÝ»wT&jQ~(?&¾üCË/ÆöÍ?e(?"ìñ|òÉÔA»Ë-kjjõA»ÊåÇßP~(?&mÞ¼¹°°0©ùÖ­[×ÞÞ>QÍ§üP~aùÅvÄ]¾|yþüù³gÏV~ ürRHº¦¦¦ÊÊÊÔ7ºækkkØæS~(?ÆµünÞ¼il/(¿ÔÛÛ/uÇÔ©SëëëÏ=97Uù¡üÍò=öAÍ1CùòËÝÝÝa·SRR´¯+,,¬««ZÊ	+¿÷ß?ïø§w$Ù÷úë¯+?P~9 T]CCCjó5aGtõêÕÌ¼ÙÊåÇh_¼±>°«üP~LTóÕÖÖ¦~êÚÜ¹szÇîGßyP*?cR~Eù¡ü;a÷²aÃÔæ«ªªjnn»¡&zè¡3f|å+_Q~(?2«ünÞ¼9þüâââø3g~õ«_U~ ü²´ùBÞ¥¾w9¬Îïþîï~úÓþýGçw>ò¹ë®»þèþHù¡üÈ ò;¦¤á½Ñ^r×®]Ê_¶&jY°`Aê ÝÚÚÚ®®®q¸±X¬¤¤ä©'Ùýü±ÇzzzÊL)¿°sììì¯9yòdX3útåÊ/óuww766¦NÔ"¬¡¡a<í¶´´¬üñìþ=ú´òCù)åïíïïÿ×2ÉòåÍv¢°~ü?i7Ú¢_[T~úÔ§FVÊåÇß¬Y³ÂrÛ¶mÑ0·7nìÜ¹3¬)//W~ ü2Sh©úúúÔæíNÔ'p½hiiéê-ñìûüç>?gÎÝåòcLÊïÄigr>~ü¸òåiºººÒNÔ2Öv(ìÖþWÑÿúÍßüÍÏþó¿¾ü×?ùÉOìP¯òCù1VåxqÑ¢EEEEyyyÅÅÅ.kÆú¦+?ÃªÔA»!7lØ0þ;ÁÛtÏ=¿÷¿·wïÞK.øzÊ±*¿	¡üP~E___sssÚæ«­­ÍÀO]-ÊåòS~(¿ÉÕ|MMM0hWù¡üÈ©ò;sæL4s4·¬¬ìÐ¡CÊßDíVTTdÈ ]åò#wÊïÈ#ñ½jT~Ñò¾û(¿qváÂ´µLì ]åò#wÊ¯¼¼<ìUÏ9/¿ãÇÉß8ÅbÚmkkTÍ§üP~aùEûÖfoîïïËùùùÊß8»Ôæ[·nÝdNåòcLÊ¯¬¬,½/*¿7onß¾=z?òå7ÖÍvÐîÖ­[Ã£uoåòcLÊ¯½½=íLÎï¼óòå7¢A»,HÚíÖÖÖ?Þ&R~(?ÆªüCË/ÆöÍ?¦KP~(¿I¨··7íD-¥¥¥Ï>ûìÕ«Wm"åòcÌËoB(?ß¤MÔ:hwöìÙöì$µ(?ÊOù¡ürkhhH;QË«¯¾:	í*? áykÎ9a_´hÑ¢°ÇQ~ üî¼ùÒNÔ²bÅÃk>åòcÊ¯­­-í³gÏ*?P~#Ý6lHm¾Õ«W·´´h>åòcÂÊ/É¹ººº§§'¼~ýú<ÖÌ5Kùòtá?©µ[¶l1QòCù1ñåíÿã³:+?P~Cl¾´vëêêÆúòå§ü*zÍïæÍñ57nÜð(¿!êîîÞ³gOjóM6íÉ'¼téòCùAå½Ï¯ºº:_8ùÑG­^½Úûü@ùÝV¨º´µTTT¨Eù¡üÈÐòr;ctØWù¡ü²Wøá;¦M´»X°`Áþýû£7£üP~dbùåÝN~~¾òåÝï½©v-[f¢åò#Êo¢(?_viiiY±bERð¬­­=ö¬æS~(?²£ü´ùâÅÊ_ôI»,Hj¾úúúqøoåÊÑ,¿°ÿò¿´rëÖ­fuI^~===÷îÿ'5_xüÀ¡üP~deùÂ»ò²²²Ë/ßüæ7£û½½Où¡ü2ßvìØÚ|sçÎmllÔ|ÊåGßÇ·^ávë³fÍ6mÚ4Ö7]ù¡ü2ÐùóçëëëÓÚmjjòf>åò#Ê/8vìX|¿ÿþq¸éÊåQÂ£cóæÍ©v«ªªÆÿ¡òCù1å÷è£F»øøÁ|PùÁd(¿¾¾¾uëÖ¥Ú­©©Ñ|ÊåG®_~~~ØËO>ýÔ©S'¼Ï¯  @ùA_h¾.^¼8íD-í*?¹Y~aGÿôÓO'­ûc!WË¯§§gÏ=³gÏN´ÛÐÐ ùÊ¿æóëBR~(¿ñwéÒ¥g6õvMÔ¢üP~Lò(Êå7ºººêëëS'jijjêííõwT~(?r¹üÂ?ñxîà'doù»ýÖ­[SíVVV¨Eù¡üP~Êr¡üBÒµµµUUUMIa¢åòCù)?ÈòÍ×ÔÔ4wîÜ´vÃ£ÀLù¡üP~Ê²¾üº»»÷ìÙÚ|&jQ~ üò)¿Ð|á1eÐ®òå§üò#Ë/ÕÖÖ¦´»gÏÍ§ü@ù)?å¹P~áþ/uÐîÚU~ ü_rùNùAÆ_xì´«ü@ù)¿aÈ»üü|åU~ÍÍÍ©Í7uêÔÍ§ü@ù)¿Ì¥üP~Ãj¾´µ´«ü@ù)?å§üÈòíVTT´«ü@ù¡ü_èëë;úôáÃ;;;_äÂi'j;wncc£æS~ üòS~Y©««ëî»ïþô¼OÿæÊßüÄ'>ñÏ|æêÕ«¹ü¢ZÚU~ÊåòS~¹¦§§çWïúÕë7ìüâÎðïéO¯X±bÃ³üÂ¢¦¦&µùÚU~ üP~Ê/>|xñâÅQöEÿ¶ÿáö3g^ºtiR_ÚZ|Ò®ò³P~(?åS^|ñÅªÏT%_ø7þü,Ê;)¿hÐîÚEù¡üP~Ê/÷MÚ×üzzzöïß:QA»(?ÊOùå¬@úÔ§Þç÷ÙÏ~6~á_øJKKÚEù¡üP~ÊoÒéêêúÕ_ýÕOÏûôoüÆo|òüÌg>E/ø«üÂïîù©ÍWYYiÐ.ÊåòS~Eð×9|øðéÓ§³.R~gÏ­¯¯6mA»(?ÊOùÅ/¿öööÍ7§NÔVj>Êå§üÈòëëëknn^·n]ê ÝÓ§OÛn(?ÊOùõå¯¥¥eÙ²eIÍ7mÚ´úúú.Øb(?ÊOùõåMÔRQQÔ|¥¥¥&jAù¡üP~Ê)¿p.))Ij¾òòòÐí¢üP~(?åG.8þü¾ð¤æ[¶lÙáÃm"ÊÉR~aÏUQQ¡üÈI§OÞ²eKê Ýªªª¶¶6¯ó¡üP~L®òëèèX¸pax"T~äp×y7%EmmmÜ0ÊåòMë×¯ïêê¤üþüÏÿüïÇ×÷¾÷½ãÇÿ=Ô/¼ðk¿ökIÁ÷K¿ôK7o~óÍ7mFÑ'¾ûÝïÚ®ýèG---¶C&ËâòûÏ8pù577_õWõãÿø<ÓÏþó¯|å+þô§ïÿïÿýÅ/~ñÛßþößþíßÚJ®üä'ííí¶£ëÜ¹so½õíÉr¹üí%óuww?ûì³³gÏNj¾¹sç¾øâÑD-CÿÜ^p´GÉ©£½ñçEåG¶Åb©µ,[¶ìàÁ8ÊåÇ$-¿´-¨üÈ.]]]µµµií>|8uÐ®òCù¡üP~ÊìvÐnHÀ6òI»ÊåòCùÝò#sôõõ577§m¾ÚÚÚX,6øÅÊåòS~dÞÞÞ¦¦¦¹sç&5_III¸^¸pa(W¢üP~(?ò#£uww§ý¤Ý°>´;DÊåòCù)?2T,«¯¯Om¾¹sç655àS×ÊåòS~dAí677øvÊåòS~d>i7¬¼óû¡òCù¡üP~Ê××××ÔÔTYYvÐnWW×¨üåòCù¡üi A»C¨Eù¡üP~(?åGhÐn4QË°í*?Êå§üÈPÚÝ³gÏX4òCù¡üP~ÊÇÿ/vÐî'jQ~(?ÊOùqÚÛÛ´ÛÖÖ6ÖÍ§üP~(?òc<°[½zuê Ý6ó]Kù¡üP~(?åÇèëë;xðàÒNÔ2ºvÊåòS~Lîîî½÷¦NÔRRRÒÐÐ0!Í§üP~(?òc]½zu'jQ~(?ÊOù1Þºººêêê¦M:h÷àÁ===p#ÊåòS~ÜÓ§O§¨eõêÕã0QòCù¡üP~ÊñÐÚÚv¢ñ´«üP~(?òc/í Ý­[·>:co¶òCù¡üP~Ê¡êíímllLm¾iÓ¦=þøãçÏÏðÛ¯üP~(?òãö®^½ºk×®ÒÒÒ¤æ+//ÏA»ÊåòCù)?FÁ¥KÒNÔ2wîÜÆÆÆ´«üP~(?òã=¶¾¾>u¢+V477gÔ ]åòCù¡ü#ÔÞÞ¾nÝºÔZ2vÐ®òCù¡üP~Ê4_È»¤æËüA»ÊåòCù)?ª¯¯¯©©iñâÅY:hWù¡üP~(?åÇíuww?qEEEVÚU~(?ÊOù1X,ÖÐÐvÊåòS~Ø|i?i7í*?Êå§üHþ©8r`Ð®òCù¡üP~ÊÿÔ×××ÜÜúI»uuu90hWù¡üP~(?åÇÿÿI»MMMsçÎM´»k×®X,6©¶òCù¡üP~Ê/7EvSpïÝ»÷Ò¥Kp(?Êå§ürÍ$´«üP~(?òtºººÒÚ]¶lYnÚU~(?ÊOùM"áo:#+ÃsæS~(?ÊOùe½í[¶léìì´ÊåòS~Yo A»¹ôI»ÊåòCù)¿ImA»¹÷I»ÊåòCù)¿I*Õ××§6_EEÅÞ½'ó ]åòCù¡ü_®©¬¬L´ÛÔÔdòCù¡üP~Ê/×´¶¶&Ú'måòCù¡ü_Îª¬¬¬©©<´«üP~(?ò¼ØU~(?ÊOùòCù¡üP~ÊÊåòS~(?P~(?òCùòCù¡üÊÊå§üP~(?P~(?åòCùòS~ÊOù¡üP~ üòS~(?ÊÏvP~ÊÊåòS~ üP~(?òåòCù¡üÊÊå§üP~ üP~(?åòåòCù)?ÊÊOù¡üP~ üòS~(?(?å§üÊåòCù)?P~(?ÊOùòCù¡üP~ÊÊåòS~(?P~(?òCùòCù¡üÊÊå§üP~(?P~(?åòCùòS~ÊOù¡üP~ üòS~(?Êå§ü@ù¡üP~(?åÊåòCù)?P~(?Ê/8~üøÒ¥Kóóó,YròäIåòåò#gËoÞ¼yï¼óNXxåWæÏZ~íííÿo|8qâÂÿFÕOúÓüä'¶£+ü75ìBmFWooo(?Û!eqù%***J-¿ç¾u|µ´´|+ªp§zë­·lF×·¿ýí°Ë²uo¾ù¦Ér¡üN8QWWçh/ö£½8ÚKÎí~½ººº§§Gù¡ü@ù¡üÈ©òò_¢/^¬­­M[ÊåÊåGv_¢öööµk×WÚsÊÊÜ)¿)	ÊÊ-¿Á)?(?ÊOù¡ü@ù¡üP~Êåòå§üòCù¡ü@ù)?å§üP~(?P~ÊOùòCù¡üP~ÊÊåòS~ üP~(?òCùòCù¡üÊÊå§üP~ üP~(?åòCù)?¹T~_úÒÏ¯7ß|ó?üáyUÇ;zô¨íÀèúÑ~tøðaÛÑõóÿüàÁ¶C&ëîîÎÍò;sæÌÎ;ÿÿ2øÑ)^öp´åò@ù üP~(¿quåÊ¤G2ÅÖcÔîW~øá¶£r¿êíí­­­-((=v»íÃ¨Ü¯wVyyy¶òË).Lz&îëë[ºt©§gFñ~uèÐ¡ðmË0º÷«Ý»w?÷Üsýýý!ûæÌc1ZÏ_~yß¾6òË)ë×¯ïêêJºÇõ«_ýÚ×¾¦üÅûUÈ¾7ÞxÃatïW-:ö¬-Ã¨?çÎ[³fí£ürt3%ÜãßÿýåËÿC+?Fñ~5oÞ¼µk×æçç×ï½gã0*÷«pzáæÌóî»ïÚ8Êý*rß÷8qÂQ~¹÷õcÇ¥ÀîI/.Y²ÄÆaTîWyyyá¿á?6£µ¿êêêZ¹r¥Í¢ü&Å=~Êÿdã0ºåØ8Êýª¬¬,¾oã0Zû«Ý»w¿ðÂ6òÐ²Q¼_Í7/|ë5¿µk×Ú8ÊýêÑGíµ×ÂÂ3g*++mFëypÕªU§N²YòÞ¯N8±páÂüüüÕ«Wø³qûÕµk×î»ï¾èý£]]]6£õ<XPPÐßßo³(?Êåò@ù üÊåò@ù üP~(?Ê@ù üP~cïßüæªU«nY³fÍë¯¿þ?ö_·dÍÞ6Ý­­¨¨¿ZOOOÒú°&??Ö¬YýýýÃ½Nåd;wNIñå/9Êo×®]aåþýûÖ¿øâaý3Ï<3ëP~@9yòdhüüü^z©ïaå©S§r¦üÎ;V.Y²$iýÂÃúX,¦üåä¾|04ÍsÏ=¸òùç+·nÝØ=Gå¢péÒ¥a9þÍôQ]]ÝôéÓÃY¥¥¥Û¶mK<¨ÚÖÖê*.ÛÚÚÔRaÍ3V­ZõöÛo555I7¬¥¥eðëÎ7):ëÈ#UÚÚµkÃúãÇÇ×¼óÎ;aMUUU|ÍîÝ»ËËËÃUÝÿý/^L-¿ÔëOZ3ÈMÀDºë®»Bµ¼ÿþû+?øà°²¢¢"±l8q":wÓ¦MIg=öØcÑYyyyi/Î½ï¾ûúûûC;ôõõsÃ×PNÅÅÅÑÛï¹°öæ¥þ¦¯¼òJbÎÆãòå_g_Ò¬Y³f¸å7ÈMÀì¦ÙgM:,±lxàë·prãÆÑ¹QèDí½t-:«ºº:z±0,wttDWxO?ýth»è`ëÃ?Ö¼ñÆa9|Ë=ôÐm¯'Tc8¾!ñ¶¥ýBMÎ93ü¾W®'Ã×pËCnÞ¼y3úY³f_áãÿ::Üòä¦Ê`âË/H[~ñõQÙDÁx1Íti89gÎGy$Û7âW¾'éU´ø¥¢|ðAüC-Åø¯a¹­­í¶×*3¼|ùrâmè=yO<ñD8ëk_ûÚÇÿuDÛ¶mß*0_èÑåËG/I·ü¹©ò`Ñ]×¯_OÓÓV³ixvuuEñøóRÂ&µTâa¹´´´   ··73âçÞözê°$gÎ"5,¯a¹³³3~nGGG¸i½ü¹©ò`ÑÝ^xáÄÑ<âï¼råJêKY§NÚµkWtì5ÑrñÃ©·í³Ç,:È¾ÖÕÕÅ×r=Ñkl~øatòòåËÃ]¹re87È&kâYÑ8ßpÖ#G®]»6xùÅ«4Úñs¹©ò`ÑüüüûöE³º¼ôÒK©£16nÜ*§··7:½Ï/zâ£Ü¶m[è¤hÔm|^´¶~z|ý ×½±/z_OOOôßâ¯Æ_9ñ¬è-á§0PùE­Z9|[(Îûï¿?ñÜAn* ü&^4ËqÝ»wÿ÷þë(âËñã¤ñAq?üpbV&:tèÐ å÷ñ­ÛëKKKr=áf$Þ°ø¸Ú~Ù7nE¿BÒ1îõë×'þ3g¯ÑÄ.×½$ujüÜAn* ü2B¨5kÖÜ²jÕªhmRù9r$6oÑ¢EÇÛÓÓ³ûöòòò¨xâÞÞÞø¹­­­Ë/A6kÖ¬ÆÆÆ¤ëL½%áªÂúG4iý@×nR4Ñà óùÅÕÕÕ%Mï¹råJuuuØÓ§O¿E,ÏPx×®]im«ªªªãÇ'ýÄAn* üP~(?Êåò@ù üP~Êåò@ù üP~(?ïÿé´j¶IEND®B`


Îw>îü¹'÷¹÷ûnúÑá&@ù üP~(?Êåò@ù(?Êåò@ù×çïù§z*ëúa¿aûòË//[¶lÜ%/¼ðÂe¯ðÊ/rS¢¢¢øþyóæ=ýôÓW±q>¿þþýûo¹åâââøéý®á¦;P~ÀH/¿':u*_ÊïGÉìÍ7ç¸¶A]d ´zòÉ'»q®z3§~nmm­òpÝ+Ü~ûíyQ~NRõùçïëë»xñb,ÄÉXÙÞÞõª~?úÜ¹s7n5QcCV~É?úè£X¾páÂºkåäwùÕÕÕÅ×_~y ¼x÷Ýwï¼óÎäPiMMMGGG¿ïÜ³gÏ¤I.]Z³wïÞ/[¶ìôéÓû÷ï7o^IIÉ+Ò__|ôÑGËÊÊÃñ#RgÔ7wßw¬O?özê©XY__õwìE2tÄb¬9¨õ5¹tWwÁÜå×ïÜ¸_æÎåÊ±ms¿:ynúÝZ[[ãÚb;Ä=g¥.[2b:Î*--]¿~OO?.P~À-¿sçÎO:52#à>§óôïìhEEEñõ;îø$ÛáÈ[n¹%â&u2*$ý¾sùòå¹ûfÚ´i±þìÙ³é+£,cå¬Y³²þ½H¿Y¼æ7öìAmÜwÕ¼òò;räHúõÄ/;ØòK¿[ÛÛÛ)N¾ùöÛoïw×®]ë0BË/^íµô×ÀÒ#àþûïåU«VtI,ÄÉXþ6mºxñbòªU²æèëë;pà@r2®9NîÙ³'£Ë&ïfÛ½w,¿÷ÞégÔ7ÅÅÅY×§_ö/2ÐéR¯vãd½UWÁoòëwÁ(¶X®©©9þ|4ªÏ®¼üÒïÖ¸X³oß¾XNîÖ5kÖ$ßáûï¿ËqoÆòøñãýqòFnùx"åxRï·~êÔ©±üÁ$'ã	>NN2%ýRç¦ÖDm¤ìêêúä?¦Ç¢¢0-Z¼ÈtÙò+))Éúd]I/ª¸lDLUUUkkkÖv%'ë­ºê^yù%ïe<útúTù¥ß­Éµ¥5ÉY.3fÌrÿB|üñÇþ²@ù#½ü"ÎJKKãù»··7ækfoz¤Ëz¹OFeö;âyÙ1ªeeeÉáéÌ¼KH¿k»´M.»Ñ®dãÔ£WwÁ+?Ú%mûÜÌ»µß7ÉY'NHâ/U---þ¸@ù#ºüÂK/½'Ss $+8ë÷êT¬¼ÈrîÜ¹Éì*÷î2»òK^L&X©©©Y¿~ýGôÌ3ÏÄÊ;ï¼3kù]ÉE®ºü»qR®úW^~Wò_ªí¢ûsoÿñãÇÇ~Ó=ztË-É!æÔ8h@ù#·ü>¹ônýÔù5ÉÈßU«V»$yjà®½ü×½ÚÛÛ£?~úé+)¿¶¶¶äU¥Å$¯ç%^íµ¬¿ã`/2¨òË½q-ÙsI¿+ìV½òKÞáûñÇGØÝvÛm]øÄOÄÆ:Î½ý[Ýß[2,Xþk&ï×<qâD#ïòFVù:u*õ®dMGGG¿÷xM:55TöZÊ/Õ")S¦Ä×ôñ­Yoó½÷ÞyäqñâÅ9~ÍA]dPåã¤&dî7óUlÕ«(¿£G¦_ÿÒ¥KÓÏí·M¢Õr_2-bÖ!/É«ªéî»ï>ü<(¿	M_ÿÎ;ïÜyç%ÔÔÔdç¸ºòëêêkëzðÁ#Räî¦¦¦yóæM6íÙgýÒ¾zÍéÚ/2¨òË½q^íµ¿âââÔxéµU¯¢üBKKË3â·ëO§§3ÇÉè³ä§WUU:tè²¯¹îÙ³gÑ¢EqmñKíØ±#µ¾§§ç¡J^L|»²··×(?âÂ÷Þï æ»ää l2X»¯¯/	ú¹sçzÀÊ Ð$ïóëç;ßù-(?BÓÓÓ³qãÆiÓ¦%tcáG±YåòP~(?Êåò@ù üP~(?åò@ù¤íµ×***/_>ØËÎ7ï¦n:qâDjM,ÇbwvÉÕÏ*w²9b(**7nÏÚµkKKKccN81Îz÷Ýw=Àå eeeÑ1/½ì£>Ý¾jÍã?k¶lÙRØå,Ç/ËkÖ¬INÖÕÕÅÉûöÅòþýûcyÖ¬Y`òFÒÞäâéèÑ£qÙeË¥ÖÜrË-±æÈ#CßaCÿ£cyÜ¸qÉÉ8yîÜ9*@ù#7ûÒkæ¡8qâøñã7oÞÜïÛ¾ô¥/-Z´¨ß5¤^2å³gÏÆr¬IÎzÿý÷«ªª".¼ymé?=÷EöÙÊÊÊøAvþüùÚÚÚ¸lÜø7&/aF.]º4®-ÖÇ5ðÁ7®ü¦O'çÎàÀ-@ù#7þåoûÛ±¼cÇh¬XøÎw¾þ=öìI½§-åþûï³yæX¯±_µxñâÝ»wÇÂï½ëËËË³^[úÈè¹^z)êêê2oüÚµkc9¾áW^G4UcçÎkooä%ÉLWQ~/^íË÷ÜsOrÖk¯½ºÂåË¿ýöÛ]òFnùUTTÄòK2Ã+ëÛÚÚâ¬U«VÅòí·ß$]êÜ£GnÚ´iÑ¢EÉ¬×Ö¯±r_$¹a'NÌ¼ìÔ©SMÑ|±2¾3.-ÛÛÛu%srÛâÊ#ûÒï>|8â5õ=ñC=ÀåÐò+..N-ÇBÌAéküøññ===ÉAÕXõôÓOÇ¥"ß~ûíôkèwmé'¯ä"Ý°H±ôñj|ùå'M¬)--M^¼.¯ùå°oß¾hÍôßÊqåW^^þ_ò²Ùes§¶¶6ÎMM­OF<äË/÷ER7,b.ó²S¦LI.ÛïæõõõíÞ½;xz±pPe°åÛ¿ üZ~É,-Ï<óLò>¿ÇüJrçå_N½xöÒK/¥Ö'yìØ±äýWR~¹/²iÓ¦ïï±pÿý÷g^ö¾ûîåçîøñãÉÞXYYYËo¿ýöéÓ§caöìÙ7®ü,XgíÝ»7[ZZbùÞïõ0BË¯¯¯/êjü%ésòå.¿?þ89Ò_ÓßôõSZZ:qâÄ7^aùå¾HkkkË-·¤¤OOOÏúõëãÜvÛmÉ0Þ¾d°ð²eËÞyçAmÌ#Â96EüÄÚÚÚ¸ñ±¦L²víÚ¸I`ò@ù üÊåò?þñO<9Ä?ô>ø÷ÿwB×%¶!vÉä/ðË_þò_þå_l¿øÅ/þã?þCù]ú§ñ7Ä?ô§?ýééÓ§=	ÿüÏÿ|âÄ	ÛO.Íö;ÛO.MKù·û·¶=öÄòCù¡üP~(?å§üP~(?ÊOù)?ÊåòS~ÊåòCù¡üòCù¡üP~(?å§üP~(?ÊOù)?ÊåòS~ÊåòCù¡üÊå§üP~(?åòCù¡üP~ÊOù¡üP~(?òS~(?Êå§üÊåòCù)?åòCù¡üP~ÊOù¡üP~(?_~ß¡C.\¼`Á#G(?Êåò+Øò5kÖo¾/¼ðÂìÙ³3Ëo÷îÝ­üä'ï¾ûîGðÑGÿï%¶!v±s°§Núáh;xã7Î=;Ä?4Ë/Ý¸qã2ËïÉ'üÉÐúÁ~ÐÖÖöøÉOZ[[ãö¶!v±s°?þñ¿ÿýïÛ$ZZZ80Ä?´ÊïðáÃõõõöâh/öâh/öìÑÞÄùóçkjjzzzÊåòCùrù:uª®®.kl)?Êåò+òkkk[±bEWWWÖsÊåòCùNùUTTÜFù¡üP~(?_Á_nÊåòCù¡üòCù¡üP~(?å§üP~(?ÊOù)?ÊåòS~ÊåòCù¡üÊOù¡üP~(?åòCù¡ü@ù)?ÊåòS~ÊåòCù¡üòCù¡üP~(?å§üP~(?ÊOù)?ÊåòS~ÊåòCù¡üòCù¡üP~(?å§üP~(?ÊOù¡ülÊå§üP~(?ÊOù)?ÊåòS~ÊåòCù¡üòCù¡üP~(?å§üP~(?ÊOù)?ÊåòS~ÊåòCù¡üòCù¡üP~¨¯¯¯¥¥¥±±Qù)?åòCù¡üVì¨7lØPQQqÓ%C¼ßV~ÊåòCùqÃõööîÜ¹³²²ò¦ÿ.*Pù)?åòCù¡üDü1ÖÕÕÝ!VF<(?å§üP~(?_~ûðÃ·oß>þüÌà;vìêÕ«[ZZzøV)?åòCù¡ü¸núúú¢®"ì"ï2ïæonjj:sæÌpÝ<å§üP~(?×AìSC7ú½ÈW[[ûÖ[oûT~ÊåòCùqõº»»wìØ9t#ëãÜørkòCù¡üP~xÒ¯­­ÍzT·¢¢¢±±qîòCù¡üP~Bggç¶mÛæÌ|cÆ©®®nmmíëë7^ù)?ÊåÇåõöö677GØEÞe6_`ä`Dáÿ-òCù¡üP~©¶¡¡aòäÉÁ+ã¬<úsS~ÊåòCùÅenìÚµkè'äS~ÊOù¡üP~(¿ë¦¯¯¯µµ5ïn(?åòCùòÜòä¡ÊOù¡üP~ ü.ï²C7FÔ|ÊOù)?ÊåwÏ¡3tCù)?Ê_¯¹¹9ïn(?åòCùòû/©¡YêVTTÄóøÈOù)?åòCù¡ürcÁÝP~ÊåòQ]~ÉÐªªª&ä+¤¡ÊOù¡üP~0JË/÷Ð¼Où)?åòCù¡üþÉ§n4tcÙ²e/¾øâhOù)?ÊåWPrêFàÖ­[~èòS~(?xù%ó³Ì93ë|Ñ---£dèòS~(?fùåþÔÊÊÊ;w~øáîå§üP~(?_KægÉ:t£´´4Î:~ü¸;]ù)?ÊåÇ¡YçgY¹resssOO»[ù)?ÊåÇåäººº¬C7æÏÏ¼ê*?åòCù)?ò»ür|´nò©mmmn(?åòS~(?ò¸üùYº±dÉ]»vyOù)?åòCùßå×ÞÞþðÃgýhÝX¹eË÷¦òS~Êåò#Ë/9ª;þü¬ò­^½º¥¥¥··×ý¨üòCù¡üÈ×òëëëknn¾ë®»²Õ½ùæ·oßî¨®òS~Êåò#¿Ë¯££cëÖ­YêN<¹¡¡ÁCWù)?åòCùßå×ÓÓóê«¯.[¶,ëQÝêêêææfGuòS~(?ù]~íííõõõ&LÈl¾3gnÛ¶­³³ÓÝ¤üòCù¡üÈãòûðÃwîÜm7Ð|­­­&äS~ÊOù¡üP~äqùEÌ<x0Â.ëÐÊÊÊ;vtww»_òS~(?y gÎhèF¬lll´R~ÊOù¡üP~äwùõõõ½úê«ÕÕÕ­ºá¨®òS~Êåò#¿ËïøñãY_ä3g¡ÊOù)?Ê¼/¿îîîíÛ·WVVf¾Ï|ÊOù)?ÊB(¿¾¾¾ÖÖÖÚÚÚÌ£ºn(?å§üP~(?¤übÇÒØØhèòS~Êåò£`Ë¯··w×®]&äS~ÊOù¡üP~lùÅ§¡¡aòäÉÝpTWù)?åòCùßå×ÙÙUm|n(?å§üP~(?¡üúúú«««3Çê¦&äëííµ%òS~(?y ºÑÑÑa*?å§üP~(?ò¸üº»»wìØaèòS~Êåò£Ë/¤LÈ§üòCù¡ü(äòé¥rÝ°ëP~Êo@]]]Êåòcëíímnnþâ¿XTTdèÊïj8p`îÜ¹ñ7£üP~(?F¬ËNÈ×ÙÙi+)?åwy·Ýv[<³æ(¿§~ú§Cë?üaäæOá§?ýÑ~ÔÖÖf;b·;Ûa´Ù¿ÿ-[fÌ|¿ök¿öÅ/~qè¤i¾ÿýïÿÃ?üÃÿÐ<.¿_ÝÄËïoþæoN­Ø¿¿óÎ;§áôé£ØØ-ÄÎÁv=Z[[«««KJJ2oÑ¢Ek×®ýÅ/~a+vïÞÝÙÙ9Ä?´ËÏÑ^íÅÑ^Ì3g¶lÙ2sæÌ&äÂUn/ö:Ú«üP~(?_ooïÎ;W®9?Ë1cV¯^>!òCù)?Ê¼wëºuëJKK3_ä>z<<y²ßEÊOù¡üP~äÎÎÎ¦¦¦%KdýÔ»îº+Ç³òCùÝ(Êåòã:êíímii°3fLfóEîÚµëÌ3¹¯Dù¡üÊåÇË[¶lÉ:!_YYYCCCû~´®òCù)?Ê¨»»ûÅ_¬¬¬¼)+WîÚµk°º¡üP~Êåòcd9xð`]]Ý	2oæÌ[·n½ê¿nåòS~(?#Â3g¶mÛuB¾¨ÀÕ«W_ûý8:Ë/¿ùÍonÜ¸±¹¹ù+?å§üP~(?nÜC7-[öÔSOøá×ågÂò[¿~ýoüÆo|qå«««gü?3þ×ÿú_âOù)?Êa¤YnL>Ã×÷'¶ò¤þìg?ûÇëÿxólÞôùÏþ±ÇóØS~ÊåòctwwïØ±#ëÐ1cÆÜsÏ=Ñ+º¡ü²ª««ûÊª¯$Ùüû?÷ü%Kx*?åòCùqcõõõµ¶¶ÖÖÖf~ÌZ3gÎöíÛ3?uCù]/éK_ýêWÓË¯áfÎéÑ¨üÊåÇÒÙÙ9ÐÐÉ'?üðÃCv¶òûæ7¿ù?ýôò»õÖ[ÿðÿÐcRù)?Êë¬···¹¹¹ºº:sèF¬õqî:ª«ü~øáoýÖoýÞïýÞ7lzxÓ¿ôåòòòãÇp*?åòCùq=÷ÆYnÌ3gÛ¶mÃrÃFáØÞ¿¯íkùÌg&L°|ùòöövOå§üP~(?®dèF´]fð;¶®®nØï39£üÊåÇ5É=t£²²2r0¢p$ÜTåòS~(?W)¯¢¢"3øbe5Òþ[~7]NQQòCù¡ü¸ÉÐªªª¬òÕÖÖ¶¶¶ÌP~lù]Nqq±òCù¡üìnvdÝP~öòvÊåò+É|YnDFæËV~Òò;wîÜý÷ß¯üP~(?rHnd/TUUý|ÊåwyÅÅÅÞçòCùqònèòCùýÊ2ÿnKKKzzÊåGºdB¾ÊÊÊ¬òä¡Êå÷+%%%ñÛÕÕUVV|ßûÞ÷b¡¾¾^ù¡üP~¤öuuu#B>åò»ÜUz±ðÞï]¼x1Æ¯üP~(¿Q®`n(?ß¯L4)þ8Ï±ðÈ#$fuAù¡üF­dB¾¬C7bM¬Ï»¡Êå÷+7nLçè÷ê½òCù¡üFø»hB¾dèFGGGÿúÊÂ/¿ðØcM2%:Q-ºÑ7]ù¡üP~#GÁÝP~(¿a¦üP~(¿aLÈauèFò©1tCù¡üÊOù1zË¯³³3öÃY'ä+È¡Êå÷_fÏÌíb&gÊ¯°åºQ`ó³(?_v³fÍJ¯½cQ~(¿B¿cî¡þ"£¢ü"òâÏþðáÃ/^Ê®üP~(¿!pæÌ&äKÍÏ2n(?ß¯ÆßÿgòCù¡ün´ØÇ®^½:ÇÐÎÎN÷¾òcÔß#Gb/°víÚóçÏ+?Ê/ß<yrëÖ­Y_äKægú¯òCù òÓ¦MËÜAáòCùåÞÞÞdB>C7Ê/3fáòCùåïíoooß°aCòÖ~be½ºòCù¥]Å%±ãâ®üP~(¿kÑÙÙÙÔÔ4þü¬GuW¯^=J>uCù¡ügêÔ©Fx üP~ùrk#æZZZrÝ8sæ»Uù¡ü²kkkÅÆÊå7bÅ#¶±±1ëQÝ	&ÚOÝP~(¿A^Åð@ù¡üF]»vUVVfÝW/Y²dçÎn(?ß*(?ßð:xð`]]Ý	²~êFìEO<éîS~(¿ü üP~(¿¬:;;/ùÔC7Êï*ÅgÌqüøqåòCù£¹W_u ¡óçÏojjrTWù¡ü®UqqqìSþ¦+?Ê/qÙ	ùboéE>åò»>å·oß¾Ø¹lÙ²åÜ¹sC9·òCù1ÊË¯»»;ùÔ¬C7-[ÖÜÜ<Ä³. ü(üò3¶åòÊòëëëkmm­­­ÍzTwúôé?ü°§òCùÝ¨ò3¶åòòëèèhll¬¨¨ð©Êå7lå7ÊQR~½½½ÍÍÍÕÕÕcÆñ©Êå§üÊÂ,¿¸ÚÉ'g_¬ô©Êå7<åwáÂÛn»mÜ¸q±3?~üwÜ1C=ÊB-¿ÎÎÎmÛ¶åÏÐåò¶òGyÖ7z¯¤üP~Xù¥nd=ª|êFD¡®üP~ÃY~sçÎ]ÒªU«Î?'Ï;wûí·ÇÅ+?ÊïJÄc)ÇÐhAC7Êo¤_IIIìÒwI.5±^ù¡üP~9ä/ÖÇ¹>uCù¡üFVùÅ*j/µ¦··7ÖÕåòË±3É1t£±±Ñ£Kù¡üFhù%GW¬Xí¯±k.üP~(¿tn(?_Þ_¤^ÖãôòCùqÿÿàþ`æÌË-û¿ø<*¿+ÏÐåòËòKèwÜqÇÄâë+bÍ¾éÊå7ª¼øâS¦LYµjUÃ5¬Y³æsû]ÝÈ/¿ãÇtTwìØ±ñ+Où¡üò¯üòCùñ¿Ê¯ÝýµÍ²9ù·áÁþô§GH6e_OOO2t#ë|n(?òS~(?zÆ©ìKþ-[¶¬©©i¤_,Ô××g¯¢¢ÂÐåòË×ò»érÊë¢££cúôéýÊï.ýO=õÔ)¿×_=vJ7ß|sÖ¡&äS~(¿¼/¿¢)?×Ýoþæo®¾cu*û¾ñÀ7Æüøñá½UÉÐ[o½5µëK7þü¦¦&C7Ê¯Êo >ø`²ËáÊëåàÁ'OþÝßýÝÿ]û¿ÿ÷ÿÓþôýÙãíèlll,++Ë¾	&444´··»×Ê¯ËïÈ#ñ_ðØëUUU¥Oì¬üP~'O~ìaî½÷ÞØËmøðÃ,Y9tÃ|(?FQùÝyçÉ¾o÷îÝCsÓÊ¡;ººº±cÇf¾È÷ÙÏ~ö0µP~~ù½òÊ+É¾oÕªUCyÓÊ!|êÆôéÓ³eK^äëèè0-ÊÂ/¿x|/^¼8Ì±oß¾!¾éÊåÇûS7fÎ9xæÌä¯âsQ~(¿<+¿'x"Ù®Y³fXnºòCùq#´··¯[·.ë|±²®®îàÁýægQ~(?¿üÌçòS~¤§§gçÎY'ä+W®ÜµkW|OÖË*?_~ES\¬üP~p­­­µµµ&LÈzTwëÖ­º¡üP~~ù;åòãZÄÝ×ØØXQQ1Ð|GuÊå§üP~äîîî;vTVVf=ª;þümÛ¶öS7Êå§üP~,ñ÷;Ð|¥¥¥ëÖ­o¸ºÖU~(?òCù1"$òÍ3'3ø®×§n(?ÊOù¡üN¹'ä¼£ºÊåòS~(?F¸_/Îºî¦üP~~ùÏå§üFÜC7bßs#~´òCùQøå>u_Öý¬ùüP~Ô|YnTTT466ÞèJù¡ü(üòKÙ»woì^ï¾ûîóçÏÇÉøzûí·Çýû÷+?7Ngggì²NÈ-ExucuÊå7 ÒÒÒØÏ¦ï^/kÊÊÊ®ñ9R^^^\<oÞ¼ûö)?ÐtTWù¡üP~¿zÃ_Ô^¿ò»ö÷ùÕÔÔ<÷Üs±ðäO®Y³&³ü~øÃö­þÛ¿ý[ôôüüç??vìí0öïßÿõ¯Ò¤IÁ+ã¬øa¹a±[;ÐÕÕÕÚÚj;xã7â?¢CüCoxùM:5v»QiñO.êùªU«bM¬¿öW/^¼¤dEEEfùmß¾ýGC+îÂÈÍÁ~ôKl!ðúë¯ßwßûÜç²$ûÂ¾ðÈ#ï»Ø9¸§ûöíkii±HÄ!CüCoxùÅ²³ðxûí·¯ñÓÇdq´GÛÝèèè	7ÕÑ^íeíï½÷Þ¬Y³Æÿÿ?~üÂÏ=íW~¼¸¤¤Dù¡üFè¹¨º0tCù¡üP~CgêÔ©ÉÛãkæ±cåò+0¹nÜÐ	ùÊå7üâöÏ>ûl,Ä×åò+TñûS7Fø¦V~(?FWù;vlöìÙãÇOÏN:õå_¾ö«=pà@YYYù¡CÊ¯ÀäøÔ1cÆTWW777÷ööü_Dù¡üEåÌäþmÉòO>yCoºòCùå¯øãhèF2!_gggý:ÊåÇ(*¿²²²ØY;v,U~å'*?é¢ç¢ê¢íº1ôÔÊåòäU,$åwñâEÛò#¥¯¯/On(?Ê¯¿d&çäu¾(¿.<ôÐCÉl[Êå7ÊÅ&h~dB¾ÂØÊåÇ(*¿¶¶¶¬39¿ùæÊå7:]vèÆHOù¡üP~Ûë-Z´(Û;nÜ¸Ù³gÁÄúÊå7å%n(?Êo¤P~(¿#ÇÐdB¾Â#åòc_j`GÊÙ³ggÏ=útåò+lÉÐ»îºk ¡»víÊ	ùÊåwõåwáÂcQ~­££cÃñ¼Âº¡üP~(¿_ÉºÇO7iÒ$åò+0ÝÝÝÍÍÍK,hB¾º¡üP~(¿_yÿý÷.IzGºÈ¾W^yEù¡üÆÁëëë³Ý9sf¾OÈ§üP~(¿+w£ì*?FIùE<´wÅ<yrË-9n¼õÖ[ÊåÇ(*¿á¢ü(¤ò½Ã3>óÏL4iñâÅÑ[ÃûÛõôô477¯2ëÐeËòùÊÑ[~.=öøñãSk¦Lòío[ù¡ü®P<?õ©Oõ«_Ýü'7=¼©ªªjæÌÑ^ÃòÅ|ÝºuYêN>ÃF4+?£ºü¦MÖoxoò$±eËåò»_øÂî¸ãÈ¾Ô¿Ïþó;vìÊ_'oþüùYl­^½º­­m4ÌÏ¢üP~(¿Ë())'öööÔ#GÄ'*?ß8aâ¦7¥ß¿üå?ú£?ß"÷|7ß|sSSSA~êòCù¡ü®R2¼÷âÅÿíz3&ùS~(¿L6í|#½ün½õÖo~ó7ôöGÏmÝºµ¢¢"3øÊÊÊâïkÎÏ¢üP~(¿Ë(//§õë×'GâA¿yóæäÉCù¡ü®ÄºuëæÏzÙoÃ~ý×ýàÁ7âfÇßiìª««³¾ÈWUUµk×.Á§üP~(¿>|8ë:¤üP~W¢§§çw~çw~ÆoG­¸uEdß·¾õ­ë~ãÖ6664tãáöÅÊåòËò§N7oÞ¸qãÆ?wîÜXs£oºò£`ÊïKï·ñÅ×¯_¿qãÆÖÖÖëx;»»»wìØuèÆ1cî¹çGuÊåT~×]ÄdmmíØ±cïÌ3îGåòCù)?åG_rT7ëÐdB¾ôø(?ÊoÐ;Læç:uêË/¿¬üP~C©···¹¹¹ªª*ëQÝêêjC7ÊåwÊoïÞ½©'¤üå'|Rù¡üæaÙÐÐuèÆ9s¶mÛfB>åòCù]·ò+++'cÇ¥ÊïÐ¡CfrFùÝhÉ§nDÛe_T`´ Q~(?ßõ/¿äæ´Ù/^¼ËÅÅÅÊåwÝ%C7rLÈ×ÜÜìcÖÊåw£ÊoêÔ©ÉìIù]¸pá¡ååò»rÝqÖHk¬üP~(¿)¿¶¶¶¬39¿ùæÊåwí	ù*++3ÿÊÆ[[[ÛÚÚjèòCù¡ü¨ü½Þ¢E±½ãÆ=vGGÇ¾éÊ/¿x¼ÕÕÕe/B0r0¢Ð¡üP~(¿¡.¿a¡ü(Ôò3tCù¡üP~ÊOùQàåLÈuèF2!¡Êåòå÷î»ïÎ1£¤¤$¢Æ7oÞ¼®®.åò»Bq=MÈ7sæLò)?Êo_kkkÖÇW~(¿ÝP~(?_þ_2sMMMOOO<þü5kbMyy¹òCùeJ&ä°3tCù¡üP~ùW~ÉÓUú+±ÕYù¡üR:;;ãqkB>åòCùåqù%¯ù]¸p!ýqï5?_Jî¡«W¯niiqTWù¡üP~ùQ~Éûüjjjâá'?úè£eËyÊ/yÀ4tcúôéñ>yò¤-©üP~(¿|*¿.çöU~ØòëîîhB¾±cÇÖÕÕº¡üP~(¿|-¿¢Ë)..V~òË=tcþüù;wî4!òCù¡üò»üòcä_,466fºQZZºaÃööv[Lù¡üP~P~MÚ|êÔ)åGaßÏþó]»veoÌ1+W®|ñÅÕU~(?_A_<É=öØcýVÞsÏ=fu¡Å#á«_ýêÄ³~êÆÖ­[;::l%åòCùæûüâÙnêÔ©gÏÏ?ÿ|òüwÞÞ§üFÝ3fLü'^äS~(?ÛB.¿O.½Â<ù'·ß~û¾éÊ!17Ð|É§n4559sÆR~ üåöïßz"|ê©§à¦+?À'6lØLW9tãk_û¡(?£®üxàä¹0õy÷Ýw+?òWww÷®]»V®u~XçFóù°5ÊQW~ÅÅÅñqâÄ£G~ö>¿åGÞ9xð`ý	²ÝG]ê¨îU|n/ÊåòËûògÄM6õ[YWWgl/y¤££cûöíÑvÁ7yòäÌçuåòCù1Ëo ùünt!)?®]__ß/¾ø¯|%sèF¬©®®nnnèS7ÊåÇh,¿á¢ü¸Æ»rÝºu'OÎ|oÎ9Û¶mëììÌÊåòc_<A¦ÏÍRù1Btwwoß¾ýæoÎ¾±cÇÖÖÖ^ùJù¡üP~(?åÇuðàÁººº¬/òUVVîØ±#¢pPW¨üP~(?òcdéììÜºukÖ¡W]oÊåòCù)?FW_µªªj ¡­­­×ø1kÊåòCù)?Ù[o½ÕÐÐp-C7ÊåòS~ÊoD;sæLSSS´ÝOÈ§üP~(?òS~ù¤¯¯¯µµµ¶¶6ëÇ¬UVVîÚµk 	ùÊåòDùå¦ü¸¡¢º+**®ûÐåòCù¡üú+ºââbåÇu×ÝÝ½cÇÊÊÊ&ä»ö¡ÊåòCù8Êo´cèÆULÈ§üP~(?òS~#Kggç¶mÛrèòCù¡üP~ÊOù]ÆÉ'_õÕ¸Szzz®ýÚz«««3'äUUUqîº¡üP~(?òS~¹|ãßøÔ§>õ/|aþüù¥¥¥×r×$C7²Õ¡ÊåòCù)?å7 ?ÿó?///ÿãõ¼ùO6Ç¿5kÖ|úÓ>yòä ®$ÇÐ1cÆåÐåòCù¡üòÐ¬Y³þï½ÿ7É¾ä_Üã?~%Í=!ßõýÔåòCù¡üò»V&LHÏ¾ø÷å/ùë_ÿzîK%C7fÎu~ºººþTªüP~(?òõ_î¡qÁ¡Eù¡üP~(?å§ü®Ô¾Ï/Ç|±räÝP~(?ÊOù)¿±½K,7o^d_[[[ê¬?ü°©©)ë|cÆ©®®öùYÊåòS~ÊopùüZ[[SÇj<8ÐÐ3gØ¡ÊåòCù)?å7§´´tØ?ZWù¡üP~(?å§ünÞÞÞ;w®2ßn(?Êå§üßÞzë­úúú¬/òïGë*?Êå§üßõqæÌ¦¦¦ªªª²©¬¬ÜµkW>ÝP~(?ÊOù)¿ÿ[~õêÕ&Lá­«üP~(?òS~W)BgË-eee=tCù¡üP~(¿áÑÕÕUQQ¡üWww÷;¾ò¯dºqóÍ7?õÔS4tCù¡üP~(¿apàÀ¹sçF[(¿áÒÞÞÞÐÐuèF¬Ü°aC|Ã¨ýV~(?Êïzºí¶Ûâ5Gùýå_þåÏVü=:tèg®µµuýúõ3gÎÌ¾¢¢¢[o½õ'xë­·~6ºí¿ägð³Ån!v¶áèÑ£o¼ñí@âûßÿþÛo¿=Ä?4ËïW7qàòknn>9´þþïÿþç?ÿùÉÂõüóÏßu×]wÍ÷Û¿ýÛëÖ­ÔI.9|øðOúSÛ»Ø9Øwß÷?øí@b÷îÝÿú¯ÿ:Ä?´ËÏÑÞëåÄQuYn&ò¡öâh/öâhïÐu^BùdèFeeå@òÅ¹===þtÊåòºT~×W___kkkmmíØ±c3¯¬¬lÝºuFù¡üP~(?åßå×ÙÙÛ­¢¢Â|ÊåòCùååwåz«««³NÈ·dÉ;vÂ	ùÊåòS~U~q&O|±ÒÐåòCù¡ü_Þ_ww÷¶mÛæÌuèÆ²eËvîÜéE>åòCù¡ü__î¡3gÎvüøqÊåòCù)¿<.¿HÆÆÆC7b[9ª«üP~(?òËãòëííÝµk×@òÍ?¿©©ÉQ]åòCù¡ü_~_¡¥¥¥Ç÷"òCù¡üP~Ê/Ë¯³³3ÇÐ¯|å+---Où¡üP~(?åßå÷ê«¯4!ßôéÓckCQ~(?ÊOùåGù­^½:ë|n(?Êå§ü­üSÍWYY¹sçÎÞÞ^TÊåòCù)¿,¿¾¾¾ùóçoØ°Á|ÊåòCù)¿/?ÊåòS~ÊåòCù¡üòCù¡üP~(?å§üP~(?ÊOù)?ÊåòS~ÊåòCù¡üÊå§üP~(?åòCù¡üP~ÊOù¡üP~(?òS~(?Êå§üÊåòCù)?åòCù¡üP~ÊOù¡üP~(?òS~(?Êå§üÊåòCù)?åòCù¡üP~ÊåòS~(?òCù¡üP~(?å§üP~(?ÊOù)?ÊåòS~ÊåòCù¡üòCù¡üP~(?å§üP~(?ÊOù)?ÊåòS~ÊåòCù¡üòCù¡üP~(?åòCùòCù)?ÊåòS~ÊåòCù¡üòCù¡üP~(?å§üP~(?ÊOù)?ÊåòS~ÊåòCù¡üòCù¡üP~(?å§üP~(?ÊOù)?ÊåòS~(?(?òCù¡üP~(?å§üP~(?ÊOù)?ÊåòS~ÊåòCù¡üòCù¡üP~(?å§üP~(?ÊOù)?ÊåòS~ÊåòCù¡üòCù¡üP~(?åòCù¡üÊOù¡üP~(?òS~(?Êå§üÊåòCù)?åòCù¡üP~ÊOù¡üP~(?òS~(?Êå§üÊåòCù)?åòCù¡üP~ÊOù¡üP~(?òCù¡üP~Êå§üP~(?ÊOù)?ÊåòS~ÊåòCù¡üòCù¡üP~(?å§üP~(?ÊOù)?ÊåòS~ÊåòCù¡üòCù¡üP~(¿¡rèÐ¡/X°àÈ#ÊåòCù¡ü¶üfÍõæoÆÂ/¼0öìÌòkkkûåÐ:|øpggç/á¿ü§ú§wÞyÇv Än!v¶áüùó­­­¶(¿øÏÀÿÐ<.¿tãÆË,¿Ç|ÏÐjiiÙ½÷Ø³çûØØ-ÄÎÁv ¼ñÆ¤¼þúëCÿC¡üâ?Óõõõöâh/öâh/öìÑÞÄùóçkjjzzzÊåòCùTùÝô§Nª««Ë[ÊåòCù¡üò»üÒµµµ­X±¢««+ë¹ÊåòCù¡ü§ü***nJ£üP~(?Ê¯`Ë/7åòCù¡üP~ÊOù¡üP~(?òS~(?Êå§üÊåòCù)?åòCù¡üP~Êå§üP~(?òCù¡üP~ üÊåòCù)?åòCù¡üP~ÊOù¡üP~(?òS~(?Êå§üÊåòCùRùë[ßjnn>9´^ýõüÇ<	'O¶µµýÝßýí@ÝBìlBüð¯ÿú¯mõWÕÑÑ1Ä?´»»»0ËïØ±c7oþSþSî#¢7y%`P~Êåò@ù üP~#EWWWEEÅm²4EEE¶Ïh~0´µµÍ;·¸¸xÞ¼y°FóáwÞY´hQIIÉwÞyîÜ9Ûgô8tèÐÂc?°`Á#GÄøZ^^ìöíÛgòÇCæîBùhñtOíyg=÷ÜsO>ù¤M4¥¥¥ï¿ÿ~,Ä×óWÍ°</^üX8~üø<`³fÍzóÍ7cá^=v,ÔÔÔÄD,ÄsÄ5kl¢QþxÈÊo$ºí¶ÛN8y½÷ÞË/·FùaÆ§NøË6Ñh~0ÄñSË&M²F§qãÆ%ÿ'¼xñb,pÁÿ	=	å7¢eÞawÜqÇáÃmQþ`8räHüaÇøêñ0Ê,8vìX,<ÿüóéÈè;úúú~ÿð`ðx($_>_Äû-·Üb³x0,^¼øøñãIzHòÃÛo¿=öìø?Àw¿ûÝä?ú*çÏ¯©©éééåô·Ø8£üñ ü¡üôÑ'xÂfñ`ð?Ìï¾ûîÜ¹smQåÔ©Suuu§ONNN:õÂÚË¶Ï(<(¿BØ¿/]ºôèÑ£6Ã-·Üàkoo_¼x±3³fÍ:räÈÅ¿ûÝï>öØc6ÎèÑÖÖ¶bÅ®®®ÔÚÚÚg6âkMMM4ÊÊ¯Ê¯¤¤$y÷.£üÁðî»ïFðÇ×X¶qFó!v÷Ó¦MC__3zTTT¤ÏöõÉ¥±eeeEEEåååÉoFóãAù üP~(?Ê@ù üP~(?Êåò@ù üÊåò¸6Ï?ÿüÒ¥KÇ]²|ùòW^yå¿í¿.É½m¶[[QQ¿ZOOO¿õ±¦¸¸¸¼¼üâÅ½NåäÍ7ßá±Ç+¤òÛ²eK¬|ê©§ú­ojjõ<òÈUòÌ#G¢i~úé¾Kyæ8+=Z0å÷ÞïÅÊô[?wîÜXßÑÑ¡üå¾»ï¾;æ;ßùNúÊÇ<VÞsÏ=éÝ³oß¾(§ÂÆrê?úè£úúú'ÆY¥¥¥ë×¯O?¨ÚÚÚugÅe÷ìÙÓ¯¥bÍ¤I.]º÷î8Y[[Ûïµ´´ä¾ä¬¸IÉY÷î¨ÒV¬Xë:ZóæoÆªªªÔG´¬¬,®jÜ¸qwÞyç©S§2Ë/óúû­ÉqSå0¦MÕòþûï§¯üàbeEEEzÙôsøðáäÜÛo¿½ßYk×®MÎjoo/**Êz©ädrîwÜqñâÅhÇ¾¾¾87¾F9?>yû]ë¬7/ó7áÒs6Ï=÷ûú]ÉòåË[~9n* üYr`7Ë>ë¦¢ÃÒËfÍ5ç/8¹jÕªäÜ$tvL^ºbKÎª©©I^,å$W~6m¶K¶Þwß±æµ×^åøË÷Þïe¯'ª1NÆ7¤ß¶¬¿QÔä)Sâ÷íêêñ5nyäæo(//Æ¯ðÉ7nÜ`Ë/ÇMÀð_ÈZ~©õIÙ$ÁN:'£.3fÌ¸ÿþû£Ø>þøãÔÄ÷ô-u©ää|úæ¨¥ÔßøË­­­½¨Ì8yöìÙôÛ6Ðò|ðÁ8ë»ßýî'ÿyDýúõéßå=ºhÑ¢ä%ÉÁ_(?a¼Ðuþüùô===±2ÎÊÑ:©.<qâD©ÐI½9/ó l¿JK%KKKKJJzãÊ'M:÷²×3PõsìØ±$Rc9¾ÆrêÜÄÈzÔøÊË/ÇMÀ0KÞëöÄO¤¯Læ:é7Â#õ^À®®®Ì²=ºeËäØk*äRS/Ûgk×®MòÆ×úúúÔú×¼ÆvúôéääÙ³gsÃ½å[âÜd"Öô³q¾qÖÞ½Ï;»üRUlÔ¹9n* üY2B¢¸¸øÉ'Lfuyúé§KJJ2Gc¬Zµ**§··79¼Ï/yÜ'Òß àúõë£Q·©yU²öY2Ø6ùémmm©õ9®'yc_ò>¿ä;sß3Ï<z5.~åô³·<¶··ÇO0Pù%­­ßÅyçw¦ã¦Ê`ø%³÷óè£þ×þë$RË©ã¤©A)÷Ýw_zV¦ùåsß'>l#Ö¦Îq=q3ÒoXj@¿ìÇ<nÜ¸äWèwû¶ÛnKÿS¦L¯ÉÄ.é×¼$tjêÜ7P~#BÔÉòåËK.Yºti2À¶_ùíÝ»76oÞ¼yû÷ïOÛÓÓóÐC%µôàööö¦ÎÝ³gÏ¢E"ÈÊËËwìØÑï:3oI¬àú­èzBÜ¸IÉD9æóK©¯¯ï7½K¢«««¦¦&¶ÀÄã·èèèHÍP~çÎ´M¶UUUÕ¡CúýÄ7P~(?Êåò@ù üP~(?åò@ù üP~(?÷ÿ¬à/	ûü1IEND®B`


Detrended Normal Q-Q Plots


iö+ûÏó»ãÛßþö³Ï>ç¥ròùüÈüî£N:µâ7Wä]@ð«¿ú«¯½öùüD~"?ò#¿²jddäù£Ïþ|eûW/^ÿ]ùq ?ùfÕ¾ðæææ/oûrtÝhþú×¿^ZüÈÏ8ÈüÈO³jbbb×®]µµµõõõáëþáÜ;ÉÈüùüÈü4·Þzë­½züÈÏ8ÈüÈOùq ?ùÈOä'òùùüD~"?ùÈOä'òùùüD~"?ùÈOä'òùùüD~"?ùÈOä'òùùüD~"?ùÈOä'òùùùÈOäG~ä'òùüD~ä7ËÉd<ommíïïÏ]400ÐÞÞµµµÈOä'òùv©Tª§§'L<xpÓ¦M¹_õÕ0qìØ±òùüD~"¿Ò®®®nrr2Ld2ÆÆÆnV]]](¿?ýÓ?^zé¥ ÑAUdö¾½o*³ÞÞÞþðÆÁÞ_*B~ñx|ÚéÜÂXtwwÊïäÉo-`gÎyã7ÞREöòË/_½zÕ8Tfýýý?ûÙÏCeöÊ+¯ÊììÙ³ÿøÿ¸?±"äÅ²ÓD¢ðwîÜI¥RétÚÙ^9Û+gål¯í-½ªþ§0]__Éd¢³½a:ï7nÜØ²eËÍ77B~"?ÈOäWbmÞ¼ùèÑ£a"|M¥RyÒZ»víèèè´+ÈOä'òùÞ°644Äb±d2900ðóÇ6u8°±±±*'òùüD~"¿üD~"?ÈüÈOä'òùüÈüD~"?ÈüÈOä'òùüÈüD~äG~ä'ò#?òùüD~"?ò#?ÈOä'ò#?òùüD~"?ò#?ÈOä'ò#?òùüD~"?ò#?ÈOä'ò#?òùi¥Óé_|ñg	_ß÷]òùüÈüÈOåÙ[o½õà>üðÃ¿ÓÙÖÖöÀtüD~äG~äG~ä§2ìüäêÕ«÷~moôçGùÈG>N§ÉOäG~äG~"?Uï¼óNý¯îÉÊ/üinnþÑ~D~"?ò#?òù©¬~øásÙþüæÿþÍçüD~äG~ä'òSYõî»ï.^¼ø+Û¿eß¯îihhxíµ×ÈOäG~äG~"?[;wî|ècmý­¿Þþëþô§'&&ÈOäG~äG~"?a÷î­©©©¯¯¯­­ýÒ¾4>>]D~ägÈüÈOä§2ì­·ÞÊê#?ùùù©â"?ò3äG~ä'òùüD~äG~"?ÈOäG~ä'òùüD~äG~"?ÈOäG~ä'òùüD~äG~"?ÈOäG~ä'òùüD~äG~"?ÈOäG~ä'òùüD~äG~"?ò#?òùùüD~"?ùÈOä'òùùüD~"?ùÈOä'òùùüD~"?ùÈOä'òùùüD~"?ùÈOä'òùùüD~"?ùÈüÈüD~äG~"?ÈOäG~ä'òùüD~äG~"?ÈOäG~ä'òùüD~äG~"?ÈOäG~ä'òùüD~äG~"?ÈOäG~ä'òùüD~äG~"?ÈOäG~ä'ò#?ã@~"?ò#?ÈOä'ò#?òùüD~"?ò#?ÈOä'ò#?òùüD~"?ò#?ÈOä'ò#?òùüD~"?ò#?ÈOä'ò#?òùüD~"?ò#?ÈOä'ò»%Éx<ÞÚÚÚßß_x¾¾¾ªª*òùüD~"¿/JõôônÚ´)oéÄÄDûLòûÏìW^	ÿÿSYx²¿ÿÆ¡2î¿uëq¨ÌÎ;7::j*³àþþç^ÈXò«««L¦±±1oéO<ñôÓOÏ$¿§zªw;uêÔK/½Ô«ÌÞ·÷½/ÿ^WòÇãÓN®]»ÖÑÑl¯í³½r¶WÎöC±X,;H$ruuu;wî¿*ùüD~"?_Võ?éúúúL&óÞÔÙÞ0=íÍ²7&?ÈOä'ò+á6oÞ|ôèÑ0¾¦R©¤X8üD~"?È¯ôµ¡¡!%Éi©G~"?ÈOäWÑÈOä'òùùüD~"?ùÈOä'òùùüD~"?ùÈüÈüD~äG~"?ÈOäG~ä'òùüD~äG~"?ÈOäG~ä'òùüD~äG~"?ÈOäG~ä'òùüD~äG~"?ÈOäWNãßxþùçß?þüäMUdaï_ºtÉ8Tfññ÷ÿ÷Æ¡2/4÷wg*³'N/äO|÷ÝwÉoÆ.^¼¸wïÞoJ$EÅOfV9+IT!$IùI$ü$ID~$I"?I$$IÈ¯|mllÌ~;00ÐÞÞÇÛÚÚOEíý/._¾<ìýÖÖÖóçÏÚûQUU~÷VÜÞ¿yófUNÆ§¢öþøøø-[Ä>xöìYò+óÂ«û²eËrÿ777¿úê«aâØ±c---¨¢ö~ßÉ'ÃDxÔÕÕ¢Úû¡ð?/ü¸÷O8^ûLeîýýû÷?ùäMMMäWæ­[·îÊ+3ý¢¯®®6D¹÷ÛÚÚQ¥íý'xâé§&¿ÜûÑ¿úT?üÿÒ¥K÷ÉÝóÛg¡zº_ôÝÝÝ§ÒöþÄÄDMMMyìØ1SQÿÚµkáßýäW¿¹¹yíÚµñx<<._¾lp*jïý~àÀêêê¦¦¦×_ü*T~wîÜI¥RétÚàT¦ûûúúêëëNEíý®®®sçÎÍôPüÝ¿qããý¶÷c±Ø#GÂD@ ?ùUâßÿð7Ë-7oÞ42ûÛ?úW Á©¨½_õý»H$NEíýÜçè¿ùýêùgÏ]»víèè¨a©À½ßÜÜ|ñâÅ÷¦.ñOS¯ýØW÷GFF¢ùû»_iÛ¶mÇoêãV®~÷hllôïþÝûCCC­­­áß|kÖ¬qÐüT9pppÙ²eáïþêÕ«þNEíýÛ·owuuEïò¼råùI$ü$ID~$I"?I$$IùI$ü$ID~$I"?I$$IÈO$Iä'I$ò$IùI$ü$IÈO$Iä'I$ò$IùI$ü$éí;ßùÎªU«ª§Z³fÍ/¼ð¿à¦*_ÇÓÝÛÆÆÆðÐÒétÞü0''ÉÉÉÉ¹nSùIRéµwïÞª¾õ­oüöíÛf>÷ÜsyóöÙ0ÿñÇÇ6%$XCCCÁ4ñxüÐ¡CS9r$|f^¸p¡läwõêÕ0³­­-oþ²eËÂüòD~Ê¿ÏþóÁ4O>ùdîÌ§z*Ìü½ßû½ô÷÷9¶··éìoÝºÕÝÝ]SSÕÕÕíØ±#÷¤ê3g®Â¢°nooo¥ÂÚÚÚU«V>:|»yóæ¼;vêÔ©âÛ»-êëëIik×®ó²s^õÕ0§³³3;gÿþýaSÕÕÕ7n¼qãF¡ü·7§È]D~ôa¶téÒ k×®åÎ¼~ýzÙØØ+¼£¥6lÈ[´ûöhÑððp,v­èÛhiWW×ääd°c"KÃ× §EEo¿+²01íÝ+|¤ÇËål===YöåmdÍ5s_»*ü$éC.:±;Í/µªªà°lÚ´éÎTa"|»~ýúhiÈÑ¡» ¶hQ*éóçÏGÉÝæ=í¢­=öXsòäÉ0¾éGô®Û	jßäÞ·iQÐä%KÂãß¯ánf2èÉd2¬Âÿsv¸ººz®ò+rW%$øòM+¿ìüH6B7nÜß3Eß¶··o¶nÝÄ666ÝH¸MÞQ´ìZÑ·×¯_ÏÞ8h)Â7|ÓgÎ¹ëv2Ã·o¿ývîé=y;wî~úé÷þçö;roä<ÚÑÑ«üÜUIä'IrÑ®;wîäÎL§ÓafXTÄ:Y^¹r%Â_:Ù7çÍ³Tîg©éºººD"1>>6^[[]z×íÌä°¼.^¼!5L¯azxx8»ôüùóáLÖxöò+rW%$ÈEïu;pà@îÌè³Nò®ðÈ¾ptt´ðPÖöíÛÍ1: =zWmß¾=:É¾vwwgçÙNtíæÍÑ·o¿ývñëpW¬XFdÀ»(ºÎ7,êëë»ûvqùeUFvi»*ü$éC.ºB"<x0úTC%Â«1Ö¯_3>>Í^½Ï/zÜ+Wrß ApÇÁIÑU·ÙÏUÖgÑÅ¶ÑO?ölv~íDoìÞçN§£[ß#G²GãÂCÎ]½åqxx8ü03É/²f°r¸YçÆs¹«ÈO>ü¢O9ÎkÿþýÿÿÜT²ÓÙó¤Ù*²=öØc¹¬ÌíÄEä÷ÞÔ¶æ×ÕÕå.²p7rïXöºÚìØØXuuuôòÎq¯[·.÷G,Y²$|>Ø%wÑ!ÉlS³KÜUIä'I÷EA'kÖ¬ILµjÕªèÛ<ùõõõE×ÚÚzîÜ¹ìÒt:½÷îHK;wîÏ.ííííèè K&ÎÛfá=	ó·mÛ7¦íÂ	w)ú Á"ç­»»;ïã]¢FGGS©Tð(FFF²P»ÍÛ·oÚFcÕÙÙ900÷ÜUIä'I$ò$IùI$ü$ID~$I"?I$$IÈO$ü$ID~$I"?I$$IÈO$Iä'I$ò$IùI$ü$ITÐÿ^rBeIEND®B`


,÷ú7nÓ«V­º>VÃÌo4gÓ¦M###Ñ¶.;v,÷Fïf;zôh¾páBî¢îp<èd×«LôFºìk¢SqïÕ´W,núÜ¹»A0Ögß$wãH/^Óá»¦çÌãD~RùÉ//z[XnaNù]ºt);çëf_?*çÑèbxúÌ±*ïh]Í_q¢Ç1<©çÍ¯««ËDá	>7oÞD7´1:=ÀÜÉd0ZZZrùe9>Óßç*¹CÖÙÚÚzüøñqm23î½ö_´r%÷&¦$¿IîÆÍÍÍábCCCkø/ÄÍ7ýVÈO*3ù©µµµ=ÝNdèb N§ê"¾êsó46;ÑüðÃãÏßÃÃÃ¹ó_3+|Ó[áãÌÅ Ì¼#·<Gµ¾¾>:<]È»<Og·0U&3Vã^a23G§·âäö¾ÿ½k»ñÀÀ@¿¬9âD~R9Éïé§Î=Ã#z-ï,ËÉ@gJëÍæêÕ«¹Kl¶øEîÀÁÃÅìg D3#å½:gâÉHîÅEE®ÒÕÕd6ùE/LF°N§·lÙrýúõûökÖ¬W~YeÚòêàdößd^óËÚ.ú¯Nñ/²¿E:ujçÎÑ!æìyÐÈOºÓåÛ¢'æì§ºDÏgá2:U¶©©)Z½Éih¬q2¬[MY¢÷iGÛÉ×¸-¾bqF¬^½:ûFþhNtæïªU«Ç¶¶iÓ¦÷/¿èu¯þþþðöîÝ;ù½ðÂÑ«Já!<y2z=/êðáÃã~§ºÊäW|pòöÜ¦:ªÓ_ôÎ¼°3Ü¼y3ÀnåÊ.Ü½wü ÃàâãËèýE¼K"?éN_a¹ç'-¿öCw£2¬[MDrO´Ì.-²Ùâ+gÄåË³ïèæ?>ï=^uuuÙ7¾ùe-5oÞ¼ì»øfíÚµß¬ÅùOi)É¯øàäí¹MuT§!¿S§NånÙ²e¹KóÆ$ùD·RdËBmýúõ~±Hä'üÂSà+ÿº×±cÇZZZ¢ÂÓùÓO?øðá0'H+÷Óì&¹î-Ù^ØxsssáÇòM´Ù[®XÑÐÜùçÎ[³fMb¬t:]x>ÇôäwíÚµ°µ°Í` ­[·eÁ]Ü7O>ùdxtáÏ?ÿþýúÔ§²¯9½ÿU¦$¿â·oä5¥QüBGihh:l?:=9>n½µµµ§§ç¯¹N´¿=ôÐCÑ©ïá[9<<ìD~t[Êd2k×®ÒgÈMc²+z­7:Ydd$ý¢Eì0ùI*­è~y=öØcFF"?IR¥544´mÛ¶ùóçGtÃÄÃ?lX$ò$IùI$ü$ID~$I"?I$$IÈO$ü$ID~$I"?I$$IÈO$Iä'I$ò$IùIºC:|øp*Çã+V¬êºUUUÙ9a:ÌijjÂo±±¦wÉ¬;Íß­Eó6oÞ[F¬¦¦&N¿ñÆÙµFFFÂüpåD"¦í]ÈOÒUÊµk×¦!]»vu¿ñodç<þøãaÎÎ;+C~ó×­[æwwwé'Néd9r$+Å£GÚ»$¤;ìÈûÀÓ©S§ÂºË/ÏÎY²dIÓ××73wræåH$ÂüÁÁÁq×zðÁÃÒèëúõëí]ÈOÒÇ¾<ôÐC555sæÌÙ±cGÞÕ>õ©Oµ´´äm!ûa¾zõjs¢E/^lmm­®®ÇãÍÍÍÑëd[Ë½õâ«ìß¿?J-]º4ÜP¡ÏnÜ¸ÑÙÙÖw~Û¶mÑKÁ¦Ë-[óÃ/]ºô>åwÏ=÷ù-:yòdÞ¢ÑÑÑ¹sçÛºyófø:oÞ¼¬§w$ü$ÝüEÓ>úh~úé§±ÂÄc=cÇEÂËmãÆaÑ¾ûÂtø¦7lØ-Z¼xqtÄóÂa~2wk¹w ø*ÁsëÖ­+¼ó7oÓáÏ=÷ØµkWjýýýa"zIr¢÷ðMdâÜë>|8;gÅ§OÎ®ü]µjU^³fMsÆ½ö:Iä'é_*Ó±á5î_xá,wV¯^.»ôÔ©SÛ·ooii	óc±Ø¸[Ë³WñU¢;VSSS¸n]]]tçGGGÃDðV®¦e§7,yõöö¡fý¶Íß²eKöb$Ô­[·¾û Iä'é6Ê/:/5;?%³æÌ®944Ðs¢E÷îk>:wy[Ë½8U&ºc¹/ÑEj<tèÐÜ¹s£9µµµÑËÍ¯È¸uwwÌåÝn7zýúõ÷Æ=éùóçìuÈOÒ/¿d2û_ô²Ù-ÔÙÙf¿fçG'C5Iù_%Ç¤×7o^´nÞÝ9zôhtNnöÅÂ)ËDEw&Üç0¥j^§Nö$òtåJË¾û¢÷ù=þøãaÐ¡C²Ð9xð`v~äÈ3gÎDïÿü¯²ûöoë[abãÆë®_¿>L8pàìÙ³ÑÑÕ0séÒ¥a:°ìÊ+abáÂïS~MMMa~WW×¿üµk×¾7ñÜùÓ¾D~n£üFFF®æû|ÅåwóæÍèHkøûq'Fµµµ555Û¶m¤ü¯rüøñ°tÉ%ÙMò>cyË-á'+WF§Ðl¥ÓéèdáåË;wnJÃRxD8l¶³³3ÜÃð`çÍ·yóæp»ïýòC­s·ýPëißI"?I$ò$IùI$ü$ID~$I"?I$ßÓK/½ôÖ[oÍä-?>ÉØóJØµ±C	»hØQCi»pá¿½VÚÞyç«W¯ö_üâ'?ùq(mo¾ùæþçßÒ×¾öµ¿¼Å^xáç?ÿ¹600ðãÿØ8°¡¡¡ïïÆ¡´ÿûßÿÙÏ~fJØo¼ñ£ýÈ8°7oþã?þ£q(m'Oüÿøò#?ùÈüÈüÈOäG~ä'ò#?ò#?òùùüÈüÈüÈüÈüÈOäG~äG~ä'ò#?òùùùüÈüD~äG~äG~äG~äG~ä'ò#?ò#?òùùüÈüÈüD~äG~"?ò#?ò#?ùùùüÈüD~äG~äG~"?ò#?ùùÈüÈOäG~äG~äG~"?ò#?ùùÈüÈOäG~%¿¾¾¾d2Ç»»»s¯[·.HÜsÏ=A]äG~"?ò#?ùwétúÀabÏ=¹víÚõØcr544ÊïèÑ£×g°ðCuéÒ¥ë*]ÿw,ãPÂ~úÓ?~Ü8¶éû·3%¬¿¿ÿµ×^3%ìòåËßýîwCiñÅß|óÍ¼ÅY!¿ÚÚÚ`»0ÉdR©Tî¢ÆÆÆ³gÏN´b_Àâf°Í'Nü@¥+`:0Å8°°Õ8ügÿûßÿ¾qð³'÷ÒK/ç;ß1¥íùçáýY!¿x<>îttq÷îÝÕÕÕ§Ov´×Ñ^9Úëh¯£½r´×ÑÞò.e§DÞ¢ûösçÎµ´´ùüÈüD~äWÞÕÕÕe2÷Æöé¼EÙé¼ÉüD~äG~"?ò+¿:;;÷ïß&Â×t:»hÓ¦MgÎYºt)ùÈüÈOäG~e?¬õõõ±X,LöôôüÏc«úïG788ØÞÞÇ[ZZÈüD~äG~"?ò¥ùüÈüD~äG~äG~ä'ò#?òùùùüÈüD~äG~äG~"?ò#?ùùùùùùüÈüD~äG~äG~"?ò#?ùùÈüÈOäG~äG~äG~"?ò#?ùùÈüÈOäG~äG~ä'ò#?òùùùÈüÈOäG~äG~ä'ò#?òùùùüÈüD~äG~äG~äG~äG~äG~ä'ò#?òùùùüÈüD~äG~äG~"?ò#?ò#?ò#?ò#?òùùüÈüÈüD~äG~"?ò#?ò#?ùÈüÈüÈüD~äG~"?ò#?ò#?ùÈüÈüÈOäG~ä'ò#?ò#?ò#?ã@~äG~äG~"?ò#?ùùÈüÈOäG~äG~ä'ò#?ò#?ò#?ò#?ò#?ùÈüÈüÈOäG~ä'ò#?ò#?òùùüÈüÈüÈOäG~ä'ò#?ò#?òùùüÈüÈüD~äG~"?ò#?ò#?òùùùüÈüD~äG~äG~"?ò#?ùùÈüÈüÈüÈüÈüD~äG~*+ùýÍßüÍ<ðð»víz÷ÝwÉüÈOäG~ä'ò«@ùðÝï½Yó¿¦¦¦ÆÆÆwÞyüÈüD~äG~"¿JßO>Ø·íO·íøêèß÷Ý·aÃò#?òùùü*M~kÖ¬ùÌÏdÙþaÝ>úÑùÈüÈOäWòûlú³¹òÛðÅäG~ä'ò#?òùU ü|òÉ,üÈö¯lÏÊoéÒ¥_üâÉüÈOäG~ä'ò«4ùÜÿý_X÷¼qùòåwß÷Ûo¿M~äG~"?ò#?_¥É/4<<üÈ#|ìcûÈG>òÀ¼õÖ[4ªäG~äG~äG~"?ò-ùùùüÈüÈüD~äG~"?ò#?ò#?ùÈüÈüÈOäG~äG~"?ò#?ò#?ùÈüÈüÈOäG~ä'ò#?ò#?òùùÈüÈüÈOäG~ä'ò#?ò#?òùùüÈüÈüD~äG~äG~äG~äG~ä'ò#?ò#?òùùüÈüÈïõõõ%Éx<ÞØØØÝÝ]x®®®ªª*ò#?ùÈüÊ¾t:àÀ0±gÏ¼¥###ÍÍÍäG~"?ò#?ùUBµµµ£££a"É¤R©¼¥>úèO<1üþú¯ÿúg°cÇýð?üg®cvÑ°£ÒöÿðÆ¡ÿ·OvêÔ©çÞ8¶®®®W_u&oqVÈ/;ºxñbKKKpáDòöÙgßÁ¾ûÝï¼¥Ò~¢zC	ûùð«Ê8¶ãÇÿøÇ?6%,HúþéC	ã7ÂQCiëîîþÑ~4·8+äÅ²ÓD"wQû'þû¡:Úëh¯íu´×Ñ^9ÚëhoTWWÉd¢£½aúWá¯F~ä'ò#?òù_y×ÙÙ¹ÿþ0¾¦Óéñª×üÈOäG~ä'ò#¿ÊÖúúúX,L&zzÆ¥ùÈüÈOäG~³:ò#?ùÈüÈüÈüD~äG~"?ò#?ò#?ùÈüÈüÈOäG~ä'ò#?ò#?ò#?ò#?ò#?ò#?ùÈüÈüÈOäG~ä'ò#?ò#?òùùüÈüÈüÈOäG~ä'ò#?ò#?òùùüÈüÈüD~äG~"?ò#?ò#?òùùüÈüÈüD~äG~"?ò#?ò#?ùÈüÈüÈüÈüÈüÈüD~äG~"?ò#?ò#?ùÈüÈüÈOäG~äG~äG~äG~äG~"?ò#?ùùÈüÈOäG~äG~ä'ò#?òùùùÈüÈOäG~äG~ä'ò#?òùùùüÈüD~äG~äG~ägÈüÈüÈOäG~ä'ò#?ò#?òùùüÈüÈüD~äG~äG~äG~äG~ä'ò#?òùùùüÈüD~äG~äG~"?ò#?ùùùüÈüD~äG~äG~"?ò#?ÍùUÝªX,F~äG~ä'ò#?ò#¿J_ìVÅãqò#?ò#?ùùUü*#ò#?ùÈüÞo7n$?ò#?òùù_¥É/JÅãqïó#?ò#?ù* MMM§wÔÖÖ'ò#?ò#?ùùUüD Þµk×êëëÃDxÎøÖ·¾&6lØ@~äG~ä'ò#?ò#¿_ô"_Ô.sæÌ!?ò#?òùù_EÉoîÜ¹Áyá'à0ñðÃG>ÕüÈüD~äG~äWiòÛ¶m[ö|Ü·ú-]ºüÈüÈOäG~äG~%¿Ð×¿þõyóæ0ØÒÒRßò#?ùÈüfKäG~"?ò#?ùùùüÈüD~ãµpáÂè³]|3ùùüÈüTÉò[°`A®ö²9·üÈüD~äG~äWiòÈæëíí-»ïùÈüÈOäG~S¨¶¶6È¯ÙG~ä'ò#?òùßÔêëëòÛ¼yó7ÈüÈüD~äG~ªdùæÏ_U3<ÈüÈOäG~äG~&¿gxùÈüÈO³B~ùúûûËñûA~ä'ò#?òùßª««sùùüÈü4+ä(ä·mÛ¶ááaò#?ò#?ù*Y~Uäò#?òùù_~ó¸9ÃüÈüD~äG~äWêR¾ùüÈüD~ä7R©TCCÃÙ³gÉüÈüD~äG~ªpùÅãñªªøµÃ¾¾¾d2îIcccwwwî¢æææ°¨©©)üÈOäG~ä'ò#¿é¤ä·sçÎÁÁÁê³]ÒéôÂÄ=:::r-X°àå_Ï<óÌÂÉüD~äG~"?ò¸Îí­­­ÐÉdR©ÔDW«®®.ß_þå_Á9rT%í;c;ª!õ³/;ê9¤3¼£ÞvùÝ	çöæÞÖD·ÛÛÛ»aÃBùÿÿ×ö½ïïÝwßý/®sçÎÿ÷ö³ý¬»»Û8¶_|1ü¿ß8°×_Ý8°ÿüçÇ7¥í¥^zçwfòo»üîr__L$W¸qãF:r´×Ñ^9Úëh¯£½r´×ÑÞò®®®.ÉDGÃtÞÒË/¯[·îÊ++ùüÈüD~ä7µ·V®]]]UU5gÎööö>Õ£³³sÿþýa"|M§ÓyÒjkk»víÚ¸+ùüÈüD~ä7µeÜ3<fòÏøa­¯¯ÅbÉd²§§çÛØgÍ¤R©ÜE~ä'ò#?òùßô[´hQÕªU«nÜ¸.®^½:ÌY¼xñÿý ?òùùüÈo%à¼ìL&æ¦ùùùüÈüÊX~±X,8/:Á"jxx8ÌÉOu!?ò3äG~ä'ò#¿_t´·­­-:Ú¾é0§¹¹üÈüÈOäG~äG~%¿@½qÏð¸~ý:ùùÈüÈü*J~Ñ¾ÒÞÞ^SSÅÂ×¶¶¶0§,¾äG~"?ò#?ùÍÈüD~äG~"?ò#?ò#?òùùü²kÞªÜ¿¥K~äG~äG~äG~äG~e,¿ØÄùùüÈüôÞl8Ú»uëÖH~Ï<óùùÈüÈü*S~sæÌ	ækmmÍý`gò#?ò#?ùùUüÖ¬Y½ÔwôèÑ2ú~ùüÈüD~ä7î¹È|«V­*»ïùÈüÈOäG~ÝE/^ÌÑÝÝ]ßò#?ùÈünÝîÝ»£ú:::Ê÷ûA~ä'ò#?òùß$Öôy~äG~ä'ò#?òÓ,_ìVÅãqò#?ò#?ùùUü*#ò#?ùÈüÈüÈüD~äG~"?ò#?ò#?ùÈüÈüÈOäG~ä'ò#?ò#?ò#?ò#?ò#?ò#?ùÈüÈ¯¼ä·páÂD"áóüÈüÈOäG~ä§ßrµçóüÈüÈOäG~ä§_@^0_ooïèèhÙ?ÈüD~äG~"?òBµµµA~åÈ>ò#?Ýnù½öÚkßþö·Ã×ò#?ò#?ò«ùõõõùmÞ¼ùÆäG~äG~Qo¿ýöýKîO¥RáëüùóúÓ½üÈüÊ^~¡ðk½ª gxùÍfù-^¼øwýÎö¯lßñÕá_kkkÀßl~åüÈüÈ¯Bä×ÐÐàò#?òËíµ×^»ûî»³ìþÿ"¾úê«ä'ò#?ò+oùEæëïï/Çïùnü9rÿûsÙþücöÙgÉOäG~äWÞò«««sùùåöÚk¯%É¼×üR©×üì]äG~äWöò	òÛ¶mÛðð0ùù_ÔÇ?þñ¥÷/Ýö§Ûùú³.]zß÷ão	ò#?òùýê&&ÈäG~³Y~a#þô§ý×ý#ùÈ¼yóV®Î;ïÌæQ%?ò#?ò«ùÅ&ÈäG~³Y~QA¯¾úêÛo¿mTÉüÈü*D~eùn«üD~äG~äG~äG~äG~ä'ò#?ò+[ùe2+WVWWWUUÍ3§½½½õ%?òùùüÈoj;Ê¸gxÅI|äG~"?ò#?ùM¡Eç­Zµ*ú»½«W¯s/^L~äG~ä'ò#?ò#¿_"ÎËýsL&Ì	óÉüÈüD~äG~äWQòÅbÁyAÙ9ÃÃÃaOu!?ò#?ùùUæÑÞ¶¶¶èhoø¦Ãææfò#?ò#?ùùUüõÆ=ÃãúõëäG~äG~"?ò#?ò«(ùEûJMMM,_ÛÚÚÂ²ø~ùüÈüD~ä7["?òùùüÈo¥R©³gÏùùüÈüTáòÇãUUåúÚ!ùÈüÈOäG~S¨»»;ÈoçÎåòGÛÈüD~äG~"?òÖ&&(ùùüÈüÈ¯¢ä äL~äG~"?ò#?ò«ù544466g²þ~ùüÈüD~äwë¢?×ûÞØÑÞ²8°K~ä'ò#?òùß4þô'zÉüÈüD~äG~ªdù%Éª¢9ÃüÈüD~äG~äW!òëíí;wnôÊ_ä<gxùÈüÈO)¿lå<ò#?ùÈüfuäG~"?ò#?ùùùüÈüD~·¹¾¾¾d2Ç»»»/ÞÜvìØñGôG_Á>ÿùÏÿÅ_üÅ×Tº6eJXØEÃjJÛÚµkÿüÏÿÜ8°/ùËò'bJØÃ?üàÒ¶nÝº¯~õ«3ye&¿t:àÀ0±gÏâ¦7'·­[·VI$UJ·]~çÎ+¡üjkkGGGÃD&I¥RÅMoùI$òîã±tinn~ã7ÞÿÖrOÎ;e¸pÑôæä¶mÛ¶úúúf°»îºkþüù÷¨tx,ãPÂÂ.vTãPÚÂ¯?û~öïðî¾ûn?ûð³ÛåÌef ÕòåËÏ??í­å~þs"(¾hzsáá9ÃÃÎð3<áñ~ù.Z´(KÀ`¬+WNc;uuuL&:8¦/Þò#?ùÈüJÐèèèÑ£G««««¦û×Û:;;÷ïß&Â×t:]|ÑôæùüÈüD~ä÷¾êïïÏÍ¯¦¦fÃÓ úúú Æd2ÙÓÓó?÷rìí¦7üÈOäG~ä'ò#¿i¾êêê/Ë÷üÈOäG~ä'ò#¿©lbìÄO~ò.»ïùÈüÈOäG~SûÙ+ßïùÈüÈOäG~³%ò#?ùÈü¦Ö3g.gÎè|ÞºººCùùüÈüÈ¯Òä×ÕÕ=Ã#_4½gÏò#?ò#?ùùUüêëëóÎ9_OOOôÁ.äG~äG~"?ò#?ò«(ùeÿ<pV~£££Ñ	¿äG~äG~"?ò#?ò«(ùÕÕÕçE¯óùe2z(L§R)ò#?ò#?ùùUüeªÆëå_&?ò#?òùù_EÉ/tåÊèÜÞêêê?¾,¾äG~"?ò#?ùÍÈüD~äG~"?ò#?ò#?òùùü~µ/677×ÔÔÄÆår¨üÈOäG~ä'ò#¿É¶~ýúª	Ú¸q#ùùÈüÈü*D~·wïÞð<Ýi>Í?vìùùÈüÈü*A~Éd2ðnß¾¢¿çæóüÈüÈOäG~äG~"¿D"xöÂEL&,W ?ò#?òùù_%È/ûGÛ&Zý%7ò#?ò#?ùùUüØüÈüÈOäG~ä'ò#?ò#?ò#?ù_Ê¯xäG~äG~"?ò#?ò«ùÅnU<'?ò#?òùù_%È¯2"?òùùüÈüÈüÈOäG~ä'ò#?ò#?òùùüÈüÈüD~äG~"?ò#?ò#?ò#?ò#?ò#?òùùüÈüÈüD~äG~"?ò#?ò#?ùÈüÈüÈüD~äG~"?ò#?ò#?ùÈüÈüÈOäG~ä'ò#?ò#?ò#?ùÈüÈüÈOäG~ä'ò#?ò#?òùùüÈüÈüÈüÈüÈüÈOäG~ä'ò#?ò#?òùùüÈüÈüD~äG~äG~äG~äG~ä'ò#?òùùùüÈüD~äG~äG~"?ò#?ùùùüÈüD~äG~äG~"?ò#?ùùÈüÈOäG~äG~äG~ÆüÈüÈüD~äG~"?ò#?ò#?ùÈüÈüÈOäG~äG~äG~äG~äG~"?ò#?ùùÈüÈOäG~äG~ä'ò#?òùùùÈüÈOäG~äG~ä'ò#?òùùùüÈüD~äG~äG~ä'ò#?ò#?òùùüÈüÈoõõõ%Éx<ÞØØØÝÝ»¨§§§¹¹9,jjjW#?òùùüÈ¯¼K§ÓöìéèèÈ]´`Á_~9L<óÌ3.$?òùùüÈ¯¼«­­L&JMtµêêêBùýÕ_ýUïöüóÏöªto,ãPÂÂ.zôèQãàgÿ/üGº»»Û8°W^yÅÏ~É;vìØ~ð¼ÅY!¿x<>îtna,6lØP(¿Ã¿=?~üÍ7ß|[¥ëµ×^;uêq(ao½õVØQCiFù×ýWãPÂN>ýÃþÐ8°/vuuÒþòüd&oqVÈ/e§DánÜ¸N§íu´Wö:Úëh¯íu´·üªúeaº®®.ÉDGÃtÞ5/_¾¼nÝº+W®nüÈOäG~ä'ò#¿2«³³sÿþýa"|M§ÓyÒjkk»víÚ¸+ùüÈüD~äW~ÃZ__ÅÉdOOÏÿ<¶±S©TUNäG~"?ò#?ùÍÒÈüD~äG~"?ò#?ò#?òùùüÈüÈüD~äG~"?ò#?ò#?ùÈüÈüÈüÈüÈüÈüD~äG~"?ò#?ò#?ùÈüÈüÈOäG~ä'ò#?ò#?ò#?ùÈüÈüÈOäG~ä'ò#?ò#?òS¥ÉïÝwß&?òùùù©åwìØ±ï½÷®»î;wîoÿöo¿õÖ[äG~"?ò#?ò#?U ü9RSSóÀìøêí_ÙÞÚÚ:þüwÞyüÈOäG~äG~ä§J_SSÓ§Û?Øý÷Ñ~ôßøùÈüÈüÈO&¿èCÛ¿²=W~¿ÿû¿ÿÅ/~üÈOäG~äG~ä§JßoýÖomüã¹òûÄòO<úè£äG~"?ò#?ò#?UüyäßüÍß|èÏØ8wîÜþþ~ò#?ùù*M~###ýìgïºë®ßýÝß]zÿÒèC¸sîùùùÈüJÜ+¯¼òäO>õÔSaÇ¸£îùùùÈüfKäG~äG~äG~"?ò#?ùùßXçÏ?öìøw¨ÈüD~äG~"?ò+ü^yåøÃ÷ÞïÂÿµð×~í×î¨7ªùüÈüD~äW2ùõ÷÷×|¨fÍ§×DNñu_¨­­=räò#?òùù_¥ÉoÓ¦MøÄ'òþAÛÿnóóC~äG~"?ò#?ò«4ùýÞïýÞvþa®ü¾ô¾tï½÷úù!?ò#?ùùUü¾ô¥/µýï¶fÍgZ[[ýüùÈüÈü*M~ýýýóæÍûüØ÷åÍ_N¥RÏ=÷ò#?òùù_¥É/ôíoû7~ã7î»ï¾ìcsçÎäGüðùÈüÈü*S~Ñ³o¸ò#GÎ?ï'üÈüD~äG~äWÉòùùüÈüÈüD~äG~"?ò#?ò#?òùùüÈüÈüD~äG~"?ò#?ò#?ùÈüÈüÈüD~äG~"?ò#?ò#?ùÈüÈüÈOäG~ä'ò#?ò#?ò#?ò#?ò#?ò#?ùÈüÈüÈOäG~ä'ò#?ò#?òùùùùùÈüÈOäG~äG~ä'ò#?òùùùüÈüD~äG~äG~ä'ò#?òùùùüÈüD~äG~äG~"?ò#?ùùùò#?ò#?òùùüÈüÈüD~äG~"?ò#?ò#?ùùùùùüÈüD~äG~äG~"?ò#?ùùÈüÈOäG~äG~äG~"?ò#?ùùÈüÈOäG~äG~ä'ò#?òùùùÈüÈüÈOäG~ä'ò#?ò#¿IÖ××L&ãñxcccwwwáºººªªªÈüD~äG~"?ò+ûÒéôÂÄ=:::ò477O$¿@±_Ì`á	uppð*]çÎ¿ýC	÷ÝwÃÿ CiñÅ¯_¿nJXø/ßë¯¿nJØ7?nJÛK/½ôïÿþï3y³B~µµµ£££a"É¤R©¼¥>úèO<1üüñc3Ø#Gþùc*]ßË8°°Õ8øÙ¿Ã;zô¨?û~öòÇããN.^¼ØÒÒh¯£½r´×Ñ^Gåh¯£½P,ËN'ÜEííí'NøïJ~ä'ò#?òù_VõËÂt]]]&yoìho÷jÙ+ùüÈüD~äWÆuvvîß¿?L¯étz")Î$?òùùüÈ¯üµ¾¾>%Éq©G~ä'ò#?òùß¬üÈOäG~ä'ò#?ò#?ò#?ùÈüÈüÈOäG~ä'ò#?ò#?òùùüÈüÈüÈüÈüÈüÈOäG~ä'ò#?ò#?òùùüÈüÈüD~äG~"?ò#?ò#?òùùüÈüÈüD~äG~"?ò#?ò#?ùÈüÈüÈüD~äG~"¿JêGyöÙgßÁ<~U½¥Òõâ/OvîÜ¹¿û»¿3¥íïÿþï_ýuãPÂN8ÑÝÝmJXø_ôßþíßÒvèÐ¡þþþ¼Åwßü&ìÌ3;vìø$IRETü`fa%IfIä'ID~$I"?I$$IÈO$Iä'I$ò+ã®]»J¥òfvuuUUåx___2ÇãÝÝÝ®$£zåÊªÝ$´È¸ÙQK>¤öÒiêðððºuëÄ=÷ÜóÂ/ØQoëÚQ§7¤¹Åî¨½Ôw±ô<yrÑ¢Ey?!###ÍÍÍ?6étúÀabÏ=F¯$£zèÐ¡ð[Ì MuHµäCj/ö¨îÚµë±ÇFihh°£ÞÖ!µ£Nû**ìao¼£öRò++W®ÈÛôÑ'x¢p·¨­­?la"É¾ ¥éjø=uøða6Õ!-2nvÔ©½tÚ£ÚØØxöìY;êÌ©uÚÏP¡.¬X±âNÛKÉï¶lÎpñâÅð.Ü-âñø¸Óz?£º`Á¶¶¶0áçÎ3nÒ"ãfG-ùÚK§=ªaÐvïÞ]]]ÝÐÐpúôi;êmR;êô4ª½½½··÷NÞ'¿ØÂ÷þÄãî¹ÿq+É¨f»|ùrSSqÒ¯ªqÇÍZò!µNTÃÞ¸oß¾0 8bG½­CjGöÏþÀÀÀ%K¯öï¥ä7@Õ¯µºººL&óÞØ«¾aÚ¸dTsóÛL)7;jÉÔ^:íQÍÝó^2±£|Hí¨ÓþÙßµk×îÝ»¯öï¥ä7£¿ýgvvvîß¿?L¯étÚ¸dT,Xpþüùè?©mmmÆmCZdÜì¨%Ré´GuÓ¦MgÎYºt©õ¶©uÚÏPË-;uêTáÕ>ð½ü>0£DÓ'O¬¯¯ÅbÉd²§§Ç¸dT-Zþçº|ùòðÛÊ¸MrHÇ7;êmRé´Gupp°½½=zçÙÀÀõ¶©uÚÏPD":ãNÞ'?I¤Y#TC ID~$I"?I$$IÈO$Iä'I$ò$IùI$ü$ID~$Iä'I$ò$IùI$ü$ID~$I"?I$$IÈO$Iä'iöôÍo~sÙ²eÕc­X±â¹çû_pcÍ¯ãñîm*mhh(o~ÇÉäèèèT·)ü$©üÚ±cGUA_ÿú×+I~;wî3zê©¼ùO>ùdÿðÃOcÈOÊ¬¾¾¾`x<¾wïÞ±öíÛ.§Nªù]¸p!ÌljjÊ¿hÑ¢0ÿüùóä'ü$U~ûÜçiì±Ü?þxùàæº§»»;È) °¹¹9Lg¯|ýúõ6ÔÔÔEµµµ[¶lÉ=¨züøñ «°(¬ìØ±<K9sçÎ]¶lÙÑ£GÃÅÎÎÎ¼;väÈâÛ»-êêêHimmma~OOOvÎË/¿æ´¶¶fçìÚµ«¾¾>lªººzÍ5/_._áöóæ¹«ÈO>ÈæÏÔrñâÅÜ.]3S©T®lòêíí®^½:oÑæÍ£Eýýý±XlÜµ¢ÑÒöööÑÑÑ`ÇD"122¯ANsæÌÞ~Wd;abÜ»WøHyæfqyàÀ,ûò6²bÅ©Ê¯È]D~ôØçZUUpX®l:::n&ÂÅU«VEK#èDv^ºb¥ÓéèÅÂ0òäÉh#¹ÛÜ¾°]t°uýúõaÎáÃÃtø¦×®]Ëí5á¹÷mÜG49oÞ¼ðx¯]».¯ánf2èÉd2¬Â¿<:]=Uù¹«ÈO>xùÆ_v~$L¡Ë/ÁLÑÅæææp±¡¡aãÆAl7oÞÌn$´ìZÑÅK.e¯´=à¾éãÇßr;AáâÕ«WsïÛDïÉÛºukXôÄO¼÷Ë#Ú[¶lÉ½BP`_ðhKKKôäTåWä®J"?Iú^èºqãFîÌ¡¡¡03,*b¬"üe¡s^áAØ<Kå~J®­­M$ÃÃÃaãsçÎÍ.½åv&rX^gÎ¦Ã×0Ýßß]zòäÉpÆ=j<yù¹«ÈO>à¢÷ºíÞ½;wfôY'ygxdßxíÚµÂ²N:µsçÎèØkÑrÙÃ©·ôÙæÍ£¼áë²ól'zíÊ+ÑÅ«W¯?wÉ%aiôA6¬¹¢ó|Ã¢®®®ÁÁÁâòËª4ìÒ"wUùIÒD<ß³gOô©.÷îM$gc¬Zµ*(gxx8:=7z_ôö¸Ü7FÜ²eKpRtÖmösUÆõYt²mtë/¼ðBv~íDoìÞç744]³üöíÛ5.<äÜEÑ[ûûûÃ­AH~5ÃÕ8×¬Y»´È]D~ôÁÊq^»víúÿ¿àÆ`Î'ÍTmýúõ¹¬ÌíÐ¡CEä÷ÞØÛókkksÙN¸¹w,^íDöæÍÕÕÕÑCÈ;Æ½råÊÜ7o^ø°Kî6£$³ENÍ.-rW%$Ý¬X±"1Ö²eË¢lóä×ÕÕl^ccã'²Kzè¡úúúHK[·nÎ.=vìXKKKY2|úé§ó¶YxOÂ¦ÂüM6åÍh;¡pgÂ]>h°ÈçùeÛ°aCÞÇ»D]»v-N¨©©	âüùóÙO¨ÉÝæàà` m4V­­­===y·Xä®J"?I$$IÈO$Iä'I$ò$IùI$ü$ID~$Iä'I$ò$IùI$ü$ID~$I"?I$$IÈO$Iä'I¤þDÞ	«bIEND®B`


³LôAºÜ>Ñ©®qïÕ´g,núøÅøÓ >ç³ÉËoOãHW®Ãaká¿X$òÊO~¹|ÑÇÂâ1EäwõêÕÜ;ÎÛUï£ÑÅðößYw´®ÈbÏ8ÑcÞÔóÆ×××ÇQxçÏ?ÑãÆmÄ/F°£¿'Ùl6X!£µµ5þØÈ/Çñþ.g¯Ò0oX«W¯>yòä¸+m2+gÜ5í'/¿èrýúõøMLI~|·´´MMMA®á¿·nÝò[E"?©Ìä¤FÖÕÕMôv;Q¢8EÞªøfªïÍÓXìDãÃC7¼ÄÇî3+üÐ[áãÌÅ Ì¼#w<Gµ¡¡!:<]È»<Oç0Y&³®Æ½ÂdVÎDÞ?Úûî]|FøËðøñã~±Hä'üyæøÑ^´¼³,')Í[|Í[o½Zd±Åg,r>.æ¾%á,oïT'¿Bâ/^»JwwwÙdäí¾`%ÉlÛ¶íÆ#7lØ0®ü&3Ë´å7ÕkÚ3N^~Ùç³]ô_"ë¿Èó-êìÙ³öì1çÎD~R©Ë/¼·EoÌ¹ouÞÏÂ;etªì%K¢IÑo7î[fy³)"Kô9­°ðh9yâw±Åg,Îõë×ç>ÈÎü]·nÝÍÛEKÛ²eË»_´ßk`` <ýû÷OF~§Nö*|æÌh^Ô±cÇÆÝ¾SeJò+¾ròñ¦ºV§!¿èyáÉpëÖ­»µk×ºpß¾aå_ÿw|	D×,rä]ùI¥"¿Ââç'-û¼îKw£oEæ-Î¦@¢ÈFñ-sS,¶øÅqíÚµÜ'º¢1/^ÌûWîCïF~9DÍ??÷©Ç"¾yðÁ7ÖÒ¥Klâ)Í2%ù_9yÏxS]«ÓßÙ³gãË_±bE|jÞ:É`>Ñ­y¾åN!ÊõÐCùÅ"Tòo«V­*üë^]]]­­­AQáíügÉ?vìX¤ÿ6»IÎG6E_ÞÒÒRøµ|-ö3gDt$4>þÂ6lHÝ.ÉÏ1=ù¥emß¾=`(îâ¾yê©§Â£|Áüä'?Ûçôîgü¯¼çF^SZ«Ó_èøñãMMMáQåGÇÓs§3ÁgÑ­¯^½º··÷ûïÜ¹3Úø6åÈÈ_,ùIÒ])Í>øàSú¹iÌRvEûz£µGGG#Ð/^¼ØF"?IR¥Î/¯ÇÜÈOTiïØ±cÁÑ!Ý0ðÈ#X-ùI$ü$ID~$I"?I$$IÈO$Iä'ID~$I"?I$$IÈO$Iä'I$ò$IùI$ü$ZÇkllL&«V­ê¼ÍÍÍUUU¹1a8Y²dÉ~Ýnz×Ì¼Óù;AñID¢ºº:ÉyI"?IåQCCCpLàËèèèTçÝ»wo÷ÿøscxâ0fÏ=e-¿"Ë	<···I"?IeòÛä]àéìÙ³aÞ+WæÆ,[¶,éïï;9»òVÃÕÕÕEÈORÙ°/®;wÖÖÖÖÔÔìÞ½;ïjüä'[[[óÛeßzë­0ÆD®²zõê£d2ÙÒÒÒÓÓ3îÒâ·^|666._¾<ÜP¡ÃÞ~ûí0o¸ó;vìva®X±",-K¾zõê]ßDôÆÆÆÂÃHÏ1Iä'©äð?öØcaøg	Æ?þxü:]]]iÛ¼ystàÀ0~áM6E.]zâÄ0péÒ¥0>N»´ø(>KðÜáÃÃÀÆïüÖ­[Ãp¸ÂóÏ?öîÝFÞsÏ=aøæÍa Ú%9îø¦*¿À»°~Âð<MÚ³gO´*öíÛyäÏ.Iä'©tå×ØØ³·+×¸<uêT´nÝº0¼~ýút¹©gÏÝµkWkkktJÄ¸KËÓUñY¢;V[[[8otçÉÂ@0_®[ZZeGFFÞÍÊî[Xx`_`e4)ÜôÂÃÝ¨©©Y´hQ¸èÙ%ü$®üÉdn8(W`VN¸æððptP5&íß¿?Ì8øÚk¯Å·´øÅÉÌ2ÑïÆÔxôèÑyóæEcêêê¢ÝïÉ>¿qNp	ÅKùI*Eù¥Óéø>¿h·Ù¹ÓÑÑ¦æ~æÆ§R©0fìv_ñYrw,`®pÞùóçGóæÝ½ÑÑÑ'NlÜ¸1¾³p+ç«âæÍõõõLfÅAÃ×¯_÷ìD~JW~Ñ·´8p úßO<1ù=z4·óìðáÃ¹ñ#Ï;þo2ò+>Ë®]»î¹0°yóæÂyzè¡0|èÐ¡óçÏGGxÃÈåËá×^-8,,Z´èîÉoÛ¶maÒÙ³gÏ9ÿ0¢$¤RßèèhÐUÍíâßÉW ·nÝ´¹½?^WWW[[»cÇIÊ¯ø,'OS-[;Ñ$~ááá`¯pÏS©ÔÚµk£Óxø2Lt²ðÊ+§z²íäå´ÿ+V`ÈO$Iä'I$ò$I"?I$$IÈO$IäW6ûÛß¾|ùòLÞâÅý-¦ìÇ?þñý×Y%¸]¦ñ§Òt·»téíRÛå?ÿó?­Rë7Þ(©í2×å÷¯|%ào&oñÔ©SÿñÿáPjóß|ûí·­Rë[ßúÖ¿ÿû¿[¥ÖéÓ§ÿíßþÍz(µÂÛYü0U"9sæ_ÿõ_ÉüD~"?òùùÈOäG~"?ò#?òùÈOäG~äG~"?òùùùÈüD~ä'ò#?òùÈüD~äG~ä'òùÈüÈüD~"?òùùÈüD~"?ò#?òùÈüÈüD~"?òùùÈOäG~"?ò#?òùÈOäG~äG~"?òùùùÈüD~ä'ò«(ùõ÷÷§Óéd2ÙÜÜÜÓÓSxîîîªª*òùÈüD~e_&9tèPèììlooÏ:::ÚÒÒ2üN8qcé¥®^½zC%VøÃ?þã?Z¥ÖË/¿l»æv¹råõPj:uêòåËÖC©õÍo~ó7ÞÉ[ò«««Ùl¶±±1oêc=öäON$¿ÅïÌ`á¿ËßQe»ìvùÖ·¾e=Z/¼ðíb»¨d·Ë_2w8þcÚÚÚh¯íu´Wö:Ú+G+¡D"N¥RñImmmáÔÿ<TòùÈüD~P6çöÑÞ0üSð§#?ùüÈOäWÞutt<x0LfüjÈüD~ä'ò«ÕÚÐÐH$Òétooï¸Ô#?ùüÈOä7§#?ùüÈOäG~äG~"?ùüÈüÈOäG~äG~"?ò#?òùÈOäG~äG~"?òùùÈOäG~"?ò#?òùüÈOäG~äG~"?òùüÈüÈOäG~"?òùùüÈOäG~"?ò#?ùüÈOäG~äG~"?ùüÈüÈOäG~ÖùüÈüÈOäG~"?ùùüÈOäG~äG~"?ùüÈüÈOäG~äG~"?ò#?òùÈOäG~äG~"?òùÈüÈOäG~"?òùùüÈOäG~"?ò#?òùüÈOäG~äG~"?òùüÈüÈOäG~"?ùùüÈOäG~äG~"?ùüÈüÈOäG~äG~"?ò#?òùÈOäG~äG~"?òùÈüÈOäG~"?òùùüÈOäG~"?ò#?òùüÈOäG~äG~"?òùüÈüÈOäG~"?ùùüÈOäG~äG~"?ùüÈüÈOäG~Öùüf ÎÎÎÕ«WøÃþßøò#?ò#?Èü*°ÑÑÑûï¿ÿîùÜg?÷;ÿ÷w>¾æã555'O$?ò#?òùüÈ¯Òú«¿ú«t:½ó÷wî~xwôïÓmnhh ?ò#?òùüÈ¯ÒzøáïÿÄý9öEÿæÏÿ<ÏÉüÈüD~ä'ò+¡¾üå/â¾OÄÙ·ëK»æÍ7<<L~äG~ä'ò+õÂÛÕ÷¾÷½7ß|üÈOøÀ~oÛïåäwÿ'îÿÈG>ò,üÈüÈOäwûÝßýÝêêêÿ³ðÿÔ××·¶¶^¾|üÈOwlÛ¶m?÷s?÷©uúLÇg>ºü£uuuäG~äG~"¿îáN§Ó_ØòèpÕ¯þê¯644¼'G¬Èü*¾o|ãûÜçî»ï¾]»v½ÿe"?ò#?òùÝ­jkk7ÿöæøÇî½÷Þ#Gùi¶"?ò#?òùÝÆêëëóÎOüØÇ>öøäG~"?ò#?ùUZóæÍõæþ-Y²Ä>?òùùüÈ¯Û±cÇÂw|qGÄ¾_ÿõ_ÿà?ø+üÈOäG~äG~"¿Ùotttýúõïßû.]zï½÷þüÏÿü÷¾÷½÷üVÈüD~äG~ä'ò+9òê«¯¾çgõùüÈüÈOä7·"?òùùÈüD~"?ò#?òùÈüÈüD~"?òùùÈOäG~"?ò#?òùÈOäG~äG~"?òùõ@~äG~"?òùÈüÈOäG~"?òù]ýýýét:L677÷ôôÄ'õöö¶´´IK,	W#?ùüÈOäWÞe2CÎÎÎöööø¤¾òÊ+aàÙg]´hùüÈOäG~"¿ò®®®nll,d³ÙÆÆÆ®V]]](¿¿ø¿ø»¬««ë»ßýîß©ÄÛ¥¿¿ßz(µ^|ñEÛÅvÑ$ëîîþÛ¿ý[ëÁvòK&ãÇëëëÛ´iS¡ü9ryûë¿þëÁÁÁË*±N<i»ævùû¿ÿë¡Ôzé¥.`=Ø.L===¯¿þúLÞâ_"È§R©Â+¼ýöÛLfxxØÑ^9Úëh¯íu´WöwõõõÙl6:Úó¦^»vmãÆ×¯_/üD~ä'ò#?_ÕÑÑqðàÁ0~f2<i­Y³fhhhÜÉOäG~"?òùßjmhhH$étº··÷[Õÿ<ºÆÆÆªXä'ò#?ùüæhä'ò#?ùüÈüÈOä'ò#?ùùüÈüÈOäG~äG~"?òùüÈüÈOäG~"?ò#?òùüÈOäG~äG~"?ùüÈüÈOäG~"?ùùüÈOäG~"?ò#?ùüÈOäG~ä'ò#?ùüÈüÈOä'ò#?ùùüÈÏz ?ùùüÈOä'ò#?ò#?ùüÈüÈOä'ò#?ùùüÈüÈOäG~äG~"?òùüÈüÈOäG~"?òùùüÈOäG~"?ò#?ùüÈOäG~äG~"?ùüÈüÈOäG~"?ùùüÈOä'ò#?ò#?ùüÈüÈOä'ò#?ùùüÈüÈOäG~äG~"?òùüÈüÈOäG~"?òùùüÈOäG~"?ò#?ùüÈOäG~äG~"?ùüÈüÈOäG~"?ÍùUÝ©D"A~äG~"?òù_%È/q§É$ùùüÈOäG~ ¿ÊüD~ä'ò#?ß»-<ù6oÞL~äG~"?òù_¥É¯±±1LúùÈüD~ªpù-Y²¤ðôºººááaò#?òùÈü*J~©T*Pohh¨¡¡!ð=÷Üsa`Ó¦MäG~ä'ò#?ùUü¢|a P/till,ÔÔÔùÈüD~äWQò7o^p^x`aàG|«ùÈüD~äWiòÛ±cGî|øGý/_N~äG~"?òù_EÉ/ôÕ¯~uþüùa ··7¶¶¶Åö ?ùüÈOä7W"?ùüÈOäG~äG~"?ùüÆkÑ¢EÑw»ø&gò#?ùüTÉò[¸pa{¹ÛK~ä'ò#?ùUüòùúúúÆÆÆÊnÈüD~ä'òBuuuA~åÈ>òùÈüD~S«¿¿?ÈoëÖ­åøÆI~"?òùÈoj-X° ª gxùüÈOäG~&¿¦¦&gxùüÈOä§9!¿È|å¸=ÈOäG~"?òùM¡úúzgxùüÈOä§9!¿@ ¿;vùÈüD~ªdùUM3<ÈüD~ä'ò#¿ü&çqsùÈüD~äWßêR¾ÈüD~ä'òBMMMçÏ'?ò#?ùüTáòK&UUïÙ¾Ãþþþt:ÙÜÜÜÓÓS|ÒôÆÄôÑG9ry;|øðë¯¿~Y%VØ.á/ÖC©õõ¯ý?øõPj=÷ÜsçÎ³J­ðvöýïßz(µ=:000·x×å8ä·gÏð_wÿÝ.LæÐ¡Ca ³³³½½½ø¤é·ûö*I¤Jé®Ëï½=··®®.âc6mll,>izcÈO$ß4oÏíÏ·ÂIÓC~$üJ¢øÂT*U|ÒôÆÄûò¿üÔSOû?ùîîîo«ÄÚ·oíRýéþé/¾h=àvéêê²J­ðvöÂ/X¥ÖýÙÍðv)3ùÕ××g³Ùèàl.>izcÛ+çö:·WÎíun¯Û;ýªÖ®][]]]UUUSSÓÖÖ6íS=:::<ÂÏL&S|ÒôÆÈüD~ä'òf·nÝ÷0óôþoXAD"N÷ööþï½¼ÐºpÒôÆÈüD~ä'òf/2[·n]ôÆvë×¯c.]ZúÛüD~ä'ò#?ßJ¥RÁy£££¹1Ùl6)<üÈüD~ä'ò#¿ò_"ÎÎ¢	c¦÷­.ä'òùÈüJW~ÑÑÞ5kÖDoágcZZZÈüÈOäG~"?ò«(ù÷ËqÏð¸qãùùüÈ/¯ááá'xbýúõ6løË¿üËøD~äWòçöé½mmmµµµD"üfMSÛüD~ä§ßo¾ù¡è[~ù·~ë·>Ýöé<ðÀðG~äWfò+ßÈOäG~IùêSú_ùÝïþíúÒ®~ðG±VÉüÈüD~äG~&¿÷¿ÿý_ÜþÅüÂ¿¯ùø;¬Uò#¿Rßÿ`püæùÈü"ùíøâ¸üî¿ÿ~ò#?ò+ù%&üÈüD~ä7nëÖ­[½zu ¿ð¿àh/ù_Èo¢¶oßÉïÙg%?ò#?ùÅ»|ùr]]ÝÇV~lóooþüÿð?,èò#¿²_MMM0_øÿÉüÈOäG~¹Þ|óÍ7óýâ/þâüÁLï¼üÈoå·aÃhWß'ÊhÈü4Ãòù_yËïùçÌ·nÝº²Ûä'ò#?ùü&Õ­[·.]ÌÑÓÓSÛüD~ä'ò#?ßÛ·o_´«¯½½½|·ùüÈOäG~"¿IÌéûüÈüD~ä'òÓ_âN%Iò#?òùÈü*A~ùüÈOäG~"?ò#?òùüÈOäG~äG~"?ò#?òùùÈüD~"?ò#?òùÈüÈüD~"?òù_yÉoÑ¢E©TÊ÷ùùüÈOä§ßÂãÚó~äG~"?òù©båÌ×××766VvÛüD~ä'ò#?ßª««ò+GöÈüD~ä'òZýýýA~[·n-Ç7NòùÈüD~SkÁU9ÃüÈOäG~"?ò«4ù5559ÃüÈOäG~"?Í	ùEæ(ÇíA~"?òùÈoÕ××;ÃüÈOäG~"?Í	ùÊùíØ±cddüÈüD~ä'òS%Ë¯jáA~ä'ò#?ùUà793<ÈüD~ä'ò#¿üVòüD~ä'ò#?ùùüD~ä'ò¯l6»víÚêêêªªª¶¶¶r9ÕüD~ä'ò#?ßºuëÖ¸gxÅ©¾ä'ò#?ùü¦ÐâÅóÖ­[½q§ÝúõëÃ¥KùÈüD~äWQòK¥RÁy£££¹1Ùl6	ãÉüÈOäG~"?ò«(ù%à¼ ½Ü0Æ·ºùüÈOäG~y´wÍ5ÑgøÃò#?òùÈü*J~áýrÜ3<nÜ¸A~äG~"?òù_EÉïÛ§÷¶µµÕÖÖ&ðsÍ5aLYlòùÈüD~s%òùÈüD~S¨±±±©©éüùóäG~ä'ò#?* Éd²ªªÈüD~ä'òB===A~öì	O¸rù£mä'ò#?ùü¦µ	J$äG~ä'ò#?ùUüäÉüD~ä'ò#¿_SSSssóððpYoòùÈüD~w.ús½ïÜ>Ú[vÉOäG~"?òùM³èÏõF¢üÈüD~ä'òS%Ë/NWÍäG~"?òù_È¯¯¯oÞ¼yÑ¿ÈyÎð ?òùÈO)¿<òùÈüD~s:òùÈüD~äG~ä'òùÈ¯$ëïïO§ÓÉd²¹¹¹§§'>©···¥¥%LZ²dI¸ùüÈOäG~"¿ò.É:t(tvv¶··Ç'-ðW^	Ï>ûì¢Eå÷o|ãÚvòäÉ/^Se»f/½ôÒ~ô#ë¡ÔÿÁþáh=Ø.L/¿üò?üÃ?Ìä-Þuù]¸paÖåWWW766²ÙlccãDW«®®.ß3Ï<óÝì^ø¿ùïªÄêêêêííµlÙ.åÛ/¾øê«¯Z¶Ë]_ô.---á¿³%¿øÉÅhÜ××·iÓ&Gåh¯£½r´×Ñ^9Ú;ýùrßÞÔµråÊ/ÎðÃkt**¼BxSÏd2eüD~ä'ò#?ß»páÂâÅsüZ»víÝ½ë?)×××g³ÙwníÃy×¼víÚÆ¯_¿^¸òùÈüD~ÓlllìÄÕÕÕ3ü×Û:::<ÂÏL&'­5kÖ;#ùüÈOäG~"¿)700ßçW[[[ø¡º»ºZ5Óétooïÿ>¶Û»ãMüD~ä'ò#?ßô¯ººº½½ýÊ+å²=ÈOäG~"?òùMe·Oì¸ï¾û.]ºTvÛüD~ä'ò#?ßÅ/s!?ùüD~ä7£ò+ëÈOäG~"?òùM­sçÎ-Z´¨¦¦&:·¾¾þèÑ£äG~ä'ò#?ùUüº»»sgxDò;;;ÉüÈOäG~"?ò«(ù544ç;w.'¿ÞÞÞè]ÈüÈOäG~"?ò«(ùå¾'/'¿±±±è_ò#?òùÈü*J~õõõÁyÑ~¾ ¿l6»sçÎ0ÜØØH~äG~"?òù_EÉ/P¦j¼^yåò#?òùÈü*J~¡ë×¯·¶¶FçöVWW/Z´èâÅe±=ÈOäG~"?òùÍÈOäG~"?òùùÈOäG~"¿îÊ+---µµµÛææær9ÔK~"?òùÈo²=ôÐCU´yófò#?òùÈü*D~G·ÿþáááhä­[·;ïêê"?ò#?ùüÈ¯äN§ï8P8)ún¾ÏüÈOäG~"?ò«ù¥R©À»[·nNÊf³aR¸ùùüÈOäG~ ¿ÜmhjôÜÈüÈOäG~"?ò«ù±ùùüÈOä'ò#?ò#?ùüÈ¯åW<ò#?òùÈü*D~;L&ÉüÈOäG~"?ò«ùUFä'ò#?ùüÈüÈOä'ò#?ùùüÈüÈOäG~äG~"?òùüÈüÈOäG~"?ò#?òùüÈOäG~äG~"?ùüÈüÈOäG~"?ùùüÈOäG~"?ò#?ùüÈOäG~ä'ò#?ùüÈüÈOä'ò#?ùùüÈÏz ?ùùüÈOä'ò#?ò#?ùüÈüÈOä'ò#?ùùüÈüÈOäG~äG~"?òùüÈüÈOäG~"?òùùüÈOäG~"?ò#?ùüÈOäG~äG~"?ùüÈüÈOäG~"?ùùüÈOä'ò#?ò#?ùüÈüÈOä'ò#?ùùüÈüÈOäG~äG~"?òùüÈüÈOäG~"?òùùüÈOäG~"?ò#?ùüÈOäG~äG~"?ùüÈüÈOäG~"?ùùüÈOä'ò#?ò#?ùüÈüÈOä'ò#?ùß$ëïïO§ÓÉd²¹¹¹§§§ðÝÝÝUUUä'ò#?ùüÊ¾L&sèÐ¡0ÐÙÙÙÞÞ7utt´¥¥üD~ä'ò#?_%TWW766²ÙlcccÞÔÇìÉ'H~þçÞ7½ðÂ¯¼òJJ,ÛÅvÑäëêêúÎw¾c=Ø.*Íí2'äL&Ç]¹r¥µµ5¸p"ù;vìf°'O¾ñÆÿ¤ë¥^ºtéõPjõôôüøÇ?¶lM¦_~ùâÅÖC©uêÔ©ýèG3ysB~D"7J¥âÚÚÚN>ý?ÕÑ^9Úëh¯íu´WöiU?)×××g³Ùèho÷j¹+ÈüD~ä'ò+ã:::<ÂÏL&3GÈüD~ä'ò+¿ÕÚÐÐH$Òétooï¸Ô#?ùüÈOä7§#?ùüÈOäG~äG~"?ùüÈüÈOäG~äG~"?ò#?òùÈOäG~äG~"?òùùÈOäG~"?ò#?òùüÈOäG~äG~"?òùüÈüÈOäG~"?òùùüÈOäG~"?ò#?ùüÈOäG~äG~"?ùüÈüÈOäG~ÖùüÈüÈOäG~"?ùùüÈOäG~äG~¥SøU>22B~"?ùüÈ¯å×ÕÕÕÔÔTWW7oÞ¼|ä#ä'òùÈü*P~ÇÿÙýÙööö]_ÚþÝÿûß÷¾÷Í5üùüÈOäG~sB~¿ôK¿´áÓv?¼;÷ï£Ë?ºeËòùüÈOäG~&¿ùÙõ¥]qù¦ã3«W¯&?ÈüD~äWiò»÷Þ7ÿöæ¸ü~í×~íóÿ<ùüD~ä'ò#¿Jß£>ú¡hçïïØ÷-_xÿûßßÕÕE~"?ùüÈ¯Òä7::ú¿ù¨ûÀÇ>ö±+VÔÔÔüÑýÑ{&ùüÈOäG~sB~Q¯¾úêSO=õôÓO?~>ÈüD~ä'ò#¿9$¿9ùÈüD~äG~ä'òùÈüÈüD~ä'òùùÈüD~"?ò#?òùÈüÈüD~"?òùùÈü¬òùùÈüD~"?ò#?òùÈüD~äG~"?òùÈüÈüÈüD~ä'ò#?ò#?ÈüD~äG~ä'ò#?ÈüÈüD~ä'òùùÈüD~äG~ä'òùÈüÈüD~ä'òùùÈüD~"?ò#?òùÈüD~äG~"?òùÈüÈüÈüD~ä'ò#?ò#?ÈüD~äG~ä'ò#?ÈüÈüD~ä'òùùÈüD~äG~ä'òùÈüÈüD~ä'òùùÈüD~"?ò#?òùÈüD~äG~"?òùÈüÈü¬òùÈüÈüD~"?òùùÈüD~"?ò#?òùÈOäG~äG~"?òùùÈOäG~"?ò#¿;ÖßßN§ÉdsssOOO|ÒÈÈÈÆS©Ô=÷ÜÔE~"?òùÈ¯¼Ëd2íííñI÷îüñÇÇÆÆ¹åÆÿ÷öòË/Wæ«ÄOðÊ´J­ ò7nX¥Vù¿üË¿X¥VùÐÐõPjÿó?ÿóLÞâ_]]]°]Èf³ñIÍÍÍçÏhÆ ¿'x¢k;~üø/¼Ð¥Ëv±]d»Ø.ªí2'äL&Ç.îÛ·¯ººº©©éµ×^s´Wö:Ú+Gí£½å]"È§R©¼I.%?ùüÈOäW~Uý¤0_Ífß¹´7Ç¯¿·;üD~ä'ò#?_ùÕÑÑqðàÁ0~f2ø¤-[¶>|8;wnùòåä'ò#?ùüÊ~µ644$t:ÝÛÛû¿íöîÀð2hkkK&­­­ä'ò#?ùüæhä'ò#?ùüÈüÈOä'ò#?ùùüÈüÈOäG~äG~"?òùüÈüÈOäG~"?ò#?òùüÈOäG~äG~"?ùüÈüÈOäG~"?ùùüÈOäG~"?ò#?ùüÈOäG~ä'ò#?ùüÈüÈOä'ò#?_%õè£9räòvøðá×_ý²J¬°]Î?o=Z_ÿú×ðX¥ÖsÏ=wîÜ9ë¡Ôogßÿþ÷­RëèÑ£3yÅÿc6×å~yíÞ½û+$IQñUvÃJ$ÍÈO$ü$ID~$I"?I$$IÈO$IäWÆ566æìîî®ªÊ_á×¯_¯eÕÍäv)²òûûûÓét2lnnîéé±êJd»x½ÌâvÙ¸qc*ºçN:åõRúÛÅëe·K|Í'z½x*¼÷9sfñâÅy/³ÑÑÑÂ×ÞÑ£GÃÖJíRdåg2CÎÎÎöövk¯D¶×Ë,n½÷>þøãcccMMM^/¥¿]¼^f÷?*¼4Âë¢¤^/ä÷Þ·víÚÁÁÁ¼gÀc=öäO>-ÂËòØ±cVÚ¬l"+¿®®.ü&Ùl¶p÷­fk»x½Ìâvinn>þ¼×Km¯Ùß]ºtiÕªU¥öz!¿»¶fcÏ+W®´¶¶-]ø´X¸pá5kÉd¸Â¬·Ü.EV~9î°fw»x½Ìâv	«ß¾ÕÕÕMMM¯½ö×Kéo¯YÜ.Qmmmy#gýõB~3ñÛþôéÓã>-r]»vmÉ%ÖÛÌ¿2Ç]ùñe¤R)ë­D¶×Ë,nð¢8pà@ðz)ýíâõ2»¿Ç-[VxµY½ßL<ª~ºfñs¶Q¸òëëë³Ùì;·÷Æaë­D¶×Ë,nø!oG×Kin¯Ùý=¶wïÞûö^mÖ_/ä7£ïdãí½xñbô²5kÖXo3¹]¬üð3ÉXo%²]¼^fq»lÙ²åðáÃaàÜ¹sË/÷z)ýíâõ2»ïû+V¬8öláÕfýõB~³&¿h¸¯¯oñâÅá?j+W®/Nëm&·Ë¸+?ºÂ3gD:îííµÞJd»x½Ìâv¹yóf[[[ô¡±ÁÁA¯Òß.^/³û¾J¥¢39ò®0ë¯ò$I3Bµ$IÈO$Iä'I$ò$IùI$ü$ID~$I"?I$$IÈO$ü$ID~$I"?I$$IÈO$Iä'I$ò$IùI$ü$Í¾öµ¯­X±¢úv«V­zþùçêÜíÊæ×ñx÷¶±±1<´ááá¼ñaL2L§ÓcccS]¦$ò¤òk÷îÝUõ«_­$ùíÙ³'|úé§óÆ?õÔSaü#<2eJ"?I*³úûûiÉäþýûGowàÀp1<ölÅÈïÒ¥Kaä%KòÆ/^¼8¿xñ"ùI"?Ißg?ûÙ`Ç<>ò'#xà¸zzz[ZZÂpîÊ7nÜØ´iSmmmTWW·mÛ¶øAÕ'O]IaÞ®®®<K1óæÍ[±bÅ'ÂÅ¼;vüøñâË&»MêîîHikÖ¬	ãsc^yå0fõêÕ¹1÷îmhhª®®Þ°aÃµk×åW¸ü¼1Eîª$ò¤ÙlÁA-W®¼zõjÙØØM^ÑÔõë×çMÚºuk4i`` H;Wt1ÚÖÖ666ìJ¥FGGÃÔð3È©¦¦&úø]åqï^á#öÙgãÍáòÐ¡C9öå-dÕªUS_»*ü$iìóK­ª*8,.ööö·oÂÅuëÖES#èDvvÝ±E2L´³09s&ZH|»ví¶¶>ôÐCaÌ±cÇÂpø|ðÁ;.'¨1Wß·qQÐäüùóÃãÃÏpÏ7³Ùltt:fá®®®ªüÜUIä'I³/¿Ð¸òËd)tíÚµp1)ºØÒÒ.655mÞ¼9íÖ­[¹ëäíEËÍ]¼zõjîÊAK¹¾ág>yòä.¾õÖ[ñû6Ñgò¶oß&=ùäïüäö¶mÛâWòmmmvINU~Eîª$ò¤Y.ÚÑõöÛoÇGaRëä88á/ÜóÂæY*þ]*a¸®®.JÏ7/7õËÈay;w.Bj?ÃðÀÀ@nê3gÂ÷¨ñäåWä®J"?Iå¢ÏºíÛ·/>2ú®¼3<r*ÜuöìÙ=öDÇ^sdvÈå§ÞÑg[·nò6mÊ/²hÛõë×£o½õVñóp-[¦F_dÀç&uwwß¼y³¸ür*ÖFnj»*ü$iÎH&Ñ·ºìß¿?J±nÝº èPlî<Üès~ÑÇãã ¸mÛ¶à¤è¬ÛÜ÷ªë³èdÛèÖO:_d9Ñû¢ÏùG×,"¿äöÆäq`` ÜJX	É/²f°r¸ZçâSÜUIä'I³_ô-ÇyíÝ»÷ÿÿ»]£Üpî8iî¤=ôPñ=ZD~ïÜþca|]]]ü@på»¿c¹ój'z°·nÝª®®BÞ1îµk×ÆobþüùágôÅ.ñeF»$sENÍM-rW%$DA'«V­JÝnÅÑ	¶yòëîî¾6¯¹¹ùôéÓ¹©ÃÃÃ;wîlhh´´ûöÜÔ®®®ÖÖÖ²t:ýÌ3Ïä-³ðEñ[¶lÉ?ÑrBáÎ»Ñ`ïóËµiÓ¦¼¯wÊd2aÔÖÖGqñâÅÜ7ÔÄyóæÍ@Ûh]­^½º··7ïÜUIä'I$ò$IùI$ü$ID~$I"?I$$IÈO$ü$ID~$I"?I$$IÈO$Iä'I$ò$IùI$ü$ITÐÿòosû»=?@IEND®B`


~±Hä'M'ùVbfmmmr²§§§´´4÷õ¸««+ü3/_¾<ÏºÙ3ÇsîÒÖÖÖÜ7«ÆÚ¯çbó¯8ò¾÷ööÖÔÔTUUÅÄÈ1¹páB¼ç^NsÑrïïÈë?~òjR($Ë¾açhQ~ßÌ=;æ_¾|yä5wîÜQß®2ìª5É~uuuü?éÇ/¿îîîÜË;;Qùs3^¶lÙ°Û¼fÍ¿X$ò¦üùa²d:NÇÉ>zôh®Ï­Ü°aÃàà`ò.Î-×ÎxÉó'o4¥ñ'cõCÅZ¹×çbó¯8êß»woî`¹ç_½zuL/]ºôÚP1'cæX÷7óäO$7,¹ä8yàÀÜ;|mÿþý1îÜ¹ÜEcÝàT*5ÖÉ®«õAºì¢QoÕ¤WÌoúÜ¹A>ë³ñËoq"ÂóçÏÇt<1=cÆ¿X$ò¦ü²;øåsòÈïÂÙ9·ûþY²W1^GñòûfÕ°½uy.6ÿcÝ÷ñ¢>l~UUUî=ø89kÖ¬±îo2'´2ÙÜÁÜ[ÉdÂ!æææÜûG~YÅôÛwHcÝÌÅ:thÔAÏàz«&½âøål!.]Ê½	ÉoqSSS3gNÈ5þ¸yó¦ß*ùIÓL~!YYY9ÖËíXFINqò¼TçñÍD_'q±cÍ»÷7^¿ûûûsç|ÏläÞFÞßñeÛãyËcT«««ÝÓ#y7ÌÓÙKÏ*ã«QÏ0ÁË£[qüoëçf|úôéYîÛ·Ï/ü¤é$¿W_5÷ä]´aGY:Z7ÿ6/_Î]çbó¯ç¼öÚkq2û(ÉÌgÃÞfâñHîÉúúúäÛU<2ü7&/XI§Ók×®½víÚöíÛcæ+FßxV´ü&:8Ù&½âøå7÷ü²¶KþÔÉ3þy¶·¤cÇmÚ´)ÙÅ=ZùI÷ºüâµ-¼0g¿Õ%y=4Ä+er¨lccc²(ùSßP£¾dæY7?²$ÓO.gØ§¸F½Øü+ægÄ²eË²äOæ$Gþ.]º´w¨äÒ|òÉÛ_ò¾WOOOÜmÛ¶G~NÞU»|ôèÑäý¼¤½÷úøNt	É/ÿàÛ6rè¨NB~É'óbc¸yófÀnÉ%#]øÒK/ÅàÁùÇÿOäó§OÎ³ç]ùI÷üF|âÈ£e³÷Ï~énòáÈÌ<ëægS(±QîÙ¥y.6ÿùqñâÅì'º9gÏö¯ªªªìoG~Y$Í5+û©Ç<¾Y¹råÈkÞ¼yyâ	­2!ùåaÛFnÕIÈïØ±c¹¿`ÁÜ¥ÃÆ$ùX×gËBí'ðE"?izÈ/^-Z4ò÷:pà@sss(*^Î_õÕìü½÷ÆVî·ÙsÝ[²)ù¼¸ð¦¦¦_Ë7ÖÅÞrÅüHöæÎ?uêÔ+ÊJ§Ó#çü®zê©§CYpç÷ÍË/¿÷.îøìÙ³wìØñØceßsºýU&$¿ü3lÛÖFuòöíÛ7gÎ¸×qùÉþôìáÌq2|âÅ;;;oùëXÛ[__ßúõë7SïñPö÷÷ûÅ"$Ý2ÌÊ+'ôrXeÚ¼×¬=00¾¾¾Þ#$©ØJ>ç7¬çÞÈHä'I*¶úúúÖ­[7öìdnL<óÌ3E"?I$$IÈO$Iä'I$ò$IùI$ü$IÈO$Iä'I$ò$IùI$ü$ID~$I"?I$¤­½÷ÖÖÖ¦R©EMtÝÓ§OgçÄtÌillÀ¯³¡&wñ¬;É_²y¯1*---//O§ÓW®õõõ­Y³¦²²2³¢¢"9sÆ&ü$ÝCUWWc/]wóæÍ±î/¾óÂ/ÄM6·üé¸ã1ÝÚÚloo1äÈ;w®LùIº~Ü;ë.0;gþüù1§»»ê6õ×VéòòòädYYYìííµQI"?I÷.ûr5³~ýú3flÜ¸qØÙì±æææaË0¦/_¾Ó1'YtþüùÅR©TSSSòfØÈKË½öü«ìØ±£¶¶öG+é°7n´µµÅºqã×­[¼6]°`AÌK¾páÂßC='ëëë=jÓD~î]ü%ÓÏ=÷¿úê«a¬xþùçsÏsàÀìgÚ²­^½:mß¾=¦ãgL¯Zµ*Y4oÞ¼ýû÷ÇÄ¹sçb~MMÍ¨ò¯íµ×b¢½½ä_³fMLÇÞxãØ¼ysVc½½½===1¼%9²IÈopp0Æ'¦üñdÑÞ½³¸hÑ¢ãÇÛº$¤W~µµµ1j$¼Fý àáÃcÑÒ¥KczÙ²e	é²K;¶aÃæææäQ/m±ò¯Ü°ëVUU%7>La¾çé¦¦¦°lÿäeäüä¶Åûrwïvuu^³ç+µI"?I÷¨üR©Tv:&âd~2ôÖ×3âÉNÕ,Ú¶m[¬<~üxî%»´ÜãYe¬Ë/QãîÝ»gÎÌ©¬¬LÞ,È~yêèèkæîø$òtÏÉ¯¦¦&÷=¿äm³[r§­­-ffç'G<5Nùå_%Ãs#×5kV²î°700°ÿþäÀÛìÊ/JngÜ$òtÊ/ùíÛ·'óáÆÃÝ»wgß<íµ×²óG8q"ùüßxä6|ç;ßÕ«W'é;w<y2ÙÃ3yä>~üø¥Kb¢®®îÎÉ¯±±1<x0¦÷íÛÓ+W®´I"?I÷¨üBW3ÊýN¾üò»yóf²§5~æ~è-ôSYYYQQ±nÝºqÊ/ÿ*¥óçÏÏh¾¾¾µk×Æ-/++[²dIro/N'/ðÔ©SóE[[[ÜøY³f­Y³&nLùI$ü$ID~$Iä'I$ò$IùI$ü¦Mo¿ýö~8×xöìÙL&cË+`W2,6ÑØPCa;wîÜþÛ7Ý²«W¯^¾|Ù8°_þò?ûÙÏCaûàþõ_ÿüî¾õ­oþ¦ò>ü_üÂ3¡>ú§?ý©q(`ßÿþ÷CaûÁ~ðóÿÜ8°3gÎüä'?1ìæÍ÷wgÛÑ£Gÿå_þüÈOäG~ä'ò#?ò#?òùùüÈüÈüD~äG~"?ò#?ò#?ò#?ò#?òùùùüÈüD~äG~äG~"?ò#?ùùùùùüÈüÈüD~äG~"?ò#?ò#?ùÈüÈüÈOäG~äG~äG~"?ò#?ùùÈüÈOäG~äG~ä'ò#?òùùùÈüÈOäG~äG~ä'ò#?òù_QÉ¯»»»¦¦&J544tttä.êïïooo/++è¡B]äG~"?ò#?ùMïÒéôÎ;cbëÖ­­­­¹6oÞüüóÏ¹æÌ3R~û÷ï¿6ÅêÂ×T¸þ×PÆ¡ôÑG2-0ýOÿôOÆ¡õôô¼÷ÞÆ¡]¼xñßûq(lo½õÖ|0×x_È¯²²2lL¦¶¶6wQCCÃÉ'ÇZ1äXüáÐ<räÈU¸ÓÁãPÀbÕ8ü¹ÿüÀ8xîßË½ýöÛßýîwCaóÍ7§ø¹_È/J:|é¥ÊËËçÌsüøqíí½½ööÚÛ+ííÞf§ËÊÊ-Ú¾L:uª¹¹üÈOäG~ä'ò#¿é]UUU&ùdhooL[öv ùÈüÈOäG~Ó¯¶¶¶;vÄDüL§Ó¹|òÉ×^-&N8ñÈ#ùüÈüD~ä7íµººº´´´¦¦¦³³óßï[É¿Ý»ÞÞÞåË§R©æææ ùÈüÈOäG~÷iäG~"?ò#?ùùùüÈüD~äG~äG~"?ò#?ùùÈüÈOäG~äG~äG~äG~äG~äG~"?ò#?ùùÈüÈOäG~äG~ä'ò#?òùùùÈüÈOäw7ºzõê=^|ñÅûöõ÷÷ùùùù_qöî»ï>ðÀóæÍ[üÿ¡>üðCò#?ò#?ò#?ù[W¯^­ªªúïéÿ¾ñéÉ¿ßýÝßùùùüÈ¯¨Ú³gOsssñoÃ77<ðÀôùùùÈüª_~yñï-Î_ü«««ûÑ~D~äG~äG~ä'ò#¿¢êÀ?üp.ûþè©?1cÆÕ«WÉüÈüÈüD~äWT444|aÁ6|sCÂ¾úßªoooOùùùüÈ¯¨úøãçw~§ªªê·~ë·fÌñøãÇ/Rò#?ò#?ò#?ù³ÿÞï½d'o6ò#?ò#?ò#?ùÝ/ùùùüÈüÈüD~äG~"?ò#?ò#?ùÈüÈüÈOäG~äG~"?ò#?ò#?ùÈüÈüÈOäG~ä'ò#?ò#?òùùÈüÈüÈOäG~ä'ò#?ò#?òùùüÈüÈüD~äG~äG~äG~äG~ä'ò#?ò#?òùùüÈüÈüD~äG~"?ò#?ò#?ò#?ò#?òùùùüÈüD~äG~äG~"?ò#?ùùÈüÈüD~äG~äG~"?ò#?ùùÈüÈOäG~äG~ä'ò#?ò#?ò#?Ïò#?òùùùüÈüD~äG~äG~"?ò#?ùùùùùüÈüÈüD~äG~"?ò#?ò#?ùÈüÈüÈOäG~äG~"?ò#?ò#?ùÈüÈüÈOäG~ä'ò#?ò#?òùùùÈüÈOäG~äG~ä'ò#?òùùùüÈüD~äG~äG~äG~äG~ä'ò#?ò#?òùùüÈüÈüD~äG~"?ò#?ò#?ò#?ò#?òùùùüÈüD~äG~äG~"?ò#?ùùÈüÈüÈüD~äG~"?ò#?òDÝÝÝ555©Tª¡¡¡££cä<XRRB~ä'ò#?òùß´/NïÜ¹3&¶nÝÚÚÚ:léÀÀ@SSùÈüÈOäG~ÅPeeåàà`Ld2ÚÚÚaKî¹-[¶%¿¿üË¿üßSØ~üãÿo®#Chl¨Æ¡°ýíßþmww·q(`ñwxÚ8°cÇ½ùæÆ¡°<xðG?úÑT^ã!¿T*5êttþüùæææpáXòýõ×?Â¾÷½ï>úC®xFuuuöÿðñ«Ê8¶Cýô§?5,$ý÷ÿ÷Æ¡9s&þD1­££ã'?ùÉT^ã!¿ÒÒÒìtYYYî¢åË9räßîª½½ööÊÞ^íí½½ööAUUUL&ÙÛÓÿéþçÈüD~äG~"?òÞµµµíØ±#&âg:ý®zÏüD~äG~"?ò+a­®®.--­©©éììzäG~"?ò#?ùÝ×ùüÈüD~äG~äG~ä'ò#?òùùùüÈüD~äG~äG~"?ò#?ùùùùùùüÈüD~äG~äG~"?ò#?ùùÈüÈOäG~äG~äG~"?ò#?ùùÈüÈOäG~äG~ä'ò#?òùùùÈüÈOäG~äG~ä'ò#?òùùùüÈüD~äG~äG~äG~äG~äG~ä'ò#?òùùùüÈüD~äG~äG~"?ò#?ò#?ò#?ò#?òùùüÈüÈüD~äG~"?ò#?ò#?ùÈüÈüÈüD~äG~"?ò#?ò#?ùÈüÈüÈOäG~ä'ò#?ò#?ò#?ã@~äG~äG~"?ò#?ùùÈüÈOäG~äG~ä'ò#?ò#?ò#?ò#?ò#?ùÈüÈüÈOäG~ä'ò#?ò#?òùùüÈüÈüÈOäG~ä'ò#?ò#?òùùéþ_É­*--%?ò#?òùù_1È¯ôV¥R)ò#?ò#?ùùü#ò#?ùÈün·ÞÞÞÕ«WùùüÈüÈ¯ØäW[[J¥|ÎüÈüD~äG~*rù566<¼£²²2^<ÈüÈüD~äG~äWTò+++ê]¹r¥ºº:&â5ã;ßùNL¬ZµüÈüÈOäG~äG~E%¿äM¾êÅÄ¹sçcbÆäG~äG~"?ò#?ò+*ùÍ93w,^câgI&|«ùùüÈüÈ¯Øä·nÝºìñ¹õäGÈüÈüD~äG~äWTòöÙY³fÅDgggL§ÅãA~ä'ò#?òùßýùÈüÈOäG~äG~äG~"?ò#?ßhÕÕÕ%ßíâÉüÈOäG~ä§bßÜ¹ssµÍ±½äG~ä'ò#?ò#¿b_ /Ì×ÕÕ5888íò#?ùÈü&PeeeÈo:²üÈOäG~ä'ò#¿ÕÝÝò[³fÍ7ÈüÈüD~äG~*fùE³gÏ.#<ÈüÈOäG~äG~Å&¿9sæ8ÂüÈüD~äG~º/ä¯§§g:>äG~"?ò#?ùM ªª*GxùÈüÈO÷ü2!¿uëÖõ÷÷ùùüÈüTÌò+#GxùÈüÈüðGÍäG~ä'ò#?ò#¿"üVéùÈüÈOäG~¨¶¶vÎ9'O$?ò#?òùù©ÈåJ¥JJîòÝÝÝ555qK:::ruvv655Å¢ÆÆÆ8ùÈüÈOäG~/¤òÛ´iSooïÝúnt:½sçÎØºukkkkî¢¹sç¾óÎ;1±k×®ºº:ò#?ùÈünã"îc+++tf2ÚÚÚ±ÎV^^>R~þç¾Û·oß~´ïel¨Ôs_6ÔsH§xC½ãò»íÍ½®±®·««kÕªU#åÿ)ìûßÿþõë×ÿ×©S§âï~ãPÀ~þówttÂöÖ[oÅßýÆ¡>úý÷ß7ì¿øÅ¡CCaûí·¯^½:×xÇåw/ûþbYYÙÈ3Ü¸q#N÷õõÙÛko¯ìíµ·×Þ^ÙÛkoïô®ªª*É$czØÒ/¶··_ºtiääG~"?ò#?ùM¬ðÖ%KÊËËKJJfÌ±|ùò)>Ô£­­mÇ1?Óéô0iµ´´reÔÉüD~äG~"?òØ2êSùßøÆ°VWWÖÔÔtvvþûú®ÚÚÚÜ[E~ä'ò#?òùßä«¯¯Q-]ºôÆq²··wÙ²e1gÞ¼y÷þãA~ä'ò#?òùß*++çdçd23êäG~äG~ä'ò#?òÆò+--ç%X$õ÷÷Ç©üVò#?ã@~äG~"?òù%[ZZ½½ñ3¦cNSSùùÈüÈüJ~A½Qð¸víùùÈüÈüJ~É¶²|ùòÒÒÒøÙÒÒs¦ÅãA~ä'ò#?òùßýùÈüÈOäG~äG~äG~"?ò#?_vÍ[ûéùùùùß4_éØùùüÈüôÉý°·÷©§Jä·k×.ò#?ò#?ùù§üº»»gÌæ[¼xqî;ùùüÈüÈ¯¨ä·bÅä­¾ýû÷O£ÇüÈOäG~ä'ò#¿	ôÆo$æ[ºté´<ÈüD~äG~"?òï&2oÞ¼ä`éøxùüÈüD~äwë^zé¥ä­¾ÖÖÖéûxùüÈüD~ä75ùùüÈütÈ¯ôV¥R)ò#?ò#?ùùü#ò#?ùÈüÈüÈüD~äG~"?ò#?ò#?ùÈüÈüÈOäG~ä'ò#?ò#?ò#?ò#?ò#?ò#?ùÈüÈozÉ¯®®®¬¬Ì÷ùùÈüÈOE.¿¹sçæjÏ÷ùùÈüÈOE+¿@^¯««kpppÚ=äG~"?ò#?ùM ÊÊÊßtdùÈüÈOäG~«»»;ä·fÍ7nùùüÈüTÌòfÏ]2"GxùÈüÈüM~sæÌqùùüÈüt_È/1_OOÏt|<ÈüD~äG~"?ò@UUUð ?ò#?ùîùeB~ëÖ­ëïï'?ò#?òùù©åW2Fð ?ò#?ùùá79#<ÈüÈOäG~äG~Eø­.Ó7ò#?ùÈüÈüÈüD~äG~"¿ÑÊd2K,)///))1cÆòåË§Ë¡¾äG~"?ò#?ùMlCõiq¨/ùÈüÈOäG~¨¾¾>·téÒäÿíííí]¶lYÌ7oùùÈüÈüJ~eeeá¼ìL&sb>ùùÈüÈüJ~¥¥¥á¼Ð^vNÌñ­.äG~ä'ò#?ò#¿âÜÛÛÒÒìí1sÈüÈüD~äG~äWTòêzÇµk×ÈüÈüD~äG~äWTòK¶åËWTTÆÏ3-ò#?ùÈüîÈüD~äG~"?ò@µµµsæÌ9yò$ùùÈüÈOE.¿T*UR2]ß;$?òùùüÈouttü6mÚÔÛÛ;]þÓ6ò#?ùÈü&ucTZZJ~äG~ä'ò#?ò#¿¢_éù&gò#?òùù_ÈoÎ9ñ1­ò#?ùÈün]òßõ~2´·wZìØ%?òùùüÈo%ÿ]oò_ôùùüÈüTÌò«©©)É#<ÈüÈOäG~äG~E"¿®®®3g&ïü%ÎsùùüÈüTòË6]G~ä'ò#?òùßùÈüÈOäG~äG~äG~"?ò#?ß®»»»¦¦&J544tttä_4¹9¹mÜ¸ñÿð¿5õ«_ýÓ?ýÓo©p­Ê8°ØDcC5måÊò'bØ7¾ñ¯ýëÆ¡=óÌ3?þ¸q(líííO?ýôT^ã4_:Þ¹sgLlÝºµµµ5ÿ¢ÉÍÉí©§*$I*î¸üN:U@ùUVVÆD&©­­Í¿hrsÈO$ßdw¥KSSÓ3gnÿÒrvÈðÈEÛºuëª««Â|ðÁÙ³g?¤ÂõëChl¨Æ¡°Å¯ÏÏý¼Ï~ö³ûEðÜ¿ãòóe´Z¸páÙ³g'i¹ßÿÑäæ8ÂÃr#<á!Gx8Âãv÷ùÖ××g	ÆZ²dÉ$.§ªª*É$;gc:ÿ¢ÉÍ!?òùùüÈ¯îß¿¿¼¼¼d²ÿ[[[Û;b"~¦Óéü&7üÈOäG~ä'ò#¿Ûª§§'÷=¿U«VMnª««C555ÿ~+>®8rÑäæùüÈüD~ä7ÉrÁW^^ÞÚÚzþüùéòxùüÈüD~ä7:°ãÑG=wîÜ´<ÈüD~äG~"?òØsoú>äG~"?ò#?ùÝ/ùüÈüD~ä7±N8QWW7cÆäxÞªªªÝ»wùùüÈüÈ¯ØäwðàÁìüé­[·ùùüÈüÈ¯¨äW]]Î;qâDV~É»ùùüÈüÈ¯¨äýï³òLø%?ò#?òùù_QÉ¯ªª*¼ÏòËd2ë×¯éÚÚZò#?ò#?ùùü2%£õÎ;ïùùüÈüÈ¯¨ä]ºt©¹¹99¶·¼¼¼®®îìÙ³Óâñ ?òùùüÈï~üÈOäG~ä'ò#?ò#?ò#?ùÈï?wþüù¦¦¦Ò¡b¢¡¡aºìê%?òùùüÈo¼=ñÄ%c´zõjò#?ò#?ùùüvïÞ oÛ¶mñ:ÝhöîÝÌ?pàùùÈüÈüA~555Á»íÛ·ün¾ÏüÈüD~äG~äW$ò+++ÞÅV2rQ&Eqò#?ò#?ùùü²ÿiÛXKÿÉüÈüÈOäG~äG~Å ¿<¶#?ò#?òùùüÈüÈüÈOäG~ä7å?ò¨üâåvÏ=/¾øbü¼~ýºgùùüÈüîùÞªT*E~ãßÇüÐC=üðÃoqccã>xòäIOò#?òùùÝò+îùá_X¸páÆ§7&ÿþàþà×ý×³_(ò#?òùù_ÈïêÕ«UUU¾¹!+¿ø7wîÜwß×süÈüD~äG~äWTòëééyøásÙÿ>ÿÛýõ×=gÈüÈOäG~äG~E%¿ë×¯êSúÆodÙ·áª««ßï=Ïò#?òùù_QÉ/Z»víoüÆo¬þ«AÀÏîs_üâ<gÈüÈOäG~äG~Å&¿hÃª¨ªª9sæêÕ«ûûû=aÈüÈOäG~äG~Å)¿¤?þØ[äG~ä'ò#?ò#¿ûB~"?ò#?ùùÈüÈOäG~äG~äG~äG~äG~"?ò#?ò#?ùÈüÈüÈOäG~ä'ò#?ò#?ò#?ò#?ò#?ùùÈüÈOäG~äG~ä'ò#?òùùùüÈüÈüÈOäG~ä'ò#?ò#?òùùüÈüÈüD~äG~"?ò#?ò#?ò3äG~ä'ò#?ò#?òùùüÈüÈüD~äG~"?ò#?ò#?ò#?ò#?òùùùüÈüD~äG~äG~"?ò#?ùùÈüÈüÈüD~äG~"?ò#?ò#?ùÈüÈüÈOäG~ä'ò#?ò#?ò#?ùÈüÈüÈOäG~ä'ò#?ò#?òùùüÈüÈüÈüÈüÈOäG~äwËïÀO?ýôºuëöìÙC~"?ò#?ù_ÑÊoõêÕ¿ök¿ö_ý¯_üâ?ûÙÏ.]ºt``üD~äG~"?ò#¿bß¾û>ýéOÿÏµÿsãÓãßú?^ÿðÃ?ûì³ä'ò#?òùÝº»»kjjR©TCCCGGGî¢ÎÎÎ¦¦¦XÔØØg#?òDííí_Zú¥É¿¯>þÕßþíß&?ùÈï.N§wîÜ[·nmmmÍ]4wîÜwÞy'&víÚUWWG~ä7ì±/ùË¹òûÚÿ÷µßüÍß$?ùÈï.TYY988L¦¶¶v¬³ß_üÅ_tMao¾ùfH´KëûCÝÑ«hooÿ/Mÿ%W~/þýßÿýbÒØD÷ïßoÓòÜ¿Ç?¤;::C÷Ýw=÷Þ~øÃNå5ÞòK¥R£Nçc±jÕªòÛ»wïÇSØ¡C>øàU¸Þï½cÇÝÑ«8uêÔg>óEýÑS´þ×?úÿ>ú«¿ú«o¿ýv±é~ªM«°QþñÿÑ8°ãÇÿøÇ?6ìüùó4-þDùÙÏ~6×x_È¯´´4;]VV6ò7nÜH§ÓööÚÛ;¹>úè£/éK¿ò+¿2sæÌ¸Ò"Rííµ·×Þ^ÙÛÏUòÅtUUU&IööÆô°s^¼x±½½ýÒ¥K#/üÈOäG~ä'ò#¿iV[[Û;b"~¦ÓéaÒjii¹råÊ¨+ùüÈüD~ä7ýµººº´´´¦¦¦³³óßïÛÐÛµµµ%9ùüÈüD~äwF~ä'ò#?òùùùÈüÈOäG~äG~ä'ò#?òùùùüÈüD~äG~äG~äG~äG~äG~ä'ò#?òùùùüÈüD~äG~äG~"?ò#?ùùùüÈüD~äG~äG~"?ò#?ùù¦ü®^½J~"?ò#?ò#?ò#¿"ßÆ+**ªªªfÎùµ¯m``üD~äG~äG~ä§"ßæÍ?óÏ¬þ«7>½ñk¾Q__ÿå/üD~äG~äG~ä§b_ÜOêS_òëÁ¾äßú?^ÿÀÄN~"?ò#?ò#?òSQÉ¯§§§®®.Ë¾äßç?ÿù=öÈüÈüÈüTTòûè£>ýéOoøæÕ××ÇóüD~äG~äG~ä§¢_ô¹Ï®¥¥%Ë¾ÿ¶ü¿UVVNÇã|ÉüÈüÈüD~äw>úè£zèá^´hQccã>øÞïMÇQ%?ò#?ò#?òùß¸nÏ=^|ñÅ×_ýúõëÓtTÉüÈüÈüD~äw¿D~äG~äG~ä'ò#?òùùùüÈüD~äG~äG~"?ò#?ùùùüÈüD~äG~äG~"?ò#?ùùÈüÈOäG~äG~äG~"?ò#?ùùÈüÈOäG~äG~ä'ò#?òùùùùùùÈüÈOäG~äG~ä'ò#?òùùùüÈüD~äG~äG~ä'ò#?òùùùüÈüD~äG~äG~"?ò#?ùùùüÈüD~äG~äG~"?ò#?ùùÈüÈOäG~äG~äG~äG~äG~äG~"?ò#?ùùÈüÈOäG~äG~ä'ò#?ò#?ò#?ò#?ò#?ùÈüÈüÈOäG~ä'ò#?ò#?òùùüÈüÈüÈOäG~ä'ò#?ò#?òùùüÈüÈüD~äG~"?ò#?ò#?ò3äG~äG~ä'ò#?òùùùüÈüD~äG~äG~"?ò#?ò#?ò#?ò#?òùùüÈüÈüD~äG~"?ò#?ò#?ùÈüÈüÈüD~äG~"?ò#?ò#?ùÈüÈüÈOäG~ä'ò#?ò#?ò#?ùùÝvÝÝÝ555©Tª¡¡¡££cä<XRRB~ä'ò#?òùß´/NïÜ¹3&¶nÝÚÚÚ:léÀÀ@SSÓXòýrÔÞÞÞ_ªp:u*~ûvýúõøÊ8¶·ÞzëÚµkÆ¡Å|ï¿ÿ¾q(`7nÜ8tèq(lo¿ýö?ÿó?Oå5Þò«¬¬L&S[[;lésÏ=·eË±ä÷Â/ÂöíÛ÷æoPáúîPÆ¡Å&ªqðÜ¿ÇÛ¿¿ç¾ç¾çþÈîù¥R©Q§£óçÏ777ííµ·WööÚÛko¯ìíµ··*--ÍNå.Z¾|ù#Gþí®ùüÈüD~ä7M+ùbºªª*É|2´·7¦G=[öÌäG~"?ò#?ùMãÚÚÚvìØñ3N%Å3ÉüD~äG~"?ò~ÃZ]]]ZZZSSÓÙÙ9*õÈüD~äG~"?ò»¯#?òùùüÈüÈüÈOäG~ä'ò#?ò#?òùùüÈüÈüD~äG~"?ò#?ò#?ò#?ò#?ò#?òùùüÈüÈüD~äG~"?ò#?ò#?ùÈüÈüÈüD~äG~"?ò#?ò#?ùÈüÈüÈOäG~ä'ò#?ò#?ò#?ùÈ¯ú³?û³×_ýÃ)ìµ×^_Uªp½õÖ[áiãPÀN:õ×ý×Æ¡°ýÍßüÍûï¿oØ#G:::C¿¢ÿê¯þÊ8¶Ý»w÷ôôLå5^¿~üÆìÄ7nü$IRQgf·a%IîÈO$ü$ID~$I"?I$$IÈO$Iä7»råJmmíÿåJKKsÏyéÒ¥Ü¥nü£zøðáúúúT*ÕÐÐpôèÑÜsvww×ÔÔ$:::Ýí©urCzêÔ©æææ²²²+VôööÚJïô¨ÚPÇSgggSSSl±æßm¨Ò»¾zb¾xÉÏQÎ;wnÝº5wÎîÝ»ÛÛÛÚ$Fµ²²òüùó1?s_¢t:C1Ú­­­FïöÔ:¹!7o^¼$ÄÄÉ'|òI[éUêx;wî;ï¼»víª««Ë¿5ÚP>¤w+%¿Â·dÉÓ§Oß¹sç-Z4lf<ü÷î5hÕ9sæx1&âgLç9388Lf`4¹!µ¡NnHã/þìôÌ3m¥wzTm¨­¼¼<ÿÖhC-øÞõ­üîØÈßòåË»ººFþ¡ÐÒÒ¿ÈO:eÜÆ?ªÝÝÝñ9ñsØÀæ¾0äNkÒCjCÜ6668q"&¾ýíoÛm¥wbTm¨*æ«V­Ê¿5ÚP>¤w+%¿)_üÍ:þü<ç¿xñbü:3nãÕyóæ<y2ñÊ°±Íý0eYYq»ý!µ¡NnH?^WWÞ²eKòN­ôªuüÝ¸q#N÷õõåßm¨Ò»¾ßÉoóæÍ/½ôRþU<©&4ªyþ¢ªªªÊd2½ÍÓÆíöÔ:éç~Ò3gêëëm¥wzTm¨ã,ÌÑÞÞ~éÒ¥[n6Ôé]ßJÉo~O-X°àØ±c#Ï6wîÜ³gÏ&MKKqÿ¨Î??ÙãÓÓÓ3oÞ¼Ü³µµµíØ±#&âgüfÜnHm¨Ò·îîîÁÁÁ-[¶<ûì³¶Ò;=ª6Ôñtøðá+W®gk´¡|HïúVJ~S$¿pòaÏagèêêJ¾GcáÂÉë5ÎQ?÷C'1tñ3¦sÏpôèÑêêêÒÒÒä@ÝæÚP'7¤ñz0öìxú¯Zµj``ÀVz§GÕ:jkk«È¨[£õé]ßJÉO$é¾ù;ÊH$$IÈO$Iä'I$ò$IùI$ü$ID~$I"?I$$IùI$ü$ID~$I"?I$$IÈO$Iä'I$ò$IùIºúö·¿½`Áò¡-ZôÆoü§_pCM_Ç£ÝÚÚÚÚ¸kÃæÇT*USS3888ÑËD~4ýÚ¸qcÉöÙbß¦Mbæ+¯¼2lþË/¿óyæI$ò¤iVwww&JmÛ¶m`¨íÛ·ÇÉyìØ±¢ß¹sçbfccã°ùõõõ1ÿìÙ³ä'ü$_ùÊWÂ4Ï?ÿ|îÌ^x!f>þøã¹îéèè9b:æk×®­Zµª¢¢"UVV®]»6w§ê¡CBW±(Ö=pàÀ0KÅ3g.X°`ÿþýq²­­mØÛ·o_þËIÅMJ<xp,¥µ´´ÄüÎÎÎìwÞy'æ,^¼8;góæÍÕÕÕqQååå+V¬¸xñâHù¼üasòÜTIä'Iw³Ù³gZÎ?;óÂ1³¶¶6W6ÃêêêJ.[¶lØ¢5kÖ$zzzJKKG]+9,]¾|ùàà`Ø±¬¬l`` ÆÏÓ3ßå¹õæ¼§»víÊål;wîÌ²oØ,Z´h¢òËsS%$Ýå»£üR+)	åÊ¦µµõÆP1'.],M Ø1yë.Ä,J§ÓÉ1ôèÑäBr/sÃa»dgëO<söîÝÓñ3¦W®ËË	5ÆÉ8Cîmõ&gÍ÷÷Ê+q2~Æ-nf2ä555±bÜOþcïpyyùDåç¦J"?IºûòF_v~"LÑÅãd)9ÙÔÔ'çÌ³zõêÛÍ7³çö.Zv­ää²g-ewøÆÏ>tèÐ-/''/_¾ÛÆúLÞSO=¶lÙòÉìÑ^»vmîB!¿ðhsssòäDåç¦J"?IºË%otÝ¸q#wf___ÌEy¬uáéÓ§üe¡ýpÞÈ°Ã,û]*1]YYYVVÖßß>sæÌìÒ[^ÎXÖ'¤ÆtüéìÒ£GÆu¯ñøåç¦J"?IºË%ué¥rg&ßu2ììg¯2ò­¬cÇmÚ´)Ù÷%còê-¶fÍd'oüjUv~ËIÞc»téRròòåËùÃ?~,M¾È&À»(9Î7<x°··7¿ü²*MF#»4ÏMD~tKH¥R[·nM¾ÕeÛ¶meee#ÆXºti(§¿¿?Ù=7ù_òñ¸Ó§Oç~@0àÚµkÃIÉQ·ÙïUÕgÉÁ¶Éµ>|8;?Ïå$ìK>ç×××3ü¶oß7.îrî¢ä#===q-1cÉ/±fX9ÎâbEîÒ<7UùIÒÝ/ùãamÞ¼ùÿþ*Qv:»4PE¶'x"¹íÞ½;ü>úÏ6b~eeeîà<7#÷e«ëÎÞ¼y³¼¼<¹Ãöq/Y²$÷*fÍ?/vÉ½Ìä-ÉlS³KóÜTIä'I÷D¡Eµ`ÁäÛaò;xð`òµyGÉ.íëë[¿~uuu¢¥§zª¿¿?»ôÀÍÍÍ²W_uØe¼%qQ1ÿÉ'6¬ËâÆÄMJ¾h0Ï÷ùe[µjÕ°¯wIºråJ:¨¨¨qöìÙì7Ôä^foooÐ6«Åwvv»Æ<7UùI$ü$ID~$I"?I$$IÈO$Iä'I$ò$I"?I$$IÈO$Iä'I$ò$IùI$ü$ID~$I"?I$èÿ°èU%ÃIEND®B`


ÊOù4ÅÎ¦¥%loÝÐå·Ò[÷Qutt|ÿûßW~ÊOùA_õ_ïÿt[i<66¦üû¡üîÜ¹ÓÚÚ:88xóæÍp6>õÔS===aüÎ;ïg»òP~îhòëêêOéB¡P¹uëVéïïuþ|òå÷ùÏÁ;wJ3¥àÿòP~Ê¤üÒétü½ÍËËËwïýÑÞ0òÎÎÎ:ÚÕÞ#Î=ë>T~@½ßÔÔÔ7âÓÓÓñ`ttTùÁÃàÉUÅUw*? ÞË/¸víZWWW*J$á4ÃLüT¯ó?æ¡üàAîû=ÝÐå×¸(?(?å(?(¿²§q"¸»ògºãK üP~ÐðåÂ.þºÄêùË(?(¿òåG-Þß°öî¹ÜÂÇl-òS~ üx@«oÛßýûuÿ|¥³'ý¯v­ûêÞ2Í/¿Fù$òåÇ²úÿáúfý|kàòú*¿AåÊåÊOùÊåÊOùÊOù)?P~ÊP~ÊOùòS~òS~Ê¼üVçoxÊOù)?hòK¬ÅßðòS~Ð$å×(?(?å(?(?å(?å§ü@ù)?@ù)?åÊOùÊOù)?P~ÊÊOùòS~ üP~Ê¿ü:;;Kgoß¾ðàÁd2¹mÛ¶ò%çææ2LE===ÓÓÓ5Î(?P~(?P~u!ËuwwMLiæøñã?üáÅbÈ¾íÛ·/Íf'&&Â`|||xx¸ÆåÊåÊ¯.ìÛ·oaa¡¼üzzz._¾|ßÛÛÛCA¡Pß&¬e¦¢ü~þóÿOàË×n½ú¾öÍ=õÓúÈcG± V~¥¥qE?ùÉOR©ÔöíÛÿð?/Vþââq-3å711qXËûï¿þú£;ûv¯û'úë-ÿ²ëïÖõ¿ýú£'O´"6KËf;|ø°µµhøòK$§N+W®ôõõ/.*Éd3öÂ:|üñÇáùäÑÜ¬­o¾ù¦±åÍåh/4çÑÞêòëèèXéM»pQ¡PäÆÕ2£ü`å÷HúñMü¤×¿þ#Êos7Ë>çÊïAßáÃß~ûí0¸téÒO<Q¾ØÈÈÈéÓ§Ã f³Ùg(?(¿:-¿7nEQÔ××·°°P¾@.K§ÓD"Éäóùg(?(¿òåòå§üXÿîÁ¸òS~ÊòÃwù*?å§ü(?å§üP~ÊOù)?P~ÊOù¡üòS~ üòCù¡üå§üÊå(?å§üP~(?P~ÊåòCùòS~(?ÊòCù)?å§ü(?åòS~ÊOù)?P~ÊÏNù)?å§ü(?å§üP~ÊOù)?P~ÊOù¡üòS~ üòCù¡üå§üÊå(?å§üP~(?P~ÊåòCùòS~(?ÊòCù)?å§ü(?åòS~Ê¯é¼OýÇÍúùÚ7÷(?P~ÊOù¡üx@|Û·ÿÝÞuÿD½å±ñÍu_ýÑÇ¾aíòS~ÊåGÃ<÷½iÊOù)?©ü¾ÒþõM<Þ÷ûòòS~< òkÙlÊOùÊOù)?ÊP~ÊOùñÏù¡ü@ù)?òS~(?P~Êå§üÊòS~(?å§üå§üÊOù)?@ù)?åòS~ÊP~ÊOù¡üP~òS~ÊåòòS~(?(?åÇ*îÜ¹óñÆ­ÿ/ùËÜÂòò²¡üP~òS~bß6ßa÷¿îäß|õ+_tÝW÷÷»¡üå§üìû±öQ~òS~öýXû(?@ù)?û~¬(?å?Ö>Êò³ï·ï·ö­(?ågßoßoí[û(?P~ÊÏ¾ß¾ßÚ·ö###O®¬åU8þ¼ûò³ï·ï·ö­ýÙlÌüc÷!(?ågßoßoí[ûÊOùÙ÷cí ü?_ÒÚoiiùÖÀ±Íúñ·ûòS~< öÙËÒí¹çÛÈ-ÌÏÏ[ÊOù)?asÓÒâé üòS~Êå§üòS~(?å§üP~(?å§üP~(?å§üP~ÊOùy(?òS~(?òS~ÊOù¡üòS~Êå üòS~ÊÏÓ@ù)?å§üÊOù)?å§üP~ÊOù¡üP~ÊOù¡üP~ÊOù¡üòCù¡üòCù¡üòS~Êê¶ÛOF±ü:;;+&§¦¦Â¡brnn.ÉDQÔÓÓ3==]ãòS~(?åWr¹äÝ¹s§···ºü²ÙìÄÄD×8£ü(¿º°oß¾ÈûÁ~ðê«¯V_±XB¡¿MXËLEù½öÚkÓÔfrrrË×ÝÄòÛ±ç¿;vÌ4XùÆW¯^íëëõV]~QUk©(¿óçÏÿ_jsåÊ¯þícúßýÙÏ~fEÀJ¾ü.P1K$¥q2¬qÆÑ^GÀÑÞ:-¿Ï/ÖÑÑQ(â#¹aòS~ üê´üV9útÓl6[ãòS~ ü©üâq.K§ÓD"Éäóùgòå÷0R~ÊòCùòS~ÊOùòS~ÊOùòS~ÊOùòS~V~-MùòS~ÊP~Êï-?G@ù)?å§ü@ù)?å§ü@ù)?å§ü@ù)?å§ü@ù)?å§ü@ù)?å(?å§üå§ü(?åòå§üòå§üòå§üòå§üòå§üòå§üÊOù)?@ù)?å(?å§ü@ù)?(?å§ü(?å§ü(?å§ü(?å×håLµþ§ÿþ?6ë§³g¯òå§ü?þñ---éG¿±î¿J¶>ÒY÷ÕÃ¿þë_ÿÚå§üá×Òâå§ü üòòS~òS~ÊP~ÊOùòS~(?P~ÊåÊOù¡ü@ù)?(?å§üÜá üòòS~òS~ÊP~ÊoóÚn#¬P~ÊOùÊOù(?å ü üòS~ÊOù(?å üòS~ÊOù(?å üòS~òS~ÊOù(?å üòS~ÊOù(?å ü@cßÒÒRgggél>ïíí¢hçÎsssåK³L&ÓÓ3==]ãò_]ÈårÝÝÝ--þÛ±cÇÅÃ`rr²«««|ál6;11ãããÃÃÃ5ÎTßÙ³gÿ7@Sh°òÛ·oßÂÂByùK¥RågÛÛÛÅbømÂZf*Êïõ×_Ï4Æ;ÚûOÿY÷+¿ÙÙÙÑÑÑò(*ÆµÌ8Ú8Ú[×åwóæÍl6»¼¼H$Jãd2Yãò_ýßâââÁ¯_¿^±XGGG¡P¸ïHn×8£üåW§å733Óßß¿´´T½ØÈÈÈéÓ§Ã f³Ùg üê´ü:;;[Ê/ËåÒét"Èd2ñGkQ~ò)?@ù)?å§üòP~Ê@ù)?å§üòP~Ê@ù)?å§üå§üòP~Ê@ù)?å·¹Þ÷Ý'xâI¦ðæo*?ò@ù üP~(?ÊåÇîòåËÏ>ûl[[[EþäO6r¯½öÚz¸-º·ÕXYióçÏÆp:44fªoê½÷Þ×§îÕf<M$©Tª··÷õ×_w¿5Çª¯åQ±Òz¯q+òc3½ýöÛÛ¶m,ál8ãÇüÚµk_Æ¶Cù5Üæ>öÖ[oíÝ»7ÏÅøq6èÝÝÝ'O¬XòÀû÷ï¯%Ü«ÍúØ¨¹råÊO<qâÄ	wWsßêë½ö­ÊM³°°"¯ú¾W_u``@ùÙÜÇÉÍ7+&?úè£t:]>¶ø[·n½uëÖ-[â­?CùÅ^Cº»ªò«Xï5n%P~l¦CWÏ=÷ÄÄDéì¹sçzzz¢(§a!8öìöíÛD¸4~W¿úÀÐÚÛÛûúúÖ¼5k¤nË/L^¾|yÍÛ	+thh(ÂiùÊ¦¦¦ÂJÿP*êïï¯åÖh ò$átdd¤üXÿ»ï¾ëà&.¿Òz¯+òc3háÙêËËd2sssaN;::fggK½÷ÆÇÃba¿^½ãç¾X,Æ[ÕoÍ©ÛòûÅ/~^¸Ð_±NNNÞ½wÜ'Ë/*½6·ß~»»»ÛÝÞLåwåÊ;wÁ'|^Ô-//ñ§~^òU¿DÓ_i½×¾@ù±ÄË¶ûéò(ð´¯Þ.TßÂÂB·flâæ~õ_ïÎ9³eË°e?pà@È»qqÁÄzoÜ¸Æag_qÀ7ÜZ>wW7_ùµ|áÂ®®®Ò.ÿÔ©Sááápû°)Ë¯z½×²@ù±ÉJïÒ­¾LüË±0nkk»ï`¥ò[ß­Q'ûmýììlØ¸¿øâL&~/6==Ýßß_:ûÔSOÿZßØØØÐÐÐÄÄÄÅ°ÑË¯Vüç=;väÈw`3ßëÍ­ÊMÖÝÝõêÕÏ»!¸ïQÝË¯ö[£Ë¯Ü;wN8^ñfþùÃèèhéÒÛ·oïß¿?ÞlÙ²å>p·7nù­y­Ð÷á©¿LÓßÆ·(?6ÙØØØJßÂUz^ØïùÕ~kÔyùÅõR¸ÇzËO|ii©½½½úí½ÅÅÅ·ÞzkÇîö&.¿l6àÀ^xÁø0_ÅVåÇæ[XXØ¶m[ØCWÌ=öøñãñxpp°üya¼oß¾u_í·F]_:®~.T]ooo<+·b0333óîEhòçwÂ«Ê0èïï_é@óß[	uáÄ;vì^³×®]åW¾óïÜºu+^ Ïçsü)ÝÙÙÙ0Îår«_kkëG¿ýS±Lí·F]ßÉ'Ã·ÞzëÓO?·æóóó###¥ýú¡CªÿnGx	æãqØúONNÆoÿ&8MV~ñïóÆ¿Ûÿ¯c¾Iù­¹@ùQ/Î;^§R©D"*ðøñã¥ì+í¿ãoàÛ¹sgùÞ¥òã7÷ÜwcQã­ñ7÷kþzoØ|?óÌ3mmmñn(#OØÄuZ`7Ì¾½%¼³ð¨·ÖþÔÔ»½)Ë/ìéË¿ÊÑ÷ù5Á¦ öMô*[	ÊåòP~(?Êåò@ù üP~(?åò@ù üP~(?À¡³³3J-//WÌ(2L±Xü|[Õ6¸X7 ü>^z)4Öë¯¿^1âÄ0ÿ½ïïsoU üêÓGkçÎóÝÝÝaþÃ?ü²6¾ÊP~^È¬|>_¹xñbyòÉ'K3ÇO§ÓQ¥R©gyfqq±<ÑÎ;·eË=öTGÛêWÑ.íííãÊïüùó¡DÃbaáðoæ?ýôÓÑÑÑ¶¶¶pQû#Gª[(??õì³Ïföïßf&&&JõÖòöîÝ[hD"UDÛW¬0;;[]~óóóñíW/688Xq/¼ð(?Ý¹sgëÖ­Q---³á4V[[[¡PÈd2!ªÞï½»ÿ|t8J'Ú/¾X,ãCÃåÑ¶æoÞáìÀÀ@uùe³ÙøÝÁ0Îårñµââ"¼zõj%[[[­P@ù¬fll,dÓ«¯¾Æ¯¼òJ9r¤|P!­BáõõõÅoò'Úµk×þ¼UýËµ«_1nÍ`qq1¹Y#a²â½Òb½½½áìöíÛ:ôî»ïÞºuËªÀ.]º'TÓ0/]ËåÚÛÛ+ò«<ÑÊ¿ù¥üÒ5¯øã(ª/­>(Ç8þJExöìYkP~kØ½w§ï~÷»á4äTùEñïù¦¦¦nÜ¸±fÀfÖ¼b| öî½CÌ+½ç×ÚÚÆ¥CÏÕ~ÿûß¿ôÒKCCCa±L&cUÊ`§N*½y6>>^~QEñ»Åbñ7Þ¨½üÖ¼âÀÀ@h¾Û·o³á´úFâ¤;räH¸PåßAÎ/þáÂÂB'I«P~k¸uëV*¹Þ¼y³ü¢ûölÝºuk8¿eõò[óqÆ¥CÌå72;;[q´÷Ì3ñEñïþùç­J@ù¬mtt´âë]bKKKÙl6L¶µµøá¥ïOY½üÖ¼âÔÔToooh¾.Ü÷FsçÎõõõ%L&sòäÉÒüòòò±cÇÒétá¸û¶õ(?ò@ù üP~(?ÊåòP~îåò@ù üP~<ÿLrùÞoòîvIEND®B`


ONEWAY Figures BY Variables
  /POLYNOMIAL=1
  /STATISTICS DESCRIPTIVES HOMOGENEITY
  /MISSING ANALYSIS
  /POSTHOC=LSD ALPHA(0.05).


Oneway


Notes	
Output Created	12-SEP-2022 22:48:15	
Comments		
Input	Data	E:\桌面\Raw Data\4. C. Cellulosae ESAs and TPx Induced Th Subpopulation Differentiation\3. SPSS statistical analysis\2. IL-4\1.  IL4--24h\1.1 SPSS statistical analysis--IL4--24h.sav	
	Active Dataset	DataSet1	
	Filter	<none>	
	Weight	<none>	
	Split File	<none>	
	N of Rows in Working Data File	20	
Missing Value Handling	Definition of Missing	User-defined missing values are treated as missing.	
	Cases Used	Statistics for each analysis are based on cases with no missing data for any variable in the analysis.	
Syntax	ONEWAY Figures BY Variables
  /POLYNOMIAL=1
  /STATISTICS DESCRIPTIVES HOMOGENEITY
  /MISSING ANALYSIS
  /POSTHOC=LSD ALPHA(0.05).	
Resources	Processor Time	00:00:00.02	
	Elapsed Time	00:00:00.02	


Descriptives	
Figures  	
	N	Mean	Std. Deviation	Std. Error	95% Confidence Interval for Mean			
					Lower Bound	Upper Bound			
Control	4	13.96800	1.355854	.677927	11.81053	16.12547			
ESAs	4	15.28575	1.075266	.537633	13.57476	16.99674			
TPx	4	15.89050	.911286	.455643	14.44044	17.34056			
LPS	4	18.72400	1.259332	.629666	16.72012	20.72788			
Total	16	15.96706	2.073655	.518414	14.86209	17.07204			


Test of Homogeneity of Variances	
	Levene Statistic	df1	df2	Sig.	
Figures	Based on Mean	.178	3	12	.910	
	Based on Median	.155	3	12	.925	
	Based on Median and with adjusted df	.155	3	11.148	.925	
	Based on trimmed mean	.171	3	12	.914	


ANOVA	
Figures  	
	Sum of Squares	df	Mean Square	F		
Between Groups	(Combined)	48.268	3	16.089	11.894		
	Linear Term	Contrast	44.240	1	44.240	32.704		
		Deviation	4.028	2	2.014	1.489		
Within Groups	16.233	12	1.353			
Total	64.501	15				


Post Hoc Tests


Multiple Comparisons	
Dependent Variable:   Figures  	
LSD  	
(I) Variables	(J) Variables	Mean Difference (I-J)	Std. Error	Sig.	95% Confidence Interval	
					Lower Bound	Upper Bound	
Control	ESAs	-1.317750	.822412	.135	-3.10963	.47413	
	TPx	-1.922500*	.822412	.038	-3.71438	-.13062	
	LPS	-4.756000*	.822412	.000	-6.54788	-2.96412	
ESAs	Control	1.317750	.822412	.135	-.47413	3.10963	
	TPx	-.604750	.822412	.476	-2.39663	1.18713	
	LPS	-3.438250*	.822412	.001	-5.23013	-1.64637	
TPx	Control	1.922500*	.822412	.038	.13062	3.71438	
	ESAs	.604750	.822412	.476	-1.18713	2.39663	
	LPS	-2.833500*	.822412	.005	-4.62538	-1.04162	
LPS	Control	4.756000*	.822412	.000	2.96412	6.54788	
	ESAs	3.438250*	.822412	.001	1.64637	5.23013	
	TPx	2.833500*	.822412	.005	1.04162	4.62538	

*. The mean difference is significant at the 0.05 level.	
