# Supplementary material for: Regulation of piglet T-cell immune responses by thioredoxin peroxidase from Cysticercus cellulosae excretory-secretory antigens
Source: Front Microbiol. 2022 Nov 18;13:1019810. doi: 10.3389/fmicb.2022.1019810 (PMC9718028; doi:10.3389/fmicb.2022.1019810)
Supplement: Supplementary file 3 [file Data_Sheet_3.ZIP › 4. C. Cellulosae ESAs and TPx Induced Th Subpopulation Differentiation/3. SPSS statistical analysis/2. IL-4/2. IL4--48h/2.3 (SPSS data export) SPSS statistical analysis--IL4--48h.doc]

EXAMINE VARIABLES=Figures BY Variables
  /PLOT BOXPLOT NPPLOT
  /COMPARE GROUPS
  /STATISTICS DESCRIPTIVES
  /CINTERVAL 95
  /MISSING LISTWISE
  /NOTOTAL.


Explore


Notes	
Output Created	12-SEP-2022 22:52:14	
Comments		
Input	Data	E:\桌面\Raw Data\4. C. Cellulosae ESAs and TPx Induced Th Subpopulation Differentiation\3. SPSS statistical analysis\2. IL-4\2.  IL4--48h\2.1 SPSS statistical analysis--IL4--48h..sav	
	Active Dataset	DataSet1	
	Filter	<none>	
	Weight	<none>	
	Split File	<none>	
	N of Rows in Working Data File	20	
Missing Value Handling	Definition of Missing	User-defined missing values for dependent variables are treated as missing.	
	Cases Used	Statistics are based on cases with no missing values for any dependent variable or factor used.	
Syntax	EXAMINE VARIABLES=Figures BY Variables
  /PLOT BOXPLOT NPPLOT
  /COMPARE GROUPS
  /STATISTICS DESCRIPTIVES
  /CINTERVAL 95
  /MISSING LISTWISE
  /NOTOTAL.	
Resources	Processor Time	00:00:00.94	
	Elapsed Time	00:00:00.85	


[DataSet1] E:\桌面\Raw Data\4. C. Cellulosae ESAs and TPx Induced Th Subpopulation Differentiation\3. SPSS statistical analysis\2. IL-4\2.  IL4--48h\2.1 SPSS statistical analysis--IL4--48h..sav


Variables


Case Processing Summary	
	Variables	Cases	
		Valid	Missing	Total	
		N	Percent	N	Percent	N	Percent	
Figures	Control	4	100.0%	0	0.0%	4	100.0%	
	ESAs	4	100.0%	0	0.0%	4	100.0%	
	TPx	4	100.0%	0	0.0%	4	100.0%	
	LPS	4	100.0%	0	0.0%	4	100.0%	


Descriptives	
	Variables	Statistic	Std. Error	
Figures	Control	Mean	15.38550	.587807	
		95% Confidence Interval for Mean	Lower Bound	13.51483		
			Upper Bound	17.25617		
		5% Trimmed Mean	15.40978		
		Median	15.60400		
		Variance	1.382		
		Std. Deviation	1.175615		
		Minimum	13.779		
		Maximum	16.555		
		Range	2.776		
		Interquartile Range	2.195		
		Skewness	-1.009	1.014	
		Kurtosis	1.456	2.619	
	ESAs	Mean	17.15925	.249889	
		95% Confidence Interval for Mean	Lower Bound	16.36399		
			Upper Bound	17.95451		
		5% Trimmed Mean	17.17117		
		Median	17.26650		
		Variance	.250		
		Std. Deviation	.499778		
		Minimum	16.469		
		Maximum	17.635		
		Range	1.166		
		Interquartile Range	.929		
		Skewness	-1.128	1.014	
		Kurtosis	1.481	2.619	
	TPx	Mean	18.64850	.537460	
		95% Confidence Interval for Mean	Lower Bound	16.93806		
			Upper Bound	20.35894		
		5% Trimmed Mean	18.61617		
		Median	18.35750		
		Variance	1.155		
		Std. Deviation	1.074920		
		Minimum	17.722		
		Maximum	20.157		
		Range	2.435		
		Interquartile Range	1.969		
		Skewness	1.313	1.014	
		Kurtosis	1.544	2.619	
	LPS	Mean	20.19100	.459280	
		95% Confidence Interval for Mean	Lower Bound	18.72937		
			Upper Bound	21.65263		
		5% Trimmed Mean	20.19389		
		Median	20.21700		
		Variance	.844		
		Std. Deviation	.918560		
		Minimum	19.041		
		Maximum	21.289		
		Range	2.248		
		Interquartile Range	1.701		
		Skewness	-.169	1.014	
		Kurtosis	1.495	2.619	


Tests of Normality	
	Variables	Kolmogorov-Smirnova	Shapiro-Wilk	
		Statistic	df	Sig.	Statistic	df	Sig.	
Figures	Control	.247	4	.	.948	4	.706	
	ESAs	.249	4	.	.936	4	.630	
	TPx	.252	4	.	.902	4	.442	
	LPS	.248	4	.	.955	4	.749	

a. Lilliefors Significance Correction	


Figures


Normal Q-Q Plots


ZûúúÔ^ÝÝÝê·nÝê÷÷íÛÇ üiùÉÄµk×|÷ùFÀ=dº¢¢âé4/e¦ïw9rdjjJíµRsöîÝëñx:::ÔrËòå7dZ*P]Wíúõë2ÿþßõÁ`ÐïÝ¼J Òy÷Îuáè>ªy_1Èüü®(Å&ÓÕÕÕÒôÞ>ùù>­r;2çæÍ2­Ö;wªoVEøàÁgS¦xqð-?!oä2-oê~ó322dúáÃêKy/W¬XáÞK½s¤6|¿þÛÃ¦¾Á199)­ QRR¢v2ÍX~ñññº?îüy·¨äº1&Én·ë.´Ù,ÝG5ï+Î¾üÔg=zäs*?ß§UÝ/£.*../srr¤WgÏñÊ(?á^~géééòþív»çk÷i?ô&I§Á¿Êô;â9ã9ªêð´6ï¼!âwk³¹J e2ãBÍÂ	Ô£ó»âìöÎ¦í_ªZý¼Qªøóakk+/.òÖå'ÞÿùÒ;©âÌoïÌM@ÿ²  @®ÒÖÖ&e6òS;&Õ+ÕÕÕxúôédæöíÛuËo6WwùÍuáxÍû³/¿Ùìóó¶tðå$süN4öÕÛÛÛÐÐ 1Ï@ùßò>ýiïùÕuæoEEÅØ4õÖ¾wïÞ/?µß«¯¯Oúãüùó³)¿öövµWIb±££CíÏS®]»¦û3Îõ*s*¿àG-I×4¿ëRGù©OæIø>öLÂnË-Ú.<sæ,|©CààË_=Béfù~Y2½zõjßS^spp0Èwð*¿ááaï'ºÔÃá÷¯ï©²/R~ÞQV¬X!ÿûßªûwïÞ­=ò¸víÚ ?æ®2§ò¾p¼2ûÆ<¥:òëííõ½ý6ø^ê·L¤ÕQ÷µWÕ×o¼Á üD@ù	u$Ôwþ½÷¶oß?­ººZ>ÇüÊottTnMnSèÍ7ßò¼oÎ=[XX·råÊwÞyçÕW_õîszñ«Ì©ü/k×®Iüïx¾æ´TçQ~¢µµ5''G~j¹u<Ý:³|)¦îÝd2uuuÍ¸ÏõÆ%%%rkòCY­Vï|ËuèÐ!µ3Uò]J·ÛÍ ü`QLNNîÞ½NcÈÍã*GU'k<ô¬0(?6ês~~N:Å@ù@´q¹r¥:¤+Çc± ü@ùP~ ü@ùòåÊ(?P~ ü(?P~ üÄk×®FÁ°qãÆ¹^·°°pÙ²eÞ92-sV¯^=Ö´ùÏl®;o£££÷îMMM³råÊC¹mÄóç@ù]R9g®×=~ü¸ôéÓÞ9o¿ý¶ÌihhôòÈ[5íþýûSSS.];Ú²eË<nª»»Ûd2Q~(?a°½xÈèííëyç¬_¿^æôôôæA.^!©¨miiY%Lù ü,VöùvÆ¡CRRR=ê÷m¯¾újIIß-xwÊôãÇeZæ¨<x`2CqqñÍ7uoÍ÷Þ_åwÞ1¥¥¥rGÚB0Ír]yðT»0¥M7lØ ·&óå>|8Ë%³zõj¹e·Û­i¥ÔÞÞ.RÂµk×´KX»$Üë'ÊÀ¢Ä>qâL[­Vi,8uêï÷Ü¸qC¯=öÈE.iù_¦kjjÔEk×®½~ýºLÜ¿_ægeeéÞï~é¹÷ß_&,öÁïÛ·O¦å>üðC8~ü¸ÌÌÎÎé±±±¾¾>P»$µ´Eb1P_JgÎQ÷%ý§~?ðbå@ùXÄòXéÉiÚðÒý `»!Ó[·nUYã½´··÷È#%%%2?..N÷Öü*'øUÔKIIÑ^7##C=ø©©)æò2]\,ih® å|)=öLMË-*?ïÏü¦X9P~±ü|s'P»øÌJJJït¹ ªÌQ?^®%9x÷î]mýè>ÙÐLôÝ§ª±¥¥%55UÍIOOW»g³Ï///OæOLLÂà|z~(?S~YYY¾» Ôn³CÄl6Ë¥Þÿ½óãããeÎÔ´Y_ð«xÄöº+V¬P×õxçúõëÅwgá9"ß/¥¨½h6Kiå7¿(?uBëÔÇÎÞ~ûíÙHKKwçÙûï¿ï5ýýýêó³)¿àWûàdbÏ=Úë¾ñÆ2éÒ¥uWfÊôÝ»w=z$yyy³cccÒa«V­zðàÔ¤Ú¹mÛ¶Y.%ßiuÄYköûÙç·Àò°åçñx¤®¦ùÉ<D=¦´ÊÿLÞù­­­éééÒ=eù¿ÝnK×¯_ï=ÑÄ÷×äÇÇÇoÙ²EÆ+ÁW]]­N.++»wïÞìÜÔ¤:-	xôèQõ¾Ù,%ßék×®ÉCReûýìó[à@ùP~ ü@ùòåÊon~ö³øN>|ø?ÿó?¬7x>=öOÓ"6ÉfAüêW¿úÏÿüOÿøÿøßÿý_Êoa|ÿûßøñ~úé§=bUø÷ÿ÷ÁÁAO(OZùOÿôO,(7nÜ_(?Ê(?P~ ü(?Ê(?P~ ü(?Ê(?P~ ü(?Ê(?P~ ü(?Ê(?P~ ü(?Ê(?P~ ü(?Ê(?P~ ü(?Ê(?P~ ü(?P~ ü(?P~ ü(?P~ ü@ùò£ü(?P~ ü@ùò£ü(?P~ ü@ùò£ü(?P~ ü@ùò£ü(?P~ ü@ùò£ü(?P~ ü@ùòòëêê*..6«W¯îéé¡ü@ùòåÊ/jËoÕªU·oßË/çååiËïúõëOCëç?ÿù_|ñxúôÓX²YËbxxøO>a9@ùøã?~â;àòó¨-¿sçÎý<´~ò´··ÿøùÏív»üfÏrÍlX?ûÙÏ>úè#ÖÖÖßi4_wwwMMGÁÑ^p´íG£öh¯211Q]]ír¹(?P~ ü@ùòæò¶X,º±EùòåÊ_ô_û¦MFGGu/¥ü@ùòåÊ/zÊÏh4.óAùòåÊ_Ô_p(?P~ ü@ùQ~(?P~ ü@ùQ~(?P~ ü@ùQ~(?P~ ü@ùQ~(?P~ ü@ùQ~ ü(?P~ ü@ùQ~ ü@ùò(?Ê(?P~ ü(?Ê(?P~ ü(?Ê(?P~ ü(?Ê(?P~ ü(?Ê(?P~ ü(?Ê(?P~ ü(?Ê(?P~ ü(?Ê(?P~ ü(?P~,P~ ü@ùQ~ ü@ùòåGùQ~ ü@ùòåGùQ~ ü@ùòåGùQ~ ü@ùòåGùQ~ ü@ùòåGùQ~ ü@ùòåGùQ~ ü@ùòåGùQ~ ü@ùòåGùQ~ ü@ùòåGùò(?P~ ü(?P~ ü@ùò£ü(?P~ ü@ùò£ü(?P~ ü@ùò£ü(?P~ ü@ùò£ü(?P~ ü@ùò£ü(?P~ ü@ùò£ü(?P~ ü@ùò£ü(?P~ ü@ùò£ü(?P~ ü@ùò£ü@ùò£ü@ùò£ü@ùòåÊò£ü@ùòåÊò£ü@ùòåÊò£ü@ùòåÊò£ü@ùòåÊò£ü@ùòåÊò£ü@ùòåÊò£ü@ùòåÊò£ü@ùòåÊòåÊòåÊòåÊ(?ÊòåÊ(?ÊòåÊ(?ÊòåÊ(?ÊòåÊ(?ÊòåÊ(?ÊòåÊ(?ÊòåÊ(?ÊòåÊ(?Ê(?Ê(?Ê(?P~g``ò£ü(?P~ ü@ùE3Ù744ÆÌÌLé?Êò£ü@ùòåm$ò$$ÊËË/_¾ì·®^½JùQ~(?P~ ü¢ÇÈÈÈÉ'Fã2ºº:Êò£ü@ùòåìvUUUrr²_ð¥¥¥íß¿¿¯¯oaïò£ü@ùòåPijjÊÏÏ×îä[·nÕj_û¥ü(?P~ ü@ù!t$ÌfsBB_ð-_¾¼ªªêÎ;zïåÊ(?,ºñññÆÆFÝ|F£QÂétàaP~(?P~ ü°¸ïÔE»OTVV¶¶¶.ì¸-åÊ(?Ûí¶Ùl¥¥¥ÚàKKK«¯¯ý£¢ü(?P~ ü@ùa!É¦¸¶¶VòNÛ|KõØ(?Ê(?P~X§¹¹Ùd2éîäM4åGùòåÊ/ÄétJèÃ¿xC´P~(?Ê(¿ñx<v»Ýï­)			f³9ôU@ùQ~ ü@ùò£ü¢%77W»OfÊEá³ò£ü@ùòåy2syy¹Ýnå-åÊòåÊoá[­Ö@ã0×××;øA(?Ê(?P~(È-&©¹¹9ÌwòQ~(?P~ ü0àã0É-åÊòåÊïÈ´¾¾><Ça¦üÀèè¨Ñh¤ü@ùòåMÔ-UUUÚàKHH°X,Ññ¢üæ¦£££  @VÊ(?P~Ñadd¤±±1Ð8Ìá<Då·è¶lÙ"ï¬AÊïüùóÖ'|"¹ù)ðé§?ýéOÛÛÛY²YË¢««ëúõë,]ÿðÿPQQç|2çÏþìÏBÿôÑ¿þë¿øN#¸ü~óß~ô£G¡%Û÷÷î==êÆrÍlXCCCmmm,_ãØ±cÙÙÙÚ|üÇ¼wïÞÏ?ÿ<Zvù5ÀétøN£¹ü8Úö£½àhoØêëë³X,ÉÉÉÚæÛ¼y³Ýnè³78ÚKùòåÊòûõ-/^f6ø233÷ïß)ã0S~åÊ(¿Ã¼nÝºæææ¨ßÉGùQ~ ü@ùòr§µµ5Ð8Ì»víêëëÍõò[H(?P~ üÓé¬¯¯×¢¥¨¨èâÅ.+×ÊòåÊ_ÄS;ù*++/_®;sÌîä£ü(?P~ ü@ùE¡¡¡'OæææêÃ|úôéhò£ü(?P~ ü£å'ï¹%!!Á/ø/_¾cÇy	x<VÊòåÊ_·Z­EEEÚ|F£±±±ñÉ'sºµ¦¦¦ï|ç;'N ü(?Ê(?P~aAÖê@C´·¶¶ÎuÙf¾ôÒK¯¼òÊk¯½öÕ_MMM½|ù2åGùQ~ ü@ùò[2Òs6Mwôôôºººyï«ûò¿¼å[~ï¨ú÷-Ë·(?ÊòåÊ_¨9úúzÝ|kÖ¬±Z­/2DÓé4Gñü¾¼xñ"åGùQ~ ü@ùòÇÓÜÜ^hY½ûúúòòò|³OþmÜ¸ñôéÓåGùòåÊoÑ9Ny3Õ9??ßjµ.à-.+11qÏ_ïñfßÃGä®ív;åGùQ~ ü@ùò[Dòj6µ;ùdÌ_¤·×Ó§OgeeÓüMÉ¾o×ýéþitCùQ~ ü@ùò[2ãããùùùºC´È«ÓépõêÕ?ø?ø?ùFýßv£ü(?P~ ü@ù-YEÃ^n·Ûò£ü(?P~ üÙå§ÆaÖ¢%--­¾¾Þápð¬Q~åÊ"»üdhf	AÍ6×qëå·l&qqq(?P~ üBIÑb2twòI²á¢üæ)n&òåÊ_h¨qC3Db±üåÊ(?Çc·ÛfP~3Û³gåÊ(¿Eât:sssµ;ùd¦N>Êo±ÊÏh4>çÊ(¿_q¢ò[ôò[½zµö·ôôôÅòåÊ1U~j%ß¯ÅÇÇË:7::)||ðLÔÔÔP~ ü@ùò[w@C´L¦ææfvòQ~¡+?µæÉ¤LÜ¿jjJ&(?P~ ü@ùÍÛí¶ÙlAÆaf+Dù-Aù¥¦¦Ê*ØÑÑ!ëL;vLM0ª(?P~ üæGÑÂ8Ì_8ßÁ½çsø­(?P~ üfOÃÌ-_Xxë­·V¬X!]]]2!XRR²ØòåÊQS~N§SÞ×òò[(?P~ôòSã0¢EæþåGùòå.¿ñññ@ã03Dåîå§Æva$gP~ ü@ùÍøVb±XòÈò[µjoíyqn/(?P~ ü¼Ô8ÌAhq8,@Ê/ÊO"OÖÚîîî©©©P>tÊ(?DDùIÒ!Z(¿H*¿ôôtYwC(?P~ÿò7~i;íÙRÒlC(¿,¿Y÷íÛ711AùòåÊodd¤¡¡AýQSh¡ü¢­üÄÊ+µ+7gxòåXÓÞÞ^^^Ì8ÌÚòËÉÉáP~ üX±l||üìÙ³ëÖ­ÓîÉÎÎ7,vòQ~ÑS~jÍîëëñC§ü@ùòÃ»sçN]]öì5DËÕ«W¢ò¶òËÈÈàP~ üX1EzNªNwäääÃ3D¢¶üÚÛÛeE?xð`OJ§ü@ùòCè©?°«ÆµðS^^ÞÒÒò£ý¥h.¿ep(?P~n·ûÊ+&Iû·7dw¹þÝ^P~W~qp(?P~CCCº;ùJKK­Vë'O¼ßLù!úËo©P~ ü@ùaQÉ»Ì;´ã0Ë]»vé>é¢¿üFcNNÎÀÀåÊ¢ú»ùùùÚ|¹¹¹N§3Ðu)?Dùy1þ¡S~ ü@ùaaÙíöÊÊJÝ|f³¹³³sÆ[ üýåwóæMyUÈï@ccc¡ÛòåÊBÃ¬»/==]ÞàfyS¢¿ü8·(?Ê/BÉg±X´§ëÍ7···ÏufÊÑ_~ÛÊåY|/99Ù;DË<P~þò[*(?P~+yÙJØiÿØ(++knn~Á?°Kùò£ü@ùòÃs»ÝRu&I|RÒ5Bå(¿ÉÉÉ-[¶$&&ÊK())iÛ¶m!8ÕòåÊ3r8õõõº;ùÔ8Ì/¸òCÌ¬åºgx,öñ¥ü@ùòC Çn·khIHH°X,ôQ~þò+((REEÅÄÄ|966¶uëV³víZÊ(?ÓélllÌÍÍÕY.ZØ|b®üâããååäÞûää¤Ìù(?P~yS0ÍÆa¶Ûís¢òå§#..N^WRÞ9n·[æ0ª(?P~ C´Fy³òÇÖ(?P~s¦önÚ´IíÿeZæS~ ü@ùaQ·ÉÆa6LÍÍÍ!ØÉGù!æÊORO÷§OR~ ü@ùaÁ¹ÝnÍVZZª;DKð×¦<Yþç[VVöÃþòå7Û¶m)))qqqòÿ¦MdÎb?tÊ(¿X#/º C´HÎ8¬Ä+WV¬XQQQQûÚ;w¾ôÒK'N ü@ùEÊ(¿áñxÃ<Ëå/·ò¯ÿåÑïUÿ¾»÷»ÉÉÉøA@ÊåÊæIììÙ³ÙÙÙÚæËÏÏë8ÌòÍÉÉñfú÷Ê+¯´¶¶R~ üfºæLâââ(?P~ ü0?AhßößápHDú_qq±¼=S~ üfåÊæçÉ'§OÖ¢EÃüGf_~ùåªmUÞì«ùvMrròÈÈåÊoÞ|óMõ½|ù2åÊfI^PÓ6_yyùBÃÜÙÙöµ¯íæo~ãßHOOÿàð§ üCå×ÓÓ¤FQòØòåÊHÏ]¹r¥¨¨H|uuuþB:pà¼UíÞ½óR~­òÛ¾»z¡^¿~=4òåÊ/¢<yR÷ìYÑ(?DùøáêZQQÊNùòå¡ÚÛÛ+++µgo¤¥¥ÕÕÕuvvFîFù!ËOÖïµk×ª9nÞ¼âNùòåY<y"îôôô¢òåÒò;sæz¹îÜ¹sI:åÊ_¤èìì¬©©Ñ½`±XÚÛÛCÿv)?P~s¼&ãùò£ü@ùår¹¬V«î-²_À?Aùò[ÜòÁ` ü@ùòMwîÜÑÝÉ'*++ív$½Aù!¦ËoÉQ~ ü@ùËe³ÙJKKµÁg4>ìp8¢	P~ ü(?P~ ü¢___ßþýûÓÒÒ´ÍWVVvõêÕ¨ù$åÊòåÊ/FËÏívÚÉ°k×®X[2(?Ê(¿($ëmm­îN>	Á+W®Dë'ù(?P~(?P~±R~§¹¹Ùd2i/99¹ªª*ô[fÊåGùòå·ÀN§lxF£î8ÌMMMQ03åÊ/ð5Ïå(?Çc·ÛËËËµl-!!Ál6ÇøN>Ê±R~¾C÷éãùòåÑÆÇÇsssµ[x)±òCW[[l^ýõ	ùRþßºu«Ì¹uëåÊ_$Çb±$$$øßòåËËËËívÑBùòÓ¡þö¶ïV`rrRýe¼å¬¬,ÁPXXxóæMÊ(¿E5>>è­Æúúú¨òå7&µçW~/þ9¿êêêK.ÉÄ¹sçvîÜ©-¿O>ùÄZÿýßÿíÏ?ÿ¼¿¿å!Ù8Dôð_üâÛßþvjjª¶ù¾öµ¯Ùl6BèÙµÛí,(üqè_;^~²iJ_tOÿé#ó_|oâÔÔJIùuS[~§OþihÉS(¹ùSà§?ýÉ4ldã¡«ñáÃth©¬¬|÷Ýwy~çäæÍ­­­,(²2È*â;]ôò»uëîwïÞÁ[ö=GD¾GÁÑ^p´wÞG q­V+gop´íèþýû«V­JLLKJJ*..~üøñß¬ïñâøøxÊ(¿|ÅÂ ¦ü@ù-õñAù_ìòåÊoöÃÌ-(¿¥g6ßyçÿ«««)?P~ üæA6²9Õîä9ÃLùòþþþ¼¼¼¤¤$u|6##£¥¥åÅo¶££#33Sn3++«««òåÊoöÑ"P§ÓÉsGùò35³ï_lSÓçÎ[ÔNùòå§KÖÌÚÚZíÙjæææfÆa¦ü@ùÍ_ff¦lPúûû½å×ÕÕ%Ó)))(?P~!ãv»¥êL&v'T ã0S~ ü/àHÎjBßÔÔ·(¿	>DÍf(äi¢ü@ù=_ÀÕ~>)¿ÉÉÉC©ÏP~ ü@ù- C´HÖÖÖ²R~ ü¾üÚÛÛuGr¾û6åÊßbollÌÍÍÕ¢q)?P~X~j«WRR¢ÎíMLLÌËËÁ§I(?P~Áò;²X,			Úq¢òå¢ò[(?ÄNù¹ÝnÍVZZªÝÉË8Ì(¿Ð÷Ä¯ÇçååeggS~ ü@ù½ @go¨!Zìv;C´P~ ü¸ü&''9·(¿!=×ÜÜ¬öÑhdÊ_¨Ë/;;YP©©©(?P~säìL&Æa¦ü@ù-Mù=xð n÷¯wøìûðÃ)?P~ üf/ÐØeÊßÒtÞbØ¥ü@ù!ËÏårÙl6Ý?°âq<yÂ³IùòS(?DzùÔÖÖ&''kh±X,!)FÊr÷îÝ©©©iii÷wÇsJùòhrr2///))É;gÅ'N ü@ùòTZV«µ¬¬L`7???ôC´üÕ_ýÕ¾ô¥ïîýîÑïÝó×V®7ó7<­(?²ð;½Wm¿(?P~ ü|Ý¹s§®®.==];DËRÃìp8ÒÒÒÖìSÿöïÛèr¹xf)?P~:âããe³Õ××çÓÓÓ#sRRR(?P~ üOïä»zõê5k´äßpÖÖÖõÿw½7ûÔ¿üü|TYÊuzïÔÔÔïÝ®f?Êb°ü$ìÑ"ÕµäC´tvv~¹Ð¯ü^~ùeÆ¤ü@ùéËÊÊMØÔÙg²Ò=zTædffR~ ü³å'UWUU¥ý$_zzz]]]øtlºå!í¨ÞáÍ¾×^-''Q)?P~úº»»uGrîêê¢ü@ù!ÖÊïÉ'gÏÍÍÍÕn×¬Y#lÙmé&þáW¾ò×^íW^zé%õR~ ü.,,LLLKJJ*((9ýÐ)?P~«ò555			ºC´Èö*÷¢ÉÊÜØØøï|çôéÓ###<§(¿°CùòC8p»Ýÿ÷¿jÕ*íN¾ÜÜòåGùQ~ üGmm­vªªª0ßÉÊßüõ÷÷«ÁÕù¼---(?D%é¹«W¯jÏÞÈÍÍMÓéd)Q~@Ô_[[w«§ÊOM;wòåh244tòäIÝ!Z¾úÕ¯Ã- ü@ù-zùeffÊV¯¿¿ß[~]]]äÊQCzÎn·ïØ±C»/--M¶EöYÈþÆ.(?P~K jó÷Ügôæ©©)6(?D4§ÓÙØØ¨;DKii©ÍfSC´ÌõïöòåÁå¡FïSå799yèÐ!6(?D(Ù¼ÍfÝ|µµµ~O=åÊ1T~íííº#9ß¾òåÈ2>>nµZóóóuwòÉEòÚkQ~ üCå§¶z%%%êÜÞÄÄÄ¼¼¼üa"ÊöÕ][[¦;sð°£ü@ù!¶ÊoIP~ üðâÜn·Íf+--Õ¹±±Qw'åÊåÊÄápÔ××kwò-_¾¼¼¼Ün·Ï~Êb«ü¾øâøøxÙh&&&R~ ü¤ç¥í´goFiÁy|Xòå*?ùÍX÷ÊÂÓéíî8Ì&IrpÞã0S~ üCå§Fr®®®v¹åÄÄÄÎ;eNVVåÊKNÃ<û!Z(?P~ üÞÄ4ßßeÚ;ª3åÊKe||<Ð8Ìùùùh¡ü@ùòFíóô]ïÙçÊKH^ª%!!AöÙl^ðMåÊ1T~ês~ÕÕÕ²ºËO>-++ãs~ üzjfÝ!ZF£l4NçbÜ/åÊ1T~Ëf²H)?P~ðêëëÓùÅÏÞ ü@ùòû=q31(?,·ÛåÊÊÊJÝ³7êëëCóQ~ üCå·T(?P~±LÃ¬;DKii©Íf(Ù¡ü@ù!Ê/Ð ÍÃÃÃ(?,Æ´ªªJöÆlþÀ.åÊßßÄ²eo½õßÌ]»v1ª(?, 'OfggëÑröìÙ¢òåÊ/)<Ùòfdd<~üX¾|ï½÷Ô¶x>ÞGùò5û÷ï×¢¥²²R6¬zöåÊ¿]»v©qVVØºuëb?tÊ_tSC´¬[·N»/33³¾¾~h¡ü@ùòÙ­[·¼å¦¦¦<tÊ_´RC´¤§§k¯¼¼Ün·òìÊ(?÷îõþ"®&^ýuÊæÄãñr¥¬¬L;Dl[êêêÂvS~ üCåg0d»ÒÛÛûÜçs~ñññ(?ÌÆÐÐ÷WG_ëÖ­t¹üø)?P~¡òMó#GüfZ,ÎíåÙíöÍ7ëÃl6ïÜ¹?åÊ1T~Æó[ìB¢ü@ùE®Ý|EEE/^!Z(?P~ üÂåÊ/ÉÈl6ëÃ#.ù-(?P~z×Ì÷xnð/)?P~1n||üôéÓºã0ËLy9?yò$r:Ê(?Ê~óª±X,Ú|bóæÍv»=wòQ~ ü@ùQ~ üð;jæüü|Ýqåõëp8¢æ¥ü@ùò£ü@ùÅ(Y,µµµiiiº;ù$Ã|Ê(?Êòå7·ÛÝÜÜl2´Á'(-ÅòåÊòå+G½îN¾ÒÒR«ÕYC´P~ ü@ùQ~(?Çn·kÇaNHH°X,±C(?ÄDùGùòVN§³±±177WûÂrQÔïä£ü@ù!æÊ/n&òåeäEg6µ;ùdÌ!Z(?P~ üÂåÊ/dÑb4åÅèt:cye ü@ùò£ü@ùE ã0L¦æææØÜÉGùòåGùai¸ÝîÎÎÎÖÖÖwXt,UÍVZZª;DK=ç5S~ ü@ùQ~µÌÌÌ/­úÒW¾ò?ú£?ú¿øIÊïEÈdÉÁ-aÊ(?ÊòÃï¸ÿ³òÿo)?ú½£òïÐÿ;TPPP[[KùÍÇã	>3eCùòåGùa)]½zµ¨¨Heú·ßþÔÔÔÐ|ò,jÊÏétÊ«Éh4j/???Æa¦ü@ùò£ü(¿pöìYÓ×M¾å'ÿ222FFF(¿YÑú×åÊåGù! öùÍÏøøxðqc|Ê(?ÊòG.ëå_æs~sZuhY¾|yUUUÌÃLùòåGùQ~áwçö®ÿõ¹½;vìàÜ^­ ã0§§§>|xhhuòåÊò£ü"¤¬	W¯^e<?-yµµµhinnfÊ(?ÊòCdô!Zêêêúúúx)?P~ ü(?Ê]~#Ð8ÌEEEMMMÑBùòåGùQ~ìò2DKBBÙl¦K(?P~ ü(?Ê_~AÆa.**²Z­¡Ýò£ü@ùò£ü@ù-"YÿÍf³îN>h¡ü@ùò£ü(?DCù¢Åh4<y|(?P~Z~£££òfFùòxfQ^^n·Û¢òåÊ/Ë¯£££  @ÞÕ(?ÄrùIÏÙl¶ÒÒRmðeffÖÕÕEô¦ü@ùò£ü~cË-ò¤ü~øÃþ2´äõÜÕÕõKà¿¼5mQïâúõëßúÖ·thùò¿|êÔ)ÖÆ0!OlX½½½ü1ËÊGt÷îÝßißobàòknn­ùùüóÏ¡¡îîîO?ýt1nù¿þë¿Þ÷Ý¯ýëqqq~Á¿÷î¶¶6XÍlX_|ñÅO~òù^6é!¾Óh.?ö"Êö<yRwüü|«ÕÊ8ÌíGÁÑÞ¨:Úë£üSåg·Û+++µgo¨qC¿ªòåÊoÉZòC´Ëå4DKnnncc#;ù(?P~ ü(?Ê_~wîÜÙµkv'ßòåËÕ-ÃLùòåååH,?ËuõêUÝ!ZFc½Ãá`ÙR~ ü@ùQ~"»üä÷ïß¯;DÉdjnnf'åÊåGù!²ËOzîÊ+eeeÚ?°+X[[Ë8Ì(?P~å/¿¡¡¡@C´2DåÊåGù!ÊÏn·kwò%$$X,òåÊò£üñå§ÆaÎÍÍÕ!Z(?P~ ü(?ÊÑP~²*ÍfÝ!ZòåÊò£üå×ÛÛÛØØ¨;³ÑhUÔét² (?P~ ü(?ÊMÖíÛ·ÇÇÇëÃÌ-(?ò£üñÜn·ÍfÓ9--q)?òåGù!ÖÖÖêÃ,!(9(QÈR¢üÊåæñxM&6øRRRÌf3ã0òåÊòCÄs:²éÃoµZ?ûì3²(?P~"Çã	2³ï-sý»½ ü@ùò£ü(?ñññÆÆFÝqe¦vfÊ(?P~"OqËËËív»î-(?P~ ü(?Dññq«Õhæh¡ü@ùòåGù!¢Åd2ÍrfÊ(?P~ÂWðq¥çTr(?P~ ü(?#§ÓY__¿°ã0S~ ü@ùò£ü^d%©ªªÒ½!s,Ë¼[S~ ü@ùò£üdÑ=Cfjh¡ü@ùòåGùQ~GÖ;vhÇa9¾ã0¿8Ê(?P~Ëå²Z­EEEÚ|ÙÙÙ²¶-ì=R~ ü@ùò£üjuuuéééÚæÛ¼y³¼êf3DåÊ(?Êò_j'_YYöÀ®T ´`__ß¢>Ê(?P~]q%m6DaåÊ(?ÊÅãñ477WVVjwò©!ZBÜa(?P~ ü(?,<ÃÑÐÐ`4uh¹xñâ'OBÿ¨(?P~ ü@ùQ~XHwîÜ1ÍºC´ìÚµ«³³sÎÞ ü@ùòåGùQ~!òäÉ¦¦¦ÜÜN>yöìY§Ó¹äòåÊåÒ××g±X´;ùDUUUkkë<þÀ.åÊ(?Êò#ãããÆaÎÌÌ<|øðÀÀ@¸=fÊ(?P~æ¼HÑb2.^¼!Z(?P~ ü@ùQ~ßbq»Ý6­´´T|ÉÉÉ555=3åÊ(?Êò[t£¾¾^w'ß5kÂç|(?P~ ü(?Êo>Ô8Ìåååºã0ïÚµ+âÞ8)?P~ ü@ùQ~ðçt:åÉÒ9;;ûôéÓãããøsQ~ ü@ùò£üð;ºã0'$$Èüööö%òåÊåGù-·ÛmµZKKKµÍ-OÃLùòåÊò£ü^ÈÀÀÀáÃµgoHÛíöÞÉGùòåÊòÃ¯wò:Ãh4Ö××;èû©)?P~ ü@ùQ~±ÅétJØé½QVVf³Ù¢i'åÊ(?Ê/FÙíö;vhwò%''ïß¿?òåÊååFFFóóóµ;ùJKKm6[Øþ±5Ê(?P~å7NNNÖÑb±Xb°(?P~ ü@ùQ~Ñ¬©©É·ù¬VkÃLùòåÊò£üÈKKKSl-ÒÇa¦ü@ùòåGùQ~3e>22ÂòåÊåÊ(?ÊåÊ(?P~åÊ(?P~åÊ(?P~åÊ(?P~åÊ(?P~åÊ(?P~åÊ(?P~åÊ(?P~@ùòåÊòåÊ@ùQ~ ü@ùòåGùQ~ ü@ùòåGùQ~ ü@ùòåGùQ~ ü@ùòåGùQ~ ü@ùòåGùQ~ ü@ùòåGùQ~ ü@ùòåGùQ~ ü@ùòåGùò£ü@ùòåGùòåÊ ü(?P~ ü@ùò£ü(?P~ ü@ùò£ü(?P~ ü@ùò£ü(?P~ ü@ùò£ü(?P~ ü@ùò£ü(?P~ ü@ùò£ü(?P~ ü@ùò[R]]]ÅÅÅaõêÕ===(?P~ ü@ùEmù­ZµêöíÛ2qùòå¼¼<mùµ··ÿ*´º»»Nç¯_ýêßþíßîÝ»ÇrÍlXv»åEÊO~ñFpùùJLLÔßÛo¿#´Z[[¯_¿~¸qã£i,Ù,ÈÆåññÇ³2ÀëÇ?þqèï4ÊO~®©©áh/8Úö£½àhoÔíU&&&ª««].åÊ(?P~QU~Ë~K9<<l±XtcòåÊ(¿È.?_ííí6mÕ½òåÊ(¿è)?£Ñ¸ÌåÊ(?P~Q[~ÁQ~ ü@ùòåGùQ~ ü@ùòåGùQ~ ü@ùòåGùQ~ ü@ùòåGùQ~ ü@ùòåGùò£ü@ùòåGùòåÊ ü(?P~ ü@ùò£ü(?P~ ü@ùò£ü(?P~ ü@ùò£ü(?P~ ü@ùò£ü(?P~ ü@ùò¦òûÛ¿ýÛæææ¡ÐúñüÙgCCíííÿüÏÿÌrÍlXò;á?þã?² ¼ûî»#Äw:>>å×ßßôèÑïà·]ÆXAùP~ ü@ùòåÊ/Fïýýýk×®5,X^¶¶¶eËx)ÅúÊðèÑ£e>X>±¼2¸ÝnÅÝÞÞÎòñõÁwËGù;i»ßí¸ßµk×dâöíÛééé,¢X^Çã)..æ¡¥¥EÞìY2¬âøñã§NìËÉÉañN¡téÜ¹s_¸Û²eËàà îSxãÆÕ«W³b|e8qâÄ~ðÊA²OýNVÂÂÂë¯û÷ïoÜ¸1!oW³[LÝ<)))2óòåË,X^<xPRR"¿ÙS~¬«V­Ú´iÁ`UâÞ½,X^d58sæLbbbNNÎÝ»wY8dØ¶m[ww7åÁOáóéOwedd°pbyeWò­[·­!Í-Ãðð0Gb|e»páLÈïòÃàààúõëÃå±ñôÌû®~±cáÄòÊ°ì÷±pØ2(ñññ,X^|wð6Áúð|ú£gÎ¡ü"õ)jU¿LtuumÚ´Ãýsöù±2LoÇóé~lb|eØ»wïûï¿ÿ|z,ÒÒRï6lèíí¥ü"õ)ìéé),,_ã6nÜøèÑ#åGù±2îîîÙ2Iü±pbyeÛ¶múÐçàà wøøø©©)Ê(?P~ ü@ùP~ ü@ùòåÊ(?P~ ü(?P~ ü@ùÀ¼¼÷Þ6lH¶qãÆ?üð÷¶_Ó"fk«÷hF£üh.Ëo¾Ì1YYYSSSs½M üD£G.Óxë­·¢©üdfSSßü³gÏÊücÇÍã6òazzz¤iÃùóç=Ó. _ÊÌÞÞÞ¨)¿û÷ïËÌÕ«WûÍ/((ùò@ù~¯¿þº4Í©S§|g¾ýöÛ2s×®]¾ÝsóæM)'Âââbö~óÓ§OkjjRRRä¢ôôôøTµÛíRWrÆ~-%sRSS7lØpýúuùÒl6û=°ÖÖÖà·£.¤.jkkTi6mù]]]Þ9·oß9&É;çøñãrSÛ·oÖööýæy¨(?XJ+W®jyðàïÌÊL£Ñè[6~º»»Õ¥[·nõ»hß¾ê¢¾¾¾¸¸8Ýk©/Õ¥Û¶mv÷x<r©ü/å¤>~ävdB÷áiÒË/ûæ¬7./]ºäÍ>¿Ù¸qã/ÈC@ùÀSvu¶YËIùÍÎ;'¦É|YQQ¡.U¡£ÚQíºbSUWW«2ÝÑÑ¡nÄ÷69"m§¶¾ñÆ2çÚµk2-ÿËôîÝ»g¼©FùR¾Á÷±éþDR+V¬wttT¾ÿåKnNNNªoÈÊÊ+Êðü·GçZ~A*Ê¾ünùyç«²QÁ$åKi&õeqq±|³gÏ)¶gÏyoD¾Ço/÷ZêËz¿YjÉÀWþi»Ý>ãíHeÊ?öl>÷æoÊE?øÁÿöö|¿A*PÊOz´¤¤Díkùy¨(?XbjG×ÄÄïLË%3å¢ ­ãíÂÁÁAÞÐñ~8OÖ¯¥|ÇRéôôôøøx·Û-7ê½tÆÛ	Ôa~úûûU¤Ê´ü/ÓÞK;::äè5ùy¨(?Xbê³ngÎñ©Æ:ñ;ÃÃûYÀÑÑQí®¬ÞÞÞuìÕj÷pê¶oß>uWþ¯©©ñÎr;jÛ£GÔ?~îúõëåR5«ïEê<_¹¨­­mll,xùy«T-ï¥A*Ê:CÂ`0;wNêrþüùøøxíÙR9n·[õ«>ç§>788èûANRgÝzÇUÑí3u²­º÷öövïü ·£>Ø§>ççr¹Ôw)¿.x÷ÆÉìúÈc__Ü,@å§ZSZY¾Msûöí¾y¨(?Xzjc?ÇÿÝök#ï´÷8©÷¤¯7ÞxÃ7+µ´´)¿çÓlCæ§§§ûr;ò0|÷¼Ú@?ì³gÏÕàwË-¾w±bÅù_ìâj¤êTï¥A*ÊÂÔÉÆã§mØ°A`ëW~mmmjØ¼ÂÂÂ[·ny/u¹ÊÌÌTµôæoºÝnï¥7nÜ()) ËÊÊ²Z­~·©$rS2ïÞ½~óÝ#I4d<?¯¿á]ÑÑÑêêjY)))òS8ï5¾·966&i«Édêêêò»Ç åÊ(?P~ ü@ùòåÊòåÊ(?P~ ü0wÿÖ´U¶ÔÀÌhIEND®B`


$ãË0ÌfQ¹!aÀ4Y ñZÂdÐÜ'ò2ð¤¼!Ã(«Ò(lCÊPÒdqFQJ(õ¨7J£òÎE,£aÈ ,fEë!ÞßËYïsw¯mÀà÷~¾BÖ¹çþññ¹çûáÏqK$IùÑ³@$ü$ID~$I"?I$$IÈO$Iä'I$ò$IùI$ü$IÈO$Iä'I$ò$IùI$ü$ID~Ìëùv;wîìvü OXO×îÙ³gáÂÅ·«¬¬|çwîøwiÄígÌñúë¯÷aæ8q¢ªªªþ#GæÏ_XX¿=ãFôÐyâ$¤¡.¿1cÆxq¸ÈïÅ_ÌvÏæÍy´ºKO´zíµ×îuæôy6N0!õkjjÈOùIz`ÀV¬X1,äwâÄªßþö·»ººnÞ¼q1F655uûP÷z_ýúõ7ÆÐØÉ/¹ãµk×b¸³³sH=5ÈOÒð_mmmüÜ³gOO¼øøãW®ì*­®®nnnÎ¸åÁÇ»`ÁÔCÍ5«°°páÂ.]:räÈ3/^¾qëÖ­åååÉnÍø©«zòÍO<ãÓ÷½F;wîk×®íöo¼×»dÿêÀb¼§Óí6¹ôúvÇÞåqm</Ó§OÙ¾dÉ·½oÌ¾6ýiâÑb>Ä3W¥îB9«ÊÊÊ6lØÐÞÞîÅ%¤!*¿ë×¯O0aüøñ1.ÄÛyºEâD+((=öØ­îvGÎ??pºI±/ã-êÝ7'NñW®²S§Níöo¼×»düê`M²ÍoÚ´i÷4szïx÷ò;yòdúãÄ¯òKZ©N8ÜxÅÓ¼~ýz/.ü$QùÅÀ¾ûÒ·¥#àé§áåË_»]ÄÅ~Ë^xáæÍÉV«dÌ3Ï<ÓÕÕuôèÑäb<rxð`û&f;pà@òÉ'éWõäÂÂÂnÇ§ß÷>ïÒÓéRÛDïuæt;U¾c/òË¸c-«««oÜ¸¦Oùìîåþ´ÆãÄÃÇpò´®^½:¹q"ÂO?ý4ãÙá/.ü$]ùEñFÃñ¦1~üøñ1|áÂäb¼ÁÇÅqãÆ¥?BêÚÔÐFúÅÖÖÖ[¿ÜmÎÎÎ°BcîÜ¹ÉF¦;Ê¯¨¨¨Û?¤Ûñ¸Kº¨â¾ªªªngÚÝÌn§ªÏw¼ù%e¼téRú¯¸'ù¥?­É£¥c«fÏ'OrÿB|öÙg^YùIêòÅûwGGGúøìmfÙzÒuû½_efìñ¼ãwTËËËÝÓÙ¼KA$ãÑîæ.=Í;Î´»9=y´ow¼û½½wc»Þ¯Í~Z3yîÜ¹)îß¿ßK"?ICZ~Ñ»ï¾SÇ@IF&8ËØ:#ï½_>zrtCÌîF~ÉÉä+ÕÕÕ6l¸víÚo¼#W®­üîæ.ß½ÎT¾ãÝËïn¶ù¥lîïþÄ/§wêÔ©-[¶$»SßD~®ünÝþ´~êüÉä¿Ë/¿~»ä­ýg¹ù%Û½Â¯¿þúÝÈ¯±±1ÙªX<zôh²=/iß¾Ýþ÷z_ï3'í·Ëx«_òÉ¼ïg°[ºti¶_õÕù¡Ã@pïó?ÂpsÜ>ædÏ5+ýÏL>¯yîÜ¹^ö¼K"?ICK~/^L¢+ÓÜÜñ¯ñãÇ§¾*?òKY$iÜ¸qñ3ýû­ÝNóO>½çñáîåÏ¼§»Üüz9©2g¹sµò;uêTúã/X° ýÚyVë]~Éa»ýÊK²U5½§zÊK"?IÃ@~Q²'4üG´råÊ¢ÛUWWg£oòkmmGÇ=÷Üs¡ÔAz÷Í;fÌQPP0qâÄ·ÞzëË_þrjÓýßåä×ûÌÙ·o_à¯°°0uH¼ôîi®öA~Ñþýû'Ou<~²?=õuæ¸>K~UUÕñãÇï¸ÍõàÁsçÎG?j×®]©ñíííÏ?ÿ|²15øOeGGD~Ô/uvv>ùä÷t¹>ÜeØìM¾¬ÝÕÕ~úôéIä'I¹Vò9¿^~ùesFùIR®ÕÞÞ¾qãÆ'&»tcàÅ_4[$$IÈO$ü$ID~$I"?I$$IÈO$Iä'I$ò$IùI$ü$ID~$Iä'I$ò$IùIR´oß¾ÂÂÂEÝëgÌ1bÄsçÎ¥ÆÄp5kÖ=¬Ån×·ÛÜÍû¸nÍ*ßÞÞ¾~ýú²²²ccÆ©®®þøãS÷êêêñqã¢¢¢¶tI"?IC«òòòJkkk¤²uëÖ¸ïöíÛSc^yå³eËÜ_öøÚÚÚøðá>räHO:5uíþýûSR<pà¥KùIb+ûÀÓ©S§â¾.L?~9yòäÀLäÀË¯¨¨(Æ_¿~½Û­Y³&®M~>õÔS.Iä'iÈ±/]9Ï?ÿü1cJJJ6oÞq³/ùËsçÎÍxÔ&Ã¾råJÇäªO?ý´ªªª¸¸¸°°pöìÙÉv²ìGKÿí½ßå­·Þª¨¨¨¬¬_í³7nÔÔÔÄcâ7nÜlÂ.X° -ÆÇ#_¸pá>å7iÒ¤?úô£GfóæÍ±cÇÆïúì³Ïâç¸qãR>îÛ4HùIêü%Ã/½ôRïÚµ+/¿ürúm</½§~:®zã7b8~ÆðÚµk«~øádç'|ã'LÐí£¥O@ïw	Ï½ûî»1P[[=ñë×¯á¸Áï½[·nMAíúõëMMM1lìé3|=8ý6ûöíKY´hÑ~ºK²ówùòå1¼råÊ1ÝN¥NùI|ùUTTÄpçí²áÕíSÜY±bEBºÔµ§NzáæÎãº´õ~dÂÆßñãÇ'óæÍoÅÈ¸eÏ=;,ÛÑÑÑ·ÙÑ'B¨)ÿÅ#'ã7lØºõ¹ç»i$òÔòK¾ïÈ `VIIIÜ²½½=Ù¡c«^ýõ¸WpðÃ?LGK¿x7wéiÂéè5îÙ³gìØ±É²²²dsàÝoóëe¾>|80¾w;Ü¿ôÚµk·nïzá'v;:Iä'iðå7aÂôm~Éf³;2¨¦¦&®MýLO¾qóvw)¿Þï°TöÇÜ7còººº8|'7µ±ðfKO%ÓÃ)ªftêÔ©>O$¤~_r7Þx#ùß+¯¼r7Ú³gO:ï¾ûnj|âÈ3gÎ$ÿ»ùõ~^xá»ßýn<ýôÓÙ÷ê©§bøí·ß>öl²w5FVVVÆp°ìÒ¥K10mÚ´ûß¬Y³bü¡Cnýò.O>ùä­pãû<D~úQ~]]]¡«Û¥¯wùöÙgÉÖø~¸QYYÙ1c6nÜxòëý.qíüùóS_4É8ÆòbÊ.]|6°U]]|YxáÂôÑ=Íì=Âñ°5551ñÇ7nýúõñoýò Öé:¨u§AÈO$ü$ID~$I"?I$$IÈO$IäwÏýøÇ?>þüÿÒ.üÇüåFQëíÌE±ZH#ýâ¿ø×ýWóAI?ÿùÏÿó?ÿüLú§øà_úÓþôÒ¥KeEÿò/ÿrîÜ9óA·nö/VænÝ>>åßþíßJ:xð`ügüÈOä'òùüÈüD~"?ÈüÈOä'òùüÈüD~"?ÈüÈOä'òùüÈüD~"?ÈüÈOä'òùüÈüD~"?ÈüD~"?òùüÈOä'òùüÈüD~"?ÈüÈOä'òùüÈüD~"?ÈüÈOä'òùüÈüD~"?ÈoxÈïøñã³gÏ.,,5kÖÉ'ÉOä'òùürV~S§NýàbàwÞ6mZ¶ü8pm`ûÉO~òñÇ_®]ûog>(ÕB¬ÌE/^üáh>(éý÷ß¿råÊÿÒa,¿ô³å÷Úk¯ýd`ûÁ~ÐØØøé'?ihhÿÙbµ+óAÑüãïÿûæöïßôèÑþ¥¹ ¿'N¬]»ÖÞ^ÙÛ+eo¯ìíÍÙ½½I7nÜ¨®®noo'?ÈOä'òËeù]¼x±¶¶¶[lÈOä'òùåü/^ÜÚÚÚíµä'òùüD~¹#¿iÈOä'òùå¬üzüD~"?ÈüÈOä'òùüÈüD~"?ÈüÈOä'òùüÈüD~"?ÈüD~ä'òùüÈOä'òùIäG~"?ÈOäG~ä'òùüD~äG~"?ÈOäG~ä'òùüD~äG~"?ÈOäG~ä'òùüD~äG~"?ÈOäG~ä'òùüD~ä'ò3D~"?ùüD~"?ùÈOä'òùùüD~"?ùÈOä'òùùüD~"?ùÈOä'òùùüD~"?ùÈOä'òùùüD~"?ùü$òùüÈOä'òùüÈüD~"?ÈüÈOä'òùüÈüD~"?ÈüÈOä'òùüÈüD~"?ÈüÈOä'òùüÈüD~"?ÈüÈOä'òùüÈOä'ò#?ÈüD~"?ÈüÈOä'òùüÈüD~"?ÈüÈOä'òùüÈüD~"?ÈüÈOä'òùüÈüD~"?ÈüÈOä'òùüÈüD~"?ÈüD~"?òùüÈOä'òùüÈüD~"?ÈüÈOä'òùüÈüD~"?ÈüÈOä'òùüÈüD~"?ÈüÈOä'òùüÈüD~"?ÈüÈOä'òùüÈOä'ò#?ÈüD~"?ÈüÈOä'òùüÈüD~"?ÈüÈOä'òùüÈüD~"?ßP+V6mêêê"?ò#?ÈOä³555­ZµjäÈ#F¨¯¯'?ò#?ÈOäÅ[ó£>/©²²üÈüD~"?_NÕÐÐPUU5â¿7zôè-[¶´··ùÈOä'òË-[a¾ÒÒÒººº|^ÈüD~"?_îoÄK,Iß·2_[[åüÈOä'òùåBa¾í|åååÛ·og>ò#?ÈOäuuuíÝ»wÞ¼yæ4iÒ¶mÛ®^½j ?òùüD~¹`¾úúú)S¦do×®]ù|Ð>ò#?ÈOä;utttk¾³÷î¸ÖóN~ä'òùümmmñn[ZZa¾ÊÊÊ0í|äG~"?È/ä×ùæÍñDùüD~R.È¯'óUUUüÛ.ùùüD~"¿~©¹¹¹¶¶vÔ¨QÌG~äG~"?rV~ù2Èc¤ùùüD~Êùõb¾¸ÊJ~]kkkEEùüD~"¿[¼.[¶ùÈouôèÑéÓ§ÇH~"?Èï¯ªª*ãÃ|£Fb>òä.]ï¬½Èïõ×_ÿéÀöÃþ0^0?~úÓýèGæ¢X-ÄÊÁ|PtüøñÙÉ÷Í/~ñæ=zôþáZ¡õGßÿþ÷ÿáþaé0ßMbÏòû¿ùK[¬ß?úè£KÒ¥K§ng>(ÕB¬ÌEçÏ?tèÐPª.ìØ±#û$¿ò+¿òüóÏÿüç?÷ÄõSñßþ¥¹,?eo¯ìí½½½ÔÓÉvKKKãm´­­ÍSfo/ùÈOä§a/¿ÌWQQÁ|äG~ä'òù)Gä#a¾P`XÐÉvÉüÈOä'òS.È¯§¯1ù'ùõùüD~"¿¤Ë/×ÕÕ1ùùüD~Êeùµµµ=ûì³£GÎ0_ee%óùÈOä§ßåË·lÙ½¯ªªjàßE~ä'òùüú¥æææn·ó-Y²ùÈüÈOä'òSÈ¯©©©¶¶6ãd»Ñ²eËéûvÛÚÚvíÚõGôG/½ôÒÙ³g=5äG~ä'òùiØÈ/Ì·jÕªló=úè£Ùï/_®¨¨ø­é¿"|dá#cÇçw<;äG~ä'òùi¨ËïìÙ³k×®Í0_\³fÍéÓ§»½ËW¾òlþÍÉ¿?¨ýÑ£G?ÞD~äG~"?¨ü;½/1_sssO÷êêêúÕ_ýÕ_Û_ü«¬¬|óÍ7=AäG~ä'òùiÈÉ¯¡¡aÙ²eÙ'Û­««kiiéý¾mmmeeeéì-Ú¾»'üÈüD~"?!ùÅ[[eee¶ù6mÚt÷'Ûýüç?ÿÕ¯¦Ø÷Â¦***B ò#?òùü4$äJ3gNùÊËË·mÛÖÞÞ~Oµ÷î²²²¯ó=ý¿9sæoÿöo;ª3ùùüD~|ùÕ××?ôÐCÙæÛµkW¹¶ÿþ¹sç~îsûÂ¾°qãÆµ£ÈüÈOä'òÓ_¨nçÎÙæKN¶ËjäG~ä'òù)ä×ÑÑæáeoæÌ1ÞnYò#?òùüòkkkÛ±cGEEEù*++ëëëü®üFÜ©òùüD~)óÅ;WiiiÆÛå9sÂfùuùÜ©ÂÂBòùüD~---ÝoáÂÇ3ÓÈoxÈoÐ#?ÈOC ÍÍÍµµµÝl·©©Éì"¿Üßõë×~úiòùüòëÖ|½lWä7läWQQQXXès~"?ÈïÍ7ßìÖ|1²íüüfÍýõ²²²þ>ùüD~RÅ»Ò#<ñ8jÔ(æ#¿_QQQ,Ù­­­ååå1àûîw¿k×®%?ÈOyb¾ªªªìíÆ[ÕÝlWä7<ä,ß1ÔO>ùäæÍ1PRRB~"?OäSò;vl,åGwÁxñÅGuùü«uuuu²ÝxOfó)å·qãÆÔ÷92GN~"?rÒ|Ù'^«¨¨7¦Xîþ¼½"¿a)¿èßøÆ¸qãbàøñã1;wnO:ùüD~È:::º5_Ixí.ÏÛ+òÞòÈOä'òÓÀÔÓ×ÒÍD~"?òùüûæ#?åü¦MÛÅE~"?å¶ù*++»5ù)_ä7uêÔtí¥òÝ^ÈOÃ±äÄk£FÊ0_UUÕßwÈO¹/¿@^¼N8qóæÍtòùüô`uö×îÒ|ä§|_YYY¼*ä'òùévöìÙ5kÖôm;ù)¿äwòäÉxm¬_¿þÆä'òùixuúôéí|q1Föá]üûò&N8"+ßðùü4ÄÍ·dÉlóÕÖÖ677÷í1ÉO¹/¿É'ûÈOäg>£í>XóòE~É¦©©i'üD~"?õÁ|Ë-ËØI5jÔ¨gö>ÍG~Êù?Þ7<D~"?óa·wïÞªªªó92Ì×ÒÒò ~ù)÷å×ØØ/7vttÈOä§!h¾ÊÊÊóÖÕÕ=ðW1ù)÷å7¢|ÃCä'òÓ ÖÕÕõæoÎ93Û|ñÒÖÖÖ¿üûò+è!ßðùü4Xæ«¯¯2eJùÊËË·oßÞOæ#?åü+òùüQ¨.lWQQa¾³cÇöööþòSîË/^N'O>ö,ùüD~¬®^½ï¥¥¥æ2eÊ®]»À|ä§|_aaa¼´~ÒÉOä'òÓ­ÛÛù6mÚ4zôèóÍ3§¾¾¾««k 'üûò;|øp¼À¶lÙrýúõ<¶ùüD~Ì÷Ío~3ßî¼yóöïß?(D~Êùùn¯ÈOäG~ÜåË·lÙ½o7Ìïµ¼ü_òóÝ^Èü¬óçÏw»owÕªU6)òS>Êo°"?È/¯jnn®­­Í8Ùnôøãü·ÉOäG~"?úkÝm¾Q£F­Y³æôéÓCjRÉOy!¿ÎÎÎ¥KÇK±¤¤ä±Ç¯zÈOäóÅz>ûd»a¾M6?~N0ù)÷åKy·ßðèïÓøÈOäoæ+--ó]¾|yÈN6ù)÷å7úôx5._¾üÆqñúõë+V¬1?ü0ùüD~º§ºººº5_ÿlüD~÷PQQQ¼&Ó¿BßÙÙcb<ùüD~ºóu²Ýaa>òS¾È¯   ^¡½Ôã¨."?îÇ|1fàOÂA~"¿;ìí]¼xq²·7~Æp=6ùüD~ê¥1ù)_äÔëö×®]#?ÈOÝÖÖÖ«ñìpôÙ|ñüîïþnÜáÂßúÖ·ÈOä×_òKôÇlÌ1ñsñâÅ1¦¿'üD~"¿_eee·óç;ß7nÜòåË×ýÑºÕ«Wþóé¥ÈOä×_òÈOä'ò^µ´´tk¾ªªªûYÇóûOüþæ?ÙüûãgþxôèÑñëÈOäG~ä'òùt=xí>Í¯ÄÉ'§Øüûâ¿¸ÿ~òù=0ù¸Sä'òù1_ÿ/õ+&M!¿Ù³gÇ.ùüüzüD~"?Å$Û|q±¦¦æ¯½ý×ýñÇO±oí®=zô êü³òë©ç.yy¿óÎ;ä'òùåaÝx-ÌlnnîßxìØ±ÒÒÒGyä«5_ýßù²²²ï~÷»ò·òH~'O,))I¶á§ØüD~"¿<éôéÓl¾TçÏß°aCüö'|rPöóòK~+W®L^áI'?ÈoètìØ±ùTä§Üßï½¼Â/_>N~"?ßPhÿþýÙæ+--­««Ë+ór_~±|?üðÃÉ9><ÀN~"?ßà¶wïÞyóæe/ÖÏmmmù¹0rV~¯¾újò"_½zõ L:ùüD~RWW×îÝ»³ÍWVVöÍo~3oÍG~Êqù9Èüoòó½ùæS¦LÉÞÎ·mÛ¶<7ù)ÇåWp§ÉOä'òËóíÜ¹3Û|ÌG~Êùzä'òù@ííí;vì4iRùzè¡°` ?ùüD~Ã¾¶¶¶íÛ·f¯¾¾¾««ËSO~"?òùürÖ|3gÎd>òùÈOä#òkiiÙ´iS¶ùæÍïaÌG~"?òùürA~/_Þ²eK¶ù.ð+[òùùüD~ýRKKË×¾öµÑ£GgoÙ²e¶óÈïö=ÏOäG~æòknn^·n]·æ³üD~ÿ­ôC÷u+?Çóùül±ôÖÖÖ92cÕ½jÕ*6ùüzëÐ¡C±²xâ'nÜ¸ãç+bÌ#GÈOä'òj>úñÇÏ0_=Ö³I~"¿;TVVkôtvvÆòòòû|ä'ON0¡°°pÆ&?Èï~ufUUUÆF¾0_MMMSSçüD~w÷·íeÈïþ?çW]]ýöÛoÇÀk¯½¶zõêlùýð?lØ;öïÿþïíRûÏ~ö³3gÎbµ+¡<±¶|äG²Í÷û¿ÿû?ÿùÏ=°ÖÖÖóAIï¿ÿ~[[ÛÿÒ~ßøñãcJÿèÜºåËÇÿ[oÞ¼P²¢¢"[~Û·oÿÑÀOa¬@$ýèG?¸ù (V±rÙ´iÓ¾ðó×ÔÔ|ç;ßñÜ=ð>¼ÿ~óAI±0Ä"1À¿´ßåwäÈn¿áñáÞç#§G$ûû"ööÊÞ^ÙÛÛS]]]õõõS¦LÉX3[ZZ<kööÊÞÞ¾÷É'L:5þYPPPRR2öì+W®ÜÿÃ¦ï/.**"?Èï~Ì«Í¶¶6ÏùühãÇO>>?³÷ÈOä^GGG·æ1»víb>òùuùÕÔÔ¼õÖ[1?«««ÉOä'òë¶P]¬³O¼æ:	ùü°üÎ93mÚ´dÿìøñã÷ìÙsÿôèÑòòòxÌ	&?~üD~"?æ#?ß Ë/9súÛá×^­_'üD~Êgùõd¾ªªªÝ»w3ùüúK~ååå±®9sæLJ~Çá1cÆÈOä÷ÀkiiéÉ|N¶K~"¿~_²ÆIùÝ¼yÓyE~"¿^sss·'Ûe>òùü#9'ÛùB~Ï?ÿ|güD~"¿h¾äÄkÌG~"¿_ccc·GrþàÈOä'òë'óÅÈ¸ÊÌ'?ß@Ë/YëÍ;7ùnoqqñ´iÓ`D~"?å¶üâ1üD~CQ~ùü«ò[UUUöI8êêêüD~,¿Ô;R]¹reÚ´i&M"?ÈïÏ×ÈOä7tå×ÙÙé»½"?óÈ/wä7iÒ¤½6vìXòùüz¯«««¾¾~æÌÙ'áØ¶móÈo¨ÈïÓO?-¸]êìéûÞï=òùüz7_Ï×ÈOä7ä*×ß;vÉOä§ß`ïêÕ«,òù×ÈOä§a'¿öööXw°ù6oÞ<fÌñãÇ;vÝºu6(È¯ïuvvN6­¤¤$5fÜ¸q/½ôùüD~©:::vîÜYQQa¾9sæìÞ½»_)¶uëÖ/|áOÿï§7ÿÉæg×?;úôßû½ßó¬È¯M81ãë½ÉêlË-ä'òùµµµõd¾½÷ö÷æ·öööâââ?~æÉ¿çÿçKKK-½ä'òëcEEE±kjjJ9yòd3fùüÏò»|ùò¦MFa¾G4V_³Ë5VÎÓ¦MK±/ù÷¥/)Ðé#?__J¾ÞóæÍÿö¸Yù#?òG~½ïôéÓ9m---/lz!]~3gÎlllôÄÈ¯/M0!Vg6lèèèHúÍ7Çòòròù)ßäKE¶ùíÖó[¿õ[¿ó?'Å¾Õ«WÿÚ¯ýïùÈ¯8q¢Û#9?~üD~Êù555­Zµ*m¾Á=Ùn,¨ãÆûâ¿ø¯|¥²²2Øgùüî«/Î1£¸¸¸   ¤¤dúôé1¦¿'üD~"¤/^<Íª¥¥eûöík×®åWÎ?ï)#?ßðüD~ôºÝÎ7jÔ¨ººº!b>ÈüÈOä§û*u²Ýló=ûì³---fùI¹,¿3gÎ$sN¾Ï;~üø=öÈO¹j¾ì¯3ÈOy!¿C¥Öüá×^üD~Êyóýë_?räY$òS^È/Öz±î;sæLJ~Çw$gr¦nÍWZZºûöööö»?o¯ÈOä7ìå¬o¥½ùæÍ1~"?ëÚÚÚbUÂË0_(p×®]qmr3òù)ä7~üøäèü:;;þù®¨¨ ?rÏ|õõõ'^#?òH~ÝÉù> ?rÛ|ä'òSÞÉ/YëÍ;7ùnoqqñ´iÓà@Vä'òÓ­¥¥¥[óUUU544tk>òù)å7(ÈOªøÏjmmmÆÁùóÝÍzüD~"?òù)÷ÍG~"?å£ü>þøãÉ'%31cFkk+ùü4ìÌl÷^G~"?åüºýÇÙ³gÉOä§!X¼2_ß>£L~"?åü#9WWW···ÇÅ7n¬^½:ÆL0üD~RÅJ£ªª*ã¿©÷c>òù)ïä¬:Ó¿õÃ©£:ÈOCÖ|¥¥¥uuu÷,òù)älóëììL_îmóùi/Ö!©pÈOäw·%ó«®®Å=.^»vmáÂ>ç'òÓàÖÕÕµ÷îþ6ùüwòq§úi·/ùüÔùêëë§LÒûÉvÉOä'òëKwª°°üD~:::Þ|óÍnÍ×Ó×ÈOä'òÈO©BuÛ·o¯¨¨`óÈOy'¿Ú|ñâEòù©¿ëèèØ¹sgöv¾ªªªÝ»w÷·ùÈOä§¼_¬a¿ñodf£ºüÔ¯µµµÅz ¬¬ì~N¼F~"?ß½ÂUíøñã¯¿ýío'+ß~úxùüæÛ¾¶ù*++;6ðÓC~"?åünÝÞÂ¬v'L¬X±¢¿'üD~yØåËëêêJKK³·óùÈOä§|_täÈÔ*xçÎ0éä'òË«ÚÚÚ¾öµ¯=:Ã|Ë-tuÈOù%¿gy&Y'çóxâ	òùéÔÜÜ¼nÝºìí|«V­Äí|ä'òSÊ¯°°0VÁcÆ9uêÔ­´ÏùÈO÷ÓéÓ§kjjF¾¸X[[Wé$?òH~±"~á2FÆzÙwE~êsgÏóeläKÌ×ßg$?È¯·z:_üD~9Ù±cÇV­Z±oôèÑa¾æææ¡9Íä'òSÉo°"?_¯¯ªªªí|£FZ·nÝåËòÈO¹/¿X#§ïÏíý"ùüÔKñZ^²dIöv¾¡o>òùüÈOä§»ª««kÿþýóæÍËÞÎ/ð¶¶¶áòÈOäG~"?õf¾úúúI&e¯¢¢âßüæÕ«W×C~"?ùüÔMmmma»lóÅøáøGÈOäG~"?e/^¶Ùd2eÊo¾ÙÞÞ>|ÿ4òùüÈOä§ÿª¥¥¥[óÍ9sçÎÃÚ|ä'òùÈOÿUsssmmmÆví|±BìêêÊ?üD~Êùõùü/Û|UUU¹d>òù)_äWp§ÉOäÇ|éæø×,ùüD~F~ùüZñÉ6_©©ÉUóÈOäG~"¿¼+^Ù'^óåíÈOäG~ä§ÿ¿öööÆÆÆxÅ?üüD~"?òSÎ¶wïÞÒÒÒÙ³géK_*))Ù¸qc·_G ?æ#?Èü4¼kjj7nÜÿZó¿6ÿÉæø÷6üßøßø¿øòKÞ½w¶ùËuuuùf>òùüÈOÃ¸uëÖ-þíÅ	ûPû?ò»õËíN2%Û|ñzlkkËÛÅüD~"?òÓ°lÙ²e_­ùjºüâßç>÷¹<óÈOäG~ÊÁlóc>òùüÈüò¥n?ç÷çþçy(¿PÝ¶mÛ²ÍcvíÚÅ|ä'òùr¡ä»½ÿcÖÿ7o^IIÉòí»½¡ºxÅLÈ6_xüD~"?ò#¿|/9ßþýûù¦jNÊïêÕ«ÌG~"?ùr a¾-[¶0ùüD~äG~Êeù]¾|yÝºu£FÊ0_UUUCCóÈOäG~R.ÈïüùóÏ>ûìÈ#³Í7ð/.òùüÈüD~ýÒÙ³gkkk³Í·páÂýû÷rÉOä'ò#?òS.È¯©©iÍ5Ùæ[²dÉ±cÇ<­ä'òùù)äwúôéeËÈêÑGµoüD~"¿!QkkkEEùüîsÕ³dÉló­Zµª©©ÉSI~"?ßèèÑ£Ó§O÷'òùõ­ªªªð9²¶¶öìÙ³DòùüPK.wÖ^ä÷­oë¶x=?~ü¤ú§#·ÓvúôéW_uÒ¤IæûÜç>·jÕªüà¾[¬bå`>(:uêÔûï¿o>(éûßÿþ~8À¿tËï¿&±gùíÞ½ûüÀö÷ÿ÷?ûÙÏÎKçÏ8qâ§?ýéPªû·ûË¿üËßüÍßÌ0_QQÑSO=kO«X9>þøãøÏù ¤Äjyi.ËÏÞ^ÙÛª««kçÎ=ôPùJKK·lÙrÇuìí½½²·wpD~"¿»¬½½=Ì7eÊóÅË¤­­ÍE~"?ß_·$?_·êvìØÂË0_EEE¿zõª§üD~"?ò#?ùuttìÜ¹³¼¼<Ã|sæÌ©¯¯w²]òùü«üzüòkkkÛ¾¶ù.|ä'òùù)GäæÅ¾´´4Ã|óæÍs²]òùüÈü#òknn^·n]¶ùªªªlüD~"?ò#?åüÂ|µµµ#GÌ>ÙîéÓ§=ä'òùù)ä×­ù¯ÅUæ?ùüD~äG~ÊùÅ]SSÃ|ä'òùù)åtUUUöI8êêêüD~"?ò#?åüz2pÈOäG~ä§_WW×îÝ»üD~"?ò#?å²üÂ|õõõÙ'Ûe>òùüÈü;òëÉ|1f×®]ÌG~"?ùrA~¡ºmÛ¶uk>'^#?ÈüÈO9"¿N¼Æ|ä'òùù)wäÇ|ä'òùù)÷å×ÜÜ×íÉvüD~"?ò#?åüz:ÙnoàZÈOäG~ä§~óÈüD~ä§Ü_SSÓªU«üD~ä'ò#?å²ü;VSSa¾¸X[[ûÀOì+òùüÈü48òÛ³gOö×ó577Eä'òùù)G^ÌóçÏ/((H7ß¨Q£üD~ä'ò#?åH]]]óæÍËØÎæöÙg[ZZÌ"òùÈüíÞ½;ûÄk¥¥¥±(^½zÕü!?óAä'ò#?û:::Â|ÙÛùÆ·~ýú¶¶6³Hä'òù]]]õõõ&MÊ0_yyùöíÛ?üðC_ÝùüD~ä§a_GGÇ¶mÛ²÷ícÁkoo¿u×çíùüD~äG~¢êvîÜYQQa¾P`úÉvÉOä'òùkmmm±P=:Ã|sæÌÙ»woºùÈOä'òùk---uuu¥¥¥æ«¬¬ìÖ|ä'òùüÈOÃ¯Ë/oÚ´);ßÝlüD~"?ùixÔÜÜ¼víÚló=úè£wùN~"?Èü4Ì÷ì³Ï5*Ã|555§O¾ûÇ!?ÈOäG~ºÅÓW[[;räÈtðÅÅ5kÖôámüD~"?ùi(ÖÔÔôøãw²Ýæææ¾=&ùüD~"?òÓÐ*²U«Velç=zôÚµkûl>òùüD~ä§!÷zË>ÙnYYY]]Ý9Ù.ùüD~"?òÓàFeeeùJKK·mÛxíD~"?Èü4huuuÕ××gl·¼¼|Çd;ùüD~"?òÓ5ß¤IvíÚõ·óÈOä'ò#?Z;wî9sfùbLÿüD~"?ùiÌWQQa¾z¨¾¾¾§íÈOä'ò#?òN]¾|9ýlóÍ9sïÞ½`>òùüD~ä§~¯­­-÷ìíÎ3§¡¡a'üD~"?ù©_jnn®««+--Í0_UUÕ±cÇl;ùüD~"?òSÿ/ûd»Ñ£>:ðÛùÈOä'òùú¥³gÏ~ík_Ë6_MMÍ±cÇòÈOä'òù@---Ù'Û1òôéÓCd"ÉOä'òù@mmméó577©$?ÈOäG~z`Ïo²oíÚµçÏSH~"?Èüô`jkk«««kii²SH~"?Èü/ÈOä'ò#?ÈOä'ùüD~"?ùÈOä'òùùüD~"?ùÈOä'òùùüD~"?ùÈOä'òùùüD~"?ùÈOä'òùÈüD~"?ùüD~"?üÈOä'òùüÈüD~"?ÈüÈOä'òùüÈüD~"?ÈüÈOä'òùüÈüD~"?ÈüÈOä'òùüÈüD~"?ÈüD~ä'òùüÈOä'òùIäG~"?ÈOäG~ä'òùüD~äG~"?ÈOäG~ä'òùüD~äG~"?ÈOä7¼äwüøñÙ³gÎ5ëäÉä'òùüD~9+¿©S§~ðÁ1ðÎ;ïL6-[~¿ØN8ÑÒÒòé¿øçþç>úÈ|P«X9nÜ¸ÑÐÐ`>()äÿà_:å^qqq¶ü^yåÛþýû8pP:xðû·3Åj!Væ¢÷ßßÂ TßûÞ÷þæüâ?Ók×®µ·WööÊÞ^ÙÛ+svooÒ7ª««ÛÛÛÉOä'òùürJ~#~YrñâÅµµµÝbüD~"?ÈoxË/½ÆÆÆÅ·¶¶v-ùüD~"?_îÈ¯¢¢bDZä'òùüD~9+¿Þ#?ÈOä'ò#?òùüD~"?ò#?ÈOä'ò#?òùüD~"?ò#?ÈOä'ò#?ùüD~"?òùüD~ùÈOä'òùùüD~"?ùÈOä'òùùüD~"?ùÈOä'òùåü¾þõ¯ïÞ½ûüÀö½ïïÿñÏKçÏ766þÝßýù (V±r0Åÿ	ÿú¯ÿÚ|PÒ_ýÕ_577ð/mkkËMù9sfóæÍ*I¤_ÖûÑ¶ÄJ$åIä'ID~$I"?I$$IÈO$Iä'I$ò´Z[[+**R;::jkk&MÔØØ~Ë'ON0¡°°pÆ6ëòya¸téÒ´Ìº|XÒñ+C·C¾-ñî0úôäåôèÑ¡¶f°fOR<aé/Î­[·¾üòË7oÞçròäÉé7®®®~ûí·càµ×^[½zµ¹ÏÃ=fZ^-©b=++C·C¾-eee~úiÄÏt5ùe¶téÒsçÎ¥?ó³gÏvãxv1ÐÙÙñì*ßX³ïÛ·ÏLË«å!éO>Y´h¡§ÁÊ!ßÉ'_¼x1âgÆ6¡°f ¿æKÚSXXXøê«¯Çó÷á¦ß,®êvXy¸0L:uñâÅq¹sç~ôÑGæ[>,I=öØ'2FZ9X¬òva8yòd¼MÄø±<5ùÝù),((xã7b ^±ñºM¿Yú9Ì·|^RÅòfÍe¾åÉü_þüùÙ7³r°0X9äíÂððÃ'ÄPX3ßÂñãÇ÷$ô¸ª³³óÖíÍ¶é7S.éy§Ï7û­[·¾úê«Ù7³r°0X9äíÂÐË½¡°f ¿;?Ï<óÌ»ï¾gÎ©¬¬L¿YMMÍ[o½ñ³ººÚ|ËçaêÔ©ÍÍÍÉë/^l¾åÉýN:3+CÞ.óçÏ÷hjjzøáÚüîü^¿~ý±ÇK>¢qîÜ¹ô=z´¼¼¼  `Â	Ç7ßòya8qâDò5þ&íU>¼Ù%×Î¸ÁÊ!o?þ8ÀÏxüá¡¶f ?I¤¼AªY ID~$I"?I$$IÈO$Iä'I$ò$IùI$ü$ID~$Iä'I$ò$IùI$ü$ID~$I"?I$$IÈO$Iä')gúö·¿½`ÁâÛ-Z´è½÷Þûoë¯ÛµmwS[QQZÆøSXX8aÂ7oÞëcJùI~mÞ¼yDVßøÆ7rI~[¶l;wîÌ¿cÇÿâ/öá1%ü$³N<¦),,|ýõ×»n÷ÆoÄÅyêÔ©ß'|#gÍ1~úôé1¾¹¹ü$¤Üï'Ó¼üòËé#_yå¹fÍt÷>|8ä(=v§n|íÚµµk×3&®*++Û°aCúNÕÐU÷=xð`¥bÌØ±c,XpàÀ¸XSS1aû÷ïïýq«b«:ÔÒ/^ã?óÁÄªªªÔ­[·ÇC¯òâÅÙòË~ü1½Lª$ò¤ÁlâÄ¡O?ý4äbdEEEºl2:qâDrí+2®Z¿~rUSSSAAA·÷J.&×>öØc7oÞ;uuuÅµñ3äTRR|ü®Çn'/û/çwÒ9ÂåÛo¿b_Æ,Z´è^å×Ë¤J"?Iä»Ý¬³F¥ËfõêÕ7nqqùòåÉµ	t;&îBlÉUÕÕÕÉÆÂ>zôhò éùÂ/í­O=õTÙ·o_ÇÏ~òÉ'ïø8¡Æ¸7H¶nÿ¢Ðä¸qãâïmmmñ3¦<¸ÙÙÙÜ`Â	qÇønýrïpqqñ½Ê¯ID~4øòº_j|"LÑÅãb)¹8öì¸8yòä§~:ÄöÙg¥$n±-u¯äâR7-¥vøÆÏnhh¸ãã2ãâ+WÒ§­§Ïä=÷ÜsqÕýÙÝúåí6¤ß òÎ;7Ù$y¯òëeR%$rÉ®7n¤looqU/ÖI¹ðÜ¹s	þRÐI8/'l¥Ò¥ÃeeeEEEñàcÇM]ÇÇéÉa9s&AjÇÏnjjJ]ôèÑn÷ß½üzTIä'IY·W_5dr¬ox¤>ØÚÚ½)ëÔ©S[¶lIö½¦ÈlKíN½£ÏÖ¯_ìäk×®Mïåqml.]J.^¹r¥÷ïáÎ??®Md`M¿*ùoèÐ¡ë×¯÷.¿J¹º¶ID~4È%ß(,,|íµ×£º¼þúëEEEÙßÆX¾|y(§££#Ùúnò9¿äãqçÎKÿ`Á6oÝ¦«Ò­Ï/Û&¿½±±15¾ÇI>Ø|Î¯½½=¹e/òã7R[ãâON¿*ùÈcSSSü	=É/±fX9nâreúµ½Lª$ò¤Á/9ÊqF[·ný¿ë¯Û%0J§ö¦¾Tê©§JgezöìéE~·nl#Æ¥ïîåqb2Ò',õ½ÚþØÏ>û¬¸¸8ù2öq/]º4ýW7.~&vIÌddªÄ©©kTIä'IC¢ÐÉ¢En·`Áä¶ò;tèPrØ¼3f9r$umûóÏ?_^^hé¹çëèèH]ðàÁ¹sçÈ&L°k×®ÇÌx¨ÿÌ3Ïdïéq¢¤ä@½Ï/ÕÚµk3ïÔÚÚZ]]s`Ì1ñW477§Pþ×¯_Ú&óªªªêøñã¿±ID~$I"?I$$IÈO$Iä'I$ò$IùI$ü$ID~$I"?I$ò$IùI$ü$ID~$I"?I$$IÈO$I÷ÞÿÃëx:Î`IEND®B`


ÅæÌóî»ïÞrý¿É°JJJÂïO2å7Þ¸sìØ±x<~ÿÐ¡C³gÏ.++÷5a7)O8Iä')ßå7räÈsçÎù½òÊ+¹îY¿~S»­ÜVÛ·o¿ÝsÇ±¦¦&¿ä'ü$Ý3`/^<$äwìØ±ªï¼óN*ºvíZÃÈ^'u»7Éºë+W®¬[·.	0ùE7¼|ùrîééÉ«§FùIÚòkjj?÷ìÙs3^|òÉ'K.644$¬ßÜ¿ÿ¨Q£æÎsàÀ©S§Õ××ñÅ2eJyyù£>¹qÓ¦MÕÕÕÑaÍpé«næ§z*Ï<öjnn#W®ëc¼ÝäÞuÀbfò¶N¯ûä2»³ö-¿¬kÃó2yòä°ØçÏmßs¯Í|ZCmmmaja9g6¾ajXÓáªªªª5kÖtuuyqIä')OåwåÊ1cÆ|öÙgáí<Ó"á7³VRR~>ñÄ×;9öìôÅ 4û²~sÞ¼yûfìØ±aü2GY&Lèõ1ÞîM²î:°&Úç7qâÄÛZ8îoØù?~<s:áÁÞ®ü2ÖèbºcÇE¿¼xñâ¬y~î¹ç¼¸$ò§òï¿ÿ~æ>°L<óÌ3axÑ¢EoÂÅ02ó7_zé¥k×®E­¢1Ï>ûl*:|øpt1L9¿nmß¾aøÌ3WÝÌ7eee½Ï¼í]Þäf¤Kï½ÝÓëñûø_ÖØÂpCCÃÕ«WéÓ>ë¿ü2Ö00æàÁa8zZ/_ýr$ÂO?ý4g31ÂK"?Iù+¿Px#ÃáM=kü1cÂðg]oðáâèÑ£3§¾6=&h#óâÅ¯ÿÇaÓLpôôô+aÌ1#ÚÉtKù÷ú@z7ÉU¸m@L<okkëu¡õgáô:Ww|ÃþË/ú,ã_|y·%¿Ì§5ZfaLtÕ´iÓÂÅñãÇ¹ÿB|ùå^YùIÊwùUUU÷ïîîîÌñ¹ûÌr?ôH×ë4û¾uÄóßQ­®®Oçò.¬©õç&7[&·ýY87óèÝ°ÿGûc»¾¯ÍZ³Ny]uúôéi¶¶¶zqIä')¯åúÑ~.¦Ïpµw*ì ú¾8yòäèì*2ëü¢Ñ	VÖ¬Ysùòå;wK.íU~ý¹ÉËïvNº;¾aÿå×~iÛ÷÷½üGÆdÑ8³'NlÜ¸1:Äþ´$ò¿ò»~ãÓúéòGc¢oþ.Z´èÊ¢·ögöîåí÷êèèþxã7ú#¿öööh¯RÀâáÃ£ýyQï¿ÿ~¯ñvor[òëáDK²ëFY¹Ý¥zò>àûå_Ø-X° ×¯¿þzXøAÁ/ÿhÃï%§Nù0£Ïk>º#ïÈOR~ÉïÜ¹séOtEcDÖg¼ÆþªìÝÈ/m¨Ñ£Gßoíu¿ýíoçy9sfó¶nr[òëá¤OÈu6æ;Xªw ¿'NdNîÜ¹×f-`µ¾å±×¯¼DU3úé§½¸$ò4äfÿøã.]Z~£ÜïsÜü.^¼¦¦ôüóÏ¥OÒ·o¶mÛ6eÊ±cÇ¾õÖ[ßüæ7Óûîþ&·%¿¾Îûï¿ðWVV>%^f·µTï@~¡ÖÖÖñãÇG¦OO9>î==zôûïß?cÆ0µð vìØßÕÕõÂ/D;SßÃSÙÝÝíÅ%$Ýzzz¾ýíoßÖ9äîà&C®è lôeíT*~òäÉVIä'IVô9¿¬¾ûÝïZ2ÈO­®®®uëÖ;6:¤^yåEùI$ü$IÈO$Iä'I$ò$IùI$ü$ID~$I"?I$$IÈO$Iä'ID~$I"?I$$eöþûï×ÖÖÍ7ïvo;eÊaÃ>:=&1S§N½ÍÙîìwúsÛ;ÙÂÞ¤Ì«JJJ***.^¼h-D~FÕÕÕÁ1/©Têvo»iÓ¦pÛ­[·¦Ç¼öÚkaÌÆ´üú~æðÀÃðòåË­EÈOÒÙÜN8n[__3öì0æøñã3+¿`å0-D~û25óÂ/9rÄë×¯Ïúµo~ó3fÌÈBza¾páBc¢«>ýôÓx<`TVV6mÚ´ö:µÌïû&o½õVmmí9sÂå:ìêÕ«á¶aæ×­[íÂ6;wnZ¦üÙgÝ'ùüñÇ%%%Ñ#ºvíZùp1´I"?Iy¿hø;ßùNÞ±cG0Vøîw¿ù;û÷ïÏýLÛ3Ï<®Ú¹sg?ÃðÊ+£«fÎ¹oß¾0pæÌ0¾¦¦¦×©eÎ@ß7	ûÑ~rgþ¹çÃáÞï½0°iÓ¦0rÜ¸qaøÊ+a Ú%ÙëønW~waùá+VDWmÜ¸1Z¯¿þzxåW¬]ÈORþÊ¯¶¶6÷Ü(^½~°½½=hÑ¢0¼xñâtékO8ñÒK/Í1#úJD¯SËÒUß7fläÈ¹·3fL4óÁda /¿§M,ÛÝÝ7'sL4oaâÑUá®'LfcÄ'N­]ÈORþÊ¯¬¬,=ÂÅ(]`VNøÍ®®®è j]õÆo[~ôÑGSÈZæÅþÜäf3(¹/Rã=F©ªªvÞ~½Á%y¬ÈOR>Ê¯¦¦&s_´ÛìÜill×¦¦Ç1×nÔOùõôÌåÞvôèÑÑm³f/JíÛ·¯©©)sgá,[.+W®3¦¡¡aîÜ¹AÃ_|ñµKùIÊ_ùEgiÙ¹sgô9¿×^­?òÛ³gOzçÙ~ô£ôøÈ§N>ÿ×ùõ^zéÇ?þqxægroûôÓOá·ß~»³³3:ÂFÎ3'ôÑGÁaa`âÄ÷O~kÖ¬	W8qâðáÃF$òòK¥RAW#nyN¾¾å÷å_FGZÃÏôÞB­­­UUU#Gn]?å×÷MÚÚÚÂµ³gÏNÑ$ówººº½Â/X° úo_CCCôeáúúúÛý²mÿå´y¹sçV0Iä'I$ò$IùI$$IÈO$Iä'I$ò~ùË_=vïô³Ï>û×ýWëBod9(6ÑÉ_¤û·ûÍo~c9(êÿùÿýßÿüîMñð7ÀwúÿøÎ§¯¨ú§:ú´å ë7Îö6®ß8-åßÿýß[Ú¿øÏùÈOä'òùùüD~"?ùÈOä'òùùüD~"?ùÈOä'òùùüD~"?ùÈOä'òùùüD~"?ùüD~ä'òùÈOä'òùùüD~"?ùÈOä'òùùüD~"?ùÈOä'òùùüD~"?ßÐßÑ£G§MVVV6uêÔãÇÈOä'òù¬ü&LðÁwßwâÄ¹òÛ·oßåíW¿úÕ'|rYº|ù×7²°q°:wîÜÏþsËAQ?ýéO/0Àw:åYEEE®ü¶oßþ«íg?ûYû¯¤_ýª­­-üÏÞrP(lÂÆÁrPè¿üåO~òËAQ­­­à;-ù;vlåÊöÊÑ^9Ú+GåhoÁíºzõjCCCWWùüD~"?_!ËïÜ¹sMMM½büD~"?È¯pä×ÞÞþè£^¼x±×kÉOä'òùüG~µµµÃ2"?ÈOä'ò+XùõùüD~"?ùÈOä'òùùüD~"?ùÈOä'òùùüD~"?ùüÈOä'òùÈOä'òÈüD~"?ÈüÈOä'òùüÈüD~"?ÈüÈOä'òùüÈüD~"?ÈüÈOä'òùüÈüD~"?ÈüÈOä'òùüÈOäg9üD~"?òùüD~"?ò#?ÈOä'ò#?òùüD~"?ò#?ÈOä'ò#?òùüD~"?ò#?ÈOä'ò#?òùüD~"?ò#?ÈOä'ò#?òùüD~"?òùIä'òùÈOä'ò+¬víÚ(ãY&?òùüÈO,¿¶¶¶éÓ§6¬®®.Jy¢ÉüD~"?©å×ÚÚ:gÎaµ´´x¢ÉüD~"?© äwäÈúúúaÿµêêê]»vy¢ÉüD~"?©@äÖçx<^ZZe¾mÛ¶uuuyÉüD~"?òS!È/¼±óeíç«­­Ýºuk2ôüùü$òS!È¯WóUUUw[æ#?òÈOä§_ggg®ù*++üÈüD~"?üDSSSÖçùb±ØæÍÏ?ïÙ$?ò#?ÈO ¿Ï~>ò#?òùüT8òc>ò#?òùüTøòëÕ|áâªU«üÈüD~"?üÎ=Û«ùÂÈÀAÏùùüD~*ù%ÉðvYYYÉ|äG~ä'òù©`å/1ùùüD~*Xù1ùùüD~*|ùõj¾Pcc#óùÈOä§ßÍÌÇþ½RäG~"?Èï¾J¥¶lÙÂ|äG~ä'òù©åÌ×ÒÒRWWÇ|äG~ä'òù©`åÇ|äG~ä'òù©(ä×ÚÚÊ|äG~ä'òù©Àå1úô,ó¶µµYòäG~ä'òù©@äÞïz5_KKK*²ØÉüÈOä'òS!È/¼ÓÕ××3ùùüD~*dù<y2goÜ¸qÌG~äG~"?G~Ë-Ë2_uuusssWWL~äG~"?A~'OdIùb±Ø-[üÈüD~"?ü:::JKK3ÍWYYùâ/&IüÈüD~"?üÂ$×|±XlóæÍÌG~äG~"?D~DbõêÕÃÏ4_¸h?ùùüD~*ùþùç¹æ+--]¹råùóç-Fò#?òùüTòK&6l¨¬¬Ì2ßªU«.]ºdùÈOä§B_ww÷-[b±Xî~¾D"aÑùÈOä§B_*Ú±cGmmmÖéZ,YÂ|äG~ä'òù©@äL&Ã»Uuuuù-[fB~äG~"?D~ÝÝÝÍÍÍ¹æ«¯¯?räE~äG~"?A~hii7nùæÌÞ¶üÉ]ò#?òùüTÕíÜ¹³¦¦&Ë|³fÍjmmµ|ÈüÈOä'òS¯¥¥¥®®.Ë|aÌÞ½íç#?ò#?ÈOR[[[®ù&M´÷næ#?ò#?ÈORxÇãYæ«©©Ù±cóüÈOä'òS!¯®®nçÎ°|D~ä'òù©Í×ÒÒJ¥úùwE~äG~"?ùb±Ø-[ÒÇvÉOäG~"?váùíÕ|á(Lfþ&ùüÈOä'òÓP-H455ÞÒ|ä'ò#?ÈOÅb>òùÈOä§b1ùüÈOä'òÓ7_¸¸aÃ[üD~ä'òùih/Wõ:ä'ò#?ÈOù[2jÕÝüD~ä'òù)¯ÍÞJb±Ø=1ùüÈOä'òS±üD~ä'òù)¿êîîÞ¸qãý0ùüÈOä'òSoëÖ­ÕÕÕY#wvvÞ«!?ùüD~ÌR©TKKK]]]®ùîùûùüÈOä'òÓ`¯¶¶vÌG~"?òùü4híÝ»w`öóÈüD~"?Z»wï4iRù¦OÞï÷]ÈüD~"?P÷î5kV®ùÂøT*53@~"?òùütßkoo¯¯¯DóÈïtñâÅÚÚZòùüÔùæÌe¾ººº]»v¤ùÈOäw·>|xòäÉáL~"?r;yòäüùósÍ×ÒÒ2ðæ#?ßÝ¶`ÁðÎÚü~øÃþz`kkk;räÈ¯¥_ÿúþá:d9(6aã`9d»víúú×¿e¾zèå_ÄûðÃúÓzõü$üÿdïtËïÿÎâÍå·gÏO¶ðNêÔ©O¥O?ýÇYÍBØ8XÓÏþóÇ¬´´4Ó|<ðÀ¦M>þøãA½ßüæ7?ûÙÏ<MÚ·oß3gøNY~öÊÑ^9Ú[<uvv®X±"Ë|ÕÕÕáí LæÉL:Ú+GÉOä'òÓ]H$²ÌÅÂAWWW^Í*ùüÈOä'òÓ^eË-Ë2_eeåæÍóg?ùüÈOä'òÓÝ/w?ßðáÃ_|ñÅ³gÏæílÈï~E~"?_AH$V¯^µ/ïüùóy>óä'ò#?ÈOý5_¯ûùV®ÏûùÈOäG~"?n£d2¹jÕª,ók×®ýüóÏÐ!?¬üÝªòùütKóíy,Ë2_SSS"rüT°ò+¹Ueeeä'òùéÌ7TíH~ùüD~g¾¡¸üD~×¯òÌ3ÏÈOä§ÌR©Ô-[²ÌÇãðï­üÂâÚ½÷ºuë^~ùå¶¶6ëùåüjkkËÊÊ|ÎOä'òSiii©««Ë5ßÀoÌüÉä×¿þõqãÆý÷ÿ÷¿Ïÿæ?ôÐC+W®ÑºD~,¿©S§æ~½£ªªê~ÿEòùü¯å×ÔÔÞa_zñ¥õ/¯ÿÖ>¿öw~çwÞ~ûmkù²üÊËËÃøâÅÕÕÕa ïÇ?þqÿ5!?ÈùÄ|÷ =ôÐ<û'û¢ßZü­õüY~ÑË8ê3gÎv-1üD~"¿¢m÷îÝ¹æcûój÷P~>ø`z_ôïÉ'p¡UüY~£F/æÃwÁ0ðÊ+¯DÎê"òùgaãÇsÍ×ÒÒRðS»ò7o^p^¦ü¦OÞd­`ä7Èò[·n]úû/ò9sæÈOäÇ|Eb¾.¿ÊÊÊ¾ð¿_XûüÚßóûµµµ.]²ß Ë/ôê«¯=:=z4Î1ã~Ï:ùüD~ÌWÀòð7oÞ¼à¿X,öøçÏ÷r#¿¼ß D~"?_>®ùTvìØQ§ q&gùüD~Y"hjj*--Í2_Ø>'ÉâÈOE!¿'Fçvq&gÈùye ?¾ü&L©½t¾Û+òù1ùüM~yáeìØ±k×®ä¬ÈOä7þùç«V­ª¬¬Ì4_ àüTDò«ªª/þfùüD~Öùóçï¢­¦ùüT;~üxØ<÷ÜsW¯^%?È¯ºtéR0ßðáÃ³>ÉÍ|ä§â_hìØ±ÃròÈoèÖÕÕµeË¬c»¡%K<yÒN~*^ù?Þ7<D~"¿B2_î±ÝÐ²eË:::<Ýä§b_´EøÍùüD~÷¶K.Mk®ù,YâÈïÿ6fÌßðùüôCèêêjnnÅb¹æëììôÈï?koo[uëÖuwwÈOä7´î7ß|³×c»>ÏG~"¿Þ&q|ÃCä'òËçR©TKKË¸qã²¶ÞñxüÈ#Vòùõ^ÉMòÈ/oÍ×ÜÜÜ«ùìç#?_F~"?ß¯µµµ®®.Ë|Ó§OoQJòùÝºÚÚÚñãÇüGÉOä'ò»­öîÝ»oÖ¬YíííAGòùõ«²²²°íøY'?È¯µµµáåîçÛ½7óÈïö:xð`ØlÜ¸ñÊ+ynòùüîÌ|uuuÌG~"¿;ïöüD~ù'¿°?~ÖyÒ¤IÍÍÍ|.òùü|·Wä'òË+ùmcùª««wìØa?ùüjä'òùå/g¯¶¶6l0»ºº<Sä'ò#?òù©ä×«ùb±Ø-[üD~÷X~===,¨¨¨#F<ñÄðUòùüBD¢±±1Ë|UUUÁ|ÉdÒ³C~"¿,¿°÷úûý	bòù©ÈåÌ×ÔÔTZZµ/lüD~÷K~'OÛE]½z5råÊâÅÃ3gÈOäÇ|ä'ò+(ù-Næ÷ÅzzzÂ0üD~"¿0ßðáÃüD~$¿°Ý	ÚKéîîcÕEä'ò»ßæÃÈpgüD~$¿èhï£>í?Ãp3mÚ4òùüî¾d2¹jÕ*æ#?_^È/P¯×ox|üD~"¿»4_ØÖÅb1æ#?_¾È/ZÑxâ#G>úhs¿güD~*`ù1ùüòW~ùüTòc>òùùüTøòëÕ|¡x<ÞÙÙi9Èo0å7ìVÈOäwæøÈOä×K%7üD~"¿~J¥ZZZª««üD~y-¿õüóÏG­wßüD~"¿¾ÍWWWÇ|ä'òò;~üø#¢ÍVæÉOä'òc>òùü.]m¹öíÛ70³N~"?9ùÝÌ|sæÌa>òùù½÷ÞÑkÑ¢E9ëä'òÓÐßîÝ»sÍÆfþésÈ/OåÖï3gF_æ8xðàÏ:ùü4Tä¶þ³fÍb>òùaù½þúëÑÆkùòå2ëä'òSþË¯½½ùÈOäWòs>?ù©ùutt,[¶¬´´4sÃX]]½mÛ6æ#?ßÐ_É­*++#?P~¹æÅbÍÍÍ]]]ùü¤ü=òù)ßäH$r÷ómÞ¼9LZPä'ò#?òù©ä·wïÞÅb±6ØÏG~"?ò#?¤D"ñäO¦ÿpeÚ|ae?ùüÈüD~*ìÖ®]µoøðá«W¯¾téåC~ùÈOÐùóç7lØPUUk¾Ï?ÿÜò!?ËAäG~"?B.]zñÅ+++3ÍW^^¾lÙ2æù©åç|~"?ò+ªº»»«««37t¥¥¥MMM~øa?ÿn¯ÈOä7Tåyê¾^åç|~"?õ³³gÏ®[·nþüùüÇÜÖÖæ«­­ÍÚÊ566&ëýþ»½"?ß_º-àSO=uõêÕp1üxqsèÐ!òùéµ··=ú¹,_¾|áÂaøÏþìÏòÇ|7o7nùâñxæöüD~*"ùEqÎücD===ÑÉKÉOä§¾@½%O,Yÿòúèß<û'#F8yòä ÏXKKK]]]ù¦O»WüD~*"ùE[Ã ½,ùùÈO·,,ÃqÿmÑ¿ßÿýßonnÎCóíÝ»·×?¹K~"?üÆ¶au»ºº-ZÆñä'òÓ-å7~üø,ùãßùÝùÈOä§¢ß¡CzýÇGD~"?Ý[±Xìú£4ûÖ>¿öÁxHõj¾0f÷îÝüD~*:ùÎ93aÂ#FL6íÂ÷ÖÉOäWµ¶¶>ðÀó±UÿkÕO>ùÕ¯~uÕªU9aKR__k¾`Á[üD~*FùJä'ò+Â+ë±ÇûÚ×¾öÈ#|ÿûßï§·îùâñøÝüD~"?òù)ß»Wæ#?Q~§N8qâ#¢ïó3fÏ=ä'òÓP1_uuõüD~*:ùEgrÎümÑðöíÛÉOä§<7_,d2y7S&?H~Ñ_±<uêTZ~GÃ#G$?ò¡D"±páÂûa>òù©èämC£H~×®]ówE~Êó555Þ'óÈOE'¿èLÎÑ~¾ ¿^x!×ÖÖÈOm>òù©èä×ÞÞÞë?øàòù©°ÍG~"?ü¢­Þ3¢ïöVTTL81lyï÷¬ÈOý1_¸F?þþÝ/ùüTÈOä§tgÏ½ùà?¢ä'òSÉ/ýÅt.8qâ¸qãÈOä§û]2[ÊÊÊA1ùüD~×zz|·Wä§1_,DóÈOÅ"¿qãÆë³Q£FÈOm>òù©Xä÷é§Ü(ý×;2ìï½÷ÈOä§[WW×Æ£ÈgÖØØ8(æ#?E~éóî÷]òù)¯¹¹¹¶¶6Ë|ñx|à7ä'òSñÊo°"?_ÔÝÝ½k×®ÜOäùÈOä§¢_OOÏÄG3zôèï|ç;ä'òÓ]J¥ZZZr÷óÍ3'OÌG~"?üÆõõÞhÓ¼qãFòùéëu?ß¬Y³Â40¯füD~*"ùÍqGGGzÌñãÇÃ#GÈOwÐîÝ»§Oe¾ººº0>ßÌG~"?ü¢¯÷^»ví¿L7ç$wPdMMMYYÙ)S<H~"¿¯µµuÎ9Yæ4iRÞüD~*:ùMó5kº»»£~ýúõaLuuõ]N¹¡¡áí·ßÛ·o_¾|9ùü¸#Gä/Ù»wo>üD~*:ù;v¬×39=zô.§íJìéé©­­Íßw¾ó¿4Ä/äßýÝßÍÚüöoÿöêÕ«û,Iº­î»üBçÎ2eJEEEIIÉ#&OÆÜýd3OÊ@ûüdßP/¼ êëë³ÌþçÇvíó~*ê~÷¯ÌOÈ¯°ÍWWW×ÜÜÜÕÕ5äùüD~÷ 1cÆôôôq´7È¯êììpaùb±Ø;Ö~>òù©xåwêÔ©èdÎÑ^º ´=öÜýdßzë­0~644ÈoH×ã?^ZZi¾êêê­[·&É!ýÐÈOä§"ßÒñH~ÑðöíÛïrÊïa555¹ß!?ßP©³³3×|k×®ÇvÉOä§¢_ÀYØ:u*-¿ 4grù)H$³ÌWUU^¿C?ùüT¤ò6å×3ÎÞ|íÚµ0ûmùùi¾p±`öóÈOE*¿1cÆDgïä×ÓÓóÂ/D§f ?_q¯©©)k?_,Û°aÃç^üD~*"ùµ··÷z&ç>øüD~EÕÙ³gsÍ7|øð0òüùóüÀÉOä§"_´Õ1cFôÝÞ'ÿôßïY'?_þL&ÃK²²²2ë«W¯.Ôý|ä'òSñÊoP"?_þ/e¯´´tÅ'O,@~"?ùüÔ|MMM°×üD~"¿Aß'|2~üøòòò°Ý¯¨¨2eÊÅÉOäWTæ566ùÈOä§¢_[[[¯ßðèìì$?_J¥ZZZ¢³xfÇþõH~"?ß È/zhhhÎÑuõêÕåË1555ä'ò+0óÕÕÕ1ùüTÔò¶þj=§ÏêL~"?æ#?È¯päíóëééÉíóù°ùæÌÃ|ä'òSÊ/ú_CCCXÝÃÅË/×××ûÈo¨^_?üpù3÷ñüD~*.ù»U÷é°/ùüîùâñ8óÈOä×K%·ª¬¬üD~ÌG~"?_!Èo°"?å¹üÂÚò­o+°©¾¾þ?øÁP4ß¸qãüD~"¿ÿÒÍNÚ|îÜ9òSÑÊ¯µµuôèÑ-Zõ¿V-_¾ü«_ýêªU«òó$f/Xtª&ÈOä1aÃ^õÕ¬+V¬pV­üR©TÓ=õGë_^ý[ûüÚ|0ßþm0_SSSiii®ùÉ¤§üD~"¿^ÂïcÆ¹páB¸øÎ;ïDï÷éãä§ü_?~üø4û¢ßøÆ7üD~"¿¡-¿ë7öðEï555ÑÀâÅï÷¬òY~ãþÛ¸,ù=òÈ#ù ¿óçÏ¯ZµùÈOä'ò»«:~·7òSÞÊïfGÁ|/¾øbeee¦ùÂUPòùüúÛ³Ï>½¤ÿûSO=E~*Zù]¿ñx O¾áL&W¯^Ý«ù§üD~"¿Û¨¬¬,¼9òÄ×3>çW^^N~*ZùEkËc=öµ¯íGùþ÷¿?(çF¹téÒæÍc±XÖWw,YÂ|ä'òùÝÑ$é¥²F655ùn¯ [0_î±ÝÐüùó;::<ä'òùÝa7;ßýùüz-LnÙ²%ýÑtË-Ë·ÓÊÈOä7ôä7XÈ/«îîî­[·æïñÇïììôÈOäwçòo'Çsû¾H~"¿ûZ*jii©­­Í2_<'òùüÈüT òæóÍ7'Me¾Y³füü$òùÈï~µk×®ººº,óM>=lk<Aä'òùù©@ä×ÚÚúðÃ÷j¾A9ùÈOäG~"¿ßÞ½gÍe¾I&2ùüD~äG~*ùµ··Ï3'Ë|uuu»víb>òùüÈüT ò«z<g>òùüS~G~"¿»ïÈ#õõõY/®ÚÚÚ7ß|ùÈOä'ò8ùÜª²²2òùÝq'O|üñÇKKK³Ì·mÛ¶îînËüD~"¿ß G~*Tùõzl·ºº:¬ó]]]<ùüD~äG~*ùõj¾ªªª­[·ÚÏG~"?ùD~¹æÅböóÈOäG~ä§Â_"hjjÊú<_0ß/¾xéÒ%ËüD~"?ò#?ünf¾°n'IKüD~"?ò#?üüD~"?ò#?¾üz5_¸¸jÕ*æ#?ÈüÈO"¿³gÏöj¾02pÐò$?ÈüÈO ¿d2V×ÊÊJæ#?ÈüÈO+¿È|±XùÈOä'ò#?òSÁÊùÈOä'ù©ðå×«ùBÌG~"?ùD~73_<øuUä'òùùé¾È/JmÙ²ùÈO"?ù©åÌ×ÒÒRWWÇ|äg9üD~ä§­³³óµ×^c>ÈOäG~*ðZ[[ÇË|"?ÈüTÈíÚµëáÎ2_]]][[C~ùüÈORXßfÍk¾T*eùå òù¡öööÇ<ëOî;öµ×^c>ÈOäG~*:::.µ¯ªªª¹¹¹³³³·Wä'òùù)¯Ïõüùó³öóÅb±-[¶$Éëýû»½"?ÈüÈOyÝÉ'rÍ·qãÆÈ|Qä'òùüÈOC¸³gÏ®Zµ*Ë|Ã_½zõ¥K²~üD~"?ù©pÌ.®]»6s?ùüD~"?òÓîÒ¥K½oõêÕÃüD~"?ùi(oíÚµUUUYæ[±bE"¸åÍÉOä'òùù¶nÝe¾Ðã?ÞÌÈOä'ò#?Ió-Y²ävG~"?Èü§¥R©;vôj¾;[ÈOä'òùòÔ|ÕÕÕYæ[¸pá#Gîx²ä'òùüÈOùU¯æ3gÎÝ¯<ä'òùüÈOyQ*zóÍ7§Oe¾Y³fÝÍ~>òùüD~ä§ü2_KKK]]]î~¾ð×Þ«;"?ÈOäG~Ê;ó=üðÃ»víºæ#?ÈOäG~ÌÚÚÚrÍ7nÜ¸Ý»wßsóÈOä'ò#?Na5ÇãYæÜµkWww÷ý»_òùüD~ä§Ûþé;¶²²rÆmmm·4_KKËÚÏG~"?ÈütòÉ'æùÏ¬yý²e<ð@kkëàüD~"?ùéÞwäÈzèÿýB`_ôïþà~ë·~+Ë|±XlË-f>òùüD~ä§ß;õ"óýÿcìØ±¹ækB2øy#?ÈOäG~º½ùæsçÎæ>3õá©_ùÊWòÄ|ä'òùüÈO÷¾_üâeeeYæ+))ÈOä'ò#?ÝËDSSSiiiù|ðÁS§NåÃÈOä'ò#?Ýóå+_1cÆ_þå_ú®>òùüD~ä§ûe¾p1WåÛÜÈOä'ò#?ÝIÉdrÕªUCÅ|ä'òùüÈOwh¾ðTÆb±!d>òùüD~ä§b1ùüD~"?òSëîîÞ¸qãÐ5ùüD~"?òÓ­ëêêzóÍ7«««³þG<ïììZüD~"?ù©÷º»»wìØÑ«ùþy$?ÈOäG~äw¿ÚµkW]]]ÁüD~"?ù©vïÞ=iÒ¤,óM>½­­m¨?4òùüD~÷¸/ÖÖÖßP¬µµ5w?ß¬Y³S©T<@òùüD~÷²ÃO<9páfòûÛ¿ýÛÿ3°:tèÔ©SÿGöÃþpÆYæ8qâ_ýÕ_9s¦`fx§?~ü¸§[¡°YËA¡D"qàÀËAQ?ýéOÿå_þeïtËoÁ§OîC~;wîüh`kkk;räÈGºI?øÁ~ï÷~/Ë|5557nüðÃìÁþÃ<éÍBØ8XÿîÛ·ÏrPTkkë'øNöÑÞÿo.?Gó§°dâñxùª««wìØQÇví£½r´Wö®9rdÙ²eYr·ªªª¹¹¹PÍG~"?Èï9/üò¿%KdíçÅbÛ¶mëîî.øO~"?Èï¾Xüò­³gÏ®X±"k?_0ßÖ­[/]ºT$üD~"?ùxDbåÊYæÛ²eËùóçjQÈOä'ò¸Èo°[½zuïç#?ÈOäG~^2Ü°aCÖ~¾pqóæÍÅ¶üD~"?ùl.]æ«ªªÊ2_ùùçùëüD~"?ùNañÆb±Ló>|õêÕgÏõb&?ÈOäG~U2LË/¯©©É~>òùüD~äW°mÛ¶­´´´±±Ñ~>òùüD~äWàuww'	/]òùüD~ä'ò#?ÈOäG~"?ÈOäG~ä'òùüD~äG~"?ÈOäG~ä'òùüD~äG~"?ÈOäG~ä'òùüD~äG~"?ÈOäG~ä'òùüD~äG~"?ÈOäG~"?üD~"?òùüD~"?ò#?ÈOä'ò#?òùüD~"?ò#?ÈOä'ò#?òùüD~"?ò#?ÈOä'ò#?òùüD~"?ò#?ÈOä'ò#?òùüD~"?òùIä'òùÈOä'òùùüD~"?ùÈOä'òùùüD~"?ùÈOä'òùùüD~"?ùÈOä'òùùüD~"?ùÈOä'òùÈOäG~"?ùüD~"?ùÈOä'òùùüD~"?ùÈOä'òùùüD~"?ùÈOä'òùùüD~"?ùÈOä'òùùüD~"?ùüD~ä'òùÈOä'òùùüD~"?ùÈOä'òùùüD~"?ùÈOä'òùùüD~"?ùÈOä'òùùüD~"?ùÈOä'òùÈOäg9üD~ä'òùüD~äG~"?ÈOäG~ýìÏÿüÏwïÞv`û»¿û»?üð¬tölû/~ñËA¡°YËA¡ðÂ¿ù¿±õ×ý×Dbï4L¦üN:µ~ýú¿$IÒÔ÷ÑaöÄJ$Iä'ID~$I"?I$$IÈO$Iä'I$òË.^¼X[[û,£Ë§WöööÉ'M2åðáÃO1¯üñ3ÊËË.]zåÊË§x:zôè´iÓÂv`êÔ©ÇcÂÏhËpðàA¨È×ÜÍùåuáí<¼µäå^õöÛooß¾Ý"*æ¡ªªêÓO?ágþ¼ª5(+ÃÌ3Ã?tvv>ûì³Qñ4aÂ>ø ¼ûî»'Ná"÷åË[DE¾>ô	òËÇ,XpúôéÜ'ìÌ3óæÍ³||e?~ü¹sçÂ@ø-¢b^ÂñÓÃ£F²³èÿ×®]===þOh¸$È/¯ËÂxâcÇY2E¾2?~<¼°ÃðÓúPä+ÃÔ©SO:ÞyçLªx+Wfý7ÀÊ`¸$Èo(É/àöìÙaæÌ­E¾2|ôÑG'NÿøÞ÷¾ýG_EÕÕ«WºººÂpæGÀËËË-"_È¯ä·iÓ¦×_Ýb±2ø½!wä'|2yòd§¨:wîÓ_|]3fLOOÏõGÃ°åSäëùÂöîÜ¹'N°X¬³gÏðuttÌ9ÓÂ)æaÂ	Ç¿víÚ÷¾÷½W_ÕÂ)ÚÛÛôÑ/¦Ç466¾õÖ[a ülhh°| ¿B_yyyôé]ùÊðÉ'ðaØÂ)æ!lîÇ6+W®L¥RNñT[[y¶¯ë7¾ËY]]]RRRSSã[Å¼>$IÈO$Iä'I$ò$IùI$ü$IÈO$Iä'I$ò$IùI$ü$ID~$I"?I$$IÈO$Iä'ID~$I"?I$$IÈOî®wÞygîÜ¹77oÞï½÷_¶_72[ÛÞæ¶¶¶6<´®®®¬ñaLYYYMMÍµk×nwD~^ë×¯Ó«¯¾ZHòÛ¸qcÙÜÜ5~Û¶maü+¯¼rÓ$ò4Ä:~üx0MYYÙo¼ºÑÎ;ÃÅ0òÄ#¿3gÎS§NÍ?yòä0>H$òTø=õÔSÁ4ßýîw3G¾öÚkaä+2ÝsðàÁ §ÂiÓ¦áô/_¾|yåÊ#GWUUU­Y³&ó j[[[ÐU¸*ÜvÿþýYcF5wîÜûöY3ÖÚÚÚ÷t¢«Â,EW8pàfJôÑGÃø£G¦Ç|ðÁaL<OÙ´iSuuuTEEÅÒ¥KÏ;+¿ÜégécV%$fcÇjùôÓO3G~öÙgadmmm¦l²:vìXtíâÅ³®zî¹ç¢«:::JJJz½Ut1ºö'¸víZ°cyyy*×AN#F>~×ÇtÂ@¯³ûHß÷ÝLÎ¦qùöÛo§Ù5yóæÝ®üúUIä'I`·mÖ°aÁa²Y¾|ùÕpqÑ¢EÑµt";F»îØ¢«¢aøðáÃÑD2§ùÒK/ÛE[~úé0æý÷ßÃágþö·¿Ëé5á2ç­×G49zôèðx/^¼.aÎ7zz¢_¨©©	7áú®¨¨¸]ùõ1«ÈO_~¡^åÉ&SèÜ¹sáb0StqÚ´iáâøñãyæ ¶/¿ü2=ð;YÑÒ·.~öÙgé_ZJð?Ãp[[Û-§.^¸p!sÞnö¼ç>½ïïúÑ^³fMæ/ùÎ1#Ú%y»òëcV%$rÑ®«W¯fìêê#ÃUX'íÂÓ§OGøKC'ýá¼Ü°YÊ<J®ªª*//ïîî5jTúÚ[NçfËêÔ©SRÃpø;::Ò×>|8Ì@¯Gû/¿>fUùIÒ Öíõ×_Ïë$ëéÏ^¼x1wWÖ'6nÜM1Ú!>zK=÷ÜsÑAÞðsåÊéñL'ÚÇöÅ_D/Ð÷÷pgÏ®NdÀyUô=ßpÕ®Ò·üÒ*FúÚ>fUùIÒ C¢¬¬lûöíÑY]ÞxãòòòÜoc,Z´((§»»;:þnô9¿èãq§OÎü`Á5kÖ'EßºMW¥WE_¶î½½½==¾éDì>ç×ÕÕýfòÛ¹sgzoÈWEyìèè÷ÂÍäY3X9üZçÒ¥K3¯ícV%$~ÑY³Ú´iÓn¿nÁ(=>NþREº§~:íÙ³§ù]¿ñÇ6ÂøªªªÌÁL'ÌFæ¥¿W³ûå_VTTD!ë÷2ïbôèÑágtbÌiF»$ÓENM_ÛÇ¬J"?IÊNæÍW~£¹sçF_°Íß¢ÓæM2åÐ¡Cék»ºº^xáêêêHKÏ?ÿ|wwwúÚýû÷Ï1#¬¦¦fÇYÓÌ0©0þÙgÍ³éÂÌYN4ØÇùüÒ­2ëô.Q/^lhhK`äÈáQ$ôj2§yåÊ@ÛhYÅãñ£GfÝc³*ü$ID~$I"?I$$IÈO$Iä'I$ò$IùI$ü$ID~$Iä'I$ò$IùI$ü$ID~$I"?I$$In¿ÿ^XüÛHüIEND®B`


µ¿¿ÿÚµka!+;::ÒÕHO2à¢/]ºôäO5¡ÆÆ¬ü¢~òÉ'a¹¯¯/«n@ù¹]~D"üÜµk×`yñþûï?ðÀÑ¦ÒÚÚÚ®®®¿¹wïÞÉ'/Y²$¹fß¾óæÍ+,,téÙ³g80gÎ¢¢¢eË¥¾¿øì³ÏVTTD5ÃE$¬ozè¡°>uÛkÐÔÔVÖ××§ýGzøEXkÂÑà¤O.ÕÍpèòpl¸]fÏùòåal~w0~lêÍ´µµsãnÙpTò¡PÃHGoØ°áÊ+ü,-¿K.UVVN:5,Ä#àã?/ç©-~s@¢÷ßÿ§é6GÞ÷Ý!nC$³oÀoÞsÏ=C÷Í´iÓÂúóçÏ§®eVÎ93íß8Ò¸è5Ñ~³fÍÑàp7ÂáßñãÇSÏ'ü±#-¿Ôµ££#:tôèÑèW­Z5à:¯[·Î¥åÞ|óÍÔ÷ÀR#àÑGË+W®üäº°©¿ùôÓO_»v-z×*ZóØcõ÷÷<x0:Î9Ü»woX6ú4Û=Âò|zÔ`SXXvêioñ$.ùèH'íµºéñ!¿'Åkkk/_¾>ÙgÃ/¿Ô5OX³ÿþ°Ý¬kÖ¬~9*Â>ú(,[3,zpò²·üðBÃúõS§NËüqt0¼ÀS¦LI=ä±É5¡6R^¸páÓ_m6M¾¾¾Ð¡0.½ÉtÃò+**Jû¤]'I-ªpÚ1555mmmim8öZÝô	_~ÑgÏ=z#*¿Ô5:·TaMtÔüùóÃÁ;ï¼3kø/ÄÕ«W=²@ùÙ^~!ÎÊËËÃëwoooêúøfñ½¤KC9`çç¨VTTD§ãyç66&7´áÎ`=zs'þÖÞá´ÝÐÇÆoÖ_yÕÙÙÅ_²[[[=¸@ùY]~Á~ðp0ù(ÑÊ(Î¼;V' >8öìèÛUöíÛÊl8å½1ÁJmmí>ùä^z)¬|àÒßpNrÓå7ÒÁIºé¿üó_²íB÷=þ¥¥¥aÍÆ©N8±yóæhsr4 üì-¿O¯Z?ùAþhM4ówåÊ®^Úì±[/¿è¯Ð/¾øâpÊ¯½½=zW)ÄâÁ£÷ó"o¾ùfÚ¿q¤'Qù=8ÑH^¹nÀtTo¢ü¢Oæð½zõj»ï½7ÞßýîwÃà:<ôøG×0tsøý0ayÞ¼y©fôyÍÎÎÎ!¶¼ÊÈ®ò;sæLò]Ñ®®®ñ:ujrªì­_²E"S¦L	?Sç·¦½Î?üp|Ëã]wÝ5Ä9¢¨üä2ø6æÕ(¿'N¤ÿ%KR0&¡Õ.¿èkÓNyÞUMõÈ#xpòr ühKhêú÷Þï(º®¶¶6>ãæÊïÂáÜÂyzüñÇC%¿dè¾Ù¶mÛ9s¦MöòË/éK_J¾çtë'Qù=8o¾ùf¿ÂÂÂäWâ¥Ñ¨ÞDù­­­wÞygø«ÃùGÛÓÓÃÁÐgÑ¥×ÔÔ9räï¹îÝ»wáÂáÜÂÕÜÜåÊ§z*z35ä¸)=¸@ù¾¾¾~xDß!w'É9ÑFØh²vô³gÏv@¾>ç7À·¾õ-#(?|såÊ'|rÚ´iÑ&Ý°ðÌ3Ï@ù üÊåò@ù üP~(?Ê@ù üP~©Þ|óÍªªªÂÂÂî¹g¤§3gÎ	:;;kÂrX3oÞ¼<]ws¿3ÓÞäìÔÖÖ^¸p!:êÊ+ëÖ­+//9iÒ¤pÔûï¿ï(? TTT	ùÒßß?ÒÓ>ûì³á´[·nM®yáÂÍ7çwùEËáËkÖ¬&ppÿþýaùÀayæÌî`ò²éÙäâéÄá´K.M®¹ûî»ÃãÇý%VË%%%ÑÁ¢¢¢pðÒ¥KîTò²7ûRkæ©§4iRiié¦MüÚ¾ô¥8ä[aùüùóa9¬úè£jjjBÎ??z3,~n©>ôI^~ùåªªªÅwØåËëêêÂiÃòÉ'£·0C.Y²$[XÎùã?½ò>z88öìºkÊÈÞø¿ùÍoåæææÐXaá[ßúVêïìÝ»7ù¶¤G4õÒK/åð3,×××GGÝu×]öì	|ðAX_YYöÜR¯ÀÐ'	=÷ü ,$ø_·n]X¿ðÆog6Yc.]êèèÑ[q7Q~×®]ã×®]õæo&Ïðîy÷ÝwÝ»ådoùUUUå¾ëâáöíííá¨+WåU«VEI<öÄO?ýôÂ£)iÏm@cèM4)~Ú©S§FW>4YXÍVßËóçÏ-ÛÛÛsÃ_]·pæ!ûR7ï=z4ÄkòwÂºÊÈÒò+,,L.ppèúôú[_¥¥¥á7¯mTk¢£^|ñÅpªï¾ûnê98·ÔÃ9É`W,¤XêÛxQ5îÚµkòäÉÑòòòèíÀ¼ç7ýû÷ÖLÝð ü¬+¿ÊÊÊÔ÷ü¢·Ín;uuuáØäÏäúhÆÃµëY~C$yÅBÌÅO;eÊè´®^ÿ=¢·É7G4,#-¿ ºáÏq¥åKËK/½Îï^NîìÚµ+ùæÙ~ðäú¨#O:þo8å7ôI~úéþðaáÑGöG	Ë¯¼òÊéÓ§£-¼aåâÅÃò»ï¾öìÙ°0kÖ¬Ñ+¿yóæ£öíÛ[[[ÃòÃ?ì(? KË¯¿¿?ÔUéu©ßÉ7tù]½z5ÚÒ~¦~è-ÔOyyù¤I|òÉaßÐ'ikkÇÞ÷ÝÉ&©¿såÊ6k^TTtï½÷FÓxCðÕÖÖF.]úÞïhXâ[puuuáÊq2eÊºuëÂUrÊå üP~(?ßXøÛ¿ýÛ?üÐMµÎ=úÅ===gÎ1dV___WWq ã~ñ_üû¿ÿ»òË?þã?ñç^µþþïÿþ>0dÖ¿üË¿¼ûî»ÆÌºråÊücã@ÆíÝ»÷ßþíßòS~ üP~(?å§ü(?ÊOù)?åÊåòS~(?(?òCù¡ü@ù¡üÊåÊå§üP~(?(?åòCù¡ü@ù)?ÊåÊOù)?åòCù¡üòS~ üP~(?å§ü(?ÊOù)?åÊåòS~(?(?òCù¡ü@ù¡ür£ü92þüÂÂÂyóæ?~)?P~(?_ÞßÌ3:^íµY³fÅËoÏ=­B¬ÿÃ?üq ³Þï½þô§ÆÌêîînkk3dÜÛo¿þüù1¾Ð.¿T%%%ñòÛ¾ûOÈVï¼óÎþýûõãÿxß¾ÆÌ:pàÀ=×ÚÚzðàÁ1¾Ð|(¿£GÖ××ÛÚkk/ØÚ­½ØÚ·[#/_®­­Kå§ü@ù¡üP~ù gÎI$gÏ¥üÊÊ/Ê¯½½Ù²e.¬òS~(?P~(¿ü)¿ªªª	)òåòCùåmùMù)?(?òCù¡ü@ù¡üÊåÊå§üP~(?(?åòCù¡ü@ù)?ÊåÊOù¡üP~(?P~ÊOùòCù¡üòS~ üP~(?å§ü(?ÊOù)?åòåòS~(?(?òCù¡ü@ù¡üÊåòå§üP~(?(?åòCù¡ü@ù)?ÊåòS~ÊOùòCù¡üòS~ üP~(?å§ü(?ÊOù)?åòåòS~(?(?òCù¡ü@ù¡üÊåòå§üP~(?(?åòCù¡ü@ù)?å§üP~(?òS~ÊÊå§üòåòCù)?å§ü@ù¡üP~ÊÏ½Jù¡ü@ù¡üÊåÊå§üP~(?P~(?åòCù¡ü@ù)?ÊåÊOù¡üP~(?P~ÊOù)?Êå§üòåòCù)?å§ü@ù¡üP~ÊOù)?P~(¿ìÑßßúôiã üÊåòËg½½½MMM3fÌ¨¨¨Cg@òCù¡üP~yèâÅáÅ·¬¬lÂ¯ìØ±Ã°(?åòCù¡üòJWWWýwÜ1á¿[»v­ÁQ~ÊåòCùåcÇ=øàñæ»ï¾ûZ[[ûûûòS~(?Ê/ç°«©©|ÅÅÅ«W¯>yò¤ñQ~ÊåòCùå¼ÞÞÞæææEÅoýúõÝÝÝHù)?ÊåózzzþùéÓ§h¾ð«ùòCù¡üP~ùàÜ¹s7n,//Ð|3fÌØºu«¯nQ~ÊåòCùåÎÎÎµk×h¾ìÞ½Ûå§üP~(?_>8|øp|G4i7¼¼j>å§üP~(?_ÎI·÷îø;î¸cÅ^XòCù¡üP~ù ¹×µxó%®®.÷å§üP~(?_Î;wîÑ¬ªªÐ|eee&í*?åòCù¡üòDWWWCCÃÄ4_¨Àð2ÚÓÓãÖW~ÊåòCùå¼ðYWWßëÚ3ZZZLàP~ÊåòCùå¼tmmmi'íá(Í§üÊåòËùòI×ÒÒvG]]WLå§üP~(?ÈòëééÙ²eK¼ùMÚU~Êåò<)¿îîîðjXVV´»qãFvòCù¡ü Ê¯««+HÄ'pL>ëÖ­/^t³*?åòCùAÎ_xí[±bE¼ùæÎkÒ®òS~(?äCù=i7æS~Êåò/¿!&í®^½º³³Ó-¨üÊå9_~===i÷º6qâÄÆÆFvòCù¡ü Êo°I»åååÏ?ÿ¼I»ÊOù¡üP~å7Ø¤Ý¹sç677û0òS~(?äCù6iwéÒ¥;wîÔ|ÊOù)?åò/¿t!ìâv£	Ö|ÊOù)?åò/¿t¯¿þú`]3iWù)?å§üP~Ê|(¿¦¦¦ø¤ÝòòòðêvîÜ97òS~ÊOùò#çË¯««kãÆñI»Ñ^×Â5qs(?å§ü(?r¾ü:::êêêâ8-Zôúë¯û0òS~ÊOùò#Ê¯½½ùòå/b2å§üòåG>_4cÑ¢EñI»DÂ8òS~ÊùP~===[·nOÚ-++/^öÀ¡üòS~ üÈò;wî|GUUUxÙEh´òÊÂ£Eù)?P~dyùuww×××O8q@óÍ1£¥¥Åå§ünìàÁ³gÏå§ü@ùµå×ÕÕÕÐÐo¾¶¶6Í§üßpÝï½Cß/¾xlµÿþ¿ù¿1dÖÂK©q ³>üöÛoßÄ	_yå/~ñ©Á^¡Þzë­þô§c|¡9 ÿy/¿¿üË¿<K¶÷õÿüçÆÌ:uêTx6dÖ/ùËwÞygD'ù¿ø%KÄ'í~õ«_=zô¨!%²gÏîîî1¾Ð|.?[míÅÖ^Ë­½½½½i¿¨Å¤]líU~ÊOù¡üÈòëééÙ²eK|O»3fÌëMÚEù)?å§üP~äCùöE-&í¢üÊåGþß±cÇDqq±I»(¿ñ§üÊF©üÂëKÈ»øºº:/=(?åòCùå×ßßßÒÒR]]= ùíiå§üP~(?ò¤ü~ô£mÙ²e°=íÀòS~(?ù »»ûÿðÓîuÍ¤]òCù¡üÈ]]]Dâ;îÐ|ÕÕÕY;i÷äÉÏ?ÿü¯âFT~Êåò/+V¬7_MMM6¿²|ïß2eJ¸_þògÏ=wÎ*?åòCùAzýýý;wîL;i7ËïÓëïöìkøzÃ¦?Øý»ûwÿþïÿ¾Uù)?ÊþæææøhÒî©S§¹÷¶qôüóÏ<Mf_ø·~ÝúÏîsn§üP~(?ø¯æ¯ñ=p¤NÚþ~ÇÑ¾ü¥/§_øWRRâ&V~ÊåòÿÀßÇ3S?!å·cÇ3g¦fßWVeáÂnhå§üP~(?nkáÕ¡®®nøvs¢üz,Xp×]wã±oì[ÿê_ûµ_koows+?åòCùq;I×ÖÖÀqÃI»9Q~Ñõlhh¨¬¬,))Y¸paøcÝèÊOù¡üP~ÜÍ×ÒÒÀqÇw$cÇÝ°¨r¢üP~ÊOù)?·µhGUUÕ­ìiWù¡üÊåGV;wîù­ïiWù¡üqÊ)((P~ÊÏ8 üÈ¸Ó§O×××Ç¿¨%>iWù¡ü2V~7RXX¨üq@ùA'Ozuü½ÅßÊvÊ/(?åòãöð-Zo¾åËßúkòCùÝªK.=úè£ÊOù·¢··7í^×î¸ãµk×<y2#¢üP~#SUUUXXès~(?jlÛ¶mñ	'NlllìîîÎìe)?ßpÍ7/þöx¬òS~ÆåÇHªmÀ1úôæææÑxqQ~(¿(**È.TTTðøùáêëëò3(?¯££#í¤ÝEÝÊåòËdùEË°®a!¼Ø_»v-,*?ågP~ÇÉ'ï»ï¾øv.]ÚÖÖ6zÍ§üP~#6yòäðà<xð`gggXxæg¢ßê¢üÊ¡¤/!ïâ8êêêN>=6WCù¡üFàÉ'LÎçðíJÊOù5ß;¦OßëÚO<1Ì½®)?ß8_ðÜsÏM2%,9r$,páh_uå§üP~ä¢ç>þE-/^û«¤üP~9@ù)?¹¥»»ýúõñ/j;wnsssooïx]1åòS~(?sòäÉÐ|ÅÅÅoÁ»wïí	Êåáò5kVôÝ.¾Éåò#Ux¢N;iwõêÕÇË+©üP~#0sæÌÔÚK2·Wù)?ßm«¿¿¿¥¥eñâÅñI»D¢³³3«®­òCù@¼ð`>zôèµk×Æòª+?åò#õôô¤ÝÓnYYÙO<Ù½®)?ß8_ôYÝ1Î>å§üP~daógæø8ªªª¶mÛÍÚk®üP~#püøñðÀ^·nÝåËÊåwêêêjhh7_uuõ¨îuMù¡üÆ¡üiÓ¦M1ÃCù)?_Þ;vìX"Oà¨©©É¡§håò;ï¼ÓÊïvCÞ¥À=vÊ/óå=Ú;::Æøª+?åòcìEv«««ã]Í7Æ]S~(¿q(¿©S§áòCùå½!&í'älÀ¡üP~,¿öööðÈòÉ'ÇxÇ;ÊOù¡ü³æK;i7T`hÁn>åòùYÂå§üP~¹®««+HÄ÷º¶xñâ´«üP~/¿Aá¡üÊ/wåÇ¤]åòË|ùå§üP~|´«üP~/¿ªªª;ï¼óôéÓÊåòË]MÚ-++khhÈÑI»Êåùò+,,OcÕòCù===[¶lÉËI»ÊåùòÛ¿xØ¼yó¥KÆò»]òCùq.^¼ßvÊ/óågn/Êåsº»»7nÜ8qâÄü´«üP~/?sQ~(¿ÒÞÞ¾zõêÛdÒ®òCùe¾üÆòS~(?¯¿¿÷îÝ-J;i·³³óvåòS~(?_èíímiiOà8qâúõëóÒ®òCùJùõõõÝï½%%%á©¤´´ôþûï©ÊOù¡ü¸aÐ455UUUÅ'ínÙ²åâÅHù¡üFìêÕ«igxön|òCù1Ó§O744Ä'íÍwûLÚU~(¿ÌßìÙ³Ã³ÉÊ+/_¾^ºtiÕªUaÍ]wÝ¥üq@ù±Ã?øàñ	-zýõ×o·I»Êåùò+**Ï)©Ï&aMX¯üq@ùåkL|¯kÁ+ÚÛÛòCù©ou	Ï,¡ökzÃßê¢üÊoD8æÎvÒîÉ'òCùe²ü¢­½Ë-¶öa9¬?¾òS~Æå7z.^¼¸mÛ¶´86nÜhÒ®òCùJùÔK;ÃãO>Q~ÊÏ8ßåwìØ±¯|å+3fÌXºté÷¿ÿý1»ÜîîîÆÆÆøÍ7;wÎ]Eù¡üF«ü>½>½÷þûï4iRAAAø¹lÙ²°f´¯ºòS~(¿ñõúë¯O2eåÊ_oX³fÍç>÷¹o~ó£¡]]]uuuñ½®úljjãN¢üP~£^~ãBù)?ß8êïïÿÑý½~oÓlþã±o ëîî¥KÏx+V¬H;i÷6ÜÓ®òCù)?ÊoìtvvÞyçÉìþþóommÍxbîÜ¹3¾×µà¾ûî;|ø°æS~(¿±(¿	7RPP üq _Ë¯««kúôéÊoþüùá>SÑÓÓÓÜÜßë=í*?ß8_ÁàÊ¼/¿à×ý×Wß¿:õÿ»~âÄ]/<ÅÅ'p?ñÄ&p(?ß8ß`üñèêµ×^S~ÊÏ8Çåwøðág¿ó;¿óµº¯ýîïþnyyùøÃ[<Ï®®®D"/þE-[·nµ§]åòË¢ò;~üxiiixª©©Iýbgå§ü /Ë/øðÃ7lØô~øá[ÜÎìØ±Ð|ñ	3fÌØ±cI»Êå]å÷ÀDORöì«®üÊ/?§²´]+ÛÚÚLàP~(¿ì*¿7Þx#zZ¹råX^uå§üP~9-$]KKKuuu|G]]ç7åòËºò»zõê]wÝMæØ¿ÿ_uå§üP~9ª§§gË-ñI»ÅÅÅDÂ^×Ê/Ëï»ßýnôTµfÍq¹êÊOù¡ür±ùÒNÚkÂúp¬Xù¡ü²´ü|Êå7|MÚ1cFss³æS~(¿l/¿),,T~ÊÏ8 üÂ3U]]]|ÒîâÅíuMù¡ür¦üÆòS~(¿ìo¾Á&ízúR~(?å§üäCù1i×^×ÊOù)?åyR~MÚ&ptww»ÊOù)?å9_~¡êÒNÚZÐåòS~ÊOùA>_4i7>£ººÚåòS~ÊOùAß±cÇêêêâ_Ôbò<,?ßçòã¶-¿öööø¤ÝhGÈA7ò<,¿Ô¯îK[~¾ÏOù)?ò¬üúûûwìØ±`Áø]S~Ïå´oß¾ðÄ÷ÐC]¾|9?W­ZÖ8p@ù)?ã@~_ooosssüZ***ìuMùÁíU~åååáé/õSÌÑâ-óñãÇ+++çÌ³ÿ~å§ü`ìËo°=íN>Ç¡Ü(Ên¯òCí(¿[ÿ_mmí+¯¼¶oß¾fÍxù½óÎ;WÈV'OüÇüGã@f½ÿþûáÿcsY?ûÙÏ¾þõ¯Ç'p|á_xýõ×/^¼èæÈÿú¯ÿúWõWÆûí·ÃÿÇøBG½ü¦NC¥]½z5úÓÊ+Ã°þÖßM¼víZUUUñòÛºuëÉVá?:ûöí3dVøÿ^x&íKùÞ÷¾÷Û¿ýÛñ1Ï?ÿOÿôOÝù'dß[o½eÈ¸ÖÖÖýû÷ñzù8p í[ß":G$>_ÄÖ^[±µw46Í,_¾|ÀóEv;::¿­½`kï/ð3gÎ,))	ÿE.--ÿ->þü­mêöâ¢¢"å§ü`Ê¯¿¿÷îÝñ½®´«ü@ù©S§F?ãÛòCùÝºhO»UUUñI»7o¾xñ¢1W~ üÆH]]ÝË/¿ÂÏÚÚZå§ü å¯±±1ívMÚU~ üuêÔ©Y³fFÛg§Nºk×®[?Ûÿvó¬¬¬<räòS~òëìì¬¯¯¾*Õ¢EvîÜÙÛÛk(¿AEßäºÇ¶hyûöí£zÕòCùÔÉ'×®]ÿ¢+Vx>Q~Æå7,áyóÔ©SÉò;räHX4iòS~Æ,)¿¶¶¶ÅÇ÷´B0ä U~Êå7ì³¸.ZÊïÚµköÛò#Ê¯···¥¥%ív7nÜøáRÊod¢orÞçå×××÷ÔSOåøw/+?åcV~íu­¢¢bË-ö´òCùÝ¤ööö´ßä|èÐ!å§üc_~]]]ñæ«®®¶§]Ê/Î=»páÂhnoIIÉ¬Y³ÆàOòCùpìØ±D"1`ÁÒ¥KwïÞÝßßoQ~(¿üÊ/)<ÔÔÔÄ'p´òCùe¸ü;Î??kÖ¬éÓ§+?ågÕòëïïoii©®®7_"°×5Êo,Ê¯¯¯ÏÜ^£Z~Ñ^×âÚ-++OÝÝÝÆåòËdùM>Â&O¬üq ãå÷Î;ï¤´[UUÖ´òCùJùôÑG×%÷Þ*dßo¼¡üq :;;×¬Y|ÚIÝÓnKK	(?ß(_Rxí»ÊOùqK;#+ÛÚÚFµù.^¼¨)(¿ñ§üy/Àv¯kuuu£ýðï|ç³ýìÔ©S'Oüµ¯­··×-¢ü@ùýÇ|Y³f&×L2åßü¦òS~ÊÖÓÓÓÜÜÀoÕªUc0i÷ÙgýßøGÿÏ£þ`Ó?±`þ¯~õ«nåÊïÓiÓ¦Þ=AoÞ¼Yù)?ãÀM4_Ú	Ñ¤ÝãÇh¿½7Ý%%%ßxì!û¢Oý¿§Âèììt)?¸ÝË¯¨¨(<)wtt$×§æ°fÒ¤IÊOù¯««+HÇ'p$÷´;üýöÞð6kÖ¬döEÿ¾ð/ìÞ½ÛÍ¤üàv/¿hÝµk×þÛùÆ¾äOù)?Lx,×ÕÕÅ÷º´;6å×ÝÝ]UUõôÆ§SËoîÜ¹ííín,å·ùUVV'è6D¾zõê¦MÂå§üCkkkÑ¤Ý±)¿à·~ë·~÷¿Ì¾ßè÷>ûÙÏ^¼xÑM¦üàv/¿£G¦ý&ç#G(?ågH+$Ý;æÎ;ÒI»cV~åååÿüç¿üå/ÿ%ÿcòäÉÞðS~ üþÓ3gæÌSRRRPPPZZ:öì°f´¯ºòS~äèKlSSS|'@ÅÅÅÃÙÓîß§×çlÛ¶­¾¾þOþäOR?ÊòÛ½üÆòS~ä/íÄ4_yyùð÷º6åòå§üP~XggçúõëÓîi÷ùçÑvÊå7þåwêÔ©èË£ù¼S§NÝµkòS~ÊÓ§O§´[]]ÝÔÔ^nGzÊåòçòÛ·o_òÙ<*¿hyûöíÊOùÛV4i7Þ|«W¯nmm½é=á*?ÊoË¯¢¢"<:u*Y~GñMÎ(¿ÛSooïÎ;-Z´H$N<yç¯üP~(¿q.¿èiýÓoo¾víZX.,,T~ÊÏ8ÜVÍ·mÛ¶ªªªø¤Ý7fjO»Êåòçò:ujôíQùõõõ=õÔSÑg·ò3·/nÞ¼¹¼¼<¾§Ýç>³ß~¬üP~(¿q.¿ööö´ßä|èÐ!å§üC~ûðÃxâøv§OÞÔÔ4¼P~(?ß8_pöìÙFsKJJfÍ©-;ÊOùN<ùàÆ¯¦¦&üoð¦'p(?Ê/Êo?åÇ¸awß÷¥´;;ºP~(?òCù1êúûû[ZZâvËÊÊ28Cù¡üP~9P~ï¿ÿþwÞYTT^JJJæÌsáÂå§ü_~èééÙ²eË34_EEÅæÍÏ;7WFù¡üP~ã mmmigx>Zù)?ãÓºººã]>úÖ­[obÊåòËùò¾É¹¶¶6z¸|ùò5kÂÊÊJå§üCêììL$ñó-Z´h÷îÝ£7Cù¡üP~Ù^~ÑëAê+AXN~«³òS~äð°ª©©¿¿|ùòlxÄ)?ÊoË/zÏ¯¯¯/¹æêÕ«ÞóCùåð¶;wÆ/ÚëÚMàP~(?_¶_ô9¿ÚÚÚ|áà'|²téRóCùåÞÞÞø²²²ð(ëîîÎªk«üP~(¿q.¿	72JòãõôôÇQ|G¨À-[¶c³ð:+?ÊoË¯àFò#«tuu544¤m¾qÀ¡üP~(¿l/¿ñ¢ü7a°I»555mmmÙÜ|ÊåòËòìKÏ9£üqÈi'í¬««Ë¡òCù¡üÆ¹üÂÇsÏ=7`åÚµk«òS~Ù ÚëÚâÅ4_qqqVMÚU~(?_n_(¼ð*2uêÔóçÏ¯¾újôº2JïS~ÊazÒnvNàP~(?_¶ß§×ßá^Q*++£U«VöUW~ÊÁäâ¤]åòCùåLùH¾º455ÁUW~Ê¸Á&íVWWgù¤]åòCùåLù=öØcÑ«K´?à¡R~ÊOù¥cÇ6i7)Êåòçò+,,¯.&M:qâÄ§)ó+**R~ÊÏ8ü´«üP~(¿Ü(¿ðóôÓOXH$ÌíU~ÊoTEv«««ócÒ®òCù¡ür£üû>¿³gÏ*?ågFCOOÏ-[òlÒ®òCù¡ür£üÆòS~·¡îîîÁ&í677çwó)?Êo<Ë/¼Ø¤nÏú òS~Ü¢®®®´8æÎ»cÇ<´«üP~(?å§üÿ1cÅñæ[ºtéîÝ»oæS~(?òS~Ê/o¤Û¹sg|Ònpß÷>|øöåòCù)?_^l¯kwÜqÇÚµkO<y;òCù¡üÊ/O¶×µ'®_¿þÃ?4DÊåòS~(¿Mà(..Ð|yÿE-ÊåòS~ÊOùÝ.ÂÝ¸®®.í×_=¼ "åòCùeEùMù)?å7þþþ¶¶¶Á&p¸o+?Ê/»Ê¯àFò3io°	D¢³³Ó)?Ê/ëÊoÜ)?ås¢	UUUñ=í®_¿>_÷´«üP~(?å§üßí%T]ccc|Ònyyy¸'_¼xÑ)?ÊOù)¿|+¿ðôÚÞÞ	·Ïtww¯_¿>>cÆMMM&p(?ÊOù)¿ü,¿Ý»w-ð_øBiiéO>ßëèèxðÁÓ6_KKËí¶×5åòCù)?åw_È )S¦üÏµÿsÓlÿþïÿû¿ùßùÎwòòo÷Ìxó-_¾ü6ÜÓ®òCù¡üò»íÊ¯¡¡aÙÿ·,Ê¾èßÿJü¯ùô'¤kmm­®®¶×5åòå§ünëò[±bÅ×ê¾Z~áßg>óüøczz¶mÛ6úôÍWVVÖØØhÒ®òCù¡üò»½Ê/_ßó;wîÜæÍãv«ªªìuMù¡üP~ÊOùÝ¦åös~ög»`´§Ý'À¡üP~ üòK?·wÁ-*--Ý°aCæQ¨ØÕ«WÇ'pÔÔÔ´µµi>åòå§üß>½¶···¶¶æâGß¢	K.Ð|á`]]£òCùòS~Ê/ôöö¶´´Ä'í'	8ÊòS~ùP~===[¶lïi·¬¬Ìåòå§üÈòëîîN»§Ý3f477k>åòå§üÈòëèè¨««K;cçÎ&p(?(?åG>_¸G-_¾<¾+V¸³)?(?åG>_ÿÎ;-Zo>8ÊßØ¹páBUUòS~£¤§§§¹¹yÆi'ptww»Êß9xðàìÙ³ÃË°òS~£Ñ|á.vÇ-[LàP~(?P~cíÞïíìì¢ü¾ÿýïÿlõ×ý×?ùÉO²ðíÙ³ç+_ùÊg>óÍ7úôçîäÉn»lvèÐ¡ðmÈ¬¿û»¿¯ÐÆë­·ÂVÇøBs¸üþó*^~;wîül^¡O8UWéG?úÑ>XPP0 ù/^ì¾+~ö³<xÐ8Y¿øÅ/öíÛgÈ¸=öüó?ÿó_h>­½¶öS¸«ÔÔÔLI$n,[±µ×Ö^lí·Î(?åwëúûûÛëZCCÃÉ'ÝLÊÊ/ëZPù)¿öº´[QQÑØØhÒ®òåòS~äCù6i·ººzëÖ­áùÝM£ü@ù¡üròS~tuu%âââøµ´´´ØëòåòS~äCùÂ`Úmoo×|ÊÊOùå7Ø¤Ý|°££Ã­ ü@ù¡ü9_~CLÚ]¿~½æS~ üP~Ê|(¿!&í>ñÄ&í*?P~(?åG>_¨º´v«ªªLÚU~ üP~Ê<)¿hÒn|GuuµI»ÊÊOù'åwøðá|0í¤]·¾òåòS~äIùGË+âvW¯^ìØ1Ã«ü@ù¡ü9_~½½½ö´òCù¡üòËóòÍ×ÔÔTUU5 ùÊÊÊÂmñâECòCù¡ü9_~]]]i÷ºÐ`¢üP~(?å§ür¾ü´[SS³sçNvQ~(?òS~ùP~áÁ`Ò.ÊåòS~Ê/ÏË/<-Z4 øB&ÎÎNãòCù¡üòËùòëíímmm7_YYYcccWWCù¡üP~ÊOùå|ùõôô¶×µ°>k¬P~(?òS~9_~i/´kÊåòS~Ê/æûâ¿XTTdÒ.ÊåòS~Ê//>i7bÛåòCù)?åúûû[ZZ,Xo¾D"aÊåòS~Ê/DÚ1cÆ»&MjhhÐ|(?ÊOù)¿|0ô¤ÝÃh¿½ üP~(?å§ü²Ñ¹sçÛÓn4cøûíåòCù)?å:::&N´ÛÖÖ:iWù¡üP~(?å§ürU»´Ú]½zõ±cÇâ¿¯üP~(?òS~9Ù|íi÷ôéÓJù¡üP~(?å§ürFôE-ñI»'N~ý¹sç>¹òCù¡üP~ÊOùåhÒnyyy|Ç-[ÂSäpÎDù¡üP~(?å§ü²ZWWWüZ,X°cÇíuMù¡üP~(?å§ü²T4i7>céÒ¥­­­7±§]åòCù¡üòË:aÓNÚ½ï¾ûÂQ7Ñ|Êåòå§ü²Koookkk|ÒîÄ:;;oñüÊåòS~Êoüõ÷÷755UUUh¾²²²ÆÆÆîîîòCù¡üP~ÊOù§hÒn¼ù***¶nÝÍàe)?Êå§üßøèîînhhOÚ;wnKKË0¿¨Eù¡üP~ ü_èèè0céÒ¥£:ÊåòCù)?å7nî»ï¾h¯kíiWù¡üP~ ü_þÜÿÄ­OÚU~(?(?åòCù¡ü@ù)?åÊåòS~ÊOùòCù¡üòS~ üP~(?å§üÊÊOù¡üP~ üP~ÊåòåòS~(?ÊOù¡üÊåòå§üP~(?(?åòCù¡ü@ù)?åÊåòS~ÊOùòCù¡üòS~ üP~(?å§üÊÊOù¡üP~ üP~ÊåòåòS~(?ÊOù¡üÊåòå§üP~(?(?åòCù¡ü@ù)?åÊåòS~ÊOùòCù¡üòS~ üP~(?å§üÊÊOù¡üP~ üP~ÊåòåòS~(?ÊOù¡üÊåòå§üP~(?(?åòCù¡ü@ù)?åÊåòS~ÊOùòCù¡üòS~ üP~(?å§üÊÊOù¡üP~ üP~ÊåòåòS~(?Êßø:räÈüùóçÍwüøqå§ü@ù¡üP~y[~3gÎ<tèPXxíµ×fÍ/¿ööö#[ýüç?ÿ§ú'ã@fýò¿<qâq ³zzzöïßoÈ¸P~W¯^ãÍáòKURR/¿^xa/Ùê­·ÞÚ³gq ³Â*ÜµõöÛo·¶¶2îG?úÑØ_h>ßÑ£GëëëmíµµlíÅÖ^líÍÛ­½Ë/×ÖÖ¥òS~ üP~(¿¼*¿	¿<sæL"8ölü7òCùòCùåvù¥joo_¶lÙÒ«üÊÊ/Ê¯ªªjBå§ü@ù¡üP~y[~CS~ÊåÊå§üP~(?P~(?åòCùòCù)?ÊåÊOù¡üP~(?P~ÊåòCùòS~(?ÊòS~ üP~(?å§ü(?ÊOù)?åÊåòS~ÊOù¡ü@ù¡üÊåÊåOå÷GôG;wîülõÎ;ïüä'?1dÖOúÓ·ß~Û8Yï½÷Þë¯¿nÈ¸?ÿó?ïêêãíééÉÏò;uêÔ¦Mþ_zèïÄÜ&ò@ù üP~(?¹çÂUUUÉï½÷ÞÂxàK.nÂ#GæÏ_XX8oÞ¼ãÇ5ágeeeX3gÎýû÷"2r¿?AFîZiïlÊ|pðàÁÙ³gOð_wª»îº+ÜãÃÂéÓ§ì1CÄM9sæ¡CÂÂk¯½6kÖ¬°P[[ûÊ+¯íÛ·¯Y³ÆûUü2r×¯Q~äï½·³³3õy3ü'¹<yòdCÄ-*))	?ËËË¯]»úúú¼CC¦îWñg0ÈÈ]kè5Êú¼9oÞ¼S§NW_5µá&=z´¾¾~Àÿ(Ü¯ÈÔý*þ¼k¥]£üÈ·ò÷ÝwgÍþóío;þ£Cîº|ùrmmí+WÂrAAArQQÁ!#÷+åÇ¨ÞµâkyX~Iï¿ÿþìÙ³7çÌ3DâìÙ³ÑÁ©S§öõõzkoX6>dä~¥ü½»VÚ;ò#ËoæÌÇ¿víÚ·¿ýíçÎàpÚÛÛ-[váÂäººº_~9,áÿÐÜ¯£t×ìÎ¦üÈÃòw÷iÓ¦Õ××÷÷÷nBUUÕ^YQQQPPPYYM[¿_)?Fé®5ØMù üP~(?ò@ù üP~(?Êåò@ù(?ÊåòW_uÉ%%×ÝsÏ=o¼ñÆþº.gmÓ]Ûªªªð§]¹reÀú°¦°°°²²òÚµk#=OåäM6Myî¹çò©ü6oÞV655X¿mÛ¶°þg¹óP~@9~üxhÂÂÂ_|±ÿº^z)+O87å÷ÁóæÍ°~öìÙaWWòÿzè¡Ð4ßúÖ·RW¾ðÂaåÚµkS»gÿþý¡BÎ??,'ùO>©¯¯4iR8ª¼¼|Ã©UÛÚÚB]£Âi÷îÝ; ¥ÂÉ'/Y²dÏ=á`]]Ý+ÖÚÚ:ôùDG«µoß¾Á*mÙ²eaý#Gk:ÖÔÔÔ$×<ûì³á¬JJJxà3gÎÄË/~þÖqUå0¦Mªå£>J]ùñÇUUU©e3ÀÑ£G£cW­Z5à¨uëÖEGutt¤=Ut0:öþûï¿víZhÇ¢¢¢þþþpløÊ©´´4úøÝçÒ^½ø_úÚk¯¥æl2._yådö8î¹g¤å7ÄUÀ86ì¦yÎ0!tXjÙ¬Y³æòua!retl:Q;FoÝbª­­Þ,ËÎ$õ<~úéÐvÑÆÖGy$¬yóÍ7Ãrø~øáO¨Æp0üBêuKû2eJø/áÜìëë~¡²²20ü	þjëpIIÉHËo«(?ñ/¿ mù%×GeSpæÌp04StpþüùáàwÞùè£b»zõjòLÂïx-yªèàÇüåPKÉ¾ágXnkk»áùÊÏ?zÝûLÞã?úö·¿ýé¯¶hoØ°!õBò=ºpáÂè-ÉßWP~ã,z£ëòåË©+¯V£hdvvvFñäóâa´Têw©åòòò¢¢¢ÞÞÞpæ'ONÃó¬Ã8uêT©a9üËÉc<®@Ú­ÆÃ/¿!®* üÆYôY·ï~÷»©+£ï:0Ã#ùYÀ.ÄßÊ:qâÄæÍ£m¯ÉdÞKnN½a­[·.ÚÈ~Ö××'×q>ÑlgÏ?~èy¸wßw86ú"¬©GEó|ÃQûöí»téÒÐå¬Òh4ÇqUå0Î¢Û·o¾ÕåÅ_,**ÏÆX¹re¨ÞÞÞhSlrnô9¿èãq©BpÃ¡¢Y·ÉïUIÛgÑdÛèÒÛÛÛë8èÑçü®ýæå÷ÒK/%ßrêQÑG;::Â¥A¬ü¢Ö­~-ç<zìWP~ã/úãöÙÿzþº.£ärr;irREÒ#<©víÚ5Dùzgayyyêà!Î'Ô+W;ØõêÕèO°ûÞïM½)S¦Ñ»¤gôdRÔ©Éc¸ªòÈ¡Nî¹ç¢ë,YM°P~ûöí¾6oÎ9HåÊ§zª¢¢"ª¥Ç¼··7yìÞ½.¬²²²¹¹yÀyÆ¯I8«°þ±Ç°~°ó	Â	W)ú¢Á!¾Ï/©¾¾~À×»D.P[[F`Ò¤Iá¯èêêJ~CMêy^ºt)¤m4V555GpC@ù üP~(?Êåò@ù üÊåò@ù üP~Üÿ2Aðó|IEND®B`


Detrended Normal Q-Q Plots


»õ¢àèèh<okk?ø?øüÁàoþðð'ø?ÁüÁàþððð'ø?ÁüÁüÁü	þàþüÁüÁü	þàþððð'ø?ÁüÁüÁü	þàOððð?øüÁüÁüÁàþàOððð'ø?øüÁüÁüÁàþððð'ø?Áß|ëÏþìÏþöoÿöýyÜùóçÿæoþæ¥ÞÞÞðËq(Nzï½÷CÔ6Åéùçë­·Czçw=Z¢ÿÑGÁß´;wn÷îÝ.IT.>ªYáu]I¤ùüI$Á$IàO$Ið'I$ø$IüI$	þtWihhÈØ¸qc"xðÁs>¸üêÕ«Y9Éd2777÷ööÌ;5ÔÙãÅ¦|Íj´ó4SþnlÇ¾£ÝßßßÚÚÆ¶¥¥%³ûîµGìbvù=hû¯XÎõõõ555eï÷îâ'&''G³¯|ìØ±ì9©TêðáÃabÿþýÆóNu¦0¼al§|Íj´ó´ÀnlÇ¾£½hÑ¢×_=L9rdñâÅöí»7Ô±9Úå÷ åÜ5k³wîðáÛo¿=åÃ~|üøñì9µµµÁ.a"Ngÿz¤O8ÔQ/^råt¯YvþØíØwo´³«¬¬´oß½¡ö]ü»´á¯üËÞ¹ãñø¾ûÂrccãÙ³gs~e_½zu¸B[[Ûùóç£+g¯h$ïÔPGµ··L7øÕhçé»n4mÇ¾£),êêê²oß½¡ö]ü»´áo~íÜ±XìàÁa"ì¬aòúW®®H$äêðçÒ¥K¾fû¨=ÝæïÆvì»7ÚQ7nÜH¥Rcccöí»=Ô±6Úeö ókç®««É¯ÑG¸r:þøÖAìõÉzïÞ½ûöí+0øí£ötC¿Û±ïÞhGÏ7n¼zõªûnµGìbv=hÃßüÚ¹·lÙrôèÑ0qîÜ¹eËe_mÑ¢E.»W¯^&:;;:&Â×ð¼¼SCZ¾|ù3g¾fû¨3¤òwc;öÝíW_5ìÀ###öí»=Ô±9Úå÷ ókçmooÞ¦0<<¦¦¦°hÅaoþøÖÉPõõõ±X,Lö÷÷É;5ÔÑoÑ[³£ò_³ÔÎÒÌòwc;öÝíÏ¿°oß¥¡ö]ÌÑ.¿mø$IOê5$Ið'I$ø$IüI$	þ$I$I?I$Á$IàO$Ið'I$ø$IüI$Á$IàO$Ið'I$ø$IüI$	þ$ItÛ~ð,_¾¼òV+W®|þùçÿËcÜ­Jæyª­mhhwmll,g~ÇÉääääloSüIRI¶÷î¼¾÷½ïþöìÙf>óÌ39ó¿ÿýïù=öØnSüIRé588XÇ8póVÃÌ3gÎþ.^¼f¶´´äÌojjó/àOÒ¼èÛßþv`ÍO<=óÉ'3ôÑlúôöö<¶¶¶éÌ¯_¿ÞÕÕU]]ÕÖÖnÛ¶-ûèjOOOVXÖíîîÎáT³`ÁåË<y2ììÌÙ°'N¾hQØ¤hÑ©S§¦ÚêÕ«ÃüþþþÌ×_=ÌYµjUfÎÞ½ëëëÃMUVV®_¿þÊ+ùøË¿ý96UüIÒ§ÜÂ]º=óòåËafCCC6nr®[·.gÑÖ­[£ECCC±XlÊµ¢ÑÒöööÉÉÉÀÇD"qóæÍ°4|xªªªÞWàvÂÄO9-Ú/>_Î¬r¶ø+°©àO>ý¢#¼S<®UTeã¦££ãÆ­ÂD¸¸víÚhidÑxmÑ¢T*½d¦ûúú¢É¾Í]»vÞEG]7mÚæ?~<L¯azÃ·½Çp1Û¦¼G555áþákØò Ît:]!LÃ]øø+++g¿*	þ$éÀ_hJüeæG¸ÌºråJ¸Ø]lmm7oÞÐ6>>¹p×Ò2kE/_¾¹rSæÈoø¦zzn;áâµk×²·mº÷çmß¾=,zê©§>þå¡ímÛ¶e_!@0à/´­­-zar¶ø+°©àO>ý¢»nÜ¸=sll,Ìp'CÃáááÈëdÞ¨46SÙ°¦kkkÄÄÄD¸ñdÞöv¦£XNçÎ¦Ã×0=44YÚ××6`ÊÃÇ3Ç_Môé½ïmß¾Ù3£@É9á#ó¾Àü´Î9³gÏè lFÑËrãª·%ÚÖ­[££½ákWWWf~Û^i»zõjtñÚµkÏÌ]ºtiXºM0kö¢èÌß°èÔ©S£££ñi4¥6UüIÒ§_tÂD<ß¿ôQ/H$ù'g¬]»6@gbb":&937zÏ_ôV¹áááì7FÜ¶m[ RtnæÃV¦$ZtúmôÝ_õÕÌü·½É/zÏßØØXtÍø;xð`æ5¹p³Eoß%Âtø¸¸®Ð¹~ýúì¥6UüIÒ=QôÈ9íÝ»÷ÿ?ÆÝ*²Qf:sÀ4sE¦M6eË2»cÇÀßÇ·þG_[[D¸ÀíÍÈÞ°Ì¶ÓÝÙñññÊÊÊè.äì^³fMö·¨©©	_£OÉ¾ÍèÉLU3Klª$ø¤¥+W&nµ|ùòèÛü:u*ú8½æææÓ§OgíÜ¹³¾¾>ÓöíÛ'&&2K»»»ÛÚÚÉÉä³Ï>sù[n*Ìß²eKÎüén'6&lRô>ç/SWWWÎg¾D¤R©0ÕÕÕá^p!ó±5Ù·9::tÕªU«úûûs¾cM$I?I$Á$IàO$Ið'I$ø$IüI$	þ$I$Ið'I$ø$IüI$	þ$I$I?I$Á$IàO$Iw¢ÿeÅOWMrIEND®B`


I$M*$IüI$	þ$I$I?I$Á$IàO$Ið'I$ø$IüI$	þ$I$Ið'I$ø$IüI$	þ¤)úSeeeuuõÂ÷ïß?ÆÕO:µlÙ²É¼«EÿÌ3Ïë&ç:thÉ%Õ7[´hÑn¹Á±¯2|hjjÚ½÷gÂûÆë¯¿;a*[ÏÛBÅ(Ý%ß8Ið'þrÛ¸qã²»Ó§O¿zõj©àïGþÍÚºuk­kÑö]»vwp&<õõõÙÛ]»v-üIò-Î¸~ýú3Ï<S]]3»ºº¦þ¢U«VþN:hõÙg¸3zzFÜÔxW¾?lÚ´)æÈ&Éï½÷^Lg2»ê[#	þ¤rÃ_Ò;ò^t9vìØ¼yóR©Ôüùó=§ìF¸Â3/^ÝìÏ9³lÙ²ää<ÐÛÛ£ÍÍÍÉíííywu´ÍÞrÅ¼;°nÝºøzèÐ¡ÑÆäÍ7ßfMrÓéôÅó®ûx9q£qÓq,YríÚµ×_½©©©ªªjùòå¹¯2nß¾½®®.9¾7]4Úþâ¿ósÂFõÙÖÖ6â÷w¼«¿éðbÌ;9®Áñ¹Ü&¶baüå-ïKì!1ìþô§cl¿F8|éwã@jdx:ÕÔÔlÜ¸±¯¯Ï/	þ¤Ã_x%f644$zz*++sO:UÉW¯^]`Ýìã¹9wikkkîKV£à+°ÙÂ+ì×¯_¯¯¯¯­­ácòöÛoÇ3zîvâyJË¼ÃowáÂÉ+©I¬üò®¹téÒÂÄ=vÌçw³æÎ;â÷w¼«äÝtÈ&yå¯±±qSxð'¼âØñ×ÝÝ»x°ãÅßwãU«VåÝç6øÅ"ÁTbøKæËét:;::bº³³3hy«'·lÙ244¼sËuã¹3õãúÉËá¤di<éÆÅXýÆÍb­ÜÛ*°ÙÂ+øØ>ûJXîõ×¯_Ó+W®|ïf1cæh7óÐC&w,ÙrzôhîLÞÙväÈ¾téRî¢Ñîp*íd×½ÍUFS]öÑñÎ÷jÂ+fîÅÜÝ X%ÚØñ7ÆÝ8AáåËc:¾1=mÚ4¿X$øJÙ#É[Är9ð÷öÛogçÜrÝì«hÉáÅx*M.Æ3hîKVyíl¶ð£=öÄñ¼7¿¶¶6÷Ås|9sæh7àÈ½ËN`î=Éd2Á@FKKKîc/¿¬ÈGúm®;¤±næ²eË;6â epF¼W^qìøKök×®åÞÄ¸ð7ÆÝ¸¹¹9.Î3'ðEô÷÷û­"ÁTzø¬ÄÌÑqGcJr1SàÙºqÆûô<Í6?r<ÞxÈ?ü³áoþxÇr1 wèóg­ÖÕÕ%Ç©/ÔÙ-e±ÕWËàFÒ­8öÃ¾·¿wq7¾páBâ¿,_|ñE¿X$øJöìÉ=á#y--ï¼Ë±Xg~åæwÞÉ]Z`³W,p<³ÌL|÷UÇ> ¹çÍ|äJàl,øK^L>u%NoÜ¸ñ½÷ÞÛ»woÌfÍøË*Æßx'ÛW;þÆòÊ_wÉ_;Æ¿ÀþtúôémÛ¶%Ç³gFK?©ðOoa ä¹9ûQ/ÉSZ¸!,gçÏ,JÞðÔw³5¬[XNZ÷lÅÆíä½£kÄÍ^±°$V­Z_2'9xåÊ×olí¡ºü%¯~õôôÄCØ½÷Xðwüøñäµ¥xÈÉ«zIñû;ÞUÆ¿Â·oä6ÞQþwéÅÎÐßß¶[±bÅpîÜ¹3?.<þ·üHÞ»yáÂà%Átáox¹g,?6ûöÿìçñ&/Ö,°na9åz]Z`³W,,«W¯fßÝÌ¹xñbÞû½jkk³o(¼üe94sæÌì; çþÍZ°`Aoñ¸VþNÞ¾ÛxGuø;útîö/^»4oL²§v+ö·ìEÙ|ðA¿X$øJñ,¸téÒáÿý×Ñ£G[ZZRñ¾gÏìüÃÇÀVî§ÜqÝ[Ê)ùx¼Øxssóðëm³·°$C¢¹óÏ?¿fÍª¥Óéá§wL½½½±µØf0èáeÍ]8O?ýt<ºxà³gÏÞ·oßg?ûÙì+O·¿Ê¸ðWxpòö¼Æ5ªÀ_ôâ/Î3'ul?9°=Á9.Ñ[_¶lYWW×-_ymëëëÛ¼ysòj>¾~±Hð'ITLæ×gËM`+yÅ79pp01ý¼yóì0üIÊ°ä=y=þøãFF?IRÖ××·iÓ¦Ù³g'ÇvcâG1,üI$	þ$I$I?I$Á$IàO$Ið'I$ø$I?C I$I?I$Á$IàO$Ið'I$øTê>|¸¡¡!J-]ºt¼ë655UTTp!;'¦cÎüùóÇñíf»ÎXÖÈ/ÙQÊ]TYYY]]N§íEàORÉTWW	ÁwÝíÛ·ÇºO>ùdvÎO<s¶mÛVÒø+°ýÜ9ñÀcºµµÕ^$	þ$Î/ÛðÓéÓ§cÝ%Kdç,0ætwwOÎ¼³ø.Çtuuµ½HüI*%ùåfóæÍÓ§O6mÚÖ­[ó®öÙÏ~¶¥¥%oÙcúwÞé,º|ùò²eËÂF©Tª¹¹¹££cÄ­åÞzáUöíÛ×ÐÐ°hÑ¢¸¡á»qãÆÚµkcÝ¸ó6mJ^È.^¼8¶ócËo¿ýö¿óçÏWVV&hhh(î|ö1Ið'énô_2ýØcÅô=Y1ñøãç^çèÑ£Ãßß¶~ýúX´wïÞ¯1ÝÖÖ,Z°`Á#GbâÒ¥K1¿¾¾~Ä­åÞÂ«é<ëÖ­~ç7lØÓq^x!&¶oß3ï»ï¾¾~ýzOOOL$/Løf¾ñâ/ãÓ_úÒEÛ¶mKbçÎ1ñÈ#Ø»$Á¤»1¹Ùpø¦ÀãÇÇ¢+WÆôªU«Õe>zË----É#n-XWIîØôéÓ¯[[[Üù`YLûbf¦³·38¹sûù,EqÓsçÎ»1mÚ´ÆÆÆ¸hïîjü¥R©ìtLÄÅÑ-¤Ököõõ%GWcN²h÷îÝ±VðÌ3¹[ÈÛZîÅ±¬2Úå¾ÀñÐ¡C3fÌHæÔÔÔ$/å¿KÎwrKüIºKñW__ûÊ_òâÙ-Å³víÚXý_UUsn6Fü^%ÇÂsÃ×9sf²nÞÝ<räÈºuër_2ÀàÜr(®_¿^[[N§/^ ¾ví½KüIº«ñ|tËÞ½÷ü=ñÄcÁß¡C²/¡<x0;?¡äÙ³g÷WÙ²eËsÏ=ë×¯¾î>Óû÷ï?wî¨7f.Z´(¦Ï9ÆÆÆ7nE§OîììÌc¢$Á¤»¬i7Ëý¬¾ÂøëïïO¹Æ×ìà¢_|±¦¦fúôé6m#þ¯rìØ±XºpáÂìy'¹×éëë~Å=¯ªªZ±bErbo/N'§/Y²d¼§ßÖÜO½Y¼xqéé±I?I$Á$IàO$Ið'I$I?I$Á_	÷üà­·ÞÌ[ü»¿û»ý×µç±¾¾¾ä8TÜuÿô/ÿò/vÔ¢wéÒ¥û·3EìÆW®Åíg?ûÙ]µ£NuüýÁüAøo¹ûéeºýþáþáÔ©SÆ¡¸½þúëÿüÏÿlØµk×ÞxããPÜ¾ÿýïVC»zõê_þå_âvüøñøóþàOðð'ø?ø?øüÁü	þàþàþð?ø?ø?ÁüÁüÁüÁüÁü	þàþðð?ø?ÁüÁüÁàþàOððððð?ø?ø?ÁüÁàþàþàOðð'ø?ø?øüÁüÁüÁüÁüÁàþàþàOðð'ø?ø?øüÁü	þàþàþð?ø¿©¿îîîúúúT*ÕÔÔÔÑÑ»h```ÝºuUUU÷Ýw_>üÁàþàOð%_:Þ¿LìÚµ«µµ5wÑöíÛüñ¡¡¡ú9sæÇ_ûI,~U]¹rå×¥KN8a[ü¼Äsq(b?ûÙÏN<iÛ«¯¾ú÷ÿ÷Æ¡]¼xñ?ü¡q(nñçßdÞâÀ_MMMð.&2LCCCî¢¦¦¦sçÎ¶bàoçÎßÄ9;Á÷U¼^yåï~÷»ÆÁjG½ôÒKá?ãPÄ;fG-uJà/J8ÞUWWÏ3çÌ3û:ì+öuØWû:ì[òUVVf§«ªªòíÝ»7&Î?ßÒÒð'ø?øüÁ_ÉW[[ÉdÞ¿yØ7¦óe§ó^?øüÁü	þà¯$[»ví¾ûb"¾¦ÓéÜE=ôÐÁcâìÙ³-?øüÁü	þà¯äëìì¬«««¬¬¬¯¯ïêêúÏÇVñ.¤µzõêT*ÕÒÒráÂø?ÁüÁàþ¦nð?ø?ÁüÁüÁüÁüÁü	þàþàþð?ø?ø?ÁüÁß¸:yòäO>ù­oë­·Þ?ø?ÁüÁà¯lñ700ðÏ|fÖ¬YúÔ§-ZôáøÀðð'ø?øü'þ~÷w÷ù7uóÖ¯m¿µî·Â'O?ø?ÁüÁà¯ñ7öìõ¿½>_òoÉ'|ýë_?ø?ÁüÁà¯ñ7ýÃÓ·ü¿-¹øûÜç>÷å/þàþð¿2Äß'>ñ/¬þB.þæÿêü=öÀüÁàþàOðWøçÁ|ä#÷ß°oóW7ÿ¯%ÿë~éúúúàþàOðð'ø+CüEÏ?ÿüìÙ³kjjfÌ±téÒ+W®÷¨ÂüÁüÁü	þ¦4þÞ÷Ý©0ªððð?øBÁüÁüÁü	þàþàþð?ø?ø?ÁüÁàþàþàOðð'ø?ø?ø?ÁüÁàþàþàOðð'ø?ø?øüÁü	þàþàþðð'ø?ø?øüÁü	þàþàþð?ø?ø?ÁüÁàþàþàþðð?ø?ÁüÁüÁàþàOððð'ø?øüÁüÁüÁàþàOððð'ø?øüÁüÁü	þàþðð?ø?ø?ø?ø?øüÁü	þàþàþð?ø?ø?ÁüÁàþàþàþàþàþàþð?ø?ø?ÁüÁàþàþàOðð'ø?ø?ø?ø?ø?ÁüÁüÁàþàOððð'ø?øüÁüÁü	þàþððð'ø?øüÁüÁü	þàþðð?ø?ÁüÁüÁàþàþàþð?ø?ø?ÁüÁàþàþàOðð'ø?ø?øüÁüÁàþàþàOðð'ø?ø?øüÁü	þàþàþð?ø?ø?ø3ðð'ø?ø?øüÁü	þàþàþð?ø?ø?ÁüÁüÁüÁüÁü	þàþàþð?ø?øKÝÝÝõõõ©Tª©©©££cøÚÛÛ+**àþð?ø+Òéôþýûcb×®]­­­yKGÃßÿùÿ|åWþæoþæç*^?ùÉO^í5ãPôõÂÆ¡=ÖZô^~ùå¿ýÛ¿5EìÇ?þñ~ðãPôõÍ7ßÌ[ø«©©L&ÓÐÐ·ô±ÇÛ±cÇhøÛ»wïI¬½½ýÔ©SgT¼N<yìØ1ãPÜ¾÷½ïÙQ¾£©CÑwÔ7ÞxÃ8±'NØQÞÑ£G'yGøK¥R#NG/_nii	:ìë°¯öuØ×a_9ìë°oà¯²²2;]UU»hõêÕñ4öþàOðð'ø¿òÖÚÚÚL&óþÍÃ¾1ý?áÿþàOðð'ø¿oíÚµûöíøN§G~¨^ù?ÁüÁàþÊcX;;;ëêê*++ëëë»ººFÔüÁàþàOð>äþàOðð'ø?ø?ø?ø?ø?ÁüÁüÁàþàOððð'ø?øüÁüÁü	þàþàþàþàþàOððð'ø?øüÁüÁü	þàþðð?ø?ÁüÁüÁü	þàþðð?ø?ÁüÁüÁàþàOððð'ø?ø?ÁüÁüÁàþàOððð'ø?øüÁüÁü	þàþððð'ø?ø?øüÁü	þàþàþð?ø?ø?ÁüÁàþàþàþð?ø?ø?ÁüÁàþàþàOðð'ø?ø?øüÁüÁq?ø?ø?ÁüÁàþàþàOðð'ø?ø?øüÁü	þàþàþàþàþàþàOðð'ø?ø?øüÁü	þàþàþð?ø?ø?ø?ø?øüÁüÁü	þàþðW4üUÜªÊÊJø?ø?ÁüÁü	þ*oU*?ø?øüÁüÁÃ¾ððð'ø?ø+/üoÖ¯_ðð'ø?ø¿2Ä_CCC*ò?ø?øüÁü©üñ7þüágÔÔÔôõõÁüÁü	þàþà¯ÜðWUUÚëíí­««0ßsÏ=mmmðð?ø?ø+7ü%/õÅDh/&.]º444Ó¦M?ø?øüÁüÁ_¹áoÆA½ÎÎÎ.ÄÄ#<Lø¨ø?øüÁü©ñ·iÓ¦ìé¹oû[´hüÁüÁàþàþÊðlßGtæÌ1ÑÕÕÁø~Àü	þàþð7?øüÁü	þàþàþàþàþàOð7zÉ¾øgø?øüÁü©Ìñ7wîÜes¶/üÁü	þàþTøçûâÙwhh¨ä¾ð?ø?Áü¯À_)ÊþàOðð'ø¿q×ÝÝøÛ°aC)þÂü	þàþð7îfÏ]1,'|ÀüÁàþàOe¿9sæ8áþàþð*øKØ×ÓÓSßø?ÁüÁàþÆWmm­>àþàOðð§©¿xH¿M6ÀüÁü	þàþTæø«%'|ÀüÁàþàOåù!Ï#æø?øüÁü©<?ê¥t?øüÁü	þào|544Ì3çÜ¹swðatww×××§R©¦¦¦ÜE]]]ÍÍÍ±hþüùq5ø?ÁüÁàþn«pUEÅ~1Nïß¿?&víÚÕÚÚ»hîÜ¹'N466Âü	þàþðw[uttþ¶mÛ¦¹SøRSSÜt&ihhíjÕÕÕÃñ÷gög?Ä^~ùåÓ§OÿDÅ+Pc'4Å­½½ýG?úq°£Þåïß;sæq(bñ·ô«¯¾jJGýÀñw7írÉh'ÄÝÖÖ6¼8;v,¾+U¼þú¯ÿ:þ¨2vÔ»¼¿ú«¿úþ÷¿o[ü9îÜ9ãPÄÂ(¯½öq(nñçôOúÓÉ¼ÅwÃÙ¾¹Ð¬ªª~7n¤Óé¾¾>öÃ¾û:ì+ö-ùjkk3LrØ7¦ïÊëÖ­ß¼ÃW?øüÁü	þà¯ôZ»ví¾ûb"¾¦Óé¼_¾|yooï+Âü	þàþð7î2Ì+ª««+**¦M¶zõêI>ó£³³³®®®²²²¾¾¾««ë?ÛÍsrßð'ø?øüÁßmÕßß?â	%ñ_ýÂü	þàþð7¾æÍÔ[¹reò²YµjUÌY°`üÁüÁàþàþÊUUUA½ÁÁÁìL&sF<ëþàþàþð¥¿ÊÊÊ ^r²mÒÀÀ@ÌÌz?ø3ðð'ø¿IÂ_rØwùòåÉO`|éÓÜÜðð'ø?ø¿rÃ_üàxÂÇï½ðð'ø?ø¿2ü¨þþþÕ«WO>½²²2¾._¾<æÄ÷þàOðð'ø¿)üÁàþàOððððð¿¼5oUee%üÁüÁàþàþÊ£ðð'ø?øS¹áo´~øá?ø?øüÁüÁ_Ùâ¯»»Ú´iÁ¾eËå~æ3üÁüÁàþàþÊkÖ¬I^ð;räH	?àþð?ø_/¼ðBÂ¾+WÜ÷þàOðð'ø¿±Ößß¿`ÁäÜRü~Àü	þàþð7¦vîÜ¼à×ÚÚZºßø?ÁüÁàþÆ¶¦Ïù?ø?ÁüÁ¦þ*oU*?ø?øüÁüÁÿÞþàþàþðð?ø?ÁüÁüÁàþàOððð'ø?ø?ø?ø?ø?ÁüÁàþàþàOðð'ø¿¢á¯±±±ªªÊçüÁüÁàþàOå¿¹sçæÏçüÁüÁàþàOå¿p^°/Jîûð'ø?øüÁßøª©©	ü¢üàþð?øwÝÝÝ¿6âO üÁàþàOðãnöìÙÃrÂüÁü	þàþTø3g>àþàOðð§©¿===¥øý?øüÁü	þào|ÕÖÖ:áþàþð*øøÛ´iÓÀÀüÁüÁàþàOe¿QrÂüÁü	þàþTò<bNø?ø?ÁüÁÊó£^J7ø?ÁüÁàþàþàþàþàþ£ÉdV¬XQ]]]QQ1mÚ´Õ«WÊÉ¿ð?ø?Áü¯þþþOø(áþð?ø_óæÍê­2ù	Ù¬Zµ*æ,X°þàþàOððå¿ªªª Þàà`vN&91þàþàOððeøQ/A½_vÎÀÀ@ÌñQ/ðð'ø?øSÙö]¾|yò_c:æ477ÃüÁü	þàþà¯Üð?x#ðñÞïÁüÁü	þàþà¯?ê¥¿¿õêÕÓ§O¯¬¬¯Ë/9%ñý?øüÁü	þàoð'ø?øüÁßøjhh3gÎ¹sçàþàþð*ü¥R©RþàOðð'ø¿ñÕÑÑøÛ¶m[¦TþW7ø?ÁüÁàþ&ºQª¬¬?ø?øüÁüÁ_~Èóùgø?øüÁü©|ð7gÎ¦¦¦¾¾¾þ~Àü	þàþð7¦ÿÒ÷ýKâ/üÁàþàOðÇ_ò_ú&ÿ/üÁüÁàþàOe¿úúú9áþàþð*üÅÓí3×ÿê9áþàþð*[üe+çÁü	þàþðçÿö?øüÁü	þàïÎÕÝÝ]__J¥:::/Øø?ÁüÁàþîÒéôþýûcb×®]­­­MlNn6mª««»o=ö*^÷Þï=÷Ücì¨vT;ªì¨wg³fÍäµÄðWSSüÁL¦¡¡¡ð¢ÍÉíá®$I*£>pü?¾øË=w$ï<á&6þ$IüÝÆãóÒÜÜüæoÞþÖr?°ªªªð¢ÍÉí÷ÿ÷·nÝú­I,nîOþäO¾¥âõGôG_ÿú×CqûÚ×¾¶÷nãPÄ~úémÛ¶â¶eË?ýÓ?5Eì©§úÆ7¾a¾£îÙ³g2oñÇ_°/+ÍT*µdÉ/NxkµµµL&9JÓMl>ß²sçÎ8áCNøpÂ>ðað1Ö¿óæÍË*°ªªjÅØÎÚµk÷íÛñ5N^4±9ðÛÉ'?ö±ýâ/þbãÇ«««ÿøÿþðð'økCCCG	CTLô¿wëìì¬««uëëë»ººþó^Þ<z=|ÑÄæÀ_¶·Þzë£ýè/¬Ùúµ­ñ¯íÿ´Í5ëÛßþ6ü	þàþàOðwzzzr_ù>z[[ÛÝÿýâøÛ´iÓO.Iäüûµ¿1wîüÁüÁàoÔrÍW]]ÝÚÚzùòåRù~Lqüæ3¹ÿþûsñÿ>ô¡Áàþàþ£oâæyþô§/]ºTrß)¿/ùËá¿­ÿíõ÷Þ/ü	þàþàOð7jEùø»#Åù_ø_"¿Í_Ýü+¿ò¿÷ð'ø?ø?Á_yælß§zê#ùÈO.Yºté=÷ÜóùÏ¾ä>ðþàþàOð¿³gÏ666N6-9Ã·¶¶öÐ¡CðWøÿæüóß|úé§cï,¹*ø?ø?ÁüM*þÚÛÛ³'|$øK¦wíÚ%¿RþàþàOð¿ººº ÞÙ³g³øëêêJ>íþàþàOððex¶oò!ÌYü%§ÃüÁü	þàþà¯ÜðW[[ÔK^íüe2Í7ÇtCCüÁüÁàþàþÊñ*FêÄðð?ø?ø+Ã³ãZKKKr¶ouuuccãÅKâûð'ø?øüÁßþàOðð'ø?ø?ø?ø?ø?ÁßH]¾|¹¹¹yúôé7¦¦¦R9æð'ø?øüÁß8zðÁ+Fiýúõðð?ø?þ:8o÷îÝÉÌþþþÃ'ó=ðð'ø?ø¿2Á_oïÞ½Ã%ÿáÏù?ø?ÁüÁÊUUU!¼þþþá2L,+ÀüÁü	þàþà¯Lðý_ÝF[üWoðð?ø?ø+üàüÁüÁàþàOððð?ø¿Å_áàþàþð*üUÞªT*ðð'ø?ø?ÿ½üÁüÁü	þàþàþð?ø?ø?ÁüÁàþàþàOððððð?ø?ÁüÁüÁàþàOððð'ø?øüÁüÁüÁüÁüÁàþàþàOðð'ø?ø?øüÁü	þàþàþð?ø?ø?øüÁüÁü	þàþðð?ø?ÁüÁüÁàþàOððð?ø?ÁüÁüÁàþàOððð'ø?øüÁüÁü	þàþàOððð'ø?øüÁüÁü	þàþðð?ø?ÁüÁüÁü	þàþàþð?ø?ø?ÁüÁàþàþàOðð'ø?ø?ø?ãð?ø?ø?ÁüÁàþàþàOðð'ø?ø?øüÁüÁüÁüÁüÁàþàOððð'ø?øüÁüÁü	þàþðððððð'ø?øüÁüÁü	þàþððW¨îîîúúúT*ÕÔÔÔÑÑ»¨«««¹¹9Í??®ð'ø?øüÁ_ÉN§÷ïß»víjmmÍ]4wîÜ'NÄÄáþð?ø+ùjjjb"É444vµêêêáøâ'Nb/¾øâw¿ûÝ£*^GQ5vT;ªUEÙQ_zé%ãPê;êÀ_*q:·S§NµµµÇ_xüß'±×_½··÷ßU¼âïÔþðÆ¡¸½öÚkï¾û®q(bW®ßBÆ¡¸Å/ðú§2Eìç?ÿùo¼aÛ«¯¾zýúõÉ¼Å)¿ÊÊÊìtUUÕð+Ü¸q#N÷õõ9ìë°¯öuØ×a_9ìë°oIVñ_Åtmmm&IûÆôð]yÝºuñwøFàþð?ø+½Ö®]»oß¾¯ét:oÄ/_ÞÛÛ;âð?ø?Áü^uuuõõõ]]]ÿùØn¾(ØÐÐPüÁàþàOðS7ø?ÁüÁàþàþàþàþàþðð?ø?ÁüÁüÁàþàOððð'ø?øüÁüÁüÁàþàþàOðð'ø?ø?øüÁü	þàþàþð?ø?ø?øüÁü	þàþàþtwáïâÅßüæ7yæIþé?øüÁüÁüi²ñ÷ío»ººú×ý×?õ©OÍ5ëóÿüÀÀü	þàþàþe¿.Ì1ã7øÍ­_Ûÿ6uó¯4þÊ¦MàOððð'ø+Cü=ùäÉD~É¿¶ÿÓvï½÷ÂàþàþàOðWøûÊW¾ò¹Ï.ñïCúü	þàþàþe¿gyæ×~í×rå÷kcîÜ¹ð'ø?ø?øü!þb³fÍZñ¿Wlù[c¾qñÀð'ø?ø?øü!þ¢ìcÿøÇ«««zê©)2¤ðððºcø÷ÝwÛÂ_ÒÅCSgHáþàþàþtð·uëÖéÓ§×ÖÖÎ1ãw~çwàÁßþàþàþàO¿íÛ·ßï½ëýÖ¯mýÊ¯Ì7ïþûï7ðð'ø?ø¿2Ä___ß?üáÿûÐÿÍgºù«?úÑ^¸pÁÂüÁàþàþÊ===y0÷O|â;ßù1?ø?ÁüÁüþ®rÏ=÷$0ý7oÞ¼ø4ðð'ø?ø¿rÃ_ô«¿ú«Ë/ÏÊï«¿PSSãÌ_ø?øüÁüÁ_yâïÊ+÷ÝwßÇ?þñ¥KÎ?Ö¬Y?úÑ üÁü	þàþà¯<ñ÷þÍÓ>¾óï<ùäÏ?ÿ<ÜÀüÁàþàþÊ?ø?ÁüÁüÁàþàOððð?ø?ÁüÁüÁàþàOððð'ø?øüÁüÁü	þàþàþàÏOüÁü	þàþàþð?ø?ø?ÁüÁàþàþàOððððð?ø?ÁüÁüÁàþàOððð'ø?øüÁüÁüÁüÁüÁàþàþàOðð'ø?ø?øüÁü	þàþàþð?ø?ø?øüÁüÁü	þàþðð?ø?ÁüÁüÁàþàOððð?ø?ÁüÁüÁàþàOððð'ø?øüÁüÁü	þàþàOððð'ø?øüÁüÁü	þàþðð?ø?ÁüÁüÁü	þàþàþð?ø?ø?ÁüÁàþàþàOðð'ø?ø?ø?ÁüÁàþ¦þº»»ëëëS©TSSSGGÇð+´··WTTÀü	þàþðW¥Óéýû÷ÇÄ®]»Z[[ó677¿W^y¥íµ×â9 _ÅëòåË'O4Å-SCÕÆ¡½õÖ[]]]Æ¡¸½úê«ÿøÿhØ¥KâÏiãPÜ:::z'ó§þjjjb"É444ä-ì±ÇvìØ1þþðÿðIì¥^zùå_Qñjoo?räq°£ÚQí¨²£ÚQ¦þR©ÔÓÑåË[ZZû:ì+öuØWû:ì[&ø«¬¬ÌNWUUå.Z½zu<ýÇC?øüÁü	þà¯t²â¿éÚÚÚL&óþÍÃ¾1=âÕ²W?øüÁü	þà¯´[»ví¾ûb"¾¦ÓéÑ°8|&üÁàþàOð¥Wggg]]]eeeWW×Ú?øüÁü	þàÏ<Ãü	þàþððððð'ø?ø?øüÁü	þàþàþð?ø?ø?ÁüÁüÁüÁüÁü	þàþàþð?ø?ø?ÁüÁàþàþàOðð'ø?ø?ø?ÁüÁàþàþàOðð'ø?ø?øüÁü	þàojõo|ãùçkûñü×éÓ§>lÛsÏ=wöìYãPÄâ	õ/þâ/Cq;xðà¹sçC?Q^zé%ãPôõ§?ýédÞbá¿Þ§:þâémëÖ­ IT.>ªYáÅXI¤©üI$Á$IàO$Ið'I$ø$IüI$	þÊªÞÞÞÿå*++s¯yíÚµÜ¥nì£züøñyóæ¥R©¦¦¦ÎÎÎÜkvww×××':::Ýí©uÂ£zþüùªªª5kÖäýï>vÔ¢©uuuu577Ç¾7þüØïvÔ¢éßQýl¿xÖgÐ¿û÷ïßµkWîC­[·Î M`Tkjj._¾ñ5÷!J§Ó1Ô1£ÝÚÚjônHí¨ÕÄ³BL;wî¡²£~ CjGcsçÎ=qâDL8p ±±±ðÞhG-úÞñþß+.0.]ZºtiÞÌøö>|Ø M`TçÌsõêÕ÷oþO1å@ÌÐÐPLd2<ÄhbCjGð¨ÆßýÙé3fØQ?Ð!µ£N êêêÂ£µèCzÇwTøûÀFvþV¯^êÔ©á+,_¾<~µ´´?Þ¸T»»»ã,æÄ×¼ÍnÈÖÔ:áQ?þÙ³gcâÙgÍÛí¨ER;êxô¶¶¶Â£µèCzÇwTø$üÅ_®.,pý«W¯Æo4ã6öQ]°`Á¹sç²ämî+«ªªÛí©uÂ£zæÌÆÆÆðô;×ì¨ÜÚQÇÕ7Òét___á½ÑZô!½ã;*üMþ¶oß¾sçÎÂ«ø¡×¨ø£ª¶¶6É¼óÅö6n·?¤vÔÛùñOzóÍ7çÍgGý@Ô:öëÖ­»víÚ-÷F;jÑôï¨ð7I¿ª/^|úôéáW;wîÅfùòåÆmì£ºpáÂäÐOOOÏr¯¶víÚûöÅD|?ÂÛí©uÂ£C×ÝÝ=44´cÇGÔú©u?~<Æ§··w,£µèCzÇwTø$üí7~æ]áÔ©SÉk,Y²$y¯½Æ8ªñG%.¾Ætî:;;ëêê*++ëëë³uCjGð¨ÆSÂìÙ³ã7@[[Ûàà õR;êkhhÈû¨÷F;ê4¤w|G?I¤©ô×!$I?I$Á$IàO$Ið'I$ø$IüI$	þ$I$I?I$Á$IàO$	þ$I$I?I$Á$IàO$Ið'I$ø¤[öì³Ï.^¼¸úfK.áþÇï¸ÌoäîmCCC<´¾¾¾¼ù1'JÕ××wàOJ²­[·VëÑG-'ümÛ¶-f>óÌ3yó~úéÿÈ#L`àOJ¯îîî`M*Ú½÷àÍöîÝcæéÓ§Ë.]óçÏÏ?oÞ¼ñâEø¦D_üâ5?þxîÌ'x"f~éK_Ê¥OGGGà)ÜÜÓÙ+¿÷ÞmmmÓ§OE5557nÌ=ºzìØ±V,u=Ç©3cÆÅ9r$.®]»6ï½øâ·,»,joojË/ù]]]Ù9'N9Ë-ËÎÙ¾]]]lªººzÍ5W¯^¿áÛÏSà®J?IºÃÍ=;àrùòåÜo¿ývÌlhhÈÅM^§NJ®Zµ*oÑE===#®®^½zhh(øXUU588KãkàiÚ´iÉ[ñl'&F¼ÃérEõåþýû³òËÛÈÒ¥KÇ¿wUüIÒ/9Â;Âïµ X.nZ[[oÜ,&ââÊ+¥u>&/àÚEét:yÉ0¦;;;änsË-Á»ä¨ë>s>Óñ5¦xà[n'àã¹÷mÄG 9sf<ÞÞÞÞ¸_ã83LrúúúX1Âûÿu¸ººz¼ø+pW%Á$ÝøFÄ_v~ÄLÑÕ«Wãb°)¹ØÜÜçÌ³~ýú@[v#q¼×Ò²k%ß~ûíìLÙ#¿ñ5¦;vËí4ãâ;ï¼ßFÞÃ?vìØñþÚÞ¸qcî¿ iKKKòÂäxñWà®J?Iºó%/wÝ¸q#wf___ÌE¸¥áÿe­£Þð£±yÊý®©©©ªªÏ1#»ôÛby=6qjLÇ×îééÉ.íìì;0âáã±ã¯À]tçKÞ÷¶sçÎÜÉ äð_`ooïð´N>½mÛ¶ä lVÉËrÙãª·$Ú£½ñµ­­-;¿ÀvWÚ®]»çw»páÂX|ºM5wQræo,joo¿~ýzaüeaFvi»*	þ$éÎ0J¥víÚ|ÔËîÝ»«ªª±råÊÎÀÀ@rL6fnò¿ä­r.³`bÁ7óp³¶2"ÑÓo[?~üxv~í$oòKÞó×××þöîÝM.rî¢äí===q+1£á/áfp9®èfMîÒwUüIÒ]QòÈymß¾ý¿ÇÝ,±Qv:À4E¶|0W¹:t¨þÞ¿ù_qÄüÜ#Â¶w#÷eÏ´íÁö÷÷WWW'!ï`÷+robæÌñ5ù´Üm&/LfK¨]Zà®J?Iº[ ,]º´êf/NN¹ÍÃ_òqzMMM¯¿þzvi__ßæÍëêê0=üðÃÙ¥Gmii	Õ××ïÙ³'oÃïIl*æ?ôÐCyóGÛNw&îRò>ç/[[[[Þg¾$õöö¦ÓééÓ§Ç£¸xñböckr·yýúõÐm2VË-ëêêÊ»ÅwUüI$	þ$I$I?I$Á$IàO$Ið'I$ø$IüI$Á$IàO$Ið'I$ø$IüI$	þ$I$I?I$£ÿ¼121lAöIEND®B`


ñ¯ÖÖÖûöÍ`áÌøµqêÔ©x¦Óé¸÷¢[¨¤2YqàOª0ü¶uëÖdå¿úúú«W¯Vþì±ñ+kçÎ%nmZ³LözØ»wïtÎc&ÉßoWWüIòÆ>DgvíÙg­««wþ¢µk×Vþúûû­>ÿüóccc7oÞ¸#Ï;7áMMwñ¯mÛ¶ÅÙ¬á/ñwÞáWV«FüIÕ¿¤=öít9yòä¢EÒéôâÅ?^§ü$q¹sç._¾¼Ä¼ù+=vÕªUÉÉ6Þi[[[2ã'êd7ÛÀÆãçáÃ'[&o¼ñÆúõëGÍf/]ºTtÍÂç;»°bÅ¡¡¡S§Nµ¶¶ÖÖÖvttîeÜ½wSSSr|3î"?i²üÐCÅøÂ°Q`=FvwwO¸~§;Ëø»/ÆxÓZ8î+lf3Æ_ÑÔX/ñÅþÀÄ²-½püÔ)¾©±$ÃÓ1©¡¡aëÖ­###6,üI¿ðJlnnN.;w.JþJîïï/¿äÊëÖ­+1oþÊñ»¹pjgggá.«Éð¸ÙÒ3î×®]Ëd210~¼ùæñ½ðvâEJ+|¾ãïwéÒ¥ÉÔ¤H^~E×reiâÌ??Æ¿ýöÛãWÖ&Ó¥è®C6É¿Nká^ø3qêø(¼x²ÓÅß_Æk×®-zÌ[¶l±aàOª0ü%ãeÉp6===1ÜÛÛ[H´¢Ù;vì¸yóf²/ç¶óÆïÎø­×Ov7©ñK7.Æì×osÞW-=ãÏýÈ#Â¯¿iÓ¦^³fÍ;·¸#'¾ÉÍ7%,¹å¸xüøñÂ'|²íØ±c1|ùòåÂI=àt:=ÙÉÏû>gìCuù=£Ó]8>ªÏXõ_Áú<Ñ¦¿)¾^¹r%cmÆð9slX$ø*ù#ÉGÄ1%ð÷æoæÇÜvÞü^´äðbü*M.ÆoÐÂ]VEíJÜlé'îã÷zÑøÆÆÆÂg¿ããâ¼yó&¾ÉGáÅäXvòI..2ÚÛÛ	üåE>Ôßç,4æ¹jÕª'ON¸Ð¦²p&|T3qêøK^!CCCw1-üMñeÜÖÖ[ZZ¯ñ¿ÑÑQ[	þ¤ÊÃ_`%F644Löw2¦$C9%~[ Ît=Ïàf'O9oü¿qãFáøñÎÆnüóÊÅfÑ¡ÏÛµÚÔÔ§/¼"Rçoa*³LeYMx©,ÉH:³§~Ø÷ý¿º¦ø2LüGáÑ£GmX$ø*Ï=÷	É¾´¢ó.§biÍ[zÏÍÛo¿]8µÄÍ±ÄxáâbþQÏöQ±xê¤ðâ¢E¯qâDàl*øKvO&ßºÍf·nÝúÎ;ïìß¿?F®_¿~BüMeãoº'ßg:þ¦²ç/Ï»ä;%×[Ò3gvíÚkÎ-	þ¤À_üz%¿ó_õüJ7Ä/ËääÙÅ'<ÜjÂß%æ--§D-Ég¶âÆÛ)úD×7[zÆÒX»vmþsýÉä5kÖUrk7o~ÿøKö~;w.Â¾û¦¿×^-Ù·O¹··7Ù«täÈ	×ïtgþJ/¢×FaÓ]ª3À_ò)½x1íV¯^=O?ýt,üb8¸ôò¿í[ ùìæàà`CðàO*#ü¯ðÅñçÏæ?þÿ>Þd7áøß%æ--§PQÂ£ÂS/óSKÜléKKâêÕ«ùOw%c.]ºTôy¯ÆÆÆüßþòI7o^þ%³aÃñ+kÉ%%Vñ´fþJ/¢×FaÓ]ª3Àß3goùòåSIþóÉî¥Äë-FQ¾~ØE?©bð¿W®þÏ?~¼½½= ¿Ñî¹üø#GÄÀVá·ÜMqÞÛÊ)ùz¼¸ñ¶¶¶ñ_×7ÙÍÞvÆÒH¿xñâúõëkoÍfÇÞ13üÇ­ÅmôÑðPÞÜ¥óÌ3ÏÄ³'>þü|æ3Éïyzÿ³L¥NÑk£¨i-Õà/:zôhKKK<ë¸ýäÀzþç¸DKîÕªU·Ýó:ÙëmdddûöíÉ.Õ|¬Ê7nØ°Hð'IV¹ÃÓún¹ÌRq%|Ó·ÇÆÆÓ/Z´ÈF?IR|æ¯¨'|ÒàOTlÛ¶mþüùÉ±Ýxì±Ç,	þ$I$I?I$Á$IàO$Ið'I$ø$IüI$ÁE I$I?I$Á$IàO$Ið'I$øTÑ9r¤¹¹9N¯rºó¶¶¶ÖÔÔæÇÄpY¼xñ46g·Ùu¦2ï7²%ï1J¥RuuuÙlvxx8422²eËXõõõ1é7ÞðÊ«¦¦¦ LflllºóîÞ½;æýÖ·¾óÔSOÅ]»vU7þáxâ1ÜÙÙ¸qcééáS§NÅð¼À$Á¤2Û¼?9s&æ]±bE~ÌÒ¥KcÌÀÀÀìSlöï1¸ÃuuuÉÅÚÚÚ¸xíÚ5/*Ið'©¬åWíÛ·×××Ï3gçÎEWûÌg>ÓÞÞ^tù1üöÛoÇpI&]¹reÕªUa£t:ÝÖÖìk÷^z477/[¶,îh<Å®_¿ÞÕÕóÆß¶m[²#3xº|ùò¸µ·üæo~xø»÷Þãâ¢Ez½´$Á¤²ö_2üÄOÄðsÏ=Ì'|²ð:ÇÏ¾-ß¦MbÒþýûc8~Æpwww2iÉ%ÇË/ÇøL&3á­>Ò³é^xáØ¸qãø¿eË+¼øâ1°÷î<È®]»vîÜ¹HvLoø»yóf,þêW¿L:räHþW®öìY¯.Ið'©¬ñ×ÜÜÃ¹[·×|íµ×bÒ5kbxíÚµêòSÏ9³cÇöööä	o­Y¥gIXýøy,`_kÆp[[[pöÆ3[,ãÇ'-n<äWx·¿¿?ü¿NÜ©$øT¾øK§Óùá¥%ôÞ­`sæÌk$GWcL2iß¾1WðìÙ³·Ptk§2Ëd,4V¸3/ãáÃçÎihhHv~ þJÔÓÓÜ,<.Ið'©ñÉd÷ü%;Ïn+®®®ÿqóVSÄ_éYò,<7~Þyóæ%ó=¼±±±cÇ%§âæwNk±LQò8ãéxI?Iå¿ä«[öïß|æï©§x>ßöÂ/äÇ'<þ|òYÀ©à¯ô,;vìøÎw¾6m?ïÃ?Ã¼páBr¨7F.[¶,Ï=;44.üðð·xñâtâÄ>zôhoØ°ÁLüI*_ü°æÜªð»úJãott49ä??jhh¨¯¯ß¶mÛñWz'OÆÔ¥KæÏ;)¼ÎÈÈÈÖ­[ã×ÖÖ®^½:9±7ÌÍfÓW¬XqñâÅi-ñK,¸Ç®®®xð±æÍ·eËxH^`àO$Ið'I$ø$IüI$Á$IàO$IðWÙýõ_ÿõ~ô£Ù¼ÇþçþÉO~âWn~·Ê¤X)ÿú¯ÿj9[±å-§¦ÒüÇZeØ?ýÓ?ýô§?¿réþàÂ³ÌMÈ(ÃÎ;.·Ê­X)±j,2Dù,o95nÞ¼ùùCöÚk¯ýû¿ÿ;üÁàOð?ø?øü	þàOðð?øü	þàþàOð?ÁüÁü	þàOð?ø?Áü	þàOðð'ø?Áü	þàþàOð'ø?ÁüÁü	þð'ø?ø?Áü	þðð'ø?ÁüÁüÁü	þàOðð?Áü	þàþàOð?ÁüÁü	þàOð'ø¿»L&N·¶¶öôô¿Â'jjjàOð?øüUCÙlöàÁ1°wïÞÎÎÎ¢©cccmmmá/ûþ÷¿ÿÖ[o]Wõ·û·?üá-r+VJ¬Ë¡ÜXlÊ,rëÝwßüYeXOOÏÐÐÐlÞã]¿øOär¹æææ¢©O<ñÄ=&ÃßÓO?ýýYìØ±cñ"ø¾Ê¬ï~÷»¯¼òåPnÅJUc9[±MåP½ôÒKB(õÕWgóïü¥Óé	£+W®´··öÃ¾ûÊa_å°oà/Jåkkk'­[·îÔ©SÿùTáOð?øüUÇbmllÌårïÝ:ìÃÿãþÏàOð?øüU|]]]øÍf'~ªöü	þàOð¿êX¬½½½MMM©T*ÉôõõM¨=øüÁàþ¾äþð'ø?ÁüÁü	þð'ø?ø?Áàþðð'ø?Áàþàþð'ø?ø?ø?Áü	þàþàOð'ø?Áßíûö·¿½jÕªO|â_úÒ.ð?øüÁ_ÕöùÏþã-ÿÊC_yä·¹ÿþûáþàOð?ø«Â=ú³?û³ÛwûÎoìLþ=ðktttÀüÁàþðWãßíååÿ¶ÛÜ¹sáþàOð?ø«NüúºÛw;üÁü	þàOðÕÙÄïwØþàOð?ø«ÒOøøµ_û5'|Àü	þàOðUÞ7¿ùÍeË-X°à×ý×ÕüÁàþð'ø?øüÁàþð?øüÁàþàþ?øüÁüÁàOð?ø?øüÁàOðð?øü	þàþàOð?ø?øü	þàOðð?ø?øüÁüÁàþ?ø?øüÁàþ,ø?øüÁàþð?øüÁàþàþ?øüÁüÁàOð?ø?øüÁàOðð?øü	þàþàOð?ø?øü	þàOðð?Áü	þàþàOð?ÁüÁü	þàOððð?øüÁàþàOð?øüÁüÁüÁàþðð'øüÁàþàþt÷âolllppðÂ1?ÁüÁü©ñ÷ê«¯~ô£½÷Þþç~îÜ¹?ÁàþàþTø3gÎÏ¯ûüÎoì¿ñÕß¨¯¯?zô(ü	þðð§*ÄßæÍW¬XÈ/ù÷ÙÏ~öþûï?ÁàþàþTøðÁ¿ÜõåBü=òÛ|ìc?ÁàþàþTøäG:îï(ÄßÖaÕªUð'øüÁüÁªñ`~æg~æ7¾úùÝ~÷ÜsÏ/¾?ÁüÁü©ñ:t¨®®®­­mÉÿZò|äÿðïÂõð'ø?ø?Ý-ø~üã¿Õ[o½uw®ø?ÁüÁüé.Âàþðð'ø?Áàþàþð'ø?ø?ø?Áü	þàþàOð'ø?ÁüÁü	þàOð'ø?ø?Áü	þðð'ø?Áü	þàþð'ø?ÁüÁàþð'ø«È2L:nmmíéé)Ô×××ÖÖ/^W?Áü	þàOðWñe³ÙÆÀÞ½;;;'-X°àôéÓïÝúsì.¿?ÿó?ÿYìßûÞøÃQõüàìÙ³C¹+%VåPnÅF,6eC¹uåÊ£GZeØ+¯¼òÆoÌæ=ÞøkhhÿñÄ@.knnìjuuuãñ·ÿþ³³Ø'úûûÏªÌzõÕW-r+VJ¬Ë¡ÜXlÊ,rëõ×_üYeØñãÇÿæoþf6ïñ®À_:p¸°ØZuww;ì+öÃ¾ûÊaß/JåkkkÇ_áúõëÙlvddþð'ø?Á_Å×ØØËåÞ»uØ7¦^½zuãÆCCCãg?Áü	þàOðWyuuu8p âg6-ZâÃÃÃÎ?øüÁà¯òêíímjjJ¥RL¦¯¯ï¿[Í>»æææàOð?øüÝ½Áàþð'ø?ø?Áàþðð'øüÁàþàþð'øüÁüÁàþ?ø?øüÁàþàþ?øüÁüÁàþàþðð'ø?Áàþàþð'ø?ÁüÁàþð'ø?øüÁàþðð'øüÁàþàþ?øüÁüÁàþ?ø?øüÁàOðð?øüÁüÁàOð?ø?øü	þàOðð?øü	þàþàOð?ø?ø?øüÁàþð?øüÁàþàþ?øüÁüÁàOð?ø?øüÁàOðð?øü	þàþàOð?ø?øü	þàOðð?Áü	þàþàOð?ÁüÁü	þàOð'ø?ø?Áü	þàOð÷â¯æv¥R)ø?øüÁàþª©ÛN§áþàOð?øsØþàþð'ø¿êÂ_l6mÚð?øüÁ_â¯¹¹9NûÌüÁàþª~ü-^¼xüÙ###ðð'ø?ÁüUþjkkCÃÃÃMMM1æûÎw¾ÝÝÝðð'ø?ÁüUþ]1ÚË/Çë/æÌð?øüÁ_µáoîÜ¹A½ÞÞÞÁÁÁxì±Ç_õð'ø?ÁªÛ¶mËÞQø±¿eËÁüÁàþðWgû>þøãóæÍ¾¾¾¶··WÄú?Áü	þàOðw?øüÁàþàþ?øüMÞÂ/|ñ%Ïð?øü©Êñ·`ÁBðås¶/üÁàþªBüóýýýñ²«¸õ?øüÁàoz544þ*Q~ð'ø?Áü	þ¦ÝÀÀ@àoË-×¯_?ø?Áü	þTåøæÏ_3.'|Àü	þàOð§*Ä_KK>àþð'øÓÝ¿º?Áü	þàOð7½ðð'ø?ÁîüÅSümÛ¶íÆðð'ø?Áª5äø?Áü	þT_ò<aNø?øüÁàOÕùU/ü	þàOð¿éÕÜÜÜÒÒráÂäd2t:ÝÚÚÚÓÓSzÒÌÆÀàþð'øyáªlb6=xð`ìÝ»·³³³ô¤)lûöí÷ÝwßªY,înÅ«TfýÒ/ýÒ²eË,r+VJ¬Ë¡ÜØ,o95~õWõü¤åPµµµêSÍüÐñ×ÓÓøÛµkWü_ðýáKCCCr#¹¹¹¹ô¤)ìÑG­$Iª¢>tü°gû&RtÊÈøI3$	þfÞ¶o!kkkKOÙÂvìØñànÅâîzè¡*³>÷¹Ïñ_´Ê­X)±j,r+6b³¼åÔTúÚ×¾ÖÑÑa9aþô§¿ò¯Ìæ=~èøû`kllÌårÉQÚ.=ifcð!'|8áCNøpÂðQ.uuu8p âg6-=ifcàOð?øü½¯r¹ÜêÕ«ëêêjjjæÌ³nÝºùÑÛÛÛÔÔJ¥2L__ß=Ê[G¯ÇOÙøüÁàþ3otttÂVÄú?Áü	þàOð7½-ZÔ[³fÍõë×ÂÚµkcÌ%KàþàOð?ø«6üÕÖÖõÆÆÆòcr¹j-üÁü	þàOð¿T*ÔKÎ¨MºqãFÙW½ÀàOð?ø+kü%;::Ã¾ñ3cL[[üÁü	þàOðÕ¿ÐÞ'|¼óÎ;ðð'ø?ÁüUáW½®[·®¾¾>JÅÏSëþð'ø?Áß]ü	þàOð?ø?øü	þàOðW4çíJ¥Rðð'ø?ÁüU	þRð?øü©Úð7Y>úh¿CÁüÁàþðWµø3gN°oÕªUßùð?øüÁ_µáoýúõÉ¿cÇUÐú?Áü	þàOð7½^|ñÅkÖ¬©¸õ?øüÁàoª.Y²$9·£§§§×ü	þàOð¿)õôÓO';ü:;;+wÀàþð'øÚ¾çþàOð?Ý=øKÝ®t:ð?øüÁ?ïð?øüÁüÁàOð?ø?øüÁàOðð?øü	þàþàOð?øüÁü	þàOð¿.¬­­õ=ð?øü©úñ·`ÁBðù?ø?Áü	þTÍøçûúûûãeWqëþð'ø?ÁßôjhhüU¢üàOð?øüM»Àß-[®_¿ð?øü©ÊñÍ?¿fø?øüÁàOU¿'|Àü	þàOð§»	û*t?øüÁàoz566:áþàOð?Ý-ø§øÛ¶mÛ7àþàOð?U9þj&É	ð?øü©:¿äyÂðð'ø?Áªó«^*7øüÁàþðð'øüÁàoòr¹ÜêÕ«ëêêjjjæÌ³nÝºJ9ùþð'ø?Áßôð8ùþð'ø?ÁßôZ´hQPoÍ5ÉßöÂÚµkcÌ%KàþàOð?ø«6üÕÖÖõÆÆÆòcr¹ñðð'ø?ÁüUáW½õ|ù17nÜ1¾êþàOð?Uíaßä°oüáÓÖÖð?øüÁ_µá/´7á	ï¼óüÁü	þàOðUøU/£££ëÖ­«¯¯O¥Rñ³££#ÆTÄú?Áü	þàOðw?øüÁàoz577·´´pþàþð'øSõã/N×ÔTêDøüÁàþÓ«§§'ð·k×®ØTÊ_u?Áü	þàOð7Ó¤T*ð?øüÁ_~Éóùgø?Áü	þT=økiiimm©èõ?øüÁàoJ%Ò÷½[+â/ü	þàOð¿ã/ù¾Éñ?ø?Áü	þTåøËd25%sÂüÁà/üÖ·¾õÈ#<óÌ3?þñáOð¿þþþ¹sç&ûÿê9áþàOðWÔþéÆ¦òS+>õÙÏ~ö¾¶ûî¹ç×_þðWøËW)Î?ÁüÍr.]úÈG>òµ_ÛùÉ¿Ï~æ³MMMcccð'ø¿Æ_E?øûðú?ù¥KæåüËd2ú÷0áþeÔÀÀ@lOÓétkkkOOOá¤¾¾¾¶¶¶´xñâ¸ü	þàoÖzî¹çî¿ÿþ"üýÂ/üB¥Ë?øüÝù²ÙìÁc`ïÞ½,Xpúôé8tèÐÂáOð³Ö~ðî¹gûïnÏË¯û·ºëêê*ýRáþw¾äÏçr¹æææÉ®ÛÜñøûö·¿ýYìøñã±ÑüÊ¬'OÆÓr(·b¥Äª©è§pÿý÷ìcÛô6ü¾ÜõåØXmÝºµÒ×KlÄbSæõYn>úèÑ£CöòË/÷ööÎæ=~èø»xñâÇ_á'ÒßßßÝÝ=ñVÅ^õÕË/«ÌÇùóç-r+VJ¬~CCC_ÿú×îç~.þóùñ|ïÞ½U°^b#2¯Ïrëßþíßfù7¦Xü'öÊ+³y:þïyikkã7îþ¿S°¶¶vü®_¿ÍfÇjqØWû:ì+öÃ¾Ó+Øÿbçt:½bÅK.ÍÆCÿïb¸±±1Ë%c¸èW¯^Ý¸qcüÿüÀàþð'øI/^hQdµµµ«W¯µ§ÑÕÕuàÀÙl¶hwttL¶þð'ø?Áßûzå;v¬®®nÿ¼[oooSSSÜc&éëëû¯çvk§`sssá?Áü	þàOð÷Áüî,ÜóW__?þì2þð'ø?Áßô*4_]]]ggç+W*eÀàþð'øæMÜ:Ïã¸|ùrÅ­øüÁàþÓë~Ãü	þàOð'ø¿ÙÆ_E?øüÁàoÚ?~áÂsæÌIÎðmll<|ø0üÁü	þàOð§*Äß'ò'|$øK÷îÝð?øüÁ_µá¯©©)¨wþüù<þúúúo?ø?Áü	þà¯ÏöM¾<9¿xý%§ÃüÁàþðWmøkllê%û¹ûöí1ÜÜÜð?øüÁ_µá/RÍD>þàþð'ø¿*<Ûwhh¨½½=9Û·®®náÂ.]ªõ?øüÁàï.þð'ø?ÁüÁü	þð'ø¨+W®´µµÕ××§n­­­rÌþð'ø?Áß4zøák&iÓ¦Mðð'ø?ÁªN·oß¾däèèè#GñÇ?ø?Áü	þà¯JðÉdBxû÷ï?)ùo¾çþàOð?UþjkkCx£££ã'år¹W?ø?Áü	þà¯Jðÿ«nMMþÔüÁü	þàOðU¿¼?ø?Áü	þðð'ø?ÁüU,þJð?øü©zðº]étþàþð'ø?Þþàþð'ø?ø?Áàþðð'ø?Áàþàþð'øüÁüÁàþð'ø?øüÁàþð?øüÁàþàþ?øüÁüÁàOð?ø?øüÁàOðð?øü	þàþàOð?ø?øü	þàOðð?ø?øüÁüÁàþ?ø?øüÁàþð?øüÁàþàOð?øüÁüÁàOð?ø?øü	þàOðð?øü	þàþàOð?ÁüÁü	þàOðð?Áü	þàþàOð'ø?ÁüÁü	þàOð'ø?ø?Áü	þàþàþàOð?øüÁü	þàOð?ø?ø³àOð?ø?øü	þàOðð?øü	þàþàOð?ÁüÁü	þàOðð?Áü	þàþàOð'ø?ÁüÁ_©2L:nmmíéé'NÔÔÔÀàþð'ø«²ÙìÁc`ïÞ½ESÇÆÆÚÚÚàOð?øüU	þâE¹¹¹¹hêO<±gÏÉð÷ÔSOÅ=úòË/O8é/þâ/6lØ°lÙ²_ù_ùßùx×lKûØ±cC¹+Å¡XlÊ,2ì¥^²Ê°¿ú?¤îü¥Óé	£+W®´··'Ã_xüÿÎb§N?~hh¨¥¥åÿâ³_üÂú/Üï½þô§òü_ÍJ÷wwéÒ%Ë¡Ü«Ær(·b#2Ë¡ÜúéOÿY²Ê°W_õÚµk³ywþR©T~¸¶¶¶pÒºuëb#õOµ¼û~å+_ùå_þåßØüÛñ;ÂôGdo¹Ã¾ûZûÊa_ö/Á766ær¹ä°oOxµüË÷ÜsÏ×ýzñïë¿ð¹ÏÎþàÏr?ÁüÁ_q]]]øÍf'Ãâøå¿~ô£Ûw!þ¾ô¥/=øàÞ3ðü	þàþëíímjjJ¥RL¦¯¯oBí9þV¯^ýÀâï¾¶ûzê)ïø?ËþðXå¿.ÜsÏ=Zñ©îßêþÍ¿yß÷µ¶¶¾ûî»Þ3ðü	þàþªÑ[o½µqãÆO|âüä'ï÷~oddÄþàþàOððWµøü	þàOðð?øü	þàþàOð?ÁüÁü	þàOð?ø?Áü	þàOðð'ø?Áü	þàþàOð'ø?ÁüÁü	þð'ø?ø?Áü	þðð'ø?Áàþàþð'ø?ø?Áàþðð'ø?ø?ÁüÁü	þàOð'ø?ø?Áü	þàOðð'ø?Áü	þàþð'ø?ÁüÁü	þð'ø?ø?Áàþðð'ø?Áàþàþð'øüÁüÁàþðð'øüÁàþàþ?øüÁüÁàþ?ø?øüÁàþàþàþð'ø?ÁüÁàþð'ø?ø?ø?Áü	þàþàOð'ø?ÁüÁü	þàOð'ø?ø?Áü	þðð'ø?ÁüÁü	þð'ø?ø?Áàþðð'ø?Áàþàþð'øüÁüÁàþð'ø?øüÁàþð?øüÁàþàþ?øüÁüÁàþàþðð'ø?Áàþàþð'ø?ø?Áàþðð'øüÁàþàþð'øüÁüÁàþ?ø?øüÁàþð?øüÁàþàOð?øüÁüÁàOð?ø?øü	þàOðð?øü	þàþàOð?ÁüÁü	þàOðð?Áü	þàþàOðü	þàþÈd2étºµµµ§§§pÒ76nÜX[[ï½÷ÆÒ?Áü	þàOðWñe³ÙÆÀÞ½;;;'íÞ½ûÉ'·D,úñøûÞ÷¾7:ýÕ_ýÕÐÐÐ¨Ê¬×_ýÿñ-r+VJ¬Ë¡ÜXlÊ,rkdd$ðg9a===ÃÃÃ³ywþw1Ëå'µ¶¶^¸pa²ßüæ7¿7Å;óW^ùÊ¬_~ù»ßý®åPnÅJUc9[±MåP½ôÒKB6û¿úïü¥Óé	O?ýt]]]KKËÙ³göÃ¾ûÊa_å°oÅJ¥òÃµµµEöïß/^loo?Áü	þàOðWÕüw1ÜØØËåÞ»uØ7¯Vx±h§ ü	þàOð¿¬««ëÀ1?³Ùlá¤Í7¿ðÂ1pþüùeËÁàþð'ø«øzR©T&éëëû¯çvk§`l¤Ö­[N§ÛÛÛáOð?øüÝ½Áàþð'ø?ø?Áàþðð'øüÁàþàþð'øüÁüÁàþ?ø?øüÁàþàþ?øüÁüÁàþàþðð'ø?Áàþàþð'ø?ÁüÁàþð'ø?øüÁàþw[¿ÿû¿ÿgög?ÅâîþþïÿþG*³N8qúôiË¡Ü«Ær(·b#6Ë[NM¥Ë/ÿñÿ±åP½ðÂÿðÿ0÷øî»ïÂß¤?~çÎ IT-Þ7_cg¬$IÒÝüI$Á$IàO$Ið'I$ø$IüI$	þªªáááæææüÅ/¶··×ÖÖ®_¿¾èÏd2t:ÝÚÚÚÓÓcÑÉzª)È¢ûPëëëkkkwÁâÅãQúá-SëÅ[æ®ñ7ï2_/wüýâ-úÁ×ÛÛ»hÑ¢ÂÕ¹dÉxYÄÀ6oÞål6ðàÁØ»wogg§¥W&ëåðáÃ7n´Ðf§>::´páÂÒïoòÞ2wp½ß¸y¿ùz¹ãïøûà[½zõàà`áúÿäçÎ[xå7oÆ@.ðÿº#ë%ÞG±Ðf¿ºººÒïoòÞ2wp½ß¸y¿ùz¹ãïøûÐlÁú^¼xñùóçcàùç/G?&é®ø?loo¿xñ¢å6;õ÷÷www~_xËçzñ¹ëeüÆÍû¥Ì×Ë¿Àßl ãìÙ³.ÿìÙ³'ùA¾T*®­­µÜÊd½ä»zõjÑr®_¿ÍfGFFJ¿/¼eÊs½xËÜÁõRÞ/å¹^îøûþfùÞxãEillÌårïÝÚ'Ã[¬Âl1g¡ØnÜ¸qhhè¶ïoòÞ2wp½Ø¸y¿çz¹ãïød,X°```àæÍöìyüñÇ¯ÖÕÕuàÀñßË­LÖKLºtéRòfîèè°Ü>Ô^íµXÈÃÃÃSy_xËçzñ¹ë¥2¼_Ês½Üñ÷üÍ2â51þüÐww÷ØØXázR©T&IÎ<U9¬þþþE¥Óé+VÄÓrûPknn.úÖ	ßÞ2å¼^¼eîàzÞ/å¼^îøûþ$Iî¢àO$	þ$I$I?I$Á$IàO$Ið'I$ø$IüI$	þ$I$I?I$ø$IüI$	þ$I$I?I$Á$IàOnßóÏ?¿|ùòº[­òÅ_üÛ¸[UÌy¢GÛÜÜOmdd¤h|I§ÓLææÍÓ½MIð'IÙÎ;kÆõøãWþvíÚ#öÙ¢ñÏ<óLì±ÇfpàO*¯`M:Þ·oßØ­öïßcä3gª/_/.¿hÑ¢éÒ%øîzè¡`ÍO>Y8ò©§_ýêWéÓÓÓx¶µµÅpþÊï¼óNwwwLjhhØºukáÑÕ'O°bRÌüøñ"NÅ¹sç._¾üØ±cq±«««è=z´ôí$â!%N81Ô:::b|___~ÌéÓ§cÌªU«òcvïÞÝÔÔ7UWW·~ýú«W¯ÇßøÛ/Sâ¡J?IºÃÍ??àråÊÂo¾ùflnn.ÄMQýýýÉÔµk×MÚ²eK2éÜ¹s©TjÂ¹ÉÔuëÖÝ¼y3øX[[;66SãgàiÎ9ÉGñJÜNLøðÆ?ÓC6ïËæåWt#+W®.þJ<TIð'Iw¾äïÛµ X!n:;;¯ß*ââ5k©u>&;ðmÉ¤l6ì2áÞÞÞäFosÇÁ»ä¨ëÃ?c9Ãñ37lØpÛÛ	8ÆÅ¸Bácð(çÍÏwxx8.ÆÏxä!ÎÉÄñÞûïÃÄuuuÓÅ_*	þ$©,ðM¿üø7¢«W¯ÆÅ`Sr±­­-.¶´´lÚ´)Ð6::¿¸NÑ¾´üÅ7ß|3åSþÈoüá'OÞövqñí·ß.|l>ïÑGIöìyï¿moÝºµðÁÀ_´½½=Ù19]üx¨àOî|Éî®ë×¯1©wò4Lü·NþzãÆqªðVb¸¡¡¡¶¶öÆqãsçÎÍO½ííLF±¢Î?85ãg;w.?µ··7À§¿UüIÒ/ùÜÛÓO?]82ù¢>ò¿CëÌ3»víJÂæÕìËW½-Ñ¶lÙíÝÝÝùñ%n'ÙÓ644ûí·K»téÒ|»MµpRræoL:qâÄµk×Jã/Ódiä§x¨àOî|É	étzïÞ½ÉW½ìÛ·¯¶¶vüÉkÖ¬	èÜ¸q#9&?37ùÌ_òQ¹ÁÁÁÂ&ÜºukP)97ÿe+-9ý6¹÷×^-?¾Äí$òK>ó722þöïßß'O¹pRòñÇsçÎÅ½ÄB	7Ëqµ@çúõë§x¨àOÊ¢äÚ½÷ÿßÆÝ*±Q~8À4E¾~¸P>|¸þÞ»õ§8b|CCCáá·£ðåÏ´ìÉÖÕÕ%O¡è`÷êÕ«ïbÞ¼yñ3ù¶ÂÛLvLæK¨Zâ¡J?I*(+W®¬½ÕòåËSnðwâÄäëôZ[[O::22²ûö¦¦¦L>úè7òS?ÞÞÞ&Ëd2Ï=÷m$qS1~óæÍEã'»(L<¤äK|Ï_¾îîî¢ï|IÎf³±êëëãYt)ÿµ5·yíÚµÐm²¬V­ZÕ××Wt%ª$ø$IüI$	þ$I$I?I$Á$IàO$Ið'I$ø$I?I$Á$IàO$Ið'I$ø$IüI$	þ$I$Iú úþ×ðö#$IEND®B`


ä~(?å üòS~ÊOù(?å üòS~ÊOù(?å(?å üòS~ÊOù(?å üòS~ÊOù(?å°	Ê¯P(ôõõÅãñÝ»wÏÍÍ=t¦,M§Óá¢ÞÞÞééé:g üÂ®]».]ºÝÝÝ)Ëf³ãããa0666<<LUùýö·¿ý_-a_¥d2¹úLggg©Tb±Édê©*¿ýìgy°YËovvvdddõx<^5®gÆÑ^ÀÑÞ&²´´ÍfW	b±XyH$êQ~òk¾uëÖ*3®®®b±xÿÁÜ0®sFùÊ¯)ÌÌÌ,..®2SËåÎ9á4ÍÖ9£üå×2L[ÎüË÷A>O¥R±X,N:g ü¶"å(?å üòS~ÊOù(?å üòS~ÊOù(?å ü üòS~ÊOù(?å üòS~ÊOù(?å ü üòòÖÙ·ou¨ü´HG[ëAù)?P~(?åÊå§ü@ù¡ü(?òåòS~ üòòS~òS~ÊP~ÊOùòCù)?P~(?åÊå§ü@ù¡ü(?òåòS~ üòòS~òS~ÊZ3ÝK8*?å¯ü~«Q?;xLù)?åÊå§ü@ù¡ü(?å§ü(?å§ü@+(?å§ü@ù)?å§ü@ù)?å§ü@ù)?å§ü@ù)?å§ü@ù)?å§ü@ù¡ü(?òå§ü üòå§üòå§üòå§üòå§üòæ-¿ÆR~ÊOùòCù)?P~(?åÐ´qðæoZÍ¼qýòS~|)Ëí[Yw«,páÂëPù)?å§üZ$<x+?å§üòCù)¿- B¡¯¯/ïÞ½nn.ÌÓt:fz§§§+®½¨å üP~Ê¯)ìÚµëÒ¥Ka011ÑÝÝÙlv||<ÆÆÆ+®½¨å üP~Ê¯é$ÉpÚÙÙY*Â X,f2Êj/ªg¦ªüN>ýG¥G±6ïæklù¹ó|-µüfggGFFÂ '+Ç½¨ªòûõ¯ý?xB:|ÿûß·6ïæklù¹ó|-²ü²ÙìòòrÇb±ò|"¨ö¢zfíp´Gím¾uëVt¶«««X,FkÃ¸rÉÚêQ~Êå§üÂÌÌÌÀÀÀââby&Ë9s&Âi6­ö¢zfòCù)¿¦Éd*¿Ï'ÌäóùT*ÅÒét¡P(ßzQ=3Ê@ù¡üßV¤üÊOù)?ÊOù)?òS~ÊOù üòS~Ê@ùyðV~ÊOù)?åòS~ÊOù(?òS~Ê@ù¡üP~Ê@ù¡üP~Ê@ù±îÍ÷DêÉuÿ$þòÛßN­ûêî<ÊOù(?O7&lýÑÑÑÜÂ_|a+(?å ü°õQ~ÊÀc?¶>ÊOùxìÇÖW~ÊýØúÊOùà±[_ù)?<öcë+?åÐjü§¡ÜýoëþùV¢ýß÷ÿÝº¯þÜ?MNNÚÊå§üÓ÷ß:Ù¨ö'þ­tP~(?åðø¼ÊOù)?å§üP~ÊOù(?åòS~Ê@ù)?åòS~ÊOù)?òP~(?òP~(?òP~(?òP~(?å§üP~(?å§üòCù)?å üÊOù)?å§ü¶ÈÝc#r¹u¨üòS~Ãïÿû6àÏþ³u¨üòS~ üòòP~òS~ÊP~Ê@ùòS~(?P~Ê@ù)?P~Ê@ù)?P~Ê@ù)?P~Ê@ù)?P~Ê@ù)?P~Ê@ùÊOù(?@ù)?å~òP~ üÊòP~ÊòP~ÊòP~ÊòP~ÊòP~ÊòP~òS~ÊP~Ê`#åò«Q?ßÙõ(?åðøÊ¯±(?å üå§ü¾éòów~ üòS~ üòS~ üòS~ üòS~ üòòP~òS~ÊP~Ê@ùòS~(?P~Õââb&)éééÇã½½½ù|¾rÉ¹¹¹t:]4==]çòP~ üBh»ÐyaSéìì¼qãFÓÊ"²Ùìøøx×9SU~¿ûÝïþ«jxù?~ÜVzl²òÛ¿ÿüü|eùíÜ¹saa!ÂiW.¢°T*A±X¢°ªòûÉO~ò!«jxùår9[ê±ùöF*é&É0Nggg+ÇãUãzfíp´ímÒòê©§®^½%àÓO?]¹X,+D3Ê@ùòkÒò[åE»®®®b±xÿÁÜ0®sFù¬c·üÿôÏúÙ±ç¿(?Ø*å÷ôÓO_¹r%._¾üÔSOU.ËåÎ9á4ÍÖ9£üÖ±[N÷¯×ýó­Dû¿Ùþu_=üë'O´`KßÇ/Ó0®Ï§R©X,N§B3ÊàñïÕ½hÊ¯ñòå§üP~ üÊò@ùòS~-.Ëí[YÛ«,páÂë6Mù¶··Á¹sçÉd<¯ýüåÐÊ'óæoZ°9ÊïàÁá6J¥Ê_ãC)?*¿Ðy³³³×®]7oAW~°¥|´1_|ñuÐìå½Â÷îÑÑÑh²òslÊÆøMP~íííaûöíèÅ¿¯ùµ]è×`Óßàà`ù)**?ï÷w~òhµòzê©X,²ïöíÛál?ûì³âÿ_ùòP~[ò:Ý»wïÓ	å÷«_ýj#·°¼¼lC(?ånO¤Oâ/¿ýWßùîº¯þõ³gÏÚ/¿+W®tww···Gïçíêê:wîò+¿_Ã?¿Õ¨¿ùûòh|ùMMMßá_4S~ ü@K_*wåÊrùêÊOù´`ù?É¹ Ñ×¸ÅãqåÊOù´TùuuuÎ^çåW,;ÆLFùòS~-U~Ñ7vÔºtéòå§üZªü[·nõ÷÷GïíM&ÝÝÝ×¯_ßÿÿÊòÛ*Ô_~d[ãÄÿu»òh|ùe2;w^½zUùAk_[£)?Æ_<ÞÛ«ü µËÏk~ÊïþôôtØ)¿öÚk·oß.JÊZµüüò»¿Òôè³ý(?åÐ:å[Orå§üZ­ü65åÊ@ù)?@ù(¿Úðw~ üÀ)¿Ê¿íûÊG0ø;?P~Ê ÅÊ¯R©TZ^^~î¹çBùÍÏÏ+?P~Ê eË/â/ß;(?åÐâåW*Bù%	åÊOù´rùÅS§NòK&Êòh©ò[é½½P~ ü@K_í·w$çnSüÿ+?P~Êo«P~ üòò«½Äãñ®®®ýèGÊZ£üÂïuÈ¯uÿdúÿq×3Ãë¾zø×@ãË¯öÛ;¢¯n+GGGlvËËËg7&ì^õÕÜÂõë×mß½÷ÚÛÛ8°´´ÎÓg¶··7ßÿý°¯ïììT~@ØøuØôå×ÝÝvèÅb±<sçÎ0300&ü|(?å÷õöæÁ½÷Ê3åà¾ÌCùÊ EÊ/JEÛ¼¼¼|ÿÁqtwvvÖÑ^@ù´NùMMM=ô½½ÓÓÓÑ`ddDùÊ Ê/¸yófwww2Åbá4ÃL´£oò/óP~ üßV¡ü@ù(?å(?åW±Åb÷WþèRå·Ý7Â4(?±üBØE×[A3ò¤«tQÞ­²À§~j*?®üZ&S<yìÇÖP~Êý|=ûV½â»Ê/_¶´ü6Ë_ò)?åÇãÜ¾199ilòÛ¤!¨ü(?åòå§üP~ üÊòCùÀ&/¿Õù´HùÅÖâ;<P~Ð"å×òå§üP~ üßö§?ý)¤[ê»½îo%ÚèJ¯ûêá_ÿÍo~cCòS~Ü§~H¶ÿÃ?ýs£~2½Ï=kCòS~£üH=9üó[úù¿Ï)?P~ÊOùÊOù)? iËoqq1ÉÏÞ½÷ðáÃDbÇ333KÎÍÍ¥Óéx<ÞÛÛ;==]çòS~ üB>ïééikûò?ìøñã¯¿þz©TÙ·sçÎÊ³Ùìøøx×9£ü(¿¦°ÿþùùùÊòëíí½zõêCîììEÅb1z°ªòûÅ/~ñß©O¨äí]ß¿ûÿùÄ6¬dßÿûÏª(¿x<þÓþ4LîÜ¹óücåb_ë©*¿ñññ«ÔgzzºÑå7üúë¯Û°M_~±XìôéÓapíÚµþþþÊÅÂEåq"¨sÆÑ^GÀÑÞ&-¿®®®^´ÅèHn´X=3ÊOùòkÒòé¥Þï½0¸råÊ=*ËårgÎ	pÍfëQ~Ê_ßíÛ·âñxÿüü|åù|>JÅb±t:](êQ~ÊßV¤ü(?åòå§üòå§üòå§üòå§üòå§üòå§ü üòòû¦Êï_ýÅ_´5òå§üSùµ5òå§üSùuüÕwþvðX£~ºvö+?P~Êï1¿óå§üòå§üòå§üòå§üòå§üòå§ü üòòS~ üÊòS~ÊòS~ÊòS~ÊòS~ÊòS~ÊòS~6(?å§üå§ü üòå§üP~ üòS~ üòS~ üòS~ üòS~ üòS~ üòòS~òS~ÊP~ÊOùòS~(?P~ÊOù)?P~ÊOù)?P~ÊOù)?P~ÊOù)?P~ÊOù)?P~ÊOùÊOù)?@ù)¿o¦üÚMùòS~ä×ÖfòS~ÊP~ÊOùÊOù)?@ù)?å(?å§ü@ù)?(?åòå§üP~ üÊòS~V8(?å§üå§ü üòòS~òS~ÊòCùòS~(?P~ÊåÊoý3LÕäÔÔTªÉ¹¹¹t:Ç§§§ëQ~Ê_SÈçó===UwïÞ½¾¾¾ÚòËf³ãããa0666<<òS~ üÂþýûççç«"ïÇ?þñ'jË¯³³³T*A±X^&¬g¦ªüÞzë­i°­pxt6Yù_*oÜ¸Ñßßê­¶üâñxÕ¸ªò»páÂÿæq	ÑGgÓßÐÐÐÅ«&#±X¬<N$uÎ8Úëh/8ÚÛ¤å×öUuuuÅèHn×9£ü(¿&-¿U&s¹Ü3gÂ f³Ù:gòå·Ê/çóùT*ÅÒét¡P¨sFù)?P~[òS~ üÊòcUûV½Mg._¾lòS~ä.µ1Ö!(?å üòS~ÊOùÊOù(?å üòS~ÊOù(?å üòS~ÊOù(?å(?å üòS~ÊOù(?å üòS~ÊOù(?å ü üòS~ÊOù(?å üòS~ÊOù(?å üòS~òS~ÊOù(?å üòS~ÊOù(?å üòS~ÊOùÊOù(?å üòS~ÊOù(?å üòS~ÊOùÊOù)?@ù)?å§üòP~Ê@ù)?å§üòP~Ê@ù)?@ù)?å(?å ü©ÅÅÅL&S>[(úúúâñøîÝ»çææ*gÓét¸¨··wzzºÎå(¿¦ÏçzzÚÚ¾üÛµk×¥KÂ`bb¢»»»rál6;>>cccÃÃÃuÎTßäääÿh	¬üöïß???_Y~ÉdåÙÎÎÎR©Åb1z°ªòûí·-aóíýÿ¬ßìììÈÈHåL<¯×3ãh/àhoSßÒÒR6]^^®Åbåq"¨sFùÊ¯yËoaaáðáÃ·nÝªZ¬«««X,Þp$7ëQ~òkÒòX\¬],Ë9s&Âi6­sFùÊ¯IË/É´U¨Ï§R©X,N§£?i¬gFùÊo+R~òS~ÊOù(?å üòS~ÊOù(?å üòS~ÊOùÊOù(?å üòS~Ê¯±>øà=öìh	gÏU~(?åò@ù üP~(?ÊGîêÕ«/¼ðBGGG<ïêêzé¥>ûì³Üà[o½µ;n»nãö++/sáÂD÷p:44fjoêÃ?×§Öj«Þ*ÏÆb±d2Ù××÷öÛo[o­±éë¹W¬´ÝëÜK üh¤÷ÞoÇÅb1§aüäOÞ¼yóQì;ß¦ÛÝGÞ÷Ý½÷R©ÝOÂ½§§çÔ©SUK:tèàÁá¹µÚª÷Úk×®íÙ³çäÉVWkßêÛ½þ½ÊWûß'ÝY¸,--UM~òÉ'©Tªr&ìñ·oß~çÎmÛ¶E¶BùECZ][ªüª¶	täÈ±±±ÚùðÈ=>>^>þüùÞÞÞx<NÃ¸rG099¹sçÎX,.^Õ¯=0tñâÅÎÎÎþþþ5oÍiÚòK$W¯^]óvÂpZ¹q©©©°ÑÃ?L&ê¹56QùEwpËå*õðÁ^náò+o÷ú÷(?)<EOÈV_&ôãpÚÕÕ5;;[ÞìÝ»7:.ëµ;0~ñÅK¥R´GXýÖl¦-¿_þòáýÕ;|øðÄÄÄýÇÂ¸ò¢òspgxï½÷zzz¬öV*¿k×®íÞ½;>ûì³ð¤nyy9?ÿüóð¯öu Z¦üÊÛ½þ½ÊFÅbk.Ú.<NW@ù@pØ_ûÚýBUùÍÏÏ×yk¶Hw÷«¿½#8wîÜ¶mÛÂýÐ¡C!ïBÆE_ê½ûvûª¾áÖUÝzå¶òÅ»»»Ëù§O~é¥Â`xx8¶dùÕn÷zö(?¬ü*ÝêËDoþqGGÇCw+ßún&ÙÝWíëgggÃÎýW^I§ÓáZÑ+|éééòÙg¶òm£££CCCããã.]ò'½ü*_áT½óg9vìØÑ£G­ÀV*¿5·û	ÖÓÓsãÆ¯»#xèQÝ:Ë¯þ[£	Ë¯Ò½÷N<ñg^|ñÅªÇò¥wïÞ=xð`ôH°mÛ¶?þØjß¼å·æµBß_íè`Z¦ü6¾@ùÑ`£££+WùYZxb÷¾æWÿ­ÑäåíÖËáê­|øââbgggíËï¾ûî®]»¬ö.¿l6èÐ¡_~ÙÜÊåWµ@ùÑxóóó;vìÐUóÇÆ¨üË¼0Þ¿ÿºË¯þ[£©Ê/JÕ¾Jª®¯¯/OOO[µ@ùEØå÷þûïga000°ÒÖ+¿5÷(?ÂÉ'wíÚ,<3goÞ¼ùÆo|ïß»sçN´@¡P¿ÏÑ_éÎÎÎq>_½üÚÛÛ?ùäèåªeê¿5ªüN:$¼ûî»þy´7¿|ùr.+?®9r¤ö;ÂS0ÃÞbb"z	°òà´XùEïçÞÛ½Ï×1ß-R~kî%P~4óçÏ§æÉd2<~üx9ûÊßÑ'ðíÞ½»òÑ¥òçw<tgQç­ñw÷k¾½7ì¾þùèËÈvñaÖØ3åOo	OÂÝ,Ü+Âm­?55eµ·dùGúÊrôy~-°+¨½Ê^åò@ù üÊåò@ù üP~(?Ê@ù üP~(?Êåð(d2d2¹¼¼fâñx:.J_o¯úÀ«óFÀ×ðÚk¯Æzûí·«æO<æð|í½ªò@súäOBcíÞ½»j¾§§'Ì_¿~ýQí| ü¿YB¡<séÒ¥0³oß¾òÌñãÇS©T<O&Ï?ÿüÂÂBe¢?~Û¶mÏ<óLm´­~ÅéééáÒ¾¾¾0^©ü.J4,ÿVyþóÏ?éèèuvv=z´ö°5òøÒÄÄDÈ¬^x¡<sðàÁ03>>^®·¶¯Ú»woe¢Åb±p:44Tmk^±Êììlmù]¾|9ºýÚÅ8Pu/¿ü²(?Ý»woûöíñx|qq1§¡´:::Åb´@:QõáÞÿÿGÉde¢½òÊ+¥R):4mk^qxxxé0gkË/ÍF¯q>®]á7Â8ü+aÜÞÞnÊ`5£££!N8Æo¼ñF=z´rP!­Báõ÷÷G/òU&ÚÍ7¿Ü«~õ@íêWZ3XXXgCnÖÞH¬za¯¼X___8»sçÎ#G|ðÁwîÜ±)å°+W®D	Æá4/_¾4ÏwvvVåWe¢U~òKå¥k^ñ+»ã¶¶x<^iíAár>ÎÏÏGñW.ÂÉÉI[P~kxúé§C<½úê«á4äTåEÑû|ÃESSS·oß^3àÊ3k^1:PÿÁ!æ^ókooãò¡çZøÃ^íµ¡¡¡°X:¶)å°Ó§O_<«¼(G¯J¥wÞy§þò[ó¡ùîÞ½ËåÂÙpZ#QÒ=z4ÜH(ÈÊÏ þÎ/ú;Âùùù0N$6% üÖpçÎd2r]ZZª¼hÿþý[·oßN£ÏgY½üÖ¼båqùsåÌÎÎVí=wîô¾J/¾ø¢M	(?µT¼Kdqq1Í&ÑÑÑë×¯??eõò[óSSS¡ùz/^¼øÐ	Î?ßßßÅÒéô©S§ÊóËËËÇK¥RQVâîÝ»¶# üP~Êåò@ù üP~(?Ê@ùYÊåò@ù üx,þ/ñI`í© IEND®B`


ONEWAY Figures BY Variables
  /POLYNOMIAL=1
  /STATISTICS DESCRIPTIVES HOMOGENEITY
  /MISSING ANALYSIS
  /POSTHOC=LSD ALPHA(0.05).


Oneway


Notes	
Output Created	12-SEP-2022 22:52:59	
Comments		
Input	Data	E:\桌面\Raw Data\4. C. Cellulosae ESAs and TPx Induced Th Subpopulation Differentiation\3. SPSS statistical analysis\2. IL-4\2.  IL4--48h\2.1 SPSS statistical analysis--IL4--48h..sav	
	Active Dataset	DataSet1	
	Filter	<none>	
	Weight	<none>	
	Split File	<none>	
	N of Rows in Working Data File	20	
Missing Value Handling	Definition of Missing	User-defined missing values are treated as missing.	
	Cases Used	Statistics for each analysis are based on cases with no missing data for any variable in the analysis.	
Syntax	ONEWAY Figures BY Variables
  /POLYNOMIAL=1
  /STATISTICS DESCRIPTIVES HOMOGENEITY
  /MISSING ANALYSIS
  /POSTHOC=LSD ALPHA(0.05).	
Resources	Processor Time	00:00:00.00	
	Elapsed Time	00:00:00.01	


Descriptives	
Figures  	
	N	Mean	Std. Deviation	Std. Error	95% Confidence Interval for Mean			
					Lower Bound	Upper Bound			
Control	4	15.38550	1.175615	.587807	13.51483	17.25617			
ESAs	4	17.15925	.499778	.249889	16.36399	17.95451			
TPx	4	18.64850	1.074920	.537460	16.93806	20.35894			
LPS	4	20.19100	.918560	.459280	18.72937	21.65263			
Total	16	17.84606	2.025965	.506491	16.76650	18.92562			


Test of Homogeneity of Variances	
	Levene Statistic	df1	df2	Sig.	
Figures	Based on Mean	.495	3	12	.692	
	Based on Median	.431	3	12	.734	
	Based on Median and with adjusted df	.431	3	9.955	.735	
	Based on trimmed mean	.490	3	12	.696	


ANOVA	
Figures  	
	Sum of Squares	df	Mean Square	F		
Between Groups	(Combined)	50.675	3	16.892	18.608		
	Linear Term	Contrast	50.599	1	50.599	55.740		
		Deviation	.076	2	.038	.042		
Within Groups	10.893	12	.908			
Total	61.568	15				


Post Hoc Tests


Multiple Comparisons	
Dependent Variable:   Figures  	
LSD  	
(I) Variables	(J) Variables	Mean Difference (I-J)	Std. Error	Sig.	95% Confidence Interval	
					Lower Bound	Upper Bound	
Control	ESAs	-1.773750*	.673707	.022	-3.24163	-.30587	
	TPx	-3.263000*	.673707	.000	-4.73088	-1.79512	
	LPS	-4.805500*	.673707	.000	-6.27338	-3.33762	
ESAs	Control	1.773750*	.673707	.022	.30587	3.24163	
	TPx	-1.489250*	.673707	.047	-2.95713	-.02137	
	LPS	-3.031750*	.673707	.001	-4.49963	-1.56387	
TPx	Control	3.263000*	.673707	.000	1.79512	4.73088	
	ESAs	1.489250*	.673707	.047	.02137	2.95713	
	LPS	-1.542500*	.673707	.041	-3.01038	-.07462	
LPS	Control	4.805500*	.673707	.000	3.33762	6.27338	
	ESAs	3.031750*	.673707	.001	1.56387	4.49963	
	TPx	1.542500*	.673707	.041	.07462	3.01038	

*. The mean difference is significant at the 0.05 level.	
