# Supplementary material for: Regulation of piglet T-cell immune responses by thioredoxin peroxidase from Cysticercus cellulosae excretory-secretory antigens
Source: Front Microbiol. 2022 Nov 18;13:1019810. doi: 10.3389/fmicb.2022.1019810 (PMC9718028; doi:10.3389/fmicb.2022.1019810)
Supplement: Supplementary file 3 [file Data_Sheet_3.ZIP › 4. C. Cellulosae ESAs and TPx Induced Th Subpopulation Differentiation/3. SPSS statistical analysis/2. IL-4/3. IL4--72h/3.3 (SPSS data export) SPSS statistical analysis--IL4--72h.doc]

EXAMINE VARIABLES=Figures BY Variables
  /PLOT BOXPLOT NPPLOT
  /COMPARE GROUPS
  /STATISTICS DESCRIPTIVES
  /CINTERVAL 95
  /MISSING LISTWISE
  /NOTOTAL.


Explore


Notes	
Output Created	12-SEP-2022 22:58:03	
Comments		
Input	Data	E:\桌面\Raw Data\4. C. Cellulosae ESAs and TPx Induced Th Subpopulation Differentiation\3. SPSS statistical analysis\2. IL-4\2.  IL4--48h\2.1 SPSS statistical analysis--IL4--48h..sav	
	Active Dataset	DataSet1	
	Filter	<none>	
	Weight	<none>	
	Split File	<none>	
	N of Rows in Working Data File	20	
Missing Value Handling	Definition of Missing	User-defined missing values for dependent variables are treated as missing.	
	Cases Used	Statistics are based on cases with no missing values for any dependent variable or factor used.	
Syntax	EXAMINE VARIABLES=Figures BY Variables
  /PLOT BOXPLOT NPPLOT
  /COMPARE GROUPS
  /STATISTICS DESCRIPTIVES
  /CINTERVAL 95
  /MISSING LISTWISE
  /NOTOTAL.	
Resources	Processor Time	00:00:01.14	
	Elapsed Time	00:00:00.86	


Variables


Case Processing Summary	
	Variables	Cases	
		Valid	Missing	Total	
		N	Percent	N	Percent	N	Percent	
Figures	Control	4	100.0%	0	0.0%	4	100.0%	
	ESAs	4	100.0%	0	0.0%	4	100.0%	
	TPx	4	100.0%	0	0.0%	4	100.0%	
	LPS	4	100.0%	0	0.0%	4	100.0%	


Descriptives	
	Variables	Statistic	Std. Error	
Figures	Control	Mean	13.95475	.329532	
		95% Confidence Interval for Mean	Lower Bound	12.90603		
			Upper Bound	15.00347		
		5% Trimmed Mean	13.95678		
		Median	13.97300		
		Variance	.434		
		Std. Deviation	.659065		
		Minimum	13.130		
		Maximum	14.743		
		Range	1.613		
		Interquartile Range	1.220		
		Skewness	-.166	1.014	
		Kurtosis	1.495	2.619	
	ESAs	Mean	15.79825	.333072	
		95% Confidence Interval for Mean	Lower Bound	14.73827		
			Upper Bound	16.85823		
		5% Trimmed Mean	15.77683		
		Median	15.60550		
		Variance	.444		
		Std. Deviation	.666143		
		Minimum	15.246		
		Maximum	16.736		
		Range	1.490		
		Interquartile Range	1.213		
		Skewness	1.353	1.014	
		Kurtosis	1.530	2.619	
	TPx	Mean	18.43575	.378331	
		95% Confidence Interval for Mean	Lower Bound	17.23173		
			Upper Bound	19.63977		
		5% Trimmed Mean	18.41350		
		Median	18.23550		
		Variance	.573		
		Std. Deviation	.756662		
		Minimum	17.776		
		Maximum	19.496		
		Range	1.720		
		Interquartile Range	1.389		
		Skewness	1.295	1.014	
		Kurtosis	1.531	2.619	
	LPS	Mean	20.51525	.329457	
		95% Confidence Interval for Mean	Lower Bound	19.46677		
			Upper Bound	21.56373		
		5% Trimmed Mean	20.52589		
		Median	20.61100		
		Variance	.434		
		Std. Deviation	.658913		
		Minimum	19.630		
		Maximum	21.209		
		Range	1.579		
		Interquartile Range	1.233		
		Skewness	-.822	1.014	
		Kurtosis	1.481	2.619	


Tests of Normality	
	Variables	Kolmogorov-Smirnova	Shapiro-Wilk	
		Statistic	df	Sig.	Statistic	df	Sig.	
Figures	Control	.248	4	.	.955	4	.747	
	ESAs	.251	4	.	.888	4	.375	
	TPx	.251	4	.	.906	4	.464	
	LPS	.249	4	.	.959	4	.774	

a. Lilliefors Significance Correction	


Figures


Normal Q-Q Plots


çÊ+ãûfþüù2`` r¦ÈRfëþ½JÔ]kÔk~Z8ñ7é+_~]]]·#?ìDå¹Z=ú2§úæõë×G=æíÛ·³q!?"2¨üdâÂ¯E"`ëÖ­2½víÚ	ùRfF~çþýûÇÆÆÔ«VjÎ¶mÛB¡P»úRnY¾¼téLÕuÕ»Ù.^¼(Ó·oß¼(oL&îüÈëNñ*±ÞH~Mt¢G÷QMúqÞäuEL×××éÃ>¿ü"W«ÜÌ¹råL«ÕºeËõÍJwîÜiY2ÃÆEüÈ¸òäåI=j~QQLß½W)OðòeAAAä-/ÏmD~988øè·M#Á1::*Va,Y²D½ÈôDùeffêþ ºó'qHQÉu1V«µ­­Mw¡gáè>ªI_qüòSïe¼ÿ~ä]LH~«UÝZd2G]TSS#_.X°@ä*B°e!?"2ºügòü#çk_3Ó¾éMH§ñ¿eFñ|âÕââbuxZË»0D¢nm<WµL¸ÐÆ³pbytrWÿÑÞñØ.þ¥ÚÕõ7ê"¯×«ðakk+ò#"CËOzë­·äËðg ¨gQ¯NÉÌñ"þ.T®rùòeÙxä§^T°R__¿sçÎ<yRfnÚ´IW~ã¹Ê¤å7ÑnÒW¿üÆó_ØvâþøË?''GæD4ìÆRÃã ùqå÷èñ»õÃoäWsÔÈßµk×=N=µoÛ¶mêòS¯y<ñÇ'Æ#?·Û­^U,¶··«×óT.ý'z	É/þÂQK2ð¸¨èRüÔ;ó¾###»5kÖh]øúë¯ËÂã/õÅÍòý²$eººº:òÇTï×ôz½q¼ò#"cÉïÞ½áwt©9>/ê=^EEEá¡²S_Ø"ªù?r|«îc~á´G.]çÇÐU&$¿ø'üÌQÆ<¥:	ùÝ¸q#òöW¬XyiÔ2«ÅúXDÝ!/êUÕÈ^|ñE6."äGD) ?I	ÿùçoÚ´)óqõõõÚñßàà ÜÜ¦h×®]¡ðÄ÷ÍÑ£G«ªª222æÏÿÆo|÷»ß¿æ4õ«LH~ñÎ&)üxMh©NB~Rkkëä§ÛWÇÓÃÃåKñºw«ÕÚÑÑñÄ×]º´dÉ¹5ù¡ZZZÂóÀÞ½Õ©ÂwYÁ`ùÍH£££/¼ðÂ>CnWI¹ÔAX5X;)Ð/_"B~DDéz_T¯¾ú*KQºöìÙ3þ|uHW&<Èb!"äGDDDDÈùò#""""äGDDDDÈ!?""""B~DDDDüùò#"""B~DDDDüùÑlëÂf³Ùd2­r¢×­ªª3g×ëÏiS]]=Öã&÷=ã¹î¤Ü¶mÛ¼yódáÌ?ïÞ½@ A;ñü¹ùÑì­¸¸X!Ê	B½îáÃåºG	Ïyíµ×dÎ¡CR]~¼òÇÝ¾llìôéÓrGkÖ¬ÄMuvvZ­VäGDÈ°¿2nÜ¸!×­­­ÏY¾|¹ÌéêêJÌ9!)Ô;w.)Kùò#¢b_¤3öîÝsàÀ¨oûîw¿»dÉ¨[¿d(Ó2-sÔEwîÜ±Z­ÙÙÙ&©¦¦æÊ+º·yïñ¯òÆoÍfÅ"w¤Òðð°ÍfëÊß³gz	SlºbÅ¹5/·|÷îÝq.êêj¹å`0¨i¥äv»åAÊB¸páv	kdâ÷ÍþÔô+¯¼"Ó---b,xõÕW#¿çÒ¥KJxmÝºU.:yò¤LËÿ2ÝØØ¨.ZºtéÅeâöíÛ2¿¤¤D÷Ö"@ü«çÞzë-°ÛíÚ¿ûvoxçwdâðáÃ2³´´T¦<L¨$µi`1½â/¥×_]ÝøOû £~öø7Å/'!?"Aù	VdzôqZxé¾ÐívËEk×®éõë×+Ö/½qãÆþýû,Y"ó322to-J9ñ¯¢X^^öºEEEêÁÉOfÊwÊtMMÐ*ÖxºÅ_ü¥422¢¦åbÉ/ü³Ç¿)~9ùÑÊ/;±ì0+''G¾3¨ª2G]tâÄ	¹pðæÍZýè>ñÖ&F¾§ÔxîÜ¹yóæ©9êåÀñ¼æWQQ!óã£0¾ðâOOn!?"ùD¾¥^6"Dl6þ?<?33Sæ=nòðÌi¯[PP ®õðB¡ÐÅíväOlÿþýòý"EíEãYJãßä8ò#¢iÐzòäIõ¶³×^m<9wîÅ³·Þz+5===êýã_ü«ÆÞ~ûmØºu«öº/¾ø¢L>ú³Ï>SGxe¦Åbé7oÞ¿_&***Æ¹XÄaåååwîÜMª#7lØ0Î¥9­8«cÍQ?ûä8ò#¢i_(]å<.ò3ùâCdddDiÿLáù­­­â=öS~ñ¯ÒÖÖ&._¾<<Ð$òÀÎ;ågff®Y³FãðÕ××«ÁÂµµµþùøÜhRË8p@½o<K)rúÂòÔeGýì[àDDÈùò#""""äGDDDDÈ!¿õ_ü¢··7Áwz÷îÝÿüÏÿä÷ÆÈ<|øå`äþýßÿå`äFFF¿¥	õßÿýßÿüÏÿÌr0x²dM!¿ééÇ?þ±à/ÁwúË_þòþýûü*¹Ï>ûì_þå_XFîÎ;ÿôOÿÄr0ròçÓÇÌr0rÿõ_ÿuùòeÁ»téÒo~óäüù!?äüù!?äüù!?B~üòC~ü!?B~Èù!?B~Èò#äüò#äGÈùòC~Èùò#äüù!?äüùòC~üòC~ü!?B~È!?äGÈò#äGÈùòC~ÈùòC~üù!?äüù!?B~üòC~ü!?B~Èù!?B~Èò#äüò#äGÈùòKùuttÔÔÔL¦êêê®®.äGÈùò#äüÒV~ååå×®]3gÎTTThåwñâÅMv·nÝzHîÆ~ú)ËÁÈýú×¿îììd9¹Û·oÿüç?g9¹þþ~QËÁà½÷Þ	¾Ó_dÙÙÙZù;vìãÄöþûï»ÝîÉÀµµµøá,#÷ÑG]¾|å`äòÅr0r¿øÅ/Þ÷]ÁkmmmooOð¦ü:;;9ÚKíåh/q´8ÚËÑÞ´=Ú«®¯¯Èò#äGÈù¥³üîÝ»g·Ûu±üù!?B~È_úÈÏív¯ZµjppP÷RäGÈùòC~üÒG~f³yNDÈò#äGÈù¥­üâüù!?B~ÈòC~Èù!?B~ÈòC~È!?äGÈù!?äGÈò#äüò#äüùòC~üù!?B~ü!?B~È!?äGÈò#äüò#äüùòC~ÈùòC~üù!?äüù!?B~ÈòC~È!?äGÈù!?äGÈò#äüò#äGÈùòC~ÈùòC~üù!?B~ü!?B~È!?äGÈù!?äGÈù!?äGÈù!?äGÈùò#äüò#äüùòC~ÈùòC~ü!?äü!?B~ÈòC~È!?äGÈù!?äGÈò#äüò#äüùòC~üù!?B~ü!?B~ÈòC~ÈòC~ÈòC~Èò#äGÈù!?äGÈùò#äüò#äüù!?B~Èù!?B~ü!?äü!?B~ÈòC~È!?äGÈù!?äGÈùò#äüùòC~üù!?B~ü!?äü!?äü!?äü!?äGÈòC~Èò#äGÈoFóz½@ù!?äüù!?B~i[(jkk³Z­sæÌ9räòC~ÈùòC~üÒÓ|N§³¬¬lÎo+--Èù!?äGÈùòKü~¿@Âl6ÏùÝ²²²<òC~ÈùòC~üÒ¡¾¾>!D~~~ùdÎîÝ»>ß4ÞòC~üù!?B~ÉITg·ÛçÎe¾²²²¿ß?í÷ü!?B~È_¢0ÔÕÕiÍg±XNç4¾±ù!?B~ü!¿ä$¤s¹ÐnT23@~È!?äGÈ/æ´«;w®Ýn÷z½yÈùò#äüùÍ`±íæçç755MïäüùòC~ü¨Nl§´+3ÌÄäüùòC~ü`¾XvgtòC~üù!?B~+Ö ]«ÕÚÖÖDó!?äGÈò#ä7=Å´«pÈµA'òC~üù!?B~/êÚÍÏÏw8	Àü!?B~Èß¤íêu-Y8ò#äGÈùòæ|>ÃáÐOàäÈùò#äüùMàiWwÐ®Õju¹Àü!?B~Èß4$Oñº8êêêÿìü!?B~Èßô§ÎºVYYe¾¬¬¬ÆÆF£à@~È!?äGÈo2ùýþææfÝA»2ÿÁ©øC!?äGÈò#ä÷;õõõéÚ-..ósÐ.òC~üù!?B~+ÖY×-[vöìY#ÚE~È!?äGÈo¼Å:ëZmmmkk«ñí"?äGÈò#ä÷ÔÅ¢ÀÑÐÐ`³®!?äü!?äGÈoòùýþí¹sçîØ±#Ep ?äGÈò#äm>ÝêííMãÕü!?B~ÈfüÔ¬¬,íS§N´_MÈùò#äü(ýå'OÐ6M;Ãb±fÏjB~È!?äGi+¿P(ÔÖÖ¦Öµ´Àü!?B~Èf£üÁ ÓéÔÀáp8Rî¬kÈù!?äGÈùòÓ©¿¿¿¹¹¹´´T;£©©)½p ?äGÈò£Ù"?QÃáÈÍÍ2_YYYKKËlÀü!?B~ÈÒ_~góæÍºgàp¹Ö5äüò#äüù=5Ãf³%þIù!?äGÈùòC~Ó:ëZee¥ö¬kv»Öà@~È!?äGi%?¿ßßÜÜ¬´/OÄr)ëùMgf³ùòC~ü(Áòëïïß·o_aa¡îÌü¦¿öööÊ/ò#äüùQÂä';Òí ]Åât:C¡ËùÍHkÖ¬ñz½qäwâÄ_&¶>ø@¸ùK2pôÑ?üÃ?°ÜÕ«W?üðCö½ÿþû,#÷ÿøï¾ûî´ß¬<±®2###Ê|Ï<óLâsÓ YG²¦|§),¿ÿ±å÷÷ÿ÷÷°ïóÏ?¿ON~éoÞ¼Ér0r~úéõë×YFîÖ­[n·å`äþíßþíÒ¥KÓx---K.ÕÚî¹ç®]»Æ/^ìëëKð¦³ü8ÚKíåh/q´£½S)=zTüüü¦¦&ír´ù!?äGÈùQ:ÈOuMwÌgòC~ÈùòC~òóz½#+++Ê|à@~Èù!?B~ÈÒD~ò,ÖÐÐ =ëÕjåÈÏè!?B~ÈòO¡PÈåréuÍn·?q4(!?äGÈò£_0t:ºgàp8à@~Èù!?B~üÒA~~¿_(Exuù!?äüùòK[ùù|>Ã¡5_YYÓé,=äüò#äGÈ/åå'ORv»]wG[[1íöõõ9rdÇòoo/òC~Èò#äGO<!êà°ÙlF´ëõzyæïïå+_ùJ1F~È!?äGÓ&¿P(ät:+++£Ìe·Û?ãßøÆï¬9ðÒõoË-¿tz"òC~üù!?ÄvºvSeG__ÙlÞ¿oX~òoÑ¢En·ù!?äGÈùò£ÿÉçó555éàhiiI¡Ì<OEEE$ûäß³Ï>þüyäü!?äGÈo¶çõzupX,T<ëZ ÈÎÎÞú·Ù·g÷Ù#?äüù!?B~³7ÝipÖµ#GüÀöaßÚÿ´¼¼N+ù!?B~ü75Ãb±híþÉüÉÉ'Óàgliiù½ßû½/éK_ÿú×ÿú¯ÿ:å^¹D~È!?äGSÍï÷bàèëëÐy	ù!?äüùò3¨ùtÏº&lnnà@~Èù!?B~È_jï¾ìvVVVù*++µ8òK´üæ<©äGÈò£'æñxbu-Ö3òC~_Æ2LÈ!?äGquÖ5 <ãÄ¹"òC~_ÒC~ü!¿-=¶¶¶V;Ãápç¬kÈùK~CCC[·nE~üù!?,:uª¸¸xg]C~È/ò3Í&÷ùò#äü(VýýýÒÚ=zt¢g]C~È/iò«®®Öï(,,?k!?B~È<Occcnn®öZ×ä.òKü233å×wppP½v-¿Áo¿ý¶LÈo9ò#äGÈùÍæ®_¿¾yófí ÝÚÚZ·Û=óU ?ä4ù©_bêÉÄíÛ·ÇÆÆd"''ùò#äüfg­­­ÚjÐnww÷Ôoù!¿¤ÉoÞ¼yòÛÜÞÞîõzeâàÁjOu!äGÈùÍ¶ü~ÿ#GJKKµ8Æ3hù!?£ËoÏ=áñ¿åùò#äüfIýýýºg]+..nnnK§÷îòKü¤_~¹  @&:::dB¸dÉ~èÈò#ägÝNcc£îvÏ=;CãòK¦üò#äüù%·îîî7j?àÂjµ¶µµMeòC~ÈùòC~üRkk«îY×l6Û´à@~ÈÏÐò«¨¨PíÂ'9ò#äüÒ¸@ püøñ²²²¨§¼ÜÜÜéÀüqåW^^©½pí%äGÈù¥MqpÈ¥	~<Èù%M~<ùÕïììKäCG~ü!¿äóùìv»öÓ-Zät:gôÍ|ÈùQ~²$Èò#ä7ÓÉ³L]]Ö|V«õüùóÉ2òC~I_WWlÛ·oF~üù!¿TOHçr¹;ù!?ãÊO?¾vL;#<ùòC~©U0t:Úùùù#8ò3®ü,XÀB~ü_Jç÷ûupÈ/í#?ä4ù©mÃãñ$ø¡#?B~ÈßÔóù|C÷N§3óa#?ä4ù1Â!?ärÉ.]wÐ®ÅbIâ ]äü.?·Û-ÛÉ=üò#äüùM"!][[v´øgäüRL~sbÄB~üÑÌ§;cîÜ¹v»Ýëõ¦ÐÏü_Òä#Fxò#äüÀa6µ8|Ö5äüR^~ÉùòC~ü¨NlBvò3ºüäO¨ÈÓ-ò#äGÈù'¯×«;Ãàvò3ºüL&lHèÈò#ä§<;ÄÀár¹>hù!?£ËïÊ+²9:thhh(íüù!?B~©%¥í"?ägtù1¶!?äÜÁàÑ£GÓcÐ.òC~Fc	ùòC~Éª¿¿¿©©)í"?ägtù%+äGÈùÑl¨nÇ¹¹¹Qæ¦ô ]äüò#äüùýÿ®_¿gÐnzà@~ÈÏÐò]³fMvv¶lx9996lHÀPäGÈùÑl<ÖÕÕißY¾zõêóçÏÏó!?ädùèðéJB~üÍù©A»Ë-Ó>×lÜ¸1Íí"?ägtù-P¶½µk×ËCCCë×¯9K.E~üù!¿©ä÷ûµvóóówìØÑÛÛ;k×òC~I_ff¦l¯±ÊüùòC~6ìçµg]+..8ò3®ü222dSíçAÃ§ºò#äü&Çã±ÛíZó-Z´höà@~ÈÏ¸òSGW­Z¥öÊÿ2-sjjj!?B~ÈoBæÛ¼y³vÐîâÅgÛäü+?¡îÙq ?B~üR(ÙÅÿûß/++³X,û÷ïOä]Ëþ|õêÕZó­[·N«ù!?ÉïÑãá½6lÈËËËÈÈÿW­Z%sfú¡#?B~È¦±³gÏ¬]»ÖñCÇ-[_yå¾ÓP($÷«ÀÛÐÐÐÝÝÍzA~ÈÏòKJÈò£iüÝþÇÏÿñ¨?Úö#áW__ßÝc0<~ü¸îY×ÀüòC~üÍ`^¯wÁaö©Ï<óLkkë´ßWoo¯îvËÊÊ9ùò3¨üæ<©äGÈ_JäóùJKK£äWSS#Ï[Ó/ºg][¼x±ËåbòC~_Fì!?B~)××¾öµ6Ù×øsssûûû§åÆew]WW§5ß,<ëòC~©*¿XíÚµKmÏgÎA~üù¥J×¯_ÏÏÏÿÖ·¾õÛ¾óï¼ýöÛS¼M!Ëå²Z­ÚãBëÖ­còC~©-¿®®®Ùe#ü`gäGÈ_JÔÛÛ»sçNÙ?÷ÜsÍÍÍS¹©`0èt:uÏºÖÔÔ$Û)Kù!¿Ôß¦MÔVñâÅÄ<täGÈùÑ5OruÖ5³ÙÌ ]äüÒA~ï¼óÚª×®]Èüù!?2ü|>ÃáÐ¯¬¬³®!?äòYºt©ÌqåÊ?täGÈùAä'ûFÝA»V«µ­­ó!?äòýõ×Õ½eË¤<täGÈùQÒå'ûaí! ÍfKü.ùòAùñy~üùÍZùB!§ÓYYYµçÏÊÊ²Ûí>eüèÑìù<?ÉdB~üù¥üÔÎºühÖÉ/é!?B~È)?Ï×ÔÔ¤;£¥¥ó!?B~È!¿t×ëÕÀa±X´üù!?B~üÒD~±pÔÕÕ1ùòC~üù¥CMMMEk>p ?B~È!¿4Éï÷·´´|ýë_×ÀÑ××Ç"B~ü!?B~)_¬ÍÍÍà@~4ÛåÇçùò#äy½Þ;vdeeEíÆ+++ÀüùýoÝ§+?>Ï!?×ÝÝm³Ù´vÿàþàØ±c,äGÈO'ùÍÝÄóÏ??<<,_Êÿë×¯9W¯^E~üù³óçÏÇÀ!;º·Í"ùÊþ"òpÀèè¨Ì)..â-wuuL¦ªªª+W® ?B~È¦ì«O:µxñâ(óåææîØ±£··WòC~übßÄãDQòúûüêëëO>-ÇÛ²eV~|ðA ±]¿~ý_ÿõ_dà>ùä_ÿú×,#wëÖ-ù»åà¾øâ^zék_ûZù¾úÕ¯þå_þå½÷"¿¹¯¯ïêÕ«,4#÷ÿñï¿ÿ>ËÁà½÷Þ~¿?Áw:ãò+**(mddD¾»v­ÌùS5qllLQÒl6kåwäÈ¬BáæGdàä,Ù²ÜåËe5±Öo¾¹aÃ/éKZóíÛ·Owikk»xñ"ÎÈ]¹r¥µµå`ðdÉJðÎ¸üäïBÝ7oÞâ-GÑáh/q´£½?ÇÓÐÐ ´»hÑ¢S§NÅym£½í%öÆëöíÛåååÙÙÙ999555S¿ÙÈãÅÈò£qÖÚÚZ[[«ý|õêÕn·ûÔü!¿$TTT¤Þ>(ÿk#?B~È¢Ò=vÑ¢Eºv½^ï8où!?B~IÈf³½ñÆ2!ÿ×××#?B~Èbå÷ûò¨[LæÈÞòÁº5äüùÅ«§§§¢¢"''G-**:wîÜÔo¶½½½¸¸Xn³¤¤¤££ùòC~¤­¿¿÷îÝÚ³®'wÖ5äüùÅLsäÛÔôLþ;ò#äüfyG÷¬kÅårMå¬kÈùòYqq±ìhzzzÂòëèèé¼¼<äGÈßLäv»×­[§ÀaµZåfêgÚE~È_ìxPòã¼½üùMB:Ý³®eeeÙíöîîîéº#äüùÅL³zOä7::ºwï^Ö~ö2ò#äGÈorùýþæææ²²2­ùGø¬kÈùòqù¹ÝnÝOr¾víò#äGÈoõõõÉîNwX°¿¿&îù!?B~ñ-Y²DíÍÎÎ®¨¨ðù|3ýÐ!?äÞÉÔn·Ï;7Ê|eee---S3òC~üR)äGÈù¥k²s«««Óoºp ?äGÈo7ñÛá***JKK!?B~ãOHçr¹wS<òC~ü*¿ÑÑQÆöò#ä7þÁ ÓéÔàÈÏÏojjJÀûgò#ä÷JKKçÄmÞ¼yÈ!¿øùý~Ýf³YæOîÈùò~ùÉ¾;ãqá³wD&ìçw!?B~±òù|Ck¾²²2§Ó7ó!?äGÈo2ófúÀ.ò#äüÒ)ÙwÙl6Ýmmm3j¾çöò#äg¸!?äZ	¹vº83½Cs¹çÏ/,,7oÞýÑÅÿ @äüùÅltt´¢¢"'''<§  àW^A~üùÍ§;Cu-8doùå/ù¹ç;ðÒ½¶÷[µßzúé§òC~ü&ü5¼WíÑ:üùÑ,Àa6µv9ãÙgÝ°a°/ü¯êU---Èùòp²óx<á9]]]2'//ùò£Y+?Ï×ÔÔkG0LäÉËÍÛ¿o¤ü¾÷½ïýð?D~ÈßSÃÇÆÆ~çv5òüùÑ,×ëÕ=ëÕju¹´;þümûQ¤ü¾ýíoÿùÿ9òC~ü&ìÑvîÜ©þ9pàÌ)..F~ühVÉÏívëà¨««Küþ*²;v,Z´(ü²ã¯|å+×¯_G~ÈßëììÔý$çäGÈfüZZZ*++uÏºø3ph/~zÁÓbÐoûÛÂ¾W_5Î÷#?äGÈ/^÷îÝ«ªªÊÎÎÎÈÈÈÉÉY¸p¡Ìéüù!¿¤Ï=»lÙ2ÝF¨ãàÁq^íC~ÈAC~ü_Õ:t¨°°P;£¹¹9Yg]®ò#äüò#ä÷?ÅÀ±lÙ²dà@~È_Bå×ÓÓ£>ÌYç-**:wîò#äGi&¿îîn]ó­^½Zöïéa>äüù=!ùÍïþüÔô±cÇ!?JùÉî»¶¶V;£¡¡!¹vò#ähùË°§§',¿>É¥üÁ î ÝÜÜÚE~È_¢å§ö">½yllL¦M&ò#äG)*?¿ßäÈõmùRòC~ü&_QQúô>%¿ÑÑÑ½÷Ê´ÙlF~ü(åä×ÛÛ»oß>íY×*++O:õàÁÙ°ò#ä3·Û­ûIÎ×®]C~ü(äwýúuÍ¦ÀQ[[ër¹|¦]äGÈùT~`hÉ%jlovvvEEEÞþüù!¿i4ÅbÑàØ¸qcZà@~È_êüù!¿)Î=«k¾Í7ËïÒ¬]GÈùòC~ÈùQúÈÏï÷Ë>Düüüûöê¬kÈò3ünÝºµ`ÁÌÌLÙofggWUU"?B~d@ùõ÷÷ËÞÃl6GoÑ¢E2?íí"?äGÈoªµµµéðé%Èòèök·Û³²²¢vV/>þ|:ù!?B~3(?õyWõõõ@@¾Þ²eÌ)))A~üÈòÕjÕþ*3Ýn7æC~ÈßDnâq»Nª3ò#äGÉìN§öYYY×¯_g] ?äGÈoÂ©×üFGGÃsFFFxÍ%Q~~¿¿¹¹¹¬¬L;ãÐ¡Cà@~ÈßäSïó«¯¯ð©ý:¯9ïó#äG¨NvÚ3pÅà@~ÈßoâIÍÐa_äGÈùEæóùìv»öN§7ó!?äGÈozäñ¤L&ò#äG3'?ÙÔÕÕiÍgµZgç8ò#ä7òKVÈß,_(r¹A»B@Íùò#ä7#òõ¡Í÷îÝC~üh&äç÷û[ZZ´8²²²ìvÎüùÑììm_~ùå¨|ª!?vù©³®ipÈÎÀü!¿DÈO'»Ý¢¢¢ùòÍ7ßT;âzò#ä7;å§phÏÀQVVÖÒÒùò#ä ù=züÚ¨õë×ÏôCG~üfüdK·ÙlÚE~üQä']½z5¼;>~üx:ò#äÞòÒµµµÅ:ë8òC~È/iòÛ¶mÚ«óyHÏ?ÿ<ò#äG:ëvÇÜ¹sív»l,CäGÈù%M~&IöÈyyy7nÜxñ>¿ÌÌLäGÈ&$?5Ãl63hùòC~ì÷ïß5SöÑí%äGã¨Îáp0hùòC~F_¬Ïói!!?B~é!?Ù¬tÏºÆ ]äGÈùQ~ÉùòKuùÉ&¬;Ãb±0hùòC~Æì#çÆÿùò£p_|ñÅË/¿É ]äGÈù!?äGÈ/móûýÍÍÍ¥¥¥Zó­[·A»ÈòC~È_:Ô××ç¬kr)ùòC~ÈùòKùÔY×´8´üù!?äüù¥I²ÖÕÕiÍg6wïÞXDÈòC~È_j§ÎÀa±X´oæ[½zµÛíþâ/&tÞ^B~üòC~üßïoiiÑuMàèîîVß6Îóöò#äü%¿ø!?B~³Ê|º8rssµg]C~ÈòK=ùe<)Éüù¥jGVVvÇ#GtßÌü!?äzòKzÈ_rÐf³Å:ëZÈùòC~ÈùòKB¡P[[îY×jkkÇsÖ5äüù!?äü(Åä'¾éîî>þ¼Çã=·µµU;cîÜ¹áOù!?B~Èù!?J%ùõõõ=ýôÓO=õÔ7ó«_ýêþá>xð j0<~ü¸Ö|YYY;vìÀü!?B~ÈÒJ~/þËïïß·ÿÀKäÿeËÕÕÕ¥åòìïïmÍl6kíîÞ½[.Äm"?äGÈù!?äG)#¿îîî§zJ±OýÛûg&Ç ÃæóùoÚÏºü!?äü¥üZ[[¿¹üaö©òëËÐãñlÜ¸Q;h×b±8Î`08ÅÛG~ÈòC~ÈRF~iüì¤tÏ´kµZ§qsC~ÈòC~ÈRF~Rõ¢ê¨÷ùç;ßIÝ¦Î´»xñbí ]»Ýîõz§÷îò#äüò£TÛ;þügVíMÑüÔ Ýâââ(óåçç755MtÐ.òC~üù!?JCù=zü:ü>?¾»»û]lÀ<x°oß>­ùÌfóp ?äGÈò£t_êæõzuÏ´[VV63p ?äGÈò#äÉ/ðÆµg]³Z­mmm	åù!?B~Èù!?B~3l,E;Ãf³%~;B~ÈòC~ÈßôN§ö¬kùùùcp ?äGÈò#äÐúûû=ª=ëo¦p ?äGÈò#ä |>ß¾û´g]S8¦~äüùò©Íf3ò#ä7ÎßO»Ý®ËeA~ÈòC~:µ··/P·!¿øÉ¶ ¼Óà¨««Küfü!?B~iÍ5^¯7üþæoþæÄöávtt|Bîç?ÿ¹Xgü°ÝÝÝ/¿üriiiù222¾ÿýï_¼xÑûÚµkô¿«FNþÊýàXßü»S¸wß÷æÍ	¾Óßÿ>ÄØòs¹MTñé§öÿêW¿Jû³§§ç¥^zê©§¢Ì÷å/y×®]ò´mä/ûÁ?þßU#÷Ùg]¹rå`ä|>ßï½Çr0x¢ó/¾ø"ÁwÎòãh/ÍÂ£½òË¯=ëZKKKrír´£½ÄÑ^âhï§B~ü¢?ñup,^¼ØårTùAò#äü_<"?åò_õºº:­ùdf"Ïºü!?B~ÈßL%¤s¹v8hù!?B~üf*äGi,?¿ßßÒÒ¢=ëZVVVSSS²Îºü!?B~Èß4OwGqq±ÌïëëKuü!?äüÍvù©YYYQæ«¬¬t:)4ù!?B~ü!¿Éo²Íf3þY×ò#äGÈùòdBº¶¶6íÉn·Ë/[º®#äüù!?äühÉOÌçt:up8îîîô^GÈùòC~ÈùÑ¬Àa6£ÌWXXØÔÔ8ò#äGÈùÑl¨Nl§´[VVvôèÑ8ëòC~üù!?B~O¨»»»¡¡A;h×b±8ÎôÀü!?B~Èf£üÜn÷ºuë´vefkkë,4òC~üòC~ò­Ù²eÚv»]á,_GÈùòC~ÈùQ:ÈÏï÷ÿä'?ÑÚÍÏÏßØYõf>äüùòC~¶òëïï5£¥¥ó!?äGÈù!?äGé ?ÇÓØØËäüùòC~¶òßÀÕ«Wëu-ñ¿Èò#äüòþB¡üîiÏº&´Ûí^¯µü!?B~ÈR^~Á`ðøñãÚ3päçç755ù|>?òC~üù!?Jyù©A»ZóÉí"?äGÈò£4Ïç³ÛíÚ3pTVV2ù!?B~ü¥ü<ÍfcòC~üù!?J[ùB¡³gÏÖÖÖFOàß2òC~üù!?JyùA§Ó©ÃÁäüùòC~òóûýò¥=g]C~È!?äGé#¿þþþûöéuÍétA)òC~üù!?Jyù©Ú³®Y­VËÅ ]äüXÈòC~é ?Ùëêê´v7nÜÈäGÈùòC~È/äN:µlÙ²(ðåææÚív¹"KùòC~üòKyùùýþ£GjíïÞ½ûÁ,7äGÈùòC~È/åå×ÛÛ«;hWØÜÜXbÈò#äü_ÊËïúõëv»]ûf¾Å3ùòC~üòKù=vÝºus4ÉL·Ûù!?äGÈù!¿/¶¶¶VUUEOu­»»Eüù!?B~Èù¥|@àøñãf³Y;hW~OúúúXDÈò#äü_Ê÷àÁûöi?¹´´ôÈ#uùòC~üòK|>ÝnÏÊÊ2_eeå_ýÕ_ñf>äGÈùòC~È/úì³ÏtÏÀ±zõêÖÖÖO?ýtBçí%äGÈùòC~ÈÏ¸ù|¾HöÉôÆ½^oØÈùòC~üòK6oÞ,æËÏÏß½woooäEÈùòC~üòK«®_¿.¿ºg]C~Èò#äüßl	ù!?B~ÈòC~È!?äGÈù!?äGÈò#äüò#äüùòC~üù!?B~ü!?B~È!?äGÈò#äüò#äüùòC~ÈùòC~üù!?äüù!?B~ÈåüòC~ÈùòC~üòC~üù!?B~Èù!?B~ü!?äü!?äGÈò#äGÈùò#äüùòC~üù!?B~ü!?äü!?äGÈòC~Èò#äGÈù!?äGÈùòC~üòC~ÈùòC~üòC~üù!?B~Èù!?B~ü!?äü!?äGÈò#äGÈùò#äüùòC~üù!?B~ü!?äü!?äGÈòC~Èò#äGÈù!?äGÈùòC~üòC~,äGÈùòC~Èùò#äüù!?äüùòC~üòC~ü!?B~È!?äGÈò#äGÈùòC~Èùò#äüù!?äüù!?B~üß,_GGGMMÉdª®®îêêB~ü!?B~È/måW^^~íÚ58sæLEEV~n·û7­³³³¯¯ï7dàzzz¼^/ËÁÈÝ¾»»»å`äÚÛÛYF.üXOä722à;MaùE­ßk¯½v)±µ¶¶^¼xñ¸wßudðdÉjb9¹÷ÞOvw,÷³ýÀ:Òòëììllläh/q´£½ÄÑ^âh/GÓöh¯jxx¸¾¾> ?B~È!?äVòóÛÔ÷îÝ³ÛíºØB~ü!?äGÈ/µåÛí^µjÕàà î¥Èò#äüù¥üÌfó!?äGÈòK[ùÅùòC~ü!?äüòC~ü!?äü!?B~ÈòC~È!?äGÈù!?äGÈùò#äüùòC~üù!?B~ü!?B~È!?äGÈù!?äGÈùò#äüò#äüùòC~ÈùòC~ü!?äü!?B~È_:Éï/þâ/WobûÙÏ~ö«_ýªÜ+WäOëèè½!ËÁÈòÉ'÷wÇr0r·nÝ:ú4ËÁàýíßþ­ÏçKðúýþô_OOÏ~LDDDD¿-þÑ9¼KDDD4KB~DDDDÈ!?""""B~DDDDüù%­ÁÁA³Ùþ²£££¦¦Æd2UWWwuuE~çýû÷çDÄ¢KÖ:êééYºt©¬£ªªªöööÈïUVRR¢.ºråÎ«MÉ ëHuùòeíZ`S2þ:b;2È:³"°ñ<!-0rU_»vM&Î9SQQùÍçÎ³Ûí,´¤¯#Ù. ²¦#¿¹¾¾þôéÓ2qìØ±-[¶°ô¹Ø²¤P($ëjÝÀ¦düuÄvdugEa;B~Ñ­Y³ÆëõÆúk);;;òKYµê².]ºT]]9G166&£££Ú¿É «MÉ8ëèW^ùéOª]qlJÆ_GlGYGqV¶#äc¹è=]uvv666FÎ)//_µjÉdZ²dÉçÎrKâ:?óòòdæ3g"çËÚÑ&C­&6%¬£;wîÈ*g&í>MÉøëíÈ ë(Î0ÂvüÆ+¿áááúúú@  ûý÷îÝz¢óË/EÎÉÈÈOgff²Ü¹Ø²6lØpõêUÝÇ¦düuÄvd´vEa;B~ãZ²òìvûýû÷ã]¡63í_Q"ÑÑÑG_Z¥JújbS2È:ó»±)¥Ö:b;2à¾.jEa;B~O^n·ÕªUÚo+//÷ù|ò=,·d­#Y===ÄZ6í7Þ	ù¿¾¾åfÌÕÄ¦d´g,íL6%ã¯#¶#ãìëb­#lGÈïÉ«Ðl6kÿÀR.4Lµµµ²YnÉZG]]]UUU²"V®~iVCqqqFFFIIåfÌÕÄ¦ddU°)¥Ê:b;2È:Ò]ÆÙÑ¬A*ùò#""""äGDDDDÈ!?""""B~DDDDüù!?""""B~DDDDüùò#""""äGDDDDÈ!?"JÞ|óÍ+Vd?nåÊï¼óÎïì¿2[½Gk6åGQóeÉd*))èm!?"J½80GÓË/¿Nò;tèÌ<~üxÔü£GÊüNâ6¥X]]]bÉtâÄÐãN<)_ÊÌ7n¤ünß¾-3«««£æ/Pæû|>äGDÈÒ¿ç^Lóê«¯FÎ|íµ×dfCCC¤®"rÖÔÔÈtø>|ØØØ'îÜ¹3ò j[[èJ.ë^ºt)ÊR2gÞ¼y+V¬¸xñ¢|i³Ù¢XkkküÛQÉCR]¾|9ÒV­Z%ó;::Âs®]»&s¬VkxÎáÃå¦²³³7mÚtïÞ=­ü´·5'ÎC%"äGDÌæÏ/j¹sçNäÌ»wïÊL³Ù)¨:;;Õ¥ë×¯ºhûöíê"Ç¡-õ¥ºtÃcccbÇÌÌÌP($Êÿ"§õö»8·#ºOû9s&³a>:Ì¾¨Y¹råDåç¡ò#"JrêÀ®Î>kÎqX¤l¶lÙ2ü8/×®]«.UÐQvT/ÝØÔEõõõêÅBnooW7yû÷ïÛ©­/¾ø¢Ì¹páLËÿ2ýÂ/<ñvDò¥|CäcÓýDòóÊò¿<ráæèè¨ú¹¢ü~t8;;¢òóPù%_~®üÂól¤÷îÉb&õeMM|¹`Á­[·ØFFFÂ7"ßõ*ZøZêË»wï¿Y´>à+ÿËt[[ÛoG)_D>¶XïÉÛµkÓþôÑohïÜ¹3òD"?ñè%KÔK_JDÈ(É©º#g)Å±NØ^¯Wá/ðó´a£,ùY*2]XXåÆçÍ¾ô·ËaQõôô(¤Ê´ü/Ó'|i»<Ý£Æã_JDÈ(É©÷º½þúë3ÕgDð¿pppPûRÖ7:¤½É¨^N¢Ï¶oß®òÊÿáùqnG½ÆvÿþõåÀÀ@üq¸Ë/KÕÙX#/Rã|å¢Ë/Å_X¥ji/óPù%95BÂd2;vLªË'233µ£1Ö®]+Ê	êPlx®zz×ë| àÎ;ÅIjÔmøsUt¦Ûªw»ÝáùqnG½±O½Ï/¨ï#¿'O_9ò"õGÇ#÷"!ü5ÅÊòm"ÎM6E^ç¡ò#"J~êS£:|øðÿß=NÁ(<>NTîÅ_dedçÎ#¿GO¶!ó#Ç¹y,<®6Ö;22­~¨cÜkÖ¬¼ù_°Kämª$Ã)§/óPù"ÑÉÊ+3·bÅ5À6J~/_VWUUuõêÕð¥@`ïÞ½ÅÅÅJK»víáK/]º´dÉYIIIKKKÔmjÜÌß¶m[ÔüX·#É¤>h0ÎçùkllúxÕàà`½,¼¼<ù)|>_øj"oshhHh«ÕjíèèºÇ8!?""""B~DDDDüùò#""""äGDDDDÈ!?""""äGDDDDÈ!?""""B~DDDDüùÑÄûcùl¬IEND®B`


*Mè³©_ðUnGvMN«?ë-[ÔUÞ»wONË_SNgffòä(?ñ[~B^Èå´¼¨,ÏËËÓ÷ïßWßÊ¼||sK¤6¿~þÅÛ¦ÁÁ166&­ QQQ¡v2MZ~F£Q÷Ñ]>«"¦ªªÊåré®´©¬Ý5ã+N½üÔg>|ü#¦U~ÁVukÁd:kùòåòmQQ«üâÙ³g<³Ê@¼ÄÉd×oÏ¼ÏLû¡7I:ÝÛü­TfÈ;QÍÏÏWoOkó."!·6«['®´©¬p=:³+NýÝÞ©´]äsµÖCÞ¨³úûûUü°££'@ùëòçÏoÇ@QUìS	Èßª£«zUÊl*å§vLª¬ÔÕÕíÙ³çÉ'gÏ6mÒ-¿©Æå7Ý0ã+N½ü¦²Ï/ÐvÒý×ff¦,	h¬···©©I½Åò¿å÷|âÓúò«%jäoMMÍèõÒ¾sçÎÙÚïåv»¥?Î93òëììT$»ººÔþ<åÒ¥Kº¿ãt¯2­ò¼rÔôN¹é®Õúdï³gÏ$ìÖ­[§íÂ'OÊÊ:¼þÕ=nËËÓåååÁ¿¦ú¼fwÞP~â«ü<xøDZ200ò¯¼¼¼ÀPÙÙ_ EÜÜ<¾U÷>¿þúëÚw_~ùå¿æ´®2­ò¼rd9óÖêÊ¯··7øöW¯^|nÈ:V ê°ºC^Ô^Õ`Û·oçÉP~ üz'4xùg¶iÓ&ãºº:íxßðð°ÜÜ¦4ÐÞ½%Ü7§N*++KKK[¼xñ;ï¼óÍo~3°ÏiöWVùE^9.]ø3CâÖZAù¢¢"ù­åöÕûéáÌò­ôúéUUU7nÜtë+W***äÖär8å^¯÷Àjgªä»ü)>O.òy166öúë¯Oër3¸JÂQoÂªÁÚ~¿_ii)$õ9¿o½õkåÉÆëõîß¿ñâÅê-]9qäÈVÊåÊ(?P~ ü@ùòåÊòåÊÄ¥KÌf³Á`X³fÍt¯[VV¶`ÁþþþÀ9-KÊËË§±0³ËLåº3Ü¶j¨å^¯w×®]&IÖXvvv]]Ýç¸ßïåra£Ñ(§yt üÄüü|)áááÊÑ£Gåº---%o¿ý¶,ijjJòÓ.·Ûí²üÚµkrúúõërzÉ%s;::¥xùòe](?q¶E<õööÊu+++KV­Z%KnÞ¼;ýò3²|ttT÷ZÛ¶msÕ×íÛ·óè@ù»ì®dgggff>|8äbßüæ7+**Bn!°ËPN?~üXNËuÖ½÷ªªª222ÃòåËÕ~2í­ÿôÈWyçwÌf³Åb¤í³§OÚl6¹®Üùýû÷«]Ò¦«W¯[årË÷ïßeùÊòÒÒÒ®®®³ÆÇÇsrräg=öL¾æææúxf÷(?óêôñãÇå´ÃáÆo½õVðe®¢/Ø;ä¬³gÏÊiù*§ëëëÕY/¿ü²zÇóîÝ»²¼  @÷Öï@ä«HÏ?^NØívíßµk|øárâèÑ£Pu»ÝrBí÷¾pM|K.¬Y³æöíÛ«¨7kjjäô¦Mä´,Ñ½<êP~b_~f³YNMÐî;;;¹³~ýzts:TQQ!ËÓÒÒto-¤½"_EÝ±ììlíuóòòÔÒ[²P.)§/_.-ëóùf¶ZBôôôH¡úOnY-ß³gOà[U¨÷îÍÊÀ<X.ßNAYrI¯×«ÞÐ%ê¬3gÎÈµ$oß¾|!·üíT®îI&ï¢SÕxáÂµÄd2©ÝSßça½]»vMb.øÝméfù¡O<y>ñÖ³^¼x±îàQòûò+((Þç§vMA6MÎ|,W!Æ'L±ü"_%pÇ$¤´×ÍÍÍU×¹~¿ÿòåËjLn`gá´VK8êÎÈÓTÑÛÛ;ãûy,?u³gÏªÏù½ýöÛSÉ .Bçüùóåª#ûúúÔçÿ¦R~¯rèÐ¡þðrbÇÚënß¾]N¿ûî»wîÜQï®ÊBÅ"§%Ë>|('.]:Ëò+//åW¯^þÅ1ýõçáp#Ëg|ò0åç÷û¥®2'/rù=öL½Ó*_w"ad2²³³÷ïß?Åò|Ë%ç®Zµ*0Ð$äËöìn4×­[§ÐJlÕÕÕ©ÁÂöÙ´Vöa¹YÍ&÷P~ÙÜÜÜ]»vÉÏþÅA­o?pPëß ü(?P~ ü@ùòå7m?ûÙÏ£üCïß¿ÿ_ÿõ_<n '° d³ ü÷ÿ÷¯~õ+Öû·ûÿùÊon|ïßøòýùÏþðáCÊÿú¯ÿÚßßÏzÀóÃþÉÆõçÇ§üçþgÖ+W®È(?Ê(?P~ ü(?Ê(?P~ ü(?Ê(?P~ ü(?Ê(?P~ ü(?Ê(?P~ ü(?Ê(?P~ ü(?Ê(?P~ ü(?Ê(?P~ ü(?P~ ü(?P~ ü(?P~ ü@ùò£ü(?P~ ü@ùò£ü(?P~ ü@ùò£ü(?P~ ü@ùò£ü(?P~ ü@ùò£ü(?P~ ü@ùòKò»qãÆòåËCyyùÍ7)?P~ ü@ùòKÚò[²dÉ§~*'>øà¥KjËïòåËO¢ëO>ùüóÏOürëB6²q`=@<xðà'?ù	ëÊÇüøñã(ÿÐ.¿`Úò;úô'Ñõãÿ¸³³óàOüÏõ!Ù8° ~ö³ôÑG¬(]]]Qþ¡ÉP~===õõõ¼ÛÞíïöwÁ»½Iûn¯òôéÓºº:¯×KùòåÊ_2ßìv»nlQ~ ü@ùòå<å×ÙÙ¹víÚááaÝs)?P~ ü@ùòKò3ÍP~ ü@ùòå´ååÊ(?P~åÊ(?P~åÊ(?P~åÊ(?P~åÊ(?P~(?Ê(?P~(?P~ üÊòåÊ(?ÊòåÊ(?ÊòåÊ(?ÊòåÊ(?ÊòåÊ(?ÊòåÊ(?ÊòåÊ(?ÊòåÊ(?Êë(?P~(?P~ ü@ùQ~(?P~ ü@ùQ~(?P~ ü@ùQ~(?P~ ü@ùQ~(?P~ ü@ùQ~(?P~ ü@ùQ~(?P~ ü@ùQ~(?P~ ü@ùQ~(?P~ ü@ùQ~ üÊ_¡ü(?Ê(?P~INRÌjµ¦§§R~åÊ(¿$äñx$|¡©©ò£ü(?P~ ü@ù%îînÍ¾à×ÍfÊò£ü@ùòåFFFÅbY¸p¡¶ù$!(?ÊòåÊ_bs»ÝûöíËÊÊZ QUUÕÞÞî÷û£vg(?Ê(?P~>¯£££ººZ|-jhhÉò£ü@ùòå¹444$I`6µÍg±XÇãÕ£ü(?P~ ü@ùan¸Í¦ý$_zzºÝng%åGùòåÊ³¢Fo¬R»¯¸¸¸¹¹9;ù(?Ê(?P~áFo,ÐjµºhÞ ü(?P~ ü@ùQ~sÏçóµ··¯X±BwôFccãÀÀ@|ÞsÊòåÊ¦jhhèàÁùùùº£7N§Da<ßÊòåÊ&á÷û].ÕjçÑåÊ(?ÊoV<Osssqq±v'_IIIlÑBùQ~ ü(?P~ üæìÖn·k'Ø]¸p¡Ífþ+>åGùQ~ ü@ùòc>ÏétZ,íN¾hN°KùQ~(?P~ üælB-ZìR~åÊ(¿¹'='U'mWìR~åÊ(¿¹aÝD½AùQ~ ü@ùò£ütÈëu¸	vwôåGùòåÊòû?ÇápÄÿ»åGùòåÊoÂÞÛ	v)?ÊòåÊßô¨	vÃÞç	v)?ÊòåÊßTIÒIØé¢%!&Ø¥ü(?Ê(?P~H	v)?ÊòåÊ_X&ØMÑåÊ(¿ä/?·Ûm·ÛuGoØl¶Ô½AùQ~ ü@ùòKÚòóù|mmmÚ7v`]Êò£ü@ùòå÷¿å57¹'Ø¥ü(?Ê(?¤zùuwwoÞ¼Y»/ù&Ø¥ü(?Ê(?¤hù=zô(ÜèeËÉYrþÖåÊ(¿Ä.¿îîn»Ý¥½±yóf97v)?Ê(?Ê]~^¯×étZ,íN>ÉÔÔÔÄèÊòåGùòCÂßÀÀÀÁÃÞèèèHÍ¹7(?ÊòåÊÉS~~¿¿½½½ººZwîúúú;wîð×¤ü(?Ê(?$vù8q"ÜèsçÎ±ò£ü(?P~ üðå'/7nLOO×Þår.£7(?ÊòåÊ]~###§NZ¶lv'_aaaKKìR~åÊ¾üÜn÷îÝ»uÑ²qãÆvòQ~åÊ»ü|>_C´466ò¢ü(?Ê(?$vùHØ;DÃáðz½ü(?ÊòåÊ	 ~¿ßårY­Öphq»Ýüi(?ÊòåÊ]~CCCá&Ø-))iiia]Êò£ü@ùòCÂ¼Úl6íN>Y"Ë£ÿúÊòåÊßóx<£¤¤D»Ïl6Ë+#ìR~åÊ¾üdÓÐÐnôF;h¡ü(?Ê(?$vù©	v¥í´Á'ØØØÈò£ü(?P~ üðå744$¯tf³YÛ|Åét2Á.åGùQ~ ü@ù!áË/Üèôôt»ÝÎò£ü(?P~ üðåçñxuGoËYL°KùQ~(?P~HøòÇÝnOOO×¢ÅjµºFoP~åÊ»ü"L°«Fo°J)?ÊòåÊ]~t'NÐ=D£7(?ÊòåÊÉP~jÝÍ7kGoH644°U¡ü(?Ê(?$|ùy½ÞÖÖÖÂÂBÝ	v£7(?ÊòåÊ	_~n·»¾¾>++	v)?ÊòåÊÉY~~¿ßét®X±BûÆ®`ÑåGùòå/¿óçÏ<xÐd2ißØ­®®îèèà-åGùòåçr¹^õÕßú­ßÒÞ¼uë«ò£ü(?P~ üØFFFN8nÝS§N1zò£ü(?P~ üðº»»wïÞ­½^__ßÙÙÉ*¢ü(?Ê(?$6¯×îÜ¹ÊÊJíN¾^zé7Þd-òááa³ÙLùòåxàv»<¨C¢¥££ãéÓ§3·å÷¿ºººJKKåEùòåØr¹7nÔ¢Åd2íÛ·/°5Ö¼½ ü(¿_³nÝ:y.E(¿¿ÿû¿ÿetÉ3¿»»ûÀ/ùÓþôúõë¬Ù,ÈÆõ~òHØýÞïýöÝòòòcÇIô_þ¿øÅÇÌzòÑGÝºu+Ê?4ËïÿßÅðåwáÂÑ%¯ô÷÷~>õ!Ù8°É¿ÿû¿¿ÿþûüÇ¬ÝÉg4ßxãk×®é^ñW¿úÕücV Ë/ß½7Ê?4ËwÁ»½àÝ^Ì-ÇÓÜÜ\ÝÉ'[ZZ=záê¼ÛÞí¥ü@ùòC?¢ÝnOOO×ÞØ¸q£¼OeîÊåÊâÏçs:E»OM°y'åÊòåÊ	@ÂÚC´ªªªöööL°Kùò/(?P~é9©:i;mðIJÎæ©Mùò£ü@ùòC×Ý	vKJJÇì'Ø¥ü@ùQ~ ü@ù!ÆäõÂf³iÑ.ËçðÕòCÒßÉ¤¥¥Q~ ü@ù!<Ãá())Ñ=DKssóìwòQ~HòKÁ` ü@ùòCL½±páB«Õêr¹f0zòCJ_ÌQ~ ü@ù!Ïç0z£±±q```^ïå-¿ÑÑÑ;vP~ ü@ù!:$é$ìtÑb±XN§DaîFj¼ÿÕ_ýÕþýûgv(Ê/ñÊÏl6>çÊ¢L:ÃårY­VÝÑv»=Ê,¿;w~ùË_~µúUù+ýAÑ+¯¼È¦übV~åååÚÿ`L&¯×KùòåyyÝù½Aùiµµµýîïþîwö|çðåß¡¾ò¯455ñøLæò3ò4ÎÏÏ|?üáåD=åÊæcSn]Í6£7(?­oûÛµ5µ*ûÔ¿?ßöçåËÊy&sù©çÔwïÞ(?P~+N°;44ó;jå·iÓ&¿àòÛñ;JJJx¸&sùåääÈ³®««K^åÄ#GÔ	êÊæDÑ3`òÇ_±|Epùík_ûÓ?ýS´É û÷ïçKEùòåSìêÞÈÊÊå»ßðz½/½ôÒýÑíÛ»ïÐÁCÖuÖßùßïCçP~1.?qìØ±ÜÜqãÆ9!XQQ1ßwòåÊ/YE`wÙ²es2Á.å7WFFF^í5	>Éñ5kÖ¸ÝnÀÉ_~1Aùòå|ÂM°+K¶mÛÖÙÙçãHÎ ü(?P~ ü0ìÊpp0!~Ê)Q~K.UÇváHÎ ü@ùaZÂM°+¬VkÂÍ	Aù!ùËoÉ%ÁµÀØ^P~ ü½¡;Á®ÉdÚ½w>¿(?$ùIäÉµ§§g||<wòåÊ/E½±bÅsçÎÍ÷P(¿YÿÉÓ5ÊÙGùòåX&`·»»;	~MÊÉ_~7oÞçí®]»>Jùòå&Ø-))³=z4¿,åä/?±xñbíó ü@ù¥¸ìZ­VË|¿2åä/¿¢¢"FxòåÇzðûý/^7z£©©)&Ø¥ü@ùÍô&&DÿÝ(?P~ñFÞÈÏÏ×½ÑÖÖXh¡ü@ùéÈËËc(?P~)¾:;;uçÞX´hÑîÝ»SgF/ÊÉ_~òlçöþýû>åÊ_J9uêT¸Ñ---q;Á.åÊo¦7#<@ùòKbÝÝÝ6Mwô,þöòå¥òK ü@ù%¯×ñâÅ+WjÿÃo6eËÄ£7(?P~±DùòåM»wïÖ`·ªª*á&Ø¥ü@ùÍü'¯¨¨èÎ;(?P~ÉÇçóµµµUWWëÞhllä@ù!µÊÏ`0Èó?úwòåÊo^=zô¨¥¥Ew]Åât:£<°òååwíÚ5Ù455FóØ.(?P~óD¶®µµµÚÑj]aCù!¥Ë±½ ü@ù%Gy½Þ¶¶6ÝC´ÈÂæææT;DåÊOcAùòKôòs»Ý»wïÎÊÊ7Á.£7(?P~1Fùòå7Kj'_eee¸Ñü)?P~@ù!±ËONÂN÷-Þ ü@ùMblllÝºu²ÉÈÌÌÜ°aCzP~ ü@ùMßïw¹«Uw'_CCjÊßärÝóýÿEÊ(¿©óx<ÍÍÍá&Øu8Þ ü@ùMIii©l8jjj>*ß®_¿^¼üòË(?P~1'÷Ín·3Á.åÊonÊÏh4Ê$xØ×ØØ,å(?P~±âñxGII	ìR~ üæ²üÒÒÒd;"µXâóùd	GuåÊ/&äÙÐÐÀ»(¿y)?õnïÚµkÕ»½òUNËåËS~ ü@ùEôT´6ø½Aùò³òÔÓáñäÉÊ(¿(Mb¸	v½AùòËòSô6dgg§¥¥É×µk×Êù¾ë(?P~²´ÙlÚC´0Á.åÊoË/&(?P~HÙò0z	v)?P~åÊIR~òtGo0Á.åÊo~ËoÁdÒÒÒ(?P~ üæÏçs:EwôìR~ üæ½üÒÂ£ü@ùò+L°KùòògïÞ½jôÁP~ ü@ùÍX¸	v½Aùòò»yófff¦:Rhð)?P~ ü¦Îëõ:ÎÂÂB&Ø¥ü@ùÅoùmÚ´Im._¾»NùòCß­[·êëëM&ìR~ üâ·ü>üðCµmª©©æ]§ü@ù!9ÊÏï÷_¼xqÅL°KùòëòÇ÷Ë/¿¬sv-ÊwòåD/?5zC»OTWWËkh¡ü@ùÅKù<yRm¶lÙ»NùòCâl¾¤ítGoÔ××»ÝnÖ*åÊ/¾Êãùò£ü0ÝòiiiÑCÊYrÖ'åÊ/Ë/m2òåÊO¹uëÍfKOO×½Á!Z(?P~ñ^~1GùòCü:DKee¥öÝüüü¦¦¦ÁÁAV åÊò£ü@ù!±ËOûöíËÊÊÒ¾±kµZ;::òåGùQ~ üØåç÷ûÛÛÛkkkÃÍ½ÁN>ÊåGùòCÂßÐÐPcc£îÜ+W®t8=b]Q~ ü(?ÊÛGôê«¯jGoÈÑBùò£ü(?P~Hx§µµU÷-f³ÑÀñü(?P~H²)°ÛíÚ|Þå¤-¿àC÷éÇóå`###GyõÕW¿ýío···'â¯ =çt:-vwâÄ	vòòCÒ_ÀÕ«We«·uëÖ§OÊ·òuýúõ²äúõë(?(n·û^øÃ?üCÉ¾ÚZÉÔØØ@÷_M°»hÑ"ÝÑ?øÁ:;;ù+òCJk<xZñ±±1uRÊ²²²µ__øÍÃêßwö|'33óÖ­[q~·Õ!Z¬V«ö-RÑ3·(¿D-?µÚ)?>çÊ×ëÍÍÍ=tðP üäßW¿úÕÖÖÖ¸½ÏCCC²1Íºì:Ç¸0åÊ)T~yyy²)¬««»ÚÄ×ÔÔ¨O½P~ ü 6&)¤ü¾öµ¯ÅgùÉÅf³iwò©	vu7;(?¤Pù]¿~]wÇíÛ·)?P~P¾ò¯¬_¿>û÷íáâª<ÃáwÙà».åÊ)T~âîÝ»K,ÉÈÈHKKËÌÌ|ùãÇçû®S~ ü<wrrr^yå±ãµ­¯ýAQCCCÜ7ù;ÊÑÞX¸p¡Õju¹c¦ü@ùòÊ_bÕUWW'ÿKråñãÇ'Í©ù¦FoTUUiwòI666Lñ¦(?P~ ü(?P~SFoX,§Ó9Ýã0S~ üZå×××·téÒÌÌL57//ïÂ(?Ä¿ßïr¹tÑn·Ûg(?¤Pù©#9ÏØ¦N>òåxàñxµ;ùd¡|Ê(¿HòóóeëÙ××(¿7nÈéììlÊb+Ü»S½Aùòå÷ë71APå7>>Î¼½ üC&Ø5ÍÓ½Aùòå÷kÔÕ~>)¿±±±¨Í+åÊQaÝªªªöööùSLùòC_gg§î?ýôSÊ¢#Âè5Áî¼þ±(?P~H¡òS[½5¶7##céÒ¥sûNåÊáE½2Á.åÊ_¢¢ü@ù¥¸pì¦§§oÞ¼YÎÚÁ¢)?P~H¡òìxüøñÒ¥K)?P~s'Ø=xð`	v)?P~ üæ¾üÆÆÆÛÊs.Ü»¢¶¶öâÅ±òåä/¿ÂÂÂåääP~ ü0&ØÍÊÊt»Ý±½(?$ùÝ»w/mB`ö`~ø!åÊ³aÝâââL°Kùòå7[ÒyóýÆ.åÊ/ÕD½±mÛ6y&Æê]ÊR½übòå|Ô»º£7Ô!Z=zwòå*¿±±±¥Kfffäææ?~òåi=¹ÂM°»qãÆ¹`òåÊo¶/^2¼Wm¯(?P~,Â»ùùù²þ!Z(?P~ ü"1²[wóæMYMùòC8tvºh±Z­ñ¼òåÔ-?5¼w||ü×nWs¿,((0eee×®]£ü@ù%yænÞ¼YwôFý;wî7¢ü@ù!ÊOâL6ÙöìQWýáÃÕ;5³¼åºººwßWN>zË-(¿¦æÞÐ=è²eËä=22 ¿åÊ)T~===ºGr¾qãÆ,oÙd2©]cccf³Y[~Çÿ¸÷ýï¿ººÚ`0l%ÒÒÒ¾úÕ¯;vìâÅ¬%ó^~âÁeee²ÏÌÌ,--%³¿ÙàÃjÈ>?°Ï/Îù|¾¶¶6Ý¹7äÿròNÑìóûüÀ>¿(	þ¤ Ñh¤ü@ù%ÁÁÁæççkOB°½½=FoP~ ü@ùEI^^ÞØØØówå4åÊ/ÎIÏuttX­VíèE544$ë£ü@ù!µÊ¯¯¯OÌYí¥J»páÂìoÖf³½óÎ;rB¾ÖÕÕQ~ üâÖÈÈHss³î»Åápx<$þõ)?P~H¡ò»zõjðçµq$çÓ§OÏò»ººòóóå6´ãE(?P~ñÀív;DÝnO¢ü@ù!ÊO§¯¯/P~RiÉ_róx<­­­+V¬Ð`·¹¹9¹wòQ~ üºå§¶õÏÞ<>>.§µ£q)?P~IàÎ;ûöíÓ`×jµÆù»(?P~³§Þ§ÊollìÀê¨(¿¤!=×ÖÖVYY©;z£±±q`` e(?¤PùuvvêÉùÓO?¥ü@ù%ÁÁAyºé¢Åb±8N5O*£ü@ù!ÊOmõ***ÔØÞ¥KFáÿ(¿yå÷û].î!ZRjôåÊ_ü@ùÍÇÓÜÜ\ÝÉWRRôh¡ü@ùò£ü(?¤DùÉ#Ün·ëÞ°ÙlÑÆQ~ ü@ùÅcùþùçEEEF£Q^!222ÊÊÊ)?P~Âçó9NÅôìR~ ü@ùÍËåÒáqçÎÊ__¡¡¡aÑ¢E)2Á.åÊßl©uuu^¯W¾úôé-[dIAAåÊ/>IÏIÕIÛi/¹'Ø¥ü@ùòõMLÞ1 §Gu¦ü@ùÅ¡¡!yîèN°ËèÊ(¿É©~cccÁöùò7ò|±ÙlºhaôåÊßT©ÏùÕÕÕÉÃ]¾òäIee%óå'<Ãá())a]Ê(¿9·7yzÛòåY¸Ñ©<Á.åÊßl¥MÆ`0P~ ü¢ÆçóE½âìR~ ü@ù%*Êú»¿û;	 ººº9ÿøA$î!Z`òåÊonÊ/ÜA<x@ù!6nÜøRÑK¯m­á/¾þõ¯L¦¹µø)?&Ø¥ü@ùò^ùÉ«Ë±cÇBnÛ¶£º :::^|ñÅß=pøÍÃê_õ+Õk×®M²ò0Á.£7(?P~ üæ¥ü¤ðäe&//ïñãÇòíï½§^xæéã¦âÍ7ßüÆ«ßdüÌÉÉIòb¸	v½Aùòå7_å÷|bzÕ)((P'Ö¯_?ßwòCäò«~¥:¸üöïÛåçóùÚÚÚtGo0Á.åÊ_ÊO~=ðÔÚÚ»Nù!ýw¿ÀïöÊÏÚ·o:p:ìR~ ü@ùÅ²üvîÜ©^/K[·n¥ü[Á#<^yåá!='«;z#++Ën·3Á.åÊ_TËÏ`0ÈPvvvooïó ÏùFÊ±õ·û·eÉ%ò'âv»çöÆç»üFFFu'Ø-..niiñz½ü)?P~ ü¢]~ò:tèÐ¡v»±½HnóW~òÀÞ¼ys¸C´0Á.åÊ_,Ë/Üñüæ»(?$Yù©	vW®»ïÄÞ ü@ùòùÅå¤)?·Û½oß>Ý¹7¬VëÅ½Aùòåãò×¤à÷s#KùòÓkkk«­­Õ¾±k2<8ç3ÎòåÊò£üíòôè<uGo¬²µµÑ(?P~å/¿îîîÍ7kçÞ`ôåÊåGù!IÊoddäÜ¹sá&ØeôåÊåGù!ÊO3»wïÎÊÊÒ6_mm­<½AùòåGùQ~Hìòóù|N§Ób±hÏd2É£÷Ñ£G¬CÊ(?ÊòCbßÀÀ@cc£î!Z*++ÛÛÛ%Y(?P~W~Q~H©òóûý.KwÝôôôÑBùòåÀå6Á@ù!Êohh¨¹¹YwôFIIÉ¹sçFFFX](?P~]~1Gù!æå÷þûïÛl6íN>Y"Ë9DåÊåGù!á©	v´;ùÌf³<29DåÊ ü(?$¼þþþÝÑUUUííí¢òåGùò£üØ¤ç¤ê¤í´Á'ØØØ8Ë	|AùòåGùQ~½¡¡¡pì._¾üí·ßæ- ü@ùò£üðäÑ¥;zCM°+ÍÛÊ(?ÊòC¼ðx<ÍÍÍ%%%ºìÊYruIÊ(?P~üqívzzºö-V«ÕårÞ ü@ùòåGù!ÁD`WÞÐ½"åÊ(?Ê	Cºmß¾ºh<zòåÊåÄx*ÖÖÖjGoH644L±ç(?P~ ü@ùQ~_§¥¥Ewô,t8Ñ(?P~ ü(?Ê/QuwwÛíö¬¬¬9`òåÊå8âóùÎ;·bÅíN¾ÂÂByÎøÆ)?P~ ü@ùQ~tº£7¬V«<g?Á.åÊ(?Ê±`WX>ÉtðàÁ9l5Ê(?P~bãÑ£GÍÍÍºì®RrpÎ'Ø¥ü@ùòåGù!Úº»»7oÞ¬;÷,ïììý»(?P~ ü(?ÄÇã9uêÔÊ+uGo477Ìë ü@ùòåGùaÞÝ¹s§¾¾^»OÞèèèó7v)?P~ ü@ùQ~*¿ß/UWUU¥Ãl6755`òåÊåGù%£7*++Nç<òåÊåGùEôËåÒ½a2vïÞçÎÞ=Ê(?P~æÇãinn...Ö`·¥¥ÅëõÆüNR~ ü@ùò£ü0Û5l·ÛuGoÔÖÖÎÉÜ(?P~ ü(?Ê/|>Óé´X,á&ØïC´P~ ü@ùò£ü(¿y'ñÔÐÐ ;ÁnUUUüìä£ü@ùòåGùa&ìjO*PZ0þ£òåÊåIÉßN÷-%%%Ããñ$Ä/BùòåÊòCXò÷²ÙlÚã0§§§Ëòèÿ5)?P~ ü@ùQ~ßóx<£¤¤D»¯¸¸¸¹¹9QvòQ~ ü@ùò£üV¸Ñ.´Z­.+nGoP~ ü@ùò£ü(¿)ñù|Fo466Fy]Ê(?P~å7÷$é$ìtÑb±XN§DaÒü²(?P~ ü(¿T¤&ØµZ­º£7ìvR¾(R~ ü@ùò£üRK	vzôåÊ(?ÊòûµU¡;ÁîÂm6[¢Þ ü@ùòåGù¥tù~ë[ßúíßþííN>³Ù,¡¡¡Y!(?P~ ü(¿ä$=»xñb£ÑpìR~ ü@ùò£ü(¿)Qì¾øâ¿ñ¿|!!&Ø¥ü@ùòåGùQ~0Áî/¼PYY¹dÉT~>S~ ü@ùò£üA¸	vó7³´´ôÏlvøÍÃo¼þFù²rÊí(?P~ ü(¿aÝ/éK_6¹á/¤ùÔ?¹Ø¡C(?¶k ü@ùò£üÌ¤ìz½ÞÚÚÚ_|ñ¯~Cüþïÿ~MMM2MÈAùòåÊòKòòS£7¦>ÁnGGÇw¿ûÝýû÷·µµ¥ÚH^Ê(?P~_¢0z#ù&Ø¥ü@ùòåGù¥bù¥æ»(?P~ ü(¿Ô*¿T`òåÊå*åa]5zOìQ~ ü@ùò£ü»ü|>Óé´X,ºìjGoòåÊåxå'I'ag2`òåÊåå§ÑR]]­½±hÑ¢T`òåÊå<å744tâÄ	ÝÑ%%%Ñ(?P~ ü(¿/¿îîî7jGoÈÍýõCùòåGùò¹ááa³ÙLùðx<çÎÓ`·¸¸¸¥¥eddçåÊ(¿D*¿®®®ÒÒR©på÷ÿøÿ]×¯_ïëëûÏØq¹vìøÒ¾¤m¾oë[²BîÞ½ûy¥¿yó&ëB6²q`=@zõåã?þÿø(ÿÐ.¿uëÖõ÷÷G(¿³gÏÞ.	¯îîîÛQ×ÓÓó7ó7åååºìÚíöú§ºèúéÖldãÀzÿ^¾|õ¥£££··7Ê?4±ßíýß»¾üRáÝ^ùïã'òóóuÑrñâEÑÂ»½àÝ^ðn/x·7ÞíMñòs¹7oÖÞ0L4åÊ(¿/¿@ß¤lùù|¾S§N-[¶L»oÅ­­­^¯çåÊ(¿d(?ÝLòëîî®¯¯×Î½n·ÛåB(?P~ ü(¿Ä.?¿ßït:+++µ;ù-[vêÔ)ÃLùòåÊ/UÊ/¤)?Ýæ«­­íèè`ôåÊ(?Ê/©Ê¯¥¥%|f³Y~¯ÁÁA-(?P~ ü(¿$,?ÇeµZÛÛÛÙÉGùòåÊòKæòSñÇÓòåÊååÊ(?P~åÊ(?P~åÊ(?P~åÊ(?P~åÊ(?P~åÊ(?P~åÊ(?P~(?Ê(?P~(?P~ üÊòåÊ(?ÊòåÊ(?ÊòåÊ(?ÊòåÊ(?ÊòåÊ(?ÊòåÊ(?ÊòåÊ(?ÊòåÊ(?ÊåÊ(?Ê(?P~åGùòåÊåGùòåÊåGùòåÊåGùòåÊåGùòåÊåGùòåÊåGùòåÊåGùòåÊåÊõÊ(?Ê(?P~ ü(?Ê(?P~ ü(?Ê(?P~ ü(?Ê(?P~ ü(?Ê(?P~ ü(?Ê(?P~ ü(?Ê(?P~ ü(?Ê(?P~ ü(?Ê(?P~ ü(?P~åÊåÊ(?P~åÊ(?P~åÊ(?P~åÊ(?P~åÊ(?P~åÊ(?P~åÊ(?P~åÊ(?P~åÊ(?P~(?P~(?P~(?P~ ü@ùQ~(?P~ ü@ùQ~(?P~ ü@ùQ~(?P~ ü@ùQ~(?P~ ü@ùQ~(?P~ ü@ùQ~(?P~ ü@ùQ~(?P~ ü@ùÅ¿þë¿noo®ýèG¿øÅ/ÁÁÎÎÎùa=@ÈfA6¬ù?áûï¿ÏzòÿðQþ¡'9Ë¯¯¯ïðáÃßÀ"¿#º=±)ò ü@ùòåÊ_ÌÍæÀ·>$ø7oÞ,((0eee×®]cÕ¥ò!ÂYHÖÇÏç³ÛíF£±°°°³³ÝT0ÿ¹ÓÒÒâmËÀC0TWWWiiiðóÂò|Ö½p]]Ý»ï¾+'N>½eËÖ^*?"d<=zô­·ÞWú¢¢"6<tlRíÁ ÙÄÛòµnÝºþþþà?¡<c/]º¤aÉ$Ïs9166ÜûHÁC³¬ù_û;wØ8ð`ü``ãjåîÝ»kÖ¬Ã-åf½ý	,Y²víZÁPQQñÙg_LêF>"d<ÈûäÉEEE·oßfãÀA÷ÁÀÆ!ÕÊzzzBÆÃòêP<xð ¼¼<xIðû÷F£õÊ©$<ÈàìÙ³rB^ÎåEÝ|¥èïï_µjöbñ°e ü¦ñb¯ý;ååå=Øm+§Yo©ü`âYH¦ÇCð³>ä¿ïlx0°qHåW£G<yR±xØ2P~ÿ	,Y200 þ»¶víÚàÙl¶wÞyGNÈ×ºº:Ö[*?"d<ìÜ¹óüùór¢¯¯Ïb±°qàÁ û``ãå·zõêÞÞ^íÅâaË@ùMþ'ìéé)--ÿÃUVVÊó6ø]]]ùùùiii7nÜ`½¥òA÷,$÷ãatttÃêó[ýýýlx0è>Ø8¤`ùF5#äñ°e üR&RY(?P~ ü@ùòåÊåÊ(?P~ ü@ùòå i¼÷Þ«W¯Î°fÍ?üð×¶_fk«woÍf³üj^¯7d¹,1ãããÓ½M ü$Ã/Ð8vìX2_SS,lmmY~êÔ)Y~äÈÜ&P~ÌÍ7¥iÃ3güÎ=+ßÊÂÞÞÞ¤)¿»wïÊÂòòòå¥¥¥²|``ò@ùH~[·n¦yë­·¾ýöÛ²pÛ¶mÁÝsíÚ5)'ÂåËËéÀ<yR__-gL¦=ö¿©êr¹¤®ä,¹î+WBZJäää¬^½úòåËò­Íf¹coG%wIuõêÕp¶víZY~ãÆÀO?ýTTUU=z4??_n*##cÓ¦M<ÐööCD¸«(?¥ÅKµÜ»w/xáýû÷e¡Ùl.===êÜõë×µk×.uÛíNKKÓ½úV»aÃññqiG£Ñè÷ûå*å©>~áväîÝÓþ¦|ðApÎâòÝwßd_È¬Y³fºåá® ü ÆÔ»:Û¬¤ÃËfË-O'È	ù¶¦¦F«BGµ£Úu'Å¦Îª««S;åtWWºàÛ<tè´z³uûöí²äÒ¥KrZ¾Êé×_ÒÛjoåÁ÷M÷7ÌÍÍßwxxX¾¯rÏ%7ÇÆÆÔäò+<ÿâÝáé_»òØÐ-¿ÀrU6*Ää[i&õíòåËåÛ¢¢¢;vH±=ö,p#r½hk©oïß¿¸°ÔRà_ù*§].×¤·#)ß>~ü8ø¾ûLÞÞ½å¬ïÿûÏ¿xGÏ=Áò­¨¨P»$§[~î*ÊbLíèzúôiðB¯×+å¬­èÂþþ~Ð	|8Oû&lHKKENL&£ÑèóùäÆsrrçNz;á:,D__T9-_å´ÛíÛÕÕ%w@÷]ã©_»òSu;yòdðBu¬Ïkweõöö655©÷^É¨vÈÞN´ÏvíÚ¥Þä¯õõõånGícøð¡úöñãÇÇá®ZµJÎU²`>Kó³®^½:::¹üUªÖFàÜwå1¦FHÓ§O«£º9sÆh4jGcÔÔÔHåø|>õVl`®úúxðUîÙ³G:IºWE·ÏÔ`[õÓ;;;Ë#Üú`ú×ëUP~gÏì_9ø,õG·Û-?EVB¸òS­)­,âÜ´iSð¹î*ÊbOå8ÄÑ£Gÿoû5AQàtàÒÀ íÛ·ge°.D(¿çmÈrÉüFpÛ»|ÇãjÃý²Ï=ËÈÈP¿BÈÜëÖ­þ¹¹¹òUØ%ø6Õ.ÉÕ©s#ÜUÄ©5kÖ'¬^½Z°)¿«W¯ªÃæ]¿~=p®×ë=pà@~~¾ª¥½÷ú|¾À¹W®¨¨ +((p8!·©½'rS²|çÎ!ËÃÝ;#wIh0ÂñüêëëCï¢×ÕÕÉÈÎÎßb`` pàÛ´UëªªªêÆ!?1Â]@ùòåÊ(?P~ ü@ùò ü@ùòåÊ(?Lßÿc­gÇ^ÁIEND®B`


ï½÷B¡ÐÄÄLÈEéñxtïjª7úÑ£££»wï9¢±ÉOÝðÁ2=>>n¨UCDÈR[~C¾vttÄâÅÍ77oÞ¬Þ*­¯¯÷ù|QßyþüùÔÔÔçp¡¢¢Âd2ÕÖÖÞ½÷Ò¥KK.ÍÌÌfMäë²X,êmMùá«bùæÅ_ùï½Jmmm2³¡¡A÷wêM´?Z°(säANiáè¾&Ùôn_~Q×Êz)//Å¾víZY¶ñ_Ô^¹Z%·Û-÷&ËAÖ¬¾¡U¤`Z®2Í»vílÈ*¿ÑÑÑ¢¢¢ÂÂBÐ"àöíÛòtiùÎ(¢eddÈ×M6=Ò;rÕªUðEQHQß¹zõêø¾Y¸p¡Ì¿wï^äL¥Ìdîï8ÕDýhazÍ¯´´tJ'>à¦ÃÉË¯··7ò~äªü"W«ÇãQÃõôô¨oÞ¸qcÔcÞ¹s'ò#"ÊO&Î=ùX$¶oß.Óë×¯ð823ò;÷íÛ711¡^µRsvìØº»»ÕE¹g¹xþüyªÛªO³;wN¦"¯åÉ¤;?ò¶OyX¤¿&:Õ£û¨¦Ã8òº¡M¦ëëë>|(¦ûlòòr?2çâÅ2­VëÖ­[Õ7+ÞºuK¦emÊtNNò#"ãÊO'r'õ¨ù2ûömuQàåbAAAä=¯ÏmD^yô«·M#Á1>>.VaTVVª(¿ÌÌLÝ_Dwþ4n)*¹­ Æf³¹ÝnÝ6£û¨¦ÃÉËOñîÝ»?bJòêÞ"9êªåËËÅE¿ccclYDÈ.?ÁÙlçï`09_ûöCoB:ÝûQõçÇ¨Z,õö´waDÝÛdnk<q¡MfáÄòèôn8ùw'c»ø×jWkÔ!oÔU^¯Wá/,ÂÎÎN6."äGDôþûïËÅð1PÔL³¨W§dædÿbyy¹:ºÊDfzaR`¥¾¾~×®]<8~ü¸ÌÜ¼y³®ü&siËoª'Ü´o8yùMæ5¿°íÄýñNNÌhY__ßÁÕ[ÌáqÐDüÈ¸òôøÓúáò«9jäïúõëG§ÚwìØñôòS¯y<ñÇ±cÇ&#¿®®.õª`±»»[½§:ö¬îï8ÕLI~ñZÇEÝÉTê4ä§>'ðØ­[·NëÂwÞyG¾èPù«G(nï%)Ó¿¦ú¼¦×ëóÎ;!?"2üîÜ¹þDãóù¢>ãUXX*û4ò[DUPP _#Ç·ê>æ^zIûÎãÊ+ãüSºÉäáÈu4æi,ÕiÈ¯¯¯/òþkjj"¯Z&bµøòSEÔò¢^Uìå_fã"B~DòÔ;¡óoÜ¸±yóæÌÇÕ××kÇsLO~###rorb W^yE0> H|ß´¶¶.]º4##cáÂï¾ûîóÏ?~Íééo2%ùÅ_8gÏüL¦ð!ñ"ÒRü¤ÎÎÎEÉo-÷¯ÞOgâ3õÓm6ÛÕ«Wøëùóç+++åÞär:áù@`Ï=êÅTá»¬Ê`0ÈÆEüf¥ñññ^ziJÇÆMR.õ&¬¬èËËËù!"äGDn©ÏùEõÆo°dù¥[@`÷îÝ.ToéÊÄX,Düù!?""""B~DDDDüùò#""""äGDDDDÈ!?""""B~DDDDÈ!?"¢ÈÎ=kµZM&ÓêÕ«§zÛ¥KÎ7ÏëõçÈ´Ì©¨¨ÂîìqÓûÉÜv:ØE^]__?22Â_!?"J,8Fø¦zÛCÉm9óæoÊ¦´üâÜäùÅezëÖ­üò#¢Ù<úúúä¶µµµá9«V­9½½½yÉXY¦³³³ù+""äGD)Ã¾HÍìÙ³'///''gÿþýQßöüóÏWVVFÝCø%C¾wïLËuÕ­[·l6ÀÈd2-_¾üâÅº÷ùÓãßäÝwßµZ­ÕÕÕò´øð¡ÝnÛÊß½·z	SlZSS#÷&óåoß¾=Kò»qãFFFú&&&äÁËEÉß!?"2þÔôë¯¿.ÓN§S%o¼ñFä÷?^û¶íÛ·ËUÇiù*Óêª+W;wN&d~QQî½E>ø7Ï½ÿþû2áp8´~çÎ2-ßðáÊÄ¡Cdfqq±Lz<P/Iê~oªòÞÉòémÛ¶©«<¨Å;ï¼#à¯W~V«U¦Ç§î»ººäªõë×ËôÆéÂ×öõõíÛ·¯²²RÐ½·(]Å¿z`yyyÚÛª/&	1ÌïéåËeÁàÓ,È9ê±Éûê*ùÑK,SZZ*ùë""äGDÆÉdOË pÂ,|g PoªÊuÕ±cÇäVÂÁë×¯GÞCÔ½E^ÌMb=0¡XäËxJ,PsÌf³z9pF^óÓMp"ß+'"B~DdDùE¾æ§^6"wìv»þ)s&7IùÅ¿Iø	æ´·-((P·zx¡PèÜ¹s#òÅÂi,'.ÑÑÑÂÂÂúúúÑðÝ»wùë""äGDÆ:JËñãÇÕçüÞ|óÍÉÈ¯££#üâÙûï¿¯Ùßß¯>ÿ7ùÅ¿É¾û¾ÿýïËÄöíÛµ·ùåeúäÉözWfVWWËôõë×Åa2QZZ:òÛµk×××ÝÝùaD""äGDF_(]å<.ò|ñå766¦Þi¯á½If³9//o÷îÝ_ü¸Ýn¹vÕªUá&ßÄ^òÈ333×­[§ñøêëëÕ`áÚÚÚ©¶¼üD«¸©©©?0"B~DDDDüù!?""""B~DDDDüù%¢ýèG	þ¡·oßþÿøþnÜ½÷<xÀr0r£££ÿþïÿÎr0rcccßÁÒúÿùþçf9<YG²¦ßÌôío[ðàúÿðOßàöÙgÿò/ÿÂr0r·nÝúéOÊr0ròß§ÿøÇ,#÷_ÿõ_.9¼óçÏÿ÷ÿ7òC~üòC~üòC~ü!?B~Èù!?B~È!?äü!?äGÈùòC~Èùò#äüù!?äüùòC~üòC~üù!?B~Èù!?B~È!?äGÈò#äGÈùò#äüù!?äüù!?B~üòC~ü!?B~Èù!?B~È!?äü!?äGÈùòC~Èùò#äüù¥ü®^½º|ùrÉTQQÑÛÛüù!?B~ü_ÚÊoÉ%/_S§NjåwîÜ¹Mv7oÞ|@®¯¯ïÿñYFîç?ÿyOOËÁÈüð?d9¹ááaQËÁàüñÇ÷îÝKðMaùE­ßÑ£GØþöoÿ¶««ëÇdàÜn÷ßýÝß±Ü'|ráÂöÉ3ËÁÈýèG?úè£X¯³³³»»;Á?4ä×ÓÓÓÐÐÀ»½Ä»½¼ÛK¼ÛK¼ÛË»½iûn¯êáÃõõõ@ùòC~üù!¿tß;w.¶!?äGÈùòKùuuu­Y³fddD÷ZäGÈùòC~üÒG~V«u^DÈò#äGÈù¥­üâüù!?B~ÈòC~Èù!?B~ÈòC~È!?äGÈù!?äGÈò#äüò#äüùòC~üù!?B~ü!?B~È!?äGÈò#äüò#äüùòC~ÈùòC~üù!?äüù!?B~ÈòC~È!?äGÈù!?äGÈò#äüò#äGÈùòC~ÈùòC~üù!?B~ü!?B~È!?äGÈù!?äGÈù!?äGÈù!?äGÈùò#äüò#äüùòC~ÈùòC~ü!?äü!?B~ÈòC~È!?äGÈù!?äGÈò#äüò#äüùòC~üù!?B~ü!?B~ÈæüÁ`WW+ù!?B~ü¥³üü~¿<5Íæùóçû|>ÖòC~üù!?JCùy<»Ý?ïWíÝ»uü!?B~ÈÒJ~W®²eËüùóçýz2uü!?B~ÈÒD~]]]6m¦/~ñ²f!?äGÈò£_ hkk+.._nnncc£×ëeÕ ?äGÈò£ßÐÐ<óF~O%sZZZü~?+ù!?B~ü¥¼ü|>ÃáÐ~oÙ²emmmù!?B~ü¥üäy¶®®Nk¾µk×vuuB!VòC~üù!?Jmù	éWuuuø[¶lIü/òC~Èò#äüf^~~¿¿¥¥¥¤¤Dûa¾ææfÏü!?B~ÈÒA~±pX­VÏùò#äGÈùQ:ÈÏëõêà())q¹ù!?äüù!?JùÉÓ¨îÑe¦ÛíÆ|Èù!?äGÈùQÊËOà(++Óà°Ûíà@~Èù!?B~ÈÒA~±pdee9p ?äü!?äGé ?Q]ss³î8ÀüòC~üòKùÉ3W¬N§ó!?äü!?äüÒA~qp´··3ù!?äüùòKyùÅÀQWWÇäüò#äGÈ/äç÷ûåÉÑjµjÍÇäüò#äGÈ/Mä788¨5chh%üòC~üù¥¼ü<Ï-[tp´´´0ù!?äüùòKùýõ_ÿµ®ùÊÊÊ8ëòC~Èùò#ä©¥¥¥ºvÀüòC~üù¥C~¿ÿÈ#±ÎÀùòC~È!¿thxxXwGnnnSS8òC~È!¿tÈãñlÛ¶Me¾Åoß¾Èù!?äü!¿tHqtp¬X±âôéÓÿùÿ9¥óöòC~Èùò#äg¸B¡À®¶¶V4f ÛíVv'Þ^B~Èù!?B~üh>Ë¥À¡äüò#äüù¥dÃÃÃ÷î5ÍQæ³X,GÔÞù!?äüù!?B~)ÏçkllÔà(++kmm±nüòC~ü!¿)ÖY×l6[ûÏÀüòC~ü!¿ÔÐÀÚµkµgàØ°aCWW×$ïù!?äüù!?B~Æ-º²²2Ý²ïÒ½!?äü!?äGÈÏùý~Ý3pÈÿþýiÜ'òC~ÈùòC~üÀ¡5_IIÉ#Gâà@~Èù!?B~È_Ê$ÏPv»];£ººúôéÓOÀüòC~ü!?£'¤koo·ÙlÚó	gðiù!?äüù!?B~IKàÐ#++Käüò#äüù¥dñpÈµ³ñCòC~Èò#äÐ|>ÃáÈÊÊÒàp:³d>äüò#äüù%4yª««ÓÀár¹~òC~ÈùòC~üê¬k|VB~Èù!?B~Èßlå÷ûN§v:×ëMðãA~Èù!?B~ÈßÌ744kGssóÚE~Èù!?B~È_R8´æ+))iiiÕÈù!?äGÈùòKP±p%fòC~ÈùòC~üf7!À®ºº:é8òC~Èò#ä7[ÁÖÖÖX8dço´üòC~ü!¿)7<<ÜÜÜlµZµ85ù!?äüù!?B~3¨®¡¡!777g]C~Èù!?B~È_¢wæk×®Õ´ër¹Á ñäüò#äüù=ùØf³iÍW[[ÛÞÞæC~Èù!?B~È_¼t.«¸¸X;cÃÀüòC~ü!¿)tÏÀÕØØø³®!?äü!?äGÈoæÚ»w¯îY×äD®Méßù!?äüù!?B~ÿÇãÙ²eöÃ|åÄ)ôa>äüò#äüùé:;;m6î8Î9æC~Èù!?B~Èæ´ütb¾+VèãÚµké·òC~Èò£9'?¿ßßÚÚª¦¦&Ãù!?ä§ßÈÈlÏÈò#ä¨®¹¹Y;Ãb±ÿÈù!?º»»ËËËe3F~ü!¿p§¡¡A;cÅ.+Íuü_ÊoÝºu^¯7üþæoþæ§Íív_¹rå§dàþþïÿ^þKÀr0rBO>ùå`äzzzD|`²Ûî¹ç222¢ÌWSSóÝï~·¯¯oî¬#ùeÏ;ÇßªÁûè£®]»àÂòûåC-¿[íÒ¥Kýýý·ÈÀýä'?ùôÓOYFNáå`ä~þóÎóxþõ_ÿõí·ß^¼x±vÝnÿá8×,?þ¿U':HðMgùñn/ñn/ïöRz¿Ûë÷û[ZZJJJtÏÀ!Ûû]G¼ÛË»½iøn/ò#äühÎÊohhH÷¬kåðáÃ÷ïßãëù!?äüù!?Jùù|>Ã¡ÀQ]]ír¹ÒéhÌÈù!?äGÈùÑÜìÕëêê´æ³Ùlßá#?B~ÆùòC~òBíííÚ³®©3px½^ÖòC~ÈùòC~òò.K;#??¿¹¹9ÏÀüòC~üÍ!ùùý~ÝV«u.ù!¿Ôß¼'üùòC~Q©YYYQÏ%%%sçÈù¥ü2ÉdB~üù!¿p²Ó¶Ûíº8Ün7æC~ÈÏÐòKzÈò£N`§;C È ]äüÒA~£££Û·oG~üùÍeùùbàhlldÇôºvíÚáÃ¿þõ¯ïÜ¹3°@_ägµZM&ó#äGÈù©ÔyvÐOH:77÷¹Úç^xáßû½ß[¼xñàà ù%T~Úáf³y¶ÿ#üù!?2 ü|>_ss³vÐ®ÀÁ8&A^vvöW_úêþoíWÿÏ>û,Kù%T~²IX,ðÿûßäGÈßÜì!uÏºf³ÙÚÛÛÀñô8qbÕªUaöÉ¿=ßÜË¾È/¡òS¶Lõdb```bbB&rrr!?B~sA~²7ÖÀQWWÇÌétþáþa¤üäÙlæÝsäPù-X°@¶ðîîn¯×+PÕ!¿ôÀQVV¦Ö5pÌxW®üç?¿çÂìûÒ¾$sX2È/¡òÛ½wx<Gä_]]üùòKKùÅÀ144Ä2¥¶lÙRRR²ýÏ·û¶Ôoyæg:;;Y,È/¡ò^íµ¸zõªL+++gû¡#?B~È/?Ï×ØØ¨ÀQ\|äÈÞvíÁà7¾ñ¢¢¢ììl! Àeü ¿¤üù!?J¤übàX¶lvGrF~ÈùòC~4óòu©®®N®bq!?sò+--UÇváHÎüù¥üÁ î8æÏßÐÐàñxXPÈæ¢ü,Y©½pí%äGÈ/Ep8ÚóÍæææfp ?ÓòäÉî §§gbb"ùòC~4ã©êp]Q8Z[[9b0ò#ä÷Hþ(;³ùòC~4³ÉÕn·kpTUU>Èß/ëíí]ÃÎ;>|üùòK­B¡P»î)Ï[uùònáÂÚ]#<ùò3rqp|ùË_þÞ÷¾Ç"B~ütZ´h#<ùòK¡Ô8´8rss|>ßTÏÛKÈæüÔþ"ñÃû!?äGSÍëõ:¬¬,Ýá3p ?äGÈ/fð äGÈÏàù|¾mÛ¶iÍWUUÕÞÞõa>äüùÅ¬««Kö»wïNðÈ/äGÈùÑd]¥ÍfÓÚ]»v­ìÀup ?äGÈ/ö]Äüù%1!ÝéÓ§-[¦Ý?ù®]»ç¶ÈùòYFáAÈ_RºÿþáÃ£Àåp8|>ßïù!?B~ùòC~ÕÐÐPSSSnnnùòóóeÀü!¿égµZ-Z$O·È!¿d588¸wï^­ùJJJNçäÍü!¿'d2dçøüù!?®¢Ö5Í¦´ü!¿§íâÅ²9xðàèèh"íüù!¿¹:ëZmm­öÁ§Ü="?äGÈ/ö]0¶!¿N§ö¬kÀü!¿éÇØ^B~üÓÐÐÐÞ½µg]êäüù¥^Èò;ÉÎG÷Ã|%%%.kÆ¤ü!?äü!¿$tíÚ5Ý3íÚl6·Û=½ÈùòªÆÇÇ×­[-;£M6%`¨ò#äüÒ;ðn6p ?äGÈoúéðíÓø"?B~È/-§Óé,++Ó~¯±±qFp ?äGÈoúË.iýúõ>£££7n9+W®D~üùM¾¡¡!Ù³Íæ(óY­ÖÀü!¿é);¦ÈÏËüùòL>ÏápÄÀ1KæC~ÈßtÊÈÈÝh/<'ÊêBÈßýX]]î8foòC~ü¦z·wÍ5êÝ^ù*Ó2gùòåÈ!?ÝÔ8tp8ÙÕ$÷á!?äGÈ/fB=Ý²ã@~üùE].öùùùÍÍÍÀü!¿§jlllÓ¦MyyyòuÍ52g¶:ò#äüR+¿ß/;.í8?ù!?B~©ò#äüR%Ï×ØØ¨5_²p ?äGÈù!?äGÈoVvºv;ù!?B~S¿åÊÈÈ@~ühnÊOHÄ3p ?äGÈoæå;äGÈæ¬üÄ|º8²²²Ap ?äGÈoÆzåWÔnîÔ©SÈÍù©V«U;h×h8ò#ä7õööæää¨Ï¯DØùò£ôÏçknnÖÀát:SË|ÈùòT7oVºsçÎ%æ¡#?B~È/éy½ÞX8ÚÛÛ;ù!?B~ÓïÃ?Tºõë×'ò¡#?B~È/ÉþGwG]]ñp ?äGÈo:­Ræ¸xñb:ò#äüÀQ]]­ÖµTÀü!¿)÷Î;ï¨ýÝÖ­[òÐ!?äÈÁ Óé,++ÓÀ144Nëù!?B~[r<?B~47ä788¨ÖµTÀü!¿)ñ¤L&ò#äG)-?ùsjjjÒà(++3æY×ò#ä7[òKzÈò½d÷²víZ­ùdfà@~ÈòC~Èß/?Ì§=pÛ¶m×®]#ëù!?B~Èù!?JgùÖÖV³Ùe¾ÜÜÜæææ4Àü!?äüÍQùù|¾W_5+++Ê|ÅÅÅN§SD8×òC~üòC~nò»råî8ªªªÒòC~üòC~4äwæÌí8¤ºº:á ëù!?B~[r<?B~jòBb¾eËip444Èkù!?B~úEºOW~Ï_'[ß×¾öµµk×îÚµËëõ_~±päççÏÁÈùò~ò'Ï_|ñáÃrQ¾nÜ¸Qæt	ùòKËÞxã!T]]Ýí_®ùBÍç>÷¹)î$Áòóù|ºgà(..¦å8ò#ä7òSÿü(ôøø¸Ì±X,È_úåñxyæí¾ÿ·ö«BÀÅP~âQÝÕÕÕs|òC~üâ.'Úó#ä9Îgk³OþíÛ»O,8ù£$@~gÎÙ°aÖ|2³««ü!¿éWXX(ûÓúúú±±±G?L³~ýz#ó!¿´ßsÏ=)?ùWPP`ùAË¥;£±±qªGD~,äGÈO§K.éð¸~ý:ò#ä~y<üüüoìúFºùOþn¯ßï×ý0Ùl~õÕWYqÈùò4SÇóX²dIvvvFFFNNÎòåËïÝ»7ÛùòKV»wïþíßþí/éK_k|áù>÷¹ÏMéx3+?Ï×ØØ¨5_YYYkkk0d!?äGÈoå!¿$vâÄ	ÍVRRòÇüÇSÝgJ~²=ÆÀqúôip ?äGÈù!?äGÉïéå'Û¾öB@»ÝøÝò#äGsN~ýýý¥¥¥999j<oaaaGGò#äG3+¿P(är¹ÊÊÊ¢Ìåp8|>Ëù!?B~³.?u$çÈ3¶©é£G"?B~4#òóûý---%%%Ú3pÈ®£1#?äGÈ/qò³X,²ÿíïïËïêÕ«2üùÑSÊohhHwÐ®(Ðétb>äüù%Z~j/¬&ü&&&8o/!?zJùy½^ÎÀüù!?ÃÉOÉY½Î'òß³gL[­VäGÈ¦!?ÝÌdò#äü,¿®®.Ý#9_¾|ùò£ÉËOà¨®®ÖÚu8ùòC~$ª¬¬Tc³³³KKK0ÈùòKùùý~§Ó©;£¹¹A»Èò3üò#äòuÖ5Q`KK8!?äg8ùv»wï^iiiqq1ò#äG±äçóùGVVö¬kà@~ü_*Éo||±½ü(VëÖ­ÓÚeò#äü+¿âââyq[°`ò#äGQ;AÝ³®9Ù¸X>Èò3®ünÝºñ¸ðÙ;"öøáÈIÁ`ðÄº8Àüù!¿_8qÞl¿±üù¥h÷ïßMÕl6G///³®!?B~È/%å¬!?#çóùsss£Ì·lÙ²·ß~÷v!?äªò/--ÍÉÉ	Ï)((xýõ×!¿¹Ù+Wìv»vÐî+ÚÛÛC¡ÐÎÛKÈò3ü.5¼Wíå<üùÍµöR]]]ä|ÈùòC~),¿ÌÌLÙ³<ðÞÞ^õ9äGÈo.NgYYYørss#wÈùòC~)/?5¼wbbâ×îWs¿i$,**2LK.½xñ"ò#äg´upÈæææX8ò#äüRX~3ÙÑïÚµKþß/ÇÆÆöïß/s,ËSÞsýÉ'eâèÑ£[·nE~üÓàà î¶¶¶@ ç¶ÈùòC~),¿Ý#9_½zõ)ïÙl6«ÇÇÇ­V«V~¯¿þú(±½õÖ[UUUácy+//ÿæ7¿ùÁ°Ø¬ËOºsçÎÒ¥K³³³åÉ ''GdÎÓßmäaµä5?â5¿D:;;Å|Úÿæ­]»vJ#¯ùññ¯ù¥ðk~³Wä'333!¿¤ÛÚÚ´'l?~CCvòC~üùM§ÂÂÂñññGßíiäGÈ/Á577[,í½÷Oïnò#äüR[~ýýýê`ÎêU:QZGGÇÓß­Ýn÷ÝweB¾Ö××#?B~	Ëëõ644hpX­Ö§?ëòC~ü_ËOþòÂÏJ~júèÑ£OyÏÝÝÝEî³¨¨H;^ùòd³ª««?¾ö¬k.+þ ]äüùQúËO½Ôßß(#9òK­B¡ÀN4f©ªªª««kòC~ü_ËO=7<8zóÄÄLkGã"?B~Ìï÷·´´hpØíöÙØÊò#äüRX~êèJ~ãããöìQB~üÜÐÐlGùùùÚ³®555ÎÒÏE~ÈòKaùuuuéÉùòåËÈ1óù|Cûa¾Àü!?JgùI¡ÊÊJ5¶7;;»´´TWfû¡#?B~Ó(ÖËðò#äüR[~I	ùò|BºöövÍ¦y^fºÝîÄù!?B~Èù!?B~³X0t¹8üI'þ!!?äGÈù¥¶ünÞ¼¹hÑ¢ÌÌLy:ÉÎÎ^ºtéÈÈò#äÜü~¿îÓÜÜd ?äGÈù¥¡üÜn·îyF~üÀÈùò£t:s½:¸ÿÃ·nÝ*s!¿'Ýn×à(++KØäüùQ:ËO=¯D>£Ètø¨ÎÈ_ÎívÇÀø-ù!?B~ô(½_óÏã5?B~	3_^¯×ù!?B~È/å§>çW__/àSûÚÚZ>çGÈo¶S8¬V«Ñp ?äGÈÒY~óÔ,½íühÎÊOT'¶ÓÚ-))iiiIúäüùQ:Ë/ãIL&äGÈo¦þ´8ª««3ù!?B~ÎòKVÈ¦*?Yò'RRRR[[ûÝï~7µ~¯+W®¤ÊäüùQ:Ë/ÖAïÜ¹üÈ8òëìì,((X¿~ã×·nÝú;¿ó;ÆÿuÔ²²²Àü!?JgùÉÐk¯½5sÛ¶mÕ#?ñS~~þ½øgû¿µ_ýõWë·~ëÚµkýEÔ³Ù¬À!óRw!?äGÈù¥°üDxòlTXXxïÞ=¹øÞï©ç§Yúxò£iÈÏëõ.Z´(Ì>õï¹çkkk3à¯àóùtp8Nãà@~È¥³ü=~O=3©7ÎöCG~4%ùÿ¿â(ù=ûì³F<N1_ªà@~È¥¹ü¤K.¥ólühòòõn¯¬P<ì+W®lÙ²%Ê|r±®®.åp ?äGÈÒ ;vìPOTê|Ò/¾üÈ8òôxÇ3Ï<cÀ²_¨ªªÒàØ°aÃlùòC~ü¦Éd'ª¼¼¼¾¾¾GóËÌÌD~dù©Õ÷Â/üîïþî³Ï>ûï|'¹oÁ¶¶6íY×²²²RzòC~ü(å'ÏUûöíép8ÛKFAÞ»w¯vÐ®ÅbiiiIoó!?äGÈù¥¼übÏo¶ü(åäçñxrss£Ì·bÅ§ÓæÈ:B~ÈòKaù%+äG)$?1v´æÌ´´ü!?J[ùÉVäû¹ñ/"?³òÍ^x§5ß|iäüò#äüòlÁ`P÷¬kjGuù!?B~ü!¿_KuMÅövò#äGÈùÑÏçkllÔ=ëZ:ù!?B~üÍiùÉÃáÐ~Ïf³¹ÝnÌü!?äü¥¼üt;áöv»=ýÎºü!?B~Èæ¢üÔÝ3p8ÏÇ@~È¥üâü(-åkÌùr-«ù!?B~òËxR&	ùQ:É/Î§Óùò#äGé,¿¤ü(aò¿4»Ý®í"?äGÈò£t®½½];CÚeòC~üù!?JùÅÀ1þ|Ã1ÇÏÀü!?B~ÈÒD~qp4773hù!?B~ü¥üDu#+++Ê|V«A»Èùò#äü(Mä'HuuuÚeeeà@~È!?äGé ?p ?B~ÈòC~é/?¿ßït:càµÌbD~Èù!?B~Èù¥¼üup4662ùòC~üòKù©3päæærÖ5äGÈùòC~È/måçñxtp¸`0ÈBC~ü!?äüR[~¡PÈívWUUéàhoogÐ.ò#äüù!?äòòtpHuuu]]],%äGÈùòC~È/å»ÿ~SSÅb_VVg]C~ü!?äüÒ¤ÁÁÁ½÷jÏÀðàA¹Eüù!?B~Èù¥|òg`·Ûµ89Â ]äGÈùòC~È/M¶¨µk×j?Ì·lÙ2p ?B~ÈòC~éP0<ú´î/~ñ'Nêy	ùòC~üò3ïßommµZ­º8Ô8&Þ^B~ü!?äüØÐÐPcc£îY×ä/A®'òC~ü!?äüR5Çc·Ûµg]+++kkkÓù!?B~ÈòC~©¬_ÝUUU±p ?äGÈùòC~È/eÒµ··kÏº6þü-[¶ìâßù!?B~ÈòC~)ßïoiiÑÚÍÍÍmjjäÑò#äüù!?ägèdêà8|øðÆü!?äGÈù!?æóùöeee'N¸ÿþTïù!?B~ÈòC~KV_]]Ö|6­³³S;hù!?B~üòC~)À!¼Óàp8^¯÷)ïù!?B~ÈòC~É/ºíüüüææfu§ù!?B~ÈòC~ÉÌï÷ëà°Z­2J8ò#äGÈù!?ägÐ|>îY×JJJW¬£1#?äGÈòC~È/µc·Ûup¸ÝîÙ0òC~ü!?äüN`§;C Uü!?äGÈù!¿DOwGVVÃá©Èùò#äüòKfjÕjÕÚñÈùò#äüòKN>¯¹¹9Öiù!?B~üòC~ÊëõêuÍf³µ··ÏÞäüùòC~ÈùÍL~¿ÿ+_ùÊç?ÿù|á_¸víöd±ëà¨««KüA~Èò#äüßtBðP±¬âë;¾¾oï>®üÊák].WYYîY×6ù!?B~üòC~3ÐéÓ§/Z,æÛÿ­ýêàï÷ÿ÷ý~KKîY×d-ê·@~Èò#äüßûÖ·¾õÂó/Ù'ÿ~ó7SwX0Ávò#äGÈù!?ä7cýÅ_üÅýÑ)óeÛW*UüÆoüFùªªªfé¬kÈùò#äüòK<6qóÂçiÚ°aÛí6þoü!?äGÈù!¿'¤pÍfíÝA¾Èò#äGÈù¥X±ÎÀ¹wïÞÁÁÁÔúuò#äüù!?ä§ÏçkllÔà(..nmm5æäüùòC~ÈùMùÑ=GUUÕ3grÖ5äüùòC~ÈùÍd¡PÈívkÏÀ¡Îº&WyÐ.òC~üù!?äü&U0t¹£1Ï?Û¶m^¯7m63äüù!?B~ÈoîÊOàÐ~Ïl6¿úê«ÃÃÃi¶!?äGÈùòC~sQ~ñp¤ôùò#äüòC~ÈïÉ±ÛíÚÕÕÕ©>ù!?B~üòC~ÿ÷G¦;#UÎÀü!?B~Èù!¿'O8QVV¾¬¬,ÃáñxæÎfü!?äGÈ/Uå722bµZ_>¬=GnnnssóÐÐÐÌò#äüù¥¤üº»»ËËË1±ä÷Áü"±]ºt©¿¿ÿÆHÌW¿úÕ(ó-^¼ø¯þê¯þéþés²¾¾¾_ûÙÏ~vùòe»qãÆ'|Âr0r¢Áûøãÿíßþ-Á?4å·nÝ:¯×G~Ç¿ØÜn÷+W®'»ÎÎÎ7fddD¯¢¢âí·ß¾>·ëêê_''ÿ©»xñ"ËÁÈýä'?¹páËÁÈúé§ôËÁàÉóu___hj¿Ûû¿1¶üæà»½Â6híÚíöÄ/Þí%ÞíåÝ^âÝ^âÝ^ä7óB!Ë¥À!ljjS8ò#äGÈù¥üÂ¦A~ªû÷ïëà0Í²æàäüùòC~é#?]ÎMùù9b±X´gàù@-ù!?B~ÈòKyù]rss£Ì·bÅ3gÎB!¶%äüù!?äüÒS~±JKùéàuuuòËb>äüùòC~È/ååN§î8¶mÛæóùØxò#äGÈù!¿ÏårG/??ÿàÁà@~È!?äüÒA~jÐ®Ùl2Åb9räü!?B~Èù¥üäkpTWW>:²© ?äGÈòC~é#?á]ä!úêêê:;;ÙBò#äGÈù!¿4tðàA1ß¶mÛ8òC~üù!?äæò»ÿþàà [òC~üù!?äþò#äüùòC~ÈùòC~üù!?äüù!?B~üòC~ü!?äGÈù!?äGÈò#äüò#äGÈùòC~ÈùòC~ÈùòC~ÈùòC~üù!?B~ü!?B~È!?äGÈù!?äGÈùò#äüò#äüùòC~ÈùòC~üù!?äüù!?B~ÈòC~È!?äGÈù!?äGÈò#äüò#äüò#äüò#äüùòC~üù!?B~ü!?B~ÈòC~Èò#äGÈù!?äGÈùò#äüò#äüùòC~ÈùòC~ü!?äü!?B~ÈòC~È!?äGÈù!?äGÈùò#äüò#äüùòC~üù!?B~ü!?B~ÈòC~Èò#äGÈù!?äGÈùò#äüò#äüù!?äüò#äüù!?B~Èù!?B~ü!?äü!?B~ÈòC~Èò#äGÈù!?äGÈùò#äüùòC~üù!?B~ü!?äü!?äGÈòC~Èò#äGÈù!?äGÈùòC~Èù!?äGÈùòC~üòC~üù!?B~Èù!?B~ü!?äü!?äGÈò#äGÈùò#äüùòC~üù!?B~ü!?äü!?äGÈòC~Èò#äGÈù!¿ÿí/ÿò/ÛÛÛÛ~ðO?ýtÜÅå¿,#wõêUÙ²ÜÏ~ö³>øå`änÞ¼yòäIÁûÞ÷¾çóùüCý~zÊ¯¿¿ÿþýß&"""¢_ÿÑy¼KDDD4GB~DDDDÈ!?""""B~DDDDüù%­«ÕúË(¢Èï¼÷näµ,ºd­£®®®òòrÉ´téÒîîîÈïìíí-**RW]¼xEgÌÕÄ¦dutãÆÊÊÊÌÌÌÍ7²)¥Ö:b;JJW¯^]¾|¹l²ÄßX°ñ<!Éî6sòäÉ£GFÎéèèp8,´¤¯#³Ù|ëÖ­GO¹êëëeÅÉ¬»­[·²ô¹Ø²V®OcqÇlJ©µØÒ%K._¾,§N*--¿±a;B~Ñ­[·Îëõjå700°zõê¨²=ôu´hÑ¢;wîÈ|éÈomLLLÈÄøøx6È8«MÉ ëÈd2§,XÀ¦Zëí(éeggÇßX°!¿ËE#¿M6õôôh¥¿fÍÙ+++oÜ¸ÁrKÖ:êíííMæÈ×¨Õ¹&C­&6%¬£þþ~xï½÷¢¶6%ã¯#¶£ä&»µø¶#ä7)ùÉ¹V­ZçûïÜ¹#[#Ë-YëhåÊö²EÔühfff&ËÍ«MÉ ëèúõë¥¥¥Bó·ÞzK½zÁ¦Bëí(=|ø°¾¾>ÄßX°!¿IÉïÐ¡Cï¼óNü°+Lâ:ó¿¨ÂÂÂñññG_Zi1Wqvwª7o³)¥Ö:b;JVBmÃq÷îÝ'n,FØß¤6³¾¾>í·-Y²Äçó©µ¾fÍ[²ÖÑªU«ÔÛgåÊßf·Ûß÷]¯ò21WAÖ¬ÞÞÞ·Þzëµ×^cSJ­uÄvºººdiLfc1Âvü&%?ùÏúHfÔ7ôôô¨£TÔÖÖª®SRÖüßW$!+B¾Êtä7tww[,¢¢"5 ¸Ø²ä9láÂ²ÇkhhBlJ©µØÕj:îÆbíùÍÿH°!?""""B~DDDDüùò#""""äGDDDDÈò#""""äGDDDDÈ!?""""B~DDDDüùò#¢´é½÷Þ«©©É~ÜêÕ«?üðÃ_Û=.eö¶zÖjµÊ¯¢æËÉTTT4111Õû$"B~Dzíß¿¦×^-äwðàAÙÖÖ5¿µµUæ8p`÷IDü(ÅêííÓL¦cÇwüøq¹(3ûúúÒF~2³¢¢"j~yy¹Ì÷ù|È¥/¾ø¢æ7ÞùæoÊÌmÛ¶EºçâÅ"'AáòåËe:üÍ<hhhÈËË«Ìfó®]»"ßTu»Ý¢+¹Jnþüù(KÉÔÔÔ;wN.Úíö¨ÖÙÙÿ~ÔUòÔU.¥´5kÖÈü«W¯ç|YæØl¶ðCY,¹«ìììÍ7ß¹sG+?íýGÍóPù%³ZnÝº9óöíÛ2ÓjµFÊ&ªuíÆ£®Ú¹s§ºÊãñdddèÞJ]T×nÚ´ibbBìäZù*rÊÉÉQ¿s?2¡ûð´¿é©S§"9ÆåÉ'ÃìºÕ«WOU~q*!?"¢$§ÞØÕÙgÍ'ÍÖ­[>N&äâúõëÕµ:Êê¥;ºª¾¾^½X(ÓÝÝÝêN"ïsß¾b;õfëË/¿,sÎ=+ÓòU¦_zé¥'Þ¨Q.Ê7D>6ÝßH4YPP ¿ïÈÈ¯òÈãããêäò+<úÕ»ÃÙÙÙS_JDÈ(ùòtå¯d£À$Ý¹sG.ÔÅåËËÅEmß¾]Ä666¾ù¨WÑÂ·Roß¾þfÑRø_ù*Ón·û÷#Ê÷îÝ|l±>÷Ê+¯ÈUo½õÖ£_½£½k×®ÈoüÄ£ê%É©Ê/ÎC%"äGDäÔ]>d¦Ç:az½^¿0tÂÎÓ¾	e©Èc©È´ÙlÎÌÌrç,_ûÄûå°¨úûûReZ¾Ê´Çã	_ÛÝÝ-@÷]ãÉË/ÎC%"äGDäÔgÝÞyçÈêX'Q#<ÂÑ¾Õ××wðàAõÞkê¹ðÛ©OôÙÎ;Õ¼òµ¡¡!<?Îý¨×ØîÞ½«.Þ»w/þ8ÜU«VÉµê@6ÖÈ«Ô8_¹êÂ£££ñåV©Zákã<T"B~DDIN0LGUGu9vìXff¦v4ÆúõëE9Á`P½«>ç§>çõz#? ¨ ¸k×.qu>®®ÏÔ`[õÓ»ººÂóãÜú`ú_ PßG~Ç¿'¿räUê#G~,XòSÖ+Ë·87oÞymJDÈ(ù©£GuèÐ¡ÿÛ=NÁ(<~4<¨"ÜË/¿ÉÊÈ:::âÈïÑãmÈ|³ÙùFpûùÀÂãjcý²cccÙÙÙêWzÝºu?¢  @¾ª»DÞ§zI2rjøÚ8!¬^½:óq555jmü. ·téÒK.¯öì±X,JK¯¼òJ0_þüùÊÊJYQQÓéºOí#»ù;vìë~$y0òÔãÏ/Ôá]T###õõõ²òòòä·ðù|á#ÔDÞçèè¨ÐV-+ÍvõêÕ¨ç¡ò#""""äGDDDDÈ!?""""B~DDDDüùò#""""äGDDDüùò#""""äGDDDDÈ!?""""zÿäÖûkjIEND®B`


ðÁrÑó(j¦ÂÏ«S2s.~1''GºÊÕ«WEfszaRÀJEEÅ¾û>|xêÔ)¹sçN¿òËM,¿ù®O¾áÜå7×ü<¶÷_ÿ			2Çg ±w7oÞlllT=ã ù~å÷èñ»õ=oäWsÔÈßòòòÇ©?íöìyrù©×½Ä'Oü¬V«zUI°ØÝÝ­^ÏS]¸pÁïï8ßÌK~ÁWZÎÇùÜÉ|×êä§Þ'ðØmÝºUëÂwßWV¾èP|ý«%7Ë÷ËéÜÜ_S½_Óf³9òNDÈô%¿Ï;ºÔ»Ýîó¯´´4ÏPÙ'Ç"ªÔÔTùê=¾Õï2¿òÊ+Ú#7nòkÎë&ó_ðãù@fOc^ÀZ]ünÞ¼éÿ7oö¾ÖgÕËO,¢ß!/êUUï^õUv."äGD ?I	õûöí;wÆ>®¢¢B;caòûíß¿_0äù@à¾iiiY·n]LLÌªU«Þï½^xÁóÓßd^ò¾r. ø3Äón^kuò:::Ö¬Y#¿µÜ¿:îÎ,Ågê§÷ôôÌúë+WòóóåÞäjmmõÌw:R/¦ßeSºv."äGD´$MOO¿òÊ+óú¹Ü$âRaÕ`m·Û­@ÃQ´¥ÞççÓ[o½Å!"äGDm9Î®ZµJÒ×_ÕBDÈò#""""äGDDDDÈ!?""""B~DDDDüùò#""""äGDDDüùò#"òîÂ&É`0lÙ²e¾·]·nÝ+l6gLËÜÜÜy<=naß3Û.ðI6èObbbâãã+**ÆÇÇÕUN§sïÞ½F£QVfRRçÎ`DüHG¥§§c/n·¾·ã7ä¶ÍÍÍ9o¿ý¶Ìillnù©iùÅez×®]ê¢Ùl]]]2íÚ5^»v-0"B~D¤§g'ÀÓÍ7å¶EEE96m9¡wXè¢XY¦ãããÕÅØØX¹811ÁéÞ9tèPRRRBBÂ#G|¾í^ÈÏÏ÷¹ÏK2=66&Ó2G]õõ×_C^^z1LoÞ?=øMÞï=ÉTXX(?Hë°ÉÉÉªª*¹­,üÁÕKbÓÍ7Ë½É|¹ç÷î-üV¯^-srrº»»yhò#"ýâOM¿ùæ2ÝÚÚ*Æ·ÞzËû®âyO§Ý»wËU§Niù*Ó555êª7^ºtI&îÞ½+ó322üÞ÷¿xî>	³Ù¬]ø½÷Ê´|Ã~(o¼ñGc2¡^Ô¶ùÍÌÌÈúéêêjuÕ<w¸eË[·nñè""äGDúÉdééÇiáå÷V«U®*//éíÛ·+Òy®½yóæáÃóóóÕ¿÷æc¬à7Q¤½mZZZx1Lùd¦|§Lçååe].×ÂVv¾Z6¹sa÷áÝÞÞ^Á«çäò#"äGD:Á`ðLËÎ G_úJHHït:ê ªÌQW<yRn%¼uë÷=øÜ÷Å¹Ü$Ð	Å¼_ÆSj<þ|rr²c4ÕËò_ºººÄÞ¾éN~Þ¯ù©ÍfåNUUùê¯F<Ì<nò~Ï	æ´·MMMU·õY<·ÛéÒ%5ðÖóbá¼VË|å'©å_!?"Ò©üÔ§´:uJ½Ïïí·ßwÎ?ïyñì>ðÌWTïÿüßäðáÃ?üáeb÷îÝÚÛ¾úê«2æÌ¯¾úJá2ëÖ­û÷ïËDVVÖÒÉ/77W®ºzõªLwttÈô+¯¼ÂéT~n·[tð8ïÏä.¿©©)u¤U¾z¿éMôc4<8Gù¿Igg§iÓ&Ï@ïïq:ûöí%Ýºu«Æ+à«¨¨Pnß¾=¯Õ¢="dUÈO¬ªªõºwï^Y$`Düùò#"""B~DDDDüùòE?þñBüCïÝ»÷ÿù<nôÜØØØÃYznbbâßÿýßYznjj*ôO°4¯þû¿ÿûþéX:O¶l)ä·8ïßüøþä'?¹ÿ>e=÷ÕW_ýó?ÿ3ëAÏýõ×ÿøÿÈzÐsòß§Ï?ÿõ çþë¿þKÜ7é¹+W®üüç?G~ÈòC~ÈòC~Èò#äGÈù!?äGÈùò#äüò#äüù!?B~Èù!?B~ü!?äü!?B~ÈòC~È!?äGÈù!?äGÈùò#äüùòC~üù!?B~ü!?äü!?äGÈòC~Èò#äGÈù!?äGÈùò#äüò#äüù!?B~Èù!?B~ü!¿È_OOO^^Á`ÈÍÍíëëC~ü!?B~È/jå·víÚë×¯ËÄÙ³g³²²´ò»téÒÃÐ&OwîÜyH:îæÍ_~ù%ëAÏýìg?ëííe=è¹»wïþèG?b=è¹ÑÑQQëAç]¾|yll,Ä?4åç]||¼V~Çÿ<´üñÇV«õsÒq~ú)ëAÏöÙgW¯^e=è9aüÅb=è¹ÿøÇ/^d=è¼îîîÿÐh_oooMMG£½í%öG9ÚµGUN§ùòC~üù!¿hßÈÈÙlö-äGÈùòC~ü¢G~V«µ¤¤d||ÜïµÈò#äüùEüL&Ó¯!?äGÈòZùùòC~ü!?äüòC~ü!?äü!?B~ÈòC~È!?äGÈù!?äGÈùò#äüùòC~üù!?B~ü!?B~È!?äGÈù!?äGÈùò#äüò#äüùòC~ÈùòC~ü!?äü!?B~ÈòC~È!?äGÈù!?äGÈò#äüò#äüùòC~üù!?B~ü!?B~ÈòC~ÈòC~ÈòC~Èò#äGÈù!?äGÈùò#äüò#äüù!?B~Èù!?B~ü!?äü!?B~ÈòC~È!?äGÈù!?äGÈùò#äüùòC~üù!?B~ü!?äü!?äü!?äü!?äGÈòC~Èò#äGÈù!?äGÈùòC~üòC~üù!?B~Èù!?B~ü!?äü!?B~ÈòC~Èò#äGÈùò#äüùòC~üù!?B~Èù!?B~Èù!?B~Èù!?B~È!?äü!?äGÈòC~Èò#äüù!?äüùòC~üòC~üù!?B~Èù!?B~ü!?äü!?äGÈò#äGÈùò#äüùòC~üòC~ü!?B~Èù!?B~È!?äü!?äGÈòC~Èò#äüù!?äüùòC~äÉívË	äüò#äGÈùEs.ëØ±céiäüò#äGÈùEa£££õõõF£qÅ/jkkC~Èù!?B~ü_T500PWW¸âÿVYYüòC~üù!¿(éÆÂ»+WúoõêÕÍÍÍN§ù!?äüùòC~Ûínkk+--]¡©¸¸ØjµÊ7ryò#äGÈùò[üGsssff¦Ö|Û¶m?ßaY*äüùòC~ü³ÑÑQ5h×|uuuv»=Ëü!?B~Èßâ444T__¯À"Hp8a_BäüùòC~ü´þþ~¿8Ö¯_¯ó!?äGÈò#ä÷¤Éþââbíù6lØÐÞÞâÈùò#äüù-~B:Å¢À±råÊmÛ¶~Ð.òC~üù!?B~Ãá¿ø&Ik>³ÙÞÈùò#äüù-N¢ºíºººááaýÿÈùò#äüùÍn>³Ù¬Àyúôéù!?äüù!?B~Küe/++Ó¯°°Ðb±èóÍ|Èùò#äüùÍ#uÖ5¿vefèÿÜ#?äü!?äGÈoñs¹íÍfÍÑ¿òC~üù!?B~ÿ´ëwGCCÎí"?äGÈò#ä7§Ú5Lº:òC~ÈùòC~üÍf4h7p ?äGÈò#äç§@g]Ñg>äüùòC~´ìähÐîÊ+«ªª"wÐ.òC~üù!?B~¿,Ð ÝÚÚÚèÀü!?B~È»üÚ²Èoñ7LÈò#äGúÝn¯­­ÕOàp¹m3!¿ùÕÝÝ#äGÈùò#=ËOkjj´vÛÚÚ¢uò[ä¶nÝj³ÙÈïäÉ?	m|òpó'¤ã>ûì³¿ÿû¿g=è¹k×®úé§¬='ìûøãYzîþá.^¼¨%ù«¿ú«oðÉÅÂÂÂÐÿÖ[²dKøF°üþwËïïþîïî6aßíÛ·ïý­[·XzîË/¿¼qãëAÏÝ¹sÇjµ²ôÜ¿ýÛ¿]¹r%¼Ëð7ó76mÒÚé¥zÙFÒ¥KCüC£Y~í%ör´8ÚËÑÞçp8;¶~ýzóFùÓ,Ðaëp´ùò#äü(âå744T__¯À.vò#äGÈùQÔÊo`` ÐY×ZZZá ]äüùòC~ò»qãFii©Ö|eeeQ|Ö5ä§Ç!?äGÈù-]çÎÓO.VWW÷÷÷³	ò#äGÈùQÄËÏétZ,í ]³Ù<44ÄÊG~È!?äG/?ÃÑØØ¨Àa4e>æC~È!?äGÑ ?ÍVWW§5_vvöéÓ§N'+ù!?B~üE¼üúûû«««ãââtèµ×^;xð`;òC~Èò#äG¡Õj-**Z¡Ifvtt,â¢îØ±ãßøÆó¿ÿ¼ôôÓO/ÁÈùò#äü(òs¹­­Ú3pÄÅÅÕÔÔÈSèâ.ç_þå_>óÌ3¾èÈkGäLüÖoýÖÑ£GòC~ü!?ZBù9ÌÌL¿8è¬k¿÷¿÷í¿­Ø§þýqõ ?äüù!?B~´$ò³ÛíµµµÚ6lXê999»ÿt··üj¿S+úD~ÈùòC~ühå'¶üumýúõ%ï·«ªªÚ²e·ü¾õ­oýáþ!òC~Èò#äG&?ùY\¬À!3­VkÈÆXØl6£ÑXZZzðÀÁCß=Tú\ôw"?äü!?B~ËQ~B:Å­Àa6ÃB®¡¡¡òòòäää±àòÙLÈùò#äühIäçp8´8S®e¥!?äü!?B~/¿ááaù¨À!lmmÅ|Èù!?äGÈ_4ÈÏn·ûÀQXX´ä·b¶bbb!?B~È¢Ï>ûlãÆZóþ#-ùÅÌÁ`@~üù!?ZÄÔÂÂBð	Ífó²<üB-¿°üù!?B~Ë'ÃÑÚÚêwGCCÝng!¿pÊobbb÷îÝÈ!?äGOn>¿8F#v_xäg2ïó#äGÈùÑ"¦pÄÅÅùüÍÎÎ>úôåËYEÈ/òËÍÍÕïÿ,éy!?äGÈ/?pUUUApÌë¼½üS~±±±òXOOO	ßøC¨©©A~üù!?n·»³³SÖ55Cþy¾ù!¿°ÉO=(eB¨'wïÞäGÈò£¹är¹à¨­­Õà@~È/lòKNNfww·Íf×_]Mð©.üù!?5ÃÑÜÜ¬Íý¬kÈùM~ôçðùqäGÈò£@ÕÖÖú=ëÅbq¹ü_Øä'=z455U&zzzdB¿Ôüù!?B~ÚÀÀ@MMÖ|ÅÅÅmmms9ëòC~á_XB~ü!¿Kþï´vKKKE	s¿äüò#äüùé4·ÛÝÖÖæ÷¬kó½CäüÂ)¿¬¬,õÙ.|3!?B~È¼s¹--F£Ñç¯d\íÏ´ü_Øä·víZoíybl/!?B~Èo97<<ÜÐÐ ´+säOØäÎòüyò8îíí	å¢#?B~È>³Ùlµµµ~Ïº¶(gÚE~È/lòS¯`Èò#ä§ÃäSYYßV«u.vòÓ»üúúúä1½wïÞÉÉIäGÈò[	éÚÛÛµ8¤mÛ¶-Å_+äüÂ&?iÕªUÚÇ:#<ùòC~Q_ ³®%&&VUU-`Ð.òC~zß5káAÈò[n;vÌd2iÏºÖÐÐ ×.éOG~È/lòSô¥ûoò#äüùé*»ÝîwÑhr¦]äü¢D~iiið äGÈù-úûû«ªª´8Ö¯_?ëvòùY­VyÐ<x0xäGÈùòYêÅÅÅÚ7µÈ_÷E´üÞå·"@ð äGÈùEz.Ëb±hp¬rÇV«5ü_Øä Fxò#äü"7Ã!hRRR´æ«©©YðY×òxù+äGÈùò[Ô­ùdNýÐÐù!¿°ÉÏd2­Y³FþÜ"?B~ü_D'Oòf³Y;#33³µµ54vòÓ»üì¡_täGÈùò[¬äß%ô8òÓ¯üºººdßhllåg» ?B~Èß&¤ØeggkÍ'ý_äü"@~í%äGÈùE£©©Éï ]³ÙöÈùéW~í%äGÈùEPv»½¡¡Áï/×FÄoü_Øä®!?äGÈo^Ùl6¿8L&SÈÎºüòC~ü!¿¥-ÐÌÌLà@~ÈO×òÞºuk||¼ìE			/¾øbz ?B~È_ðÔÂÂB¿8:;;#Ñ|ÈùY~SSS~Gx,õi|!?äGÈ/P£µµ5Ðy2ômü_Øä#ûRyyùää¤Ø¾»ÌÙ¸q#ò#äGÈùÞ|~Ïº&sjkk#eòC~ú_ll¬ìQÞ/OOOËüùòC~!KTg6ãââ´æ¸ÈùéW~111²_ö<sÌáS]ùòC~¡i`` ÐY×,ËR¿ûùÑò:Ú[RR¢öÊW9yyyÈ!?ä·ÔYüàhkkÜÈùéW~B=¿#<äùò#äü"!]»Ö|+W®,++ÓóY×òxù=z<¼÷Å_LJJ¯%%%2g©ùòC~´å§pFóýÊ¯üÊ¯ÿú¯Fñë|ÈùéE~a	ùòC~´¬ä7::ÚØØ¨´PPPPûÚ#¯YµjU[[ò#äüò#äG*¿ºººÄÄDóÅÄÄ<WòÜÁÅ|êßóÏ?/ßüù-¾üVÌìÈ!?ä÷$Éó|YYvÐîúõëO>ýÔSOØÀÃ>ùWúw¿û]äGÈoñå8äGÈòÔív³®ûÛß~öÙg=ì;8íÿ¥ÉµÈßâË/Pû÷ïWûçÙ³g!?B~Èo^¹ g]ó9Çyæy^zé¥/î0Lò'²L¶òC~á____BBú%ïvF~üù!¿YÆµ´´Þ|Ì~àÀ@g]s:o¾ùæe/lß¾ýûßÿþ2Øü_øå·sçNµ^ºt)4üù!?ùêjjj´8233[[[Åvläüt$¿?üPí¢ååå¡äGÈùQ¤ËOvØêêjí6>zù¼ü_dÈojjjãÆj0GWWWùòC~¹ò§Ó²²2íçBvtt`>äüt'¿wßWí¥»víË¢#?B~È"Q~ò·Px§5ÙlçXV>òC~:çGÈò.Ëb±dggûü±«««³Ùl¬väüt-¿Ù2È!?ä§Î´«=ëÑhù<`#?äòÈò#ËÏn·×ÖÖjÍÙÜÜìr¹XÕÈù!?äGÈùQÄËO-Íf³vÐnaaa;8òC~Èò£h<3û=ëZUUUè´!?äü!?Z|ù¹Ýî@8´g]#äüò#äü("åçp8´gÚMII'j¹õüòC~üE¼üýÚUg]Ã|Èø<?äGÈ_4ÈÏf³Àa±XÀü(åçýÑ~åÇçùò#äòó;Cà@~Íòó$<Ùç_~ùåÉÉI¹(_·oß.s®]»üùòù©ÚA»f³3p ?ZFò3²ó¿¶?==-sÒÓÓðûúú222Ãºuëººº!?äG¡Ãáhmmõ;£¡¡A»ÈüÔShÏG~Oþ>¿3gÎÈÄñãÇwíÚ¥ß'|âm7nÜø×ýW'é¸/¾øâg?ûëAÏÝ¹sGþ_ÇzÐsÃÃÃ×®]ù³?û³äädó­Y³æ/þâ/äZVTûÿø?þõ ó._¾,ÿwñ]rù¥¥¥É(mjjJ.Ê,//92ÿÉ_MQ4LZù577Úd7?#'ÿÁgCÖ»zõªl&ÖûÁ~ðÜsÏýê¯þªù~ã7~£¾¾¾³³Uöººº:::X:O¶l©ÿÐ%ü¿Ðï[·n=á=Ñáh/q´£½´èÉójUUvÐ.88ÚKíýewïÞ]»vm|||LLLBBB^^ÞØØØß­÷ñâØØXäGÈùÑÒÕÙÙé÷¬kf³YúX?Èß¦Þ>(_µÇ!?äGOÛínmm-((ð1_bbbmm-8!¿ÐUUUõÞïÉ|­¨¨@~ü-bãØ±c~í¾öÚk¨ùòØàà`VVVBB:>vþüù'¿Ûîîîôôt¹ÏäGÈùÑ¢4::Z__¯>Ëç¬kÅårÍë¼½ühyÉO³÷ÛÔôñãÇtÑ!?äGóÍf³UVVÆÅÅù¯¨¨¨­­MÌ§¾ù!?B~KOOgÁÁAüzzzd:))	ùò#ä§umÛ¶mÚçRäüù¾Ç©	%¿ÎÛKÈr»ÝmmmÚ+W®¬­­ð+äüùL³zOä7==èÐ!Ö~ö2ò#äGÈ/d9@g]~¦]äüùÌjµúý$çë×¯#?B~üBßèè¨<CjpùD³ÞòC~ü%ÊÏÏWcããã³²²BðPÈò#ä¹×l6'&&úoýúõ§Oöà@~È_äüù!?òtãÆ²²2¿g]³Z­n·^÷ü!¿ÀwñÆÆÆ²²²V¯^üùò[Òtò§¥°°ÐÇ|r±²²²¿¿awü!¿yÈozz±½üù-iN§Ób±Èÿ±^ä3ÍÃÃÃOrçÈùòóMûtãSrr2ò#äGÈoÑmjjJIIñyÖMOO?vìØp ?äGÈo!ÏÝ1ó½Ã;aß~üùò[Äìv½Ö|ÙÙÙ­­­N§s±~òC~ü&Î[ê»Èò[æÉÓße¾8æ%?Ã1÷AÁü(úå®!?äõ©3phÏº&¬®®^ºç@%?«ÕúÌ3ÏÆäääßþíßþáÏüh¹Èozz:+++!!Á3'55õÍ7ßD~üù-8Ëe±X´gàP8umåwîÜ9ßöíÛ×ÏÿþóBÀ'8BÈùEüV­Zå3¼W==566"?B~üæÃáç7íùL&ÌðàAAä÷ÜsÏë[ß:òÚÏ¿g7=øða6ò£å.¿ØØXyJòþh__ÌIJJB~üùÍ=»Ý^[[«5_ffæR¼/¸ü~sõoîþÓÝÞòû£ª?*--e3!?ZîòSÃgffþÏýj>äùò#ää¹Ål6û=Gggg(Íç_^^Þ·_þ¶·üþ`û¼ôÒKl,äGË]~òô´oß>5økjjêÈ#ê¥!?B~Áç1¿8ªªªBÿç-¿ºººgÖ<sè»ûì?ðo|ãìÙ³l2äGË]~½½½~?É¹§§ùò#äç7·Ûm±X²³³ýà°Ûíá]<5¶·¬¬L´÷üï?_úSO=õï|'ô¯>òC~º422²nÝºøøø³Ôüù!¿HLà0L>æKIIùrÅ8ïÜ¹sûöí;xð`gg']äGÈ/!?B~È/²²Ûí~p´¶¶êÄ|Þòã±üù!?äüùÍ;ÍæwÇù!?B~K+¿ÁÁAõaÎj<oZZÚùóç!?B~pqòC~ü<ò<OgJ~júøñãÈ-Où©ZóéaòC~ü^zzº<zä×ÓÓÃ'9ò£å)?ÃÑÚÚª=ëÀ)g?C~È_à»xÜ#¯OoiÁüùÑò¨ÎïY×DMMMºÀü!¿¦>½OÉozzúÐ¡CêÈ-ùÙív¿8²³³õ9ù!?B~Ïjµúý$çë×¯#?B~Ýòg¡²²2¿g]Óóäüù=Q¡üü|5¶7>>>+++ï_F~ü_¸r»Ýmmm~íÍfy¢ômü!?Ýüù!¿Ðçr¹,vG\­þí"?äGÈù!?äGÈoöÔY×´8ÒÓÓ;6::MÛù!?B~ÁºsçÎ5kbccåI0>>~ÝºuãããÈEüìvmm­Ö|«W¯>qâÓé¾mü!¿uvvúá!!?hùÉ.ïwÐnAAA»ËåÖmü!¿©Or®¨¨PÿñÜµkÌÉÈÈ@~ü(åçv»åÿ´ÚRiié7"ñZò#ä÷h?ÉÙûyP¦=êüùQÉOuM;cåÊUUUËd!?äGÈ/`ê5¿ééiÏ©©)^ó#äG%?5Ãd2ù/11±®®nhhhYm#äüùL½Ï¯¢¢BÀ§/x!?ùÙíöí£Ñ(Ï3Q9ù!?B~Op³µD!?ä÷Ùl6¿8V¯^ÝÚÚYgÚE~È_ä3[ùò#]ÉO=üà(,,ìèèúÈùò¼!?ä7ßÔáÖ|Û¶m³Z­läüùÍR mA~üH'òó577¯^½zÚE~ÈßßÅGõY]]Í§ºò#=ÈïÁÇÓ~PKJJÊ¢ì¬kÈùò[rùðä94--mllL.¾ÿþûêYuÞÞüù!¿¹?jjjµæ.çÈùò¢ª««ÕóiFFØ¾ûR/:ò#äüe³ÙvìØ¡´yâÄ	Ìü!¿'íÚµkçVybÁ¢#?B~ÈO[gggYYÖ|ÅÅÅrvò#ä·íÙ³G=·ªóyH/¿ü2ò#äG!ËåjmmÍÎÎö;#ôÏÈE­ü<½&%%Ý¼yó×ûübcc!?ZjùùF£ùâââÌf³Ýng#?äGÈo1å'Ï°ö)O¸í%äGK*¿¡¡¡­ùRRRäù7ó!?äGÈoIäèóüZHÈß²_¿ß³®eff.ó³®!?äGÈoÉå®!¿e(?ÙëüuÍb±0ù!?B~K%?yªõ>ü"ò#äGO"?!Ý¹sçüu­¸¸ÈùòC~ÈùQ4ÈÏáp477kÏÀ±råJ³Ùl³ÙXÈùòC~ÈùQÄËoxx¸®®.%%Åï¹5üëù!?äü_ÄËohh¨¾¾^ÖµÌÌÌ¦¦&p ?B~Èù!?B~Ñ ?Ù©·mÛ¦´ÍäGÈù!?äGÈ/ä'¤Ø1ùòC~Èùòfù9ÖÖÖ@8dcu!?B~ÈO/òò#äGAä'æ½ØïÚÚZÎºüù!?É/f¶ò#äGÚº»»+++µæ3LuùòC~:_ØC~ü".ÙuÈòC~È_$kqq±ßùòC~Èù®å'Xéïïooo`µYK%;;[û>àªª*í"?B~Èù!?ù?óÌ3O?ýô³ê©§¾ùÍo>xðï] A»qqq/^d!?B~Èù!?ùmØ°áw÷pýá#¯¯eee¬|ùÚU8æuÞ^B~üòC~Nùõ÷÷?ýôÓêß¡ïJMM]æ+ßn·Íæ¸¸8íÖÖVÏ ]äüù!?äü(bä×ÑÑñì¦g=ìSÿ²²²óÇ´[XX¨´ü!?äüEüxÍÏ;¿veeeöPäüù!?äü(bä'å®ÏõyßóÏ?¿¬Ös A»ê¬kÁÏÀü!?äüEüÔØÞU«VýÎïüÛ»|^ðs8MMMÚA»j¬Yïù!?B~Èù!?$ù=zü¢<ÚÛÛûûûÉùµvEbÁ¹uù!?B~Èù!?0ù-«ìvû´v³³³pÖ5äüù!?äüùé1ÙË*++µv¼"?äGÈù!?äGÈO_uttùÀñ_ü!?äü!?]ät:O8¡´8ë ]äüùòC~ü"£áááúúzíôôôyà@~È!?äGÈO¿ÙíöÄÄDí ÝÓ§O/úÈeäüù!?äç¿ññqÉüù-Q7nÜØ±cßKôi5ÈùòC~ÈÏOÝÝÝ999òGùò[ôd÷ñ;£²²r©O@ü!?äüü´uëVÍD~ý×ýEhûôÓOzz¾ ÷£ýHLÃzTÿÑ£G³²²|ÌóÒK/ôÑG!Xë×¯öÙgl='úÿäOX:ß/]ºÄzÐy/^¼uëVhËï1°üÚÚÚB¨âË/¿"'4ÿéOÊzÐ688¸ÿ~Éäc¾ÔÔT/éC¶$ò<øùç³EôÜW_ÕÕÕÅzÐsv»ýòåË¬':ÿùÿÐhG£½sIüÚÍÌÌ<qâÓéñòp´£½ÄÑ^ör´÷ÎS!?B~²6ª««µ8ÛÛÛ].WXù!?B~Èù³ ò#ä7ßÔ ÝÊÊÊ:;;Ã»lÈùòC~Èùò[Ünwaa¡øâââª««eåèa!ò#äüß¼C~ü¼s8MMM>æKIIihhÖÏ¢"?äGÈù!?äGÈoêdwÐà0ÍÍÍxÖ5äüùòC~üÂÝn7ÍÚ6lX³®!?äGÈò#ääÁ_VVæ÷¬kº5òC~üòC~üæ®­­Mx§=ëÙl¶Ùlñ[ ?äGÈù!?äGÈ/X.Ëb±Àa·Û#èwA~ÈòC~Èÿß&IæëpòC~üù!?B~óÎn·×ÖÖú=ëÅbÑùùò#äGÈùòSòØ®ªªÐÈùò#äüùÍN`çw@0ôxäüùòC~Èù-ùüà3Í5ù!?B~ü!?ÿ©&I;h7Bp ?äGÈò#äçÝnohh4ÃårEë6B~ÈòC~Èül6ß³®·µµEúäüùòC~üþ'yÐúÀQVV58ò#äGÈùÑ²ËårÖµ(Àü!?B~È©üÄ|'N4cxxxn#äüù!?äü(Úä7::êwÐnfffSSSTÚE~È!?äGËN~ê¬k>æ[¿~Duù!?B~ü!¿_&É;vhínØ°¡½½ó!?äGÈù!?äGÑ ?«Õh<l0òC~üòC~ñòS8²³³µg]«­­µÙlläüù!?äü(âå7::ÚÔÔ¤ÀÞØØ¸<í"?äGÈò£hÝn?pàvGvvö'N'[ù!?B~üE¼ünÜ¸á÷¬kÚE~È!?äGÑ#?y°®Ð$3ÏY×ò#äGÈùQ4ËÏårY,íêêêåvÖ5äüùòC~òðàAKKß³®ÕÕÕ²ªò#äGÈùQÄËÏn·744ð|Ì·zõêææfp ?äGÈò£hl_¿88òC~üù!?ùÉcI©¬¬Ìjµb>äüùòC~ñòÒÉn©À¡ÎºÖßßÏúD~È!?äG/?uÖ5¿8XÈù±!?äü"^~£££õõõÚ¢À!ëùòC~üòxùÙíöêêjíY×8ò#äüù!?ä=òÊ;´v´üù!?B~ÈùEütçÎó;h×l6ËÖdu!?B~ÈòC~/?§ÓyìØ±ÌÌLí Ýºº:ÍÆB~ü!?äü">yTìÛ·O;h711±±±ñÁ¬"äGÈùòC~È/â8pàÀ¯ýÚ¯iírÖ5äGÈùòC~È/JGBee¥vGAAA[[8!?äGÈù!¿/Ð8Ô ]¹ó!?B~ÈòC~Ëå²X,ÚÒ7¿ùÍ>úUüù!?B~ÈùEC²Ýµgà9uuuCCCó:o/!?B~ÈòC~ºÎápx#==½±±Qfªkò#äüù!?äUÕÔÔù233[ZZ|í"?äGÈùòC~È/ªÞY,Ëå÷*äüù!?B~Èù-"?äGÈùòC~Èùò#äüù!?äüùòC~üòC~ü!?B~È!?äGÈò#äGÈùò#äüùòC~üòC~ü!?B~Èù!?B~È!?äü!?äGÈùòC~Èù!?äGÈùòC~Èùò#äüù!?äüùòC~üòC~ü!?B~È!?äGÈò#äGÈùò#äüùòC~üòC~ü!?B~Èù!?B~È!?äü!?äGÈùòC~Èùò#äüù!?äüùòC~üòC~üù!?B~Èù!?B~È!?äGÈò#äGÈùò#äüù!?äüù!?äüù!?äüù!?B~üòC~ü!?B~Èù!?B~Èò#äòëééÉËË3¹¹¹Èò#äGÈùE­üÖ®]ýúu8ölVVV~V«õç¡­··wxxøç¤ãm6ëAÏÝ½·¿¿õ çÆÆÆº»»YzÎétüX:Oä755âÁòó.>>^+¿·ß~ûJhëèè¸téÒÒq/^dé<Ù@²XzîòåËòtÇzÐyô+m¤-ä×ÛÛ[SSÃÑ^âh/G£½ÄÑ^öFíÑ^ÕäädEEÓéD~ü!?B~È/ªä·â©###f³Ù/¶!?äGÈùòlùygµZKJJÆÇÇý^üù!?B~È_ôÈÏd2­ðùòC~üù!¿¨_ð!?äGÈùòC~Èù!?äGÈùòC~Èùò#äüù!?äüùòC~üòC~ü!?B~È!?äGÈò#äGÈùò#äüùòC~üòC~ü!?B~Èù!?B~È!?äü!?äGÈùòC~Èùò#äüùEüþüÏÿ¼­­m(´ôÑG?ýéOHÇuuuÉ	Xz®§§GYzî/¾øÛ¿ý[Ö»sçÎ3gX:ï?øÝnñu8Ñ)¿ÁÁÁ#G|~Qð#¢+x%hü!?""""B~DDDDüùò[ããã&ÉsñöíÛùùù±±±;wîðþÎ¾¾¾Á°nÝº®®.V·Ñýû÷WxÅªY===yyy²wäææÊ|aWÒÿ6bWÒÉ6Ò>²EÐ6ÒÃ~ÄÞë[wwwNN÷öØ¸q£l×GO¶gÏïo®¨¨8sæL?~|×®]¬=n£óçÏÍfVZè[»víõë×eâìÙ³YYYÁ÷v%ýo#v%l#í ûQm#=ìGÈÏ·­[·Úl6ï&÷L'''³Ñhééi¿º§°o#ÙÇ.ÀJoñññÁ÷v%ýo#v%l#í ûQm#=ìGÈ/ÀzñÚ`¹¹¹2ñþûïÃ>WN¶üo¬¤¤Dfæççß¾õúzkjjï/ìJúßFìJ:ÙFÚ'@ö£ÚFzØßìª¸uëVVV@þwÞQ÷ãe½épy ²ÞBÜäädEEÓé¾¿°+é±+édQûþ·ö#ä7»*<Ý¹s'''ÇNZZÚôôô£Ç/­Ë4ëMÛÈ;C<µÍæû÷ïÏº¿°+é±+édyd?Òÿ6ÒÃ~üfWÅÚµkûúúfffÞyç£Gz[UUÕï½'òU°ÏzÓá6«ìv»Ú3KJJXo!ËjµÊËþÂ®¤ÿmÄ®¤mDìGúßFzØßìªºjÕ*±yMMÛíöþîîîôôô5¶ô¶zsrrCQQìi¬·e2|>¼ÀïþÂ®)Û]I'ÛÈ¯*Ø"eéa?B~DDDDË%äGDDDüùò#""""äGDDDDÈ!?""""B~DDDDü!?""""B~DDDDüùò#""""äGDDDDÈEOï¿ÿþæÍã·eË?üðÿ<=.bmý-­Éd_ÍétúÌ9!##cfff¾÷IDü(ò:räÈMG&ù566ÊÌ'NøÌoiiù¯¿þúîùQÕ××'¦1'Ot?îÔ©SrQfÞ¼y3jäw÷î]ë3?''GæÛíväGDÈ¢¿_~YLóÖ[oyÏ|ûí·efuuµ·ºººDNÂ¼¼<ö|óÃkjjä*£Ñ¸oß>ïª¢+¹JnåÊKÉäääÍ7_ºtI.VUUù,XGGGðûQWÉ"©«®^½Hi%%%2¿§§Ç3çúõë2§¸¸Ø3ç7ÞHOO»ß¹sçÈÈV~Úû÷dQù³U«VZ¾þúkï÷îÝ&É[6>õööªk·oßîsÕÞ½ÕU111~o¥.ªk_|ñÅ±cll¬Ûíkå«È)!!A½ý.ÈýÈßÅÓþ¦gÏõæ¬gÎñ°ÏçN¶lÙ2_ùYT"B~DDaNØõóµb8Ì[6»ví|LÈÅòòru­²£zéNÄ¦®ª¨¨P/Êtww·ºïû<|ø°ØNlõÕWeÎdZ¾Êô+¯¼2ëýå¢|÷²ùýD©©©òûËEù*K.ÜVß!7_áÑ/ÇÇÇÏW~AQøå'ùg¾422"ÅLêb^^\³fÍîÝ»ElSSS;ïñyÍs+uñÞ½o-yøÊWîììõ~DrqllÌÙ½'oÿþýrÕ;ï¼óèG´÷íÛçý¢@x4??_½$9_ùYT"B~DDaN½Ð599é=ÓétÊL¹*u<.´ÙlèxÞ§=ëc)ïÏRi£Ñër¹äÎ=×Îz?æÓàà BªLËWðÝÝ-à÷¨ñÜådQù9õ^·wß×¦ú¬÷k_Êºyófcc£:öê!£zAÎs8uVíÝ»Wä¯555ùAîG½Ævÿþuqll,ø8ÜM6Éµêl¬ÞW©q¾rÕÕ«W'&&ËÏ£Rµ6<×YT"B~DDaN0ÇWêròäÉØØXíhòòrQËåRb=ãpÕûüÔÛãl6÷÷íÛ'NR£n=«â×gj°­úéV«Õ3?Èý¨7ö©÷ù9NõAäwêÔ)Ï«qò+_¥Þò800 ?EVB ù)kåÛD;wîô¾6È¢ò#"êSzã7~ùüõ8#Ï´ç8©gP§W_ÕÞ?>ü=>ÙÌ7ÞÜ,÷yÆÕúe§¦¦âããÕ¯àsëÖ­Þ?"55U¾ªvñ¾Oõ¤'åTÏµA.lÙ²%öq7oVläwõêUõ±yëÖ­»víçZ§ÓyèÐ¡ôôt¥¥ýû÷»ÏµW®ÏÏedd´¶¶úÜ§vIä®dþ=|æºIFIÐ`ÏóóTSSãóñ.ªñññYIIIò[ØívÏ'ÔxßçÄÄÐV­«âââdQùò#""""äGDDDDÈ!?""""B~DDDDüùò#"""B~DDDDüùò#""""äGDDDDÈÍ¿ÿ XîYwK9¼IEND®B`


Detrended Normal Q-Q Plots


,,S§ü®sÛyÓûÏ¢£á4ºÞ>3wVe­Ë³Øü3NõØ£@oêYãkjj2QxçÏ?ÕãÆÚÈ¼Â`æ=I¥R¡Ba455e>ö<åÎñ©2ý=Î¹JÃ¼ae®ZµêäÉ®´é¬IïÕ¬g~ùEÏk×®eÞÄÊoOãÆÆÆp±¾¾>kø/Äèè¨ß* ü ÀÊ/JY]]=ÕÛíT]ç­:OßÌô½yj|xÈáñ÷ï±±±Ìñ¹ûÌr?ôûx§s1TfÖÏÛ£Z[[ÎÍ»¬N/a:³Lg]Mzé¬©ztv3NÿhïvMói<88Å_º_|ñE¿X@ùA!ß³Ï>yG´-ë,ËéÎæÍ¿Ïæí·ßÎg±ùgÌs;.¦¿%ÅYÖÞ©¬&þÉ¼¸xñâèÛUº»»CM§ü¢Ñ¬$ÉmÛ¶Ý¸qãðáÃaäºuë&-¿éÌ2ëòéÊIõÓ/¿éìóK·]ô_<ë?Ïó-rîÜ¹=öDÓçAÊ>èåÞÛBEoÌéouÞÏB4wÊèTÙ%KD¢9Ü2é[fyógS,Ñç´ÂÂ£ådkÒÅæ1F¬]»6ýAþhLtæï5knÞ-mË-ï½ü¢ý^á!:th:åwêÔ©h¯RxÈgÎöçE?>éöé,3*¿ü+'ë¹i¦kuå2/<FGGCØ­^½:·÷ïßV~¨ÃÁù×ÿm_Ñç5óy|PÊ/Wæù¹gË¦?ïþÒÝhaî[fyógSH¢¨2O´LOÍ³Øü3æÏ«W¯¦?ÑÊúWMMMúCï¥üÒ-?~úSyúæGÉÝXK.Í³g4ËÊ/ÿÊÉzndéZEù;w.sù+V¬ÈµNÒ'Ou+yoéSÒ6nÜè(?(òoÍÍÍ¹Ý««««©©)TTx;öÙgÓã?ÆÒÊü6»iÎÛl¾/,¼±±1÷kù¦ZìmgÌÑÐÌñ/^n]ù-Éd2÷|ÙßððpXZXfh íÛ·Jwþ¾9pà@xtá/X°àÈ#?üpzÓeFååd=7²Ìh­Î¢ü_|±¾¾><ê°üèxzútæp1ôYtë«V­êíí½í>×©o###;wîv¦|rllÌ/P~wD*zäGfôr³¥àDûz£µÇÇÇ£ _¼x±'(?Mô9¿,O>ù¤5Êb322²cÇDtÃÀc=fµò@ù üP~(?Ê@ù üP~(?ÊåòæÞñãÇëêêâñxssóLçmhh(++L	ÃaÌ%KfðëÙ]g:óÎÚððð-[æÍVÎvîÜ9222G¿ÄïäãPºjkkCdÊé¼÷îó>ýôÓé1O=õT³gÏB/¿yo¹téÒÄÄDGGG¸¡Õ«WÏbQ«V­R~ò>¿/ÞCd;w.Ì»råÊôeË1ýýýss'ïEQÛÙÙyWÖ°òp§²/³3vîÜYUUUYY¹÷î¬«=üðÃMMMYKHï2Ão¿ývc¢I/_^µjUEEE<ollìééti·#GÔÕÕ-_¾<ÜPn!½óÎ;íííaÞpçwìØíÂmºbÅ°´0>,ùÊ+ÓK,	KtjµtêÔ©p'ÃJ8~üxîÎ]yåù	(?àÄ_4üÄOág64VxòÉ'3¯ÓÕÕ^¦Í7IÃágÞ´iS4iéÒ¥'N.]ãÄ¤KË¼ùg	=wìØ±0°aÃÜ;¿uëÖ0®ðüóÏ½÷<ð@¾yóæÀÀ@vIæÊ]-!§j¯ükiÿþýÑmþË½Y=ÿ¢<9åÜÁò±S·ä×¤<uêT´fÍ0¼víÚ(kÒSÏ;·k×®¦¦¦0>Mº´¬ÊÉ?KtÇªªªrç­©©îüÄÄDÍFkáÆÆÆVSíÀTòË¿FGG£á°©Ê/ýØó/ÊP~À,¿ÌÜª]²Ìª¬¬×ª1Ñ¤C¹B¾þúë¹õ3éÎ,SÝ±»ñ¢jììì7o^4¦ºº:Ú8~-ãßyçüQ¿ðòÏn(?àý)¿D"¹*ÚmvÛiooSÓ?ÓãËËËÃ[¦Y~ùgIß±s¹óÎ??7ëî8qbÃ;ok×®]áú¡s'Mg-M³üf·Âð>_tBëáÃ£=õÔSÓ	ÎÎÎôÎ³cÇeeÍùóç£ÏÿM§üòÏjìßüfØ¼ysî¼7nÃ.ðË/Ã¯¿þúµk×ÂÀ¢E¦¹ZnÞ¼:láÂ/_5ílmmæZÊ8GÇ³ûìV8òÞòuUyKæwòåÑÑÑèHkø)=þÅ_¬®®Ý³cÇi_þYN<¦.[¶,¢IæuFFF¶mÛîyyyùêÕ«£ÓxCð%Éèdá+W^¼xqúk&ÜJ¨ÉèXvHÀÝ»wGáÎZÊ>~üx¸KÑeg=öÙ­på üP~(?Êåw|ûÛß~ë­·æòR©g^iúÑ~ô¿ÿû¿ÖCiºtéÒþ(E¶õÿçþÇz(Mo¾ùæjëzùå+_	ñ7·xêÔ©ÿþïÿöJ(Mÿðÿ0éßø¢|ë[ßú¯ÿú/ë¡47Ì/°¤¤9sæ?ÿó?òCù¡üP~(?å§üP~(?ÊOù)?ÊåòS~ÊåòCù¡üòCù¡üP~(?å§üP~(?ÊOù)?ÊåòS~ÊåòCù¡üòCù¡üP~(?å§üP~(?å§üòS~(?Êå§üÊåòCù)?åòCù¡üP~ÊOù¡üP~(?òS~(?ÊåWTå×ßßH$âñxCCCOOOîº»»ËÊÊÊåòCù¼d2ÙÑÑ<ØÖÖ5u||¼±±qªò;qâÄ9ôÊ+¯rå%)ü·äßÿýß­Òôê«¯Úú%+üÿ­·Þ²JSø?ÿo¾9·XåW]]=11R©T]]]ÖÔ'xbß¾S_ÅïÌ¡§Oþ%ÉÖ/ñ­ÿ­oËz(M/½ô­oëÏ(¿x<>éppùòå¦¦¦Ðöâh/öâh/öX,.//ÏÔÚÚzúôé=TåòCù¡üP~E ¦¦&J½ëhoþ¥GøËÊåòCù¶ööö#Gð3LNþPíóCù¡üP~(¿âX­µµµ±X,HôööNzÊåòCù¡üJòCù¡üP~(?å§üP~(?ÊOù)?ÊåòS~ÊåòCù¡üòCù)?å§üP~ÊOù¡üP~(¿"uýúu[_ù)?å§üP~(¿b622òÙÏ~ö¾ûî«©©©ªªú¿øå§üÊåò+Bããã>ºýÑÝ_Þ½é7øÃþÚ×¾¦üòCù¡üP~ÅæäÉ,Øõ¥]!û¢°áî¿ÿþPÊOù)?ÊåWT8ÐÜÜÎ¾è_MMÍO~òå§üÊåò+*/¼ðÂÒ¥K3³ïÑíÎ7Ï>?å§üP~(?_±¹~ýúý÷ßÿÉßKßò,ÿÔ§>U²[_ù)?å§üP~(¿böÚk¯Ýï½K.mnnþõ_ÿõßüÍßüéOªüòCù¡üP~ÅéúõëÏ=÷ÜÓO?ýÂ/òÖW~ÊOù)?Êå§üÊåòCù)?åòCù¡üP~ÊOù¡üP~(?òS~(?Êå§üÊåòCù)?åòCù¡üP~ÊOù¡üP~(?òS~(?Êå§üÊåòS~ÊOù)?åòCù¡üòCù¡üP~(?å§üP~(?ÊOù)?ÊåòS~ÊåòCù¡üòCù¡üP~(?å§üP~(?ÊOù)?ÊåòS~ÊåòCù¡üòCù¡üP~ÊOù)?¯åòCù¡üòCù¡üP~(?å§üP~(?ÊOù)?ÊåòS~ÊåòCù¡üòCù¡üP~(?å§üP~(?ÊOù)??388øôÓOþó?pàÀõë×Êå§üÊ¯8½ðÂóæÍûØÊò|°ñÁèCÊåòS~ÊåWl~üãßwßäs»¿¼;ú÷É?ùk¿ökãããÊåòS~ÊåWTþîïþî·~ë·ÒÙý«­­R~(?òS~(¿¢ràÀU«VeßoüÆoüÓ?ýòCù¡üòCùðú]°`Á®/íJgß¶~áîQ~(?òS~(¿bóÑ~ôÁÆÝþhÈ¾Í´9àþéNgFå§ü¬å§üÊ322²~ýúyóæÕÔÔÜï½?þøtNïP~ÊOù)?å§üP~°ë×¯O³ùÊOù)?åò£´(?åg=(?å§üP~(?ÊOù)?ÊåòS~ÊåòCù¡üòCù¡üP~(?å§üP~(?ÊOù)?ÊåòS~ÊåòCù¡üòCù¡üP~(¿»¨¿¿?HÄãñÌI½½½aÒ%KÂÕÊåòCù¶d2ÙÑÑ<ØÖÖ9iáÂgÏG]´hòCù¡üP~(¿ÂV]]=11R©T]]ÝTW«¨¨È-¿¿ýÛ¿ýç9ÔÕÕõ½ïï)Iaë÷÷÷[¥éå_¶õKVww÷w¿û]ëÁÖ%Q~ñx|ÒáL6mÊ-¿çî­9ô÷ÿ÷oQN<iëòÖÿùë¡4½òÊ+/^´JSOOÏo¼1·XåÅÒÃååå¹WxçwÉäÈÈ£½8Ú£½8Ú£½­¦¦&JEGÃpÖÔ«W¯nØ°áÚµk¹3*?Êåò+0íííG	ág2Ì*­áááIgT~(?ÊåWx«µ¶¶6%ÞÞÞ?¶²=ºººº²ÊåòCù¡üJòCù¡üP~(?å§üP~(?ÊOù)?ÊåòS~ÊåòCù¡üòCù)?å§üP~ÊOù¡üP~(?òS~(?Êå§üÊåòCù)?åòCù¡üP~ÊOù¡üP~(?òS~(?Êå§üÊåòCù)?åòCù¡üP~ÊOù¡üP~(?òS~(?å§üÊOù)?ÊåòS~ÊåòCù¡üòCù¡üP~(?å§üP~(?ÊOù)?ÊåòS~ÊåòCù¡üòCù¡üP~(?å§üP~(?ÊOù)?ÊåòS~Êå§ü¬åòS~ÊåòCù¡üòCù¡üP~(?å§üP~(?ÊOù)?ÊåòS~ÊåòCù¡üòCù¡üP~(?å§üP~(?ÊOù)?ÊåGé_ÙíÄb1å§üP~(?ò+òÝN<W~ÊåòCù)¿b(¿â üP~(?Êï½OñÍ7+?åòCù¡ü_±_]]]<÷9?åòCù¡ü(òò[²dIîéÕÕÕ###ÊOù¡üP~(?åWTåW^^Roxx¸¶¶6àûæ7¿6mÚ¤üÊåòS~EU~ÑN¾0R/tibb"TVV*?åòCù¡ü_Qß¼yóBç688ì±hÀ·º(?Êå§ü­üvìØ>#ó£~Ë/W~ÊåòCù)¿¢*¿à«_ýêüùóÃ@oooØÔÔTÛCù¡üP~(?_©P~(?Êå§üÊåòCùMfÑ¢EÑw»ø&gåòCù¡ü(æò[¸pafí¥9·Wù¡üP~(?åWlå"/4___ßÄÄDÁmåòCù¡üP~3P]]Ê¯³Où¡üP~(?ßÌô÷÷òÛºuk!¾¥)?ÊåòåpòCù¡üP~Ê¯ØÊ¯¾¾ÞÊåòCùQå5ßÀÀ@!nåòCù¡üP~3PSSãåòCù¡ü(ò)ÊoÇcccÊOù¡üP~(?¹üÊ¦àåòCù¡ü_~ó¤á¡üP~(?ò+Âou)åòCù¡üf ®®®¾¾þÂÊOù¡üP~(?¼üâñxYÙû¶ï°¿¿?He644ôôôä4»1üñçî­9tìØ±7Þxã-JRØúáÿHÖCiúÆ7¾ñüÀz(Máæûßÿ¾õP:;;æòïxùå·gÏð÷þÝ.Éd²££#<x°­­-ÿ¤ÙÉ´ûö2bqÇËïý=··ºº:ÊÇT*UWWÒìÆ(?@ùÍÒûnoæÈ4»1ÊP~ËËËóOÝLögvàÀoÏ¡¿üË¿ìîîþ6%iÿþý¶~ÉúÚ×¾öòË/[¥)¼Ñ¼ôÒKÖCiú«¿ú«9ÞúV~555©T*:8óOÝçöâÜ^Ûsqnïì¨Z½zuEEEYYYeeekkë¬Oõhoo?räH?ÉdþI³£üP~(?ÊoFGG'=Ì<»?ãVPmmm,K$½½½?¿·ZçNÝåòCù¡üP~³´xñâPfkÖ¬ÞÒÂíÚµaÌÒ¥K?øÛCù¡üP~(?ßÎOI¥RaLîéÊOù)?å§üP~(¿Â.¿X,:/:"266ÆÌî[]ÊåòCùpË/:ÚÛÒÒ½¥a8illT~ÊåòCù)¿¢*¿ðN6é7nÜP~ÊåòCù)¿¢*¿woÞÛÚÚZUUÅÂÏ0¦ ¶òCù¡üP~(¿R¡üP~(?ÊOù)?ÊåòKÏy;0Wù)?òS~ÊåWÀåòS~(?ÊwKáhïöíÛ£ò;zô¨òS~(?ÊOùgùõ÷÷WVVæ[µjUæ;+?åòCù)?ëAùUù­[·.ÚÕwâÄÚÊåòCù¡üfàùçoÍ5·=ÊåòCùMËèèèÒ¥K£9zzzq(?Êåò»½ýû÷G»úÚÚÚw(?ÊåòÆ¾ÏOù¡üP~(?J¤üb·ÇòCù¡üP~Ê¯Ê¯8(?ÊåòS~ÊåòCù¡üòCù¡üP~(?å§üP~(?ÊOù)?òS~Êå§üÊåòCùVù-Z´¨¼¼Ü÷ù)?ÊåGßÂ3kÏ÷ù)?ÊåGÑ_¼Ð|·=ÊåòCùÍ@uuu(¿BÌ>åòCù¡üP~3ÓßßÊoëÖ­ø¦üP~(?Êof,XPÃÊåòCù)¿b+¿úúzgx(?ÊåGI_Ô|¸=ÊåòCùÍ@MM3<Êåò£$Ê/¤L(¿;v)?åòCù¡ü(æò+3<ÊåòS~EøMÎròCù¡üP~Ê¯¿Õ¥p)?ÊåòS~ÊåòCù¡ü&J¥V¯^]QQQVVVYYÙÚÚZ(§ú*?ÊåòÑÑÑIÏð(SÊåòCùÍÀâÅCç­Y³&zKOîµk×1K.U~ÊåòCù)¿¢*¿òòòÐyãããé1©T*	ãòCù¡üP~Ê¯¨Ê/Îµ366ÆøVåòCù¡ü_qímiiÞÒÂÏ0Æ466*?åòCù¡ü_Q_x'ô7n(?åòCù¡ü_Qß»·Nïmmm­ªªÅbágKKKSÛCù¡üP~(?_©P~(?Êå7uuuõõõ.~ÊåòCùQäåÇËÊuß¡òCù¡üP~(¿èéé	å·gÏð´.?Ú¦üP~(?ÊoVB,S~ÊåòCù)¿¢*¿Ø|³òCù¡üP~Ê¯HÊ¯¾¾¾¡¡add¤ ·òCù¡üP~(¿Ûþ»·öÄ]åòCù¡üP~³ý¹ÞèOô*?åòCù¡ü(æòK$ey9ÃCù¡üP~(?åW$å×××7oÞ¼hÏ_ÔyÎðP~(?2½õÖ[_üâzè¡ÏîsûöíS~ÊåWÀåV(§üP~Ì¥ÁÁÁï½wÙ²eë×¯ÿÄ'>ÿüÏÿÜjQ~(¿/¿¦üP~Ü!Ë/oùxËî/ïþýñ?®¬¬9hÍ(?òS~(?ÊØØØ÷Ý·ëK»Òåþ­X±âg±rÊOù½WýýýD"744ôôôdNêíímll,Y®¦üP~Ìêêê¬òkþXó¬åòS~ïU2ìèèlkkË´páÂ³gÏ£G.Z´(·ü^xá«sèäÉCCCW)I¶~I©¯¯ÿTë§ÒÙ·ãÑ¡_zé%k¦õôôüð?´JÓ«¯¾ú¯ÿú¯syw¼ü.^¼x×Ë/ü>©Tª®®nª«UTTäß³Ï>û½9~ïÿã?þã÷(I]]]½½½ÖCxægî¹çüãÿhóúõëåW~eÍ5VKizùå_í5ëÁÖw¼ü¢¯tillÿ¡¹[åyrñT'÷õõmÚ´ÉÑ^íeÎ|÷»ßµ÷«¿ú«ýèG¿ð/Ü¸qÃ:q´Gþhoh¾ô·7êZ¹råÐÐÐ?Ìo.//Ï½Bx»M&¹eXù¡ü¾ÉYùYÊ¯HÊ/rñâÅÅ§0ä×êÕ«ïì]ÿ0J¥Þ½u´7g]óêÕ«6l¸víZîBÊåòCùÍÒÄÄÄ'***æø¯·µ··9r$Éd2«´ZZZ'Qù¡üP~(?ßdîó«ªªÊýPÝ]­µµµ¡5DooïÏÛ­ÝuuuMXù¡üP~(?ßìe_EEE[[ÛåËe(?ÊåòÉ"nØñÐC]ºt©à¶òCù¡üP~(¿¸_æ¢üP~(?ÊoNË¯ )?ÊåòóçÏ/Z´¨²²2:·¦¦¦³³Sù)?Êå§ü­üº»»ÓgxDå<xPù)?Êå§üªüjkkCç?>]~½½½Ñ»(?åòCù¡ü_Q_úòÒå711ð«üÊåòS~EU~555¡ó¢ý|¡üR©ÔÎ;Ãp]]òS~(?ÊOùUù)ÌÙ³gòCù¡üP~Ê¯¨Ê/¸víZSSStnoEEÅ¢Eb(?Êåò+ÊåòCù¡üòCù¡üP~(¿_vùòåÆÆÆªªªØ-a ¡¡¡Põ*?Êåò®7MaóæÍÊOù¡üP~(?åW$å×ÙÙEÞ¡CFFF¢£££ÇÆwuu)?åòCù¡ü_1_"ywøðáÜIÑßsó~ÊåòCù)¿")¿òòòw£££¹R©T® üÊåòS~ÅP~é?Ú6ÕÔè/¹)?åòCù)?å§ü¡üò´òS~(?Êå§üòCù¡üP~X~ù)?åòCù¡ü__ìvâñ¸òS~(?ÊOùCùåòCù¡üP~ÊOù¡üP~(?òS~(?Êå§üÊåòCù)?åòS~ÊOù¡üòCù¡üP~(?å§üP~(?ÊOù)?ÊåòS~ÊåòCù¡üòCù¡üP~(?å§üP~(?ÊOù)?ÊåòS~ÊåòCù¡üòCù¡üP~(?å§üP~ÊOù)?òS~(?Êå§üÊåòCù)?åòCù¡üP~ÊOù¡üP~(?òS~(?Êå§üÊåòCù)?åòCù¡üP~ÊOù¡üP~(?òS~(?Êå§üÊOùYÊå§üÊåòCù)?åòCù¡üP~ÊOù¡üP~(?òS~(?Êå§üÊåòCù)?åòCù¡üP~ÊOù¡üP~(?òS~(?Êå§üÊåòCù)?å§üòCù¡üòCù¡üP~(?å7MýýýD"744ôôôä^¡»»»¬¬Lù¡üP~(?_ÁK&aààÁmmmYSÇÇÇÊåòCùêêê0J¥êêê²¦>ñÄûöíªüþú¯ÿºo½ôÒKgÏí»KÂÏç?ÿùìcÿøÇwíÚõÚk¯õQ2[[»¥««ë;ßùõ`ëÏ(¿x<>éppùòå¦¦¦ÐSßñãÇ2N<ùæoþän¸xñâ<ÐØØ¸~ýúOµ~êÃþpè¿ÿøÇ?a®¼òÊ+.]²JSOOÏ~ô#ë¡4½úê«CCCÖCi:uêÔ¿ýÛ¿Íå-DùÅb±ôpyyyæ¤ÖÖÖÓ§Oÿì¡üÑÞÏ~ö³ùÈGvywôo×vü¿ù»âíÅÑ^íÅÑÞº²_Ã555©T*:Ú'½ZúÊ%[~úÐÝþhºüÂ¿O¯ûôïþîïzY*?ÊåWHÚÛÛ9ÂÏd29U)æ,©ò»ÿþûw~qgfù­_¿þø¥òCù¡üP~¶Zkkkc±X"èíí4õ_ssóïüÎïdß>öØc^ÊåòCù*¿î¹çr×výÉ¶?ùíå¿]WWwýúu/KåòCù¡ü_±_ÍÍÍ¡ÿî½÷ÞOúÓ·=åòCù¡ü_¡ÊåòCù)?åòCù¡üP~ÊOù¡üP~(?òS~(?Êå§üÊåòCù)?å§üòCù¡üòCù¡üP~(?å§üP~(?ÊOù)?ÊåòS~ÊåòCù¡üòCù¡üP~(?å§üP~(?ÊOù)?ÊåòS~ÊåòCù¡üòCù¡üP~(?å§üõ üP~(?å§üP~(?ÊOù)?ÊåòS~ÊåòCù¡üòCù¡üP~(?å§üP~(?ÊOù)?ÊåòS~ÊåòCù¡üòCù¡üP~(?å§üP~(?ÊOù)?åòCù¡üòCù¡üP~(?å§üP~(?ÊOù)?ÊåòS~ÊåòCù¡üòCù¡üP~(?å§üP~(?ÊOù)?ÊåòS~ÊåòCù¡üòCù¡üP~ÊOù)?å§üP~(?òS~(?Êå§üÊåòCù)?åòCù¡üP~ÊOù¡üP~(?òS~(?Êå§üÊåòCù)?åòCù¡üP~ÊOù¡üP~(?òS~(?ÊOù)?åç üP~(?òS~(?Êå§üÊåòCù)?åòCù¡üP~ÊOù¡üP~(?òS~(?Êå§üÊåòCù)?åòCù¡üP~ÊOù¡üP~(?òS~(?ÊOù)?åòCù¡üP~ÊOù¡üP~(?òS~(?Êå§üÞíïïO$ñx¼¡¡¡§§'sÒØØØÊËËxàP]ÊåòCù¡ü[2ìèèlkkË´wïÞ'|rbb"$Wnùñÿ7^õÕðúÿ?JRx²×¿õPB÷ß¸qÃz(M§O¶JSèþÿøÿË[,ò«®®mR©T]]]æ¤.L5c(¿§zªk½øâ/½ôR%ÉÖ·õ­[[ÿN+òÇãG÷ïß_QQQ__ÿúë¯;Ú£½8Ú£½8Ú[Øb±Xz¸¼¼<kÒáÃÃÀÅÊåòCù²_Ã555©TêÝ[GÃpæÕ2/fíT~(?ÊåWxÚÛÛ9ÂÏd29iË-ÇçÏ_¾|¹òCù¡üP~(¿_­µµµ±X,Hôööþü±ÝÚ^l­­­ñx¼©©ippPù¡üP~(?_R~(?Êå§üÊåòCù)?åòCù¡üP~ÊOù¡üP~(?òS~(?å§üÊOù)?ÊåòS~ÊåòCù¡üòCù¡üP~(?å§üP~(?ÊOù)?ÊåòS~ÊåòCù¡üòCù¡üP~(¿bòøã?÷ÜsoÍ¡cÇ½ñÆoQÂÖ¿páõP¾ñoüà?°JSx£ùþ÷¿o=¦ÎÎÎ¹¼Åþô§ÊoJçÏß½÷WBþevÃå üP~(?ÊåWÀëêêÒÏ?¿téÒx<ÞÐÐpæÌÌkö÷÷'hROOUWR[ÿÚµke¬ºâÛúîîîÜíëµ_Ê[ßk¿è·~M|×_ûpï¿ðî¾xñâÌ-¶îñãÇÃÀÙ³g«««3¯L&;::ÂÀÁÛÚÚ¬½Úú6l°Òxëããã¹ïî^û¥¼õ½ö~ëçÙÄwýµ¯üÞ«W¯ô¿q]]]K,ÉR`bb"¤R©Üÿ,RÜ[?ü^¢"ÞúO<ñÄ¾ûr^û¥¼õ½ö~ëçÙÄwýµ¯üîØÍù_UUUyôèÑÌññx|ÒaJaë/°¥¥%l÷¦¦¦/ZoÅ·õ/_¾6nø-ûÞïµ_Ê[ßk¿è·~M|×_ûÊoÞû#ÝÝÝ555cb±Xz¸¼¼Üz+©­võêÕ¬ÝÇÖomm=úô¤O	¯ýRÞú^û¥ó?wßõ×¾òÓg@nàH¥RïÞÚë;UP¬[?÷þ¢Üúe¿ÌkßÖ÷Ú/ÁßüYø®¿öß.þüù0ÐÛÛÛÒÒyµööö#Gð3LZo%µõÃ¤¡¡¡è?Y(²ßþ¹#½öKyëíÂoþ©6ñ]í+¿¹xô÷÷744Äãñæææk×®e^áÌ3µµµ±X,H2°ÞJjë÷õõ-^¼8LZ¹reøí`½È¿×¾­ïµ_ô[ÒMüyí+?)T«@ù üP~(?Êåò@ù(?Êåò@ù üP~(? t|ýë__±bEÅ-ÍÍÍÏ?ÿü/ý»¥`~OvoëêêÂCÉÆÄãñD"1111ÓeÊ ðìÞ½»,ÇW¿úÕb*¿=öÏ<óLÖøñ=öØ,	(?Óßß&:thüÃaä¹sç¦ü.]ºF.Y²$küâÅÃø¡¡!å(? øæ3	MóäOf|ê©§ÂÈßÿýßÏìPN!ÃpúÊ7nÜØ´iSUUUT]]½mÛ¶Ìª'Ou&y»ºº²Z*7oÞ+N8.¶··gÝ±_|1ÿr¢Iá.Eº»»§ª´0¾··7=æìÙ³aÌªU«ÒcöîÝ[[[UQQ±nÝº«W¯æ_îò³Æä¹«ò¸,XªåòåË#¯FÖÕÕeM¾¾¾hêÚµk³&mÝº5400Å&+ºMmmmíX^^>>>¦¡*++£ßåYNôîå>Ò£Gfæl:.;::ÒÙµæææ_»(?»,:°;É/µ²²ÐaeÓÖÖöÎ-a \³fM45¨£]w¡Ø¢IÉd2ÚYÏ9-$s»vímlÝ¸qcsüøñ0~áGyä¶Ë	Õ.+dÞ·IQ¨ÉùóçÇ;<<.áÜL¥RÑD1<wqt¸¢¢b¦åç®Êàî_0iù¥ÇGeSpõêÕp14St±±±1¯¯ß¼ys(¶ÑÑÑôBÂu²ö¢¥ç.^¹r%åPKé¾ág>yòäm*3ûí·3ïÛTÉÛ¾´oß¾wqDÛ¶mWÊ/ôhSSS´Kr¦åç®Êà.vt½óÎ;#GFFÂÈ0)Oë¤»ppp0¿tè¤?6«¥2¿K%WWWÏ7/=õ¶ËªÃ²?>Ô0~áôÔ3gÎ;0éQãé_»(?»,ú¬Ûþýû3GFßuuGú³ÃÃÃ¹»²Î;·gÏèØk:£réÃ©·í³­[·FyÃÏM6¥ÇçYN´íÚµkÑÅ·ß~;ÿy¸Ë-S£/²	Á9):Ï7Lêîî¾yófþòKWi´6ÒSóÜU@ùÜeÑñxüàÁÑ·º:t¨¼¼<÷l5kÖÊÅ¦ÏÃ>ç<npp0óQnÛ¶-tRtÖmúU&í³èdÛèÖO:g9Ñû¢ÏùD×ÌS~Nï9sRôÇp+a%LU~QkVWÅ¹nÝºÌ©yî* üî¾è[³ìÝ»÷ÿÿ»%£ôpú8iú¤´7ffe¦ÎÎÎ<å÷î­?¶ÆWWWgÎ³p72ïXú¼Ú©ìèèhEEEô²q¯^½:ó&æÏ~F_ì¹ÌhdZÔ©é©yî* ü>B477ß²bÅèÛ¬òëîî¾6¯¡¡áôéÓé©###;wî¬­­jiûöícccé©]]]MMM!ÈÄ³Ï>µÌÜÆoÙ²%küTË	Â	w)ú¢Á<ßç¶iÓ¦¬¯w'É°ªªªÂ£JCMæ2oÞ¼Ò6ZW«V­êííÍºÅ<wP~(?Êåò@ù üÊåò@ù üP~(?9þ'¼ùa$¸IEND®B`


ûòã9ÆÒÝ4ç½e6E_ÞÖÖVüµ|S-ö3ÎèHh|üÖ­[ÍfÏç]ù¥eÚ¶m[¡|pî_|1<ºðÀ,XpàÀÇ,¿ÏéãÏ2£ò+½rf´VgQ~ÁÑ£GÃ£Ë§çOgCE·¾råÊÞÞÞ[îsêù6<<üäOF;SC¾M9::ê(?Û"Ë­_¿~Fß!7YÊN´¯7:Yll,úOP~Tès~î9kfxx¸»»ÁÑ!Ý0ðÔSOY- üP~(?ÊåòP~(?Êåò@ù ü»Ä#GÉä+f:okkkMMÍàà`~Lc/^<ßbfwéÌ;Ëß­E¢ñÃÃÃ[¶lI§ÓaÕÕÕe³Ùwß7?×ØØX®J¥Â°g ü»Kccc(¡¡¡YÊ®]»Â¼üÇóüóÏ1;wî¬ò+¿aÃ0¾§§'>:/0?õèÑ£ùR<vìg ü»ìÈÇ§³gÏy/_³dÉ0¦¿¿nîäÜ_*ãoÜ¸1éüâÃÔèçÆ=»åÜuÙ¯'|²®®nÞ¼y;vì(¸Úc=ÖÞÞ^°ü.Ã0üÁá0&tùòå+WÖÖÖ&É¶¶¶h?YñÒâ·^z455-]º4ÜPqÝ¼y³««+Ìî|www´3´é²eËÂÒÂø°ä+W®|Ìò»ÿþûÃø3gÎL?~¸­ð³¾¾>ßÇ³»Ê¸-ñ?ûì³axß¾¡±ÂÀsÏ=¿ÎñãÇ£ÂÛ¼ys´ÿþ0~áM6Exàèç¥KÂøL&3éÒâw ô,¡ç:6lØP|ç·lÙÃ^õÕ0°k×®|¨Ý¸qc`` D»$§úßTM¿Î#GòcV¬XñÎ;ïägþ®Y³&¯[·.1ÞÏ:@ùw¾üÂpnBqxMúAÀS§NåsgíÚµQÒå§=vûöíííía|"tiíUzèÕÕÕÏÛÐÐÝùñññ0z+×Ãmmm¡eGGGg·ZôõõBÍ÷_Xr4~ëÖ­ùQ¡nÛ¶íãÜåÜÆòÎKÍoA!³æÍ®9<<Ðc¢I÷îs|çwâK(XZüâtfêLï¢ªñðáÃóçÏÆ¤Óéhwàô÷ùXo===!æâG·C7½~ýúGÃð&½uòî|ùe2ø>¿h·Ù-3¨««+LÍÿÌN0Íò+=Kþ*·¾¾>·àî;v,:'7¿³pF«e*Ñ	÷9çSµÀÙ³ggpË/úýû÷Góþùç§AÎÎ¡Còã£<wîù¿é_éY¶oßþÍo~3lÞ¼¹xÞ7á?>:ºF.]º4,»víZX´hÑÇ,¿Åñ'NøèGßá²~ýú¦þ0~Ö÷@ù·±üÆÆÆB]ÍÿN¾Òå722i?ã_wÂ(N×ÕÕuwwO³üJÏròäÉ0uÉ%ùM¾cyëÖ­á§R©Õ«WG§ÐØÊf³ÑÉÂË/¿páÂVKñá°Ø®®®pÃ­¯¯ß²eK¸Ý~ô¥Öñåç¿ÔzÖ÷@ù(?Êåò@ùÝßùÎwÞÿý¹¼Å/ær9Ï¼êôýïÿ¿þë¿¬êtéÒ%­·þþçZÕé½÷Þ»«¶~µßÓO?âo.oñÔ©SÿñÿáP¾õ­oÝ¼yÓz¨Nßþö·ÿýßÿÝz¨Ná&þ]T3gÎüÛ¿ýòS~(?Êå§üÊåòCù)?åòCù¡üP~ÊOù¡üP~(?òS~(?Êå§üÊåòCù)?åòCù¡üP~ÊOù¡üP~(?òS~(?Êå§üÊå§üòS~ÊåòCù¡üòCù¡üP~(?å§üP~(?ÊOù)?ÊåòS~ÊåòCù¡ü*ªüúûû3L2lmmíééOÝ°aC*ºÿþûCu)?Êåò+oÙlöàÁa`Ï=ñI»vízî¹çÆÇÇCr577ß±cÇ®Ï¡×_ýÊ+×©Já¿%ÿôOÿd=T§7ÞxÃÖ¯Záèý÷ß·ªSø?ÿï½7·XåN§CÛ×ÔÔÔÚÚzþüù©fåbñ»s(æéÓ§¿KU²õ«|ëûÛß¶ªÓk¯½fëÛús¦*Ê/LN:]Ü½wmmmssó;ï¼ãh/öâh/öâhoyK$ùáT*U0iÿþýaàÂíííÊåòCù¡üÊ[CCC.ûhâho..Ø¨üP~(?Ê¯ütuu8p Ùl6>é'8tèP8wîÜÒ¥KÊåòCùýjmllL$L¦··÷­æ]x±utt$ÉöööÁÁAåòCù¡üP~UJù¡üP~(?òS~(?Êo6'ØîÊOù)?å§üÊ¯½ýöÛ/N§Óõõõ-°õòS~Êåò«@óçÏ_»víößÛ¾ãk;>÷Øçî½÷ÞþçV~ÊOù)?åòCùUÇü¡Íÿ÷+KeûöíÊOù)?å§üP~(¿JÓÒÒ²ù·7ÇËï7»~óáV~ÊOù)?åòCùU|0¤^¼ü~mÍ¯=þøãÊOù)?å§üP~(¿Jóâ/.X° û«ÝQöuÛWæg~æÏÿüÏòS~ÊOù¡üP~èw÷wï¹ç|ð³ýì§>õ©;vØúÊOù)?åòCùU¬&¼õÖ[6½òS~ÊOù)?åòCù)?åòCù¡üP~ÊOù¡üP~(?òS~(?Êå§üÊåòCù)?åòCù¡üP~ÊOù¡üP~(?òS~(?Êå§üÊåòCù)?åòCù¡üP~ÊOù)?Êå§üÊåòCù)?åòCù¡üP~ÊOù¡üP~(?òS~(?Êå§üÊåòCù)?åòCù¡üP~ÊOù¡üP~(?òS~(?Êå§üÊåòS~ÊOù)?åòCù¡üòCù¡üP~(?å§üP~(?ÊOù)?ÊåòS~ÊåòCù¡üòCù¡üP~(?å§üP~(?ÊOù)?ÊåòS~ÊåòCù¡üòCù¡üP~ÊOù)?å§üP~(?òS~(?Êå§üÊåòCù)?åòCù¡üP~ÊOù¡üP~(?òS~(?Êå§üÊåòCù)?åòCù¡üP~ÊOù¡üP~(?òS~(?ÊOù)?åòCù¡üP~ÊOù¡üP~(?òS~(?Êå§üÊåòCù)?åòCù¡üP~ÊOù¡üP~(?òS~(?Êå§üÊåòCù)?åòCù¡üP~s¥¿¿?É$ÉÖÖÖâ+8q¢¦¦Fù¡üP~(?_ÙËf³öìéìì,:66ÖÖÖ¦üP~(?Ê¯¤Óéñññ0Ëå¦>ûì³/¼ðÂTå÷§ú§;?þ7ó7KU[¿¿¿ßz¨NõWeëW­'N¼ýöÛÖ­?7ª¢üÉä¤ÃÁåËÛÛÛCNU~¯¼òÊûsè¯ÿú¯ß§*<yÒÖ¯æ­ÿÿðÖCuzýõ×/`=T§ïïsyUQ~D"?J¥â:::N>ý¿ÕÑ^íÅÑ^íÅÑÞÐÐÐËå¢£½aøÇáS~(?ÊåWÞººº8ÂÏl6;ùCµÏåòCù¡ü*cµ666&L&ÓÛÛ;iê)?Êåò«jÊåòCù¡üòCù¡üP~(?å§üP~(?ÊOù)?ÊåòS~Êå§üòCù)?åòCù¡üP~ÊOù¡üP~(?òS~(?Êå§üÊåòCù)?åòCù¡üP~ÊOù¡üP~(?òS~(?Êå§üÊåòCù)?åòCù¡üP~ÊOù¡üòS~(?å§üP~(?ÊOù)?ÊåòS~ÊåòCù¡üòCù¡üP~(?å§üP~(?ÊOù)?ÊåòS~ÊåòCù¡üòCù¡üP~(?å§üP~(?ÊOù)?ò³ÊOù)?ÊåòS~ÊåòCù¡üòCù¡üP~(?å§üP~(?ÊOù)?ÊåòS~ÊåòCù¡üòCù¡üP~(?å§üP~(?ÕS~5·H$òCù¡üP~Ê¯Ê/q+ÉdRù)?Êå§ü*¡ü*òCù¡üP~(¿+<Å7oÞ¬üÊåòS~V~MMMÉdÒçüÊåò£ÂËoñâÅÅ§w¤Óéááaå§üP~(?ò«¨òK¥R!õÃ@¾o~óa`Ó¦MÊOù¡üP~(?åWQåíä!õÂÀ¥KÆÇÇÃÀ¼yóòCù¡üP~Ê¯¢Êoþüù¡óÂO=õT4à[]ÊåòS~V~ÝÝÝùó9âõ[ºt©òS~(?ÊOùUTùÏ<óLèíí¡ÛÛÛËb(?Êåò«ÊåòCù¡üòCù¡üP~(¿É,Z´(únßä¬üP~(? .×^sÊåòS~V~!òBóõõõÝöP~(?Êå7ét:_9fòCù¡üP~(¿éïïå·eËr|KS~(?Êå73,¨)âåòCù¡ü_¥_ss³3<Êåò£*Ê/j¾rÜÊåòCù¡üf ¡¡ÁÊåòCùQåR&_ww÷èè¨òS~(?ÊJ.¿)8ÃCù¡üP~(?åWßä<)gx(?Êå§ü*ð[]ÊòCù¡üP~(¿hjjjnn>þ¼òS~(?Ê/¿d2YSs÷ö÷÷g2pOZ[[zzâzÛÚÚÂ¤Å«)?Êåò½PZ¡üvîÜÖwê»]²ÙìÁÃÀ=:;;ã.æo_~yÑ¢EÊåòCù¡ü>Æ"îsÓét¹©©iª«ÕÖÖßýÑCG=Fµ²õmªvëÿå_þ¥õ`ëÏÛ^~wÃ¹½ñÛêvûúú6mÚT á?âÿ3Þxã?üð¨J§Nÿï·ªÓ·¾õ­ðÿ~ë¡:>úúõëÖCuúÎw¾óü`.oñ¶ßÝ ¾1J_áæÍÙlvxxØÑ^íÅÑ^íÅÑÞòÖÐÐËå¢£½a¸`êÕ«W7lØpíÚµâÊåòCùÍLè­Õ«W×ÖÖÖÔÔÌ7¯££cOõèêê:pà@?³ÙlAi­ZµjhhhÒÊåòCùÍÀÈÈÈ¤gxÌåñ«µ±±1Hd2ÞÞÞ>¶ïijjß+åòCù¡üP~³×ÒÒjÍ5Ñ[Zxr¯]»6yàîþí¡üP~(?ÊoR©Tè¼±±±üÆLz¦òS~ÊÏzP~(?__"`cæò[]ÊåòCùÍEùEGW­Z½¥a8ikkS~ÊåòCù)¿*¿ðN6é×¯_W~ÊåòCù)¿*¿&Nïíèè¨««K$áçªU«Â²ØÊåòCù¡üªòCù¡üP~(?å§üP~(?Ê/?ç­Äÿ®òS~(?å§üÊ¯Ë/15å§üP~(?UÃÑÞmÛ¶Eå÷òË/+?åòCù¡ü_e_ÿ¼yóBó­2þÅÎÊOù¡üP~ÊÏzP~U~ëÖ­võ;v¬¶òCù¡üP~(¿xõÕW£æ[³fMÙmåòCù¡üP~Ó222òÀD'sôôôãöP~(?Êåwk»wïvõuvvïöP~(?Êå79òCù¡üP~TIù%n%L*?åòCù¡ü_%_eP~(?Êå§üÊåòCù)?åòCù¡üP~ÊOù¡üP~(?òS~(?å§üÊOù)?Êåò+¯ò[´hQ*ò~ÊåòCùQáå·páÂxíù>?åòCù¡ü¨Øò¯¯¯o||¼ì¶òCù¡üP~(¿H§Ó¡üÊ1ûÊåòCùÍL(¿-[¶ã[òCù¡üP~(¿Y°`AMgx(?Êå§ü*­üá¡üP~(?UQ~QóãöP~(?Êå7ÎðP~(?Êª(¿2¡üº»»GGGòCù¡üP~TrùÕLÁÊåòCù)¿ü&çI9ÃCù¡üP~(?åWßêR¾ÊåòCù)?åòCù¡üP~Éår«W¯®­­­©©7o^GGG¹ê«üP~(?ÊoFFF&=Ã£,NõU~(?Êå7---¡óÖ¬Y½¥'÷ÚµkÃx@ù)?Êå§ü*ªüR©Tè¼±±±üÆñÊOù¡üP~(?åWQåH$BçÚËc|«òCù¡üP~Ê¯2ö®Zµ*zK?ÃpÓÖÖ¦üÊåòS~U~álÒ3<®_¿®üÊåòS~U~MÞÛÑÑQWWH$ÂÏU«V1e±=ÊåòCùUåòCù¡üP~3ÐÔÔÔÜÜ|þüyå§üP~(?^~Éd²¦¦*?ÊåòP~;wîOërù£mÊåòCù¡üfµ)$	å§üP~(?ò«¨òKLÁ79+?Êå§ü*¤ü[[[Ëz(?Êåò»µèÏõ~4q´·,ì*?Êåò¥èÏõF¢Wù)¿Ò^|ñÅ+WþÂ/üÂç?ÿùåòCù¡üÊ¬ü2LMIÎðP~Í7ßwß?þøçË>òèOþäO<yRù¡üP~(¿r*¿¾¾¾ùóçGþ¢Îsò+öÖ[oÝsÏ=_Ùú_ÛýûßhllT~(?Ê¯Ê/¯OùÝûöíðÁóÙý«¯¯ÿðÃÊåò+¿ò+kÊïvûú×¿þàò+¿í¿·þüùå~V¸òCù¡üP~ÊOùºxñâOýÔOmþíÍùòôGù¹:oÊOùYÊå§ü~L&I&­­­===¥'ÍnLÜ;~ë·~ëé9ô¥/éþà®&<òHmmí§?ýé_ú¥_jjjúø-[¶<]Ö¯_ÿû¿ÿûOcëSeÂMx»±ªÓ¾öµ¯Íå-Yùe³Ù=ötvv4»1qÛ¶m«¨·½ü.ð	_:¹©©©ô¤ÙQ~òíã¯tikk÷Ýw?þÒâ§	2ivcâº»»ïC÷Þïî§*'­oëcëcëßV·½üBóå33¤ÕòåË/^¼8ë¥Å¿ÿ9J4»1ÎðÀ8ÃgxàÌ·¥¥%¡±V¯^=å444är¹èàl.=ivcÊåòCùÆÇÇ;V[[[3Û¿ÞÖÕÕuàÀ0~f³ÙÒf7Fù¡üP~(?ßÇ200ßçWWW·iÓ¦Ù­ ÆÆÆPL¦··÷÷râãÅf7Fù¡üP~(?ß,Å¯¶¶¶³³óòåËå²=ÊåòCùÍd'v<üðÃ.]*»í¡üP~(?Êo>/sQ~(?ÊåWåWÖÊåòCùÍÌ¹sç-Z4oÞ¼è|ÞÃ+?åòCù¡ü_¥ß'ògxDåïÙ³Gù)?Êå§ü*ªüCç;w._~½½½Ñ»(?åòCù¡ü_E_þÏçËo||<:áWù)?Êå§ü*ªüBçEûùBùår¹'|2755)?åòCù¡ü_E_HÉ¼ùæÊOù¡üP~(?åWQåv­½½=:··¶¶vÑ¢E/^,í¡üP~(?Ê¯Z(?ÊåòS~ÊåòCù¡ü~ÜåËÛÚÚêêêÂ@kkk¹êU~(?Êå7]7n¬ÂæÍòCù¡üP~Ê¯BÊïðáÃQäíÝ»wxx8922räÈhüñãÇòCù¡üP~Ê¯Ê/É¼Û¿ñ¤èï¹ù>?åòCù¡ü__*y722R<)ËIáÊOù¡üP~(?åW	åÿ£mSMþòS~(?òS~Ê¯Ê¯DÛ)?åòCù¡üP~ÊOù)?ÊåWåWòS~(?ÊOùUHù%n%L*?åòCù¡ü_%_eP~(?Êå§üÊåòCù)?åòCù¡üP~ÊOù¡üP~(?òS~(?å§üÊOù)?ÊåòS~ÊåòCù¡üòCù¡üP~(?å§üP~(?ÊOù)?ÊåòS~ÊåòCù¡üòCù¡üP~(?å§üP~(?ÊOù)?ÊåòS~Êå§üòCù)?åòCù¡üP~ÊOù¡üP~(?òS~(?Êå§üÊåòCù)?åòCù¡üP~ÊOù¡üP~(?òS~(?Êå§üÊåòCù)?åòCù¡üP~ÊOù¡üõ üP~ÊOù¡üP~(?òS~(?Êå§üÊåòCù)?åòCù¡üP~ÊOù¡üP~(?òS~(?Êå§üÊåòCù)?åòCù¡üP~ÊOù¡üP~(?òS~ÊOù)?ÊOù)?ÊåòS~ÓÔßßÉdÉdkkkOOO|Rooo[[[´xñâp5åòCù¡üP~å-Í<x0ìÙ³§³³3>iáÂo¾ùfxùå-Z¤üP~(?Ê¯¼¥Óéñññ0Ëå¦ºZmmmqùýÉüIßzíµ×BöQl[ßz¨NÇÿîw¿k=Øús£*Ê/LN:ÖÅ¦MËïÈ#ÿ2N<ùÞïýUéõ×_¿téõPzzz¾ÿýï[Õé7Þ¸xñ¢õPN:õÿøsyUQ~D"?J¥¯póæÍl6;<<ìh/öâh/öâhoù©ù0ÜÐÐËå¢£½a¸àW¯^Ý°aÃµk×¢üP~(?Ê¯Ìtuu8p Ùl¶ ´V­Z5444éÊåòCù¡üÊoµ666&L&ÓÛÛûÃÇ6±;°©©©&Fù¡üP~(?_R~(?Êå§üÊåòCù)?åòCù¡üP~ÊOù¡üP~(?òS~(?å§üÊOù)?ÊåòS~ÊåòCù¡üòCù¡üP~(?å§üP~(?ÊOù)?ÊåòS~Êåw×ù³?û³~xáÂûÜçÞzë-åòCù)?åò«LÝÝÝ÷Ýwßã?þåßùòÿYý>õ©OUFü)?åg=(?å§üP~üþéþÊÖ¯ìøÚèßç×þç~îçÊå§üÊ¯ÒìÛ·ïÁÌg_ô¯¾¾~xxXù¡üP~ÊOù¡ü*­ü>»ì³ÊåòS~ÊåWùî¹çGQ~(?å§üP~U!~Ççû3<P~(?å§üP~ìë_ÿúÊ+?ýéO?úè£süU~(?òS~(?Êå§üÊåòCù)?åòCù¡üP~ÊOù)?Êå§üÊåòCù)?åòCù¡üP~ÊOù¡üP~(?òS~(?Êå§üÊåòCù)?åòCù¡üP~ÊOù¡üP~(?òS~(?Êå§üÊåòS~ÊOù)?åòCù¡üòãûÁ~ðÔSO=òÈ#?þø+¯¼¢üP~(?òS~i`` Næ3éììüõµ¿~ß÷mØ°allLù¡üP~(?å§ü*MkkëC=´ãk;¢_ÝöÕï½÷Üó§üõ üP~ÊOùqW®¯¯ßþÛóåþ=´ê¡îînåòCù¡üò«´òK§Óå÷è£*?Êå§ü_úÅ_üÅµk×æ³ïÉÿûäÏþìÏ:ÚòCù¡üò«@o¿ýöüùóõWuóooþÒ¿ôó?ÿókÖ¬qÊåòS~Ê¯2f³Ù~æ3yægFGG?Á+?åg=(?òS~Tå§ü¬åòS~ÊåòCù¡üòCù¡üP~(?å§üP~(?ÊOù)?ÊåòS~ÊåòCù¡üòCù¡üP~(?å§üP~(?ÊOù)?ÊåòS~ÊåòCù¡üòS~ÊOù¡üP~ÊOù¡üP~(?òS~(?Êå§üÊåòCù)?åòCù¡üP~ÊOù¡üP~(?òS~(?Êå§üÊåòCù)?åòCù¡üP~ÊOù¡üP~(?ßmÓßßÉdÉdkkkOOOñN8QSS£üP~(?Ê¯ìe³Ù=ötvvLkkkªüBý÷zã7Âëÿ¿©JáÉ^ÿÖCuÝýúuë¡:>zhhÈz¨N¡ûÿõ_ÿu.o±*Ê/N×ÔÔT0õÙgá¦*¿çþø:zôèk¯½vªdëÛúÖ­­»UEù%ÉIË/···.t´Gq´Gq´·$üp*Oêèè8úôÿ>TåòCù¡üP~eªæGÂpCCC.ûhâhoôjù++?Êåò+c]]]ág6ªG*?Êåò+¿ÕÚØØH$2Looï¤©§üP~(?Ê¯ª)?ÊåòS~ÊåòCù¡üòCù¡üP~(?å§üP~(?ÊOù)?òS~Êå§üÊåòCù)?åòCù¡üP~ÊOù¡üP~(?òS~(?Êå§üÊåòCù)?åòCù¡üP~ÊOù¡üP~(?_%ùÃ?üÃW^yåý9tèÐ¡ïïïSÂÖ?þ¼õPþâ/þâïÿþï­êÞhþîïþÎz¨NË[üðÃßÎ;·cÇ§*Bé5vÃT	å üP~(?ÊåWÆò¯]»V¿f&I&­­­===V]Umý¨­?::ºaÃT*uÿý÷:uÊkßÖ÷Ú¯­ß¾Dâ®zíÂòÎ9ÓÒÒ1>|8¼þ'½r6=xð`Ø³gOgg§µWU[¿Ä$*cëïÚµë¹çoüÍÍÍ^û¶¾×~lý¼ð2¯ñ»êµ¯ü>y«W¯?Â+üÈ#^9Nßa ËÅÿ»@5lý¨­þOþüy¯[ßk¿Ú¶~äÒ¥K+V¬¸Û^ûÊï¶­ÙØ3`áÂ«V­J&ííí._-tjØú%&Q[?lÜÝ»w×ÖÖ677¿óÎ;^û¶¾×~lýHGGG___ÁÈ;þÚW~s÷®^½ºxñâøøáÿT*e½UÕÖÎ$ÊzëøþýûÃ@xwïñ^û¶¾×~õüædIñÕîøk_ùÍéñfnhhÈårMìõÃÖ[UmýiN¢|·~üE]ð¯ýjÞú^ûÕð×®]»wï.¾Úí+¿¹x,ðâÅÑïV­Z¿ZWW×Â@øÍf­·ªÚú%&Q[ÿ'8tèP8wîÜÒ¥K½öm¯ýê)¿eË=¶øjwüµ¯üæâÐ×××ÒÒþÏ·|ùòð:_áÌ3D"ÉôööZoUµõ'D%mý7ntttDçôÚ·õ½ö«§üR©Tt&GÁîøk_ùTM¡ZÊåò@ù üP~(?Ê@ù üP~(?Êåò@ùÕãßøÆ²eËj'¬X±âÕW_ý±_pÊæ×ñd÷¶©©)<´áááñaL2Ìd2ããã3]& üÊÏ;j<óÌ3T~;wî#_zé¥ñ/¾øbÿÔSOÍbò(3ýýý¡iÉäÞ½Ç&ìß¿?#Ï=[1åwéÒ¥0rñâÅã[ZZÂø/*?@ùï_øBhç.>òùç#¿øÅ/Æ»§§§'SÂ¶¶¶0¿òõë×7mÚTWW&¥Óé­[·Æª<y2ÔUæ=~üxAK1óçÏ_¶lÙ±cÇÂÅ®®®;vôèÑÒË&»M:qâÄT¶jÕª0¾··7?æÍ7ßcV®³k×®ÆÆÆ°¨ÚÚÚuëÖ]½zµ¸ü_0¦Ä]À´`ÁP-/_¼råJÙÔÔ/ÑÔµk×LÚ²eK4i`` HL:Wt1ÚÑÑ1>>Ú1J©ág(§yóæE¿+±00éÝ+~¤/¿ür<góqyðàÁ|ö,dÅ3-¿wP~wXt`w_j55¡ÃâeÓÙÙysB×¬YMB'jÇh×](¶hR6vá3gÎD/sûöí¡í¢­7nc9ÃÏ0¼~ýú[.'Tc¸®¿o>¢Põõõáñág¸ç!7s¹L&fá£®­­iù¸«ò¸óåLZ~ùñQÙDÁz5Í]lkk7oÞmdd$¿p½hù¹¢W®_9ÔRþoøO<yËåÊ?øàøê3yÛ¶m^xá~tDëÖ­ñ+åz´½½=Ú%9Óò+qWåpE;ºnÞ¼9<<FI%Z'ßQüåC'ÿá¼â°-ÿ.0N§S©ÔèèhXøüùóóSo¹©:¬À¹sç¢HÃágÈO=sæL¸5~ù¸«ò¸Ã¢ÏºíÞ½;>2ú®3<ò*ÞuöìÙ;wFÇ^óÉíËN½emÙ²%:È~nÚ´)?¾Är¢l×®].~ðÁ¥ÏÃ]²dIMÖø¤è<ß0éÄ7nÜ(]~ù*ÖF~j»(?;,:C"LîÙ³'úV½÷¦R©â³1Ö¬Y*gtt4:?7ú_ôñ¸ÁÁÁø£Üºukè¤è¬Ûü÷ªLÚgÑÉ¶Ñ­:u*?¾Är¢öEó®Y¢üöïßßr|RôÇp+a%LU~QkVWÅ¹nÝºøÔwP~w^ô-ÇvíÚõÿÁMÂ(??N?©"oãÆñ¬;|øpòûhâmñét:~ ¸ÄrÂÝß±üyµS=ØÚÚÚè!ã^½zuü&êëëÃÏè]âËvIæEZâ®Êà®êdÅ©	Ë-N°-(¿'ND_×ÚÚzúôéüÔááá'|²±±1ª¥mÛ¶æ§?~¼½½=Y&Ù·o_Á2ïIXTÿÄOj9A¸3á.E_4Xâûüò6mÚTðõ.¡¡¡l6Ö@]]]x/^ÌCM|7nÜi­«+WöööÜb»(?Êåò@ù üP~Êåò@ù üP~(?Ê"ÿ:Út²V°IEND®B`


oÂbÇ9o¼½ÏlôÞF?Þeñ¼ë9ª---ÉáéÑ¼+ñta	e"ëjÌ+LdåçÑ©Í8ñ£½ïþÙ5Á§ñÅüDxäÈ¿X$ò*I~ûöí+>Ã#ÙVråD 3©yËï³yë­·§YlùËÜç..¾%à¬dïT'¾B/.X° ùvãÇÌ&"¿dÇdò+¹Ë-7nÜØ¿nÝòÈ,SßdWN¡)Ï8qùMd_ÁvÉuÊ¬ÿ2Ï·¤³gÏnß¾=9ÄZùI3]~ñÞJÞßê¼â29UváÂÉ¤äCNwó-³Ì¼åÙ%ùV,<YNÉ§¸ÆùË3bÍ5ò'c3W¯^óNÉÒ6mÚôîåì÷êïï°wïÞÈïäÉÉ^¥xÈ§ONöç%>|xÌí;ÙY&%¿ò+§ä¹QÜd×êä|2/·oßØ­Zµj´wïÞ+?t.¿þïúH>¯yñâÅ2GÞ%4Sä7ºâóG-[ø¼áKw£ß2ËÌ[MA¢ÄFÅ'Z¦YlùË3âÚµkOt%c.]ºTò¯æææÂßüI3gNáSe|óðÃÞX-*³'5Ë¤äW~å<7ìZüÎ=[¼ü¥KO-Y'ÌÇ»2Ï·Â)D6lØàD~ReÈ/Þ/_>ú¯;v¬££#oçûöí+?|øp	iÝç½+¯Á···þZ¾ñ×Ë3"9Z<þÂëÖ­«¿S.>ÇÔä700KeyäÀPÜåóµ¯-]<ð¹sç8pà3ùLaÓ»eRò+¿rJ%Mj­NA~Ñ#GæÍ:O/ÎÃgÉ­¯X±¢··÷®û¾>úè£ÉÎÔàlÊ¡¡!¿X$ò¤÷¥|>ÿðÃOê;ä¦0KÅìëMNÖN@¿`ÁOü$IÕVò9¿zê)kF"?IRµ588¸uëÖ¹sç&tcàñÇ·Z$ò$IùI$ü$ID~$I"?I$$IÈO$ü$ID~$I"?I$$IÈO$Iä'I$ò$IùIi>|8Í¦ÓéåËOvÞ¶¶¶ººº/ÆÄpY¸pá$~Ýij×È¼Sù;NÅR©TCCC.ð,D~*£pLðexxx²óîØ±#æý½ßû½Â;wÆíÛ·W´üÊ,¿xL<ðîììô,D~*ä·É»ÀÓÙ³gcÞeËÆ,^¼8ÆôõõMÏü`åVáÏ"Iä'©bØW¬G´±±qÖ¬YÛ¶m+¹Úg>ó%vÆð[o½Ã1&tåÊ+VÒétOOÏK+¾õò³8p Í.Y²$nh´ÃnÝºÕÕÕóÆßºuk²3lºtéÒXZ%_½zõßR©TòFFFâÎÇÅé9&ü$Í8ü%ÃO>ùdïÛ·/O=õTñu;6ú3m7nIû÷ïáøÃÝÝÝÉ¤E=z4._¾ã3ÌK+¾åg	Ï=÷Üs1°~ýúÑw~óæÍ1Wxáb`Ç1ò¾ûîá7oö÷÷Ç@²KrÌðMV~Á»X?1ü/|!´ûödUìÞ½;üqÏ.Iä'iæÊ/ÍÆpþN£á5æO<V¯^ÃkÖ¬IHWzöìÙÇ¬££#9%bÌ¥èªü,Ékll=osssrçÃd1æqÍnooË½S<&¹o±ð`_°27=þü¸³fÍjmm]ÈOÒÌ_:.Ç@@Y¸æàà`rP5Æ$öîÝs_íµâ%,­øâDfïÅwã%j<tèÐìÙ³1MMMÉîÀ÷dß%'¸DÅÇÊ%ü$ÍDùe2â~Én³»r§««+¦~Æ×××Ç;MP~åg)Ü±ÀÜèyçÌÌ[r÷=º~ýúâSX9w]7oÞlnnÎårK._¿~Ý³KùI¹òK¾¥eÿþýÉçüvîÜ9ù:t¨°óì¹ç+OyîÜ¹äó_ùYì±¯ýë1°qãÆÑónØ°!<xþüùäodI¿öÚká°hmmÿä·eËtöìÙÓ§OQÈOÒLßððpèjÖ¿¯¼ünß¾i½EGijjjllÜºuëåW~'NÄÔÅN4)¾Îàà`Ø+îyýªU«Óx|¹9YxÙ²e=Ùvâò­ÁÍÒ¥Kãb¿'$ò$IùI$ü$IÈO$Iä'I$ò$IùULßþö·ß|óÍé¼ÅK.ù[L5Û~ðý×µj³Ë/OáÏ¾©j¶þ¿üË¿XµÙo¼1£¶~­Ëï«_ýjào:oñäÉÿüÏÿìPóß¼uëõPë[ßú§ú'ë¡67â¯¨TMuúôéüÇ$?òùüD~"?ò#?ÈOä'ò#?òùüD~"?ò#?ÈOä'ò#?òùüD~"?ò#?ÈOä'ò#?òùüD~"?ò#?ÈOä'ò#?òùüD~"?ò#?ÈüÈüÈüD~"?ÈüÈOä'òùüÈüD~"?ÈüÈOä'òùüÈüD~"?È¯ªä×××ÉdÒét[[[OOOñ¤¡¡¡õë××××ßwß¡.òùüD~"¿Ê.Ë<x0öìÙÓÙÙY<iÇO=õÔÈÈHkÞ¼y£åwôèÑÓØK/½tõêÕªÉâ¿%ÿ÷o=Ôf/¿ü²­_³ÅÐo¾i=Ôfñþ7Þxc:o±&ä×ÔÔ¶|>Íf'µµµ?~¼C~ÅïLcÍS§NG5­_ã[ÿ[ßúõP½øâ¶¾­?mÕüÒéôÃÉÅÝ»w744Ì7ïµ×^s´WöÊÑ^9Ú+G+»T*U®¯¯/´ÿþ¸páBGGùüD~"?_e×ÜÜÏçß¹s´7K&KvÈOä'òùU^]]]øËå'mÚ´é¹çsçÎ-Y²üD~"?È¯âWkKKK*Êd2½½½?yluÿöèâÅ¶víÚt:ÝÑÑqñâEòùüD~"¿üD~"?ÈüÈOä'òùüÈüD~"?ÈüÈOä'òùüÈüD~äG~ä§êOþäOxàùóçÿÂ/üÂ/¾H~ä'òùüTmÚ´ég~ægzè¡ÿùÿóÁlllùüD~"?U[ßýîw?òüï-ÿÛ·%ÿ¾ø/þÔOýÔàà ùÈOä'òSUµoß¾_üÅ_,°/ù÷ÑÿòÑþþ~ò#?ÈOä§ªêÿøqY©üîûÙûÎ?O~ä'òùüTU]ºtéÃþðÆ_ßX`ß¯ä~åÞï&?òùüD~ª¶~ÿ÷ÿîyðÁÿ[×[öée³gÏwÿrßÈüD~"?ÞûÞÝÝýÀ|îsëíí9wüÈOä'òùéýÊ79ùÈOä'ò#?òùüD~"?ò#?ÈOä'ò#?òùüD~"?ò#?ÈOä'ò#?òùüD~"?ò#?ÈOä'ò#?òùüD~"?ò#?ÈOä'ò#?òùüD~äG~äG~ä'òùüÈüD~"?ÈüÈOä'òùüÈüD~"?ÈüÈOä'òùüÈüD~"?ÈüÈOä'òùüÈüD~"?ÈüÈOä'òùüÈüD~"?ùùy%ÈOä'ò#?òùüD~"?ò#?ÈOä'ò#?òùüD~"?ò#?ÈOä'ò#?òùüD~"?ò#?ÈOä'ò#?òùüD~"?ò#?ÈOä'ò#?òùüD~äG~ä'òùüD~äG~"?ÈOäG~ä'òùüD~äG~"?ÈOäG~ä'òùüD~äG~"?ÈOäG~ä'òùüD~äG~"?ÈOäG~ä'òùüD~äG~"?ùùùüD~"?ùÈOä'òùùüD~"?ùÈOä'òùùüD~"?ùÈOä'òùU`L&N·µµõôô¾ÂñãÇëêêÈOä'òùü*¾wðàÁØ³gOgggÉÔáááöövòùüD~"¿j¨©©idd$òù|6-úäOîÚµk<ùýÑýÑ_McÇûË¿üË¿RM[¿¯¯Ïz¨ÍþüÏÿÜÖ¯Ù?þ½ïÏz°õ§§_:s8ºråJGGG¸p<ù=ÿüóoNcñqñâÅ7U8qÂÖ¯å­ÿ·û·ÖCmöÒK/]¸pÁz¨Ízzz¾ÿýïOç-ÖüR©Ta¸¾¾¾xÒÚµkO:õoÕÑ^9Ú+Gåh¯í­óù|r´7ÿÓ#üÏÈOä'òùUv]]]øËåÆ~¨öùüD~"?_u¬ÖT*ÉdzÇ¤ùüD~"?_MG~"?ÈOäG~ä'òùüD~äG~"?ÈOäG~ä'òùüD~äG~"?ò#?òùùüD~"?ùÈOä'òùùüD~"?ùÈOä'òùùüD~"?ùÈOä'òùùüD~"?ùÈOä'òùùüD~"?ùÈüÈüD~äG~"?ÈOäG~ä'òùüD~äG~"?ÈOäG~ä'òùüD~äG~"?ÈOäG~ä'òùüD~äG~"?ÈOäG~ä'òùüD~äG~"?ÈOäG~ä'ò#?ëüD~äG~"?ÈOäG~ä'òùüD~äG~"?ÈOäG~ä'òùüD~äG~"?ÈOäG~ä'òùüD~äG~"?ÈOäG~ä'òùüT;ò«»[©TüÈOä'òù_5È/u·Òé4ùÈOä'ò#¿j_uD~"?ÈOä÷n§øÆÉüD~"?ùUü²Ùl:ö9?òùüD~ªrù-pôéMMMäG~"?ÈüªJ~õõõA½ðýë_îînò#?ÈOäG~U%¿d'_õbàòåË###10kÖ,ò#?ÈOäG~U%¿Ù³góâ]¼x1üñdÀ·ºÈOä'ò#¿jßÖ­[çsÔoÉ%äG~"?ÈüªJ~ÑO<1gÎèííP`GGGElòùüD~"¿ZüD~"?ÈüÈOä'òùüÆªµµ5ùnßäL~"?ÈOÕ,¿ùóçk¯sÉOä'òù_µÉ/æ;sæÌÈÈHÅmòùüD~"¿IÔÔÔò«DöÈOä'òùM®¾¾¾ßæÍ+ñ-üD~"?ÈorÍ;·nTÎð ?ÈOäG~Õ&¿yóæ9ÃüD~"?jB~ùúûû+qÈOä'òùM¢ææfgxÈOä'òSMÈ/(òÛºuëÐÐùÈOä'òS5Ë¯náA~"?ÈüªðÇÌä'òùüÈ¯¿Õ¥r#?ÈOä'òDÙlvÞ¼yçÏ'?òùüD~ªrù¥Óéººxßa___&ÒÖÖÖÓÓS<©···½½=&-0®F~"?ÈOä7õBZ!¿íÛ·ÇÓúúnwðàÁØ³gOgggñ¤ùóç¿òÊ+1ðì³Ï¶¶¶ÈOä'òù½EÌstæóùl6;ÞÕFËïw~çwNcG9ªZÍÖ·õU³[ÿÏþìÏ¬[zzßå7Îí-¾­ñn÷Ì3ÝÝÝ£åÿÿÓØË/¿üöÛoÿ?Õd'Oÿ÷[µÙ7¿ùÍø¿õP:uêÆÖCmöíoûÇ?þñtÞâû.¿PñþÅúúúÑW¸uëV.t´WöÊÑ^9Ú+G+»æææ|>íá©×®][¿~ýõë×GÏH~"?ÈOä7¹Â[«V­jhh¨««5kÖÚµk§ùT®®®Ä@üÌår%ÒZ¹råÀÀÀ3ÈOä'òùM¢Û·oyÇtþßX­---©T*Éôööþä±Ýù®l6[|¯ÈOä'òùü¦ÞBT«W¯NÞÒâÉ½fÍ³hÑ¢¿=ÈOä'òùü&Q8oxx¸0&ÏÇ1Ï´ ?ò#?ëüD~"¿_*ç%'X$ÅéüVòùüD~"¿é_r´wåÊÉ[ZüáÓÞÞN~ä'òùüÈ¯ªäïdcáqãÆò#?ÈOäG~U%¿wîÞ»víÚÆÆÆT*?W®c*bÈOä'òùÕJä'òùüD~äG~"?ÈOäWónÿ-]ò#?ùùü*X~©ñ#?òùüD~z§ö>òÈ#üöYò#?ÈOäG~Õ)¿¾¾¾Y³fùV¬XQüÅÎäG~"?ùYäWUò[·n]²«ïèÑ£´=ÈOä'òùü&Ñ/¼oõêÕ·=ÈOä'òùü&ÔíÛ·-ZÌÑÓÓSÛüD~"?ÈïîíÞ½;ÙÕ×ÙÙY¹ÛüD~"?Èosú>?òùüD~ªù¥îV:&?òùüD~äWò«ÈOä'òùüÈüD~"?ÈüÈOä'òùüÈüD~"?ÈüÈOäG~äG~"?ò#?ÈOä'ò«,ùµ¶¶Ö××û>?òùüD~ªrùÍ?¿X¾ÏüD~"?ªV~¼0ß3gFFF*nÈOä'òùM¢¦¦¦_%²üD~"?Èorõõõü6oÞoiä'òùüD~kîÜ¹u£rùüD~"?ò«6ùÍ7Ïä'òùüTòKÌ×ßß_ÛüD~"?Èo577;ÃüD~"?jB~AßÖ­[ÈüD~"?ªY~uãäòùüD~äWßä<fÎð ?ÈOäG~Uø­.ùüD~"?ùÈOä'òùU>_µjUCCC]]Ý¬Y³Ö®][)§úÈOä'òùM¢Û·oyGEêK~"?ÈOä7,XÎ[½zuòOî5kÖÄEùüD~"?ò«*ùÕ××ócòù|ñäG~"?ÈüªJ~©T*Ú+1¾ÕüD~"?ùUçÑÞ+W&oiñ3cL;ùÈOä'ò#¿ª_¼yÇ7ÈüD~"?ùUüÞ¹szïÚµkS©Türe©íA~"?ÈOäW+ÈOä'òùM¢l6;oÞ¼óçÏùüD~"?U¹üÒét]]¥î;$?ÈOä'òD===!¿íÛ·ÇÓºRþhùüD~"?ß1N©TüÈOä'òù_UÉ/5N¾ÉüD~"?ùUüæÍ×ÖÖ688XÑÛüD~"?Èïî%®÷;G+âÀ.ùüD~"?ßKþò'zÉüD~"?ªY~L¦®lÎð ?ÈOäG~U"¿3gÎÌ=;Ùó8Ïä'òùüTò+T)È#?ÈOä'ò«éÈOä'òùüÈüD~"?Èï®¯¯/É¤Óé¶¶¶ò¦6¦¸mÛ¶ýê¯þêW§±/~ñ¿ýÛ¿ýUÕd?üðoýÖoY¶¾j­x£·ë¡6[¿~ý¿üåé¼Å_.;xð`ìÙ³§³³³ü¤©)îG©$IªÞwù]¸pá=_SSÓÈÈHäóùl6[~ÒÔÆ$I"¿©0¾ó.ííí¯¿þú»_ZñiÂ%§4µ1ÅmÝºµ¥¥å¾iìÞï;wîªÉâÉfëÛú²õeë¿¯½ïòó´Z¶lÙ¥K¦¼´âï®¯¯/?ijcá!gxÈráñnù.X° @À0ÖªU«¦°æææ|>áò¦6üD~"?Èï=hddäèÑ£uSýëm]]]øËåÊOÚòùüD~"¿wUñ>¿ÆÆÆîîî©­ Pc&éííýÉ½¼óqÅÑ¦6üD~"?Èo¯¡¡¡³³óÊ+²=Èoôê«¯~ãßÃÃÃä'òùüf®ü;xàË/WÜö ¿¼þðZü©l6?çÎÛÖÖcÈOä'òùÍPù½'_æB~µÙðððG?úÑO/ýôc_zlÛ·Å¿+VÜwßä'òùüf¢ü*:òû`õÕWög¶À¾äßÜ¹s¿ûÝïÈOä'ò¡ò;wîë¬Y³óy:D~äw×9ò©Å*f_üûä'>ùüóÏÈOä'òò;~üxáD~Éð=ÈüÊwþüùâ~1|ï½÷¾úê«ä'òùüf¢üâ;wîÜ¹üz/v!?ò»k<ðÀýí÷oýÍ­Á¾GÿÏ£øÄ'/_^­gøùYä'ò«xùþ<pA~###É	¿äG~wmppðsûÜ?üáìcsæÌYµjÕüãj°äG~Öùü*^~ÍÍÍá¼d?_È/Ï?úè£1ÍfÉü&XhïßûÞ~ô£ê~äG~Öùü*^~Aº±zåWÈüD~"?ùUü¢ë×¯wtt$çö644´¶¶^ºt©"¶ùüD~"?_­D~"?ÈOäG~ä'òùüD~ÿ¹+W®´··766¦îmmmr¨üD~"?Èo¢mØ°¡n6nÜH~ä'òùüÈ¯JäwèÐ¡y÷îLFÞ¾ûðáÃÉøcÇùüD~"?ò«ùe2àÝþýûGOJþïó#?ÈOäG~U"¿úúúàÝíÛ·GOÊçó1)®@~ä'òùüÈ¯äWø£mãMMþùÈOäG~äG~Õ ¿2¶#?òùüD~"?ò#?òùüD~(¿òùüD~"?ò«ù¥îV:&?òùüD~äWò«ÈOä'òùüÈüD~"?ÈüÈOä'òùüÈüD~"?ÈüÈOäG~äG~"?ò#?ÈOä'ò#?òùüD~"?ò#?ÈOä'ò#?òùüD~"?ò#?ÈOä'ò#?òùüD~"?ò#?ÈOä'ò#?òùüD~"?ò#?ÈOä'ò#?òùùÈüÈOä'òùüÈüD~"?ÈüÈOä'òùüÈüD~"?ÈüÈOä'òùüÈüD~"?ÈüÈOä'òùüÈüD~"?ÈüÈOä'òùüÈüD~äg=ÈüÈOä'òùüÈüD~"?ÈüÈOä'òùüÈüD~"?ÈüÈOä'òùüÈüD~"?ÈüÈOä'òùüÈüD~"?ÈüÈOä'òùüÈüÈüÈOä'ò#?òùüD~"?ò`L&N·µµõôôOêíímooI.«ÈOä'òùUv¹àÁ1°gÏÎÎÎâIóçÏåWbàÙgmmm%?ÈOä'ò«ìFFFb Ïg³Ùñ®ÖÐÐ0Z~ðpfñÅC¢gTÙú¶¾õP;vì;ßùõ`ëOO5!¿t:=æpq±.º»»GËïðáÃ?ÆN8ñÆoüH5ÙK/½tùòeë¡6ëééùÁ~`=Ôf/¿üò¥K¬ÚìäÉ÷w7·XòK¥RáúúúÑW¸uëV.t´WöÊÑ^9Ú+G+¯º/óù|r´7K®yíÚµõë×_¿~ôBÈOä'òùü*¬®®®Ä@üÌår%ÒZ¹råÀÀÀ3ÈOä'òùUÞjmiiI¥RL¦··÷'íÎîÀl6[WùüD~"?_F~"?ÈOäG~ä'òùüD~äG~"?ÈOäG~ä'òùüD~äG~"?ò#?òùùüD~"?ùÈOä'òùùüD~"?ùÈOä'òùùüD~"?ùÈOã688ø»¿û»ýìg×­[·oß¾ááaòùüÈüD~UØÐÐÐ¢E>Öú±_ÉýÊ/¯ûåûî»oÕªUSÆùõ@~äG~"?ÍÜ¾ò¯|üãìKmûò¶ø¿?üÃ?$?ÈüÈOäWmâøµõ¿°/ù÷Ëë~ù³ý,ùüD~äG~"¿jëþûïïþÝÅòè¡|ðAòùüÈüôÈïí·ß~úé§ã7~ãÉ'<þ¼5öÁ¶iÓ¦%ZR,¿ûÛïß¹s'ùüD~äG~z·ò»xñâ½÷Þûó?ÿó¿ôK¿ôé¥þÐ>Ï+íì?üa6ümØ°á×ÖÿÚý÷ßßÖÖ:'?ÈüÈOïV~?÷s?·ê¿®*ì^úïÿï÷ÜsÏ¡÷Á¾|üãÿÒ¾4888åEùYäG~ä'òûaüôOÿtá4Òä_Ãn¿ªüÈÏz ?ò#?ßOêïïomm-f_üûä'?ùo|Ãz#?ÈüÈOU%¿ÁÁÁèCÿkÓÿ*°ïÑÿóè=÷Üã<òùüÈüTmòvìØÍf7þúÆ`ßo>ò÷·ßÿÐCYiä'òùù©åíÜ¹ó#ùHssóìÙ³;;;¬4òùüÈüTòKúÑ~4å?+òùüÈüTIòùüD~äG~"?ÈOäG~ä'òùüD~äG~"?ÈOäG~ä'òùüD~äG~"?ÈOäG~ä'òùüD~äG~"?ÈOäG~ä'òùùùÈOä'òùùüD~"?ùÈOä'òùùüD~"?ùÈOä'òùùüD~"?ùÈOä'òùùüD~"?ùÈOä'òùùüD~äG~äG~ä'òùüD~äG~"?ÈOäG~ä'òùüD~äG~"?ÈOäG~ä'òùüD~äG~"?ÈOäG~ä'òùüD~äG~"?ÈOäG~ä'òùüD~äG~"?ùùùüD~"?ùÈOä'òùùüD~"?ùÈOä'òùùüD~"?ùÈOä'òùùüD~"?ßÌ¬¯¯/É¤Óé¶¶¶ÑW8~üx]]ùüD~"?_ÅËå<öìéìì,:<<ÜÞÞ>übÿwùåãõÿUÅ-^ÿÖCmî¿qãõP:uj``Àz¨ÍÂýÿðÿ0·Xòkjj|>ÍfK¦>ùä»víO~;wî<69räÅ_<¦ÌÖ·õ­[_¶þû]MÈ/N9]¹r¥££#h¯í£½r´WöVC©Tª0__<iíÚµ§Nú·J~"?ÈOäW¡Õý1ÜÜÜÏçß¹s´7Ç¼ZáÊä'òùüD~×b ~ær¹ñ¤8z$ùüD~"?_å­ÖT*ÉdzÇ¤ùüD~"?_MG~"?ÈOäG~ä'òùüD~äG~"?ÈOäG~ä'òùüD~äG~"?ò#?òùùüD~"?ùÈOä'òùùüD~"?ùÈOä'òùùüD~"?ùÈOä'òùùüD~"?_5õ¯|åùçsî¹ç¾ÿýï¿©,¶þùóç­ÚìOÿôOÿæoþÆz¨Íâæ¯ÿú¯­ÚìÐ¡CýýýÓyo¿ý6ùÛ¹sç¶mÛöUI¤ª¨üÁÌ:»a%Ij$ò$I"?I$$IÈO$Iä'I$ò$IùUpÙlö?ÖrQ©Tªø×¯_/jÕUßÖ?yòäÒét[[ÛéÓ§¯Ù××ÉdI===V]Mm¯ýªßú.èè¨¯¯_·n]ÉððÚ¯å­ÿ¿öýºyïßïñ»~ÌÍyðàÁ=ö9tèÐúõë­´*ÞúMMMW®øYü!ÊårñxVtvvZ5µõ½ö«~ë/Z´¨··7Î?¿iÓ&¯[¼öÉï½oÕªU/^-¿Ë//_¾¼ddlþÃ[iU¼õçÍwíÚµ1å`ÁÈÈHäóù¨ê·¾×~Õoýt:]=¶×¾­?C^ûä÷¾­ÙQò[»ví3gJFÎ?åÊñéèè¸páõV[¿¯¯¯¡¡!ÆÄÏ'@ñ¯âaÕÂÖ÷Ú¯ú­¿páÂsçÎÅÀ3Ï<Sò÷Ú¯å­ÿ¿öÉoäÿX¼xqë_»v-(Ö[õmýE?>A@És øCõõõÖ[Mm¯ýªßú¯½öZkkk ×®]ñÓkßÖ!¯ò&ùíØ±c÷îÝågñú¯Ê­_æ?÷ÍÍÍù|þ;G|bØz«©­ïµ_¿ù^ýõxíÛú3äµO~ÓôXºtéÙ³gG_mþüù.]Jì¿råJë­ú¶þâÅþýýý-*¾ZWW×b ~ær9ë­¦¶¾×~ÕoýØÄ###»vízâ'¼ömýòÚ'¿i_¸>ù<oÉÎ9|éÃ²eËO«Ê¶~ü/ÞòcÇÏ.¾ÂéÓ§[ZZR©T&IÎSíl¯ýªßú'O;wnüòïîîöÚ·õgÈkü$IjF¨V$IùI$ü$ID~$I"?I$$IÈO$Iä'I$ò$IùI$$IÈO$Iä'I$ò$IùI$ü$ID~$I"?I$¤ÚégYºtiÃ/_þÂ/ü§_pwª_ÇcÝÛl6mpp°d|I§ÓLfddd²ËD~TymÛ¶­nTO<ñD5Éoûöí1òé§.ÿµ¯-Æ?þøãSX¦$ò¤«¯¯/LN§÷îÝ;|§ýû÷ÇÅyöìÙªßåËcäÂKÆ/X° Æ_ºtü$¤êïóÿ|æ©§*¹sçÎù/|¡Ø====!§@a®|ãÆîîîÆÆÆÔÔÔ´eËâª'N]Å¤÷Ø±c%1³gÏ^ºtéÑ£GãbWWWÉ;räHùå$â.%?>ÒV®ãc^yå³bÅÂ;v´´´Ä¢Ö­[wíÚµÑò½ü1eîª$ò¤²¹sçZ®R<òêÕ«12ÍË¦¤3gÎ$S×¬YS2ióæÍÉ¤þþþT*5æÅdêÚµkGFFÂõõõÃÃÃ15~fÍ|ü®Ìrb`Ì»7ú>ûì³Å-àòàÁö,dùòå_»*ü$é.9°;Æ/µººpX±l:;;oÝ)ââêÕ«©	t;&»îBlÉ¤ì,áÓ§O')^æc=¶K¶nØ°!Æ>|8ãg?üðÃw]N¨1.ÆïÛ(49gÎx¼q1~Æ=næóùäL&fðÎ¿nhh¬üÊÜUIä'I¼ü¢1åWÈ&StíÚµ¸fJ.¶··ÇÅyóæmÜ¸1ÄvûöíÂBâ:%Ñs%¯^½Z¸rh©pÀ7~Æð'îºPfë­·ïÛxÉäGbÒ®]»Þù÷#Ú[¶l)¾B(0äíèèHvINV~eîª$ò¤¸dG×­[·GÆÈTÆ:^¼x1Á_:ç>[b©âïRá¦¦¦úúú¡¡¡XøìÙ³SïºñVÒ¹sç¤ÆpüáþþþÂÔÓ§OÇó¨ñÄåWæ®J"?IúK>ë¶÷îâÉwáQø,àÀÀÀè]YgÏÝ¾rìµ@Ædpê]¶yóæä oüìîî./³dÛõë×o½õVùóp/^S/²	°OJÎóIÇ¿yófyùT¬ÂÔ2wUùIÒD:Þ³gOò­.÷î­¯¯6ÆêÕ«C9CCCÉ¡ØÂy¸ÉçüÇ]¼x±ø	·lÙNJÎº-|¯Ê>KN¶MnýäÉñe|°/ùßàà`rÍ2òÛ¿aoäâIÉGûûûãVb%'¿Äaå¸ZsÝºuÅSËÜUIä'I|É·´cÇÿøw§FáÂqÒÂI6lØPÌÊâ:TF~ïÜùc1¾©©©ø@påÄÝ(¾cójÇ°·oßnhhHBÉ1îU«VßÄ9sâgòÅ.ÅËLvIJZZæ®J"?IN/_^§¥K&'ØÈïøñãÉ×æµµµ:uª0uppðÑGmiiI´ôÈ#¦;v¬££#@ÉdöíÛW²ÌÑ÷$ã7mÚT2~¼åDqgâ.%_4Xæûüuww|½KÒÀÀ@.5ÐØØâÒ¥Ko¨)^æÍ7¶ÉºZ±bEoooÉ-¹«ÈO$Iä'I$ò$IùI$ü$ID~$I"?I$$IùI$ü$ID~$I"?I$$IÈO$Iä'I$ò$IùI$iTÿÒFVí­Oû@IEND®B`


÷ú?Xÿ¥û¿ôO|âîµß¬J¥Rãããa Í644LvµÂòû¿øE]]]ßÿþ÷ÿ¶~¿õP^zé%[¿buwwÿÃ?üCÙ<¿ù¿¹÷Þ?úÑ677?üðÃØ·ÛÖ¯òK&Çõõõ­[·®°üî¹7fÑßþíß¾AE:vì­_É[ÿþé¬ÊôòË/?Þz¨L===¯¾úêlÞbE_"ÈWWW^áúõëLfddÄÑ^íÅÑ^íÅÑÞÒVWWÍf££½a8oêåË×®]åÊÂÊåòCùÎÎÎýû÷ð3ÉäVûððð3*?Êåò+½ÕZ__H$ÒétooïO[Õÿ>ºªåòCù¡üP~Jù¡üP~(?òS~(?Êå§üÊåòCù)?åòCù¡üP~ÊOù¡üòS~(?å§üP~(?ÊOù)?ÊåòS~ÊåòCù¡üòCù¡üP~(?å§üP~(?ÊOù)?ÊåòS~ÊåòCù¡üòCù¡üP~(?å§üP~(?ÊOù)?òS~Êå§üÊåòCù)?åòCù¡üP~ÊOù¡üP~(?òS~(?Êå§üÊåòCù)?åòCù¡üP~ÊOù¡üP~(?òS~(?Êå§üÊåòCù)?åòS~ÖòCù)?åòCù¡üP~ÊOù¡üP~(?òS~(?Êå§üÊåòCù)?åòCù¡üP~ÊOù¡üP~(?òS~(?Êå§üÊåò£rÊ¯êVòS~(?ÊOùCù%n%L*?åòCù¡ü_9_yP~(?Êå÷n§øúõëòCù¡üP~Ê¯ÜÊ¯¡¡!LúòCù¡üP~yùµ´´ÞJ¥FFFòCù¡üP~Ê¯¬Ê¯ºº:¤ÞððpÁ÷­o+¬[·Nù)?Êå§üÊªü¢|a ¤^¸páÂøøx3gòS~(?ÊOùUùÍ;7t^x`a`ûöíÑouQ~(?ÊOù[ùmÞ¼9w>Gü£~/V~ÊåòCù)¿²*¿à±Ç7o^èíí¡ÛÚÚJb(?Êåò«Êåò±üä'ccc6®òCù)?åò£Ëï¯þê¯î¸ãyóæÍ;÷îyóÍ7mbåG_cccôÝ.¾ÉYù¡ü(ûòÙ÷K©_úÒý_Úöµm[¾ºå3K?sçwÄw¸*?ßP~.×^sÊ²,¿;î¸#Ê¾Ü¿æO4ïÝ»×VV~TDùÈÍ×××7>>^rÛCù¡üP~ÓòöÛo§R©xö_øÂzè![YùQå~ò+ÅìS~(?ßtèCÚüÍñò»ûî»¿þõ¯ÛÊÊ(¿þþþP~6l(Å·4åòCùM×¾ð%Kä²ïáþð?ü½ïÏVV~TDùóçÏ¯*àåò£,ËïÍ7ß¼óÎ;?ÞøñÏîswßwÈ¾'xÂ&V~TJù-X°ÀÊåGå_0::ºwïÞ~xûöíöö)?*«ü¢æ(Åí¡üP~(?Êoêêêá¡üP~(?Q~!eBùmÞ¼yttTù)?ÊåG9_Õ$á¡üP~(?ò+Ãor3<ÊåòS~eø­.¥Kù¡üP~(?ß4444,X°àÜ¹sÊOù¡üP~(?Ê¼üÉdUÕ¶ï°¿¿?Ne677÷ôô4³1q>úèsÏ=÷Æ,:tèÐ«¯¾ú)lýð$ë¡2óßüáh=T¦ðFóüÀz¨LÍ[|ßË/äT(¿;vÿÐ¼ûïvÉd2»wïîèè(>ifcâ6mÚTP.Þ÷òoÏíM¥RQ>f³Ùâf6FùÊoÞÛsãså-¡pÒÌÆ(?@ùÝâ«««OÙ¸¯ýëO?ýôwfÑüÉtwwôÔSOÙúëOÿôO_zé%ë¡27_|Ñz¨Lög6Ë[¿ÄÊ¯®®.ÍFgÃpñI3ãÜ^Ûsqn/Îí¹U+V¬¨©©©ªª3gÎêÕ«g|ªGggçþýûÃ@øÉdOÙåòCù¡üP~3tãÆ	3ÏìÏøT__H$ÒétooïOïåÍÖf6Fù¡üP~(?ß5552[¹reôÜ«V­c-ZtûoåòCù¡üP~ÓP]]:oll,7&Í1§S(?å§üòCù¡üJ»üDè¼è,Èèèh3³ouQ~(?Êåwû_t´·½½=zK?ÃpÓÚÚªüÊåòS~eU~álÂ3<®^½ªüÊåòS~eU~ïÜ<½wõêÕµµµD"üloocJb(?Êåò«ÊåòCù¡üòCù¡üP~(¿Ü·ÿ¹ÊOù¡üòS~(¿.¿ÄäòCù¡üP~¼S	G7mÚßÁòCù¡üP~Ê¯<Ë¯¿¿Î9¡ù/_ÿbgå§üP~(?åg=(¿²*¿5kÖD»ú=ZBÛCù¡üP~(?ß4<ÿüóQó­²ä¶òCù¡üP~(¿)¹qãÆ¢E¢9zzzJq(?Êåò»µ§z*ÚÕ×ÑÑQºÛCù¡üP~(?ßæô~ÊåòCùQ!å¸d2©üÊåòS~åP~åAù¡üP~(?òS~(?Êå§üÊåòCù)?åòCù¡üP~ÊOù¡üòS~(?å§üP~(?Ê¯´Ê¯±±±ººÚ÷ù)?ÊåGßÂãµçûüÊåò£lË/D^h¾¾¾¾ñññÛÊåòCù¡ü¦!Jò+ÅìS~(?Êå7=ýýý¡ü6lØPoiÊåòCù¡ü¦gþüùUá¡üP~(?ò+·ò[°`3<Êåò£"Ê/j¾RÜÊåòCù¡ü¦¡®®ÎÊåòCùQåR&ßæÍGGGòCù¡üP~sùUMÂÊåòCù)¿2ü&ç	9ÃCù¡üP~(?åWßêRºÊåòCù)?åòCù¡üP~Éf³+V¬¨©©©ªª3gÎêÕ«KåT_åòCù¡üP~ÓpãÆ	Ïð(SÊåòCùMCSSSè¼+WFoiáÉ½jÕª0fÑ¢EÊOù¡üP~(?åWVåW]]:oll,7&Í1a¼òS~(?ÊOùUù%Ðy¡örcFGGÃßê¢üP~(?ò+Ï£½íííÑ[ZøÃÖÖVå§üP~(?ò+«òïdáqõêUå§üP~(?ò+«òçæé½«W¯®­­M$ágSÛCù¡üP~(?_¥P~(?Êå7,8wîòS~(?Ê2/¿d2YUUªûÊåòCùMCOOO(¿;v§u©üÑ6åòCù¡üP~3ZÄ$òS~(?ÊOùUù%&áÊåòS~eR~,hnn)éí¡üP~(?ÊïÖ¢?×ûÎÍ£½%q`Wù¡üP~(?ßE®7ú½ÊOù¡üP~(?Ê¹üÒétUQÎðP~(?ÊOùIùõõõÍ;7Úóu3<Êåò£<Ë/§T"Où¡üP~(?_ES~(?Êå§üÊåòCùÝúûûÓét2lnnîééOêíímmmZZZÂÕÊåòCù¶L&sàÀ0°÷îø¤:u*<x°±±±°üþú¯ÿúò,:vìØÐÐÐe*­_É^~ùåýèGÖCeêééyíµ×¬ÊôÊ+¯üó?ÿólÞâû^~çÏÿÀË/Jl6ÛÐÐ0ÙÕjjjËoïÞ½ßE/¾øâßÿýßÔÕÕÕÛÛk=ØúT^zéßûõ`ëÏ÷½ü¢¯timmÿ¡ù Ê/~rñd'÷õõ­[·ÎÑ^íÅÑ^íÅÑÞÍûöæP]K.åÿÖèêêêÂ+·ÛL&SøWÊåòCùMÛùóçr	òkÅïï]ÿ0Ífß¹y´7ç]óòåËk×®½råJáBÊåòCùÍÐøøøÑ£Gkjjfù¯·uvvîß¿?L&¯´ÚÛÛ'Qù¡üP~(?ß´Ä÷ùÕÖÖ~¨î]­õõõ¡5ÓétooïOÛÍÝñ¿&¬üP~(?ÊoæâÁWSSÓÑÑqñâÅRÙÊåòCù¡ü¦³'vÜï½.¹í¡üP~(?Êo>À/sQ~(?Êå7«åWÒÊåòCùMÏÙ³gçÌÏ[WWwøðaå§üP~(?ò+·òëîîÎá_4¼÷nå§üP~(?ò+«ò«¯¯wöìÙõööF_ì¢üÊåòS~eU~¹ïÉËßøøxtÂ¯òS~(?ÊOùUùÕÕÕÎöóòËf³[¶l	ÃÊOù¡üP~(?åWVåR¦j"§NR~ÊåòCù)¿²*¿àÊ+mmmÑ¹½555CCC%±=ÊåòCùUåòCù¡üP~ÊOù¡üP~(?ßÏ»xñbkkkmmmâ¦0ÐÜÜzÊåòCùMÕ>X5õë×+?åòCù¡ü_ßáÃ£ÈÛ³gÏÈÈH4òÆGÆwuu)?åòCù¡ü_9_:y·oß¾ÂIÑßsó~ÊåòCù)¿2)¿êêêw7nÜ(ÍfÃ¤på§üP~(?ò+òËýÑ¶É¦FÉMù)?ÊOù)?åWåW¤íòCù¡üP~(?å§üÊåò+Áò+Nù)?Êå§üÊ¤ü·L&òCù¡üP~Ê¯Ê¯<(?ÊåòS~ÊåòCù¡üòCù¡üP~(?å§üP~(?ÊOù)?òS~Êå§üÊåòCù)?åòCù¡üP~ÊOù¡üP~(?òS~(?Êå§üÊåòCù)?åòCù¡üP~ÊOù¡üP~(?òS~(?Êå§üÊåòCù)?åòS~ÊOù¡üòCù¡üP~(?å§üP~(?ÊOù)?ÊåòS~ÊåòCù¡üòCù¡üP~(?å§üP~(?ÊOù)?ÊåòS~ÊåòCù¡üòCù¡üP~(?å§üP~ÊÏzP~(?å§üP~(?ÊOù)?ÊåòS~ÊåòCù¡üòCù¡üP~(?å§üP~(?ÊOù)?ÊåòS~ÊåòCù¡üòCù¡üP~(?å§üP~(?ÊOù)?å§üÊå§üÊåòCù)¿)êïïO§ÓÉd²¹¹¹§§§ðÝÝÝUUUÊåòCù¡üJ^&9pà@Ø½wGGGÞÔ±±±ÖÖVåòCù¡üP~å Jl6ÛÐÐ7õñÇßµk×då÷çþç³èÅ_<uêTÉÖ·õ­ÊÔÕÕõÝï~×z°õgGE_2p8¸xñb[[[èÂÉÊïÈ#oÎ¢cÇ½þúëoR^~ùå.X©§§çÇ?þ±õP^yå¡¡!ë¡2?~üG?úÑlÞbE_"ÈWWWÇ'­^½úÄÿûPíÅÑ^íÅÑ^í-QU?ëêê²Ùlt´7OxµÜÊåòCù°ÎÎÎýû÷ð3ÉLV#ÊåòCùÞj­¯¯O$étº··wÂÔS~(?ÊåWÑÊåòCù)?åòCù¡üP~ÊOù¡üP~(?òS~(?Êå§üÊOù)?åòS~ÊåòCù¡üòCù¡üP~(?å§üP~(?ÊOù)?ÊåòS~ÊåòCù¡üòCù¡üP~(?å§üP~(?ÊOù)?ÊåòS~ÊåòCù¡üòCù)?å§üP~ÊOù¡üP~(?òS~(?Êå§üÊåòCù)?åòCù¡üP~ÊOù¡üP~(?òS~(?Êå§üÊåòCù)?åòCù¡üP~ÊOù¡üP~(?òS~(?åg=(?òS~³gllì¹çÛ¼yó×¾öµcÇyA*?Êå§üÊ³üFFF>õ©OÝ¹àÎ/|þíw·ßqÇ=ôPhA/KåòCù¡ü_¹ß¿üå­¸uÛ×¶_Ùô|ä#ô²T~(?ÊOù[ùÍ?ÿá²/ú÷+ã¾ûîó²T~(?ÊOù[ùÕþÚÜ¿èßïvþî½÷Þëe©üP~(?ò+·òûµ_ûµßZý[ñòûôâOoÝºÕËRù¡üP~(?åWnåé/üÂ/Üwß¡ù¶þáÖ_ÿì¯§R©ý×õ²T~(?ÊOù[ù]]]óçÏÁðWõW½&ÊåòS~åY~·ß~ddÄ«Qù¡üP~(?åWþåòCù¡üP~ÊOù¡üP~(?òS~(?Êå§üÊåòCù)?åòCù¡üòS~ÊOù¡üP~(?å§üP~(?ÊOù)?ÊåòS~ÊåòCù¡üòCù¡üP~(?å§üP~(?ÊOù)?ÊåòS~ÊåòCù¡üòCù¡üP~(?å§üP~(?òS~ÊÏ+Aù¡üP~(?å§üP~(?ÊOù)?ÊåòS~ÊåòCù¡üòCù¡üP~(?å§üP~(?ÊOù)?ÊåòS~ÊåòCù¡üòCù¡üP~(?å§üP~(?òS~ÊåòCù¡üßô÷÷§Óéd2ÙÜÜÜÓÓ4::ºvíÚêêêìc¡ºÊåòCù¶L&sàÀ0°÷îø¤;w>ñÄããã!¹,XPX~aüÿÌ¢W^y%¼þÿláõo=T¦ÐýW¯^µ*Ó'­Êºÿßþíßfó+¢üR©Th»0ÍfâÏ;7Ù¡ü|òÉ®YôÂ/¼øâ]T$[ßÖ·llý÷[E_2p8ºøÔSOÕÔÔ,X°àÌ3öâh/öâh/ö¶D"®®®Î´oß¾0pþüù¶¶6åòCù¡üP~¥§êgÂp]]]6çæÑÞ0¿ZübÞî@åòCù¡üP~¥§³³sÿþýa üÌd2ñI<òÈ¡CÂÀÙ³g/^¬üP~(?Ê¯äWk"H§Ó½½½?l7wÛêÕ«Éd[[Ûàà òCù¡üP~(¿¥üP~(?ÊOù)?ÊåòS~ÊåòCù¡üòCù¡üP~(?å§üP~ÊOù)?òS~(?Êå§üÊåòCù)?åòCù¡üP~ÊOù¡üP~(?òS~(?Êå§üÊåòCù)?åòCù¡üP~åäÑGî¹çÞEzõÕWß "­îÜ9ë¡2óßüáh=T¦ðFóüÀz¨LÍ[|ûí·ß¤Î=»mÛ¶oâ3«ì¨Ê@ù üP~(?Ê¯744ä.?¾­­­ººzÍ5y_ã~åÊª«®Ôõöö¶¶¶&Éþþþ0&üL§ÓaLsssOOOüÊE&Qö[ßk¿ì·~á×¾­;¼öýºyï<y²©©)¾9-ZaàÜ¹s<òHüÊ^»v­V6.êÔ©0pðàÁÆÆÆ0Éd8vïÞÝÑÑ¿rIýÖ÷Ú/û­_ø^àµoëß¯å÷Þ[±bÅàà`|ÿäçÎ¿rØüG±ÒÊRMMMøJ¥ÆÇÇÃ@6Íûÿ_IýÖ÷Ú/û­_ø^àµoëß¯å÷¾­ÙØönii9ölxöÙgãýG¡½½=lkk;þ¼õV6úúúÖ­[×ýy[¿È$Ê~ëíýÖ/|/ðÚ·õo×¾òò;sæLcccøÀ®]»¢ÿº|ùrDë­<~=ÉáD"_]]¿ZIýÖ÷Ú/û­_ä½ßk¿·þþÚW~³Q~9¯½öZSSÓd³xýðb^»ví+W¢uuuÙlöuÂpüE&Qö[ßk¿ì·~÷¯ýJÞúøk_ùÍFù-°¿¿|||×®]=öXüjaÒÐÐPô¤ioo·ÞJÝñãÇÃvÎéììÜ¿?ÃãW.2²ßú^ûe¿õ¼÷íWòÖÿÀ_ûÊo6Ê/<'æÏÒ~Ýºucccñ+ôõõ555%É¥K'õVêòN×?yòd"H§ÓÑ)Þ¹­?á$*dëíýÖð½ßkßÖÿÀ_ûÊ R(?åò@ù üP~(?Êå üP~(?Êåò@ù üòì³Ï.Y²¤æ¦eË=ÿüó?÷î¦ùu<Ñ½mhhmdd$o|L&Óéôøøøt	(?Ò³mÛ¶ª=öX9ß;ÂÈgy&oüÓO?Æoß¾Ë@éïïML&÷ìÙ3vÓ¾ûÂÅ0òôéÓeS~.#[ZZòÆ755ñCCCÊP~@ùûâ¿æ'|òÉ'ÃÈûï¿?Þ====¡B¶¶¶áÜ¯^½ºnÝºÚÚÚ0)JmÜ¸1~PõØ±c¡®Â¤0oWWW^K1sçÎ]²dÉÑ£GÃÅÎÎÎ¼;öÂ/_N4)Ü¥hRww÷dÖÞÞÆ÷ööæÆ:u*Y¾|ynÌÎ;ëëëÃ¢jjjÖ¬YsùòåÂò+ Þ"wP~¤ùóçj¹xñb|ä¥KÂÈxÙäéëë¦®Zµ*oÒ¢IDbÂ¹¢ÑÔÕ«Wv¬®®SÃÏPNsæÌ>~Wd9a`Â»WøH<ÏÙ\8p yY¶lÙtË¯È]À,:°;Á/µªªÐañ²éèè¸~SW®MB'jÇh×](¶hR&vá'OF/sëÖ­¡í¢­>ø`säÈ0~áxàË	Õ.+ÄïÛ(Ôä¼yóÂãÃÏpÏCnf³Ùèét:ÌÂ;?;:Ýò+rWåðÁ_0aùåÆGeSpùòåp14St±µµ5\°`ÁúõëC±Ý¸q#·p¼½h¹¹¢.]Ê]9ÔRîoø;vËåÊßzë­øì3y6mvíÚõÎÏhoÜ¸1~P¡üB¶µµE»$§[~Eî* ü>`Ñ®ë×¯ÇGaRÖÉuáàà`¹ÐÉ8¯ð l^KÅ¿K%§R©êêêÑÑÑ°ð¹sçæ¦Þr9uX³gÏFÃÏ0<00zòäÉp&<j<õò+rWåð>ëöÔSOÅGFßuwGî³ÃÃÃ»²N>½cÇèØk.£r¹Ã©·ì³6DyÃÏuëÖåÆYN´íÊ+ÑÅ·Þz«øy¸wÝuWMÖø¤è<ß0©»»ûÚµkÅË/W¥ÑÚÈM-rWåðÎH&»wï¾ÕeÏ=ÕÕÕgc¬2TÎèèht(6wnô9¿èãqñF!¸qãÆÐIÑY·¹ïU°Ï¢m£[?~üxn|åDì>ç722]³HùíÛ·/·7.<äø¤è#áVÂJ¬ü¢Ö­®sÍ5ñ©Eî* ü>xÑ·çÙ¹sçÿÿwSF¹áÜqÒÜI9>ø`<+ã>üÞ¹ùÇ6ÂøT*?ánÄïXî¼ÚÉì7jjj¢wÅñ7o^ø±K|Ñ.É¨SsSÜU@ùÜB,[¶¬ú¦%KD'Øæ_wwwôµyÍÍÍ'NÈMÙ²eKTK6mÍMíêêjkkAN§÷îÝ·ÌÂÆ?òÈ#yã'[NîL¸KÑù>¿uëÖå½Kdxx8É5P[[ÅÐÐPîjâË¼víZHÛh]-_¾¼··7ïÜU@ù üP~(?ÊåòP~(?Êåò@ù üP~ø8%¬øUIEND®B`
