# Supplementary material for: Regulation of piglet T-cell immune responses by thioredoxin peroxidase from Cysticercus cellulosae excretory-secretory antigens
Source: Front Microbiol. 2022 Nov 18;13:1019810. doi: 10.3389/fmicb.2022.1019810 (PMC9718028; doi:10.3389/fmicb.2022.1019810)
Supplement: Supplementary file 3 [file Data_Sheet_3.ZIP › 4. C. Cellulosae ESAs and TPx Induced Th Subpopulation Differentiation/3. SPSS statistical analysis/3. IL-5/1. IL5-24h/1.3 (SPSS data export) SPSS statistical analysis--IL5--24h.doc]

EXAMINE VARIABLES=Figures BY Variables
  /PLOT BOXPLOT NPPLOT
  /COMPARE GROUPS
  /STATISTICS DESCRIPTIVES
  /CINTERVAL 95
  /MISSING LISTWISE
  /NOTOTAL.


Explore


Notes	
Output Created	12-SEP-2022 23:10:07	
Comments		
Input	Data	E:\桌面\Raw Data\4. C. Cellulosae ESAs and TPx Induced Th Subpopulation Differentiation\3. SPSS statistical analysis\3. IL-5\1. IL5-24h\1.1 SPSS statistical analysis--IL5--24h.sav	
	Active Dataset	DataSet1	
	Filter	<none>	
	Weight	<none>	
	Split File	<none>	
	N of Rows in Working Data File	20	
Missing Value Handling	Definition of Missing	User-defined missing values for dependent variables are treated as missing.	
	Cases Used	Statistics are based on cases with no missing values for any dependent variable or factor used.	
Syntax	EXAMINE VARIABLES=Figures BY Variables
  /PLOT BOXPLOT NPPLOT
  /COMPARE GROUPS
  /STATISTICS DESCRIPTIVES
  /CINTERVAL 95
  /MISSING LISTWISE
  /NOTOTAL.	
Resources	Processor Time	00:00:02.70	
	Elapsed Time	00:00:01.65	


[DataSet1] E:\桌面\Raw Data\4. C. Cellulosae ESAs and TPx Induced Th Subpopulation Differentiation\3. SPSS statistical analysis\3. IL-5\1. IL5-24h\1.1 SPSS statistical analysis--IL5--24h.sav


Variables


Case Processing Summary	
	Variables	Cases	
		Valid	Missing	Total	
		N	Percent	N	Percent	N	Percent	
Figures	Control	4	100.0%	0	0.0%	4	100.0%	
	ESAs	4	100.0%	0	0.0%	4	100.0%	
	TPx	4	100.0%	0	0.0%	4	100.0%	
	LPS	4	100.0%	0	0.0%	4	100.0%	


Descriptives	
	Variables	Statistic	Std. Error	
Figures	Control	Mean	8.47700	.108943	
		95% Confidence Interval for Mean	Lower Bound	8.13030		
			Upper Bound	8.82370		
		5% Trimmed Mean	8.47228		
		Median	8.43450		
		Variance	.047		
		Std. Deviation	.217885		
		Minimum	8.263		
		Maximum	8.776		
		Range	.513		
		Interquartile Range	.406		
		Skewness	1.052	1.014	
		Kurtosis	1.500	2.619	
	ESAs	Mean	9.13300	.113601	
		95% Confidence Interval for Mean	Lower Bound	8.77147		
			Upper Bound	9.49453		
		5% Trimmed Mean	9.13222		
		Median	9.12600		
		Variance	.052		
		Std. Deviation	.227202		
		Minimum	8.862		
		Maximum	9.418		
		Range	.556		
		Interquartile Range	.421		
		Skewness	.184	1.014	
		Kurtosis	1.500	2.619	
	TPx	Mean	7.19625	.197837	
		95% Confidence Interval for Mean	Lower Bound	6.56664		
			Upper Bound	7.82586		
		5% Trimmed Mean	7.18439		
		Median	7.08950		
		Variance	.157		
		Std. Deviation	.395674		
		Minimum	6.855		
		Maximum	7.751		
		Range	.896		
		Interquartile Range	.725		
		Skewness	1.305	1.014	
		Kurtosis	1.505	2.619	
	LPS	Mean	14.08825	.274110	
		95% Confidence Interval for Mean	Lower Bound	13.21591		
			Upper Bound	14.96059		
		5% Trimmed Mean	14.08422		
		Median	14.05200		
		Variance	.301		
		Std. Deviation	.548219		
		Minimum	13.456		
		Maximum	14.793		
		Range	1.337		
		Interquartile Range	1.021		
		Skewness	.392	1.014	
		Kurtosis	1.501	2.619	


Tests of Normality	
	Variables	Kolmogorov-Smirnova	Shapiro-Wilk	
		Statistic	df	Sig.	Statistic	df	Sig.	
Figures	Control	.250	4	.	.944	4	.680	
	ESAs	.250	4	.	.954	4	.744	
	TPx	.250	4	.	.903	4	.446	
	LPS	.250	4	.	.961	4	.785	

a. Lilliefors Significance Correction	


Figures


Normal Q-Q Plots


úªU«s9²dÉüüüÕ«WôÑGÇ[´hQAAÁ5kß_|ægJKK£aÍð#gÖ7=ôP<öìß¿?Ì¬««Kù;ÞîUþèaNXÈÛZ9)ßK6¼+¦/¿~çûeáÂaµ¯]»6¬Ûôï<7ùnb±X¸µ°Â=ÎJjX!¦ÃY%%%[·níîîöÇÊ§åwíÚµ²²²3gðáóä	ìhyyyáû<ðIªáÈ+W¸IÈ¾~¼çÒ÷Í¬Y³Âü?þ8yf(Ë0sÞ¼y)ÇÛ½J¿²&zÏoþüù·µrÒÜ°¯8ôò;uêTòí_övË/ùnmooN&<y2ºðý÷ßßo7oÞì0NË/L¼þúëÉï%GÀ£>¦×­[wõ¦0NÉ|òÉ'ûúú¢w­¢9=öX<?~üxt2Ür8ÙÚÚ¦CF×>ÍöÖ[oé÷ß?ù¬Áú&???åüäëÞáUû ]â=ÑÛ]9)jØWLó!¿~WÅ¦«««¯_¿>ÑgC/¿ä»5ÜNsôèÑ0Ý­7n.á|¦Ã½¦§Nê0~Ë//äa:¼¨÷?sæÌ0ýáF'Ã|89cÆä[Hj#ùäåË?ùÙ°irpôööV±|ùòèM¦[_AAAÊ_$åüa¹¨ÂuCÄTVVÆb±+m(+'åRûC/¿è³ôQò¸­òK¾[£[KæDg-]º43gN(×ð/Ä7üeòÆù8+))	¯ß===Éó¾g6ðCo!éRÞfú¡2ûxÞrÕÒÒÒhxz`Þ%B¤ß­å*­[®´¡¬ÁztxWúhïPÚ.ý¹ïÖ~»¼Îêèèâ/Q---þ¸@ùãºüoë[ádb(ÑÌ(Îú½;f% Òpa´w#G2JùEoLF;X©®®ÞºuëÕ«W_xá0sÃ)Ëo(WvùÝîÊIö^~CyÏ/Ñv¡ûÓ¯ÿ©S§9ý64Nvúôé]»vECÌí åßòûäæ§õäæD[þ®[·îÚMÑKûc=vçå½ïÕÞÞúãùçJùµµµEï*X<~üxô~^äõ×_Où;ÞîUn«üÒ¯hMvßÔïFnw­£ü¢Oæð½qãF»ï½w`~ãß+?Ôaàôë?ZÂÐÍáòaMé%K$ÿÑç5;::Ò¼Ê_åwñâÅÄ'º¢9çÏï÷¯3g&6½òK´HdÆáòö­)ùá8ò¸bÅ4¿æm]å¶Ê/ýÊIì¹ßÞ±VQ~§ON¾ýU«V%ÛoVK_~ÑnSnò½«ìGñÇÊÈò¢Ðäùï¾ûînª®®¸=ÇðÊïòåËáÖÂmzüñÇC%v¾oöîÝ»hÑ¢¼¼¼Y³f½øâÿüçï9ÝùUn«üÒ¯×_=Ä_~~~bxÉnk­£ü9sæß:Ü~4Ø9ýôÊÊÊ'NÜò=×ÖÖÖåË[¿TSSSb~ww÷O<½ò=Ü===þ¸@ùÞÞÞ~ø¶ö!7«dh6ÚX;GA¿páB@ùäès~ý|ík_³fåkº»»·oß>kÖ¬hH7L<ýôÓV üP~Êåò@ù üP~(?Êå üP~(?àõ×_///ÏÏÏ¿çn÷º-4iRGGGbNs,YrÏb7ï2C¹î0[æwwwoÞ¼¹¤¤$¬±iÓ¦UWW¿÷ÞkÅãñ0?   LtÊ_JKKC©|y¥òÌ3ÏëîÙ³'1ç¹çsvíÚå7p~mmmôèÑ0ìØ±0=oÞ¼Ä¹---R|ë­·<ºå³';§Ó§Oë®^½:1gåÊaÎ©S§Fg!G¿üÂük×®¥¼Ö¦MÂ¹Ñ÷GyÄ£P~À¸Ë¾äÊyâ'¦M6uêÔ;wö»Øç?ÿùåË÷»Ä[aúã?ÓaNtÖ|PYYYXX¿téÒè²·üÓÓ_åÅ_,//¯¨¨?h`]¿~½¦¦&,üöíÛ£·0C®Zµ*ÜZnùÃ?¼Ãò=v¿páÂãÇ÷;«¯¯oúôéágÝ¸q#|1cF¢·Êø¦¿úÕ¯é¦¦¦ÐXaâk_ûZòeZ[[£ÂKöè£³^xá0¾éºººè¬+VD#ï¿ÿ~_VVòÖ ýUBÏë[ßµµµ~óæÍa:µ×^Ï<óL"Ô®]»ÖÞÞ&¢·$ûß`M|×_=1çîyçwW×­[¦7lØ¦ÃËàQ(?`ìË¯¼¼<L÷Þ40¼R~°­­-;÷ßtsO>ýäO._¾<ÌÏËËKykýÚ+ýU¢6mÚÀëÎ93Zø¾¾¾0z+ÌÓK.-ÛÓÓ3¼ÕÒÏÉ'C¡&ú/Ür4ëÖ­Q¡>þøãw²ÊÁò¶KMÌ'oA!³¦N.ÙÝÝh9ÑYÏ?ÿ|¸VÈÁwÞy'ùúÝZòÉ¡°ü]T¯¾úêôéÓ£9%%%ÑÛCÏ/Íz;zôh¹äÑíÐÍá^½zõCÏazÖ¬Y)Á£P~ÀØ_YYYò~ÑÛf·Ì pnâb~´1DßMC,¿ôWI,X©×1cFtÝ~Çßzë­hÜÄ·µZ-LXæ0HÕ~N>=ìeP~À_´^x!úßsÏ=7zõÕW¡ó­o+1?êÈ³gÏFÿJù¥¿ÊO>ùío;L<úè£¯ûÈ#é^zéÜ¹sÑèjYQQ¦CôÑGabþüùwX~K,	ó9òÉÏöáòðÃ2ønÂüa/òF°üâñx¨«©7%ï/ùÝ¸q#ißwwÂ¨¤¤dÚ´iÛ·obù¥¿J,ç®2±¡I¿,oÝº5,yAAÁ½÷ÞmBb«ºº:ÚXxõêÕï¾ûîm­#ÂáfkjjÂ_vÆ7o?÷íÔ:ùö;µö2(?åò@ù üP~(¿Ûöñ.åúáþË¿üÇuåÊú§²È¬ý×ýà¬2«¯¯ïÇ?þ±õ üÆÀoÿöoøåú×ý×ôÇõÞïýýßÿ½õ@f]¾|ù/ÿò/­2ëßþíßÞzë-ëAù)?P~(?ÊOùòCù¡üP~ÊÊåòS~(?P~(?òCùòCù¡üÊÊå§üP~(?P~(?åòCùòS~ÊOù¡üP~ üòS~(?Êå§ü@ù¡üP~(?åÊåòCù)?P~(?ÊOù¡ü@ù¡üP~ÊåÊåGß'.]¿dÉS§N)?(?9[~óæÍûí·ÃÄ+¯¼2þüå|WG×~ðð"2ª½½ýÌ3ÖõÓþôØ±cÖþ£xóÍ7­ñ,Ë/YaaáÀòÛ·oßF×þé¶µµý2êßû^,³È¬ïÿû­­­Öuüøñëa<Ëò;yòd]]Ñ^öÑ^ö³£½ë×¯WWWwww+?(?¹ /^¬­­M[ÊåÊåGî_[[Û5kÂWÊsÊÊÜ)¿òòòIIÊÊ-¿ôÊÊå§üP~ üP~(?åòCùòS~ÊOù¡üP~ üòS~(?(?å§ü@ù¡üP~(?åÊåòCù)?P~(?ÊOù¡ü@ù¡üP~ÊåÊåòS~(?P~(?òCù¡üÊå§üP~(?P~ÊOù)?ÊòS~Êåòå§ü(?Êå§ü@ù¡üP~(?åÊåòCù)?(?ÊOù¡ü@ù¡üP~ÊåÊåòS~(?òCù¡üÊåÊOù)?åòCùòS~ÊOù¡üP~ üòåòCù¡ü(?Êå§ü@ù¡üP~(?åòåòCù)?(?ÊOù¡ü@ù¡üP~ÊåòS~(?òCù¡ü@ù)?å§üP~(?P~ÊOù)?Êr¼üâñxÎÿÕ(?åòCùÁD/¿óçÏ®(--<yò¥KòS~(?äZùuvv655-[¶lRÆÆFå§üÊå9R~ñx<­_¿~òäÉxðÁòS~(?dùµ··×××¾¢¢¢ÚÚÚ¡òS~(?dkù]ºt©±±±ß¨nBeeåÁ»»»'Â=¨üÊå¹Y~===!éÖ¯_?eÊÁ7wîÜÐ.P÷ òS~(?äZùÅb±ÚÚÚ<ª«üÊå9^~çÎkll`Q]å§üP~(?ÈÍòëêê:tèÐÚµkSn«;öì	8ª«üÊå9U~===mmm6mJ|ÅÅÅõõõáÛ½¦üÊåY ;vì(--|¡«ªªCº¿òCù¡ü [Ë¯³³3ÍÇøÂüpn¸»Iù)?Ê²µüzzz«ªªê*?åòåGÎ_xÙìFuòCùò#Ê/ý¨nEEQ]å§üP~ üÈîòK?ª[^^ÞÐÐÐÑÑá^P~ÊåÊ,.¿4£ºS¦L©©©ÅbóÊOù¡ü@ù#å×ÑÑÑÐÐ0öìÁFuººº¬vå§üP~ üÈÖò1.]Êà3ª«üÊY_~ñx<ÕÔÔL2Å¨®òS~(?P~äfùE£ºååå)wÎRYYiTWù)?(?²»üÒê:äòS~(?P~dù¥ÕuÈå§üP~ÊåG._û¶mÛÕuÈå§ü@ù¡üÈúò»råÊ=-[fTWù)?P~(?r³ü¢CnÜwßFuòåò#gËïÌ3uuu%%%)Gu|ðA£ºÊOùòCùÝºººþèþhñâÅ)«û¹Ï®±±ñÂVòS~ üP~d±h[Ý¢¢¢Ü¨¯¯÷`S~ÊÊìÖÞÞ¾cÇÒÒÒÜX¿~KKCn(?åÊåGëììÜ»wï`Ûê~ö³ç:äòS~ üP~d·X,¶~ýúÛêÎ=Û¶mgÏöqQ~ÊÊ±wþüùÁFu7mÚÔÖÖêï¸½(?åÊåÇöÀ<Ø¶ºUUU¾téRòUòS~ üP~dÁöÀ<öì]»v?>åuòS~ üP~dð*V___\|ËQÝÁ(?å§ü@ù¡ü×:;;,Xr[Ý£ºÊOù)?åòCùM¢ãê°ìát°Q]å§üòCù¡üÈiFuËËËÚÛÛwËÊOù)?P~(?Æ4£ºS¦L©©©ÅbwxÈå§ü(?c)ý¨nEEESSS¦¹¡üß?s+?(?úÇã±X¬¦¦&åÎY¢QÝÌþPå§üFÐñãÇ.¾ÊåÊÐs¡êBÛÜ¨®òS~càÞïî4å÷üóÏÿõèúîw¿ró¯!£ÚÚÚþìÏþÌz ³~ð9rÄzÈ±ç§zjñâÅ)wÎæsÃeFtN<ÙÒÒâ¾Ï²¸üþ/¿?ù?ùhtì÷Ýw?zçwN:e=Yá?çïÿûÖCøðÃÿøÿø(((|sçÎâ'Þ~ûíÑY/¾ùæîñ,ËÏh/FÁhïÄÕ-..®¯¯¿å<FQ~Êåòcììljjª¨¨|'O®ªªjnnîééeS~ÊOùòCùéwÎ²`ÁÆÆÆc»ÊOù)?P~(?îôÅe°CnÕ¨®òS~ãòCùòËji¹1æ£ºÊOù)?åòCùC9äÆê*?å§üÊåÇ¾6ª;BÜP~(?åòCù1ª.0Ø¨îHrCù¡üÊåÇhèêêlç,QÝpìú¥Q~öìÙ²eKø"ØCù)?òCùåx<Åjjj¦L½£º*¿ð üùÿù_ù_ùÂ¾°råÊ0=ú¯Ý(?åòCùyi¹u£º)¿OúÓÜ¸ó©Ñ×ÆCüeÝ;ÊOùòCùñïÒêF;g9xð`Î´Îm_x.úåEì¾/^ÜÖÖæa£üÊ_6I?ª;N¹1¶å×ÒÒ²òîýÊïî»ï>|ø°ÇòS~(?P~Ù¡½½°QÝñvÈ±-¿óçÏO:õË[¿È¾íÛ¶Ï1ãÜ¹sEÊOù¡ü@ùk]]]È®Cnmù¿ù¿ùÙÏ~ö7þáû¼yójkk=òCù)?ßøÕÚÚº~ýú	5ª©ò¾ùÍoàûÔ§>ðw÷w³å§ü@ù¡ürÓ3g¶mÛ61Gu3X~(?åÊå7~]ºtiÿþý)GuÊÊÊæü¨®òS~ÊOù¡üP~9.6ª;wîÜðº¢p¯"å7qËoÒ­äåå)?P~(¿ñïÌ3uuu¥¥¥_ËJJJ&æ¨®òS~ýåÝJ~~¾òåò·.]º´gÏ£ºS¦L©ªª:|øpww·¥üß¸ üP~ ü!ÄÜÁSr#óCÕU~ÊïövíÑGU~ üP~ãD<Ïá[¶l)..N9ª»cÇV~ÊoHÊËËóóóÎÊoêèè/³gÏN9ª[[[ÛÚÚj'sÊOùÕ%KRþó4ÒP~(?P~itvv<xpíÚµ'ON9ªÛÔÔÔÕÕå¡¢üßí)((Bá)&Ú**ß·¿ýí0QWW§ü@ù¡üFYOOOsss³444?Þ#Dù)¿áÞÄMa"¤^xÿý÷ûúúÂÄÔ©S(?ßh>E×××§ü_QQQ8+¼jÕU~ÊïNM>=üQ?~¼££#L<ýôÓÑ½ºòCùÎÎÎÆÆÆ;g<yò÷Ý×ÜÜlTWù±òÛ¾b~¡P~ üP~#$Õ­ªªJù1¾ÅïÞ½ûÂ	Ê_ðì³ÏÎ1#L8q"L|ùH/ºòCùÁÄ,¿4£ºa¦Cn(?F¼üÆòCùÁ*¿ô£ºUUUÍÍÍ===îzåòS~(?_¶êêê:xðà`£º!C(t+?F¯üæÏíÛÅAù¡ü2"Çb±;g1ª«ü³ò7o^rí%Ø¶Êo:::ÊËËê*?ÆcùÈ'OìëëÍEW~(?È¥òëêêjjjª¨¨Q]åÇx)¿ð79ÊÙ§üP~ågTWùeåwêÔ©ðÇ¹yóæë×¯+?P~(¿!2ª«üÈÊòfÍ5ðïÖ üP~E;g1ª«üÈÖò3g-<@ù¡üÒKÈ£ºÊ¬)¿è¶½½]ù¡ü +Ê/Í!7ê*?²¯üfÎiP~(¿~ÒrÃ¨®ò#Ë¯­­-üoß¾ÿcS~(?å~T·¨¨¨¶¶öÜ¹sî åG¶ß¤AØÂªüÒêÞwß­­­FuY_~y°(?&Bùutt'äÁFu-[vàÀ+W®¸G9R~cEù¡ü`Ë/ý!7JKKwíÚ5úÿ¡üñò+//3gÎèhCù¡ü`ôËïÜ¨««kii1ª«üÈÙòËÏÏí£¿èÊå£Y~·<äFSSQ]åGîßÑ£GÃý®]»®]»6ûvQ~(?òK?ª»`Á!­påÇD)?Ûöò#÷Ê/ý¨nIII]]Ýè?	£üûò³m/(?r©ü:;;ÃlQÝÃûòcâßXQ~(?È`ùuuu:t¨¢¢"å-[¶ÿþp+Vù)?å§ü@ù­åÇÛÚÚjkkSêÖÕÕ9sÆúDù)¿ÿ«··÷Þï-,,OS§NàFaSåò;)¿4£º¡«ªªìå§üR¸qãFÊ-<FúùBù¡ü`å~T7Ìojj2ªòS~Z¸pax²X·nÝõë×ÃÉk×®ÝÿýaÎ+(?ÆIù¥Õ-//·sòð¬SszÃ0_ùòcÌËïÜ¹siFukjjb±Xòs8(?åN^^^xúµÓÓÓæØ«(?ÆÐO~òmÛ¶ÕEùáòF×¬Yöïa:ÌYºt©òåÇ(Çã---6mdê¢üÈpùÔK¹ÇÕ«W(¿,ÒÓÓóÌ3ÏÜ÷Ý/þÒ¾táÂìZþsçÎv£º(?2V~ÜÜ¼÷6mZ^^^ø¾fÍ0g¤]ù¡üÈ Dk×®ý¥9¿ô¥¾ôèÿûèê_]]\ÜÞÞ>þüÊ+û÷ïO9ª«ªªíåG&ËoL(?tøðáÏ|æ3Oü¯'v>µ3úZóÿc×çTFuSn«»`Á]»v½ñÆîYÊOù¡üèï©§úo¿þßÙ¾BÃEODõõõ)GuÃYáÜÁqAù)¿T×¼¼¼<åÊ/[|å+_ùõµ¿ ÛßBjü,agggccã>ßN<yà¨®òCùÉòËòåuÂóÉ/üÂ/|yëå÷_*þËÆ|ÁBÌ¤arç,!C(xEåò#å7Ç<z>zåW(¿,òØcýâ/þâëîûâ¿¸lé²òòòK.áòD£ºÅÅÅ)wÎÕòCù1²åwêÔ©©S§§¤ÊÊÊä;+?P~ÙâàÁ!ûªªª~úé+W®É2¤Õ½­³(?#X~6lFí¡üP~äô£ºÃ8äòCù1"å÷Úk¯EOLëÖ­ÍEW~(?r@<ÅbµµµêûÊåGËïÆ+V¬6æ8zôè(/ºòCùÕBÏè!7ÊLß7¾ñèjãÆc²èÊåG6êêêjjjª¨¨H¹?¬aê*?£Q~öçÊ¡FukjjRrcöìÙÃÕU~(?F£üòn%??_ùò#Í¨nò!72Nù¡üÈdù9åòc<K3ªòÊåòS~(?²L<I·~ýú£ºi¹¡üP~(?åò#kçmÛ¶ò¨®òCù¡üÊQÒÙÙ¹wïÞÏîsc5ª«üP~(?åòcdkiiY»vmÊCnµQ]åòCù)?#¥½½½®®®¨¨h`ðnÙ²%NöÀ¬üP~Çò³??P~ÊùóçæÎrT÷ÁÅbc2ª«üP~Fù%ïº/eùÙ(¿péÒ¥¦¦¦µk×¦ÜVwñâÅ»wïK®üP~d²ü9þzè¡ë×¯áûý÷ßæ;vLùòËRÑ!7jkkS~¯¸¸xÛ¶m£¼­®òCù1.Ê¯¤¤$<&¨¥··7ú¼ËÞò©S§ÊÊÊòóó-ZtôèQåòcttt§èmà¨îúõë[ZZÆÕ¨®òCù1ªå=!ÚëW~wþ9¿êêê^z)LìÛ·oãÆËï»ßýn÷èO£ÿðÿÐuîÜ¹¿ýÛ¿µÆÖÅÿ÷ÿî»ïNùñ_þå_þ½ßû½üä'Yôýã?þãñãÇÝ³dÖ?ÿó?ò³Æ³/¿3g§ÅPi7nÜ'Ãn]æßù»QJ,¿=öüÙèúÎw¾róÏ £þô&ëaLÄb±ßùß©¬¬üÔ§>50ø~îç~®¦¦¦©©)Kµðí.&ãÞxã+a<ñò;vìXÊßyç;¼åämDn/b´£½Üá¨nCCCø¯ràÓWQQQ¾ÖÖÖñ°s£½íe|öï¿ÿþ¼yóóòò¦NºtéÒ?þøÎo6y¼¸  @ù¡ü¸s,Hù/keeeSSÓ-KÊ	]~#dæÌÑÇÃ÷cÇÊåÇÐõôô477WUU¥ÜVwñâÅ!Ï?K¿²òCùeåWSSóâ/ð½ººZù¡üÞmqqqÊ³lÚ´)«GuÊÑ.¿³gÏÎ?êÔ©ÑøìÌ3_õÕ;¿ÙãÇÛ,++;qâòCù1tiFu§L/7FuÊQ-¿hOÎÉGl¦÷íÛ7¢®üP~~T·¢¢¢©©©««k"¬åòcDÊ¯´´4<=6Q~'NÓÓ¦MS~ üFóÏs°QÝòòò	µBÊ)¿è5Ê¯¯¯ÏqAùhç,³gÏlT7åäÇøÊ±)¿hOÎÑû|¡üzxâèlåÊotuu555UTT¤Ü9ËÕU~(?FµüÚÚÚR>ó¾ýöÛÊ_fÅãñX,VSS3eÊ£ºÊåÇ_bhùòåÑ¶½óçÏÝb)?ßæÅÅÅµµµvTWù¡üíòÊå7¤Õ<yrUUUsssOO¥üP~Rù%6ìHøøãçÏ?ölåÊoxBÌ¥Õ]°`Acccgg§¥üP~ùõööÚ¶ß°ÿÊÒr#.`-)?£]~)÷¡lúôéÊß¥9äQ]åòcìËï>È»)qôd!û^í5åÊ/½ôÜ0ª«üP~òK7Ò»ÊåFyT7$fn¥Wù¡ü¥ò+ÊåFT÷Â¿ú«¿:úôÏ|æ3­­­Êò¾ÞÞÞùóçO:51gÆ_ýêW(¿±Õ?÷~éV­ZµÛöOíüâ¿x×]wÅb1åÊOùÓ¬Y³úmÞ=ïÚµKùÁ/¿ôÜmu8°`þÐ|¯ÿ¾á¿/_¾òS~ÃTPPÁÛÛÛsN:æL6MùÁ-¿4ÜÍmu·nÝúÏ!¹üÂWaa¡òå§ü)Ú¼·¯¯ï?Üîü)?åæc²­îîÝ»+++³oËæ-þô§(?å7Leeeá	=ücýû~ãÆ;w9¥¥¥Ê&Hùù¨î`Î93cÆúÿ¯>Q~ÿyù~øá(?å7L'OLùÏý'ä|ùQÝ4î¹»îºë×~í×ÂÂÌ;wÅ]]]Êò¾/.Z´¨°°0//oêÔ©.sFzÑÊo]ºtiê¦+¾ò¯lÙ²åÀç@ ÊåÇHßP~(¿Ñê®_¿¾¨¨ÈquÊå§ü 7ËïÜ¹s[¶lÏ£º(?£T~gÏvæmÏ;sæÌW_UùAß¥K-[£º(?#^~GI¼DåMïÛ·OùA_OOOKKË>ò¥¥¥uuuFuÊX~á5 ¼=6Q~'N°'gÈÒò;sæÌ-[_QQÑúõë»»»ÝkÊåÇ-¿è%á¤½7÷õõéüü|åÙR~éGuW¯^½wïÞ	²3åÊOù¥3sæÌhïQùõöö>ñÄaº¼¼Á8/¿C­]»6å¨îÜ¹swìØqþüy÷òå§üþ][[[Ê7	Þ~ûmåã³üâñxhÚÚÚÁFuÃv¸GùòS~ýZ¾|y´moaaáüùóGáMåòÝ»wÏ;7å?l2ª«ü@ù)¿qGù¡ü.ÄÜÞ½W¯^2ø/^¼k×®.¸/(?å§üÙZ~Ñ!7|ðÁÁ¹QWWgTWùòS~·ýº5gÎðZRXX¸hÑ¢ð£ü`Ë/<VÓrcõêÕFu(?å7±X,åøÑ¹sçrùuvv666.X° å_åÜ¹swíÚåÊò¾hOÎÕÕÕÑÎ]¯_¿¾qãÆ0§¬¬LùÁè_OOOsssUUUÊ³oÛ¶M(?P~dlOÎÉÓ½:+?ÑòÉúúúÜ2eJhÁPFu(?2V~Ñ~½½½97nÜðhù¥Õ­¨¨hjjºråu«ü¬.¿ès~ÕÕÕ!øÂÉ«W¯Fð9?Èxù¥Õ-//ohhèèè°JQ~(?Fªü&ÝÊû*?&NùÅãñðhO3ª[SSþ³sÊ/¿¼[ÉÏÏW~0¼òëèèhhhH¹sÄ¨®ñ¡üP~^ùåG_¹t!ìRQ]Ê1+¿ÁvÚ|ñâEå·U~Ñ!7jjj¦LbTåòc<_xAzöÙgûÍÜ´i½ºÀÐË¯µµu°QÝÉ'WUU<xÐ¨.ÊåÇØ_(¼ðâ4sæÌ?þ8|ùå£«úxò#D£ºË-K9ª»`ÁÆÆFÜ@ù¡üGå÷ÉÍwø¢ª²²²hâþûïéEW~d¯ô£ºÅÅÅõõõáÁfE¡üP~Çò;xÝÚ¿ÿ(,ºò#¥ÙV7Õmnnîéé±¢P~(?Æoù=öØcÑKWt<à¡R~~[Ý9sælÛ¶Í¨.ÊåG_~~~xé6mÚéÓ§?Iú_AAòcâ¨î·ÊQ-¿ðöäOöY[[kÛ^&²4£º¡×¯_01ª«üP~(?²¦üÛßHòcJ?ªû¹ÏnïÞ½wÎ¢üP~(?²¦üÆòcüH?ª[^^¾mÛ¶4ÜP~(?ã½üÂëYòxnúÊ~[Ý|°¥¥åÜP~(?ÊOù1~¥ÕóSê*?Êå§üÈéGuçÎ»÷î3gÎÜîÍ*?Êå§üGÒêmÚ´)<o9ª«üP~(?òcüJ?ª[YYyèÐ¡¡ê*?Êå§üwÒêÎ=×®].ÔS~(?ÊOù1ÚÛÛwìØ1Ø¨nmmmx)ö¨®òCù¡üÈâòKOùE:;;/^òÁÕÜÜÜÝÝ=B?]ù¡üP~÷òË»üü|åÇ8Ç:TYY9yòäÁ·lÙ²=öti¤Cù¡üP~÷òsÊ;	¾¶¶¶ºººÜhhhHsÈåòCù¡üYàüùóöì=öÀà2eJMMM,ËøÇøÊåòS~£§³³³µµ54Í+W&æ½»»»©©iíÚµ)Gu+**Â¹w¾såòCù¡üßê©§fÌ±òîK.-**úö·¿=¡âgÎ©¯¯/..£ºÊåòCù)¿ôÍo~³¬¬ìË[¿¼ó©áëÖþÏPá·ÈùGö¹sçvïÞ=ÞFuÊåòS~#hÞ¼y¿ñðoDÙý×5ÿµ®®.WÐ]]]·£ºÊåòCù)¿TTTáëÔüÊÊÊÜ(Çb±õë×§Õ]°`Accc¹¡üP~(?òrþ=¿Ðv/T`Vm+?Êå§ü2 W?çæ'O¹ÑÓÓ-¿òCù¡üP~Ê/3zê©»îºëî»ï^ú²~ÛÞs!éBØ¥ü_4ª¢0ë~/åòCù¡ü_ÆjiiimmÍÞýù5?ØÎY²hTWù¡üP~(?åGºfmllLù1¾)S¦dÝ¨®òCù¡üP~ÊþÒêÛ³(?Êå§ü¸½5<þ¹¡üP~(?òcøBÏ;t°QÝñvÈåòCù¡ü·­«««©©©¢¢bR*¹7ª«üP~(?òpâñx,«©©2eÊÕU~(?ÊïÏ_Î¹îÓ9ª«üP~(?ß ?¾páÂÐÊ/Û¥ÕÍÆCn(?Êåy÷ÞoGGGòûÃ?üÃ¿]ßûÞ÷N8ñ·Í3gþàþ ÜúÔ§6ßìÙ³·nÝÅ¬¨ð?Ìÿù[dÖÉ'¿ûÝïZdÖøÃëa<Ëâòû÷E¼ü/®ðòüw÷w¸#G|éK_*--|wÝuW8ë7Þ°N:õWõWÖõ£ý¨­­Íz ³Þÿý7ß|ÓzÏr¹üö7.]Ú¿Ê³Õ5ÚÑ^öb´7EçE_Çãá¾X¿~QQÑÀæ!ØØØØÙÙiE)?ÊåwëT~ãVX!uuu%%%¯¸¸¸¾¾>ZR~(?ÊOùe±®®®ÆÆÆeË¥Ü9Ëúõë>lTWù¡üP~(¿Q~£¯»»ûÐ¡C«W¯N¹æFuÊåòS~Y/¼~lÙ²%åÇøJKKwìØqæÌNÊåòCù)¿,ÖÙÙ¹wïÞÛêFÜhiiéîîö¤üP~(?òËV]]]¨¨¨<yrÊQÝÝ»wËøûQ~(?ÊOùe±3gÎÔÖÖ§<äFCCC»?åòCù¡ü_;wîÜ;Rr#ÚV7Åãq0ÊåòS~ÊOùe«h[Ýªªª£ºöì¹té¿åòå§ü_¶êééinnÁrç,¥¥¥õõõþ<ÊòS~Y aùCÕ¥üßäÉ×¯_ßÚÚjTWù¡ü@ù)?åÅå×ÙÙÙØØrç,muê*?(?å§ü²¸ü£º)?ÆW^^^__o[]åòå§ü_v_QÝhÌ¶ÕU~(?P~ÊOùewù¥Õ­¨¨hjj²fåòå§ü__¹tê644ØVWù¡ü@ù¡ü²¸üâñx,«©©I¹s£ºÊå§üP~(¿¿òòr£ºÊÊååê°K|Ñquê*?P~(?__úQÝâââúúú°`ÍÊÊåÅåfTwòäÉUUUÍÍÍ===ÇÊÊå­å~ç,a~87ÃWùòCù¡ü²µüâñxCnÕU~ üP~(¿¿sçÎÕU~ÖÊåòËåòFu/^òM¾£ºÊÊåÝå×ÓÓfT·´´tÇFu(?Ê/»ËïÌ3õõõÅÅÅr£¥¥Å!7(?Ê/ËïÂ»wï;wî`ÜØ»wï¥K<(?Ê/[Ë¯»»ûÐ¡Ck×®M9ª[^^î(?ÊåõåmëêêRêmÚ´)ÕEù¡üP~(¿,.¿mÛ¶¤Õ­¬¬<xðà+W<ìP~(?Ê/[Ë¯»»;$]»£ºsçÎmll¼páGÊåòCùeqùµ··×ÖÖ¥Õ¹aTåòCù¡ür¡üÚÚÚ6ßÚµk>ÜÕÕåáòCù¡üP~¹S~ñx<qÈµìÝ»×!7P~(?Ê/7Ë/µW__oç,(?ÊåûåÊåòCù)?P~(?ÊOùòCù¡üP~ÊåÊåòS~(?P~(?òCùòCù¡üÊåg= üP~Êåòå§üòCù¡ü@ù)?å§üP~(?P~ÊOùòCù¡üP~ÊÊåòS~ üP~(?òCùòCù¡üÊÊå§üP~ üP~(?åòCùòCù)?ÊòS~Êåòå§üòCù¡üP~ÊOù)?P~(?ÊOùòCù¡üP~ÊÊåòS~(?P~(?òCùòCù¡üÊÊå§üP~(?P~(?åòCùòS~ÊOù¡üP~ üòS~(?ÊOù)?åÊåòCù)?P~(?ÊOùòCù¡üP~ÊåÊåòS~(?P~(?òCùòCù¡üÊåÊå§üP~(?P~ÊOù)?ÊòS~ÊåòCùYÊOùòCù¡üP~ÊÊåòS~ üP~(?ß¿;qâÄÒ¥Kóóó,YrêÔ)åòåò#gËoÞ¼yo¿ývxåWæÏ?°üÚÚÚþ÷è:yòdggçÿúñü£ýÈz ³Â¿©á)Ôz ³zzzBùYãY_²ÂÂÂå÷ÜsÏµ®ðoª7ß|Óz ³¾óï§,ëã7¬ñ,ÊïäÉuuuF1ÚF1ÚKÎöF®_¿^]]ÝÝÝ­üP~ üP~äTùMúèäÅkkkSÆòCùòCùÝå¬­­mÍ5áÉ+å¹ÊåÊåGî_yyù¤$ÊåÊåGÎ_zÊåÊåòS~(?P~(?òCù¡ü@ù)?å§üP~(?P~ÊOù)?ÊòS~ üP~(?òåòCù¡ü(?Êå§üP~ üP~(?åòåòCù)?(?ÊOù¡üP~ÊåG.ßoýÖo577_]o¼ñÆßüÍß:vìØÑ£G­2ë?üááÃ­2ë§?ýé¬ñ¬««+7ËïìÙ³;wîüm~&ýè$oí@ù üP~(?Êo4´µµ-0??Ñ¢EÇâYpKï¾ûîòåË6lØpíÚµä³N:UVV=´=j]ÇÕ'.]WK,	1ë<®"G4IK(¿PRRòÁð½¼¼|gÁ-­X±"¼sçÎ=öØcÉgUWW¿ôÒKabß¾7n´®ÈÈãjÞ¼yo¿ývxåWæÏo]ÇUÇÃ?ÊOùå9sæx1LïazgÁ-åçç'¦§OÞï¾¾¾0ÑÛÛë2õ¸JVXXh]©ÇÕW¿úÕ¯ýëÊOùåS§N§ÈðßO<9Ä³à,YröìÙ0ñòË/'?«öíwûq¯êêê¬+2ò¸úà/_þYU~Ê/G¬X±âÜ¹sQç­rgÁ-½óÎ;óçÏÿ6ÿû½.((°®ÈÈã*rýúõêêêîînë<®xàcÇýÿ%¡ü_nHóî7fÈ÷ÞoáÂÉsfÎÙÛÛûÉÍÑÞ0mÇÕ'7?R[[ûÑGY?dêq5é?²_Ö[¹reôwû+xÜÒ¼yóN:Õ××þ~öÙgÏª©©yñÅÃDø^]]m]ÇU[[Û5k._¾l-ÁÇUrZQÊ/Gþ¿	U¾éäÇwÊ³`ÂËð¬Y³êêêâñxòCëøñã¥¥¥yyyeeeÑötpç«òòrïÍñÇòS~(?Êåò@ù üÊåò@ù üP~(?ò³Ê`T¼üòË«V­*¼éîyíµ×þÃó×MYóljiËËËÃ¯ÖÝÝÝo~_VVÖ××w»·	 üì³sçÎI<ûì³¹T~»ví3÷ïßßoþÞ½Ãü§~z·	 ü,sêÔ©Ð4ùùùÏ?ÿ|ü¦^x!3O>3å÷þûïK,é7áÂaþùóç üÜ÷ÐC¦ùÚ×¾<ó¹ç37mÚÜ=Gå¢péÒ¥a:qá«W¯ÖÕÕM6-URR²uëÖäAÕX,ê*®ÛÚÚÚ¯¥ÂéÓ§¯Zµê­·Þ'kjjú-XKKKúÛÎuäÈÁ*mÍ5aþ'sÞ~ûí0§²²21çg)--7UXX¸aÃ/,¿·ßoNEÀX5kV¨>ø yæ~f'M?'OÎ½ÿþûûµyóæè¬ööö¼¼¼×NFç>ðÀ¡âñx87|å4uêÔèãwin'L¤¿é+¯¼³¸|é¥Ù×ïFî¹çÛ-¿4(?1ì¦xÎ4)tXrÙlÜ¸ñúMa"n]tn:Q;FoÝbÎª®®Þ,ÓÇn$ù6|òÉÐvÑ`ë#<æ¼þúëa:|Ó?üð-o'Tc8.¼l)£P3fÌ¿ïåËÃÉð=,yÈÍÞÞÞèeeeááWøäg£Ã·[~iP~c_~AÊòKÌÊ&¦àâÅádh¦èäÒ¥KÃÉ9sæ<úè£¡ØnÜ¸¸p~ï¢%®üðÃµðßÃt,»åíÊ'?þøãäeì3y?þx8ëë_ÿú'?ÑÞºukòBò=º|ùòè-ÉÛ-¿4(?1½ÑuýúõäÝÝÝaf8+Më$º°££#¿Dè$>7p¶_K%ïK%Lôôô>zâÜ[ÞÎ`ÖÏÙ³g£HÓánooOüøñ°)G^~iP~c,ú¬Û7¾ñäÑ¾Númáø,àåË¾uúôé]»vEc¯dÞK§Þ²Ï6oÞòïuuuùin'zí£>N~üñÇé·Ã]¹re87ÚMÖä³¢í|ÃYG¹víZúòKTi´6ç¦YT@ù±hüüüûöEuyþùçn±nÝºP9===ÑPlb;Üès~ÑÇã:::? àÖ­[C'E[Ý&ö«²Ï¢m£ÞÖÖæv¢öEóëîî.¦ü^xáÄ»qáWN>+úÈcø)a%V~QkVÅ¹aÃäsÓ,* üÆ^´ã~yæÿûüuSFéÄ8ib£Gy$9+½úê«iÊïÛóKJJÓÜNXäKlW;Ø/ãÆÂÂÂèWè7Æï½÷&ÿ3fïÑ]o3zK2!êÔÄ¹iP~ãB¨î¹§à¦U«VEØö+¿#GD»Í[´hÑ±cÇçvww?ñÄ¥¥¥Q-=þøã===s[[[/_¬¬¬¬©©©ßmpSaþc=Öoþ`·	íh0ÍþüêêêúíÞ%rùòåêêê°¦M~óçÏ'öP|×®]i­«ÊÊÊ'NôûiP~(?Êåò@ù üP~(?åò@ù üP~(?·ïÿº§Ã,ñ´ÛIEND®B`
