# Supplementary material for: Regulation of piglet T-cell immune responses by thioredoxin peroxidase from Cysticercus cellulosae excretory-secretory antigens
Source: Front Microbiol. 2022 Nov 18;13:1019810. doi: 10.3389/fmicb.2022.1019810 (PMC9718028; doi:10.3389/fmicb.2022.1019810)
Supplement: Supplementary file 3 [file Data_Sheet_3.ZIP › 4. C. Cellulosae ESAs and TPx Induced Th Subpopulation Differentiation/3. SPSS statistical analysis/3. IL-5/2. IL5-48h/2.3 (SPSS data export) SPSS statistical analysis--IL5--48h.doc]

SAVE OUTFILE='E:\桌面\Raw Data\4. C. Cellulosae ESAs and TPx Induced Th Subpopulation '+
    'Differentiation\3. SPSS statistical analysis\3. IL-5\2. IL5-48h\2.1 SPSS statistical '+
    'analysis--IL5--48h.sav'
  /COMPRESSED.
EXAMINE VARIABLES=Figures BY Variables
  /PLOT BOXPLOT NPPLOT
  /COMPARE GROUPS
  /STATISTICS DESCRIPTIVES
  /CINTERVAL 95
  /MISSING LISTWISE
  /NOTOTAL.


Explore


Notes	
Output Created	12-SEP-2022 23:13:43	
Comments		
Input	Data	E:\桌面\Raw Data\4. C. Cellulosae ESAs and TPx Induced Th Subpopulation Differentiation\3. SPSS statistical analysis\3. IL-5\2. IL5-48h\2.1 SPSS statistical analysis--IL5--48h.sav	
	Active Dataset	DataSet1	
	Filter	<none>	
	Weight	<none>	
	Split File	<none>	
	N of Rows in Working Data File	20	
Missing Value Handling	Definition of Missing	User-defined missing values for dependent variables are treated as missing.	
	Cases Used	Statistics are based on cases with no missing values for any dependent variable or factor used.	
Syntax	EXAMINE VARIABLES=Figures BY Variables
  /PLOT BOXPLOT NPPLOT
  /COMPARE GROUPS
  /STATISTICS DESCRIPTIVES
  /CINTERVAL 95
  /MISSING LISTWISE
  /NOTOTAL.	
Resources	Processor Time	00:00:02.47	
	Elapsed Time	00:00:00.82	


[DataSet1] E:\桌面\Raw Data\4. C. Cellulosae ESAs and TPx Induced Th Subpopulation Differentiation\3. SPSS statistical analysis\3. IL-5\2. IL5-48h\2.1 SPSS statistical analysis--IL5--48h.sav


Variables


Case Processing Summary	
	Variables	Cases	
		Valid	Missing	Total	
		N	Percent	N	Percent	N	Percent	
Figures	Control	4	100.0%	0	0.0%	4	100.0%	
	ESAs	4	100.0%	0	0.0%	4	100.0%	
	TPx	4	100.0%	0	0.0%	4	100.0%	
	LPS	4	100.0%	0	0.0%	4	100.0%	


Descriptives	
	Variables	Statistic	Std. Error	
Figures	Control	Mean	10.24625	.152976	
		95% Confidence Interval for Mean	Lower Bound	9.75941		
			Upper Bound	10.73309		
		5% Trimmed Mean	10.24067		
		Median	10.19600		
		Variance	.094		
		Std. Deviation	.305952		
		Minimum	9.932		
		Maximum	10.661		
		Range	.729		
		Interquartile Range	.572		
		Skewness	.915	1.014	
		Kurtosis	1.505	2.619	
	ESAs	Mean	11.13300	.167054	
		95% Confidence Interval for Mean	Lower Bound	10.60136		
			Upper Bound	11.66464		
		5% Trimmed Mean	11.13539		
		Median	11.15450		
		Variance	.112		
		Std. Deviation	.334109		
		Minimum	10.704		
		Maximum	11.519		
		Range	.815		
		Interquartile Range	.622		
		Skewness	-.382	1.014	
		Kurtosis	1.500	2.619	
	TPx	Mean	9.13300	.061437	
		95% Confidence Interval for Mean	Lower Bound	8.93748		
			Upper Bound	9.32852		
		5% Trimmed Mean	9.13222		
		Median	9.12600		
		Variance	.015		
		Std. Deviation	.122874		
		Minimum	8.990		
		Maximum	9.290		
		Range	.300		
		Interquartile Range	.229		
		Skewness	.339	1.014	
		Kurtosis	1.500	2.619	
	LPS	Mean	14.66325	.229231	
		95% Confidence Interval for Mean	Lower Bound	13.93373		
			Upper Bound	15.39277		
		5% Trimmed Mean	14.66322		
		Median	14.66300		
		Variance	.210		
		Std. Deviation	.458463		
		Minimum	14.102		
		Maximum	15.225		
		Range	1.123		
		Interquartile Range	.842		
		Skewness	.003	1.014	
		Kurtosis	1.500	2.619	


Tests of Normality	
	Variables	Kolmogorov-Smirnova	Shapiro-Wilk	
		Statistic	df	Sig.	Statistic	df	Sig.	
Figures	Control	.250	4	.	.954	4	.742	
	ESAs	.250	4	.	.961	4	.785	
	TPx	.250	4	.	.960	4	.778	
	LPS	.250	4	.	.945	4	.683	

a. Lilliefors Significance Correction	


Figures


Normal Q-Q Plots


F£QÙ¿)?"~Q*â¼øâ2?q'¬tèÐ!ÙÖÖ¦ù;Nö&ê-^9ò 'µp4GæÚÓã/éRY/uuu²ØW­Z%Ë6ý¡úÒÄÕ*ù|>¹7Y²få¢ø©²$ÅÓrQEEÅÖ­[#O."ðGDúÅßððpUUUee¥L¨pûömyEOäIiyyyòuÝºu´öK._¾ÿV _Ò5þùôÄY°`Ì¿ÿ~âLÁ¥Ìx±æï8Ù$ýh2òWSS3©ÞpS¾áÄñwíÚµÄû_v²øKÊ·ñz+¯]»6é1oÙ²'ø#"ýâO&Î98èM6Éô5k<N&ä[xÍÝ»w?|øP»RælÞ¼9uww+ßÊ=Ë·]]]2-Tn«¼³íüùó2óæÍÄRÇ`0hÎO¼íÞ$Õêâ#£]8jÊ7Ló¿¤ÚdºµµuddDX'ÚÄñ¸Zå~dÎ¥KdZY­7nT®¬ ðÖ­[2-kS¦yr?"Ò5þ$y-iy]O_YY)Ó·oßV¾×xùvþüù÷¿4>GÀøíààà£ßì?M4ÇØØpAÑÔÔ¤5¿Í_Dsþn*¹­8Æb±ø|>Í6£ù¨¦|ÃãOy_ãÝ»wÄ¤ð¸ZKLæ(-Y²D¾]´hàUþ1::Ê3üQàO|VQQ!/áÑh4q¾zäLý8Qæ¦ÿV ´ësÜO­Fe?µZxq$ÝÛDnj»Ð&²pRtj7ønßð.ý¥êÕtå¢@  ø/ÂsçÎñä"D¤wüIï½÷|?02SñYÒÌ!Ò[WW§råÂ³àOTºÒÚÚºuëÖ9rDf®_¿^¹Éñ7ÙoÊ78þ&2òçÐ?ýò/..9I=Nìúõë÷îUö5Ç?MDàt¿Gß¼_¿2Gù,ð5k§¼ºoÞ¼ùÉñ§~õõõ	A><üùý~elI¼ØÝÝ­ê)9sFówìM&¿ôGYÇ%ÝÉdêð§¼KOì;::*¶[½zµ/@§_þÊ#:ËõeIÊtcccâ¯©¼w3¤ÙODàt¿;wîÄßÝ¥Ì	Iï÷ª¬¬xöIðçÒüùóåkâ'^5óË/¿¬Þ¹lÙ²4¿æ¤n2)ü¥_8ñc5'¨yKuø»~ýzâý¯X±"ñÒ¤e"?åpQÆVåWxr?"ÊüIÊ.ÑÄùöÙúõë×ÚÚªþxÇÔð788(÷&÷)Ú¶mx(~ôÄéìì¬¯¯ÏËË[°`Á;ï¼ó­o+>òôä7þÒ/3gÎÿCüPyMj©NÒ¹sç-Z$¿µÜ¿²c=þgùV¦ütÅÒÓÓ3îÈkWWWSSÜüRn·;>?ìÜ¹SRÁËªF£<¹ÀÑL566öòË/OêØrS¸IÖ¥ìU>¾ÅÓ×ÕÕñCDà(SÞóÔo¼Á!"ðGDE";v,X°@Ù·+¯¾ú*À?""""DDDDàÀ?""""DDDDþüø#""""ðGDDDDàÀ?""""ðGD¦3gÎL&ÁðüóÏOö¶õõõóæÍñ92-s'±ÁzÜÔ®3ÛN¹ÁÁÁÍ7ÉÂY°`ÁÎ;#H6â3ùø#¢9Ñhgtb±Ødo»oß>¹mGGG|Îo¾)söîÝíøç-~ÜÍ7>|xìØ1ùA«W¯Â]õööZ,ðGDàô±½xg~]nÛÜÜ³|ùrsíÚµÌ<ÈCâÚ'OÎÊDþhåH;wïÙ³'éjßúÖ·î!>p(Ó÷ïßi£ëÖ-ÅRTTd0,YréÒ%ÍKüééoòÎ;ïL&³Ù,?H¤Í&·¿cÇe SxºbÅ¹7/÷|ûöí	.ÆÆF¹çh4ªyi¥ä÷ûåAÊB8sæz	«d»âïÀÍÿé×_]¦Ýn·0K&ÞxãÄëtuu)ÈKlÓ¦MrÑ#GdZ¾Êt[[rÑ²eËÎ?/7oÞùUUU÷øÒßDH÷ÞïÉÝnW?ø-[¶È´Ô©S2±oß>¹páBîëë	e`Rz±Sñ+ýR:pàò³êô»§¿+þ8üÑÌâO¼"ÓcSÛKóM~¿_.Z³fL¯]»VMüÒë×¯ïÞ½»©©IæçååiÞ[tÒßDy`¥¥¥êÛVVV*þáÃ2!ìrM^²dè*Õ0fið~)*Ór©ðÿÝÓßDþhfñ(T|IJ¤U\,×D"ÊÞU£øða¹ðÓO?UHóLä&©H1q0OãÉ'ËÊÊ9Ê àDFþjjjdþÈÈHz¦G^úé©-p""ðGDÓ¿ªªªÄ(eðlØl6¹4þ5>¿  @æ<|Üñþ&ñ&SßvþüùÊm^,;þ¼ÝnO2·Ý»wËõê&²&¿©-p""ðGDÓ?å#®GQÞöæoNÄ"'O¡½÷ÞI²¹qãò^Àà/ýMdï¿ÿ¾LlÚ´IÛW^yE¦;Ößß¯ìêf³Y¦?ýôÓ»wïÊDMMÍËðð°PlñâÅ·nÝP*CëÖ­àRJVv=+;~÷©-p""ðGDÓ¿X,&À*~±úÒ[dttTÙå*_ÅLñùçÎ«¨¨úìØ±cøKÏ'._¾<þ¹ÄëD"­[·Ê#/((X½zµòÁ^1_kk«òñáæææÏ>ûlâKF~RÙ©-Ü³gò~¾,¥Äé3gÎÈCR¡ô»Om?""""ðGDDDDàÀ?""""DDDDþ¦¡üä'þ¡¿üå/õ«_ñw£çþó?ÿS9ré¹ý×ýïÿþo»sç;ýoîB¡ËAÿ;ð7mýÅ_üø/Ã?ôÓO?ý÷ÿwþõÜÝ»wúÓ²tÞ| >Áéªú§âÿQ:ï¿üåµk×X:ïÜ¹szû¿.øàÀ?ðGàü?ðGàü?ðGàü?ðGàüø#ðþüøþü?þÀ?ðþÀ?àÀøàÀø#ðGàü?ðGàüø#ðþÀø#ðþü?ðþÀø#ðþü?àü?þÀ?ðþÀ?àÀøàÀ?ðGàü?ðGàüø#ðþüøþüMO===K,1ê¿oðGàüø,ðSø[¼xñ+Wdâøñã555jü]¸pa$³*þíßþmtÜ_|qõêUÎüÃaëííýüóÏY:ßÜõôô°tàïW¿úUhã/±¢¢"5þ8ðãÌÖÕÕåóù~L:îâÅ~ø!ËAç= óØÜé?YAlîØÜiøÿ¶µµ±ÛØíËn_b·/±ÛÝ¾¹¼ÛWidd¤µµ5?àÀ?ðãø»sçÝnsõEàÀø#ðþXà/§ðç÷ûW®88¨y)ø#ðþü?øË)üL¦y	?àÀ?ðËøKø#ðþü?øàü?ðGàüøàüø#ðþü?ðþüøþÀøþÀøþÀøþÀ?àÀ?ðGàÀø#ðGàüøàüø#ðþü?ðþü?þÀøþÀ?àü?àÀøàü?àÀø#ðþÀø#ðGàüøàüø#ðþü?ðþüøþÀøþÀ?àÀ?ðGàÀø#ðGàüø#ðþüøþÀøþÀ?àü?àÀ?ðþÀ?ðGàÀøàÀø#ðþü?ðþüøþÀøþü?àü?þÀ_NvíÚÁøàÀ?ð³E£QÁVKKK~~þ¼yóÄàü?ðGàÀøËÁó*//D"àü?ðGàÀøË¢Ñ¨Çã±X,ÊP_üNç½÷ÀøàÀ?ðõ±Éd§J,xôèQq¡'øþü?S/»Ýn³Ù¬6_yyy¿®0øþü?SI¶íC§f_ss³×ëÕÉPøþü?S/ýPÓéÔÛPøþü?SI6æv»½°°0É|ùùùV«U·Càüø#ðþüM¢p8ìr¹jkkÕC&I^ýC¡Pý:àüø#ðþüi'/ë6-ÕPÏçÅbY÷K?ðGàÀø#ð÷[B¡TCÕÕÕrQvõ?ðGàÀø#ð§Q,óù|6M|æÂÂB¥Càüø#ðþüýV¡PH^Ä5Ï[ër¹ÂápÎ¬&ðþüø4GñÅ¼^¯ÕjM5ÔùuðþÀø#ðGàüMÁ`0Õ©ØjkkÝnw.õ?ðGàÀø£9¿h4j¨¯¼¼ÜápÌÍ5øþü?Êü§Ó©y*6³ÙÛCàüø#ðþh®à/z<T§bs8Â¹¶Àø#ðGàüQâOT'¶K5Ô'"ÌS±?ðþÀ?ðGà/]ápØívkõF§Ó9úÀø#ðGàüQâO¶´©ú,Ë'"ëü?þÀe7þ¡>ÍS±Æ]»v1ÔþÀ?àr²iµÛí©úràTlàü?ðGàüÑ_8v¹C&iûöíýýý¬ðþüøÝøÅb>Ïf³iõY­ÖÓ§OÏÙð?ðGàÀø£ÜÁ_(r¹ÕÕCòâË»úÀø#ðGàüQÖãOêÓ<ôÂ/bxWøþü?ÊzüB!yI5Ljó-°££C®ÀÒàÀ?ðGÙ¿X,æõz5údNKKËéÓ§êàü?à²Á`pûöí©úCàü?ðGàüQÖã/>º¥¥Eý^e¨O^Uù/øàüøõøµ¹ûvÍS±UWWwttÃa'øàüøà/»ñD<ÅbQO èp8d-³ÁøàÀ¿¬Ç¬¾¶¶¶ÍS±y½^vï?ðþÀ?Y?!ÛínhhÐês:ü?ðþüøËü]½zµ­­­¢¢BýI«ÕÊPøàüø#ðø»xñâ¡Cjkk5OÅæt:Á ü?ðþüøËúdC÷â/iõù|>ÏþÀøþüeCCC.Kó]&I^9>3øàüø#ðùý~Í¦þo~~¾Ìg¨ü?ðþüøË¡¾¥KjùÏþìÏêàü?ðþüe±XÌçó½ðÂêS±ÉÍ&¯S>·/?ðþÀ?z):NÉ¤ê«­­u»ÝñS±?ðþÀ?ðGà/[Åb^¯×jµæçç«úìv»zËþÀøþÀ¿ì+¤ê3ÍCàü?ðGàüøËÖ¢Ñ¨×ëµX,§bs8ãnÊÀøàÀø#ð±Os¨ÏãñLðTlàü?ðGàüøÓoB:ð.ÕP pRwþÀøþÀ?=&¥TCÅëõNp¨ü¿9¿ÁÁAÉþü?YW8v»ÝCòÒæt:';ÔþÀ_îã¯»»»®®N$àÀø#ðEÉVÈn·«ÏoµZ½^ï´ü¿ÄßêÕ«åEið÷þûïßÌlÂMÙ Þ$÷ÏÿüÏ?þñY:O¶_|ñËAÏÉæNþ»ËrT»wï~ægÔCF£Ñáp|yz7wÿðÿÀb×ygÏÍüæ.ñ÷¿15þÞyçg6¿ßßÓÓósÒq½½½.]b9è<Á¼L²ôÜßÿýß³¹xòzdµZÌ÷õ¯ýðáÃ?ûÙÏ¦ý~üñÇlî²bsùËøc·/±ÛÝ¾ÄnßY,¹ÚÚZõP_uuµW¹În_vûæàn_ðGàüøÓg>¯¥¥EóTl6M.wõ?ðþÀ?ðGào6SúÔCµµµrQªS±?ðþÀ?ðþÀ_ÖÅä%Üf³hõeþõü¿_ªÀ?ðGào¦èèè0Cn·;Càü?ðGàüøb±XWW×ªU«Ôïê9/½ôÒÕ«W3ð®>ðþÀø#ðþüÍlÁ`pûöí&Is¨ïèÑ£CCC:y¨àü?ðGàüøbápøÄV«UZc·Û3ó^ðþÀø#ðþüÍlýýý»ví*//Wõ=÷Üs3¬¾ìÅßÐÐ¸««ëÞ½<_Àø#ðGàüé½h4zúôi³Ù¬6_IIÝnðfþ~ô£=ÛôìÒ¥K8ÀSü?þÀN©újkk;::fñ¼Y¿®®®/ùËþtÓìï;~úi4Ïðþüø:*¸ÝîÍÓrØíö¾¾¾ìúfßüæ7¿³ö;ü-ëZ,Ïðþüøº¨¿¿¿½½=Õ»ú<~>Àø«­­u|ß?ùö+_ùÏðþüø³Y8îìì©¨Í'Ü°aC¶o+fqä¯e]K"þ¾»þ»ü?ðGàÀøb±Ïç³Ùlê¶HGÍwõéïù;uêÏðþüø-¹êêjÍ¡¾íÛ·ËÆAoÇêËFü=züißåÓ¾eeeo¿ý6Oðþüø*>Ô§þ$d±X:;;sc¨O?øôø8]ÓíqÁøàÀåþòZ y*¶òòrÃÑßßÃë3|?ðþü?øÅb^¯×jµ¦êK£ÑhÎ¯#ðþ2¿yãþüøÓX0t:©úä¢@ 0wÖøÆ_ÞxðGàÀøò¢Ñhª¡>#óçÈPø³¿YüøóøN§SóøÌ&I.sv?ð§/üoÚ´	üø#ðþ¦P4õx<f³Ym>e¨ÏçóåÒA[ÀøË>üÉ¿ïù#ðGàü=aòüu8©údËÏEÀøü566ª¢Hüø#ðþ&R8v»Ý©úl6Càüéòä42!æÿý÷e¢­­üø#ðþÒf¨¯¶¶Öår1ÔþÀîð§<EeB´'7oÞmºL?þÀfÊPðNm¾ÂÂBÍù-<ø#ð7ÑÊÊÊä¹ÚÝÝdâÕW_U&8Ô?àOlºÅv"<Í¡>aNü¿Âß;âïH|ÍfðGàÀøSÒ¹TCv»g.øY?éµ×^?¾LôôôÈ@°©©i¦:ø#ðþHÿøÅb>/ÕPÙlf¨ü¿¬Äß¬þü?Ò3þB¡Ëåª®®Öêé¥xª?ðþÀ?ðGY?e¨OóTlÒÒ¥K:ÄPøY¿å/äÀ¿9¿P($[fÉ¤6_III[[[__ü¿ßâÅÁOûø#ð7ðÅ¼^oª¡>³Ùìñxfú°ÿàÀ_Fñ'Î§wooo7åàÀø£ÙÅ_ p:CC®Àà/ñ'Ïpyg~;þü?üE£Q¯×k±XæiÕÜÜ,2Ôþ(ñ'yòlß²eËÈÈø#ðGà/ñRÍh4îÚµ+²èÀå>þ¤¨7|àÀ¿ÜÀ_4õx<f³Y½©ÏÏÏ·Z­òò&×a¡?+ø[´hø ðGà/'ñ'Ï¦TC&I¶Ã¡PeþhÎáOÙdþüàÀø£*¾þúëÏ>û¬æP_KKKWWW,cA?£ø«¬¬äþüåFò?ùöövÍ¡¾êêêýû÷±ÀÍuüùý~Ù(ìØ±#ÃoøþÀMWHäèÑ£K.Õ<Ífí-CàÀßoî"E|àÀ?ýwõêU±O½æg:::8ø#ðøÀ?ºm`` ³³Ss¨¯¼¼Üáp;vljçö%ðG¹¿Ùüø4dËùÂ/¤:ÛíVú¦pn_4Wðg2-ZÔßßþüøÓm¡PhÿþýF£1ÕP_Òü?)3²íÈüCþÀM¤«W¯nØ°!ÕPÇãÑü¸øþRvéÒ%ÙìÝ»wxx8[sðGàüQN§ÉdÒê@?ðGà/õ]ði_þtS4Í¡>Åâõz'rd.ðþü¥Oûø#ð§îÝ»·wï^Í¡>9îPøþôø#ðþèÑã¡¾'NÍfõPÌ±Z­^¯wÇgàÀøàÀ¾»víÒ<Édd(ò?ðGà/]ccc«W¯.**-Nqqñºuë2°YþÀßÜ,»Ýn³Ù¬6_~~¾Ífóù|O~*6ðþü¥lttTó3ª_ðGàüÍµäoÛáphõUWW»'êàÀßD«««íÎ5kFFFäÛáááµk×ÊeË?þ¼4CÓ5ÔþÀ¿VPP  ÄíÎØØÌùàÀ¿'Iþív»OÍ¾ÚÚZË¥m&àÀ_Êòòòd3$àÏF£2C½ø#ð7µt;áæPp0äàüøK²ÛwåÊÊn_ù*Ó2gÉ%àÀ¿I%5Íj¨ÏívÏÜPøþ&hOó<þ(óøóûýg`` [H(J5ÔW^^îp82ÿWþÀ¿t®[·®´´4//O¾®RæÌôCþÀ_R±XÌjµ>ýôÓ¿ÿû¿o6KJJÞÿ=/yÀ>Ïf³iM~Lõ?ðGàOï?à/©þðÕÕÕ;ÿïÎ=?Ø#ÿþÄþ'eee:¡PH6b§bSú&u*6ðþü?ðþh.âOä·éO7)òSþ£ù¯¿þº~~ñX,æõz­Vkª¡>Ç3ÓIàÀßTo9^yyyàÀe%%%òßþö·¿÷½ïéáWN§3ÕPëCàüø§¼Ô?4+øklhüCÛ&âïÙ¦g;::fñ7F£©údÌKu2ÔþÀ¿)¶mÛ6e»vüøqðGà2¿Ó§O¹âËmßkûßa¿oûé§ß1¶··kÍd29N¹×øþÆOþe»f±XùþüQfð'utt<õÔS_ýêWçw~çgÉü§=¢Ñè'V­Zj¨o&NÅþÀ¿YÀßúõë­Ûùóç3óÐÁ?ð§Y$¸Ák``ÀétFÍ¡>ÙdB¡,ZGàüøKÙ©S§­Û5k2ùÐÁ?ð§ÄgéÒ¥C6-[úÀø#ð7~£££Ë-S>ÛqéÒ¥?tðGàüÍnéßÕuCàüø§(Û¸7ÎÊCþÀß¬:d6ÕCÙ;ÔþÀ¿ñnÉqþüÑÃ____IIzW]]ÝÙÙ9[,àÀ_&ð7^üø£À_wõnØ°Áï÷çÀPøþôø#ðþf:ù[jkká©úÄ÷îÝËÕuþÀ?ðþÀÍü)ïêÞiÍápr~?ðGàü?ðG¹?ÙªlØ°As¨¯ººÚívçÈ:àÀøàrjó´··gþL!àÀøàÀøæb±XWWWKKæP_ss³0ÌÍuþÀ?ðþÀåþÓé4LïêÛ¾0ãëü?ª[r?møF£^¯×b±hnµ¬Vë'réXàüøNü%ÒOs3Êqþü~ð§õiÍh4¶··ËX/àüøP.­ç/¾822"ßÊ×µk×ÊË/?4»øF£Çl6«ÍoµZsãTlàüøË(þ***d3¸õSþ3ý÷,ÓUUU¡¾¾þÒ¥KàÀøxògàp84úª««].W(bE?ðGàoJwñ8_þü=­­­ÇnÜ¸üøãÝnwª¡>ÍÆPøþ´ÊÊJÙªÔFGG=>	æ5kdÌÂ®¨¨P^D&I?ÙÄ_Ïl/^ìîî¾N:îÿñÿîïþå óÎ=ûÉ'LïÊÿ¿ûÝïªÙ·páÂ-[¶|ôÑG,y6w9¶¹ÿÌ°ô¿¹Ëüqü]¾|Yó~úéÞsâGFÔü:u*Ù®Ò××"×ßßÿüå ó>øàÛ·oOË]öÙgo¾ùfuuµz+TPPðï|'óÜH`ñóÿå çþå_þÍþ;wîÜtmî&ÞãOºyóæâÅòòò,Yrÿþý'¿ÛÄÇ²g·/±ÛÝ¾ÉÀf³i¹¶¶vNÝ¾ìö%vûft·ïÌUYY©¼P¾ªw"?sòZË%¼SO h·ÛùàÀ_¶âOþOÿÎ;ïÈ|mmmþæ2þb±ÏçÍB~~¾f³¡>ðþüe7nÜ¨©©)..VvÔVVV<yòÉï¶»»Ûh4ÊVUUõôô?sÊPæ»úÊËËkü?ÅrçÄSº)ÓÑþüå6þ¡>«Õj¨ÏãñD£Q&øþ2?£Ñ(â7nÄñ×ÓÓ#Ó¥¥¥àÀMÁ`ÐétL&Í¡>¹S±?ào6ñ§l	²MçÜ¾þh²øÅb^¯7ÕPÅbKêþÀßìãO9È³2Ú'øÛ¹s§L«Ëþü&þ@ª¡>ÉPø#ðþô?¿ß¯yç+W®?ÑhÔëõZ,ÍS±Y­V¹S±?àOwøS^nOûÕÔÔÁ~èàÀ_ööWõWßÿþ÷ËËË5úäÙ=îê	üø³¿Y	üøËº¢Ñ¨Çã1Í©ú|>CàÀøÓ;þâówÿþý?¤$+ÅáphõUWW»úÀ?ðÅøãÓ¾þHÃn·[s¨¯°°Ðf³1Ôþü¿¬ÁßÂç¥­¬¬üø³ÉZ°Ûí"<õÆ¡¶¶öþèþã?þ¥þü¿lÂß­[·ò?·Gb"¿S§N?s­p8ìr¹w©ú§íÎíKàÀøeüÅêÍô^ðGàOÿÉóQlj¨ÏívãWàÀøËbüÍVàÀßÅººº:::N8qïÞ½)ÜC(J5ÔW^^îp84WøþÀ_ãoll¬¦¦¦¸¸8>gþüù¯¿þ:ø#ð§óDõõõÕÏT£ùË]VZZê÷û'~sYÎ6MóTlf³9i¨ü?à/wð·`Á¤ü*[ÿ½÷?zîë_ÿzssóìQþýÁüøoÜñ¿H$"°[ºtiª¡¾ü?à/ñWPP ý¾¾¾øù[9ò*þüé¶¡¡¡ÊÊÊÝ»vÇñ'ÿ=»ìÄ©nâóù6lØPQQ¡9Ôçñx¢Ñè:øþÀ_ãOùÀoÒv}ð?ðGàOWÉØ¾úÕ¯&ÊOþãßp¹&vtthÝI èt:'2ÔþÀ?ð;ø«ªª×­[·*ÿéÝ³gÌ1àÀnÃ%%%í[ÚñW]]úôéøu®^½ÚÒÒ¢ù^Åâñx"ÈÔ~:øþÀ_ã¯··Wó Ï===àÀÛ¶m[õ3Õmßkömß¶Ýü5sCCü/Nÿ~ÍwõL&§Ó900ð?ü?à/ñ'Ý¹s§¾¾¾¨¨(//¯¸¸¸®®NæÌôCþ¼7ß|³´´´²²²¬¬lÝºuó7c·ÛKJJÌoµZ½^ïtü?à/»ñ7+?ÓÕgvàÀÍwõÉLË¦÷'?ðGàü?ðGàoJu^e¨ïôéÓÓ5ÔþÀ?ðSø»qãrgå¾'Oþt[8gæP_CCÃLõ?ðGàüåþ.ÙPð§L<xüøÓU±XL6CàÍÏÏùréõ?ðGàüåþF£¼rÜ¸q#¿òLàOWÁTgà]ºtiggçÐÐP&øþÀ_ãOyýxp`gÙ¦Ë´Á`þf·X,¦CÞÂÂBù'øþÀ_Öã¯²²R9ª¿±±±;w*Çþf«@ àt:åij¨oÜsø?ðþÀ?íü~¿æA¯þüe¸h4êõz-ú)YRRb·Ûå]ü?à/ñ§¼Ü655)ö-**ª©©	3ýÐÁ¿Ä¡¾òòr5ûÌf³Ûíò©ØÀø#ðGàO?õy<áÚ|AÃ!(ÔáÃàÀøàÀßä_Sl§9Ôg±X¼^¯¸P·ü?à/»ñ÷ùç/Z´¨  @^uêëëÁ¿(»ÝîTCN§SCàüø¹?Ï§ùþþ~ðGàoK5Ô§MçCàüø¹?å Ï­­­ÊÛÉGFF6nÜ(sªªªÀ¿'OêÓ<>³Édr:ø|øþü%ÜÅãO	%Óñ>?SNþÂm6æ©Ø¬V«ÏçËÌ©ØÀø#ðGàï·RFþÆÆÆâsFGGù#ð7åB¡PªS±L&ùË+dûjàÀøËbü)ïùkmmóÉ·<hnnæ=þ&r*6Í¦>ÌùY=ÔþÀ?ð;ø7^3´ÿüQÎàOê«®®V?jkkå¢êàÀøËüåÁ`þ4óûýV«U=ÔWXXh³Ù2ÿþü?úüQöâohhhÿþý.Ôês»Ýáp8·WøþÀ_ã/ÕñïÜ¹þü%F½^ï/¼ þ¯Ì±ÛísçÄtàüøY?yÝzíµ×f¾ôÒKêÀ_¼þþþýû÷L&õPÙlCàüø¹?A¼UVVÞ¿_¾÷Ýw´z«ø£,Â2Ô·jÕ*õ»úJJJìv¿«ü?ô(ßó÷ÒK/)¯gUUUÊÄÚµkgú¡?Ò3þúûûrÌ¤î¹ÎÎÎ¹6ÔþÀ?ðSø._¾m;tèP:ø#â/=zÔl6k~W88wÞÕþÀ?ÊeümÞ¼Yyys¼øâàæþúúúRõ544¸Ýî¡¡!ÖøþÀ_.àÏ`0ÈË[iiéõë×%¼ç¯  üQÎãOHwôèQÍã3ûûûsæ´àüø#ð÷ø.æÍÛ½wÒL»ÝÎ§)·ñ'/§bSÞÕçv»#«ü?àoçO^ÁåþB¡Ðþýû5úFc[[ÛLÕüø#ðþf³ø£ãOÀ---êã3ççç777>:²üÁø#ðG9?yÍKÜ±þ[ðGÙ¿¡¡¡C544¨ú.k×®;øþü?ÊnüÅb1Ïg³ÙÔCÒªU«äùÌ'9Àø#ðGàüQÖã/É_æ©Ø.Ý»wåþÀ?à²ÊPÕjÕ<>sKKâàÀ?ðGY¿`0èt:5újkk;::êàÀ?ðGY¿h4êõz5úÊËË9øàüøà/GðN§O=Ôg6Ýnw8fI?ðþÀ¿dü¥üÞð§õY,õ+CàÀø#ð7Nyãe0Àé@@lj¨Ïãñp|fðGàüøÓàÆÅ_8v»ÝÂ;Í¡>§Ó)(d?àÀøY?)ÕPÅbñz½õ?àÀøY_8~ë­·~÷wWm>ÉÄPø#ðþü?ð÷DB¡öövù:»§¸ýéOj·ÛÕ§bËÏÏ·Z­^¯S±?àÀøOßïÿÒ¾ôµ¯íÛßþöòåËeZædø1ÃaËU[[«9Ô'$ÂSÖø#ðþü?ð÷¤E"ÒÒÒ6îùÁåßÆËËË3s<åTl6Ms¨ïßü¦Pø#ðþü?ð7mùýþººº¸üõÿ§¾««kFn(r¹ÕÕê¡>)ýìg?ãpàÀø#ðþÀß4çóù/_¿çNþÊgâÇÅúÔ§b+,,ùñ¡¾)ÛÀ?ðGàü¿qBÅÅÅ¾õÏãòi3í§$ëÚd2©újkk].WÒfðþü?àüÍH?øÁªªªþøå?ùÉ×¯|å+íííÓuç±XÌëõZ­ÖTC©Ö>øþÀ?ðþfªýèG/~ê©§D~o¿ýö´|Æ":Îõ?ðGàüøà/ûF£SêàÀø#ðþÀ_6N§æ©ØjkkÝn÷¤þÀ?ðGàü?=F=ÙlV¯°°Ðn·OÍpàüøþÀøÓW3Ã1]CàüøþÀøÓcB:Ý´õ?ðGàüøàO_ÍèPøþÀ?ðþt®££Cx7£Càüøþr&	üe]b»5ûÌfóôõ?ðGàüøËüuww×ÕÕ	À_¶$¤;tèÐÒ¥KÕæ+//w8`øþÀ¿lÅßêÕ«@ü½ýöÛ¾Ìvþüù>úÈGªd]üÞïýÞSO=¥ù®¾ööö¿ýÛ¿ÍÌ#$«5¢óÎ=ËBÐylîô;6w©ÊbüýïCL¿/þWfûäOÁàÑoØ·oß3Ï<£6ß¾ô¥¶¶¶îîî?¤[·nõôô°jtÞ|ðë_ÿå ç®]»öÅ_°t¾þøãY:ïÜ¹s£££þ¡¹?vûÎbòÂÂB5û,ËéÓ§£Ñè¬<0vû²ÛØíËn_b·o6á/ð§ÏB¡Ðþýû«««5ßÕ·ûöþþþÙàüøþ²	³^,;ú´ÕjÕêùòÇ=[CàüøþÀø¶Á Óé¬¨¨POfîÚµ+èê?ðGàüøËnü¥üÍhÊPßªU«òóóÌ's^xáýõ?ðGàüøàoê¥êäz~üàüøþÀø¿H$âñx5úZZZ|>_,Óÿ/þÀ?ðGàü¿tõõõµ··«úd9B¡,úuÀø#ðþü?ð§Q8v»Ýf³Ym¾ÂÂÂ6øýþ¬êàÀø#ðþÀ_ºÄFv»]ó -.ëÞ½ÙûÛ?ðGàüøàïÒ	ìjkk5úl6ÛÕ«W³q¨ü?àÀø¿`Èáp¨Ù'ìèèÈê¡>ðþü?àüýOCCCJ3Ôç÷ûsïiþÀ?ðGàüÍ-üÅb1Ï'¶Óê[ºtiggg8ÎÕ§øþÀ?ð7WðÉdRO h·ÛekóO3ðþü?à/ÇñÅººº¬V«úøÌÙlöx<Hd<ÍÀø#ðþü¿Å_¿ÓéÔê«¨¨Ø¾ kO3ðþü?à/×ðÅäIóTlÌ÷z½sg¨ü?àÀøËYüÁíÛ·WTT¨Íg4N§?ÍÀø#ðþü¿¬Ç_$9qâÄªU«ÔC2§¥¥Eþ¶¢Ñ(Ï1ðþü?à/ëñ×ÑÑQ^^®ê«®®v¹P§øþÀ?ð;øóx<êã3û|¾8øþü?ðþF£ÊÈr|æ¡¡!Kàüø#ðþÀ_ÎâOr»ÝW¯^å)þÀ?àüÍ	üøþü?ðþü?àÀøàü?àÀøàÀ?ðGàü?ðGàÀø#ðþÀø#ðþÀø#ðþÀø#ðþüøþü?þÀ?àÀøàÀ?ðGàü?ðGàüø#ðþÀø#ðþüøàüøþÀøàüøþÀ?ðþÀ?àÀøàÀ?ðGàü?ðGàÀø#ðþÀø#ðþüøþü?þÀ?àÀ?ðGàÀø#ðþÀø#ðþüøàüøþü?ðþü?þÀøþÀ?ðGàü?ðGàÀø#ðþÀø#ðGàüøàüø#ðþü?ðþü?þÀ?àÀ?ðGàÀø#ðGàüø#ðþü?ðþü?þÀøþÀ?àü?àÀ?ðþÀ?ðGàüøàüø#ðþü?ðþüøþÀøþü?àü?àÀ?ðGàÀø#ðGàüø#ðþüøþü?àü?àÀ?ðþÀ?ðGàÀøàÀø#ðGàü?ðGàüøþÀøþü?àü?þÀ?ðþÀ?àÀøàÀø#ðGàü?ðGàüø#ðþüøþü?þÀ?ðþÀ?ðþÀ?ðþÀ?ðGàÀøàÀø#ðGàüÍüõôô,Y²Ä`0466ªÿ¾Á?ðGàü±À_NáoñâÅW®ãÇ×ÔÔ¨ñwñâÅÑÌ&O³`08J:n``@þÛÀrÐy¿H$ÂrÐsò¨/¾øå ç~ñ_|üñÇ,'øûõ¯áÅøK¬¨¨H¿¿üË¿¼Ù>üðÃ>úè"é¸.?å óÎ=ËBÐylîØÜQönîr½½½mmmìö%vû²ÛØíKìöe·o.ïöUimmD"àÀø#ðGàüåþæý&åÛ;wîØívy9W_üøþÀËüe=þóûý+W®Ô¼üøþÀËüåþL&Ó¼À?ðGàÀøËeü¥üøþÀËü?ðþÀø#ðþü?ðþüøþÀøþü?àü?àü?àü?àÀ?ðGàÀø#ðGàüø#ðþü?ðþüøþÀøþÀ?àü?àÀ?ðþÀ?ðGàü?ð7×ð÷ÃþÐëõd¶?üðã? ÷É'=å óþú¯ÿúæÍ,='»ÞÞ^ù	,X:ïÈ#¿øÅ/2üCÃápnâïÆöìù""""J(ý®ÑyÇÍÀø#""""ðGDDDDàÀ?""""ºë³Ï>kjj*((X¿~ýððpâE~¿¿®®Î`0Ô××www³¬f¥ÁÁAÉÿöÚµkUUUÊJ¹téRâ5Ó:YG===K,9c>×ÒæÍãC¿«)ÚívyåZ¸p¡¼T±èt¸ô@Ã)[¶l¼ ÉDÿæÍ/ª¨¨¸uëLÈ×ÄMK0òäI|jmm=vìL<xpãÆWNséd-^¼øÊ+2qüøñ×ÅÄèàOÏ«iß¾o¼ñÆÃE-béépé<S&*O%^$Ï¨;wîÈ|åÙ5+­^½:$>Óäé¤+vll,ééæ"ÒÉ:J¬¨¨¥§Ïuôúë¯¿õÖ[àOÏ«©¾¾¾¿¿¦çu¤BðNYccã7dâÝwßMà£ÇÃ¹òú$«Y¾ööö²¬f«ÄgZâ:JZ_i."¬£xòjkkc¹épÝºu«©©I^ÏÀÎ7w×&QÅ§~ÊrÓá:Ò!x§L6555²näºICË-Sþk%«pùòå,+=<ÓòòòâÓWKséd)´¶¶F"×Ñºuë._¾tÒáæîÈ#~ó¶u×Ásxü>ÿüóººº¤ÿZ;A~¦UVV=z<Æ.ÓWKséd=z¼Än·ß½¦Ïu4ï·c¹évsÇËÎ×Á8e/?|øð­·Þzíµ×/ª+ûúúð,+=<Ól6Û;ï¼#òµµµ5ñji."¬#¿ß¿råÊÁÁAn×æõIo«ióæÍï½÷LÈÙlf¹épé<S&¯F,(((hkkÅb«öóÏ?&`¯2Í²ÒÃ3­»»Ûh4æååUUU)Ó_Aó"ÒÕ:2L*é|¿¬XMÃÃÃëÖ­§¦¦¦@ ÀrÓá:Ò!xÍ%§²À?""""DDDDþüø#""""ðGDDDDàÀ?""""DDDDàÀ?""""DDDDþüø#¢9Þ»ï¾»bÅ¢Ç=ÿüó§Nú­í×ã²fk«õhM&üjH$i¾Ì1UUU>ì?"ÊÊöìÙ3OÕk¯½KøÛ»w¯Ì<tèPÒüÎÎNÿê«¯Ná>Àe_×®]ÖÃÇwäÈùVf^¿~=gðwóæMÙØØ4¿®®NæAðGDàæD/¾ø¢°æ7ÞHùæoÊÌ^z)>.]<,Y"Óñ+?xð ­­­´´T.ª¨¨ØºukâÞUÏ'Àä¶]]]I9eee+V¬8þ¼|k³ÙØ¹sçÒßr<$å¢.¤ÚÊ+e~OOO|Î+WdÅbÏÙ·oÑh»***Z¿~ý;wÔøSßÒ4ÀÑ,·`ÁË­[·gÞ¾[fL¦DÜ$ÕÛÛ«víÚ¤¶lÙ¢××§y+å[åÒuëÖ=|øPøXPPÅäRù*x*..VÞæ~dBóá©ÓãÇ'6îËcÇÅåt'Ï?ÿüdñæ¡ø#"ý=¼Û¬yób¸Ù¸qãÈãdB¾]³fr©bÊ M¹¨µµU2éîînåNïs÷îÝÂ;e¯ë+¯¼"sÎ9#ÓòU¦_~ùåqïGà(ßÊæo$ ?¾ü¾ò­|G.âS®PUU%7_áÑovMi*?""]àOÒÄ_|¾ÅLÒ;wä[aòí%KäÛEmÚ´IÐ6::¿¹NÒXZüVÊ··oß_YÀßó+_eÚçó?Mùöþýû-Õûó¶mÛ&½õÖ[~³këÖ­Wþ¤MMMÊÀädñæ¡ø#"ýá®ÄHDfÊEi¸§a Pü·Nüzê½±IJ<ÀLWTTD£Q¹ó²²²ø¥ãÞO*%uãÆÅ©2-_eº¯¯/~iww·<ÍÝÇÇ_JDàhöSÞ÷vàÀÄÊP>ð_ààà z@ëúõë÷îUvÂÆÕ¨ËÅ÷«K´-[¶(åk[[[|~ûQFÚîÞ½«|ÿþýôÌ]¾|¹ÝFÌxòÉ_¹èÂÃÃÃéñ©²4â¦y¨Dþf?åáàÁÊ¡^> þpÆ5k:ÑhTÙ'ÿd®ò?å­r@ ñÍ·nÝ*TR>?Ø&Ñß*?Ýï÷Çç§¹åM~Êþ"rÍ4ø;räH|LN~åÄ·?öõõÉO7Ër5Açúõë/MóPüé"åÈIíÛ·ïÿo¿§Ø(>ßaÿE¼W^y%Q<y2þ=>Ì¯¨¨HÜ#æ~äa$>°ø'mSý²£££EEEÊ¯´³õêÕ?bþüùòU9ÚKâ*ñªÆ/MóPüé%ÊóÏ?_ð¸+V(¹MÂßÃéÕ××_¾|9~i$Ù¹s§ÑhTÀ´mÛ¶h4¿´«««©©ILVUUåv»îSýHä®dþæÍæ§ºI<$åió¯­­-é/J­­­²JKKå·ñÃÖ$Þçðð°èVYV¥§§'é'¦y¨Dþüø#""""ðGDDDDàÀ?""""DDDDþüø#"""DDDDþüø#""""ðGDDDDà2Õÿ?5i§Ã¶IEND®B`


s:¶Ü<®¢»ÔÑXu÷mEQTÓWTTðCDà(S?ó×ë¯¿Î!"ðGDÃá£G®^½ZÛ_~ÕBDàÀ?""""ðGDDDDàÀ?""""DDDDþüø#""""ðGDDDDàÀø#"kW®ÍaóæÍs½neeå+@tLËªªª9<=l~ß3ëÎó¹U:?<xÐh4Ê+,,¬««ûòË/£×REæË7çääÈ4¿]Dþ(í2L±±±y`åÔ©SrÝÓ§OGç¼ñÆ2§©©)3ð§ït:e~WWL_»vM¦KKK£¶··G±xõêU~»üQú=<äºµµµÑ97n9ýýýK³K¿?>>ðZ÷îKÕÿ÷ïßÏo?"JGùÅBçØ±cùùù'Oû¶ç¾¦¦&î¢oÊôÝ»weZæ¨ýõ×6-//Ï`0TWW«ïio-ö§'¿ÊÌf³Õj¤%ÚÄÄÃáëÊÂ=zT#SxºiÓ&¹5/·|ûöíGÄß5kd~EEÅõë×ã.Z¹r¥ü¬ÉÉIùÕªUQ"ÏoÀ-ÿÔéW_U¦Ýn·0K&^ýõØïéèèPÛä¢óçÏË´ü/ÓõõõêEë×¯W>oÝº%óKJJÞZì$¿î½÷Þ	§Ó©]øÊ´|ÃåËeâÔ©SQ«ÊúÆätçÅ±ßsåÊèÍ7öÙgÑ«¨£ÀÛ¶mé]»vÉ´ÌI¸üÖø#¢´ÀÙléûÓÚ+áý~T<;vìPU½t``àÄ5552?+++á­Åñ+ùUÔ+,,Ô^·¸¸X]ø©©)rÉLùN®®®ÎF"ù­¸z©QÊ-«ó:ýREêáÃeÀ-.þÔ=U£óåË%$ÒÊÏÏïÃêÈ¦ÌQ/:wîøÙgÅÞBÜ­Å~9«L·`"ÅØ7êT8^ºtiåÊê£Ñ¨¾)8ûwþ¬·®®.ñ0·ÐY~è½÷<éÕ«W'~ëüQZà¯¤¤$ö?õÍ³%äp8äÒèÿÑùê¾S%þ_%º`b)íuW­Z¥^7nñE¹zõªºnô-Ã9­éRFY¦£Zk```ÞË@Dþhqñ§ºåüùóêgþÞxãÙHèÒ¥KQë¼÷ÞÑù*%oÞ¼©~p6øK~'N¼ÿþû2qàÀíu÷ïß/Óï¼óÎÐÐ:Ì*3­V«LÌFFFd¢¬¬ìñWUU%ó;;;üêÀ.ûöí0ýQodþ¼üÑââOQVþÃbÕê«ü±Ñh,,,<zôè,ñü*>O.Ý¸qct¿¸Ã/:tH<''gëÖ­êNµâ­ºº:u÷áÚÚÚÏ?ÿ|N«E;4,7ëp8d	åÎ®ZµêàÁòsüêx×±·=Þõ¼ü?""""DDDDþüø#""""ð·0ýä'?ùÅ/~Á&LI_|ñÅÿ÷³ôÕ×_=99ÉzÐW£££±¿!]$lddõ ¯~ùË_.Tè?üáÅü¾¦¤>úèþýû¬õé§þû¿ÿ;ëA_ýüç?¿uëëA_!~ö³±ôÕüÇ~ü?àÀ?ðþÀ?ðGàÀøàüø#ðGàü?ðþüøþÀøþü?àü?þÀ?ðþÀ?ðGàÀøàÀø#ðGàüø#ðþüøþÀøþü?àÀ?ðþÀ?ðGàÀøàÀø#ðGàü?ðþüø#ðþÀøþü?àoõôôTWWªªªþþ~ðþü?þ2¥¥¥òr%/^,++Óâ¯³³sRQGGÇ½÷XúJþXú×ýWÖ¾øüóÏYú*üô§?e=è«Û·oÿýßÿÆÜã/¶¼¼<-þÞzë­S*joo÷ûý¬uõêÕO>ùõ ¯>úè£?þõ ¯dÉ_È¬ÕÕÕ%Osw2½½½õõõû2ìKû2ìKûÃ¾<ì«611QWWÁø#ðþüøËpüÝ¹sÇéth/àÀø#ðGà/£ðç÷û·lÙ266ðRðþü?þ2f³yELàüøþüe2þþÀ?ðGàÀø#ðGàüø#ðþü?ðþüøþÀ?þÀ?ðGàÀøàÀø#ðGàü?ðþüø#ðþÀøþü?àü?þÀ?ðþÀ?àÀøàÀøàÀøàÀø#ðGàüø#ðþüøþü?þÀ?ðGàÀ?ðGàüø#ðþÀø#ðþüøàü?þü?ðþÀ?àÀøàÀ?ðGàü?ðGàÀø#ðþÀø#ðGàüøàüøþü?þÀ?àÀ?ðGàÀø#ðþüø#ðþü?þÀøþÀ?àü?ðþÀ?àü?ðGàÀøàü?ðGàÀø#ðþÀø#ðGàüøàüø#ðþü?ðþü?þÀ?àÀ?ðGàÀø#ðGàüøþüøþÀ?àü?àÀ?ðþÀ?ðGàÀøàüø#ðGàü?ðþüøþÀøþü?àü?þÀßÒ¥(çaàü?àÀe2þ].Ùl^±bEQQQ(àüøþ(ñwãÆÃQPP°"&·ÛþÀø#ðþüQFáÏëõÚl¶jkkÛÛÛÁøþÀ?ÊüÉc_^4×¬Yg¾ììì½÷òbþÀ?ðGà27nÜp:¹¹¹qì3ò2ÊsøþÀ?Êüù|>»ÝÇ>ÅòöÛo+ÂàÀø#ðGºÇ_8nnnái?Ø·ûööövØþÀ?ðGà2@àÈ#qûðJ2§±±qppMþÀ?ðGà2íííÓð>:³QÀø#ðþüîñ'ªkii)//gü?þÀe2þäG9r¤¨¨^ðþüøÉøëèèØ¹s§öÐ-ð?ðGàÀø£ÌÁ_(r»Ý	Gx­Vk[[#¼àüø#ðþ(ð].v7;;Ûétöõõ±ÂÁø#ðGàüQ&àO^ãv^³Ù,¯ÃÃÃ¬jðþüø¤üE"ÇcµZðÊEð?ðGàÀø£LÀßðð°¼´1ÂþÀøþÀe8þvÂ;í¯@^ðþÀø#ðþ(Cð§(×ëµÙlÚÞòòrÇDXàü?ðGàüîñ-v×ápðêþÀøþÀeþÓéÔ¥¹¨¨ÈårAV øàüø¤ü)âóùðZ,·ÛXuàü?ðGàü?ÝãO=9vW9tøàüø#ð	øÚC·ÈXWàü?ðGàÀ_&àï¯ÿú¯ív»öÐ-¥¹¹^ðþÀø#ðGà/ROÎñäO&áõz½ð?ðþÀ?Ðèè¨ËåÒðææærrðþÀø#ðGà/s¬¯¯×ºÅl6Ëë#¼àü?ðGàÀ_&¤(JÂC·ÔÔÔ¼òÊ+ð?ðþÀ?P$9sæLyyùt'çÇ¹	ü?ðþüøK»@½Ñhc_AAÓéü?ðþÀ?úÎï÷?÷ÜsÚC·kÝþÀø¤ÆÆÆÌf3øþÀ-áp¸¥¥E;Â«º¥­­-áûÀøóO6CEE<ÆÀø#ðþh)À?~{èÁÁÁä×àüÍ³­[·$øÿý÷oQ*ºzõê_~ÉzÐW]]]7oÞd=è+!ûOúSÖÃR&¯,7oÎÊÊÒºåûßÿþ?þã?ÎxÂÖ¤¾úçþçO>ù$cîñ÷¿8=þ.ðsJE?ù»õ ¯|>_?ëA_ýøÇ?ÿ± AöéÓ§-v÷÷ÿ÷å"ùYÞTww·l8V©î~>þøã¹;?ö%ö¥GlxxØårL&íÉ9öîÝæ1dÌ°/Ã¾û?ðGàÀ_ÚÕÝÝ½sçNíÉ9MMMó~¼?ðþÀø#ðGà/R¥µµuÝºuÚÞ6ÈEHäQnü?ðþÀ?iÑèè¨ËåÒ¥9;;÷îÝrN6ðþÀßbþÀ?ðG³L^/vW xäÈAáþ,ðþÀøþü¥&EQ<ÕjÕð®[·N.zÄ^ðþÀøþü¥E¡PH^&Ìf³Ãï÷/È/øàü?þR_ÓéÔð¹Ùb/øàü?þ=EQ¼^¯ÍfÓ¾ÕW^^¾H#¼óÀ,Fwwwû< Hàü?ðGàüÑÿð677kOÎm·Ûþ"	þ%%%OU>óÌ3«V­Ú¿ÿâ>øþÀøV0lhh(**ÒðÊ|¹4%K5þeÁöìÞsò'åßÑ#GKKKß|óM¶#øàüø#ð,EQ|>ÝnÏÎÎcÅbq»Ý¡P(7þN>ýÍo~SúïO÷ýéïýÞï±AÁøàÀ¿ÄêÄvÚ^Éf³Óau:ü566~ë[ßÅüì±ÇØ¬àü?ðGàÀ_|Á`ÐåriGxsssNgZí<ä¿gy&V~ßu~wþÀøàÀ¿_K=9v×l6ËAjGxç¿ùûÑ~Äï9øàüø#ð÷?Dñx<ååå	Gx½^oÚî'|oß'xâ©§zfã3?þø¾ûØÛü?ðþüÑrÇßðð°<ÃkGx³³³Ng___/ÿÇù¯¶¶6óþÀøþh¹ãO=9vW (ÏùB]ÜÎðþÀøþüÍßï¯­­ÕðZ­Ö¥<9øà/é5g*++ü?þÏ9³fÍí¯ÃáÐé<ø¿¬2àüø#ð°$'çp¹:9øà/­àÀøÓc~¿ûöíéyrðþÀßü?pàøþü©Ãa±Õjîäúú`øËf³Ù`0ð?ðGàÀ6õÐ-òJ¡=9Gcccæñü¿ÌÇ_UUöÏ8£Ñ(ä?ðGà3þº»»ëëëâ^#L&SSS^ÝþÀø/''GÉcccò`	1ßûï¿/òàÀ-Cü)ÒÞÞn³Ù´olØ°¡¥¥e±ßþêãY&D2!ÏSSSS2þÀ?ZVøß±Ó§O«ïhÝâ÷ûÃVà/óñ·råJy`Ë*2ñòË/«êüø£å¿7nìÝ»777Wû) ¦¦&]ºü?ðßÑ£G£»wÄü?ñøëèèH8ÂûôÓO9sfþÖ?ðùø^yåU«VÉDOOLkjjÑÁø#ðþRØèèhKKÅbÑ²oçÎ"BEQçVàoYà/%?ðGàü¥¤ÁÁA§Ó©=JsAAÁÞ½oÜ¸±Ì·øàü?	øSEáÕ²oÝºu---prðþÀßl+++SøÂAÁ?Ê<üê¥Y=9G;[ü¿å¿ÒÒÒXðEco_ðGàô¿@ àt:µûð444dÞÉ9Àø³J'O½½½SSSK¹èàüø¢(>/á>¼f³Y~áàoYãÏh4ÊÓÁËü?ào1Õ577'ÜWáD"lðþ;þúûûåIáàÁàüø#â/644Å/77×ét2ÂþÀøûµV¯^­ý>À?Òþä¹Ôn·k÷áeü?ð¸µk×²Ãø#ðGºÃ_$ñx<åååÚ¿Þ­V«lÒþÀøé&688¸ÄþÀ?ð7¿Á ËåÒðfgg;ZÁø3T\ÌàÀéò´)¼ÓðBÖ9øàoæü~¿<q=ztwàÀøe¢x<«ÕªáµX,n·ö?ðþærÓÄàÀ¥ÃÃÃINÎáóùø`ø#ð7ç²¦>À?J!þúúúN§vC·?zü?àO¢(^¯s??y6Y»víÐÐøþ(øKrrÝþü-$þ<³,ý¢?ðGàü©ÉßGÉÍÍåÐ-àÀßRà¯««KbÆÇÇò/àüøò4¸ûvÝþü-)þØÛüø£%Æ_spèðGàoÑñÇÞ¾àÀ-þÁàñãÇM&v»C·?K¿TþÀ¿e¿¾¾¾;wjGxeÎÞ½~¯;àü?ðGà_õUkkë¦;tüøKþÄ[·nÍËË'£üüü^xa	öüàÀ_f7::zâÄßú­ßÒ²¯¶¶¶­­^ðGà/5øL¸ÃÇbêü?Z hhh(((Ðð:ÎîînVø#ðJüUTTÈSÒ¶mÛ&&&äËñññ;vÈõë×?ðGàæÏç³ÛíÚ?§M&Sssóèè(«üøK=þrrrä)vôAX sd>øþh6ÃaÇóôÓOkÙ÷ÔSO½ýöÛ=Bàü¿9%OO±')Ã¡^À?±`0xäÈ¢¢¢8óåææ:.ÌõÜ¾þü-:þÔaß-[¶¨Ã¾ò¿LËêêjðþüÑtÉ3XÂC·æxn_þ¢½;|Ü»wü?:MQÓ§O·¶¶.ì'í"ÜfÂs<ýôÓ'öäàüøKGü=x¸Ãï/¼PXX%ÿoÙ²Eæ,ö¢?ðGàoÃëÖ­[½zõ³µÏ®ÿõò´æ÷ûýfEu¢I³Ù¬eßÎ;ûúú´WàÀ_â/%?ðGàoúÎw¾³®zÝã'Nþà¤üûö·¿-þ÷ÿÓéÌÍÍÕð?~îàüøàüø[ô~ó7óÈá#ªüÔëÿ`kkëÏg³Ù´oõY,æææØ^ðþü¥;þVÌTVVøþt¬¸¸8V~òïÙg«ÍéFÜn·OûÜ(ôûý³<9øþÒYÓþÀ?]÷øãø³±ø+--mkkÍuÁ`CCöÐ-2GæÎiIÀø#ðFø®Ã«Ït/^àÀóÍ7Ífó¿ôçÂ¾ÇO<ûì³O>ùäYg$»Ý®=tË,GxÁø#ð§?üõ÷÷ççç«K ðþü-^òwìc=öÒo¯_¿>N÷BÇðÐ-òdèõzg9ÂþÀ?áo×®]êÝÕ«WfÑÁø#ð·¨B¡¾¾¾áááéô&ÉQÂs8ÎnàÀ_&àïòåËêóÝ¶mÛrÑÁø#ðªäÉÇáphGxÍf³<5Íoü?:ÀßäääúõëÕ;ºººxÑÁø#ð·Ä)âñx¬V«vWfÊE2ÂþÀ¿tÇß[o½¥>åíÙ³'%þÀ¿%+ÉsöäÙÙÙcñÀø#ðFøã8àõþCÓÃår%Ùü?¿¬2àüøÓi¢x½Þ'ç(//÷x<3ùü?¿þÀ¿Å(577kOÎm·Ûøiü?àü?U HrrÅáàÀøàÀßR¤(ÏçK8Âk±XÜn÷ºü?àü?)KT'¶Óðª'ç.ø¡[Àø#ðþÀø#ðÁ Ëåîä@ Mü?àü?<oØívíÉ9,KsssGxÁø#ðîøã8àõþtT$ñx<ååå	Gx½^oÊGxÁø#ðîø=¤_Büq?ðGà/§ôáàÀ_ºã/Zgg§<¾øâò¥ü¿cÇsíÚ5ðþü¥0õäÚ^£Ñèr¹Òmü?ºÁ<Êiì°@æL¦G¼åþþþÁPYYÙÕÕþÀ¿Ù$OGÇjµjG$Ö­[çv»Ãá°^îøþÒêSj,Tü=úgþêêêÞyç8öì=Àø#ð¼P($Ïf³YríÛ·§Ã¡[Àø#ð	ø+..çVÚää¤|)RoÛ¶MæÈüG¼e£Ñ855¥jRÍµø¿à(ðÁýýý¬ÕÙÙÙÝÝ©÷îòåËÂ»ÇLr?ù??Wtz¿þîïþîÚµküöê«üä']]]¬ÕÓÓ£ß'm?ybJ¸ÃÇgö·»Ëv÷Á<ÝS*úðÃåO[Ö¾òûýÿò/ÿawJ~Ï;÷øÚ§ ÊÊÊ7Þxã/¾Ðõ¤ú§â·W_ýìg??´Xú*ÈßZswÒ­[·JKKóòò²²²òóó«««ïÞ½ûè7;pÃ°/Ã¾Ä°oìoss³öäúáeØa_bØW7Ã¾Wqq±*ù_;þÀ-OüÁí¡[Fcccãò?þÒ7ÃqáÂÿëêêÀø£e¿éNÎ±fÍææfíÃþÀ?ÝãïæÍeeeùùùê@mqqñ¥Kýfe3L&¹Íðþhyâ/ùÉ9ÚÛÛ3cü?ºÁzçØSº©ÓgÏ]ÔEà2Á`ÐåriGxNçàà`Æo5ðþü¥#þL&<ß¼y3¿.,,àÀßüG÷îÝ»ðÊc_'çàÀ_ÆâOFV'TüMMMqn_ðGàoE"·ß~Ã	Gx½^oð?ðGàO7øSò¬¾Û'ø;vL¦µeàÀßt?~]dÜ¡[Ng__ßòÜjàüøKGüùýþyWðþüÍXww·ÃáÈÍÍÕCævèðþüeþ¤uoß¼¼¼²²²`0¸ØþÀéH¤­­-á¯Õjõx<òl5ðþü¥)þRø¤SüÉb455Ífí¯ÃáàqþÀ¿tÇ_t?hwïÞ-++[³føþbÚC·ÈËµ#àÀ¿EÁ°½Á¿h¢´µµ=÷ÜsÚC·X,·Û½|ÝþÀ?ãoÍ5+¶råJðþhãOT×ÒÒ"ÂÓðÚívÏ·ÜÝþÀ?ãïë¯¿ÎzXôÜ±ü._¾þÀ-[üÁí¡[d>#¼àüøÓþ¢	õü?Òþä¹ûö#¼ÍÍÍð?ðGàO÷øKUàüQZáOTçv»µ#¼Kyrp8ÜÖÖvúôiù?3	þÀ¿tÄ  ¬¬,???:gÕªU¯¾ú*ø´Lð].vÞÜÜÓæÊú«ÿßSOÙþÈVUUõÄO?þ«W¯ÛáWÞojjà2òt8Ú^³Ù,Ð¥|ïMQ'|Ò¾Õ~ò'ÕßúÖ·~çw~'?þ999ò88Óßß/sÁø£Ä_$ñx<V«uºs,ý>¼7nÜøFé7¢òSÿvww?þê¿SSS¿v»ÿ?ðG¿ááayèiGx³³³Ng___ªî]ûâð÷Íßôz½àÀ¿Æ_II<õ:tH=çäääÉ'eÉdà2;ávW (FAajïÝÐÐÐã?~ôÈÑ¨üN?!ÏB7nÜþü-0þzä¹§§ü?Ò;þEñz½6Mû///÷x<ê_éÐóÏ?_YYÙð½_ãÁÆuÕëìv»Þ"þÀ¿tÄtçÎyÎÍËËËÊÊÊÏÏ¯¨¨9½èàüÑ¢â/577'<9ÃáHÃG8ïèÑ£ÅÅÅ+W®lhhH?ðGà/ÓðÀø£EÂ_ p:¹¹¹Ú^Ëþ'çÍ³Æ?ðGàü?ðG?ÏçK8Âk±XÜn7'çþÀøûßnÞ¼©çYÝÃ·¸¸øÒ¥Kàü^ðüä"Ây#üø#ð·øëìì¾N¨øS§Ï=þÀ¥9þÁ`CCöÐ-2Gæ/ÙÉ9ü?zÂÉd7oFñ×ÓÓÃAÁ¥9þäác·Ûµn±X,ÍÍÍð?àüMs`ç©©)6àüQºáO=9GyyyÂ^¯×Ë/ø#ðþÀß«GõSñ'&8vìzrOðþ(úàN8¡áÍÍÍMíÉ9ü?:ÃßïOxçO?ýü?Jòòòâ¤ò<áþüÍ¹uo_y)++[#?ðGÉD"n·»¶¶VûÁ>«Õêñxáþüé)ðþhºFGGF£^NÎAàüøàüÑ|ª¯¯×~°O (ìã	üø#ð·0øûòË/×®]#¯1yyycccàüÑRæ÷ûº¥¼¼ÜãñD"¹ÛÀ?ðþçóùîð144þÀ-v£££òXHxrÚÚZ¯×+ìS¿ü?þ,àAëêêÂá°|911±gÏSRRþÀ-^ê>¼qæ9N§Srðþüø°yÝmP¦£|à<¿ß¿ûö'ç8sæúg6ðþüø°ïüÅR`rrwþÀ-x¢:·ÛðäµµµmmmÑ^ðþüø[Dü©ù«««óÉ÷îÝ×!>óþha_K9¢=tKAAÃáåÉ9Àø#ðGàïÁû&iÆÁø[	ìwÚöL¦×^mN'çàÀ¿Á_ÖLðþhNE"Ç³nÝ:í_S2³µµ5ù/øþü-"þRøÙðððt'çØ»wïààà¼oü?þÓÏùÎ;àüÑìßgÃ¡ÝW (G¼ðþüø°PùåWâfîÝ»C½?M¢x<«Õªá---Óºü?þR?A¼Jß½W¾|÷ÝwÕ×­Eú¨øS(_c³Ù¬eßöíÛü×ü?þ,ÔgþöîÝ«¾b¨;vìXìEàO¿õõõ9ÎÜÜÜ8ó566ÁÅø¡àüø#ð÷`wø¸víZô¬¥¥e	ü?Ý¥(×ëµÙlÚ·úÊËË=Ï<öáàÀ¿àï¥^LxñÅÁø£h¡P¨¹¹Ùb±h÷áµÛíKóþÀ??Á ¯ab>óþÀÁ¢¢"í¯Ì_¤^ðþüø[DüÉËØ'âf:NööË9EQ|>Ýn×ºÅb±¸Ýî9ü?þÒÓçoddü¿e¨Nl§ál6PàÀ¿áÀß].v777×étÔ.øþü=þä%-v`7ùàüevÓÃl6Ë/êÒð?ðGàÀø#ð·ðE"ÇS^^p×ëõ¦jü?þÀ¿lxxX~µ#¼ÙÙÙN§³¯¯/ü?þÀ¿9§C;Â+ßIAaÚ.9øþü?³MQ¶¶¶ÚÚZí¯Õj]ìs?ðGàÀøào0½öÚkkÖ¬Ñð:ý?ðGàÀø#ð¬@ ÐØØXPP áu¹ðþüøKü%ü?ý6ÝÉ9~úéü?þR¿¬2àüé«p8,¶Kxè;w¦ðäàüø#ðzü¥<ðþ°`0ØØØðÐ-ãÆpÁø#ðGàüøÐÝÝðä¥¹¹9´þÀ?à/þ"Hkk«ÕjÕðnØ°¡££C×#¼àüø#ðþi¡P¨¥¥åßûÞ«¯¾:44þ<<9GSSÑhÔðîÞ½[~©2àüø#ðþËÙl~ªâ)»Ýþlí³+W®|çw3þ¦á5Lòë$«+³Àø#ðGàüexôG´iÓ¦?8©þû®ó»rt:áO=9ÍfÓð·¶¶¦ÿÉ9Àø#ðGàü¿Ð¬òè£QüÉ¿g6>ÓÒÒ²|ð'Ö9ú´öäÝn÷ûý:ÂþÀ?àoÙxâXùÉ¿Í79sf9à/644$<9G½îNÎþÀ?àüÍÜêÕ«¿ýíoGåwìûÇ~¿?ñ§(Jkk«ÍfËÍÍcß5kN>-&^¶¿àüø#ðþ2¼ßøßØµs×ã'üÙò²òíÛ·/È@gâ/É¯ÙlÖðÊ½ÖûÉ9Àø#ðGàü¿YÕÞÞþÔSO=öØc¿û»¿ôèÑzß+­ðN§ö­¾¢¢¢Ì89øþü?ðÊÒ¢ø|¾ûðL¦æææP(ÄàÀ?ðGºÇ¨Nlg±X´Gi^ûð?ðGàÀø£ÅºoQQQÂÞå¹/øþü?Ê@üÉ¶¶ÛíÚsX,FxÁø#ðGàüQà/x<òòríûl6×ëeü?þÀeþÁ ËåÒðæææ:Î@ ÀVàÀ?ðG?Ù¬C;Âk6e£3ÂþÀ?¿±±1yÉË¢x<«Õªár#¼àüø#ðáøÍPQQ!¯ýà/³ñ7<<ðäÙÙÙ­þÀëüø[.øÛºuk H¿ýèG>JEíííüñ£ßÎ3gþøÿ8+++ùùùßùÎwZ[[YÕØ|ÐÙÙÉzÐW~øaGGëA_É&ÇzÐWòô(Oswt¿ÿ]Äéñ÷É'üR<µMLLÌûêÿõ_ÿõ·û·7oÖð?~||¼àÉµwîÜa=è«Ï>ûì/¾`=è«¯¾új``õ ¯FFF®]»1w'ñÇ îÊ°/1ìË°/1ì»ì£¿@ àt:sssµ'çp¹üøþ)þrüé¢ø|>Í¦áµX,n·C·?àÀø?QØN;Â«CDÈ¡[À?ðGàüÍ!ð¶øüøþÀøËpüÉ±ÛíÚsL¦Ó§O#ðGàüøà/ðD<Oyy¹vwÃmmmò¬4ðGàüøàO÷øSOÎp×áptwwóÁ>ðGàüøà/ðç÷ûwÚ^£Ñèr¹¬%ðGàüøàO÷)2Ý¯ÌlmmÃ¬%ðGàü±Àøº/É:7ÍZö©'ç`üø#ðþÀøËúúú£  àÈ#CCC¬"ðGàÀøàO÷)âõzã·û·ÿê¯þjttµþüøàüé¾P(ÔÜÜðäµµµmmmW¯^ë¹	üøþÀøK»¦;9Gtwöçö%ðGàüøà/»qãÆöíÛº¥¹¹9nü?þÀøºLQÖÖÖuëÖiGx­V«ÇãIxrðþüøàüé¬P(ôÚk¯iÝíp8ü~ë?ðGàÀøàO7õõõ566j?Øg±X´#¼àüø#ðþÀøÓeê¯ÕjÕðÚl6¯×;û£4?ðGàÀøà/SÝ²fÍí¯Óéìëëë?ðGàÀøà/ëîî>räv×l6ËßÍ?ðGàÀøà/½êèè¨­­nÞG</øþü?ðþÒ¢H$âv»ËËËîÃ»Pkü?þÀø)npp°¡¡Áh4Æ±¯¨¨ÈårÁüYàüø#ðþÀøKY>ï¹çÓ£¼¼ÜívB¡ÿàüø#ðþÀø[ê"ÈÛo¿ýôÓOkGxívû¢®ðþüøàü-]CCC&I;Â+óvü?þÀø©IQÏ·ûví¯zrÅáàÀ?ðþÀßR'ªs»Ý"¼'ç>â¡[Àø#ðGàü?ðAË¥=Jsnn®Óé)Y*ðþüøàü-prïvW=9Çð?ðGàÀøàoD"GfiÃ^¯wGxÁø#ðGàü?ð·(ËÒðfggïÞ½»¯¯/ØþÀ?àü¿GM`çt:µ#¼Á¦¦¦%8tøþü?ðþ"Ïg·Ûµ#¼ëÖ­;sæL8NÏÅàÀ?ðþÀßD"---	?Øçp8Òÿ~?ðGàÀøàoV?~Á>£ÑØØØªC·?ðGàÀøàoëëëÛ½wÂs¤ó/øþü?ðþæP$q»Ý6lHxröööôÙü?þÀøóottÔårÍæ'çÔïÊàÀ?ðþÀßÿ%°s8qì3L)?9øþüøY½öµµµ^¯W_ìàÀ?ðþÀßÌuttÄð:]ð?ðGàÀøàoÖ¬Yc4e	3ò÷ü?þÀøÿW D"üþÀ?àü¿eøþü?ðþÀ?þÀøàÀ?ðGàü?ðGàÀø#ðþÀø#ðGàüøàüøþü?ðþü?þÀ?àÀ?ðGàü?ðGàÀø#ðþüøàüøþü?ðþü?þÀøàÀ?àü?ðGàÀø#ðþÀø#ðGàüøàüø#ðþü?ðþü?þÀøþÀ?àÀ?ðGàÀø#ðGàüø#ðþü?þü?àÀ?ðþÀ?ðGàÀøàüø#ðGàü?ðþüøþÀøþü?àü?þÀ?ðþÀ?ðþÀ?ðþÀ?ðGàÀø#ðGàüø#ðþüøþü?àÀ?àÀø#ðGàü?ðGàüø#ðþÀøþüøàoáêéé©®®6UUUýýýàüøþüe2þJKKååJ&.^¼XVV¦Åß'|2I©¨££ã?ÿó?YúJþXf=è«Ï>û,°ôÕ_~900ÀzÐWÿöoÿvíÚµ¹;:Æ_lyyyZü½ùæP*joo÷ù|¬uõêÕ?þõ ¯>üðÃ>úõ ¯dÉc=è+yzüà2æîdþzëëëöeØöeØö%3yØWmbb¢®®.?ðGàüø#ðiø[ñ«Ô/ïÜ¹ãt:GFF´ß	þÀ?ðGàÀîñßïß²eËØØXÂKÁø#ðþüøË(üÍæ1?ðGàüø#ðÉøKøþÀ?àÀ?ðGàÀø#ðþÀø#ðGàüøþüøþÀ?àü?àÀ?ðþÀø#ðGàÀøàüø#ðþü?ðþüøþÀøþü?àü?àü?àü?àÀ¿å¿¿ø¿ðz½¿ Tô7ó7_õëA_]¾|Y^Xúêã?þøÖ¾êîî¿YújppðÒ¥KswB¡PfâïæÍ'Oü!Å|htïô-Àø#""""ðGDDDDàÀ?"""".Íæèýýý%%%¡²²²««ë×~bÊÊÊ9###±3Y©Új	çL·AlbJ­ÖÓÓS]]-Û¨ªªJ¶5½l8ífâáþ[m9¼´ñA¿Öõë×+**b¹ëêêÞyç8öì=^K¾A.K.9NVcÊ·vN:ML©Ýj¥¥¥~ú©Lx±¬¬Ç^6v3ñpKÿ­¶^ÚÀýZ[·n±£Ñ855%÷ïßOøGÒ­[·6oÞ¬NËÃãÊ+¬Æo5í$tÆML)ßj±åååñXÓËÓn&nzy¸eöKø£D¿1Áp:Ú/¼ÐÛÛbË-òm555þ9k2U[mº9	7èR¾Õ¢Éc­¾¾Ç^6v3ñpÓËÃ-³_ÚÀÍðQ?ñ ÷òÓÆµ·pçÎªª*Öd>¯i7hòMLéój411QWWy¬éNíÑÍÄÃM[-ã_ÚÀÍð)..¾ÿþ2÷§Nzë­·ÞÏkéù¼¦Ý É71¥É«¼ê8ÎkzÄ_t3ñpÓÅVËø6ðG3<BÇdBþ¯««ûÎM6D¿,--êÕ-[Xiø¼¦Ý É71¥ÃVóûýòã±¦¯§ÝL<Üt¿i4Ã#äúõë&)++«¤¤¤§§'îäo õÃËj½½½¡¶¶V$¬É´z^S¿ÔnÐÒj«Íæ¸#MðXÓÅÓn&né¿ÕÃKø#"""ZNf?""""DDDDþüø#""""ðGDDDDàÀ?""""DDDDþÀ?""""DDDDþüø#""""ðGDË¼wßwÓ¦MyÛ¼yóåËíùëaºy¶M´´f³YîZ8/sCIIÉÔÔÔü.;yòäM¯¼òJ&á¯©©If¶´´ÄÍ?sæÌùåçqDDàôW¿°Æ`0;wNyØùóçåK9001ø»uëÌ¬ªª_QQ!óÁ ø#"ðGDË¢_|QXóúë¯ÇÎ|ã7dæÞ½céÓÕÕ%xVWWËtôïÝ»W___XX(ÆCÅ®ú|>×íèèãÌY¹rå¦M®^½*_:¸kooO~;êE²HêEÓAmË-2¿§§':çÓO?96-:çÔ©S&In*//o×®]wîÜÑâOûqs,*?"¢·zõjË×_;óöíÛ2Ól6Çâ&®ÞÞ^õÒ;vÄ]tðàAõ¢ÁÁÁ¬¬¬×R¿T/á¦¦¦999¢È¥ò¿à)??_ý(^Û§½§/^mÔï¼óNT~q7²yóæ¹â/É¢ø#"Jêoç¬+b±¸Ù³gÏÄÃdB¾Ü¶mz©jêx6õ¢ºº:õ-C¾~ýºz#±·yâÄ	á:êºÿ~såÊÿezß¾3ÞÀQ¾o]¶÷H@¹jÕ*¹¿cccò¥ü/K.â¼ÿ¾ú%%%rE¹~5L7Wü%YT"DDi?)!þ¢óUÜ¨fîÜ¹#_Ô/«««åËµk×8p@Ð699½ù¸÷Ò¢×R¿¼ûvôLÑ_ù_¦>ß·#Ð/ïÞ½»lÓ>ïðáÃrÑ_þå_>øÕÐö¡Cb¿A (øÖÔÔ¨oLÎIÀQêSßîe¦;QÕQëD?¨§ãTìVdÚh4æääD"¹ñ+WF/ñv¦£XoÞT*Óò¿LF/½~ýº,@ÂáãÙã/É¢ø#"JêçÞÞzë­ØêPâvø~.pllLûÖÀÀ@SS:U£ú¶¢<xPíÿëëë£óÜúNÛÈÈúåÝ»wï»qãF¹T=º5ö"uÏ_¹¨³³s||<9þ¢0U×FôÒ$JDà(õ©;L³gÏªz9wîvçmÛ¶	t":&Ý3WýÌúQ¹@ ûaAÕ*©ûáF¶hêî·êO÷ûýÑùInGýú¿p8¬~gü?>úÜåØÔ?ÊO0þTnåÛ»ví½4É¢ø#"JÔ ÇuêÔ©ÿþzj£èttÀ4ºE´ýû÷ÇÊ2¶K.%Áß§âùF£1vD8ÉíÈbÄ.XtOÛéîìääd^^zâ»·nÝû#V­Z%ÿ«G½MõÉh*U£&YT"DDéeóæÍ9Û´iºËmþ:;;ÕÃéUVV^»v-zi8>vìÉdRÁtøðáH$½´£££¦¦FLVRRâv»ãnS»$rS2ÿ¥^?ÝíH²0²Hêç/ZÜ1_ÔÆÆÆêêêdÊ½ÑÃÖÄÞæøø¸èV]W6­§§'î'&YT"DDDDþüø#""""ðGDDDDàÀ?""""DDDDþü?""""DDDDþüø#""""ðGDDDDKÕÿ!ç+#4µWIEND®B`


ñâÅ69dÃ¿è|òIîîîÕ¦2µµµáãîÝ»ûÆûï¿¿råÊèiuuu[[[Êg644L8qÑ¢E9;wnnnneeåGtèÐ¡Ù³gçåå-^¼8ù(ãSO=U\ß_"qW_Ä¹çÂüä°¡úúú0³®®®×ïq°IÿÒÁaNXÉAN¯Gæ»¾ö¿Ãv5kVö;ï¼3mÿÇÓïMÞ¬¡ÆÆÆ°´0aË»H#<î***Z¿~gg§_.	þ$Å/^,))<yrHwÀ~Ñ9>3Ei999áã]wÝõioç%.|¸ _ÊgÞ~ûíýgÊ)aþg3fÌèõìCR¾tMtä¯¬¬lPÓ¿á®ûÇßñãÇ¾ÙÁâ/y³¶´´D7;v,úä+V¤¬ó>èK?IñÅ_xë­·%;àþûïÓË-ûäja"Ü3?ó±Ç»råJtì*óÀôôô>|8ºn644éÁè±Ñ+Ûöïß¦O>|W_ÄÉÍÍíu~òcoð!½¨.qdt°ÓëZ]÷ûyÁ_ÊÚÂtuuõ¥KëD8þ7kXNsðàÁ0mÖÕ«WG¡ðìÙ³a:lÍ0]PPàK?I±Æ_(<éð¼2òäÉaúÃ?nçøpsÒ¤IÉKHÜÀ|³½½ýÓÿ>lîîîÀùóçG®¿¼¼¼^¿^ç_ÇCQSUUÕØØØë dpz]«ë~àÀñ½®ñ£>JþÂ_òfÝ5oÞ¼psÚ´i¯á/_ö%Á¤À_ðYQQQxïêêJ~ä,ýpAu½.³ÿ)§>¯yÕjqqqt:]x	¤,m ékL®9h¾Hzøißð®ÿÓ7kÊûàDwµ¶¶FþK pß¾~¹$øwüÞxãp3ñÆ(ÑÌÈg)Ç¨ÂÌ¢ÿ³fÍÞråÀgÁ_tx2z×êêêõë×òÉ'Û·o3W®+þòëÆß`'Ñu?pàøÈ¿ïýûÿ0'åÒãäN8±iÓ¦èâÊhIð')Öøûôê÷¯ëæD×/[¶ìâÕ¢g÷xàÆñýjii	yé¥¿¦¦¦èØRðâáÃ££zQo½õV¯ßã`2(üõ?8ÑHv^-e!ÕëÀ_ô*½`ßË/Û-Y²$/¾øbüÄààþÇ?ZÃ@çðùa$ÃôÜ¹s¿Íèµ­­­ýb¿óçÏ'^ÝÍikkKy½×äÉÏÞþ4iRø|Åk¯ë|ï½÷¦`A?ßæ 2(üõ?8÷jNy£æëÕëÀß'¿hÑ¢äSÆ$p­üEoØë0Ñ±Õäî»ï>¿üIÊü¢S¢Éóßï½+Wæ]­ºº:ýòëÃ_XZXf`ÐC=<xþ³eËÙ³gçääL2åW^ùâ¿8òtãþú·Þz+ø/777ñVyÉjT¯¡ûöM6-|×aùÑõÄÎáf ZôÕ«ªª¯yäµ¡¡aþüùaiáÚ¶m[b~ggç#<R²««Ë/4Tuwwßï½zo¹ëxHÆ.ßîééL?kÖ,?0àO²°è5)=÷ÜsFFüIRÖÙÙ¹aÃ)S¦DçvÃÄO<aX$Á$IàO$Ið'I$I?I$Á$IàO$Ið'I$ø$IüI$	þ$I$I?I$Á$IüIÒôÖ[oæææÞ~ûíììÙ³ÇÓÚÚ¦Ã¹sçbvµëû<özv²|WNNN~~~uuu»"Ið')c*..	éééìczê©ðØÍ7'æ<ÿüóaÎ¦M2ý,?yNøÆÃôêÕ«ýI?I³C¹?8q"<¶²²21gáÂaÎñãÇg%GËa:??ßO$øIòKÍ#<2aÂ7¦|Ú¿øÅùóç§,!qà0L_¸p!L9Ñ]gÏ­ªª6ÊÍÍ7oÞÁ]ZòWïÿ!¯¼òJiiiEEEøBé»téRMMMxlXù6D2O-Zæ%øáC¿÷Þ/'''ú®V>Ü3ýI?Iqô_4ýÌ3ÏémÛ¶fç.ùsÒ_ßvÿý÷»¶oß¦ÃÇ0]WWÝµ`Áýû÷Ó§Où%%%½.-yúH Ýo¼&jkkÓWþÁÓáÞ|óÍ0ñÔSOS§NÓ/^lii	ÑÉ^_Ì7Xüáñ	ÓkÖ¬îÚ´iS4/¾øbxâ'ütI?I±Æ_iiiî¾Zº½zQ`SSS¸kÙ²eazÅê÷8qâ±Ç?~tD¯KKVÿVlÂ	é<yr´òea"°/Ì¦çÍ8ÛÕÕu#<'Z·°ð ¿ Ëè®ð¥gÌV£   ¬¬,ÜôÓ%	þ$Å¹¹¹é0nöe DAZÁ:á3;;;£³«aNt×K/½Døî»ï&/!eiÉ7ò¾V,h,ù`^ÇÝ»wO81STT¼)Gþz-ºÞ%|ÒàORLñWRR|ä/:xvMñÔÔÔóóòòÂ+W þúHbÅçÒ;iÒ¤è±)«×ÓÓ³ÿþÚÚÚäC×18×/N<¹ººzÑ¢EÄô.Ið')ÖøÞºeûöíÑkþþùào÷îÝCho¼ñFb~DÉ'OF¯þúÈc=öío;LÜÿýé½ï¾ûÂô«¯¾zêÔ©èToYQQ¦ß÷Ý@±0QVV6tø[¿~¸ëÄN~a¢$Á¤â¯§§'«àjÉïÕ×?þ._¾r/íÛ·¯¨¨hÂ	6l þúHccc¸wáÂëN?§³³3ð+¬y^^Þ%K¢ùª««£Ë+++ùíÀñÀü®7-7[ZZüI?I$Á$IàO$Ið'I$I?I$ÁßÈôÃþðÌ3ÃüEö³ýâ¿ðsç~ùË_FoÌ¡8÷/ÿò/ÿùÿiâÜùóçíîâ¿»;wîqÿîþnZñü7Ì_ôÝwßý÷ÿw?Êqî£>úøãó¾óï¤ÿýÅªÿøÇþó~ö³?~Ü8Ä¼ûöÅíÿºðð'øüÁàþàþðð'ø?ø?Áü	þð'øüÁàOð?Áü	þàþàOð'ø?ÁüÁü	þàOð'ø?ø?Áü	þðð'ø?ÁüÁüÁü	þàOð?ø?øü	þàOðð?Áü	þàþàOð'ø?ÁüÁü	þàOð'ø?Áàþ¿Sssó¼yórssçÎþó?øüÁq¿¬Âß39&vîÜYVV¿Þ*þõ_ÿõbÜ|pôèQãóþ:::C;vìØûï¿ob¾»knn61/àï¿øÅ0ÑÆ_rùùùéøñÅ¿?¼544466~_1îí·ßþîw¿kbÞÞ½BÌ³»aÙÝÙÝõZ6à/ü´®®Îi_9íë´¯öÓ¾Nûfóiß¨K.UWWwvvÂàþ?øËrü?¾¶¶6<§ß?øüÁq¿¬Â_SSÓâÅÛÛÛ½þð'ø?ãY¿ÒÒÒ1IÁàþ?øËfüõü	þàOðÆþàþàþàOð?ø?øü	þàOðð?Áü	þàþàOðð?ø?øüÁàOð?Áü	þð'øüÁàþàþ?øüÁüÁàþ?ø?øüÁàOðð?øüÁüÁüÁàþð'ø?ø?Áàþðð'øüÁàþàþ?øüÁüÁàþ?øü	þàOð'ø?Áàþ?øü	þàOðð?øü	þàþàOð?ÁüÁü	þàOð'ø?ø?Áü	þàOðð?Áü	þàþàOð'ø?ÁüÁü	þð'ø?ø?Áü	þð'øüÁàOð?Áü	þð'øüÁàþàþð'øüÁüÁàþ!üuuu[ø?ø?Áü)ûñ×ÖÖ¶fÍòòòÃðð'ø?e-þÎ;WWW7vìØ1WÛ³gá?ø?øüÁ²mmm555	öE^ø?ø?Áü)«ð6q:ûÂÍºººS§N^ø?ø?Áü)Kð×ÒÒ²|ùòtö­[·®µµÕÀÂüÁü	þàOY¿°L?Ú7nÜ¸µk×¶µµRø?ø?Áü)KðwôèÑU«V¥³oÝºugÎ1ðð?ø¿,Á_SSSEEÅßlüøñ?þø¹sç#üÁüÁàOð%ø;zôèòåËÓÙ÷è£büÁüÁàOð=øÏ)ì+,,OØðð'øüeþöìÙ3gÎtömÚ´©££Ã ÁüÁü	þY¿¦¦¦tö?ûì³Øðð'øüeþöìÙ~IG`_x¢ììì4Pðð?Á_6|òÉô£¥¥¥Øðð'øüeO===;vì:ujûÂÍ7cüÁüÁàOðUì>:öÁüÁàþ£3gÎÜ²eKWW!?ø?øü	þ²¡^x¡Wö=ùäþàþàþàOð%ìOvéìýõ×ÃóÑÀÿ¶¯àþàþ¿ÌcßôéÓwíÚíàßöüÁüÁàOðyì«¨¨Ø³gOòkûàþàþð'øËNö@¤>üÁüÁàþYÅ¾ÊÊÊ@¾.é?ø?øüÁà/KØWQQÑÐÐÐÿ¼ðð?øüe<ûªªªÂ³Û@ÞÀþàþàOð¿¨­­míÚµéì»óÎ;µû?ø?øüÁà/îì«­­;vlû.]zôèÑÁ.þàþàOð¿cßòåË®oðð?øüeûî¾ûîÖÖÖY2üÁüÁàþ1êÌ3éì7oðð?øüÅ¨ñãÇ§°oÕªU§NºY_þàþàOð¿.ì/_ÞëkûnÊÑ>ø?ø?Áü	þâ2bK.M?É[[[æÌ¡øðð?øü@---±¯­­mè¾.üÁüÁàþÃZSSS`_ÊÞa`üÁüÁàþ#Ï¾ÂÂÂÇ|Øðð'ø?Áß0uôèÑ^Ù:::sMàþàþð'øZöUUU¥¼¶oDØðð'ø?ÁßÖÐÐPYYöÁüÁü	þàOð7TÏÜåååq8Éðð'ø?ÁßÐ>gWTT¤°¯´´4ì?ø?øüÁàïf>[§í>ú;zzzâ³ðð?øü]váyzæÌñgüÁ_6ã¯½½½´´þð'øRöíÙ³gÎ9Â>ø¿¬ÅßáÃgÍ~ýàOð¿!bß®]»n½õÖÌbüÁ_ÖâoÉ%­­­ýàïÛßþöéá-p3ìO+ÆýÓ?ýÓ÷¿ÿãóÂÞð>0q.ìîÂw³øü·û·oë[¿ó;¿Â¾Ïîsa~¸7#vw?øÁü¬Æ¼½÷ÿî.ñ÷ëUì¯¼òÊO·¦¦¦æææ(Æ;vìàÁÆ!æüµ´´8÷w÷wÙº»ûÇüÇgyæ³ýlûÊËËÿiåFzçwìî2bw7ü_4ñç´¯öuÚWNûê$ï;¦OÂ¾ªªªáBqÚ×i_§áOð¿¡ª«««WöUVVf"ûàþàþð'øë½ð|QXX~´ïèÑ£1¿¤þàþàOð¿eßòåË³`Áüe-þúþð'øû.]¹'yáþàþð'ø(û/_ø?ø?Áüiôâ¯/öÝyçáÉÊmðð'ø?Füõs´¯¥¥%·üÁüÁàþ4ºð×ûV­Z¡ìëììljjjhh8sæüÁüÁàþ¿îÜ¹séì;vl]]]æíì0aÂïÿþïßvÛm6lèÿhàþàþð§ìÇ_[[[mmmp^ûjjjN:¹8;qâÄUÕ«6~mcø÷ÖÿßýÝßýÖ·¾ðð'ø?RüõÊ¾qãÆö»2À7oÞ|Ûm·EòþýÙ½üðð'ø?:üõu´/Ì<wîøºuë¾ô¥/%ã/üûÌg>ðð'ø?"üµ¶¶öÊ¾µk×fû¢6oÞü/|!Y~ÿ«ö9òð?øÓhÁ_°Ý5kÆÂ¾uëÖfß÷ú¿o~óðð?øSã/Ø.áæÃ?¯íë§èjßY³f¬_¿ÞÕ¾ðð'ø?e3þÂ]¾|yú%k×®ÈûÞeAÑûü.¹ðð?øS¦â/|¹U«V¥°¯°°0ìêþóÛðð?øSàïèÑ£éGû"öuttØð1Âßk?ÁüõÃ¾»ï¾ûàþ29×*77þ?øëõé°¢¢"åöÁüÅ#ü	þàO¿=öTVV¦°oúôéÛ¶mÃ>ø¿ÌÆßÅï¿ÿ~øü	þà/ª¡¡¡¼¼<;vìèÿMðGüæææzÍàOð)ØíÙ³gÎ9Øð=ø;wnúÕEEEð'øüfüöäaüÁ_¶á////ü2···`¾oûÛa¢®®þ¿Q¿»_~yæÌØÊNüE¿Òa"h/L>:ìÓÃDAAü	þ£]]]õõõöÁ²'N¿ØnmmO<ñD4á­^¿Ñ¿ÎÎÎ-[¶D§°þåøÛ°aCâòä_øøü	þ²]]]/¼ðBaa!öÁFþBO?ýô¤IÂDsss?þP¯:ü	þàO#¿°+.**Ja_UUUcc#öÁ>õ&Ïð'øüeþ"ö¥íìþ=3ü	þàþàþàOC?ì?ÁßUVV½á7yü	þ²Ø¿_7cÆdð%rµ¯àOðøkkk[»vm:ûÊËË±þ4ñvÇæ]9ü	þàOC¿À¾ÚÚÚ±cÇ¦°oéÒ¥áÍ%ð§Q¿è:¯áßÃàþ4tíÝ»wõêÕéìsþ¼°;xðÁ/]º?Á_¦íÎê$·|ùòã¿ÿjÊ)cÒrÁàOðìK9Ún.]ºôÇ?þ±ñ?Áß¯6m>¿ìc_èî»ïwøüýæ"®6üçàOð"öôGô½ïÏøÀà¯&OìÁà/kØf»þ·uøkjjû6tuuÁàOðÿÂÎséÒ¥)ì7n_â4üÁà¯ïEô>¿²¯ªª*ewí;wîgÂü	þú,§!øüÅãÆøá½¤þàOð»àOðºnö;vÝºu)Gûàþª´´tÚ´i§N?Áà/þì?~ü¦M®ù.ð¿>ËÍÍá_uøüÁÅ¾qãÆõ´þàOð7 <ö)áÿ/^Î½9ü	þàOd_aaá³Ï>ûñÇ|9ð¿¾áj_Áà/566öÊ¾°·ìèèìÒàþæj_Áàod;zôèÝwß³Øð'øið'ø?544TVVÞÁü	þàþàOð»úzmß³þàOðwíº»»,Yv=wÝu×0ìÖáOð£°]»v§°¯´´ôf±þàOðw._¾ÜëCý§~áOð£­9sæ¤ìl§O¾cÇÂûµàþ6kÖ¬°÷Y¶lÙ¥KÂÍ/®X±"ÌY°`ü	þ7^Ýë¯¿>lì?øü]£¼¼¼°JÞûtww9a>ü	þ7þÌ1uêÔádüÁàïåää=Q_bNWWWã­^¿ë.ìH_~ùå¼ágüÁàïE§/^öÃt3oÞ<øü	þ[]à]iiéH±þàOðwöz½àãO>?Áâ¿ð£R__¿mÛ¶S§NÅaXºººÂúàÑ>ø?Áß@»|ùò]wÝ5aÂðqñâÅaÎP¯:ü	þàïºZµjÕ-·ÜRUUõ?+þg~~þ«¯¾:²ìáâÀ>ø?Á_L?Áü]wO>ùdpÕ#_dã×6÷åþ'¶´´ÿtttlÞ¼9ý$oEEÅH±þàOððÊ6üùðEòþýaå>óÌ3ÃÌ¾°+,,La_UUÕðïÜàþxäµÊÉÉ?Áâ¿ñãÇ'Ë/üûÒ¾ôçþçØð'øë³¾?Áb¿¹sæþIÍ$ãïæÿÁæÍ±þàOð7èzè¡h_¶sçNøü)øÛ³gÏÿ(úu^Éï®»îºå[~þóÝW<wî°þàOð7ÐÂ`AAA´/K~Ïgøü)nømÛ¶müøñe¿WöÙÒÏëÑjkk«­­;vl¦°þàOð7 V®íÎöïß?<«?ø»ÁºººZZZN:5DÕöÅ¾8³þàOðwÞ|óÍhw¶lÙ²áøüÁ_lëË/ºCð¿!ÇßåË,X]ÛqðàÁa^uøüÁöÁüø>ü½øâÑmõêÕ#²êð'ø?ì?ø3ð7|øó>?Á_?ì7nÜÚµk3ð¿ÞË¹V¹¹¹ð'øSvã¯/ö­[·îÜ¹sý­Áü	þbü	þào?féì7ÃÌ3gÎdÁ6?øüÁüÁàï¿ªªªºDìkkkËmð'ø?ø?vüõÊ¾ÂÂÂM6eûàþðð§Ñ¿¾ØvDY¹àþðð§Ñ¿£G6öÁü	þàþàO£á©²²2ýJÞG4»Ùð'øëãÞçOð§,Å_Ø·ö¥ìÓ7oÞüñÇmð'øK-ù-ýzÅ÷ùü)ãð×ÔÔTQQÑ+û:;;GÕ6?øüõÙÂÎñî¹téR¸>®X±"Ì9tèü	þ)økhh(//ïõµ£ð¿kTTTv===9ÝÝÝÑÿopÉágº¤¤$77wöìÙ?ÁüÝôÂ¾kÇ3gÎtÀü	þ®wWàKÁß¿æ¯ººúÕW_[·n]½z5ü	þàïæ¶mÛ¶éÓ§cüÁàopM<9ì.Ô._¾nvvv.[¶,Ì	óopÉEEEÑÓCÐdiii:þÂûÄðööÛo>|øbÜßÿýßÿíßþ­qy÷îýÑ~4+ðWõW³fÍJaß	¾ò¯üà?°ìî2ew×ØØhâ¿»þ/:äø;tèP¯|¼ûî»7¸ääKFÒ/	øóÍ7ÏoGiii9§wêÔ©þðÆ!æç;ßùðÃÿë9sfëÖ­ÿüçSöWS§Nþùçßï=&QÅO~òãçþùÿÙî.þíÛ·oøwwC¿ÐéÓ§gÌSPP0oÞ¼.ÜøbOçåå9í+§ö½î¢×ö¥äsÂüäW-Ëi_§å´ï5yòäè¥ácúIdøüÁß@êêêêõµØð'ø]555¯¼òJ«««áOðª³³³¾¾¾´´ûàþ7'O,+++((NÔN<y÷îÝ7¾ØÃe477ÃàþÎ¾°(,,Ä>ø?ÁßÍÇ_ô&ÏÉÒ-Þºuë®:ü	þà/½èµS§NÅ>ø?ÁßPá¯¸¸8ìXO<À_sssô¦	ð'øÓ°á¯««ËI^ø?Áßpà/Ú½FþÂ>ÝßöüiØð×ÑÑñì³ÏbüÁàoð½Ést´/à¯»»ûG	ÓéoË?ÝöõúÚ¾ªªªÆÆFì?øü	þzç#GÀàOC¿~Ø7üøüitá/zº?~tµo~~~YYY[[ÛP¯:ü	þF'þ°þð7òøàOð7Úðð'ø¿Xà/qG¢.M:þº)økkk[»v-öÁàþb¿îînWûþtSðØW[[;vìXì?Áü0þÒßC5¥'ÂàO×¿^ÙnÖÔÔ`ü	þàoðwöìÙ«%þ¶GrA~o¾ù&ü	þtøëaæ0&ø?Áß5Ôê3¼ð'ø%øÃ>øüÁ_ào¤?Á_6áïºÙwæÌ;vÔ××;ð'ø&üuww$æL4ég?Á¿9Ú÷úë¯çççá_¸ã;n¹ååËwuu^ø?ÁßÐâoÊ))üFûîM6ÁàOýà¯Wö7n'y[[['Nøg÷þÙÆ¯mÿùê#3ËfnØ°ÁðÂü	þyyyaÝÒÒ~Ã	&ÀàO½öÜsÏ-Y²$á÷º££cÙ¼yóVþa$¿èßý_¹ÿ³ý¬á?øü-þ¢~S^¾þæð'øS(üÚVUU¥¼3Ô`ÙµnÝº¥K&ã/üûÌg>cáþC¿°ï^¿~ôRË/oÜ¸1Ì)..?ÁQõõõÿüçå÷ÇüÇ¿÷¿g¨áþC¿cÇõú&ÏÍÍÍð'øÓP°/*<¶´´´êªoòû?ùíßþí]»vpø?ÁßÐâ/tþüùÙ³gççççääÌ5+ÌêU?Á_æ²¯¦¦æ?þã?n|ùgÎ¹ã;&NXTTtË-·¬1?øüþF$øüÅ¹öM>Ç]]]ÿÛ¾),ðF þàOððº!ö÷Ê¾èsn.þð'ønü<y2zçèßÉ'ïÞ½þ£ª»À»3g¦°¯´´4ð?øËxü8p ±ðMoÝºþ£Ó§OOa_qqñæÍ;;;Óð'ø¿Æ_Ø¿½üÉ'øknnö&Ï¿ÑÌ¾0gË-½²þàOð¿h_ÿiÒ;zÎÍÍ?Á_·k×®^Ù~þàOðY¿É'Gïêá¯»»ûG^è¿ì««««¾¾þºÙð'ø¿Ç_SSS¯oò|äÈøüeûÂÿëR~ÙçÌ3pöÁü	þà/ãñ=ÝÎ??ºÚ7??¿¬¬¬­­m¨Wþ#Î¾[o½uÏ=büÁàþ²#ü	þ2ð?ø?øü"öÁü	þà/ð÷þûïO6-///<1äççÏ=»½½þZÝ¶mÛÒÙWUUu³~éàþðÁøkllìõS§NÁà/³êèè¿YEEECÇ>ø?Áüe<þ¢7y®®®ÞÐõÒ¥K«W¯sJJJàOðYì+,,LaßwÞ9¿hð?øË`üEÏÉ/Ó7|?Á_²oùòåáé¾(üÁàþ2Ñ¿îîîÄË/;ò'øÃ>ø?Á²ù5ÕÕÕÁ|áæ'|RYYé5¿cßØ±cï¾ûî¡füÁàþ2c®Õÿ?ÁßuÔÖÖ¶víÚtöÕÖÖÃ³Ãü	þà/ðs­rssáOðöáç,ûàþðñø©àOðì?øüÁ_Æã¯¯÷s>þ<ü	þ°þàOðÙ¿ðôÓO§Ìf·züaüÁàþ²ó5áIeòäÉ.7_íµèif^ê¿þß~:ûüñ8°þàOð¿O¯ç`JJJ¢+VõªÃà/¥ðQUUr¹`_øeéèèÕªÂü	þà/³ñ:tèPâÉ¦¾¾~VþÈ>ø?ÁüeþxàèÉ&úk¡î¹þØð'ø¿,Ä_nnnx²0aÂ'>MzÍ_^^ü	þ°þàOðÙ¿ð|óØc¥Ì¬­­uµ¯ào(êééillÌÁü	þà/ãñ××ûüç`øüÝíØ±cúôéÍ>ø?Áüe<þF*øÓèÁ_6±þàOð¿ðÄ|b·ÿð'ø»î^~ùåtö9Û¶mËDöÁü	þàþàOð×öì9sf:ûvìØÑÓÓÑßüÁàþàþ¿±³ÈVöÁü	þàþàOð÷ÿzýõ×³ð?ø?øüýW»víJg_iii6±þàOððÕø°Û³gO¯t¼üòËÙÇ>ø?ÁüÁüiâ/À.ð®´´4ÙÊ>ø?Áüe0þúþÕÕÕµeË©S§¦üÖïÚµ+»Ùð'ø¿LÅ_ÎµÊÍÍ?Á_:ûêëëGçÑ>ø?Áüe6þF<øSfá¯³³3üÐ¥°¯¢¢¢©©iô°þàOððÊrüuttlÙ²%ýh_UUÕ¨:Úð'ø?ø?e9þûÂjaaaû*++ÿ§þð'ø?øËÂþú×¿¾aÃ¼ÑqLð÷ñÇ?úè£éì[ºté¨=Úð'ø?ø?]»§zê·~ë·ªªªfÌ±`ÁÿüçqÆ_`£ð?Áüéz:zôhßÚÿoíÆ¯mþýÁü?øò¿Oüö­]»vìØ±)ì[¾|yøYu´þàOððºF_ÿú×ï¸ãüÂ¿u®+,,þÚÚÚjkkÇ~7øÕv?øüÁüÁÔ×¾öµ/ñKÉøìÑÇ±â¿½íÏ¶ üÁàþàþ4^ýõÏMû_K.½í¶Ûâ¿^Ùn®Y³&³þ¸0ü	þàOððzzz¾ð/Ì3÷?ð¿üÆß?­ýíkmmµÕàþ?øÓõ×ÑÑñ§ú§·ÜrK`ßm·ÝvMØ)þú:Úf9sÆÆ?øü	þàOÃÝá/ü°-]º´Wövø?Áàþ%ø?fUUU)gx±þàOð'ø?eþ°þàÏ8ÀàþàoTà¯Wö7ûàþ?øSVá¯Wö·Ãð'øüÁ²Ø?øüÁüüaü	þàOðÚÛÛKKKáOC?ì?Áü	þbÔáÃgÍáO7===;vì9s&öÁàþ1jÉ%­­­ýàïßüfãð¶ÿþïïqaÍÔÏ½_ýêWKJJRØWPPðå/ùoþæoàð´wï^óìî2w§Ñ¼»Ë`üýzûÆßÛo¿ý·ýèGmmmÿW1îìÙ³ÍÍÍéóùË_nß¾ýsûû&Nøõ¯ýã?6tÃÙw¾ó_ýêWÆ!Î?~ü>0qîÌ3ï¼óqyûöí»|ùò0ÑlÆÓ¾àiß;vL>=¥¥¥Nò:í+§öÓ¾#O½(øÓã¯§§çõ×_///Oa_`à`¸×pÁàþ±þtø°ùåÓöaü	þàOððU§«ð³1gÎì?Áü	þ2J)z©S§bü	þàOððÍuuumÞ¼¹´´4·Þzk¸×ÁàþðYÂ¾-[¶¤í«¨¨Øµk£ð'ø?ÁüÁ_ö°¯×£,øË¿üKì?Áü	þàþ²¤^x!åååáwlPÛWð'ø?ÁüÁ_ÜÙW\þÚ¾¦¦¦èsàþð'ø?øËö=ûì³éì+//?zôhògÂü	þàOððì«¬¬ìKmüÁàþðÙÇüðÃ?>wÞygØô]Òð'ø?ÁüÁ_°oéÒ¥×öõüÁàþðÓ¹sçêêêÒÙ·|ùòðT4%Àü	þàOððÁì«©© ûàþð'ø?øËÚÚÚÖ¬Y3nÜ¸tö]ãàþð'ø?ø/ûjkkÇl¾ps°Gûàþð'ø?øËTö:uêFð'ø?ÁüÁ_jii	ÂKgßÝwßÝÚÚzãË?øüÁàþà/.ì[µjUûBµµµmmm7ë«Àü	þàOððGöëÖ­»)Gûàþð'ø?ø/ûÆÿðÃßÄ£ð?øüÁüÅÑÑ¾sçÎÝ×?øüÁàþàoX;zôhú%a)ûàþð'ø?øÖÂ©ªªJ¹£¸¸øñÇÿøãgàþð'ø?øöÍÔÙÙ9kð'ø?ÁüÁßp³¯¸¸xóæÍ]]]Ã¿>ð?øüÁüû¦OØ7ÌGûàþð'ø?øÂÒÙWZZºeË]7ø?Áü	þàþnZåååéGûvíÚ5âì?øüÁàþàohÙwë­·nÛ¶-&ì?øüÁàþàïæT__Â¾3gîÙ³'Vì?øüÁàþàïæÔÑÑQXX±¯¼¼|øGþàOð'ø?ø¿aíñÇ¯ªªjhháÑ>ø?Áü	þàþnr17üÁàþð£1ø?Áü	þàþàOð'ø?ÁüÁü	þð'ø?ø?Áàþðð'ø?Áàþ?øü	þàOð'ø?Áàþ?øüÁüÁàþ?ø?øüÁàOðð?øü	þàþàOð?øüÁüÁàOð?ø?øü	þàOðð?Áü	þàþàOð?Áü	þð'øüÁàOð?Áü	þð'ø?ø?Áü	þðð'ø?Áàþàþð'øüÁüÁàþð'ø?ø?Áàþðð'øüÁàþàþ?øüÁüÁàþ?øü	þàOð'ø?Áàþ?øü	þàOðð?øü	þàþàOð?ÁüÁü	þàOð'ø?ø?Áü	þàOðð?Áü	þàþàOð'ø?ÁüÁü	þð'ø?ø?Áü	þðð'ø?Áàþ?øü	þàOð'ø?ÁüÁü	þàþàOðð?øü	þàþàOð?ÁüÁü	þàOð¿lÂ_ssó¼yórssçÎþó?øüÁq¿¬Âß39&vîÜYVV¿·ß~ûòð~ÍÚÚÚ.+Æ9s&ü·Á8Ä¼¿ÎÎNãçÂ¢>øàãç~úÓ¾óÎ;Æ!æüýêW¿æ/ÁøK.???ßúÖ·ÞÞ¾ûÝï~ïß[1îÀû÷ï71oïÞ½!æÙÝÙÝ)swwÙ¿cÇÕÕÕ9í+§öÓ¾rÚ×ißl>íuéÒ¥êêêÎÎNøüÁàOðÙ¿1ÿ]tóüùóµµµáé<ý3áOð?ø3ðñøK®©©iñâÅííí½Þ?øüÁq¿¬Â_iié¤àOð?Áüe3þúþð'ø?ãððð'ø?ÁüÁü	þð'ø?ø?Áàþðð'ø?ø?ÁüÁü	þàOð'ø?Áàþ?øü	þàOðð?Áü	þàþàOð?ÁüÁü	þàOð'ø?ø?Áü	þàþào´áïÉ'Üµk×áí»ßýî;ï¼sF1îG?úÑÞ½CÌûë¿þëÓ§O8vwÇ3q.È/ÀÂ8Ä¼íÛ·ÿô§?æ/ÚÑÑø;yòäÆÿB$IIõjtã±$I£'ø$I?I$Á$IàO$Ið'I$ø$IüÅ®¦¦¦Y³fåææÎ=ûðáÃÉw½÷ÞóçÏÏËË[¹råÅÕÖÏ¶8~üxIII´<h¬b¸Bííí¥¥¥F)¶Û¨¹¹yÞ¼yá÷hîÜ¹þDl7ÓÉ',XÐëâ³»8p`Ì§üõYQQÑÙ³gÃDøòä~ÇÂ1L:uê0V#X?Û¢ººúÕW_[·n]½zµ±á6ÏRá¿XqØÚFm£3f9r$LìÜ¹³¬¬ÌXÅs3ó½õÖ[a"l¬ðäe¬b¸B===á¿Rðë¦Mvþüù0>éä»Âÿ®Ó'N4V#X?Û"ì£¿ÛÝÝíØR<·Ñ%KZ[[á/ÎÛ(¹üü|cóÍÔÐÐ0wîÏmôÌ3Ï|ãß¿Xwüøñ°§)|<vìXò]áWëäÉaâµ×^KÞÒþúÙÉ7m¦xn£_ïà/öÛ(vuuuÆ*¶©§§gÂ	á·içÎÆ*ÛèìÙ³óçÏ¿råüÅº:u*RàÂïz÷ÝwËÊÊáýWxdëg[äää$¦óòòU·üeÊ6ºtéRuuugg§±ófúôêKÊ&Ol¬b¸îºë®CÅdgÛg9nôþûïÏ5ËXÅ¡ômöÝÝÝ^=íkoÏm±Î?_[[ûÑGÿ*õÿ¥ÝFc~3øi.Þ¶´´,X° ù®3f?~üÊ+öO?ý´±ÁúÙ555¯¼òJ«««U·üÅ555-^¼¸½½Ý(Åy3»¢'¬æææ°½Ulww:ò¶GWÎa:y½á)SòòòêêêzzzÕÖë¶6ÓáÃsrrJJJ¢Ë¯·mñßF¥¥¥ñ9õµ8fÏ°n¿ývÇhã¼»?I$Á$IàO$Ið'I$ø$IüI$Á$IàO$Ið'I$ø$IüI$	þ$I$I?I$Á$IàO$Ið'I$ø$I?I$Á$o¯½öÚ¢Eò¯vûí·¿ùæ¿±ÿºZÆìm[ÛÒÒÒð­uvv¦ÌsrssKJJ®2ØeJüIÊÈ6nÜ8&­§~:ð·iÓ¦0³¾¾>eþ-[Âü'xâ:)Ið')ó:~üx`MnnîK/½ÔsµíÛ·aæ'²§O3çÎ2Ö¬Ya~[[üI?I£¢î¹'°æ¹çKùüóÏkÖ¬I¦ÏÁçÍ¦üÉ'ÔÕÕM0!ÜUTT´~ýúä³«Xá®ðØN9'NhÑþýûÃÍÛ·o_ÿËî«ÝuàÀ¾ ¶xñâ0¿¹¹91çÈ#aNUUUbÎSO=U\¿råÊóçÏ§ã/ù)súYUIð'I#Ü)S=<óÃ?3KKKqÒ±cÇ¢W¬Xr×>ÝÕÒÒÓë£¢Ñ½wÝu×+WóòòzzzÂ½ácÀSAAAôR¼~&z]½ôïtçÎÉ¢MøòÕW_MÈ/e!·ß~û`ñ×ÏªJ?Iù¢3¼½ì³Æ	KÆÍêÕ«/]-LË-î¬ñ1:ÐÝU]]2Ó¼ÌÇ,ð.:ëzß÷9o½õVÃô½÷ÞÍå8á×­×ï(rÒ¤Iáûmoo7ÃÇ°æAÝÝÝÑ'oáÓÿ>M?Xüõ³ªàOb¿P¯øKÌp)tþüùp3°)º9oÞ¼psÚ´i÷ß@ÛåË	r,-ñ¨èæ~øä¦Äßð1L766^s9áæ×­¯×ç=ôÐCá®o|ãþ÷©íõë×'B`À_ éüùó£Å_?«*	þ$iäw]ºt)yfgggîê;	¶¶¶FþKX'ñB½ô³±)J~0]TT×ÕÕ>qâÄÄ½×_KéäÉSÃtø¦[ZZ÷>|8¬@¯§¿~VUüIÒÈ½îíÅ_L½JÊ×¶··§Ð:qâÄ¦M¢°	5FåçU¯I´|0:Û>ÖÕÕ%æ÷³èHÛGÝ¼páBÿWæ.0Ü½»M0kò]Ñ¿á®x±ü%`FâÞ~VUüIÒÈ]0»uëÖè­^^zé¥¼¼¼ô3-[ ÓÕÕM½æ/z©ò#®_¿>P)º7ñf+½-ºü6úêMMMùý,'z_ô¿ÎÎÎè3ûÁßöíÛÇäÂ·|WôòÇðUÂ ô¿ËáÓ:W®|o?«*	þ$)EoÒSO=õÿö_WlN0Mè¾ûîKer»wïî^ýSa~QQQòá~V#yÅWÚöõÍ^¾|9???úRNv/Y²$ùKL4)|Þí%yÑÉDU÷ö³ªàOâRÊí·ßwµEEÜ¦àïÀÑÛéÍ=ûÐ¡C;;;yäâââL=ôPWWWâÞùóçlÛ¶-eékæ?ðÀ)óûZN(¬LX¥èûy¿Duuu)ïùÕÞÞ^]]F`Â	á»hkkK¼mMò2/^¼tUUUUsssÊWìgU%Á$IàO$Ið'I$ø$IüI$	þ$I$I?I$Á$IàO$Ið'I$I?I$Á$IàO$Ið'I$ø$IÒpõÿ$Ñy¬Q!®IEND®B`


S~Dø¬åç¥^õê°¢««KV655E½«½Hä^5r#W58QßS[ÛcÇæØëêêdlc¿Gy®úaG®MÆAY9+|ATIéi9Ëh4?~<ðË7þ<xPTTTXX(ðÕW_É_tuÈwj*-##C¾8pàq´í»ví¾		æ;÷ìÙ;q¶nÝ*ëïÝ»§^)q)+KJJ¢ÞÇÕ^Dó£¥lwþ¶oß¾ªÁÝpk¾àÊãobbB=rgWêujjJ9666¦|óþýû5·ùØ±cürÄä?Y¸zõªú0u¼òÊ+²¼oß¾ûOÈêï<uêÔÒÒòÞ²æÕW_BÃÃÃÊI¹f9év»eYBP¹¬2³íÚµk²|çÎõYË%Á`º^Ùu^d¹IuáwFW;8QoÕ/cÂæm²l·Û>|(YN´Çúaë5²¬<¬V¾YÂ»wïÊ²<²Ë/@üHêøò·åïºfaa¡,õÕWÊIù/'·lÙ¢¾ð¹á5êóóóÿcû©º9=z$¹ QUU¥¼ÕôÔøËÊÊzG¢®_ÃEÔQ%±Ùl'ê ­dp¢Þª5_påñ§ÌkSÿUÅúaU®MMÖ(gíÜ¹SNnÛ¶MâUþ±¸¸Èo@üHø>3ò'<ª×G¾s9Nª.êuÆ>)¡©ÙôùÔ½VM&²:²ðÂ-¢¹¶¹1yê ­dpKÒµ]påWw±Ï|X5ÇÁQÎQú/ýýýürÄd?ñÑGÉÉðQJiÞ£+iØ'KKKC®H­$þ·'£®ØíöãÇß¿ÿÂ²òÐ¡CQão%Ysü­vpÂÖ|ÁÇßJÞùç¤ìñÏÍÍ5]ÕnÜ¸ÑÞÞ®lkïøÔñ÷øÉäýð¼~e²/ð¾û<¡üuõÕW×Ê»_SSS çÏ_Iü)ï-I/+ïê)®^½õ>®ö"«¿Ø£dà	Í¬vT×Ê,=ißÅÅEi»½÷F¦áï½'/(ü[(é,ß/#)Ëê»©ÌÝ±	ñ éâovv6<»KYãõz5ó½Ã;Ï®'þÂ9¢Ø²e|Uïñõ6¿üòË þùwsUYUüÅð±5j^Ã¨®!þnÜ¸¡¾þÝ»w«ÏÕäZìøSuå½Uµ£GòËR#þ²IT½þÖ­[ÊzÂn·GîÞ±¶økëzíµ×¤ÂG	8gÏ-++ËÈÈØºuë¥K^xáð;Oë¿Èªâ/öàzUúÏ`0§¶ªQ]CüþþþmÛ¶É½ëW6¬wphÊO·Ùl£££OçÕívWUUÉµÉr¹õ@àõ×_WÞR2òË°Q=zôòË/¯êØrk¸HÊQ¶Æ*»oB!¥éKKKyÂ þ@9ï¼ó#ø'OÜºu«²mWÞxãñâÄñâÄ? þ@üøñâÄñkvõêU³Ùl0öìÙ³ÚËmÚ´iff&¼FeMEEÅ*^ÎXÛ÷¬ä²k|ùEFFFNNÝnWÎÇ32ùùùrÖíÛ·y þ$É$)#VÙ7ß|S.ÛÙÙ^óî»ïÊöövÇ²,w>¬t:rrppP¯_¿.Ë%%%<ÁìÕdýtãÆ¹lMMMxÍ®]»dÍÄÄDüS,þ?QrYsrrYYYròÁ<©ºüÔAóúë¯ççççææ>Zóm/¼ðBUUæÂoÊò½÷dYÖ(gÝ½×f³I;w*oE^ú§Ç¾È¥KÌf³Õjb>t8rY¹ñ'OTÞÈ<Ý½·¬kþê«¯6.þådiiéðð0O-Ä¤î?eùí·ßeË%%ï¼óúÜnwx~[Ø+¯¼"g]¸pAå«,755)g=ÿüó×®];wîÈú¢¢¢¨×¦¾±/"I÷ÑGÉÓé¼ñÇeù+W®ÈÂo¾²LMMÉòÆd¤5ÄßÒÒ,9rD9ëêÕ«á+Ü³gÏ_|Á³ñ ©ãÏl6Ëò£'"Û+ê¤À¡¡!9kß¾²¼ÿ~¥êÂçÞ¸qãÔ©SUUUÊQ¯MY±/¢Ü°üüüÈË*7^²L$ûd¥|§,ïÜ¹Sr6®mX"×+·M®O½wllLú5ü=òCy þ$oüð²,ÈÉØ%ôøÉ`¹¹¹ò@@Ùº*k³Î?/"üâ/Ô× ¹6õÉ¹&5¦~3O	ÇÞÞÞÍ7+kF£ò¦à3yç/ÁÁAÉMõp þ$cü©ßùSÞ<jñ897ü5¼^ÙbéÆ_ìoôe·lÙ¢óB¡Ðµk×]qÃo®jXVB¹rwx þ$oü)n¹pá2çïÝwß]IñôöößBûè£Âë¼yó¦2p%ñû"§NúéO*¯¼òJäe=*Ë/_V6õÊJ«Õ*Ë_|ñÅÜÜ,lß¾ãâ¯¢¢BÎåþþ~Y~ùåy þ$oüB!	¬Ü'ÔÇêÊ&Wùª'd4óóóO<¹ÂøÇ#çîÚµ+¼ßúÀñãÇågeeíÝ»WÙ±WÏn·+»×ÔÔÜºukUÃ¹i8ÆPÈOt8rãe¶lÙrìØ1¹I<Á þ@üø þ@üøKF¿øÅ/~ûÛßòèÆßïcp³³³>dp·oßþ·û7ÆÄ_<üùÿ¹ônIpÿú×¿fp7nÜø§ú'Æ	÷·û·=b@üÄ@üø?âø?ñGüñâ þ?â þ@üÄñGüÄ?øñâ þâÄ?øñGüøñ þ? þâÄñâÄ@üø#þ?øñGüø#þ?øñÄñ þâø#þâÄ@üÄ@üøtóóóf³9rýÜÜÜ&âø?@ÊÇßððpiiidÛÞÞ^§Ó¹Ü%þ"¾fff~õ«_1H¸_þò_~ù%ãs»Ý÷ïßg)÷îR~W¯^ï½÷ÞÿF|úé§òJÇ8 áäyèñx$ÿÐÐãøKíÍ¾ÿ~£Å_IIImm­Á`¨ªªºuëÙì°ÙlöÅúÉ«G `³o2Æ_ØììlEEñGüÄ?¬4_[[[^^Þ3g¿¤?Eüñâkæv»-²ivv¶¾_CRx³¯×ëüä¿ÚÚZâø?XM¿ëìÙ³Ä_Årrll¬´´Ô`0È&ýGüñâ«ý³ÕØØ©Î¾ÊÊJ·Û¿Cü þXN0ìììÌËËSgÉdr¹Î>âÄ?øK/ÒvÝÝÝÅÅÅêìËÌÌloo×ýN¾Ä?ñ^¤ÊËË5Óû§¦¦Òj? þâOç¼^o½&û¬VkzÖñâÄ@üéßïokkÓìÕa6ûúúÒazñâÄ@ü¥i;ËUPP Î>9yöìÙôÞGüøñi!rz_ff¦Óéôù|ñâÄ@üéGÔé6mzzÁ!þ@üø?ýðûýÍÍÍéÅãñ08Ä?ñ§ËMïK6ÄñâøñF<ÅbÑLïknnöûýñâÄ@üéGÔé²FÖ38Ä?ñ§Q§÷ó×øñâ þt%êô>ÉÔÝÝÍô>âø#þ@üÄ®DÞ×ÖÖÆô>âø#þ@ü þt%êô>ÃÁô>âø#þâÄ®DÞgµZùCOüÄ@üøÓP(ÔÑÑ¡ÞW\Ìô>âø#þâÄÞDNï¿ï@Á!þ?â þ@üéÇôô´ÍfÓLïs:>Á!þ?â þ@üéÇÂÂBSSfzàÈÈCüÄ@üøÓP(ÔÙÙ©Þg±Xzzzâø#þâÄ®íØ±C3½¯££½:?âø?º233ÓÐÐ Þ×ÜÜÌA?âø?º"¿¤éõõõñGüñâO?Áà3gòòòÔÙg4/^¼ÈàÄñ þt¥¿¿ßl6«³/;;»½½í¼ÄñGüÄ?]<z_cc£üí`p?âø?ºúë §É>ÞGüÄ@üøÓ`0ØÑÑ¡Þg2.^¼Èa?âø?ºâr¹¤óÔÙ'xöìY>ø#þ?øñ§+CCCV«5òÃyùÅ$þ?â þ@üéüêÕ××k²¯®®éÄñGüÄ?]ñûýíííç5Ín·gñGüñâOWº»»#§÷µ¶¶2½ø#þ?øñ§+Q§÷555ù|>ÄñGüÄ?]½æ744DNïç¡'þ?â þ@üéÇÂÂBKKæèÅÅÅLï#þ?â þ@üé*þB¡PGGf¯ììì3gÎ0½ø#þ?øñ§«øëéé±X,êìËÌÌlmm]XXà±&þ?â þýÄßôô´ÍfüpÞ©©)eâø#þ?~âÏçó9ÎÌÌLuöíØ±?²ÄñâÄ «ø:½Ïd2¹9ø#þ@üøôQ§÷µ´´°WñGüøñè*þÆÇÇ#§÷544ÌÌÌðÄ?~â/êô¾òòòJâøñâÐOüAù»©Þg4Ï=Ëô>âøñâÐUüEÞ×ÞÞî÷ûy¿Ä7Íë'&&CYYÙàà ñGüÄ¿UYnzGï#þixx¸´´TgÙíöË/ËÂ¹sç>Lüñâo¢Nï«®®æO'ñx÷îF£qiiIä÷*ò­A?Ëõ+Ä×õë×$Üg&ÿudpüñøøxRÝ¤¦¦¦üü|uöÉÉÓ§OñéFÇß×71Zü¨ËáøëëëE|MMMÉËãò»yó&ãûäOîÞ½<·çüùóÅÅÅêìËÊÊjnnþÇüG,Ñgüeddå¹Ëf_6ûlöuzÓéä7Í¾©Êo|eâø?Q_#§÷UVVñ)ãÒ¥K² _ív;ñGüÄ?5åèyyyêì+..îîîæèÄ_ÅrrxxØd2eddÄ@üøÂ<z_[[ÎÇäÄ?@Oñ7==]SS£Þçp8¼^/? þýÄßÂÂBsssäÑûÆÇÇy,@üøñè'þÁ`gg§æÃyM&ÓÅÞâÄ?@Wñ×ÓÓ£9z_fffKKÎâÄ?@Wñ7>>nµZ#§÷É«1âÄ?@?ñ·°°yô>	AþöøñâÐUüB!Ëe28z?âø?è<þ$û4Gï+((?y½ÄñGüø#þ «ø?jÎëóùRzÁ åJüÄ@üøûÖj¦÷Ùl¶?þã?þæ7¿i4î9>kø#þ?øCºÇ_(joo×|8¯ÅbéééIõYXXæû?ùS­§Nÿ¯ÓöF9955Åsø#þ?øCÆ___æèúØ«ã7ÞØµkd_øß¯ûïâ9CüÄ@ü!íâojjÊf³iÚÜÜÜ¬§6¿ðÂßÿþ÷Õñ×ü?-Ïâø#þâiQ?WBpzzZg#ãt:_|ñEuüýð¥VWWó!þ?â þñ;::4Îk±X<.G¦¿¿ÿþè~|üÇJùj=µcÇ·ÞzçñGüñÇ_(êìì4é.Kßm>~üø·¾õ­º?©«¯¯ÿö¶o×þ·ZRMüÄ@üAçñ722¢9z_fff[[¦÷Å ©ÿôOÿ´¥¥åâÅÁ`'ñGüñÝÆ¼`:Íô>Yãõz7ÄñÐOüùýþ¶¶6ÍÑûÊËËù³âø#þâº¿P(är¹L&:ûädww7ÓÝ@üÄ@üAWñçv»5Óû²³³å¯ULïñGüñt¿©©©úúúM¿Ëét2½ÄñGüÄtåg?ûÙÑ£G5uTWW08 þ?â þ +ÝÝÝ£÷L¦¦÷ø#þ?ø®X­VÍÑûZZZÄñÐÆÆFÞâø#þâ:ç÷ûO8¡É¾ï|ç;ccc?âø?èòá¼é]]]×®]êgûÄñGüÄRFOOÅbÑ½¯µµU9zßS?Û þ?â þÆÇÇ5u(GïWÂð÷ þ?â þò|>_ä^åååGóÄ?âø?¤°å>÷âÅQÞGüø#þ?øCªr»Ý;vìPg_AAÁ3gÀr!þ@üÄ@ü!õLNNÚl6Íô¾§>Í?ÄñH%>¯©©I3½Ïjµ®ðOñâø#þâ©!uttäåå©³Ïh4º8/ñâø#þâ)Rx£÷I¶¶¶®öÃy?ÄñHjò·£¼¼½¯±±Qô>âÄñâÄR×ë­¯¯×dÕj_óu þ?â þtü~[[f¯;vtww¯|zñâø#þâÉNÞWPP 9z_GGG0õ þ?â þ,"§÷eff:NÏ÷¬~ñâø#þâuzÍf~¶?øÞâoÓÓdddÄ@ü!yøýþææfÍô>Åâñx6âÇÐ[üe<Á` þ?øC2XnzßªÚLü!Ýã/Ä?ÆaGsÐæÌÌÌææf¿ß¿¡?øCzÅß^yåâø?$PÔé²FÖÇá§ÐsüÍfÁÀ?âøñ$uz_yyy<ÿ:ÐmüUTTDîía4ñGüÄâ,êô>É´þ6 þ¾%¿Wóóóò«%Ò|?ýéOe¡©©i×<11QTTd0ÊÊÊÕgÍÍÍ©«?â þðxémmm=½øCzÅ_8¿¤ödáÎ;KKK²»Îk¶Ûí/_sçÎ>|XVoo¯Óéÿñ533ó«_ýq@Âýò¿üòË/tóÿðuuuÍP²>Q7ÉívÿË¿üâoÃãoóæÍò6<<,ûeá7ÞPÖ¨£Ñ()ò?'³Ù¬>KÊïêÕ«1âï/ÿò/_~ú©¼Ò1H8yz<Æ!ô÷÷744ddd¨³¯´´4áäýüç?çBümxü<y2¼úÏjµ®óÕù¨IÉÚÚZYYUUuëÖ-6û²Ù`³oBéÅÅÅñÞÇf_¤×f_ñÖ[omÙ²EFGGeABPlýW«Þ_8+++ê÷ÌÎÎVTTÄ@ü¥ÈéRòúÀÝ?¤QümÂÂBå×F¾ÊòrßÙÄñâqÐ±ééiÍ¦Þçt:>_RÝNâÄßª9K.É|µÛíê³JJJCtÎÎÎÖÖÖÄ@ü¥¿ßßÚÚª9zàÈÈHÞZâz¿íÛ·+|y¶y6Lr=EEE£££_ßâ'é?âø?B]]]é¥§§'io3ñÝÆ_II:øÂÖ¿·ïÄ?ÆAO<Oee¥fz_GGG2ìÕAü!ãOÙ»~llL9,K2 þ?n^X4mnnnNÈA?_3òÛ<åGü þ?P¦÷ååå©Ë¯¾¾~zz:UîñÝÆßÄÄüB;vìáÃÄñÇ8øÃúuww+¶cÇ·ÛZ÷ønãOlÝºuSõïðAüñÙW^^®þkb4].WOï#þ^ñ·mÛ6vøñâë422bµZ5ï#8q"%¦÷H¯øS~?§¦¦ç^Ä?Æ!Èãåp84Gï«©©LéûEüA·ñWXXÈ þ@üa¼bG~8oOOO*nç%þ.ñ744$¿«'OÄñÇ8øÃõõõIçiÞ×ÙÙ<ÎKüø[æ*ÁÄ@ü!ªññqÍô>åèÉöá¼Ä¿è2ÁÄ@üAÃëõFNï³Ùlú»³ÄtIø#þ@ü1É&9sFÊÑût0½øCzÅÙlÞ¶m[Rrø#þ@ü1IåÃ?Þ×ÑÑ<Å?«`0ä×8©îñGüøcÛí®®®ÖLïkiiYXXÐý'þ Ûø_æööö$É_?âÄã¯	õõõ¼^oñÝÆûøñ5¿ßßÖÖ§þ£`±Xôqô>âÄûøñ¯IÛutthÚ¬½OßÓû?¤Wü%!âøñÇ8Ä_OOÅb<z_ê~8/ñâøñâQLOOÛl6Íäúúú¤úØwâxñ'Oî½÷æääÈonnî»çñGüøcâÃçó9NÍÑûª««âº¿ÅÅÅ¨;|$pzñGüøc6ÚrÓûWZíÕAü!ã¯´´T~á÷íÛ÷ðáC9ùàÁýû÷Ëçø#þâO¢NïkkkKÛéÄÒ+þ²²²ä×^ýÿ<y®ËYOüñ§3ãããQ§÷¥ÏÑû?ÿ~¨ùÍW?¿Á ¬áP/Ä@üéIÔéååå¼ÞH»øS6ûÖÖÖ*å«,Ë;wÄ@üéü^^WÞGüøûÔ^Ô>îß¿OüñêÞGüøbqqñÀùùùòµ¶¶VÖ$ð^Ä?Æaý¢Nïs8Lï#þ@ü%âøñÇ8¬Óû?Ä?i!êô>ÉÔÝÝÍô>âé&##ø#þâ/0½øñKÆò?â þRÓû?k÷Úk¯)¯|ðñGüÄ_òÿæ2½øñ·F¹¹¹òª!ÿLìÓø#þ@ü1O¥LïËËËczñâo-:¤¼pv-á÷ø#þ@ü1±Iá1½øñ·FW®^8öíÛ$÷ø#þ@ü1Ë¯©©azñâo-þyeßÁÁÁä¹WÄñâq$ÃÒÒÒ¢ÞgµZyÁ$þ@ü­Èï½§¼p>|8ÙîñGüøcÔB¡PGGÓû?à1ÇùñâO÷>üðÃââbõësvv¶¼N2½øñ·:Oc0?â þhdd¤ººZó?ó¦÷ þôø#þ@ü¥ù HÞ9ÎììluöUVVó!þ@ü þ@üéG <zÙlfzñ þ@üé´ËåÎSg_AAAgg§!Oâ þ@üøÓÇSYY©9h³Óéôù|<%?øñâO?fff<¨Ù«ÃjµNMMñd þâÄ?ýP¦÷iöê°X,n·éÄðìããüøñ(Òv/^4Lé.ì#þ?õ!ý¢ÆÇù#þâo#Èë[äô¾ÖÖVpCüaòÒóÒK/=|øPNÊ×ýû÷Ëë×¯Ä@ü­Ó~ðÜÜo¶|µÙlÿi×ÕÕ1½øâF£Q^Ôä¹®|jä:¯ybb¢¨¨È`0®ð,âøñ§ø³Z­[¶üÿÇ×NTW|ãßÐìÕáv»yÄ? Þñ§¼©ßJü­ÎÝn¿|ù²,;wîðáÃ+<ø#þ@üé#þäüÃùññ¿øâ¹¹¹é]]]Lï#þÄÄ_aa¡¼I-..>~²÷Ù¾ûd¬_ç5Æ¥¥%¥&ÍfóÏRâÏårÝ@|Ip2H¸Ï>ûlxxXw¤¹¹Ùüf;oYYÙßýÝßñ@'¿?þxbbq@ümxü]¿~=ê_|ñÅ:¯Y½Ëf÷g)ñwåÊB|ýú×¿apR~7oÞLõ1>>þï|GóºZRRb2N8Á£>ùäßþö·âoÃãOÜ¹sG^rrr)É;wî¼wïÞú¯V½á8++kg±ÙÍ¾`³oJoöõûýmmmçæûã?:ò£ÌÌLI[e6ûÜì»q_ùªÙã,âøñ¢ñº»»5GïûÆ7¾ñÜsÏ½ðÂUUòÝ·ß~øtãÒ¥K² _ívûÏ"þ?©òªU^^®9z_KKËððpCCCiiéÞ½GFFx|? )âïæÍÛ·oWDõøÉÛr½½½ë¿ZyÉÿËu~7mZî,âøR1þ¼^o½fzßÁåwGø1þ<«?ÒMY>wîîñGüøKê÷û333ÕÙg³Ù8h3ñ$uü)ÓSnÞ¼¿ÑÑQYÎÏÏ'þ?ø*¹Í^ÝÝÝ<Äìñ§¼f)Jü---ñÙ¾Ä@ü-ÇãñX,uöeggËW àá#þ?å ÏÊ»òDýõ×e9òØËÄñ¤yüMOOÛl6Í^N§éÄJñ744õ Ïþ9ñGüÄÂçóIäi¦÷Y­ÖÉÉI2âH±øsssUUUÊÞ¾999Û·o÷z½	¼WÄñâ/ynO(êèèÞ×ÓÓÃó@ªÆ_²!þ?Irc¤ð"§÷uvv2½øR8þÂûyÝ»woûöíÅÅÅÄñ¤müGÞØ# þ?y®³·/ñ¤müÉïBäô¾êêêééiâHáø+..ÞÓæÍ?âH«øóûýòú§~1Ü±cG__Óû? åãïîÝ»O?ÛCMÊïÊ+Äñ¤Oüuww+½+((àèÄãýÄ_¤^·ðÄðøÍÖ#G,,,ðptIø#þ@üÅçgy½^Ã¡É¾úúzÞâz?yfoß¾=777¼fË-o¿ý6ñGü:?¿ßßÖÖ¦Ù«Ãb±0½Äô[·nÕìð«¼¶··Ä Ëøs¹6ËÉ²ÄÒ"þ²²²äojj*¼fbbBÖäççÄ ³øóx<ÕÕÕí¼MMM~¿ñt?eß¥¥¥ß¹ÞÿÄÒñçóù"§÷5440½ÄÒ.þäðøñãÁ`PN...>ZùäJâøt~¿¿½½]sô>ÅÒßßÏhøC:ÆßØØXÔ<ÄêñwñâÅÈ£÷utt(ÿÝ?¤cüÙÙÙ²²²ÜÜÜÒÒRYÀEü þÖ=§¼¼<òÃy>âéÉø#þ@ü­ç¼^ïÁ5q±Ùl|8/?Ä?è*þ#ÞçñxX þþÓÍ7ã<+øöööÄZñõè²£÷øñ÷;Â/Jü)ËçÎ#þ? %â/òèÍÍÍ½Ä¿(ýànÞ¼¿ÑÑQòLü)^¯·¾¾>òÃye=#	âÄß2WñÄcÕdÙ`0Ä´ñ·°° ¯£÷óêâÄßS*GõSâOè¯¿þº,Ífâø0þB¡PWW&ûL&Sww7Óû@üøº¡¡¡¨yþüóÏ?âH¶ø¬ÊÊJÍô¾¶¶6¦÷øñ·sssUUUÊÞ¾999Û·oOìtâøñ¹^^l6æ¿©é þ@ü¥<âøñ§^ã÷ûÛÚÚ4Ûy­V+/ þ@ü þ «øB£÷3½Ä¿u¹ûö¶mÛ²²²äU5''§¬¬l~~ø#þÄÆ¼h¦÷IÊëC `@üø[;Çu~&ñGü!ÍãO^"§÷9NÏÇøøñ·^ÊAív»òé>|XÖÄgwßûÞ÷4Î+!822Âàø?Ã<«gÏÈrøÏÄñÄGÔ£÷Y,Äð,ãOyçOýü^\ä?â'·Û½cÇÍô¾öêñ<ûøSæüÙívi>9yÿþýæü@|ÈKMuuµ:û222~ô£qÐfÀFÅß¦§ÿö_âøC:ðù|'NÐLï¼råÊJ>Û þ@ü­QÆÓâø¡@  ¿æéååå¡Pè©í þôø#þ cn·Ûb±Ä8zñâØØø[îxÎ³³³Äñ<Cò­L)V;räÈÂÂúÛ?ÀÆÆ¼ø¾õÖ[òrÌ¡^?àY¼WMö544LNNF~3ñâØØøÈWáÂÂÂ÷îÉÉ÷ß_y]ÿT?âøþ(Óû4Îk±Xúúú»ñâØØøüä>å¹¨¨HYØ¿ïñGüAºººÌf³:ûòòòÎ9cøñlxüë×¯_åõ:±÷ø#þêFFF"§÷µ´´¬¤ê?ÀÇß«¯¾ª¼4+ö!^zé%âøÖÀëõ666jÞWWWuzñâH@üyiÎÏÏÜÇª9YYYÄñ¬lmmÕdÅbñx<«ºâÄ°±ñ'¯Î§NÒ¬t:ìíKü+¢Nï;öì>øñllü-w¿¹¹9âøÊãñhÚ¹ÂéÄ? ñ·q&&&CYYÙàà ¦,Õ*?â©(êô>Í6==½«%þ@üò­Þ°ûäÚØíöË/ËÂ¹sç>¬>«··×ét.wA?Çóÿ_·oß?ºVâÿùO>­Î¾mÛ¶½ÿþûë¿ò±±±ßüæ72NâÏï÷3¿?£Ñ¸´´$ò?'³Ù¬>KÊïêÕ«1â¯³³sñ%/s|ò	ãØ>ýôÓ£GnÞ¼Y3½OV~üñÇÏäGÈóp``¡FÂýìg?ûì³ÏÄ_Çú3B4RRRR[[++«ªªnÝºÅf_6û"%ôôôh¦÷	ù¿Ïç?Í¾`³/ªñ§¾å3;;[QQAüHrÓÓÓ6M«:zñâÐgü©÷á(,,T~mä«,/wÈ.$þ?$Ïçt:5u»ÝîúÄ? âOÍáptIä«ÝnWURRâõz?yç¯¶¶ø#þB¡PGGGAA:ûä¤ËåZÃÑû?þãoxxØd2Éõ¯Y¾é?âøC²ÞÙÖÖæ÷û7úG þ¿Øøâéi||<rz_½òn þ¿§ÑìKüÐ½å¦÷Åù÷øñlHü%3âøCAù½ÿô>âÄ@üÄâ-Óû?ñGü¨ÓûGÜ¦÷ þâø#þI2½øñÄñuzÉdêîîÿô>âÄ@üÄ6P²Mï#þ@üÄñGüaC$çô>âÄ@üÄý#´Óû?ñGüxféyyyI;½øñÄñgC/ù§÷ þâø#þ°^555)1½øñÄñµóù|yéV«5µ~¡?ñâOºººFcjMï#þ@üÄñGüaÕ"Þ-¿GÉ?½øñÄñU©®®ÖLïs:©2½øñÄññù|---ÙÙÙêì«¬¬ÔÁïñâ þ@üá?Èç5Í===)7½øñÄñXt:ûòòòRwzñâ þ?âÑMMME=zþ:øñ þÒý¡9räæè6mrrR÷øñ þÒßïommÕ|8¯Ùlöx<:¾×Ä?øñúúúL&:ûFcggg Ð÷'þ@üÄ¿ô"Ï«ÕªÎ¾ÌÌL§Ó&IDüø?ébff¦±±Q³WG]]¬OA þ@üÄ?ý[XXhnnÖìÕ±cÇþþþtâÄ@üøÓ³P(är¹F£:ûL&SWWî§÷ þâø#þÒK__ÅbÑLïknnÖÓA?ñGüQtww×ÕÕ¼øâ###ºðÉÉÉúúzÍô¾¯×æOEâÄ@üAÿñwòäÉçîûßÿ~óÿl®ß[ÿÍo~SÇý'Cíp84Óû*++õô>âÄ@üÄß×¦¦¦þàþàÇÇ|úVþïÐ÷¾ýíoëoý~¿<½5m.((¸xñb(âIHüø?¤Eü¹ï~÷»áòSþmÙ²Eg»;twwkÚÝÞÞ¾°°ÀÓøñÄ_zÅßÝý_uò|®©©ÑLïkllL«£÷ þâø#þ¾655õû¿ÿû-ÇZÂåwàÀÅ¢Qu8ì³Z­é°Gñâ þ?âoYï¾ûî·¾õ­úúú8~ðÝïþáþáäädJgÔéÅÅÅÝÝÝLï#þ@üÄñîñ÷øÉ¶Ñ¦¦¦ºººW_uzz:uGRÚ.êô>yb§çA?ñGüº%OÝòòrÍv^§ÓÉÑû?ñGüº"yÇô>âÄ@üÄþùýþ¶¶6ÍAÞGüø?âøÓ¨ÓûÞGüø?âøÓå¦÷ù|>øñÄñ§Q§÷Ùl6¦÷ þâø#þte¹é===ñâ þ?âO?ÞGüø?âøKLï#þ@üÄñGü¥¯×[__Ïô>âÄ@üÄÎ1½øñGüø[µùùy³Ù¹~bb¢¨¨È`0Ä_R	B.«  éÄ?ÆÄß*Ê_ÍÈ³ìvûåËeáÜ¹s&þ¿äáñx4Óû233ÞGüø¿§Û»wïÌÌLÔø3KKK² ¿WoJü]¸páÄÄúÏþót¿ù¿ùîw¿«Þ÷üóÏÿõ_ÿ5OxüüóÏ$Üµk×ä¿"â/ãïë-þCÔåpüÉÛÿøúû¿ÿù÷jjêÈ#ÓûºººxbÄß/~ñyD$Äß;wÄ>ã/###¼Åf_6û&ÐÙ³g#§÷uttB!lö°Ù÷é©§Êo|eâøKêêjÞGüÄ¿g+Ç¥KdA¾Úívâøÿ'¡f;¯Ífç@üÄ¿gÊÉááaÉQTT4::Jüqã÷û[[[óòòÔÙg±XÜn7Ïâ þ@ü%ñGümåèÎæÌÞGüÄ?âøÓyFY­VÍÑûZ[[ý~?=ñ þ?âO?fff4Óûêêêd=:ñ þ?âO?çÍÎÎVg_ee%Ï.â þ@üÐUüB¡®®.£Ñ¨Î>9)+9zñ þ?è*þÜn·ÅbÑLïkiiYXXàQ&þâÄñýÄ×ë<z_ccãÔÔ/ñ þ?è'þ£÷1½ø?ÄtÊÑû4Î[\ÜÝÝÍô>â þ@üÐUüy<ÊÊJuöåååµ··sÐfâ þ@üÐUüMNNÖÕÕmú]MMMDñ þ?è*þü~ss³f¯«Õ:>>ÎHüÄ?âú¿P(ÔÑÑ¡Þg2>üðC¦÷ñâø®â¯§§Gsô>©À3gÎ0½ø?Ätããã6MsÐf§ÓéóùxÔ?øñGüA?ñ'y'§Þ'!8==ÍãEüÄ?âú¿`0(OÍô>Åâñxx¤?øñGüAWñ×ÝÝ9½Ïår±Wññ]Å<î555émmm~¿ø?ø~âO~ºÃáÐ´¹ººéÄ@üÄt~¿_ñ¼¼<uöÍæ¾¾>¶óñÐOüIÛuwwL&Íóvvvrô>âqñÐUüÉC^®Þ×ÒÒÂ_ þâº?¯×[__¯ÞWWW799É£âÄ@üA?ñç÷û5mÞ±cÓû@üø?âOWñ'mçr¹4m6Lïñâ þ?½ÅÇãÑ´9;;»¥¥çñâ þ?]Åßôô´ÍfÓLïkhhaÀAüø?âO?ñçóùN§fzÎâÄ@ü:¿ÈérRÖ`0È8øñÄ~âÏãñTWWkÞçt:a þâøÓOüÉ_ëÍô>ÍÆô> þâøÓUüùýþÖÖVÍóZ,¡¡!FÄ?ø#þtÎ+'].mñâ þ?]ÅÇã±Z­é'Nà Í þ@üÄñ§«øóz½<zßÔÔÃâÄ@üú¿@ 9½¯¦¦fddñâ þ?ýÄ_(:ö¬ÙlÖ½OV2½Ä?ø#þtCCC;vìÐ|8okk«ßïgÜ@üø?âO?ñçõz#Þçt:ùÛâÄ@üº¿'Ndgg«³¯²²£÷øñÄ®â/uuuFuöwww3½Ä?ø#þt§²²R3½¯³³£÷øñÄ®TWWk¦÷<xÐëõ28 þ@üÄñ§~¿¿¹¹9##C6m||Áñâ þ?ýçíïïgp@üø#þ@üº"§9hóïýÞïÉà3½Ä@üø#þô&©ÛùÃþÐãñ02 þâÄñ§O2ÈJùY­Ö|¶/@üø¿XæççÍfsäú¹¹9õÖFâ/QN8Ñ××§½øñ þÖexx¸´´4²íDoo¯ÓéöÙ"âëöíÛòGq@Âýæ7¿apn·û_ÿõ_Ä_ÇßÞ½gff¢ÆßÕ«WcÄß_üÅ_|øÿã~òÉ'N®¿¿ßãñ0¿ÔÞìûï71ZüÔÖÖªªª[·n±Ù7°ÙlöØì6ûnTüÍÎÎVTTÄ@üøR2þ"÷á"++ø#þâÄñ5#W(;;;[[[KüñâÐgü)'ÇÆÆJKKCMMôñGüÄ?@'ñ·Äñâqñ þ@üÄ?âÄ?øñGüøñ þ? þâÄñâÄ@üø#þ?øñÄñ þâø#þâÄ@üÄ@üø?âø?ñGü þ?@ºÅßýÙõôôüñ522"¯tîÚµkãããî¯þê¯¾üòKÆñç÷ûÓ.þnÞ¼yúôé?H?±·nâÍO6ûøñâÄ?H:óóóf³Y³r```Ó&íÓibb¢¨¨È`02tHÈópnnnC*Æx¾ñâ)ixx¸´´TóvîÜùgÕn·_¾|YÎ;wøðaF	yööö:Nñy*Æx¾ñâ)iïÞ½333¿¯o¿ýöO~òÈ?ºF£qiiI=zù&ç¡ü%¾zõ*ø<c<ßxIñT~Þ¨^éîÞ½[UU%¯htCÔe ÏÃÚÚZyÊ7ÜºuqÃ>c<ßxIñ¼Ò8pàúõëðrVVã<Ãfgg+**7lèS1ÆóDÐÉ+Ý¦ß¥þ¶ÂÂÂG=~²C7$äy¨Æ_-þ"o¼$øÞ^é"W:K.É|µÛíò<,))ñz½¼S[[Ë¸aC1o¼$øÿè*ËÃÃÃ&)##£¨¨httqCBccc¥¥¥¡¦¦Fþ3nØÐ§bÔç/ þ@üøñâÄ?âÄ? þ@üøñâÄ?âÄ$÷ß÷îÝ9OìÙ³çÊ+¿óröDÊ¼øF»µf³YîZ Ð¬5¡¨¨hiiiµ×	ÄtúôéMÞzë-=Å_»¬ìêêÒ¬?ö¬¬ã7Öp@üH=5áüùó¡'. 'eå7twîÜõ¥¥¥²Þëõ?iá¥^¬yçwÔ+ß÷]YyäÈuúJ<IîÜ¹SÃß|ÿþý¦¦¦üü|9Ëh4?~uÕãñH`ÉYrY·Û­É)Y³yóæÝ»w_»vMN:Íëïï=ÊYr³µÚÚZY?::^óùçËÍ^óæoL&¹ªCÍÎÎFÆ_äõkÖÄ¸©?H°­[·J¸Ü½W½ò«¯¾f³Y7cccÊ¹û÷ï×uìØ1å¬©©©¨RN*ç8p`iiIò1+++É¹òUâ)77Wãzd!êÍ¼§|ðºhÃyùòåpùi®dÏ=«¿7ñ§láò¶i¤:n>üð	YûöíSÎUZGÉGå<6å,»Ý®¼e(ËÃÃÃÊ¨¯óÔ©SwÊV×£GÊ«W¯Ê²|å_~ù©×#á('åÔ·-ê= Ü²eÜßùùy9)_åKq>zôHù¢¢"¹ ÜÇÿ±8''gµñã¦ þ )âOD¿ðz%nf³³³rR²I9¹sçN9¹mÛ¶W^yE¢mqq1|%ò=÷ÒÂRN~õÕWáo`où¯²ìñxz=ròÞ½êÛ¶Üü¼×^MÎúÉO~òø?6m?~¤UUUÊ«¿7ñ§¼ÝõðáCõÊ@  +å¬¹NÃ¥ÿÂ­¨¹5VSê¬È²ÑhÌÊÊrå7oûÔëY.Å4nÞ¼©tª,ËWY;<<,7 êæãÇ_øÄSæ½½÷ÞêÊP4;|çÎÏÏG¾¡uãÆööve#l¸·åÂÛUhÇS¶öÊ×¦¦¦ðú×£¼Ó677§¼wï^ì=swíÚ%ç*G·fU¥ìù+g<xð vüÃTð¹1n*âOÙaÂ`0;wN9ÔËùóç³²²"wÎØ·oN0T¶É÷ÌUæü)SåfffÔ<~ü¸¤²nø`+QMÙýVùéCCCáõ1®Gä§ÌùÊwÆ¿.ß»¬>Kþ855%?Ea¹øSrSrY¾M¢óÐ¡CêscÜTÄ$åÈo¾ùæ¾=¡´Qx9¼Á4¼EØÑ£GÕe©ÖÛÛ#þ?ù(Yo4Õ[cÜõïi»Ü]\ÌÉÉQîfc÷Þ½Õ?bË-òU9Úú:7&ÃTã¦ þ YH ìÙ³'ëÝ»w+»Üjâo``@9^YYÙõë×Ãç×_Ýd2)ÁôÚk¯Áð¹n·»ªªJ¬¨¨Èåri®3òÈUÉúW_U³~¹ërcä&) q¿°¦¦&Í1_óóóv»]F ??_î×ë¶F<ºUÆÊf³j~bøñâÄ? þ@üøñ@üøñâÄ?Ø0ÿh9ssß°UIEND®B`


Detrended Normal Q-Q Plots


ÑÞºu«	þ¤i¿d~°,Îf³ñkgggLwuu­hõä×;wÞ¹s'Ùs×uã½3ÞõãòÉîÆpR²4Þtã×Xýöp±Vám¸ÚÒ+úØ?^¸'¬ðò7oéuëÖ½?Ä¯1s¬ÇÌÙ²eËÐÐPrÇk_O:UøO¶<y2¦¯]»V¸h¬;N§Çz ùu?â*c¨.¿gt¢3ê½ô¥Y_økáÓ X'Úøñ7Î§qÂë×¯ÇtlÍ5k	þ¤i¿ü¾ä#bÅøë­·òsîºn~/Zrx1ÞJ_ã´pUÑa»W[zÅ±bÄx_/_WWWøâ=>~;wîX7à(ü59<ÀÂËåæææÂÇ^y%õ¸JáÆº1«V­:úô¨6Áõ^MzÅñã/yÜ¼y³ð&&¿q>â×ùóç^ãÿ"¼ªHð'M?üVbfmmíXï¸c1%ù5SâÝºq&úö<«k~<äx¼ñ>88X8ä³ùxÇók@³èÐç]ÏZ­¯¯OS^©ó×0UÆ3V£^`<3I'·âøû~ôg×8ÆW®üGá'¼°Hð'M3ü<x°ðd_ZÑyã±ÎÖ-½çæÝwß-âjK¯Xâ=z4~Í1J23ñYÑ>ª"@]´hQò+³ñà/Ù=|ëJ6Ý¶mÛûï¿èÐ¡¹aÃQñ7U&¿N¾I¯8~ügÏ_wÉÿíÿÏ·¤óçÏïÞ½;9Ö?3ZüIÓñöJÞó_õ¼¥âÍ29yvñâÅÉ¢äOýÃú®YbÝÒrJÔ|f+®<¹¢Otzµ¥W,-õë×ç?×ÌIÎ^·nÝ­ákÛ²eËGÇ_²÷ëâÅñ80ü9s&Ù·¹««+Ù«tüøñQ·ïDWþJNÑs£°ê$ð|J/a»µk×¤á¾ûbðáàÒã×?ä³W®q^üIe¿±8òüÙüÇÿóßÇì&ù®YbÝÒr%<*<õ2¿´ÄÕ^±´$nÜ¸ÿtW2çêÕ«E÷ª««Ë ð£à/Ï¤¹sçæ?Y8=öØÈµdÉxB«L¥§è¹QØDGuø;þ|áõ/_¾¼piÑäO9ëVJ<ßògåüñÇ½°Hð'MüÅ»àÊ+Gþó_§NjnnHÅ;úÁóó?s[ßr7Îuï*§äëñâÊF~]ßXW×KK"9$Z8ÿ7ÞØ°aCõpÙlväéÃ____è'åÍ]8ßøÆ7âÑÅ7oÞáÃôÑü§¾ÊðWzpEMhT'¿èÄóçÏG×XÏà¿Ñ[_µjUww÷]÷¼õ|ëïïß±cG²K5rppÐt¯Êår=öØ¾[n«L»=¾ÉéÛCCCé-Zä	#Á$©K>óWÔ3Ï<cd$ø$U`ýýýÛ·o7o^rl7&|òIÃ"Á$IàO$Ið'I$ø$IüI$	þ$I$I?I$ø3$Ið'I$ø$IüI$	þ$I$I?IeÞñãÇÒéôÊ+'ºncccUUÕ+Wòsb:æ,^¼x/XÃMî2ãYwÒõõõmÙ²eÎ918óæÍÛ±cGÿ½ßËÇ%	þ$ÍèêëëÃ¡¡¡®»gÏX÷ÿðósöÙ³÷îé¿pÞá®]»vçÎööö¸¡µk×NâªzzzV­ZàORy¼^|g?>Ö]±bE~ÎÒ¥KcNooïÔÜÉ¤ÄµÇ»/#àOÒ=_!5vìØ1öìY³fíÚµ«èb>úhsssÑ5äwÆô»ï¾Ó1'YtýúõU«VÕÔÔ¤Óé¦¦¦ÎÎÎQ¯­ðÖK¯røðáeËÅDÒíÛ·[[[cÝ¸óÛ·oOvdO/_×óãßzë­qÌâÅãG]ZbÎ9w2áøñã#GxäH¸*ÏOIð'é^ù/~úé§cúàÁÁ¬xæg/sêÔ©ymÞ¼9:t(¦ãgL·µµ%,YròäÉ¸víZÌÏd2£^[á(½JîèÑ£1±iÓ¦w~ëÖ­1xñÅcbÏ=1óáé[·n]¼x1&#9,áÅ±øUzöíÛÜVpä,zì¥¯ÊSüIº·ø¯Ätn¸öõCgÎEëÖ­éõë×'²É/=þüÎ;c~*õÚ SzäÍ=äºuuuÉ¿sçNLûbf¦BWcíÆµø+=JÉtXøË?öÒWåÉ)	þ$Ý[üg,¾Ò5kV¿¿?9ºsEµB.	 QïÀxVëwæ%p<vìØ9s9µµµÉNÁñìù[¸paÌ¿ûviF^ééÉ¸$Á¤L¦pGT²óì®imm¥ùùùÕÕÕ1çÎpãÄ_éUòw,<7rÝ¹sç&ëÝ½¡¡¡'OnÚ´©pá]Û¹sg°8rÑxFiøÜKüIúxðâzèÐ¡ä#hÏ>ûìx,rìØ±ü.´£GÉæÒ¥KÉgÇ¿Ò«È¾õ­oÅÄæÍG®ûøãÇtûåËC½1sÙ²e1áÂ7oÆÄÂÇ9,·nÝ-X°àúõëÊddKKË8G©p:9ôt.zìpI?IþX³+ü®¾ÒH¹ÆÏ0S~þ'jkk>Û·o'þJ¯rúôéXºtéÒüy'éïïß¶m[Üóêêêµk×&'öù²Ùlrúð+ÞxãñLÜJ29¨ÜµkWòy¾ñRáôñãÇã.%ß¡]ôØ'7à$Ið'I$ø$IüI$	þ$I$I¿ré¯þê¯~ô£Må-þã?þã¿üË¿xæ[7oÞ,üæI±QÞyçãPnÅØ¿rj<ýÛ¿ýÛ+WCöÿð?þñá¯ÝßýÝðßs2Ê°/ËC¹%6q(COñ+§ÆÓ;wþò/ÿÒ8agÎùçþgø?Áàþðð'øüÁàþàþð'øüÁüÁàþ?ø?øüÁàþð?øüÁàþàOð?øüÁüÁàOð?ø?øü	þàOðð?øü	þàþàOð?ø?ø?øüÁàþàþ?øüÁüÁàþ?ø?øüÁàOð3½½½L&N766vvv.Ü´iSuuõÃ?£?øüÁàoÚÍfÛÛÛcbÿþý7n,gÏgy&þ$bèçÏ?·§°ï~÷»o¿ýömYßÿþ÷øÃr+6JlãPnÅX¼rë>ü2¬³³óæÍSy3µµµÁ»Èår/_¾<Ö¿ûöw;yòd<	¾«2ëå_~åWC¹%6q(·âE,^ÊCöÒK/2,Pþê«¯Nå-Îü¥ÓéQ§_w555óçÏ¿páÃ¾rØ×a_9ìë°¯öö¥R©ütuuuÑ¢CÅÄo¼ÑÜÜ?øüÁàoÚWWWËå>>ìÓEòÓE;áOð?øüMËZ[[>ñ3Í.Ú²eËÑ£GcâÒ¥KË-?Áü	þàOð7íëêêª¯¯O¥RL¦»»ûß[UUò"ÕÒÒN§¯?øüÁàoæ?øüÁàþàþ?øüÁüÁàOð?ø?øüÁàOðð?øü	þàþàOð?ø?øü	þàOðð?ø?øüÁüÁàþ?ø?øüÁàþð?øüÁàþàOð?øüÁüÁàOð?ø?øü	þàOðð?øü	þàþàOð?ÁüÁü	þàOðð?Áü	þàþàOð'ø?ÁüÁü	þàOð§òÂßï½wúôéS§N½óÎ;6üÁüÁàþTÉøû£?ú£9sæ455-ùÜO|âûöí³-àþàþð§ÊÄß©S§>õ©OmþÍ»~kWü÷ÕßüêC=ôñ6üÁüÁàþTøäG¾Ôò¥D~É¿´áV­ZesÀüÁü	þàO¿û¹ûêo~µñëÏüÌÏØðð?øSâïGùÅõ¿X¿/µ|É?ø?ø?Áü©2ñ7êgþ^|ñEþàþàOðª@ü8|¶ï'>ñÏ5.9Û÷þàlø?ø?Áü©bñ÷áð÷üîí·ß¶!àþàþð§Çàþàþð'øüÁüÁàþðð'øüÁàþàþ?øüÁüÁàþ?ø?øüÁàþàþàþð'ø?ÁüÁàþð'ø?ø?ã?øüÁüÁàOð?ø?øüÁàOðð?øü	þàþàOð?ø?øü	þàOðð?Áü	þàþàOð?ÁüÁü	þàOð'ø?ø?Áü	þàOðð'ø?Áü	þàþàþàOð?ø?øü	þàOðð?ø?øüÁüÁàþ?ø?øüÁàþàþ?øüÁüÁàOð?ø?øüÁàOðð?øü	þàþàOð?øüÁü	þàOð?ø?Áü	þàOð7ÝêííÍd2étº±±±³³sä:::ªªªàOð?øüUBÙl¶½½=&öïß¿qãÆ¢¥CCCMMMcáïÏÿüÏÿiûÎw¾óÃþðTf½öÚk.åVlØ4Æ¡Ü±x)3åÖõë×O8aÊ°W^yåÍ7ßÊ[ø«­­ÿã×ÐÐP´ôé§Þ»wïXø;tèÐ)¬£££§§çÊ¬W_µ««Ë8[±QbÓr+^Äâ¥Ì8[¯¿þzàÏ8a§Núë¿þë©¼Å¿t:=êtÿ'ÔÜÜ4tØWû:ì+öÃ¾¿T*®®®.ÒÒröìÙÿ÷PáOð?øüUÆ°ÖÕÕår¹ûÆôxÿ1øüÁàþÓ¾ÖÖÖÃÇDüÌf³£?Tþð'ø?Á_ekWWW*Êd2ÝÝÝ£jþð'ø?Á/y?Áü	þàOðð?Áü	þàþàOð'ø?ÁüÁü	þàOð'ø?ø?Áü	þàþàþàOð?ø?øü	þàOðð?øü	þàþàOð?ÁüÁü	þàOð?ø?Áü	þàOðð'ø?Áü	þàþàOð'ø?ÁüÁü	þð'ø?ø?Áü	þðð'ø?Áàþàþð'ø?ø?Áàþðð'ø?ø?ÁüÁü	þàOð'ø?ø?Áü	þàÏ8ÀüÁàþð'ø?øüÁàþðð'øüÁàþàþ?øüÁüÁàþ?ø?øüÁàOðð?øüÁüÁàOð?ø?øü	þàOðð?øü	þàþàOð?ø?ø?øüÁàþþªîV*?ø?Áü	þà¯Bðº[étþàþð'ø?áþàOð?ø«,üÅKÃæÍáþàOð?ø«@ü544¤Óiù?øüÁàO¿Å<Û£¶¶¶¿¿þàþð'ø¿JÃ_uuuh¯¯¯¯¾¾>&Â|ßúÖ·b¢­­þàþð'ø¿JÃ_²«/&B1qíÚµxþÅÄ¬Y³àþàOð?ø«4üÍ3'¨×ÕÕuåÊxòÉ'	_õð'ø?Á*Û·oÏÞQø±¿eËÁüÁàþðWgû>õÔSsçÎîîî677Oí?øüÁào?øüÁàþàþ?øüÝÂ/|ñ%Ïð?øü©Âñ·`ÁBðås¶/üÁàþª@üó===ñ´vÛþð'ø?ÁßÄª­­üMGùÁàþð'øp½½½¿­[·Þ¾þàþð'øSã/7o^Õðð'ø?Á*óçÏwÂüÁàþ)øKØ7M_ áOð?øüM¬ºº:'|Àü	þàOð§¿xH¿íÛ·ÂüÁàþªpüU>àþð'øSe~Éó¨9áþàOð?UæW½LßàOð?øüM¬ùóç_¾|ù>>ÞÞÞL&N§;;;uww755Å¢ÅÇÅàOð?øü¤ÂUUU÷yb6mooýû÷oÜ¸±pÑÎ;GY¸p!ü	þàOð¿Tgggào÷îÝñrp¿¾ð¥¶¶6¹é×ÐÐ0ÖÅjjjFâïOÿôOÿÏöÊ+¯?þÿ¨ÌúÞ÷¾÷Úk¯r+6JlãPnÅX¼rëÒ¥K'N0eØË/¿|áÂ©¼Å¿r8Û·ðä±N4éééikk¿£G^ÂN>[åªÊ¬®®®ïÿûÆ¡ÜÆ8[ñ"/eÆ¡Üúû¿ÿûÀq(Ã:::~ðLå-ÞsüÃÙ¾Ð¬®®yÛ·og³Ùþþ~å°¯Ã¾rØ×a_9ì;í«««ËårÉaß.ZzãÆM6Ý¼ysäð'ø?Áü	þ¦_­­­øÍfF|õêÕ£®?øüÁàoÂår¹µk×ÖÔÔTUUÍ5«¥¥eÏüèêêª¯¯O¥RL¦»»ûßÛð9ÈD?Áü	þàOð÷õiñOýÂàþð'øX-ê­[·îöíÛÉÂúõëcÎ%KàþàOð?ø«4üUWWõòsr¹õ¬[ø?øüÁàþ¦7þR©TP/9Ù6ipp0æLåW½Ààþðð7EøKû®^½:9ì?c:æ455ÁüÁàþðWiøízÂÇûï¿ð?øüÁ_~ÕËÀÀ@KKËìÙ³S©TüzuÌÛþð'ø?Áßþð'ø?ÁüÁü	þð'ø+Zón¥R)ø?øüÁàþ*©±?ø?Áü	þTiø«'x"Áß#GàþàOð?ø«XüõööÎ5+Ø·jÕªÂï|?ø?Áü	þà¯Òð·aÃdßÉ'§Ñö?Áü	þàOð7±^|ñÅëÖ­vÛþð'ø?ÁßxX²dIrnGggçtÜð'ø?Áü	þÆÕ¾û~7n¾Ûþð'ø?ÁßøÖô=ð?øüiæà/u·Òé4üÁü	þàOðþy7ø?øüÁàþàþ?øüÁüÁàþ?ø?øüÁàOðð?øüÁàþàOð?øülø[¸pauuµïù?øüÁàO¿Ï÷üÁü	þàOð§JÆ_8/Ø×ÓÓO»i·=àOð?øüM¬ÚÚÚÀßtü	þàOð¿	×ÛÛøÛºuëíÛ·áþàOð?U8þ¢yóæUÈ	ð?øü©ñ7þ|'|Àü	þàOð§¿Óôþð'ø?ÁßÄª««sÂüÁàþ)øøÛ¾ûàà üÁü	þàOð§Ç_Õ9áþàOð?Uæ<>àþð'øSe~ÕËôþð'ø?ÁüÁü	þð'ø»·víÚªªªY³fµ´´LáOð?øüM¬QOø'ÿÂàþð'øX-ê­[·.ù·ãEaýúõ1gÉ%ðð'ø?ÁüUþª««zCCCù9¹æÄ|ø?øüÁàþ*ð«^z¾üÁÁÁã«^àþð'øSÅö]½zurØ7~ÆtÌijj?ø?Áü	þà¯ÒðÚõ÷ßþàþð'ø¿üªÙ³g§R©ø¹zõê3-¶ü	þàOð¿ü	þàOð¿ÕÐÐ0þüË/ÃüÁàþª|ü¥Óéªªéºþð'ø?ÁßÄêììüíÞ½;^¦Ë¿ê?øüÁào²W1F©Tþàþð'ø¿üçQó%Ïð?øü©rð7þüÆÆÆþþþi½=àOð?øü«äôýpø°ï´8Â?øüÁàoòøKþIßäñ?ø?Áü	þTáøËd2U%sÂüÁàþªüõôôÌ3'ÙÿPÏ	ð?øü©bñoº8þð'ø?ÁÛþð'ø?Áßý«··7É¤ÓéÆÆÆÎÎÎÒ&7þð'ø?Á_¹ÍfÛÛÛcbÿþý7n,½hrsÛ¾ýÃSXÜÜ¼yóVÿÐÐÐ`Ê­Ø(±iC¹/bSüÊ©qVWWgÊ°zhßú§þjkk 8ËÅë~éESØO<Q%ITAÝsü½ñÆ#þÏ):dä¢ÉÍ?IáÈñð÷¼455½ùæýÚ¿°ºººô¢ÉÍ)ì·û·wíÚõÍ),nîÿø¿©2ë÷~ï÷¾þõ¯r+6JlãPnÅØ¿rj<µ··ík_3eØÎ;<8·xÏñìËK3N¯X±âêÕ«¾¶ººº¥éÒ&7Ç	rÂ>ä'|È	ÃÁßEåX]]½víÚIëáÃc"~f³ÙÒ&7þð'ø?ÁßÇöÌ;yòdMMMÕdÿy·®®®úúúX7Étwwÿû½>z=rÑäæÀàþð'øûÞ;÷üÍ=»­­­ü·ü	þàOð¿Uh¾7^¿~ºløüÁàþ¼áó<Ö¬YsíÚµi·=àOð?øüM¬å^àOð'ø?ÁüMüMëàOð?øüM¸K.-pÖ¬YÉ¾uuuÇ?ø?Áü	þTøëèèÈðà/Þ¿?üÁü	þàOð¿úúú Þ¥KòøëîîN¾íþàþð'ø¿<Û7ùæ<þâùð?øüÁ_¥á¯®®.¨ìíüår¹;vÄtCCüÁü	þàOð¿xHU£uîÜ9ø?øüÁàþ*ðlß7o677'gûÖÔÔ,ðêÕ«ÓbÀàþð'øAÁàþð'ø?ø?Áàþ£uýúõ¦¦¦Ù³g§ÆÆÆérÌþð'ø?ÁßzüñÇ«ÆhóæÍðð'ø?Á*ÇKwàÀþþþdæÀÀÀñãÇù§N?ø¿Jêßüæ5k,XðÅ/~ñµ×^?øüifá/Éð:4rQò¾ù?ø¿Jjûöíþô§õWõ«¿ùÕ/¬ýÂ>8þ?øüÁ_á¯ºº:7000rQ.Eqø?ø«ñùä'?ùµm_Ûõ[»ÿ~iÃ/ýìÏþ,üÁàO3ùÕm¬¥É?õðÐÁ?ÿùÏçåü7wîÜüG>àþø+Á;ø?ø«0üýÂò_?Áü	þàþào¦Ï<ð?¶þ¼üZZZ>óÏÜëÛ?øüÁ_yá¯tððWI=ûì³úÔ§¾ð/üZë¯~Åçzè¡×_þàOð§¿ÔÝJ§ÓððWIÅ³·­­mÍ5[¶l¹|ùòÜ"üÁàþÊü	þÊ9ø?ÁüÁü	þàOð'ø?ø?Áü	þàþàþàOð?øüÁü	þàOð?ø?øü	þàOðð?Áü	þàþàOð?ÁüÁü	þàOð'ø?ø?Áü	þàþàOð'ø?ÁüÁü	þð'ø?ø?Áü	þðð'ø?Áàþàþð'ø?ÁüÁàþð'ø?ø?ø?Áü	þàþàOð'ø?ÁüÁü	þàÏ8Ààþàþð'øüÁüÁàþðð'øüÁàþàþ?øüÁüÁàþ?ø?øüÁàOðð?øüÁàþàOð?øüÁü	þàOð?ø?øü	þàOðð÷±öÞïÁàþððWáíÝ»÷üd]]Ý9s~åW~eppþð'ø?ø¿ÊlÛ¶mþô§7ÿÆæ]¿µë>ñ?þç~Í5ð'ø?ÁüÁüU`|ðAMMÍßòßC~É;þ×|ðõ×_?Áü	þàþà¯Ò÷òÏ~ö³yù%ÿý×ÿò__xáøüÁàþàþ*­÷Þ¯®®nçÿÞY¿¼öÚkð'ø?ÁüÁüU`¿-ÿ¼ü~qý/f2>øþð'ø?øW½½½¡t:ÝØØØÙÙY¸¨»»»©©)-^¼8.åÐ;ï¼³páÂÏ~ö³+W®üÏMÿù§ú§ïû<üÁàþÓ©l6ÛÞÞû÷ïß¸qcá¢;w.&9à¿2ihhèÄ¿ÿû¿ÿgögo¿ýö¿?ð?øüM§jkkãI¹¡¡a¬ÕÔÔÄß³Ï>jñ|ûÛß>¥2+^1O<iÊ­Ø(±iC¹/bñRfÊ°^zÉ aSÿÖ?#ðN§G.¬§§§­­m$þÂãÿ:=¶¯¯ï_UfýÍßüÍÕ«WC¹%6q(·âE,^ÊC¹õãÿ8þgÉ8a¯¾úê­[·¦ògþR©T~ºººzänß¾ÍfûûûöÃ¾ûÊa_å°ï´¬ê'Åt]]].KûÆtÑ%oÜ¸±iÓ¦7o¼øüÁàþÓ¯ÖÖÖÃÇDüÌf³E#¾zõê¾¾¾QW?Áü	þàOð7ýêêêª¯¯O¥RL¦»»ûßÛðNÁªàOð?øüÍÜàOð?øüÁüÁàOð?ø?øü	þàOðð?øü	þàþàOð?ÁüÁü	þàOðð?Áü	þàþàOðð?ø?øüÁàOðð?øgßüæ7yä<úè£ð'ø?ø?U2þ~úé|ðK-_úêo~õ_üâ<4Úð?ø?øSâïí·ß®©©ÙüwýÖ®ä¿_ÿÊ¯ÿÔOýT¿?øüÁüÁ*§OþçòòKþûÌÏ~Æáþðð§Ä_¼777áïáÿôðåË8üÁàþàþTiøëïïýÙ¿þ_ÏËï³¿üÐCpø?ÁüÁü©Òð½ðÂ<ðÀêÿ¶ú×ZmÅsæÌf£ð'ø?ø?U&þ¿Ä¶¶¶5kÖlÙ²Å_ø?ÁüÁü©Âñ'ø?ÁüÁüÁü	þàOðð?Áü	þàþàOð?ÁüÁü	þàOð'ø?ø?Áü	þàþàOð'ø?ÁüÁü	þð'ø?ø?Áü	þðð'ø?Áàþàþð'ø?ÁüÁàþð'ø?øüÁàþðð'øüÁàþàþðð'ø?ø?Áü	þðð'ø?ÁüÁü	þð'ø?ø?Áàþðð'ø?Áàþàþð'øüÁüÁàþð'ø?øüÁàþð?øüÁàþàþ?øüÁüÁàOð?ø?øüÁàOðð?øü	þàþàOð?ø?øü	þàOðð?ø3ð'ø?ø?Áü	þðð'ø?Áü	þàþð'ø?ÁüÁàþð'ø?ø?Áàþðð'øüÁàþàþð'øüÁüÁàþ?ø?øüÁàþàþ?øüÁüÁàþàþðð'ø?Áàþf:þz3L:nllìììyªª*øüÁàþP6mooýû÷oÜ¸±héÐÐPSSÓXøûÎw¾30ïß»yóæÊ¬×_ýïþîïC¹%6q(·âE,^ÊC¹Õßßø3eXggg__ßTÞâÀ_mmmüOLär¹¢¥O?ýôÞ½ÇÂß×¿þõïLañùÊ+¯|GeÖ·¿ýí_~Ù8[±QbÓr+^Äâ¥Ì8a/½ôA(Ã¦þ­Fà/N:]¿~½¹¹9hè°¯öuØWû:ì++©T*?]]]]¸¨¥¥åìÙ³ÿï¡Âàþð'ø¾CYõbº®®.Ë8|Ø7¦G½XþÂð'ø?Áü	þ¦w­­­øÍfÇÂâÈð'ø?Áü	þ¦_]]]õõõ©T*Étwwª=øüÁàþ¾äþð'ø?ÁüÁü	þð'ø?ø?Áàþðð'ø?Áàþàþð'ø?ø?ø?Áü	þàþàOð'ø?ÁüÁü	þàOð'ø?ø?Áü	þðð'ø?Áü	þàþð'ø?ÁüÁàþð'øYýÎïüÎ/¼ð£),nîoÿöo¤2«££ãÜ¹sÆ¡ÜÆ8[ñ"6Å¯O×®]û?ùãP=zô?øÁTÞâ|cvéÒ¥]»vý®$IR¥Tzß|±$I3'ø$I?I$Á$IàO$Ið'I$ø$IüU`o¼ñFsssuuõþU3gÎ,Z´(N766vuu«©©¯¯¯¡¡!ÿkooo&I¶Bgggá%K,ÒýÝ4ÝÝÝMMM±hñâÅq1CW&Û%©£££ªÊÛJmÁÁÁM6ÅÛÐÃ?ï;®L¶Ë7¿ÒØ%Kâ*&._¾¼eËÂEµµµ×¯_øYø½+þÀâ­ð)Í¶··ÇÄþýû7nÜXxát7ÍÎ;GY¸p¡Ñ+íËá¯¬¶Ë=yæ;wî6æÏoôÊd»Üwø+½êóÓsæÌ)7nÜøérjZ»ví+Wÿ2ãÏ/^c"ËýùX¤û»i«©©1zå³]~úé½÷Â_YmÆÆÆË/´rÛ.÷ÝþJïa/¾téRL<ÿüóüpxop¼oÅ³$~öôô«©Æüen¢Tbîï¦É8mmmÆ­L¶Ëõë×ã­þÊí¥lß¾ñFÂ¸páq+írßà¯ôi.Mÿ7bÉ%ÉÿÅ3`éÒ¥Æê¾üe¦R©ütuuuáÅJ,ÒýÝ4I·oßÎf³ýýýÆ­L¶KKKËÙ³g.¯rx);tèÐ?ùºq+írßà¯t*zóÍ7-ZTôcwÝ±¡ýYWWËå>Þ'Ó+±H÷wÓ|8| dÓ¦M7oÞ4hå³]ªþcÆ­|^Ê¼×áv¹ïð'z[°`A þÎ;÷îê©§ô#Â/^ÿ0V÷å/³µµõðáÃ1?³ÙláÅJ,ÒýÝ4gÎY½zu__+«í2êåuß·Ë-[=ñ³lÙ2ãV&Ûå¾À_é=,Þ¥æÍW]]ÝÖÖ644TøÌxóÍ7c÷ãgL«ûòÙÕÕU__J¥2Lr^vþ£.R9lÊp»À_yn[·nµ´´ÄMssó+W[lûn¥$I3	©@$	þ$I$I?I$Á$IàO$Ið'I$ø$IüI$	þ$I$I?I$ø$IüI$	þ$I$I?I$Á$IàOîÚóÏ?¿|ùòáV®â/þ×¸á¦Í+òh÷¶¡¡!ZÑüN§3Ì;w&zàO¦e»víªÑSO=UIøÛ½wÌ|î¹çæãßùO>ùä$®SüIÒô«··7XN§804Ü¡Câ×yþüùÁßµk×bæâÅæ/Z´(æ_½zþ$Á¤Ñ¿üå`Í3Ï<S8óÙg_ùÊWéÓÙÙx655ÅtþÂï¿ÿ~[[ÛìÙ³cQmmí¶mÛ®>:bÝS§Nq*æÌ3gùòå'O_[[[îØ'J_O²(îR²¨££c,¨­^½:æwwwçç;w.æ¬Zµ*?gÏ=õõõqU5556l¸qãÆHü¼þ¢9%îª$ø¤ûÜ¼yó.×¯_/ùÖ[oÅÌBÜÕÓÓ,]¿~Ñ¢­[·&.^¼J¥F]+ù5YÚÒÒrçÎàcuuõÐÐP,§Y³f%Å+q=11êÝùH9R(Ú¼/ÛÛÛóò+º+WN%îª$ø¤û_rw×µªª X!n6nÜx¸_×­[,M¬ð1ÙhKe³ÙdaLwuu%WRx;wîÞ%G]üñsüøñ1ýØcÝõzñkð¾úsçÎÇÛ××¿ÆÏ¸ç!ÎÉÄñ>üÉaââ¯Ä]TøFÅ_~~ÄLÑ7â×`SòkSSSü:þüÍ7ÚòW)Ú_+ùõ­·ÞÊ_8À?ò?cúôéÓw½füúî»ïÞ·±>÷ÄOÄ¢½÷~øCÛÛ¶m+¼@@0ð$mnnNvLN%îª$ø¤û_²»ëöíÛ3ûûûcf,*Á<¯ø/oüõF-âTá¬Ätmmmuuõàà`9sòKïz=cQ¬¨K.%NéøÓ/^Ì/íêê;0êáãñã¯Ä]tÿK>÷¶oß¾ÂÉ ðÿ__ßÈZçÏß½wr6¯Æd·¸ê]¶uëÖähoülkkËÏ/q=É¶7o&¿¾ûî»¥ÏÌ]ºti,M¾Ý&ÌZ¸(9ó7uttÜºu«4þò0MF#¿´Ä]tÿKNH§Óû÷ïO¾êåÀÕÕÕ#OÎX·n]@gpp09&?37ùÌ_òQ¹+W®~X0±à¶mÛJÉy¸ù/[hÉé·É­9s&?¿Äõ$òK>ó×ßßþ:ß'¹pQòñÇ/Æ­Ä ¿Áå¸X sÃKKÜUIð'IeQòÈEíÙ³çÿ¿ÆØ(??`?Ç"ßã?^(ËÂ;VÿS1¿¶¶¶ðpë»QxÇògÚõ`jjjPt°íÚµ71wîÜø|ÛKáu&;&ó%TÍ/-qW%Á$K+WV·|ùòäÛ"üutt$_§×ØØxöìÙüÒþþþ;vÔ××'`zâ'óKO:ÕÜÜ&Ëd2,ºÎ÷$®*æoÙ²¥hþX×Å»|aïùË×ÖÖVô/IÙl6F`öìÙñ(®^½ÿÚÂë¼uëVè6«U«VuwwÝb»*	þ$I$I?I$Á$IàO$Ið'I$ø$IüI$	þ$IàO$Ið'I$ø$IüI$	þ$I$I?I$Á$I>þ/wu%_ÙàIEND®B`
