# Supplementary material for: Regulation of piglet T-cell immune responses by thioredoxin peroxidase from Cysticercus cellulosae excretory-secretory antigens
Source: Front Microbiol. 2022 Nov 18;13:1019810. doi: 10.3389/fmicb.2022.1019810 (PMC9718028; doi:10.3389/fmicb.2022.1019810)
Supplement: Supplementary file 3 [file Data_Sheet_3.ZIP › 4. C. Cellulosae ESAs and TPx Induced Th Subpopulation Differentiation/3. SPSS statistical analysis/3. IL-5/3. IL5-72h/3.3 (SPSS data export) SPSS statistical analysis--IL5--72h.doc]

EXAMINE VARIABLES=Figures BY Variables
  /PLOT BOXPLOT NPPLOT
  /COMPARE GROUPS
  /STATISTICS DESCRIPTIVES
  /CINTERVAL 95
  /MISSING LISTWISE
  /NOTOTAL.


Explore


Notes	
Output Created	12-SEP-2022 23:25:17	
Comments		
Input	Data	E:\桌面\Raw Data\4. C. Cellulosae ESAs and TPx Induced Th Subpopulation Differentiation\3. SPSS statistical analysis\3. IL-5\3. IL5-72h\3.1 SPSS statistical analysis--IL5--72h.sav	
	Active Dataset	DataSet1	
	Filter	<none>	
	Weight	<none>	
	Split File	<none>	
	N of Rows in Working Data File	20	
Missing Value Handling	Definition of Missing	User-defined missing values for dependent variables are treated as missing.	
	Cases Used	Statistics are based on cases with no missing values for any dependent variable or factor used.	
Syntax	EXAMINE VARIABLES=Figures BY Variables
  /PLOT BOXPLOT NPPLOT
  /COMPARE GROUPS
  /STATISTICS DESCRIPTIVES
  /CINTERVAL 95
  /MISSING LISTWISE
  /NOTOTAL.	
Resources	Processor Time	00:00:01.24	
	Elapsed Time	00:00:00.91	


[DataSet1] E:\桌面\Raw Data\4. C. Cellulosae ESAs and TPx Induced Th Subpopulation Differentiation\3. SPSS statistical analysis\3. IL-5\3. IL5-72h\3.1 SPSS statistical analysis--IL5--72h.sav


Variables


Case Processing Summary	
	Variables	Cases	
		Valid	Missing	Total	
		N	Percent	N	Percent	N	Percent	
Figures	Control	4	100.0%	0	0.0%	4	100.0%	
	ESAs	4	100.0%	0	0.0%	4	100.0%	
	TPx	4	100.0%	0	0.0%	4	100.0%	
	LPS	4	100.0%	0	0.0%	4	100.0%	


Descriptives	
	Variables	Statistic	Std. Error	
Figures	Control	Mean	10.53225	.206516	
		95% Confidence Interval for Mean	Lower Bound	9.87502		
			Upper Bound	11.18948		
		5% Trimmed Mean	10.52511		
		Median	10.46800		
		Variance	.171		
		Std. Deviation	.413033		
		Minimum	10.103		
		Maximum	11.090		
		Range	.987		
		Interquartile Range	.772		
		Skewness	.873	1.014	
		Kurtosis	1.504	2.619	
	ESAs	Mean	11.33325	.151130	
		95% Confidence Interval for Mean	Lower Bound	10.85229		
			Upper Bound	11.81421		
		5% Trimmed Mean	11.32928		
		Median	11.29750		
		Variance	.091		
		Std. Deviation	.302260		
		Minimum	11.004		
		Maximum	11.734		
		Range	.730		
		Interquartile Range	.565		
		Skewness	.683	1.014	
		Kurtosis	1.504	2.619	
	TPx	Mean	9.90350	.141489	
		95% Confidence Interval for Mean	Lower Bound	9.45322		
			Upper Bound	10.35378		
		5% Trimmed Mean	9.90033		
		Median	9.87500		
		Variance	.080		
		Std. Deviation	.282978		
		Minimum	9.589		
		Maximum	10.275		
		Range	.686		
		Interquartile Range	.529		
		Skewness	.588	1.014	
		Kurtosis	1.493	2.619	
	LPS	Mean	15.57100	.150800	
		95% Confidence Interval for Mean	Lower Bound	15.09109		
			Upper Bound	16.05091		
		5% Trimmed Mean	15.57339		
		Median	15.59250		
		Variance	.091		
		Std. Deviation	.301599		
		Minimum	15.182		
		Maximum	15.917		
		Range	.735		
		Interquartile Range	.562		
		Skewness	-.422	1.014	
		Kurtosis	1.500	2.619	


Tests of Normality	
	Variables	Kolmogorov-Smirnova	Shapiro-Wilk	
		Statistic	df	Sig.	Statistic	df	Sig.	
Figures	Control	.250	4	.	.956	4	.756	
	ESAs	.250	4	.	.962	4	.792	
	TPx	.249	4	.	.964	4	.801	
	LPS	.250	4	.	.962	4	.790	

a. Lilliefors Significance Correction	


Figures


Normal Q-Q Plots


ûvªàï¥^RÓçÀ.-ª³ÓÕ#G¢icQQQàz-ø#"ðGD3i,iýúõ)¿ÞÞ^E«o¿ý¶ßïùRVj^T´g	¹êÑÑÑ½÷ÊYÜð§ñþýû²<11T¿"Dòø³Z­òÿ©S§Â	ãÓO?Ý´irÌ´®®nhh(ä;;;;çÍ·råÊÀ®®®%KdffÖÔÔÜ¹sçÊ+-ÊÊÊZ½zuð^ÆFåø¦à¤pÄÙ²e¬>+µ´´ÈÊúúzÍ1Ú³¨¯Z¼(käFF58æÞ#ã/äTù½TTTÈ°¯Y³FÆ6ò>Bõ©Á¿VÉétÊ¥É8ÈoVNQ*#)víÚåõzyp?"J^üÊÚ·nÝgô`Èw(-##Cþß°aÃ­ã+V¬ß¾äò«V­LââbYïÞ½àKYYZZªù3F«Ù(þÊÊÊ¢Èöõã¯¯¯/øräÁ¿ÖÁÁAåË@½½½Ê7¯_¿>ä6ïØ±ø#¢äÅ,>:xOX°¶mÛ&ËëÖ­»ÿ0Y/eeðwîß¿rrRÙw¥¬Ù¾»ßïÿà/ååËÎÎNY*çU^ÙvîÜ9Y¾qãFðIá©¹>ø¼xp/ªìvp4oÕ´Ïá!g´Ér]]ÝØØ°>@4ýøþµÊåÈîînYV~­7oV¾YAáÍ7eY~²ÃüQRãOçrYçõõ²|ëÖ-åKy/çÏ|	SkÁ_?øÍñÓ`sLLLUUUÊ®¦)ñ¥ùh®ÆYQ%çÇÍf§Ó©9hzGóVMûúñ§¼®ñÎ;ÁWþ­Ê¥'k*++åË^å¯ññqYDàRâ³y÷ù|ÁëÕÎÔ/Õi^fä/!>§|×ªÑhTS«°HÈ¥é9K¸1rÐôN8Nïúûêá]äSÕ¿Öypâ¿Ï=ËüQ²ãO:yò¤|EY©ø,d¬ÔcÈ_VTT(S®tuu	ÎôàOÙ=©ÌºRWW·k×®û÷ï=zTVnÚ´IzÎ2müE;8¦FýøÓ³ç/À;¡äñÏÉÉ5!o=®¿¿¿±±Q9Öxg4?"Jjü=xøâýÀëú5Ê×­[7ú0åÙûöí?eï×àà äÍ7ßÔ¿Ë/+ûÄ|ð²WOéôéÓ?c´gGIïÃB.$ÚQþWéÇÇÇÅvk×®UÓððáÃ2øDqpäñWn¡ÐY¾_FR,Yüc*¯Ýt¹Áø#¢¤ÃßíÛ·¯îRÖ¼Þ«°°0ðæÙGÁ_#JóçÏÿßñªyþyõ!ÈeËEø1£:KTø<8¹C&jÆ¨NýýýÁ¿råÊàSCÆD¸ÊtïQö­÷Â/ðà"DøC¢Áë?ùäM6e=¬®®NýöéáoxxX.M.S´÷nñP`ÈÄinn^´hQFFFqqñ[o½õõ¯=°çéÑÏþ"ÎéÓ§Å©òjT§?éìÙ³.Z._9°x³|)DS®Ýl6÷ôôL¹çµ³³³ªªJ.M~(ÃXïõz÷íÛ§ìRÁË¯Òçóñà"DD±jbbâùçjn¹i%åRÆ*oßöûýé+**¸Ãø#"JÃ×üôê«¯22DþÒ0¯×»wïÞââbåØ®,¼ôÒK?""""DDDDþÀ?""""DDDDþüø#""""ðGDDDDàÀ?""""DDDDà(B§O6L«V­ö¼-3gËå¬eY³dÉ(6XÞ÷è9ï´Þ¾û¼yódp÷íÛçõzã´åÏEDàfuF£Q!ÐñûýÑ÷àÁrÞ¦¦¦À×^MÖ466¦:þÄy¥»qãÆäääñãÇåÖ®];êíí5ÍàÀ%ÇöâÑßß/ç­©©	¬Y±b¬éëëÏ×:u*!#þüQåLûöååååää8p äÛ¾þõ¯WUU`Ç¡,ß»wOerÒÍ7Ífsvvvfffeeeww·æ¥_ä³¼õÖ[&©ººZ®H¤±±1Å"ç¿wï^eG¦ðtåÊri²^.ùÖ­[:GfÉ%rÉ>OóÔ£tùòe¹2§OV°z$#÷O"D+ÿ)Ë¯¼ò,;a,¼úê«ÁßÓÙÙ© /¸mÛ¶ÉIGeù_ëëë-[vîÜ9Y¸qã¬/**Ò¼´àù,Bº'OÊÕjUßø;vÈ²|Ã»ï¾+,åÑÑÑÁÁAYPvLªSx1¿"ÒáÃëªodÈÏù¢¸sø#¢ØâO¼"ËSÛKóE/_Ö­['Ëë×¯Wd8µ¿¿ÿþýUUU²>##CóÒB ù,ÊËËËS·°°P¹ñ² ìò²)º·O³ø<JãããÊ²ü~öÈÅÀÅÁâ	ÇDZ999ò^¯W9º*kÞ|óM9ð£>RHóè9K¸&RÞ§ÀñÔ©SóæÍSÖ(;õìù+++õccc]y§7àDDàfEEEÁ;¢gSZÄb±È©ÿë³²²dÍäÃtâ/òY7L<§>ïüùóóÜ<¿ßîÜ9«Õ¼ËpÊöïß/ß/XT¤gtâozNDþhfð§¼ÅõèÑ£ÊKÐ^í5=9uêT`ÚÉ'Cdsýúuåµzðù,²wÞyG¶mÛ¦>ï/¼ ËÇÿøãC½²²ººZ?úè£;wîÈBYYÎaÞ¼yS@©ìÜ°aÎQ^V=+C~öé8ø#¢Áßï`å<,x®¾ÈW¹Êÿb¦Àú³gÏöîÝ«Ïât:åÔ+VÞwü=^¯w×®]rË³²²Ö®]«¼±WÌWWW§¼¸¦¦æO>Ñ?2r-Jå ¶(ðÀÊëùôRðòéÓ§å&)shüìÓp""ðGDDDþüø#""""ðGDDDDàÀßô½ïïóÏ?óþô§?ýùÏÎýFO£££wîÜaôôÿùöã ³üä'ÿõ_ÿÅ8èéÖ­[¿øÅ/=Ý¿ÿg?ûã §_þòñþMÝ~üã¿ëÛßþ¶ø/ÎWúÑGýÛ¿ýwe=É¦á?ü!ã 'yz¾|ù2ã ³ÎÎÎ_ýêWþáþ¿Átö/ÿò/ü1ã §ÿ÷ÿà=ö¿ÿû¿ÁøþÀøàü?ðþÀ?ðþÀøàü?àü?ðþÀøàÀøàü?ðþÀ?ðþÀøàü?ðGàü?ðþÀøàÀøàü?ðþÀø#ðþÀøàü?ðGàü?àü?ðþü?ðþÀøàü?ðþÀ?ðþÀøàü?ðþÀøàü?ðþÀ?ðþÀøàü?ðÇ8?ðþÀ_â¯§§§²²233sÉ%àü?àü?ðÎø+--½zõª,8q¢¬¬L¿®®®±ø&[Òüä'c¤#Ëõá2z§çîînÆAgçÏçÆAO×®]ûì³Ï=üþñÿqÐÓ­[·¾ûÝï2:üýüç?ó¦0þËÎÎVãïðáÃïÇ·ÎÎN§Óù>éèâÅ2¾óï¼÷ÞÎdKzùòeÆAOåK.1zêêêºpáã 'ùcõÜ¹sÎÎ9ÿ+MüõööÖ××sØÃ¾ö%ûrØÃ¾öå°o:öU«««óz½àü?àü?ðæø»û¶ÕjÕÜx?ðþÀ?ðþÀ_ZáOW¯^=<<¬y*øàüøàü¥þL&Ó ÀøþÀøà/ñ9ðþÀø#ðþÀøàü?ðGàü?ðþÀøàüøàü?ðþÀø#ðþÀøàü?ðþü?ðþÀøàü?ðþÀ?ðþÀøþÀøàü?ðþÀøàÀøàü?ðþÀøcÀøàü?ðþÀ?ðþÀøàü?ðGàü?ðþÀøàÀøàü?ðþÀø#ðþÀøàü?ðGàü?ðþÀøàüøàü?ðþÀø#ðþÀ?ðþÀøþÀ?ðþÀøþÀø#ðþÀøàü?ðþÀøàü?ðþÀø#ðþÀøàü?ðþÀøàü?ðþÀø#ðþÀøàü?ðþðþÀøàü?ðGàü?ðþÀøàüø´|>_ð	ü?ðGàü?ðþÒö¯±±Ñd2F¿ßþÀø#ðþÀøéÓéÜ¸qãÜ¹sçü¦ÀVü?ðGàü?ðþÒ$·ÛèÐ¡òòò9ªöìÙþÀø#ðþÀøil´-KnnnùæÎ+ë7éàü?àü?ðªù|¾æææ¥KªwõÆÆÆÆ»wïü?ðGàü?ðþR/¹Ô××¨Ù·fÍcÇy½^Í3?ðþü?ðþÀ_Êäóù:::ª««ÕæËÏÏß¹sçwðþÀ?ðþÀøKg±=öF5ûjjjZZZÂíêàüøàü¿Éï÷ËFÆl6ÏÛ¢d0l6Ûàà`TþÀø#ðþÀøÉÛíZL&õ®¾òòòææfÇ3àüøàü¿$Êï÷;ÎÚÚZõ®>eÞÑ[àã:Àø#ðþÀøà/Uóx<v»½¤¤D½«Ïd2iÎÛþÀ?ðþÀø)ùÐ°Z­A½«¯¶¶¶££ãQvõ?ðþü?ðþÀ_Räñxæ§±L¦¡¡¡¿RðþÀ?ðþÀøw.Ëf³åçç«Ùg6ÛÛÛgpWøàÀøàü%&Ï'°ÞiNÑ,Æú6?ðþü?ðþÀ_ÌjhhÐÜÕW]]íp8¦7oøþÀøàü%Qæm1V«Uq¾Iàü?àü?ð7óE¢¹¤¤Än·ÇmWøàÀøàüÅ0ÁÅb	7E³ÓéÝ9Àø#ðþÀøà/NE·Eàv»a¸ÀøþÀøàïQïØ¦hé¼-àüøàü?ð§tÕÕÕó¶ÄhfðþÀøàü?ðoü¹Ýn±ÑhÔ·¥µµÕçó%íp?ðþü?ðþÀ_÷ÚÚZõ^eæøÏÛþÀ?ðþÀø3ßÝ»wåY¾¤¤D½«¯¼¼<S4?ðþÀøàü¿âÏétZ,ÜÜ9¶nÝzíÚµ¤z3øþÀøàüM§æææÅkNÑÜÒÒr÷îÝ.ðþÀ?ðþÀøû­éÎ;5wõY,ÛÕþÀø#ðþÀø¡y½Þ¶¶6Íy[L&ScccrÎÛþÀ?ðþÀøÑår¹^|ñÅüü|5ûjkkÓ`WøàÀøàüýÏ®¾ÖÖVÍ]ÂAyÖHËáàüøàüÍ.ü¹;wÛÕ×ÖÖ&.LãáàüøàüÍüÉÓ´Åb;w®æ§±	gÃp?ðþü?ðþÒ###MMMåååê]/>vì|Ãì.ðþÀ?ðþÀ_ÚâOÙÕ§ùilöì%»úÀøþÀøi?Çc·Û5wõ¥Ü§±?ðþÀøàüQXüÉÌjµªwõåææîÜ¹S¦ÓlÞðþü?ðþÀßlÄßG¸]---³yWøàÀøà/­îTñ¡þ46Á`±Xâÿ¼þÀøàüø3Ïç7EsIIÝngWøþÀøà/r¹M=EóÜ¹skkkN'¯êàÀøàü¥|>¯½½Ýl6«wõmÙ²ehhQàÀøàü¥|¢ºÍOcö³Mã³ÁøàÀøà/òûý»ÚÚZÍOc³Ùl)£ýl_ðþÀø#ðþÀøK¢Ün·<¥L&õ®¾êêjõÍàü?àü?ðzùý~§Ó©¹«Ï`0X­V¹ÿhü?ðGàü?ðþR)·Ûm·ÛKJJ4§hrÞðþÀ?ðþÀøKäyÓb±¨wõÉýS4?ðÛM&øàÀøÓÎãñØívÍOc'Yy>u»Ýú/ü¿&÷­¹k?ðþü?ð7½ûÕj5S4···OcfðþbØÚµk].Wü½óÎ;7âps``àé¨¿¿_¶>ùä®®.ÆAgçÎçiÆAOï¿ÿ¾üÆ8èItíÚµ´ùq~üã¿ñÆUUUê]=öÍf»råÊ´/üÿù/]ºÄFggÎùì³Ïâ|¥)¿_ßÄðøë­·þ)¾]¾|¹§§çHG²'ÆAOòÅ½÷ÞÆAOÝÝÝ½½½¾ÿýïÒà9öìæÍóòòÔì[¾|yCClñ*>üðÃ/rÑÿÿ¦3þ8ìËa_ûrØ8ìËaß¿¢YóÓØÃÖ­[åÌº.ûrØüøàü¿aæ¥K:thdddf¯ü?ðGàü?ðþâ])%Y»§HðþÀ?ðþÀø_Ê¼-á¦h>tèÐÐÐPLoø	ü?ðþüÍ*ü)S4«çmjjj:::¦1oøàü?ðþÀøK®<ÃáÐ¢Ùh4666Æóö?ðþü?ðþÀ_Lr¹-??_óÍmmm>/þ·ü?ðGàü?ðþf2!]kkkuuµÚ|¹¹¹ÂAÙbÄç/øàü?ðþÀøíSÝ4wõ´´´x<ßÈdÀØw`` ££#Î¼Áø#ðþÀø3C§Óùì³Ïªçm5[·nç»îêK6ü¹'öÕ_ýâ¿øôÓOÏøàü?ðþÀø1Éãñ455iÎÛb4å.	YXüy½ÞÇ¯][àäßþ÷/_¾¼¶¶ü?ðþÀø#ðÔøs:7nTá;wî³Ï>+Op	y3Gòã¯££ãË_Vä§üÛ÷÷=öØcwïÞàü?ðþÀøK:ü	éÂÍÛb2ä©Íív'ùX%ÍÍÍæ§ÍÁøeeeroàü?ðþÀøK"üØl¶õ®¾ÚÚZ§Ó<¯êKfüutt,^¼8dÏßüùóÙóþÀøàÀ_RàOH×ÖÖ¦9oK~~~CCC¬?-Íðçõz¿ô¥/¼æïÿøv¸ÀøþÀø-øÕ:tÈd2©Ù'lmmMÚWõ%3þ<|·ïã?þÄO|å+_QÞí´»ýÀøþÀøKüùýþÍ]6MìÒc$óüÉÝ[Æy`` É?ðþü?ð¶ø9tèÐÔì+//w8É0Eszà/àüøà/ñ'á5kÖ¨§h6%þÏVàüÅs¦*##ü?Æü?ðÆøóz½áæm)))±Ûíé±«ü¿_1Uàü1àü¿´Äßµk×,æÍ©5oø©øàüø3þ<ÃáX¼xqêNÑþÀ_¬ð7::ºmÛ6ðþðþÀ_zàOÓf³©wõIf³¹½½=wõ?ð§ü¹Ékþü?ðþÒ>¯µµ5ÜÍi0oøÓiÉ%êDAA×ëàqàü¥(þ4wõ¥ôÍàüÍþ²²²ä0<<l4eAÌ÷Î;ïÈB=øøà/µðç÷ûÛÛÛkkk5çm±Z­2ª³|¬Àøûõ/² Ú7nLNNÊBNNøøà/Uð÷þûïË3æ§±¥ÓÍàüÍþæÍ'¹¸Yxé¥¦zàü?ðÉ³Éºuë222Ôó¶¤ßÍàüÍþöîÝxGÈK"ÀøcÀøIÇã±ÛíS4Ïy[Àøf/¿üòüùóe¡§§GUUU±¾éàü?ðGàoÚ#cµZÃ,¢ü?&yàü?ð7[R¦hÖ·å±ÇûË¿üË¡¡!F	ü?ðþÀøà/ås¹¦hVæmùøaøz+++S&|agàü¿äI·Ål6O9Es´íþY¿ÒÒÒ`ðâÝ¾àü?ðþ2E³þy[ÀøQ¤¼7¾··wrr27ü?ðþü©s:Ó¢ü?ðEò ³üÀøàÀ_È6§¹¹YsÞ»ÝyfðþÀ_õõõÉCkÇcccàÀøà/ÎX,õ®¾¨æmàüEWqq±ú/-ÞðþÀøà/v455iÎÛ2)ÁøQ´páBÞðAàü?ð·Di;wîÌÍÍU³oùòåíííÓ¢ü?ðÍE<lpp0Î7ü?ðþhVáÏëõ:poæxg"ðþÀ_òàü¿nO÷$lkk»÷î#^øà/äIK~÷îõù|àÀøào¦LÑ¬ÞÕkµZS4?zàü¿h."L¼áü?ðþÀß´7 7Esss³×ëÙkàüEQFxÃøàü¿h7E³¬Ù¸q£<R¦ñfðþÀßã/Q?ðþÀ¥þ|>ÃáÐ¢yÁQÍÛþÀø-þL&ÓÂãÿàü?JülÝºÕ`0hNÑ,O1ÚÕþÀø~ò(ÿMàü?J]üy½Þ¶¶6Í)Fã=dÏÛþÀø¢îîny¬666ÆsÂðþÀø£TÄËåÚ¹sg~~¾æ¼-Çñ7s?ðþf¼ÛÀøàoÊ|>2oúùÂ`0Øl¶Þ<ðþÀ_ñn_àü¿544hîê[¾|yKKËÈÈHÂo$øà/àü?Jfüùýþó¶X­ÖØÍÛþÀøàü?ðþâÛí¶Ûí%%%ê]²RNzôOcàü%k×®ÍÎÎvNNÎâðÎðþÀø£dÃl-æ®>Yït:gWøàoúk¾á#ÖõþÀø$øóx<á¦h6L²¹õÍàü¿¸â¯¢¢BÞëÖ­/GGG×¯_/k-[þÀãþÀ_zãO®Âf³©ßÌ¡LÑÌ»úÀøÓ/++KçÁï	Y#ëÁøcÀøKKüù|¾ÖÖVÍ)M&SCCÃÐÐPjøà/222äÑ.àÞ(È¦zàü¿ôÃËå7oÙlnooO]àü¿é§ö]½zµrØWþeYSYY	þÀãþÀ_zàOHnf pPPÒcþÀø"Ñæ>îß¿þÀãþÀ:ùu755µ¶¶Æúãkgn·[¶·&I½¯®®"Öoïàü%þ<|Ãïòòò222äÿÕ«WËXßtðþÀøK¹¼^ï×¾öµâââ§~zÅ¿û»¿ñâÅäÄ2E³æ¼-ùùù6-Õwõ?ðþR/ðþÀøK¹î¹¥K÷¿¸ÿÀ7È¿ç¶<»§?ÇnæêêjÃ!ß~÷+ðþÀøàü¿ï÷~ï÷öìÞ£ÈOù·ì-kkkKüÉ÷[­VÁb>Y#ëåÔ4¾_?ðþtsª222ÀøcÀøäñxå'ÿþèþÈn·'¦héº«ü?ðuáþÀøÓì÷ÿ÷·ýÕ¶`üvtt$á¦h6%þÛXðþÀ_Rã/wïV¶'Nàqà/¸7ÞxÃd2cû7ù­þ?«ÿðÿ0vï¿S4´´´Ì]àü¿¨¯¯/''Gç3xÎgðþü¿@ÌÎÎ~òÉ'FãO<ÓOÂPãÏårû4¶gVî):E3øà/øÛ´i²9wîn:øàü¥h^¯w``@Økiðçóù"LÑ¼gÏXÏ8þÀøK+ü½ûî»ÊdÝºuñ¼éàü?ðGSâ¯··7Ü§±ÕÔÔÓcfðþÀ_ð7>>¾lÙ2å½ÝÝÝq¾éàü?ðGáR>­ººZ=E³Á`°Ùløà/º>¬lG6oÞþÀø¤.Â§±-_¾¼­­m¾ü?ð73øc?àü%OÊ§±ÕÖÖjîê«¯¯`Àøb4ÏRff&øø±Îívû4¶ÂÂÂûö0Jàü¿À_Âàü¿Yl-zW_`Þi|¶/ø#ðþÀøàü%QÇn·k~ÑhlllÌÛígû?Æü?ðþÀøIT¸OcSæmçÁøàü?ðþÀ_êåóùÚÚÚ4?­  `çÎn·;Áøàü?ðþÀ_Ê$ªkhhÐ·¥¦¦Æápx½Þgàü?ðþÀø)ßïSsÞüü|Íær¹ôøàüÍ$þçÀø3ÞÈÈH¸]ÕÕÕ#ª)Áøào&ñ<¥&þçü?ðþô'´7jNÑlµZqÓ¸LðþÀøIüêêêÍÓ-[ÆÆÆäKùýúõ²æÊ+àü1àüEndd¤¹¹YsfYi·ÛåÓØÀøà/&ø+((TðüÊtSxÉEEE-êîîàüQ:áOdfµZ5§h¶X,N§3dÞðþÀøKü)[+_þý5uuuÇ#GlÞ¼ü?ðGi?¯×ÛÚÚº|ùrõ®>É$[¶pó¶?ðþÀ_²à¯°°P¶Yµññqe»¶nÝ:å3%ñ&''MÊ6Q?ÃÑß.]º$÷ø~ÒÐ¼»»qÐÓ~ðóçÏ3:-i___*Þò'Oþùÿù¾ð5û-[öê«¯Õfö/^¼øýïû¾ûÝïÊß`zzz.À8èìÌ3ñ¿ÒãïÊ+oøøè£ñß2¢~ûàïÝwßuÇ·«W¯ºIG?üá¯]»Æ8èihhÈét2:(ß¼y3nðgö·û·O=õz;ùØcíØ±CþRÑUË%üñÇÜgô400ÐÛÛË8èÉårç;ßat&¯Þºu+ÎWsüI7nÜ(--ÍÎÎÎÈÈÈÉÉ©¬¬¼wïÞ£_lðã¬¬,ûrØÃ¾ZõF£QsÞÖÖVÏÓÀa_ûrØÃ¾±Â_*,,T^J(ÿ«"?ðþÀ_ræ÷ûÛÛÛ#LÑ<½y[Àøà/ýñg±XÞzë-YÿëêêÀøäøs»Ý²iÒ¢¹¼¼<Ú)Áøà/yñwýúõ²²²å@maaá©S§ýbå¾e4å2zzzÀøøóûýN§SsW2oKü7Vàü?ðCü)<¤²|äÈÞtðþÀøKxÇn·G¢yçmàü¿dÁòZæë×¯ð×ÓÓ#Ëyyyàü1à/]ñ§LÑl0Ô»újkkgdfðþÀøKRü)Û;eAÁßää$íKàü¥%þ<Ãá(//×¢¹¡¡ahh(yÆü?ðþb?egeoàobbbß¾Êvü?Æü¥þÍfËÏÏW³Ïl6···'Ã®>ðþÀøþäIKsç«W¯?ðÇ8¿TÇÏçØ	ïÔ[9eÞAaÒøàüÅlªªwûfggÅáÀøàüÅ4efÍ]ÕÕÕñ·ü?ðþ		ü?ðþbQy[ÕjÛÍàü?ð¤ø¼Ï#Ð½÷ÊÊÊ,XþÀãþR¦hVæmIþ]àü?ðüMLLðn_à/ð'Ånæ$·ü?ðþ¿ÌØ¼yóÀøcÀ_2ã/ò¼-²IìÍàü?ð»yófÆÃíÈïÝwßàqÉ¿ÈS4'ç¼-àü?ð`üêÅú/øàfBºó¶$ÛÍàü?ð¤øKTàü¿ÙÙï½'Ó¦¦¦¶¶¶»wïê¿C666hÎÛÒÚÚêóùÒo¬Àøà/&ø(++ËÉÉ	¬?þ+¯¼þÀø3hïñÇ²ôÉU«V-]º4//opp0òYÂÍÛ¢LÑBó¶?ðþÀ_²à¯¸¸8ä¿ÊUþÈàq3Û×¾öµÿåoPþýéú?ýÒ¾äõz5ØÔÔTRR¢ÞÕ·|ùò¢ü?ðþYYY²1þã»¯¯OÖÈ_äàü1ào),,Üÿâþþä_iiéµk×¿M¾´X,ê7sH7nùfðGàü¿¨SÞð;99ù[«üü?üùäOËOþeùWÚÛÛåT¯×ÛÒÒ²xñbµù,X`·ÛSÞðþÀøKüÉ¶u×®]ÊË¥ÇÇÇ8 kF#øøÁ<OnnîÎ;òÛÿâ~ÙÔ?~Üf³ÉIS4wvv¦Ç¼-àü?ð,øëííÕä¹§§ü?ÆüÍl»wï~üñÇ·ýÕ6ßvÛöâââüü|ÍOc;tèÐ,ÜÕþÀøñÀtûöíEegggddäääTTTÈXßtðþÀßììùçÏËËûÂ¾ð;¿ó;oæH)Áøà/yñÀø³-Ï'Ñ¥KjÎÛR__fS4?ðþÀøàüÍÒäY¹¡¡AsæÅ;vLs¶àü¿XáïúõëÊ<ÏÊ;|O:þÀøßïïìì|öÙgÕ»úrss-ËÀÀ£þÀøñÆ_WWW`s¬àOY>räøøöýÇn·/X°@½«Ïd2½þúëìêàü¿áÏh4Êæøúõëüõôô0É3?ð7½äqm±XÔ»ú¤gyæÚµk¿úÕ¯(ðþÀøKþò '''e933ü?ÆüéÌãñØíöòòrµùä/ÌÆÆÆÀ9Àøàü%Ê¬~þ&&&öíÛ§àqzbµZ5?Íl6Ëv3ä/øàü¿ãO´4'y¾zõ*øøÏçkmm­®®Vo=òóó_|ñÅàOàü?ðDødãRUU¥¼Û7;;»¬¬,³m?ðþR4Ëe³Ù4?C,("T>+2ü?ðþ¿þÀøK­ü~»ÙlÖÜÕ'ê¹ðþÀøàü?ðÔ¹ÝnyÀL&Í]Ããñè¿4ðþÀøÇß§~ºpáÂ¬¬,Ùggg/Z´hxxü?ð7Ëñç÷ûNgmm­zÞÁ`µZ&Ó¸XðþÀø	ÆlÜ5ßðëGøà/iSæm)))QoÊËË£ÝÕþÀøà/¹ð§Lò§LÇ066¶yófYSTTþÀã0Ûð§LÑ¬·EÖÈúyÌ?ðþÀøK0þ-»ßï¬åÀÏàüÑlÀÇãq8S4ØíöGÙÕþÀøà/¹ð§ìù¬gÏ¿Y¿pó¶Ì;×b±8Îà¿Áøàü¥þ×üÕÕÕùäËû÷ï×ÔÔð?é¿S4L&yxºÝî]5øàü¿ãoÎTÅèø/øà/!¹=öhîê«­­Å®>ðþÀøÉ¿©ÊÌÌàRB:ÙiÎÛb2âð¹>àü?ðþü?ðFFF:¤9E³Ùlnooõ®>ðþÀøÉ¿pó9ß¾ü?Æ!¥ñ'ÌÚºu«zÞÆþÀøà/Ýð'Ï/¿ürÈJyÂ`ªðþR§©©IsÞÖÖVeRÏü?ðþÀ_"ñ'È§ÂÂÂ÷îÉo¿ý¶ò$£ú?ðþb×ÀÀÕjÍÍÍU¿£¾¾>IÁøàü%îçS!õë×Çú¦?ðþf*¯×ÛÒÒ²téRõ9,X 5ùm&Ïp?ðþÀøK<þ¤+W®-äY$7ü?ð÷èÉ³ÍfÓÜÕ·qãFaVßÌþÀøà/5ð·ûvå	Cù´iË-àü¿¤Å®£££¦¦&%võ?ðþÀøK.üeffÊF^^^ÿ ×üeee?ðÇ8$þ)5çmY³fMrîêàü?ðçýû÷¬´Z­¼Ûü¿äÁ_)sss·nÝ*JHáàü?ð`üç/Ö[ðþÀ"LÑ¼téÒÇZÃþÀøà/ÁøKTàü¿ÈZ­VÍ]²>uçÀøàü%ò|`7òàüQÜðçñxÇâÅÕ»úJJJRqWøàü?ðþÀøÓ~¦·Ùlùùù!æ37n«Hþ7s?ðþÀøàü¿)òù|­­­ÕÕÕ»úRWøàü?ðþÀøûæ®¾¹sç>óÌ3N§S~ÃþÀøàü?ð7»ð'¤koo7Íê]&In·;ü?ðþÀøào¶àohh¨¡¡AsW_mm­ÓéLWõ?ðþÀøàüÍjü	éÚÛÛ5§h³g¸Àøàü%àü=:þÜn·ÜÕ5§h®®®nmmMËWõ?ðþÀøKFüeLUff&øÃôðç÷ûNg¸]6ÍårÍÚáàü?ðü%<ðþÒn·Ûn·¨wõ;4·ü?ðþÀøàoâOîÏE½«Ï`0ÈúøßÛÁøþÀøàoæóx</¿üòüÁhNÑl·ÛÙÕþÀøàü?ð7Í|>ß±cÇl6Û·¾õ-y^Lø³Õj5³yÞðþÀøàü¿Þ¤'xâË_þ¯ÿÉÓO?ûw÷wñ¿ÇápÏÎ)Áøàü?ðþâoåÊ¾y@ù·í¯¶ÿâ9C<k~TYYÙÞÞÎ®>ðþÀøàü¿ÉçóÍ7oï½üÉ¿¯®øjKKK®ºµµµººZm>eÞ¹_Mï³Á?ðþÀøàO;¯×ûÅ/~1X~òoÕªUÍÍÍ±»ÒpÆ2Es´íþÀøàü?ðþ¦®¸¸øÏþìÏòÛÿâ~á`,ÔáÓØÕjy¯	øàü?ðþÀø	ì±M7	û¾±ýO=õÔ3Ï<3³/³ðil¦hàü?ðþÀø1Iî3UUUÙÙÙ/¼ðÂ~0n¸)eÍS4?ðþÀøàü¿ÔÈãñØíöG·ü?ðþÀøàü¥ÀÓêLMÑþÀøàü?ðþ4eæpó¶444Lcâ@ðþÀøàü?ðt¹pS4ÏÛ2Àøàü?ðþÀ_²¤ÌÛb6ÃMÑ,(|Ä«àü?ðþÀøO¢9Úy[Àøàüøà/År:ú§hàü?ðGàoÆ6LàüÅ§æææÅ«wõØíöÚÕþÀøàü?ä¾UQQ!Ï»àüÅºÁÁÁ;wæææ>ú¼-àü?ðþüM³µk×ºøû¿ùg|;wîÜ¤#y>þ|ßHù644<õÔSê]Ï=÷[[nÆÅßï=î3:-©ãÀ&kn²¤®®.6Yú;sæLü¯4ñ÷ë.]úe|ûðÃ~I:?£ûûûöæÉøßúÖ·æÍ§fßÊ+=ú_ü"n7fxx¸»»ûÎä:¿®§§çæÍ>ùäÁÁAÆAOwîÜ¹råã 3ùu||<ÎWÎøã°/£Íï÷·¶¶Ífõ9y[dëÿ[Åa_ûrØÃ¾öå°ïì=ìx&àoÆoÏ¡CJJJÔ»ú/_>ó¶?ðþÀø#ð÷¨àïQûÉ5k4çm©¯¯OÈ®>ðþÀøàü?ð7Ã¹Ýn»ÝaÞ»wï&ÉX?ðþÀøàoêÀø×ÀÀÀÎ;zÞg6Öó¶?ðþÀø#ðþÀ_<òx<MMMK.Uïê[°`Á¡CFFFs¬Àøàü?ðþÀ_îÙ³'??_Í>³ÙÜÞÞl»úÀøàü?ðþÀ_Ô	é;¦¹«¯  àÅ_t¹1Vàü?ðþÀøà/R¢º=öL&Í]mmmI¾«ü?ðþÀøàüMÏçkooÞ©Íg0¬VëÀÀ@*øàü?ðþÀøû­ÜnwCCæ«ú.]ÚÒÒÀ)Áøàüø4cøs:%77WsWßµk×Ò`¬Àøàü?ðþf;þFFFZZZÊËË5çm±Ûí)½«ü?ðþÀøàüýÿ§+Í]I;E3øàü?à¢ÆÇãq8Ë/Wïê3I;E3øàü?à¢ÀßÐÐæÍsçÎægäaãóùÒ¬Àøàü?ðþÒ~¿¿­­mÍ5<õÍn·øàü?ðþÀ_:ãON_O·¦¦¦½½ÝëõÎª±àü?ðþÀøKCüùý~yhîêËÍÍµZ­©òilàü?ðþðþ(þîÞ½ÛÔÔTRR¢ÞÕW]]íp8fÛ®>ðþÀøàü¿ôÄßàà ÕjÕ¢Ùb±È©øàü?ðþÀ_:ôöÛoi~[sss:MÑþÀøàÀø£çÏÙÕ·qãÆôø46ðþÀøøÚçþå/YØW^^n·ÛïÞ½Ë?ðþÀøàü¥3þ¦àü?ðþÀø-øÞgû?àü?ðþÀøþÀøàü?ðþÀ?ðþÀøàü?àü?ðþÀøàÀøàü?ðþÀøàü?ðþÀøàÀøàü?ðþÀøàüøàü?ðGàü?Æü?ðþÀøàüøàü?ðþÀøþÀøàü?ðþü?ðþÀøàü?àü?ðþÀøþÀøàü?ðþÀ?ðþÀøàü?àü?ðþÀøàÀøàü?ðþÀ?ðþü?ðþÀø#ðþÀøàü?ðþÀøþÀøàü?ðþÀøàü?ðþÀøþÀøàü?ðþÀãþÀøàü?ðþü?ðþÀøàü?àü?ðþÀøþÀøàü?ðþÀ?ðþÀøàü?àüøàü?ðGàüøàü?ðGàü?àü?ðþÀøþÀøàü?ðþÀ?ðþÀøàü?ðþÀøàü?ðþÀ?ðþÀøàü?ðþÀøàü?ðþÀ?ðþÀøàü?ðGàü?ðþÀøàÀøþÀøàüøþÀøàüøàÀøàü?ðþÀ?ðþÀøàü?ðGàü?ðþÀøàÀøàü?ðþÀø#ðþÀøàü?ðGàü?ðþÀøàüøàü?ðþÀøàü?ðþÀøàüøàü?ðþÀøþÀ?ðþÀøþÀø#ðþÀøàü?ðGàü?ðþÀøûízzz*++333,YÒ××þÀø#ðþÀøé¿ÒÒÒ«W¯ÊÂ'ÊÊÊÔø»téÒx|>ýôÓþþ~ÆAOÃÃÃÝÝÝÎÎ??66Æ8èIþ?Ã=ýèG?¿W=ýô§?½råã 3ÁßüÇÄùJSÁegg«ñ÷Æooò¬sáÂK¤#(.ÆAO/^|ï½÷ÉÔét2z:wîã sÕÙÙÉ8°ÉñÎ9ÿ+MüõööÖ××sØÃ¾ö%ûrØÃ¾öå°o:öU«««óz½àü?àü?ðnøó/oß¾mµZ57^àü?ðGàü?ðòøNW¯^=<<¬y*øàüøàü¥þL&Ó ÀøþÀøà/ñ9ðþÀø#ðþÀøàü?ðGàü?ðþÀøàüøàü?ðþÀø#ðþÀøàü?ðþü?ðþÀøàü?ðþÀ?ðþÀøþÀøàü?ðþÀøàÀøàü?ðþÀøcÀøàü?ðþÀ?ðþÀøàü?ðGàü?ð7Ûð÷×ý×íííÇ·óçÏÿà?øttíÚµ.0zúÑ~tòäIÆAgÿ÷?44Ä8èéÌ3~ø!ã §ïïÝÝÝO:Å8èìèÑ£ÿú¯ÿç+õx<é¿ë×¯8pàÛDDDDTäC£sØKDDD4DDDDàÀ?""""DDDDþü%]|òIUUUVVÖ¦MFGGOº|ùrEEEffæ¢Eå|xxØd2¾ìëë+**R§»»;ø;#ÄXHOOOee¥´dÉù6ÆjÊ;OWW×9sxF+ÏgµZe³¶`ÁYû¡2úMÈXi®aóíX%ÃæümÙ²eò?þxûöíÁ'Ü¼ySäÍ_íìI6²q~Ò­««;~ü¸,9rdóæÍÁßá$Æ*d@JKK¯^½*'N(++c¬"ßyü~¿lLg'þ¢«¾úê«Â2koêÕc¥^Ãæcwð6Qy`yÞ¼yÁ'ÉFóöíÛ² ÿÏÎh µk×ºàû·l.å©E&&&B6Nb¬"Hvv6cy¬^yå×_vâ/ª±Z´hÑ,ÿøÚ¨koêÕc¥^Ãæcwð¶%K~]Þ~ûí`>x¸[~aòKÿ«àûwðX[«p"w°úúzÆ*ÂXÝ¼y³ªªJxfíaß¨Pæ£>b¸"ú±·ÍTcwð6Ù2Écþõ×_±ù²eË¿eÓ°bÅÆ*øþXÎÊÊþ¶'1V266VWWçõz«cµaÃ+W®L¹e¬=úà7¯if¸"zý aó-þ»yS÷é§VTTð'Î|aaáÄÄÄ;ÿe9øÛ"ÄX©äöíÛV«õÎ;Ü¯"Õß±üdÛ¥¸ØÔë÷¨ððÍ;ø[ii©üµ799ùúë¯¿üòËÁ'ÉÊáÁÁAùÓ±¾[,·ÞzKäù³&øÛ"ÄXÈåËW¯^=<<ÌýJÿ=SÕöíÛO<)²«®®f¸"zý aó®¬aóþÂ&¿âââ¬¬¬úúz¿ßüüôÓOeC Êÿ²ÌX¼ÅÉh4fdd)o|æIæXL&öfé+ð§¬FGG7lØ Û®ªª*ËÅpE.6õz@Ãæ=Ú±JÍ;ø#"""MNeÀ?""""DDDDþüø#""""ðGDDDDàÀ?""""DDDDàÀ?""""DDDDþüø#¢YÞÛo¿½råÊì­ZµêÝwßý­í×ÃRfk«ukM&üh^¯7d½¬ÉÌÌ,**ö2À¥d£êå_N'ü566ÊÊõÍÍÍ²þ¥^Æe?"J½úúú5o¾ù¦ÿaG/eeÚàïÆ²rÉ%!ë+**dýÐÐø#"ðGD³¢-[¶k^õÕà¯½ö¬Üºuk0º»»OâÂÊÊJY|óýû÷ëëëóòòä¤]»v]u:,9IÎÛÙÙÂ)Y3oÞ¼+W;wN¾´X,!7ììÙ³/G9InrRWWW8¨­^½ZÖ÷ôôÖzUÖÍæÀF¹¨ìììM6Ý¾[?õå¬pSü%¸ââbËÍ7WÞºuKVL¦`ÜÔÛÛ«º~ýúvìØ¡488¡y.åKåÔ6LNN³²²ü~¿*ÿrrrâE¸YÐ¼yêôÄÁ¢øòøñãùªU«¢Å_JDà(ñ)Gx5¶YsæÅq³yóæ±É|¹nÝ:åTÅ:x6å¤ºº:e¡,ðÁÊ_æþýûwÊQ×^xAÖ>ZåY~þùç§¼£|)ß|Û4"åüùóåç/å¹å"Î	åäò#<øÍaâìììhñá¦ø#"JüIø¬Wp£Iºû¶|)lR¾¬¬¬/.mÛ6AÛøøxàBäBö¥Î¥|yëÖ­À7G~åYv:S^@S¾¼wï^ðm÷ú¼Ý»wËI¯¿þúßÚÞµkWð7BÒªª*eÇd´øpSü%>ew×ØØXðJ¯×++å¤Ü	ÐÐår)þX'ðB=õÑØNO°"ËYYY>O.|Þ¼yS§¼péúõëSeYþåÁÁÁÀ©|ðÜÍÃÇúñá¦ø#"J|ÊëÞ>¼R%ä×«whõ÷÷766*ajTvË«NI´;v(GåÿúúúÀú£ìi»sçòå½÷"¿3wÅrª2»5ø$å¿rRWW×èèhdü`ªFàÔ7ÀQâSÞ0yäÈeª7ß|3++KýæuëÖ	t|>rL6ðÎ5ÊKåWðîÚµK¨¤¼70Ù&Ñ·ß*×~ùòåÀú£¼ÈOyÍ×ëU¾3þ=Ø''?rðIÊËåZdÂáOá¦pY¾MÐ¹iÓ¦àS#ÜT"DDI2rHüÿÛ¯)6,Þcè^ep§N¿?CÖp9r3oXà¶á~ØñññììlåG9Ø½víÚà«?¾ü¯Ìö|ÊÉ@U§F¸©Dþ%ÊªU«²¶råJå-·!øëêêR¦Ó[´hÑ+W§z½ÞûöFL»wïöù|S;;;«ªªÄdEEE#ä2Õ·D.JÖoß¾=d¸ËäÆÈMR& 0Ï_ úúú9_ëêêdòòòä§L[|£££¢[e¬ÌfsOOOÈ5F¸©Dþüø#""""ðGDDDDàÀ?""""DDDDþüø#"""DDDDþüø#""""ðGDDDDàâÕÿÌX/[¦4IEND®B`


1DïgÌr¥¡¡!à¬/øÎºRYY¹aÃ.ìÚµ+¬fMJüõå&Æ_'ÖoØwüõå¿ïýÿÑ£G5	o=ïøñã555Ñ±æØ;£%Á¤¬Æßõ/Þ½®?Z½xÅo=º?øà·¿èÙ¯@gy¦/økjj[^<räHô¬^ÔË/¿òìïMú¿Þ'ÉÎ%ÜIGuø^¥ìåÊ`»eË%Óðé§ÜûøG[è>?dX5kVü·½v³µµµCðàORÖáïÜ¹s±WwEkÚÚÚ^ï5aÂØgo1D?>|ÇkÊm¾ï¾ûAÎ7¯o³_7éþzØ¹NÔ<Qþ?ÿwÜqGüµ	c¸Ö;þ¢Ó%¦|LôÜj|÷ß¿?.	þ$ü¢C¢ñë¿ÿýï¯Y³¦àFÉoïþ:::Â½ûzäGbg	é8_ùÊWfÎ7qâÄÝ»wîs=ótë7éþz_~9ø/???vª¼øú5ªÀ_èW^<yrø®ÃýGÖcopÑ¢¯^QQÑÜÜ|Óg^<8wîÜpoáª««­ïìì|ôÑG£§TàÃ²««Ë®®^½zß÷õëÜr¸É°+:½»»»;2ý3üÂH?IÊÁ¢×ü%ôäOIð'I9Xggç¦M&NÛ=öa$I?I$Á$IüI$	þ$I$I?I$Á$IàO$Ið'I$ø$IüI$	þ$I$Ið'Iýíå_.//ÏÏÏ_¼xqo;sæÌQ£Fµ¶¶ÆÖå°fÖ¬YýØÝh`ÓÛpßT´¾³³sýúõ%%%aÄÆ[YYùÎ;ïÄnÕÝÝÖO.((Ë~»$Á¤¬«´´4`¥££cXÙ¾¸mmmmlÍ¿øÅ°¦¦¦&7ð¼¾ªª*¬?tèPX>|øpX:ujìÚW^y%Åøí²o'r~:~üx¸í¢Ebk,XÖ;v,3yüõ/^Ly«ï½7¼ÿþûývI?IÙ(¿xè<úè£cÇ=zôÖ­[>ísûÜÜ¹sî!öÄaX>þ|Xk¢«N>]QQQTT?öìèÙ²äÿê½ßd÷îÝååå._(h/_^»vm¸mØøM6EOdÞqÇáÞÂúpÏgÎ¹EüM4)¬1cÆ#G®ºvíÚ¸qãÂ×ºråJø8~üø¶Òå¿hùñÇËuuuYaáÉ'ÿFÈïWíÚµ+,a¹ºº:ºjÞ¼yÑ¡Ï÷Þ/¬/++KyoñÐûMé^|ñÅ°PUU¼ñë×¯Ëá^zé¥°°ûöÕ.^¼ØÒÒ¢'&z=_O,ÿ_~9¶fñâÅo½õVì&ÑQà+Vå5kÖå°&å6ø­²åååaùêíòEMMM1ñ¬Zµ*R]ìÚãÇoÙ²eîÜ¹a^^^ÊKàWï76lìØ±É·0aB´ñ×®]øÌ°<öìÀÙ®®®KBGH0Üs´~Ã±Ryä[ÙI?IéÅ_ôNÕØúpñ¦Ò=ztøÌÎÎÎèÈfX]õÌ3Ï[¾õÖ[ñ÷poñûr6,H1þº÷î7n¦¤¤$zR°ïÏüõ2n?Ìè¾è®ß8'NrüÖI?IY¿²²²øgþ¢'Ïn*¡µk×kccë£÷FQñ×ûMb,|ÛñãÇG·MØ¼îîîDïÒ=eØ¯aé©hcÂ6åV:~üø·AàORzñºe×®]Ñkþ¾øÅ/öEB÷îYçÅ_­(yòäÉèµÁ_ï7Ù²eË×¿þõ°ðÀ$ßöþûïËÏ=÷Ü©S§¢Ã¬aåÂÃrÙ|¦Mvø5kVXßÐÐpýg'v¹ï¾û®÷|Ö°~ÀÛ Ið')½øëîîÀ£øsõõ¿+W®DÇøs ;vÓ¦MÄ_ï7ill×.X° ö¾Ó/oØ°!lyAAÁ²eË¢7ÕoUVVFo^´hÑ÷¿ÿý~Kò¡áp·k×®[¾ÙñãÇ¯_¿>|Ýë?;ßuüýÇÎw=àm$ø$I?I$Á$IàO$Ið'I$ø$IüNßúÖ·ÞÿýÑýèGÿò/ÿâ÷&M9sæ§?ý©qHS§O¾råqHSï½÷¹×ÒW[[Û¿ýÛ¿4õÃþðßÿýßCúÁ~ÖýÑÿeø¾õÖ[ÿôOÿäW9Mç;ßùàCzã7þùÿÙ8¤©¿ù¿ñ?ÃôõÍo~Ó]Ò×ÁÙ:½òÊ+ÿñÿð'ø?øüÁüÁüÁüÁü	þàþàþàOððððð'ø?øüÁüÁü	þàþðð?ø?ÁüÁüÁü	þàþàþàþàþð?ø?ø?ÁüÁàþð'ø?øüÁàþàOððð'ø?øüÁüÁü	þàþððð'ø?ÁüÁüÁü	þàþðøknn=v~~þ¬Y³;ð'ø?øüÁ_.ãoêÔ©áá*,<ÿüóÓ¦MKÆ_CCÃåÌtòÃþð²ÒÓo¾ùî»ï4þ³þëbÒTSSÓ~ô#ã¦üã4õÚk¯ýä'?1i*à/üÏ0Ã_tã/¾¢¢¢dü=ýôÓÙÂÂ>è¯Â(üÿÞ8¤©Þôõê«¯:tÈ8¤©ððùWõWÆ!Ãþ÷bÒÔþýû3ÿEsG­®®vØ×a_9ìë°¯Ã¾rØ×aßìuùòåÊÊÊÎÎNø?ÁüÁàþrçÎ«ªªJ	ø?ÁüÁàþrMMMK,éèèHy-üÁàþàOð9¿òòòQqÁü	þàþðËøë=ø?ÁüÁàþàþàOðð'ø?ø?ø?ø?øüÁüÁüÁàþàþàþàþàOðð'ø?ø?øüÁü	þàOð?ø?Áü	þàþðð?ø?ÁüÁüÁàþàOððð?øüÁüÁüÁàþàOððð'ø?øüÁüÁü	þàþððð'ø?ÁüÁüÁü	þàþðð?ø?Áü	þàOðð'ø?ÁüÁàþð'ø?øüÁüÁü	þàþððð'ø?ÁüÁüÁü	þàþðð?ø?ÁüÁüÁàþàOððð?øüÁüÁüÁàþàOððð'ø?øüÁàþðø³£?ø?ø?ÁüüGÕEM2¥»»Û°ÀüÁüÁàþr³W_u÷îÝ·ß~û¨µoß>Ãðð?øËµºººêëë?ö±úùÖ®]kpàþàþàOð¹Ó¥KÂÃhqqqûJKKwîÜPhàþàþàOð¹Ï¾p­!?ø?ø?Áüå2û>ö±íÞ½Û³ððð'ø¿©­­­ªªª°°0.¬¯¯õÕWçþàþàþð;ì»í¶ÛØWQQugø?ø?øüÁß`üÁüÁüÁàþáÁqùòå	ìÃÊððð?ø®ì«¨¨Hxª/°¯ªªª­­­§[ÁüÁüÁü	þàoD°þàþàþàOðÃ©îîîúúúéÓ§'°¯°°°/ì?ø?ø?øüÁßpbß)SØW\ûu®fø?ø?ø?ÁüöÁüÁüÁü	þà/¦è(//O`_X30öÁüÁüÁü	þà/Ù<3Û)Sêëë»»»oåÎáþàþàþð-?ÛmÛ¶¦ððð?øËÚÛÛ×­[ülßôéÓððð?øúÚÚÚûÆsÓÙàþàþàOðð7;uêTuuuò¼óçÏOßCüÁüÁüÁàþ2]KKË]wÝÌ¾E½ùæiýÒððð?øË±lõêÕ	ì×®]öØø?ø?ø?Áüe¢ÆÆÆ¥K&OÈï½÷:u*cððð'ø¿´ ¢¢"yBÞêêê>NÈðð?ø¿l/mÎ9	ì3fLMMÍÙ³gd«àþàþàþð7øì«««»ýöÛ'äâ'ÚÛÛpÛàþàþàþð7huuu=ûì³S¦LI÷©§Ê¿Jø?ø?ø?ÁüB.]¼ÈK`ß¤Iêêêqø?ø?øüÁü=û'äôÙàþàþàOðð7µ··oÞ¼9yBÞEíÛ·/ÛØððð'ø¿þ¾¶mÛ<!ïÒ¥KÓ=EüÁüÁü	þàþ2W[[[UUUaaaû***2ÿ8ðð?ø¿4îµû'ä]¾|y?Ûððð'ø¿~§èÝu×]'N^Ãððð'ø¿þ±ï¶ÛnìËä¼ððð'ø?øKcÝÝÝ)ÙWUUù	yáþàþàOðð.öÕ××'OÑQ\jB^ø?ø?øüÁüe.]Êá?ø?ø?øütüuuu¥d_yyy.±þàþàþàOð7Òñ`_§èÌÂÙàþàþàOððððð'ø?øë¹³gÏ¦d_EEEccc³þàþàþàOð7²ðÍÌ<EÇpþàþàþðg_¸¸|ùòÃ>ø?ø?ø?Á_îã¯µµ5%ûû¹áþàþàOðð÷sõ43ÛeüÁ_6ÖÑÑQ^^ð'ø?øtöoÛ¶m$³þà/ë:räÈ3Âß'üÁàþàopÙçj?øËü-[¶¬µµµüýë_/³È'ÞSzúë¿þë·ß~Û8¤©C<yÒ8¤©×_ýïÿþïCúÆ7¾úuøøò¿üO|"¥¥¥?üpKKQuàÀ0!Míß¿ÿÝwßÍðÆøû¿Ø3þvïÞý?3[SSSssóÿTz:9zô¨qHSÇ3iª¡¡áøñãÆ!M½öÚko½õV?ùïþîïüñüã	ìkÂúp­ñLèÕW_ÿñ6iêW^ÉüÍeü9ìë°¯öuØ×aßXÝÝÝ)'äÍù):öuØ7wûÂü	þàOÁ_43[yyyûæÌððð¿ÜÁ_OòöíÛ·ûàþàþàþ9¿Ø·páBì?øÞøë)ø?ÁüLüµµµ­[·.#jB^ø?ø?øüÁ_îã/å¼Øðð?øË5üïßKf_aaáC=tâÄ	Cðð?øËÂ®~þüù)ÙwöìYãðð?øËöõ4!oN²/ü666µ··ÃüÁüÁàþ°/'äýÓ?ýÓqãÆjî§æÌSTTôÕ¯~þàþàþðãESt,0þðì±_Ä¯_ø_xàÿ`ëïmÿª·ú#ùHØ%ÂüÁüÁàþrÉ3³ÖÖÖÏ¾Lï6|ûìg?ûk«~-_ôï¿Vü×êêjø?ø?øüÁ_®ÕÕÕµsçÎäÙ&MØ×ÙÙy½Ïsûß¦O¾î¿¯Çß[ûß***àþàþàOð¹SûæÍÏÕ<eÊºººQ9¿Ï~ö³«ï¿_Yò+ù?ø?øüÁ_téÒ¥mÛ¶3¦òæ<þ¼æþàþàOð¹Y[[[uuuÊÙ7çñwýÆ»£wû7n×®]¤'üÁüÁüÁàoðÙ7àÙFþ®ß8ÏßÁeø,ððð¿Á,ì«W¯^À¾p1X°µµµ/÷0Bð7TÁüÁüÁü	þÉçjØ×ÖÖÖ÷û?ø¿¬Àß¨?ø¿Ë¾¥KÞ:ûàþà/ðw³òóóáOðð7%1þü3³ø¥lððøòàþð=uuu¹Øw3³ÁüÁß0ÀßÅxàøüÁüö¥¢cPØðY¿ð7ï5?ø¿UgggJöEStûàþà/ñ7kÖ¬äwÄÏÌ?ø¿ÀnóæÍÉStöÕ××÷t®fø?øËü?øÒÒÒ°Ì÷õ¯=,¤RBø?Áü	ûÂî7å¼é`üÁüe#þ¢?û°´Þï½k×®Ñ£GÃàþà/g¦è(,,ì×Ìlððø7nã?räHkkkXxì±Ç¢§züÁüåû63üÁüå þ6mÚGüNaáÂð'ø?øîCË>ø?øËFü¾ð/?>,477Á¹sç¦Óáþð¾z-ü©fxcàþà/ëð7$Áü	þà/ÃìÀÌlððð'ø?øËÆº»»S²¯ººúý÷ßÂm?ø¿¬Ãß´iÓ¢¾8É³àþào8²¯¾¾~Ê)	»ñÂÂÂzhhÙðÙ¿©S§Æ/wûþàþ)û7oÞÜÞÞ%Û	ðÙ¿à¼°§8zôèµk×2¹éð?øp]]]=±ï'ÄÙàþà/ñWRRöüÁàþVOSt?õÔSÙÆ>ø?øËFü;v,ì5Ö¯_ùòeøüÁü;öM>çÎéþàþr¡'JÊ>ðYÒÙ³gS²oÒ¤Iiþàþr'OöÁüÁ_vâ¯§ÙæÏ¿gÏìgüÁüe#þ¢ýHKKK7þàOð`ßÒ¥KÃýpaüÁüe#þ&Là?ø¿ìÁ_kkkOò8qb8/üÁüeþÂ>eÓ¦M]]]ð'ø?øÂRÎÌºë®»2?!/üÁ®çöa_oøüÁüeû¢	yO:5Üþàþ²y=ä?ø¿ÔÓ¼Ù03üÁüåæ©^$ø?ÁßÇß=æÌ<!ïºuërìgðÙ¿òòòÉ'gþ°üÁàoÄâï^¸ýöÛÙ·qãÆ³gÏæÞðÂüÁ_vá/???ìt2¿éð¿¿®®®gvÒ¤IÉò]bÿ|áþà/»ðwèÐ¡°ë©©©¹xñb&Oøð'ø9ø»téRmmmiiiû¦LµòÂüéºwûÂàþàoì»»döÕ××¯s5ÃüÁ_àÏ»ð§tà/°oÛ¶mÉò(öÁüÁ_6âo¨?øüå*þÂOjãÆÉì«¨¨hllQì?ø?ø?øüå2þN:µnÝºÂÂÂdöe~ð'øë±«W¯.[¶¬¨¨(ì¡Fçwfàð¿_KKKuuuÂ¼áâÚµkG2ûàþà/ñþ S¾á#ÝSýÂü	þr'N¸ë®»ÙWUUÕÖÖfláþà/ëð7cÆ°Z±bÅåËÃÅ/®Zµ*¬7oü	þàþzÿ_¹r%öÁüÁß0Ã_AAAØ[Å¿ùêÕ«aMX?ø¿566Î??å¹srø?øË)üåååV_lMWWWXãT/?ø¿ÂÿÃ#ÁÂS²/çÏÕð9¿è°ï%K¢Ã¾ácXkfÏ?ø¿ûöìÙ3gÎöcüÁü3üí¥|ÃÇàOð¬³³sß¾µµµác9Õþûêëë§Lâðð#ø»~ã¿wÞyçØ±cóòòÂÇ%Kdà¯þàOð_kkëø>9ãÿ_Å¬Y³>úÑ:ujhñúÔSOaüÁüå þ$ø?Á_¬©O|âË-ßú[£ÿüçñ±³³sHðMÈkø?Áü	þà/-8qâ?OýÏ1ùEÿ¦Núæof½°Ï¹áþàoxãoÔÍÊËË?Áüel÷ú_üüzþ§÷ìÙ1ü½ýöÛÛ¶mKfßÊ+Ãï¶ß@ø?øöøËë9øüÁ_;uêTiiéÍ[bòËýèGO8¯ÞÖÖ¶lÙ²s5ÌÌð§qØ÷GözÏ?ÿ<ü	þà/c-]ºtÎì96nòô<:þüÅ§ûÕuUUU)§èÈ;áOðC¿cÇ=:ziKü9áOð¨³³sõêÕþð§O>~üøeË¥õû¿®Éì3fÌºuëÌÌð§¿5kÖDû¾dfÓáþÉï1ü"µ··§ïK=Oø/nò«xà3³ÁüiDàï¥^ö+V¬Èä¦Ãü	þ2öÖÔÔìß¿¿_sûþàþ%þÂâ¼yó¢ÿò:t(Ãð'øËLÑÌlÉì+..®­­eìãÜ¾?ø¿a¿§~:ÚýÝ÷ÝC²éð¿°/åÌlåååuuuñ3ÈÁüÁrÎó'ø¿È¾I&ÕÖÖ&OðÊüåÝ¬üü|øüÁß°ëÒ¥Kuuu)'äöÙg:wüÁü)÷ñ7äÁü	þ)gf»ýöÛ÷íÛ×û)áþàOð?øöì?~÷3ðð'ø?ÁüÚÛÛ7nÜ8fÌöUTTGÄ¾Oð?øüÁ_V×ÒÒòÐC%³oåÊMMMý½7ø?øüÁàþ²´°ëX¾|yò¼k×®ð¼ðð'ø?Áüe]ÁvÉçjì«ªªºÅ	yáþàO¹?çùüÁß0ª±±1åÛ¶m»EöÁüÁFþâOéÎó'ø¿!¯»»û^?~2ûÂ>$~ø?øüõµ°'½ç._¾.«V­k>?øÂöíÛ|®æAgüÁüiÄá¯¤¤$ìOãOpõêÕ°¦´´ôïùØ±ceeeùùù3gÎ<tèüÁà¯/uuuÕ××O>=y§zjÐÙðqøöª|	ø»õ×üUVV>÷ÜsaaÇwß7üÁà¯÷Â7X[[[^^Ì¾ÀÁ¾´þàþ½5aÂ°oPþ2;;;W¬XÖõ·xÏ%%%×®]4öæÉø«««;ÙÂèÈ#Ç^ýõ¿ýÛ¿5iª¡¡áÍ7ßÌÕï.Øë·û·?ô¡%°ïãÿøöíÛÃÿ+Ò½ááóÛßþ¶_³4uàÀüGlA'Ç3ijÿþýÿ¢iÇßáÃS¾áã­·ÞºÅËHòÛGþ^zé¥³í7Þhii9«ôô­oëÔ©SÆ!M555ýà?È½ïëí·ß~øá?üá'ì>õ©OýÉüÉûï¿Íÿ3|çwü¥©ð_wß×8¤©×^íôéÓÆ!M[9s&Ã_4íø½÷ÞS§N-**ÊËË=zôìÙ³Ï?ëwà¸  Àa_å°o|mmmÕÕÕ)gfËüÎÁa_öÕ:ì¾&L½0|L>ð§¿ÖÖÖ»îº+a¡büÁü	þ­µk×îÞ½;,ð¿S§NUUU¥m¨Øðø;yòä´iÓF¨0aÂÞ½oýn9RZZî³¬¬¬¹¹þàO#áïåÊÉì»õÙàþàþà¯E'yÒ-ZÞ±cGZ7þàO#á/=MòÂü	þào ñÉ'cøknnËcÇ?Áü¸îîîçj...^·n]ö°þàþ4âðí£×®]3·¯àþnþàþàþúWtçèÙ¾¿«W¯>úè£a9ù´Ìð'ø¿ÜcüÁüiÄá¯©©)åIÃ#ü	þà¯Ø?êäÙÂ,güÁüiÄá/°çÎ½Û·¨¨hÚ´ix9üÁrû3?!/üÁàþSðîøËöÁüÁFþbïóuþüùiÓ¦M4	þð²¶¶¶uëÖ%³oúôéÃðð'øû?³±y·¯àþzb_òC;3üÁüÁüõ©I&êµqãÆÁàþrðð§¿Ó§OçÝ(6·G|A~/½ôü	þàïú):/_«ì?ø?üÅÔK÷^ø?Süõ23[kkk./üÁüiáo¨?øSvâ¯¥¥¥®®îá7o^OÈð'ø¿tõêÕiÓ¦=:¶füøñ?þ8ü	þF þyä1cÆ;6¹Ê>ø?øÓÃßÄÞðíëkjjàOð7rð×ÝÝý»¿û»É/ì+**StÀüÁà¯¯ýKKKlÍ±cÇÂð_øüüuuuÕÕÕ%OÈv3gÎpáH^ø?øÓÂ_ôßk×®ýÜý&üþ¹¿îîîÚÚÚä	yû>óÏl|dãºÿ¾î~éàOððSø+++ûú6ÿýáïsëÖ­aMii)ü	þráïýÙgM~¶ïCúÐç?ÿùGÿÇ£[okø÷ùÏþWõWáOððSø;zôhÊ<777Ãà/÷ðwéÒ¥ÏöM>½¦¦fÜ¸qkV¯Ù²yKß¯¯ùõ|ä#o¾ù&ü	þàþr¡sçÎÍ9³¨¨(//oôèÑ3fÌkÒ½éðÊ0þÚÛÛÃß]ò¼sæÌÙ·o_4!oø!~ò£O|â#D~ðð§¿!	þàOÃ_`ßæÍ§èhjjØ_çFÔðÂüÁàþ¹¿cÆÉáÙàþàþà¯ß<y2:Ïsôß	&ìÝ»þÃ§Nºë®»í[¹reü©ðqøkhh=*DøwìØ¿á¿'NTUU%°/\½zu¸ÊHÂüÁF:þJKKÃÃÉ'cøknnvgÁßpÄ_øª¨¨H9!ïÙ³g!üÁü	þnÜÅ®ÇØùÚµka9??þÃa÷´téÒö¯[·.'ä?øüÁß@0aBtV¿W¯^ôÑGÃryy9ü	þ²a¿?þüönÜ¸1ç'ä?øüÁß@jjjJyçðH¿¬Å_WW×/¼Ì¾ÒÒÒmÛ¶9Èð¿ÞØsçÎÞí[TT4mÚ´'?øÓÀð×ÝÝ]WW<EGqqqmmíH;?üÁàþMðê/þÞ÷Ý§zjúôé	ì2eÊW¾òyáþàOðð9RÝïüÎï7.&Mª¯¯O¢Cðð'øë­wÞygòäÉá±¤¨¨hæÌð'øËÎ=ÛÓ¼òÂü	þà¯ß566¦|ÃÇ©S§àOð7µµµUUUÝvÛm	·ß~û¾û<Ûð'ø¿ä¹²²2záòåËwßwXSVV¿¬bßg>óð¿5ì?øüÁß­ÝÅâNÂrìÏð'øËd­­­)ÙWQQ±cÇ~Íí+ø?øü¥.zæïêÕ«±5áOÔ3¿×ËÌla`¯÷gn_ÁüÁà¯·¢×üUVVF.´h×ü	þ²ñgÜ?ø?Áü]ÄÃ¾½¦ã¿ðJÉ¾ÂÂÂöÁüÁàþy7+??þXwwwò¹ÃßEOçj?ø?Áü]wgø¿áÈ¾)S¦ôðð'ø¿ÁÄ_Oçs>wîü	þ«®®®ìkêêêú23üÁü	þàïú`½æï_øBÂÊï½×©^R]Ê):ûú53üÁü	þàïú`½æ/<M0áüùóáâ×¾öµè)M/õ?øÃ¾éÓ§`B^ø?øüÁßõÁzÍß½÷Þ=&E«V­J÷¦Ãü@öUTTø×þàþðwßðqøðáØãÓÎ;3°éð9Y43[aaá ²þàþð7ÈøðÁ£Ç§h¶Ð=÷Ü¿°/af¶pqíÚµò«ð?øüåçç¨±cÇ?~üzÜkþàOð×Z[[ðíK¢þàþðøR[¶lIX´¼ÛWð×oyùòå	ìëiø?øüÁ_Và¯§óü¥ûQþàoX×ÔÔ´zõê¼9W3üÁü	þàoñ7TÁüÓ.]<EÇ¶mÛÒÄ>ø?øüÁß à/<Øíý"ü	þº»»_xá&°¯´´´¦¦&­ì?ø?ÁüÁüÁ_¦Ù7þüö×ÕÕuvvff3àþàOðððvöÕ××O4)aM&Ùð?ø?ø¿ôÖÕÕx7úôöÍ?Ï=ýþàþàþàþàþ²´ÎÎÎÚÚÚØ	ÏcÍ3gß¾CÂ>ø?øüÁüÁü~V555Éì[¾|yccãoüÁü	þàþàþ§öööÀ¾âââö­²©©)K6þàþðw«øë=øÓHÀßÙ³g7nÜ8fÌägûZZZ²jSáþàOð·¿¼Êaüµµµ­[·.Ñ¼­­­Y¸Áðð'ø¿ëfø?ø@ÁvAx	3³Á>!/üÁàþàþàþ¬'N¤wãÆíííY>¼ðð'ø?ø?øëká­¢¢"ácÆyâ'²ðð'ø?ø¿,Åß_þå_þÁüÁ¦Mêëëðx±¢):RNÈ[[[;ð?ø?øËÆ*++KJJ~åW~eùòåS§N7oÞb%bß)SgfÛ¹sggf?ø?ø?ø?øËµ^xá	&<¼áá­¿·5ú÷©¹úÍßüÍÌoIWWWJö5þàOðððýÆoüÆÊ+còÿZÿPqqq&·áÒ¥Ká÷*ùYrþàþðð#­Y³&ø/[6o3fÌÐ²¯¢¢¢±±q¸³þàþððu=þøã¿<ëãñ·|ùòOúÓéþºÑ¹S²/ó¿`ð?ø?ø¿RgggyyùÞððÍ[üÆóï|'­ìK>WsN²þàþðð566þú¯ÿúG?úÑÀ¾OúÓé_OStiå&üÁàþàþàOÿ¯ä9å¹#öeóÌlððððððð'ø?ø?øûùº»»÷ìÙÌ¾âââmÛ¶öÁüÁàþàþr=MÑØ~y.]º4ÒþàþððøëiË>ø?øüÁüÁ_nâ¯):êêêF,ûàþàOðð¹¿Àþàþàþào¤à¯£££¼¼þà¯.]º´mÛ6ì?ø?Áüü9rdÆáQþà/egÏMÉ¾=ö`üÁü	þàoáoÙ²e­­­½àïÿø3Û¾ño4j¨÷ÏþìÏ~í×~­¨¨(¿üË¿ùßáÒ«¯¾ÚÐÐ`ïp,<|¾þúëÆÁðÇöïßù/:ñ÷7±güÿþ¯ÌöÝï~·­­í)=577>º÷Ïiiiù­ßú­ä	y/^ùßáÕ#GÎ;gÒTSSÓücã¦ÂÙO~òã¦^íµþô§Æ!M[_¹r%Ã_4ñç°ï:ìêÔ©ä	yC+W®Ìá	yöuØWû:ìë°oö=¢ÃüÅ:qâD^ûÂÅï½7ÜàþàOðÃ)9#É3³VWW¨ÙàþàþàþàþrK.M`ß1cjjjÎ=k¬àþàOð9¿¿_ø;>zò³=ôP»Q?ø?ÁüÁüåB]]]¿ÿû¿rB^ÏöÁüÁüÁüÁüåûêëë'MÀ¾òòòÚÚÚ>!/üÁàþàþà/w°ê©§òØ7út3³Áü	þàþàþr§ 'x"ûØÇjkk»ººüÁàþàþà/ºtéÒC=4fÌöÝ~ûí555gÎ1Dð?ø?ø¿­­-°¯¸¸8a×ÓÝÝÝû?ø?ÁüÁüZ[[SÎÌ¶|ùòø"üÁü	þàþàþwág<EGôl_ò¼ðð'ø?ø?øË5ö­2ðð'ø?ø?ø®566._¾<Á|·Ýv[UUUïòÂüÁàþàþàoíAæÏrf¶¾LÑð?ø?ø¿aPwwwJöE3³½ÿþû¼ø?øüÁüÁüeu]]];wîL>Wsiiiøõðð'ø?ø?øfì2eJmmmggçîþàþððYWÊ	yÃººº[þàþððÙÅ¾Í7'ÏÌ6eÊûöÝûàþàOðððE4¤dß9soðð'ø?ø?øËÚÚÚª««'ä]ºtiÿÁbüÁü	þàþàþ)'ä]½zuFþàþððCÐo¾Ì¾pqíÚµ'NHß×?ø?ÁüÁüÁ_FüJ·°°ð¦3³ÁüÁüÁü	þàoøµsçÎÍÌððð'ø¿áWWWWIII43[áöööL~uø?øüÁüÁüeº'xâ©§ºtéRæ¿4üÁü	þàþàþFPðð'ø?ø?ø?ÁüÁüÁüÁüÁàþàOððð'ø?øüÁüÁü	þàþððð'ø?ø?ø?ø?øüÁü	þàþàþð?øüÁàþàOð?ø?Áü	þàOðð'ø?ø?øüÁü	þàþàþàOð?ø?ø?øüÁü	þàþàþð?ø?ø?ÁüÁàþàþàþð'ø?ø?ø?ÁüÁàþàþàOðð'ø?Áü	þàþð'ø?øüÁàþð?ø?ø?ÁüÁàþàþàþð'ø?ø?ø?ÁüÁàþàþàOðð'ø?ø?øüÁü	þàþàþàOð?ø?ø?øüÁü	þàþàþð?øüÁàþàOð?ø?Áü	þàOðð'ø?ø?øüÁü	þàþàþàOð?ø?ø?øüÁü	þàþàþð?ø?ø?ÁüÁàþàþàþð'ø?ø?ø?ÁüÁàþàþàOðð'ø?ø?øüÁü	þàOððgàþð'ø?øüÁüÁü	þàþðððð?ø?ø?øüÁüÁüÁüÁü	þàþðð?ø?ÁüÁüÁüÁüÁàþàþàþðððð?ø?Á_¦knn=v~~þ¬Y³;ð'ø?øüÁ_.ãoêÔ©áá*,<ÿüóÓ¦MKÆ_´;ÈdÁ mmmW÷ßÿã¦ÂÎ=kÒTSSÓùóçCjll¼páqHS¯½öÚåËCøû×ý×Ña¿øñ÷å/ù-ü|ãßø¦ÒÓCú÷õ×_7iêÕW_5¼é+<|ÿÃ;Û¿æ¿h.àïèÑ£ÕÕÕû:ì+öuØWû:ìË£._¾ÙÙÙ	ð'ø?øüÁ_®áoÔÏ.;w®ªª*%àþð?øöø¯©©iÉ%)¯?øüÁü	þà/§ðW^^>*.ø?ÁüÁàþr½ð'ø?øüÁüÁü	þàþðððð?ø?ø?øüÁüÁüÁüÁü	þàþðð?ø?Áü	þàOðð'ø?ÁüÁàþàþàOðð'ø?ø?øüÁü	þàþàþàOð?ø?ø?øüÁü	þào¤áïÿð÷ìÙó~fíµ×¾ýío¿¯ô´ÿþï~÷»Æ!M½ôÒKo¿ý¶qHSññ½ïÏ8¤©?ÿó?ÿÁ~`ÒÔW¿úÕ¶¶6ã¦víÚõÿøþ¢.]ÊMü<yrëÖ­$I¤¸z?4:Êó±$I#'ø$I?I$Á$IàO$Ið'I$ø$IüeQååå±Ç+++ËÏÏ9sæ¡Câ?³©©iÆÑUG1tÞkn:òºõámnn=vÞY³f¡6t;¼Q£FÙÍþðvuuUUUL4)ìÝà¯¶[Þ^v°ÙðÐf¯XøE¿ôñûëÊÊÊç.,ìØ±ãî»ïÿäÓ§Oð±õ2¼Ékú2òºõá:uêo¼þùiÓ¦½ÁÞPwwwØûÃ_:wûöíO>ùäµk×S&Olôwx=´Ýúðö²Í6¥Ä-[ÖÚÚÿ#a®^½ðgv:çÎá£ÐÀ7yM_F^·>¼ñ½AÞÇüK_úü¥cxgÎyêÔ)¦áõÐvëÃÛË6Úìz¸a~~~Êåë7¼?ÔðÉáãÑ£GÛ·§5½¼nxc_Ýêêjã6¸Ãúôé¹sç]<ü¥içðôÓOo É[o½eÜwx=´Öð¦ÜÁfÃC½ÒÍyyy±åøO7o^ô¿Ïð§²`Áã6¸; ^F^¿Ë/WVVvvv·ÁÞ;ï¼óðáÃ7·¼sØµkWXøþ÷¿mÜwx=´Öð¦ÜÁfÃC½ÒÍ&L¸zõêõÏÐålóïzyþÎ;WUUõÁ´AÞQ?qôoú×CÛ oO;Ølxh³KºùpíÚµ»wïác |ü§ÿ<y2,´´´ÿ*·ÁÝõ2òºõámjjZ²dIGGK­ûr­6¼>øà/¾ÂxáÂÆmp×CÛ­o/;Ølxh³WºùðÈ#¥¥¥yyyeeeÍÍÍñðÎ;ï?ð£ð1,·ÁÚES¼kxËËË=5¾á¿´ïÅï¼óÎ°ï;wnkk«qÜáõÐvëÃr=möJ$I#É©@$	þ$I$I?I$Á$IàO$Ið'I$ø$IüI$	þ$I$I?I$ø$IüI$	þ$I$I?I$Á$IàOÒïk_ûÚwÜQt£Å¿ôÒK?·ÿºÑ°ÙÛ¦ÚÚòòòð­uvv&¬kòóóËÊÊ®]»Ößû$ø4,Ûºuë¨¤¾ð/äþjjjÂÊ;w&¬ÿÊW¾Ö?öØc¸OI?IÃ¯cÇÖäçç?óÌ3Ý7ÚµkW¸V?~<gð÷Þï³fÍJX?cÆ°¾­­þ$Á¤Ñ=÷ÜXóäOÆ¯üâ¿VÞï½ñô9tèPÀSpáìÙ³Ãrì/P]]=vìØpUIIÉâ®666`«Âm<À©°fÜ¸qwÜqÇÂÅµk×&lØ+¯¼ÒûýDWM®jhhè	jK,	ëckÞxã°¦¢¢"¶fûöí¥¥¥á®Ö¬YsîÜ¹dü%ßÂ^6UüIÒ7qâÄÓ§OÇ¯<sæLXY^^=]»jÕª«Ö¯_]ÕÒÒòVÑÅèÚ;ï¼óÚµkÝÝÝáÚð1àiôèÑÑKñz¹°ró¿Óç>^´1_>÷Üs1ù%ÜÉâÅû¿^6UüIÒÐáM±Ï5*P,7wß÷åpqÅÑµu">FOà´EWUVVFOå#GDw[¶l	¼ºÞÿýaÍË/¿ÃÇ°|ß÷Ýô~ÃÅð	ñÛò; ?~|ø~;::ÂÅð1lyçÕ«W£O(++7ßÂõ&.**ê/þzÙTIð'IY¿PJüÅÖG¸Ì:wîØ]=v¸8yòäx  íÊ+±;	ðìVÑÅ3gÎÄ>9)vä7|Ë7½ÍpñüùóñÛÖÓëóyäpÕ¾ô¥ë?;´½aÃøOø$;wnôÄdñ×Ë¦J?Iú¢§»._¾¿²³³3¬WõÂ[[[#ÿÅ¬¡^òÑØNÅ`%,tuu;7nÚÞOOKèäÉSÃrø[ZZb×9r$l@ÊÃÇÇ_/*	þ$iè^÷öôÓOÇ¯NðØë;::Ð:~üxMMMt6¦Æèi¹ØqÕmýúõÑÑÞð±ºº:¶¾ûiûà¢çÏïý¹,×Fg·	f¿*zço¸ª¡¡áâÅ½ã/Óh4b×ö²©àO¾èùùù;vìNõòÌ3Ï$¿9cÅ:]]]Ñ1ÙØ;s£×üE/kmm±`dÁ6*EïÃl%%Ñ¢·ßF_½©©)¶¾û^ä½æ¯³³3úÌ^ð·k×®Øsrá[¿*zùcKKKø*azÂ_ÄÍÀåðikÖ¬¿¶ME'@Nhûöíÿoÿu£ÈF±åØÓØ,bÝÿýñ²oïÞ½½àïú©8Âúø#Â½ÜOØø½Ó¶§oöÊ+EEEÑ·p°Ù²eñ_büøñáct¶øûQ5vm/*	þ$)[@Y¼xqÁî¸ãè-·	økhhN§7sæÌÃÇ®íìì|ôÑGKKK#0=òÈ#]]]±k<8wîÜ`²²²²ºººûLÞpWaý>°¾§û		°óüÅª®®N8çKTGGGeee±cÇï¢­­-vÚøû¼xñbÐm4VÍÍÍ	_±M$I?I$Á$IàO$Ið'I$ø$IüI$	þ$I$I?I$Á$IüI$	þ$I$I?I$Á$IàO$IêÉ4?(jIEND®B`


ä>¼ÒîÃþÀqñ7ç?àÀßÔ»Ý9Â+lhhHã?ðg,üoÛ¶üø#ðþ:JsóðúuÀøKüY­VÉÄgþüøÆ,Úojjjmmm[[qöáà/ð·dÉÈÿBÍæÙþ,ø#ðþüÅNHçv»«««uGxåudnÒþÀ_¢â/--MEê¨Hb¾3gÎÈD]]ø#ðGàüÍIj·°°PwÞÄáàÏXøSÏ%íÉÄ½÷d.àÀ?ðç|>ÓéÔáu8É´àoÎð#Oª¯×+PêÀ?ð·bðZ­ÖDáàÏXøÛ½wh÷°÷ÕÁ?ào¶illÔá­ªªjmmMÄ9Àø34þ¤·ß~áÂ2ÑÕÕ%ÁÊÊÊÙ^tðGàüÑ<Ç×ëÕáMOOO²^ðþ¿9	üø4?ñ[[[«ªª"ßêS#¼###óá1àü?àóvü?Ãá¯¸¸Xð<ø#ðþf#y^;ôôtÝ^¯×;#ðþæEEEZðbo_þÀß41Â[XXØØØ8OFxÁø3þÄyò$ìîîó¦üøÄøëïï?räHAAî¯ÛíW#¼àüf³Yñß?à===C÷lN§s~ð?ðg,üÉ_<'wìØ1::þüøS+¶´´,]º4.k>ð?ðg,üI-|[>üøéÑ£GMMMV«^ðþ/fþÀßêéé±Ûíº#¼;wîeààÏøSOTyÇyÑÁ?ðG	¿@ ÐÚÚºvíZÝÞææfFxÁø34þòòòØáÀ?ð7dë­öÔ&´ÛíçÏgü¿ÀÇãçíîÝ»åràÀ?ð§[ggç"ÒUWWÇ>¼àü%þD>üø~¿ÿüùó6-òebéÒ¥'N`ü¿ÄÃ_JØáÀ¿ù¿G544äææF²¯ººxÁøK`üÍUàÀø#câO6ÎÂ»È^Å"nÏÇêà/±ñgµZ/^ÜÛÛþüøÏø'NX¾|yä[ååå.Ëï÷³bÁøKüL&ybÇÑÁ?ðGÁ_ÿ¡C"÷áMOOß²eKgg'#¼àü%þ®^½*ÏðáááxnÍÁ?ðGs?!ÛíÞE®O ([éG±&Áøco_ðGàÀ_ÂãoddÄårêîÃËyxÁ%9þØÛÀ¿ù?¯×ët:#÷áMMM­­­åÙþh^ào®þÀÅj·ªª*ò­>«ÕzèÐ¡¡¡!Vø#ðþüøKxü466êð[[[ÙüÑ|Äßøøøºuë222d[¹iÓ¦8lÖÁ?ðG³?ÙÆ:ÈÃõÉÏüÑüÅßØØî³ª_ðGàüÑl[[[-[¹a/,,llldgðGó¥¥¥²EX¿~ýèè¨Þ¸q£Ìø#ðGà/büøPiii²]ÐnÆÇÇeÌþü%Dò¬ÑáÍÍÍu:^¯Uþüý_)))²ðæÃ¡^üø3xjWwÞ?þã?®¯¯güøÓIû®Y³FûÊW9àÀ?cÖßß/[Q«Õª;Âëv»E=·/?/øíéîðñäÉðGàæþü~ÿùóçä«ß0S#¼'dáÔ¹	üÑüÂß³ç;ünÚ´);;;%%E¾®Y³FæÌö¢?àÏ8¾±èåååUÿ¯ª¬¬ìk_ûZoo¯¡0´´´Øl6Ýx#OÈþÀ?Ãþü?¿ùÍoV¯«Þÿãýêß+¯¼òõ¯Ýï÷añÔ¯î	Ùª««Ýn·î>¼àüøàüøÓOô­¢oä§þuvvÎíÉFÒn·ëðÖ××û|¾Ø¿øþþð/*%%üø£ù?Ù¸Å·Ãð÷òò[[[çdybðDð?ðGàoB¥Düø£y¿ÞÞ^Å²oï¾üdúk_ûø)ÎKc×n·Oj	þÀ¿öæoªmÍ©S§À?øÖ®]»´béî·vüöühÏòåËW¯^ÏóaÄØ÷#¼àüøbò'©¥=æ3ø#ðGI?¿ßÿê«¯.°¤¤ä«_ýêºuëâðsÅ1Fx§|Ððþü½ Í7«ÍÍåËã³èàÀø3Z>ùóôèQ~V´£4§¦¦Ceó8Í÷Áø#ðµsçÎ©-Îúõëã¹èàÀøEáÍÊÊª¯¯ÎÈOàÀNcccË-Sûvz5Îþü¿yUÞòòrË5³àÀ_xV­[·ÎÉ¢?ào7ÚQÁø#ð7Ãøã8þüÍv1öámhh©^ðþüM¨e2À?S(¶¶¶VUUéðNg^ðþüqz7ðþü(Q]´Þ-[¶Äs£þÀ?ðþÀ¿YLð¦§§GðÊæ®¯¯/ÎËþÀ?ðþÀ¿Ï#¼àüøàüøõÔoAAA´óðÆóìpàüøàüø­zzzvîÜÆ>Å2GiàÀøàÀß×ÖÖ¶jÕªÈÞÂÂÂ'NÌìQÁø#ð7Ãøã8þüM°¾¾¾#GDðVWWçvü?JH?]üq?þPµµµºGiv:>ÏÈKþÀ?ýÚÛÛeCöÚk¯ÊEùºqãFsíÚ5ðGàæ-þÚÚÚtÏÃ[RRâr¹æj^ðþüÍþÌf³lÎ´cãããêËÓ¼gùÎÏÏ7LeeeW¯^þÀñóù|º#¼ñßj?ðGàoæñ§¶k¾0üMÿ3555~ø¡L=ztëÖ­àÀø3rv»]w·¾¾ÞÈ#¼àüø²u¨ÉE¿ß¿~ýz#ó§yÏf³Y½<&­Vk$þ×­øvåÊ[dàþùÿùþáXïâÅöYrü.òÆÆÆòòòÈÞvuu%âïÅæ.!6wn·õ`üÍ]üè¬ãïÚµkº;|Ü¾÷¬Ýe$r÷Áß¹sçúãÛõë×zzúÉÀõööþò¿d=¼O>ùäÁþ[tvvÖÕÕé÷»ßýnü7P3ÀâW¿ú«FîßÿýßÙÜ¿K.Ås7ëøîÝ»WTTYQQñøñãéß­và8--a_bØa_Fxö%öûaßÙ+//OP¾F"?à/ÎÁóçÏ/_¾<r¬Ãf³µ´´¤yÀø#ð7É¬O<)òµ¦¦üøsÏçs:º#¼ê<¼É÷?ðGà/VwîÜ)..ÎÌÌTµyyygÏþÝvttX,¹Ïüüü®®.ðGàüÅ¹`0èv»7lØ9Â+­AÎÃþÀ¿¸âOäYJ75ôèÑY]tðGàüÍ^###.+òp¡^ü?qÂÅb­á;wBøëêêéììlðGà^¯×étæææ/==ÝápÌ¿.ðþüE¿ç©	?Ù¦sn_XøS#¼UUUoõ©^ãü?qÂ:È³z·Oð7>>¾gÏµ¹þÈøøÕ566êð[[[~ü?ËãñèäùúõëàÀ^¯×áp¤§§Ïó^ðþüMåå¶²²RííQ\c?àojÁÖÖVÝÞÂÂÂÆÆÆù6ÂþÀ¿Äüø^ðþüÍÌÚó°I?....((þÈ ø¿ÝÞÜÜÓéõzyPÀø#ð7uü³·/?2þáàÀßá¯  `AÌrrrÀ?+üõ÷÷ËV"òlj×ív3ÂþÀ¿ÉuÿþýçÎí¡MäwîÜ9ðGàâ?5ÂyB6FxÁø#ð÷lFz³=Âþü¿ZZZl6î¯Ëåbü?ÏØÛü?Jü©ÞÈ²ÉM«««áàÀßão||¼¸¸83334gáÂï¼óø#ðG³?ÙØíöÈ^ÿGôGgÎam?ðGàoñ·hÑ¢°~ÕÆ·¡¡üø£ÙÀ_Þ¯|å+k×®ëÍ·öÿxÿ_9þ*''§§§þÀ¿Ä_ZZlpµWù[9ÙÙÙàÀÍ,þ¢ð¦¦¦Úíö¯ýëÛþz°/ôïÏWýy"À?_øS;ümÇ#þþüÑtðcÞúúzuJÉ¬¬¬÷iñ÷Ê+¯üà?`?ðGào&ñ/Ûß]»v¹866¶ÿ~c±XÀ?&þÁ`´Þ¦¦&í>¼ú§º¥fò=¬pðþüÍ$þº»»uòÜÕÕþüÑñã(Ík×®õx<ûðÊFÖl6ÿ¥ý/~´gÍ5ßøÆ7XáàüøIüIeee)))¥¥¥2g¶üøKVüÅáÝ¹sgì8Z[[ÍÏËÉÉY½zµµþÀ¿Çßþü%þbððÆnhhHÀø#ðþÀø#Ãá/Æouuµ£4?àÏXø»sç:Î³ÚÃ7//ïìÙ³àÀ½ÑFxÓÓÓwîÜ©öá%ðGàüíííÚ)=ûò ÏGþH·.9s¦ªª*ò­>ÅÒÔÔÄ^àÀø3.þdK-Ûë;wîð×ÕÕÅA	ün###òäé¥"Ò¼aÃ·ÛÍgõÀ?ðgtü©÷3Í>*Ó&	üø£Pj7===YYY2^ðGàü%þòòòÔQýþÆÇÇ÷ìÙ#ÓV«üø£`0ØÚÚª;Â[XXØÐÐ0ñx	üøÀÇãÑ=Èóõë×Á¿ùáÕÝwùòå~ú)ûð?à/!ñ§^n+++ÕÞ¾ÅÅÅqÁþ[´^ãt:zz¦pn_þÀð7'?F+öQ9:ßøþÀøþ¸hGiNMM­®®</øþÀ_bãïîÝ»/NKKmFFFYYÙàà ø#ð7§¡Ýn×=¯Óéö	ðþü¿ÆÛíÖÝá£··üøKÖ@´ÞË^ðþü¿Æ:ÈsMMßï£££[·n9ùùùàÀ_ò¥FxsssuGx'ø¬àÀøK`ü©í¾ö=2:à3ø#ð4Åá­¯¯Ô>þàüø	?õÎßøøxhÎØØïüøKÔoIIî¯²?ðGàü%0þÔgþjjjÄ|rñÉ'«V­â3þ ÏW__¯;Âk·Û§óàÀøK`ü-xQ³4þþüÍ^òäª®®^ðþü¿dÃ_Ê2LàÀ_B422âr¹tGxm6ÛÔFxÁø#ðþsø#ð7ù|>§Ó9#¼àüøÉ¿hÇsþÇãÑáµZ­ò,ü?þ¾¼Þ~ûí°µµµêÀ§O^¾|y´Þ°²?ðGàÀßÿ&ÈW¼¼¼ÇËÅ>úH½~ÌÒGýÀ¿iæõzëêêÌfsùÒÓÓG|ÖøþÀ_ãïÙó÷ùÔG~~¾Ø¸qãl/:ø#ð7ÙÜnwÞØ'dàÀ¿?èÚµk¡æææ8,:ø#ð7Á@ÞÖÖÖYáàÀøKBümß¾]½¨³H¯½öø#ð7çõööÖÕÕ¥§§GîÃ[[[ÿgøþÀ_2àÏd2ÉkIvvö­[·i>óþüÍUn·íÚµ#¼f³¹¾¾¾¯¯onü?à/ñ'/'ûöíép8ØÛÀ_ü:qâDyyyäïÒ¥Kåªx~°ü?4¿ó'¯ÁàÀ_ÜêííÿtémïÞ½òBÿö?ðGà8Ãø#ð7óµµµéð¸¼ÕþÀ?ððøíÀnìàÀß×ßßßÜÜ¬ÞêêjÇc¨·úÀø#ðþÀø#ð7õWeÝÞ¬¬¬ºººÞÞ^ãÿàüøàüøAÁ`°¥¥Åf³E¾Õg±X9ÂþÀ?ðþÀ¿É544ÔÔÔTXXy¸¾W_õôéÓ@ ±~#ðþü?ðþüé$äv»=ò(ÍYYYN§Óçó%èïþÀ?ðþÀ¿ÿ+DáµZ­MMM	4ÂþÀ?ðþÀ¿¨ù|¾úúúÜÜÜÈ^»Ý~þüy#ïÃþÀ?JrüÅüøTj7ÚQwü?à/Iðò¢L&ø#ð÷ÂFFFîáúdfKKKÂíÌþÀ?JNüÍyà±Gxãÿçþü?àü¿YIþt«««uGxå»¿¿?é&ðþü?ðþ(ùñc×f³%ë/øþÀø4ÝúûûvîÜ)_ûúú?Ïçt:uGxÇ<91øþÀø4<ÏK/½ôíoûW^yùåETò*eLüA·Û­;ÂkµZçÉ/øþÀø4õü~vvöÖ-[÷ÿx¿ú÷Ýß]´hQèèwÁßÈÈHcccä	ÙB#¼Éq¸>ðþüø4»y<ÒÒÒüÔ¿üüüÞÞ^àÏëõêð¦§§ÏÏ^ðþü?ðþhê¹Ýî+Váï[ßúVOOÏÜâ/¶¶¶VUUéMþýlàüø#ðþhêïïÏÌÌüá®ä·í¯·eddøýþ¹Â_^± pð?ðGàü?ðGÓêÇ?þq~~þ÷_ÿ¾Èïµßé>ø tm<ñçõzGzzºî¯þÀ?ðþÀÍ@?ýéO¾ò¯üÉüÉáÃµWÅ1FxáàÀ?ðGqjVñ×ív3ÂþÀ?à±÷áeü?þÀ%þÔQuGx9Â/øþü?JüÅá]»v­<çÏyxÁø#ðGàüQ2ã/ÚoVVÌPÀø#ðGàüQã/öï¡CáàÀ?ðGÉ?öáþÀ?£488hµZÁÍþ|>_xCç&ðþüøG¥¥¥òJþhfñ§Fx«««SSS#Ò|äÈG±&ÁøàÀ_¼[·n×ë¿¿û»¿sÇ·Ë/ÿüç?wH¦h×þýßÿýÎ;óóó#Gx/_^__Ïã.^¼ÈJ0xlîsGóysÀøûßE¿+W®üw|ûì³Ï|>ß»ÿ~WWWäüÞÞÞºººÈxÕ/«.òÉ'¿ÿýïYFîæÍ_|ñëÁÈõõõÝ¸qõ`ð.]º466çÌøcØ&2ìÛÙÙ©;Â[PPàr¹ü~?+a_bØa_bØw.©§4Mü'NèîÃÛÖÖÆ>¼àÀø#ðgÄÀM/^¬¯¯·X,aæËÍÍë­·ØüøþÀøKÁ üIüÅ_üEJJJûÌf³üÁpfðGàüøã Ïà/Õ555Y­VÝÞÖÖVÎÃþü?àü%C===#ÚQ§y_àõþü?ðg<ÏÚµku÷áÝ¾;#¼àÀø#ðþÀ_2$ª;tè/Ú¯¼ø#ðþü?ððõööîÝ»7rWæ8N¯×«¾mRçö%ðGàüøàÏpµµµÙl¶È·úÃFxÁø#ðþü?ð=zô¨¡¡!òpj×ívë¥ü?àÀø	:oVVVì^ÝÀø#ðþü?ðÁÖÖÖåËëðºìÃþÀ?ðGàü?£÷èÑ£úúz^ùRSS«««£ð?ðGàü±Àø	<v»====rW8èóù&àüøþÀø3@ ¥¥EwÞ	ð?ðGàü±ÀøFÏçóÕ××G®/55Õn·Oÿ1àÀø#ðþÀ!R#¼'dò/øþÀëü?ðg¸FFFWIIIäoyyù#GÀþ8ðþü?àüÍM1Fx_õÕÎÎÎïÃþÀ?àü?ã&°«®®áµX,÷î©^ðþü?Öøào.­­­6-%%%---~¿¶ü?àÀø³:J³ÕjÕá§ÆÀø#ðþü?ð7´ìväyxÍf³p°¿¿?ÎËþÀ?ðGàü¿/¸ÝóðÊÌÙØü?þÀøñ®¿¿?Ú¯Ãáe®ØþÀ?ðGàü¿ÌívoÙ²Ew^y8â?ÂþÀ?ðÇzàüÍJ---º#¼6­µµ5ûð?ðGàÀøà/®k[k¾ôôôÚÚÚø¯ðþüøàüÅ£ÑFxÁø#ðþXàü¿Ùª©©éüùó3^ðþüøàüøþü?ðþü?þÀøþÀ?ðGàü?ðGàÀø#ðþÀø#ðGàüøàüø#ðþü?ðþü?þÀøþÀ?àÀ?ðGàÀø#ðGàüøàüøàüøàüøþü?ðþü?þÀøþÀ?ðGàü?ðþÀ?ðGàü?ðGàÀø#ðþÀø#ðGàüøàüøàüøàüøþü?þÀ?àÀ?ðGàü?ðGàÀø#ðþÀø#ðþüøàüøþü?ðþü?àü?ðþü?àÀøàÀ?ðGàü?ðGàÀø#ðþÀø#ðGàüøàüøþü?þÀ?àÀ?ðGàÀø#ðGàüøàüøþü?ðþü?þÀøþÀ?àü?àÀø#ðþÀø#ðGàüøàüø#ðþü?ðþüøþÀøþÀ?àÀ?ðGàÀø#ðGàüø#ðþüøþÀøþÀ?àü?àÀ?ðþÀ?ðGàÀøàÀø#ðþü?ðþüøþÀøþü?Æ¬«««¢¢Âd2-Y²$òïüøþÀëü%þ®_¿.§N*..Äß+WÆâ<Í|>ß¸¾¾>ùoëÁà	þü~?ëÁÈÉ¢¾øâÖûÍo~sãÆÖÁüýþ÷¿óM`üiËÈÈÄß|p%¾úé§?ÿùÏ¯koo¿|ù2ëÁà]¼x`ðØÜ±¹£ÄÝÜ%þº»»ëêêö%ö%a_yØW5::ZSSã÷ûÁ?ðGàÀøK6ü-ø2uq``ÀápÈËyäw?àÀøc=¿Ç6Ç³fÍÁÁAÝkÁ?ðGàü±À_RáÏjµ.Ðþü?þÀ_2ã/vàÀø#ðþXàü?ðþÀ?ðGàü?ðGàÀø#ðþÀø#ðGàüøàüøàüøàüøþü?þÀ?àÀ?ðGàü?ðGàÀø#ðþÀø#ðþüøàüøþü?ðþü?àü¿ù¿¿ýÛ¿mmmío~úé7úÈÀöÙg/^d=¼ýìg÷îÝc=9ÙÜuww³ÈO`Áz0xÇÿÍo~ç:22ø»sçÎþýûBDDDDb.àýX"""¢ùø#"""DDDDþüø#""""ðGDDDDàÏpþùçiii7oüöööXÆÃá«<ëÊ<.¥¥¥&©¬¬¬££uÿ­VkèâÍ7óóóÕ#rõêUíwÆ¸ó0uuuUTTÈUK,áäÆ|@ìµeËÉ³H&z·oßvm0'ø3òÃtðàÁwß÷éÓ§"Å³®øÍæû÷ïË|Õn7)>	¸ßÚíXMMÍ~(GÝºu«öcÆy®_¿.§N*..fíð12!°KÔì¡é°kßyç÷ßüùaÿo6XEF~å2!_zü[·n×ëÕnÇãêËãããaqçaÒÁÚ3æc4çÀ.Q[²dÉ;wdâ£>Ò¾z©w)*++åqF~äâáÃeó'ª¸û6ëÊÑÍ7åç|íîîf]ÍÍËf;¦"PÑ®"ã<L¡ä	UWWÇz3àcdB`¨	å5Ixöÿ§M6]»v-ì&£=L)))ÇöågÎXW|-[¦Þ®X±u5ç¯Xò¬	M§¥¥=¡¢]EÆyT£££555~¿õfÀÇÈÀ./îîÝ»¥¥¥a°6V1¦¼¼<Þ¨0øcÄIFÅgÍøøø³çcUÚgPì«È8Ó³ç¢p8>d¥ó12!KÔnÞ¼ùôéÓ÷ßÿí·ß~á#MF¶oßþñÇËÄ;wl6ëÊÑ+ÔpOOÏ²eËXWsþe·ÛO<)òµ¦¦Fûm1®"ã<LgÍ5¬1Ã>FF v<-ZVWWu*ðgäixxxÓ¦M&©²²Òëõ²®øÝ½WÌ'|iÖÕ¿buttX,üü|µvèt¯"£=LV«)?FàÀ?""""DDDDþü?""""DDDDþüø#""""ðGDDDDàÀ?""""DDDDþÀ«üø#"côÑÊ+3·zõêsçÎýÁöëy	³µÕ[Z«Õ*¿ßï/sL&S~~þÓ§O'DDà²ýû÷/èí·ßN&ü544ÈÌæææ°ùGùÂ?"J¼nÞ¼)¬1LÇ>ïøñãrQfÞºu+iðwïÞ=¹dÉ°ù¥¥¥2ßçó?"D4/zíµ×5ï¾û®væï½'3kkkµô¹zõªàI!Ó¡o~òäI]]]vv¶wíÚ¥]u»Ý,¹JnÛÖÖÆ)³råÊË/ËE»Ý¶`.]?ê*Y$uU4¨­Y³Fæwuuæ~]æTUUæ<xÐb±È]eddlÞ¼y`` ÷6'Æ¢ø#"ã-Z$p¹ÿ¾væd¦ÕjÕâ&¬îînuíÆÃ®Ú±cºª§§'%%E÷Vê¢ºvÓ¦MO>>¦¥¥A¹V¾233ÕGñbÜLè.^äozêÔ)­hC¾üðÃCò»Õ«WO1ÀÑÜ§Fxu¶YÅ´¸ÙºuëèódB.®_¿^]«¬£ø¨ÞÀ´©«jjjÔ[2ÝÑÑ¡îDûöíÞ©Q×7ÞxCæpA¦å«L¿þúë/¼£oÐ.îo$ p¡ü¾rQ¾Ê8ÇÇÇÕ7äççËåWxöå0qFFÆdñcQü.þBón¤¹(lR+**äââÅ·mÛ&hÝ|OØi¡[©<³)4ò+_eÚív¿ð~rññãÇÚeöù¼7ß|S®zÿý÷9´½k×.í7BÒÊÊJõÆädñcQüÍêí®ÑÑQíL¿ß/3åªÜ	ÑÐëõ*ÿ¬ú ^ähl§´Xi³ÙäÎsrrB×¾ð~¢Q,¬;wî(§Ê´|éÐµ²ºÃÇÇ_E%"ðGD4÷©Ï½>|X;S%lÐç#ßÐºuëVCC©Q½-W!ÑvìØ¡Fåk]]]h~ûQï´=|øP]|üøqì=sW¬X!×ª£ÛYµW©=åªöööáááØøÁT­Ðµ1ÀÑÜ§v0LGUz9vìXZZZäÎë×¯è5&Ú3WæOTÎëõj?,¨,¸k×.¡Ú7t°]¢©ÝoÕO÷x<¡ù1îGÈOæÏï÷«ï¿ãÇÞ_YúøcOOüY	Ñð§¸)otnÞ¼YmE%"ðGDdÔÃ:xðàÿm¿§lö±õÆohe©íìÙ³1ð÷ìù©8d¾ÙlÖÇ¸Yíö´öËedd¨_!l°ÝºuÚ±páBùªö¢½OõÆd(EÕÐµ1ÀQ ¬^½:íy+W®T»Üá¯½½]N¯¬¬ìÚµk¡ký~ÿ=,Óo¾B×¶µµUVVÉòóó].WØF.ÜÌß¾Øüh÷#ÉÂÈ"©Æ8Î_¨ººº°c¾¨kjjddggËoáóùB­ÑÞçðð°èV­«ªªª®®®°cQüø#""""ðGDDDDàÀ?""""DDDDþüø#""""ðGDDDþüø#""""ðGDDDDàÀÅ«ÿ³¢ä4êIEND®B`


út¤ÕýèGDâ³Ï>ábÙÜÜñ¡úßúÚµk6msÈÑ?ýôÓ0ÝÝÝ«§FüIÊyüÕÖÖ¯û÷ïïM|ðÁ>í3­®®nkkëqË#G?þÞïMÎ9zôèìÙ³/^|éÒ¥'NÌ5«¨¨héÒ¥©£Û·o///öoo¼ª7â<üðÃa~êNØÐ®]»ÂÌººº?cïþ­Ã°ýZ9GæRØ³ã¯Çµáy9sfXíË-ë6ûaúµ©Ok¨±±1<ZXáW%ïÖdðt¸ª´´tÃ^üI/þ®]»VQQQVV&ÒðñÇ¿è©	·ì¡´ðõþûïÿ<Ó~Éß$/$å×ãK,ÉNI&ùW®pfN6-ãÏØß»ôøÖA6ÑÈßôéÓûµr²nÀwì;þÎ9ú8áí/þRÖæææèb²Ó§OG7^µjUe^·nâ¿0qðàÁÔ°T<öØcazåÊÞ,Lafê-·lÙòÙgEcWÑÇ<H<y2º9räHî½³íðáÃaú£>J½ª7âfzßÛ¼KooªKöwådß1ËþzÜ1 -LWWW_¿~=°>I´¾ã/õiæ;v,LGOëêÕ«£G(¼páBÏf.))ñâàOR¬ñËÃtø»Þc~YYYþøã£áo|¸8qâÄÔGH^ÀzñêÕ«ÿvÿiª9º»»2æÍ5ÝEEEópTTûÇTUU566fY9jÀwì;þ¢÷5^ºt)õ[ô©Okôh©9ÑUsæÌ	§LðþqãÆ¯,	þ$åþÏJKKÃð®®®Ôùé#géoªËøÙ/höØõyË£VËËË£ýÔéÂKZ¤Ç£õå.½­[®´¾¬ÞH:°;ö·o_xýÚô§µÇyp¢«Z[[#ÿ%QxèÐ!/.	þ$Å¡×_=%ù¬ÇUÙCd¿8sæÌè+G8ëþ¢áÉè¬+ÕÕÕ6løôÓO÷ìÙf>øàñ×»ý]9É|Ç¾ã¯/#IÞúg_ÿ%%%aNCS;öì¶mÛ¢ÍÉ#£%Á¤XãïóoÞO¾¯?¼råÊk7þº?þøã·¿hô«¹¹9ä¥^êþ?-/<y2Õ:xð`Æ±¿wéþ²¯hMvÞ¬Çôw­Ñ»ôoÜ¸l·|ùòt¾ðÂaå g_ÿÑ:Û5¦gÏúcFïÝlmmÍ²^üIþ.^¼|wW4§­­­Çû½ÊÊÊÏÞþ8qbøzÄkÆe~äGÒwAÎ??ËÙ¯»ôÙWNò=NÔ<µ:ü=6õñï½÷ÞÔk¬ÀµìøNñhl5µGÔK?I¹¿P´K4uþûï¿ÿàÝ¬ºº:ýðáïêÕ«áÑÂc=ñÄÁCÉ³d'ÎÎ;gÍUPP0iÒ¤_~ù[ßúVräéöïÒ/üe_9þ+,,L*/µ~­Õà/tèÐ¡)S¦:<~´c=ys¸÷ªªª¦¦¦[¼9rdÞ¼yáÑÂµ÷îäüÎÎÎ'|2ROeWW4Xuww?òÈ#ý:·ÜîsEc£Ã·Ddú3gúEïùëÑ³Ï>kÍH?IÊÃ:;;7mÚ4iÒ¤hßnxê©§¬Ið'I$ø$IüI$Á$IàO$Ið'I$ø$IüI$	þ$I$I?I$Á$IàO$Ið'I4à<XYYYXX¸dÉþÞwÖ¬Y£FjmmMÎ	ÓaÎìÙ³û±9»ÙÀnÓûp#õ;«««¯^½]ÕÙÙ¹nÝºÒÒÒ°2Ç®úàüI?Iñª¼¼<P&&Hô÷¾Û·o÷þùçsî¹0gÛ¶mù¿h:üàazõêÕÑÅÚÚÚpñØ±caúÄazÚ´i~Á$Á¤mMnÃOgÏ÷]¼xqrÎÂÃ3gÎ=Åþ;.éâââèbQQQ¸xíÚ5¿TàOR¬å'|rÜ¸q%%%[·níq³oë[óæÍëñÉÃ0åÊ0æDW]¸p¡ªª*Ø¨°°pÎ9ÑXú£¥~÷ìwyùå+++-Z¾Q:Å®_¿^SSî~Ó¦MÑ@fàé½÷Þ-ÌüñÇþ&O.Î9óäÉ~µ$Á¤Xû/~úé§ÃôîÝ»³ÂÄ³Ï>z#G$ßßì±ÇWíÙ³'L¯aº®®.ºjþüùôQ_QQñÑR û]é^ýõ0Q[[¾ðëÖ­Óáo¾ùfØ¾d×®]knnÑÀdzÀßgÖO^³fMtÕÁ¸dÉ÷ÞÏo$øküUVVéî¥Û+ã?®Z¹re^µjU¤ºäµgÏÝ²eË¼yó¢#$2>Zfe¿K´`ãÆK¿oYYY´ðea"°/Ì·ÓsæÌ	íêêØjI-[xð ¿Ôý¼§O~MÞ&|S¿`àOR|ñWXXábv	~s¬¤¤$Ü²³³3Ú»æDW½ôÒKá^Aï½÷^ê#ôx´Ô¹Ko4:ÁqÿþýãÇæFwdä/KÇÜLÝ.Ið')ø«¨¨HùÏn)pmòkr~tÄg7ë#þ²ß%¹`Ásé÷8qbtßH$>2ì×jé/þBÑrÇ/$ø_üE§nÙ³gOô¿ç®/âÙ¿ríõ×_OÎ(yîÜ¹è½Á_ö»lÙ²å7Þ=öXúôÑ0ýÊ+¯´´´D»zÃÌEé÷ÞïÒ¥Kabúôé¿Ù³g«=¦:¦yä¿`àOR|ñH$°Jnz®¾ìø»qãF´Ë5|MPiié¸qã6mÚÔGüe¿Kccc¸váÂÉãNRoÓÙÙ¹aÃ°äEEEË/ìæ«®®^¼xñûï¿ß¯Õ¾k8Ëªß±¦¦&,|X'Nn]X$¿`àO$Ið'I$ø$IüI$Á$IàO$Ið7lýüç??þüÓ_ýêW¿þõ¯ýÞÄ¹û·ÎÍ¡8÷ÏÿüÏÿþïÿn=Ä¹/ÚÜÅs×ÞÞn=Äsw¬?þã?þâoúÞïýë¿þ«_å8wéÒ¥¿û»¿³bÞüãôÐP¬úû¿ÿÿy¿úÕ¯Î9c=Ä¼CÅíÿºðð'øüÁàþàþðð'ø?ø?Áü	þð'øüÁàOð?Áü	þàþàOð'ø?ÁüÁü	þàOð'ø?ø?Áü	þðð'ø?ÁüÁüÁü	þàOð?ø?øü	þàOðð?Áü	þàþàOð'ø?ÁüÁü	þàOð'ø?Áàþ¿;SSSÓ9sgÏþû?øüÁõy¿iÓ¦:u*L¼úê«Ó§OOÇßÑ£G¯mAÿò/ÿr]1îÃ?|çw¬ð×ÑÑa=Ä¹Ó§OðÁÖCÌ7wMMMÖCÌøûõ¯=Äß4ñZqqq:þ^xá¿Ú9ÒØØø×qo¿ýöO~òë!æ½õÖ[VBÌ³¹á	²¹³¹ËX>à/ü´®®În_Ùík·¯ìöÝ¾vûæónß¨ë×¯WWWwvvÂàþ?øËsü]¼x±¶¶6ü9O¿þð'ø?ëþòÇ_ºtéÕ«W3^?øüÁõy¿ÊÊÊQ)Áàþ?øËgüeþð'ø?ëþàþàþàOð?ø?øü	þàOðð?Áü	þàþàOðð?ø?øüÁàOð?Áü	þð'øüÁàþàþ?øüÁüÁàþ?ø?øüÁàOðð?øüÁüÁüÁàþð'ø?ø?Áàþðð'øüÁàþàþ?øüÁüÁàþ?øü	þàOð'ø?Áàþ?øü	þàOðð?øü	þàþàOð?ÁüÁü	þàOð'ø?ø?Áü	þàOðð?Áü	þàþàOð'ø?ÁüÁü	þð'ø?ø?Áü	þð'øüÁàOð?Áü	þð'øüÁàþàþð'øüÁüÁàþ?ø?øüÁàOðð?øüÁàþàþ?øüÁüÁàOð?ø?øü	þàOðð?øü	þàÏ¯2ü	þàOð'ø?Áàþ?øü	þàOðð?ø³àOðð?øü	þrµ®®.ø?Áàþy^"8pàÀ<°`Áø?Áàþy[[[ÛÆ'O<ê·µ¶¶Âü	þð'øË«ºººkjjF=ê?·~ýzø?ÁàþyR[[ÛóÏ?_YY9*­03àäòåËð?Áü	þr»®®®#G,[¶,¨/ÌY±bE@^"H¿#üÁàOð¿µµµ¾¾¾´´4¨¯¼¼<þüù,w?øü	þàOðuuu544TUU¥oôèÑ<ð@cccú]àþ?øüåXmmmõõõ&LHgßäÉ·mÛÖÞÞÞ÷G?øü	þàOðÇDCCÃ+2¾«ï¾ûîëËPüÁàOð¿Xõe<wêÔ©;vìè×PüÁàOð¿8¨¯¦¦¦±±1ã¼ð?Áü	þr©ööö ,çê»¡>ø?Áàþ±(H466ö6Ôæß¡>ø?ÁàþÃÜPõÁü	þð'øe¨þàOð'ø?ÁßP¨¯¾¾¾­­mhþàOð'ø?Áß`õÕÔÔô6Ô×ÐÐ0ØCð?Áü	þ½ööö;vL:uØúàþ?øüV1ê?øü	þàOðwçË2Ô7aÂaê?øü	þàOðwÇÊ2ÔªªªjhhÀ'ðÂüÁü	þàþà/^eê+//¯¯¯ommábÃü	þð'øëGYÎÕZ¼xñ¾û÷]ð?Áü	þî@YÎÕWZZºqãÆæææøÿð?Áü	þ²uËåØ½÷'|+?üÁàOð¿Ìeêó»úàþ?øüõ£ìïêÎÕ×ÙÙ£?üÁàOð¿ÿ(ËP_t®¾àþ?øÓHÇ_"hhhèm¨oÁL±:WüÁüÁàþ©­­­¾¾¾·wõ=óÌ31ùXø?ø?Áü	þn«,CË-kllÌ¡>ø?Áàþ4rñwùòå;wÎ;7ãPßæÍsñ^øÜ®^½ZYY	?øü)·ðþF×ÖÖ;6÷Ýwßk¯½¯Cðw[<yræÌá·þð'øSNà/ËP_iii«þ®åË_,øã7>Ú7Ãñ#Å¸øøë¿þkë!æ­á~h=Ä¹°¹ÿÝµb¾¹û¿ùø,Oø£]ý;¿ó;=ÌWPP°dÉ½÷¶´´À§é­·ÞúÍ]ãï?±wü½üòËÿ8´?~¼©©éãN>ìØ1ë!æü577[qî¯þê¯lîbÞ»ï¾Í]øÿö-[***Òúî¾ûîµk×ðmrØÜý7ÍgüÙí+»íöÝ¾vûWá¯ðC=4fÌÀÛØØH$<MvûÂàOðÊmüuttìÞ½òäÉéCõõõùw®>ø?ø?Áüi$â/l]kkkêkhh0Ôð'øüÁrÑPß3õÁß0?øüÁß ~ÞúBUUUúàþ?øS>à¯££cÇú&L0ÏÕðð'ø?å'þÂÖ,C#äc9àþàþð§|Æ¡>ø?ø?ÁüÁßÀ_nõyçw§r£ð?Áüiøñ¨oíÚµ1ÔUKKËÝwßýµi_ûÝßýÝ»îºë;ßùNNì?øü	þàOÃ¿,ð.Z´hß¾ñUggçW¾òËWlýîÖðïÉÿ÷É3g¤ÂüÁàþð¡,çêíP_jøúÌ¯Gòþ­_·~üøññ?×üÁàOðRüåèP_vîÜYõß«Rñþ]¾|þàþàþàOð9?Ô×£ÜsÏ=Fþàþð'ø¿m`°]^Nõõ¨³³³âÿªð?ø?Áü	þàï?úïòõ}ÿçhßÿÿÑ¾=ô£áþð'øøË2ÔgË~ð§ÿÀÎóð'ø?Áß+è»ßýnõåGð?Áüé°oíÚµ_üâ/ü)þFÝªøü	þàOwª@ºûöê¿aÃ_Á­*,,?ÁàþtûEC#á]ðküð'ø?Á_~e¨¯²²rýúõña-Á_wíÚµÇþ?øÓêm¨oôèÑ+V¬hhhH$ûl_ÁßÁ_øÿGaa¡÷ü	þð§Û)ûP_[[[òÆðÃ¿Ù³g§ÿvvvÂàOðêKYÎÕ·lÙ²ôÏ1?ø6ü_Í«W¯`¾7Þx#LÔÕÕÁàOðÊRåU×¯_ßÒÒÒÛáþÑïhÚôQØ¦øü	þàOË2Ô·`Á×^í;ÐàþãÇ¿©'OlmmO=õT4áT/?Áü©GYúÆ[WWÖ|þàoØð·iÓ¦äá©¿Äá7þ?øSTö¡¾½÷ö÷½òðÃ¿Ð÷¾÷½'¦¦¦0 8oÞ¼Á^tøüÁà/þeê3fLà`ßúàþb¿a	þð'øsYúwîÜ;ðð'ø?ÁßðõÍ1#ãPß5k<Ôð#üM>=:á<þð7bÚÚÚ ¼töÍ;÷öúàþâ¿iÓ¦¥/£?ø	eê;vì5kÞyçôS4Ãüå0þóÂï÷éÓ§xS?øüoYú¦Nºk×®;;Ôðüßò¡ßÃàþÃÒ-ßÕ¶B1Ôðüß¼ðë¾nÝºë×¯ÃàOðyxï¹ç ÂË/ÙÂÀüþB&MJ8àCð'ø¿ü(Ë¹úe³ð7lø2e>?øËË²«/pPßÕðSüE¯æææ!^tøüÁàoêêêÚ·o_Üúàþâ¿²²2|þðµ¶¶Æv¨þà/.ø;~üxxIlÚ´)ü?	þ?øËÅn9ÔP·e?ø6üê%|þðÿ²õñÐüÁ_à¯ ð!øüÁ_lËÅ¡>ø¿¸ào¸?Áü	þPP].õÁüÅS¦Liii?Áàþb[ ]CCCUUUîõÁüÅá3ô?øüõ¥¼ê?øþ;^?Û¶m»víÚPnÍáOð¿,eêÌ¹¡>ø¿¸àÏÑ¾?ÁüÅª,CÁA9:Ôðü9ÚWð'ø¿8e¨¯¼¼<úàþâ¿áþð'øÊr®¾ÅïÚµ+?úàþàþð§¿[«/üyüÁü'þº»»/_^\^o%%%÷ßÿlÖáOð±økooß¸qcoC»wïþäOòþ9?ø6üÝ¸q#ã=Æ?øÓHÃ_"8räÈ+F>Ô8ØÜÜ<r#ø¿aÃßÌ3Ã«nåÊ×¯_¯]»¶jÕª0gþüùð'øüÁß©½½½¾¾¾²²2¬aÁ#d¨þà/.ø+**¯½ð¿±äîîî0'Ì?Áàþn§,CcÆY»vmx½§þ?ÁßPà¯   ¼øsºººÂ§zü	þàoÀµ´´ô6ÔwÏ=÷ìÞ½ûòåË#ü9?ø6üE».]íö_Ãt3gÎøü	þà¯_EçêË2ÔßðÂüåþö2ðñé§ÂàOðgøöÃ>ÿüó¯½öÚË>Ô·wïÞø®>ø¿âïóüÞÿýãÆ+((_.]æö¢Ãàþ4Äøëìì;wî¤I¾¹øó¿1?lð?~ß¨££c÷îÝÏÕ7vìØÚÚÚ¡ßÔÃà/¦Áàþ4Äøûýßÿý¹sænÙ¼eëw·ßùÎwÿ<þ^ª½,ÇÔ©SwìØa¨þàþàOðNüéK_ÚøÄÆH~Ñ¿ùßÿÚk¯õë³|,ÇèÑ£kjj¼áþb¿Q·ª  þ¿¼Á_GGGYYYªüÂ¿o~ó;vìèãcfùÞ3fìÚµËPüÁ_¬ñWÐð'øüåþBwÝu×cÿÏc©ø6mÚ²?T¡¾1cÆÔÖÖzÍÂüåþzë'^Ò¯¾ú*ü	þù¿üàÿëñÿØ·eóo~ó_ýêW³|gö¡¾Ý»wwttXÕð9¿ð+XRR^ÒUUU©ç|?Áà/?ðýÿ_øÂ×¦­¬¬lþüùmmmé·ÎÕþê?å3þ|ðÁèøðá¡YtøüÁß|ó_xµ··§®ZkkkÆ¡¾Eê?øËü½ùæÑåÊC¹èð'ø?þÒË2Ô ¯Uðø»qãÆüùó£c;;6Ä?øÓ°ãÏPüiáï^^Þ«W¯E?Áüi¸ðH$õÁFþçOð'øøkkkëíxõÁò·ª°°þ¿üÀ_4Ô·bÅÑ£Gê?Püð'ø?þõÁàþàþù_PÅýÙeêkmmµàOð?Á_ÎõeêÛ·o_ôü	þàOð'øË¢sõê?ÁüÁü	þò¼ìçê3Ô?ø?øüåCÙ?£ººzè·±?ø5þçOð'øËÑúò±ýúx7ÁFþROéÎó'øüÅª~/üÁà¯×=6?üðõë×ÃÅðuÕªUaÎ'àOð'øCø^ø?Á_¯ÍG"HÎéîîsÊËËoóÃïtEEEaaá¬Y³;?øS¿Ê2Ô7fÌÚÚÚ,/ø?Á_ïq³¾ø»ý÷üUWW¿òÊ+aâÅ_z5ü	þàO¬µµuíÚµúfÌÑå?øüõZt"Ðµ7n+W®sÂüÛ|äÒÒÒèÏCÐdeee:þÂöëìÐööÛo<yò¬bÜßþíßþìg?³bÞ[o½õ_üÂz¸ã555mß¾ýîI7ß¾ðûî»/üÚæ.6wÖCü7wCÿM'NÈxÀÇï½wzÈHúá#o¾ùfûÐvêÔ©ææævÅ¸ÿüçÖCÌûñüñÇ[w°ðkÿð_üâÓ·ÆS§Nî¹çÞÿý~=`Å?þã?Z±qîþélîâß¡C~s7èøôÑGÓ¦M+...((())3gÎ+WnÿaSwÙí+»íöUºººöíÛ·hÑ¢¼«Ïn_»e·oì*++ÞJ¾¦ïD?Áüä²¿«oÇ·|WüÁà/vÕÔÔ¼üòËa"|­®®?Áü)H444ô6Ô6wjÃð'øËÖ¹sç¦O^RRí¨-++Û¿ÿí?ìÉ'ËËËÃcVTT455ÁàþFr---õõõéì3yæÛê?øüõµè$Ï©éM¿øâºèð'ø¿Pt®¾+V=:÷Ýw_Ø¦iþàOð7èø+// sçÎ%ñ×ÔÔ¦Ç?ÁßË2Ô7cÆgy¦­­mð¾;üÁà¯÷¸Y4á/lÓ¶¯àOð7°Ä#G2õ9555áoÉ`õÁü	þúZtçh´/à¯»»ûÉ'Þ?Á_ßkooïm¨oêÔ©Û¶m»|ùò-üÁà¯×?ñ$Ï§N?Áàï%ÆÆÆz(ãP_äÈ!ê?øüõïÏí¼yó¢£§O>¨ïD?ÁüåGaS¹mÛ¶É'§ÿÿ9Ì[¹O>ùd¸þàOð»àOð9Zt®¾,ð?~|èúàþ~ßçìÊ+Ó§Oÿm?Áà/µööö°íÊø®¾òòòmÛ¶µ´´ÄdQáþýÀ_ww·£¿dÑ»úzêó80ìCð¿[ñM*©?þáøË2ÔfnÞ¼yÞ!ð'ø»3ø»páBÁÍíZßo¾	?Lüeê»ï¾ûb8Ôð'øëkz½þð+eê<yòæÍÏ??üÁà/vÁàþâSxÃ0?CxþùçÃ×ø?ÁßÄ_ww÷ôéÓKJJs&NøôÓOÃàO#mmm½,G®úwõµ¶¶þIÿåë3¿^õß«fÏ÷Ýwà bø?Á_¯M4©Ç¿ÑVoÛ¶mð'øS¾â/nC©öÕ¯~uÅò[¿»5ú÷íoûË_þrgg'üÁàïÎà¯¨¨(lìsÂïb3nÜ8øü)ÿðÃ¡¾hûÚ´¯%åý6mÚ;ï¼ð'øûüNð6y=¶ãé'ÿ?Ár·êkllÃ¼á/Ízàï¿.ø¯aááþwaÛ·aÃ®®®pñÆ[·nÎV?åþ²õmQ|¶¥¥å®»îÚ´qSR~[6o	ä9ø?ÁßÁßéÓ§3ä¹©©	þÊ]üÿÐæÄP_zßúÖ·fÍµö×ù­_·~î¹aiû»¨ð¿l]¼x1lhJJJfÎæö¢Ãàþ©ÖÖÖúúú	&äÄP_zÁy6m;vlYYÙøñã×®]í?øüî<ð¿dÑP_UUUºùb>Ô×[/_ðÃü	þàþàOy¿êàþÙ:wîçèß²²²ýû÷ÃàO1Ç_ö¡¾ê?øüþ=Üø¦_|ñEøü)øË2Ô7uêÔ;vÀ¡>ø?Á__+//ËsçÎ%ñ×ÔÔä$Ï?ÅD¢±±qÅúàþ·ñ7û<åÄÎa¦áOð§à¯½½½·sõM<yóæÍ/_ö¼Àü	þúTYYYtV¿ÝÝÝO>ùdô.iøüixñH$9ÒÛ¹úî»ï¾ãÇê?øüõ¯°éÌxçS§NÁàOÃ¿[õ?Þð'øøÛyóæEGûO>>×þð¡>ø?ÁßPàoX?Áü¥e¨/Ì4Ôð'ø?ø?å	þ¢xÓúBË-×àÃÍð'øËÖ|0eÊ¢¢¢°©-..5kÖÕ«WáOð§AÅßùóçyæ©S§¦¯¼¼<}ð¿AÁ_ø_uÆ>ZZZàOð§;¿D"6¦<ð¡>ø?ø¿áÁ_tçêêêÎÎÎpñúõë«W¯s***àOð§;?Cð'ø¿Xà/Úò¦=¦'|?ÁnÑÇrÔÔÔdê[¼xqCC¡>ø?ÁßÐá/ùëîîNÎ¹qã?Ánííí;vìÈ8ÔWZZZ__?g?Áà¯gÑþª««ùÂÅO?ý4üGÜþ0þ²õÍ;wß¾úàOðÃ¿Q·jöÿÂà/ÿðe¨oÂ	ÎÕ?øþnUaa!ü	þÑP_oçê«ªªò®>øüÁ_ð7à/ð×ÞÞ^Ë?cÂ	õõõ­­­Vü	þà/^øëí|Î/^?Á2vðàÁýìgúàOð9¿°¥þÞ÷¾×cæ5kêEð§ô¢¡¾»îº+ã'ðê?ÁüåþòÂV»¬¬ìÊ+áâ~ô£h;>Hoõ?Á_.å]aNßÐÐzºPÁàþâ¿ÏoóEñhbÕªU½èð'øË²¼«/ês®>øüÁ_îá/tâÄä×®]C°èð'øsD¢¡¡¡·¡¾o|ão¼ñ¡>øüÁ_®âïñÇO~¼f4ñðÃÃàodÖÖÖV__¨o`í+øüÁ_,ðWXX¶éãÆ;öìç)ïù+**?ÁßÈ)ûP_wõÁü	þà/ñ¶ì[¶lé1³¶¶ÖÑ¾¿R_úzÜþàOð9¿ÞÎóþÃà/»åP_cccoïê?øüÁ_ão¸?ÁßpýÞpU¸AöG?øüÁ_îá/låSwìf¿¿<èçêË2Ôð'ø?ø?Á_ÎtûCð?ø?øüÅ½;8Ôð'ø?ø?Á_|kooß±cÇÔ©Séc9àþðð'øþ¢¡¾Áþ^ø?ÁüÁü	þ³Áê?øüÁüÁàoøË2ÔªªªºSCð?øËüeþq®£££·¡¾	&Ô××·¶¶Þw?øüÁ_îá¯àVÂà/NMMÍ1czêëêêìe?øüÁ_îáoØ?Á_¿úfÌ1,Cð?ø?øüQqê?øüÁüÁàopûäOvïÞ½`ÁCk×®Ê¡>ø?ÁüÁü©uttìÚµëÿð~úéìç¿ÐñãÇkjjÆÎ¾EíÛ·oèúàþðð§¾ÖÜÜ|÷Ýwãßøö·¿ýßîýoÁ4ðÞ'|²sçÎ¹sçfê«««Æ¡>ø?ÁüÁü©¯M<yùÿ½|ëw·Fÿ~ÿáßéèè¿dáG®­­Íx®¾î¹gïÞ½±Z`ø?ÁüÁü)smmm_þò·lÞÄ_ø7ëë³9/_îm¨¯´´tóæÍ---ñø?ÁüÁü)s­­­3¦ÏH_ø·`ÁðrÉøknn®­­Íø®¾ÅïÚµ+nCð?ø?øSêêêÄ©ûuIùm|bcIIIoo_ËoüuttìÜ¹3ã¹úJKK×¯_Û¡>ø?ÁüÁü©¯íÞ½ûK_úÒÿ¨ùA~AÓ¦MÊéíÆù¿æææººº	&dêíµ×b>Ôð'ø?ø?õ£Ì9ó_øÂW¾ò?ù?I$#|òÉÞ½-[n¾òòò7Æç^ø?Áàþ4åþ×®]ñ]UUUak[Cð?ø?øüe¨££cïÞ½/N?oKô®¾ð'9&ø?ÁüÁüi¤ã¯µµuíÚµ½½«oß¾YvvÃàþððuuuØ-Z´(Ý|7nÌþqvð'ø?ÁüÁüåþ²õ-[¶ìÀù4Ôð'ø?ø?Püeê<yòæÍÏ?÷OüÁàþàþÿøq¨oôèÑ+V¬hhhÈã¡>ø?ÁüÁ_ºzõjee%ü)§ñ×ÑÑ±÷îÞÞÕW__ïê?øü	þúÝÉ'gÎþ@Âr½õEçêQCð?ø¿[·|ùòÖÖÖ,øûÓ?ýÓÆ¡íðáÃ?ýéOãÂ¦á]¿üË¿~ÆOà-))ù½ßû½þð#üizë­·ü®Æ<;;åîæ.ñ÷Ø;þÞ~ûíÿ=´ýâ¿hkkûßq.®ï~òäÉºººñãÇ§³oÉ%þç~íÚ5ÏQèÇ?þño~óë!Î9sæÃ?´âÜùóçß÷]ë!æ:tèÆCüMóvû*&»³¼«oÂ	õõõ9ú	¼vûÚík=Øí+»zQð§À_wõîÛ·¯««Ëð'ø?øëáOqÃ_4Ôñ]úàþ?øS>à/<xmmí1cõÁü	þCüi(ñg¨þàOð'ø?ü½óÎ;555úàþ?øS>ã/.:wôèÑúbþ	Âð'ø?ÁüÁüõ©ìðvttXÏð?Áü)·ñ½«oÁéæ3fÌC=ÔÜÜlõÂü	þð§Ç_P]]]]Æ¡¾3f^¾|Ù?øü	þàO¹¿ÎÎÎûöÍ;7Ý|£GH$¬Rø?ÁàþÛøçwzêÜ¹s§¡>øüÁàþà/çñõ-^¼8Ý|cÇ]»vmø2Ô?øüÁüå<þ7nÜÎ¾ê?Áü	þàþò»víÊø®¾	&¬Y³fèð'ø?ø?Ýyüµ¶¶®]»6ãPß=÷ÜóüóÏ;Wü	þàOððóÒÿûß¼ëí}>þð'ø?øËZ[[ëëë3À;uêTCð'ø?ÁüÁ_>ÔÕÕÕÐÐPUUÕÛ¼---à?Áü	þàþr¾,CS¦LÙµk¡>øüÁàþà/çË2Ô ¸qãÆS§Nycü	þàOððóEðfê".ü|@í+øüÁàþà/.ÒíÛ·oÑ¢EúêëëSoð'ø?ÁüÁ_Ne¨/X00êëüÁàþð¹Tö¡¾ÀÁCð?øüÁüådê?øüÁàþà/ºÍ¡>ø?Áü	þàþr£ ´,C»wïÀ¹úàþð'ø?øWtvYúnGoð?øüÁüÅ¥Áê?øüÁàþà/^êPüÁàþðq)ËPß3îÈPüÁàþðÃxÇS[[;x>?øüÁàþàoèÊr®¾Aê?øüÁàþào¨Ë>ÔWSS3dOüÁàþðX¡¾¹sçîÚµk°úàþð'ø?øôºººªªªzêknn?øüÁàþàïNÖÚÚZ__ßÛPßÎ;/_¾<ð'ø?ÁüÁß(ËPß?WüÁàOððÃVö¡¾!8þàOð'ø?ø¿A/Hô6Ô7vìØ5kÖcÛÄm±áþð'ø?øë_mmmõõõ91Ôð'ø?ÁüÁß;pàÀ+F~ïúõëë^ø?Áàþàþ¥x û-Zó¡>ø?Áü	þàþXcccd¾ÒÒÒøÀð'øüÁüÁß`USS³wïÞÎÎÎ]~ø?Áü	þàþFPð?øüÁüÁàOð?ø?øü	þàOðð?Áü	þàþàOð?Áü	þð'øüÁàOð?Áü	þð'ø?ø?Áü	þðð'ø?Áàþàþð'øüÁüÁàþð'ø?ø?Áàþðð'øüÁàþàþ?øüÁüÁàþ?øü	þàOð'ø?Áàþ?øü	þàOðð?øü	þàþàOð?ÁüÁü	þàOð'ø?ø?Áü	þàOðð?Áü	þàþàOð'ø?ÁüÁü	þð'ø?ø?Áü	þð'øüÁàOð?Áü	þð'øüÁàþàþð'øüÁüÁàþ?ø?øüÁàOðð?øüÁàþàþ?øüåþæÌSXX8öìôßoøüÁàþ¬øË+üM6íÔ©SaâÕW_>z:þÞ~ûíC[xµµµÝP;þ|øoõóþ:;;­8þõáZqî¿üå»ï¾k=Ä¼¿ßüæ7CüMs©§ãï?øÁÛCÛO~òþô§o+Æ=zôðáÃÖCÌë­·¬gsgs§ÜÝÜåþN>]WWg·¯ìöµÛWvûÊn_»óy·oÔõë×«««;;;áOð?ÁüåþFý¶èâÅkkkÃóô[Âàþðg=À_Îã/µãÇ/]ºôêÕ«¯?Áü	þàÏz¿¼Â_eeå¨àOð?Áüå3þ²?øüÁõððð'ø?ÁüÁü	þð'ø?ø?Áàþðð'ø?ø?ÁüÁü	þàOð'ø?Áàþ?øü	þàOðð?Áü	þàþàOð?ÁüÁü	þàOð'ø?ø?Áü	þàþào¤áïþèÎm?ùÉOÞ÷Ýóq¿øÅ/Þzë-ë!æýð?üè£¬86w§O¶â_õóöìÙóË_þr¿iGGG~âïÜ¹s[·nýcI$¥×è(ã±$I#'ø$I?I$Á$IàO$Ið'I$ø$IüÅ¨«W¯VVV&/^ºtiTJ©·<sæLEEEaaá¬Y³;fÕÅóijjj3gNxfÏíløñ|¢=q¾âðuuuÕÖÖM<ùøñãV]<¦ðÔÌ93ú«tòäI«n¸£,¯8ÈÁv¶gáÕ^9©/§ýû÷§0ã«««_yå0ñâ/®^½ÚÚçÓ4mÚ´S§NW_uúôéÖ^£P"F¿Ø>GÛ·oöÙg?ûì³ðlÊ)Ö^<¦ÒÒÒ.ð5Õ"âç(Ëë%r°íÙòåË[[[SÂð;xð`o/³èóI»»»½Ìbû4¥V\líÅó9zúé§¿ÿýïÃ_l£Y³fµ´´Xi13.^¼&ÂWFÆç(Ëë%r°íe½¤<Ó¦M[ºtiaaá¼yóÞÿýÔ§«§)ÙéÓ§ëêê¬·>G.3Ãþâ¼¹áÂ)Þï=ë-OÓ3gÂsn¾-õ6Q×Kä`;ë§0YøÔìÙ³Sç$§¬·x>MQ×¯_¯®®îìì´ÞbøÝÿý'NèíöÉænÏ=a"h#ÃzçÓ4þühÀ)(páÂÖÛp=GY^/qíl?^féÏSYYYww÷ç7oÃ´õÏ§)ÚDÖÖÖ^ºtÉJçs4ê?g½Åss7ìÃ¦[>MöGÅä9ÊòzldoýN6­­­-ÄÒ¥KSoVSSóòË/ðµººÚzçÓtüøñ0çêÕ«ÖXl£[þÓ°?G?þøë¯¿&Î;·hÑ"ë-OÓÂÃ&çÏo½×såõ9ØÎÞú)<úttäüâÅ£7Ò&opòäÉòòò¦¦&ë-OSee¥Q¥?GðÿçèÚµk÷ßô>³ÖÖVë-OÓ|Ì®_Ã´õ6QÆ×K|ä`;+I4jH$Á$IàO$Ið'I$ø$IüI$	þ$I$I?I$Á$IàO$Ið'I$I?I$Á$IàO$Ið'I$ø$IüIáýèG?º÷Þo¶dÉ7ß|ó?m¿n3[ÛLK[YY~´ÎÎÎóÃÂÂÂÏ>û¬¿)Ið')'Ûºuë¨´¾÷½ïåþ¶mÛfîÚµ«Çü;wùO=õÔSàORîuæÌÀÂÂÂ^z)q³=öaæÙ³góôQ9öìógÎæ·µµÁ$ø4"zøáköÙÔÏ=÷¹fÍTú;v,à)¸pÎ9a:yãO?ý´®®nÜ¸qáªÒÒÒ6¤î]mllÀWû9r¤§ÂñãÇßï½kjjz,Ø¡C²?NtUX¤èª£Göµ¥KùMMMÉ9§Nsªªªs¶oß^^^ª¸¸øÁ¼xñb:þÒ¿Ç,*	þ$i4iRËRg~üñÇafeee*nztúôéèÚU«Võ¸jÝºuÑUÍÍÍï]®½ÿþû?ûì³ÀÇ¢¢¢D"®_JJJ¢·âey0qñÒÒW_5U´I_¾òÊ+Iùõx%KôYUüIÒðíáÍ°Í5*P,7«W¯¾~³0.®2º6²NÄÇh/ -ºªºº:2Ó'O$õ1·lÙxíuôÑGÃéð5L?òÈ#·|Çp1Ü uÙ2þD'N?ïÕ«WÃÅð5,ygwwwtpÇð#|þÛÝÄÅÅÅýÅ_Eü2â/9?ÂMd¦ÐÅÃÅÀ¦èâ9sÂÅ)S¦<öØcm7nÜH>H¸M±´ä½¢üqòÆLÉ=¿áknll¼åãhW®]¶ÞÞ÷ÄO«¾ÿýïþÛ]Û6lH½A`À_ é¼yó¢Éþâ/Ë¢J?Iþ¢á®ë×¯§Îììì3ÃUY¸¤akkkä¿¤uoÔKßÛS©'X	Ó¥¥¥EEE]]]áÁÇ¼öÓÅztîÜ¹È©a:|ÓÍÍÍÉkO< ãîã¾ã/Ë¢J?Iþ¢÷½½ðÂ©3£ ô8à#ù¾À«W¯¦h=vÛ¶mÑNØ¤£a¹ä~Õ[mÝºuÑÞÞðµ®®.9?ËãD#m.].^¹r%û¹.×Fg·	fM½*:ò7ôèÑk×®eÇ_¦ÑÚH^eQ%Á$Ñ/¾øbtª^z©¨¨(ýà+WètuuEûdGæFïùÞ*×ÚÚúfÁÈ6lTÃMl%#Ñ¢Ão£ï~üøñäü,½É/zÏ_gggtË,øÛ³gOrL.üÈ©WEolnnß%¬Þðq3p9Ü, óÁL½6Ë¢J?IEÑ	´ûöÿ³ýºYd£ätriòd>úhª,SÛ¿ü~ó£8ÂüÒÒÒÔ=ÂY',Fê%´íí½qãFqqqô#ôØÙ½|ùòÔo1qâÄð5:ÛKêcFÉ"ª&¯Í²¨àOâRÊ%Knvï½÷FÜöÀßÑ£G£ÓéÍ5ëÄÉk;;;|òÉòòòLO<ñDWWWòÚ#GÌ7/¬¢¢b÷îÝ=3IÂCù?þxù½=N(,LX¤èYÎó¬®®®Ç9_¢®^½Z]]ÖÀ¸qãÂOÑÖÖ<mMêc^»v-è6ZWUUUMMM=¾cE$I?I$Á$IàO$Ið'I$ø$IüI$	þ$I$I?I$Á$IüI$	þ$I$I?I$Á$IàO$ICÕÿ¡2vOwòIEND®B`


Detrended Normal Q-Q Plots


º¡¡¡¼¼|Í5wïÞ]SSß7^4Õþüç?æg¬íííþ|gºJîM/9áNÎhp&Ý3ÙìVÌ¿¬¥áç¶0ìüä'ÃØæßG»tq@jÉàé°¨ªªª££cddÄRá/x%Ì¬­­¾Åb/ÉgÏÍ¿èÂ---yÖM_8¼6g.mmmÍÜe5Õ¾<WÅÜÇ~íÚµD"Q]]&rÇäÍ7ß¯è×.¥´ÌÇ»+V¬ö¤F¤åuÉÕ«Wç'ÎâÅÃü·ß~;÷µdÉI¾3]%ë¦l¢=õõõ3ü?ë§¿þþþÌë	v¦øæf¼~ýú¬û¼eËO,üIE¿h~`Y4L&Ã·===aº··7hY«Gßnß¾bb"ÚsÓuÃkgxÕv7'EKÃnø6¬~ýFa­ÌÛÊsµùWô±;v,sOXæå7mÚ¦×­[÷îÂDø6ÌêñFs6oÞ<>>Ý±èÃ·]]]0zgÛ'ÂôåË3MuãñøT$½î-®2ÕêÒFg:8Þ«Y¯õßfnõi¢MÓÜ#éðÓÓ,ðÄ"ÁTøKéÞ"Yo¾ùfzÎM×MïE/ÒèÛð¹Ë*ë°]«Í¿âT=2bx]Ï_]]ùÂk|øvÑ¢ES=ÞhNGæ·Ñ±ìèfÞT*¸ÑÔÔùØóà/-ò©¤~«diX7fssóÉ''´éÎ¤÷jÖ+NÑrõêÕÌþ¦¹766oëêê^Ãÿ"FGG=«Hð'þVÂÌªªª©^q§bJômPNWë<ÄéËó,®vªùá!Ç^ÂÇÆÆ2ççî9ËãÎ·Y>ozÖjMMMt:WxY¤N_ÃtVÎXMzéÎT$ÝÓ?ìë[×47ãÁÁÁÈi?~ÜRáïÀ'|DûÒ²Î»uf´nþ=7o¿ývæÒ<WÅ<wàÈ#áÛô£D3#eí£Êbñô$óÛ¥KF¹ÒÝÝp6üE»'£O]I&ï¾ûîÁÃÌ6L¿é¬2küÍtpÒÍzÅéão:þÒ¼þ·güóloQçÎÛµkWt¬9f´$øáå-(zmNÔKôÜ^,£g-[-Þð4r£I_5ó¬_NZ¢÷l+®'ë]^mþóKbýúõé÷õGs¢s×­[wíFÑµmÞ¼ùÖñíýaÿþýÓÁß©S§¢Ká!÷ööFõ¢;6éÏw¦«Ìù'kÛÈl¦£:üEïÒÃèèh°ÝÚµksi¸wïÞ0øÁÁùÇÿ¦¿Ñ7óÒ]¿Ü2ÏXÌ=6ýöÿôçñF»	s_5ó¬_NAE2O½L/ÍsµùWÌ/+W®¤ßÝÍ¹téRÖû½ª««Óo(¼ü¥9µhÑ¢ô; óçÁÌýa-_¾<ÏxF«Ìù'kÛÈl¦£:ü;w.óúW­Z¹4kLÒ§Ou+y¶·ôEézè!O,üIE¿ð*¸zõêÜ?ÿÕÕÕÕÔÔ ^Ñ8ìØ±0'`+óSî¦¹îMå<^¸òÆÆÆÜëêjoºb~IDD3ç¿öÚk6l(¿Q2Ì=½cvø×®30èáJ;?qxâðèÂ_¼xñ¡C>óÏ¤÷<Ýú*3Â_þÁÉÚ6²Ñ¨Î¡ãÇ×ÕÕG®?:°>Á9|Ýzsss__ßM÷¼Nµ½lÛ¶-Ú¥~cccX$ø¤ÛU*zðÁgôÙr³X¥èöøF§oG¦_ºt©F?IR	½ç/«ÇÌÈHð'I*ÁFFF¶nÝºxñâèØnØ¹s§aàO$Ið'I$ø$IüI$	þ$I$I?I$Á$IüI$ø$IüI$	þ$I$I?I$Á¤»¼cÇÕÖÖÆãñÕ«WÏtÝ²²²ÁÁÁô0æ,[¶lOX7Ýe¦³î¬Þ¼yseeeÅoÛ¶mdddÄoçãæu555Á:ããã3]w÷îÝaÝ?üÃ?LÏÙ³gO³k×®bÇ_pÞ]¾|ybb¢³³3ÜÐÚµkgqUgÏmnn?Ið'éîx¾¸g;w.¬ÿý÷§ç¬X±"Ìéïï;yû¹öèÑ£wdáOüIºòË¤Æ¶mÛ.`Á;vd]ì3ùLSSSÖ5¤wé·ß~;L9Ñ¢¡¡¡æææx<ÞØØØÓÓ3éµeÞzþU:T[[»råÊpC¹Hº~ýz[[[X7Üù­[·F;2OW­Z®-Ì×üæoNsd-[®ylllÒ¥yFéÔ©SáNA8vìXîçd«²J?I·ËÑô£>¦8&ì±ÌËtuuEÈËlÓ¦MaÑÁÃtø¦ÛÛÛ£EË/?qâD¸|ùrH$&½¶Ì;@º#G7æÞù-[¶épg6LìÞ½;Ì¼÷ÞÃôµk×ÂD´c2·Üa	^_ùGiïÞ½ÑmæÞÉ¬ÇÿªlàOÒíÅ_ðJNÝ(×^¾)ðÔ©SaÑºuëÂôúõë#Ù¤;wnûöíMMMa~,ôÚ² è-0wÝêêêèÎOLLÀ¾03L766]MµoÒòà/ÿ(FÓá¦Â_ú±ç¿*§$øtñ)©øUÖÂ%GFF¢£«aN´hÿþýa­ ÂóçÏçhÒ;0U¦ºcA;ó"8=z´²²2SUUíÎ¿úúú0ÿúõëù]yù§g7à¿D"¹#*ÚyvS´µµ¥é¯éùåååaÎÄ¦¿ü«¤ïXðº-ÖÍºããã'NØ¸qcæ.Ã¶ûöpùÅÜEÓ¥iâov.Ið'©0øNq=xð`ô´=öLÇ"GMïB;räHl.½p:øË¿JÙ3Ï<&6mÚ»îC=¦;;;/^¼ê3W®¦Ï?õêÕ0Q__?Ía¹víZ Ø%K(£]---Ó¥ÌéèÐstÐ9ë±ÏnÀ%	þ$ãããXnùYù-2::r_Òó?^UUè³uëÖiâ/ÿ*'OKW¬X>ï$ó2###á¯]»6:±7/LF§ßÿý¯½öÚôG&ÜJetP;(pÇÑûù¦3JÓÇw)úí¬Ç>»$ø$I?I$Á$IàO$Ið'I$ø$IüÝ-÷»ßã7æòÿáþáßÿýßmyldd$ú¨vCÑ8ÓMû·û7jÁ»|ùòüÇvýúõ·ÞzË8¶ýèGwÕ:ßñ÷ðÁsÌÍÌ7Ó­÷OÿôOgÏ5íôéÓÿú¯ÿjØÕ«W_yåãPØ¾óïLúõ4ë®ò×ý×Æ¡°:u*ü÷þàOðð'ø?ø?øüÁü	þàþàþð?ø?ø?ÁüÁüÁüÁüÁü	þàþðð?ø?ÁüÁüÁàþàOððððð?ø?ø?ÁüÁàþàþàOðð'ø?ø?øüÁüÁüÁüÁüÁàþàþàOðð'ø?ø?øüÁü	þàþàþð?ø¿ù¿þþþD"Çzzz2mÜ¸±¼¼üÞï£ð'ø?øüÁ_ÑL&;;;ÃÄ¾ûZ[[3íÞ½û±ÇC_WW¿îîîësXxªzë­·®«p]¾|ùÌ3Æ¡°ßð`Ø~ô£_~Ù8¶_|ñÿñC»téÒ÷¾÷=ãPØzzzÂÿæòçþªªªïÂD*ª­­ÍÐÐpñâÅ©VøÛ»wïwæ°'Nà;*¼ðÂ·¾õ-ã`Cµ¡ÎÃî¹à?ãPÀN<iC-u^à/O:xWQQQWWwþüyöÃ¾û:ì+ö-úb±Xzº¼¼<kÑÁÃÄk¯½ÖÔÔð'ø?øüÁ_ÑW]]J¥Þ»qØ7Lg-JOgí?øüÁü	þà¯(kkk;tèP_Édæ¢Í79r$LpaåÊð?ø?Áü½½½555±X,Hôõõý÷c+û¯G¤ÕÒÒÇáþð?ø¿Áü	þàþððððð'ø?ø?øüÁü	þàþàþð?ø?ø?ÁüÁàþàþàþðð?ø?ÁüÁüÁàþàOððð'ø?øüÁüÁüÁàþàOððð'ø?øüÁüÁü	þàþðð?ø?øüÁüÁü	þàþðð?ø?ÁüÁüÁàþàOððð?ø?ø?ÁüÁàþàþàOðð'ø?ø?øüÁü	þàþàþàOðð'ø?ø?øüÁü©Øñ÷ê«¯~ík_ëèè8pàÀÈÈüÁüÁüÁüÁJßøÆ7-ZÔÜÜüÀ444Üwßo½õüÁüÁüÁüÁJ¯¾újßïþïßÝñåÑ¿ßÿñÕ«WÃüÁüÁüÁü©ñ÷µ¯-P/-¿ðoÛÿÙVYY966ðð'ø?øS©á¯££ãÏ<¿ð¯ººúwÞ?ø?øüÁü©Ôð÷ôÓOdéG2å÷Û;à¯¸FþàþàþàOðÓjllì¾ûî[µjÕÖ/mä÷ªüÉü	üÁüÁüÁüÁJÑü<PYYY£§zªèFþàþàþàOð3k||¼¸Þçððððó4ø?ø?ø?ÁüÁüÁàþàOððð'ø?øüÁüÁü	þàþððð'ø?øüÁüÁü	þàþðð?ø?ÁüÁüÁàþàþðð?ø?ÁüÁüÁàþàOððð'ø?øüÁüÁüÁàþàþàOðð'ø?ø?øüÁü	þàþàþð?ø?ø?øüÁü	þàþàþð?ø?ø?ÁüÁàþàþàOððððð?ø?ÁüÁüÁàþàOððð'ø?øüÁüÁüÁüÁüÁüÁàþàOððð'ø?øüÁüÁü	þàþððððð'ø?ø?øüÁü	þàþàþð?ø+öúûûD<ohhèééÉ½@wwwYYüÁàþàOð¥P2ìììûöíkmmÍZ:>>ÞØØ8þþâ/þâÇsØ/¼ðw÷w?VáúÁ~ðÒK/o¨Æ¡]¸pÁZðþù¿ÿû¿7ìûßÿþw¿û]ãPðõõ×_Ë[ø«ªª©Tª¶¶6ké£>úøãO¿Ãº»»Ï=^ëå_>yò¤q(lßþö·m¨ßP©CÁ7ÔW^yÅ8°3gÎØP^WW×o¨óñx|ÒéÐÐÐPSSS ¡Ã¾ûÊa_öÃ¾ûþb±Xzº¼¼<sQKKKxû¯ð'ø?øüÁ_ikuuu*zïÆaß0ýSð§?øüÁü	þà¯èkkk;tèP_ÉääÕ?øüÁü	þà¯4µ···¦¦&%¾¾¾Iµð'ø?øüÁy?øüÁü	þàþàþàþàþàOððð'ø?øüÁüÁü	þàþðð?ø?ø?ø?ø?øüÁüÁü	þàþðð?ø?ÁüÁüÁàþàOððð?ø?ÁüÁüÁàþàOððð'ø?øüÁüÁü	þàþàOððð'ø?øüÁüÁü	þàþðð?ø?ÁüÁüÁü	þàþàþð?ø?ø?ÁüÁàþàþàOðð'ø?ø?ø?ÁüÁàþàþàOðð'ø?ø?øüÁü	þàþàþððgàþàþàOðð'ø?ø?øüÁü	þàþàþð?ø?ø?ø?ø?ø?øüÁü	þàþàþð?ø?ø?ÁüÁàþàþàþàþàþðð?ø?Áüe7+ÁüÁü	þàþà¯Dð»YñxþàþàOððûÂüÁüÁàþà¯´ð|³iÓ&ø?ø?ÁüÁü þjkkãñ¸÷üÁüÁàþàO¥¿eËåíQUU522ðð'ø?ø¿RÃ_yyyÐÞððpMMMæægÂD;üÁüÁàþàþJÑ®¾0´&._¾<11&,Xðð'ø?ø¿RÃ_eee ^ooïàà`Ø¹sg4á£^àþàOðð§ÄßÖ­[Ó§wd¾íoåÊðð?ø?ø+Á³¿úÕ¯.Z´(LôõõÁ¦¦¦¢øyÀü	þàþð7?øüÁü	þàþàþàþàþàOð7uõõõÑ¾øgø?øüÁü©Äñ·dÉLð¥s¶/üÁü	þàþTøÎì¯¾E÷ó?øüÁü	þàofUUUü£üàþð?øqýýý[¶l)Æß@ø?ÁüÁàþfÜâÅËrrÂüÁü	þàþTø«««sÂüÁü	þàþ4_ð±o`` ð?ø?ÁüÍ¬êêj'|ÀüÁàþàOóá!ümÝºullþàþàOðð§Ç_Ù9áþàþð*Íy4'|ÀüÁàþàO¥ùQ/ÅüÁàþàOð3«¶¶¶®®îâÅwðaô÷÷'x<ÞÐÐÐÓÓ¹¨¯¯¯±±1,Z¶lY¸üÁàþàOð·TpUYÙÞL&;;;ÃÄ¾ûZ[[3-Y²äÌ3aâðáÃõõõð?ø?ÁüÝR===»ví¦¹SøRUUÝt*ª­­êb¹øûÓ?ýÓÌaÏ?ÿü¹sç~ Â^PÃFh[ww÷«¯¾jl¨wyßþö·Ï?oXø¿ô/¾hC½íø»ÎöÍ<¹dªMÂÝÞÞ¿#GÃN<~*T¸þöoÿ6ü§Ê8ØPïòþæoþæ;ßùq(lá¿Ó/^4,å¥^2-üwú?üáâmÇßÝp¶o&4ËËËs/pýúõd2922â°¯Ã¾rØ×a_å°¯Ã¾E_uuu*ûéÜMyãÆá7wEø?ÁüÁàþ¯¶¶¶Cð5Lfø5k']þàOðð'ø¿J¥Ö®][QQQVV¶`Á9>ó£···¦¦&%¾¾¾ÿ~l7ÎA®­­Í|'"üÁàþàOð·Ôèèè¤'|Åú?øüÁü	þàof-]º4PoÝºuÑo`ÍúõëÃåËÃüÁü	þàþà¯ÔðW^^¨7>>J¥ÂIÏº?ø?ø?ÁüÁ_qã/êE'ÛF9sùQ/ðÆþàþð7Gøû®Y³&ú_ÃtÓØØðð'ø?ø¿RÃ_øÅôwßþàþàOðð%øQ/£££---.Åbáë5kÂ¢øyÀü	þàþð7?øüÁü	þàþàþàþàþàOðµæÍÅbðð?ø?ø+üÅ¦þàþàOðð§RÃßT=üðÃþ>ðð'ø?ø¿Å_ÿû3?óþàþàOðð¥¿6D;üN8QD?ø?ÁüÁàþfÖ³Ï>±oÝºuE÷ó?øüÁü	þàoº._¾<:·£§§§ð?ø?ÁüM«½÷F;üZ[[÷çð'ø?øüÁßôÖô9ðð'ø?øÓüÁ_ìfÅãqø?ø?ÁüÁüùónððð'ø?ø?øüÁü	þàþàþð?ø?ø?ÁüÁüÁüÁüÁü	þàþðð?ø?Áüõõõååå>çþàþð*ü-Y²$|>çþàþð*eüçöWß¢ûyÀü	þàþð7³ªªªþQ~ð?ø?ÁüÍ¸þþþ¿-[¶ão üÁàþàOð3nñâÅe99áþàþð*AüÕÕÕ9áþàþð/øØ700P?ø?ÁüÁàþfVuuµ>àþàOðð§ù¿ðþ¶nÝ:66ðð'ø?øSã¯lðð?ø?æ<O>àþàOðð§Òü¨âþàOðð'ø?ø?ø?ø?ø?ÁßÔ¥R©µk×VTT-X° ¥¥¥XNþ?øüÁü	þàofNzÂGQüð'ø?øüÁßÌZºti Þºuë¢ßÀ õë×9Ë/?ø?øüÁüÁ_©á¯¼¼<Po||<='J9a>üÁüÁàþàþJð£^õøÒsÆÆÆÂõð?ø?ìaß5kÖD¿áksáþàþððWjø¿xðñî»ïÂüÁü	þàþà¯?êett´¥¥eáÂ±X,|]³fMS?ø?ÁüÁàþæQð?ø?ÁüÍ¬ÚÚÚººº/ÂüÁü	þàþTúøÇãeeÅºþàOðð'ø¿ÕÓÓð·k×®`bù«nð?ø?ÁüÍö*¦(ÁüÁü	þàþà¯?äyÒ|È3üÁü	þàþT:ø«««khh)êüÁàþàOðÓ*ú¾ïÝ8ì[Gxáþð?ø=þ¢?éý_ø?ø?ÁüÁJD¢,oNø?ø?ÁüÁJáå¶²²2ÚÿQÏ	ðð'ø?øSÉâ/]±8þàOðð'ø?ÛþàOðð'ø¿;W"Çã===ùÍnüÁàþàOðwKÉd²³³3LìÛ·¯µµ5ÿ¢ÙÍÉlëÖ­555÷Îaáæ/^|¯×>ô¡û¹36TªU6Ô»³ýÙãµÈðWUUýàT*U[[ÑìædöðÃI$P·¯½öZñyîHÖy$¹f7þ$IüÝÂãóÒØØøúë¯ßúµe~:`yyyþE³ÙïÿþïïØ±ãÏæ°psüÇüg*ôGô¯|Å8¶/ùËû÷ï7ì'Øµkq(lÛ·oê©§CûÆ7¾ñÈ#o¨Ë[¼íøìKK3ßÿý.]õµUWW§R©è(mÎ¿hvsðá9áÃ	Nø>ðQ¿K.M+°¼¼|íÚµ³¸¶¶¶Cð5Læ_4»9ð?ø?Áü¦'NTTTÍöÏ»õööÖÔÔuD__ßßËG¯sÍnüÁàþàOð·ÚÀÀ@æ¿¶··ßý?ø?ÁüÁàþfV¦ù***Z[[åçð'ø?øüÁß¯âÆyüä'/_¾?ø?ÁüÁàþfVA>áþð?ø¿âÀ_Qð'ø?øüÁß»páBý¢3|«««=ðð'ø?øS	â¯»»;ÂG¿hzß¾ðð?ø?ø+5üÕÔÔê]¸p!¿¾¾¾èÓ^àþàþððWgûFÂÆßÄÄDt0üÁüÁàþàþJÕÕÕzÑÞ¾¿T*µmÛ¶0][[ðð'ø?ø¿RÃ_xHeuæÌø?ø+Þ§ª¯ýë6lø_ù=öø?øüýÔZSSSt¶oEEEý¥Kâçð§Üõ>òÔÿú_Oþzø~£?ñOø?øüðÊíGYúá¥Ûÿïö_Þý»¯î¾§zÊÈÀüÁàþàþJ°~ô£¿õào¥åþ¶å³¿ú«¿jdàþàOð÷ÞÐÐPccãÂc7ÅrÌþàOö¿øþ×¦Lüîsûô§?mdàþàOó=ôPÙmÚ´	þàþ´7~ìcËÄß/5ýÒÎ;üÁüi^ãïèÑ£óöïß?22Í=vìX4¿««þàþ±·Þzëîiþåæ­_Úþ­^½úçþçßyç#ð×øK$AxÌ]ýÁ7óðW¼=óÌ3úÔ§+++?ñOÑ9àþðw»ðW^^7::»(JEáððW¤ùgø?øüå¬ù¿ê6ÕÒèO½ÁüÁü	þàþà¯Dðwðð?ø?ÁüÁüÁü	þàþù?ø?øüÁü©tð»YñxþàþàOððþ¼üÁüÁü	þàþàþð?ø?ø?ÁüÁàþàþàOððððð?ø?ÁüÁüÁàþàOððð'ø?øüÁüÁüÁüÁüÁàþàþàOðð'ø?ø?øüÁü	þàþàþð?ø?ø?øüÁüÁü	þàþðð?ø?ÁüÁüÁàþàOððð?ø?ÁüÁüÁàþàOððð'ø?øüÁüÁü	þàþàOððð'ø?øüÁüÁü	þàþðð?ø?ÁüÁüÁü	þàþàþð?ø?ø?ÁüÁàþàþàOðð'ø?ø?ø?ãð?ø?ø?ÁüÁàþàþàOðð'ø?ø?øüÁüÁüÁüÁüÁàþàOððð'ø?øüÁüÁü	þàþðW¬ø?øüÁü	þà¯Äñ÷Æo|ìc«¬¬¬ªªúà?ØÕÕð'ø?øüÁ_iâollì¾ûî[µjÕÖ/mÝñåûÜçÞÿþ÷<yþàOðð'ø»+êïïO$ñx¼¡¡¡§§'sQ___cccX´lÙ²p1øNO?ýôë?Øþ÷k~­©©	þàOðð'ø»+J&abß¾­­­,YræÌ0qøðáúúzøN|æLüð?ø?Áß]QUUÕÄÄDH¥RµµµS],/öìéÃ?þ­o«ëîî_øÂêÕ«3å÷Å-_hÑÝyoO8FµKóoC-®l¨6ÔbÙPî9ãPìê¼À_<t:³³gÏ¶··çâ/xü?ç°Ó§OÿçÝÝ+¯¼rÏ=÷üîÿþÝ4þ~©é~ó7óî¼·áÿ©ßûÞ÷þSí¥^zçwCë­·Â³q(lá	ü_þå_Cûñ^CañÅ¯]»6·8/ðÅÒÓååå¹¸~ýz2ÌýÔ§jÏ=ïÿûùùÓþô/üÂ/,_¾ü®=è°¯Ã¾û:ì+öË~R®®®N¥RÑaß0»)oÜ¸1<óæ^	üå¿«_ùÊW¾øÅ/>ýôÓcccwíý?ø?øüÁß¼À_fmmmák2Ìñ5kÖOº"ü@ðð?øwøëíí­©©ÅbD¢¯¯ï¿ÛµµµeÁü	þàþð7?øüÁü	þàþàþàþàþàOððð'ø?øüÁüÁü	þàþðð?ø?ÁüÁüÁü	þàþàþð?ø?ø?ÁüÁàþàþàOðð'ø?ø?ø?ÁßMûË¿üËeÿsYEEÅ>ô¡;wÃü	þàþàþà¯4ñwàÀ|à¿Ñö;¾¼£ýwÚï»ï¾ßû½ß?øüÁüÁüÁ_	âollìï_0__ôïKiÁ.]?øüÁüÁüÁ_©áoppðÃõNË/ú÷Ñ~ôøñãð?ø?ø?ø+5ü]ºtiñâÅYø[¶lYWWüÁàþàþàþJð=üà?ÛòÙ´üÚ§ýï_ØàþðððWøùåï¹çüã¿ÑöúÔ§~æg~æÈ#ssÓððð?ø»½ñÆÍÍÍ>øàÜð?ø?ø?ø?ø¿yüÁüÁüÁàþàOððð'ø?øüÁüÁü	þàþðð?ø?øüÁüÁü	þàþðð?ø?ÁüÁüÁàþàOððð?ø?ÁüÁüÁàþàOððð'ø?øüÁüÁü	þàþàþàÏoüÁü	þàþàþð?ø?ø?ÁüÁàþàþàOððððð?ø?ÁüÁüÁàþàOððð'ø?øüÁüÁüÁüÁüÁàþàþàOðð'ø?ø?øüÁü	þàþàþð?ø?ø?øüÁüÁü	þàþðð?ø?ÁüÁüÁàþàOððð?ø?ÁüÁüÁàþàOððð'ø?øüÁüÁü	þàþàOððð'ø?øüÁüÁü	þàþðð?ø?ÁüÁüÁü	þàþàþð?ø?ø?ÁüÁàþàþàOðð'ø?ø?ø?ÁüÁàþàþàOðð'ø?ø?øüÁü	þàþàþððððð'ø?øüÝ]õ÷÷'x<ÞÐÐÐÓÓîîî²²2ø?ÁüÁàþJ¡d2ÙÙÙ&öíÛ×ÚÚµt||¼±±q*ü½ðÂ£sØK/½^FU¸^~ùeãPØÂkjPµq(`o¼ñF__q(l/¾øâ?ÿó?vùòåðßiãPØzzzçòçþªªª&&&ÂD*ª­­ÍZúè£>þøãSáïë_ÿúsØsÏ=÷üóÏ¿ ÂÕÝÝâÄ	ã`Cµ¡ÚPeCµ¡FÍüÅãñI§CCCCMMMû:ì+öuØWû:ì["øÅbééòòòÌE---áeì¿*üÁàþàOðÅ;e?)LWWW§R©÷nöÓ^,aø?ÁüÁàþ»¶¶¶Cð5LNÅÜð?ø?Áü_½½½555±X,HôõõMª=ø?ÁüÁàþ|È3üÁàþàOððððð?ø?ø?ÁüÁàþàþàOðð'ø?ø?øüÁüÁüÁüÁüÁàþàþàOðð'ø?ø?øüÁü	þàþàþð?ø?ø?øüÁü	þàþàþð?ø?ø?ÁüÁàþæW<òÈÿù¿1ûþ÷¿ÿ×¹sç;fÛ3Ï<sáÂãPÀÂê_ýÕ_ÂväÈ/þòÜsÏo¨?üáçòóÿï¾ã/¼¼íØ±ã$IJ¥üG5Ëì$I?Á$IüI$	þ$I$I?I$Á$Ià¯¤®­­MÛßßH$âñxCCCOOOæ%ó,Ò¬Gµ¯¯¯±±1,Z¶lY¸¡»õ!êîî.+ó¤Q°QÛ¸qcyyù½÷ÞêÔ)CwëCqéÒ¥Ñ¢ÞÞ^C7ýQt×©Û4¤wüEÊóxáÏ8áÙ'ó52Lvvvûöµ¶¶f^8Ï"ÍzT,YræÌ0qøðáúúz£wëCÏVðWÀQÝ½÷c=611ÈRWWgônH«ªªÂDø:é®¦ÕÜ9^§nßÞñ)ÏãoíÚµ?ïð|ßÃD*Êz>Ê³H³ÕÌ***^AôÑGüñÇá¯£ÚÐÐpñâEVÀ!¾råÊ7þ@-OÏhTsçxºCzÇ_¤<ß¶ÍøyÇãñI§ó/Ò¬G5ÝÙ³gÛÛÛÛ­éÐÐPSSSx¿ÂþúïÝ»7<õ¦?Þ¸Ýúö÷÷ñ_Ã¯¿qþ¨N5ÇëÔíÒ;þ"åy|.¶X,.//Ï¼XEõ¨F]¿~=L·[ÒÓ§OßôL3ýõ?xð`xíµ×­Û­éòåË£©A+V¬0n×©Û¿;ø"åy|.¶êêêT*õÞæa:óbyiÖ£úÞ>7n¼zõªA+ÈýtÆ­P¿þv¨vHí£ºRñ:u;ðwg_¤<ÏÅÐÖÖvèÐ¡0¾æg^,Ï"ÍzTO:µfÍááa#V¨!Îÿb5ÓQÝ¼yó#GÂÄV®ÜnHW¬X3L,_¾Ü¸D*^§>¤wüEÊóø½½½555±X,Hôõõe^`ÒEºÅQ­­­µª°C·cT¯]»ÖÒÒÇÛ­éë¯¿Ì4|ÓÆí¥âuê6éò<.I4j$IàO$Ið'I$ø$IüI$	þ$I$I?I$Á$IàO$Ið'I$ø$I?I$Á$IàO$Ið'I$ø$IüI$	þ$é¦óßjUÅV¯^ýì³ÏþÔsÜæy²[[[ÚÈÈHÖü0''^§$ø¤¢lÇe9õ«_-%üíÚµ+Ì|òÉ'³æ?ñÄaþÎ;gqàO¯þþþÀx<¾ÿþñ<x0|f;w®dðwùòå0sÙ²eYó.]æ_ºt	þ$Á¤yÑç?ÿùÀÇ,sæ=ÂÌ/|áôééé	x.lllÓé¿ûî»ííí.ªªª:::2®<y2+,ëvuueq*Ì©¬¬jÕ'Â·mmmYwìøñãù¯'ZîR´¨»»*¨­Y³&ÌïëëKÏ9sæLÓÜÜ³÷îpU6l¸råJ.þr¯?kN»*	þ$é·xñâ¡¡¡Ìo¾ùfY[[¬Î=-]¿~Ö¢-[¶Db±Ø¤kEßFK[ZZ&&&ËËËÇÇÇÃÒð5àiÁÑ[ñòôîå>ÒÃg6íËÎÎÎ´ü²®dõêÕ3Å_»*	þ$éÎáäy­¬,P,7­­­×o&Â·ëÖ­FÖøíÀh%Éhaîíí®$ó:·oßxuè¡ÂcÇéð5L?øà7½Çðm¸@æôP.Z´(<Þáááðmøîyg*.H$Âá!¼÷ÃÄ3Å_»*	þ$é®À_hRü¥çG¸ÌºråJø6°)ú¶±±1|[WW·iÓ¦¶ÑÑÑôËdíKK¯ûæo¦/À>ò¾é'OÞôz4Ã·o¿ývæêýy?üpXôøã¿÷CÛø$mjjvLÎyîª$ø¤;_´»ëúõë3GFFÂÌ°(wÒ4ü¶Núz¹Gc³8ù+aºªªª¼¼|ll,éM¯g*euáÂÈ©a:|Óé¥½½½áLzøxúøËsW%Á$Ýù¢÷½íÝ»7sfô(Y'|¤ß8<<»CëÜ¹s»víÂ¦ÕíKW½)Ñ¶lÙí_ÛÛÛÓóó´§íêÕ«Ñ·o¿ývþ3sW¬XFnÌ¹(:ó7,êîî¾víZ~ü¥aFzi»*	þ$éÎ0Ç÷íÛÔËþýûËËËsOÎX·n]ÎØØXtL6fnô¿è­ro,ØÑÑ¨þ°I~Ýú©S§Òóóô&¿è=###Ñ%óàïàÁérá!g.Þþ800n%ÂTø¸¸.Ð¹aÃÌ¥yîª$ø¤»¢è³Ú½÷ÿ»Qd£ôtúiút=ôP¦,3;zôhü½wãOqùUUUGó¸w,¦íTvtt´¢¢"zY»×®]y-_£OÉ¼ÎhÇdºªé¥yîª$ø¤»¥Õ«WßhÕªUÑ)·Yøëîî>N¯¡¡áôéÓé¥###Û¶m«©©ÀôðÃ¥vuu555%d]gî=	WæoÞ¼9kþT×w&Ü¥èó|Î_ºööö¬Ï|N&a.Å¥KÒ[y×®]ºÆª¹¹¹¯¯/ëóÜUIð'I$ø$IüI$	þ$I$I?I$Á$IàO$Ið'I$I?I$Á$IàO$Ið'I$ø$IüI$	þ$ITþ/úW¶IEND®B`


öÃº¡y¤^¸paõ-óçÏ?xðàm8úY>÷ìÙ33îçÆ©S§âIN§ãÖP5»dÃâ&YüÚ¸qãGdwOüM:õòåË%þ¶mÛ6tcmÝºµÄÒÆ4ËHÏÝ»wuå5f³Ùüí¶´´?À>ÂÎ¸~ýú³Ï>[]]#;::*!þÂòåË'Eüuvv&µúo|c```pp0âbìîîvQceèóaóæÍ1&lÂâ/ñÚµk1|óæÍ»jÓâÊ-þ»ví*ÚérâÄÙ³g§Óé9sæ?~¼(òIâÓ¦M[°`AyóW>sæÌâÅ#?þxOOOá655%3¶¶¶ÝÕÛîÀêÕ«ãçáÃGZ'çÏ_¹rers¹Ü®Yøx1q£qÓq.åÊS§N566f2%KîeÜ±cG]]]r|3n"?i¤;ü/|!Æë1ríÚµÃnß±Î2ô¦£cLÜÉ1­a÷Ìß¥ã¯hjlxÄjè¡bÝÞG8tê(Æ©±&£§cRMMÍÆýbñ,þ¢Wbdr±»»;J¾%wvv¿äÊ+V¬(1oþÊñÞuÕªU»¬F:ÀWb±¥gúØ¯_¿Ífkkkc`è:y÷Ýwã½p9qÍ¢J+|¼CowÞ¼yÉÔDH¾ü®¹hÑ¢Ò3cÆõêÕ¡kæÌÃnß±ÎRtÓQ6É¿Y³fiå^ùãqôñ×ÕÕU¸x°c¿Q>/_^t7lØà?dñ,Ks¹\lkkáöööÂD+=¹¸eËÁÁÁd_Îmç÷Îx×ë'»£©ñ¦cö·Ä·Ub±¥gö±9r¤pOXáõ×­[ÃË-»vKÄÅ9ÒãMÆ¬_¿~`` ¹cÉãâñãÇ`òÉ¶cÇÅðÅ'tÓéôH$?ïÎ2ÒêòFÇºr½Wã±tÖ^,|DÖçmôñ7Ê§q.]áØ1<eÊ¿X@üÁ¤¿ü¾ä#bbLø÷Ýwócn;o~/Zrx1ÞJñZ¸Ëªè°]Åq¤Ç4b¼¯¯­­-|Dñ§O>ÒãMÆDp^Le'°ðÜ¼y3r!"£¹¹¹ð±¿|Têw8Ká*yce.^¼øÄÃ®´Ñ¬aïÕ¸gü%Ï+W®ÞÄâoOã¦¦¦¸ØÐÐñEôõõù­â&_üE¬ÄÈÞqGÊäbTNwë3Ö·çq,v¤ññãñÆ[xáø¡Î~nèãÍÅÍ¢C·=kµ®®.9N=´ð:¿ÑÌ2u5ìF³rFJÒñÍ8úÃ¾wþìåÓøÜ¹sIÿå£ðèÑ£~±øI÷î-<á#ÙVtÞåhZgLóÞssõêÕÂ©%[zÆwàÐ¡Cq1ÿÅ(ÉÈ¤ÏöQeñèWHáÅÙ³g'_¹ÒÚÚq6øKvO&ßºËå6nÜxíÚµûöÅÈ+W£eÜñ7Ö7îG£ÙóÏ»ä¯ë¿Äó-qúôéíÛ·'ÇógFâ&AüÅÛ[4PòÞÿªä--º!Þ,gçÌLJ>ðÔË°ï%æ-]NIµ$Ù'Ë)úD×°-=céX¾|yþsýÉäeË]¿%YÚúõëï<þ½_ÝÝÝñöìÙ3ø;yòd²o)r²W/qäÈa·ïXgSü^9EÏBc]«ã¿äSzñdèëë¶[ºtéÐ4|úé§cåG F^ÿ·	$Ý<wîCðø»(þ*<cqèù³ùÿç¿7ÙM8ô]³Ä¼¥Ë)ª(É£ÂS/óSK,¶ô¥KâòåËùOw%c.Pôy¯ÚÚÚüï$þò9>zþ%çñÇº±æÎ[bi1Å_éSôÜ(4Öµ:ø;útáò,XP8µhäO9éVJ<ßògå­Y³Æ/0iâ/Þ-Z4ô¿ÿ:~üxsssT¼£ïÝ»7?þÈ#1&b«ð[îF9ïmË)ùz¼XxSSÓÐ¯ëi±·±tI$DÇ¿ýöÛ+W®ÌÜËåÞ1¾øëéé¥Å2#6mÚ=oîÒóÌ3ÏÄ£>cÆýû÷?òÈ#ù=Ow>Ëâ¯ôÊ)znÓZGü£G644Ä£å'Öó'8ÇÅH´äÖ/^ÜÑÑqÛ=¯#=ßzxâdj|lÊþþ~¿X@ü|TnÞ¼ùøãé»åÆ1Ë¤ìñMNßH~öìÙ0 þ(CÉgþìÜ¹Óñ@êííÝ¼yó3c»1°mÛ6«Äâñø@ü þ?ñg?Äâñø@üÑ#GêëëÓéô¢EÆ:occcUUÕ¹sçòcb8ÆÌ3g¿ÅnßuF3ï8·ïííÝ°aCMMM¬±©S§ær¹óçÏççñqåL&Ã]øî:uuu+===ã;vÄ¼_ýêWóczê©³ûöò¿¡ãW¯^ãÛÚÚbøÔ©S1<sæÌüÔ£GæcñØ±c]øî¾_"wÐO§Oy.3oÞ¼ÓÕÕ51wrâã/ÉÄøë×¯;×c=SkÖ¬ñìÄp7_aè<ñÄS§N2eÊÖ­[®öÈ#477-!¿ã0¯^½Ã1&téÒ¥ÅWWW§Óé¦¦¦doÙÐ¥ÞzéYöïß___?þü¸¡¡vãÆ7îüæÍ§,¥ÅøXò»ï¾ñ÷ÀÄøÙ³g···M6mZÜV___ü>z>ÇwÄðQõ_2üäOÆðÞ½#³b`çÎ×9~üxyÖ­[öíÛÃñ3×®]L;wnrèóâÅ1>Í»´Â;PzHºCÅÀêÕ«Þù6Äp^;vä[íúõëÝÝÝ1ìéó|#eqáu9³hÑ¢3gÎägI/[¶,W®Ã1fØûàY?à®¿úúú¾yËÐööC'OÌÏòåËªËO=úô-[c|*viEùUzäM:uè¼µµµÉH®×á¦¦¦ÈÙþþþñ­"©ù%'ã7nÜ¿Dê¦Mîä>?à£¿äLÕüø¸xÛÒ2eJ··79²cIöì¹¢Ï9S¸¢¥^Í,#Ý±(ÅÂuI8>|xÚ´iÉd§àè÷üXommmÑs¹#ãF¯]»öÁ­cÐ1<cÆaïg þ»"þ²Ùlá¿dçÙmK¨¥¥%¦ææÇ'çFÞ2Êø+=KþEKwúôéÉ¼Ewo``àØ±cÉYºù]cZ-#IîLÜçÎ×jÓ§Oû>?à£¿ä«[öíÛ|æï©§M	>|8ß:ÊORòìÙ³ÉgG¥gÙ²eË7¿ùÍX·nÝÐy×¬YÃxóÍ7Ã¬1rþüù1evåÊ5kÖÆß9sb|kkë?ûbÇü¿õ&Æû>?à£¿¬)·~W_éøëëëK¹ÆÏÂï@6ª©©:uêæÍG¥g9qâDL7o^þ¼¢¯_Þ¸qcÜóL&³téÒä¤Úè­>¼páÂ·ß~L«eè¡áXlKKKÜÃx°Ó§Oß°aCÜî?û¾ëÂåç¿ïzÜ÷@ü?Äâñø@üÝE~ð¼óÎ;yÿðÿð¯ÿú¯yéêÕ«Éÿß@úçþçäëc¨@ÿþïÿ~þüyë¡býøÇ?þ·û7ñw·ø?ùè¿	ÎÍÂo2£¢¼ùæÿ÷o=T¦+W®üð?´*ÓÍ7¿ûÝïZëäÉñçø?Äâñ'þÄâñøCü?ñøCü!þâOü!þ?Äø?Äâñ'þÄâñøCü?ñøCü!þâOü!þ?Äø?Äâñ'þÄâñøCü?ñøCü!þÄøâOü!þ?ñ'þ?ÄâOü?ÄâñøâñøCü!þ*7þººº²Ùl:nlllkk+Ôßß¿zõêL&óÀÄÚ?Äâñ7éår¹ÄÀîÝ»W­ZU8iÇ;wîUßÐÐ04þZ[[oL W^yå½÷Þ»AEúÑ~ýg=T¦ÿøÇ¯½öõP®]»vüøqë¡bµµµÅy555wÉ_Wõõõãív¤#þ~úéW&Ð±cÇâIð)þô?6¬ÊôòË/ç;ß±*ÓÉ'=j=T¬_|ñßûÞDÞbEÄ_:v8¹yW]]ÝÐÐpæÌqØqØ'½T*Îd2EöíÛo¿ývss³øCü!þ¿I¯¶¶6^ZÉ,&åv?ÄâñøZZZöïßñ3ËNZ¿~ý¡CbàìÙ³óçÏ?Äâñ7éµ··×ÕÕ¥R©l6ÛÑÑñ­ê?]Ö+Òétssó¹sçÄâñøCüU.ñøCü!þâOü!þ?Äø?Äâñ'þÄâñøCü?ñøCü!þâOü?Äâñ'þÄâñøCü?ñøCü!þâOü!þ?Äø?Äâñ'þÄâñøCü?ñøCü!þâOü!þ?Äø?Äâñ'þÄâñøCü?ñøCü!þâOü?ñ'þ?ñ'þ?ÄâOü?ÄâñøâñøCü!þÄøCü!þ?ñ'þ?ÄâOü?ÄâñøâñøCü!þÄøCü!þ?ñ'þ?ÄâOü?Äâñøâñ'þÄøCü?ñøCü!þâOü!þ?JúéOúÆoÄOñ'þÄâñø+g½½½ÿüç§O>û¿Ì¾ç.]úþûï?ñ'þ?Ä_yzøá­é×6ióÖ/oâ÷øõ_ÿõÏ~ö³âOü?ÄâñW¿Hëêê¶üÁ(¿ä_ßï½o¼ñøâñøCü£GþÆ¼ßÈ_òïÁ_ðùçâOü!þ¿róÆoüÊ¯üJá¿ø×ÐÐðÚk¯?ñ'þ?Ä_¹ø[²dI¾ÿú­xà»ùñ'þÄøCü!þ¿÷ÞoîÜ¹úÔ§|ðÁúûëßyç»ù?ñ'þÄâñÇzã7¾õ­oÅËj``à.¿«âOü?ñøCüQAÄøâñøCü?ñøCü!þâOü!þ?Äø?Äâñ'þÄâñøCü?ñøCü!þâOü!þ?Äø?Äâñ'þÄâñøCü?ñøCü!þâOü!þ?ÄøâOü?ÄâOü?ÄâñøâñøCü!þÄøCü!þ?ñ'þ?ÄâOü?ÄâñøâñøCü!þÄøCü!þ?ñ'þ?ÄâOü?Äâñøûxtuue³Ùt:ÝØØØÖÖ6ô­­­UUUâñøCü!þÊA.;pà@ìÞ½ÕªUESF¿¿ø¿øÇ	ôòË/ÿíßþí?Rþò/ÿòõ×_·*ÓÙ³g¿ÿýï[éâÅÇ³*ÖK/½tþüù¼Å¿ÁÁÁä¯«úúú¢©O>ùä®]»F¿ûö@­­­g¨H¯¼òÊ~ðë¡2½öÚkñ·õPN>ñg=T¬ãÇÿð?È[¬øK§ÓÃK.577G:ìÃ¾8ìÃ¾8ì[&ñJ¥òÃL¦pÒ+N:õUü!þ?Ä_y¬ÖÚÚÚxi%/°þ¹GøóÄâñøCüMz---û÷ïøËå¨öü!þ?Ä_y¬ÖöööºººT*Íf;::­=ñøCü!þ¾äYü!þ?Äø?Äâñ'þÄâñøCü?ñøCü!þâOü!þ?ñ'þÄø?ÄâOü?ÄâñøâñøCü!þÄøCü!þ?ñ'þ?ÄâOü?ÄâñøâñøCü!þÄøCü!þ?ñ'þ?ÄâOü?ÄâñøâñøCü!þÄøâOü!þâOü!þ?Äø?Äâñ'þÄâñøCü?ñøCü!þâOü!þ?Äø?Äâñ'þÄâñøCü?ñøCü!þâOü!þ?Äø?Äâñ'þÄâOü?ñøâñøCü!þÄøCü!þ?ñ'þ?ÄâOü?ÄâñøâñøCü!þ>´ø«ºT*%þÄâñøe©ÛI§ÓâOü!þ?ñç°¯øâñøCüWüEß¬[·Nü?Äâñ'þÊ0þêëëÓé´Ïü?ÄâñGùÇß9síQSSÓÛÛ+þÄâñøåL&j¯§§§®®.¢ù¾ùÍoÆÀÚµkÅøCü!þâ¯Üâ/ÙÕQ1pñâÅÁÁÁ2eø?ÄâOü[üM6-R¯½½ýÜ¹s1°mÛ¶dÀW½?ÄâñGÆßæÍó§w~ìoþüùâOü!þ?ñWgû~å+_>ztttÄ@`ssó¤ØâñøCü!þ*øCü!þ?ñ'þ?Äâod³fÍJ¾ðÅ<?ÄâñGÇßÌ3/ÏÙ¾âñøCüQñÙ×ÙÙ9888é¶øCü!þ¿±©©©øå'þ?ÄâoÌººº"þ6lØpãÆñ'þ?ÄeaÆUC8áCü!þ?Ê0þð!þ?ÄIöuwwOÆí!þ?Äâoljkkð!þ?Äñ"þ6oÞÜßß/þÄâñø£Ìã¯jNø?Äâòüça9áCü!þ?Êó«^&/ñøCü!þcS__ßÐÐïjãÃèêêÊf³étº±±±­­­pRGGGSSSL3gNü!þ?Äß®ªªú÷ ær¹ÄÀîÝ»W­ZU8iæÌ¯¾új<xpÖ¬YâñøCü!þîH[[[ÄßöíÛ£i>®/|©©©In:^`õõõ#]­ººzhüík_û	ôÒK/>úo¨Hþñ§õP¢üâ·¥õPº»»;f=T¬Hÿ3gÎLä-~äñw7í[xrÉH'tvv®]»vhü:tèÂ:qâDlT¤(¿xZéG?úÑ+¯¼b=T¦¿û»¿ø³*Vkkë[o½5·øÇßÝp¶oahf2¡W¸qãF.ëííuØqØqØwÒ«­­Vòá¢©/_^½zuüæ:£øCü!þ¿É§¥¥eÿþý1?s¹_²dIOOÏ°3?ÄâñøÏÓzéÒ¥ÕÕÕUUUS¦LY±bÅùÑÞÞ^WWJ¥²ÙlGGÇ>¶[ç ×××~Qü!þ?ÄßéëëöIñ_ý?ÄâñøÙ³gGê-[¶ìÆq1Êfùòå1fîÜ¹âOü!þ?ñWnñÉd"õå1fØ³nÅøÖøCü!þ&wü¥R©H½ädÛDÈ¯z?Äâñ7Añö]²dIrØ7~ÆpijjâñøCü¿r¿¨½aOø¸víø?ÄâOüáW½ôõõ­X±bêÔ©©T*~.Y²$ÆLí!þ?Äâ¯?ÄâñøâñøCü!þæ¼T*%þÄâñøe©?ñøCü!þ(·øÉ¦Mø;xð ø?ÄâOümüuuuM2%²oñâÅßù,þÄâñ'þ¬ñWnñ·råÊdß±cÇ&Ñö?Äâñ76/¼ðBË-tÛCü!þ?ÄßhõõõÍ;79·£­­m2nñøCü!þ£òôÓO';üV­Z5y·øCü!þ¿ÑÍéþÄâñø£râ/u;étZü?Äâñ'þü÷nâOü?Äâñ'þÄâñøCü?ñøCü!þâOü!þ?Äø?Äâñ'þÄâñøCühñ7kÖ¬L&ãþÄâñø£üãoæÌÁçþÄâñø£ã/:/²¯³³spppÒmñøCü!þcSSSñ7ËOü!þ?ÄßuuuEümØ°áÆâOü!þ?Ê<þÂ3ªpÂøCü!þaü5448áCü!þ?*%þìëîîÛCü!þ?ÄßØÔÖÖ:áCü!þ?*%þâ!EümÞ¼¹¿¿_ü?ÄâñGÇ_Õð!þ?Äåù%ÏÃrÂøCü!þçW½L^âñøCü!þÄøCü!þ¿Oë¥KVWWWUUM2eÅåä_ñøCü!þcÓ××7ì	âä_ñøCü!þc3öìH½eË%ÿ·oÍòåËcÌÜ¹sÅøCü!þâ¯Üâ/ÉDê>ËcLâñøCü¿2üªH½xfçÇô÷÷Ç_õ"þ?ÄeØwÉ%ÉaßøÃ1¦©©Iü?Äâñ'þÊ-þ¢ö=áãÚµkâOü!þ?ñW_õÒ××·bÅ©S§¦R©ø¹dÉ3)¶øCü!þ¿"þ?Äâolêëëâ]Mü?ÄâñGùÇ_:®ª¬ÅâñøCüM[[[ÄßöíÛ£i&Ëÿê&þ?Äâo¼A*âñøCü¿2üçaùgñøCü!þ(økhhhllìííÔÛCü!þ§å>X]]Íf×®]Ûßßoc?Äâ¯Xò_ú~pë°ï¤8Â+þÃzþùçï¹çÿkå?ØòëoöìÙ-²±ÄâñW,ù/ÿÆWü?&oüÝï½>úèÖ/oMþEÖÖÖ8qÂö?ÄßÏÉf³U%9áCüq÷Çßûï¿_SS/¿äß[øß¾úÕ¯Ú^âñøû9Ó¦MKöÿ%©çñÇ¤¿O~ò¿´¹0þæÏÿÜsÏÙ^âñøÞdé<ñøÖç>÷¹äËoÝï¬2eÊl/ñøCü!ñøûÉO~òéOú¿Îþ¯?üðg?ûÙî¹Çn?ñøCüM®®®l6N§ÛÚÚJOßñø+Òßß¿wïÞßýÝßÝ¶mÛk¯½fK?Äâoâär¹ÄÀîÝ»W­ZUzÒøÆÚ¼ys]]Ý(nnÆPâïûï¿ßz¨L¿üË¿|ß÷YéSúÔ½÷Þk=T¬ØúüÖ?Éâ¯¦¦&ùãï¤úúúÒÆ7¦Ð¦MªÊÈGo¿ýöçG2tÒøÆ?@üÝÁã[ßóÒÔÔtþüù;_Zá·f2ÒÆ7¦ÐþánÝºõë(nîÏÿüÏ¿NEÚ¹sç®]»¬ÊôÌ3Ïlß¾Ýz¨LÏ=÷Ü¾ô%ë¡bmÙ²eïÞ½yyüEöåK3N/ðN¾$¢¶¶öæÍÉQÚ.=i|cð>pÂNøÀ	ÂÁßÙ³gç+0É,]ºtËiiiÙ¿ÄÏWzÒøÆ?Äâñøûp;v¬ºººj¼ÿ½[]]]ÌÍf;::þó^Þ:z=tÒøÆ?Äâñø»SÝÝÝþ¦NºvíÚ»?ÄâñøÂæ«®®^µjÕ¥K&Ëö?Äâñ7ÆEÜ:Ïã¡ºxñâ¤ÛâñøCü!þÆæCùñøCü!þ#þ&5ñøCü!þcvöìÙY³fM2%9Ã·¶¶öðáÃâOü!þ?Ê0þZ[[ó'|$ñïÞ½[ü?Äâñ'þÊ-þêêê"õÎ=¿äÛ^ÄøCü!þâ¯ÏöM¾9É)ÀâOü!þ?ñWnñW[[©ìíø§øO<ÃõõõâOü!þ?ñWnñ©j8¯¾úªø?ÄâOüáÙ¾ñK­¹¹99Û·ººzÖ¬Y.ÛCü!þ?Ä_?Äâñ'þÄâñøCüçÒ¥KMMMS§NMÝå¯øCü!þ¿1X³fMÕÖ­['þÄâñø£|âïðáÃIçíÙ³§··7Ù××wäÈdüñãÇÅøCü!þâ¯Lâ/ÍFáíÛ·oè¤ä?|ó=âñøCüQ>ñÉd¢ðúúúÇ¤¸ø?ÄâOüIüåÿW·¦&ÿÕø?Äøâ¯Lâ¯DÞ?ñøCü!þâOü?ñ'þ¿I¥?ñøCü!þ(øKÝN:âñøCü?ÿ½øâñøCü?ñøCü!þâOü!þ?Äø?Äâñ'þÄâñøCü?ñøCü!þâOü!þ?Äø?Äâñ'þÄâñøCü?ñøCü!þâOü!þ?ÄøâÏz?Äø?Äâñ'þÄâñøCü?ñøCü!þâOü!þ?Äø?Äâñ'þÄâñøCü?ñøCü!þâOü!þ?Äø?Äâñ'þÄâñøCü?ñ'þÄøCü!þÄøCü!þ?ñ'þ?ÄâOü?ÄâñøâñøCü!þÄøCü!þ?ñ'þ?ÄâOü?ÄâñøâñøCü!þÄøCü!þ?ñ'þ?ÄâOü?Äøâñ'þÄâñøCü?ñøCü!þâ¯®®®l6N§ÛÚÚ'utt455Å¤9sæÄÕÄâñøCüMz¹À1°÷îU«VN9sæ«¯¾5køCü!þ¿I¯¦¦fpp0yÕ××tµêêê¡ñ÷ÔSO@GýÎw¾sôâ/;vÌz¨L±éãåo=T¬oûÛVBÅø·þ¿t:=ìp¡ÎÎÎµk×¿èñÿ;N:ÕÓÓó©HgÏ=wîõPÞï½ø-d=T¦ùùîw¿k=T¬ïï×¯_È[¬øK¥RùáL&3ô7nÜÈår½½½ûâ°/ûâ°/ûNJU?ÃµµµñÒJ^`1ÍË/¯^½:~ó]øCü!þ¿É§¥¥eÿþý1?s¹_²dIOOÏ°3?Äâñø|ÚÛÛëêêR©T6íèèøÏÇvk§`UñøCü!þKü!þ?Äø?Äâñ'þÄâñøCü?ñøCü!þâOü!þ?ÄøâñøCü¿¿þþþ¡ÿãâñøCü¿r¿×_ý3ùÌ'?ùÉûï¿ÿ¶ÿ3âñøCü¿É/¾øâ/þâ/þÖoýÖ?Ø²õË[ôÑî¹çÄ^âñøCü¿2¿ÇlÞó"ûòÿ>û|ss³×øcLúûûwìØñàþê¯þê¿øÅwÞyGü!þâïn¿ùóç?úè£ñÿª««½6Ä£700ðÐCºáÓ_üÂ×ýÎº¿¹ðøDww·øCü!þÄß]<òÈç>÷¹Âòû½õ¿wï½÷zm?Fï[ßúÖý÷ßÿÄï?-ùïKøCü!þÄß]ú§:úôÿ½á'ïX[þ`KccãêÕ«½6Ä£÷å/ùüöÿ(ü#*Bð~áÄâñ'þîºø½çþæÂEÝwß<üH¿×øcôþèþè·úíÂøûÒ¦/â?Äø»ãïúõë.î¹çyæxBxa?Æú:ú¥_ú¥ÿ³ñÿäãï3ó?³råJñøCü¿»4þ¼Äwhýúõ÷Ýwßÿ?ôÑ_kúµúúúüä'âñøâñW¶¾þõ¯Gù=üðÃÛ¶mûéOzß[ñ'þ¬ñ'þÄâ"þÄõ þÄøCü!þ?ñ'þ?ÄâOü?ÄâñøâñøCü!þÄøCü!þ?ñ'þ?ÄâOü?ÄâñøâñøCü!þÄøCü!þ?ñ'þ?ÄâOü?ÄâOü?ñ'þÄWøCü!þâOü!þ?Äø?Äâñ'þÄâñøCü?ñøCü!þâOü!þ?Äø?Äâñ'þÄâñøCü?ñøCü!þâOü!þ?Äø?Äâñ'þÄøCü!þâOü!þ?Äø?Äâñ'þÄâñøCü?ñøCü!þâOü!þ?Äø?Äâñ'þÄâñøCü?ñøCü!þâOü!þ?Äø?Äâñ'þÄøâñøâñøCü!þÄøCü!þ?ñ'þ?ÄâOü?ÄâñøâñøCü!þÄøCü!þ?ñ'þ?ÄâOü?Äâñø»»tuue³Ùt:ÝØØØÖÖ6ô­­­UUUâñøCü!þÊA.;pà@ìÞ½ÕªUESF¿_~¹oÿûß÷>*Ò_ýÕ_½õÖ[ÖCezçw:::¬ÊôOÿôOÇ·*V[[[OOÏDÞbEÄ_MMÍàà`ò×UÑÔ'|r×®]#ÅßýÙ½<^|ñÅ^zée*Rüö¿þ­ÊÔÚÚzìØ1ë¡28qâèÑ£ÖCÅø·þ¿t:=ìp¸téRsss¤¡Ã¾8ìÃ¾8ìÃ¾e©T*?Éd'­X±âÔ©SÿñPÅâñøCüMÞUYõ31[/­äÃÃ^-eñøCü!þ[KKËþýûc ~ær¹bqèHñøCü!þO]]]*Êf³ÃÖøCü!þ?_ò,þ?ÄâOü?ÄâñøâñøCü!þÄøCü!þ?ñ'þ?ÄøâOü?Äâñ'þÄâñøCü?ñøCü!þâOü!þ?Äø?Äâñ'þÄâñøCü?ñøCü!þåÿøþùw&PÜÜ_ÿõ_¿CEjkkú·*Óë¯¿þíoÛz¨LñWß×¾ö5ë¡b:tè­·ÞÈ[|ÿý÷ÅßÎ=»uëÖ?(¥jVÙP9Äø@ü þ?Ä_Yééé©¯¯/=&ÑÕÕÍfÓétccc[[UWQ[¿£££©©)¶þ9sâ`ÕUÚ ÑÚÚZUåWqÅmýþþþÕ«Wg2xàäÉV]EmýØâ³gÏNÞúÛÛÛÅ_9µð·ùÐ1y¹À1°÷îU«VYµõgÎùê«¯ÆÀÁgÍeíUÚ Äâ¯·þ;vîÜ988ÐÐÐ`íUÔÖ¯©©¹téRÄÏ¿ÉdéÒ¥çÎ+ÜÞCÇ>âÅÿÁ­ÿùñcyð1nýBÕÕÕÖ^>|òÉ]»v¿Üúo¾ù¦V[?rÿòåË1??ô÷ç#[³C¶÷°Ït:=ì0°õó:;;×®]k½UÚ þèonn?ÿÄ_eþòúé§ã¯¾xï?sæõVQ[¿««+6Lñû_üUÜ3 Jå3õVñwãÆ×ÛÛk½UÚ`Å§Nºíßë/ÿûöÅÀÛo¿XoµõçÎì÷7oø«¸g@mmíÍ7?¸uØ7­·J¿Ë/¯^½úÊ+VZ>ª~õVi¿üóÃûTÚÖÿØúùuó1?ZZZöïßñ3ËYoµõO<¹dÉk¬bë4S)Ë­¿~ýúCÅÀÙ³gçÏo½UÔÖ7o^l÷èîî;w®ø« g@r±½½½®®.Je³Ùë­¢¶~½?ü¼õ¯_¿¾bÅt:ÝÜÜ|îÜ9ë­¢¶þùóç£ùbëÇÏ?Äâñ þ¬ñø@ü þ?Äâñø@ü?Äâàcõo|cÁÕ·,Z´è^ø¹ßq·LßÈÃÝÛúúúxh½½½EãcL:Îf³c]& þ&¥­[·Vñ¯|¥âoûöí1òÙg-ÿÌ3ÏÄømÛ¶cø|ººº"kÒéô=nÙ·o_§O.ø»xñb3gNÑøÙ³gÇø.?@üá_øBdÍÎ;G>õÔS1ò±Ç+L¶¶¶§èÂ¦¦¦Î_ùÚµkk×®:ujLª©©Ù¸qcáÑÕ'ND`Å¤÷øñãE9c¦M¶`ÁcÇÅÅ¢;vôèÑÒËI&Å]J&µ¶¶jK,ñù1¯¾újY¼xq~Ì;êêêbQÕÕÕ+W®¼|ùòÐøºü¢1%î* þ>f3fÌp¹téRáÈwß7FÖ××ÆMÎÎÎdêòåË&mØ°!ÔÝÝJ¥+¹L]±bÅàà`äc&©ñ3âiÊ)ÉGñJ,'½CéÁ6ßÈ_ÑB-Z4Öø+qWñðñKðó­ª*R¬0nV­ZuãË-K¦&­äc²/¢-Ëå]1ÜÞÞ,¤p[¶l¼Kº®Y³&Æ9r$ãg?þøã·]Nc+Þ·aQåôéÓãñöôôÄÅø÷<óæÍÉ²ÙlÌá&®®®kü¸«ø¸+â/ùñIÜ$Í._¾#MMMq±¡¡aÝºumùÄuö¥åçJ.¾ûî»ù+G0åüÆÏ>qâÄm¡¯^½ZxßFú|Þ¦MbÒ®]»>øÙ¡í7^!B0â/´¹¹9Ù19Öø+qWñðñKvwÝ¸q£pdoooI%r'çÎKú/ß:ùê=[S_°Ã555L¦¿¿?>mÚ´üÔÛ.g¤+röìÙ¤Sc8~Æpwww~jÜa>þJÜU@ü|üÏ½=ýôÓ#/@):á#ÿ¹À¡;´N>½ûöä l¾Ýrùãª·M´6$GãçÚµkóãK,'ÙÓvåÊäâÕ«WK;oÞ¼|»M4ká¤äÌßÔÚÚzýúõÒñÓdmä§¸«øøø%'L¤ÓéÝ»w'_õ²gÏL&3ôäeËEèô÷÷'Çdógæ&ùK>*wîÜ¹Â&-¸qãÆH¥ä<Üü­hÉé·É­<y2?¾Ärù%ùëííM®Y"þöíÛß'¹pRòñÇîîî¸X	#Å_ËqµÎ+WN-qWñpWH¾¹È;þÿï¸[6ÊçæÏ±È[³fMaY:|øpøûàÖÅãkjjXNÜÂ;?Óv¤Û××W]]<¢ÝK.-¼éÓ§ÇÏäÛ^ìÌKR5?µÄ]ÄÀÝ"eÑ¢E[,Xr[­­­É×é566:u*?µ··÷'¨««KiÓ¦Mýýýù©Çonn&Ëf³÷î-ZæÐñë×¯/?ÒrBÜ¸KÉø¿¼µk×çK¢§§'ËÅ:uj<.ä¿¶¦p×¯_ºMÖÕâÅ;::n±Ä]Äâñø@ü þ?Äø@ü þ?ÄâñÀáÿ*kþAZYnIEND®B`


üÞ½sÕG$Iªª$IüI$	þ$I$I?I$Á$IàO$Ið'I$ø$IüI$	þ$I$Ið'I$ø$IüI$	þ¤ý+HTWW¯^½úÈ#³¿¿ýúõySoàÑügyæF£¹§Ç¿çª¯µfÍ£G^w³_eêó¡±±ñÀóy?7^íµð$L&á»l¡jnNüI%¿x»víúDAvëàoñâÅï½÷^©àï±Çú`íÞ½»ÈÖæ´ÊLÏÎÎÎ¹Î¼1Nç¿o[[üIò[ú122òÌ3ÏTWW¿ÐæÍKýýýVî¹ÉÉÉ0.Ónj®«L>ttt9d¿hÅ?ü0Lçr¹[ê¡R¹á/jß¾;]N>½bÅd2¹råÊS§Nà)¿h"É%k×®-²nþÊçÎ[¿~tDrëÖ­ÃÃÃñoÚÔÔ­ØÕÕUpSgÚìuW,¸Û¶m_?>ÓpaË-Ñ-Ìf³/^,¸füþFsÂ7ß:Üî¹çÊ+¯½öZccc*Ú°aC|/ãÞ½ëêê¢ãá[äÍt¿üå/ùñ°¡õ0³½½ÚÇw®«LýÖÁaN¸si÷ÌÅßÅñW°4<.áýÞïc[|áÔ¥³|¤jjjvíÚ5::êRá/x%ÌÌd2ÑÅÁÁÁD"Iîïï/¿èÊ---EÖÍ_9¼6Ç¶¶¶ÆwYÍt¯Èf¯8õ¾¤ÓéÚÚÚ01uLÞyçðßN¸fÒâ÷wê÷]½zu´'5*@$/¿k®[·®8q-[æðÁS¬åËOûøÎuodíùkhhÓàüy¯8üÄ·îì7Ë§ñæÍnóÎ;ý`àO*1üEóË¢él6.vwwéÞÞÞ8ÑV.>úè£Ñ¾ë®^;Ã«~¸~´»18)Z^tÃÅ°úÕkµâß«Èf¯8í?qâD|OXüúÛ·oÓ6múðZa"3gº¿Ñ;vLLLD7,Úr¸xêÔ©øÞÙöÒK/éK.ÅÍtÉäLw$¿îÇ¦7Õå÷Îup¦½Uó^±8ëããOÀú<Ñf¿Y>#^¾|9LG3L/Z´È	þ¤Ä_þH_ô±xaNü½óÎ;ù9×]7¿-:¼^J£á4¾Ëªà°]Í_q¦û1¼®Ì¯­­ß£ð..]ºt¦ûÍ	à_eGw0~Kr¹B@Fsssü¾Á_^ä3Iýc®Ò°nÌõë×>zÚAÍàL«æ½âìñ=C®ÿsÂß,ÆMMMábÀkø-bllÌO	þ¤ÒÃ_ÀJYSS3Ó+îLL.åyµ.B¹¾<Ïc³3Íw9Üßð>>>?uÏÙÔ7ÀM½¿³¹ Ypèóºg­ÖÕÕEÇ©§¯Ôù-ÌfÙÕ´WÍàÌDÒù­8ûÃ¾ÿÙ5Ë§ñÐÐPä¿<O<éRáïàÁñ>¢iç]ÎÆ:sZ·ø>ø ¾´Èf¯Xä;v,0J43òYÁ>ªÏ~@âW¬XäJWWWÀÙlðí>u%ÍîÚµëÃ?<tèP¹eËiñ7Uæ¿¹N¾y¯8üÍfÏ_wÑo;EÆ¿Èó-êìÙ³öì5çÏR	à/¼¼E¯Íùz^ÒÂetòìÊ+£EÑF¯5í«fuË)RKô­°ñh;ïèv³ÅW,.Í7çß×ÍÎÞ´iÓÈµ¢­íØ±ããã/Úû588îÂf¿hßR¸Ë½½½Ñ^½¨'NLûøÎu9á¯øà<7âÍuTç¿è]záÉ066l·qãÆ©4Ü¿üÄààâãÝÿÑ7Ò-¿©ÅÏXzþlþíÿùÏãvNÕ,²nq9E<z_Zd³ÅW,.÷Þ/ÿî®hÎÅÞïU[[CáÇÁ_#QK.Í¿²q¶nÝ:õÁZµjUxN«Ì	Å§à¹o®£:ü=6¾ýµk×ÆIþó¾Kç[þ¢|>ø ,üI%¿ð*¸nÝº©þëÔ©SÍÍÍRáýàÁùù'Ns¶âr7Ëu¯+§èãñÂÆ¦~L½îÅ%Ïë­·¶lÙºV6zzÇüð7<<¶¶ôðÃåÍ]8ßÿþ÷Ã½w|Ù²e¾ï¾ûò>þ*sÂ_ñÁ)xn4§QþB'O¬¯¯÷:l?:°?Á9D¾ûúõëûúú®»çu¦çÛèèè#<íRåøø¸,üIÒ'U.Ûºuë>[n«ßèôíÈô+V¬ðàOTEïù+èÉ'42üIÊ°ÑÑÑeËEÇvÃÄc=fX$ø$IüI$	þ$I$I?I$Á$IàO$Ið'I@$	þ$I$I?I$Á$IàO$Ið'©Ô;qâD&I&ëÖ­ëºUUUCCCù9a:ÌY¹rå~¢]k~×Íºóù!;CñED¢ºº:ÍI?I%S]]] LÌÄÄÄÝ»woX÷ßû^~ÎSO=æìÙ³§¤ñWdûñ9áéÖÖVÏ"Ið'©t~ |?=6¬Ï=÷äç¬^½:ÌXysñ¸¦«««=$Á¤R_4<òÈâÅ-Z´÷î«ÝwßÍÍÍ[Èï8Ó|ðAs¢E/_^¿~°Q2ljjêîîvkñï^|Ãg25kÖo4bW¯^mkkëßÑÑíÈ<]»vmØZ¶üÎ;ï|Bøë­·Dt&''ÃÃLÏ1Ið'éVô_4ýÄOéf'|2~S§NMÛöíÛÃ¢Céð5L···GV­ZõÒK/K.ùétzÚ­Åo@ñUé;&¶mÛ6õÆïÜ¹3L+¼ðÂabïÞ½aæ]wÝ¦GFFÃD´crÚ7óÍAxa|Âô<-Ú³gO4û÷ï=ög$øtKã/ÉéÜµ¦ÚkÚ7öôôE6mÓ7oT_zöìÙG´¹¹9:CbÚ­«ø*Ñ[¼xñÔukkk£X&ûÂÌpÍ0ÝÔÔ8;>>þq'>'ºmaãA~AÑ¢ð­/_nÆ¢EÂEÏ.Ið'éÆ_2ÌOpq&åÒÖ	×®9Ñ¢µÏ;ßBÁÖâg³ÊL7,h,¾3/ãñãÇ,YÍ©©©vÞ=Óï4$øtâ/NÇ÷üE;Ï®+¶¶¶°4ÿ5??J9×%þ¯¿aÁsS×]ºti´nÁÍxé¥¶mÛße8Á¹îPÔÖÖf³Ùµk×_¹rÅ³KüIº¥ñtË¡C¢÷ü=õÔS³ÁßñãÇó»Ð;QòÍ7ßÞ8ü_åÑGþùçÃÄöíÛ§®ûàé#G?>:Ôf®Y³&L;w.P,L444|røÛµkWXtöìÙÞÞÞø%	þ$Ý¢øÀZt­øgõÇßØØXtÈ5|Í¿.tòäÉÅwttÌÅW9útXºzõêüy'ñë~[J¥6nÜØÌÍf£Óï¹ç¹~;ü°Æ?õfíÚµáâàà '$ø$IüI$	þ$I$Ið'I$ø$Iüpý×ýöÛo,Xï¾ûîÕ«WCeöoÿöoï¿ÿ¾q¨ÌÆÇÇÿå_þÅ8TlCCCS?5]ðwsúö·¿üçy°`ýÝßýÝï½g*³þç~óÍ7Ceöïÿþï¯½öq¨Ø^zé¥ÿüÏÿ4ðð'øü	þð?ÁàOðð'øü	þð?ÁàOðð'øü	þð?ÁàþàþàOð'øüÁü	þ?ÁüÁàOð'øüÁü	þ?ÁüÁàOð'ø?ø?ø?ÁàOð'ø?øü	þ?ø?ÁàOð'ø?øü	þªPü¤Óéd2ÙØØØÝÝ_4>>¾mÛ¶T*u×]wõôôÀü	þ?ø+ù²Ùì#GÂDgggkkk|ÑÞ½|òÉÉÉÉ ¿úúú©øëêêºªêoÿöo/^¼h*³üÇ|ã7Ce~ë?CÅð~0VEà¯¦¦&ð.Lär¹L&_ÔØØxþüùVøÛ¿ÿO´P½üòË§O6Ù«¯¾úÊ+¯Ê¬»»;¼üíG?úQÐ¿qX°*ÉdrÚéèbà]uuuý¹sçöuØWûÊa_9ìë°oÉH$òÓ©Tª`Ñ¡CÂÄ[o½ÕÜÜð'øü	þà¯ä«­­Íår];ì¦å§vÂü	þ?ø+ÉÚÚÚ>&Â×l6_´cÇcÇðª³fÍø?ÁàOð%_ooo]]]"H§Ó¿¸oUÿïÞ´´´$Éæææ¡¡!ø?ÁàOðüÁàOð'ø?øü	þ?ø?ÁàOð'ø?øü	þ?ø?ÁàOð'ø?ø?ø?ÁàOðð'øü	þð?ÁàOðð'øü	þð?ÁàOðð'øü	þð?ÁàþàOð'øü	þàþ?ÁàþàOð'øü	þàþ?ÁàþàOðð?ÁüÁàOð'øüÁü	þ?ÁüÁàOð'øüÁü	þ?ÁüÁàOð'øüÁü	þ?ø?ÁàOð'ø?øü	þ?ø?ÁàOð'ø?øü	þ?ø?Áàþü	þàþ?ÁàþàOð'øü	þàþ?ÁàþàOð'øü	þàþ?ÁàþàOð'øüÁü	þ?Á_a?üáï½÷ÞåËßwßeø?øü	þÓÔÑÑñéOºµµõ¡¯=tÿý÷ßvÛmù	ð'øü	þTø¼ã;¾±ë»¿¹;ú÷¾rûí·Ãü	þ?þ<øÅ/~1/¿èßg?ûÙóçÏÃü	þ?!þ¾°öø»ûî»áþ?ÁÊ·ß~»Ã¾ð'øü	þTøûhº>þù2x8àþàOð'øüMß³Ï>»~ýúÏ~ö³_úÒzzzÊãá?ø?ÁàOðWAÁüÁàOð'ø?øü	þ?ø?ÁàOð'ø?øü	þ?ø?ÁàOð'ø?ø?ã?ÁàþàOð'øü	þàþ?ÁàþàOð'øü	þàþ?ÁàþàOð'øü	þàþ?ÁüÁàOð'øüÁü	þ?ÁüÁàOð'øüÁü	þ?ÁüÁüÁü	þ¿ßÀÀ@:N&ÝÝÝS¯ÐÕÕUUUð'øü	þà¯Êf³G	­­­K'&&fÂß_üÅ_üU ö?üÃ?Êì7Þèëë3ÙÐÐÐücãP±<yòí·ß6VEà¯¦¦frr2Lär¹L&S°ô'Ø·oßLø;tèÐ9-Tá§ÿ3gCeèßÓÓc*³þþþW^yÅ8Tl/¾øbøõÏ8,X¿d29ítèòåËÍÍÍû:ì+å°¯öUà/Hä§S©T|QKKKôþàOð'øü©LðW[[Ëå>ºvØ7Lÿø_?øü	þðWòµµµ>|8L¯Ùlvú»jÏü	þ?ø+»ÑÛÛ[WWH$Òét__ß´Ú?øü	þò!Ïð?ÁàþàOð'øü	þàþ?ÁàþàOð'øü	þàþ?Áàþàþü	þ?ø?ÁàOð'ø?øü	þ?ø?ÁàOð'ø?øü	þ?ø?ÁàOð'ø?øü	þð?ÁàOðð'øü	þð?ÁàOðð'øü	þððð'øü	þàþ?ÁàþàOð'øü	þàþ?ÁàþàOð'øü	þàþ?ÁàþàOð'øüÁü	þ?ÁüÁàOð'øüÁü	þ?ÁüÁàOð'øüÁü	þàþàOð'ø?øü	þ?ø?ÁàOð'ø?øü	þ?ø?ÁàOð'ø?øü	þºaø«º^Dþ?Áà¯Lð¸^Édþ?Áà¯LðWÁü	þ?ø»lß¾þ?Áà¯ñÉdÉ¤÷ü	þ?Á_ùãoåÊSÏö¨©©?ÁàOð'ø+7ü¥R© ½áááººº0Ì÷üóÏöövøü	þ¿rÃ_´«/LíK.MNNEÁàOð'øüþ,Y¨×ÛÛ;44&ì±hÂG½þ?Á_â¯££#zGümkÖ¬?ÁàOð'ø+7üüñ¥K¾¾¾0 ØÜÜ÷þàOð'øüÁ_ð'øü	þàþ?Áàþf®¡¡!úÀò,øü	þe¿åËÇÁÏÙ¾?ÁàOðWøÎìëïï,¹ûð'øü	þàonÕÔÔü¢üàþ?ÁüÍ¹¿;w^½zþ?Áà¯ÌñZ¶lYÕð!øü	þe¿úúz'|þ?Á_¥à/bßàà`)Þø?ÁàOðs«¶¶Ö	?ÁàOðW)øëéé	øëèè?ÁàOð'ø+süUÍ>?Áà¯ñ!'|þ?Á_â¯¤?øü	þð7·2LýùóçoâÝH§ÓÉd²±±±»»;¾¨¯¯¯©©),Z¹re¸üÁàOð'ø¿UpUUÕMÞÍf9&:;;[[[ã/_~æÌ0qôèÑø?ÁàOð«îîî¿=öÜ¬|©©©¾u.Ëd23]­ººz*þ~ðüo-TõWÕßßo*³ðkXxù7Ùo¼ÑÕÕe*¶'OßýÃõãïV8Û7~rÉL's´··OÅß±cÇ.j¡úéOzîÜ9ãPüÍßüq¨ÌÂ«ÑéÓ§CÅöâ/^¸pÁ8,X8þn³ãÐL¥RS¯põêÕl6;::ê°¯Ã¾rØWûÊa_K¾ÚÚÚöÓK6¶mÛvåÊ©+Âü	þ?ø+½ÚÚÚ>&Â×l6_ÔÓÓ³aÃáááiW?øü	þð7çr¹ÜÆ««««ªª-ZÔÒÒ²Àg~ôööÖÕÕ%t:Ý××÷ûvíäL&'"üÁàOð'ø¿ÕØØØ´'|Äú?øü	þð7·V¬X¨·iÓ¦«W¯###7osV­Z?ÁàOðWnøK¥Rzù9¹Ìö¬[ø?ø?ÁàO¥¿D"¨l5>>æ,äG½Àü	þ?-þ¢Ã¾6lû¯a:Ìijj?ÁàOð'ø+7üíMÂÇ~?ÁàOðWnøûèÚ	¿---/N$áëÂ¸ÿð?Áàþ*(ø?ÁàOðð'øü	þðW°æõJ$ð'øü	þe¿ÄÌÁàOð'øüþfêáðwôèQøü	þ¿²ÅßÀÀÀ¢EûÖ¯_ÿÌgøü	þàþàÏ8þ¶lÙíðp	Ýø?ÁàOðsë^Ø·iÓ¦»ÿð?ÁàþfÛØØØªU«¢s;º»»KñþÃü	þ?øUû÷ïvøµ¶¶îý?øü	þð7»5ÎàOð'øüUþ×+LÂàOð'øü	þÊ#ø?ÁàOðð'øü	þð?ÁàOðð'øü	þð?ÁàOððð?ÁJ©TÊçü	þ?Á_ùãoùòåqðù?ÁàOð'ø+güçöõ÷÷ONNÜý?øü	þð7·jjjþJQ~ð?ÁàþæÜÀÀ@ÀßÎ;¯^½?ÁàOðWæø-[¶¬jJNøü	þ¿2Ä_½>?Áà¯Rð±opp°ï?üÁàOð'ø¿¹U[[ëÁàOð'ø«üõôôüuttÃàOð'øü9þªfÈ	?ÁàOðWøKÌ>?Áà¯ñWÒÁü	þ?ø?ÁàOð'ø¿Ëår7n¬®®®ªªZ´hQKKK©üð'øü	þàonMÂGIüð'øü	þàon­X±"PoÓ¦MÑßöÙ¼ys³jÕ*øüéæâobb"°ÃàÃàO7©T*P/üÍÏÉåraN?Ý,ü½ÿþû¿÷¿÷«¿ú«wÞygMMÍýÙyàOð§nÔG½êðåç9>êEð§¿ðSè3ùÌÚµk;þWÇîoîþÊ_ùµ_õÕW=ð'øÓ;ì»aÃè°oø¦Ã¦¦&øüé¦àïÙgýÜÏöåÿý~ö÷û·Û£?ÝüíMÂÇ~?ÝüíÚµëþûîã/ü«®®ö(ÀàO7æ£^ÆÆÆZZZ/^H$Â×69%qÿáþTøûÎw¾³~ýú¸ü¾¾óëwÞy§Gþò!Ïð*Cü½ñÆK.èkåñ·jÕª­[·zàOð§¿L&S__þüyøüéÁ_èé§þûÝßýÝûï¿ÅûÜç|æü	þtcðL&«ªJu"üÁÊ]Ûÿ÷ï|ç¡:xð ùÁàO7ÝÝÝöì)¿êð§JÀàOð§OU3H$àOð'øü	þÊò!Ï?ÁàOðW>ø«¯¯oll-éûð'øü	þàoVEÒ÷£kKâ/üÁàOð'øÓüñýIßèÏøÂàOð'øü9þÒétUÑð!øü	þå¿þþþ%KDûÿ"ê9áCð'øü	þÊùJÅyð?ÁàOþ¶/üÁàOð'ø¿×ÀÀ@:N&ÝÝÝÅÍoüÁàOð'ø¿[¥l6äÈ0ÑÙÙÙÚÚZ|ÑüæÄëèè¨««»KÕ¯ÿú¯ÿÆoüq¨Ì2LøMÌ8TfË-óÃ¶«­­5Yá¯¦¦&úÁ¹¼T_4¿9ñ~øá*I¤2êÇß[o½uñ?w¤à<©æ7þ$Iü#Ç×>ç¥©©éÂkñOL¥RÅÍoN¼?þã?Þ½÷µPë[ßzúé§CeöÝï~÷'0Ù¾ùÍoíßøÆ~ðã°`âøìËK3LÞsÏ=/^÷Öjkks¹6L_4¿9NøpÂð!'|È	Nø¸W¬XW`*Ú¸qã<¶ÓÖÖvøðá0¾f³Ùâæ7þàOð'øüÁßirr2<ÀÕÕÕUóýón½½½uuuaÝt:Ý××÷[yíèõÔEóð'øü	þàïã688ßó·xñâööö[ÿþÃü	þ?ø[qóUWW·¶¶^¾|¹Tî?üÁàOð'ø¿9nâÚy÷Þï¥KJîþÃü	þ?ø[7ä^àþ?ÁJ%üÁàOð'ø¿9~ 744,Z´(:Ã·¶¶öøñãð'øü	þe¿®®®ü	þ¢éÎÎNøü	þ¿rÃ_]]] ^øÇ____ôi/ð'øü	þå¿üÎãorr2:þ?Áà¯ÜðW[[¨ííøËår<òHÎd2ð'øü	þå¿ªé:sæü	þ?Á_¹á/tåÊæææèlßêêê/Äý?øü	þðWAÁü	þ?ø?ÁàOð'ø¿éº|ùrSSÓâÅ×¥rÌþàOð'øüÁßzðÁ«fhûöíð'øü	þå¿ãÇGÎ;pàÀèèh4sllìÄÑüS§NÁàOð'øü	þÒétÞ¡C¦.þàÏùü	þ¿òÁ_*Âº(ËEáð'øü	þe¿ü_uiiô§ÞàOð'øüÁü	þðþ?Áàþàþàþ?,þ?ÁàOðW>øKd2	?ÁàOðW&ø+àþ?ÁüÁàOð'øüÁü	þ?ÁüÁàOð'øüÁü	þ?ÁüÁüÁü	þ?ø?ÁàOð'ø?øü	þ?ø?ÁàOð'ø?øü	þ?ø?ÁàOð'ø?øü	þð?ÁàOðð'øü	þð?ÁàOðð'øü	þð?ø?øü	þàþ?ÁàþàOð'øü	þàþ?ÁàþàOð'øü	þàþ?ÁàþàOð'øüÁü	þ?ÁüÁàOð'øüÁü	þ?ÁüÁàOð'øüÁü	þðgàOðð'øü	þð?ÁàOðð'øü	þð?ÁàOðð'øü	þð?ÁàþàOð'øü	þàþ?ÁàþnÁÒét2lllìîî/êëëkjjV®®ð'øü	þà¯äËf³G	­­­ñEË/?sæL8zôhCCüÁàOð'ø¿¯¦¦frr2Lär¹L&3ÓÕª««§âï©§:¥êÅ_?Ceúð0ÙË/¿|òäIãP±ýèG?2YEà/LN;¯¿¿¿½½*þzzzþª×_ýç?ÿ¹q¨Ì.088h*³ý×ýéOj*¶ð»ßøø¸qX°*D"?J¥¦^áêÕ«ÙlvttÔa_å°¯öÃ¾ûæMÿeaº¶¶6ËEÃtÁ56¶mÛvåÊ©?øü	þðWzµµµ>|8L¯Ùl6¾¨§§gÃÃÃÃÓ®ð'øü	þà¯ôêíí­««K$étº¯¯ï÷íÚNÁL&SþàOð'øüÁ_åð'øü	þàþ?ÁàþàOð'øü	þàþ?ÁàþàOð'øü	þàþàþàOð'øüÁü	þ?ÁüÁàOð'øüÁü	þJ³·ß~»££ãÞïýêW¿zúôiøü	þàþe[OOÏwÜñÅ/~ñÚþàK_úÒm·Ýöì³ÏÂàOðð'ø+Ã&&&.]ºålÙýÍÝÑ¿öÿÙþ©OêÝwß?ÁàþàOðWnÝõßîÊË/ú÷[¿õ[§N?ÁàþàOðWø«¯¯/Àßªÿ¾þ?ø?Á_611qûí·ÿáÿ0/¿?ÚñG¿ò+¿òöÛoÃàOð¥Ú»ï¾û½ïïë_ÿzø:íKüÁ_%ÀÉ'o»í¶M6=ôµZ[[3Ìw¿ûÝ¹ïðððWýìg?»ã;~çw~çþûïÿüç?ûí·Âà/Þë¯¿þÜ÷Ý_øÂþôOÿ´rî8üÁüÁü[ãããwÞygëï·æiµ´´|æ3?Áàþàþà¯ÜúÙÏ~Öø¹Æ7³úÓ¾xñ"ü	þððåÖÉ'W~uþ~ó7ó7Þ?Áàþàþà¯Üºxñâ¢E¾±ëyù=ôµª««GGGáOð'ø?ø?ø+Ãzè¡eËuëWü¾òÀWÂôã?^pø?ã?ÁüOË/ÿÔ§>u÷Ýw?ýôÓS¯ðgàOð'ø¿þàÏ8ÀàOðð'øü	þð?ÁàOðð'øü	þð?ÁàOðð'øü	þð?ÁàþàOð'øü	þàþ?ÁàþàOð'øü	þàþ?ÁàþàOðð?ÁüÁàOð'øüÁü	þ?ÁüÁàOð'øüÁü	þ?ÁüÁàOð'øüÁü	þ?ø?ÁàOð'ø?øü	þ?ø?ÁàOð'ø?øü	þ?ø?Áàþàþàþàþ?ÁàþàOð'øü	þàþ?ÁàþàOð'øü	þàþ?ÁàþàOð'øüÁüÁü	þ?ø?ÁàOð'ø?øü	þ?ø?ÁàOð'ø?øü	þ?ø?øü	þ?ø?ÁàOð'ø?øü	þ?ø?ÁàOð§JÅßÀÀ@:N&ÝÝÝS¯ÐÕÕUUUð'øü	þà¯Êf³G	­­­K'&&fÂßüã1-Týýý.]2Ù?ýÓ?ýýßÿ½q¨ÌÞÿýüä'Æ¡bñÅÿã?þÃ8,X¿ÉÉÉ0Ëå2LÁÒ'xbß¾3áïOþäO~¬*üò×ÕÕe*³W^yåå_6Ù«¯¾^þCÅvòäI°UþÉä´Ó¡Ë/777:ìë°¯öÃ¾rØWe¿D"N¥RñE---ÑOø?ÁàOð§Æ_Õ/Óµµµ¹£kÃô´WË_þàOð'øüÁ_i×ÖÖvøðá0¾f³Ù°8u&üÁàOð'ø¿Ò«···®®.H¤Óé¾¾¾iµð'øü	þäCáþ?ÁüÁàOð'øüÁü	þ?ÁüÁàOð'øüÁü	þ?ÁüÁüøü	þð?ÁàOðð'øü	þð?ÁàOðð'øü	þð?ÁàOðð'øü	þà¯²úÖ·¾õçþçok¡zñÅ_ýuãPõöö>Ú8TfÁýÏ?ÿ¼q¨ØöÙðëqX°Â¯[ð7cáçÑîÝ»¿-IT.?ªYeç§$IÃ¾$I?I$Á$IàO$Ið'I$ø$Iü©Të­·S©Ô-[FFFâÆÇÇ·mÛÝu×]===ÆªÒáA_±bE2lllìíí5VåÔððp&É_H§ÓÑcÝÝÝ¿fE*ûG¿¯¯¯©©),Z¹re¸¡?I«V­ÿ½ÃÄùóçwìØ_´wïÞ'|rrr2  ¾¾ÞXUÚ ¦¦æòåËa"|¿T¨Ô¬¯ªúÿ¯,ÙlöÈ#a¢³³³µµ5~å"TöþòåËÏ9&=ÚÐÐ`ôàOeRø.?½dÉø¢ð[`!ªØ'@ô'ÃWú/§6nÜ844ùÐ¿æWý"Tö~¼êêj£*V®æoç.îû÷ïÿáÃÿ¹sçU¥=Â£^$Â×þþ~cUn¯+±ÿøC?õçÀLTö~¾ð ½½Ý¸ÁÊ¤ ºðê¾oß¾_ìÄ¡C>úåÛÂU¥=V­Zíúzµ±*ãÿð=?J¥~Ì´HeÿèG]½z5Í7øS¹uáÂ+VÄçÔÖÖú¿vùTÎËøÏËå>ºvà/þ¿ø"ý£ÿÑµwlÛ¶íÊ+þT>-_¾|```rrrß¾?þx|Ñ;;&Þ|óÍ5kÖ«J¬^½::"<88¸jÕ*cUÆ/ÿmmmák6_­È"ý£ßÓÓ³aÃááa#*«ÂÿíeË¥R©öööøÏd2ÙÜÜ<44d¬*í	páÂ`¾ð_Ã´±*ãÿÞÞÞºººD"N§£³¿óWv*äÑÏd2U±üI$	þ$I$I?I$Á$IüI$	þ$I$I?I$Á$IàO$Ið'I$ø$IüI$	þ$I$I?I$ø$IüI$	þ$éæõÜsÏ­]»¶úZëÖ­áþËÏ¸kÌOäénm&	wmtt´`~L&Óéôäää)	þ$©$Û½wÕüñrÂß=ÂÌgy¦`þ÷¿ÿý0ÿ±ÇÇ6%Á$^5ÉdòÀ×:tèP¸f=¶lðwéÒ¥0såÊóW¬Xæ_¼xþ$Á¤èË_þr`ÍO>ùÔSO<ð@>ÝÝÝOÁMMMa:å?ü°½½ñâÅaQMMÍ®]»âGWO>uO:UÀ©0gÉ%k×®é¥ÂÅ¶¶¶vòäÉâÛ-êêê	j6lóûúúòsÎ9æ¬_¿>?gïÞ½uuuaSÕÕÕ[¶lyï½÷¦âoêöæ¹©àOnrË-p¹|ùr|æ;ï¼ff28nêïïnÞ¼¹`ÑÎ;£EDbÚµ¢ÑÒÉÉÉÀÇT*511¯O-ÞWd;abÚ7õ=z4.Ú¼/9_ÁFÖ­[7Wü¹©àOn~ÑÞi~®UUÅqÓÚÚzõZa"´iS´4²NÄÇh^@[´(ÍF»Ãtooo´ø6ôÑÀ»è¨ë>æ8q"L¯azëÖ­×ÝNc¸®¿mÓÞ£Ê¥Kû;<<.¯áqær¹èét:¬îÂG¿<L]=Wü¹©àOn	ü¦Å_~~ÈL¡÷Þ/l.655õõõÛ·ohËo$`_Z~­èâ;ï¼¿rSþÈoø¦O>Ýíh|ðAü¶Íôþ¼~8,Ú·oßG¿<´½k×®øþI£sÅ_*	þ$éæíîºzõj|æèèháNCCCÿòÖÉ¿QoêÑØNÅ?`%L×ÔÔ¤R©ñññ°ñ%Kä^w;3Q¬ 7ß|3rj_Ãôàà`~iooo¸Ó>=þÜTIð'I7¿èoû÷ïÏ>¥àüû§îÐ:öì=¢°y5F»åòÇU¯K´;wFGÃ×öööüü"Ûö´]¹r%ºøÁ?3wõêÕaiôé6Á¬ñEÑ¿aQWW×ÈÈHqüåaF~i*	þ$éæ0L&;;;£z9pà@*zrÆ¦MtÆÇÇ£c²ù3s£÷üEo¿Y0²à®]»¢ópó¶2-Ñ¢Óo£ïÞÓÓ_d;Ñü¢÷üF×,¿Cå÷É»_½ýqpp0|03á/âfàr¸Z@ç-[âKÜTIð'I·DÑ ´wïÞÿÿ3îZòÓù¦ùs,ò=øàqYÆ;~üxütíOqù555ñ#ÂE¶nFüåÏ´éÎUWWGw¡à`÷ÆãßbéÒ¥ákôi/ñmF;&óETÍ/-rS%Á$Ý* ¬[·.u­µk×F§Üà¯««+ú8½ÆÆÆ×^-¿tttôG©««ÀôðÃç:uª¹¹9,N<x°`SoIØT¿cÇù3m'nL¸IÑù¿|íííù5<<ÍfÃ,^¼8Ü/æ?¶&¾Í Ûh¬Ö¯_ß××WðÜTIð'I$ø$IüI$	þ$I$I?I$Á$IàO$Ið'I$I?I$Á$IàO$Ið'I$ø$IüI$	þ$It#ú¿:Ê,²åG&IEND®B`


)ÆBê1rË-nßéÎ2þ®1&ä´VÎûäÙÅå7fjlxÄjè¡bÝß;8~ê_Æ!ÔXéT[[»ûö¡¡!¿X$òÊI~ÙÐÐïïO¥RïÇ½½½EäÜ¸­­­È¼ùÇsáÔöööÂU×+²Øâ3î·oßÎd2uuu10~~=ÞÎ·C´Âç;þ~W®ìCMäÙ7æk×®-îEÅøwßwüÆZ²dÉÛwº³¹ë`M²ÏoéÒ¥ÓZ9ÅWþgºüúúúOvºòâËxÃcó¶mÛübÈO*'ù%ãÃdÉp6«ÝÝÝ1|öìÙB=¹úÄO&qî9o¼qÆ[~Ü>ÙÑHJ¦Æ;nÙß¿[ÌUx_E[|Æ	ûñãÇ÷Þ~ëÖ­1¼~ýú[w¸#'¾ÉÇldd$y`ÉãjgggáL>ÍvòäÉ¾zõjá¤Ép:ìäçý³LöAºü>Ñé®	Õg,núÂ«/0ÞgSß_Æ¯]»Ã±5cxÞ¼y~±Hä'üòøcÈïúõëù1÷7¿ÿ,9ªï£ÉÕxû,ÜY5æh]Åq²ç1ÞÔÇ¯««+|FñW.ÙóMÆ6¯&°'XøHr¹!ÑÜÜÜÈ/ÏñÉþ!g)1o¬ÌS§NM¸Ò¦²r&|T3qêòK^!7oÞ,¼iÉo/ã¦¦¦¸ºxñâküâÎ;~«Hä'üB*1²¶¶v²·ÛÉây«.âé¾7Ï`±§Ï7Þ¿Çßg6þCoãïT®2Çñ¼ç9ªõõõÉáéñ¼ãéü¦2ËTÖÕ7ÊÊâÈ)Î8õ£½þÕ5ÅñÀÀ@¿¼O8áD~R9ÉïÀgx$ÑÆe9èLkÞâûlÞ÷ÝÂ©E[|Æ"àèÑ£q5ÿ(ÉÈgcöN1ñÔWHáÕeË%ß®ÒÕÕ2üÉ¬d³ÙíÛ·ßºuëàÁ1rãÆÊo*³ÌX~Ó]9ùf<ãÔå7~yÛ%ÿÔ)²þ¼ÞÎ?¿÷îäsþ<hIä'ºüâ½-¼1ç¿Õ%y?4Ä;erªìòåËIÉî6á[fy³)!Kò9­Xx²1âp±Åg,Î6ä?ÈIÎü]¿~ýí»%Kì±Ç>¼üý^ýýýñöïß?ù>:Ù«OùìÙ³Éþ¼¤ãÇO¸§;Ë´äW|åym6Ýµ:ù%ÌÃ;wvëÖ­ïÂ½÷ÆÊ¯ÿþ$×(rä]ùI¥"¿ñ8þlÙüçýó_ºì ÿYdÞâl%6*<Ñ2?µÈbÏX7nÜÈ¢+såÊ1ñª««ËðÃÈ/o¤æ?õXÄ76m¿±V¬XQdOkiÉ¯øÊóÚ(lºkuò;þ|áòW¯^]8uÌ:É`>Ù½y½åO!Ê÷è£úÅ"Tò·Àµk×ÿë^ÍÍÍ¡¨x;?pà@~üñãÇcLH«ðÛì¦8ï=Ù|^,¼©©iü×òM¶ØÎXÉÐÂñ/_Þ¸qcõÝ²Ùìøó9f&¿ÁÁÁXZ,3ôøãòà.îç>]<ñE:tèsû ÓeZò+¾rÆ¼6Æ4­µ:ùE'NX¼xq<ëX~r<=:s%÷ÞÒÒÒÓÓsÏ®½ÞvîÜìL¾Ç¦öE"?IúXÊår6mÖwÈÍ`²+Ù×¬=22~Ù²e^0ùI*­äs~czæg¬ü$IÖÐÐÐ;-ZÒ'|ÒjÈO$Iä'I$ò$IùI$ü$ID~$I"?I$ò#?I$ò$IùI$ü$ID~$I"?I$$IÈOR©uüøñt:½víÚéÎÛØØXUU500Ã1fùòåS_HÕÝfv©Ì;³ßcJ¥jjj²Ùìàà`2ihhhÛ¶mµµµ±2çÏÞ|óM/0Iä'©ª¯¯Ç_FFF¦;ï=bÞ?üÃ?ÌyöÙgcÌîÝ»+[~Ép<ñnooO®nÞ¼9®vwwÇð3gbxÉ%^`ÈOR	õaðtþüùwÍ5ù1+W®1³ï°Ù¿Ç°r×ÔÔ$W«««ãêíÛ·½¨$¤Òe_¡fvîÜ9þüyóæíÚµkÌÍ>÷¹Ï577YB~a¿ûî»1cI×®]kii	¥Óé¦¦¦dgØø¥ÞñY:ÔÐÐ°jÕª¸£ñÿý÷;::bÞxð;vìHvaMW¯^Kñ±äë×¯|òàâê²eËÎ=ë¥%ü$.þá§~:8Ægy¦ð6ùÏ´åÛºukL:xð`ÇeoÙ²%´bÅ'OÆÀÕ«Wc|&pi ø,á¹£GÆÀæÍÇ?ømÛ¶ÅpÜàå_=öä5vûöíþþþHvIoòõÃ_þòIÇÏ/píÚµ.êD~JW~1»ÛxxMøAÀÓ§OÇ¤õë×ÇðÒå§?þ'hnnNNpicU|äÍ?ü¼uuuÉÅ@/FÆ-c¸©©),;<<<³Õ2~|òØbáÁ¾ÂÃ»½½½×ümâN½À$¤_:ÎÇ@Î îîú7o^Ürhh(9¨cIû÷ï¹.¥^Ê,=° Xán¼DÇ[°`A2¦¶¶6Ùøìó+RwwwX³ðÀ·$¤_&)Üçì6»'w:::bjþ2?>9ãaônS_ñYò,07~Þ&óyx###'OLN¼Íï,Öj®ü¢äqÆÓñD~JT~É·´<x0ùß³Ï>;î;v,¿óìèÑ£ùñ#/^¼|þo*ò+>ËO<ñâ/ÆÀÖ­[ÇÏûè£ÆðáÃ/]ºá«V­á.Ü¼y3.]úñÉoùòå1©««+O8Ã6mòD~JT~###¡«yw+üN¾âò»sçNr¤5.?ôú©­­?þ;¦(¿â³:u*¦®2¢Iám¶oß¼ººzÝºuÉi¼¾l6,¼fÍË/Okµ?"Ä=vttÄõ°páÂmÛ¶ÅCòD~$I"?I$$IùI$ü$ID~$I"¿²é;ßùÎÛo¿=÷xåÊçõWjýøÇ?þ÷ÿwë¡·Ë´þTf§«W¯Ú.¥¹]þíßþÍz(µÞzë­Ù.ä÷ÿúßùÀßlÞãéÓ§ÿõ_ÿÕOB©õ­oëý÷ß·J­oûÛÿò/ÿb=ZgÎùçþgë¡Ô·³Â/ÂTtöìÙú§"?òùüÈOäG~äG~"?ùüÈüÈOäG~"?ùùüÈOäG~äG~äG~"?òùÈüÈOäG~"?òùùÈOäG~"?ò#?òùüÈOäG~äG~"?òùüÈüÈOäG~"?ò#?òùüÈOäG~äG~"?ùüÈüÈOäG~"?ùùüÈOäG~äG~äG~"?òùÈ¯¢ä×××ÉdÒétcccww÷øtuuUUUÈüD~ä'ò+û²ÙìáÃc`ß¾íííc¦455M&¿'OÞÅ^íµë×¯ßRÿ`øøë¡Ôzýõ×mÒÜ.×®]³J­Ó§O¿ýöÛÖC©õ­oë­·ÞÍò«­­×ÐÐ0fêÓO?ýÜsÏM&¿Àâwg±füsù»*±lÝ.ßþö·­RëW^±]lìvòK§ÓGñÓæææp¡£½r´×Ñ^9Úëh¯í­ù¥R©üpuuuá¤¶¶¶ø%ä'ò#?ùü*A~uuu¹»Gc¸pRÕüD~ä'ò#?_y×ÑÑqèÐ¡Ël6;ámìóùÈüD~¿[«µ¾¾>Je2	©G~"?òùÈÏ79ÈüD~ä'ò#?ò#?ÈüD~äG~ä'ò#?ÈüÈüD~ä'òùùÈüD~äG~ä'òùÈüÈüD~äG~ä'ò#?ò#?ùüD~äG~ä'ò#?ùüÈüD~ä'ò#?ùùùüÈOäG~äG~"?ùüÈüÈOäG~"?ùùüÈOä'ò#?ò#?ùüÈüÈOä'ò#?ùùüÈOä'ò#?ò#?ùüD~äG~ä'ò#?ùüÈüD~ä'ò#?ùùùüÈOäG~äG~"?ùüÈüÈOäG~"¿Yïoÿöoï÷~oûöí"?ò#?ùüÈ¯2ñÅçÍ÷×üçÏþóË/ÿÔ§>õÎ;ïùÈüD~äWiò.~s×Ww%ÿ×®]K~äG~"?òù_¥õo|cÕªUyöÅ;kçÉüÈOäG~"?ò«¨þùBùÅuuuï½÷ùùüÈOäG~Õ÷¾÷½û¹Ûù[;óìû/ÿÆ'?ùÉR~ÌäG~äG~"?òùÍ°~xÙ§mýï[_Ì~±®®îßøùùüÈOäG~ØðððöíÛ3LMMMsssggg?`ò#?ò#?ùüæJäG~äG~"?òùùÈOäG~"?ò#?òùÈOäG~äG~"?òùüÈüÈOäG~"?òùùüÈOäG~"?ò#?òùüÈOäG~äG~"?ùüÈüÈOäG~"?ùùüJ±·ß~ûGùÙýÙ<üðÃqüÈOäG~äG~"¿ìwÞ©­­miiÙñ?wÄkÖ¬ùùÿù÷ÞüÈOäG~äG~"¿JkóæÍk×®ÝõÕ]ùÿ~é~éÉ'$?òùùÈ¯Òçmýï[å÷ë¿þë¿ò+¿B~ä'ò#?ò#?_¥õÏ|æ77ýf¡ü¾ÐöGyüÈOäG~äG~"¿Jë©§jll|â=ß<ðçþçäG~"?ò#?òùUZÃÃÃ¿üË¿üé¥þböñß%K|ðÁò#?ùùü*°pÞóÏ?ÿÈ#üê¯þêïÿþïMw	äG~"?ò#?òùÍÈüD~äG~ä'ò#?ÈüÈüD~ä'ò#?ò#?ÈüD~äG~ä'òùÈüÈüD~ä'òùùÈüD~äg=ùÈüD~ä'ò#?òùÈüD~äG~ä'òùÈüÈüD~äG~ä'ò»¯õõõe2t:ÝØØØÝÝ]8©§§§©©)&-_¾<nF~"?òùÈ¯¼Ëf³ûöµ··NZ²dÉ¹sçbàÈ#K.%?ùüÈOäWÞÕÖÖÆ@.khhìf555ãå÷µ¯íÏbßÿþ÷ÿ·J¬Ø.ÖC©õê«¯Ú.¶¦XWW×ßüÍßX¶Ë_:p¸°ÞÞÞ-[¶ßK/½ôö,öWõWo«Ä:uêíRÛåïþîï¬Rëµ×^»|ù²õ`»h*uww¿ñÆ³ysB~©T*?]=þï¿ÿ~6r´Wö:Ú+Gí£½å]]]].KöÆð©7nÜØ¼yóÍ7ÇÏH~"?òùÈ¯Ìêèè8tèPÄe6#­ÖÖÖÁÁÁ	g$?ùüÈOäW~«µ¾¾>Je2ddUUU44TD~"?òùÈoF~"?òùÈüÈüD~"?òùùÈüÈüD~äG~ä'ò#?ÈüÈüD~ä'ò#?ò#?ÈüD~äG~ä'òùÈüÈüD~ä'òùùÈüD~ä'ò#?òùÈüD~äG~"?òùÈüÈüD~"?òùùÈü¬òùùÈüD~"?ò#?òùÈüÈüD~"?òùùÈüÈüD~äG~ä'ò#?ÈüÈüD~ä'ò#?ùÈüD~ä'ò#?òùÈüD~äG~ä'òùÈüÈüD~ä'òùùÈüD~"?ò#?òùÈüÈüD~"?òùùÈüÈüD~äG~ä'ò#?ÈüÈüD~ä'ò#?ùÈüD~ä'ò#?òùÈüD~äG~ä'òùÈüÈüD~ä'òÓÜ_Õ½J¥RäG~ä'ò#?ùUüR÷*NùÈüD~äW	ò«ÈOäG~"?òùØâÅ·uëVò#?òùÈü*M~étÚçüÈüD~ä'òSËoùòåãOï¨­­"?ò#?ùüÈ¯¢äW]]Ô¬¯¯ß/¾[¶l!?ò#?ùüÈ¯¢äìä ^zutt4æÍG~äG~"?òù_EÉoÁá¼xb1ðäO&¾ÕüÈOäG~"?ò«4ùíØ±#>GáGýV­ZE~äG~"?òù_EÉ/YÖÂc §§'BÍÍÍe±=ÈOäG~"?òùÍÈOäG~"?òùùÈOäG~"¿ZºtiòÝ.¾ÉüÈOäG~"?U²ü,YR¨½|Îí%?òùÈü*M~¼0_ooïèèhÙmòùÈüD~Ó¨¶¶6äWì#?ùüÈOä7½úúúB~Û¶m+Ç7NòùÈüD~ÓkÑ¢EUãrùÈüD~äWiò[¼x±3<ÈüD~ä'òÓ_b¾þþþrÜä'ò#?ùü¦Q]]3<ÈüD~ä'òÓ_P&ä·cÇááaò#?òùÈO,¿ªIrùÈüD~äWßä<aÎð ?òùÈü*ð[]Ê7òùÈüD~Ó¨¡¡añâÅ.]"?ò#?ùüTáòK§ÓUUUÕÃêëëËd2±ÌÆÆÆîîîâf6¦°§zê¥^z;zôèo¼ñ¶J¬Ø.ñ¯ë¡Ôúæ7¿ùÃþÐz(µ^|ñÅ/Z¥V¼ýà?°J­cÇõ÷÷Ïæ=~ìòNüvïÞÿÔøðßíÍf>ûöíkoo/>ifcüñÇ«$I*¥]~í¹½µµµ	s¹ñI3C~$üfØGnoá0~ÒÌÆ$I"¿¨pOauuuñI3SØoÿöo?ÿüóßÅþèþ¨««ë;*±öîÝk»`üÇüê«¯Z%¸]:;;­R+ÞÎ^yåë¡Ôú?ùYÞ.e&¿ºººáâf6Æ¹½rn¯såÜ^çöÊ¹½3/PµnÝºªªªyóæµµµÍøTCÅ@³Ùâf6üD~ä'ò#?ß»sçÎgög|cÕ××§R©L&ÓÓÓLZ4³1ä'ò#?ùüfØ²eËBfë×¯OÞ8ãe·aÃ³bÅÒßä'ò#?ùü¦Quuu8odd$?&ËÅñ§SùÈüD~äWÞòK¥Rá¼ä,¤ááá3³ou!?ÈüD~äWºòKö¶¶¶&oqÃ1¦©©üÈüD~ä'ò#¿_¼_NxÇ­[·ÈüÈOäG~"?ò«(ùp÷ôÞ¶¶¶ùóç§R©¸lmm1e±=ÈOäG~"?òùÍÈOäG~"?òùùÈOäG~"¿tÏ?sÉüÈOäG~"?ò+cù¥&üÈüD~ä'òÓsáhïã?ÈïÈ#äG~ä'ò#?ùU¦üúúúæÍækii)übgò#?òùÈü*J~7nLvõ<y²¶ùüÈOäG~"¿iôòË/'æ[¿~ÙmòùÈüD~SêÎ;+V¬HNæèîî.ÇíA~"?òùÈïÞíÝ»7ÙÕ×ÞÞ^¾ÛüD~ä'ò#?ß½ó~äG~"?òùi®È/u¯Òé4ùùüÈOäG~ ¿ÊüD~ä'ò#?ùùüD~ä'ò#?ò#?ùùüÈüÈOäG~"?ùùüÈOäG~äG~"?ùüÈ¯¼ä·téÒêêjßçG~ä'ò#?* K,)Ôïó#?òùÈO+¿@^¯··wtt´ì¶ùüÈOäG~"¿iT[[ò+GöÈüD~ä'ò^!¿mÛ¶ã'ùüÈOüþôOÿ´¥¥åÓþt6½téE~ú RÏðX´hQÕ¸áA~ä'ò;òûÂ¾ð©Åú¯_ú¯_ù_yðÁkkk¬+òSÊoñâÅÎð ?òùÍeù8qâüäÎßÚ¹ë«»ÿúìC­­­Öù©å¯¿¿¿·ùüÈO^~_ýêWþÿÎ³/þ.X°Àº"?U üêêêáA~ä'òãòè³ÊoÇÿÜA~ä§Ê_P&ä·cÇááaò#?òùÍAùM|´÷AGÉO´×äG~"¿9+¿þãýìgáA~ªXù¥&ÉäG~"¿¹#¿èþàV­ZµdÉ_ûµ_+ÓÈ¯Â#?ùé£ÈOäG~äG~"?òù_eÉ/Ë­[·®¦¦¦ªªjÞ¼ymmmårª/ùüÈOäG~"¿itçÎ	Ïð(SÉOäG~"?òùM£eËóÖ¯_¼qÆËnÃ1fÅäG~ä'ò#?ùUüª««Ãy###ù1¹ÆÄxò#?òùÈü*J~©T*ÚË1¾ÕüÈOäG~"?ò«Ì£½­­­Égpijj"?ò#?ùüÈ¯¢äïáqëÖ-ò#?òùÈü*J~Ü=½·­­mþüù©T*.[[[cLYlòùÈüD~s%òùÈüD~Ó¨¡¡añâÅ.]"?ò#?ùüTáòK§ÓUUUeº=ÈOäG~"?òùM£îîîßîÝ»ãW.´üD~ä'ò#?ßLª¤T*E~äG~"?òù_EÉ/5I¾ÉüÈOäG~"?ò«ù-^¼¸±±qhh¨¬·ùüÈOäG~"¿ü¹Þîí-»ä'ò#?ùüf(¿äÏõ&¢üÈüD~ä'òS%Ë/ÉTÍäG~"?òù_È¯··wÁÉ¿ÄyÎð ?òùÈO)¿|å<òùÈüD~þn/ùüÈOäG~"?ò#?òùüÈOäWjõõõe2t:ÝØØØÝÝ]8©§§§©©)&-_¾<nF~"?òùÈ¯¼Ëf³ûöµ··NZ²dÉ¹sçbàÈ#K./¿¿üË¿¼1:uêÊ+7TbÙ.¥Ùk¯½ö£ýÈz(µâØo¾ù¦õ`»h*½þúëÿ÷?÷ø±ËïòåË÷]~µµµ£££1Ëå&»YMMÍxù8pàû³Ø+¯¼ò×ý×ßWÕÙÙÙÓÓc=Ø.²]Ê·W_õßûõ`»|ìòK¾Ò¥©©)þ©q¿äWxrñd'÷öönÙ²ÅÑ^9Úëh¯íu´WöÎ¼0_þÛC]kÖ¬¹råÊ,?Âo®®®xSÏf³ãÿÊ0ùüÈOäG~"¿iwùòåeËå	üZ·nÝÇúò÷Ãuuu¹»GcxÌ-oÜ¸±yóæ7o_ùüÈOäG~"¿6::zòäÉYþëm¸Ìf³c¤ÕÚÚ:888áä'ò#?ùü¦]á>¿ùóçÿPÝÇºZëëëÃL¦§§'ìlhh(ükÂä'ò#?ùüf^!øjjjÚÛÛ¯]»V.ÛüD~ä'ò#?ß4JNìxè¡®^½ZvÛüD~ä'ò#?ß4º_æB~"?òùüÈoVåWÖÈüD~ä'ò^/^té¼yóóyëêê;F~äG~"?òù_¥É¯««+G"¿dxß¾äG~ä'ò#?ùUüêëëÃy/^ÌË¯§§'ùbò#?òùÈü*J~ùïÉËËott49áüÈüD~ä'ò#¿_]]]8/ÙÏòËår;wîáò#?òùÈü*J~Aª:wîùùüÈOäG~%¿èæÍÍÍÍÉ¹½555K.½råJYlòùÈüD~s%òùÈüD~äG~ä'òùÈï?víÚµ¦¦¦ùóç§îår¨üD~ä'ò#?ßTôÑG«&iëÖ­äG~ä'ò#?ùUü; oÿþýCCCÉÈ;wî?~<ßÙÙI~äG~"?òù_%È/Éï<8~Rò÷Ü|ùÈüD~äW!ò«®®ÞÝ¹sgü¤âäG~ä'ò#?ùUüò´m²©É_r#?ò#?ùüÈ¯äWÄväG~ä'ò#?ÈüÈüD~ä'ò#¿2_ñÈüÈOäG~"?ò«ù¥îU:&?ò#?ùüÈ¯äWÈüD~ä'ò#?ò#?ÈüD~äG~ä'ò#?ò#?ùùüÈOä'ò#?ò#?ùüÈüÈOä'ò#?ùùüD~ä'ò#?ò#?ùüD~äG~ä'ò#?ùüÈüD~ä'ò#?ùÈüD~ä'ò#?ò#?ÈüD~äG~ä'ò#?ëüD~äG~ä'ò#?ÈüÈüD~ä'ò#?ò#?ÈüD~äG~ä'ò#?ò#?ùùüÈOä'ò#?ò#?ùüÈOäG~ä'ò#?ùüÈüD~ä'ò#?ùùüD~ä'ò#?ò#?ùüD~äG~ä'ò#?ÈüÈüD~ä'ò#?ò#?ÈüD~äG~ä'ò#?ò#?ùùüÈOä'ò#?ò#?ùüÈOäG~ä'ò#?ùüÈüD~ä'ò#?ùùüD~ä'ò#?ò#?ùüD~äG~ä'ò#?ÈüÈüD~ä'ò#?ò#?ÈüD~äG~S¬¯¯/É¤ÓéÆÆÆîîîñ7èêêªªª"?ùüÈOäWöe³ÙÃÇÀ¾ûÚÛÛÇLijj"?ùüÈOäW	ò«­­×ÐÐ0fêÓO?ýÜsÏM&¿?û³?ëÅ^yåsçÎõªÄ²]lM½ÎÎÎï~÷»Öí¢ÒÜ.sB~étzÂáèÚµkÍÍÍáÂÉäwüøñÿ3:uê­·Þú?*±^íµ«W¯Z¥Vww÷ücëÁvÑTzýõ×¯b=Z§OþÑ~4÷8'äJ¥òÃÕÕÕÚÚÚÎ9öÊÑ^Gåh¯£½r´·Wõb¸®®.Ë%GcxÂåoL~"?òùÈ¯ëèè8tèPÄe6LãGÈüD~ä'ò+¿ÕZ__J¥2LOOÏÔ#?ùüÈOäçÉOäG~"?òùùÈOäG~"?ò#?òùÈOäG~äG~"?òùüÈüÈOäG~"?ò#?òùüÈOäG~äG~"?ò#?òùù¦öð+_ùÊC=´ûöò#?ùüÈü*³^ziÁ­¶þÿ²fÍ.²­ÉüD~ä'ò#?ò+×~æg~æ7¾ü»¾º+ùïÙ/~â!?òùÈüÈ¯¢õÜÜÜg_òßÿéK.ùüÈOäG~äWQ:uê3ùÌùýâ/þb?ùÈüD~äG~Õ;ï¼óS?õS_ù_É³ï¿mþo?ýÓ?=44D~ä'ò#?ù_¥õôÓOâøBÛÂÿüçkkk¿öµ¯Mvcò#?ùüÈüÊ»¿ø¿h°õ~á~øáXóEnI~ä'ò#?ùßüÈOäG~"?ò#?òùüÈOäG~äG~"?òùüÈüÈOäG~"?ùùüÈOäG~äG~"?ùüÈüÈOäG~ÖùüÈüÈOäG~"?ùùüÈOäG~"?ò#?ùüÈOäG~äG~äG~"?òùùÈOäG~"?ò#?òùÈOäG~äG~"?òùüÈüÈOäG~"?ò#?òùüÈOäG~äG~"?òùüÈüÈOäG~"?ùùüÈOäG~"?ò#?ùüÈOäG~äG~äG~"?òùùÈOäG~"?ò#?òùÈOäG~äG~"?òùüÈüÈOäG~"?ò#?òùüÈOäG~äG~"?òùüÈüÈOäG~"?ùùüÈOäG~"?ò#?ùüÈOäG~äG~ÖùüÈOäG~äG~"?ùüÈüÈOäG~"?ùùüÈOä'ò#?ò#?ùüÈüÈOä'ò#?ùùüÈOä'ò#?ò#?ùüD~äG~ä'ò#?ùüÈüD~ä'ò#?ùùüD~ä'ò#?ò#?ÈüD~äG~ä'ò#?ÈüÈüD~ä'ò#?ò#?ò#?ùüÈüf__&I§ÓÝÝÝ7oÞ]ýÀºÈOäG~"?òùwÙlöðáÃ1°oß¾öööÂIöìyægFGG//¿ÿg±×_=~2ÿ¯J¬xÄO¦õPjÈoÝºe=Z!ò÷ÞÏz(µBäÖC©"ÿÇüÇÙ¼Ç9!¿ÚÚÚ°]är¹ÂI.]lÆß³Ï>Û98qâW^éTe»Ø.²]lUÆvòK§Ó'W÷îÝ[SS³xñâ.8Ú+Gí£½öÊÑÞò.Jå«««ÇL:xð`|¹¹¹üD~ä'ò#?_ùUõb¸®®.Ëp÷hoÞ¬ðêÝä'ò#?ùüÊ¯CÅ@³ÙÂI=öØÑ£GcàâÅ«V­"?ùüÈOäWö«µ¾¾>Je2dd²;0~ÚÚÚÒétssóÀÀùüÈOäG~"¿9ùüÈOäG~"?ò#?òùüÈOäG~äG~"?ò#?òùùÈüD~"?ò#?òùÈüÈüD~"?òùùÈOäG~"?ò#?òùÈOäG~äG~"?òùÈüÈOäG~"?òùùüÈOäG~"?ò#?òùüÈOäWI=õÔS/½ôÒÛ³ØÑ£Gßxã·UbÅv¹téõPjóßüáh=Z/¾øâÅ­R+ÞÎ~ðX¥Ö±cÇúûûgó'ûùý¿â×®]»~G$©"ì`&ùI$ÍÈO$ü$ID~$I"?I$$IÈO$IäWö644ä¯Þ¼y³ª Â[öõõe2t:ÝØØØÝÝmÕÈvéééijjí²|ùòØFV]l¤®®®	Çë~máááÍ7WWW?ðÀ§O¶êJd»Ä¶X¶lYòþröìY«n6·Kûø¾O~Wñ?l?ÇWÀ7Îf³ûöµ··[%²],YrîÜ¹8räÈÒ¥K­½Ù.ÑÈÈH üJj»ìÙ³çgw¸Å[%²]jkk¯]»qYÍÂv)òCqß÷ÉïãjÝºu¯ø±<~üød?ñÊç'³t¶Ka555Ö^él§~ú¹ç#¿Ú..]²ÒJm»8nÜ¸qIä³¼]üPÜÇ÷òûx+|,Y²¤µµ5N777_¾|¹ðf1rÂaÝßí¯··wË-Ö[lk×®ÅÈø¥I~¥ölïÞ½ño¤àÅ¬·Ù.±QâÆq¿Ê¬·ÙÜ.E~(îãû>ùÍÞ+ _üÃkùòåcR©T~¸ººÚz+íôþûïg³Ù¡¡!ë­D¶K[[Û3g&»½îãï±Æ@È#üa½ÈvY±bE²Û)¸råJëm6·Kûø¾O~÷á'süf®««ËårÜÝëÃÖ[lä×èæÍoÞ¼i¥Îv©úYo¥óì~íÃ°]lÇîãv)òCqß÷Éoö^K,¹råJÖÖÖÂutt:t(â2ÍZo%²]N>c­±Ú.÷|çÓÙ.=öØÑ£GcàâÅ«V­²ÞJd»¬2¶Hô÷÷¯X±ÂzÍíRäâ>¾ïßì½zSë×¬Y|Þ6³gÏÖ××§R©L&ÓÓÓc½Èvihh°o©·ùæv¹ûv[[[òQ³ë­D¶Ëo¾àIqÃÖÛln	(îûû>ùI$ÍÈO$ü$ID~$I"?I$$IÈO$Iä'I$ò$IùI$ü$IÈü$IÈO$Iä'I$ò$IùI$ü$ID~$I"?I$¤9××¿þõÕ«W×ÜmíÚµ/¿üráÔª»ËsðÑ644ÄS3>Æ¤ÓéL&3:::ÝeJ"?I*¿víÚU5®Âßi ¿Ý»wÇÈ^xaÌøç>Æ?ùä3X¦$ò¤2«¯¯/LN§÷ïß?r·ÆÕyþüùßÕ«WcäòåËÇ_¶lY¿råùI"?Iß¾ô¥0Í3Ï<S8òÙg_þòÝÓÝÝr655ÅpþÆ·nÝÚ²eËüùócRmmíöíÛª:u*tbÞÎÎÎ11,X½zõÉ'ãjGGÇvâÄâËI&ÅCJ&uuuM¦´ÖÖÖßÓÓsîÜ¹ÓÒÒ³gÏúúúXTMMÍÆoÜ¸1^~ã?fL*ü$é~¶hÑ¢PËµk×G^¿~=F644ÊfL½½½ÉÔ6´mÛ¶dR*p®äj2µ­­mtt4ìX]]=22Sã2ä4oÞ¼äãwE>¼ñÏôÈ#ÍãòðáÃyöYÈÚµk§+¿"UùIÒ.9°;~|Ê¦½½ýý»Å@¿~25NbÇd×]-Íf1|öìÙd!Ë|â'ÂvÉÁÖG4Æ?~<ã27mÚtÏåãjÜ ð±MøB.ç;88Wã2yp3Ë%7Èd21c<~rt¸¦¦fºò+òP%$ÝùEÊ/?>M¦èÆq5Ì«/ÞºukíÎ;ùÄmÆìEËÏ~ýzþÆ¡¥üß¸áS§NÝs9¡Ì¸úî»ï>¶É>÷øãÇ¤çîÑÞ¾áB!¿ðhsss²Krºò+òP%$Ýç]ï¿ÿ~áÈ¡¡¡X'ïÂyèä?7þ ìK~J×ÖÖVWWÇÂ,XzÏåLæ°1]¼x1AjÇe÷÷÷ç§=6ÀG§.¿"UùIÒ.ù¬ÛÞ½G&ßu2æügÇïÊ:þüîÝ»c¯y2&;äòSïé³mÛ¶%yãrË-ùñEìc»yófrõÝwß-~îÊ+cjòE6ÖÂIÉy¾1©««ëöíÛÅåWi²6òS<TIä'I÷¹ät:½oß¾ä[]öïß_]]=þlõë×rC±ùópÏù%(ü`ÁíÛ·³nóß«2¡Ïm?út~|å$ìK>ç744Ü²ü<ßO¹pRòÇþþþ¸X	É/±fX9nâÜ¸qcáÔ"UùIÒý/ùã1íÙ³'dL£üpþ8iþ¤|>úh!+;vìXùp÷mÄøÚÚÚÂÁE£ðåÏ«ìÉÞ¹s§¦¦&ycq¯[·®ð..É».3Ù%/qj~j*ü$©$¬]»¶ún«W¯NN°#¿®®®äkóÏ9:44´sçÎúúúDK?þøððp~jgggsss,É8p`Ì2Ç?XTì±ÇÆl9Q<xHÉù>¿|[¶lóõ.IÙl6ÖÀüùóãYr%ÿ5Ë¼ûvÐ6YW---===cî±ÈCD~$I"?I$$IÈO$Iä'I$ò$IùI$ü$IÈO$Iä'I$ò$IùI$ü$ID~$I"?I$4òkiiù]I$Ut	ùª¬I¤¹ÐÙ³gÿzÅÄÕ@IEND®B`


aÐÛÛ¢"ùýæ7¿ù_«vìØaª¶;wþýï·ÕÙ·¾õ­7oZêìÛßþöØØu³üÖ­[7::Z(¿K.=pãúúú Â0Èf³ÑËåÌÉï§?ýé æª·ÞzË"Tm?ÿùÏßï=ëP:tèÜ¹sÖÁÞ×´øÎöþ¿ÿ¬ùÅãñ×_=L.[¶ìücáfáª¢q93Îö:Û+gål¯íu¶wÊ/<x0._¾ÜÖÖV¸Y¸*?N$eÎùüD~"?ò[ òkhhÈ^´We³Ù÷ÏäF3C~ä'òùüÈoÊoÇÇ/®ZµªèäÐ¡Ca.3L3äG~"?ï£ölúÑ~dÉïÿañx¼­­mtt´pÁÁÁT*ÅÒéôÐÐP3äG~"?ï¿î5/âÝ4>ÚV»üæ,ò#?ÈOO55öì±äG~ä'òùüD~äG~"?ÈOäG~ä'òùüD~äG~"?ÈOäG~ä'òùüD~äG~"?fìðìòþùçgs###öùùüD~+ÔÔ|ñ«]üÑØöü²ÿþÂ#ß<|õð`m/ùÈOä§9ßÝ¯ëØM~äG~ä'òùüD~äG~"?ÈOäG~ä'òùüÈüÈüD~"?ùÈOä'òcò#?òùüD~äG~äG~"?È¯Òä÷ô¿>;_µOýùùÈOä§¹ßüF~äG~ä'òùüD~äG~"?·üüùÈOä'ò#?ò#?òùüD~äG~äG~"?ò³ä'ò#?ò#?òùüD~äG~äG~"?ÈOäG~ä'òùüD~äG~"?ÈOäG~ä'òùüÈüD~"?&¿¯½òûùúhZõ"ùùÈOä§¹_êó_xäÏ%jÿiiý#ß<|õüÈüD~"?-8îÙ³Ç:ùÈOä'òùùüD~"?ùÈOä'òùùüD~"?ùÈOä'òùùüD~"?ò#?ÈOä'ò#?òùüD~"?ò#?ÈOäW555ÕÌ¢Ã[Cò#?òùüD~"?ò#?ÈO§-[¶|ôÑGÖüÈüD~"?ÈüÈOä'òùüÈüD~"?ÈüÈOä'òùüÈüD~"?ÈüÈOä'òùùüD~"?ùÈOä'òùùüD~"?ùÈOäG~äG~"?ò#?ÈOä'ò#?òùüD~"?ò#?ÈOä'ò#?òùüD~"?ò#?ÈOä'ò#?òùüD~äG~"?ÈOäG~ä'òùüD~äG~"?ÈOäG~ä'òùùÈüÈOä'òùüÈüD~"?ÈüÈOä'òùüÈüD~"?ÈüÈOä'òùüÈüD~"?ùÈOä'òùùüD~"?ùÈOä'òùùüD~ägÈOäG~ä'òùüD~äG~"?ÈOäG~ä'òùüD~äG~"?ÈOäG~ä'òùüÈüÈüD~"?ùÈOä'òùùüD~"?ùÈOä'òùùüÈüÈOä'ò#?òùüD~"?ò#?ÈOä'ò#?òùüD~"?ò#?ÈOä'ò#?òùüD~äG~äG~"?ÈüÈOä'òùüÈüD~"?ÈüÈOä'òùüÈüD~äG~ä'òùùüD~"?ùÈOä'òùùüD~"?ùÈOä'òùùüD~"?ò#?ÈOä'ò#?òùüD~"?ò#?ÈOä'ò#?òùüD~"?ò#?ùYòùü>ÕøøxcccÑdMMñíÒét<oii(süÈOä'òùßhpp°¹¹¹ySSS­­­¥òËd2aÐÛÛ¢ò#?ÈOäG~¢uëÖ!ï?øÁþýûKåW__ËåÂ ÍF/3S$¿üä'g5Wýâ¿°U[xö»ßýÎ:Øûª¶>|æÌë0g-2ùýÿÿ¬ä]»v­­­-è­T~ñx¼hLü~ûÛßþoÍUÛ·o·UÛ7¾ñ	ëPóß·ÕYww÷ßþö7ë0g-zùuvv;w®h2*åÇD¢Ìgí³½r¶WÎö:Û»@åWóé7khhÈf³ÑÜ0.süÈOä'òùßß4áäÐ¡Ca.3L3äG~"?Èüü¢ñàà`*Åbétzhh¨Ìò#?ÈOäG~ÕùÈOä'ò#?òùüD~"?ò#?ÈOä'ò#?òùüD~"?ò#?ÈüÈüD~äG~"?ÈOäG~ä'òùüD~äG~"?ÈOäG~ä'òùüD~äG~"?ÈOäG~ä'òùüÈüD~"?ÈüÈOä'òùüÈüD~"?ÈüÈOäG~äG~"?ùÈOä'òùùüD~"?ùÈOä'òùùüD~"?ùÈOä'ò#?ò#?òùüD~äG~"?ÈOäG~ä'òùüD~äG~"?ÈOäG~ä'ò#?ò#?ÈüÈOä'òùüÈüD~"?ÈüÈOä'òùüÈüD~"?ÈüÈOä'òùùù> ?ÈOäG~ä'òùüD~äG~"?ÈOäG~ä'òùüD~äG~"?ò#?òùüÈüD~"?ÈüÈOä'òùüÈüD~"?ÈüÈOä'òùüÈüD~"?ùÈOä'òùùüD~"?ùÈOä'òùùüD~"?ùùùüD~"?ò#?ÈOä'ò#?òùüD~"?ò#?ÈOä'ò#?òùüD~zRòëèè¨­­ãÇ'Éx<ÉdÈOä'òùü*M~7n¬©©Åb¹¦ Í7ÈOä'ò#?ò«(ùÕÕÕç_¾|9¯_¿aüD~"?ù_EÉ/z/V¯^ÝÝÝÑd,#?ÈOäG~äWQò«­­Î^ü;sæ×üD~"?È¯å×ÑÑÿÙ¾T*uï?^ôs~"?ÈOäWiòéK_ÅbáÓ0^»ví¢øÿ'?òùüD~äW-ùüD~"?ò#?ÈOä'ò#¿uñâÅ+VÔÖÖF¿ÏÛÐÐpüøqòùüD~"¿J_þ7<"ùEãÞÞ^òùüD~äG~%¿T*wñâÅ¼ü¼«ÈOä'òùU üòïä_ôgÜâñ8ùüD~"?ò#¿_CCCp^ô:__6Ý½w766ÈOä'ò#?ò«(ùE±£´óçÏÈOä'ò#?ò«(ùnÜ¸ÑÖÖýno2bÅ+WÅÿ?ùÈOä'ò#¿jüÈOä'òùßg¨±±qÙ²e.]"?ÈOä'ò«pùÅãñèwÉOä'òùü* A~÷îÈårä'òùüD~+¿½·ùüD~"?ò³#¿ØCòNÎ"?ÈOäWiò[ÔùüD~"?ò#?ÈOä'ò#¿Ò»ðs~"?ÈOäW%ò+üÙ¾Bùù9?ÈOä'ò«4ùËå&''î¹ ¿ÑÑQòùüD~äG~+¿¨¿ ¿¦¦&òùüD~äG~.¿äH$ÈOä'òùùU²ü²ÙìüÉ$ùüD~"?ò#¿ßÃ~·wýúõä'òùüÈü*J~¥½#H<÷ÜsâÿüÈOä'òù_µD~ä'òùüÈüD~"?ÈüJïâ!Åãñïÿûä'òùù*D~¥½#úÓmùqww7ùüD~ägÈO ¿©©©ÚÚÚõë×ß¾;|.×®]ÛÒÒÆï¼óN_=ùüD~ägÈO ¿+VÞe³ÙüÌ;wÂLÀüÈOä'òùßg¹ûMMMågòàþùüD~äG~ä§_*Þ·yrròÞý?ÚÆÑIÞááagE~"?ùYÊ_ÿ·w`` lÛ¶üD~"?ò³ä§J_èúõë+V¬H&±X,q¹wÿDðÿcäG~"?Èüª%ò#?ÈOäG~ä'òùüD~ä¿åý·k¾÷ð¿á]K~"?Èü¬Ã¢_]ôv-±´ßÌüÈOä'òù_ÕE~ä'òùüÈüD~"?Èüò·?ÉG~ä'òùüôå·H!H~ä'òùüÈüD~"?ÈüÈOä'òùüÈïqÉo||¼±±1ÿéÐÐPkkk<_¹rå·¦ÓépUKKËÀÀ@3äG~"?ÈüüÃ½åg/_~þüù08zôè+7Îd2aÐÛÛ¢ò#?ÈOäG~Y~Ó÷hnÝèèh¡üK&Ö××çr¹0Èf³ÑËåÌÉïí·ßþ@sÕw¾óPµíÙ³çêÕ«Ö¡:ûîw¿åÊëPÚ?ÿùÏÖaÎzRòÍÔlþÇå7<<¼mÛ¶ÂÂ/Ë)ß[o½õï«^í5PµýøÇ?þÓþdª³×_ýøu¨ÎzzzÞÿë0g=)ù=Ù"KäwûöíL&399Y¤Ïü8H9ãl¯³½r¶WÎöÊÙ^g®üÆÆÆÂ÷Í76khhÈf³ÑÜ0.süÈOä'òùßß3gÚÛÛÇÇÇø@rèÐ¡0L¦Ìò#?ÈOäG~T~¿8R¸Áàà`*Åbétzhh¨Ìò#?ÈOäG~ÕùÈOä'ò#?òùüD~"?ò#?ÈOä'ò#?òùüD~"?ò#?ÈüÈüD~äG~"?ÈOäG~ä'òùüD~äG~"?ÈOäG~ä'òùüD~äG~Êìêéé±ä'òùùiqôÞ´E¼f?üÐÈOäG~ä§8ÜÔÔ=Ö:ÈOäG~ä'òùüD~äG~"?ÈOäG~ä'òùüD~äG~"?ÈOäG~ä'òùüÈüD~"?ÈüÈOä'òùüÈüD~"?ÈüÈOäWyýõ¯tKþüñ¹DíSéG¾yøê¿úÕ¯ìòùüÈüôÄûàÉÚ¯½òûùúhlYøða;üD~"?ò#?ÍüJ=³ág7æëã_í"?òùüÈüD~"?ÈüÈOä'òùüÈüD~"?ùÈOä'òùùüÈüD~"?ò#?ùÈOäG~ä'ò#?òùùùÈüÈOä'ò#?òùùüD~äG~gù%µ5óWü?ÕùüD~äG~#ùÕÌwäG~"?ùæH~^óùüÈüD~Õ"??ç'òùùüÈüD~"?ò#?ùÈüÈüÈüD~äG~"?ùÈüÈOä'ò#?òùùüD~äG~"?ÈOäG~ä§Ç)?ïä,òùùüTÞá¦¦æìÙ³ÖüD~"?ò«¨G÷ÙtòäIkH~"?ÈüGk¦-âÝ4XCòùüD~äW!ýüD~"?ùÈOä'òùùüD~"?ùÚþM===ÖüD~"?ò#?-Þ¶ööö³gÏN³Á~hÉOä'ò#?òS%ÔÕÕõÉ'XòùüÈüD~"?ÈüÈOä'òùüÈüD~"?ÈüÈOä'òùüÈüD~"?ùÈOä'òùùüD~"?ù_õ599yxvù½üòË³¹+W®Øä'òùüÈï÷Áºñ«]üÑØöüòg7<òÍÃWø³#ÈOä'òùßï©Ô3~vc¾>þÈüD~"?ùÈOä'òùùüD~"?ùÈOä'ò#?òùüD~"?ò#?ò#?ÈOäG~äG~ä'òùüÈüÈüD~äG~ä'ò#?ò#?òùüD~äG~äG~"?ÈüÈüÈOä'òùùüD~"?ùÈOä'òùùüD~"?ò#?ÈOä'ò#?ò#?òùüD~äG~U!¿®ÿ¯kþç¯çëãÿíùüD~"?ò#ùÕÌwäG~"?ÈüÈOä'òùüÈï±ÊÏÏùüD~"?ò#?ò#?ÈOäG~äG~ä'ò#?ò#?ùùÈOä'ò#?ò#?òùüD~äG~äG~"?ÈüÈOä'òùüÈüD~"?ÈüÈOä'òùùüD~úl'³kÍ5ÖüD~ä·àäÐüøÏÿåþü#ßÜ_o#?-^îÝ»×:ÈüSSSSÌ®pôûí·gsvùüD~"?ò[GN~"?ÈüÈOä'òùüÈüD~"?ÈüÈOä'òùüÈüD~"?ÈüÈOä'òùùéqßgÓÉ'­!ùüD~"?ò[­¶§~zúFFF¬!ùiaÒ-õù/<òÇçµÿ´ôéG¾yøê===öùüÈoµaÃ@~Z¤òûÚ+¿¯¦U/Ãµ½@~"?ò#?æH~óø7»ÿ­c7ù*V~ãããùO/N§ãñxKKËÀÀ@á¥W3C~ä'òùüÈoA488ØÜÜ1ùL&Ó××½½½En(½ªò#?ÈOäG~¢uëÖÊ¯¾¾>ËA6-|-ðW3S$¿W^yå¤æª_ÿú×ÁÞ×blÞå÷â/Ú´'NX¹lÉ/ÿä2?Çã?ðªrfäwîÜ¹ÿ£¹*»-BÕ¶qãÆ©©)ë°H[¯ùÙ´¯ýëÖaÎZôòÅbùq"(Ü¬ôªrfíu¶WÎöÊÙ^9Ûëlï_CCC6N×qáf¥W3C~ä'òùüÈoÊ/<Z:t(Âe&)z )ºªò#?ÈOäG~T~©T*¥Óé¡¡¡ÂJ¯*güÈOä'òù_5F~ä'òSòßÈüD~äG~"?ÈOäG~ä'òÓãßÓÿúì|Ô>õ/äG~"?ò#?æN~~ÎOäG~ä'òùùüÈüD~"?ò#?ùùüD~äG~"?ò#?ÈüÈOäG~ä'òùüD~äG~"?Íü¿æëÃûùÈüÈOä§9=ZÎ¦@·Õ«WÏæ«ÉOäG~ä'òÓ"y°©©Ù»w¯u ?ùÈOä'òùùüD~"?ùÈOä'òùùüD~"?ùÈOO²¦¦¦YtøðakH~"?ò#?ÈOä'ò#?òùiñ´iÓ¦?þØ:ÈüÈOä'òùüÈüD~"?ÈüÈOä'òùüÈüD~"?ÈüÈOä'òùùùüD~"?ò#?ÈOä'ò#?òùüD~"?ò#?ÈOä'ò#?òùùÈOäG~ä'òùüD~äG~"?ÈOäG~ä'òùüD~äG~"?ÈOäG~ä'òùüÈüÈüD~"?ùÈOä'òùùüD~"?ùÈOä'òùùüÈüÈOä'ò#?òùüD~"?ò#?ÈOä'ò#?òùüD~"?ò#?ÈOä'ò#?òùüD~äG~"?ÈOäG~ä'òùüD~äG~"?ÈOäG~ä'òùüD~äG~"?ò³ä'òùùüD~"?ùÈOä'òùùüD~"?ùÈOä'òùùüD~"?ò#?ÈOä'ò#?òùüD~"?ò#?ÈOä'ò#?òùüD~"?ò#?ò#?òùüD~äG~"?ÈOäG~ä'òùüD~äG~"?ÈOäG~ä'òùüD~äG~"?ÈüÈOä'òùüÈüD~"?ÈüÈOä'òùüÈüD~"?ÈüÈüD~"?ÈüÈOä'òùü`'NXµjÕI¤èðáÃä'I$ò$I"?I$$IÈO$Iä'I$ò$IùI$ü$ID~zâ]ºtiÓ¦Muuuñx¼¡¡aÇ7oÞÍ¾öÚkò[ã[wþ/¿ÍéÓ§×¯_ËÎÎÎ0SzWï¾ûn¸U¸´ªúýPøi,K&­­­o¼ñu«]_ÎwÅÃöG	æ³cÇ555=z4ÍOÃe?óÌ3×¯_Çò[tû¨#G¬^½zhh(ËEß'áÞÜÜ|àÀ¢-7oÞ¼qãÆðªVê÷FéÌåËW­ZÕÓÓc¹*[~Óï÷ò"?Í[£££y¥¯ðíß¿¿££üîóïÛ·oM^½z5JÎ#þÒ¥KïÜ¹³dÉèè¯j_ðÒrUüöG	æ³íÛ·÷ööÎGî¾¾¾ü§§NjiiÇãá2'OlY,×F¯ê:wî}[[Û÷f,Xù%K.Íx?avvvA¸,Ü¹¡þþþ°ÓÃJ&íííåÜü¢opÙÕÕUx®ÿÄ^®`ùå÷ùG	æ³ð-<!~à¹t:áÂ0ÃÃÃùÁêÕ«£óÂa³ð¸^zã­[·ær¹è0ý½Ù#V~¿üå/Ã÷ýé7Û²eËÑ£GïÝ?ïÆWåocÇ577[öJßåËW®7oÞOê&''ÃøÖ­[á)_éë@ªùå÷ùG	æ³X,6ã6ÁváqºPùÁá@þÙä7::Zæ½Ù#óx¸þ×;BÇ_²dI8²oÞ¼9ð.0.|¾èTïÄÄDû¢¾áÞ,uåÉ/ìåsçÎ­X±"ÿðàÁ;vÁÂUÖ°"åWºßË9Jü4Ïå_¥~è?¢Â¸®®îÉïÑîMäp_t¬÷^z)N[E¯ðE´··ç?]»vmá¯õuwwwvvöõõ?Þ.vùþ	¯_¿¾èW8öÙÝ»wïÚµËVüfÜï3%D~ç¯]»öY<«[¦üÊ¿7-@ù655ÕÓÓñçg¶nÝZôØ°mÛ¶üµwïÞÝ¸qcôH°dÉ¿üå/ñÊoÆ[ßÚÑÀªùÍþ(!òÓ<×ÝÝý°wáÊ?KOìãk~åß¸ü¢ÃzîÑ©ÞÂß¯¯¯/yollìÈ#Ë/·ì,¿L&³yóæ;wZÀj_ÑQBä§ùott´©©)<BÍ<yrß¾Ñxýúõ?ÆëÖ­dùoZPòK¥R¥¯ÒÕµ¶¶Fã°s63gÎy"ZòçwÂ³Ê0hooØ7*O~3%D~Zõôô,_¾<,<3^¿~ýÕW_ýÊW¾rçÎh¡¡¡ðï9ú)Ýááá0^~µµµW¯^^þ)Ú¦üÓßÂ#GÜºu+:tuuå×·oß^úw;ÂS0ÃÑÿèÑ£ÑK¿	®_ôû¼ÑïöF¿çëoÈoÆ£ÈO¥S§N§æÉd2îÛ·/Ï¾üãwô|+W®,|tüÞ|óÍÄýx°(óÞ4Çû½7¾_xáºººè7uttäß'âÃ>-=±fòïÞo³ð]î3ìýþþ~Ë^òôoåèýü*àPPþ!z£ÈO$Iä'I$ò$IùI$$IÈO$Iä'I$ò$IùI$ü$ID~$I"?I$$IÈO$ü$ID~$I"?I$$IÈO$Iä'I$ò¤'Qccc2,3ñx<Nçr¹ÏvT½ß,7+óN$ü$é3´wïÞ`¬7Þx£h¾§§'ÌïÙ³ç3UÉOùIÒÂìêÕ«ÁX+W®,onnóW®_òD~4÷µ··fågÎ?fÖ¬YÙ·o_*ÇãÉdò^+$Ú©S§,Yòì³Ï¢mútk[[[Ãøaò;úthØ,l¾V~þÖ­[Û¶m«««WÕ××ïÚµ«ô´µ$$ý££GfmÚ´)?³qãÆ0Ó×××[Í§[½zu!Ñb±X¸ììì,BÛ7,jxx¸T~###Ñýn¶~ýú¢Ø¹s§*ü$é¡MMM-]º4OÃeV]]]66H§ÓUï¾ûî½ÿ8;L&öÒK/år¹èÔp!Úf¼ánß/Â§¥òËd2Ñ«a<88Ý*º*áµk×Â8|0®­­µC%$MWwww`ÓþýûÃøÕW_ã]»vnh×ÖÖ½ÈWH´ë×¯ÿã¨úéµÓß0²fhll,|¸Yz'a²è½üf­­­áÓeËmß¾ýÄwîÜ±+%$ÍÐÅ#Bq¸ãüµõõõEü*$Zá;¿^;ã?u8®©Çã¥×Îóqtt4Â_^'O´7%$ÍÐ¿üå§_~9N^ýo¸ª¿¿bbbFÀågf¼at¢öÞýSÌÍ¯¶¶6ó§Kÿý÷÷îÝÛÙÙ6K§Óv¥$ò¤:xð`þÅ³ÞÞÞÂ«âñxô*`.óÍ7Ëß7ìèèæ»÷nWWWø4ÞIDº]»v;	,|èçü¢#ãD"aWJ"?I¡;wî$ÉèëíÛ·¯Z·n]áÉÖ¥KËèýY¦ß7hçO1ÞÉððpÑÙÞãÇGWE¿RØÖ­[íJIä'I3·mÛ¶¢·wÏd2D¢®®®»»ûÊ+ù÷O^~3Þ°¿¿¿µµ5¯¥¥åÜ¹s¼Ð©S§ÚÚÚb±X:>pà@~~rrr÷îÝ©T*beøwïÞµ%$IÈO$ü$ID~$I"?I$$IÈO$Iä'I$ò$IùI$ü$IÈÏH$$IÈO$Iä'I$ò$IùI$iNú¿ÿ»]Z£óºBIEND®B`


ONEWAY Figures BY Variables
  /POLYNOMIAL=1
  /STATISTICS DESCRIPTIVES HOMOGENEITY
  /MISSING ANALYSIS
  /POSTHOC=LSD ALPHA(0.05).


Oneway


Notes	
Output Created	12-SEP-2022 23:25:31	
Comments		
Input	Data	E:\桌面\Raw Data\4. C. Cellulosae ESAs and TPx Induced Th Subpopulation Differentiation\3. SPSS statistical analysis\3. IL-5\3. IL5-72h\3.1 SPSS statistical analysis--IL5--72h.sav	
	Active Dataset	DataSet1	
	Filter	<none>	
	Weight	<none>	
	Split File	<none>	
	N of Rows in Working Data File	20	
Missing Value Handling	Definition of Missing	User-defined missing values are treated as missing.	
	Cases Used	Statistics for each analysis are based on cases with no missing data for any variable in the analysis.	
Syntax	ONEWAY Figures BY Variables
  /POLYNOMIAL=1
  /STATISTICS DESCRIPTIVES HOMOGENEITY
  /MISSING ANALYSIS
  /POSTHOC=LSD ALPHA(0.05).	
Resources	Processor Time	00:00:00.02	
	Elapsed Time	00:00:00.01	


Descriptives	
Figures  	
	N	Mean	Std. Deviation	Std. Error	95% Confidence Interval for Mean			
					Lower Bound	Upper Bound			
Control	4	10.53225	.413033	.206516	9.87502	11.18948			
ESAs	4	11.33325	.302260	.151130	10.85229	11.81421			
TPx	4	9.90350	.282978	.141489	9.45322	10.35378			
LPS	4	15.57100	.301599	.150800	15.09109	16.05091			
Total	16	11.83500	2.307209	.576802	10.60558	13.06442			


Test of Homogeneity of Variances	
	Levene Statistic	df1	df2	Sig.	
Figures	Based on Mean	.165	3	12	.918	
	Based on Median	.157	3	12	.923	
	Based on Median and with adjusted df	.157	3	10.913	.923	
	Based on trimmed mean	.165	3	12	.918	


ANOVA	
Figures  	
	Sum of Squares	df	Mean Square	F		
Between Groups	(Combined)	78.549	3	26.183	241.878		
	Linear Term	Contrast	37.464	1	37.464	346.092		
		Deviation	41.085	2	20.543	189.772		
Within Groups	1.299	12	.108			
Total	79.848	15				


Post Hoc Tests


Multiple Comparisons	
Dependent Variable:   Figures  	
LSD  	
(I) Variables	(J) Variables	Mean Difference (I-J)	Std. Error	Sig.	95% Confidence Interval	
					Lower Bound	Upper Bound	
Control	ESAs	-.801000*	.232647	.005	-1.30789	-.29411	
	TPx	.628750*	.232647	.019	.12186	1.13564	
	LPS	-5.038750*	.232647	.000	-5.54564	-4.53186	
ESAs	Control	.801000*	.232647	.005	.29411	1.30789	
	TPx	1.429750*	.232647	.000	.92286	1.93664	
	LPS	-4.237750*	.232647	.000	-4.74464	-3.73086	
TPx	Control	-.628750*	.232647	.019	-1.13564	-.12186	
	ESAs	-1.429750*	.232647	.000	-1.93664	-.92286	
	LPS	-5.667500*	.232647	.000	-6.17439	-5.16061	
LPS	Control	5.038750*	.232647	.000	4.53186	5.54564	
	ESAs	4.237750*	.232647	.000	3.73086	4.74464	
	TPx	5.667500*	.232647	.000	5.16061	6.17439	

*. The mean difference is significant at the 0.05 level.	
