# Supplementary material for: Regulation of piglet T-cell immune responses by thioredoxin peroxidase from Cysticercus cellulosae excretory-secretory antigens
Source: Front Microbiol. 2022 Nov 18;13:1019810. doi: 10.3389/fmicb.2022.1019810 (PMC9718028; doi:10.3389/fmicb.2022.1019810)
Supplement: Supplementary file 3 [file Data_Sheet_3.ZIP › 4. C. Cellulosae ESAs and TPx Induced Th Subpopulation Differentiation/3. SPSS statistical analysis/4. IL-10/1. IL10-24h/1.3 (SPSS data export) SPSS statistical analysis--IL10--24h.doc]

EXAMINE VARIABLES=Figures BY Variables
  /PLOT BOXPLOT NPPLOT
  /COMPARE GROUPS
  /STATISTICS DESCRIPTIVES
  /CINTERVAL 95
  /MISSING LISTWISE
  /NOTOTAL.


Explore


Notes	
Output Created	12-SEP-2022 23:29:41	
Comments		
Input	Data	E:\桌面\Raw Data\4. C. Cellulosae ESAs and TPx Induced Th Subpopulation Differentiation\3. SPSS statistical analysis\4. IL-10\1. IL10-24h\1.1 SPSS statistical analysis--IL10--24h.sav	
	Active Dataset	DataSet1	
	Filter	<none>	
	Weight	<none>	
	Split File	<none>	
	N of Rows in Working Data File	20	
Missing Value Handling	Definition of Missing	User-defined missing values for dependent variables are treated as missing.	
	Cases Used	Statistics are based on cases with no missing values for any dependent variable or factor used.	
Syntax	EXAMINE VARIABLES=Figures BY Variables
  /PLOT BOXPLOT NPPLOT
  /COMPARE GROUPS
  /STATISTICS DESCRIPTIVES
  /CINTERVAL 95
  /MISSING LISTWISE
  /NOTOTAL.	
Resources	Processor Time	00:00:01.14	
	Elapsed Time	00:00:00.86	


[DataSet1] E:\桌面\Raw Data\4. C. Cellulosae ESAs and TPx Induced Th Subpopulation Differentiation\3. SPSS statistical analysis\4. IL-10\1. IL10-24h\1.1 SPSS statistical analysis--IL10--24h.sav


Variables


Case Processing Summary	
	Variables	Cases	
		Valid	Missing	Total	
		N	Percent	N	Percent	N	Percent	
Figures	Control	4	100.0%	0	0.0%	4	100.0%	
	ESAs	4	100.0%	0	0.0%	4	100.0%	
	TPx	4	100.0%	0	0.0%	4	100.0%	
	LPS	4	100.0%	0	0.0%	4	100.0%	


Descriptives	
	Variables	Statistic	Std. Error	
Figures	Control	Mean	29.94575	.833107	
		95% Confidence Interval for Mean	Lower Bound	27.29443		
			Upper Bound	32.59707		
		5% Trimmed Mean	29.96428		
		Median	30.11250		
		Variance	2.776		
		Std. Deviation	1.666214		
		Minimum	27.759		
		Maximum	31.799		
		Range	4.040		
		Interquartile Range	3.114		
		Skewness	-.584	1.014	
		Kurtosis	1.496	2.619	
	ESAs	Mean	39.28625	.641338	
		95% Confidence Interval for Mean	Lower Bound	37.24523		
			Upper Bound	41.32727		
		5% Trimmed Mean	39.26711		
		Median	39.11400		
		Variance	1.645		
		Std. Deviation	1.282676		
		Minimum	37.916		
		Maximum	41.001		
		Range	3.085		
		Interquartile Range	2.399		
		Skewness	.767	1.014	
		Kurtosis	1.505	2.619	
	TPx	Mean	32.99000	1.556909	
		95% Confidence Interval for Mean	Lower Bound	28.03522		
			Upper Bound	37.94478		
		5% Trimmed Mean	32.97983		
		Median	32.89850		
		Variance	9.696		
		Std. Deviation	3.113818		
		Minimum	29.271		
		Maximum	36.892		
		Range	7.621		
		Interquartile Range	5.758		
		Skewness	.176	1.014	
		Kurtosis	1.503	2.619	
	LPS	Mean	48.44700	1.817102	
		95% Confidence Interval for Mean	Lower Bound	42.66417		
			Upper Bound	54.22983		
		5% Trimmed Mean	48.34944		
		Median	47.56900		
		Variance	13.207		
		Std. Deviation	3.634204		
		Minimum	45.140		
		Maximum	53.510		
		Range	8.370		
		Interquartile Range	6.712		
		Skewness	1.226	1.014	
		Kurtosis	1.520	2.619	


Tests of Normality	
	Variables	Kolmogorov-Smirnova	Shapiro-Wilk	
		Statistic	df	Sig.	Statistic	df	Sig.	
Figures	Control	.250	4	.	.963	4	.800	
	ESAs	.250	4	.	.960	4	.781	
	TPx	.251	4	.	.953	4	.737	
	LPS	.251	4	.	.921	4	.544	

a. Lilliefors Significance Correction	


Figures


Normal Q-Q Plots


,CN(dAiÊRê"ô#Whî)=¹·tP D'FoÇér¢Nâ¯p½ pÀåM/iºewîÍÇ`$%4£ñRZñûoë¹~l?~ÿy¿¢'cÇù?~Å_?Ï3ï!¥Mó""""ðGDDDDàÀ?""""DDDDþüø#""""ðGDDDDàÀø#""""ðGDDDDàÀÍÁãùQa×Ïùté©S§êêêrU[[âÄÇÞàÔ¯2/¨ùúªªª×^mÓ××g6gðã_¹reÅA¾È-ÌPüÇø#¢$Àßüùó/¼ð>ûöírkÓºJ$]9rdº3ãa,))	|_ÅþüQ,%­_¿>)ð×××§´úÆoøýþÉÉIYOeåÀÀ@ØîUB¾õØØØ®]»d,nøSW¼ÿ¾,OLL$Ô?"JzüY­VùxêÔ©HÂøøã7mÚ¤æLÜnwÈW^ºt)??åÊ5]]]ÕÕÕ¡®®îîÝ»W®ªªÊÊÊZ½zuð«(..Vóò-E"Î3Ï<#ë'a¥YÙÔÔögîU´ßZ¼(käNNkpÂ¾2ÜÌ®!ÊÿKee¥û5kdl£¿F¨½4ø¿Ur8rk2ò?+®(HOËE;vìðz½<¸À%.þÆÆÆJJJdAë;wîÈ3z0Gä+C!7lØð0Ü¼ä+Ä7O"ù|åªU«¢gáÂ²þÞ½Á+²²¬¬,ìÏ8Ý«|kzå¯¼¼|ZÝp3¾âÔñ×ßß|;òÃNÁÿ­êÓ@ê×¯_r·oßÎüQââOÎ=üJX°¶nÝ*ËëÖ­»ÿ(YOeeðWîÝ»wrrR½v¥ÖlÛ¶Íï÷www«OååÓK.É²@P]W½³íÂ²|óæÍà"Ç`0]|ÝY^%Òê¯NwpÂÞ«_1ÊþB®(håññqahSÇ_ð«Ü¬¹|ù²,«ÿÖ-[¶¨/V(¼û¶,Ëÿ¦,çææòà"DÐøä¹åy=dQQ,ß¹sG*Ïñòéo!pi`#øÓÑÑÑ¿?6ÇÄÄpAQSS£^jz,þ²²²Âþ a×Ïà*Á¨ëcÌf³Ãá;hS°÷jÆW:þÔûïÞ½ü-¦¿àÿVukÁÉuÑ%KäÓE	^å¯ðÈ"DøÊS¸Ïç^¯åLû8Q]ØÛþ©@3dêó±­«yj­ð	¹µ©Ò<vÐ¦28H:³+NÚw*¼~©ö¿5ä88ê"Ë¥ü@áùóçyp?"JtüIo¾ù¦|80Z©|ò¬!¢ZYY©¹ÒÕÕ%8þÔËê¨+;vì¸ÿþÑ£Geå¦MÂâo*W1þ¦;8f|Å©ão*¯üx'ô>þ¹¹¹²&d×ãà®^½ºÿ~5×Ø3À%4þ>zó~àýjÚxÝºucRÏîÛ¶m=þÔ«_B×^m*øs:êµ%ñbww·zUOuöìÙ°?ãt¯2-üE5ÞGÜÈtGuøSïÒû>xð@l·víZ->,/@GuÎòõ2²]ücª÷nº(SðDþ(áð7<<xwZãv»CÞïUTTØyv6øpDµ`Áù¼ÇkØûüì³Ïj§ -[åÇÖU¦¿è8VsÈg0ª3ÀßÕ«WoåÊÁp-:þÔáÃî£^[î¹çãÁEþ(9ð'©)Ñàõ~øá¦M²ÕÐÐ Ý½cfø[Û=ÿüóâ¡ÀQB¢§½½½ªª*##cáÂ¯¿þúSO=xåiöWþ¢ÎÙ³gÅ!p¨¼à¦5ª3ÀtþüùEÉO-·¯&Ö;8Ë§B4õÝÍfsooïc_y½téRMMÜüP6-°ÞëõîÞ½[½¤*ÿJÏÇüéÕÄÄÄ³Ï>;­cËÍà*IU»oûý~eúÊÊJ~aü¥`ê=!½üòË?"¢ÌëõîÚµkáÂjnW^xáÀ?""""DDDDàÀ?""""DDDDþüø#""""ðGDDDDàÀ?""""ðGD¥³gÏFÁ°jÕªé^·ªªjÞ¼y.+°FeMuuõ46XÙ×Låº3ntttÛ¶mùùù28.Ü½·×ëÓFÀ¥uÅÅÅâßïîu8 ×=tèP`Í+¯¼"köïßìøç=êæÍÇo´víÚÜT__ÙlDþ(1¶³pÆÕ«Wåºuuu5+V¬5ýýýñ¹ú!I¹öÔ©Ss2ÂàÀé(¿`jìÞ½þüù¹¹¹ûöíù²§zª¦¦&ä/Êò½÷dYÖ¨nß¾m6srrÃ%K._¾öÖ¿ô«¼þúëF£±¶¶V¾IãããE®+w~×®]êLáéÊ+åÖd½Üò;w¦82ÕÕÕrË>/ì¥QFÉétÊA8ö¬vµ#å¦øý$"ðGDzùO-¿ôÒK²l³ÙY²ðòË/Í¥KòÛºu«ôèQY²ÜÔÔ¤.Z¶lÙdáæÍ²¾¤¤$ì­ßèWÒ½ùæ²`µZµw~ûöí²,_púôiY8pà¬,--å±±±YP/LjÓx1¿¢ÒáÃÕ÷jïdÈÏý¦øå$"ðGDúâO¼"ËÒÚ+ìN§nÝ:Y^¿~½MàÒ«W¯îÝ»·¦¦FÖgdd½µèD¿ºcóçÏ×^·¨¨HÝùÉÉIYöÉJùJY^²dè*ÒËxa¿è£ôàÁµ,·	=úMñËIDàôÅ_°x"ñ%$Vnn®|¥×ëU³«²F]ôÚk¯ÉµD×¯_×(ìÊU"Ý1bðy§NÊÏÏWkÕSyå¯¼¼GwatäE_Ù?"þJJJ_R/=Ö"E.|¬ÏÊÊ5"þ¢_%pÇÄsÚë.X°@]7äîùýþ.X­ÖàÛÞ½åëÚ¦2JSÄßÌüQlð§vq=zô¨zÚ+¯¼2:u*ðÚo¾"7n¨÷NÑ¯" ë­·daëÖ­Úë>÷Üs²|üøñÁÁA5Õ++kkkeùúõëwïÞòòò)ËØØP¬¬¬ìöíÛJõä¦8JÁËjêYM:üì3p""ðGD±Áßï`å>*øXÑ-òàÁ5å*ÅLõçÏ/,,úìÚµkø~Ã!®X±"°ßIð×x½Þ;vÈ=ÏÊÊZ»v­Ú±WÌ×ÐÐ v®««ûðÃ§>2ò]jR[¸oß>õ~¾©RðòÙ³gå.©chüì3p""ðGDDDþüø#""""ðGDDDDàÀ_úáxëÖ­8ÓýìgÿñÿÁïNÝ¹sç¿ü%ã S·oßVÇ!=ºyóæ´NGÓÊívÿ×ýã S?ýéOÿû¿ÿqÐ©>úüÅ¬ï|ç;â¿8Óë×¯ÿÛ¿ý¿Ê:õÿðwïÞetêý÷ßÿ÷ÿwÆA§þþïÿ¿õë?øºè×¥K°µ~?þþçÀø#ðþÀ?ðþÀøàü?àü?ðþü?ðþÀøàüøàÀøàüøàÀøàüøàÀøàü?àü?ðþÀøþÀø#ðþÀøþÀø#ðþü?àüøþÀø#ðþÀøþÀø#ðþÀøþÀø#ðþÀøàÀø#ðþÀøàÀøþÀ_â¯··wÉ%¡ººº¿¿ü?àüø©¿²²2yº'Nkñ×ÕÕ5ßD'?ýéOÇIzzz>ùäÆA§ä%ùÓqÐ)§Óù³ýqÐ)ÃñóÿqÐ©/þâ¿`tJð'Æù&1þËÉÉÑâïðáÃïÅ7ùóH¶Aï>ÉHþ¾gtêÂ¯~½ýöÛ/_ftJ>ÿîïþqÐoxå¯ÆA§Î;ÿoøëëëkjjbÚi_bÚi_¦i_¦SyÚW5>>ÞÐÐàõzÁø#ðþÀ?ðâø¶Z­aAþÀ?ðþü¿ÂÓézõèèhØKÁø#ðþÀ?ðRø3óàÀøþÀ_*ã/zàüøàÀøàüøàÀøàü?ðþÀ?ðþÀøþÀøàü?ðþü?ðGàü?ðþü?ðGàüøþÀø#ðþü?ðGàü?ðþü?ðGàü?ðþü?ðGàü?ðþÀ?ðGàü?ðþÀ?ðþü?ðþÀ?ðþü?ðþÀ?ðþü?ðþÀø#ðþü?ðþÀø#ðþÀ?ðþÀø#ðþÀ?ðGàüøàÀø#ðþÀ?ðGàüøàÀøàüøàÀøàü?àÀøàü?àüøàü?àüøàü?àüøàü?ðGàüøàü?ðGàü?àü?ðGàü?àÀø#ðþÀ?ðGàü?àÀø#ðþÀ?ðþÀø#ðþÀ?ðþÀøþÀ?ðþÀøþÀø#ðþÀøþÀø#ðþÀøþÀø#ðþÀøàÀø#ðþÀøàÀøþÀøàÀøþÀ¿ÊàüøàÀø#ðþÀ?ðGàüøàÀøàüøàÀøàü?ðþÀ?ðþÀøþÀø#ðþÀøþÀø#ðþÀøþÀø£9Àßïg<Áø#ðþÀ¿ÇÏn·WTT:tñàÀøþRn·»¥¥¥°°pÞ£yñü?àüøKAüX,ÌÌÌy¿ÜCþÀ?ðþü¥þ|>ß±cÇjkkµì«««ëììä?ðþü?ðGà/ðçñx<h4CÌ'´X,×®]c$Áø#ðþÀ¿TÀßààà=BØWXX¸ÿþ[·n1àüøàÀ_*àO.ª­­§iùòåÇóz½øþÀø#ðôøóx<b;É¤e_ü|ÁøàÀø¤þÜnwSSS^^^ùdMss³Ëåb¸Àø#ðþü¿TÀßµk×6oÞ¬Ý·¢¢¢££Ããñ0PàüøþÀ_ÒçóùþüÏÿ¼¼¼Ã[WWwæÌÝþÀøàÀøKÜnwkkkàä²³³eÌ?ðþÀø#ðþR!yör£ÑxðàA6àü?ðþü¿TÈï÷Ûíö°n©ªªáàü?ðGàü¥BCCCò¤öä7o>xðà¯~õ+F	ü?ðþÀ?ðÛU«Õªá-((§QAáÃiÛÀø#ðþü¿Ëï÷wvvÍfíoEEÝn÷ù|/àü?ðþü¿dÍãñ´µµiOÎéäàü?ðþÀ?ð|¹æææíokk«ÛítEðþÀøàÀøKü~¿Ãá;Ãk2l6ÛcOÎþÀøàüøI¨Nl§áÄ"Â)ºü?ðþÀø#ðþ:uríovv¶Õju¹º5ðþÀøàÀøKÐä¯¾¾^èÉÔÖÖöØ^ðþÀøàÀøK|>Ýn¯¨¨;ÃÛÙÙ9s?ðþÀøþÀ_¢¤NÎ¡áÍÌÌ´Z­²Ùý·àü?ðþü¿¹o`` ±±Q;Ãk4'çIàü?ðþÀ?ð795kÖhÙW[[k·Ûg3ÃþÀøàüøÏç;vìXØsX,ýòÀøàü?à/®X­ÖÂÂÂöÉè'çà/ñ7::j4Áø#ðþÀ_êý ¿ÿÌ3agxKKK<8³C·?ðÄøëîî®¬¬ÇøþÀøK¥ÈëõF:9G]]p!æoìà/9ð·víZËo½õÖÍø&Ü¼víÚMÒ§÷Þï>`têòåË7nÜ`têÝwßýçþgÆA§Þyçy:HåG?úÑ7¾ñOúÓ!æËÈÈhhhp8ñ¿K.ù~ÍtêÜ¹s|òI¿iãï×w12þ^ýõoN§³··÷ID'NÉJ?ã S]]]W¯^etêâÅ×¯_OöB³¾üå/gee°¯  @8(?ÌÕûí·åo~Ítêüùóñÿ¦©?¦ö%¦öeÚ7ÁSûð=9Ç5k.]º$_0·÷i_¦ÁøàÀøACCC­­­Úx333¯]» ÷ü?ðþÀø#ðþfUOOÅbÉÎÎÖÎðîß¿?'çàü?ðGàü¿9Ëï÷wvvÖÖÖjgxM&ÍfÏ¡[ÀøKzüEü?àü%H###ò¬¤áÍÎÎnll[<ÝþÀøàÀøzuíÚ5«Õöä6ÃþÀøàüø3IÍðÍæ°3¼Çóz½É2¼àü?ðþÀ?ð1ÇÓÖÖ¦=9GffææÍR<ÃþÀøàüøSÍív777hgx÷ïßëÖ­xÞ¯×ët:Åm³Yàü?ðþü¿ßJnêëë333µ3¼ñá=sæ´¦¦æsû ~þ_þå_?ðþÀø#ðþÀßlóù|v»=ìÉ9Ìf³ÃáÞ|­ñkû¾½Oþµlo1ÂAðþÀøþÀøaCCCò,£áÍÎÎ¶Z­.kG£¹¹Yè©ä§þmÜ°QÖ?ðþÀø#ðþÀßL6DÂ;í¯ÑhçD8JsýW-_Æ_ó7ÿàþü?ðþÀ?ðþ¦ZC·ÔÖÖÚíöÄÙ·¹¹yõWãïO6ý	¯ü?ðþÀ?ðþ¦TC·X,ø?Ñ<¶°ïù;ú4øàü?àüE+Ò¡[dMkk«°¢öö]ºtéòåËìíþÀøþÀøVC·Øl¶DxcßcSÇùXÌþøàü?ðþÀ¿ÔÄ_bºeÎàü?ðþü¥þùÐ-àü%þæ=®ðGàü?[ü%þ¡[ÀøKüe<.Áþü?ðGs?Ã±fÍÄ?tøI¿9ü?àüióz½ItèðþRccc[·nþÀø£¸áÏív·´´$ã¡[ÀøK>üFÁÀþü?ðGs?§Ó¹yóæ¤>tøÉ¿êêjí;*½^/ø#ðþÀé?ÏwòäÉÅ=t7ö?ð§þ²²²äa6::Z\,b¾·ÞzKÀ?ðþHüìß¿?ì¡[ZZZ4ðþtÄz¼ÉhOnÞ¼999)¹¹¹àÀø[üÉÂb±ò´nioogü¿xà/??_uÝÝÝ.K^xáµÀ¡^ü?ðG±ÂßøøøÉ'ëêê´3¼kÖ¬çWfxÁøþvíÚØ½#ä@JàÀø4ËFFF~ÿ÷_»ossóÀÀCþÀ_¼ñ'½øâ,ÞÞ^YÖÔÔè×Áø#ðþR»ÁÁAaö¥¥¥mmmBü¿9ÃßþÀ?ðÂ1ÍÚÞ¥K<y^ðþÀøþÀ_*äõzm6[EEöÀ±°ü¿DÁ_yy¹:ày&ðþÀÍìávÞïï38·/?ð§þÊÊÊÁ½	ü?ðGÑóûýgÎ	»oðï´ÎíKàüé?q<Jûúú&''ãy×Áø#ðþ7ÇÓÖÖf2´Gi^³fÓéáàü%þåágù?ðGàü%in·»¹¹Y»o^^^KKËÐÐö*àü¿ÄÂ_¿<h·oß>>>þü?ðGvfffû/^|ðàÁ('çàü%þ¤jß®ÁþÀø#ÉçóÙíví>¼Ùláàü%þ-ZÄþÀøÚd[­áÍÎÎ¶Z­.k·þÀøK,ü©GrüO°þÀ?ðÈgáv×h4ÊÖ;Ê/øà/	ðWTTÄþÀøº¥³³3ìÉ9jkkívûÌÒþÀøK,ü9NyTïÚµËçó?àü¥gÝi±Xf¹¹àü%þæE>ü?ðE:t¬immKgÿ-Àø¿±Ã?ðþR»Hn1L6mºoìàü%þæ*ðþü¿9)ú¡[ÇÌÞØþÀøKüÆE?àü¥v±:tøà/¹ñg0äÿ»þÀ?ðÏgÝþÀøKnü]¾|Yÿû÷ïç_Àø#ðþôN§C·?ðþìíKàüQêáÏãñÈfÖh4êqèðþÀ_rã½	ü?J%ü¹«Õ­ß¡[ÀøÉ¿¹ü?à/ùý~Ãv7ænàü?ðGàü¿9+ÒÉ9¤ºº:=ÝþÀøKzüMLL¬]»6''G¶¹¹¹6lÃàüø³,ÒÉ9233u=tøà/¹ñ'È°;|èª_ðþü¿éä¥¥¥²uM´_ðþÀ_bá¯²²R¶ëÖ­OÇÆÆÖ¯_/k-[þü?ðPE99Ç5kN<9ç3¼àü¿$À_VVl5·²FÖ?àü%Hn·»µµ5ìÉ9¯]»Èwü?ðXøËÈÈÍ/ø/KYÃ¡^ü?ðÉ¦Òb±hgxÛÚÚFFFxÁø?5í»zõj5í+eYÖ,Y²üøào®òûýv»½¶¶V;Ã»téÒ'Oz½Þd^ðþÀ_báO´vû÷ï?àüÍ>QÚ3g:$§r°½¡¡¡H'çØ¸q£ÓéLÌ7ö?ðþíð»aÃùóçgddÈÇÕ«WÇáQþÀ¿tÀßÈÈHiiég?ûYóÌÕÕÕO<ñÄàà`Õj;Ã»gÏAa/øà/áð7'?ðGà/ð÷ÇüÇuuuû¾½OýûÊW¾òÏ|&dÆÖï÷wvv=9ÇâÅívÒ½ÔþÀøàüøKGüÉYQQÑÞ=øeee===/rÌÌLÅât:ScxÁø	¿y+##üøào6TVTËOþnùç:;;å"«Õ²í5­­­n·;ü?ðøËø#ðþÀ_Lòx<ùùù;ßßîoí^°`Á²eË´oì3LJ¢xÁøI¿H=ÿüój3tâÄ	ðGàü¿Y¶mÛ¶²²2ñüûbÝÕqõC2Íòìoìàü%þúûûsssÕf(øÏàÀø3NH÷ÔSOÀ¼J ææfËòÃþÀøKDümÚ´Im.»þÀ¿ÇÃáØ¸q£ö¥>ÉÔÖÖ6cþ?à/öø;ú´Ú­[·.wü?©?Ïg³Ù/_v·³³3gxÁø	?y(.[¶LíÛqùòå8ßuðþü¥þäÿhÏ=!æËÎÎ¶Z­òIÏáàü%þ>¬6I[¶l»þÀ¿TÂ_OOOØsFÙÜ¥Ï/øà/qñÇqþü?=þü~¿Ýn_ºt©v+Z[['çàü¥ÅqþTüøà/R###mmmaOÎÿíøþ=ðþü%)þ"£   õNÎþÀøàÀøKSüù|>y0ÍÚ7öUTTØívùFü?ðþÀø#ðôøóx<mmmF£Q;Ã[__Ï/øàü?ðGà/Eðçv»µnQ'ç`ü?ðþÀøà/Eð'¨úúzí¯Éd²Ùli~èðþÀøþÀ_àÏçóÙíö°'çp8ºü?ð¬øã8þÀãï£>íR¤s¸F	ü?ðÜø>¤_Xüq?à/~u×®]ËÉ9Àø£Ç_ ®®.ÙÆ=óÌ3ãããò©|~½¬¹råø#ðþR8¿ßßÙÙi699ø^øS§ÞÆMLLÈâââYÞrIIÁ`¨ªªº|ù2øþ$uèÉÄÉ9Àø£tÄÚä	øBð7û÷ü544?~9²eËðþüÍyÝ·k×.ÝþÀ¥þdÃ'PSL¯×»nÝ:Y#ëgyËJF£Q?Ív5¾É¨»»û*éÓ»ï¾û£ýqÐ©®®®ÆaÆÉç_øö]ÎO>ùdkkë©S§~üã3J:uáÂùãqÐ)ÑI?ã SçÎÿ7ÕW®»ÃÇõë×gyËÁ»hwü>z(¾½ÿþûC¤O?üár:ôã0Ý>ùäï~÷»Ú^I,xòäÉ[·nÉÉ_ü1Ã¥Sò§üG0:uñâÅÛ·o3:%¶¾sçN¿©îønÞ¼YVV#çææ.Y²äÞ½³¿Ùàã¬¬,¦ö%¦ãl@§~è©ÛöeÚißöÕ¯¢¢"õVBù¨DàÀ~¿Â»iºü?ðGà/Y,×_]äcCCøþtm6nàüQzáïÆååå¹¹¹j¢¶¨¨èÔ©S³¿Ùîîîââb¹ÍÞÞ^ðþüéÔìÝþÀø£4Â:Èsð)ÝÔò#Gt½ëàüøaÝ"kZ[[§~èðþÀ¥þeCyãÆþzeyþüùàÀøKØzzz6nÜ¨cÉd²ÙlÓ='øàÒjs©þ&''9·/?ðùýþcÇ-^¼XûÆ>³Ùìp8fvN6ðþÀ¥þÔAÕ«¿Ý»w«ÝâÀ?ð8îÜ¹SMV<öÐ-àü?s:aò,Ï4àÀøK"ÍðF9tøàÀ_´ä	»¦¦FííS^^?ðGà/z>Ïn·WTTá'¼Íð?ðþüÍMàüøÔÐÐÐÁKKKCÌ×ÒÒ2Ë^ðþÀ¥;þûyºwï^yy¹lvÁ?ðçzzzy!ì-R[[×ëÕéû?ðþ(­ñ711ÁÞ¾þÀ_<óûýgÎ©­­rÎÎNýØþÀø£tÁv>%¤üü|ðGàüéÝÈÈÈ¡CÂîÃÛØØ800»þÀø£ÔÇßíÛ·38·Gp"¿Ó§O?àO¿WKK OrÙ>ÅóÎ?ðþ(õñH¨§÷/øþB«êëëµì[¼x±Ífóù|ñ¿Kàü?J#üÍUàüQºáÏãñ´··L¦óeffnÜ¸±§§'nÛí>vìXGGGÈöü?ðGi¿òòòÜÜÜÀ¼ôÒKàÀøUöì)((Û¡[ÂöÝï~÷ÓþôÊ+¿ô¥/=ñÄO?ýtàUFðþÀ¥þ.²Ã¯Ú(ïß¿üø³Éï÷;1öä6MïxÎïþîïnýÆÖßÞ'ÿvkwEyÅ®]»ÀøvøËÊÊqð.uýýý²fþüùàÀøYGl§áêëëecÿ»ôÍo~S¾µú'|òÉ'ÁøvøS;üNNNþÖíjþþü¿©äv»[[[µ3¼q>t6ßþéãOþêSàüQÚá¯¤¤D¶Ë;vìPïÇç¾ûdMqq1ø#ðþ¦<¢-v×h4:thdddnïÞ·¾õ­+WËO,øÙÏ~ü?ðGi¿¾¾¾°yîííþÀßc¿ívEEv3b6;;;ã°ïTºuëVaaáº¯¬Ûý­Ý"¿¯Z¾ú¿÷r÷Àøvø«ªªrrr222rss+++eÞwü?JvüÉY;ÃiµZå÷!ÑÆÐårá_ÈÏÏ>ñÄÁo=àüQzáoNàò-¼ÓÎðÎÉÉ9¦Ïçóx<!+ÁøþÀ?ðßïïìì4ÍÚÞÚÚZ»Ý>''çIàü?J/üÝ¸qCçYíá[TTtêÔ)ðGàüòx<ò5Ú^ÅÿÇ2øþÀßÌëêêlÇþÔò#GÀ?ðçr¹¬V«ö<¼­­­n·;5ü?ðGi¿ââbÙß¸q#¿ÞÞ^òLà/Íñ§NÎv×d2Ùl6íÛæÀ?ðþjkþ0èÀÎ²l0À¿4Ä¨®­­MrÌÌÌúúzaºü?ào©£ú)üMLLìÞ½[üøK+ü¹Ýîææfí¡[d¬O^ðþÀ¥;þNgØ<Ë3ø#ð&øc½öÐ-&©­­-ÅfxÁøîøä	»¦¦FííS^^?ñÁø£9Ç×ëµÙlrðþü¿ãoNàæNÎmµZ].Wº/øàÀø#ðøÿ&Å¢=tÑhGe:Ìð?ðþü=üøã-Z%O999UUU£££àÀ_*áÏï÷Ë&ìé§Ö¾±O#fxÁøîøs8awøþR^¯·½½½´´Tû0¯¯¯w:-øàÒê Ïò!oÙ²EÖ?I¿íûòòò¬Vkjºü?ðGà/òM<*xÆG|þ===uuuÚúä½49tøàÀ_ÄÔ+5òå?É?Ïg³Ù.]ªeXðÌ3ò$øàÒê=êyÿþõïù#ðDø:xð ö7o¾víþÀø#ð÷xÍÿ?ðG1Á_OOÕjÕîÃ·sçÎøüøà/ðñ¸ø#ðhøóù|v»Ýl6kÿ`«¨¨8yò$3¼àü?ø43üy<£Ñ¨eßÓO?ít:Óüàü?)ÒñÁ¿Âß|°gÏ¼¼<í9Ùdü?àoF71oÞ/¾²²±±C½øKäÁòùÏ^ûÆ¾CñÆ>ðþÀãþ¦ OBîÝ»'¾ñÆêIE§·ú?ðGSÏãñØl6É¤á5ÍÌð?ðGàüÍ¤ÆÆFõtRRR¢Ö¯_¯÷]à¢äv»[[[ÃC°===øþÀß¬ºråJàÙ¥££#wü?<.,v7??ûöí###øþÀßlÛ¶m[àÔOjágþâ:tKmm­vwéÒ¥rÑï½ÇûÀø#ðþb?Á Ï.óçÏ¿zõêÃ ÷üeee?qhhhHÚÞÌÌL«Õ*£ª¾lZçö%ðþÀ¿È71oÞÞ½CVÊSûøÃ=9@P Âà/àüøu=ÎÞÏâàü¥m~¿¿³³3ÒÉ9ìvØs?ðþü¿áü¿äÊãñ´µµiÝi±X¢?"ÀøþÀß¬ð'Ï7Á»Ñ?þfËå²Z­ÙÙÙÚÞÖÖV·ÛýØ[àüøàü¿DÏï÷;°3¼&Éf³y<)ÞøàÀøàü%nÑOÎ!"îÉ9ÀøþÀøà/s»ÝÍÍÍÚC·ÈYïr¹fv³àü?àü?ðXÉ¯t½öÐ-&©­­mê3¼àü?àü?ð¸©sTTTáíììî/øàÀøàü%bNÎ|rþÀø#ðþf¿è?QºvíZØsFùÝå/øàÀø=þ2Á`þBR'çX¾|¹öï¥ÚÚZ»Ý^ðþÀ?ðüÍyàü%W§½½½´´t'çàü?ðþÀøI:9G^^^û[ZZ¦rrðþÀøàü?ð95kÖhgx+**ÚÛÛ½^o<ïøàÀøào&y<o~ó/½ôÒàà øÓæóùdÂº¥®®N6ú½±ü?ðGàü?ðËxâ?ú£?úÊW¾òÅº/æçç?~üÚ¹sg¤C·Ämü?ðGàü?ðJKK×þ¿µû¾½Oýûºõëyyy±2MRãOîüÆÃºeÏ=###s~ÁøþÀøÓK÷Ï|fï½üÉ¿Ï¯ø|GGGÚâOºeéÒ¥Ú^YyòäIÏ wü?ðGàü?ð7½WEyE°üäßªU«ÚÛÛÓCCC4ZömÜ¸q`` Ñî0øàÀøàozù|¾¼¼¼¦?kÈo÷·v?ñÄN§3­ð'÷3ì¡[dÍÎ;aü?ðGàü?ðl6ÛïüÎï|ÕòUßÖol­(¯xúé§cµïjãO~Ì3gÎD:tÝn×ãlàüøàü¿9NTYYù©OêÉ'ÜµkWV°ø±££CrIì+¿srèðþÀ?ðþÀ_røS3¼ÙÙÙÚs²555%àûÀøþÀøàoÚ©xÍf³ö¥¾ÒÒÒ9tøàÀøàüÍ6ÇÓÖÖf2´ì[¾|¹ÝnO^ðþÀ?ðþÀøLn·»¹¹YrÌÌÌ$zcøàÀøàü=&ùEª¯¯×C (¿fCCC©1¼àü?àü¿´ÆÏç³ÛíÝ8'çàü?ðþÀø3ohhH~ÂÎðÖ××Çÿ÷ü?àü¿_7::j4Áøá·°Z­agx[[[Ýnw/øàÀøKtüuwwWVVÊ3ø³,Ê¡[L&ÍfKðs?ðþÀøi¿µk×º(øû«¿ú+G|»páÂ;ï¼ã äÞ¿ýÛ¿ýú×¿^RR¢eßþá<x0þCß~ûí®®.~ÍÞdL>ß÷]ÆáMÆÎ;ÿoÄøûõ]?ù[ðWñí'?ùÛíþéSooïíÛ·cukMMMùùù!æËÎÎþÚ×¾600nÃÛÝÝ=<<Ì¯N9ÎÿüçNÉÙ/~ñÆA§.^¼øË_þqÐ)±õâüMSLû2í¶Hn1Lmmmé0ÃË´/Ó¾LûÓ¾Lû&þÏÓàüM·(n1ÍÉ~fðþÀ?ðøËAðþ¢åÐ-V«Un±àüøàü¥þäwÀb±hgxFc*ü?ðþÀøK#üEü¥3þü~¿Ýn¯­­ÕÎðÊJ¹(ÍgxÁøþÀø)?Ç#ÿõF£Q;Ãk±XRõäàü?ðþÀøi?urììì4<9øàü?ðþÀ_ºà/ÊÉ9/^ÜÞÞîóù:ðþÀ?ðþÀ_ÒãÏãñ´µµL¦°n­oìàüøàü¥þÜnwss³öÐ-ÙÙÙMMMÌð?ðþü?ðþR¡ÞÞÞïÿûNÎÑÞÞ>22Â(?ðþü?ðþ>Çc³Ù´ûðJuuu^ðþÀãþÀø©Ûínmmræææk×®1Dàüøàü¿T(úÉ9`øþÀøà/òù|v»½¢¢B;Ã[VVÖÞÞîõz%ðþü?ðþÀ_Ò744$ÿkagxÕÉ9fpn_àüøàü%äÚÞÂÂÂÀ¡[ÀøþÀøà/óûýgÎ©««ÓÎð.]ºôØ±c!3¼àü?àü?ðê:::JKKµìúé§GØk?ðþü?ðþÀ_år¹ZZZ´oì5²þÖ­[Q®þÀø#ðþÀøISOOÏæÍÃãÐ¡Cç±·þÀø#ðþÀøßï?yò¤O;Ãk6§urðþÀ?ðþÀøKÜFFFZ[[µçdËÎÎ¶Z­.kº7þÀø#ðþÀø(­©©IöäSáàüøàü¿äØFÍfíomm­Ýnú/øàÀøàü%nÝ89GL¾øàÀøàüÍq;wîèÖÖÖÀÉ9Àøàü?à/¹rèÍ6ã7ö?ðþü?ðþÀ_å÷ûívEEÅìÝþÀø#ðþÀøÛÈÈÈ=cuèðþÀ?ðþÀøKÄzzzZZZòòòbèðþÀ?ðþÀøK Ôomm­Nnàüøàü¿hhhHPrØºü?ðGàü?ðþæ8ÕjÕîÃ«Ç¡[ÀøþÀøàoÎr8õõõÚÞÝþÀø#ðþÀøñÎçóutthÝ)_ÊÄ¹«àü?àü?ð7ó÷ìÙöä;wî¼uëV¢ÝaðþÀ?ðþÀøI#ÒÉ9:°àüøàü¿i÷Ì3q>tøàÀøàüéR¤Þìììææf¹4)þOÁøþÀøàï1Eá---çÉ9Àøàü?àOÇü~ÿÉ'ÃÃl6ÛívÏtÿ§àü?àü?ð<ËÏ^XX¨áµZ­×®]KÞÿSðþÀ?ðþÀøû¿zzzÂÃh4¶¶¶$û#ü?ðGàü?ðþ~ýÈ4ÍÚÞºº:»Ýàûð?ðþü?ðþÀßôáÌðnÜ¸±§§'Å!àü?àü?ð÷¿ùýþâââÀïÐÐPJ>BÀøþÀøàï×TÊÌð?ðþü?ðþÀ_ºþÀø#ðþÀøàÀøàü?ðþÀ?ðþü?ðþÀ?ðþü?ðþÀ?ðþü?ðþÀø#ðþÀøàü?ðGàü?àü?ðGàü?àÀø#ðþÀ?ðGàü?àÀø#ðþÀ?ðþÀø#ðþÀ?ðþÀøþÀ?ðþÀøþÀø#ðþÀøþÀø#ðþÀøþÀø#ðþÀøàÀø#ðþÀøàÀøþÀøàÀøþÀ?ðGàü?àÀøþÀ?ðGàü?àü?ðGàü?àü?ðþü?àü?ðþü?ðGàü?ðþü?ðGàü?ðþü?ðGàü?ðþÀ?ðGàü?ðþÀ?ðþü?ðþÀ?ðþü?àÀøþÀ?ðþü?àÀøþÀø­··wÉ%¡ººº¿¿ü?àüø©¿²²2yº'Nkñ§6ñLêv»>	÷oÝºÅ8èü±444Ä8èÓé¼wïã SãþýûN]¼xq||qÐ)ÁßþçÆù&1þËÉÉÑâï¯ÿú¯ßäòÎ;ïüôéÂ]]]~Ãûî»ï2:õöÛo3¼ú%Oâ?ÆáMÆÎ;ÿoøëëëkjjbÚi_bÚi_¦i_¦SyÚW5>>ÞÐÐàõzÁø#ðþÀ?ðjø÷Ô§ÃÃÃV«5,Àø#ðþÀ?ðôøÎét®^½ztt4ì¥àüøàÀøK)üÆyA?ðGàü?à/ñ=ðþü?ðGàü?ðþü?ðGàü?ðþÀøàÀøàü?àü?ðþÀøþÀø#ðþÀøþÀø#ðþü?àüøþÀø#ðþÀøþÀø#ðþÀøþÀø#ðþÀøàÀø#ðþÀøàÀøþÀ_ºáï/þâ/:;;oÅ·/þøÇ?¾EútîÜ¹üä'N>ú>`têûßÿþ?ýÓ?1:õ½ïï£>btêoþæoÜn7ã SGý×ý×8SÇø»qãÆ¾û¾CDDDDAEÇë±DDDDéø#"""DDDDþüø#""""ðGDDDDà/Aëíí]²dÁ`¨®®îïïÿß!*##!íð:ÎÊÊJYSUUÕÝÝÍÅvx?üðÃ¬¬¬M61D³éÆË-þ]A.))Qk._¾ÌÅvx¥ÑÑQ£ÑÈàè1¼ÚÍÅpxÃþ>¿Ä­¬¬ìý÷ß'N_tüøñ#G0D±ÞÂÂÂÛ·oË|d+óá­lâeapppÛ¶mÑløÙ³g>:oüÞÊBCCldA¶[¶lab;¼ò)Ê_ÝÃåÉf?¼Ú5à/iÊÉÉ	,ß¼ysÕªUIÌwÑ¢EÃÃÃ² eaíðÊß5ùùùKLºtéRuuµúÓerrR&&&øÓ%æÃ»víZËþtÞHOvÛá;àà/qëëëkjj|ºaÃYÃ°Ä|xûûûe»#ÛwùÈÇ|xe£sãÆYxã7!H3Ëï÷Ï?_~]O8bk7æÃûë'-ð§çðjì(VÃiÀÁ_â6>>ÞÐÐàõzÕ§ò×ç+=wÙ²eJrÌ÷úõëåååëW_?îcUWWWQQ,¿	8++íð¿8oÈÅvxÃ®	Úðð°Õj½÷n`Í>ÌÈè1¼¼v¢÷o¯êã?®¬¬d|bú]múÄÄÄÃGÓ¾°O±ázo¤ÍÅê·7qÚx=&§Ó¹zõêÑÑÑà+W®¼zõ*£Çð®X±BÍK,[¶!íðõ÷÷ONN¾úê«/¾ø"C4d0Õïjoo¯³,X,×_]äcCCCÛáºoØ';ÕðFú	Ñh>¶Z¥ÞÖM1Þ?þXí/e!íðÊöáÂòÜÔÔä÷û¢Ù$®ªªßÕU«V©Kº»»322JJJÔ^ÕÃáºoØ';ÕðFúDDDDþüø#""""ðGDDDDàüø#""""ðGDDDDàÀ?""""DDDDþüø#""""ðGDDDþüø#"Ò¹7ÞxcåÊ9ZµjÕéÓ§kûõ¨¤ÙÚ»·F£Q~4¯×²^ÖÉÉÉéÞ&ø#¢¤lß¾ó4½øâ©¿ýû÷ËÊõííí²þ^Ám?"J¾úûû5áµ×^ó?êèÑ£ò©¬¼zõjÊàïæÍ²²ºº:dee¥¬w»ÝàÀ¥EÏ<ó°æå_^ùÊ+¯ÈÊÆÆÆ`ú|Yð$.d,¾øþýûMMMóçÏwìØ<»êp8XrÒ¥K!5ùùù+W®¼pá|j±XBîØùóç£ßºHîº¨««+ÔV¯^-ëkÞÿYc6k8P\,7³iÓ¦ááa-þ´·²&Ê]%"ðGD4Ç-PàrûöíàwîÜF£17!õõõ©K×¯_rÑöíÛÕEa¯¥>UnØ°arrRøå÷ûåRù(xÊÍÍUoÅr;²öîiÒ'N6àËãÇär#«V­.þ¢ÜU"DDsá³Í7O(-[¶?JäÓuëÖ©KuÕx6uQCCzÉP»»»ÕßæÞ½wjÖõ¹ç5gÏeù(ËÏ>ûìcoGà(Êß·°?rÁòóÊ§òQî¹sbbBAII~¿&ÎÉÉ.þ¢ÜU"DD	?),þën¤ááaùTØ¤>]²d|ºhÑ¢­[·Ú<x¸ù×Ò×RÞ¹s'ðÅ¦ÀÌ¯|eÃñØÛhÊ§÷îÝ¾oÞ÷üóÏËE¯¾úêÃßLmïØ±#ø?!iMMzarºørWüÍêå®ñññà^¯WVÊEQ¸ ¡ËåRþX'ðF=íll§°"ËYYY>On<???péco'ÅBºqãrª,ËGYÝÝ-w ìôñÔñå®ø#"ûÔûÞ>¼R%dÀûGGGµ/h]½zuÿþýj6 Fõ²^õ±DÛ¾»íMMMõQnG½Òv÷î]õé½÷¢ï»bÅ¹TÝFÌ|ÚóW.êêê¿LÕh.rWüÍj	ÁpäÈu¨×^-++K»sÆºuë:>OÍÉöÌUïùSos¹oTÜ±cPIí8ØJX¢©ÝoÕww:õQnG½ÉO½çÏëõª¯¿£G^9ø"õöÇù.2ð§¸)/tnÚ´)øÒ(wÀQB¤Òþoûõ(e£Àr`Â4°E ç.XÁ:u*þ>:¬/,,r;r7ïX`OÛH?ìrrrÔ2Ù½víÚào±`Áù¨ö|êÉ@ªK£ÜU"DDeÕªUYZ¹r¥Úå6]]]êpzUUUW®õzwïÞ]\¬ÀôüóÏû|¾À¥.]ª©©Øl¶ÛÔÞ¹)Y¿mÛ¶õnG;#wI0Êqþ555óE5::ÚÐÐ #0þ|ù)Ünwà°5Á·966&ºUce6C¾c»JDàÀ?""""DDDDþüø#""""ðGDDDDàÀ?""""ðGDDDDàÀ?""""DDDDþ(^ý2Ú	¬IEND®B`


QEEÅ¹sç^z¿È<¿äóKJJN:5ÓÝÝmµZ§ñíß¸qcÅ&I¾zÀ5ÌAî8"DøËÈÈü½÷Þzú<x0ÌµMé"¡tuòäÉ©nioÆÜÜëÖÔÔ?"D4Æ6lØøëîîVZýäO|>ß³gÏdANÊÊÞÞÞ W5Õ|é½÷ÊYÄð§.øôéSY0Ô]CDàb6M>^¸p!0îÝ»·yófuÌ´ººÚív|fûV®­éèè(--5L=ºqãFIIIrròêÕ«ýe<|ø°ÙlVÇ7åKhg"Î[o½%ëýÂJMMM²²®®.è÷8Õè¿´xQÖÈÒÆ	úÌÓ»`xü+÷Kqq±lö5kÖÈ¶ÿ¡þ»Ur8rm²ä³´ReK§å¬ìììÝ»w<~¹À###¹¹¹999² wÀÃåÝ#òJKJJ7n|ì¸ä+Ä7ÚI&¿ÏjUxâ,Z´HÖ?yòÄ¥àRVý§z/-²QÏüNiã7Ü´/8yüÝ¾Ûÿzä*þüïÖÞÞ^uR«»»[ònóÎ;ùå"DdÉÂ¥Kü	ówÀöíÛeyÝºuO_$rRVúæ=¦»RkvìØáóù:;;ÕI¹f9ÙÞÞ.ËAuYõÊ¶«W¯Êòýû÷ýÏEÉt½ÿegxP/ªÓêÆ	z«¦Á0/ø¸  M«««ÇÆÆõÑ&?ÿ»U®GÖ~]ÕÝºuëVõÉ<e¹7e9--_."ðGDÆ$å²,ëësrrdùáÃê¤<ÆËÉ¬¬,ÿkÐÎÕÖ8üO=ÿíñSsLLLåååê©¦â/999è7tý4.â*¹¬8Æjµ: m2'è­ö'?õºÆGù)áÏÿnU×æ¬QgÉÉÅ^å¯ññq~³ÀÅþÄgÙÙÙòîõzý×ë9Ó¿NTô:Ãhú|é»VÍf³:N­fkÌEBmn´ÉlP$Þ'Øw2¼®þn£Îr¹¯Â/ø#"£ãOúôÓOå¤6E­T>xJVNÆáO«+³ÉàO==©¦®TWWïÞ½ûéÓ§§O7o¿ÉÚøêÆÑö'¿É<ó§ñNè~û§¥¥É·û×ÓÓS__¯5kï&"ðGDÆßó/Þ×^×¯Ö¨÷¯[·näEêÑÇ3Çzö«··WrêÔ©ÉàÏétªçÄêY=Õ¥K~S½Èð~ã¨-éyQÀLu«NêUzbßññq±ÝÚµkõ4<~ü¸l|¢88üöW·Pè,/[RKKKý¿MõÚMËæ<?"2þµWw©5n·;àõ^999Úgg?#ª¬¬,ùèÿ× ·ùí·ßÖlYosJþÂomVsÀ æilÕià¯§§ÇÿúW®nÀ6®Çô0ê¹Uÿ¶mÛÆ/ø#¢ØÀ¤ú¯ÿúë¯7oÞü¢êêjýÛ;¦¿¡¡!¹6¹NaÐ=ÄCÚðÄ9qâDIIIRRÒ¢EÎ9óÆohÏ<Íü"SÂ_øséÒ%ñÉdÒFåù7¥­:üIW®¼x±|×rýêÀºög9)DS_Ýjµvuu½ô×öööòòr¹6ù¦µõgß¾ê)U¼Ü^¯_."ðGD4WMLL¼ýöÛS-7Äh¬zû¶ÏçS¦/..æÀQ¦^óÐ|À!"ðGDy<½÷.Z´HÛ÷ÞÍBDàÀ?""""ðGDDDDàÀ?""""DDDDþüø#""""ðGDDDDàÀø#"j.]²X,&iÕªUS½lIIÉ¼yó¶FeMiiéöb/ÞçLæ²ÓÜ·êRë=ÏÎ;³³³eeddTWWß»wO»ÏçõòÉÉÉÉ²ÌO?"2³Y°2444¬>|X.ÛØØ¨­9vì¬©¯¯üé×Ûl6YýúuY¾qã,hç^¹rEÃâÕ«Wùé""ðGDÆÛÌÀO===rÙÊÊJmÍ+dÍíÛ·#s##¿äädY?22ôRµµµr®ú¸mÛ6~ºüåçûöedd¤¥¥<x0àÓÞxãòòòkÐ8å'OÈ²¬Qg=xðÀjµ¦¦¦L¦²²2õlþÚü¿zø9sÆb±TTTÈÒmll¬¦¦F.+7~ïÞ½êLáéÊ+åÚd½Ãg¿¼¼<Y_\ÜÙÙpÖ³gÏ,X _k||feeiDÞm "D4WþSËï¿ÿ¾,777³dá>ðÿööv<ÿ¶oß.g>Zå£,×ÕÕ©³-[¦Þ¿_Öçææ½6ÿþ"BºO?ýTl6þÆïÜ¹Så.^¼(Ö¬622ÒÛÛ+êÉP¯çÅbÿÏ¹té¶fÕªUwîÜÑ.¢¯[·N7oÞ,Ë²&èmà§ÀE'^¤·WÐ:NM<6lPªÓÎíéé9pà@yy¹¬OJJzmüuÃ222ôÍÉÉQ7þÙ³g² äò²&õz½ÓÛ,uwwR5Ê5«õ»wïÖN*¤îÙ³g&·üÑÜâO½SU[/'_*!VZZ|¦ÇãQG6e:ëÔ©Sr)á;wü¯!àÚüONæ"¡nHÑÿ:Ç.,X°@­ÉÎÎVONþ¿0Ûíúõëâ9ÿÃÜBgù¢O>þâ´,/Z´(èmà§À¹¹¹þÏü©'Ï^*¡9Wû¨­Wïxö¢Iâ/üE´&Ò_6++K]6àæù|¾«W¯ªwéjONi³JÝ¹Í²¬i5 iß""ðGDs?5ºåôéÓê5Ç. YçÓO?ÕÖ+JÞ½W½p2ø|öÙg²°ûvýe·mÛ&ËgÏíëëSYeeEE,Ì=z$3Ä_ii©¬ïèèxþÛÁ.o¿ýöóÐSodý´oø#¢¹ÅÏç`¥½ÈV_xü«C®òÑØ(;;;##cïÞ½Ä_ø89wÅÚûNÆ/ïÞ½[nyrròÚµkÕjÅ[ÕÕÕêíÃ_ýõ6þÐ°ÜBùf³²²vîÜ)_÷ùoç]û_¿6ïzÚ·ü?""""DDDDþüø#""""ð7;ýô§?íïïðýå/ù«_ý#÷_ÿõ_jH¹ÿøÿøÿù¶dwgüÝÝÀÀÛÁø»;ð7kýð?ÿEøÞ¹sç?ÿó?ùQ6r=úçþg¶ÁûüóÏõÿªùáï(÷Ë_þòöíÛlwåÊ£ý­þÀø#ðGàüøàüøàüøàüøþü?þÀ?àÀ?ðGàü?ðGàÀø#ðþÀø#ðþüøàüøþü?ðþü?àü?ðþü?àÀøàÀ?ðGàü?ðGàÀø#ðþÀø#ðGàüøàüøþü?þÀ?³SWWWYYÉd*--Õÿ|?àÀøc;¿¸Â_AAÁÍ7eáÜ¹szüuttE6QÅÏþó12pß|óÍ­[·ØOð7::Êv0rÝÝÝ÷îÝc;|w×ÕÕÅv0x¿_ýêWþ¢1?ÿRSSõø;~üøO"[»Ãáø	¸k×®ñÅlwùòe6ÁcwgüäbwÇî.hñ?ù´®®Ã¾Äa_ûÃ¾öçÃ¾ª±±±êêjÇþü?þÀ_ãoppÐf³ÉÃ¹þ,ðGàüølðWøs:«W¯z.ø#ðþü?¶ø+üY,y~?àÀ?ðÏøø#ðþü?¶øàü?ðGàüøàüø#ðþü?ðþüøþÀøþÀøþÀøþÀ?àÀ?ðGàÀø#ðGàüøàüø#ðþü?ðþü?þÀøþÀ?àü?àÀøàü?àÀø#ðþÀø#ðGàüøàüø#ðþü?ðþüøþÀøþÀ?àÀ?ðGàÀø#ðGàüø#ðþüøþÀøþÀ?àü?àÀ?ðþÀ?ðGàÀøàÀø#ðþü?ðþüøþÀøþü?àü?þÀ?ðþÀ?ðGà/T^¯ûü?þÀÅ?þÜnw]]]~~¾ÇãáîàÀ?ðGq?yüÝ²eËüùóç½¨©©»ü?þÀÅ!þnÝºUUU5ïw[¾|9wøþü?üy½Þ¶¶6A^ûRRRìv»ÛíæîàÀ?ðGñ?ÇÓÒÒb±XØY__?00ÀþÀ?àâ£££òP+ÈÓ³¯±±QÎå.àÀ?ðGñ?·Ûm·Ûõì3ÍòàûÀø#ðGàüQàOØg³Ù´·ñjåçç7773Òü?þÀÅ	þä!µ¦¦FÏ¾åË·¶¶ú|>68øþü?üÉ©Õj§KVFþqü?ðGàüø£9ÁÏçkmmÕ³oþüù6Íår±ÁøàÀø£xÀ°¯¥¥%??_?´OØÇÐ>ðþÀø#ðþ(Nðçõz²/33·ñ?ðþÀ?ðþâ¡öY,ØþÀøþüÅþB±/??¿¥¥·ñ?ðþÀ?q?5´/%%%°ü?ðþüøüÕÌôðþÀø#ðGà/®ð'UUUì555°ü?ðþüøüÕ¬ö1½ü?ðþüøü©¡úé-àü?ðGàÀ_$GGahøàü?ðGà/Îóx<MMMð ÞÒÜÜûÀøàÀ¿8ixx¡àü?àÀ_ü700°ÿþôôtýôÃûÀøàÀ¿8ÉårÚ÷'ò'û·Ëöàü?ðþüÅI555zömÚ´I§ôûøàüø#ðgÜÇ5kôCûêêêzÕç?ðþÀ?ðGà/¶óù|çÏ×íöíÚµ+`høàüøþb5¯×ÛÜÜ¬Ú¾ÿþááaýEÀøàÀø#ð644X,öÍfy¤óx<¡.þÀøþÀ¿XêñãÇõõõú¡yyyaØþÀøþÀ¿XjxxøÐ¡Cú¡K.=þü$ö?ðþÀ?ðGàÏèØíöý¬f§Ó9¥YÍàü?ðGàüø3nn·»¶¶V?´oýúõÓÛk?ðþÀ?ðGàÏ sË-ìSCû¦·?ðþÀ?ðGà/Ç©õë×ëÙW[[;ó=øàüøþÏçkmmµZ­GxSRRô³ÁøàÀø#ðÃìkiiÑÏjÎÌÌ¬¯¯Å¯þÀøþÀ¿¨¥f5ëÙ§öÉ¹³þÁøàÀø#ðöÉC~V³@°¥¥eJÓ[ÀøàÀø#ðgÜÜn·Ýn×³Ïjµ:¹cøàüøþ"Í>Í¦Ú'ìØcøàüøþæ<yè©ªªÒOoFxçþÀøþÀ¿¹e~zbßlMoàü?àÀ_SÓ[ôÓ[:ö?ðþÀ?ðGàoöóz½¡öÉcÐàü?àÀ_5½Åb±àü?ðGàüø¢2´ü?ð÷äÏ,ðGàüøëBMo)**2ûÀø[üuvvË/ø#ðþüÍ]²ÓúÐ>ðþÀßÿ¶víZËöÙýÈ&Ù!Þ'÷oÿöo?ùÉOØOöß|óÛÁÈÉîNþÜûoóÜ¹s¯¿þú<]²2ò1ÓØÝýã?þ#?«ïòåËßÝÅ0þ~sCãïÌ3?lN§³««ëgdàº»»¯_¿Îv0x¿ÞÞ^¶ûøøÞÝ566¾öÚkæKJJúó?ÿsùùoáË/¿dw»»ÈÑxÆÃ¾ö%ûN)Ï×ÖÖf¨YÍöå°/Á?ðGàoöóx<ÍÍÍ¡öÄÜwþÀøþÀ¿ ÞÝÐÐ`¡àü?ðGàüøö566Íæöååå	ûäÜþîÀø[üüøþBÕßß¿ÿþôôtý¬æææfíàü?àÀß4où-[ôCûÖ¬Yãt:ãàü?ðGàüøÞ××W[[À>9¹iÓ&a_üÝGàü?ðGàüQâOl·fÍ=ûjjjDñz?ðþÀ?ðG?Ï×ÒÒRTT¤Ú·ÿþØÚ7ïýÇ?þñ±cÇzùqàü?ðþ(Îñ§f5ëöÍfytèïïïÇår÷»ßoÙ÷ýþïÿ~UU×ëåü?ðþüQâÏãñ455ååå°Ïb±8q"$[àE¯T­­:øîAùw`ÿ¥eKív;?´àü?ðGàâ£££GÕí[ºtikkk<½7|mmm¯¿¦ä§þíÚ¹kÁ³ÀøþÀÅ9þ?~_¯ÿ/:ä5ÑÐsâÄ	ë[ýñ'ÿrrrd+ñsþÀøþ(¶ñçv»ëêêô³­V«ÃáHÌçºÚÚÚ,YÂ3àü?àâòl³ÙôÓ[jkküwÜãñ|ç;ßá5àü?àâ²o·Z­ótmÙ²%¾§·L>ËõÊ+¯äççïß3Í¼Ûü?ðþÀø£ØÃÏçkmmÕ³/%%Ån·w¸S6Wû±cÇäâ/øàü?ðG±?5«Y?´/33SvõÃÃÃÜAãøàüøcømhhÐ³OÖ477Ë¹Üàüó^VRRø#ðGàübìÆõÓ[---ÇàÏøKzY&	üø#ðþr»Ýv»]Ï>«ÕP³Áø=üE=ðGàüQláOØ§Þ¢Øùý9ø#ð7Ëlß¾üø#ðþ¿ÞRUU¥ÚWSSûÀøIüY,ÉÄkþüøzöé§·ûl6CûÀøUüêßííñxÀ??5½¥¨¨H?´ö?ðóøKNNßç¡¡!³Ù,b¾Ï>ûLêêêÀ??¯×fhÓ[Àøü©ßjYíÉÂýû÷e.iiiàÀ¿ÄÁ_¨é-°ü¿xÃßäw»³³ÓårÉÂï½§õBàÀ_ào`` ÌÐ>þÛYðþâ÷îÕÞÞáÿ;_QQþüøoüÞÂ¬fðGñ?éÈ#YYY²ÐÕÕ%Áòòò¹¾éàÀø£(â¯···¦¦&èÐ>ÃûÀÅ9þ¢ø#ðþ(*øïúõëÕþü?þâò0jh¿wàjàC	üø?ü©¡Ë/×³¯¶¶¡àþàÓâÝ¾þüÅ:þ<Oss³~h_zzºÝn`C?JDüódGÐÝÝá]9ø#ðþhîr:ï¿ÿ¾ÅbÑÿNíèøì"¿þÀÍEÃÃÃ²ýÖ·¾À>`ccã×þÀ_àO~òd§°sçÎ±±1ðGàÀ_ìæv»wíÚ¥oQQCûÀ¿ßiÑ¢Eótñþb%ËµeË=û,YÒÚÚûÀ¿ßiñâÅ¼áÀ¿íÖ­[úé-Ò÷¿ÿýÏ>ûíþü»õööFø¦?àfREEEÀ³jzüMéÿö%ðG¿ÞðAàÀ_¬¤ö-]º4à©¾»Ý®íàÀ_ÈN§ì5öîÝëõzÁ?MöÒMMMK,	`_ffæ¡Cö?ðGà/ôU7|ø#ðgFGGôCûÌfóÑ£GíàÀ_ÈBÄ>üø3ûdo©g_sss¡àüø3Àø£0eßòåË[ZZ^úBðþüÌb±,^¼¸¯¯üø#ðgÜn·ÍfÓí«¬¬<þü$ö?ðGà/d&Iö)¿éàÀø£ä?(û6mÚ$ÒU?ðGà/d×¯_=KýÈÈH$÷æàÀø#-Ùêg5ÅÓ;2þÀ¿ÐWÁ»	üøR>¯µµUÏ>5½EÚþÀ¿ÙÄïö%ðGà/*ìkiiÉÏÏ×³OvA§·?ðGàï9ïöàÀ_Ì¥öéÙ'kdýÌÙþÀ?ðþÀ?£°/èôa_KKË$ßÆþÀ¿YhbbbíÚµ©©©²JKKÛ¸qcvëàÀøKÜn·Ýn×³¯¢¢bÖÙþÀ¿4>>ôsý_ý?à/AØtzÕjÓ øþBV\,»¡uëÖÉÉ6ÈeË?þ¦ìâªªª"Ï>ðþü½¤äädÙùt5²üø#ð7=öÚçr¹"sÀø#ð²¤¤$Ù+	ø´5^¯WÖ0êÀ¿)¥¦·eßLö?ðGào6ñ§û®^½Zö²,kÊÊÊÀ?Iþf3´o`` ò7	ü?!íÃÇÓ§OÁ?2þ<O[[[cc£|­Ix³R¨é-³5«ü?³¿ç/Þð»qãÆ¤¤$ù¸zõjY3×7üøÌår½²è×_³þ±µ´´táÂÓûïng·PCû£.Tðþü.ðGàüM&Ï÷Ýï~·jmÕÁwªo¾ùæw¾óÇ­o?Ôô¹ÕþÀ?ðþÀ%þ1¯¼ªÉOý+((¸uëVä¿qËjh_kk«AØþÀ¿|YIIIàÀE²çÅëøû£å$Øä·tz´fÍÈïÊÀø#ð7Bþüqð×××g6ì? ÉO.¸.û¶lÙ±ÛþÀ¿YÀ_¨öìÙ£ökçÎþ(êøÖ¬Y³´léÞwöüöýßË/_µjÕc3´ÏàìàÀßdÁ´´4õâÿÏàÀEgÓ¦Mßþö·bYYYk×®köéö¥§§ïÚµ«¿¿?&î#ðþü½¤Í7«½ÛÕ«W#sÓÁ?ð7¥|ò³ñøñã¹û£££zöeggËzCÍàÀßô»xñ¢Ú»­[·.7üøÆ)Ô¬f³Ù|ôèÑØbøþB6>>¾lÙ2õÞë×¯Gø¦?àÏ¹ÝîCÕüñÇGq øþfÇW;¸­[·Få¦?à/êì:´¯²²²½½ÝPCûÀø#ð7øcÎ?JXüÉÏX(öÉ>ÖÙþÀ¿à%½,ÉþüQá/èÐ>5½Å³Áø#ð7køzàÀøX>ÏápèÙn·ÛÝnwüÝGàüøàüQ"â/ÔÐ¾Í6§cÀ?ðþÀ?ðQöµµµÚ'û9þü?ðþü¿È5::zôèQÅÀ>YÓØØCûÀø#ðþÀø#ð¤áááýû÷ggg°oÉ%1=´ü?àü?¿ÓÀÀÝnOII	`Õj¡àüøü1çÀÅþä¦¦¦&`hq6½ü?ÏgkÎ_Pü1çÀN§sÓ¦MzöÙl¶¸ÞþÀ¿ç³rØ·££Cvo½õÖØØ6l57nÜþÈøÝqEEEÀ¬ñ:´ü?³?õÊhÿÄLLLÈ³Ù<ÃkéÜÜÉTRRrýúuðGàüÍ¼óçÏ/Y²DÏ>Ù¥$ÈÛxÁø#ð7ã«x/3Í_uuõÙ³geáäÉ[·nþÀß´Ø8q"//O?½ö?ðGàojåääÈT 6>>.'=Ïºuëd¬á5ggg«Ñ¤ì õøknnîl×®]ëììì!÷OÿôO?þñÙïòåË_õUd¾Ãá°ÛíìûÃ?üÃÃËÜìîbww'?Þlãïî"ÿEç7nÜú;wîÌðýß2¢ûàïâÅíæÍ½½½dàúúú~úÓ²ÞçþðáÃ¹þ*»ººº´´´½S^^ÞGÔßßÏ&ÅÏ~ö3¶û÷ÿwvwÆïÊ+ØÝ4çøîß¿_PP$ûÙ²²²'OÌüjý'''sØ8ìËaßIær¹l6[zzº~hÃáHÀ¡öå°/qØwñ7Gåää¨ÊGýAdðGàüé¿ªªJ,BØÈCûÀø#ðø«©©9sæ,ÈÇêêjðGàüÉétðÌ§öñ#þÀ¿YÆßÝ»wÓÒÒÔÚ.Ìüj;;;Íf³ø#ðþôù|¾ÖÖÖåËeCûÀø#ð7ûøSCýÿK7µ|òäÉ9½éàÀ_ãÏëõ¶´´äççëö:tö?ðGào®ðg6eo÷î]]]]²þüÑottTö<f5?ðÇvQÀÚçª?Ù§óûø£¹À_(öåçç744À>ðþüEjÈ³z¶Oð711±oß>57üø£ÙÂÛí¶Ùl)))zöµ´´0½ü?ÃÓé:äùæÍàÀÍóçÏØÉ,]º´µµö?ðGà/ÒøS·åååêÝ¾©©©x©5ø#ð÷øßñªª*=û¬Vk;ìàü¿¨á/*?q?ùíÖí6mÚÄ¬fðGàüEÚû<´<yRXXþüÑð§öÕ,aÓ?àÏøàÝ¾þhJøöÚn·ÛÚþü¿èã///o^Ø,XþüÑKñ7::ÚÜÜ¬g_vvö®]»ØÀø3þ<xô"íÿöðOäwñâEðGàÂà/ÔÐ>a¬÷x<l(ðGàüZB½¹>ÂþüÅYÿ÷øðá ³x/ø#ðþ¿hþü9ÇÓÖÖÖØØ(ýÿã5´O;h UTT$	ûÀ?ðø(,,LKKÓÖdee½ÿþûàÀ_ÂöøñãW^yåÕW_µþ±µ´´táÂ.+è¬æÊÊJ¦·?à/ð·hÑ¢7üªz=ø#ðUTT|¿âûß=¨þ½þúëÉÉÉú·­_¿^î,íþÀ_áOíÓµ5ò³(k222À¿lxx8''çÀþÂ¾­[·ª¿ö­^½ú_ÿõ_ÙVàÀøIü©×îìÒÿþ$ùS°  `ÓÆMf³9)))»víêëëÒÿíKàÀø3þrsse¾÷n¯×+'ÇÇÇ<(kd¿þü%Z²øè£~ï÷~/²Æf³iCûÀø#ðþbÝÝÝA<wuu?Óèèhcc£ÅbÑÏ?ÿôOÿÔÿµàüø1?ipp°¤¤$555)))--­¸¸XÖÌõMþÒãÇ:¤Ú';oûÛ?»Ý®?ðGàüÅ	þ¢ø#ðõÜn÷®]»ÒÓÓØgµZÕ¯§¸0èyÁø#ðþÀø#ðKõööÖÔÔíUUUùÅàÀømüÝ½WÍyVïðÍÉÉ¹páø#ðÝºukýúõzöÙl6·Û=É+àÀøaüuttø¿Äçùo<<yüø§NgEE~zËØþÀ?ðóøS£¼îÞ½«á¯««!Ïþâ© ÏöeffÊ¯¡ÿÿÛþÀ?Jü©ç~e.Ë&	üøõÚÛÛõÏöÍàüø1¿5ÕOáobbbß¾²l±XÀ¿Íçó?>???²¦¹¹y&ìàÀøyü9Î CoÞ¼	þüÅ§©©)//OÏ¾ s[Àø#ðG?õp[^^®ÞíZXX8ÕW?QoxxøèÑ£úYÍV«µµµu¶ØþÀ?ðøJàÀßlÕßß¿ÿþ0³g=ðþü?ðþüE!·Ûm³ÙôCûjjjæô÷ü?à/¶ñwïÞ½Å'''ËÃFjjjIIÉÐÐø#ðgää×§ªªj³Áø#ðG?ÃôàÀ1ÙgµZõÓ[ìvØþÀ?ðóøSC«««=Ûºu«¬ÉÍÍþÏçkii)**õ¡àüø£ÄÂzüð' ,kÁ¿¨çõzú¡%òìàÀøyü©gþ&&&´5ããã<óGàÏ	ìä7E?½evö?ðGàê5ÕÕÕb>9ùôéÓÊÊJ^óGà/ºe_QQQtÙþÀ?ðóø÷²æèø/ø#ð´ Ó[æthøþ(±ðô²L&ø#ðä7>ûÀø#ðþbÑüøÓ:½Eís¹¼Áàüø1¿PóÁ¿9Íçóµ¶¶b_Äö?ðGàòHsäÈµµµz!ð7§ì:½%33óÐ¡CFføþÀ_ÌãO'9999O<|òz£ú?Jpü644e_´ö?ðGàÏ_<Ï§~rssÕÂæú¦?J4üÚ'ö?ðGàüÅþ¤7nhEMMM¸éàn·Ûn·pV3øþ(Añ·cÇõP¤þ·é­·Þþf^___mm­~zKEEE²ü?à/æñg2ä¡(##£§§ç¹ßkþÁ¿<ônÚ´I?5ÝCûÀø#ðG?y4:pà@ÀJÍÆ»	üM¯[·n­_¿>àÙ>9YUUìàÀøyüó'ÁàÀßjkk[ºtiÌíàÀ%þ¢ø£¸ÁÚ·dÉö¥§§ÇûÀø#ðþbòÈä`7üIðGà/h^¯÷ÄyyyìËÎÎ¹é-àüø#ðþülxxX~yì3ÍÍÍÍqÌ>ðþü?ðþ(±ð÷øñcùéMOO`_QQQ[[×ëM»	ü?àü?ü©YÍzö­Y³Fö	u7?ðGàü?ðGñ?¹I[¶lÑÏj^¿~½ÓéLÀ»	ü?àü?OüÝºu+(ûl6<¸&ìÝþÀ?ðþÀÅþÚÛÛ+++õCûìv»ËåJð»	ü?à/&ñ>ðG	?a~Vsvvö;ï¼óøñcî#ðþü¿Ä_ÒË2Là>ïã?ÎÏÏ×Oo×¸ÞþÀ?süE=ðGÆÁÀ®±±QÀ>`SSÇãá~àÀøàâÂ>ùQÌÌÌ`ß%KZZZdhøþü?ü©¡zöUTT8îðþüø4£Wcc£xëÄÃÃÃQÄ°Ïf³ÚùIðGàüøà/s8ßJýÖë¯¿þæo~¯ü.5$oNñ'?rUUUì555<´ü?þÀÍf'##cë­ß=¨þ½ùÆ¯¼òÏçþäÍjµ<Õb³ÙÜn7÷øþü?µNgqq±&?õ/77·¯¯o®ñ'¾lii)**`_ff&Ó[Àø#ðGàüÑäp8V¬X¿W_µ··wîðçõzú¡°ü?þÀÍmiii¹û/5ùÕýºÔÔÔ óóf¿PÓ[LoàÀ?ðGèÝwßÍÍÍý·ÿBä÷,ð£>ú3Á(3û¾ÄÀø#ðGàüÑtøðáÅ§¦¦=6Ô§M¡¦·X­VÃûÀø#ðGàüA*þäC±¡àü±À?ðþâA§·¨¡°üøþÀøüù|¾ÖÖÖ ìchø#ðþü?ð?øSCûNo±Ûí°üøþÀøü6440´üøþÀøsüÞb±X`ø#ðþü?ð?øö½óÎ;íþÀ¿XÂßÐÐÅb4%ü]¸pÁn·§¤¤°¯¨¨ö?àÀqëìì,..ÇlðG¬···ºº:))¡àÀø#ðø[»v­Ëå¿¿þë¿vD¶«W¯þèG?rñ:vìØòåËçéÿ9¡vùòe6Ácwgüä»íÀîN_ãï7714þ®]»ößí«¯¾r»ÝÿMFêÒ¥Kög¦Ú÷ü ««ícÌ>ÿüó_ÿú×l#wûöío¾ùí`äúûû¿üòK¶Á»råÊøøx¿h<ãÃ¾Ïçkkk[ºtiûÒÒÒ¶lÙ"ûD6Ã¾ö%ûÆþ´pðG¡òx<ÍÍÍEEEì3ÍòSñóÿ|Jÿ·/?àÀá8þHåõzO8¡Þ×ØØ((|>ÿÛÀ?ðGàü?6<<ÜÐÐ`±XØ·téÒé-àüøþb¡	Ëåª««Ó?ÛWYYÙÖÖ¦ÚþÀ?ðGàü¿¬¿¿ÿþýúYÍkÖ¬	s×?ðGàüøà/ÆºuëVMMMûæÏ_[[+Há/þÀ?ðGàü¿ÉápX­Ö§úDuuun·2×þÀ?ðGàü¿Ø`ß5kØ¾k×®É_øþÀ?ðþÏçkii	:´ïèÑ££££S½Bðþü?àü1¯×tzÿÐ¾iþÀ?ðGàü?cõøñã ì[²dÉÇ¬ÞþÀ?àü¿lxxxÿþýéééÚþÀ?àü¿ÌívÕåp8fñ?ðGàüøà/ÝºuËf³Í?_?´o.þÀ?ðGàü¿èÔÛÛ«Þ"ìÛµkW__ßQðþü?àüEºöööåËëg5:thJCûÀø#ðGàü?ðgÜÔÐ>=û233=:×ìàÀø#ðþÀ_òz½Â¾üü|ý¬æ'NLcV3øþü?ðþÀNîýÛxóòòÎ?/(ðíàÀø#ðþÀßôøñã£GêÙ·dÉÖÖÖiÿàüø#ðþÀø3VAg5WTT8ÎYÕþÀ?àü¿è×××·eËýÐ¾õë×ËCn!øþÀ?ðþf!ÙÚ6mÒ³OVºÝnãÜNðþü?àüÍ´ááá¼)))6­¿¿ßh7ü?àÀø³Ð;ï¼£Ø-ËÚþÀ?àü¿èÔ×××ÐÐÉ¡àüø#ðþÀøZÑ/øþü?ðþü?àÀøàÀ?ðGàü?ðGàÀø#ðþÀø#ðGàüøàüøþü?ðþü?þÀ?àÀ?ðGàÀø#ðþÀø#ðþÀø#ðþÀø#ðþüøàüøþü?ðþü?àíþÀøàüøþÀøþü?àü?þÀ?ðþÀ?ðþÀ?ðþÀ?ðGàÀø#ðGàüø#ðþüøþÀøþü?àü?àÀ?ðþÀ?ðGàÀøàÀø#ðþÀøàÀø#ðþü?ðþüøþÀøþü?àü?þÀ?ðþÀ?ðGàÀø#ðGàüø#ðþüøþü?þÀ?ðþÀ?ðGàÀøàÀø#ðGàü?ðGàüø#ðþÀø#ðþü?àü?þÀ?ðþÀ?àÀøàÀ?ðGàü?ðGàüø#ðþüøþü?þÀ?àÀ?ðGàü?ðGàüø#ðþÀø#ðþüøàüøþü?ðþü?àÀøàÀ?ðGàü?ðGàÀø#ðþÀø#ðGàüøàüøþü?~Á?ðGàÀø#ðGàüø#ðþüøþÀøL]]]eee&©´´Tÿóþü?àíþâ7oÞsçÎêñwíÚµñÈ&¿fn·¿üÙÀv0x?ÇÃv0ròGÔ7ß|Ãv0r¿øÅ/¾üòK¶Áüýú×¿ðaüùªÇßßüÍßl_|ñÅ~ô£kdà:::®^½Êv0x/_f#<vwìî(vwwñ¿îîîºº:û9ìKö%ûrØ7ûªÆÆÆª««=ø#ðþüøñ¿y¿M´Ùlòp®ÿLðGàüølðóøóÏét®^½zhh(è¹àÀø#ðþØà/®ðg±Xæùþü?þÀ_<ã/|àÀø#ðþØàü?ðþÀ?ðGàü?ðGàÀø#ðþÀø#ðGàüøàüøàüøàüøþü?þÀ?àÀ?ðGàü?ðGàÀø#ðþÀø#ðþüøàüøþü?ðþü?àü¿DÃß_ýÕ_µ¶¶öG¶/¾øâË/¿ì'÷ÕW_]¾|í`ðþîïþîþýûl#'»»îîn¶ù	,ØïôéÓ¿øÅ/"üEGGGãwïÞ=xðàÈ¯ðFçñ|,QâþÀ?""""DDDDþüø3hwïÞ]¶lÉd*))éììüßMæWRRÈ÷Óé,..ö_C¼¾þúëòòòäääÍ7°RGGìÜÔòíÛ·sssÕ½výúu6ï#ihhÈb±°Y|7uuuÉ¯Rii©þSð÷dwéÒ%Y¸yófvv¶ÿYgÏ=yò$È÷||ðà,ÈGö½Ä²C¾¾¾;v°Ïç(í«ººZvt² ûº­[·²xÉRò·®¿ÉwSAAìúdáÜ¹sà/fjoo°k'ïß¿¿jÕ*61ï£ÅÊ|e¶1ï&ù#X[¹`Á¶zÿý÷?üðCíK®þæ	þ2æ´víZËþ~7ùþbÃïrØµ7nìîîfãó>ºû¶üvÉIùÈÝdØ»Ix÷î]YøäOü!HÑêÁååå¢=íËÿ~á>2æôÇrðw$Iuuuà/fêèèÈÉÉQËògÖ+Ø&½-[Ö××§È=eØ»éÎ;tùûØüMãÆIø¿¬999MdÀûüÅÐÝ466V]]íñxÀ_,¥ýáøðáãÇ³AñtE¬ü*©îÝ»W\ÌfþãÁï&kéÏ_öÕþú%CÝGà/Vî¦ÁÁAÍöèÑ#CÜBî¤ð¨#S]]]«W¯V+W®ÓÓÃÆ1ì´bÅµ¦··wÙ²el"cÞM²æöíÛÏ=ûðÃ9Â&2ÔCZ¨©©9sæ,ÈÇêêj¶ï#ðwÓéýÞÐÐQn÷MøäÁ©¤¤Äd2­ZµJrr²z4ó>ºwï*"eMdÌ»Iö-ß¦ºº:ÏÇ&2à#Vgg§ÙlNJJÊÍÍUoÍ&ðGÓ¸,KÐ§lÁ?""""DDDDþüø#"""DDDDþüø#""""ðGDDDDàÀ?""""DDDDþü?""""DDDDþæ¸O>ùdåÊ©/ZµjÕÅgÿõ¢ÙÛ»µE¾5Ç°^ÖL¦ÜÜÜgÏMõ:ÀÅd§ëÈ#ñ¿úúzYÙÔÔ°þÄ²þ½÷ÞÆu?"½nß¾-¬1L§Nò½èôéÓrRVöôôÄþîß¿/+KKKÖËz·ÛþüQBôÖ[o	k>øàÿÇµµµþô¹~ýºàI&ËÚ'?ú´®®.##CÎÊÎÎÞ½·ÿÑUÃ!À³ä²ííí5,X¹råÕ«WådMMMÀ»råJøëQgÉMRguttÚêÕ«eWW¶ææÍ²Æjµjk>l6åªRSS7oÞ<88¨ÇþúÖ¹©Dþ¢Ü¢E.<ð_ùðáCYi±XüqPww·:wÃgíÜ¹SÕÛÛôRê¤:wãÆÏ=>&''û|>9W>ÒÒÒÔKñÂ,½yúïôÜ¹sþ¢Õ|yöìYM~W²jÕª©â/ÌM%"ðGDýÔÞ û¬yóbþ¸ÙºuëØdAN®[·N«¬£ø¨À´©³ª««ÕS²ÜÙÙ©®Äÿ:8 ¼SG]·mÛ&k.]º$ËòQß~ûí^ÀQNÊ'øß¶ ß2++K¾ß¡¡!9)å8'&&Ô'äææÊå[xþÛÃÄ©©©SÅ_JDàÈøâO[¯p£Ì$ÊIa:YVV&'/^¼ûvAÛøø¸v%ò9Ï¥iR'>|¨²I;ò+eÙáp¼ôzròÉ'þ·-ÔëóöìÙ#gøáÏh÷îÝþ ü	IËËËÕSÅ_JDà(ú©§»ÆÆÆüWz<Y)gáFCË¥ü§YG¡þhl§ü¬Èrvvvrr²×ë+_°`vîK¯'Åº÷®rª,ËGYîííÕÎíììôðñäñæ¦ø#"~êuoÇ÷_© ¼áC]àÐÐþ	­úúzuVS£zZN;®úR¢íÜ¹SíuuuÚú0×£iôè:ùäÉðïÌ]±b«¦ÛYýÏRïü³:::FFFÂãO©ÚÚ¹an*?"¢è§Þ0a2N<©F½:u*99YÿæuëÖ	t¼^¯:&«½3W½æO½TÎårù¿XPYp÷îÝB%õ>ØJP¢©·ßª¯ît:µõa®G½ÈO½æÏãñ¨Ï¿Ó§OkÏÉÉ·ìzùcoo¯|Ù¡ð§¸)OtnÞ¼ÙÿÜ07À!R:|øðÿß½HÙH[Öjï±ÐÚ¶m¿,ý»páBü=ñ_qÈúììlÿ#Âa®GnÿÓÞiêOMMUßBÀÁîµk×ú¬¬,ù¨¦½ø_§zbRKQU;7ÌM%"ðGDd(«V­J~ÑÊ+Õ[nð×ÑÑ¡ÆéÜ¸qC;×ãñìÛ·Ïl6+0íÙ³Çëõjç¶··Érss®SKäªdý;ÖºInÜ$50Ì?­ººº/ª¡¡¡êêjÙò]¸ÝnmlÿunÕ¶²Z­]]]_1ÌM%"ðGDDDDàÀ?""""DDDDþüø#""""ðGDDDDàÀø#""""ðGDDDDàÀ?""""DDDD©þmZÛÀsóVIEND®B`


N2àÀ?ð7ëÔ¤-%ôõ%99Ù(§ßø[²d	>üøsUäÓïÆå¤-àüê	ét:£|×Á?ðGñ?5iæHÔÔT«Õý>ø#ð§]NN>üø³kÊÓï&Â¤-àü]]]òüÜ¿Y?à¿ÙkäÈÊÊª¯¯ç¹þÀNñ· Lø ðGàüáô»êP_kkk¢MÚþÀÁð&|ø#ðþs¹Fr¨I[g~fðþ¿Xþü?2þ<Okk«æéwýó3'ø¤-àüòßµ%Kôõõ?þÀ_`N§Óf³eddhêÝ8¶?ðgHüL&yGÿ®?àô?ÇÓÜÜ¼|ùòp¶0?3øÆÆßµk×äùÜÐÐ0::Í½9ø#ðþHWøÏÉëµæHâââ¦¦&&mà/ðÇh_þn·ûèÑ£¶Øl6Ù]s¨ü¿øÁ£	üøKLüE´eåÊÍÍÍêàÑ¾àÀ?Ãã¯¯¯Ïn·kÎÏ,+÷íÛçv»ÙàÀø#ðGàÏØøóx<çÏ_·n]è¡>Y³uëÖöövÞÞø~ZZ<ÿÓÓÓ7oÞÝ:ø#ðþ(jø§U]]æüÌùùùLÚþ(ð711¡9àc¾OÎþü?oü©ù-Kè¡¾mÛ¶Ý¼yCàK.½ÀÆÇÇåÓÑÑÑM6É+V?þÚßüÍßÔÖÖjÎÏÀHðG	¿ÙþÏorrRÖÈzðGàÀ±hll,..Ö´e÷îÝN§CàIII²Sðù×x½^YÃT/þü%ñÃá°Z­#9ªªªÎ9ãñxØPàÀß/Roû®]»V½í+eYÖ?þtËå7iËoþæoÊEòl%ðGàï×íiøxüø1ø#ðGñ?ÇÓÞÞ~üøqùh¬¿Só3[,ÍÓïVWW:u¹úÀ¿°MLLlÞ¼9333))I>®]»VÖÌ÷]þÀ_ls¹.~±´´Ôò¿,%%%_þòûúúô·å	R__¯9iKqq±@V)vçö%ðG	¿þü¿æóù^zé¥ªõUÞ8¤þãßøßùÝþa®¥¥%ÜHá`Ðü?àü?¿f£_öËOý+,,¼yó¦ÞªFr¤¦¦²Ïb±´µµiNËþÀ¿kNURRø#ðGñ?Ù¡eÕWð÷+ÿ@,¥¨&m)((Ð<ýî#9Àø#ðøÀ?üõõõåææ|ý _~²üå/YÌÛ;¦FrTUUiNÚbµZÇt&êàÀßtÛ»w¯ÚË;wüø£xÅ´nÝºåeË÷ïÛ/ò;ðgV®fÍN¬&m	7c¦§ßàÀßÔÉ¯`zzºú#À9Á?Küy<êêêE	­^xáõë×Dÿn¨¶Øl¶Ù=#Àø#ð7E[¶lQû«W¯Fç®?àO	øäWnhh(úßZ¾¯ØNs$XPDø<S?ðGà/l/^Tû6Dó®?à/1|úÝ¹:'øþ4X±bÛqíÚµ(ßuðGàü%TjÒp#9d¸I[Àø#ð77ø;qâÚéÔÖÖÆä®?à/ApúÝäàÀßl¯É<þüÍg^¯·µµ5ÜHéOÚþÀ¿¹Á_ÒTL&ðGàÀß,púÝçÉþÀ?£þü¿8KHn$´%;=ðþü?ðþüÍKO¿»råÊ3gÎDAðþü?ðþüÍq²+ÓÉQ__ÃÅ?ðGàü?ðGàonpú]u¤3gÎÌá¤-àüøàüøM.+ÜHÜÜÜ¾¾>ÜUðþü?ðþüÍ²§ßªªªÚÛÛçoÒðþüÍþçÀ¿)ÝT¸ÅÅÅGÉIÁø#ð7§ôÓÄóüø£Å_Óï&''oÛ¶­££CoúÀø#ð7Ý:;;ewöÊ+¯Ë§òqÓ¦M²æúõëàÀ%þÄsFr655Ea~fðþüÍ/þ²³³e§ø_ØÉÉIõÇËÏyËò;g2JJJ®]»þü?ÝÖßßüøqÙï/++«®®N~-u~¨ü?Ó¾g	øð÷üóWSSóÎ;ïÈÂÉ'kkkÁ?ð§·<O[[[ee¥æ¡>YßÜÜ,_cÄü?aËÉÉ@mbbBí7lØ kdýsÞrvv¶zyMÍæPüµ´´ÜnôQww÷mÒqÿüÏÿüÿðlwùòåO>ùÄÐ?ÂßýÝßýÑýÑoüÆohêûæ7¿)(4ôÈîÎ»;ÃÁvÐÿî.úßtÞñwýúuÍwîÜyÎ[2:|DðwñâÅèvãÆ§Ó9@:®¯¯ï?øÛAçðÁ<0â=ÿÉO~rôèÑ¢¢"ÍåÔ©Sò5qð	,~üãó»ªçþíßþÝþ»råJôwwó?éÞ½iiiIIIéééeee=zþ|ã8%%··yÛ7Vù|¾7onÝº5Ü¤-SîpyÛxÛ·ãämßù+''Gý)¡|üøQÈívìòóóCÍ'´ÙlñúûþÀ¿dµZÏ=+ò±¦¦üøQËëõ¶··´¥¢¢¢¥¥Å(¶?ðGàoîñw÷îÝ¢¢¢ôôtõFmNNÎÿf»»»ssså6óòòzzzÀ?ðNçë¯¿®&±Éa·Û].W"<Fàüøä9ðnjùäÉóz×Á?ð7555­Rs$GUUU[[×ëMÇü?aSÞ½×¿YÎÌÌþHÿø=IVVV(ûÌf³ìjâl$øþû&¥þdÎ¹	üÎñáô»©©©V«ÕápëàüøþÔ$ÏêhàorròÀêÌàÀéâ9ÙoÝºUs$X0îGr?ðGàïyëêêÒäùÆàÀé.Ën·ËÿK5GrÔ××óþÀ¿¼Ü«Ñ¾iiiEEEn·¾ï:ø#ðþ¦×ëmmm­¨¨ÐüoªÅbI´àüø3jàÀøü&DÉ8¶?ðGàoñççáïÑ£GEEEùùùàÀEccc---#9ÉþÀ¿¹Çßää$£	üQôñ'û±]â~ü?ðþ¢?Í3]¶páBðGà¢¿¶0ü?s¿û÷ï'=ËnÀD~/^þhþðçóùG"~ü?ðþ¢?B½ù~üø¹Ýîp¶0ü?ó¿Xþü%þ¼^o[[æ¡>ÿéwçp$ÜTGGÇñãÇÏ??44þüøûeEEEéééþ5-zë­·À?+ü¹p¶ÌÇHÑ^IIIÁK_«üÚß_ÙÕÕþüøûE/ð«öÈàÀ=þÔ¤-ó3Ï÷éw¿úÕ¯VVVzãú÷­oKüPÇÿÀø#ð¶Ù;Nÿù]5²£þhvø0?sFräää|ý òoÅï¯8þ<ø#ðGàï©ð´üüø£)ñ'êÒÃ¤-ò¿Ù_~9P~òïk_ûÜ7ðGàÀßÓ¼¼<Ù/ïÙ³GsbbâÐ¡C²&77üø£éàO­¨®®Ù?#§ßËÈÈØ½kw þÚÛÛÁ?O5'yîééþ(2þìvvvv¸I[ÜnwLîáÞ½^*¨ûv°oßÞ_©(--&@Á?ð§_üI%%%iiiIIIéééK.5ó×Á?ãvéÒ¥Ë/oÝº5ôPß|ä~ÇËÌÌÌÉÉY¸pauuõÈÈHB=FàüøÓ]àÀëëëÛ·oß¾ô%ÍI[õvN¡¡¡3üøàüø3X###MMMË/×´¥ººº««+1þü?CâïîÝ»jg5Â7''çÂàÀI¢:ÍjÒãÇsú]ðGàüþ]¹ÂZ>yò$ø#ð°E8ÔWUUõ£ý­þü?Câ/77WöæwïÞõã¯§§I	ü%f>Oötáõ­òüùóccc³;·/?àOøSûô§;Ë>]M&ø#ð89ÎûöiNÚ"+å¢¾¾>ÿ?ðGàü999jV?¿ÉÉÉ¨9ºÀ¿¸ÏãñWª¬¬lmm¯	ºøþÀñ×ÕÕ¥¹Ó¿qãø#ðÇ©I[Ô~UZZ*"w]ðþü?ãO½Ü«Ñ¾iiiEEEQüøI###ÍÍÍú·mÛ6I[Àø#ðþ¿þüE3ñÃá°Z­#9òóóå)Ùßß?Í[àÀøàÀNs¹»Ýl6¹££c¦ó3?ðGàüöÙ%KRRRäÅ --­¤¤dxxüø3t^¯·­­Íb±Ìh$øþ(þñçp84_§uþlÀúúú¬¬¬Ðçµ¬´ü?pøScýjjjÔ1ñññÚÚZYþü¨±±±ââbÍUUUmmm^¯wN¾øþÀñ§^ÿâGý>?:/òHÆÆÆ¶?ðGàêÈßää¤ÍÄÄGþüé?!ÀNx§9C8((éHðþüQüãOýÍ_MMO>üøqee%óGàO·©UUUÉÉÉ¡ì«¨¨hii×ûþÀ?ðg`ü-ªyzÿüøijÒp#9êëë£¶éÀø#ðþ¿¤©2LàÀ_S#9***4ÿf±Xæp$øþ(þñ«À¿)Ma³Ù4GrÍf»Ýîr¹brÇÀø#ðþ¿pó9?1Iä7iË¼äàÀÅ?þäåäÍ7ßZ¹mÛ6¦z!ðåÔ¤-áFrçcÒðþüQÂáO'¯+999=Oß÷]õJ3Oêþüæv»Ã~7Ê#9Àø#ðGñ¿§Ïó©¼¼<µ°iÓ¦ù¾ëàÀ&màÀ%"þ¤ë×¯û_r£p×Á%2þW¸ÓïÆv$øþ(!ð·sçNõª£Îö!½òÊ+àÀßaÒuúÝäàÀÅ?þL&¼ðdffÞ¾ûiÀßü¥¤¤?süPáõåççëg$øþ(þñ'¯=Zi³ÙíKàïù7i:ýnWW!õ?ðGàüÅþÂÍó'¯ÁàÀßìR¶í4çg^¾|ySSÓÐÐq@ðþü?ã/V?Kü©ù2iøþ(Qð'/EoìFþüøÏç3è¤-àüø#ðþüÍ Ën~æüüü§ÓøþÀøXøSó3[,ÍÕÕÕ²¿¯×	ü?àü?JüE´E~×Ð#9Àø#ðGàüøûEægV¶Dÿ÷üøþÀøsÜÍnÒæææxÉþÀ?àjÒÍù322êêêâr$øþ(ñ9ðG?¯×ÛÞÞ^YY:i:ýn[[ÇãIð	ü?àÏxøK*Éþ(¡ðçt:÷íÛ§9£  Àp§ßàü?úüNð722ÒÒÒ²råÊp#9O¿þÀøþÀø¿VWWW]]]FFæ¤-qvNðþüø øinn.--ïÓï?ðþÀ?ðþ~YGGÇo¼±ÿþöööÄÁÏç¼ªªJsÒø;ý.øàüøàïmß¾ý·~ë·þÏºÿóø/¾øâtu²ùÀËåß·ììlÍCv»]¾øþü¿8ìüùó¿ýÛ¿ý§þôÐäß?;ðòË/744Ä%þ<O[[ÛºuëBÍç´%O¿þÀøþÀøú­okãJ~êß«Û^]Vº,Îð'·nÒ³Ùl·ÛÝn74ðþüøñß-[ÄøÛñwÇþÔéw5'mINNfÒðþüø	×[o½µ¼ly þ*¿ZùÍo~ÓÐøÏêÄv#9¶ÌÏþÀ?à/Aóx</½ôÒW¾ò÷ø³kÿ÷Ú¬¬,]½:#ü	éäÉl69ú¿càÀ?ðþÀ¾0mÜ¸QÌ·páÂµk×êm ëtðçõzÛÚÚªªªBO¿Ë¤-àÀø#ðþÀ?¡j½æHægþÀ?ðþâj$GEE¶?àÀøñ?Y¶Ùl#9´üøþÀøü466ÉÁ¤-àÀø#ðþÀáÏýýßÿEEæH5i#9À?ðGàü?Ãçr¹ìv»æ¤-äþÀ?ðþâ$¯×ÛÚÚª9I[À?ðGàÏxø6ÍàBûánÒ»Ý®·		ü?àüMQww÷Ò¥Kåµü¿±±±p#9Ô¤----===l(ðGàüø3þÖ¯_ïr¹"àï/ÿò/ÑíêÕ«ßûÞ÷þú¯ÿzíÚµééé¡ìûÝßýÝ?þã?>þ¼|<@ò0±¹tÞåËÙ:ÝþcwÇî.Æß/ïbxüôÑGÿÝ>ùä·Ûý_Åd9rä¥^Ò´¥¶¶6è×àþýû===l7÷Áüüç?g;è¹[·nþùçl=×ßßÿñÇ³tÞ+W&&&¢üMã¼íßÉÿ]l6ÛLO¿;sûoûoûò¶/ñ¶¯¨§	ËåjhhÈÍÍ5¬¬««<ü?àÀð§ÉAðy½ÞóçÏ[,ÍC­­­gÊÛàÀø#ðþÀ®ëëëÛ·o_vv¶æ¡>»ÝÞßß?ý[àÀø#ðglüü½±±±ÍùSSS×­[çp8¼^ïLoü?àÀøúJp¶oß>Íù/_."õ?ðGàüøàO	é?.¼5_FFF]]ÝÍ7ÿ»?ðGàüøà/Æ9­[·FÉ1·wÁø#ðþÀøàO_?~<Ü¤-3ÉþÀ?àü?&ÍºuëRSSCO¿»qãÆ¶¶¶éLÚþÀ?àü?]çr¹ìv»æ¡¾üü|yÈg$øþü?ðþtQäI[¶mÛM?ðGàüøào¾fÙl¶Ð·w¥+W655i~üøþÀøFj`` ±±±¸¸8Ô|YYY»wïv:±ºoàüøþÀø|>Ãá¨ªª´EÖÈú¶¶¶9´ü?þÀø±Iä0Í¡úd¥v»urWÁø#ðþü?ð7Ë¼^o[[Åb	5_rr²Õju8>OW÷ü?àÀø3NüT__¯yúÝâââÆÆÆÞsðþü?àüM75iæHÔÔT«ÕýmþÀ?àü¿9NäÛiNÚRQQ!"þ¤-àüø#ðþÀøãdKjäÈÊÊª¯¯7¤Àø#ðþü?ðÉ¡9i:Ô×ÚÚóI[Àø#ðGàü?ð÷¼¹p#9Ô¤-òþÁø#ðþü?ð÷CçÏ×<ý®~f½MÚþÀ?àü¿aÒ³Ù,Q·¶?ðGàÀøàoº455´Eó3?ðGàÀøàoÆuttTWWkä2iøþü?ðþÂæv»eäççkêÛ¶mÛÍ7ãòPøþÀ?ð@øÏuttX,¶%ÎÓü?àÀøOü¹»Ý®9?svvv½Ñ'màÀ?ðþÀ_¤I[¤7¶··<?ðGàüøq¿)'méïïçiþÀ?ðGàü	;iøþü?ðXø0iKqq±Póü?àÀø36þ|>_¸I[²²²vïÞít:y:?ðGàü?ðþâRqqqè¤-gÎñz½<Àø#ðGàü¿xÃ_SS2_nn®Ýnw»Ý<Àø#ðGàü¿¸Å_UUüÆp¨ü?þÀøüøþü?ðþü?þÀøþÀ?ðGàü?ðGàÀø#ðþÀø#ðGàüøàüø#ðþü?ðþü?þÀ?àÀ?ðGàÀø#ðGàüø#ðþü?ðþü?þÀøþÀ?àü?àÀ?ðþÀ?ðGàüøàüø#ðþü?ðþüøþÀøþü?àü?àÀ?ðþÀ?ðGàÀø#ðGàüø#ðþüøþÀøþÀøþÀøþÀ?àü?àÀ?ðþÀ?ðGàü±Àøàü?àÀøàÀ?ðGàü?ðGàÀø#ðþÀø#ðþÀø#ðþÀø#ðþüøþü?þÀ?àÀøàÀ?ðGàü?ðGàüø#ðþÀø#ðþüøàüøþÀøàüøþÀ?ðþÀ?àÀøàÀ?ðGàü?ðGàÀø#ðþÀø#ðþüøþü?þÀ?àÀ¿×ÓÓSVVf2-[úûþü?àíþâ7nÜsçÎâï£>nò4s»Ý¤ãúûûå¿l'øóx<l='ÿúüóÏÙzî/¾øøãÙ:Oð÷óÿ<ÊßÔÀø,---õWõQtûðÃ¿÷½ïD:®³³óêÕ«lwùòe6ÎcwÇî»»üõööÖÕÕñ¶/ñ¶/oûoûoûò¶o<¿í«¯©©ñx<àÀø#ðGàüÅþü*õéàà ÍfóÐ¯þÀ?ðÇvÇ_`]]]k×®Ö¼üøþÀÛüÅþÌfóÀ?ðGàÀøgüEüøþÀÛü?ðþÀø#ðþü?ðþüøþÀøþü?àü?àü?àü?àÀ?ðGàÀø#ðGàüø#ðþü?ðþüøþÀøþÀ?àü?àÀ?ðþÀ?ðGàü?ðhøû¿ø¶¶¶þèöá~üñÇý¤ã>ùäË/³tÞßþíßÞ»wí çdw×ÛÛËvÐs"?ÛAç>ú/¾ò7OüÝ½÷Ð¡Cß!"""¢"¿5ºã±DDDDø#"""DDDDþüø#""""ðGDDDDàO§õôôL¦eË©Ô»ºº.]*kJJJº»»ÙD1ïîÝ»+V¬|DäÊËËSk®]»Æ&ÒçÃúä"½=FªÎÎÎx±Ðécäõzm6[JJJ~~¾¼<±ôù0éM<§¨°°ðÆ²pîÜ¹¢¢"YÈÎÎ¾ÿ¾,ÈG³ÙÌ&yòté,È#%,ÔÔÔ¼óÎ;²pòäÉÚÚZ6>¦Ð'éí1|>üéö1:räÈÛo¿ýäÉáÅ%KØDú|ô&Ï3(--M>Ê³kppPä#Ï4]ÕÑÑ±lÙ2õ4SçèºBÃÇè­·Þúîw¿þtû3úúúØ :ô&ÏÓ­···®®îé³·åJvòQV²eôÏçËÌÌåÜ¹sò©Édò_¸LºzB·Çèþýûåååò_)ð§çÝÝ'äõHHqçÎ¶>&½Éçó´¯©©ñx<²¼bÅõß,y,W­ZÅÆÑO999²ä_ÂÑçÃúä"½=F7o¾~ýú/^*Àww§OO?ýT¤ÎÑçÃ¤79ð|ºÁÁAÍöðáCõ)GôzDäÉ699ùôÙÛ¾Î ]=L¡O.ÒÛc´à×c³èswÇKþ&½É'óuuu­]»vxxØ¿FÌ~÷î]Yp:by6QÌ+,,THOO<X²`µZÏ=+ò±¦¦M¤Ï)ôÉEzþç¥ùéõ1Ú¹sçï½÷ôÙÓ6>&½ÉçóÍæ ÿø~öÙgj·|e6QÌ»uëVII<"kÖ¬QÇº»»sssòòòä¹Ç&ÒçÃúä"½=FàOÿÑèèèæÍeMyy¹ËåbéóaÒx>%Pàüø#""""ðGDDDDàÀ?""""DDDDþüø#""""ðGDDDþüø#""""ðGDDDDàÀ?"JôÞ÷ÝÕ«W§=kÍ5/^üµý×³³·Õº·f³Y~4Ç´^ÖL¦¼¼¼'OÌô6À²C-éÍ7ß'ü544ÈÊæææ õMMM²þðáÃ³¸M""ðGDÆëÖ­[ÂÉtêÔ)ß³N>-ÊÊÛ·oÇþîÝ»'+-[´~éÒ¥²Þív?"D½òÊ+Â·ß~;på±cÇdå¶mÛésíÚ5Á¸°¬¬Lý_üøñãºººÌÌL¹(;;Ï=ï®:×íèèâ¬Y¸páêÕ«¯^½*Z­Ö ;våÊÈ·£.»¤.êììµµk×Êúÿ7nÈÅâ_säÈÜÜ©´´´-[¶â/ôöÖD¸«DþbÜâÅ.÷ïßàÁYi6qToo¯ºtÓ¦MAíÚµK]ät:4¯¥>UnÞ¼ùÉ'ÂÇÏ'ÊGÁSzzºúS¼·#w/ô'=wîhý¾|çwüòº5kÖÌî*?"¢Ø§ÞáÕØg-X ÄMmmíø³dA>Ý°aºTYGñQÀ´©jjjÔ!CYîîîV7xÞ©w]·oß.k.]º$ËòQ_íµ)oGà(ÊÞ7ÍH@¹hÑ"ùyåSù(÷999©¾ //O®(?ÂÓ_½M6SüE¸«Dþt?Iþõ7ÊLÒàà |*lRÉ§K,Ù±c mbbÂ#ò5AÇÒü×R>xðÀÿÅ&ÿ;¿òQÇ·#ÐO=zxßÂýÞÞ½å¢ï~÷»OõÖö=¿@ (ø«3Å_»JDà(ö©Ã]ããã+=¬"pÇOCË¥üç·ÿõBßâTà+²âõzåÆ.¿tÊÛ	G± îÞ½«*ËòQN§ÿÒîîn¹oOî*?"¢Ø§þîíÄ+Õ(A>ü8<<z@ëöíÛêMX¿Õa9ÿûªSm×®]êÝ^ùXWWç_ávÔ¶ªO=zydîªU«äR5»5ð"5òW.êìì??LÕÖð_á®ø#"jÀÉd:yò¤êåÔ©S)))¡36lØ Ðñz½ê=YÿÈ7êOåWà*îÙ³G¨¤Æáú'[Ñ$~«¾WWÛQä§þæÏãñ¨¯¿Ó§OûÉÉxúóG§Ó)ßE6B8ü)nåË[¶l	¼4Â]%"ðGD¤ÔÈA9räö_ÏR6ò/ûß0õ±ð·ûö@YváÂøúìT²>;;;ðá·#w#ðùGÚûa'&&ÒÒÒÔôf÷úõë¿Å¢Eä£í%ð6ÕIªþK#ÜU"DDzI²fÍg­^½Z¹Â_gg§N¯¤¤äúõëþK=ÏrssöîÝëõzývttÉòòòZZZn3ôÈMÉú;w­w;Ü¹KjÂóüù«««óE5<<#[ 33S~·Ûí¶&ð6GGGE·j[Y, ïá®ø#""""ðGDDDDàÀ?""""DDDDþüø#""""ðGDDDDàüø#""""ðGDDDDàÀ?""""Vÿ<s¦rXñIEND®B`


ûö°C½*,çîÝ»çÍwúàþ£7nðûðÃ?>///ü¿hÑ¢0g¨þàþ¿ãÇö³ðÁ«¿ù?>WæÐõÁü	þF5þF$ø?øæºººÊÊÊ«ylãmþéK_ÌÀ²M3Ô7nÜ¸2Ôð'ø?ø?å8þöíÛ¨_ø·aýÀÁ5xvOJ3Ô7mÚ´mÛ¶uwwÅ÷?øüå>þÆÜª¼¼<ø?åþvìØQýåêDüS§N=yòä/Û-úúéþàOðûøË8ø?å$þ~ó73Q~kY[TTÔÕÕ5Kè9"Cð¿Q¿zæg¢§Ý=öÀü)ðÅfÌñÅù_Ü°~C$¿éÓ¦×ÕÕÈÂÒ544àPüÁàþn:uª¨¨(<óVWW'óþàO¹¿Ð¥K~ë·~«¬¬lÚ´iáñ¾bÅa^ê?øüvü=úè£ÑïÛo¿=<ð#HÀ3gÎóÞÞX,ÖØØ¸xñâê?øü^ü½ùæÑóïÒ¥KsÑáþàoÔÖÖ¶~ýúÒÒÒC#2Ôð'øø»víÚ>ÛqèÐ¡a^tø?øËíb±Ø¾û¾öµ¯;6y¨¯®®.2?øü"ü½ðÂÑ³ðòåËGdÑáþà/W;þ|xgøPüÁàoÔáÏyþàOðwoÅbág_²dIòP_ô	¼áÒü4ø?ÁßhÁ_Þ­ÊÏÏ?øüÝNííí6mJ9Ô7iÒ¤íÛ·gàPüÁàoÔáoÄ?ø¿¨¹¹yñâÅ)úV¬Xã;Ôð'ø?ø?ÁßíÖÑÑ±uëÖÉ''õÍ9sçÎ#û©!ð?ø?øüÝyäqãÆ%À[__½¿±ð?ø?øüýWáÉzíÚµÉCsæÌÙ¾û¥K²ú?øüÁüÁàïFooïk¯½ò]ÅÅÅ=öØñãÇ³å]ð?ø?øüØùóç7mÚT^^r¨oÇÃÿ¡Àð'ø?Ýp?øüÝÛ¢sõ¥ùX3gÎääV?øüå>þOéÎóUøÎÕò]3gÎ|ùåsx«Áü	þrñ<Üüñ«W¯/ÃÿË-s9ð§Ç_,kllLù±ÅÅÅ«W¯ÎwõÁü	þàï?NÊøäÞ××æßå=:uª¢¢"??Æ?ø¿*ÍPß9s^íµÌÿXø?ø?ø»£»¸Y_?üÝýþjjj^õÕ0ñâ/._¾þàþ2¡ÞÞÞúJJJV®«ïê?øüÁßTVVôÔ®]»¾ìééYºtiæßå=^¿~=Òdeee2þNkà~ð¼óÎ;ÖCÖõÖ[oeæ½ùæð0~üøïêÛ²eKKKË¨ÝjMMMáç·7»úÞ÷¾wôèQë!»h>|ØzHßãïÈ#)øQÝå='2|øHÀ_x)êÐÀµ··777[Y×>ùäÌYýìg»víúíßþíäù¯üÊ¯üþïÿþûï¿o«F|ôÑGÖCvuìØ±ÖÖVë!»:sæÌ'¬ô9þBçÎ2eJaaa^^^QQÑìÙ³/_¾|÷w¸ã¸  Àn_»íöæÚÚÚ6mÚTRRÌ¾yóæíÞ½;·àµÛ×n_Ùík·ïpWVV½0ü¼þàþ¨è]ÕÕÕÉæ(´àþðwï«­­åWÂDø¿¦¦þàþº4CÁAúàþð÷ï=vêÔ©EEEÑÚ²²²½÷ÞýÝ=z´¼¼<ÜgEEEKKüÁüQúàþð7¢<'~¤[4ýâ/é¢ÃüÁßÝT·råJCð?øDÑ·=6¿0=~üxø?e&þévïÞ=oÞ<CðÖüÁßàïâfÑD¿ë×¯ûl_øSfâïÌ3úàOððwWE'yFûþúúúöÙ0|Zfø?þzzzöíÛ·`ÁäåUCð?øD)Oò|ìØ1ø?8þÎ9³víÚâââ~Ð À%K466&~0·àþðw[]¼x±ªª*:Ú·°°pêÔ©íííC½èðð¦è]sæÌIþÃ,ê)ü	þ9¿	þàþRëëëÇ7Ð»úõÁàþàïîîâ?ówùòå©S§N4	þàOÃ¿;w¦|W_qqñêÕ«½«þð§¡Â___£áOÃ¿è])à3gÎË/¿ÜÕÕe­Âàþt·ø4iÒ´M0þàOC¿îîî;v¤|W_qqñÊ+[[[­LøüÁîþ>þøã¼Å?Û#± ¿7ß|þàOC¿ð¿zõêCK,Ù·o¡>øüÁî=þâêõ^ø?ø»qs¨ïå_~àRÀ»yófðÂàþ4ø©àþîUa-mß¾÷îÝçÏÏLü_õºººà×÷±ð'ø?þúúú¦NZTT3qâÄo~óð_OOÏ¿øÅûî»ïË_þòÜ¹sßxãÌÁ_tïÌ3ÍWZZºvíZÏð'ø?þÂg¿~£§Í7Ãüex¿÷¿÷Àì6¬ß°ñ6_¯ûú	Fü8¿ýèGõõõ)?cñâÅ>þð§Ä_AAAxMJ|½<uêT3~üxø¿ïWõW×>³6_ôïK¾4ãÖÕÝÝÝÐÐýAÕ¯À?Á_Fà/:à÷úõë¿t¿I'ÿ?øË´´JKKåþõ«_ýÃ?üÃá_'O¦|W_4Ô÷òË/ê?Áà/SðWQQ^¢Ö¬Y½8]»vmãÆaNyy9üÁ_WRRòõº¯'â¯ªªjûöíÃ	ÐiÓ¦%õGÐæÍ;::l&øü	þ2'NHyçø¿oÇá¯ÈÏþ?Ï.Z´èsûÜð$/ÍPß9sÞï=Cð'øüe(þB.1cFaaa^^^QQÑôéÓÃ¡^tø¿ÒîÝ»Ko6aÂõH[ww÷¶mÛRõM<9àÎ>ÛWð'ø?þF$ø¿XWW×P´_×ÚÚÚCa~sss,®	ð'øüÁüÁ_¶f¨¯²²2ü'5Âü	þ¿³gÏFçyð-++Û»w/üÁß(/ÍPß%Kúàþ¿lÂßÁã¯jþ¢é_|þàovCð?Á_6á¯¼¼<¼ª=6¿'y¿QØõÁü	þÙ¿èåíFÂ¯_¿¦óóóáþFCiú6mÚÔÞÞ>¨;?øü	þ2eeeÑYý"üõõõ=ûì³ÑËüÁ_ÅkkkÇr¨¯±±ñ6úàþ¿lÂ_àEÊ<;vþà/'ú&O|¯úàþ¿lÂ_èâÅUUUÑÑ¾S§N½Ë?ø¿lèúàþ¿,Ãßð7l¥ê+))¹û¡>ø?ÁàþàþF¾4C¡êêêÆÆÆ¡øø?Áà/Óñ÷ÑGÝÿýá±°°pÆðÙÛ-úÚÚÚî»Ãü	þ¿æææ|õ¶?ø»çECK,æ¡>ø?Áà/ðä¹¦¦¦§§'|yõêÕåË9ðÙRGGGøª¬¬¡>ø?Áà/ð½@&ä¦ã'|?øËä2d¨þàOð'øË&üE#ñ9×®]3ò^¡¾è}Ã9Ôð'øüeþ¢÷üÕÔÔó/?ýôÓxÏüefiúîí¹úàþ¿Åß[5DûáþÕ-úáÌäð?Á_.à/ïVåççÃüT±X¬±±1Ãúàþ¿lÂßHð¾öööM6eÅPüÁàOðMøè|Î.?øþ²q¨þàOð'øË&ü×Ôo|ãýf®X±Â©^ào»téRø­Hù±>Ôð'øüeþòÂkYYÙåËÃßùÎw¢Û!z«üÁ_¿z/^<ÐP_sssõÁü	þÙ¿7Çù¢×ÚhbÙ²eC½èðçÏß¾û@ïê¿!ÙøsÁü	þ¿Ð#Gâ¯»;wîE¿Q¿ÞÞÞÀ£4ïêËº¡>ø?Áà/Ëð÷ôÓOG/½Ñ§üqø¿^kkëúõëKJJ²ý]ð?Á_ã/???¼ô?þôéÓ7ÞóWPPðw¯jjjJù®¾qãÆ=öØcÇÏê¡>ø?Áà/ð^7lØÐof]]£áïîðÕ«WO4)y¨oÚ´iÛ¶mëîîÎ½ð'øüe4þ:ÏßÅáþî¬X,öÚk¯tï#<6.õÁü	þÙ¿þrÑ'ð¦ê3·nÝÚÕÕóþàOð'øËDüãÄ»é¿?øK_ooïîÝ»çÍl¾qãÆík_knn=þàOð'ø?øËYüEÀ[ZZÌ¾3g­|éÒ¥Ñ¶áàþ?ø¿_OOOCCÃ@ðÖÕÕeû¹úàþ?ø?øû÷ÚÚÚÖ®]r¨oÚ´i;vìCð?ÁüÁ_®á/Í»úÆûØcæ¡>ø?Áàþà/wð×ÖÖ¶råÊË1yòä­[·ê?øü	þàþ²·êkjj2Ôð'øüeþÒ£'Oh¨/úX[þàOð'øË2üåÝªüü|øUøëîînhhè}µµµ¶üÁàOðÅøñà/sðwË¡¾ü^øüÁàþàotáÏPü	þàOðð7*ð~¨/ÐPüÁàOððøëèèØ¾ûêÕ«ÃÿçÏÔ¦ê«««(´Úáþ?ø¿LÁ_kkkIIÉ¾ð¯~õ«ÁpýìgoshÐPü	þàOððøûÜû¿ó?6þÑÆèßòåËçÒ¸-ê¼3Ô?øüÁüeþÚÛÛý×Ãúqü3þMMMÉ·ª¶Â3Ô?øüÁüe%þÚÚÚ¦M(¿ðoÎ9ñëtuuíØ±ÃPü	þàOððõøëíí-..®ÿÃú¸üþ×ÿUTTPxãæÛW®®Ì¾yóæê?Áü	þàþ²¡]»vM,ø»µ¿ä÷õº¯ÿÆoüFý;æÌl¾ÀACð'ø?ÁüÁ_¶â/ÔÐÐ0eÊÏ|æ3sçÎ5Ô?øüÁüå2þ¢wõ¥êb¡>øüÁàþà/ðwæÌúúúC<ðÀÎ;õÁàþðY?Cð'øüÁàoTôýïÿ+_ù¡>øü	þàOðËEC&MJ6_iiéÚµk[[[­%øü	þàþà/ëKó®¾ìÞ½»§§ÇZ?Áàþàþ²»®®®mÛ¶¥ê0aÂÚµk=NàOð'ø?eþ:;;+++áo Òõ544¼óÎ;~ËáOð'ø?eþ=:úôàøëWOOÏîÝ»'O<Ð»úÚÛÛop?ÁàOðð¡=ôÐCmmmið÷§ú§Í£©wßwëÖ­_þò?óÏ$³¯ªªêgyë­·â×ï½÷¾ûÝï6+ÛJÜÊÂcíàÁÖCvõöÛoçUë!»jjjzçw¬ôe1þþcÆß÷¾÷½ÿ=:êèèøó?ÿóÏþó½«/üý|«ÎÎÎCýoe[¸víõ]>|ø¿øõ]ýð?loo·²«?ü°µµÕzH_.ã/çwûÆb±à÷ÚÚÚÎÕ÷òË/§9×n_»e·¯ìöµÛW¿¸lF9þÒ«¯¤¤dõêÕmmm·¼ø?ÁàþéøKÉÁQ¿ðC=òÈ#cÇMfß9snÿc9àþ?øüeh.]Ú±cÇ´iÓR~oý|,üÁàOðÊ>üTÎà/ü=öØ¸qãÙ7oÞ¼;þXø?ÁàþRww÷@ËQ\¼råÊ'OÞå·?øü	þàOð7ÂÅàM9Ô÷Àê]ð?Áü	þ2´èÞÊÊÊð®òÞÕð'øüÁà/ãJs®¾ÁÀð'øüÁõZWW×öíÛïí¼ð?ÁüYðquwwt®¾»<þàOð'ø?ëþ2±~~÷ê^ø?Áàþ¬øËÄ¶nÝ:ðÂü	þðg=À_&véÒ¥¡8þàOð'ø?ëþð'øüÁàþàþ?øüÁàOð'ø?ø?øü	þàOðð?Áü	þàþàOð'ø?ÁüÁü	þàOð'ø?ø?Áü	þðð'ø?ÁàþàþàþàOð'ø?ø?øü	þàþàÏ&?øü	þàOð'ø?Áàþð'øü	þàþàþ?øüÁüÁàOð?ø?øü	þàOðð?ø³àOðð?øü	þàþàOð?ÁüÁü	þàOð'ø?ø?øü	þðð?Áü	þð'øüÁàþ?Áü	þàOð'ø?ÁüÁü	þð'ø?ø?Áàþðð'ø?ø?ÁüÁü	þàOð'ø?ø?Áü	þðð'ø?ÁàþàþàOð'øüÁüÁü	þð'øüÁàOð?øü	þð'ø?ÁàþàþàþàOð'ø?ÁüÁü	þð'ø?ø?Áàþðð'ø?Áàþàþð'øüÁüÁàþ?ø?ø?ÁàOððð'øüÁàOð?Áü	þàþàOð'ø?Áü	þ?ø?ø?Áàþðð'øüÁàþàþ?øüÁüÁàþ?ø?øüÁàOðð?øü	þàþàþàþ?ø?ø?Áàþàþð'øüÁàOð?Áü	þàOð'øüÁüÁü	þð'ø?ø?Áàþðð'øüÁàþàþð'øüÁüÁàþ?ø?øüÁàOðð?øü	þàþàþ?Á_®ã¯¥¥eöìÙùùù³fÍ:uêüÁü	þð¹¿)S¦;v,LìÙ³gêÔ©ÉøûÞ÷¾wM×ÙÙyèÐ!ë!ëøûùë!»h]¾|ÙzÈ®N:ÕÞÞn=dW?ùÉOþöoÿÖzH_ã/±ÂÂÂdüýÙýÙ÷4pï½÷Þw¿û]ë!ëzë­·¬¬+<ÖÂ#ÎzÈ®Þyçwß×zÈ®Â&kjj²Òø;qâD½Ý¾vûÚí+»e·¯Ý¾vûæònß¨«W¯ÖÔÔôôôÀüÁàOððkøóE_^¸p¡®®îâÅÉ×?ø?ÁàþõøK,ÀeÑ¢E)/?ø?ÁàþSø«¬¬üÁü	þð¹¿ôÁüÁàOð?Áü	þð'ø?ÁàOð?øü	þàOðð?Áü	þàþàOð'ø?ÁüÁü	þð'ø?ø?Áü	þðð'ø?Áàþàþð'øüÁüÁü	þ?ø?ø?Áàþ4Úð÷ÇüÇç5p?ùÉO^ýuë!ëzé¥þáþÁzÈ®þò/ÿòG?úõ]½óÎ;?øÁ¬ìêý÷ß?tèõ¾îîîÜÄßÙ³g7nÜø'$IJ(ý®Ñ1?%Iìö$IüI$	þ$I$I?I$Á$IàO·ÕÁÇù­yñâÅ1	%^íÔ©Sùùù3fÌ8tèõ9[-qååå%^-ÍÕp¼!Ò< <Ö2v«µ´´Ì=;lY³fÍä±-ÎKüéÅbá¹,þ`Ø»wo]]]ÊkÖÔÔ¼úê«aâÅ_|¹U9[-^Ø@aë$ÎI³A5%o4(µÝjS¦L9vìXØ³gÏÔ©S=Ö²eÃyi?ýRßüæ7¿ýíoÇû÷ïOyÍÒÒÒë×¯¾¾¾ÊÊJ«.s¶ZÔ¹sç.ïi6¨³äæå±±[-±ÂÂBµlÙp^ÚàOÿÕÇ~ïã×.Z´(???ÌÿðÃ¯f¦Öoµ¨~øÄý®fj8KÞiPk»ÕâÇZ½ÇZ¶l8/mð§_âÂ#GnÜ|ÓX¿.0kÖ¬Ä9o&+((°ö2j«µµµÍ;7Í­7¨F¤øHóòXËØ­uõêÕµìÚp^ÚàO7nüòÉþë÷0(++ëëë»qsl<L[µÕ¶lÙòÂ/¤¿¡çµ)ÚiPk»Õ"=ÔÕÕ]¼xÑc-»66øSODS¦Liooà-ZxÚÚÚW^y%LÿÃ_½VZælµÐüùóO>|4TÃYòHóòXËØ­vøðá0ÑÙÙé±]ÎKü)#N81úôüüüIâ¥G-//ÏËË«¨¨hii±Ò2áoÙè=Ëý.M¹A5ü%o(µßjÉãîk¿á¼´Á$IàO$Ið'I$ø$I?I$Á$IàO$Ið'I$ø$IüI$	þ$I$I?I$Á$IàO$	þ$I$I?I$Á$IàOßw¾óùóçÞláÂo¾ùæ/=Ý,kmS-meeeøÑzzzúÍsòóó+**®_¿>Øû$ømÜ¸qLRßøÆ7r	7o3wîÜÙoþ;ÂüçîîSàORöuêÔ©Àüüü]»vÅnöÒK//ÃÌÓ§OçþÎ;fÎ5«ßüéÓ§ùíííð'	þ$üñÀoë[3þù0sÅô9tèPÀSpáìÙ³ÃtüÊ~úiýøñãÃE¥¥¥kÖ¬IÜ»ÚÜÜ.·mjjêÇ©0gÂ	óçÏûí·ÃµµµýìÀéï'º(,RtÑÁÚ¢EÂüøcÇ9ÕÕÕñ9[¶l)//wUXXøè£^¸p!É÷ßoNE4ÂÝwß.üqâÌO>ù$Ì¬¬¬LÄM¿N8]ºlÙ²~­Zµ*º¨µµ5///å­¢/£K~øáë×¯>Äb±piø?à©¨¨(z+^û	)/ù'Ý³gO¢hã¾|õÕWãòëw'.,þÒ,ª$ø¤/ÚÃâ9kÌ@±DÜ,_¾üêÍÂDøréÒ¥Ñ¥u">FxmÑE555Ña>zôht'÷¹aÃÀ»h¯ëO>æìß¿?LÿÃôO<qËû	p_+$.[Ê(râÄáçíìì_ÿÃqöõõEW¨¨¨7?ÂÿÜMXü¥YTIð'I¿PJüÅçG¸ÌºpáBø2°)úröìÙáËûï¿ÿ©§h»víZüNÂuú¥ÅoùÉ'Ä¯ÀßóþÓÍÍÍ·¼ÍðååËm ÷ç=óÌ3á¢oûÛ7þs×ö5k¯ ðHZUULiUüIÒÈw]½z5qfOOO.JÃ8ÛÚÚ"ÿÅ­£^òÞØ~J<ÁJ.---((èííw>aÂø¥·¼(Ö¯³gÏFNÓáÿ0ÝÚÚ¿ôèÑ£aRî>¾ü¥YTIð'I#_ô¾·^x!qft~|ÄßØÙÙ< uúôéÍ7G;aãjåâûUoI´U«VEÃÿõõõñùiî'i»xñbôååËÓ;wîÜpitv`ÖÄ¢#ÃE¼råJzüÅa­ø¥iUüIÒÈ0ÿâ/F§zÙµkWAAAòÁK.ÐéííöÉÆÌÞó½U®­­-ñÍ×¬Y¨?ÙJJ¢EßFßýðáÃñùiî'z_ô¿èið÷ÒK/ÅÇäÂxQôöÇÖÖÖð]ÂJ7ÃÕ:ôÑÄKÓ,ª$ø¤(:r¿¶lÙò_Ï_7lï0cïÉ'Leb÷îM¿7?#Ì/--MÜ#æ~Âb$.XüHÛ~Øk×®F?B¿Ý=ôPâ·8qbø?:ÛKâFñ"ªÆ/M³¨àO2¥ÜlþüùÑ!·ýðwðàÁètz3fÌ8räHüÒg¶¼¼<Ó3Ï<ÓÛÛ¿´©©©ªª*¬¢¢¢¡¡¡ß&/I¸«0ÿé§î7 û		0ÍyþâÕ××÷;çKTgggMMMXãÇ?Eü´5÷yåÊ Ûh]UWW·´´ôûiUüI$	þ$I$I?I$Á$IàO$Ið'I$ø$IüI$	þ$I$Ið'I$ø$IüI$	þ$I$I?I$Wÿ?-?ACgIEND®B`


Detrended Normal Q-Q Plots


7ÚÚÚ¬xäÈ¢»:ÚÕÞvÅ¢;°zõêøzàÀÑÆä½÷Þ[¹rer³ÙìÅ.Yøx9q£qÓq.õêÕwÞy§¹¹¹ººzÉ%·oß^__ßÈ/í?õÔS1¿ð lXkÖ¬qûwá7^9q'Ç58#î+lb+Æ_ÑÒØ.ñaÿÒ¾c[záð¥c|Rc$ÃÓ±¨¶¶vÃýýý^X$ø¦þÂ+1³¡¡!ùöÌ3©TªðWòÉ'Kà/¹ð+J¬¿pün.ÞÞ^¸Ëj´|%®¶ôÃûµk×2L]]]L>ø ~£^OHiwøíÎ7/ÙÉË¯è-*MY³fÅü>úhøÆ=öÛw¼«ÝtÈ&Ùó×ÔÔ4®Á)=ø^qìøëíí-¼x°ãÅßÆË//ºÏë×¯÷Â"Á4ÅðÌ%ÓÙl6¾íêêéîîîB¢­|»eË7o&ûrn»nüîßúqùdwc8)Y¿tãÛXýÆ­b­ÂÛ*qµ¥Wñ±<x°pOXáå×®]ÓË-ûøV1ßÆÌÑo2gÝºuCCCÉK®9¾íìì,|É;Û>Ó.]*ÚN§Ó£=üºrÑÞTß3:ÞÁñ^MxÅÒ¬/ü¶ði¬ÏmìøãÓ8AáåËc:¶fLO6ÍÒÄ_þH_ò±ÂbN	üðÁù9·]7¿-9¼¿Joã7há.«¢Ãv%®¶ô£=öÄñ½h~]]]á#ßññíÌ3G¼ÉGá·É±ìäÞd´µµ>öøË|4©ÊU4ÖÁxñÑ£GG´±Î÷jÂ+É3äêÕ«71.üñiÜÚÚß666^ã^U$ø¦þ+1³¶¶v´ß¸£1%ù6Sâ·u	â÷×ó®v´ùñãñÆ¯ðÁÁÁÂùÃ÷ÜðÇ;oE>oÖjrz¸ðH¿±¬2±ñcÑH:±Ç~Ø÷Ó?»Æø4¾páBâ¿<:äE?iáoÏ='|$ûÒÎ»uÆµné=7ôQáÒW[zÅwàW^oóÌL|V´ªÅcÂoçÌ|äÊ#GgcÁ_²2ùÔl6»aÃ?þxïÞ½1såÊ#âo,«Lã|^qìøË¿<ïÿíÿÏ·¤S§NmÛ¶-9Ö?3ZüISñë-ünÎÔKò+-Ü¿,g[ZZEÉúo5âoÍëS¢ä=[qåÉõ½£kÄ«-½biI,_¾<ÿ¾þdNr.ð²eË®Ý*¹¶uëÖzü%¿Î9a÷îÝcÁß±cÇKñ»»»½zIqûwqá¯ôà=7ï¨NÉ»ôâÉ000¶[ºtépîÜ¹3?.=þ·ýHÞ»yáÂà%ÁTFø^áÃÏÍ¿ý?ÿy¼ÉnÂá¿5K¬[ZN¡¢G§^æ¸ÚÒ+Ä+WòïîJæx±èý^uuuù7~üå94sæÌü; Kçé§¾±æÎ[bkqá¯ôà=7ï¨N§N*¼þ.-ü)ç£ÝJç[þ¢|Ï<ó	þ¤)¿ø-¸hÑ¢áþ«³³³­­- ¿Ñ÷ìÙðàÁØ*ü»1®[9%WÞÚÚ:üãúF»ÚÛ®XZÉ!ÑÂùçÏ_¹reõ­²ÙìðÓ;&¿¾¾¾¸¶¸Î`ÐÆÃCys&Îw¿ûÝxtñÀgÍµoß¾¯~õ«ù=O~qá¯ôà=7×¨NÑ¡CãQÇõ'Öó'8Ç·A´äÖ/^ÜÓÓsÛ=¯£=ßúûû7oÞìRÁÇ¦ôÂ"Á$Ý­r¹ÜÓO?=®ÏÀ*S®dorúöÐÐPbú9sæxÂHð'IªÀ÷üõÂ/	þ$IXÿ¦MfÍÛgÖ°Hð'I$ø$IüI$	þ$I$I?I$Á$IàO$	þ$IüI$	þ$I$I?I$Á$IàORwðàÁt:½hÑ¢ñ®ÛÜÜáÂü9---ãxÁºÕÄ.3u'_ßºuëfÌ3kÖ¬Í7÷÷÷OÒøÝ|àOÒ]8# 3444Þu·oßë~ç;ßÉÏyñÅcÎ¶mÛ¦:þÂy³ouéÒ¥7ovttÄ-]ºtWuòäÉÅÃ$øT¯Â§Nu.3oÞ¼ÓÛÛ;9wòî!)qíîÉÃ$øtåWHÍ7O>Ú´i[·n-ºØW¿úÕ¶¶¶¢kÈï8é>ú(¦cN²èòåË/®©©I§Ó­­­]]]#^[á­^eß¾óçÏ¤7n¬Zµ*Ö;¿iÓ¦dGfðtÁqm1?®ù>ãÈ´´´Ä5¸´Ä(;v,îdÂÁðð,qUàOÒÝò_2ýüóÏÇô=Y1ñÂ/^¦³³3A^ak×®E÷îéøÓkÖ¬IÍ;÷ðáÃ1qéÒ¥ÉdF¼¶Â;Pz Ý+¯¼«W¯~ç×¯_Óq×^-&¶oß3~øá¾víÚ3gb"Ù19¼áÃ^_¥GiçÎÉmßÉ¢Ç^úª<9%Á¤»¿ðJLçn5Ü^#¾)ðØ±c±hÙ²e1½|ùòD6ù¥§NÚ²eK[[[ÌO¥R#^[tJ¯Ü±éÓ§_·®®.¹ó7oÞ`_ÌKÆtkkkèj´Ýx#V¥Gi`` kùÇ^úª<9%Á¤»¿BñÆ¢BZÓ¦MKö÷÷'GWcN²h÷îÝ±VðôéÓÃ4âË*£Ý±báÎ¼1cF2§¶¶6Ù)8=MMM1ÿÆ¥]Xy¥§'6àîþ2Lá¨dçÙm-²jÕªXÿ_]]snÞjø+½Jþç¯;sæÌdÝ¢»744tøðáÕ«Wî2¼m[¶lË/Ë(pI?IwÉ)®÷îMÞöâ/Å"ÈïBåWdsöìÙä½cÁ_éUdßûÞ÷bbíÚµÃ×ægbº££ãÜ¹sÉ¡Þ9þü>úôÕ«Wc¢©©iÃríÚµ ØìÙ³/_¾ LvI®X±b£T8zN:=ö¸$Á¤;¿¡¡¡Ö´[~V_i$k)?ÿÐ¡CµµµAM6¥W9zôh,7o^þ¼ÂËô÷÷oØ°!îyuuõÒ¥KÃ|Ùl69xáÂçÏûÈÄ­(Ú¡À­[·&ïçË(N<x0îRòÚEb.Ið'I$I?I$Á$IàO$Ið'I$ø+þæoþæý÷ßÌ[üçþçû·óÌ+·®^½ZøÉ#*b£|øáÆ¡Ü±I~åÔXú÷ÿ÷.2ìþé~ö³Á_¹ôøá¿Iæ&dagÎ	r+6JlãP(äWN¥7oþÕ_ýq(Ã;ö¯ÿú¯ð?Áü	þàþàOð'ø?ÁüÁü	þàOð'ø?ø?Áü	þðð'ø?Áü	þàþð'ø?ÁüÁàþð'ø?ø?Áàþðð'øüÁàþàþð'øüÁüÁàþððð'ø?ÁüÁü	þð'ø?ø?Áü	þðð'ø?Áàþî_üõööf2t:ÝÜÜÜÕÕ5üG©ªª?Áü	þàOðW	e³ÙØµkWÑÒ¡¡¡ÖÖÖÑð.¼1ýð?üÉO~rCeÖßþíßþøÇ?6åVlØ4Æ¡Ü±x)3åÖõë×Æ¡ëêêºzõêdÞâ¿ÚÚÚøOLär¹¢¥Ï?ÿü;FÃßÎ;8>|8?Tõýïÿ­·Þ2åVlØ4Æ¡Ü±x)3eØo¼aÊ°@ùÛo¿=·x_à/N8]¾|¹­­-hè°¯öuØWû:ì++©T*?]]]]¸hÅï¼óÎ<TøüÁàþ1¬uuu¹[cú?=Âÿü	þàOð¿)ßªU«öíÛñ5ÍüPíùüÁàþ1¬ÝÝÝõõõ©T*Éôôô¨=øüÁàþ>äþð'ø?ÁüÁü	þð'ø?ø?Áàþðð'ø?Áàþàþð'ø?ø?ø?Áü	þàþàOð'ø?ÁüÁü	þàOð'ø?ø?Áü	þðð'ø?Áü	þàþð'ø?ÁüÁàþð'ø?ø?Áàþðð'øüÁàþàþð'øüÁüÁàþ?ø?øüÁàþàþ?øüÁüÁàþàþðð'ø?Áàþàþð'ø?ãð?øüÁàþàOð?øüÁüÁàOð?ø?øü	þàOðð?øü	þàþàOð?ÁüÁü	þàOðð?Áü	þàþàOð'ø?ÁüÁü	þàOð'ø?ø?Áü	þàþàþàOð?øüÁü	þàOð?ø?ø?øüÁßýÝ»ï¾ûGôG6lØ³gO?üÁüÁàOðÛÿù?ðÀO,|âÉ'liiyôÑGßÿø?ø?Áàþ*°p^MMÍ×þúÖ?Øü~á_?ø?øü	þà¯û³?û³yóæååÿ6ÿÞæxàü?ø?øüÁà¯¼Ú³gÏ¯ýÚ¯â/þÕÖÖ^¿~þàþàOððÖ'zè¡Í¿·9/¿ßüÍß9wäÊáþàþS£sçÎ½þúëñKqppþà¯âûÚ×¾ö«¿ú«kÿ¿µ!¿¯e¿ö+¿ò+?ø?øüÝö|òÉÏ|æ3sçÎ=v&)Üðú´ÿæ7¿Ïö¶¶¶ÎÎÎ;uÍðð¿rïw~çwôÑM¿»)9þ@ðÃ??øüÁüÁà¯1cÆÿZ÷¿ßùþ¹Ïî/þâ/àþðð'ø«´®_¿^WWWtÚãO<ñÇüÇð?ø?øüU`>ø`ò¶÷ü¿Ù³g¿þúëð?ø?øüU`ßþö·#¿[~ËO<ñè£æÏù?øüÁüÁà¯ÒÚ¸qã/ýÒ/=6û±ººº¹sç^¼x1¿þàOðð¿ìúõë?úÑ~òÎ?øüÁüÁàï>þàOðð?øü	þàþàOð?ø?ø?øüÁàþàþ?øüÁüÁàþ?ø?øüÁàOðð?øüÁàþàOð?øüÁü	þàOð?ø?øü	þàOðWÞõööf2t:ÝÜÜÜÕÕU¸¨§§§µµ5µ´´ÄÅàOð?øüMù²ÙlGGGLìÚµ«½½½pÑìÙ³?û÷ïojj¿¿üË¿üßØ~ðÿøÇÿ[eÖ'N>mÊ­Ø(±iC¹/bñRfÊ­Ë/:tÈ8ao½õÖï½7·x_à¯¶¶6þÇ¹¡¡a´ÕÔÔÇßÞ½OObG9yòäiYo¿ývww·q(·b£Ä¦1åV¼ÅKq(·Þ÷ÝÀq(Ã:;;ô£Mæ-ÞøK§Ó#N¯VkÖ¬qØWû:ì+öÃ¾S¾T*®®®~7nd³Ùþþ~øüÁàþS¾ººº÷É­Ã¾1]´ôÊ+«W¯¾zõêðáOð?øüM½V­Zµoß¾¯Ùl¶hÄ,YÒ××7âð'ø?Áü	þ¦^ÝÝÝõõõ©T*Éôôôüü±UýÇ£khh¨*þð'ø?Áßýü	þàOð?ø?øü	þàOðð?Áü	þàþàOð?ÁüÁü	þàOð'ø?ø?Áü	þàþàOð'ø?ÁüÁü	þàþàOðð?øü	þàþàOð?øüÁü	þàOð?ø?Áü	þàOðð?Áü	þàþàOð'ø?ÁüÁü	þàOð'ø?ø?Áü	þðð'ø?ÁüÁü	þð'ø?ø?Áàþðð'ø?Áàþàþð'ø?ø?ø?Áü	þàOðð'ø?Áü	þàþàOð'ø?ÁüÁü	þð'ø?ø?Áü	þðð'ø?Áàþàþð'ø?ø?Áàþðð'øüÁàþàþð'øüÁüÁàþ?ø?øüÁàþwU·+JÁüÁàþðW!øKÝ®t:ð?øüÁÃ¾ðð'ø?ÁüUþâ¥aíÚµðð'ø?ÁüU þÒé´÷üÁü	þàOð§ÊÇ_KKËð³=jkkûûûáþàOð?ø«4üUWWöúúúêëëc"Ì÷½ï/&Ö¬Yð?øüÁ_¥á/ÙÕ¡½¸téR<ÿbbÚ´iðð'ø?ÁüUþfÌÔëîî¾páBL<ûì³Éz?øüÁàO¿M6åOï(|ÛßüùóáþàOð?ø«À³î¹3gÆDOOOLÛÚÚ¦Äö?Áü	þàOðw?øüÁàþàþ?øü^SSSò/>äþàOð?U8þfÏ]¾|Îö?øüÁàO¿p^°ïäÉñ´rÛþð'ø?Áßøª­­üMEùÁàþð'øw½½½¿õë×ß¸qþàþð'øSã/5kVÕ°ðð'ø?Á*Nø?øüÁàO÷þöMÑhøüÁàþã«®®Î	ð?øüé~Á_<¤Àß¦MáþàOð?U8þªFÉ	ð?øü©2?äyÄðð'ø?Á*ó£^¦nð'ø?Áü	þÆWCCCccã¹sçîÈêííÍd2étº¹¹¹«««ô¢Í?Áü	þàOð7ñÂUUUwlb6íèè]»vµ··^4±9mÞ¼ùóÿüâI,nnáÂUf=þøãóçÏ7åVlØ4Æ¡Ü±I~åÔXúâ¿ø¹ÏÎ8a­­­O<ñÄdÞâ]Ç_WWWàoÛ¶mñÁOÿ/µµµÉär¹Ò&6§°7VI$UPwwölßÂÓDN¾hbsàO$ÁßÄ»³gû±ºººô¢Í)lË-_ùÊWVObqsO=õÔjY¿þë¿þµ¯Í8[±QbÓr+^Ä&ùScéë_ÿú%KCöå/ù·~ë·&óï:þîluuu¹9JÓ¥Ml>ä'|È	Nø>Ê¥U«VíÛ·/&âk6-½hbsàOð?øüªr¹ÜÒ¥Kkjjªªª¦M¶bÅ	ùÑÝÝ]__J¥2LOOÏÏïå­£×ÃMlü	þàOð¿7000â§Äú?Áü	þàOð7¾æÌÔ[¶lÙ7åËÇ¹sçÂüÁàþðWiø«®®êåçär¹3üÔZø?øüÁàþ¦<þR©TP/9£6ipp0æLì£^àOð'ø?Áü5þÃ¾K,IûÆ×9­­­ðð'ø?ÁüUþB#ðññÇÃüÁàþðWõ200°bÅéÓ§§R©øºdÉ3%¶ü	þàOð¿û(øüÁàþðð'øSáï§?ýé»ï¾_>üÁß½Ä_ÕíJ¥Rðð'ø¿OÓÐÐÐoüÆo<ðÀsþË3g.]ºôúõë6üÁß½Á_jôàþàOðw¤uëÖ=öØc¿»ñw·þÁÖÍ¿·ùñÇÿâ¿hÀüÝüÖÆüíß¿þàþð7á®_¿>cÆD~É¿-¿¿å¡z÷Ýwmø¿²À_ooï´iÓ/.üÌgø?øüÁßÄ~0çü9yù%ÿþëãÿõÕW_µàþî=þV®ìð;|øðÚð'ø?-þ~úÓ>øà[~K!þO8a+ÀüÝKü½öÚk	û-[6å¶ü	þàOe¿èÑ/äå÷äO~ö³uÎüÁß=ÃßÀÀÀÜ¹ss;ººº¦âö?Áü©ñ÷á>üðÃ=öØ¢EZZZzè!O	ø¿¿;w&;üÚÛÛ§îö?Áü©ñõ÷÷¿þúëßùÎw^õUûüàþî%þ|ÎüÁàþºðº]étþàþð'ø?Þþàþð'ø?ø?Áàþðð'ø?Áàþàþð'øüÁüÁàþð'ø?øüÁàþwMMMÕÕÕ>çþàOð?U>þfÏ]>óð'ø?Á*á¼`ßÉ'ãi7å¶ü	þàOð¿ñU[[øò?Áü	þàOð7îzë×¯¿qãüÁü	þàOð§Ç_4kÖ¬ªa9áþàOð?U þðð'ø?Áîü%ì¢/Ðð'ø?Áü	þÆW]]>àþð'øÓý¿xH¿M6ÂüÁàþªpüU>àþð'øSe~Èó9áþàOð?UæG½LÝàOð?øüÁüÁàOð¿ÑËårK.­©©©ªª6mÚ+¦ÊÉ¿ð'ø?Áü	þÆ×ÀÀÀ'|LáOð?øü¯9sæõ-[ümßxQX¾|yÌ;w.üÁü	þàOð¿êêê ÞÐÐP~N.91þàþð'ø¿ü¨ ^/?gpp0æø¨ø?Áü	þT±,Yö¯1sZ[[áþàOð?ø«4üöF<áãã??ø?Áü	þà¯?êe```ÅÓ§OO¥RñuÉ%1gJløüÁàþ÷Qð'ø?Áü	þÆWCCCccã¹sçàþàOð?U>þÒétUÕTÝ?øüÁào|uuuþ¶mÛ/Så¯ºÁàþð'øèUR*?ø?Áü	þà¯?äyÄ|È3üÁàþªü566677÷÷÷Oéí?øüÁàoL%Ò÷[§Ä^øüÁàþÇ_ò'?ãð?øü©ÂñÉdªJæø?Áü	þT9ø;yòä3ý	õðð'ø?Á*ù¦óàOð?øüùÛ¾ð'ø?Áü	þ¦Z½½½L&N777wuu.êééimmE---q1øüÁàþS¾l6ÛÑÑ»víjoo/öìãÇÇÄþýûàOð?øüMùjkk?+ËåF»XMMÍpüýÉüÉI¬³³3^4O¨Ì:zôhüpr+6JlãPnÅX¼rëøñã2eØo¾ÙÝÝ=·x×ñwþüù¿Â3NF;ûääÉkÖ¬¿øQéÄÞ~ûíK.õ©Ì§ÇÙ³gC¹%6q(·âE,^ÊC¹õ/ÿò/üMc,þùòåÉ¼Å»¿äs^Z[[ßï½¿ÂÏ¬®®~7nd³ÙáÎa_9ìë°¯öuØWû¯`_þÓéôÂ/^¼8wýÅt]]].KûÆtÑ%¯²zõê«W¯¿øüÁàþéüùósæÌÉ¬ººzéÒ¥ö0V­Zµoß¾¯Ùl¶hÄ,Y2Ú.PøüÁàþêwøðáIþónÝÝÝõõõqL¦§§ççíÖNÁÂ?:?øüÁàïÎüî,Üó7úôágWað'ø?Áü	þÆW¡ùjjjÚÛÛ/_¾<U¶ü	þàOð¿q^Å­ó<¾ô¥/]ºtiÊmøüÁàþãë~Âü	þàOð'ø¿ÉÆßþð'ø?Áß¸;ölSSÓ´iÓ3|ëêê8ð?øü©ñwäÈü		þé]»vÁüÁàþðWiø«¯¯ê=6¿äÓ^àþàOð?ø«À³OÎã/É)Àðð'ø?ÁüUþêêêzÉÞ¾À_.Û¼ysL744ÀüÁàþðWiøT5RÇ?ø?Áü	þà¯Ïö½zõj[[[r¶oMMMSSÓÅ§Äö?Áü	þàOðw?øüÁàþàþ?øüÔåË[[[§OºUL477Oc¾ð'ø?Áü	þÆÑ3Ï<S5Jk×®?ø?Áü	þT9ø;pà@â¼Ý»w÷÷÷'3<Ìïìì?ø?Áü	þà¯BðÉdBx÷î¾(ùo>çþàOð?Uþª««CxÃår¹X?ø?Áü	þà¯Bðÿ«n£-MþÔüÁü	þàOð¿¼?ø?Áü	þðð'ø?ÁüMYüþàþð'øSåà/u»Òé4üÁü	þàOðþ¼üÁü	þàOðð?Áü	þàþàOð?ÁüÁü	þàOð'ø?ø?Áü	þàOðð'ø?Áü	þàþð'ø?ÁüÁü	þð'ø?ø?Áàþðð'ø?Áàþàþð'øüÁüÁàþðð'øüÁàþàþðð'ø?ø?Áü	þðð'ø?Áü	þàþð'ø?ÁüÁàþð'ø?ø?Áàþðð'øüÁàþàþð'øüÁüÁàþ?ø?øüÁàþàþ?øüÁüÁàOð?ø?øüÁàOðð?øüÁüÁüÁàþð'ø?øüÁàþððgàOð?ø?øü	þàOðð?øü	þàþàOð?ÁüÁü	þàOðð?Áü	þàþàOð'ø?ÁüÁ_©z3L:nnnîêê~#GTUUÁàþð'ø«²ÙlGGGLìÚµ«½½½héÐÐPkk+ü	þàOð¿Á_mmm<éc"Ë544-þùçwìØ1þ^|ñÅÎIìÐ¡Co¾ùf§Ê¬xÅ<|ø°q(·b£Ä¦1åV¼ÅKq(ÃÞxãPMþ¯þûétzÄéèòåËmmmAÃÑðÿ?Ø;ï¼Ó××÷TfýÝßýÝÅC¹%6q(·âE,^ÊC¹õ³ý,þ³dÊ°·ß~ûÚµky÷þR©T~ººººpÑ+âEê?ªÃ¾rØ×a_9ìë°¯öºCYõbº®®.Ë%czÄå/?øüÁàoj·jÕªûöÅD|Íf³£aqøLøüÁàþS¯îîîúúúT*ÉdzzzFÔü	þàOð?ò?øüÁàþàþ?øüÁüÁàOð?ø?øüÁàOðð?øüÁüÁüÁàþðð'øüÁàþàþð'øüÁüÁàþ?ø?øüÁàþð?øüÁàþàOð?øüÁüÁàOð?ø?øü	þàOðð7	]¼xñÜ¹sð?ÁüÁüUr'Nøìg?ûÈ#4=ÖôË¿üËð?ÁüÁüUfï¿ÿþ>¸ò¿­Üú[ãßÿXý?jkkïÒö?øüÁàþàï·iÓ¦'x"_òïÉ'üò¿ð'øüÁüÁ_ö¯|å¿¯úïøûÆÿüÆ#<ð'øüÁüÁ_öo|ãËÿÏñ×ÞÞ¾páBø?Áàþàþ*°'NÌ1cÍÿ»&ß77|óGÙ¿?üÁàOððÙ«¯¾úÏ|æóÿüã?üÖ·¾unþàOð?ø¿²¨¿¿?~l:tñâÅ»w+ð?øüÁüÝGÁü	þàOðð?Áü	þàþàOðð?ø?øüÁàOðð?øüÁüÁàOð?ø?øü	þàOðð?øü	þàþàOð?ÁüÁü	þàOð?ø?Áü	þàOðð'ø?Áü	þàþàOð'ø?ÁüÁü	þð'ø?ø?Áü	þðð'ø?Áàþàþð'ø?ø?Áàþðð'ø?ã?ø?øüÁàOðð?øüÁàþàOð?øüÁü	þàOð?ø?øü	þàOðð?Áü	þàþàOð?ÁüÁü	þàOð'ø?ø?Áü	þàþàOð'ø?ÁüÁü	þàþàOðð?øü	þàþàOð?ø?ø?øüÁàþð?øüÁàþàþ?øüÁüÁàOð?ø?øüÁàOðwoêííÍd2étº¹¹¹«««pÑàààêÕ««««~øáøüÁàþS¾l6ÛÑÑ»víjoo/ûö^x!~$bèãï?øÁÀ$ö×ý×W¯^Põî»ïþã?þ£q(·b£Ä¦1åV¼ÅKq(·úûûÆ¡ëêêêëëÌ[¼/ðW[[¼×ÐÐP¸¨¹¹ùÜ¹s£­øûö·¿ýI,~2ßzë­¨ÌzóÍ7¿ÿýïr+6JlãPnÅX¼2ì7Þ0eØäÿê¿/ðN§GN¾Ý¹sgMMMccãéÓ§öÃ¾ûÊa_å°ï/Jå§«««íÝ»7&Î?ßÖÖ?øüÁàoJVõbº®®.Ërë°oL^¬ðÛ¢ð'ø?Áü	þ¦d«V­Ú·o_LÄ×l6[¸hÝºu¯¼òJL=vþüùð'ø?Áü	þ¦|ÝÝÝõõõ©T*Éôôôüü±ÝÚ)/R+V¬H§Ómmm.?Áü	þàOðwÿ?øüÁàþàþ?øüÁüÁàOð?ø?øüÁàOðð?øü	þàþàOð?ø?øü	þàOðð?ø?øüÁüÁàþ?ø?øüÁàþð?øüÁàþàOð?øüÝoë[ßzõÕWßÄâæþþïÿþYG9~ü¸q(·b£Ä¦1åV¼Mò+§ÆÒ¥KþôOÿÔ8a¯¼òÊ?üÃ?Læ-^¿~þFíìÙ³[·nýCI¤J©ô¾ù*;c%IîàO$	þ$I$I?I$Á$IàO$IðW±õôô´¶¶¦ÓéÞÞÞÿôR©!*írìØ±9sæÄæææîînCT&ÛåüùómmmÕÕÕ+W®ô×qîUgÏ;wnáOGlL&Ìéêê2De²]¢¾¾¾SnføüUr³gÏ>~üxLìß¿¿©©©pQGGÇ®]»QlÚÚÚË/ÇD|õÒY>Û%^@ãE3&Î;·nÝ:CtO_`Ø:ñÙl6^Áb"^ÄÚÛÛQlpFü'¶ªÊ/÷²Û4%0^MMM~úÒ¥K-2&å³]¯ñ5¦Klør~Î3Ë½­³³³¥¥%ùÏÒÍ7c"ËùÏRùl¥K^¸pþÊpÓø«ðN<¹fÍü·+V¬9¥|¶Koooü@Æf|µiÊg»ÄëæÙ³gcâå_. &¹¡¡¡éÓ§ÇÈþýûPn»Ïvùù¯vø+×M3ðWÉÝ¸q#Íö÷÷'ßÆÿÌæÍgXÊj»Ì;÷Ü¹smòÙ.§OnjjïØ±ãÿwYG©««Â÷+WWW2Ù.ðWæ¦ðWÉ]¹reõêÕW¯^ÍÏÙ¾ûÎ;LYm2Êöç%é½÷Þ3gñ¹ç%?ñû,Ërë°oáï6ÝÛíå¼iFq¿ìØ±cK,éëë+¹`ÁS§N²Ú.óæÍK/9sfîÜ¹¨L¶ËìÙ³oÞ¼¹cÇçÎÝb+$?===±bbÕªUûöíøÍfQlø+ÛM3"à¯bkhh(üldfuuuò^iÏvyï½÷3óãkL¢2Ù.ñ9kÖ¬øY³fÍÐÐ!º'¿ã§cÑ¢EÉ~îîîúúúT*ÉdÓ±UÛþÊvÓø$IüI$	þ$I$I?I$Á$IüI$	þ$I$I?I$Á$IàO$Ið'I$ø$IüI$	þ$I$Ið'I$ø$IüIRôòË//X° æV-zíµ×þÓkÜ­¦Ì+òH÷¶¡¡!ZÑüN§3ÌÍ7ÇàO¦d[·n­ÖsÏ=WIøÛ¶m[Ì|é¥æ÷»ßùÏ>ûì®SüIÒÔ«··7XN§wïÞ=t«½÷Æ·1óÔ©S¿K.ÅÌ¢ùsæÌù/^?Ið'é¾è©§Ö¼ðÂ3_|ñÅùÛ¿ýÛôéêê<[[[c:á?þxÍ5Ó§OEµµµ6l(<ºzôèÑV,u;;;8sfÌ±`ÁÃÇ·«V­*ºc*=É¢¸KÉ¢#Gµ%KÄüüãÇÇÅççlß¾½¾¾>®ª¦¦fåÊW®¿á×_4§Ä]t5kVÀåòåË3?øàÙÐÐP¢N<,]¾|yÑ¢õë×'Î9J¥Fù6YºbÅ7o«««bi|<M6-y+^ëïÞðGºÿþBÑæÙÑÑ_Ñ,Z´h¼ø+qW%Á$Ýû#¼#¼®UUÅqÓÞÞ~ãV1ß.[¶,YX'ác²/Ð,Êf³É.ÃîîîN®¤ð:·lÙ¼Kº>óÌ31çàÁ1_cúé§¾íõãÛ¸@áñ(gÎ·¯¯/¾¯qÏC¹¹@&ã!|òÃÄ555ãÅ_»*	þ$©,ð¿üü7¢+W®Ä·Á¦äÛÖÖÖø¶±±qíÚµ¶üÄeö¥å×J¾ýàò0åüÆ×>zôèm¯' ß~ôÑG÷m´÷çmÜ¸1íØ±ã_ÚÞ°aCá¿ i[[[²cr¼ø+qW%Á$ÝûÝ]7nÜ(Ùßß3cQ	îäixáÂÄyëäß¨7ühl§?`%¦kkk«««ãÊgÌ_zÛëbE=6qjLÇ×>sæL~iwwwÜ%îª$ø¤_ò¾·;wÎL>¥èüûûúúïÐ:uêÔ¶mÛ°y5&»åòÇUoK´õë×'Gãë5kòóK²§íêÕ«É·ôQé3sçÍKO·	³.JÎüEG¹víZiüåaF~i»*	þ$éÞ0N§wíÚ|ÔËîÝ»«««±lÙ²Îàà`rL6fnò¿ä­r.|³`bÁ6ópó¶2"ÑÓo[?vìX~~ëIÞä¼ç¯¿¿?¹d	üíÝ»7¿O.rá¢äígÎ[A	7Ëq±@çÊ+¸«àOÊ¢äÚ¾ûÿÿw«ÄFùéüÓü9ùyæBYvàÀøûäÖâùµµµGKÜÂ;?Óv´;00PSS<¢ÝK.-¼3gÆ×äÓ^¯3Ù1/¡j~i»*	þ$© ,Z´¨úV,HN¹-Âß#GÓknn~çwòKûûû7oÞ_iãÆù¥mmma²L&³gÏ¢ë~Oâªbþºuëæv=QÜ¸KÉø¿|kÖ¬)úÌ¤¾¾¾l6#0úôx/^ÌlMáu^»v-tÕâÅzzn±Ä]$I?I$Á$IàO$Ið'I$ø$IüI$	þ$I$Ið'I$ø$IüI$	þ$I$I?I$Á$IàO$Iw¢ÿ	ÂJ5hÈfIEND®B`


9÷¼wwýpûhwcpR´4¼é«aõ;SµrVÍ^qÆÇ~êÔ©Ü=a¹·ß¾Þ¸qãí©ÂD¸fÎöx£9;vìîX´åpµ««+÷Flã7Âôõë×sÍvÉäl$^÷>WíCuñÑùÎ÷jÁ+fîÕÜA`L´¹ão/ã7nÜÓáÙÓK,ñE?©(ñé>"[Sï¾ûn<çëÆÑ¢Ãá­4ºÞAswYå¶+°ÙÂ+ÎöØ##÷õ¼ùuuu¹(¼Ç«555³=ÞhNGîÕèXvôsïI6ÈhkkËìð|6©ßç*¹CÖ¹nÝº3gÎÌ8hsïÕW;þ¢WÈÍ7sÄ¼ð7Çqkkk¸ÚÔÔðþó[E?©øð°fÖÖÖÎö;S¢«A9Þ­g¾oÏØìlóÃC7¼çÎ¾çlúà¦?Þ¹ÐÌ;ôyÏ³Vëëë£ãÔÓGêxsYe.c5ãæ28³ta+Îý°ïý¿ºæø2ü£ðôéÓ~±Hð'þ^|ñÅÜ>¢iyç]ÎÅ:óZ·ð?ü0wiÍ^±ÀxùåÃÕøQ¢ÏòöQå±xîµ¹¹9úÊîîî³¹à/Ú=ëJ&Ùµk×íÛ·=fnÞ¼yFüÍeão¾·àç¿¹ìùyýµS`ü¼Þ¢.pàÀèXs|f´$øáí-(zo¿ê%zKnoÑÉ³Ë/ExjÆwÍëS¤è3[aãÑvò>Ñ5ãf¯XX6m?×ÍÎÞ¸qãÈTÑÖvìØqÿøö~päÈ¹à¯··7Ú·r___´W/êÔ©S3>¿ó]e^ø+<8y¯Üæ;ªÀ_ô)½ðb¶Û°aÃt>÷Üsaðÿ=ÿ>»9<<¼$ø üM/÷ÅéçÏÆÿ¿7ÚM8ý]³ÀºåTñ(÷ÔËxiÍ^±°$ÞÿýøÓ]Ñk×®åÞ«®®.þ@áýà/æHTMMMü	ÈÄyâ'¦?Y+V¬(ðÏkyá¯ðàä½6rï¨..ÝþêÕ«sæI|Êùl?¥Àë->£(nëÖ­~±Hð'þÂ»àÚµk§ÿó_]]]mmmRáýÅ_ç:u*Ì	ØÊý»9®O9E_6ÞÚÚ:ýëúfÛì=W,,èhîü+W®lÞ¼95U&~zÇÂðwëÖ­°µ°ÍÀ Ý»wÅæ.Lç><ºðÀ;öøãÇîyá¯ðàä½6ò×¨.¡Ó§O755G¶XOpWÑ¢¾nÝºþþþîyíõ6::ºwïÞhj|x*ÇÇÇýbàO>©²ÙìO<1¯ï[À*EW´Ç7:bb"2ss³¤,úÌ_^Ï<óàOTîÙ³§±±1:¶&|òIÃ"Á$IàO$Ið'I$ø$IüI$	þ$I$I?I$ø3$Ið'I$ø$IüI$	þ$I$I?IÅØ©S§ÉäÚµkç»nKKKEEÅððp<'L9Ë/Ço±©v¹¬»Àß­ÓæîÜ¹³¶¶6XUUU&¹zõj¼ÖÄÄDnJ¥Â´W$øôÀU__°rëÖ­`åàÁaÝ?üÃ?ç<ûì³aÎJÓçoÙ²%Ìïéé	ÓgÏÓK.>:Æâo¼áÕ%	þ$=x¿DîÃO.ë®Y³&³råÊ0gpppqîäâã/Jù###3®õ¯|%,.·nÝêÕ%	þ$=òËÎÞ½«ªª,Y²ÿþ¼=þøãmmmy[wé?ü0L9Ñ¢7n¬[·®²²2L¶¶¶FË¦o-÷§^åØ±c«V­?h:ÑîÜ¹ÓÙÙÖw~Ï=ÑÌÀÓÕ«W­ùaËï¾ûîâïG	óûúúòMNNVWW566.kjjb"/ì>HüIú¤üM?ýôÓaúÅ_ÌÏ<óLîmººº"äå¶ûö°èèÑ£a:émÛ¶EV¬Xú¼~ýzN§gÜZî(¼J ÝË/¿&¶lÙ2ýÎïÜ¹3L¼úê«aâàÁ±ÕFFFÂD´cr¶ÏóÍÆâÜÛ:u*³víÚK.Å«DG7nÜ¦7oÞ¦ÃïW$øô@à¯¡¡!Lg§n¯?ØÛÛgÓ¦Mêâ¥.·o_[[[H$fÜZ¿¯Ý±ªªªéëÖÕÕEw~rr2Lráaºµµ5pv|||aÃ×ÀÀ@@jLÀ°åhþ®]»â«RwïÞ?÷AàOÒ'¿èLÕx~¸zO	i-Y²$Ürtt4:²æD9Ö"¼téRîò¶u.«ÌvÇswÔEp<yòduuu4§¶¶6Ú)8÷=Æ­§§'x.÷0w sø¡·oß¾;u:L766Îx¼ê$Á¤ét:wÏ_´óìêììKãËx~tnÄäTsÄ_áUâ;,5ÝhÝ¼»711ñÆoDgéÆ»ç5,³ÝpÃt¬Õ¼.°àû Ið'éÅ_ôÕ-G>ó÷ì³ÏÎEB'O­óòË/Çó#J¾ýöÛÑgç¿Â«ìÛ·ïW^	Û·o¾îÖ­[ÃôñãÇ/_¾f3W­Z¦ÌnÞ¼&-[vø[¾|yßÝÝ÷§_ìòÄOÜý[oÂüßI?I,þ&&&°Lû]ñ766r¹ßlT[[[UUµgÏ9â¯ð*gÎ	KW®w÷õË»ví÷<JmØ°!:©6x+ÉD§¯Y³æÊ+óéÃf;;;Ã=¶¦¦fçÎáçÞýé÷]çn?þ¾ëßI?I$ø$IüI$	þ$I$I?I$ÁßÔ_ýÕ_½óÎ;^Öï½wçÎãPýë¿þë|`Ê³ñññþç6eÛðððôoPü:ýÁüAð×Á¢õ·û·ï¿ÿ¾q(Ïþéþéí·ß6åÙ¿ýÛ¿=Ö8mo¼ñÆüÇø?øü	þ?ø?ÁàOð'ø?øü	þ?ø?ÁàOð'ø?øü	þ?ø?ÁàOððð'øü	þàþ?ÁàþàOð'øü	þàþ?ÁàþàOð'øüÁüÁüÁàOð'øüÁü	þ?ÁüÁàOð'øüÁü	þ?)þÓét2liiéééÉ]4>>¾eËT*õÈ#ôööÂü	þ?ø+ú2ÌñãÇÃÄáÃ;::r<xðgòkjj¿îîî;Z¬þæoþæÚµkÆ¡<ûø/ò,üÕ~	²-à/ü`­²À_mmmà]Èf³¹ZZZ._¾<ÛÏ=÷Ü_j±zóÍ7Ï9cÊ³üào½õq(ÏzzzÂÛ¿q(Û¾ÿýïýE«,ðL&g®ÞUVV655]ºtÉa_å°¯öÃ¾ûD"N¥Ry=&®ÒÖÖð'øü	þà¯è«««Ëf³w§ûé¼EñtÞNAø?ÁàOðEYggç±cÇÂD¸Ìd2¹vìØñòË/ð®³jÕ*ø?ÁàOðE____"H§Óýýý?ylÿÿ£iooO&mmmÃÃÃð?ÁàþÊ7ø?ÁàOðð'øü	þð?ÁàOðð'øü	þð?ÁàOððð?ÁàþàOð'øü	þàþ?ÁàþàOð'øü	þàþ?ÁàþàOð'øü	þàþ?ÁüÁàOð'øüÁü	þ?ÁüÁàOð'øüÁü	þ?ÁüÁàþàþ?ø?ÁàOð'ø?øü	þ?ø?ÁàOð'ø?øü	þ?ø?ÁàOð'ø?øü	þð?ÁàOðð'øü	þð?ÁàOðð'øü	þôÓÂì£>J¥ÒéôöíÛÇÇÇ	üÁü	þ*Íºººzè¡ÍÿÏæ_Û÷Õ_ýüç?ÿ¿ùþàþ?f¿ó;¿³ÿëû£ÿ~øáÞÞ^#ð?ÁJ­ÑÑÑ@½X~Ñk×®þùçüÁü	þ*µÆÇÇ«««÷þþÞý×Uÿõ^08ðð'øü©ûâ¿¸þ¿­å÷Õ_­¬¬¼|ù²?ø?ÁàO%Ø;ï¼óK¿ôKÿüçý×ý±Ç«®®þÎw¾cXàþàOð'øSÉ6>>þâ/>þøãßøÆ7~üãø?øü	þTúùgø?øü	þ?ø?ÁàOð'ø?øü	þ?ø?ÁàOð'ø?øü	þ?ø?ÁàOð'ø?øü	þð?ÁàOðð'øü	þð?ÁàOðð'øü	þððð'øü	þàþ?ÁàþàOð'øü	þàþ?ÁàþàOð'øü	þàþ?ÁàþàOð'øüÁü	þ?ÁüÁàOð'øüÁü	þ?ÁüÁàOð'øüÁü	þàþàOð'ø?øü	þ?ø?ÁàOð'ø¥ÁÁÁt:L&[ZZzzz¦ß »»»¢¢þàOð'øüÁ_)Éd?&>ÜÑÑ·tbb¢µµu6üýùÿù¿h±Ôþû¿ÿãP]¼x±¿¿ß8gÃÃÃ?üáCÙvúôéwÞyÇ8,Ze¿ÚÚÚÉÉÉ0Ífò>ýôÓG½¤Å*üö?wîq(ÏýCy600ðÖ[o²íõ×_þE«,ðL&gÝ¸q£­­-ÐÐa_å°¯öÃ¾*ü%x:Jå.joo~ãÀü	þ?þêêê²ÙìÝ©Ã¾aú?=ÂÿüÁàOð'ø¿¢¯³³óØ±ca"ª=ð'øü	þà¯4F___"H§Óýýý3jþàOð'øüÉ<Ãü	þ?ø?ÁàOð'ø?øü	þ?ø?ÁàOð'ø?øü	þ?ø?ø3ð'øü	þàþ?ÁàþàOð'øü	þàþ?ÁàþàOð'øü	þàþ?ÁàþàOð'øüÁü	þ?ÁüÁàOð'øüÁü	þ?ÁüÁàOð'øüÁüÁüÁàOð'ø?øü	þ?ø?ÁàOð'ø?øü	þ?ø?ÁàOð'ø?øü	þ?ø?ÁàOðð'øü	þð?ÁàOðð'øü	þð?ÁàOðð'ø?ø?ÁàþàOð'øü	þàþ?ÁàþàOð'øü	þàþ?ÁàþàOð'øüécÃ_Å½J$ð'øü	þ%¿Ä½J&ð'øü	þ%¿ÒþàOð'øüÁßÇÐÈÈÈöíÛáOð'øü	þJÉdÒgþ?Áà¯ôñ·|ùòégÔÖÖÂàOð'øüþR©TÐÞ­[·êëëÃD0ß+¯¼&¶mÛ?ÁàOðWjøvõ ½0qýúõÉÉÉ0±dÉøü	þ¿RÃ_uuu ^__ßððpxòÉ'£	_õ"øü	þ%¿=öÄ§wä~ìoÕªUð'øü	þ¥¿ÐSO=USS&úûûÃD`[[[Q<~ø?ÁàOðeüÁàOð'ø?øü	þ?ø½eËE_øâK?Áà¯Äñ·téÒÅ9ÛWð'øü	þJÁyE÷øáþ?ÁüÍ¯ÚÚÚ¿büÁàOð'ø¿y788ð·sçÎ;wîÀàOð'øü8þBÓrÂàOð'øü þð!øü	þå¿CCCÅøøáþ?ÁüÍ¯ºº:'|þ?Á_¹à¯··7àoÏ=ãããð'øü	þ%¿YrÂàOð'øü þ³äÁàOð'ø+Aüuð?ÁàþæWCCCSSÓåË?Å188N§ÉdKKKOOOî¢þþþÖÖÖ°hùòåáfð?Áàþî«àªOyb&9~üx8|øpGGGî¢¥K;w.L8qbÙ²eð?Áàþî«¿|Z_øR[[ýèl6ÛÐÐ0ÛÍ*++§ãï»ßýîÿÖbõñÆ¡<·ãP]¼x±»»Û8m§OûEëÇßp¶oîÉ%³hÌ±mÛ¶éøùå¯i±úÑ~téÒ%ãPþõ_ÿµq(ÏÂ»Ñ3gCÙöúë¯_½zÕ8,Z8þ³s¡J¥¦ßàÎ;LfttÔa_å°¯öÃ¾ûuuuÙl6:ì¦ómlÙ²åæÍÓW?øü	þðW|uvv;v,LËL&»¨··wýúõ·nÝqEø?ÁàOðó.ÍnØ°¡²²²¢¢bÉ%ííí|æG___"H§Óýýý?ylSç 744ä~þàOð'øüÁß5666ã	EñOýÂü	þ?ø_ÍÍÍz7n¼sçN¸:22²iÓ¦0gÅð'øü	þ¥¿T*¨711ÏÉf³aÎgÝÂüÁü	þ*nü%@½èdÛ¨ñññ0g1¿êþàOð'øüiðö]¿~tØ7é0§µµþ?Áà¯Ôð´7ã	·oß?ÁàOð'ø+5üÝ:á·½½½ªª*HËõë×9Eñøáþ?ÁüQð?ÁàþàOð'øü	þà/oÍH$àOð'øü	þJÙ?ÁàOð'ø+5üÍÖîÝ»#ü8qþ?Áà¯dñ788¸dÉÀ¾uëÖå~ç3ü	þððgJ7ovø'¸?üÁàOð'ø¿ùõê«¯FìÛ¸qcÑ=~ø?ÁàOðsmlllÅÑ¹===Åøøáþ?ÁüÍ©ç.Úá×ÑÑQ¼þàOð'øüÁßÜÖô=?ÁàOðW>øKÜ«d2	?ÁàOðW"ø+àþ?ÁüÁàOð'øüÁü	þ?ÁüÁàOð'øüÁü	þ?ÁüÁüÁü	þ*vü-[¶,Jù?ÁàOð'ø+ü-]º4|¾çOð'øü	þJÁyE÷øáþ?ÁüÍ¯ÚÚÚ¿büÁàOð'ø¿y788ð·sçÎ;wîÀàOð'øü8þBÓrÂàOð'øü þð!øü	þå¿CCCÅøøáþ?ÁüÍ¯ºº:'|þ?Á_¹à¯··7àoÏ=ãããð'øü	þ%¿YrÂàOð'øü þ³äÁàOð'ø+Aüuð?ÁàþàOð'øü	þàoö²Ùì*+++**,YÒÞÞ^,'ÿÂü	þ?ø_ccc3ðQ'ÿÂü	þ?ø_ÍÍÍz7nþmßM69+V¬?ÁàOð'ø+5ü¥R©@½xN6sÂ|øü	þ¿RÃ_"Ôàç9¾êEð'øü	þJÑaßõë×GÃesZ[[áOð'øü	þJA3ðqûömøü	þ¿RÃßÝ©~ÛÛÛ«ªªD¸~SþàOð'øüÁ_ð'øü	þào~544455]¾|þ?Áà¯ôñL&+*u"üÁàOð'ø¿ùÕÓÓðwàÀbùWÝàþ?Áü-t³H$àOð'øü	þJYò%Ï?ÁàOðW:økjjjii-êÇð'øü	þàoNEÿ¤ïÝ©Ã¾EqþàOð'øüiáøþIßèñ?ÁàOð'ø+qü¥Óé9áCð'øü	þJÕÕÕÑþ¿zNøü	þ¿Å_8þàOð'øüÉ¿íð'øü	þàïÓkpp0N'ÉÂ6þàOð'øüÁßR&9~üx8|øpGGGáEÛ=êëëÑbõó?ÿó¿ð¿`Ê³ðq(Ïý²-çêêêÂbVdø«­­þàl6Þ*/ZØÜvïÞ]!ITBâø»råÊÇ¿ÜsGòÎ#¾hasàO$Áß9úÖÖÖ«W¯ÞÿÖr¿0J^´°9¹ãßØ¿ÿÿ«ÅêßüæýÑÒëÿø.]úÐC¥>ý3?ó3kÖ¬ùÓ?ýÓ¼Û|ë[ßzúé§UyväÈ¯ýëÆ¡lûÝßýÝï~÷»ÆaÑúÄñØK3L_ú×®][ðÖêêê²Ùlt6L^´°9NøpÂ>Þ&&&~õWõ«¿¸ïkûöÿïíþ½æ_iÞ²e>ä9á£Oø¸råJsss¬ÀT*µaÃl§³³óØ±ca"Â6þàOo]]]ûÜçûâÿÿ,YòÑGÁàOðW²øOpeeeÅBÿy·¾¾¾úúú°n:îïïÿÉ½:z=ÑÂæÀüéãíùç_÷Øºÿ-[vþüyøü	þJCCC¹þªªª¶mÛöà?~ø?Ý¯½öÚ¾ðíûÚ¾û¹ï½÷àOð'ø+Aüå¯²²²££ãÆÅòøáþtÿôÑG555ííí±üVM511?Á_	â/:ÏãK_úÒõë×îñÃüécéòåËýìg?·ôs>úèÃ?üØcðÁy·?ø3ð§ÁßÇò/ð*ö&&&Î?ÿÚk¯]¼x1oü	þàþJEüÁ-ø?ã*ü_èË-[²dIto]]ÝÉ'áOð'øü	þJÝÝÝñ	þ¢éÃÃàOð'øüþêëëõÂïôýýýÑ·½ÀàOð'øüþâB8Æßäädt0ü	þ?Á_©á¯®®.P/ÚÛðÍf÷îÝ¦àOð'øü	þJ½½½3uîÜ9øü	þ¿RÃ_èæÍmmmÑÙ¾Ë-»víZQ<~ø?ÁàOðeüÁàOð'ø?øü	þ?ø©7n´¶¶VUU%¦---ÅrÌþàOð'øüÁß<ÚºukÅ,mß¾þ?Áà¯tðwòäÉÈyGf:u*ßÕÕ?ÁàOðW"øK§ÓAxG¾(úß|ÏàOð'øüþR©TÞØØØôEÙl6,7?ÁàOð'ø+üÅÿªÛlK£êþ?ÁüÁ_à¯ïàOð'øü	þàþàþàOð'øSÑâ¯pð'øü	þ¥¿Ä½J&ð'øü	þ%¿ÒþàOð'øüÁü	þ?ÁüÁàOð'øüÁü	þ?ÁüÁàOð'øüÁüÁüÁàOð'ø?øü	þ?ø?ÁàOð'ø?øü	þ?ø?ÁàOð'ø?øü	þ?ø?ÁàOðð'øü	þð?ÁàOðð'øü	þð?ÁàOðð'ø?ø?ÁàþàOð'øü	þàþ?ÁàþàOð'øü	þàþ?ÁàþàOð'øü	þàþ?ÁüÁàOð'øüÁü	þ?ÁüÁàOð'øüÁü	þ?ÁüÁàOðÆþð?ÁàOðð'øü	þð?ÁàOðð'øü	þð?ÁàOðð'øü	þàþ?ÁàþàOð'øü	þàïlpp0N'ÉÜEýýý­­­aÑòåËÃÍàþ?ÁüLæøñãaâðáÃ¹.]zîÜ¹0qâÄeËÁü	þ?ø+újkk'''ÃD6mhhífÓñ÷ì³Ïvi±zýõ×Ã¯ãP§>¼Cyöæo>Ú8mßÿþ÷ÂbVøK&3Nç600°mÛ¶éøëííý?Z¬Î?ÿ/ÿò/Æ¡<»zõêÐÐq(Ï>úè£ýèGÆ¡lûE«,ðH$âéT*5ýwîÜÉd2£££û:ì+å°¯öuØ·8ïúOÓuuuÙl6:ì¦ón´±eË7oNßüÁàOð'ø¿â«³³óØ±ca"ÜE½½½ë×¯¿uëÖ+Âü	þ?ø+¾úúúêëëD:îïïÿÉcÚ)ØÐÐPüìôÑG/^ð'øü	þàïAþî§ßþíßþÌg>Óü+Í5556l¿ßáOð'øüÁüf;vìøÜç>÷»oÿ×÷ïýý½¿ök¿öØcÁàOð'ø?ø+Í_åÕÕÕü¢ÿömßÃ?|ñâEøü	þð¥ÖÐÐPó¯4Çòþô×ýÞ÷¾?Áàþà¯Ôúè£jkk÷þþÞ555ýøÇ??ÁàOððW­Y³æÑGÝ÷µüþûúÿþÙÏ~¶ð9ðÆþ?ø+âßæ?üp¸llllnn~çw¯ðgàOð'ø¿âîâÅ¯½öÚùóç'&&îycø?ã?ÁüQðÆþ?ø?Áßbwþüùßú­ßúå_þå5kÖ|ç;ßñìÀàþàOð§ÅßéÓ§kjj6nÜø¿þçÿêèèxøá¿ýíoàOðð'øS	âobbâgögÿÇÿGüE_ÝñÕÏ|æ3ï½÷çþð?þò¾|ÅYÑÕÕå9?ÁüÁàO%¿G>ûHþ¾ð/Àü	þàþ*AüÍvØ÷ßR)øüÁü	þT|ø»;uÂÇC=ðÑÐÐð­oË?ø?ÁJw§¾êå7~ã7~ññ_üâüÉxvàOðð'øS)ãOð'ø?øü	þ?ø?ÁàOð'ø?øü	þ?ø?ÁàOð'ø?øü	þàþàOðð'øü	þð?ÁàOðð'øü	þð?ÁàOðð'øü	þð?ÁàþàÏëþ?ÁüÁàOð'øüÁü	þ?ÁüÁàOð'øüÁü	þ?ø?ø?øü	þ?ø?ÁàOð'ø?øü	þ?ø?ÁàOð'ø?øü	þ?ø?ÁàOð'ø?øü	þð?ÁàOðð'øü	þð?ÁàOðð'øü	þððð'øü	þàþ?ÁàþàOð'øü	þàþ?ÁàþàOð'øü	þàþ?ÁàþàOð'øüÁü	þ?ÁüÁàOð'øüÁü	þ?ÁüÁàOð'øüÁü	þàÏ8ÀàOðð'øü	þð?ÁàOðð'øü	þTîøL§ÓÉd²¥¥¥§§gúº»»+**àþ?ÁüBLæøñãaâðáÃyK'&&Z[[gÃßøÃ1-V×¯_7åÙ?þã?þÝßýq(Ï>øà¿üË¿4eÛë¯¿þïÿþïÆaÑ*üÕÖÖNNNl6ÛÐÐ·ôé§>tèÐløûö·¿ýC-Vá¿îînãP½õÖ[o¾ù¦q(Ï~ð·ãP¶>Ú ,fe¿d29ãtèÆmmmû:ì+å°¯öUà/HÄÓ©T*wQôþàOð'øü©ñWñÓÂt]]]6½;uØ7LÏx³øÆð?Áàþ»ÎÎÎcÇpÉdfÃâôð?Áàþ¯¾¾¾úúúD"N§ûûûgÔüÁàOð'ø/y?øü	þð?ÁàOðð'øü	þð?ÁàOðð'øü	þððgàOð'øüÁü	þ?ÁüÁàOð'øüÁü	þ?ÁüÁàOð'øüÁü	þ?ÁüÁàOð'ø¿òêßüæ÷¾÷½w´X½þúëçÏ7åY__ß3gCyÜÿÊ+¯²íÏþìÏÂÆaÑnÁß¬ßGû÷ïÿI¤R©ðQÍ;?%Iö$IüI$	þ$I$I?I$Á$IàOÅÝÛo¿½bÅd2ÙÒÒÒ××æôöö677çÎQù<ûW®¥R7o1D%_wwwEÅOÞhÓétôzèéé18åöÝºu«¡¡Á°ÀJ¼ð[þÔ©SaâÜ¹sµµµa"¸q#LK¿ÊíÙìïï/_Þ±c!*í&&&Z[[ã÷þL&süøñ0qøðáãSn/ð`øË?×?x]]]Ë/MMMÑ?ï.Ã´)«g?LÆ3«««Li÷ôÓO:t(~³LNNl6ë¿2|lØ°axxþàOåòÇ_UUUøþÄw§ýTVV«ár``ÀøÕ³øöÛo^z)*½nÜ¸ÑÖÖ´¿Ùç>ãý2|üð*º»»ëêêîNø»|ùr¤À+W²zö/]º´lÙ²àþCK#SÂµ··=6÷Í>HÄKS©!*·üÁÊ±èoýó³wõêÕææfÃRÊï.ÿ¹0'üÍfïNöþPY½àþT.-]º4:Ì×ßß¿~ýú0±råÊhÎÐÐÐ+QY=ûaÎàààäää¡Czê)CT&&:;;;&Âe&12åö?øS¹Þé[ZZÉäÚµkoÞ¼ywjOôõá2L¢²zöS©Ô¶mÛ&&&QY½÷÷õõÕ××'t:ô-øüI$	þ$I$I?I$Á$IàO$	þ$I$I?I$Á$IàO$Ið'I$ø$IüI$	þ$I$I?I$ø$IüI$	þ$éÓî¥^Z½zuåTk×®õÕWÿÓï¸©æ7òL÷¶¡¡!<´ÑÑÑ¼ùaN2L§ÓóÝ¦$ø¤¢lÿþýÓzê©§J	3_xá¼ùÏ?ÿ|ÿäO.`àO¯ÁÁÁÀd2yäÈ©=®.ü]¿~=Ì|yÞüæææ0ÿÚµkð'	þ$E_þòkyæÜÏ>ûlù¯|%>===OÁ­­­a:¾ñíÛ··mÛVUUÕÖÖîÚµ+÷èê3g°Â¢°nWWW§ÂêêêÕ«W¿ñÆájgggÞ;útáíDÂ]uwwÏµõë×ùýýýñsçÎ9ëÖ­ç<x°¾¾>lª²²róæÍï¿ÿþtüMß~ÞwUüIÒ§Ë7rg¾ûî»afCCC.nònÚ´)oÑÎ;£ECCCDbÆµ¢«ÑÒöööÉÉÉÀÇT*511Ë§%KDÅ+°01ãÝþHO8+ÚØÇå·µk×Îîª$ø¤O¿èï¿×**ÅrqÓÑÑqgª0®nÜ¸1ZY'âc´/ -ZÉd¢]aº¯¯/ÚHî6÷íÛxuÝºuksêÔ©0.ÃôO<qÏí8«á¹÷mÆG@YSSï­[·ÂÕpîyg6nN§Ãá!ÜýéaâÊÊÊùâ¯À]ô@à/4#þâùn"3Þÿýp5°)ºÚÚÚ®655mß¾= mll,ÞH¸MÞ¾´x­èê»ï¾ß8)>ò.Ãô3gî¹ÍpõÃ?Ì½o³>o÷îÝaÑ¡CîþôÐö®]»ro ðHÚÖÖí/þÜUIð'I~Ñî®;wîäÎ3Ã¢Üi8<<ù/¶NüA½éGcó8û+aº¶¶6JWWWÇKï¹Ù(×Ûo¿95LË0=44/íëëw`ÆÃÇsÇ_»*	þ$éÓ/úÜÛsÏ=;3ú¼>âÏÞºukú­.8p :«1Ú-W½'ÑvîÜíÛ¶mçØN´§íæÍÑÕ?ü°ð¹+W®K£o·	fÍ]ùuwwÆ_Óh4â¥îª$ø¤O¿èd2yøðáè«^9J¥¦±qãÆñññèl|fnô¿è£rÃÃÃ¹,¸k×®@¥è<ÜøËVf$ZtúmôÓãù¶È/úÌßèèhtËø;zôh¼O.<äÜEÑÇÂO	0þ"n.tnÞ¼9wi»*	þ$é(úä¼<øÇMÙ(ÆçXÄmÝº5W¹<y²þîNýSa~mmmîáÛ	w#÷ÅgÚÎö`ÇÆÆ*++£w°Ã¹?¢¦¦&ßö»ÍhÇdÕxi»*	þ$éA)eíÚµ©©V¯^r¿îîîèëôZZZÎ=/Ý»wo¦Ý»wÇK»ººÚÚÚÉÒéô/¾·Íé÷$l*Ìß±cGÞüÙ¶w&Ü¥è|Ï_Ü¶mÛò¾ó%êÖ­[L&@UUUx×®]¿¶&w###A·ÑX­[·®¿¿?ï'¸«àO$Ið'I$ø$IüI$	þ$I$I?I$Á$IàO$	þ$I$I?I$Á$IàO$Ið'I$ø$IüI$éãèÿÜQÀ®´/IEND®B`


öÙºººÙßß;à/Z»vmEàïÔ©SV¿õ­o^¿~=âb<öìî,ãÛ¶m1²YÃ_2ãï½Ã¹¬6$øªIO?ýtÑNãÇ/Z´(N/^¼¸»»»Où$q¹sç._¾¼Ä¼ù+9s¦££#9"ùàÞh[[[2cOOOÑ]l±7±èlØ°!~>|x²uòæo®_¿>¹ÙlöÂE×,|¼É¸Ñ¸é¸+V¬¸råÊ'Z[[kkkW­ZU¸qçÎMMMÉñÍ¸ü¤Éîðg?ûÙ_x6¬ÇÈ7N¸§;Ëø/Æ¸ÓZ9î+lf3Æ_ÑÔØ.ñÕ~ï½÷Æº-½püÔ)>©±&ÃÓ1©¡¡aË-CCC^X$ø*áÙÜÜöl**|K>uêT	ü%W^·n]yóW÷æÂ©»¬&;ÀWb±¥gÿØ¯^½Édc`ü:yûí·ã½p9qÍ"¥>Þñ·»téÒdOjR@$/¿¢k®²4qæÏãß÷ÝñkÁnßéÎRtÓ!dÏßÂ§µrJ¯üÏ8uü.'ìtñ7Å§ñÚµkîóæÍ½°Hð'UþñÁ²d8ÍÆÅÞÞÞîëë+$ZÑìÉÅÇìúõëÉ¾Îïñ®×Ov7©ñ¦cökcÅ·Ub±¥gð±9r¤pOXáõ7mÚÃkÖ¬yo¬1r²ÇyäGFGG;,9.vww>ÀämÇá/Nì§ÓéÉH~Þ9ËdªËïîÊð^ÍxÆÒ¬/¼Xø4Öç6uüMñi ðÒ¥K1[3çÌãE?©"ñ?Ò|D¬°So¿ýv~ÌçÍïEK/Æ[ir1ÞAwY¶+±ØÒ3NöØ#ÆûzÑøÆÆÆÂGïñqqÞ¼y=ÞdL£ðbr,;y÷$Ëííí½þò"LêrÂUóÆÊìèè8~üø+m*+gÂ5ã§¿äråÊÂþ¦ø4nkk---×ø_Äðð°W	þ¤ÊÃ_`%F644Lö;S¡ïÖ%3Ý·ç,v²ññãñÆ[øÈÈHáøñÎÆnüãÊÅfÑ¡ÏµÚÔÔ§/¼"Rç0Y¦²®&¼ÂTVÎd$ÙS?ìûá]S|?>ñ_GõÂ"ÁTaøÛ»woá	É¾´¢ó.§biÍ[zÏÍ»ï¾[8µÄbKÏXâ:t(.æ¿%ø¬hU§¾B/.Z´(ùÊÀÙTðìL¾u%ÍnÙ²å½÷ÞÛ·o_~ýøÊ,3ÆßtWN¾Ï8uüMeÏ_wÉÿvJ¬ÿÏ·¤Ó§OïØ±#9Ö?3ZüI¿x%ïÍù¯zIÞÒÂñf<»xñâdRò§¡±&|×,1oi9%jI>³OSô®	[zÆÒX»vmþsýÉä5kÖ+YÚ#<òáñìý:öl<=öL¯¼òJ²o)r___²W/éÈ#nßéÎ2-ü^9EÏÂ¦»Vg¿äSzñdÛ­^½z<wíÚ+?.½þoø+|vóüùó%ÁK?©ð7¾Â3Ç?ÿøþûxÝãß5KÌ[ZN¡¢G§^æ§XléKKâòåËùOw%c.Pôy¯ÆÆÆü?þòI7o^þ%óàßXK,)±§5Ë´ðWzå=7îZþN>]¸üåËN-Z'ùSÎ'»Ï·üEùzè!/,üI¿xråø?ÿÕÝÝÝÞÞwô½÷æÇ9r$Æ¶¿ånóÞPNÉ×ãÅÂÛÚÚÆ]ßd½á¥%-ÿÆo¬_¿¾v¬l6;þôáopp0ËmÝº5<7wiâ<óÌ3ñèâÏ?ÿþý÷Ýw_~ÏÓeZø+½rEMk­ÎÑÑ£G[ZZâQÇòëùãb-¹õþþþîyìù644ôè£&»TCð±)GFF¼°Hð'IU¹ÁÖwËÍ`+Ùã¾=::~Ñ¢E0üIª°ä3E=ùäÖ¤*lhhhÛ¶móçÏOíÆÀã?nµHð'I$ø$IüI$	þ$I$I?I$Á$IàO$	þ¬I$ø$IüI$	þ$I$I?I$Á¤JïÈ#ÍÍÍétzåÊÓ·µµµ¦¦æüùóù11c/^<W´±fv©Ì;ÙI*J¥êêê²Ùìàà g$øT1555eB0£££ÓwçÎ1ï×¾öµü§z*ÆìØ±£¢ñWbùcâÇpgg§g$øT9/(ÂO§OyW¬X³téÒ3000;wòÖâ/¸ÃuuuEàOR%É¯4>úhý9s¶oß^tµûî»¯½½½h	ù1üî»ïÆpI&]ºt©££#lN§ÛÚÚz'á­eÿþýÍÍÍË-O±k×®uuuÅ¼qç·mÛìÈ._¾<ãcÉo¿ýöG¿7Þx#J%èúõëqçãbôÊÑÉðO<Ã÷îfÅÀO>YxîîîñoÛ´iSLÚ·o_ÇÏÞ¸qc2iÉ%Ç/ÆøL&3áÒï@éYt6¿ó7oá¸ÂóÏ?;wî÷ÜsO_½zõìÙ³1ìðÃ|ÓÅ_/ÖO?ðÀÉ¤;v$«b×®]1ðøãvI?Ie¿æææÎ5Þ^~(ðW^IkÖ¬áµk×&ªËO=úôc=ÖÞÞ!1áÒUzäÕ××·±±1¹óÁ²öÅÈ¸f·µµgGFF>ÌÊ)Ü·XxÈ/dL^°`AÜ9sæ,0.zvI?Ie¿t:¸8ò´Â:qÍ¡¡¡äèjI&íÙ³'æ9s¦p	EK+¼8Y&»c¡±Ây	><wîÜdLCCC²Sð¦ìù°ä|¨ð ¹$Á¤2Å_&)Üóì<»¡xºººbjþg~|mmm¹>ÖñWzüÏwÞ¼yÉ¼EwottôØ±c6l(Üe8sÃUqõêÕÆÆÆl6»|ùòñ+W<»$Á¤²Æ_òÕ-ûöíK>ó÷ÔSOMÎïB;tèP~|BÉsçÎ%þJÏòØc=÷Üs1°iÓ¦ñó>ôÐC1|àÀ×_=9Ô#-[ÃgÎ	ÅÀÂ?:ümÙ²%&>º¯¯¯ðÊ£££¬9c~W_iü'gþpÑÑ£Gêëë·mÛ6Eüåøñã1uéÒ¥ùóN¯344ü^[[»zõêäÄÞ0_6MN^±bÅtO¿:þ¬ßz³|ùò¸xöìYO0Ið'I$ø$IüI$	þ$IàO$Ið'I$ø«àþú¯ÿú­·ÞÍ[üçþçû·óÌ+·®RøÅ"*b£¼óÎ;ÖC¹/b³üÊ©©ôÿñçÏ·Ê°ú§úÉO~åÒüÁÿfQ=6=[±Q|¿]y¢|_95®_¿þWõWÖCöÊ+¯üë¿þ+üÁàOð?ø?øü	þàOðð?øü	þàþàOð?ÁüÁü	þàOð?ø?Áü	þàOðð'ø?Áü	þàþàOð'ø?ÁüÁü	þð'ø?ø?Áü	þðð'ø?ÁüÁüÁü	þàOðð?Áü	þàþàOð?ÁüÁü	þàOð'ø¿ÛL&N·¶¶öööNÙ°aCmmí=÷Ükþð'ø?Á_ÅÍf8»wïîìì,´sçÎ'|2~%bÕ·´´Ç_OOÏµYì»ßýî~ô£k*³^õÕþðÖC¹%6õPnÅX¼YåÖûï¿ø³Ê°ÞÞÞ+W®Ìæ-ÞøkhhÞÅ@.knn.ÔÚÚúúë¯O6cào×®]ßÅ;OïªÌzñÅ_zé%ë¡ÜÆz(·âE,^Ê¬2ì^°Ê°@ùË/¿<·x[à/NO8ÞÕÕÕµ´´9sÆa_9ìë°¯öuØWûV|©T*?[[4iß¾1ðÆo´··Ãàþð'ø«øs¹Ücc¸hR~¸h§ ü	þàOð¿¬««kÿþý1?³Ùlá¤GyäÐ¡C1pîÜ¹eËÁàþð'ø«øúúúR©T&éïïÿ¯ÇVS¼H­[·.N···?þð'ø?Áßíü	þàOð?ø?øü	þàOðð?Áü	þàþàOð?ÁüÁü	þàOð'ø?ø?Áü	þàþàOð'ø?ÁüÁü	þàþàOðð?øü	þàþàOð?øüÁü	þàOð?ø?Áü	þàOðð?Áü	þàþàOð'ø?ÁüÁü	þàOð'ø?ø?Áü	þðð'ø?ÁüÁü	þð'ø?ø?Áàþðð'ø?Áàþàþð'ø?ø?ø?Áü	þàOðð'ø?Áü	þàþàOð'ø?ÁüÁü	þð'ø?ø?Áü	þðð'ø?Áàþàþð'ø?ø?Áàþðð'øüÁàþàþð'øüÁüÁàþ?ø?øüÁàþð?øüÁàþàþàþð'ø?ø?Áàþðð'ø?ëþðð'ø?Áàþàþð'ø?ø?Áàþðð'øüÁàþàþð'øüÁüÁàþ?ø?øüÁàþð?øüÁàþàOð?øüÁüÁàOð?ø?øüÁüÁàþàþð'øüÁüÁàþððð'ø?ÁüÁü	þð'ø?ø?Áü	þðð'ø?Áàþàþð'ø¿Jéµ×^ûò¿¼eË½÷ÁüÁàþàþTµøûú×¿>oÞ¼ûï¿Ñ¢E¿Øúï¿ÿ>üÁü	þàþàOU¿×^-ä÷ðo?¼ýw·'ÿ~réç?ÿyø?øüÁüÁª_þò;::òò_Øü»ï¾þàþðð§*Äß-[î¿ïþBüÅ¿ºº:ø?øüÁüÁªò'ò¡P~YÿüãðSm`` É¤ÓéÖÖÖÞÞÞñWèéé©©©?Áü	þà¯ù¹û¹åËoûâ¶ßçøãñãÇáþ¦Z6=pà@ìÞ½»³³³hêèèh[[Ûdøû¿øÅ¾óïüð?üYßÿþ÷Ï9c=[±QbÓXåV¼ÅKõPn]ºtéèÑ£õ¾jÕª;ï¼óÿõGôGÕºi^zé¥7ß|s6oñ¶À_CCCü'r¹ÑÔ'xâé§ûöí;3õôô:uêÊ¬_~¹¯¯Ïz(·b£Ä¦±Ê­x2ë¡Üzíµ×w·_õÕï~÷»Õ½iº»»ÿæoþf6oñ¶À_:p8ÿ	µ··öÃ¾ûÊa_å°oà/Jåkkk'­[·îÄÿùPáOð?øüUÇjmllÌåröáÿõÿwð'ø?Áü	þ*¾®®®ýû÷Ç@üÌf³?Tþð'ø?Á_u¬Ö¾¾¾¦¦¦T*Édúûû'Ôü	þàOð?_ò?øüÁàþàþ?øüÁüÁàOð?ø?øüÁàOðð?øüÁüÁüÁàþðð'øüÁàþàþð'øüÁüÁàþ?ø?øüÁàþð?øüÁàþàOð?øüÁüÁàOð?ø?øü	þàOðð?øü	þàþàOð?ÁüÁü	þàOðð?Áü	þàþàOðð?ø?øüÁàOðð?øüÁõð?øüÁàþàOð?øüÁüÁàOð?ø?øü	þàOðð?øü	þàþàOð?ÁüÁü	þàOðð?Áü	þàþàOð'ø?ÁüÁü	þàOð'ø?ø?Áü	þàþàþàOð?øüÝ4üÕÜ¨T*ð?øüÁ_à/u£Òé4üÁü	þàOðûÂüÁàþðW]øM6ÁüÁàþðWøknnN§Ó>óð'ø?Áª/¶GCCÃÐÐüÁü	þàOðÕ¿ÚÚÚÐÞàà`SSSùî¹Ø¸q#üÁü	þàOðÕ¿dW_öbàâÅñü9sæÀüÁàþðWmø;wnP¯¯¯ïüùó1ðøã'¾êþàOð?U!þ¶mÛ?½£ðcË-?ø?Áü	þà¯ÏöýÒ¾4oÞ¼èïï`EløüÁàþ·Qð'ø?Áü	þàþàOð'ø?Áßä-0ùÂ_òð'ø?Áª,(_>gûÂü	þàOð§*Ä_8/ØwêÔ©xÚUÜö?Áü	þàOð7½(?øüÁàþÓn`` ð·yóæk×®ÁüÁàþªrüEóçÏ¯>àþð'øSâ¯¥¥Å	ð?øüévÁ_Â¾?Áü	þàOð7½ðð'ø?ÁnüÅCümÛ¶mddþàþð'øSã¯fðð'ø?ÁªóK'Ì	ð?øü©:¿ê¥r?Áü	þàOð7½[ZZ^ýõ[ø02L:nmmííí-ÔßßßÖÖ/^W?ÁüÝÄº»»í×~mÁ÷ÞïóÏ??ÝøWÕÔÜâ=ÙlöÀ1°÷îÎÎÎÂIñ¢|òäÉ8xðàÂáOð7«^x¡¾¾~Í5ÿöÃYÿ»ï¾ûë_ÿ:ü	þTýøëííüíØ±#^nÕ¾444$7Ëå'»Z]]ÝxüýéþéßÏb/½ôÒéÓ§ÿ^eÖ÷¾÷½ïÿûÖC¹%6MyÞ·PéwÞùýÍí¿»=ùü©ú©x¯úí/bñRæùYn;wîèÑ£ÖCöâ/9sf6oñ#Ç_9í[xrÉd':ujãÆãñwèÐ¡³ØñãÇc«Õ××÷ê«¯ZåVlØ4åyßâÿ½?û³?_òï~é¾ñoTýv±x)óü,·þñÿ1ðg=a===?øÁfó?rüÃÙ¾Ð¬­­k×®e³Ù¡¡!å°¯Ã¾7¥óçÏ·´´áoÉ'tww;ì+UåË¡ÆÆÆöá¢©/_Þ°aÃ+WÆÏ?øY£££wÝuWÑaß;î¸ã­·Þ?Áàï#¯««kÿþý1?³ÙlÑ_µjÕààà3ÂàþfÂÇý÷ß?áã+_ùÊí°]àþÿ¹¿mõêÕuuu555sæÌY·nÝ,ùÑ×××ÔÔJ¥2Lÿ=¶±s??ø»%_õò±¬££ÃW½þt»àoxxxÂ>*âOýÂàþð'ø^-ê­Y³æÚµkÉÂÚµkcÌ%KàþàOð?ø«6üÕÖÖõFGGócr¹ð¬[ø?øüÁàþ*©T*¨l422cfó«^àOð?ø?ø%ü%W­Zö1cÚÚÚàþàOð?ø«6üö&<áã½÷Þ?ø?Áü	þà¯¿êexxxÝºuõõõ©T*~®Zµ*ÆTÄö?Áü	þàOðw?øüÁàþàþ?øüÍy£R©üÁü	þàOðU¿ÔäÁüÁàþª6üMÖÖ­[ü<xþàþð'ø¿ªÅßÀÀÀ9sßùð?øüÁ_µáoýúõÉ¿cÇUÐö?Áü	þàOð7½þùkÖ¬©¸í?øüÁàoª/Y²$9·£····ü	þàOð¿)µk×®d_gggånøüÁàþSÓ÷üÁü	þàOð§Û©N§áþàOð?øóçÝàþàOð?ø?øü	þàOðð?øü	þàþàOð?ÁüÁü	þàOð?ø?Áü	þàOðwÓð·páÂÚÚZßóð'ø?Áª,(ïù?øüÁàOÕ¿p^°ïÔ©Sñ´«¸í?øüÁàoz544þ*Q~ð'ø?Áü	þ¦ÝÀÀ@àoóæÍ×®]?ø?Áü	þTåøæÏ_3.'|Àü	þàOð§*Ä_KK>àþð'øÓí¿ú?øüÁàoz566:áþàOð?Ý.øøÛ¶mÛÈÈüÁü	þàOð§*Ç_Í$9áþàOð?Uç<O>àþð'øSu~ÕKå?øüÁàþàþ?øüM^.[½zu]]]MMÍ9sÖ­[W)'ÿÂàþð'ø^ÃÃÃðQ'ÿÂàþð'ø^-ê­Y³&ùÛ¾ñ¢°víÚ³dÉø?øüÁàþªµµµA½ÑÑÑücb<üÁü	þàOðUøU/A½_~ÌÈÈHñU/ð?øü©jû®Zµ*9ì?c8Æ´µµÁüÁàþðWmøíMxÂÇï½ð?øüÁ_~ÕËðððºuëêëëS©TüjU©í?øüÁàï6þð'ø?Áßôjnnniiyýõ×áþàOð?U?þÒétMM¥îA?Áü	þàOð7½z;vìJù«nð'ø?Áü	þfºIJ¥Rðð'ø?ÁüUá<O/y?øüÁàOÕ¿ÖÖÖ¡¡¡Þð'ø?Áü	þ¦Tò'?;ì[GxáOð?øüÍÉôMþ/üÁü	þàOð§*Ç_&©)>àþð'øSõàïÔ©SsçÎMöÿ%ÔsÂüÁàþªZüå«çÁàþð'øó·áOð?øüÝº2L:nmmííí-=ifcàOð?øüKÙlöÀ1°÷îÎÎÎÒf6¦°mÛ¶555Ý3ÅÍÍ?ÿYñ?ææfë¡ÜÆz(·âEl_95Å­2ìî»ïå·þÃ_CCCòs¹î4³1mÝºµF$©úÈñ÷ÆoÜDü;RtÉøI3$	þ>Äã±ïyikkóÍ7?üÒ¿°¶¶¶ô¤)ì÷~ï÷¶oßþÍY,nîÿø¿©2ëÿð¿úÕ¯ZåVlØ4ÖC¹/b³üÊ©©tàÀßùß±Ê°ÇlïÞ½³y9þyi¦Óé+VpaÆKkllÌårÉQÚ.=ifcð!'|8áCNøpÂðqþ.Z´(¯ÀÚÚÚÕ«WÏ`9]]]û÷ïøÍfKOÙøüÁàþ7íwìØ±ºººþy·¾¾¾¦¦¦7Éô÷÷ÿ×½;z=~ÒÌÆÀàþð'ø»	ïþêëë7nÜXþÛþð'ø?Áßô*4_]]]ggç¥K*eÀàþð'øæ"ÆÎó¸÷Þ/^¼XqÛþð'ø?Áßôº)ßð?Áü	þà¯2ðWÑÁàþð'øvçÎ[¸pá9s3|>ð?øü©ñ×ÓÓ?á#Á_2¼÷nø?øüÁàþªMMMA½sçÎåñ×ßß|ÛüÁü	þàOðUx¶oò%ÌyüÅó/9þàþð'ø¿jÃ_cccP/ÙÛøËår>úh777ÃüÁàþðWmøT3Q'O?ø?Áü	þà¯Ïö½råJr¶o]]ÝÂ/PÛþð'ø?Áßmü	þàOð?ø?øü	þàOð7Q.]jkk«¯¯O­­­rÌþð'ø?Áß4zè¡j&iÓ¦Mðð'ø?ÁªN·gÏ¡¡¡däððð#GñÝÝÝðð'ø?ÁüU	þ2Loß¾ã'%ðÍ÷üÁü	þàOð§êÁ_mmmoxxxü¤âðð'ø?ÁüU	þòÕm²©Éz?ø?Áü	þà¯JðWwðð'ø?Áàþàþð'ø¿Å_éàþàOð?UþR7*NÃüÁàþðçÏ»ÁüÁàþðð'øüÁàþàþð'øüÁüÁàþ?ø?øüÁàþð?øüÁàþàOð?øüÁüÁàOð?ø?øü	þàOðð?øü	þàþàOð?ÁüÁü	þàOðð?Áü	þàþàOðð?ø?øüÁàOðð?øüÁàþàOð?øüÁü	þàOð?ø?øü	þàOðð?Áü	þàþàOð?ÁüÁü	þàOð'ø?ø?Áü	þàþàOð'ø?ÁüÁü	þð'ø?ø?Áü	þðð'ø?ÁüÁüÁü	þàOð?ø?Áü	þàOððÖü	þàOðð?Áü	þàþàOð?ÁüÁü	þàOð'ø?ø?Áü	þàþàOð'ø?ÁüÁü	þð'ø?ø+ÕÀÀ@&I§Ó­­­½½½úûûÛÚÚbÒâÅãjð'ø?Áü	þ*¾l6àÀØ½wgggá¤<y2<¸páBøüÁàþ_CCC<éc Ë577Ovµºººñøê©§ºg±£G~ûÛßîV¯Ç³Ê­Ø(±i¬r+^Äâ¥Ìz(Ã^xá+¡ý·þÛétzÂáÂN:µqãÆñøÿû,vâÄÁÁÁWõ·û·.Ê­Ø(±i¬r+^Äâ¥Ìz(·~òÄ¬2ìå_¾zõêlÞâm¿T*®­­k×®e³Ù¡¡!å°¯Ã¾rØ×a_9ì[Õüw1ÜØØËåÃ¾1ÍË/oØ°áÊ+ã?øüÁà¯òêêêÚ¿ÄÏl6[´ÆW­Z5888áð'ø?Áü	þ*¯¾¾¾¦¦¦T*Édúûûÿë±ílnn®)þð'ø?Áßíü	þàOð?ø?øü	þàOðð?Áü	þàþàOð?ÁüÁü	þàOð'ø?ø?Áü	þàþàOð'ø?ÁüÁü	þàþàOðð?øü	þàþàOðeØüããÇwww¿óÎ;ð'ø?ø?Áªßüæ7ëï¨ÿøÇ?¾äKî¸ã]»vÁàþàþªNüÅïûÜ¹s?÷Àç¶ÿîöø÷ðo?|÷ÝwïÝ»þðð'øSâïá^õ+«ù%ÿ>³þ3+V¬?ÁüÁü	þTøûô§?ý]¿Q¿ûáìcð'ø?ø?ÁªÛ¶mûÔ§>U¿ûï¿ÿWõWáOðð?U!þÞzë­þé^ÿëëùîÏÝyçUÀ&ø?ÁüÁü	þ&Ñ7oÞ=ÿ÷ÿù¿ë®»î¹*Ø.ð?ø?øüMÚèèèëcTÇv?øüÁüÁàï6þàOðð?øü	þàþàOð?ø?ø?øüÁàþàþ?øüÁüÁàþ?ø?øüÁàOðð?øüÁàþàOð?øüÁü	þàOð?ø?øü	þàOðð?Áü	þàþàOð?ÁüÁü	þàOð'ø?ø?Áü	þàþàOð'ø?ÁüÁü	þàþàOðð?øü	þàþàOð?ø³àþàOð?øüÁü	þàOð?ø?øü	þàOðð?Áü	þàþàOð?ÁüÁ_~ô£ýÙýÙ3Ï<süøñÑÑQøüÁàþàþª¶¿üË¿;wî'?ùÉ_éøî¹çùßÿøüÁàþàþª°óçÏü>ÿàç·ÿîöäß'Ú?ñë¿þëð'ø?ÁüÁüUa_ûÚ×>µâSyùÅ¿/nýbp°LþÂü	þàOððw3ûÂ¾ðéOºñ¯±±ñwÞ?Áü	þàþà¯ÚzöÙg/^¿ÿoã]wÝeÏàþððW½ÿþû?ó3?ó©Oê[¿òû­¿ÕÜÜü¯|¥LîüÁàþð7¹wÞyç¾OßwÇw4õì³ÏÏ?øüÁàþàï#ittôÇ?þq¹Ý+ø?Áü	þàþn£àþð'ø?ø?Áàþðð'ø?ø?ÁüÁü	þàOð'ø?ø?Áü	þàþàOð'ø?ÁüÁü	þð'ø?ø?Áü	þðð'ø?ÁàþnOüd2t:ÝÚÚÚÛÛ;þ===555ð'ø?Áü	þª¡l6àÀØ½wgggÑÔÑÑÑ¶¶¶Éð÷ï|gxûÞ÷¾wåÊaY¯½öÚ?üÃ?XåVlØ4ÖC¹/bñRf=[CCC?ë¡ëííÍ[¼-ð×ÐÐÿã×ÜÜõ'xúé§'ÃßW¿úÕïÌbñùÒK/GeÖ·¿ýí_|Ñz(·b£Ä¦±Ê­x2ë¡á¬2lößúoü¥Óé	£K.µ··öÃ¾ûÊa_å°oà/Jåkkk'­[·îÄÿùPáOð?øüUîª¬ùïb¸±±1Ë0vØ7'¼ZþÊð'ø?Áü	þ*»®®®ýû÷Ç@üÌf³aqüHøüÁàþW___SSS*Êd2ýýýjþð'ø?Á/y?Áü	þàOðð?Áü	þàþàOð'ø?ÁüÁü	þàOð'ø?ø?Áü	þàþàþàOð?ø?øü	þàOðð?øü	þàþàOð?ÁüÁü	þàOð?ø?Áü	þàOðð'ø?Áü	þn¯~ÿ÷ÿÏÿüÏßÅâæþîïþî-Y==='O´Ê­Ø(±i¬r+^ÄfùSSéâÅßøÆ7¬2ìÐ¡C?øÁfóßÿø´sçÎmß¾ý$Iª¥Òûækì$Iº?I$ø$IüI$	þ$I$I?I$Á_ÕÖßßßÖÖN§/^<00ðÁØ7/Z´(Æ´¶¶öõõYE·¤sçÎ-Y²¤p+ÄÖÉd2ÉÞÞ^«¨L¶Ëøß ÃvIêéé©©ñ>RFÛedddÃµµµ÷ÜsO¼×XEå³iÊê­ß/íGÞN<pa444t)âgss³UtK_¿#GÄ@lØ"1Íf8»wïîìì´Êd»ÿR9lhtt4eµ]vîÜùäO^¿~=¨ÑÒÒbÏ¦)«·~¿´³Z]]]ü_ÈË/Ç@üôËyËëîî^¼xqò¯1Ëå ¼|¶Ëøß Évyâ'~úiø+«íæxýõ×­2Ü4eõÖïvö:uêÔÆ?;¼ïañ?c¤5s«­¯¯qðàÁ¸N§óuk·Ëøß Ãv¹téRü	þÊíul×®]ñæ¼8sæõS>¦¬ÞúýÒÎR×®]Ëf³CCC1¼dÉäfñTXºt©skëééillT*Y[[kÍÉvÿ¤rØ.ëÖ­;qâÄ¾À_½íÛ·/ÞxãÐ¹5S>¦¬ÞúýÒÎF/_Þ°aÃ+Wö0[ÉVßÏ÷ÁØaßBvèÖnñ¿A*íRó¿³ZÊçuÌûKyn²zë÷û÷Ê+¯¬Zµjpp0?&ÈîÜ¹8ölüWÀ*º%-X° Ùýýý±b ««kÿþý1?³Ù¬UT&ÛeüoÊa»üÏ»ùÓvyäG:ôÁØÙ¦Ë-³ÊgÓÕ[¿_Ú¼æææ¢ÿ¿ùæÉàñ3­¢[ÒÀÀ@kkkl+W&»úúúR©T&_W«¨L¶Ëøß Ãv¿òÜ.W¯^]·n]ioo?þ¼UT>¦¬ÞúýÒJ$ÝFÁ$IüI$	þ$I$I?I$Á$IàO$Ið'I$ø$IüI$	þ$I$Ið'I$ø$IüI$	þ$I$I?I$Á$Ý¸oë[Ë/¯kåÊÏ?ÿüÿz«b^'º·ÍÍÍñÐÆÇt:Éd®_¿>ÝeJ?IªÈ¶oß^3®/éKÕ¿;vÄÈg¶hü3Ï<ãüñ,SüIRå500¬I§Óöìkß¾q1F>ºjðwñâÅ¹xñâ¢ñ-ñ.?Ið'é¶è³ýl°æÉ',ùÔSOÅÈx >½½½§pa[[[ç¯üÞïmÜ¸±¾¾>&544lÙ²¥ðèêñãÇX1)æíîî.âT;wîòåË;»ººîØÑ£GK/'w)ÔÓÓ3ÔV­ZãûûûócN<c:::òcvîÜÙÔÔª««[¿~ýåËÇãoüòÆ¸«àOnqóçÏ¸t©päÛo¿#qSÔ©S§©k×®-´yóædÒÙ³gS©Ôs%©ëÖ­»~ýzð±¶¶vtt4¦ÆÏÀÓ9sâXNLx÷Æ?Ò6ïËäåW´+WN%îª$ø¤[_rw×µ X!n:;;¯qqÍ5ÉÔÄ:	x¶dR6MvÆp___²Âe>öØcÁ»ä¨ëC=c9Ãñ3|ðÁ.'àã÷mÂG 7o^<ÞÁÁÁ¸?ã8s¹L&3ÆCøà¿×ÕÕM%îª$ø¤²À_4!þòãÜ$f._¾MÉÅ¶¶¶¸ØÒÒ²iÓ¦@Ûððp~!q¢iù¹o¿ývþÊ¦üßøÃÇ¿árqñÝwß-¼o>oëÖ­1éé§þà¿moÙ²¥ðÁÀ_´½½=Ù19]ü¸«àOnÉî®k×®1©wò4<þ|â¿¼uòÔ4¶S_°Ãµµµ###±ð¹sçæ§Þp9Q¬¨sçÎ%NáøÃgÏÍOíëë;0ááã©ã¯Ä]tëK>÷¶k×®ÂÉ ðÿàààøZ§OÞ±cGr6¯Æd·¸ê¶yóæähoüÜ¸qc~|å$Ú®÷ÝwK»téÒ|»MµpRræoLêéé¹zõjiüåa¬üÔwUüIÒ­/9a"NïÞ½;ùª=öÔÖÖ?9cÍ5älþÌÜä3ÉGåÎ?_øaÁÄ[¶l	*%çáæ¿leB¢%§ß&·þÊ+¯äÇXNò!¿ä3CCCÉ5Kàoß¾ùrñ'%<ölÜJ¬Éðp3¸Wt®_¿¾pj»*	þ$©,J¾¹¨;wþÏkÜXòÃù¦ùs,ò=ôÐC²,ìðáÃ%ð÷ÁØâñGK,'îFáËi;Ù®««KBÑÁîÕ«WÞÄ¼yóâgòm/ËLvLæK¨Zâ®J?I*(+W®¬kùòåÉ)·EøëééI¾N¯µµõÄù©CCC>úhSSS¦­[·ä§vww···É2ÌÞ½9þÄ¢bü#<R4~²åDqgâ.%_@XâþòmÜ¸±è;_³Ùl¬úúúx.mMá2¯^½ºMÖUGGGÑ-¸«àO$Ið'I$ø$IüI$	þ$I$I?I$Á$IàO$	þ$I$I?I$Á$IàO$Ið'I$ø$IüI$éfôÿ6«Èó±IIEND®B`


ØªÆ¨D6$òÊI~mÞ¼ù®j¬tä7sæÌ+W®üüñkÛ¶mE6¡YÆz=ìÜ¹s¢+gÒ«1Éäï·½½ü$òt·qãÆ^x¡¦¦&FvuuMùE«W¯.ùuww'TýÎw¾388844q5Föööº¨Î2òõ°eË2ù%3^¿~=s¹Iä'Uüyæa»[=:þüt:½`Á#GS~!É@Ü`Ö¬YK,)2oþÆ§NZ¾|yr ríÚµwÚÜÜÌØÑÑ1ì¡µØ;Î8ì¬[·..80Ö:yï½÷Ö¬Y<Âl6þüùa·,|¾É¸Ó¸ëxK.½zõêñãÇª««W¬XQ¸qÇõõõÉaÍ¸ü¤±ðW¿úÕ_xì5©ÇÈõë×º':ËÈ»,ÆxZ9£î+lr3ß°©±]â«ý_øB¬ÛâGNçË8k20jkk7oÞÜßßïD~R9É/°#«½½½©Tªðý¸»»»ü·¶¶7ãxc.ÚÖÖV¸³j¬ãzE[|ÆÏýÆL¦®®.F®Ë/ÇÛyárâÃVø|GÞï¢E¨I¡<ûÝrÙ²eÅ3gÎÿáÜX£nßÎ2ì®5É>¿yóæMhå_ùqüòëéé)<ÙÊo/ãÕ«WÌ6mòE"?©ä%ÃÙl6®vvvÆð'6löäêÖ­[½8w7Þ8ã-?nìh$%Sã7®Æì7osÞWÅqÔç~ðàÁÂ`·ß°aC¯Zµêúíb ®ÆÈ±o2fãÆÉKW9RøO³>|8/P8i¬N§Çz"ùy?æ,c.¿Ot¢+gÔG5é¾ðjáË L÷Ùøå7Îq"ÂK.ÅplÍ1c_,ùIå'¿ü¾äcaÅ"ò»|ùr~ÌçÍï?K*Æûhr5Þ>wV;ZWd±Ågë¹'@7õaãëêêQ¼ÁÇÕÙ³gõ|1¡Â«É!ìä	>Va´´´>÷"òËs|,¦ÌYWiÌ+sùòåGu¥gåú¨&=ãøå¼B®^½Zxß8_ÆÍÍÍquîÜ¹!×ø/Ä­[·üVÈO*3ùTbdmmíXo·c%¹Ä)òV]Ä7oÄbÇO9o¼¹ÏläÞF>ßñe;âyÇsTëëëÃÓ#y7ÌÓù%gñ¬«Qo03G'7ãøö~üW×8_ÆçÎKðá¡CübÈO*'ù½øâgx$Ñe9èLhÞâûl>üðÃÂ©E[|Æ"`ÿþýq5ÿ(ÉÈgÃöN3ñøWHáÕùóç'ß®ÒÑÑ2üÉ¬d³ÙÍ7_¿~÷îÝ1rÍ5£Êo<³LZ~]9ù&=ãøå7~yÛ%ÿÕ)²þ¼ÞN<¹ûöäsþ<hIä'ºüâ½-¼1ç¿Õ%y?4Ä;erªìIÉúo7ê[fy³)!Kò9­Xx²aâu±Åg,ÎÕ«Wç?ÈIÎü]µjÕÛ%KÛ¸qãÇ_²ß«··7Â®]»Æ#¿cÇ%â)8q"ÙtðàÁQ·ïDgü¯a¯Â&ºV'!¿äyñb¸uëVÀnåÊ#]øÜsÏÅÊ¯ÿ;þ$×<wî#ïÈO*ù¬ðüÄgËæ?ïÿÒÝdáÈ·Ì"ógS(±Qáù©E[|Æâ¸råJþ]ÉóçÏûW]]]þCG~y$Í=;ÿ©Ç"¾Y»víÈµpáÂ"xB³LH~ÅWÎ°×Fa]«ßÉ'¿dÉÂ©ÃÖIþó±î¥Èë-Q¾~Ø/ü¤ò_¼.[¶lä_÷:räHKKK(*ÞÎ_|ñÅüøÆVá·ÙsÞ;²)ù¼XxssóÈ¯åk±w±8##¡ãÏ=»fÍêÛe³ÙçsLN~±´XfèG	åÁ]Ü7Ï?ÿ|<»xâsæÌÙ³gÏC=ßçôñgü¯a¯aMh­NB~Ñ¡CæÎÏ:OÏÎWÃgÉ½/_¾¼««ëûz½õ÷÷?úè£ÉÎÔàlÊ¿X$ò¤»R.[»ví¾Cn³]É¾ÞädíÁÁÁôóçÏ÷ÈOTi%óÖSO=eÍHä'Iª´úûû·lÙ2gÎän<þøãVD~$I"?I$$IÈO$Iä'I$ò$IùI$$IÈO$Iä'I$ò$IùI$ü$ID~$I"?I¥ÖÁÒéô²eË&:oSSSUUÕ¹sçòcb8Æ,X°`¿În7¹ÛgÞIþ-zQ*ª©©Éf³É¤þþþM6ÕÖÖÆÊ9sfLzï½÷¼À$¤ª¾¾>|è¼;vìyöÙü§~:Ælß¾½²åÇá¶¶¶äêºuëâjggg?~<½À$¤Rúmò1ðtòäÉwéÒ¥ù1-1===Sï°©¿Ç°r×ÔÔ$W«««ãê7¼¨$¤Òe_¡fôÑ3gÎ1cÛ¶mÃnöÐCµ´´[B~aøá1cI.]Z¾|yÀ(N777';ÃF.­ðÞÏ²gÏÅÇtØÍ7ÛÛÛcÞxð[¶lIvaM,YKñ±äË/ß=ù=ðÀquþüù'NðÒD~JÉðO>Ã/¾øb+zê©ÂÛ9r$ÿ¶|6lI»wïá¸áõë×'.øðá¸páBÏd2£.­ð%<·ÿþX·nÝÈ¿iÓ¦¼òÊ+1°cÇ¼ÆnÜ¸ÑÛÛÉ.ÉMB~CCC±~bøk_ûZ2éàÁù.[¶ìÔ©S^]ÈORéÊ¯¡¡!s·	¯Q?xìØ±´jÕª^½zuBºüÔ'OnÝºµ¥¥%9%bÔ¥3VñY6sæÌóÖÕÕ%>La¾·áæææ°ìÀÀÀäVËÈñÉcûïvww^ó·;õD~JT~ét:?qµ8>º½ëkÆqËþþþä jI&íÚµ+æ:uªp	ÃVxu<³õÀb»ñ58p`Ö¬YÉÚÚÚdwàOe_:;;Ã¾%ü$ü2Lá>¿d·Ù¹ÓÞÞSóùñÉC·§üÏ`¹óÎ=;wØÃ<|øprâm~gáVËDå%3$òT¢òK¾¥e÷îÝÉçü~úéñpçÀùgû÷ïÏOyúôéäóã_ñY¶nÝúÒK/ÅÀFÎûðÃÇðÞ½Ï9á/áS§N]½z5æÍw÷ä·`ÁÔÑÑÃáµk×zI"?I%*¿ÁÁÁÐÕÛ~'_qùÝºu+9ÒzýÔÖÖÎ9sË-ã_ñY=S-Z?Ñ¤ð6ýýý7oG^]]½råÊä4Þ_6MN^ºtéÙ³g'´ZF.²*âÛÛÛãÁÇz=ö¦Mâ!yI"?I$$IÈO$ü$ID~$I"?I$_ÙôÝï~÷âÅSyçÏÏår^y¥ÖøÃÿøÿ°Jp»LèO¥ijºpáíRÛåßÿýß­Rëý÷ß/©í2Ýå÷ío;ð7÷xìØ±û·óPj½ùæ7oÞ´J­·Þzë_ÿõ_­Rëøñãÿò/ÿb=ZñvVøE*N8ñÏÿüÏäG~"?ùüÈüÈOä'ò#?ùùüÈOä'ò#?ò#?ùüÈüÈüÈOäG~"?òùùüÈOäG~"?ò#?òùüÈOäG~äG~"?ùüÈüÈOäG~"?ùùüÈOäG~äG~"?ùüÈüÈOä'ò#?ùùüÈOä'ò#?ò#?ùüÈüÈüÈOäG~"?òùUüzzz2L:njjêìì,400°nÝºêêêx ÔE~"?òùÈ¯¼Ëf³÷î;w¶µµNÚ±cÇSO=544ä;wîHù>|øúöúë¯_¾|ùºJ¬øÃ?þã?Z¥Öo¼a»æv¹téõPjÅÛÜÅ­RëÍ7ß|ÿý÷§ò§üjkkÃv1Ëå'5559sf¬C~ÅïMaÍøïò÷TbÙ.%»]Þzë-ë¡Ôzíµ×lÛE%»]¦üÒéô¨ÃÉÕç®¦¦fîÜ¹§Nr´Wö:Ú+Gí£½å]*ÊWWW´÷î8ölKKùüÈOäG~"¿ò®®®.Ëtûho¶;üD~ä'ò#?_ùÕÞÞ¾gÏËl6[8iãÆû÷ïÓ§O/^¼üD~ä'ò#?_Ù¯ÖúúúT*Édººº~ôÜªþóÙÅAkkk:nii9wîùüÈOäG~"¿iùüÈOäG~"?ò#?òùüÈOäG~äG~"?ò#?òùùÈüD~"?ò#?òùÈüÈüD~"?òùùÈOäG~"¿»Úµk×ÈüÈüD~"?ò«äúûûý×Ö¬Yuuu÷ÝwßüÁùùüD~äW~æ3ùTó§¾µù[ÛÛ¶á77Ì3gãÆäG~äG~"?ùUZG	êmý­Á¾äß77³¦¦æ.ù%?ò#?òùÈïõüóÏ/[¶,Ï¾äß¼yóÞyçò#?ò#?Èü*ªW_uáÂìÛú;[ëêê>øàò#?ò#?Èü*ªk×®ýÜÏýÜ³_ÎËïsûÜ§?ýéÁÁAò#?ò#?Èü*­ïÿû÷Ýw_KKË²eË|ðÁóçÏß¥û"?ò#?òùÈïwíÚµ_~ùÙgõÕWûûûïÞùùüÈOä7]"?ò#?òùÈüÈüD~"?òùùÈüD~"?ò#?òùÈOäG~äG~"?òùÈüÈOäG~"?òùùÈOäG~"?ò#?òùüÈOäG~äG~"?òùüÈüÈOäG~"?ò#?ò#?òùÈüÈüD~"?òùùÈüD~"?ò#?òùÈOäG~äG~"?òùÈüÈOäG~"?òùùÈOäG~"?ò#?òùüÈOäG~äG~"?òùüÈüÈOäG~"?ò#?òó@~"?òùùÈOäG~"?ò#?òùÈOäG~äG~"?òùüÈüÈOäG~"?òùùüÈOäG~"?ò#?òùüÈOäG~äG~"?ùüÈüÈOäG~"?ùùüÈOäG~äG~"?ùüÈüÈOä'ò#?ùùüÈOä'ò#?ò#?ùüÈüÈüÈOäG~"?òùùüÈOäG~"?ò#?òùüÈOäG~äG~"?ùüÈüÈOäG~"?ùùüÈOäG~äG~"?ùüÈüÈOä'ò#?ùùüÈOä'ò#¿i/¿L&N§:;;GÞ £££ªªüD~ä'ò#?_ÙÍf÷îÝ;wîlkk6upp°¹¹üD~ä'ò#?_%T[[;44¹¡¡aØÔ'|ògK~ò'ò7SØ#Gþú¯ÿúoTbÅvééé±J­¿üË¿´]l³wÞyÇz°]¦üÒéô¨ÃÑ¥KZZZÂcÉïå_¾8ýÕ_ýÕ¹sç.ªÄ:zô¨íRÛåïÿþï­Rëõ×_?ö¬õ`»h<uvvþà?ÊòK¥RùáêêêÂI­­­ÇÿÏ§êh¯íu´Wö:Ú+G+ ºººíáx?ùüÈOäG~"¿ò®½½Ï=1Ùlvô§jÈüD~ä'ò«ÕZ__J¥2LWW×¨Ô#?ùüÈOä7­#?ùüÈOäG~äG~"?ùüÈüÈOäG~äG~"?ò#?òùÈOäG~äG~"?òùùÈOäG~"?ò#?òùüÈOäG~äG~"?òùüÈüÈOäG~"?òùùüÈOäG~"?ò#?ùüÈOäG~äG~"?ùüÈüÈOäG~ÖùüÈüÈOäG~"?ùùüÈOäG~äG~"?ùüÈüÈOäG~äG~"?ò#?òùÈOäG~äG~"?òùÈüÈOäG~"?òùùüÈOäG~"?ò#?òùüÈOäG~äG~"?òùüÈüÈOäG~"?ùùüÈOäG~äG~"?ùüÈüÈOäG~äG~"?ò#?òùÈOäG~äG~"?òùÈüÈOäG~"?òùùüÈOäG~"?ò#?òùüÈOäG~äG~"?òùiúÈ¯êN¥R)ò#?òùÈü*A~©;N§ÉüÈOäG~"?ò«ùUFä'ò#?ùü>nñâÛ°aùùüÈOäG~&¿t:ís~äG~"?òù©Âå·`Á§wÔÖÖö÷÷ùÈüD~äWQò«®®êõõõÕ××Ç@ï¥^õë×ùÈüD~äWQòKvòÅ@P/.0443fÌ ?ò#?ùüÈ¯¢ä7kÖ¬p^<±sçÎÅÀã?øVò#?ùüÈ¯Òä·eËüùõ[¼x1ùùüÈOäG~%¿è'=vtuuÅ@(°¥¥¥,¶ùüÈOäG~"¿éùüÈOäG~"?ò#?òùüÈOä7ZóæÍK¾ÛÅ79ùüÈOä§J_ccc¡öò9·üÈOäG~"?ò«4ùòÂ|ÝÝÝCCCe·=ÈOäG~"?òùM ÚÚÚ_9²üD~ä'ò#?ßÄêéé	ùmÚ´©ß8ÉOäG~"?òùM¬9sæTÈäG~"?òù_¥ÉoîÜ¹Îð ?òùÈOÓB~ùzËqÈüD~ä'ò@uuuÎð ?òùÈOÓB~Aß-[ÈüÈOäG~"?U²üªÆÈäG~"?òù_~ó¨9ÃüÈOäG~"?ò«Àou)ßÈOäG~"?òùM ¹sç9süÈüD~ä'òSË/NWUÝã===L&ISSSgggá¤®®®æææ´`Á¸ùüÈOäG~"¿ÉÒùmß¾=^p÷ê»]²ÙìÞ½c`çÎmmmß~ûíØ·oß¼yóÈOäG~"?òùEÀ¹½µµµ	:s¹ÃX7«©©)¿?ú£?:<:tè°J/ÛÅvÑ¶Ë_üÅ_X¶Js»ÜuùÂ¹½÷5Öývww¯_¿~¤üÞzë­ÿ;½ñÆñåÿ«ëØ±cñeë¡ÔzóÍ7ãÿÊÖC©¿6mìøñã×¯_·J­ï~÷»×®]Ê¼ëò+÷/VWW¼ÁÍ7³Ùl¿£½r´×Ñ^9Úëh¯í-ïêêêr¹´7M½råÊºuë®^½:rFòùÈüD~+¼µråÊªªª3f´¶¶Nñ©íííöì¸Ìf³Ã¤µbÅ¾¾¾Qg$?ùüÈOä7nÝº5êSùg|cµÖ××§R©L&ÓÕÕõ£çvû»fùüÈOäG~"¿É7þüÕªU«7ÎxÙ­^½:Æ,°ô·ùüÈOäG~"¿	T]]ÎÌÉår1fÔ3-ÈüÈOäG~"?ò+cù¥R©p^rEÒÀÀ@Êou!?ùüÈüÈo*äí]±bEòÆ1cÉüÈOäG~"?ò«(ùÅûå¨gx~üÈüD~ä'ò#¿ßG·Oïmmm9sf*Ë+VÄ²Øä'ò#?ùü¦Kä'ò#?ùüÈüÈOä'ò#?_~Î;Uø·tÉüÈOäG~"?ò+cù¥ÆüÈüD~ä'òÓGÓáhï#<Èoß¾äG~ä'ò#?ùU¦üzzzfÌæ[¾|yá;ùÈüD~äWQò[³fM²«ïðáÃe´=ÈOäG~"?òùM W^y%1ßªU«ÊnÈüD~ä'òW·nÝZ¸par2Gggg9nòùÈüD~wî¹çKvõµµµïö ?ùüÈOä79ùÈüD~&òKÝ©t:M~äG~"?òù_%È¯2"?ùüÈOäG~äG~"?ùüÈüÈOäG~äG~"?ò#?òùÈOäG~äG~"?òùùÈOäG~"?ò+/ùÍ7¯ººÚ÷ùùüÈOä§_ccc¡ö|ùÈüD~ªXùòÂ|ÝÝÝCCCe·=ÈOäG~"?òùM ÚÚÚ_9²üD~ä'ò#?ßÄêéé	ùmÚ´©ß8ÉOäG~"?òùM¬9sæTÈäG~"?òù_¥ÉoîÜ¹Îð ?òùÈOÓB~ùzËqÈüD~ä'ò@uuuÎð ?òùÈOÓB~Aß-[ÈüÈOäG~"?U²üªÆÈäG~"?òù_~ó¨9ÃüÈOäG~"?ò«Àou)ßÈOäG~"?òùùÈOäG~"¿ÑÊår+W®¬©©©ªª1cFkkk¹êK~"?òùÈoÝºukÔ3<ÊâT_òùÈüD~hþüùá¼U«V%oñ²[½zuY¸p!ùùüÈOäG~%¿êêêpÞàà`~L.11üÈüD~ä'ò#¿_*çöòcbou!?òùÈü*óhï+7Î¸áÓÜÜL~äG~"?òù_EÉ/Þ/G=ÃãúõëäG~ä'ò#?ùUü>ºzokkëÌ3S©TX±"ÆÅö ?ùüÈOä7]"?ùüÈOä7æÎæÌò#?òùÈO.¿t:]UU®ûÉOäG~"?òùM ÎÎÎßöíÛãW.´üD~ä'ò#?ß¤1F©TüÈüD~ä'ò#¿_j|3ùÈüD~äW!ò;wnSSSYoòùÈüD~w.ùs½Ý>Ú[vÉOäG~"?òùM²äÏõ&¢üÈüD~ä'òS%Ë/ÉTÍäG~"?òù_È¯»»Ö¬YÉ¿ÄyÎð ?òùÈO)¿|å<òùÈüD~Ó:òùÈüD~äG~ä'òùÈï.×ÓÓÉdÒétSSSgggñISØ¶mÛ¾þõ¯ûßøßû½ßû¶J¬µk×þîïþ®õ`»h<Å¯Íøåi=Ø.OëÖ­ì±Ç¦òËL~ÙlvïÞ½1°sçÎ¶¶¶â&7¦°Gy¤J$©Rºëò;öìOQ~µµµCCC1ËåOÜò$Iä7ÙÆ·¿Ò¥¹¹ù½÷ÞûøK+<MxØ)Ã#'MnLa[¶l©¯¯`»ÿþûçÌóJ¬xØ.¶lÛE°]îºüÂ|yf­.]zþüùI/­ðû«««OÜgxÈÎð3<á!gx|Üc¾óçÏÏ0µråÊI,§®®.Ë%gc¸ø¤É!?ùüÈOä÷ShhhèðáÃ555Uýëmíííöì¸Ìf³Å'MnùüÈOäG~"¿Uoooá>¿3g®_¿~r+¨¾¾>ÔÉdººº~ô(oä¤É!?ùüÈOä7ÉÁWSSÓÖÖvéÒ¥rÙä'ò#?ùü&²Û'v|á_¸páBÙmòùÈüD~è§òe.ä'òùÈüÊ@~eùüÈOäG~"¿uúôéyóæÍ1#9·®®îÀäG~ä'ò#?ùUü:::ògx$òKwîÜI~äG~"?òù_EÉ¯¾¾>wúôé¼üººº/v!?ò#?ùüÈ¯¢äÿóÀyù%'üùÈüD~äWQò«««ç%ûùB~¹ÑGáò#?òùÈü*J~AªÑzûí·ÉüÈOäG~"?ò«(ùEW¯^miiIÎí­©©7oÞùóçËbÈüD~ä'ò.ÈüD~ä'ò#?ò#?ÈüD~?Ù¥KgÎº]455Ë¡^ò»K]¼xñÏþìÏ^xá)^·äG~"?ò³Èï.Êïá®£6ßôßÞ½kjj~åW~åóÿüýu÷?ô¥ÈOäG~"?ò+où8p AÞ®]»úûû·nÝ:xð`2þÈ#ä7Ýä÷î»ïÎ1ãëk¿¾í±mñïÑÿõhcccüüD~ä'ò#¿ò_&	ÞíÞ½ä¤äï¹ù>¿i(¿?üÃ?üÜÒÏ%ìKþmøÍ÷ß?ùüÈOäG~å-¿êêêàÝ­[·FNÊår1)n@~ÓM~7oþïý÷BùÅ¿òùÈüÊ[~ù?Ú6ÖÔä/¹ß´ßþéþùÿ¥_Î~ùSúùüÈOäG~e/¿"¶#¿é)¿|pÉ%[~K°ï+_ùÊ'>ñ£GÈüD~äG~äW]¼xñóÿü¬Y³jkkï¿ÿþCÅÃ&?òùÈïò+ùMOù%×ùÈüD~ÅJÝ©t:M~ÓV~eùÈüD~ùüÈOäG~"?ò#?òùüÈOäG~äG~"?ò#?òùùÈüD~"?ò#?òùÈüÈüD~"?òùùÈOäG~"?ò#?òùÈOäG~äG~"?òùÈüÈOäG~"?òùùüÈOäG~"?ò#?òùüÈOäG~äG~"?ò³ÈOäG~äG~"?òùüÈüÈOäG~"?ò#?òùüÈOäG~äG~"?ò#?òùùÈüD~"?ò#?òùÈüD~äG~"?òùÈüÈOäG~"?òùùÈOäG~"?ò#?òùÈOäG~äG~"?òùüÈüÈOäG~"?ò#?òùüÈOäG~äG~"?ò#?òùùÈüD~"?ò#?òùÈüD~äG~"?òùÈüÈOäG~"?òùùÈOäG~"?ò#?òùÈOäG~äG~"?òùüÈüÈOäG~"?ò#?òùüÈOäG~ä7Îzzz2L:njjêìì,ÔÕÕÕÜÜ,X7#?ùüÈOäWÞe³Ù½÷ÆÀÎ;ÛÚÚ'566¾ýöÛ1°oß¾yóæÈüD~ä'ò+ïjkkb Ë544u³òûã?þãî)ìµ×^v«Ä²]l¿#G|ïß³lævòK§Ó£ëbýúõ#åwðàÁÿ==zôý÷ßÿß*±^ýõ.X¥VggçøCëÁvÑxzã7Î?o=ZÇûø©¼Çi!¿T*®®®y7of³Ùþþ~Gåh¯£½r´×Ñ^9Ú[~Uý¸®««ËårÉÑÞvË+W®¬[·îêÕ«#B~"?òùÈ¯Ìjooß³gOÄe6&­+Vôõõ:#ùüÈOäG~"¿ò[­õõõ©T*Étuuýè¹ÝÞØÐÐPUùüÈOäG~"¿iù©ÔäwèÐ¡õë×?ô¥ì±>øüÈüD~äG~ä§ÊßO<ñ³?û³_úÒ~­ý×>»ø³µµµï¾û.ùyÈüÈüTiòëííýÄ'>±á77ll[òïÿí¿ôK¿D~^ä'ò#?ò#?Uü^|ñÅÿºä¿æÙÿ¶þÎÖ°àÈo#"?ÈüÈüTöòûÜç>W(¿ø7ölòóú$?ùù©Òä×ÛÛß÷kó·òìû­ÿãÁÎÛüÈOäG~äG~ªLùEßøÆ72ÌW¾òßú¿õÅÿöÅùùþ÷¿O~^ä'ò#?ò#?U ü¢çþ³ýlccã5k¦ù½äG~"?ò#?òSËOäG~"?ò#?òùßO³k×®Y½äG~"?ò#?òS%ËopppëÖ­³fÍª««Ëoë[1Æz&?òùù*P~«W¯þä'?ùßØöØ¶¸lllÜ¸q£õL~ä'ò#?ò#?UüÎ9sß÷mùí-ùïÍùæ¦oÖÔÔ8òK~ä'ò#?ò#?Uü:ôÏ|fØweÏ7ïwÞ±ªÉüD~äG~ä§ß»ï¾ûÉÆOûûxuuu|ðUM~ä'ò#?ò#?Uüññ_üb^~¿ú«¿úéOÚIäG~"?ò#?òS¥É/:wîÜ/üÂ/üò/ÿò²eËæÏÿó?ÿó/^´ÉüD~äG~ä§_Ôßßÿê«¯>ûì³/¿ü2¾ùüÈüÈO,?ùüÈüÈüÈOäG~"?ò#?òùüÈOäG~äG~"?òùüÈüÈOäG~"?ùùüÈOäG~äG~"?ùüÈüÈOäG~"?ùùüÈOä'ò#?ò#?ùüÈOäG~ä'ò#?ùüÈüÈüÈOäG~"?ò#?òùüÈOäG~äG~"?òùüÈüÈOäG~"?ùùüÈOäG~äG~"?ùüÈüÈOäG~"?ùùüÈOä'ò#?ò#?ùüÈOäG~ä'ò#?ùüÈüÈÏz ?ùüÈüÈOä'ò#?ùùüÈOä'ò#?ò#?ùüD~äG~ä'ò#?ùùüD~ä'ò#?ò#?ùüD~äG~ä'ò#?ÈüÈüD~ä'ò#?ùÈüD~ä'ò#?ò#?ÈüD~äG~ä'òùÈüÈüD~ä'òùùÈüD~äG~äG~ä'ò#?ùùüD~ä'ò#?ò#?ùüD~äG~ä'ò#?ÈüÈüD~ä'ò#?ùÈüD~ä'ò#?ò#?ÈüD~%^OOO&I§ÓMMM#oÐÑÑQUUE~"?òùÈ¯ìËf³÷î;w¶µµ:88ØÜÜ<übÿgã7â'óÿ¨ÄAüdZ¥VüúõëÖC©"¿víõPjÈûúú¬R+DþOÿôOSyÓB~µµµCCC1ËåMòÉ'yæ±ä÷ôÓOÂ:ôÚk¯Qe»Ø.²]lUÆvòK§Ó£G.]jii	:Ú+Gí£½öÊÑÞJ(Jå«««'µ¶¶Æ/©ÿ|ªä'ò#?ùüÊ´ªÃuuu¹£ÛGcxÔåoL~"?òùÈ¯kooß³gOÄe6K#GÈüD~ä'ò+¿ÕZ__J¥2LWW×¨Ô#?ùüÈOä7­#?ùüÈOäG~äG~"?ùüÈüÈOäG~äG~"?ò#?òùÈOäG~äG~"?òùùÈOäG~"?ò#?òùüÈOäG~äG~"?òùüÈüÈOäG~"?òùùüÈOäG~"?ò#?ùüÈOäG~äG~"?ùü*©ßÿýßùå/Naû÷ïÿÁ~pQ%Vl3gÎX¥ÖÿùÿÝßýõPj½ôÒK§O¶J­x;ûÛ¿ý[ë¡Ô:pà@ooïTÞcñÿMwùÅ/¯mÛ¶[$©"*~0³ÊnXI¤iùI$$IÈO$Iä'I$ò$IùI$ü*ªªªªâcto·ËÀÀÀºuëª««xàcÇY9%²]b[Ì??N7558qÂÊ¹']½zµª ÓÓÓÉdíÒÙÙiÈvéêêjnní²`ÁØFVQlzß'©hpp0~·÷È1ºçÛeÇO=õÔÐÐPPcîÜ¹ÖOlÚÚÚK.Å@44X?÷¤ÄÿÇd³Ù½÷ÆÀÎ;ÛÚÚ¬¢Ù.o¿ývìÛ·oÞ¼yVQlzß'©èÉ'|æg·÷È1ºçÛ¥©©éÌ3VK©mPø+Wb .ü^oc,"ÿ&Å@.#òÒÙ.ÕÔÔXE¥³]Jç<îz.]jii_ùí=rJa»¤Óéç.~W/N:eÈvéééWã²»»Û*º'566®X±"~Fbë=6ùyÉO-Ö½Ý.ùâeýúõVQlzß'»^kkëñãÇÿs]ÿx£RØ.©Tj÷îÝ1?¨ñ#jÈvY¸pa²/6¸hÑ"«èÞvåÊ$?/ùÕÕÕÖLl¤7of³Ùþþ~k¦D¶KI½ïÇÝ_Å?Ù¨cTÛ¥®®Î>Ü.ö-Zóâç%ËtûhoáÏîívI´±nÝº«W¯Z'¥³]Jê;¦ô-íct·ËÆ÷ïß§O^¼x±5S"ÛeÑ¢E±Eb ··wáÂÖÌ=©±±ñüùó*V¬Xíííöì¸Ìf³VQlcÇÅ@__SRÛ¥¤Þ÷Éülm7n´¶¶&Ì8wî5S"Ûå½÷ÞðÅvË¶fîIÝÝÝÉwë,]º49áæÄõõõ©T*ÉtuuYE%²]S*ÁíB~$I"?I$$IÈO$Iä'I$ò$I"?I$$IÈO$Iä'I$ò$IùI$ü$ID~$I"?I$$IùI$ü$ID~$I"?Iºç;ßY²dIÍí-[öÊ+¯üÄ/¸ÛÍ¯ãÑmCCC<µþþþaãcL:Îd2CCC]¦$ò¤òkÛ¶mU#zâ'*I~Û·o/¼ðÂ°ñÏ?ÿ|üñÇ'±LIä'IeVOOO&NïÚµkðv»wï«1òäÉ#¿.ÄÈ?þüþüyòD~*¿¯~õ«a§zªpäÓO?#¿öµ¯º§³³3ä(lnnáü¯_¿¾~ýú3gÆ¤ÚÚÚÍ7T=zôhè*&Å¼Gf©3kÖ¬%K>|8®¶··`*¾dR<¤dRGGÇXJ[±bEïêêÊyûí·cÌòåËócvìØQ__ª©©Y³fÍ+WFÊoäò)òP%$ÝËæÌj¹téRáÈË/ÇÈBÙ«»»;ºzõêa6mÚLêííM¥R£Î¦¶¶¶«««cjfÌ|ü®Èrb`Ô7òîÛ·¯³yÝ»7Ï¾aY¶lÙDåWä¡J"?IºÇ%vGù¥VU+M[[ÛÍÛÅ@µjU25NbÇd×]-Íf1|âÄd!ËÜºukØ.9ØúðÃÇÆpðÚµkï¸Pc>¶QQhröìÙñ|ûúúâj#nær¹äL&f§ðÑ×ÔÔLT~Eª$ò¤/¿hTùåÇ'²IÀ]¹r%®«ÍÍÍquîÜ¹6l±Ýºu+¿¸Í°½hù¹«/_Îß8´?à1|ôèÑ;.'W?üðÃÂÇ6ÖgòyäôÌ3Ï|ôã#Ú7o.¼A(0ämiiIvINT~Eª$ò¤£ëæÍ#ûûûcdL*b¼Ï;à/üóFf©ÂïRáÚÚÚêêêXø¬Y³òSï¸±6¬Ó§O'Há¸áÞÞÞüÔ'NÄõ¨ñøåWä¡J"?IºÇ%uî¹çG&ßu2ìügûúúFîÊ:yòäöíÛc¯y2&;äòSïè³M6%yãrýúõùñEìc»zõjrõÃ?,~î¢EbjòE6ÖÂIÉy¾1©££ãÆÅåWi²6òS<TIä'I÷¸ät:½sçÎä[]víÚU]]=òlU«VrC±ùópÏù%;wînÞ¼9uÿ^QlÜû±cÇòã,'ù`_ò9¿þþþäEä·÷îüÞ¸xÊ<öööÆ½ÄJK~5ÃÊq³ç5k§y¨ÈOîÉ·kÇÿÿÜíåóÇIó'UäøáYYØÈï£Ûl#Æ×ÖÖ.²x,^íXOöÖ­[555ÉSvåÊw1öì¸L¾Ø¥pÉ.É|SóS<TIä'I%QèdÙ²eÕ·[²dIrí0ùutt$_×ÔÔtüøñüÔþþþG´¾¾>ÑÒ#<200zäÈY&yñÅ-sä#EÅø7?Ör¢x0ñ/,ò~ùÖ¯_?ìë]úúú²Ùl¬3gÆ³8þ|þjyãÆ m²®/_ÞÕÕ5ì<TIä'I$ò$IùI$ü$ID~$I"?I$$IÈO$ü$ID~$I"?I$$IÈO$Iä'I$ò$IùI$ü$I4¢ÿ÷M/ÍyIEND®B`


3ÎöÎöVãkJ¤[KKK.Ïäq3ÊP~Uñ N/..ÞºÌ/É¯Óßß?22á2ÍV8£üåW¥å7;;ÛÖÖEÑÁCü/N&D"JÍÌÌT8£üå·)?@ù)?å§üòP~Ê@ù)?å§üòP~Ê@ù)?å§üå§üòP~Ê@ù)?å§üòP~Ê@ù)?å§üå§ü üòS~ÊOù(?å üòS~ÊOù(?å ü üÇ ü*ÕÝÝÝØØãããQe³YåPkåwøðáºººD"Ïçë9rDùÔTù555Î]XX¶¶¶+W®AW~5U~ñ¾08tèPÆDBùÔTù566Î»víZ|ðoiiéÜ¹sùÔ`ùuww^ÛL&oýù( ×ùÔZù÷ß"ÙwíÚµðm?üðÃ;âÿ_ùÊo·P~òS~Êo-óóóû÷ïollßÏÛÒÒ2>>®üj­ü&''ïðË/+?*¿d2:o~~¾P~333>Õ Ë¯ðIÎòÿ[EÊ ¦Ê¯¥¥%t^|/_.;~üx·¶¶*?*¿ø/v»páòÝµCÙ?; üååå®®®ø½½û÷ï_\ÜÿÿÊòÛ-ÜÍ.ôã°ãË¯µµõ¾ûîûè£ üj¼ü¢(ßÛ«üåPãå755vè'N¸víZ>W~ò¨Ùò»ÓµãÏöS~ò¨òKÜOr@­ß¦ü@ù(?å(?å·ÖÞÜëüå°+Ê¯øµÅå·×ùMNN>)fnn.J[koo*^­|Q%3Ê6áOúÓc=½ýO<µé¯¯Õ7þ§ÿü½M_ýõþæ7¿±!¶¹üåóùG4tÛ¥K6w#«««òËf³£££a0<<Ü××W¼fù¢JflÂ'|~*82´]_Ñ¿j|ë­·l**¿X¿ð±oß¾Í]ýW^9yòd¡üã	Ìår­­­Åk/ªd¦¤üB^6òÞïýÍ7¾Ù÷óåíúJèûáhC¬oÊ/Vè¶úúúMòåË]]]ñ-Ä3ÅgKÎ /ªd¦¤ü~õ«_ý	ØÈïÿû¯ÿÛomcùí?ø§N²!Öw·Ë/Ë>:t[CCÃ&®ÞÛÛþüù[·_Ï¿S¤¤&ËU2ãl/lîlï=É·±ü¾ý÷ýÎölÿÙÞ;½··§§gë·fZZZBMÆMÆÅ+/ªdFùòP~Tþ×;êëëôÑ­e<èïï	pÍf×)_TÉòå üªì¿ìÏå7==L&CP¦R©â¥å*Q~ üßn¤ü@ù(¿/swEQKKËO~òåÊOùÔHùÿõøO·ÆÊò¨ò[]]mllìéé¹~ýzø6üðÃíííaüÞïòknnV~ ü@-ßþýûCÞÅ»qãFÉd2arÀWùòS~UT~ñ)ÝÕÕÕÂL!øâ?Å¡ü 6Ê¯n»)?í/¿d2nóÊÊÊ­Û´7ã¼³³³ÎöòS~µS~kî£§¦¦âÁÑ£GÔ@ù9Û üþÙ+WöïßßÐÐH$Âe[·Ooýy(?P~Ê ÊoçR~ üòò+öÇ5ßºóßð*?P~Ê`Ç_»øãZwPÍæ¢ü@ù(¿]GùòP~Ê(-¿ºººÿþ?núëoZÛ[¿ý6uç°å·S^É§üà+qóæÍ·&ì4¶r~ú©På·CCPùÁÝÛÝÔÕùqP~ÊÊOùò@ù)?P~(?åÛfÃwxÄ?qwòÅ_¸ª·üÖçoxÀ®Û¡lÍÐÐû JË/±Ã FÊ¯6(?@ù)?å§üòP~Ê@ù)?å§üòP~Ê@ù)?å§ü¾¿ýío?ÜO?ýÔÊOùì§­éïïwòS~5Òaë~å§ü üòòP~òS~ÊP~Ê@ùFå§ü(?åòå§üP~ üÊòP~òS~ÊP~Ê@ùÊOù(?@ù)?å(?å ü@ù)?å ü@ù)?(?åòå§üP~ üò_±ùùùûï¿?¢öööééé0³¼¼¤xå¹¹¹T*¯<55UáòP~ üªBè³0¸páBsss¬¹r6ááá¾¾¾gòåW]Î=ÛÑÑ!ûâ,Ò0ÏA.kmm­p¦¤ü^íµ`]ÿkBùõöönå~ùË_ÚPW~«««MMMa7166¾M§ÓL&¢®®®â5ÃdÉ¸ò;wîuò×÷nú«þ_½éëÉM_=üë'N° ;õßäädKKKñÌÒÒR|° HÆõõõÎ8Û°Óµ?_Þ®¯ïtw²jülïèJÒ-¤aÜ¶á2ÎÄJfòåWÒéôüü|ÌÌÌd2xfqqñÖíc~ñLAÿÈÈHËl6[áòP~ üªÂÜÜ{EZ^^3³³³mmmaæàÁ!þû p9==L&D*¥XáòP~ üv#å ü@ù)?(?å ü(?å ü(?å ü(?å ü(?å ü(?å üå§ü üòòP~ üÊòP~ÊòP~ÊòP~ÊòP~ÊòP~ÊòP~òS~ÊP~Ê@ùòS~Ê òÛ^ÊòP~òS~_uùãßØ®¯Æ¾¥ü@ù)?»W~^çÊOù(?åÊOù(?åÊOù(?åÊOù(?åÊOù(?@ù)?å(?å üå§ü(?åòå§üòå§üøKnÍ_|á>å§ü@ù)¿óô°CCCîCP~Êò«'w8(?åÊOù)?@ù)?P~ÊOùÊOùòS~ÊP~òS~ÊP~òS~ÊP~ üÊP~ üÊüð*?P~ÊoYYYykkÂÇ/¾¸[X\´!`[Êï#CÛõÕxÏ·(?åw·òÉ'aïÿí¿ïßôWk×cé¾zø×CüÙp÷õüCoÿOmúëkõ×õÀ¦¯þèc½gÎ±@ù)¿»]~÷$ïÝÆ3>!þìÈ'º:í@ù)?å§ü@ùÊOù)?åÊP~ÊOùÙ üå§ü üå§ü üå§ü ü@ù)? ü@ù)?å§üåÊOù)?å(?P~_Âüüüý÷ßEQûôôtK¥RñÌÔÔTñÊå*Q~[)¿ºí¦ü@ùµS~¡Ï&&&ÂàÂÍÍÍaÍfGGGÃ`xx¸¯¯¯xåòEÌ_ÈÁTæøÃ¶ßo¾iCÀ~xüã»à.ØaåWpöìÙ0ýÏçÃ Ëµ¶¶¯S¾¨ò;yòäÿ¤2o½õÖæonãÙÞôÿòüÀ'ßO<á~»`çßêêjSSSØMo£(*,*¯¹¨g·r¶×ëügÁÙÞ¯ÞäädKKK$Âdñ:å*Q~ÊP~ üªN|.ô_.»uûtmÜå*Q~ÊP~ üªB:L&ýýý###a.³ÙlñÊå*Q~ÊP~ üªÂÜÜ{EZ^^3ÓÓÓÉd2H¤R©È*Q~ÊP~ üv#å§üåÊOù¡üåÊOù)?å(?P~ÊOù)?ØÍm·Ï?ÿ¼ûòS~Êv¡¡¡·àâÅîCP~ÊOù)?P~ÊOùÊOù)?@ù)¿¯ üêêê¾Ó|»¾Â¿®ü@ù)¿»á/¾xykBº=õÔS^ÊOùí;òS~òS~ÊP~ÊOùÊOù)?@ù)?åÊOù¡ü@ù)?(?åòå§üP~ üòsòS~ÊP~ÊogµÝVØ üòòP~Ê@ù)?@ù)?å§üòP~Ê@ù)?å§üòP~Ê@ù)?å§üå§üòP~Ê@ù)?å§üòP~Ê@ù)?å§üòòP~Ê@ù)?å§üòP~Ê@ù)?å§üò¨Òòéìì¢¨££cnn.Ì,//×)^9¬J¥ÂÊíííSSSÎ(?@ùUt:áÂ0Û¿¬¹r6ááá¾¾¾gJÊïÌ3ÿ &ì°ò+ÖÐÐ.CöMLL¬¹Bsss>×ÚÚZáLIù:uj &ìÔò=zôh|0ÉDQÔÕÕµ°°P¼N,W2ãl/àlo¹~ýz6]YY)êèè(I$q3ÊP~Õ"ÞÀÀÀòòrù¢tkiiÉår·nÉãg üªÂ¹sç2ÌÕ«W3étzqq1.Â°¨xåþþþ0Ùl¶Âå(¿ªÐÚÚZò.³³³mmmQ<x0Äßÿÿï¾½hzz:L&T*¿¤±å(¿ÝHùÊOù(?å üòS~ÊOù(?å üòS~ÊOù(?å(?å üòS~ÊOù(¿í511ñà>PÞzë-åòP~(?Êåò@ù üø«ûè£|òÉ¦¦¦(ZZZöÙÏ>ûl+7øÓþt3Ü:ÝíÛkÜYa>ø §§'~ËÞÞÞ0S~Sï¿ÿ~¸V¸t¯Öêã¡øÛD"ÑÐÐÐÙÙyêÔ)÷[mlúJwÚîî%P~l§wÞygß¾ccc¹|.ÃøÞï½råÊ_cß¡üvÜî>ööÛo:thff&ÏÇ°Cokk;útÉG9|øpø]Â½Z«ò|phhÈÝUÛå·þv¯|/òcÛt)D^ù¾'Ovww+?»ûð8¹~ýzÉäÇL&gÂïÞ½7nÜØ³gO¼÷g7_áwHw×®*¿í^á^åÇvzægËçÃ3÷èèháÛ³gÏ¶··GQ.Ã¸xGpæÌûî»/H¥ñQýòCçÏonnîêêÚðÖlª-¿úúú>úhÃÛ	´··7ÂeñÆ&''ÃFÿPCCC&©äÖØAå?HÂeñ¹þ	k¸üÛ½ò½Êí~E¿­¿Nè¹T*577Æá²¥¥evv¶°#8tèP|^8¬×ËwaüôÓOçóùx°þ­Ù"U[~ï¾ûnøÅ=þú«ÝºÞ'~7wÞy§­­ÍÝ^Kå·°°ÐÑÑöYø¥nee%?ÿüóð+_ùq j¦üÛ½ò½ÊíH$6x..Âà°#?öåûò»téR·flãî~ý·wãããöì	ö#G¼|A|ª÷ÚµkaìKNø[qW×^ù­|þüùýû÷òßxãg6úúúÂ"÷aM_ùv¯d/òcÒ­¿NüæX755­¹#¸SùmîÖ¨ÝÉ¾~vv6ìÜ_xáT*®áMMMe2Â·?üpñÛúGGG/à%;½üá·p8pàøñãÇsÖRùm¸Ý7ÜK üØfmmm/_þ²;5ÏêVX~ßUX~ÅVWWÂoü§~ºä¹áèÑ£¥7oÞ<|øpüL°gÏ?þñîö[~^+ôøÑS3å·õ½Êm688x§Oá*ü~±ûùU~kTyùÅ»õB¸Ç§zß'~õêÕæææòÃKKKo¿ýv:v·×pùe³Ù#G<÷ÜsîÀÝ %	ÛïÒ¥KûöíÏÐ%ógÎyé¥âqOOOñ+óÂøGÙtùU~kTUù%Éò£t¡ê:;;ãñÔÔTØ¸%+sçÎÏ"ìòï½÷ÂoaÉdîô öÊoÃ½Êª044N§CßÌÂ·W®õÕW<xãÆxðó¿Jwvv6§§§×/¿ÆÆÆ?þ8>üS²Nå·FUßéÓ§Ã/	o¿ýöçïÍ/^¼Øßß_x^ægÊÿnGø"ÌÇã°÷¿+¿øý¼ñã÷ù:ç»KÊoÃ½ÊjqöìÙð«yCCC"øÒK/²¯ðü_GGGñ³ûÊïõ×_¯¿mÍE·Æ]ÞÝoøöÞ°û~üñÇâ?ÜÔÝÝ]øD°Û´üÄn)|zKøe <ÌÂ£"ÜfØúîö,¿ðL_üQ>Ï¯vï¢×ÙK üP~(?ò@ù üP~(?Êåò@ù(?Êåò@ù üþZ[[VVVJæÃLE©T*Ï¹½êm[ÂP~_Â'Bc:uªd~hh(ÌÿèG?úÒUå(?êôñÇÆêèè(okkó­¯òÀÝÉdBfÍÌÌf.fzè¡ÂÌK/½L&£(jhhxüñÇíìÙ³öì9pà@y´­Å©©©aigggß©ü>øàP¢aµ°rø·óþùÑ£GÂ¢æææcÇ¶P~ÿbll,dÖO>Y9|øp-Ô[Ý_:tèPq¢%pÙÛÛ[m^±Äììlyù]¼x1¾ýòÕzzzJná¹ç³AåpG«««÷î¢èêÕ«áÛpJ«©©)ËÅ+¤R©Uï¿ÿþ­?nhh(N´^x!ÏÇ§£mÃ+öõõ]¿-Â·ÝÝÝååÍfã£a<==_+^áåËÃ8ü+aÜØØhÊ`=!N<Æ¯¾új;v¬xP!­BáuuuÅùíÊ+ÿ²WýËµë_1nÍ`ii)|r³üFÂdÉ½ÂjáÛûî»ïg¸qãM	(?ÌÏÏÇ	Æá2/^¼XX:==ÝÜÜ_ÅVüÉ/ÅK7¼â_ìëê¢(*_Z~R¸.]ã¯PgÎ±5å°ï~÷»!^|ñÅprªxQü>ß°hrròÚµkÃ+Æ'joÝ>Å|§c~aõw¿ûÝ'zÃj©TÊ¦ÀÞxãÂÁ³áááâEQÅGóùüë¯¿^yùmxÅîîîÐ|7oÞìïïßËòîØ±cáFBAMü:¿øu.]ãúúzP~¸qãFCCC|ÊõúõëÅyäâ­÷îñç³¬_~^1NÃÂ¸p¹øFfggKÎöÇâ÷úé§mJ@ùlìèÑ£%ï»zõj6­¯¯ojj\|~Êúå·á''';;;Cóµ··?~Í	Î=ÛÕÕH$R©ÔéÓ§ó+++ÇO&qVâæÍ¶# üP~Êåò@ù üP~(?Ê@ù¹Êåò@ùpWü?I¾WÚr	~IEND®B`


ONEWAY Figures BY Variables
  /POLYNOMIAL=1
  /STATISTICS DESCRIPTIVES HOMOGENEITY
  /MISSING ANALYSIS
  /POSTHOC=LSD ALPHA(0.05).


Oneway


Notes	
Output Created	12-SEP-2022 23:29:48	
Comments		
Input	Data	E:\桌面\Raw Data\4. C. Cellulosae ESAs and TPx Induced Th Subpopulation Differentiation\3. SPSS statistical analysis\4. IL-10\1. IL10-24h\1.1 SPSS statistical analysis--IL10--24h.sav	
	Active Dataset	DataSet1	
	Filter	<none>	
	Weight	<none>	
	Split File	<none>	
	N of Rows in Working Data File	20	
Missing Value Handling	Definition of Missing	User-defined missing values are treated as missing.	
	Cases Used	Statistics for each analysis are based on cases with no missing data for any variable in the analysis.	
Syntax	ONEWAY Figures BY Variables
  /POLYNOMIAL=1
  /STATISTICS DESCRIPTIVES HOMOGENEITY
  /MISSING ANALYSIS
  /POSTHOC=LSD ALPHA(0.05).	
Resources	Processor Time	00:00:00.00	
	Elapsed Time	00:00:00.01	


Descriptives	
Figures  	
	N	Mean	Std. Deviation	Std. Error	95% Confidence Interval for Mean			
					Lower Bound	Upper Bound			
Control	4	29.94575	1.666214	.833107	27.29443	32.59707			
ESAs	4	39.28625	1.282676	.641338	37.24523	41.32727			
TPx	4	32.99000	3.113818	1.556909	28.03522	37.94478			
LPS	4	48.44700	3.634204	1.817102	42.66417	54.22983			
Total	16	37.66725	7.673609	1.918402	33.57827	41.75623			


Test of Homogeneity of Variances	
	Levene Statistic	df1	df2	Sig.	
Figures	Based on Mean	.862	3	12	.487	
	Based on Median	.776	3	12	.530	
	Based on Median and with adjusted df	.776	3	8.049	.539	
	Based on trimmed mean	.852	3	12	.492	


ANOVA	
Figures  	
	Sum of Squares	df	Mean Square	F		
Between Groups	(Combined)	801.290	3	267.097	39.099		
	Linear Term	Contrast	484.276	1	484.276	70.892		
		Deviation	317.014	2	158.507	23.203		
Within Groups	81.974	12	6.831			
Total	883.264	15				


Post Hoc Tests


Multiple Comparisons	
Dependent Variable:   Figures  	
LSD  	
(I) Variables	(J) Variables	Mean Difference (I-J)	Std. Error	Sig.	95% Confidence Interval	
					Lower Bound	Upper Bound	
Control	ESAs	-9.340500*	1.848135	.000	-13.36724	-5.31376	
	TPx	-3.044250	1.848135	.125	-7.07099	.98249	
	LPS	-18.501250*	1.848135	.000	-22.52799	-14.47451	
ESAs	Control	9.340500*	1.848135	.000	5.31376	13.36724	
	TPx	6.296250*	1.848135	.005	2.26951	10.32299	
	LPS	-9.160750*	1.848135	.000	-13.18749	-5.13401	
TPx	Control	3.044250	1.848135	.125	-.98249	7.07099	
	ESAs	-6.296250*	1.848135	.005	-10.32299	-2.26951	
	LPS	-15.457000*	1.848135	.000	-19.48374	-11.43026	
LPS	Control	18.501250*	1.848135	.000	14.47451	22.52799	
	ESAs	9.160750*	1.848135	.000	5.13401	13.18749	
	TPx	15.457000*	1.848135	.000	11.43026	19.48374	

*. The mean difference is significant at the 0.05 level.	
