# Supplementary material for: Regulation of piglet T-cell immune responses by thioredoxin peroxidase from Cysticercus cellulosae excretory-secretory antigens
Source: Front Microbiol. 2022 Nov 18;13:1019810. doi: 10.3389/fmicb.2022.1019810 (PMC9718028; doi:10.3389/fmicb.2022.1019810)
Supplement: Supplementary file 3 [file Data_Sheet_3.ZIP › 4. C. Cellulosae ESAs and TPx Induced Th Subpopulation Differentiation/3. SPSS statistical analysis/4. IL-10/2. IL10-48h/2.3 (SPSS data export) SPSS statistical analysis--IL10--48h.doc]

EXAMINE VARIABLES=Figures BY Variables
  /PLOT BOXPLOT NPPLOT
  /COMPARE GROUPS
  /STATISTICS DESCRIPTIVES
  /CINTERVAL 95
  /MISSING LISTWISE
  /NOTOTAL.


Explore


Notes	
Output Created	12-SEP-2022 23:32:50	
Comments		
Input	Data	E:\桌面\Raw Data\4. C. Cellulosae ESAs and TPx Induced Th Subpopulation Differentiation\3. SPSS statistical analysis\4. IL-10\2. IL10-48h\2.1 SPSS statistical analysis--IL10--48h.sav	
	Active Dataset	DataSet1	
	Filter	<none>	
	Weight	<none>	
	Split File	<none>	
	N of Rows in Working Data File	20	
Missing Value Handling	Definition of Missing	User-defined missing values for dependent variables are treated as missing.	
	Cases Used	Statistics are based on cases with no missing values for any dependent variable or factor used.	
Syntax	EXAMINE VARIABLES=Figures BY Variables
  /PLOT BOXPLOT NPPLOT
  /COMPARE GROUPS
  /STATISTICS DESCRIPTIVES
  /CINTERVAL 95
  /MISSING LISTWISE
  /NOTOTAL.	
Resources	Processor Time	00:00:00.94	
	Elapsed Time	00:00:00.85	


[DataSet1] E:\桌面\Raw Data\4. C. Cellulosae ESAs and TPx Induced Th Subpopulation Differentiation\3. SPSS statistical analysis\4. IL-10\2. IL10-48h\2.1 SPSS statistical analysis--IL10--48h.sav


Variables


Case Processing Summary	
	Variables	Cases	
		Valid	Missing	Total	
		N	Percent	N	Percent	N	Percent	
Figures	Control	4	100.0%	0	0.0%	4	100.0%	
	ESAs	4	100.0%	0	0.0%	4	100.0%	
	TPx	4	100.0%	0	0.0%	4	100.0%	
	LPS	4	100.0%	0	0.0%	4	100.0%	


Descriptives	
	Variables	Statistic	Std. Error	
Figures	Control	Mean	31.80400	1.290675	
		95% Confidence Interval for Mean	Lower Bound	27.69650		
			Upper Bound	35.91150		
		5% Trimmed Mean	31.85983		
		Median	32.30650		
		Variance	6.663		
		Std. Deviation	2.581350		
		Minimum	28.263		
		Maximum	34.340		
		Range	6.077		
		Interquartile Range	4.811		
		Skewness	-1.049	1.014	
		Kurtosis	1.486	2.619	
	ESAs	Mean	37.57775	1.052305	
		95% Confidence Interval for Mean	Lower Bound	34.22885		
			Upper Bound	40.92665		
		5% Trimmed Mean	37.59639		
		Median	37.74550		
		Variance	4.429		
		Std. Deviation	2.104609		
		Minimum	34.849		
		Maximum	39.971		
		Range	5.122		
		Interquartile Range	3.927		
		Skewness	-.470	1.014	
		Kurtosis	1.496	2.619	
	TPx	Mean	41.86500	1.286776	
		95% Confidence Interval for Mean	Lower Bound	37.76991		
			Upper Bound	45.96009		
		5% Trimmed Mean	41.90278		
		Median	42.20500		
		Variance	6.623		
		Std. Deviation	2.573552		
		Minimum	38.429		
		Maximum	44.621		
		Range	6.192		
		Interquartile Range	4.816		
		Skewness	-.755	1.014	
		Kurtosis	1.492	2.619	
	LPS	Mean	57.22775	2.621279	
		95% Confidence Interval for Mean	Lower Bound	48.88567		
			Upper Bound	65.56983		
		5% Trimmed Mean	57.28478		
		Median	57.74100		
		Variance	27.484		
		Std. Deviation	5.242558		
		Minimum	50.357		
		Maximum	63.072		
		Range	12.715		
		Interquartile Range	9.802		
		Skewness	-.572	1.014	
		Kurtosis	1.487	2.619	


Tests of Normality	
	Variables	Kolmogorov-Smirnova	Shapiro-Wilk	
		Statistic	df	Sig.	Statistic	df	Sig.	
Figures	Control	.249	4	.	.945	4	.682	
	ESAs	.249	4	.	.963	4	.797	
	TPx	.249	4	.	.961	4	.786	
	LPS	.249	4	.	.964	4	.804	

a. Lilliefors Significance Correction	


Figures


Normal Q-Q Plots


/ñöNúýìÁo_N"B~D´ò¬ÈòôlZx|# Ýn¶mÛ&Ë;vìP¬ñ^zãÆcÇÊúøøø·æ§àWQw,55UÝÌÌLuçgffdAÌ'+å+e¹¤¤Dh5×x"¿à£455¥åæ÷g~Sürò#¢/wæ²_Â¬äädùJË¥ªÊuÑùóçåZÂÁ7ojõð,ä*sÝ1a¢ïËxJW®F£z9p!¯ùåååÉúÉÉÉà(.¼àËKp""äGDË#¿ììlß ÔËfóB¤²²R.õ~ô®OHH53³-P~Á¯â½c9íu322ÔuýîÇãioo·Z­¾/ÎÛ±cÇäëEÚ2JßÒùÑ2ÈOÐzáÂõ¶³S§N-"W®¾xöÎ;ïø±æÖ­[êý_ð«Æ~ðÈÂþýûµ×Ý·o,_¼xñóÏ?WGxe¥Ùlå7o>|øPòòò8,ãããâ°µk×Þ¿_4©^Ü¹sçGÉwYqVÇý~ö¥8ò#¢eÇã]%Ïæ;'_pLMM©#­òQÀä]ßÖÖf4Å=GY ü_¥««K.Ý¸q£÷Dß¯q¹°uëVu¯¯¢¢B,ûöí|Ñ¤:-<~ü¸zßBFÉwùêÕ«rÔDÙ~?ûÒù!?""""B~DDDDüùò#""""ä·¸~üãß»w/ÄßôÁÿõ_ÿÅïüø1ã çÆÇÇÿã?þqÐsSSS¡ßÁÒ¢úÿùùatl#ÙRÈoyúîw¿+øñ7ýéOúðáC~õÜçþ¯ÿú¯»ÿþ?ÿó?3zNþûôüqÐsÿýßÿÝÙÙÉ8è¼_þòÈùòC~ÈùòC~ÈùòC~üù!?äüù!?B~üòC~ü!?äGÈù!?äGÈò#äüò#äGÈùòC~Èùò#äüù!?äüù!?B~ü!?B~È!?äGÈò#äüò#äüùòC~ÈùòC~üù!?äüù!?B~üòC~ü!?äGÈù!?äGÈò#äòëíí-))1ÅÅÅýýýÈò#äGÈùE­üÖ®]ýúuY¸téR^^V~íííCìïÜ¹ótÜ7>ûì3ÆAÏýüç?ïëëcôÜÝ»wô£1znddDTÁ8è¼÷ßtt4Äß4åç[RRV~çÎûIhûàìvûOHÇuuuôÑGûøã;;;='ìg,ÆAÏýøÇ?~ï½÷×ÖÖÖÓÓâoòëëë«ªªâh/q´£½ÄÑ^âh/G£öh¯jrr²¢¢Âår!?B~È!?äÍò¶Z­±üù!?B~È_ôÈÏn·oÙ²ell,à¥Èò#äüùEüL&SOÈò#äGÈùE­üüù!?B~ÈòC~Èù!?B~ÈòC~È!?äGÈù!?äGÈò#äüò#äüùòC~üù!?B~ü!?B~È!?äGÈò#äüò#äüùòC~ÈùòC~üù!?äüù!?B~ÈòC~È!?äGÈù!?äGÈò#äüò#äGÈùòC~ÈùòC~üù!?B~ü!?B~È!?äGÈù!?äGÈù!?äGÈù!?äGÈùò#äüò#äüùòC~ÈùòC~ü!?äü!?B~ÈòC~È!?äGÈù!?äGÈò#äüò#äüùòC~üù!?B~ü!?B~ÈòC~ÈòC~ÈòC~Èò#äGÈù!?äGÈùò#äüò#äüù!?B~Èù!?B~ü!?äü!?B~ÈòC~È!?äGÈù!?äGÈùò#äüùòC~üù!?B~ü!?äü!?äü!?äü!?äGÈòC~Èò#äGÈù!?äGÈùòC~üòC~üù!?B~Èù!?B~ü!?äü!?B~ÈòC~Èò#äGÈùò#äüùòC~üù!?B~Èù!?B~È!?äü!?äGÈòC~Èò#äGÈù!?äGÈùòC~üòC~üù!?B~Èù!?B~ü!?äü!?äü!?äü!?äGÈò#äGÈùò#äüùòC~üòC~ü!?B~ªäü!?äGÈ¢ ]]]åååqqqQ?äüùòC~üx<¶¶6³Ù÷«öîÝüò#äüùQTÉÏív777çææÆýf999ròC~Èò#äGÑ ¿	yîNOO÷3_VVV½Ëå¾Íü!?B~ÈbN~CCCÍg±XÚÚÚ¢Ò|Èùò#äü(æäçt:­VëªU«´æý8òC~Èùò#äüVêSk>ù´²²2Ìü!?B~ÈbB~òìl±Xü^äó	NgLm&äüùòC~òóx<---Zó¥§§×ÖÖÆùò#äGÈùQtÊOÌp¢1<YOLLÄìfB~È!?äGÑ#?QÝéÓ§µæ5±l>äüùòC~=òkr>1_ss³Çãa!?äGÈò£Óé´Ùl'çëêêÂ|Èùò#äü(äãó!?äGÈò£<ák'ç:6òC~üù!?ù/111'çC~È!?äGÑ)?5QK~~>µ ?äü!?äGQ+?·ÛÝØØ¨¨Åd2a>ä·âÉ¯ò#äüùÑJËOTWWW'O»LÔüÂSOOOAAüÎ!?B~È­üFFFNÔb61ò][·nu8Aäwþüù¶?üP¸ùSÒqüñ?þã?2zîÚµkôã ç|ðã çþéþé½÷ÞúÛ¹råÊ-[¾ð/ø¯¤¤$ôO²Ñl#ÙR!þ¦,¿ÿ»sËïþá6aßíÛ·_ú7o2zî³Ï>ûäO=wçÎ»ÝÎ8è¹ÿ÷ïèèx[üÁüvr¾M6=å-·ööö¡¡¡ÓhG£½í%ör´w	 Íf³ù«ªª£½Èù!?B~È¢D~/_Ö´^SS344Ä¨"?äü!?äG/?·ÛÝÐÐãg¾¬¬,1ß£GOäüò#äü(âå722"OÚvsssùERÈò#äüìkjjRRRüÌWTTÔÒÒÂD-Èù!?äGÈ_4ÈoppÐjµjOÚµX,ÌüòC~üùEüäiqûöíqdåÀÀüòC~üùE¼ü<OKKËüÀ·jÕªÝ»w;Nù!?äüùòxù¹ÝîÆÆÆµÔÖÖÎ;QËÀÀÀÉ':$7âr¹Uäüò#äüHþéþ©Édò3_JJo!'í~ÿûßÏÈÈ°X,_ýêW_xá¦ôC~Èù!?B~Èôø,àD-F£±¡¡aóûlß´ÿÎqõïKe_Ú¼y3ÃüòC~üé"§Óð¤]ÉÔÜÜ¼¨Ãµ'O´X,^öÉ¿£~4--Íív3ÎÈù!?äGÈùQ8gæ[·n]KKË¸vèÐ¡¯¾ôU_ùÉ¿ÌÌLþòC~ÈùòC~¶äÎb±h'jÙ¼ys]]Ý_¢kjjZ»v­/û¾±÷Ï>û,üòC~ü:5QÖ|«V­²Z­òd´¨¿Û«MÈ¸nÝºõë×ëÀ·»+vgff^¾|G~Èù!?B~ÈBj¾ææfíD-b>ïä|O)?ÉårÙl¶ììì¤¤¤ÒÒR1üòC~ü¨Ó§OOòü&jyzùòC~Èùò#äó¨E(89òC~ÈùòC~ü",§Ói³Ù¯¹¹ÙãñÌuEäüB-¿¸ùG~üù!?Ë|'j±X,]]]AÌü_xä?_ùò#äüÈ/yò*//h¾?¯!?äjù=äGÈùò8óÍ5QÃáXÔM!?ä§/ùïß¿ùò#äüHMÔ¯=i×f³y'jA~È/bäg2ïó#äGÈùon·;àä|'jA~È/2äW\¬=½Ãh4.êOG#?B~È_4d¢ÆÆÆ§1òC~a_BBü*eeeÉï?ø,TUU!?B~ü_¬544Ð|ùùùÁ'jA~È/2ä§~¡eA¨'wïÞäädäGÈòLÔ²ìOXÈùM~iiiòkÝÓÓãp8dáµ×^SÌêBÈòäÙAk>ù´²²rªòü9â=Ã÷7Þl6#?B~ü_tp¢ÄÄÄªªª¥´üÞå'½þúë²ÐÛÛ+¢ÀÒÒÒ¾ëÈò#ä®<OKKK@óÕÔÔ­ô@~È/òKÈò#äóÍ5QK]]]ÌüòC~ü!¿obbâôéÓZóåää,ËD-ÈùEüòòòÔÜ.ÌäLÈòJó¨EÌW__¿Òó×"?ä§/ù­]»ÖWÞ8·!?äé9NÍ¦5Ùlnmm]®Éùò$ù	òä1Ð××733Ê»üù!?B~+j¾ómØ°AÂ~÷òüF£<BÌ>äGÈùò[¡äÉ¥¼¼9ßîÝ»ív_çC~ÈOòëïïÄÁ'''!?B~È/¢Í§¨%%%Åf³­èä|ÈùEü¤Õ«WÇiâB~ü_D¤&jÉÏÏ×NÎW]]=22¢Ãûü_Øä·fÍÎð äGÈùEbn·;àä|YYY'O|ôènï9òC~azø®#?B~Èßs¹'jó544e¢äü"C~áAÈòFFFêêêDx~æËÏÏojjÒ¿ùò³üìv»<`9âv»!?B~ÈOÏû½÷¨E¡urÒ.òC~z_Üq!?B~ÈO'òÉ'ÚZ¤Ý»wGùò³üâç3<ùòC~a¯¥¥Ål6ûOhµZew¹?òC~a_¸B~ü!¿¹Rµ¬[·ÎÏ|éééµµµzù!¿HÉdZ³fMèÿçüù!?B~Ú&&&êëëå¹Ik>yâK£c!?ä6ùyDþ®#?B~Èo=§õ7EËÍÍmlló!?äfùuwwËãª®®n||<s» ?B~ÈjhhÈf³%&&jÍ×ÜÜ'p ?ä§_ùqn/!?B~È/X­VíI»¥««+*Íü_åÇ¹½üù!¿Ð'OÂ»æý³ò£_¸B~üÅ ü<Okkëµ8ÙFÈù!?äGÈùQ4ËOÌ×ÐÐPTTäg¾ÄÄD1_LÔü_ÄÈozzzëÖ­IIIòLNNÞ¹sgNõ@~üÅü&&&Î=õµ ?äòxÇJÿ_äGÈùQÔËodd¤¦¦Fç÷#Yó!?äfùÈpÛ¶mòéøøø;dÍúõë!?B~Èoi9NÍ;µ ?äòKHH¢ïpzzZÖÈzäGÈò[Â6àD-f³ó!?ä~ùÅÇÇËR´ç]ãv»e³ºò#äü¨Eûö¡¨ù!¿È:Ú»eËu´W>Ê²¬)))A~üù!¿yóx<---Zó­Zµª²²ó!?ä§/ù	õá!;äGÈòn¾æææÜÜÜ¥MÔÒÐÐ ^ÌÏÏÿÚ×¾688üùB~OfOïÝ¹sgjjj||¼|Ü²e¬Yé»üù!?MLL>Zk¾´»oß¾çîë_ÿºí¶¯üÑWäv»ùòüÂò#äü(å'ª¸v¢¬¬,±àÐÐÐBnd``@náÏýÙñïWÿ^Þùò/¼üù!?äüù.ä§&jÑ¯¨¨¨¾¾Þår-ü¦¿ô¥/yÙ§þeddÄÎÈùZ~qóüùòC~Ê|'jóµ¶¶.a¢¦¦¦/ýüÕKKK[!¿E?wÈ!?ä§uyy¹Ö|Ån·/yr>¡ä3Ï<³ÿÿí÷Êï+ô/~ñ±³òµüæêðáÃêQéÒ%äGÈ_ÌÊ/àä|BÀ½÷.ËÞû¯ÿú¯û·[Xù'òæß3ùòüúûûÕÿä|'vF~üùÅüÔD-ùùùÚZ*++ç¨e±O«V«õÅ_<vìØ½÷bj!?äfùíÚµK=¶ÛÛÛCs×!?äGºÛí89_JJJuuõOÚ%äüô.¿wßW=¶·mÛÊ»üù!?ÒüLÔ²Øv	ù!?ýÊojjjýúõêdîîîßuäGÈùQØå744Ð|¹¹¹ÍÍÍù!¿èß3gÔÃÏ=a¹ëÈò£0Êo®ZÌf³oÉ'íòC~:óùò#äò=aeeeÀZB¿[F~üB$¿øù2È!¿hÝnùåµæ15£ò£X_ØC~üF~§µµÕl6k'j±Ùlò#äüùòxùù._¾¬¨%==]vÂ=b¬!?äGÈ_ÄË¯­­­¾¾>''GÒnccãÄÄ£üù!?B~ü"¾&%%¨v!?äGÈ_4ät:«ªªRRR´'ívuua>äGÈùò#äòÉ''çc¢äGÈïW×d>?B~ü¢âiÃ~;p!àK/½ÄD-Èß¯óº/ üÏ!?Ý¦&jÑ/11Ñjµ,êïöò£è7ùÍÅ+¯¼299)ÊÇ;vÈk×®!?B~üôÖÄÄÄéÓ§óóóNÔ¢NÚ]øßí%äG1'?£Ñ(»ßwþNOOË¬¬¬§¼åþþþììlÁPXXØÝÝüù!?z=ztòäIùL&×|*äüùÍ³öüä÷ôïó«¨¨¸xñ¢,;wnÏ=Zùøá®ÐöÉ'üÛ¿ýtÜ§~úóÿqÐswîÜÿ×1¡ì¿øÅáÃýÌ·fÍ¿ù¿óùýÐÐÐµk×7=÷ÿù|ðã óÞÿíãk¥[qùeffÊîC655%Ê·Ü¶m¬õOÿjâÌÌ¢¤üT+¿úúúClBáæÇ¤ãä?X²7dô§l&Æ!4ÿûßÿÃ?üCíä|555]]]¯%ëÛÛÛ==×ÝÝÝÖÖÆ8è<ÙF²¥BüMW òÿÂgxÜ¼yó)oÙ÷íù"í%ör´$HÅ¢Ý9/d¢ör´8Ú¬»wï®]»VþO::úô7ë¼8!!ùòC~4o§¥¥Ek¾U«VUVV.p·ü!¿0©Þ>(µÇ!?äG~æknnÎÍÍÕÏjµ:ÎßòC~üÂü÷ô­·ÞùXQQüù!?¨Ek¾ôôôÚÚÚEù!?B~ówëÖ­¼¼¼äädu|633óÊ+O³===YYYrÙÙÙ½½½Èò#­ùdg¨¨Åwr¾%ü!¿9S39ûþÅ6µ|îÜ¹½ëÈòåN§ÍfÓ/77·±±qÉæC~Èß<eeeÉ¾æÖ­[^ùõööÊrjj*ò#äGÈo%ÌgµZW­Z¥5_ss³ï¼úÈùò[~ù©=ZPòáïöò#ä·ìÉN¯¼¼>ÅÒÕÕµ,æC~Èß<©Õë|"¿ééé£Gª?üùò[.ó-yr>äüù-§üìvÀ¯_¿üùòÔD-ùùù'jq8+ôò#ä,ÁPii©:·7)))//o	 ?B~È¼¹Ýîó%&&.vr>äüù-³üÂò#äü¢²¨ù!?B~Oëß?³&æåååää ?B~üÞÐÐP@óL¦ù!?B~ßôô4çöò#ä·ðB0QòC~üJ~999qAKKKC~üùÍ»W	h>³Ùó!?äGÈoÎwülÞ¿Þá°ïÝwßE~üùÍU'jA~Èße9Ú+Î[é»Èò¦ÚÚÚÊÊÊ´µTVV®ènÍív»äüùEdÈò¬^­­­6l89ßNÔrïÞ½ßÿýßOKK3Ï=÷<!?äGÈoMOOçåå%''×ddd¼ñÆÈ!?¯ùL&v¢ÚÚÚO¾û/¼°iÓ¦#ß>rü;Ç¿þõ¯?óÌ3]]]Èùò[J«W¯ö;½WíÑêêê!?qùMLLÈÎÐh4ú/+++dµ455åçåù¼ÿ¾¶ëk¥¥¥Èùò[J			²ô®éïï5©©©ÈÅ¬üNgMMvr¾¢¢¢úúú¾ßnY:tèÐW_úª¯üä_RRòC~ü:½wffæ7nW3Éò#äG1"?Ã±wïÞÄÄDíD-ò4úZN<i±X|ÙW°úÙgE~ÈßRÊÎÎ=üÒívË§SSSÇWÇ2!?)ùÉ." ù^~ùåpÝ+ùÖ¶oÚ¼òûbé_õUäüù-¥¾¾¾39÷öö"?B~#òkkkÛ¾»ßÌê¤]Ãö»wêÔ©gyæË_þryyynnîúõë¿Åù!?B~Á.,,LJJONN.((5+×!?äö<OkkkQQßm6ÛJ´»¨dùñÕÕÕMMMêòC~ü")äGÈù1Ë%ÊÍÍõ3Ñh<yòäÈÈHDÿtÈùòC~ÈùòûßÜnwVVv¢´üãüÂ#¿[·n©ÉÕù¼W®~ü(Êä7×ä|EEEóBE~ü_4ÈO~ó¼»?%?µ|îÜ9äGÈ¢C~Ãjµj'ç³X,]]]¡¨ùòC~a:äqëÖ-¯üzÉEüÄ|~'íJååå¡ß!?B~È/üòS;Á'>³7ÏÌÌÈ²Á`@~ü(rå'µTUUÉ¯Yto#äüùÍYff¦½OÉozzúèÑ£²l2!?8ùy<ææf³Ù¬¨¥ººúÞ½±°ò#ä7gv»=àLÎ×¯_G~ü(äçv»Å|ÚZÒÓÓkkk#¢äüù=Y®sC¥¥¥êÜÞ¤¤¤¼¼¼Ì_üù!¿åjbbBv)Ú8L&ÓÙ³gÿ¹äGÈbN~a	ùòC~Oü7µ¶¶Vk¾ÜÜÜæææè;iù!?B~Èù!?Eùù´­µ ?äGÈoÙäwçÎ5kÖ$$$ÈN3))©°°pllùò#ÊOvåååÍÅµ ?äGÈoyä'ÿ9xÇJÏzüù!¿%Oxç·³Z­VÃÁ¦A~Èßü©+**Ô_«Ü³g¬ÉÎÎF~üHòSµäççk'jóà4äGÈùEüÔÔ÷=1²ìÕùò£0Êobb¢±±1àD-²Ávò#ä÷´©×ü¦§§½k¦¦¦xÍW~A&jÁ|Èùò[zê~>µ¿(++ã~ü(s:6Zò#ä·"ò¯:ìüù!?¿GÀZÌf3æC~ÈßòÈ/~¾ò#äG+*¿'í2QòC~ü_~áùòC~§¥¥%àD-ù!?B~Ë/¿¹&mF~ühä§&jÑ´«&çc¢äüù­üdWûúë¯û­Ü»w/³ºò£_Zjkk1òC~üVV~"<ÙçfffÊ§o¿ý¶Ú¯ÐÛû!¿ßÈÈØNÒ.ó!?äGÈ/tò2ûÚÿfgg«;v¬ô]G~übG~b¾íD-ù!?B~!tíÚ5ï¾¸¡¡!wùòùZ­V­ù¨ù!?ÆùG~Pûbõ÷<¤W^yùò£§o÷îÝÚÉùÖ­[×ÚÚùòC~È/<ò3²/NMM½qãÆ÷ù%$$ ?B~´Xù	éÚÚÚÊËËýÌ§NÚÇ æC~ü_8å'äcÇù­4çöò£EÉo®Zm6Ãá`è!?ä~ùÍ5ßJ	ùòù¹æKOO¯®®bÐ!?ä§ù+äGÈ/äçv»O<©¨Åh4Ö××.äGÈùéB~²kö=üSäGÈüä§&jñæ7QË£G(äGÈù!?äGÈ/âëééùÆ7¾¡¨Ål6755qò#äüò#äÍ5QKYYYèÎü!?äü­HòhµX,Zómß¾]8Èø ?B~Èù!?B~Çãiiióùï_øÍfÃ|ÈòC~È_/àD-F£qß¾~ø!Cüù!?äüùE|§OÖ/++KMÔ²¨¿ÛKÈòÓüüùÅ ùä©OØÔÔäù!?B~È/òä?_ùòN§ÍfÓ¯¬¬¬¹¹Ùo¢äüù!¿È_ØC~ütb>«Õª=i×b±ÌõE~ÈòC~È_%Àòòr?óÉ§AÃäÈùòC~ÈùQØäçñxZ[[ddáæÓNÔ(æs:ó^ù!?B~Èù!?üÇ³ýîÆßöÙg¿üå/óc¹¹¹9??ßÏ|éééòxXàí ?äGÈù!?äGaËåz~õóå[Ëç¸ü;VslÃååå£_n·;àä|&iQæC~ÈòC~ÈÂ&¿ÖÖÖ¢¢"Å>õïèÍÈÈa$UA&jÑ´ü!?B~Èô+¿³gÏZ¾lñüËËËÍH4Ùl^²ùò#äüò£°É×ü¶Zò#äGÈùÞåçr¹î9¿÷ùå+_ÙÑßp­ùäÓÊÊÊe|¸!?äGÈù!?äGaßÙsþù^xáw~çwÔ¹½±ù_ÀZÔä|¨ù!?B~üEüÌNV"[¶µµu``àiÞ¾ÉÏÛÒÒ¢5_zzzmmí²ù!?B~Èù!?³üb31_SSv¢ÅNÎü!?B~È~s¹999ÚZWÔ|ÈùòC~ÈùòQ=ZöÉùò#äGÈùòÓWÃf³FíD-]]]!~w#òC~üòC~üVÊ|÷îMLLô3_yyyèAÈùòC~Èùò[ìvûîÝ»ýÌ·jÕ*Y900Æ;ü!?äü766f2!¿E%²²2¿ù6m&jA~È!¿§­§§§  @±!¿äñxNÔRSS344¤ûü!?äü´uëVÃD~û·ûihûè£z?%÷£ýHþKk?µ<Ií´µù¾ùÍoÊ¥ºº·×¯_ÿøãù]Õsò¿Ü?üqÐsíííÎï½÷nÞ¼âoÁòû¿»8·üZZZî6QÅgvtÐüg?ûYìü¼ò ¯®®ÎÈÈð3ßóÏ?ÿWõW·oßÖáý `ßU=÷ùçwww3zÎét¾ÿþûÎÿâ¿ñ7fùq´bùh¯ì÷Å|)))ÚÉù._¾ìv»uÏ9ÚËÑ^âh/G9Úûkç©!¿¹´Z­«V­ò3ßd/£ÿ?=ü!?äüYùòSÉï¼ÅbÑOVkr>äüùòC~ü¹¢¢¢8MUUUõ³ ?äGÈù!¿Eü(äçr¹´µ¤¤¤|ûÛß6(äüù!?äüùýFòK®óÉùò#äGÈùò[zN§Óf³iÍg2N>-"ômü!?äü!¿ÿ5_Àvô<QòC~üù!?B~M~ËËËµæ[·n]KKþ'jA~È!?äGÈoAæ³X,~à#h¢äüùòC~üæÌãñ477çççkÍgµZNgo#äüù!?äü(Väçv»Å|ÚZÒÓÓkkk£Û|ÈùòC~ÈùQ¬È/ÈD-²>NÚE~È!?äGÈïÉÐÐP@óåææ677GÍI»Èùò#äü(¦å7×D-f³YÌe'í"?äGÈò£ü¶4ÅbÖvò#äGÈùQÌÉo®Z*++cÜ|ÈùòC~ÈùQÈÏãñ´´´4_ÔOÔü!?B~ÈbE~jr¾µÈ/êÐÐÛù!?B~Èù!?xùMLL>Zk>ÉSµ ?äGÈò£hß£GN<9×D-±yÒ.òC~üù!?6ùÔÔÔ¤¤¤hOÚmiiÁ|Èùò#äü(äçt:m6[@óqÒ.òC~üù!?ùÉ£w÷îÝ~ó©vÃü!?B~È¢A~ò¸Ý°aß|ÕÕÕLÔü!?B~È¢A~Á'jyôèCü!?B~È"^~n·»±±Qk¾úúz&jA~È!?äGÑ ?59Édb¢äüò#äü_ÔÊOÌ'¿Q'çkllt¹,òC~üù!?xùîÝ»Wk¾uëÖµ´´¸Ýnù!?B~üE¼üä÷çÅ_ô¨EMÎg·Û9¶üãüù!?äòëêêÞùOXYY900À"?B~ÈòC~Ñ ¿ÖÖÖ¢¢"­ùª««ùòC~üòù©ÉùòóóýÌg4kkk7äGÈùòC~È/âåçv»Å|999ÚÉùN>ÍI»Èò#äü_4ÈOMÔb4ýÌöìYNÚE~ü!?äü¢A~òaµZµµ´µµqÒ.ò#äüù!?äþñÿñoýÖoùoûöíLÔüù!?B~ÈùEùvïÞí79µ ?B~ÈòC~Ñö¸2Í~æKLL´ÙlN§ñA~ü!?äü">ÇÓÔÔ´aÃ¿»©©©ßúÖ·¨ùòC~üòWsssnn®ù²²²êëë¯_¿¾¨¿ÛKÈò#äüSµ¤§§ûO(Tµ,üïöò#äüù!?ä§ÇNç·¿ým­ùÖ­[×ÒÒâÒ.òC~ü!?äü"Ø|ÕÕÕ~'pHeee'jA~Èò#äü_äp¢59_Zò#äüù!?äa_~Y;9Õju8Á¯ü!?äGÈù!¿Èãñ´µµi'jIII©®®^àD-ÈùòC~üòÓ»ùæ¨¥®®nbbbá7ü!?äGÈù!?¦&j1L~æ5õõõ.k±7ü!?äGÈù!?&ÑÏ|EEEÍÍÍÚvò#äGÈù!?äñy'jimm]²ùò#äüù!?ä§÷._¾¼ûv»Ý¾,·ü!?äGÈù!¿X	ù!?B~ÈòC~È!?äGÈù!?äGÈò#äüò#äüùòC~üù!?B~ü!?B~È!?äGÈò#äüò#äüùòC~ÈùòC~üù!?äüù!?B~Èq@~Èù!?äüù!?B~Èù!?B~ü!?äü!?B~ÈòC~Èò#äGÈùò#äüùòC~üù!?B~ü!?B~ÈòC~Èò#äGÈù!?äGÈùò#äüò#äüù!?B~Èù!?äüù!?B~Èù!?B~ü!?äü!?B~ÈòC~Èò#äGÈùò#äüùòC~üù!?B~ü!?B~ÈòC~Èò#äGÈùÅ´üzKJJCqqq?ò#äüùòC~Q+¿µk×^¿~].]º§Ýnÿehëëëú%é¸[·n9ÆAÏÝ½w``qÐs£££===s¹?ÆAçü¦¦¦BüM#X~¾%%%iåwêÔ©ÐÖÖÖÖÞÞÞA:î½÷Þcé<Ù@²=÷þûïËîqÐy?üá¶¶h____UUG£½í%öG9ÚµGU.ùòC~üù!¿¨_Ü¯R[­ÖØB~ü!?äGÈ/²åçÝnß²eËØØXÀK!?äGÈùòùL¦8!?äGÈòZùùòC~ü!?äüòC~ü!?äü!?B~ÈòC~È!?äGÈù!?äGÈùò#äüùòC~üù!?B~ü!?B~È!?äGÈù!?äGÈùò#äüò#äüùòC~ÈùòC~ü!?äü!?B~È_4Éï/ÿò/[ZZî¶þð?ûÙÏîëîîÿ0z®··WöûôÓOÿþïÿqÐswîÜ¹xñ"ã óþîïþÎétøNLLD§ünÝºuüøñïÑ¯~D4Wbb$äGDDDüùò#""""äGDDDDÈ.êíí-))1ÅÅÅýýý²Æív[­Ö»ÝÎépÉv)((5===QØ»uëÖúõë·l©ììlµ¦»»!Òá6Ò>²HoÛHÕÙÙÇ³¹~7ÞØÀïÊ<­]»öúõë²péÒ¥¼¼<Y8qâÄo¾933#ÛoÍ5·Ñh¼ÿþÙ¿f2¢°'À«W¯Êl)Ù:²PQQqñâEY8wîÜ="n#í#ô¶$Ç#@G~zÞLzc¿+())ImÔÏ?ÿÑÐó6Öðð°,ÈGt®«:::Îe?(ÓÓÓèÛHûÈ"n£7ÞxãßûòÓófÒø]YhUUU²`0Î9#ûA!ÅÍ7n£þþ~Ù@²+²ÑC'55U6Ê¥KÔãÈï2égiY¤·mtÿþýÒÒRùOòÓùîNWlàweAMNNVTT¸Y¿pá,Ü¾[r·ÑúõëÕ°7ndpôSgggff¦zyW&$$02:ÜFÚGémíÜ¹óÚµkÿûüô½»Óø]¿ááa«ÕúðáCõ©ï>×*ô¹x=IÏ©-"£ééé'³GS¤m¤dÞ¶QÜoÆ°èvw§«§$~QæÉn·oÙ²ellÌ»æÀï¼óÎÙówÌf3C¤Ãm´qãFÙ:²088¸~ýz(ì­]»VmÞÞ^ÙX²PYYùÖ[oÉ|¬¨¨`t¸´,ÒÛ6úõs9ìÓñfÒø]'Éä÷?ªñññ;wÛKKKC¤ÃmtçÎuR½|e(ìõ÷÷ÊÙ¼y³z©§§'+++>>>;;[ö·öEzÛFÈ/"6ÞØÀïQ¬ü!?""""B~DDDDüùò#""""äGDDDDÈò#""""äGDDDDÈ!?""""B~DDDDüùò#¢èéí·ßÞ´iSÒl7o~÷Ýwcÿ5[ÄìmÝ[É$?Ëåò[/kCvvöÌÌÌboùQäuüøñ8M¯¿þz4É¯®®NV644ø­?ö¬¬íµ×pDDÈ"¬þþ~1Á`8þ¼g¶.È§²òÆQ#¿»wïÊÊââb¿õ²Þét"?"B~Dý½òÊ+b7ß|Ówå©S§dåÞ½ÝÓÝÝ-rÈ²÷?~*ÆCùTíêê]ÉErÝ?KÉ´´´M6µ··Ë§~w¬­­-øí¨ä.©:;;çRÚ-[doo¯wÍõë×eÅbñ®9qâDVVÜTRRÒ®]»µòÓÞ¾ß wQ8[½zµ¨åþýû¾+<x +M&¯lüêëëSîØ±Ãï¢ªããã^Kª.Ý¹sçÌÌØ1!!ÁãñÈ¥òQä¬Þ~ävd!àÝÓþ¤.]òå¬/^ô²ÏïF6oÞ¼Xù¹«DüÂ:°`'óÍ=&gùtÛ¶mêReGõÒM]TQQ¡^,åu#¾·yìØ1±:Øºoß>YsõêUY²üê«¯Î;¢FùT¾À÷¾üDòóÉ§òQî¹pszzZAvv¶~'¿::´Xù¹«DüÂ/?) ü¼ël¤ááaùTÌ¤>-))O×¬Y³ÿ~ÛÔÔ÷Fäkü^Eó^KúàÁï¼|å£,wuuÍ;¢LùtttÔ÷¾Íõ¼ÃËEßûÞ÷üêö¡C|¿@(òª$+¿ wQS/tMNNú®t¹R.b¯Â:Þ7çiÂúYÊw.Y6			n·[n<--Íé¼·3ÃüºuëBª,ËGYô^ÚÓÓ#w àQãË/È]%"äGDæÔÝÎ9ã»RÍuâw÷½cccÚ²nÜ¸QWW§½zÉ¨^óN×gTyåcUUwÛQ¯±=|øP:::ü<Ü7Ê¥j"«ïEê<_¹¨³³s||<¸ü¼*U£á½4È]%"äGDæÔáÜ¹sjVóçÏ'$$hÏÆØ¶m(Çív«C±ÞópÕûüÔÛãï:$NRgÝzçU	è3u²­úîv»Ý»>Èí¨7ö©÷ù¹õAäwáÂï«qò#û^¤Þò888(ßEa.ù)kåËD»víò½4È]%"äGDþÔ,Ç~8qâ×û¯Ù¼ËÞã¤Þ*¼íÛ·Ï¾]¹r%üÌþ±Yo4¹¹¾wÌ^íìÔÔTRRúüqoÝºÕ÷[dddÈG5±ïmª$½)§z/rWùé"ÑÉæÍfÛ´i:ÁÖO~jÚ¼ÂÂÂk×®y/u¹ÍÊÊRZ:|ø°Ûíö^ÚÑÑQZZ* ËÎÎnllô»Mí=õð[?×íHrgä.©Ìçç­ªªÊozÕØØXEE@jjªüN§Ó;CïmmÕXY,ÞÞ^¿ïä®ò#""""äGDDDDÈ!?""""B~DDDDüùò#""""äGDDDüùò#""""äGDDDDÈ!?""""Z|ÿtÉª8ë»IEND®B`


tÑ¢E¢-ï½÷^_^^öÖ óUBÏ½òÊ+a¢®®.uá|ðÁ0~àµ×^?þx"Ô.^¼ØÑÑ&¢!Éöá¨fÿþý9Ë-ûí·W6þ®X±"L¯Y³&L9iÁ£P~ÀÈ_EEEî½&5¼ÒîØÞÞÈU«VEI¸ôøñã[·n]¸pa_PPöÖúµWæ«D6aÂÔëN<9Zø¾¾¾0z+Ì?¦çÏZ6ÝÚjéçèÑ£¡Pýn9¿iÓ¦Ä·Q¡>üðÃ·³ÊÂòKMÌß^7Bf7.üdOOO´A3Ì.zöÙgÃµB¾ýöÛÉ·ÐïÖ¿½«´`!è¢j|õÕW'NÍ)--o|Ì/Ãz;xð`¹ä­Û¡Ã/½páÂÕkÃô)SÒ.G ü/¿òòòä1¿hØìºT[[.M|MÌè»æË/óUB*õº&M®Ûoñâñøo¾,¼©Õ2haÂ2éDªösüøñ[^åaùEgiyî¹ç¢ýü~úéÉ W_5:¯¼òJb~Ô'Oöÿ»òË|­[·~ûÛß6lH½îý÷ß¦_|ñÅS§NE[WÃÌÊÊÊ0²ìÃ?3gÎ¼Íò7o^àÀ«??Ë÷ÝwuàÜù·¼ÊÂòÇã¡®Æ]|N¾ÌåwåÊhKkø|ºF¥¥¥&LØ²eË_æ«´µµK/^8Ð¤ß97mÚ¼¨¨hùòåÑ!´!¶jjj¢.]úÎ;ïÜÔjIÝ"n¶¶¶6,aøc'Môàßõç'µN¾ýÄI­oyò@ù üP~(?ßMûë¿þëÓ§Oó/ýàþíßþÍãÁuþüùù±þïÿþþûï[®¾¾¾þô§Öò¿÷¿âoéßýÝßøá7®wß÷þá¬×¹sçþæoþÆz`pýÇüÇo¾i=(?åÊåòCù)?P~(?ÊOùòCù¡üP~ÊåÊåòS~(?P~(?òCùòCù¡üÊåÊå§üP~(?P~ÊOù)?ÊòS~ÊåòCù¡ü(?Êå§ü@ù¡üP~(?åÊåòCù)?(?ÊOù¡ü@ù¡üÈÃò;räÈüùóçÍwìØ1åòåò#oËoÆo½õVxé¥fÎZ~áÁwaxýà?oÒ`Putt8qÂz`pýìg?;tèõÀàÿ£xã7¬lÃå¬¤¤$µüvíÚõáõñííí?Aõ½ï¯­­Íz`pÿûßommµnii±²Y>ßÑ£GëëëmíÅÖ^°µ[ÉÛ­½Ë/×ÔÔôôô(?(?ù gÎ©««K[ÊåÊåGþ_û]wÝ^¼Ò^ªüP~ üP~äOùUTTI¢üP~ üP~ämùe¦üP~ üP~(?åòåòCù)?ÊòS~Êåòå§üòCù¡ü@ù)?åÊåòCù)?P~(?ÊOùòCù¡üP~ÊåÊåòS~(?P~(?òCùòCù¡üÊå§üP~(?åòCùòS~ÊOù¡üP~ üòS~(?(?å§ü@ù¡üP~(?åÊåòCù)?P~(?ÊOù¡ü@ù¡üP~ÊåÊåòS~(?P~(?òCù¡üÊå§üP~(?P~ÊOù)?ÊòS~Êåòå§ü(?Êå§ü@ù¡üP~(?åÊåòCù)?(?ÊOù¡ü@ù¡üP~ÊåÊåòS~(?òCù¡üÊåÊOù)?åòCùòS~ÊOù¡üP~ üòåòCù¡ü(?Êå§ü@ù¡üP~(?åòåòCù)?(?ÊOù¡ü@ù¡üP~ÊåòåòS~(?(?å§üÊåÊOù)?åòCù¡ü²¯üÎ=ÛÚÚê®Q~ÊåòS~äsùutt¬_¿¾¸¸xìØ±¡ÿÜ;ÊOù¡üP~oåwþüùÝ»w/X°`L¦¦&÷òS~(?äOù8q¢¶¶¶¸¸xLºº:÷òS~(?ä|ù]ºtiçÎwÞygjðÜ¸qcGG»Fù)?ÊOùÛåÞèä!øòË/(t§(?åòåG_è¹ÆÆÆY³f¥ßøñãëêêN:å¾P~ÊåÊÜ.¿ð>Â.í _eeåÎ;ò)?åòåGn_è¹=ö¤ä»ã;6nÜxâÄ	+_ù)?(?r»ü2òíÝ»7YíÊOù¡ü@ùÃåwÝA¾ÎÎNk[ù)?(?r»üÂ;i»wiùBÚOù)?(?r»ü¢A¾ÐvòwX«Wù)?(?r»üò)?åÊåG_A¾âââºº:|ÊOù¡ü@ùóå×ÙÙ9Ð ß¬Y³ò)?åòS~(?r¾üb±ØÞ½ò)?åÊåG>A>å§ü@ù¡üÈóò3È§ü(?ù_~ùò)?åÊåGnÅbög6wîÜ´|µµµÃÿ¾òS~(?P~²ÎÎÎùvìØaOù)?P~(?r[,knn®ªªJ¾ñãÇ¯^½º½½=[QÊOùòCùÃºººäûä'?iOù)?P~(?r^<onn®®®;vljóùØ·o¥ü(?9,ä«¨¨H¾0³©©éìÙ³WoésQ~ÊÊ¬yoåÊá­6yO>å§ü(?¹'Ã ßôéÓwïÞòõ£üòåò#gÄãñ¶¶¶´|aÎúõëûò)?å§üÊåGîéîîoiù¢sò¸î(?å§ü@ù¡üÈ^ùjkk[ZZnü|ÊOù)?P~(?²QwwwccãôéÓútÝäS~Êo¸_¹***Ê7(íÛ·oéÒ¥iùÖ®];¼¡üß:|øðìÙ³Ã#Uù¡ü@ùq]>]wÁ»wï>þümþå§üÐòåËÃ8Cù=ûì³7¼¾ûÝïÜü;Táÿßùi=0¸~ð8pÀz9òðûÜçR?]wÍ5Ï?ÿü`ý®£G¶´´XçÙ,Ëï¿qàòûó?ÿóWÈ¾wÞyçCTo¿ýö±cÇ¬Wøó÷¿ÿë!¿ýð?Ü¼yó'>ñÔA¾EãßøÇüÇÁýgÎyã7¬ùlÏågk/¶ö­½£P,kmm­®®N¾âââºººëüØÚkk¯òS~(?ÙîÔ©Sáí¯¬¬,íáºöì¹téÒ.òS~ÊÊ¡ÅöîÝö|C=È§üòS~(?Ã¤³³sóæÍ#8È§ü_¶P~(?P~ù*ïÛ·o A¾ÚÚÚáT~ÊOùòCù1Èººº¶oß>Ð _ccã0ò)?å§üÊåÇ ùV®m|ÊOù)?åòCù1h¢Þ¨¨¨ÈÎA>å§üòCù¡ü¸]±X¬¹¹¹ªª*5øÆ=|ÁÙ³gwïÞýÐC555ýìg?S~ÊOùòCùq£ºººútÝéÓ§766vwwgÏÒvvvN4ésûÜ¾ô¥ÊÊÊO|â¿ÿû¿ïNT~ÊÊLâñxsssÚÃu£A¾¶¶¶ð3Ù¶Ø¿ôK¿´üË·[ôoÝºu'NÌªÐ(?åòåE¢A¾´òeá _²°`a±·>º5Q~áßg?ûÙöövw«òS~ üP~ü·äKÖÑÑ1sæÌäìÿ.oß>÷¯òS~ üP~ü§îîîð&v/Ìeí _?===%%%~kC"û¶lÞòñüÔ©Sîeå§ü@ù¡üFµx<ÞÖÖ6Ð _ý|©ÊËË³ö7Cöýºÿ3cÆ»ï¾Û­ü(?ßè7|i=ÿüóóæÎûØÇ>ö¿ø¯¿þº)?P~(¿Q'/ù2s&gå§ü@ù¡üFÌ|]]]yù+?å§ü@ù¡üFh¯¶¶v A¾æææ<äS~Êïç×¼åÊåº»»§O>Úùòû/×SXX¨ü@ù¡ür]x¯Í|ÊOùeåòå7tzzzvïÞ=kÖ¬ÔA¾;î¸côò)?åwC.^¼¸aÃåÊåsN8Q___ZZÚ|UUUÍÍÍ±XlÔ®å§üþSEEEaa¡ýü@ù¡ürWOOÏ=,X|¡:;;­%å§ü®Î7/í$<(?_ökkk[»vmÚA¾;ï¼³¥¥e4ò)?å×_QQQxn²²²0ïÛßþv¨¯¯W~ üP~Y«»»Çi×?~üÆ;::¬%å§üRnâ0R/L¼÷ÞabÜ¸qÊÊ/·Õ«W§6ßÒ¥KGù|ÊOù]ÇÄÃSåðáÃaâ±Ç&ÕÊ/«?~çÎsçÎM»ÒÆÃ¼µ¤üßulÙ²%q<Gò³¨²²RùòCùe'N<ôÐCãÇOm¾»ï¾»¹¹y¨÷MW~äOùO<ñÄ¤IÂÄ#GÂD¨Àõ¢+?(¿ÌBÏíÝ»7í _´'_(Bå§üròCùòÈ©S§6oÞ|Çw¤6_Á_~Ù|ÊOù)?åòCùå¶Ðs¡ê*++íÉ§üÂò9sftngråò§Na_,u¯©©éÒ¥KÊA(¿3f$×^cAù¡üZè¹=ö¤ä»ã;zè!çäS~rùÈO°£Göõõç¢+?æò/È7nL»'ßò)?ªü¢»æìS~(?åÅöîÝvoìØ±õõõ×U~mù;v,<ß|ðÁË/+?P~(¿!ÒÙÙ9Ð ß¬Y³vîÜÙÝÝí~W~yùS¦LI:ÂÊïöeä+..®««s¸®òcXËoÚ´iðåòtùöìÙcO>åÇ_ô$þ§Êò²üb±XsssUUA>åG6ßäÉáÊåwû:;;ÒòUVVäS~dEùµ··çä-[ùpÊò£ü2òôÁÊì*¿1p(?_fù¹W~p(?_ZñxÜ ò#WËo¤(?äuuu544TTTäS~äjù'ð´iÓN:¥ü@ù¡üÒùª««ÇkOùÛåWXXºÃ¿èÊåÙ_~ùùV~OàíÛ·_¼xq8Ïí¢üP~µåwÝA¾ÎÎN÷ò#'ËÏ±½ üP~	ùöîÝ;Ì§Cù1ÈåçØ^P~(¿è|òÕ××äS~äIùåòl(¿çä;wîóÏ?ßÓÓãîP~(?åÊ.¿¼1~üøºººá?çÊa*¿ÞÞÞåË'ü¸qãî¹ça8ÔCù¡ü`DÊ/Ã ß¬Y³ò)?ò¹ü®ö¡ÞWù¡ü`8Ë/Ã _qqñÚµkO8aµ+?ò¿üfÏö+V¬¸|ùrøöâÅ«V­s-Z¤ü@ùåaoúôéÎÉ§üEåWTTüñx<1§··7Ì	ó(?r·ü2ïÉW__µòcË¯   ¼ÚK~±sÕ9Z~7nL;Èwçw655äS~Þò¶öÞu×]ÑÖÞð5L9óçÏW~ üÈ¡òÿoß»woeeeÚ=ùêêêÿUåGÖ_H½´GxpAùòcètww×××Ï;wÁ_ÿú×ûWwSå×ÕÕ5Ð _¸ñÆÆÆð»¬pòû/W®ç&LPPP¾Þu×]aÎP/ºòCù1:uª´´ôWþÊßÚðû¾2kÖ¬_ûµ_KÞåúË¯­­míÚµ©¼æÔÖÖ¶¶¶&ß&(?å7bÊÑlùòåUUUÛ¾¾-ñïÓþtssó_wwwx-++Kä«¨¨?ÞJFù)?å§üP~dO~ò[6oI.¿/|á=ôPæòÇã­­­µµµ©|ÁÊ+ÛÛÛò¡ü_ºk^OAAòåÇùÔ§>µùáÍÉåw÷¯Ýý;¿ó;ßÙ³g§Nö7ÂEá¬UòPÁÀ(?Ú¿üåÏþóìÛúèÖÐmmm©å×ÞÞ¾råÊÔA¾âââÚÚZë¢üßmyøá£×^zIùòc?þ3ùÌù~ã7~cõ=«§LR__ü?þñû·;í Þ@ù)¿ApìØ±qãÆ×ªªªä;+?P~'|òÕ_jÕÿñGûç¯ííí×mkk³'ÊOùÝ®5kÖD¯,ÃöP~(?HvöìÙ;vTTT4Èç|(?¡ü^íµèeÅÃ¹èÊåW¯òµ´´¤=  À ÊA+¿+W®,Z´(zq9xðà0/ºòCù1Ê>º©©iúôéiÏÉ·uëÖ×_ÝZBù18å÷Ì3ÏD¯/ëÖ­EW~(?F­¶¶¶Õ«W§îÉW]]òÝÂçöòS~_Óùü@ù1¼º»»wìØ1kÖ¬>x#yO>åòc0Ë¯àz(?n_<>]7ó _¿k)?Y~#Nù¡üÈÑ§ë¦=uOù¡üP~ÊåGîùª««Ó/Ìonn¾îáºÊåòS~(?²Zwwwccã@ë644tuuÝàM)?ÊOù¡üÈFÑ _ÚsòÝø òCù¡üÊ¬6|ÊåòS~(?²TxK;Èúù-ò)?CX~ÎçÊ[péÒ¥ÆÆÆ´çä»ã;:;;å)?Y~É§îK[~ÎçÊdÑ _ê9ù|±XlòCù1åpàÀð²uï½÷^¾|9|¾®Zµ*Ì9tèòåÇ°ò)?ÃQ~¥¥¥áõ+yÞÞÞ0§¬¬ì6oùØ±cåååsæÌ9xð òCù[2òUVVîÝ»wpùÊá(¿èU,Ô^¿ò»ýýüjjj^|ñÅ0±k×®uëÖ¥ßw¿ûÝá^Fÿéþ©Õ©S§þþïÿÞzÈgÎùÃ?üÃÏ~ö³©Á7qâÄ¯~õ«?úÑa1þùÿùðáÃî×¿þë¿ò³²ÙßäÉÃËY¨´+W®oÃ¯bEæßþhb___©å×ÔÔôÃë;ßùNÈÍ¿Aõ×Xy ü7õ_øÂÇ>ö±Ôæ=ö£>:wt[[[xv§0è^ýu+!yù:t(ío¿ýömÞrò1"©ÇØÚ­½dX,¶gÏöäÛ¸qãíÉgk/¶ö2[÷ÞoÆ%%%ãÆ?þGtû7¼½¸¨¨Hù¡üÈ6á5aóæÍeee#²'òCù12å7D&Oí>¾¦n;V~(?FJOOÏ=æÎ;Ð _x­ñT~(?r¬üjkk_xá0¾ÖÔÔ(?#®³³3]È»´|!/]º%ªüP~Uù<yræÌãÆ¶ÏN<ùÕW_½ý=|øpYYY¸Íòòò#G(?#¥§§g÷îÝUUU©¶6~üøõë×gÃ òCù1åÉ9ùÛ¢é]»vé¢+?ÃàÔ©SõõõÑKûY°`AÈÁìäS~(?£ü¢]O<(¿#Gé	&(?P~9êüùó/¿ürÚA¾P7oîèèÈò?Aù¡üò^£¨üúúú|n/(¿nãÆãÇOä[ºtéÞ½¯¢Tåò#Ë/:s4ÎÊ¯··÷G	Ó©ç^V~ ü²V,UWUU|ÅÅÅ7o>uêTnýEÊåÇ_Ú39¿õÖ[Ê_öëììlhhÿYM!øüóÏ?>ÿ.åòcHÊ/1´páÂèØÞ3gvuuõ¢+?·#¿üòËK.M»'ßÆO8Ó òCù1Tå7"Ê[ÓÝÝýè£¦ýà---#øÁÊåG¶_âÀ>úhæÌS§NU~ ü²Gè¹PuÕÕÕÅÅÅ9z¸®òCùå×ÛÛëØ^P~Ù#Ú/í9ù*++wïÞ+ë*?#V~S§NÑÄ(¿ÅÓ®;~üøºººaØ#Yù¡üÈòÿý÷®I|zG²¯½öòå7"¢A¾´®[]]ýòË/gío(?YZ~	¡ózÃ®òCùq#2òþ=ùÎ?ÇÊ¼*¿¢üP~$tvvnÜ¸1í _ÁÃ|¸îÞ½?õ©ON8ñî»ï3*?CR~½½½3gÎ7nÎ¤I|òIåÊo¨E¼QYYv¯¡¡!áð/UX¤ÿUú¿¾rßW¶Û#¿óHåç+?ÿùÏ+?yR~S¦Léwxoô²»ûvåÊodä!ÚkÏÉ÷©Oê¯ÿß!ûÿ~á~¡­­Mù¡üÈò+**/µÉÏ;v,Ì0aòå7¸2òA¾d.]*--MÎ¾ðoÙ²eMMMÊåG>_txo__ßÿ¸Ýü)?P~·#ùÅãñO|â[6oI.¿°Ï?ÿ¼òCùåW^^^y7mÚ½ì^¹reÛ¶maNYYòåw²/Õ¾ô¥%K$²oÃom?~üð;Pù¡üò;zôhÚ399rDùò»§[A¾=ödí9ùÎ=ûÏ|æfÿRuuõ¯þê¯N8ñÅ_þÅP~(?¤ü3gÎÌ3§¤¤¤  `Ü¸q³gÏszÑÊ//Ua/<³ÿ¯Åbá¯KûØcT~)?CU~#Bù¡üòLîòe'åòCù)?_ÖÉA>åòcßÉ'£9GÇóN<ùÕW_U~ ü®ûhoÖ¬YùÊl,¿$^¬£ò¦wíÚ¥ü@ù¥Êp¸nqqq]]A>åò#Ë¯¬¬,¼^<y2Q~Gq&gP~©2oúôéùÊ(¿èUûjÒÙûúúÂtaa¡òåw5ã ßøñã×®]ËñxÜ#Aù¡üÈò<yrtö¾¨üzyä0]QQ¡ü`_A¾Y³fíØ±Ã òCùcå×ÞÞöLÎo½õòÑY~±X¬¹¹¹ªª*í|ë×¯7È§üP~äjù!.Û[RR2sæÌaø"åòËBíÉ·sçÎîîn÷¸òCùÛå7"Ê/däV®;ùÊå§ü ·Ë/Ã ßÜ¹sÃóôüùóîbåò#¯Ê/¼oM6­¨¨(¼ÖÌ3'¼â(?ÈãòÇãò;¶®®.4A>åò#Ë¯­­-íöS§N)?È¿òëêêjhh¨¨¨H¸ncc£=ùÊ|.¿èLÎ555===áÛË/¯[·.Ì)//W~7åòUWW;6õpÝÕ«WåòcT_ôÒ¼Y'L'Îê¬ü ×ËïìÙ³MMMS§NMä[°`A¸È9ùÊQT~Ñ_ooobÎ+WùA®_ø/ËÊ+Sùêêêp©=ùÊQW~Ñ~~555!øÂ·.ºt©ýü wËïìÙ³;vìH»'_ùè£Ã	;Q~(?²´üÆmöU~(¿ASk×®Mäsª««÷íÛgOù¡üßh/¿ë),,T~Íåò¥Ý¯¢¢"<×®«ü@ù)¿¦üP~·/ó _[[A>åÊOùõqI;ÿÌ3Ê²³üÎ=»sçÎY³fäS~ üßMÞÄ1O<ñD¿ë×¯wVÈÂòkoo_½zuÚA¾ÚÚZ|Êò»PxámcòäÉôQøö[ßúVôF2D»÷)?ß-8þ|cccÚ=ù¦Oî7(?åwÖ¯_½G«V­êEW~(¿qâÄð?~¼A>åÊA;ÂãÐ¡CwÝ»wÃ¢+?_ùP~(?ªüxàè%ú<àÞïU~0"å¥ùP~(?ªüÃÊ	?~5i?¿¢¢"åÃV~.]Ú½÷ò¡üP~aù÷­[·öYWWçØ^òOÚÚÚâââ~ÁæäS~ÖÊA.¿Îç7Ô¤üååwéÒ¥ÆÆÆ´çä3ÃEá¬[åg= üäò)ÊQ[~ùÿyòCùÿåÞf·çfþVùÁí_ww·A>Êå§üÈçòÇãmmmµµµ©¼aåòCù)?ò¤ü¢A¾éÓ§t¸®A>Êå§üÈíòË0Èç|(?ÊOù'åwÝA>çäCù¡üP~ÊÜöÓþôßü¦A>Êå§üÈgÑ ß§?ýéÔA¾ð 7ÈòCù-åòDòUWW§äóò¡üP~dWù¡ò~º»»ÃC·¢¢Â ÊåG.ßS~ä|¿ò+¿òÍo~Ó ÊåòS~ä¶Ó§OïÜ¹3ío$ùnísAù¡üP~Êo¨tuuíÛ·/¬óÏ|´t¸nuuusssbOù¡üP~(?åE¾öµ¯üãÿå_þå¹sçú¸°Î=ÛÔÔTYYv¯¡¡!4t¿«(?Êå§ü²ÅýÑÿßMÿwÛ×·ëÖ­ûä'?yúôiÏ~N8QWW7~üøÔæ!<È§üP~(?òËR3fÌøÊ_²/ú:æé§öüt©±±qéÒ¥©Á*pãÆoAù¡üP~(?å-B¾$g_ø÷¥/é«_ýªçÏ©S§êëëKKKS¯ººzß¾çÏ¿ÛQ~(?ÊOùec~ýôôôìÙ³géÒ¥©Go|ôÑGovS¸òCù¡üP~Ê/[ØÏ/áÄ7o¾ã;Rù,XðòË/Çb±[¸YåòCù¡ü_ùÚ×¾6nÜ¸;ï¼sÎ9!ûÚÛÛGÕéÒ¥;wÞÎ|ÊåòCù)¿Ï¯­­-dÐèù«;;;äÛ»wï ÝPù¡üP~(?åÇ	=2÷Î;ïL¾âââÍ7ßæ òCù¡üP~Ê®;Ð9ùvïÞë*?Êå§üÈR.]Ú³gÏÜ¹sÓîÉZpHßAÊåòS~ÓZWWW\vO¾;wÃ~ÊåòCù)?P4È7kÖ¬´|µµµÃùèR~(?ÊOù1TwâÆÓ®B°©©iø^V~(?Êï¿^¹***·/ä«¬¬L¾P¡Ã;RË¦üP~(?ßÕÃÏ=;¼1+?nó^h/`ÈÁ?C¡òCù¡üP~W/_ÞÙÙ¡üþäOþäï×÷¾÷½#Gü=¹àØ±cO<ñÄ¼yóRïcûØ¯ÿú¯ÿéþi,jø?Ì_ýÕ_¹Ëýîw¿k=0¸~ô£µ´´XÙ,Ëï¿qàòknn>=¼ÂÛóüãÓd·ïÿû_þò?þñ§6ßg>ó§zêäÉYµÀ!RÿöoÿÖÇàúÉO~ÒÞÞn=0¸Þï½7ÞxÃzÈfù ¶ö,íÝ»7í|ÅÅÅuuu#¸'­½ØÚ­½ØÚ¾ó"Ê×ÙÙápÝlØOù¡üP~(¿mAåGZùù|ÊåòCù)?DgggCCÃ@|Y>È§üP~(?ßÍQ~£P,knn®ªªÊõA>åòCù¡üÊ³A>åòCù¡üýeä;vlîò)?Êå§üøo]]]òM>½±±±»»;oþXåòCù¡üßhÇ«««Çv¯­­-üLýÕÊåòCù)¿Ñ%ä«¨¨Hä+++wwø|ýÛÊåòS~£BæA¾µk×¶¶¶Æb±ü^	ÊåòCù)¿<×ÝÝîÄ´|S§NmllÌãA>åòCù¡üß¨ÇÛÚÚ2ìÉ×ÚÚò)?Êå§üF|eeeMMMgÏkFù¡üP~(?å'2ò¯2÷ò)?Êå§üò=ùvìØOçäS~(?ÊOùFÑ _mmmÚA¾0¿½½´íÉ§üP~(?òË7ÝÝÝÓ§OèÓuGí|ÊåòCù)¿üîººº´|a~~|º®òCùòS~ÊoT;út¸#ÊÊÊä³'òCù¡üP~Ê/·]÷|---öäS~(?ÊOùå¶îîî;v¤=Ö¬Y»wï>þ¼çòCù¡üP~Ê/ÅãñÖÖÖÕ«W§Ý/¬O'åòCù¡ü_n;ölA¾=ötÉIù¡üP~(?åÛÚÛÛW¯^=Ð9ù®«üP~(?òËy]]]ò566äS~(?ÊOùå¶x<ÞÒÒ²råJ|ÊåÊOù)¿¼-¿èÓuÓoúôéùÊòS~9ïºçä:'òCùòS~Ê/·e8'_4Èç7ÊòS~¹]~ÎÉgOù¡ü@ù)?å'å×ÙÙ9ÐáºùÊòS~ùP~=ùRoìØ±a¾A>åòå§ü_Î_wwwcccÚA¾03¬:|ÊåÊOù)¿Ü.¿hO¾´ëùÊòS~ùP~§OÞ¾ÚA¾ÒÒÒÎÎNwåòå§ü__ôÁi?]7¸ûî»Ã¥ùÊÊ/·ËïôéÓ;vì>zÚ=ùÂx|+?õòCùåvùµ··ß÷Ýi?x£ººÚ òåòCùå|ù=6üÉS§NMä3·oßîp]åÊåòËíòÅbÑ|©¼TUUíÛ·/ü²òåòCùåpùuww¿ÑáºÊÊå·åðFmmí@ë677÷ôôxì*?P~(?_n_øÒò=úè£]]]²ÊÊå'åwéÒ¥ä¡¾ÄáºöäS~ üP~(¿|+¿`íÚµÑ9ù®«ü@ù¡üP~y^~'NpN>åÊåòåòåòCù)?(?ÊOù¡ü@ù¡üP~ÊåòåòS~(?(?å§üÊåÊOù)?åòCù¡üòS~ üP~(?òåòCù¡ü(?Êå§üP~ üP~(?åòåòCù)?(?ÊOù¡üP~ üP~Êåòå§üòCù¡ü@ù)?å§üP~(?òS~ÊÊåòS~ üP~(?òåòCù¡üÊÊå§üP~ üP~(?åòåòCù)?ÊÊOù¡üP~ üòS~(?(?å§üÊåòCù)?P~(?ÊOùòCù¡üP~ÊÊåòS~(?P~(?òCùòCù¡üÊÊå§üP~(?P~dù9rdþüùóæÍ;vìòCùòCù·å7cÆ·Þz+L¼ôÒK3gÎL-¿öööÿ7¼=ÚÝÝýÿ`Pýô§?ýÉO~b=0¸ÂSÃK¨õÀàÅb¡ü¬lÃå¬¤¤$µü~úéÖáÕÒÒñ­0¨Âê7Þ°ùÎwÂKõÀ ýõ×­låwôèÑúúz[±µlíÅÖ^òvkoäòåË555===ÊåÊåG^ß¾=sæL]]]ÚØR~(?P~(?r»üµ··ßu×]áÅ+í¥ÊåÊåGþ_EEÅ$ÊåÊåGÞ_fÊåÊåòS~(?P~(?òCù¡ü@ù)?å§üP~(?P~ÊOù)?ÊòS~ üP~(?òåòCù¡ü(?Êå§üP~ üP~(?åòåòCù)?(?ÊOù¡üP~ÊåG>ßïþîï677^¯¿þúøÃÓ0¨:tðàAëÁõ£ýhß¾Öëg?ûÙóÏ?o=d³K.ågù<yrÛ¶m¿ÀÏeÞ":Æ°'­½(?Êåò@ù'O.Z´¨°°pÎ9NÌ?pàÀ1Öö¸ÅbuuuEEES§Nmoo·ÇUx,Í=;õnVò»Þ±cÇÊËË£ÇÕÁ­åWÂÃzÿþýaâ­·Þ*--fÆãñùóç+?ñqõøã?õÔSá­zÚ´iVò¸_ßÿý0¾VTTXEÜ~ïz555/¾øbØµk×ºuë¬åZ[[çÍM?ùäßøÆ7ø¸ïÙ§N²BÜÇUø_Ä3gÂDøêÜ²~ïzáá¿©a¢··×ÿ(_~þ_gÂ	áÿÒK/Eÿu^¸paxÐ+?ñqUXXøÌ3Ï·ç·ß~ÛúaPWÇªðmøzôèQë[ú®^¯&O£üòÊ&O&î¹çCýçºS~Þãª  à¹çï¼óNxµfÇÕ¢E¢±ä/¶f¸©ïzáõ*qiQQU¤üòVô?1ÿÕÂ <®¢÷iÿfpWÆfPHy×¯W½½½W¯míM~íBùå3f<y2L9rä®»îê÷d°~¬ÇÕ<ðÊ+¯vxfee¥UÄ <®/^ÍéèèX´hUÄm&`4Q[[ûÂ/ðµ¦¦ÆQ~yåØ±csæÌ	ÿW^¶lÙ~¨ü¢ÇÕÅï¹ç0gáÂVò¸z÷Ýw£ó¼¯aÚ*bPÊïðáÃeeeåååá¿Öò@ù üP~(?Ê@ù üP~(?Êåò@ù üÊå0¾õ­o-Y²¤äeË½öÚkÿãõëyµM·´áOëééé7?Ì),,,//ïëë»ÙÛP~@îÙ¶mÛO<ñD>ßöíÛÃÌÝ»w÷¿sçÎ0ÿ±Ç»ÛP~@9vìXhÂÂÂg6~ÍsÏ=¾3?7å÷ÞïóæÍë7öìÙa~WWòÿî½÷ÞÐ4O=õTòÌ§~:Ì~r÷<x0SÂùóçéÄ_¸p¡¾¾~Â	á¢ÒÒÒM6%oTmkku.×mmmí×RaÎÄ,Yòæookkkû-XKKKæÛ.]tàÀ*í®»îó9óÖ[o9UUU9?þxYYY¸©5kÖ9s&µüRo¿ß(?4eÊP-ï¿ÿ~òÌ>ø Ì¬¨¨H.~=]ºjÕª~=øàÑEi¯]zÏ=÷ôõõv,**ÇãáÒð5Ó¸qã¢Ýï2ÜNH»x©éK/½³¸|ñÅÙ×ïF-[v³åaQå0Â¢»i^³Æ	ëÖ­»|Mß®X±"º4¨£¡»PlÑE555Ñ`a>|øpt#É·¹uëÖÐvÑÆÖûï¿?ÌÙ¿_Ãô÷ÝwÝÛ	Õ¾?¼liÿ¢P&Mï¹sçÂ·ákXò½½½Ñ+?áêÏ·ÜlùeXT@ù|ùiË/1?*(3gÎoC3EßÎ??|;mÚ´6b»råJâFÂÏôEKúö>Hüp¨¥Äßð5L·µµ]÷vBeo?úè£äeh¼~8o|ãêÏ·hoÚ´)ùBò=ºpáÂhHòfË/Ã¢Ê`E]/_NÙÓÓf2´N¢;;;£øKNbç¼Ô°ýZ*ùaº´´´¨¨(8qbâÒëÞÎ@ÖÏÉ'£HÓákîèèHøðá°i·ßxùeXT@ù°h_·gy&yft®~Gx$ö<wîPÖñãÇ·oßmM$c4 ØzÝ>ðÁ£¼ákb~ÛÆØ>üðÃèÛ>ú(óq¸/F'²	Á|Qto¸èÀ/^Ì *ÖFâÒ(?!QXX¸k×®è¬.Ï>ûlQQQêÑ+V¬Å¢M±ãp£ýü¢Ýã:;;wBpÓ¦M¡¢£nçUIÛgÑÁ¶ÑooooOÌÏp;ÑÑ~~===ÑOf(¿ç.1þää¢];::Âo	+a òZ3´rø±PkÖ¬I¾4Ã¢Ê`äEg9îçñÇÿï×¯k¢0JL'¶&ªH¸ÿþû³2Ù«¯¾¡ü®^û°0¿´´4yCpÛ	¼`ãjúc¯RRRý	ý¶q/_¾<ùWL4)|Nì|ÑdBÔ©K3,* ü²B¨eË]³dÉèÛ~åwàÀè´ysæÌ9tèPâÒGy¤¬¬,ª¥~8%.mmm]¸pa²òòò=öô»ÍÔ%	7æ?ðÀýæt;AX°HÑ3Ï/¡¾¾¾ßé]"çÎ«©©	k`Â	á¯èêêJ¡&ù6/^¼Ò6ZWUUUGé÷3,* üP~(?Êåò@ù üP~Êåò@ù üP~(?nÞÿÞÔ2LXc5IEND®B`


e+Ê¶ÿÑöýßØÿÊË¯¤§§±ÀøÓþ"õòË/«gò3gÀ?àOåóùJJJBØe6½ü×÷½ºOÉ¯¨°Èn·³	ÀøüÉCPþ¯¦þÀbðÏàÀ¿DÆßÈÈÈáÃµöåää466Êµ_øÂ²³³åudÛ¶méþÀ_àoË-êÉ|åÊØ,:ø#ðþHçøØÉ¾:++KË>Ã"<! üPö?ðø;þ¼z2¯_¿>þü?Ò-þúúúÂ²¯¼¼¼¹¹Ùçó±ÁøKü­RÛqíÚµ/:ø#ðþHøóx<v»=ä4^©²²òìÙ³ÚþÀ_ãïèÑ£êù¼uëÖyYtðGàü®ð'O½°ìÛ¼y³Ü«ü¿¸Çãüø#ðþT²7¶Z­ÚÑ[Ä===¬Lðþ¿ä2LàÀ?ãÏï÷·´´hÙU__ïñxXàøónàÀ?#àOÕ¯eìGFFXàÀø#ðGàÏøSöiÙ'sìþÀ?Á_¤AûÍÍÍÆþü?þ?ÇSWW§eÕju:°üøþüíöÅ~Làü?ðGàüÑ×ý×"¼ö©Ñ[Ün7ëüQ"âqþüø3daíKMMö1zø£Æ_ð~añÇ8þüÅQjôÂÂBFoþ&¨­­Mö/¼ðÂèè¨¯7n9×¯_þüé?ÏvÐ¾Ï~ö³°üøÙlDðÙ^ããã2'''g÷,éÜÜÉT\|íÚ5ðGàüÑìeôÿÉ'°À¿pwñ4_þfþ¿êêê7ß|S&?¾uëVðGàüÑlÕ××jÐ¾)ým_XøËÎÎý@mllL.z½Þõë×Ë?Ã6ÍêåA4i±X´øs8·bÛÕ«W;::nûÞ÷¾÷ÿð¬wéÒ¥>øõ0/]¼xqÃÚk¯2x§Êî..vwN§õ ÿÝ]ìèãïúõëaOø¸ûöï9øíé#¿óçÏ÷Å¶7n¸>Òq===ßýîwY:ïÝwß½ÿ>ë!Æ]¹reëÖ­ÚÑ[¾üå/kw§ïÿû¬4=÷oÿöoìîôßåËc¿»süIwïÞ]¶lYZZüW2==½´´ôáÃ3¿ÛàÇ)))ö%ûrØ¦W¤Ñ[jkk#ÞÂa_ûç¡ììlõQBùª=þü?ßïoii	Ë¾W_5úÛàüøl6ÛéÓ§eB¾VWW?à&Ï¾°£·dee544LxTü?tçÎôôtu 6;;ûÜ¹s3¿Û¹ÏÜÜÜ®®.ðGàüÑ>|XË¾¼¼<Ã1ùAûÀø#ð15ÈsðtSÓÇÓEþÀ°/ìè-Â¾#Gx½Þ)Ýøþ"#;;wîð×ÕÕ%ÓàÀ¿äñxêêêÂÚwáÂàAøÁø#ð7k<«	?Ù§ó·	üøûìvÈè-RyyùàüøäY½Û'øß»w¯LkeþüÍV²¬ªªÒÚWSSÓÞÞ>½wûÀø#ð7©d/vç7n?þæÚÑ[222êêê"ÚþÀ¿ÙÄz¹-++Sgû¦¥¥Ìâüøôä'£·jíÛµk×ÀÀÀìþ8ðþüé.ðGàü%H>/ì 999CCCsñCÁø#ðù.~rG äåå?þf×ë;z°¯©©iª£·?ðGào®ð7>>ÎÙ¾þüÍ¤¡¡¡ÆÆFÅÂ¾ÂÂÂS§NÍ)ûÀø#ð¾¼¼¼¤¨-Z´üø#ð7Õzëêê222´£·´¶¶Îü4^ðþüM³÷î%?-ð·=ù?üø#ð7ùÍfÓ²¯¦¦&ìàÀß	õæú/ø#ðþìÍ6lØb¾ØíöyY$ðþüé.ðGàü §Ó©´/++«¾¾~NÇÌ;?uêTSSS¤)øþ"6>>^PP³xñâ×_üø#ð¥³gÏhÙ';·9ýÑñ±páÂ/U~é7ó7é~é÷ÿ÷µÇÁø#ð±%Kð«vaàÀ¿vMMMÚsæòóó><×ìòô³oûmßÿýòoÏ+~ùù/ÿò/Áø#ð7ÙRRRd·år¹sä±(s233Á?ÁìÞåäähÙ×ÜÜ³ó9¾úÕ¯VUU)ù©Ï?ÿ|ee%øþ&:á7d?®üüø£ÅßÀÀ@]]v¬æX²O%òíã¯î«u¿ò+¿þÀ¿É+»°Ý»wû|>¹866¶ÿ~5=ø#ðG	?!Ô¶mÛRSSCØgµZc¿Sýë__½zu0þ¿ó;¿þÀ¿ÉÖÝÝvç®®.ðGà555,ÐûT½½½f³yýsë÷½ºOóýìg?+KþÀ¿)Ôßß_\^TT$sæzÑÁ?ð§ÏZ[[«ªªÂÚçv»õ°²¿ñ¿ñ¿ø¢ÀÏ|æ3N§Sû=àüøÓ]àÀøÓU~¿_öòaÙ7§öM/Ïåäbðþü?ðþüETTØÑ[Ô ñøK?ðGà/ZwîÜQã<«3|³³³Ï;þüáñ§Fo±X,ó2V3øàüÍþÚÚÚû;?5üøqðGà¿¾¾>ÙiGoÉÏÏw8qÍ>ðþüM°ôÎ;üuuu1È3?2*þ<Ýn×Æã±Á?ð7oøS½'A;Ë>]¦M&ø#ðGFÂ<´Â²Ïjµ¶´´àüø ììl5ªÂßøøøÞ½eÚb±?düÉÞF§ÐtÞíàõþæíííay¾qãø#ðGq?¿ßßÒÒ6lþæàÀ%þÔËmYY:Û7--­   #Z?àoNÙ×ÜÜb¾ÔÔÔºº:á·øþtø#ðþæ"5zYYY¯¼òJoool#ðþü?ðþÈàøö½Åb±Äû àüøeüôÑGK.MII½dZZZqqñàà ø#ðGñ?ÇSWW§e_NNÎ#G¼^on#ðþüEÌét=á£§§üø#ã/Ò .ù|	»Àø#ð15ÈsuuµúÏñèèèÖ­[eNnn.ø#ðGºÅì@ªªªÂÚ'ÿ§5Ø àüøMü©ÝeðR¦>?¤7üE´oóæÍ<xÀø#ð7qê¿ñññÀ±±1Þù#ðGzÃ½¥°°P;zÍfKÑ[Àø#ð÷d?óW]]-æ=ª¬¬ä3þH?øóù|aíËÈÈ°Ûí3zøþÌâaß(ÍÑñ_ðGàüMX¤Ñ[ÌfsÞþÀ¿ÙÁ_òDL&ðGàb¿¡¡¡°ìS£·$òi¼àüø×À?ð¶¾¾¾W_5###%%%ÍÍÍ9høþfÆsîïïþ(øs»Ý»víÒ²Ïjµ¶´´0zøþÌÖgþ^íµÛ¶mc¨3üuvvÖÔÔ´/ö;ðGà?Aìa³³³>|(ßzë-µÏ£ú?à/ÚAûD6ö?ðGàoNð÷äéû|j«&6nÜ8×þü%8þZ[[+**´ì³ÛíþÀ¿9ÄtýúõÀÎ·©©)þü%&þÔè-%YYYõõõ°ü?±Àß;c(¨^xüø£ÙÅßÀÀ@ØÑ[dö?ðGà/vø3L²óÍÌÌ¼uëÖ Ïü¥¤¤?4+øö½%??ßápÀ>ðþüÅ²ÿÝ·o_ÈL»ÝÎÙ¾þhæøóx<µµµaÙ×ÜÜÌè-àü±Àß<à/Ò8òþüÑ´ñçr¹l6[ØÑ[N'ìþÀß¼áo¾þ?aö?àOwøqðÝèÁ?>¯¹¹¹°°0ìè-n·õþü?ðþüÂ¾¦¦&íè-©©©Úþü?ðþü###aícôðGàü?ðGàÏ8ø§RØAû°üøàüø3NgíÚµÚó9½üøàüø3Tn·Ûn·kÙWQQûÀ?ðþÀ?ã$ÏV«Õ¤Ñ[À?ðOøø#ðGØ'ûçö?à/ð<Q&	üøKØü~AûÇþ¶/?àoþñ7ï?zf_~~¾vôúúz5hßäÿ¶/?àü?:mddäðáÃaÙ2zøþÀøþâaí:í àüøàü®ñ'/»wïÊx½^Ös ÇS__QFoàÀøàô?ALFFÆ*¿ôÜsÏ-_¾üWõWYÕÂ¾°öY­V§ÓÐ>ðþü?ðþH§øç¥¥¥ýÁ°ÿûÕ?Qà¿øÅD^É²#±oÏJðþü?ðþH§ø;uêÔªU«ò¿¾7###1þ´OÞâv»'?àüøàüNñçp8~ë·~+òÏl6kOb0p~¿ßétjÙ*ìS£·L)ðþü?ðþH§øëììüÌg>³÷ëòþùçeNâ¬ÒHöÞþÀ?à?©¦¦Fô³ý¶üjªk.OfÃ¯I¿ßêÔ©+V°Ïb±ÌàüøàüÞñçóùþøÿ8777--­¬¬¬µµÕØëphhèÈ#999S½ü?þÀÓÀÀ@Ø±+**fàüøàüøÿÜnwmm­½ü?þÀ¿8¨³³³¦¦F;hßænM?ðGàü?ðGà/Öµ¶¶VVVjGoÙµk×íàÀ?ðGàOï;£òòòöedd¼òÊ+±¿ü?àü?s½E;hÙlnllå°ÕàüøàüøÃ¼^ï±cÇòòòBØWXX(ó>_ü?àüoppÐb±?Óndd¤±±Q;h_IIÉÙ³ggqôðþüøiEEEò*þüM#ÇSWWvÐ>ÙÍûÀø#ðþÀ_ÄÖ­[çv»£àïÏþìÏ±íÊ+ßþö·¤ãdÉfJðàp8~û·;999_øÂ:¤%¼téUÇîÝÅïî.ñ÷ãE¿«W¯þOlûà<Ïÿ»wï^WWWÂþúò»?ûì³!ö¥¦¦þÞïýËåÒÏr¾ûî»?úÑx¸ê¹7o~üñÇ¬=×ÛÛûþûï³tÞåËÇÆÆbüC?ûµ··?óÌ3ÚAûjkkåBoKËa_û9ìËaßROþüM&¿ßáÂöeeeÕ××ès±Áø#ðþÀ_4?Ú|>_ss³vÐ¾#GÄrÐ>ðþüøþæ0<þµ§ñæåå	c?høþüÅ"ðG	¿¾¾¾°ì+//öÍïè-àüø#ðþüÍZÇn·Æ+Y­V§ÓGìàÀøàÀ_´ä×Ñ²O.Úl¶Ø?Àø#ðGàüø«änµZCÞêö=Ojàüøàüøûq~¿¿¥¥EË>5zK¼³ü?àü??e_ØÑ[ò×ùè-àüø#ðþüM6ÝáÃµì9ÃHìàÀøàíöÅ×è-àüø#ðþüEËãñÔÕÕiÙ§£·?ðGàÀø#ðíßÑ[Àø#ðGàüøM´UUUÚAûÄn·;q6øþÀødpü´/55Õö?ðGàÀø#ð÷ãÔè-½ü?þÀ%4þ|>ÃáÐÞb±XàüøàüÑð'°khhä%Ôè-àüø#ðþ(áð×ÛÛûÊ+¯hGo©¨¨àüøàüqð×ÓÓ#ìËÈÈa_eeeÞþÀ?àò8´ÙlaíëììäÝ>ðþü?ðþÈ øs:7o1(Pfº¶øþü?2þü~ÿÙ³gËËË´ü?þÀ>ïÔ©SÚÑ[»víêëëcý?ðGàÀø##àOØ×ÔÔ¤½%++«¡¡ö?ðGàÀø#àoddDlf³9ì BÖ9øþü?2þêëëµö­X±AûÀø#ðGàüqð×ÙÙYSSªeß`øþü?2þV«5IìàÀ?ðGÂ_kkkEEvÐ>»Ýîv»Y±àüø#ðþÈøóûýÍÍÍ%%%aÙÇ àüø#ðþÀAð722âp8"ÚûÀøàÀøÁÏç;|ø°ö4ÞW_AûÀ?ðGàü?àOÞ¢«9??ßápx½^V ø#ðþü?ðgüõõõ½úê«aÇjæ4^ðGàüøàÏ øs¹55,ÐÞÒÒÒûÀ?ðGàü?à¯³³Óf³iÙWYYÙÞÞûÀ?ðGàü?àOÚ±E7o4Öø#ðþü?ðgü~SSÓç?ÿyíþÀ?ðþÎ¾ææfí »víbôðGàüøàÏ ù|¾¦¦¦¼¼<í ###¬"ðGàüøàÏ;vL;h_vvö#G´üøþÀø3ûÂÚWRR"ìêêb?àÀøFÈívÛív-û*++/à÷û'ÿ·	üøþÀøÓ;û´ömØ°¡½½=ðmàüøþÀøïzzzÂÚg³Ù´ö?ðGàüøà/¾óx<Áoø¥¦¦ÖÕÕE´ü?àÀøq_MM°/++«¡¡!ú àüøþÀøû:;;92444áw?ðGàüøà/àÀø#ðþÀø#ðGàüøàüø#ðþü?ðþüøþÀøþÀ?àÀ?ðGàÀø#ðGàüø#ðþüøþÀøþÀ?àü?àÀ?ðþÀ?ðGàÀøàÀø#ðþü?ðþüøþÀøþü?àü?þÀ?ðþÀ?ðGàÀø#ðGàüø#ðþüøþü?þÀ?ðþÀ?ðGàÀøàÀø#ðGàü?ðGàüø#ðþÀø#ðþü?àü?þÀ?ðþÀ?àÀøàÀ?ðGàü?ðGàüø#ðþüøþü?þÀ?àÀ?ðGàü?ðGàüø#ðþÀßOëêê*--5LË/×>¾Á?ðGàü±À¡ð·lÙ²7nÈÄ3g´ø»zõêXl§Çã#×ÛÛ+ÿm`=è<Á×ëe=è9ùOÔÇÌzÐs|òÉûï¿ÏzÐy¿ýèG1þ¡q¿àÒÒÒ´øûó?ÿó«±í½÷Þûö·¿t[Û+WX:ïÒ¥K¬ÇîÝÅïîÎøëîî®­­å°/qØÃ¾Äa_â°/|ØW5::Z]]íõzÁ?ðGàÀø3þ~ºØßßo·ÛååàÀø#ðþXà/îñ{ûÚµkÃ^þü?àõþ?Åø#ðþüøFÆ_ôÀ?ðGàü±Àøàü?àÀøàÀ?ðGàü?ðGàÀø#ðþÀø#ðþÀø#ðþÀø#ðþüøþü?þÀ?àÀøàÀ?ðGàü?ðGàüø#ðþÀø#ðþüøàüøþÀø¿?ýÓ?miiémï½÷Þûï¿ßK:î>¸téëAçýÍßüÍÝ»wYzNvwÝÝÝ¬='òX°tÞÉ'?ùäÿÐcâïÎ;û÷ïÿ&ýÐhïÇ%Nàüø#""""ðGDDDDàÀ?vçÎ+WL¦âââãóùìvJJJ^^^;«HÛH¶KQQQðÒImmmII?ÞíÜ¼y377Wm¦k×®±rt¸ºººJKKe-_¾¿$¡Ïmiéj3éM<V&H^.^¼(7nÜ0Í2qðàÁ7ÞxãñãÇ²ý.]Ê*Òá6¯÷îÝ	ùj±XXE:Éï÷$Ãêêê7ß|S&?¾uëVÖ·Ñ²eËäi%gÎ)((`ýèpCzÛLzÉÖÚÚ*ÿ÷UÔèééaèyÉS«¿¿_&ä+@×O¯¿þú·¾õ­ÀÞP®þ¼ïøø8F×ç6.--õ£Ïme«N6ÞäÀceR~ÏÌÌM(ÿ÷&éèÑ£²UÜ¾õ£ÃmtóæMÙ@rQ¾vww³~ôÐ½÷ÊÊÊD½¡<×O~¶Q yÕÖÖ²t¸¢l5ÒÕîNWrà±2ÙÚÚÚ²³³e"99ùäÉ2ñáÊÖeÍèp­RýK¸jÕ*ÖÚ´iÓõë×ÿ§ó½¡<×¦¤¤°t¸T£££ÕÕÕ^¯U¤Ãmi«Þvwº)¤ÞP¼àí=o#ÞRÒaI?z*?yzØ7øiEúÙFO~vÂn·?xðõ£Ïmv«wwºzUâ2AË-»sçÎ§g½­]»V&vìØñöÛo?yziEE«HÛhÕªUjËåZ¹r%«Ho»E5a³ÙN>-òµºº5£ÃmÔÞÞ.Ï©ÁÁAÖn·Q9¤Í¤79ðX 7oÓ×¬Y£þï;<<¼iÓ&SVVæv»YE:ÜFôüE¾Ê4«HÃäääÜÜ;kFÛÈb±ð®ø£o&½ÉÇQ"©U@DDDþüø#""""ðGDDDDàÀ?""""DDDDþüø#"""DDDDþüø#""""ðGDDDDàÀ%xo½õÖêÕ«Ó¶fÍóçÏÿÌþëiq³··´E~5¯×2_æL¦ÜÜÜÇOõ>ÀÅeû÷ïOÒôÚk¯	2³©©)dþ±cÇdþ¦qDDàâ¯7okL&Ó'üO;yò¤·nÝ2þîÞ½+3/_2¿¨¨Hæ<ðGDà¢^xAXóÆoÏ<tèÌÜ¶m[0®]»&xÊtà=zT[[)WÍæÝ»w]u:,¹JnÛÚÚÂ)³hÑ¢Õ«W_¹rE.Úl¶»|ùrôûQWÉ"©«ÚÚÚ"AmíÚµ2¿««+0çÆ2Çjµæ<x0''Gî*--mË-ýýýZüiï?dNE%"ðGD4Ï-Y²DàrïÞ½à÷ïß%7!uww«k7nÜrÕÎ;ÕU.+999ì­ÔEuí¦M?~,|LIIñûýr­|<¥§§«âE¹»xÚßôÌ3Á¢øòÍ7ßÈ/äNÖ¬Y3UüEYT"DDó:Âf$ÆÍÖ­[G&rqýúõêZeÅGõ M]U]]­Þ2éu'Á÷¹oß>á:êúÒK/É/Ê´|é_|qÂû8ÊEùàeû	(/^,¿ïàà ¯²ä"Îññqõ¹¹¹rCùüä0qZZÚTñeQüéRXüæ+Ü(3IýýýrQØ¤.ÊÅ¥Knß¾]Ð666¸ù÷Ò·Rïß¿øfSàÈ¯|i§Ó9áý4åâÃ-Òçó^~ùe¹ê[ßúÖÚÞ½wð7BÒ²²2õÆäTñeQüÍêí®ÑÑÑà^¯WfÊUQ¸ ¡ÛíVþX'ðA=íÑØN°"Óf³9%%ÅçóÉ/Z´(pí÷b!Ý¹sG9U¦å«L»Àµ²aOQÀÑü§>÷vôèÑàj>Ô¾¡uëÖ­u6 Fõ¶¸êDÛ¹s§:Ú+_kkkó£Üz§íÁêâÃ£»jÕ*¹Vn#f¾Jù+WµµµGÇ_¦jm®²¨Dþæ?uÂÉd:~ü¸êåÄ)))Ú3Ö¯_/Ðñù|êlàÌ?õQ9·ÛüaAeÁÝ»wÔy¸ÁVÂM~«~z`~ûQòSùóz½ê;£àïäÉ÷ääW¾JüÑårÉO	Âeù6Aç-[¯²¨Dþt9¤þtÿõ4e£Àtàià@/½ôR°,;wîü=yú§8d¾Ùl>"å~d1,p¦m¤_vll,--Mý!»×­[ü#/^,_Õh/Á÷©Þ¤¨¸6Ê¢ø#"ÒK5kÖ¤<mõêÕêÛüµµµ©áô¯_¿¸ÖëõîÝ»7''Géå_öù|k[[[ËÊÊÄd¹¹¹#ä>µK"w%ówìØ2?ÒýH²0²HjÂ(ãüª­­óE588X]]-k 33S~Ç¶&ø>E·j]Y­Ö®®®eQüø#""""ðGDDDDàÀ?""""DDDDþüø#""""ðGDDDþüø#""""ðGDDDDàÀÅªÿ51íiñ;IEND®B`


ûöþËÏÏU^¢A­Õ!Ä_°ÿþéÓ§¿:,?Ú±àND®½ººúØ±c·çµ©©iÑ¢EaiájhhÏïêêzæg¢·TCÁ»²»»ÛÄÀpéééyì±ÇõÝrC¸Èío÷ööFM?gÎ@üd¡è3I^xákY¨««kÓ¦MS§NöígÖjÄâñ þ?Äâñø@ü þ?ñ0dûöí«¨¨ÈÏÏ_²dÉ`/;wîÜqãÆµµµÅçé0gþüùØÝ2´ßÈe¸í÷¼¼¼¢¢¢+W®Dguuu­_¿¾¤¤$¬Ì'³>üðC0@ü£KYYYHP0½½½½ìsÏ=.»cÇø¯ýëaÎ¶mÛ²;þ¢éðé5kÖD'c±X8yèÐ¡0øðá0=sæL0@ü£lkrýtêÔ©pÙªªªøï½7Ì9yòdæS,ó×r9LE'ÂÉk×®yPâÕå4Ï<óÌÄ'L°uëÖ¤_ðÁ-Z´øaúòåËa:ÌÎúè£ª««Cåçç/X° zK,ui×ÞÿE^õÕÊÊÊpE©)výúõÚÚÚpÙpã7mÚ½òô¾ûîKóÃÏ??|ñ7mÚ´prÎ9GñÐÄ0ªû/þÚ×¾¦Bf^x!ñwâoâ'ÂY;wîÓág^»vmtÖ=÷ÜsàÀ0qîÜ¹0¿¼¼<íÒo@ÿ	I÷ÆoX,zã×¯_¦Ã/¼õÖ[aâ¹çÙµk×Z[[ÃDôÆdª!ÄßÍ7Ãú	Ó>úhtÖ¾ûâdÉûï¿ïÑ?`TÇ_EEEî¹%µ½Ò~(°¥¥%µ|ùò0½råÊ¨êâç:ujË--FH¤]ZRfõèM81õ²¥¥¥ÑY&Böá7ÃôBÎvwwmµ¤În[Xx(¿Äý¼Çýÿp¥`øFoüåççÇ§ÃD8Ù	rë°	&ßìêêö®9ÑY¯¼òJ¸T(Â÷ß?q	IKK<9ôuÃB%¾ã=&MÍ)))Þ¼+ïüõãÐ¡C!7÷?`4Æ_yyyâ;Ñg·-ÚÚÚpnüg|~4âæ-¿þ/¿a¡çR/;eÊè²I7¯··÷ÀÑPÜø[Z-¿ ºáÏñÄ0zã/úê;wFùûú×¿>âÙ³gOü-´7Þx#>?JÉ3gÎDHüõ-[¶¼ùæaâ'H½ìã?¦_íµ³gÏF»zÃÌÊÊÊ0ýþûï_¼x1LÌ5køâoþüùá¬éýû÷éÇÌÀè¿ÞÞÞXnIü®¾þãïÆÑ.×ð3ñp!JJJ&N¸iÓ¦Æ_ÿinnçÞï½ñq'¿ÓÕÕµqãÆpËxàh`oh¾høpUUÕ|0¨Õºk¸U®±¶¶6Üø°¦L²~ýúp<Àñø@ü þÄâñ7b¾ÿýïÿô§?Íðþìg?û×ýWÿüç2j;wnP#]»víâÅÖ¿û»¿wÍýÑþËð¾ÿþûÿôOÿä¡Lð/ÿò/G±üÕ_ýÿ	ÿ-üÑ~d=Ù¿ÿ¿ÿû¿?ñøCü!þâOü!þ?Äø?Äâñ'þÄâñøCü?ñøCü!þâOü!þ?Äø?Äâñ'þÄâñøCü?ñøCü!þâñâñø?Äâñ'þÄâñøCü?ñøCü!þâOü!þ?Äø?ÄâññwìØ±äççÏ?ÿäÉâñøCü!þ²9þfÎùÞï×_Ö¬Y©ñwðàÁëuâÄ¿ÿû¿¿×¯?>¼Þ[DZZZ~ö³Ymmm?üá­"!þÂÿ3|¥c8þ¥Æß/¾ø½Ìjjjjnnþ|ï:pàõ@äÛßþvxHXßýîwÃõ@äí·ßÎüfCü?~|íÚµvûb·/vûb·/vûfónßÈõë×kjjºººÄâñøCüeyü]¸p!]¼x1õ,ñøCü!þY---K.½råJÚsÅâñøCüeUüUTTK þ?Äâ/ã¯âñøCü!þÄøCü!þ?ñ'þ?ÄâOü?ÄâñøâñøCü!þÄøCü!þ?ñøñøCü?ÄâñøâñøCü!þÄøCü!þ?ñ'þ?ÄâOü?ÄâñøâñøCü!þÄøCü!þ?ñ'þ?ÄâOü?ÄâñøâñøCü!þÄâÄâñ'þ?Ä?ñøCü!þâOü!þ?Äø?Äâñ'þÄâñøCü?ñøCü!þâOü!þ?Äø?Äâñ'þÄâñøCü?ñøCü!þâñ'þ?ÄøCü!þâñ'þ?ÄâOü?ÄâñøâñøCü!þÄøCü!þ?ñ'þ?ÄâOü?ÄâñøâñøCü!þÄøCü!þ?ñ'þ?ÄâOüy(#þ?ÄøCü!þ@ü!þÄâñøCü?ñøCü!þâOü!þ?Äø?Äâñ'þÄâñøCü?ñøCü!þ¸SW¯^âOü!þ¿,wöìÙX,6~üø6?ñ'þ?Ä_Ö:zôè+Bö»¥°°°³³Sü?ñøCü!þ²MSSSeeå¸_*°¹¹Yü?ñøCü!þ²Dww÷®]»fÌ7oîèè¸Ãå?ñøCü!þ:;;þù¤ì+))©««»óìâñøCü1*twwì<yrRöÕ××wuuÝÅëâñøCü1b.]ºTWWWRR/Þµk×ÝÍ>ñ'þ?Ä#¦­­míÚµÅÅÅIÙ·lÙ²aÍ	ñ'þ?ÄuôèÑ~8þí-Ë>ñ'þ?ÄÕÔÔT]]Ô|ÅÅÅ7onooÏÌmâñøCü1¼zgÏ6l¸[ÃxÅøCü!þÄâouvvîØ±#õÛ[ÂíÛ·ßá±:ÄøCü?Äâo´hoo¯««KýöÙ³gßõooâñâñ7bÚÚÚb±XêxêêêæææÞÞÞ½yâOü!þ?î©ã9¢ì;zôè(¹âOü!þ?îHooïîÝ»S³oüøñ±XììÙ³£êÖ?ñøCü!þ¢îîîÆÆÆ3f$eßäÉCdx¯ø?ÄøCüÎÎÎðº:#àHãâñø¿»¯½½Ýºui³¯±±qÄÇs?ñøCü!þÄßÝ1Êñ?ñøCü?ÄßÝvo¨ÀÚÚÚÌ¿ô?ñ'þ?Äß°ËVYY±X,cGãâOü!þ¿áÕÿ0ÞÑ<Cü?Äâñ'þaìãâñø¿AèkïìÙ³ÇÄ0^ñ'þ?ñø'Nô5wÌçâñøCü¿>µ´´dÍ0^ñ'þ?ñøK¯··÷[ßúVjöéa¼âOü!þâñ¬«««¡¡aöìÙÙ7Wü+W®TTT?Äâñ7¶zuÇeee©ÃxCæBö¿¡¯¬sæÌ	ñøCü!þÆööö§~º°°0)ûæÍ²/kñ¿añÀ´µµõo¾ùæ¹Ì¹yúôéspîÜüãwß×z òÝï~÷oÿöo­S§Nÿææßþñ>ø`^^^RöýÖoýÖ®]»~òäà:yûí·ÿáþ!ÃW:ãï7±ïøõÕWÿ&³ZZZ;ö7ð7óÃþ0¼Þ[D<^ò­£G~ïßË©?ù¯ÿú¯wìØñùÏ~%K¼ùæ¹üxØ¿æ¯4ãÏn_ìöÅn_ìöA½½½÷îMÏã«ÂãÁn_ñøCü!þ²Aww÷K/½eeeuuu.]òHâñøCüe¾Ær°««Ëc@ü?ÄâñÚÚÚ~úéâââÔa¼ÝÝÝîñ7¼ÄâñøËÓ§O¯^½:õh¼UUUÍÍÍ¹öí-âOü!þ@üµñ^jCá¥=ï'dø?Äâ/â/:oeeeê0Þ6d÷ÑxÅø?Ä9]]]õõõÓ¦MK4Þw±ø?dCüuvv¦ÆB0GãÙÔÔÔÜÜ|õêUñ'þÄâñø»ûÚÛÛc±XêÑx.wïÞL~°ï+_ùÊ)SîýÍçÏ_\üæo?ñ'þ?Äß]^IkkkSñVWW·´´døÆüéþiyyùÿÛøÿ¶~ekø÷¿bÿ+à'Äø?Äâï.d_(¼´ÃxO>="7iæÌ¿ÿèïGåýûBõÖ®]+þÄøCü!þCÔÛÛÛØØzX¶É'¯[·ndñ'_ø÷?kÿg(Tñ'þÄâñø´ÎÎÎíÛ·Ï1#í0ÞçèÇÌ3¿ôØãï,ýÞùâñøCüN?GãUeó?ñ'þ?Äßñ¦ç¨¬¬Ù7Ïñ¯|å×~í×~ó7sÞ¼yFû?ñøCü!þªa¼õLMMM¾çOü?Äâñw½½½ÍÍÍiñÆb±¶¶6wøâñø#â/Æv<G]]£ñ¿»ãn'//Oü!þ¿á6úñ¿,¿¼ÛÉÏÏ?Äâoøa¼â/KâoÄ?Äâ¿¾ñVWWïÞ½ãÙ×®]â'Äâñø»»Âkß²eËÆâ0^ñUñWQQï3?Ä_üÆ+þFKüÍ??u´GIIIWWøCü!þw¨··w×®]/6Wüø+((Á+W®Ð|o¾ùfîñ?Äâì¿ðúüóÏ§ã­¨¨0WüdüEÄ0j/L;wîæÍabÂ	âñøCüÁ¥K6oÞ½«:×xñ7Âñ7iÒ¤ðp¯mmmaâÙg&|Õâñø¬ðÅ²¯ªªjïÞ½²OüøÛ´iS|xGÒA£Åâñø 'N¬^½:õcô+V¬hnn¶bÅß(¿à«_ýê)SÂÄ±cÇÂDÁE÷M?ÄY½½½¡*++¯°°Ð0^ñ7zãoD?Äâ1ÝÝÝ»víJÏ²oÃV¦øâñø#â/d_EEEêxK.Yâo´Çß¬Y³¢/|ñ%Ï?Äâ¯øËV\l¯øÃñ7sæÌÄà3ÚñøCüÅµ¶¶ÖÖÖ¦mÅñ¿1¡óÂc÷øñã7oÞÌäM?Äc"þ=úðÃ§Í¾Ó§O[câoìÅ_IIIxg¸üÄâñÇè¿©Gã-,,zµìc8þN<Êë×¯¿~ýºøCü!þá1íxÉ';,øËø¦Nú¥| þävO=õT<ÙG6ÇßôéÓø@ü!þÄ_koo¯««KÍ¾îÝ»·»»Û*ÙÑ»µµ5Ã7]ü!þÑÑxSÇsDÙg¯øËÂø+--5àñø9(¼¥çÈËËûÝßýÝÖÖVÙGÖÆ_KKKx¬oÚ´)Ãïi?Äâªn÷îÝ©Ù7~üøX,¶:¶/âoìÅß¸>ðøCüeº»»SÆçÆ;ðcû"þÆjüåõÁÄâ¬ÑÙÙ^wRÇsÜ¾â0^ñGöÇßH?ÄÐÞÞ¾nÝº´Ùöh¼âì¿éÓ§=Vü!þd¾ñVWW777÷5Cüýñ	¿éâñøc¤Æ*°¶¶ö¶/=âì¿C§Ä¶mÛ®]»É/|?ÄwWoooccceeeêÑxc±Xû@"þÈþø3Úñâo¬ëï Ë&þÈþø3Úñâoìø0^ñøaâñøãNô5wöìÙiñ?ÄøCü?ÄßtâÄ¾ñÞù+ø#'â¯§§ç(**Ï	&¬Zµ*#?ÄâñÇ`µ´´y¯øCüýÂ7ÒøîCý?ÄâA½/^¼øNñ?Äß/Ì3'</_~ýúõpòÚµk+W®sî¹çñøCü1²¢a¼Ó¦M»óa¼âñ÷áYøÁØ0'Ì?Ä#åêÕ«ãmhhìäJüåååçR¾Äÿf9¾êñøcD´µµ=ýôÓ%%%IÙ7oÞ¼]»vy¯øCüýB´ÛwéÒ¥Ñnßð3L9,?ÄtúôéÕ«W&eßâÅ÷îÝ;ÜÙ'þÈøµvÀÇÇ,þ?¦««+äÚ;ÂÏì¢mnn®ªªJ1Z¶lY_,ÄÙÜð»jÕª'æååK.sû¦?Äâ/[µµµý©ÿeÞ¼yÕ¿]ý_çü×²²²PTi³»»û[ßúVê0ÞÉ'oÞ¼ùìÙ³¿ñâ¿!þ¿¬ÔÛÛû¹ÏnÙË¶~ekôï·üö¢EvÚìÛ±cGê0ÞaÆ+þâñ'þwßéÓ§cæoÄË/üÛ²yKiiiGGGôaâé§.++KÆ[__ßÕÕ5²·_üµñ7îvòòòÄâñÇ^,ëÞßJ¿ðoÖ¬Y'NhmmMX¶yóæK÷ÁÄ¹y?ÄCvöìÙ²²²-·ÄËïé§.,,¬­­MÍ¾ûï¿¿©©)3ÃxÅ¹yê©§¢'äë¯¿.þ? $ÝÂ7=½)ßªU«¢cÇ'÷áÎü«øCüý'ON0!<'«««¿óYü!þJWW×êÕ«ÃkÊ§>õ©Ô£ñ®[·®¯Á¿âñ¹øè¡¢§å2sÓÅâñ­åW__?cÆÔa¼7o¾téÒ(¿ýâì¿·Þz+zZ._¾<7]ü!þY¦³³3íÑxËÊÊFÃ0^ñøûïv¾ç¢±ÊðM?Ä_ÖhooÅbiË¶÷îQ2Wüëñ÷â/FÏÌ5kÖÈM?Ä_[ò´Ãx-[6Úñ?r=þ|ÏâOü!þî0ûª««Sñ®^½úÄc÷ïdmüåÝN~~¾øCü!þHÒÛÛÛØØ8öìÔ£ñ®[·®½½¬ÿâ¬¿'þ¿±¥³³sûöí©ÃxCöìÑxÅâOü?ÄâïnêèèH;7`ccãØÏ!þâñâ¯OÑ0ÞÔñ!ûÆâxñø?iô5·ººztMü!þÄøCü!þ­···¹¹9í0ÞX,ÖÖÖõk@ü!þÄâñ+Ù×ØØv<G]]]ãäzüù?ÄøCüErd¯ø#×ã/ñ+ýÒÆïùCü!þ²^NãäzüÅ<x0<Ïyäë×¯áçÊ+ÃÃ?Äâ/[õ5·ººz÷îÝY9Wü!þ~¡¤¤$<Ûç===aNYYÙ.ùäÉåååùùùsçÎ=tèøCü!þF°í]¶lYã¿_.â|Iñwçù«©©yíµ×ÂÄË/¿¼fÍñøCü¬¦¦¦ªªªÆ+þ¿PZZü!ÔnÜ¸Nvuu-_¾<Ì	óïpÉ%%%7oÞj²¢¢"5þNeÖ»ï¾^ïOÁ©SÇçw¬"¡~ðdñ£ý¹ç6mZRöM8ñK_úÒ¾û<ÿûß?tèõ@äí·ßÎüü>|8í÷ßÿ8d$uøH¿·Þz«#³Þï½ÖÖÖèèhkkûË¿üKëHøá~×|PWW÷ë¿þëI[ø0gÓ¦MáªýèGGµìß¿ÿüùó¾Òa¿àÜ¹s3gÎ,**ÊËË0aÂ._¾|çMÜq`·/vûb·oÆt)d_ê0ÞyóæeëaÙìöÅnßWZZ0üLÝ,þ¿ápöìÙµk×&eßý÷ßßÜÜ,ûÄâoÕÖÖ¾úê«a"ü¬©©?Äß°:zôèÃ?voÈ>÷²øCüý§3gÎÌ5kÂ	ÑÚÒÒÒ=öÜùbÃ+kYYYXfyyù±cÇÄâñ7zwïÞ:·°°píÚµñ?Ä_²èKéM¿üòËÃzÓÅâñwº»»RË²¯®®îêÕ«îYñøK£¬¬,l)Î9¿cÇE_ þ¿Ñ)ÝóÏ?K¢°IóeøCüõ»[¢(þnÞ¼éØ¾?Äß¨MÍ7'eßÂsöh¼âñ78Ñ<GïöøëééyægÂtê×2?Äâoµ¶¶>úè£©ã9*++ÃæÔ0^ñø¨´_òüÞï?Äâo4[Ëûï¿?5ûV¬Xáh¼âñ7/^hQ4Ú·¨¨hÖ¬YíííÃÓÅâñ×¿ÞÞÞðÂ³xñâ¤æ+..Þ¼ys6Ôâ|ÏøCü!þ2©GãÙ·aÃÛý	ñøëw¿çwùòåY³føCü!þ2¬³³óùçOÍ¾íÛ·sÝYâñw÷ã¯§§Çh_Äâ/ÃÚÛÛÓwöìÙõõõ]]]î&ñø»£øKýoeI&?Äâ/ÚÚÚb±X_e3Wü!þîNüôÑGy·Äí(ß[o½%þ¿a6¡ðRÿûf=zÔý"þw3þâBê÷^ñøCü%ÆãÇÅbgÏu?Äß0ÆßH?r0þº»»SÆ;yòä°UÖa¼aáßúÖ·^zé%»Å¹===³fÍ0aB|Î)S¾öµ¯?Äâï.êììÛ½Ôñ!30wïÞ½&Mª¬¬üBõ***¾ð/8ø#wãoêÔ©I~£íÑ¶mÛÄâñwçÚÛÛ×­[6û3ð&[[(¿/=ö¥­_ÙþmÙ¼eÞ¼y6mr¿?r4þÂ6¨µµ5>çäÉaÎÄÅâñwÕ5ñîØ±ã¿Wý÷¨ü¢Oüï'>ûÙÏºßÅ9Ñß7oþÊrS¾üOü!þvo¨ÀÚÚÚÌoú6lØ°lÙ²Äøÿ>õ©O¹ßÅ9åååa´qãÆèó7nÜØºukSVV&þ¿AËVYY±Xl¤Æ[__ÿùÏ>±ü~ï÷~ï7~ã7Üïâ¿ãÇ§ýçcÇ?ÄâoúÆ;²e×^QQQýÛÕÞÊï÷ýýpr÷îÝîwñGÆ_páÂ¹sçåååM0aÎ9aÎpßtñø#;âodñÐ¥K¾øÅ/N4©´´ô3ùLèTwºø#§ãoD?Äc=þúÆ;öìÌã¬p®^½êî?ñøCü.þN8Ñ×0ÞÌoÙ¿A;sæLô=ÏÑßÒÒÒ=ö?Äâ/Õ¨ÆøCüÅÁãÛ¯(þ¢é_~Yü!þhïìÙ³GÕ0^Äâo(ÊÊÊÂöëÌ3ñø;vì/yFü!þ"]]]Û·oÃx¿!-âO¾ØùæÍa:??_ü!þÈåø»téRÈ¾Ôa¼²Oü!þÆjüFßêÅ_OOÏ3Ï<¦ÃöNü!þÈÍø;öìæÍ²¯²²r×®]£p/âñ7---i¿äù½÷Þ?r-þZ[[~øáÔa¼UUUÆs?Ä_Ä_pñâÅEE£fÍ/?Ä£*þöìÙSYY+V¬8qâU$þY#Bü!þz¦N:wÃ¡¬"ñøâñG6èìì|þùçSñùVø¬¿?üpúôéa«WTT4wîÜ+W®?ÄYüêöh¼a~WWU$þ¬²9þÓø8ö¬øCüeZ[[ÓmÖ¬Yögf/â¿èKkjj¢ÿì^¿~Í5aNyy¹øCü5Ò-~4ÞÁÛñøÃñmþÿ¿¦ã_ø,þu÷îMý`ßøñãc±X|øCüCñ½ó×ÓÓsãÆïü!þëº»»ëëë.a¼âñGÅ_ô¿Ð|áäÇå3?Æ®K.-LêaÙÂÿu·mÛv¯øCüCñ7îviÿ¯øCüq×µµµÅb±ÂÂÂ¤íØ´iÓvìØÑÏ0^ñø#â/ïvòóóÅâQ®¯ñ3fÌhll¼í0^ñø#âo¤?Äw.T]h»ÊÊÊÔì[±bÅÑ£G»»»²ñø#â¯¯ïs¾páøCü1jªÙwÛa¼$þäPümåW¿úÕ¤>ú¨¯zAü1:uvv¦=>G³nÝºööö!,Sü!þÈ¡ø6¥¥¥/_'¿ùÍoFÑaú¨øCü1d!ìBÞ¥Í¾°I	Q8ä%?Ä9Üz/ÚG+W®î.þÜ'Ò-Ï1Àö?Äâï?>|8¾1­¯¯ÏÀM?¢¯a¼Æ+þ¿4|òÉø7 F<òøCü1úÆ÷î^øCüCñ6¦'N<uêÔ'	ù+((?2¯³³³¡¡¡¯a¼mmmÃq¥âñGÅ_Ø¤nÙ²%ifØÂíø#óÙ××0Þººº¡ã?Ä_²¾¾çïâÅâñGfß0^ñøCüâñÇ'Ã?Wü!þ¿¼ä¸q;vû?)þÜuã3wïÞ·ñ?ÄâOü?ÄßHñÎ=;3ÃxÅâñ'þÄâodÈ0^ñøCü?ñøì©a¼âñøâñ9!ìb±XaaaRöUTTdf¯øCü!þÄøCüeBÿÃx3<Cü!þâñ'þK?GãÝ»wïhË>ñø#â¯âñÇ ô5wüøñ+V¬Ï÷ÑâñG®Ä_Þíäçç?ÄÑÙÙ¹ûö´Ãx×­[wöìÙÑÿ'?ÄÙ#Nü!þ²#ûÒã-))©««»zõêXùCÄâñ'þô§a¼/½ôÒèÆ+þ?ñ'þw$<skkkSñ.^¼xã?Äø(þº»»wíÚµnÝº?ø?8qâ5öIßÃx«ªªFí0^ñøCü?ñÇíã¯³³sáÂþ¹/>øÅ/|áþô§_xá]Wýã]½zõÑ£GÇtö?ÄâOü!þ>ÅbóçÏß²yËÖ¯lÿÖýu¡ÿ2ÿqã-,,Ü°aÃÆ+þ?ñ'þ¸üæ3ù¿Oþß¨ü¢ÕÕÕ_þòsgýtttôs4ÞK.eÙß+þ?ñGNÇß§?ýéøÛ~Ñ¿eËmØ°!ÖL[[[ÚÃ²M6m,ã?ÄøÜ>þ,Yj/1þfÌ±k×®ì^'ííí>úhê··,^¼x÷îÝYðÁ>ñøCü?Ä_z­­­ÅÅÅ_|ð[6oÙ°~Ã[ôßî¹çîîîl]§O^±bEê»÷ßKKKvgøCü!þÄâï?=z4_QQQiié¾ô¥lÝÝÙÔÔTUUúí-!ûBæÎãAü!þâ¿ìÖÝÝÝÐÐöÛ[b±X®­ñøCü?Ä_Öfß®]»***RñgeGGGn>Äâñ'þÙæÒ¥Kuuu%%%IÙ7cÆíÛ·gë~mñøCü?ñGÎÅßéÓ§×®][\c÷h¼âñøâñ¬¹¹9íÑxÃÌpì?ÄøCüesö?¾¶¶6S'þ?ñøËÎøÆsÌ7/õh¼¹9Wü!þuåÊñøË«W¯§Uêxhoç?Ä_&WÖ9sæ×ñøV­­­ç0Wü!þóÀ´µµõüÇÜYxçw¡¹ùàÁßþö·Çú_ÑÐÐð;¿ó;yyyIÙ÷ÙÏ~öË_þ²GûÀCxHXMMMßùÎw¬"o¿ývæ¯tÇß/nbßñ÷î»ïþ<³~øÃ¶··ÿ~þó/>|xìÞþðôYºtiêÑx,YùgVhiiùçþgëà'?ùÉ©S§¬"û÷ï¿qãF¯4ãÏn_ìö¬ÞÞÞÆÆÆÔññÚíÝ¾Øí;b©¿»«³³3<kRËf¯øCü!þF]?ÄßaWWW7yòdÃxÅâñ'þÄÙ!ûb±XêûfÌÑÐÐ ûÄâñ7?Ä_?Â³cÙ²e©Ù7ölGã?ÄødIüªÛ½w_Gã5Cü!þâOü%ñ×ÝÝÝØØ8cÆÔa¼±XìÄî2ñøCü?ñG6Ä_47u<a¼âñøâ¬?ÃxÅâñ'þÄ9mmmñ?ÄâOü?²?þÂÃ>íxÊÊJÃxÅâñ'þÄYñ?ÄâOü?r"þã?ÄøäDüõ57ÌY·na¼âñøâ,¿v!ïã?Äødyü=öÑG5Wü!þâOüåñwúôéÕ«WÆ+þ?ñ'þÈòøkii©®®N·Ï0^ñøCü?ñGöÄ_ooï®]»fÏvo[[,þ?ñ'þÈøëêêª¯¯OýöââbGã?ÄødOüuttlÞ¼9uoIIÉöíÛç?ÄødIüµ··¯]»¶¸¸Ø0^ñøCü?ñG6ÇßÑ£GW¯^v<GØ(Æ+þ?ñ'þÈøÑªªªÔooY¶lYKKu(þ?ñ'þÈøëíímllp¡a¼âñøâl¿èh¼Ó¦MKÆ[WWg¯øCü!þÄø#Kâ/lîãÖâñ'þÈþøëëh¼³gÏ~é¥º»»­+ñøCü?ñG6Ä_Ú£ñ.Ð0^ñøñ'þÈ¡êöîÝ»xñâÔì[±bÅÑ£GeøCüY?ñG6èììlhhH=,Ûøñã×®]ÛÚÚj??ñGd_xÈ¥ç(..Þ°aa¼?ÄâOü%BØÅb±ÂÂÂ¤ì+--òÉ'ãEü!þâ,qâÄ©Ãx££ñ?~PÇöEü!þâOü1JVuuuÚa¼»wïÆsüØ¾?ÄâOü?F£è°l³gÏNÍ¾ÐIÃxÅâñøUÛ·oOÆ[XXX[[v<øCü!þâ±§»»»¾¾¾¬¬,)û&OüôÓOwttôuAñøCü!þÄcÉ¥KÒ·¢¢b Gã?ÄâOü16´¶¶®[·®¸¸8u<GCCCWW×@"þ?ÄøcdßÃ?úí-ÕÕÕ=¯øCü!þâÑ«©©©²²25û¢£ñaâñøCü?FîîîÅ§ãÅba=ä%?Äâñ'þE®^½Z___QQ%%%uuuw~X6ñøCü!þÄ£ÅöíÛSÇsL6mÇÏ!þ?ÄøcFbö-^¼¸±±qPã9ÄâñøâoÌèîî¾ÀoÅÆ+þ?ÄøcÏîÝ»O>=|Ë?ÄâOüCÄâñø?ÄâOü!þÄâñøCü?ñøCü!þâOü!þ?Äø?Äâñ'þÄâñøCü?ñøCü!þâOü!þ?Äø?Äâñ'þÄâñøCü?ñøCü!þâñø³?ñøCü!þâOü!þ?Äø?Äâñ'þÄâñøCü?ñøCü!þâOü!þ?Äø?Äâñ'þÄâñøCü?ñøCü!þâOü!þ?ÄøCü!þÄâñ'þ?ÄâOü?ÄâñøâñøCü!þÄøCü!þ?ñ'þ?ÄâOü?ÄâñøâñøCü!þÄøCü!þ?ñ'þ?ÄâoD;vlÁùùùóçÏ?yò¤øCü!þ¿l¿3g¾÷Þaâõ×_5kVjü½ûî»72+4hû¸qãg?ûÙáÃ­"---/_¶>üðÃS§NYDBüýÛ¿ý[¯tÇ_¢¢¢¢Ôøû?ùw3ë;ßùÎ;ï¼ó.¼ûîw¿ûÝoûÛÖð`	ë ¼LëÈÛo¿ù+Íø;~üøÚµkíöÅn_ìöÅn_ìöÍæÝ¾ë×¯×ÔÔtuu?ÄâñøË¶ø÷KÑÉ.Äb±/¦þ¦øCü!þ¿1ZZZ.]zåÊ´ç?ÄâñøËªø«¨¨@ü!þ?Ä_6Ç_ÿÄâñøCü?ñøCü!þâOü!þ?Äø?Äâñ'þÄâñøCü?ñøCü!þâñâñø?Äâñ'þÄâñøCü?ñøCü!þâOü!þ?Äø?Äâñkñ÷ø»wïþifç;ßùÁ~ðSøéO[[[÷ìÙc=ùó?ÿóÿøÇÖÁÑ£GßyçëÈÎ;ÿñÿ1ÃWÚÙÙñwæÌ­[·þ	úß5:Îû±¹Cü?Äâñø@üj7îëêâÅãX9¹#õ®?yòdyyy~~þÜ¹s:dåøãÁÆ!guwwÇb±iÓ¦µ´´Ø8x<$=FÛÆÁæéöz,X¿·öìÙîT«%¥Þõ555¯½öZxùå×¬YcåøãÁÆ!g=÷Üs/¼ðÂÍ7Ã+ýôéÓm<£mã þnïk_ûÚ7¾ñxüûoß¾VKJ½ëKJJÂÓ;LôôôTTTXE9þx°qÈYsçÎ=ö¬=FÛÆAüÝÆG´hÑ¢ðÇßÌ3.]æðÁVQîH½ëÃtüÜÄiróñ`ã³Âþâ/M>ýý÷ß·qðxHz<¶ø»U«V>|ø?ÖTÊNú.Ì?ß*ÊAñ»>///>³  ÀÉñÇCÎ;wðº^Ým<£mã þn·~UÒ¹Ò9+ºëKKKzz>¹µg'L[-9þx°qÈYOÿè>ÄÇÃhÛ8¿AT`41sæÌööö¨ß.]jÍäÔ»¾¶¶öÕW_ágMMUãõäO¾ñÆaâÌ36IÑ¶q¿ãÇÏ3'´|UUU¸­Üz×9r¤¬¬,//¯¼¼üØ±cVQ?lrÖµk×V­Z¢«­­ÍÆÁã!éñ0Ú6â ?ñø@ü þ?Äâñø@ü?Äâñø@ü þÍo~ó¾ûî+ºeÉ%o½õÖ¯l¿n3[Ût·¶¢¢"üi]]]IóÃüüüòòò7ovâ¶nÝ:.ÅW¿úÕl¿mÛ¶õõõIó_zé¥0ÿÙgÂ2Ä0ö<y2dM~~þ+¯¼ÒËÎ;ÃÉ0óÔ©SYçÎ3çÏ4Î9a~»øÄyä5/¼ðBâÌ¯ýëaæ£>>ñºpÁa:þËüñÚµk'NÎ*))Ù¸qcâÞÕæææXá¬pÙ¦¦¦¤s&Mtß÷8p ¬­­Mºaû÷ïï9ÑYá&Eg<x°¯P[ºtiìØ±ø÷Þ/Ì©®®Ïyî¹çÊÊÊÂ¢zè¡.¤Æ_êòæôsSñ0Â¦NÂå£>Jyþüù0³¢¢"1n?~<:wåÊIg­_¿>:«µµ5///í¥¢Ñ¹«V­ºyófÈÇÞÞÞpnøâiÂ	ÑGñúYNHóRÿÒ×_=±hãùÚk¯ÅË/i!K,lüõsSñ0ò¢=¼i¶YãÆK5kÖ%LË/ÎZ'ÊÇè¼mÑY555Ñ[aúÈ#ÑB¹eËwÑ^×Ç<ÌÙ·o_?Ãôc=vÛåp'Ã/$Þ¶´QÊ)S¦¿÷Ê+ádøny(ÎèÊËËÃÃðÉ/w6þú¹©øñ¤¿øü(n¢f.NlN.X° >úO<¢íÆñßIz/-~©èäùóçã¿)¾ç7üÓÍÍÍ·]NÍpòòåË·­¯Ïç=õÔSá¬o|ãür×öÆ!`¿¤-ÞlüõsSñ0ò¢·»®_¿8³««+Ìgõ;ñ4lkkú/Þ:ñê¥îMÊ©Ä/X	Ó%%%ÝÝÝaá&MÛåôbIÎ9uj?ÃtkkküÜ#Gv÷ñÀã¯?îíÅ_LJÒøç¯úÖ©S§¶mÛíWcô¶¿êmmýúõÑÞÞðsíÚµñùý,'z§íâÅÑÉË/÷?2÷ÞïçFßn5ñ¬häo8ëàÁ×®]ë?þâa­ø¹ýÜT@ü¼hÀD~~þË/¿ÕË+¯¼RPP:8cùòå!tº»»£²ñ¹Ñgþ¢Êµµµ%~X0jÁ7TÆáÆ¿l%m¢EÃo£koiiÏïg9Ñü¢ÏüuuuE¿ÙOüíÜ¹3þÏ>þØÚÚ®%¬¾â/ÊÍËá×Bt>ôÐCçösSñ0*D_ä¹çûÏí×-QÅ§ã;Lãc,âüñÄ²L´gÏ~âï[âóKJJ÷÷³p3oX|¤m_ì7¢?!ig÷<xS¦L	?£oIôÆdªñsû¹©ø-B ,Y²¤àûî»/r¾NoîÜ¹ÛÕÕõÌ3ÏEÁôÔSOuwwÇÏmjjZ´hQh²òòò¤e¦Þ°¨0ÿÉ'Lß×rpcÂM¾°ïù[»vmÒw¾D®RSSÖÀÄÃ_ÑÞÞÿÚÄe^»v-Ôm´®ª««;týÜT@ü þ?Äâñø@ü þâñø@ü þdÊÿù,ø¾LIEND®B`


Detrended Normal Q-Q Plots


úTT*UWW·jÕª¾¾¾Y®><<ÜÙÙy7ê¸b2ÿ¹ç¯¾ÑÝy¦G]³fMÝ~øÈ#wÜàìW)ÝÚÚÚ<8Á÷¾qúôéØ	Óét|÷¢-ÔÌàùÁâ*,þmÛ¶í²'þêëë¯]»V)ñ÷ÄOþ°vïÞ]fksZe¦ýáÀsyc&ÉßÞÞ^ñøÞÇÎ¸uëÖsÏ=WWW3îø6l¨øNjõ«_ýêÄÄÄäädLÄÍ9222í¦æºJéþ°cÇAv×â/YñæÍ1Ëåî© þ Úâ/ñôÓOt9uêÔòåËÓéô+N<YOù$qÕ«WY7ç×^­³³39#ùè£Þ¸q£ð¶··'+ö÷÷=Ô6ÇÀ¦MâëÑ£Gg7ß|sãÆÉ#Ìf³/^,ºgáóMæÄ7o`Í5×¯_?út[[[mmíºuë2îÝ»·¹¹99¿ß"¿h¦üÈ#ÄüÂ°!b=fnÞ¼yÚï)ýÖÑ1'ägÚ#sæ·bùø+Z?ØCbØ?ùÉOÆØ?FXºt»qDjdôt,jllÜ¶mÛØØPañ½3[ZZ###©TªðWòððpøKîÜÝÝ]fÝüãwsáÒÂCV3à+³Ùò+>÷[·ne2¦¦¦(+W®ÄoôÂíÄ=*­ðù~ßU«V%GR"ùò+ºçÚµkË'Î%KbþÛo¿]úÃZºté´?ß¹®Rô­£l#Ë-Óàüy¯8ûø;ölávâÉÎ5þf¹oØ°¡è1oÝºÕ?¨°øKæG%ÓÙl6nÄô3g­hõäæ®]»&''c9w~wÆoý¸r¸1:)Y¿tãf¬>:%Ö*ü^e6[~Åiû±cÇÞË-1ÝÕÕusJLÄÍ9ÓóMæ<þøãÉK¶7O<Yøw¶8q"¦/]ºT¸h¦N§gz"ùu¸ÊLoªËëàLû¨æ½bù¬/¼Y¸DÖçmöñ7ËÝ8ÂË/Çtü4czÑ¢E^X@üAEÆ_þL_ò±B1§Lü]¹r%?çëæ¢%§ãWir3~²*:mWf³åWé¹'¿×æ755>£ø7/^<ÓóMæDpÞLÎe'O°ðär¹ÈÂç^&þòE>S©/pÂ!uc0;;;O:5í Ífp¦Tó^qöñì!×¯_/üs¿YîÆíííq³µµ5â5þqûöm¯* þ òâ/b%f666Îôw¦LInFåùm]&qæúëyi~<åx¾ñ+|||¼p~é³Ò7À>ßÙÜÐ,:õyÇ«VóÔ¥WÔù-ÌfÙÕ´wÍàÌ¤ó[qö§¾wÍr7¾páBÒù(<~ü¸Pañ÷üóÏ^ðK+ºîr6­3§uË¹yûí·ÙlùË<_|1næ?%ôYÑ1ª¢,ýÞ|yò+ýýýg³¿äðdò©+ÙlvÛ¶m7oÞ<tèPÌÜ¸qã´ñ7Uæs¼y¯8ûøÍ¿|Þ%ÿÚ)3þeö·Ä¹sçöìÙkÎ_?¨ø_oÑ@ÉïæüG½$¿Ò¢âerñì+EÉÆ¦Lû[³ÌºåË)©ä=[±ñd;Eïèv³åW,_6lÈ¿¯?ÜÕÕukJ²µÇ|áñý§pðàÁÙÄßàà`rl)ò3g£zcÇMûóë*s¿òS´oë¨Î#þwéÅÎpûöíh»õë×¦áþýûcð#£ËÿÿHÞ»yáÂ2§àñ÷Pü*¼b±ôúÙüÛÿóÇ&,ý­YfÝòåUäQá¥ù¥e6[~Åò%qíÚµü»»9/^,z¿WSSSþ¿|$/^dÄyôÑGKX+W®,ó#Ó*s¿òS´oë¨Î#þÎ;W¸ýÕ«W.-ü%ç32û[þ¢¼ÇÌ?¨øßk×®-ýó_'OìèèßèÏ?ÿ|~þ±cÇbNÄVá§ÜÍrÝ;Sòñx±ñöööÒëi³w|I$§Dç¿ñÆ7n¬ÍfK/ï_üÝ¸q#¶ÛÚ¾ôP¾¹Ë'Î³Ï>Ï.ø%K>üéO:äiá«Ì)þÊNÑ¾QdN£:øÇommgÛON¬ç/phÉwïììºã×ö·±±±;w&T£àãG9>>îÄÀû%Ë=úè£súl¹y¬Rq#¾ÉåÛIÓ/_¾Üâ*¼ç¯ÈO>id@üPÆÆÆvìØ±dÉäÜnL<ñÄÄâñø@ü þ?ñgÄâñø@ü þÜ±cÇZZZÒéôÚµkçºn[[[MMÍòsb:æ¬X±b/XSæwÙ¬;o7nÜxüñÇbp,Y²sçÎ±±±»ô"þ~>/@ü÷µæææè¹®»wïÞX÷gÉÏyê©§bÎ=*=þ¢óN¹téÒääd___|£õë×ÏcSÃÃÃâÀ½ñz±Î8wî»fÍüU«VÅ³gÏÞùþERÒµGý@FXüâxË¯05vîÜY__¿hÑ¢Ý»wÝíÓþtGGGÑòcúí·ßé,º|ùrggg]]]:noovkß½ü*niiyøáãFÒèèhooo¬~ÇÉÌÈÓÕ«WÇÖb~lùÊ+³+VÄÇÇÇ§]ZfãAÆ ;v¬tKG²Ì¦ìøÞ¯þK¦÷íÛÓÏ?ÿ|dVL<ùä÷9yòdy¶lÙ:Óñ5¦7oÞ,Z¹rå'bâÒ¥K1?ÉL»µÂP~Hº_|1&6mÚTúà·nÝÓq^z)&öîÝ3?òÄô­[·FFFb"90YªtX¢gÊ¯ò£´ÿþäE>È¢ç^~SvN@üïoüE¯ÄtnJiMû¦ÀÁÁÁXÔÕÕÓ6lHÊ&¿ôÜ¹s»víêèèù©TjÚ­NùUV___ºnSSSòà'''c"²/fÆ=cº½½=êj¦ÃxÓ*åGéöíÛÉtla¦øË?÷ò²sâxã¯°xfÊ"QZ-%gWcN²èàÁ±Vák¯½V@Ó>Ù¬2ÓR,<ãÑ£G9ÉAÁÙù[¶lYÌ-ßå#¯üôü@üïYüe2ÂQÉÁ³;¶Hooo,ÍÍÏ¯­­9SfåWÉ?°è¹Òu/^¬[ôð&&&N8±iÓ¦ÂCw´k×®¸Äbé¢ÙÒ,ão~ þ÷&þK:¼í©§M=z4íÅ_,*óçÏ'ïMü_%ìk_ûZLlÙ²¥tÝÇ,¦ûúú^ýõäToÌ|øácúµ×^»~ýzL,[¶lÃrëÖ­H±¥K^¾|929$ÙÝÝ=ËQ*NN=''ûü@üïMüMLLD`-RøYå[äöíÛÉ)×øÍüøñÆÆÆH;vÌ2þÊ¯rêÔ©XºjÕªüu'÷Û¶m[<òÚÚÚõë×'öFóe³Ùäòá5kÖ¼ñÆ³ø.ÉIí¨ÀÝ»w'ïçÍ(N;v,RòÚEÏ~ þÄâñø@ü þîßùÎwÞzë­»ùðüû¿ÿ»=¸ûö]Þo©JW¯^ý·û7ãÀBüøÇ?þÇüGã þ*ÉýÙEÿÝåÜ,üx3ý×=sæq`Î=ûÏÿüÏÆþ[ôßøqâOü!þ?Äø?Äâñ'þ@ü!þ?ñâñøCü??ÄâOü!þ@ü!þâñâñø??ñ'þÄâñâOü?ñøCüøâOü!þ?ñ'þÄøCü!þ?ñ'þ?ÄâOüøCü!þâÄâñø»ÃKX&I§Ómmm¥wèïï¯©©??ñW²Ùl___L8p §§§héÄÄDûLñ]8zë[ßºzõê(,À+W¾ýíohhhèþéñÃþðÔ©SÆá^s_Ä_ccãäädLär¹¢¥ûöíúé§g¿ýû÷ë.:qâÄÀÀÀ·`bÉ8°@/¿ürüÚ6,Ä7¿ùÍãÇÍétzÚépùòåHC§qÚöÅi_ª$þR©T~º¶¶¶pQww÷éÓ§òTÅâÄâOüUÇÓhjjÊårïNöéy?Kü!þ@ü!þÄ_Åëíí=|øpLÄ×l6;ýSuäñâñ'þªãiÄ/ÂæææT*Éd¦­=ñøñøÃ<?ÄâÄøâñøñ'þÄøCü!þ@ü?ñ'þ?ÄâOü?ÄâñøâñøCü!þÄ?Äâñ'þ@ü!þ?ñø3?ÄâOü!þ@ü!þâñâñø?Äq@ü?ñ'þ?âOü?ÄâÄøâñøCü!þÄøCü!þ?ñ'þ?ÄâOüøCü!þâÄâñø?ñøCü!þÄâÄâñ'þ þ?ñøCü?Äøâñøñ'þÄøCü!þ@ü?ñ'þ?ÄøâOü!þ?Äø?Äâñ'þ@ü!þ?ñâñøCü??ÄâOü!þ@ü!þâñâñø??ñ'þÄâñâOü?ñøCüøâOü!þ þÄø?Äâñ'þÄâñøCü??ÄâOüøcÖ&&&ÞyçñøCü?ÄU.¯«««¡¡áÃS¾ò¯?ÄâOü!þ¨ZkÖ¬ùèG?úí_ØýÅÝ¸é±ñçÚâñ'þÄøCüQùy×ÿÙåüý×ÔÔ$þ?ñø£=ûì³ùòKþøûÑ~$þ?ñø£Ú¼ðÂ>ø`aùíüãããâñø?ªÍÕ«Wþçþs|.«W¯þßù9mDü!þÄø?*Æàà`ôßÊ+;?ÑùÀ<ôÐCs:ç+þâOü?ÄæêÕ«/¼ðÂ3Ï<sêÔ©9ð?ñ'þÄâûøCü?ñ'þ?âOü?ÄâÄøâñøCü?ñ'þÄâñøCü?ñøCü!þâÄâñø þ?ÄøñøCü!þÄâÄâñ'þ þTiüÅKX&I§ÓmmmÚÛÛcÑ+ânâñâñ'þ*^6íëëôôô.Zºté+¯¼GY¶lYiüýõ_ÿõÿ½âïÿûÿ v¡øGq`N>ý½ïÏ8°/^üÛ¿ý[ãp¯¹/â¯±±qrr2&r¹ËLw«««+¿C½võ÷÷¿ðÝï~7v$ãÀÅ¿E_õUãÀB=öå_6÷û"þÒéô´Ó"¹6oÞì´/NûÓ¾8íë´oÅK¥RùéÚÚÚÒ;f³Ù±±1ñøñø¯©©)Ë½;uÚ7¦^»vmÓ¦M×¯_/]Qü!þ þÄ_åéíí=|øpLÄ×l6[¸hpppÝºu7nÜvEñøCüø'~677§R©L&344ôÏ­æ'Ï®¥¥¥¦øCüøCü¿ûøCü!þ@ü?ñ'þ?âOü?ÄâÄøâñøCü!þÄøCü!þ?ñ'þ?ÄâOüøCü!þâÄâñø?ñøCü!þÄâÄâñ'þ þ¨Èø»zõê3Ï<óÇüÇñõ­·Þ2àâOü!þ@üÝÙÉ'¿øÅ/îØ±ãoþæoüÜ+(þ^õÕ_ø_xè¡~ÿ÷ÿÁüÐ>422bÌÅøCüøÑøøøg>ó_ú¥_úäùäïýÞï=ðÀ]]]1ÓOÿÞ¿ø1øÃîù¯=»¿¸;ù¯»»ûWõW'&&»ø?ÓÛ·o_äÂÎ?ÙÔCLüÚ¯ýÚ=üôïýøõÕWÛþS[¾üÿ¢ã/^¼hØÅøCüøÞoþæo~îÏÖÃûü[ñWøéßûñwüøñU®*¿_ÿõ_ÿøÃ.þÄâÄßô>ö±mù[ë!nF@øéßûñwñâÅEýïmÿ;ÿ³û_ÿóÕÕÕvñ'þ þ¦·iÓ¦¯ùxaü­ùí5ýìgýôïýøôGô+¿ò+ÿýÑÿ²éûösñ'þ þftõêÕÆÆÆÎÎÎ_Ø±óOv®ûuúÐ¼i¬Râ/|ùË_^ºtéÏýÜÏEùýÅ_üâñâïÞzë­®®®h¾uëÖ]¸pÁ¾âñ'þ@ü!þ?ñøñøCü?Ä?ÄâOü!þ@ü!þÄø?Ä?ñ'þÄâñâOü?ñøCüøâOü!þ?Äø?Äâñ'þ@ü!þ?ñâñøCü??ÄâOü!þ@ü!þâñâñÇ5wJ¥ÄøCü!þTIü¥î$N?ñøCü!þ¨ø«âñøñ'þÞÑ7[¶lâñøCüQñ×ÒÒN§½çOü!þ?ª?þV¬XQzµGccãØØø?Äâj¿ÚÚÚ¨½7n477ÇD4ß×¾öµØ¼y³ø?Äâj¿äP_LDíÅÄ¥K&''cbÑ¢EâOü!þ?ª-þ"õâWÑbâ'H&|ÔøCü!þTaüíØ±#yGáÛþ~øañ'þ?ÄÕáK_úÒâÅcbhh(&";::*âù?ÄâÄø»?ÄâÄøâñøñ'þf¶lÙ²ä_|È³øCü!þTyü-]º´0øò+þ?ÄUÑyÃÃÃ÷üÅâñâOüÍMcccÄ_%øCü!þâOüÍçå#âoëÖ­£££âOü!þ?ª<þÂ%KjJ¸àCü!þ?ª0þZ[[]ð!þ?Ä÷Kü%Ù722RÏ_ü!þ þÄßÜ455¹àCü!þ?îøøÛ±cÇøø¸ø?Äâ*¿¸àCü!þ?ª0þR3pÁøCü!þTaüU4ñøCüøsÓÒÒÒÚÚúúë¯¿W/FL&N·µµ_4¿9âñâñ'þæ/ºª¦æ=;ÍfûúúbâÀ===åÍoN¡;w~ìcë¼âÛ­Y³¦à·û·ïò~KUúßøßú­ß2,ÄÇ?þñ~ô£Æá^ó¾ÇßÀÀ@Äß=nÝºµð|illL6ËåZZZÊ/ßBÛ·o¯¨"ïü½·Wû^&RtÉHé¢ùÍø¿÷öjßÂd¬­­-¿h~síÚµëSúÔ¦»(¾Ý#<²	àþàîò~KUêêêúìg?kXÏþó¿û»¿kî5ïü½·r¹6¦Ë/ß|àpÁ.øpÁÇ½¢··÷ðáÃ1_³ÙlùEó#þ þâoAr¹Üúõëëêêjjj-ZÔÝÝ=ï+?âWZsss*Êd2CCCÿñ(§Î^.ßñøñøúÁOûfÃøS¿âñøñ'þæfùòåz]]]£££q3ÊfÃ1gåÊâOü!þ?ª-þjkk#õ&&&òsr¹)½´Vü?ÄâñGÅÇ_*ÔK®¨MÇùÔøñøCüqOÇ_rÚwÝºuÉißøÓ1§½½]ü?ÄâñGµÅ_ÔÞ´|Ü¼ySü?ÄâñGµÅ_ò³ïîî®¯¯O¥RñuÝºu1§"¿øCü!þ@ü¿ûøCü!þ@ü?ñ'þ?â¯hÍ;I¥RâOü!þ?ª$þR3âñøCüQmñ7íÛ·'ñwäÈñ'þ?ÄUñ:²hÑ¢È¾ÎÎÎÂÏ|âñøCüQmñ·qãÆäß'*èù?ÄâÄø^z)É¾®®®þâñøñ'þæð#_¹rermÇÀÀ@%>ñøCüø³²ÿþä_OOOå>ñøCüø³[Óçü?Äâñ'þîøKÝI:âñøCüQ%ñWÄâñâOü?ñøCüøâOü!þ þÄø?Äâñ'þÄâñøCü?ñøCü!þ¨ôø[¶lYmm­Ïù?Äâê¿¥KÏù?Äâj¿è¼È¾áááÉÉÉþâñøñ'þæ¦±±1â¯ËOü!þÆñ'þæóòñ·uëÖÑÑQñ'þ?ÄUaÉ%5%!þ?ÄU­­­.ø?Äâû%þì©Äç/þ?âon!þ?Ä÷KüFüíØ±c||?ÄâñGÇ_Í!þ?ÄU©¸àCü!þ?ª0þ*øCü!þ@ü?ñ'þ?âof¹ýúõuuu555-êîî®ÅâñâOüÍù?íqñ¯øCü!þ@ü¿¹Y¾|y¤^WWWò·£l6lØsV®þÄâñø£Úâ¯¶¶6Robb"?'ËÅ/þÄâñø£Úâ/JEêEðåçÇõ"þ?ÄUÉißuëÖ%§ãkLÇöövñ'þ?ÄÕQÓ^ðqóæMñ'þ?ÄÕÉÏ¾»»»¾¾>JÅ×uëÖÅxþâñøñ'þî#âñøñ'þæ¦¥¥¥µµõõ×_âñøCüQýñN§kj*õ¢øCü!þ@ü¿¹øÛ³gO4M¥üU7ñøñøóÝÄR©ø?Äâj¿Ô|È³øCü!þTOüµ¶¶¶µµUôó?Ä?ñ7+Éôwê´oEá??æÉôMþ¯ø?Äâ*¿L&SS>Äâñø£zâoxx¸¡¡!9þ¤>Äâñø£jã/¯R:Oü!þ@ü!þð·ÅâñâOüUàKX&I§ÓmmmÚÛÛcÑ+ânâñâñ'þ*^6íëëôôô.Zºté+¯¼GY¶løCüøCü¿×ØØüYá×ÒÒ2ÓÝêêêJãïË_þò«wÑÉ'£ÿ^8útìHÆêïïÿÖ·¾eXøè'Ã½æ¿7Þxã¿Â+NfºúdxxxóæÍ¥ñwüøñwÑ7¿ùÍK.Ý]hppÐ8°@ÿ÷áÂãÀBz5þaî5ïü%óÒÞÞþæo~PñWøµµµ¥wÍf³¥Îi_öÅi_pÚ×iß¹ìË°s:^³fÍÅïÆCÿ©njjÊårÉiß.ºçµk×6mÚtýúõÒ?ÄâÄø7Þxcùòåù «­­]¿~ý]½½½øÍf®[·n¦C âñøñ'þæorròÄuuuwùÏ»Å/ÂæææøLfhhè?ÛÔAÁÂ?:'þ þâï=022Rxä¯¾¾¾ôêøCü!þ@ü¿¹)l¾ºººË/WÊó?Ä?ñ7ÇML]çñÉO~òÒ¥K÷üÅâñâOüÍÍø	/âñâñÇÝ¿&þ?âoÎÎ?¿lÙ²E%Wø655=zTü?ÄâñGÆ_þ$þé?ñøCü!þ¨¶øknnÔ;þ|>þOâñøCüQmñÿðä|üMNN&?ñøCü!þ¨¶økjjÔKöEüår¹;wÆtKKø?Äâj¿ÁÁÁé¼òÊ+âOü!þ?ª-þÂõë×;::«ëêê-[vñâÅxþâñøñ'þî#âñøñ'þÄøCü!þ@ü¿é|¹½½½¾¾>5%&ÚÚÚ*å¯øCü!þâOüÍÁc=V3-[¶?ñøCü!þ¨ø;zôhÒyËïÇKæ<yRü?ÄâñGÄ_&Â;tèPé¢ä¾ù?ñøCü!þ¨ø«­­Âzé¢ââOü!þ?ª$þòÕm¦¥ÉzâñøCüQ%ñW&ïÄøCü!þ?ñ'þ?Äå?ñøCü!þ¨øKÝI:âñøCüQ%ñWÄâñâOü?ñøCüøâOü!þ þÄø?Äâñ'þÄâñøCü?ñøCü!þâÄâñø þ?ÄøCü?Äâñ'þ þ?ñøñøCü?ÄâOü!þÄø?Ä?ñ'þÄâñâOü?ñøCü!þâOü!þ?Äø?Äâñ'þ@ü!þ?ñâñøCü??ÄâOü!þ@ü!þâñâñø??ñ'þÄâñâOü?ñøCüøâOü!þ?ñ'þÄøCü!þ?ñ'þ?ÄâOüøCü!þâÄâñø þ?ÄøCüøCü!þÄâÄâñ'þ þâOü?ÄâÄøâñøñ'þîÍ°L&N§ÛÚÚJïÐßß_SS#þ þâ¯d³Ù¾¾¾8pà@OOOÑÒöövñøñø£Jâ¯±±qrr2&r¹ÑÒûö=ýôÓ3ÅßSO=uò.:~üøË/¿| v¡Øôõ¯ýÄÆ/GÕç¾¿t:=ít¸|ùrGGG¤áLñ788øã»èôéÓ7nÜø1,ÀøÃï|ç;Æ¾råq`!FGGO:eî5÷Eü¥R©ütmmmá¢îîîè­<U§qÚöÅi_§+ø¡ÿTL755år¹ä´oLO·üÅâÄâOüU¶ÞÞÞÃÇD|Íf³3ÅbéLñøCüø'~677§R©L&3444mí?Ä?Ä>äYü!þ þÄø?Ä?ñ'þÄâñâOü?ñøCü!þâOü!þ?Äø?Äâñ'þ@ü!þ?ñâñøCü?Äq@ü!þâñâñø??ÄøCü!þâOü?ñøCüøâOü!þ þÄø?Äâñ'þÄâñøCü?ñøCü!þâÄâñø þ?ÄøCü?Äâñ'þ þ?ñøñøCü?ÄâOü!þÄø?Ä?ñ'þÄâñâOü?ñøCü!þÄøâñøCü!þÄøCü!þ?ñâñøCü??ÄâOüøCü!þâñâñø??ÄøCüøCü?ñWñ711ñÎ;ïØÅ?Äâ¯Êã/ï3ùLCCÃ§|å+_±+??Ä_ÕÆß'>ñµìÛ¿°û»ÿpÓþbã/ê?ñâñø«ÎødÉ®ÿ³+Ê/ù/ú¯©©ÉÞ,þ@ü!þUÏ>ûlggg¾üÿ"þ~ô£Ù¡Å?Äâ¯Úâï^xðÁËoçìlhh·C??Ä_µÅß¿üË¿Ô××îÏåãoõêÕúÔ§ìÍâÄâñWñ÷îÔÛþ¢ÿV®ùÎxà¡rÎWüøCü!þª6þÂÕ«W_xágyæÔ©SNø??Ä_ÇâÏ8 þ?ñøñøCü?Ä?ÄâOü!þÆñ'þÄøCü!þ@ü?ñ'þ?âOü?ÄâñøâñøCü!þÄøCü!þ?ñâñøCü??ÄâOü!þÄâñø??ÄøCüøCü!þÄâñ'þâOü?ÄâÄøâñøñ'þî±°L&N§ÛÚÚoÚ´©¶¶ö#ùÈàà øCüøCü¿ÍfûúúbâÀ===öîÝûäONNNFùµ¶¶Æ_ìµ·ï¢oûÛ×¯_¿¿°O>mX ï~÷»?øÁqóæÍS§NÍw1ËåZZZµµµ½þúë3­ñ÷çþçß¸¾þõ¯ÿÝßýÝ7`bÉ8°@'Nèïï7,DßñãÇÃ½æ¾¿t:=ítrsÿþýuuu­­­¯½öÓ¾8íNûâ´¯Ó¾/Jå§kkk:t(&ÞxãñøñøùÐ*¦r¹Ü»S§cºðn7?ÄâÏ8 þÄ_Eêíí=|øpLÄ×l6[¸èñÇñÅcâüùó?ü°øCüøCü¿¿S©T&úç6uP0J«»»;NwttpAü!þ@ü!þÄßýKü!þ þÄø?Ä?ñ'þÄâñâOü?ñøCü!þâOü!þ?Äø?Äâñ'þ@ü!þ?ñâñøCü?ÄøCü!þâñâñø??ÄøCü!þÄâOüÝoþôOÿô¯þê¯ÞºâÛïß`ddäèÑ£Æ:~üxôq`!¾ÿýïÿå_þ¥q¸×¼óÎ;âoFçÏß½÷Tòg5küpÚñø@ü þ¿744ÔÞÞN§W¬XqöìÙ388¸|ùòÓÖÖæï40çÏ_¹reá>ûR&Iæ"æ·#¾@Á¢DM¨LYºté+¯¼GY¶lYL466^¾|9&âkKK!ââEöØ±c1ûRì?1ÍfûúúbâÀ===ùíH¥/P0×½(LLLÄ¿"ÄøcuuuñµµµõÚµk1_cÚ°0'ObEòOÉÉÉÈårþ	Á¼w¤Ò(Ç^´oß¾§~Zü?oÞ¼ùÝ©vñ"ÿÄ×idøuì6Gét:¿¨pæ´#¾@Á¢Ë/wttÄ?GÅøãgf³Ù±±±^¹råë¯¿TàªU«³×ßßßÔÔ©T*?³¶¶ÖÈ0¿©ôæºuww>ú'I!þÄy×®]Û´iÓõë×Ù°É>¯¹¹Ý©Ó¾¿ÅaN;RéÌu/ªùYEüñk×­[wãÆüU«V?>&FFFV®¸£¥K&ûÌÐÐPìN1ÑÛÛøðá¯ÙlÖ1¿©ôæºýÿ¤P~âDKKKÑ?Þ|óÍä:ùøÓ;:öl[[[ì3k×®MÐ9s¦¹¹9Je2x	6DÌoG*¹îEâOü þ?Äâñø?Äâñø@ü þ?Äø¨d_ýêWW¯^]7eíÚµ/½ôÒÏ¼ÆM©WäémKKK<µ±±±¢ù1'Ng2ÉÉÉ¹ni÷îÝ5%¾ô¥/USüíÙ³'f>÷ÜsEóöÙÿÄOÌcø¨<gÏ¬I§ÓrèÐ¡¸3Ï;W5ñwéÒ¥¹bÅ¢ùË/ù/^øî<òHdÍO>Y8ó©§ÿüçÓg`` â)º°½½=¦ów¾yóææÍëëëcQccã¶mÛÏ®:u*+Åº'O,Ê©ÓÐÐ°zõê'NÄÍÞÞÞ¢vüøñòÛIÅCJõ÷÷ÏjëÖ­ùCCCù9¯¼òJÌéììÌÏÙ»wossslª®®nãÆ×®]+¿ÒíÍ)óPñð[²dIËåËg^¹r%f¶´´ÆMááádémÝº5Y422J¥¦]+¹,íîî|¬­­¥ñ5âiÑ¢EÉ[ñÊl'&¦x¥ÏôÈ#EïË¾¾¾|ùmdíÚµs¿2¼äï4¯k55bqÓÓÓ3:%&âfWWW²4i$xmÉ¢l62é3gÎ$)Üæ®]»"ï³®=öXÌ9vìXLÇ×~ôÑGï¸Ç¸w(|lÓ>£ÊÅÇó½qãFÜ¯ñÈ£8s¹L&+ÆSx÷§§ëêêæe* þîøÓÆ_~~7I3k×®ÅÍÈ¦äfÜlmmÝ²eKDÛíÛ·óûKË¯Ü¼råJþÎLù3¿ñ5¦O:uÇíDhÆÍ·ß~»ð±Íôþ¼íÛ·Ç¢§~úÝÚÞ¶m[á"#þ"I;::s¿2¼äp×èèháÌ±±±ÊäN>/ô_¾uòoÔ+=[S°Óµµµããã±ñüÒ;ng¦+rþüù¤Sc:¾ÆôÈÈH~é3gâLúxöñWæ¡âà¼ïmÿþý3@)ºà#ÿ¾À7nÐ:wîÜ=°ùjLËåÏ«Þ1Ñ¶nÝí¯7oÎÏ/³äHÛõë×o¿ývù+sW­ZKO·f-úûûoÝºU>þòaF~i?^rÁD:>pà@òQ/¬­­-½8£««+Bg||<9'¿27yÏ_òV¹.¾Y0iÁmÛ¶E*%×áæ?leÚDK.¿M¾ûàà`~~í$oòKÞó766Ü³Lü:t(L.rá¢äí###ñ]bf¿$7#ãn7n,æ¡âà|r½÷þÿ×¸)Iå§ó'Ló×Xä=öØceYèèÑ£eâïÝ©?ÅóÏÙN<Â¿Òv¦'ûöíºººä)ì^¿~á·X¼xq|M>í¥pÉÉ¼$UóKË<T@üÜ+"PÖ®][;eõêÕÉ%·Eñ×ßß|^[[ÛéÓ§óKÇÆÆvîÜÙÜÜÓöíÛÇÇÇóKO<ÙÑÑMÉdþù¢m>ØTÌüñÇæÏ´&Ròe>ç/oóæÍEù¸qãF6¨¯¯gqñâÅüÇÖnóÖ­[Q·ÉXuvvÇ2?Äâñø@ü þâñø@ü þ?Äïÿ9£*É~£ÊIEND®B`


òÉ'«ªªbfggç ¿héÒ¥B~]]]	U~úéþþþ³1³§§gØ«ë*C_7n9¡±	_²âåËc:ÍÞVO$òÊJ~I[·n-ØÜrðàÁY³f¥ÓéÙ³g·µµÈ)w%ÉDÊ)óçÏ/²nîÂo¿ýö¢E÷ßÿ¥Kòo´¹¹9Y±½½½à®tµ7à¬2N÷îÝ;Ò>zùòåÉ=Ìd2gÎ)¸dþãMæÄÆMÇX°`Á>ÜÔÔTYY¹xñâüí[¶l©¯¯OvkÆMät¿ò¯Äüü¯QH=f®^½zØçw¬«½éÀbÌ;9¦Áv ã[±¸üÆó¯öï½7Æ¶øÖÁ¡KGù2¡ÆH¦cQmmíúõë¯^½êD~Òd_`%f666&gzzR©TþûqWWWù%^¶lYus7æü¥+V¬ÈßX5Ò~½"W[|Å¡½···¡¡¡®®.&É|oçù×, Zþãz»sçÎM¶¡&Brì+¸äÂûfÚ´i1ÿâÅC¬3fûüuÖ$ÛüfÎ9¦Á)>øã^qôòëîîÎ¿x°cß(_ÆK.-¸ÏëÖ­óE"?i2É/&K¦3Líèèé#Gäû¬`õäì¦M­87Þ8ã-?.lh$%Kã7ÎÆêWµòo«ÈÕ_qØÇ¾oß¾üm`ù_³fML/Y²äò`1gcæH7³víÚþþþä%×gÛÚÚò`òi¶ÄôÙ³gótÓéôH$·îM®2ÒérÛDÇ:8ÃÞ«q¯XÜôùgó_aúÏF/¿Q¾;w.¦ãÙéêêj¿X$ò&ür;øåsÈï>ÈÍ¹áº¹ígÉ^ÅxMÎÆÛgþÆª½uE®¶ø#=öñ¦^0¿®®.ÿÅ|:uêH7ÚÈ?ìÂN`þ=Éf³aFKKKþc/"¿ÇGbúM®?¤±næ¢E<8ì fp½Wã^qôòK^!.¿1Éo/ãæææ8;úôkü	qíÚ5¿U$ò&üB*1³¶¶v¤·Ûây«.â±¾7ãjG9o¼÷õõåÏºÍlèÞ>ÞÑeìñ¼á1ªõõõÉîé¡¼+ðtîF³ÊhÆjØfpFòèøVýÞÞuòe|êÔ©9îß¿ß/ü¤É$¿;väálE+8Êr4ÐÓºÅ·Ùx1i«-¾b;°gÏ8ûdf³­S&ýä5kVòí*ííí!³ÑÈ/Ù0|ÁJ&Y¿~ýåËwîÜ3/_>¬üF³Ê¸å7ÖÁÉ5îG/¿ÑlóËÙ.ùS§Èøy½%;vlóæÍÉ.æÜqÐÈOºÝåïm ä9÷­.ÉûY !Þ)CegÏ,J>ätu°aß2¬[M	YÏiÅ'×Sð)®a¯¶øÅ±téÒÜù9É¿K,é,¹¶µk×Þ¼üí^===ñzê©ÑÈïÐ¡CÉV¥xÈGI¶ç%íÛ·oØçw¬«I~Å§àµßXGuòK>/k×®ìî»ï¾¡.Ü¶m[~è0üoø#|^óÔ©SEö¼K"?évßÐòOz´lîóþ¹/ÝM6Ë,²nq6åh[Zäj¯XçÏÏ¢+sæÌÏxÕÕÕå>Dx3òËY$iêÔ©¹O=ñÍý÷ß?ôÉ3gN§xL«I~Å§àµßXGuò;vìXþõÏ??iÁä0éV¼ÞråZµj_,ùIC~ñ¸páÂ¡ÿ»W[[[KKK(*ÞÎwìØ¿oß¾ÒÊÿ6»Q®C6%_WÞÜÜ<ôkùFºÚ®XÉÐüù'O|yå`Lfèñãß¥KâÚâ:Ã@6låÀ]Ü7O<ñD<ºxàÓ¦MÛµk×¾ðÜ6§_eLò+>8¯Æ4ªã_´ÿþéÓ§Ç£ëOö§çg³á³äÖ-ZÔÙÙyÃm®#½Þ®^½úÀ$SïñTöõõùÅ"$,e³Ùûï¿Lß!7U&]É¶Þä`íþþþô³fÍòÈOTn%ó+è±Ç32ùIÊ­«W¯nÜ¸qÚ´iÉ.Ýxè¡D~$I"?I$$IÈO$Iä'I$ò$IùI$$IÈO$Iä'I$ò$IùI$ü$ID~$I"?I·IûöíkllL§Ó.ëºMMM§NÊÍé3öì1ül|ÍºãüÝ:¤dþÕ«W×­[W[[#VSSÉdN>[«¿¿?æÇ+++cÚ«KùIº½ª¯¯©tiRÙ²eK¬ûÍo~37çñÇ97o.ù¿råÊßÑÑÓé3fäîß¿?'ÅxuI"?I·Ù/ÀÓ±cÇbÝäæÌ;7ætwwOÌxùUVVÆüÞÞÞa×úêW¿KÓU«VyuI"?I·ûòóÀÔÔÔTWW?øàûÂ¾ÐÒÒRp¹M1ñâÅ9É¢sçÎ-Z´¨ªª*N777'ÛÉ^[þ­_e×®]óæÍê³+W®´¶¶Æºqç7nÜlÂÎ??®-æÇ5ðÁ7)¿»ï¾;æÏ5ëÈ#¦L·uíÚµ8:ujÎÇã»D~>ü%Ó>úhLïØ±#=öXþeÚÚÚáå·fÍX´sçÎÓ^½zu²hÎ9ÉÏ³gÏÆüa¯-ÿ_%<·gÏX¹råÐ;¿nÝº¼øâ1±eËÔzzzb"Ù$9ÒgøF2qþeöíÛ³páÂ·ß~;·J²ówÉ%1½|ùò9ÃÞ¯:Iä'éÖË¯±±1¦³×°<tèP;K.MH[zìØ±M6µ´´ÄüT*5ìµØ«ø*É«©©ºn]]]rçb"¼3ã1ÝÜÜíëëß°ÔÕÕBÍù/®9¿~ýúÜÙD¨6l¸û Iä'éc_r~½!YÕÕÕqÉ«W¯&;4cN²è©§µo¿ývþ5þÙÑ¬2Ò&æo¢KÔ¸wïÞ)S¦$sjkkÍ£ßæWdÜ:::sù·ÃÍq£/_¾>¸ë9¦§M6ìðªD~n½üò·ù%ÍnÈ ÖÖÖX;ÍÍOlò+¾Jî¤®;uêÔdÝ»×ßßàÀäÜÜÆÂ1ËH%w&îsLç¨ZÐ±cÇÆ$ü$òK¾¥eçÎÉçüüñÑ0hïÞ½9èìÙ³'7?qäñãÇÏÿF~ÅWÙ´iÓóÏ?kÖ¬ºîªU«bz÷îÝ'NHö®ÆÌyóæÅt°ìÂ11sæÌßìÙ³c~ûõ_|Ëý÷ßä/¸ùã¾D~>Fùõ÷÷®ªËÿN¾âò»víZ²§5Nó¿î$`T[[[SS³qãÆQÊ¯ø*¥sçÎÍhRðËë×¯^YYyß÷%Ð¶2Lr°ðN<9¦aºG8®¶µµ5îa<Ø©S§®[·.n÷ú/¾Ô:ÿús_j=îû Iä'ID~$I"?I$$IÈO$Iä'I$ò»5½þúëï¿ÿþDÞâ3g²Ù¬W^	»4q(añªq(mgÏõ¯¶ýìg/^4%ìç?ÿù»ï¾kJÛï½÷¯ÿú¯äw»ô'ò'¿¼ÅCýË¿üvêÔ©þðÆ¡]½zõþáCiûÎw¾óÏÿüÏÆ¡>ú?øq(a×®]ûö·¿mJÛ#GþéþüÈOäG~ä'ò#?ò#?òùùüÈüÈüD~äG~"?ò#?ò#?ò#?ò#?òùùùüÈüD~äG~äG~"?ò#?ùùùùùüÈüÈüD~äG~"?ò#?ò#?ùÈüÈüÈOäG~äG~äG~"?ò#?ùùÈüÈOäG~äG~ä'ò#?òùùùÈüÈOäG~äG~ä'ò#?òù_YÉ¯»»»¡¡!N755utt½@EEùÈüÈOäG~¾L&³÷îØ¾û+ö÷÷777$¿Àâê>¸¬Òõ¿3%ìÃ?<xð q(méüÇ4%¬§§ç­·Þ2%ìüùó¯¾úªq(m¯½öÚï½7·xGÈ¯¶¶v`` &²ÙlcccÁÒGtëÖ­#É/°øÝ	, yøðáïªt¦)Æ¡ÅK4^¨Æ¡ä?ûßùÎwýÛ¹×_ýïÿþïCiùå'øgÿ_:v::wî¸ÐÞ^eo¯½½ööÊÞ^Ë¡T*®¬¬Ì_´lÙ²ÃÿÛC%?òùùüÈ¯ª««Ëf³×÷öÆô/=Â_üÈOäG~ä'ò#¿É]kkë®]»b"N3ÌðÕ6?òùùüÈ¯<µ¾¾>J544tvvK=ò#?ùÈüîèÈüD~äG~"?ò#?ò#?òùùüÈüÈüD~äG~"?ò#?ò#?ùÈüÈüÈüÈüÈüÈüD~äG~"?ò#?ò#?ùÈüÈüÈOäG~ä'ò#?ò#?ò#?ùÈüÈüÈOäG~ä'ò#?ò#?òùùüÈüÈüÈOäG~ä'ò#?ò#?òùùüÈüÈüD~äG~"?ò#?ò#?ò#?ò#?ò#?òùùüÈüÈüD~äG~"?ò#?ò#?ùùùùùüÈüD~äG~äG~"?ò#?ùùÈüÈOäG~äG~äG~"?ò#?ùùÈüÈOäG~äG~ä'ò#?òùùùq ?ò#?ò#?ùÈüÊ§ýìgo½õÖGD~äG~äG~ä'ò#¿²-´÷»¿û»S§NõfUWW¯òàÁäG~äG~äG~"?ò+Ã>ÿùÏÿÇæÿ¸ñ6>øõÿhÃÿ¾ð/ùùùüÈ¯Ü:qâDý¦ÿµ)ØüüýÊ¯üÊ»ï¾K~ä'ò#?òù_Yµÿþÿ4÷?åØüûõ_ÿõò#?ùÈüÊª·Þzë3ùLþ6¿þÕ_ýÕ'NùüÈüD~äWVõ÷÷ü/^à/NçÍ×ÔÔôÓþüÈOäG~ä'ò#¿rëý÷ß¿ç>ýéOö³½ë®»~çw~çïþîïáA~äG~äG~"?ò+Ïúûûßzë­^z)NcÚ÷ùùùÈüîÈüÈüÈüD~äG~äG~"?ò#?ùùÈüÈOäG~äG~ä'ò#?ò#?ùùÈüÈOäG~äG~ä'ò#¿±öÆo<ùä;vì¸þ7ò#?ò#?ò#?ù¸¾¾¾/~ñwÝu×¢E~ÞoWWWÿéþ)ùÈüÈüÈOe(¿¯ík÷ÜsÏÿóäÿ!ýÃµø©Oê¹ç#?òùùù©Üä7mÚ´5ÿcMî ÿüç¿ô¥/ùüÈüÈüTnò«ùDMòßÏçþ¹õË÷Þ/ùÈüÈüÈOå&¿Ï~ö³ÿmÙËßoÏûíM6ùüÈüÈüTnò_üä'¿ô¥/ù6ý¯Mû¯«­­ýðÃÉüD~äG~äG~*7ùEmmmÓ¦Mðó73~ènE~äG~äG~ä'ò#¿«>ú(^·Ïý!?ò#?ò#?òùßùùùÈüÈOäG~äG~ä'ò#?òùùùüÈüD~äG~äG~ä'ò#?òùùùüÈüD~äG~äG~"?ò#?ùùùùùùüÈüD~äG~äG~"?ò#?ùùÖÝÝÝÐÐN§:::òuvv677Ç¢Ù³gÇÅÈüD~äG~"?òÜe2Ý»wÇÄöíÛW¬X¿hÆGgvæÌäG~"?ò#?ùMîjkkb"Í666t±ªªª¡òû¿ùÿ3µµµ½ùæÿG¥ëð`Æ¡ÅK4^¨Æ¡´½òÊ+ÝÝÝÆ¡ÅßíáiãPÂ;öòË/ÒÖÞÞþÆoLä-ÞòK§ÓÃNç×ÕÕµzõê¡òáÞÀ^õÕS§N½¯Ò?Qñäö£ý(~UÒvðàÁþðÆ¡¤¿ÿýïvúôéøÅ8¶üàywüR©Tnº²²rè®Éd®^½jo¯½½²·×Þ^eo¯½½»ºººl6ìíé¥çÏ_¹rå®H~ä'ò#?òùß$«µµu×®]1§L¦@Z/¾téÒ°+ùüÈüD~ä7ùµ¾¾>J544tvvþûc«ø·G×ØØXùÈüÈOäG~whäG~"?ò#?ùùùüÈüD~äG~äG~"?ò#?ùùÈüÈOäG~äG~äG~äG~äG~äG~"?ò#?ùùÈüÈOäG~äG~ä'ò#?òùùùÈüÈOäG~äG~ä'ò#?òùùùüÈüD~äG~äG~ä'ò#?òùùùüÈüD~äG~äG~"?ò#?ùùùùùùüÈüD~äG~äG~"?ò#?ùùÈüÈüÈüÈüÈüD~äG~"?ò#?ò#?ùÈüÈüÈOäG~ä'ò#?ò#?ò#?ùÈüÈüÈOäG~ä'ò#?ò#?òùùüÈüÈüÈÏ8ùùÈüÈOäG~äG~ä'ò#?òùùùüÈüÈüÈüÈüÈOäG~ä'ò#?ò#?òùùüÈüÈüD~äG~"?ò#?ò#?òùùüÈüÈüD~äG~ºsäWq£R©ùùÈüÈüÊA~©N§ÉüÈüD~äG~äWò+ÈüD~äG~"?ò»Ùz×¬YC~äG~ä'ò#?ò#¿r_ccc:ö9?ò#?òùù©Ìå7öì¡wÔÖÖÆùùÈüÈüÊJ~A½K.Õ××ÇD¼g<ÿüó1±zõjò#?ò#?ùùü|1Ô³gÏÄDuu5ùùÈüÈüÊJ~S¦L	çÅ7àxè¡	ßêB~äG~"?ò#?ò+7ùmÜ¸1w<GþGýæÍG~äG~ä'ò#?ò#¿²_ôÈ#L:5&:;;c"ØÒÒ2)ò#?ùÈüîÈüD~äG~"?ò#?ò#?òùùükæÌÉw»ø&gò#?òùù©å7cÆ|íårl/ùùüÈüÈ¯ÜäÈóuuuLºçüÈOäG~ä'ò#¿1T[[òì#?òùùüÈoluwwüÖ­[wåÊò#?ò#?ùÊY~Ñ´iÓ*äò#?òùù_¹Éoúôéð ?ò#?ùîù%æëééÏùÈüÈOäG~c¨®®ÎäG~ä'ò#?òÓ!¿ LÈoãÆäG~äG~"?ò#?³ü*FÈäG~ä'ò#?ò#¿2ü&çasùùüÈüÈ¯¿ÕeòF~ä'ò#?òùßjll>ú'ÈüÈüD~äG~*sù¥Óém;ìîînhhëljjêèè(¾h|sòøá_xá÷'°=öÄ¯ª÷Uº^íµð´q(a'O|î¹çCiûÖ·¾õÎ;ïvøðáø­nJXüýÌ3ÏÒ¶wïÞ¼Å]~ñòÛ¼ysooïÍ·K&Ù½wLlß¾ÅÅoN~6l¨$I*>vùöØÞÚÚÚÙl¶±±±ø¢ñÍ!?ID~ã¬´Çöæ¯UpCoùI$ò»-ÊßRXYYY|Ñøæä÷ÇüÇO<ñÄëØÿù···¿®ÒõÌ3Ï<ýôÓÆ¡½úê«ßüæ7Ciû¿øW^yÅ8°çîoÿöoC	ûö·¿ýgögÆ¡´ýå_þåË/¿<·8ÉäWWWÍf³1]|Ñøæ8¶×±½rl¯cÛ+Çö:¶wüªî»ï¾ªªªêêêeËûPÖÖÖ]»vÅDf2âÆ7üÈOäG~ä'ò#¿ñ¿PÝÍ<¾ÿÆ7¨¾¾>J544tvvþû½Üi=tÑøæùüÈüD~ä7ÎfÍ2[²dÉ+WâlooïÒ¥KcÎ9snÿçüÈOäG~ä'ò#¿1TYYÎëïïÏÍÉf³1gèáäG~äG~ä'ò#?òÜòK¥Rá¼ä(¤¾¾¾3¾ou!?ùÈüÈïö_²·wñâÅÉÞÞ8éÓÜÜL~äG~ä'ò#?ò#¿²_PoØ#<._¾L~äG~ä'ò#?ò#¿²_òZY¶lYMMM*ÓÅÇIñ|ùüÈüD~äw§D~ä'ò#?òùùùÈüÈOä[óFåÿ¹äG~äG~äG~äG~ä7å9ò#?ò#?ù®ß	7lØÈïÙg%?ò#?òùù_yÊ¯»»»ºº:Ì·hÑ¢ü/v&?ò#?òùù_YÉoùòåÉ¦¾L¢çüÈOäG~ä'ò#¿1ôâ/&æ[²dÉ¤>ÈüD~äG~"?òíKdÎ9ÉÁñù ?òùùüÈïÆmÛ¶-ÙÔ·bÅÉû|ùüÈüD~ä75ùùüÈütÈ/u£Òé4ùùÈüÈüÊA~åùÈüÈOäG~äG~äG~"?ò#?ùùÈüÈOäG~äG~ä'ò#?òùùùù­¿þë¿1cFUUU~ãß ?ò#?ò#?ù_yöõ¯½¡¡á¿õ¿?øõåïæ3Y¿~=ùùÝ.ò9sfee¥ïó#?ò#?ßÍ÷á~òüÚú¯û1]]]æÌò#?ò»õò1cF¾ö|ùùün¦Î;7Ç¾äßoýÖoíß¿üÈün½üya¾®®®I÷|ùüÈïv+~OþÆoüFüfÏÝÖÖF~äG~·^~µµµ!¿ÉÈ>ò#?ùÝ¯ÀOÔ$òKþýþÊßÿÄ'>ñüüÈün½üº»»C~ëÖ­»råùùÈïæé¥¦NºhÑ¢/·~ù¿,þ/1ýüóÏüÈünü¢iÓ¦UÉäG~ä'òw===ðø[½zõo¼1ôäG~äwkä7útGxùÈo#?ò#¿[#¿Ä|ñÇÙd|>ÈüD~äG~"?òCuuuð ?ò#?ùîùeB~7nìëë#?ò#?òùù©åW1Bð ?ò#?ùùá79#<ÈüÈOäG~äG~eø­.7ò#?ùÈüÈüÈüD~äG~"¿áÊf³÷Ýw_UUUEEEuuõ²eË&Ë¡¾äG~"?ò#?ùí2ìâP_ò#?ùÈüÆÐ¬Y³ÂyK,IþßÞÞÞÞ¥KÆ9sæùùüÈüÈ¯¬äWYYÎëïïÏÍÉf³1'æùùüÈüÈ¯¬äJ¥Ây¡½Ü¾¾¾ã[]ÈüÈOäG~äG~å¹·wñâÅÉÞÞ8éÓÜÜL~äG~ä'ò#?ò#¿²_PoØ#<._¾L~äG~ä'ò#?ò#¿²_òZY¶lYMMM*ÓÅÇIñ|ùüÈüD~äw§D~ä'ò#?òùßjll>ú'ÈüÈüD~äG~*sù¥ÓéÉºíüÈOäG~ä'ò#¿1ÔÑÑòÛ¼ysooïdùOÛÈüD~äG~"?ò×UP*"?ò#?òùù_YÉ/5B¾ÉüÈüD~äG~äW&ò>zSSS¼CLêçüÈOäG~ä'ò#¿üw½×÷öN»äG~"?ò#?ù³ä¿ëMþ^ò#?ò#?ùÊY~EsùùüÈüÈ¯Lä×ÕÕ5eÊdË_â<GxùÈüÈOå)¿yäG~"?ò#?ùÝÑùüÈüD~äG~äG~ä'ò#?òùÝuww744¤Óé¦¦¦üEÍÍÍ±höìÙq1ò#?ùÈü&wLf÷îÝ1±ûö+Vä/1cÆÑ£GcâÙg9sæPù½ôÒKç'°9sæ¼J×öãÿøÕW_5¥-ÞPß÷]ãPÂÞzë­7ÞxÃ8°³gÏ¶··ÒHÿèG?È[üØåwòäÉ[.¿ÚÚÚÈf³#]¬ªªj¨üvìØñæöòË/ÿûßS¥ëµ×^;tèq(añªq(mmmmÆÁÏþí×CiåW¾÷½ïMä-~ìòK¾Ò¥¹¹ùôéÓ·J~ùt q¼ W¯^mo¯½½²·×Þ^eo¯½½ã/ÌûöæP×Î93Á#ÿ[£+++^àÊ+Lfèÿ2L~ä'ò#?òùß;yòä¬Y³r~Ýwßï]ÿE1]WWÍf¯îíéK?~åÊ.z%äG~"?ò#?ù³TUUMðÿÞÖÚÚºk×®ÓL&S ­Å_ºtiØÉüD~äG~"?òs===ùÛüjjj~¨îcÖúúú°fCCCggç¿?¶ÁÍùÿ0ùÈüÈOäG~ã/|UUU+V¬8wîÜdy>ÈüD~äG~"?òËUØqï½÷=vÒ=äG~"?ò#?ùígoò>äG~"?ò#?ùÝ)ùüÈüD~ä7¶?>sæÌêêêäxÞººº½÷ùùüÈüÈ¯Üä×ÞÞ;Â#_2½ûvò#?ò#?ùùüêëëÃyÇÏÉ¯³³3ùbò#?ò#?ùùürßßÀÀ@rÀ/ùùÈüÈüÊJ~uuuá¼d;_È/Í>ðÀ1ÝØØH~äG~ä'ò#?ò#¿²_P¦b¸=J~äG~ä'ò#?ò#¿²_táÂäØÞªªª3g9sfR<äG~"?ò#?ùÝ)ùüÈüD~äG~äG~ä'ò#?òùýrçÎknn®©©IMMMeW/ùÈüÈOäG~£mÕªU#´fÍò#?ò#?ùùüöîÝ ï©§÷Üfß¾Éü¶¶6ò#?ò#?ùùüw;wîº(ùÿÜ|ùùüÈüÈ¯LäWYY¼WÉÐEÙl6ÅÈüÈüD~äG~äWòËý§m#-Mþ'7ò#?ò#?ùùüØüÈüÈOäG~ä'ò#?ò#?ò#?ùß$_ñÈüÈüD~äG~äW&òKÝ¨t:M~äG~ä'ò#?ò#¿r_yD~ä'ò#?òùùùÈüÈOäG~äG~ä'ò#?òùùùüÈüD~äG~äG~äG~äG~äG~ä'ò#?òùùùüÈüD~äG~äG~"?ò#?ùùùüÈüD~äG~äG~"?ò#?ùùÈüÈOäG~äG~äG~"?ò#?ùùÈüÈOäG~äG~ä'ò#?òùùùùùùÈüÈOäG~äG~ä'ò#?òùùùüÈüÈüÈüÈüÈOäG~ä'ò#?ò#?òùùüÈüÈüD~äG~"?ò#?ò#?òùùüÈüÈüD~äG~"?ò#?ò#?ùÈüÈüÈüùùùüÈüD~äG~äG~"?ò#?ùùÈüÈüÈüÈüÈüD~äG~"?ò#?ò#?ùÈüÈüÈOäG~ä'ò#?ò#?ò#?ùÈüÈüÈOäG~ä'ò#?ò#?òùùüÈüÈüÈOäG~äG~ä'ò#?òùùß(ëîînhhH§ÓMMMC/ÐÞÞ^QQA~ä'ò#?òùß¤/ÉìÞ½;&¶oß¾bÅ¥ýýýÍÍÍäG~"?ò#?ùCµµµ1Íf>úè£[·nI~õWÕ5½üòËGíRéúÁC	èýÛ¼øCº££Ã8°ïï~öK^[[Ûw¿ûÝ¼Å;B~étzØéèÜ¹s---áÂä·oß¾L`|ï½÷~¢ÒõÖ[o;vÌ8°÷ß?^¨Æ¡´Q~üãööÛo¿ùæÆ¡Å;f»q(mñ'Ê»ï¾;·xGÈ/Jå¦+++ó-[¶ìðáÃÿöPííµ·WööÚÛko¯ìíµ·wVñbº®®.Í&czØå.L~ä'ò#?òùß$®µµu×®]1§Lf$)I~ä'ò#?òùßäÖúúúT*ÕÐÐÐÙÙ9,õÈüD~äG~"?ò»£#?òùùüÈüÈüÈOäG~ä'ò#?ò#?òùùüÈüÈüD~äG~"?ò#?ò#?ò#?ò#?ò#?òùùüÈüÈüD~äG~"?ò#?ò#?ùÈüÈüÈüD~äG~"?ò#?ò½ñÆO>ùä;N8A~äG~"?ò#?ò#¿ò¬¿¿ÿ_üâ¯ÕþÚþóüÿ]ýo|üÈüD~äG~äG~eØÃ?|Ï=÷<ð?xðëÆ¿?úÔ§ÚÚÚÈüÈOäG~äG~äWnûVÿÿ«ö%ÿ>÷¹ÏýÞïýùùüÈüÈüÊ­O|âùì_nýò¢EÈüÈOäG~äG~äWnÍþÿfõòå·`Áµk×ùÈüÈüÈ¯Üzé¥~­ö×r;|¿ùâ)StäG~ä'ò#?ò#?ò+ÃvìØQUUuÏ=÷|úÓ¾ë®»Fÿ¼ùùüÈüÈoòÕ×××ÓÓsâÄþþþÑ¯E~äG~ä'ò#?ò#¿;%ò#?ò#?ùùÈüÈOäG~äG~ä'ò#?òùùùüÈüÈOäG~äG~ä'ò#?òùùùüÈüD~äG~äG~"?ò#?ò#?ò#?ò#?òùùùüÈüD~äG~äG~"?ò#?ùùùùùüÈüÈüD~äG~"?ò#?ò#?ùÈüÈüÈOäG~äG~"?ò#?ò#?ùÈüÈüÈOäG~ä'ò#?ò#?òùùùÈüÈOäG~äG~ä'ò#?òùùùüÈüD~äG~äG~äG~äG~ä'ò#?ò#?òùùüÈüÈüD~äG~"?ò#?ò#?ò#?ò#?òùùùüÈüD~äG~äG~"?ò#?ùùÈüÈüÈüD~äG~"?ò#?ò#?ùÈüÈüÈOäG~ä'ò#?ò#?ò#?ã@~äG~"?ò#?ò#?ùÈüÈüÈOäG~ä'ò#?ò#?ò#?ò#?ò#?ùùÈüÈOäG~äG~ä'ò#?òùùùüÈüÈüÈOäG~ä'ò#?ò#?òùùüÈüÈüD~äG~"?ò#?ò#?òùùüÈüÈüD~äG~"?ò+'ùuww744¤Óé¦¦¦üE+W®¬¬¬¼ûî»C]äG~"?ò#?ùMî2ÌîÝ»cbûöí+V¬È_´eËÇl`` È5úô¡òù?Àâµ··÷ç*]'OßþÆ¡ôÑGñq(m¯½öÚåËC	?ùÞyçãPÂ®rðàAãPÚ^ýõþô§ywüjkkÃv1Ífó5558qb¤C~?þxÛ¶ÿþ_~¹M¥ëï3%,^¢ñB5~öoó8àgßÏ¾ý¡ÝòK§ÓÃN'g·mÛVUU5úô·ß~ÛÞ^eo¯½½ööÊÞ^'w©T*7]YYY°hçÎ1qòäÉò#?ùÈü&_¿(¦ëêê²ÙìõÁ½½1±ü³ÉüD~äG~"?ò|µ¶¶îÚµ+&â4Éä/Z»ví=bâøñãóæÍ#?òùùüÈoÒk*jhhèììü÷Ç6¸9°··wÙ²eétº¥¥%@~ä'ò#?òùßùÈüÈOäG~äG~äG~"?ò#?ùùÈüÈOäG~äG~ä'ò#?òùùùùùùÈüÈOäG~äG~ä'ò#?òùùùüÈüD~äG~äG~ä'ò#?òùùùüÈüD~äG~äG~"?ò#?ùùùüÈüD~åÔÃ?üÂ/¼?íÙ³'~U½¯ÒõÚk¯§C	;yòäsÏ=gJÛ·¾õ­wÞyÇ8°ÃwttE?óÌ3Æ¡´íÝ»·§§g"oñ£>"¿;~üø>ø'$IeQñ6ÃJ$Ý!$IùI$ü$ID~$I"?I$$IÈ¯L:~üø9sÒétSSÓ#GróÛÛÛ+*iôÂy¢Ò¾¾¾+WVVVÞ÷Ý2D%ÕüWi*2D7?¤ñâ5kÖÐ_°÷<y²¥¥%~ö/_ÞÛÛkÆ]þ»|wwwCCC2ÎäWæÅÓ¼oß¾8zôhmmm2³¿¿¿¹¹QJ5¤÷î¦é-[ì±xg>º!*ÕÏ~ÒîÝ»·oßnn~HãôÜ¹s1§èæ4 ØÙÙ'NX»v­!_ïòL&~êc"~ðW¬XA~wJmmm³gÏN¦ôÑ­[·_©4ØüòR©4Þâ÷¾)ùÏ~töìÙiüYrþüùS¢dHÓétnæ)SÌø*xUÇÒ1Ífoù(ä1Aö¯©©WÀ³Ï>ümÚÒÒ/ò+ÕÎ1cñâÅñ+öäÉÆçæ4sÛ¶mUUUñnúöÛojÒ²eËºººNI´»»;^¥q6NjI4üwüøñxúé§ó¨Ñ7ô]>$où¨ÇÄÕÞÞ^WWüÞ?|øðõÁýi®ø»?ãÆ=¤©TjçÎ×ñ¡#Sªê©S§æÎkLJ5¤sæÌI6NlI4þÒ9sfHzëÖ­qjdÆÑÐwùüÏõVVVßT"ý_Î°ÜüæwË¨ÊcHóIíïþ¾P·lÙ²mÛ6Rª!½­6¥Ù¯ÓÓ§OÏ5Ë°VCÞåã7j6½>¸··`ùa3fÌH¶wvv.^¼¸àÅa|J2¤1çÌ3×·ù²Æ7¤k×®Ý³gÏõÁCÿæÍgJõ³?þücÇRéÜ¹s9===sæÌ1D%ùuÚÝÝ=00°uëÖGyÄÝ$ÖÖÖ]»vÅDf2ò+óâG¨©©)þZ¸páÈïãÒ®®®ä,X|Ü[79¤½½½Ë-K>:yêÔ)CTªýÊÊÊäÞ*É>:ùR8iCtóCzèÐ¡iÓ¦ÅuõêÕýýý¨$ò;räH*jhhH&?I$$IÈO$Iä'I$ò$I"?I$$IÈO$Iä'I$ò$IùI$ü$ID~$I"?I$$IùI$ü$ID~$I"?I$$®§~zþüùU-ðÅ_ü¥_pM_ÇÃÝÛÆÆÆxhW¯^-sÒétCCÃÀÀÀX¯SùIÒäëÁ¬Ò#<RNòÛ¼ysÌ|òÉ'æ?ñÄ1ÿ¡ÇuJ"?IduwwiÒéôSO=Õ?ØÎ;ãlÌ<vìXÙÈïìÙ³1söìÙógÍóÏ9C~ÈORù÷¯|%LóØcåÏ|üñÇcæW¿úÕ|÷tttÍÍÍ1»ðåËW¯^]SSjkk×¯_¿SõàÁ¡«Xë¶µµX*æL2eþüù³­­­wlÿþýÅ¯'Yw)YÔÞÞ>Ò/^ó;;;ss=s-Z³eËúúú¸ªªªªåË?~¨ü^Á"wUùIÒ­lÚ´i¡sçÎåÏüàbfccc¾lêêêJ.]º´`ÑºuëE===©TjØµ³ÉÒeË+++ûûûciª««ß¹öî¤Ï>ûl>gs¸Ü½wW²páÂ±Ê¯È]D~tKvìóK­¢"/+V,&âì%K¥	t;&îBlÉ¢L&l,é#G$W6mÛ%;[W­ZsöíÛÓqÓ÷ßÿ¯'Ôgãù÷mØG:uj<ÞK.ÅÙ8ÜÌf³ÉbÅx×±w¸ªªj¬ò+rW%$ÝzùEÃÊ/7?M¦èüùóq6Ìmnn³Ó§O_³fMíÚµk¹+ËlEË­ýàr-åvøÆiL<xð×Ê³/^Ì¿o#&oÃ±hëÖ­×±GýúõùüÂ£---É&É±Ê¯È]D~tK6t]¹r%æÕ«Wcf,*bO:à/Üóî-°Tþw©Ätmmmeee___)SrKox=#9¬ ãÇ'Hé8éÜÒ#GÄv¯ñèåWä®J"?IºÅ%uÛ¶m[þÌä»NðÈðÒ¥KC7e;vlóæÍÉ¾×r¹Ý©7ôÙºuë¼qºzõêÜü"×lc»páBröâÅÅÃ;wn,M¾È&À¿(9Î7µ··÷öö_N¥Éhä¹«ÈOnqÉétzûöíÉ·º<õÔSCÆX²dI(§¯¯/Ù;7ù_òñ¸S§Nå@0àúõëÃIÉQ·¹ïUÖgÉÁ¶É­:t(7¿Èõ$ìK>çwõêÕäEä·sçÎÜÖ¸xÈù<öôôÄ­Ä $¿Äaå¸XsùòåùKÜUIä'I·¾ä[Ú²eËÿû7X£Ütn?iî V­Êge~÷î-"¿ëÿÙFÌ¯­­Íßzânäß±Üqµ#=Øk×®UUU%¡`÷÷ÝS§NÓä]ò¯3Ù$+qjni»*ü$é¶(t²páÂÊÁæÏ`[ ¿öööäkó>[zõêÕx ¾¾>ÑÒúúúrKÛÚÚZZZd;vì(¸Î¡÷$®*æ¯]»¶`þH×Å»|Ñ`ïóËµzõê¯wIºtéR&¨©©GqæÌÜ7Ôä_goooÐ6«EuvvÜb»*ü$ID~$I"?I$$IÈO$Iä'I$ò$IùI$$IÈO$Iä'I$ò$IùI$ü$ID~$I"?I$$Iô¤èøÉzú¾ÝIEND®B`


ãüùó±¦ÓéøìãÖP1yòRùÚ³gÏZcó§üª««¯_¿^*å÷È#Lübíß¿¿ÈÚf´ÈTûC[[ÛL7Î¬7c&ÉÞÖÖVåÊø°"c``àé§®ªªÝÝÝ¡üÂ-[J¢ü.^¼¤ê·¿ýíÑÑÑÄÍØÛÛ;éªfºÈÄýaïÞ½1%jì®_²àï½ã7¯¾4ò²*¿Äã?>îpËÙ³gW¬XN§W®æÌqå_I2;,^¼xíÚµEÍßùµ×^Û°aCr"ò¸uëVá'mjjJìèè÷P§Zí÷¶oßO<9Õ6yë­·¶mÛ<Âl6Ûßß?îÏ74>u<uëÖÝ¸qãüùó7n,<¾xðàÁºººä´f|ü¬©ð¿øÅ^xî5D©ÇÄ;wNúõé"?uÄbL9£3é1¹B³[°xù_ØCb³ö³m[üèàÄ¹ÓÜ£PcKFLÇ¬=öúÆÊJ©ü"Vbbr³··7Jþ<¾xñbòKî¼uëÖ"Ëæï?ç¶´´¬ê¼^Õ_pâsÈd2µµµ1¸M®]»?Î×÷hÏwâç]½zur5Ï¾q÷~ñ¾YºtiL¿yóæÄ/Ö²eË&ýúÎtq:²&9æ·|ùòmâÖN¿üzzz×Ov¦å7ÍÝxË-ãóîÝ»cå¥T~Éôh²dÍfãfggg»ººûlÜâÉÍûö&Gqî¸lüàùqÿä@cDR27~âÆÍXüöXªðsYmñ'î§N*<Vxÿ]»vÅxóæÍïAÜS=ßdÊ>822<°dÍqóÌ3O0y5[¯R8kªN§§z"ùeç¸ÈT/¤ËéÆôQÍzÁâM_x³p7¦Ï÷ÙôËo»qRW¯^q|5c¼hÑ"ßX@ùAé_þ_ò²°B1¥Hù]»v-?åËæ%gãçhr3~|¬w¶®Èj/8ÕsO1~¨^[[[øâ|ÜdÉTÏ7µQx39<ÁÂGËå¢¢0òËçøT>ÇE7i,sÃgÏt£MgãLú¨f½àôË/ÙCnÜ¸Qø)fT~ÓÜâfCCCkü144ä»(?(±òR555Sý¸ªQ8E~TéþlÅj§O9oüü.>ñÙÄ½M|¾Ó¹9îç¯Q­««KNOOÌ»q=_ÃtÎ¶ôÓÙ8SõèìþÙÞ¹ï]ÓÜûúúøËáéÓ§cå¥T~Ï<óLáÉQ´qWYN'tf´lñc67oÞ,[dµÅ,òN87óïLLâlÜÑ©qM<ýRxsÅÉ»«tttDM§üÉ¬d³Ù=ö¼÷ÞGÛ¶m´ü¦³È¬Ëo¦'oÖN¿ü¦sÌ/ßvÉ¯:E¶ý-qéÒ¥$§ó×AÊæùÅÏ¶ äsþ]]gñ2¹TvåÊÉ¬äENc&ýYdÙâÙ$Kò:­Xy²q¯âtµÅ,[¶lÉ¿?»yóæ1ÉÚ|ðÁ¹_rÜ«··7ÂáÃ§S~çÎK*ÅSîêêJç%N:5é×w¦Ì¨üoqûF¡nÕY_òÊ¼Ø"ì6mÚ4±|òÉØøQÁÅ·ÿÿ$¯×ìëë+ræP~0_Êo¢Âë'^-½þMwdY¶x6E%mTx¡e~nÕ_°xF~=ÿ®dJÿ¸×xÕÖÖæ_D8òË·HbÉ%ùW=éx`âkÕªUE¾Ä3ZdFåW|ãÛ7Ít«Î¢ü.]ºT¸þµk×Î·MòOõYìoùKòvìØá(?(òë×¯ø×½Î9ÓÜÜ?ÎyæüôS§NÅ(­Âw³æ²wÌ¦ämðbåMMMßoªÕÞqÁâ	-þæonÛ¶­rL6x=ÇìÊïÖ­[±¶Xg4ÐC=1îâóÔSOÅ³'¾téÒ£G~þóÏsû"3*¿âgÜ¾1Î¶ê,Ê/>º¡¡!u¬?9¿9nF%ÃÝÝÝw<æ:Õþ688øðÃ'S#ßãK9<<ì(?E.àfôr³X¤ä$ÇzµGFF _±bå&yß8=ö-Êr388¸wïÞ¥K&§tcðÈ#Ø, üP~(?ÊåòP~(?Êåò@ù üùæÔ©Sõõõétzýúõ3]¶±±±¢¢¢¯¯/?%Æ1eåÊ3øv6fv÷Î²³ù;ÂY©Tªªª*ÍÞºuË^(? 4ÔÕÕEÇD¾ÌtÙÆ²O<ñD~Ê¡CbÊJºü¬¿pJ<ñ·´´ØåÈw9ÄÓ¥KbÙuëÖå§¬^½:¦ôôôÜùÑ_´r«ªªìEòJ&ûkæá®®®^´hÑþýûÇÝíóÿ|ssó¸5äÆøæÍ1)É¬«W¯nØ°!Â(N755uvvNº¶ÂÏ^|£GÖ××¯Y³&>ÑÄ»ûvkkk,~ïÞ½É!ÌhÓµk×ÆÚbz¬ùÚµkRù½ùæ©T*yF£££ñàãfL´ÊwñôÑ?óÌ3ÑX1xì±ÇïsæÌ¯iÛµkWÌ:räHãcwîÜÌZµjU®Ó3Ì¤k+|Å;qâD¶oß>ñÁïÞ½;Æqç>ÿøÇc<00ÐÛÛää¤/àiùEÞÅöñ¾ô¥dÖMñäOÆàG±wÊ¿åW__ãÜá5éÏ;³6oÞã-[¶$IéÒ¥ûö577'DLº¶quU|äUWWO¶¶6yðÑd1æqÏ755EËÏeãNI[¬<²/²2zÙ²eñ0-Z´|ùò¸iï0Ë/NçÇ1SP^dVNÜspp09©SY¥"_íµÂ5[[áÍé,2Õ+<TãÉ'/^L©©©IþVùM*¹À%+P~À|,¿L&SxÌ/9lvÇÜimm¹ùùé1etÌ4Ë¯ø"ù17qÙ%K$Ëx###íííÛ·o/<X8sÇM100P[[Íf×®]5|ãÆ üù[~É»´9r$yß¡C¦S~'OÌ<;qâD~zÒ/_N^ÿ7ò+¾È¾ûöÙìÚµkâ²;vìñ±cÇ^ýõäoLfM_íµè°,_¾üÃ+¿=öÄ¬K.uuu¾@ùó±üFFF¢®)|O¾âå744iù½Ó§O×ÔÔTWWïÝ»wåW|³gÏÆÜÕ«Wç/4)¼Ïàà`´W<òÊÊÊM6%ñFðe³ÙäbáuëÖÍôbÛé_ÔjáÜ¬]»6nöööÚÁåò@ù(?ÊåW2¾ÿýï¿ýöÛwó3ö÷÷û[LÌÑÍ7ßï=Û¹ºËßý(Kÿó?ÿc;(¿ño|#âïn~ÆsçÎý÷ÿ·=¹xýõ×úÓÚÌEüòðüÀv`Î9ó_üÂvP~ÊOù¡üP~(?òS~(?Êå§ü@ù¡üP~(?åÊåòCù)?(?ÊOù¡ü@ù¡üP~ÊåÊå§üòCù¡ü@ù)?å§üP~(?P~ÊOù)?ÊåòS~ÊåòCù¡üòCù¡üP~(?åÊåòCù)?P~(?ÊOù¡ü@ù¡üXXå×ÓÓÉdÒétcccggçÄ;tttTTT(?(?ò+yÙlöØ±c1hkkkii7wdd¤©©iªòkooï.ú×ý×k×®½spéÒ¥ÿøÿ°+W®¼òÊ+¶sôÒK/Ý¼yÓvWDùÕÔÔÆ ËÕ××ûè£>þøãS_Äâî¢ÍóçÏÿæàìÙ³ñ+íÀöÅÏlÛ9:útWWí0¯,òK§ÓÃÕ«W£íÅÙ^p¶gí-©T*?®¬¬,µuëÖóçÏÿò©*?(?ò+µµµ¹±³½1þgøÊÊOù¶ÖÖÖ£GÆ >f³ÙÉªc~(?P~(?åWºººêêêR©T&éîî4õÊÊOù-hÊåòå§üòCù¡ü@ù)?å§üP~(?P~ÊOù)?ÊåòS~ÊåòCù¡ü(?Êå§ü@ù¡üP~(?åòS~(?ÊOù¡ü@ù¡üP~ÊåÊåòS~(?(?å§üÊåÊOù)?åòCùòS~ÊOù¡üP~(?òS~(?Êå§ü@ù¡üP~(?åÊåòCù)?òCù¡üP~ÊåÊåòS~(?P~(?å§üÊåÊOù)?åòCùòS~ÊOù¡üP~ üòS~(?Êå§üÊåòCù)?P~(?ÊOùòCù¡üP~Êåg; üP~(?åòåòCù)?(?òS~Êåòå§üòCù¡ü@ù)?å§üP~(?òS~ÊOù¡üP~(?òS~(?Êå§ü@ù¡üP~(?åÊåòCù)?(?ÊOù¡ü@ù¡üP~ÊåÊå§üòCùQâ;ÌO<ñOÿôOo¿ý¶òCù¡üÊò488xÿý÷ìcûÌg>³fÍî¹çÙgU~(?òCùQþìÏþì¾¦ûömßþ¿Þÿ¾¼ýË/îííU~(?òCùQn¢ó¾ºç«Iö%ÿî_ÿ£>ªüP~(?åò£¬¼ÿþûµµµÙÿ¾ð/üùÿ¹òCù¡üÊró¿÷»þbWaùªùSO<ñòCù¡üÊrów÷wõõõõà_ýêßç¿ððï¾û®òCù¡üÊ2tðàÁßùß¹÷Þ?ö±â(ryòCù)?å§üP~¼ÁÁÁW_µ¿¿dd¤ø=°ü~þóïÚµkå'WÞwßûöíÿ/ÊOù)?òc¡ßûï¿¿téÒOêS;vìøòö/òljjZñ§üÊåå_~_ùÊW¯Z±bÅ¡CòS~(?[ùÝwß;vì(,¿?ù?Q~ÊOù¡üP~ üòS~(?fù9Û«üÊå¥ü¡üÊå¥ü>WÈ¾+VDöík_ó®.ÊOù¡üP~P¶åòS~(?(?å§üÊåÊOùÍO===L&N766vvvÎêîînjjY+W®»)?(?ò+mÙlöØ±c1hkkkii)µlÙ².ÄàøñãË/W~(?P~(?åWÚjjjFGGcËåêëë§º[UUÕÄòûÇüÇÜEñäÇ?þñO`^yåøÅv`..^¼ØÑÑa;0G/¾øâk¯½f;Ì+¢üÒéô¤ãBñmnçÎËï¹çû.úù¾¾¾·aº»»ã÷Û¹xã7^~ùeÛ9jooÿÙÏ~f;Ì+¢üR©T~ñ·oßÎf³ß¹ÇÙ^íÅÙ^p¶×ÙÞS[[Ëå³½17÷úõëÛ·o¿qãÆÄÊåÊOùÖÖÖ£GÆ >f³Ùq¥µqãÆ[·nMº òCù¡ü@ù)¿ÓÕÕUWWJ¥2Lww÷¯[Å/]EåòåòS~òCù¡ü@ù)?å§üP~(?P~ÊOù)?ÊòS~ÊåòCù¡üòCù¡üP~(?åÊåòCù)?P~(?ÊOù¡üÊåòS~(?P~(?òCùòCù¡üÊåÊOù)?åòCùòS~ÊOù¡üP~ üòS~(?Êå§üÊåòCù)?P~(?ÊOùòCù¡üP~Êå§üP~(?òCùòCù¡üÊÊOù)?åòCùòS~ÊOù¡üP~ üòS~(?(?å§üÊåòCù)?åòCù¡üP~ÊÊåòS~ üP~(?òCùÙ(?ÊOù¡ü@ù¡üP~ÊåÊå§üòCù¡ü@ù)?å§üP~(?P~ÊOù)?Êå§üNùUÜI*R~ÊåòCùQåºt:­üÊåò£Ê¯<(?Êò«]»v)?åòCù¡ü(·ò«¯¯O§Ó^ç§üP~(?e^~+W®xyGMMÍàà òS~(?Ê²*¿ÊÊÊH½[·nÕÕÕÅ ïÙgÁÎ;òCù¡üP~Uù%ùb©+W®Æ`Ñ¢EÊOù¡üP~(?Êªü/^×ÕÕÕ××Gy$xWåòCù¡ü(·òÛ»woþzÂú­Y³Fù)?ÊåGY_øæ7¿¹dÉtwwÇ *°¹¹¹$¿òCù¡ü@ù)¿Bù¡üP~ üòS~(?(?å7åË'ïíâÊåò£ËoÙ²eµçÚ^åòCù¡ü(·òÈæ»xñâèèhÉ=åòCùòS~3PSSåWÙ§üP~(?P~Êofzzz¢üvïÞûömå§üP~(?å aéÒ¥¸ÂCù¡üP~(?Ê­ü¡üP~(?¢üæëíí-Åç¯üP~(?P~Êojkk]á¡üP~(¿ßè'xâ;ßùÎ;ï¼ã¥ü_å)å·wïÞááaå§üP~(¿¹Ôkll¼÷Þ×¯_ß÷UWWè9%åGÙ_Å¡üP~(¿ºÿþû×­[·ÿ¯÷'ÿþÏÿÉdÉó¥üRSpòCù¡üfäÝwß­­­Ý÷µùòË-ûáèK¦ü/åWÒÊå7ôööÞï½ÙÿþøýñsÏ=çK¦ü/åW__ßÐÐ?òCù¡üæâý÷ß¿ç¾²û+ùìÛ÷µuuu¯¾úª/òc¾_:®¨ø­;ìééÉd2±ÎÆÆÆÎÎÎâ³f7¥Ð×¿þõøUòí»èÄo¼ñÆÛ0±'Ço,¶sñüä»ßýî<|`_þòÿðÿðO[ÿô/ÿï_îøò+W~æ3ùÙÏ~æK6?ë[ßêïï·æ½üâPßæþÞ.ÙlöØ±c1hkkkii)>kvS=ôÐCåâC/¿ßîµ½555I>ær¹úúúâ³f7EùÊo~»×ö.5ngÍnòß¼Px¤°²²²ø¬ÙM)ô7ó7O=õÔ÷ï¢¿ÿû¿ïèèø>ÌÁ±cÇ¾óïØÌE[[íÀ:tèÜ¹s¶Ã¼RbåW[[Ëå³1.>kvSkÁßíÅµ½®í½ªM6UUUUTT,Z´hëÖ­³¾Ô£µµõèÑ£1Ùl¶ø¬ÙMQ~(?P~(?å7KCCCfÝñíêêª««K¥RL¦»»ûWrì¤õÄY³¢üP~ üP~ÊoV¬Xe¶yóæÛ·oÇÍ-[¶ÄU«VÍÿç¯üP~(?P~Êo*++£óFFFòSr¹x9òS~(?ÊÒ.¿T*)³WåÊåòcþ_r¶wãÆÉÙÞøãÒÔÔ¤üÊåò£¬Ê/RoÒ+<âÛòS~(?Ê²*¿Æ.ïÝºukuuu*7n)%ñüÊåÊOù-Êåòå§üòCù¡ü@ù)¿üwRøsòCù¡üP~pù¥¦¦üÊåò£¬Êo*=ôPR~ÇW~ÊåòCùQå×ÓÓ³hÑ¢h¾6¾±³òS~(?Ê²*¿mÛ¶%úÚÛÛKèù+?Êòç>i¾Í7ÜóW~(?(?å7-CCC«V­J.æèìì,Åç¯üP~(?P~ÊïÎ|òÉäP_KKKé>åòCùòS~ÓXÒûù)?Êå§üHù¥î$N+?åòCù¡ü(ò+Êåòå§üòCù¡ü@ù)?å§üP~(?P~ÊOù)?ÊåòS~ÊåòCù¡ü(?ÊÒ*¿åËWVVz??åòCù¡ü(óò[¶lYaíy??åòCù¡ü(ÛòÈæ»xñâèèhÉ=åòCùòS~3PSSåWÙ§üP~(?P~Êofzzz¢üvïÞûömå§üP~(?å aéÒ¥¸ÂCù¡üP~(?Ê­ü¡üP~(?¢üæëíí-Åç¯üP~(?P~Êojkk]á¡üP~(?¢ü"e¢üöîÝ;<<¬üÊåò£Ë¯b®ðP~(?Êr+¿Ô¡üP~(?åV~%Mù¡üP~ üòS~(?(?å7·iÓ¦ªªªEmÝºµT.õU~(?(?å7CCC^áQú*?Êò+VDçmÞ¼9ù»½[¶l)«V­R~ÊåòCùQVåWYY722ËåbJLW~ÊåòCùQVåJ¥¢ó¢öòScwuQ~(?Êr+¿älïÆ³½ñ1Æ1¥©©Iù)?ÊåGY_¤Þ¤WxÄ·å§üP~(?eU~]Þ»uëÖêêêT*7nÜSJâù+?Êò[(ÊåÊOùÍ@CCCü(R~ÊåòCùQæåN§+*JõØ¡òCù¡ü@ù)¿èììò;pàÀÀÀ@©üÑ6åòCù)?òÕ*¦J¥òCù¡üP~Uù¥¦àÊåò£LÊ¯¡¡¡±±qpp°¤¿òCù¡ü@ù)¿;KþcgKâÄ®òCùòCù)¿YJþò'zòCù¡üP~sùe2¢¡üP~(?eR~/^xqrä/é<Wx(?ÊåGy_^©DòCùòCù)¿Mù¡üP~ üòS~(?(?å7/õôôd2t:ÝØØØÙÙY8«»»»©©)f­2î¦üP~ üP~Ê¯´e³ÙcÇÅ ­­­¥¥¥pÖ²eË.ãÇ/_¾|bù½ðÂ×ï¢³gÏö÷÷_9ø·û·W_Õv`.úúúâQÛ9jooÿ¯ÿú/Ûa^ùÐËïÍ7ßüÈË¯¦¦ftt4¹¾¾~ª»UUUM,¿gyæÇwÑK/½ô£ýèÇ0/¿üò+¯¼b;0?üá¿÷½ïÙÌÑ/¾¿ÚóÊ^~É[º455½õÖ[Uù^ÕÆ/^Ü¹s§³½8ÛÎöâl¯³½³Í÷æ¨®uëÖõ÷÷ßå§Qø®Ñïpûöíl6;ñ¯+?Êò±7ß|sÅùüÚ´iÓûÐ-Æµµµ¹±³½1wÏë×¯oß¾ýÆW¢üP~(?P~ÊoFGGÛÛÛ«ªªîò_okmm=zôhâc6WZ7n¼uëÖ¤*?Êò±ÞÞÞÂc~ÕÕÕ_T÷áéêêª««ÖÌd2ÝÝÝ¿zncëëëÿ°òCùòCù)¿Ù+¾ªªª«W¯ÊóW~(?(?å7U]ØñÙÏ~öÊ+%÷üÊåÊOùÍÀGøf.ÊåÊåÇ]-¿¦üP~(?P~Êof._¾¼|ùòE%×óÖÖÖ<yRù)?ÊåG¹_GGGþ¤üq[[òS~(?Ê²*¿ºººè¼Ë/çË¯»»;ycå§üP~(?eU~ù÷ÉËßèèhrÁ¯òS~(?Ê²*¿ÚÚÚè¼ä8__.øác_¯üÊåò£¬Ê/R¦b2.~ÊåòCùQVånÜ¸ÑÜÜ[UUµ|ùòþþþxþÊåòå§üåòCùòS~ÊOù¡üP~ üßoºzõjSSSuuujLKåT¯òCù¡ü@ù)¿éÚ±cGÅvíÚ¥üÊåò£LÊïäÉIä>|xpp0844têÔ©dzìÊOù¡üP~(?Ê¡ü2LäÝ#G&ÎJþ÷óS~(?Ê2)¿ÊÊÊÈ»¡¡¡³r¹;(?åòCù¡ü(òËÿÑ¶©æ&ÉMù)?ÊåG9_¶S~ÊåòCù¡üòCù¡üP~`ù§üÊåò£LÊ/u'étZù)?ÊåG9_yP~(?(?å§üÊåÊOù)?åòCùòS~ÊOù¡üP~(?òS~(?Êå§ü@ù¡üP~(?åÊåòCù)?òCù¡üP~ÊåÊåòS~(?P~(?òCù¡ü@ù)?å§üP~(?P~ÊOù)?ÊòS~ÊåòCù¡üòCù¡üP~(?åÊåòCù)?P~(?ÊOù¡üÊåòS~(?P~(?òCùòCù)?å§üP~(?P~ÊOù)?ÊòS~Êåòå§üòCù¡üP~(?å§üP~(?ÊOùòCù¡üP~ÊÊåòS~(?ÛåòCù)?(?ÊOù¡ü@ù¡üòS~(?(?å§üÊåÊOù)?åòCù¡üòS~ÊåòCù¡üòCù¡üP~(?åÊåòCùMSOOO&I§ÓïÐÑÑQQQ¡üP~ üP~Ê¯äe³ÙcÇÅ ­­­¥¥eÜÜ¦¦&åòåòS~å ¦¦ftt4¹¾¾~ÜÜGôñÇªüþáþáâ]ôÒK/]¸pá"ÌAgggüa;0]]]ßûÞ÷læèôéÓ?úÑlyeA_:t®^½ÚÜÜ]8Uù:uê»èìÙ³ÿùÿùÌAOOÏ¿ÿû¿ÛÌÅOúÓøýÁv`^zé¥ÿüç¶Ã¼² Ê/JåÇ³¶nÝzþüù_>Ugq¶íÅÙ^gKõ¡ÿZkkks¹¶7ÆÞ-gåòåòS~%¬µµõèÑ£1ÙlvªR8Qù¡üP~ ü_éêêª««K¥RL¦»»ÒÔS~(?P~(?å· )?ÊòS~Êåòå§üòCù¡ü@ù)?å§üP~(?ÊOù)?ÊåòS~ üP~(?òåòCù¡üÊOù¡üP~(?åòåòCù)?(?ÊOù¡üP~ üòS~(?(?å§üÊåÊOù)?åòCù¡üP~ÊOù¡üP~(?òåòCù¡ü(?Êå§üP~ÊåòCù)?(?ÊOù¡ü@ù¡üòS~(?(?å§üÊåÊOù)¿Ò+¿W_õ^èííµß+?P~(?å§üÊ¶ü">ñOÜ»ìÞO¯þôïÿþïîs¶÷+?íòS~Ê¯ÜÊïÝwß­©©ùÜ¦Ïíÿëýñoß×öÝ×t_KK½_ù¡ülòS~åV~/¼ðÂ'?ùÉ$û_Ùýî¹gddÄåòå§ü_YßSO=µáo(,¿øW[[ûÎ;ïø üP~ üò+«òÿ÷Þoaöíú]¿û»¿ëòCùÙ(?å§üÊ­ü¢ðï_ÿ¾¯íìûê¯.[¶ìoÿöoíýÊåg; üò+·òï¼óÎ§?ýéÚÚÚ?ú£?ºçvíÚåòCù)?òS~åY~ùþõÕWß÷]û½òåòS~Ê¯ÌËåÊå§üòCù¡ü@ù)?å§üP~(?P~ÊOù)?ÊòS~ÊåòCù¡üòCù¡üP~(?åÊåòCù)?P~(?ÊOù¡ü@ù¡üP~ÊåÊåòS~(?P~(?å§üÊåÊOù)?åòCùòS~ÊOù¡üP~(?å§üòCù¡üP~(?å§üP~(?ÊOùòCù¡üP~ÊÊåòS~(?P~(?òCùòCù¡üÊÊOù)?åòCùòS~ÊOù¡üP~ üòS~(?ÊOù)?å§üP~(?ÊOù)?ÊåòS~ üP~(?òåòCù¡üÊÊW~===L&N766vvvÎÞ¾eeåÇ?þñ¨.åòåòS~¥-Í;v,mmm---³<øØcFr544L,¿þ»èå_øÌÁåËûúúlæâæÍ]]]¶så744d;Ì+¢üjjj¢íbËåêëëg566¾þúëS-åwèÐ¡3wÑéÓ§_zé¥30/¾øb»íÀ7¢øvd;0GÿüÏÿl#Ì7¢üÒéô¤ãäæO>YUUÕÐÐðÚk¯9Û³½àl/Îö:Û[ÚR©T~nÖ#Gbðæo677+?(?ò+Áþk1®­­ÍåríqáÝo;¨üP~(?P~Ê¯ô´¶¶=z4ñ1ÍÎzðÁO8Ë/¯Y³Fù¡ü@ù¡ü_iëêêª««K¥RL¦»»ûWÏmìpàÀÀÀÖ­[Óétsss__òCùòCù)¿Jù¡üP~ üòS~(?(?å§üÊåÊOù)?åòCù¡üP~ÊOù¡üP~(?òåòCù¡ü(?Êå§üP~ÊåòCù)?(?ÊOù¡ü@ù¡üP~Êåòå§üòCù¡ü@ù)¿ròõ¯ý¹çû.:qâÄo¼ñ6ÌAgggüÆb;0?ùÉO¾ûÝïÚÌÑ·¾õ­þþ~Ûa^yÿý÷ß._¾¼ÿþoâ'3+öp¶åò@ù üP~(¿RrùòåU«V¥ÓéÆÆÆ®®®ÆþÇ+§ÀttttTTüêÿlOOO&Iö¢ÎÎNYìEÝÝÝMMM±­2ö(ÙíHSMAù-PñùÔ©S1¸páBMMMâãÕ«Wcëëëm"¦cdd$~Hç¿±f³ÙcÇÅ ­­­¥¥ÅöaÑ²eËâûR?¾|ùrÛÙíHNAùñË?n¿XÇ ¡¡áúõë11¶eGôñÇÏcßFGGcËåüþÀìö¢BUUU¶³ÞìZ(¿úëQuuuü_¬?;Oßdãf|¼xñ¢íÃ]½zµ¹¹9R/ÿ5NççaúQ^|#Ú¹s§MÄìv¤"»ÊoAëèè¨­­ÁªU«^ýõ$W¯^mËpG[·n=þü/ÿÇþúk*ÊÏ­¬¬´Å^¸ûv6´Ý4Õ®òãWÇf­aÆÿQSLß"r¹Ücgß(`¦Ñc¯9Ù¾û7lf½#Mºk¡ü®eË]¾|ù±Ëè6nÜÕ«W'SzW­Ze1£ï¹É µµõèÑ£1ÙlÖaÑ¹sçâÒ­[·læ²#ò[zzzÓéôúõë_¬ßzë­ä^âcm"fñ­¶«««®®.Je2ø¥ÂaQ½C5(?åò@ù üP~(?ÊåòP~(?Êåò@ù üP~(? ÄûÛß^»vmÕõë×?ÿüó¿ñnLÉ|;ìÑÖ××ÇS7=¦¤ÓéL&3:::ÓuÊ ôìß¿¿bo~óåT~O?ýô¸éO=õTLäGf±N@ùht:øðá1G1ñÒ¥KeS~W®+W®7Å1½¿¿_ùÊ(_üâ£iì±Â_úÒ»§³³3Ê)¢°©©)Æù;¿÷Þ;wî¬®®Y555öì)<©zöìÙ¨«Ë9sfÅÅ¯]»¶½½=n¶¶¶`§O.¾dV<¤dVGGÇT¶qãÆÞÝÝráÂ²aÃüÖÕÕÅªªªª¶mÛvýúõå7qýã¦y¨òø(-]º4ªåêÕ«¯]»ëëëËf/&s·lÙ2nÖîÝ»Y½½½©TjÒ¥ÉÜ­[·F;VVVÄÜøå´hÑ¢äåwEÖIÞÄgzüøñÂÍÇå±cÇòÙ7n%ë×¯iùy¨òø%'v'ù¦VQVX6---·ÇÄ nnÞ¼9NÒÉ¡»(¶dV6MÆ¸««+YIá:÷íÛmlÝ±cGL9uêTãcxà;®'ª1nÆÛ¤Ï(jrÉ%ñ|oÝº7ãc<òÈÍÜ!ÉÄñ>øõÙáªªª_(?¾üÂ¤åMLáúõëq3)¹ÙÔÔ7víÚÅ644_IÜgÜQ´üRÉÍk×®åïµ?ác|öìÙ;®'*3nÞ¼y³ð±Mõ¼z(f=þøãüúö=ïå=ÚÜÜiùy¨òø%ºnß¾]8qpp0&Æ¬"­ïÂ¾¾¾$þò¡qÞÄ°ãZªð½Tb<<+_¼xq~î×3Usùòå$RccÜÛÛÛÕÕ`Ò³ÆÓ/¿"P~±äµnO>ùdáÄä½NÆ]á-à­[·&ÊºtéÒs¯ùdLÈåO§Þ±ÏvïÞä;wîÌO/²äÛ77oÞ,~îêÕ«cnòF6¬³ë|cVGGÇÀÀ@ñòËWi²5òs<T@ù|Ä+$Òét[[[ò®.®¬¬x5ÆæÍ£rS±ùëp×ù%/ëëë+|`öìNJ®ºÍ¿¯Ê¤|ösçÎå§YOòÂ¾äu~É=ß#GòGãâ)ÎJ^òØÛÛ%6ÂTå´f´rÜ-sÛ¶ms<T@ù|ôw9çàÁÿÿÜ$òãüyÒüEy;vì(ÌÊB'O,R~ý±^SSSx"¸Èzâa>°üuµS=Ù¡¡¡ªªªä);Ç½iÓ¦ÂO±dÉø¼±Ká:CyI§æçy¨ò¢NÖ¯__9fíÚµÉ¶ãÊ¯££#yÛ¼ÆÆÆóçÏçç>üðÃuuuI-=ôÐCÃÃÃù¹gÎinn Ëd2Ï<óÌ¸uN|$±ªþà>ÕzB<xHÉy?¿¼;wÄ­[·²Ùllêêêxýýýùw¨)ÀÀ@¤m²­6lØÐÝÝ=î3y¨ò@ù üP~(?Êå üP~(?Êåò@ù üàÿpTÊSÅ1MQIEND®B`


ûn»Á¯2zhllÜµk×gÊûÆ'b'L§ÓñÛGl¡bî?8Iä'M'ù¶aÃ»ª±G~3gÎ¼råÊtßSO=5úkóæÍE¶6©UÆÛvìØ1ÙÁò0f2üïmmm%?ü$Ý-dô÷÷ïÜ¹³ªª*fvuuÝòV®-ä×ÝÝPõk_ûÚÐÐÐððpLÄÍyæÌ175ÙUFï7n9¡±É/Yñúõë1Ëåî©?Iä'üî¹[;ÖÐÐN§çÏäÈrÊo$;Ì5kÑ¢EEÖÍßùôéÓK.MND>öØc×®]+ü¥MMMÉGñPÇÛìmWñÖ¬Y?80Þ¼ûî»«W¯Na6íëëqÏÂçÌ_¿:ÀâÅ¯^½zâÄÆÆÆÊÊÊeË_ÜºukmmmrZ3~E~Ñxøsû/<÷Ôcf[[Û¾]eô¯,Æx1É6µËoÄÒøs=$ý3ùLmñ££Np7¡ÆH¦cQuuõ¼°Hä'M'ùVbf]]]róÌ3©Tªðý¸»»»ü;¯ZµªÈºù;ÇsáÒÂUã×+²Ùâ+~îýýýL¦¦¦&&FÉåËãí¼p;qÏD+|¾£ïc¨I¡<ûFÜsÉ%Å3gÎÿþûïþÃ;wî¾]eÄ¯Ö$ÇüæÍ7©Á)>øS^qâòëéé)ÜN<ÙÉÊo»ñÊ+G<æõë×aÈONòKæÉél67;::bº³³³Ðg#VOnnÚ´ixx89sÛuã3ÞòãþÉÆ@R²4Þqãf¬~ãV±Váï*²Ùâ+ùÜ<Xx¬ðþëÖ­é+VULÄÍ9ÞóMæ<þøãCCCÉK¶79RøO³½úê«1áÂÂEã=àt:=ÞÉ¯û!Wïtùc¢1ÕW,núÂ»A>ï³Ëo»q"ÂK.ÅtüiÆô3¼°Hä'M?ùåOð%+,æßåËósn»nþøYrV1ÞGñöYx°jÄÙº"-¾âxÏ=b¼©_SSSøâ>nÎ=¼çÌ	mÞLNa'O°ðär¹°B£¹¹¹ð¹_ãã1ýC®R8¤±næÒ¥K;6æ MdpÆ|TS^qâòKö«W¯þIÉo»qSSSÜ¬¯¯¹Æ_!nÞ¼éUE"?iÉ/¤3«««Ç»Ï(ÉÍ N·ê"¾ìó6;ÞüxÊñ|ãýpp°pþècf£?ô6úùNäf(sÄÏÛ^£Z[[Í»Îoa"«Ld¬Æ¼ÃDg<NmÅíýð×wãÞÞÞy:tÈD~ÒtßîÝ»¯ðH¢¸Êr"ÐÔºÅÙ¼ÿþûKl¶øEÀþýûãfþ;P	ÎFaâHáÍäÛU=2üÉ¬d³Ù6~Ï=1sõêÕcÊo"«LY~|S^qâòÈ1¿¼í¿êÿ"û[Ò©S§¶lÙbÎ_-ü¤]~ñÞJÞóßê¼â2¹TvþüùÉ¢äCN·ó-³ÈºÅÙ%ùVl<ÙÎOq¹Ùâ+gÄÊ+óäOæ$Wþ®X±¢ÿVÉÖüñ/¿ä¸×3gâ)ìÚµk"ò;~üxrT)rgggr</éàÁcþùNvIÉ¯øàØ7ì¨NA~É'óbg¸yófÀnùòå£]¸ûöüÐa ¸øøßöäó½½½EÎ¼K"?é^ßè¯Oµlþóþù/ÝM~Ë,²nq6^h_Zd³ÅW,Î+W®ä?ÑÌéëëñ¯ü?üòI=vþSE|óØcþÃzäGüOjIÉ¯øàØ7ì¨NA~§N*Üþ¢Eüæãý"û[þ¢|k×®õÂ"4=äoK,ý¯9r¤¹¹9oç»wïÎÏ?xð`Ì	i~Ý×½-¯Á755þZ¾ñ6Û3"9Z8ÿüùó«W¯®¼U6=ÇÔäwíÚµØZl3ôÄOòà.îç>]<ñ9sæìÝ»÷³ýlþÓ_eRò+>8#öMjT§ ¿èÐ¡Cõõõñ¬cûÉùôüåÌq3|üö¥KvuuÝöëxûÛÀÀÀO>L¾Çåàà ü$é®Ëåì±IÜVv%ÇzµÐ744Øa$ò$[ÉçüFôì³Ïü$IåÖÀÀÀÆçÌÒ§zÊ°Hä'I$ò$IùI$ü$ID~$I"?I$$IùI$ü$ID~$I"?I$$IÈO$Iä'I$òt¯uðàÁºººt:½dÉÉ®ÛØØXQQÑÛÛÓ1gþüùx9»ÕÔî3u§ø"[ô7F©Tªªª*Í^»v-Y400°~ýúêêêÌ3gÆ¢wß×&ü$ÝCÕÖÖc/CCC]wëÖ­±îW¾òümÛ¶Å-[¶·üéxâ1ÝÒÒÜfMÜìèèé'NÄôÜ¹sí`ÈOÒ½ôjò!ðtêÔ©XwñâÅù9,9===¥wXécX9¦«ªªq³¿¿ßN%ü$Ý»ì+ÔÌO>9sæÌ3flÞ¼yÄÝ>ûÙÏ677ØBþaL¿ÿþû1sE.]ZºtiÀ(N755%ÃFo­ð·_eïÞ½uuu._4Úa7nÜhmmuãÁoÜ¸196]´hQl-æÇ/_¾|÷ä÷àÆÍÎÎN»$òtïâ/~ægbz÷îÝa¬xöÙgïsäÈügÚò­[·.íÙ³'¦ãgL·µµ%yäW_5&.ó3Ì[+|ÅW	Ïíß¿?&Ö¬Y3úÁ¯_¿>¦ã/¿ürLlÝº5¯±þþþ3gÎÄDrHrtSßððpOLþóO<x0¿Á%K>ÚÞ%ü$Ý»ò«««éÜ­FÃkÌ?~<­X±"¦W®.¿ôÔ©S6mjnnN.sk#U|äÍ9sôº555ÉÅD/fÆ=cº©©),;888µa=?yl±ñ`_áéÝîîîÀkþ>ñKí`ÈOÒ=*¿t:¸YAÜ:ô5cÆ¸çÀÀ@rR5æ$víÚkO>]¸[+¼9UÆ`A±ÂÃx80kÖ¬dNuuur8ðó+RGGGX³ðÄ·$¤N~L¦ð_rØì¶Üimm¥ùùùÉÃ· ü¯`¹ÑëÎ=;YwÄÃzõÕWoó'5,_<Îx:v0Iä'é_ò--öìI>ç·mÛ¶pçÀùgû÷ïÏÏOyöìÙäó_ñU6mÚôâ/ÆÄºuëF¯»víÚnoo?wî7f.0¦O>õêÕ7oÞÝßüùócÑÑ£GcúÐ¡C1ýØcÙÁ$¤T~CCC¡«·*üN¾âò»yófr¦5~~è-ôS]]=sæÌ7NP~ÅW9vìX,]°`AþBÂûlØ°!yeeåòåËËx|Ùl6¹XxñâÅçÏÔ°>#(â7¶¶¶Æq=öúõëã!ÙÁ$$IÈO$Iä'ID~$I"?I$$IÈoÚôæo^¼x±¿±¯¯/ËÙóJPÿÕ«WCizçwþû¿ÿÛ8 ùù×ýWãPþë¿þëþéCiºråÊ¿ýÛ¿ò»ëýÑýQà¯¿ñøñãÿþïÿnÏ+Aßÿþ÷Ï9cJÓ«¯¾oÆ¡ýã?þã»ï¾kJÐüÇ$ß§­ôw÷wÉwgüÈOäG~"?ò#?ùüÈüD~ä'ò#?ùÈüD~äG~"?òùùùÈüD~äG~äG~ä'ò#?òùùùüÈüD~äG~äG~ä'ò#?ùùùüÈOäG~ä'ò#?ùÈüD~ä'ò#?òùÈüÈOäG~"?òùùüÈOäG~ä'ò#?ùùùüÈOäG~ä7êééÉd2étº±±±££côâÿÞò#?ùüÈü¦Ùl¶½½=&vìØÑÒÒ2béÐÐPSSÓxòw¬ë%ìµ×^ûºî~çÎûö·¿mJÓ+¯¼ríÚ5ãPâ2þJcJÐ~ð#GÒÔÕÕõÎ;ï;Ò!¿êêêáááÈåruuu#>óÌ3Ï=÷Üxò,~«4O8ñ-Ýý:::¾ñoÒtèÐ¡ÎÎNãP;4%èÍ7ß¿ÒÒtôèÑ×_Ý8Üîù¥Óé1§£K.577íu¶WÎö:Û+gíu¶·J¥RùéÊÊÊÂE«V­:qâÄÿ<Uò#?ùüÈüÊ ÷Á­³½1ýÏð'#?òùÈüÈoz×ÚÚºwïÞÙlvì§êùüÈOäG~äWuvvÖÖÖ¦R©L&ÓÕÕ5&õÈüD~ä'ò#?ò»¯#?òùÈüÈüÈüD~äG~äG~äG~äG~"?ò#?ùùùüÈOäG~äG~äG~"?òùùüÈOäG~äG~äG~"?òùùüÈOäG~ä'ò#?ùüÈüD~ä'ò#?òùÈüD~äG~"?òùùùÈüÈOäG~äG~ä'ò#?òùùùÈüD~äG~äG~ä'ò#?ùÈüD~äG~äG~ä'ò#?ùÈüD~äG~"?òùÈüÈOäG~"?ò#?ùüÈüÈüÈOäG~"?ò#?ò#?òùùüÈüÈüD~äG~"?ò#?ò#?òùÈüÈüÈüD~ä'ò#?òùÈüÈüÈüD~ä'ò#?òùÈüÈOäG~"?òùùüÈOäG~ä'ò#?ùùùüÈOäG~äG~äG~"?ò#?ùùÈüÈOäG~äG~äG~"?òÓG(¿þð;wîüÝßýÝgyæÜ¹süÈüD~ä'ò+OùÅ+ÿ<ððÃÿÊ¯üÊ//þåY³fµ··aò#?òùÈ¯åWWW·üWoþÒæä¿/¬ùBUUUoo¯A&?ò#?ùüÊJ~¿ð¿°éòòÿ>µàS;wî4ÈäG~ä'ò#?_YÉ¯··÷¡y²/þ[²dÉóÏ?oÉüÈOäG~"¿²ßàààÏüÌÏ´ýv[OþÁ<ð@¼ùdò#?òùÈ¯¬äíÞ½ûç~îç~ó73Ø·îwÖ=4ï¡GthhÈ ùÈüD~å&¿èë_ÿzCCÃOýÔOüãß¸qãÀÀ&?ò#?ß=Ñï½×ÖÖöO|âáÞ´iÓmß¢ÈüÈOäG~"?ò]¼x±ººúÓ>½víÚ/¬ùBø¯©©©8þÈüÈOäG~"?ò­X±béÒ¥!644lÛ¶üÈüD~ä'ò#¿rëçþçÿß/ß¯gýÑG%?ò#?ùüÈ¯å·ñ÷7Ê¯¥¥üÈüD~ä'ò#¿2ÌÙ^òùùüÈï~)GÛo·¹ÂüÈÏ8ùüÈ¯Ìï½÷Ö¬YóÐCû¾øÅ/úVò#?ùüÈOäG~ä'ò#?ùÈüD~ä'ò#?òùÈüÈOäG~"?ò#?ò#?òùÈüÈüÈüD~äG~"?ò#?ò#?ùÈüÈüÈüD~ä'ò#?ò#?ò#?ùüÈüD~ä'ò#?òùÝ®L&N§;::uuu555Å¢ùóçÇÝÈüD~ä'ò#?òÞe³ÙöööØ±cGKKKá¢¹sç<y2&öíÛ7oÞ<ò#?ùüÈü¦wÕÕÕÃÃÃ1ËåêêêÆ»[UUÕhùýÅ_üÅwKØ#GÞ~ûíïêî÷­o+mJÓ¡C¾óïôÆoÄßWC	Ä_iCiêèèøÛ¿ý[ãpGº/äN§Ç.¬»»»­­m´ü^zé¥%ìßøFooïEÝýâUûäÉÆ¡4½òÊ+.%è­·Þêéé1%¨¯¯ïðáÃÆ¡4uvvÆßÃé¾_*ÊOWVV¾Ã7²ÙìÀÀ³½ÎöÊÙ^gål¯³½ÎöNïjjjr¹¶7¦G,½råÊ5k®^½:zEò#?ùüÈü¦Y­­­÷îøÍfGHkÙ²e×®]sEò#?ùüÈü¦Yµµµ©T*Étuuýè¹UüÏ³««««(üÈOäG~"?ò#¿û4ò#?ùüÈüÈüÈOäG~äG~äG~äG~ä'ò#?òùùùÈüD~äG~äG~ä'ò#?ùÈüD~äG~äG~ä'ò#?ùÈüD~äG~"?òùÈüÈOäG~"?ò#?ùüÈOäG~ä'ò#?ùùùüÈüD~äG~äG~"?ò#?ùùùüÈOäG~äG~äG~"?òùùüÈOäG~äG~äG~"?òùùüÈOäG~ä'ò#?ùüÈüD~ä'ò#?òùÈüÈüÈüD~ä'ò#?ò#?ò#?ùÈüÈüÈOäG~ä'ò#?ò#?ò#?ùüÈüÈüÈOäG~"?ò#?ùüÈüÈüÈOäG~"?ò#?ùüÈüD~ä'ò#?ùÈüD~äG~"?òùùùÈüD~äG~äG~ä'ò#?òùùùüÈüD~÷ü*nW*"?ò#?òùÈ¯äº]étüÈüÈOäG~"¿r_yD~ä'ò#?ùß­¿¿ÝºuäG~äG~"?òùüêêêÒé´ÏùùÈüD~e.¿ùóç¾¼£ººz``üÈüÈOäG~"¿²_eeePïÚµkµµµ1àñÅc¢­­üÈüÈOäG~"¿²_r/&z1qáÂááá1cùùÈüD~e%¿Y³fó:;;câ©§J&|«ùùüÈOäWnòÛ¸qcþzÂú-üÈüÈOäG~"¿²_ôå/yöìÙ1ÑÕÕ¡ÀæææiñüÉüD~ä'ò#?ò»_"?òùÈüÈüÈüD~äG~äG~ä7VóæÍK¾ÛÅ79ùÈüD~å,¿¹sçj/kÉüÈOäG~"¿r_ /Ì×ÝÝ=<<<í?ùÈüD~äG~¨ºº:ä7ÙG~ä'ò#?ùßäêéé	ù­_¿þÆäG~äG~"?òù³ü¢9sæTÊäG~ä'ò#?_¹É¯¾¾ÞäG~äG~äG~"¿ûB~ù¦é[,ùÈüD~äG~¨¦¦ÆäG~äG~äG~"¿ûB~AßÆÉüÈüD~ä'ò+gùU+<ÈüÈOäG~"¿r_jA~äG~"?òùü¦uäG~"?òùùM¢ºººúúúsçÎùùüÈOäWæòK§ÓwìØaOOO&m666vtt_4µ9=ýôÓ/½ôÒÅ¶ÿþxá¾¨»ßÉ'ãUÛ8¦^xáßûq(Añ:vâÄ	ãPBØíííÆ¡4>|¸»»Û8Üîºüâe(ä·eËþþþÿÝ.Ùl6þO;v´´´_4µ9=ñÄ$IåÒ]ß½¶·ºº:ác.«««+¾hjsÈO$ß»³×ö®5b£MmùI$ò»'*<RXYYY|ÑÔæöøÏ?ÿü%ì«_ýêÑ£GßÔÝïÅ_|áCiúã?þã7ÞxÃ8 ¿üË¿ü«¿ú+ãP^ýõçÎ8¦?ÿó?ÿë¿þkãpGfò«©©ÉårÉÉÙ.¾hjsëÚ^¹¶×µ½rm¯k]Û;õUË/¯ªªª¨¨1cÆªU«¦|©GkkëÞ½c"~f³Ùâ¦6üÈOäG~"?ò#¿)vóæÍ1O3Oíñíìì¬­­M¥RL¦««ëGòÖIëÑ¦6üÈOäG~"?ò#¿)ÖÐÐ2[±bÅ7âfÿÊ+cÎ#<rï?ò#?ùüÈü&Qeee8ohh(?'ËÅÑSùùüÈOä7½åJ¥ÂyÉUI1gjßêB~"?òùùéÞ_r¶wÙ²eÉÙÞøÓ1§©©üÈüÈOäG~"¿²_PoÌ+<®_¿N~äG~ä'ò#?_YÉï[÷®ZµjæÌ©T*~.[¶,æLçO~ä'ò#?ùßýùÈüD~äG~äG~ä'ò#?ò#?ò#¿ü·«ðÌ%?ò#?òùÈoË/5~äG~äG~*ùýÙýÙÜ¹s«ªªâçüÉÈï¾ßx=ñÄüöíÛG~äG~ä§i*¿/éKLæ·û­Í_Úüÿ>ÿÿbú«_ý*ùüÈïëéé1cFoéÒ¥_ìL~äG~ä§é%¿÷ÞïcûØïmø½`_òßºßYWUU500@~"?òûV¯^ê7iôüÉüD~ä7ºcÇ-X° Ï¾ä¿_üÅ_¼¯þ"?ò#¿±ùåó­X±bÚ=ò#?ùùJõK¿ôK#ä÷ñüÜ¹sä'ò»åwóæÍGy$¹£££c:>ò#?ùn``à§ú§ÿoëÿÍ³ï×VÿÚ<044D~"¿ûT~Û·oOõµ´´LßçO~ä'ò#¿ñ^¬fÍõ©OêWõW?ùÉO~ìcûí·ï«¡&?ò#¿÷ùùüÊW~ÜºÎã+_ùJ[[Û¶mÛ.^¼x¿5ùùýD©ÛN§ÉüÈü4åwG~äG~eùÈüD~äG~äG~ä'ò#?ò#?ò#?ò#?òùùüÈüÈüÈOäG~"?ò#?ò#?òùÈüÈOäG~"?ò#?ò^ò7o^ee¥ïó#?ò#?ùüÊ sçÎ-Ôïó#?ò#?ùüÊV~¼0_ww÷ððð´þäG~"?òùùM¢êêêßtdùÈüD~äG~«§§'ä·~ýú7nùùüÈOäWÎòæÌS1*WxùÈüD~å&¿úúzWxùùùüîù%æ¦o±äG~"?òùùM¢WxùùùüîùeB~7n$?ò#?òùÈ¯åW1N®ð ?ò#?ùüÊM~©qrùùüÈOäWnòÖùüÈOäG~äG~äG~"?ò#?ò#?ò«·|ùòªªª3f¬ZµjºK~ä'ò#?ùß$ºyóæWxLKÉüD~ä'ò#?òDá¼+V$ÿnoÿÊ+cÎ#<B~äG~ä'ò#?_YÉ¯²²2744ËåbNÌ'?ò#?òùÈ¯¬äJ¥Ây¡½üÁÁÁã[]ÈüÈOäG~"¿r_r¶wÙ²eÉÙÞøÓ1§©©üÈüÈOäG~"¿²_PoÌ+<®_¿N~äG~ä'ò#?_YÉï[÷®ZµjæÌ©T*~.[¶,æLçO~ä'ò#?ùßýùÈüD~äG~¨®®®¾¾þÜ¹säG~äG~"?òù¹üÒétEÅt=vH~ä'ò#?ùß$êèèùmÙ²¥¿¿ºü£mäG~"?òùùMiãJ¥ÈüÈüD~ä'ò++ù¥ÆÉ79ùÈüD~e"¿úúúÆÆÆiýüÉüD~ä'ò#?ò»É?×ûÁ­³½ÓâÄ.ùÈüD~äG~S,ùçz¢üÈüÈüÈüD~å,¿L&SQ4WxùÈüD~e"¿îîîY³f%Gþç¹ÂüÈüD~ä'ò+Oùå.È#?òùÈüÈï¾üÈOäG~"?ò#?ò#?òùùùß=YOOO&I§ÓºººbÑüùóãnäG~"?òùùMï²ÙlLìØ±£¥¥¥pÑÜ¹sO<ûöí7oÞhùýë_¿RÂ;Ö××wEw¿ï~÷»á~ãP^yåxÕ6%èÛßþv¼GtñâÅ#GÒô­oëìÙ³Æát×åwþüù ÕÕÕÃÃÃ1ËåêêêÆ»[UUÕhùíÞ½ûívøðá·ÞzëmÝýN8ÑÑÑaJÓ¡CzzzC	:~üø7¿ùMãPÙñWãP^íµ7ß|Ó8Üîºü¯tijjúO@^ÞÆÝÝÝmmmÎö:Û+gí³½Îö:Û;õÂ|ùoou-^¼¸¯¯¯ÄO£ð[£+++GßáÆÙlvô¿2L~ä'ò#?ùß¤;þ|CCCÁ¯åËßÝþãbº¦¦&ËpëloL¸ç+WÖ¬YsõêÕÑ!?òùÈüÈoÇ@UUUÿõ¶ÖÖÖ½÷ÆDüÌf³#¤µlÙ²k×®¹"ùÈüD~äG~.ÞbùÍ9sôêî^µµµaÍL&ÓÕÕõ£çvëp`]]]á¿&L~ä'ò#?ùßÔ+_UUUKKË¥K¦Ëó'?òùÈüÈo2¸uaÇg>ó.L»çO~ä'ò#?ùß$Ö¯AäG~"?òùùÝ/ùüÈOäG~ä7¹Î=;oÞ¼3f$×óÖÔÔ8püÈüÈOäG~"¿r_ü¿Â#_2½cÇò#?ò#?ùüÊJ~µµµá¼³gÏæå×ÕÕ|±ùùÈüD~e%¿ü÷äåå7<<K~äG~ä'ò#?_YÉ¯¦¦&çùår¹'|2¦ëêêÈüÈüD~ä'ò++ùe*ÆêäÉäG~äG~"?òùü¢«W¯677'×öVUUÍ7¯¯¯oZ<ò#?ùüÈüîÈüD~ä'ò#?ò#?ò#?ùùùýd.]jjj9sfêV1ÑØØ8]NõùüÈOäG~ä7ÑÖ®][1NëÖ­#?ò#?òùÈ¯LäwàÀy»víHfÞ¼yóàÁÉü#GùùüÈOäWòËd2Á»=ö^ün¾ÏüÈüD~ä'ò+ùUVVïnÞ¼9zQ.Eqò#?ò#?ùüÊA~ù´m¼¥É¿äF~äG~ä'ò#?_9È¯íÈüÈüD~ä'ò#?ò#?ò#?ùü¦¡üG~äG~ä'ò#?_È/u»Òé4ùùÈüD~å ¿òüÈOäG~"?ò#?ò#?òùùùùùüÈüD~äG~äG~ä'ò#?ùùùüÈOäG~ä'ò#?ùùùüÈOäG~ä'ò#?ùÈüD~ä'ò#?òùÈüÈOäG~"?òùùüÈOäG~äG~äG~"?ò#?ùùÈüÈOäG~äG~äG~"?òùùùÈüD~äG~"?òùùùÈüD~äG~"?òùùüÈOäG~"?ò#?ùüÈüD~ä'ò#?ò#?ò#?ùüÈüÈüÈOäG~ä'ò#?ò#?òùùüÈüÈüÈOäG~"?ò#?ò#?òùÈüÈOäG~"?ò#?ò#?òùÈüÈOäG~"?ò#?ùüÈOäG~ä'ò#?ùÈüD~äG~äG~ä'ò#?ùùùüÈüD~äG~äG~"?ò#?ùùùüÈOäG~äG~äG~"?òùùüÈOäG~ä'ò`===L&N766vtt¾Cüß[QQA~ä'ò#?ùß´/Í¶··ÇÄ;ZZZF,jjj"?òùÈüÈ¯ª®®WWW7bé3Ï<óÜsÏ'¿?ýÓ?í.a>yòd·î~ßüæ7_í5ãP:ôÖ[oÔÑÑ4%¨««ëW^1¥éØ±co¼ñq¸#ÝòK§ÓcNG.]jnn'¿þ ÅÎýýïÿºûýýßÿXÄ8¦xüçþgãPÞ~ûíÓ§Otùòå#GÒtòäÉsçÎ;Ò!¿T*®¬¬,jÕª'NüÏSu¶×Ù^9Ûël¯íu¶×ÙÞéúÐ×ÔÔär¹äloLy·üÉüD~ä'ò#?òÆµ¶¶îÝ»7&âg6O£gùüÈOäG~ä7Íêìì¬­­M¥RL¦««kLêùüÈOäG~äw_G~ä'ò#?ùùùüÈüÈüÈüÈüD~äG~"?ò#?ò#?òùÈüÈüÈüD~ä'ò#?òùÈüÈüÈüD~ä'ò#?òùÈüÈOäG~"?òùùüÈOäG~ä'ò#?ùüÈüD~ä'ò#?ò#?ò#?ùÈüÈüÈOäG~ä'ò#?ò#?ò#?ùüÈü¦$¿¡¡¡;w®^½úÑGÝ¶mÛàà ]üÈüÈüÈOäWòçêSzhÞC¿ýõ¦¦¦-ùùò#?_¹Éïé§þÄ'>±é6isò_Üüâ¿h¯%?ò#?ã@~ä'ò+7ùòüÂ/äÙÿÅÍùo¯%?ò#?ã@~ä'ò+7ù=üðÃm¿ÝV(¿u¿³î¡²×ùq ?òùüÖ¬YóéE.ßâO/þßø-ùùò#?_¹Éï½÷Þ«®®^ºtéÆßßøä<¹ìÿ,ûÙýÙ¾¾>-ùùò#?_¹É/ºxñâ+|³fÍZ¶lYoo¯]üÈüÈüÈOäWòùÈüÈOäG~"?ò#?ùüÈOäG~ä'ò#?ùÈüD~äG~äG~ä'ò#?ùùùüÈüD~äG~äG~"?ò#?ùùùüÈOäG~äG~äG~"?òùùüÈOäG~ägÈüD~ä'ò#?òùÈüÈOäG~"?òùùüÈOäG~ä'ò#?ùùùüÈOäG~äG~äG~"?ò#?ùùÈüÈOäG~äG~äG~"?òùùùÈüD~äG~"?òùùüÈOäG~"?ò#?ùüÈüD~ä'ò#?ùÈüD~äG~"?òùùùÈüD~äG~äG~ä'ò#?òùùùüÈüD~äG~äG~ä'ò#?ùùùüÈOäG~ä'ò#?ùÈüD~ä'ò#?òùÈüÈOäG~"?òùùüÈOäG~ä'ò#?ùùùüÈOäG~äG~äG~"?ò#?ùùÈüÈOäG~äG~äG~"?òùßÝ©§§'É¤ÓéÆÆÆÂEkÖ¬©¬¬|ðÁC]äG~"?òùùMï²ÙlLìØ±£¥¥¥pÑÖ­[öÙááá WýhùÅüÿ,a¯¿þzÿêî×××÷ï|Ç8¦_ü-Ë8 øxçwC	ùÒôöÛo_¼xÑ8ÜîùUWWíb"ËÕÕÕ.jll<wîÜx+ü¶mÛv¤:tèðáÃGt÷¼òÊ+Æ¡4ýÍßüA(M±WÇ¾mìØvl×!¿t:=ætrsûöíUUUõõõ§Ov¶×Ù^9Ûël¯íu¶×ÙÞé]*ÊOWVVX´gÏ8þ|ss3ùÈüD~äG~Óð¡ÿ¸®©©ÉårÜ:ÛÓw+¼9âp ùÈüD~äG~Ó¯ÖÖÖ½÷ÆDüÌf³üñýû÷ÇÄÙ³g.~ä'ò#?ùßô®³³³¶¶6Je2®®®=·[ûûûW­ZN§ÉüD~ä'ò#?ò»O#?òùÈüÈüÈüD~äG~äG~äG~äG~"?ò#?ùùùüÈOäG~äG~äG~"?òùùüÈOäG~äG~äG~"?òùùüÈOäG~ä'ò#?ùüÈüD~ä'ò#?òùÈüD~äG~"?òùùùÈüÈOäG~åÔÓO?ýÒK/],aû÷ïîºû<y2^µCizá¾÷½ïÔÑÑqâÄ	ãPBØíííÆ¡4>|¸»»Û8Ü~øÃß¸=vóæÍ$IT?Yá°§$I³½$I"?I$$IÈO$Iä'I$ò$IùÝÓ]½zµ¢ ÓÓÓÉdÒétcccGGGá,ÒÔF»«««©©)tþüù1¼Åï¬3ÔEÆÓgºðf*²WßÁ×¬YSYYùà?~Ü+v)ÚË5ùI»pN6moo;v´´´Lp¦6ÚsçÎ=yòdLìÛ·oÞ¼yÅï¬3ÔEÆÓg:_j©½ú¶uëÖgvxx8,R__ï»CíåüÊ¤ØY<X8§ºº:öõÈåruuu©vaUUU¿³&;ÔEÆÓ7öê.,Y²dRÿè¶566;wÎ+öG2Ô^®É¯L¿Ä,[¶,N777?>æÄt~iátñEÚhçëîînkkà5¡.2vì»±W¯Zµ*öêÿ/ C·ûö`GýéÓ§½br¨½_¹uåÊùóçÇDáçr*++ïSd¦6ÚI7nÜÈf³¹³>ÌP9ÇÇº··wÁúsÑDuÏ=1¼dxÅ.åP¹&¿2,yi¨©©ÉårÜ:AÓw(²HSíäbÍ5W¯^Èõaz¼9vì;>Ô[·nÝ¾ûdÿÛ÷Ïä0Wìµkò+æÎÛ××ìÐË-ÖÖÖ½÷ÆDü¿ÙÞ¹È"Mm´?×®]ÈõaºÈxÚ±ïìPG-:uê½ú÷øãïß¿?&Î=»páB¯Ø¥j/×äW&uww744Ä_h/^»lÌéìì¬­­M¥RL¦««ëGCë2õ1éÃv]]ÝèïH&FßYf¨ÇO;öÝêä°GrmÁÿ¾|Û«ïDýýý«V­J>OÖÛÛë»Cíåü$ID~$I"?I$$IÈO$ü$ID~$I"?I$$IÈO$Iä'I$ò$IùI$ü$ID~$Iä'I$ò$IùI$ü$ID~t'úÚ×¾¶hÑ¢ª[-Y²äå_þ¸[Mã±m]]]<µócN:Îd2ÃÃÃÝ¦$ò¤é×æÍ+Fõå/¹ä·eË¹sçÎóþùÿÔSOMaÈO¦Y===at:½k×®¡[íÙ³'nÆÌS§Nü.3çÏ?b~CCCÌïëë#?Iä'©üûÜç>¦yöÙggnÛ¶-f~þó/tOGGGÈ)PØÔÔÓù;_¿~½­­mæÌ±¨ººzÃ'U;ºE±î#GFX*æÌ5kÑ¢E¯¾újÜlmmñÀ:T|;É¢xHÉ¢£G§´eËÅü®®®ü'OÆ¥KæçlÝºµ¶¶66UUUµzõê+W®ßèíSä¡J"?Iú(3gN¨åÒ¥K3/_¾3ëêêe3¢îîîdéÊ+G,Z¿~²èÌ3©TjÌµÉÒU«V+++biü9Í1#ùø]íÄÄoô3Ý·o_!gó¸looÏ³oÄF,Y2Yùy¨ÈO>â»c¼¨UTÃeÓÒÒrãV17W¬X,M Ø19tbKe³Ùä`aLwvv&)Üæ¦MÂvÉÉÖµk×ÆÆtüéÇì¶Û	5ÆÍ¸Cácó&gÏÏ÷Úµkq3~Æ#nær¹äL&V§ðÁÏWUUMV~Eª$ò¤^~ÑòËÏOd)ºråJÜ3%7âfýºuëBl7oÞÌo$î3â(Z~­äæåËów-åOøÆÏ>vìØm·Êï¿ÿ~ácï3yO<ñD,zî¹ç>øñí6Þ!ò677'$'+¿"UùIÒG ëÆ3bf,*b¼üå¡ÿpÞè°#,Uø]*1]]]]YY9885kV~ém·3ÃFtöìÙ©1?cúÌ3ù¥ñÆ<k<qùy¨ÈO>âÏºmß¾½pfò]'#®ðÈðÚµk£e:ujË-É¹×<rùÓ©·õÙúõë¼ñ³­­-?¿ÈvclW¯^Mn¾ÿþûÅ¯Ã]°`A,M¾È&ÀZ¸(¹Î7=z´¿¿¿¸üò*MF#¿´ÈCD~ôN§wìØ|«Ë®]»*++G_±bÅPÎàà`r*6nò9¿äãq½½½L ¸aÃpRrÕmþUÆôYr±mòÛ?_d;ÉûÏù$÷,"¿=öäÆÅS.|äñÌ3ñ[bÆ_bÍ°rÜ-Ä¹zõêÂ¥Eª$ò¤¾ä[G´uëÖÿ»U£ütþ<iþ¢|k×®-dea("¿nýc1¿ºººðDpíÄÃ(|`ùëjÇ²7oÞ¬ªªJÂsÜË//ü³gÏÉ»n39$/qj~i*ü$é(t²dÉÊ[-Z´(¹Àvü=|m^ccã'òK|òÉÚÚÚDKO<ñÄàà`~é#GdLf÷îÝ#¶9úÄ¦bþã?>bþxÛâÁÄCJ¾h°È÷ùåkkkñõ.I×®]Ëf³13gÎgÑ××ÿÂmö÷÷m±ZºtiWW×ßXä¡J"?I$$IÈO$Iä'I$ò$IùI$ü$ID~$Iä'I$ò$IùI$ü$ID~$I"?I$$IÈO$Iä'I¤Qý¾â$7|IEND®B`


ýÆ/_¾f¦¦¦R©T<oooË_¹xQ)3Êò«Gýõ¯ÍfCömÚ´)Ì¤Óéááá0êïïÏ_¹xQ)3å÷?üá°²ßÏþs¡tUV~ííí7nÜÈijj!L¦¥¥eùE¥ÌßçþÀÊ^~?ûÙÏìÒUYùÅãñßþö·DbÓ¦Mþó£ü¥+Kq¶íu¶·"Äb±'OÁÌÌLggg4[Z___°rÁ¢Rf(?åWsãè%º0Éd>;]¿ô¹JQ~ ü_E8pàÀ¹sçÂ`zzzëÖ­a000pêÔ©0ét:åâE¥Ì(?P~Ê¯"ÌÏÏ÷õõÅãñÎÎÎÙÙÙ03>>L&c±X*ÈÝ»¨åÊOù½(?å§üå§ü(?å§ü(?å§ü(?å§ü(?å§ü(?å§ü(?òåòS~ üP~ÊòS~òS~ÊòS~ÊòS~Êª¶üB~ëëµÖ)?å§ü`íÊ¯¼òS~ üP~Ê^vùù=?å§ü@ù)?å§ü@ù)?å§ü@ù)?å§ü@ù)?å§ü@ù¡ü(?òåòS~ üòòS~ üòS~ üòS~ üòS~ üòS~ üòS~ üP~ÊÊOùòCù)?P~ÊOùÊOù)?P~ÊOù)?P~ÊOù)?P~ÊOù)?P~ÊOù)?P~ÊOù)?P~(?åÊå§ü@ù¡ü(?å§üå§ü(?å§ü(?å§ü(?å§ü(?å§ü(?å§ü(?òo»ü¾ÜðÂ_õÿðÿ|á«]ù)?åkäÖêt-üõ¯µòjH/Ú)?åÊå§ü@ù¡ü(?òåòS~ üP~ÊòS~òS~ÊP~ÊOùÊOù)?P~ÊOù)?P~(?åÊå§ü@ù¡ü(?òåòS~ üòòS~òS~ÊP~ÊOùòS~ÊOùòCù)?P~(?åÊå§ü@ù¡ü(?òå§ü üòòS~òS~ÊÊOù@m´Ýjlß¾Ý6|uËïÞ½ù÷0355J¥âñxûØØXþÊÅJQ~ò«###öìÉI§ÓÃÃÃa044Ôßß¿ü¢Rf ü*BÈ¾óçÏçÏ455e³Ù0Èd2---Ë/*e¦ ü~÷»ßý@M¨²òkmmíîîÇã333a&sKóÇÏ]TÊLAùÜ¨	UV~9wïÞÝ²eKÄb±Üdþ:ÅJq¶p¶·âD¡ÖÜÜÉd¢Óµa¿Bñ¢Rf ü*BkkëÜÜÜÓg¯ùuwwÁÀÀÀ©S§Â Óéü2£üåW&''ÛÚÚâñø[o½â/Ì'ÉX,J¥&&&þÿ÷³|)^TÊòß«HùÊOù(?å üòS~ÊOù(?å üòS~ÊOù(?å(?å üòS~ÊOùÔFùõôô444ÁÈÈH"ÇãétZùÔZùíÜ¹³®®.e³Ùº<»wïV~5U~¡ó&''gffÂ ­­íÎ;aæ@M_ô_tuuÁàà`4Å@M_CCCè¼ùùùèÅ¿»wï^¾|Ùk~5X~===¹ßíK&OÿûU@¿çPkå¼ùæ±X,dßüü|ø6wìØQÿÿÊP~¯å(?å ügzzzóæÍÑûyGFF@­ßèèhîQùEã¡¡!åPSåL&CçMOOçÊobbÂ§ºÔ`ùå>É9W~ÑqÇãÊ ¦Ê¯¹¹9t^ô:_(¿L&sèÐ¡0niiQ~5U~Ñ_ì(võêUåPSåÜ»w¯³³3zoo"Ø¼yóÜÜüÿ+?@ù½* ü¾M6Ý¸qCùÔxùÅãñè½½Ê ÆËoll,ß#Gæçç³Ù¬ò¨Ùò«[BôÙ~Ê vÊ/¶äPkåWÕ üò+¾	¿çð_þïöåßó¨µòËÍfÞyçP~³³³Ê fË/â/ßÆ@_6åW__¯üj¹ü2Ì'Bù%	åPSå·ÔÀ+âÚµk_­Âýû÷mC¨ò+þëõõõï¼óNUüÿ+?óp²:¶!TGùU5å°6]·¶(?å üê)¿¥^ºÇãÍÍÍ¿üå/òS~P#åWü×;¢?Ý*?åg;@-ßââbCCCooï£GÂ·árÇíííaüÅ_ö¦¦&å ül¨òÛ¼ysøÎd2¹Çîîî0YáÀWù(?P~ßìç9X\ÌÍä/úcÊ@ù)?¨òK&Ñç6/,,<öGÃ8:É;99él/Êj§üFGGûÞÞ±±±h°oß>åPÕ¾ZðXðÓþt5·pëÖ-*¢ü;wîlÞ¼9HÄb±pÆa&zWáÌCùôhQW·ñ_ÿý¿¾ÛÒü_o¾ðÕ½dU~ÕKùX~ýÝ+××=(?å ü5)¿èã.ý7<¢¥Ê@ù)?¨úòa	üa.Ê@ùòå(?åÊOù ü@ùåªâ7ùò^rùUi*?åÊOù ü@ù)?å§ü@ù)?å§ü@ù)?ª/¿òR~Pþò[¿á üÔHùÅVâox(?å5R~µAùX~~ÏòP~ÊòP~ÊòP~ÊòP~ÊòP~òS~ÊP~Ê@ùÊOù(?P~ÊåÊOù(?åÊOù(?åÊOù(?åÊOù(?åÊOù(?åÊOù(?@ù)?åTsù£L4J¥Rñx¼½½ll,µâE¥Ì(?(¿íïÿ±¯ý`ò-¿ÅÅÅ¥Óéááá0êïïÏ_³xQ)3ÊàzÃÆûQ×Åÿ~Ýëÿü¾ú?½þýÓ§OÛPå÷«_ýêØ±c¹òkjjÊf³aÉdZZZò×,^TÊLAùýæ7¿ù?ÀËP·:Gµkø¾±k×.ÛÖ@ßíÛ·;;;C«åÊ/çæ»¨òÛè1ð2üxYQÞ-³Âµk×lÃZvý~h;À¨²òëëë»råJô;%ÑL,Ë-­¯¯Ï_¹xQ)3ÎöByN1ÔÕùq÷¾_ÔgtÈf3Ltº6óW.^TÊòåòåWqh000pêÔ©0ét:âE¥Ì(?P~(?P~Z~ãããÉd2¥R©ü¥ÅJQ~ üP~ ü^EÊÊòÊòÊòÊòªwÿþý×7|¿«mËÿ^Íßïºté¡üå§üà[wëÖ­ðà½ýý?ëkªÕ_nU~òS~°Få÷½äþÏîëë_~< ü ü(? ü(? ü(?^zÛ­ÆÀÀmÊOùòS~ÕáÚµk_­Âýû÷mCP~ÊòP~ÊÊOùò@ù)?P~ÊOù%ßßç;ue¥üò5*¿ºrS~ÊOùÁ_ãw_£çP¹¾7u*?å§ü`ÊÏïù(?åÊOù(?åÊOù(?åÊOù(?åÊOù(?åÊOù(?åÊå§ü@ù ü(?å§üå ü(?å ü(?å üÔRùÕÕÕüzá¯ÎÿhÝÖÿÂWÿºòP~ÊÖÂÂÂÂéÕ	évøðáÕÜÂÜÜ üTÃá¦®ÎòS~ üP~ÊÊOùò@ù)?P~(?åÊ@ù)?÷P~ÊOùÊ@ù)?@ù(?å(?å§ü@ùùqP~Êj¥íVãÂ¶!òS~P¶¯Îõë×mCå§üòP~Ê@ù)¿où.µ:§O¶@ù)¿ªÙ¤ËònnÝºeòS~5qón üòòS~òS~ÊP~ÊOùÊOù­ÊÂÂÂéÕ	åwøðáÕÜÂÜÜÊOùënÝºÒí_~<ðÂ_-ÿÑº­ÿ¯îÿ@ù)¿µ+¿ï%7ôv¯!þ(?å§üå§ü üòå§üP~ üòS~ üòS~ üòS~ üòS~ üòS~ ü_Ëïï¾óº²R~ üß_]¹)?P~ÊoÊ¯ñ»¯½Ñs¨Í:(?å·Fåç÷ü@ù)?å§ü@ù)?å§ü@ù)?å§ü@ù)?å§ü@ù)?å§ü@ù)?å(?å§üå§ü(?åòå§üòå§üòå§üòå§üòå§üòå§üÊOù)?@ù)?åT`ùMOO¿ùæñx¼½½||<ÌLMM¥R©hfll,åâE¥Ì(?åÊ¯">;þ|zµ©©)Òéôððpõ÷÷ç¯¨ò»téÒÿ¥4333uuuoô*×Wø×?ÿüs;ReåsñâÅ-[¶Aè¿l6L¦¥¥%âE¥ÌßÇ<FiBïZn?ùÉOVsöK©¾ò[\lllpöìÙðm<Ï-Ê?wQ)3ÎöóEæº:í-4::ÚÜÜ±X,7Y__¿Nñ¢RfòåWq¢èBÿe2§ÏN×F-S¼¨å§ü@ùUÖÖÖééé0èîîS§NA¸L§Óù+/*eFù)?P~ajjª½½=wuuÝ»w/Ì'ÉX,J¥Bæâ¹JQ~Êß«Hù)?P~ÊåÊOù¡ü@ù)?(?åòå§ü üòòS~òS~ÊP~ÊOùÊOù½ü¶[»òS~òS~ÊOù(?å(?å üòS~ÊOù(?å üòS~ÊOù(?å ü üòS~ÊOù(?å üòS~ÊOù(?å üòS~òS~ÊOù(?å üòS~ÊOù(?å üòS~ÊOùÊOù(?å üòS~ÊOù(?å üòS~ÊOù(?å(?å üòS~ÊOù(?å üòS~ÊOù(¿¥?~ëÖ­ÛjÂéÓ§Ê@ù üP~(?Êåòã[wãÆ]»v566Æãñæææ|ýõ×«¹Á?þøEî¸uîºå;j,-·Î¥Kz£ûI¸ìëë3Å7õå_kK[µVïùßÆb±D"ÑÑÑñÉ'Ønµ±ëK¹W,µßK<J ü(§sçÎmÜ¸ñìÙ³L&|.ÃxÃwîÜù6Ê¯ê÷3gÎtuuMLLd³Ùè~èmmm'N(Xs÷îÝ;wîÏ%lÕZ½oÏÌÌÌlÝºõøñã6Wmßòû½ô£Ê²Wüß±cÇzzzÃN¸<zô¨`òæÍÉd2&ñ×¯_ÿøñãuëÖEG^ò" <´¹^©ò+Øï%%P~ÓþýûçÃ#÷ððpîÛ/¶··ÇãñpÆù.lÚ´)¥Ñ«úÅ'®ÒÔÔÔÙÙ¹â­Ù#[~õõõ7nÜXñvÂíëëp¿sÑÑÑ°ÓÃ?H$º»»K¹5ª¨ü¢;I¸È?×þüy/×pùåöéG	å¢'dË¯z.JMMMq¸lnnÌººº¢óÂaµð¸^|ã½÷f³Ùè°ü­Ù#[~¿ÿýïÃ÷úË¯¶gÏ³gÏ>vÞ'óå;Ã¹sçÚÚÚlöZ*¿-[¶Á×_Ô-,,ñÃÃS¾â×¨òËí÷Ò(?Ê)­¸Nh»ð8_¹Áá@~ìå7;;[â­Ù#e<Ü/ÿö`dddÝºuáÈ¾÷îw!ã¢ÏNõÎÏÏqx°/8ánmbbÂ¦®½òùÊ+7oÎ=ä<yòÀaÐßßÙ5Y~Åû½£Ê2Ë½J·ü:Ñ?"aÜØØøÜÁRå÷b·FîõáàþÁ¤R©p­è¾ÈØØXwwwîÛ;vä¿­opp°¯¯oxxøêÕ«~°ÚË/_øîíí-xç¶mÛ:tðàA°ÊoÅý¾âQåGµµµÝ¾ûV·Äò+ýÖ¨ÀòË·¸¸xüøñð?7³wïÞÇûöå>yòdçÎÑ#Áºuëþò¿ØìÕ[~+^+ôøÑ^¦fÊoõG	e688¸Ô§på¥'v/ñ5¿Òo/¿è°÷èToþûÄ<xÐÔÔTüòÞÝ»wÏ9ÓÚÚj³×pù¥ÓéÝ»w¿÷Þ6à« G	å7;;»qãÆð]0áÂ£GFãÞÞÞüßÌã·ß~ûË¯ô[£¢Ê/L¿Jª®££#[°B¹|ùòK|¡*Êï/¾Ï*Ã »»©;µW~+%P~TãÇ·¶¶ÏÌÂ·wîÜùè£Þzë­ÇG+LLLçè·t'''Ãx|||ùòkhh¸yófôòOÁ:¥ßU~'NOÎ9óðáÃèh~ýúõÜãúþýûÿnGxæ£q8ú=6z	0ÿàÔXùEïçÞÛ½Ï×9ßW¤üV<J ü¨/^OÍD,xôèÑå¿£OàÛ²eKþ£ûRå÷é§Ö?óÜE·ÆîW|o8|¿ûî»ÑnêééÉ"O8ÄZ|b7Ìä>½%<w³p¯·öþèè¨Í^åéó?ÊÑçùÕÀ¡ ôCô2G	ÊåòP~(?Êåò@ù üP~(?åò@ù üP~(?À·¡¥¥%H,,,Ìx<J¥²Ùì7;ª>³ÊÕJ¼åð9r$4Ö'|R0üøñ0ÿá~ã£ªò@eºyófh¬-[¶Ì·µµù¹¹¹oëà«üå°öº»»CfMLLäf®^½f¶oß9zôh2ÇãDâÝwß½÷n~¢]¼xqÝºuÛ¶m+¶å¯866¢3,íèèã¥ÊïÒ¥K¡DÃjaåðoåæ>|¸oß¾ÆÆÆ°¨©©éàÁÅ§­Àÿ8ölÈ¬]»våfvîÜfsõV÷·ºººò-Ë¾¾¾h[ñ&''ËïúõëÑí¯ÖÛÛ[pï½÷(?%-..®_¿>?xð |.Ci566f2hT*¢êË/¿|úßgD~¢ðÁÙl6:5m+^±¿¿ÿÑ3a¾íéé).¿t:½:ÆãããÑµ¢EQÞ¾;Ã¿Æv( ü388²éØ±caüÑGñÁóWÒ*^gggô"_~¢Ý¹sçª¢vù+F­Ü½7|r³øFÂdÁ¹Õ:::Â·6mÚ¿ÿùóç?~lWÊ`ÓÓÓQBq¸ãë×¯ç755äW~¢åòKþÒ¯ø7ãººx<^¼´ø¤p.ggg£øËáìM@ù¬à?üa§ÃËSù¢÷ùE£££óóó+Å+F'j>;Å¼Ôk~a;õOúÓ#GúúúÂj©TÊ®ÀN<ñlhh(Q<^Ìf³~úiéå·âzzBó=yòd`` |.o$Jº	ÿ4ÑïùE¿G8;;Æõõõv% üVðøñãD"rôèQþ¢·ß~;ÿdëúõëÃeôù,ËßWÒ07ÎbÎ¿ÉÉÉ³½###Ñ¢è!ùöîÝkWÊ`eûöí+øxÈÒétccãàààÜÜóS/¿¯8::ÚÑÑ¯½½ýÊ+Ï½àâÅ±X,J8q"7¿°°pèÐ¡d2eeø'<yb?Êå üP~(?Êåò@ù üM üP~(?Ê5ñÿSFg,fIEND®B`


ONEWAY Figures BY Variables
  /POLYNOMIAL=1
  /STATISTICS DESCRIPTIVES HOMOGENEITY
  /MISSING ANALYSIS
  /POSTHOC=LSD ALPHA(0.05).


Oneway


Notes	
Output Created	12-SEP-2022 23:32:57	
Comments		
Input	Data	E:\桌面\Raw Data\4. C. Cellulosae ESAs and TPx Induced Th Subpopulation Differentiation\3. SPSS statistical analysis\4. IL-10\2. IL10-48h\2.1 SPSS statistical analysis--IL10--48h.sav	
	Active Dataset	DataSet1	
	Filter	<none>	
	Weight	<none>	
	Split File	<none>	
	N of Rows in Working Data File	20	
Missing Value Handling	Definition of Missing	User-defined missing values are treated as missing.	
	Cases Used	Statistics for each analysis are based on cases with no missing data for any variable in the analysis.	
Syntax	ONEWAY Figures BY Variables
  /POLYNOMIAL=1
  /STATISTICS DESCRIPTIVES HOMOGENEITY
  /MISSING ANALYSIS
  /POSTHOC=LSD ALPHA(0.05).	
Resources	Processor Time	00:00:00.02	
	Elapsed Time	00:00:00.01	


Descriptives	
Figures  	
	N	Mean	Std. Deviation	Std. Error	95% Confidence Interval for Mean			
					Lower Bound	Upper Bound			
Control	4	31.80400	2.581350	1.290675	27.69650	35.91150			
ESAs	4	37.57775	2.104609	1.052305	34.22885	40.92665			
TPx	4	41.86500	2.573552	1.286776	37.76991	45.96009			
LPS	4	57.22775	5.242558	2.621279	48.88567	65.56983			
Total	16	42.11863	10.188376	2.547094	36.68962	47.54763			


Test of Homogeneity of Variances	
	Levene Statistic	df1	df2	Sig.	
Figures	Based on Mean	.738	3	12	.550	
	Based on Median	.705	3	12	.567	
	Based on Median and with adjusted df	.705	3	7.044	.579	
	Based on trimmed mean	.737	3	12	.550	


ANOVA	
Figures  	
	Sum of Squares	df	Mean Square	F		
Between Groups	(Combined)	1421.444	3	473.815	41.930		
	Linear Term	Contrast	1297.934	1	1297.934	114.861		
		Deviation	123.510	2	61.755	5.465		
Within Groups	135.601	12	11.300			
Total	1557.045	15				


Post Hoc Tests


Multiple Comparisons	
Dependent Variable:   Figures  	
LSD  	
(I) Variables	(J) Variables	Mean Difference (I-J)	Std. Error	Sig.	95% Confidence Interval	
					Lower Bound	Upper Bound	
Control	ESAs	-5.773750*	2.376982	.032	-10.95275	-.59475	
	TPx	-10.061000*	2.376982	.001	-15.24000	-4.88200	
	LPS	-25.423750*	2.376982	.000	-30.60275	-20.24475	
ESAs	Control	5.773750*	2.376982	.032	.59475	10.95275	
	TPx	-4.287250	2.376982	.096	-9.46625	.89175	
	LPS	-19.650000*	2.376982	.000	-24.82900	-14.47100	
TPx	Control	10.061000*	2.376982	.001	4.88200	15.24000	
	ESAs	4.287250	2.376982	.096	-.89175	9.46625	
	LPS	-15.362750*	2.376982	.000	-20.54175	-10.18375	
LPS	Control	25.423750*	2.376982	.000	20.24475	30.60275	
	ESAs	19.650000*	2.376982	.000	14.47100	24.82900	
	TPx	15.362750*	2.376982	.000	10.18375	20.54175	

*. The mean difference is significant at the 0.05 level.	
