# Supplementary material for: Regulation of piglet T-cell immune responses by thioredoxin peroxidase from Cysticercus cellulosae excretory-secretory antigens
Source: Front Microbiol. 2022 Nov 18;13:1019810. doi: 10.3389/fmicb.2022.1019810 (PMC9718028; doi:10.3389/fmicb.2022.1019810)
Supplement: Supplementary file 3 [file Data_Sheet_3.ZIP › 4. C. Cellulosae ESAs and TPx Induced Th Subpopulation Differentiation/3. SPSS statistical analysis/4. IL-10/3. IL10-72h/3.3 (SPSS data export) SPSS statistical analysis--IL10--72h.doc]

EXAMINE VARIABLES=Figures BY Variables
  /PLOT BOXPLOT NPPLOT
  /COMPARE GROUPS
  /STATISTICS DESCRIPTIVES
  /CINTERVAL 95
  /MISSING LISTWISE
  /NOTOTAL.


Explore


Notes	
Output Created	12-SEP-2022 23:35:49	
Comments		
Input	Data	E:\桌面\Raw Data\4. C. Cellulosae ESAs and TPx Induced Th Subpopulation Differentiation\3. SPSS statistical analysis\4. IL-10\3. IL10-72h\3.1 SPSS statistical analysis--IL10--72h.sav	
	Active Dataset	DataSet1	
	Filter	<none>	
	Weight	<none>	
	Split File	<none>	
	N of Rows in Working Data File	20	
Missing Value Handling	Definition of Missing	User-defined missing values for dependent variables are treated as missing.	
	Cases Used	Statistics are based on cases with no missing values for any dependent variable or factor used.	
Syntax	EXAMINE VARIABLES=Figures BY Variables
  /PLOT BOXPLOT NPPLOT
  /COMPARE GROUPS
  /STATISTICS DESCRIPTIVES
  /CINTERVAL 95
  /MISSING LISTWISE
  /NOTOTAL.	
Resources	Processor Time	00:00:01.13	
	Elapsed Time	00:00:00.86	


[DataSet1] E:\桌面\Raw Data\4. C. Cellulosae ESAs and TPx Induced Th Subpopulation Differentiation\3. SPSS statistical analysis\4. IL-10\3. IL10-72h\3.1 SPSS statistical analysis--IL10--72h.sav


Variables


Case Processing Summary	
	Variables	Cases	
		Valid	Missing	Total	
		N	Percent	N	Percent	N	Percent	
Figures	Control	4	100.0%	0	0.0%	4	100.0%	
	ESAs	4	100.0%	0	0.0%	4	100.0%	
	TPx	4	100.0%	0	0.0%	4	100.0%	
	LPS	4	100.0%	0	0.0%	4	100.0%	


Descriptives	
	Variables	Statistic	Std. Error	
Figures	Control	Mean	32.47850	.978101	
		95% Confidence Interval for Mean	Lower Bound	29.36574		
			Upper Bound	35.59126		
		5% Trimmed Mean	32.52522		
		Median	32.89900		
		Variance	3.827		
		Std. Deviation	1.956203		
		Minimum	29.776		
		Maximum	34.340		
		Range	4.564		
		Interquartile Range	3.635		
		Skewness	-1.130	1.014	
		Kurtosis	1.490	2.619	
	ESAs	Mean	38.26675	1.780448	
		95% Confidence Interval for Mean	Lower Bound	32.60057		
			Upper Bound	43.93293		
		5% Trimmed Mean	38.27528		
		Median	38.34350		
		Variance	12.680		
		Std. Deviation	3.560896		
		Minimum	33.831		
		Maximum	42.549		
		Range	8.718		
		Interquartile Range	6.581		
		Skewness	-.129	1.014	
		Kurtosis	1.498	2.619	
	TPx	Mean	33.49875	1.614742	
		95% Confidence Interval for Mean	Lower Bound	28.35992		
			Upper Bound	38.63758		
		5% Trimmed Mean	33.43211		
		Median	32.89900		
		Variance	10.430		
		Std. Deviation	3.229485		
		Minimum	30.281		
		Maximum	37.916		
		Range	7.635		
		Interquartile Range	6.023		
		Skewness	1.013	1.014	
		Kurtosis	1.514	2.619	
	LPS	Mean	50.37000	2.252504	
		95% Confidence Interval for Mean	Lower Bound	43.20153		
			Upper Bound	57.53847		
		5% Trimmed Mean	50.39767		
		Median	50.61900		
		Variance	20.295		
		Std. Deviation	4.505008		
		Minimum	44.621		
		Maximum	55.621		
		Range	11.000		
		Interquartile Range	8.381		
		Skewness	-.329	1.014	
		Kurtosis	1.494	2.619	


Tests of Normality	
	Variables	Kolmogorov-Smirnova	Shapiro-Wilk	
		Statistic	df	Sig.	Statistic	df	Sig.	
Figures	Control	.249	4	.	.936	4	.628	
	ESAs	.249	4	.	.953	4	.733	
	TPx	.251	4	.	.947	4	.700	
	LPS	.249	4	.	.960	4	.782	

a. Lilliefors Significance Correction	


Figures


Normal Q-Q Plots


O(nZÀ?""""DDDDþüø#""""ðGDDDDàÀ?""""DDDDþÀ?""""DDDDþhÏÏjiiÑïw,Ð©çÏ¯¬¬LyVEEÅÙ³g§½Â_dW			rþ¢¢¢cÇ18===UUUA|û7nÜX±bÉd[÷¹?"ü-ðÁÑ¿_~Ù>Ô¹¶Y]$®=:ÛÁ	zsrr<·k±XÀ?"¥±¤õë×GþzzzVÏ9ãv»§¦¦dA¾W5ÛøÜôØØØÞ½e,løS|üø±,ONNFÔÀE=þjkkåÿóçÏÆ;w6mÚ¤æLkjjÎyíÚµôôô+WzÖ´··/[¶Ìd2UVV>|øðÆEEEIII«W¯ö~ñÐ¡CÙÙÙj~SnÂsR âlÝºUÖOÂJ---²²®®NóíEüoZ¼(käNÎjp4_ó.¸êãÏçTù¹Ê°¿ðÂ2¶ú¯úêýcìv»üdå$Ï©2âi9)33s÷îÝN§ø#¢ÈÅßØØXNNNVV,ø;àþýûòîÍ9§Òäÿ6<ÕbøÆó¥@Ä#?s®ZµJ8/õ=ò^)¸yyyßãl/âsÓ"õÊ_~~þ¬GßpA_pæøëííõ¾ùfg?ïk__úÒSOO:óúõëîóÎ;yp?"ÉÂÅ½_	óvÀöíÛeyíÚµ%ò¥¬ô>ç¦¦¦ÔkWjÍ;Ünwgg§úR®Y¾¼ví,ÕeÕ;Û®"ËwïÞõ>)qL&æzïËÎñ"ÞTçyet¶£y¯¾ Îþ|.(hå	a½h3Ç÷U®GÖ~]ÕuË-êÌ÷îÝeùiÊrjj*."ðGD?IËeY×ÖgeeÉòýû÷Õò/_.Z´Èû<§zÖ8¼¿úùSosLNN¥¥¥ê¥¦iñ¤ùh®â"Þ¨Ëcªªªìv»æ Ídp4ïUÐ9þÔû>|è³Â÷U]w²FTRR"_.Y²Dð*E<yòGø#¢(Àø,33SÂ].÷zÿWÎüß'ªÓ¼Ný/>SÓ~j5;;[ÍSûÏckÉEÉ´6Á	DÒà.8óißðNÿTÿ«Ï~pÔICùÏÂË/óà"DéøÎ;'_zv¢V*ù¼F%+gbý/Õ.WÚÛÛg3ÁzyRíu¥¦¦f÷îÝ?>~ü¸¬Ü´i&þfr ñ7ÛÁñôg¿¼òçáÐ_üSSSeÏG½»yófcc£kö|2ÀE4þ>ó¾çýjú,ðÚµkÇ¥ÝwìØ1wü©W¿úúú Ç	þ:::ÔkKâÅÎÎNõªêâÅßãl/2+üéIç³|®d¶£þÔ»ôÄ¾O<Û­Y³ÆGÁ õÇ_ÝC¡³_FR-[æýmª÷n:)x"DqøðàçÝ]jÍàà Ïû½²²²<þ<Q-Z´Hþ÷þÄ«æ~ñÅý§ ËÊÊt¾ÍY]dVøÓÏ¾vÔÄ¨¿7oz_ÿÊ+½Oõá>þÔî5?£^[õî¥^âÁEþ(:ð'©)Qïõ~øá¦MUSSãÿñàð7::*×&×)Ú³gxÈ³â477%$$,^¼øäÉ_ùÊW<¯<Íý"³ÂþàxQüg2<»ÊónV£þ¤Ë//Y²D¾k¹~5±îù³|)DS·^UUÕÝÝ=í+¯×®]+--koÊf³yÖ;Îûö©TEðò£t¹¸ÀQMNN¾øâ³Ú·ºÔl¬úø¶ÛíV¦/,,äÀQ¦ÞóçÓë¯¿ÎÈø#"ÁNçÞ½/^¬æveáå_fXüø#""""ðGDDDþüø#""""ðGDDDDàÀ?""""DDDDþüø#"""DD:]¼xÑl6L¦U«VÍö²EEE,p85²,k-[6Ö³;ÏL.t£££;vìHOOÁY¼xñ¾ûNg6âF~_Dþ(®ËÎÎgtÜn÷l/èÐ!¹ìáÃ=kÞxãYÓØØíøçå=ëîÝ»SSS§NZ³fMWÕÓÓSUUþüQdl/æà7oÊe+++=kV¬X!kzÃs'CríùóççeÁ?"2P~ÞÔØ·oßÂSSS<ès¶¯|å+¥¥¥>×àyáP=z$Ë²FtïÞ½ªªªÉTRRrýúuÍkó¾uý<yÒl6WTTÈù#ibbÂb±ÈeåÎïÝ»W½)<]¹r¥¬k¾ÿþGfÙ²erÍ.KóTQêèè;)pñâEÿöI«â÷Àå?µüê«¯Ê²ÍffÉÂë¯¿îk×®)äy·ûv9éøñã²,ÿËr]]:©¬¬ìÊ+²p÷î]Y£ymÞw@ÿ"BºsçÎÉBmm­ÿß¹s§,Ë. ¹¹¹²<66Ö××'êIÿüE¼_ú£täÈu[B@ÿ;éó½ë_¿DþÈXüWdyòYþöÒ|S`GG´víZY^¿~½çÔ7o8p ´´TÖ'$$h^tô/¢îØÂý/¥îüÔÔ,ûd¥SKJJDW^ÆÓLú£ôäÉµ,×ï]ÿªøå$"ðGDÆâÏ[<øâH+55UÎét:Õìª¬Q';vL.%"¼û¶?4ïÀL.è½_ÌSp<þ|zzºZ©^É+ùùù²~bbBßúÈÓ_nÀÀ999Þ/D©Ï¦µÅbS=ÿÖ'%%É©gÍúñÜ1ñÿe-Z¤.ës÷Ün÷+Wjkk½_2¶Èùþ'Ídf¿àüQhð§>âzüøqõ´7Þxc&9þ¼ç%´sçÎùÈ¦¿¿_½p&øÓ¿ìí·ßíÛ·û_ö¥^åS§N¨©^YYQQ!Ë·oß~øð¡,äççÏpXÆÆÆbyyy÷îÝPª$7lØ0ÃQò^VSÏjÒÙçnÀÀn·[ú,ïõé[äÉ'jÊUþ3yÖ_¾|933Sè³wïÞâOÿ"v»]N]±bçs'Þçq:»wï´fÍõÁ^1_MMúøpeeå~8ó[PªImQàÁÕûùf2JÞË/^»¤ö¡íó½7àDDàüø#""""ðGDDDDàÀ¿ô­okhh(Ì7ú½ïï?þã?ø½1¨û÷ïÿð?dêÞ½jÿ#dDwïÞÕAáhVþ÷ÿ7ã`Pßýîwÿçþq0¨>úü¬¯ýëâ¿0ßèíÛ·ÿíßþ_eúø2õþûïÿû¿ÿ;ã`P÷wÇ_Æõo|?]ëÚµkØÚ¸._¾ü¿ÿû¿àüøàÀøàü?ðþÀ?ðþÀøþÀøàü?ðþü?ðGàü?ðþü?ðGàü?ðþü?ðGàü?ðþÀ?ðþÀøàü?àüøàü?àüøþÀ?ðþü?àüøàü?àüøàü?àüøàü?ðGàüøàü?ðGàü?à/ñ×ÝÝ]RRb2-[ÖÛÛþÀ?ðþü¿XÆ_^^<]ÉÂÙ³góóóýñ×ÞÞ>ÞD'ßýîw'Èººº>þøcÆÁ ä%ùÓq0¨ïïAÙíöïÿûA]½zõ?øã`P?ùË0Ì7Åøó.%%ÅGùfx?dôM2&ÙÉß÷A]¹rá5®wÞyçúõëAÉÓçßþíß2Æ¯üõÂ8Ô¥KÂ£±¿ºº:¦ö%¦öeÚöeÚ7§U555N§ü?àüø1¿ÔÖÖjü?àüø1¿Õ«WjþÀ?ðþü¿ÂÙl^àøþÀø#ðþbú?ðGàü?àü?ðGàü?àü?ðþÀøþÀøàüøàü?ðþÀø#ðþÀ?ðþÀø#ðþÀ?ðGàüøàÀø#ðþÀ?ðþÀø#ðþÀ?ðþÀø#ðþÀ?ðþÀøþÀ?ðþÀøþÀø#ðþÀøþÀø#ðþÀøþÀø#ðþÀøàÀø#ðþÀøàÀøþÀøàÀøþÀ?ðGàü?àÀøþÀ?ðGàü?àü?ðGàü?àü?ðþü?àü?ðþü?ðGàü?ðþü?ðGàü?ðþü?ðGàü?ðþÀ?ðGàü?ðþÀ?ðþü?ðþÀ?ðþü?àÀøþÀ?ðþü?àÀøþÀøàÀøþÀøàüøþÀøàüøàÀøàüøàÀøàüøàÀøàü?àÀøàü?àüøàü?àüøü*?ðGàü?àÀøþÀ?ðGàü?àü?ðGàü?àü?ðþÀøþÀøàüøàÀøàüøàÀøàüøàÀøàü?ðþÀ?ðþÀøþÀøcÀøàüøàÀøàüøàÀø#ðþÀøàÀø#ðþÀ?ðþÀø#ðþÀ?ðþÀø#ðþÀ?ðþÀøþÀøàü?ðþü?ðGàü?ðþü?ðGàü?ðþü?ðGàü?ðþÀ?ðþÀøàü?àü422râÄ¹l<Áø#ðþÀ?ðÙívÅ¶`Áðþü?àüÅ`£¡¡!77wWåååàüøþÀø+++h%ëN'øþÀ?ðþ¢;ËÕÚÚZ]]èo¾âââ¹ÜøþÀø#ðþ"âÇjµfddø/--­®®Náv»ç~CàüøàÀø·üÍ¸yóæÓ§OvS	þÀ?ðþü¿p§?½[QQa³ÙÚB?ðGàü?à/¬Ï,¦wÍfsCCÃá0ô?ðGàü?àÏðMï&''[,»Ý·ô?ðGàü?àoÞ·ÙllQÓ»rpÞ%ðþü?ðGàü8·Û­È<_Ó»àüøàÀø3<u@áÝ¼Oï?ðGàü?àÏ¨t¦w«««O>æé]ðþü?ðGàü8ýéÝ¦¦¦áááºÏàüøàÀøæ	"ÐônFFÕj3Dæ=³nttT~ÒàüøàqCüé£ºººµµÕårEòð¿ÙÕÙÙYXX(?`ðþü?ðÇ8ÄþôÈÓ»à/4­Y³Æápèàïí·ß¾Þ·nÝºKÆôÍo~ó;ßùã`P×¯_ïïïgê½÷ÞûçþgÆÁ Þ÷]y:`êÊ+ÿò/ÿ!wæoþæo¶nÝúÉO~Òß|²RN3D×ð^ºtéã?óF1þ~|ãïäÉÿÞ:::º»»ÿItÒÓÓÃ8Ýnïííeª½½ýæÍA]½zõöíÛA½óÎ;ò÷¼oÿÿàþàsû¿ùª««;öÁDãð^¾|9ü7ËøcÚi_bÚi_¦)z§õ§wçåLû?ðþÀ?ðþBÛígp«Õiäàü?ðGàü¿ùäàü?ðGàü¿¹¦s@Þáü?àüQ$ãOÿ16½þÀ?ðþüÅ/þt¦wÕ9ä<Æ¦wÁø#ðþÀ¿¸ÃÎôn´ü?ðþÀ?ðþ¦Éív·µµÞ®r?ðþÀø#ðþÀÞv»¡¡!777Ðô®A·nÝ/öõõ?ðþÀøàÀ_¤ãohh¨¹¹yùòåALï:ÏþóÏå=÷üçá~AÎÛÁàüøàÀ_´âO&Ù¸q£æ9f2½ët:?»ø³Õkª~í ü;°ÿÀòåV«ü?ðþü??Ù>×ÕÕi¾¥/333Ðô®mmm_(üú·kç®ôôôþä/øþÀø#ð5ø|íµ×.]ª9½»qãFÁÜ¬&m«~¥Êò/++kddü?ðþü?ó?ñÍfÓ|KÞ=|øppÞ,óÊøþÀø#ðø³Ûí7oÎÎÎö7¬lhhãçsNç§?ýiÞóþÀ?ðþüÍ'þG éÝäääuëÖÉyBõâÜÖg?ûÙçòûÒ¾$ äÓ¾àü?ðþÀ¿0åt:m6Û%K4§w+**äT#Þ'~[[Û­[·bþ oàüøàÀßüg·Û×­[áo¾ÜÜÜúúúF	ü?ðþÀ?ðÝÝºuK=5§wµµµòÄoÇÞ³¹ät%$$?àüø÷z÷_øÂ¹sçÆÇÇ(ð7M	Óe2À?ðþüÍW.«µµµºº:Ð9^íµ¡¡¡¹ÛâóøþÀø£@S«Õªù¾ÌÌÌúúzïr?ðÆÆÆ¶oßþü?ðGà/l9yr,((´Ç·ÞzËÿ-àüÙl6L¼çÀøþÂßøø¸Íf«¨¨ÐÙcÎ[úÀøuË-Ó|UÙét?àüø3(·Ûm·Û-Krr²ÿ±Ùlnhhp8Ó^ø³.))I~ÉFGGÕÑ`Ä|o¿ý¶,ÔÕÕ?àüø3bkiµZwÓ»ÂAAáÌwþÀßì¯âY² Ú»wïNMMÉBjj*ø#ðþÀ¿P¥öØ¢ù¾Lï?ð2ü¥§§Ëï§Ãá_~Y-°«àüøúlùô.ø!ÃßÞ½=ïðùüøàÀ_p©·ôÕÖÖjî±%é]ðþB?éW^Y´h,twwË@°´´Ôè»þÀ?ðþb2ÃÑÐÐ ù¾ÄÄÄêêêÖÖÖü¿¨	ü?àüÅRúl)((hjjùí?ðþÀøþÀ_øò¼¥Os-V«ÕûàüEþòóóÕ_ØÉ3?ðþüÍ|£è lFLï?ð2üåååyÏö%ðþÀ?ÿô÷ØbÜô.ø!Ã8O~Yzz¦¦¦Ây×Áø#ðþÀ_¥¿Çììl£§wÁøþ233å·6ÌòàÀøQ´q4½¼qãÆÓ§OÏ-àü½½½òë»sçÎ	ðGàü?ªiÈæé]ðþB?iñâÅþ¿Ö|àÀøøSl©ªªÒÞ]ºtiý­[·"ç?ð7ë,YÂ>ü?ðþâêEs-²rÛ¶mÂ¬yÞà/døS¿Öa¾ëàüøà/Ò9 TYYi³ÙFFF"öþ?ð7ë²²²øÀ?ðþÀ_¼áOÿÙÙÙ¯½ö¸0òü¿Y×ÑÑ!¿å÷îÃ(Áø#ðþÀßü6éÝ®®®0?'?ðVü-ø ðþÀÅþô§w×­[×ÚÚêt:£nxÁøu	âþÀø£À<pt¦wÃ@ðþæóøþÀø3.·Û-OÛ7oÖÞÍÌÌ¬¯¯ïêêáàoÖÍæ%K?àüQàÏápìß¿?Ðônuuõ'¢qzü¿áÏd2É!üwü?àü°¦¦¦åË: GKKK$ï±ü¿ðáïúõëò¨hllç_Àø#ðþÀßÜs¹uëÖiÃl6744DÅ[Àøþø´/?ðGà/ñ'Û«Õ­¹ÇÅb·Û#ðàüÍ?þø´/?ðGà/ð744ÔÜÜ\hz×f³ÇÏð?ð5?ðGàü¿';útUUæ§wãazü?ðþÀø#ðøëêê²X,q>½þÀ_(ñ799¹fÍy ¥¦¦nØ°!üàÀø:9ÆÆÆÜÜó%&&VUUÅÛô.ø!Ã< 5ß6aôaÁø#ðþÀòÞÜÜÃäàoþñWXX(§µk×NLLÈcccë×¯5eeeàÀøü©=¶lÛ¶Mó-V«U62&ø!À_RR<®¼ß-199)kd=ø#ðþÀ?µÇÍ·ô%&&VWW·¶¶=þ(¾ð 0÷_²]½øàÃßððpSSSAAÓ»àüjÚwõêÕjÚWþeYSRRþü?ðG¡ÅËåjmm­®®Ö< Ó»àü¢=Í¿º?~þü?ðG¡ÂÎô®ÚcKH¦wßzë­æææøÙÿøÁ$É6,0!!Aþ_½zuÀþÀ?ðÆ¾úÕ¯>÷Üsa8 <­È³XYYÙ¯VýjnnîóÏ?ûà/jàÀøáô§w8 <@²²²6×l>øµêßK¿¸qãFðGàü?ðþüÛí¶Ûí%Ð[jkkmkk+++óÈOþÕï©OOOùÉ_ðþf|ÉéJHHþÀø£æp8Ìf³æÊ¯ýÚ¯½Çæææª_©òÆüËÊÊþ~TBàÀ?ðþhÛl6ýr;w.o%=÷ÜsÞòÛþ·gddðÊ¿iÚ³gzÄ=üøà4ÓÞUoéóì±%¸cûq¾¼òËöùíÚ¹kñâÅüÇó?ðþ¯··755U´UUUÞû|þÀø#O:Ó»j-þoéþ>ûdqqqqVVVaAaZZÚâao/àüÙ¦MÔC÷Ê+á¹ëàüøQÔÈÈÎô®þ[Â?ÕÐÐÐ­[·NgühÀøu.Ýµk×ó®?ðGàüE~.Küjkk5÷ØRPP ói÷ØfüÅ[àüÍ"y(©Ïv~=Ìwü?à/ÂÑ¦wg6ðþÀ_DàïÈ#ê1¼eËy¹ëàüøØàà`sssyy¹¿ù«««Øcøà/"ðÇ~þü?Äso½õÖºuë4§wÅ6mxx8¸+àüEþ¦Ëd2?àüÅ|]]]V«5;;Ûß|jzwàüEþæ=ðþü¿yldd¤±±±  @szwãÆòªý¤?ðþÀøþÀßü¤¦w+++5wÎ¼|ùòæææ §wÁøàü?à/`±XÒÒÒ4ÈaµZûúúºiðþÀøàÀøS:äPÞáô.øàü?ðGàüÍOòÛÒÒÒRYY©¹?òòò¦¦¦Oï?ðþÀøþÀ_¸³ÛíuuuÓ»¹¹¹Óü?Mü±?àb	òÜµkæ[kkkekôô.øà/¢ñç½K?Mü±?àüE~ÃÃÃMMMl*++O81222¿wü?ðøóÔÞÞ.­[·NLLÈòÿúõëeÍ7À?ðþ"3ËÕÚÚZ]]­y@âââ×^-üÓ»àü¿èÀ_ff¦l)¼ç&''eMvvö¯¹··7''Çd2]¿~ü?à/$4«Õ¡¹Çýû÷ÏýàüQãOm2|>øûþjjjN:%GÝ²eøþÀ_ÐéLï&''[,»ÝiæàüE(þ²²²dÛ!PSL§Ó¹víZY#ëçxÍSSSJò÷¨?þl6ÛÍð& ÎÎÎdLï½÷Þßÿýß3ÕÞÞÞÕÕÅ8<~ûÛßÀ;ÖÝÝýúë¯ÿò/ÿ²æû³D®>¼W®?ù53(ÑIoo/ã`P.]ÿ¿7nh¾MøöíÛs¼fïø|DðwáÂáðöþûï÷õõ1ë[ß`ª££ã£>bJþ2¼sçNäÜ¡¡!ÙBþÎïüÎ'?ùIÿíó/þâ/îÜ¹SqÑ2¼ò§ËÇÌ¯A]½zõÞ½A­ïß¿æ5ÒÝ»wóòòRRRä/ËÔÔÔGÍýj½'öeÚöeÚwÚtÈùÓ»Lû2íË´otLûWVVz+¡üï?þÀ?ðçi||Üf³UTThNÅÈz9UÎ¥ÃþÀøüÉ_¨'Où¿¦¦ü?àÏ'·Ûm·Ûek¬ùéÝy9 øûøëïïÏÏÏOMMUµYYYçÏûÕvvvfggËuæäätww?ðGàüyÒÞÍÈÈ°Z­óx@ðþ(Æñ§vòìH7µ|ôèQCï:øþâ:Ó»ÕÕÕ­­­.+Æü?ðYøSìïï÷à¯»»[.þü?ðÔ9Mï455Mû?ðGàü=áNÕÂßÔÔÇö%ðþÀ_¨È¡¦wå1?¼àü¿ÈÂÚÉ³zµOð799¹oß>õFcðGàü¿àÒ9 GOï?ðþ¢gðGàü¿Y¥¦wÅv"¼8ÞàüEþ$yÂ.--UöMIIÉÏÏ4ú®?ðGà/ð§3½3làüÅþæ%ðþüÅþt¦w£÷àüQãÏó9O=ÊÏÏÏÍÍþÀøÓl||üôéÓ¦w£ýàüQÜáorrOûøàÏ¿89 øøËÍÍ] [zz:ø#ðþÀJçLï?ðþ¢÷îÝKxçØÞü.þü¿8ÇÎ9ÞàüEþ<	õáàÀ_táOzWí±ehhqàüE%þæ+ðþüE þt¦wç÷òÓokk;|ø°<EÝÞ¡Áø¿ÉÉÉüüüÔÔTÏE½úê«àÀøÄU²QÒÞrtuuýüÏÿ|YYYÕ¯Tîs+**®¹fðþÀ_dáoñâÅ>øUÛ»ÆÆFðGàüÅvêëÖ­KIIØrÈÏ=++ksÍæ_;¨þÿÆoüø#ðþ,))I¶q5½½½²fáÂàÀøÕºººêêê233#mz×¿¶¶¶²²2üä_ýúôôô(úp1øà/²ð§>ð;55õ3×ë·ó?ðGàüÅ@²ñ)..Ìé]ÍW­Zå?ù522þü¿`ÊÉÉ­ÞîÝ»ÕöN5ÙÙÙàÀøÔ[*++5È±|ùòßÿýß¿sçNÄ>µ/Y²äÀþùÕýn]FF¯üøAÖÓÓ£¹ÿªîînðGàüEuj-7nÔÜcükµZ»ººälAÛ7<ßðtÅVÔï©Wòûüç?ÿæoFÑü?ðYø<xPTTZXX(k¾ëàüø3®[·nÚcKZZÚæÍeûãýÊY$ãOr:¿õ[¿ñ'ò'ÑõãàüEþæ%ðþü¼¡¡¡¦¦¦Í	åË: GãÏüDãAäÀøàü?¡Ìår>ºªªJó-K.ÝµkÃáÐ¹hÁ_þÀø8üõ÷÷«ý<«Oøfee?üøÚcKvv¶¿ùkkkg¸àüQá¯½½Ý³­TøSËGþÀ_d600ÐØØhz·¢¢âÄ³.ðþÀÅþÔ_Ìýýýüuww³gà/>|ø°ØNsz·¸¸X6,Áøàâj£ùÔkÇÎSSS²l2À?ð	©°UWWk/--­¶¶Ví±%èàüQá/++KíÕOáorrrß¾²l6Á?ð7ï¿TV«5##Có-ÂÁ§Ó9÷àüQá¯££CóM3òLþü¿yixxXg-K.mnnóðÁøGøä	»´´TÚ7%%%??ppÐè»þÀ?ô§wÍfs]]ü±£;ðþÀÅþæ%ðþüyÿòèLïZ,»ÝnèÎÁøþÀ?ðgxúÓ»ÈþÀ?ð7§îÜ¹³dÉ¤¤$ÙÚ¦¤¤?àÏ Äs¢º@ä0ÍúäàÀø>»Ý®ù7÷ÀÀø#ðþBÛíÅbINN¯é]ðþÀ¿ïä¹¦¦Fí.abbbË-²&''üø!Éáp444ÍæyÞàüøûñN½ÿÔeÏÁ?ðtjzWl§i¾ÜÜÜðOï?ðþüýø¿ÉÉIÏyòÊ?ðtúÓ»V«U~["ê>?ðþ(ð§ÞóWSS£?®¬¬ä=þÀ_éLï&&&VWW·¶¶º¼çàü?#ü-.æÁø£Áþ[d½Úr?ðGàü_ÂtL&ðGàüù§@ÈÞàüø·Àø£èÅÎ9"|zü?ðGàïi ý9?xðüøÞéOïGþô.øàÀßÞó÷Ê+¯ø¬Ü¶m»z!ðþTúÓ»f³yÿþýó²sfðþü¿Y'ÈÍwVVÖ£GäË3gÎ¨ºAoõà¢:Ó»iiiëÖ­ÍbTü?ðGñ¿§Ï^çS[óµ°~ýz£ï:ø±øSlÉÍÍ4½k³ÙÔ1b&ðþÀÅþ¤7nx¶ì---a¸ëàüQ¤áoÚrÈÃÖè=?ðGàü;vìPwu´iëÖ­àÀ_àOÿ²²¶¶VÎÓ»àü?OM&lß.óæÍ§^ïùKJJþb:ä¢q-àü?3¸8à³RþÐçÓ¾þbúÓ»Ë/onnÒ=¶?ðþüM_ ýüý,þÀúÓ»ÙÙÙ»vír?ðGàüE_àüQØð×××W__ïyS¯Ï[ú6nÜØÖÖÛoéàüøûÑl¯÷Ä®þàÀ_4âoddÄf³UVVjî¹¼¼¼¹¹YÎÃØ?ðþü?Q?§ÓÙÖÖVUU¥i¾úúú¾¾>ü?ðGàüønü	é#99Ùb±ÄÏ§wÁøþÀø1¿ÁÁÁÍOïVTTØl¶ññqÆü?ðGàüøbü¹Ó§OWUUiÏl67448ü?ðGàüønüÉP×ÕÕeffj¾¥/ÈþÀ?ðþÀøüIòÀÑ4_bbbü?à/üéþüEHN§ó­·Þú¥_ú%ÍOï455ÅÛ9Àø#ðþf]ÂtL&ðGàos¹»×®]iiiÞÒ·äàÀø¾Àø£@Ö××ïí±·ô?ðGàü?ðþ¢¾ááá'NTTThNï>|=¶?ðGàü?ðþ¢;ËÕÚÚZ]]è-ò`q8s9¶/?ðþü¿xo||¼¥¥å÷~ï÷^õÕð7_t@Y)'y¿¥ü?ðGàü?ðdúÔ§¾øÅ/þú¯ÿúW~9==ýÔ©Sà/l755iCg-àü?àü¿ ËÍÍ]óÖüÚAõï«µ_MKK¦?½«Â¦³ÇðþÀ?ðþÀ_0	ò>ýéOØÀ?ù÷üç[ZZÀAéLïÎü làü?àü¿`gäxËOþ­Zµª¹¹ü¶¡¡¡@Ó»Aì±ü?ðGàü?ðL.+--­îwë<òÛ÷ÿöêSêèè!i||Üf³UTTh;GMï±ÇðþÀ?ðþÀ_	>~îç~î·-¿-òä¬[·nî;süÉÚívÅ<é]ðþÀ?ðþÀ_èkkk+,,üÄ'>ñÏ|fïÞ½N§sî×·øÒ	ì> øàÀøà/²7üéLïª=¶>:äàüøàü¿yHz·   ©©Ig-àü?àü?ðéLïúü?àü?ðþ¢²áááåËûïYçàüøàü?ðMçÔô®æ9Þàüøàü¿°vëÖ-«Õ9/Ó»àü?àü?ðVMMMåååÞµX,mmmaÞàüøQ¿ÑÑQ³ÙþÀ_$çv»Eu/¼ðæônqqqKKËÐÐÐ¼ßOðþÀ?ðéøëìì,,,§Oðþ"3ùÜµkWFF¿ù²³³­VëÈþÀ?ðþâkÖ¬çNüýéþ©=¼]¹råÝwßµS|ï3g~ó7ó3ù¿ù>ñO<ÿüóøßÈ;ï¼ÓÞÞÎ¯ÃÉÓçï½Ç80¼ÑØ¥KÂ£Q¿ßÅÀø¿ÿ+¼ðÁÿEÆÔÝÝïÞ½½ÃÃÃñ±råJÍéÝ+Vüùÿ¹'bïggçø53¨ïÿûAÉÙ~ðÆÁ ®^½úÃþq0(±õ'OÂ|£±?¦öCn·»««+Ð[rssoÝºùÃË´/Ó¾LûÓ¾LûF"þ<Ï©àüÍBº@äHKKÛ¸q£l1Ñ2¼àü?àï)¯ü?ðçßøø¸Íf«¨¨X Uyyyss³'êü?ðGàü?ðþ~ËåjmmµX,ÉÉÉþæ3Íû÷ï¨Oï?ðþü¿ØÄ_ ÀøáíZ­VÍ=¶·mÛ&Ý(Þàüøàü?4§w«ªªZZZ¢qzü?ðGàü?ðþ~Þ­®®ÖüôîÒ¥KcoxÁøþÀøñ?é]³ÙÜÐÐÕoéàüøàü¿¥3½l±Xìv¼¥ü?ðGàü?ð×ø?út éÝÍKoéàüøàüÅ#þÜn·Ýn×ÙcKÌOï?ðþü?ðþâBº@ä«é]ðþÀ?ðþÀ_,ãOÿq8½þÀø#ðþÀøAüéOï4551ªàü?àü¿èÆÎônFFÕjëa0ÁøþÀøÑ?µÇÍéÝÄÄÄêêêÖÖVËÅ0?ðþü?ðþ¢úäPÓ»âBFü?ðGàü?ðÝøÓ9 Ó»àü?àü¿ÁÃán r0½þÀø#ðþÀøÆÇÇ¿öµ¯±Çðþü?ðþÀ_ÌÆ9Àø#ðþÀøqzKäàÀøàüÅrj-oéÊÊÊÞàÀøàüEúlQÓ»çÏË±	ü?ðGàü?ð7Ï©·ôÕÖÖjî±Ågz7¸cûøàÀøàoþÓ9[ r?ðþü?ðþÀ_5>>n³Ù4Â6í9ÀøþÀøà/:r»Ý­­­7oÖÜcËÈþÀø#ðþÀøÞÀÀÀ®]»rssg>½þÀø#ðþÀøQÖÈÈÍf+..bzü?ðGàü?ðþ¢#·ÛÝÑÑa±X4÷Ø2Ãé]ðþÀ?ðþÀøôÔô®Î§wÛÚÚNçü?ðGàü?ðþæ95½[QQ¡ùR¬oii	Õ9ÀøþÀøào~R;gÞ¼y³¦ùúúúB£àü?àü?ðîtvÎQ__/DSäyàü?àü?ð¦[ZZ/_®ùR_UUÍf3àü?àü?ðgln·»­­mãÆæËÍÍb-àü?àÀø¸dôvíÚáo¾ììl«Õ*g0hzü?ðGàü?ðþÂÔàààk¯½¶téRÍ3WVV¶´´ÌðàüøàÀøÐäY¿©©©  @Ó|åååÍÍÍaÞàüøàü?CRl±X,iiiþæËÌÌ´Z­]]]roÁøþÀøà/Èúúú½¥OZ·nü:Î×ô.øàÀøàü&yvonn^¾|y éÝ¦¦¦ÁÁÁÈ¼óàü?àü?ð7£üzmÜ¸199Ysz×Ð3?ðGàü?à/Lì²³³ýÍ'¶¶¶F¸ùÀøþÀøàoõ?½k³ÙÆÇÇ£ëgþÀø#ðþÀø?Ëåjkk«®®t@úúú(ý?ðþü?ðþÀßOadµZ5?½[Þàüøàü¿Ù544ÔÜÜ\o¾ÄÄÄhÞàüøàü¿Éårµ¶¶¾ðÂÓ»K.mllÆØ#ü?ðGàü?ðwøs»Ý>½Q[[+ßu´|zü?ðGàü?ðþ¦¯¼¼ÜÕÕÕ'Nö·ô?ðþü?ðþÀF6Í3½øðáøyÂàüøàüÅ#þFFF¬Vk___¼=BÀøþÀøñ¿¸ü?ðGàü?ðþÀ?ðþÀøàü?àüøàü?àüøàü?àüøàü?ðGàü?ðþÀøàÀøþÀøàÀøþÀ?ðGàü?àÀøþÀ?ðGàü?àü?ðGàü?àü?ðþü?àü?ðþü?ðGàü?ðþü?ðGàü?ðþü?ðGàü?ðþÀ?ðGàü?ðþÀ?ðþü?ðþÀ?ðþü?àÀøþÀ?ðþü?àÀøþÀøàÀøþÀøàüøþÀøàüøàÀøàüøàÀøàüøàÀø"üuwwL¦eËõöö?ðGàü?à/ñ'OW²pöìÙüü|ü©ÍA8>!cî1%,3ÕÑÑñèÑ#ÆÁ ìvûãÇºzõêÄÄã`P¿ÿüÏÿóF1þ¼KIIñÇßýÙ#¼É#äÝwßýÓ+WÚÛÛã÷½÷ÞcêwÞaxK>ÅÃ]ºt)ü7øëéé©««cÚi_bÚi_¦i_¦cyÚW511QSSãt:Áø#ðþÀ?ðkø[ðÔ<¨­­ÕøþÀø#ðþ¢Þutt¬^½zttTóTðþü?ðGàüÅþÌfó¯Àø#ðþÀ?ðËøÓü?àüøàü?àüøàü?ðþÀø#ðþÀøàÀøàü?ðþÀ?ðþü?ðþÀ?ðþü?àÀøþÀ?ðþü?ðþÀ?ðþü?ðþÀ?ðþü?ðþÀø#ðþü?ðþÀø#ðþÀ?ðoøû£?ú£ÖÖÖ¡ðvõêÕoûÛCdL.]úàºpáÂw¾óÆÁ þú¯ÿúþéú«¿ú«>úq0¨¿üË¿dêøñãÿú¯ÿæMüõ÷÷<xðëDDDDäþÔè^%"""Àø#""""ðGDDDDàÀ?""""ZwwwIIÉdZ¶lYoo¯¬ùðÃKKK6mÚ466ÆÍ¥þþþ²²2Þ¢¢¢ÎÎNY#£Ö~!íðJ£££f³Á1bxý7ÂáÕü¦n¤öööAè÷áÃ¼^^^Þûï¿/gÏÍÏÏùÊ&^vìØÁÍ%y`xñé³#effÊBMMÍ©S§dáèÑ£[¶laB;¼²*,,dãnÐðúo.(Ãë¿B8¼Ûí¿^Ø>1¼çÏ¯­­ÈÏx¥¤¤ÈÿbyÏôôt%$]»vmÙ²e² ©©)Yäªï5kw×sAF¯æÓÜ÷ÕW_óÍ7Ù>1¼"?ÅAðeõôôÔÕÕÉü ûûûeáÌ3Þ¤à¿5.³gÏúØáùðþøaÏÆÝÈáõÞ7ÐÓÜ÷Þ½¥¥¥ò·7Û#7//oõêÕò¤&üá¿èhbb¢¦¦ÆétÊòíÛ·óóóåÏzù?îCUVV,$$$xV&%%12¡^ðáõÞÈWsÍx7lØpãÆ¶Fÿö>xð ^·æg<ò£ª­­øð¡Ïú;wî2>¡J½Î'ÉÉÉ§Ï¦Ù¾|xÁÑÃhsA¡úí´æ8¼~6Å¸ßÞHx]ð4utt¬^½zttÔ³&//¯··wjjêÍ7ß|åW¢¹$©æÐ»»»eeÁb±<yRäÿ(´Ãþ^ÿÍpxý>S7lûíTFÂo/?ãi2Í>ÉöñâÅ"÷ºº:·ÛÍÍ%atQQüa´jÕ*õbIgggvvvBBBNNúT5pxÙ¸:¼þáðú¦nØ>4¼===²¦²²Rüþüø#""""ðGDDDDàÀø#""""ðGDDDDàÀ?""""DDDDþüø#""""ðGDDDDàüø#""""ðGD®Î9³råÊg­ZµêÂ?³ýzVÔlmµî­ÙloÍétú¬5&)''gjjj¶×IDþ(*;xðà¿^yåXÂ_cc£¬liiñYßÜÜ,ë_~ùå ®üQôÕÛÛ+¬1LÇs?ëøñãò¥¬¼yófÌàïîÝ»²rÙ²e>ëeýàà ø#"ðGDqÑÖ­[5¯¿þº÷Ê7ÞxCVnÛ¶Í>×¯_<KJJdÙsæÇ×ÕÕ-PNÊÌÌÜ½·÷ìªÝn`ÉIrÙk×®ùpJÖ¤§§¯òÊ+ò¥Åbñ¹c/_Ö¿uÜ%uR ¨­^½ZÖwwwÖ¼ÿþû²¦ªªÊ³æÐ¡CÙÙÙrU)))6mzðà?þü¯ßgÎ]%"ðGD4Ï-^¼XàrïÞ=ï÷ïßf³Ù7>õôô¨S×¯_ïsÒÎ;ÕI			R_ªS7lØ055%|LJJr»Ýrªü/xJMMUoÅÓ¹YÐ¼þßéÙ³g½Eëñå©S§<òó¹U«VÍ:wÀÑü§fx5¶YÅ¼q³eËgÉ|¹víZuª²â£zOÐ¦Nª©©Q/Êrgg§ºïë<pàðNÍº¾ôÒK²æâÅ²,ÿËò/¾8íõåK9÷Óü-ïwttT¾ÿå8'''Õrrräò-<ýÉ4qJJÊlñ§sWüEþ$MüyÖ+Ü(3I</MêËùrÉ%Û·o´=yòÄs%r×Ò<R_Þ¿ßsfgæWþe»Ý>íõ4åËGyß·@ïÏÛ³gôæo>ýÉÔöîÝ»½Ï ü	IKKKÕ³ÅÎ]%"ðGD4ÿ©»&&&¼W:NY)'épÇCCÃ¡üç±çzþ³±>òÞÁ,gff&%%¹¹òôôtÏ©Ó^O ùÔßß¯*Ëò¿,÷õõyNíìì; 9<süéÜU"DDózßÛ#G¼Wª ø|àÃó¾ÀÑÑQÿ´nÞ¼ÙØØ¨&a=jT/ËyæU§%ÚÎ;Õl¯ü_WWçY¯s=ê¶ª/=z¤ÿÉÜ+VÈ©jï6bVïÔ'å¤ööö±±1üy`ªFÃsªÎ]%"ðGD4ÿ©LL¦£Gª]½;v,))ÉÿÃk×®è¸5'ëùd®zÏz«Ãáð~³ ²àîÝ»Jês¸­hMüVÝzGGg½Îõ¨7ù©÷ü9NuNü?~Üó|ËÞ'©·?öõõÉ­È Ââ¦pYÎ&èÜ´i÷©:wÀQD¤vìÓ¡C~ºýz²gÙ3aêù§^zÉ[Þ?^OCÖgffzÏëÜï;æù¤m oöÉ')))ê[ðì^³f÷M,Z´HþWñ¾NõÂ¤'EUÏ©:wÀQ¤$@YµjUÒ³V®>rë¿öövµ;½¢¢¢7nxNu:ûöíËÎÎV`Ú³gËåòzíÚµÒÒR1YNNÍfó¹Nÿ"W%ëwìØá³>ÐõHrgä.©êìçÏS]]Ï>_T£££5552.ïbppÐ³ÛïëÝª±ªªªêîîö¹E»JDàÀ?""""DDDDþüø#""""ðGDDDDàÀ?""""ðGDDDDàÀ?""""DDDDþ(w¡8¬n¼ÜIEND®B`


Ió»à/ÒèèèêÕ«Á?àÀ¥þ¤ðGàÀø#ðGzÆ_aa¡ßï¿ÿà¿ðGàÀø#ð7«FFF8Á_²ãO9)/±EEEÁf³ÿÀ?àÀßB¼¼<AE]]øÓCàÀ?ðGà/¶p8|âÄ	J999·oßàüøþHWøö¹«ÕôôtÃ122þÀø#ðþü~ðçóùÊËËüvUUUÊøTü?àÀéÂ»;v¤§§G³O ÊÛ>øþÀ?Ò!þäÙ¸®®.'''V«ÕårÃáT^/àüøþHWøB*öåååÉÌgøþÀø¤7üµ´´X,Õ¨úúú@ ÀàÀøàô?ÇSVV¦Õáp8RsTøþü?Ò-þâæµÛí½½½¬ðþüø¤üÆæµZ­^¯åþÀ?àô?e0off¦jTGKK£:Àø#ðGàü~ð'¶Ì+WFu?ðGàÀø#ýà/Á!ÚÕþÀ?àt?ÙZKJJbGu²¨Áø#ðGàü~ð×ßß/ÈS±O ÈöþÀ?àt¿Ã¡Ì[PPÀ!ÚÀø#ðGàü®ðbÌ'/ñÁ`eþÀ?àt?e0¯8O5ªÃét2ü?þÀémmm±£:jjjÌþÀ?àt?Ùm6eeel¤àüø#ðþHWøóûý555ªQ&Éív3ªü?þÀøÓþnß¾àÀÕ!Ú'NB,@ðþüøàO'øÃMMMªQ999uuu"BøþüøÓ	þ§O.((Pæ­­­f¡?ðþÀ?úÁÇãÌ»eËÑþÀøþüé>¯¼¼¾µk×ööö² ÀøàÀ?ýàodd$v0ïªU«Þzë-ó?ðþÀ?úé³Ï>Û¹s§êmF£±©©	ö?ðþÀ?úIl×ÜÜl6Uy8À!ÚÀøàÀ?]åv»­Vkì`^ÁøàÀ?]Õßß;ª£²²Òçó±pÀøàÀ?ý÷mEEE,ðþÀø#ðGàO?Õ!Ú,Ëå5cûøàüø#ð§B¡¼àÆ¢­©©)ÞÁ±	ü?ðþüøÓ@ápØårÅê8pàÀÈÈHäbàü?ðþüøÓ|²ÕÄ¢mÛ¶mCCCªK?ðþÀø#ðGàOÃõ÷÷ÛívûdÌyðþÀøþüi2¿ßïp8TyKJJoAàü?ðþüøÓX@ ¾¾^Å¾ËõÐC´?ðþÀø#ðGàO3íUyå¤¼È*yøàü?þ´Á¾¸yNç¬ÌþÀøàÀ¿d/î`Þ¿ß?Ûàü?ðGàÀ_ò688w0ï7ðþÀøþü%cÃÃÃ±y­V«Ûí~àü?ðGàÀ_räE3''G5ª£¥¥å¡yÁøàÀø#ð§âæMOO¯¯¯Õ¨ðþÀøàüøKöbóJc£:Àøàü?ðGà/yßyÍ;ª£··÷±ÿ,ðþæÒøø¸Åb?00`6CqqqWWø#ðGàüQâü~UUV«ÕëõÎÓOàoÖõôôÉ¯fìYÕÕÕgÏ'OîÜ¹üø#ðþhºâæ5L39DøÚ¦MââÏh4NMMÉÄäädì[¿Õ~ç_I;ðÁòLÇrÐJ½½½ï¿ÿ>ËMLÇõôôÔÖÖfeeE³/77Wf~ôÑG°uvv²>ãï×w1þCÜéþÚÚÚFµßý×?~´Ó~þó³´Òõë×»ººXêã?¾víËa&Ý¼y³©©é«_ýªj0ïýÑù|¾¹CCC~ø!ëbáÓ'þÒÒÒ"Óìö%vû»ÙíK<Oì!Úªªªï`^vû²ÛwAñ?99©ìöiðGàÀø#éÊ+±£:ÊËËåWü¿Ç¿3gÎÈü_]]þüø)ÞÈÈ¼8ªFuX,ùÕþÀß¼ãO9ÙÓÓc2ÒÒÒÌfs__ø#ðGàü¥l@ ¡¡Au¬9ÙÔÔñ?ð·p?þÀ_*M&S4û233÷îÝÏEàü?ðþüølÅ=DÛ;FFFä?ðþÀø#ðGàï1äóùÊËËcóÎÇ!ÚÀøàÀø#ð·hùýþ]»veffªÑÖÖÖ÷ü?ðþÀ?s,ìÝ»7ömn·±ó?ðþÀ?ðGàïñäu-v0occ£0ï9øàü?þfWKKÅbQ¢Íétjâ©ü?ðþÀ?3M~9m6[ì`Þ¡¡!­<ðþÀøþü=¼þþ~»Ý®b_eee²æ9þB¡Ðâ~Ý4?ðGàÀøKÆä	¤®®N5·¤¤ÄãñhñáþÞzë­o|ã¿ó;¿c4xâîîn~ÁøàÀ¿_³/'''&ÁSÒæh×¯_ÏËËûÖ·¾u¸îðì¨Þ!ÈçóñþÀøþ(uñ'¶kiiQ±ONÖ××ß¾[ÓíàÁ_/ûº°/òïÛßÞ¾;¿Éàü?ðGàR±hKOO¯­­ÕÇ3IeeåÿñGãÏùgNy¼ü&?ðþÀ?J9üé`0ïCûþ÷¿ÿøÑøîÙçÊÊÊøMàü?BøóûýUUU*ö	57÷¡½ùæùùùÿwßÿUäw¸îðªU«;Æo2øàüø£À<?8Õ!ÚJJJ:::´;ª#AwîÜ©®®þÝßýÝÊoUwdÅ7+tùHÁøàÀ¿ß*Ä¢Íd2¹cHù?ÙÊþüÏÿ|ïÞ½§OBü?ðþÀ?Ò3þÄv"<qêÈ¼òRäGæã÷ü?ðþüQªàÏëõÆæu8)òDþÀøàÀ¥þâ¢­ªªÊï÷§Îúàü?ðGàô¿áááØQååå)øËþÀøàÀéÊ¨Õ±:¬V«¾Gu?ðþÀøàRÊ!ÚTyådccc*³	øàü?¤Cü¹ÝîØQõõõºÌþÀøþÀ¥þä÷'vTGMMMJêàü?ðþÀéÂ;AA~£Àøàüø#]á/8NÕ`^«Õêv»YAàü?ðþü~ðbÑ&'[ZZ8^-øàü?¤ü)hSêÈÌÌLC´?ðþÀ?ðG©?ù%)))=D£:ÀøàÀø#]áÏçóUUUÅêd]?ðþÀ?ðGúÁ_ÜC´ðþÀøþÀé@ ®®Nu¶=Døàüø¤OüíN8;W^SÁ ü?ðþü?ÒþÚÚÚTy¥½÷2ü?ðþü?Òþäw ¬¬LÅ¾-[¶rþÀøþÀéÛ¶mS±Ïf³Á>ðþÀø#ðþHWøí´¶¶6ömêàü?àt¿`0ØÐÐ ÌË!ÚÀøàÀø#½áOl'Â=DXQàü?ðGàü®ðçv»Uh8Døàüø¤7üù|>Í¦b_UUU?Ëü?ðþü?ÒþvìØ¡Õ±víÚÞÞ^$øsºæÃJKKàüø£ÇlN§SÅ>ÅÒÖÖÆ¨ðGsÇ_ÚÃ2àü?´øBªC´)£:8Dø£GÅ_2þüøK5üÃáææfÅ¢ÚU[[;22Â¢4ïø§ÔÝ»w?ðþüÑàÏãñ¬]»C´?Z8üÉ_ZÏü?à²ív»êH ØÑÑÁâ4_ø[½zuìh£Ñ¸®þüé~¿¿¦¦FõêcµZßzë-Fu?_üeddÈö6>>n2dBÌ÷üDùÅ#ÞòÀÀÙl6ÅÅÅ]]]ÑgEoíàÀ¿ÔÁ_ Ì×ÜÜûÀ-þ"üíÉÄ7¦¦¦d";;ûo¹ººúìÙ³2qòäÉ;wFÕÚÚêp8¦»¢à¯³³óÿi¿Ï?ÿüÃ?ü¤.^¼øé§²´ÒèèèGÄrÐPÝÝÝû÷ï_ºti4ûD?øÁFFFX>ÉÖ­[·.ÀrXøæÊFØÓÓ344$/¿ü²2ñè_õb4Å2199i±X¢Ïùµ··'Àß_ÿõ_wk¿®®®sçÎuvzï½÷¼^/ËA+?MLC+ëðáÃ_ùÊWTûy¿óïüýßÿ=ËM¢wü<x02¼#z,//Ä[æ£2³´´ôÚµkìö%vû»u¬¦Ø#óÚívÕÁn_ZÝ¾Ò±cÇ-[&2!=úÍFÎÈÈÑÑÑÕ«W?þtÙàà`ì`^«ÕêõzY8àóT~~þäääý»ezºÅºüø#ð§õjTÇW¾ò'O2ªünñWSSsæÌÿ«««£Ï*,,ôûý÷¼óWQQþüøÓM@@ÆsrrTyeæG4cûø£yÇßÊ+/|y¼_òÜÓÓc2ävÌfs__ß¯ïñÅò[TTd0l6øüø#ð§Âá°ËåR¾8,z0¯ÓéÞÁ±	üÑBà¯°°0|´ïþüi.¯×kµZUï#ÔÔÔ(ûyÀø£¤À8O¶OyÍS¾%þüi¨þþþØQ2'ví?ðGI?£Ñ([iòÈüø#ð§ü~ì¨yÁø£¤ÀßÀÀl«öì¹wïøàü?I@ ¾¾^Å>Éär¹æàÒòåËÄôè>ÀøþôØ®±±1///v0¯2ª#AàüQRàoÅøþÀÍ$Ë¥Õîp8f¸á?ðGI?eëõù|Éó¨À?É,jÍx0/ø¤üåçç3àüø4]Â»ªªªæà´¿îînÙ<ÀøàüQ$ÙNgì`^·Û=·àK¦àüøKÙB¡ÐñãÇcGu´´´<ÊyÁø£¤À_Ú41àü?)ØÎív[,9æà´¿$üø#ð·(y½Þ9æà´?ùónÅàüø)Ïç«¬¬Õñx_Àø£¤ÀÁ`-<©ø#ðGàoÁÞíØ±C5ªcíÚµÓ¢ü?Ò<þºººd;ohh§Ñ$ùÂðGàÀßêêêTì³X,n·ûQFu?ðGÉ?Fû?à/ÕBMMMªÁ¼bÁGÕþÀ%;þíþü¿êôéÓªÁ¼éééµµµ###óý£Áø£¤À_þüødq©öólÛ¶mVhàÀøàÀ_<ÏK/½tðàÁ¶¶¶E¿3½½½v»]Å>ÍÖßß¿wü?JüMNNnÚ´)++K²³³~úéÅùþüÖñ·mÛ¶'xâ;ßþÎ·+¿ýÕ¯~uóæÍó4â¡É¯ôT£:L&ÓéÓ§þ.?ðGI¿¸>ñP¿àÀi?þñ|òÉC?8tä¥#òO&zê©cÇ-ðÝ¸ûv]]]NNêXMMMÁ`pQøø+**§ù«ôÞ½ÊéÖ­[eÎºuëÀøþæÐüÁ<÷ìsü²ëOÊÊÊìÄ=2offfý¼æà´¿yR~órrRæÈ|ðþÀ¿¹ýQ½ûÿìÆóÏV«ua~ºËå¥Ìët:`0/ø¤ü¥¥¥ÉS/úOFÃW½?ðGàonÕÔÔlÜ¸1ßüæ7yæùþ¹'v0oyyù+WdÉ?ðGI?e·oEE²ÛWþi³fÍðþÀ¿9444d4+++8(ÿ*¿U)'çõê~¿?öÈ¼«V­jkk[¬&àüQòâO´wÀ¬rðþÀ¿¹5<<¼yóæ¼¼¼¥KÊ_ÔÂÁùcÃá=DËåJ*ö?ðGI¿ûü>ýôÓ¹¹¹iiiò¿<OÉE|TàÀiP ¨¯¯W±oqó?ðGÁ_²þüøKP8nllTævíÚuûöíd¾çàüøþüÍ®ØÁ¼Ò-[gTø¤ø[ò°ÒÒÒÀøþ'y°6-ömZàüÑbâ/múÀøþ*¿ß_UU¥b_AAÛíNÂQàüQâoºöïß¯<­¼ùæàü?üBÆÌÌ£:Àø#Íào`` ;;[Vìvôw>?ðþü-|@@cÑÖÐÐ Eö?ðGI¿íÛ·+Ï,çÎ[ôGþüQ*ã/»É¤ÚÏëp8ámàüæñ÷Î;ï(O+7oNGþüQÊâOQIIIì¨þþ~<:ðþhñ711±nÝ:elGWWWò<*ðGàRv»=ÚÕþÀ%)þ^í5åeçÎÉö¨À?J)üÇêÐâ`^ðþ(©ñÇ÷ü?àoÑSFuääähèmàüVñö°øàÀß<[ZZTyÓÓÓN§	Áø£ÅÄ_2þü¾ñçv»cÑV[[;88¨ïUþÀ?ðGàRrãêÐÇ`^ðþü?ðþüý:¿ß_SS£bßªU«ÚÚÚt6ªü?àÀ¥4þÓéTæÍËË;qâDê°ü?àÀéqGu(yoß¾«ü?àÀé)5ü?Jjüñ=àÀø§<ÏªU«TOª[¶lJñUþÀ-&þ¢¿Ò/.þø?ðþüÍ¶+W®Äæ92õþÀ-2þ"uvvÊsÓ³Ï>ïÞ=9)ÿoÝºUæpü?ðGàoÅ=D[II>þÀéF£Q¡¢GMNNÊÉô·<00`6CqqqWW×ÏþH[øªC´¸TÌþÀiÊóO¿GÿÌ_uuõÙ³geâäÉ;wîáYàÀiÊ`^Å¢Ì+Obº<2/ø¤üåççË³hlbbBùvóæÍ2Gæ?â-Æ©©)Eòä8Ã³üÉóéeí'Osç2i§>øàâÅ,­Ô××÷þûï/ÖOÿñüÔSO©ÆÉ=óÌ3ò'«MLõöö.â&ÊÍ;þ.wÀÇ'|ò·=dD5|$ÁYþÞyçíwýúõóçÏvÍappå þó?ÿ³««ká®l×±£:¾÷½ïÉ+%+%q"ãÿüç,­444ÔÝÝÍrXøæÒ7³²²äÏÖììì5kÖ<·å£wgddÌð,vû»)iwûÊ¯Gmm­jT@Qìöe·/ii·ïü¯|PþWíDNpø#ðGI?ù[|×®]Ñì³Z­n·þÀ¿_WSSsæÌÿ«««gxø#ðGI¿`0X__¯Ì×ÒÒÂ`^ðþH«ø»zõêÊ+³³³½±ùùù­­­~³===&InÓl6÷õõýú/Y2ÝYàÀ%þÄvÍÍÍªÁ¼ééébÁ@ Àòà´?åK£é¦L<yr±ø#ðG?¯×[RR¢Õáp8ü~?Kü?Ò6þL&<£]½z5¿¾¾>ÎÍÍàüQâopp°²²2öm½½½,sðþHøS×	SSSÛü?JAüÄ¢Íjµz½^6ø¤ü)_ò¬¼Û'ø<tèLÇ~÷2øàô¿`0(Ï<ªQ&C´?ðÇrÐ!þº»»ã~ÉóÅÁø¤üíN>÷mêàüéÒØØXii©2Ú7++kåÊûfðGàG5ª#==ÝápðkþüéÉø#ðGó¿þþþØC´UUU1üøÓ?þ"ã<"É¹råÊðþÀéCCC;vìPê(//çmàÀ_êâorrÑ¾àüþð'+×étÆæeTø#ðø+((X°¥K?ðþHø;×h4655B!$ø#ðø»yófÚ"ÇöNä÷Î;ï?ðþHëøãmàÀøS'Ô[Ä=¼àÀÍþÜn·ÕjUíÖØ²eÏçcé?©¿$üø£GÄß+WÊËËUì³Ùlýýý,7ðGà/Õñ799¹råÊìììÈeË½òÊ+àü?Ò"þdîÝ»W5ªcíÚµmmmêþÀß¯Z¾|¹jÀ¯òÐÐþÀø#á/î¨¼¼¼Øþü¿ÿ-##C£?300 srssÁø¤	üÅÕyàÀFu?àO2àwjjê·n7æËÿÀøøkkkSêPÑ6<<Ì"þÀ_Ìf³<WîÛ·Où²«#GÈÉþÀø£dî½÷Þ[¹reì`ÞÁÁAø#ðþ½ÚÅýç¾¾>ðþÀ%gÂ»mÛ¶©µÊÊÊÌþü¿5::Z\]TT$sñQ?4]ÃÃÃC5×d28qbáGuÁîînÇÃ.fðþHcøK¶À?-ÔÕÕ©óÊÉãÇÂþþ´µµååå~ýë_?;Æ:àÀøàCápØårL&Õ¨?ýÓ?moo_»äóù-[ö'»þäÈKGäßÞ=Ífó"üø³ëêÕ«Ê÷<+#|óóó[[[Áø	ûTïSóÎäØ¾óÓé¬øf"?åß3Û±Ûí¬/ðþHøëìì<*øS¦O<	þÀø£ELl6Q¿ªªªï×|?Î?sþÞïýkü?Òþ=)W¯^à¯¯¯/yà1¿ß/ºÌëõz£/¶¸ïüÙíöhümzïü?ðGÚÀò¬z?ê§¦¦dÚ`0?ðþhå;wÕªUn·;v0ï"âÏü?ðGÆ_~~¾ò­~þ&'':$Óü?ðGV m?//Oõ..kºïpYDüÝÿÍhßµk×éK_ZÄã¡?àovuwwÇýç/?ðþh;WOÌ»¸ø»ÿïùëèèðûý¬GðþH3øÆÆÆJKKÑ¾YYY+W®'2ðGà/uòz½qÌ;±èø#ðþH«øK¶À¿T¨¿¿ßn·«ö9TUUÍüOðþü?ðþüi ¸h+//íþÀ?ð7Ç®_¿¾bÅyþÍÊÊ*..àüÑcOÕ¡:DÕjM0ªü?ôñçõzãøàüÑãJl×ÒÒ¢Ì+'çÀ>ðþü¿¹§°«®®V~ïÞ½;wÊ³ÙþÀø£ÇÛíÕQ__x0/øþh^ð§<Gÿå-Ó/|àüÑ£$Ûrì¨ÇòàüøsIyçorr22gbbwþÀø£GLx'ÈS±O ø·nðþü¿¹¤|æ¯ººZÌ§¬iåHê|æü?[@ÀétªóZ­V·ÛýxøþÀßnâa-üþ_ðGàO£B¡ØC´ÉÉ9êàÀ=fü¥=,ÁþÀø£Ä)hSêÈÌÌ|è!ÚÀø#ðG¿$üøÓV²ÁÄ¢m¾	þÀ?ð7¦û>çÑÑQðþÀ%nhh¨²²2vTÇÂ|hü?àoN7±dÉ±cÇT3wíÚÅW½?ðG	òûý±h+))YÈü?ào.	òä);??_Ù ßxãåI|á?êþüi¢`0ØÐÐÍ¾¹¢ü?´Ðø»ÿà>åéÛl6+[·n]ÄGþü%mÍÍÍE5W¶YåAøþÀßÜ»páBä©Ü÷Q?IÇã)++Sêp:¸ÄÀø#ðþæØ/¾¨<+GûöYðþÀ)]¹rEÅ>iÇ>oqïøþÀßò<ùòåûQùËÈÈàü¥x<Õ¨Í$[(øþÀßnbÉÃ«f:Fû?ðÊÉr8pà«V­jkk[àQàüø£Ç¿é¾çollü?ðÁºººhöL¦æææEÕþÀ?zÌø¿Ìf³Á`(..îêêRÉ2úuüøKÂáðéÓ§Uy333ëëëçïmàüø£Â<§GïØM|rnUWW=V&N<¹sçÎè³Z[[ÇtWüy½Þ_j?aDww÷/I;õöö§æcî÷ÿ÷cGu~=iïóç._ñ«¡.^¼xóæMV¿¯Xñg4§¦¦dbrrÒb±D%òkooO¿¦¦¦.í'¯¦ï¾ûni§sçÎðÁ©ö¨_íµ¯ík*ömØ°áÌ3lbÄ&ÊÉÊb[4¿èc¨RXXXQQ!3KKK¯]»Æn_b·ï¢¤¢Mu¬²²²ÞÞ^MÜvû²ÛØíËnßäÂ_ô-L÷Å1£££«W¯þ¸@ ;×jµ&Õ`^ðþüð=#??rròþÝ¾2=ÝUb]þüÍ_¡PèøñãF£Q5ª£©©ICìàÀøKüEWSSsæÌÿ«««£Ï*,,ôûý÷¼óWQQþü-L.K57==ïÞ½###Z|8àüøÉ¿É$·c6ûúú"·¬¼ÄÍ&þþæ»¶¶¶òòrÕ¨Ã¡ü¦ÑÀø#ðþf¿ÄqðþôlMv»]µ¯]»Öãñhý¡?ðGàüÍ¢´¥þÀøÓ ¿¿¦¦FÅ>«Õêv»5÷ñ>ðþüÑ£â/þ±@ àt:UyóòòN>­ö?ðGàü?ðGàïWB!ÙÄyºÕþÀ?àÀ_Âá°Ëå²Z­:ÕþÀ?àÀ:ÙdJJJTì³ÛíZ9Vøþü?3jpp0v0¯Õjõz½º__àüøàüQáoxxØáp¨Fu¸=êàÀ?ðG©¿@  INNj0¯Ì©³¾Àø#ðþÀø#ãOÕa2TyN§0ÕÖøþÀø¤güy½ÞØÁ¼555zÌþÀ?àRýýý±£:dNÓü?àü?Òþü~ì¨ÌþÀ?àR@ ¾¾^Å>É:yÁø#ðGàüQJàOl×ØØ¨:D27Gu?ðGàÀø#=ã/öméééCëþÀ?ðGàï·_ÍÆ`^ðþüø¤sü	ïªªªÌþÀ?àt?ùqN§3v0¯Ûífu?ðGàÀø#ýà/?~<vTGKKyÁø#ðGàü®ðçr¹,yÁø#ðGàüÎñ×ÝÝ]VVÆ`^ðþüø¤sünÙ²E5ªCæ±äÁø#ðGàü~ð÷m6­··eþÀ?àô?åm999ªC´¹ÝnFu?ðGàÀø#ýàOl×ÔÔ¤Õ!dTø,ðGàüÞð×ÖÖ6§Ó922ÂBàåþü?ÒþúûûcÑÆ¨ðGàüø¤7ü	ïbó®]»?àÀøÓOó7c·ÛW­ZõÌ3Ïø|¾ÔÄßððpMMj0¯,ËÅ¨ðGàüøúiÛ¶mO®xò¹gsþ³ò[999ZÿîÙâ/Ææ5§OàÀø#ðþtUGGÇO<qè¼tDù·íémO>ùdàOl×ÒÒb2T£:öîÝË`^ðGàüø:ì¥^úÎ·¿òoÙ²eÁ`P÷øs¹*öÕÖÖúý~À?ðGàüéßªL5üõööªÌ+Ùív|Þüø#ðþÀøKTGGÇ¿üåFä·yóf«ÕªéW®±cjTGAAÇãáüøþÀþñ'íÞ½ÛòeËæ-ß¯ùþ7Ê¿!!é~¿?v0¯°ïôéÓ~üø#ðþÀøummmÏ=÷åáÃµþpTøSÌ«b_ff¦üÂ>ðGàüø©?Á_8nllÌËËS¼Ïápp6ðGàüøàOWøs¹#óJ»víd?àÀøúéoÿöo×¯_;·¿¿þü?àüé'¿ß_UU¥bÕju»Ý,ðGàüøàO?ýâ¿p8ªQyyy---¢üø,ðþÀ~òË¦Õñ¥/Ifr6ðGàÀøàO?ÃaËdÞÍ7_½zåþüøàüé'ùí*))Q¼¯ªªÊï÷ÏðØ¾þü?àüi ÁÁA»Ý®b_yyyä÷ü?þÀøzhxx8vTÕju¹£:Àø#ðGàü?ð§íQ9993ÌþÀ?àï·X,±óÌf³Á`(..îêê4íDxªÁ¼Êy§ÌþÀ?àoîõôôÉËmìYÕÕÕgÏ'OîÜ¹üÑcÏív«Ñîp8ü~k?ðGàÀø6m?£Ñ855%±oþN:õöëïïÿý÷?¡M~yÖ­[§Õ!s~úÓ>ôº^¯···e¨ë,%X__ËA+	ÖÙÄ%ãï×w1þCÜéþäuú3í'ögºÏh¡¿P¿÷½ï©ØWZZ:ó_§?þøg?ûKR+ýÇüÇùóçYê£>bÓP×®]ûðÃY>ñÎÈÈ`·/=b@Àét>t0ïCc·/»Ý¾Än_vûÎzJñ?99©ìöiðGs.?~ªCN666ÎáÈ¼àüø#ðþcgÖÔÔ9sF&äÿêêjðGs«­­-vTGýÌþÀ?àï1ãO9ÙÓÓc2ÒÒÒÌfs__ø£Ùæñxl6Ûló?ðGàÀøKÆÀ%ÈçómÛ¶M5ªÃn·>ú?ðGàÀøà/YºûöÞ½U£:JJJ:::×àÀ?ðþÀßâFc4ûää'æ0ªü?þÀøIØîôéÓ*öeff644ÌyTøþü?ðþ±¶¶¶²²2ÕÇûÇððð<ýDðþüøàü-BW®Ûí*öUVVú|¾yý¹àüø#ðþÀø[ÐFFFvìØ¡b_II×ë]þÀ?àü¿*Ö××gffF³Ïb±Ìömàüø#ðþÀøKêÄv'Nç©ÑvüøqáBÞðþüøàüÍommm%%%ªcu8ÎEAøþü?ðþæ«îîîòòrÕÇûvìØñhàÀ?ðþÀ_r%¼Û²eAÇ³¸wü?þÀø³@ PWW§:DÛªU«ÚÚÚlTøþü?ðþæ=±]ccc^^^4ûL&Ssss2°ü?þÀø§O^µjêmõõõ<ü?þÀøó×ëµZ­ª÷9Î$¼·àüø#ðþÀøcýýý±hÛ²eË|¢ü?þÀøßïw8ªQk×®]C´?ðGàÀøàoN§SÅ>«Õêv»gTøþü?ðþµP($+N5WN666jàüø#ðþÀøQb;Ë¥Õ)ë1hë±?ðGàÀøà/Q²¦TGæ¶mÛ¶hàÀ?ðþÀßã/î`Þ²²²+W®h÷A?ðGàÀøàOÝtyämàüø#ðþÀø<úúzûL&Ó'´Î>ðþüøàüýoqÌÓÔÔlhàÀ?ðþÀß#±/î`Þ½÷&ç!ÚÀø#ðGàü?ð7ÇâæµÛíÉ|6ðþüøàüÍºÁÁÁ¸yõýZþÀ?àü¥þcóZ­VË¥Qàüø#ðþÀøûU@@~NNêmºÌþÀ?àü¿_5Ý`^-¢ü?þÀøòz½ªÁ¼ééécxx8Õ¶(ðþüøàOÏøóù|åååªQ6M¯yÁø#ðGàü¿ÅßÈÈ^ÑþÀ?àü¿ÿ-ÔÕÕ©FuL¦Tføþü?ð§7üíT£:233ëëëõt6ðþüøàüÝw»ÝêàÀ?ðþô?ÇSVV¦Õ±eËÕþÀ?àüéÂ;A^ª¢ü?þÀøK9ü`jkkcÑæv»ÕþÀ?àüé@`ïÞ½ªÁ¼F£±©©)±Í?ðGàÀø:Á_8nnn;7¥ÑþÀ?àüémmmæàÀ?ðþô?¯×k·ÛU£:Ö®]ÛÝÝÍþÀ?àüé>oÛ¶m*ö¼õÖ[êàÀ?ðþô?ÊÞ½Uyóòò8Vøþü?ð§üB¡ãÇ«ófffÖÕÕ1ªü?þÀøÓþÂápcc£ÑhTíçu8###làüø#ðþ¿ññqÅ;ll,/ào&Åê¨ªªºråøþü¿¤¨§§§¨¨(ÖvRkk«Ãáî¿óçÏOh?yëîî~öw÷wöík_ÿý÷'èq×ÛÛóæMV?#åï+ºxñâgÆrÐJ£££ò÷ËaáÓ0þ6mÚ444"¿öööøû«¿ú«óÚï>x÷Ýwã>õÔS_þò_zé¥ÎÎÎó4;weÊ±X2¤íÝ¾¿ºñðWXXXQQa0JKK¯]»Ænßæ÷ûÌËn_b·/»Ý¾ìöÕþ"®^½üø#ðþü&ñ;#1þ¤ðGàÀø#ðGÄ_ÆÎ,,,ôûý÷¼óWQQþüøþHøSNÊKlQQÁ`°Ùlâ?ðGàÀø#ðG:Áßþü?þÀ?àÀøàÀø#ðGàü?ðGàüø#ðþÀø#ðþüøàüøþü?ðþÀ?àÀøàÀ?ðGàü?ðGàÀø#ðþø5þü?àÀ?ðGàÀø#ðGàüø£TÃß_üÅ_¸Ýîaí÷³ýìþáI;µ··_¾|å þýßÿ]Ï©Ó?þã?^¹rå þíßþí§?ý)Ëaá)¿«W¯9rä/R¯Äû4ðæ'»üø#""""ðGDDDDàÀ?ýwõêÕuëÖâââÓ××·fÍ³zõêè-¥ë+ÁJÕg6wuu±ôdE¯¯´´46±$¬³³3²ülGlbÉ¹Êxôä9«½½]&.^¼h4e¢°°P¦eâÍ7ßreô[[[-©ÖWR]]öìY8yòäÎ;YzI²Ê"ÉÚUÃ&lÃa¡CD	¶#6±ä¼?iGþHRÍÌÊÊ>)Ûò2FÉ³¾¬¡ÆÔÔLLNNZ,ZRmb7nÜØ¸q£ê2lbÉÐ+¯¼ò£ý("ÛXr®2^ÅÀÍèo¦ÜÜlä¤èù.]ª­­#NUTTÒÒÒk×®±èa%X)23î4%Ã&öôÓOË&¦º$Ø¢wóæMYøBº$lGlbÉ¹Êx4Ó:;;óóó#'ïÝ»W]]ã^xtt4ömBZÄõw¥D,##<«lhhhýúõ	.Ì&¶X	Ê/pÿÁG3º±%ç*ãUüÑ,üå*[ÃáKpaégM·R÷ìRIw=zôµ×^K|a6±ÅyYúíoGlbÉ¹Êxô¯^½zÿÁð¨èîîñññ¸öûýÊv¥%X)555gÎ	ù_þfé%É*6lØpùòe6±$'ÅC·#6±ä¼?zHÅÅÅaãÆÊI%ö¯(eâÒ¥KEEEraÍ&[K/ÖWÜ¢¬¯Éf6E,½$YeÊ[Ê@ÕëXJ"îvÄ&Ì«W1ðGDDDDàÀ?""""DDDDþü?""""DDDDþüø#""""ðGDDDDàÀ?""""DDDDþü?"¢¤ì7ÞØ°aCÖ6nÜøÎ;ïüÖÓÙ4óäïÞZ,yhÁ`P5_æ³Ù<555ÛÛ$"D¤É9²$¦cÇé	2³¹¹Y5ÿÄ2ÿå_Ãm?"Ò^ÂÁðúë¯têÔ)9)3/_¾¬üÝ¸qCf®^½Z5¿¨¨Hæûý~ðGDàR¢gVXóê«¯FÏüá(3wíÚM®®.Á¸pÍ52¹ð;wjkkssså,£Ñ¸oß¾è½«^¯W%gÉu=S2géÒ¥6l8wî¬©©QÝ±Ä·£%wI9«³³s:¨UTTÈü¾¾¾È/Ê»ÝsôèQÉ$7µûöÑÑÑXüÅÞ¾jN»JDàh[¾|¹ÀåæÍÑ3oÝº%3-K4nT]ºtI9wëÖ­ª³öìÙ£åóùÒÒÒâ^K9©ûôÓOOMM	322Âá°+ÿ²³³â%¸÷béo¾-Ú/Ï=êF6nÜ8[ü%¸«Dþ?eo§°%KbÑ¸Ù¹sç½ÉÜ¼y³r®bÊx6å¬êêjå-CîééQn$ú6>,¼Söº¾ðÂ2§½½]¦å~þùçz;G9)¾oqrÙ²eòxÇÇÇå¤ü/÷999©l6Ëå!ÜÿÍnâ¬¬¬Ùâ/Á]%"ðGDøââ/2_Áb&ittTNkÖ¬+V¬Ø½· mbb"r#rÕik)'oÝº¹°)²çWþi¯×ûÐÛhÊÉ/¾ø"ú¾M÷ù¼ýû÷ËY?úÑîÿf×ö¾û¢/ ü	IKKK7&g¿wÀÑâ§¼ÝuïÞ½èÁ`PfÊY	¸¡áÐÐâ¿u"ÔÝ«âTô¬È´ÑhÌÈÈBrãK.ûÐÛbª®^½ª8U¦åöù|szzäÄÝ<sü%¸«Dþ?åso¯½öZôLåPT>"CëòåËÊNØ·å"ûUJ´=ö(åÿÚÚÚÈü·£¼Ó666¦üâ/Ì]¿~½«|»5ú,eä¯ÕÙÙy÷îÝÄøÀTYsÜU"DD2`Â`0<yRùª×_=###vpÆæÍ:¡PHÙ'«|æOù¨ÜÐÐPôîÛ·O¨¤Ã|ÙJ)ÃoÞÝÝàvù)ùÊ%àïÔ©S÷ää!G¥|üÑçóÉO0þnåbÎíÛ·Gà®ø#"J/@VuôèÑÿ:b£ÈtdidE¤^x!ZÑµ¶¶&ÀßýâùF£1zpÛ»Ç"#m§°YYYÊCPíìÞ´iSôX¶lü¯|ÛKôm*oLFR¨97Á]%"ðGD,	P6nÜñ 6(CnUøëììT¾N¯¸¸øÂsÁà¡CL&¦ýû÷B¡È¹§´´TLf6[ZZT·Oä¦dþ/¾¨?ÝíHrgä.)_@àþ"ÕÖÖª¾óEi||¼ººZ@nn®<¿ßùÚèÛ¼÷®èVYVv»½¯¯OõÜU"DDDDþüø#""""ðGDDDDàÀ?""""DDDDþÀ?""""DDDDþüø#""""ðGDDDDóÖÿ|P¯ÅÇ-ú¿IEND®B`


¾Yð2¦ø#""""ðGDDDDàÀ?""""DDDDþüø#""""ðGDDDDàüø#""""ðGDDDDàæáçy¢'OFÜ?ïwl²KÏ=[[[7Ñh<úô78ý«,)++K>¾ªªêÍ7ßÅàÜ¸qÃd2ÍâË¿~ýú5kt:|ö°[X0IIòGDàR©¿_~YK#GD¹µ]e2]8qb¦3ëa,++~^³ÙþüQ,%mÙ²%%ðwãÆ¥Õwß7Ë¼+;].WÄéUÂ>õÈÈÈd,aøSW|øð¡l%Õ?"JyüY,yöìÙÉñÙgmß¾]½fZ__ïñxÂ>òêÕ«.vmpOGGGuuµN§«­­½ÿþõë×«ªªrrrÖ¯_ú,ãÑ£GKKKÕëò)MF^xAö¾+<yRv666Fügzí§/Ê¹3ÏÌ6»+FÇ_Ø¥òÿ²lÙ2ö6ÈØFPiè«äp8äÖdäV.^Q*#)÷îÝëóùøá"D¼ø)+++))­îÝ»'è¡SZVV¼ÝºuëãH¯K®Y³F||W _ØG®[·.:q/^,û<xºSp);.]ñkéUÂ>µÈF=óWQQ1£Án¸Y_qúøëéé	½ùbg¿ÐÿVË¥ÞvãÆõÁ[¶l	»ÏöìáüQòâO6.úLX¨víÚ%Û6mz8lÈ»²3ô#><>>®»RvïÞ:;;Õ»rËòîÕ«We[ ¨®«f¶]¾|Y¶ïÜ¹zÑdÄÑét÷^wWlR]ðÑNÄ5ë+FðvEAl×××ëD>þBÿ[åvdÏµk×d[ý·îÜ¹S°BáÝ»we[þ7e;??."ðGDI?IËe[×ÃöÈö½÷Ô»ò/ï.Z´(ô÷8Bß~üË×OCÍ166&dÔÔÔ¨§¦Ä_NNNÄ/$âþYUr]qÉdr8m:ñ^ÍúÓÇ×xÿþýÐO1#üþ·ª[Mö¨V¬X!ï.Y²Dð*¿E<zô,"ðGD)?ñYqq±<ûýþÐýÚgÎ´àDuo3ú»Í°>§<kµ´´T½N­^Ð"a·6«L6&SÚtg2ÎîÓÙw:¼~©ö¿5luÛíVþ¢ðÒ¥Küp?"JvüIï½÷¼EíT>JvNÇÑß]¶lZr¥££Cp6ü©§'Õª+õõõ÷îøðá©S§dçöíÛ#âo:W5þf:8ÁfÅéão:Ïüy'ô>þùùù²'ìÔãÐnÞ¼ÙÚÚª^kMDà'&ïçõ«=êM6L¤ÝwïÞ=wü©g¿äÍ7ßþN§znI¼ØÙÙ©ÕS]¸p!â×8Ó«ÌÑG¤o¢°é¨ÎjØ÷Ñ£Gb»7jixìØ1|¢88úø«(tíêêêÐ/SÍÝt»ÝQ^'"ðGDI¿ÁÁÁàì.µÇãñÍ÷*))	<;ü9¢Z´h¼=ã5â~ñÅµ/A®Zµ*Ê9£«ÌÑ'¸VsØBÍ³ÕYàïæÍ¡·¿víÚÐKÃÆD¸j¹ÄgÀ¨çVCé¥øá"DøÔK¢¡û?ùäíÛ·çLT__¯=½cvø[ÛíÛ·O<$:q?^UUµxñâ·ß~ûk_ûZð§¹_eFø>8.ÿétºàRy¡ÍhTg?éÒ¥KK,¯Zn_½°<ÁYÞ¢©Ïn2º»»§|æõêÕ«555rkòEÙl¶à~ÏwðàAõª^þ+ý~??à(^½øâ3Z[nWI¹Ô«±êôí@  L¿lÙ2¾aü¥ajÎ_X¯¿þ:#CDà(óù|X¼x±zmW6^~ùeÀ?""""DDDDàÀ?""""DDDDþüø#""""ðGDDDDàÀ?""""ðGD4. ×ëu:ÝºuëfzÝªªª¸ÝîàÙ=ÕÕÕ38¢M4»Îugs¤Ð²²²òòòêëëù.""ðGD)Sii©PFfzÝ£GÊuÛÚÚÞxãÙÓÚÚÒørû¡äí;wò]DDàRç2?Ý¼yS®[[[Ü³fÍÙÓÓÓ;9¿ø.Ëv^^ßEDþ(äæçç9r$ìÃ¾öµ¯ÕÔÔÝBðCÙ~ðàlËuÑÝ»wM&ØH§Ó­X±âÚµko-ô³G¿ÊÛo¿­×ëF£|"-ÅFGGÍf³îüÔÂÓµk×Ê­É~¹å÷îÅ	|òIVVúÆÇÇåÎË»²ï1"DþSÛ¯¾úªlÛl6al¼þúë¡sõêUíü¶]»vÉE§Nmy+Ûê¢U«V]¾|Y6îÜ¹#ûËÊÊ"ÞZè~!Ýï½'Eç÷ìÙ#ÛòçÎ£GÊÎòòrÙq¹¡8o¦øáÉøÈvCCº¨µµUÅ±cÇdãå_æ»À%5þôz½lM¤µWÄIN§S.Ú´iloÙ²E©.xéÍ7>£ÎxkaÀ~uÇµ×-))Qw^X&Â>Ù))Û+V¬Îúýþ¹NèußäÆE~"Ku|ê¥KÊÝÈÏÏ¯¨¨wùî""ðGDI?NÜyw2iuä#>zuUö¨Þ|óM¹ðÖ­[¡·vk¡ïNç*Ý1ÑXèygÏ]¸p¡ÚS\¬É3Sç»H¡/?"JRü>ó§<R<f³Y.¾îÏÉÉ=ãMÑ¯¼câ9íu-Z¤®v÷ÀåË-KèS³)bdd¤¤¤¤¾¾~íÚµâû÷ïóÝEDàjéS§N©9o¼ñÆtðwöìÙàShï½÷^p¿¢äíÛ·Õéà/úU>üþûïËÆ®]»´×é¥dûwÞéëëS/õÊN£Ñ(Û·nÝÉFEEEüð·wï^¹èæÍ¡À%)þ+¢Ðµú¢ãïÑ£Gê%Wy']ºt©¸¸¸°°ðÀÓÄ_ô«8¹tÍ5ÁóNB?Æçó	¿äçäälÜ¸QØ+æ«¯¯W§×ÖÖÎôôÛéãOÀºêÍÚµkå]ËÅ7?""""DDDDþü?""""DDDDþæ§üàýýý	þ¤?ýéOÿã?þï8uïÞ½_üâCº÷î£G8uçÎYüÍ7fç¿þë¿8õüä¿ÿû¿8õé§¿õï|GüàOzëÖ­û·ã[9Nýýßÿ=É ~üñÇÿþïÿÎ8Ä©¿ýÛ¿å7Ãøõ½ï_]â×Õ«W±uüºtéÒÿüÏÿ?ðGàü?àü?ðþÀøþÀøàüøàü?ðþÀø#ðþÀ?ðþÀø#ðþÀ?ðþÀø#ðþÀ?ðþÀøþÀøàü?ðþü?ðGàü?ðþü?ðGàüøþÀø#ðþü?ðGàü?ðþü?ðGàü?ðþü?ðGàü?ðþÀ?ðGàü?ðþÀ?ðþü¿4Ä_ww÷+t:]uuuOOøþÀø#ðþÒK.+Ù8útEE£MtòüdâSWW×çÎ8Ä)ùeI~uaâÓéüéOÊ8Ä)Ãñ³ýqSW®ùÏÎ8Ä)ÁüfàOÂø-//O¿cÇ?±É¯Grú>Å'9Éï÷Cº|ù2Ã¿>øàk×®1qJ>ÿæoþqßðÊo/Cºxñbâ?i:àïÆ¼ìËË¾ÄË¾¼ìËË¾ÄË¾¼ìÎ/ûªFGGëëë>øþÀø#ðþÒ%"Àø#ðþÀ?ðVøs:ë×¯x)øþÀø#ðþÒz½~AHàüøàÀøKgüEü?àüøàü?àüøàü?ðþÀø#ðþÀøàÀøàü?ðþÀ?ðþü?ðþÀ?ðþü?àÀøþÀ?ðþü?ðþÀ?ðþü?ðþÀ?ðþü?ðþÀø#ðþü?ðþÀø#ðþÀ?ðþÀø#ðþÀ?ðþÀø#ðþÀ?ðþÀøþÀ?ðþÀøþÀø#ðþÀøþÀø#ðþü?àüøþÀø#ðþü?àüøàü?àüøàü?ðGàüøàü?ðGàü?àü?ðGàü?àü?ðGàü?àü?ðþü?güy½Þ¶¶69ò?ðþÀ?ðGà/ñçñxöïß_\¼`ÁÉþÀøþÀ¿ôÄ_oo¯ÙlÎÎÎ^Ûíàü?àÀ_úà/V¯^½@Ó¶mÛúúúÀøàÀø#ðøóz½6Í`0/;;»©©©··7íü?àüøËü:t¨¨¨(ÅÅÅò9#ðþü?ðGà/Íñ×ÛÛ»cÇÜÜÜ0ö¶¶6¿ßQÃþÀ?ðþü¥-þä¦jkkÃÎçP§ô?>dàð?ðGàü?é?¯×òäÉòòríÄ¾Í7'þÁü?ðþü?ðGqÁ_kkkiiiû^ðþü?ðGà/ð×××g±X´ûmmm^¯àÀøþÒòñ&I»bßòåËív»ÏçcHÁø#ðþÀ¿ÇßïÛUVVjÙWWWáûÀø#ðþÀ¿ôÁßÀÀ<æjWìËÍÍ5Í°P3øàü?à/#ðçv»-vé`kkk?£þÀ?ðþü¥þä6âÄ>µP3ðþü?ðþÀ_:à/L6±Ïh4fìBÍàü?ðþü¿tÃ×ëV½^¯]¨Ùl6s>øþÀ?ð&ýùÿù®]»´çsÈ¦¦&ÇÃ?ðþÀø#ðþÒ!y5ÍYYYaìÓëõò ËBÍàü?ðþü¿t(´··G<£²²Òn·3±ü?ðþÀ?ðy½^«Õj0´ì2±ü?ðþÀ?ð&y<íÄ¾ìììßû½ßëëëcÀøàüøirhl¡fy$éßö%ðþü?à/éc²m6[ð|ðþÀøàÀøKáü~¿Ýnlb0ì|ðþÀøàÀøKÉÔBÍ'öY,9ÆF¼øàü?ðGàü¥XÂ»ÜÜÄ¾è5?ðþÀøþÀ_Êär¹Ìf³ö|Á`µZ§³P3øàü?ðGàü¥@Ãh4j'öÉÎ-ÔþÀøàüøÉÏç³Ùl+W®ÔNì«««Å##øàü?ðGàü%cò#lµZµçsäææ644DØþÀøàüø)À®¹¹¹   ¥¥¥ÇÎÄ>ðþÀøàÀøKºººÌf³ö4Þ+W¶··û|¾¹ðþÀøàÀøK4ÔÖÖjÏçÆöáü?ðþÀø#ðþæ-¯×ÛÖÖV^^®Ø×ÜÜìr¹bþÁøàü?àohii)..c@p+ö?ðþÀø#ðþÀ_ÔÛÛk±X´5¯Òf³Ådbøàü?ðGàüÍs@@àe2´ì]]]Ó_¨ü?ðþÀ?ðþ7¿ßo³Ù*++µû,ÛíNäàü?ðþü¿x500 _ÚEEE¯½öÚÐÐPâïøàü?ðGàüÅ¾¾¾>Å¢]¨yùòåv»Ýï÷Ï×àü?ðþü¿ÚÛÛM&vÅ¾ºº:Ãàü?ðþÀ?ðß¼^¯Íf3ÿo<Vìàü?ðGàü¿yHMì+**Òþ)^Ù/&Õ½àü?ðþü¿Yæv»#®Øg0°bøàü?àü%(yH8±Ov&ÃÄ>ðþÀøàÀøA¢:»Ý®]±/;;Ûb±Èa-ùü?ðþÀø#ðþ¦ÎëõÊ#^¯×®Ø×ÒÒâñxRexÁøàü?à/Z»¦¦&íùÁjµSkxÁøàü?à/rò¸SWW§=Ãh4ÚíödØþÀ_ÊàoxxX¯×?ðGàü¿y¼jbO;±O,ø#ðþÒË-.ðþü?ð7/ÚëõZ­Ö5[,ØþÀ_jàoãÆn·;þÞÿý;M¸ÙÛÛâÓ÷¿ÿýÿøÇCºvíÚíÛ·8õÑGýó?ÿ3ã§>üðCy8Hð'½~ýzSSÓO<¡]¨ùßü¦ËåJá½|ùò¿üË¿ðm§.^¼øùç'ø¦0þþ÷.N¿·ß~ûÓéìîîþGO¢7n0qÊápôôô0qª££ãæÍCºråÊ­[·öéN>ýõ¯=+++O=õÔ«¯¾úÿði6¼|ðüâÍ·YºtéRâ?i:ãyÙxÙyÙ7Vùi²Sb/ûò²/øàüø¿bÝn×NìS5»Ýîô^ðþÀøàÀ_¦ào``àøñãåååÚåQF.Íáàü?ðþü¥?þÜn·ÅbÑ®Øg0l6[Ê-ÔþÀ_Záo²Àø#ðþÀß,êííÝ±c&©½½=Ejàü?ðGàü¿_Ëï÷GYÚ¼ysÏþÀøàüøË,üù|¾'OêõzíBÍi>øàü?ðGà/Sð'°íµ×´çs´¶¶ö÷÷3¶àü?ðþÀ¿tÀ_WWWÄåååmmmu>GXçÏññûýàü?ðþÀ¿ÔÆ_ ÊDyõêÕv»=Ïç-[¶­ZµjéÒ¥O>ù¤Ëåàü?ðþü¥$þÔùÚûÔù]]]Ì>ÕÄ|ö8ò­#òï¹çûò¿ìóùÀøàü?©?ùÆGíùÅãñ0*¦?jRòSÿ~ë·~«½½ü?ðþÀø#ðøëëëÞåææ±O ØÚÚÉû´ÉhÊOþ=ûì³V«ü?ðþÀø#ðìøãÿæÍµçs¬òÌ3¼Â±~c÷7BñW]]Í3àü?ðþü%/þü~¿<®^½:âÄ>AìRÄ9^¯ü?ðþÀø#ðtøÕjÕNì+**jnnf¡æéz¶¯Á`xòÉ's¶/øàü?IÇãÙ´iÓÂÃØWZZ*Lìi.«½½½««Ëçó©=àü?ðþÀ¿¤Hòf³y²A»Ðàü?ðþüÍg@ ½½=âBÍ6lp:Lìào~ð·`ª²²²À?ðþhúy½^Íf0ÂP~ã7~£¡¡ûÀøgüeMN§þÀø£é400 Gõ¢¢"íù²ÿ¯ÿú¯gú·	ü¿ØãoÞàÀøKÜn·ÅbÑNì3v»Ýï÷?Éßö%ðþæ###»víþÀø£ÉcxÄ²ÓápNìàü%þôz½N§cÎ?ðþhÊü~¿Ýn¯¬¬ÔÈÎÎ¶X,rÐ^ü?ð«®®ÖþÞV\ïðÁø#ðþR+¯×;ÙÄ¾(çs?ðþ999ò£;<<*b¾÷ß_6Á?ðþèñÄBÍ%77W;±ÏjµN¹P3øà/¹ð§~eC´'wîÜüü|ðGàü¿OÔuuuÚó9F£Ýnæàü¿äÂú;n·[6^~ùeµÁR/þÀøËØDub;vbÙléÑü?ð;pà@ðô°ßêÀ?ðþ2-¯×kµZµ5555Ín¡fðþÀ_ráOzåW-Z$ÝÝÝ²!¬©©÷]àÀøKªv---Úó9ôz½±§ØþÀøK%üÍKàüøÉóÃÛØØ¨ØWYY9ýàü?ðþÀ?ðìuuuM¶PsÑàü¿¤Ã_EEZðE	ü?ð	©ó9&[¨ÙívÇöÓ?ðþK._0Îö%ðþÀ_ú544tèÐ¡5Ëay`` ü?ðçÉý7ÆÇÇy×Áø#ðþËåjll,((Ð.Ôl³Ùær>øà/ÅðW\,?ü	øþÀ_ÂêêêÚ±cÇjàü¥þzzzä°gÏÑÑQðGàü¿´Éï÷?~õêÕÚuuu<?ðþÒâÅµ§zqÂ?ðþR'OÔëõaöY/ÔþÀøKü-Y²>ü?ðøØ577«ù<¡¶µµù|¾y¹Wàü¿äÂ:.¸ßuðþü¿&GÔºººÜÜÜ0ö­Òn·ÏûÀøÉ¿Nø ðþÀ_â/´··oØ°A;góæÍò¸ó9Àø)?§Ó)Çøý~ðGàü¿TIMì3aæËÍÍµX,½½½ÉsWÁøÉ¿Ä	þÀøKÎäª=£´´4~5?ðþÒYÄ	þÀøK¶<ÅbÑ.Ô¼råÊãÇÏïÄ>ðþÀ_Êào¾àÀø~ê|5?>&ö?ðþRz½~É%àÀøÉ¨În·WVVjj6Í??ðþÒ:N#¿ëàüøQòz½'öÍËBÍàü¿ôÁßµk×ähÒÚÚ:22È_Àø#ðþ"&°Þ	òÂØg0¬V« 0åü?ðãl_àrT4Í'öÙíö$ØþÀøKüq¶/?ðGó?µP³ÉdÒNì«««Kàü¿Áß|þÀ?ðçõzm6Ûd5§ÖÄ>ðþÀøàÀø´9j'öÉÙûÀø©¿±±±7æååÉq'??ëÖ­	8óü??ùI±X,ÚÁn·'ø/m?ðG?ùxÂG¼@àüøË(üÃ¡Ø'ÉN¹(uÏçàü¥þ-[&M6Ê»###[¶l=«V­þÀøò»´Ýn×NìËÎÎ¶X,òöÃþÀøK.üåääÈ1(ô7Î±±1Ù#ûÁ?ðþæZ¨9âÄ¾´9ü?ðbøËÊÊ#/ôTÙÃR/þÀøu;Å«Øg³ÙÒì|ðþÀ_áO½ì»~ýzõ²¯¼mÙ³bÅðGàü¿&G¶ºº:íù&©½½='ö?ðþR¢½'|<|øüøÉËå²Ùl'OìííMBüêìv»ÑhÔNì3Íi°P3øà/ð÷xâß­[·feeÉÛõë×'à§ü?ÓïÀ_úÒöÙgy¦¨¨¨¹¹9¶O¡Í^¯×jµjÏçûÙÔÔ9ûÀø©¿y	ü?ÓÌn·?ùäßÜûÍ#ß:"ÿöïÛÿ¯|åÏþìÏæCCCû÷ï×Ï¡×ëÓo¡fðþÀøþÀ_Ú°aÃóÏ?¯ä§þÉ»µµµó?Ëe6ÂØWYY)TÍÌàü¿dÇß©ÊÊÊþÀ_2$¢jú£¦PüÉ»O=õÔ¼àOhF£ö|ÁhOìàü%;þ²&üøIÕ×¿þõç.òîïÿþï'~¿ÿäÉÂPí¯Ê;vìp»Ý|?ðþµoß>u8;ú4ø#ðþä[ë'øþ@ÉïþPÞí$þdØåxU\f¾ÒÒÒCõ÷÷óÍ	þÀøKUüõôôäçç«Å¨B×|þÀ_2,YòÔSOÉÆûï¿ÛÛ?µP³öÞòòò¶¶6ÏÇ·%øà/ñ·ûvuP»|ùrbî:øþfT èÈï÷ÇüÆÃð'G§6hÙg4ßzë-Îçàü¥6þÎ;§j6mJä]àÀ_ò¤ð'¬loo×®Ø'mÛ¶-K?à/¡øÅU«V©s;®]»à»þÀ¿äéüùóV«U¯×/77·¹¹¹¯¯!àü¥<þ;¦m;wî»þÀ¿dÈãñ:t(//O»P³pphh!àü¥	þXçÀøËðä´cÇíÄ¾åË³P3øøË*Nþü¿ôKT×ÞÞ^[[«ý¥×d2±P3ø¶ø÷Àø#ðà¼^¯ÕjÕÏûÜsÏÁ>ðþü?à/McNQQQûdOkkkÿLÿ¶/?ðþÀø#ðþ1·Ûq¡æÒÒÒ¶¶6¯×«>ü?ðGàüø©aL&vbßNgØJÑàü?àÀøKÉÝn¯¬¬3_vvvCC|F¼øàÀø#ðþR,¯×+íBÍEEE---'ÊuÁøþøc?à/mØ555iÏç0V«58±ü?ðG¿Ð%ý"âuþü¿äO#f³Y>ÑhÑBÍàü?Jüëèèå/¼0::*ïÊÛ-[¶Èë×¯?à/9S5kÏçÖÕÕÍâØþÀø£Â_qq±1C?Së ÌñzzÊÊÊt:]UUÕµk×Àø#ð7÷¢,Ôl±X¢Oìàüø¸|aøû¿úúúwÞyG6N8±sçNðþüÍ%]KKKÄåx2àü?_TRR"GOúÉôù|6m=²·\>>®4©×ëµø³Ùl7:;;oR|úè£þîïþqS]]]ùµË¯7oÖNPþÊW¾rôèÑîîî¹yøüáÈ·Yº|ù²ürÈ8Ä)ÑIOOã§.^¼øOwü]¿~=â	·nÝã-2¢=DðwîÜ¹ÄöñÇ»O?øÁúúú8åt:?ýôÓúûûûÏ9ó;¿ó;Úìäbõ¹ä7ÃÏ>ûo³8%¿º|þùçCºråÊÝ»w8%¶¾wï^?iÜñ'Ý¹sgéÒ¥yyyòu~~þ+<x0÷á8''yÙxÙwùý~»Ý®Øm±XÜnwÌ?#/ûò²//ûR½ì¿JJJÔTBy«ü?ÚÔBÍMìòbðþÀ¿ùÌl6¿ýöÛ²!oëëëÁø#ð%Çc±Xrssµ5Ûl¶9ÏþÀø#ð÷kÝ¾»¢¢"??_½P[RRröìÙ¹ßlgggii©ÜfYYYww7øþ"&ºº:íBÍ&©½½ú5?ðþüM+µÈsètSÛ'Në]à2¢:»Ýn4µûÌfsàü?Ê üÊÑööíÛAüuwwËvaa!ø#ðþâÑd5555Íz¡fðþÀ¿éÝÄDjCáo||¿íKàüÅ£ÉjÖëõÂÁxOìàüøû"µÈ³z¶Oð766vðàAu,þÀ_¿=,vbßÊ+m6Ïçß»þÀø£ÂÓé¸È³<Ò?àoÃa2´Ù)ÇÄÏþÀø#ð÷kÉvMM:Û7//¯¢¢"snÀø£ôÆßd5çæænÛ¶-5?ðþü%uàüQºâo```^jàüøÞMüò<`<¨¨¨(//þÀßr»Ý'ö%f¡fðþÀ¿Yâoll³	ü¿%?Î'öÆóçÏ'ÉÄ>ðþÀe4þÊËËDmáÂàÀøZ¨¹²²R»PsCCCâÆÁøþ&íîÝ»Yÿ¶Gh"¿sçÎ?ào²¼^¯üëõú0ö477'Ûùàü?¿J¨ïWxÁø£tÂÇãijj¸Ps[[Û¼¯ØþÀø#ð¤?ðGqÅ_ ÑØ3gbrägÖl6kÏçX½zµÝnOþàü?_466VQQÜ³hÑ¢W_üøK]üöªªª_5<[ûìªß^UXXèt:çøèR[[«¼yófÃÒìàüQÆáoñâÅa'üªczkk+ø#ð¢øægÄjG¾uDýþùçÅ³xþÏçóÙl6íù¥¯¯/mü?ðG¿9»àÙ#àÀ_*âO>¬¤¤äð¡ÃAüÉ¿U¿½êÌ3Óÿtò vb_iiiKKK2¯ØþÀø#ð7Eêßñññ_»]ÍâàÀ_ªàO~ûÍßüÍPùÉ¿gÖjµNóê-ÔÜÖÖ~ìàüQÆá¯¬¬Lë÷îõûýò®ü|9rDý~þü¥"þÄg_¬·²§9B·óçÏG¹V hoo_½zµvbÉdJàü?_tãÆ<www?©?iß¾¯ÿO£°oÿ¾ýÆ§Ë/W¿àEdÝnjj¶X,òß	ÃþÀø£Â488XUU¿lÙ2Ùï»þÀÅÒo¼QXXXRR²páÂmÛ¶E¼îÐÐÐ¡C´ûdOKKÇãÉáàüQfáo^àâ¿ ï"¾r¹´û¬VkºNìàüøþÒ'F£v¦ìLõÁøþ¦ÛíÛ·Õ:Ïêß³gÏ?é?¯×ÛÖÖ¶|ùríÄ¾ºººÄÿ0?ðGàüÍþ:::jûÄàÀ_àohh¨¥¥E;±/77×b±dÔÄ>ðþÀ¿/*--Û·oñ×ÝÝÍ"ÏþÒò?²ÿ~íÄ>½^/?8±ü?ðGàoâ&&z²°óøø¸lët:ðGà/Eñçt:M&vb_eee&Oìàüøû¢µªÂßØØØÁÕsàÀ_jáOý)Þ+WFàü?ÿûAÄEåüøKüÈÏvbZ¨Ùív3àü?¿J°kjjÔÙ¾yyy	þÀÅ»W (?eBü?ðGà/)àæ?ù	8±Ï`0Øl6Îçàüøàü¥þÔâ­¬¬8±¯½½ó9Àøþ¦è³Ï>[²dINN<xäååUUU?É¿Ï?ÿ pôz½vbÙlæ|ðþÀ¿iåp8"ðÑ××þü%IgëÖ­.ÔNìkjjb¡fðþÀãþfZä¹¾¾ÞçóÉ»£££;wî=eeeàÀß¼'?&f³Y>5?ðGàüÍö&&$$ÛÁÁ¿yI~ÛÛÛY¨ü?à/öøSÏü÷È(Ïüø¯¼^¯Õj5Zö=óÌ3LìàÀøkjÎ_½úÉ|øðamm-sþü%>ÇÓÒÒ2ÙBÍï½÷ÞLÿ¶/?ðþüEº©Óë¿àüQèÐM¹Póþ¶/?ðþüMZÖTét:ðGà/Ã1ÍÁøþÀßcyà/Eóûýv»=âÄ>± P>øàÀøþ&[Ïyppüøy^¯W¾ù'Ø'c8ÙÁøþÀßãXÍùåWÂv644°Ô¿Øæñxw¹¹¹Ú---S.ÔþÀø#ðþÇjÎ<ü<xð@Þ÷ÝwÕR¦ú?ð#³yófíùÁjµNs¡fðþÀ?ð÷8VsþÔCQYYÚØ²eK¼ï:ørÔX½zµvbÑhéBÍàü?àïqOø¸~ýzðaéäÉ	¸ëàü¥q~¿_~*++µûêêêf÷þÀø#ðþb¿Ý»w«G&õ×>¤^xüøEýýýjÎÍÍmhhrbøàÀø;þt:<2Þ¼yóqÈ¿ðGàoúõööF<C~§ïöiNìàüøqÇ<8>|8l§<q¶/¿iæt:#ÏQ[[;Óàü?à/îøl¿x??ðêêÚÛÛ#Ï1ëàü?à/îø¯ÀøKÝa¯½öZyy¹ö|ÞÞÞx|RðþÀ?ð7'üÉUè»ÑßþTn·»©©©   ²§¹¹¹¿¿?~ü?ðGàü?ðþ|»FØ·råÊ·ÞzËçóÅû?ðþü?ðþÀ_ÜSûF£vbÉdr8±:ü?ðGàü?ðþæ3¯×ÛÖÖqbß;äMðýàüøàü¿¸ÔßßèÐ¡5755Íe¡fðþÀ?ðþÀøK¢ºººÌf³v¡f`kkëÀÀÀ<Þ7ðþÀ?ðþÀøMjbÉdÒNì+//okkûßçàü1àüÍ?þ¢þ(ð'ª³ÙlAû#°yóf§Ó°ó9ÀøþÀ_|ñ5U:üQão``@¾	µû²³³-ÛíN¶;þÀø#ðþó>ÀøE;ávÅ>à¡CæwbøàÀøàüÅ,ù8±Ï`0Øl¶dØþÀø#ðþÀøs-ØíöÊÊÊy_¨ü?ðGàü?ð¡øëíííµ×öîÝk³Ùâ÷·Ñ¼^¯|§éõúû¿P3øàÀøà/ñ÷Æo,Z´Èd2=÷ÜsUUU_ýêWûûûcg<OSSö|ÙÓÒÒ2_5?ðþü?ðþ2]]]O<ñDÓ5ùÖõïÙÚgyæXÝù¾ª««ÓÏûÀøþÀøé¿?þã?þÝßýÝ üäßÁÿ°  `/þªF£1âÄ¾öööØþÀø#ðþÀøK7üë[ßzîkÏâOþÏú99¹¢ÕjÕ.ÔûÀøþÀøé¿3gÎ|uÉW:ßóÏ?ÿå/yÑãñ´´´¤ÓÄ>ðþÀ?ðþÀ_ºá/<ýôÓÕË«¿±û"¿õ;xâ	ùééç¸PsªOìàüøàü¥þ$Ï×ÔÔTVVWSS#G®i~£Ãá¸PszLìàüøàü¥!þfßï·Ùl'öÍæÄ/?ðGàü?ðþÀ_'âikk+..Îàü?àü¿ÌÅÛínnnÎÍÍÕNì³Z­i9±ü?ðGàü?ðøs:;vìÐÏöûÀøþÀø¿óçÏGyóæÍ]]]<¼àü?àü¿ôÁ×ë=~üøÊ+ÃÌk±XÜn7ÃþÀø#ðþÀøKüµ¶¶jÏç=òMwÀøþÀ_*áoxxX¯×?ð1ËÕÐÐñ|·ÞzkêüøàÀ_¢ëììl<?ð§ýYÚ¼y³ö|£ÑxþüùÌ<ü?ðGàü¥<þ6nÜèv»£àïOþäOíòåË~ø¡æixåÒÃkÏçxúé§?ÎFé>èèè`ÞTL>?úè#ÆáMÅ.^¼øOÂøûß»89þäwÁÿØ~ô£y<ÿGñ©»»ûîÝ»/:zôè¾ô¥0óÉýû÷Ë/	Þuvv2qÊétþìg?câ<ýüç?gâÔ+W~ñ_0qJlýèÑ£ÒtÆ/ûfÂË¾;á]QQQÄó9ØÇË¾¼ìËË¾ÄË¾¼ìÚ/ûÚÁøl¡f£ÑøÖ[oeÚßçàü1àü¥'þ"rüeþÀd5744$þ?üøàÀø£¸ät:¿ýíokÏçÈÎÎ6Í½½½øàÀøKüMøK§<OSSSAAAûôz=5?ðGàü?ðþÒ'ùO¬««8±ïÌ3¬ØþÀ?ðþÀøKÃÍ´ûL&5?ðGàü?ðþÒ$¿ßo³ÙV¯^f¾íÛ·_¿~!àÀøàü¥CCCC---z½>EEE²```¦ÛÀøþÀøÉËåx>Geeeèàü?àü?ðÚ9ÉÎçÐNìàüøàü¿LTwæÌÉqÅ>A^Äk?ðþü?ðþÀ_åõz_í5Á =£¹¹y`` ÊuÁøþÀøà/eòx<û÷ï/**c_iii[[Ûtþ/øàÀøàü¥@]]]E;±oùòåv»ÝçóMóvÀøþÀøà/Ù¿§µû¤ºººYüï?ðþü?ðþÀ_2æ÷ûív»v¡æìììÆÆÆÞÞÞÙÝ,øàÀøàü%WCCC2òÚKKK[ZZæhðþÀ?ðþÀøK®ìvûÃñãÇ§?±ü?ðGàü?ðþR¦@ P^^®ØW[[ëp8ÂjàüøþÀ_ZeµZ·mÛ6ÙBÍàü?àÀø#ðþÀ?ðþÀø#ðþÀ?ðþÀøþÀ?ðþÀøþÀø#ðþÀøþÀø#ðþÀøþÀø#ðþÀøàÀø#ðþÀøàÀøþÀøàÀøþÀøàÀøþÀ?ðþü?àÀøþÀøàÀøþÀøàü?ðþü?ðþÀø#ðþÀãþÀøàÀøþÀøàÀøþÀøàü?ðþü?ðþÀø#ðþÀøàü?ðGàü?àü?ðGàü?àÀøàü?àÀøþÀøàÀøþÀøàÀøþÀøàüøàü?ðþÀø#ðþÀ?ðþÀø#ðþÀ?ðþÀø#ðþÀ?ðþÀøþÀ?ðþÀøþÀø#ðþÀøþÀø#ðþü?àüøþÀø#ðþü?àüøàü?àüøàü?ðGàüøàü?ðGàü?àü?ðGàü?àü?ðGàü?àü?ðþü?àü?ðþü?ðGàü?ðþü?ðGàüøþÀø#ðþü?ðGàüøþÀø#ðþÀøþÀø#ðþÀøàÀø#ðþÀøàÀøþÀøàÀøþÀøàÀøþÀøàüøþÀøàüøàÀøàüøàÀø#ðþü?ðGàüøàÀø#ðþü?ðGàü?ðþü?ðGàü?ðþÀ?ðGàü?ðþÀ?ðþü¿4Ç_ww÷+t:]uuuOOøþÀø#ðþÒK.+Ù8útEEêpÈÄ çÅ'á~?ã§ä¥Æ!N9Î0qÊáp<|øqSW®eâàï?ÿó?üIS¡åååiñ÷§ú§ßKlòòá~âÓåË;::øïGÄ8Ä©>øá_òð)þcÞTìâÅÿ¤é¿7n466ò²//û/ûò²//û/ûò²o:¿ì«­¯¯÷ù|àüøàÀøK7ü-øeêÝÁÁAÅàüøàÀøKyüæt:×¯_?<<ñRðþü?ðGàü¥þôzýÀø#ðþÀ?ðÎøøþÀø#ðþÀøþÀø#ðþÀøàü?ðGàü?ðþÀ?ðþÀøàü?àüøàü?àüøþÀ?ðþü?àüøàü?àüøàü?àüøàü?ðGàüøàü?ðGàü?à/Óð÷ío»½½½?±]¹rå?üa?Å§/þèG?bâÔ¹sç~üã3qê¯þê¯þéþqSùùé§2qê/þâ/<ã§N:õ¯ÿú¯	þ¤^¯7=ñwûöí#G|BþÒè%"""ÊÀø#""""ðGDDDDàÀ?""""IZww÷+t:]uuuOOìq:Ë-=UUUÑûöªU«BS¹¬¬Lí¹víCÛáÕ~?SWÕÑÑ±`ÖØ¯ßï·X,999ååårfb;¼<´Å¼ÐCA²=´q¢¥K~üñÇ²qúôéÙ(..¾÷®lÈ[½^ÏÍ%ù1¸páÂã¿<&+õõõï¼ól8qbçÎQlWûýL1^)¯Á_<÷èÑ£¯¿þúøø¸0eÉ%Ql¶Øv(H¶6P3(//OÞÊAgppP6ä- XuõêÕêêju»lqùðj¿)¶Ãûê«¯~÷»ßñ^ñJ__§áå¡-¶í¡#Ôt»qãFccãã'oåQSþGå­ìddæþëQaa¡çéÓ§å]N¼(tb2¼ÚïgáðÞ½·¦¦Fñà/NcÇÉWhrëÖ-Æ'¶ÃËC[Óí¡#Ô´­¯¯÷ù|²½jÕ*õÛ§ü¨¬Y³ÁI%%%²ÜÃÈÄvxµßÏÃáÝºuëõë×¿8°¿øN:%|ò<²22±^ÚböPlm¡¦nppÐb±Ü¿?9ý6©ÁÃÐØØØãçÆC½B1^í÷3ÅpxüzKÌã7¼<´ÅVCA²=´qx"§Ó¹~ýúáááàùèöíÛ²ár¹äW%h.-]ºTfww·³lÍæ·ß~[6äm=CÛáÕ~?S7ôÐÏøÄ|xwïÞýÞï=8SÕh42D±^Úâ¤@µlm¡¦H¯×ùý³Ï>SgÈË[ÙfæROOOUUæºuëÔsQ¥¥¥YYYeeerTbb;¼Úïgáð¿¸ïÈÈÈÖ­[eOMMÛífb;¼<´ÅÉöÐÆ(TÊ?""""DDDDþüø#""""ðGDDDDàÀ?""""DDDDþÀ?""""DDDDþüø#""""ðGDÞ»ï¾»víÚ¼Ö­[wîÜ¹_;~M2GÛH÷V¯×ËæóùÂöËNWVV6>>>ÓÛ$"D9rd¦W^y%ð×ÚÚ*;O<¶ÿøñã²ÿå_Åm?"J½zzz5:îÍ7ßLtêÔ)yWvÞ¼y3mðwçÎÙY]]¶Ù²e²ßãñ?"D½ðÂÂ×_=tço¼!;BésíÚ5Á¸pÅ²üà666ÊEÅÅÅ÷îuÕáp°ä"¹îÕ«WÃ8%.víÚË/Ë»f³9ì]ºt)úí¨ä.©:::&ÚúõëewwwpÏÇ,L&SpÏÑ£GKKKå¦òòò¶oß>88¨ÅööÃöD¹«Dþæ¹ÅÞ½ºóÞ½²S¯×â&¬7n¨K·lÙvÑ=ÔE.++++âµÔ»êÒ­[·srroOùùùj*^ÛwOû>:T´A_¾óÎ;AùÝÈºuëf¿(wÀÑü§^ápÌZ°@(;wN$òî¦MÔ¥Ê:ê	<Aº¨¾¾^=e(ÛêFBoóðáÃÂ;õªëK/½$. ÛòV¶_|ñÅ)oGà(ïÊÞ·_rÑ¢EòõË»òVî¹sllL@YY¾Ç¿|8//o¦ørWü%þ¤øîW¸Qfå]azwÅòî%KvíÚ%hôèQðFäcÂK^K½ïÞ½à¯üÊ[Ùv8SÞ@SÞðàAèl~Þ¾ûä¢ï~÷»ùÒöÞ½C?@ (øÖÔÔ¨'&g¿(wÀÑü§îÝéóùd§;AºÝnå¿ uõ´¯Æq*tÙ...ÎÉÉñûýrã.^:åíLF±°nß¾­*ÛòV¶].WðÒÎÎN¹_>>þ¢ÜU"DDó÷vìØ±Ðj°>óµOhÝ¼y³µµU½T£zZ.øºêDÛ³gzµWÞ666÷G¹õLÛýû÷Õ»<~fî5käRµº5ô"uæ¯ÑÑ122AªÑ^å®ø#"ÿÔ	:îÄj©7ß|3''GrÆ¦M:~¿_½&<3WÍùSSåÜnwèdAeÁ½÷Ôy¸ÁÅV"M~«>»Óéîr;jóçóùÔGFÁß©S§ÏÉÉzþèr¹ä³È L?ÅMá²| sûöí¡F¹«Dþ"µrXGýÕñk"e£àvðÓà9Á^zé¥PYvöìÙ(ø<ñ§8dqqqè+ÂQnGîFèi;ÙûèÑ£¼¼<õ%½Ø½qãÆÐO±hÑ"y«V	½MõÄd0EÕà¥Qî*?"¢dI²nÝºÖ®]«N¹Ã_GGZN¯ªªêúõëÁK>ßÁKKKöíÛç÷û^½zµ¦¦FLVVVf³ÙÂnSOä¦dÿîÝ»ÃöOv;Ü¹KjÂ(ëükll[óE5<<_/#PXX(_Çã	.[z###¢[5V&©»»;ì3F¹«Dþüø#""""ðGDDDDàÀ?""""DDDDþüø#"""DDDDþüø#""""ðGDDDDàÕÿm*¥.~¶cIEND®B`


ûö¾¾>¿þÌ¿«W¯Î=»´´4,$wÀGÑs$|æ JËËËzè¡OS½.yï½÷¾!/¿A¹jÕªôs×]wõ/_Nâ2¬?~Êïq¤ô¥CÙD¿hã¤o¸Q_pøñwöìÙÄë	ßìHã/ñÇÚÞÞ;sæLôÉëÖ­t·mÛæÄ¹ñ>8	KìÇ,,¯]»öÂB8V&~æ®]»nÜ¸Í®¢5[·nÅb'ONk'=CFöl;räHXþàÏ*qòóóS®O¼ìm^d¨êâÑn·jÔL³Ãß hËÕÕÕ×®]YO´áÇ_â5üøñ°ýX7mÚr~øaX?Í°äÄÑñçò°×­/--ËôQt2<Ç³fÍJ¼ø¹ñ5!8O^¹råÓÿ|ý4±9B.ÈX¶lY4jºeü¤üFR®ÅE£*tLeeekkkÊ6òVúÃ¿h¿Æ?þ8ñK(þ¬Ñµ%k¢³,YNÎ;7Äkø_Äõë×ýfø² þB§ðþþþÄõÉ³äàBÕ¥¼Îô'ChzéóG­E¯S'^¼E]Ûp.2Ô6¹åFÎÆ*IGwÁá¿ì;¼KnòuÐûàDguttDýÂ¿þL¿àG?úQ8cheÔgfTaåp"ýÉFo¹rìØ±gÃ¿h<½ëJuuõöíÛ?ùä^z)¬Ü°aCÊøÎEF#Ý8q£¾àðão8¿xÞôO¿ýÂA':wîÜ=¢×ãGFâÈèøûôæÎûñýú£5Ñ±Àk×®½zSôì¾uëÖÛ¿húÕÞÞäÅ_NüµµµE³¥Ð'O¦zÃ§üGzÅ_úmÉ¾]ÉH·ê(â/ÚK/´ïõë×CÛ=ðÀÉiøüóÏ1tpúíÝÂÎáóÃË/Nü6£7;::Ò¼? ãâïâÅñ½»¢5ö÷*--<;ñÏÈ¬Y³ÂÇÄ#^SÞæGy$ù%Èî¹'Í·9¢(þÒoø5z£æQlÕQÄß¹sç¯ÿ¾ûîK<wÐ6	¹>þ¢·KLyL4[Môè£úåñdGüÑK¢ëö³mØ°¡à¦êêêäÃ;FW®×®3dÐã?z(þ.!égß¾-ÊËË»ë®»^yå¯~õ«ñÉÓí_dDñ~ã>|8ô_~~~ü­òh«"þ¹sçï:ôÂzüçp2$ZôÕ+++O>ËÉëÑ£G-[®-|SñõO<ñD4R~ýýý~¹@üGydDï-7dèÕØèðíX,5ýÂÝañ¢þùÞ÷¾gËâ õõõíÜ¹ó®»î^ÛO=õÍ?Äâ@ü þ?Äâñø@ü þâ`Ô>^¿jÕª^vÑ¢ES¦Léèè¯	ËaÍâÅGðpvÓè>g8ålÚ¯äååM6­ººúÊ+ÑYÛ¶m+))	³¸¸8õþûï»âÈ,eee!eBÁÄb±^öé§î¹çâköÙ°fÏ=¹ÑrøÆÃò¦M¢µµµáäñãÇÃò'ÂòüùóÝÁñdØ£ÉmôÓ¹sçÂeW®_sï½÷5gÏÿÿ¯r9,O6-:YPPN^½zÕ@F_bÐ<ñÄÅÅÅEEE»wïôi_ýêW-[6èâÃ°|ùòå°ÖDgøá¡òóó,YÄ¯-ñ«§¿È+¯¼R^^¾bÅðSìÚµk555á²áÆïÜ¹3d<½ï¾ûÂµõá?úè£±¿9sæ.<yò¤» þî¿hùg	Ë!³ÂÂ÷¾÷½ÄÏ9zôh|ÿ¶¸Ç,õÒK/åð1,×ÕÕEgÝsÏ=G	|ðAX?öì×xÒ_$$Ý~ô£°P[[|ã·mÛÃ'¼þúëaáé§ÙÕ«WÛÛÛÃB4L6ø»qãFØ>ayóæÍÑY_áªU«Þï=÷.@üåååayà¦äöJ¹S`[[[8kíÚµayÝºuQÕÅÏ=wîÜ®]»-[!òÚeVúD7¬¸¸8ù²¥¥¥ÑYBöá3Ãò%KBÎö÷÷n³$¯n[¸òP~¯ó9s&ôküsÂuÄ¹ñ_ádúúôæ¬¨¨(|f___ôêjXõâ/K"|ï½÷¯aÐµ%ÎEºa¡ÆyQ8<xpÆÑh(xG&i?~<äfâ+àâÈÄø=vâä/Ý²xjjjÂ¹ññõÑ7nfü¥¿HüK¾ì¬Y³¢Ëºy±XìÈ#Ñ¡¸ñá6ËHã/ngøvÜÁñdnüEoÝòÒK/Eûü=ûì³Ã)ÆGh?úÑâë£<þ|´/àpâ/ýEvíÚõãÿ8,<öØcÉôÑGÃò«¯¾záÂè¥Þ°rÅaù½÷ÞûøãÃÂÆ.þ/^Î:vìXXnii	Ë<ò; þÌ¿X,«è¦Ä÷êK×¯_^rwTRRR\¼sçÎaÆ_ú´¶¶sï½÷Þøq'Ó××·ûöpËxàèÀÞÐ|ÕÕÕÑáÃ+W®üÙÏ~6¢ÍüÒpM¾bMMM¸ña;Ì5kÛ¶má&¹âñø@ü?ÄâoÂüä'?éêêò#Ì%ôÑÏþsÛlôÁèÏ¾Aæøøÿ÷·Ä_øýßÿýÐ~¹äoþæo>þøcÛlôùÿú¯ÿj;=úoÿöo¶ø??ÄøCüøCü!þÄâñ'þ?ñøCüøCü?ÄâÄâOü!þ þâOüøñøâÄ?ÄâÄ?ÄâÄ?ÄâÄâOü?ñøñøCü?Ä?ÄâOü!þ@ü!þâñøñøÓ§O/Y²$??ñâÅgÏâÄ?r9þæÏÿÎ;ï×^mÁÉñwìØ±käS§Nýã?þ£í@6jkkû§ú'ÛlôæoþË¿üíK²8þM6-9þþù¿  ·ß~Ûv ½ñÆÇ·ÈF---á/¶C.Éø;sæL]]½ì^ö/ûË/ûF®]»V]]Ý××'þÄ?äxü]¼x±¶¶6e?ñâÄ9mmm«W¯¾råJÊsÅøñâ¿òòò)	Äøñâ¿ôÄøñâñøñâñøñâñøñøâOü!þ@ü!þâñâñø??ÄøCü!þ@ü!þÄâñâñ'þ??ñ'þ@üøCü?ñâÄâñâÄâñâÄâñâÄø??ÄøCüøCü!þÄâÄâñ'þ??ñøCüøCü?ÄâÄâOü? þâOüøñøCüøñøCüøñøCüøñ'þÄâÄâñ'þ þ?ñøñøCü?ÄâÄâOü!þ þâñøñøâÄ?Äø þ@ü!þ þ@ü!þ þ@ü!þ þ@ü?ñøñøCü?Ä?ÄâOü!þ@ü!þâñøñø?Ä?ÄøCü!þ@ü¿ÎÎÎ_~Yü?Ä?Ä_.ÅbÍÍÍkÖ¬rSH@ñ'þ þ9¨££ãÉ',))`ÇâOü!þ@ü!þrG,;pà@eeåÔ©S§üW3gÎÜ»w¯ø?¿áÂ;vÂ¤ªªª¥¥¥··w2ÿÄÅâÄâ/â/$]ccã+GÓ§Oß²eKGG·øCüøCüeü§ÚÚÚÂÂÂäQ_hÁP|Ô'þ þ â/õUTT$7ßÔ©S7oÞVü|ÅâÄdüç-[¶¤Ü«oùòå/¿ürOO¬øCüøì¿ø^ÉÍB0ä QøCüø¿4£>õ?Ä?ÈøëïïojjJ9ê+,,¬­­5ê?ñ×ÑÑ1Ô¨¯¢¢Â¨Oü!þ@üA.ÄQøâÄLø3êâOüøÜ¿ô£¾ÏõâOü?ñ×ÑÑQ__?Ô¨¯¡¡Á¨Oü?ñâ²>þúûû+++úÄøCü!þ ãÏ¨Oü?Äâr?þÒú¦NjÔ'þÄø?ÈøëììjÔ7oÞ¼îînÛ_ü?ñ'þÝñÅ«ªª¦NrÔ×ÚÚ>Çâñøì¿hÔW^^<ê+++OÜálpñ'þ?ÈîøK?ê[¿~ý¡Cúûûmjñ'þ?ÈîøëîîOÇ)GsæÌÙ³gOWW-,þÄâñÙ±X¬µµ5å¨/ë[ZZìÕ'þÄâñYiF3gÎg]ºtÉ&âñøì¿ô£¾+W677fñ'þ?ÈúøK?ê«¯¯¿pám(þÄâñÙÑ¨¯¦¦&å¨oÍ5p¯ø?Ädüuww744Ì7/åõíÙ³Ç¨Oü?Ä?r!þN:U[[;Ô¼MMMàâñâ¬wðàÁ?üÃ?tiróÔÕÕõ?ñøñG.x÷Ýw·lÙRTTwß·QøW®//âÄÜ)/¿ürÊQßÌ3|òÉðh,ûÄßÄ8yòäÂÃQü?pû¢QßôéÓ³¯²²²¹¹Ù¼âo=ðÀiâïÇ?þñä¿ø¿øÛ¿ý[ÛlôÖ[oýýßÿ½í@fºpáÂ³Ï>»lÙ²¼¼¼AÍ÷Ï|¦ººúí·ß¶rIÇß/nâÐñ÷Ê+¯ürÈñãÇÏ9c;;vîÜ9ÛLó§ú§555ÅÅÅÉ£¾/éKÏ<óÌOúÓ7Þx#üÇÛ¶Ê%¹^öõ²/xÙõ÷÷755­X±"å^[¶léèèòþ¶/^ö?döööoë[¡ð³/´`(Âä½úÄø?dhÔ·råÊáúÄø?d«.ìØ±c¨Q_cccoooúkâ/k?ñâI«¿¿ÿÀ+W®LþklÑ¨/<¢óªÄø?d®hÔWVV6êQøâñâL.]x?êâOü!þ@üÑ¡íRîÕWQQ1Qøâñâæ½úkkkG=êø»páÂ¡CN:åïÅ?ñøCüÁ-ttté¨oLã¯¯¯¯ªªª¬¬ìË_þòÜ¹sõWµ½½ÝÏTü?Äâ·QßÆßúõë.]ºëÉ]»wwø÷µ¯ísûB?_ñ'þ?øqõ]üõôô|æ3Ù¹cgT~Ñ¿/~ñÍÍÍ~ÊâOü!þLvýýý¡*++SújjjÆçiîÆ_ûÂåþÝÿý~ÜâOü!þL^õõõCúB*é¨oìâ¯§§gÖ¬Y&/6ùâñøc2ÊQßØÅß§Ñ>Kïó7-øCüøcâ¥õÍ7oGckÖ¬I<Ú÷ÝwßuâñøcRÅbCú¦NZSSÓÚÚ>gboäX¼Ï_øÆO:å8_ñ'þ?&ÎÎÎúúúòòò¡FÝÝÝrSýñ'þ þ¥hÔWUU5uêÔõ?ñ7ÚKÞJ^^øCü!þ$ºººÂÓ¼yó2Ô'þÄß(åÝJ~~¾øCü!þÈm±XìÐ¡Cë×¯O9ê«ªªÊÀQøÙJü?L îîî½÷¦õ'©õ?ñ7&®^½úØc?ÄâÜÓÖÖVWW<ê*++3Ô'þÄßþo?ÄâvéÒ¥½÷VTT$7_IIIWWW6~_âOüØâÅSþõ[û?ñâñÑÖÖ¶eËéÓ§'ïÕ·fÍCe×¨Oü¿ÛUPP~®RVVBóýøÇ?uuuâñø#]ºt©¡¡áî»ïNqÌ3gÇ9ðm?ñ7ò«¸),Ú|ðÁ7ÂBQQøCü!þÈF§NÚ¸qcaaarö­Y³¦µµµ¿¿?g¾Yñ'þFlÆááäÉaá©§¼ÕâñGv¹téRccãP£¾'|2K÷êÜáøÛ¹sgüðÄß+V?Äâ¬ðî»ïÖÖÖ&ú¢÷êkjjÊê½úÄw8þï~÷»³fÍ§O!-[6Ö7]ü?Ü¦ÞÞÞûö¥<·¼¼¼¾¾¾££#ç7øYCü?Ú©S§êêêRîÕ·|ùòõV?ÄøCü!þs½½½û÷ïO¹WßôéÓkkksã^ñÇÇß¢7|ñ&Ï?Ä)<eÔÔÔ¤õ­X±âå_Î¥xÅcóçÏO¾8Gû"þL¸ÞÞÞ¡þ,Ç-[Þ÷ÝI¾Äø±ÐyáWèÌ37nÜÏ.þÄ?Ò?¤¤<7X¹råË/¿<yöêÜáøÿs¿Hã âOüø#¥ÞÞÞÆÆÆ£¾3gõ?î@ü=6üFmÛ¶íÚµkâñøcCõ­X±¢©©iÒîÕ'þ¸ÃñÜu×]É¿iø@ü!þ·õM÷êküÍ;×?ÄøÐv¡ðRúB.´Äw>þ¢_³öööq¾éâOüø¢Q_È»¡FáÁÄVaü:àñøãó(aÔ'þøøkkk¿u;wîçiÅøñ7I¤õÖÖÖõ?Æ5þ¦Á?Ä·©££c¨Q_EEQøcbâ/oø@ü!þþþþ¦¦&£>ñGÆßDâÄ_î1êdAüÏ;÷ÂâñøctúÄÙùùùásüoºø þr@úQ_CCQø#ãâïøñãáWtÏ=W¯^Ï7|âÄ_öêïïonn®¬¬L9ê«©©ñ/þÈÜøs´/âÄßðuttÔ××§õÍ7oÿþýFâL?Gû"þ@üÝRQßÔ©S7nÜØÖÖÅl(ñGÄßDâÄ_VèììjÔW^^¾gÏ®®.[Iü!þÄøñÝb±XsssUUÕÔ©S÷êÛ¸qã©S§úÄÙ<ðÀ´iÓÂ¯tQQÑC=4G~?ñâ/3E£¾òòòäQ_YYÙÞ½»»»m%ñGÇßõë×Sð1ÖêWü?%Í¨/¬Y¿~[[Û8ÿxÄc.¿Øk×®½víZ8yõêÕuëÖ5÷ÜsøCü!þ&ô£¾=öõ?r*þÂ¯wâ~aMX/þ¿ù[[[õ­Y³¦¹¹Ù¨Oüñ~ÏCðÅ×_õ°Æ[½ þ¹ª»»;<§õÍ3'ÕÓÓã^!þÈÙø^ö]½zuô²oøÃ%K?Äâ/Ç5êÖ¬Y2Â¼âÜ¿P)øøäOÄâñ7Ö¿5û÷ïoll¼páÂ~¡K.544Ì7/å^õõõîâÉÞ<à÷¡*..ÎËËW¯^ÖõMâ&yüýÎïüÎÌ3ï¿ÿþÿvß+**úÁ~pÇ¿D,kiiY¿~aaaröUVV:tÈ^âÉBü?Ìñ÷ê«¯~þóÿööoïþÝÝáßÿÞú¿?ûÙÏgñ;uýiFåååõõõþ,øCü?Äâoü¬Y³æë_ÿzT~Ñ¿ªªªo~ó·yµÑ¼555Ó§OO>7|ææfõ?&iüM¹¼¼<ñøCü-ÿïÄøû5ÿ£²²rÔWØÓÓ³oß¾9sæ5ê³Wøc²Ç_ÞÐÄâñ7ÖÖ­[÷µ¯-1þV®uëÖQ,Ý¸qcÊxCMõ?Äß-<þøãÑCÆk¯½&þ¿±û)..þÆÃßÊocõÆ3ftttÿ¢QßÒ¥KoæÌõõõ#º6Ä1þÎ=[TTýO1ñ=Åâñ78ðË¿üËsçÎýµ_ûµÏîsÃHöêê^Cü!þeÃÑÇ#GÆç¦?ñ<þ>½ùÚÛÛ/0Wfzz/_nÔø·åõ×_;Ö®];7]ü?ÃtêÔ©ºººäx+V455õ?ÛAüËõë×ï¹çèØãÇóMâÄ_z/¿ürÊ÷ê9sæ-[úâoþùèdÓ¦MrÓÅøñæ·©¶¶Ö¨ñÇ?ïóøL¿ÞÞÞÆÆÆ£>Äw>þòn%??_ü!þãöëS[[ò^£>Äw&þ&ø þn9ê¿V~:?Äâ²>þÂïKh»Px)G¡Cú¹ þ?ÈîøF!ïú?Äärüõ!þâOüAîÇ_SSSÊQ_aaamm­Qâñø¿¡FF?Æ;þ¼ÏâÆ"þúdhü%¾¥_Êøó>?Äßõ!þÈèø;vìXx`zøá¯]»NëÖ­kN8!þ¿[J?ê«©©ñXø#³â¯¤¤$<BÅb±ø°¦¬¬ì6¯ùìÙ³³gÏÎÏÏ_´hÑñãÇÅø¿úúú¡FF?21þ¢Ç©|âïö÷ù«®®~õÕWÃÂ/¼°iÓ&ñ'þ 7â¯¿¿¿¹¹¹²²Ò¨ñGVÆ_iiixÀ¡výúõp²¯¯oíÚµaMX×ãÆ¨&ËËËã¯±±ñ9ä­·Þú«¿ú+Ûl>ÿú¯ÿúïßüfqqqröÍ3gÛ¶m!"mLÆYKKËÙ³gmæñwâÄ|¼÷Þ·yÍ$>âïõ×_ï&üä'?¹páí@6zûí·ßÿý4ÐÔÔôë¿þëS§NôPÖüÖoýG3&Ðo¾ùáÚ¹dÌã/øàæÏ?mÚ´¼¼¼¢¢¢%K|ùö¯6ñã/ûzÙ²îeß®®®ð`UVVü?äyóæ544Üòa¼ìKÆ½ì;vJKK£]	ÃÇäÅø¿X,P|ðÁÂÂÂäQ_MMMkkkâqr þÿ!<>¾òÊ+a!|¬®®â2?þ:;;ëëë£·AHy¯Qâ¿óçÏ/X° ¨¨(z¡¶´´ôàÁ·µ'O,++×9öìÓ§O?ñáqoóæÍ)÷ê«ªªjmmµäHüEoòø'Ý¢å^xaLoºø	ºººRîÕw÷Ýwõ!þÈÁøòÎ?¿Ó§Oåââbñø# 7&úÃúS§NÙ«ñGnÆ_ô`-DñwãÆÛñG®êîînhh3gNò¨/ügxïÞ½F?r<þ¢7y¦!þxâ°ü¶ÌâñGVkmm­ªªJ¹Wß¿üå?þã?îïï·ä~üµµµ¥|çwÞyGü!þÈ===Ï=÷ÊQß=º»»G÷·Aüñ'ìeËEGûN6mÁcÓÅø±6Ô^ÁÊ+ã£>ñøcrÅßâÆHwwwxI¹W_XÎêéétñøcÅ_ü8¸Ë//X° <D?ÄY$µ¶¶ÖÔÔ¤õ­Y³&<Gu¯øCü1©ão``ÀÑ¾?²Htï¼yó¯¼¼|Ç·ÜEü!þÈýøKùH¢3f?Ä.<5ê[¹rå¡Cù^âñGîÇß~wSüo$å÷úë¯?Ä©···¡¡!å¼3gÎüÖ·¾ÕÑÑ1¢+?r?þâBêõ+¼âOüÁú³¯²²2ñ^ñøCüeñ'þ`øÒúêëëG:ê»øëéé9tèÐsÏ=×ÒÒâ¡d_ü,X° ¨¨(¾fÖ¬YÏ<óøCü!÷«ÚÚÚ£¾+V455Ýü~ü:uê³ýì²eË*£rþüù_øÂºººüdSüÝu×]øU÷ìÙ#þLÞÞÞÆÆÆ¡F[¶l¹ÍQßèâ¯§§§´´tcõÆÝ¿»;úwÿý÷ßsÏ=Ã<¬ÄáÁ´½½=¾æìÙ³aMqq±øCü1!w¤qõ.þ:R/^~áß®'wìîîöCü5ñðãÆÿr½Ioþ'þ©qõ.þöíÛ·jÕªÄøÿ,Xîü~?²&þfÏ[·oßýgúúõë»wïþÞ¹øCü1>÷4£¾P¡Çú63þÂ³ìÜ¹sw=¹+^~ßÞþíéÓ§'ÿ½8dnü9s&å<>Zü!þ;Ñ¨/äÝP£¾ñ§3þb±ØÒ¥Kïýò½¡ùBù=ö¿?þÖ­[ý4dSü/^hÑ´iÓòòò.ÖõMâÉ|W	moG£¿ ¯¯ïë_ÿúôéÓKKKÃ·ðï|ÇÑ?²/þ&øL65êuüÅõôôÈ>ÄâOü?ñGêûÆP£¾	õÝ~üø#ãïüùóÑû<GGø<xPü!þ¸MýýýMMM)Gµµµs¬øCü1âïØ±cñã(þ¢å^xAü!þ¡FË/ÏQøCü1yã¯¬¬,<?>§Oö&Ï?Fá£¾S§Neæ-?&QüEË&¼±ó7Âr~~¾øCü1LiFK.Ý·oß¥K2ùö?Ä(þJKK£wõâo``à'ËåååâñGzýýýÍÍÍÉÍ7uêÔÍ7:u*+?&Qüµµµ¥|çwÞyGü!þJGGGÊQßÝwßÝØØá£>ñøcòÆ_°-[í;mÚ´tvvõMâlfÔWXX¸~ýúð<ßøCü1¹âoB?ñGvI?êkhhèîîÎÞïNü!þâñÇÅbiöê«©©	O<9ð.ÄâÉï¿ÿþÜ¹sÂ£ù´iÓ-ZtåÊñøä:;;ëëëËËË³oÞ¼yû÷ïÏ®½úÄâñ÷­­­)ø¸páøCüMBÑ¨¯ªªjêÔ©É£¾õë×·µµåÞß´?&QüEoò]Ý××N^»vmÓ¦MaÍìÙ³ÅâoR	ÿåK3êÛ»woWWW®~ïâñÇ$¿è=ñÿña9þÏâñó¢xSú|°¥¥%÷FâñÇä¿hò700_sýúu?ÄßdÐÕÕµwïÞ9sæ$7_xd¿¤ãð®OâÄãÑ>ÕÕÕ¡ùÂÉO>ùdåÊöùCüå°X,ÖÒÒ²~ýúõUUUçh?ÉCü!þDñ7åVÆèõ_ñ'þ]]]áW¯¤¤$ù7½¼¼<ÕïÕ'þ¿aÉ»üü|ñøËjÑ¨oÍ5CúZ[[s~¯>ñøCüM0ñ'þ.]Ú·o_´koò¨¯¾¾~òìÕ'þ¿_êý/^¼(þÙ+ü~mÜ¸±°°0å¨¯¹¹yúÄâÉáÉà»ßýî 7oöV/¿lúRÀkÔ'þ¿ÿ"/<+^¾|9üá=OÑ®~âOü1FõFâñøû¿6oÞ=CÌ=;ZX·nÝXßtñ'þ¸#zÃoSÊQßÌ3ëëë;::l%ñøCüvâÄøÆþýûÇá¦?ñÇm¿A555iFýýý¶øCü!þRØºukü=ý£~Xü!þ2SoooCCCEEQøñ'þF#???<g;wîÓþÄâ/£õ?Ü£wíÚ5hemm­£"ý¨oË-Fâñg;¿êþÆúY?³IÃÿÄRúV¬XÑÔÔdÔ'þ@ü¿¬!þÄCéíímll4ê þ¸ñE_ØMRü!þÆmõ??Ä_3ê þ¿É²ÑBÛÂK9êEºÐV þ¿ìúBÞ5êÓV þ¿JFâÄâñãÒúkkkúÄ?Äâ/ttt5ê«¨¨0ê þ¿øKOü!þnGSSQøñG¦Ä_Þ­äçç?Äß(õ?dbüM8ñ'þrQøñøâoRH?êkhh0ê þâOüe½X,ÖÜÜ<Ô¨¯¦¦Æ=øCü¿,~.¿÷¿·sçÎ¦¦¦Ð7â/ÙêëëËËË³/¬Ü»w¯QøñøÙaëÖ­¿ò+¿òß×ü÷ªªª¹ÿÏÜßüÍßìïï°)Ã:uê æk6oÞîÒ#ÊeÄ?ÄéÀÿüç¿½ýÛ»wwø·ëÉ]_üâ÷ìÙ#þÒú/_ÞÐÐÐÝÝíþ#þ@ü!þÄ_ùú×¿þàÚ£òþýÏÍÿsñÝ'müÅb±ð¨:Ô¨¯¦¦¦­­ÍÝFüøCü¿lµaÃÐñ÷Øÿz¬¢¢bÆ_ww÷P£¾°Aî¹wñâñ'þ²Û3Ï<³tÉÒÄø»ÿþûû·òÄ_,kmmÝ¸q£½úÄ?ÄøË_øÂ¾ò¯ìx|Ç®'wU=Põ¹Ï®³³s2Ä_ø6÷ìÙ3gÎäQßÝwßmÔ'þ@ü!þÄ_nóo|#4ßôéÓ¿rïWÞ÷Ýá_6ã/z¯¾ôõõ??ñGÖÇ_www¸õ^öìéêêò3 þâì¿h¯¾£¾ ¬oii1ê þâ¬¿[ú¼Wø³?ñGÖÇ_úQß>xèÐ!£>Äâñ'þÈúøëîînhh7o^róÍ3çÉ'´WâñødüE£¾ðVUU577õ!þ?ñGÖÇ_Q_yyyýðß³ñâñ7W®OºâOüµp_J9ê*++ú?Äßx8yòäÂÃ³¯øc¤···¡¡¡¢¢"¹ùfÎY__ßÑÑáçøCü!þÆÉ<zÓÄß~ðVrÈ#GþüÏÿ||¾V¸óüÆoüÆ/ýÒ/%gß¾ô¥áÆø0|o¼ñÆ±cÇl²QKKË[o½e;ä,¿_ÜÄ¡ãïí·ßþÿÈ!§OþðÃÇôKtégY°`AróÍ1ãÉ'loo÷`ÚÚÚþùÿÙv ½ùæ?ÿùÏmËñçe_/û_´W_aaarö­X±¢©©©¿¿ß/ûâe_¼ì;©âïèíímllj¯¾-[¶Ø«ñøâ/ãrPü¿Ñ][mm­QâÄâ?£>Ä?²;þ"þÄß ï¾û®½ú þäxü¤kllyüÍÑ¨/møñø#ëã/|~h»Px)G¡mUÄ?ÄÙyÜ|7nloo·1 þdüª«««K9ê«¨¨hll¼téÍøñø#»ã¯¿¿¿¹¹yéÒ¥ÉÍ7uêÔá±XÌDüøCüÝñ×ÞÞ¾cÇ²²²äì-¸oß>£>Ä?ÄYMMMÉðN>Ë-áGoÔøñø#ëãïwÞ©¯¯<ê[¾|¹QâÄâßßßÔÔt÷Ýw§ü³7oö³FüøCü¿ÕÕ5Ôõ|î¹ç¼WâÄâOüe½èÞªªªä½ú¢÷êóg9 þâ/tttÔ××$úÊËË¿óïõ!þ@ü!þÄ_ÖFSR©ªªjkk;úô(þ¶/??2HgggýPõíß¿¿§§'úÌþm_ þdX,6Ô^aÍúõë[[[½WøCüøCü¿ìúÊËËGóæÍkhhèîîNyAñøñøY#ý¨¯¦¦&yÔ'þ þâ/ûtwwGÊQ_XÎjÔ'þ þâ/kÄb±ÖÖÖ¡Faý-Gâñâñ'þ²Àõ?Ä?ÄøË|Ô'þ þâ/¥õÕ××wvvÞþW??ñ7¢Q_MMÍP£¾æææÛõ?Ä?ÄøËÝÝÝóæÍÓQøCüøCü¿4Î£>ñøñø£§§gÿþý)G3gÎ£QøCüøCü¿qõM>=9û*++ûûûÇçÆ?Ä?Äø+===ûöí3gÎP£¾q¾Iâñâñ'þî¼°ÝÖ¯_¼W_°|ùòÆÆÆÞÞÞ	¹aâñâñ'þîK.íÛ·¯¢¢"¹ù¦O^WW×ÞÞ>±·Pü!þ@ü!þÄß6ÔÆ³oÅp;ÅâÄâOü^oooCCCÊ½ú¦O¾eË[uÅâÄâOüXüÞ£¾¥KNà^âñâñ'þîèÞwæÌ[¶lð½úÄâÄâOüÝiÞ«obà??ñwÇôôô<÷Üs|¯øCüøCü¿; ··w¨÷êË¨xÅâÄâOüÝ~y¯øCüøCü¿;cïÞ½¯øCüøCü¿;ãÒ¥K¯øCüøCü?ÄâÄâOü?ñøñøâÄ?Äø þ@ü!þ þ@ü!þ þ@ü!þ þâOü?Ä?ÄâOü!þ@ü!þâñâñø?Ä?ÄøCü!þ@ü!þÄâñâñ'þÄ??ñ'þ@üøCü!þ@üøCü!þ@üøCü!þ@üøâñâñø??ÄøCüøCü!þÄâñâñ'þ??ñøCüøCü?ñâÄâOü? þ? þ? þ? þÄøCüøCü!þÄâÄâñ'þ þ?ñøCüøCü?ÄâÄâOü!þ þâOüøñÇd¿Ó§O/Y²$??ñâÅgÏâÄ?r9þæÏÿÎ;ï×^mÁÉñ÷öÛo_'Üïêê²ÈFmmm/_¶ÈFo¾ùæµk×lÅñhÚ´iÉñ÷ðoC9rìØ1ÛlôÆo¼õÖ[¶Ù¨¥¥¥µµÕvÈ%¹gÎ©««ó²¯ÁË¾àe_rùeßÈµk×ª««ûúúÄøñâ¿)ÿ):yñâÅÚÚÚA þÄ?dü%jkk[½zõ+WR+þÄ?äTüOI þÄ?ärü¥'þÄ??Ä??Ä??Ä?Äøâñâñø??ÄøCüøCü!þÄâñâñ'þ??ñøCüøCü?ñâÄâOü? þlñ÷ï|§¹¹¹ògög?ýéOm²ÑüÉüÝßýí@6ú£?ú£ÎÎNÛ!ôööæfü?~÷îÝ¿@ô/N1üð²/âñø@ü þä¾cÇMò»âÇ<%CÆJ¾¯=vöìÙùùù-:~ü¸MDÝ=ö??±XlÉ%ñÇÖÖÖÚ,d¾äûjuuõ«¯¾^xáM6ÙDdÑ½×c¯øñóÌ3Ï|ÿûßÇ_xô9|ø°ÍBæK¾¯Ü¸q#,ÛDdÑ½×c¯øqòá.[¶,<_Æãoþüù«W¯ÎÏÏëö³ÙDd¬äûjX¸ïõØ+þ`<ôÐC'Nø;bÒ.&/^x±MDæßWóòòâ+l²èÞë±WüÁxÝÿþ«Açzú$[D÷ÕÒÒÒOo¾ìm²èÞë±WüÁT`´0þüÎÎÎè«W¯¶eÈXÉ÷ÕW^y%,ÕÕÕ6YtïõØ+þ`ÂâïÌ3.ÌÏÏ_¹rex²eÈXÉ÷Õ'OåååÍ=ûôéÓ6YtïõØ+þ?Äâñø@ü þ?ñø@ü þ?Äâñä¢þð÷Ýwß´V­Zõúë¯ÿÇ¯²æÑ6Õ­-//ßZ__ß õaM~~þìÙ³oÜ¸1Òë@VÚ½÷$ßýîws)þöìÙVîß¿ÐúûöõO=õÔ(®@üÙçìÙ³!kòóó_|ñÅØM/½ôR8V;w.gâï>+/^<hýÂÃúÎÎNñ?`RxøáCÖ|ïßKì³Ï7oNLãÇx]¸dÉ°ÿäO>ù¤®®®¸¸8URR²ûöÄWW[[[C`³Âe=:(§Â3fÜwßG	'kjjÝ°ô×nRtÖ±cÇµÕ«Wõ§O¯yçwÂÊÊÊø§~º¬¬,´iÓ6lØpñâÅäøK¾þAkÒÜT@üL°»îº+Ë~¸ò£>+ËËËãf3gÎDç®[·nÐYÛ¶mÎjooÏËËKy©èdtîC=tãÆ±X,>x***vÅKs=a!åÍKþN_íµÄ¢÷å«¯¾/¿AW²jÕªÆ_?½Ââ1kÊbq³iÓ¦k7príÚµÑ¹QëDùðB´EgUWWG#Ã°|òäÉèJ¯s×®]!ï¢W]ôÑ°æðáÃa9|Ë<òÈ-¯'c8>!ñ¶¥üBPÎ5+|¿W®'ÃÇpËCqD0öìpÁð-|ú/O6m¤ñæ¦â #â/HñõQÜDÍx1ÙdI89wîÜÇ,DÛõë×ãW>gÐ,-~©èäGÿäLñW~ÃÇ°ÜÚÚzËë	¡N^¾|9ñ¶µÞã?Îúþ÷¿ÿé¾´½ûöÄO!â/$é²eË¢ÁäHã/ÍMÄÀÄÆ]×®]K××V³ÒäN<;::¢þ·N|G½äWcåTâ¬åþþþpå3fÌËë*Å9þ|Ô©a9|ËíííñsO<n@Êin* þ&^´ßÛóÏ?¸2zA|Ä÷¼råJò@ëÜ¹söì^Wc4¿®zËDÛ¶m[ôjoøXWW_æz¢IÛÇ¼|ùrú#sï½÷Þpnôî6¡YÏüg;vìêÕ«éã/¦ÑÖæ¦â`âELäçç¿ðÂÑ[½¼øâÉg¬]»6NôlüÈÜh¿hW¹Ä£Ü¾H¥è8Üø­¤L´èðÛè«·µµÅ×§¹h'¿h¿¾¾¾è3ÓÄßK/½Éo9ñ¬h÷ÇöööðUÂF*þ¢Ü¹>-DçÏMsSñ¢7@äé§þ¿_7Em_¿`?Æ"îÑGM,ËDLÞüSaIIIâ+Âi®'ÜÄ?Òv¨oöúõëÓ¦M¾A/v?ðÀ_bÖ¬Yácôn/×&ã¢Tæ¦â S@YµjUÁM÷Ýw_tÈí ø;vìXôvz-:qâDüÜ¾¾¾'x¢¬¬,¦Ç¼¿¿?~îÑ£G-[löìÙ®3ù«ë·nÝ:hýP×nRôiÞç/®®®nÐ¾D®R]]¶@qqqø.:;;ão[xW¯^um«ÊÊÊÓ§Oúin* þ?Äâñø@ü þ?ñø@ü þ?ÆËÿpÀ&r¨IEND®B`


Detrended Normal Q-Q Plots


£17n,ºçðm[[[öFïl;zôh>wî¤8O´"yßã,½©.sdtªgÜ¥öùYýmöÃ °>C´Éãoã.ÃaoáY³fùÅ"ÁTøËéÞ"]/^Ì¹å¼£hÑéÅðR^A³Yå¶Ës·ùghÝ##×õñÕÕÕÙk^ãÃ·­o4&#ûÛè´ÙKN§2³×=þ2"HêïqìMæsÙ²eÇw£Mfã»TÓqòø!/_ÎþSÂß$ÆáÛººº×ðWÄÐÐß*üIÅ¿0²ªªj¢WÜçÕ:q¦úò<»h|Xå°¾á%|xx8üØ#gcß7v'ómfÎ©Ï[^µZSS§+¼Rgîa2³Lf[ÉlH:½'Ú÷½?º&ù0îïïüAá#GübàO*2ü=õÔSÙ|DÇÒr®»u¦4oþ#7W®çnóÏgyæðmæQ¢ÏrQå°xò$ûÛùóçG¹ÒÞÞp6üE'£O]I¥R7o¾víÚ¾ûÂÈÕ«W¿ÉÌ2müMuãdöÇßdüexýµgûçy¼E<yrçÎÑ¹æÌÑàO*ü·` èµ9óQ/ÑKZpCx±.]°`A4)zÃÓàÍÆÕÌ3o~9EjÞ³î<ºwt·ùgÌ/U«VeÞ×®^¹råÀÍ¢Û¸qãÇ_tôëôéÓaöîÝ;üuvvFÇÂ*wuuEGõ¢><îþê,SÂ_þóØÈnª[uøÞ¥CCCÁv+V¬KÃÝ»wûßò)½w³¿¿?Ï)xIð'þÆÅâØëg3oÿÏ|otpì«fyóË)¨(âQö¥©yî6ÿù%qéÒ¥Ì»»¢1gÏÍy¿WuuuæïDUVVfÞ8kÖ¬»³.gOi)á/ÿÆÉyld7Õ­:ü<y2ûþ,Y=5gd.9è§äy¼e®(Ê´nÝ:¿X$øáU°©©iìÿÕÖÖÖØØ ^Ñzê©ÌøÃ1[Ùr7Éyo)§èãñÂ744ý¸¾îö3æDtJ4ü3gV¯^¸Y*yÇôðwõêÕpoá>¶lÙ<1w~â<ñÄaíÂÏ3gÿþý<ð@æÈÓeJøË¿qr9Mi«N¡#GÔÕÕµ÷XÏ¾D~ú²eËº»»oyäu¢ÇÛààà¶mÛ¢CªAðaWûÅ"Á$P¥Óé5kÖLé³å¦1KÑñ.ßL?þ|	þ$I%Xô¿ôQ[F?IR	688¸uëÖ9sæDçvÃÀÃ?l³Hð'I$ø$IüI$	þ$I$I?I$Á$IàO$	þlI$ø$IüI$	þ$I$I?I$Á¤bìðáÃµµµñx¼©©iªóÖ××õ÷÷gÆá0fÁSø-v³éÝf2óNówë¢ñ6mªªª[¬¢¢"J½õÖ[¹FFFÂøpãD"=º$Á¤«¦¦&`åêÕ«ÓÀÊ®]»Â¼ßúÖ·2cì±0fçÎ¥¿±ã×®]ÆwttáãÇá¹sçf¦9r$Å£GztI?I÷Kä=øéäÉaÞ¥KfÆ,Z´(éíí½=yûñH$Âøqçúâ¿¦F_×­[çÑ%	þ$¢ü²¡³mÛ¶Y³fíØ±#çf<ð@cccÎ=dá+W®á0&táÂeËÇãñèhÙØËþéùgÙ¿mmíâÅÃK´ë×¯·¶¶yÃÂoÝº5:xºdÉpoa|¸ç/¾Güä#	ãçÏßÕÕ3itttöìÙág¯"Oo$	þ$Pþyä0üÔSOfG4û6mmmò²Û°aC´oß¾0¾áõë×G.ú<wîL&Ç½·ìÈ?K Ý3Ï<Ö®];vá7mÚÃöÙ0°k×®ÕN>¢½o"gßæðáÃ1MMM§NÊÌ^¹re^½zucÆ]:Ið'© ðW[[Ó7k¯qßØÙÙÏªU«"Õe¦<yrûöía|,÷ÞrøhÁ***ÆÎ[]]-üèèhä#Ã-ÃpCCCàìðððô6KN===©ÆoÞ¼9ómÔ-[¶¼e$øôÁâ/ºR53>|K	iÍ5+Ürpp0:³ÆDöîÝæ"<uêTö=äÜ[ö·e¢RÌ>PÁñÐ¡C³gÏÆTUUE'ä/ÏvëèèË>Íè~èµk×nÜ<çÌ3î2xÔI?I¿d2ä/:xvK	µ¶¶©¯ñÑµ£7$þòÏY°`©±óVVVFóæ,ÞÈÈÈÑ£G£«t3§´Y&*Z°Ìa8£ÕN<9íe$øôÁâ/úèûöEïùì±Ç&#¡Ce¬óÌ3ÏdÆGìëëÞ8üåeûöíßýîwÃÀÆÎ»nÝº0|àÀ7ß|3:ÍF.^¼8]¾|9Ì7ï=âoÁa|û_°Ë5knLü©7aü´AàOÒ¿¬Y7Ëþ¬¾üøN¹¯ÙlTUUUQQ±uëÖIâ/ÿ,ÇS-Z¹î$çã7oÞ<H¬X±"º¨6x+JE/]ºôÌ3SÚ,cO»mmmKV¶²²rÓ¦MáçÞøõç]gßæó®§½$Ið'I$ø$IüI$	þ$I$I¿ê?üáùóç-Þ~ûmæÂïÊ+Ñÿß ïâÅ?ÿùÏmÂïÜ¹s¿øÅ/lÂ¯¿¿ì'¨þîLßøÆ7ÿ-þõ_ÿõôéÓÍßo¾ù/ÿò/¶Cá÷£ýèòåË¶Cá÷ýïÿúõë¶CáwôèÑÿùÿ±àþàþ?øüÁüÁàOð?ø?øü	þàOðð?øü	þàOð'ø?Áàþ?øüÁüÁàOð?ø?øüÁàOðð?øü	þàþàOð?ÁüÁü	þàOðððð'øüÁàþàþ?øüÁüÁàOð?ø?øüÁüÁf(þzÉd<¯¯¯ïèèööö²²2øü	þàOð¥P*:pà@Ø³gOKKKÎÔð½Èûçþç×_ýº¾ÿøÇÁ¶Cá÷Úk¯ýìg?³¿W_õ?þã?lÂ/àïÝwßµn[3UUU£££a N×ÖÖæLäGüñð·÷îïy/¿üòK/½ô|a7?6lÂïÅ_|åWlÂï^þ³¿ç¾³³Óv¸mÍüÅãñqC.4tÚWNûÊi_§å´¯J±X,3H$²'577?~ü«?Áü	þà¯4V£ºº:Nß¸yÚ7ÿÆþfð'øüÁàþ¾ÖÖÖýû÷ð5J¿ªü	þð'ø¿ÒX®®®X,L&»»»ÇÕü	þð'øy?Áàþðð'ø?Áàþàþð'øüÁüÁàþºþÞyç7ÞxãÝwßµAàþàþ¿Rîé§^²dIuuõüÍ///ß¸qãÈÈÍðð'øü`ÃÃÃ÷ÜsÏ§|jû×¶ïøú/oþòÜ¹s¿úÕ¯Ú2ðð?Á_	ÖÖÖö»¿û»þÏ»îºËÁ?ø?ø?Áà¯â'î»ï¾lüÕÕÕo¿ý¶ðð'øüZÏ=÷Ü¼yó²å÷¥M_=¶#ðð?Á_	688XUUuÿû#ùmÿÚö7|ü¡²eàþàþ¿ÒlïÞ½þðÿ×Üÿuï½÷ÖÔÔÜÿýÃÃÃ6üÁüÁàOðW=zô¿øExf=÷Üso¼ñ¾ðð?Á_ãÏÿððð'øüÁàþàþ?øüÁüÁàOð?ø?øüÁüÁàþ?øü	þàOð'ø?Áàþ?øüÁüÁàþ?ø?øüÁàOðð?øü	þàþàOð?ÁüÁüÁàOð?ø?øü	þàOðð?Áü	þàþàOð'ø?Áü	þð'øüÁàOð?Áü	þð'ø?ø?ÁüÁü	þàþàOð?ÁüÁü	þàOð'ø?ø?Áü	þððð?øüÁüÁàOð?ø?øü	þàOðð?Áü	þàÏ£þð'øüÁàOð?Áü	þTøÏååÿùoýÖoÝ÷ÝûÜçÞyç»þàOðð*Mü'ò]wÝõ>³ýkÛ¿¼ùËî]´páÂááaþàOðð*Aü555Ý¿âþ_ßù÷Ñ~ô;ßù=ð'ø?ø? þî¾ûîm_Ý¿ûï¿ÿK_ú=ð'ø?ø? þî¹ç¯lùJ6þ>ýÇþêW¿jÀü	þàþàO%¿/|áüä'3òÛþµíÇ³Gàþðð§Äß;ï¼ó¿÷oøø>ø§Í:gÎõë×Ûð?ø?øSiâ/488øÈ#<pÿ«V­úö·¿=22bwÀü	þàþàO%?Áü	þàþàOð'ø?ÁüÁü	þàþàOð?Áü	þð'øüÁàOð?Áü	þàþàOðð?ø?øüÁàOðð?øü	þàþàOð?ÁüÁüÁüÁàþï¹ÞÞÞd2Çëëë;::²'uww744I,7?ÁàþðWô¥R©=ö´´´dO;wî'ÂÀÁçÍ7ÿðÿðoEÞ©S§^íµSÁ÷ÿø¯¿þºíPøýà?èëë³¿_~ù§?ý©íPø9räüùó¶ÃmkFà¯ªªjtt4¤ÓéÚÚÚnV^^>ûöí;Uäuuu½úê«§Tðÿûßÿáh;~¯¼òJøÊv(ü^zé¥ýèG¶Cá÷Â/¼ñÆ¶ÃmkFà/;]OOÏúõëöÓ¾rÚ×i_9íë´oÑÅ2ÃDbìÂoT*588?Áü	þà¯è«®®N§Ó7nöÃ9S/]º´víÚqÃàOð?ø+¾Z[[÷ïßÂ×T*=©³³sùòåW¯^wFøü	þàOðÅWWWWMMM,K&ÝÝÝ¿Z·²_®]mmmYVð'øüÁàþfnð'øüÁàþàþðð'ø?ø?Áü	þðð'ø?Áàþàþð'øüÁüÁü	þð'ø?ø?Áàþðð'øüÁàþàþ?øüÁàOð?Áü	þð'øüÁàOð?ø?øü	þàOðð?øü	þàþàOð?ÁüÁü	þàOð'ø?ø?ø?øüÁàþàþ?øüÁüÁàOð?ø?øü	þàOðð?øü	þàOð'ø?Áàþ?øüÁüÁàOð?ø?øüÁàOðð?øü	þàþàOð?ÁüÁü	þàOð?ø?øü	þàOðð?Áü	þàþàOð'ø?ÁüÁü	þàOð'ø?ÁàþzñWv«b±üÁàþ¿Á_ìVÅãqø?Áü	þ%¿Òþ?øüÁßûÐÀÀÀàþð'øü þjkkãñ¸÷üÁàþ¿ÒÇßÆ^íQUU588ð'ø?Áà¯ÔðH$ö®^½ZSSù¾ûÝïõë×Ãü	þàOð'ø+5üEúÂ@Ð^8wîÜèèh5küÁàþ¿RÃßìÙ³õºººúûûÃÀÃ?ø¨øüÁàOðWøÛºukæòì·ý-^¼þàOð?Á_©á/ôÍo~³²²2twwÁÆÆÆ¢Xøü	þàOð3(øü	þàOðð?ø?øüÁßÄÍ7/úÀò?øü	þJsçÎÍ_&WûÂàþ¿Ä_p^`_OOÏèèhÑ­?ü	þð'ø¿©UUUðWò?Áàþð7åzþ6mÚTÏ@øü	þàOðSnÎ9ecrÁü	þàOð'ø+AüÕÕÕ¹àþð'øüÍüEì+R|ÀàOð?øZÕÕÕ.ø?Áü	þ3[·n?øüÁàOðWâø+ |Ààþ¿Ä_l?øü	þJEü	þð'ø¿©U[[[WW^ÕÞêííM&ñx¼¾¾¾££#ÿ¤é?Áàþð7ý«ÊÊÞ·#©TêÀa`Ï=---ù'MoLvÛ¶mûøÇ?¾¬È[¼xñ'>ñe*øî½÷ÞO~ò¶Cá÷øK,±¿¥KÚßüÁüÑýípÛúÀñ×ÑÑð·sçÎ÷þ/UUUÑ¤ÓéÚÚÚü¦7&»-[¶I$P8þÞß«³/É¹ddì¤é?IÓïý½Ú7D"ÿ¤éÉnûöí÷ßÿÚ"ïsûÜg?ûÙµ*øÿìÏþÌv(üV®àÚß+¾ð/Øßÿñÿå_þ¥ípÛúÀñ÷þV]]N§£³´a8ÿ¤éqÁ!|¸àC.øpÁG¡ÔÚÚºÿþ0¾¦R©ü¦7þ?øüÁß*N¯X±¢¼¼¼¬¬lÖ¬YÍÍÍÓ¾ò£«««¦¦&%Éîîî_-åÍ³×c'Moü	þð'ø¿é7444îâ¿ú?Áàþð7µæÏ¨·råÊè800°jÕª0fáÂð?øü	þJD"Podd$3&N1c/­?ø?ø?Áü©èñÅõ¢+j£ÃéÔüÁü	þð§Æ_tÚwùòåÑ30|ÃaLCCüÁàþ¿RÃ_xâÁÇµk×àþð'øüþnÜ¼à·¹¹¹¢¢"¯Ë/cbýáOð'ø?ÁüÍ àOð'ø?ÁüÁü	þàþàOð9sÞªX,ð'ø?Áà¯Dð8ø?Áü	þ¥¿Ú²eK¿Âü	þàOð'ø+YüõööÎ5+°oÙ²eÙùð'ø?ÁüÙ¥¿Õ«WGüÂ.¢õ?Áàþð7µöÙ+W®,ºõ?Áàþð7Ù.]ÛÑÑÑQë?Áü	þàoRíÞ½;:à×ÒÒR¼ë?Áü	þàorsú?øüÁàOð7sð»UñxþàOð?Á_à¯4?Áàþðð'ø?ø?ÁüÁü	þàOð'ø?ø?Áü	þðð'ø?ÁàþàþàOð'ø?;þæÍH$|Îü	þàOð'ø+üÍ;7|>çþð'øü2þóûzzzFGGnýáOð'ø?ÁüM­ªªª¿bü	þð'ø¿)×ÛÛð·iÓ¦b|ÂàOð?ørsæÌ)>àOð?Á_	â¯®®Îð'ø?Áào¦à/b_âþ?øüÁßÔª®®vÁü	þàOð'ø)øëììøÛºuëðð0üÁàþ¿Ç_Ù¹àþð'øü þbäøüÁàOðWø+êàOð'ø?ÁüÁü	þàþàOðN§W¬XQ^^^VV6kÖ¬æææb¹øþ?øüÁßÔ÷¢¸øþ?øüÁßÔ?~ ÞÊ+£gàÀÀÀªU«ÂÂü	þàOð'ø+5ü%@½Ìt:Æñð?øü	þJ±X,P//3fxx8ñQ/ð'ø?Áà¯ñö]¾|yô_ÃpÓÐÐð'ø?Áà¯Ôðxã^ðqíÚ5ø?Áü	þ¥¿7/ømnn®¨¨ÅbáëòåËÃ¢Xøü	þàOð3(øü	þàOðS«¶¶¶®®.¼ªÁü	þàOð'ø+üÅãñ²²b=?Áü	þàojuttüíÜ¹s`` XþW7øü	þàOðÓ½	Åbð?øü	þJ±	ò!Ïð'ø?Áà¯tðWWWW__?88XÔë?Áü	þàoREÿ¥ï§â/ü	þð'øÓôñý¾Ñãð'ø?Áà¯ÄñL&ËòæøüÁàOðW:øëéé=vtü/¢>àOð?Á_Éâ/S±8þ?øüÉÿí?Áü	þà¯ØêííM&ñx¼¾¾¾££#RwwwCCC´`Áp3øü	þàOðE_*:pà@Ø³gOKKKö¤¹sç8q"<xpÞ¼yð'øSñâoppð¹çûÖ·¾¾¾ûî»öüÁf.þªªª¢ÿV8N×ÖÖNt³òòò±øû»¿û»×¼ÎÎÎcÇ½¦ïW^yõÕWmÂ¯½½ý?øA¡-UxùüÐ>TWW×t_Óïÿþïß÷Ýÿ÷?Ã÷Ô/¾þ÷-ü9râÄ	Ûá¶õãïÌ3wÙWLtõIOOÏúõëÇâ/<"¯yaí®ªàýõ×òØ_WWW¡-Õ¢E~jé¯ïþæ3I~(yþüù¼§ÂT.-ü^xá+W®Ø·­Ñç¼444¼õÖ[wÙ)H$ÆÞàúõë©TjìFç´¯öUQöçwî¾ûîí_ÛÁ_øWWWwË?ñöÓ¾zÿñØù`çx<¾téÒ³gÏÞEÿua¸ºº:NG§ÃpÎ-/]º´víÚqÃàOE¿ðÿ±ùÙòÿîýÄ½ßûÞ÷àÏ#þt»ñuæÌùóçg@H$V¬XqÛV£µµuÿþýa |M¥RÙ:;;/_>Ñ!Pøü©(ð÷î»ïÎ=û+[¾ßö¯m¿çÞxãøó?ÝüE^~ÿ·®®®ðÉdww÷¯ÖíæAÁÚÚÚìÿtþ*Fü6nÜ8wîÜÈÛ¾ºíøDSSÓÈÈüyÄÂîþ~yJ"ëÈ_EEÅØ«+0øü©Xð÷çþçwÝu×Ç>ö±ÊÊÊ+V¼óÎ;3|OÁüéÎà/Û|ååå---.õ?ÁQ|añÞ~ûm»	þàOwÑuþô§Ï;Wtë?þð§;¿;ø	/ðð'ø?Ûþt»ñWÔÁàOð?øróæÍ5kVtouuõ¡Càþð'øü þÚÛÛ3|Dø÷ìÙð'ø?Áà¯ÔðWSS¨×××Á_wwwôi/ð?øü	þJOÎàott4ºþàOð?Á_©á¯ºº:P/:ÚðN§·mÛkkkáþð'øüþ:;;ËÆëÄð?øü	þJ¡ð[²±±1ºÚ·¼¼|Þ¼ygÏ-õ?Áàþð7?Áàþðð'ø?ø?Áü×***b7õõõÅrÎþ?øüÁßZ·n]ÙmØ°þàOð?Á_éàïÐ¡CóöîÝ;88:|øp4¾­­þàOð?Á_à/LáíÛ·oì¤è?|ó9ð'ø?Áà¯tðH$ðÆNJ§ÓaR¸üÁàþ¿Á_æuhjô_½Áü	þàOððW"øËÃ;ø?Áü	þððð?øSÑâ/ð?øü	þJ±[Çáþð'øüþJ#øü	þàOðð?ø?øüÁüÁàþ?ø?øüÁàOðð?øü	þàþàþ?øüÁüÁàOð?ø?øü	þàOðð?Áü	þàOð'ø?Áàþ?øü	þàOð'ø?ÁüÁü	þð'ø?ø?Áü	þðð'ø?Áàþàþð'øüÁüÁüÁü	þàOðð?Áü	þàþàOð'ø?ÁüÁü	þð'ø?ø?Áü	þð'øüÁàOð?Áü	þàþàOð'ø?ÁüÁü	þàOð'ø?ø?Áü	þðð'ø?Áàþàþð'ø?ÁüÁü	þð'ø?ø?Áàþðð'øüÁàþàþð'øüÁàOð?Áü	þð'ø?ø?Áàþðð'ø?Áàþ°ÞÞÞd2Çëëë;::ÆÞ ½½½¬¬þôeø³ýìG?úÑ¥K~ûÛß?Áàþ7R©ÔÂÀ=ZZZr¦444À_1väÈÊÊÊ+W>ôöw~çwzè!øü	þàO3UUU£££a N×ÖÖæLäGüñð÷ØcµyáIõÂ/´¥îºë®/|þ;¾¾#ú÷-_=ößüÍßïÕ¦/üÕaOËzñÅmÂïùç·ng3ñx|ÜáÐ'Â_ggçygÏýñüß%×o¾YWW_ôï¾ûîûÛ¿ýÛ"]£¾¾¾þþþÿVÁ×ÓÓóïÿþï¶Cá~ÿ×ýíPø¿¦mÛÖÀ_,Ë'ìIÍÍÍÇÿåª:í[l'äÃÉÁß§>õ©'|Òi_9í+§öÕ;í[öëÂpuuu:Nûáqo¹1üQ555>ø`F~_Úô¥ÊÊÊ@(øü	þàO3Ùµ¶¶îß¿?¯©Tj",	^ggçÝwßß÷ýEë_üÉüÉoÿöo?üðÃÅ»:ð?øü½?uuuÕÔÔÄb±d2ÙÝÝ=®öà¯H;þüæÍ-[¶fÍ[¾þð'øÓÀß´?Áàþðð'ø?ø?ÁüÁü	þàOð'ø?ø?Áü	þðð'ø?ÁàþàþàOð'ø?ÁüÁü	þð'ø?ø?Áàþðð'øüÁàþ?øü	þàOð'ø?Áàþ?øüÁüÁàOð?ø?øüÁàOðð?øü	þàþàOð?ÁüÁüÁüÁàþðð'øüÁàþàþ?øüÁüÁàOð?ø?øüÁàOð?Áü	þð'øüÁàþàþ?øüÁüÁàþ?ø?øüÁàOðð?øü	þàþàOð?øüÁüÁàOð?ø?øü	þàOðð?Áü	þàþàOð?Áü	þð'øüÁàOð?ø?øü	þàOðð?øü	þàþàOð?ÁüÁü	þàOð'ø?ø?Áü	þàþàþàþ?øüÁüÁàOð?ø?øü	þàOðð?ø³àOð?Áü	þð'øüÁàþàþ?øüÁüÁàþ?ø?øüÁàOðð?øü	þàþàOð?ÁüÁüÁàOð?ø?øü	þàOðð?Áü	þàþàOðð?øü	þàOð'ø?Áàþ?øü	þàOðð?ø?øüÁüÁàþ?ø?øüÁàOð·¹ÞÞÞd2Çëëë;::²'¯]»6H|ä#éìì?ÁàþðWô¥R©=ö´´´dOÚµk×£>:::äWWW7¯¼òÊP÷Óþô7ÞRÁ÷üäþélÂ¯»»ûüùó¶Cá÷ê«¯þçþ§íPø½ðÂ?ÿùÏmÛÖÀ_UUUà]H§ÓµµµÙêëëß|óÍføûë¿þëW¼^zéÅ_|E_[[[ØY¶CáwôèÑöövÛ¡ð¤xùåmÂïÈ#6ÂílFà/;»÷îòòòºººS§N9í+§å´¯Ó¾rÚ×iß¢/eDÎ¤ûö3gÎ466ÂàOð?ø+ÎEÿua¸ºº:Nß¸yÚ7gß,ûÛð'øüÁàþ²ÖÖÖýû÷ð5JeOÚ¸qã3Ï<úúú/^?Áü	þà¯èëêêª©©ÅbÉd²»»ûWëvó àÀÀ@sss<ollìïï?Áàþð7s?Áàþðð'ø?ø?ÁüÁü	þàOð'ø?ø?Áü	þðð'ø?ÁàþàþàOð'ø?ÁüÁü	þð'ø?ø?Áàþðð'øüÁàþ?øü	þàOð'ø?Áàþ?øüÁßLë¯þê¯¾÷½ï/òN8ÑÞÞ~^_GGGøcÃv(üþù×_Ýv(üyæð7íPøç;ßùÙÏ~f;Ü¶Þ÷]ø°¾¾¾;v|C$©TÊV³ÌÁOI$§%I$I?I$Á$IàO$Ið'I$øÓ¯êëë[¸pa<¯¯¯ïêêcº»»ÂôööfßøòåËeYÙzwp7åÙa¯%ÉèÆ¶ÞÝSÙ»)yBTííí-çã9UPÊüé½~>|88q¢ªª*Ì;7Î7/ûÆZ»v­V»)Ï¾H¥Röìiii±õîìÊvJØ#PÓÈÈH0Dy8Sµ§¼HÁÞ·ÚÚÚÂ_Q9#ËËË³¿OªèMw|7åÙÁ£££a N×ÖÖÚhð:wîÎm<¡îl<òÈã?!E'çTAí)/Rð§÷çªð¼EeïééY¿~öð÷ÖòåËãñxccã3glº;¸òì0rÜaÝÁ'TsssxBåÜÒêváÂ°Ùé2¤ÈóÄñ*¨=åEþô¾ÕÞÞ^]]ùöúõë©TjpppÜ_ºtiìaBÝþÝ4î¾È~cY"°Ñîøêïï_´hQBÝþÇ?~ãæ2oùÄñ*¨=åEþô~ù6<mÖ®]ùòå<7öðï¦öE0G:¾qóUuGöÔ®]»vïÞÿÆP·ûUê7ËÿÄñ*¨=åEþô^;wn__ß×O-_¾<tvv«W¯ã³gÏFO¼èÆºS»)Ï¾hmmÝ¿_Ã_Æ¶ÞÝS¡%K<yÒª`mqË'çTAí)/Rð§÷Zooo<ojjþª­­ûgV4ÐÓÓ3þüpã¥K§­wwÓ¸û"ÚM]]]555±X,LØzwvOEG ¢Ër^Æ<¡ã>q<§pOy?I$Á$IàO$Ið'I$ø$IüI$	þ$IàO$Ið'I$ø$IüI$	þ$I$I?I$Á$IàO$Ið'I$ø$I?I$Á$CO?ýô%KÊoÖÔÔôì³ÏþÆï¸Íoäñ¶¶¶6¬Úàà`Îø0&'ÉÑÑÑ©Þ§$ø¤¢lÇecúæ7¿YJøÛ¹sgùäOæâ'Âø~x÷)	þ$©øêíí¬Çã÷î¹Ù¾ûÂ·aäÉ'KçÎ#,X3~þüùaüÙ³gáOüIþó¬yôÑG³G>öØcaä¿øÅlúttt<644áÌ¯]»¶~ýú0©ªªjóæÍÙgW;&yÛÚÚr8ÆÌ=É%Gß¶¶¶æ,Ø#GòßO4),R4©½½"¨-_¾<ïîîÎ9qâD³lÙ²Ì]»vÕÔÔ»*//_½zõ¥KÆâoìýçÉ³¨àOîpsæÌ	p¹páBöÈ/µµµÙ¸É©§§'ºjÕªI6m&>:;Wôm4µ¹¹ytt4ð1H©ákÀÓ¬Y³¢·âå¹00îâ]Óf6ãËdäs'MMMSÅ_EtçÎðó­¬,P,7---×oÂ·+W®¦FÖøÀh&¥R©èaîêêî$û>·oßxu]·n]søðá0¾á5kÖÜò~Ã·áÙË6îPVVVõ½zõjø6|KÄN§£$É0cX¿>M^>UüåYTIð'I¿Ð¸øËp)téÒ¥ðm`SômCCCø¶®®nÃmCCC;	·É9+úöâÅ0eÎü¯aøØ±c·¼Íðí+W²m¢÷çmÙ²%LzüñÇoüúÔöæÍ³o ðHÚØØ*þò,ª$ø¤;_t¸ëúõëÙ#ÃÈ0)w24ìïïü±NæzcÏÆæp*ûVÂpUUU"w>öìÌÔ[ÞÏDË©¯¯/rj_ÃðéÓ§3S»ººÂúxòøË³¨àOî|ÑûÞvïÞ=2ú>2ï¼zõêØZ'OÜ¹sgt6£Æè°¼ê-¶iÓ¦èloøº~ýúÌø<÷i»|ùrôí+Wò_»hÑ¢05út`ÖìIÑ¿aRûÀÀ@~üe`mÌÔ<*	þ$éÎ]0Ç÷ìÙÔËÞ½ÄØ3V® 3<<Í½ç/z«ö#nÞ¼9P)º7óa+ã-ºü6úéñyî'z_ô¿ÁÁÁèyð·oß¾Ì1¹°ÊÙ¢·?>:ü°&Â_ÄÍÀåp³ÎÕ«WgOÍ³¨àO¢èsÚµk×ÿÿw³ÈFáÌ	ÓÌ5Ö­[-Ëì:7nþWa|UUUöá<÷#Á2WÚN´²CCCåååÑ*äì^±bEö¨¬¬_£OÉ¾ÏèÀd¦ª©yUüIR¡ÒÔÔ¸Ù%K¢Knsð×ÞÞ^ýñãÇ3S·mÛVSSiË-ÃÃÃ©mmmÁdÉdò©§Ê¹Ï±Kî*ß¸qcÎøî'&,Rôy>ç/Óúõës>ó%êêÕ«©T*l°gÏÍ|lMöÝFÛjÙ²eÝÝÝ9?1Ï¢J?I$Á$IàO$Ið'I$ø$IüI$	þ$I$I?I$ø$IüI$	þ$I$I?I$Á$IàO$Ið'I¤÷£ÿvM¹]	NIEND®B`


ûïP6*ø@ü þ?Äâñø@ü þÄâñø2ýÊJ¥ªªª,YräÈ)®~îÜ¹+WÎæUýWLæïß¿ÿº Ù¹¥Ç_¾|yÕK.=zôè]78õUÆ?80ÁñcãìÙ³ñ L§Óqéc¶P1äÄYüåÛºuëd÷NüÍ;÷êÕ«ÅO=õÔø;kÇ¶6­U&<ìÛ·oº3ãaÌd2¹Ëmkk_lø;ãæÍû÷ï¯ªª===åaíÚµEçÎKjõë_ÿúÈÈÈèèhLÄÉÙ××7á¦¦»ÊøÇÃ¶mÛbNÙ¬Å_²â7búöíÛ÷Ô]?(µøKìÙ³gÌ.§O^´hQ:njjêèèO¹$qyóæ-[¶¬Àº¹3_¸paåÊÉÉÇ|`` ÿB;;;ÇÉ6×Ç6ÄÏãÇO6&o¾ùæúõëkÍf/]º4æù·7W`ùòå×®];ölccceeåªU«òßeÜµkWmmm²3."·h²+ü¹Ï.æçïë1³½½Âûwº«¿èèÅWrZ3á;sùf¶báø³4îxÄ°?úè£1¶ß#¿tãÔÉèéXT]]½uëÖÁÁAO, þ Èâ/z%fÖÕÕ%'ûúúR©TþKò¹sçÄ_ræuëÖX7wæxmÎ_ÚÚÚÿÕd;øl¶ðãoûÍ73LMMML·ß~;^Ñó·çSiù·wüå.Y²$y'5!+¿1çbEáÄY°`AÌ¿~ýúø;káÂÞ¿Ó]eÌEGÙ$ïü544LkpþWzüõööæo'nìtãoãµk×¹Î[¶lñÄâ,þùeÉt6]]]1ÝÝÝhcVONnß¾tt4y/ç®ëÆkg¼êÇù·£¥ñ¢'cõ[wÄZùU`³Wð¶8q"ÿ°üóoÚ´)¦×¬Ysã1s²ÛÌÙ¼yóÈÈHrÅ-ÇÉü|²íÔ©S1ùòåüE]át:=ÙÉ­û>WìCu¹wF§;8^«¯X8ëóOæ?"ës6õøâÃ8Â+W®ÄtÜ1=gÎO, þ (ã/·§/ùX¾S þÞ~ûíÜ»®-Ù½/¥ÉÉxÍËjÌn»-¼âd·=iÄx]3¿¦¦&ÿÅk|?þd·7Á2ÙÜÀükrûöíÈüÛ^ þrE>Y©¿ÏUò4ÖÁråéÓ§'´©Î×jÆ+N=þGÈµk×ò/bZñ7Åqsss¬¯¯x¿"<«øâ¿Y]]=Ù+îdÊ)ðj] q¦ûò<ÍN6?nrÜÞx	Î?þ³ñ§r2BsÌ®Ï»µZ[[ì§_xc:·©¬2±ðSÉtf+N·ïûtMñaÜßßô_.O<éÄYü<x0ÿä½´1Ç]N¥u¦µnáwn®_¿¿´Àf¯Xà;v,Næ¾%ôÙ÷¨ÆdñÔ$ÿä¢E¯ìì8Jü%oO&ßºÍf·nÝzãÆCÅÌõë×OSYeÆñ7ÝÁÉñS¿©¼óË»ä¯ã_àñ8þüÎ;Í¹#£ñEñò¼6ç¾ê%yInËäàÙ¦¦¦dQò§Á;&|Õ,°nárJª%ùÌVl<ÙÎOtM¸ÙÂ+.µk×æ>×ÌI^³fÍÍ;­mÞ¼ùýÇ_òîW___ÜL%þÎ9¼·7¹»»;yW/qâÄ	ïßé®2­ø+<8cù¦;ª3¿äSzñ`¶[½zõø4Ü»wo~btpáñ¿ë¯@òÙÍþþþ»àñ÷PüÄâøãgsÿÏoò6áøWÍë.§¨¢$ò½Ì--°ÙÂ+.«W¯æ>ÝÌ¹téÒÏÕÔÔä>Pø~â/#ùóçç>Y qüññwÖâÅÜÅÓZeZñWxpÆ<6òMwTgçÏÏßþ²eËòÜ!ç]JÇ[î¢7zbñEñ*¸bÅñÿýWGGGKKKT¼¢<x07ÿÄ1'b+ÿ[î¦¸î]Ë)ùz¼Øxssóø¯ël³w]±pI$»Dóç¿ñÆë×¯¯¼#Í?¼cfñ700[mF=ñÄÑC¹æ.8_þòãÖÅ_°`ÁáÃ?ûÙÏæÞyzÿ«L+þÎÇÆÓÕÄ_8yòdÜêØ~²c=wsDK.åÊ===wçu²ÇÛàààO>¼¥wåðð°'Û·o?þøãÓún¹¬Rtw|Ã·GFF¦_´h?JPò¿1vïÞmd@üP·mÛ¶`ÁdßnL<õÔSÄâñø@ü þ?ñgÄâñø@ü þbwâÄºººt:½bÅé®ÛØØXQQÑßßÓ1§©©iÏhwÌì<SYw&O²È_J¥ªªª²ÙìÀÀG þ¢Q[[)3222ÝuwíÚë>ûì³¹9Ï<óLÌÙ¹sgQÇ_íçÏÓ­­­Eøç	åôÓùóçcÝåËçæ,Y²$æôööÎÎühã/r9¦«ªª<ñSùåÍO>9wîÜ9sæìØ±cÌÙ>ûÙÏ¶´´ÙBîÃ¾~ýzLÇdÑ+WV®mN§»ºº&ÜZþ¥^åðáÃuuuK.b·nÝjkkuãÊoÛ¶-y#3òtÙ²e±µ[~ûí·?¤øã7R©TrFGGãÊÇÉé1?à^ì¿dúé§éFfÅÄîÝ»óÏÓÑÑ1þóm6mEéøÓíííÉ¢Å:u*&._¾ó3Ì[Ë¿W¤;vìXLlØ°aüß²eKLÇî¹ØµkWÌ|ðÁcúæÍ1¼19áù¦Qx1>1ýùÏ>Y´sçÎd(öîÝO=õG þ:þêêêbúöãÛkÂ9s&­Y³&¦×®]T]néùóç·oßÞÒÒ!1áÖÆVáU+6wîÜñëÖÔÔ$W>²,&"ûbf3¦#gßÏàäÏI®[l<Ê/Ê2Y½páÂ¸sæÌihh]øîéøK§Ó¹é5PNV´Nspp0Ù»sEµ¢/¿1[Ë?9U&»bQcùoæ%áxüøñyóæ%sª««7?wþ&ïòw?à¿L&ÿÎ_òæÙ]§­­-æ~ææWVVÆÑ;¦WÉ]±è¹ñëÎ??YwÌÕ9uêÔòß2ÁàÜu(nÞ¼YSSÍf-[A|íÚ5.@ü÷tü%_ÝrèÐ¡ä3Ï<óÌTâïøñã¹·Ð;¤äÅÏN%þ¯²ûöo|ã1±iÓ¦ñënÜ¸1¦9òÚk¯%»zcæÒ¥KcúÂb1ÑÐÐðáÅßÖ­[cÑùóç»»»ó? þ4þFFF"°æÜÿ]ãohh(Ùå?s'O¬®®;wî¶mÛ¦W9út,]²dIî¸üóF~Å5¯¬¬zur`o4_6M^¾|ùt¿züE°æëÍ²eËâd__ þ?Äø@ü þØ~ð·Þzk6/ñoÿöoÿéþÉ#¯<]¿~ýÆÆ¡<ýÃ?üCò1¡ùyóÍ7CÙúÉO~òÏÿüÏâï^ñGôGÑ³ùß^FYyíµ×þæoþÆ8§k×®ýð?4åéöíÛßýîwCÙ:sæLüù'þÄâñøCü?ñøCü!þâOü!þ?Äø?Äâñ'þÄâñøCü?ñøCü!þâOü!þ?Äø?Äâñ'þÄâñøCü?ñøCü!þâOü!þ?ñ'þÄø?ÄâOü?ÄâñøâñøCü!þÄøCü!þ¿ò¿ÞÞÞL&N§»ººòoØ°¡²²òÁÑ?ÄâñWô²Ùì#Gbbß¾­­­ùvíÚµ÷îÑÑÑúúúúññ×ÙÙyk½ôÒKï¼óÎ-ÊÒüãè?ãP~ò¼òÊ+Æ¡<Ý¸q£££Ã8­®®®øóo6/±,â¯ºº:ò.ùëª®®.Qccc¼ÜN¶bÄßÞ½_E§NÁK¥øÓ?þØ0åéÅ_üÎw¾cÊÓ3gN<iÊÖóÏ?ÿ½ïo6/±,â/NO8¼«ªªª¯¯¿páÝ¾ØíÝ¾ØíÝ¾E/Jå¦+++Ç,:tèPL¼ñÆ---âñøCü!þ^MMMüj%¿`1=fQnzÌâñøCü!þR[[ÛáÃc"~f³ÙüE7o>vìXLxqéÒ¥âñøCü!þ^wwwmmm*Êd2===ÿvÛ*þõÖEi­[·.N·´´ô÷÷?Äâñø+_âñøCü!þÄøCü!þ?ñ'þ?ÄâOü?ÄâñøâñøCü!þÄø?ÄâOü?ÄâñøâñøCü!þÄøCü!þ?ñ'þ?ÄâOü?ÄâñøâñøCü!þÄøCü!þ?ñ'þ?ÄâOü?ÄâñøâñøCüÁÁÁoë[Ï>ûlü|÷ÝwÅø?ñ'þÄ%«¿¿ÿ>ñÉEÿ¬ljjºÿþûã	_ü?ñøâOüQFFF~ùù3«?³ãv$ÿ~ã7~ãâOü?ñ'þW_õ?-üO¹òKþ-ðW^âOü?ñ'þ'O>òÈ#câï??ò¿ùÍo?ñ'þÄø?Jð¹ýcûØö/lÏ_Lßÿý¯¾úªøâÏø?JÐ£>ú©æOmûýmQ~OþO>òÈ#+V¬âOü?ñ'þ ÁÁÁßþíß¾ï¾ûzè¡ùóç¯^½úïþîïrKÅøâñø£EðÅ¯ùOúÓ1óÅøâñø£?ñ'þÄâñøâñøCü!þÄøCü!þ?ñ'þ?ÄâOü?ÄâñøâñøCü!þÄøCü!þ?ñ'þ?ÄâOü?ÄâñøâñøCü!þÄøCü!þ?ñ'þÄø?Äø?Äâñ'þÄâñøCü?ñøCü!þâOü!þ?Äø?Äâñ'þÄâñøCü?ñøCü!þâOü!þ?Äø?Äâñ'þÄâñøCü?ñøâOü!þÄøCü!þ?ñ'þ?ÄâOü?ÄâñøâñøCü!þÄøCü!þ?ñ'þ?ÄâOü?ÄâñøâñøCü!þÄøCü!þ?ñ'þ?ÄâOü?ÄâOü?ñ'þÄâñøCü¿éííÍd2étº±±±««kü:;;+**ÄâñøCül6äÈØ·o_kkë¥###ÍÍÍÅßýÙýïYôâ/þÕ_ýÕÿ¦,ýùÿù~ô#ãP.^¼øýïß8§Ë/:uÊ8­^xáÍ7ßÍK,ø«®®Mþºª««³ôé§Þ³gÏdñwèÐ¡³¨³³óÜ¹s(K/½ôÒ~ðãP^yåøÛÏ8§óçÏGü²ÕÑÑñÃþp6/±,â/NO8®ÒÒÒih·/vûb·/vûb·oÄ_*ÊMWVVæ/Z·nÝÙ³gÿõ¦?Äâñø+a­©©_­ä,¦ÿÃ-üÄâñøCü½¶¶¶ÃÇDüÌf³ßTïü!þ?Ä_ikwwwmmm*Êd2===ÖøCü!þ?_ò,þ?ÄâOü?ÄâñøâñøCü!þÄøCü!þ?ñ'þ?ÄøâOü?Äâñ'þÄâñøCü?ñøCü!þâOü!þ?Äø?Äâñ'þÄâñøCü?ñøCü!þâOü!þ?Äø?Äâñ'þÄâñøCü?ñøCü!þâOü?ñ'þ?ñ'þ?ÄâOü?ÄâñøâñøCü!þÄøCü!þ?ñ'þ?ÄâOü?ÄâñøâñøCü!þÄøCü!þ?ñ'þ?ÄâOü?Äâñøâñ'þÄøCü?ñøCü!þâOü!þ?Äø?Äâñ'þÄâñøCü?ñøCü!þXüUÜM*âñøCü¿¿ÔÝ¤Óiñ'þ?Äø³ÛWü?ñøCü!þJ+þ¢o6mÚ$þÄâñø%uuuétÚgþÄâñø£ôã¯©©iüÑÕÕÕâOü!þ?ñWjñWYYµ700P[[Ñ|ßøÆ7b¢½½]ü?Äâñ'þJ-þ·úb"j/&._¾<::sæÌâñøCü¿R¿yóæEêuww÷÷÷ÇÄSO=LøªñøCü!þ(ÁøÛ¶m[îðüý-]ºTü?Äâñ'þJðhß/éKóçÏlii)ûCü!þ?Ä_?Äâñ'þÄâñøCüM®¡¡!ùÂ_ò,þ?Ä%.Ì¾Gû?ÄâñG	Æ_t^dß¹sçFGGîþ?Äâñ7=ÕÕÕÅX~âñøCü!þ¦­··7âoË-·nÝâñøCüQâñ,XP1>Äâñø£ã¯¾¾ÞâñøCüQ.ñd____1ÞâñøCü!þ¦§¦¦ÆâñøCüQ.ñ7)âoÛ¶mÃÃÃâOü!þ?J<þ*&áñøCü!þ(Í/y>Äâñø£4¿ê¥x?ÄâñøºººúúúxUûoFooo&I§Ó]]]ùzzzcQSSSMü!þ?Äßû]UQñ¿Íf9ûöíkmmÍ_´páÂ_~9&=ÚÐÐ þ?ÄâïéêêøÛ¹sg4ÍGõ/ÕÕÕÉEÇ/X]]Ýdg«ªª_ýêWÿ×,záÎ?ÿ¿(Kþñ§q(OQ~ñliÊS__ß©S§CÙô¿páÂl^â÷ÂÑ¾ùLv É¹sçÚÛÛÇÇß±cÇ.Í¢Ó§OÇ½r²åCãP~üã¿ôÒKÆ¡<ýõ_ÿuÄq([¯¿þúl^â÷ÂÑ¾ù¡YYY9þ·nÝÊf³vûb·/vûb·/vû½øÕJ~ÁbzÌÒ«W¯nØ°!yÇ¯(þ?Äâ¯ø´µµ>|8&âg63â«V­pEñøCü!þ3yX¯^½ºªªª¢¢bÎ9ëÖ­å#?º»»kkkS©T&éééù·ÛvçäºººüO"?Äâñø_&<à£(þ«_ñøCü!þÓ³hÑ¢H½5kÖÜºu+NFÙ¬]»6æ,^¼Xü?Äâñ'þJ-þ*++#õFFFòå1gÂ£nÅøÆAü!þÅ©T*R/9Ø61<<sfó«^ÄâñøCüÍRü%»W­Zìö1sÅøCü!þâ¯Ôâ/joÂ>nÜ¸!þÄâñø%øU/CCCëÖ­;wn*«V­9Eq?Äâñø+#âñøCü!þÄøCü!þ¿1kÞM*âñøCü¿¿ÔäÄøCü!þZüMæ'HâïèÑ£âOü!þ?ñW²ñ×ÛÛ;gÎÈ¾+Wæç³ø?Äø3â¯ÔâoýúõÉ~§N*¢ûCü!þ?Äßô<÷ÜsIö­Y³¦èîñøCü!þS544´xñâäØ®®®b¼?ÄâñøCüMÉÞ½7üZ[[÷þ?Äâñ7µ5ÏøCü!þOü¥î&N?ñøCü!þÄÿÞMü?ñøCü!þÄøCü!þ?ñ'þ?ÄâOü?ÄâñøâñøCü!þÄøCü!þ¿,þ*++ÏøCü!þ~ü-0?ø|ÏøCü!þrüEçEö;wntt´èîñøCü!þÓS]]ñWå'þ?ÄâoÚz#þ¶lÙrëÖ-ñ'þ?Ä%aÁã8àCü!þ?J0þêëëð!þ?ÄåIöõõõãý!þ?Äâozjjjð!þ?Äåq"þ¶mÛ6<<,þÄâñø£Äã¯bø?ÄâÒüç	9àCü!þ?Jó«^øCü!þ?ñ'þ?Äâ¯àÃzõêÕUUUsæÌY·n]±ü+þ?Äâoz&<à£(þ?Äâñ7=-Ô[³fMòûFÙ¬]»6æ,^¼Xü?Äâñ'þJ-þ*++#õFFFòå1'æ?ñøCü!þÄ_	~ÕK¤^<²ssc¯z?ÄâÝí»jÕªd·oüéÓÜÜ,þÄâñø¥QðqãÆñ'þ?Äø+Á¯zZ·nÝÜ¹sS©TüjUÌ)ûCü!þ?Ä_?Äâñ7=uuuõõõñª&þÄâñø£ôã/NWTë;âñøCü!þ¦§««+âoçÎÑ4Åò¿º?Äâñøé&&J¥ÄøCü!þâ¯¿äyB¾äYü!þ?J'þêëëúþ?Äâñ7%ÉéûÞÝ¾E±Wü!þ?ÄßÌã/ù/ÿÆWü?ÄâñGÇ_&©(ÈâñøCüQ:ñwîÜ¹yóæ%ïÿ%©çñøCü!þ(ÙøË)Î?Äâñçÿö?Äâñ÷ÑéííÍd2étº±±±«««ð¢Í?Äâñw¯Èf³Gûöµ¶¶^4³9ù¶mÛV[[ûà,[°`Á¥ø;ä0åéãÿø/þâ/òôO|âþûï7e+îýY~é/²ø«®®Nþàø;©®®®ð¢ÍÉ÷ÄOT=þÞxã0þòsÉøE3#þñ÷>ößùæææ7ß|óýo-ÿÛ+++/Ù|ø¸cÇ¯Í¢¸¸?ýÓ?ýei÷îÝöì1åéË_þòÎ;CyúÊW¾òû¿ÿûÆ¡lmß¾ýàÁ³yzüEöåJ3N/_¾üÒ¥K3ÞZMMÍíÛ·½´1]xÑÌæ8à|àð>>¿-ÊU`eeåêÕ«g°¶¶¶ÃÇDüÌf³ÍløCü!þ¿Æèèè©S§ªªª*fúß»uww×ÖÖÆºL¦§§çß®å½×ãÍløCü!þ¿÷«¯¯/ÿ¿¹sç¶··ßû÷øCü!þ¿éÉo¾ªªªÖÖÖ+W®Ëý!þ?Äâo¸sÇ£>zùòå¢»?ÄâñøCüMÏò/âñøCü!þ#þøCü!þ¿i»xñbCCÃ9s#|kjj?.þÄâñø£ã¯³³3wÀGÉô¾ûÄøCü!þâ¯Ôâ¯¶¶6RïâÅ¹øëééI¾íEü?Äâñ'þJðhßäKsñ7::,þÄâñø¥555zÉ»ñòÉ'cº®®Nü?Äâñ'þJ-þâ&ULäå_âñøCü¿<Ú7ÔZZZ£«ªª.]ºT÷øCü!þ¿2"þ?ÄâOü?ÄâñøÈ+WçÎº#&e¯øCü!þ¿iØ¸qcÅ$6mÚ$þÄâñø£tâïøñãIç8p`pp0944tâÄd~GGø?ÄâOüHüe2(¼C_üo¾çOü!þ?J'þ*++£ð&|Ç¢8ø?ÄâOüHüåþW·É&ÿÕø?Äøâ¯Dâ¯@Þ?ñøCü!þâOü?ñ'þ¿¢¿ÂÄøCü!þNü¥î&N?ñøCü!þÄÿÞMü?ñøCü!þÄøCü!þ?ñ'þ?ÄâOü?ÄâñøâñøCü!þÄøCü!þ?ñ'þ(ÊøÙ¿ÿúõëó7óg6¶âñøâÒ¿H½_ù_y¨á¡ÿý/ñoÑ¢EÍÍÍWü!þâOüQñ÷Å/~ñáÞþí;þ`Gò/N~á_0¼âñøâ¿Gyä¿oøï¹òq²éá&Ã+þ?ñ'þ(ÁøûÔ§>Õþÿ¶çÇß¦ßÝôÐC^ñøCü?ñG	ÆßÆþÊÒüø[¶lÙc=fxÅâñ'þÄ%ï¼óÎ'>ñè¿w,]º4N¾õÖ[Wü!þâOüQñÞ÷Ý­[·6=ÜôðÃ···ÿô§?5¶âñøâ?Äâñ'þÄâñøCü?ñøCü!þâOü!þ?Äø?Äâñ'þî±ø÷Ýw÷ïßÿ¿÷O?ýtÄ_*ñøCü!þÄ_ÉÆ_ÿý÷ßÿ©Oê×ý×?½üÓóæÍ;zô¨ß+ñøCü!þÄ_iÆß'?ùÉÕ¿¾:ÿ?uýÙýY_ð+þ?Äø+Áøçwêêê¶aþíµtéÒ¯|å+~µÄâñø¥ùåÿV¬Xñì³ÏúÕ?ÄâOüZüVUUmúÝM¹òÛþíuuu§Oö«%þ?Äø+µøÏ>ûl&yì±Ç¢ü¢~øá_ûµ_ñ«%þ?Äø+ÁøßúÖ·-Zô3?ó3ÿøÇ·mÛ688è÷Jü!þ?ñW²ñøCü!þâOü!þ?Äø?Äâñ'þÄâñøCü?ñøCü!þâOü!þ?Äø?Äâñ'þÄâñøCü?ñøâOü!þÄß¥··7É¤ÓéÆÆÆ®®®üE===ÍÍÍ±¨©©)Î&þ?Äâ¯èe³Ù#GÄÄ¾ûZ[[ó-ðå_£G644?Äâñø+zÕÕÕ£££É/X]]Ýdg«ªªÏ<óLÇ,:yòäw¾óÊÒóÏ?êÔ)ãPâ®_ãP¶¾ýío²5û/ýeétzÂé|çÎkooÑãÿg=v``àÿP.^¼ØßßoÊÓ;ï¼ÏBÆ¡<ýã?þãw¿û]ãP¶¾÷½ïÝ¼ys6/±,â/Jå¦+++ÇáÖ­[ÙlvppÐn_ìöÅn_ìöÅnß¢Tñïbº¦¦&~µ_°sÎ«W¯nØ°!yÇoDü!þ?Ä_ñikk;|øpLÄÏl6;fÄW­Z5000áâñøCü!þOwwwmmm*Êd2===ÿvÛî¼)XWWWGü!þ?Ä_ù?Äâñ'þÄâñøCü?ñøCü!þâOü!þ?Äø?Äâñ'þÄøCü!þâOü!þ?Äø?Äâñ'þÄâñøCü?ñøcBÜ¿ÿ«¯¾*þ?ñ'þ¥lÛ¶m¿ð¿ðéOúWõWï»ï¾?þã??Äø¿Òôµ¯íø[ÿç?ØÿþÇÿñ±¬££Cü!þâOü!þJÐ£>úØc%åüûÌg>ó;¿ó;âñøâñWzè¡M¿»)?þþkÛ]¹r¥øCü!þÄøCü ßú­ßúÌg>Ë/ß¼y³øCü!þÄøCü è­ûî»ï¿î¿%å÷ØcýüÏÿ|¿øCü!þÄøCü¦'OþÜÏý}ý/ýÒ/ÅÄGr´øâOü?ñøcö¼vÇðððGuÄø3âOü?ÄeDü?ã þÄøCü!þ?ñ'þ?ÄâOü?ÄâñøâñøCü!þÄøCü!þ?ñ'þ?ÄâOü?ÄâñøâñøCü!þÄøCü!þ?ñ'þ?ÄâOü?ÄâOü?ñ'þÄßñøCü!þÄøCü!þ?ñ'þ?ÄâOü?ÄâñøâñøCü!þÄøCü!þ?ñ'þ?ÄâOü?ÄâñøâñøCü!þÄøCü!þ?ñ'þ?ÄâOü?ñøCü!þÄøCü!þ?ñ'þ?ÄâOü?ÄâñøâñøCü!þÄøCü!þ?ñ'þ?ÄâOü?ÄâñøâñøCü!þÄøCü!þ?ñ'þ?ÄâOü?ñ'þÄâñ'þÄâñøCü?ñøCü!þâOü!þ?Äø?Äâñ'þÄâñøCü?ñøCü!þâOü!þ?Ä_QèííÍd2étº±±±««kü:;;+**ÄâñøCül6äÈØ·o_kkë¥###ÍÍÍÅß/¾84¾ÿýïÇkÀeé/þâ/^ýuãPÞzë­ãPþþïÿ¾££Ã8­®®®Ù¼Ä²¿êêêÑÑÑä¯«ººº1K~úé=öLò'òâ,zþùç_xá)KñìýòÔÙÙyêÔ)ãPN>òäIãP¶fÿ¥¿,â/NO8®ÒÒÒih·/vûb·/vûb·oÄ_*ÊMWVVæ/Z·nÝÙ³gÿõ¦?Äâñø+Þ¡¬øw1]SS¿ZÉ/XLOx¶ÜÅâñøCü·¶¶¶ÃÇDüÌf³ÅâøâñøCü!þOwwwmmm*Êd2===ÖøCü!þ?_ò,þ?ÄâOü?ÄâñøâñøCü!þÄøCü!þ?ñ'þ?ÄøâOü?Äâñ'þÄâñøCü?ñøCü!þâOü!þ?Äø?Äâñ'þÄâñøCü?ñøCü!þåå_üâ7¿ùÍ·fQ_þå_¾EYêêêú7åéG?úÑ·¿ýmãPâ¯¾¯~õ«Æ¡l;vìõ×_ÍK|÷ÝwÅß¤.^¼¸cÇ?(÷jVx3 |?ñø@ü þ¿uñâÅÅ§ÓéÆÆÆîîîÓÛÛÉd9]]]¨¬îýæææÓÔÔCTV÷~¢³³³¢ÂsoÙÝûÃÃÃ6l¨¬¬|ðÁÏ9cÊíwú¢EÆ?!¿Òwó'bâå_®®®l6äÈØ·o_kk«!*«áÂ1Gmhh0Deuï¨ñW÷þ®]»vïÞ=::P__oÊí?¯ñ³®®Nü¦¦¦ä¿ÿïÝùß?òG³|ïç«ªª22åvï?ýôÓöìexïG¼öÚk¤lQüW¯^øù×¿' ÙëÏ;7î='ÓétnQþ4åpïç;w®½½ÝøÕ½ñ·´´Äß~â¯<ù÷îÝòÅÿO¹=zãÞñ3ÿÅ_¹èìì¬©©T*YYYidÊêÞOÜºu+Í²º÷×­[wöìÙæeùÌèÐ¡xã7âo#SnÅ'oýF.Y²Düä¾xÜ¾û½;»ór¸÷ß»óÿ®]»fLÊíÞ¯øK¹=ó6 |÷ÔN?Ï>º^¼xñ½;y®Zµ*&ÚÚÚ>ñ3Í¢²º÷Ï9§ïýÿûÌ«üÊïÞß¼yó±cÇÞ»sèÒ¥KQ¹=,YÌéëë[¼x±ø+q½½½ù+V¬HÞìéîî®­­M¥RL&¨¬îýºº:ïýí½/þÊùÞ¿yóæºuëbNKKK¿!*·Ào¾|ùKüiñø@ü þ?ñø@ü þ?Äâñø@ü?Äâàñõ¯Ù²eUw¬X±â¹çûÏqwÍ3òD×¶®®.nÚàààù1'Ng2ÑÑÑénEiÇã|éK_*¥øÛ¹sgÌÜ¿ÿù_þòcþSO=5mâ øôööFÖ¤ÓéÜqèÐ¡83Ï?_2ñwùòåÙÔÔ4fþ¢Ebþ¥KÄ þ²ð¹Ï.²f÷îÝù3yæùùÏ>?ººº"¢c:wæ7n´··Ï;7UWWoÝº5ïêéÓ§#°bQ¬ÛÑÑ1&§bÎ¼yó-[vêÔ©8ÙÖÖ6æ<y²ðvEqEÚªU«b~OOOnÎË/¿sV®³k×®ÚÚÚØTUUÕúõë¯^½:>þÆoÌW±D¸r%æÛo¿3ëêêòãfsçÎ%K×®];fÑ-[E©TjÂµÉÒuëÖF>VVVÄÒøñ4gÎä£x¶^½ñ·ôèÑ£ùEëË#GäÊoÌFV¬X1Ýø+pUñðÑKöðNð¼VQ)7­­­·î8¹fÍdiÒ:I>&oàE´%²ÙlòaLwww'ÉßæöíÛ#ï½®7n9'NéøÓ?þø]·á'ãù×mÂ[A9þü¸½q2~Æ5â¼ûvrL&+ÆMxïßwWUUM7þ@üÜñ&¿Üü$nfW¯^MÉÉæææ8Y__¿iÓ¦¶¡¡¡ÜFâ<cÞKË­|ûí·sg`Êíù1úôé»n'B3N^¿~=ÿºMöù¼'x"íÙ³ç½ßµ½uëÖü3DFüE¶´´$oLN7þ@ü|ô·»nÝº?spp0fÆ¢¹KÃþþþ¤ÿr­û Þø½±cr*ÿVbºººº²²rxx86>oÞ¼ÜÒ»ng²ãâÅI§Ætüé¾¾¾ÜÒîîî¸î>zü¸ªøøè%Û»woþÌäPÆðûÀÀÀø7´Î?¿sçÎd'l®·årûUïh[¶lIööÆÏöööÜüÛIÞi»víZròúõëÌ]²dI,M¾Ý&5Qräo,êìì¼yófáøËi2¹¥®* þ>zÉétzß¾ÉW½8p ²²rüÁkÖ¬ÐNöÉæÌM>ó|T®¿¿?ÿÃInÝº5R)97÷e+&ZrømrégÎÉÍ/°äC~Égþs¿CåÞ¿(ùøc___Âdñäfär-¢sýúõùK@üÜ/@c×®]ÿ÷9î¤rÓ¹¦¹c,r6nÜ_ù?^ þÞ»ó_qÄüêêêü=Â¶W#ÿå´ìÆUUU%7aÌÎîÕ«Wç_Äüùóãgòm/ùÛLÞÌIR5·´ÀUÄÀ½"eÅw,[¶,9ävLüuvv&_§×ØØxöìÙÜÒÁÁÁ'|²¶¶6	¦'xbxx8·´£££¥¥%,É<xpÌ6Ç_ØTÌß¼yóùm'Ä«|aïùËiooó/l6#0wîÜ¸.]ÊmMþ6oÞ¼uÕÊ+zzÆ«?Äâñø@ü þ?ñø@ü þ?ÄâÂÿ¡3Ï­l2åIEND®B`
