# Supplementary material for: Regulation of piglet T-cell immune responses by thioredoxin peroxidase from Cysticercus cellulosae excretory-secretory antigens
Source: Front Microbiol. 2022 Nov 18;13:1019810. doi: 10.3389/fmicb.2022.1019810 (PMC9718028; doi:10.3389/fmicb.2022.1019810)
Supplement: Supplementary file 3 [file Data_Sheet_3.ZIP › 4. C. Cellulosae ESAs and TPx Induced Th Subpopulation Differentiation/3. SPSS statistical analysis/5. IL-17/1. IL17-24h/1.3 (SPSS data export) SPSS statistical analysis--IL17--24h.doc]

EXAMINE VARIABLES=Figures BY Variables
  /PLOT BOXPLOT NPPLOT
  /COMPARE GROUPS
  /STATISTICS DESCRIPTIVES
  /CINTERVAL 95
  /MISSING LISTWISE
  /NOTOTAL.


Explore


Notes	
Output Created	12-SEP-2022 23:39:09	
Comments		
Input	Data	E:\桌面\Raw Data\4. C. Cellulosae ESAs and TPx Induced Th Subpopulation Differentiation\3. SPSS statistical analysis\5. IL-17\1. IL17-24h\1.1 SPSS statistical analysis--IL17--24h.sav	
	Active Dataset	DataSet1	
	Filter	<none>	
	Weight	<none>	
	Split File	<none>	
	N of Rows in Working Data File	20	
Missing Value Handling	Definition of Missing	User-defined missing values for dependent variables are treated as missing.	
	Cases Used	Statistics are based on cases with no missing values for any dependent variable or factor used.	
Syntax	EXAMINE VARIABLES=Figures BY Variables
  /PLOT BOXPLOT NPPLOT
  /COMPARE GROUPS
  /STATISTICS DESCRIPTIVES
  /CINTERVAL 95
  /MISSING LISTWISE
  /NOTOTAL.	
Resources	Processor Time	00:00:01.14	
	Elapsed Time	00:00:00.88	


[DataSet1] E:\桌面\Raw Data\4. C. Cellulosae ESAs and TPx Induced Th Subpopulation Differentiation\3. SPSS statistical analysis\5. IL-17\1. IL17-24h\1.1 SPSS statistical analysis--IL17--24h.sav


Variables


Case Processing Summary	
	Variables	Cases	
		Valid	Missing	Total	
		N	Percent	N	Percent	N	Percent	
Figures	Control	4	100.0%	0	0.0%	4	100.0%	
	ESAs	4	100.0%	0	0.0%	4	100.0%	
	TPx	4	100.0%	0	0.0%	4	100.0%	
	LPS	4	100.0%	0	0.0%	4	100.0%	


Descriptives	
	Variables	Statistic	Std. Error	
Figures	Control	Mean	9.16675	.133853	
		95% Confidence Interval for Mean	Lower Bound	8.74077		
			Upper Bound	9.59273		
		5% Trimmed Mean	9.17072		
		Median	9.20250		
		Variance	.072		
		Std. Deviation	.267707		
		Minimum	8.809		
		Maximum	9.453		
		Range	.644		
		Interquartile Range	.501		
		Skewness	-.763	1.014	
		Kurtosis	1.505	2.619	
	ESAs	Mean	8.71325	.110816	
		95% Confidence Interval for Mean	Lower Bound	8.36058		
			Upper Bound	9.06592		
		5% Trimmed Mean	8.70661		
		Median	8.65350		
		Variance	.049		
		Std. Deviation	.221632		
		Minimum	8.522		
		Maximum	9.024		
		Range	.502		
		Interquartile Range	.406		
		Skewness	1.305	1.014	
		Kurtosis	1.510	2.619	
	TPx	Mean	3.97800	.206049	
		95% Confidence Interval for Mean	Lower Bound	3.32226		
			Upper Bound	4.63374		
		5% Trimmed Mean	3.97533		
		Median	3.95400		
		Variance	.170		
		Std. Deviation	.412098		
		Minimum	3.499		
		Maximum	4.505		
		Range	1.006		
		Interquartile Range	.766		
		Skewness	.346	1.014	
		Kurtosis	1.500	2.619	
	LPS	Mean	28.46550	.786725	
		95% Confidence Interval for Mean	Lower Bound	25.96179		
			Upper Bound	30.96921		
		5% Trimmed Mean	28.44983		
		Median	28.32450		
		Variance	2.476		
		Std. Deviation	1.573450		
		Minimum	26.695		
		Maximum	30.518		
		Range	3.823		
		Interquartile Range	2.938		
		Skewness	.526	1.014	
		Kurtosis	1.499	2.619	


Tests of Normality	
	Variables	Kolmogorov-Smirnova	Shapiro-Wilk	
		Statistic	df	Sig.	Statistic	df	Sig.	
Figures	Control	.250	4	.	.960	4	.781	
	ESAs	.250	4	.	.903	4	.446	
	TPx	.250	4	.	.960	4	.779	
	LPS	.250	4	.	.963	4	.798	

a. Lilliefors Significance Correction	


Figures


Normal Q-Q Plots


999¦q¢fðþüø!øØíÞ½Ö¬YqÇsp`øþü?ÊüÉ)îçàõþü?ðQøkooË>ÙÚÚÊxðþXàÀø¿`0xêÔ©=ûV¯^ÍxðGàüøà/Cð×××·ÿ~ýxY³f544ðùàÀø#ðþÀ_àÏçói¦ÏQRRâp88°üøþÀøËü%ú|¶¶6ìþÀ?ðþ2ÆsÔÖÖÊ³ö?àÀø¿ýìgÍÍÍúÏçÈÉÉÙ´iÓò/?ðGàÀø£©Éï÷755éÇsÈf``Uþü?àüeBgÛ¶múñåååï¾ûn0d?àÀøòUWW§?°¯¦¦Æårq`ø#ðþü?ð	êNç¢EôìûÖ·¾uõêUVø#ðþü?ð	ÃëOÔÜØØxåÊt?àÀøßïÞéÇs[ZZÔÇôÙ¾þü?àü1Çc·Ûõã9,Ë©S§¢ìàÀø#ðþÀ_çv»­V«þó9jkk[[[õã9Àø#ðþü?ð~AË÷DÍ¦õôô$º"øþÀ?ðþÒ©¾¾¾êì92ßï÷'¿:øþÀ?ðþÒ#ÇÓÔÔ¤?°¯¤¤¤¹¹yÏþÀ?ðGàü?£×ÑÑ±nÝ:ý555qìàÀ?ðþÀ_ú%ªs¹«5Æ|¢@±àÕ«WÇñùàüøþÀø3`PîA³Ù¬g¦i^¯wÜ·þÀ?ðGàü?å÷ûwïÞ­ÏQPP ÷éSÇs?ðGàÀøà/m^K6lØ ?°Ïb±´´´Á	ù-àüøþÀøÊÂá°<ôm6Û]-Æq`øþü?ðþW(jiiÑ¨YZ·nÝ$ÝàüøþÀøKuáp8îxY³f%ÿ|ðþüøàü¥e1ûyä.åÁø#ðGàü?ð~÷Èçs8ÎÏþÀ?àü?#Ö­[×ÖÖ6±ã9Àø#ðGàü?ðGàüøþÀøþü?àü?þÀ?ðþÀ?àÀøàÀø#ðGàüø#ðþüøþü?þÀ?àÀøàÀø#ðGàü?ðGàüø#ðþÀø#ðþüøàüøþÀ?ðþÀ?àÀøàÀ?ðGàü?ðGàÀø#ðþÀø#ðþüøàüøþü?þÀ?àÀ?ðGàü?ðGàü?ðGàü?ðGàüø#ðþÀø#ðþüøàüøþÀ?ðþÀøþÀ?ðþÀ?àÀøàÀ?ðGàü?ðGàü?ðGàü?ðGàüø#ðþüøþü?þÀ?ðþÀ?àÀøñêêêªªª2L.Ô?¾Á?ðGàü±À_Fá¯´´ôÊ+2qòäÉ²²2=þ>þøãáÔ&O3Ï7L®··WþÛÀz0x¿`0Èz0rò¨Ï?ÿõ`ä¾øâk×®±àï×¿þuiã/ºÜÜþ~ô£Ú>úè£ÿøÇ»xñâXïÜ¹s¬ÇæÍ¥ïæ.ð×ÝÝÝÐÐÀn_b·/»Ý¾Än_vûfòn_ÕÐÐP0þÀ?à/Óð7ãËÔ·÷îÝÓ4M^Îõ?	þü?àõþÒÑutt,_¾¼¿¿?î¥àÀø#ðþXà/£ðg6gDþü?þÀ_&ã/yàÀø#ðþXàü?ðþÀ?ðGàü?ðGàÀø#ðþÀø#ðGàüøàüøàüøàüøþü?þÀ?àÀ?ðGàü?ðGàÀø#ðþÀø#ðþüøàüøþü?ðþü?àü¿é¿¿ù¿immíMmôÑµk×zÉÀòÉ'çÎc=¼¿û»¿»û6ëÁÈÉæ®»»õ`äD~ÖÁ;vìØ_|â_2·nÝÚ»wï(ªä»Fgð~,Ñô	ü?""""DDDDþüø#""""ðg¸:::***L&SeeegggôE¡PHÓ´ìììù1Ö1ï¦$Q*ûôÓO«««åù²~ýúÁÁÁè®_¿^\¬î£K.±®xuuuUUUÉ´páB>LÂ°wêâÅ3fð²nÐûÈPràQ°;wîÈ|5ÍÑ8pàÍ7ß|ôèÜóçÏg]ónJr¥²Å d¢§§gË-ÑÕ××8qB&9²qãFÖï£ÒÒÒ+W®ÈÄÉ'ËÊÊXWÆ¼¤p8,L½%%	ûæÞ½2!_cî§ÊÊJ¹_YE¿©Ìd2E¦gÏtõ	¿###Ý÷Qt¹¹¹¬+ÃÞMo¼ñÆÛo¿þJ<JvýúuÙÒÉI¾vwwÇÜ»ùB7o²®y7%¹RÙÂoÝº%ï½÷^ô1fCsä>$O¢Ö1ï¦;wîTWWË¥À7wÆ-^¼X!]±dÉè²²²;öøË½û¬+cÞMI.¢T&¹²²2Ùä½ýöÛ1oÉS)2Íº2à¤ª¯¯¬+cÞMk×®½|ùò¿¨?oî#%	KòDaa!oWÿnâ]%£õÙgUTTÄ<FFF?Ùíý´"ãÜG8¡iÚýû÷Y?½füßXEÆÜÜç%HÂ,Y¢Þ¼õx</¾hË-ï¿ÿ¾LÈX­VÖ1ï¦$Q*+--½~ýú£Gä¿Â¯¿þzôEv»ýøñã2!_ëëëYW¼:::/_ÞßßÏZ2òÝ­@V1ï#CÉGI2¶çòU¦£Wk×®ª««½^/ëÊwSÜ(õæÍÝÐÐ£ï£ÎÎÎ¢¢¢¬¬¬ââb5DvÍfÞR2þÝþJ<J¦Qàüø#""""ðGDDDDàÀ?""""DDDDþüø#""""ðGDDDþüø#""""ðGDDDDàÀ?"î½÷ÞK.ÍÒ²eËÎ9ó¶_OJ­m¼¥5Íò§Áù2Çd2?zôh¬·IDþ(-Û»wï]¯¿þz&áoÿþý2³¥¥%fþáÃeþk¯½6Û$"D~]¿~]Xc2=~Ò±cÇä[yãÆÁßíÛ·eæÂcæWTTÈ|ÏþüÑ´èå_Ö¼ùæÑ3ßzë-¹iÓ¦hútIð$.¬ªªéÈ?|ø°¡¡!??_.*((Ø¾ôÞU·Û-Àäºííí19³gÏ^ºtéä[»Ý³`çÏO~;ê"Y$uÑÅAmùòå2¿««+2çÊ+2Çf³Eæ8p ¨¨Hn*77wýúõ÷îÝÓãOû1s,*?"¢)nÞ¼y;wîDÏ¼÷®Ì4ÍÑ¸©»»[]ºfÍ¶nÝª.òx<YYYq¯¥¾U®]»öÑ£GÂÇìììp8,ÊWÁS^^:/ÉíÈDÜÅÓÿ¥'OmÄ'NÈ/æF-[6Vü%YT"DDSÚÃg5cP,77nzLÈ·«V­R*ë(>ª7ðmê¢úúzõ¡Lwvvª¾Í=öïÔ^×W_Uæ=V¦å«L¿òÊ+O½£|+?½lqÿ"åÜ¹såïíïïoå«,¹sddDý@qq±þÇ_î&ÎÍÍ+þ,*?""CàO¿È|e&éÞ½ò­°I[UU%ßÎ?óæÍ¶áááÈÈÏÄ¼¹úöîÝ»0EöüÊWv»ÝO½¦|ûàÁèeKt|Þ;ä¢·ß~ûñ»¶·oßýAÁ´ººZ½19Vü%YT"DDSz»khh(zf0rQîDhèõzÿ"Ö¨§ßÃ©è¬ÈtAAAvvv(=väÒ§ÞN"ÅtëÖ-åT¯2íñx"vvvÊÄÝ<zü%YT"DDS:îíÐ¡CÑ3Õ	Pb|Dìïï×¿¡uãÆýû÷«°5ª·å"ûUJ´­[·ª½½òµ¡¡!2?Éí¨wÚîß¿¯¾ðàAò¹K,KÕÙmÄ¬Ñ©¿rÑÅã/Sµ6"&YT"DDS0a29¢NõrôèÑììlýàU«V	tB¡Ú'«ùSÊy½Þè·oß.TRãp#'[K45üVýöÈü$·£òSÇüAõIðwìØ±Èrò'G_¤ôx<ò[d%$Ââ¦pY~LÐ¹~ýúèK,*?""C¤NÓþwûõ$e£ÈtdidE¤W_5ZÑ>:	þ?ù(_PP½G8ÉíÈbD/Xd¤m¢?vxx877Wý	1;»W®ý+æÎ+_ÕÙ^¢oS½1IQ5riE%"ðGDd(Ë-Ë~ÒÒ¥KÕÛü]¼xQN¯²²òòåËKÁà®]»vìØ"¶··WWWÉNgÌmêDnJæoÙ²%f~¢ÛdadÔ	ç/RCCCÌ9_Týýýõõõ²òóóå¯ðù|ÓÖDßæàà èV­+ÍÖÕÕó,*?""""DDDDþüø#""""ðGDDDDàÀ?""""DDDDþÀ?""""DDDDþüø#"""¢Tõÿ¤TH,oÜIEND®B`


xùòå®Ý7lØpëñ½úÕÖÖäÅ_Hü8p zm)ôâ¡C¢Wõ"o¿ývÊßq°gTü¥ß9Ñì¾®Ïv¯ÞDüEïÒíåÊÐv÷Þor>ÿüóaç@~ÿG[Ò9ü|Øayþüù¿fôÞÍööö4Cðø2.þÎ=wW´¦£££Ïû½JJJâ½øçHdêÔ©ákâ'^SnóC=<yÇw¤ù5uAÅ_ú«¹ÏDÍ7±Wo"þ?xùwßwâ©öIÈµôñMò0Ñk«~øa7.@vÄ_&®ÿè£V­ZUp]MMMòÇ;n.þ..-È G4ôP|ô³sçÎ¹sçæååM>ýW^ùú×¿åéÖÏ2¨øK¿sÞ~ûíÐùùùñ©òj¯ÞDü---3gÎ¿u¸üh`=þçðmH´è¯ªª:räÈ_yÝ»wï¢EÂ¥_j×®]ñõÝÝÝ?þxôj(øð§ìééqãñ0zè¡AÍ-wgÉ:ÑhlôñíX,5ý9sñ¢÷üõñì³ÏÚ3øÈAÝÝÝ=öØôéÓ£±Ý°ðÄOØ-ø@ü þÄâñø@ü þ?Äâñ þëí·ß.++ËÏÏ_ºté`Ï;wîÜqãÆµ··Ç×å°fþüù¸»îæ~f ç½ÉûÖ$Ñúîîî7=6iÒ¤?þ8~®X,Ö.((Ë®]ø2Niii.ÜD¬<ùäá¼;vì¯ùÎw¾Ölß¾=7â/y]]]X¿ÿþ°|ðàÁ°<kÖ¬ø©---ñX|çwñdÞÈ-ôÓñãÇÃy,Y_s×]w5Çùø+((ë/]ºòÖ¬	§F_~øa×.@üX~¡óøãO4iÂ	Û¶mëóc_ÿú×-ZÔçâ/åóçÏå°&:éÓO?­ªª***ÊÏÏ_°`AôjYò¥%þïéÏòÊ+¯UVVÿ(9Ñ._¾[Î6þ±Ç^Èyz÷ÝwKëÃ%9sæãoÆaý9s:Ôç¤«W¯N<9ü_W®_§NOäÛñWÿEËO?ýtXÞµkWÈ¬°ðì³Ï&þÌÞ½£ÈK´nÝºpÒK/½Ã×°¼víÚè¤;î¸#úüäOÂúiÓ¦¥¼´ÄHto¾ùfX¨««KÞø7åðo½õVXxòÉ'ã­véÒ¥¶¶¶°½0ÙßûùúËâÄyûí·ãk.]úáÆÏ/_¾<,¯Zµ*,5)·Áµ@FÄ_YYYXî½.¹½R¾)ðÀñâY¹reTuñS?¾uëÖEõyyy)/­O~¥?K´a&MJ>oIII´ñW¯^!¹ÂÊðayÁ!gzznn·ôqôèÑ©ñ­ß´iSüÛ(RôÑ[ÙñoüET¯ßÞ°BiM0!üdwww4²ÖD'½øâá?üðÃÄKèsißä,ýmX(ÅÄê¢pÜ½÷äÉ£5ÅÅÅÑå/Í~Û¿è¹ÄaîÎá?ýüóÏ¯]ËÓ§OO¹®uø2"þ¦MøÊ_ôâÙK¨¶¶6ÿ_6âêu¿ôgoXh©äóN:5:oÍÅbï¼óNô)ÝøKÚ-ý6&lsX×jÇ¿émÀðÆ_4uËK/½½çï;ßùÎ@Jh÷îÝñÖyóÍ7ãë£<yòdô^ÀÄ_ú³lÝºõûßÿ~XX·n]òy~øá°üê«¯:u*f++++Ãr(³Ï>û,,Ì=ûãoþüùaý¾û®ýzbzèZÿ³Þõ7½âÞøÅb!°&8W_úø»råJ4ä¾&ÎÚ¨¸¸xÒ¤I=öØã/ýYZ[[Ã©wÝuWüs'¦_Þ´iSØòï½7úPmè­èãÃK,ùè£µ[ÃÅÖÖÖ-¿ìÔ©S7nÜþßk¿ï:ñòãó]ßô6?ñø@ü þ¿¡ñw÷w§Oáÿô_þå_þýßÿÝõ¡õ_ÿõ_¿üå/íÜ3gþã?þÃ~`hýçþçÈ?þ"þ~åOÿôOCÿðúáþó?ÿ³ëC+<<8pÀ~`ÈðÁöýÀÐú·û·CÙâOüøCü!þâÄâñø þ?ÄøCüøCü!þÄâÄâñ'þ þ?ñøñøCü?ÄâÄâOü!þ þÄø?Ä?ñ'þÄâñøâOüøCü!þâÄâñø þ?ÄøCüÙ?Äc=þ9²`Áüüüùóç;vLü!þ@ü!þÈåø5kÖûï¿^ýõÙ³g'Çß¾û.¬pOú_üâ2©ðð¼ÿ~û!wøðá_þòöCëÌ3û·k?d²,¿DEEEÉñ÷üóÏÿxdíÝ»·µµõÇ0¤~ô£ýÍßüýÀ÷ÝwøÃÚ­ðdõwÞ±2Y.ÄßÑ£G×®]kØÃ¾`ØÃ¾äò°oäòåË555ÝÝÝâñâñGÇßÙ³gëêêRÞy?Ä?Ä9ááðî¹páBÊSÅâÄâ¿²²²q	ÄâÄâ¿ôÄâÄâñ'þ þ?ñøCüøCü?ÄâÄøâñøñ'þÄøCü!þâOü??ÄâOüøCü!þâÄâñø?ûñøCü?Ä?ÄâOü!þ@ü!þâñâñø?Ä?ÄøCü!þ@ü?ñ'þ?âOü?ÄâÄø þ?ÄøñøCü!þÄ?Äâñ'þâñøCü?Ä?ÄâOü!þ@ü!þâñâñø?ÄýøCü?ÄâÄøâñøñ ½½ýÔ©SâOü?ÄâñãZZZª««Ç¿bÅñ'þÄâñøËMÝÝÝóæÍ÷k¡ÿ:::ÄøñøCüåPxë×¯8qâ¸ÿnÊ)­­­âOüøCü!þrDÈh·Oö-pçÎ]]]9öâñø±===MMMã,Y²$Ü-Çb±ü?ñøCüÁØ¿ÎÎÎPS¦LéÓ|'Nvm¸áäö_Pü?ÄâÆJüE]]]òoYYYèÜáâñø±±X¬¹¹¹ªª*y·²²²©©)WGxÅøCü!þ`lÅ_WWWCCCyyyæ?~|mmíÈø þ¿aMÝüÆ¾°¦¾¾>Ç¦îâñâ±ýMÝR^^¾k×®1òÆ>ñ'þ?ÈñøK3uKUUUkkëzcø?Äälüõ7uKaaa]]]»¿ø?ÄäBüºEü?Ä?r?þLÝ"þÄâÄc"þLÝ"þÄâÄc"þÚÛÛMÝ"þÄâÄ9±X¬µµ5å¯©[ÄøCüø#wâ/T]h»ä^S·?ñøñGNÅ_GGG½©[ÄøCüø#Çã¯¿smß¾Ý¯ø?äBü¥98Ç%Kð?ñøñG.Ä_çV¬XqâÄ	;?Ä?r!þú;8GÁð¢Ð®âñâ¬¿X,ÖÔÔTYYüR_EEÅË/¿ÜÝÝm'?ñøñGÖÇ_WWWxì.++K9Âøðaoìâñâ¿öööºººÂÂBçâÄâ¿X,ÖÜÜÜßÁ9ð?ñøñGÄ_WWWCCCÊsTWW755áâñ'þäBüutt¬_¿>åÁ9Ö®]kêñ'þ@ü!þÈøëïàååå¦nâÄâ¿è)§n	+ßxã#¼âOüøCüñwñâÅÆÆÆä©[Æ_[[ÛÖÖf_?ñâñG.Äß÷¾÷½GydâÄ²/`xt>wî½$þÄ?ÄY/µ´´|ík_ËËËKáõ^ñ'þ@ü!þÈ===óæÍK9Â;òÇ?ñøñÇ°èììÜ²eKii©s?ñâñG.²µµµÉS·üîïþî·¾õ­®®.»Hü??²^OOÏo¼QQQ<uKUUUkkë¿þë¿öØ¾?ñâñGÆ9wîòÔ-uuuíííÑüØ¾?ñâñG&jkk[»vmS·ôáâOüøCü­«ªªR£¿©[Äø þdîîîÆÆÆ3fÜÄÔ-âOü??²FGGÇæÍGx>uøâÄâ,4«««§n)//ßµk×À§nâOüøCü¹zzzÒLÝ2Øc²?ñ'þ@ü!þÈDá±rÊ)é§n,ñ'þÄ?Ä÷§y<Ârêñ'þÄøCü!þÈJ±Xì&¦nâOü?Äâ,ÓÕÕÕÐÐP^^~S·?ñ'þÄâñGÖèèèX¿~òû>uøâOü!þd¡ºEü?ñ'þ?2×OÝ"þÄø?Äh¦nâOü?ÄâûSßÔ-âOü?ñøCüFfêñ'þÄøCü!þe#9uøâOü!þºEü?ñ'þ?FA¸©[Äøâñøcäôôô¶[¼xñhMÝ"þÄø?Ä#áôéÓÏ<óÌ3Fwêñ'þÄøCü!þ^'NÈ©[ÄøË,.·ñøñb±Ø=*++Gx,Y2ZS·?ñAÂukÎ9á&Ñ_üýÕ_ýÕg#ëðáÃ'OüÔ'|ÒÚÚj?0äÂ3ä>úÈ~È?ÿùÏ¿ýíoÿÎïüNæ+((øÆ7¾þeÑïòOÿôO?úÑüM3YÇß½÷ÞÛÞÞ&þ^|ñÅFVx>xðà0¤Âwß×~`Èýà?ýg?®×_ùòå¿õ[¿Õ'û¾ð/lØ°á½÷ÞËºßèïÿþï³q³ÇìöýÕ&ö1ì3v·¿s,^¼8[FxûöâñøãÎ;×ÐÐPQQòà'NÈö_Pü?ñâñÇ¯>úGI>8GqqqCü!þÄâñÇ7áeË%OÝRQQñòË/gøÔ-âOü?ñøCü1 ¡êBÛ'ðVWW©ìcøHü!þ@üööö-[¶$ð5ë×¯?uêTÿîâOü?¿1¤µµõ(,,ì3fÌØ¹sgð?ñ÷ßÏy#yyyâÄâ/7ôôô455Í7/ùñnÙ²e---áÆÈ®c7þòn$??_üøCüe»ÎÎÎð<Â[XXX[[ÛÖÖ6Övø»ñ7êÄâÄßpïÀºººäÏðÇ /ÍÝ"þÄ_j.]Z·nøñøË:iÎQYYÙÔÔÔÝÝ=÷ÏPÅ_¨ç'NwIæfüç@ùùùÞóâñÕB444¤º¥¶¶vätr5þÎ;÷Õ¯~µ¤¤dNÅ¢¢¢6ää8¹óçÏO~nT\<ÜOÄâÄßPéèèX¿~Ê©[réà!B^õî¯nÝ²uÛ··sÓ7gÍõÍo~ÓÍ¦ø+((7.ðGýþ÷¿Ö®]+þ@ü!þ2(©®®N~c_]»vòøÛ³gÏí·ß²/þoÝÿY7qâD/þeSüE7°j/,|òÉ'W¯^&L þ)º¥¢¢"yðªªªªµµUSüíÜ¹³êkUñþ;wÎ¾Íø<yr¸©ëAXxâ'¢S½øCüe 4S·ÔÕÕ0»hXãoÏ=óæÍK,¿G6>ZBmgSü=öØcñwôùHøñøË¨fê#¼#ÝÝÝ_úÒªï­Êoë­,~½Mñ<õÔSS§NG	!-Z4Ü.þ þâS·xÍi$ãïÚõ##ùË_¾ÖíwÞygiiiuuõØ9>JîÄß¨?éº%3ã/*òpýÜ³gÏ'Ä·ø?·ÊÔ-düÍ=;ðÅ$Ï þ£ËÔ-âa¿Y³f%_OûøCüîîîÐv¦nDüÎ7­£G^½zu$7]ü!þ@ü]3uøcäã¯¸¸8ÜÆF¸üÄâÄ_økkkCäºEü1¢ñwìØ±pKÛ¸qãåËÅ?ÄßpÅb---+V¬H~c©[Ä#ÁôéÓßcá þC«»»çÎ3fÌH~Ð©®®v;PüÍ9Ó>@ü!þU[[Û#<üÆ¾'õ¦nhüE7¿p³áM?ñwøðá%K$¿ÔW^^ÞØØèâQ¿øñøZ===»víZ¸parö-[¶lÏ=ÞØ'þµøZá¦øØcðùÄâr2þ:;;yæä7ö®Y³æÔ©Sþ²âQ¿qýð¿A9|øp]]]ògxMÝ"þÈ øËë|øCüDOOOSSSUUUòë/niiá%ÄøËPâñÙ]]]eeeÉÙwÿý÷í÷GdbüíÌ3GþMâñÙ§OÞ²eËÄû4ß)S¶oßîlâ¿üüüpsùM?ÈÆøwÝÕÕÕÉoì«¨¨0uø#;âoÿþýáF¨]ºti$'|?È¢øU·k×®òòòçhnn6uø#kâÏ§Aü!þÒèèè¨¯¯Oyp5kÖ8qÂ_JüeñçÓ¾ þ)éÚÚÚS·lÙ²åÜ¹sþFâ¬¿Ñ"þñMÝRYY<(´páÂp©[ÄâOü!þÄ¹áÎ9ywüøñuuu¦näNüõööÞï½EEEá>aÂûî»o>ù!þ9ñþ¯ç!î®Cús?r'þ®òÃýª¾øCüÁ¨Ç_,knnNyp#¼âÜ¿9sæùòåË/_¾¾½téÒÊ+Ã;î¸Cüø#Wã¯«««¡¡!yêñãÇ×ÖÖüý3â¿pkO©··7¬	ëÅ?r/þÚÛÛëêêGxëëë;::ìyñGÇ_^^^¸Íà¯ééé	kLõâ¿ð$¿µµ5åoyyù®]»Cü1Vâ/ö½ç¢aßð5,5, þÈøKspÐ¡Cü1¶â/Ô^Ê||þùçâÄYë×¯Oº%¬	ëÛÛÛídñÇX¿k×?ðß÷M4)///|½çÂáÞtñøá¿p[]]<uKyyyCC^ñg?õøâñCÑÁ9***Rð677áEü?ñâ¿þÎQXXèà?ñwý7'þ@üùñwâÄç(++÷´FxâïÿÉëøñGæÇ_tpÅ'?¯¬¬ljj2Âøòè£F÷¯¿þºøñGÆ_WW×Î;gÌáà?ñwK;6aÂèMÁs>?dHüEç8qbì+..~äGñ'þaÕªUÑ=È;ï¼32.þ0ðøkmm]¶lYòoEEÅÎ;»»»í.Äø¨·Þz+ºY¾|ùHnºøCüÁãïôéÓ)§nY²dIKK7ö!þÄß rå;î>Û±ÿþÞtñø4:;;¿ñoÜvÛm)§n1ÂøöüóÏG÷#«W¯M?Hé>¸ÿþûSNÝ²eËsçÎÙE?ñwSç4Ï?2I4uËÂïÃÊ7Þx£§§Ç^Bü1,óüEòóóÅ?F@ggç3Ï<SVVÕÕÕmmmvâ!¿Q'þðÁ¤º%¬Ù¼yóï½7ØcûøâOü!þÈ8±XlÏ=ýMÝÒÔÔmPÇöñ'þÄøCüÙÚ.åûV¬Xî§n?ÄøCü­:::êëëKKKÉ¶víÚoì?ÄøCüÂ]mmmòÔ-3fÌH?uøCü!þÄâ¬ðVVV&ð.^¼8tÃs?ÄCæùñÇ0éììwqÉS·?>ùâñÇÅ_â~)ãÏ< þFKWW×'BBeÝGS·$ðN2%Üéö7?2þâöíÛî|ðÁË/oÃ×+W5 þFX,[·nÝm·ÝVQQñ/|áÎ;ï¼xñbVlvsssUUUS·ÜÜÁ9Äâa¿âââp÷8ÑÛÛÖ?#ì(ÿ½òÍnÞöím[·lý?øùóæptTtuu544'ðVWWßâ½øCü1,ñÝOàëÞóâo]¼xñWG¶¸^~Ñ¿Ð_,ùbh ÜÚöööõë×O2%y·¾¾¾££ãÖÿñøcXâ¯¤¤$Ü[ÕÔÔr%|ÛÝÝ½|ùò°&¬ þFR[[Û9ñòþÝ¹øÎæææÌÙÈX,ÖÚÚr·¼¼|×®]ÑÁ9øCü1,ñwðàÁø$þ@ü¤/N:õ±Í%ÆßôéÓ3ä¿Pu¡íGxÐ¡|xZü!þø>ùäY³fåååM0aÁçÏîM?Ýÿý,ÜºekT~ÿãÿ1gÎû´ÄÎ<Â[XXXWW×ÞÞ>Lÿ¯øCü17*ÄâdÝÝÝË-+--½óÎ;gÎùå/ùôéÓ£¸=ánªºº:yêòòò!á?ÄøCü]mmmÍÍÍ-8*ÐÓÓÓÔÔTQQr7lÛÈ|Yü!þ®ø;yòäìÙ³'LÂ·¤¤d÷îÝâÄß#ywüøñuuu#üîCñøcXâ/ä9ñnÑò/¼ þ@üý£¬¬ì&Î!þdnü·'OÆãïÈ#ayÒ¤IâÄ_ÎKspÊÊÊ¦¦¦QbZü!þøîã¢(þ®^½êØ¾ þr^sÔÖÖüøCü1BñMò½Úâ¯··÷ñÇF:Ä¿ÔÑÑ1Üç?27þÂVÊIßÿñâ/Çô7uË¼yóvîÜ9ê?Ä#A¸sY´hQôiß¢¢¢Ù³gÀ_ñøcÄ¤º¥¥¥eßØ'þBüñøc¤º¥­­-7^ü!þøÎ#îüùó³gÏ1cøñ½úº%Ü¹544ÊÔ-âñGÆ_oo¯OûøËRé§n	'ÖQCÄâQ¿ðÜw'O þ²HS·2uøCü1ñ÷é§æ]?¶G¢P~o½õøñúº¥¸¸xóæÍ§OÎÒßKü!þÊø©7Ü#¼âñÇ0éoê3fìÚµ«««+«;ñøcXâo´?Ä7-ÍÔ-Ë-Û³gOfNÝ"þdDüõööÎ=Â	ñ5S§Núé§Å¿ÔßÔ-µµµ>uøCüñ7úô>øîI·oß.þ@üevkÖ¬Iá-++Û±cÇ¹sçrïW?%þÂ½gâÓåcÇ5&M þF]OOÏÞ½-[/nnnÎ^ñøcäâ/úÀïÕ«WÿÛå&MþwBDN6-??îÜ¹û÷ï?åâÅÏ<óLqqqòÁ9î¿ÿþÃçpö?ÄÃ¡ÏÂé¦M¢#_¹reÛ¶maMiié-^rMMÍ«¯¾^xáÕ«W?ÄÔÞÞ¾eË'öÉ¾)S¦õ#pðqñø#ãïèÑ£)'y>räÈ-^rx¾½ ØÛÛ[VVßûÞ÷~9²Bn8qâ0¤>úè£ûöÙC"<cüÊW¾|ôå/ùé§þÇüÇ1µ7~üãµ_ð³ýì?ü¡ýÉ=þ³gÏÎ;·¨¨(//oÂ	sæÌ	knýb§LJ0Äßw¿ûÝ#ëÀ!jOÂÏ(Þï=ûáVæ·¾õ­çX¼xñ_þå_»eÿþýáù¹«Cë§?ýé~ðû!DüÄwöÅ°/É.^¼ØßÔ-uuuííícyçöÅ°/Y%%%½½½Ñ°oX?úÍ<"/yêpÿíç?2:þN<Íó½VBm÷îÝ·~±µµµ¯¼òJX_kjjÄâk¿º¥ªª*y·²²²©©)ç?Ã+þrüíÛ·/~ÏÅ_´üÂ/Üâ%ëViii¸ÌiÓ¦%|Dü!þÆ®®®²²²ä©[ÂsÅ¿C?Æhü>w¾'OÇ_5<øBmmmk×®M9uKýØºEü!þÈøî¯%Lì|õêÕ°üùâo°>üÀ$£¼¼|×®]ÞØ'þBüD³úEñ×ÛÛûøãGï¹ þnNOOÏË/¿rêªªªÖÖVoì?F-þÂVÊIßÿñâo°N>]__½ÄÔ-âñG&Æ_î-ZÚ·¨¨höìÙ#ðñøË1á][[<Âkêñø#ãâoT?Ä_nÅbMMM¦n?ÄøCüå²®®®pC6uøCüñ÷ñÇÏ9³   ÜkÍ;÷ÂâÄ_(©««K>8©[Äâ,¿ÖÖÖø8uêøñ(577§<8GEEESSSOOëøCüéñ(¯¦¦¦»»;|ùòåÕ«W5Ó¦M þ"ÑÁ9§n?~|uuµ^ñø#â/ºO|GvXOø,þ`Ç_GGÇúõë§L<ÂÖá?²/þ¢Wþzãk®â?ãñ¶¶¶VWW;8øCükñ½ç¯¦¦&4_øöóÏ?_²d÷üÁ¿Pu¡íCü!þÈÙøw#Ã4þ+þ¦£££¾¾>y×Á9Äâ¿¼ÉÏÏÛñçàâñÇ¿Ñ"þÈR/^<qâDbeoüõôô455UTT¤ámnn6Â+þä`üõ7óÙ³gÅ$:wîÜW¿úÕ9s6lØµQ6Æ_ggg¸&ð?¾®®.4?·øCü³ñîîzê©>+×¬YcªH:ï÷ÿ÷¿z÷W·nÙºíÛÛ¾¹é³fÍú³?û³¬¿èàÉ#¼!Ã­2D¡¿µøCüãñ"/Üï?>|ûÚk¯EÃôV?ñGÚ»wïí·ß²/þoÝÿY)DaVÄ_sTVV:8øCü1âïÚõ×ù¢ÇiÓ¦E+W®îMd;wV­*1þÂ¿ð¬éÜ¹siÎQ[[ëàâñÇX¿ààÁñÆÆÆØtñGvÙ³gÏ¢EËoó£'OÉ¯ü¥98G½s?Äc7þ6lØ=$DGû|ðAñ.^¼ø/|áâñWùÊûï¿ÿZF¾ç/Ü¸R£¢¢ÂÁ9ÄâOüõøËÏÏ	&M:~üøµ÷ü?Htøðá)S¦ÜqÇK.-//7o^TQi¦n	-ØÒÒâ(þ@ü¿_ÚwëÖ­VÖÕÕù´/$»xñbssó;öìÙÓÝÝ­ÌøK3uË5kLÝ"þ@ü¿ÿ¯¿yþûGü3F7þúº¥´´´¾¾>Ôª?øñ'þ2øCüÝ4S·,°±±1þÂ$âÄø»~ÎqãvÓ+þ sâ¯¿©[âoìsL6ñâOü?ñG.Ä_©[¶lÙbêñâOü?ñGÄ_S·ïØ±ÃÔ-âÄøâ¿4S·,Y²$dWüøâOüñ×ßÔ-uuuÉ&þ@ü?ñ'þÈøK3uË;LÝ"þ@ü?ñ'þÈøK?uK8©§§Ç® þ¸¥øKOüÁÈÄ_©[V¬Xqøðaoì þøË»üü|ñÃi¦n	7sçÎÙ·âOü!þ²øuâ±i¦nyùåñøCü?r!þÒLÝRUUÕÚÚjñøCü?r!þÒOÝÒÞÞn7"þ?ñG.Ä_S·[s þ?ñG.Ä_©[*++ñøCü?r$þúºeüøñµµµÎøCü!þÄ9i¦n©¯¯§ÚW?ÄâOüñ÷çþçýMÝ²k×.oìCü!þâMÝrûí·ºñøCü?r©[?Äøc¬<ºñøCü?rÛ»woÊ©[î¼óÎ­[·áEü!þâÕÕµcÇ²²²þ¦n¹cûøCü!þÄçÔ©Së×¯/..N?uøCü!þâ,ÅZZZxàNÝ"þ?Äø#+ES·$#X±bÅ=R¾±Oü!þ?ñG9úô-[§n8qâ§n?ÄâOü5öîÝr·´´tÇºEü!þ?ñG¦ëîî~ã7/^òàÍÍÍ===¼(ñøCü!þÄëÜ¹sÛ·oO9Âÿý÷8qb°(þ?Äø#>|¸¶¶¶°°°Oö¼és?Äâñ'þÈ ÑgxScÉ%ÍÍÍÝÝÝ·rùâñøCü?2BWW×3Ï<òà+V¬ªk øCü!þâÑÔÜ¼ysòûÂíÛ·>zÿ/ñøCü!þÄ£#íÙ³§²²2ywÞ¼y;wî¼Å^ñøCü!þÄ¡«««¡¡!åÁ9ª««õÊ&þ?Äøcättt¬_¿>åïæÍO:5Ü þ?Äøc$«PuuuòÁ9fÌ±cÇ/Ìf?Äâñ'þFÑÔ-)ÎÑÚÚÅFrÄâñøÎÎÎpÍIá-,,¬««koo­?ÄâOü1ô!ïGxËÊÊÂué¦Î!þ?Äø#Äb±æææç¨¬¬ljjá^ñøCü!þÄÃ¢¿©[Æ_[[;òWñøCü!þÄÃ"ÍÔ-õõõáÔLÛ`ñøCü!þÄ7£¿©[ÊËËwíÚ5ºoì?ÄâOü142mêñøCü!þÄÃ"3§n?ÄâOü1h§NêéééïQ-c§n?ÄâOü1mmm¡á¾ô¥/Í¾vQQÑ_üÅ_ÄOÊ©[ÄâñøTWWWIIÉÿúÿkë­Û¾½míÿ^ûÅ/~ñ7ÞëÃ=DaæOÝ"þ?Äøc ÿ`Ñìÿ[þGËo»í¶ÂÂÂlºEü!þ?ñÇ@=òÈ#ôGoë­«W¯>zòoOÝ"þ?Äøc vìØñ¯|åkK¿6uêÔäì[¶lYæOÝ"þ?Äøc@:::jkkã7~£Oóýæoþæ5kN8¿µøCü!þâoÌéïà!ûþðÿ°³³3wñøCü!þÄßXæà¿÷¿÷øãÿâ¿Èù þ?ÄøËõõõÉçV¬Xb(ÇÞØ'þ?Äø£Â®¶¶6y·¸¸8üY/^¼8ÖvøCü!þâ/Åb±¦¦¦ÊÊÊäú***»»»Çæ?ÄâOüåÎÎÎçªªªöìÙ3vFxÅâñø9þØSWW<Â;eÊ-[¶äÀÁ9Äâñøüj·¹¹¹ªª*ù¥¾ÒÒÒ98øCü!þâoLëîîmW^^K,yùåÇìûÄâñø9åÜ¹sÛ·o/--íÓ|ãÇ¯««koo·Äâñø».¿ÌY³fMÊ7öÕ××çöÁ9Äâñø2áº5gÎÐâ/3õôô477§º¥¼¼|ÇÞØ'þ?Äß Üï½íííiâï»ßýîÏFÖüãüä'?óÂNøö·¿ýÅ/~19ûî¼óÎçþÄöÒÀ'ï½÷ýÀÛ¿è?û¡uüøñüàöC&ËâøûØü½ùæ#ëàÁáJß1½ûî»üÇ|Ûm·õi¾ßþíßëÃ#M÷óÿ|ß¾öC.<_ýøû¡uòäÉþðöC&Ëåø3ì;8P]]üRß3vìØ1ÉfØÃ¾öÅ°ïÐ¤^DüeîîîÆÆÆyóæ¥º¥¹¹yCü!þ¿¡ÏAñ7*:;;·oß|L¶'®Y³æÄn?âñøCü¿øðáx °°°Oö»Ý¯øCü!þìñ7ÄßÅb---Ë-Ká]¼x±^ñøñ'þÄ_¸xñbÊc²?~Í5âñâOü¿ÑÑ±yóæäÞ)S¦=|úôi7ñøñ'þÄ_.µ±bÅäc²-°©©©§§ÇÍCü!þ@ü?ñ#ñòµµµ!Að?Ä?ñ'þr-þyæÄÞÍ7á?âOüålü;w®°°pÞ¼y;wî4Â+þ þÄøËñøN:eWü!þ@ü?ñ7Vâñøñ'þÄøCü!þ@ü?ñ'þ?âOüøCü!þâÄâñø þ?ÄøñøCü!þÄâÄâñ'þ þ?ñøñøCü?ÄâOü!þâñøñ'þÄøCü!þ@ü?ñ'þ?âOüøCü!þâÄâñø þ?ÄøñøCü!þÄâÄâñ'þ þ?ñøñøCü?Ä?ÄâOü!þ þÄø?Ä?ñ'þÄâñâOü?ñøCü!þâÄâñø þ?ÄøñøCü!þÄâÄâñ'þ þ?ñøñøCü?Ä?ÄâOü!þ þâñøñ'þÄøCü!þ@ü?ñ'þ?ÄøâÄâñø þ?ÄøñøCü!þÄâÄâñ'þ þ?ñøñøCü?Ä?ÄâOü!þ þâñøñ'þÄøCü!þ@ü?ñ'þ?âOüøCü!þâÄâñø þ?ÄøCü?Äâñ'þ þ¿_û?ùæææÓ#ëÝwßýÉO~rÔÏþó7ß|Ó~`Èýõ_ÿõOúSû¡ÕÖÖ¶÷nû!uuuåfü<yrÛ¶m@ôC£ã¼ø	`Øñø@ü þ¿QsàÀ9sæäççÏ;7åTæûöí7Îd(¯Z===uuu3fÌpÌêzuÃ3Hã£>Z´hQ¸_ZµjÕ¥KO:vìØ´iÓ¢«Öþýûí+ñõ?ýôÓ°¾õ95-X°@ü1´W­'|òÙg½zõjx´9s¦ÅÒßAzwÜqÇ#GÂÂ©S§6lØxRMMÍ«¯¾^xáÕ«WÛWâ/ëÞ³gÏð5ùaøé§~î¹çÄCÕÏÃÝ«]ÄÐ^¯ÒßAzùùùñåÉ'÷y^¬ÞÞ^Ï+Ä_.8vìXQQQÈ»ðõèÑ£'gÏ-×xñÇÐ^µÂìóÏ?ÖGè?üÐ¾bH®WiN?þÉ'ÃÂk¯½º°ÏI¿¬tÇwD¯ÁûÍ»îº+ñ¤ûî»ïàÁ¿Úâ!½jååå½ôÒK×~ý&û!¹^¥9	n(<=vxæðÜsÏ¯î²âËöøËziÐûïì+êªURRâi4C~½òòCâã?3gN»¬ÞÞÞk×ï¾Ù*<?^ènkkÏSï>åÇ^µ6lØðæoðöCr½È½ôgÖ¬YÇ»zõêsÏ=÷ÔSO%T[[ûÊ+¯ðµ¦¦Æ¾¹ð'ÜKgÉákXNYâ¡½j]ºté¾ûî'-Z´¨½½Ý¾bH®W)O:pàÀôéÓÖ®]Å¯Z*--ÍËË6mZô`Äâñø@ü þ?ñø@ü þ?Äâñø@ü?LòÚk¯Ý÷ÝE×-]ºô­·Þúo÷_×eÍ½mª­-++¿ZwwwõaM~~þ´iÓ®^½:ØË@VÚ¶mÛ¸$O=õT.ÅßöíÛÃÊÆÆÆ>ëwîÜÖ?ñÄ7qâÈ>ÇYÿâ/Æ®é¥Â·aåñãÇs&þ>ùä°rþüùÖÏ3'¬ïèèøÆ|0dÍ³Ï>¸ò;ßùNX¹fÍÄôÙ¿§Ð,ËñþüóÏ×®];iÒ¤pRqqñ¦MGW[[[C`Ây÷îÝÛ'§ÂÉ'ß÷Ýï¼óNø¶¶¶¶Ïµ´´¤¿è¤°IÑIûöíë/Ôî¹ç°þÈ#ñ5ï¿ÿ~XSUU_óäO***ZµjÕÙ³gã/ùòû¬I³©øeÓ§Oáòé§&®<sæLXYVV7=z4:uåÊNÚ¸qctR[[[^^^ÊsEßF§ÞwßW¯^ùXPPÅÂ©ák§	&DoÅKs9a!åæ%ÿ¦¯¿þzbÑÆûòÕW__Yºté`ã/Í¦â`ôE#¼)î³Æ)7«W¯¾|]Xß._¾<:5j(£ðB´E'ÕÔÔD/åCEx[·nyº>üðÃaÍÛo¿Ã×°üÐCÝðrB8oÃ$n[Êß(åÔ©SÃïáÂðmø¶<goooôÓ¦Mg¿Âµ_6þÒl* þ2"þñ_ÅMÔLÁÙ³gÃ·!¢o,X¾9sæºuëB´]¹r%~!ágú¼?Wôí3gâ?)>ò¾åÖÖÖ^NÍðíùóç·­¿÷ç=úè£á¤çîÚ¯¶7mÚø!Cü$]´hQôÂä`ã/Í¦â`ôE/w]¾|9qewwwXNJ;ñ4looú/Þ:ñ7ê%ÆöÉ©Ä	VÂrqqqAAAOOO¸ðÉ'ÇO½áåôb<y2êÔ°¾å¶¶¶ø©røxàñfSñ0ú¢÷½=ÿüó+£	Pú|à#þ¾À.$¿ uüøñíÛ·G°ñj^«Þ0Ñ6nÜö¯k×®¯Os9Ñ+möYôíùóçÓ2÷®»î§F³ÛfM<)úäo8iß¾.]Jñ0öFüÔ4?Ñ`"??ÿ^¦zyñÅ?±|ùò:===Ñlü¹Ñþ¢·Êµ··'¾Y0jÁM6T>l%e¢E¿þ÷Ä×§¹èM~Ñþº»»£L/½ôRü5¹ð+'½ý±­­-ü/a'ôQn?¢sÕªU§¦ÙT@üdhä>|òÉÿÿu]ÔFñåøiü3q?üpbY&Ú½wø»výPaqqqâpË	¸añOÚö÷Ë^¹r¥¨¨(úúvßï½ÿÅÔ©SÃ×h¶ÄË^R5~jMÄ@¦²téÒëî¾ûîè#·âoß¾ÑtzsçÎ=xð`üÔîîîÇ¼´´4¦G´§§'~êÞ½-ZlÚ´i»víêsÉ[.*¬ß°aCõý]N6&lR4ayþâÖ®]ÛgÎÈjjjÂ4iRø-:::âÓÖ$^æ¥KBÝFûªªªêÈ#þÇ4?Äâñø@ü þ?Äâ@ü þ?ÄâòpMÉÂxIEND®B`


ík_[´hÑ kH¼q/^¼Ãè¬sçÎUWW6ÊÏÏ_°`Á#GÒ^[òOþ"¯½öZyyyUUUøA©)võêÕÚÚÚpÙpã7mÚ½òtéÒ¥áÚÂúpÍôÑ(Åß~ô£¼¼¼è7º~ýz¸ñádXé1? û/ZþÖ·¾BfoûÛÉßsèÐ¡Ôñmë×¯g½úê«a9|ËõõõÑY<ðÀÁÃÂ~Ö¥½¶ä0üEBÒïßuuu©7þé§ËáÞzë­°ðüóÏ3gÎËW®èèÑió4þBáí×­[µûöhS¼üòËaá¹çóèÄÑñW^^nHm¯´ÛÛÛÃY«V­Ë«W¯ª.qîéÓ§·nÝºhÑ¢èi¯mP`èM6-õ²%%%ÑYBöá;ÃòBÎö÷÷ßÉÆI^Ý¶på¡üBYFg=kÖ¬p3¦L2öìpÒ£@FÇ_~~~b9,C5PB(­Ð:á;ûúú¢½«aMtÖ+¯¼.ð?üaò5º¶ä·r¡nX¨±ä7ó¢pÜ¿ÿôéÓ£5ÅÅÅÑwå¿´¢Ï»É;ÍÄ¡ñWVVüÎ_ôæÙM§¶¶6øX_PPÖáãoø$nXè¹ÔËÎ1#ºì Ç<XWWüámln+W®ÔÔÔ,]º4ñÇìÑ? £ã/ºåÕW_Æü½ôÒK·û÷ïO¼ö½ï/±>JÉ³gÏFco%þ¿ÈÖ­[ß|óÍ°°~ýúÔË>ùäaùõ×_ïììvõUUUaù?üaH±°0öìÑ¿7³N>ìØ±äâÈÐøÇã!°¦Ü<WßðñwíÚµhkø´¶¶O6mÓ¦M·Ã_¤­­-»dÉÄçN¿§¯¯/äW¸å=ôPôÁÞÐ|555ÑÇ-[6ÒßÞzü`MõféÒ¥ádGG þ?Äø@ü þÆÇ÷¿ÿýîînwa.ùè£~òØd£?üð6ùàoÿöoÿíßþÍvYà·û·Cÿ¹sÉ~ðG2 KýÙýÙ?ÿó?Ûd£Cýë¿þ«í þÄâÄâñ'þ þ?ñøCü?ÄâOü!þ þâñøñø?Ä?Äø þ@ü!þÄøñâñøñâñøñâñøñøâOü!þ@ü!þâñâñø??ÄøCü!þ@ü!þFæÄ,ÈÏÏ?þ©S§Äøñâ¿Y³fðÁaá7Þ=vjü>|ø*9äøñãÿ÷o;ÚÛÛÿáþÁv ½ûî»ÿôOÿd;ä,¿dEEE©ñ÷òË/ÿ)9$<½ÿþû¶ÙèwÞ9räí@6jmmÿ±rI.ÄßÉ'ëëëíöµÛìö»ÉåÝ¾«W¯ÖÔÔôõõ?ñâÄ9çÏ¯««KâOüøñGNÅ_ûòåË/]ºö'þ@üø#§â¯¼¼|Rñ'þ@üø#ãoxâOüøñøCüøñøCüøñøCüøCü?ñ'þ þ?ñøñøCü?Ä?ÄâOü!þ þâñøñø?Ä?Äø þ@ü!þÄøñâñøñâñøñâñøñâOü?Ä?ÄâOü!þ@ü!þâñâñø?Ä?ÄøCü!þ@ü!þÄâñâñ'þÄ??ñ'þ@üøCü!þ@üøCü!þ@üøCü!þ@üøâñâñø??ÄøCüøCü!þÄâñâñ'þ??ñøCüøCü?ñâ&vüõööºkÄø þ Çã¯¯¯oÏ=óæÍ«®®v×?ñ'þ@üAÎÆ_,Û²eË=÷Ü3é?9sÆ½#þÄøñ¹!òêêê&O<é¿-èÞâOüø¿þþþ,[¶,5ûV®nU<wï?ñ'þ@üAÖÇ_ooox///Ô|ëÖ­³·Wü?ñâr$þb±ØöEfÎ¹eËwøâÄäBüµ¶¶®X±"uoEEEss³=¼âOü?ñ×ßß¿oß¾ÅOJñðÃ···Ë>ñ'þÄøCüA.Ä_OOOccciiiêÀ¾[^ü?Äâr!þ?vê=ö|Ù6âñø¬¿x<ÞÒÒR]]ºwñâÅö?ñøCüAÄ_ooï;***R÷ð®Y³&<·ÛÈâOü!þñÅR§n:uêLÝ"þÄâñ9á%xåÊiöíÝ»·¯¯Ïâñø¬¿x<ÞÜÜ:°¯ººº­­ÍÀ>ñ'þ?Èøæluuu]]]¶¤ø?ÄäBüÅb±w!òe_ÁðZ¢Ð6âñø¿¡öUVVºEü?ÄâOü#ñ×ßßÚ.Þ æZÐ¯ø?äHüõôô×Ô©[ÂX,fs?ñøñG.Ä_WWWÚc²Ø'þÄâÄ¹#¿ðÂ¿ôK¿:uKUUâOü!þ@ü#zRÉ6yòäÚÚZ¯°âOü!þ@ü#b±XcccÚa½âOü!þ@ü#ÂëfmmmêÀ¾/|á;vì0°Oü?Ä?rA<?tèPuuuÚc²mÝºõ_þå_l%ñ'þ þÈz/_Þ³gOê1Ù&Ou?á±âñâLÔÝÝÝØØ8uêÔÔá´§§'ñâOü?Ä?²Xxq|ôÑGSöÍ9sçÎ©ûÄø?dþþþûö-0íÀ¾ÖÖÖ¡fìâOü!þ@üM.°k×®ÔuuugÎþââOü?Ä?²CGGÇ³Ï>:°¯´´tûöíÉûÄâñâlÇ÷íÛ·råÊÔóæÍÛ»wo__ß­_øâñâÕÛÛ»k×®Ôc²+V¬¸½Eñ'þÄâÄ§««ëÅ_L=&[aaáîälâOü?Ä?2H[[Û5kR÷ðîØ±ãÂwxýâOü?Ä?Æ____KKËâÅS÷ðVUUíÚµkDûÄâñâÅS÷ðN<yÍ5wýµOü?ñøñÇ¸=ÖÕÕ¥îáfì»âñøñG¦Çã---ÕÕÕ©xKKKÓMü!þ þÈ>¡êvìØvêªªªô÷÷ömâOü!þ@ü1êb±XCCCÚëÖ­;~üøÝñ'þÄâÄ£(¼xÕÖÖ¦ì!ØØØ8JûÄY.]*//âÄÙ.777WUU¥îá­¨¨hjjÕâì¿cÇÍ3'üU?ñâìª.¼f§f_uuu[[[èÂq¼yâOüez¨««køóÍ7?$üéþé_ýÕ_Ùd£÷Þïoþæol9zôèã?þÏ|fPóÔÔÔüÉüI&ÜÈþÝßý;+dqüýô&¯½öÚÿ#9rääÉ¶ÙèðáÃ§O¶H¯P_úÒòòòeß½÷ÞÛÐÐpüøñÌ¹©ï¼óNø·»,ärüÙík·/ØíKFéïïonn®¬¬L;uK8k|÷ðÚíøCüøãîö¥º¥¶¶6_°Äø?@WWW]]]aaaLÝ"þ?Üñx¼­­-í1ÙÆwêñGÖÇßPÄøñÇ¸UÚ.í1Ù2aêñøCüøãîèééyñÅSöÖÕÕuuueã/%þÄøCüø#ÍTÈ»Ôc²×£lÙÃ+þ?Ü¼/^ES·?ÄâÄ#Ößß¿gÏÔ?uËmÄ_wwwøÚÖÖvùòew½ø?K,Û°aCqqqNÝ2RO<ñÄÔ©Sÿû¢ÿ¾`Á°ðæoz?ñøñ7!+W¦ÎØ]S·È¡C>ûÙÏ®ÿõõÛ~s[ø÷¿ëþ÷3Â³±ø?9+777Ï7/íÔ----90°o(_ýêWÿ×êÿ_ôï+Õ_©¯¯÷¨âñâ/]¸paË-¥¥¥©S·¬[·n"¼ÊTVV6üÉñ÷ÚoäõØâñâ/§tttlØ°aêÔ©²/`xééé Ûá«_ýê£<ÿsùÿôÎø?¹£­­íÑGM±oáÂ»víêëëP[Ã?ñ'þ þrSooïÎ;ÓíánmmÍáÃâ'-Z´à>í+þÄâÄ_öëééillêlgÎàÛ'ç/äoX0Ïø?Y¬³³3í1Ù&ÚÀ¾Æ#|?ñøñõA3ÔÀ¾æææ6°OüqwâoÒÍäåå?Äâ±ÔßßÚ®²²2íáUcÂìÜøË»üü|ñøCü16zzzÂëÂPûºººl"ñÇÆß¸âÄÑSGÚåååá"'É&þÈÄø»råÊúõëÅâñÇ(Çã---ÕÕÕ©x«ªªíábüÿ]åççóøCü1zwìØ:cßäÉkkk½4?F=þæÏú¿®âââÑþ,ø þ&X,ÖÐÐ:°/¬illçÚDâ±¿ðwéÒ¥èÀØ¡ùÞ|óÍ°0ÚGâÄßÄðW®:°¯¢¢¢©©ÉÀ>ñÇÆ_ôçBí?üðúõëaaÊ)âñøãN?uK[[âq¿éÓ§?ÂcÇuuuç.Z0ÕâñÇm3uø#sãoÓ¦Mwú°øCü!þ¸§S·?2:þ^xaÆaáÄa!à¢EFû¦?ñâ/ºEüMñ7.ÄøñLÝ"þâOüøLÝ"þÈÖø=v4áI?n©[ÄY³fÍJ¾öEü!þÄÔ-â¿ÐyáöäÉ×¯_Ë.þÄ¿,bêñGîÄ_qqqøëãòâÄ_ýiºEüSñwêÔ©ð7üôÓO_½zUü!þDLÝ"þÈÙøî»ï¾Ô¿mø@ü!þ&¦ÞÞÞ¦¦¦´S·ôÔ-þÈúø»ÿþûàñøÞº¥±±1íÀ¾6ôôôØDâ¿è»££coºø þ2GxB®­­jê.x?r'þJJJ|àñøñ7ÌÀ¾°rïÞ½ö?r0þÚÛÛÃù¦MúûûÅâñ7AuL¶àá>sæGø#gãoÒ|àñøËIÃ-<3_¾|ÙãAüãñ7ø@ü!þrÌPÇd[¸p¡c²?&Püñ'þ@üx<ÞÜÜ:uË+8``øcbÅ_yyùý÷ßßÙÙ)þ¿ÓÛÛlÃó|jöÕÕÕÅb1w½øc"Æ_~~~x"û.þÄ¿ÑÓÕÕò®°°pPöîÜ¹ÓÔ-â	G	OÛ·o¿råÊXNø"þÄ¿ÑÐÖÖvêeËíÙ³§¯¯ÏÝ-þèñçÓ¾?ÈøUvêÉ'×ÖÖ?~Ü½,þ?åÓ¾?ÈêøëîîÞ²eËÔ©SÓìçºÅâ/#?ñâïuL¶ÒÒÒíÛ·;¯øCü?ÄäBüõ÷÷·´´,0uÄNUUUssó±	ñGöÅßÀÀÀC=TTT8¦LòÈ#Á'?Äøñ7R½½½i§n©­­õ¤*þ·äÚµki?ð1ÚÿqâÄß­ëìì¬¯¯OL¶fì¶ø9sæ§U«V]½z5¼råÊêÕ«Ãx@ü!þðÒþè£¦ì«¨¨pL6Äø»áI$ùØ>aMX/þ¿ñªnÏ=3gÎLÝ3S]]ÝÖÖæl?ñwòòòÂSI¾Äþþþ°ÆT/?Äß¸èééillLÝÃ[XXXWW×ÕÕåþBü¿;íö]¾|y´Û7|ËaÍÄâñ7ÆDµµµ©3öçLxÜøµö|òøCü._¾|àÀ;w¶¶¶õQ¶ñ¿x<¾oß¾´Çd¦n±ñÇÝ¿Oo|à÷G6mZ^^^øº|ùò°f´oºø0:;;ï¹çyóæUÿRõ¬Y³¾ð/¤=âÅxÅ____¨ÒââbS· þÓøâOüÁhÇãÿüçùyÛonþùË_~àRßHûøëêêzöÙgS÷ðºñøCüÁm:sæÌõßåþmÝ²µ¤¤$õ¸gcá©oåÊ¦nAü1Öñ7éfòòòÄâ¬ÖÚÚú?üäøÿfÏ«cýýýÍÍÍ¦nAü1>ñ74ñø#7tvvÞï½[·lMß³Ï<;eÊË/eüõôôg<S· þçøÊ3Ï<=+½ñÆâñG¶«®®?þ¦g7Eå7§rNè­Ôo¥øáÇ¥îá5uâ¿S§NÿG; ç|?²W¬eËÍ1£²²2<Å­[·®¯¯o´ã/·µµºñGFÇßc==1<xplnºø0f.pæÌÔ½½w=þBk655UTTºñGæÆß[o½=7­Zµj,oºø9î<þb±XÚc²ºñGÅßµk×xàè³Gã.þÄäFü5°ÏÔ-?2+þ^~ùåèéiíÚµãrÓÅø¬¿x<ÞÒÒv`_XÎ2°ñGfÅyþpñ×ÛÛ»cÇ´ûêêêR'ñGFÄ_ÞÍäçç?Äâ/Y,khhH;°/<§¥8ÄãNü?È¢øÏWµµµö!þâñG.Ç_<onn®ªªrL6ÄâOü!þÈåøëííOSåååÉøCü?Ä¹!ïBä¥Øg/âñ'þäHü?~üÑGMØWYYÙÜÜÜßßoC!þâñGÖÇã7o3gNêÔ-+W®ôLø#GâÏ<?èîîÏE3gÎLÝÃÛÐÐàl?r*þ§ôKæùCüÃ:::Ò­¼¼üÅ_4°ñGÆ_ÂáÃÃóÝã?~õêÕp2|]½zuXsôèQñø#ÇÄãñ¤ºeþüùá,S· þÈýø+..ÏzÉÏwaMiié^ó©S§ÊÊÊòóóçÎäÈñ'þ`]¾|y×®]©ûjkkÿ÷¤ÇöñG¶Æ_ôôoPüÝù¿×_=,ìÞ½íÚµâOüÁ¸èîî~öÙg£ÿè&kÂúèl#:¶/?²;þJJJÂ`µk×®«V­kÂú;¼æðÄzýúõ¨&ËËËSã¯©©é49ä½÷Þûó?ÿsÛÌñ¿÷K,IÙü¹ÏnÓ¦MÇK|gxùü¿ø[lÔÚÚzêÔ)Û!zü=z4í>~øÃÞá5'd$õã#!þÞzë­rÈ÷¿ÿýÎÎNÛq×ÝÝ½÷î/~ñ©Ïl¿ø¿øÚk¯ot÷ßÿÇ?þ±MG6z÷ÝwÏ;g;äQ¿àÃ?5kVQQQøÿñ)S,XpñâÅ;¿ÚäÇvûÚí£íÂÛ·oêlC]Ðn_ìöeíö=%%%ÑPÂð5u'²øpw~6lH=&[qqqX¢pø?Äâï.¨­­íµ×ÂBøZSS#þÄÒ+_uuuêÞ]»võõõÝÊ?Ä+þÎ=;öì)S¦D;jKJJöïßçWìØ±ÒÒÒpeee'Nâî¢ÞÞÞÐv¡ðR³/´àFt(^ñøcÅ_4Ésò!Ý¢åÝ»wêMânO,Û²eË=÷Ü:c_]]Ý3gnã:Åâ	¥¥¥áIóìÙ³ø;qâDX6møCüQÂÆÊ+SÉV\ÜØØxÓÈ?ÄâïÆUÜ-DñwýúuÇöEü9úûûSÎÌ7oïÞ½w~(^ñøcÅ_4Ésôn_¿Í7GG7?ÆWOOOx®HÝÃ<üðÃ­­­wëP¼âñÇ¿ööö´<ðÁâñÇxéêêª««KÝÃÍØÎ½»?Nü!þ@ñìEEö-**=v,í.þÄ¤Çãmmmi§n)//Ïw¾Wü!þãCü?Hª®©©)íÔ-UUUÍÍÍwk¯øCü1Ñã/ñ9/Î=æÌâñÇj`ßäÉkkkÇæBü!þÐñ700àÓ¾?Ææ1v`_ÁÆÆÆ1"þL ø9sæ¤aM>]ü!þñx¼¥¥e¨c²555ÒÀ>ñøcBÇß¹sçònHÛ#Y(¿·ÞzKü!þ¸»BÕíØ±c¨c²µµµêÀ>ñøcBÇ_BH½ÑÞÃ+þÄÄb±ÆÆÆÔ£4uøCü!þ2øLá/½¶¶6u`ß-NÝÒÑÑÑÔÔ´gÏÎÎNñâ»³gÏ2eJbÍ3¾õ­o?Äw"777WUUÝÉÔ-6múù/ùË_úÒ>óÏ|óß þÄßºï¾ûà7zvÞ¾»øCüq._¾þÀËËËïpê?÷s?÷6þm¿¹-üÛðôýÙ¯sâÄø»#áI¹££#±æÔ©SaÍ´iÓÄâéììÜ°aÃÔ©SïÊÔ-+V¬øú×¿_ôoåÊ¿ök¿&þ@ü¿;à÷úõëÿåzS&ÿ?qüøñÐj©ûîdêÊÊÊõ¿¾>9þ¾QûêêjñâOüÝ²²²ð½qãÆþþþpòÚµkÛ¶mkJKKÅâáÅãñûö¥Í¾;ºåGùå¯þrrüyÙzê)ñâOüÝ'O¦äùÄâñÇP._¾¼cÇÔéâCÖ××ß©[Â#júôé=úØÖ-[Cù¼÷ÞGiRñøcÅ_pþüù¹sçåååM2eÎ9aÍhßtñ'þÈÞ;º®®®°°0u`ß/¾xáÂ»û³~á~aÆaáÌ3£ôK?Ä+þÆød¤=&[eeessóè­ïQýÕÄâñ'þüTÿP3ö=úè£áy¼É&þ@ü¿ÛtöìÙhçè¾%%%û÷ï?zzz5ßÔ©SÆýlâÄø»N<¡Gñ-ïÞ½[ü!þ&¬¡É¶sçÎÑÛÃ+þ@ü1êñWZZÐÏ=¿'Näñ7aµ··§ºeÙ²e÷îí±wâÄ£ÑÓú§I;_¿~=,ççç?ÄßÄ·²²2uêuëÖåü_«øCü1â¯¤¤$Õ/¿Í7G;wÄâo"èêêª¯¯O;°¯®®®»»"lñøcÅ_ÚI?øàñøËmÇ_³fMÚc²åäÀ>ñøCüýTxÁ^´hQôiß¢¢¢Ù³gôøëâñEâñøÒNÝ²xñâpVLÝ"þ¿#þÄc¯··wÇiö?~|føCü!þÄâ/Åb±î¹gPöoß¾ì?ÄßOýøÇ?¾ÿþûÂ+AQQÑÜ¹s/]º$þ¹!ü¡­2u`ß¼yó&ÚÀ>ñøCüý»¶¶¶´øèìì¿ì-uê ºº:¼ZLØ=¼âñÇD¿hçhîÖ«W¯®]»6¬)++¿lÔÓÓþ¾R÷ðÖÕÕåÒ1ÙÄ?ñw[WqCòa91á³øCüe×]ò.í1ÙÂ_=¼âñøûwÑ;5×®]óÎâ/ÿ°µ´´TWW§îá­ªªjnn¶Wü!þÿ)óWSS/üäO-[fÌâ/+3uKmm­?1ñøCü¥»¥ý¿âOüq'º%¬illyÚÅ?²5þòn&??_ü!þ2ÇPS·TTT455Ø'þ¿%þÄ#2üÔ-mmmö?Äâï5óùóçÅâ/ºEüøãnÆ_x	yá­n©^°IMÝ"þ@üqã/D^x-)))¹xñb8ùÝï~7zu¥¡~âOüqS¦n þÅøûôÆû|ÑëJYYY´°zõêÑ¾éâOüêòåË;wî9s¦©[Ä?F1þ£G&^föìÙ37]ü?uwwoÙ²eêÔ©¦n þõøê©§¢èhÁã?.þc£½½ýá6uøñÇÅ_~~~x6mÚéÓ§?MóWPP þ£§¯¯/´]Ú©[V®êñâÑ¿ðJ³uëÖA+ëêê|Úñ7J.vêÉ'¯Y³ÆÔ-âÄ£CÍó7Ú¯ââOüM@!ìÖ­[WXX8(û»»»m"ñâQ¿ñ"þÄßrüøñÚÚÚÔûöíëïï·Ä?F7þÂ«NòÝáO?ÄßíÇã¨ªªJ;°/<eØ'þ@ü!þ¹`ûìá þ¿ÑÙÙY__:c_iiixØË>ñâñøËííí+V¬HØ·xñâûÄ?Äø¹`øûBØ'þ@ü!þÄøË===©3ö5õõõÉ&þ@ü!þÄøËgÎY·n]êÞ3gîÜ¹³¯¯ÏãAüø#ãâoxâñ*·´´TWW§þÉ,pß¾öð?dhüåÝL~~¾øCü%ôööîØ±£¢¢"uêuëÖßÝ@üø#£ãoÜ?ñ-b±XCCCêÀ¾âââ6Ø'þ@ü!þÄøËá!ºråÊÔ÷î5°OüøCü?ññ×ßßßÜÜvêêêê¶¶6ûÄ?ÄøÐºººvîÜù«¿ú«¿ó;¿sùòåìýEzzzÂ#3uoaaa]]]ø5Ý×âÄâOüMtûöí>úùW~åW¾øÅ/þüÏÿ|6Áì?øAÈ»Ô=¼åååá±ÚÛÛë þâOCçýÚ¿¶í7·Eÿ/_þ¥/)[nÿ0S·TUU577ÛÃ+þ@ü!þÄÿiïÞ½K,I_ø·ù76O:5ó?1ÌÔ-µµµâÄâOüFSSÓW¾òäøÿ3y?éPS·5¦n þâ!?~üÞïÝüå÷Úo5ykº%T¬âÏv@ü!þÄ7·fÍOë(¿¯ýë%%%­­­uMÝøCü!þÄwM(§çîóÿ|aaá¾ðûöeÎm3uâñøäÙÔ-?ÄâOüûñgêÄâñ'þñgêÄâñ'þñgêÄâÏvâ	CMÝRYYiêÄâñ'þÈøÇãÍÍÍUUU©ûBºñøCü?r$þzÃ#§¼¼<u`_½©[?Äß8»téRxâïÎÅb±ºººÂÂÂÔ[¶l1°ñøCü¿cÇÍ3'¼<?ñw'Ø7oÞ¼=öØøCü?ñ)zè¡®®®aâïw÷wÛÈ!üã?þã»xm¿ñ¿ñ¹Ï.u`ßâÅ_zé¥»ø³àwÞ9|ø°í@6jmmï½÷lÅñ÷Ó8tü½ÿþûÿB9qâÄ¹sçîüzb±ØoýÖoM>=íÀ¾ÎÎN»®½½ýÿñm²Ñ»ï¾ûüÄvÈ%¹vûÚí;HWWWÚc²766vwwÛÈØívûÚíq©âïÖÅãñ¶¶¶´Çd7oÞÞ½ûúúl^Ä?>õÎÙ½½½MMMiÉ¶fÍööv3ö!þ@ü!þÈøÅb©Çd:uê-[ìáEüø#[ão(âoÂÆ_ggçPû¶oßÞÓÓcK"þ@ü!þÈúøÇã---ÕÕÕ©Ù·xñbû þäHüõööîÜ¹3íÀ¾ÚÚZÄ?Ä96lHØÖ466:&âÄâ¿¶¶¶+V¤îá­¨¨hjjrL6Ä?Ä¹ÝÝÝ÷î7o^êÕÕÕ¡MÝøñø#ìâ'>ûÙÏj¾ÂÂÂººº®®.ñâñ'þrÁ3gjkkS÷ðÞsÏ=á^¶ñâñ'þrDûÃ?ÍÍÍýýý6âÄâOüe½¾¾¾¦¦¦Px///ï+_ù»ñâñ'þrDwwwÚc²Eû80Òcûøñø#9sfÝºu©xËËËûFtl_ þdx<¾oß¾ªªªÔ©[ÂÊæææä©[ÄâÄâOüe«.¼øâååå·~L6ñøñøÙ'Ü/!ïS§nihhælâñâñ'þ²IKKKÚ=¼;vì¸éâñâñ'þ²@ÿ=R§nÉðÉ&þ þâ/£uvvnÙ²%uêÉ'×ÕÕÑµ?Ä?ÄøËPmmmkÖ¬Iº¥¸¸øÙgíéé¹ë??ñYº¥¢¢b×®]·åâñâñ'þ2Eooï/¾/íÀ¾Üù¡xÅâÄâOü¿úúúÔS§NmhhéÀ>ñøñøª½½½ºº:õ­¾âââ;vÜÞÀ>ñøñø¥¿¿¿¹¹yñâÅ©Ù·páÂ½÷ÞâÔ-âñâñ'þ2ÚÂML¶+W>JÙ'þ þâoLutt¬Y³&õlaÍðÇd þ?ñ5âñx[[Û²eËR÷ðÎ93läMüøCü!þÄ_U×ÔÔvê677ßùÔ-âñâñ'þ2EØziÉvæÌñºIâñâñ'þFQâó¼÷ÜsOccã]ºEü!þ@ü!þÄ_vû¯c9°Oü!þ@ü!þÄßøß9·Gü!þ@ü!þÄß"þ þâOüøñøâÄ?ÄâÄ?ÄâÄ?ÄâÄâOü?ñøñøCü?Ä?ÄâOü!þ@ü!þâñøñø?Ä?ÄøCü!þ@ü!þÄøñâñ'þÄ??Ä??Ä??Ä?âOü!þ@ü!þâñâñø??ÄøCü!þ@ü!þÄâñâñ'þ??ñ'þ@üøCü?ñâÄâñâÄâñâÄâñâÄø??ÄøCüøCü!þÄâÄâñ'þ??ñøCüøCü?ÄâÄâOü? þâOüøñøs? þ? þ? þÄøCüøCü!þÄâÄâñ'þ þ?ñøCüÙ?ÄøCü!þ@ü!þÄâñâñ7È',X?þüS§N?ñâÄ¹³fÍúàÂÂo¼1öìÔøÿý÷¯CBîwwwÛd£ööö/Úd£wß÷êÕ«¶C.ÉâøKVTTÿ÷ÿþß÷É!<|ø°í@6zçwÞï=ÛlÔÚÚÚÖÖf;ä¿'OÖ××Ûík·/ØívûË»#W¯^­©©éëëâÄ?r-þ&ýèäùóçëêêÒø þ@üõñ¬½½ùòå.]J®ø þ@üSñW^^>)ø þ@üËñ7<ñ'þ@üøCü!þ@üøCü!þ@üøCü!þ@ü!þÄø??ÄøCüøCü!þÄâÄâñ'þ??ñøCüøCü?ÄâÄâOü? þâOüøñÇD¿o~ó---Ýä?ú£?úË¿üKÛlôøý×m;þàþ Ù¹¤··77ãïìÙ³Û¶mûm¿kt7?ìö@ü þ?ÄâÜtöìÙx ??îÜ¹ÇK>ëã?Ä¶"3>|8õñyêÔ©²²²èäÈ[,zôzî0ºÂKãÛo¿>øàâââä³öïß_WWgÉâñøR_ kjj^ýõ°°÷îµk×ÚPdÑ£×s¯ø1rèÐ¡ùóç'¯	Ï>QBÆúÖ·¾õï|'õå3üOæúõëaa`` ¼¼Ü"½ÅÅÿ>§MÞxãäõ³fÍZ¾|y~~þ¢E~ô£ÙPdsçÎg¼ÔÏð¸M»ÿèõÜ+þ`>|¸¤¤$íYçÏô¦ dGyäèÑ£ÿþòòX.((°­È¢G¯ç^ñcg7H¼|ÏÿUòYá2ÞØí;Ôÿj 3½Å®Y³f=6,8qbùòåÎÅbÑÿ>ö::hMmmík¯½Â×,zôzî0ºN:5wîÜüüü|ðã?N~2:yòä9sÂYË-ÏA¶Yñò-;v¬´´4//¯¬¬,üÇÆ&"½Åâñø@ü þ?Äâñ þ?Äâñø@ü þóÝï~wéÒ¥E7<øào½õÖyþº!kmÓÝÚòòòð«õõõZÖäçç]¿~¤×	 þ¬´mÛ¶I)^xá¿íÛ·öì´~×®]aýsÏ=w×	 þìsêÔ©5ùùù¯¼òJüW_5+O>3ñ÷áóçÏ´~Î9a,ø&Ç<dÍ·¿ýíä/½ôRX¹nÝºäô9räH§Ð,ËoþäOêëë§MÎ*..Þ¸qcòÞÕ¶¶¶Xá¬pÙCÊ©°fúôéK.=xð`8Y[[;èµ¶¶=ÑYá&Eg>|x¨P[¾|yXâÄÄ>ø ¬©®®N¬yþùçKKKÃU=öØcçÏO¿Ôë´f?qvß÷p9wîÊ>ú(¬,//OAN<»zõêAg=ýôÓÑYyyyi/ÎäG®_¿ò±   sÃ×OS¦Lâs=a!íÍKýMßxãä¢Môåë¯¿(¿AWòà4þ¹©øÑÞ4ÏY&Kµk×^½!,«V­ÎZ'ÊÇè¼mÑY555Ñ[aùØ±cÑ$_çÖ­[CÞE]|òÉ°æí·ßËákX~â'nz=!ÃÉðÉ·-íorÆá÷½téR8¾[s`` ú²²²pÁð+|ú»FÃÜT@üdDüiã/±>¨óçÏ!¢,'ï¿ÿþõë×h»víZâJÂ÷z/-q©èäGøæL=¿ákXnkk»éõÐ'/^¼|Û÷Ì3Ï³¾óï|ú»¶7nÜü!Cü$]´hQôÆäHão?ñ½ÝuõêÕäae8kÜI¤aWWWÔÖIÔKÝ;(§'X	ËÅÅÅýýýáÊ§O8÷¦×3TröìÙ¨SÃrø;::ç;v,Ü´»o=þ¹©øÑ¸·_~9ye4Ê |$Æ^ºt)õ­Ó§Ooß¾=Ú	¨Æèm¹Ä~Õ&ÚÓO?íí_ëëëë¹è¶?þ8:yñâÅá?»dÉpn4»MhÖä³¢Oþ³>|åÊáã/¦ÑÖH;ÌMÄÀø>0¿÷îhªW^y¥   õÃ«V­¡ÓßßíM|27óëêêJ,µàÆC*EÃML¶6Ñ¢ßF?½½½=±~ëùEcþúúú¢ï&þ^õÕÄráWN>+þØÑÑ~JØCÅ_!Ã·è|ì±ÇÏæ¦â #D òüóÏÿçó×Q%;L±HxòÉ'Ë2Ùþýû¿Oo#¬/..NÞ#<Ìõ|Ã´ê½víZQQQô+ÚÙýÐC%ÿ3f¯Ñl/É×½1¥jâÜan* þ2E|°à¥KF¹¦Ó;wîÑ£GçöõõmÞ¼¹´´4¦gy¦¿¿?qî¡C-Z¬¬¬¬©©iÐu¦ÞpUaýSO=5hýP×nR4á0óü%Ô××ó%réÒ¥°¦M~X,¶&ù:¯ê6ÚVÕÕÕ'Nô¹©ø@ü þ?Äâñø@ü þÄâñø@ü þ+ÿ?v.¬UvX©IEND®B`


Ä·u=û±óß~l¿ßBèñãØqÇ_ñãï×sQÒ4M@DDDþüø#""""ðGDDDDàÀ?""""DDDDþüø#"""DDDDþüø#¢<_tôèQÍõ1¿aáÎ=þ|UUUÆÌfóéÓ§Ç½Â_dN@)))òõ.<vìØ6NOOÅbÂýúõ¥KùîA×0'L:¹ãüQà/;;`` ^ðwàÀPúìß¿?ÂµMê"átuäÈÉn)oÆüü|ÿ÷µZ­àÀÍ¤±¤µk×ÆþzzzVßÿÏ÷ìÙ3Y²Òáph^Õd/ô­wïÞ-kdQÃºàÓ§OeyllLWw?"üÕÖÖÊÿçÏ'÷îmØ°A3­©©éëëúÊ¶¶¶eËù×´··ªªªG]¿~áÂiii+V¬|ñàÁyyyêø¦|ÿYáóê«¯ÊúÀ°ÒÑ£Gee]]æÏ8Ù~kñ¢¬9©£ùÊS»`dü+÷Kii©lö+WÊ¶üaè¹w«d·ÛåÚd;È=+gù/(H-)³FãÎ;=."ðGDúÅßððp~~~nn®,:àáÃòÈùÊ ¥¥¤¤ÈÿëÖ­®uéÒ¥âÿI_~A_¹|ùòÈÄ?¾¬òäIàJÁ¥¬,**Òü' o-²Q¯üOjãD6Ü/8qüÝºu+ðzä,þïVÃ¡NúëééQ_¼víÚ Û¼ûvàô?Y¸téRà+aØºu«,¯^½úédANÊÊÀ¯Ü·oß³gÏÔkWjÍ¶mÛ|>_gg§:)×,'ÛÚÚdY ¨.«ÞÙvõêUY¾ÿ~àYác04×^v	÷¦:ÿ+£Ý8·jÊð¿ Úd¹¦¦fddDXï'ÚÄñx·ÊõÈk×®É²º[7mÚ¤¾X¡ðÁ²,÷¦,gffòà"D¤küIòËò¼´>77W>|¨NÊs¼7o^à5øÏõ¯p|þ«ã§æ.2*++ÕKMãâ/--MóÑ¢J.+±X,v»]s£MdãhÞª)_pâøSïk|ôèQà·þïVumÉuVyy¹`àUþåEþ(ð'>3òîõz×¾rú8QæuF>)Ð:ô9î¨Õ¼¼<u:Tx~]ÛD.n»Ñ&²qÂtjøaßð.ò¹¡wkÐ<8ê,§Ó©üçGá+Wxp?"Ò;þ¤³gÏÊIÿÄ(j¥òYÐkT²r"|²´´TM¹ÒÞÞ.8þÔËjÖ;w>úôøñã²rÃøÈE¦¿ÉnS¾àÄñ7Wþü¼úGÞþ²&hèq`·oßnllTÇý#£ü®ñ÷üÅ÷ýïëWkÔXàÕ«W¿H=»oÛ¶múøS¯~9!È±cÇ&¿õÚx±³³S½ª§ºtéæÏ8ÙL7Ú]Éd·êð§Þ¥'öÛ­Zµ*/@GÞþêåëeKÊrYYYà©Þ»ét:#'"ðGDºÃßÀÀÿÝ]jM___Ðû½rssýg§??GTóæÍÿG¼jÞæ-[¶xqsRþ"oÿA5Oa«N·oß¼þeË´Mkñ§¦KÔ£^[ìõ×_çÁEþ(>ð'©C¢ë?ùä6¤½¨¦¦&txÇÔð788(×&×)zã7ÄCþYB"§¹¹yáÂ)))óçÏ?yòäW¾òÿ+OÓ¿È¤ðyãtIüg0üSå6©­:üIW®°`üÔrýêÀº³¢©ïn±Xº»»Çåµ­­­²²R®M~(Íæ_ïñxöìÙ£^RÁË]éõzyp?"¢ÙjlllË-[n»ÔÑX5|Ûçó)ÓòCDà(SïùêwÞaËø#"JÀ<ÏîÝ»çÏ¯íÊÂØ,Dþüø#"""DDDDþüø#""""ðGDDDDàÀ?""""DDDDþü?"¢)wéÒ%Éd0/_>ÙË.pÎ9N§Ó¿FeMYYÙ$vg/Ú×Lä²SÜÉFüRJJJFFFMMÍàà :Ëãñlß¾Ýh4ÊÆÌÎÎ³îÝ»Ç/?"ÒWyyyBÏçìe<(=tèÍ»ï¾+kjY~pYÞ´i:Y[[+'¯]»&Ë×¯_å¢¢"~ÁüÎö&ÓðÓíÛ·å²UUUþ5K.5·nÝ>Å¢ÿË²¡N¦¥¥ÉÉááa~©ü®å=ödgggffîß¿?èË¾ò¯TVV]ÿCY~òä,ËuÖ,ØÈ`0«ÄB¯-ð»G¾ÈÉ'M&ÙloJ±«Õ*¿÷nõB¦ðtÙ²erm²^®ùáÃ³¿9YZZÚÙÙÉ¯?"ÒµÿÔòw¿û]Y¶ÙlÂ,Yxçw¿¦­­Íÿþ6[·n³?.Ëò¿,×ÕÕ©³/^|õêUY¸ÿ¾¬ÏÏÏ×¼¶Àù"Bº³gÏÊBmmmèß¾»,ËpA<èÙðð°ÃáõÂdhSÀß³gÏdûÈòæÍÕY.]ò_áòåËïÜ¹Ão?"Ò5þL&,½(Ô^oìèè³V¯^-Ëk×®UªóûöíûöUVVª×Ä¬ÈQ7,;;;ô²¹¹¹êÆËdAØ'+å+e¹¼¼ëõz§¶YB×«Û&W.ò<ÎÛÓÓ#~õ|S~Áü~ñg0üË² '#KèùÀ233å+=:º*kÔYÇKïÜ¹xA×xr"	wÃDc/æ)8?>''G­1êEÁyå/B×®]n'"D¤Güåçç¾ò§^<W<V«UÎõÿï_¯@<Ññù"þ&½ì¼yóÔenÏç»zõªëÉpRe²øÔí_0"D¤_ü©©[?®Þó÷î»ïND<çÏ÷¿vöìYÿzEÉ»wïª÷N/²oß¾sçÎÉÂÖ­[C/ûúë¯Ëò©S§zÕ¡^Yi6eùÎ;=âââÙÃ_YYÕÞÞ.ËW®å-[¶ðFDàô?Ï'ÀÊ|Qà}ñ7::ª¹ÊÿoÆìììÝ»wO/b·ÛåÜ¥KúÇ~ÇãÙ¹s§Üò´´´U«V©½b¾5|¸ªªêO>Ôf	=4aSÈw´Z­rãe;Ì7oûöírø#"ðGDDDDàÀ?""""ðGDDDDàÀ_ÌúÑ~Ôßßåoú³ýìç?ÿ9¿7zî¿þë¿ÔÜ¤çþíßþíþçØzn``Ýþww.í ÿÝø±¾óïÿ¢üMïÜ¹óÿùü*ë¹GýøÇ?f;è¼>ø ô#4HWýÓ?ýGé¼ýìg·nÝb;è¼+W®èío]ðþÀ?àÀøàÀøàÀøàÀø#ðGàüø#ðþüøþü?àü?þÀ?ðþÀ?ðGàÀøàÀø#ðGàü?ðGàüøàü?ðGàüøþÀøþü?àü?þÀ?ðþÀ?àÀøàÀø#ðGàüø#ðþüøº»»ËËËCYYYèï7ø#ðþü?¶øK(üÝ¸qCN>]\¿öööè&ªø÷ÿ÷Òq~úiWWÛAç	þÜn7ÛAÏõôôÜ»wí óÝ]ww7ÛAç	þ~þóGùÆ1þËÈÈÅßáÃÝÚÚÚìvûIÇôÑG~ø!ÛAç]¾| óØÝé?¹ØÝ±»Ó,ð'ÖÕÕqØ8ìËa_â°/qØÃ¾|ØW522RSSãñxÀ?ðGàÀøKpüÔÖÖÊÓyèYàÀø#ðþØà/¡ð×ÑÑ±bÅÁÁAÍsÁ?ðGàü±À_BáÏd2Í	üøþü¿DÆ_äÀ?ðGàü±Àøàü?àÀøàÀ?ðGàü?ðGàÀø#ðþÀø#ðþÀø#ðþÀø#ðþüøþü?þÀ?àÀøàÀ?ðGàü?ðGàüø#ðþÀø#ðþüøàüøþÀøàüøþÀ?ðþÀ?àÀøàÀ?ðGàü?ðGàÀø#ðþÀø#ðþüøþü?þÀ?àÀ?ðGàÀø#ðþÀø#ðþüøàüøþü?ðþü?þÀøþÀ?ðGàü?ðGàÀø#ðþÀø#ðGàüøàüø#ðþü?ðþü?þÀ?àÀ?ðGàÀø#ðGàüø#ðþü%Lýýýàüø#ðþü%r>O0·fÍÔÔÔðþüøþ3Ã±k×®¼¼¼9MäÅ?ðþüøþâ&·Ûm³ÙÌfóRSSÁø#ðGàüøû|>Ýn·Z­ééé¡ì«¨¨xûí·Å¹*ðþüøþôËå`L&Íú6oÞ,NüÁø#ðGàüøÓ]^¯·µµµººZÊ¾%K:thhhh×þÀ?àÀr:õõõsçÎ5¬³¦ùþÀ?àÀ_ì<£ºººµµÕëõNÿ?ðGàÀø#ðËä9¢¶¶Vs$GaaaSSËåÁoþÀ?àÀ_Ò	ìJJJBÍ'´Z­v»R#9Àø#ðGàüøÓ]jÒp#9Ìf³Ífà¤-àüø#ðþüé7§ÓÙÐÐ 9i¬³ä¢p3Àø#ðGàüøÅ¼^oKKKä³qxü?þÀ¿¨&;ÿp¶ÌøHðþüøþbÛí7cF&êàü?àÀ_ìüñ»QÉþÀ?àÀ_4ÒÉHðþüøþf±#9fu¢>ðþÀø#ðþüEµqGrèáð.øþü?Ó*Âgrèa$øþü?3ÏçkmmÕüLÿD^¯7^~ðþüøþ´w$G___ÜýPàüø#ðþüý¯Ün·Íf»àüø#ðþüM.y×ÿDàü?ðGàüøVãäÐÕDàü?ðGàüøJ^¯7Fr?ðGàÀø#ð§Ý¸õ¹D½Àø#ðGàüQ²àohh(òHè??ðþÀ?àoæëèè¨­­ÍÊÊJààüø#ðþ(Ùñ×××÷öÛo¨/Fr?ðGàÀø£$ÅÇã9sæÌÊ+CGrHÕÕÕq=Qøàüøþ~YWWW]]æHÂÂÂC%ùÝþÀ?àâ.ëí·ßÖ¨/==½¶¶V ö¥>ðþüø8øgÞp/õ-Y²$©Fr?ðGàÀø£ÅÓé§]ÍF£±¾¾^v¼¼ÔþÀ?àâçÄEs$GUU+_ÃþÀ?àâv»óæÍwóòòvíÚ¶?ðGàÀø£DÃúL%KDøLÄûø]ðþÀø#ðGà/¹ðçóùÚÚÚÄvÉ±hÑ"Fr?ðGàÀø£DÀÓélhh0¡æ2ü?ðþü?Jü¹ÝnÍf6çh¥FrðRøàüøßøóù|v»Ýjµ¦§§ÉÑÛÛË¶àü?àâ.«©©Is¢¾ÔÔÔêêê/2ü?ðþü?oüçZ[[ÅvõÅlLðþÀø#ðþÀ_|ãOöõõõõÉJ5mþôØàà ÉdþÀ?þÔHÍ¥µµÃ»àO¿uvvÊ/+ø#ðþüQüEÉa2øLðø[µjü¦FÀß¹sçîG7á¦ìïûçþçþðl'ÃO?ýí çdw'î²t¾»ûø¿ÿû¿¯¯¯ÏËË5_ZZÚ5kZZZþã?þÍ«._¾ýÝ]ãï71<þN<ùèÖÑÑÑÝÝýÒq===×®]c;è<ÁÃá`;è9!»;=÷ñÇÿùÿù¾ðÍÃ»²þÛßþvWWJ»»èÓDÆÃ¾ö%û&[äà°o"öþÀ?R©ú4Gr¨úÉþÀøþüÅ>Oôn¢>ÉÄDàü?ðþÀ¿D¨¿¿¿±±Qxj¾ôôt«ÕzöìÙ7o²¡À_â/Àø#ðxy½ÞÅ¢ùRÙl¶Ùln·ûù>ÛÀøàÀ?½a$æDàü?ðGàüø¿Ünwssó¢E"|&Ïç½ øàüøþâ&ñ[Ûúõë5?£  @æúúú"øàüøþâ §ÓÙÐÐPXX¨9ipPP¨ùR_àÏårÉ°ÛíCCCàü?àÀ_¼æñx.^¼¸råJÍ-jnnÔ¤-	¿7ß|sÞ¼yK¿¸´¬¬,++ëÜ¹sàü?àÀ_¥Fre4Gr466ÊLä¥¾Çß_ýÕ_åççkç·ö¿¹_þýqíãþÀ?à/Y:tèæHÔÔÔ7Úíöé|&Gâá¯¨¨è¿¡ä§þ½ly¹®®ü?ðGàüøÓo>¯££cóæÍwfäÝl¿¬¬¬@ùÉ¿¯[¿n±XÀøþÀ?=ÖßßßÐÐ ùÂá`WW×ï&þ^ÛòZ þþ`ÅðÊøþÀ?%»xñâÆ5_ê«¨¨hiiñx<3þyÏøàÀø#ðÕÔ¤-/õÉÊ½÷:Ùûî:Ú÷3ùÌ¿øÅE1Úü?àÀ.r»Ý6Íl6käX³fÍÅgððnRáïùyþLmmmÌóþÀ?ðGà/ÆÉóÕjÕüLÂÂÂ'NDÓ+|ÂøàÀø#ð7+¹¦¦¦PóÍ;·¾¾~jõ?ðgø3^)))àÀ?ð¿y½ÞÖÖÖêêêÐ²FÖË¹Ó¨ü¿8Ã_ÊxðGàÀøÇÔgrÌ;7ô¥¦¦¦Iø	¿þü?3[éééV«5úÏ;àüÅþ·nÝþüøúÏçóÙíöp#9Ä"Bq¡Þn6ø±ÄÉd2¼çÀ?ð_õõõE¨OÎr:º½ñàüÅeee¡£Ñ8³?àÀßôw$Ýnþè]ðþâiiiòhÌËË1ß¹sçda¶?üøþ&[äàOïøSYíÉÂýû÷e.àÀ?ð§&2Q_ÜýPàüÅ999òàéììt:²pàÀµÀT/þü¿ØæÉzxWähiiáDàüÅ+þvïÞíÞô þü¿ät:ãw$øzÇôÖ[oÍ7Oº»»eA XYY9Û7üøþw¢¾¸ÉþÀ_à/&?àÀ¿È#9ô9Qøàüøþ&WBäà/ðW\¬&|agþÀ_0Qd±XäÜøÉþÀÞñWTT>ö%ðGàüÍxj$æáÝÉþÀÞñ'ÎwåàÀø£äÁ_©©©	6ü?½ãÏh4Êc/úûqðGàüQÂãÏ?Q_zzz|&ø	?ùÍGàöíÛGFFÀ?àoFR#95'm©­­åþÀ_Ìð'Í??ôÁÉþÀßd<Ãl6'ä¤-àüÅþ,XÀþÀß40Q¬Lªàüéêép8¢|ÓÁ?ðG	¿Ç8qBs¢¾ÔÔÔêêêd´ü¿8Ã_nn.>üøMöáV«5+++É<ü?½ã¯££C¨»wïò_fàÀø£xÄ_¿À®   >~üQ"öeÀ?à/B^¯WÉþÀ_"à/%Lø ðGàü©«ª¯¯WÂ&ÛÇï?JØ©^bø#ðþHÏøs»ÝZ²dæÑ!³ÙÜÒÒÂHðþâ&iÁ½½½àÀ?ð§>cãÆ#9íãwÁ%&þ<£ÓÁ?ðGºÂ_¿ìóòò4Gr»ººÉþÀ_"àïÚµkòÀnllæÞüø¤ü¹ÝîÖÖVÅ¢yx·¢¢¢¹¹àü%þíKàÀ_râÏápûL£Ñ(gÉ°%Áøc´/ø#ðGà/¾ñ÷øñãæææÉqñâEFr?JdüÅ*ðGàüQ4ñ'kkk[¿~½æD|&ø#ðþüøü9Î½÷jäPõuuu±ÅÀ%þÆÆÆV­Z!;ÌÌÌuëÖEa·þü?Uüýë¿þ«Íf3Íoì®ªªsØVà£££ûÙ~Ãø#ðþh½ëþá¦¥¥îÛ[	üQòâ¯´´Tv«W¯ÃÃÃk×®5/þüÅQ.«©©)ÜH«Õ*OcLÔþü=WîÆÆÆd¬þüé?Ù·¶¶VWWkä?~Ìþ~YJJì|þ5^¯WÖ0Õ?:Ïét644L¦PóeggoÞ¼Gø#ð§:ì»bÅuØWþeYS^^þüøÓaò'zKKæH5Q_kkëÍ7§öÙ¾þ(ññ'ÚÓðñôéSðGàò;vôèQÍÖÛÛw·<ÜgrL¦¾¾>õSøl_,øþbÀïºuë²³³SRRäÿ+VÈÙ¾éàÀøI[·n<ùË_þÒ²/eff~ïßÓÿmv»ÝáFr¤§§[­V»Ý4ü?ºüøÑ¯¥¥å·û·¿µó[ûßÜ/ÿþtÛþÖoýV[[>o­xNT'¶á²Ïl6Ûl6q¡æeÁø#ðþÀø#ð÷|åÊ_ûÚ×üÔ¿êêê×^Mo·³¯¯/ÜHõN§3ò5?ðGà/äãþüQá¯¤¤¤þõøûºõëE'7Ïëõ´Å?c3ð?ðGà/¸ð?¨øûêW¿úÊ+¯â¯ªªjÛ¶m1¿aFr¨ú×¤®ü?í7ÞP»Ó§O?`øß®Ï|æ3ôê)ùm¬Ù3î!ÔÙËívûø]5cÊûIðþüüfffÊÇb±ÎùþüQÂàO:sæÌoþæo.X°à³ýìK/½ýÑóéäàÀßÌ´aÃµÓ¹zõjtn:ø#ðþb×ëu8½½½Ñÿ Û#9ÔD3õ2$øþÂváÂµßY½zu4o:ø#ðþ§#9¤+WNÔþÀ¿ÇßèèèâÅÕØk×®Eù¦?à/0Ãh4Êphhh6¾/øþ;|ø°ÚûlÚ´)&7üø	Ëå÷ÁºººY=èþÀ¿K2Ï?3$æá]³Ù|èÐ¡iäàÀßK/Áþüø`N§3ÜH¼¼¼úúú(ÿb?ðGàOw?à/0QT]]-Ï'ú7ü?àü?3YyyyMMM1;ü?àü?3SYYYV«Õn·OðãwÁøàü?àO§E¨O,xèÐ¡ÞàÀøàÀßL¦Fr¨¯®®®··W7ü?àü?hhhèÄ%ÜHè:øþçü?3Ýn¯««ËÊÊÒ|©oïÞ½ýýýúÿ)Àø#ð~øc?TøS#9C÷©©©ë×¯×ÉHðþüM·öövÙµ½úê«###rRþ_»v­¬¹~ý:ø#ðG	?ÏwæÌÅn$Pw?øþÂf4eøÎ±±15OÕ4¯Y~§óóóÃÂ¯]»þü?]åt:wìØ¡ö¡¿ýÏäàÀ_ð§öt¾ üMÿ=555§N#GlÚ´	üøzÈår=z´¢¢Bó¥>ÅÒÚÚGwÁø#ð7érsse'PgõêÕ²FÖOóåïiõô 4L¡ø³Ùl·£ÛGÔÙÙytÜ?þã?þÝßýÛAç]¾|ùã?¯Û,Tüõ_ÿõ5k4ßè,¼×^íÂ	s±»ÝÝng;èwýo:ëø»~ýºæ;wîLóüÉ~ÖÝnÜ¸áp8ãzô£±tÞ|ððáÃx¹µ]]]ò'hè.--í«_ýê3gúûûì>Xüä'?áwUÏýË¿ü»;ýwåÊèïîfÒýû÷222äâÌÌÌòòò'OLÿjËÃ¾Äa_ûF3¯×a$Ùl¶Ùln·;Qï#ûrØ8ìrssÕ[	åÿÐÈàÀø=÷Ô××'êHðþüé7«ÕzòäIYÿkjjÀ?ð7«y<Í¶dÉÍúª««c$øþf »wïgffªµ¹¹¹çÏþÕvvvæååÉuæççwww?ào6òù|v»Í5ééé¡ì+,,ÓúÀø#ð7[øS<~¤Z>räÈ¬ÞtðGàüM3§ÓÙÐÐ 9C hµZzþø]ðþÀøþòòòdGy÷î]?þº»»e9;;üø#âÏëõ¶´´ÍfÍ~$øþ¦/R²Oç³	üñ'¿õõõsçÎ5Édjhhp:Ü;àüø'5É³zµOð766¶gÏµ'þHøs»ÝMMM%%%Fr$íá]ðþüMºÍC'7nÜþ(øS#9¬V«æH±`räàÀßÌ<ÝVVVªÑ¾ÅÅÅ³ÓÁ?ð.!ì"4Gr$ÏDàüøEüÅ$ðGàüåõz[[[«««ó39Àø#ð%üùÇyøòäIqqqAAø#ðGÑÁÓéd$øþb¿±±1Fûø£(àÏívÛl6ÍI[¨ü?3¿9ËÉÉþhð'ÿÈ#98¼þÀ¿ÆßR^äÿlÀD~.þhfñçr¹ÂMÚÂHðþüÍ.þü	õfû/ø#ðäøóù|áFrø'êóz½lXðþüE±üøKü9Ny°FÍÛ·oÿ¿øææfÞÛþÀ¿èáoll¬¸¸833Ó¿fÞ¼yßýîwÁ?2þ<OKKË¢E"ä8þ|NNÙl~Ùò²@ðå_æÅ?ðþüEóçÏð«vÐàÀMr÷ÕÕÕiä¨Ïétü^ÛòÚþ7÷Ë¿÷wïÞÍ¶àÀß¬ã/--MvÊÃ¿F~eMvv6ø#ðGÄËåzûí·óòòBÍg4wíÚ4Qß¡C¾e%?õoëÿÝú;¿ó;l[ðþüÍ:þÔß ýxèäàÀâOüîúõë5?cåÊ/^ôx<¡×°cÇêêê@üÉ¿ßøß`Û?ðGàoÖñ/ûè;wªwÛîß¿_ÖÈ_ðàÀÃß'|²wï^ÍMMM.+Â5=zô_øB ü¾öµ¯þógÛ?ðGàoÖñ×ÓÓ£9Ésww7ø#ðGA¹ÝnqÛç>÷9Í7nåDÆíÊõL&Ëÿ±ìÞµ[ä÷Íß­­­laðþüÍ:þ¤fdd¤¤¤dffÊÙ¾éàÀ_|ÕÕÕî39-Zd³Ù?~<©+¯åWrrrrss_zé¥62øþ¢¿þüÅEýýý&)Ô|YYY;vìp8Ó¢O.;44ÄvàÀøàb×ë÷Riié¹sç4Grø#ðþâwïÞUó<«¾¹¹¹çÏþ÷î;wn¨ù].×?ÛÀ?ðüµ··û÷ìjùÈ#àÀ_ò$¤kjj*))Ñ<¼»~ýúÿá]ðþü¿8ÆõîÝ»~üuww3É3¿$IMÔgµZCïÊªªªææfõ?ðGàüÅ1þÔ^þyÀÄÎ²OeÁþü%pN§3ÜHù°±±±¯¯/ÜeÁø#ðþâ¹¹¹jV?¿±±±=öÈ²<%?Ûí¶Ùlf³Ys¢>uxWMù!ðþü¿8Æìè5'y¾qãø#ðHÉ­¯¯×ÉQRRrôèÑÈÉþÀ?ð øSO·j´oFFFqqqÃ=àÀ_|a$@P88þÀ?ðßøIàÀß¬y¢>Å"çxü?àü?zOäÐ<¼k2ä,ùi~ðþü¿øÆß½÷,X&Ï.þâ«#9RSS­V«ÝnÎç°?ðGàü%þäù@sÀGoo/ø#ð§ÿüõ¥§§kähjjøHðþüQâãOMò£>¦sdddÓ¦M²&??üøÓsj$Gaa¡æ¤-µµµ³·%Áø#ðþâê©"ð`,û'|þôVäf³Ùf³~&øþüý2õÊßØØÍèè(¯üøÓa&ê32ü?øøSïù«©©óÉÉ§OVUUñ?úIäÐ¨/55µººz:¶?ðGàsÆkÿ?<£°°p6Fr?ðGà)ãe0À¿h¦&ê3L#9fvÒðþüQÒá/V?A©Eó5øèäàÀ%>þÂÍç<00þüE§È#9¦öñ»àü?ðGà/ÌUÌóÖ[o­Ü¼y3S½øíÔDºÉþÀ?J|ü	òä&77÷É'ròý÷ßWÏ=³ôV?ðGàOä7Q_lGr?ðGàÏ_¼Î§uòóóÕÂÚµkgû¦?JBüé$øþ()ð']¿~Ýÿ$tôèÑ(ÜtðGÉ?5QÙlÖÿHðþüQRàoÛ¶mêIHÚôê«¯?ÓO~ÚÚZÍúL&S4?ü?þ~Á`ç¡ìììÛ·o?xÏ_ZZø#ð7µ&2#.ï?ðGàü%æhßûö­¬­­e´/¿É¦&ê7C,¨ÿàüø£ÄÇ_¸yþä9üø`#¾&êàÀ%/þbø£À×ë½xñb"äàÀ%,þä)ðÀnäàÀ_P£¶¶6+++ÁFr?ðGàÀø#ð÷ë+**â¢>ðþüøþ"ÕÕÕµqãFÍI[ÔH<¼þÀ?ðþÀ%þúúúÞ~ûíÂÂÂp#9GÞMàüøàüQBáÏçó]¼xqåÊ¶X,'N$ÕKàüøàüQbâÏápìØ±Ãh4¯   ¡¡¡¿¿»	ü?àü?oü©K,ÑüL«ÕÚÑÑÁ½þÀ?ðßøø£$Á¨.Ü¤-r¿?ðGàüÅ=þRÆË`0?J`ü¹p#9òòòv$øþ(añóÀÅj$Guuµæ¤-+W®lmmõx<Üàüø#ðþ(¾ñçp8víÚ¥9£°°P~!ûúúØþàüø#ðþ(¾ñçv»m6æÇïfeeÕÖÖ&Égr?ðGàÀø£àNç¡Cêëëc5Êa¦ð'ÕY­VÍÃ»"Âd¨ü?þÀ_²wæÌ/-ûÒ+¯¼ò¿÷ÒK/ÉG<â¯¯¯¯¡¡Ád2iäØµkWL~.ðGàÀøàOGõ÷÷gdd¼¶åµýoîWÿ^ùÊ+ýìg£<tÊøóz½­­­ÕÕÕ¡É!kd½+_ÃþÀ?à8qbéÒ¥~ù©ùùù½½½úÇ|ýÜ¹sC_ê+))ijjr¹Åàüø#ðþè×Ùl¶_~9ÿüç£?×ÝÄñ'¤Ø	ïBÍ'êá3Á?àü?=ÖÕÕõÒK/íùüò«ûºèÏx7.þü#9BïJf³¹¥¥Ã»àÀø#ðþÀß8mÜ¸±°°PÌ'òûºõëbÁ¿üË¿þÍ?§Ón$¬³ä¸Á?ðGàü¿	åóù8ð¹Ï.##£¨¨èÔ©S1¹¡ø0Q_zzºÕje¢>ðGàüøà/^Ä_äLÔþü?àü%þ®^½ÊHðGàüøà/ÁSõ½üòË)))LÔþü?àü%l½½½áïL&ùUa¢>ðGàüøà/îóx<j$Gè¤-äþÀ?ðþ§ÿøÇuuu/õ-^¼øÍ7ßd$ø#ðþü?ð÷õ÷÷Ëý^QQj>£Ñ¨&êògûø#ðþüÅ¾ÁÁAÉþ<5£ºº:ôð®¬Y³f<´ü#9Àø#ðþüÅ+þ:;;KKKå	ü%m½½½yyy¡/õÉÊ¦¦¦¡¡¡ ?ðGàüøWü­ZµÊétFÀß÷¾÷=t»zõê~ð;Írû·ûÍo~³¨¨(tÒYóû¿ÿûßþö·å¾Ð¼¬ÜAáÎ"ýtùòe6Îcw§ÿØÝ±»Wãï71<þ>úè£ÿnüq__ßÓ¬%÷é7¾ñôôôÐú~÷w÷ûßÿþ¸ÛÿÁÝÝÝlI÷Áüâ¿`;è¹[·núé§l=×ßßóæM¶Î»råÊèèh¿i"ãÃ¾	Ëåjjj*((ÐÉ±k×.Ã1Á«â°/Ã¾ö%ûÆþüOùà/0CZ¹reàHðþüø	?M¿ÄËápìØ±Cs¢¾¼¼¼I½ÔþÀ?ðGàü?æñxN8¡9Q_jjêúõë§ðRøþÀ¿Â_¸À_åóùìv»ÕjÍÊÊeX°¹¹9tÒðþüøàüÅYN§³¡¡Ád2O 8Ã»àüøàü?ð§Ün·Íf3Ís´Z´hQssóãÇgã[?ðGàüøà/z	¼jkk5'ê3;vìñúÀø#ðþü?ðíÔD%%%#9ª««[[[§9ü?þÀø1.òDbA¡¸07	ü?àÀø3Óé¬¯¯×¨OVÊY±øþÀ?ðþf¬È#9d½+_Ã[þÀ?ðGàü¿éæ¨Os$Édjhhp:z¸©àüøþÀøz.K6µæDAá  Ph¨þÀ?ðGàü¿I7±=¼þÀ?ðþÀø3ÈI#9Àø#ðGàü?ð7c¹ÝnLÔþÀ?àü¿YlÜqôã?ðGàüøàO£É¡Ïàüø#ðþÀøtGrèa¢>ðþüøàüÍ@cÇ#9F£~&êàÀ?ðþÀßÔjnn®¨¨ÐüLªªªÖÖVÇ03ðþü?à/Iñ'Z¿~½æáÝÂÂBÙñ5ü?þÀø=~ü8ÜKq=ü?þÀø¿ÎëõÊoØúõë5'm1ÍG&üÃü?àÀøKpü9Î¼¼¼PóeeeíØ±Ãáp$ÏÃü?àÀøKLü¹ÝnÍf65?cÍ5gÎI¤àüø#ðþÀ_â¯««+Âgr466º¤?ðGàüø	¿¿+Ü¼y³ 0Gr?ðGàÀøÉ?§ÓÊ¾ææædÉþÀ?àü%þ$ÅxÉþÀ?àü?íl6[â&øþü?ðþü?þÀøþÀ?àü?àÀø#ðþÀø#ðGàüøàüø#ðþü?ðþüøþÀøþÀ?àÀ?ðGàÀø#ðGàüø#ðþüøþÀøþÀ?àü?àÀ?ðþÀ?ðGàÀøàÀø#ðþü?ðþüøþÀøþü?àü?þÀ?ðþÀ?ðGàÀøàÀø#ðGàüø#ðþüøþü?àü?àü?àü?àÀ?ðþÀ?ðGàÀøàÀø#ðþü?ðþÀø#ðþü?ðþüøþÀøþü?àü?àü?àü?àÀ?ðGàÀø#ðGàüø#ðþü?ðþüøþÀøþÀ?àü?àÀ?ðþÀ?ðGàü?ðþÀ?ðGàüøàüø#ðþü?ðþüøþÀøþü?àü?àÀ?ðGàÀø#ðGàüø#ðþüøþü?àü?àÀ?ðþÀ?ðGàÀøàÀø#ðGàü?ðGàüøþÀøþü?q¿îîîòòrÁPVVúûþü?àíþEEE7nÜÓ§Oâï£>nò0ëëë%×ßß/6°tàÏãñ°ôüõé§²ôÜOúÓ7o²tàï¿øE¿iã/°Püÿûßÿ(ºøá?øÁ>"×ÞÞ~õêU¶Î»|ù2Aç±»cwGñ»»KüõôôÔÕÕqØ8ìËa_â°/qØÃ¾|ØW522RSSãñxÀ?ðGàÀøK4üÍùUêäÀÀ@mm­<~%ø#ðþü?¶øüÖÑÑ±bÅÁÁAÍsÁ?ðGàü±À_BáÏd2Í	üøþü¿DÆ_äÀ?ðGàü±Àøàü?àÀøàÀ?ðGàü?ðGàÀø#ðþÀø#ðþÀø#ðþÀø#ðþüøþü?þÀ?àÀøàÀ?ðGàü?ðGàüø#ðþÀø#ðþüøàüøþÀøÉ¿?û³?kmmín~øáÍ7ûIÇüñÇ/_f;è¼¿ù¿¹ÿ>ÛAÏÉî®§§í çD~¶Î;~üøOúÓ(S·Ûø»÷îþýû¿CDDDDE>4:×c'ðGDDDþüø#""""ðGDDDDàÀNëîî.//7eeej"u¯×[[[VPPÐÑÑÁ&Òá4' 6>ï&yøÊvvv²txòÉ'²»Û°aÃðð0(æÝ½wñâÅ¹§òóóÕk×®±ôy7I&	üÅGEEE7nÜÓ§OËÂÁßyçgÏÉS×ØD:¼ü:uêÈ#l"ÞMF£ñÁ² ÿëgÈxÉPz·mÛÆ&yK.ÉÜSòÙÑÉìë6mÚÄ&ÒçÝ$¿uçÌÑºÀß$ÊÈÈPwªìÙz¾T÷ïß_¾|9ÛD·wüí400 ò?Géó>2þ5999lýÔÖÖVVV¦þR=66ÆQº½V­Zåt:Á_üÕÓÓSWW§ö=£<]Ý¹s-£ÃûHµnÝ:YÃfÑíÝtëÖ-yÉÞPþçÒç$Ï[wïÞ÷ß?Ã|>_vv¶<pN>tî#ÝÞM¿$ø¯FFFjjj<,§¤¤?~üù¯ÞÃÆÑá$ÉßXK.e³èùnZ¼x±z]È¥ÏûHþ¾-..¿÷Þ/«SÌkooÏÍÍUOIþiiilÞMà/þ¨­­ôè:xGòg>ï£ç/Þyøða¶ï&^±êÞ½¥¥¥l]¥5ò466öüÅaßÀ§'ÒÕÝþâ¬+Vú×lÛ¶íìÙ³Ï_ç1Íl"ÞGÒ²eËnß¾ÍÆÑóÝ´téRuHÑáp,^¼M¤Ãû¨¨¨èÖ­[Ï=ï½÷Þzë-6QÌD=jº»»åÎ«ÕzòäIYÿkjjØDú¼À_e2ç5ÃÃÃëÖ­ËWVV:N6ï£ç/¨7Anï¦÷î©ÙäYféð>Î?_Muuu>MóÄâ.GÍòåËÕ´yyy)))ùùùjh6éðnDDDDþüø#""""ðGDDDDàÀø#""""ðGDDDDàÀ?""""DDDDþüø#""""ðGDDDDàüø#"Qï¿ÿþ²eË2^´|ùò.ü¯ý×âfo«ukM&üh'h½¬1ùùùÏ=ìu?"Ëöïß?'¤·Þz+ð×ØØ(+=´¾¹¹YÖ8p`×IDþ(þºuë°Æ`0;vÌ÷¢ãÇËIYyûöíÁßýû÷eeYYYÐúÒÒRYß××þüQRôê«¯kÞyçÀï¾û®¬Ü¼ys ®]»&xË²ÿ>ZWW-gÆ;w]µÛí,9K.ÛÖÖÄ)Y³lÙ²«W¯ÊI«ÕtÃ®ùzÔYrÔYíííá ¶bÅYßÝÝí_sãÆYc±Xük<'W±aÃPü^Ð7ÀQ?¾ÀåÁ+>|(+M&S nêééQç®]»6è¬íÛ·«³GJJæ¥ÔIuîºuë=&|LKKóù|r®ü/xÊÌÌToÅp=² yóBÒÓ§OÖïËS§Nùåt%Ë/,þ"ÜT"DD±OáÕØgÍ#ÄÍ¦MF^$rrõêÕêÅGõ MUSS£^2åÎÎNu%×¹oß>á:êúúë¯ËK.É²ü/Ë[¶l÷zrR¾ ð¶iþDÊyóæÉÏ;88('å¹å"Î±±1õùùùrAùÿê0qFFÆdñá¦ø#"Òþ$Müù×+Ü(3IrRØ¤NËÉlÝºUÐ6::ê¿ù ×ÒüR'>|èÿbÿÈ¯ü/Ëv»ÜëhÊÉ'OÞ¶pïÏã7ä¬÷Þïù¯mïÜ¹3ð?!iee¥zar²øpSüÅ>õr×ÈÈHàJÇ#+å¬ÜñÓÐét*ÿù­ã£^èÑØ NN°"ËF£1--ÍëõÊçääøÏ÷zÂQ,¨»wï*§Ê²ü/ËÃngg§ÜÍÃÇÇ_JDà(ö©÷½>|8p¥%hÀÿ¡/hÝ¾»±±Qõ«Q½,ç?®:.Ñ¶oß®öÊÿuuuþõ®G½ÒöèÑ#uòÉ'Gæ.]ºTÎU³ÛYÏR#å¬öööáááÈøóÃTmÿ¹n*?"¢Ø§L#G¨©^;:8cõêÕ¯×«ÉúGæª÷ü©·Ê9ÎÀ7*îÜ¹S¨¤Æáú'[Ñ$~«¾GGëQoòSïùóx<ê+#àïøñãþ×ääG<K½ýÑápÈwÂeù2AçÏpSüé"5rPüõþëEÊFþeÿSÿ¯¿þz ,;þ|ü=ñQ²Þh4p=r3o¤m¸vtt4##CýA»W­Zø-æÍ'ÿ«Ù^¯S½0éOQÕnJDàH/	P/_ö¢eË©!·AøkooWÓé-ðúõëþs=Ï=òòòÞxã¯×ë?·­­­²²RLo³Ù®3ôÈUÉúmÛ¶­w=Ü¹IjÂóüù«««óE588XSS#[ ;;[~¾¾>ÿ´5×9<<,ºUÛÊb±twwÇ7À?""""DDDDþüø#""""ðGDDDDàÀ?""""DDDDàÀ?""""DDDDþüQ´úÿ¥Ø¦wMIEND®B`


Detrended Normal Q-Q Plots


yuªÍÞrÅ¼°nÝºðõàÁSÉo¼±víÚèf2óçÏçÝ3þ|£9áoÀ²eË®rìØ±T*µråÊøQÆ]»vÕÕÕEç7Ã·È-ê?úè£a~ü$lb=Ììîîôç;ÓU¿uèÅ0'<ÈÎ¤Gæâf·bñøË[~.a	ÃþÐC±-~°pé4wã©a$COE5557oõÄXü^	3ëëë£ÃÃÃD"þ<44T$þ¢;wttY7wçðÚ_ÚÙÙ?d5Õ	¾"-¾bás¿~ýz:®­­créÒ¥ðßN¸g^¥Åoá÷]²dIt$5B$W~y÷|yñÄY´hQÿöÛoþ°'ýùÎt¼oÊ&:ò×ÔÔ4£Á)>ø³^qúñwâÄøvÂiüMs7^³fMÞcÞ´i_, þ Äâ/²,Îd2áf>~üx<ÑòVnnß¾bb":sËuÃkgxÕ÷7NÝp3¬>rSX+þ½l¶ø>÷CÅÅï¿aÃ0½zõêk7p3ÌêùFs6nÜ8>>=°hËáæ#GâO0zgÛáÃÃôâ¦zÀÉdrª'[w«Lõ¦ºÜÑÎ¤jÖ+ÏúøÍøn²>hÓ¿iîÆQ^¼x1Lf®ªªòÄdüåÎôEosÄß¥Krsn¹nî(Ztz1¼F7Ã+hüUÞi»"-¾âTÏ=jÄðº7¿¶¶6þÂk|¸¹páÂ©o4'Güft.;zñGÍfC.Èhoo?÷"ñ+ò©J«Ä4¬sÅGtÐ¦38>ªY¯8ýøö+W®Ä¿Åâo»q[[[¸ÙÐÐâ5ü+âÆ~«øÒ¿+afMMÍT¯¸SeJt3TNWë"3ÓçYlvªùá)ç^ÂÇÆÆâó¾®ðùNçfÍ¼S·¼jµ®®.:O]XxyIÛÂtVÎXMzéÎTI:»§Úwî×4wãsçÎEýÂÞÞ^¿X@üAÅßóÏ?¿à#:wÝåtZgFë?róöÛoÇÙlñ<_|1ÜÌ0J43ê³¼cTyY<ýßlnn>r¥¯¯/ÄÙtâ/:<êJ&Ù¼yóµk×öíÛf®]»vÒøÎ*³¿NÎ¬W~üMçÈ_.ï¢íÿ"û[ääÉ;wîÎ5ç®Ä@ü·Ð@Ñksî£^¢´ÐáÅ2ºx¶µµ5Z½áiô¦I_5¬[¼¢jÞ³6m'ï]n¶øÅKbÍ5¹÷õGs¢kW¯^ý¦hk7nüEG¿ÃSØ»wïtâo`` :¶òñãÇ££zCMúóé*3¿â·oÄÍtTgÑ»ôÂÎpãÆÐv«V­*LÃÝ»wÁ:¸øøßòè½çÎ+rpÅ_¡ø×ÏæÞþû<Þè0aá«fuS¨¢(â^æÙlñÄåËsïîæ?>ïý^µµµ¹7Î%þr9Y¸paîEç±Ç+üa-^¼¸ÈxF«Ì(þNÞ¾7ÓQEü<y2¾ý¥KÆæIîó©¾Ký-wEQÎúõëýbñ%áUpùòåþëÈ#ííí!¤Â+úóÏ?èÐ¡0'ÄVüSî¦¹î-Ë)úx¼°ñ¶¶¶Âëj³·xID§DãóÏ=»víÚÔML¦ðòÙÅßÕ«WÃÖÂ6CmÙ²%ôP®¹'Î7¾ñðìÂ_´hÑþýû~øáÜ§¹¯2£ø+>8yûFê,â/èíímhhÏ:l?:±»À9Ü÷+VÞòÈëTûÛèèè¶mÛ¢Cª¡àÃrllÌ/·K6ì±ÇfôÙr³X¥äDG|£Ë·ÇÇÇ£¦onn¶Ãø EïùËóÔSO¡ÑÑÑ­[·.Z´(:·&üqÃâñø@ü þ?Äø3âñø@ü þÀ]îÐ¡CõõõÉdrùòå3]·¥¥¥¢¢âÜ¹s¹9a:ÌimmÁ/¬fwé¬;kW¯^Ý¸qcuuuEmÛ¶mttôý¿ÏÀ¼VWW:#ÎøøøL×ÝµkWX÷ÙgÍÍyúé§Ã;wzüÎk¼éÂ===á­ZµjZ±bøÄpwü¾Cg<y2¬»lÙ²Ü%K9'N¸3òöERÔµü@FXüâ¸åOmÛ¶-X° ªªjÇywøáÛÛÛó¶;p¦ß~ûí0æD.^¼¸bÅÊÊÊd2ÙÖÖÖßß?éÖâß½ø*û÷ï¯¯¯ðÁÃ7*¤®®®°nxð[·nd<]ºtiØZ¶|éÒ¥iLkkkØòØØØ¤KÒÀÀ@xa:T8Â#YdSöO@ü·«ÿ¢é'|2L?ÿüó!³ÂÄSO=¿Ï#G¢ÈÛ°aCX´oß¾0¾éîîîhÑâÅ>&.æ§ÓéI·ÅW	I÷â/uëÖ>øM6ép^z)LìÚµ+ÌüÈG>¦¯_¿><<&¢%ôâTùU|vïÞ¯2ï¹ßÀí¿Ð+a:SaMú¦À°hõêÕazÍ5QÙä<yrûöíííía~"tky¡S|è-X° pÝÚÚÚèÁOLLaf¸gnkku5Õa¼I¿â£tãÆh:laªøË=÷â²sâ¸½ñ/©ò%O(­ªªªpÏÑÑÑèìj-Ú»woX+á©S§hÒ0U¦z`¡ãó¢p<xð`uuu4§¦¦&:(8#MMMaþÈÈHñ.,yÅ§g7àâxßâ/NÇDEÏnÙ"]]]aiîkn~*s&nfü_%÷ÀBÏ®»páÂhÝ¼7>>~øðáuëÖÅÞÒöíÛÃýC,.Î(M3þf7àâxâ/ºÄuß¾Ñ[Ð~úéé´ÈÁsÐ^|ñÅ¼²9útô^ÀéÄ_ñUBóß6l(ýúõaº§§çÌ3Ñ©Þ0óÁÓ§NºråJhjjæ°~=¤XccãÅCPF$;::¦9JñéèÔstÒ9ï¹ÏnÀÄðþÄßøøx¬ªâÕW¼EnÜ¸r_C3åæ÷ööÖÔÔôÙºuë4ã¯ø*GK,Y»î$~ÑÑÑÍ7GJ¥V­Z]Ø/ÉD/[¶ììÙ³Óð]BPF'µCîØ±#z?ßtF)>èÐ¡ð¢ÏÐÎî³pñ þ?Äâñw·øÁ~ðæoÞÉïøÓþôÿþïÿìyÌÅ7îð~KYzë­·þ÷ÿ×80?ÿùÏÿýßÿÝ8¿Ròõ¯=ôßÎÍøÇÁ,üÏÿüÏñãÇstâÄÿú¯ÿ2Ìñß¢ßýîwøâñøCü!þÄøCü!þ?ñâñøCü??ÄâOüøCü!þâñâñø??ÄøCüøCü?ñ'þ?âOü?ÄâÄøâñøCü?ñ'þÄâñøCü?ñøCü!þâÄâñø þ?Äß-~¥Óéd2ÙÒÒÒßß_x¾¾¾ñøñøå Éôôô=ötvvæ-okk*þBÜA¯¼òÊ[o½5spéÒ¥ïÿûÆ9üÿøãÀìg?;zô¨q¸ÛÌø«©©Ùl¶¾¾>oéO>ùÌ3ÏL»wï~å:|øpÿ+0a;q`^~ùåð²mïï½½½Æán3/â/LN:x±½½=¤¡Ó¾8íNûâ´/eD"7J¥â:::;ö§*þ þâ¯<Fmmm6ïæiß0ýkÏð×?Ä?Äø+y]]]û÷ïák&ü©:òøñøåñ4Âa]]]"H§ÓÖøCüøCüáCÅâñâOü?ñøCüøâOü!þ þÄø?Äâñ'þÄâñøCü?ñøCü!þâÄâñø þ?ÄøCüÄâñ'þ þ?ñøñøCü?ÄâÏ8 þÄø?Ä?ñ'þÄâñâOü?ñøCü!þâOü!þ?Äø?Äâñ'þ@ü!þ?ñâñøCü?ÄøCü!þâñâñø??ÄøCü!þÄâOü?ñøCüøâOü!þ þÄø?ÄâOü?ñ'þ?ÄâOü?Äâñø þ?ÄøñøCü!þÄ?Äâñ'þ þ?ñøñøCü?Ä?Äøâñøñ'þÄøCü!þ@ü?ñ'þ?âOü?ÄâñøâñøCü!þÄ?yçwÆÇÇÅâñ'þâ¯Ìýã?þã=÷ÜSSSS]]ýø¡Åâñ'þmù¸æÃúØîøÚm_Ýö»¿û»øÄ'Äâñ'þ§î¹'*¿Ü÷ÝwßÑ£GÅâñ'þwß·¦¦&^~á¿åË?ûì³âñø?ÊÍøøxuuõW¶|%K,ùøñøCü?ÄeèGYºti®ü6üÙªªªóçÏ?ÄâOü!þ(Cÿýßÿÿý÷ÿvóoîsû?øßøß¸ýÄâOü?ñøãn166öüóÏÿùÿùã?þÚk¯Ý¦ï"þâOü?Äóø£¼ãoô&?#ñ'þ þ(óøûÑ~ÔÚÚZSS³páÂüã¯¿þºø?gü;w®ººzÍ5Ûÿzû¯íxäáGî¹ç·ÞzËKü?ÄâOüQñ÷ùÏþÓþtüÃ~oÉïmß¾ÝKü?ÄâOüQñ×ÜÜ¼áÏ6Äãï]_xè¡ü°ÄøCü!þÄeúÔ§¾øèãñ÷Gkþèóÿ¼ø?Äø£ãïïþîïîo¸ÛW·Eå&-ZtàÀ?,ñ'þ?ñGÆ_ðÇüÇ÷Þïg?óÙÏ<ôP~ëÖ­÷Ãâñøgü½½½_ýêW·nÝ&ø?yü!þÄâÄâñ'þ?â¯D¥Óéd2ÙÒÒÒßß_488ØÖÖµ¶¶»?Ä?Äø+yL¦§§'LìÙ³§³³3¾¨±±ñÕW_hjj*¿þçþÏ;(üOòüä?aÂ.þc£cÇýøÇ?6ÌÅùóçÿå_þÅ8ÜmæEüÕÔÔLLLl6[__?ÕÝ*++ãoß¾§î ¾¾¾¡¡¡S0?üáÃd£ðoÑ×^Í80'NxùåÃÝf^Ä_2t:.$Www·Ó¾8íNûâ´¯Ó¾%/Hä¦S©TáFFF2Ìèè¨øCüøCü¿W[[Ífß»yÚ7Lç-½|ùòºuë®R¸¢øCü!þ@ü¿ÒÓÕÕµÿþ0¾f2ø¢+W^½zuÒÅâñâOüðBXWWH$Òéôààà/[Å/]EøCüøCü¿ùKü!þ þÄø?Ä?ñ'þÄâñâOü?ñøCü!þâOü!þ?Äø?Äâñ'þ@ü!þ?ñâñøCü?ÄøCü!þâñâñø??ÄøCü!þÄâOü?ñøCüøâOü!þ þÄø?Äâñ'þÄâñøCü?ñøCü!þâÄâñø þ?ÄøñøCü!þÄâÄâñ'þ þ?ñøñøâOü!þ þÄø?Ä?ñ'þÄâñøâOü?ÄâñøâñøCü!þÄ?Äâñ'þ@ü!þ?ñâñøCü?Ä?ÄâOü!þ@ü!þxã¯âVø?Äâ2¿Ä­$Iñ'þ?ÄeåAü!þ þÄßû ôÍÄøCü!þaüÕ××'Iïù?Äâò¿ÖÖÖÂ«=jjjFGGÅøCü!þ[ü¥R©PW¯^­««¡ù¾ùÍoîînñ'þ?ÄåÑ¡¾0j/Lpabb"LTUU?ñøCü!þ(·ø«®®©^Î;&üñhÂG½?ÄâñGÆßÖ­[swÄßö÷à?ñøCü!þ(·øxâÁÁÁ0B°½½½$¿øCü!þ@ü¿yDü!þ þÄø?Ä?ñ7µ¦¦¦è_|È³øCü!þyü566Æ/ÇÕ¾âñøCüQñ:/dßÐÐÐÄÄDÉ=ñøCüø3SSSâ¯ËOü!þÆñ'þfóë#Äß¦MFFFÄøCü!þyü-ª(àñøCü!þ(ÃøkhhpÁøCü!þÌø²oxx¸¿øCü!þîÑÑÑ×_ýÍ7ß4ìâr¿ÚÚZ|?Äâo*Ï>ûleeeÓGî½÷Þûï¿ÿÌ3_üQÚñ700âoëÖ­cccâOü!þqßþö·?üáw¹Ç×vÿyøî¹çwÞ1þâ¿)¸àCü!þ­kýB×¢òþ ýgÖø?J8þSpÁøCü!þ>ô¡ÅËïÿyäË_þ²ñpü4ñøCüÝVë×¯Çß'~òÉ'4þâ¿úúú÷ë¼áQ:N&---ýýýÅÍnøCüÁ¿]»vÝwß_Ùò¨ü¾øè«««Kô"âïBWUT¼oG3LOOOØ³gOgggñE³·mÛ¶ßùßYqo·lÙ²0üä'ïð~KYzà~ÿ÷ÿ|£ð/ðD"±`ÁªªªðÑÖÖfðËÆ§>õ©üãÆánsÛã¯¿¿?ÄßÎ;¯_¿>÷|©©©6Ífëëë/Ý¸-[¶TÛïïÕ¾ñËDò.)»9â³÷þ^íOÆT*U|ÑìæÄmß¾ýsûÜº;(|»GtÌÁ¾ð;¼ßRV¯^ý'ò'Æ¹øÒ¾ôÏ|Æ8Ümnü½¿jkk³Ùlt6L_4»9.øÀð¿í>qèêêÚ¿_3LñE³#þ þâoN²ÙìªU«*+++**ªªª:::fåGxI«««K$étzppðòæÙëÂE³#þ þâoN?øIßlXêWü!þ þÄßÌ477Ô[½zõÈÈH¸ÊfÍ5aÎâÅÅøCü!þ[ü¥R©zããã¹9Ùl6Ì)¼´Vü?ÄâñGÉÇ_"©]Qsf÷Q/âÄâñÇ]Ñiß+WF§Ã×0æ´µµ?ñøCü!þ(·øµ7é×®]âñøCüQnñýì;::,Xýéî+W9%ñüÅâñâOüÍ#âñøñ'þÄøCü!þ@ü¿¼5o%H?ñøCü!þ(øKLMü?ÄâñG¹ÅßT¶lÙÅßÄøCü!þmüß#UUU!ûV¬XÿÌgñ'þ?Äåk×®ø>|¸¿øCü!þ@ü¿yé¥¢ì[½zuÉ=ñøCüø3ø/^¼8º¶£¿¿¿¿øCü!þ@ü¿iÙ½wtÀ¯³³³t¿øCü!þ@ü¿é­ésþÄâñøó'þ·L&ÅøCü!þIüñøCüøâOü!þ þÄø?Ä?ñ'þÄâñøCü?ñøCü!þâOü!þ?J=þR©Ïù?Äâò¿ÆÆÆxðù?ñøCü!þ(çø²ohhhbb¢ä¿øCü!þ@ü¿©©©	ñWå'þ?ãø³ùõâoÓ¦M###âOü!þ?Ê<þEUpÁøCü!þaü544¸àCü!þ?æKüEÙ7<<Ï_ü!þ þÄßÌÔÖÖºàCü!þ?æKüøÛºuëØØø?Äâ2¿)¸àCü!þ?Ê0þSpÁøCü!þaü4ñøCüøâOü!þ þÄßÔ²ÙìªU«*+++**ªªª:::Jåâ_ñøCüø3þÁOzÁGI+þ?âofCê­^½:úÛ¾¡lÖ¬Yæ,^¼Xü?ÄâñG¹Å_*©7>>ÍfÃ0_ü?ÄâñG¹Å_"©/7gll,ÌñQ/âñøCüQñö]¹retÚ7|ÓaN[[ø?Äâr¿P^ðqíÚ5ñ'þ?ÄåÑÏ¾££cÁD"|]¹reSÏ_ü!þ þÄß<"þ?âofêëëÎ9#þÄâñø£üã/LVTêDñøCüø3ÓßßâoçÎ¡iJå¯º?Ä?Äøí&¦H$ÄøCü!þ[ü%¦àCÅâñø£|â¯¡¡¡¥¥ett´¤¿øCü!þ@ü¿iþ¤ï7OûÄ^ñøñøcöñýIßèÏø?ñøCü!þ(óøK§ÓE¹àCü!þ?Ê'þª««£ãQê¹àCü!þ?Ê6þrJ¥óÄâÄâÛWü!þ þÄ_	þK§ÓÉd²¥¥¥¿¿?¾hpp°­­-,jmmw??ñWò2LOOOØ³gOggg|Qccã«¯¾&8ÐÔÔ$þ þâ¯äÕÔÔDV8ÍÖ××Ou·ÊÊÊÂøûû¿ÿû×î #GþæàØ±caG2ÌQ__ß+¯¼bðÑÃ»Ím¿³gÏ~àñ¿âdª«Oº»»ã¯··÷êô½ïïÂWaÂ.400`£û·;wîq`.Þzë­ð¯ãp·¹íñÎK[[Ûo¼ñAÅ_ü3S©TáFFF2Lá£sÚ§qÚöuÚwfBöå>Ø9L.[¶ìüùówâ¡ÿJ®­­Íf³Ñiß0wÏË/¯[·îÊ+?Ä?ñ7gÏmnnÎY*ZµjÕ]]]û÷ïák&/X¹råT@ÅâñâOüÍÞÄÄÄáÃ+++ïðw/uuuá;¦ÓéÁÁÁ_>·ëëëãtNü!þ@ü!þÄßû`xx8~äoÁWWÜÄâñâOüÍL¼ù*++;;;/^¼X*Ï_ü!þ þÄß7qó:zèÂ%÷üÅâñâOüÍÌø	/âñâñÇ¿&þ?âoÆN>ÝÔÔTUU]á[[[ðàAñ'þ?Äe¹>¢ø¦÷ìÙ#þÄâñø£Üâ¯®®.¤ÞéÓ§sñ788Úø?Äâr¿Ü'çâobb"ºXü?ÄâñG¹Å_mmmH½èh_¿l6»mÛ¶0]__/þÄâñø£Üâo`` b2¯¾úªø?Äâr¿àÊ+íííÑÕ¾MMMçÏ/ç/þ?âo?Ä?ñ'þÄâñâOüMæâÅmmm,HÜ&ZZZJå¯øCü!þâOüÍÀúõë+¦°aÃñ'þ?Äå:oïÞ½£££¹àÐ¡CÑü#G?ñøCü!þ(øK§Ó¡ðöíÛW¸(úo>çOü!þ?Ê'þR©T(¼ðS/ÍfÃ¢pñ'þ?Äe¹¿ê6ÕÒèO½?ñøCü!þ(ø+wâOü!þ?Äø?Äâ¿âÄøCü!þOü%n%L?ñøCü!þ(ø+âñøñ'þÄøCü!þ@ü?ñ'þ?âOü?ÄâñøâñøCü!þÄøCü!þ?ñâñøCü??ÄâOü!þÄâñø??ÄøCüøCü!þÄâñ'þâOü?ÄâÄøâñøñ'þÄøCü!þ?ñ'þ?ÄâOü?Äâñø þ?ÄøñøCü!þÄ?Äâñ'þ þ?ñøñøCü?Ä?Äøâñøñ'þÄøCü!þ@ü?ñ'þ?ÄøâOü!þ?Äø?Äâñ'þ@ü!þ?ñâñøCü??ÄâOü!þ@ü!þâñâñø??ñ'þÄâñâOü?ñøCüøwç¯°t:L&[ZZúûûïÐ××WQQ!þ þâ¯d20±gÏÎÎÎ¼¥ãããmmmâñâñGÄ_MMÍÄÄDÈf³õõõyK|òÉgyfªøúé§ÜA½½½/¿üò°É80GßùÎw>lðë¨üÌøK&N/^looi8Uüüü:vìØÕ«Wsð³ýì?øq`.]ºd£G»Í¼¿D"N¥RñE¡·~ñTöÅi_pÚ§ö-áþ+aº¶¶6ÍF§Ãô¤wËÝYü!þ@ü!þÄ_iëêêÚ¿_3ÌT±X8Sü!þ þÄ_é	/uuuD"NNZâñâñy?Ä?ñ'þÄâñâOü?ñøCüøâOü!þ?Äø?Äâñ'þÄâñøCü??ÄâOüøCü!þâñg?ÄøCüøCü!þÄâÄâñ'þ?ãøâOü!þ þÄø?Ä?ñ'þÄâñøCü?ñøCü!þâOü!þ?ÄøñøCü!þÄ?Äâñ'þâñøCü?Ä?ÄâOü!þ@ü!þweü½óÎ;öHÄâñø+óøß¾uuummmøúWõWa]ñøCü!þÊ3þÖ¬YóÑ~ô/6þÅ¯í_7oÞl×Dü!þ¿2¿sçÎýæoþæÖ¯låý÷þòCúSÀ?ÄâñWñ×ÛÛûO|"W~ÑMMM?úÑì?ÄâñWnñ÷úë¯´ñ£ñòÛþ×Ûkkkßzë-'âñøCü[üßwßýìgsñ÷éOúpÍâñøCüaü½wóm÷ÞïÇ>ö±åË777ÿÖoýÖo¾i×Dü!þ¿ò¿`ttôÛßþö³Ï>û­oëÝwßµ_"þ?Ä_9Ç?Äâñ'þ@ü!þ?ñø?ÄâOü!þ@ü!þâñâñø?ÄøCü?ñ'þ?âOü?ÄâÄøâñøCü?ñ'þÄâñøCü?ñøCü!þâÄâñø þ?ÄøñøCü!þÄâÄâñ'þ þ?ñøñøâOü!þ þÄø?Ä?ñ'þÄâñâOü?ñøCü!þâOü!þ?ÄøñøCü!þÄ?Äâñ'þ@ü!þ?ñøñøCü?Ä?ÄâOü!þ@ü!þÄø?Ä?ñWj¿ÂÒét2liiéïï/[·n]*úÈG>200 þ þâ¯äe20±gÏÎÎÎø¢]»v=õÔS¡üã/ìµ7î ïÿûW®s^°;f£þð?ýéOsqíÚµ£G»Í¼¿wa"ÍÖ××Çµ´´9sfªCüýíßþíwï ï|ç;ÿú¯ÿú]°É80Gîëë3ÌE(¿ÞÞ^ãp·ñL&'nîÞ½»²²²¡¡áÔ©SNûâ´/8íÓ¾Nû¼D"N¥RyöíÛ&Î=ÛÞÞ.þ þâ¯4ú¯éÚÚÚl6ûÞÍÓ¾a:~·øÍ¼âñø3?ñWºººöïß&Â×L&_´qãÆ_|1L>úÁ??ñWòÂa]]]"H§Ó¿|n7ÒêèèH&íííçÎ??ñ7?ÄâÄøâñøñ'þÄøCü!þ@ü?ñ'þ?ÄâOü?ÄâñøâñøCü!þÄ?Äâñ'þ@ü!þ?ñø?ÄâOü!þ@ü!þâñâñø?ÄøCü¿ùæoþæo¾õ­o½yo÷ãÿøMáááæ¨··7ôq`.~òüÓ?ýq¸Û¼ûî»âoJ§OÞ±cÇ×ÊEñ³~8íø@ü þ?Ä_	hnnN&---y¡È"ÈsöìÙöööT*µvíÚ¼?sâÄt:íHýýýÆYìEmmma/jmm±bv;R¤¯¯¯¢BZ¿y¬¦¦æâÅa"|­¯¯æ"È³xñâðò&Î9³qãÆø¢L&ÓÓÓ&öìÙÓÙÙi¬Å^ÔØØøê«¯455+f·#ãããáâOüÍk/_ákæ"ÈL&sÓÕÕÕyÿÙlÖ¿"Ý^WYYi¬õôäO>óÌ3âOüÍk'N¿IÃÿáëÐÐÐ4AÖÖÖÓ§O^x!þ7ïqÞ"æ^~uww+f·#]¼x±½½=üsTü¿ymñâÅgÎRoÉ%ÓyN:ÕÔÔþþIw`&Hä¦S©±bQddd$É+f·#utt;vìa!þÄß|Vä¨6ÌÂo¼ÑÜÜS[[Ífß»yÚ7L"f±½wóý'ëÖ­»råñaÖ;RÅ¯3DâoZ²dItx|xxxñâÅÓyO8111þ©ýÄOÄuuuíß¿?L¯LÆX1½h```åÊW¯^5JÌeGW óúF!ìÉdø¦ãÿWLº&^-ZJ¥º»»ÇÇÇã;ÒñãÇëêêD:.ÁîEõõõØ0÷Iü?Äâñø@ü þâñø@ü þ?Äâñø¥é^XºtiåMË/é¥~íwÜM%óy²G[__ÚèèhÞü0'L¦Óén%iÇxâr¿;wÏ=÷üo|ãaþã?>mâ ô8q"dM2Ü»wïøMûöí7ÃÌ'OMü]¸p!ÌlmmÍßÜÜæ?^üâôÑ5O=õT|æÓO?f~éK_§O§Ðmmma:wçk×®uww/X° ,ª©©Ù¼ysüìêÑ£GC`EaÝ#GäåTS]]½téÒÃ]]]y¬···øv¢Eá!Eúúú¦µ+Wù¹9¯¾új³bÅÜ]»vÕÕÕMUVV®]»öòåËñW¸ý¼9E* þ>`-árñâÅøÌK.õõõñ¸É344-]³fMÞ¢M6EÄ¤kE7£¥!S©ÔøøxX¾xªªªÞWd;abÒWøL8/ÚöôôäÊ/o#Ë/iüy¨øøàEgx'ù½VQR,7#7psõêÕÑÒ¨u¢|àhe2èa>~üx´ø6·oßò.:ëº~ýú0çÐ¡Ca:|Ó=öØ-·Â1Üw?¶IQÊçõêÕp3|<g6îN§Ãá)¼÷«ÓÄ3¿"wEüÆ_n~7Q3/_7C6E7ÛÚÚÂÍ6h»qãFn#á>yÇÒrkE7/]º»s¦Üßð5L=zôÛ	¡n¾ýöÛñÇ6Õûó¶lÙ=óÌ3ïýêÔöæÍãw!â/$it`r¦ñWä¡âàîÏ3Ã¢"¹KÃsçÎEýkÜõÏÆæåTüVÂtMMM*¯®®Î-½åv¦J±<§O:5L¯azxx8·ôøñãáLzúxúñWä¡âà½ïm÷îÝñÑ ä]ð_àÕ«Wh<yrçÎÑIØFårçUoh6mÎö¯ÝÝÝ¹ùE¶i»råJtóí·ß.~eî%KÂÒèÓmB³ÆEWþE×¯_/¹0F#·´ÈCÄÀ/º`"LîÙ³'ú¨½÷¦R©Â3V¯^Bgll,:'»27zÏ_ôV¹sçÎÅß,µàæÍC*E×áæ>leÒD.¿¾ûÀÀ@n~íDoòÞó7::Ý³HüíÛ·/wL.<åø¢èíÃÃÃá»A*þ¢Ü¹î¢síÚµñ¥E* þîÑ çÙµk×ÿÿwSÔF¹éÜ	ÓÜ59ë×¯eÜÁÄß7ÿG_SS?#;áaÄXîJÛ©ì7*++£§w²ÕªUño±páÂð5ú´ø6£9Qªæy¨ø¸[@Y¾|yê¦¥KFÜæÅ____ôqz---ÇË-Ý¶m[]]]L[¶lË-=räHh²t:ýüóÏçm³ðMù7nÌ?Õvð`ÂC>°ÈçüåtwwçæKäêÕ«L&ÀÂ³8þ|îckâÛ¼~ýz¨Ûh¬V¬X188÷<T@ü þ?Äâñø@ü?Äâñø@ü þ¼þEQÉàõIEND®B`


ØæpðñùYÏ=Ï=ÏÝýöü<÷I$éÁ¨ÂH$$IÈO$Iä'I$ò$IùI$ü$ID~$I"?I$$IùI$ü$ID~$I"?I$$IÈO*¨¼R©TUUÕ¬Y³vïÞ=ÌÕÛÛÛçÏ?wµ+&ó_|ñÅRÝÐØ<Ò½÷Î;·ª¿Ù³g¿òÊ+wÜàðWø|hhhØ±cÇ(gÔÏ£GÆ0NÇ­l¡bîo$òÆüò[·nÝ=ÕØý#¿'^¾|y¼Èïé§øÍÚ´iS­h¡Û·oéàz3Lîv[ZZÈO"?I÷]]]/¾øbUUUÌlkkä-^¼x¯½½=¡êË/¿ÜÛÛÛ××q1fvvvº©®2ðù°aÃ3ù%+Þ¸q#¦³Ùìõ­D~RYÉ/iëÖ­»[><úôt:=cÆÈ)·d"®0iÒ¤9sæY7wå'OÎ??9¹|ùòk×®åßhccc²â¡CîêP½ãw`ÅñuïÞ½CÉ¹sç.]ÜÃæææóçÏÿñ&sâFã¦ãÌ;÷Ê+Gmhh¨¬¬`AþþÅ-[¶ÔÖÖ&5ã&rºÃO<ñDÌÏ?öÔcæªU«ýþt7X9q'G48îËot+_ÁÒø¾Ä3$ýóÿ|mñ½óiBLÇ¢êêêuëÖuwwûÅ"4äXuuuÉÅÎÎÎT*ÿzÜÞÞ^D~É,YRdÝÜã9é²eËòwVuÈf¯8ð±wuue28&.]óüíÄ5ÿxÞî¬Y³¨I¡û®9oÞ¼â¾2eJÌ¿zõêÀoVý ßß®RpÓÁdß´iÓF48ÅÔ+_~ùÛ;Rùói¼xñâû¼víZ¿X$òÆüùa²dº¹¹9.¶¶¶Æô±cÇòV°zrqãÆÉ^;®/ñ×Ov4¥ñcõ[ýÅZù·Ud³ÅWô±ïÛ·/XþõW¯^Ó-ºÑ_LÄÅ9ÔãMæ¬Y³¦··7¹cÉãâÁó`òn¶ÄôòuÓéôP$·î]®2ÔérûDG:8Þ«Q¯XÜôùóaúÏ/¿a>^¼x1¦ã»Ó&LðE"?iüÉ/w/y[X~1§ü.]ºsÇusûÏ£ñ:ÏüUGël¶øC=öñ¢^0¿¦¦&ÿÅ|<yòP7ÚÈ¿ÂN`þ=Éf³aFSSSþc/"¿Çbú]®?¤±næüùó><è gp½W£^qøòK!W®¿ÉoOãÆÆÆ¸8uêÔkü	qûöm¿U$òÆüB*1³ººz¨Û¡ây©.â¾6b³CÍ7^¿zzòçÜg6ðMoïp.2xÞñÕÚÚÚäðô@Þx:·á¬2±ôÃ¡<:º´÷î]Ã|=6Á_Nû÷ï÷E"?i<Éï¥^Ê?Ã#ÙVpåp 3¢uï³¹zõjþÒ"-¾b;°gÏ¸ûdf³½S&þä_>zòé*G~ÉÉäV×­[wãÆ;wÆÌ¥K*¿á¬2jùtprzÅáËo8ûür¶KþÔ)2þEoIÇß¼ysr9w´$òîwùÅk[(yaÎªKòzhWÊäTÙ3f$79u÷7èKfu³)!Kò>­Øx²wqºÙâ+gÄâÅsoäOæ$gþ.Z´¨«¿dkkÖ¬¹ù%û½:;;ã!ìØ±c8ò;räH²W)ò±cÇýyIûöíôû;ÒUF$¿âSðÜÈo¤£:ù%ïÌ'ÃíÛ·v.èÂmÛ¶ÅàÁÅÇÿ?Éû5Ï=[äÈ»$òîù,ÿüÄgËæÞïûÐÝdáÀÌ"ëgS(±Qþ¹¥E6[|Åâ¸|ùrî]ÉóçÏ¼Ç«¦¦&÷&Â»_Î"I'OÎ½ë±o/_>ð5sæÌ"ßâ­2"ùçF~#ÕQÈïøñãùÛ3gNþÒ1É`>Ô­y¾åN!ÊµråJ¿X$òÆüâ%pÞ¼yÿw¯655¢âåü¥^ÊÍß·o_Ì	iåÝ0×½#Á766üX¾¡6Ç3"9?ÿÌ3K.­ì¯¹¹yàù£ßµk×bk±Í0ÐúõëC9p÷Í7¾ñxtñÀ§L²k×®Ç<·ÏéîWüNÁs£ ê(äíß¿êÔ©ñ¨cûÉñôÜéÌq1|ÜúüùóÛÚÚî¸Ïu¨ç[ww÷SO=ìL¾Ç·²§§Ç/ü$éÍf/_>¢ÏÅ*ã®d_or²voooúéÓ§ÂHä'I*·÷ùôÜsÏü$IåVww÷¦LÒ§~Ú°Hä'I$ò$IùI$ü$ID~$I"?I$$IùI$ü$ID~$I"?I$$IÈO$Iä'I$òt´oß¾ºººt:=oÞ¼®ÛÐÐPQQqöìÙÜ93fÌÁo±þFwá¬;Êß­Jæwww¯]»¶ºº:FlâÄÍÍÍçÎË­ÕÛÛóãÊ1íÙ%ü$Ý_ÕÖÖT®]»6©lÙ²%Öársþù³yóæòßÀù+V¬ù­­­1ôèÑ®¯¯Ï-Ý¿NðìD~î³_"w§ãÇÇºsçÎÍÍ5kVÌéèè;9öò«¬¬ù]]]®õäOÆÒäëÊ+=»$¤ûùÊyê©§&N8aÂM6ñÇojj*ØBnaL_½z5¦cN²èâÅóçÏ¯ªªJ§ÓÉ~²[Ë¿õâ«ìÚµ«®®nöìÙqCvëÖ­X7îü]aÓ9sæÄÖb~lùÒ¥Kw)¿G4æO>ýØ±cúúú&M·uûöíø:yòäGw$ü$Ýü%ÓÏ>ûlL¿ôÒKa¬xî¹çò¯sðàÁDxù­^½:íÜ¹3¦ãkL¯Zµ*Y4sæÌäçb~&tkùw ø*á¹=öÄÄ+Þùµk×Ætõ×_-[¶ä ÖÕÕÕÙÙÉ.É¡ÞÃ7ó¯³oß¾Üyóæ<y2·JrðwÑ¢E1½téÒ9ÞÏ:Iä'éã_]]]Lgû¯AßxäÈw/^.·ôøñã7nljjù©TjÐ­Ø«ø*É8qâÀukjj;ß××á­×éÆÆÆ°lOOÏè¥ öööjÎ±ådþºuër¡®_¿þnî$¤(¿ä¼ÔÜü¸xG³&L×ìîîNhÆdÑ;b­ààÉ'ó·P°µüÃYe¨;LÌßE¨qïÞ½&MJæTWW'»¿Ï¯È¸µ¶¶æònãFoÜ¸ñQÿ¡ç2eÊ ÷Á³NùIúøåÉdò÷ù%»ÍîÈ XûÑ×ß0åW|ÜHòäÉÉºw¯··÷ÀÉ9¹¹#¡JîLÜçÎQµ ãÇú>HùIºòK>¥eçÎÉûüþùá0hïÞ½9èìÙ³'7?qä©S§÷ÿG~ÅWÙ¸qã7¿ùÍX½zõÀuW®Ó»wï>útrt5fÎ=;¦eW®iÓ¦Ý¥üfÌó:ôÑ/>Ãeùòåý71Ô÷AÈOÒ=_oooèjBùÉW ·oßN´Æ×ü;	UWWO8qÃÃ_ñU>KgÍ;Ñ¤à3×­[÷¼²²ráÂÉ)´­æææädá¹sç9sfDÃ2ðpl¶¥¥%îa<ØÉ'¯]»6n÷£_|¨uþösj=êû Iä'ID~$I"?I$$IÈO$Iä'I$òûxzë­·Þÿý±¼ÅóçÏg³YÏ¼v­?ãPÂâ)OTãPÚ.àÿ^+m×¯_¿zõªq(a?ûÙÏ~üãÒöÞïýÇüùÝ/ýë_üå-9rä§?ý©vöìÙýèGÆ¡uwwç;ß1¥í»ßýîO~òãPÂÎ;÷ÃþÐ8°Û·oÿã?þ£q(mÇû÷ÿwò#?ùÈüÈüÈOäG~ä'ò#?ò#?òùùüÈüÈüÈüÈüÈOäG~äG~ä'ò#?òùùùüÈüD~äG~äG~äG~äG~ä'ò#?ò#?òùùüÈüÈüD~äG~"?ò#?ò#?ùùùüÈüD~äG~äG~"?ò#?ùùÈüÈOäG~äG~äG~"?ò#?ùùÈüÈOäG~e%¿L&N§Z[[óõôô¬X±¢²²òÑGuùüÈüD~ä7¾knnÞ½wLlß¾Ù²eù¶lÙòÜsÏõõõ¹¦N:P~¸1ÅÕ¥Kn¨týïþC	ûà>lJ[`ú_ÿõ_C	ëìì<qâq(a/_þö·¿mJÛo¾ùÞïå->ò«®®ÛÅD6­««Ë_ÔÐÐpúôé¡Vù¿74=ú=®Àt0Å8°xÆÕ8ügÿ»ßý®qð³?÷Ö[oë[ß2¥í7ÞãýB~étzÐéäâ¶mÛªªª¦NzòäIGí£½ö:Ú+Gíß¥R©ÜteeeÁ¢;wÆÄ3gÈüD~äG~"?òßÕÔÔd³ÙúöÆtÁ¢ÜtÁî@ò#?ùÈüÆ_---»víøÚÜÜ¿hÍ5öìS§NÍ=üÈOäG~ä'ò#¿q?¬µµµ©T*É´µµýü±Uüç£ëêêZ²dI:njj"ùüÈüD~ä÷F~ä'ò#?òùùùÈüÈOäG~äG~ä'ò#?òùùùüÈürÝ¼yóÄ|ðùùüÈüD~e+¿ÞÞÞ5kÖL4éüä'>ñÙ³g_¿~üÈüD~äG~"¿2_°ï±Çûòú/oúê¦_Ù8kÖ¬ßú­ß"?ò#?ùÈ¯ÜäwóæÍz(a_ò/ð÷ðÃ8qüÈüD~äG~"¿²_ggçc=c_òï7?ó¯½öùùüÈüD~e%¿ë×¯×ÔÔlüÊÆ|ùÕ××¿óÎ;äG~ä'ò#?òùü¢ÏEs>cßï-þ½GyäæÍäG~ä'ò#?òùü>üðÃiÓ¦=öØcóæÍûtã§'OÜÙÙù`*ùùùÈ¯Ìå÷Qÿ»ìß¿ÿ/þâ/^õÕù#ýÈüÈüÈüD~å/?ùùùüÈüÈüD~äG~"?ò#?ò#?ùÈüÈüÈOäG~äG~äG~äG~äG~"?ò#?ùùÈüÈOäG~äG~ä'ò#?ò#?ò#?ò#?ò#?ùÈüÈüÈOäG~ä'ò#?ò#?òùùüÈüÈüÈOäG~äG~ä'ò#?òùùùüÈüD~äG~äG~"?ò#?òùùùüÈüD~äG~äG~"?ò#?ùùÈüÈüÈüÈüÈüD~äG~"?ò#?ò#?ùÈüÈüÈOäG~ä'ò#?ò#?ò#?ùùÈüÈOäG~äG~ä'ò#?òùùùüÈüÈOäG~äG~ä'ò#?òùùùüÈüD~äG~äG~"?ò#?ò3äG~äG~ä'ò#?òùùùüÈüD~äG~äG~"?ò#?ùùùüÈüÈüD~äG~"?ò#?ò#?ùÈüÈüÈOäG~äG~"?ò#?ò#?ùÈüÈüÈOäG~ä'ò#?ò#?òùùÈüÈüÈOäG~ä'ò#?ò#?òùùüÈüÈüD~äG~äG~äG~äG~ä'ò#?ò#?òùùüÈüÈüD~äG~"?ò#?ò#?ò#?ò#?òùùùüÈüD~äG~äG~"?ò#?ùÛ:::2L:nhhhmmxCUTTùüÈüD~ä7îknnÞ½wLlß¾Ù²eKÉüD~äG~"?ò+ª««ûúúb"ÍÖÕÕ,öÙg·nÝ:üþæoþæÿaüÁ~ðTºögJX<EãjJÛ?üÃ?ttt·§C	;~üøo¼aJÛ¡C¾ÿýïå->òK§ÓNG/^ljj%¿×^íý1ìÛßþöÙ³gßWé¨öövãPÂþùÿ9~UÒvøðáýèGÆ¡¤ÿéþÉ8°sçÎÅ(Æ¡´µ¶¶þð?Ë[| äJ¥rÓù,YrôèÑÿ|¨ö:Ú+Gíu´Wö:Ú[ÕÔÔd³ÙähoLÿGø_#?òùùüÈo|×ÒÒ²k×®¯ÍÍÍ?TûüÈOäG~ä'ò#¿òÖÚÚÚT*ÉdÚÚÚ¥ùÈüÈOäG~täG~"?ò#?ùùùüÈüD~äG~äG~"?ò#?ùùÈüÈOäG~äG~äG~äG~äG~äG~"?ò#?ùùÈüÈOäG~äG~ä'ò#?òùùùÈüÈOäG~äG~ä'ò#?òùùùüÈüD~äG~äG~ä'ò#?òùùùüÈüD~äG~äG~"?ò#?ùùùùùùüÈüD~äG~äG~"?ò#?ùùÈüÈüÈüÈüÈüD~äG~"?ò#?ò#?ùÈüÈüÈOäG~ä'ò#?ò#?ò#?ùÈüÈüÈOäG~ä'ò#?ò#?òùùüÈüÈüÈÏ8ùùÈüÈOäG~äG~ä'ò#?òùùùüÈüÈüÈüÈüÈOäG~ä'ò#?ò#?òùùüÈüÈüD~äG~"?ò#?ò#?òùùüÈüÈüD~äG~zpäWq§R©ùùÈüÈüÊA~©;N§ÉüÈüD~äG~äWò+ÈüD~äG~"?ò»ÛºººV¯^M~äG~ä'ò#?ò#¿r_]]]:ö>?ò#?òùù©Ìå7cÆ§wTWWÇùùÈüÈüÊJ~A½k×®ÕÖÖÆD¼f|óßU«VùùüÈüÈ¯¬äìä ^Lp¡¯¯/&&L@~äG~ä'ò#?ò#¿²ß¤IÂyñÀâ8&~úédÂ§ºùÈüÈüÊM~6lÈÏÿV¿Ù³gùùüÈüÈ¯¬ä=óÌ3'O¶¶¶655ïùÈüÈOäG~JäG~"?ò#?ùùùüÈüD~5mÚ´ä³]|3ùùüÈüTÎò«¯¯Ï×^.çöùÈüÈüÊM~¼0___ß¸û~ùüÈüD~ä7ª««C~ãäG~"?ò#?ù¬ßÚµkoÝºE~äG~ä'ò#?òS9Ë/2eJÅáA~äG~"?ò#?ò+7ùM:ÕäG~ä'ò#?òÓ!¿Ä|ãñûA~ä'ò#?òùßª©©qùùüÈüô@È/(òÛ°aCOOùùÈüÈOå,¿!rùùüÈüÈ¯?ÉyÐáA~äG~"?ò#?ò+ÃOu¿ùüÈüD~ä7êêê¦Nzúôiò#?ò#?ùÊ étº¢âcÞwØÑÑÉdâ444´¶¶æ/jkkkllE3fÌ«ùüÈüD~ä7úBZ!¿Í7wuuíÒÜÜ¼÷îØ¾û²eËòÕ××¿ýöÛ1ñÊ+¯L6üÈOäG~ä'ò#¿»ØÄpnouuuÎl6[WW7ÔÕªªªÊïÏÿüÏaû÷ï? ö­þ'ª!õ³/OÔûsHÇøzÏåw?Û[CÝnûªU«Ê/þÿ¿cØw¾ó7oþ_®3gÎÄßýÆ¡ýä'?imm5¥íÍ7ß¿ûC	;öì»ï¾kJØOúÓÃÒöÖ[o]¿~,oñËï~(ÿbeeåÀ+Üºu«¹¹¹»»ÛÑ^Gåh¯£½öÊÑ^GÇw555Ùl69ÚÓK/_¾¼bÅ+W®üÈOäG~ä'ò#¿ÞZ¸paUUUEEÅ	,Y2Æ§z´´´ìÚµ+&âksss´,XpíÚµAW$?òùùüÈodOAÏðËÿÆ7µ¶¶6Je2¶¶¶?¶þÏ©««Ë¿WäG~"?ò#?ù¾éÓ§¨-ZtëÖ­¸ØÕÕµxñâ3sæÌûÿûA~ä'ò#?òùßª¬¬çõööææd³Ù3èäG~äG~ä'ò#?òÇòK¥Rá¼ä¤3êB~ägÈüÈOäG~c!¿ähï£½ñ5¦cNcc#ùùÈüÈüÊJ~A½AÏð¸qãùùÈüÈüÊJ~ÉseÉ%'NL¥RñuÁ1g?ÈüD~äG~"?òP"?òùùüÈüÈüÈOäG~ä'òË­y§òÿ/]ò#?ò#?ò#?ò#?òÇòKùùÈüÈO=G×¯_ÈïW^!?ò#?òùù_yÊ¯££cÂ	a¾ùóçç°3ùùÈüÈüÊJ~K.Mvõ8p`?ÈüD~äG~"?òA¯¿þzb¾E»ïùÈüÈOäG~ÃÌ939£µµu<~?ÈüD~äG~"?ò»sÛ¶mKvõ-[¶lü~?ÈüD~äG~"?òÆ>ÏüÈüD~äG~z@äºSétüÈüÈOäG~äG~å ¿òüÈOäG~ä'ò#?ò#?ò#?ùÈüÈüÈOäG~ä'ò#?ò#?òùùüÈüÈüÈüÈüÈüÈOäG~ä'ò#?ò_ò6mZee¥Ïó#?ò#?ùÊ õõõùÚóy~äG~ä'ò#?òSÙÊ/ækooïëëwßò#?ùÈüFPuuuÈo<²üÈOäG~ä'ò#¿ÕÑÑò[»ví­[·ÈüÈüD~äG~*gùES¦L©3<ÈüÈOäG~äG~å&¿©S§:ÃüÈüD~äG~z ä¯³³s<~?ÈüD~äG~"?òA555Îð ?ò#?ùùeB~6lèéé!?ò#?òùù©åW1DÎð ?ò#?ùùá'93<ÈüÈOäG~äG~eø©.ã7ò#?ùÈüÈüÈüD~äG~"¿ÁÊf³.¬ªªª¨¨0aÂ%KÆË©¾äG~"?ò#?ùì2èãâT_ò#?ùÈüFÐôéÓÃy-JþßÞ®®®ÅÇ3gùùüÈüÈ¯¬äWYYÎëííÍÍÉf³1'æùùüÈüÈ¯¬äJ¥Ây¡½ÜãS]ÈüÈOäG~äG~åy´wÁÉÑÞøÓ1§±±üÈüÈOäG~äG~e%¿ Þ gxÜ¸qüÈüÈOäG~äG~e%¿ä¹²dÉ'¦R©øº`Á3.¾äG~"?ò#?ù=(ùüÈüD~ä7êêê¦Nzúôiò#?ò#?ùÊ étº¢b¼î;$?òùùüÈoµ¶¶ü6oÞÜÕÕ5^þÓ6ò#?ùÈüFµ!J¥RäG~äG~"?ò#?ò++ù¥È'9ùÈüÈüÊD~S§NmhhWqýý ?òùùüÈïÎ%ÿ]ïGýGÇÅ]ò#?ùÈüFYòßõ&ÿE/ùùÈüÈOå,¿L&SQ4gxùÈüÈüÊD~ííí&MJöü%ÎsùùüÈüTòË5^G~ä'ò#?òùßùÈüÈOäG~äG~äG~"?ò#?ß=®££#É¤ÓéÖÖÖâF7'¿M6ýáþá×Ç°/~ñú§úu®µýOÑx¢Ò¶|ùò?ù?1%ìK_úÒÿñöôÓO?ùäÆ¡´­X±â«_ýêXÞâ8_ssóîÝ»cbûöíË-+¾htsò[¿~$IR¹tÏåwæÌÊ¯ººº¯¯/&²Ùl]]]ñE£C~$üFÀ¸ÿ#]Ï;w÷[Ë?M¸àáF7'¿6ÔÖÖ>:=üðÃS¦LyT¥ëþC	§h<QCi_5~öýìßçýÚ¯ýý2øÙ¿çòóå´;wîùóçG½µüÏ®¬¬,¾htsáá9ÃÃÎð3<áq·Ç|§O#`káÂ£ØNMMM6MÎÆtñE£Sòëíí=xðà/¼ðê«¯~øáäG~äG~ä'ò#¿1_R__ßªªª*Fû¿·µ´´ìÚµ+&âksssñE£3ÞåÔkhhxì±ÇæÍ÷éOzâÄäçùùüÈoìäøÈßçYµjÕè¨¶¶6ÔÉdÚÚÚ~~/ûß®8pÑèæwùîs;wî¦¯nJþýÞâßäGâ¥üD~äG~"?ò»çòË_UUÕ²eË.^¼8^¾ãN~×¯_¯©©Ùø9ùÅ¿úúúwÞyüD~äG~"?ò»çòKNìøüç?áÂq÷ýwòëìì|ì±ÇòÙÿ~ó3¿ùÚk¯ÈüÈOäG~÷ %ù0òf7oÞ|è¡¾¼þË9ömüÊÆ~øÄä'ò#?òùß=ß¸n<¾ÏoÍ5õõõ	þ³gÏ7o^oo/ùüÈüD~ä7ò;uêÔ´iÓ&LÏ[SS³wï^ò»Gòä­^½ú¡úä'?ùO|"äwýúõùüÈüÈOäG~c'¿CåÎðHäLoß¾üîünÞ¼ùýïÿ>x÷öùùò#¿±_mmm8ïÔ©S9ùµµµ%ìB~÷N~"?ò#?ò#?ò#¿A~¹ÿ8'¿¾¾¾ä_ò#?ò#?ùùüjjjÂyÉ~¾_6ê©§bº®®üÈüÈOäG~äG~e%¿ LÅ`½ýöÛäG~äG~"?ò#?ò++ùEW®Îí­ªª6mÚùóçÇÅ÷üÈOäG~ä'ò#¿%ò#?ùÈüÈüÈüD~äG~"¿ÿÚÅ'Nê/&ÆË¡^ò#?ùÈüÛÊ++hõêÕäG~äG~"?ò#?ò+ùíÝ»7AÞ;âu"÷¤Ù·o_2ÿàÁäG~äG~"?ò#?ò+ùe2àÝÎ;.Jþ?7çG~äG~"?ò#?ò+ùUVVïâY2pQ6Eqò#?ò#?ùùürÿiÛPKÿÉüÈüÈOäG~äG~å ¿"¶#?ò#?òùùüÈüÈüÈOäG~ä7åW<ò#?ò#?ùùüRw*NùùüÈüÈ¯äWùüÈüD~äG~äG~ä'ò#?òùùùüÈüD~äG~äG~"?ò#?ùùùùùùüÈüD~äG~äG~"?ò#?ùùÈüÈOäG~äG~äG~"?ò#?ùùÈüÈOäG~äG~ä'ò#?òùùùÈüÈOäG~äG~ä'ò#?òùùùüÈüD~äG~äG~äG~äG~äG~ä'ò#?òùùùüÈüD~äG~äG~"?ò#?ò#?ò#?ò#?òùùüÈüÈüD~äG~"?ò#?ò#?ùÈüÈüÈüD~äG~"?ò#?ò#?ùÈüÈüÈOäG~ä'ò#?ò#?ò#?ã@~äG~äG~"?ò#?ùùÈüÈOäG~äG~ä'ò#?ò#?ò#?ò#?ò#?ùÈüÈüÈOäG~ä'ò#?ò#?òùùüÈüÈüÈOäG~ä'ò#?ò#?òùùüÈüÈüD~äG~"?ò#?ò#?òùùùüÈüD~äG~ä7Ì:::2L:nhhhmmÍ_ÔÖÖÖØØfÌW#?òùùüÈo|×ÜÜ¼÷îØ¾û²eËòÕ××¿ýöÛ1ñÊ+¯L6üÈOäG~ä'ò#¿ñ]uuu___Ld³Ùººº¡®VUU5P~ý×Ý>½ñÆ!Ñv®ïôgJX<E8`üìßçÅÒ­­­Æ¡½óÎ;~öKÞÁ¿÷½ïå->òK§ÓNçc±jÕªòÛ·oßcØáÃßï½UºN8qüøqãPÂÞÿýx¢ÒFùùãPÂN<ùüÀ8°/:tÈ8¶øåÇ?þñXÞâ!¿T*®¬¬x[·n577www;Úëh¯íu´×Ñ^9Úëhïø«âÅtMMM6MöÆtÁ5/_¾¼bÅ+W®ÜùÈüÈOäG~ã¬]»vÅD|mnn.Ö®]»6èäG~"?ò#?ù¿a­­­M¥RL¦­­íç­w`]]]E^äG~"?ò#?ù= ùüÈüD~äG~äG~ä'ò#?òùùùüÈüD~äG~äG~"?ò#?ùùùùùùüÈüD~äG~äG~"?ò#?ùùÈüÈOäG~äG~äG~"?ò#?ùùîüâÇçk_ûÚþöoÿ¶··×ÐùÈüÈüÊS~ëÖ­û_ùÿöùÿö/|aê¯Oýíßþmø#?ò#?ùù¡üöïßÿ«¿ú«ÿkÝÿÚôÕMñoãW6~êSzægùùüÈüÈ¯Üä·bÅß]ô»	û_|òùÌgùùüÈüÈ¯Üä÷øãÿÁüA¾üþçÿø¿ñ¿aôÈüÈOäG~äG~å&¿¯íkMMMùò?þïÿþï=ò#?òùù_¹ÉïúõëS¦Lû¹¹_^ÿå_Ùø/|¡¶¶öôéÓFüÈüD~äG~äWnòKð÷ÄO<üðÃ=ôÐ¬Y³N8aèÈüÈOäG~äG~å)?ùÈüÈüÈOäG~ä'ò#?ò»»zzzÞyçýû÷õÈüÈOäG~äG~äW¶ù2LÃ§>ûÙÏN<yåÊ¹ÿ.üÈüÈOäG~äG~åÓ|ðË¿üËËþû²äÓC6|yCý_þå_ùùò#?ò#¿rë^øìg?ÿÑq´â~ý×üÈüÈÏ8ùù[_úÒ~çw~'_~ñï~éÈüÈüùù_¹õâ/~vÖgþXûüÈüÈüÈüÈüÊ°?üpòäÉwñÆ¯lLþØ`ß_ýÕ_ùùò#?ò#¿2,x÷©OêGþÉé=ôÐ-[òùùüÈüÈ¯Üzÿý÷O8.)@!ùùÈüÈüÈüÈüD~äG~äG~"?ò#?ùùÈüÈOäG~äG~ä'ò#?ò#?ùùÈüÈOäG~äG~ä'ò#?òùùùüÈüÈüÈüÈüÈOäG~äG~ä'ò#?òùùùüÈüD~äG~äG~äG~äG~ä'ò#?ò#?òùùüÈüÈüD~äG~"?ò#?ò#?ùùüÈüÈüD~äG~"?ò#?ò#?ùÈüÈüÈOäG~äG~äG~"?ò#?ùùÈüÈOäG~äG~ä'ò#?òùùùùùÈüÈüÈOäG~ä'ò#?ò#?òùùüÈüÈüÈüÈüÈOäG~äG~ä'ò#?òùùùüÈüD~äG~äG~"?ò#?ò#?òùùüÈüÈüD~äG~"?ò#?ò#?ùÈüÈüÈüùùüÈüÈüD~äG~"?ò+/ùuttd2t:ÝÐÐÐÚÚ:ðª¨¨ ?òùùüÈoÜ×ÜÜ¼÷îØ¾û²eËööö666%¿ ØÏÆ°xAíêêúJ×3gâ·¿q(a7oÞ¿ CióÍ7oÜ¸aJXüÉ÷î»ïvëÖ­ÃÒöÖ[oýÛ¿ýÛXÞâ!¿êêê¾¾¾Èf³uuuKöÙ­[·%¿çþà¶ÿþ7Þxã J×·ú3%,¢ñD5~öïó8àgßÏ¾ý=òK§ÓNG/^ljj:Úëh¯íu´×Ñ^9Úëho9J¥rÓù,YrôèÑÿ|¨äG~"?ò#?ùÓ*~QL×ÔÔd³ÙúöÆô WË]üÈOäG~ä'ò#¿qË®]»b"¾677%Å3ÉüD~äG~"?òÃZ[[J¥2L[[Û Ô#?òùùüÈïüÈOäG~ä'ò#?ò#?ò#?ùÈüÈüÈOäG~ä'ò#?ò#?òùùüÈüÈüÈüÈüÈüÈOäG~ä'ò#?ò#?òùùüÈüÈüD~äG~"?ò#?ò#?òùùüÈüÈüD~äG~"?ò#?ò#?ùÈüÈüÈüD~äG~"¿rêÏþìÏ^íµ÷Ç°=öÄ¯ª÷UºÞ|óÍð´q(agÎyõÕWCiûû¿ÿûwß×8°£G¶¶¶EÿÝßýq(m÷îíììË[¼yó&ùÙ©S§6mÚôuI¤²¨øÁÌ»a%IÈO$ü$ID~$I"?I$$IÈO$Iä7î;räÈôéÓÓétCCÃ±cÇò]¹r¥"/cUQíééY±bEeeå£>W3Vw?¤ùÏÒT*e¬î~H,Ò¨GõÌ3MMMñ³¿téÒ®®.c5Ì[GGG&IF»µµÕXÝýF×®]«««#¿r®ººúâÅ1_¾Ù÷î£¢Òê-[î¹¾¾¾x:uª±ºû!Íµ÷îíÛ·«»Òá¶F:ª3gÎlkkÓ§O¯Y³ÆX³"ãÖÜÜ?õ1?øË-3Vw?¤ñçJüér?ìë!¿XàãòåË1_ ìÛ·o!*í¨Æß¦ñÃfJ8¤I.7o*ÉÞq´5QM§Ó¹éI&«aVdÜÂÙñWtLd³Y¢dH.öìYò+ó:::ªªªâÛ_ÛÛÛóÕ××/X° "MMMgÎ1V%ÕÏmÛ¶ÅüxU8yò¤±ºû!MZ²dÉ ó5!½ãhk£:cÆS§NÅÄË/¿ÿÒ«â·ü´$Cúsu_y7sæÌdTüÂ5kÖ ×?^ãb¬J2ª©TjçÎýâÆª$OÔø#u¨g¯F1¤Ãùµ jü¥7mÚ´áÖ­[ã«±fEÆ-ÿ½Æêîüù7ªRjMM?RKþDÝ²eË¶mÛR©Ô®úõÜ¹sÓ§O7V#mà¸Å¯Ól6ûQÿÑÞü_­õßQüUìõíìì¿VóÕ××?þ£þ~,0V%Õ5kÖìÙ³'&â³gÏ6Vw?¤Ñ9s?nJ5¤ÅG[£þÚÑÑÑ××·uëÖgyÆX³"ãÖÒÒ²k×®¯ÍÍÍÆêîüòÇ¯§øÛ4¾Ætþw½½½=ùx¹sç&ïYÖÝjWW×%KwO=ÖXÝý~Ô¿O:y£·J2¤.Ò]ê#G¦LÏÕU«Võöö«a6è¸%CzìØ±ÚÚÚT*ÉdUuCJ~$I"?I$$IÈO$Iä'I$ò$I"?I$$IÈO$Iä'I$ò$IùI$ü$ID~$I"?I$$IùI$ü$ID~$I"?Iºg½üòËsæÌ©êoÞ¼y¯¿þúù×ß¸ùu<Ø½­««ÖÝÝ]0?æ¤ÓéL&Ó××7ÒmJ"?ImÚ´©b@Ï<óL9ÉoóæÍ1óÅ_,ÿo|#æ?ýôÓ£Ø¦$ò¤qVGGG&NïØ±£·¿;wÆÅyüøñ²ßbæ3æO>=æ?ü$¤òï'Ó<÷Üsù3þùùäOæ»§µµ5ä(llléÜoÜ¸±jÕª'Æ¢êêêuëÖåT=|øpè*Åº,°TÌ4iÒ9s8[ZZîØþýûo'Yw)YtèÐ¡¡¶`ÁßÖÖóöÛoÇùóççælÙ²¥¶¶66UUUµtéÒË/ßÀíÌ)rW%$M2%ÔrñâÅü.]uuuù²)¨½½=YºxñâEk×®Muvv¦R©A×J.&K,YÒ××v¬¬¬ìíí¥ñ5ä4aÂäíwE¶Þ½ôW^Éçl»wïÎ±¯`#óæÍ©üÜUIä'IsÉÝA~©UTÃòe³lÙ²[ýÅD\´hQ²4NbÇd×]-YÔÜÜì,écÇ%ÉßæÆÃvÉÁÖ+WÆûöÅt|éåËßq;¡Æ¸WÈ¿o>¢ÐääÉãñ^»v-.Æ×¸çÁÍl6ÉÄñ>úÅÑáªªªÊ¯È]D~ôñË/T~¹ùl0E/_a¦äbccc:uêêÕ«Cl·oßÎm$®S°-·VrñÒ¥K¹+r|ãkL>|øÛ	eÆÅ«W¯æß·¡Þ·~ýúX´uëÖ~qDÝºuùWüÂ£MMMÉ.ÉÊ¯È]D~ô1ìèºuëVþÌîîîX'çÂ³gÏ&øËA'÷æ¼a,ÿY*1]]]]YYÙÓÓ4iRné·3Ã:uêTÔ¯1ÝÙÙ[zìØ±¸5¾üÜUIä'IsÉÝ¶mÛ?3ù¬3<rï¼víÚÀ]YÇß¼ysrì5GÆdpê¶víÚä o|]µjUn~í$ûØ®zõjñópgÍK²	°æ/JÎóEêêê*.¿JÑÈ--rW%$Ì%gH¤ÓéíÛ·'ê²cÇÊÊÊgc,Z´(ÓÓÓÍ¼Ï/yÜÙ³góß @pÝºuá¤ä¬ÛÜçªê³ädÛäÖ9_d;Éû÷ùuww'×,"¿;wæöÆÅCÎ_¼å±³³3n%a(ù%Ö+ÇÕBK.Í_Zä®J"?IúøK>å¸ -[¶üÿ_pý%0ÊMçæNªÈµråÊ|Væ·wïÞ"òû¨ÿ?ÛùÕÕÕùl'îFþËW;Ô½ûvUUUòq/0ÿ&&O_vÉßf²K2WâÔÜÒ"wUùIÒQèdÞ¼yýÍ3'9Á¶@~J>6¯¡¡áèÑ£¹¥ÝÝÝO=õTmmm¢¥õë×÷ôôä<x°©©)@Éd^zé¥m¼'±©¿fÍùCm';w)ù Á"çkÕªUïtíÚµæææ'Æ£8þ|îjò·ÙÕÕ´MÆjþüùmmm·Xä®J"?I$$IÈO$Iä'I$ò$IùI$ü$ID~$Iä'I$ò$IùI$ü$ID~$I"?I$$IÈO$Iä'I¤ý?lÁ¤9"®½IEND®B`


*mñùK§ùcF2x:,ª¯¯ß²eËØØ_,üIe¿à0³©©)º8<<H$â/ÉEð]yýúõEÖÍ^9¼6ÇvttÄwYMu¯Èf¯ßGGGS©TCCCÈ7Þx#¼¢Ç·®£´øýÍÿ¾K.ö¤FdåsÍ+W'ÎÂüË/ç?X-*øøÎtodíùknnÑàüY¯8üÅ·îìLñ7ÍãuëÖåÜæÍ7ûÅ"ÁTføæEÓét:ííÓýýýq¢å¬]Ü¾ûääd´/ç¦ë×Îðª®ínNÝp1¬þöÂZñïUd³ÅW,xß>ß¿þ¦MÂôÚµk¯Þ(LaæT÷7sï½÷NLLD7,Úr¸xìØ±øÞÙvôèÑ0ýÚk¯ÅMuÉäTw$»î-®2Õê²Fg:8oÕ¬W,ÎúøÅøA`hÓÇß4#^¸p!LG3L×ÖÖúÅ"ÁTøËéÞ"/Ì)¿7Þx#;ç¦ëf÷¢EÃKit1¼ÆwYå¶+²Ùâ+Nuß##×õùñ^ãÃÅùóçOu£9ñÑ±ìèÆoI&	Èhooß÷"øË|*©ßâ*ñ!ëÁjÕñãÇÚt§à­õÓÇ_ôréÒ¥ø·þ¦ùcÜÖÖ..0à5ü/âúõë~«Hð'þVÂÌúúú©^q§bJt1(§È«uâÌôåyj~¸Ëáþðñññøüü=gùoË¿¿Ó¹ sèó¦g­666FÇ©óCêì¦³ÊtÆªà¦38Stv+Nÿ°ï­ÿtMóÇxdd$ò_GñE?©Ìð÷ÄOÄOøö¥åw9ëÌhÝân._¾_Zd³ÅW,r<.f?%ù,gU§? ñ---ÑG®ôôôMÑîÉèSWÒéô-[®^½ºoß¾0sÃñ7Uf¿N¶Y¯8üMgÏ_wÑÿv·¨S§NíÜ¹3:Ö=3ZüIe¿ðò½6g?ê%zIn/ÑÉ³/Eox»QÁWÍ"ëS¤è=[aãÑvrÞÑUp³ÅW,.uëÖeß×ÍÎ^»víè¢­Ýï½·¿hï×ððp¸÷îþúúú¢Ká.÷÷÷Gõ¢>ñé*3Â_ñÁÉùÙ7ÓQþ¢wéë×¯Û­Y³&»wïüoúÞ»922Rä¼$øJùÅÏXÌ?6ûöÿìçñF»	ó_5¬[â§^fÙlñKââÅÙwwEsÎ?ó~¯ìoYDÍ??ûÈ"Ä¹ûî»ó¬%Kyg´ÊðW|pr~6âÍtTg¿S§NÅ·¿|ùòøÒ1Ér>Õw)òó=£(Û=÷ÜãRÙà/¼®2ÿÏ;v¬½½=@*¼¢?ñÄÙùs¶âr7Íuo*§èãñÂÆÛÚÚò?®oªÍÞtÅâÆç;wnÃÕ7J§Óù§wÌW®[ÛÚºukðPÖÜÅóØcîøöïßÿÏ|&»çéÖWþNÎÏFN3ÕYà/täÈ¶XÏà.¢EßÕªU7Ýó:ÕÏÛØØØ÷ÝíRåøø¸_,üIÒU&¹ûî»gôÙr³X¥ìöøF§oOLLD¦oiiñ#Á$©ÞóÓC=dd$ø$U`cccÛ¶m[°`Atl7L<ðÀE?I$Á$IàO$Ið'I$ø$IüI$	þ$I$Iðg$IàO$Ið'I$ø$IüI$	þ$IÊ½Ã755%É+WÎtÝÖÖÖªªªì0æ,^¼x¿Ñn4»ëLgÝÙü¢ø¢D"QSSN§¯â§HüI*e`&&&fºî®]»Âº>úhvÎÃ?æìÜ¹³¬ñWdûñ9áé?EàORùüB¹?:u*¬»bÅì¥K9CCC·çF¾¿øÓ555~$Á¤r_4÷Ýw_]]]mmí;r®öÏ|¦½½=gÙaúòåËa:Ì]¸paÕªUÁFÉd²­­­···àÖâß½ø*û÷ïojjZ¶lYøFùûí·;;;ÃºáÆoÛ¶-Úxº|ùò°µ0?lù7ÞxðwîÜ¹D"Ý£ÉÉÉpãÃÅ0ÓÏ$øTþ¦|ðÁ0ýÄOfz(~cÇå¿¿mÓ¦MaÑ¾ûÂtø¦»ºº¢EK,9zôhxíµ×ÂüT*UpkñP|@º7æßøÍ7ép§z*LìÚµ+Ìüð?¦GGGÃD´c²àùf¿ ¼0>aúóÿ|´hçÎÑPìÞ½;L<ðÀ~º$Á¤Æ_SSSÎÜ(ß^ßØ××­]»6L¯[·.R]vé©S§¶oßÞÞÞ!Qpk9À*¾JtÃêêêò×mhhn|`Yì3Ã5Ãt[[[àìøøø­N|NtÛÂÆü,£Eá[/Z´(ÜÚÚÚæææpÑO$øTÒøK&Ùé0.Ne lAZÁ:ácccÑÑÕ0'Z´wïÞ°VáéÓ§ã[ÈÙZüâtVêÅwæEp<tèÐ¼yó¢9õõõÑNÁweÏ_Á¢ó]BñæJ©T*¾ç/ÚyvSñtvv¥Ù¯ÙùÕÕÕaÎä¦¿â«doXðºóçÏÖÍ¹yGÝ¸qc|á,ç¦C1::ÚÐÐN§/_@|éÒ%?]àORIã/úèûöEïùøá§¿Cew¡<x0;?¢ä3g¢÷NÅWÙ¾ûO>&6mÚ¿î=÷Ü¦»»»Ï=ê3-[¦O>(&ß;ümÙ²%,:uêTüJXµ7V_qü]¿~=:ä¾fß:räH]]Ý¶mÛ¦¿â«?~<,]ºtiö¼øuÆÆÆ¿Â-¯®®^³fMtbo0_:N^±bÅLO¿>þXãz³|ùòpqxxØ$ø$IüI$	þ$I$Ið'I$ø$Iüqßýîw_ýu?ùMNNÒï?ÿó?_í5ãPúýìg?>2F%ÞÕ«W/_¾lJ¿þô§ñüM·¯íkÁ~òû¯ÿú¯£GÒïí·ß~é¥CéwéÒ¥ïÿûÆ¡ô;þü~ô#ãPúýð?ô__ø?ø?Áàþðð'øüÁàþàþ?ø?øüÁàþàþàþàOð'ø?ÁüÁü	þð'ø?ø?Áàþðð'ø?Áàþàþð'øüÁüÁàþ?ø?øüÁàOððð'øüÁàþàþ?øüÁüÁàOðð?øü	þàþÊ£¡¡¡T*L&[[[ãÆÇÇ7nÜX]]ýá¸¯¯þàþ?ø¿²/Nwww=ötttÄíÚµë¡ò[¸pa>þzzzÞV^×®]ø3¥ß¿ýÛ¿ûÛß6¥ßO~ò_~Ù8~gÏ=ú´q(ýN:uîÜ9ãP°9¿úúúÀ»0ÉdâZ[[Ã3yªþvïÞýòV~ægCéä÷ì³ÏÒïÅ_|î¹çCé÷Â/<ÿüóÆ¡ôSx°CÁæþÉdÁéèbà]MMÍÂÃæöuØ×a_9ì+öuØ·ìK$ÙéêêêEûöíçÎkoo?ø?Áàþà¯ìkhhÈd2ïÜ8ì¦se§svÂüÁàOððWuvvîß¿?L¯ét:¾èÞï=xð`8sæÌ²eËàþàOð'ø?ø+ûúûûD*øù«úï7::º~ýúd2ÙÞÞ>22ð?ÁüÁßÜþàþ?ø?øüÁàþðð'øüÁàþàþ?øüÁüÁàOð?ø?øüÁàOðð?øü	þàþàOð?ÁüÁü	þàOðððð'øüÁàþàþ?øüÁüÁàOðð?øüÁq?ø?øü	þàOðð?Áü	þàþàOð'ø?ÁüÁü	þàOð'ø?ø?Áü	þðð'ø?Áàþàþð'øüÁüÁü	þð'ø?ø?Áàþðð'øüÁüÁàþððð?Áü	þàþàOð'ø?ÁüÁü	þð'ø?ø?ÁüÁü	þàþàOð?ÁüÁü	þàOð'ø?ø?Áü	þððð?øüÁüÁàOð?ø?øü	þàþàOð?ÁüÁüÁàOð?ø?øü	þàOðð?Áü	þàþàOð'ø?ÁüÁü	þàOð'ø?ø?Áü	þðð'ø?Áàþàþð'ø?ÁüÁü	þð'ø?ø?ÁàþàþàþàOð'ø+SüÇëé§/åcccÆþàþàOð'ø«Ø^yå6ÜyçÿøÇ?úÑÖ××A?ø?øü	þ*¶/|á¿ýÛ¿½í«ÛvÜ¿#üëèè|ýõ×üÁüÁàOðWÕÖÖþÉþ$_ôoéÒ¥?ü°?ø?øü	þ*­k×®Õ××ÇåþýÁüÁÿñø?ø?Áà¯«««ûò½_ãoÙ²eöüÁüÁü	þ÷üÁüÁü	þs¨èlß|àwÝuWkkk___a?ø?øü	þ*³øçü?~üÚµkÆþàþàOð'ø«püø?ø?Áàþðð'øüÁàþàþ?ø?øüÁàþðð'øüÁàþJ¤¡¡¡T*L&[[[ó¯ÐÓÓSUUð?ÁüÁ_%N§»»»ÃÄ=:::rNLL´µµM¿ú§ú?Êëõ×_?räq(ý~üã¿ðÂÆ¡ô;sæÌw¾óãPú:uj``À8~/¿üò~ðãP°9¿úúúÉÉÉ0Édr>øà<òÈTøÛ·oßiåQÏ>û¬q(ý¾ÿýï?ÿüóÆ¡ô/T/¾ø¢q(ý¾ûÝï¾ôÒKÆ¡ôëëëëïï7øK&§C.4tØ×a_å°¯öuØW¿D"®®®/Z¿~ý'þû®ÂüÁàOððWw£¡¡!É¼sã°oþ¥øËÁüÁàOððWöuvvîß¿?L¯étºð]µçþàOð'ø?ø«»ÑßßßØØH$R©ÔÀÀ@AíÁüÁàOðò!Ïðð'øüÁüÁüÁü	þàOðð?Áü	þàþàOð'ø?ÁüÁü	þð'ø?ø?Áü	þðð'ø?Áàþàþð'øüÁüÁàþðgàþàþ?øüÁüÁàOð?ø?øü	þàþàOð?øüÁüÁàOð?ø?øü	þàOðð?Áü	þàþàOð?ÁüÁü	þàOð'ø?ø?Áü	þðð'ø?ÁüÁüÁüÁàOð?ø?øü	þàOðð?ÁüÁü	þàOðððð'øüÁàþàþ?øüÁüÁàOð?ø?øüÁüÁàþàþð'øüÁüÁàþ?ø?øüÁàOððð'øüÁàþàþ?øÓ»¿ªH$àþ?øüUþ7+LÂü	þð'ø«üUFðð'øüÁü½nÚ´	þàOð'ø?Á_â¯©©)LzÏü	þð'ø«|ü-^¼8ÿlúúú±±1ø?Áàþ¿êêê ½+W®466`¾'|2LtuuÁü	þð'ø«4üE»úÂDÐ^Äääd¨­­?øü	þàOðWiø7o^ ^ÿÈÈHxà¢	õ?Áü	þ*Û¶mËÞÛß²eËàþ?øüUþB_ÿú×çÏ&ÂD`YÜø?øü	þàþæPðð'øüÁüÁàþð'ø¿©knn>ðÅ<ÃàOð¿Çß¢EâàËæl_øü	þàOðWøÎì,»ûð?ÁüÁßÌª¯¯ø+GùÁüÁàOðð7ãþ6oÞ^àþ?øüU8þB,¨ÊË	ð'øüÁà¯ñ·páB'|ÀàOð¿¹¿ÃÃÃåxÿáþàOð'ø?øYNø?ÁàþsÛ¶m?øü	þàOðWáø«"'|ÀàOð¿Ä_bð?Áü	þ*eüÁü	þð3«©©iáÂgÏïÆÐÐP*J&­­­½½½ñEmmmaÑâÅÃÕàþàOð'ø?ø»¥«ªªÞç=étº»»;LìÙ³§££#¾hÑ¢E'Ohnn?ø?ÁàþàïêííøÛ¹sçèèèûõ/õõõÑ·Îd2MMMS]­¦¦&ßüæ7¤¼Î9säÈãPú>úùç7¥__ømiJ¿'O8qÂ8~ßùÎw^~ùeãP°÷¥p¶oüä©N4ìêêÊÇßÁÏ+¯W_õÙg5¥ßÙ³g_xáãPú½òÊ+/½ôq(ýÓÿCé÷½ïohhÈ8ì=Ç_)ífuuuÁCcétzllÌa_öÃ¾rØ×a_Ë¾L&öÓ9K/^¼¸qãÆð7Eø?øü	þàþÊ¯ÎÎÎýû÷ð5NÇõõõ­^½úÊ+W?ø?ÁàþàoÆe25kÖÔÔÔTUUÕÖÖ®_¿þ6ùÑßßßØØH$R©ÔÀÀÀÏïÛsâïD?ø?Áàþàïº~ýzÁ>ÊâOýÂüÁàOðð7³ZZZõÖ®]^ÂÅÑÑÑuëÖ9K,?øü	þàOðWiø«®®ÔÈÎÉd2aNÁ³náþð'øü7þD ^t²mÔøøxs;?êþàþ?øÓmÂ_tØwõêÕÑaßð5L9mmmð?Áü	þ*áµ§à	W¯^?øü	þàOðWiøçÆ	¿ë×¯¯««K$áëêÕ«Ã²¸ÿðð'øüÁüÍ¡àþàOð'ø?ø?Áü	þàOð9kÞ¬D"ð'øüÁà¯Bð:ø?Áàþ¿©Úºuk¿Àü	þð'ø«XüÕÖÖö­Zµ*þÏð¿illìé§~ôÑGÃ×k×®ÁàþàoömØ°!ÚáW^b?ø¿¹ÓÈÈÈ|è£ýèªÿ¹ê#-ùà?øúë¯ÃàþàoÆ=õÔSûÖ®][v÷þàþæH¿õ[¿õé5Þqÿèß'>ñ%KÄÿ.9ü	þàþnÒõë×Ã¯ÎèÜÞÞÞr¼ÿðð7GúÁ~ð;~'+¿ðoûmohhxóÍ7áOðð7­vïÞíðëèè(ßûðs¤#GÜu×]qüÍÍÍ·dð¿²ÇÏù?Á_uöìÙ;ï¼sÛW·eå÷-_¹ã;Þzë-øüÁüM«ÄÍJ&ð¿ÒéÓþtKKËïýr_øÚò?Z¾ô¥/Ý¶ïð'ø+üUFðð7wØºuë¼yóÂ×?ýÓ?½mgÀü	þàþàOð÷¾õÓþôv²þàOðð¿9üÁàþàþð'øüÁüÁàþ?ø?øüÁàOðW*øknn®®®ö9ð'øüÁà¯òñ·hÑ¢8ø|Îü	þð'ø«düçöNNNÝý?ø?ÁàþàofÕ××ü£üàþàOð'ø?øqCCC7o/Bð?Áü	þ*¡Tååøü	þàOðWø[¸p¡>àOð'ø?Áß_Ä¾ááár¼ÿðð'øüÁüÍ¬'|ÀàOð¿¹¿¾¾¾¿mÛ¶Ãü	þð'ø«püUM>àOð'ø?Á_â/1ENø?Áàþ¿²þàþ?ø?øüÁàþð7uLfÍ5555UUUµµµë×¯/áþàOð'ø?øY×¯_/xÂGYüð?ÁüÁßÌjii	Ô[»vmô·GGG×­[æ,Y²þàOð'ø?Á_¥á¯ºº:Pobb";'É9a>üÁàOð¿JÃ_"ÔàËÎs|Ôü	þð'ø«@üEW¯^ö_ÃtÓÖÖð'øüÁà¯Òð^ðqõêUø?Áàþ¿wnð»~ýúºººD"¾®^½:Ì)ûð?ÁüÁßþàþ?ø¿ÕÔÔ´páÂ³gÏÂü	þð'ø«|ü%ÉªªrÝð?ÁüÁßÌêííøÛ¹sçèèh¹üU7ø?øü	þàþf»)J$ð?Áü	þ*)ò!Ïð'øüÁà¯rð·páÂÖÖÖ±±±²¾ÿðð'øüÁüM«èOú¾sã°oYá?ø?Áàþ4üEÒ7ú3¾ð?Áü	þ*©TªªhNø?Áàþ¿ÁÁÁyóæEûÿ"ê9áþ?øüU,þ²óàþàOð'ø?ùÛ¾ðð'øüÁü½¥R©d2ÙÚÚÚÛÛ[|ÑìæÀüÁàOððW*¥Óéîîî0±gÏâf7'Þ¶mÛ?¬B544ÒoÁ~Ë¢ßüÍßüà?hJ¿ßøßH¥RÆ¡ôSSSq(Xá¯¾¾>úÁL&<¨ÅÍnN¼­[·VI$UPï9þÎ;÷.â/~îHÎy$ùf7þ$IüÝÂãóÒÖÖöê«¯ÞúÖâX]]]|ÑìæÄûó?ÿó;vüoåõÍo~ó+_ùq(ýþîïþnûöíÆ¡ôì±ÇvîÜiJ¿¿þë¿þË¿üKãPú=øàßøÆ7CÁÞsüöe¥L&W¬XqþüùYo­¡¡!ÉDGiÃtñE³ã'|8áCNø>ðáwáàoKKKVÕÕÕkÖ¬Åv:;;÷ïß&Â×t:]|ÑìæÀüÁàOðð÷î499ÄPSSS5Û?ïÖßßßØØÖM¥R?¿7^ç/Ýø?øü	þàþnµáááø¿ººº®®®Ò¿ÿðð'øüÁüÍ¬¸ùjjj:::.P.÷þàþ?ø¿nâÆyúÔ§Êñ1?ø?Áàþàof½+ðð'ø?Áà¯<ðWÖÁüÁàOðð7ãÎ9ÓÜÜ[áÛÐÐpèÐ!ø?Áàþ¿ì	þ¢é=öÀü	þð'ø«4ü566ê9s&¿èÓ^àþ?øüUþ²B8¿ÉÉÉè`ø?Áàþ¿@½ho_À_&¹ï¾ûÂtSSüÁàOð¿JÃ____U¡N<	ð'øüÁà¯ÒðýRkooÎö­©©innO²¸ÿðð'øüÁüÍ¡àþàOð'ø?ø?Áü	þàOðºpáB[[[]]]âFa¢µµµùÂüÁàOðð7î¹çª)Ú´iüÁàOð¿ÊÁß¡C"çíÝ»wll,yýúõÃGó;ð'øüÁà¯BðJ¥ðöíÛ¿(úo>çþ?øüUþª««ð®_¿¿(ÉEáð?Áü	þ*Ù¿ê6ÕÒèO½Áü	þð'ø«üáüÁàOð?ø?øüÁàOðW¶ø+üÁàOð¿ÊÁ_âf%Iø?Áàþ¿Êþàþ?ø?øüÁàþðð'øüÁàþàþ?øüÁüÁàOð?ø?øüÁàOðð?øü	þàþàOð?ÁüÁü	þàOðððð'øüÁàþàþ?øüÁüÁàOðð?øüÁq?ø?øü	þàOðð?Áü	þàþàOð'ø?ÁüÁü	þàOð'ø?ø?Áü	þðð'ø?Áàþàþð'øüÁüÁü	þð'ø?ø?Áàþðð'øüÁüÁàþððð?Áü	þàþàOð'ø?ÁüÁü	þð'ø?ø?ÁüÁü	þàþàOð?ÁüÁü	þàOð'ø?ø?Áü	þððð?øüÁüÁàOð?ø?øü	þàþàOð?ÁüÁüÁàOð?ø+ÁR©T2lmmííí/hkk/^®ð?ÁüÁ_ÙN§»»»ÃÄ=:::â-ZtòäÉ0qàÀææfø?øü	þàþÊ¾úúúÉÉÉ0Éd¦ºZMMM>þ~øác*Ô3Ï<cJ¿çîÈ#Æ¡ôÿòHË#õì³ÏÒ/<LáÁ2øK&§ãvuuåã¯¯¯ïÿ*¯ñññð¤2¥ßüÇø.Þ|óÍð[È8~?þñøÃÒï_þå_þõ_ÿÕ8lNà/Hd§«««K§Ócccû:ìë°¯öÃ¾û:ì[7ýéL&öÓ9×¼xñâÆÃoÞüÀüÁàOððW~uvvîß¿?L¯ét:¾¨¯¯oõêÕW®¸"üÁü	þðåWccc"H¥R?¿o7v655UÅ?ø?Áàþàoîð?ÁüÁü	þàOð?ø?øü	þàOðð?Áü	þàþàOð'ø?ÁüÁü	þàOð'ø?ø?Áü	þðð'ø?Áàþàþ¥Ô[o½511ð'ø?ø?Á_×ÝÝýë¿þëõõõóæÍûä'?ð'ø?ø?Á_eö7ó7wÞyçïþâûwÜ÷¿î»ë®»>òLµþàOðð¿òîø@$¿ì¿èCO?ý4üÁàþàþÖµk×êëëãòÿ~ÿ÷ÿ¯þê¯àþðð'ø«À~í×~í«[¿Çß-ùøø?ÁüÁü	þ*°Ïîs¿Ûö»Ûÿl$¿Ïösuuuo¾ù&üÁàþàþØØØØïýÞï-X°`åÊÿøÇåW~åøñãS]þàOðð¿J(ïÑGâ'þàOðð¿9üÁàþàþð'øüÁüÁàþ?ø?øüÁàOðððð'ø?ÁüÁü	þð'ø?ø?ÁàþàþàþàOð'ø?ø?ø?øüÁàþàþ?øüÁüÁàOð?ø?øü	þàOðð?øü	þàþàOð?ÁüÁßmïÍ7ßòóÖ[oøüÁàþà¯»ÿþûõWµýwÛ?Òò;î¸ãÉ'4&ð'ø?ÁüÁ_eö·û·©Tê+[¾²ãþáßmü£ùóçÅ?Áü	þàþ*³Eñî/Fòþrõ'»ººü	þàOððWÝqÇqùØù«V­22ð'ø?ÁüÁ_¶hÑ¢/|þqübÕ'ìù?Áü	þàþ*3ïù?ÁüÁüÁüÍ­î¿ÿþÚÚÚìc---Îö?Áü	þàþ*¿7ß|óðáÃ;wîô9ð'ø?ÁüÁßÈ_ø?Áü	þàþàOð'ø?ÁüÁü	þð'ø?ø?Áàþðð'ø?Áàþàþð'øüÁüÁàþ?ø?øüÁàþàþàþàOð'ø?ÁüÁü	þð'ø?ø?Áàþàþð'ø?ÁüÁü	þð'ø?ø?Áàþðð'øüÁàþàþð'øüÁüÁàþ?ø?øüÁàOðð?øü	þàþàþ?øÓÁßÐÐP*J&­­­½½½ùWèéé©ªª?ø?Áàþà¯J§ÓÝÝÝabÏ=9K'&&ÚÚÚ¦Âß/¾x]yýìg?öÙgCé÷ïÿþïßþö·Cé÷úë¯ÒïÜ¹s¯¼òq(ýN>=22b6'ðW__?99&2LSSSÎÒ|ðGßøÆ7^T¡9bJ¿^x!0Ý8~===G5¥ßóÏ?ìØ1ãPú=÷ÜsáÁ2øK&§C.4tØ×a_å°¯öuØW¿D"®®®/Z¿~ý'þû®ÂüÁàOððWÆ7ýéL&óÎÃ¾aºàÕ²W?ø?Áàþà¯¼ëììÜ¿_ÓéôTXÌ	ð?ÁüÁ_ùÕßßßØØH$R©ÔÀÀ@AíÁüÁàOðò!Ïðð'øüÁüÁüÁü	þàOðð?Áü	þàþàOð'ø?ÁüÁü	þð'ø?ø?Áü	þðð'ø?Áàþàþð'øüÁüÁàþðgàþàþ?øüÁüÁàOð?ø?øü	þàþæVññÿø¯+¯üä'ÿ÷oJ¿³gÏ<xÐ8~ÿüÏÿüÌ3ÏÒ¯¿¿ÿÅ_4¥ß/¼ð½ïÏ8ìÚµkð7egÎÙ±cÇ×$I*¥âG5«ìü$IrØW$Ið'I$ø$IüI$	þ$I$I?½9sfÉ%Éd²µµµ¿¿?¾èÒ¥KU±Â¡¡¡T*]¹··×èÈ#DÁÇN·¹üÏyNæ#500ÐÖÖÅÇÈsª4&/Rð§Y!'O¬¯¯/:tèÐÆãsÒétwwwØ³gOGGÑ+G*[xtÂCSð±Óílbb"Ð!ÿµ*ÿä9UÔ¢EÂ-L8p ¹¹Ùsª4&/Rð§[íØ±cáÿ¸ñ9áIi#[0ÇäädÈd2MMM­D©¨×^måÊS=vº=øà<òHþkUþ3Èsª4©x555S¥ù0y?ÝÒªêêêÂó*ü7çÿ¾«W¯N&íííçÎsÂtvi|Zïï#µ~ýúÁÁÁ©;Ý¶.=¼å¿Vå?<§JóÊS]]]S¥ù0y?Ýj===]¼x1ÚÕ½,ªººÚ Î#522²téÒ"n[Aá'NxçÆ1så?<§Józûí·ÓéôØØçT)?L^¤àO·Tÿ'EÏ¢`L&óÎ=êSIQïË#µk×®Ý»wyìtû~¥þrñEùÏ Ï©Ò|¤"OlÜ¸ñÒ¥KS¥ü0y?Í¦E9sæg·­^½:gÑùóç£_Ñ¢ÎÎÎýû÷ð5üØèÈ#Z¾|ù©S§<vz_^´ræä?<§JóêëëO+W®xNòÃäEþ4ËZ[[ÉäÊ+³ÿÇc---aÑ+ÂS+ÌéïïollL$©T*ÄèÈ#ý¯7zsTþc§÷÷µ*ÎyNæ#ÕÔÔ³·Ésª&/Rð'I$ø$IüI$Á$IàO$Ið'I$ø$IüI$	þ$I$I?I$Á$IàO$Ið'I$I?I$Á$IàO$Ið'I$ø¤w©oë[Ë/¯¹ÑÊ+zê©_úw£²ùÖ655»6663?ÌI&©Tjrrr¦ÛTíØ±£*¯¯ýë¿;w?þxÎüÇ,Ìàf±MIð'Iå×ÐÐP`M2Ü»wïÄöíÛ.§Nªü½öÚkaæâÅsæ·´´ùçÏ?Ið'iNôÙÏ~6°æ¡Ï|øáÃÌÏþóqúôöö<¶µµéì¯^½ÚÕÕUWWÕ××oÙ²%~tõøñãXaQX÷Ø±c9sæÍ·|ùò£G97ìÈ#Å·-7)ZÔÓÓ3ÔV¯^ædç<y2ÌYµjUvÎ®]»Ã¦jjj6lØpñâÅ|üåo?gN*	þ$énÁ..Ï|ã7ÂÌ¦¦¦8nr®[·.gÑæÍ£EÃÃÃD¢àZÑÅhéúõë'''«««'&&ÂÒð5à©¶¶6z+^í7/ÿ8p .Ú¬/»»»³òËÙÈÊ+g¿"7UüIÒû_t·Àïµªª@±8n:::Þ¾Q×®]-¬ñ1ÚÐ-J§ÓÑ.Ã0Ýßßm$¾ÍíÛ·ÞEG]ï¹ç0çðáÃa:|Ówß÷M·à.+Äo[Á@9þüp¯.¯áqf2è©T*¬îÂ;¿8LSü¹©àOJ¡øËÎp)tñâÅp1°)ºØÖÖ..pÓ¦Mm×¯_Ïn$Zv­èâo¼½rSöÈoø¦?~Óíh/_ß¶©Þ·uëÖ°èGyç¶·lÙ¿B`À_ i´cr¦ø+rS%Á$½ÿE»»Þ~ûíøÌ±±±03,*Â,GFF"ÿe­£^þÑØNÅ?`%L×××WWWÏ7/»ô¦Ûb99s&rj_Ãôððpvi¸OEnª$ø¤÷¿èo»wïÏ>%çìû¯¿CëÔ©S;wîÂfÕíËW½)Ñ6oÞí_»ºº²ól'ÚÓvéÒ¥èâåË»téÒ°4út`Öø¢èÌß°¨§§gtt´8þ²0F#»´ÈMôþ0L&÷ìÙÔËÞ½«««óOÎX»vmÎøøxtL6fnô¿è­r###ñ7FÜ²eK RtnöÃV-:ý6úîÙùE¶½É/zÏßØØXtÍ"øÛ·o_vËñEÑÛÃw	0þ"n.«tnØ°!¾´ÈMTEÓ®]»þÿï¸E6ÊNgfÏ±ÈvÏ=÷ÄeïÐ¡CEð÷Î?Åæ×××ÇÙN¸ñ=Óvª;ýúõè.äì^³fMü[Ì??|>í%¾ÍhÇd¶ªÙ¥Enª$ø¤R)eåÊÕ7Z¾|ytÊmþzzz¢Ókmm=qâDvéØØØ÷Ý×ØØiëÖ­ãããÙ¥Çkoo&K¥RO<ñDÎ6óoIØTï½÷æÌj;¡pcÂM>°ÈçüeëêêÊùÌ¨+W®¤Óé0uuuá^?>û±5ñmÝFcµjÕªïXä¦J?I$Á$IàO$Ið'I$ø$IüI$	þ$I$I?I$ø$IüI$	þ$I$I?I$Á$IàO$Ið'I¤w£ÿå@i;¥¿IEND®B`


UC-^¼xÿþý·]àøgù|hjjÚ½÷$VÎ¤'O'a:[¶1*'ü¤é$¿Â6mÚô±j¬täW]]õêÕé"¿'xbäÆÚºuk¥Mh±»víèÊôjÌd2ùÛmkk#?ü$¸qãÆóÏ?_UU#;;;gü¢U«VMù>:¡ê/¾800088q1Fvwwº¨Î2òù°yóæ2ù%3^¿~=s¹Iä'üvìØ1lwËñãÇÓéô=:LNù$qÙ³g/Y²¤È¼ù+=vÙ²eÉÈµk×öööÞhsss2ã±cÇÝÕ±ÛÝuëÖÅÏµNÞyç5kÖ$÷0Í^¼xqØ5o2&n4n:îÀ÷ÝwíÚµ'O655UVV._¾¼pÿâöíÛëêêÃqùIcÝáz(ÆBê1²½½Ôí;ÑYFÞt`1ÆÄÐÊu±¸üMíÏXí÷ß¬ÛâGNçÓ8k20jjj6mÚÔ××çE"?i:É/°#ëëëÝÝÝ©TªðýøôéÓEäõêÕEæÍ_9Þ§¶¶¶î¬ë¸^Åqäc¿qãF&©­­ëäÊ+ñv^¸¸æ0¢>Þ·»hÑ¢djR($Ï¾a×tiqßÌ;7Æ¿ÿþû#7ÖüùóGÝ¾eØMk~Z9ÅWþ¤g¿üºººv¢òçÓxÕªUÃîóÆ½°Hä'M'ù%ãÃdÉp61|êÔ©B=¹¸eËÁÁÁd/Îmç7ÎxËë';IÉÔxÇ1ûÍ¡b®ÂÛ*²Øâ3úØ:T¸¬ðúë×¯á+W^*âbëñ&c6lØ000Ü±dÉqñèÑ£0ù4Û#GbøÒ¥KÆºÃétz¬÷#Î2ÖéòûD'ºrF½W±¸é/>Âôy_~ã|'"¼|ùrÇÖáY³fyaÈO~òËàK>VX)"¿+W®äÇÜvÞüþ³ä¨b¼&ãí³pgÕ°£uE[|Æ±ÄxS6¾¶¶¶ðÅ|3gÎX7Ú(¼ÂN`á=ÉåraFKKKác/"¿<ÇÇbúG¥pÆ¼±2-[vüøñQWÚxVÎ¨÷jÒ3_~É3äÚµk71!ùóiÜÜÜçÍrÿBÜºuË«D~Ò4_H%FÖÔÔõv;QA"oÕE|3Ñ÷æI,v¬ññãñÆûwáøûÌF~èmäãÏÅPæ°#·=Gµ®®.9<=wÃ<_ÂxfÏºõãY9cytr3ÿhïGvóiÜÓÓà//ÂÃaÈONòáÏðHö¢;Ër<ÐÐ¼Å÷Ù¼ÿþûS,¶øEîÀâbþ;P	Îífâñ¯ÂÉ·«;v,d6ù%;&/XÉf³6mº~ýú=bä5kFßxf´ü&ºròMzÆñËo<ûüò¶Kþ«Sdýy¾%9sfÛ¶mÉ!æüyÐÈO*uùÅ[(ycÎ«Kò~hwÊäTÙ$9õ5ê[fy³)!Kò9­Xx²aâu±Åg,ÎU«Vå?ÈIÎü]¹rå¡¥mØ°á£Ë/ÙïÕÝÝa÷îÝãß'½JñO:ìÏK:tèÐ¨Ûw¢³LH~ÅWÎ°çFa]«_òÉ¼x2Üºu+`·bÅ.Ü¹sg¬üÐa ¸øú¿í¯@òyÍ"GÞ%T*òYáù#ÏÍÞ?ÿ¥»ÉÂoEæ-Î¦ Qb£Â-óS,¶øÅqõêÕü'º1/^ö¯ÚÚÚü?üòI3gNþSE|³víÚkáÂE6ñfü¯aÏÂ&ºV'!¿3gÎ.É%S­ü	æcÝJç[þ¢|<òü¤é!¿xtéÈ¿îuôèÑPT¼¿ðÂùñ1!­Âo³ç¼·eSò5x±ðæææ_Ë7Öbo;cqF$GBÇ_¸paÍ5Ce³ÙçsLN~½½½±´XfèÑGåÁ]Ü7Ï=÷ºxàsçÎÝ»wï<ßçôÑgü¯aÏaMh­NB~ÑáÃçÍ:OÏÎÃgÉ­/[¶¬³³ó¶ûz¾õõõ=öØcÉÎÔàlÊþþ~/,ùIÒÇR.[»ví¾Cn³L»½ÉÉÚ	è=a$ò$[ÉçüõôÓO[3ùIÊ­¾¾¾Í7Ï;79¤O<ñÕ"$IÈO$Iä'I$ò$IùI$ü$ID~$Iä'I$ò$IùI$ü$ID~$I"?I$$IÈOR©uèÐ¡úúút:½téÒÎÛÔÔTQQÑÓÓÃ1fÁx9jr×Ï¼|-zQ*ªªªÊf³½½½É¤¾¾¾7ÖÔÔÄÊ¬®®Iï¼ó'$òTBÕÕÕc/wûöí1ï³Ï>óÌ3ÏÄmÛ¶·üáxà1ÜÚÚ\·n]èèá'OÆðüùó=Á$¤Rz5ùx:sæLÌß÷åÇ,Z´(ÆtuuM½Ã¦þÃÊ1¬¬7nÜð¤D~Jyì±Çª««gÍµuëÖaWàZZZ-!¿Ë0ßÿý1É¤Ë//[¶,`N§a#VxëÅgÙ»woýâÅãF:ìæÍmmm1oÜùÍ7'»0Ã¦K,¥ÅøXò+W>>ùÝsÏ=q±±±ñÔ©SZÈORéâ/~ê©§bø^cÅÀÓO?]x£Gæ?Óoýúõ1iÏ=1?c¸½½=´páÂ#GÄÀ¥Kb|&uiw ø,á¹ÄÀºuëFÞù7ÆpÕW_íÛ·ç5vãÆîîîHvIlòõÃ_ýêWIÊ/péÒ¥gÏõìD~JW~õõõ1j$¼Fý à'bÒÊ+cxÕªU	éòSÏ9³eËäQ6ÌXÅgIîXuuõÈykkk;&0_kÆpsssX¶¿¿r«eäøä¾ÅÂwO>xÍ_'nÔLùI*Qù¥ÓéüpÄÅâúph××¬Y³âÉAÕLÚ½wÌ<ölá-­ðâxfëÅwã%j<xðàìÙ³1555ÉîÀË>¿"utt5|KùI*9ùe2Â~Én³Ûr§­­-¦ææÇ'g<5Nù%Çs#ç3gN2ï°»700päÈäÄÛüÎÂ	­Ê/Jîg<O0Iä'©Då|KË=Ïù=óÌ3ãáÎÁó;Ï88òÜ¹sÉçÿÆ#¿â³lÙ²åå_õë×÷Gáûö?>9Â#/^ÃgÏ½víZ444||ò[°`AL:vìX>|8×®]ë	&ü$¨üBW³*üN¾âò»uëVr¤5~~è-ôSSSS]]½yóæqÊ¯ø,Ç©-ÊhRx¾¾¾M6Å=¯¬¬bEro/Í&'ßwß.ÐjyD¸Èª[lkk;ëaÎ97n»ä	&ü$ID~$I"?I$ò$IùI$ü$ID~Ó¦ï|ç;ï½÷ÞTÞâÅs¹g^©õýïÿ?þã?¬Ü.úSi.]ºd»ævù÷ÿwë¡Ôz÷ÝwKj»Ìtùë[ßüMå-8qâßþíßü&Zo¾ùæÍ7­Rë­·Þú×ýWë¡Ô:yòä¿üË¿X¥V¼~¦J¤S§Nýó?ÿ3ùÈOäG~"?ò#?òùüÈOäG~äG~"?òùüÈüÈOäG~"?ò#?ò#?òùÈüD~äG~"?òùÈüÈüD~"?òùùÈOäG~"?ò#?òùÈOäG~äG~"?òùùÈOäG~"?ò#?òùüÈOäG~äG~"?òùüÈüÈOäG~"?ò#?ò#?òùÈüD~e%¿®®®L&N§:::'õ÷÷¯[·®²²òî	uÈüD~ä'òÞe³ÙûöÅÀ®]»Z[['mß¾ýé§rÍ7o¤ü9rýõ×¯r]%Vüáþá¬Rë7Þ°]Js»|Ùz(µâmî½÷Þ³J­7ß|óÝwßÊ[ò«©©	ÛÅ@.«¯¯/ÔÔÔtþüù±fù¿;4ã¿ËßUe»ìvyë­·¬Rëµ×^³]lìvòK§Ó£'wîÜYUU5oÞ¼³gÏ:Ú+Gí£½öÊÑÞé]*ÊWVV´gÏ¸páBKKùüÈOäG~"¿é]mmm.ûpèho¶;üD~ä'ò#?ßô«­­mïÞ½1?³Ùlá¤68p Î;·xñbòùÈüD~Ó~µÖÕÕ¥R©L&ÓÙÙùÃÇVñß.~V¯^N§[ZZzzzÈOäG~"?òùÍÐÈOäG~"?òùùÈOäG~"?ò#?òùùÈüÈüD~ä'òùùÈüD~äG~ä'òùÈüÈüD~"?òùùÈüD~"?ò#?òùÈüD~äG~"?òùÈüÈOäG~"?òùùÈOäG~"?ò#?òùõ@~"?ò#?òùÈOäG~äG~"?òùùÈOäG~"?ò#?òùùÈüÈüD~ä'òùùÈüD~ä'ò#?òùÈüD~äG~"?òùÈüÈüD~"?òùùÈüD~"?ò#?òùÈOäG~äG~"?òùùÈOäG~"?ò#?òùùÈüÈüD~ä'òùùÈüD~ä'ò#?òùÈüD~äG~"?òùÈüÈüD~"?òùùÈüD~"?ò#?òùÈOäG~äG~"?òùùÈOäG~"?ò#?òùõ@~òëïïïëë#?ò#?ùüTÎòï½÷,Y2öìL&óÚk¯ùÈüD~*CùÅ³ë§ú§ÿââÍßØ¼õñ­>ø`uuõ+¯¼B~äG~"?òù©Üä÷üóÏ766ùòÿ~-ûk?û³?K~äG~"?òù©Üä÷õ¯ýK_úR¡üâß'>ñ	ò#?òùÈOå&¿gö÷¡ëcý§?ýiò#?òùÈOå&¿Ù³gmí×ö=öûlÃg¿ñoùÈüD~*7ùE/½ôRUUÕ¢E.]z÷Ýwå+_éïï'?ò#?ùüTò.^¼øÇüÇÏ=÷oLßB~äG~ä'ò#?ßLüÈüÈOäG~"?ò#?òùüÈOäG~äG~"?òùüÈüÈOäG~"?ùùüÈOäG~"?ò#?ùüÈOäG~äG~"?ùüÈüÈOä'ò#?ùùüÈOä'ò#?ò#?ùüÈüÈüÈOäG~"?ò#?òùüÈOäG~äG~"?òùüÈüêêêÊd2étº©©©££cä;VQQA~"?òùÈoÚÍf÷íÛ»víjmm6u`` ¹¹üD~ä'ò#?_9TSS388¹¾¾~ØÔ§zjÇcÉïþèþz;zôè_þå_þµJ¬Ø.]]]ÖC©õgög¶í¢qvìØ±¿ø¿°l!¿t:=êptùòåpáXòåWÞÂþüÏÿ¼§§ç=XÇ·]Js»üÝßýõPj½þúë.l§ïïSy3B~©T*?iõêÕ'Oüïêh¯íu´Wö:Ú+GË ÚÚÚíáÿñÿgä'ò#?ùü¦wmmm÷îøÍfG¨öùüÈOäG~"¿òX­uuu©T*ÉtvvJ=òùÈüD~3:òùÈüD~äG~ä'òùÈüÈüD~äG~ä'ò#?ò#?ùüD~äG~ä'ò#?ùùüD~ä'ò#?ò#?ÈüD~äG~ä'ò#?ÈüÈüD~ä'ò#?ùÈüD~ä'ò#?òùÈüD~äG~ä'òùÈüÈüD~äg=ÈüÈüD~ä'òùùÈüD~äG~ä'òùÈüÈüD~äG~ä'ò#?ò#?ùüD~äG~ä'ò#?ùüÈüD~ä'ò#?ùÈüD~ä'ò#?ò#?ÈüD~äG~ä'ò#?ÈüÈüD~ä'òùùÈüD~äG~ä'òùÈüÈüD~äG~ä'ò#?ò#?ùüD~äG~ä'ò#?ùüÈüD~ä'ò#?ùÈüD~ä'ò#?ò#?ÈüD~äG~ä'ò#?fü*nW*"?ò#?ùüÈ¯äº]étüÈüD~ä'ò#¿r_yD~"?òùÈï£O¾õë×ùÈüD~äWnò«¯¯O§Ó>çG~ä'ò#?Ê ,yzGMMM__ùùüÈOäG~e%¿ÊÊÊ ^ooo]]]ø^~ùåhoo'?ò#?ùüÈ¯¬äìä ^tipp0fÍE~äG~"?òù_YÉoöìÙá¼x`===1ðÄO$¾ÕüÈOäG~"?ò+7ùmÞ¼9>GáGý/^L~äG~"?òù_YÉ/zòÉ'çÌ1liiÛüD~ä'ò#?ßLüD~ä'ò#?ùùüD~ä'ò­ä»]|3ùÈüD~*gùÍ?¿PùÛK~ä'ò#?ùüya¾Ó§ON»íA~"?òùÈoÕÔÔü¦#ûÈOäG~"?òùM¬®®®ßÆ§ã'ùüÈOäG~"¿5wîÜ9ÃüÈOäG~"?ò+7ùÍ7ÏäG~"?òùiFÈ/1_ww÷tÜä'ò#?ùü&Pmm­3<ÈüD~ä'òÓ_P&ä·yóæþþ~ò#?òùÈOå,¿1rùÈüD~äWßä<jÎð ?òùÈüÊð[]¦oä'ò#?ùü&Pý¼yóÎ?O~äG~"?òù©ÌåN§+*îð¾Ã®®®L&÷¤©©©£££pRgggsssLZ°`AüD~ä'ò#?ßäiü¶mÛO¸;õÝ.Ùlvß¾1°k×®ÖÖÖÂIóçÏûí·c`ÿþýä'ò#?ùü>Â"JàÜÞ¹¾¾~¬«UUUßüÁÂ>|D¥íb»hBÛåOÿôO­ÛE¥¹]>vùÂ¹½·5Öí>º½½¤üÞzë­ÿÂÞxãø¿ò©Ä:qâDü_Ùz(µÞ|óÍø¿²õPjÅË¦íR<yòúõëÖC©õï|ç>Ê[üØåWî_¬¬¬y7of³Ù¾¾>Gåh¯£½r´×Ñ^9Ú;½«­­ÍårÉÑÞ6õêÕ«ëÖ­»víÚÈÉOäG~"?òùM¬ðÖ+ªªª***fÍµzõê)>Õ£­­mïÞ½1?³Ùì0i-_¾¼··wÔÉOäG~"?òùM [·nzÇTþßX­uuu©T*Étvvþð±×Lá½"?ùüÈOä7ùCT+W®LÞ8ãi·jÕª³páÂÒßä'ò#?ùü&Peee8o`` ?&ËÅQÏ´ ?ò#?ùüÈoË/Jó,úûûcÌT~«ùüÈOäG~äG~S!¿ähïòåË7ÎøÃ1¦¹¹üÈüD~ä'ò#¿²_¼_zÇõë×ÉüÈOäG~"?ò++ù8tzïêÕ««««S©Tü|yÛüD~ä'ò#?ßLüD~ä'ò#?ùùüD~ä'òËÏy»ÿ.ùùüÈOäG~ÓX~©±#?ò#?ùüôáL8Úûè£&òÛ¿?ùùüÈOäG~å)¿®®®Y³fù-[VøÅÎäG~ä'ò#?ùüÖ¬Yìê;räÈ4Úä'ò#?ùü&Ð«¯¾oåÊÓnÈüD~ä'òW·nÝZ¸par2GGGÇtÜä'ò#?ùünßÎ;]­­­ÓwÈüD~ä'òÇ¾ÏüÈOäG~"?Íù¥nW:&?ò#?ùüÈ¯äWÈüD~ä'ò#?ò#?ÈüD~äG~ä'ò#?ò#?ùùüÈOä'ò#?ò#?ùüÈüÈOä'ò#?ùM/ù544TVVú>?ò#?ùüTæò?~¡ö|ùÈüD~*[ùòÂ|§OvÛüD~ä'ò#?ßª©©	ùMGöÈüD~ä'òX]]]!¿7NÇ7NòùÈüD~kîÜ¹#rùÈüD~äWnò7o3<ÈüD~ä'òÓ_b¾îîîé¸=ÈOäG~"?òùM ÚÚZgxùüÈOä§!¿ LÈoóæÍýýýäG~ä§R_åùçÿÍßüÍ§zêüùóÖ*ùÈï£-báA~ä§;.¿»ï¾ûçþç¿üå/ÿÒ_úä'?¿V,ùÈoò¥ÆÈäG~ºãò»÷ÞWüÊ­oMþ=üÐÃ?õS?E-äG~"¿ùüÊX~ÿøÿX__¿åw¶äåÿ~îç~În?ò#?ùù_¹É¯»»»¡¡¡ñï~á¾ýío[·äG~"¿ÉËåV¬XQUUUQQ1kÖ¬Õ«WOSÉOäWÆòëëë×¥õ¿±>Ï¾ÍßØ<gÎçyùü&ß­[·F=ÃcZêK~"¿2_ôì³Ïf2ÿÝö¿íÿ§ýÞï]·nK~ä'ò|á¼+W&oñ´[µjUY¸p!ùùéÎÊ/úö·¿/SøÄ'>ýéO?þøãV,ùÈoòUVVó_Ls¹ñäG~ä§;.?ùü~òK¥Rá¼Ð^~Lñ­.äG~"?òù_yí]¾|yòÆ?c8Æ477ùÈüD~äWVò÷ËQÏð¸~ý:ùùüÈOäG~e%¿Nï]½zuuuu*Ë/1ÓbÈüD~ä'ò)ÈüD~ä'ò@õõõóæÍ¦ßJ~"?òùÈo¥ÓééºïüD~ä'ò#?ßêèèùmÛ¶-pÓå¶ÈüD~ä'òÔ"Æ(JùÈüD~äWVòKor&?òùÈüÊD~óæÍkjjêëëÖÛüD~ä'ò#?ßíKþCG§Å]òùÈüD~,ùs½Éè%?ò#?ùüTÎòËd2EsùÈüD~äW&ò;úôìÙ³=óáA~ä'ò#?ÊS~ù¦òÈOäG~"?òùÍèÈOäG~"?òùùÈOäG~"¿¹®®®L&N§:::OÜÂ¶nÝúµ¯í[SØ¯ÿú¯ÿÞïýÞ·Tb­]»öw÷w­ÛEã)^6ãÅÓz°]4Ö­[÷øãOå-N3ùe³ÙûöÅÀ®]»Z[[OÜÂôÑI¤récß~ò«©©W___|ÒäÆ$I"¿É0úJæææwÞyç£/­ð4áa§4¹1mÞ¼¹®®î)ìî»ï;wî=*±âi`»Ø.²]lÁvùØåæË33huß÷]¼xqÒK+üþçÊÊÊâ&7Ær3<ägxÈõoccca¬+VLb9µµµ¹98ÃÅ'MnùüÈOäG~"¿CG©ªªªì_okkkÛ»woÄÏl6[|ÒäÆÈüD~ä'òûHuwwîó«®®nooÜª««5f2ÎÎÎÞË¡+4¹1ä'ò#?ùü&Y!øªªªZ[[/_¾<]¶ùüÈOäG~"¿,bèÄûï¿ÿÒ¥KÓnÈüD~ä'ò@?/s!?ÈüD~ä7ä7­#?ùüÈOä7±Î;×ÐÐ0kÖ¬ä|ÞÚÚÚùÈüD~äWnò;vìXþD~Éð®]»ÈüÈOäG~"?ò++ùÕÕÕóÎ;_gggòÅ.äG~ä'ò#?ùüò8/¿ÁÁÁä_ò#?òùÈüÊJ~µµµá¼d?_È/Ë=öØc1_O~äG~"?òù_YÉ/(S1Zo¿ý6ùùüÈOäG~e%¿èÚµk---É¹½UUU/^ÛüD~ä'ò#?ßLüD~ä'ò#?ùùüD~ä'òû]¾|¹¹¹¹ºº:5T455MC½ä'ò#?ùüÆÛ#<R1Fë×¯'?ò#?ùüÈ¯LäwðàÁy»wïîëëKFÞºuëÐ¡CÉø£GùÈüD~äWòËd2Á»=öü=7ßçG~ä'ò#?ùü*++w·nÝ9)ËÅ¤¸ùùüÈOäG~å ¿ümkjòÜÈüÈOäG~"?ò+ù±ùùüÈOä'ò#?ò#?ùüÈoÊ¯xäG~ä'ò#?ùüR·+NùÈüD~äWò+ÈOäG~"?òùùÈOäG~"?ò#?òùùÈüÈüD~ä'òùùÈüD~äG~ä'òùÈüÈüD~"?òùùÈüD~"?ò#?òùÈüD~äG~"?òùÈüÈOäG~"?òùùÈOäG~"?ò#?òùõ@~"?ò#?òùÈOäG~äG~"?òùùÈOäG~"?ò#?òùùÈüÈüD~ä'òùùÈüD~ä'ò#?òùÈüD~äG~"?òùÈüÈüD~"?òùùÈüD~"?ò#?òùÈOäG~äG~"?òùùÈOäG~"?ò#?òùùÈüÈüD~ä'òùùÈüD~ä'ò#?òùÈüD~äG~"?òùÈüÈüD~"?òùùÈüD~"?ò#?òùÈOäG~äG~"?òùùÈOäG~"?ò#¿qÖÕÕÉdÒétSSSGGGá¤ÎÎÎæææ´`Á¸ùüÈOäG~"¿é]6Ý·o_ìÚµ«µµµpÒüùóß~ûíØ¿CCùüÈOäG~"¿é]MMÍàà`är¹úúú±®VUU5R~øxzíµ×B¢§UbÙ.¶ÆßÑ£G¿ûÝïZ¶Js»Ìù¥ÓéQuÑÞÞ>R~úÁvüøñwß÷*±^ýõK.Y¥VGGÇ÷¿ÿëÁvÑxzã7.^¼h=Z'Nøû¿ÿû©¼Å!¿T*®¬¬y7of³Ù¾¾>Gåh¯£½r´×Ñ^9Ú;ýªøQ1[Ëå£½1<ìW¯^]·nÝµk×F.üD~ä'ò#?ß4«­­mïÞ½1?³Ùì0i-_¾¼··wÔÉOäG~"?òùM¿ÕZWWJ¥2LggçÛÐîÀúúúÈOäG~"?òùÍÐÈOäG~"?òùùÈOäG~"?ò#?òùùÈüÈüD~ä'òùùÈüD~äG~ä'òùÈüÈüD~"?òùùÈüD~"?ò#?òù_ÉÔßß?òoÈüÈüD~*+ùõôô|þóÿÉüÉùßzòùùÈOe(¿>ø À÷Å/~qËïlÙúøÖ_ËþÚ]wÝÕÝÝM~"?ò#?òù©Üä÷ÄO,Z´(Ìÿ÷+¿ü+¿ú«¿J~"?ò#?òù©Üä÷À<øàòû­ßü­Ï|æ3ä'ò#?ò#?ÊM~ëÖ­ûò¿¿zøsûùüÈüÈOä§rßáÃ?õ©Oýö¦ßNØ·åw¶Üï½O>ù$ùüÈüÈOä§r_´iÓ¦»îºëþ/Þÿ¥/é3?ó/~ñä'ò#?ò#?ÊP~Éð7¿ùÍ¯ýë/½ôR±üÈOäG~äG~"¿ùÈüÈüD~ä'òùù£^zéáþÊW¾²ûö;òB~ä'ò#?ùßT´aÃOêS_Yù|ð5ÿ¯úúúüàä'ò#?ùùáKÕ]wÝÿø÷ùÅú?x@~ä'ò#?ùßÇÞ7¿ùÍ_¾ÿ¿öö~ã'~â'ÈOäG~"?ò#?ò+·üñ/­øR¡ü¶üÎO~òä'ò#?ùù[/½ôÒÜ¹sCÞþý"ùüÈOäG~äG~åÖÀÀÀý÷ßßÐÐððC¯ÿõ_øÂî¾ûî¿ú«¿"?ùüÈüÈ¯ëïïê©§>÷¹Ïö³ðÁ/^¼8õ÷üÈOäG~"?ò#¿ùÈüD~äG~ä'òùÈüÈüD~ä'òùùÈüD~"?ò#?òùÈüÈüD~"?òùùÈü¬òùùÈüD~"?ò#?òùÈüD~äG~"?òùÈüÈüÈüD~ä'ò#?ò#?ÈüD~äG~ä'ò#?ÈüÈüD~ä'òùùÈüD~äG~ä'òùÈüÈüD~ä'òùùÈüD~"?ò#?òùÈüD~äG~"?òùÈüÈüÈüD~ä'ò#?ò#?ÈüD~äG~ä'ò#?ÈüÈüD~ä'òùùÈüD~äG~ä'òùÈüÈüD~ä'òùùÈüD~"?ò#?òùÈüD~äG~"?òùÈüÈü¬òùÈüÈüD~"?òùùÈüD~"¿)®««+É¤Óé¦¦¦W8vìXEEùüÈOäG~"¿i_6Ý·o_ìÚµ«µµuØÔæææ±äûÏ)ì7ÞßÌÿTOøÍ´J­ùõë×­R+DþÁX¥V¼··×z(µBäÿôOÿ4·8#äWSS388¹¾¾~ØÔ§zjÇcÉïg9:>|øµ×^;ªËv±]d»Ø.*í2#äN§G._¾ÜÒÒ.t´Wö:Ú+Gí£½åP*ÊWVVNZ½zu¼Hý÷C%?ùüÈOä7M«øQ1[Ëå>:ÚÃ£^-eòùÈüD~Ó¸¶¶¶½÷Æ@üÌf³cIqäHòùÈüD~ÓoµÖÕÕ¥R©L&ÓÙÙ9*õÈOäG~"?òùÍèÈOäG~"?òùùÈOäG~"?ò#?òùùÈüÈüD~ä'òùùÈüD~äG~ä'òùÈüÈüD~"?òùùÈüD~"?ò#?òùÈüD~äG~"?òùÈüÈOäG~"?òùùÈOäG~"¿rê÷ÿ÷_yå÷¦°|ïßO%VlóçÏ[¥ÖüÉüíßþ­õPj½üòËçÎ³J­x;û¿ùë¡Ô:xð`ww÷TÞbñÿÍtùÅ×Ö­[¿%IT?Ya7¬$IÒü$IÈO$Iä'I$ò$IùI$ü$ID~eRgggsss:^°`AWWWéïï_·n]eeå=÷ÜsâÄ	«¨D¶KEA©TÊ**í¿#1¦©©éÔ©SVQl.´´´ÄëØ5küÝ;Õ¹sç.øÛ['É$c:::¬¢Ù.Qooo=ùÍæÏÿöÛoÇÀþýûb`ûöíO?ýôàà`¼¥Í7Ï**íoß¾»ví²Jd»ÔÔÔ|9âg¼nÚ.1olÁÁ8þü¬¢;RÀâÐ¡C1['~Sb ÍÆ+XÄXkk«UT"Û%üÿ­¨(	tßVUU<'âµÒÚ(µítéÒ¥¥KZ'¥³]âGW¯^øéJ¥³]Òét~ÌìÙ³­;ÛÑ£G,XüOipp0r¹ÿ)ÎvY±bEOOùÍ¸N>ÝÞÞ¼bîÜ¹3^=ãmììÙ³ÖLl¤Õ«WÇ«¥t¶KWWWü²Ä+fü´iJg»ÄûÙ¹sçbàÅ_,T ¦¸êêêøÙ¿ÿ0Û.¥³]~H.òQÝ¼y3ÍöõõÅp*Ú³gÏ?ú S"Û%ÿ-Z´Èj)©í²páÂdyÐÖ)íÿkmhhïØ±£p¯¹îHÇ«­­MÞ_ò#+++­Ù.ä7ãºzõêºuë®]»|ø?Yél>¹sçNk¦¤¶%ûûôÎ;ï466Z?w¼ä·#Þ_r¹ÜCGßktg·ùÍ¬N8±|ùòÞÞÞü68pàÃ¡Ó/^lÈv,YræÌ+§¤¶Ë¢E£ÝÝÝ.´Jd»Ì?¿««kpppÇO>ù¤UtG­üvtvvÆ¶¶¶½÷Æ@üÌf³VQlòYÕ××~]H¹qãÆêÕ«ãÿ---===VQl$VélwÞy'ù~øÃVQl°àÜ¹sãW¦½½``À*º#¾â·céÒ¥ÉîØS§NÕÕÕ¥R©L&|­RØ.ä'I$ò$IùI$ü$ID~$I"?I$ò$IùI$ü$ID~$I"?I$$IÈO$Iä'I$ò$IùI$$IÈO$Iä'I$ò¤³_|qÉ%UC-]ºôÕW_ý/pCMãÑîm<´¾¾¾aãcL:Îd2]¦$ò¤é×Ö­[+FôäOü¶mÛ#þùaãî¹ÿÄOLbÈO¦Y]]]at:½÷î¡öìÙcä3gÊF~.],6¾±±1Æ_¼xü$¤òï¡Ó<ýôÓ#yæùÕ¯~µÐ=!§@asssç¯|ýúõöööêêêTSS³iÓ¦ÂªÇ]Å¤÷èÑ£Ã,cfÏ½dÉ#GÄÅ¶¶¶awìðáÃÅL»L:vìØXJ[¾|yïììÌyûí·cÌ²eËòc¶oß^WWªªªZ³fÍÕ«WGÊoäò)rW%$ÝÉæÎj¹|ùráÈ+W®ÄÈúúúBÙëôéÓÉÔU«V´qãÆdRwww*u®äb2uõêÕaÇÊÊÊ?CN³fÍJ>~Wd910êÝùH÷ïß_ÈÙ<.÷íÛgß°,]ºt¢ò+rW%$Ýá»£¼¨UTÃeÓÚÚzs¨+W®L¦&ÐIììº±%²Ùl²³0O:,¤p[¶l	Û%[yäsèÐ¡1¼víÚÛ.'Ôã÷mÔG3gN<ÞÞÞÞ¸?ã7s¹L&3ÆCøðGG«ªª&*¿"wUùIÒ_4ªüòãÙ$`®^½ÃLÉÅæææ¸8oÞ¼õë×ØnÝº_HØ^´üÅ+W®ä¯ZÊð1|üøñÛ.'ßÿýÂû6ÖgòôÑ´cÇtDÓ¦MWüÂ£---É.ÉÊ¯È]D~tKvtÝ¼y³pd___IE¬waOOO¿<tòÎyv¥¿K%kjj*++ûûûcá³gÏÎO½írÆrØ°Î; 5ãgwwwç§:u*îÀ¨GÇ/¿"wUùIÒ.ù¬ÛÎ;G&ßu2ìügGîÊ:sæÌ¶mÛc¯y2&;äòSoë³7&yãg~|å$ûØ®]»ÿý÷»hÑ¢|MµpRroL:vìØ7Ë/¯Òdmä§¹«ÈOîpÉétz×®]É·ºìÞ½»²²räÙ+W®åô÷÷'bóçá&óK>×ÓÓSøÁ6m'%gÝæ¿WeT%'Û&·~âÄüø"ËI>Ø|Î¯¯¯/¹fùíÙ³'¿7.rá¤ä#ÝÝÝq+±Æ_bÍ°rÄ¹fÍÂ©Eîª$ò¤;_ò-ÇÃÚ¾ûÿ*Q~84RE¾Gy¤<x°ü>úc1¾¦¦¦ð@påÄÝ(¼cùójÇz°·nÝªªªJÂ°cÜ+V¬(¼9sæÄÏä]ìÌ85?µÈ]D~TN.]Z9Ô%KlÉïØ±cÉ×æ555<y2?µ¯¯ï±Ç«««K´ôè£ö÷÷ç§=z´¥¥%@Éd^xáaËyObQ1~ÃÃÆµ(îLÜ¥ä|_¾öööa_ïÔÛÛÍfcTWWÇ£¸xñbþjyãÆ m²®-[ÖÙÙ9ìÜUIä'I$ò$IùI$ü$ID~$I"?I$$IÈO$ü$ID~$I"?I$$IÈO$Iä'I$ò$IùI$ü$I4¢ÿ²Uô¥¬¼IEND®B`
