# Supplementary material for: Regulation of piglet T-cell immune responses by thioredoxin peroxidase from Cysticercus cellulosae excretory-secretory antigens
Source: Front Microbiol. 2022 Nov 18;13:1019810. doi: 10.3389/fmicb.2022.1019810 (PMC9718028; doi:10.3389/fmicb.2022.1019810)
Supplement: Supplementary file 3 [file Data_Sheet_3.ZIP › 4. C. Cellulosae ESAs and TPx Induced Th Subpopulation Differentiation/3. SPSS statistical analysis/5. IL-17/2. IL17-48h/2.3 (SPSS data export) SPSS statistical analysis--IL17--48h.doc]

EXAMINE VARIABLES=Figures BY Variables
  /PLOT BOXPLOT NPPLOT
  /COMPARE GROUPS
  /STATISTICS DESCRIPTIVES
  /CINTERVAL 95
  /MISSING LISTWISE
  /NOTOTAL.


Explore


Notes	
Output Created	12-SEP-2022 23:42:09	
Comments		
Input	Data	E:\桌面\Raw Data\4. C. Cellulosae ESAs and TPx Induced Th Subpopulation Differentiation\3. SPSS statistical analysis\5. IL-17\2. IL17-48h\2.1 SPSS statistical analysis--IL17--48h.sav	
	Active Dataset	DataSet1	
	Filter	<none>	
	Weight	<none>	
	Split File	<none>	
	N of Rows in Working Data File	20	
Missing Value Handling	Definition of Missing	User-defined missing values for dependent variables are treated as missing.	
	Cases Used	Statistics are based on cases with no missing values for any dependent variable or factor used.	
Syntax	EXAMINE VARIABLES=Figures BY Variables
  /PLOT BOXPLOT NPPLOT
  /COMPARE GROUPS
  /STATISTICS DESCRIPTIVES
  /CINTERVAL 95
  /MISSING LISTWISE
  /NOTOTAL.	
Resources	Processor Time	00:00:00.98	
	Elapsed Time	00:00:00.85	


[DataSet1] E:\桌面\Raw Data\4. C. Cellulosae ESAs and TPx Induced Th Subpopulation Differentiation\3. SPSS statistical analysis\5. IL-17\2. IL17-48h\2.1 SPSS statistical analysis--IL17--48h.sav


Variables


Case Processing Summary	
	Variables	Cases	
		Valid	Missing	Total	
		N	Percent	N	Percent	N	Percent	
Figures	Control	4	100.0%	0	0.0%	4	100.0%	
	ESAs	4	100.0%	0	0.0%	4	100.0%	
	TPx	4	100.0%	0	0.0%	4	100.0%	
	LPS	4	100.0%	0	0.0%	4	100.0%	


Descriptives	
	Variables	Statistic	Std. Error	
Figures	Control	Mean	11.64800	.437311	
		95% Confidence Interval for Mean	Lower Bound	10.25628		
			Upper Bound	13.03972		
		5% Trimmed Mean	11.66522		
		Median	11.80300		
		Variance	.765		
		Std. Deviation	.874621		
		Minimum	10.456		
		Maximum	12.530		
		Range	2.074		
		Interquartile Range	1.633		
		Skewness	-.974	1.014	
		Kurtosis	1.500	2.619	
	ESAs	Mean	13.53025	.267350	
		95% Confidence Interval for Mean	Lower Bound	12.67942		
			Upper Bound	14.38108		
		5% Trimmed Mean	13.52228		
		Median	13.45850		
		Variance	.286		
		Std. Deviation	.534701		
		Minimum	12.959		
		Maximum	14.245		
		Range	1.286		
		Interquartile Range	1.000		
		Skewness	.767	1.014	
		Kurtosis	1.502	2.619	
	TPx	Mean	5.46225	.276971	
		95% Confidence Interval for Mean	Lower Bound	4.58081		
			Upper Bound	6.34369		
		5% Trimmed Mean	5.44894		
		Median	5.34250		
		Variance	.307		
		Std. Deviation	.553941		
		Minimum	4.936		
		Maximum	6.228		
		Range	1.292		
		Interquartile Range	1.029		
		Skewness	1.136	1.014	
		Kurtosis	1.503	2.619	
	LPS	Mean	40.41125	.606229	
		95% Confidence Interval for Mean	Lower Bound	38.48196		
			Upper Bound	42.34054		
		5% Trimmed Mean	40.42172		
		Median	40.50550		
		Variance	1.470		
		Std. Deviation	1.212459		
		Minimum	38.841		
		Maximum	41.793		
		Range	2.952		
		Interquartile Range	2.261		
		Skewness	-.459	1.014	
		Kurtosis	1.502	2.619	


Tests of Normality	
	Variables	Kolmogorov-Smirnova	Shapiro-Wilk	
		Statistic	df	Sig.	Statistic	df	Sig.	
Figures	Control	.250	4	.	.951	4	.719	
	ESAs	.250	4	.	.960	4	.781	
	TPx	.250	4	.	.935	4	.624	
	LPS	.250	4	.	.962	4	.792	

a. Lilliefors Significance Correction	


Figures


Normal Q-Q Plots


ú¸î¿ÿ~ñ'þÄâñøËC§Njll4iÒ¸ÿª»»[ü?ñøCü!þòD<ß³gOMMÍ¸4K.Ý±cG__øâñøCüy½½½Û¶m«ªªÊ9u´ø?Äâ[×ÝÝÝÔÔt×]w¥4ß	Ö¯_ßÓÓ[ü?ÄâñÇÐûÚÚÚñãÇ§d_UUUkkkîìáâñøCüqëb±Xh»Ù³g§ïá­©©iooÇã¹ÿ]?ñøCü!þ¸ð*qoCCCWW×ú^ÄøCü!þèøñã!ïÒ÷ðVVV×ýÞÞÞ1÷?ñøCü!þHeèêêêÖÖÖ1±Wü?ÄâñÇM4tËøñãëëëspèñ'þ þ·b ¡[Â­[·æÎ):Äø?Äâï¶dº¥¥¥e,Ø'þÄâñø#U~Ý"þÄâÄâï&òièñ'þ þÊ¿¡[ÄøCüøCü¥Çã­­­ÕÕÕù7tø?¿ÿÔÛÛ^ +++óuèñ'þ þÿ_WWWCCÃ	òèñ'þ þ(èøËrr¼ºEü?Ä?4þ²cÕªUííí±XÌóAü?Äâñ7ætrI&544ì^ñ'þ?Ä_¾Érr;wø^ñ'þ?Ä_U×ÒÒñäK.Ý·o_áÝ"þÄâÄùÝÝÝ[·nMßÃ¬Y³&¿OÎ!þÄøCü!þ( ø/²õõõéx+**6mÚ¾M?kñ'þ?óñÅ:9ÇÂwïÞÝ××ç§,þÄâñ'þóñ×ÓÓ^[Ó÷ð?þ¡êèèp`ø?ñø#âïøñãéxC®_¿>D¡«ø þäCütrîÜ¹Ó^ñ'þÄâñG>Ä_,m/=ûjkkÝ"þÄøCü!þÈø»páÂ¦M***ÒOÎÑØØxêÔ)?Añ'þÄâñG>Ä_ggç5k&L~r'çâOü!þäCüEC·,^¼8oMMM»=¼âOü?Äâ|¿.×ÊÊÊÊæ0aBCCs?ñ'þ?ò$þ:;;CÞM4)%ûBWOxÅø?Äymmm555é#öUWW·¶¶ÚÃ+þÄøCü!þÈøëíímii=vúÉ9êëëGþåñ'þ?Äß°èîîÞºukú9ÙÂ0?'"þÄøCü!þÈø/õõõéxÝ"þÄøCü!þÈøn©®®6tøâñø#ã¯§§'¼ö¥ïá5tøâñø#¯âïøñã!ïÒ÷ðºEü?ñøCü?ñÇ÷ìÙSSS¾×Ð-âOü?Äâü¿ÞÞÞmÛ¶UUUºEü?ñøCüÏñ×ÝÝÝÔÔdèñ'þÄâñGÇ_xQ«­­5tøâñø#ã/º%ýänâOü!þäUüºEü?ñøCüQñgèñ'þÄâñGþÇ¡[Äø»¹K.?ÄâñÇ?C·?ñ7(áuÎ9áwCü!þÑø3tøC°bÅ®®®,ñ÷íoûìÈ¹yêÔ©³pöìO~òwÞyÇz ò½ïïïþîï¬'O¿ÃDxZ¶lYQQQJöúÓ~î¹ç:;;­«BðÖ[oýã?þãßé¿ÿxÇß+¯¼ò·#«££ãèÑ£û·?úÑÂë½õ@äàÁá%ßz øþ÷¿¿yóæÏ|æ3éö-^¼ø¥^ú¿ùk©pìß¿äï4ãÏn_ìöÅn_rG4tËoüÆoº»ÅâñG>3tâOü!þä?C· þÄâñGA¸|ùòöíÛ§O>tËêÕ«ÿüÏÿÜ*BüÝyâñøcäuuu544L0a ¡[zn_Äø?Ä¹èÈ#õõõéÙWUUÕÒÒ8°Oü!þÄâñÇÖ××·k×®Ù³g§ØW[[ÛÞÞr`øCü?Äâ1©»»û©§4iRJó9YNÎ!þâñøc9räÈ5kÒ÷ðÎ7oÇYn[PñVEGGG[[[ø®=mÄøCü!þ)³÷îêêêôÏðfÜÃ[àñ·oß¾»îºkÑ¢EûÜç&MôÇüÇBâOü!þ.)~r¦¦¦3gÎ~QS§Ný_ëþ×/o	ÿÖ?±¾²²2ä çø?Ä¹1ëÖ­KßÃ;úôíÛ·ßÂÉ9$þB×ÔÔDåýèÁÂÏ(ñ'þ?rQ<ß½÷ÂÓ?Ã»téÒðË'ç(ø«­­ýõÿ39þþwÓg>óO-ñ'þ?rËåËî¹ôsL0á¡:uêÔm.¿pÞù[þß'ÇßÿxøxçOü?Äârüøñõë×§Ý£§§çÜK!ó÷Æox?ñøCü1úÚÚÚjjjÒ÷ðÎ7¯µµ5ÝÁû*´Oû.pñâÅS¦LùÊW¾â&þÄâñÇhêííÝ¹sgUUUzö­ZµjC·¿,¢qþBß4ä5âOü!þÐ"MMMéC·9O=õÔ°3| þÄâñÇÈ	/µµµãÇOÉ¾ªªª;vÜÂÐ-âñ÷ë[ÞLQQøCü!þ±X¬µµuöìÙé¯G÷ßÿíÝ"þÿ¡èfÅâñÇpëéé	¯OÎÑÐÐÐÕÕ5ÂGü·ñ7êÄâñWà?ò.oeeex=¼âñ÷®òØc?Äâ;.ïÙ³'ãÐ-ÕÕÕ­­­#¶WüQ¸ñþÆ*..vÌâñÇ°êííÝ¶m[úÐ-ãÇ¯¯¯ùñGÆßüùóÓÿö*++ëëë?ÄwD¡[¶nÝSÌ?ò?þJJJÂ¯ß¥K***ÂDh¾oûÛa¢±±Qü!þÜ¦,C·´´´Öâ¿è0LÚgÏ½~ýz8q¢øCü!þ¸5Yn©©©¦s?Äß L2%ü*WÁ®®®0ñÌ3ÏDzAü!þ¸¹6tøCü¥Ú¸qcâã)¹?ÄC·?Ä__ýêW§N&=&B.Z´h¸ºøCü!þòCÝ"þ¹Bü!þcÝåËsèñøâñø»]ë×¯4iRîÝ"þÌ5+ðÅ Ï?ÄÙuttdÜÃ³C·?Ä_ª3g&_Oû"þ$Äb±Ý»wgÌ¾ºEü!þRÎ¿ºÇ»~ýúH>tñøCü	.[ìÊÊÊô¡[rèñøKUVV~G¸üÄâñû?^__"/%û***¶mÛvùòå¼ü®Åù'N¿ÉO<ñÄÕ«WÅâñG°k×®¦ïá7oÞîÝ»c±Xïâü¿àîIÿ÷Äâ¯Ðôõõµ´´ÂKºeÝºuÇ/ þÈÿø1c| þ]]]MMMéçd+++knn=T8«BüÿñýzwvvðC?Ä_.âÚÚÚôs²Í7o×®]¶BÄùååå>ðøCüZüõöö¶«®®N?ì'´`GGGÞÝ"þ©ÂoxøUß¸qãÀ+þ¿QqáÂ­[·¦ïá4iRSSÓ>9øCünðÄâ/Ïtvv666¦múôéÛ¶mËs?Äß À>¿üÇ÷ìÙ³jÕªöíÞ½»`÷ð?4þFøCü!þ[ooïöíÛÓOÎ*pÍ52tøCü¥3f9sFü!þy£«««¡¡!ýäeee6m*¨¡[Äâ/UqqqØüC?ÄßÇÛÛÛkjj2cçÎ8tøCü¥:tèPØ(477_¹re$|?ÄßÔÛÛÛÒÒRUUqè¶¶6ö?Äß¯áÓ¾?ÄßX¿îîî'çsÂü?ªGü!þr=þ|Úñc4þ:9GUU¡[Äâ/ç?ÄâïÖÄb±ÖÖÖÙ³g§ï´©©©Ù³g=¼âñ'þÄâ|¿°ñLßÃ;aÂC·?Äß`õ÷÷¯X±¢´´4lA&NøàÀ'?Äâñ7xaY__¾·²²2lNíá¿!¸víZÆ|÷©~ÅâñwSñx¼µµµºº:+f«ìá¿!3gNØ¬òêÕ«áâ+WV¯^æÜï½âñø-½½½a;ñäõõõ#¿ý¿ü¿°5IþÛ±¿¿?Ì	óÅâñ7ò?ñäwÝu×Ö­[»»»ý¼Äâï¶mJ¾ÄX,æêñøIáð=öd<9ÇìÙ³[[[ûhÄÑnßåËG»Ãÿa:ÌY°`øCü!þF@ooï¶mÛÒOÎ1~üøÚÚZxÅâïÇ_¨½øøøãÅâ±ëòåËûöíÛ¾Øvßô=³Ñ¿ì'ç°Wü!þ%þ>¹ñß|pòäÉEEEáÿåË9ÃýÐÅâásäÈ»ï¾ûÞï­ù½ÏÞ^Ñs*þ:::V­Zñä---n¿á¿Q!þË/¯©[³åË[¢¿û»¿B0Ë¨(#á1¶èäííín?ñ'þÍ¾ûBê%Ê/üÛ¼isÈÁQ¿èäOÎÑÕÕå'þÃãn¦¨¨Hü!þvìØQó5ÉñþÍ5+ËÙÏ5þÂýtrmÛ¶ÙÃ+þ#E?Æ´¶¶¶ßú­ßJ.¿§|jâÄ/_ÉøÇãáe£¦¦&=û/^¼k×.Ù'þ#yòÉ'£mÓk¯½&þE¡ºæÎû%_Ø¼isT~sfÏihhÈr;;vì>zúNÚÚÚ#Gø?Ä_NÄß'Â_ÆÑAÇÉc>?ÄcÎ>ÿùÏÏ=;lÙÖ­[lâ¯««ë©§4iRJó577ºEü!þr(þ~øáhuàÀyèâñÇ$à©S§²ìí½ñ7ÐÐ-Ó§Oß¶mÛ`âñ7Bñ÷ÆoD[¨+WäC?rÇ-Ç_,Û³gOuuuúÞ¶¶¶ºEü!þr(þ®]»vï½÷Fí8tèÐ?tñøcLÇ_OOÏ¦MÒn	zè¡,,Fü!þF'þ^xáh#µvíÚQyèâñÇ¿ÎÎÎ¦¦¦ö4bøCüåhüçñ'þRüÅãñûöÕÔÔ¤o0«ªªvìØqÓ3#þ£E7S\,þ¿OnÝ²sçÎçd«­­¯ì¿1£Nü!þÈýøëîîÎ¸wÂ	§N²êÄâOü?ÄùaU[[ñlÛ·o7tøCü?ñø#â/µ¶¶fÜÃ[SSÓÞÞîÀ>ñøâñG>ÄßÏ~ö³°]ºë®»RoÒ¤IgÎ±ÄâOü?Äùàøñã+V¬È¸7l©z­"ñøâñÇÇ÷ìÙqèêêj'çðqþÄâüÐÛÛ»mÛ¶ªªªíÞøñãëëëG~ÓøCüDü%é1þóø#ÿDC·d<°oãÆáZ«ñGÞÆ_ÂÁÃïG¹zõj¸þ_½zusøðañø#o4tKUUUKKËzn_Äâo¬Æ_YYYØü%ÚÒßßæTTTÜæO81mÚ´âââ¹sç:tHü!þy7º%ÚúéÜ¾?ÄßØ¿h#/%þnÿ¿ºººW_5L¼øâk×®?FROOOÆ¡[&LÐÐÐÐÕÕüÅâñGÅ_yyyØP»víÚ'7Na¹råÊ0'Ì¿Í%]¿~=ªÉÊÊÊôøkii99²ÞyçðzN<zôèÛo¿m=ä¥ðgçªU«Òhµ?ø?~¶¶¶þðVÁ~ðCYDÞzë­¿Óa¿ÃgüÀÇûï¿KNþÈHúÇGBü½ñÆ=#ë½÷Þëììì®®®¿ú«¿²òÉÏþó^zéw~çwÒ7h¿ýÛ¿ý§ú§áºmøËð>°	~üã9rÄz ²ÿþ>úhïtØã/8öìÌ3KKKÃÊ'N`ÁÅo±É;KJJìöÅn_ÉíÝb·/vûR@»Oyyyt(aø?'²øCüqûººº2ÝælÝºuðC·?Äâïp¿òÊ+a"ü_WW'þÜ)ñx¼½½=ãÉ9¢¡[zN6ñø£°âïôéÓ³fÍ8qb´£¶¼¼|ïÞ½·¿ØðÊZQQ9mÚ´£G?Ä·/T]h»ô=¼)C·øCüQ@ñò|J·húÅ_Ö.þIww÷Ö­[9tøCü!þTQQ¶§ONÄßÑ£GÃôäÉÅâÐÉ9*++Ãd¨xÅâB¿hMDñwýúuçöEü1ê²cñâÅáª[ÛÃ+þzüE<Gïöøëïïúé§£?©ÅâQ1ÐÉ9¢¡[N:uÇïQü!þ( øëèèÈ8Èóï½'þ°ãÇ744¤ïá!øÔSOWåaº_ñø£â/8þü¢E¢OûÎ5kðc?Ä·/·¶¶VWW§ÿ!:öìpU__ß°>ñø£°âoT?ÄÜº%l*++Ó³¯¶¶¶££cdøCüQ@ñøGÂÅgÍ5útñøcøtuu544L0!èõë×ßæÐ-âñøBüõ÷÷û´/âaÇ÷ìÙñä;vì¸#C·?Äâ/ÕôéÓÇe5eÊñøãU·mÛ¶'ç¨®®[Ø;8tøCü!þRøáE7$Îí,ßo¼!þÜÝÝÝMMMÝÒÙÙ9êPü!þÈÿøK©7ÜxÅâ¯`9rd s477÷ôôäÈã?(þFøCü¾¾¾ûömß¾=ü?ÇÒEöeº%Ì¡[Äâñ7 þþþY³fM811gêÔ©_ûÚ×Äâo¬ëêêúô=þosþ[ÍïÕÌ?ÿSúÔ3gûNCbîØ±#ý¨âñãÇ¯Y³fÄn?Äßî¹çüF[êææfñø»âñøg?ûÙÚµ[¾¼%ú÷Å/~ñ7ó7ï-·îîîÆÆÆI&¥d_³~ýúá;9øCü!þ¦¤¤$l¸>qâD3yòdñø»N:õ[3+Q~Ñ¿3g9räWf[[Û5kÒìöðÆb±Ü_]âñGÅ_ôßë×¯ÿå¦þ'þcnµxñâøûÜâÏíÙ³çNÝE__ßÎ;3îá­¯¯ù_pñøCüÊ´iÓÂÆzÃÑ_ç×®]Û²eKSQQ!þc×3gî¾ûîOmLßæMÃïõ©S§ná===ÍÍÍéC·9[·n?ÄâïÖ;v,ã ÏG¿1í;wnÓÿnå·þõ,¬­­½ÍÛÛÛÒ÷ðVUUµ´´ÊÉ9Äâñ7dçÎ¯¥¥¥EEE'N3gN3Ü]ü!þ[è¼7N4©¼¼|Ê)MMM·|ø]XÔîÝ»çÍþbMMM(ÂQ<9øCü!þÆñø1.å8ëííî¹çÒì0aBCCCWWW~¬"ñøCü?Ä_¡;uêTcccúá÷wìîá?Äß'§OÆy>á[^^¾wï^ñø+XmmmYNÎ1Ö÷ð?ÄLlÙ£ø¦_|ñEñø+(±Xl×®]UUUy0tøCü!þTQQ6î§ONÄßÑ£Gòø+(gÎijj¶ù1tøCü!þ^Ä$ì|ýúõ0]\,þy/ü®Zµ*ÿn?ÄßÊËË£Qý¢øëïïúé§£cºÅâ/_Åb±ÖÖÖÙ³gçëÐ-âñøPGGGÆAßï=ñøË?===á·/ý3¼y6tøCü!þ²9þü¢E¢OûÎ5kñ¿vüøñ'çÈË¡[ÄâñsÄâodÄãñ=öÔÔÔÔÐ-âñøâ¿ÞÞÞmÛ¶æÐ-âñøËæ>1cFIIIxU(--;wî¥KÄâoìêîînjjJ?°¯pn?ÄßÚÛÛ3~àãÌ3âñ7æ_«ÚÚZC·?Äâo@Ñ°®uuuáâÕ«W×®]æL6Mü!þÆC·?ÄâoÐ¸!ù!L'|¿gèñøCüMôÎ_bÎµk×¼óøËn?Äß­ù«««Í.~üñÇK.uÌâ/gºEü!þ··¦ý¿âñ7Tn?Äß¿¢)..¿ÑeèñøCüÝ±ø-âñ7n?Äß¿Æs>wîøCü¾¾¾]»v-^¼ØÐ-âñø»Ãñ^K¾úÕ¯¦Ìn¡^£âÂÍÍÍn?ÄßpÅ_¼ðºR^^~ñâÅpñ[ßúVôJ3Lú?Äß@ÂoÄ5kBäºEü!þÃÜx/z6mZ4±zõêá~èâñn©®®NßÃ»páÂÝ»wÛÃ+þ¿;ÁáÃ¯7;wî.þ.î¹çÊÊÊÒ÷ð®Zµª££Cö?ÄâoXâïñÇ^r¢³<òøCüèä&MJÉ¾áWãòåË~@âñø®ø+../9'O>yòä'IÇü?ÄßÅÚÚÚ2cöìÙÛ·oïëëó£?ÄßðÆ_xÕÙ¼ysÊÌöEüÝAÑÉ9*++Ó³¯¶¶ÖÉ9Äâñ7rñ7Ð8çÏ¿Û×ÙÙþÊxrM6÷I´?Ä_®äwüÅãñöööU«V¥cÞ¼yn?ÄßHÇ_xJÞ±ý¢øCü^,Û¹sgUUUúÞÅïÛ·ÏgxÅâñ'þÄùÝÝÝOÍ;9øCü!þÄø#?ã¯½½½¾¾>ýäUUU;vìèéé±ÂÅâñ'þÄc>þb±XkkëìÙ³Ó÷ð.]º4¯øCü!þÄø#â¯§§'<Ó?Ã;~üøuëÖ:uÊ?ÄøäCüE'çHÿoEEÅsÏ=wáÂëVü!þâOü1æã/ïÙ³'ãÉ9.]ºsçN'ç?Ä_®Ç_vâñNÎ>tËøñãëëëCü!þc#þn¦¸¸XüQàñ×ÝÝÝÔÔñä[·n×Zâñø3ñ7êÄ¹áÉY[[~`_UUUKKs?ÄâOü?ò!þ²ÝRSSÓÞÞnèñøCü?ñG>Äß@C·89øCü!þÄøc4EºÝ¸qã¿üåöööÛ¿n©¬¬ÏRxÅâñ'þÄ£&¤Øç?ÿùÏÎøìøâòÿ¾üî»ïnnn¾µEýò¿|öÙg3ÝR]]íäâñâOü1úæÏ¿yÓæ-_Þþ=õäS¡ÿúDnùÌg>cèÄâñ'þÈiúÔ§þÏãÿ'*¿è_MMÍþáòænAü!þâ±äî»ïN¼íý«­­]¿~ýMohèÄâñ'þ-[.9þB½íÚµk ¯Åb»wïÎ8tË¾ðo~óìCü!þâÜÕÙÙ9iÒ¤/>ðÅÍ6ÿßÿ÷¾ÏÝwï½÷ÂKÿÊ.dºe¨çöEü!þâOü1:ý·lÙ²!ìÖ¬Ysùòå/8uêTýMn?ÄâOü1Åãñð+ºxñâAÝ"þ?ÄøcLêííÝ¹sgeeån?ÄâOü1ÆtwwoÚ´iÒ¤I·0tøCü!þâ±!:ÃÛý÷ß¾wðC·?Äâñ'þÈu¡ê¶oß/=ûjjjÚÛÛ?tøCü!þâÜíáÍ2tËP(þ?Äø#µ··¯Y³æ¦C·?Äâñ7ú.]º^¡Å· íÚµkðC·?Äâñ7ÊÂ+ë9sÂKµøcH._¾ÜÜÜ<Ô¡[Äâñøe+V¬èêêÊò'Ò>²8ðöÛo·«þâ/þ¢¶¶¶¨¨(%û&Nøû¿ÿûáÚ;x_üÎw¾c	Oð°ÚÚÚ¾ûÝïZDÞzë­¿Ó1ÿñ¿wÞyçW#ëG?úQww÷¯È=o¾ùæêÕ«Óìûìg?ûgög.ã÷xþüùÃ[óD:::~ùË_ZÿðÿpòäIëÈþýû¯]»6ÂwÏñg·/½½½---³gÏ¾ý¡[ìöÅn_ìöÅnßÑI½ø#»­[·ÞÁ¡[ÄâñøË$;~üxú9ÙnsèñøCü!þÄøË!±XlÏ=Ã7tøCü!þ9Mü,C·<ôÐCÇG%þ?ÄøãëììlhhHÿoYYYÈÁQ|lâñøCü?îx<¾÷îô=¼óæÍÛ±cG,õ)þ?ÄøãvõõõíÜ¹3^zöÕÖÖëÐ-âñøCü?ñ7r:;;zê©ôÏð9ë×¯¡[Äâñøâo$´µµ=ôÐCéöUTTlß¾Än?ÄâOü¿aåäÕÕÕûöíËûÄâñøâïvuwwtruëÖåà^ñøCü!þÄ·âÈ#µµµ¹9tøCü!þâOüÝYNÎ±páÂÖÖÖßÃ+þ?Äøcº»»Óßí«¯¯?räÈØý¦Äâñø(1bsÎÝ"þ?ÄøwÌ¾ûªªªZZZrvèñøCü!þÄø»câ7äÓw$þ?Äø£?Äâñ'þ?ÄøCü?ÄâñøâñøCü!þÄøCü!þ?ñ'þ?ÄâOü?ÄâñøâñøCü!þÄøCü!þ?ñ'þ?ÄâOü?ÄâñøâñøCü!þÄâñg= þâñøCü!þÄøCü!þ?ñ'þ?ÄâOü?ÄâñøâñøCü!þÄøCü!þ?ñ'þ?ÄâOü?ÄâñøâñøCü!þÄøCü!þ?ñøCü?ÄâOü!þ?Äø?Äâñ'þÄâñøCü?ñøCü!þâOü!þ?Äø?Äâñ'þÄâñøCü?ñøCü!þâOü!þ?Äø?Äâñ'þ þ?ñøCü!þ@ü?ÄâñøâñøCü!þÄøCü!þ?ñ'þ?ÄâOü?ÄâñøâñøCü!þÄøCü!þ?ñ'þ?ÄâOü?Äâñø?ñøCü!þÄâñø?ñøCü!þâOü!þ?Äø?Äâñ'þÄâñøCü?ñøCü!þâOü!þ?Äø?Äâñ'þÄâñøCü?ñøCü!þâÄâñø?Ä?ÄøCü!þ?ñ'þ?ÄâOü?ÄâñøâñøCü!þÄøCü!þ¿¼¿£G.X° ¸¸xþüù'N?ÄâñÏñ7sæÌ÷Þ/L¼öÚk³fÍJ¿wÞyçÚÈÚÝÝ®]ûÅ/~qøðaëHGGÇÅ­>øàäÉÖÿöoÿ6Âw:ã/Yiiizüóß|gd÷»ßûí·ßwÞùÞ÷¾÷ï|Çz á)a=ðba=yë­·FþNó!þ;ÖØØh·/vûb·/vûb·o>ïözµ®®®¯¯Oü!þ?Ä_¾Åß¸_.;w®¡¡áüùóé_)þ?ÄâoÌÇ_²åË_ºt)ãµâñøCü!þò*þ*++Ç%?ÄâñÏñøCü!þ?ñ'þ?ÄâOü?ÄâñøâñøCü!þÄøCü!þ?ñ'þ?ÄâOü!þ@ü!þâñøCü!þÄøCü!þ?ñ'þ?ÄâOü?ÄâñøâñøCü!þ-þ¾ò¯ìÙ³çç#ë»ßýîøÃÃÏÞÙÙ¹wï^ëÈ_þå_þä'?±9òöÛo[D^~ùåú§á;íííÍÏø;úô-[þ$Ùwó~,@áâñø@ü þ9êàÁãÆ¥®¥óçÏKb-,?ô'NL6­¸¸xîÜ¹²®ùÉ`ãPb±XCCCIIÉôéÓ;::l<2>rjã`ÛM<_°`AúiïÞ½á§ký,?ôºººW_5L¼øâk×®µ®ùÉ`ãPöÙçþúõëáÅ~Æ69µqÙ|ík_ûÆ7¾áç÷æoZ?%Ë½¬¬,üªþþþÊÊJëª6hîÜ¹gÎ±q û!§6âo@~øá¢EÂïmzüÍ9sùòåÅÅÅá~úÓZW Ë=ÌÌ8M>lPøq¿ðÂ¥¥¥3fÌxÿý÷m<2>rjã þôà>|øÿ¯£÷Í;wnþüùÖUAIÿ¡%¦KJJ¬¢B~2Ø8 °xùåÃDxE¯ë69µq¯ÿj /óûR~èåååýýýÜØ³¦­B~2Ø8 äßú·÷l<rsã þU)sfÎÙÝÝõûòåË­¢Bå^__ÿÊ+¯ð]]uUÈOôøã¿þúëaâôéÓÕÕÕ69µqC¿húØ±csæÌ	Q¿téÒðS´AÆzô|x÷Ýw+**¦MvôèQëª6èÊ+>ø`t,WWW'CÆ'CNmÄ@âñø@ü þ?Äâñø?Äâñø@üî[ßúÖ%KJoX¶lÙo¼ñ_¶_7­m¦G[YY¾µ¾¾¾ùaNqqñ´iÓ®_¿>Ôe?`LÚ²eË¸4_ýêWó)þÃÌ;w¦Ìß±cGÿÌ3ÏÜÂ2Ä0ö8q"dMqqñK/½¿áå_ÃÌ'OæMü=6Ì?~Êü9sæùÝÝÝâ@AxäGBÖ<ÿüóÉ3¿þõ¯ëÖ­KNCx]¸`Á0øâ?þ¸±±qòäÉáª²²²6$ï]moo®·mkkKÉ©0gÊ)K,9pà@¸X__òÀöïß9ÑUá!EW<xp P[¾|yôèÑÄ÷Þ/Ì©©©IÌyöÙg+**Â¢JKK~øásçÎ¥Ç_úòSædy¨øe÷ÜsO?ü0yæGfVVV&ÇMcÇE×®^½:åª'x"ºª³³³¨¨(ã­¢Ñµ>øàõë×C>Äãñpmø?ÄÓÄ£Cñ²,'Ld|xéßék¯½¾|õÕWå²eË5þ²<T@ü¾homÖ¸q!ÅãfíÚµWoáâÊ+£k£Öò1z/D[tU]]]ôa~÷Ýw£$/sóæÍ!ï¢½®>úhóæoéðþÒ¾tÓåpÃ$?¶ßQÊ©S§ï÷Ò¥Kábø?<òPýýýÑL6-Ü0|üz7qiiéPã/ËCÄ@NÄ_1þó£¸)8wî²)º¸`ÁpqÆ=öX¶k×®%¾&å½´Ä­¢ôQâC0%öüÿÃtûMB3xñbòcèø¼'|2o|ã_ïÚÞ°aCòñtÑ¢EÑC¿,£/z»ëêÕ«É3ûúúÂÌpUÜI¤aWWWÔÖI¨¾76%§X	Óeee%%%±X,,|Ê)koºR,ÅéÓ§£NÓáÿ0ÝÙÙ¸öÝwß ãîãÁÇ_?Ñ÷öÂ/$Ï@IùÀGâ¸ÀK.¥¿¡uòäÉæææh'l¢£·åûUohO<ñD´·7üßØØe9Ñ;mçÏ.^¼x1û'sï»ï¾pm4ºMhÖä«¢Oþ«<xåÊìñÓhm$®ÍòPñ0ú¢L¿øâÑP//½ôRIIIú3V®B'EûdÌùëêêJ>X0jÁ6T>l%c¢E¿î½££#1?Ër¢ü¢cþúúú¢¯Ì/¿ürâ=¹ð-'_þØÙÙî%¬â/ÊÍËáËBt>üðÃÉ×fy¨øÈ	ÑÈ)öÙÿÜ~ÝµQb:±Ã4ñG4¹,íÝ»7KürãTa~YYYòá,Ë	#ù%>i;Ð7íÚµÒÒÒè[HÙÙ½bÅä»:ujø?í%yÑ	Qª&®ÍòPñ+B ,[¶¬ä%KD¹M¿FÃéÍ;÷ðáÃkûúú~úé(|òÉX,¸¶­­mÑ¢E¡É¦MÖÒÒ²ÌôGæ?þøã)óZNLxHÑYÆùKhllLó%réÒ¥ººº°&O¾îîîÄ°5ÉË¼råJ¨Ûh]ÕÔÔ=z4å³<T@ü þ?Äâñø@ü þâñø@ü þÿíÝZÀIEND®B`


?1cFtËõÑîÉè®+5557nüì³Ï^|ñÅ0rÕªUñ×]8ýÂþã¯?þ¼ôïù1)'wòäÉíÛ·GÇWFK?I±Æßõ'ï'ÎëÆD×/[¶ìÊ¢w÷uëÖÝ:þ¢½_ÍÍÍ /¼ðBð×ØØí[^<räH´W/jß¾¿Ç¾d@øëáDK²ãF)èRþ¢³ô;;;í.]NÃç.,üÄàà¾4ÎáóÃÃ³fÍJþ6£s7[ZZú8/	þ$Å.ÝimmM9ß«´´4qñì­à/Á¨I&ÉW¼fç|0ýä¼yóúø6ôá¯ï¸WsÊ±T¿'O&OÑ¢EÉÏ¦,Àµ¾ñÝ.1ã0Ñ¾Õäzè!¿üIÊü¢C¢Éã?üðÃU«VÜ¨¦¦&ýòÁá¯­­-L-L30èÑGJÜ%¤oâìÜ¹sæÌyyy'O~ùå¿ô¥/%ö<ÝúK¿¾Î¾ûÿòóó·ÊKn@Kuøíß¿êÔ©á»Ó¬'.pÑ¢¯^]]ÝÔÔtÓ=¯õõõsçÎSßÔîÝ»ã;::ì±hj|X]]]~¹$ø¤áª»»ûÁÐ½åñ¬+:]¾ÝÓÓ~Æ~`$Á$å`Ñ9)=ýôÓ$ø¤¬££cóæÍ'Oí'xÂb$I?I$Á$IüI$	þ$I$I?I$Á$IàO$Ið'I$ø$IüI$	þ$I$Ið'Inß¾ùùùUUUíÌ3Ç×ÒÒÃY³f`sv£ÁN^;Èl_1WXXXSSÓÖÖ=ÕÑÑ±~ýú°0ÃSô0Ið')^ÊÁôôôôµO>ùdxí;cyæ0fûöí¹¿h8|ãaxõêÕÑÃÚÚÚððÐ¡CaøðáÃaxÚ´i~À$Á¤mMnÁO'O¯]¼xqbÌÂ'N<ÅFþ+.áÂÂÂèaAAAxxåÊ?TàOR¬åÇ¬¸¸¸¨¨hÛ¶m)ö¥/iîÜ¹)SHì8Ã.]ÃaLôÔ¹sçª««òóógÏíKZòWïû%/¿ürEEÅÂÃJ§ØÕ«W×¬Y^f~óæÍÑÌÀÓE©ñaÊ|òÉðáoÊ)áá39âGKüIµÿ¢á¯íkax÷îÝYaàé§NþúúúÄùm~øáðÔ/¾ÃÇ0=5oÞ¼3gÎñååå§<¿$îÍ7ßµµµé3¿~ýú0>áí·ßO>ùddW®ÑÉô¿k×®å×®]=µoß¾Ä«ªª>øà?]àOR¬ñWQQ»on¯'666§-[W¬X©.ñìÉ'·nÝ:wîÜèSKaVß/f¬¸¸8ýµ¥¥¥ÑÌÀ¾02|f=vàlWW×àKúøhÞÂÄüó?~<ø5ñ9áúâ¿üüüÄpûÐõ;ÀÂgvttDGWÃè©^x!¼*ð>HBÊÔöç%½ÍXÐXòÎ¼öì0aB4¦¤¤$Ú)8$þúèÐ¡CÉGÀ%	þ$ÅåååÉþ¢g7Ï5kÂ³ñÑ×nÔOüõýÄÏ¥¿vÒ¤IÑkSf¯§§çÀÑ¥¸]Z,Å_(Ïðíøâ¿èÖ-/¾øbtÎß3Ï<ÓñìÙ³'±íÍ7ßL(yêÔ©èþà¯ïlÝºõÛßþvxøáÓ_ûÐCáW^yåôéÓÑ¡Þ0ráÂaø>øôÓOÃ@eeåðáoÖ¬Yá©áýû÷á|Ð$ø_üõôô`Ý(ù^ã¯³³3:ä>&TRRR\¼yóæ~â¯ï444g,X¸î$ùs:::6nÜæ¼  `éÒ¥Ñ½Á|555ÑåÃ/þðÃ´XÒ÷±(ÂWfMù°&M´~ýú0K~À$Á$IàO$Ið'I$ø$I?I$Á$IàoÔúÁ~pöìÙþ¢?ûÙÏ~þóû¹s¿øÅ/¢s(ÎýøÇ?þ·û7Ë!Î]¸pÁæ.þ»óçÏ[ñßÜÁßõgögÁ#üE?øàùñ£ç>ýôÓø°bÞw¾óô?¡¡Xõÿøþó~ö³8qÂryû÷ïÛÿuáþàOð'ø?ÁüÁü	þàþàOðð?øü	þàOð'ø?Áàþ?øüÁüÁàOð?ø?øüÁàOðð?øü	þàþàOð?ø?ø?øüÁàþðð'øüÁàþàþ?øüÁüÁàOð?ø?øüÁàOð?Áü	þCSSSÓìÙ³óóógÍþó?øüÁå9¿iÓ¦=z4¼þúëéø;xðàÕ-¨â'?ùÉUÅ¸?þøØ±cCÌøkoo·âÜñãÇ?úè#Ë!æ»¦¦&Ë!æüýüç?á/ÅøK®°°0Ï=÷Ü÷F¶úúúï)Æ½÷Þï¾û®åóÞyç!æÙÜÅ¿°lîlî2øÿ­««sØWû:ì+å°¯Ã¾¹|Ø7êêÕ«555ð'ø?Áàþr.­­oçéOÁàþðg9À_Ná¯±±qÉ%mmm?Áü	þàÏr¿Â_EEÅ¸¤àOð?Áüå2þúþð'ø?ËþàþàþàOð?ø?øü	þàOðð?Áü	þàþàOðð?ø?øüÁàOð?Áü	þð'øüÁàþàþ?øüÁüÁàþ?ø?øüÁàOðð?øüÁüÁüÁàþð'ø?ø?Áàþðð'øüÁàþàþ?øüÁüÁàþ?øü	þàOð'ø?Áàþ?øü	þàOðð?øü	þàþàOð?ÁüÁü	þàOð'ø?ø?Áü	þàOðð?Áü	þàþàOð'ø?ÁüÁü	þð'ø?ø?Áü	þð'øüÁàOð?Áü	þð'øüÁàþàþð'øüÁüÁàþ?ø?øüÁàOðð?øüÁàþàþ?øüÁüÁàOð?ø?øü	þàOðð?øü	þàÏ2ü	þàOð'ø?Áàþ?øü	þàOðð?ø³àOðð?øü	þàþàOð?ÍÍÍ===üÁüÁàOðãø+qùòå·ÝvÛÞ½-.ø?ø?Áà/gñ6+W®ìw£éÓ§Ûùðð'øüå þûÆ%5qâÄææfKþàþàOð'øËüU¶råÊqÿ±À¾;vtuuYð?Á_àïôéÓ÷öaüÁüÁàOðSøùåÏíK°ïë_ÿz»Eðð'øüåÎºçòòò°þàþàþàOðã«&ã¹Øðð'øüåÚJI?·¯¤¤äßøöÁüÁü	þ¹Sx¿Kgß¯ÿú¯å+_Á>ø?ø?ø?Á_N±¯ºº:ý ï-[ZZZüm_ø?øüÁà/÷ÙííëçßöüÁüÁàOðÝì?ø?øüÁà/7ÙÞòÒÏí?ø?øüÁàoL°þàþàOð¿1Ä>ø?ø?Áü	þÆûàþàþð'øCì?ø?øüÁào±þàþàOð¿1Ä>ø?ø?Áü	þÆûàþàþð'øËöÝu×]»wï¾õ¿Éðð'ø?Á_ÜÙ÷ê«¯öôôÉ?ø?øüÁàoL°þàþàOð¿1Ä>ø?ø?Áü	þF³cÇ$ûàþàþð'ø/_~Ûm·$ûàþàþð'øé;vï½÷ðÞ>ø?ø?Áü	þFº>·þàþàOð¿Ñ©¡¡aáÂ)ì+++Û½÷H²þàþàOð¿agß=÷Ü¾·ï7ÞèèèY?øËMüµµµUTTÀàþ£È¾ô¼Ó§O½ð9¿#GÌ1#üÁàþ£Â¾»ï¾;oßK/½4ºì?øËYü-]º´¥¥¥üûÛß>3²nâÅ¸ú§úÞ÷¾g9Ä¼°5üøã-86wá¿»cöÛÿË¿üËßþíßNa_EEÅ³Ï>ûãÿ8>»ïÿû~VcÞ;ï¼3ò»,Æß¯f±wü½üòË?Ù~¤wüøñCY1/à¯¹¹Ùrs÷w767wññ/|!¿ñ¿ñçþçaó«Yÿý÷mî²bs7ò_4ñç°¯öuØWûáá¹ùóç§°oÊ)¯¾újWWWgØa_sð°/ü	þàOð72oÏ/Ngß(^Éðð'ø?Áß×ÓÓx7gÎôsûÂøxîí?ø?øüÁào0íÝ»7ýJÞøïí?øøë-øüÁào½úê«½±/þûàþàþð'øëWÑAÞìÉûàþàþð'øö½úê«yéçöú_é?ø?øüÁàoÙw×]wåÌÞ>ø?ø?Áü	þÀ¾ÿ¼õõõgÏ?ø?øüÁà/gÙwýÆ5Å'N=ö¿øÅ¢¢¢Í7áÀüÁü	þàOc½±/	ãGþ ossó¤IþÇÚÿ±í«ÛÂ¿ÿ½ñÿæoþæ³Ï>ðð'ø?Á_N±/êG©®®äýûþÏà?ø?ø?Áü	þrQ÷Þï_óßñþýÚ¯ýüÁüÁàþ9Å¾¨GydÉ^,¿ÿUû¿ìù?ø?Áü	þrQÏùûÖ·¾ðð'ø?Á_N±/QtµïfaþüùEEE7ntµ/üÁü	þàOðìKÝç/ µµuh§ðð'ø?å þ²ÃüÁüÁàþSøëÕÕÕcð£¿q7+//þ?øZöüþ¿*ïfåççÃàOðØð#øõàOðÊ%üaüÁ_vãïÊ+?ü0ü	þð×Þxãì?øË&üUTTäçç;çOð'ø¿ÕÓÓ³k×®»ï¾ûàþ²	³fÍJ¿Ú£¤¤¤££þ?øë-yáþ²á×µ­­­¬¬,óûÛßuuuð'øüÁ_J]]]»wï3göÁüe+þ¢_Ú0´Î9¶éa ¨¨þ?øK]ÒQQQÂ¾ÅcüÁ_6áoÂ	áW÷È#---aà'ÜêEð'ø¿¨®®®;wF3gÎÞ½Ý®þà/Ëð·yóæÄåÉ¿Ò.?Áàoã/Àî¥^2eJûæÏÿÖ[oaüÁ_Vâ/ôÔSOM4)455Á¹sç÷¬Ãàþgüõ¶·/°ÏÞ>ø¿¬Çß¨?øS<ñØñÜ¾»ï¾ûàþàþð§ÜÁ_`ß;Òòö·@ì?øËüUVVF7|qgÁàolâ¯££#°/ý /öÁüå þ¦M¾D®öü	þÆþ"öe<ÈÞö,Iø¿Ä_p^ø%?~üøoÊáOð]üöíÜ¹3.é?å8þJJJÂ¯úÈoÇáOð-üEû&NÂ¾bü)÷ñ~òÂ/üúõë¯^½?Á_nã/º¤#ýÜ¾Å;·þ4Vð<yò¸´!øüåþÚÛÛÃ7:Ú²·¯¾¾ûàOcS§NuÁàOðÃøØ~wþüùØsø6ÍÍÍ#<ëð'ø?7þzcß9s°þ4vñWZZêÁà/Ç:|øðæÍÛÛgùÀÆ4þÃæ l#ºººàOð'øËö¢½ÅÅÅÏí³|àOðw¹àCð'øËFö¥ïí«®®ùí­àþâ¿¼^rÁàOð?øËÍ[½Jð'ø?ûæÍ÷×ý×ü	þ2TQQ1uêÔÓ§OÃàOðìööõÿoûþ4æð6#?ëð'ø?û¢Ï?øüõÚ¡CÂ&cûöíW®É­9ü	þàOÃÁ>ø?ÁßÍ&áj_Áà/Øð'ø»I®öü	þrð¿?øÓ ØwÛm·ÕÖÖ¶´´ôýZø?ÁüÁü	þr­­­ýüÁà¯¯º»».]ZXX6.EEE÷ÝwßlÖáOðöÁü	þnRgggÆ>ûOýÂàþ4ì?øüÝ¤3fíË²eË®^½^¹reÅÑÝááOð'øË:öÁü	þnRAAAØÊôôô$Ætww1a<ü	þÃÍ¾]»v!ûàþ7)///lkøcºººÂ·zü	þ¯Ë/oÙ²eüøñCÎ>ø?ÁßMû.Y²$:ì>á0föìÙð'øüyÃqþàOð7ö2^ðñÙgÁàOð¨§§§¾¾~Ço¼ñÆÅ1ðª¯ýëé·k^³fÍ²þàOðwó:;;ï»ï¾âââ¼¼¼ðqÉ%aÌpÏ:ü	þà/n9sæoýÖoUUUÍ3'l*ôòô¼¡åËÓ<üÁà/vÁàþ²¨ßýÝß]¼xñ¶¯nþýþßÿüç?ßÑÑqÓ¶··oß¾=oßý÷ßúôéáaø?ÁüÁü	þÙåËKKK·nÙÀ_ø7mÚ´cÇõñª/fü¼+W®VöÁü	þzyåÍÊËË?Áà/ÔÜÜ<cúdù_ÿÅ·Þz«7ömØ°!ãAÞ,?øü%þòzþ¿äÚÛÛ'L°éÑM	ùmÝ²õÎ;ïÀJùÌóçÏoÚ´éöÛoá¼ð¿Á÷è£Fª×_þ¿¨uëÖM6-ò_ßÂ«ªªoùòå6d<·oÄööÁü	þVø,**ªêêêä>ÃàOð÷ðÃ?~úôéwÜqGÀ_Ð^ôT´·¯¤¤$é»áOðqÁßªU«¢­ÕFfÖáOðYWøùÚöù6¤ä]¹rå(²þàOðwÞ~ûíhkµlÙ²uøüÁ_öííK¿¤ãþûïïû`øüÁßhâ¯³³sÞ¼yÑµáY?Áüec­­­uuuÙ7êûàþõÜsÏE¬Õ«WÊ¬Ãàþ²«ÚÚÚÛn»-æì?øüõòJ÷ùü	þúWkkkoìá¸Àü	þå>Qùùùð'øÓÇ_oì¿Òð'øJüzð'ø¿ldßHþø?ÁüÁü	þFáaòÂü	þàþàOð7dì#ÃSÙõ½Àü	þàþàOð7&Øð'ø?ø?ÁßbüÁàþàþcð¿^^é>?Uüå6ûàþK¾¥_Fü¹ÏàO¹¿±À>ø?ÁßM:xð`Øö=ðÀW¯^ÃÇ+V1?Árcð¿TRR¶===1ÝÝÝaLYYÙ-N9üLçççÏ9óÐ¡Cð'ø¿ø°ïöÛoÏUöÁü	þn6ð¥àïÖÏù«©©yåWÂÀóÏ?¿zõjøüÁ_Ø7qâÄ°ýiooÏíuð'øëµÒÒÒ°5Pëìì;::-[Æñ·8åèí!h²¢¢"»wï>9²½÷ÞG9©÷÷ÿ÷û·k9Ä¼wÞyç?üaçpß¾Ë/O9§¹¸¸øÿø¿ÿýïuds»Ë!þ»ÿ¢Ã¿Ãg¼àã>¸Å)'_2~ùHÀßÛo¿~d;zôhssóyÅ¸Ó§Oÿà?°bÞw¾óO>ù$óvìØ±Õ«W§ìíûÜç>·yóæ?üpì¬£ýèG~VãÜ?ÿó?ÛÜÅ¿ýû÷üænØñ:sæÌ´iÓÃÿfÏéÒ¥[lòãå°¯Ã¾ò:ì+öýÃ¾ÃWiiit*aø~þððüåþÖ¬YóòË/ð±¦¦þðð'ø¿XàïÔ©SEEEÑÚÒÒÒ=öÜúd9RVV¦Y^^ÞÔÔ?øÂÎ=ð¿ÁÝä9ùOºEÃÏ?ÿü°Î:ü	þàop577¯]»vüøñØð'øLeeea»yêÔ©þ¢"ÀàO±Â_`ßý÷ß~»æ6xÑz?øüõo7"ümº¿í+øS¬ð×ûêêê°þàOð7°¢<Gûþº»»ì±0~[føüiäñ×ÒÒñ³mÙ²å¦wÃ?ËþjllÌxç£GÂàO£¿À¾µk×ç¥³ïòåËÖüÁàïÞnçÎ]í[XXXYY9ìþð×[aÎ¾Ûn»ÍA^ø?Áß¸ì]ð'ø¿ôz;ÈF=Ö?øüÍÉ-téÒ¥ÊÊÊ)S¦ÀàO#¿è oÆsûä?øü/þº»»]í+øÓá/0%ýJÞñãÇoÚ´ÉA^ø?ÁßáoÊ)ãúlÂ	ð'øÓ°â/eùòåîÛð'ø	ü;w.ïF¿íßÛo¿?þzc¼ð¿áÂ_¢@½á>Â?øKÖïÊ+ä?ÁüþF+øü5üüAÞË/ïÝ»wÇaóÝÕÕ?Áßÿ_wwweeeQQQbÌ¤I¾öµ¯ÁàOC¿ðË^]]rbñÄ·lÙ2|û;vÇwÌ7¯ú÷ª§þæÔ»ï¾»½½þ¿ë'ON¹à7Ú.oß¾þºEüedßøñãûb/_.--½¿æþm_Ýý[ø;W®?Áßõ°-nnnN	?aLqq1ü	þ4hüedß]É»wïÞyóæ%äþmztÓ	zzzàOð§±¿èßíxúÍÿàOð§~âoTò¦´sçÎªªªdü¥¥¥cçø?Á_¯íòÆ£³¡;;;·mÛÆÁàOÂ_oì¿ï#|¾]ýÔ©S·nÙ_ÝÕ9±çOð'ø»~üøñ7ynjj?Áú¿ø°/* oÎ9¾¸`Ó£"ùUþVå³Ï>;vÖüÁà¯¯.0sæÌÂÂÂ¼¼¼¢¢¢3f1Ã=ëð'øËüÅ:::¾üå/O0¡´´4ÌÏ3Ï<3¦ÖüÁà/vÁà/Ûñ[ö¥tùòå±s´þàOððú~úéßû½ß?ûÆrð¿¾:uêTtçèßÒÒÒ=öÀàOéeËÞ>Áü	þzíàÁ-x¿høùç?Á°þðkø+++[ðS§N%ð×ÔÔä&Ï?aü	þø¶ã×nì|íÚµ0?ìËÈ¾5kÖüë¿þ«å?øËJüFwõð×ÝÝýØcáøüa_Æ½ýÿÛ¾?ÁüÅoò|ôèQøüa_Æ¼ð?øËbüEo·sçÎ®ö-,,¬¬¬lmmîY?Á_6²þàOð¹¿Q	þÙÈ>ø?ÁüÁü	þÆûàþðøûè£¦NZPP6ú3gÎlkk?ÁöÁü	þà/ñ×ÐÐñÓ§OÃà/'«¯¯_¼xqÊ¯|IIIÿïÛð'ø¿,Æ_tçððêÕ««W¯cÊËËáOðì?~úÞ¾Ç|@·k?øüÁ_ã/Úú÷ôô$ÆáÄáOðÃìÐÞ>ø?Áüåþ¢=ÝÝÝ1öü	þr¦½÷Þ÷Ý)ì¿ø»víö÷"ø?Áüe1þ¢sþjjjùÂÃÏ>û,:È9¿Øfe<È»sçÎA³þàOðY¿q7kÿÂàoøÚ»wï9sR~+**víÚ5¼ð?øË)üåÝ¬üü|øüeÑvjÈòÂü	þà/§ð7ZÁàohÛ»woúAÞhoß²þàOðY¿Þîç|áÂøüeË¶iáÂ#Ã>ø?Áüe=þÂÄSO=2ríÚµnõ"øÏí8qâð±þàOðY¿¼ðnQZZzéÒ¥ððµ×^Þ?éT?øüÕö(ýÜ¾!¼¤þàOð§ë9|ÎßÚµk£wòòòh`ÅÃ=ëð'øynüÁàþröÃ'ÞEÂ[ÈÌ:ü	þTOOOÆÛ5O2å¥^1öÁü	þà/ð·nÝºÄý ¢xþñaß«¯¾z×]wîÞ>ø?ÁüåþòóóÃ»HqqñÉ'¯'óWPP¿x²oúôé£Å>ø?Áüe=þÂÉÖ­[SFÖÖÖºÚWðCöUTTìÜ¹3<;º³ð'ø¿,Æ_o÷ùïÁð'øûÂÑÝÛð'ø¿Áßh¿þ³/õ½ð?øËnüwä»??ÁöÁü	þàþàOð7æØð'ø?ø?ÁßbüÁàþàþcð?ø?øü!öÁü	þàþàOð7Øð'ø?ø?ÁßbüÁàþ²?ì?øüÁ_îà/ïfåççÃàûàþð#øõàO9¿güÁàþàþcð?ø?øÓXÇßbüÁàþàþô+½õÖ[7oþêW¿ÚÐÐ0Fð×ÕÕµk×®±Ã>ø?ÁüÁüézûïüÎïL2å¿ý×ÿvÏ¹çÎ;ï¬««²/½ôÒdüÁàþàþt½¶¶vÖ¬Y[·lÝöÕmáß¦G7þóåWrÑAÞ»ï¾;Ó§Oì?øüÁüÁ®ßyç_Y÷H~Ñ¿ß_ñû¡Ã_oçöUTT¼ñÆ]]]cgÃü	þàþàoLwÇw$vûEÿ¾üå/ßï½9¿èÜ¾)S¦¤ïíkì?øüÁüÁ®WUUê%ãoÎì9O<ñDà/ÀnçÎ%%%)ì?~ý9Èð'ø?ø?ýÇïÒû?mztÓZø***._¾Õø°Û±cGúÞ¾»îºë­·Þ³ì?øüÁüÁ~å¿ªªª@À'þÁüÁÅ78à¯££ã¥^J?·oÎ9cyoüÁàþàþ4ô.þºººvìØQVVÂ¾ÚÛð'ø?ø?åþz;·oÎ9÷îÅ>ø?ÁüÁü)Gð×ÑÑqoßüùóßxãì?øüÁüÁr;wîLÿ+sæÌ	¿íØð'øüÁrííí½íísIüÁàOðÊüEtdÜÛØgùÃü	þð§Á_oûÜÀþàOð'ø²ÚÚÚ***àO£¿À¾ðó6qâÄöUWW766büÁàOð749rdÆá-þ4ZøëÎí?øü	þ¾¥K¶´´ô¿oë[#Û¾ûÝï6(ÆVÓ­Oçoþæoþðÿ°¨¨(Ó§OægüÜbï¼óólîÆÎæN¹·¹ËbüýjÇßï½÷G¶þð­­­ÿW1îÜ¹sMMM·2/þÉüÉ	RØWUUµoß¾_üâò­÷ï|ç¿ü¥åçN8ññÇ[qîìÙ³ï¿ÿ¾åóöïßßÙÙ9Â_4ñç°¯ö°oçöüÃ¾rØWû:ì;&û&ÞnáO#?ì?Áü	þbÄAøÓðáûàOð?ø¿1?ì?Áü	þ²)øÓ ñð'ø?ÁüÁßÀöÁàþðcØ?øüÁü	üaü	þàOðð7&ðð'ø?ÁüÁßÀöÁàþðc¢üä'ôGð'ø?ÁüÁ_ííûÜç>ð'ø?ÁüÁ_î³ÏÞ>øüÁàþà/ÇëêêÚµkWII	öÁàþð¹{û×¿þõö-Z´è^°|àOð?ø¿éòåËéyçÏ~$úÿ·?øüÁüÅº®®®;w?>/¿]===×û÷·?øüÁüÅºöööo|ãéy«««;üð?øüÁüe7û2^É»|ùòÓ§O§>üÁàþðYYtn_úÞ¾ûï¿¿ÞÁü	þàOððìÛ¾úûÂRß¯?øüÁàþà/kjooüñÇÓ/éX¾|ysss¦ð'ø?ÁüÁ_v°oûöíÏíë'ûàþð'ø?øË.^¼¸aÃy[[[:5ø?Áü	þàþbÚùóç3îí»ç2^Éð'øüÁüÁ_VííK?·¯ï+yáþ?ø?øË²Î?ÿÈ#Ü~ûí¸þàOð'ø?ø¿¬©µµuÃ)ì»í¶ÛÖ®];$ì?øüÁàþà/.ì«­­ÎKaßý÷ß?èsûàþððð».^¼¸iÓ¦öV®ÒÒ2_þàOð?ø¿Ñaß-[Ò/é¨­­&öÁü	þàOðð7>:#ûÖ¬Y3äyáþð'ø?øµZ[[ðÒÏíäGuoüÁàþð#Í¾ôK:n¿ýö`ÁAüø?Áàþàþ²áa]]ÝùóçG~~àþð'ø?ø¢¿Ò¾·ïGsûàþððð7rìK¿¤#(°¶¶öìÙ³£;oð?øüÁü;û6lØ0êì?øüÁàþàoÈjooÏÈ¾¿¤þàOð'ø?ø¿a,ØîñÇ8qbúåø?ÁàþàþoàRWW«½ð?øüÁüÝRçÏÕþ?ø?ø2^Òqûí·oÚ´)æì?øüÁàþào`µ´´¤³/7p?øü	þàþàoX?~òboüÁàþð,,ÕñãÇ?þøã±½¤þàOð'ø?ø¿¡ìòåËÙ;óð?øüÁü¡àþð'ø?ø?Áàþðð'øüÁàþàþ?øüÁüÁàþ?øü	þàOð'ø?Áàþ?øü	þàOðð?øü	þàþàOð?ÁüÁü	þàOð'ø?ø?Áü	þàOðð?Áü	þàþàOð'ø?ÁüÁü	þð'ø?ø?Áü	þð'øüÁàOð?Áü	þð'øüÁàþàþð'øüÁüÁàþ?ø?øüÁàOðð?øüÁàþàþ?øüÁüÁàOð?ø?øü	þàOðð?øü	þàOð'ø?Áàþ?øü	þàOð'ø?ÁüÁü	þàOð'ø?ø?Áü	þðð'ø?Áàþàþð'ø?Á_à¯©©iöìÙùùù³fÍJÿù?Áü	þàÏr¿Âß´iÓ=^ýõÊÊÊtü½÷Þ#[ø5kmmíT;öløoåóþ:::,8þõñÇ[qî§?ýéûï¿o9Ä¼¿_þò#üE³É¦ãïÙgod÷Ýw¿ûÝï¾§wðàÁX1ïwÞ±bÍÍ²wsø;~üx]]Ã¾rØ×a_9ì+öÍåÃ¾QW¯^­©©éèè?Áü	þðkø÷ïE/P[[ÞÎÓ?þð'ø?Ëþ²É566.Y²¤­­-ã³ð'ø?ÁüYðSø«¨¨ü	þàOð'ø¿_ßÁàþðg9ÀüÁüÁü	þàOðð?Áü	þàþàOð'ø?ÁüÁü	þàþàOðð?øü	þàOð'ø?Áàþ?øüÁüÁàOð?ø?øüÁàOðð?øü	þàþàOð?ø?økøûÓ?ýÓ·ÞzëìÈöî»ï¾ÿþûgã~øÃ¾óÎ;CÌû«¿ú«3gÎXq.lî?n9Ä¹ ¿Ë!æ½øâ?ýéOGø¶··ç&þN:µmÛ¶?$IRRg¬$IÒØ	þ$IàO$Ið'I$ø$IüI$	þ$I1íÔ©SóæÍËÏÏ9sæ#GÂÆÆÆ3f$QÜÖÑ~8wîÜU«V]¹rÅ"O7îW'NG+îÐ¡CN×Q¨­­­¢¢Âbí:jjj=vø=5k¿öÛÕþ>±.¬§ûö£GðñÜ¹sa |´Mç:¿caN>½nÝ:(&õôôw©ÄÖ°¦¦æW^	Ï?ÿüêÕ«-®£ð.þ¯lAÅmM6-lúÂÀë¯¿^YYiùÄs5¥¿OÁ_vT__þ_¦NzáÂ0>aK&ë(üï*1rÂ	LLúÚ×¾öÍo~3±5[ÀèÏûvwwûT<×ÑÒ¥K[ZZà/Îë(¹ÂÂBË'æ«)ñ>Yà÷âââ°Ãÿ«®ß8V~ÁÂÃðñøñãO×QøÕ:uêTxíµ×!¨QìÜ¹ssçÎÚKlWÕÏuô«÷	øý:÷£ºº:(¶«)åþ²£^¿qHñôéÓ,X`ÉÄpðÁAçá?^þ+î»ï¾Ã'K"///ñlAAEÃuÙ²®^½ZSSÓÑÑaÅy5%¿OÁ_Öí°»"þë(ÑG4cÆ%û1aØÝÝýÆaß8l¾à/+ÖÑjkk?ýôSË'æ¿Jñ_é4mÚ´èbSSÓ%KÂÀ¢1ÍÍÍóæÍ³b¸Â'NvíßüæSO=eÅm³¬Y³æå_ácMM%Ãuñ_Ga»×ÖÖfÄy5¥¿OÁ_¬9sfpzUUUôÿª>ú(º`;|ÃQ×QØN<¹   ®®®§§Ç"çÖðÈ#eeeyyyåååÑÕÙ?tUTTô±I1YMéïSð'I$ø$IüI$	þ$I$I?I$ø$IüI$	þ$I$I?I$Á$IàO$Ið'I$ø$IüI$	þ$IàO$Ið'I$ø¤éµ×^[´hQáªªªÞ~ûíÿ°ýºQÖlm3ÍmEEEøÖ:::RÆ1ùùùååå×®]è4%	þ$eeÛ¶mÖSO=KøÛ¾¹k×®ñ;wîãxâALSàORöuâÄÀüüü^x¡çF/¾øbxF<y2gðwæÌ0rÖ¬Y)ãgÌÆ·¶¶Â$ø4&zàk~úéäÏ<óL¹víÚdú:t(à)¸pöìÙa8ñÉöY]]]qqqxª¤¤dãÆÉGW°ÂSáµõõõ)c&L°hÑ¢kÖ¬I±ýû÷÷=è©0KÑSìjK,	ãc=ÆTWW'Æ<ùäeeeaR«V­ºpáB:þÒ§2¦Y4ÊM<9ÀåÜ¹sÉ#?ùä0²¢¢"7)?~<zvÅ)O­_¿>zª¹¹9///ã«¢Ñ³÷Ýwßµk×zzzÂ³ácÀSQQQt*^Ó	g/ý;ýõ×Eðå+¯¼_ÊDªªª¿>fUüIÒèáÍ°Í7.P,7«W¯¾z£0.[¶,z6²NÄÇh^@[ôTMMM´Ë09r$Hò4·nÝxuè¡Âûöáð1?øà7Ncx>!yÞ2~G&Mßo[[[x>9âìîî>¡¼¼<¼0|×ÿý0qaaá@ñ×Ç¬J?IþBñá&2SèÂáa`SôpöìÙááÔ©S~øá¶ÎÎÎÄDÂç¤ìKK¼*zøÉ'$>9)qä7|Ã7NfxxéÒ¥äyëíü¼G4<õÍo~óú¿ÚÞ¸qcò'üÎ;7Ú19Püõ1«àOF¿hw×Õ«WGvttá©>¸ aKKKä¿u'ê¥MáTòVÂpIIIAAAWWWø	ÏÞt:½Q,¥S§NENÃácnnnN<äÈ0÷Ìª$ø¤Ñ/:ïí¹çKÝ%åÄymmmé;´N<¹ûöè lBÑn¹ÄqÕmýúõÑÑÞð±®®.1¾éDÚ>ýôÓèá¥Kú¾2wÁáÙèî6Á¬ÉOEWþ§<xåÊ¾ñi´4Ïö1«àOF¿èüüüç>ºÕË/¼PPP~qÆ²eËtººº¢c²+s£sþ¢SåZZZO,¸qãÆ@¥è:ÜÄÍV2-ºü6úêñL':É/:ç¯££#úÌ>ð÷â/&öÉo9ù©èôÇæææðUÂBè7Ã§t®Zµ*ùÙ>fUüIR,nÒO>ùÿ¶_7lN0Mè¡Jeröìé×oü)0¾¤¤$ùpÓ	³<c+mûf;;;£o!å`÷Ò¥K¿Ä¤IÂÇèn/ÉÓvL&¨x¶YPªªªn´hÑ¢èÛü<x0ºÞÌ3>x¶££ã±Ç+++Àôè£vuu%­¯¯;wn0YyyùîÝ»S¦>'aRaüºuëRÆ÷6P0KÑû¸Ï_¢ººº¾DµµµÕÔÔ%P\¾ÖÖÖÄmk§yåÊ ÛhYUWW755¥|Å>fUüI$	þ$I$I?I$Á$IàO$Ið'I$ø$IüI$	þ$I$Ið'I$ø$IüI$	þ$I$I?I$TÿíÜ¸;éeIEND®B`


Detrended Normal Q-Q Plots


ðð'ø?ÅAÿÏÆÝßÙýûæ¦o~êSúàìø?ø?Áü)nø¾ó³wfäýûßùpOìø?ø?Áü)ø«««ËÁß/­?ø?ø?Áü)ø¼ýöÛ¿ñÀ72òøO¾í¶ÛÞï=»þàþàOðþBÇÿßøuëÖöýáÆ?¼ã;þâ/þÂ¾?ø?øüÁâ¿èû~õ«_ýíßþíçÞ?ø?øüÁâ?ÁüÁü	þàOð'ø?ø?Áü	þàþàþàþ?øüÁüÁàOð?ø?øü	þàOðð?Áü	þàþàOð?ÁüÁü	þàOð'ø?ø?Áü	þð'ø?ø?ÁüÁü	þàOð'ø?ø+S©T2lhhèééÉ^ÔßßßÔÔ566«Áü	þàOð'ø+ùÒétggg8pà@[[[ö¢eË½þúëaâÈ#Ë/ÏÇß_ÿõ_ÿªð¤úéOj¿W^yexxØ8]]]ï¾û®q(òÎ;öq(þ~ö³~1Ø¿ªªª©©©èWµµµ3]­¢¢"zK·ªð¤7ÅßË/¿<00`¿_|ñÔ©SÆ¡Èû(àÏ8áç^www6dAà/LN;]Ø£û:ì+öÃ¾rØ·äK$éòòòü+¦Óé±±1ø?Áü	þ%_uuuxjEO°0³4hcÓ¦M/^Ì_þàOð?Á_éÕÞÞ~øðá0¾¦ÓéìE½½½­­­###Ó®ð'ø?Áà¯ôêëë«©©I$©Tª¿¿ÿWÛVöß[W[[[üÁàþ¿üÁàþ?øüÁüÁàþ~Uooï¾û~øÃ¾÷Þð?øü	þb¿ÉÉÉûî»ï;îøò¿¼råÊÛn»íÙg?øüÁàOðOü÷»ßýüç?¿óÏwîþÎîðï¾¹xñâ¡¡!ø?Áü	þ1Ä__Ç·:"ùEÿþ÷ªÿýè£Âü	þàOð'ø!þn»í¶lù÷ßÿ·¾õ-ø?Áü	þ1Ä_ãÿjüzû×³ñ÷¥æ/íÛ·þàOð?Á_ñ÷Â/üVÕoeüÞÿµûï¸ãK.Áü	þàOð'ø!þBûöíûÔ§>u×]wæ3ùÜç>WêgÀüÁàþ*¿ÐØØØo¾yöìÙÉÉÉl&üÁü	þàOð§BøYðð'ø?Áàþðð'ø?ø?Áü	þðð'ø?Áàþàþð'øüÁüÁüøüÁàþàþ?øüÁüÁàOð?ø?øü	þàOðð?øü	þàþàOð?ÁüÁü	þàOð'ø?Áàþ4.]ºðð'ø?Á_ÌÜµk×m·ÝV]]½xñâ~8Ì?ø?øüÁà/­_¿þ®»îzøOÞýÝ¶õÏêëëï»ï>ø?ø?Áü	þbØÛo¿ûí·ïøö ¿èßÎ?ßùéOúÍ7ß?ø?øüÁà/n?~üî»ïÎÈ/ú÷»wÿîsÏ=ðð'ø?Á_ÜzóÍ7ïZvWþ-[öüþàþàOð¿¸599ùÙÏ~ö«_ýjF~÷ßÿg>ói8ððð'øü|ÃÃÃA_¨ÿÂ5kï¸ã¡¡¡i¯	ðð?Á_áöíÛ÷ÜsÏxÊÀüÁüÁàOð·?ø	þÊ®W"?Áü	þðü%®W2?Áü	þðüÅ#ø?Áü	þ7fø6oÞ?øü	þà/ø«­­M&Þó'ø?Áàþâ¿ÆÆÆü³=ªªªÆÆÆàOð?ÁüÅåååA###555a"ïÙgð'ø?Áàþâ¿è¥¾0´&Î;755&-Z?øü	þà/nø[¼xq ^__ßððpxäG¢	õ"ø?Áàþb¿;vdNïÈ~ÛßÊ+áOð?ÁüÅ¡ïÿûK,	ýýýa"@°¹¹¹$¶þàOð?ÁßþàOð?Áü	þàþàOðð7sË/>ðÅ<þàOð'ø¿ãoÙ²eÙàËäl_Áü	þðCüçöLMMÜöÃü	þàOð'ø[UUU¥(?ø?Áü	þsnpp0àoëÖ­£££ð'ø?Áàþb¿ÐÒ¥KËòrÂàþ?ø!þêêêð!ø?Áàþþ"öâöÃü	þàOð'ø[ÕÕÕNøüÁàOð½½½;vì?Áü	þðsüÍ>ð'øüÁ_ñ!'|þàOð'ø¿â¯¤?øüÁàOð7·jkkëêêÞ~ûírS©T2lhhèéé)¼h~sàþð'øüÍ¿àª²²öb:îììhkk+¼h~s²Û¹sç¿øÅÝª¾ô¥/ýÞïýq(þÂóbõêÕÆ¡økllüò¿l¼5kÖ=e¿ðs/*¸éøëéé	øÛ³gOðòÇÿÀªªªèFÂÿjkk/ßì¶oß^&I£n:þnìÙ¾Ù§ä2¿h~sàO$Áßü»±gûf±¼¼¼ð¢ùÍÉn×®]÷Ýwß&ÝªþàþàÿøCñ<ðq(þ¾ò¯<øàÆ¡ÈûèÞï5Å_ø¹ÜtüÝØª««¯^½¥ÓÍo>ð!'|8áCNø>¥öööÃð5N^4¿9ð?øü	þ>îÃzíÚµeee-Ú°aÃ¼Ïüèëë«©©I$©Tª¿¿ÿW÷òÚÑëüEóð'ø?ÁàoþOûfÃøS¿ð?øü	þæV ÞºuëFGG£[¿~³bÅøüÁàOðqÃ_yyy Þäädö£<ÌÉ?µþð'ø?Á_Éã/HêEgÔFMLL9óû¨ø?ÁàþE¿è°okkktØ7|ÓaNSSü	þàOð'ø¿¸á/hoÚ>._¾?øü	þà/nøûèÚ	¿6l¨¬¬L$ákkkkSÛð'ø?Áàoð'ø?Áàþðð'ø?øËYóz%	øüÁàOð1Á_bæàOð?ÁüÅ3µûöG?Áü	þð[ü.Z´(°¯¥¥%û3áOð?ø3ð7ümÜ¸1zÁ¯«««¶þàOð?ÁßÜzþùç#ö­[·®ä¶þàOð?Áßl_±bEtnGOOO)n?üÁàþ¿Yµÿþè¿¶¶¶ÒÝ~ø?Áü	þ³[Óçü	þàOð'ø¿¿ÄõJ&ð'ø?Áàþb¿xð'ø?Áàþðð'ø?ø?Áü	þðð'ø?Áàþàþð'øüÁüÁüÁü	þàOðWêø[¾|yyy¹ÏùüÁàOðñÇß²eË²Áçsþð'øüÁ_ñØ700055UrÛð'ø?ÁàonUUUü¢üàþð'øüÍ¹ÁÁÁ¿­[·Âàþ?ø9þBK.-ËË	?øü	þà/ø«««sÂàþ?ø[(øØ744TÛð'ø?ÁàonUWW;áCð?Áü-üõööüíØ±cbbþð'øüÁ_ÌñW6CNøüÁàOð1Ä_bð!ø?Áàþb¿þàOð?Áü	þàþàOððWða½víÚ²²²EmØ°¡TNþ?øüÁàOð7·ÆÇÇ§=á£$Nþ?øüÁàOð7·êëëõÖ­[ýmß0pë×¯sV¬X?øü	þà/nø+//ÔÌ~9a>ü	þàOð'ø¿¸á/HêGvfÎÄÄDã£^ð'øüÁ_ñömmmû¯a:Ìijj?Áü	þð7üíMÂÇåËáOð?ÁüÅ];áwÃD"|mmmsJbûáþð'øü- àþð'øüÍ­ÚÚÚººº·ß~þð'øüÁ_üñL&ËÊJõDø?Áü	þs«§§'àoÏ=aÈJå¯ºÁü	þàOð'øïMÌP"?Áü	þð7ü%fÈ<þàOð'ø¿øà¯®®®¡¡all¬¤·þàOð?Áß¬þ¤ïG×ûÄ^ø?Áü	þóÇ_ô'£?ã?øü	þà/æøK¥ResÂàþ?øþ/^½þQÏ	?øü	þà/¶øËT*Î?øüÁàOðçoûÂü	þàOð'ø+µS©T2lhhèééÉ^ÔßßßÔÔ566«Áü	þàOð'ø+ùÒétggg8pà@[[[ö¢eË½þúëaâÈ#Ë/?øüÁàOðWòUUUEV8<ÁjkkgºZEEE>þþò/ÿò'ºUuww¿úê«Æ¡øé¥ÂO@ãPü½øâáÿ·Æ¡Èûø°§Cñ~îÿPÅ`Cn:þÎ9óã/ûÎ>èèèÈÇßñãÇGt«¿¥~ö³âïïþîïÞ÷]ãPüuuu]¼xÑ8yaÿPâïÜ¹sá§_6ä¦ã/ú¦¦¦wÞyçÂ_ögç_att4Nçÿ1:öÃ¾ûÊa_9ì;·û2ìL&W¯^öìÙ[q×]®®®O­è	¦s®´±iÓ¦ð¯ü?øüÁàOð7Î9S__YyyùÚµkoÙf´··>|8L¯ét:QoookkëL/Âü	þàOð'øSSS]]]·øÏ»õõõÕÔÔïJ¥úûûµm×^¬­­Íþ£sð?øü	þn@CCCÙ¯üUVVæ]QÁü	þàOð'ø[Ùæ«¨¨hkk;þ|©l?üÁàþ¿9ÞÄµó<î½÷ÞsçÎÜöÃü	þàOð'ø[à'¼Àü	þàþàOðw«ñWÒÁü	þàOð'øs§O^¾|ù¢E¢3|«««=?øü	þà/øëîîÎðá/>pàü	þàOð'ø¿¸á¯¦¦&PïôéÓüõ÷÷Gö?øü	þà/nøË|xrSSSÑ)Àð'ø?Áàþâ¿êêê@½èÕ¾¿ðß¹sg®­­?Áü	þð7üõööM×ë¯¿?øü	þà/nø]¼x±¹¹9:Û·¢¢bùòågÏ-í?øüÁàOð·?øüÁàOð?ø?øüÁüM×ùóç*++×¥rÌþàOð?Áßzè¡ÊfhóæÍð'ø?Áàþâ¿£GFÎ;xðàØØX4s||üØ±cÑüð#	þð'øüÁ_LðJ¥ð:¿(úo>çOð?ÁüÅåååAxãããÓ>ÊÃ¢pøüÁàOð1Á_æ¯ºÍ´4úSoð'ø?ÁüÁüÅx?øü	þàþàþàþð'ø+Yüþð'øüÁ_|ð¸^Édþð'øüÁ_Lðàþð'øüÁàþàþðð'ø?Áàþàþð'øüÁüÁàþ?ø?ø?ø?Áü	þàþàOð'ø?ÁüÁü	þð'ø?ø?Áàþðð'ø?Áàþàþð'øüÁüÁàþ?øü	þàOðð?øü	þàþàOð?ÁüÁü	þàOð'ø?ø?Áü	þàOðð?Áü	þàþàOð'ø?ÁüÁü	þð'ø?ø?Áü	þðð'ø?Áàþàþð'øüÁàOð?ø?øüÁàOðð?øü	þàþàOð?ÁüÁü	þàOð?ø?øü	þàOðð?Áü	þàþàOð'ø?ÁüÁü	þàOð'ø?ø?Áü	þðð'ø?Áàþ?øüÁüÁàþ?ø¿"lpp0J%Éü+twwÁü	þàOð'øCétº³³3L8p ­­-géäädSSüÁàþ¿à¯ªªjjj*zÕÖÖæ,ôÑGxâð÷øãÐ­êÅ_ìêê2ÅßñãÇ_zé%ãPüýÍßüA°§t£?÷ÂO¿lÈÀ_2v:tþüùæææ@Ãð×ÛÛûKÝªÞxãÿüçÆ¡øûñ|éÒ%ãPüòãããÆ¡Èûÿø_~Ù8###¯½öZ6dAà/Hd¦ËËË³mØ°!ìÈÿÞTöÃ¾ûÊa_9ì[Âwý×éêêêðÔ`azÚ«e®ð'ø?Áà¯´koo?|øp_ÓéôLXÌ	ð'ø?Áà¯ôêëë«©©I$©Tª¿¿ZíÁü	þàOð'øó!Ïð?øü	þàOðð?ø?øüÁàOðð?øü	þàþàOð?ÁüÁüÁq?Áü	þàþàOð'ø?ÁüÁü	þð'ø?ø?Áàþðð'ø?Áàþàþð'øüÁüÁàþ?øü	þàOðð?øü	þàþàOð?ÁüÁü	þàOð'ø?ø?ø?øüÁàþàþ?øüÁüÁàOð?ø?øü	þàOðð?øü	þàþàOð?ÁüÁü	þàOð'ø?Áàþðð'ø?Áàþàþð'øüÁüÁàþ?ø?øüÁàþðð'øüÁàþàþ?øüÁüÁàOð?ø¿âèÒ¥Kð?ÁüÁüÅ¼±±±o|ã¿ù¿Y]]]YYùüþàOð'ø?ø¿x699y÷Ýw±éßÞþíÝßÙÝñ­ÚÚÚ'xþàOð'ø?ø¿xþâYºté®ÿ»+È/ú÷ÍMß¼ýöÛáþ?ø?ø[O>ùä5k2òþUWWÿâ¿?øü	þàþà/n½ðÂ+V¬Èß··ñâÅ^ù?ÁàþàþbØ¥K>ýéOÿQú2ø»ûî»×¯_?Óõáþð'ø?ø+í~òÜ~ûí+V¬X³fÍç?÷ù/|áÐð'ø?ÁüÁ_ÉwéÒ¥çnß¾/¼ðÂØØXkÂü	þàOðð·?øüÁàþàþ?øüÁüÁàOð?ø?øü	þàOðð?øü	þàþàOð?ÁüÁü	þàOð'ø?Áàþðð'ø?Áàþàþð'øüÁüÁàþ?ø?øüÁàþðð'øüÁàþàþ?øüÁüÁàOð?ø?øüÁàOðð?øü	þàþàOð?Áü	þð'ø?ø?Áü	þðÅ×àà`*J&===Ù&&&6mÚT^^~çwöööÂü	þàOð'ø+ùÒétggg8pà@[[[ö¢½÷>öØcSSSA~uuuùøåWÆu«8wîq(þ^õÕ_üâÆ¡øé¥FGGC÷ïÿþïéÆ¡ø»xñâüãlÈÀ_UUUà]ô¿«ÚÚÚìEo¿ýöL+üýà?xE·ª®®®îînãPü½øâû·k¿ãÇ<yÒ8ya=e¿ðs/üôÁ,ü%Éi§£û÷ï¯¨¨¨««ë­·öuØWû:ì+å°oÉH$2Óååå9:&Î9ÓÜÜð'ø?Áà¯4ïú¯ÓÕÕÕá©=ÁÂtöÕ²/æ¼(ð'ø?Áà¯$koo?|øp_Óétö¢-[¶<óÌ3aâôéÓ+W®?øüÁàOðWòõõõÕÔÔ$T*Õßßÿ«m»ö¢`Ø6lH&ÍÍÍÃÃÃð?øü	þnð?øü	þàOðð?ø?øüÁàOðð?øü	þàþàOð?ÁüÁüÁüÁàþðð'øüÁàþàþ?øüÁüÁàOð?ø?øüÁàOðð?øü	þàþàOð?ÁßBë»ßýîsÏ=÷nU/¾øâo¼a¿göôéÓÆ¡øû«¿ú«³gÏ"ï_þå_Â2ÅßOúÓx¨ ðÿÞ:þÂ¯·Ý»wO$).~ýr¡ãO$iA$Ið'I$ø$IüI$	þ$I$I?Å§ÚÚÚÌÅÁÁÁT*L&zzz²¯yñâÅ²¬Ý'»§¦sÝ¨¢ÚSSEµ§úûûÂ§±±1<<§JqOÅò9åGnpõõõÙÏt:ÝÙÙ&8ÐÖÖå£GnÚ´É ÉÊ3¨¢ÚSSEµ§-[öúë¯#G,_¾Üsª÷T,Sð§ÜÚµk³TUUUSSS]ûë9¯UgÔ±cÇZì©ü9³Ù*ª=å9UT*»Ï©RÜS±|NÁnÎ+ëIL&§þ³ÕÚÚf6779sÆ¸²j¦9w¢jOyNátttxNâåsþtÓTD"3]^^>íõ/ÐØØhÜ³Ù*=å9Ujtt4NyNâåsþtÓTÕÕÕW¯^ýèÚÑ0=Ó*~ü3)f¹U<øó*=Ä°iÓ¦/zNèåsþtÓTíííákøOUöÕ-[vöìÙèY×ÚÚjÜv¢jOyNÕêííaddÄsªt÷T,Sð§þ¤êëë«©©I$©Tª¿¿?ûõõõÉdrõêÕáyeÜÑÅiw¢pOyNÕª­­ÍÿÏ©ÒÚS±|NÁ$IÒB¯!$I?I$Á$IàO$Ið'I$ø$IüI$	þ$I$I?I$Á$IàO$	þ$I$I?I$Á$IàO$Ið'I$ø¤ëö£ýhÕªU×Z³fÍóÏ?ÿ?~Æ]«d~"OwokkkÃ¦åÌsÉd*ëmJ?I*ÉvïÞ]×÷¿ÿý8áoÏ=aæSO=3ÿÉ'óyäyÜ¦$ø¤Òkpp0°&L<xpòZÃÌS§NÅçÎ3sæ×××ùgÏ?Ið'iAôÀÖ<öØcÙ3üñ0óOþäO²éÓÓÓðÔÔ¦3W¾|ùrGGGeeeXTUUµmÛ¶ì£«'OÀÂº'NÈáT³xñâU«Vuuuííí9wìøñão'ZîR´¨»»&¨µ¶¶ùýýý9¯¿þzÓÒÒ³wïÞpS7n¼páB>þòo?gN»*	þ$énéÒ¥.çÏÏùþûïµµµÙ¸Éi`` Zº~ýúE[·n%i×.FK7lØ055øX^^>99¯O-ÞWàvÂÄ´w/K9-Ú/;;;3òË¹5kÖÌîª$ø¤O¾èï4?×ÊÊÅ²qÓÖÖ6z­0.®[·.ZY'âcô^@[´(NG/é¾¾¾èF²os×®]wÑQ×z(Ì9vìX_Ãô>xÝÛ	pÃ²ïÛ´[@¹dÉ°½###ábøîyçÕ«W£+¤R©°bØ~¸¢¢b®ø+pW%Á$þBÓâ/3?ÂMd¦ÐÂÅÀ¦èbSSS¸XWW·yóæ¶ñññÌëä¼Y+ºøþûïg®À9ò¾é'O^÷v4ÃÅ?ü0û¾Íôþ¼íÛ·EO<ñÄG¿>´½mÛ¶ì+ü677G/LÎîª$ø¤O¾èå®ÑÑÑìcccafXT;GþËX'óF½ü£±9Êþ0]UUU^^>11n|ñâÅ¥×½(ÓéÓ§#§éð5Leöõõ;0íáãÙã¯À]ôÉ½ïmÿþýÙ3£@É9á#ó¾Àü´N:µgÏè lFÑËrãª×%ÚÖ­[££½ákGGGf~Û^i»xñbtñÃ?,|fî=÷ÜFnÌ½(:ó7,êîî¾råJaüe`Ffi»*	þ$é/:a"L8p ú¨ç±nÝºèlæÌÜè=Ñ[å³ß,YpÛ¶mJÑy¸[hÑé·ÑwïííÍÌ/p;Ñü¢÷üE×,¿Ce^½(zûãÐÐPø.afÂ_ÄÍÀåpµÎ7f/-pW%Á$EÑ ç´wïÞÿÿ3îZ2Ó¦s,2=ôÐCÙ²ÌîèÑ£ð÷Ñµ?ÅæWUUe.p;ándß±Ì¶3mìøøxEEE´	9»×®]ý-,Y¾FöÑ"ªf¸«àO¥5kÖ_kÕªUÑ)·9øëîî>N¯¡¡áµ×^Ë,Û¹sgMMM¦íÛ·OLLd8q¢¹¹9,J=ýôÓ9·OÂMù[¶lÉ?ÓíÂ	w)úÂó©££#ç3_¢FFFÒétÊÊÊ°gÏÍ|lMöm^¹r%è6«þþþïXà®J?I$Á$IàO$Ið'I$ø$IüI$	þ$I$I?I$ø$IüI$	þ$I$I?I$Á$IàO$Ið'I¤Ñÿä ]RðMw?IEND®B`


¼EG)EyKÇ¡)ª[UÊùh9ÊQ6eÑ¦	[E4í*í²Q¡°F-Ë¨ëXà/eëÒE,B¥	bS!y§^+ïoý,Ï;;¶Û		3þ|¬ggñ3?þx?ã·%I4mª1$Ið'I$ø$IüI$	þ$I$I?I$Á$IàO$Ið'I$ø$IüI$Á$IàO$Ið'I$ø¦éWTQL¦®®nÑ¢E÷îàê'O|ù¹©ïâÉü§zêÝúDïÍ3Ý¿ÿÒ¥KëFZ¼xñ¾ûnù_eôþÐÒÒòôÓOOap¦¼o;v,vÂl6½äjÆéùRá¯¸Í7ßVÝ9ø«¯¯¿|ùr¥àïáýµûö26©UÆÛvíÚ5ÙÁò0ær¹ôó¶··Ã$_ØÒmtFÿSO=UWW3zz¦þ¢Õ«WWþN<hõ»ßýîÐÐÐððpLÄÍÙ××7æCMvÑûÃÖ­[cNì=Ã_²âµk×bºP(ÜQÿ5àOª6ü%=ñÄ%/º=ztþüùÙlvÁ.ÁSú ÉDÜaæÌK,)³nzçÓ§O/_¾<9#ùÀzµø¶¶¶&+9r¤dSÇØ[®X²ë×¯û÷ïoLÎ?¿víÚdóùüJîYü|9ñIãSÇ,]ºôÊ+Çkii©­­]±bEñ«;vìhjjJÎoÆ§H·Á_üâc~ñIØ(°3;::Æüÿì*£?ux1æÄFNjpÆ|e®¸©­X%Kãÿ%ööûï¿?Æ¶ük£Np7¤ÆH§cQCCÃæÍX$ø*áÙÜÜÜìëëËd2ÅßO<YÉ×¬YSfÝôÎñ½¹xéºuë_²ï_-¿âèçÞßßËåcbô¼öÚkñ½øqâ%J+~¾£?ï¢EWR"©üJî¹lÙ²òÄ=vÌã7FÿgÍ;wÌÿßÉ®Rò©C6É+óæÍÔàü)¯8qüõöö?N<ÙÉâo»ñêÕ«K¶yÓ¦M,üI¿d~°,Îçóq³³³3¦»»»V²zrsÛ¶mÃÃÃÉk9·¾wÆwý¸òrc8)Yßtãf¬~¤X«øsyØò+ùÜ8PüJXñý7lØÓ«V­º6RLÄÍ9ÞóMælÜ¸qhh(Ù°äãæáÃ`òmé//o³ÙìxO$]÷®2Þ/Õ¥¯NvpÆÜª)¯XõÅ7w`J´ão»qÂK.ÅtüoÆô3X$ø*é¾äWÄ9eð÷Úk¯¥sn¹nú*Zrz1¾&7ã;hñKV%§íÊ<lùÇîãûzÉüÆÆÆâgßããæ¬Y³Æ¾ÉGñÍäò·¤P(mmmÅÏ½þR'õw¸JñÆº1Ë/?zôè6Ás«¦¼âÄñì!W®þÂßwãÖÖÖ¸9gÎÀküqãÆG	þ¤ÊÃ_`%f644÷w<¦$7C9e¾[!Îd¿=OáaÇO9o|,?ú³Ñ¿7úùNäf@³äÔç-¯ZmjjJÎS^	©ÓGÈ*«1ï0Á¤S[qâ§ßùÞ5ÁÝøÜ¹sÿR<xÐE?©Âð÷Ì3Ï_ð¼VrÝåD¬3©uË¿róÆo/-ó°åW,³Ï=÷Lß%ø¬ä5ªO|@oÎ??yË#GÎ&¿äåÉä]WòùüæÍ¯]»¶÷î¹víÚ1ñ7U¦¿ÉNÚW8þ&òÊ_Ê»ä§2ã_fK:uêÔ#<kN¯Rà/¾½ïÍé[½$ßÒÂñÍ2¹xvÁÉ¢äFó»fuËË)QKò;[ñàÉãüF×[~ÅòX½zuúýÉäZàU«Võ<ÚÆß9þW¿úúúâ)<ýôÓÁ_WWWòÚR<åîîîäU½¤ùÿ;ÙU&¿òS²o7ÙQþßÒáÆa»+W¦áO>@ÿ[~	$¿»yîÜ¹2§à%ÁtáotÅW,¾~6ýõÿôýx	G×,³ny9_z.-ó°åW,/Ë/§¿ÝÌ¹páBÉï566¦¿PøNðr$iÖ¬Yéo@!Î<0ú?káÂeþ'µÊ¤ðW~pJöâ&;ªSÀß©S§É%ÅKKÆ$½ä|¼ÏRfK¯(JðÁX$ø*ñ]pÙ²e£ÿü×áÃÛÚÚRñýgIç8p æ¶ßånëÞRNÉÛãÅ·¶¶~»¾ñö+DrJ´xþÙ³g×®][;R>yÇÔðwõêÕx´xÌ`Ð-[ÂC©¹Ëç;ßùN<»xâ³gÏÞ³gÏg?ûÙô§w¾Ê¤ðW~pJö&5ªSÀ_tðàÁ9sæÄ³ÇON¬§8ÇÍ ZòÙ/_ÞÓÓsËW^ÇÛßzè¡ä%Õ|üW:°Hð'I·«B¡ðÀLê½å¦°JÅ¼â=44~þüùv	þ$IUXò;%íÜ¹ÓÈHð'IªÂ¶nÝ:öìäÜnL<üðÃE?I$Á$IàO$Ið'I$ø$IüI$	þ$I$Iðg$IàO$Ið'I$ø$IüI$	þ$I*±477g³ÙeËMvÝsçÎ¥sb:æ,X°`G±¦v¬;Åcë¨ù6mjhh«¯¯ÏçóçÏO×ùqçÚÚÚ¶wI?Iw«W¯N+;vìuÿôOÿ4óøãÇGy¤:ð7zþúõëc~gggL;v,¦çÎ.=xð`ÅCÙ»$Á¤;ï òütêÔ©XwéÒ¥éEÅÞÞÞ÷f#ßüÕÖÖÆüþþþ1×úÒ¾K>ø ½KüIºåWz¨¾¾~ÆÛ·o/¹Ûg?ûÙ¶¶¶GH_8é7Þx#¦cN²èÒ¥KË/¯««Ëf³­­­É«e£­ø³_eÏ=ÍÍÍ/O4h×¯_oooucã·nÝ¼<]²dI<ZÌG~íµ×Þ!þî½÷Þ?þüîîîEÃÃÃ3gÎÏuãÆø8kÖ¬ÈSÛI?I·ËÉôc=ÓÏ<óL0+&vîÜY|Ã'È+nÃ±h÷îÝ1cº££#Y´páÂäÔçÅc~.óÑ7 ü*Aºç.&Ö¯_?zã7mÚÓqç>&vìØZ­¿¿¿¯¯/&&Çû¾ñX¤s-[vúôétä,ðªU«bzíÚµ1sÆÜ$øtGà¯¹¹9¦#¶×¿ØÕÕgõêÕêÒ¥§NÚ¶m[[[[ÌÏd2c>Z	¿Ê¯lXýèu WÌÆtkkkpvpppjÃRÒÉ'©)ãù7oNo&HÝ²eË;ÙI?I·Éªéü¸yK	´fÌ÷HÎlÆdÑÓO?kO>]ü%V|s"«·a!Åâê8îß¿æÌÉäEÁ¿òWfÜ:;;ÃsÅ§¹ÎñI¯]»ööÈ9è=öÛ`¯îüår¹âWþÏn)¡öööX~Lç'×F4Aü_%Ý°°ÔèugÍ¬[²yCCCJ®ÒM_2Ô°W²1±Í1jµ¤S§NMy$	þ$Ý^ü%oÝ²÷îäwþüñHhÿþý©uî¹t~BÉ3gÎ$¿8ü_eÛ¶mßÿþ÷cbÃ£×ðÁczïÞ½¯¾újr5f.^¼8¦CfW®yóæ½Cü-X° æ9räíoìòÀ¼=þ»ÞÄü)o$Á¤Û¿¡¡¡Öß«¯<þnÜ¸rÅï6jhh¨¯¯ßºuëñW~£GÆÒE¥×¼ýòæÍcËkkkW®ÞÊçóÉåÃK.=öì¤eô©áxØöööØÂx²³fÍÚ´iS|Þ·o¾ßuñã§ïw=åm$ø$I?I$Á$IàO$Ið'I$ø$IüÝA½ôÒK¿úÕ¯*kÿå_þåüùóöÝ´ê¾¸«#´_ÿú×ÉÛ©() q1I¿ýíoÿñÿÑ8¤]¿~ýõ×_7i¿ùÍo¿»ÕÓýÑÿ*kÿùÿ9yX%ýÓ?ýÓÉ'CÚ?üÃ?;wÎ8¤Åî;qHHFCÒ7~øÃ´Ë/ÿÝßýqHõÕW«ïÇø?ø?ø?øüÁüÁüÁüÁüÁüÁüÁüÁüÁüÁüÁüÁüÁüÁüÁüÁüÁüÁüÁüÁüÁüÁüÁüÁüÁüÁüÁüÁüÁüÁüÁüÁüÁüÁüÁüÁüù?ø?ø?ø?ø?øüÁüÁüÁüÁüÁü	þàþàþàþàþàþàþàþàþàþàþàOððððððððððð÷^×ÛÛËå²ÙlKKKgggñ¢ÁÁÁõë××ÖÖÞï½]]]ðððððWñåóù½÷ÆÄ®]»Ö­[W¼hÇ;wîùÍ3g4þâ8x½¢zóÍ7_|ñÅëºÙÅ?nÒúúú~þó´Ø=b'1iqÃqHúÍo~sôèQãváÂ_~Ù8¤ýýßÿø¯â6Zà¯¡¡!xB¡¹¹¹xQKKKü··bàïÉ'üqE=xðàu³ø©ý^0iGF2i±ÄNbÒâãô£ýÈµ¸ °#jqñÃR%Q§þ²ÙìÓÉÍà]]]Ý9sN>í´¯Ó¾Nû:íë´¯Ó¾Nû:íë´oÅÉdÒéÚÚÚE»wï³gÏ¶µµÁüÁüÁüÁüÁ_Å×ØØX(Þ9íÓ%Òéáþàþàþàþà¯"kooß³gOLÄÇ|>_¼hãÆÏ=÷9sfñâÅðððððWñuww755e2×ÓÓóoÏ­æ_]ÿ5k²Ùl[[ÛèïðððÆþàoðððgàþàþàþàþàþðððððððððððððððððððððððððððððððððððð?ø?ø?ø?ø?ø?ø?ø?ø?ø?ø?ø?ø?ø?ø?ø?ø?øüÁüÁüÁüÁüÁüÁüÁüÁüÁüÁüÁüÁüÁüÁüÁüÁüÁüÁüÁüÁüÁüÁüÁüÁüÁüÁüÁüÁüÁüÁüÁüÁüÁüÁüÁüÁü	þàþàþàþàþàOðððððð'ø?ø?ø?ø?ø?ø?ø?ø?ø?ø?ø?ø?ø?ø?ø?ø?ø?ø?ø?ø?øÞøzôÑG?òÔÕÕÍ?ß¾ðððððWµøûüç?òëøÛÿ`ûÚ¿pÏ=÷üùÿ9üÁüÁüÁüÁüU!þN8ñÁ>ôûü¡Àºººø?ø?ø?ø?ø«6ü=óÌ3üä'Sù%ÿî»ï¾¾¾>ø?ø?ø?ø?ø«6ü=ûì³XúüÝûáã¡àþàþàþàþà¯ÚðwáÂ|àþ×T~Ëîî?ø?ø?ø?ø¿jÃ_ôíoûî»ïþô§?ýö/,]ºtæÌ]]]wÎ?ø?ø?ø?Áß»¿(tÑÑÑqÿý÷oÜ¸ñÎ9áðððð·wrðððð'ø?ø?ø?ø?ø?ø?ø?ø?ø?ø?ø?ø?ø?ø?ø?ø?ø?ø?ø?ø?ø?ø?ø?ø?ø?ø?ø?ø?ø?ø?ø?ø?ø?ø?ø?ø?øó	ððððð'ø?ø?ø?ø?ø?øüÁüÁüÁüÁüÁüÁüÁüÁüÁüÁüÁàþàþàþàþàþàþàþàþàþàþàþàþàþàþàþàþàþàþàþàþàþàþàþàþàþàþàþàþàþàþàþàþàþàþàþàþàOððððððððððð?ø?ø?ø?ø?ø?ãðððððwR½½½¹Í¶´´tvvy°«©©?ø?øüÁüÁ_5Ïç÷îÝ»víZ·n]ÉÒ¡¡¡ÖÖÖñð÷WõW¿®¨.^¼øÂ/üZ7ûÅ/~ñüÄ8¤½òÊ+ñãqHÝ#vã8¤.¼øâÆ!íç?ÿùK/½dÒ^~ùåÐpÅmö´À_CCÃððpLæææ¥=öØO<1þvïÞº¢½ðÐ¡C§u³'N=zÔ8¤ýd$ã»Gì$Æ!- q1IñRhØ8¤?~ü?ü¡qHûñ®¸ÍøËf³cNG.]jkk:íë´¯Ó¾NûÊi_§öuÚ·JðÉdÒéÚÚÚâEkÖ¬9vìØ¿>Uø?ø?ÁüÁüUÇÓhll,oöé÷ÿðððððWñµ··ïÙ³'&âc>û©zåþàþððWO£»»»©©)Éär¹1µðð'ø?ø?oòððð'ø?ø?ø?ø?ø?ø?ø?ø?ø?ø?ø?ø?ø?ø?ø?ø?ø?ø?ø?ø?ø?ø?ø?ø?ø?ø?ø?ø?ø?ø?ø?ø?ø?ø?ø?ø?øó	ððððð'ø?ø?ø?ø?ø?øüÁüÁüÁüÁüÁüÁüÁüÁüÁüÁüÁàþàþàþàþàþàþàþàþàþàþàþàþàþàþàþàþàþàþàþàþàþàþàþàþàþàþàþàþàþàþàþàþàþàþàþàþàOððððððððððð?ø?ø?ø?ø?ø?ãðððððððððððððððððððððððððððððððððððððððððð'ø?ø?ø×ðWs«2üÁüÁüÁüÁüU	þ2·*ÍÂüÁüÁüÁüÁ_à¯:?ø?ø?ø3ðïBýýý6l?ø?ø?ø?ø¿*Ä_sss6õ;ððððÕ¿¾Ú£¡¡a``þàþàþàþàþªµµµ¡½«W¯655ÅDïûßÿ~LtttÀüÁüÁüÁüÁ_µá/y©/&B1qñâÅááá1cüÁüÁüÁüÁüUþfÎÔëîîï71ñðÃ'Þêþàþàþàþà¯ñ·uëÖôòâ_û[¼x1üÁüÁüÁüÁüUþ¢o~ó³fÍ¶µµUÄó?ø?ø?ø3ðÓ(ø?ø?ø?ãððððð'ø¿yóæ%oøâMáþàþàþàþªsçÎ-_«áþàþàþàþªá¼`_^+îùÃüÁüÁüø¿ÉÕÐÐø«DùÁüÁüÁüÁüÁß¤ëííümÚ´éúõëðððððWåøfÏ]3*|ÀüÁüÁüÁüU!þæÌãø?ø?ø?ø¿é¿øüáþàþàþüÁßäjlltÁüÁüÁüÁüÁßtÁ_WWWàoëÖ­ðððððWåø«'|ÀüÁüÁüÁüU!þ2ãäø?ø?ø?ø¿*Ä_Eðððgàþ&Wssó9sbtÞÇ§ÑÛÛËå²ÙlKKKgggñ¢ÖÖÖX´`Á¸üÁüÁüÁüÁü½£ÂU55ïó+ù|~ïÞ½1±k×®uëÖ/;wîñãÇcbß¾óæÍ?ø?ø?ø?ø¿wTgggàïGéïï¿Þð¥¡¡!ùÔB¡¹¹y¼»ÕÕÕÆß_üÅ_ü¢¢êëë;tèÐ/t³W^y%vBãÖ=qHÝ#vã8¤ýìg6iñÃÒ~ô#ãöÒK/?~¼â6û¶ãïN¸Ú·øâñ.4º££c4þî¹Õ¹sç^xáºY»»ººCZOOÏË/¿lÒb÷Ä8¤Å$#Æ!éìÙ³?ãvúôéüä'Æ!-ä~¨¸Í¾íø»®ö-fmmíè;~=Ï8íë´¯Ó¾Nû:íë´¯Ó¾Nû:í[ñ566ä´oLÞ×¯_åÊÑ+ÂüÁüÁüø¿Ê«½½Ï=1óù|ñ¢®®®+VzuÌáþàþàþüÁß¤++W®¬«««©©1cÆ5kÞã+?º»»2L.ëééù·ç6rrsssño"ÂüÁüÁüÁüÁß;ýÊóøS¿ðððÆþàorÍ??¨·jÕªë×¯ÇÍþþþÕ«WÇÂüÁüÁüÁüÁ_µá¯¶¶6¨744Î)1gÌ«náþàþàþàþà¯²ñÉdzÉÅ¶I1ç½|«ø?ãððð÷á/9í»bÅä´o|éÓÚÚððððÕ¿ÐÞ|vþàþàþàþàþªÉÏ5kêëë3L|bEÌ©çðððgàþ¦QðððÆþàþàþàþàþàOðW²æ­Êd2ðððððW%øËüÁüÁüÁüÁüUþÆkË-	þöíÛððððU¿ÞÞÞ3fû/_^üÏðððððWmø[»vmòß¡C*èùÃüÁüÁüø¿ÉõüóÏ'ì[µjUÅ=ø?ø?ø?ãð7¯&×vtvvVâó?ø?ø?ø3ðêÉ'L^ð[·n]å>ø?ø?ø?ãð7±5½ÏüÁüÁüÁüÁßôÁ_æVe³Yø?ø?ø?ø?ø«üUGðððÆþàþàþàþàþàOðððððððððððððððððððð¿yóæÕÖÖz?ø?ø?ø?ø¿êÇßÜ¹sÁçþàþàþàþàþªá¼`_^+îùÃüÁüÁüø¿ÉÕÐÐø«DùÁüÁüÁüÁüÁß¤ëííümÚ´éúõëðððððWåøfÏ]3*|ÀüÁüÁüÁüU!þæÌãø?ø?ø?ø¿é¿øüáþàþàþüÁßäjlltÁüÁüÁüÁüÁßtÁ_WWWàoëÖ­ðððððWåø«'|ÀüÁüÁüÁüU!þ2ãäø?ø?ø?ø¿*Ä_EðððgàþàþàþàþàþãW(V®cÆ5kÖTÊÅ¿ðððÆþàoÒ_9c^ðQÿÂüÁüÁüø¿É5þü ÞªU«¿íÛßß¿zõê³páBø?ø?ø?ø?ø«6üÕÖÖõÒ9B!æÄ|ø?ø?ø?ø?ø«6üe2 ^/388s¼ÕüÁüÁüÁüÁ_â/9í»bÅä´o|éÓÚÚððððÕ¿ÐÞ|vþàþàþàþàþªÉÏ5kêëë3L|bEÌ©çðððgàþ¦QðððÆþàor577Ï3'FþàþàþàþàþªÙl¶¦¦R_A?ø?ø?ø3ð«³³3ð÷È#ô÷÷WÊ_u?ø?ø?ÁüÁßTb2üÁüÁüÁüÁüUþ2ãäMáþàþàþàþªsæÌiii¨èçðððgàþ&Tò'ß9í[gxáþàþðð7uü%Ò7ù3¾ðððððWåøËår5esÁüÁüÁüÁüÁ_õà/§3gÎL^ÿK¨çø?ø?ø?ø¿ªÅ_Z¥8þàþàOððþ¶/üÁüÁüÁàþÞ¿zs¹miiéìì,¿hjsàþàþàÏ8ÀüÁßR>ß»woLìÚµkÝºuåMmNq[·nmjjº·¢úð?|Ï=÷Ü«ýîïþîïüÎï´dÒb÷Ä8¤Å$#ÆÁÕu"år¹J<¢Vþ?Ë/Úâ¶lÙR#ITEÝvü=ö]Ä_ñµ#%×^4µ9ð'Iàï9yÖÖÖóçÏ¿óG+~wÀÚÚÚò¦6§¸?üÃ?Ü¾ûÿ­¨öÙ­[·þ_ÝìÏþìÏ¾ño´oë[?þø4|â_ûÚ×>øÁÞu×]uuuþðÓAÝ#v;FZ@â0bvïÞýÐC´oûÛ>ú¨qHÛ¹sçO<QqÛñìK¥Íf.]záÂ)?Zccc¡PHÎÒÆtùESã|¸à£ú/ä|àëÖ­ÛöõmñïÓÿï§^ýu|¸àÃ.øpÁÇíú¿³gÏÎ??U`mmíÊ+§ð8íííöìøÏçË/Úø?ø«¾>þñ¯ýk·ÿÁöôß^ü·mÛðð·IÃÃÃª«««êwëîînjjus¹Ï¿måÈÙëÑ¦6þàþª¯ú»ê·[1þ¾Ðþûï¿þàþàþnþúúú_ù«¯¯ïèè¸ó?üÁüUG³gÏþÊ¿R¿ÿ¶ê¿þó?ø?ø¿wÅæ«««[·nÝ¥K*åùÃüÁ_uôû¿ÿûÿqþL_üû?ÿÏ>ô¡¿þë¿?ø?ø¿wÉu÷ßÿÅ+îùÃüÁ_u488ø¹ÏîüÄ'>±dÉ3f|ë[ßJÁüÁüÁß»¿wå^àþàþÞy'NøÎw¾óÔSOÅñ:	ððï2þ*:ø?ø«úàþàþàïÝÇß3gæÍ7cÆäßÆÆÆýû÷ÃüÁüÁüÁüÁ_â/#é	þé]»vÁüÁüÁüÁüÁ_µá¯©©)¨wæÌ===É»½ÀüÁüÁüÁüÁ_µá/ýÂ)þKáþàþàþàþà¯Úð×ØØÔK^íüz(¦áþàþàþàþà¯Úð×ÕÕU3VÇ?ø?ø?ø?ø¿jÃ_tåÊ¶¶¶äjßºººyóæ]¸p¡"?üÁüÁüÁq?øFÁüÁüÁüø?ø?ø?ø?ø?ÁßX]ºt©µµµ¾¾>3RL´´´TÊ9_ø?ø?ø?ø?øD>ø`Í8mØ°þàþàþàþàþªû÷ïO÷ôÓO¤_EHæ>|þàþàþàþàþª¹·÷î1,Þçþàþàþàþà¯ªðW[[Â/ÑB,;ÀüÁüÁüÁüÁ_à/ý«nã-MþÔüÁüÁüÁüÁüU	þÊðþàþàþàþàþàþàþàþàþàþ*å?ø?ø?ø?ø¿êÁ_æVe³Yø?ø?ø?ø?ø«üUGðððÆþàþàþàþàþàOððððððððððððððððððððððððððððððððððððð'ø?ø?ø?ø?ø?ø?ø?ø?ø?ø?ø?ø?ø?ø?ø?ø?ø?ÁüÁüÁüÁüÁüÁüÁüÁüÁüÁüÁüÁüÁüÁüÁüÁüÁüÁüÁüÁüÁüÁüÁüÁüÁüÁüÁüÁüÁüÁüÁüÁüÁüÁüÁüÁüÁàþàþàþàþàþððððð?ø?ø?ø?ø?ø?ø?ø?ø?ø?ø?ø?ø?ø?ø?ø?ø?ø?ø?ø?ø?ø?ø?ø?ø?ø?ø?ø?ø?ø?ø?ø?ø?ø?ø?ø?ø?ø?ø?ø?ø?ø?ø?ø?ø?ø?ø?ø?øüÁüÁüÁüÁüÁüÁüÁüÁüÁüÁüÁàþàþàþàþàþàþîÀzs¹miiéìì,^ÔÓÓÓÚÚ,Xw?ø?ø?ø?ø¿/ÏïÝ»7&víÚµnÝºâEsçÎ=~üxLìÛ·oÞ¼yðððððWñ544ÇD¡Phnnïnuuu£ñ÷øã®´þæoþæ°nvèÐ¡´¿É8¤Åî;qp³^xÁ¤äêRrD­ÄÈ´À_6sºä§ÿÑøëêêúmE500?¸ÿV7S_~ùeãöË_þòìÙ³Æ!-vØICZ@â0b®_¿~ôèQãöë_ÿúW^1igÎ9wîmö´À_&I§kkkGß!¾¼óù|ïöuÚ×i_§öuÚ×i_§ö­ÌM¿YL766ä´oLÞ×¯_åÊÑðððgàþ*¯ööö=öÄD|ÌçóÅºººV¬XqõêÕ1W?ø?ø?ø3ðWwwwSSS&Éår===ÿöÜF^lnn®)þîØÞzë­þô§¯¿þ:üÁüÁüÁüÁßíþî6nÜ8sæÌßû½ß»ûî»/^üæoÂüÁüÁüÁüÁ_uâ/äwß÷mË×¶ÿÁöm_ß¶hÑ¢O~òðððððWøë­·îºë®D~É¿ðß=÷ÜóÓþþàþàþàþàþª÷Ýw_*¿äßÇÿÿà??ø?ø?ø?ø¿jÃßo¾ÙØØ¸íëÛñ7wîÜ'NÀüÁüÁüÁüÁ_þÎßüT~ÿõÿÐ>ôÖ[oÁüÁüÁüÁüÁ_â/¾1Ï7ï¾ûî[¶lÙÇZ?6kÖ¬¾¾¾I­ððððÔÐÐÐÁÿøÿøßûÞdßêþàþàþàþàþ¦Qðððððð§ÊÃß'-[VWWËå:::àþàþàþàþà¯:ñò»ë®»þëgÿë¶¯oûê¦¯þ§¶ÿôÑÞÿÁüÁüÁüÁüÁßûß²eËB~Åïyô|äÙg?ø?ø?ø?ø¿*Ä_ý]õ%oxùÏ|æË_þ2üÁüÁüÁüÁüU!þfÏý¿7þïbüêSúÆ7¾ððððU¿¯~õ«ýèGÓÿ¾òå¯Ü÷ÝÿS7ððððª$ü|ìcûsþÃg>óOêS!¿;wÞOððððwDßûÞ÷¾ò¯<üðÃ·ã5?ø?ø?ø?ø?øvÁüÁüÁüÁüÁüÁàþàþàþàþàþàþàþàþàþàþàþàþàþàþàþàþàþàþàþàþàþàþàþàþàþàþàþàþàþàþàþàþàþàþàþàþàOððððððððððð?ø?ø?ø?ø?ø?ãððððð'ø?ø?ø?ø?ø?ø?ø?ø?ø?ø?ø?ø?ø?ø?ø?ø?ø?ø?ø?ø?ø?ø?ø?ø?ø?ø?ø?ø?ø?ø?ø?ø?ø?ø?ø?ø?øüÁüÁüÁüÁüÁüÁüÁüÁüÁüÁüÁüÁüÁüÁüÁüÁüÁàþàþàþàþàþàþàþàþàþàþàþàþàþàþàþàþàþàþàþàþàþàþàþàþàþàþàþàþàþàþàþàþàþàþàþàþàOððððð?ø?ø?ø?ø?ø?ÁüÁüÁüÁüÁüÁüÁüÁüÁüÁüÁüÁüÁüÁüÁüÁüÁüÁüÁüÁüÁüÁüÁüÁüÁüÁüÁüÁüÁüÁüÁüÁüÁüÁüÁüÁüÁüÁüÁüÁüÁüÁß¿ÖÛÛËå²ÙlKKKggç»ø?ø?ÁüÁüUCù|~ïÞ½1±k×®uëÖ,jmmñe£¢zë­·^|ñÅºÙ¥KN8aÒÎ9ó_üÂ8¤Åî;qHHFCÒµk×=jÒ.^¼?/´ýìg¿üå/+n³§þc¢P(477,ì±Çxâñð÷'ò'?¬¨â8uðàÁêfG9tèqH;<qHÝ#vã8GTGÔ	Qãç¥Ûìi¿l6;æt?ñ·µµöuÚ×i_§å´¯Ó¾Nû:í[%øËd2étmmmñ¢5kÖ;vì_*üÁüÁàþàþ*xÓoÓBáíÓ¾1=æÝÒ;ÃüÁüÁq?ø¿Ê®½½Ï=1óùüxX=þàþàþàÏ8ÀüU^ÝÝÝMMML&Ëõôô©=ø?ø?ÁüÁüygø?ø?ø?ÁüÁüÁüÁüÁüÁüÁüÁüÁüÁüÁüÁüÁüÁüÁüÁüÁüÁüÁüÁüÁüÁüÁüÁüÁüÁüÁüÁüÁüÁüÁüÁüÁüÁüÁüÁüÁ/Hø?ø?ø?ø?ø?ÁüÁüÁüÁüÁüÁàþàþàþàþàþàþàþ¦W>úè~ð_UTçÏß»wï¯t³S§N8pÀ8¤uuuýøÇ?6i±ÄNbÒâã?,ýå_þ¥qHåWþöoÿÖ8¤uvv¾ôÒK·Ùo½õüÛ3g¶oßþG$IÕRù³5^Ñ$I>Á$IüI$	þ$I$I?I$Á$IàO¥]½zµ¹¹9½ÙÓÓÓÚÚÍf,XÐÛÛ[|Ï®®®ùóçÇ¢îîîi2 gÎY¸páÏ:Æ'Ë%:;;§ùhÙs¦ç$9r¤¦¦fú@ÊÈàààúõëkkkï½÷Þ8éyD-óu1¨eF£:¨ðwG8úïvsçÎ=~üxLìÛ·oÞ¼yÅwnhh¸téRLÄÇÑ»oµH81,1ÅwÎçó÷î]»v­[·nF=gzH444ï*Æß¤dÇ;wîôÌ3ÇLÏ#j¯ixD-3ÕqD¿;´+W;wn¼oNuuuÅ7ãxùòå·Gþ&cµ»ËÈáÃã'°cw|'B¡PÇîIF=gÚÈc=öÄOT1þ&5 Á W_uÚQGÈ´=¢÷u1=¨9JTîþîìÿ±v»'OvttÏéíí]0îcéôøÉ¬¾¾>fÆO`Åó³ÙìÓÓs4Êì9Ós@.]ºÔÖÖßÌªû´ï¤¾^|òÉ8ztN>m@¦çµÌ×Å4<¢Nä(QÑGTø«0ü]¿~=ÏÏpaò³-Z4Ý4|äÈÆÆÆâ9L&®­­æ£QfÏ²fÍcÇwÿéùõ²÷î8öl|Ã3 ÓóZæëbQoy¨ô#*üUÒ¡êòåËë×¯¿råÊôü±¬Ì×aÉ³Cy¡Pxä$ÅV£QfÏRóï3 Å_# ÓöZæëbQË%ªà³/vuu­X±âêÕ«£ï?9s&&úúúâgÖi2 sçÎMuOOOLñÝÚÛÛ÷ìÙñ1~8æ£QfÏrKL·Ù¸qãsÏ=÷öÈ°/6 ÓóZfæ4<¢YGTø«±¹¹yô"ÉÄùóç÷,1=M¤···¥¥%õ²eËÒÀ;tww755e2Göi>cî9Óy@¦'þÊHÿ5kbQ[[Û¹sçÈô<¢9sÚQËFuQáO$i$Ið'I$ø$IüI$	þ$I$I?I$Á$IàO$Ið'I$ø$IüI$Á$IàO$Ið'I$ø$IüI$	þ$Itë¾ûÝï.Y²¤n¤eË=ÿüóÿî7RÅÇÚÚæææxj%ócN6ÍårÃÃÃLIð'IÙöíÛkFõÍo~³ð÷È#ÄÌ§zªdþw¾óÿðÃOá1%Á$U^½½½Ál6ûôÓO´÷î¸3O:U5ø»xñbÌ`AÉüùóçÇü.À$ø4-úâ¿¬Ù¹sgñÌÇ<f~éK_*¦Ogggà)ÚÚÓé¯]»ÖÑÑQ__6oÞvõèÑ£¬Xë>|¸S1gæÌK,9tèPÜloo/Ù°dQlR²èÈ#ãAmÅ1¿§§'süøñ³|ùòtÎ;â¡êêêÖ®]ùòåÑøýø%sÊlª$ø¤÷¹Ù³g]ºT<óµ×^ÍÍÍÅ¸)éäÉÉÒÕ«W,Ú´iS²¨¯¯/É¹Vr3YºfÍáááàcmmíÐÐP,§3f$¿WæqbbÌÍýL÷íÛW,ÚÔ÷îMåWò Ë-,þÊlª$ø¤÷¿äïÇµ X1nÖ­[w¤«V­J&ÖIø¼hKåóùä%ÃîîîN¤ø1·mÛ¼KÎº>øà1çÀ1cú¸åããfÜ¡xÛÆ|FÊY³fÅó½zõjÜ±å!ÎB¡Ü!ËÅñÞ¾y¸®®n²ø+³©àOîüEcâ/à&1Stùòå¸lJn¶¶¶ÆÍ9sælØ°!ÐvãÆôAâ>%¯¥¥k%7_íµôÎ¦ôÌo|é£GÞòqqó7Þ(Þ¶ñ~?oË-±è'xûæ©íÍ7ß! ø¶µµ%/LNe6UüIÒû_òr×õë×gÄÌXT;)Ï;ø/µNúz£ÏÆpªøVbº¡¡¡¶¶vpp0|æÌéÒ[>Îx+éÌ3Sc:>Æt___º´»»;6`ÌÓÇÇ_MôþüÞÛO>Y<3y>Òß¼zõêè´N:õÈ#$'aS5&/Ë¥çUoI´M6%gãcGGG:¿Ìã$¯´]¹r%¹ùÆo¿2wÑ¢E±4yw0kñ¢äÊßXtäÈþþþòøKaFº´Ì¦J?IzÿK.Èf³»víJÞêåé§®­­qÆªU«:É9ÙôÊÜäwþ_;wî/&Ü¼ysP)¹7³1 |ö®®®t~ÇI~É/ù¿äeð·÷îô5¹xÊÅ_ìëëÏ0þnãnÎµk×/-³©àOî7@.iÇÿÿ1n¤ÄFétzÂ4½Æ"íÁ,eqû÷ï/¿·GþGÌohh(>#qb37,½Òv¼'ãÆºººä)ì^¹reñ§5kV|LÞí¥ø1&Óª¦KËlª$ø¤;¥Ê²eËjGZ²dIrÉm	þ9¼^KKË±cÇÒ¥=ôPSSS¦-[¶¦K>ÜÖÖ&ËårÏ<óLÉcÞx¨¿qãÆùã=N¼a÷ùKëèè(yÏ¤«W¯æóùúúúx.ß¶¦ø1ûûûC·ÉX-_¾¼§§§ä3ÙTIð'I$ø$IüI$	þ$I$I?I$Á$IàO$Ið'I$I?I$Á$IàO$Ið'I$ø$IüI$	þ$IônôÿÊ6IEND®B`


FÒë¯¿ÏçíeîJ59xð`LBB5>µùpZf¨ÍÓ;Þ)ªãüîúÿ¡ÌïdÅ<5557ÆOÄtFòô¶yóæë×¯×æ§d@ÊÜjöþRË %£Q§eöÚ|8-§¨GTò«$Ì_L/ñkÃÂ/_¾Ü	×¬YS¼hãÆø¿ÕøhôôôÄ7oÖÔÝ¤Ì¹+ÕælÛ¶íèÑ£1#Ë/¯ñÑ¨ÍÓ2û@>jzD%¿_2ýÒK/Å#Tü^_cºFÆ¡¿¿?ùð+W&ïÈN¤···¹¹9Éär¹¾¾¾|«ÌÔ¦üÊÈÐÐPgggò¦·K.ÕøhÔæÃé¤û@Í>jzD%?I¤Zü$IÈO$Iä'I$ò$IùI$ü$ID~$I"?I$$IÈO$ü$ID~$I"?I$$IÈO$Iä'I$ò$IùI$ü$ÕP_ÿú×W¬XÑ0ÞªU«yæx¯b'»¶---qÓKæÇl6ËåÆÆÆfºMIä'I×®]»ê&ô¥/©ä·gÏùÕ¯~µdþW¾ò¿÷îYlSùIR500¦Éf³ïàÁq1f;w®jäwåÊ¹dÉù/ù/_&?Iä'©úûÌg>¦yì±Çg>þøã1óþûï/vOwwwÈ)PØÞÞÓé7ßºukË-sçÎE;vì(>¨zæÌÐU,uO:Ub©3oÞ¼+V<y2.nÜ¸±ä8q¢üvEqE§OJikÖ¬ùé^x!æ¬^½:³wïÞæææØTCCÃ®]»6Q~·_2§ÌUD~ôN¶`ÁPËÕ«Wg¾òÊ+1³¥¥¥X6%õ÷÷'K×¯__²hûöíÉ¢ÁÁÁL&3éZÉÅdiggçØØXØ±¾¾~tt4Æ×Ó9s·ßÙNLLzõ&ÞÒ#Gs6ÅåáÃSöldÕªU3_«*ü$é.9°;ÉZ]]8¬X6]]]¯qqÝºuÉÒ:îBlÉ¢|>¼XÓ½½½ÉF·ùÈ#í­>ø`Ì9~üxLÇ×Þ´iÓ·jñÅ×mÒ[?~ÜÞ7oÆÅø×<¸Y(oÈår±bÜ7Þ<:ÜÐÐ0Sù¹ªÈOÞyùEÊ/È&StíÚµ¸fJ.¶··ÇÅÖÖÖ­[·Ønß¾n$¾§äU´t­äâ+¯¼~sh)=à_cúÌ3wÜN(3.Þ¸q£øºMõ¼z(=ñÄo¼yDÇÅßùG;::$g*¿2WUùIÒ;B×ë¯¿^<sxx8fÆ¢2ÖI]xéÒ¥)tÒ7çM<[b©âÏRéÆÆÆúúúØø¼yóÒ¥wÜÎT+éÂ	Rc:¾Æôàà`º´··7®À¤G§/¿2WUùIÒ;^·ûöÏL>ë¤äô½7oÞøRÖ¹sçöìÙMÉ¼ N½£Ï¶oßä¯[¶lIçÙNòÛõë×7nÜ(î²eËbiòA6ÖâEÉy¾±èôéÓCCCååª4ti«*ü$é.9C"Íîß¿?ùTÔ××O<cÝºu¡äPlznò>¿äíq.]*~`Á;v³nÓÏUÔgÉÉ¶ÉOïééIçÙNòÆ¾ä~ÃÃÃÉwßÁÓWãâ&/JÞò888?%a*ù%Ö+Ç·87lØP¼´ÌUD~ôÎ|ÊqI÷îýÿpã%0J§Óã¤éIi>ø`1+;vìXù½1þÇ6b~cccñà2Û«Q|ÅÒój§º±·oßnhhHnBÉ1îµk×ÿùóçÇ×ä]·¼$85]ZæªJ"?Iº+¬Zµª~¼+V$'ØÈïôéÓÉÇæµµµ=6]:<<üðÃ777'Zzè¡FFFÒ¥§Nêèèår¹'|²d¯Il*æoÛ¶­dþTÛâÊÄUJ>h°Ìçù¥mÙ²¥äã]nÞ¼ÏçcæÎ·âòåËé'Ôoshh(hÕêÕ«ûúúJ~b«*ü$ID~$I"?I$$IÈO$Iä'I$ò$IùI$$IÈO$Iä'I$ò$IùI$ü$ID~$I"?I$$I&ôÿ0îaB8gIEND®B`


ôS©ÔÀÀ@Î-³o4&Üi¸ëð/_~óæÍ3gÎ466&U«Veï_Ü³gOMMMtX3ÜEfÒdøsûì5¥F¶µµM¸§;Ëø»±Æ9­3á>¹l31ùåLÛ%¼BÂjÿä'?Ömþ½ã§Nñe5¬ÉÓaRUUÕÖ­[ü`åT~!VÂÈÚÚÚèj___,Ë~?îééÉS~Ñ×­[gÞÌÃsöÔìU×Ë³Øü3î·oßN&ÕÕÕa`ü:¹~ýzx;Ï^N¸eN¢e?ßñ÷»dÉhj$TH&ûrn¹bÅü³`Á0þý÷ß¿±.áöî,9w²&ÚçW__?­åÏxÆ©_oooörÂnùMñe¼víÚÇ¼eË?X@ùA!_4>4Y4J¥ÂÕ®®®0|öìÙì>Ë=ººsçÎÑÑÑh/Îçoá-?Ü>ÚÑ")ÞqÃÕ0û1a®ìûÊ³Øü3NøÜ?½,ûö6mÃkÖ¬¹5&«aädÏ7³yóæèEKW;::²`ôi¶'Oá+W®dOìÇãñÉHfÞ8Ëd¤ËìîÊðQÍxÆüM5ûe>ÓgS/¿)¾£"¼víZ[3WTTøÁÊ¯ü2ø¢ecòßõë×3cî;ofÿYtT1¼FWÃÛgöÎª£uyÆÉáM=g|uuuö3oðáêüùó'¾ÑPÙW£CØÑÌ~$ét:´B(æææìç§ü29>Y¦ÀY²Wi7¬Ì+W:ujÂ63á£ñS/¿èróæÍì»VùMñeÜÔÔ®ÖÕÕr¿BÜ½×OP~P`åJ%¬ªªìív²F®ÄÉóV§o¦ûÞ<ÅN6><åð|Ãû÷ððpöøñûÌÆèmüóÊÕP9G<ïjMMMtxz|Þåôtf	Se*ëjÂLeåLÖ£3qêG?ø«k/ãþþþ(þ2ExâÄ	?X@ùA!ßÁ³Ïðö¢åe9ÐÖ¼ù÷Ù¼ÿþûÙSó,6ÿyÀÑ£GÃÕÌw D#£8ËÙ;ÓÄS_!ÙW¢oWéììe6òvLF_°J¥¶nÝzëÖ­Cë×¯°ü¦2ËËoº+'cÆ3N½ü¦²Ï/ÓvÑ¯:yÖ×[äüùó»wï1gÎÌõòïm!¢7æÌ·ºDïg!Â;etªì¢E¢IÑÆLøgÞüÙ%Kô9­°ðh99âp±ùgÌk×®Í|?ù»fÍÛc¢¥mÞ¼ù_´ß«¯¯/<L¥üN>íUOùìÙ³Ñþ¼ÈñãÇ'Ü¾ÓeZååä¼6²Mw­Î ü¢OæÃÝ»wCØ­^½z|¾ðÂaå:ýß÷¿@ôyÍþþþ<GÞås¥üÆË>?qüÙ²Ïûg¾t7ÚA8þ-3Ï¼ù³)$QÔFÙ'Zf¦æYlþógÄ72èÆä|Æ«ºº:ó!ÂR~Ì??ó©Ç<³aÃñkñâÅy6ñ´fVùå_99¯lÓ]«3(¿óçÏg/Ù²eÙSsÖIæóÉî%Ïë-sQÆSO=å(?(òo+V¬ÿ×½:::CE·ófÆ?~<	¥ýmvS÷¾Ù^XxSSÓø¯ål±÷1FDGB³Ç_¾|yýúõ1©Tjüù3+¿ÁÁÁ°´°ÌÐ@Û¶m1	îüóâ/gø>ü©O*³ÏéÏ2­òË¿rr^9¦µVgP~Á'êêêÂ³Ë§gNgWCE÷¾råÊîîîûîsìõ644´cÇhgjÈ÷°)ý`åð@¤Óé6Lë;äf0KÁöõF'kDAßÐÐàÊbÎ/ÇsÏ=gÍò Ømß¾ÁÑ!Ý0ðôÓO[- üP~(?ÊåòP~(?Êåò@ù ü¹æøñãµµµñx|ÅÓ·±±±¬¬¬¿¿?3&1-Æ³13»ÍTæáÙ¼÷Äb±òòòT*588MÚ²eKUUUXaÒ;ï¼ã(?`©©©	òedddºóîÙ³'ÌûüóÏgÆìÝ»7Ù½wq_4xnii®nÜ¸1êêÃgÎ	Ã.ô0~|x:þ|wùòå1K,	czg¿ÃfÿC+áòòòèj"Woß¾íE(?`îf_vÍìØ±£²²²¢¢b×®]97ûÔ§>ÕÜÜ³Ì.Ã0üþûïá0&tíÚµ+W0ÇãMMMÑÎ°ñKË¾÷ü³>|¸¶¶véÒ¥áÆwØ;wZ[[Ã¼áÁoß¾=ÚÚtÙ²eaia|Xòõë×=þøãájCCÃÙ³g½´åÌÝøöÙ0|ðàÁÐXaà¹çË¾MGGGæ3m6m:Ãenkk&-^¼øäÉaàÊ+a|2piÙ ÿ,¡ç=6nÜ8þÁoÙ²%¼úê«a`Ï=»ûv___vI7òë'þó&?~<³À+VpÁ«P~ÀÜ-¿ÚÚÚ03>¼&ü àéÓ§Ã¤5kÖáµk×FIzþüù;w677G§DL¸´ÆÊ?KôÀ*++ÇÏ[]]=øÐda 4_nBËÏlµ=¶°ðÙwzzB¼fnîÔP~À-¿x<ájþº7¶ë«¢¢"Ürhh(:¨ÆD8æ9xáÂì%ä,-ûêTfìËÞUã±cÇæÍ©ªªv~(ûüòèêê­à@ùs®üÉdö>¿h·Ùs§µµ5LÍÆGg<bùå%óÀBÌwþüùÑ¼9oddääÉÑ·ÓZ-Ó-¿ záéxÊ£åKË¡C¢ÏùíÝ»w*¹sìØ±ÌÎ³£GfÆGyñâÅèóS)¿ü³ìÜ¹óßüfØ´iÓøyzê©0ÜÞÞ~éÒ¥èo¹téÒ0|áÂ7oúúúW~-:;;Ãð'Âð¼ÀåÌÑò	uU1&û;ùòßÝ»w£#­á2ûCo¡~ªªª*++·oß>ÅòË?Ë©S§ÂÔ%KdN4É¾ÍÐÐÐÖ­[Ã#O$«W¯NãÁJ¥¢/_~ùòåi­ñGó¬p­­­áÁõ0þü-[¶ä(?Ê@ù üP~(¿ñíoûêÕ«³yétÚ+¯4ýèG?úÿùë¡4]¹reZö"Ûúÿýßÿm=¦wßwNmýR/¿¯ík!þfóO>ý_ÿõ_þ'¦ø¸sçõP¾õ­oýçþ§õPÂMöWTRRÎ=ûÿñÊOù¡üP~(?òS~(?Êå§üÊåòCù)?åòCù¡üP~ÊOù¡üP~(?òS~(?Êå§üÊåòCù)?åòCù¡üP~ÊOù¡üP~(?òS~(?òS~ÊOù)?ÊåòS~ÊåòCù¡üòCù¡üP~(?å§üP~(?ÊOù)?Êåò+ªòëííM&ñx¼±±±««kü:;;ËÊÊÊåòCù¼T*ÕÞÞöïßßÒÒ3udd¤©©i²ò;yòä­Yôæo^¿~ý%)üZòoÿöoÖCizë­·lý~ç¿zõªõPÂïüï¾ûîlÞcI_UUÕèèhH§Óµµµ9SöÙûöMV~!¿3Bh9sæ;$[¿Ä·þ·¾õ-ë¡4½þúë¶¾­?kJ¢üâñøÃÁµk×C:Ú£½8Ú£½8Ú[b±Xf8HdOZ·nÝ3gþï©*?Êåò+ÕÕÕétúÞØÑÞ0üÏð)?Êåò+l­­­á2JMüTíóCù¡üP~(¿âX­555±X,LvwwOzÊåòCù¡üJòCù¡üP~(?å§üP~(?ÊOù)?ÊåòS~ÊåòCù¡üòCù)?å§üP~ÊOù¡üP~(?òS~(?Êå§üÊåòCù)?åòCù¡üP~ÊOù¡üP~(?òS~(?Êå§üÊåòCù)?åòCù¡üP~ÊOù¡üP~(?òS~(?å§üÊOù)?ÊåòS~ÊåòCù¡üòCù¡üP~(?å§üP~(?ÊOù)?ÊåòS~ÊåòCù¡üòCù¡üP~(?å§üP~(?ÊOù)?ÊåòS~Êå§ü¬åÇñüDù)?åòCù¡üÜ¿üåÊÊÊêêêyóæýñÿñÈÈòS~ÊåòCù¡¯|å+=öØ¦?Ú´ëË»þdË444|æ3Q~ÊOù¡üP~(¿b344T^^þ¥Í_ÙýÛñç;yäþþ~å§üÊåò+*õõõìþâxíµ×òS~(?Ê¯¨¼÷Þµµµ;ÿbgvùìcoÍÊOù)?ÊåWl~ç¿É¾Gôç«üòS~(?Gÿ#<òñæ?ùäK.Ù÷@wø)?å§üòS~(?¦÷ÞïùçokkÛ»wïÕ«WôÝ)?å§üÊåG©P~ÊOù)?Êå§üÊåòCù)?åòCù¡üP~ÊOù¡üP~(?òS~(?Êå§üÊå÷Áýä'?±eÊOù)?Å íííýèG«ªªæÍ÷Û¿ýÛ?þñm_åòS~ÊåGß×¿þõGô7üá®/ïÚñç;XöD2| üJù¡üòCù¡üGy$Ê¾ì?xÿüóÏÛÄÊå§üÊ¢*¿þô§UUUÙÙþ=ùä_üâmbåòS~ÊåGQ_ðK¿ôK¶íÏ²Ëï·ÿÖ³Ï>k+?òS~(?­ü~ÿ÷ÿ7~cç_ì²ï¿0oÞ¼¾¾>Xù¡üòCùQlå744ôÄO,X°`ÅøÄ'>ò9rÄöU~(?å§üP~aùEN:õüóÏ<xp``ÀÆU~(?å§üP~sù¡üP~ÊOù¡üP~(?òS~(?å§üÊOù)?ÊåòS~ÊåòCù¡üòCù¡üP~(?å§üP~(?ÊOù)?ÊåòS~ÊåòCù¡üòCù¡üP~(?å§üP~(?ÊïáéííM&ñx¼±±±««+RwwwSSS´hÑ¢p3åòCù¡üP~-Jµ··ýû÷·´´dOZ¸pá¹sçÂÀ#GêëëÊåòCù¶ªªªÑÑÑ0N§kkk'»Yyyùøòû»¿û»EßûÞ÷þ¶~oo¯õPÞxã[¿duvvþÓ?ýõ`ëÏ(¿x<>áp¶¶¶¶ñå÷Ê+¯Eÿ÷ßßßtêÔ)[¿·þ¿üË¿X¥éÍ7ß¼|ù²õPºººÞ~ûíÙ¼Ç(¿X,N$ãopçÎT*544äh/öâh/öâhoa«®®N§ÓÑÑÞ03õÆ7n¼yóæøÊåòCùÖÖÖÃpJ¥rJkÕªUÎ¨üP~(?Ê¯ðVkMMM,K&ÝÝÝ?neÿ÷ìjkkË²(?Êåò+QÊåòCù¡üòCù¡üP~(?å§üP~(?ÊOù)?ÊåòS~Êå§üòCù)?åòCù¡üP~ÊOù¡üP~(?òS~(?Êå§üÊåòCù)?åòCù¡üP~ÊOù¡üP~(?òS~(?Êå§üÊåòCù)?åòCù¡üP~ÊOù¡üòS~(?å§üP~(?ÊOù)?ÊåòS~ÊåòCù¡üòCù¡üP~(?å§üP~(?ÊOù)?ÊåòS~ÊåòCù¡üòCù¡üP~(?å§üP~(?ÊOù)?ò³ÊOù)?ÊåòS~ÊåòCù¡üòCù¡üP~(?å§üP~(?ÊOù)?ÊåòS~ÊåòCù¡üòCù¡üP~(?å§üP~(?¥S~e÷ÅòCù¡üP~Ê¯Ê/v?ñx)?Êå§ü¡üòCù¡üP~(¿*¼Ä7mÚ¤üÊåòS~ÅV~µµµñxÜçüÊåò£ÈËoÑ¢EãOï¨ªªR~ÊåòCù)¿¢*¿D"Ropp°¦¦&àûæ7¿ÚÚÚòCù¡üP~Ê¯¨Ê/ÚÉBê+W®å§üP~(?ò+ªò7o^è¼ðÄúûûÃÀÓO?øVåòCù¡ü_±ßöíÛ3çsdÔoéÒ¥ÊOù¡üP~(?åWTå<óÌ3óçÏÝÝÝa T`sssAlåòCù¡üP~¥Bù¡üP~(?òS~(?Êå7úúúè»]|³òCù¡üP~sù-0»ö2Û«üP~(?ò+¶ò¯§§gtt´à¶òCù¡üP~(¿i¨ªªåWÙ§üP~(?ÊozzCùmÙ²¥ßÒÊåòCùMÏÊÆqòCù¡üP~Ê¯ØÊ¯®®ÎÊåòCùQå5____!nåòCù¡üP~ÓP]]íåòCù¡ü(ò)ÊoûöíÃÃÃÊOù¡üP~(?¹üÊ&áåòCù¡ü_~óá¡üP~(?ò+Âou)åòCù¡ü¦¡¶¶¶®®îÒ¥KÊOù¡üP~(?¼üâñxYÙ¶ï°··7Le666vuuå4³1Ù¾úÕ¯¾òÊ+WgÑÑ£Gß~ûí«¤°õÃïHÖCiúÆ7¾ñüÀz(Máæûßÿ¾õP;Ö××7÷øÀË/äT(¿Ý»w_h>øw»¤R©ööö0°ÿþüf6&Û¶mÛÊÅ/¿÷ÜÞªªª(ÓétmmmþI3£üå7Cî¹½Ùså,aü¤Q~ò²÷&üf6&ÛW¾ò_|ñÛ³è¯ÿú¯;;;¿MIzálýõõ¯ý7Þ°JSx£yýõ×­Òô7ó7³¼õ¬üª««Óéttp6ç4³1ÎíÅ¹½8·çöâÜÞQµzõêòòò²²²uëÖÍøTÖÖÖÃpJ¥òOÙåòCù¡üP~3t÷îÝ	3ÏìÏøTSSÅÉdww÷ÏåØAëñf6Fù¡üP~(?ß5442[³fMô^Ük×®c/^<÷·òCù¡üP~(¿iH$¡óFFF2cÒét3þtå§üòS~(?_a_,Ecfö­.ÊåòCù¡üænùEGW­Z½¥Ë0Æ455)?åòCù¡ü_Q_x'ð[·n)?åòCù¡ü_Qß½±Ó×­[WYYÅÂåªU«ÂØÊåòCù¡üJòCù¡üP~(?å§üP~(?Ê/3çýdÿÁ§üP~ÊOù)?__lrÊOù¡üP~(?îÂÑÞmÛ¶EåwäÈå§üP~(?ò+Îòëíí­¨¨Í·råÊì/vV~ÊåòS~Öò+ªò[¿~´«ïäÉ´=ÊåòCùMÃ«¯¾5ß5kn(?Êåò»wï.^¼8:£«««·òCù¡üP~(¿ûá¢]---»=ÊåòCùMaNßç§üP~(?%R~±ûÇãÊOù¡üP~(?åWåWÊåòCù)?åòCù¡üP~ÊOù¡üP~(?òS~(?Êå§üÊOù)?åòS~ÊåòCù¡ü«üêëëïóS~(?ßwðàÁ_ûµ_+//_¸pá_ýÕ_)?ßÜ*¿ð?3»ö|òCù¡üfìÀóçÏÿlëgwy×6~áWõW·nÝªüP~s¨üBäæëéé-¸í¡üP~(¿¹cxxø#ùHÛÛBöEÿþtëVTT(?ß¿ªªªP~ÊåòSúûû½þ×3ÙýûÍßüÍ'N(?ß¿ÞÞÞP~[¶l)Ä·4åòCùÍ,È)¿Eutt(?ß¿ üG-ÇÊåò®ÇìÓk>É¾?øüTVVþøÇ?V~(¿¹R~uuuÎðP~(?ß¢¯¯ïGùxóÇ|òÉ¥K>úè£§N*ô'¥ü_Q_Ô|áÿj!nåòCùÍ5ï½÷ÞóÏ?ßÖÖ¶wïÞ«W¯Á3R~Ê¯¨Ê¯ººÚÊåòCùQåR&ßöíÛòCù¡üP~sùMÂÊåòCù)¿"ü&ç	9ÃCù¡üP~(?åWßêR¸ÊåòCù)?åòCù¡üP~I§Ó«W¯.///++«¨¨X·n]¡ê«üP~(?ÊoîÞ½;áqª¯òCù¡üP~(¿ihhh·fÍè--¼¸×®]Æ,^¼Xù)?Êå§üªüDè¼Ìt:ÆñÊOù¡üP~(?åWTåÅBçÚËc|«òCù¡üP~Ê¯8ö®Zµ*zKa8ijjR~ÊåòCù)¿¢*¿ðN6á·nÝR~ÊåòCù)¿¢*¿c§÷®[·®²²2ËU«V1±=ÊåòCùåòCù¡üP~ÓP[[[WWwéÒ%å§üP~(?E^~ñx¼¬¬P÷*?Êåò®®®P~»wï/ëBù£mÊåòCù¡üf´IÄb1å§üP~(?ò+ªòMÂ79+?Êå§ü¤üêêêz(?Êåò»¿èÏõÞ;Ú[vÊåòCùÍPôçz£?Ñ«üÊåò£Ë/LåååòCù¡ü__OOÏ¼yó¢=Qç9ÃCù¡üP~(?³ü2%òÊåòCù4åòCù¡üP~ÊOù¡üP~(?ßÔÛÛL&ãñxcccWWWö¤îîî¦¦¦0iÑ¢EáfÊåòCù¡ü[*jooû÷ïoiiÉ´páÂsçÎ#GÔ××/¿×^íÆ,:uêÔÀÀÀJ­_ÊÞ|óÍþðÖCiêêêzçw¬ÒôÖ[oýë¿þëlÞã/¿Ë/?ôò«ªªétº¶¶v²/¿~o½þúëÿøÿø=JRGGGww·õ`ëSjÞxãï~÷»Ö­?;xùE_éÒÔÔ~¡yXårñd'÷ôô´µµ9Ú£½8Ú£½8Ú;s¡ù2ßÞªkùòå³ü4²¿5:H¿Ax»M¥RãÿÊ°òCù¡üP~(¿i»|ùrCCC&C~­^½úÁ>ôÃÕÕÕétúÞØÑÞ0sË7nlÜ¸ñæÍã¢üP~(?ÊoFGGO<Y^^>Ë½­µµõðáÃa R©ÒZµjÕààà3*?Êåò¶¾¾¾ì~ã?T÷@WkMMMhÍd2ÙÝÝý³ç6¶;°¶¶6û¯	+?Êåò¹ìà+//oii¹víZ¡låòCù¡üP~ÓYÄØüä'¯RpÛCù¡üP~(?ß4<Ä/sQ~(?Êå7«åWÐÊåòCùMÏÅëëë+**¢óy«««;¦üÊåòS~ÅV~3<¢ò÷ïß¯üÊåòS~EU~555¡ó.^¼)¿îîîè]òCù¡üP~Ê¯¨Ê/ó=yòNøU~ÊåòCù)¿¢*¿êêêÐyÑ~¾P~étzÇa¸¶¶Vù)?Êå§üªüBÊMäÜ¹sÊOù¡üP~(?åWTåÜ¼y³¹¹9:··¼¼¼¾¾~``  ¶òCù¡üP~(¿R¡üP~(?ÊOù)?ÊåòûE×®]kjjª¬¬	r¨Wù¡üP~(?ßT=õÔSeØ´iòS~(?ÊOùIù;v,¼E#ïÞ½üøñh|GGòS~(?ÊOùCù%Éw?)ún¾ÏOù¡üP~(?åW$åH$BÞÝ½wü¤t:&(?åòCù¡ü_1_æ¶M65úKnÊOù¡üP~ÊOù)¿b(¿<m§üÊåòCù)?å§üP~(?__~ÊOù¡üP~(?åW$å»x<®üÊåòS~ÅP~ÅAù¡üP~(?òS~(?Êå§üÊåòCù)?åòCù¡üP~ÊOù¡üòS~(?å§üP~(?ÊOù)?ÊåòS~ÊåòCù¡üòCù¡üP~(?å§üP~(?ÊOù)?ÊåòS~ÊåòCù¡üòCù¡üP~(?å§üP~(?ÊOù)?òS~Êå§üÊåòCù)?åòCù¡üP~ÊOù¡üP~(?òS~(?Êå§üÊåòCù)?åòCù¡üP~ÊOù¡üP~(?òS~(?Êå§üÊåòCù)?åòS~ÖòCù)?åòCù¡üP~ÊOù¡üP~(?òS~(?Êå§üÊåòCù)?åòCù¡üP~ÊOù¡üP~(?òS~(?Êå§üÊåòCù)?åòCù¡üP~ÊOù)?å§üP~(?å§üP~(?ÊOùMQooo2Çã]]]ãoÐÙÙYVV¦üP~(?Ê¯à¥R©ööö0°ÿþ©###MMMÊåòCù¡üAUUÕèèhH§Óµµµ9SöÙûöMV~û·Û3^ýõsçÎõPl[ßz(MßùÎw¬[vDùÅãñ	k×®577.¬ü?þãYtêÔ©wß÷Ç¤7ß|óÊ+ÖCiêêêúÑ~d=¦·Þzk``Àz(M§Oþá8÷XåÅ2ÃD"ÒºuëÎ9óOÕÑ^íÅÑ^íÅÑÞUösa¸ºº:NGGÃð7ËÜXù¡üP~(?_kmm=|øp©Tj²R?Rù¡üP~(?_á­ÖX,L&»»»'L=åòCù¡üP~%Mù¡üP~(?òS~(?Êå§üÊåòCù)?åòCù¡üP~ÊOù¡üòS~(?å§üP~(?ÊOù)?ÊåòS~ÊåòCù¡üòCù¡üP~(?å§ü×^mÑÇÿÊ¯üÊÎ;ÊåòS~Ê¯<xðù?óÏìüþhÓ¯×ÿú§?ýiåòCù¡üò+6ÃÃÃåååm_lÛõå]Ñ¿¾ã£ýhØpÊåòCù)?åWTúûû.É¾èß+^|ñEåòCù¡üò+*?þxNù=±ì^zIù¡üP~(?å§üÍc=öë~/_Úü¥K.)?Êå§ü_±ùîw¿ûÈ#,bùg[?û»¿û»>úè_þå_~(KV~ÊÏzP~(?å§üs®^½ºuëÖ+WnØ°¡££ãÃZ¬òS~ÖòCù)?åG©P~ÊÏzP~(?å§üP~(?ÊOù)?ÊåòS~ÊåòCù¡üòCù¡üP~(?å§üP~(?ÊOù)?ÊåòS~ÊåòCù¡üòCù¡üP~(?å§üP~(?ÊOù)?å§üÊå§üÊåòCù)?åòCù¡üP~ÊOù¡üP~(?òS~(?Êå§üÊåòCù)?åòCù¡üP~ÊOù¡üP~(?òS~(?Êå§üÊåòCù)?å§üòCù¡üòCù¡üP~(?å§üP~(?ÊOù)?ÊåòS~ÊåòCù¡üòCù¡üP~(?å§üP~(?ÊOù)?ÊåòS~ÊåòCù¡üòCù¡üP~(?å§üõ üP~(?å§üP~(?ÊOù)?ÊåòS~ÊåòCù¡üòCù¡üP~(?å§üP~(?ÊOù)?ÊåòS~ÊåòCù¡üòCù¡üP~(?å§üP~(?ÊOù)?åòCù¡üòCù¡üP~(?å§üP~(?ÊOùýLooo2Çã]]]Ù7nÜH$üñP]ÊåòCù¡ü[*jooû÷ïoiiÉ´gÏçntt4$W]]Ýøòãÿw½õÖ[áÿÿÿRÂ-üÿ·JSèþ[·nY¥éÌ3ÖCiÝÿïÿþï³y%Q~UUU¡íÂ@:®­­ÍÔØØxéÒ¥Éfå·wïÞYtâÄ×_½dëÛúÖ­­ÿ DùÅãñ	£«/¼ðByyy]]ÝíÅÑ^íÅÑ^í-l±X,3H$r&:t(|¹¹¹Yù¡üP~(?_á)û¹0]N§ïíÃÙ7Ë¾³;Pù¡üP~(?_áimm=|øp©T*ÒæÍ=.^¼¸téRåòCù¡üP~¿Zkjjb±X2ìîîþÙsÛþ³­[·.777÷÷÷+?Êåò+QÊåòCù¡üòCù¡üP~(?å§üP~(?ÊOù)?ÊåòS~Êå§üòCù)?åòCù¡üP~ÊOù¡üP~(?òS~(?Êå§üÊåòCù)?åòCù¡üP~ÊOù¡üP~(?òS~(?ÊåWL¾úÕ¯¾òÊ+WgÑÑ£Gß~ûí«¤°õ/]ºd=¦o|ã?øÁ¬ÒÞh¾ÿýï[¥éØ±c³y?ýéOß¤.^¼¸k×®¯ü3Ëì(Ê@ù üP~(?Ê¯HxqñâÅñx¼±±ñìÙ³÷ÆþGCCCöJgë_¾|¹¹¹9H¬_¿ÞwúÎÎÎ²²ý¤íííM&Ñë¡««ËÊ)©­ÖÖÖZ-%¸õ»»»ÂÿýEÊ¯ÈñÇçÎ«ªªáòÚµka )Pj[?`ø.]º´yóf«¨¸÷þ©Tª½½=ìß¿¿¥¥Åú)©­~÷¿óg ¥³õ.ÞÂÀ#Gêëë_©èèè±êêênÜ¸Âe¶fJjëßù2#çÍgÍ·gvß¾þ¡þGGGÃ@:ö[_©mýÕ«W÷÷÷+¿ÒÜúÙÊËË_I´eeexØ¿7vÄ'løp5ôôX?%µõCÿ]¼x1¼üòËÙHñ¹víZsssH½ÌOÿì-nëÚÖÿÙ®ò+á­7ý¶¶6åW*:;;«««ïï»téRK,±fJjë_¸p¡¾¾>Døuð¡ÿæÇµnÝº3gÎd¿ÙÇb±ÌÔD"aÔÖW~¶þ;wR©ÔÐÐò+!Ñoù~ï/å­ñÎ;ï444X-E¬ì1¡þÓéô½±£½ÑoÎÖW~%¾õoÜ¸±qãÆ7o>üg=h.îuww¯Zµ*,Y²$Ó××·xñb«¨¤¶~ÓÛÛ;::ºoß¾gyÆ**·h µµõðáÃa _ý­ÚúÊ¯·þéÓ§Ã[ÀàààxT6ÌÞæãñø+¢Øçw¢oúaØ**©­þÿ/X° H´µµXE%õÓÿìÙ³555±X,LF§x£ü(­_[[;á>`åò@ù üP~Êåò@ù üP~(?Êå üP~(?ÊàÃðòË//[¶¬|Ì+^õÕ_ø7¦`~OôhkkkÃSÊÆÄãñd29:::ÝeÊ ðìÚµ«lgy¦Êo÷îÝaäK/½3þÅ_ã~úé,P~¦··74M<?pàÀÈC«aäùóç¦ü®F.Z´(g|CCC?00 üå¿Ïîs¡iî¹ì÷î#?ÿùÏgwOWWW(§MMMa8sã[·nµµµUVVIUUU[·nÍ>¨zêÔ©PWaR·££#§¥Âyóæ-[¶ìäÉájkkkÎ;qâDþåDÂC&uvvNVi«V­ã»»»3cÎ;Æ¬23fÏ=555aQåååë×¯¿qãÆøò¿ü1y* ü¦j¹víZöÈë×¯µµµÙe£§§'ºvíÚI[¶l&õõõÅb±	ç®FS×­[7::Ú1H©á2SEEEôñ»<Ë	>¼ñÏôÈ#Ù9ËöööLöå,dÅÓ-¿<P~Yt`wjee¡Ã²Ë¦¥¥åÎ0®®Y³&NÔÑ®»PlÑ¤T*í,ÃgÏ½Ì;w¶¶>õÔSaÌñãÇÃp¸Ã6l¸ïrB5«áÙmÂgjrþüùáù«á2<òét:ºA23§pïçGËËË§[~y* ü~ù_f|T6Q07nÜWC3EWÂÕºººM6b»÷nf!á69Ñ2sEW¯_¿¹q¨¥ÌßpO:ußåÊWßÿýìÇ6Ùgò¶mÛ&íÛ·ïÞÏhoÝº5û¡CùmnnvIN·üò<T@ù<dÑ®;wîd#Ã¤<­éÂþþþ(þ2¡ùpÞø°9-ý]*a¸ªª*HÏ7/3õ¾Ë¬Ãrx1Ô0.Ãp___fêÙ³gÃð¨ñÔË/ÏCÀCÖí^È×IÎÏßuþüùÝ»wGÇ^3ÉíËN½omÙ²%:È.ÛÚÚ2ãó,'ÚÇvóæÍèêûï¿ÿ<Ü%K©ÑÙ`Íç&uvvÞ¾;ùeª4Z©y* ü²èx<¾ÿþè[]8H$Æ±fÍP9ÃÃÃÑ¡ØÌy¸Ñçü¢Çõ÷÷g@0Á­[·NÎºÍ|¯ÊlÝûéÓ§3ãó,'ú`_ô9¿¡¡¡èyÊïÐ¡C½qá)gO>òØ××î%¬ÉÊ/jÍÐÊáf¡8×¯_=5ÏCÀÃËq=öüÿpc¢0ÊgfNªÈxê©§²³2Û±cÇòß½±?¶ÆWUUeÎ³ð0²Xæ¼ÚÉìÝ»wËËË£§sõêÕÙw1þüp±Kö2£]Q§f¦æy¨òB¬X±"1fÙ²eÑ	¶9å×ÙÙm^ccã3g2SvìØQSSÕÒ¶mÛ3S;::C%Éæ,sü#	ã7oÞ3~²åáÁÑ`ïóËhkkËùzÈàà`*k ²²2<Ì7Ôd/óöíÛ!m£uµråÊîîîÌóPåò@ù üP~(?Ê@ù üP~(?Êåò@ù0ÎÿÏ¸ó]H+ûIEND®B`
