# Supplementary material for: Regulation of piglet T-cell immune responses by thioredoxin peroxidase from Cysticercus cellulosae excretory-secretory antigens
Source: Front Microbiol. 2022 Nov 18;13:1019810. doi: 10.3389/fmicb.2022.1019810 (PMC9718028; doi:10.3389/fmicb.2022.1019810)
Supplement: Supplementary file 3 [file Data_Sheet_3.ZIP › 4. C. Cellulosae ESAs and TPx Induced Th Subpopulation Differentiation/3. SPSS statistical analysis/5. IL-17/3. IL17-72h/3.3 (SPSS data export) SPSS statistical analysis--IL17--72h.doc]

EXAMINE VARIABLES=Figures BY Variables
  /PLOT BOXPLOT NPPLOT
  /COMPARE GROUPS
  /STATISTICS DESCRIPTIVES
  /CINTERVAL 95
  /MISSING LISTWISE
  /NOTOTAL.


Explore


Notes	
Output Created	12-SEP-2022 23:44:43	
Comments		
Input	Data	E:\桌面\Raw Data\4. C. Cellulosae ESAs and TPx Induced Th Subpopulation Differentiation\3. SPSS statistical analysis\5. IL-17\3. IL17-72h\3.1 SPSS statistical analysis--IL17--72h.sav	
	Active Dataset	DataSet1	
	Filter	<none>	
	Weight	<none>	
	Split File	<none>	
	N of Rows in Working Data File	20	
Missing Value Handling	Definition of Missing	User-defined missing values for dependent variables are treated as missing.	
	Cases Used	Statistics are based on cases with no missing values for any dependent variable or factor used.	
Syntax	EXAMINE VARIABLES=Figures BY Variables
  /PLOT BOXPLOT NPPLOT
  /COMPARE GROUPS
  /STATISTICS DESCRIPTIVES
  /CINTERVAL 95
  /MISSING LISTWISE
  /NOTOTAL.	
Resources	Processor Time	00:00:00.89	
	Elapsed Time	00:00:00.84	


[DataSet1] E:\桌面\Raw Data\4. C. Cellulosae ESAs and TPx Induced Th Subpopulation Differentiation\3. SPSS statistical analysis\5. IL-17\3. IL17-72h\3.1 SPSS statistical analysis--IL17--72h.sav


Variables


Case Processing Summary	
	Variables	Cases	
		Valid	Missing	Total	
		N	Percent	N	Percent	N	Percent	
Figures	Control	4	100.0%	0	0.0%	4	100.0%	
	ESAs	4	100.0%	0	0.0%	4	100.0%	
	TPx	4	100.0%	0	0.0%	4	100.0%	
	LPS	4	100.0%	0	0.0%	4	100.0%	


Descriptives	
	Variables	Statistic	Std. Error	
Figures	Control	Mean	10.69375	.454709	
		95% Confidence Interval for Mean	Lower Bound	9.24666		
			Upper Bound	12.14084		
		5% Trimmed Mean	10.70039		
		Median	10.75350		
		Variance	.827		
		Std. Deviation	.909418		
		Minimum	9.525		
		Maximum	11.743		
		Range	2.218		
		Interquartile Range	1.693		
		Skewness	-.390	1.014	
		Kurtosis	1.501	2.619	
	ESAs	Mean	14.43475	.496842	
		95% Confidence Interval for Mean	Lower Bound	12.85358		
			Upper Bound	16.01592		
		5% Trimmed Mean	14.47178		
		Median	14.76800		
		Variance	.987		
		Std. Deviation	.993684		
		Minimum	13.030		
		Maximum	15.173		
		Range	2.143		
		Interquartile Range	1.774		
		Skewness	-1.409	1.014	
		Kurtosis	1.502	2.619	
	TPx	Mean	4.02600	.161481	
		95% Confidence Interval for Mean	Lower Bound	3.51209		
			Upper Bound	4.53991		
		5% Trimmed Mean	4.02467		
		Median	4.01400		
		Variance	.104		
		Std. Deviation	.322962		
		Minimum	3.643		
		Maximum	4.433		
		Range	.790		
		Interquartile Range	.599		
		Skewness	.222	1.014	
		Kurtosis	1.500	2.619	
	LPS	Mean	41.37150	.602370	
		95% Confidence Interval for Mean	Lower Bound	39.45449		
			Upper Bound	43.28851		
		5% Trimmed Mean	41.40667		
		Median	41.68800		
		Variance	1.451		
		Std. Deviation	1.204741		
		Minimum	39.685		
		Maximum	42.425		
		Range	2.740		
		Interquartile Range	2.213		
		Skewness	-1.287	1.014	
		Kurtosis	1.504	2.619	


Tests of Normality	
	Variables	Kolmogorov-Smirnova	Shapiro-Wilk	
		Statistic	df	Sig.	Statistic	df	Sig.	
Figures	Control	.250	4	.	.961	4	.785	
	ESAs	.250	4	.	.847	4	.215	
	TPx	.250	4	.	.956	4	.753	
	LPS	.250	4	.	.908	4	.471	

a. Lilliefors Significance Correction	


Figures


Normal Q-Q Plots


æÕ«W³²²Ö®]ë]ÓÕÕU\l0***îÝ»wýúõ+V¤¤¤¬_¿ÞwñèÑ£999jÿ¦|	ïE¡óâ/Êzß°Rss³¬¬©©	ú=Îö*_Z¼(käNÎjpÎÌù6·+jãÏïRù¹É°oØ°AÆV0ðRß«dµZåÖdä'+y¯(HOËEF£qÿþý.ø#¢èÅßØØXnnnvv¶,:àîÝ»òÝ#ò~JKJJÿ·nÝú8Ø~É5kÖo¼D¼òóûÌuëÖi'//OÖ?xðÀw¥àRV._¾<è÷8Û«øiù+((ÕàhnÎW9þúûûoG¾ÙÙâÏ÷Çj·ÛÕÞúúúÔ'oÙ²Åï>ïÝ»ø#¢èÅ,xÑw&Ì×»wïåM6=|,È²Ò÷3><55¥æ®Ô=öx<îînõ¡Ü²|xõêUYªëª#Û:;;eùöíÛ¾"Á`ºÞ÷ºó¼J¨ê¼3£³ ÷jÎWÔ8àÏï6Y®®®Ö6süùþXåvdÍµk×dYýXwîÜ©>Y¡ðÎ;²,?MYNOOçÁEþ(ªñ'ÉïrYßë~ë³³³eùîÝ»êCù/.Y²Ä÷¼z×8|?ü»ý§¾æ.2JKKÕTÓ´øKII	ú]?«ø¢J®+©¬¬´Z­Am&ô^Íù3Ç:®ñÞ½¾_bVøóý±ª[óMÖ¨JJJäÃeË	^å¯GñÈ"DøFùîv»×Î'ªzÚ4ývNÖjNNÚO(<¯Eünm&W	5&ÓÚL'IçvÅïö	ï´/ü±ú½ºÈáp(ÿyQxùòeà¢Òï½'z_E­T>ó£31öEEEê%Wºººg3ÁT¯ºR]]½ÿþ:uJVnß¾=(þfr9ão¶ãmÎW9þf2óçåÐ_üÓÓÓeß©Ç¾Ý¸q£¡¡AíköMDà¢¼ï=®_­QçoÚ´iìIê·û=æ?5ûe·Û 'O	þl6[/vww«Y=ÕÅ~³½Ê¬ð§=8j$]Oò»Ùêð§Òû>zôHl·qãÆ@?~_(ÖuÎòù2²\ûmªc7Æ.x"DuøñÝ¥Ö8N¿ã½²³³½'ÏÎ^¨,Y"ÿûñô>¿ôÒK» W¯^­ñmÎê*³Âöàx_«Ùïç0ªsÀß7|oíÚµ¾úpMêå£æVùåyp?"üIj¨ïúO>ùdûöí)Oª®®<½cnø[Û½òÊ+â!ï«h§©©iÅIIIyyy§O~á¼3Oó¿Ê¬ð§=8/^ÿïKåù6«Qþ¤Ë//[¶L¾k¹µcÝ³|(DS_½²²²··wÚ×«W¯Ê­É7ÕÒÒâ]ïr¹<¨¦TEðò£t»Ý<¸À^MNN¾ôÒK³zm¹9æRcÕéÛG¾¨¨ÀQ¦ùóë­·ÞbdüÅa.ëÀyyyjß®,¼öÚk?""""DDDDþÀ?""""DDDDþüø#""""ðGDDDDàÀ?""""DDDDàH£/L&Á°nÝºÙ^wÅ-r8Þ5²,kgñõ¤¹ÎL®;çFGG÷ìÙ%wðàAË¦'q=¿/"DÐåää3:g¶×=zô¨Ø±cÞ5o¿ý¶¬ihhuüó?éöíÛSSSgÎ/´qãÆ9ÜT___ee%ø#"ðGDÑñ|1gÜ¸qC®[QQá]³fÍYÓßß;©k;::"2ÂàÀé(?_j<x0333==ýÈ#~öÂ/úÝwâP<x Ë²F]tçÎÊÊÊ´´4ÁPRRríÚµ ·æûÕµ¯rúôiÉT^^._(If³Y®+wþÀj"SxºvíZ¹5Y/·|÷îÝLqq±Ü²Ûíz©Æ(Ùl6¹2/^áÀÔ¸)¶O"D¤ÿÔòo¼!Ë---Â,Yxë­·|?çêÕ«y¾íÞ½[.:uê,Ëÿ²£.Z½zugg§,Ü¾[Öçææ½5ß; !Ýï½'%ðÎïÝ»WåÎ?/GK.å±±1»Ý.jb2°Àa/âö(?~}-!`àôûÞµoÀé?ñ,O>)Ð^A´ÙlrÑ¦MdyË-J6ÞKoÜ¸qøðáÒÒRYôÖü £uÇ233¯­îüÔÔ,ûd¥|¦,®BMãMÚ£ôèÑ#µ,·Þï]û¦Ø8ü¾øóO(¾ø%ÒJOOÏt¹ïª¬Q<yR®%"¼yóf ÞÔ)úNæ)8vttdee©5F£QMÎdæ¯  @ÖOLLh»PyÚËsp""ðGD¿ÜÜ(5y6­EÌf³ýß»>%%EÖL=iøÓ¾÷ç¯»dÉu]¿»çñx:;;-ïá´>|X>_°xÑLFiøÛ?"Zü©S:¥Aûí·gbïÚï½ç'[·n©cg?í«ÈÞÿYØ½wàu_~ùeY>sæÌàà ÚÕ++ËËËeùæÍ÷îÝËØØPlùòåwîÜPª)É­[·Îp|Õ®gµÓÙïÛ?"Züy<Vú|_«OÛ"=R»1wýåËF£ÐçÀ3ÄöU¬V«fÍïy'¾ãr¹öïß/÷<%%eãÆêÄ^1_uuµ:¸¢¢âO>ùÈÈWPªÚ¢À#G¨ãùf2J¾Ë/^»¤^CÛïÛ?""""ðGDDDDàÀ?""""DDDDþ ÿøÇCCCaþ¢¿üå/ÿýßÿíF§îÞ½ûßüqÐ©;wî¨×!=ºûö¬ÞfÓéüÏÿüOÆA§~ñ_ü×ýã S~ú)ø[°¾ÿýïÿÂüEoÞ¼ù¯ÿú¯lÊ:õÓþôÞ½NüñÇÿöoÿÆ8èÔßýÝßñ¡~ýð?äOýºzõ*¶Ö¯Ë/ÿ÷ÿ7øþÀø#ðþÀøàü?ðGàü?ðþÀ?ðþÀøàü?àüøàü?àüøàü?àüøàü?ðGàü?ðþÀøàÀøþÀøàÀøþÀ?ðGàü?àÀøþÀøàÀøþÀøàÀøþÀøàüøþÀøàüøàÀøCüõööâââþþ~ðþü?ðGàüÅ3þ/_.¿®dáìÙ³øëêêo¢_üâ¤O===þ9ã SòÇüéÂ8èÍfûå/É8èÕjýÕ¯~Å8èÔ+W~ýë_3:%ø¿ÃüEc¾¥¥¥âïøñã?oòç<ýôIäïÆA§:;;^ýúà®]»Æ8èüúüÛ¿ý[ÆA¿á¿^ºtéRø¿h<à¯¯¯¯¦¦Ý¾ìö%vû²ÛÝ¾Än_vûÆón_ÕÄÄDuuµËåàÀøþÀ_ãoddÄb±øþÀø#ðþâ6mýúõ£££A/àÀøþÀ_Ïd2-ò	ü?àüøñ?íÀø#ðþÀ?ðþÀø#ðþÀ?ðþÀøàü?àü?ðþü?ðþÀøàüøàÀøàüøàÀø#ðþü?ðGàüøàÀøàüøàÀøàüøàÀøàü?àÀøàü?àüøàü?àüøàü?àüøàü?ðGàüøàü?ðGàü?àü?ðGàü?àÀø#ðþÀ?ðGàü?àÀø#ðþÀ?ðþÀø#ðþÀ?ðþÀøþÀ?ðþÀøþÀø#ðþÀøþÀø#ðþÀøþÀø#ðþÀøàÀø#ðþÀøàÀøþÀøàÀøþÀ?ðGàü?àÀøþÀ?ðGàü?àü?ðGàü?àü?ðþü?àü?ðþü?ðGàü?ðþü?ðGàü?ðþü?ðGàü?ðþÀ?ðGàü?ðþÀ?ðþü?ðþÀ?ðþü?6eðþü?ðGàüøàÀø#ðþü?ðGàü?ðþü?ðGàü?ðþÀøàÀø±W[[Ûðð0øþÀø#ðþâ¹ÚÚÚE544?ðGàü?à/s¹--|ÊÉÉq»ÝàüøàÀøvíÚ¼è'k¶mÛ¸çü?àüø±×øøxss³ßTjåÊr:A¯þÀ?ðþü¿Xª§§Çl6§¦¦NõíØ±cZ?ðGàü?à/'ÌÆÆÆüüüÀ©>É$Íðü?àüøÑÇã±Z­7o<ª/55µ¶¶V.<«ü?àüø1Öðð°ÐÂd2=ª¯©©éþýûs¸Yðþü?ðGàüEQjª¯ªª*pªÏh4Ífù9ß>øþÀø#ðþ¢"©¾U«Vµ´´¸ùðþü?ðGàüE2ÇÓÞÞtªoñâÅµµµòËh¿øþÀø#ðþ"Óé¬¯¯:ÕW^^ÞÒÒ2>>¾à_ü?àüøa-ÌSàüøàÀøLêàÀøþÀ_XvªÏápíÎ?ðGàü?àO¯´§úÚÚÚfõúÌàü?ðþü?ð	é¢gªü?àüøz%ª«¯¯áEÏTøþÀø#ðþ85ÕWYYh¾Oõ?ðGàü?àoÁ¬««æ©>ðþü?ðGàüÍ7!Ý6lØô¨¾úúúèêàÀøþÀßÜs:ÊÉÉ	ê«¬¬loo¶©>ðþü?ðGàüÍ:!Ý¹sçÀk4£yªü?àüø³HTWWWjª¯­­ÍårÅÐð?ðGàü?à/Hêm9ÀzèÐ!§ÓÃþÀ?ðþü¿ÿÝnß·oÑhdßÊ+îß¿»ÃþÀ?ðþü¿ÿIÕW^^h¾ÚÚZAa/øþÀø#ðèøSGõ­¾²²²ÖÖÖxz²àÀøþ.«¥¥¥¢¢"ð^uT_¬ÀþÀ?ðGàü?­ÔQA§úòóóãøÙ	ü?àüøKü¹Ýî¶¶¶ GõíÚµ«§§ÇãñÄ÷ð?ðGàü?ñ?©¾ÂÂÂ¦¦¦Øz­>ðÖFGGM&øþÀøc¢ããã---AOàMMMµX,ñq/øÓ±îîî¢¢"ÙbÀø#ðþÀãÍø'ðÚÚÚP'ð¶··'ÎTøW7nt8øÿý÷o7áæÀÀÀmÒ§ýèG?ÿùÏºvíÚ­[·úè£þéþqÐ©?üP~0:ÕÙÙùÏÿüÏs»®ÝnóÍ7KKKÍ÷ôÓOïÝ»WdàÃéÒ¥Ï?ÿ<Ì_4ñ÷Û»§Oþðf³ÙzÿôItÒ××Ç8èÕjíïïgtª««ëÆN]¹råæÍNðÁò÷l¯uöìÙ;wfffú/))é«_ýê;ï¼Ãó¹êòåËáÿ¢ñ?vû²ÛØíËn_vûR8wûª£ú§úL&üjfHÙíþü?ðGà/æñ'OÑ%55ÕÏ|ÉÉÉUUUV«5î_´ü?àÀøüiOõÕ××;NÆü?àüøyüiOõµ··3ÕþÂøþÀø£ÇSàü?ðþü¿À_¨©>©²²©>ðþü?à/êèèxë­·Nõ-^¼¸¾¾Þáp0JàüøþÀ_Ì'¿FÍfóSO=jªÏív3Jàü?ðþü¿Øn||¼±±1¶¦ú<ÏÀÀÀbîÝÁø#ðþÀ¿¥¦ú4êÎ©¾áááçîÙgýò/?óÌ3_ÿú×cèÙü?àüøwÚS/¼ðÂàà`4ßÿU«V¥ü+>òÝ#òYYYUUøàüøàüÓê+//okks»Ý³z·ð700`2üÔ¿¯|úé§ïß¿þÀøþÀø£ÿixxXcª¯¶¶Ö÷¨¾(ÇàéËk¾ìúWPP ¿2ÀøàÀø	Çã±Z­f³999YcªÏïZÑ?ó÷ì³ÏúÍü-Y²?ðþÀ?ðþ75Õ?©¾ØÂT¼²Øï¿o|ã±ò£àÀøþ¬¹MõÅþÔÙ¾yyyø¨Îöi?ðþü?ðGàoÁ<¤1Õ7«×ê~ü)æÊï.ÄÖ»Ì?ðGàü?ó2ÆTßÜ^«/&ð»?ðGàü?si§úÀøë5§+))	üøàÀß|RSUUU8ÕþÀßK.Áþü?ðGàonËo:É¤÷;ð?ð3?ðGàü¿¸Ìf³é:ÕþÀ.íÞ½üøàÀß_mAßÃd2-àTølcþü?ðGào¶¹ÝîP'ðÊªªªööv]_Üü¿YW\ø7Ñht¹ÀøþB%ÉcÇ=ªOMõ9Î0Üðþf]JJl¦£££999² æÿý÷e¡¦¦üøàÀ_`êÞÔÔÔPGõóuÁøýM<ID²pûöí©©)YHOOþÀø#ðçÍáp444­>YÙØØ©>ðþæ[VVlµÝÝÝ²MËÂk¯½¦x©àüøüä¨¾sçÎmØ°!ð¨¾ÔÔTÅbµZ#øeàüÍºxOïð?iðGàü?Jdü744Æ Sr|BÄï$øséõ×__²d,ôööÊ@°´´Tï»þÀ?ðþ¢6Ueee ùRSS7oÞþß_àü-0þ"øþÀø¶êëëÕ~-]º´±±q||<þÀøàüø³Èãñ´··WVV­>³ÙÙ£úÀø[xü¨|áE	ü?ðG	¿ÁÁÁ¥Kê;tèÐÐÐP/ø³nùòå¾àóÆÙ¾þÀø£xÅÛí_êAß755Õl6¨¢vªü¿ù&Îm½¯¯ojj*wü?àü?ÃQ__ô^Y%'ð?ð§/þÔ ÌòàÀøáÌív«£úkóæÍ6-V¦úÀøoýýý²ÝïÝ»wbbüøàâjªoñâÅ¡NàõGøs)///ð!Á	þÀø£ØÅÆT_rrò;.£SàüÍ·eËqÂ?ðþÀ_ÜàÏápÔÖÖêSïÀsGõ?ð·ÀøS»Ýæ»þÀ?ðþ0·ÛÝÖÖV^^tª/Ê_«ü¿°â/;;>ü?ðþb6ÕþÀß|³Ùlòð8pàüÍþü?ðG±?©>õZñ:ÕþÀß¼o"DðAàü?NüiLõFç;ð?ðEøK'|øà¢ÓNõÿ×ø1¿HþÀ?ðþfþôÂTø?É´lÙ²ÁÁAðGàü?*ü	éZZZêàoñg0äQþ»þÀ?ðþ4OêàO/ü]»vMKcccá|Áðþü?ðçÛí>wîSàüé?Îö%ðþüEuuu¡¦úZZZêàoÁðÇÙ¾þÀ¿HáOÀ[YY8Õg±Xäàoñ©Àø#ðþ¡ê[¹r%Sàü?ðþÀøñ?Ë¥êêËÈÈÿ½æÁ%"þ&''7nÜ&½ôôô­[·áÌðþü¿Â¨®®®Îh4r/øÆ< ð¡÷[ý?ðGàü%B.kÿþýkÖ¬	üE³xñbêà/ø+**Gà¦M&&&äÃ±±±-[¶ÈÕ«W?àüÑ³Ûí¡êËÏÏokk2JàüE)))ò8ôx<Þ5²FÖ?àüÑlÒ	ìÊÊÊÍ±k×.ÍæûKÀø7þä)àó®q»Ý²z!ðþÀÍöI ¶¶VÈ¾µk×¶¶¶²?ðøS»×¯_¯vûÊÿ²,kJJJÀ?ðþhÚW¨wàU'ðûæöÞ¾þÀ.øí=áãáÃàÀø±þ¨lnnêqûGõåK«xçðÞ¾þÀøüäß­[·fff&%%Éÿë×¯Ã£ü?àO¿<Ï;¾hübEEÅW×~5==ý?øÁBÝ¸N@¹jÕª ïÀ[WWçgMðþÀ_Ôá/"?ðGàüé×÷¾÷½üüü¯<òÝ#òïÏöüÙ¾ðQÂ<oV·Íf³/¡êàü?ðþü?ùíþön%?õ¯ªªê[ßúÖÜnm||¼±±±°°0Ôkõõôôhü?ðø[4]IIIàÀø1ZFF¯üäßÿ¤²²r¶·£1ÕWVVÖÞÞ>×êàüEþBþü?ðë¯,íùâ¯¢¢bÏ=3¼úðð°ÆTßlßü?ðøÕ+¯¼¢ÞgÏþÀøÑ.ðEãkþOßêYYYCûZÇjµÍæäää 'ð¶µµÍáÍ?ÁøQ¿þþþôôtuÐ®ïk>?àüÅb---iiiùùùÏ>ûì3Ï<£ý«¦úäCMõMGðþÀ_áoûöíêAÞÙÙ»þÀ?ð§wn·Ûn·z;5¦úÀøQ¿óçÏ«ù¦MÂy×Áø#ðþ"öT_ý|¦úÀøQ?y(®^½ZÛqíÚµ0ßuðþü¿ð§=ÕWYYÙÞÞ>ÿ©>ðþÀ_4âïøñãê¡¾sçÎÜuðþü¿pÎ©>ðþÀ_4â×ù#ðþ(ð§¦úªªªÂ9ÕþÀøFü%MÁ`þÀøÝåÖd2ªü?ðøxàüøzSàü?ðþÀ?ð§S²2Sàü?ðþÀ?ð§WS²FÖ···z?ðþü?àüÅLÓNõ9Îh¸àü?ðþÀ?ð7÷<O4Oõ?ðþÀøþÀßät:ëëë£ªü?ðøãuþü?üÅÜTøà/ñçû~AñÇëüøà/âÅèTøà/ñç­««KD^|ñÅ	ùPþß²e¬¹~ý:ø#ðþÀ_DvªÏjµFóTøà/ªñg4åÙÄ÷IdrrRÖäääÌóûûûsssÃ+®]»þÀ?ð7mÚSòÌ9<<sÃþÀø.ü©çþæÌ_uuõ3gdáÄ;wîàÀøÛí§©>ðþÀ_Tã/;;[jêér¹6mÚ$kdý<oÙh4NMM)MÊ¬økii¹Þä	¨»»ûéÓGô÷ÿ÷Nuuuõôô0:%¿>òDäK?þ[ßúVfffàT<ûÛßþðÃcx;;;åC63ô÷÷3:uéÒ¥ðQÝñwýúõ '|Ü¼ys·ìÊHàé#?yÊoü±Ýn&úñ<88È8èÍfûôÓO¿?ûì³p~ÅÏ?ÿüäÉ_ùÊW~+++Ï;744Ã+ºÈ÷Ëf¦SW®sçã Sbë»wïùê?éöíÛË/OKKKJJJOO/))yðàÁüoÖwÇqJJ»ÙíKìöe·¯äp8êêê/^OGõ±ÛÝ¾ìö¥Ý¾ú­%ÿw"?ðGà/¡ðçv»å·H¨£úÌfsLÕþÀøÿ<>Zäÿêêjðþü%&þGÐ©¾üüüÆÆÆøêàüE;þnÝºUPP®vÔfggwttÌÿf»»»srrä6sssÁø#ðPøs»Ýmmmååå	8ÕþÀøjü©yöK7µ|âÄ	]ï:øþâv»]cª¯©©)î§úÀøQ¿y>ºuë½½½²	þü?ð7óÔT_EEE ùRSSwìØ!¿EäspxÁøÑ?õÄ¤þ¦¦¦xo_àüÍ<»Ý~èÐ¡ SeeeMMMããã<¼àü¿èÂzg5Û'ø<xð zÅðGàü?ÜnwkkkÐx322,KøåÀø#ð76-è<ËoðGàü¿ ÖÖÖª÷Fê;vìXOõ?ðþ¢üÂ.--Ugû¦¥¥8N½ï:øþbçÜ¹sAOàMMMeªü?ð3øHàüø!üÉÄûöízTßªU«áµúÀøñ?ïyÞ<xPPP°téRðGàü%8þWYYYÐx-Kâ¼Vøà/ñ799ÉÙ¾þÀ_ão`` ¾¾>èQ­­­ü8Àø1¿¥K.Ò,++üø¿ñññòòòÀgÅäädÅb·ÛêàüÅ$þîÜ¹ô$ïø&ò;þ<ø#ðþ6­¶¶6èQLõ?ðþâÞzzïáàÀ_Ô¦¦úþù S»víêééaªü?ðWøTàüøø«1Õ×ÜÜæw8­­­òuÀ?ð§#þ&''ÒÓÓ½k,YòÆo?à/.SSÂ»P'ðÊþõöÛo?ýôÓ_­øÚ×¾ö/|aß¾ñ4ÝþÀø.üåååùð«À?ðg¨ØNÈ>y&ü¿øH½-<~ñ_ÜýíÝG¾DþÕ½R÷¥/é/ÿò/Á?ð§þRRRäÏn·×ô÷÷ËÌÌLðGàüÅA3êÛû.TßùÎwªªªüÔ¿o~óàÀøÓêß©©©ÿu»/þþü¿XÜ&CMõ½SÅÈO´ç¿ÚïÔþÁüø#ðþtÁ_nn®<îß¿ßívËòø<rä¬ÉÉÉþÀ_,¦1Õ·xñâÚÚÚÀ£ú"¿W_uíÚµ¾øû£þèßøø#ðþtÁ____ÐyîííþÀ_Ìm3ê*üÆM´éð¡Ã"¿?ÞþÇ¿÷¿×ÓÓþü?]ð'¬X±"---)))==½¨¨HÖè×Áø#ð·P	ég5ÕUøìvû5k<é¹ç'ù?ðþ¢	ü?óOFÌfó¦ú¢*×âïÇþÀøàüøWóêNüÅkàü¿¨Ãß­[·Ôë<«3|³³³;::À?ðÙÜêàüøûm]]]Þ§N?µ|âÄ	ðGàüEIò½;vLcªÏápÌçöÁø@øËÉÉgÏ[·nyñ×ÛÛË<øÑ³]mÛ¶-999èT_[[zªyþÀø£ÂzìóÂÎSSS²l0À?ð©îß¿ßÔÔ´jÕª@óÆûöÍsªü?ðG¿ììlõª~eÉþü¿0çñx¬Vk¨xÅ­­­2ÕþÀø£ÄÅÍfú"ÏòüøaëþýûùùùA§úêêêõûêàü?J üIò»´´TíVPPàt:õ¾ëàüøSõôôÍæ@öUTT´´´áàüQbá/"?ðG	?©>à¾ûÂðW(øàÀø#ðþÂñ»jÛ¶mAêÛ°aC[[[øßåü?ðG¿Ï>ûlÙ²e)))òÌ¶bÅÑÑQðGàü-lÃÃÃ¡Þ###£®®îþýûºoàü?J üY­Ö '|èzl5ø8øóÀøZ²fÛ¶m.ã^ðþÀ¿à©y®®®VûY&&&vîÜ)krssÁ?ð7Þ|óMÉøç¥¬¬««[Ø×êàüøÙM<Iþ4÷ý3ÝûÏàÀømjª/ÔQUUUòdñ©>ðþÀ%.þÔÌßää¤w<Dù#ðþæÐðð°<´CMõ566Fð¨>ðþÀ¿ß¦ù«®®VÌVTTpÌ?ð7óÔT_UUUÐwà-++kmmæáàüQáoÑté´ÿü?üiLõÆCÙíö^ðþÀ%þ¦Ë`0?àÏ7ÇÓÞÞtªOÖÈz¹4ÚêàüøpàüQ,âÏétÖ××:ªO.çÛr?ðGàüÍ¥P¯ç<22þü?ÕL¦ú|_4üøà/zñ'ÏÝ¯¿þºßÊ]»vñR/þÀßãøêàüQââO'OâÙÙÙ<ß÷]õ´®Ó¡~àüQLàoÚ©>«ÕÓSàü?J=~2Ï§ÓsssÕÂ-[ô¾ëàüQtâOªO¹ÃÃÃñ7¼àü?J,üI×¯_÷>¿777á®?ðGQ?·ÛPSàü?JhüíÙ³G=Å«wû^|ñEðGà/Aðçp8êëë/^PSàü?JyÏÌÌ¼qãÆccþRRRÀ¿8Æê«¬¬4_"Lõ?ðþ(qñ'Oôö[i±X8ÛÀ_¼â©>ðþÀ%4þB½ÎÞ¿ÅÁø£0ãOªÏl6'ÔTøàü?þ4¦úòóópªü?ðG	?yÒ÷Ý±«ý!ø#ðøcªü?ðGàü?ðøs8µµµLõ?ðþü?ðþân·»­­­¼¼©>ðþÀ?ðþÀ_<ã©>ðþÀ?ðþÀ_üãOcª/55©>ðþÀ?ðþÀ_àOcª¯°°°±±q||AàüøàüÅ6þ¦êÿÃüøà/fð§ø#ðUµ´´X,¦úÀø#ðþæXÒtðGà/â	éLõ?ðGàüÍü?v<9ªü?àü?ðçi¼-GJJSàüøàü¿8ÉápÔÕÕÆ SüüóÏ%ðþü?ðþ¢ãããÍÍÍßùÎwÞxãÁÁAG#ïT_rrràQEöñÞÛÀøþÀøþ~ÿ÷ÿù¢ç«ªª¾Vñµ¬¬¬3gÎ0>	ëëëÕWVVÖÒÒâTøàÀøà/Jñ·nÝºò/ùîõïO-át:"êÛ°aCàT¬ÙµkWOOOàµÀøþÀøÑ?MVVÖº^üÉ¿/¯ùrss3C¤êËÉÉ	ê[¹reKKËýû÷C]ü?ðGàü?ðøs¹<ó¯üäßºuëvddL4^«Ïb±Ìä±þÀø#ðþÀøFüÉB^^Þ7¿ùM¯ü¾zP8h³ÙpLìvmmmFFFÐx[[[gî9ðþÀ?ðþÀ_âO233·mÝvøÐáÝßÞ]XP¸yófÇ8Cáv»[ZZ***Í'TGõÍv@ÀøþÀøQ?õhyþùçzê©/éKp¹3%èTßªU«;6ç·åàüøàüE/þ-Ñmss³ð.èQµµµóßÚÁøþÀøà/òzÞ+W655-ØÀøþÀøà/b;v¬¬¬,Ð|Aá z[ü?ðGàü?ðþ"l·f³9555åååMMMs>ªü?ðGàü?ðþ¢%!]cccaaaÐ£úöíÛ700 ëàüøàü¿p¤1Õ·jÕ*í·åàÀøàü¿Øhxx8ÔT:ªopp0÷ü?ðGàüÅþFGGM&ø1Çã±Z­f³9999èQmmmn·;üwü?ðGàüE;þº»»ä÷%ø1êËÏÏ5Õçp8"x÷ÀøþÀ_´ãoãÆòËR?øÁ¬á­³³óÃ?´ÃëÜç7ß|óë_ÿzRRRÐwàõÕWå[øýüàºººØÌÞXL~~ôÑGÃ]ºt)ü_4ñ÷Û»ò·àÿo?ûÙÏNçÿ%êíí½sçNÝaÙÞxãç.Ð|YYY²ÛíÑso»»»GFFØÌtÊf³ýêW¿btJ~ýú×¿ftêÊ+¿ùÍo[?zô(Ì_4ñÇn_vûF$í£ú*++ÛÛÛ#rT»ÙíËn_b·/»co·¯÷7(øQöQõõõ=ªü?ðGàüÅþrü¿È¦¦úªªªbkªü?ðGàü?ðþf×ðð°li&)§úÀøþÀ_Lâ/TàüéW|Lõ?ðþü?ðþÀß4×××ê±5ÕþÀø#ðþÀøÁóÀág¾äääªªªöövù^ðþÀ?ðþÀø~ªÏétÆÇð?ðþü?ðþG7ozT_UUëSàü?àü?ð÷xhhèÍ7ßúZjªopp0.ü?ðGàü?ðXøGà§úÔQçÎs¹<¼àü?àü¿ÀÓé­eéÒ¥A§ú:tÿþýD^ðþÀ?ðþÀ_<ãÏívËCnÛ¶mAê«¨¨KcîµúÀøþÀøàÏ?§ÓY__tª¯±±qhh(ü?ðGàü?ðWøs»ÝíííAß#55uÇ6ÕþÀø#ðþÀøOüÕ××/^¼8pª¯°°°¡¡!AêàüøàüÅ3þ4¦údÍæÍm6[½VøàÀøà/ñçp8êëëFc¨£úPøàÀøà/æñ§¦ú*++K½-G"ÕþÀø#ðþÀøü©©¾ Gõ­²©©ixxàüøàüÅ6þ4¦ú·mÛfµZ9ªü?ðGàü?ðóøs8µµµA§úòóóêàüøàüÅ<þÚÚÚÊËËNõÍf¦úÀøþÀøñÃáØ¾ûÓO?ÍTøþÀøà/ns»Ý¡¦úRSSêàÀøàüÅIGõ6663Jàüøàü¿ØNcªï©§Úºukø¬àÀøøàoáûéOª=Õg³ÙæöÞ¾þÀø#ðþÀøÆÇÇ[ZZ4êóþgûÞ¾þÀø#ðþÀø¢¦êó;ªü?ðGàü?ðþb/Ë5Ã©¾@,?ðþü?ðþÀ_Ì¤=Õ'"Ô>ü?ðGàü?ðþb ËÕÚÚZVVtªÏb±êfhGðþÀ?ðþÀøÞzzzjjjNõ§êàüøàü¿Èårµµµ/##C8(ÃÛr?ðþü?ðþÀ_t%cn±XDxì[µjUssó|Þü?ðGàü?ðþ¢"õZGõÌÿ«?ðþü?ðþÀ_³Z­f³Y§ÇTøàÀøàüEE¢í©¾ÿ¢àü?àü?ðîl6ÙlzTX°©©i§úÀøþÀøà/2	vùùùæ[¼xq]]Ýàà Þ÷ü?ðGàü?ðþtOqÇÉÉÉì+//okks¹¹'àü?àü?ð§cgåÊA§újkkGïøàÀøàüéÛ®]»üêkmmÕï¨>ðþÀ?ðþÀød2jªoß¾v»=²wü?ðGàü?ðþt¯½½ÝívGÃ=àüøàü¿ü?ðGàü?ðþÀ?ðþÀøàü?àüøàü?àüøàü?àüøàü?ðGàü?ðþÀøàÀøþÀøàÀøþÀ?ðGàü?àÀøþÀ?ðGàü?àü?ðGàü?àü?ðþü?àü?ðþü?ðGàü?ðþü?ðGàü?ðþü?ðGàü?ðþÀ?ðGàü?ðþÀ?ðþü?ðþÀ?ðþü?àÀøþÀ?ðþü?àÀøþÀøàÀøþÀøàüøþÀøàüøàÀøàüøàÀøàüøàÀøàü?àÀøàü?àüøàü?àüøþÀ?ðþü?àüøþÀ?ðþü?ðþÀ?ðþü?ðþÀø#ðþü?ðøëíí-))1ÅÅÅýýýàüøàÀøgü-_¾ ]ÉÂÙ³gñ§ÂÔét>"î1:%,3:e³Ù<xÀ8èÕjøð!ã SW®`tJð÷ÿñaþ¢1?ßÒÒÒñ÷çþç?oòùðÃHúÔÙÙÙÕÕÅ8è7¼ôã S|ðÃ«_òëSüÇ80¼±Ø¥KÂÿEã555ìöe·/±ÛÝ¾ìö%vû²Û7wûª&&&ª««].øþÀø#ðþâ~úpddÄb±øþÀø#ðþb¾Ùl¶õë×½ü?àüøq?É´È'ðþü?ðGàüÅ3þ´àÀøþÀøàÀøþÀøàü?ðþü?ðþÀø#ðþÀøàü?ðGàü?àü?ðGàü?àÀø#ðþÀ?ðGàü?àü?ðGàü?àü?ðGàü?àü?ðþü?àü?ðþü?ðGàü%þ¾÷½ïµ···+W®üä'?"ºtéÒÏ~ö3ÆA§Î?ÿóÿqÐ©¿ù¿ùÇüGÆA§þú¯ÿúÓO?etê¯þê¯N'ã S§Núù0ÑñññøÄß­[·9ò""""òI×è"æc'ðGDDDþüø#""""ðGDDDDàÀ_ÔõÉ'¦¤¤lß¾llÌ÷¢þþþÜÜÁ°bÅk×®1V;¼÷îÝ[äc5«FGGM&ÓL¶U6c]ÍxþÃt[o­wþÃÛÛÛ[RR"hqq±l®Ñ¶õòCÙêÕ«å'öìñ½¨ººúÌ3²pâÄ;w2V;¼!CÝÝÝEEE¾OÖÛ*±®ÃËf<ÿáÖáeëÿð._¾üã?³gÏDÛÖþB&*÷.geeù^d4§¦¦darr2èM4á'/2DshãÆÃ÷	Hc[e3ÖuxÙç?¼kØzÃ3¼l½ó^ßÒÒÒ¢më!+..¾uë,¼ûî»¾RñßE4ÿá?Ö¯_/+KKK?ùäÆjÖj' mÍX×áe3ÿðZÃÖ«÷ð²õ.ÔðJ555Ñ¶õ¿Ý¼y³  @ÀþÎ;ïø±=))É»ÂX-ìðz#2VóyÒØVÙu^6c]uÂÖ«ëð²õ.ÔðNLLTWW»hÛzÁßôöÙgEEE¾k²³³'''?³eha×7Öçù¤±­²ë:¼lÆºê­7<øcëÏð-Ë½÷¢pë![¾|yÿÔÔÔ;ï¼óúë¯û^d6O>-ò¿ ±ZØáN§zä¬_¿±ÏÆ¶Êf¬ëð²ëª¶^]­wþÃk³ÙdèFGG£së!^üÑSSSãñx|´ÝÝÝ999III¹¹¹êUZÀáíëë+**2òÔÃXÍç	(è¶Êfáe3ÖI'l½a^¶Þù¯Éd|¹èÙzÁQ"9! """DDDDþüø#""""ðGDDDDàÀ?""""DDDDþü?""""DDDDþüø#""""ðGDDDDà¼wßwíÚµiOZ·nÝùóçÿ×ó×bæÙ6Ø½5Lò­¹¿õ²Æ`0äææNMMÍö6ÀÅdGYÐë¯¿OøkhhÍÍÍ~ëdýk¯½6Û$"Dõ÷÷kÃÉ'=O:uê|(+oÜ¸7ø»û¶¬,..ö[_TT$ëN'ø#"ðGD	Ñ/¾(¬yë­·|W¾ýöÛ²r×®]¾ô¹víàI"ËÞO~øðaMMMff¦÷ïßï»wÕjµ°ä"¹îÕ«Wý8%k²²²Ö®]ÛÙÙ)Íf¿;vùòeíÛQÉ]RuuuÚúõëeoo¯wÍÇ,k*++½k=#7¶ûö@üÞ¾ß»JDà(Âååå	Ü¹ã»òîÝ»²Òd2ùâÆ¯¾¾>ué-[ü.Ú»w¯ºÈn·'%%½úP]ºuëÖ©©)ácJJÇãKåÁSzzº:Oãvd!èÝüNÏ=ë+Z¯/Ï9ãß¬[·n¶øÓ¸«Dþ"ÚÃä9kÑ"¡/nvîÜ9ñ$Y7mÚ¤.UÖQ|Tx6uQuuµ2åîînu#¾·yøðaáÚëúòË/Ë/Ê²ü/Ë/½ôÒ´·#på|ï[ÐïH@¹dÉù~GGGåCù_î¹srrRBnn®¾Ç¿ÛM6[üiÜU"DDQ?)(þ¼ën¤ùPØ¤>,))-[¶÷nAÛ£G¼7"ã7æ½úðîÝ»ÞO0y÷üÊÿ²lµZ§½¦|øàÁßûêø¼W^yE.zçwÿn×öþýû?A (øªÉÙâOã®ø#"|jºkbbÂw¥Ëårw¼4t8Ê^ëxÔÜëÇ)ßXe£Ñâv»åÆ³²²¼N;¡(æ×­[·SeYþe»Ýî½´»»[î@ÐÝÇ3ÇÆ]%"ðGDùÔqoÇ÷]©^Åïïq£££Z7nÜhhhP;a½jTÓrÞýªÓmïÞ½jo¯ü_SSã]¯q;j¦íÞ½êÃh»fÍ¹T½ºÕ÷"uæ¯ÕÕ566¦?/LÕhx/Õ¸«Dþ":aÂ`08qB½ÔËÉ'SRROÎØ´i@Çív«²Þ3sÕ1êP9Ãá° ²àþýûJê<­%:ýVuÍæ]¯q;ê ?uÌËåR©¿S§Nyçää[ö½Hþh·Ûå«È Ââ¦pY>MÐ¹ûvßK5î*?"¢¨H½²_GýÿÏ_OR6ò.wzÏ±ðöòË/ûÊÒ·ü=~òV²Þh4úîÖ¸¹¾wÌ¦m¨oöÑ£Giiiê[ðÛÙ½qãFß/±dÉù_½ÚïmªIoªÞK5î*?"¢hI²nÝº'­]»Vrë¿®®.õrz+V¬¸~ýº÷RËuðàÁ¦W^yÅív/½zõjii©,77·¥¥Åï6ïÜ¬ß³gßúP·#É»¤^Pãuþ¼ÕÔÔø½æjtt´ººZF 33S¾§ÓéÙßÛÝª±ª¬¬ìííõûwÀ?""""DDDDþüø#""""ðGDDDDàÀ?""""DDDDàÀ?""""DDDDþüQ¸úFpÃñ(ºIEND®B`


9ÏÍ%YÒúçã<û ur5G©#»%u¡´4®).NàL:WõyÆù÷Oª=$dþHâô~ôHz_=zùyô%w(eZÂ?""""DDDDþüø#""""ðGDDDDàÀ?""""DDDDþÀ?""""DDDDþhÏÓ555¿èl¦kÛÚÚÊËËÓ§+++;uêÔ]8û»,Qe0äöEEEÇÇàôõõÙíöyüø.·nÑhï®YÂÒÉ/À%þ²²²FGGO>ùd(8aisºËLºjllëàÌ­VkðûÖÕÕ?"DMcI7oNüõõõ)ZñÅÀÔÔLÈE988vQs½æ[ONNîÝ»WæÈâ?å7nÜi¿ß¯«_?"Jxü9NùÚÖÖ60ÞÿýmÛ¶)ÇLÇãÑÜÒåregg¯_¿>8çüùóÅÅÅF£±¼¼üúõë.**2Lê½¶X,ÊñMùÁ«f"ÎC=$óÕa¥¦¦&Y__ögë]B¿µxQæÈÓàÝ3§n~w?Íµò),,a¯¬¬±¼0ôZõ¯Ur»Ý²4ùÍÊUÁ;Re$ÅÓrÙlÞ½·ÏçãÉEþH¿ø´Z­¹¹¹2ê>úH^ÑÕ[jf0äë-[î;.¹nÝ:ñMð¢@$(?Í-7lØ8Ë/ùü±z¦àRfæççýçzÍ·Ù(þæ487ï;ÎýýýêåÈ;Wü©­ÊÅ`Ê7oÞ¬yÌ;wîäÉEþH¿øöövõ0µvìØ!Ó6mº1LÈE©¾åþýû§¦¦WÊÇ,tww+eÉrÑårÉ´@P¹¯òÎ¶³gÏÊôÕ«WÕWÍD£Ñv¾ú¾¼ËLoªîëàTó¾c7üiî(hiÃqóæMah³Çú×*Ë92­üZkkk+(¼víLËoS¦322xr?"Ò5þ$y-iy]×ÌÏÍÍé>úH¹(¯ñrqÙ²eê%¯Îp¨/ßùÝñSµ9ü~¿pAQZZªìjº+þL&SØ$ìüyÜE*¹¯8Æn·»Ýî°6Á	û¨æÇÙãOy_ãõë×ÕßbNøSÿZ¥©9ÊU%%%rqåÊWù_Ä­[·xf?"JüÏÌf³¼ß¾[=?tÏYèàDuaù¢@Ssèó®g­Z,å8u¨ðÑ,m6wiLî:h³H:¿;Îþ°ïlxùÚÐ_«æsp«®¢ø/Âàô?éå_ÁFQf*>Óì£³1DäÊG®?^p6ü)»'O]q8»wï¾qãÆñãÇeæ¶mÛÂâo6w7þæ:8ÁæÇÙão6þ¼úGÿ£9õXÝ¥K:¤kMDàt¿;ÓoÞ¾¯_£¼iÓ¦ÉéW÷ÇláøSö~A;6üuuu)ûÄÝÝÝÊ^=¥ööö°?ã2'üEe$Ói2×QþwéoÝº%¶Û¸qc(_xá|¢88òø+Pè,·éââbõ©¼wóÊ+Áø#"Ýáott4øî.eÇãÑ¼ß+777xòìBðäÒ²eËä«ú×°ùá=¹fÍ?æî2'üEàg5k>¨y£:ü]ºtI½üõë×«¯Õp-2þK²oUÝ£>ÊüQbàORªç_¾|yÛ¶m¦éGèéóÃßøø¸,M)züñÇÅCÁO	L£GåË<yòîyZø]æ¿ÈÓÞÞ.þ3ÁÊS7§Qþ¤+WÊO-ËW¬OpB4å»ÛíöÞÞÞ»îyu¹¥¥²4ù¡ó>ß¾û]ª"xùUÞ¾'ø#"U~¿ÿáÓgËÍã.	r4V9;(¦/,,d!"ðGD)ïùÓôì³Ï22Dþ0Ï·wïÞåË+ÇveâÉ'dXüø#""""ðGDDDþüø#""""ðGDDDDàÀ?""""DDDDþüø#"""DDs­½½Ýf³Æ6Ìõ¾EEEK,¹råJpLËâââ9lÅ¦ßmfsßyn[CRæû|¾;wÍf±¬¬,Ãñþûïïd¾ÜØd2É4k?"Ò]E°2>>>¬>|XîäÈàçNæ:t(9ð:ßétÊüÎÎN¾páLççç¯íèèbñìÙ³¬]DþHøéÒ¥KrßòòòàuëÖÉþþþø<ÈøãÏd2ÉüÉÉÉ°÷Ú¾»|ôÑGY»üå§Î¾û²²²2228 ¹Ù<PZZªYBpÇ¡LüñÇ2-s«®]»f·ÛÓÓÓFcII²·,tiêïù.'O´ÙleeeòBvóæÍºº:¹¯<ø½÷*;2§ë×¯¥É|YòG´@ü­X±Bævwwk®ÊÎÎïuëÖ-ùºlÙ² ç÷ÀÅÊÊôÓO?-ÓÍÍÍÂ,xöÙgÕ·q¹òÔíØ±C®:~ü¸LËW®¯¯W®Z³frèóêÕ«2ßjµ]úD¾îå_	§ÓúàwîÜ)ÓrW^yE&>´Úäääàà L(;&gz?ßL,Vß¦½½=8gÃo¿ývð.ÊQàM6Éô¶mÛdZæ¬uDþHø³Ùl2í.Ô^aßØÕÕÏæÍÕ¯½téÒþýûKKKe¾Á`»4¿"ßEy`YYY¡÷ÍÍÍUüÔÔL¹d¦ÜR¦KJJ³·oßß°hêëë¤	(KVæïÞ½;xQAêã?¾Ç@Dþ(¶øSÎTÎwH+##Cnéóù#2G¹êØ±cr/áÛo¿­^fiê³¹ËLL¤¨ÞQ§À±­­-;;[c6³ßóaÜ:;;ÅsêÃÜBgù¦7nÜ¸3Z¦/_ö1°Öø#"]àÏjµª÷ü);Ïî*¡ºº:¹6ø58_97bjºYâ/ò]L,zßeË)÷Õ<¼@ pöìYå,Ýà.Ã9ËL)F³LµªéÒ¥Kó~DDàb?å£[?®¼çï¹çÚÚÚÖyùåóJ)ïþ"ßeÿþýßûÞ÷dbÇ¡÷ôÑGeº¥¥å¿ü¥rUfÉ´Èìúõë2QPP°@üËüóçÏßùÝ»<üðÃwfþÔ?ïÇ@Dþ(¶ø¬éÔÕ·nÝR¹ÊWõg ÌfsVVÖÞ½g¿Èwq»Ýríºuëçh>~y÷îÝòÈM&ÓÆjÅ[C9¸¼¼üòåËsÐCÃ²Øºº:yòÃ.[¶lçÎòïüîó®ÕË~Þõ¼ø#"""DDDDþüø#""""ðGDDDDà/æ½ñÆÃÃÃñüÿó?ÿóþûï³>Å¢Ð?KQéßÿýßÃþÉ5Z`¿ùÍoO¡¨÷áþö·¿e¢Þÿ÷<Æ!üñÇÊ_î±í[ßúø/ßñ¿þë¿Om¥¨wåÊ_ýêWC,úáÈßE×¯_ÿéOÊ8Ä¢üä'òRÊ8D½[·n½öÚkC,úå/)ÿÓàÀøþÀøàÀøþÀøàü?ðþü?ðþÀø#ðþÀ?ðþÀø#ðþÀ?ðþÀø#ðþÀ?ðþÀøàü?àü?ðþü?ðGàü?ðþü?ðGàü?ðþü?ðGàüøàÀøàüøàÀøàüøàÀøàüøàÀøàü?àüøÉ¿ññqÍ:¿¿¿ßjµÆ¢¢¢ÎÎNðþü?ðGàü%<þº»»,	ó GKKL466ÖÖÖâO(v3MLL;wî&Å _üâC,§üÿqz|ðAOOãÞ|óÍ_ÿú×CÔûÿø·ÛÍ8DH^äÿuó¸ãÏ~ö3ñ_mãoãÆW®?³Ù<55%~¿?t× àï^x=uuuutt¼N1èütC,VV]Æ!ê½öÚk¯¾ú*ãd`ex¨÷£ýW±úîw¿+ 1;vìÇÝÏ;ÿW±Ä>ìû¿1þFcØiûrØ8ìËa_û£Çãq:iiiK¦³X,>Ã¾?xpÚd2?ðGàü?1b_0·Ûþ¹¹¹~¿ÿÎôa_àÀøþbÄ>¹XWW7000¿¨áO~'O	ùêp8Àø#ðþÀ¿X°¯¦¦fpppÞQÀr±»»Ûb±«ÕÚÛÛþÀ?ðþüÍ/1CUUU(ûÄ"Â.üÅ)ðþü?ðGào6ì³Ûí7öEàüøàÀøK!ö?ðGàü?àO§ì[ºtiÔÙþÀ?ðþü?Ý±/''GØàõzcôMÁø#ðþÀ?ðìàÀøþÀ_±ü?àüø)Ä>ðþü?ðGàüÅ¼@ ÐÚÚºjÕªEgøþÀø#ðþ~oó~äÈ£GNLLDyyy:aøþÀø#ðþ>éÌ3ÙÙÙ_*ÿÒ>¸ºdõ=÷Ü³?¡¦Oö?ðGàü?àïùÔ§>õÈÃøæåß<ø'ò'b¸h±Ïf³-:ûÀø#ðþÀ?ð÷¿½ôÒKeeeAù)ÿ>óÏÌé3'N¸÷Þ5ìçáHðþÀøþÀøIGµÛíü­Zµj```ìkjj=¥CoìàÀøþÀßÿÖÕÕõÇüÇûþn_P~ÿ¯!==ÝçóÝGÍìàÀøþÀß'Ýÿý;þvÈïku_³Z­O=õTdö555Y,Ð÷öéàüøàÀøû?Ì<xðóÿ|zzz~~~KKËL·ÍTØ½«V­zé¥ô¿àÀøþÀß¬òù|G1Í¡ìkmmMÍøþÀø#ðþîlþùÌÌLûV¯^æÌÄÚp?ðGàü?àoÆ|>ß3Ï<Ê¾8ÈþÀ?ðþü¿Y511qèÐ¡Ð½yyyz>¥ü?àü?ðþæÐÈÈÈÁCÙW^^ÐìàÀøþÀßï566vo°¯§§'9Æü?àüøwÃîí³Ûí]]]¾·ü?àüøäñxöìÙ³téÒPöÍòÏ»?ðþÀ?ðþ(ð'ì«¯¯OKKÓ°oëÖ­q~Õàü?ðGàü¿âoll¬¡¡!ÕØþÀ?ðþü¥þ<Ï®]»4ïínß¾=E&àüøàÀ_JàOØçt:C÷öUVV¦ÎØ?ðGàü?I¿ØW]]RìàÀøþaÙ'kjjäªÔ[ðþü?ðGà/	ñ'ë|XöÉÌáááT[ðþü?ðGà/©ð'/¸v»]sWa_ÊîíàÀøþ°ü?ðþÀ?ðøàü?ðþü¿À_Xöåää<xö?ðGàü?É¿Ø'¯¹^¯1àÀøþ°ü?ðþÀ?ðøàü?ðþü¿äÇ_ 8ú4ìàü?ðGàü%9þ­­­yyy°ü?ðþÀ?ðÌøW1ØþÀøàÀøKþÀñãÇ­V+ìàü?ðGàü%9ûN>º·Oæ477Ã>ðþÀøþÀ_ò°Oxg³ÙBÙ×ÚÚ*×2Dàü?ðþü¿dHõMMM«V­Ò°ÏjµÂ>ðþÀøþÀ_òäóùfÚÛwüøq^ÅÀøàüøIR hjjeß½÷ÞâÄ	¹vöÛÀøàüøúMãÏ<óÙlÝÛ÷ÒK/ò?ð§úûû­V«Ñh,**êììÔl[Õ+1øþÀø#u^¯÷ùç÷Ì3÷ö?ð§GKKL466ÖÖÖª¯jkks:3Ýü?àü¥2û=£aßÚµk»ººÂÒþÀ^2ÍSSS2á÷ûåÿ.ê«D~íííð×ÜÜüVýÙ³gß¢ôúë¯ËÖqE²Òöõõ1QïÍ7ßt»ÝC,ÿ±Èð26õõõYYYö¼ðÂïrÕ«¯¾ÊÆ"y	ûñçoÀø3a§¥üüüYZZzùòåPü9sf4ËÓfb²3±ÈårÉªË8D=ùþX$/¢²ÍgBûÕ¯~ÕÐÐ¡aßºuë?~íÚµÈw¿zõê¹sçÆX$°ó7M`üà´Édù	9ìËa_â°/9ìíÙ³'ô oeeåì_9ìËa_½ë÷ûÃ¾2=ÓÍB]þÀ?ðþ¾ááay½ËÌÌÔ°Ïn·ÏuàO/ÕÕÕ<yR&ä«ÃáP_ïñxîLïù«¨¨àÀø©ÓØØØO<±téRûª««ç·î?ð§º»»-Á`°Z­½½½<âévéëë+,,4ååå¡Ç¶Áø#ðþÀ_R622ât:CÙWWW'[Ëy/ü¿ü?àü%Y§¡¡As7--mûöí_ßÀøàü?àO/íÚµK§aÓéÊ·àü?ðþü¿ÅÏãñ=È»uëÖÁÁÁ(~#ðþÀøàÀø[|öîí«©©.ûÀøàü?àOwìSNéÖA^ðþÀøàÀøÓ)û½~kðþÀøàÀø_òUUUöåCmcøàü?ðGàüÅv»]s7ìàü?ðþü¿Ec_NNÎÁãÉ>ðþÀøàÀø[öÉ×ë]þÀøàüø)Á>ðþÀøàÀøK!ö?ðþÀøþÀ_±ü?ðþÀø#ðþbÈ>Í¦+ö?ðþÀøþÀß­­­«V­Ò°///OæËµ:|Ìàü?ðþÀ?ð7Oö	òàü?ðþÀ?ðBìàü?ðþü¿bøàü?ðGàüÍ§OeßªU«ÑÅßÄÄDbýààü?ðþü¿»'O¦¦&Í¦aÝnó«~ð'Þ½çÌfsvvvee¥(Õü?àüQÂãO9È»bÅ¤a_Tð'còió§yøß<°ïïö±ì_ü"«+øþÀø£Æ<Y9b6QÁß=÷Ü£È/øïsûÛífàÀøxøé ïêÕ«Æ7Á×ë«å'ÿ6lØ VfàÀøHøÖÈÅbÑ°¯ººº§§'ÎlXþd²³³÷<¾G¿uëÖ8q5ü?àüQbàOÏ?ÿ|NNNèA^a_òìû×a]P~ãü?üÃ?ôx<¬±àüøàô¿±±1a_èAÞòòò$xo_ðçõzÿò/ÿrùòå6løÂ¾ òs¹®àüøàt?å offfèAÞÁÁÁäØ¨|ÎÛí>räHss3ûüÀø#ðþÀécccÏ<óLèA^aßÀÀ@*,áü?ðþÀ¿À°ï'ÝÛWYYïíàü?ðþÀøKQü=È»ûö<j	þÀøàüøKZü	ûNgZZZÊäàü?ðþü¥þ¡yE555)Ë>ðþÀøàÀ_âÏãñîí2SSÁøàü?É?±Ý®]».]ªa_CCl^RðþÀøàÀ_à/ìÞ>Q Ì×cü?ðþÀø#ð$øË¾ÌÌÌ0àü?ðþÀ¿$ÁßLì«¯¯ç½àü?ðþÀøÉ?óPöåääÈ+×ëeèÀøàüøI?ÙàÛívÍöÁ>ðþÀøþÀ_²áö?ðþÀø#ðþR°ü?ðþÀ?ðøûþ÷¿Ê¾¼¼¼ææfØþÀøàÀøKdÃ^ZZÊ¾ÖÖÖ@ Àø?ðsü-¹[üøà¢Â¾°û`øqÅánFðGàü?àü%	þôøþÀ_B'°;út(ûl6[cc#ìàOøÜ±cø#ðþÀÍ­­­yyya÷ö]¼xqöÛÀø!þä¿bF£÷ü±?ðGà/FìSööÍòoûø±Å_qqqèÙf³Ùçó?àüQTØ§þÀøÓþL&<EÇÇÇ-Lù¾÷½ïÉDýÜßßoµZFcQQQggç,¯àÀøKtöÙív·ÛúÞ>ðþÀ.ð§<QeB´'W¯^.Ùáp´´´ÈDcccmmí,¯Rð'ûM»qãÆ¹sç~C1è¿øÅàà ãd¥`¢lzzÈMNN677¯RÃ¾/éK6ào¾ùæðð0£õÆÇÇåC,úÙÏ~&þó79þ²³³åéÚÝÝåÊxòÉ'ÔÙlGÊßï·Ùl³¼JÁß?ýÓ?uÅ±ýèG]ÎOÇ8Ä¢üà²ê2QÏív¿úê«C'õþýû­V«%%%wÝtËÀÊð2Q¯³³S6C,ÿfÇÿU,æøÛ»woðôõÓ¸¬¬lKVóQCÉWqØÃ¾Äa_ûê3¯×+ÛçÐ¼³ÜhsØÃ¾öÕÅa_é©§Z¶lLôööÊ@°´´táU/l2fyøþÀ_ò±ü?ð§/üÅ¨ÜÜßgúØ®LÏò*ðþü¿äcøà/%ðWWWwòäI¯cW?ðGàü%ûÀøúÂ_AAò/ÑýçîînÅ"Ë±Z­½½½<âé3Ã^þÀ?ð§göUWW÷ôô,dÉàü?]à/??_¾`?ÛwÞ?ðGàü-JcccT>öUÃ¾/ü?ð§üóäÝ××§|öàÀø?ûxâÌÌÌ±ü?ð§#üÍfyzëG~àüøÉÇ>ðþÀð×ßß/Oò;wÞ¼yüø:ø'ûÀø:Â´|ùò%!-üðGàü?ØþÀøÓþÔ>ü?Jnü644hØæt:åE.Ößü?ð§ü)Ï|Ùèç§àÀøûjjjÄy¡ìóx<ñyàü?]à/77>ÀøÄøÓûÀø:Â_WWlöîÝ«Wðþü¿¨$¶áiØ'mß¾=ÎìàüéKf>ü?ðdì[ºté®]»ëQ?ðþt?ÃqÂ?ðþ###ûØÀøºÀàÀøûrrrd£êõzõðÁøºÀÍf[¹reÎðàÀøQ²Í¬ªªÒ3ûÀø:ÂÑhm®~*ðþü¿Ù³Ïn·kÞ´­Cö?ðþt¿ÎÎNÙR:thrrR'øþÀ?ðdìàüéíþÀø£ÄÂ_XöÙl6=³ü?ð§#üq¶/øàaÙ×ÚÚô?¶àü?]àO?ðGàü%ûÀøàü?à/ØþÀøÓþü~ÿÆÓÓÓe±eËÅ=óü?àOÿìg÷W¿úÕüüü²²²øÍCàüé²Æ=ác_TÀø#ðÊøE	ïV­Z¥ç½2DÙÙÙ_þòwüí¯oÿúþénÚ´é®ü?ð§üÊ6E´7oÞ7o9kÖ¬þÀø?ûyú?Èûgögòbqàûþnßç>÷¹Ó§O?ðþ&I¶,êmßï92üøàöæóùÌfóþ'öñ'ÿî¿ÿþ½÷?ðþA¶/¾ày99|Ô?ðþeeeº=¥Cð·lÙ2þî«¼ü?ðøSûVTT(å«LËðGàü¿ø³Ïn·Çy8|àAõaßÏ~ö³öà/1ð'ÚÂÇ7À?ðþ`_Øsss¿¸î_ýêW¿²ù+¶ÏØ¾þõ¯sÂø?e¥Ù²eKVVÁ`¯2g*ðþü%+þÁ&&&¾ùÍoVVVþÕ_ýÕ©S§ø¨ðþ	zü?É?ùe%ûæøàü?ðGà/ùñçõze³£a_ee¥nÿ0øø[r·ø#ðþÀ_,Øg·Û].WÂýq6ðþÀ_bãÏ0sàÀø1b_UUUêäàüé3õøã+[¨S§N?àüE555)>¶àü?á¯¿¿?##C9¡þÌgðGàü¿°O6ª===,øàO_øÛ¶m²:öì¢ÿTàüøK,üÍÄ¾êêjöö?ðþt¿W^yEÙHmÚ´I'?øþaÙæt:eåg0ÁøúÂ¬+kÖ¬QÎíèììÔÏOþÀ?ýãoddd¦½òjÁ0?ðþt¿^xAÙNÕÖÖêm Áø#ð§güy<§ÓÆÞ>ðþÀ_"áÏùàüÑK.Ýµk×ðð0CþÀøÓ5þwËh4?àüE`²·ö?ðþzü?úÁ¬Ãa÷ö544.ðþÀøþÀ_àopp°¦¦&toß=FFF(ðþÀøþÀ_àïØ±c[·nÕ°/33÷ö?ðþÀø#ðþ*Ùø¬_¿^srJÇØØãþÀøàÀøK«««5ìËÉÉ-ÒÄÄãþÀøàÀøK×ÚµkÃ²Ïëõ2>àü¿$ÁóþÀø#ÙÔ²ïþè¾ñoÀ>ðþèNÎ_Xüñ9þÀ_²Z[[ËÊÊÂîíï½÷fÿ·	ü?ð0ø&èMÞC=tóæM¹(_7oÞ,s.þü¿ä«££#//OÃ>ÓÜÜ¬ìíåßö%ðþÀ_¢âÏl6ËOþã÷ûeÅbYàûûû­V«Ñh,**êììT_%ÛVõfü?±N¶raßÛ'ìkmmUoÁøäøS6>þþ?ÃÑÒÒ"µµµê«ÚÚÚNçLwàÀ_tÙ×ÔÔd³ÙîÊ>ðþÀ¥þrsse#(PUG.ú|¾M6É¿À%Íæ©©)E²ÙU_%òkoo¿æææKq¬¿¿ÿ?øÁ%A¯OÇ8Ä¢³gÏQÉø>|ø³ý¬2GæÏ4zo¾ù¦ÀÑE2°/^db±ªËqE]]]?þñãüMc¿.=áãí·ß^àÕ§hNÉÏÏ¯¨¨¥¥¥/_Åß+¯¼2Ç~ýë_»Ao½õØqE²ÒÊªË8í½÷Þ-É+4[6óï|gxx8Âå?úo¼ñcäEôÝwße¢Þ|pþüyÆ!õõõÄùÆÒÕ«WEcééé!##£¤¤$*»åÕM&SØÛsØÃ¾Äaßh%c2§¼¡qØÃ¾ö¥$?ìå¤:#77Wy+¡|p9ÔàüøG>oìàüø[Puuu'O	ùêp8ÔWåçç<;Óþ***Àø#ð·¼^¯l7233È>ðþÀ¥þ222µ¹¹¹mmm_lww·ÅbeZ­ÖÞÞÞOñôNÁ¾¾¾ÂÂB£ÑX^^.þàÀßBØ£aÝnw»ÝseøàRÊ<«ÿ¤2ÝØØ¸X?øþÂ¾n@Àøäø³X,²¹â¯··W¦³²²À?ðRìàüQJà/x~FSSSüm_à/ÙþÀø£Àò!ÏÊÞ>Áßïß·oLk>üøÇãihhe_YYYÔ7àü?Jrüuuuýç/?àOìs:iii¡ûdó5¿S:ÀøÒøS¶t¥¥¥ÊÙ¾éééÊç°?àOì)ÎÀøüøÓ[àüQão&öUUUÅeàü?JrüÏó&ÏÌ+V?àO'ì«®®Ûfü?ðG)?¿ßÏÙ¾þÀNØWYY)ßx>ðþÀ%'þV¬X±$bÙÙÙàÀø[DöÕÔÔ,ÂÀøø»víaºàßöP'òåWÀ?ð·(ì«®®GþÀø£äÄ_0¡Þ"áàRò¯ªªe_]]¬3ûØÀøäøÓaàüQãOÝv»]c>Q ÓéóûÀø¢øóûýÁ9Ë-úé§Á?ðöI[·nÖÏãàüQãoùòå~Íñ¡CÀ?ð;ö¥¥¥íÚµkq?Rü?ðG©?É$[aõ«ûûûeNVVø#ðþl[ªªªÂäÕ!ûÀøøSNøú½å|øø#ðþæÊ¾Í)K.öèy`Áøäø³Z­²EÞ½·ò*"+ÐdÅbþÀßüØWYY©ÙÛ'ìÛµk×ØØþü?ðGI¿¾¾¾°òÜÛÛþü¿9¥¼·O³·/''çÐ¡C:ßÛþÀø£Â4::ZTTn0222eÎ"þTàüQÂáO´úÞ>a<½^ob,øàzü?J üÉ³µ¬¬,9ØþÀø#ðþü¿s¹×®M&ö?ðþ(Uð744¤|Î³ronnn[[ø#ðþÂN>Ê>ÍèìàüQJàOÐÜv+øS¦Á?ð§ÉårÝï½öåååµ¶¶c`Áøäø³X,²íâ¯··y&ðþÔ	ìwIÏ>ðþÀ¥þ-øÕ;OMMÉ´ÑhþÀ|áÝªU«Ràü?J	üåææ*ê§àÏï÷ïÛ·Oyûø#ðÊøSööÉ¦ uØþÀø£À_WWWØy¾xñ"ø#ðøÅ=z4ÙþÀø£À²¥+--UÎöMOO/((XÜ?¸þÀ-þ¼^¯°oÅ©É>ðþÀ¥þôøKnüMLL¸ÝnË%~ðçóùÌfs*³ü?ðGàüQñ÷ÏÿüÏÙÙÙ^úç«W¯ÎÌÌüùhÑñçõzåI'¿ûÊÊÊRàü?Jü½ÿþû+W®4L²¹OOO/**uü¹OúÓ;þvÇoõß¨7Í¼Ê."þöåäähØg·ÛãüàÀøþÜnwØ>ä§]üÝwß[·lUä§üûrÅëëë¥øãö?ðþ(uñ§|È³Ãáðù|rñæÍµµµ2Çjµ?.þV­ZÕðÿÔøûZÝ×DR<ñûÀøêøS¶ûê·õÈtðÁE÷ßÿW6E?þâ¿°ì«¬¬àü?J!ü)þü~¿zbÏÅ.Ël6?òð#üþú¡¿þÔ§>Å«lðçñxNgZZUUU===$øàRÊþ¬:rñÆååå¼çb?©££#''gåÊÿüçï¹çá CSüÍÄ>yËï1àüQ*âoÉÝÿñ_ðÄø»3ý¾_N¿KÊøÈü?ðG)?ÃÝ2à¢?5þ"°o÷è?àüé:ü?þfbÝnç½àü?4Óç9?¿ØWSSÃÞ>ðþÀ¿ß_Ä%O=õfæöíÛù¨	¿ì`Àøþ´	òäu"77WyB¾øâÊ+GüßêþÀÍ©ï|ç;_ÿú×aøþÀßÛ¾»òaµZÍ7/âOþÀEN v»]c>Q`=yÁøþfÕ/!MMMûS?ðGseünppñàüøU=öò¢üµé¡þN§sxxñàü1ào¶Fy	ÉÊÊºtéÒÕþL&ø#ð§göÝwßï½÷ãþÀ?ð7ÇE,Y²ÿ~ÍL§ÓÉÙ¾þtË>eoßìÿ¶/?ðþüý_3ÎlþÀ¿Åª«««ªªJÃ¾¥K644LLL(·àüøºKdi³ÙBç÷÷÷[­V£ÑXTTÔÙÙ	þÀs¹¡ìÛ³gÏÈÈúàü?ào.÷D`7òÅùÕÝÝ]XX(½Êáp´´´ÈDcccmmm(þÜn÷oãØäää¹sç~K1èwÞbfSGGÇ_üÅ_hØýäO~øá¡·ÿ±üçþ'ãõXtñâÅk×®1Qobb"Î¯©ÓÏþówß7Îß4ñ·qãÆ+W®ÅÙl	¿ßºkPðwäÈÎ8&ÿa×ÝNA®éÈ=ýôÓÊÿÔeffnß¾½½½¦ÉJ+«.£õDÕgÏebìøCÆW±Äz;wî¿iã/¸¨Ðê?ú§D8ìËaßÔéôéÓk×®Õ°/''G^¯7ò9ìËa_û9ì0øS/<ô3eÀøKúÀ3gæÍ>ðþÀ?ð§ü_Ã"ã/77×ï÷ß>ì+Óàü¥ûZ[[W¯^­a_^^^ssó,ÙþÀø#ðþô¿°ß(tf]]ÝÉ'eB¾:ðþR/½ô /ÂA¹v®àüø	?åbww·ÅboaµZÁøKîh'N"ûÀøþÀß|L9þÂ¿¨°Oxg³Ù¢Ë>ðþÀ?ð7çw+ô$Gào®ì[±bE,ØþÀø#ðþ*ðþ:¯×ÛÔÔ£½àü?àü?ð§|>°Ïb±Äàü?àü?ð·MLL=zÔl6Çàü?àü?ð·8y½^Ycsrr4ì³Ûín·;Fìàüøàü?±/>+0øàÀøàü¥ûÀøþÀøà/ØþÀø#ðþÀø±Íãñ444²¯²²Òív/ÊCàüøàü¿°Ïét¦¥¥éàü?àü?ð?öUUUÅÿ /øàÀøàüÅÕÕÕ===:yàü?àü?ð+öUUUêê¡?ðþü?ðþÀ_Ù'·nÝª7ö?ðþü?ðþÀ_ôÙWSS#OÝ>lðþÀ?ðþÀøûd¦<r-øàÀøàüÍ6YÇªªªBÙW__¯ç½àü?àü?ð7göÙívÍùÂ¾]»véoøàÀøàüE'áÆü?ðGàü?ðþæÀ¾¥KûÆÆÆtlÁøþÀøàoVìËÌÌLhö?ðþü?ðþÀß¬Ø³gÏ$[ðþÀ?ðþÀøûåååIvü?ðGàü?ðþÂ°¯¬¬,toßO<Lìàüøàü¥:þÂîíöÉêäõzrlÁøþÀø©¿®®®°ûàü?àü¿TÄ_GGÇ½÷Þìàüøàü¥þ×êÕ«5ì³Ùl)Â>ðþÀ?ðþÀ_Jà/9s&to_^^^kk«RcþÀø#ðþÀøKZü	ìw+V¬àü?àü¿dÆìàüøàü¥þ`øàÀøà/%ð'¦9qâìàüøàü%9þ|>ßÑ£G-ìàüøàü%3þÄ1MMM°ü?ðGàü?ðäøóz½G	e_YYìàüøàü%þòëÎÉÉÑ°Ïn·ÇyþÀø#ðþ(øàü?àü¿Àìàüøàü¥þFFFÂ²¯¼¼ö?ðþü?ðþÇét¦¥¥îíëééáðþÀ?ðþÀ_ÔÙÙ¹mÛ¶PöUWW»ÝnÆü?ðGàü?ð$)û[·níééa|ÀøþÀøIÅ>ÍÞ>¹(ìã5ü?ðÇ8?ðþÀ_³O9È;88Èø?ðþÀøàü%9û|ðÁsçÎ1>àüøàü¿dfòýßö%ðþÀ?ððø·Ùla·­j(¿M^ Ã²o×®]òlQnþÀø#ðþÀ_ªà¯»»»°°0ÔvR[[ a¦;?ý'¿ »Ý®9Â»téÒíÛ·<õ-ÁøþÀøKümÜ¸Q^øÃâOä×ÞÞÇ;½õÖ[gÏfüjÖ¬Y£aßüÁlÞ¼ùÕW_½ý§cÜb¬´²ê2Q¯§§G^GXäv»¨×ßßvLïõ×_ã7âüM<ÄpøËÏÏ¯¨¨0¥¥¥/_Åß¿þë¿~Ç®^½*O)bòK)++Ó°Ïd2=òÈ#²Ýé^rÕOúSF/¹>øqzCCCò?Æ!Éëè;ï¼Ã8D=ÇsîÜ9Æ!ýä'?ÿfÇù&'þsØWç=È»gÏ¯×ù¾öå°/Ã¾öå°o2ö=#2þ$Éþt¼nÝºUsJGffæ¡Cg³ðþÀ?ðþa9:3??_9-`tt´¢¢üé°êêjûrrrä·3222ûå?ðþü?ðÒøS.öõõÆòòrñøÓU.K~/¡yxâ±±±¹.ü?ðGàü¿ÔÂß<UØp1Íß2ÁøþÀøàOwuuu­]»VÃ¾çÞçó-dÉàü?àü?ð§£ÜnwèÞ>ÅräÈyïíàüøàü?ÝuæÌ¼¼<ûl6ÛÂ÷ö?ðþü?ðþÀ^/½ôRèA^³Ù|äÈ(²ü?ðGàü?ðþgÎ¹÷ÞCò8qB®Å7àüøàü¿x'Dhnn=È+sd~Ô÷ö?ðþü?ðþÀßâZ[[W¬X¡aßªU«=£½àü?àü?ðï|>_SSSXö	ãÀ>ðþÀ?ðþÀøGaÅb	=ÈæÌ¸±ü?ðGàü?ðþbÏçægÌf³«W¯îèè3ûÀøþÀøà/V	ìl6eeeñ<ÈþÀø#ðþÀøñH ßêÕ«ãü?ðGàü?ðþâTSSÂ¾µk×ÆyÁøþÀøà/Þjjj×¢ïíàüøàü¿ü?ðGàü?ðþÀ?ðþü?ðþÀ?ðþü?ðþÀ?ðþü?ðþÀøàüøàü?ðGàü?àü?ðGàü?àü?ðGàü?àÀøþÀ?ðGàü?àü?ðGàü?àü?ðGàü?àü?ðþü?ðGàü?ðþü?ðGàü?ðþü?ðGàü?ðþü?ðGàü?ðþÀ?ðþü?ðþÀ?ðþü?ðþÀ?ðþü?àü?ðþü?àüøàü?àüøàü?àüøàü?ðGàü?ðþÀøàÀøþÀøàÀøþÀøàÀøþÀøàüøàÀøàüøàÀøàüøàÀøcàÀøþÀ?ðþü?ðþÀ?ðþü?ðþÀ?ðþü?ð¢øëíí-))1ÅÅÅýýýê«ä¢Õj«:;;Áø#ðþÀ?ððøËÏÏ¿xñ¢L:uª  @ÃáhiiÆÆÆÚÚÚPüÉJ|+y½ÞsçÎÝ¢ôÎ;ï1±HþÇ299É8D½ááaù¿+ãäEáÃ?d¢Þ7Ün7ã~þó¿ûî»qþ¦	?uéééêf³yjjJ&ü~¿ÍfÅß?þã?¾Çä9ÓÑÑñÅ ×tC,VV]Æ!êªÏ=Ë8Ä"X^ÆW±Äz;wî¿i2à¯¯¯¯¾¾^=Çh4æ°/Ã¾öå°/qØÃ¾¿7o:Ï§i0Ó&	ü?àüø	¿%¿K¹8::êt:eKª¹Ynn®ßï¿3ØW¦Áø#ðþÀ?ðøS×ÕÕUQQ1>>zU]]ÝÉ'eB¾:ðþü?ðGàü%<þl6ÛU<âéîînÅb0¬Vkoo/øþÀø#ðþóü?àüøàü?àüøàü?ðGàü?ðþÀøàÀøþÀøàÀøþÀøàÀøþÀ?ðþü?ðþÀ?ðþü?ðþÀ?ðþü?ðþÀøàüøàü?ðGàü?àü?ðGàü?à/Õð÷÷ÿ÷§Ocï¿ÿ~KKË0Å ×_½««qE­­­ï½÷ãõÞzë­ïÿûC,ú·û·Æ!êÉÿ±¿ûÝï2±¨³³ó7Þó7õz½)¿¡¡¡|(õ|üs	ûcR'ðGDDDþüø#""""ðGDDDDàÀ_b4>>n³ÙKJJFcqqq¿ú×¯__¢¡ëØ­Y³FÆ¶¨¨¨»»[Kj«ÕªÙÙÉÐEk`Yi8¶JçÏ=VÚ,+íÇ6Â²ÒÆh`õ°ÒòTCòJYXX¨þUåçç_¼xQ&N:UPP ¾q[[ÓédÐæ=¶²¹ioo	a³Ù¬¾±ÃáhiiÆÆÆÚÚZF/ZËJ»À±ü0tÎJ£e¥]àØF@VÚ¬VZð76nÜxåÊ®¾(¿Zå%8¶.«¸¸X=GÈ255%~¿?t÷Í`Yi>¶O?ýô·¿ýíÐg¥ÑÀ²Ò.pl# +mV+-øûíëë«¯¯WÏÉÏÏ¯¨¨0¥¥¥/_fÜæ1¶òý¬¬,yêÔ)õ|Õ°Ó´Àe¥]àØ^»vMN^/C·¬´1XVÚmd¥ÑÀêa¥QÀßÍ7ÏçûÑÑQÍþ¬Ï?«c0Ó&qÖÀ²Ò.pl·lÙráÂ°ÎJ£e¥Ö!tYic4°zXiÁßB»òËs:×¯_p6yæþ§SÈâ÷ûïL/4e¥]àØ.ùýXiã0°¬´QÜ h6F«ü-è·ÛÕÕUQQ1>>z³üü|Ç£èPnÃ¸ÍuleîLR­Àººº'OÊ|u8[´6ZûÐ¬´1XVÚof@VÚ¬VZð· ß®Ífýÿ¨2Ñ××WXXh4ËËËåÌ¸Íulûûûd7lØÜ±ªÜ »»Ûb±«Õ*aÜ¢5°¬´±0+mLvcvYic:°zXiÁQ*9! """DDDDþüø#""""ðGDDDDàÀ?""""DDDDþü?""""DDDDþüø#""""ðGDDDDàHÝ/¾¸~ýúôé6lØðÊ+¯üÞælºÙø´6M~4Ï§/sF£Õjë2À%dXÒSO=Lø;tèÌljjÒÌ?zô¨ÌòÉ'ç±L""ðGDW¿°Æh4;v,0ÝñãÇå¢Ì¼téRÒàïêÕ«2³¸¸X3¿°°Pæ<ðGDàR¢zHXóì³Ïªg>÷Üs2sûöíjútvvÄ%%%2¼ñ7êëë³²²ä*³Ù¼÷nõÑU·Û-À«ä¾.KÃ)½~ýú³gÏÊÅºº:Íëèè¼å*yHÊUçÏ	j2¿··78çâÅ2Çn·ç>|Øb±È¢ÒÓÓ·mÛ6::¿ÐåkæDx¨Dþ¹åË]»¦ùÑGÉLÍ¦Æ¦¾¾>åÚÍ7k®Ú¹s§rÕàà Á`/å¢rí-[¦¦¦&)ÈµòUð¡¼/Ârd"ìÃýIO:¥mÐ---Aùi²aÃ¹â/ÂC%"ðGD´ø)GxÃlÂ,©qS[[s:6mR®U¬£ðQÙ'hS®r8Ê.CîîîV¢^æþýûwÊQ×GTæ´··Ë´|é~ø®Ë8ÊE¹ú±ýË-w||ÊWyä"N¿ß¯ÜÀjµÊåG¸ó»ÃÄééésÅ_JDàHøÂâ/8_Áb&ittT.%%%rqåÊ;vì´Ýºu+¸¹f_Zð^ÊÅ>ú(xcSðÈ¯|i·Û×å4åâÇ¬~l3½?ïñÇ«¾ýíoßùÝ¡íÝ»w«o ü	IKKKsÅ_JDàhñSvwÝ¼yS=ÓçóÉL¹*w4¼råâ¿ uoÔ=«áúVdÚl6L¦Û·oËÂ³³³×Þu93QLÓÐÐâT¯2=88¼¶»»[@ØÃÇ³Ç_JDàhñSÞ÷öÂ/¨g*¢9á#ø¾ÀñññÐZ.]:tèr6¨Fe·¸ê]¶sçNåh¯|­¯¯Î°eOÛõë×üqä3s×­['×*n#fU_¥ù+W?~rr22þ0UF#xmJDàhñSN0ÊG½;vÌd2±iÓ&ÎíÛ·c²Á3s÷ü)o»råúÍwïÞ-TRÎÃ~ØJX¢)§ß*ß½««+8?Âr7ù)ïùóù|Ê-#àïøñãÁrò#«¯RÞþ888(ßEa&ü)Ü.ËÍÛ¶mS_á¡ø#"ÒEÊ k:|øðÿmÎ¦Sl0cìÑGUËR][[[üÝþS2ßl6«GX<õi;ÓëÖ­ôôtåGÐìÞ¸q£ú[,[¶L¾*ö¢^¦²c2BÕàµ*?""½$@Ù°aiºõë×+§Üjðwþüyåãô.¼ÖçóíÛ·Ïb±(`züñÇoß¾¼ÖårÉ¬Vkss³f¡D%óì1Íü#É¤|aÏùV__¯ùÌ¥ññqÃ!#%?Çã	~lz¢[e¬ìvoo¯æ;Fx¨Dþüø#""""ðGDDDDàÀ?""""DDDDþü?""""DDDDþüø#""""ðGDDDDàbÖÿÛ$àiÿIEND®B`


Ê*ªIþoëÞïã¸Ç,Y~KzYÏ÷ùzÎC""""Jæ°	À?""""DDDDþüø#""""ðGDDDDàÀ?""""DDDDàÀ?""""D4çGµ´´Äëw,Þ¥§NªªªÊ~TeeåÉ'G½Á±_eNDòùeeeGÀÆéîî6øö/]º´jÕ*N'_=êæÄ)Iv?"Òþòòònß¾­üíß¿_Mûö%¸µq]%®><Þ3áÍXTTþºf³üø#¢©4´qãFMà¯»»[Ñêo¾FFFdA>n·;æM÷*Q_úÁ»ví5²ÃrÅû÷ïËòððpRí"D¤yüY,ùÿÔ©Sññé§nÞ¼Y9fZSSãõz£>ÓápÌ7oõêÕá5íííË-ÓétUUUýýý.]*++ËÌÌvmä»(,,ToÊ_8O?ý´¬<+µ´´ÈÊúúúßãx¯¢þÒâEY#wr'æ;sMìñu©ì%KÈf_·nlÛÄïª/Ü­Óé[í V._Q*[R<-åççïÜ¹3ðà"D¼øðàAQQQAA,¨pëÖ-yEä|fÒ222äÿM6=uÕªUâð°ü¢>sÍ5³páBY÷îÝÈKYY\óïU¢¾´ÈFyç¯¤¤d'±á&|Å±ã¯§§'òvä/þ"w«ÛíV>×ÝÝ­|òÆ£îóöíÛyp?"J^üÉÂÙ³g#ß	tÀÖ­[e¹ººúþ£dA>¹wïÞå½+eÍ¶mÛB¡Pgg§ò¡Ü²|èp8dY ¨ÙváÂY¾~ýzäEñ£Óéb®¼î$¯oP]øÑñn÷jÂWL0à/ê6Y®©©Ö6vüEîV¹YÓÑÑ!ËÊnÝ²eòÉoÞ¼)Ë²7e9''ø#¢¤Æ$¯å²,¯ëQëdùÖ­[Êò/.X° òÂ×8"?xø«ã§æ.2***·FÅ_fffÌo$æú	Ur]qÑht:17ÚX6NÌ5á+Ê¸ÆþþþÈ/1.üEîVåÖ"5ÊEåååòáâÅ¯ò[ÄÐÐ,"ðGDÀø,??_^ÂÁ`äzõ;gêp¢º·øCfÔ¡ÏQÏZ-,,TS«¶HÔ­å*ñ¶É¨m,'I'vÅ±öï_ªÞ­Qóà(y<Åa?ø#¢dÇôÖ[oÉáQÏ¢Þ£c1Dâ,Y¢L¹ÒÞÞ.8þ·'YWjjjvîÜyÿþý£GÊÊÍ7ÇÄßX®2aüwãðÇ¿±¼óæÐ?ñöÏÉÉ5Q§GÖÛÛÛØØ¨kMDàÞëWÖ(çWWW?xòê¾mÛ¶ÉãOy÷ËívA92ü]¼xQyoI¼ØÙÙ©¼«§töìÙßãx¯2.ü%Þ8Ê<*êFÆ»U'?eØwhhHl·~ýz5:$_(N¼ý(tÏ-)ËË-ü6±'Á!x"Dtø»ûvxt²ÆëõF÷*((<;ü9¢´`Áù?ò×÷ùgQbEosþoðQ5O`«N½½½·¿zõêÈK£¶p-1þéc£¼·ÙsÏ=Çü6ð')D#×_»vmóæÍª©©QÞ11üÈ­ÉmþyñPxÄÄyã7ÊÊÊ222.üøñ'x"üÎÓä¯2.ü%Þ8gÏÿétºðTyk«NÒùóç/^,ßµÜ¾r`=|³|(DS¾ºÑhìêêõWÃQQQ!·&ßÍf¯»wïVÞRÁË®<¸ÀÑt5<<üÌ3Ïkn¹	)GcÓ·C¡bú%KðCDà(SÆüEõê«¯²eü¥`@`×®].TíÊÂþýûÙ,Dþüø#"""DDDDþüø#""""ðGDDDDàÀ?""""DDDDþü?"¢ÉtöìY½^¯ÓéÖ¬Y3ÞëÍ3Çãñ×È²¬Y¶lÙ8Ñ5±ÏËu'ò$§È222²³³kjjø)""ðGD©°°P(#	Bã½îäºÍÍÍá5¯½ö¬illÔ4þÜ~äùÆeyË-üø#"í<¡LÂO½½½rÝªªªðU«VÉ¹³?á²,gggóSDDà´$¿HÐìÞ½;///''gß¾QöÄOTTTDÝBøCY¾÷®,Ëå¢7oF±N§+//ïèèyk_=ñU?®×ë+++å©)688h6åºrçwíÚ¥¼)<]½zµÜ¬[¾uëÖ4áïÚµkÊw422"w^>üø#¢dô²|ðàAY¶ÙlÂ,YxõÕW#?Çáp¨Ç·mÝºU.:zô¨,Ëÿ²_¯bÅ.ÈÂõë×eQQQÌ[¼¯"¤ë­·dÁb±¨ïüöíÛeY>áôéÓ²pàÀY¹hÑ"Y~ðàÛíåÉùÆ?lY®««S.jllT6Å¡Cdaÿþýütø#¢¤Æ^¯åáG©ísPàÅå¢êêjYÞ¸q£¢ºð¥½½½÷î­¨¨PÎykQÀJ|åååå©¯[PP Üya,ûd¥|¦,gÁàd6Näå¾ÉüDÊEò¥ånäääÈütø#¢¤ÆN§/Ë|Ï@áDZbùÌ@  ]5ÊEGk?þøãÈ[ºµÈÇrxwL4ùfÇS§NÍ7OY¯¼)8%ïüÅL9ßE<hNDþ(IñWTTùÎòæÙ¨â1ÍriøÿðúÌÌLY3ò¨1â/ñUÂwL<§¾îëFÝ½P(táÂÅùá6Î¨âÁ555«W¯÷÷÷óÓEDàÊÔ-GUÆü½öÚkcÁß©S§Âo¡½õÖ[áõ%¯^½ªþ_eïÞ½o¿ý¶,lÝºUÝçNO8ñãÿX9Ô+++++eùã?ÉBIIÉôáoçÎrQooogggäÀD""ðGDI¿P($ÀÊyTä}ñ744¤rÿÃà¤óçÏççççååíÚµkøK|§Ó)®Zµ*|ÞIäçáÜóÌÌÌõë×+'öùjjjÓ«ªªÆúíØñ'`õfõêÕò¡ÛíæÀ?""""DDDDþÀ?""""³Ó~xãÆvaþçþç'?ù	ÛAsýë¿þ+As]¿~óf·þô§ÿõ_ÿÅvÐV÷ïßÿÙÏ~ÆvHSüç;ßÿ±ôóÿüâÅlÍuþüùÿýßÿe;h«üà0BsüñÇÿùÿÉvÐVÿþïÿþãÿíþü?þÀ?ðþÀ?àÀø#ðGàÀøàüø#ðþü?ðþüøþÀøþü?àü?àÀ?ðþÀ?ðGàÀøàÀø#ðGàü?ðþÀø#ðGàü?ðþüøàü±Áø#ðGàüø#ðþüøþÀ?þÀøàÀ?ðGàüÍ~]]]ååå:nÙ²e===àü?þÀøKeü_¾|YN<YRR¢Æ_û Å¯¿¿¿££í ¹Â¶¶_´~úÓ²´Ëåú·û7¶¶ùýð?d;$NÃø,;;[¿Cý_ß?þã?¾óÎ;lÍuîÜ96æÇü®ÅvÐVÃét²´Uûï½ÇvHà¯»»»¾¾Ã¾öå°/qØ8ìËa_û¦òa_¥ÁÁÁ@ þÀø#ðGàü¿ÇßíÛ·-K¿ú"ðþÀ?àRµk×Ä¼ü?ðGàÀø£Â^¯øàÀ?ðþRàüø#ðþüøþü?àÀ?àÀø#ðGàüøàüø#ðþü?ðþüøþÀøþü?àü?àÀ?ðþÀ?ðGàÀøàÀø#ðGàü?ðþüø#ðþÀøþü?þÀ?àÀø#ðGàÀø#ðþüøàü?ðþüøþÀøþü?àü?þÀ?ðþÀ?ðGàÀøàÀø#ðGàü?ðGàüø#ðþÀøþüøàü?þÀ?àÀ?ðGàü?ðGàÀø#ðþüø#ðþÀøþü?àü?þÀ?ðþÀ?àÀøàÀø#ðGàü?ðGàüø#ðþÀø#ðþüøàü?ðþüøàü?þÀøþÀ?i?·Ûíp8Ø8àÀø#ðGà/ñìvUUÕÜ¹sÛü?ðþüøKAüùýþÁ0'¢ÖÖV¶øàüø#ðRôÑGßúÖ·æÏ?ç×+,,<vìÛü?ðþüøKÜnwmmmfffû/_ÞÚÚÙDàÀø#ðGàOóB!±Ñh;wnûL&¼ÌÉ'°À?ðGàÀæóûýMMMQû¤ÚÚZ&|þÀ?)×ëµZ­ê²æ/ÿò/;::ØDàÀø#ðGà/r¹EwÑ¢EMMM~¿IÁøàÀ?ÍN§Ñh£JV:ðÀ>ðþÀøþüi¸`0h·ÛÕûæÎk±X<OÔç?ðþÀø#ðGàOùý~y)9°OÖû|¾×àü?ðGàÀÆòz½%+++Áf³	ü?ðþÀ?I^¸M&ú|£ÑØÖÖ6ûÀøàüø#ðìêìvii©z`Ùl×«9øàü?þ7e`^¯Wìkhhðz½ã½AðþÀøþü%c;áú| ¼'ØþÀøàÀ?Í$¯Îf³Y=°¯²²Òn·OòOñ?ðþÀø#ðGà/)Õµµµ©'jL¦©zÉàü?ðGàÀß,ç÷ûm6z¢æ¬¬,Å2àü?ðþüøKÆîÜ¹ÓØØo¢æ	ìàü?ðGàÀ_råõzëëësssÕ5Ûíö`08M_ü?ðþÀ?3Ëåª­­9Q³Óéäùàü?ðþüøKÁ Ífy>ÅbÎÌÝàü?ðGàÀßôvïÞ½W^y%???¹¹¹;vìÚó9Àøàüø#ð7kÝ¸qcÏ=ê-jjjÎü]àü?ðGàÀßÔçr¹êêêbNÔÜÖÖ6ÝûÀøàüø#ð7C9NõÀ>Éd2]¹reÖïøàü?þ¦ P(ÔÚÚºtéR5û6lØ</¸àü?ðþüøT7nÜ°Z­z½^>G½ÇãIªþÀøàÀ¿	æv»cì655MÇßçàü?àÀß,$/ ëÖ­S³oåÊ­­­³x>øàü?þ¦¬`0xìØ1z¢f³Ùçs?ðþÀø#ðGàoòûýÍÍÍ-ù÷9nÜ¸¡oü?ðþÀ?òz½õõõê>¶¾ðþÀøþüÅN^%M&SÌ[[[Á ¿)ðþÀøþüýZ¡PÈn·ðÔ3öFË¥éïü?ðþÀ?å÷ûCù²²²êêê´Î>ðþÀøþü×ëµZ­óçÏWÏØ'/÷îÝK½þÀøàÀ¥5þÅbQì+--µÛíÉ<cøàüøþÆ¨ÎétÆûRøõü?ðþÀ?J/üA»Ý®Ø§ÌØlü¿q700 ×ëÁøþüù|>yíSì5²^s3ö?ð£ÎÎÎ%KÈ5øàÀ¥3þ<OÌÁf³ùýþôÙkà/Åñ·~ýzùqO¿·ß~û:ÅïÚµkííílÍuîÜ¹üä'lmõþûïòÉ'lmõáöõõ%ùWºßû½ßSì	¥Û^s¹®§7qÆßgw1>þ?þÿ(~ò¤öÞï±4×ùóçÙK~Ñêííe;h«/vuu%çûáxðàÁßùß2_FFÉdJç¿ú§ºté?½KeüqØÃ¾ö%ûRöõûýò§×ëÕû¬V«×ëMó½Æaß?ìþÀø#ðGé?]CCú|ÁÐÔÔVûÀøàüø£TÆ¼Ífõù)9Q3øàüøøÕµµµ©'jL&^ãÀ_úâ/^àü?¤Qüùý~Í¦¨9++Ëb±0°ü?ðþÀ?Jü%¨àü?ðþüQà/ÁDÍv»=²GÀøàüø£TÀ¼Z©öI+W®lkkã|ðþÀøàüQ*àOTg·ÛKKKÕçsX,­Æçó9§ÓyïÞ=ðþÀø#ðþ(­ño¢æ¬¬¬8ãÅ_`Áª¯®Z¶lYnnîÛo¿þÀøþÀ¥#þLÔl³ÙRã|¿ù¿)**ú«µïÅòïÏ-.t¹ü?ðGàüQáO^L&ú|åË§ØDÍÅÅÅß¬û¦"?åßÿ°¾¾ü?ðþü?Jü)û*++ÕûÌf³<Sï|ÜÜÜHùÉ¿§ÌOFðþÀø#ðþ(ñç÷ûbNÔ¼cÇ¨¹¸¸øÙgÄß××~wþÀøþÀ¥,þvV«U=°oÑ¢E>/µ·cþÀøþü¥þ81'j^¾|ykkkúÌØ÷â/>öØc_ýêW.]ÊÙ¾àü?àR¢:§Ó©¨Yh2Î95û|>y²r8ÌóþÀø#ðþ(uð[[[cìKû4øàü?4-ø»sçÎ/¼ Ø'kÒa`ø±®9Zàüø¤9ü¹Ýn³Ù«>£¹¹95&jào"eN§àÀø#áÏáp¨öI+W®lkkKÃàü%Wàü?4%øS&jáÅ¨×ðþÆÔ¶nÝþÀ?ðGÉ?a¼èõzõÀ>Îçà/Qò°ÑétùþÀi%Ä7¿ùÍÇ,êÅK^Ñäõàü%jÙ²eêáùùù@ü?à­+W®ÍfõDÍ¥¥¥v»àü^ff¦<feAÌ÷öÛoËÂtÿ?ðþÀ?WC=°O2¼ ?ð7x,ödáúõë###²þÀ?ðG³ßïoiiY´hzxÒ-[<ü¿ñ5oÞ<yuvvÊãGöïß¯,0Õø#ðþhvóù|V«5æDÍò"òþûï÷oûø¿l×®]áÓ;"Zàüø4+Ô5Í¦Ï1ö¿íKàüE÷òË//X°@ºººdA XQQ1Ýwü?ðGàÔ]¹reÝºuêó9F£Óé<ü?ð§±Àøþ(7Q³Åbq¹«?ðþÀø#ðGàOùýþ¦¦¦5[­Ö5?ðþ&^II2á<?àf,]CCú|Á u¢fðþÀß+.._8ÎöþÀMSòüs¢æÊÊÊ±OÔþÀø`â<y¼uwwÌä]àüøK·DummmF£Q=°Ïd2÷Eü?ð7Áòóóå7ÃòàüøK«ü~¿Íf3QìËÊÊ²X,	ö?ðGS¿yømß¾ppü?à¦6Ï'Oøñ&ju`ø4õø.þSðþü?LÇb±¨ö»Ý'yûàü¿	¶xñbNøþÀøÂäé]=°/æDÍàüÑ,àOy@ºÝî¾ëàü?)2QsiiéØ'jàfðþü?ð7ü~¼'jàfÂy|îÚµkòÃ/Àø#ðþÒ-ÅbÉÊÊØDÍàüÑ,àoN8áüø y7L¨ü?üeÄ>À?ðGêDub;z`ÙlÉ'vðþÀÆàüøÓV~¿¿©©I=Qóüùó¦i`ø4õøÓëõ/ù=þÀø#ð§vV«U>¼Lr¢fðþhð§Óéä<ówü?ðGà/ùs»Ý1'j.--àüÑÔã¯££CÆ<É	_Àøþ9yþy>ÑhLgoðþÀßDo³Á?ðþ~ßïåW.]s¢fÇ<wü?ð7Á8Ûüø#ð'Ý¸qcÏ=êùùùòíóùí?ðþ4øàÀ_Ô××s`ßòåËßxã@ wü?ðþÀ?ãËápTUUÅØ'?É³>ø4ø^¿~vv¶<àsrr6mÚ4g~?ðþüÍV@Àn·«gìSö¹M|àü¿	644óéþS¿àü?3ÏçåWÔûdMrìà¦K,uuõàà |øàÁ7Ê+V?ðGàü¥Ln·»®®N·°°°©©i¶&jàfòàØ1<<,kd=øþÀ_äp86lØó|¶¶¶¤=ü?.üeddÈS/¼&Ê¦zþÀ¦óûý---¥¥¥ê=bAËäçs?ðGÓ?å°ïÚµkÃ¾ò¿,Ëòòrðþü?-vçÎyUìSÎçp»Ý©ñm?ðþ&h/æ	÷ïßàÀøÊårµ´´Øl¶ä|SöåææÆ¨ùÞ½©´×ÀøohhhÓ¦MyyyòÿÚµkeÍtßuðþÀi¡P¨¶¶öù_¨ªªúÕóúë¯'Ï· Ï¨ëÖ­Sì+--µÛíZ?ÂþÀM1þf%ðþÀi/½ôÁ`Øý×»÷½¸OþÛ·üqÃ1»÷<Æ±O9ãÌ3)É>ðþÀøþhÚñ'ÀÚú[ù)ÿL&Ó³Ï>;[÷Ùï÷ÇØõäOöõõ¥ü^àüó£þÀ?ð.777R~òï)óSF£qæï­×ëµX,<õDÍ/¼ð¶&jàfñàÀøS·lé2Ñ^$þªªª¶mÛ6÷S6M&z`Á`ÐèDÍàüÑÌá/^Ï?ÿ¼òTròäIðþü¿pgÎùBþê¿U¯È¯¶¦vÞ¼ygî^(²Ûíê£4²2UÏçà¦===999òTb4#ç|àÀøS²ÙlÙÙÙáK_úÒ¿øÅxóûýMMMêó9æÎk2ÒüYü?ð7©6oÞ¬<¡pafî:øà4¿Î®u»Ýòê5Ýo¶y½^«Õó|Å"²×ÀøìôéÓÊJuuõLÞuðþÀi3ËåÞ©ö	å3Ýö?ðGS¿¡¡¡+V(çvtttÌð]àüø,µµµFõÀ>Á`·ÛÁ ü?8þ:¤<§lÙ²eVî:øàÀßï·Ùl1'j:Îô<ü?bü1Ïø#ðG³?Ïs¢æ¹sçZ,ËÅ®à¦£¥ÓéÀø#ðþ¦)Ço`Õjå|ðþhêñ7ë?ðþ(=ñ'OñöÙl6ÎçàÀø#ðG©?e¢æÒÒÒûÚÚÚØþÀ?ðþÀ¥þü~¿<ãéõzõÀ>³ÙÌ3!øþü?Jüy½Þõù²FÖ3°ü?þÀ¥þâMÔ¬×ë¨ü?¶ø#ðþ(EðNgÌó9JKKív;ûÀø³?æùþhñÅvñ&jæéüøüENéÌóþü¿±¤Ï¡Øe±X<üøKükoo'©§~zppP>ÿ7nÜ(k.]ºþÀ?ð xçsäççïØ±ãÎ;lXðGà/ñ'ORòT9exxXÖNòzzt:]YYYGGøàRòôe6Õçs,]º´¹¹9°IÁ¿äÅò%àÂßäÇüÕÔÔ8qB>¼eËðþÀiòr[[[Ìó9L&ìzÎçþ4¿yÚ¨Éòkuuµ¬õ¼åüüüEz½^?ÍÖKñûè£Þ÷]¶æ:wîAs9yÄ%øÑáöíÛû·;Ê|¿ñ¿±iÓ¦¿ÿû¿gÎ||ðAgg'ÛA[ÉCéâÅlÄM;þ.]ºóùj·yÊúôÁßéÓ§?¯×ët:Ùëüùó·nÝb;h+aÄ§~ó¢+W®ûì±¨'IYc±Xäi­7[]¾|Ùív³´U___ww7Û!qÓ?éúõëÅÅÅÙÙÙ999åååwïÞüÍF8ÎÌÌä°/9ìKÚ:ìo¢æE1°Ã¾Äa_ö¾¡ò¿ú 2øà	ö­òÌ3ìþÀ_ìÌfóñãÇeAþ¯©©àüQãÏï÷Ûl6õDÍYYYµµµl(ðGà/EðwõêÕå@mAAÁ©S§&³rEEE]]]àü?JÚN<¹k×.õò$ÆâþRÊ$ÏÒMY>|øð´ÞuðþÀ%CÊÀ>õß:2ü)^ðGà/5ñ'¿×ÊÓÜÕ«WÃøëêêå¼¼<ðþü¥j¢:§Ós`¬t8°üøKYü)OvÊ¿þ¶/ø#ðªA»Ý®Ø7wîÅÂÀ>ðGà/õñ§Lò¬¼Û'øÞ½·,«§eàÀ¦óûýòÌ£Ø'kêêê~ô£±À¿´Àð"æ$Ï/_àÀ_jäõz-KVVz`Íf÷oûø#ð§aüIýýýÊÙ¾ÙÙÙ%%%òD9Ýwü?ðG3<ÏL&õDÍF£±­­-<°ü?é¿Y	ü?ðGÓ¨În·WVVªöÍfõøþÒáó<ÂÝ½·¤¤dÑ¢EàüøÓ ¿¿©©I>ÇüùóâÖàÀ_Zãoxx³Á?Í%°Þ©ÏçÐëõ£NÔþÀ¿ÔÇß¢Eæ$lÞ¼yàüøÓDòdb6ÕûJKKÇ8Q3øþR7oÞÌxTøoD&ò;ú4øþ9Q][[[¼ÇõþÀ¿ÔÇ_8¡ÞtáàüÑÔæ÷ûm6z`_VVÅbñx<ã½Aðþü¥þf+ðþÀM Ïo¢æQö%¨¥¥åßû%ðGà/õñ7<<^³`Á?ðGà/©òx<E=°Ï`0Øíö`08±BµµµùùùÆÿcüýÊß'Ã×_­þü¥2þ.uÂ¯ò|ÚØØþÀ¿dèÊ+ñö9Î±Ï ^zIø¸û¯wïqüûö¶o?þøãÍþü¥,þ233å9Ôív×ôôôÈ¼¼<ðþüÍngÎ)--UOÔl±X×|	ßÖ¿ØªÈOùg2öY6>ø#ð²øSNøùµÛUMþþÀ¿ëÎ;ÍÍÍê©æÏoµZ§öïOæææîÝ³7O2ìðGà/eñWTT$O©;wîTFÌíÛ·OÖ?ðGào»qãÆ=dêMMM>#A_þòkkj#ñ÷µª¯mÛ¶þü¥,þº»»cNòÜÕÕþÀ¿ËårÕ××«ÏçX¹re[[Û$ö%Þ_ùùùOöíÝ³÷øcùpÅø#ð§üI·oß.++ËÎÎÎÈÈÈÉÉY²d¬î»þÀø£ðá©5L?c,9ÂÂÂÇü±ÇûÊW¾üÀ¿ÔÇß¬þÀøKóÁ`KKËÒ¥KcNÔ<µûFí?ø0"°_À?ðþÀ¿)ÎçóY­ÖÂÂÂ(öÉ^xA.ù»Ä_øþÒW¯^UæyVÎð-((8uêøþ¦<yÒ7Q³ÍfÅ7ÞÀø#ðFøkoo?ÿ*øS>þÀ¿©Jï1'j®¬¬Öó9Àø#ðþ¢S¼z5¿®®.&yþ¦¤`0xìØ1õùsçÎòÉ'§j¢fðþü¿ñÜÄ£FLì<22"Ë:ü?Îï÷¿òÊ+ê³²²êëëgø|ðþü¿Ï+((PfõSð7<<¼÷nYÖëõàüø@wîÜÙ³gÏüùóÕçs477OÇDÍàüøãHxsçË/?ðGào¼Ï'ëÖ­ËÊÊz>)--µÛíÊJÎÀø#ðFøúûû+**³³³³KJJfàøà/eB­­­ê_#7lØ<ûÀø#ðþf3ðþÀ_ä÷ûCùrssëêêúúú´ò?ðGàü?ðGà/Q^¯×jµªöåçç766&çÀ>ðþü¿ÏúôÓO/^)OÜÙÙÙeeeàüøËå9QóòåË5úÒÀø#ðFøs:1OøîþÀøÓV¡PH.bNÔ,+µþpàÀ_áOä¹¦¦FùpppË-²¦¨¨ü?MÔl·ÛÕûæÎk±X<O|àüøK#ü)Oâ[IÃ>?ðGé?¿ß/UõÀ>Y#ë>_Êì5ðþü¥þwþÃkxçüQãÏëõZ,õÁf³iî|ðþü¿ÏSÆüÕÔÔùäÃû÷ïWUU1æüQÚâO&I>Ñhlkk<JþüøÓ$þæÖ4ÿàü%U¢:»Ý^ZZªØg6SþÑþÀ¿4Â_Æhét:ðþ(ñ§ìÓëõê3ð÷~À?ðG3¿Ùü?ð7ë	ìwêó9òM½àüø¿,Þ|Î·oßàRòè3Íêv»=Uö?ðGàü=º9s^~ùå¨uuuLõþ(õð'ªkkkSOÔ,4LéüàÀ_áO'OýwïÞß|óMåÅ`ú?ðþf%¿ßo³ÙÔ5geeY,4ØþÀ?ð÷YuuuÊË@QQ²°qãÆé¾ëàü¿ÉçóY­Öx5§ÕÀ>ðþü¿Ï»téRø%¡¥¥eî:øàoºs»Ý¹¹¹êív0dg?ðGà/Mñ·mÛ6å%AùkÒÓO?þÀiòàZ·n]ÌNgÏþÀ?ð÷y:N^òòòzFùËÌÌà´¿`0(_=æDÍÅår±wÀø#ðþ~y¶ïÞ½£VÊëgû?ÒþâMÔÕÐÐàñxØ/àüøo¿þþ~ðþ(ùñx¢æ÷î±GÀø#ðþ"ðþÀß$s¹µµê+W®lmmìðþü¿kÎy`7ñàüQòà/íÔ5K&ðþü?ðþ(EðZZZâÏÁDÍàüøàüQàÏçóíÙ³G=cßüùóeý;wØìàüøàüQ*à¯¯¯Ïb±¨öþ>ølðþÀø£ÁÃá0L1'j>sæ5?àü?ðG©¿@ `·Û.]ªØW[[Û××ÇþÀøàRwîÜijjRÏØ'k¬V«ÏçcÛ?à&¿Ä?ðG3?¯×»gÏ57773°üø4øË-NþÀM7þäQs`ßÒ¥K;ûÀ?ðGS¿Yü¿tÆ_(²Ûíê7Ýkkkå¡ÁùàÀø#ðþ(ðç÷ûú|³ÙÌùàÀø#ðþ(Eðçõz­VkÌòp¸wïüøþÀ_:âÏáp¼øâ»ví:sæLjàÏårÅ¨¹´´Ôn·süøþ(Mñ7lØð[¿õ[ô?úÆºoüæoþf]]ælÆÜs§Ói4Õûd%?ÿàÀø#ðGé¿þîïþîî¿Þ½ïÅòO.âÄ	Íáï¿øÝn9°Ïb±x<~¼Á?ðGàÀßÃ+WþÙÓ¦ÈOù÷'ÿDÒÐ·àóùþôOÿ4æDÍò3ÏDÍàÀøàÀßç-_¾|ë_lÄ@Êd2iâÎ<ûÍfcÆ>ðGàÀøà/:ÁÓ×ª¾¿¯T|eÿþýI~·å'9ÞÀ¾¶¶6Îçþü?ðþbçóùòóóÅL»^Ø%ÿÖ¬Yó¥/)iç@Q&j.--UÿiD³ÙÌ7ø#ðGàü?ð7z7nÜ¨®®?þ¼yó¾þõ¯½Þ$¼~¿_~õz½z`ÕjýÛ¿ýÛñþm_þÀ?bçdL0ÚÐÐ >Ã`0455)ûÆõ·	üøþü%còãj6ÕçsTVVFMÔþÀ?àü?­&ªkkkSÏ!4L1Áø#ðGàü?ð§½ü~¿ÍfSOÔe±XFàÀ?ðþÀòù|òóo¢æQgìàÀ?ðþÀ6J0Q³Ýnc¹ðþüøàü%ñ&j^·nÝ3gÆ5Q3øþü¥/þôz=øI[¼³²²|òÉ¾¾¾	Ü&øþü¥)þ:;;,Y"¯£àü%añ&jÎÍÍµZ­>oÂ·þÀ?i¿õë×<øýõ×¿÷ßÿwÞa;Ly÷wW]]Å¾/~ñõõõÿðÿ0ÉÛ?wîYsÉc­½½í ­.ðÞï±´Ãáx÷ÝwÙÓ0þ>»ññ÷Áüâ700ÐÑÑÁvÂäGîßøú|/ùËßÿþ÷þóOÉW9þüÐÐ[[[]¼xñg?ûÛA[ýó?ÿ³×ëe;h«k×®¹Ýn¶CâRöå°ïÌ¤ì«¬¬TÏa6¯2µ_Ã¾ö%ûÓè°oø5ü¿dÈï÷755©'j;wnýÄÎçàÀ?ð7Áøù¼^¯ÕjUOÔ(ëïÝ»7_ü?þÀø3Ëå7Q³ÍfÓÀø#ðGà/­ñ/ðþ¦¶P(ät:cNÔÞÁø#ðGàü?ð¤ÁÖÖVõÀ¾ÉLÔþÀ?àü¿¤Ëï÷777«öM~¢fðþüøàü%Qn·Ç¼(ö-Z´H8((ÅûþÀ?àü¿)K~lL&ú|+W¶¶¶ÎØÀ>ðþüøàüMc¢º3gÎ¨'jÍæ¤úYàÀ?ðþÀßÄ6M>Çüùó¼^o²ÝaðþüøàüM$ÇS__Å>½^/??³;°ü?þÀøSÖ+WjkkÕûJKKív2ìàÀ?ðþÀßäp8bNÔ,+µòþÀ?àü¿QRöªÏç°X,GCßøþü?ðþâæ÷û_yå½^¯>C~Hfk¢fðþüøàüMq7nÜØ³gúïsÍ´çs?ðGàÀøào|9uëÖ©Ïç0N§3ÉÏçàÀ?ðþÀßÕÙíöxûWj|àüø#ðþÀ_ºãÏï÷777ÇØgµZp¢fðþüøàüM$]CCCÌMMMÚØþÀ?àü¿_Kö¯ÉdRì«¬¬LþÁø#ðGàü?ð7¦"<õÀ>±`:ìtðþüøà/-ðç÷ûCûrssRl`øþü?ð¾ø7°¯°°Ðjµjq¢fðþüøàüÅHv¢ÙlVì3Çi¸ãÀø#ðGàü¿TÃ_¼ÒòåËS`¢fðþüøàüý²xûûúúØqàüø#ðþÀ_*à/ÞÀ¾¨ü?þÀøK_üÅØ§×ëSr¢fðþüøà/íðÚÚÚF£z`_UUUjOÔþÀ?àü¥þâì6lØ2qüøþÀøKwüy½^«ÕªØµcÇ7n°_Àø#ðGàü¿TÀ_¼²³ØþÀ?àü¥þÔcû/_n³ÙÒs¢fðþüøà/Åñçp8ÂìÊÏþÀ?àü¥,þzÁl63cøþü?ðúøSüÇfàÀ?ðþÒþÀ?àü?àÀ?ðþÀ?ðGàÀøàÀø#ðGàü?ðþüø#ðþÀøþü?þÀ?àÀø#ðGàÀøàüø#ðþü?ðþüøþÀøþü?àü?àü?àü?àÀ?ðþÀ?ðGàÀøàÀø#ðGàü?ðþüø#ðþÀøþü?þÀ?àÀø#ðGàÀø#ðþüøþÀøþü?àü?þÀ?ðþÀ?àÀøàÀø#ðGàü?ðGàüø#ðþÀø#ðþüøàü?þü?ðþÀ?àÀ?ðGàÀø#ðþüø#ðþü?þÀøàü?þÀ?ðþÀ?àÀøàÀ?ðGàü?ðGàüø#ðþÀø#ðþüøàüøþü?ðþÀ?þÀøàÀ?ðGàÀø#ðGàüøàüø#ðþü?þü?ðþÀ?àÀøÍºººÊËËu:Ý²eËzzzÀøþü?ðÊø+..¾|ù²,<y²¤¤D¿>ø`â700ÐÑÑÁvÐ¿ÿþïÿf;h+ùEëîÝ»lmÕÓÓãõzÙÚêO>ùù¶Câ4¿È²³³ÕøûÞ÷¾÷Åïý÷ßçwØëÜ¹slÍ%5yÄ±´Õ»ï¾ûÞï±´ì2ÃÁvHà¯»»»¾¾Ã¾öå°/qØ8ìËa_û¦òa_¥ÁÁÁ@ þÀø#ðGàü¿TÃß_¥|xûömÅÒßß¯þLðþÀ?à4¿È.k×®y)øàÀ?ðG)?½^?'"ðþÀ?àü¥2þþÀø#ðGàüø#ðþüøþÀ?þÀ?ðGàÀø#ðþÀø#ðGàüøàüø#ðþü?ðþüøþÀøþÀ?àü?àÀ?ðþÀ?ðGàÀøàüø#ðGàü?ðþüønøé¥ÚÚÚnPü>ùä·Þzí ¹=úÿñlmõýïÿG?úÛA[½ûî»ôÛA[øálÄùýþÔÄßÕ«W÷íÛ÷""""(ñ¡Ñ9¼ùIDDDÄa_""""DDDDþüø#""""ðGDDDDàFéêÕ«+V¬Ðéteeeõ÷÷ÏHÖôôô)ÜÑÑÁÖKÂ½¹Ë222bîGÅÚÛÛÕAýÈâ±ü­«««¼¼Ñ²eËdñXÓÊã¥üÑCùY?ö¬,|9???ò¢S§NY,È5555'NÃoÙ²­-ì)ÙM1÷#ÍV¡PH¸ ~5R?²x¬%ÿ^+.. ,<y²¤¤ÇVv/mà>ÏápÈï¯käá¡#8cddDõz=-	÷Òõë××¬Yo?ÒluðàÁï~÷»êW#õ#ÇZòïµÈ²³³y¬ieÇñÒþè³_òòòä"¿¿Fý^»víZNWQQqíÚ5Y#ËáK#)yöÒ¦Mº»»ãíGnÞ¼)»@^cÔ¯FêGµäßkáä±V__ÏcM+;6ðG×ÞÞ^PPó¢Û·o+o/)cÈ233ÙhÉ¹×<ÏªU«ìGDä.]zøhÔEêGµäßkJ555@Ç¶v/màFÿGy<3>zo<iÖ÷Ú:`?Òì<]þz©Y<Ö¯)z°X,ýýý<Ö´µãxié^qqñÕ«W>:smíÚµQy½^å	N¹Èl6?~äùe­MZ½zuoooýH³þ²µFýÈâ±üíâÅòà±¦­ÇKø£_â^VV¦ÓéÖ¬YþýUy´tww/Y²D.ªªª¬éìì,,,ÌÈÈ(**v°õp¯)¿Ë*ÔûçÕHYV?²x¬%ÿ^ÓëõQï-ñXÓÄã¥üø#""""ðGDDDþüø#""""ðGDDDDàÀ?""""DDDDþüø#""""ðGDDDþüø#""""ðGDDDDàhÌ½ùæ«W¯Î~Ô5kN>ýkÏ_ÒÌ³m¬«×ëå[QëeN§+**ïm?"Òdûöí£êå_N%ü566ÊÊ¨õo¼ñ¬ß¿ÿnüöêééÖètº#GuôèQùPVööö¦þ®_¿.+-[µ~É%²Þëõ?"D=ýôÓÂW_5råk¯½&+ëêê"éÓÑÑ!xËrøïß¿___'åççïÜ¹3òèªÓé`ÉEr]ÃÅ)Y3oÞ¼Õ«W_¸pA>4ÍQwìüùóoG¹HîrQ<¨­]»VÖwuu×|YÖÆðÊMeggoÞ¼ùöíÛjü©o?jM»JDàh[¸p¡ÀåæÍ+oÝº%+õz$n¢êîîV.Ý¸qcÔEÛ·oW.r»Ý1¯¥|¨iÓ¦ácfff(KåÁSNN2/ÁíÈBÌ»§þNO<)Ú°/O8_Ô¬Y³f¼øKpWüÍ~ÊÞÏYsæÅ"q³eËÁGÉ|X]]­XGá£ò M¹¨¦¦FyËP;;;¼Í½÷ï£®Ï=÷¬9ö¬,Ëÿ²üÌ3Ïz;GùP>!ò¾Åü,ïw``@>ÿå8O(**+Ê·ððW³³³Ç¿wÀQRàO¿ðz7¤Û·oËÂ&åÃòòrùpñâÅ[·n´oD>'ê½´ðµoÝºþdSøÈ¯ü/ËN§sÔÛhÊwïÞ¼oñÆç=ÿüórÑw¿ûÝ¿:´½sçÎÈOþ¤ÊãÅ_»JDàhöSÞîd¥;az<Åaëê©ÆFq*rYÎÏÏÏÌÌrãóæÍ_:êíÄ£XTW¯^U*Ëò¿,»Ýîð¥rb>;þÜU"DD³2îíÐ¡C+	P¢NøP¿¡ÕÛÛÛØØ¨«Qy[.|¢mß¾]9Ú+ÿ××××'¸å¶þþ~åÃ»wï&>3wÕªUr©2»5ò"åÌ_¹¨½½ýÁñ©²5Â&¸«Dþf?å	Nwøðaeª#GdffªOÎ¨®®èAåløÌÌ2TÎãñDT,¸sçN¡rnx²DSN¿U¾úÅÃëÜ2ÈOóÏL¿£Gßo9ò"eø£Ûí¯"!þnåÓ7o¼4Á]%"ðGD) GuàÀÏ¿¥Ø(¼>`>Ç"ÜsÏ=)ËÈN:ý)YyD8ÁíÈÝ¼cá3mã³CCCÙÙÙÊ·u°ýúõ_bÁò¿2ÛKäm*oLS¨¾4Á]%"ðGD,	PÖ¬Yù¨Õ«W+§ÜFá¯½½]N¯¬¬ìÒ¥KáKÀîÝ»0=ÿüóÁ`0|©Ãá¨¨¨Ùl¶¨ÛTß¹)Y¿mÛ¶¨õñnG;#wI0Á<áêëë£æ|Q¨©©-'ß×ëO[y<Ý*ÛÊh4vuuEÅwÀ?""""DDDDþüø#""""ðGDDDDàÀ?""""DDDDàÀ?""""DDDDþüÑLõÿÆµ¨¯<*?IEND®B`


ÄñGüøñ#ÄT´î<´ c¼ÄñGüøñÄÚ-î<Guu5óÄñGüøñÄç°Z­Úìcø#þ? þ`øs»ÝõõõÚ1^5ÏÁþ9?âøñâ¿¾¾>ÝÝ²Ùl¶öövæ9?âøñâ?y8Óç(--eø#þ? þ`øSó¹¹¹º»ecø#þ? þ`øóx<ºc¼òè688Èf$þ?âÄ?!þ$ìtwË¶téRÆx?âøñâÆ?µ[6í<Ç+Z[[ç þ?âÄ?$þäÑª´´TÅÅÅgÏ%û?âøñâF?©ºööví¯Ø´iÓµk×ØVÄñGüøñ#Äß?þã?¶´´hç9L&SUUÙGüÄ?0Ç³wïÞ´´4ío]]c¼ÄñGüøñPó&I»[¶°?âøñâF Fv»];Ï¡öÆË<ñGü þ@üÁ¤êN§î<Gaa!c¼ þ? þ`ìkkkÓÎs¨1Þ?þxºûöñGü þ@ü!y½^yô±Z­Ú1Þµ7Þ©ïÛÄñGüøñ(5<<ÜØØ¨Í>í/ñâøñâ1LÂNò.555$û,Ë±cÇ´ã%þ@ü þ@ü!&¹ÝnÝ1Þ+V´µµy½^ÝS þ? þcäñ¥¤¤D6Íétã%þ@ü þ@ü!6¨1ÞÕ«W4T Ãáp¹âÄñâÄ¢ÇãijjÒ~zKFFF½ã"â¿O@üøñ('­&(y!bV«UÖkç9?Äoü%<Mbb"ñâÄ¢Ûí®««ÓÝï³ìøaãoÞ þ@üaf***f6ÏAüøÓ7::ºsçNâÄ?D)¼ìv»¶S?ÄEüY­ÖÄÄDÞóâÄ¢ÏçknnÖñL&Ã1­yâÄßã+Wj§=Ìfód~IüøñÖÝoFFÆÌæ9?ä¯hddÄb±È4ßOúSY¨ªª"þ@üøÃ|GínÙÔ<Ïç£KüÁøñ§þdAjOnß¾=11!)))Ä?<Ëåp8tç9ÚÛÛqøñ÷8==]þ¢äQPþØdáàÁjzñâ&RxÚýsFì!øñãoß¾ñà?¶¢¢"âÄ?DÚ-[nn®înÙfwøñ÷ÿ>¼hÑ"Yèéé	Á¹¾èÄ?ðx<º»eÁÁÁÈ_$âqóøñâ/©yínÙ$%çbøñGüø#þ@üÍ·Û­;ÏQXXyð<xPSSóÇüÇÛ·oÃÃÆ_NNúÀ>äÄ?Ì¹Û/--ÕãíèèßìSå·dÉUß^%å÷ýÍßÿæ7¿ùñÄãoùòåÁÁÀ´/?RuíííÚ1^QQQ100%óÕW_ý£?ú£oPÿ^ßûú7¾ñ§ÓÉ5Hü-þ¤óäÏ¯··wbb"øñâÏð|>_[[î<GMMÍ_~UVRO/ò¯ä»%o¼ñ×#ñg´ø3Íòwáò#þ@üø36Ç£;Ïa±Xæh·lÏî¹ç«£68þJKK%R¹6?£Å_¿ü5îÞ½ûÑ£GÄ?xFjÃd2iÇx[ZZæzÇñÏâOþäOJJJå·¿n¿ÖÖV®SâÏhñ'/^¬ þ@üaZä^Ýn·kç9V¯^öìÙyçxª7oÍæç~çwþ ò¿ÿû¿ÿÝï~wîv"âoÞâoÙ²e|øñGüÍTÓéÔÝ-Û¦Mä®>ú³/Àårïß[²dIaaááÃ)?3þÔhä­? þºó&©²²2Â»e-|È3|øñGüMç°Z­Ú1ÞºººyÙ-ñâoªºººäÏuß¾~røñâ/¹ÝîúúzÝ1Þ£GFç/ñâï×Ïb|øñìÓÝ-[nnîÙ³góö8âÆ¿I0ðâÄ+W®èñGþøñ«? þ¢ßï?útaa¡v÷å_îëë3äoMüÁøñgµZ-[vóæMâÄ?(>¯µµUw·luuu1:ÆKüøûZbb¢ü=Gþ¢ þ@üE!ÇsôèQí¯`cc£æ9?/^¼(Õ£££üÀâÄ¿¨2<<§ãÍÍÍmkk¡j&þ@ü=í,öñâ/¾ãÏívWUU¥¦¦<Øl6ãÍs þöñÄoüõõõUTTñÊA»ÝÙGü!^âo¾ þ@üÍ#Ý½ñL&ÃaìyâÄñâ£øóx<ºc¼r·óÄ¿ÿ7>>¾qãÆäädùûOIIÙ²eK&?? þ"ixxXw·l---dñ8¿±±1Ý¹ÞQñâÄ_d¨Ý²L¦8ã%þ@ü-//OîÊÊÊ=z$GGG7oÞ,kÖ¬YCüøñÓ®]»¦»[¶øã%þ@ü-))Iîÿç7>>.kd=ñâÄ_Òçt8FÝ-ñâoªäA/°ÆçóÉ>êÄ¿3ÙÞxã%þ@üýzÙwÃêe_ù*Ë²fÕªUÏxÎýýýYYYùùù/^$þ@üø;ñ þ¦JjOwàãáÃÏxÎåååï¿ÿ¾,?~|Û¶mÄ?sappPîQã%þ@üMÃØØØ-[ÒÒÒäëdÍ³­ÙlV3>>nµZµñ÷ãÿøóÈºpáÂ§~ú9ðùçöÙÏ~ö3¶¹DÌ]lùö/¼ Þ½2Æ[[[ÛÙÙÉ5;ò0ñÉ'° |üñÇÿ¡¿9ü®Aí;%þä×¬þçv»ÝcÀØØW_ÕÝÝÍvòw÷w÷ïß¡,÷Ú1Þç>òw­sëÖ­«W¯² Èÿñ_DøÆpüÉFËÚÙa^ö/û§Ëï÷···O6Æër¹¸*yÙ¼ì;ù)&8Ýf&33SËWY&þ@üø1Ï×ÖÖ¦;ÏQ__Ï/ñâïé&7[ñg·Ûßï=Y¯åååÄ?3àñxtç9¬V+c¼Ä¿Y°wï^u·rêÔ©g<+ydµX,YYY===Ä?Óâv»«««uÇxÙ-ñâoô÷÷§¤¤¨Ýþæó!þ@üøÜ=N¶[6§ÓIö þfÁÖ­[Õ=Ë"sÑ? þBHÕM¶[6iAöÆKüøøûè£ÔKYYY$/:ñâÄ_pöéÎs°[6âÄßlÆßØØØ5kÔlv÷kÄ? æ9¬V+»e#þ@üÍmü½ûî»êþE»ã5âÄ¿p»ÝõõõÍsø|>®âÄßlÆ_>çøñâOËår8ºóíííÌs þæ$þF»C6âÄ¿g$÷ºó¥¥¥Ìs þæ6þæñâñ~¿ÿôéÓÅÅÅº»ecøñGü þ`øóz½íMîÙøÄ?âøñ#Äßd»ellldøñGü þ`øs»ÝUUU©©©ìøñGü þ`äø»råÊdc¼ìøñGü þ`øëêêÒÎs)B¶0ñâoþãÏùñGüáÙãOí-77W;Æ[YYyíÚ5¶-ñâ/Zâ/ø#ýtãÏùñâ/¯×ÛÔÔd6µã­««cøñuñÐÙÙ)÷V¯¼òÊ£Gä |Ý¼y³¬éîî&þ@üøÓ°«©©ÑfÕj=zôè"ù;::Þ|óÍûöÉï,$þ@ü=ºÿ¾³5øñâ/ØÍ7+++M&vw¾vËæóù6mÚôÍo~óÅ^|¡äßùßKHÿ þÂÅ|!ñÇþ@üøP»eÓñÏïÞx:ô»¿û»µoÔxóüÅÿÍßü×)ñâoRrÿU^^>66öøÉ»XÊÊÊd¬'þ@ü!ÎãOª®µµuÅÚ7FôõõÍû%/,,|õWUù©ßßüÁuJüøTww·îÀtñâqjcéÒ¥!÷©©©ÕÕÕ.+J.ùêÕ«wþpgpümß¾½´´ëøñÎíÛ·/_²jÕªû÷ïÏõE'þ@ü!:ãoxx¸¦¦F»[6É>Y/ÇFÕ%w8Ï?Pðä:%þ@üEâÄ¢-þ´v#77·¹¹9:÷Æ;88h6m6Û¾×÷É¿õë×/Y²d^&?Äñâ1ã¯ÿú¯u÷Ï±bÅèß¯ÄJYYYFFFzzú÷¾÷=·ÛÍJüø7näää¤¤¤¨	ßÌÌÌ3gÎ þ`xjÿEEEºó]]]|fñâÏ°ò¼K7µ|üøqâÄÊçóIöeggkwËVQQù' þ"EîïnÜ¸¿YNKK#þ@üÁx<Üÿhç9ÒÓÓëëëyÍÄê^O-¨ø`ß¾ þ`<vÕÕÕÚì³Z­;vìàÎÄâ%þÔ<«gû$þÆÇÇkkkÕ½!ñâÆÐ××çp8´ûçÈÎÎVóÓÚ·/?±]]]ºò|ùòeâÄbÚ-ö.NV:ÎÀ<ñâqbhh¨  @Mû&''çääDà]/Ä?ÌÉÆx.·Ûµw>Ä?ÄWüÍâÄæÇãiiiÑñL&Ã1Ùÿl?£øÌyÜ¿?''géÒ¥Ä?ÄVöéñÊY~ÿÄ?Äuü3íâ1d²1Þììì©ìøñãÇßÒ¥KNüøCl×f³µ··OÿÄ??þîÜ¹ðD`ßÁ¤ü>úè#âÄ¢î¯T`iiéî[?0~üHêÍõ+¼Ä?Ì5Æ«Í¾0óÄ?óøñiñz½ÍÍÍÚ1^5Ï188ø,gNüøCÅßøøxNNNJJJ`Í¢E9BüøCô<×××Ífí<GccãTæ9? þ~eñâÅ!¿ê^µ¡¡øñùÕ××g·Ûµó¹¹¹j·l³õ?£øKJJÒÀþþ~YFüøÃ|éêê*..Ö~AIIÉu þGñ§~'&&~í|5þGüøCd8N)¼gûä`eeåÜÝi þGñ%w¬öìñù|rpllìÀ²Æb± þ1rÔÚÚªãMMM­ªªr¹úÓ?£øëííÕýçâÄ"`xxXî´ó²¦®®NÀe þ@ü!âOÜ»w/???999!!!%%%//OÖÌõE'þ@üáæÍÕÕÕ©©©Ú1Þ¦¦&õrDd þ_ñ7/?ñ¬¯¯oÓ¦MÚ1Þâââ³gÏÎâ/ñâÄñGüøçÒÂÂBíþ9***¤çëR þ_ñwãÆõ9ÏjÂ733óÌ3Ä?Ì"Ï§»[6ÉT]]=×óÄ?¿ÒÙÙ¸Vñ§?NüøÃ³óx<%$ûdÜ<xð .$ñâqêùÆøëééáCAüaVD_ýuíïÒ¥K[[[ge·lÄ?Ó?'°óÄÄ,'&& þ03.«²²R;Æ[XXxúôi¯×møñ8¿ÌÌLõ©~*þÆÇÇkkkeÙjµ þ0]òw]ZZªã]]]Q±?£ø»cÝy¾|ù2ñâSä÷û'çp8ó>ÏAüøñ÷kÔ´orrrNNÛíëNüø35Ïò8¿ñâñóøñëåY"O;ÏqìØ±¨ç þ@üø#þ@ü3çv»ö¹¹¹mmmß?ñâÄßôÜºukÙ²eIIIrß?22BüøÖdó6-òÑÄ?3át:u>nÞ¼Iüøâ÷ûÛÛÛ¥ð´»es8ó¸[6âÄ¿iSò^®>yëÑ£GÛ¶m5YYYÄ?¨Ý²iç9ÔoÃ? þf9þÔýxðtd9ðÏÄ¿¸åñxtç9d¬­yâÄ¿_QÏüÖñÌ¿x¦æ9L&SHöegg·´´,û?»øSïù+//æ>,..æ= þâüUÚíví<GQQQñ þ@üiÎâiæèõ_âÄ_ôªÿjç90ÆKüøñ*ái?Î>ÝyÃñ þ@üEâÄßüRóV«U;ÏQ]]m1^âÄ¿Pó½÷?$aW__'c¼Ä?³X°àðáÃ!++++ù¨ÆÓ××WUU¥ç0ð/ñâÄ_(<¹ëÏÌÌ¼ÿ¾üàÔÁ½Õøñ7/®¢;Ïaø1^âÄ?êa ++K-lÞ¼y®/:ñâ/2:::¤ðâvøñâO_wwwà!¡¹¹9øñ7§|>ü-/]ºTw×årq þ@ü!~ão×®]êQAííC¼òÊ+Ä¿5<<ÜÐÐ ;Æ[__oc¼Ä?¡åQ!--íêÕ«ÞóDüø-òòÿºÆÆFB®wâÄ¿ÿöÝ¿ÈJÃÁ´/¿ríÚ5ù³Õñ®^½º¥¥Åëõr þ@üm²Ïù"þ0o¾ùæ¾ûÎ=KüEüéñ···3ÆKüøñ-?Cúó?ÿs³ÙüBÉ/¾øâ%KÊÊÊ|>ñ7ÔnÙV¬X¡ç¨¬¬døñâOsÊ_ØøÃTH|ãßøË=yàÍò¯öÚßû½ßÓ¾©øFñL¦æ9? þ?â/B¶oß¾©l*?õï?X¹b%ñ7[=ªãµX,ò588Èøñâø#þ"gëÖ­ÒÁñ·ó;sss¿Yy4ª««Óã=vìóÄ?Äñ79òío;8þ¿SügögÄß³p»Ýºc¼6ÍétFí[*? þ?âÏø¼^ï·¾õ­?üÃ?|ïëµoÔnøÓQû´è¿ââbÉÄnÙ? þ?â/JIó¥§§oØ°!÷!µñ~yâÄ?âøAâÏãñ466fggdÙl®©©aøñâïYã/<âÄ_Ä¨Ý²edd0ÏAüøñ7Wñð4Ä¿¹6Ù<GQQQkk«Çãá"þ@üøøwÄâ<þäö_ZZª;ÆË<ñâÄñGüÁ ñç÷ûÛÛÛµãt8Ñ<"CüøñGü þ¦Áçóµµµiç9222êëëã%þ@üø#þ?$þ<î<Õjõ¼±øñâø#þ`øs»ÝÕÕÕÚìËÎÎnkkóûýÄ?Äñ#ÄÜ¼ívûd»e#û? þ###V«øCüÄT´î<´ c¼Ä?F?ydÍËË=âñj·lÚyÉäp8ç þ@üø3~ümÜ¸Ñår¿#G|Ä¾Ó§Ooß¾ý¹çÉ¾Y/Ç²SÃñ÷õEä?÷?·Û]__?Ù<Ïçc;óÌxæ<óGÏü0pü¹ÝÝ²Ùl¶öövæ9? þâ%þÄr»Õç(--eøñâ/îâO7? þü~ÿéÓ§uwËÆ<ñâÄñGüÁ ñçóùW¬X¡Ý-ÜÙÄ?ÄßÓþøÖÝ-[vvvcc#»e#þ@üø#þ?$þWUUUjjjHö2ÏAüøñGü0NüõõõUTThÇxKJJ:::ØnÄ?ÄñÄ´î¯´àÀÀ[øñâø#þ`øóù|­­­ÚÝ²IöUUU¹¶ñâÄñGüÁñçt:åvh±XB²Ïl6×ÕÕ³? þ?â¹s¯©©ÑÎsäææ655=xðMDüø?âF000PYY©ç(**joogo¼Ä@üø#þ`N§S;Ï¡öÆËnÙ@üøñGüÁ ÔnÙ´ó6l`Ä?ÄÂãñ455-]º4$ûRSS«««åÆ0­ûøñâø#þ¥<x »[6Å"ëÕnÙ¦¾o_ þ@üÄ¢Ëår8&IÍÍÍ^¯7ðÄ? þ?Ä0¹9éÎs¬^½º­­M;ÆKüøñâøCìñûýÒvÚyµ7Þ®®®ÉNHüøñâøC,ñù|º»e«¨¨èëërâÄ?ÄbÇãÑç5õõõn·*gBüøñâøC´°«®®ÖfÕjñNñâÄ?âÑ«¯¯ÏáphwËÝÖÖæ÷û§Ä? þ?D£ÉÆxe¥ÓéAö þ@üø#þu&ã]¸p¡ÝnöñâÄ?âQÁãñ´´´hÇxM&Ãáâ<ñâÄ?âøìlwºóÄ? þ?â/zM6ÆÝÒÒ2»ÙGüøñâøÃ¼l×f³µ··ÏxøñâÄñGüEÝ1^©ÀÒÒÒÜ6? þ@ü5Æ«Í¾Yç þ@üøñGüóÌãñ466jÇxÕ<Çàà`$/ñâÄ?âsEÂNwWBPrp.æ9? þ@üÄß<p»Ýºó¹¹¹3Û-ñâÄ?âøFrýj³¯¤¤$òW=ñâÄ?âsÂï÷···ëî×n·»(¹Ä? þ?<Ï×ÖÖ¦»[¶ÊÊÊk×®EÕ¥%þ@üøñGüa&Û-Ùlõ<ÂËLüøñâøÃ´M¶[¶ÜÜÜÖÖV¯×µøñâÄñi«Ïn·kç9V¯^=¿c¼Ä? þ?âoÖHÕ9NÝyÒÒR9*ú³øñâÄñ)eî<ÇÂ«««£gøñâÄñGü=5ÏaµZµc¼UUUrG¿ñâÄ?â¡Ünw½vÃb±466Fç/ñâÄ?âø6Ë¥»[¶ÂÂÂ(ã%þ@üøñGüÓpåÊÝy¢¢¢ÃüÄ? þ¿x'm'§;Æã%þ@üøñGü³Àçó577çææ4_jjj]]]Îs þ@üø#þ¿P§¡¡Áb±hwËV__?88hàßøñâÄñ_÷nuuu©©©ÚyöövÏgø-@üøñâø×®]ÓãµÙl±²âÄ?Äñ÷N§³´´T6m"·­Aüøñâø3&¿ß/·ÝÂÂÂæËÈÈ¨®®6ê<ñâÄ?â/îâÏëõ677k÷Æk±X<O<ÿ= þ@üø#þcxxX¶°ÙlÉ>	Á¦¦&ìøñâÄñGü·USS£ã]±bE[[[<ñ þ@üø#þâ"þ®]»VQQa2´ûçp:ñ3ÆKüøñâø3xüuuuñÊA»Ýc¼Ä? þ?cÆßï?ú´v·l&©¦¦ÆívóGKüøñâø3Büy<¦¦&möÍfÙ°ÆÞ-ñâÄ?â/îâO6]Hö-]ºyâÄ?Ä1ãO«ì[½zõÙ³gç þ@üøñGü9þN>m³ÙºººøË$þ@üøñGü?þ@üøñâø#þ@üøñâø#þ@üøñâøñÇvñâÄñâÄ?ø#þ@üøñâø#þ@üøñâø#þ@üøñâø#þ@üøñâø#þ@üøñâø#þ@üøñâø#þ@üøñâø#þ@üøñâø#þ@üøñâøñGüøñâøñâÄñâøñâÄ?âøñâÄ?âøñâÄ?âøñâÄ?âøñâÄ?âøñâÄ?âøñâÄ?âøñâÄ?âøñâÄ?âøñâÄ?âÄ?øñGüøñâÄñGüøñâÄñGüøñâÄ_ÜÆ_OOÏªU«W®ßßOüøñâÄãoùòå/_S§NåäähãïóÏ?,iP·Û=õÕWÝÝÝl(]]]÷ïßg;@ÜºuëêÕ«l(¿øÅ/"üCc8þ%''kãïÇ?þñçõÉ'|úé§þÙgýìg?c;@Ü$Øò0!l(üqä¨â¯···ªªÁË¾àe_ð²/xÙ×È/û*=*//÷z½Ä? þ@ü-þü:xïÞ=Ã144¤ýNâÄ? þb>þuuumØ°addD÷XâÄ? þV«uAâÄ? þá þ@üøñGü þ@üøñGü þ@üøñGü þ@üøñGü þ@üøñGü þ@üøñGüø? þ? þ@üø#þ? þ@üø#þ? þ@üø#þ? þ@üø#þ? þ@üø·øû«¿ú«ööö/#ëO>ù§ú§//¿8sæÛÊßþíßþüç?g;@råÓO?e;@9yòä¿ÿû¿Gøz<cÆß78ð#	ÿÒèÄñâÄ? þ¢Ô7Ö¬Y¯vçðÅ_$%%mÝºuttMo:;;,øú§¿¿?++KÝ<.^¼ÈÆóÛ±Z­ln===«V­;+WÊ'ÎoÚ þ¢çÎË/ÍfYëOþªeáæÍ»víbÅ¿ß/wè¿çòòò÷ß_?¾mÛ6¶Oßä>=///¸·7åËË£,:u*''íç·mK±¡££Cþ'Rîééél¸räÈwÞy'ð÷,Ã²0>>Îó=Ü6nÜèr¹?n!Ù>ÜBZø~OKK«Pþ'åj»qã,|ðÁÁ!Ã»sçNAAÔ^àï9øÀÛÃ×÷ªÄ7 ½½½UUUl"n!-AüÅÎÎÎÌÌLY¸~ýzNNüON¢ÿÏÅ-[¶tww?º'$$MJJbÅùíøãÆrí?zô¨¼¼Üëõ²¸=´ñ3BÚ¹uëV^^%~,øu²FþÇÇÇ?yÙ7þ1¿·âCðáÞ½chhíÃí!LKÑhùòåêEÞ6¨5ýýýï¼óÎáÃÙDñù­ìvûï½'òUþÏóÛñÀµßÕÕ%###lnº-AüE5é¼üü|éôõë×«ÿÀÉôâÅªªªü~?(ÿ/]ºd±X²²²Ô8?â°Z­aûA¼Ý´-AüøñâÄ?âÄ? þ@üøñâÄ?âÄ?>ø`ÝºuÉO¬_¿þ£>úµû¯'bæÞVïÒZ­VùÕ¼^oÈzY5111Ýóâ@L:pàÀÃ)þdesssÈú¦¦&YðàÁ'bO¿dMbbâ'üO<yRÊÊ«W¯&þnß¾-+W®²>//OÖ»Ýnâñ .¼òÊ+5o½õVðÊ·ß~[VVVV§ÏÅ%¤W­Z%Ëo~øðaUUUZZe6÷ìÙüêªÓéÀ£ä´!9%kÒÓÓ×­[wáÂ9h·ÛC.ØùóçÃ:J.:ª³³s²PÛ°a¬ïéé	¬¹|ù²¬±Ùl5²X,rVÉÉÉ[·n½wï6þ´ç²&ÌE@üÀ<[¼x±Ë;wWÞ½WVZ­Öà¸	ÑÛÛ«Ý¼ysÈQ»wïVG$$$èJTÇnÙ²ebbBò1))Éï÷Ë±òUâ)%%E½/ÌùÈîÅÓþ¦§N.Ú@_¾ÿþûò9õë×O7þÂÄÌ?õ¯ÎÖbÁq³mÛ¶GOÈ,++SÇªÖQù¨ÀhSG«§eùÒ¥KêLÏsÿþýwêU××^MÖ;wNå«,ïØ±ã©ç#á(å/îo$A¹hÑ"ùGFFä |K.Å9>>®¾!++KN(¿Âã_¾L<ÝøsQñ'tã/°^Åj&qïÞ=9(Ù¤®ZµJ.[¶lçÎmccc3ï	y.-p*uðîÝ»o`¼ò+_eÙét>õ|$4åàýû÷/ÛdïÏÛ»w¯õÎ;ï<þåKÛöì	þ	A?IÒõÄätã/ÌE@üÀüSOw=zô(x¥×ërTÜ	¤¡ËåRýhÀõ´¯ÆäTð¬È²ÙlNJJòù|ræééécz>¥X7n¨Neù*Ëc/]º$@÷åã©Ç_øù§Þ÷öî»ï¯T2ðx_àÈÈö	­«W¯644¨aÕ¨¼®úÔDÛ½·zµW¾VUUÖ9õLÛÐÐ:xÿþýð¹k×®cÕ§ÛH³¥&å¨ÎÎÎÑÑÑðñSµ5Ç¹¨?j`"11ñøñãê£^N8¤Î(++Ðñù|ê5ÙÀd®zÏz«Ëå~³ jÁ=öH*©9ÜÀ­è&¿U?½««+°>Ìù¨7ù©÷üy½^õaâïäÉçääW>J½ýq``@~lÉâOå¦ä²|DçÖ­[sQÔ 8tèÐ¯î¿PmX¼`±xíµ×Ë2Ø3gÂÄßã'»âõf³9øá0ç##ø&m'ûeÇÆÆÕ¯òb÷ÆÄ¢Eä«ú´àóTOL¨Tæ¢ þ ZH ¬_¿>éuëÖ©ÛøëììT§ßÝÝ8ÖëõÖÖÖZ,L÷îõù|c;::¤É²²²ZZZBÎSIä¬dý®]»BÖOv>B.õa>ç/ ªª*ä3_òòrÙiiiò[¸ÝîÀÇÖçèè¨Ô­ÚV6­§§'ä'¹¨? þ@üøñâÄ? þ? þ@üøñHù?õép*^fIEND®B`


Detrended Normal Q-Q Plots


hãÇß8Æ	¯^½Ã±5cxÖ¬YÞX$ø¦%þòGúcJàïÝwßÍ¹ã¼ù½hÉáÅøUß »¬ÛXléÇzìã÷zÑøºººÂG¿ããâÜ¹sÇz¼ÉGáÅäXvòïI..2Z[[	üåE>Ô?æ,«4æÙÖÖvòäÉQWÚxVÎ¨÷jÒ3É3¤¯¯¯ð&&¿q>[ZZâbcccà5þ188è]E?iúá/°#kkkÇú;S¡¿­Kg¢¿'±Ø±ÆÇCÇ¿ÂÇÜs6òp#ïx.4Þñ¬Õúúúä8õHá:¿ñÌ2u5êÆ³rÆ"éäfÿaßÿìçÓøÒ¥Kÿò(<vì7	þ¤i¿^x¡ðd_ZÑyã±Îæ-½çæý÷ß/Zb±¥g,q^zé¥¸ÿbddâ³¢TE,ÿ)¼¸páÂä+WN88þÝÉ·®d³Ù­[·~øák×®ãeÒøèÊÉ7éÇ¿ñìùËó.ùßNõ_âùtæÌ]»v%ÇógFK?ià/~½ßÍù¯zI~¥âeròlsss2)ùÀÓÀíFý­YbÞÒrJÔ|f+,§è]£.¶ô¥%±zõêüçú1É¹À«V­º~»di7oþøøKö~;w.ÂþýûÇ¿S§N%ûâ!wuu%õ=:êöè,Â_éSôÜ(l¢kuøK>¥OÁÁÁ°ÝÊ+GÒpïÞ½±òáàÒëÿ/ä³.]*q^üIe¿±8òüÙüÇÿóßÇì&ù[³Ä¼¥å*JxTxêe~jÅ±´$®]»ÿtW2æòåËE÷ª««Ë ðãà/Ï¤¹sçæ?Y8=öØÈµhÑ¢xB³L¥WNÑs£°®ÕIàïÌ3Ë_ºtiáÔ¢u?å|¬[)ñ|ËQoÃÞX$ø¦þâ·àòåËGþù¯ÖÖÖTüFáòã=c[ßr7Îyï(§äëñbá---#¿®o¬ÅÞqÆÒH¿xñâÚµk«oÍfGÞ19üõ÷÷ÇÒbÁ Ç<<7wiâ<÷ÜsñèâÏ7ïàÁ?üp~ÏÓÇeBø+½rEMh­NÑ±cÇãQÇòëùãb-¹õ¶¶¶îîî;îyëù600°ûödj>6åÐÐ7	þ$énËåì±	·Ü$fv%|Ó·Ó/ÐF?IR|æ¯¨§~ÚàOTlÛ¶mÞ¼yÉ±Ýxâ'¬	þ$I$I?I$Á$IàO$Ið'I$ø$IüI$ÁU I$I?I$Á$IàO$Ið'I$øTæ=z´¡¡!N/_¾|¢ó655UUU]ºt)?&cLssóÞ°n7¹ëgÞI×ßß¿yóæ9sæÄÊ7oÞöíÛ¦èMün>.Ið'iFW__ÎèOtÞÝ»wÇ¼Ï>ûl~Ì3Ï<cvíÚ5ÝñÎ»+W®ÜºuëÐ¡CqC+W®Ä¢zzzÚÚÚàOüI*÷á3gÎÄ¼Ë-ËY¼xqéíí;y÷¸öÈ#÷dÃ$øtåWHíÛ·Ï=Ö¬Y;wî,ºÚÃ?ÜÚÚZ´üÃ~ÿý÷c8Æ$®^½ÚÖÖVSSN§[ZZ:;;G]Zá­åàÁK,¤7o®[·.æ;¿mÛ¶dGfðtéÒ¥±´K~÷ÝwÇ¹fcÉCCC£N-±N:w2VÂÑ£GG®ák²Ä¢<?%Á¤»å¿dø©§á^x!O?ýtáu:::ä¶iÓ¦tàÀ1¼qãÆdÒ¢E?W®ñLfÔ¥ÞÒ³é^zé¥X¿~ýÈ;¿eË+¼üòË1°÷îyß÷Åðõë×Ï;ÉÉáÅ±øUz-íÝ»7¹­ àÈ;YôØK/ÊSüIº»ø¯Äpîv#í5êO:V­ZÃ«W¯NdzæÌ;v´¶¶ÆøT*5êÒ SzäÍ=ä¼uuuÉ¿uëVûbd[ZZBWcíÆµø+½áXÂXøË?öÒòäî.þÅ3_iÍ5+®900]1É¤ýû÷ÇÂ³gÏÐ¨w`<³uÇB;ó89rdÎ9ÉÚÚÚd§àxöü-X° Æß¼y³´K#¯ôðäV¸$Á¤OL¦pGT²óìY·n]LÍÿÌ¯®®1·n7Nü%ÇÂs#ç;wn2oÑÝ>~üøúõëwÞ±;vÄõ#'g-[á>ü%§¸8p ùÚ3Ï<39r$¿í¥^*ÍùóçÏ¥g	ç;ßM6wÃ1|èÐ¡.$zcä%KbøìÙ³1°`Áq®ë×¯ÅæÏõêÕe²KrÍ5ãÃÉ¡çä sÑcÜ$øôÉàoxx85ëvßÕWÚ"É!×øfÊ?vìXmmmÐgÛ¶mãÄ_éYN<S/^?ï¤ð:[·n^]]½råÊäÄÞ0_6MN^¶lÙÅÇ¿fâVÉAíPàÎ;Ïóg-=z4îRòÚEr+àO$	þ$I$I?I$Á$IàO$IðW.ýõ_ÿõ~ô£©¼Åþçþ÷ÿwÏ¼r«¯¯¯ðGT&ÅFyï½÷¬r+ÞÄ¦øSãé?þã?.]ºd=aÿôOÿôüþÊ¥?ú£?ÿM17!£;wîÜz(·b£Ä¦±ÊåSüÎ©ñtëÖ­¿üË¿´Ê°S§NýÛ¿ýüÁàOð?ø?øü	þàOðð?øü	þàþàOð?ÁüÁü	þàOð?ø?Áü	þàOðð'ø?Áü	þàþàOð'ø?ÁüÁü	þð'ø?ø?Áü	þðð'ø?ÁüÁüÁü	þàOðð?Áü	þàþàOð?ÁüÁü	þàOð'ø¿¿ÞÞÞL&N§:;;G^áÄUUUð'ø?Áü	þ*¡l6èÐ¡Ø·o_ÑÔááá±ð.¼9ýà?øñ|SeÖßþíßþð?´Ê­Ø(±i¬r+ÞÄâ­Ìz(·nÜ¸ø³Ê°ÎÎÎ¾¾¾©¼Å¿ÚÚÚøOär¹¢©O=õÔ=ÆÂßÞ½0?~<?Põ½ïïõ×_·Ê­Ø(±i¬r+ÞÄâ­Ìz(Ã^õU+¡¿ñÆSy3étzÔáèêÕ«­­­ACå°¯Ã¾rØ×a_9ì[!øK¥RùáêêêÂIkÖ¬yóÍ7ÿó¡Âàþð'ø«ÕZWWËå>ºØ7ÿÛ#üïÁàþð'øö­[·îàÁ1?³ÙìèÕ?Áü	þàOðW«µ«««¾¾>Je2îîîQµ?øüÁàÏ<Ãàþð'ø?ø?Áàþðð'øüÁàþàþð'øüÁüÁàþððð'ø?ÁüÁü	þð'ø?ø?Áü	þðð'ø?Áàþàþð'ø?ÁüÁàþð'ø?øüÁàþðð'øüÁàþàþ?øüÁüÁàþ?ø?øüÁàOðð?øüÁüÁàOð?ø?øüÁüÁàþàþð'øüÁüÁàþð7­Â~ô£?ø?xðÁ¿þõ¯<yþàþð'øSÅâïÒ¥KsçÎ]¼xñoþæo>ôÐCþô§ÿøÿþàþð'øSeâïK_úÒW¾òßÞüûýÍ¿?kÖ¬!üÁü	þàOð§JÃßÐÐÐÏÿüÏïøÖ<þâß¯.ýÕçþàþð'øS¥áo`` ¶¶¶Ë/î¹çàþàOð?Uþ¢yóæµ··çå·ý¶æ3	6ÁüÁàþª@üuttüìÏþìÚÿµvÇ·vlúÝM_ðù¯~õ«ÃÃÃðð'ø?Á*Ñ±cÇ¾ð/üÌÏüÌ/þâ/nÛ¶m`` 26üÁüÁàþ3(ø?ø?Áü	þàþàOð'ø?ÁüÁü	þàÏz?ÁüÁü	þàOð'ø?ø?Áü	þàOðð'ø?Áü	þàþð'ø?ÁüÁü	þð'ø?ø?Áàþðð'ø?Áàþàþð'øüÁüÁ*?þñ7oÞÜüÅæûï¿Çóççáþðð'ø+îÆýìgXôÀ~gýï|ñ_liiQþ?øüÁüÁfþ¶nÝúËüòÎoïÌÿ[¸pá3Ï<?ÁüÁü©ñwÿý÷oØ°¡ííí_ýêWáOð'ø?ø?U&þ~gýïâïì#ð'øüÁüÁ*ßüæ7ÿgëÿ,Äß~Áa_ÁàþàþTø»qãÆý÷ßà$ûÈÿ^û¿ÿGãÿøÊW¾âÁàþàþTøzÏ>ûìÃ?¼zõê?û³?QÛþàOððYøáÁü	þàþàOð?ÁüÁü	þàOððð?øüÁüÁàOð?ø?øüÁàOðð?øü	þàþàOð?øüÁü	þàOð?ø?Áü	þàOðð?Áü	þàþàOð'ø?Á_ÔÛÛÉdÒétSSSgggá¤îîîÔÜÜW?Áü	þàOð7íËf³ûöµ··N?þéÓ§càðáÃ,¿¿ø¿ø)ìûßÿþøÃQõÖ[o=Öz(·b£Ä¦±Ê­x·2ë¡Üºzõê±cÇ¬2ìõ×_çw¦ògþjkkã<1ËåÆºZMMÍHü8pàìvâÄ³*³Þxã®®.ë¡ÜÆz(·âM,ÞÊ¬rëí·ßüYeXGGÇßüÍßLå-Îü¥ÓéQw«7:ì+öÃ¾ûÊaßi_*ÊWWW¼ÂÍ7³ÙìÀÀü	þàOð¿i_]]].ûèöaß.zíÚµõë×÷õõþð'ø?ÁßôkÝºuøÍfÖø+úûûGþð'ø?Áßô««««¾¾>Je2îîîÿzlUÿùèª?Áü	þàOð7s?Áü	þàOðð?Áü	þàþàOð'ø?ÁüÁü	þàOð'ø?ø?Áü	þðð'ø?ÁüÁü	þð'ø?ø?ÁüÁü	þàþàOð?ÁüÁü	þàOð?ø?Áü	þàOðð'ø?Áü	þàþàOð'ø?ÁüÁü	þð'ø?ø?Áü	þðð'ø?Áàþàþð'ø?ø?Áàþðð'øüÁàþàþð'øüÁüÁàþððð'ø?Áü	þàþð'ø?ÁüÁü	þð'ø?ø?Áàþðð'ø?Áàþàþð'øüÁüÁàþðð'øüÁàþàþ?øüÁüÁàþ?ø?øüÁàOðð?øüÁàïÄ_ÕJ¥Rðð'ø?ÁüUþRw*NÃüÁàþðç°/üÁü	þàOð¿xkØ´iüÁü	þàOð¿t:í3ð?øü©òñ×ÜÜ<òlÚÚÚø?øüÁàþ*ÕÕÕ¡½þþþúúúóç;ß7ÂüÁàþðWiøKvõÅ@h/®Ï¿5küÁü	þàOð¿9sæõººº.]ºO<ñD2à«^àþð'øSâoÛ¶mùÓ;?ö·dÉø?øüÁàþ*ðlß'|rîÜ¹1ÐÝÝÁÖÖÖi±=àOð?øüÍ àOð?øüÁüÁàOð¿±[°`Aò/¾äþàOð?U8þæÏ_¾|Îö?øüÁàO¿p^°¯§§'vÓnÀàþð'øXµµµ¿é(?øüÁàþ®··7ð·eË7oÂüÁàþªpüEóæÍ«>àþð'øSâ¯±±Ñ	ð?øüi¦à/aß4?Áü	þàOð7±êêêðð'ø?Áfþâ!þ¶mÛ644ð?øü©ÂñW5FNø?øüÁàOù%Ï£æø?Áü	þT_õ2?Áü	þàOð7±/ðÜ¡ÞÞÞL&N§:;;KOÜøüÁàþ/õíAÌf³ûöµ··4¹1mß¾ýþûïoÂâæ-[Ö¦2ëX²dõPnÅFMc=[ñ&6ÅïO_þò¿øÅ/ZeXKKË¯ýÚ¯Må-ÞuüuvvþvíÚÿüø_øR[[,$Ë5444¹1=þøãU$IÔ]Çß'¶oái"E§4¹1ð'Iàoò²gû±ºººô¤É)lÇ=ôÐú),nîÑG]¯2ëk_ûÚ#<b=[±QbÓXåV¼Mñ;§ÆÓ×¿þõ+VXeØ¯ÿú¯ÿÖoýÖTÞâ]Çß'[]]].KÒÆpéIã9áÃ	rÂ>äriÝºuøÍfKOÜøüÁàþ«·råÊªªªY³f­Y³fÒg~tuuÕ××§R©L&ÓÝÝý_÷òöÑë&7þð'ø?ÁßäõÃÓâOýÂàþð'øX.ê­ZµêæÍÉÂêÕ«cÌ¢EàþàOð?ø«4üUWWõócr¹yj-üÁü	þàOðÓ©T*¨Q444c&÷U/ð'øüÁàþÊÉaß+V$ãgÇø?øüÁàþ*¡½QOøøðÃáþàOð?ø«À¯zfÍìÙ³S©TübEÛþð'ø?Áßþð'ø?ÁüÁü	þð'ø+óN¥R)ø?øüÁàþ*©±?ø?Áü	þTiø«Ç<ÁßáÃáþàOð?ø«XüõööÎ5+Ø×ÖÖVøÏðð'ø?ÁüUþÖ®]ìð;~üø4Úð'ø?Áü	þ&ÖË/¿°oÕªUÓnÀàþð'øo-JÎíèììÛþð'ø?Áß¸Ú»wo²Ã¯½½únøüÁàþãÓ÷üÁü	þàOð§¿ÔJ§Óðð'ø?Áüùónðð'ø?ÁüÁü	þð'ø?ø?Áü	þðð'ø?Áàþàþð'ø?ÁüÁàþð'øûÄð·`Áêêjßóð'ø?Á*óçÏ/ïù?øüÁàO¿p^°¯§§'vÓnÀàþð'øXµµµ¿é(?øüÁàþ®··7ð·eË7oÂüÁàþªpüEóæÍ«>àþð'øSâ¯±±Ñ	ð?øüi¦à/aß4?Áüg|ðÁ+¯¼òì³Ï;vlhhþðWFø«««sÂüÁàïì­·Þúô§?½hÑ¢¶/·ýÒ/ýRSSÓ7àOðå¿xH¿mÛ¶ÛÿMáOðÓ±>ø þSýHößÞüà¾öµ¯ÁàþÊUcäø?Áß$zåWZ[[óòÿçñÿ3gÎááaøüÁ_Yà/5FNø?øüM¢ç®íËmøuuuï½÷ü	þà¯,ð7­?Áü[ûÜçå·éw7ýÜÏý=?ø?øüÁ_ÈkjjúÕ¥¿ºã[;B~ßÜúÍÆÆÆÝ»wÏ=?øüËåV®kÖ¬5kÖLáOðeØï½÷+¿ò+uuuÿüç?õ©OmÜ¸±|vûÁü	þ>õiqò/ü	þà¯	øöÛoðÁåvÇàþ4Óñ·páÂ ÞªU«¿ío«W¯1-?ø?Á_åð§¿êêê ^á!cb<üÁü	þàOðøU/A½_~ÌÐÐPñU/ð?øü©bû®X±"9ì?c8Æ´´´ÀüÁàþðWiøízÂÇ~ð?øüÁ_~ÕËààà5kfÏJ¥âç+bÌ´Øð'ø?Áü	þfPð'ø?Áü	þ&VCCCccãàþàOð?U>þÒétUÕtÝ?øüÁàobuvvþvíÚoÓå¯ºÁàþð'øì"Æ(JÁüÁàþðW_ò<j¾äþàOð?Uþ¦õö?Áü	þàOð7®?éûÑíÃ¾Óâ/ü	þàOð¿Éã/ù¾Éñ?ø?Áü	þTáøËd2U%sÂüÁàþªüõôôÌ3'ÙÿPÏ	ð?øü©bñoº8þð'ø?Á¿í?øüÁàoºÕÛÛÉdÒétSSSgggá¤îîîÔÜÜW?Áü	þàOð7íËf³ûöµ··N?þéÓ§càðáÃ,?Áü	þàOð7í«­­Mþ¬p.khhëj555#ñ÷§ú§oMañ¦ùÊ¬'OÆÓz(·b£Ä¦±Ê­x·2ë¡Ü:úô±cÇ¬2ìµ×^ëêêÊ[¼ëø»xñâ=Ç_á'cÒÓÓ³qãÆøJÿöÆor¥_eV<=Î?o=[±QbÓXåV¼Å[õPnýë¿þëÿFÓ8ÿÄ^½zu*oñ®ã/ùwÞyç^á¯ð;«««G^áæÍÙlvä£sØWû:ì+öÃ¾+Øÿbçt:½lÙ²Ë/OÅ]ÿi1ËåÃ¾1Ík×®­_¿¾¯¯oäBàOð?øüM¦/.0²êêê+WNÙÃX·nÝÁc ~f³Ù¢5¾bÅ±vÂàþð'øûXÏ¼ãÇ×ÔÔLñwëêêª¯¯[Ìd2ÝÝÝÿõØnïlhh(ü£sð'ø?Áü	þ>ßþfÏ=òì2þð'ø?ÁßÄ*4_MMMûÕ«W§Ëö?Áü	þàOð7ÁEÜ>ÏãÁ¼råÊ´Ûð'ø?Áü	þ&Ö=üøüÁàOðS¿iü	þàOð¿	wþüùÌ5+9Ã·®®îÈ#ðð'ø?Á*'NÈðà/Þ·oüÁü	þàOð¿úúú ÞùóçóøëîîN¾íþàþð'ø¿<Û7ùòä<þâùð?øüÁ_¥á¯®®.¨ìíüår¹íÛ·ÇpCCüÁü	þàOð¿xHU£uúôiø?øüÁàþ*ðlß¾¾¾ÖÖÖälß|yZløüÁàþ3(øüÁàþðð'øüÁào´®^½ÚÒÒ2öìÔíb ©©iºó?Áü	þàOð76lØP5F6m?ø?Áü	þT9ø;räHâ¼ýû÷$#=ïèè?ø?Áü	þà¯BðÉdBx9)ùo¾çþàOð?Uþª««Cx#'år¹W?ø?Áü	þà¯Bðÿ«ncMMþÔüÁü	þàOð¿¼?ø?Áü	þðð'ø?ÁüM[üþàþð'øSåà/u§Òé4üÁü	þàOðþ¼üÁü	þàOðð?Áü	þàþàOð?ÁüÁü	þàOð'ø?ø?Áü	þàOðð'ø?Áü	þàþð'ø?ÁüÁü	þð'ø?ø?Áàþðð'ø?Áàþàþð'øüÁüÁàþðð'øüÁàþàþðð'ø?ø?Áü	þðð'ø?Áü	þàþð'ø?ÁüÁàþð'ø?ø?Áàþðð'øüÁàþàþð'øüÁüÁàþ?ø?øüÁàþàþ?øüÁüÁàOð?ø?øüÁàOðð?øüÁüÁüÁàþð'ø?øüÁàþððg=Ààþðð'øüÁàþàþð'øüÁüÁàþ?ø?øüÁàþàþ?øüÁüÁàOð?ø¿Rõööf2t:ÝÔÔÔÙÙ9ò'N¨ªª?Áü	þàOðW	e³ÙCÅÀ¾ûÚÛÛ¦·´´Ààþð'ø«üÕÖÖÆ>r¹ÑÔ§zjÏ=cáïgéÂ;öÚk¯u¨ÌwÌãÇ[åVlØ4ÖC¹obñVf=a¯¾úªPMý¯þ¿t:=êptõêÕÖÖÖ áXøÿß)ìÍ7ßìïïÿ¿*³þîïþîòåËÖC¹%6õPnÅX¼YåÖO~òøÏõP½ñÆ×¯_Ê[øK¥RùáêêêÂIkÖ¬7©ÿ|¨ûÊa_å°¯Ã¾rØwú®ÊªÃuuu¹9ìÃ£^-eøüÁàþÓ»uëÖ<x0âg6#GÂàþð'ø~uuuÕ××§R©L&ÓÝÝ=ªöàOð?øüùgøüÁàþðð'øüÁàþàþ?øüÁüÁàþ?ø?øüÁàþàþñ700ðÊ+¯<ûì³ñóÆ^!ð'ø?ÁüÁ_Åâï½÷Þûìg?û¹Ï®íËmÍÍÍùÌg.àE?øüÁüU&þ,Yò¥%_ÚùíÉ¿ßøßø_ø¯øüÁàþà¯Òð÷ÁÔÕÕíøÖ<þâßüùóßzë-¯øüÁàþà¯Òð¿Ò>÷¹ÏÊ/þýò¿üÝï~×ëþð'ø?ø«4üÝ¸qãSúÔ7¶|#/¿ßÚQ__ÿöÛoÀàþð¿èßøÆ÷Ý·éw7üB÷·ÜÿÐCÀàþð¿èÛßþöìOÍ®««3gÎïýÞïyÀàþð¿¤÷ÞÏ?øüÁàþào¦àOð'ø?ÁüÁü	þàþàOðð?øü	þàþàOð?ø³àþàOð?øüÁü	þàOð?ø?øü	þàOðð?Áü	þàþàOð?ÁüÁü	þàOð'ø?ø?Áü	þàþàOð'ø?ÁüÁü	þð'ø?ø?Áü	þðð'ø?ÁüÁüÁü	þàOð?ø?Áü	þàOððð?øüÁüÁàOð?ø?øüÁàOðð?øü	þàþàOð?ø?øü	þàOðð?Áü	þàþàOð?ÁüÁü	þàOð'ø?ø?Áü	þàOðð'ø?Áü	þàþð'ø?ÁüÁü	þð'ø?ø?ÁüÁü	þàþàOð?ÁüÁü	þàOðð^	ð'ø?ÁüÁü	þð'ø?ø?Áü	þðð'ø?Áàþàþð'ø?ÁüÁàþð'ø?øüÁàþðð'øüÁàþàþ?øüÁüÁàþ?ø?øüÁàOðð?øüÁüÁàOð?ø¿ñÖÛÛÉdÒétSSSgggá¤¡¡¡õë×WWWßwß±öáOð?øüMû²Ùì¡Cb`ß¾ííívïÞýôÓOÇK"VccãHüÿûßÂþê¯þª¯¯oPeÖÛo¿ýÿøÖC¹%6õPnÅX¼YåÖÀÀ@àÏz(Ã:;;ûûû§ògþjkkw1Ëå'555]¸pa¬ò'òý),^¯¿þú÷Uf½öÚkßûÞ÷¬r+6Jlë¡Ü7±x+³Ê°W_ÕJ(Ã¦þWÿÀ_:u8¹¸wïÞÆÆÆ³gÏ:ì+öÃ¾ûÊaßi_*ÊWWWM:pà@x±µµþð'ø?Áß´¬ê§Åp]]].ûèöaß.¼ZáÅ¢ð'ø?Áü	þ¦eëÖ­;xð`ÄÏl6[8ióæÍ/½ôR?~É%ð'ø?Áü	þ¦]]]õõõ©T*Étwwÿ×c»½S0Þ¤Ö¬YN§[[[/]º?øüÁàoæ?øüÁàþàþ?øüÁüÁàOð?ø?øüÁàOðð?øü	þàþàOð?ø?øü	þàOðð?ø?øüÁüÁàþ?ø?øüÁàþð?øüÁàþàOð?øüÍ´þðÿð»ßýî¦°¸¹¿ÿû¿ÿÊ¬'N>Úz(·b£Ä¦±Ê­xâwN§+W®üùÿ¹õP½ôÒKÿðÿ0·xãÆø³óçÏïÜ¹ó$I*¥Òûæ«ì$I9Á$IüI$	þ$I$I?I$Á$Ià¯»xñbkkkuuõÚµkþªG___UAÖÕÔÔßßßÐÐ¿ØÛÛÉdÒétSSSgggá5KLÒ½Ý4^;÷p»:ÆK¦·×Ë=Ü.ÝÝÝ---ñ¢hnnHY½^<îb-m.¼ysá¤#G¬_¿Þ*Êººº.øöÍf:ûöíkoo/¼rIº·Ækçnc¼dÊ|»x½ÜÃí2þüÓ§OÇÀáÃ,XPV¯ø»êóÃsæÌ)¯Æ£GZESÙÊ+/]ºTøÊ¬­­½uëVär¹¢ÿ4¤»i¼vîáv9ÆK¦Ì·×Ë=Ü.ÕÔÔÕëþîbÍÍÍçÏ_|±ÉV¬X#[[[/^¼h]MÝ3¾àY¸Q6PIº·Ækçn±ÆxÉívñz¹çÛ%êééÙ¸qcY½^àï.vöìÙ÷÷ìÙS¤þ|×®]#ZW÷äJ¥òÃÕÕÕW+1I÷vÓxí'2¼dÊs»x½ÜóíróæÍl6;00PV¯øÞyç5Õå½zeÖÕÕår¹nïxáÂ«¤»i¼vÊ^2e?¯²]ÂÜë×¯ïëë+·×üÝÅæÏßÛÛëÖ­=ö<ùäE._¾<3V¬Xa]ÝWæºuë<ñ3þgVxµto7×Ny"ÃK¦<·×Ë=Ü.§NuÞßß_¯ø»Å7o^ügkãÆÃÃÃÏ¦ÓéeËÅkÒºº'¯Ì®®®úúúT*Édó²óWuÊaÓxí2¼dÊy»x½ÜÃíÒÐÐ0òvÊäõ$I3	©V$IüI$	þ$I$I?I$Á$IàO$Ið'I$ø$IüI$	þ$I$Ið'I$ø$IüI$	þ$I$I?I$Á$Ý±_|qéÒ¥5·[¾|ùË/¿üßÞãn7mÞG»·ñÐÆÇt:ÉdnÝº5ÑeJ?IíÜ¹³jDO>ùd%áo×®]1òùç/ÿÜsÏÅø'xbË4ýêííÖ¤Óéýû÷ßîÀq1F9s¦bðwåÊÙÜÜ~áÂ1þòåËð'	þ$ÍôÑ`ÍÓO?]8òg¿ýÛ¿]HÎÎÎÀS¸°¥¥%óWþðÃ7nÜ8öìT[[»uëÖÂ£«'O`Å¤·£££S1fÎ9K.=~üx\·n]Ñ;vìXéå$â.%N81ÔV¬Xã»»»ócN>cÚÚÚòcvïÞ]__ª©©Y»víµk×FâoäòÆ¸«àOîqóæÍ¸zµpä»ï¾#qSTOOO2uõêÕE¶lÙL:wîu®äb2uÍ5·nÝ>VWWÇÔøx5kVòQ¼ËQïÞÈGzøðáBÑæyèÐ¡¼ü²|ùòâ¯Ä]tïKðò¾VU+ÄMûÍÛÅ@\µjU25±NÂÇd^ -Íf]1ÜÕÕ,¤p;vìÞ%G]7lØc=Ãñ3ì±;.'àã÷mÔG ;wn<Þþþþ¸?ã8s¹L&3ÆCøè§kjj&¿wUüIRYà/ùñ	n3E×®]Á¦äbKKKÜ´iS mpp0¿¸NÑ¾´üÅwß7åSþÈoüá'OÞq9Í¸øþûïÞ·±>÷øãÇ¤=ö|ôÓCÛ[·n-¼B@0ð$mmmMvLN%îª$ø¤_²»ëæÍ#bdL*Á</]ºø/oüõF-âTá¬ÄpmmmuuõÐÐP,|Î9ù©wX+êüùóSc8~Æð¹sçòS»ººâzøxüø+qW%Á$ÝûÏ½íÝ»·pdò(E'|ä?Øßß?rÖ3gvíÚÍ«1Ù-?®zG¢mÙ²%9Ú?7nÜ_b9É¶¾¾¾äâûï¿_úÌÜÅÇÔäÛmÂ¬3cÒ'®_¿^y&k#?µÄ]tïKNH§ÓûöíK¾êeÿþýÕÕÕ#OÎXµjU@ghh(9&?37ùÌ_òQ¹K.~X0±àÖ­[JÉy¸ù/[hÉé·É­:u*?¾Ärù%ùH®YÈï)ùøã¹sçâVb%¿Áå¸Z síÚµSKÜUIð'IeQòÈEíÞ½ûÿ¿ÇÝ.±Q~8À4E¾6Ê²°#GÀßG·ÿG¯­­-<"q7ïXþLÛ±ìàà`MMMòv¯²ð&æÎ?o)²c2_BÕüÔwUüIR¹@Y¾|yõí.]r[¿'N$_§×ÔÔôæoæ§lß¾½¾¾>Óã?>44ÚÑÑÑÚÚ&Ëd2/¼ðBÑ2GÞXTß¼ysÑø±Å»|aïùË·qãÆ¢ï|IêïïÏf³±fÏâòåËù¯­)õë×C·Éºjkkëîî.ºÅwUüI$	þ$I$I?I$Á$IàO$Ið'I$ø$IüI$Á$IàO$Ið'I$ø$IüI$	þ$I$I?I$ý?"±(ØuIEND®B`
