# Supplementary material for: Regulation of piglet T-cell immune responses by thioredoxin peroxidase from Cysticercus cellulosae excretory-secretory antigens
Source: Front Microbiol. 2022 Nov 18;13:1019810. doi: 10.3389/fmicb.2022.1019810 (PMC9718028; doi:10.3389/fmicb.2022.1019810)
Supplement: Supplementary file 3 [file Data_Sheet_3.ZIP › 5. Supplementary Material/1. TPx gene synthesis/TPx gene synthesis.docx]

| **Certificate of Analysis** | | | | | | | | | |
| --- | --- | --- | --- | --- | --- | --- | --- | --- | --- |
| **Gene Name** | TPx | | | | | **Cloning Vector** | | pCDNA3.4 | |
| **Cloning Sites** | EcoRI(GAATTC)-BamHI(GGATCC) | | | | | **Insert Size** | | 678 | |
| **Competence** | Top10 | | | | | **Vector Resistance** | | Amp | |
| **QC Results** | | | | | | | | | |
| **Test Items** | | **Specifications** | | | | | | | **Results** |
| **Insert Sequence** | | Insert sequence results consistent with target | | | | | | | Pass |
| **Vector Sequence** | | Flanking sequence consistent with expected | | | | | | | N/A |
| **ORF Across Junction** | | Correct and consistent with target | | | | | | | N/A |
| **Restriction Digest** | | Expected fragment sizes observed | | | | | | | N/A |
| **PCR Amplification** | | Correct without non - specific bands | | | | | | | Pass |
| **DNA Quantity/Quality** | | Actual yield (by A 260 ) | | | | | | | 2μg/2μg |
|  |  | Concentration (n/a if lyophilized) | | | | | | | N/A |
|  |  | Purity (A 260/A280 = 1.8 - 2.0) | | | | | | | Pass |
|  |  | # of Tubes | | | | | | | 2 |
|  |  | Matrix | | | | | | | TE (lyophilized) |
| **Endotoxin Test** | | Verified, <0.1 EU/µg (Endo-Free Preps Only) | | | | | | | N/A |
| **Appearance** | | Clear, no visible particles | | | | | | | Pass |
| **Label** | | Correct and white | | | | | | | Pass |
| **Comments** | | - | | | | | | | - |
| **Restriction Digestion Map** | | | | | | | | | |
|  | | | **M** | **1** | **2** | |  | | |
| 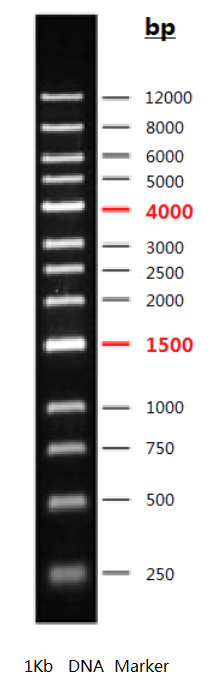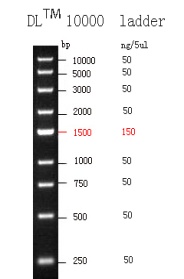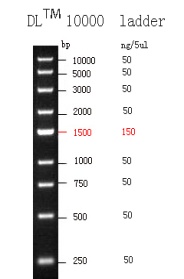 | | | 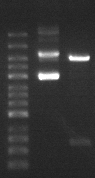 | | | | **Lane 1：**Plasmid  **Lane 2:**Plasmid Digested with EcoRI-BamHI  **Lane M**: DNA Marker | | |
| **Certified by：m** | | | | | | | **Date:20210702** | | |

**Gene Sequence:**

GAATTCGCCACCATGAAGTGGGTGACCTTCATCAGCCTGCTGTTCCTGTTCAGCTCCGCCTACAGCATGGCTGCTGCTGTGATCGGCAGGCCTGCTCCTGGATTCACTTGCAAGGCCCTGGTGGATGGCGAGCTGAAGGACGTTAGCCTGAGCGACTACAAGGGCAAGTACGTGATCCTGTTTTTCTACCCTATGGACTTTACCTTTGTGTGTCCTACCGAGATCATCGCCTTTAATGATAGAGCCGGCGAGTTTCACCAGAGAGGCTGTCAGCTGCTGGCCTGTTCCACCGATAGCGCTTACTCCCACCTGGCCTGGAACAACGTGAGCAGGAAGGAGGGCGGCGTGCAAGGAATGAAGATCCCCATGCTGGCCGACACAAACCACAGAATCAGCAGGGATTACGGCGTGCAGATCGAGGAGCAGGGCGTTGCTCTGAGAGGCCTGTTCATCATCGACGACAAGGGCATCCTGAGGCAGATCACCATCAATGATCTGCCTGTGGGCAGGTGTGTGGATGAGGCTCTGAGGCTGCTGGATGCCTTCCAGTTCACCGATAAGCACGGCGAGGTGTGTCCCGCTAACTGGAGACCTGGCTCCAAGGCTTTTAAGCCCAATGCCGGCGACCTGAAGTCCTTTATGAGCAGCAGACACCACCACCACCACCACTGAGGATCC
