# Supplementary material for: Regulation of piglet T-cell immune responses by thioredoxin peroxidase from Cysticercus cellulosae excretory-secretory antigens
Source: Front Microbiol. 2022 Nov 18;13:1019810. doi: 10.3389/fmicb.2022.1019810 (PMC9718028; doi:10.3389/fmicb.2022.1019810)
Supplement: Supplementary file 3 [file Data_Sheet_3.ZIP › 5. Supplementary Material/1. TPx gene synthesis/TPx gene synthesis.pdf]

Certificate of Analysis

**Gene Name**	TPx	**Cloning Vector**	pCDNA3.4
**Cloning Sites**	EcoRI(GAATTC)-BamHI(GGATCC)	**Insert Size**	678
**Competence**	Top10	**Vector Resistance**	Amp

QC Results

**Test Items**	**Specifications**	**Results**
**Insert Sequence**	Insert sequence results consistent with target	Pass
**Vector Sequence**	Flanking sequence consistent with expected	N/A
**ORF Across Junction**	Correct and consistent with target	N/A
**Restriction Digest**	Expected fragment sizes observed	N/A
**PCR Amplification**	Correct without non - specific bands	Pass
**DNA Quantity/Quality**	Actual yield (by A 260)	2μg/2μg
	Concentration (n/a if lyophilized)	N/A
	Purity (A 260/A280 = 1.8 - 2.0)	Pass
	# of Tubes	2
	Matrix	TE (lyophilized)
**Endotoxin Test**	Verified, <0.1 EU/μg (Endo-Free Preps Only)	N/A
**Appearance**	Clear, no visible particles	Pass
**Label**	Correct and white	Pass
**Comments**	-	-

Restriction Digestion Map

	M	1	2	
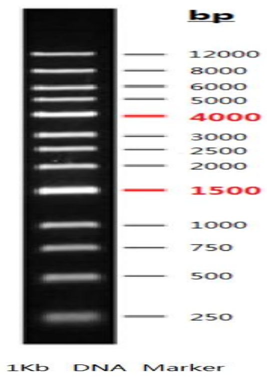	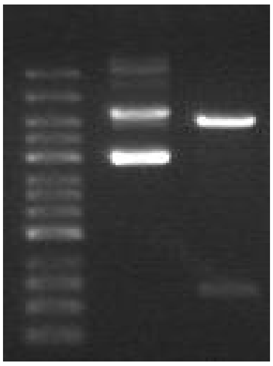			**Lane 1**：Plasmid<br>**Lane 2:**Plasmid Digested with EcoRI-BamHI<br>**Lane M**: DNA Marker

**Certified by：m**	**Date:20210702**

Gene Sequence:

GAATTCGCCACCATGAAGTGGGTGACCTTCATCAGCCTGCTGTTCCTGTTCAGCTCCGC
CTACAGCATGGCTGCTGCTGTGATCGGCAGGCCTGCTCCTGGATTCACTTGCAAGGCC
CTGGTGGATGGCGAGCTGAAGGACGTTAGCCTGAGCGACTACAAGGGCAAGTACGTG
ATCCTGTTTTTCTACCCTATGGACTTTACCTTTGTGTGTCCTACCGAGATCATCGCCTTTA
ATGATAGAGCCGGCGAGTTTCACCAGAGAGGCTGTCAGCTGCTGGCCTGTTCCACCGA
TAGCGCTTACTCCCACCTGGCCTGGAACAACGTGAGCAGGAAGGAGGGCGGCGTGCA
AGGAATGAAGATCCCCATGCTGGCCGACACAAACCACAGAATCAGCAGGGATTACGG
CGTGCAGATCGAGGAGCAGGGCGTTGCTCTGAGAGGCCTGTTCATCATCGACGACAA
GGGCATCCTGAGGCAGATCACCATCAATGATCTGCCTGTGGGCAGGTGTGTGGATGAG
GCTCTGAGGCTGCTGGATGCCTTCCAGTTCACCGATAAGCACGGCGAGGTGTGTCCCG
CTAACTGGAGACCTGGCTCCAAGGCTTTTAAGCCCAATGCCGGCGACCTGAAGTCCTT
TATGAGCAGCAGACACCACCACCACCACCACTGAGGATCC
