# Supplementary material for: Regulation of piglet T-cell immune responses by thioredoxin peroxidase from Cysticercus cellulosae excretory-secretory antigens
Source: Front Microbiol. 2022 Nov 18;13:1019810. doi: 10.3389/fmicb.2022.1019810 (PMC9718028; doi:10.3389/fmicb.2022.1019810)
Supplement: Supplementary file 4 [file Data_Sheet_4.zip › 3. C. Cellulosae ESAs and TPx Induced the Increase in the Number of CD4+CD25+Foxp3+ Tregs in PBMCs/2. SPSS statistical analysis/2.1 SPSS statistical analysis--Foxp3+/2.1.3 (SPSS data export) SPSS statistical analysis--Foxp3+.doc]

EXAMINE VARIABLES=figure BY Variables
  /PLOT BOXPLOT NPPLOT
  /COMPARE GROUPS
  /STATISTICS DESCRIPTIVES
  /CINTERVAL 95
  /MISSING LISTWISE
  /NOTOTAL.


Explore


Notes	
Output Created	01-OCT-2022 14:50:03	
Comments		
Input	Data	E:\学习\1.文章\4.SCI（1）\Raw Data\3. C. Cellulosae ESAs and TPx Induced the Increase in the Number of CD4+CD25+Foxp3+ Tregs in PBMCs\2. SPSS statistical analysis\2.1 SPSS statistical analysis--Foxp3+\2.1.1 SPSS statistical analysis--Foxp3+.sav	
	Active Dataset	DataSet1	
	Filter	<none>	
	Weight	<none>	
	Split File	<none>	
	N of Rows in Working Data File	19	
Missing Value Handling	Definition of Missing	User-defined missing values for dependent variables are treated as missing.	
	Cases Used	Statistics are based on cases with no missing values for any dependent variable or factor used.	
Syntax	EXAMINE VARIABLES=figure BY Variables
  /PLOT BOXPLOT NPPLOT
  /COMPARE GROUPS
  /STATISTICS DESCRIPTIVES
  /CINTERVAL 95
  /MISSING LISTWISE
  /NOTOTAL.	
Resources	Processor Time	00:00:02.52	
	Elapsed Time	00:00:01.52	


[DataSet1] E:\学习\1.文章\4.SCI（1）\Raw Data\3. C. Cellulosae ESAs and TPx Induced the Increase in the Number of CD4+CD25+Foxp3+ Tregs in PBMCs\2. SPSS statistical analysis\2.1 SPSS statistical analysis--Foxp3+\2.1.1 SPSS statistical analysis--Foxp3+.sav


Variables


Case Processing Summary	
	Variables	Cases	
		Valid	Missing	Total	
		N	Percent	N	Percent	N	Percent	
figure	Control	4	100.0%	0	0.0%	4	100.0%	
	ESAs	4	100.0%	0	0.0%	4	100.0%	
	TPx	4	100.0%	0	0.0%	4	100.0%	
	LPS	4	100.0%	0	0.0%	4	100.0%	


Descriptives	
	Variables	Statistic	Std. Error	
figure	Control	Mean	2.026667	.0209497	
		95% Confidence Interval for Mean	Lower Bound	1.959995		
			Upper Bound	2.093338		
		5% Trimmed Mean	2.027407		
		Median	2.033333		
		Variance	.002		
		Std. Deviation	.0418994		
		Minimum	1.9700		
		Maximum	2.0700		
		Range	.1000		
		Interquartile Range	.0783		
		Skewness	-.890	1.014	
		Kurtosis	1.500	2.619	
	ESAs	Mean	2.673333	.0047140	
		95% Confidence Interval for Mean	Lower Bound	2.658331		
			Upper Bound	2.688336		
		5% Trimmed Mean	2.673704		
		Median	2.676667		
		Variance	.000		
		Std. Deviation	.0094281		
		Minimum	2.6600		
		Maximum	2.6800		
		Range	.0200		
		Interquartile Range	.0167		
		Skewness	-1.414	1.014	
		Kurtosis	1.500	2.619	
	TPx	Mean	2.390000	.1592168	
		95% Confidence Interval for Mean	Lower Bound	1.883301		
			Upper Bound	2.896699		
		5% Trimmed Mean	2.378333		
		Median	2.285000		
		Variance	.101		
		Std. Deviation	.3184337		
		Minimum	2.1500		
		Maximum	2.8400		
		Range	.6900		
		Interquartile Range	.5700		
		Skewness	1.405	1.014	
		Kurtosis	1.500	2.619	
	LPS	Mean	2.910000	.0081650	
		95% Confidence Interval for Mean	Lower Bound	2.884015		
			Upper Bound	2.935985		
		5% Trimmed Mean	2.910000		
		Median	2.910000		
		Variance	.000		
		Std. Deviation	.0163299		
		Minimum	2.8900		
		Maximum	2.9300		
		Range	.0400		
		Interquartile Range	.0300		
		Skewness	.000	1.014	
		Kurtosis	1.500	2.619	


Tests of Normality	
	Variables	Kolmogorov-Smirnova	Shapiro-Wilk	
		Statistic	df	Sig.	Statistic	df	Sig.	
figure	Control	.250	4	.	.956	4	.752	
	ESAs	.260	4	.	.827	4	.161	
	TPx	.250	4	.	.852	4	.233	
	LPS	.250	4	.	.945	4	.683	

a. Lilliefors Significance Correction	


figure


Normal Q-Q Plots
