# Supplementary material for: Regulation of piglet T-cell immune responses by thioredoxin peroxidase from Cysticercus cellulosae excretory-secretory antigens
Source: Front Microbiol. 2022 Nov 18;13:1019810. doi: 10.3389/fmicb.2022.1019810 (PMC9718028; doi:10.3389/fmicb.2022.1019810)
Supplement: Supplementary file 4 [file Data_Sheet_4.zip › 3. C. Cellulosae ESAs and TPx Induced the Increase in the Number of CD4+CD25+Foxp3+ Tregs in PBMCs/2. SPSS statistical analysis/2.2 SPSS statistical analysis--CD4+CD25+Foxp3+ Treg/2.2.3 (SPSS data export) SPSS statistical analysis--Treg.doc]

SAVE OUTFILE='E:\学习\1.文章\4.SCI（1）\Raw Data\3. C. Cellulosae ESAs and TPx Induced the Increase in '+
    'the Number of CD4+CD25+Foxp3+ Tregs in PBMCs\2. SPSS statistical analysis\2.3 SPSS '+
    'statistical analysis--CD4+CD25+Foxp3+ Treg\2.2.1 SPSS statistical analysis--Treg.sav'
  /COMPRESSED.

Error # 61 in column 14.  Text: E:\学习\1.文章\4.SCI（1）\Raw Data\3. C. Cellulosae ESAs and TPx Induced the Increase in the Number of CD4+CD25+Foxp3+ Tregs in PBMCs\2. SPSS statistical analysis\2.3 SPSS statistical analysis--CD4+CD25+Foxp3+ Treg\2.2.1 SPSS statistical analysis--Treg.
The filename is not valid.
Execution of this command stops.
EXAMINE VARIABLES=figure BY Variables
  /PLOT BOXPLOT NPPLOT
  /COMPARE GROUPS
  /STATISTICS DESCRIPTIVES
  /CINTERVAL 95
  /MISSING LISTWISE
  /NOTOTAL.


Explore


Notes	
Output Created	01-OCT-2022 15:18:52	
Comments		
Input	Active Dataset	DataSet0	
	Filter	<none>	
	Weight	<none>	
	Split File	<none>	
	N of Rows in Working Data File	39	
Missing Value Handling	Definition of Missing	User-defined missing values for dependent variables are treated as missing.	
	Cases Used	Statistics are based on cases with no missing values for any dependent variable or factor used.	
Syntax	EXAMINE VARIABLES=figure BY Variables
  /PLOT BOXPLOT NPPLOT
  /COMPARE GROUPS
  /STATISTICS DESCRIPTIVES
  /CINTERVAL 95
  /MISSING LISTWISE
  /NOTOTAL.	
Resources	Processor Time	00:00:02.42	
	Elapsed Time	00:00:01.51	


[DataSet0] 


Variables


Case Processing Summary	
	Variables	Cases	
		Valid	Missing	Total	
		N	Percent	N	Percent	N	Percent	
figure	Control	4	100.0%	0	0.0%	4	100.0%	
	ESAs	4	100.0%	0	0.0%	4	100.0%	
	TPx	4	100.0%	0	0.0%	4	100.0%	
	LPS	4	100.0%	0	0.0%	4	100.0%	


Descriptives	
	Variables	Statistic	Std. Error	
figure	Control	Mean	1.93000	.043205	
		95% Confidence Interval for Mean	Lower Bound	1.79250		
			Upper Bound	2.06750		
		5% Trimmed Mean	1.93222		
		Median	1.95000		
		Variance	.007		
		Std. Deviation	.086410		
		Minimum	1.810		
		Maximum	2.010		
		Range	.200		
		Interquartile Range	.160		
		Skewness	-1.190	1.014	
		Kurtosis	1.500	2.619	
	ESAs	Mean	3.33333	.057927	
		95% Confidence Interval for Mean	Lower Bound	3.14898		
			Upper Bound	3.51768		
		5% Trimmed Mean	3.33481		
		Median	3.34667		
		Variance	.013		
		Std. Deviation	.115854		
		Minimum	3.180		
		Maximum	3.460		
		Range	.280		
		Interquartile Range	.217		
		Skewness	-.666	1.014	
		Kurtosis	1.500	2.619	
	TPx	Mean	3.05000	.047081	
		95% Confidence Interval for Mean	Lower Bound	2.90017		
			Upper Bound	3.19983		
		5% Trimmed Mean	3.04778		
		Median	3.03000		
		Variance	.009		
		Std. Deviation	.094163		
		Minimum	2.960		
		Maximum	3.180		
		Range	.220		
		Interquartile Range	.175		
		Skewness	1.121	1.014	
		Kurtosis	1.500	2.619	
	LPS	Mean	3.92000	.021602	
		95% Confidence Interval for Mean	Lower Bound	3.85125		
			Upper Bound	3.98875		
		5% Trimmed Mean	3.92111		
		Median	3.93000		
		Variance	.002		
		Std. Deviation	.043205		
		Minimum	3.860		
		Maximum	3.960		
		Range	.100		
		Interquartile Range	.080		
		Skewness	-1.190	1.014	
		Kurtosis	1.500	2.619	


Tests of Normality	
	Variables	Kolmogorov-Smirnova	Shapiro-Wilk	
		Statistic	df	Sig.	Statistic	df	Sig.	
figure	Control	.250	4	.	.927	4	.577	
	ESAs	.250	4	.	.963	4	.795	
	TPx	.250	4	.	.937	4	.635	
	LPS	.250	4	.	.927	4	.577	

a. Lilliefors Significance Correction	


figure


Normal Q-Q Plots


QÄ×ÈDUUó5kd0Ã/ìÖ&ûÜüàò_)ñx!D&ø----..5nÝºUTTnùÊ¥eeeÉÇ7>vØtÅâÐ§âü"¾rÕªU±ñ·hÑ"Y÷îÝðKYY^^õg÷*ßúÞ½Ê¿¸'6þ¦wÅØøëíí¿T~ºxñþÿØßß¯|ª§§ø#¢tÀ,=6|OX8¶mÛ&ËëÖ­»÷0YOeeøWîÛ·obbBÙw¥¬Ù¾0ìêêR>[O/^¼(ËAåºÊ¯?^oÜ¸~ÑdøÓétQ×_wWìoþBFã¨[5í+Fþ© MÇÆÆÄñ6láÿr;²æÒ¥K²¬ü?nÝºø#¢4Á$/í²,/óëeùÖ­[Ê§7oÞO.~¡KCkÄá<øÕñÓpþÄµµµÊ§Gâ/;;;êuý4®>¹n^^Édr¹m*u«¦ÅøSþñöíÛá·þÂÿ[OÖð!D>ø-^¼8¯Wï9Sÿ¨n*:øT qè3TJJJãÔjáhqkS¹J]Åþ©Îd$ÞcçTxûRõÿcÄoxÈ?"JüIo¼ñ|:1²RñYÄ>*Y9RÄþ´ªªJ9åJgg§àl*øSvO*g]illÜµk×½÷=*+7oÞS¹Ê´ñïàög¸ç/Ä;±~ìÏËË5L(&DÎø6lØú3e2xÝºu£Sþ°lûöí3Ç²÷«¿¿_Dòê«¯Nn·[9þ(^ìêêRöê)=6êÏïUâÂ_ìÁQFÒÿ°wT§2Ê_é	vïß¿/¶[»v­Ñ |c¸²Ibeùz:Y®®®æ!Cþ(Ýð7<<úc/e×ëøó¯âââÐäÙà/¤¥ÊÇð¯Q·ùé§V|y3®«Ä¿Ø:!sÄ§1ªSÏ«W¯ßàÊ+Ã/ìììØøSÎuÊ?"JüIÊ!Ñðõ~øáæÍ³ÖØØ¨Þ1=üÈ­ÉmöYñcÇGìðáÃK,ÉÊÊZ´hÑk¯½öÄOfÏü*qá/öà=Vü§ÓéBgÎ/®Qâð¾õÖ[/SnP9Ñ,>óÌ3Ê·3LÝÝÝÜÕzñâÅÚÚZ¹5ù)ìv;"ðGD?ýôÓµ»Jò§eVækAñUUUÜCü¥aÊßüEôâ/22DþÒ0¿ß¿gÏE)Çveá¹çcXüø#"""ø#""""ðGDDDDàÀ?""""DDDDþüø#""""ðGDDDDàüEíìÙ³z½^§Ó­Zµ*Þë.Y²dÞ¼y'´FeMuuuOXÞ×LåºÓndddûöí28-Ú»w¯ßïOÐ¸??"ÊèJJJÄ`0ïu8 ×moo­yé¥dM[[[ªãOWþ°7nLLL?~ÑÚµk§qS===&	üø#¢äx¾3®^½*×­¯¯­Y±b¬éííMÌFj$Åµ§NDþHCùScïÞ½,ÈËËÛ¿Ä=ñÄµµµ·Úq(ËwïÞeY£óæMÉ«Óé-[véÒ¥¨·þÝc_åµ×^ÓëõF£Q¾Icccf³Y®+¿gÏeG¦ðtåÊrk²^nùÖ­[Sêêj¹å@ õÒ£äv»e#eÎ=«aõHÆ¸)îDþH+ÿ)Ë/¼ð,Ûíva,¼øâá_sñâEyámÛ¶M.:zô¨,ËGYnnnV.Z¾|ùùóçeáÆ²¾´´4ê­o@ì«éÞxãY°X,êß±c,Ë>Z8 +ËÊÊdytt´¿¿_êÔÃ"^_±GéÐ¡CÊ÷ª72âgSÜ9ü¶ø¯ÈòøÃÔöúGn·[.Z·n,oØ°AMèÒ«W¯îÛ·¯¶¶VÖgeeE½µèÄ¾²a,P_·¸¸XÙø	YöÉJùJY^¶lèj²ÝxQ¿Ø£tÿþeYna2ü~öØ7ÅÀi¿pñLÆDZyyyò~¿_9º*k^õU¹ðÚµkjEÝ©²)ïÌSàxêÔ©eMQQ²Sp*þ***dýØØXlÆF^ìåé8ø#¢YÃ_iiiø(eçÙ#-b6åÒÐÇÐúììlY3ñ°)â/öUB&S_wáÂÊu#6/?Þb±ï2|dûöí¯,ª/Ê(MÓp""ðGD³?eëÑ£G?Aé¥¦bS§Nv¡½ñÆ²¹~ýºò·SÁ_ì«ÈÞ|óMYØ¶múºÏ<ó,?~|``@9Ô++F£,_»víöíÛ²PQQ1Åaß¼yS@©ìÜ¸qãG)|Y9ô¬tøÙ§7àDDàfÁ`P÷°ðsõÅ¶Èýû÷C®òQÌZÿÖ[o	öìÙ3EüÅ¾ËåKW¬Xwþ5~¿×®]²åÙÙÙk×®U&öùéÃõõõ~øáÔGF¾R9¨-Ü¿¿ò÷|S¥ðå³gÏÊ&)çÐøÙ§7àDDàüø#""""ðGDDDDàÀ¿YèÇ?þñàà`¿éÏþóÿøÿà~£Q·nÝúì³Ïºyó¦rþÒ¢7nÄõ¦pW^¯÷¿ÿû¿úÙÏ~ö?ÿó?FôÑGàoÖúþ÷¿/þKð7½víÚ¿ýÛ¿qWÖ¨ø¸û6ã Qï¿ÿþ¿ÿû¿3õw÷wüf¨]?úÑøÕE».^¼­µë­·Þúßÿý_ðþü?ðGàü?ðþÀøàÀøàü?àü?ðþÀøþÀø#ðþÀøþÀø#ðþÀøþÀø#ðþÀøàÀøàü?ðþÀ?ðþü?ðþÀ?ðþü?àÀøþÀ?ðþü?ðþÀ?ðþü?ðþÀ?ðþü?ðþÀø#ðþü?ðþÀø#ðþÀ?ðøëîî^¶lN§«®®îííàÀøþÀ_:ã¯¼¼®dáÄjüuvv%6ÑÉÏ~ö³1Ò¦+W®|òÉ'FÉ/Kò«ã Qn·ûç?ÿ9ã Q.ë¿øã Q.ôÓOüÉo	þ¦)¿ðrssÕø;tèÐM~=ç ÷Hä	H~¿g4êüùó¯v½ýöÛ.]b4J^>ÿöoÿqÐnxå·ÆA£Î;øoøëééinnæ°/Ã¾öå°/qØÃ¾é|ØWill¬±±Ñï÷?ðGàü?à/Íñ7<<l±X¢ü?àüøi?·Û½zõê¨?ðGàü?à/­ð§×ëçþÀ?ðþü¿tÆ_ìÀø#ðþÀ?ðþÀø#ðþÀ?ðþÀøàü?àü?ðþü?ðþÀøàüøàÀøàüøàÀø#ðþü?ðGàüøàÀøàüøàÀøàüøàÀøàü?àÀøàü?àüøàü?àüøàü?àüøàü?ðGàüøàü?ðGàü?àü?ðGàü?àÀø#ðþÀ?ðGàü?àÀø#ðþÀ?ðþÀø#ðþÀ?ðþÀøþÀ?ðþÀøþÀø#ðþÀøþÀø#ðþÀøþÀø#ðþÀøàÀø#ðþÀøàÀøþÀøàÀøþÀ?ðGàü?àÀøþÀ?ðGàü?àü?ðGàü?àü?ðþü?àü?ðþü?ðGàü?ðþü?ðGàü?ðþü?ðGàü?ðþÀ?ðGàü?ðþÀ?ðþü?ðþÀ?ðþü?îÊàüøàÀø#ðþÀ?ðGàüøàÀøàüøàÀøàü?ðþÀ?ðþÀøþÀø#ðþÀøþÀø#ðþÀøþÀø#ðþÀøàü?ðGàü?ðþÀ?ðþðþÀøþÀø#ðþÀøþÀø#ðþü?ðþÀø#ðþü?ðGàü?ðþü?ðGàü?ðþü?ðGàü?ðþÀ?ðþÀøàü?àüøàü?àüøàü?àüøàü?ðGàü?ðþÀøàÀøþÀøàÀøþÀ?ðGàü¿¤l`` ?ðþÀø#ðþ(ñ'à;yòdýüùó].øàü?àÒ^¯÷àÁ%%%ó~USSøàü?àÒW®²eËüùóç¾üü|¿ßþÀøàÀø£tÀÀîäÉuuuóT-]ºÔn·ß¹sgn·ü?àüø³×ëÝ¹sgaaaùæÏ¿eËù¡æ|ªøþÀø#ðþfg7mÚ¤>Â[VVÖÒÒ288T[þÀ?ðþü¿éäóùl6[MMú¯¬<yòdìêàÀøþÀßòz½%'''Â|²FÖ<dÞxðþü?ðGàüÅ±mF£QwéÒ¥íííCCCÉ?¼àüøàÀøD,++SOæhjjJÉàüøàÀøQòÊn6Õ»úôzýîÝ»m2øþÀø#ðþ¦Ïç³ÛíêÉõõõ#võ?ðGàü?àoÒ<Õjzº¾äÌþ4iddD¯×?ðGàü?Æ!ðN§ÉdRïê«¬¬ìèèHç+ð_]]]UUUr?àÀøCzàohhH^Áõz½zWÙlNü+;øK®Ö®]ëñxbàïÍ7ß¼ØäNÙ××w´é½÷ÞûéOÊ8hÔ¥K®_¿Î8hÔ»ï¾ûÏÿüÏF½óÎ;òrÀ8hÔùóçÿå_þ%ßH^µ×¯_Á¾ÇÌjµ^¾|9-÷Ü¹s|òI¿iãïÿ6qrü½öÚkÿØÜnwww÷?6Nzzzr¹½½Fuvv^½zqÐ¨.vqÐ¨·ß~[~ñÖîö?øà^xá·û·ÕGx¿üå/ËEòi<¼o½õVâ¿i:ãÃ¾ö%ûrØÃ¾´½^okk«z2òÎò	ÃËa_ðGàü?i¿`0èr¹¢®O^¾>_æ/øþÀø#ð¶øÕÙl6Á >Âk2Ngê®ü?àÀø·X,999æ+,,´Z­ipº>ð?ðGàü?[üÃa4Õ»úÝnÏ¨#¼àüøàÀ_Úâo²ÉóçÏohhp¹xü?àüøKCüÉoÔÉAá  !àÀøþRòb·ÛëêêÔGxF£Ãá&øþÀø#ðòøhnnzWyC6ynq¹;wîÜa<Áø#ðþÀ¿Ä2#ÆéúäË^yå¯Ô~¥¦¦&77÷øñã)øþÀø#ðJøçE=Â~º>¹îoþæonûÎ¶ýßÝ/ÿ¿Ý!oÝþÀ?ðþÀøKyü¹Ýn³Ù¯>ÂÛÔÔ¤~Ùýæ7¿ùû~_òïwM¿ÛÜÜÌ¨?ðGàü?É¿Ï>ûÌáp,]ºT½«OVÚívå¯ºÊÊJë[Ãñ÷-ó·L&£þÀ?ðþü%cO<ñDIIë×¯w»Ý±O×÷Ío~sÓÆMáøû½Õ¿Ç?ðþü?ðGà/¹Ò9s¦¾¾^=£¨¨¨¥¥eóvù?ðþü?ðGà/©S&sèõú¨9:::ü~øÊ+¯äçç+³þò/ÿAàÀøþæ¾¾¾>Åa¾ßøßxæg®2P^|ØdþÀø#ðþÀ¿å÷û;::jjj&;]ßøÃi¼·/?ðGàü?ðþ+Ç³÷î¨çmihh(9âo_ÊÍTYYYàÀøþN<·~ýzõ®¾ÂÂÂÁÁÁð¯àoªe=*Nþü?ðGà/aÉcÔÉ555Çóù|êk?ð2?ðGàü?=Z,õ®¾¦¦&¹4ÆéúÀøFGG·mÛþü?ðGàO»Ãá0ê]¡­­m*§ëào:éõzNÇßüøàÀ_bòz½­­­Q's9sFÅà/îª««Õ¿pÅHðGàü?L^ûwQ'sñÞ øq-÷¹åÅ|o¾ù¦,hýÖ~àüøà/sòù|v»½²²R½ÃEVÊEQ's?ð§	þ,ödáÆ²þü?ðGàoy<«Õõ¯ÙlùK!øqWPP wÁ®®.¹wÊÂsÏ=§,pªàüøvÁ`ÐétL&õ®>å9fë½ÔÀø»=ö¦wD¼?4ø#ðþÀ¿xóù|6Í`0¨Ù'¯­#Æy[ÀøKþ¤ç~áÂ²ÐÝÝ-ÁÚÚZ­7ü?àü¥ßÓ ÅbÉÉÉQ®OÖË¥Z|SðþR&ðþü?ðÅ>]ÍfödðþÀøàÀøK¢7dSOæL&ËåÝ#¼àüÍþ***¾pgàüøJòf6£®Ïjµz<m	øqW^^¾PÌö%ðþÀ¿ü~¿F§ëà/qøçÉ]¶§§gbb"þÀ?ðþR(¯×«ééúÀøKþä¾`ù?ðGàü¿zù_³fÍ,¾!øs¿ÞÞ^¹ïØ±cllüøàÀÒÐÐÍf+++ìt@ ¶ü¿é´hÑ"õ=	þÀø£ÌÄrº>õ®>Y³eËN×þÀ_âð·xñb&|øàü?¿ßïp8.]ªÞ!RRRrðàÁ;wî$áf?ðÿM<¬¿¿?ÁþÀ?ðþ$¯×ÛÒÒ¢ü¼útòò$GxÁøü3áÀøà/3ñçt:&Ìø=#àü%n·[îåöìIð¯5àüøào®ºsçÍfzº¾ºººÄ®ü¿ÄáoÞ$1áÀø~øëëë:#''§©©)1oÈþÀßã/kðAàü?JüõÎÁÐÖÖ688¢ÃþÀ_ÊþÀ?ðþ¨.ê;s(9Î9r»úÀøiz½~ñâÅàÀø6øÒÉËöúõëÕGxvîÜ9çïÌþÀßáO§ÓÉ#!ñþÀ?ðþ´Èçóµ··õ®¾Ãá÷ûÓixÁø»K.Éã¡­­mtt4'|àÀø³þ,d±XrrrÔïÌÑÔÔäv»Sý/øíþÀø#ðþÃá0ê×2½^ßÚÚêñxÒxxÁø;fûøþR^¯Wlu2G¿3øs¿¹ü?àüM¯`0èr¹&g«ÕïÌþÀøàÀøÈçóÙl¶¨9*++ív½3øs¿ñññµk×æææÊ'//oãÆ	ùþÀ?ðþâzl2ÙlNüø)?y@Fð¡õßI?ðGàü¿GN§Éd:C^J2|xÁø»ªª*y­[·nllL>Ý°a¬Y¾|9ø#ðþÀÍþDuòJ!ÂúÎ"Â´<oøÀ_vv¶<ÂBããã²FÖ?àüQâñ'¯f³y²ÉéÞðþ¿¬¬,yD	øBk¬áT/þÀø£DâÏçóÙíöÊÊJõ®>Á9ÀøÓÊaßÕ«W+å£,ËeË?àüQðçñx¬V«útóçÏohhp¹áào6ñ'Ú:áãÞ½àÀø¤þbLæ¶¶¶z½ÞYÜy9s¦½½=ÍNþþÀßtÇäÆ,X%W¯^7aàÀøËXüÅÌa4Ç¬ãl```áÂK.5ÃT^^þ¥/ippüQæâoNàÀøË@üM6#''Çb±ÈÓEköw¿òïë_ÿúòåËÓãh2øàü?ðGà/éð÷é§:É&sØl6M'sôõõýNùïä'ÿöµì+..Ns?ð7åk>ª¬¬,ðGàü?a^¯÷þàæp2¼~uÅWÃñ'ÿ***4ÚÑþÀ_â/kòÀ?ðþh	évÂ»¨§ëõÉ±())Ù×²/$¿ÝÏîÎËËKÇø3íÙgU'NþÀø£xóù|6Í`0$l2ÇTZ³fMÍ²=»÷(ò«ª¬²X,é1ààüM¿ÞÞ^ù5HyÛðs>?àüÑTìtYYY[·nMüs~I¿þõ¯/°²²R^ìü~?ø£ÆßæÍèùóç³éàüøéQÓõéõzy¶ã7p±©tçÎ¾¾¾4(?ðw§OV¢ëÖ­Kä¦?ðGàü¥z1N×'*9âo_àO+üÉCqùòåÊùK.%xÓÁø#ðþRúñn±X&;]Çã	ÿbðþÀ_RàïÐ¡CÊuëÖ­s²éàüø)W p8F£1®Óõ?ðþçÀø#ð7õ¼^okk«z2ÇTN×þÀøKüe=*Nþü?ðGòÃÓõ?ðþsøþÀ_2çóùìvÔ7drÑÔßü?ðþÀø#ðþ·¨§ë?¾ÙlÆS7øàü?ðGàü%c.kÍ5ê#¼Êéú¦w³àü?ðþÀ?ðDù|>yN.++ìÙbLæàü?ðþü¿I¢®OÔ³ò]Àøàü?ào.óûý'O¬©©ìÙ¦>ü?ð2øã<þÀe þ[ZZ¢NæØ´i0bGxÁøÉ¿ðSúEÅçù#ðþÀ_:%O¹Q'sìÞ½ûÎ;ÚkðþÀ_Rà/Tgg§<øzê©±±1ùT>nØ°AÖ|üøà/Õóù|4ê_òëêêG ÐzÀøÉ?åï|Ã÷ó+¿ÎðKKKu:Ý%K.]ºþÀ?ðÈúúú¢Næ?¾Õj¬GxÁø)?å¹@À¿ÿÍ_ccãñãÇeáÈ#[·nàÀøK@@ÀápÔ××«ðöööÄßÀøÉ¿ââbyF¨)L¿ß¿nÝ:Y#ëgxËòëæÄÄ¢I½^¯ÆÝn¿Øä	¨««ë*iÓ»ï¾û÷ÿ÷Fuvv^¹rqÐ(yùüÉO~ê?ÅÙ³g¿óï(Ïêø¾öµ¯	ûä7´9Ù°óçÏÏÕ·ÎD'½½½F;w.ñßTsü]¾|9êk×®ÍðÃ§¨§þN>=ØÞÿýþþþ!Ò¦ÿøÇF¹Ýî>úqÐ(ùÍðã?NÝí?yò¤üÞñLþ/|áþèÞï½¹Ý<ùÕåO>án¦Q.yó&ã Qbë[·n%øj?éÆååå¹¹¹òÛa^^Þ²eËîÞ½;ó?p,OIöå°/qØÃ¾³ßï·ÛíQ's,]ºôØ±còÉ°öå°/ë°¯v+J(ÕÁø#ðþ¦]ÿÎ;srrÔ9,<»&l2øàüý:³ÙüÚk¯É|lllàÀøaÊd£Ñ¨ÞÕWVVf³ÙyÀü?¿ìúõëyyyÊÚâââS§NÍüf»ººJJJä6KKK»»»Áø#ðþ¦×ëmmmU¿3T__æÌ¤ÚÕþÀøKjü)'yK7eùÈ#n:øþÀß#Ò¹õy[òóó-ÇãIþáàü%þJJJäIäúõë!üuwwËòÀ?ðþæ*Ïg³Ù¢Næ¨¬¬´ÛíI2ü?ðzøSJ¼·/?ðþæð¡d±X¢Næ0Íæàü¥þÓ*ûããã÷îeõiÁ?ðþ´+ÆdyBçÌäÌþÀøK=ü¹Ýî¨'yWðGàü¿$ª§Ä¨9L&ÓéLæÉàü¿ÔÃ$/ØµµµÊlßÜÜÜ¯×«õ¦?ðGàüÉÓ ÙlVOæZ­ÖÌþÀøKIüÍIàüøËXüù|>»Ý^YY9Ùdùt^ðþÀ_rá/4Ï#ÔÝ»w+**ÊÊÊÀ?ðþf7ÇcµZÕGxSw2øà/ð7>>Îl_àüÍbÁ`ÐétL&õ®>`kkkþØü?ÊtüÍYAAø#ðþÀßS&sèõzõÓ¬Ñht8@ íü?ðø»yófÖÃBïíÈïôéÓàÀøÓn²É999E/3¼àü¿¤À_(¡ÖGxÁø#ð9ø:Ã`0Øl¶4ÌþÀøK=üÍUàüøK3ü¶´´©'s444¸®Ov»``ü?WTTäååÖ,ð^þÀøb»5kÖD=]_'s/·lÙò[¿õ[&ékÆ¯åææþà?àÀßçZ´hQÄ_å9«­­üøà/F>ïàÁK.MÉßûÞ÷ÃÞÿ·wÿw÷Ë¿mßÙöØcõ÷÷?ðGàï×eggËóTøSCoo¯¬Y°`ø#ðþÀ_ÔóóóÕGx·lÙ2§ëù	øù)ÿ¾^ÿõÌàü¥þ	¿»]ÕÉÿÀ?ðþÓõ­_¿^·¤¤¤µµupppnñè¾áøòÉ'¿ýío?ðGàï×ÊÓÖ®]»ÃòøÜ¿¿òDþü?ð§äóù>õ©uuu'OôûýÉð|ùË_ÞÒ¸%555íííàüøûu===QOòÜÝÝþü?ð××××ÔÔõtsþçtêª¢¢¢o¿%ìÛ×²oÍï­ùâ¿8ç÷ðþÀ_ráO^²dInnnVVV^^^UU¬ÑzÓÁø#ðÌø£¾¾^ý»qYYÙÁö¿C(°hÑ"!àc=ö¯|eÎD?ðþsøþwîÜikk+))Q³oÍ5òJ°ÓõÍ$Ï$G¢Áøàü?I¿+W®ÍfõÞüüüææfÇÃ@?ðþÒ×¯_WÎó¬Ìð-..>uêø#ðþ2¤`0ØÖÖöøã«wõÕÔÔ8ÎÄ®üøâ¯³³3ô4§àOY>räø#ðþÒ»þþ~Åõt	~C6ðGàü%Êßµ~=¿îînNòLàü¥qBº3gÎL¦¨§ë³Ùl>QàÒÊóÝ°;OLLÈ²N§þÀ_544$O>z½^·¶¶Ön·'Ï$	ðGàüi¿ââbå¬~þÆÇÇ÷îÝ+Ëòäþü¿´IpÌf³zWrº>Ã1÷ö%ðþÀ_JâÏívG=É³¼Ò?à/Õóù|v»½²²Rý,'+[[[ÄÿÞ¾þÀøKaüIò][[«ÌöÍÍÍ­¨¨ðz½Zo:øþ4ÍãñX­ÖÂÂÂ¨9ä÷ÞðÉàü?Ê,üÍIàüøÓ"!Óé4Lê]ÁÖÖÖ¨¿Ü?ðþü?à/Å1Ãh4:§ëàüQfáïã?^¼xqvv¶<Eæææ.Y²dddüø)tß³X,MæKyàü?Ê ü¹¨>À?ðÌÃa4ÕÏ`!®Óõ?ðþ(ð§ä¹±±Q9»ÕØØØÖ­[eMii)ø#ðþ3¯×ÛÚÚ:Ùdi¼3øà2Ê3fø¥,Nøþü¿äIvÂ;õÞ9Àøþ>²ço||<´F¢ìù#ðþ*Ïg³ÙCÔÓõÙíö¾!øà2Êßü566*Ì÷îÕ××ó7þÀ_òÜ¯,KNNú¯Ùl­§ðþÀeþæ=*ÿ?ðGà/F1&sèõzyQÞc¶àüQá/ëQét:ðGàü%¬É&sH&ÉétÆ;ü?ðGà/)àÀ_Dò0Ùd«Õêñx´ûÖàü?Ê üMv>çááaðGàü% Ïg·Û+++5ÌþÀø#ð÷ù7ïùçXÙÔÔÄ©^ü?­óx<V«5êéúfq2øàÀßçäÉSmqqñÝ»wåÓ×_]yòÕèOýÀø#ðN§Éd:c÷îÝ	Þ$ðþÀeþ<ÜÏ§<í*6lÐzÓÁø£Ä_ÓõÕÕÕ9-&s?ðþüEéòåË¡§àl:øQøìtùùùV«Uë?ðþü®íÛ·+ÏÂÊ»HO=õø#ðþf^ÓõÉó@&s?ðþü.N'ÏÂ,¸zõê°¿ùËÎÎþÀß´yÔÓõ544¸¹:ÂþÀø£LÇ<ïÛ·/b¥Åba¶/?ð7½ä¡m6Õ§ë+))±Z­I¸Íàü?Ê üMv?­_ÅÁø£4Ã_Óõ¹Èï÷'íÆ?ðþ(ð7W?ðGi?¯×;ÙéúäTGxÁø¡øçåð»±?þÀ:!Ëåúlz½¾¥¥%ñ§ëàüøàü¿Ù/öéúNgòïêàüøþÀß£ëïï·Z­EEEêÓõíÜ¹Óãñ¤èð?ðþü?àï×)§ë«©©zº¾ööö$9]øàÀøàüÍ(¯×ÛÒÒõtë×¯GqÊáàüøþÀ_dB:yÊ:Ã`0Øl¶;wî¤Óð?ðþü??QØ®¬¬L½«Ïd2%Û;s?ðGàüÅ¿Ø?¿+W®ÍægÂÂB«Õº9Àøþ~YÖ£ÒétàÀ_&à/;v,êdÊÊJ»Ýê9Àøþ"ðþhÎñ744$D½^¯~g³ÙøG(øþÀøà4Á<ú¶lÙ¢ÌQXXØÚÚêõz3mxÁøþÀ¿4ÄÏç³Ûíê#¼F£ÑápÌ^ðþÀ?ðGs¿¾¾¾îÚµëØ±c~¿!Eüõ÷÷ïÜ¹Sº¾Å"ÿw>¼àü?à¿^ziáÂ&éÉ'¬ªªZºdiò¼mêâ/<yÒh4ªwõéõzyfÂdðþÀ?ðGI¿+W®<öØcÖ?¶îÿî~åßÇWüáþ!£:müµµµ©ßW9]ÓéLËÓõ?ðþü?ðøû³?û³o|ã!ùÉ¿;~yQþäe6£NæHûÓõ?ðþü?ðøûîw¿ûäOão_Ë¾üü|Fuêø1Ã`0dÈéúÀøþÀøKü<yòK¿$àáïÉ'|üñÇÕ©àÏãñX­Võdùóç744¤ë²?ðGàü?ðÂø|õ«_­^Zý'ÛÿDä·iã¦/|áLA?4§Ói2Ô»ú2ötàü?àü¥þ$¿ßoµZKKKsssüq·ÛÍN¿ÉÞÓõ?ðþü?ð2ø£©ào²É®ü?ðGàü?ð>?ýÓ?-//:Ãf³1ü?ðGàü?ðy½ÞÖÖV&s?ðGàü?ðþÒ9!ÀNxõtLæàÀøàü¥I>Ïf³õÞºº:&s?ðGàü?ðþÒg-KNNú¯Ùl>räovþÀ?ðþÀøKùÃá0ê]z½^)CCCâo_àüøFFFäÕü?Úd9$Éät:Ã's?ðþü¿dÇ_WWWUU¼?ðGáÅÌaµZ=úZàü?à/Ùñ·víZy¿?ÿó?w%¶óçÏ¿óÎ;.bxç¨¿þë¿Þ¹sgYYzWß¿øE¹èoþæo&»îÛo¿ÝÙÙÉjÃ«iòòùî»ï2o*vîÜ¹ÄÓÆßÿmâäøßÿ+±ðÁ^¯÷¿Hº»»oÞ¼É8Dm``Àjµæçç«'slÝºu*®®®ááaFR£Ün÷/~ñÆA£äÅìÓO?e4êÂöã Qbëû÷ï'ø¦3þ8ìËaß9d°fÍõÞðÉSÃ¾öå°/qØÃ¾É¿ÐøÞ;w>õoMMÍÉ'ãgðþÀ?ð÷=àü%ç8D=]_~~þ¦MÜn÷ôÞü?ðGàü?ðþ¨@ àt:M&SÔÓõ<xÐçóÍäöÁøþÀßNòþÀ_2488h³ÙDxjö5448éíêàüøàü¿äJîÃf³9êéúvïÞÝ××7ßü?ðGàü?ðþæ¦@ ÐÑÑQWW§ÞÕWYYÙÞÞ>Ã#¼àü?àü?ð¶´´D=Â+Ï³rü?ðGàü?ðþæ¸+W®D=]_QQpÐëõj½àü?àü?ð§yâ­¨9êêêä"¿ß-àüøàü?mæææÂÂBõ²Y,oøàÀøàüÍ~@ÀápFõ®¾ööö¹øàÀøàüÍf^¯·µµU½«O2L.KþÀø#ðþÀø³P0Ø544¨'säääìÜ¹Óãñ$Ãv?ðþü?ðþÀßòù|6Í`0D=]ß±cÇ[àü?àü?ð7ýMµX,999êÉf³9ñ÷LðþÀ?ðþÀøýbLæÐëõrJÚàüøàü¿©&ª»Üd9N§vïÌþÀ?ðþÀøKîff³Y=C hµZd2øàÀøàüÍ(Ïg·Û+++£NæäRkxÁøþÀøà/JÇjµFg¤ÌþÀø#ðþÀøñN§ÉdRïê¶¶¶z½Þ^ðþÀ?ðþÀøûeÊd½^¯fÑht8sûÎàüøàü?ð7;M6#''Çb±Èö¤Óð?ðþü?ðþ2@àØ±cQ'sÍr9ÀøþÀøà/J­­­EEEêÉ.+ùO×þÀø#ðþÀø(<yrÓ¦MQO×9ÀøþÀøàï	wÚÛÛÓ~2øàÀøà/Óñ700ÐÜÜõti6ü?ðGàü?ð¡øgÎY³fú¯^¯·Ùl ðþÀ?ðþÀ_Záo`` ¥¥¥¬¬L·¡¡A~sü?ðGàü?ðÎøs¹õîÞ½[PÈØ?ðþü?ðþR>¯½½Ý`0¨wõÕÔÔttt¤åéúÀøþÀø?åtêÉ[¶lIüüøàÀø£ÙÇrº>£Ñu2Ü%ÀøàÀøà/ð744ÔÖÖ¦~g©¾¾ÞétfødðþÀ?ðþÀ_àÏív777O6£¯¯qàüøàü¥<þÉuuuê]²òðáÃLæàüøàü¥þä£ÅbÉÉÉQOæ0LLæàüøàü¥CÁ`ð^X¹r¥zW_IIIKKËÐÐ£þÀø#ðþÀøKùDuò¿©×ë£áu:âBF	ü?ðÇ8?ðþÀ_Ê'ÿf³Y=#??ßjµz½^ü?àü?ðòÃQYY©ÞÕ'+ÛÛÛÌþÀ?ðþÀøK¼^ïdïÌ±iÓ¦W^yåÖ­[øþÀøà/µ.«¡¡A·¤¤d÷îÝçAüïíKàü?àü¿äÊçóÙl6Á >Âk4ßï1øàÀøàü¥jÇjµªðæääX,qú*àü?àü?ðbA§Ói2Ô»úl6[Óõ?ðþü?ðþÀ_ÊãtõõõrïäéúÀøþÀøà/RÞ-êéúvîÜÙßß?õÛàüøàü¿$M9]Ñhzº>»Ý>ü?ðGàü?ðþR»ºº:õéúÖ¯_?íÿðþÀ?ðþÀøKÞGød9ÀøþÀøà/µuuu¢ÀGNæàüøàü¿ov1þÀø#ðþÀøøàÀøàü?àü?ðþÀøþÀø#ðþÀøþÀø#ðþÀøþÀø#ðþÀøàÀøàü?ðþÀ?ðþü?ðþÀ?ðþü?àÀøþÀ?ðþü?àÀøþÀøàÀøþÀøàüøþÀøàüøàÀøàüøàÀøàüøàÀøàü?àÀøàü?àüøàü?àüøþÀ?ðþü?àüøþÀ?ðþü?ðþÀ?ðþü?ðþÀø#ðþü?ðþÀø#ðþÀ?ðþÀø#ðþÀ?ðúøëîî^¶lN§«®®îííàÀøþÀ_:ã¯¼¼®dáÄjü)OLêõzï6	÷_r»ÝwïÞe4ÊårÝ»wqÐ¨.1%øûÏÿüÏÓÆ_x¹¹¹jüýÅ_üÅ<BÞyç6?¾³³qÐnxß÷]ÆA£Þ~ûmW»äåSüÇ80¼©Ø¹sçÿMÓ===ÍÍÍöå°/qØÃ¾ö%ûrØ7û*566úý~ðþü?ðGàü¥þæý*åÓááaÅàüøàÀøKyüçv»W¯^=22õRðþü?ðGàü¥þôzý¼°Àø#ðþÀ?ðÎøøþÀø#ðþÀøþÀø#ðþÀøàü?ðGàü?ðþÀ?ðþÀøàü?àüøàü?àüøþÀ?ðþü?àüøàü?àüøàü?àüøàü?ðGàüøàü?ðGàü?à/Óð÷½ïÏét&¶.üä'?$m:wîÜ|À8hÔéÓ§úÓ2õÃþðþéú«¿ú«>úqÐ¨üà^¯qÐ¨£Gþë¿þk¿©ÏçKOü]¿~ÿþýß'"""¢°bÇþX"""¢Ì	ü?""""DDDDþüø#""""ðDèõú(VVV¬¹ûvøJn&Ãëv»«ªªt:Ý%KºººdMoooii©²æÒ¥KÝì/÷ÞYÞ?ü°¶¶6;;óæÍ£££Üµ^î½ñÖÝÝ½lÙ2¹CVWWË3ü"õ¯¦Ã÷^3Éë¢¼FÆþÿ8~üø#GdáÔ©SAá-**ºyó¦,ÈGå ±±QZd´·nÝÊèÍîðrïÅá]¾|¹<ûËÂÀÀÀöíÛ¹÷j=¼Üã­¼¼üý÷ß'NTTT_¤¾¯rïÕtxáÞþ"[»v­Çã¿7n¬ZµJYÿ¿³gÏ2h³2¼/ù(ËW&&&da||<êþÉðrïÅá_ëCËÜµ^î½3)777â7Ãû*÷^M7î½àoq7nìéé	aõêÕòÄT[[ûá2n3ÞÞÞ^yÌÈEòQáðgüðeáåÞ;Ã[]]ýúuYxýõ×û*÷^Mï´ssód¶æÞáM/øòé+ÔëåéqÉð._¾|``@a2ÈÊV*egg3n³;¼Ügqx¯]»VQQ!°~ùåßû¹÷j:¼Ü§×ØØXcc£ßï_©¾¯rïÕtxáÞþâÃß:õ"!3^õ¯GÅÅÅãããî-eÆmvïì>9(üñÇUUUÜµ^î½ÓH¨a±Xnß¾±^_åÞ«éð&Ã½üÅ÷´råÊ«W¯>-//÷z½ÊÿúêÕ«·ï+#;ýýýË/³ÙüÚk¯É|_§·Ù^î½³8¼2½½½/¿üòóÏ?Ï½WëáåÞon·[jddDú¾Ê½WÓáM/øÒPèSAºòJ===Êé3êëë¿¦§i¯üN/(Á²üàáì¿¬¬¬ÒÒRe®ÍâðrïÅá§þEÉóCsss0äÞ«õðrï7½^¯>½² ¾¯rïÕtxáÞþ2é·,üø#""""ðGDDDDàÀ?""""DDDDþüø#""""ðGDDDþüø#""""ðGDDDDàÀ?"Êð^ýõ+Wæ>lÕªU§OþÜó×ÃRæÙ6ÚÖêõzùÑü~ÄzY£ÓéJKK'&&â½M""ðGD)Ùþýûç©zþùçÓ	mmm²²££#býáÃeýsÏ=7Û$"Dzõöökt:Ý«¯¾|ØÑ£GåSYyõêÕ´Áß7deuuuÄúªª*YïõzÁ?"Êzê)aÍ/¾¾ò¥^MMMáô¹téàIlÙ2Yñ½÷,X íÚµ+üèªËå`ÉErÝ/FpJÖ¬òüùóò©ÙlØ°·Þz+öí(É&)uvvNµÕ«WËúîîîÐ÷ß_ÖL¦ÐÈMåæænÞ¼yxxX?õíG¬±©Dþæ¸E	Þ¼¾òÖ­[²R¯×ã&¢åÒ6DcÇå¢þþþ¬¬¬¨×R>U.Ý¸qãÄÄð1;;;Ê¥òQð§ü)^Û¨§þIO8.Ú/?_Ä¬Zµ*^üÅØT"DDsr7ÊsÖ¼yB±pÜlÝºuìa² ®[·N¹T±ÂGe M¹¨±±QÙe(Ë]]]Êßæ¾ûwÊQ×gyFÖ=Vå£,?ýôÓ¼£|*_¾mQ"åÂåçOå£l¹s||ÒÒR¹¢ü~u8777^üÅØT"DDI?)*þBëÜ(fåSaòé²eËäÓÅoÛ¶MÐvÿþýÐÈ×DìK]KùôÖ­[¡/0üÊGYv¹¼¦|z÷îÝðmìïóöY¹èå_~ð«CÛ»víÿ àOHZ[[«ì16ÀÑÜ§ìî_é÷ûe¥;!z<Å!ëþPO46Sá'Xå¢¢¢ììì@  7^PPºô·3Å"º~ýºâTY²Üßßº´««K6 êáã©ã/Æ¦ø#"û¿;tèPøJå(>B822¢Þ¡uõêÕ¶¶6å lHÊn¹ÐqÕGmÇÊÑ^ùØÜÜZãv=m·oßV>½÷nì¹+V¬K³ÛYÃ/RfþÊE£££±ñ©2¡Kcl*?"¢¹O0¡Óé9¢êåÕW_ÍÎÎVOÎX·n@'(ÇdC3s¿ùSþTÎãñÿ± bÁ]»v	y¸¡­D%2ýVùîn·;´>Æí(ä§üÍßïW¾2þ=Ú''?røEÊ?ö÷÷ËwA7ËòeÎÍ7_cSü%EÊ	#:pàÀ¯¿¦Ø(´:`cêg	ex§N¿ßCÖq;²ái;ÙÿþýÜÜG8Ø½víÚðo±páBù¨í%ü6¡ª.±©Dþ%ÊªU«²¶råJeÊmþ:;;Óé-Y²äòåË¡Ký~ÿÞ½KJJ0=ûì³@ téÅkkkÅd¥¥¥v»=â6Õ["7%ë·oß±~²ÛdcdÆ8Ï_¨æææs¾(466Ê,X°@~¯×:mMømn±2LÝÝÝß1Æ¦ø#""""ðGDDDDàÀ?""""DDDDþüø#""""ðGDDDDàüø#""""ðGDDDDàÀ?""""JTÿým	[*IEND®B`


Ä_" þ@üø[l^¯×f³éõzí9ìv__qâøñâÄ_ÊÇ_(r»Ý&I»ªoõêÕ'NáoMü þ@ü)>Ïn·F5^¯·Ùl^¯?1ñGüø?¤|üMNN¶´´Íæáe¿-Äñ þ&ñ700 ©F£Q»ßÅÒÞÞÁr/½½½­­­iö=BâøñâÄ_²Q«ÕªÝoKaaa÷Ûâóùxà¯ýáßwß¡Pø#þ? þ@ü-@ àr¹*++cî·Åív'8¼Áà×V~Í²ÕrôGåßÃGÖ®Yk·Û?âøñâÄß¼$ç`ÖÖÖ?©úU~êßþû/_û$þ? þ@ü%Z(n0ÑhÏÓ¥ÌqâÄ	ó£æÈøEEECCCÄñGüøñâoü~¿ÃáÐ®êÒRÉ°v­µµuõêÕ¬ù#þ? þ@üÍ:¯Åb9Ãn·û|¾äcÁ`ð«_ý*ßù#þ? þ@üÍZðVVVºäÜ]Äè×¾öµ¯ý¡*..f´/ñGüøñâï¦;ovv¶ÕjMü'ælMNNÊShmmíííM¾ÄñâÄ¿ç ¼j0ÇÀÀâø#þ@üøCÊÇ_Jæ@ZÅß²ÉÊÊ"þ@üøñ·°âæÈÏÏO¶ÁH«øËºNGüøñâo¡ÄÌ!v0ñÇf_âÄ?³3Ý`QWW×ÑÑÁ^âoéãotttïÞ½Ä? þæL.Î9Ü3ø[øEP§Óñ? þ¿ùWmöL&·ÛÍª>âo)ã¯¦¦F»h`0HüøñâoVä£ÍjµÆÌa³ÙúûûÃÄßÒÇ_NN,ÃÃÃÅÅÅrBïí·ßÄ? þf"¸ÊÊÊ9?>22Â¼%þ%þÔ¢)'¤öäÄ7¦¦¦äD^^ñâÄ¿ø|>Ýn¹»>«ÕÚÞÞÎ^â/éâoùòå²Ê§ ,¾râùçW'ØÕ? þ¦g0Gqq±Ãá`wÄ_òÆß¡CÂÃ;¢¾Jüøñâ/JÁë×¯g0ññ'^xá+VÈ®®.9!!¸nÝºÅ~èÄ?H­øëííÝ³gv0^¯ß¿__søKø[Ä?Hø­­­&I»ª¯´´#sÄñâÄÒ$þ|>ßþýûµ9²³³ëêê<[x¿T¿µÃvòâÄ¿ÞÞÞÉ;í^	ÁÃûý~fñÂñW^^|aöñâfddäûßÿþý÷ß?Ý9B¡søKùøÎeúÚµkSSS|èÄ?H½½½V«U¯×ksØl6¯×Ë,"þÒ'þ,Ü	.?âÄ?$ÉÉI·Û½zõêGæp:æ þÒ0þº»»eß·oßØØñâÄ2Ïç;|ø°:´iÔWÞ7nÜÈ9Îñ'V®ý>@üøCú¤°ÛµkWÌÁCÞæpl_©«V­bÀ?Ä_Úinn.++?c¶Çöñzñ§ûÄïøñâáõzòóóµ»ë³Z­QFÄÒ?þðâÄñ~B¡Ð3gjkkµ«úF£|h¯Eü!ýã¯££C^Jð¾? þ°H¥ð´Ùg6[ZZâæ þþñ·løñâ©EæØ²eKÌÁv»ÝçóÝóF?¤üeM þ@ü!U§Ós0Gee¥Ëåùîú?¤ü-âÄ?Ì×ëµÙlÚ#sÄÌAüøû£Ñ¸jÕªþþ~âÄ?¤P(äv»M&Ó¬s þ~C§ÓÉK%ñøñâsà÷ûGaaás þ~ãÒ¥KòillMä_? þ0sj0ÅbÏ`âÄßÝ`´/?Ä_[ÀÁÄ¿ß`´/?øKN>Ïn·k·ðÎy0ñâo) þ@ü!¦ÉÉÉ³Ù¼à9?Ä?øK"Ruòé0·#s þfabbbëÖ­¹¹¹òêÊËËÛ±cGF~ þ@ü!L>¬V«v0^¯·Ùl5øñ÷ããã1|,ö¡~? þWee¥öc¨¬¬Ìét.ì`âÄßoTUUÉklÛ¶mcccrvtttûöí2åÁ$þ@üøÃ"3Ãb±´··'`/ñ¿y±E¾Æ&&&dL'þ@üøÃÂ3CBÐápøýþ¥ÄÒ?þ²²²ä%'Ád»zñâUòÎ_ZZªÍ>Éäv»ûëFÄ¿ßR7oÞ¬6ûÊO9-SÖ¬YCüøñùwû]»vM7Ãëõ&[¤HóøÚ9àãÎ;Ä?£···¹¹Ùårõ÷÷k/9~üxRæ þ@üýÖøøø;²²²äçæÍeÊb?tâÄ¿¶wïÞÂÂÂo~ól|$//ïG?úQø¢¾¾>«Õª×ëµÙ·´9?Køñâ/u¹Ýî¯~õ«à¯~ï¨üûî3ßý£?ú#Ç#aWWW§Í>ÁpðàÁ+?ÄñâÄ_²Û²eË·¾õ-U~*þÊËË´«úÖ®]ÛÜÜSå©HÛø[v/YYYÄ?©²²ÒþWvÉ¾o[¿-Ùs0ÕjMüû<ñâoZYÓ#þ@üøC|¥ªªê>Ã1sÈ;|ræ þÑñ7gV½zO>Müøñ(½½½û÷ïWò§ú§òÆÌ9?_ÒÝÝ'¯^³Ù¹ÏgâÄ?A·Û½zõê_¿û»¿KgJü!SâoçÎê5|áÂÄ<tâÄ¿à÷û´á<òÈ±cÇ>úè£T_ÛGü!³âïwÞQ¯ámÛ¶%ò¡ þ@ü%9ÇóÄOÄÌ*ûm!þ@üÉøøø>¨ÖØ_ºt)Áøñâ/ië§©©)æAx×¯_âÄ¡¡¡ô~úÄÒ3þ^yåõJÞ½÷<tâÄ¿d#oËV«U»ªO¦ìÚµËãñ¤Óæ]âìçÄñâO	.+æAxCccãÈÈHæÌâiY÷¢Óé? þÒÏç³Ûí1sÔÕÕÉÇ^(Ê´yBü!mãoÉ þ@ü-ÉÉÉ³Ù¬m>	ÁöõõeìÂ@üø#þ@üøKòök4µÙg2Ünw®ê#þ@ü þ@ü¥¡ésèõzÍæõzY?Ä?)/¹Ýî9ÊÊÊNgêøñGü þ@üý~¿ßáphsdgg[,öööLØoñâø#þ@ü!ÍãONÂNòN»WBPrP¢¿8ñL?öóâøCÅ_ p:eeeæ þ@üÅ¹K¿ñÇ~þ@üøK^¯×f³éõzí^«Õø7[âÄ_2Æ_X[[¼A<ùäcccrV~nß¾]¦|øñâ/©Á&Iûx£Ñ(o³üq?Ñ¼MD~íwbbB¦Ïó»»»KJJt:]uuõ¥K? þÊt9Ùlniia0ñâoú¸K/*þæÿ¿úúú7ÞxCN¼úê«»wï&þ@üø§ø9ìv»Ïçã¯Iüø»¢¢"y×P³Á`pÛ¶m2E¦ÏóÃÔÔªI£Ñ¨?ËÕX/^Ïû §§««ë>`>@ñx<?ûÙÏùJîÛ·ïþûï×®ê+--u8òüÄOúÓK.1 ¼ûî»¿ÓE¿Ë/ÇðññÇÏó#hHü½óÎ;uõêÕ¾¾¾``ÀçóýÃ?üóüÏðÓO?MÎÇváÂÝ»wçäähsüÙýYâßHÓÞÏþóÎÎNæóçÏß¼y3Áwºèñ'nÜ¸Q^^·fÍÛ·oÏÿf#7ËÛÁf_°ÙwæÌÁf_°Ùwão©¯ÊOíFdâÄ¿âæØ²eÇãa0ñâ/IY­Ö×_]NÈÏúúzâÄ¿8âæÐëõäÈÄ¿¿ë×¯WTTäåå©µEEEgÏÿÍÊ'kqq±ÜfIIIWWñâÄ_LqÌQYYyüøñþFÄ¿?µçÈCº©Ó¯¾úê¢>tâÄ?Ïg·Ûcnáâ'ØÂKüø[ø+..wë×¯ã¯««KN þ@ü-Iº³Ù¬m>Ápøðaéþ(Ä¿Å?õv£N¨øâØ¾ þ@ü-y÷3Úì«­­u¹á%þ@ü-zü©<«µÏ=÷ÚñâÄßBw<«ÕªÌo³Ùúûûù+ þ1wò|õêUâÄ¿y.«²²Rû6[VVÖÜÜ,¿Àü'þ@ü%4þÄàààºuëÔhßÜÜÜìPøñô¿89¶lÙ"(æ þ@ü-Yü-	âÄÒ2þâæÜ¿?[x?Káqa·oß®¨¨(--%þ@üø¹¦¦¦»ë«­­=qâD0dV þ1þ&&&íâÄßÌuvvÚl6í`bµZÿ^âÄ_¥¥¥ËâZ¾|9ñâÄ_Á`Ðív¯]»VûZ\ìt:·Ä¿d¿Ï>û,ë®ð±="Iù½óÎ;Ä?1ùý~y3ÚU¥½½=1W?Éaz½øñ´?ÇSWW§ÝÂ[XXèp8°« þRñâ)CCC'NùÉäv»¶ªOúòÔ©SÍÍÍ|øñ7Gyyyá)+V¬xñÅ? þD__ÍfËÏÏj>½^/Ó½^o"ç3grss~øáGô+_ùÊO<ÁöeâÄß¬­2jÀ¯z_kll$þ@ü!cãO¢Êív¯_¿>æ9NgâÌáóù/_þOýåÑïÏýßç*+*:Äøñ7;999ò^&ÿµOéîî)Ä?d`üùýþÃO7c©ÌqüøñoÖ~Sú·÷ÿì½ÿþûùó þfGøúÒíjvþGüøCÚÇ_æØ¿¿ÔgdüÉ¿ßû½ßãÏGüøyk;pàúâÈøøøÑ£GÕNª?È@ ¡¡áë_ÿºvoee¥ËåJüÞ¿ñoDß·¾õ-yØü?³síÚµ;yîêê"þ@ü!½y½^Í¦×ëSâÈÒ F£Ñü¨ùÐÁCR~ß¶~ûÿø[ZZø; þfíÖ­[ÕÕÕ¹¹¹YYYyyyUUU2e±:ñâKEæ0LÚÿ÷J]É»SÒCºäGY¾|¹Á`øÊW¾"Kü5?)øñÄóûý£°°Pµµµ---K5c¶ñ$[¢?ÄñâÉH®½½Ýb±ÄÌa·ÛÝn÷íâÄ_ªÆßõë×Õ~Õß¢¢¢³gÏ þÓé,++?c¶Çöñâ/ã¯­­-üV¨âO~õÕW?H]³ÌAüøCÅÚéõë×Ãñ×ÕÕÅNAü!EÍm0ñâê=ñ;OMMÉiNGüøC3Ãl6ÇÌAüøCÅ_QQÚ«¿çNýøñ o&qsø|¾Þñâ1wò|õêUâÄY p¹Úw0É4«#s þAñ'×­[§FûæææVTT$àÄ?ÌÏç³ÛíÚ-¼z½Þf³y½ÞÙÞ ñâKøñÙlii1ÍÚUeeeN§sÎ»>&þ@üø#þ@ü!ÈÑhÔî·Åb±´··ÏóÈÄ?dVüúé§«V­ÊÉÉwÒÜÜÜêêêááaâÄ¼QX­Ö9ÇBGøñ?ùsÌýýýÄ?,ø9Ünw(ZÀ»#þ@ü!âOíä¹¾¾>ÊÙ±±±Ý»wËâÄÏï÷/ì`âÄ¿/ßÄ]_Óá> þ0Çl6k·ðFw þAñ§ÖüMLL§³æÄfhh¨±±Q½Ýó ¼Ä?ó¥¾óW__/Í'gïÜ¹S[[Ëwþ@ü!:;;òóóg~^âÄ¿yßÄ½,Òö_âÄ_Æ'NX¿~½ögíÚµn·ûm!þ@üø'ë^t:ñâ¢··÷àÁ1s444x½ÞÄgñâKøñ9$éZ[[M&vU_qqqccc"·ð þéñ7ÝþoÝºEüøÃ<©#sí`ººº3gÎ,ìîú? þfpË½ðÂQ÷ìÙÃ®^@üa>:;;­V«^¯Ê¾üü|»Ý¾PGæ þ@üø5<y;.**ºû¶óÍ7Õô"ÕøñÞÁà©S§bC&677/êîú? þfdÏ=ê­¹¤¤DØ¾ûb?tâÄ_Wô®]»´ûm)6­½½©s þ@üÅpùòåð;µü×<øñÁàtá-..v:É¹ªøñ¿gy&üN­N<ùäÄ?Ä×ßß¿ÿ~í~[ÉdJÁÄ?_¢Óéäº   §§çïüåää þ 599)ïE^Áàp8zSîI þAñ'ï×Gh³ÙíâQÔ~[F£vUßúõëO:Sô© þAñ7Ý~þ?PäÕjµZµ«úÄO<ÑÙÙêOøñ¿¥BüøK~@`ºÁ¥¥¥N§Óçó¥Ç3%þ@ü!ýãOÞ»#7ìÆ?KüøË4Ruv»];#;;Ûb±x<dÞoñâÄñGüøIº³Ù¬]Õ'!èp8íÈÄ?Äñâo.âæ0Ln·;öÛBüøñGü þ¦åõzm6v0^¯éri&ÌâÄ?âÄ_Bn·Ûd2iWõ¥Ê9? þ?âÄß=øý~Ã1Ý`$?/ñâÄñGüøI:	»GæHïÁÄ?¿Í»ø?i#8Î²²2í+½²²ÒåreÔ^âÄ24þ²îE§Ó þRÌ¡×ëµ[x­Vkâ_Ä?KKøñ·xâæ0òê` þ@üø#þ@ü¥<¿ßßÔÔ¤Ì!ÌfsKKKæ þ@üø#þt?5c×®]1sØíö´9/ñâÄñGü¥ÞÞÞ¦¦¦:u*3KKKÌAüøñGü©äå_^±bÙl~üñÇ«ªªVW¯!þâðù|ÁÄ?Äñb:;;ÿàþÀþWö£ß;ªþmxhÃw¾óâOkrròÌ3µµµÚ-¼ÅÅÅòÊÊäÝõ þ@üÄ_jø¿ùG4 òoÿ¾ýÄ_¤¡¡¡ÃKái·ðnÙ²¥µµu±· þ@üÄÆ÷¾÷½Ç<2þ>Oü)òb±X,ÚÝõI<x°··Eøñâø#þRÉ3gXõ_8þüñz(Ãã/Î9Ö®]ÛÔÔÄ`âÄ?âøKI?üpÍêï>ó])¿ºuøèõz36þúúú>¬Ý]_vvö=ÚÛÛÙ]ñâÄñGü¥¶`0h·ÛKJJrsszè¡E½»ä?Iº'xB;£´´ÔápÈÇñâÄñGü!åãÏï÷766Fí^ÅÂ9? þ?âi6mº#sôõõñÇ"þ@üø#þ?¤|üB¡S§N­_¿^»ª¯´´ôøñãæ þ@üø#þ?¤CüÅ92G]]9? þ?âéj0ÙlÖ®êt8øñâø#þñ700 |ÌÁk×®"äÈÄ?Äñt?YÈ­V«v0^¯·Ùlü!? þÒ*þF#ñL¿@ àr¹*++µ«úÊÊÊN'9? þÒ0þäµªªJ>í?dNüù|>»ÝóÈÁÄ?é[·nÂ8ñ÷£ý¨=±.ðÁ´íímmmï½÷ÞBÝ,WãßøvU_^^Þ_üÅ_üíßþ-ó<ÉÂ óÂãñ¼ÿþûÌ(ï¾ûnâï4ãï·qúø»xñâ¯ë£>òûý¿~ýëÁÁÁË/Ïÿv^|ñÅx@6l8yòäèè(s;ùuttüçþ'óâ_ÿõ_zzPÎ??>>à;Mçøc³/Rz³¯×ëµÙlz½>æ`¹Ìf_°ÙlöMÿÍ¾áÏ?âé¡PÈívL&s þ@ü±søCzÄßïw8ÚÁÂl63øñâø#þñ'I'ag±X´»ë´Ûí>ùIüøñGüÝñä¿@ àt:ËÊÊ´«ú*++].[x? þ?âéÓæÈÎÎ¶Z­_tAüøñGüXøø3Ãh4ÊB;00À¬#þ@üø#þ?¤|üÅÌÑÒÒÂ`âÄ?âøCÊÇ9@üøñGü!#ô÷÷ïÝ»7æ`È`âÄ@üH²àY­Víª>²k×.v×Güø#þ@üH@ÀårUVVjWõË944ÄþâÄñçóùìvÌÁµµµ---¡P¹Dü þ@üHmvf³YÛ|j0Ûíf.øñâøCÊÌh4j³oõêÕ'NÃ±AüøñGüH.qsÔÕÕy½Þðo þ@üø#þªÔAxcæ0ònuâÄ?ÄRÏtáUGæít9? þ@üHqÂo·ÛûúúâßñâÄ?â)`dd¤©©)æ~[Ö®];ó#s þ@üø#þÔ:;;­V«vovv¶LÅiVGæ þ@üøñGü!ÁS§NÕÖÖjWõ:Î9Ü,ñâÄ?âÉ¥¯¯ïðáÃÚ-¼j¿-gÎÏAx? þ@üHêÈwÚÝõFYræ¶ªøñâÄñä244ÔÔÔTVVs¿-RóYÕGüøñâøC²èììlhhÈÏÏy^Ï·à÷HüøñâøC¢ÅÌQYY9óý¶ þ@üø#þ¿¤æóù¦Ì¡öÛ²ØøñâÄñDðx<µµµ:øñâÄñGü-±¡¡¡ÆÆÆââbí^Éäv»p0ñâÄ?âø[2ÓC¦Øl6¯×»$øñâÄñ×êÕ«µ«úÊÊÊNçâæ þ@üøñGüãóùv0ÅbiooOð^âÄ?Ä$¼HÌf³vU_aa¡ÃáðûýÉóh? þ@ü£¡¡!§Ói4§Ìí1 þ@üø#þ0kV«Ud¥ÌAüøñâø#þR0t»Ýk×®Õ®êS»ë[ÚÁÄ? þ?âoaô÷÷744hÌ!Ìf³ÇãIÁÄ? þ?âo^B¡¼êêê´Gæ0(L­gDüøñâøCCCCòÇ9cõêÕÉ9øñâÄñGüÍüvíÚ¥=2Gvvö=¼^oªlá%þ@üøñGüaZ@ÀårUVVjWõÊnhh(&ñâÄ?â/Óõ÷÷<xP»ªOæH#s þ@üø#þ¿y	B---E;£°°ððáÃ>/ý5ñâÄ?â/ã466ÆÌ±víÚæææäß]ñâÄ?âø»7©ºûm)6­··7íçñâÄ?â/³Dé(--u:òô? þ@ü¥©©)<£µµ5s þ@üø#þ¿hCCCCÞæ2óé þ@üø#þ@üøñGüø#þ@üøñâø#þ@üøñâø#þ@üøñâø#þ@üøñâø#þ@üøñâø#þ@üøñâø#þ@üøñâø#þ@üøñâø#þ@üøñâø#þ@üøñâøñâùâÄñâÄ?ÄñâÄ?ÄñâÄ?ÄñâÄ?ÄñâÄ?ÄñâÄ?ÄñâÄ?ÄñâÄ?ÄñâÄ?ÄñâÄ?Ä?Ä?Ä? þ@üÄ? þ@üÄ? þ@üÄ? þ@üÄ? þ@üÄ? þ@üÄ? þ@üÄ? þ@üÄ? þ@ü-®®®5kÖètºîînâÄ? þÒ9þÊËË¯^½*'N>]QQ¡¿/'4¨ßïÆÇ?ÿüóË/3 tttÜ¾ùñé§öôô0 HüýÏÿüOï4ã/Rnn®6þ~üã_L¬÷ßÿ>¸øá¾÷ÞÌ(²0È"Á|	ù°`>@y÷Ýw§é×®]khh`³/Øì6ûÍ¾`³o:oöUÆÆÆêëëÁ ñâÄ?éË~G½uëÍfÔþ&ñâÄ?):::6oÞ<<<óRâÄ? þÒ*þFã²Ä? þ@ü¥süÅGüøñâÄñGüøñâÄñGüøñâÄñGüøñâÄñGüøñâÄñGüøñâÄñâ þ@üø#þ@üøñâø#þ@üøñâø#þ@üøñâø#þ@üøñâø#þ@üøñâ/Óâïûßÿ~KKË¯ëý÷ßÿÙÏ~ö+àW¿êëë;ö,óÊßÿýßÿò¿d>@tvv~ðÁÌ('Oü÷ÿ÷ßi HÏø»~ýúÑ£Gñ7.c,@æ þ? þ@üøñt®_¿þàêtºêêjíF#såAtuu­Y³F.ª©©éîîf^eòÂÿøa!ÚÚÚ-ãÓ6ÓÁÁÁe¿$%¹sçÎÉ«W¯Èä/ZUUÅåA)//râôéÓÌ«L^âÄää¤üçÏ³gÏÚl¶$y,÷æñxjjj"§lÝºÕçóñbfyÐÊÍÍe±0Üs9Aæ,/¾øâøC>/X¤üTÉNþÇVPP /ÚÓ§OÇw¼Y¾ìÚµkÌ¨_î¹ sÏ>ûlÝºuSSS|^°0oÞ¼Y§ÓÉ"ñÉ'É®­­­¨¨øCüåall¬¾¾>2Xâ_ÌYvìØqùòe>/X"ÝºukÉ7°8Î¤:ñ8Ë¼m6Ûàà 3a&!Ce_ÆüáÍAÉÉÉ!þTyyùõë×¿¸;sóæÍÄËÃtËCGGLf.±0ÜóøaÁçºÈï÷«KþæÀâ8­îîîêêj)÷M6×èD¾y1³<¨eÀh4òõ§y2üÃÏ/î~)¼ªªJ.ª­­þ#þ@üøñâÄñâÄ? þ@üøñâÄñâÄ$Äo¾¹qãÆÜ»6mÚôÎ;ï|éýë®y·õhF£<µ`05]¦ètº©©©ÙÞ&RÒÑ£Gi¼ðÂé2±¹¹9jú'dúóÏ??Ûâ@êéîî¬Ñét¯½öÚä]'O³2±§§'mâïÆ2±¦¦&jzUUL÷ûýÄâ@FxòÉ'%k^zé¥È/¿ü²LÜ³gOdútIâIºpÍ5r:üËwîÜihh(((Ã"·®¶··K`ÉEr]ÇS2eùòå7n¼páµZ­QìüùóñoG]$I]ÔÖÖ6]¨mÞ¼Y¦wuu§zU¦ÍæðcÇËMåææîÜ¹óÖ­[ÚøÓÞ~Ô8ñKlåÊ.öYäÄ7oÊD£Ñ7Q®]»¦.Ý¾ÔEûöíSõõõeeeÅ¼:«.Ý±cÇÔÔäcNNÎää¤?%òòòÔWñâÜùð´ÏôôéÓEîË7Þx# Q7²iÓ¦ÙÆ_ø¥§¶ðÆxÏZ¶LR,2nvïÞ=v³Û¶mSªÖQù¨VàI´©êëëÕ*C9åÊu#·yäÈÉ;µÕõé§)çÎÓòSN?õÔS÷¼	G9+¿ùØb>#	Ê+VÈó³òS¹çÄÄú¹¢</~·877w¶ñç¡ þ )âOÄ¿ðt7ªÄ­[·ä¬d:»fÍ9»jÕª½÷J´oD~'j]ZøZêìÍ7Ã¿,ÁÞò+?åtû=oGBSÎÞ¾;ò±M÷ý¼gV.úáøÅï6m8p ò$%þ$I×­[§VLÎ6þâ<TÄ,=µºkll,rb0rQÜ	§¡ÏçSýnðõ´[c£r*r+rÚ`0äääB!¹ñåË/½çíLbQ®_¿®:UNËO9Ý××¾ôÊ+òbn>yüÅy¨?Xzêo¯¼òJäDµ¨áïkWhõôô466ª°ájT«åÂÛUïhûöíS[ågCCCxzÛQkÚÕÙÛ·oÇ»aÃ¹TíÝF5ò"5òW.jkká0Us#|iø¥§LètºW_Uíêåµ×^ËÉÉÑÎØ¶mN(RÛdÃ#sÕwþÔWå|>_äU8p@RIÃïl%f¢©á·êÞ;::ÂÓãÜúúÎ_0T¿'þN<^''O9ò"õõÇ¾¾>¹	ÓÅÊMÉeù5Î;wF^ç¡ þ )¨ G9vìØÿÿºKµQøtxixEØÓO?YÎ='þ¾¸(n0"·Ç¹y,<Òvº';>>«BÔÆî­[·FÞÅ+ä§ÚÛKämªa*UÃÆy¨?H(6mÊ¹kãÆjÈmTüµµµ©ÝéUWW_¾|9|i0|î¹çU0=ûì³¡P(|©ÇãY·n4YIIËåºMí#éÏ<óLÔôénGÈ¤v@g?aQû|QëëëeÈ³ðûýáÝÖDÞæèè¨Ô­Wf³¹««+êã<TÄ? þ@üøñâÄ?Ä? þ@üø@¢ü?ÄÉ¥B¡ ÙXIEND®B`


÷khhHäÔ©Ss¿ë×¯«Ç¥oÞ¼©¾«§º|ùrØïq¾7WüEÞ8êô>r'óÝªsÙê(=Ý©©)i»­[·jÓðøñã²µ%%|#opõ!I+ËçË¦åÕ«Wó+R-þ>|ì¥®q¹Ã¿'Ï.&þu¢Z±b|>ã5ìc~åW´G$×­[áÛ×Mæ7N`Bæ°Uç²=oß¾|7n¾6d#deeE?u~Ä°§¼ þ¤Nü	õhðúÏ?ÿ|çÎYÏÔÕÕiOïXXüÉ½ÉJ½öÚkÒCrÕ¾û[`'N(//ÏÈÈX¹råÙ³g¿þõ¯Î^üMæ7ÎåË¥ÿt:]`æ¼`óÚªsÜ¼W®µj|rêôÀÍrñÕW_U¿Åbéëëî[­]]]roò]Øl6~_âÂôôô+¯¼¢VcìnøÔ£ÌêùÚ~¿_ø²²2!?HAê¿o¿ý6[ñ)Èëõ<xpåÊê±]Y8räñâøc þ@üøñâÄ? þ@ü°0/_6L:nÓ¦Mó½myyù²eËNg`,ËÕ«WÏcöÌÂ>g.·]ÈNvÁWedddgg×ÕÕñ,@üHF£QRFÆï÷Ï÷¶GÛ¶··Ö¼óÎ;²¦µµ5©ã/Âý¯owíÚÅ³ñ yv(è§Û·oËmkjjk6lØ kâó 6þ$e9;;gâ@2_pÐ¼ñÆyyy999ù´¯ýë!÷xãP?~,Ë²F½êþýûEÚH§Ó­Y³¦§§'ì½õÈ79ö¬Édª®®/¤M±ÉÉI«Õ*·ðàAõLÉÓ7Ê½Éz¹çÄ(þ>ÿüóõ;/e%Ï1ÄDì?uùûßÿ¾,Ûl6É,Yxûí·?§««K;¾mÏ=rÕéÓ§eY>Êrcc£zÕºuë®^½*÷îÝõaï-øD¾$Ý| ¢hü¾ûdY>áâÅ²pôèQYYTT$ËCCC² ¾1v0ß|ãOO¶,744¨Wµ¶¶ªâøñã²päÈ]?	&I§Ñ¶WØA×¯_«¶mÛ&ËÛ·oW«.pííÛ·:TYY©!öÞB+òMÔ§½mAAúà%ËdA²OVÊgÊò5k$g>ßb6Nðõ±ÉKùIYªWÉ...SRR"yv þ$tüétºÀ²,ÈÅÙ(@JKZG>ÓëõªGWezÕ©S§äVRwîÜ	¾¾8ÌöÀ¤ÆßÌSÃñÂË/W×õMÁ¨¼óz¾>hÄ¿ÂÂÂàwþÔ7Ï[<V«U®|¬ÏÊÊ53ÏÌ1þ"ß$ðÀ¤ç´·]±bzÛç÷û¯^½ª(Jð[Ø8ÏÝuuu7n ~ôèÏ.Ä?uêÓ§O«cþÞyç¹Äßo¡ðÁõjJÞ½W8ø|Cøá²°gÏím_õUY~ï½÷ÕC½²²ººZïÜ¹#)&%%%±¿ÈU·oß¾yófðÀD þ$hüùý~	¬gçêSSSê!Wù'®b0òòò<8Çø|Ã!×nØ°!pÞIðçx½^É/yäYYY[·nUOìæ«««SO®©©ïé·s?	ÖàYo6nÜ(x þ@üøñ@üøñ·4~ö³Äùþò¿üÿù7®ÿýßÿýÅ/~Áv@Ô=xðàW¿úÛÑõë_ÿ:þ¯¿ þ~ãßûô_¿è;wþë¿þç¢K^¯_¿Îv@Ôýó?ÿ3ÿ;¢î¿ÿû¿oÞ¼Év þ?øñâÄñ þ@üø#þâÄ?Ä?øñâøñ þ@ü þâÄ?âÄ@üøñGüøñ þ? þâø#þ? þâø#þ? þ@üÄñ þ@üø#þâÄ?Ä@üøñâøñÇvñâékÖ¬Ñét«W¯ þ@üÄ?¤rüßºuKÎ;WRR¢¿îîîÉø=éþçNQ%/Ï===lD]ooï/~ñ¶¢ëÁ?ýéOÙ,ã/Xvv¶6þ?þøêêêr8?¢êÇ?þñGÄv@ÔüñÇ×®]c; ºäÕ«W¯²Y*Ä_cc#Áa_Ã¾à°/Rù°¯jrr²®®Îëõ þâÄR<þ>|¨(JØñâ þ@ü!¥âO^7oÞ<66öZâÄ@üøCJÅÉdZøñ þÊññâ þ@üø#þ@üÄ?Ä?ñâøñâ þ?âøñâ þ?âøñâÄñGüñâÄ?â þ@üøñGüÄ? þ?l þ@ü þâÄ?âÄ@üøñGüø? þ? þâÄñâÄ@üÄñâÄ@üÄñâÄ@üÄ@üøñâø? þ@üñâÄ?âÄñâÄ?âÄ@üøñGüø? þ?ñâÄñâÄÛ1?¿ßéÒ¥úúz¯×ËÆ!þ?øñ?ÇÓÑÑa6=sþüy6ñGüÄ?¤`üýèG?WáüüüeAªªªØ8Äñ þR²³³ý>³Ùl·ÛÙ>Äñ þ"d7U__/½ô<Óü~?ø#þâÄÏç;þ|UUUHóI644²?â þ@ü!¶´´ÆìáEkÙDÄñ þrssC²¯¨¨¨½½ýÁÌóGüñâ©@^X_zé%íÀ>Åâp8ÔLòLüñâÉMªÎn·köéõz«ÕòjKüÄ@üøC²òx<Ç+**ÒfßþýûÝn·ö&ÄñGüÄ?$ËÕÔÔ2Q³0L­­­ããã³Ýø#þ?øñd"¯V«U;°oíÚµv»ý¹3öÄñ þ¤ê:;;-Ë2ÚÚÚ+W®Ìq¢fâø#þâÄÇã±Ùlf³Y;°OQË5¯#þ?â þ@ü!A¹Ýny¹Ôì5²^¢p÷IüÄ@üøCÂq:¢höÍf»Ýîóù|ÏÄñGüÄ?$yq;°/x¢æÅ þ?â þ@üaéù|>»Ý^ZZÒ|¢È!Z_ø#þ?øñ¥äñxfØ×ÒÒ2ßó9?âø#þ@üøC°SE¯×köµµµ-ì|âø#þ? þpä°¶¶V>Guuõj&þ?âøñâI@ªNÚNO;°ÏjµÆçeø#þ?øñóx<mmmÚóóó¢>°ø#þ?âÄ?,	»íù&iÁ5ÄñGüøñÓÛÛkµZµûJKKc=°ø#þ?âÄ?ÄT]WWWØó9,Kü_û?âø#þ@üøCLx½^»Ý^TTv¢f§Óø#þ?øñÅmkk;Q³¼Þ¹ÝîÄy¨ÄñGüÄ?,Üðð°¢(¹¹¹Úó9b7Q3ñGüÄ?+¿ßïp8Âì«ªª:þ¼ÏçKÌGNüÄ@üøÃ<HÕuvvVTT,ÓØ²eKWW×RÆKüÄñâÄ¢ÉëõvttL¦æÓëõ;vìLïø#þ?øñçp¹¢Hä=#öÄñGüøñ;QóÚµkívreñGüñâ³êêêªªªÒìì»téRì#þ?âøñâs¢NÔl6y¢fâø#þ? þ°X£££---!$ûddËåJoø#þ?øñîµ5Æööv¯×Jß,ñGüñâ/É+ÅbÑì#a'j&þ?âøñâóà÷ûívii©v`d_ü_?Ä?ø	Ç#/@a'jÞ¿¿ÛíNù-@üÄ@üøK.«©©)???$ûÌf³ÍfMí@üÄ@üøKqòrv¢æêêj»Ý¼3öÄñGüøñßªëììÔÏ!X[[Úû?âø#þ@üøK#Çf³i'jÖëõ©4cñGüÄ?éÎívËëv`¬õÉø_ñ þ? þ~s)¢Øg6ívJÎØGüÄñâÄ_Úñûý#ÂDÍév>ñGüÄ?©ÉçóÙívíÀ¾ÌÌLEQdë±?âø#þ@üøKêDÍaöµ´´¤ùùÄñGü þ@ü¥	;EQôzØ9ø#þÞØØÉd"þ@üÄß"ÉKFmm­ö|ÅÒÙÙÉÀ>âøKòÜ*++ßLâÄ@ü-TÝn¯®®Öì³Z­i;Q3ñGü%¨­[·:Îñ÷áÞ/ÙMÞ¢êóÏ?ïîîf; ê~òüË¿üKÚ~ûÿñÿqìØ±/ùË!Ù÷ø»wï¾qãÏù·û·k×®±YÇßÿ=ÄÙãïìÙ³ÿ_×¯_ïëëûW ªä/O>ùí¨ëéééïïOÃoüÓO?mjjÊËËÉ¾_|QÖ÷ööòÜXÏ>ûL¶0Û!¥rüqØö8ìLþ²Z­Ú¥¥¥v»öñGüøñ¤ê:;;g¨Ä?âÄ¿ÔÉ>ÍVTTÒ|z½Þjµ2Q3ñâøñâ/E;vÌh4d_nnnKKÛíæ	@ü!Yão6Ä? =ãÏår566Jäi'jîèè`¢fâÄñâÄ_p8[¶lÑÏQ]]éÒ%ÏÇøñGüøñô¤êìvEEv¢æúúzö þ?@Äßèèhkkk~~~HöÉ×_xx2ñâøñ¤Bü9Î¦¦&íÀ>£ÑØÖÖÆÀ>¯×+/]]]###Ä?âÄÄñ'[QíÀ¾Í&ÑÃOVöyyy_ùÊWÖ¯_sðàÁxÎ_MüÄ@üøÉóçÏÏ6QsWWÿ?ÊívçççïªßuøÍÃòïÛ¾ý¥/é?ü!ñâøñâ/9âO=cíÚµÚó9¨Y«½½ýúõjù©ÿ¾ùÊ7¥ÿ?Ä?n·[væ!ìÀ¾ÑÑQ~Zû÷ïÿÆ7¾òïþà?Ä?N§3ìÀ¾µk×Úívfì ½½ý«_ýjpùýò7¼óâøñ$hüÉ®;ìÀ¾ÃÁÀ¾ç;æï?øñâøñâ/âOªÎn·jö544ôööòó;õlß²²²ªªªp¶/?âÄ(ñçñxdm2´5·´´¸~Ràõze¿qåÊøo@âø#þâÄ_xÒ%MMMÚÿÃl63Qsò"þ?â þ@üý³ÕjÕÏQ]]m·ÛØGü!)ãoÙódddñ´?©ºÎÎNíùRµµµñßiøC4ã/ãyt:ñHøóx<mmmf³9$ûôz½¢(ì#þñ·ä?@"Ä]KKv`¬5û?¤KüMLLìÙ³ø?¤püÉý¨Ùl63Q3ñ?É¤ÓéóHøóûý#ìDÍ²?¤~ü­^½Zûûo0¼^/ñH¥øóù|v»];°/33SQ§ÓÉ6'þñ%¿ùcccF£Q¤ù>üðCYhll$þâ©êDÍ³ìs»Ýlmâiêï¿,HíÉÂ½÷fffd!''ø?$ü¹EQôz½v`Ífã|âéË/½<N§,9rD]`ªøCRÇ<-ëëëµçsX,ÎÎNöHßø;xð`àôiÜ?øCÒÅÏç¶[»v­v`Õje¢fÄßo¼õÖ[+V¬¾¾>Y¬¬¬õC'þ@üÑ?u¢fÉ¤Ø×ÔÔÄDÍ þ¿%Füø¢N§³¹¹Y>Ñhd`?âø?¤HüÉçìØ±C;°¯¢¢âÌ3LÔâø¯¤¤DðIâI~¿¿³³³ªªJ;Q«¬ìêêâ|Äß¬/³â	>Ïf³UTT4^¯g¢fÄßHçÉ^£¿¿ff&øñÌ+þÜn·ì9ö|ÖÖÖ¶?âoNÔýHËøñÌ=þEÉÍÍÉ¾ÒÒÒ3gÎ³­@üó000 ûöMNNñ¿üÇ´X,Ú555.]b`?âoV®Ý³pÂ@üa©ø|>»Ý¾jÕ*íDÍ/½ôRoo/Äñ·pÁ;Nø?,­ññqÙ=¨¹¹¹ þøSw+CCCq~èÄ? :°O¯×d`[[û@ü!jñWPPÀ	ñ%ÔÛÛ[__¯¨yÕªULÔâQ?yÑ]ÌÁã¼!þ@ü!Í©5=£¶¶VTóú¿âøÛ]Ì>â1âñxl6ÙlÖNÔÜÐÐ088¨~ÚÜÿo_ø#þæ!cð:u¢æüü|íÀ¾æææÑÑÑàO&þ@ü!&ñ·T?H+N§SQíÀ>³Ù<ÛÀ>âÄbòçæªU«?øC,È¾.ìÀ>Yép8"LÔLüøCLâO§ÓÉ>(þøñÔ&Ug·ÛKKKµ5+"a÷Ü þ@ü!&ñ×ÓÓ#;£ÖÖÖxNøBüøCªòx<³MÔÜÒÒâr¹æx?Ä?Ä$þ8Û þ-vMMMÚó9Ìfs[[Dá¼îøñÄgûÄOvhµµµÚó9ª««ívÄ?Ä;þñâ)@Ø'§Ø'-¸È½ñâÄñâÂãñ´µµ¨YQ¹ì#þ@üa	âozzzëÖ­ÙÙÙ²ÛÊÉÉyùåãpæñâIJÂ®¥¥%ìDÍ²gïÀ>âÄâSSSaOøõõKüøCÒ;Qsiiéö þïø+++=×¶mÛ&''åâÄÄÄöíÛeÍºuë?øÃÓgûÇl5ÇnWFüøCLâ/++Kö_Á°NOOËYOüÄ_óù|v»];°O¨ÙétÆô« þøËÈÈ_ðÎNÖ0Õ@ü¥3u¢fíÀ>Y#ëÝnwñâ1?õ°ïæÍÕÃ¾òQeÍ5k?øKC.KQ½^¯¨Ùf³Eñ|âÄ&þ¤öÂðñäÉâ þ¢Èï÷wuuµ··?>	5w³MÔl±X:;;£~>ñâKOðûòË/çååeddÈÇÍ7ËX?tâÄ_Z-//ÿã?þãM6­]»vÅCCCS¥a'j¨øñD¿%AüøK+ögVSSsøÍÃê¿¿ÜþôGäõzöQE¨yÿþý±>øñâøñÆÇÇ5Äü+..îíí]ª4ÛDÍF£±µµ5AJ þÍø[ö<Ä@üEÅÐÐPYiYpùÉ¿õUë;;;¤¨ÂNÔæÌøì#þ@ü!Nñ1;â þ¢Ëãñ,_¾üõ×^ß¡æC/¾øâàà`ÜT´fØkkkGBeñâÑ¿Ù¼öÚkêÞðÜ¹sÄ@üEËÞ½Õþò«®®Þ´iS|zKÒÓf³ØgµZv£ þÛøÈÉÉQ'5óø¿ÅÎÛ³gOnnniié/¼ðµ¯m||<Ö_Ôív¨Ù`0ÈúÑÑÑßhÄ?Ä0þvîÜ©î¯^½NüøKCG&ÿ=Óé;°OÒóÄK~1ñâK/^T÷Û¶mçC'þ@ü!dÇv`_MMÍ+Wp`ñâñ¿©©©uëÖ©çvôôôÄù¡ þEêDÍ¥¥¥!ÍÙÐÐ GüøCôãïøñãêq×®]KòÐ?ññqÙL¦ìËÏÏß¿ÿÈÈHò~kÄ?D3þç þ]kk«ö|¢¢¢öööÄüï?X²øËxNGüÄ_bêííÝ±cö|êêj»ÝîóùRãÛ$þ@ü!ñ·ä?/¿ßþüùíÁ-[¶ÄBüøñGüøCL;v¬¨¨H;Q³¢(.+%¿kâÄ?âÄ_Úq:û÷ïÏÍÍÕÏ!»ØGüøñGüøÃoôööÖ××köÍæTØGüøñGüøKkêÀ¾êêjíÀ>Åâp8qÆ>âÄ?âÄBù|¾öövíÀ¾ÌÌLEQ¤Òm þÍøc?øK^¯×f³ÍfíÀ¾T=øñ¸Æ_ð~aãyþâ/Ünwss³v¢f	Á¶¶¶Ô>øñ¸Æ_@ww·ìgwïÞ=99)åãöíÛeÍ7?øÞÞ^«Õ:ÛDÍé3°øñxÇÁ`½mð~vzzZÖÆEÞóÀÀ@aa¡N§+//ïéé!þ@üáé³ó9.]ºd±X´û¤So¢fâÄ.þÔÝ®_Hü-~Ì_]]Ýï½''OÜµkñâ/ÍEØ×ÔÔ¶û?wüÈÎWBmjjJÝ;oÛ¶MÖÈúEÞ³Á`QkÒd2iãO^nÇ×µk×äªÿüçü1Û!ÃñÍo~3///$ûd?ó­oë§?ý)(¬O?ýôþéØ®¾¾¾O>ùíÈb7nÜÂÇ;wyÏÁ§hOø»xñ¢;¾nÝº544ä¢ÊårIÜ°Âºzõê®]»²²²´çsüÝßýÝÈÈ(ýìgÃÃÃlDÓéüñÌvHd1?qïÞ½âââììì5kÖ<~üxñw|àXvýöÓßï ÖìS'jf`Áa_,åaßØ)((PÊGíAdâÄ_Jòù|v»];°O¨Ùét²?HÙø³Z­gÏùXWWGüøKmG~¯µ3öÉYÿÜ þ@ü!~ñw÷îÝõ@mAAÁ·òÜ2rÄ¿Tår¹EÑëõÚ6-Í'j&þ@ü!áâOä9ø¿tSO<ÓNüøKò[[«¨Ùb±tvv2Q3ñâF£QöÔwïÞÄ___,çååñTÝn¯®®f¢fâÄ/þÔ]¶º ÆßÌÌÿ·/@üåñxÚÚÚ¨øñ$?ugõÝ>¿ééé7ÞxCµÓ2@:Çäö|ÙWÈ¯3û?Hø­°<ßºuø¿§ÏöY­VíÀ¾ÒÒR»ÝÎÀ>âÄ,þì++Õ³³³³KJJâpìøñà¤ê:;;¨øñ¿%AüøKXÇf³iöéõz&j&þ@ü!éã/pGÀãÇKJJ? ÝâÏívG¨Ä?¤füMOOs¶/nñçt:EÑì3Ív»Ýçóñ&þ@ü!¹ã¯¨¨hYDË/'þt?ùMm`Ãáà|âÄR$þîß¿ñLàÿö&åwñâEâHáøóù|6M¢Hvð%þ@ü!¥â/@R/ÖGx?	e||üÄ&I;°¯¥¥?Hñø[*Ä?°K.µ··_¹r%>ê$ìöïß«Ø×ÖÖÆùÄ?¤EüMOOäääÖ¬X±âûßÿ>ñÄ4þz_xáÊÊJË×,ÅÅÅ_þòGFFb÷ å×­¶¶V¯×d_EE5 þ^ñ·råÊ~ÕÖÖVâQüÔ×Õ~ó°úïÏÿüÏ×­[õóù|gÎ©©©Ñì³Z­ò;Hö þvñ%¯CCC5²&//øb.]Ôü;Ô|HrÐívGëQy<ùÎ`0hö5773°øñô?õßß»_ÍäÄÅø;qâÄ¦MãOþDå[)ÈÆÆFmöÉcÇ1°øñGü¥üÊ«ÂÔñæSSS5F£øb]]]«V­:Ô|(P~ß>ðíÜÜÜñññÅ<ÞÞ^EQ´çsTTT9sÆëõòÃ"þâøÚßßvç¾¾>âQüùýþµk×nX¿AOÊoÏ·öïÝ»wa@îíÊ+a'jÞ²e&û?ø#þ~ÏÃËËË³³³322rrrÊÊÊdM¬:ñt?áõzÿê¯þ*77·   ??ÿ»ßýîMnréÒ%³Ù¬Í¾;vóÓ!þâøKÄÒ<þÆÇÇ³Ï!5ÙÔÔät:ù¹ñGüÄ1þækpp0ìù¥¥¥ÏAüÄñ÷|wïÞUçyVÏð-((¸páñ$ZüõööÖ××gffd_UUû?ø#þæª»»;ð¢Æº|òäIâHøp>Gmm­|i²ø?âoF£¼Ü½7Lò$Büy<¶¶6íùz½¾±±?ø#þtÏ<ØyffFu:ñ,UüIØ555åççkÿÉÁENâÄÛ!­ã¯  @ÕO¿ééé7ÞxCM&ñÄ?þä÷ÂjµjöIöq>ñÄßbÉVØIoÝºEüq?¿ßßÙÙv`¬ä|â þµø²s©¬¬TÏöÍÎÎ.))ÃX"âÄj¶¢0cñÇvñèÇß þ@üÉ_Y---aöÉ/ÛífÛÄ?ÄR!þfØg6m6û@üøClãï/¾XµjUVV¼ödgg@Ôã/òÀ>¹ þ@ü!æñçp8ÂðëÿøCZÅ_V«5þ¿ þ@ü!ãOä¹®®ÎëõÊÅÉÉÉ]»vÉÂÂBâX|üEØ×ÔÔÄDÍ þ@ü!Þñ§¾låÀÏÄ°àøWnEQ´ûL&<ÿØâÄ&þÔwþ¦§§k¦¦¦xçXpüEØW]]m·ÛØâÄ2þÔ1uuuÒ|rñÉ'555ùÇf³ØW[[ËÀ> þñ·ìybtüøC*Åß?üÃ?Ø§×ëEa`?H øËxNGü³¬¯¯ß°53°Ä?$-âÉÎáplÙ²%ìDÍv»Ýçó±@üøC"Æßló9?|øø´¤êÎ??ÛDÍRÏâÄ:þäë­·ÞYÙÐÐÀT/@ñññöövõùó9þâ/þB^§ÙD þ@ü!	âO«TPPðøñc¹øþûï«¯g1êGü!I³¯¥¥%77W>GSS<¯æûûÄ?,Yü=ö>úJVXX¨.lß¾=ÖøCR´Z­y!ÙWTTÔÞÞ®Ï1÷ÿÛ þ@ü!!âOÜ¸q#ðªÖÑÑNü!ùýþK.=£ªªêÌ3Áû? þdñ·wï^õU-0i÷îÝÄÒÏç³ÛíEEEÚ;vìÈÓÏAüøñd?N'/lyyy·oß~4æ/++øCZí|üüüæææÙnHüøñd?ym;tèPÈJEQ8ÛéÃårIÞiÏç0s¨øñâÉ³Íóë=ñD OÂ°3ö­]»öÌ3s¨øñâÉKøÃòûýçÏ¯ªª;Qó+WæuoÄ?Hø¹à»/H£££íííÚó9rss­VëÐÐÐîøñâÄñ322v`z>DáïøñâÄñ"Ï´°3öµµµ=÷|âÄ?Äßï·Ûí¥¥¥ÚUUUÚû? þ@üH>G`&Iõõõa'j&þ@üøñGü!ù¸¦¦¦üüüæÓëõÍÍÍÃÃÃ±ø¢Ä? þ?Ä<¬V«v`Éd'Ûøøxì¾4ñâÄ#þ"#þü~gggØKKKívtð þ@ü!Yã/ãyt:ñDæñxl6Ùl;Qs<]Ä?Hø[rÄÌívËóG;°/33SQ§ÓçÇCüøñâøC¬^%ï´û$å%Q¸$øñâÄñhòûý#ìÀ>³Ùl³Ù?Q3ñâÄ?â!?Öï|ç;ÊÉ>Oîg¶Q¨øñâÄñykmmÍËË,«­­-..^·nÝçXQ'j;°ÏjµÆÿÉCüøñâøÃïéííòkúMß<¬þûÓÊ?ýë¿þëùÞËåRE¯×köµ´´Èµö þ@üø#þÒÑw¾ó¯íkòû÷íbû=ÈS¢¶¶V>Ùlnkk[ÚÄ? þ?ü7ß|ó_ÿFpüj>ûÜúý~»Ý^]]­Ø'+ã3Q3ñâÄ?âósþüù/éK|ø«­­]¿~x½Þ¢¢"íÀ>¹mBì#þ@üøñGüá÷øýþªªªò¯ÿíÞ¿zËÍÍW¯°<22ÒÒÒ¢=C¯×+û? þ@üåñx^|ñEÉ¾õë×-?Y)3ÛDÍ	;°øñâÄñùm¢æ»Ýîóùôû"þ@üøñGüáw¤ê:::ÂNÔ[ÛÕÕàçs þ@üø#þ0'^¯·¥¥Å`0h³ï¥^Jó9? þ@üxÑÑÑcÇL¦ælmmM®ó9? þ@üÕððpcc£öÿç06Íëõ¦Þ·LüøñâøKGcË-ÚÓxÕ÷|âÄ?Ä~GªîÌ3ÚRf`ñâÄ¿ç3LÄ_ª;°O¯×¿þúë###i²? þ@üý<·ÊÊÊ$¿Ôãt:sssC²OB°­­M¢0­¶ñâÄ¿ßØºu«$BøûÁ~à¯«W¯~òÉ',Âßÿýßõ«_ÍÈÈÉ¾U«Vµ´´¤çæýôÓO?úè#`¤ÐÝÝÍ.+Á%qüýßC=þ®]»öëøúì³Ï×¯1¿úÕ¯N>ý'ò'Úû6oÞ,¯Oò	i»qÆÆÆzzzx êúúúîß¿Ïv@t=zôèÆlDÊñÇaß¤àv»å¥¨933SQ§ÓÉ&â°/8ìû"ûÊøKÉ;íÔ-ùùù­­­l"âÄ?¤üÍAâ/¹øý~Ãa±X´GxÚÛÛ=[øñâÄñÙ×ÙÙi6µÙWUU%WÉ'°? þ@ü-ñPÆÇÇ;::´3ö;vÈ+øñâÄñÜnwss³vÆ>YÓÔÔÄùÄ? þ¿Ôy]©¯¯×Ïa4ØGüøñâøK~¿¿«««ªªJ·¢¢Ân·½^~µ? þ@üI/òÀ¾øÿ8?øñâøÙöeff655ò»DüøñâøKÃÃÃaöI¶¶¶ó[DüøñâøK²y·lÙ¢Í¾ÒÒÒ'N0°øñâÄñü~¿Ýn;QsMMÃá`¢fâÄ?¶ñGü¥ññqÙªA;°ÏjµÊ«ÙGüø?âøKííí³ìs¹ª þâø#þRÄGpöIòKBüø?âøKMê8?ÅÒÙÙÉ^âÄ@üÄ_Ç_WW	Büø?âøKøñâ þ?âøñâ þ?â þ@üøñGüÄ? þ?øñâÄñ þ@üø#þ@üÄ?Ä?øñâøñ þ@ü þâÄ?âÄ?ø#þ?âÄ?ø#þ?âÄ?ø#þ?âÄ? þ?øñâÄñ þ@üø#þâÄ?Ä?øñâøñ þ@ü þâÄ?âÄ@üøñGüøñ þ? þâø#þ? þâø#þ? þ@üÄñ þ@üø#þâÄ?Ä@üøñâøñ þ@ü þâÄ?âÄ@üøñGüø? þ? þâÄñâÄ@üÄñâÄ@üÄñâÄ@üÄ@üøñâø? þ@üñâÄ?âÄñâÄ?âÄ@üøñGüø? þ?ñâÄñâÄ@üø#þ@üø?âø#þ@üø?âø#þ@üø?âø? þ@üñâÄ?â þ@üøñGüÄ? þ?ñâÄñâ þ@üø#þ@üÄ?Ä?Ä?Ä?ñGüÄ?ñGüÄ?ñGüñâÄ?â þ@üøñGüÄ? þ?øñâÄñâ þ@ü!=ã¯¯¯oÍ5:nõêÕÄ?øñT¿âââ[·nÉÂ¹sçJJJ´ñwíÚµ©øu¹@Tõôô°uò'ôÈÈÛÑõË_þòÆlDÄñ,;;[?üá¯Å×ÇüÉ'¢êÓO?ýè£Øº«W¯vww³À.+Ý¤Büõ÷÷766rØö8ìû"ûª&''ëêê¼^/ñâ þ@ü!ÕâoÙo©>|¨(JØñâ þ@ü!éã/¼nÞ¼yll,ìµÄ?øñ?É´,ñâ þ@ü!ã/2âÄ@üøñGüø? þ? þâÄñâÄ@üÄñâÄ@üÄñâÄ?âø#þâÄ?Ä@üøñâø? þ@ü þØ þ@üø#þ@üÄ?Ä?øñt¿ï~÷»#ñõñÇÿüç?¢êßÿýß?øà¶¢îG?úÑgÆv@t]¸píÈ<OjÆßÝ»w>ü=|hto~pØÄ? þ@üø[2kÖ¬Ñét«W¯¾êóÏ?¯¬¬ÌÊÊÚ¹sçÄÄÛór÷îÝuëÖÉS«¼¼<d|y¦ªWõôô°­ç3Ll%D÷©áÄ_R*..¾uë,;w®¤¤$ø*ù5g¼,ïÝ»myèåËeA`!øªººº÷ÞON<¹k×.¶¢ò¼ì²²²eËØá#ÊO­/ þ^vvvðEù+'°¼|ùr¶¦««Kþ^#;ÖYæDëyµuëV§ÓIü!êO­/ þ[cccðyöß½WÞÿýàæÈï÷çååÉ±ü¹<Ûß<µ­çÕÿíî?Äæ©öÄ_¬««óz½Á+ïÜ¹SRR"å¼ûî»ü­ëîî.((^XÎÊÊb!*Ï+â1j¡ñ¬>|¨(Ê£Gfû/¾ø¢¬¬yOv¬ÓÓÓOö»ð¼"þ»§Ös_(Aü%ë×¯oÞ¼yllLUqqñÀÀÀÌÌÌ»ï¾ûÖ[o±­0/òüQôõõÉs,ø*«ÕzöìYYò4ÛQy^ÑS+Â%¿¤d2	ÞÊÓåÊYYY~¿my¿ÊËËåèM6þZ7oÞ4êåÀâWÄbôÔûB	âÄ? þ@üøñ@üøñâÄ? þ@üøñ@ü@Âxÿý÷7nÜýÌ¦M.^¼øû¯gfoîÑL&ùÖ¼^oÈzY£Óégffæ@üHJ^¦ñÖ[o¥Rüµ¶¶ÊÊõ'NõGYÀñ ùHÖètºS§Nù9ú´·oßNø»wï¬zuÈú²²2Yïr¹?Ä´°÷nÉ·ß~;xå;ï¼#+Ó§§§GâIºpÍ5²øä'O466æååÉUáÀÁGW·íêêÉ)Y³|ùò7^½zU.Z­ÖvåÊÈ÷£^%I½ª»»¶PÛ¼y³¬ïëë¬¹uë¬±X,5G5rWÙÙÙ;wî|øð¡6þ´÷²&ÂC@üÀ[¹r¥Ëýû÷W>xð@VL¦à¸	Ñßß¯^»ûö«öíÛ§^544öVêEõÚ_~yffFò1++Ëï÷ËµòQâ)''Gá~d!ìÃÓ~§çÎ.Ú@_¾÷Þò¹M6Í7þ"<TÄ,=õoÖ²ebÁq³k×®ÉgdA.nÛ¶M½Vm5Õ7ð$ÚÔ«êêêÔ·eùæÍêßç¡C$ïÔ£®¯¾úª¬¹|ù²,ËGY~åW?rQ>!ø±ý$(W¬X!ßïØØòÈ¥8§§§ÕO(,,Ê·ðô·³³³ç*â"þDØø¬WãFm&ñðáC¹(Ù¤^f\µjÕ=$Ú¦¦¦w"ò^ZàVêÅ>Y)päW>Ê²ÃáxîýHhÊÅÇ?¶ÙÆç½öÚkrÕ»ï¾ûô·¶8ü	¤êó¿ñKO»krr2x¥×ërUÜ	¤¡ÓéTû/Ð:zÚ£±!9<Á,¬¬,Ï'w¾|ùòÀµÏ½ÙR,ÄÝ»wÕNeù(ËCCCkoÞ¼) ìáã¹Ç_ø¥§;~üxðJu>ãÇÆÆ´ohÝ¾»µµU=¨Fõm¹ÀqÕç&Ú¾ûÔ£½ò±±±1°>Âý¨ï´=zôH½øøñãÈgænØ°A®Ug·f¾J=óW®êîî0U·FàÚñKO=aB§Ó<yRêåÔ©SYYYÚ3¶mÛ&¡ãóùÔc²3sÕ1êP9§Ó<XPmÁH*©çá&[	hêé·êW¿~ýz`ûQù©cþ¼^¯úâïôéÓ÷ää[¾Jþ844$_E6Âlñ§æ¦ä²|DçÎ;¯ðPÔ	C=zôwû¯gÔ6,Î±xõÕWË2Ø"ÄßÓgÿ¬7ÁG#Ü<à8Óv¶ovjj*;;[ýBvoÝº5øK¬X±B>ª³½ß§úÆdªk#<TÄ$	M6e=³qãFõÛøëîîV§Ó+//¿qãFàZ¯×ûÆoF5^í5Ï¸¶«««²²R¬°°Ðf³Ü§öÈ]Éú½÷¬í~<yHêæùhllóE566VWW'[ //O¾Ë¶&ø>'&&¤nÕme±XúúúB¾bøñâÄ? þ@üøñâøñâÄ?ÿÞé_8 ¿IEND®B`


íÚµþ5ÍÍÍË/7L¥¥¥ýýý­­­EEEëÖ­ÜËxäÈììluS¾ÿ¢plÚ¹s§¬<+ÕÕÕÉÊêêjÍqªW	ýÖâEY#wrJ´ã0ôMã¡üGÊ8¯_¿^3ðÒp·îÿQr:rkòË¥ïø#¢8ÁßÐÐPNNNVV,²àþýûæ¯RZBB|Ü²eËgZM×¬Y#¾ñ*.ñË/è+ËÊÊ"ãoñâÅ²þáÃ+²réÒ¥?ãT¯ô­=z¤öüåççOip"ãozW¿®®®ÀKå§*þÿ].úÔ_GG¿2Dàâ²påÊÀ=a,Øµk,oÜ¸ñÑãdA>_yèÐ¡	µïJ­Ù½÷øøø7Ô§rËòéÕ«WeY ¨®«^¾ÖÔÔ$ËwîÜ	¼(þL&æúÀëÎð*á^óçß3:ÕÁÑ¼WÓ¾bÐEÚd¹¢¢bddD¿yóæ©â/ðÿQnGÖ~]Õÿã;ø!D'øä©]åi>hVV,ß¿_zïÞ=ùtÑ¢E·à¿Ô¿FüøéÀÀÀg¿;~H±±1Á£¸¸Xíyz"þ5ÍõÓ¸J øäº)))ÅétjÚdGó^Mûð§^ÈØßßxSÂ_àÿ£ºµÀd¿2Dàââ³ÌÌÌ%Kø|¾Àõ¡ÎB_'ªN>hú Uvv¶:N*<?Mnm2W «È_0Á	GÒé]1ÂxNw/ýø_"ðGDñ?éwÞOý£¨ÊgAû¨dådHùÓÂÂB5åJss³àl2øS»'Õ¬+ûöíôèÑéÓ§eå¶mÛ4ñ7«LSÓ¾â÷üùy'Ö<à)))²ÀÅ3þ¤Í7û_æ¯Ö¨s7nÜ8ô8õÂ²Ý»wÏjïËå:uj2økiiQÇÅ7nÜPõTW®ü§z)á/òà¨ô>.èF¦:ªOõ*=Áîèè¨ØnÃ¡4<~ü¸¶QàyÀÕ]+Ë×ËÐÉòòåËù!Doøëëëó¿ØK­ñx<A/ÿÊÊÊò<;üùu¢Z´h|<ãUó>¿ðÂ¡G$W­ZáÇÒU¦¿Èã9h¢æiêdÆóÖ­[7¸víÚÀK!1112þÔü§¼ø#¢øÁ¤®ÿøã·mÛø¸ÐÓ;¦¿¹5¹MaÐK/½$öìÙóD8q¢¨¨(!!añâÅgÏýö·¿í?kxæWþ"Î+WÄ&É?s^`SÕIoccã%KäÇTGÒýg4Ë§/¾ø¢úv¥½½ý»Z¯^½Z\,·&?E=¿/DàÈ½ðÂJswã§2«óµÇÇÇâyø#"ÃÔkþzã7"DDq×ë=pàÀâÅÕ±]YxåW"DDDDþÀC@DDDþüø#""""ðGDDDDàÀ?""""DDDDþüø#"""DDÓèÊ+f³Ùd2MõºEEE,p»Ýþ5²,k/_>ÍÙã¦÷5¹î47²¿£ .òz½öìÉÌÌÁLKK>ùä`DþÈXeggeD0ãããS½î#GäºÇó¯yóÍ7eMmmm|ãO-Ë.Ë;vìPVUUÉ§×¯_åÖÖVY^ºt)0"Dd°­ÉütëÖ-¹nii©Í5kdMWWþÓÿ;e999Y(ñ ""ðGD_ h<røðá /ûö·¿]\tþ²üðáCY5ê¢÷îY,±ÉdZ±bÚ%zkß=òUÎ=k6KJJäRlddÄjµÊuåÎ8p@íÈ®]»VnMÖË-ß¿îð+Þ¸q?"2´ÿÔòë¯¿.ËõõõÂ,Yxã7¿æêÕ«þ×·ùÛµkúôiY²]­.ZµjUSS,Ü¹sGÖçäähÞZà|!Ý;ï¼#UUU¡w~Ï=²,_péÒ%Y8rädCCC.KÔÉÐ¦¿	Y®¬¬T]¹rÅeee·oßæÑEDà?³Ù,Ëcµæ[ZZä¢7ÊòæÍêüÞºuëÐ¡CÅÅÅê	Í[bVä«¨;zÝ¬¬,uçe² ìò²¼bÅá¬ÏçÞ°®W÷Mnx·££Cüêÿù¦<Àüqñg2üË² FÐgw¥¤¤ÈWz½^utUÖ¨N:%×Þ¾;ðn-ðÓÉÜîÌSp¼xñbzzºZ©vÎÊ¿]¿~]¸xüñ¸çOí<¢x¬V«ÿè_¯NxÜ$ñù*þ;&½î¢EÔuîÞøøxSS:×¿ËpJÃ2UüIê~ÊÃÀjêÓ§O«×ü½ùæÏÅý»ÐÞyçÿzEÉõZÀÉà/òU:táÂYØµkWèu_|ñEY~ë­·zÕ¡^YYRR"Ë·oßîïïüüü¹ÃßòåËå¢ææfYnllå^x?"2.þÆÇÇX)«/2þFGGÕ!Wùø8PfffZZÚ&¿ÈWq:ré5küç~×ëÝ·oÜóÄÄÄ6¨Å|êôáÒÒÒ?þxJÃzh8ÂPÈw´Z­rçe-Z´gÏ¹K<Àüø#""""ðGDDDDàüø#""""ðµþéþéîÝ»üJÎýö·¿åþíßþíþçûÍo~óÿùÎôõõ1:711áv»ÁûÑ~$þã1*üñÇòtÈ8èÜüÇÜ¸qqÐ¿«W¯þ÷ÿ7ã s·oßþ÷ÿwÆAç>ýôÓ[·n1:7>>þÿøàü?àü?ðþÀøàÀø#ðþÀøàÀø#ðþÀøàÀø#ðþÀøàÀø#ðþÀøàÀøàü?ðþÀøàüøàü?ðGàüøàÀø#ðþü?ðGàüøþÀø#ðþü?ðþÀø#ðþü?ðþÀøàü?àü?ðþü?àü?ðþü?àü=¡ööö+VL¦åËwuu?ðþü?ðGà/ñ·téÒ7oÊÂ¹sçòóóCñ×ÜÜ<B##¿øÅ/zzz»ÿþ~È8èlEÞÎuvvÊÎýêW¿úùÏÎ8èÜÐÐlgbúGaü¿ãÇÿ~ö³æÇ1:wýúõ¦¦&ÆAÿ[ZZ»zõªÓédtNÆqÐ9ÙÂüÃ?üCLÿñ¿êêjûrØÃ¾Äa_ûrØ8ìÏU###^¯ü?ðGàü?q¿¾¾¾ªªªþþþÐÀøàÀø#ðWøkiiY·nÝÀÀæ¥àü?ðGàüø+üÍæ?ðþü?ðGà/ñ9ðþÀø#ðþü?ðGàüøþÀø#ðþü?àüøþÀ?ðþü?àü?ðþü?ðGàü?ðþÀ?ðGàü?ðþÀ?ðGàü?ðþÀ?ðGàü?ðþÀ?ðGàü?ðþÀ?ðþü?ðþÀø#ðþü?ðGàüøþÀø#ðþü?àüøþÀ?ðþü?àü?ðþü?ðþÀøàü?àÀøàü?àÀøàü?àÀøàü?àÀøàü?àü?ðþÀøàü?ðGàü?ðþÀ?ðGàü?àÀø#ðþÀ?ðGàüøàÀø#ðþÀøàÀø#ðþÀøàü?ðþü?ðþÀø#ðþü?ðþÀø#ðþü?ðþÀø#ðþü?ðþÀø#ðþü?ðþÀø#ðþÀ?ðGàü?àÀøþÀ?ðGàü?àÀø#ðþÀ?ðGàüøàÀø#ðþÀøàÀøþÀøàüøþÀøàüøþÀøàüøþÀøàüøþÀøàüøàÀøàü?àÀøþÀ?ðGàü?àÀø#ðþÀ?ðGàüøàÀø#ðþÀøàÀøþÀøàüøþÀøàüøþÀøàüøþÀøàüøþÀøàüøàÀøàü?àÀøþÀ?ðGàü?àÀø#ðþÀ?ðGàüøàÀø#ðþÀøàÀøþÀøàüøþËåàüøþÀ_7<<_¿lÙ²¸ÝnðþÀ?ðGàüÅgâ«Õ´àwÕÖÖ?ðþü?à/ÞÆÇn·,iåÊàü?àÀøgN§³¼¼|áÂAæ5²ÞápÈ×?ðþü?à/¶óx<6Íl6îê+((°Ûí2b³KðþÀ?ðþÀøZ>¯¡¡AsW_RRÕj]?ðþü?à/:¹Ýîp»úgý?ðþü?àO×Ô¤-%%%¡æKJJªªªêìì»ïþÀø#ðþü?ÕÛÕ'ÎÅ®>ðþÀ?ðGàüé.Ü¤-AáàîêàüøþÀNÌr:Aó3ë¿«ü?ðGàüøsþ£Ùíö¼¼¼p»úfòþlàü?àÀø3Dâªp¶HE.õù|Q¿à/ÍfðþÀ?ðþb:·Ûn~fY)EqWø3PòÌZXX(ðþÀ?ðþb15?³Åb	5z+6¹t&oÅþâ6l¿"àïÂwèÎöööÆAçþå_þåÚµkþ555ýêW¿btNþØîîîftîÿù[[[côÎÿô§?Ý¹sçSO=Ê¾ìììÃþhçÝwßéGNãïÿîbxü=öôË_ÊFùÆÎôÑGï¿ÿ>ã ²QþÅ/~Á8èüÉ8èù|[÷¹­­íÕW_ýêW¿j¾¯ýë§NÔùG-lgbúÏøã°/9ìËa_â°/RgggUUæ¤-yyyv»]~øA8ìþÀ?ðGàüEºáægZ­V§Ói´Wõ?ðþü?àoÊBRó3kNÚ"ÊüÌàü?àÀøý%OÁ¶èÿVlà/>ñ.ðþÀø#ðþtKMÚn~æh½øàÀø#ðþf95?sFFæ®>CÍÏþÀø#ðþü¿iæóùGIIIù£þVlàü?àÀøi555»úÔ[±y<8vðþÀ?ðGào^àoxxøÄ¶,PMÚâõzÛÚÚãìP/øàÀø#ð7ð'Ï§b;Í]bAÿüÌ½½½Ï>ûì/ýã¯íkO?ýôóÏ?Ïa_ðþÀø#ðþ(fð788xôèÑÜÜÜpó3>Ïz½Þ/~ñåÊÿà°ü;ø7kjjÀøàüødtüÉïQee¥æ[±´åòåËRø'J~êßÞ=ÓÓÓcëÝ;ÀøþÀ¿y?¹ÁÚÚZÍWõeffFùÄoXñ'ÿ²²²<xþÀøàÀø#áO4ÓØØh±X4wõ­_¿Þápx½ÞÈ7rùòåeË±çü?ðþü?2.þz÷îÝ«ùVl²²¶¶vò¶s¾ÃkþÀøàÀø#Ãáoxx¸¡¡Áb±h¾[yyùåË§±Çîó³×üÿ³·oßÎÙ¾àü?ðGàüQ4ñ×ÙÙ¹wïÞÔÔTÍ]ÇáKôDòä+vd?ðþÀø#ðþ(jø®¯¯_½zµæüÌò/Îàüøþæþä©pëÖ­ó3ëêêâïl5ü-xR			àü?àæwïÞµÛíyyy¡Ï¿©©©&m!ð7ÍÉdàüø4ø|ÙjµjNÚ¢võÎÏLàovðõÀøàÀßüÁÛí¶Ùl¶dddÈEÝÝÝø&þvíÚþÀø#ðþh&økooW¶h¾ÂjýúõçÏgWøþäÉÄkþÀø#ðþh¶r»ÝöìIKKÓ|+6Íær¹%ðü-_¾qùÄ7àüøÏçs8%%%¶Ë¯@­þb	òÈÎÎ1ßd¡ººü?ðGàüÑ$ëìì¬©©Ñ´%//Ïn·úé§ø3þÔãRD²pçÎ	YHIIàüø95?³æ®¾ÄÄÄoë[N§ùÁ±ð.Pyt»Ý²ðÊ+¯¨¦zàÀø£EØÕWPP "ÍûôÞÛÀßÜâïÀþÓ;¸òGøàÀø£ Ô®>áæ¤-ÂAÿüÌÓxo_zàOzíµ×-Z$ííí² ,..ë»þÀøþb+yÚ7?sII0hÒðþ¿¨þÀøþb"1Ýn·«Ïf³¹ÝîpWàü?ðþÀø1c§ÓiµZ.9iKCCCäI[Àø3.þòóóÕ/LòþÀ?ðGjW_^^^è®>³Ùl³Ù<Ï$oü?#âoéÒ¥àóÇÙ¾àüøóN§³¼¼Õjê¤-àüâ<yXwttLLLèy×ÁøàÀAòx<6Íl6kNÚ2íùÁø3(þ233åÁ­³üÀøàÀ_Ôóù|»ú¬Vë§Àø3(þºººäQ¾gÏðþÀ?ð7r»ÝáægÖ´ü¿¸Â´xñâÐG?'|?ðGàüÅYÞ-))iûöí.k¿øÅß%K8áü?à/¾ðVlË-s8³²«ü¿ØÀzèÏîß:àü?àÏ	éÂÍÏ´ÿþ9úàÏ øËÊÊâðþü¿8úWó3k¾ÛúõëæbWø±¿ùM8pà@äiÊÁøþÀñâüÌzéàÏ ø[&NøàÀø¡çûp¶È­[·Ê¥^¯W?ðgDü%>Àø#ðþÛí0?³_­ûþÀAñ­ÀøàÀß´Só3[,Í39¶nÝ*´¨ßIðþ?ùkiÉ%½½½àü?àÏøE´¥  @ü¿ØÆÉd_ýï:øàüø|<8qâÄÊ+CÍZUUeÀçðþ¿ë×¯ËoNmmíÐÐ¾?ðþÀ¿É$OÛ·o×´¥´´´®®nppÐ÷ü?â³Áø#ðþÇãgÜÜÜÐg¨ÍÖÝÝmðü?â³Áø#ðþÏçkllÜºu«æ¤-ååår©q^ÕþÀ_Lâ/Z?ðþÀ¿À<ÏË/¿º«///Ïn·¥bkØÁøàü?à/8¯×ëp8,æ®>«Õêt:åé<ü?ãâolllÃÉÉÉò²eËÎüàü?çøSó3§¦¦jNÚ»úÀøüjð1×oõþÀø4?ñ'Ï/£¤¤Ds~f«Õ7Ïàüòû¶qãÆùthhhóæÍ²fÕªUàü?ào<?s¬ÉþÀ_lã/11Q~ë_N166&kd=øàÀøyB:ðNsW_UU 0.ü?â/!!A~ý|þ5>OÖ0ÕøþÀßí¼ÕjÕ¹¤¤$þvõ?ðøS×­[§ûÊGY5+V¬àüøÓCÝn×ÜÕQSS¯»úÀøüö4OøxôèøàÀøÒ®Óé,//´Eíês8sq6¡Ëåª¯¯¯««3Ú~?ðgPüöøß-[¶¤¥¥%$$ÈÇuëÖÉ¹¾ëàü?ðGq?Çc³ÙÌfs¸·bs»Ýsô­ß|óÍ§zªôë¥Ï=÷ÜÓO?½wï^ãLþÀqñÀøàb>¯¡¡!Ü®>Å"ÎéÄaò<òÌ3Ïìú«]pXþíiÿ¿øÅüä'àü?ðþü?ð7øs»Ýá&m1Ísº«/°ïÿûBO%?õïùç/--àüi]óI%$$?ðþü¿ÀÔ¤-ó3/PÖÐÐ çQWùßµ~75ß¯ùò¿þÀøÓ(!|àü?à/¨ó3ÍfÙ¤Gå­Ø8ðÜsÏâï;ßùÎ7¿ùMðþÀßzé¥Ô/ó¹sçÀøþæ9þÃMÚ¢ÞÍétFñ»wï>ýôÓÛ¶nSòû^å÷zê)ã<¹?ðgtüuuu¥¤¤¨èÎùþÀø#ð7ßð§&m	7?³XPDhù[ZZ-Zû¥Ü¼¼¼.gØÁø34þ¶mÛ¦~¥ô¹ëàü?ðGÄY CoÅ&Oö½Ó3Áøü]ºtIýVoÜ¸QÏ»þÀødüÉóeI[æÃ[±?ð7/ð7::ºjÕ*unÇõë×u¾ëàü?ðGFÀÛí0?sMM>¶?s¿ãÇ«ßí;vDå®?ðþÀEj~fÅ¢9Ûó3?zãyþÀøào~â/ò¤-ºÍÏþüé¿'e2Àøþâÿú¯ÿêp84wõ©ù£;iø#ð7çøzàü?ðGúÔÖÖögögøÊ¾¼¼<»ÝùÁ?ðþü?ÍÔüÌ«W¯6æüÌàÀøþÀÍNmmmb;ÍWõååå1iøàü?ðGà/7?sZZZeeeww7£þÀøàüøí|>_KKË¦M4çg^¹rå'd;½÷ö%ðþÀøàÀQr»Ýv»]s~æìììêêêÞÞ^õª¾©¾·/?ðøc?ðþÀøÑ|>ßåËKKK5wõÉúÐùÁøàï÷æùÓÄóü?ðGàÏhõööîÝ»WóLìììÚÚZÇ£yEðþÀøû¼ææfÙjìÜ¹sddD>7o5­­­àü?FhppÐápÛÕ·~ýz§Óù­ØÀøàïó233eÛ8ÕÓØØú#r·ÜÕÕc2®_¿þÀø#ð7Õd;YSSj¾ÜÜÜÉÏÏþÀø7ñ8_þfþ¿·ÞzKN<¹cÇðþÀ¿I6<<|ìØ±Í·bÛ´il?§4?3øàü^VVlMj£££ò©×ëÝ¸q£¬õ3¼åÌÌÌ	¥I³Ù¿úúú[tëVKKËÏ~ö3ÆAçÚÛÛßï=ÆAÿ»ººÍ:;;å¯å?ýÓ?ý?øPöå+_±ÙlN§s·|íÚ5ùkÖ9syÆAç>úè#ÙÎÄô0çøkmmÕ<áCþLá-2zúàïÒ¥KÒ§ª'BÆAçÜn÷|À8èüE~ïÞ=Æ!¨¶¶¶=ö|á_Ý'&&~ïßCÌäöoÞ¼ér¹gëééÿ1:'[¦¦¦þæÒ;w.]²bÅÎüfËÆÃ¾öå°/qØ7ôøTCCCyy¹æ«W¯>þü¬¼9ìËa_ûêQVVz)¡|=þÀøó9·Ûm³Ù4çgÎÈÈzgñÛ?ðþÀY­Ö³gÏÊ|¬¨¨àüøóù|EóLòòòÐùÁø#ð7ûøëééÉÏÏOIIQj³²².^¼8ógÖììl¹ÍöövðþÀÍgü¹ÝîÍùÍf³l'9iøþfä9ð-ÝÔòÉ'çô®?ðþÀß|Èçó9Pó%%%Y­V§Ó9¥I[Àø#ð7Ó²³³eÔÓÓãÇ_»,§¥¥?ðþüM»ÎÎÎp»úìvû¬ÉþÀ¿©ßÄãÔÂßÄÄïíþÀ¿é%¤«¯¯·«¯ªªJP¨ÿ½àü¿ÏS<«½¿±±±ª× ?ðþüM>QØNÊ>± PÏ]àü?ð¶ÍIoÞ¼	þÀø#ð÷Ätv»]ó­Ø222jjjÜnwÔï$øàüý^ýýýÅÅÅêlßäääüü|Ç3×wü?ðþb=ÙY­VÍ]e&màÀ_¬þÀø±ûnWÙl¶ÙlFØÕþÀøáoâwçyøøða~~~nn.øàÀ_à3Óé´Z­¡oÅ¦ægÖgÒðþüÍ>þÆÆÆ8Ûü?Oá²ÉÒ|+¶¼¼<»Ý>§ó3?ðGàovð» béééàü?ÏøSoÅV^^º«OÏùÁø#ð7;ø»wï^ÂãüïíÈïÒ¥Kàü?øs»Ý6-ÜüÌÑ´ü?3M¨7×GxÁøøðVl©©©mmm±²«ü?ðþøàü§oÅ¶zõêºº:ýwõ	4;&½÷.øþfcccùùù)))þ5-zýõ×Áø÷øSoÅ¦9iKjjªp°»»;*O]åååÏ>ûì7¾ñ¹'.àÀß¬áoñâÅA'üª_mm-øàâæg^½zõ3g¢øª¾þðyyyÿæàáQõééé.ü?³¿ÄÄDÙØnVºººdMZZøàâægä­ØD~»þjú÷s3< þÀø§Nøø½Ûüü?ðG±?5?³æ¤-ÒúõëÏ?o·bKMM=ôò¡@üç;ßùË¿üKðþüÍþrrrdÛ·oß>µá=|ø°¬ÉÎÎàüQ¬ãÏãñØl6Íùe+W[[«Ã[Oµ¯~õ«Û+¶â¯¸¸øØ±càüøüutthNòÜÞÞþÀø£Å_ùeÍ¦MängW_Pßµ~WØwðo®[·îK_úÒàà øþ>­©^úúúRRReÍuðþÀøº»»ÃMÚ[[[kü·bD®/¦§§Íü>?ðþÀ_ôàü¿YÌëõÖ××jîê³Z­òcn~æÁÁÁÙÚ=	þÀøàü?ð'økkkÛ¾¸I[êêêfxÀ4>àü¿ß«§§GÍó¬ÎðÍÊÊºxñ"øà?yZ=qâD¸ù«««[ZZbô­ØÀø#ð7·øknnöo1þÔòÉ'Áød@üÉ-lÝºUóðnyyyCCaÏäàÀ!ð-Í?þÚÛÛäü?2þäyT¶¶ÈJ¹(&ÎäàÀ_ôñ§6Lì<11!Ë&	ü?ðGQÇÏçkll7i¬w:ÞàÀßÊÊÊR³ú)ü<xPý%þÀø£(âÏív×ÖÖª£AåååÙívvõ?ðGào:µ´´hNò|óæMðþÀé?Ïçp84'mIJJ²Z­ìêàÀßLëïï/..Vgû&''çççëð~Gàü?ðËåª©©ÉÌÌýs´  Àn·3àüøÕÀøàOåõzÏ9³råÊPó©]l+Àø#ðþÀ¿xÀl*++SSS5wõÕ××³«ü?s¿O>ùdÉ%²ÁMNN.**àüÑá¯¿¿ÿØ±cË-5_FFFMMMgg'þÀ¿¹ÂÓéÔ<á£··ü?ðG³»ENYYæ[±°«ü?zàOM£PQQáõzåÓ;vÈðþÀÍÖS`¸ùÕ®>·ÛÍ(?ðGàO'ü©íoà¼	²ìðü?ðGÓÎçó544hÎÏ¬võ9Þü?zãOíùó¯eÏø4Ün·ÍfËÈÈ5_JJÊ~ðvõ?ðGà/jøS¯ù«¨¨óÉ§=*--å5àüÑ4Ró3O½[CCÃ+W¦÷Þ¾þÀ¿Ïfñ°oæèø/øà/êìì¬©©ÑÜÕg6m6êø©¾·/?ðGàoñð¤L&øà4®¯¯/((ÐÜÕ§ùVlàü?QÆ_´àüÅtòû+¶Ó´E½<íi^ü?ðGà/Êø7s__øàBÌv»ú&ùVlàü?QÆl²_íµ LõþÀnLNgI[&??3øàÀ_ñ'ÈmwVVÖÃåÓ·ß~[mÍçè¥~àü¿ØÊãñØl¶ó3Oõ­ØÀøþ¢¿ÏïçSòµ°yóæ¹¾ëàü?#7wó3?ðþüERkk«³^WW§Ã]àü³ÞÞÞÈ¶Ìp~fðþÀ¿èão÷îÝjË®ÞíCÚ¹s'øào^5<<,ø­2ÂüÌA¶?ðGàüÅ$þL&lÜÓÒÒÔÔÿ¿ÄÄDðþÀß|Èår½üòËávõÉ¯j¸I[Àø#ðþb²?tèPÐÊªª*Îöà/¾<qâÄêÕ«ÃMÚ:?3øþÀ_<à/Ü<ýýýàü¿¸¬»»ëÖ­ó3çååÕÕÕMrÒðþü¿Ä_´àüéÚÕ§ùª> p°¥¥e.võ?ðþü²¹<°ùSðþÀ_LoÃíê+))9vìØîêàüøàü?=zðàAmm­ÿ,þ ]555.KÿþÀø#ðþÀø³¼á°«oõêÕçÏ×sWøàÀøàüÍI.kÿþý¡ærQoooÔï$øàÀøàüÍ(¯×îL©´´ÔápÈ×äÞ?ðþü?ðþÀß4ëííÝ¿¿æ«ú-[&"ÝùÁø#ðþÀøà/Fx+¶ÊÊJ&màÀø1üEü?ðgÀ-Óé´Z­gräææÊ¯ÕÝ»wþS?ðþüE	OÊd2?ðþó"¿2f³9ÜüÌ"*Ãîêàüø3þ¢øào20§ÓY^^¾páBãÉþÀ?ðþÀøÓÌãñØl6Í]´ü?àü?ðþfÏçkhhÐÜÕ'kd½_Ó#þÀø#ðþÀáð'ÿ5¯¾úêG¬¼,Öñçv»m6[FFFè®>³Ù,y<øyðþÀ?ðþ¿Ý»w?óÌ3ßÍòòò/ùËëþßºXßÕddü©]EsÒ«Õêt:ãÌßàü?àüçÏÿÂ¾ð×ûþúðË¿C/*((¨­­³Ûí®©©ÑÜÕ'cn·Û8?3øþÀøñ¿ç~ÓÆMJ~êß÷*¿·|Ùrð7[×××hNÚbµZãþ÷ü?ðGàü?áoÛ¶mâ¿@üíú«]àoæuvvÛÕ'çÃü?ðGàü?áïõ×__¹be þJKKàoÚ©]hÍI[ÂyõàüøàÏ@øóz½_ùÊW¾öµ¯íiÿ¡ë[ßzægâæ<Sñ'ª«ªªÒ|+¶ÃgÒ?ðGàü?ðKøÿüÏÿüÙgMMM]³fMwwwûÜáoxxØn·kîêS¶¸ÝîùüàüøàIãê­Ø¬Vkè®>ÿüÌñ:i"øþÀøàoáO¶ïv»=//OsW<ÔãuÒðþü?ðþÀß<Âl_Â½´%þægàÀøàüÍGü©·b3Í¡»ú-[vâÄy2iøþÀøà/ñá­Ø*++ÛÚÚØÕþÀ?ðþÀøyüEYíêðàCþÀ?ðþÀømüEx+65?3aðþü?ð÷Ífðþbæg^½zõ3gxUøþÀøNYåÉü¿XÁ´%Ü[±íÝ»×år1tàüøàO»6¸ÝîøûÛ¿ý['9ò§CÆAçß÷]µüÞï=zôßøFBBB(ûm6ÛOúSmVjll|ÿý÷kjjÇ9ã s2æ2òCT¶31ÿcÿwÃãïÚµk¿¥ßþ¶§§§··qÐ¹þþþÖÖVÇóê«¯þÑýQ¨ùöÙýû÷ó_3ëÉ_;ÿõ_ÿÅ8èÜG$vÆAçîÜ¹ÓÙÙÉ8èlaÄÜ1ý#Ä3þ8ìËaßhåóùÎ9³fÍÐù¥òòòË/Ë×0Pöå°/qØÃ¾ö2õTàüv[[«9iKnnîÑ£GÝn7£þÀ?ðþfÄAðþ°-8þ¼Åb	ÝÕ'k¶nÝ*"a~fðþü?ðþÀ_Ì×ÝÝî­Ø²³³ív»l%ðþü?ð7ç?ð7§:eËoáÂÛ·oK?üðCü?àü?ðÛ¹êêêÔÔÔPöøwõMã½	ü?àü?ðg<xPWW§9?sRRÕjz¼?ðþü?ðþÀ_Læt:·oß®9iKIII½æ[±?ðþü?ðþÀ_mU;§ùVl555®þÀø#ðþÀø1ÏçkhhX¿~¸]c2ó3?ðþü?ðþÀ¡óx<6-;;;Ô|f³Y.ÒüÌàü?àü?ðgÄ¼^¯ÚÕ§9iKyy¹ùÁøþÀøàÏX©I[4ßÍl6ËCh&ó3?ðþü?ðþÀ!®¯¯/))	7iÓéù[±?ðþü?ðþÀ_]Eub;^¸ù5'màÀø#ðþÀ_mv¶«ªª"OÚþÀ?ðGàü¿Øøýlhh(//êüÌàüøþÀø¥Ün·Íf3ÍáægÒ¤-àüøþÀø3bj~fÅ²@+Y/Nf~fðþü?àüºÎÎÎp¶Lu~fðþü?àü±¶¨ùgeÒðþü?àüE¹ÎÎÎªª*ÍI[òòòìvûLægàÀø#ðþÀQ¶t»9ü?àÀøQþMSó3kNÚ"ÓI[Àø#ðþü?ð§ß¦Mþ+#LÚ2ó3?ðGàüøàO×Ô¤-Ñü?àÀø:åñxöîÝ«9i¬Ê¤-àüøþÀøå|>ßùóçKJJBwõ©I[ôü?àÀøsÇã±ÙlÙÙÙáæg/Åaàüøàü¿Ïóz½cÙ²eó3jÒðþü?àüM?Ëî­Ø2?3øþÀ?ðþfÚàà`]]ÝÊ+??3øþÀ?ðþ¦_[[[UUæ¤-Ë-3þ¤-àüøþÀørá^ÕZYYÙÒÒO»úÀøøàüÍSüê¶oß¤9?ó3gârWøàqàü¿ù¿=zTsW_vvöþýû].×üvðþÀ?ðþÀ_|âO~äévÓ¦M¯ê³X,ÃëõÎ·aàüøàüÅþîÞ½k·ÛóòòBÍ[[[+oÅþÀ?ðGàü¿Hy~Ýºukè«ú.ëãøLðþü?ðþÀß|ÁÛí¶Ùlf³YsÒ£GÎç]àü?Æü?ðþâ^¯·¾¾¾¤¤$ÜüÌìêàü1àü?ðóøëíí·«OMÚ288È?ðGàü?ðþbB:µ«/ôÞÌÌLá wÁø#ðþÀøñ¿ÎÎÎÔÔÔÐ]ë×¯ohh¶?ðGàüøñ¿ÁÁA»Ý^PP 9?³Ífóx<$øþÀ?ðóøkkk«®®ÎÈÈ´eÓ¦M>1àÀø#ðþbwïÞ=zô¨æüÌf³YFO¶)øþÀ?ðÛølccãÖ­[CÏäPó3Ë'¶?ðGàüø1?ËµÿþÌÌÌÐ]+W®<zôè+ðþü?à/¶óù|?ùÉOî¹PóeddÔÔÔtww3JàüøþÀ_Ì§võeggkÎÏ_?<<Ìï0øþÀ?ðÕÕÕiîêùÕàÀø#ðþâªÒ±fÍÃÁüÌàüøþÀ_ÜVYY©Þí½÷ÞöûøþÀ?ðª]Óxo_àÀø#ðþb5ðþÀ?ðGàü?àÀø#ðþÀ?ðGàüøàÀø#ðþü?ðGàüøàü?ðGàü?àü?ðþü?àü?ðþü?àü?ðþü?àü?ðþü?àü?ðþü?ðGàü?ðþÀ?ðGàü?àÀø#ðþÀ?ðGàüøàÀø#ðþü?ðGàüøàü?ðGàü?àü?ðþü?àü?ðþü?àü?ðþü?àü?ðþü?àü?ðþü?ðGàü?ðþÀ?ðGàü?àÀø#ðþÀ?ðGàüøàÀø#ðþü¦ööö+VL¦åËwuu?ðþü?ðGà/ñ·téÒ7oÊÂ¹sçòóóCñwíÚµQýå/ÙÛÛË8èÜo~óÖÖVÆAÿd£<22Â8èüîñxûõ¯ÝÑÑÁ8èlabúGaü¿¿û»¿»F×®]ã sï¿ÿþ»ï¾Ë8è_cc£ÓédtNÌýÞï1:×ÜÜ,#Ï8De;Ó÷?ð'÷TWWsØÃ¾ö%ûrØÃ¾Äaßx>ì«©¨¨ðz½àü?àüø7ü-ø]êÓ¾¾¾ªªªþþþÐ¯àü?àÀ_Ìã/°uëÖh^þÀøþÀ¿¸ÂÙl^øàÀøþâàü?àÀøþÀ?ðGàü?àÀø#ðþÀ?ðGàüøàÀø#ðþÀøàÀøþÀøàüøþÀøàüøþÀøàüøþÀøàüøþÀøàüøàÀøàü?àÀß|ÃßøÃ»t÷î|ðá2:çr¹.^¼È8èßßÿýß<ÆAçä¹ðç?ÿ9ã sMMMÎýú×¿>ölLÿÃÃÃñ¿Ãÿ|htwæOàüø#""""ðGDDDDàÀ?Ò¨§§gÕªU&©¨¨(èÍ$ZZZ/êïï_¬éêêÊÉÉQ_sýúuÆsF>pØ4ÿ/hÃþñÇ'&&nÛ¶mhhHóáÍ^aooo_±b|ñòåËeÀy´ë6òlá£2ìñºyçÕÐÉCóÊ+²póæÍÌÌÌÀäÓ÷îÉ|4Í²pñâÅªªªÀ¯©¨¨xë­·dáäÉ;vì`<çhäýÉhËPkþ_Ð]6ÖÂYèííÝ½·æÃ¼Ã¾téRù2Y8wî ~>vÝF-|T=^7ïà/6ºzõªü¸fÉ%² eYäá¨ÐL±±± ¦Ð,¼êÎ;eeej9ôÿf8ìògº9==]óáÍ^a,99G»n#Ï>øøÛ¼?£7>>¶`Áù;;pWWlye½|ìèèP¯[·N»ÅÅÅüqÐã8pfwäU[¶lñúA3vÙF÷ôôÈÂÛo¿­Ì¡oð:»?y´WWWóh×mäÙÂGñwð577gee®YµjUoo¯²È5k/êëëSÍ¨(¨Æ¹y·Ûô¿ôA3öÛ·oççç¸üã«N¡oð:»jdd¤¢¢Âëõòh×yäÙÂë?ìq¹y1SÐvÿæSyXöø @ÐCfwä9rüøqÍ«³Qa÷÷É'j>¼yÀë0ìê9¯ªªª¿¿G»Î#Ï>*Ãwðgè.]ªvA···¯[·.ð"ùCD]är¹V­Z¥¾Øãñ¨M³úb«ÕzöìYYòg:ã9G#/­]»öÖ­[Wú¿ »ÕÕ511!¿öÚkoð:KK|ÍÀÀvG-|T=^7ïàÏÐÉ£°¨¨Hþ4)++óÿ­Î-¿KÔÉêòQ?ü5Iii©:#áÆÙÙÙ			999ê&Wÿ©W^«Bÿ/hÃ.æX¼x±suuõøø¸æÃ¼Ãn6æ¹àÑ®ÏÈ³Ê°ÇëæüÍ£Àø#""""ðGDDDDàÀ?""""DDDDþüø#""""ðGDDDDàüø#""""ðGDDDDàÀ?""""D4ßûí·×®]ü¸²²²K.ýÞöëq1³µÕº·f³Y~4¯×´^ÖL¦©Þ&ø#¢ìðáÃBzíµ×â	µµµ²²®®.hý'dý+¯¼2Û$"Duuu	kL&Ó©S§ÆwúôiùTVÞºu+nðwçÎY¹|ùò õ²Þãñ?"D4/Ú¹s§°æ7ÞæoÊÊÊÊÊ@ú~]ð$.b,û¿øÑ£GÕÕÕiiirQffæ¾û®:N×½zõj§dMzzúÚµkäS«ÕtÇ#ßºHîº¨¹¹9ÔÖ­['ëÛÛÛýknÞ¼)k,Í#G²³³å¦·mÛÖ××¿ÐÛZá®ø#"r/¸Ü»w/påýû÷e¥ÙlÄMPêÒÍ7]´gÏuËåJHHÐ¼úT]ºeË	ácbbâøø¸O)))ê¥xnG4ï^èOzîÜ¹@ÑúùÖ[oùåt#eeeSÅ_»JDà(ú©#¼Û¬b¸Ù±cÇÈãdA>Ý¸q£ºTYGñQíÀ´©***Ô.CY¾qãºÀÛ<tèðNuñÅeÍ+WdY>Êò/¼ðÄÛ8Ê§ò÷Mó'P.Z´H~ÞùT>Ê=q©/ÈÉÉ+ÊðÙï'''Oî*?""CàOÒÄ½Â2Ô××'Ô§+V¬O,Y²k×.AÛèè¨ÿFäkö¥ù¯¥>½ÿ¾ÿLþ#¿òQNçoG )>|ø0ð¾ÞK/½$ýøÇ?þìw¶÷íÛøAÁ´¸¸Xí*þ"ÜU"DDÑOíî	õze¥;~ºÝnå?¿uü/Ô=Ä©À	Vd933311ÑçóÉ§§§û/âí£XP===Ê©²,eÙårù/½qãÜÍÃÇÇ_»JDà(ú©×½?~<p¥%èÿëBwhÝºu«¶¶Võ«QíóW"ÑöìÙ£öÊÇêêjÿú·£ö´õ÷÷«O>|ùÌÜ5kÖÈ¥jv1kàEêÌ_¹¨¹¹yhh(2þü0U£á¿4Â]%"ðGDýÔ	&éäÉjªS§N%&&±qãFÏçSÇdýgæª×ü©Ê¹ÝîÀ*îÛ·O¨¤ÎÃõO¶¢I4uú­úî---þõnG½ÈO½æÏëõª¯¿Ó§Oû÷ÉÉxzù£Ëåï"Âeù2Aç¶mÛ/pWü"5rPGù|ûõ8e#ÿ²ÿ©ÿ/¾øb ,»xñbüöø­8dfffàá·#w#ðùÏ´÷Ã&''«!è`÷¿Å¢Eä£í%ð6ÕIªþK#ÜU"DDFIRVVø¸µk×ªSnð×ÜÜ¬¦Ó+**jmmõ_êõz<­ÀôÒK/ù|>ÿ¥W¯^-..åääÔ××Ýfè=õ»wïZîv$¹3rÔæùóW]]4çj`` ¢¢BF --M~Çã¶&ð6D·j¬,KÐwpWüø#""""ðGDDDDàÀ?""""DDDDþüø#""""ðGDDDþüø#""""ðGDDDDàÀéÕÿ36¢sDbIEND®B`


Detrended Normal Q-Q Plots


ÏzßëØNbÎçÇSÈúøó¹ÏýyîÎ>ÎUïH$iÞTe$IàO$Ið'I$ø$IüI$	þ$I$I?I$Á$IàO$Ið'I$ø$I?I$Á$IàO$Ið'éÿ½ÌòJ¥R555«V­êîîâê­­­³yWßÃùßúÖ·Þ«GzäÈµk×ÖÜnõêÕ¾ãNâçCSSÓf083~n>:ét:n½àÝõ-%	þ¤¹¿üvìØñ¾¬|ð·páÂk×®Íü=þøãÅk÷îÝ%®mZ«Lö|Ø¿ÿtgÆÃÉdr·»yóæàO?IïÖ7nÜøÖ·¾USS3ûûûçþ¢7Î	ü$Zî¹çÆÇÇoÝºñmÌ<wîÜW5ÝU;wî9¡®YÃ_²âõë×c:ÍNq$ø4sgìÝ»·`§ËÉ'/_N§W¬XÑÓÓS§Ü$qE­Y³¦Äº¹=¶µµ59"ùðÃçßhsss²â'îêdWÇîÀ-[âë#G&×_Ó¦MÉ=ìèè¸|ùrÁ%óo2'n4n:îÀÚµkN>ÝÔÔT]]ÝÖÖ¿qÏ=õõõÉAÌ¸Ü¢ÉîðC=óóÂFõÙÕÕ5áöî*Å7^9q'§58ÅÏf°âdÏ·ÜbCÄS"ÆùÓþtfþÒÉ®m²íXú	&	þ¤JÃ_x%f644$ß;w.JåÿÜ(ñÃ8¹pusÕùK;;;ówYWú.ÝqÅâÇ~ãÆL&SWWÅcòÆoÔÖÖæ_O@iù·øvW­ZìIMääWpÉuëÖÆß%Kbþ[o½U¼±.]:áöî*7ýúõdÏß²eË¦58¥f+Æßàà`þÒxtÓÅß·àOª@ü%óeÉtGGG|ÛÛÛÓùD+X=ùö±Ç»uëV²/çënÜ¸1~êÇåÝá¤diüocõ·µòo«ÄÕ^qÂÇ~ôèÑü=aùßºukLoØ°áúíb"¾=ÞdÎ¶mÛÆÇÇ;|ÛÓÓÿ__;~üxL_¹r%Ñdw8NOö@rë¾ËU&û¿ÜÑéÎ÷jÆ+Nø|+Þîáøx^MS|ÞJ?©bñ;ÒüX~1§Äã7Þx#7çëæö¢%S©TòíòwYÅ+qµ¥Wì±'FóóëêêòÑÕ«WãÛÅOöx9áüocÙÉÌ¿'Ùl6ðæhiiÉì%ðùdR«äi¬ÙÚÚzòäÉ	m*3á½ñ%ð<%ò¯sZøâóVüI¿ÀJÌ¬­­loÐdLI¾åØT8Óýi=«l~<äx¼cccùó÷ÿãÊ·ÍC%RrºXx¤Î]ÃTVÊXMx©Îd$Ù%ÆóÝ?¦ø¼RâïÙgÍ?á#Ù6áÉ¥­3­uKïÈyë­·ò¸ÚÒ+¸Ï?ÿ||û`dfâ³T,úä»|ùòä#WN88þÝÉ§®tttìØ±ãúõë6mSYeÆøîàäñïrÏ_wÉoJx'$ø*ñÓ.ülÎÔKòÛTáøÙ<»bÅdQòKñ#·ðhuKË)QKò+åÉõü×W[zÅÒÐÙ¸qcî×ü9É¹À6l¸q»äÚ¶mÛöîñìý:wîL§NJ?ÆCîëëKöê%=ztÂí;ÝU¦¿ÒSðÜÈoº£:ñL~K/¶þèèhØnýúõÅ4Ü·o_v1à[zÀK<Á$ÁT	ø+nûöí¹Ë?ûõÿÜî&»	X·´BE	òÏÄÌ--qµ¥W,k×®å~Ù+sùòå_ÿª««ËýBá»Á_N'I/Îýd	ý<üðÃÅkåÊ%6ñ´VþJNÁs#¿éêTÆóÕW_Í¿Â5kÖä/-Ü9æÝb'$ø*ñCqÝºuÅþ«§§§¥¥% ?ÑöÙÜü£GÆÀVþ§ÜMqÝ;Ê)ùx¼¸òæææâëìjï¸biè$Dóç_¼xqÓ¦MÕ·ëèè(>½cføkë=úè£á¡¹KìßøF<ºxàK,9tèÐç>÷¹ÜYÃï~iá¯ôà<7Ö¨Nqx;ÖØØ3®09;£9¾äGkmmíïï¿ã®ÖO0Ið'Iw­l6ûðÃçï©?V)ÿ]¼ÉùÚããã	â/_î"Á$©K~ç¯ §~ÚÈHð'IªÀFFFvîÜ¹dÉäØnL<þøãE?I$Á$IàO$Ið'I$ø$IüI$	þ$I$Ið'I$ø$IüI$	þ$I$I?I$Á¤²íèÑ£étzÝºuÓ]·©©©ªªêÒ¥K¹91sV¬X17¬ÛÍì2SYwÆoÛ¶mÑ¢E18K,Ùµk×ÈÈÈ,½¿KüI×Õ××3:ãããÓ]wÏ=±î_üÅ_äæ<óÌ31ç'ëøç-½Ý+WnÝºÕÝÝ7´~ýúÀÀ@kk+üI?Iåñ~ñ.ñê«¯Æºk×®ÍÍYµjUÌ;ùþ!)qí#GîÊÃ$øô>Ê/»víZ¸pávïÞ]p±Ïîs---×ÛqÓo½õVLÇdÑÕ«W[[[kjjÒétsssooï×ë¥W9tèPCCÃêÕ«ãtóæÍÍ7ÇºqçwîÜìÈ®Y³&®-æÇ5¿ñÆS+VÄ5M¸´Ä(:u*îdÂÑ£GG¸x$Kç§$øô~ù/~ê©§búÙgfÅÄÓO?yùmÝº5<x0¦ãkLwuu%V®üøñ¸råJÌÏd2^[þ(½Jîùç-[¶ßùíÛ·Çt^=öÄÌèC1ãÆsçÎÅD²c²¸âa	/NÆ¯Ò£´oß¾ä¶Åw²à±¾*ONIð'éýÅ_x%¦³·+¶×¿xêÔ©X´aÃÞ¸qc"ÜÒW_õ±Çkiiù©TjÂk+NéU;¶páÂâuëêê;ëÖ­öÅÌ¸dL777®&Û7a%ðWzFGGé¸Éðì¥¯ÊSüIzñ/ÉøRPHkÁqÉäèjÌI8p Ö=¶@Þ©¬2Ù)æïÌKàxäÈE%sjkkSÙó·lÙ²óæÍÒ.,¼ÒÓ3pI?Iïþ2Lþ¨dçÙ-²yóæXû_]]snÝnø+½Jîç×]¼xq²nÁÝ?~üø-[òwÞ±Ç,.X,^4Q"þf6àÞü%§¸<x0ù´gyf*9räHnÚóÏ?_ óçÏ'¿8ü^%@öío;&¶nÝZ¼î#<ÓÝÝÝ.õÆÌÕ«WÇôÙ³gbbÙ²eS7nÅ.]zõêÕe²K²½½£?zN:<ö¸$Á¤÷ããã¬·Ëÿ¬¾ÒM¹Æ×0Snþ±cÇjkk>;wî"þJ¯ròäÉXºjÕªÜy'ùÙ±cGÜóêêêõë×''öù:::Ó×®]ñâÅ©LÜJ29¨Ü½wòû|S¥üé£GÆ]J>C»à±ÏlÀ%	þ$IàO$Ið'I$ø$IüI$	þ$IåÒßÿýßÿô§?Í[ü×ý×ÿøÿðÌ+·ò?yDeRl7ß|Ó8[ñ&6ËïJÿùÿyéÒ%ãPýË¿üË/~ñø+þìÏþ,ü7ËÜ2ìÜ¹sárãPnÅFMcÊå³üÎ©©tëÖ­¿ýÛ¿5eØ©S§þýßÿþàOð'ø?ÁüÁü	þð'ø?ø?Áü	þðð'ø?Áàþàþð'ø?ÁüÁàþð'ø?øüÁàþðð'øüÁàþàþ?øüÁüÁàþ?ø?øüÁàþàþàþð'ø?ø?Áàþðð'ø?Áàþàþð'øüÁßüÅßàà`&I§ÓMMM½½½Å8qâDUUü	þàOð¿J¨£££»»;&öïßßÙÙY°t||¼¹¹y2üoÎb?øÁ~ö³ÝTõãÿø'?ùq(·b£Ä¦1åV¼Å[q(·Þ~ûíÀq(Ãzfóçþjkkã<1Íf>õÔS÷îûöíûÁ,vüøñxü@eÖ÷¾÷½_~Ù8[±QbÓr+ÞÄâ­Ì8a/¾ø¢A(Ãå¯¼òÊlÞâ¼À_:p:ºzõjKKKÐÐa_9ìë°¯öuØWûVþR©Tnººº:QûéÓ§ÿë¡Âàþð'ø«a­««Ëf³ïÜ>ìÓ¿ò5øüÁàþs¾Í7:t(&âkGGÇÄÕ?Áü	þàOðWÃÚ××W__J¥2LÿÚ?Áü	þàOðçCáOð?øüÁüÁàOð?ø?øü	þàOðð?øü	þàþàOð?ø?ø?øüÁàþàþ?øüÁüÁàþ?ø?øüÁàOðð?øüÁàþàOð?øüÁü	þàOð?ø?øü	þàOðð?Áü	þàþàOð?ÁüÁü	þàOð'ø?ø?Áü	þàþàOð'ø?ÁüÁü	þàþàOðð?øü	þàþàOð?ø3ðð'ø?Áü	þàþð'ø?ÁüÁü	þð'ø?ø?Áàþðð'ø?Áàþàþð'øüÁüÁàþðð'øüÁàþàþ?øüÁüÁàþ?ø?øüÁàþàþàþð'ø?ÁüÁàþð'ø?ø?ø?Áü	þàþàOð'ø?ÁüÁü	þàOð'ø?ø?Áü	þðð'ø?ÁüÁü	þð'ø?ø?Áæ+þ~þó?õÔSùÌg>ÿùÏç;ß?ÁüÁü	þà¯bûÙÏ~öÁ~ðãÍù=¸ñÁßøßØ²eËøø8ü	þàþàOðØïÿþïÿÎïüÎî?Ùüûê£_ýÀ>Pûÿàþðð'ø·Ýwß¾þâßým÷ïÜ¹þðð'ø¿ÊÄßÎ¯îÌÇß<?ø?øüÁ_e¶aÃÖÖÖüvm×?øAðð'ø¿Êì§?ýé=÷Üó©µÚúÇ[¿ôG_úèG?tÂàþàþðW±]ºtiË-K.ýÄ'>ñäOçý?øüÁüÁàoð'ø?ø?Áü	þðð'ø?ÁüÁüÁü	þàOðð?Áü	þàþàOð?ÁüÁü	þàOð'ø?ø?Áüi¾ã/îÛ'?ùÉL&ÓÕÕ522?ø?Áü©2ñ×ÓÓók¿ökííí»¾¶ë+Û¿òÛ-¿½råÊ²ý¸lø?ø?ÁàþÞUK,éììÌýäø÷|ä¯þê¯àOðð'ø?UþFFFjkkû_åãï³ýìW¾òøüÁü	þàO¿±±±ï½·¾ÿÓ_ûÚ×àOðWiøÌd2étº©©©··7Qsss,Z±bEþð§JÅ_ô»¿û»÷ßN~_ô«øÀN<	¿JÃ_GGGwwwLìß¿¿³³3ÑÒ¥KÏ9^¶lY1þþæoþæÏbßÿþ÷òüoY?üáÏ=kÊ­Ø(±iC¹obñVV÷íôéÓ÷ÞïÊ+?ÿùÏ?¸ñÁL&óÅ/~ql«W¯;vÌó³ùå_ýõÙ¼Åy¿ÚÚÚøOLd³ÙÉ.VSSS¿ÅN8100pVeÖ+¯¼Ò××gÊ­Ø(±iC¹obñVV¶wï?øÁ¿üåÕ«W?ðÀO=õÔüãy²]^íµÀçgÖÓÓó£ýh6oq^à/NO8_¼[uuu9ì+öUöÏ9ìë°ïü:ìJ¥rÓÕÕÕÅ¸yófGGGñGÂàþð'øÕÕÕe³Ùwnöé¥×®]Û²eËÐÐPñð'ø?Áü	þæ^7o>tèPLÄ×okkpEøüÁàþs¯¾¾¾úúúT*Édúûûÿû±Uý×£khh¨Êþð'ø?Áßüþð'ø?ÁüÁü	þð'ø?ø?Áàþðð'ø?Áàþàþð'øüÁüÁàþðð'øüÁàþàþðð'ø?ø?Áü	þðð'ø?Áü	þàþð'ø?ÁüÁàþð'ø?ø?Áàþðð'øüÁàþàþð'øüÁüÁàþ?ø?øüÁàþàþ?øüÁüÁàOð?ø?øüÁàOðð?øüÁüÁüÁàþð'ø?øüÁàþðð'øüÁàþàþ?øüÁüÁàþ?ø?øüÁàOðð?øüÁüÁàOð?ø?øü	þàOw/_þîw¿ÛzddÄðÂüÁü	þàO¿±±±/|á÷Ýwß'?ùÉåËßsÏ=¯½ö?ø?øüÁ*_úÒ>üáïüêÎÝ²;þýAûÿÞ|óMðð'ø?U þ-Zô?·ýÏD~É¿ìcý×mç;þªîT*?ø?Áüináïí·ß®««Ë_üûÔ§>õçþçy¾ã/u§Òé4üÁü	þàOsÑ÷Ý·õ·æãoéÒ¥ßýîwò|Ç_e?øüÁ_A_ÿú×rG~ï¿ÿþøÃcccþîðÖ°uëVø?øüÁæþ¢=öÔÔÔ|ô£­¯¯ÿÈG>rùòe#¿Rüÿ Nû?ø?Áü©2ð¼öÚkÁ¾ññqÃ¿Ò+Ïö¨­­		?øüÁàozUWWöëëëc"Ì÷ío;&ºººàþàOð?ø«4ü%»úb"´W®ç_L,X°þàþð'ø¿JÃß¢Ez.]Ç<ðQ/ð?øü©ñ·sçÎÜéù¿ö·zõjø?øüÁàþ*ðlß'|rñâÅ1ÑßßÁ9±=àOð?øüÍ£àOð?øüÁüÁàOð¿É[¶lYò/>äþàOð?U8þ.]¾ö?øüÁàO¿p^°o`` vsnÀàþð'ø^µµµ¿¹(?øüÁàþÓnpp0ð·ûö7oÂüÁàþªpüEK,©*Ê	ð?øü©ñ×ØØèø?Áü	þ4_ð°o¾AÃàþð'ø^uuuNø?øüÁàOóñ;wî?ø?Áü	þTáø«$'|Àü	þàOð§Êüç	sÂüÁàþªÌz»Áàþð'ø^.îÐàà`&I§ÓMMM½½½¥Ílü	þàOð¿®ªªzÏö vtttwwÇÄþýû;;;K/ÙüvíÚõñ¼u[»vm«Ê¬O|â«W¯6åVlØ4Æ¡Ü7±Y~çÔTú½ßû½ìcÆ¡knnþÔ§>5·ø¾ã¯··7ð÷ÄOÄÿßý¾ÔÖÖ&WÍfJ/ÙüôÑ*I¤êÇß¶oþi"§/Ùø$Ið7óÞÛ³óÉX]]]zÑÌæä÷Øcö³Ý2ÅÍ=ôÐC[Tf=øàøhÊ­Ø(±iC¹ob³üÎ©©ôå/¹­­Í8a<ðÀ¿øÅÙ¼Å÷ïmuuuÙl69JÓ¥Íl>ä'|È	Nø>Ê¥Í7:t(&âkGGGéE3?øüÁàï]Íf×¯__SSSUUµ`ÁöööùÑ××W__J¥2LÿßËÛG¯Ílü	þàOð¿7:::á/Î?õ?øüÁàoz-_¾<¨·aÃ7o&o7n9+W®?ø?Áü	þà¯ÒðW]]ÔÏÍÉf³1§øÔZø?øüÁàþæ<þR©TP/9£6ill,æÌì£^àOð'ø?Áü5þÃ¾mmmÉaßøÓ1§¹¹þàþð'ø¿JÃ_hoÂ>®_¿ð?øüÁ_~ÔËèèhûÂS©T|mkk9sbÀàþð'øGÁàþð'ø?ø?ÁàþkÞ©T*ð?øüÁ_à/5yðð'ø?Á*õè£&ø;|ø0üÁü	þàOð¿ÁÁÁûZ[[ó?óþàþð'ø¿JÃß¦M~ÇCÛþð'ø?ÁßôzáömØ°aÎmøüÁàþSmtttåÊÉ¹½½½sqÀàþð'øRûöíKvøuvvÎÝí?øüÁàojkú?ø?Áü	þ4ðºSétþàþð'ø?Þþàþð'ø?ø?Áàþðð'ø?Áàþàþð'øüÁüÁàþð'ø?øüÁàþïþ-[V]]ísþàþð'øSåãoéÒ¥ùàó9ð?øü©ñÎöÄÓnÎmøüÁàþÓ«¶¶6ð7å?øüÁàoÚþ¶oß~óæMø?øüÁàO¿hÉ%UE9áþàOð?U þðð'ø?ÁæþöÍÑ7høüÁàþÓ«®®Î	ð?øüi¾à/RàoçÎcccðð'ø?Á*Uäø?Áü	þTò<aNø?øüÁàOùQ/s7øüÁàþðð'øüÁàoò²Ùìúõëkjjªªª,XÐÞÞ>WNþ?Áü	þàOð7½FGG'<ácNü?øüÁàoz-_¾<¨·aÃäoûÆÂÆcÎÊ+áþàOð?ø«4üUWWõÆÇÇss²ÙlÌùðð'ø?ÁüUàG½õ|¹9ccc1ÇG½Àü	þàOð§=ìÛÖÖö¯1sáþàOð?ø«4üö&<áãúõëðð'ø?ÁüUàG½¶··/0JÅ×¶¶¶3'¶ü	þàOð¿yü	þàOð¿éÕÐÐÐØØxáÂø?øüÁàO¿t:]U5W÷ Âàþð'ø^½½½¿'x"ÞæÊ_u?Áü	þàOð7Ó«¤T*ð?øüÁ_~Èóùgø?Áü	þT9økllljjÓÛþð'ø?ÁßJþ¤ï;·ûÎ#¼ð'ø?Áü	þf¿äOú&Æþàþð'øSã/ÉTÌ	ð?øü©rð700°hÑ¢dÿ_B='|Àü	þàOð§Å_®¹â<øüÁàþþ¶/ü	þàOð¿¹Öàà`&I§ÓMMM½½½ùúûûcÑ+âbð'ø?Áü	þæ|ÝÝÝ1±ÿþÎÎÎüEK.=sæLL>|xÙ²eð'ø?Áü	þæ|µµµÉÎf³]¬¦¦¦ßüæ78õôôÄæUf<y2^Æ¡ÜÆ8[ñ&oeÆ¡Ü:sæÌ±cÇCöÒK/õõõÍæ-¾ïø»xñâ]Ç_þ'200ÐÕÕU¿x©Ïb¯¼òÊ+WUfÅÓãüùóÆ¡ÜÆ8[ñ&oeÆ¡Üú·û·Yþ¦)ÿ½zõêlÞâû¿äs^_ýõ»¿üÏ¬®®.¾ÀÍ7;::ÿÃ¾rØ×a_9ìë°¯ö^Á¾Ü;§Óéµk×^¾|y6îú/éºººl6öéK^»vmË-CCCÅW?øüÁào&]¼xqùòå9UWW¯_¿~ÖÆæÍ:ñµ£££`ÄÛÚÚ&Û?øüÁàï]=ó?^SS3ËÞ­¯¯¯¾¾>n1Éô÷÷ÿ÷c»½S°¡¡!ÿÎÁàþð'øo~væïù[¸pañÙeü	þàOð¿éo¾ÎÎÎ«W¯Îí?øüÁàoWqû<OúÓW®sÛþð'ø?Áßôºð?øü	þào¶ñ7§?Áü	þàOð7íÎ?¿lÙ²$gøÖÕÕ9rþàþð'øSâïÄ¹>ü%Óû÷ï?ø?Áü	þà¯ÒðW__Ô;þ|ýýýÉ§½ÀüÁàþðWgû&Ã_<ÿSáþàOð?ø«4üÕÕÕõ½¿l6»k×®nhh?ø?Áü	þà¯Òð©j¢Î9ð?øüÁ_í;44ÔÒÒí[SS³lÙ²Ë/Ïí?øüÁào?øüÁàþàþ?øüMÔÕ«W.º]L455Íc¾ð'ø?Áü	þ¦Ñ#<R5I[·n?ø?Áü	þT9ø;räHâ¼$3GGG=Ìïéé?ø?Áü	þà¯BðÉdBx,^üÁ7óð'ø?Á*ÕÕÕ!¼ÑÑÑâEÙl6ÅàþàOð?ø«üåþªÛdK?õð?øüÁ_à¯ïàþàOð?ÁüÁü	þàOðs¥?ø?Áü	þT9øKÝ©t:ð?øüÁ?ïð?øüÁüÁàOð?ø?øüÁàOðð?øü	þàþàOð?øüÁü	þàOð?ø?Áü	þàOðð?Áü	þàþàOð'ø?ÁüÁü	þàOð'ø?ø?Áü	þðð'ø?ÁüÁü	þð'ø?ø?ÁüÁü	þàþàOð?ÁüÁü	þàOð?ø?Áü	þàOðð'ø?Áü	þàþàOð'ø?ÁüÁü	þð'ø?ø?Áü	þðð'ø?Áàþàþð'ø?ø?Áàþðð'øüÁàþàþð'øüÁüÁàþððð'ø?Áü	þàþð'ø?ÁüÁüøüÁàþàþ?øüÁüÁàþ?ø?øüÁàOðð?øüÁüÁàOð?ø?øü	þàOððWªÁÁÁL&N§z/pâÄªª*øüÁàþPGGGwwwLìß¿¿³³³`éøøxss3ü	þàOð¿Á_mmm<éc"Í644,ê©§öîÝ;þyæYìØ±c/½ôRÊ¬xÇ<~ü¸q(·b£Ä¦1åV¼Å[q(Ã^|ñEPÍþþy¿t:=áttõêÕ ádøÿYìôéÓÃÃÃÿGeÖ?üÃ?|Ù8[±QbÓr+ÞÄâ­Ì8[¿øÅ/â?KÆ¡åWnÜ¸1·8/ðJ¥rÓÕÕÕùÚÛÛãMê¿ªÃ¾rØ×a_9ìë°¯ö»CYõËbº®®.Í&czÂå.?øüÁàon·yóæCÅD|íèèÅ3áOð?øüÍ½úúúêëëS©T&éïïPð'ø?Áü	þ|È3ü	þàOð?ø?øü	þàOðð?Áü	þàþàOð?ÁüÁü	þàOððð?øüÁüÁàOð?ø?øüÁàOðð?øü	þàþàOð?øüÁü	þàOð?ø?Áü	þàOðð?Áü	þàþàOð'ø?ÁüÁü	þàOð'ø?ø?Áü	þðð'ø?ÁüÁü	þð'ø?ø?ÁüÁü	þàþàOð?ÁüÁü	þàOðÆþàþð'ø?ÁüÁàþð'ø?ø?Áàþðð'øüÁàþàþð'øüÁüÁàþ?ø?øüÁàþàþ?øüÁüÁàOð?ø?øüÁàOðð?øüÁüÁüÁàþð'ø?øüÁàþððð'ø?ÁüÁü	þð'ø?ø?Áü	þðð'ø?Áàþàþð'ø?ø?Áàþðð7~þó<y²§§çÍ7ßôê?øüÁq?ø«düóßhÑo·üöÇ?þñîîn/0ø?Áü	þà¯2ñ×ÓÓóë¿þë[ÿxëî?Ùÿºþ¿®ÚÚÚýèG^cð?øüÁ_âï3ùÌ´ÿA"¿äßým÷wuuyÁü	þàOð¿ßú­ßúÿÿÿÈÇß6¡µµÕkþàOð?ø«@ü=m|0!?þàþð'ø¿ÊÄßÉ'kkk¿üðùñ¡/Þï½¯½ö×üÁàþðWø;vÏ=÷466þæoþfLôôôxÁü	þàOð¿h||üÂíÆÆÆ¼ºàþðgàþ*?øü	þàþàOð?øüÁü	þàOð?ø?Áü	þàOðð?Áü	þàþàOðð?ø?øüÁàOðð?øüÁüÁüÁàþðð'øüÁàþàþð'øüÁüýjL&N755õööæ/Û²eKuuõ>ô¡øüÁàþs¾îîîØ¿gggþ¢=ö<ýôÓñ¡oll,Æß÷¿ÿýÑYìïþîïFUf½öÚkÿüÏÿlÊ­Ø(±iC¹obñVfÊ­Àq(Ãzgóçþjkkw1Ífò555]¸pa²_ÿú×¿?Å+óå_þ¾Ê¬^zéßûq(·b£Ä¦1åV¼Å[q(Ã^|ñEPÍþþy¿t:=átòí¾ûjjjÏ=ë°¯öuØWû:ì+ç|©T*7]]]]°èàÁ1qñâÅøüÁàþs²ª_ÓuuuÙlöÛc:ÿbùßì?Áü	þàOð7'Û¼yó¡Cb"¾vttä/Ú¶mÛóÏ?çÏ_½z5ü	þàOð¿9____*Êd2ýýýÿýØnï7©öööt:ÝÒÒréÒ%øüÁàþó7øüÁàþðð'øüÁàþàþ?øüÁüÁàþ?ø?øüÁàOðð?øüÁüÁàOð?ø?øüÁüÁàþàþð'øüÁüÁàþð'ø?øüÁàþð?øüÁào¾õ§ú§ßùÎw~:ÅÍýã?þãOUf8qâÌ3Æ¡ÜÆ8[ñ&6ËïJW®Ë¿üKãP=ÿüóÿôOÿ4·øöÛoÃß¤?~÷îÝ&IT)Þ7_eg¬$IÒü	þ$IàO$Ið'I$ø$IüI$	þ$IÕððpCCCñüS§N-_¾<N755õõõÅÁÁÁL&Ìéíí5te²]ªò2twkÓx±¥¥¥ººzÓ¦MÉÆñ)Ïíâ%3kõ÷÷777ÇK`ÅñrÈ_Tüêðz)Ïír×_/^¢ï¡Ä³¶¶öêÕ«1_·ÔîîîØ¿gg§Ñ+íräÈ-[¶´»¾iV®o©1qáÂmÛ¶yÉívñµ.]zæÌ8|øð²eËò¿:¼^Ês»Üõ×ü½÷­_¿þÒ¥K¾c666^»v-&âkL'ì¸uëVLd³Ù	ÿ­»²]âeyôèQv×7Mü_97½hÑ"/²Ý.^2w¥ÿÇ¼:¼^Ês»Üõ×ü½o#;Ñ;æàà`<'bQ|(xÍÖÝÝ.ñ¸¶¶¶Ø"---/^4nwkÓ¬X±âüùó1ñÜsÏ%//òÜ.^2³_¼YuuuMr¯rÞ.wýõ³ú¹råÊ.$ÚXµjUL¤R©ÜÒêêjãV&Û%×µk×âçq»[æìÙ³Ë-ïÝ»7ùÏ´Lyn/YîæÍ###ù3_^/å¹]îúëþfõ³ÿuuuÙlöÛcÚ¸ÉvÉÏ;æ]Ü4¹^ýõåËÉívñÍB[¶l*_üêðz)Ïír×_/ð7«ï«V­J;wnåÊ1±yóæCÅD|ÿ.·2Ù.K.½|ùròbnkk3nwkÓÄ¼uëÖÞ½|òI/²Ý.^2³Ö©S§b¿:¼^Ês»Üõ×üÍÒ;fòmü/9lN§ãkL¿sû|ºúúúT*Éd³çTÛe`` ùðµk×&çè®lx?]²dIüÏ¸««k||ÜK¦l·Ì¬ÕÐÐPü)!ÉDñ«Ãë¥<·Ë]½À$IÒ|úO!$I?I$Á$IàO$Ið'I$ø$IüI$	þ$I$I?I$Á$IàO$	þ$I$I?I$Á$IàO$Ið'I$ø¤;öÜsÏ­Y³¦ævëÖ­á~å=îvsæy¢ÛÐÐmdd¤`~ÌI§ÓLæÖ­[Ó½NIð'Is²Ý»wWõäOVþxâù­o«`þ7¾ñÿøãÏà:%Á$Í½5étúÀã·;xð`|3_õÕÁß+Wbæ+æ/_¾<æ_¾|þ$Á¤yÑC=¬yúé§óg>óÌ31óþèòéÓÛÛx677ÇtîÂ×¯_ïêêZ¸pa,ª­­Ý±cGþÑÕ'O°bQ¬ÛÓÓSÀ©³hÑ¢5kÖ?~<¾Ý¼ysÁ;vìXéëIÅ]J8qb2¨µµµÅüþþþÜ3gÎÄÖÖÖÜ=öÔ××ÇUÕÔÔlÚ´éÚµkÅø+¾þ9%îª$ø¤»Ü%K.W¯^ÍùÆoÄÌ|Ü400,Ý¸qcÁ¢íÛ·'Î;J¥&ù6YÚÞÞ~ëÖ­àcuuõøøx,¯§$¿WâzbbÂ»WüH>/Ú/»»»sò+¸uëÖM%îª$ø¤»_rw÷µªª X>n:;;oÞ.&âÛ6$Kë$|LvàÚEÉ.ÃîëëK®$ÿ:ì±à]rÔõG9GéøÓ?üð¯'àßÆòïÛ(@¹xñâx¼ÃÃÃñm|âÌf³É2L¬á_&®©©.þJÜUIð'Ie¿hBüåæ'¸IÌ]»v-¾6%ß677Ç·[·n´æ®$.S°/-·Vòío¼»p)wä7¾ÆôÉ'ïx=Íøö­·ÞÊ¿oý~Þ£>öîÝûÎ/mïØ±#ÿÁÀ_´¥¥%Ù19]ü¸«àOî~Éî®7oæÏ±¨wr4¼téRâ¿ur¿¨W|4¶Sù°ÓµµµÕÕÕcccqå-Ê-½ãõLF±Î?85¦ãkL;w.·´¯¯/îÀ§¿wUüIÒÝ/ù½·ûöåÏL>¥àÜïïÐzõÕWxâä lNÉn¹ÜqÕ;mûöíÉÑÞøÚÕÕ_âz=mCCCÉ·o½õVé3sW­ZKO·	³æ/JÎüE'N¸qãFiüå`Fni»*	þ$éî0N§÷ïß|ÔËª««OÎØ°aC@gll,9&;37ù¿äWå.]ºÿËwìØTJÎÃÍØÊDKN¿MnýÔ©S¹ù%®'ù%¿äwþFFFKÀßÁsûäâ!ç/J~ýñÜ¹sq+1á/áfp9.èÜ´iSþÒwUüIRY|rAöìùïq·KlÎ0ÍcëGÉe~G)¿wnÿ)_[[D¸ÄõÄÝÈ¿c¹3m'°£££555ÉC(8Ø½~ýúüX¼xq|M>í%ÿ:¹ªæ¸«àOÊ¥Êºuëªo·fÍäÛü8q"ù8½¦¦¦Ó§OçìÚµ«¾¾>Ó£>:66[ÚÓÓÓÒÒ&Ëd2Ï>ûlÁuß¸ª¿mÛ¶ù]Ow&îRò%>ç/WWWWÁg¾$wttÄ,0ÅåËs[7nÜÝ&cÕÚÚÚßß_p%îª$ø$IüI$	þ$I$I?I$Á$IàO$Ið'I$ø$I?I$Á$IàO$Ið'I$ø$IüI$	þ$I$Iz/ú¿~ø«Þ©HsIEND®B`


§:KÎCß¹s':ò·hÑ¢)mÂz3¿ÞÞÞì©áÙM5þ&¹ßâJ0þ¢ñ!Ë¢áT*nvvvá®®®ìDË=º¹sçÎÑÑÑèXÎç]»vmx×÷7N¦÷àp3Ì~wL+û±,¶ðã>÷'Of	Ë¾ÿæÍÃð5kî	áf9ÑóÆ<óÌ3÷îÝV,Zr¸ÙÞÞý£¯>:_¿~=ÒD+Ç'z"ye¢ÏüeNuã»VÓqÜý-ÿuö«©Æß$÷[@üAÉÆ_æL_ô±laL7ã7ofÆ<pÞÌQ´èôb,nÎ7/ûUÎY¼-<ãDÏ=jÄð63¾ªª*ûÝ¸q#Ü`ÁDÏ7ú#ûft.;zÙkN§CüæhjjÊ~îâ/Säz³doÒ0oØ«V­:sæÌ¸m2gÜµöâ/Ú%úûû³9¥øä~?(Áø±FVVVNt4h¢LnÊ)p$©@âLõÝzh|xÊáùÖÖÖdÏ?rÿ¸üç;!4sNxîêêêè<u~áå$uf	e2ÛjÜ;LfãL¤Ó±Àö,~wä~?(Áø;tèPöÑ±´q/®,Ü:S·ðÛ·ogO-°ØÂ3XãÇ/FFFs*''¿A²o.^¼8úÊg¿èðdô­+©TjëÖ­wîÜ9|øp¹nÝºqão2³L;þ¦ºq2¦=cGþ2yýó¦À/°âJ*þÂ»]h è½9óU/Ñ§©B7÷ÎèâÙhRô¡ø¡1ã¾·p9EÕ+,<ZNÎ¼Æ]láÎÚµk3óÆD×¯Y³fpL´´gy¦øø~õõõ§pðàÁÉÄßÙ³g£óá)wuuEGõ"'O÷õê,S¿Â'gßÈ6Õ­:íJ/¼úÃÃÃ¡íV¯^û÷ï[;bßÂ¼À?(øË·eËÌò¯Í|ü?ó¥»ÑaÂü7Ñó.§PEQe_Z`±g,:·nÝÊ|Ø+síÚµUUUe>PXLüeê$²`ÁÌ' ÔÏúõëó_¬%Kx§4Ëâ¯ðÆÉÙ7²Mu«Nf^¸p!Ë/Ï³2×Oôv0@üAIÅ_xSreþÿjooojj!ÞÑ:òäÉ0&ÄVö·ÜMrÞSôõxaáù_×7Ñb8cáÐNf¿råÊºuëcR©TþåÓ¿°´°ÌAÛ¶m=iîÂöâ/gøÂ9ò©O*sÕpñ³L)þo#Ç¶ê$7ï©S§jkkÃÓÎ¤g®h77nÜ=ÜªU«º»»x¨µÀ?÷M:^¿~öÚ÷b/:Ä]¯ïÞ½(â/^lñ@	>óãùç·e@üP¶oß¾páÂèÜnxî¹çl?Äâñø@ü þÄâñø@ü þÀìròäÉx<¾råÊ©Î[___VVöæofÆá0¦¡¡a¿ÅÆLï>w¿[óDã¶lÙRYY¶XEEE*ºzõjf®÷îñáÎD"Û»ñÌ8ÕÕÕ!V¦+öì	ó~å+_ÉyáÂÝ»wFüåß°aCßÙÙÏ;ëêê2SO:ÅÓ§OÛ»ñÌ¼_"EôÓÂ¼+V¬ÈYºtiÓÛÛûpVòáÇ_"ãÇë©§S£7n´wâå:;vì¨¨¨7oÞ®]»rîö©Oª©©)g	aøöíÛa8&Ý¸qcÕªUåååñx¼±±1:Z¿´ìG/<Ë#Gjjj-[(?ÑîÞ½ÛÖÖæ+¿ûöè@fÈÓåË¥ñaÉ7oÞ,2þ>üáñ/îêêÊ4:::þüðXÃÃÃáç2<½uÀÕÑðÞ½Ãð¡CBfç>û>íííQäeÛ¼ystøðá0~áM6E,Yú¼~ýzL&Ç]Zö%$ÝñãÇÃÀòW~Ë-a8ÜáW^	öìÉ´Úàà`___LNôy¾²8û>'OÌY¹råÅ3³Dg×¬Y×­[Ãq×Á^?`FÄ_MMMNÉo¯q?xöìÙLñ¬]»6ªºÌÔ.ìÜ¹³©©)Åbã.-'¿Ï­XEEEþ¼UUUÑÊ¸gnll9;222½Í£§§'Dj&Ã£ñ[·nÍÜ"uÛ¶mÅ¬øÞÛø®TÍ7XB¡´æÍî944Ùc¢Is"¼xñbörs2³L´b¡³ÔEáxâÄùóçGc*++£?òW`»uvvË>ÍÒ9<è;wîÃ.wìuøfDü%Éì#ÑÁ³P[[[ù]1:fñWxÌÊwÁÑ¼9«wïÞ½Ó§OGWéfNi³L$Z°Îa8S«9.0íuÀÑW·>|8úÌß/¼0:qâD¦u?¥ä¥K¢ÏN&þÏ²sçÎ_~9lÞ¼9Þ7á£G^¾|9:ÍF.[¶,2ëïï-*2þÂøû?ûbõë×ßø[oÂøi¯øÞÛø»wï^¬yc²¿«¯püG§Ïìï@	mTYYYQQ±ûöIÆ_áYÎ9¦.]º4sÝIÎ×/oÝº5¬y"X½zutQmè­T*]>¼bÅ+W®Li³ämkkkì¶lÙ÷þÏ¾ï:ùï»ö:?ñø@ü þ¿äÿñô£Ùf·ß~Ûv Ho¾ùfþ76Ãö÷÷Ûé­·Þúÿùñ7S|éK_ýg¿Qþýßÿ=¼mÛé[ßúÖ¸M&ïG?úÑ¿þë¿ÚéìÙ³ÿõ_ÿ%þÄâñøCü?ñøCü!þâOü??ÄøâÄâñ'þÄøñøCü?ñ'þ þâOü?Ä?ÄøCü!þâñ'þ?Ä?ñøCü!þ@ü?ÄâñøâñøCü!þÄøCü!þ?ñ'þÄ?ÄâOü?ñâñø»ñ×ÛÛL&ãñxgggþ:::ÊÊÊÄøCüøCüT*uôèÑ0pàÀÖÖÖ©á ±±q¢ø]x¤¯¯ïßþíßlâoppÐv á¢ÿüÏÿl;P¤ÎÎÎþþþùs"þ*++GGGÃ@:®©©ÉºwïÞûöMû÷ïÿ.3IÇÛ"óßÿÚ¶(Æk¯½ÖÞÞn;P¤W_õõ×_8'â/;Ü¸q£©©)¤¡Ó¾Nûâ´/8íÓ¾%±X,3H$²'µ´´;wî'OUü?Ä?Ä_ilÖªªªt:ì´oþ¹gøóÄøCüøCüÍzmmmG	ág*ÿ©:ò'þ þ¥±Y»ººª««c±X2ìîî·öÄøCüøCüùgñ'þ þâñøCü?ÄøCü!þ þÄâñøñ'þ?ÄâOü?ÄâñøâñøCü!þÄø þ?ñ'þÄ?ÄâOü?ñâñøâOü!þ@ü!þÄø??ñøCüøCü?ÄâñâOü!þ?âñøCüø?Äâñ'þÄâñøCü?ñ'þ@ü!þâOü??ÄøâÄâñ'þÄøCüøCü?ñ'þ þâñøñø?ÄâÄøCü!þ þÄâñøñ'þ?ÄâOü?ÄâñøâOüÙ?ÄâOü?ñâñøâOüøCü!þÄø?ÛñøâOü!þ@ü!þÄâñâñ'þ?Ä?ñøCü!þ@ü?ÄâñâOü!þ?Äø?Äâñ'þÄøÌùø¿~úéÇ|ëÖ­þw%iddäòåËa÷ÿHâñø+äèÑ£>úhó'?ÛöÙ+VÌ?¿½½Ý*þJÉ¡CÂ½hÑ¢_ù_©®®~ã7ÄøâOü1Gãïí·ß.//ÿüSßõg»¢ÿ>útEEÅÈÈ×Tü¿ÿû¿ÿÀ>°é¢=¼¥¥åü`ØóÅøâæbü9s¦±±1S~ÑúÐ._¾ì5¥¡á£mûlöþëM¿þ¯|Eü?ñ'þ`Æ_x#Ì¿ÇLü¿ñÈ#äìáO>ùä¾ðñ'þÄø¹ÑißÍ¼9ó¾øù§>ÿ¿ðNû¿QWW·qãÆìøûíå¿½wï^ñ'þÄø¹Á_ýÕ_-X°à÷[~ÿé/>ýäO>úè£/¿ü²Tü=ö|èCúÓmßmø£ùóç÷õõ?ñ'þÄÌÑøþöoÿöøÄc=öÄO·¯¦ø+1Ï>ûløWÍoþæo~ô£äG^íµÒ~¾âOü!þ¿¹îÇ?þñ©S§ÚÛÛKû:_ñ'þ?Äsø?Äâñ'þÄâñøCü?ñ'þ@ü!þâOü??ÄøâÄâñ'þÄøCüøCü?ñ'þ þâñøñø?ÄâÄøCü!þ þºÞÞÞd2Çëëë;;;³'uww766IánâOü!þ@ü!þf½T*uôèÑ0pàÀÖÖÖìIuuuçÏÇ[´hQ~üýÝßýÝÿe&ùþ÷¿2Ýv H¯¾úêüÇØã_þå_Â;í@^íµ«W¯>ÌGñWYY9::ÒétMMÍDw+//Ï¿Ã_d&ù1¶Eñ÷ÆoØ£««ëõ×_·(Rû÷¿ÿýùs"þâñø¸ÃÙzzz6mÚä´¯Ó¾8íNûâ´ï¬Å2ÃD"ÿwïÞM¥RCCCâOü!þ@ü!þf½ªªªt:ì´oÎzëÖ­6ô÷÷çÏ(þÄâÄâoöikk;räH?S©TÎonnwFñ'þ þ³OWWWuuu,K&ÝÝÝ?ne?yv555eYÄøCüøCüÍ]âOü!þ@ü!þÄâñâñ'þ?Ä?ñøCü!þ@ü?ÄâñâOü!þ?Äø?Äâñ'þÄøñøCü?ñ'þ@ü!þâOü??Äøâñâñ'þÄøCüøCü?ÄâÄâOü!þ?âñøCüø?ÄâÄøCü!þ?ñ'þ?ÄâOü?ñâñøâOüøCü!þÄø þ?ñ'þÄâÄâOü?ñøñø?Ä?ÄøCü!þ þÄâñøñ'þ?Ä?ñøCü!þâOü!þ?ÄøâOü!þ?ñ'þÄ?ÄâOü?ñâñøâOü!þÄâñ÷.Æ_ÙÄb1ñøCü!þ%±ÇãâñøCü!þö?ÄâñWZñ788¸yófñøCü!þ%555ñxÜgþ?Ä~ü544ä_íQYY944$þ?Äâ¯Ôâ/HÚ¨®®¡ù^~ùå0°iÓ&ñøCü!þ¥Ñ¡¾0j/~tt4Ì7Oü!þ?Ä_©ÅßüùóCêuuu7ì0ðÜsÏE¾êñøCüA	ÆßöíÛ3wdìoÙ²eâñøCü!þJðjß/ùË,ÝÝÝa `SSÓ¬x=ÄøCüøCüÍ!âOü!þ@ü!þÄâñâñ7±EE_øâK?ÄxüÕÕÕe_«?Ä`üÎÙ×ÓÓ3:::ë^ñ'þ þSSYYâo6ø?¿)ëííñ·eË»wï?Äâñ%ÁÂËò¸àñøCüA	Æ_mm­>?ÄÌø²¯¯¯o6¾âOü!þ@ü!þ¦¦ªªÊ?ÄâæJü§âoûöí###âñøCüAÇ_ÙøCü!þ 4¿äyø@ü!þP_õ2?ñøñøÚÚÚË/¿++ÔÛÛL&ãñxgggáIÓ#þÄâÄâoúBW½kGS©ÔÑ£GÃÀZ[[OÞl;vìøµ_ûµUÌ$KÇØ©¡¡áãÿ¸í@1-[ö¿ñ¶EjllüØÇ>ö0ñ=¿ÎÎÎ»wï,þ_*++£¤ÓéÂ¦7&Û¶mÛÊJÈïîÕ¾Ùä?izcÄ þ¦ïÝ½Ú7;DáIÓmçÎüä'70¬c;P¤ßùßY¿~½í@1>ýéOÿÞïýí@xâ?ø?xøÇß»«ªª*NGgiÃpáIÓã|àpÁ.ø)ÚÚÚ9ÂÏT*UxÒôÆ?ñøñø+J:^½zuyyyYYÙ¼yóZZZ¦åGWWWuuu,K&ÝÝÝ?]Ë±³×ù¦7Fü?ÄÓ322ò'ò'.|äGÎ9#þ`.Æßððð¸6êWü?Ä÷»¿û»=öØæ?Þ¼ëÏv:õéGôë_ÿºø9/©·fÍ»wïk×®c,Y"þ¿Rzûà?¸ãÙ¡ü¢ÿ>óÏTVV?sñH$BêeÿM§ÓaLþ¥µâñø½^|ñÅU«VeÊ/úï~éÞ~ûmñs+þb±XH½èÚÈÈÈH3½¯z?ÄßÌôõ¯Ù²eÙå·ãÙóçÏò¿w3þ¢Ó¾ÍÍÍÑißð31âñø+?þñCêýáú?ÌÄßÇV|låÊ%üÅâo|¡öÆ½àãÎ;âñø+%ßøÆ7yäÐ|O>ùdÃG~õW5¡ø9÷Ç.ømii©¨¨Åbágsss3+^ñ'þLÉo¼ññO?ýô¡CÞyçÒ~²âñWÄøCüøCü?ÄâÄâ/gÎÅbâñøCü!þJ$þb?ÄâJ-þ&²mÛ¶(þ;&þ?Äâ¯dã¯··wÞ¼y!ûV­ZýÏâñøCü!þJ-þÖ­[ð;úô,z=ÄøCüøCüMÍ+¯¼eß5kfÝë!þÄâÄâo²,Y]ÛÑÙÙ9_ñ'þ þ²ÿþè_kkëì=ÄøCüøCüMnNßóøCü!þ`îÄ_ìAâñ¸øCü!þ?ÞMü!þ?Äø?Äâñ'þÄøñøCü?ñ'þ@ü!þâOü??Äøâñâñ÷®Åß¢EïùCü!þPúñWWW|¾çñøCüA)Ç_è¼===£££³îõâñâñ75!þfcù?ñøñø²ÞÞÞ[¶l¹÷®øCü!þPâñ,°,>?Ä`üÕÖÖºàñøCüÁ¿(ûúúúfãë!þÄâÄâojªªªøCü!þ`®Ä_xJ!þ¶oß>22"þ?ÄxüMÀ?ÄâJóKÇåÄâñ¥ùU/³ø??ñøCüøCüM,N¯^½º¼¼¼¬¬lÞ¼y---³åâ_ñ'þ þS3<<<î³ââ_ñ'þ þS³xñâzkÖ¬þ¶ïàààÚµkÃ%K?Äâñø+µøK$!õ²É¦Óé0&?ÄâñW_õR/_fÌÈÈHã«^?Äìißæææè´oøÃÆÆFñøCü!þ¥¡öÆ½àãÎ;âñøCü!þJð«^[ZZ***b±XøÙÜÜÆÌ×Cü?Ä?Äß"þÄâÄâojjjjjkk/_¾,þ?Ä~üÅãñ²²ÙzQü?Ä?ÄßÔtvvøÛ½÷àààlù«nâOü!þ@ü!þ¦»	Äb1ñøCü!þ%ø%Ïãò%Ï?ÄâJ'þjkkëëëfõë!þÄâÄâoR¢?éì´ï¬8Ã+þÄâÄâoúñýIßèÏø?Äâñ%Éd²¬ | þ?(øëéé?~tü/J=| þ?(ÙøË-'þÄâÄâÏßöâñâñ7Ûôöö&Éx<^__ßÙÙ=©»»»±±1Ljhhwâñâñ7ë¥R©£G´¶¶fOª««;þ|8vìØ¢EÄøCüøCüÍzÑN§Ó555Ý­¼¼<?þ¾úÕ¯~¤sí@N:þág;PðæÌÛ"vuu=ÌG|ÏãïÊ+ïüe_q2ÑÕ'===6mÊ¿ð1ÀLòÆoxÑv H§OþÏÿüOÛbüà?ø§ú'Û"BÜ¸qãa>âÑ÷¼466^½zõý¿ìïL$ùw¸÷n*ÊÿctNû:íÓ¾à´/NûNMÈ¾Ì;Çãñ+Vvía¬úÏáªªªt:öÃ9÷¼uëÖúûûó"þÄâÄâo:®²xñâL%Õ«W?´§ÑÖÖväÈ0~¦R©-ÞÜÜ<Ñ!Pñ'þ þÓ7::zúôéòòòüçÝºººª««Ã#&Éîîî>·±555ÙtNü?Ä?Äß» ¯¯/ûÈ_EEEþÕ3ø?¿©Én¾òòòÖÖÖ7nÌ×Cü?Ä?Äß1vÇã?~ýúõY÷z?ñøñø÷ñ^ÄøñøCü=ìøÕÄøCüøCüMÙ¥K-Z4oÞ¼èßªªª'N?Äâñ%>¢ø8 þ?Äâ¯Ôâ¯ºº:¤Þ¥K2ñ×ÝÝÛøCü!þ¿¼Ú7úòäLüF?Äâñø+µø«ªª©íñN§wìØkjjÄâñøCüZü§T6óçÏ?Äâñø+Á«ûûû¢«ËËË-ZtíÚµYñz?ñøñøCÄøCüøCü?ÄâÄâo<7nÜhll¬¨¨	õõõ³å¯ø?¿)Ø¸qcÙ6oÞ,þ?ÄNü8q"ê¼E#O<ooo?ÄâñW"ñL&Cá>|8Rôß|ÏâñøÒ¿D"oxx8R:ÂÄâñøCüHüeþªÛDS£?õ&þ?Äâ¯Dâ¯@Þ?ÄâñâOü!þ?Äß¬¿ÂÄâñøÒ¿ØÄãqñøCü!þþ¼øCü!þ?ñ'þ?ÄâOü?ñâñøâOüøCü!þÄø þ?ñ'þÄâÄâOü?ñøñø?Ä?ÄøCü!þ þÄâñøñ'þ?Ä?ñøCü!þâOü!þ?ÄøâÄâñ'þÄøñøCü?ñ'þ@ü!þâOü?Ä?Äøâñâñ'þ??ñøCü!þ@ü?ÄâñâOü!þ?âñøCü!þÄøCü!þ?ñ'þÄ?ÄâOü?ñâñøâOüøCü!þÄø??ñ'þÄâÄâOü!þ þâñøCüø?ÄâÄøCü!þ þÄâñøCü?ñøCü!þâOü?ñøCü!þÄ_!½½½Éd2×××wvvæß¡£££¬¬Lü?Ä?Ä_)H¥RGhmmÍÞÅøCüøCüHüUVVt:]SS3uïÞ½ûöí(þ^xávfWÇØéßü¦@N>í×Å;uêTøçèÃ|Ä9ñx|ÜáàÆMMM!'¿Ðãÿä?üá+Wl~Õþ÷ÿ·í@1Þzë­/Úéõ×_|8'â/eDö¤sçÎýä©:íë´/NûÓ¾8í;7eÙÏáªªªt:öÃãÞ-sgñ'þ þ³[[[Û#GÂ@øJ¥&ÅüâOü!þ@ü!þf®®®êêêX,L&»»»Ç­=ñ'þ þ¾äYü?Ä?ÄøCü!þâñ'þ?Ä?ñøCü!þ@ü?ÄâñøâñøCü!þÄøCü!þ?ñ'þÄ?ÄâOü?ñâñøâOüøCü!þÄø??ñ'þÄâÄâOü!þ þâñøCüø?ÄâÄøCü!þ þÄâñøCü?ñøCü!þâOü½Ï®]»ö7ó7/½ôÒCÞDâñøCü?ñ÷°ýõ_ÿõ£>ú±ûøÇ?þ|àóÿüC?ÄâOü¿*l|ÿxó®?ÛþÛþ§Ûk?RûÕ¯~Uü!þ þÄ_	úâ¿øÉO~2*¿è¿Ï|æ3+V¬?Ä?ñWBùÚË¿§¿øôG>òñøCüø%èÙg]¾|yvü=ù©'xâ	ñøCüø¥ùû®²²rÍkvþÑ9ß_þå_þÞ÷¾'þ?â¯4üú­ßú­_üÅ_X]]æÌöÐâñøCü?ñ÷þyçwò?Äâñ'þÄß"þ?ÄøâÄâñ'þÄøñøCü?ñ'þ@ü!þâOü?Ä?Äøâñâñ'þ?ÄøCü?ÄâñâOü!þ?âñøCü!þÄøCü!þ?ñ'þ?ÄâOü?ñâñøâOüøCü!þÄø þ?ñ'þÄâÄâOü?ñøñø?Ä?ÄøCü!þ þÄâñøñ'þ?Ä?ñøCü!þâOü!þ?ÄøâÄâñ'þÄøñøCü?ñ'þ@ü!þâOü?Ä?Äøâñâñ'þ??ñøCü!þ@ü?ÄâñâOü!þ?âñøCü!þÄß»¢··7LÆãñúúúÎÎÎìI###6lH$þðÃÖâñâñ7ë¥R©£G´¶¶fOÚ³gÏóÏ??::6mmm~üç;ßf&¹téÒ~ðÛ">úîÝ»¶Å¸zõêlÔÙÙ900ð0qNÄ_eeeÈ»0N§kjj²'Õ××_¾|y¢Cüýå_þåwIÚÇØéÔ©S6Eúö·¿ý­oËv H¯¾úêk¯½ö0qNÄ_<w8º¹ÿþòòòÚÚÚ/:íë´/NûÓ¾8í;ëÅb±Ìp"Ètøðá0påÊ¦¦&ñ'þ þ³RÙÏáªªªt:ì´oÎ¾[öÍâOü!þ@ü!þf¥¶¶¶#Gð3JeOzæg?.]º´lÙ2ñ'þ þ³^WWWuuu,K&ÝÝÝ?nc[ZZâñxSSS~R?ñøñøCÄøCüøCü?ÄâÄâOü!þ?âñøCüø?ÄâÄøCü!þ?ñ'þ?ÄâOü?ñâñøâOüøCü!þÄø þ?ñ'þÄâÄâOü?ñøñøkþüÏÿüßøÆIÂÿ$ßýîwmtäÈ·ÞzËv ßûÞ÷¾ýíoÛéøñã?üáæ#¾óÎ;âoB.]Úµk×JEá³eÆÌâ@ü þ?Äâ¹èÒ¥KK,Çãõõõ]]]Ùº»»Ã¤ÞÞ^ÛéíH&Áäw¤HGGGY÷M¦¹õ÷÷eÌQáÿ'OóçÏWVVfOª««#ÃÀ±cÇ-Zd[1½©À$üÜ»w/üTü1í½èÄ6l	+i'fFhooohhhjyy¹MD;Rá¸·ìÝ»wß¾âiïE¡ü¢.ÌuáÓá÷é±cÇÆ½COOÏ¦Ml(¦½#=pî-7nÜhjjL/ª««knnÇãa_ºråøc®ëèè¨ªªÊ÷îÝT*544dQÌTx<poiii9wîÜOÞ5ÅEÿ:ºuëÖû"ÂNÌLþ1ÿ¿ÇúûûmÙ&3	ï-e?Ïö¡È_GDBü1GÕÕÕ]ºtéþØµ½ÍÍÍÙÎ=ÆØJ³#ß²+ÐbÚ¿®]»vìÐÆûûëÈNÌû©···¾¾>üÛhåÊ#|ÑïÖÿÎ¦øiÜI0ÕIüQü^ÔÓÓ³xñâ0iÅ¡ÿÄâñø@ü þÄâñø@ü þ?Äâñ þ?ák_ûÚòåËËÇ¬òW^ù¹ßqcfÍoäñÖ¶¦¦&<µ¡¡¡ñaL<O&£££S]& þf¥]»våùò¿ñ·÷î0ò¥^Êÿâ/ñÏ=÷Ü4	?Ù§··7dM<?xðà½17ÃÈ.Lü]¿~=lhhÈ¿xñâ0þÚµkâÀð¹Ï.dÍóÏ?=ò^#zê©ìôéììñº°±±1gî|çÎM6UTTI[·nÍ>»zæÌXaR·½½='§Âùóç/_¾üôéÓáf[[[Î:uªðr¢Ia¢IZsssßÝÝsþüù0fÕªU1öì©®®*//_·nÝ­[·òã/ù9c¬* þÞg.árãÆì7oÞ#kjj²ã&GOOO4uíÚµ9¶lÙMêëëÅbãÎÝ¦¶´´|L$÷îÝSÃÏOóæÍ>W`9a`ÜÕË¦ÇË.ÚL_=z4S~9Y¹råTã¯Àªâàýáç÷ZYYH±ì¸imm½;&kÖ¬¦F­åct/D[4)JEÃpWWW´ìeîÜ¹3ä]tÖuãÆaÌÉ'Ãpø×¯_ÿÀåp7Ã²×mÜgrÁáùágXóPét:ºC23§pÿg§ËËË§V3"þqã/3>¨[·n!¢áfmmíæÍC´gîs,-3WtóæÍ;`Êù?Ãð3g¸áæíÛ·³×m¢ÏçmÛ¶-LÚ·oßýÚÞºuköBøIÚÔÔjüXU@ü¼ÿ¢Ã]wïÞÍ944FIr'o¾ùfÔÖÉ|P/ÿllNNeÁJ®¬¬L$###aáóçÏÏLàr&J±.]:5a¸¯¯/3µ««+¬À¸§'Vï¿èsoû÷ïÏJÎÏäÐºpáÂîÝ»£°jËeÎ«>0Ñ¶lÙí?7mÚ_`9Ñ¶þþþèæíÛ·_»téÒ05úvÐ¬Ù¢+Ã¤ÁÁÁÂñ	Óhkd¦XU@ü¼ÿ¢&âñø¢¯z9xð`"È¿8cÍ5!tFFF¢s²+s£ÏüEóÍ7³?,µàÖ­[C*E×áf¾leÜD.¿ýìÙ³ñÈ/úÌßÐÐPtÏñwøðáÌ1¹ð³'Eìëë6ÂDñåfÈåp·ëÖ­ËZ`Uñ0#D_cÏ=ÿû;nLÔFáÌ	ÓÌ57nÌ.Ël'N(÷ÇþG_YYF¸ÀrÂjd¯XæJÛìððpyyyôrNv¯^½:û!,X~Fßö½ÌèÀdFª©V3E+W&Æ,_¾<ºä6'þ:::¢¯Ó«¯¯?wîêÐÐÐ;ª««£`Ú¶mÛÈÈHfjSSSh²d2yèÐ¡eæ¯IXTÿÌ3Ïäh9AX°JÑø¿M6å|çKd`` J-PQQÅµk×2_[½ÌÁÁÁP·Ñ¶ZµjUwwwÎ#XU@ü þ?Äâñø@ü?Äâñø@ü þ¼þ?1¶q$gÕIEND®B`


<lÙ²%æºfÉ/_é7ÅEàOÒÍ;c×®]Eoº=ztñâÅétzÉ%===ExÊ_H2g7oÞòåËË¬?ó©S§V­Zì|ðÁ¯´µµ5Y±···è¦Nv±7èlØ°!~<xp²1yã7Ö¯_ÜÂl6þüù¢sÞßdNÜ+V;v¬¥¥¥¶¶¶½½½ð]Æ;w666&;1ã*ò&»Á_øÂb~áNØ(°3»ºº&Ü¾Ó]¥ôªÃ1'nä´§ô±QÔM¬8Ùã-Øñq¾÷Þc0NvimÇò0Ið'UþÂ+1³©©)9yúôéT*Uø÷Äe~'g^·n]uóg_ÕK;::ß²*­üMºá¥÷ýÊ+L¦¡¡!&JÇä­·Þª¯¯/¼8gÒïoéõÞsÏ=É;©Iá¼üÎ¹råÊòø[°`AÌçwJ7ÖÂ'Ü¾Ó]¥èª/_¾¼ó·hÑ¢iNùÁ¿¹Ëãopp°piÜ»éâo[Ið'U!þùÁ²d:ÍÆÉ¾¾¾îïï/$ZÑêÉÉ­[·^»v-y/çë®]»6~ëÇù·ÃIÉÒø'cõ«×µ¯«ÌÅ_qÂû~èÐ¡ÂwÂÏ¿qãÆ^³fÍåëÅDÝßdÎÃ?<>>Ü°äãdOOOáL>¾väÈ¾páBá¢Énp:ìä×«Lö¿ü;£Ó	oÕM¯8áã­t»ããq5]üMñq+	þ¤ªÅ_~O_ò±ÂbN_Æo½õV~Î×Í¿ì^L¥RÉÉ9sæ¾eU´¯ÌÅ_q²û1~ÍÍohh(¼G/^óçÏìþ&sÂ'ÙÉ,¼%¹ðæhkk+¼ïeðùdR«i¬¹jÕª£GN8hS	oÕM¯XÉCbhh¨ð2§¿)>n%ÁTø¬ÄÌúúúÉÞ)ÉÉPNwÊgº¿­oâb'w9îossóØØXáüÒwÎJ?Wz§r2 Y´ë³ÌOjllLöS¯ÔùKÊ*S«	Ï0Á¤7·bñ|ÿ§)>n%ÁTøægøHÞKðàÊòÖÖºåßÈyçw¹Øò+¹ù/FIf&>+zªÅSÂ/N¾r¥··7p6ü%oO&ßºÍf7oÞ|ùòå½÷ÆÌõë×O¿©¬rÓøîàä»éßç;yÞ%ÿ½)3àe`àOª*üÅo»0Pò»9ÿU/É§©Âñ»39xvÉ%É¢äCñ#×ðhuËË)QKò®¸ðär>à5áÅ_±<tÖ®]ÿ2'9xÍ5W®Ã?üþñ¼ûuúôé¸öìþ^~ùådÿcÜåþþþä]½¤CM¸§»Ê´ðW~pMwT§2É§ôbëíV¯^]JÃ§z*F;ð-?àe`àOªü¶iÓ¦üyJÍü?ÿ¥»ÉÛ¥¿DË¬[^N¡¢GGbæ¹Øò+Î¥KòöJæ?¾èã_ù¾üåu4þüü' ËèçÁ,ÝXK.-³§µÊ´ðW~pMwT§2'O,¼ÀåË.-ü1æ]c$øªñKqåÊ¥þ«§§§­­- ¿ÑyæüüCÅÀVá·ÜMqÝÊ)ùz¼¸ðÖÖÖÒ¯ëìbo¸byè$»Dç=výúõµ×Ëf³¥wÜþãÒâ2A<òHx(oîòûæ7¿÷.îøöíÛ÷¹Ï.Ôðû_eZø+?8E¢¦5ªSÞÃ777ÇÝLö¤çh=ôPru«V­¸á[­e`àOn[¹Á,|§öV¬Rù%oñ&Çk'_¼x±G¤*,ùÌ_QO<ñàOTlÙ²eÁÉ¾ÝØ¾»aàO$Ið'I$ø$IüI$	þ$I$I?I$Á$IüI$	þ$I$I?I$Á$IàO$Ið'iövèÐ¡¦¦¦t:½råÊé®ÛÒÒRSSsîÜ¹ü9K,Æ+Úõnî<SY÷f^d'©pQ*ª««Ëf³ÃÃÃEàOÒ¬©±±1(îº;wîuÿäOþ$?çÉ'9;vìÕø+sùsâÇtGGG$ø4^PÞN<ë®X±"?çî93s#o/þË1]WWçQ$	þ$Í&ùæÑG;wî9s¶mÛVt¶ÏîsmmmEã0¦ßyç9É¢/®Zµ*lN§[[[ûúú&¼´Âk/¿Ê¾û-[WTJ±«W¯vvvÆºqã·lÙ¼<]¾|yÌK~ë­·nþÎ=J¥tíÚµ¸ñq2fzI?Iè¿dúñÇégy&O<ñDáyzzzJ?ß¶qãÆX´wïÞ1ÝÕÕ,Zºté#GbâÂ1?ÉLxi7 ü*AºÄÄJoü¦Mb:ÎðÜsÏÅÄÎ;cæÇ>ö±¾råÊéÓ§c"ycrÂóM!¼þâ¿,Ú±cG2O=õTLlß¾Ý£KüIªhü555Åtîz¥öðC/¿ür,Z³fML¯]»6Q]~éÉ'·nÝÚÖÖ!1á¥«ü*É;wnéºÉÅD°/fÆ9cºµµ58;66ö~§pNrÛâÂC~!ËdQÂãfÌ3gÑ¢EqÒ£KüIªhü¥ÓéütLÄÉÉ/¤Ös$WcN²hÏ=±VðÔ©SPti'§²Êd7,4Vøf^ÇÎ7/S__¼)ø¼ó7aÉñ.QáNsI?I¿L&SøÎ_òæÙÅÓÙÙKó?óókkkcÎµëMåWÉß°ðºóçÏOÖ-ºyãããGÙ°aCá[7187+W®444d³ÙåË<º$Á¤Æ_òÕ-÷îM>ó÷äONÌ¿vàÀüügÎI>8ü_eëÖ­ßùÎwbbãÆ¥ë>ôÐC1ÝÝÝýúë¯'»zcæ²eËbúÔ©SA±X´hÑ­ÃßæÍcÑÉ'ûûû?(Ið'©Bñ7>>Às½Âïê+¿ÑÑÑdküÌ.:|øpýÜ¹s·lÙ2Eü_åèÑ£±ôîÉwRxàWÜòÚÚÚÕ«W'öù²Ùlrøð+¦øíÔñ`-üÖåËÇÉÓ§OI?I$Á$IàO$Ið'I$I?I$Áß,îoþæoÞ|óÍ¼Æþçþ÷ÿw¼Jkhh¨ðET!ÅFyûí·C¥/b3üÊ©©ôÿñçÎ3Ø?ýÓ?ýô§?¿JéÿðÃ3ÌMÈ¨ÀN>.7VlßoW(áWNM¥k×®ýõ_ÿµq¨À^~ùåû·?øü	þàOðð?Áü	þàþàOð?ÁüÁü	þàOð'ø?ø?Áü	þàOðð'ø?Áü	þàþð'ø?ÁüÁü	þð'ø?ø?Áàþðð'ø?Áàþàþð'ø?ø?ø?Áü	þàþàOð'ø?ÁüÁü	þàOð'ø?ø?Áü	þðwçâopp0É¤Óé¾¾¾ÂEccc6l¨­­ýØÇ>£?øüÁàoÖÍf»»»cb÷îÝvîÜùÄOÄS"¾¹¹¹½½½Wg°ïÿûÿú¯ÿzUÖøÃÿøÇÆ¡ÒÆ8TZñ"/eÆ¡ÒúÉO~ø3X__ßÐÐÐL^ã¿úúúà]Lär¹¦¦¦ÂE---¯¿þúd+þzê©ïÏ`GÁ÷Ua½ðÂ/¾ø¢q¨´b£Ä¦1V¼ÅKq¨ÀþyPÊ_zé¥¼Æ;étzÂéädð®®®®¹¹ùÔ©SvûÊn_»e·¯Ý¾²ÛwÖJ¥òÓµµµEöîÝgÏmkk?Áü	þàOð7ëkhhÈårï]ßíÓEòÓEoÂàþð'øuvvîÛ·/&âg6-ðÃ8p &Î9³lÙ2øüÁàþ³¾þþþÆÆÆT*Éd~vßjj©uëÖ¥Óé¶¶¶sçÎÁàþð'ø»s?Áü	þàOðð?Áü	þàþàOð'ø?ÁüÁü	þàOð'ø?ø?Áü	þðð'ø?ÁüÁü	þð'ø?ø?ÁüÁü	þàþàOð?ÁüÁü	þàOð?ø?Áü	þàOðð'ø?ÁüÍÚÆÆÆüñeËýÂ/üÂ<ðæoÂüÁàþðWµýú¯ÿúÇ?þÀØø»Wü÷wÝu×¹sçàþàOð?ø«Â>üÑ~ôÑ¯>ºíëÛí¿ÚÁüÁàþðWýë_¿÷×îÍË/þmùÊyóæÁüÁàþðWøûìg>[¿G¿ú(üÁü	þàOðÕÙÄ»Õn_ø?Áü	þà¯JsÀüÁàþðwÇÕË/ýÒ/â¸ÿþûÏ?_±7þàþàOð¿;(ø?ø?Áü	þàþàOð'ø?ÁüÁü	þàÏ8Ààþàþð'øüÁüÁàþð'ø?øüÁàþð?øüÁàþàþ?øüÁüÁàOð?ø?øüÁàOðð?øü	þàþàOð?ø?øü	þàOðð?ø?øüÁüÁàþ?ø?øüÁàþàþàþð'ø?ÁüÁàþð'ø?ø?Áàþðð'øüÁàþàþð'øüÁüÁàþ?ø?øüÁàþàþ?øüÁüÁàOð?ø?øüÁàOðð?øü	þàþàOð?øüÁü	þàOð?ø?ø?øüÁàþàþ?øüÁüÁàþ?ø?øüÁàOðð?øüÁüÁàOð?ø?øü	þàOðð?øü	þàþàOð?ÁüÁü	þàOð?ø?Áü	þàOðWf2t:ÝÒÒÒ××WzÞÞÞøüÁàþÕP6íîîÝ»wwtt-ommõWõ/3Ø÷¾÷½ÿøÇÿ¢ëW^9uêq¨´b£Ä¦1V¼ÅKq¨´.^¼xøðaãP½øâo¼ñÆL^ã¿úúúøOLär¹¦¦¦¢¥?þø®]»&ÃßÞ½OÍ`½½½'N8¥ë¥^êïï7VlØ4Æ¡Ò±x)3Ök¯½ø3XOOÏ«¯¾:×xGà/NO8ÅÿÚÚÚvûÊn_»e·¯Ý¾²Û·JðJ¥òÓµµµÖ­[wìØ±ÿwWáOð?øüUÇ°644är¹÷®ïöéÿrÿkð'ø?Áü	þfûöíøÍf'¾«ÞùüÁàþÕ1¬ýýý©T*ÉL¨=øüÁàþ¾äþð'ø?ÁüÁü	þð'ø?ø?Áàþðð'ø?Áàþàþð'ø?ø?ø?Áü	þàþàOð'ø?ÁüÁü	þàOð'ø?ø?Áü	þðð'ø?Áü	þàþð'ø?ÁüÁàþð'ø?ø?Áàþðð'øüÁàþàþð'øüÁüÁàþ?ø?øüÁàþàþ?øüÁüÁàþàþðð'ø?Áàþàþð'ø?ãð?øüÁàþàOð?øüÁüÁàOð?ø?øü	þàOðð?øü	þàþàOð?ÁüÁü	þàOðð?Áü	þàþàOð'ø?ÁüÁü	þàOð'ø?ø?Áü	þàþàþàOð?øü`ø«¹Q©Tþàþð'ø¿*Á_êF¥Óiø?øüÁàþìö?ø?Áü	þà¯ºð/7n?ø?Áü	þà¯ñ×ÔÔN§æþàOð?U?þ,YRz´GýÈÈüÁü	þàOðÕ¿ÚÚÚÐÞððpcccLù¾óïÄDWWüÁü	þàOðÕ¿ä­¾íÅÄâñsæÌ?ø?Áü	þà¯Úð7oÞ¼ ^ÿ¹sçbbûöíÉ¯z?øüÁàOU¿-[¶äï(üØß²eËàþàOð?ø«Â£ì±ùóçÇÄÀÀ@LÛÚÚfÅö?Áü	þàOðw?øüÁàþàþ?øüMÞ¢E/|ñ%Ïð?øü©Êñ·páÂBðås´/üÁàþªBüó'NÝ¬Ûð'ø?Áü	þ¦Wào6Êþð'ø?Áß´ümÚ´éêÕ«ðð'ø?ÁªÑjJrÀüÁàþªBü577;àþàOð?Ý)øKØ7K_ áOð?øüM¯|Àü	þàOð§;q[¶l?ø?Áü	þTåø«$|Àü	þàOð§êüç	sÀüÁàþªÎ¯z½Áàþð'ø^MMMÍÍÍ¯¿þúm¼L&N·´´ôõõ.hmmEK,³Áàþð'ø_«jjnó;Ùl¶»»;&vïÞÝÑÑQ¸háÂÇýû÷/Z´þð'ø?Áßûª¯¯/ð·cÇx9¸]_øR__.kjjìluuu¥øû¿øÿ=½øâ'Oüßª°~ð¼òÊ+Æ¡ÒÆ8TZñ"/eÆ¡Ò:sæÌáÃCöÂ/:uj&¯ñã¯ö-<¸d²MN8ÑÕÕU¿Á=[å¼*¬þþþþðÆ¡ÒÆ8TZñ"/eÆ¡ÒúÇüÇÀq¨Àzô£Íä5ÞrüUÂÑ¾Ð¬­­-=ÃÕ«W³ÙìÈÈÝ¾²Û×n_Ùík·¯ìöõ544är¹d·oL-½téÒJW?Áü	þàOð7ûêììÜ·o_LÄÏl6[4âíííÃÃÃ®?øüÁàoÚår¹Õ«W×ÕÕÕÔÔÌ3gÝºu3|äGccc*Êd2?»o×Anjj*ü$"ü	þàOð¿÷Õèèè|Ì?õ?øüÁàoz-^¼8¨·fÍ«W¯&/k×®9K.?ø?Áü	þà¯ÚðW[[ÔÏÏÉår1gÂ£náþàOð?øÝøK¥RA½ä`Û¤±±±3_õ?øüÁüÁßá/ÙíÛÞÞìö1sZ[[áþàOð?ø«6üö&<àãòåËðð'ø?ÁüUáW½®[·nîÜ¹©T*~¶··ÇY±=àOð?øüÝAÁàþð'ø?ø?ÁàþEkÞ¨T*ð?øüÁ_à/5yðð'ø?ÁªõÈ#$øÛ¿?üÁü	þàOðU¿ÁÁÁ9sæûV­ZUøÏðð'ø?ÁüUþÖ¯_¼áwäÈY´=àOð?øüM¯ç.aß5kfÝö?Áü	þàOð7ÕFGG.]ÛÑ××7·ü	þàOð¿)õÔSO%oøuttÌÞí?øüÁàojkú?ø?Áü	þtçà/u£Òé4üÁü	þàOðþ¼üÁü	þàOðð?Áü	þàþàOð?ÁüÁü	þàOð'ø?ø?Áü	þàOðð'ø?Áü	þ>0ü-Z´¨¶¶Ö÷üÁü	þàOð§êÇßÂÁçþàþð'øS5ã/ì;qâD<ìfÝö?Áü	þàOð7½êëë³Q~ð'ø?Áü	þ¦Ýàà`àoÓ¦MW¯^?ø?Áü	þTåø,XPS>àþð'øSâ¯¹¹Ùð?øüéNÁ_Â¾Yú?øüÁàoz5448àþàOð?Ý)ø»øÛ²eËØØüÁü	þàOð§*Ç_Í$9àþàOð?Uç<O>àþð'øSu~ÕËìþð'ø?ÁüÁü	þð'ø¼·zõêººº9sæ¬[·n¶ü?øüÁàozNxÀÇ¬8øþð'ø?ÁßôZ¼xqPoÍ5Éßöµk×Æ¥KÂüÁàþðWmø«­­êççär¹óáþàOð?ø«Â¯z	êøòsÆÆÆb¯z?øüÁàOU»Û·½½=Ùí?c:æ´¶¶ÂüÁàþðWmøíMxÀÇåËáþàOð?ø«Â¯z]·nÝÜ¹sS©Tüloo9³bÀàþð'ø»?Áü	þàOð7½_ýuø?øüÁàOÕ¿t:]S3[ßA?Áü	þàOð7½úúú;vìÙòWÝàOð?øüÝìELR*?ø?Áü	þà¯¿äyÂ|É3üÁàþªü577·´´Ìêí?øüÁàoJ%Ò÷½ë»gÅ^øüÁàþ7¿äOú&Æþàþð'øSã/ÉÔÍð?øü©zðwâÄyóæ%ïÿ%ÔsÀüÁàþªZüå-Î?Áü	þàOðçoûÂàþð'ø»f2t:ÝÒÒÒ××W~ÑÍÍ?Áü	þàOðW)e³ÙîîîØ½wGGGùE77§°-[¶466~l«[°`ÁÇTaÅÿC¥%6q¨´âEl_95ÅBö|dõÏ2üÕ××' 8ËÅë~ùE77§°Gy¤F$©ºåø;öì¿ÂcG#)]tssàO$ÁßûØs|ý^Z[[ßxã÷ißX[[[~ÑÍÍ)ì÷ÿ÷·mÛöí,®îÏþìÏ¾­ëþè¾ñoJ+6JlãPiÅØ¿rj*uwwùË_6ØÖ­[yæ¼Æ[¿`_^étzÅçÏ¿éKkhhÈårÉ^Ú.¿èææ8àCøpÀðá9àãØù»xñâ¼kkkW¯^ÓÙÙ¹oß¾Ùl¶ü¢?øüÁàïä9r¤®®®æfÿ¼[ccc¬Éd~v+¯ï½.]tssàOð?øü¿;ßù;wnWWWåoøüÁàþÓ«Ð|uuu/^-Ûþð'ø?Áß4/âúq÷ÞïfÝö?Áü	þàOð7½>ox?Áàþð7;ð7«?Áü	þàOð7íÎ9³hÑ¢9sæ$Gø644<xþàþð'øSâ¯··7ÀG¿dz÷îÝðð'ø?ÁüUþzgÎÉão`` ù¶ø?øüÁàþªðhßäKóøÇ_r0üÁü	þàOðÕ¿ ^òn_à/Ë=úè£1ÝÔÔð?øüÁ_µá/îRÍD?~þàþð'ø¿*<Úwhh¨­­-9Ú·®®nÑ¢EçÏÛþð'ø?Áßü	þàOð?ø?øü	þàOð7Q/^lmm;wnêz1ÑÒÒ2[öùÂàþð'øF=ôPÍ$mÜ¸þàþð'øSõàïàÁóöìÙ322Ì=tèP2¿§§þàþð'ø¿*Á_&	áíÝ»·tQòß|ÏüÁàþªüÕÖÖðFGGKår¹Xg?ø?Áü	þà¯Jðÿ«n-MþÔüÁü	þàOðU¿2¼?ø?Áü	þðð'ø?ÁüÍZüþàþð'øSõà/u£Òé4üÁü	þàOðþ¼üÁü	þàOðð?Áü	þàþàOð?ÁüÁü	þàOð'ø?ø?Áü	þàOðÕ¿wß÷èÑ£===o¿ý¶güÁàþüÁ_5ãï[ßúÖ¼yó~±í?ùÉOÖÕÕuwwÁü	þàOðÕ¿øÃwã¶¯o]ÿ««¾¾þÕW_õ?øüÁàþªùÌg>¿îóü¿Öþk]]]cð?øüÁ_âïøÄïé÷ñ÷Û¿½jÕ*Ï1ø?Áü	þà¯ñ÷ÙÏ~ö7ÖþF!þB~Þù?øüÁàþª===õõõÿóÁÿÈï/<ðó?ÿó>óð'ø?ÁüU'þ¢Ãßu×]ÍÍÍwßwL=Áàþð'ø¿ªÅ_4>>þúõÆÆÆ<»àþðgàþª?øü	þàþàOð?øüÁü	þàOð?ø?Áü	þàOðð?Áü	þàþàOðð?ø?øüÁàOðð?øüÁüÁüÁàþðð'øüÁàþàþð'øüÁüÁ*ï¾ûî<ð|dÞ¼yþô§_í5Ãð'ø?ø?U'þFFF>þñÿò/ÿò7yë×¶ÞwßúÐ^õU#ð'ø?ø?U!þüñÿ¶ä¿mûú¶ü¿ðß§>õ)#ð'ø?ø?U!þÖ¯_ßÑÑQ¿­_Ûú¡ÈÃü	þàþàOU¿ûï¿ÿ7×ÿf!þ¾òÈWîºë.#ð'ø?ø?U!þ¾ýíoß÷Ý[¾²%¿O/ûôúõë0üÁàþàþTø>ÿùÏÿ>¿îó÷ßÿ'[?ÙÔÔôöÛoaø?ÁüÁü©:ñ÷Þõ÷ÿ~ë·~ë¾ûîÛ¾û»ï¾kxáþðð§jÆàþ?ø?øüÁàþàþ?øüÁüM·ÁÁÁL&N§[ZZúúú´¶¶Æ¢%KÄÙàOð?øüÍú²ÙlwwwLìÞ½»£££pÑÂ?û÷ï_´hü	þàOð¿Y_<èc"Ë555Mv¶ºººRü=ùä=3ØáÃ¿ûÝïö¨ÂWÌ#GJ+6JlãPiÅX¼ìùç7ØÌÿê¿#ðN§'.ìÄ]]]¥øÿìØ±cÃÃÃÿGÖßýÝß?Þ8TZ±QbÓJ+^Äâ¥Ì8TZ?ýéOã?KÆ¡é¥®2×xGà/Jå§kkkKÏpõêÕl6;22b·¯ìöµÛWvûÚí+»ge5ÿYL744är¹d·oLóÒ¥K6l*½øüÁàþ³¯ÎÎÎûöÅDüÌf³E#ÞÞÞ><<<áð'ø?Áü	þf_ýýý©T*Éüì¾]S°©©©¦ øüÁàþwnð'ø?Áü	þàþàOð'ø?ÁüÁü	þð'ø?ø?Áü	þðð'ø?Áàþàþð'ø?ø?Áàþðð'ø?ø?ÁüÁü	þîÎ;÷·û·Æþðð'ø«~ö-[¶ì®»î?þG?úÑ^zÉÀàþàþÕÙo¾ìkÿÕö­_ÛºíëÛþGöÔ××¿òÊ+Fþðð'ø«Â¾úÕ¯~êSöåÿæÞÏ|ö³52ð'ø?ø?Á_vß÷Ýÿýøû½/ýÞÝwßmdàOðð¿*ìK_úRø¯aÁ+VøüÁüÁà¯§É?üá¿»1ß¯li¾»ù[ßú?ÁüÁü	þª³?ýÓ?ý¹û¹åËÿÊ_illüßùññqÃ?ø?øüUmçÏÿã?þã¯íkñºI~ð'ø?ø?Á_õç/|Ààþàþð'øüÁüÁàþððð'ø?Áü	þàþð'ø?ÁüÁü	þð'ø?ø?Áàþðð'ø?Áàþàþð'øüÁüÁàþðð'øüÁàþàþ?øüÁüÁàþ?ø?øüÁàOðð?øüÁàþàOð?øüÁüÁüÁàþðð'øüÁàþàþðgàOðð?øü	þàþàOð?ø?øü	þàOðð?Áü	þàþàOð?ÁüÁü	þàOð'ø?ø?Áü	þàOðð'ø?Áü	þàþð'ø?ÁüÁü	þð'ø?ø?ÁüÁü	þàþàOð?ÁüÁü	þàOððð?øüÁüÁàOð?ø?øüÁàOðð?øü	þàþàOð?øüÁü	þàOð?ø?Áü	þàOðð?Áü	þàþàOð'ø?ÁüÁü	þàOð'ø?ø?Áü	þ·ªÁÁÁL&N§[ZZúúúJÏÐÛÛ[SS?øüÁà¯Êf³ÝÝÝ1±÷î¢¥ããã­­­áïßûÞèöü`hhhTÖk¯½öÿðÆ¡ÒÆ8TZñ"/eÆ¡Ò	ü¬¯¯oxxx&¯ñÀ_ü'&r¹ÑÒÇ|×®]áïßøÆ÷f°xf¾øâßSõÝï~÷^0VlØ4Æ¡Ò±x)3ØóÏ?o*°ÿÕGà/NO8]¼x±­­-hh·¯ìöµÛWvûÚí+»«©T*?][[[¸hÝºuÇûwþð'ø?ÁßìÊÿ,¦r¹Ü×wûÆôgËþð'ø?Áßì®³³sß¾1?³ÙìdX,	?øüÁàoöÕßßßØØJ¥2ÌÀÀÀÚ?Áü	þàOðçKáOð?øüÁüÁàOð?ø?øü	þàOðð?øü	þàþàOð?ø?ø?øüÁàþàþ?øüÁüÁàþ?ø?øüÁàOðð?øüÁàþàOð?øüÁü	þàOð¿;«?ø?øË¿üË7g°¸º¿ÿû¿SVooïñãÇC¥%6q¨´âEl_95.ðçþçÆ¡;pàÀ~ô£¼Æüä'ð7igÎÙ¶mÛJ$UKåß¯ñf¬$IÒüI$Á$IàO$Ið'I$ø$IüI$	þª§ÖÖÖt:½dÉÁÁÁÂEgÏmkk«­­]¿~òw>j2z·®3gÎ,]º4¶KKKKá¢ØLL&YÔ××7áUÈ¦ñ©í755ÙRªíâùR	Û¥T·ýùâ¡ðÁ·páÂãÇÇÄþýû-ZT¸(ñ ×_ýánØ°Á Í@ñ;tèPLÄÖ©¯¯/Íf»»»cb÷îÝÎQlOJØ.ñ»mñâÅð©ÌíâùR	Û¥T·ýù·¶ºººÂÁüüô¼yóâg<-f¬øïWáx¢^»v-&r¹æÒ9ªMã)S	ÛeõêÕçÎ+D§LenÏJØ.¥*¸íÏø»8q¢«««pN< Î9Ï>ûlÁøAL·µµ=Ö ÝÒÆÇÇçÎ¯ñß¯ÉPLÎQlOJØ.?ûýQOÊÜ./³]UpÛ/ðw«ºzõj6)yêÔ©EüwíÚUô¦à¥KÊü_A`½½½sR©T~º¶¶vÂ9ªMã)S	Û¥2¹]<_*g»ªà¶?_àïO³6Mv7ÞxcñâÅE3½bÎXEÿÓ'j.ïú;ðÉ¶t*dÓxÊTÂv)E§LenÏÙ.E*¸íÏøûàùåÛÛÛK-pppðÚµk»vízì±Ç9çÏO±Ñ»uÅP'ûÜº³³sß¾1?ãfÎQlOJØ.¥Èð©ÌíâùR	Û¥T·ýù|MMM¥Ö'ñX°`Aü÷«««k||ü½ëX¼xqü/aÅñä4z·®`wKKKõÊ+óÿýJ¶Kccc*Êd2ÉáØ¥sT!ÆS¦¶Ké´§LenÏJØ.¥*¸íÏø$Iº?I$ø$IüI$	þ$I$I?I$Á$IàO$Ið'I$ø$IüI$	þ$IàO$Ið'I$ø$IüI$	þ$I$I?IºqÏ>ûìòåËë®·råÊçî¿¼Æ]oÖ¼"Otkâ®Í9ét:Évmº)	þ$iV¶mÛ¶ì±jÂß;bæÓO?]4ÿßüfÌß¾ûM$ø¤Ù×àà`°&NïÙ³güz÷î1óäÉU¿.ÄÌ%KÍ_¼xqÌ?þ<üI?IwD_øÂ5O<ñDáÌ'|2f~ñ_,¤O___à)ÚÚÓù3_¾|¹««kîÜ¹±¨¾¾~óæÍW=ÀE±nOOO§bÎ¼yó/_~äÈ8ÙÙÙYtÃ>rEqE½½½A­½½=æäç?~<æ¬Zµ*?gçÎqQuuuë×¯¿téR)þJ/¿hN*	þ$é6·`ÁËÅg¾õÖ[1³©©©7E8q"YºvíÚ¢E6mJ>:JM¸Vr2YºnÝºk×®kkkÇÇÇciü<Í3'ù(^Ë	o^é=Ý¿¡hó¾ìîîÎË¯èBV®]ü¹©àOnÉÞ	^×jjb¸éèè¸z½kÖ¬I&ÖIø¼hKe³Ùä-ÃîïïO.¤ð2·nÝ¼Köº>ôÐC1çÐ¡C1?cúÁ¼áåãd¡ð¶MxóçÏû;<<'ãgÜòg.KÎÉdbÅ¸ïýçnâºººéâ¯ÌMTø&Ä_~~ÄLÑ¥Kâd°)9ÙÚÚ'7nÜhÍ_H§è½´üZÉÉ·Þz+æS~Ïoüé£GÞðrqòwÞ)¼m>ïGE»vízï?wmoÞ¼¹ðÁÀ_´­­-ycrºø+sS%Á$Ýþ·»®^½Z8sdd$fÆ¢2ÜÉÓðÜ¹sÿòÖÉP¯tol§¿`%¦ëëëkkkÇÆÆâÂçÍ_zÃËbE9s&qjLÇÏ>út~iÜ	wOenª$ø¤Û_ò¹·§zªpfò(E|ä?8<<ÖÉ'wìØìÍ«1y[.¿_õDÛ´iS²·7~vuuåç¹ä¶¡¡¡ää;ï¼SþÈÜî¹'&ßnf-ùz¯Ry&£_Zæ¦J?Iºý%L¤ÓéÝ»w'_õ²gÏÚÚÚÒ3Ö¬YÐKöÉæÌM>ó|TîÜ¹sL,¸yóæ RrnþËV&$Zrømrí/¿ür~~ËI>ä|æodd$9güíÝ»7ÿåÂEÉÇO>×0þnãlÎõë×.-sS%Á$UDÉ µsçÎÿÿw½ÄFùéüÓü1ùzè¡BYvðàÁ2øïúâùõõõËÜÂ?Òv²;;::ZWWÜ¢Ý«W¯.¼ùóçÇÏäÛ^/3yc2_BÕüÒ27UüIR¥@Y¹reíõ/_r[¿ÞÞÞäëôZZZ;_:22òè£666&`zäGÆÆÆòKzzÚÚÚÂdLæg)ºÌÒ[ó~øá¢ù]N7&nRòe¾ç/_WWWÑw¾$g³Ù¹sçÆ½8þ|þkk/óÊ+¡Ûd¬V­Z500Ptenª$ø$IüI$	þ$I$I?I$Á$IàO$Ið'I$ø$I?I$Á$IàO$Ið'I$ø$IüI$	þ$I$Iú ú¿1ÓKIºIEND®B`


ûösõS§N-[¶l*êÿÒK/Ý©;gzðàÁ%KÔÞjÑ¢Eû÷ï¿íÒ×CKKË®]»&18~m8q"^ét:îÄ-Xt×·$ø¦7þÛ¸qãÇ²ÊÁ_]]Ý+W¦þ~úéÒµuëÖ2·6¡UÆz=ìÜ¹s¢3éaÌf³ûíìì,³þ$øôQqýúõ^z©¶¶6föööÞøV­Z5-ðwêÔ©D«/¿üòðððÍ7c".ÆÌ3gÎzS]¥ôõ°yóæê2ü%+^»v-¦óùü8I?IwÆöíÛGìt9vìXsss:?þÑ£GGà©p#ÉDæÌ/.³náÊ§O^¶lYrDòñÇïïï/¾ÓÖÖÖdÅ®®®u¬½í#À5kâëÁÇwÞygõêÕÉ#Ìår/^qÍâçÌ;»°dÉ«W¯8q¢¥¥%É,_¾¼x/ã¶mÛqEc=àG4æë1síÚµ£nß®Rz×áÅrBSúÚÑ$VëõV¸BlxIÄ8ö³Á,^:Ö­µË¿À$ÁTmø¯ÄÌ¦¦¦äâ3gR©TñÏÝS§Nùa½½½Ìº+Çêâ¥Å»¬J+ÿn»bés¿~ýz6mhhÒ1y÷Ýwëëëo'®9BiÅÏ·ô~.ìIMä7âK.-¿Ù³gÇü÷ß¿tcÍ;wÔí;ÑUFÜõµk×=óæÍÐàüÉ­XÅKãÙMã|ÝJ?©ñÌ%Ó¹.vwwÇtOOO1ÑF¬²eËÍ79·]wÕªUñS?®ìn'%KãgpÕoÜ*Ö*¾¯27[~ÅQû¡C÷_Ýºu1½råÊk·¸3Çz¾Éõë×',¹å¸xôèÑâ'üúÚ#GbúÒ¥KÅÆzÀétz¬'RX÷#®2ÖïüöNtpFT^qÔ×[évÇÇëj¢øçëVüIU¿Â¾äWÄ9e~¿ûî»9·]·°-9¼J¥3fÌ(Þe5â(^-¿âXÏ=1bü1¿¡¡¡ø]¾|9.Î5k¬çÌ	_Le'O°øäóùÀ_£­­­ø¹Á_AäcIý#®R<¤±næ²eË;6ê gpFT^±þÄÕ«WosBøçëVüIU¿ÀJÌ¬¯¯koÐXLI.rÊìI*Cþ´ÄÍ5?r<ß9sæÏ/ÝsVúp¥Ïw<#yîIÉqêRá uáÆ³ÊxÆjÔ+gpÆ"éäV,3ýå4Î×­$øª»wï.>á#Ù6êÉå­3¡uËïÈyÿý÷¹Ùò+yFIf&>±jÇ? ÅêêÉîÉäSWr¹ÜÆ¯]»¶gÏ¹zõêQñ7U&¿N¡I¯ø÷üxü÷¦ÌyI?©ªð?íÂ@ÉÏæÂG½$¿MnÉÉ³óçÏO%¿?p«QY·¼µ$¿Â7ÜÎ_ðõfË¯X:«V­*ü2'9xåÊ×oÜÚúõë?:þ½_gÎ§°k×®ñàïøñãÉñÇxÊ===É^½¤Cº'ºÊðW~pF¼6è¨g<ßÒ­?88¶[±bE)wìØ£@øð2/0Ið'UþJÛ°aCá:¥çÏ~ý¿ð¡»ÉnÂÒ¢eÖ-/§PQÂ£â31KËÜlùËCçÊ+_öJæxqÄ¯544~¡ð£à¯ ¤Y³f~²~üñÒµ`Á2xB«LågÄk£¸êxÆó­·Þ*¾ÁÅ/1sÌÇºÇ2/0Ið'UþââÒ¥KKÿü×Ñ£GÛÚÚRñ÷îÝù9­âO¹çº·Sòñxqã­­­¥×7ÖÍÞvÅòÐIÏ?þüêÕ«3·Êår¥§wLýýýqkqÁ M6æ./°_|1]<ñÙ³gïÝ»÷_øBá¬á¾ÊðW~pF¼6F4¡Qçð>|xÎ9ñ4ã#é3ãâO<ÜÝ²eËzo»«µÌLüIÒ]+Ï?þøãÅj?U*¿dor¾öððpøææf¯	þ$IUXò;#zþùç¤*l```óæÍ³gÏNíÆÄÓO?mX$ø$IüI$	þ$I$I?I$Á$IàO$Ið'I$I?I$Á$IàO$Ið'I$ø$IüI¦:t¨©©)N/]ºt¢ë¶´´ÔÔÔp¡0'¦cÎüùó'ðvv«É]g<ëNòM¶ì=F©Tª¶¶6Ëõ÷÷'6lØP__YWWÞyç/0Ið'©²jllÊ`'ºî¶mÛbÝßÿýß/ÌyábÎ3Ï<SÝøK¦ãÇtGGGrqÍ5q±»»;¦O8ÓsçÎõ*ìÝä#øé­·Þu,YR³páÂÓ××7õú.Çtmmmr1ÉÄÅë×¯QI?I-¿bÐ<ùäuuu3fÌØºuë«á_hkkq1ýþûïÇtÌI]¾|yÙ²ea£t:ÝÚÚì+½µâ/¿ÊÞ½-ZwTJ±7ntvvÆºñà7oÞìÈ.^¼8n-æÇ-¿ûî»þ|ðÁ¸ØÜÜÜÓÓã¥%	þ$U´ÿéç.¦wïÞÌç¾ø:G-ü~[¡uëÖÅ¢=öÄt|éµk×&,XpäÈ¸téRÌÏf³£ÞZñ(¿JîÀ1±fÍÒ¿aÃ+¼úê«1±mÛ¶È®_¿~æÌHvL6	üÝ¼y3Æ'¦¿üå/':T¸Á¥K>Ú«KüIªhü555ÅtþV¥öõ?V®Ó«V­JTWXúÖ[omÙ²¥­­-9CbÔ[Á¬ò«$¬®®®tÝäÁËb"Ø3ã1ÝÚÚÜ°ÎO[ÜxÈ¯ø8ï©S§Â¯ëÄzI?I¿t:]¸X^BÞÚ6cÆ¸æÀÀ@rt5æ$víÚkO>]|#n­øâxVëÆwæ%p<xðàÌ39õõõÉNÁ;²ç¯LÝÝÝÁÍâ#à*Ùl¶xÏ_²óì¶âéìì¥¯ùÉ	7o5Nü_¥ðÀÂs¥ëÎ5+YwÄÃ>räHr*naáe¢øÇOÇLüIª%Ý²gÏäwþ^xáñçàÁ]h(ÌO(yöìÙäwÇ¿ò«lÙ²åW^uëÖ®ûÄOÄô¾ûÎ;ê-éÓ§O_½z5&æÍ÷ñáoþüù±¨««+¦>Ó?þ¸$øT¹ø`Í¸UñgõÇßàà`rÈ5¾ÿ¨¾¾¾®®nóæÍãÄ_ùU;K.ï¤ø:7nGÉdV¬XØæËårÉéÃK,9þü¥ôÐp¡ìììã0kÖ¬6ÄCò$I?I$Á$IàO$	þ$I$I¿éÝ_ýÕ_ÿûßÊü§ú§û·óÊ«´®^½ZüÙ"ªb£üà?0V¼Mñ;§ÆÓüÇpÁ8T`ÿøÿøÃþþ*¥ßû½ßÿM17!£;sæL¸Ü8TZ±QbÓDù¿sj<Ý¼yó/þâ/Cvüøñý×?øü	þàOðð?Áü	þàþàOð?ÁüÁü	þàOð'ø?ø?Áü	þàOðð'ø?Áü	þàþð'ø?ÁüÁü	þð'ø?ø?Áàþðð'ø?Áàþàþð'ø?ø?ø?Áü	þàþàOð'ø?ÁüÁü	þàOð'ø?ø?Áü	þðwïâ¯¯¯/Í¦ÓéîîîÒ+tuuÕÔÔÀàþð'ø«r¹Ü¾ûbbçÎ#·¶¶¿pá)ì;ßùÎï½wCÖßüÍß|ïß3VlØ4Æ¡Ò7±x+3Ö|ø3Xww÷Õ«W§òï	üÕ××Çÿxb"Ï755XúÜsÏmß¾,üíØ±ã;SØ#GâEðUXßúÖ·ÞxããPiÅFMc*­x·2ãP½öÚk¡¿ùæSy÷þÒéô¨ÓÑåËÛÚÚûÊa_å°¯Ã¾rØ·JðJ¥ÓL¦xQû'þó©Âàþð'ø«amhhÈçóÞ:ìÓÿãþÏàOð?øüMû:;;÷îÝñ5ËþTíùüÁàþÕ1¬===©T*Íöööª=øüÁàþ>äþð'ø?ÁüÁü	þð'ø?ø?Áàþðð'ø?Áàþàþð'ø?ø?ø?Áü	þàþàOð'ø?ÁüÁü	þàOð'ø?ø?Áü	þðð'ø?Áü	þàþð'ø?ÁüÁàþð'ø?ø?Áàþðð'øüÁàþàþð'øüÁüÁàþ?ø?øüÁàþàþ?øüÁüÁàþàþðð'ø?Áàþàþð'ø?ãð?øüÁàþàOð?øüÁüÁàOð?ø?øü	þàOðð?øü	þàþàOð?ÁüÁü	þàOðð?Áü	þàþàOð'ø?ÁüÁü	þàOð'ø?ø?Áü	þàþàþàOð?øüÁü	þàOð?ø?ø?øüÁàþàþ?øüÁüÁàþ?ø?øüÁàOðð?øüÁüÁàOð?ø?øü	þ*¸Ã?öØc-zê©§Þï=?ø?øüUmÏ=÷Üý÷ßÿùÏþW:å/úßõõõo¿ý¶a?ÁüÁü	þªóÛäøÄº_[·õ©­É¿ÏýßÏ=üðÃFþðð'ø«ÂvïÞýó¾ ¿ø·åÿm	øüÁüÁà¯ñ÷È#ã/þÍ5þàOðð¿êü6¹ï¾û~ãoä÷Åö/>ôÐCFþðð'ø«Î6nÜø?ù_úÒ~ã×ãsÿ÷s?þã?þÝï~×°ÀàþàþUÛ/¾øs?÷s?õS?µzõj§úÂàþàþÕy?ÁüÁü	þàOð'ø?ø?Áü	þàþàþàOð?ø?øü	þàOðð?øü	þàþàOð?ÁüÁü	þàOðw/öøsçÎÍd2ñõþàüÁü	þàOðWµ=õÔSÙlö+eëS[ûòcMMMüð?øüUgï½÷Þ3ÿæòº_[W[[;00`pàþàOð¿jëØ±cùÌgòKþýôOÿ´ïø?øüÁà¯:aÑÖÖ6þ¯Ï;gpà¯ñ×××ÍfÓétKKKwwwñ¢ÞÞÞÖÖÖX4þü¸ü	þàOðWMÔýxÝc_~¬ ¿/éK<ðÀðð°Á¿jÆ_.Û·o_LìÜ¹³£££xÑÜ¹sO<û÷ï7o^)þþìÏþì§°oûÛßûÞ÷þYÖw¿ûÝÓ§OJ+6JlãPiÅX¼Êi÷îÝ÷Ýwßòÿ³üW:eÙ²eøÄ'¦øGÊ÷Æo¼óÎ;Sy÷þêëëoÞ¼ù|¾©©i¬«ÕÖÖâoÏ=§§°®®®S§NVõæoöôôJ+6JlãPiÅX¼êW^Éår?û³?ûÅ/~ñOþäOHEuôèÑ¿þë¿Ê¼'ðN§G..Þ­Ö®]ë°¯öuØWûVe>äÙaßë°o**Lg2Ò+Ü¸q#þKTzÞ;ü	þàOð¿éWCCC>ÿðÖaß±ôÊ+kÖ¬¹zõjéð'ø?Áü	þ¦_÷îøËåFøòåËûûûG]þð'ø?Áßô«§§§±±1Je³ÙÞÞÞÿzn5ÿùìj?Áü	þàOðwï?øüÁàþàþ?øüÁüÁàOð?ø?øüÁàOðð?øü	þàþàOð?ø?øü	þàOðð?ø?øüÁüÁàþ?ø?øüÁàþð?øüÁàþàOð?øüÁüÁàOð?ø?øü	þàOðð?øü	þàþàOð?ÁüÁü	þàOðð?Áü	þàþàOð'ø?ÁüÁü	þàOð'ø?ø?Áü	þàþàþàOð?øüÁü	þàOð?ø?øü	þàOðð?Áü	þàþàOð?ÁüÁü	þàOð'ø?ø?Áü	þàþàOð'ø?ÁüÁü	þð'ø«Î;÷Õ¯~µ»»hhþàOð'ø?Á_ÕÚûÅ_üÅûî»¯µµõáÎf³òð'ø?ÁüéÎ÷Øc=ôÐCgóÖ§¶Æ¿?øÁàþ?øüUa3gÎüÍõ¿È/ù÷©OêOÿôO§1þjnW*?ø?Áü	þîÁ>øàbùÅ¿Gyäk_ûÚ4Æ_êv¥Óiø?øüÁàïÞìþûï_÷këñ7wîÜ?ÿó?Æø«àOð?øÓÇÑ×¿þõ¦¦¦äÈïÿ·åGyè¡*áßï­aÝºuðð'ø?Áß=Û¦M~ìÇ~ìüäOüÄO,X°àâÅð¨îþ¶étÚïüÁü	þàOð§â>øào|ã.®tð7þüÒ³=êëëàþàOð¿¼*üL&´×ßßßØØa¾W^y%&Ö®]ð?øüÁ_µá/ÙÕ¡½¸téR¼þbbÆðð'ø?ÁüUþfÎÔëéé¹páBL<ýôÓÉz?øüÁàOU¿Í7Nï(þµ¿EÁüÁàþðWmøöÙY³fÅDoooLÛÚÚ¦Åö?Áü	þàOðw?øüÁàþàþ?øüÝ¼yó|ñ!Ïð?øü©Êñ7wîÜbðr¶/üÁàþªBüó§NÝ´Ûð'ø?Áü	þ&Vào:Êþð'ø?ÁßëëëümØ°áÆðð'ø?ÁªÑìÙ³kJrÂüÁàþªBüÍ3Ç	ð?øüé^Á_Â¾iú?øüÁàob5448áþàOð?Ý+ø§øÛ¼yóÐÐüÁü	þàOð§*Ç_Í9áþàOð?Uç<>àþð'øSu~ÔËôþð'ø?ÁßÄjjj3gÎ¹sçîÈêëëËf³étº¥¥¥»»»ü¢ÉÍ?Áü	þàOð7ùÂU55wlb.Û·o_LìÜ¹³£££ü¢ÉÍ)îÉ'üô§?½l»[²dÉ2UXùÌg-Zd*­Ø(±iC¥obSüÎ©ñô¿ðúÔ§CÖÚÚúÈ#Lå=~ìøëîîü=óÌ3ñÁþ/õõõÉäóù¦¦¦ò&7§¸M6ÕH$UQ;þîìÙ¾Å§8e¤tÑäæÀ$I¿ÉwgÏö-&c&)¿hrsÛ²eËç?ÿù5SXÜÝ£>ºFÖ/ýÒ/ýò/ÿ²q¨´b£Ä¦1V¼Mñ;§ÆÓW¾òåËìsûÜ¯þê¯Nå=~ìø»³544äóùä(mL_4¹9Nø>ð!'|8áCNø¨:;;÷îÝñ5Ë_4¹9ð'ø?Áü	þ>Rù|~Åµµµ5553fÌhooô===©T*Íöööþ×£¼uôºtÑäæÀàþð'ø|£þ²á´øS¿ð'ø?Áü	þ&VsssPoåÊ7nÜHÞV­Zs,Xð?øüÁ_µá/Éõsòù|Ì)=µþàþð'ø¿i¿T*ÔKÎ¨M9û¨øü	þàOð¿ä°ïòåËÃ¾ñ5¦cNkk+üÁü	þàOðÕ¿ÐÞ¨'|vþàþð'ø¿*ü¨ÁÁÁöööºººT*_/_s¦Åö?Áü	þàOðw?øüÁàþàþ?øüXóv¥R)ø?øüÁàþª©±?ø?Áü	þTmø«M6%øÛ¿?üÁü	þàOðU¿¾¾¾3fû-[VüÏðð'ø?ÁüUþV¯^ìð;räÈ4Úð'ø?Áü	þ&Ö«¯¾°oåÊÓnÀàþð'øo,HÎíèîîÛþð'ø?Áß¸Ú±cG²Ã¯££cúnøüÁàþã[ÓçüÁü	þàOð§©ÛN§áþàOð?øóçÝàþàOð?ø?øü	þàOðð?øü	þàþàOð?ÁüÁü	þàOð?ø?Áü	þàOðwÇð7oÞ¼L&ãsþàþð'øSõãoîÜ¹Åàó9ð?øü©ñÎö:u*^vÓnÀàþð'øXõõõ¿é(?øüÁàþ®¯¯/ð·aÃ7nÀüÁàþªrüE³gÏ®)É	ð?øü©ñ7gÎ'|Àü	þàOð§	û¦é4ü	þàOð¿ÕÐÐàø?Áü	þt¯à/RàoóæÍCCCðð'ø?Áª5cäø?Áü	þTò<jNø?øüÁàOÕùQ/Ó7øüÁàþðð'øüÁàoìòùü+jkkkjjfÌÑÞÞ>]Nþ?Áü	þàOð7±G=ácZü?øüÁàob577õV®ümßxSXµjUÌY°`üÁü	þàOðÕ¿L&Ô.ÌÉçó1'æÃüÁàþðWõÔðæÅõð'ø?Áªö°ïòåËÃ¾ñ5¦cNkk+üÁü	þàOðÕ¿ÐÞ¨'|vþàþð'ø¿*ü¨ÁÁÁöööºººT*_/_s¦Åö?Áü	þàOðw?øüÁàob555Í3çÜ¹sðð'ø?Áªétº¦fºîA?Áü	þàOð7±º»»Ï<óL¼L¿ê?øüÁào²71F©Tþàþð'ø¿*üçQó!Ïð?øü©zð7gÎi½=àOð?øü«äOú~xë°ï´8Â?øüÁàoòøKþ¤oòg|áþàOð?U9þ²ÙlMÙðð'ø?Áª§N9sf²ÿ/¡>àþð'øSÕâ¯Ðtqü	þàOð?Ûþð'ø?Áßt«¯¯/Í¦ÓéîîîâE½½½­­­±hþüùq5øüÁàþÓ¾·oß¾Ø¹sgGGGñ¢¹sç<y2&öïß?oÞ<øüÁàþÓ¾úúúäÏçóù¦¦¦±®V[[[¿o|ãßÂ=oßUuìØ±øæ4VlØ4Æ¡Ò7±x+3ÖÉ'>l*°×_½§§g*ïñcÇßùóçï:þÏ8ëìS§N­]»¶ñ­Ò?½ùæ.]êW/³gÏJ+6JlãPiÅX¼Jë_þå_¦ø'ÆYü'öòåËSy;þÏyimmçwîþ?S0É^áÆ¹ôÑ9ì+öÃ¾ûÊaßì+|°s:^²dÉÅ§â¡ÿ¨nhhÈçóÉaßqÍ+W®¬Y³æêÕ«¥7?øüÁào2?¾¹¹¹²L&³bÅ)÷îøËåFøòåËÇÚ?øüÁàï#½ò9R[[;ÅÞ­§§§±±1î1Íöööþ×s»µS°©©©øÎÁàþð'ø»3?;÷üÕÕÕ]QÁàþð'øXÅæ«­­íèè¸|ùòtÙð'ø?Áü	þ&x·Îóøìg?éÒ¥i·=àOð?øüM¬»ø	/ð'ø?Áàþ¦Ó:øüÁàþîìÙ³óæÍ1cFroCCÃÁáþàOð?U!þººº'|$øK¦wîÜ	ð?øüÁ_µá¯±±1¨wöìÙþzO?ø?Áü	þà¯ÏöM><¹¿xý%§ÃüÁàþðWmøkhhê%ûù|þÉ'é¦¦&ø?øüÁàþªñjFëäÉðð'ø?ÁüUáÙ¾W¯^mkkKÎö­­­7oÞÅ§Åö?Áü	þàOðw?øüÁàþàþ?øüÖåË[[[ëêêR·érÌþð'ø?Áßzâ'jÆhÝºuðð'ø?ÁªL·k×®dæààà¡CùG?ø?Áü	þà¯JðÍfCxöì)]üÁ7óð'ø?ÁªL&788Xº(ÏÇ¢¸üÁü	þàOðU¿Â_ukiò§ÞàþàOð?ø«üáüÁü	þàOð'ø?ø?Áü	þàoÚâ¯|ðð'ø?Áª©ÛN§áþàOð?øóçÝàþàOð?ø?øü	þàOðð?øü	þàþàOð?ÁüÁü	þàOð?ø?Áü	þàOðð'ø?Áü	þàþàOð'ø?ÁüÁü	þð'ø?ø?Áü	þðð'ø?Áàþàþð'ø?ø?Áàþðð'ø?ø?ÁüÁü	þàOð'ø?ø?Áü	þàOðð'ø?Áü	þàþð'ø?ÁüÁü	þð'ø?ø?Áàþðð'ø?Áàþàþð'øüÁüÁàþðð'øüÁàþàþ?øüÁüÁàþ?ø?øüÁàþàþàþð'ø?ÁüÁàþð'ø?ø?ã?øüÁüÁàOð?ø?øüÁàOðð?øü	þàþàOð?ø?øü	þàOðð?Áü	þàþÊÕ××ÍfÓétKKKwwwéºººjjjàOð?øüUC¹ß¾1±sçÎK[[[áOð?øüU	þêëëãEù|¾©©iÄÒçnûöícáï^8:>|øõ×_?ª+Þ19b*­Ø(±iC¥obñVf*°×^Í T`Sÿ£ÿÀ_:u:º|ùr[[[Ðp,üÇÿ;qâDÿ¿«ÂúÛ¿ýÛ/J+6JlãPiÅX¼Jë?üaügÉ8T`o¾ùæõë×§òï	ü¥R©Ât&)^ÔÞÞoRÿùTöÃ¾ûÊa_å°ïôÊÓù|>9ìÓ£^­peøüÁàþÓ»ÎÎÎ½÷ÆD|Íårca±t&ü	þàOð¿éWOOOccc*Êf³½½½£jþð'ø?Áy?Áü	þàOðð?Áü	þàþàOð'ø?ÁüÁü	þàOð'ø?ø?Áü	þàþàþàOð?ø?øü	þàOðð?øü	þàþàOð?ÁüÁü	þàOð?ø?Áü	þàOðð'ø?Áü	þàþàOð'ø?ÁüÁü	þð'ø?ø?Áü	þðð'ø?Áàþàþð'ø?ø?Áàþðð'ø?ø?ÁüÁü	þàOð'ø?ø?Áü	þàÏ8ÀüÁàþð'ø?øüÁàþðð'øüÁàþàþ?øüÁüÁàþ?ø?øüÁàOðð?øüÁüÁàOð?ø?øü	þàOðð?øü	þàþàOð?ø?ø?øüÁàþð?øüÁàþàþàþð'ø?ø?Áàþðð'ø?Áàþàþð'øüÁüÁàþðð'øüÁàþà¯ªñwñâÅgöõ×_ðÍ?øüÁüU-þì±ûï¿ÿÓþôÏüÌÏÌ5ëí·ßöý?øüÁüU'þ6oÞ<wîÜÍ¿³yëS[ãßÛ¿øÀØÿ?øüÁüU'þfÎùë3_òoþüùßüæ7KÀàþðÕ¿>ø ¡¡¡X~ñïGùÚ×¾æ[þð'ø?ø«6üE÷ßÿoüúoã¯¹¹Ù?øüÁàþà¯:ñ÷»¿û»?ôðomø­D~_øüüÎü	þàOððWø¶mÛV[[ûÉO~òxøá/^¼èûþð'ø?ø«ZüEôGtúôéááaßð'ø?ÁüÁ_ãïCáþð'ø?ø?Áàþðð'ø?ø?ÁüÁü	þàOð'ø?ø?Áü	þàÏ8ÀüÁàþð'ø?øüÁàþðð'øüÁàþàþ?øüÁüÁàþ?ø?øüÁàOð÷ñÖ××ÍfÓétKKKwwwñ¢¡¡¡5kÖd2|0Fþð'ø?Áß´/ËíÛ·/&vîÜÙÑÑQ¼hÛ¶mÏ?ÿ||KÄÐÏ3§ßþö·§°¿üË¿¼zõê *¬·ß~ûþáC¥%6q¨´âM,ÞÊC¥500ø3XwwwÿTÞã=¿úúúà]Läóù¦¦¦âE---çÎkÅÀß×¿þõoOañùÆo|[Öë¯¿þ­oË8TZ±QbÓJ+ÞÄâ­Ì8T`¯½öA¨À¦þGÿ=¿t:=êtrqÇµµµsæÌ9ú´Ã¾rØ×a_9ìë°¯öö¥R©Ât&±hÏ=1qþüù¶¶6øüÁàþÓ²Óù|þÃ[cºøjÅGì?Áü	þàOð7-ëììÜ»woLÄ×W¼hýúõ³gÏ.Z´þð'ø?Áß´¯§§§±±1Je³ÙÞÞÞÿzn·vÆT:nkk»páü	þàOð¿7øüÁàþðð'øüÁàþàþ?øüÁüÁàþ?ø?øüÁàOðð?øüÁüÁàOð?ø?øüÁüÁàþàþð'øüÁüÁàþð'ø?øüÁàþð?øüÁàï^ë«_ýê7¿ùÍïOaqw÷w÷UX]]]'O4VlØ4Æ¡Ò7±)~çÔxºtéÒÿñìÀÿ÷?÷øÁÀß=vëÖ­¿'IT-ß7_cg¬$IÒ½üI$Á$IàO$Ið'I$ø$IüI$	þª§³gÏ.X° N·´´ôôô/:~üxsssñ¢¡¡¡5kÖd2|0½Ù.¥stW6ÍùóçÛÚÚâdõêÕÉÆéëëËf³É»»»^lÞÞÞÖÖÖ¸òüùóc½Ù.I]]]55~ÜWÐv¹ë?ú½î|±á:'O¬¯¯/^/_¾ñµ©©)&¶mÛöüóÏß¼y36ÿ9s^lÒ9º+&ÞLC1qîÜ¹õë×ÇD.Û·o_LìÜ¹³££ÃèUÈv;wn&öïß?oÞ<£W!Û%Ã_Em»þ£ß«ácìèÑ£ñàâ9±¯ñ5ÙÞñrW±ª´íR:GweÓÄ£Ó3gÎLï1Ïç¹¼r¶Kqµµµ­r¶ËsÏ=·ûvø«¨ír×ô5|,Åÿ´êêêâ-þ¿¯¯/Þc~|=uêTòØ±cGa>ÚÐUÈv)£»²iâ=ôìÙ³1ñòË/'o Åo£ÅÓº»Û¥P|¿¬]»ÖÐUÈv¹|ùr[[[ü	þ*íìîþè÷jøëêêjhh(³`ÁûaÆD*Ú³gÏ?úV!Û¥tîÊ¦·ÅyóæÅ[äöíÛýIñ-SXÉdZl¤7när¹V!Û¥½½ýÄÿùÃþ*ììîþè÷jøxñâÒÅ/»1*g»Ø½T!¦Ð;ï¼ÓÜÜ|Ëäóùoöñ«»¸]>¼õ;kÖ¬¹zõªáªíRó?3bó>vwÄx)ÜùæÎìàííí]¾|yñ¢&Î9³`ÁX¿~ý>¼u¢Ð¢E^lÒ9º+&õõõÝ¼y3þÇüì³ÏÆÎÎÎ½÷ÆD|ÍårF¯B¶ËñãÇã:ýýýÆ­¢¶Ëÿ°'¿JÚ.wýG¿WÃ/¶qKKKX~éÒ¥ÿ'ßx¡þäTðøÓ1çúõëííí1§­­íÂF¯B¶KéÝM¤=v&Y»víððpÌéééillL¥RÙl69N°]ìaªÀí¹]îú~¯I¤(ø$I?I$Á$IàO$Ið'I$ø$IüI$	þ$I$I?I$Á$IàO$	þ$I$I?I$Á$IàO$Ið'I$ø¤Û÷òË//^¼¸öVK.õÕWÿÇÜ­¦Í;òh¶©©)ÚÀÀÀù1'Ng³Ù7oNô6%Á$MË¶nÝZSÒ³Ï>[MøægbæK/½4bþ/¾ó~úéIÜ¦$ø¤éW___°&NïÚµkøVöì1ó­·Þªü]ºt)fÎ?ÄüæææñâEøîôÑ`ÍóÏ?_<ó^_þòéÓÝÝx¶¶¶ÆtáÊ×®][»vm]]],ª¯¯ß¸qcñÑÕcÇ°bQ¬ôèÑ93gÎxñ#Gâbggçvøðáò·,,êêêjË/ù½½½9'O9Ë-+ÌÙ¶m[cccÜTmmíêÕ«¯R¿ÒÛ1§ÌCt=vÀåòåËÅ3ß÷ÝÙÔÔT:u*YºjÕª6lØ,:sæL*u­äb²´½½ýæÍÁÇL&3<<KãkàiÆÉ¯â¹õá>Óýû÷¶àËûöä7âF.]:Qüy¨àOî~ÉÞQÞ×jjbÅ¸éèè¸q«+W®L&ÖIøìÀ´%r¹Ë0¦zz)¾Í-[¶ï£®O<ñDÌ9tèPLÇ×~üñÇo;Ç¸W(~l£>£å¬Y³âùö÷÷ÇÅø<ÄÏç+d³ÙX1Â?:L[;Qüy¨àO*Ñ¨ø+ÌOp)ºråJ6%[[[ãâ9sÖ­[h,ÜHÄ¾´ÂZÉÅwß·påSáÈo|écÇÝövqñý÷ß/~lcý~Þ¦MbÑöíÛ?üÑ¡í7_! ø¶µµ%;&'¿2UüIÒÝ/ÙÝuãÆâ13áN.üW°NáõJÆàTñ¬Ät&9sfaémog,èìÙ³Sc:¾Æô3gKzzâzøxüø+óP%Á$ÝýßÛ±cGñÌäPFðQø½ÀþþþÒZo½õÖ3Ï<-¨1Ù-W8®z[¢mØ°!9Ú_×®][_æv=mW¯^M.¾ÿþûåÏÌ]¸pa,M>Ý&ÌZ¼(9ó7uuu]¿~½<þ0MF£°´ÌCt÷KNH§Ó;wîL>êe×®]L¦ôä+Wtc²3sßùK~UîÂÅ¿,XpãÆA¥ä<ÜÂ­J´äôÛäÞ?^_æv_òK~ço`` ¹füíÙ³§°O.rñ¢ä×Ï9÷0þnãjÎÕ«W/-óP%Á$UDÉ hÛ¶mÿýw«ÄFéÂÓÂ9xâbYwðàÁ2øûðÖâùõõõÅGËÜN<âV8Óv¬';88X[[<»W¬XQ|³fÍ¯É§½ßf²c²PBÕÂÒ2UüIR¥@YºtiæV/NN¹¿®®®äãôZZZN8QX:00ðäO666&`Ú´iÓÐÐPaéÑ£GÛÚÚÂdÙlv÷îÝ#n³ôÄMÅüõë×?ÖíDñ`â!%@Xæsþ­]»vÄg¾$õ÷÷çr¹ºººx/^,|lMñm^¿~=tÕ²eËzGÜc*	þ$I$I?I$Á$IàO$Ið'I$ø$IüI$	þ$IàO$Ið'I$ø$IüI$	þ$I$I?I$Á$IîDÿàBM-D0IEND®B`


ë.´`>éÆ]XÉøØ¾¿ë|üÉÛ>!ÙN·õïÿñÿzð¿ÙPÏñ×ÞÞ>??¹É.<yòdól6[áøØÎ§UÖ¿íêê¢hïÞ½KKKÅG8J¥RD"N4¬pFü? zãï»ûömæx«[ þÄøÄøâ@ü?ñ'þñ'þÄøâ@ü?ñ'þÄø¨ÑøïèèhjjJ$áÇÖÖÖÓ§O?:¿âhã/?z¿T*Ro~~¾¹Å@½Å_ü>_<ã¯P(qEâ Þâ¯µµ5¤^ün_¿|>äÈ0nkkõçÏoXÏôô´ø¨·øzãOû666vttt©ÿoÅ þî"âÕÖÖ¶÷îwßWüÔüEQÚWüÔüMNNøþùç?þøãB¡ þê9þn"þÎ?ñPWñ¸	_òPñWCÄ þÄø«ü&üÎÀÝ¥¿çW~ç ã¯T¡PX^^~äGBü-,,?z¿Xè¿»víõB!Ä_2uù|þÄ!þÅ@½ÅßÍ>íÛ××'þê-þÖþmd2ùÈ#Táÿ­øÄß]DüâOü¿Êoâ&¢(jmmýÑ~$þê'þÖþmøo»Çu+++MMMW¯^?óz¨««+ß|óÍ---â Nâ¯££#^>/Îv-Ìd20YUäWüâoÓ7±jee¥8Sl¾øO?ú¿T*¥óòòòÕ?ìÆñÑÞê*þ&&&Öý´ïääd<uÁåË;::D8ã0scõpUý©ñ¿»øÄø^sõoÜü/|ÄKÅ@=Ä_h»ø;7Q=ßð"þ6»wïîêê¿Þ¥V?@üÝ¦d2ÙÐð§«Wç^ñðyÆ_¾×¯_õétºá|à ~âofffÇñûqêùÀ@ÝÆ_Quvø¸#ñWCÄ þÄøâOü?ñ þÄøâOü?ñ þÄøâOü?ñ'þÄßÈår===QuwwÏÎÎ.ZZZj(fÂÒét¸pWW×äääº3â zã¯½½zz:ÆÆÆ:::J>zhh¨t&ÍÁÈÈÈÀÀÀº3â zã¯Tcccé¡üÆÇÇKgZZZBäóù¶¶¶ugÊâï¥^¨_µ333ÃÃÃeof2(z/^¼fÂ¸¸4¯)¿sçÎýúUñwõêÕl6»¼¼¼îÒÅÅÅîîî0H$ÅÉd2¹îÃ¾U¡ínq8ìZ[[óù||7×ÕçÏÏd2W®»¨½½ýÒ¥KqËÁàààÉ'Ã g³ÙugÄ@õÆ_[[[Ù÷¹üé_¹:éìì¢hÏ=¡ÿÂÌÔÔT*J$ét:Ë­;#þª7þî(ñ?ñ þÄøâOü?ñ þÄøâOü?ñ þÄøâ@ü?ñ'þÄøâ@ü?ñ'þÄøâ@ü?ñ'þÄøâOü?ñ þÄøâOü?ñ þÄøâOü?ñ'þÄøâ@ü?ñ'þÄøâ@ü?ñ'þÄøââ@ü?ñ'þÄøâ@ü?ñ'þÄøâ@ü?ñ'þñ'þÄøâ@ü?ñ'þÄøâ@ü?ñ'þÄøâ@ü¿íËåzzz¢(êîî-]~L§ÓaQWW×ääd3âïß6ghhÈ:»7þÚÛÛ§§§Ã`ll¬£££tQ6gÊâ/áu>Wa=ï½¹¸ðnq×^Í:ÏQÅ_©ÆÆÆÒ[ZZBäóù¶¶¶gÊâïØ±cÿ-ÊïñÇ·`ËÔjüÍÌÌÎDQT6®dÆaßÍøãÿøÝ¾þÁÇ¼íÓMÿ®÷þÛ¾ú£ßûþ?úuØ7võêÕl6»¼¼H$ãd2YáøÛ÷ß¿¡¡áþÇ·ë´ëïÿëÓaC@=ÇßâââÐÐÐÒÒRÙ|kkk>¿±zH7+¿/§îøéÒvö=û«ûÿf¯uçÏÏd2W®»hppðäÉaÎ³Ùl3âOüÕmmm¥ßòÏÿÊÕÁÔÔT*J$ét:ËU8#þÄP½ñwG?ñâOüq«økØn÷ìúÄø»[â/Ýv¯âOümQüýyËW÷=û«í:ýõ÷þÇ7ïÐñ'þ¶(þüÎ?ñ'þÄ?ñ'þÄ?ñ'þÄ?ñ'þÄ?ñ'þÄ?ñ'þñ'þÄ þÄøÄø»øK66ç~³]§ûïùæ¿·!@ü¿­ðûßÿ¾¡¡!õµoûôdÓv~å¶¯þgÉ/îùö´!@ü¿¹54Õn=øââOüâOü?@ü?ñ?ñ'þñ'þÄ?ñøñ'þ¨8ï6c×®]Ö!?ñW3Þyç·7áý÷ß·@ü?ñ'þÄøâ@ü?ñ þÄøâOü?ñ þÄøâOü?ñ þÄøøâOü?ñ þÄøâOü?ñ þÄøâOü?ñ þÄøâ@ü?ñ'þÄøâ@ü?ñ'þÄøâ@ü?ñ þÄøâOü?ñ þÊr¥­­míüÒÒRC03;;N§£(êêêüToüMMMuvvÆmWæôéÓCCC¥3Ùlvtt4FFFÖ)¿_þòõ«Æâïá^XXX7þBùÎ´´´0ÈçóñkgÊâïÄ¿¨_µwØ÷Oÿ¬õâ¯½½=ÉDQÔÛÛñâÅ0ÆÅ¥ñxíÃ¾5EÝÝÝaH$ÉdrÝñPÛñW»ÖÖÖ|>cõ o¯;#þj2þÚÛÛ/]ºtcõ¿L&'OpÍf×5ñ333QíÙ³'þ$ËÔÔT*J$ét:Ë­;#þª=þîñ?ñ þÄøâOü?ñ þÄøâOü?ñ þÄøâ@ü?ñ'þÄ_àöÔ¯S§N?Äø@ü þ?ÄâñÇz÷Ýwxâæææ(Z[[~úé?üp37øÒK/ÝÎ=µÁu»w7W¼Ì¹sçúúúâK8ïïï3koê­·Þ×çÖjß%JL$===/¿ü²õVOwJî7Ûúî.lµ7Þxc×®]cccù|>üÎÃøî¹|ùòØ_¿ÝÑÇ^ýõ½÷ær¹B¡ß[Â®¼³³óÄe<pàÀc=^TX«u|÷X;sñâÅxàøñãV×Ý·Þúï.l©Ðykßç;vìØþýûÅpo¹zõjÙäï½J¥JgÂ¾~çÎ×®]Û±cG¼ßç.¿¸ÂëI«ë.¿²­_áîñÇV;xðàÈÈÈÚùð´=::ZüñìÙ³]]]Qó0.ð9sf÷îÝD",ßÏ_`èÂ---½½½Þ-RåñL&ß÷Ýo'lÖþþþ0ç¥8>ü3L%·FmÅ_|?	ç¥ÇýÇÇÇ½ñWÜúï.lµðÊ,¼»õeBÒ¥ÓéÙÙÙ0ç­­­333ÅÿÞ½ãÄábáIíN!zê©B¡ïnk¶HÇßÏ~ö³ðª=ÿ­/644466vcõ¸O.*¾Hw7Þx£³³Ój¯³ø»xñbwww|øááÞòòrôÑGáåßÚ÷¨³ø+nýÊw?¶Z"Øð2!ïÂtéÓñpxðúÚAYü-,,Txk¶È¶ïèoýiàôéÓ;vìûôÂ%§|Q|Ì÷ã?ãðd_vä7ÜZ.³ªë2þÂ¾páBGGGñùþÕW_úé§Ã``` ,²ë8þÖnýJv?¶Añ½º[_&þ,H,×ðß,þnïÖ¨ªÙ^~ff&ìÖî¹t:®¿ÏÌd2Åzè¡ÒÏ÷>|¸¿¿tttzzÚ¯ÖAüå¾¾¾²s>øàG9tèXñ·áÖßpwøctvv~ðÁõÁ¿îáÝã¯ò[£jã¯ÔÊÊÊñãÇÃËýâÌSO=Uö¬0<<\zýúõÇ,~Ø±cÇï~÷;«½¦ãoÃkÊñø`ê,þ6¿»@ü±>|³/å*¾8¯ç>Çwþ*¿5j"þâz±àãc¾¥¿råJKKËÚ7ù_ýõööv«½¾ã/Í8pàg±Å_ÙîñÇöXXXØµkWxz.?sæÌÑ£Gãq___éoéñÃ?|ÛñWù­QñJ¥Ö¾WÂ®§§'ONNM0sþüùÏñùZ¿7ß|3¼ÂL&s³ûõî.lãÇ···/ÈÂ/_~ñÅ÷ìÙsíÚµø¹<ãßÒ	ã©©©[Ç_SSÓï½¿ýSvÊo*¿'NW¯¿þúGïÇçææÏëW=Âk0Ã~ll,~#°ôâÔ_üÅð?íò×Áß»*þ6Ü] þØNgÏ¯ËDÁ£GË¯øä3_wwwéSûÍâïW^I®ZwQá­±-;ú?ðvÜ>úhsssüöïß_ü²°s[víÞ0SüJðª ÜÙÂ#Üf¸LLLXíõái¾ô[Ï_Ýì*ßcßbwø@ü þ?ñø@ü þ?Äâñø@ü?Äâñø¨*mmmËËËeóa&¢t:](>ÛþtÕ&/Vá?ÏæùçõòË/Í?~<Ìÿð?üÌûSñ?ªõÞïÌêîî.ïììó.]ºS»]ñ?mÉdBiår¹âÌôôtÙ·o_qæèÑ£©T*¢ÆÆÆGtqq±´ÒÎ=»cÇ|pm·Ýú¡;ÃÒ0¾Yü;w.Äh¸X¸pøoç?úè£áááæææ°¨¥¥åÐ¡Ck_?O¥õÄOgì±03::Z¸OÛ»woi¥%pÞßß_Öm^±ÌÌÌÌÚøoíÅúúúÊnág±Añp++++;wî¢èÊ+áÇpb«¹¹9ÏÇH§Ó¡«Þzë­ÿï0qccci¥=÷ÜsB!>Fm^q``àêª0?îß¿müe³Ùø=Â0¯/£ð>ãð_	ã¦¦&8|øp(§cÇñ/¾Æ*½@ÁPW!òzã·úJ+íòåËÿúé#¶·¾bÁââbø1çÚ	eoï/ÖÓÓ~Ü½÷ÁÇÇÇ¯]»fSâ`cóóóqEq8ã¹¹¹âÒ©©©²+­´Ò¯)]ºá?µ#nh¢híÒµG¹°°÷_1Ï9ckâ`cßúÖ·B?ýà?ç¡¨JÅü&&&>þøã®8³áã#¶7V5ßì¿¦¦¦0.^ë·¿ýíóÏ?ßßß.N§mJ@ülìÕW_-¾622Rº(¢ø½ÀB¡ðÊ+¯T^qÿþý!û®_¿>88~çko$®ºC	YúÅ4ñïüÅ¿S¸°°ÆÉdÒ¦ÄÀÆ®]»ÖØØ½zõjé¢~¸ô¨ëÎ;Ãyü¥-·¿¯×aqÖ#333eO>/?&Rê©§²)ñPááá²ï|]¹r%Í&ÉæææÃ_ºt©ø¥*·¿¯811ÑÓÓ²¯««ëÂëÞHpöìÙÞÞÞD"N§O8Q_^^>räH*Ë2ü'®_¿n;âñ þ?Äâñø@ü þ?ñø@ü þ¨zÿg-D0îIEND®B`


ONEWAY figure BY Variables
  /POLYNOMIAL=1
  /STATISTICS DESCRIPTIVES HOMOGENEITY
  /MISSING ANALYSIS
  /POSTHOC=LSD ALPHA(0.05).


Oneway


Notes	
Output Created	01-OCT-2022 15:19:13	
Comments		
Input	Active Dataset	DataSet0	
	Filter	<none>	
	Weight	<none>	
	Split File	<none>	
	N of Rows in Working Data File	39	
Missing Value Handling	Definition of Missing	User-defined missing values are treated as missing.	
	Cases Used	Statistics for each analysis are based on cases with no missing data for any variable in the analysis.	
Syntax	ONEWAY figure BY Variables
  /POLYNOMIAL=1
  /STATISTICS DESCRIPTIVES HOMOGENEITY
  /MISSING ANALYSIS
  /POSTHOC=LSD ALPHA(0.05).	
Resources	Processor Time	00:00:00.03	
	Elapsed Time	00:00:00.01	


Descriptives	
figure  	
	N	Mean	Std. Deviation	Std. Error	95% Confidence Interval for Mean			
					Lower Bound	Upper Bound			
Control	4	1.93000	.086410	.043205	1.79250	2.06750			
ESAs	4	3.33333	.115854	.057927	3.14898	3.51768			
TPx	4	3.05000	.094163	.047081	2.90017	3.19983			
LPS	4	3.92000	.043205	.021602	3.85125	3.98875			
Total	16	3.05833	.751002	.187751	2.65815	3.45851			


Test of Homogeneity of Variances	
	Levene Statistic	df1	df2	Sig.	
figure	Based on Mean	.520	3	12	.676	
	Based on Median	.465	3	12	.712	
	Based on Median and with adjusted df	.465	3	9.395	.713	
	Based on trimmed mean	.520	3	12	.677	


ANOVA	
figure  	
	Sum of Squares	df	Mean Square	F		
Between Groups	(Combined)	8.365	3	2.788	352.714		
	Linear Term	Contrast	6.468	1	6.468	818.113		
		Deviation	1.898	2	.949	120.015		
Within Groups	.095	12	.008			
Total	8.460	15				


Post Hoc Tests


Multiple Comparisons	
Dependent Variable:   figure  	
LSD  	
(I) Variables	(J) Variables	Mean Difference (I-J)	Std. Error	Sig.	95% Confidence Interval	
					Lower Bound	Upper Bound	
Control	ESAs	-1.403333*	.062871	.000	-1.54032	-1.26635	
	TPx	-1.120000*	.062871	.000	-1.25698	-.98302	
	LPS	-1.990000*	.062871	.000	-2.12698	-1.85302	
ESAs	Control	1.403333*	.062871	.000	1.26635	1.54032	
	TPx	.283333*	.062871	.001	.14635	.42032	
	LPS	-.586667*	.062871	.000	-.72365	-.44968	
TPx	Control	1.120000*	.062871	.000	.98302	1.25698	
	ESAs	-.283333*	.062871	.001	-.42032	-.14635	
	LPS	-.870000*	.062871	.000	-1.00698	-.73302	
LPS	Control	1.990000*	.062871	.000	1.85302	2.12698	
	ESAs	.586667*	.062871	.000	.44968	.72365	
	TPx	.870000*	.062871	.000	.73302	1.00698	

*. The mean difference is significant at the 0.05 level.	


SAVE OUTFILE='E:\学习\1.文章\4.SCI（1）\Raw Data\3. C. Cellulosae ESAs and TPx Induced the Increase in '+
    'the Number of CD4+CD25+Foxp3+ Tregs in PBMCs\2. SPSS statistical analysis\2.3 SPSS '+
    'statistical analysis--CD4+CD25+Foxp3+ Treg\2.2.1 SPSS statistical analysis--Treg.sav'
  /COMPRESSED.

Error # 61 in column 14.  Text: E:\学习\1.文章\4.SCI（1）\Raw Data\3. C. Cellulosae ESAs and TPx Induced the Increase in the Number of CD4+CD25+Foxp3+ Tregs in PBMCs\2. SPSS statistical analysis\2.3 SPSS statistical analysis--CD4+CD25+Foxp3+ Treg\2.2.1 SPSS statistical analysis--Treg.
The filename is not valid.
Execution of this command stops.

SAVE OUTFILE='E:\桌面\2.2.1 SPSS statistical analysis--Treg.sav'
  /COMPRESSED.
