# Supplementary material for: Regulation of piglet T-cell immune responses by thioredoxin peroxidase from Cysticercus cellulosae excretory-secretory antigens
Source: Front Microbiol. 2022 Nov 18;13:1019810. doi: 10.3389/fmicb.2022.1019810 (PMC9718028; doi:10.3389/fmicb.2022.1019810)
Supplement: Supplementary file 4 [file Data_Sheet_4.zip › 3. C. Cellulosae ESAs and TPx Induced the Increase in the Number of CD4+CD25+Foxp3+ Tregs in PBMCs/4.2 Flowjo analysis data export-- Foxp3+.pdf]

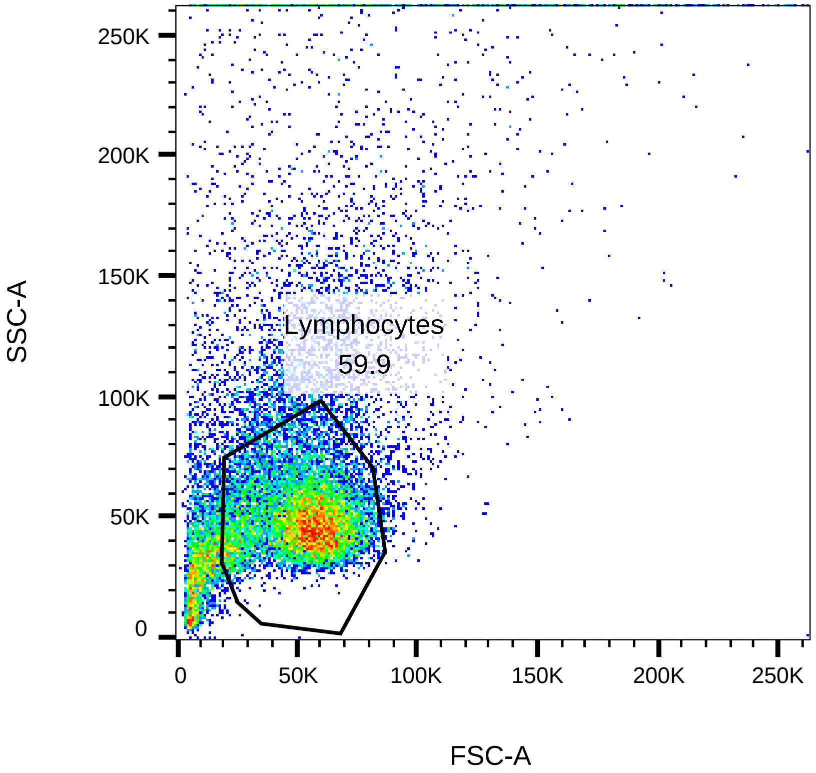

Specimen_003_1640-1 10000_005.fcs
Ungated
20394

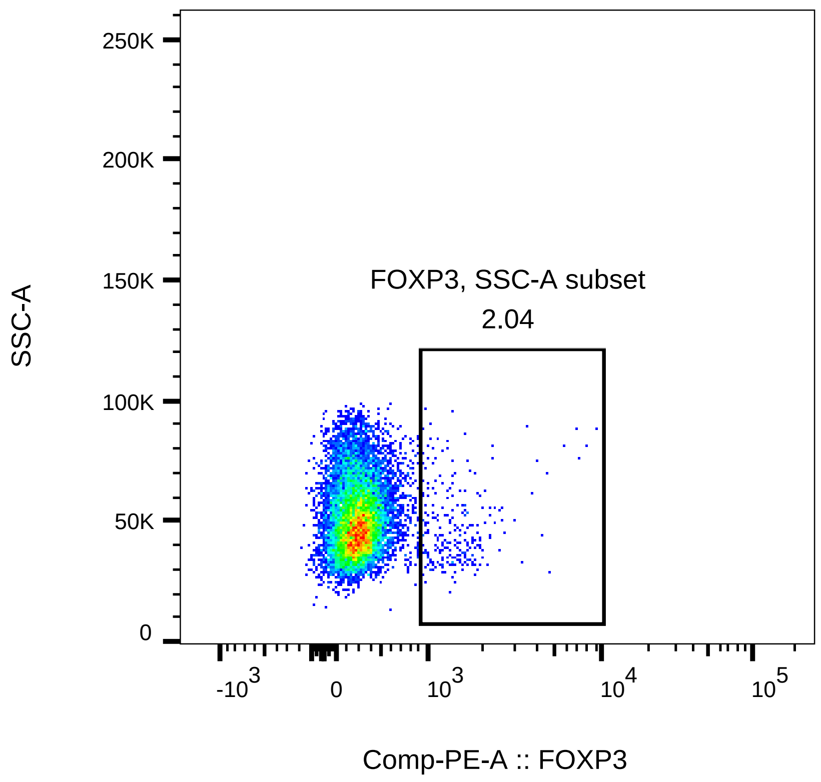

Specimen_003_1640-1 10000_005.fcs
Lymphocytes
12225

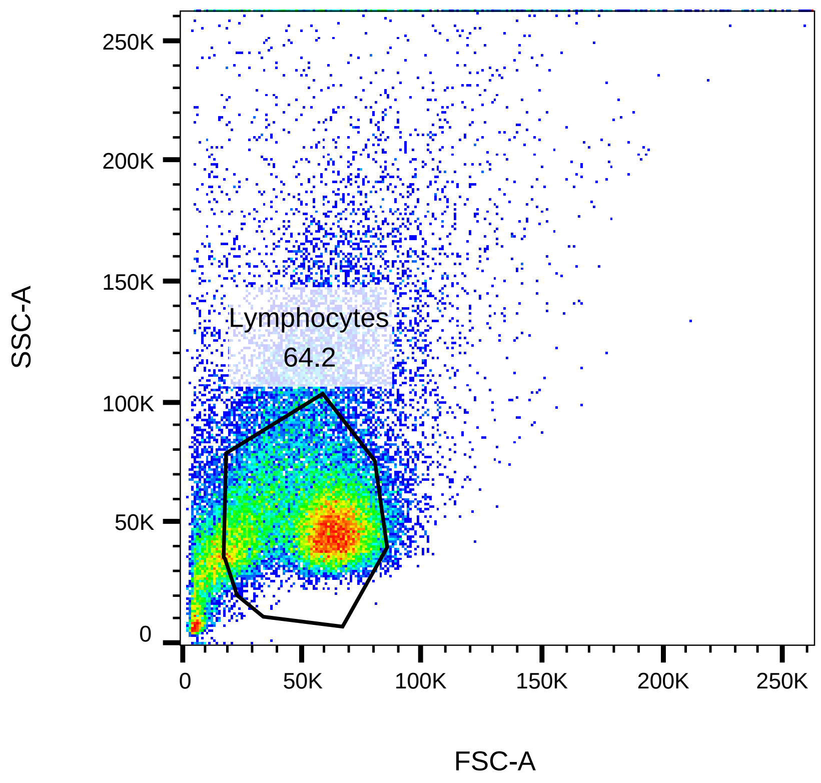

Specimen_003_1640-2_007.fcs
Ungated
39093

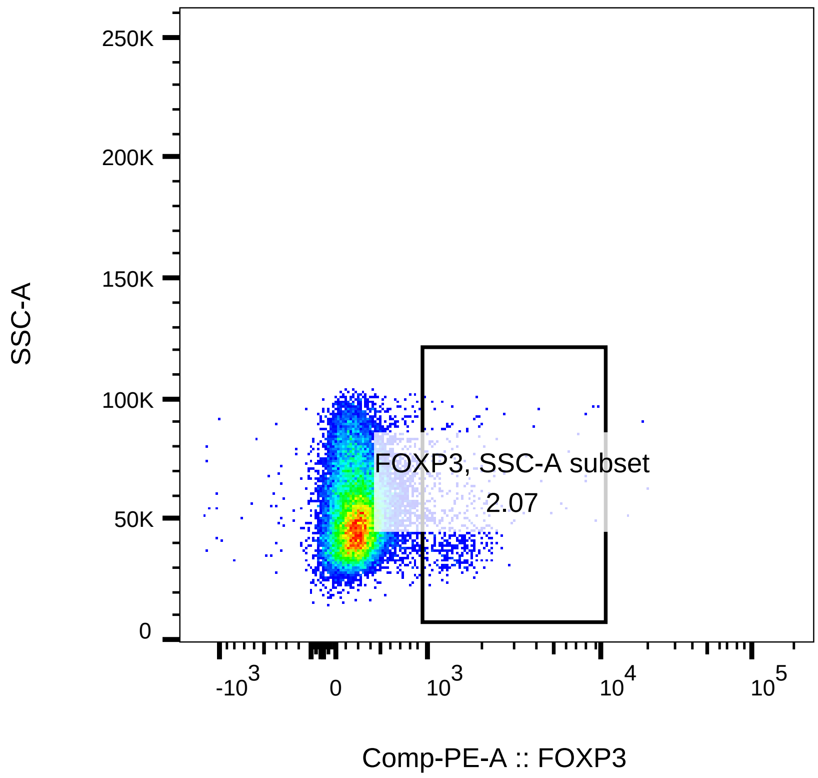

Specimen_003_1640-2_007.fcs
Lymphocytes
25099

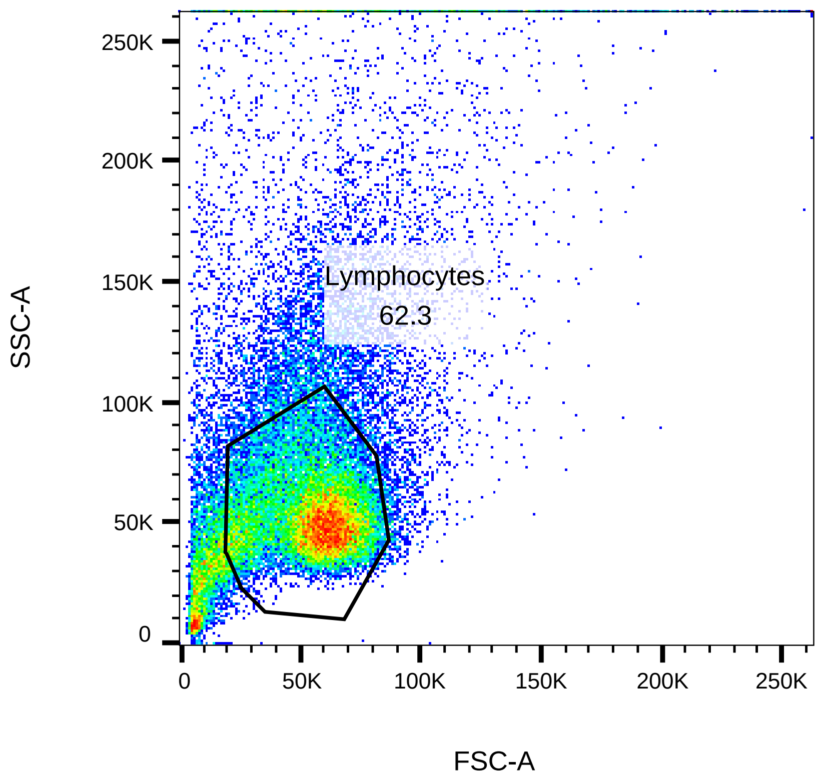

Specimen_003_1640-3_008.fcs
Ungated
39470

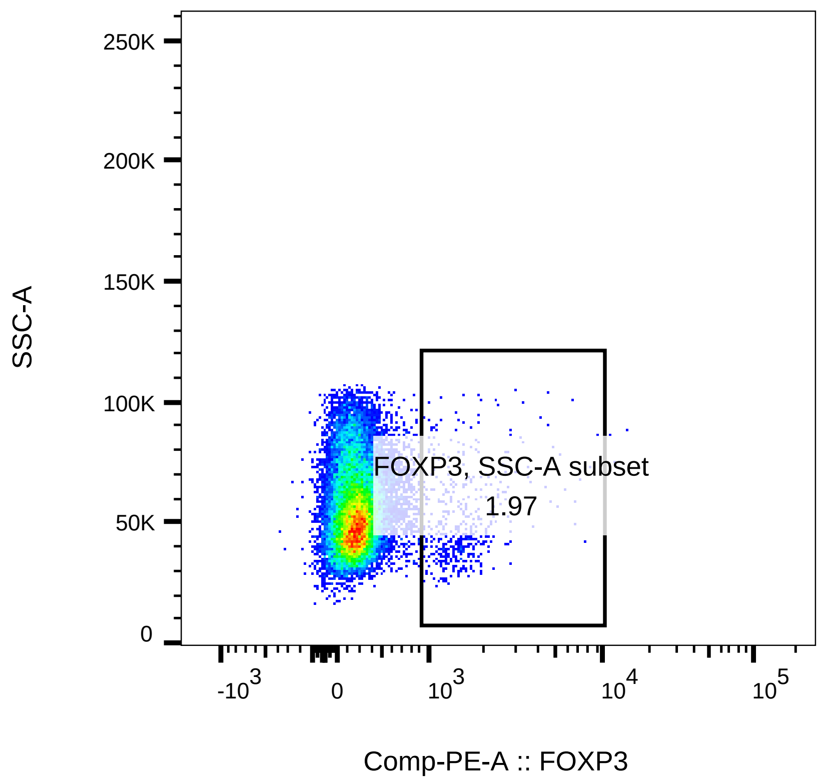

Specimen_003_1640-3_008.fcs
Lymphocytes
24586

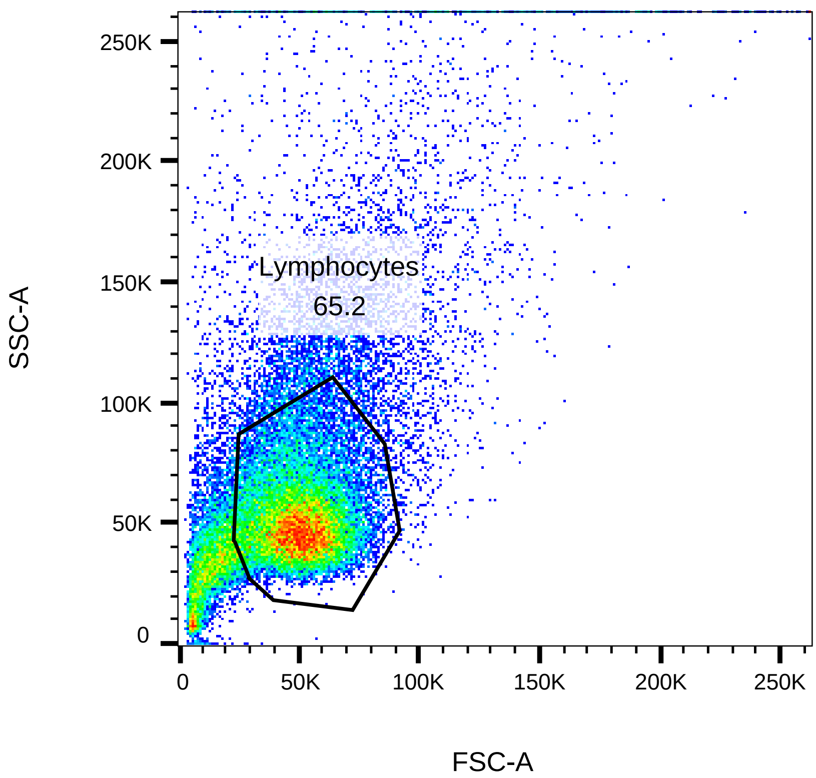

Specimen_003_ESA-1_009.fcs
Ungated
38575

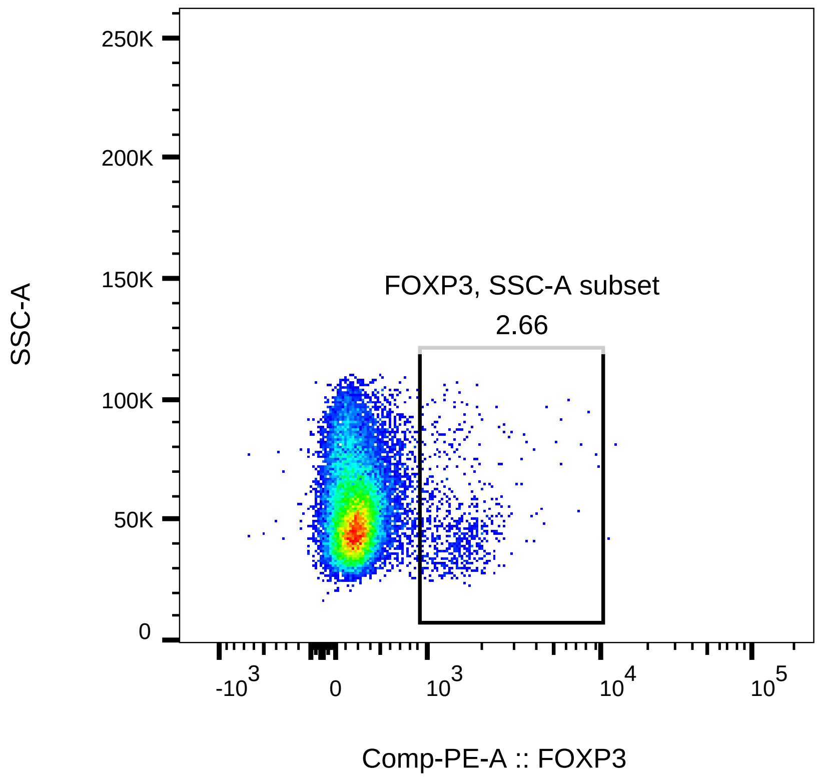

Specimen_003_ESA-1_009.fcs
Lymphocytes
25159

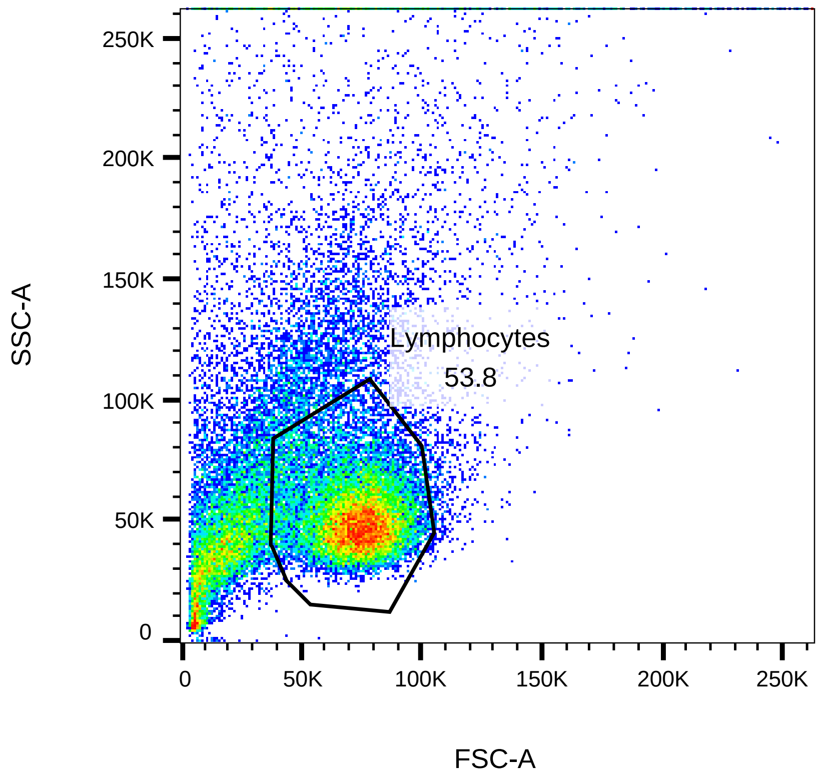

Specimen_003_ESA-2_010.fcs
Ungated
36327

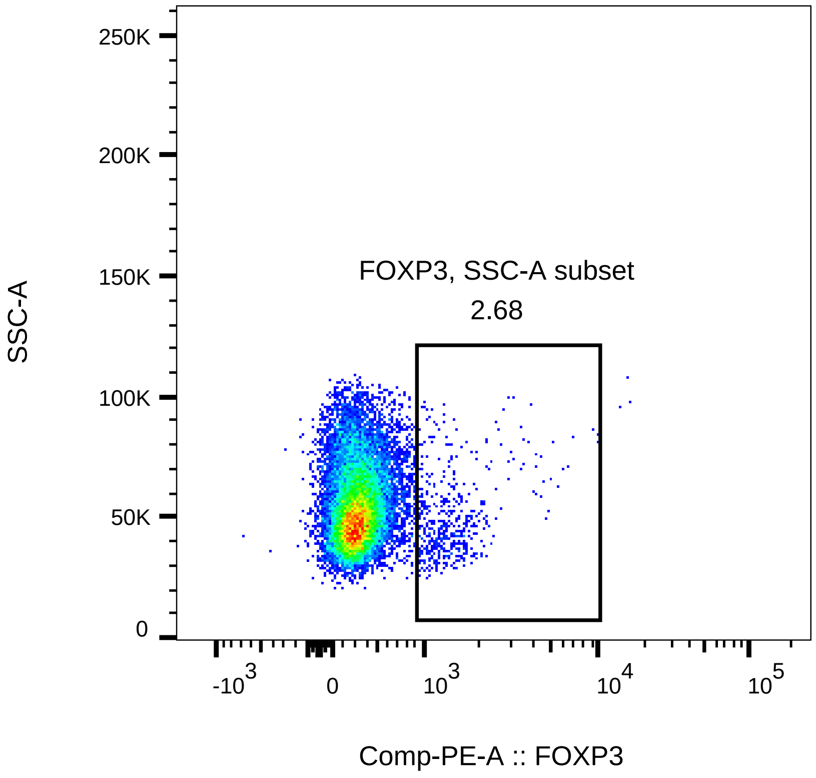

Specimen_003_ESA-2_010.fcs
Lymphocytes
19530

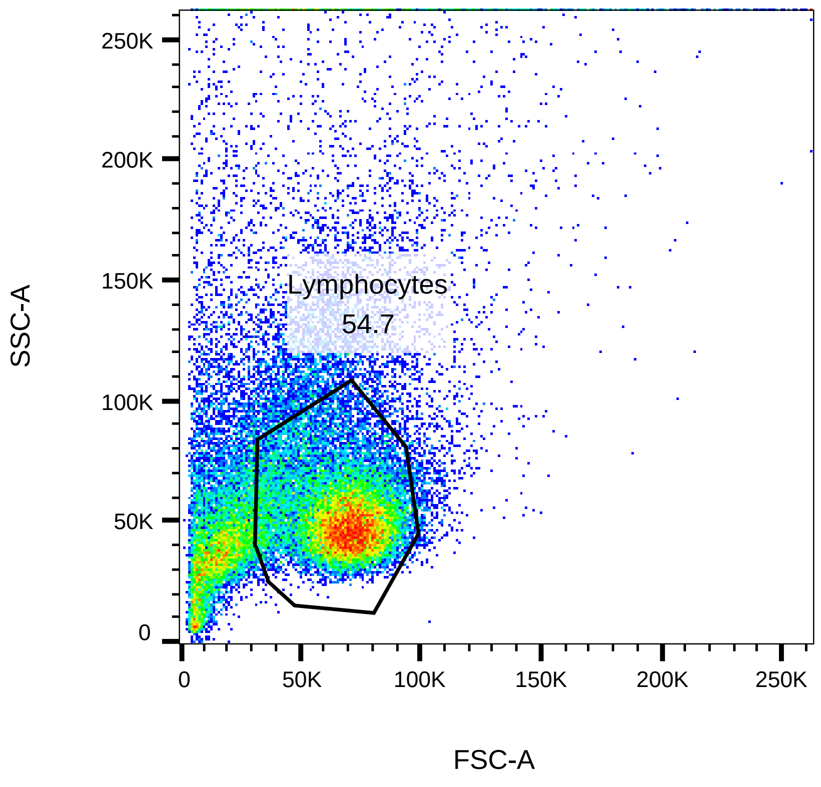

Specimen_003_ESA-3_011.fcs
Ungated
38213

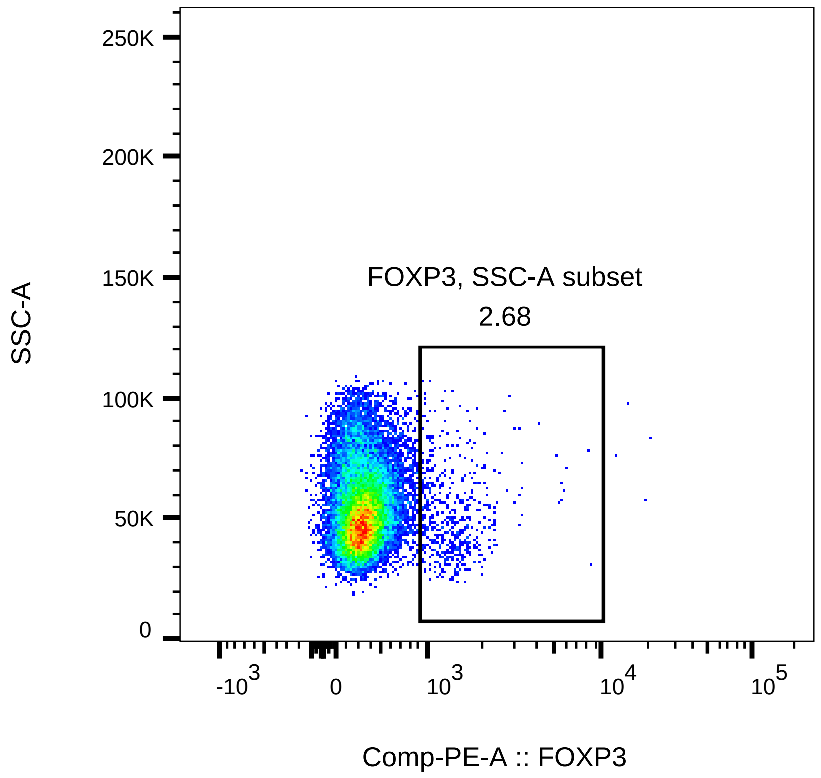

Specimen_003_ESA-3_011.fcs
Lymphocytes
20901

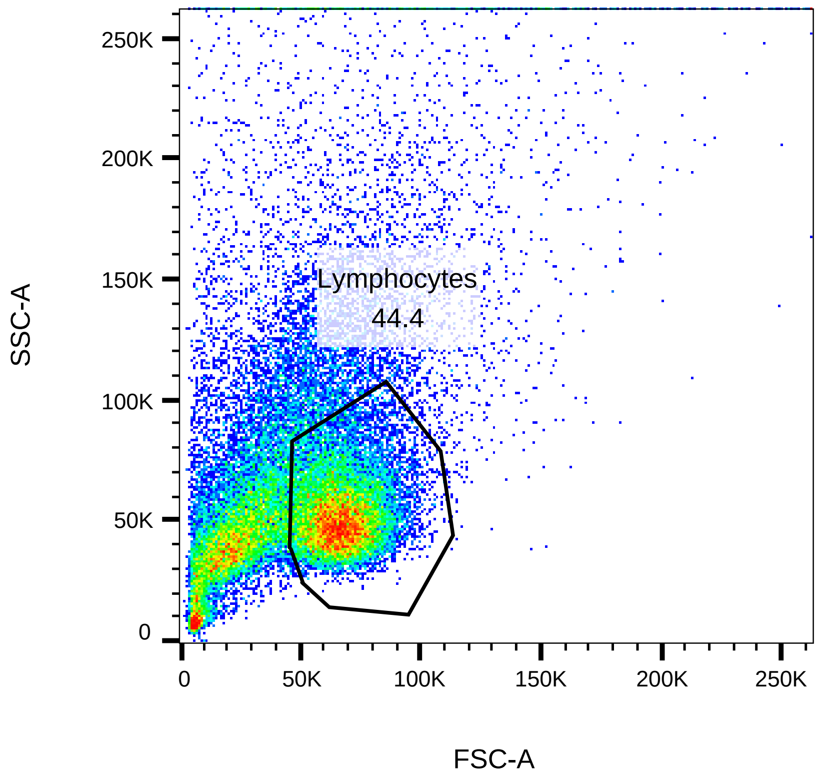

Specimen_003_TPX-1_015.fcs
Ungated
39309

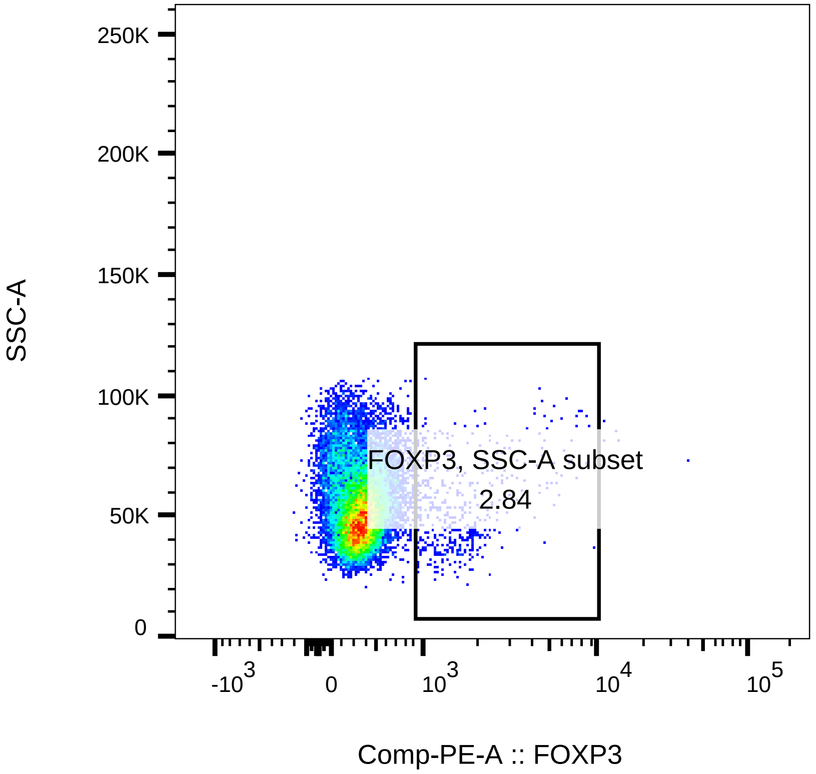

Specimen_003_TPX-1_015.fcs
Lymphocytes
17467

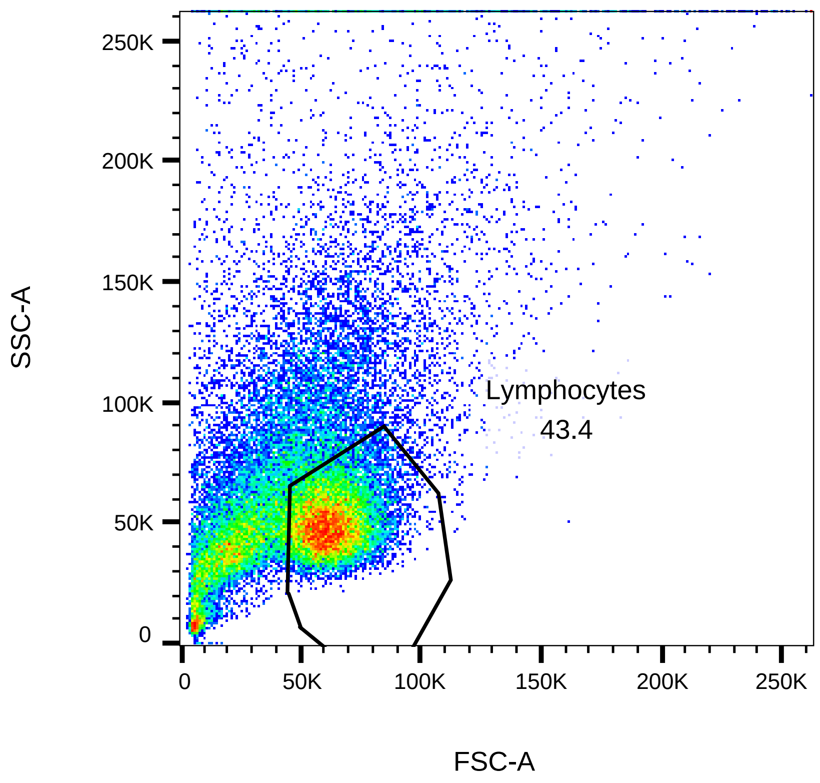

Specimen_003_TPX-2_016.fcs
Ungated
36845

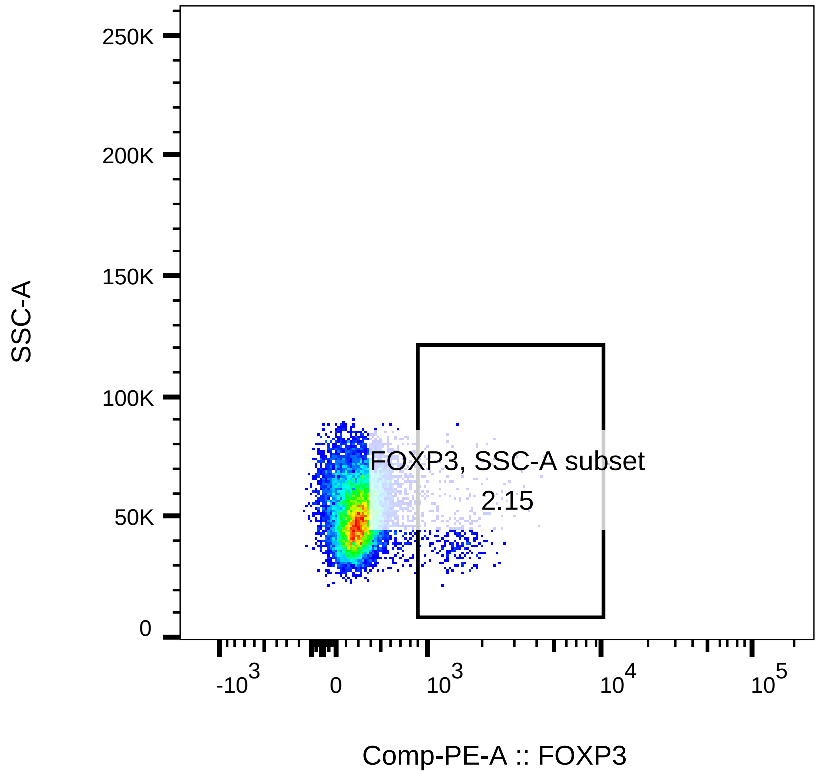

Specimen_003_TPX-2_016.fcs
Lymphocytes
15996

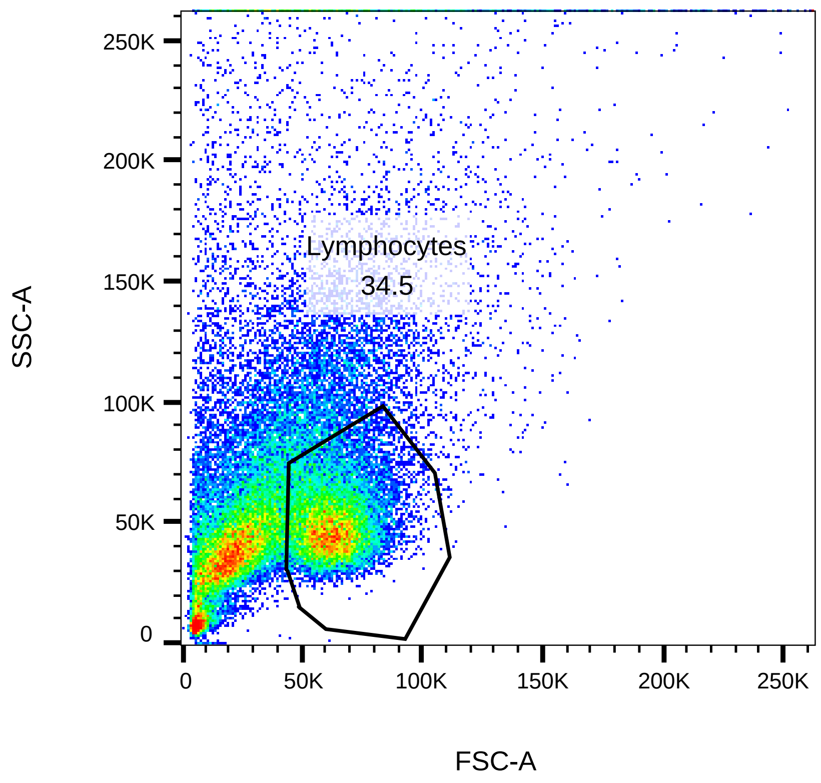

Specimen_003_TPX-3_017.fcs
Ungated
47800

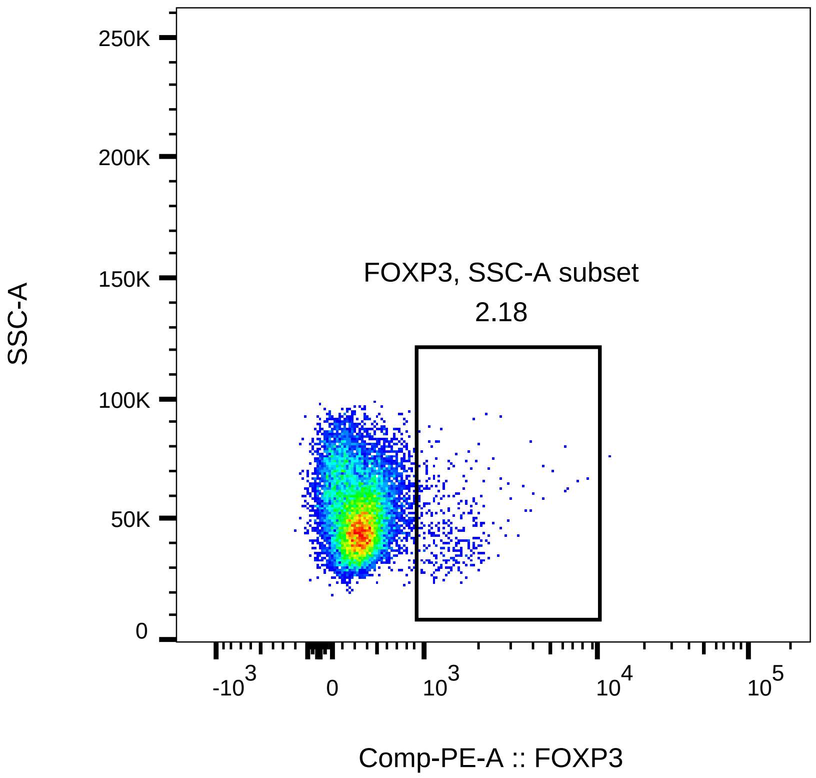

Specimen_003_TPX-3_017.fcs
Lymphocytes
16513

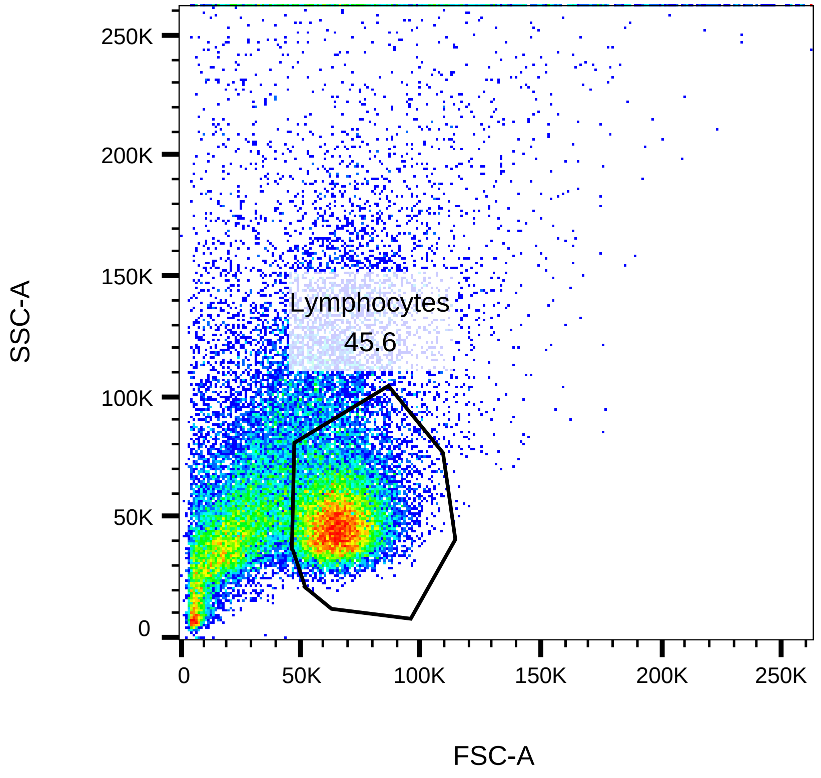

Specimen_003_LPS-1_018.fcs
Ungated
38431

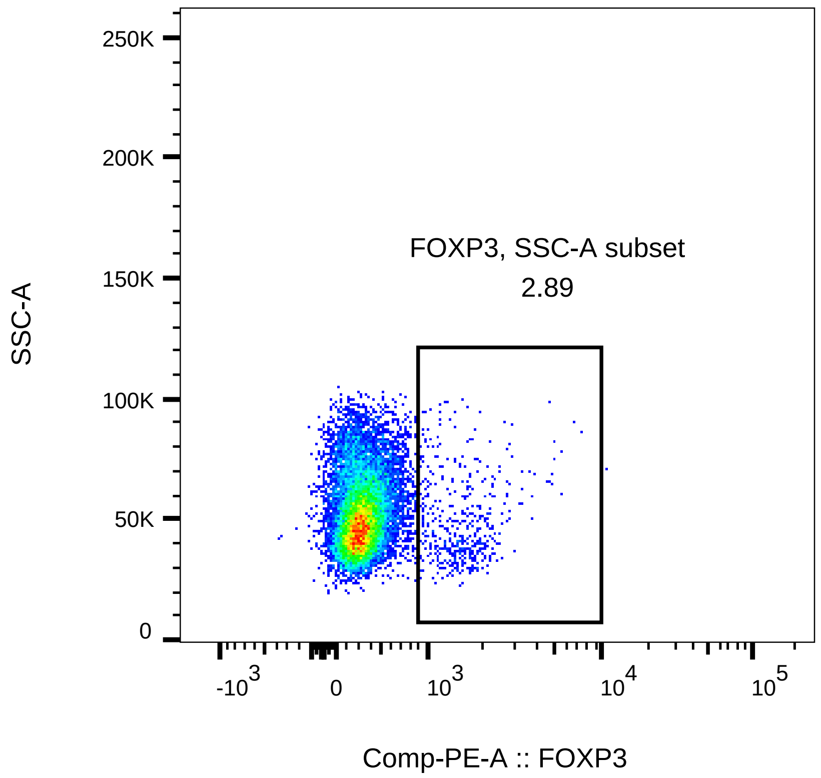

Specimen_003_LPS-1_018.fcs
Lymphocytes
17521

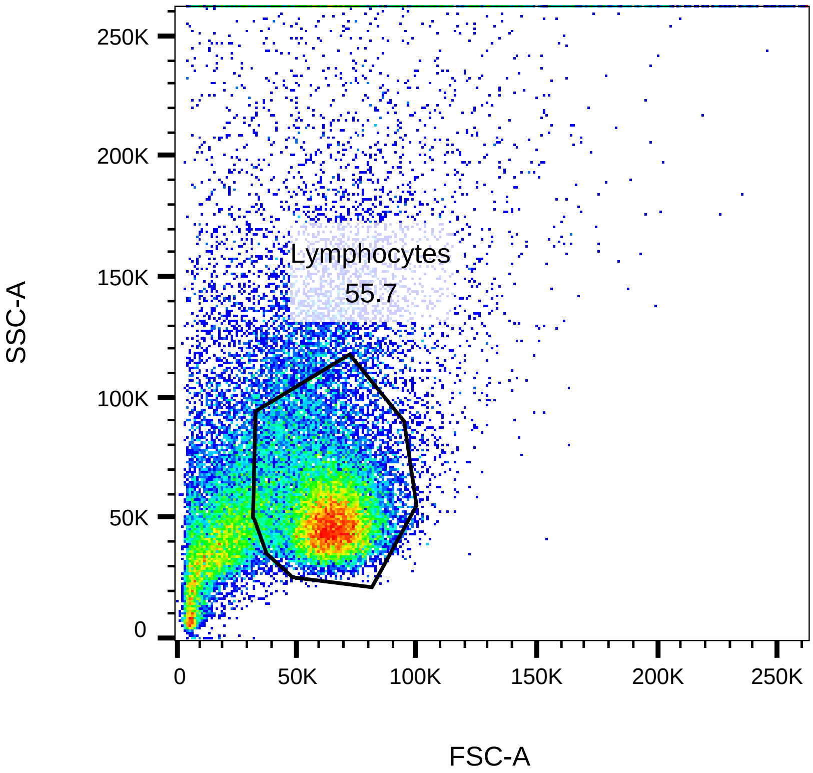

Specimen_003_LPS-2_019.fcs
Ungated
39479

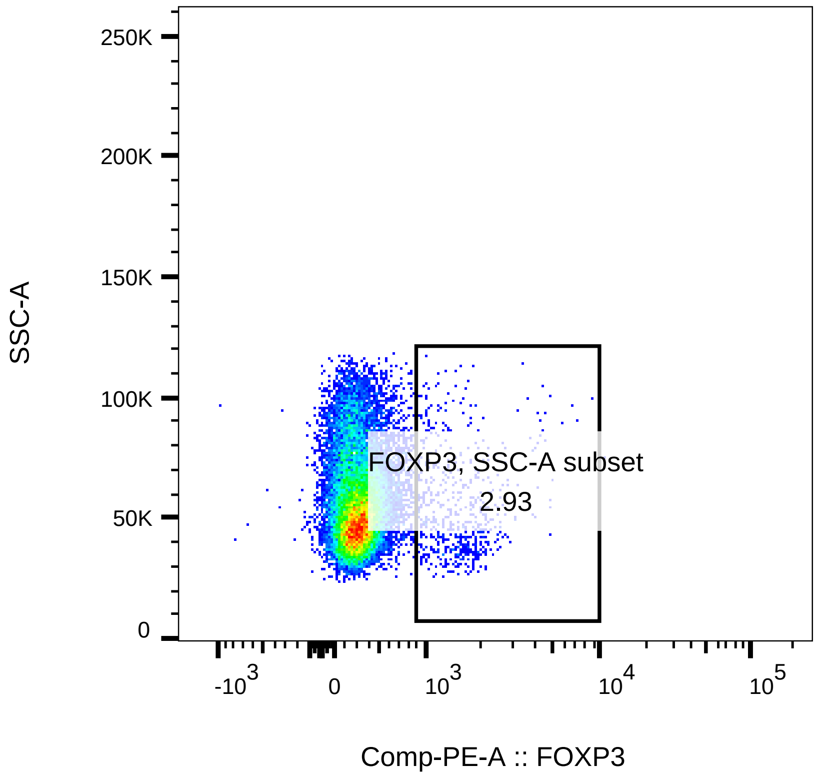

Specimen_003_LPS-2_019.fcs
Lymphocytes
21978

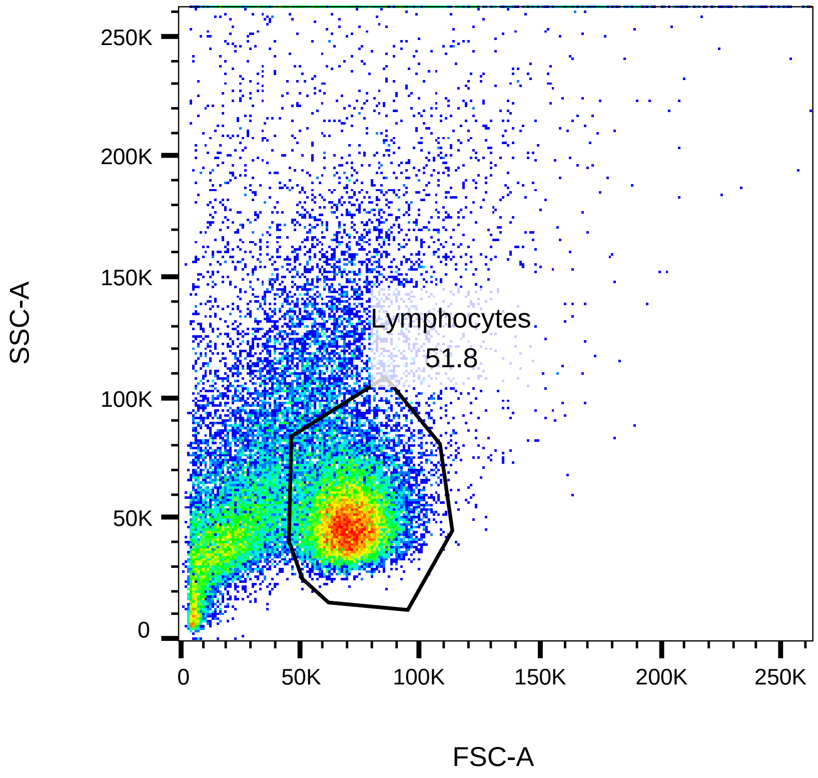

Specimen_003_LPS-3_020.fcs
Ungated
35657

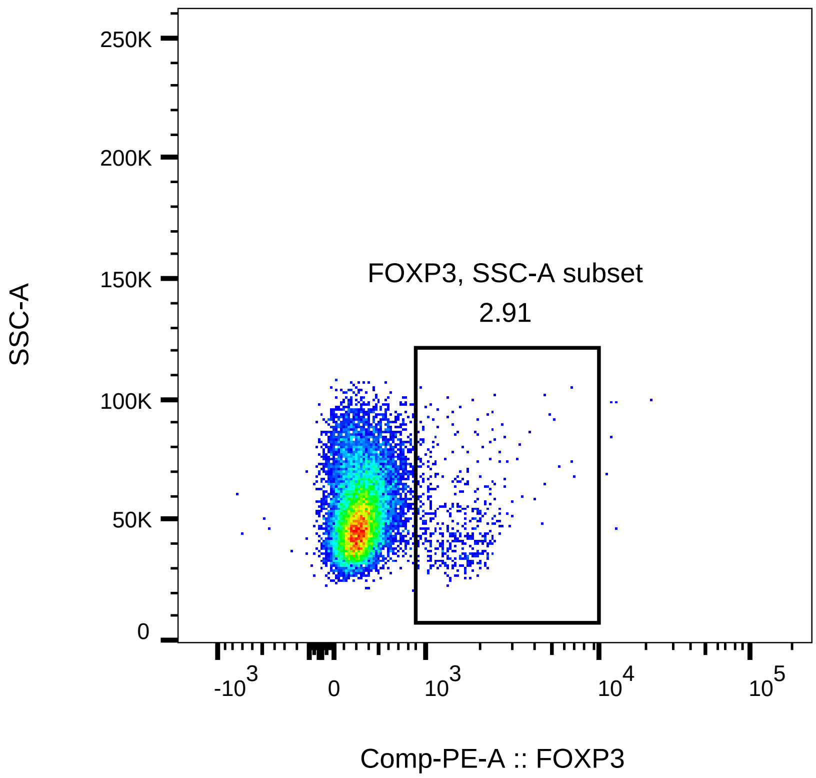

Specimen_003_LPS-3_020.fcs
Lymphocytes
18479

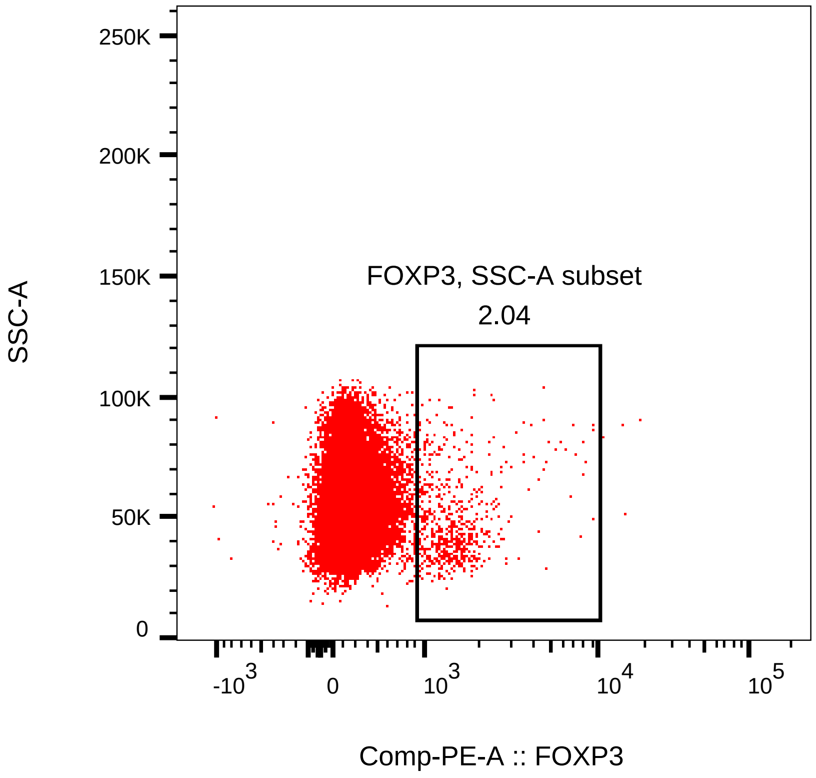

	Sample Name	Subset Name	Count
	Specimen_003_1640-3_008.fcs	Lymphocytes	24586
	Specimen_003_1640-2_007.fcs	Lymphocytes	25099
	Specimen_003_1640-1 10000_005.fcs	Lymphocytes	12225

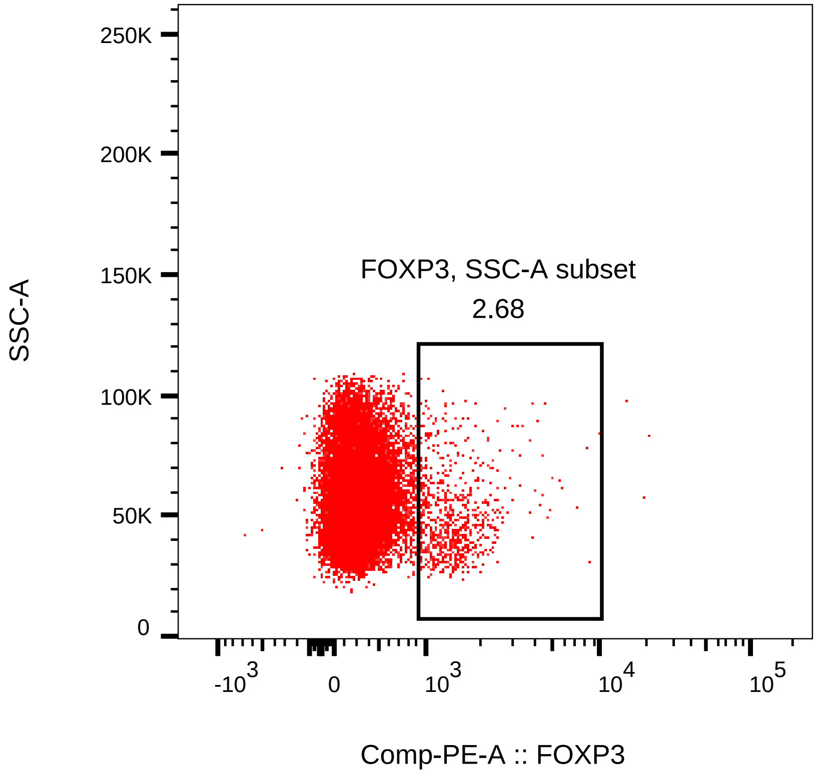

	Sample Name	Subset Name	Count
	Specimen_003_ESA-3_011.fcs	Lymphocytes	20901
	Specimen_003_ESA-1_009.fcs	Lymphocytes	25159
	Specimen_003_ESA-2_010.fcs	Lymphocytes	19530

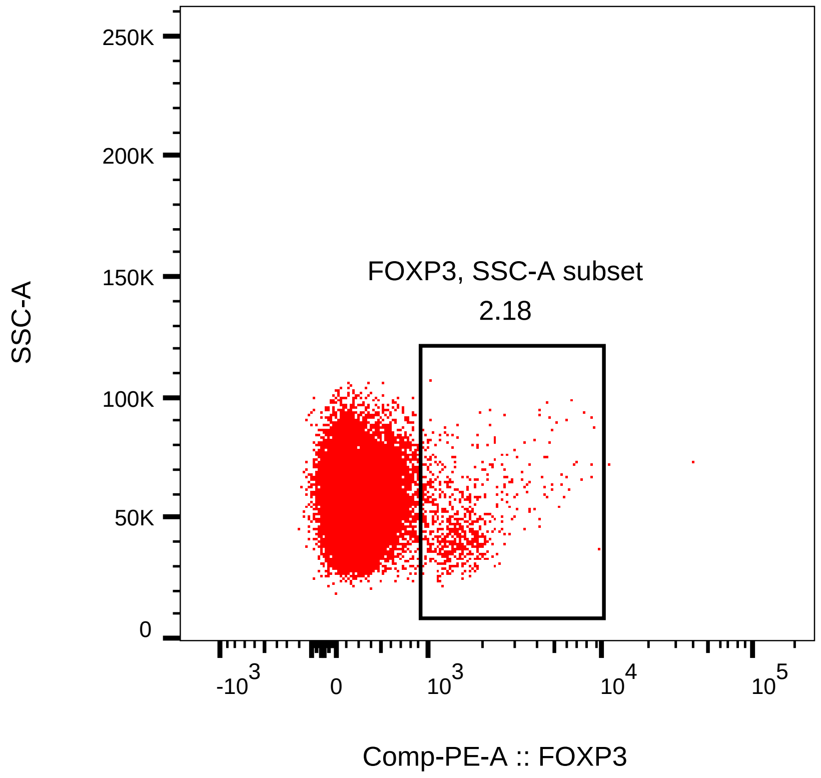

	Sample Name	Subset Name	Count
	Specimen_003_TPX-2_016.fcs	Lymphocytes	15996
	Specimen_003_TPX-1_015.fcs	Lymphocytes	17467
	Specimen_003_TPX-3_017.fcs	Lymphocytes	16513

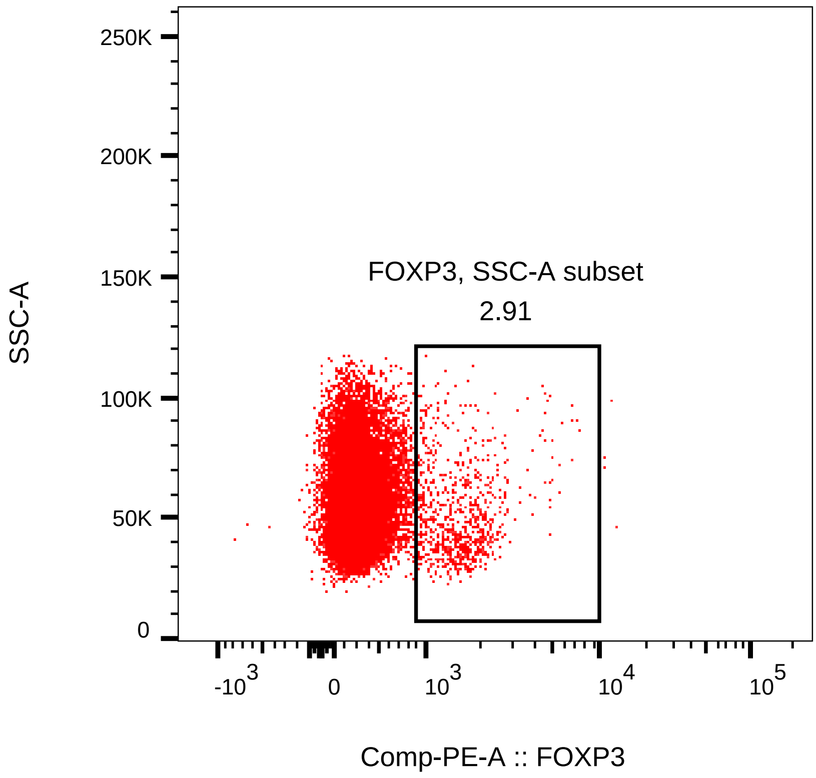

	Sample Name	Subset Name	Count
	Specimen_003_LPS-2_019.fcs	Lymphocytes	21978
	Specimen_003_LPS-1_018.fcs	Lymphocytes	17521
	Specimen_003_LPS-3_020.fcs	Lymphocytes	18479
