# Supplementary material for: Regulation of piglet T-cell immune responses by thioredoxin peroxidase from Cysticercus cellulosae excretory-secretory antigens
Source: Front Microbiol. 2022 Nov 18;13:1019810. doi: 10.3389/fmicb.2022.1019810 (PMC9718028; doi:10.3389/fmicb.2022.1019810)
Supplement: Supplementary file 4 [file Data_Sheet_4.zip › 3. C. Cellulosae ESAs and TPx Induced the Increase in the Number of CD4+CD25+Foxp3+ Tregs in PBMCs/5.2 Flowjo analysis data export-- CD4+CD25+Foxp3+.pdf]

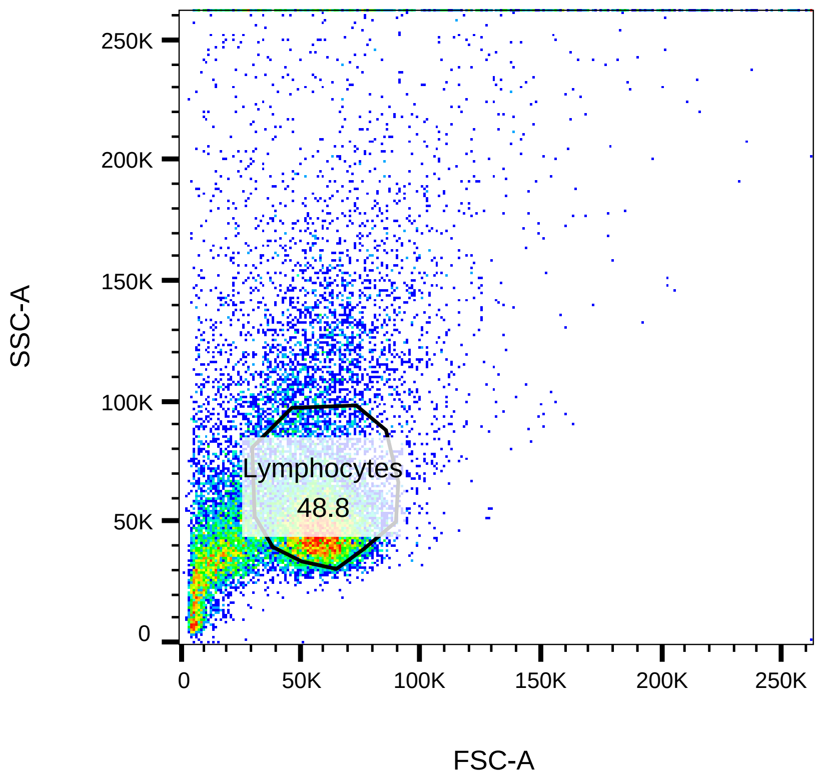

Specimen_003_1640-1 10000_005.fcs
Ungated
20394

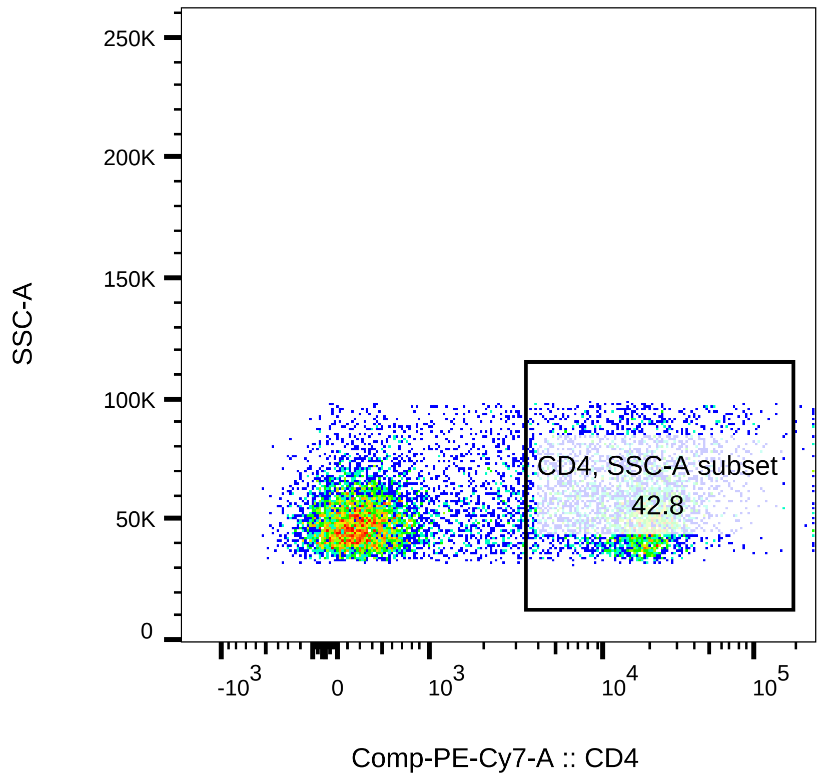

Specimen_003_1640-1 10000_005.fcs
Lymphocytes
9945

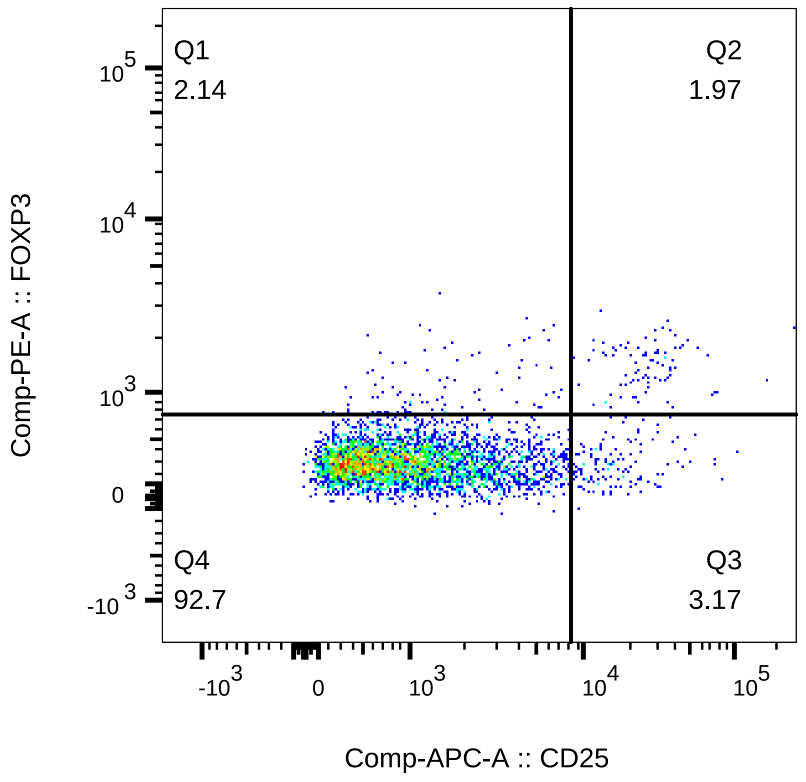

Specimen_003_1640-1 10000_005.fcs
CD4, SSC-A subset
4254

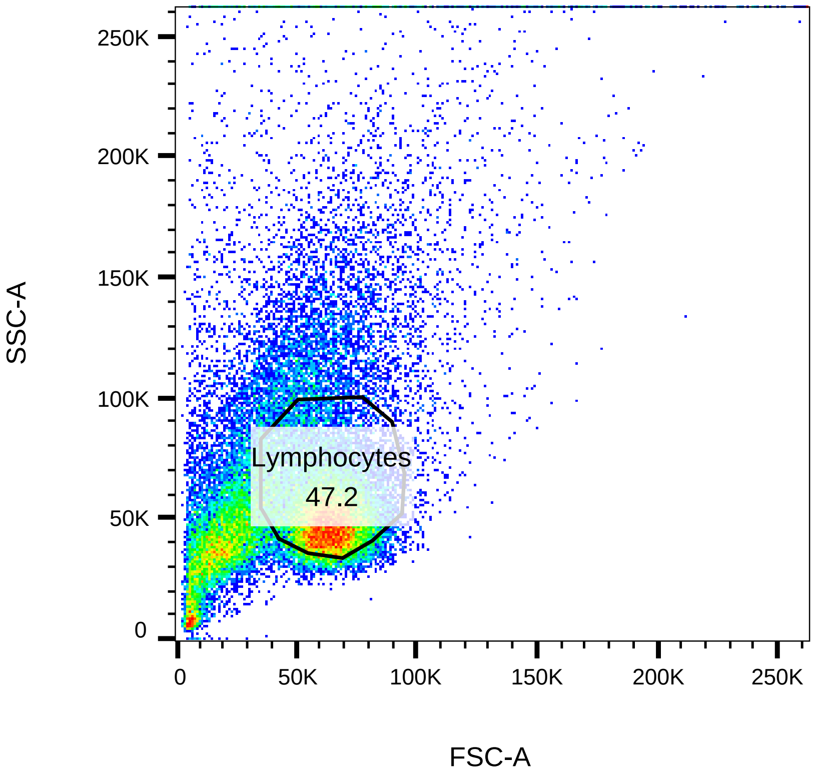

Specimen_003_1640-2_007.fcs
Ungated
39093

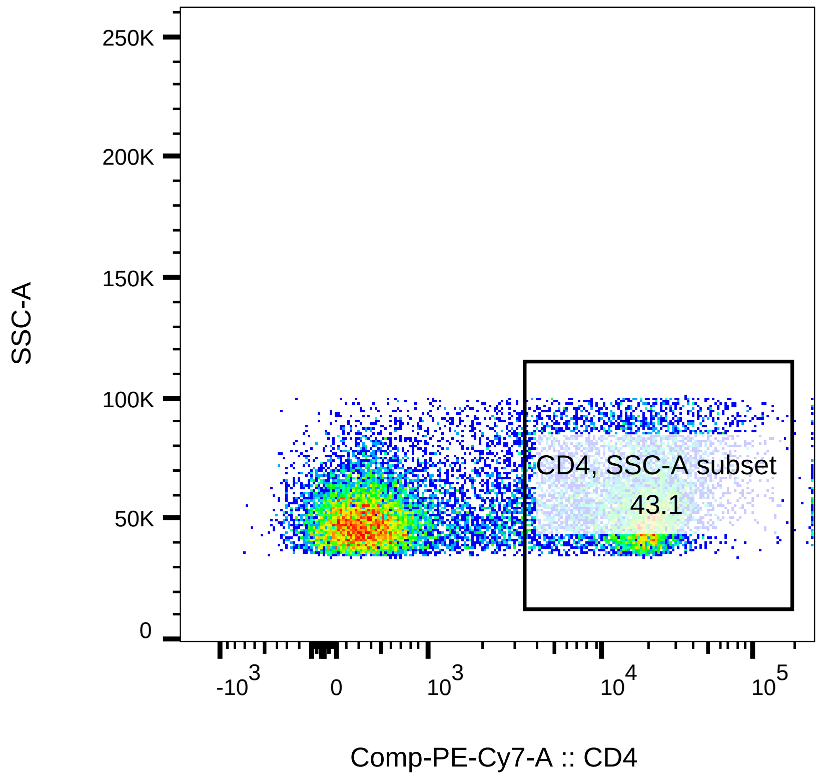

Specimen_003_1640-2_007.fcs
Lymphocytes
18471

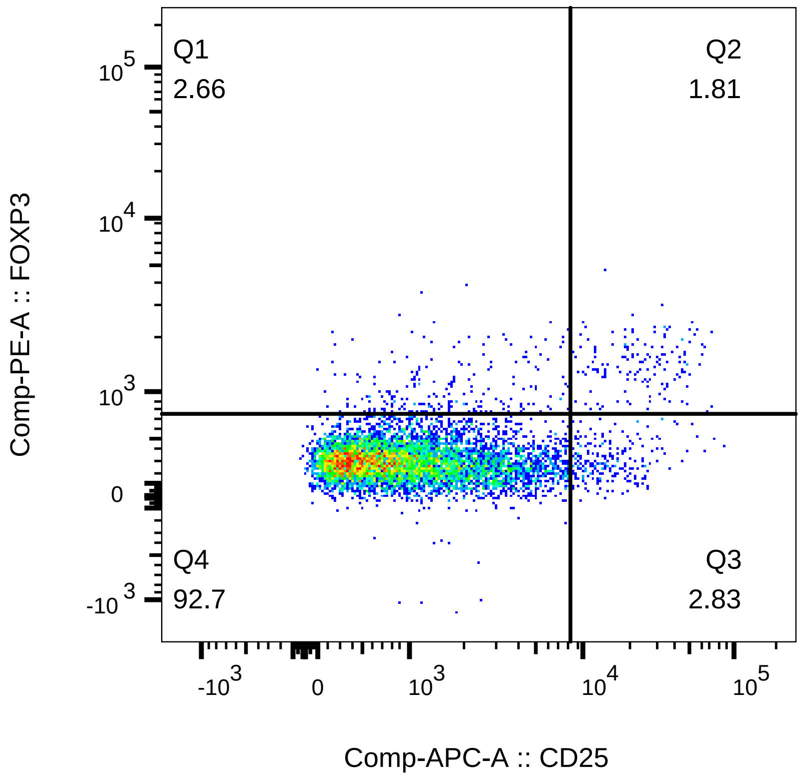

Specimen_003_1640-2_007.fcs
CD4, SSC-A subset
7955

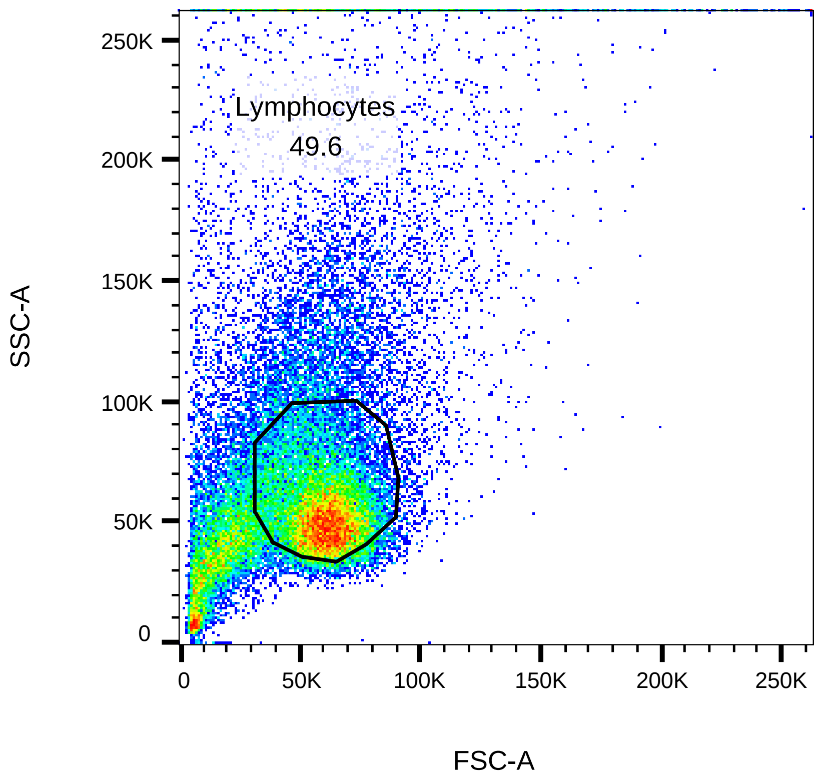

Specimen_003_1640-3_008.fcs
Ungated
39470

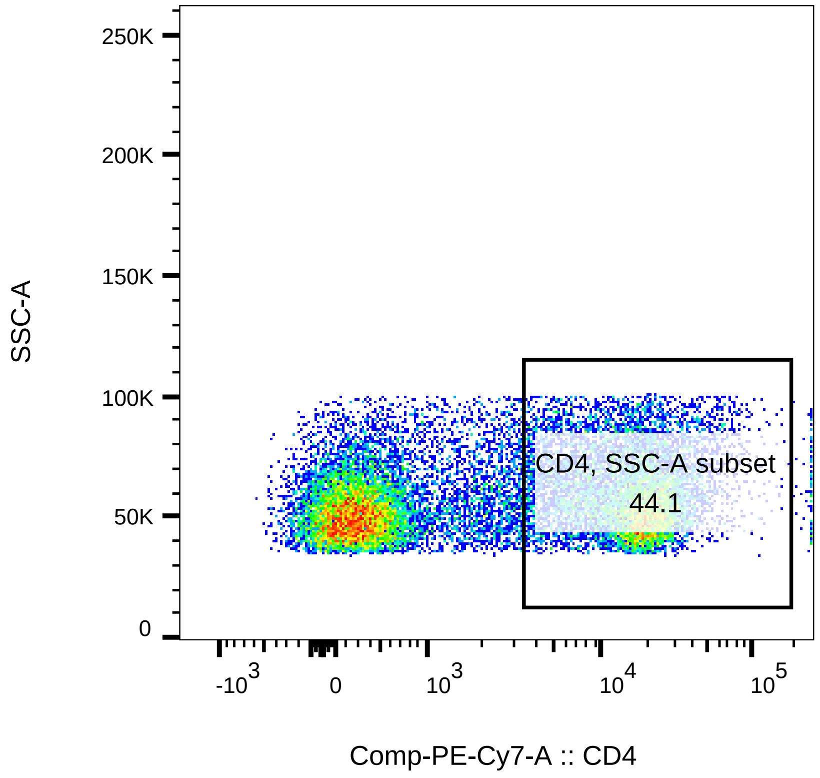

Specimen_003_1640-3_008.fcs
Lymphocytes
19579

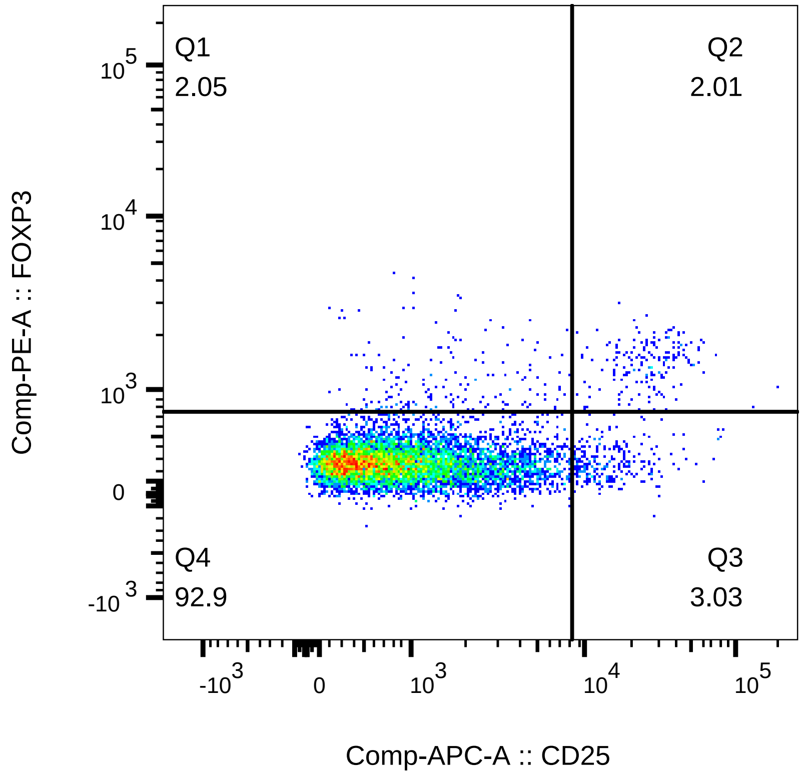

Specimen_003_1640-3_008.fcs
CD4, SSC-A subset
8628

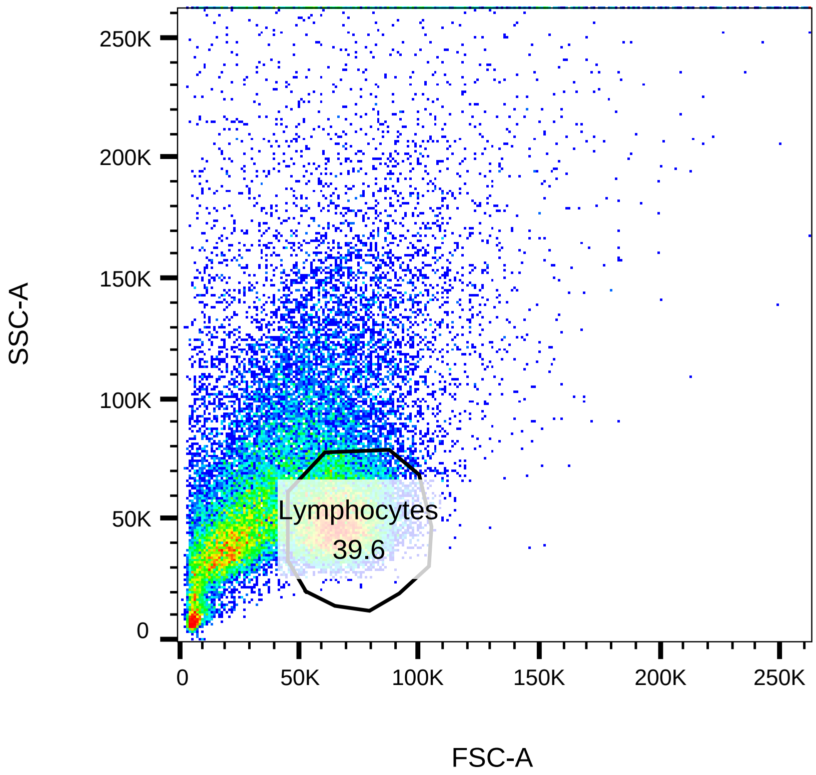

Specimen_003_TPX-1_015.fcs
Ungated
39309

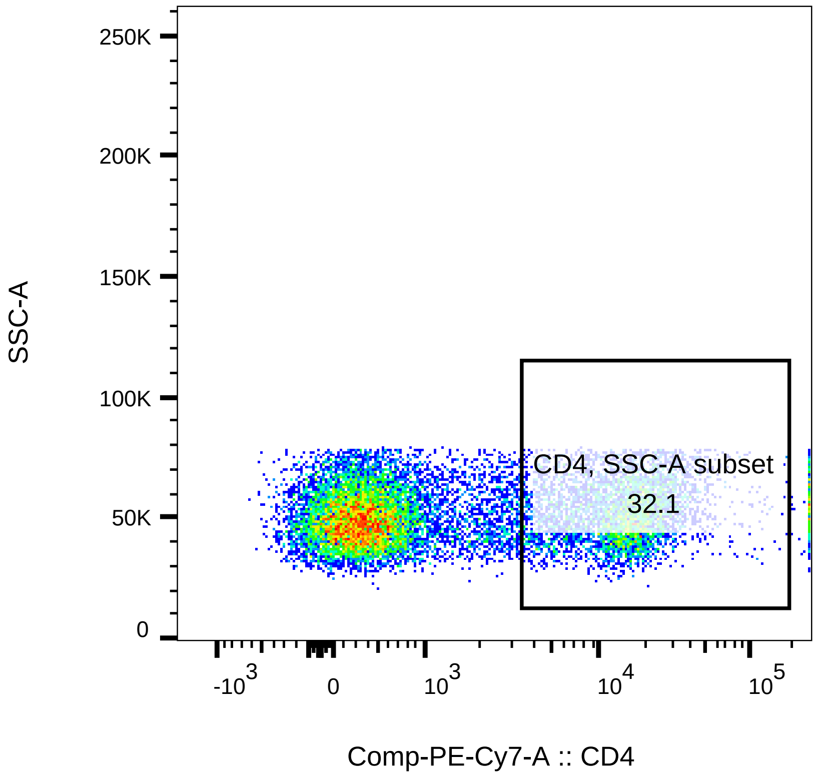

Specimen_003_TPX-1_015.fcs
Lymphocytes
15557

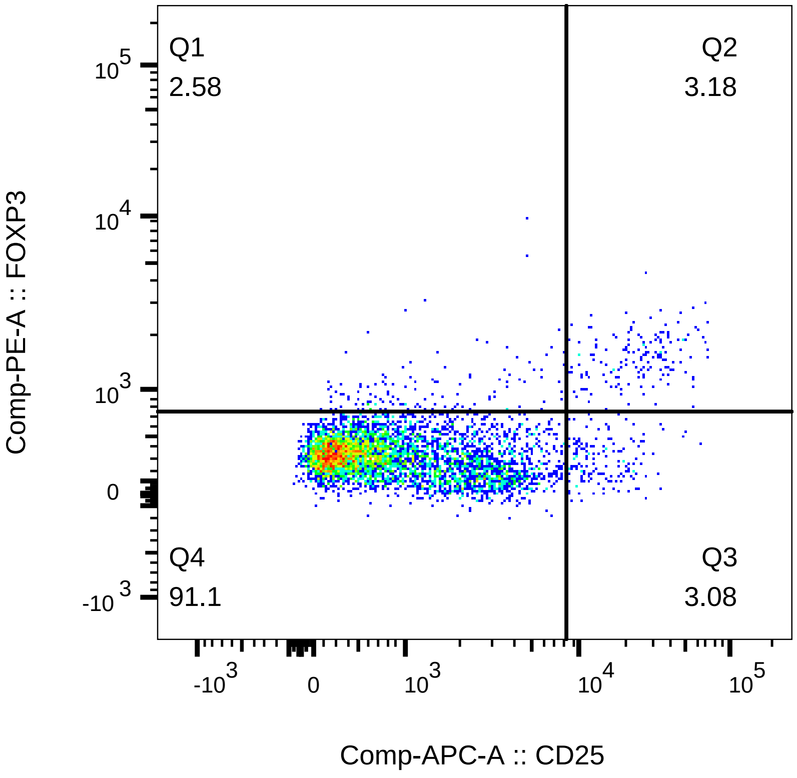

Specimen_003_TPX-1_015.fcs
CD4, SSC-A subset
4993

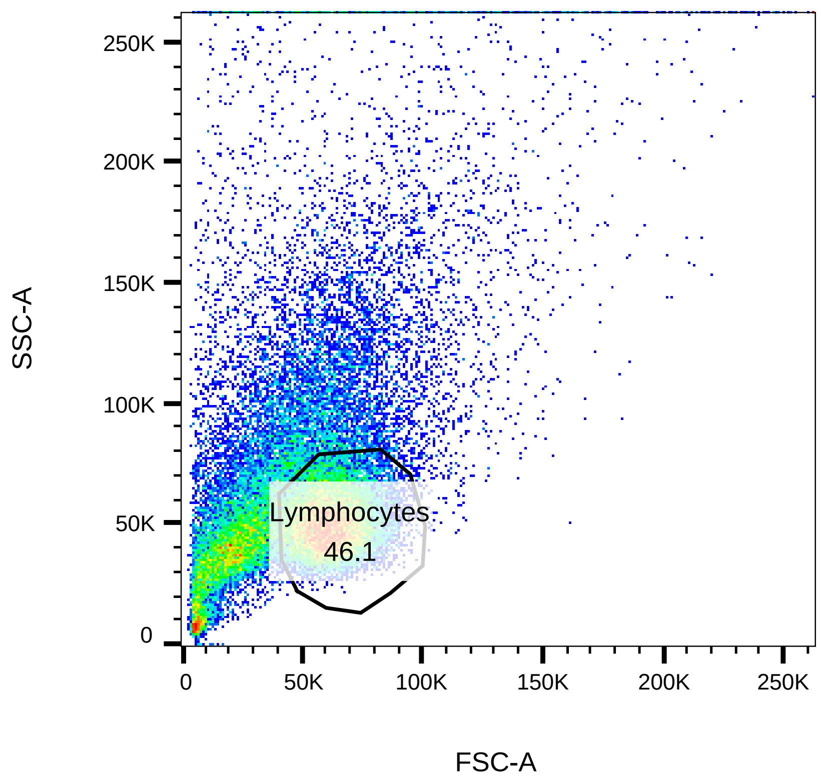

Specimen_003_TPX-2_016.fcs
Ungated
36845

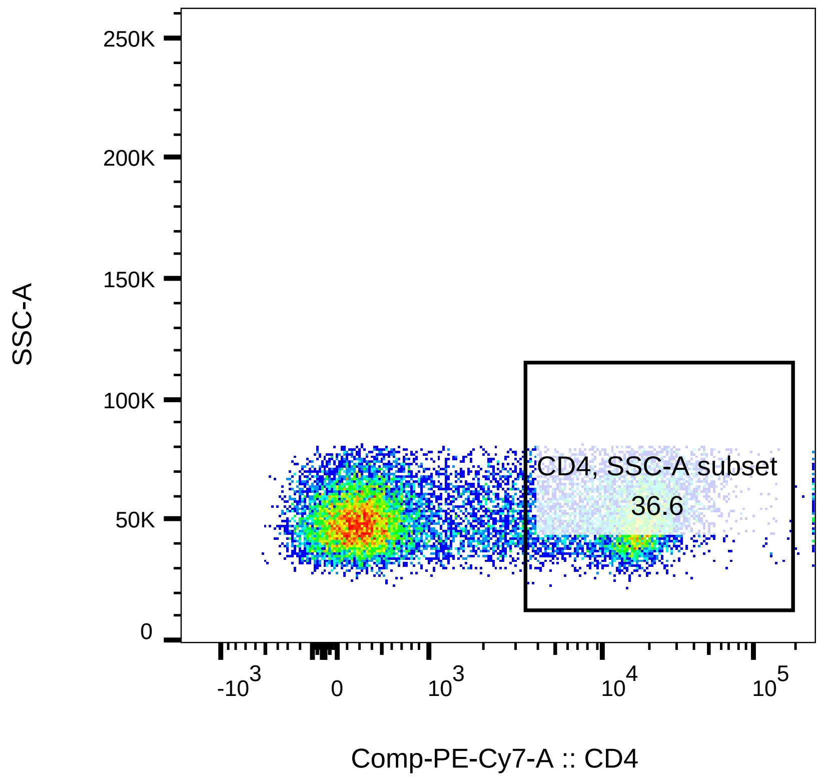

Specimen_003_TPX-2_016.fcs
Lymphocytes
16994

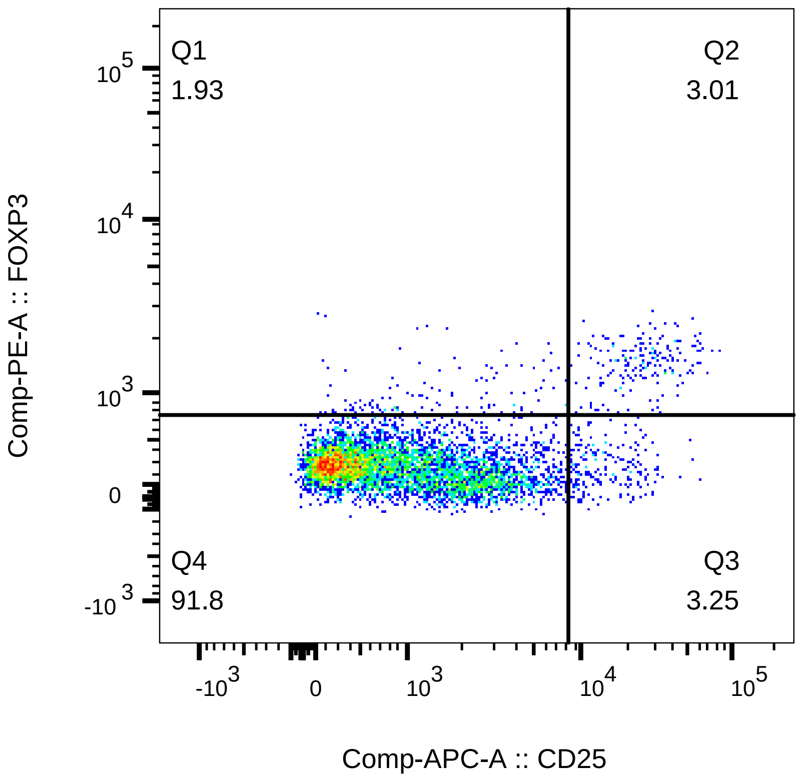

Specimen_003_TPX-2_016.fcs
CD4, SSC-A subset
6213

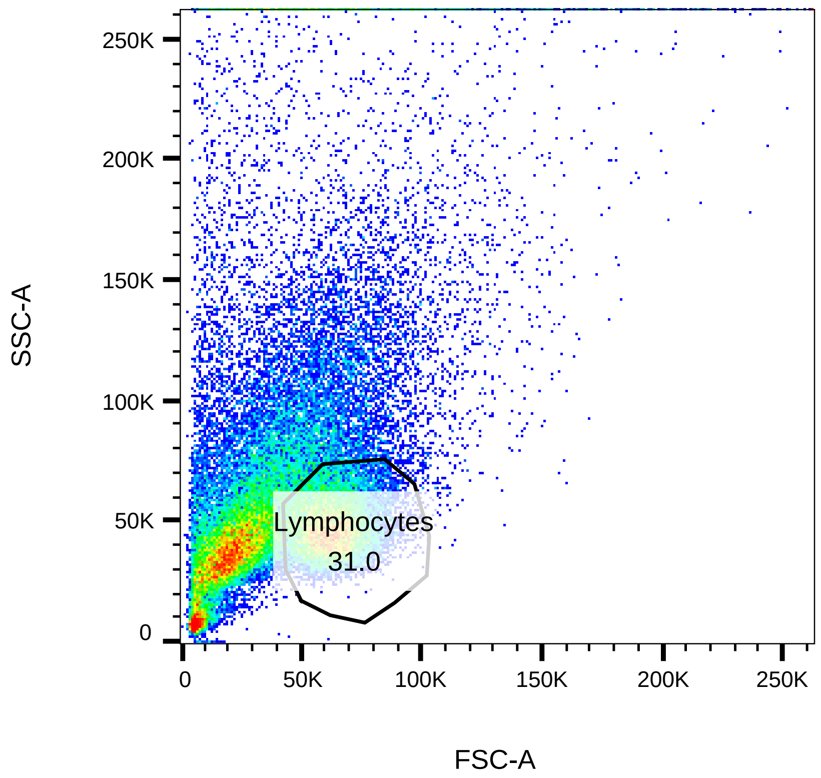

Specimen_003_TPX-3_017.fcs
Ungated
47800

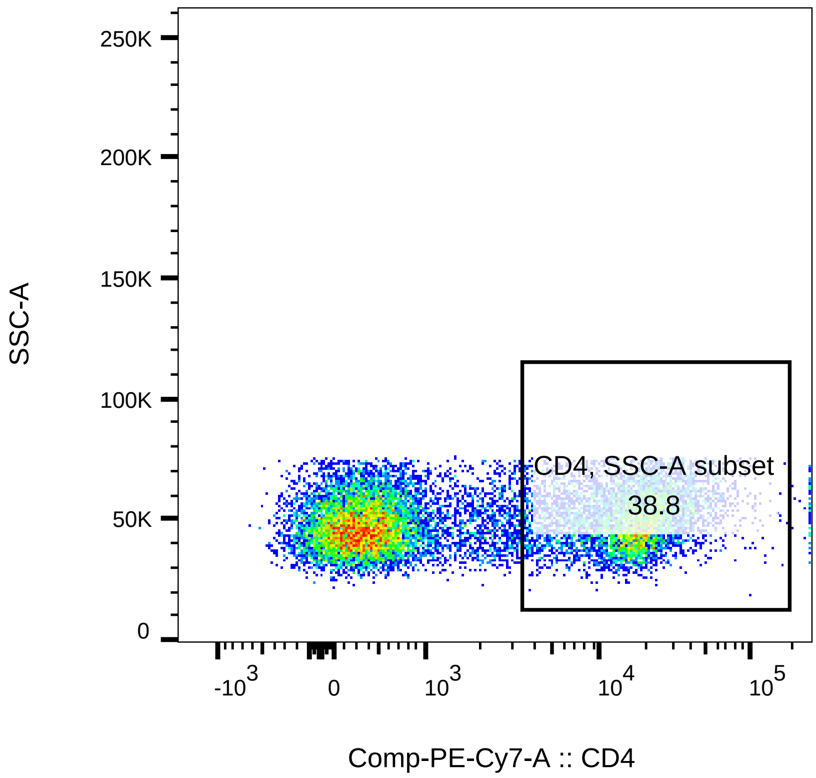

Specimen_003_TPX-3_017.fcs
Lymphocytes
14823

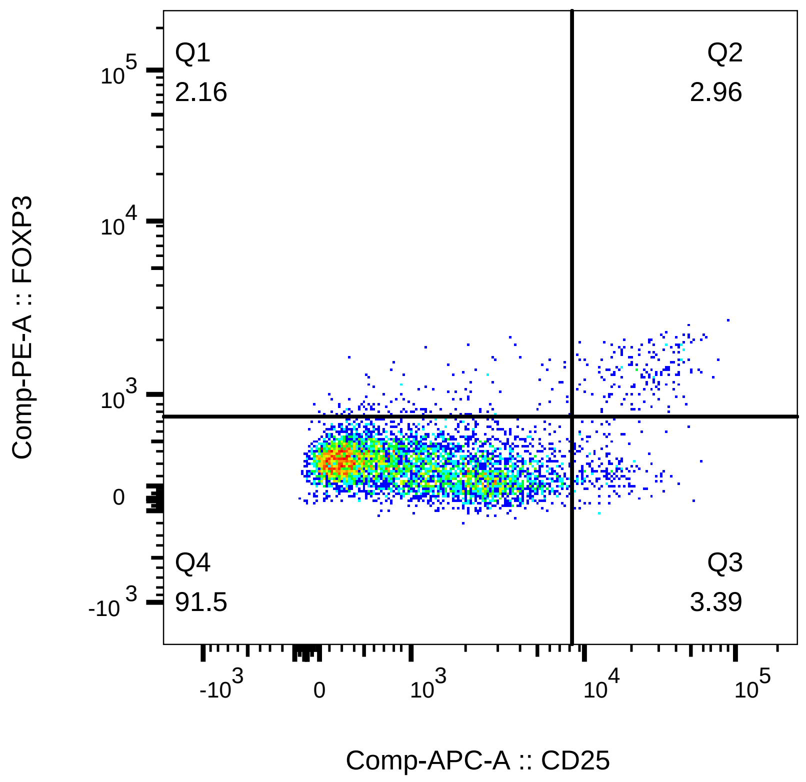

Specimen_003_TPX-3_017.fcs
CD4, SSC-A subset
5744

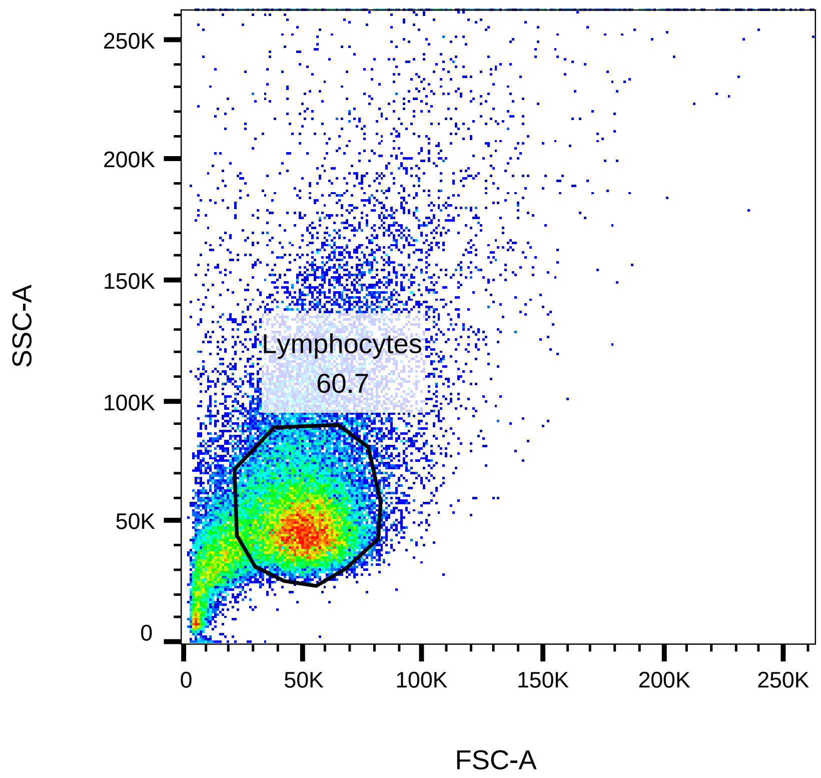

Specimen_003_ESA-1_009.fcs
Ungated
38575

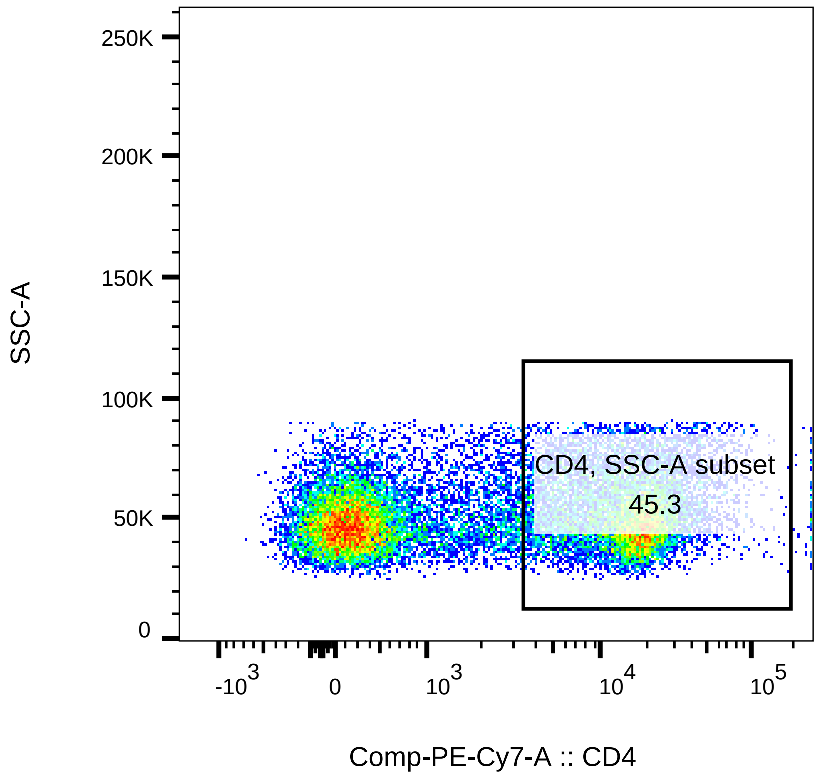

Specimen_003_ESA-1_009.fcs
Lymphocytes
23408

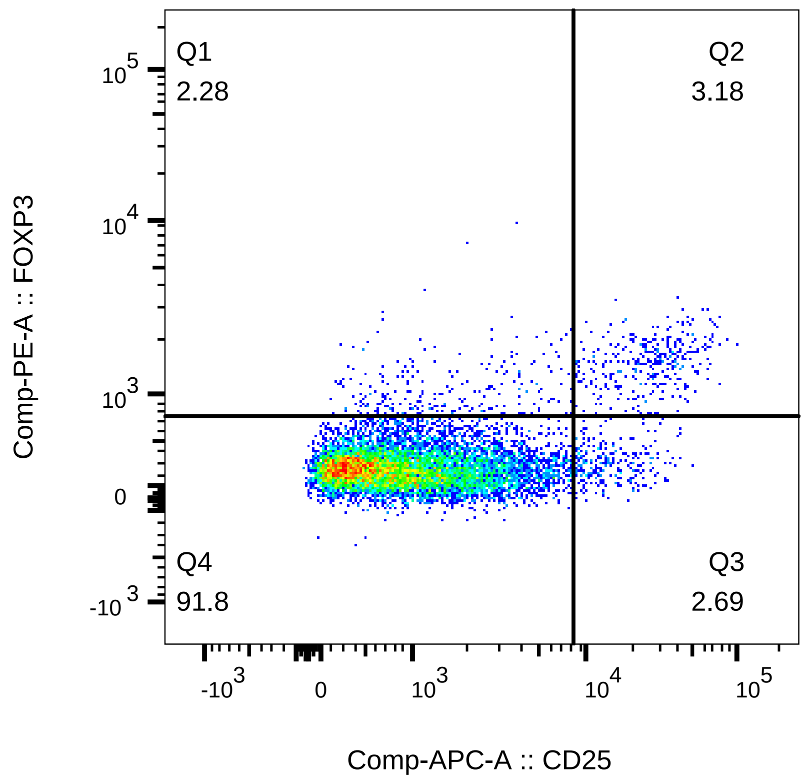

Specimen_003_ESA-1_009.fcs
CD4, SSC-A subset
10598

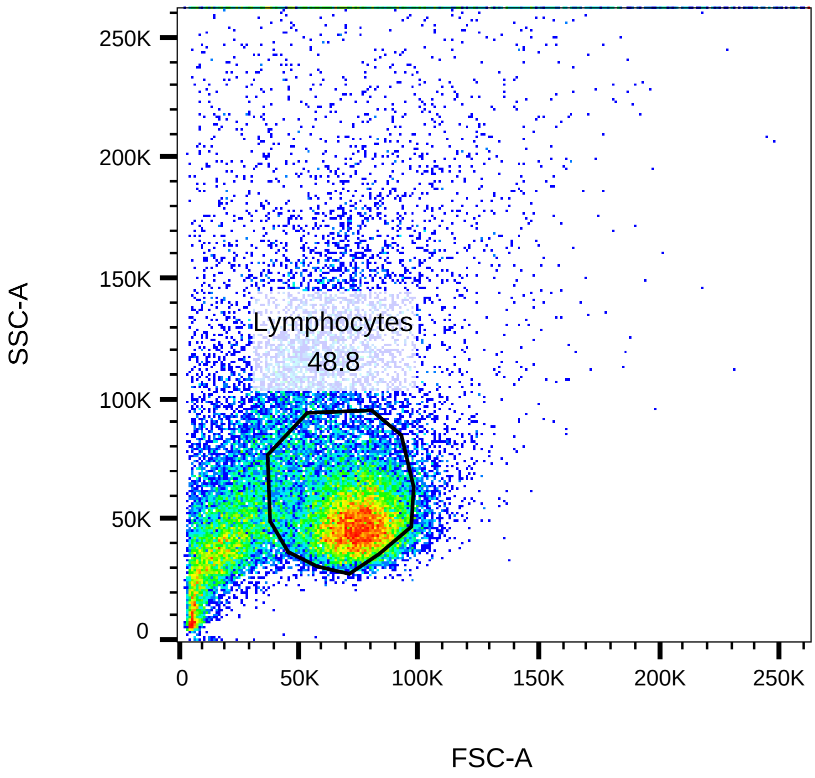

Specimen_003_ESA-2_010.fcs
Ungated
36327

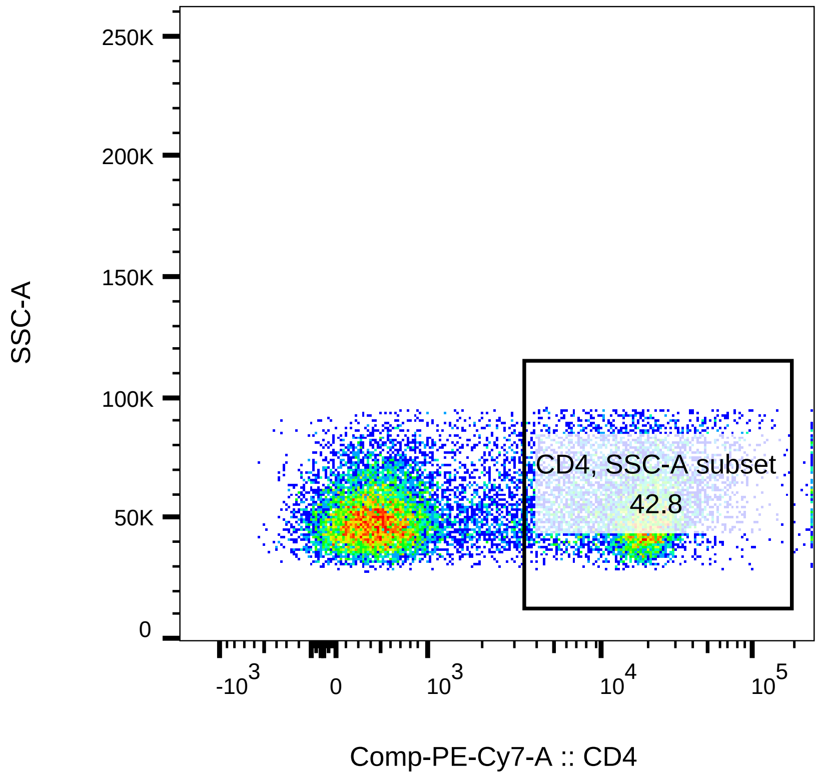

Specimen_003_ESA-2_010.fcs
Lymphocytes
17723

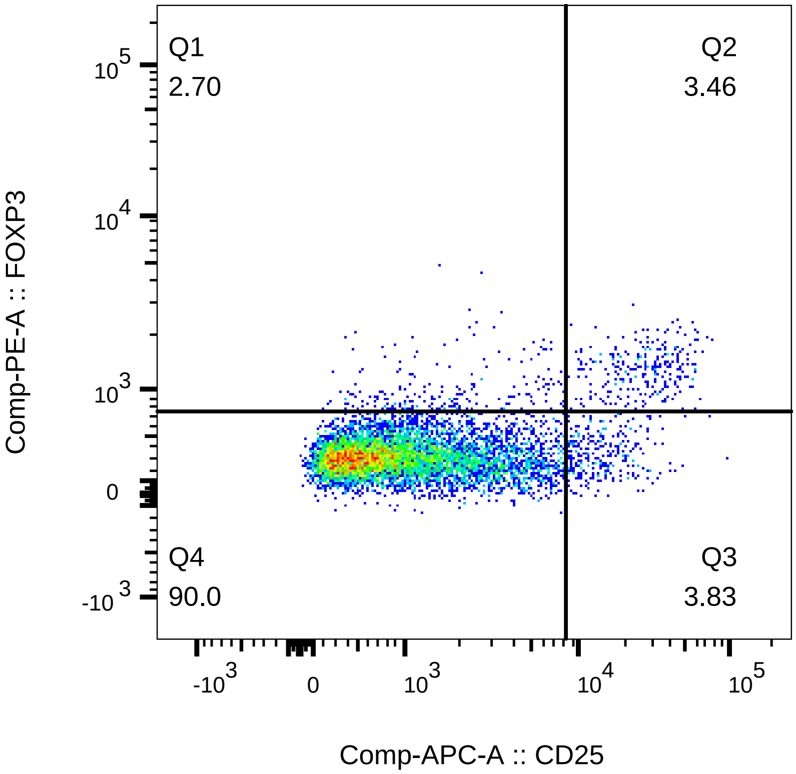

Specimen_003_ESA-2_010.fcs
CD4, SSC-A subset
7592

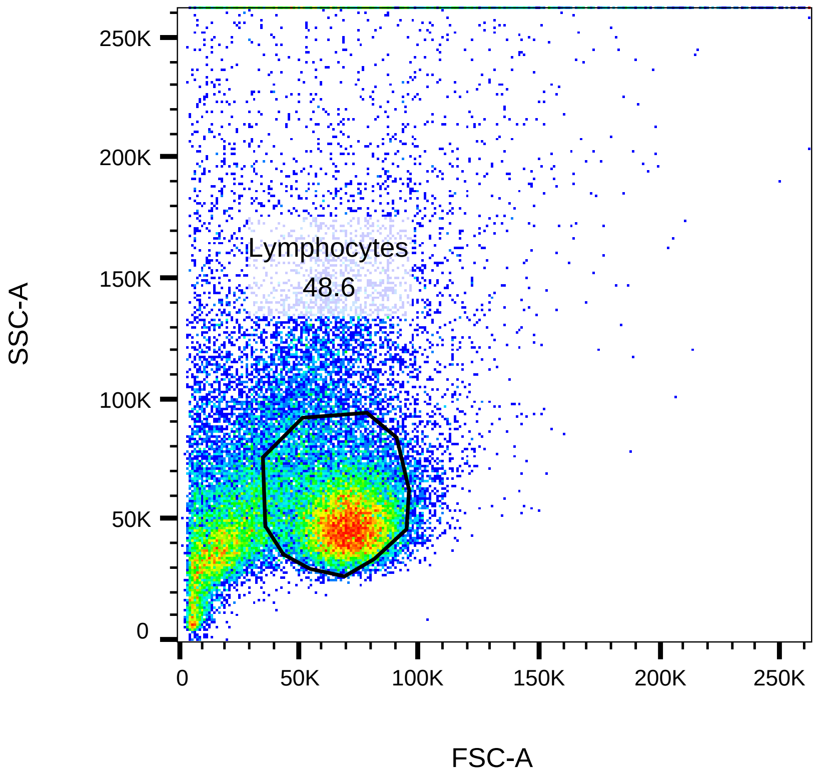

Specimen_003_ESA-3_011.fcs
Ungated
38213

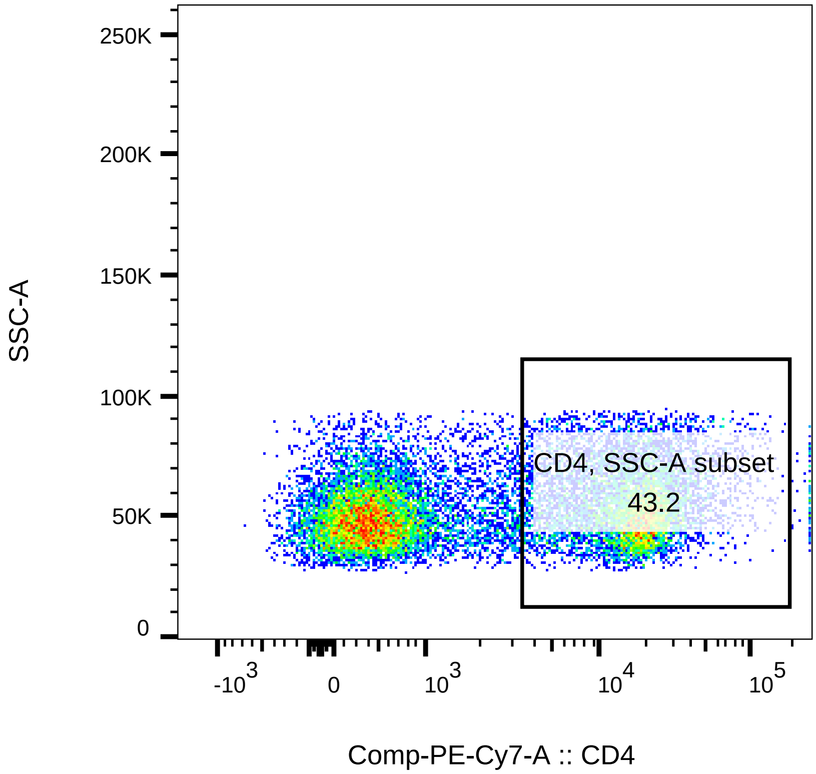

Specimen_003_ESA-3_011.fcs
Lymphocytes
18567

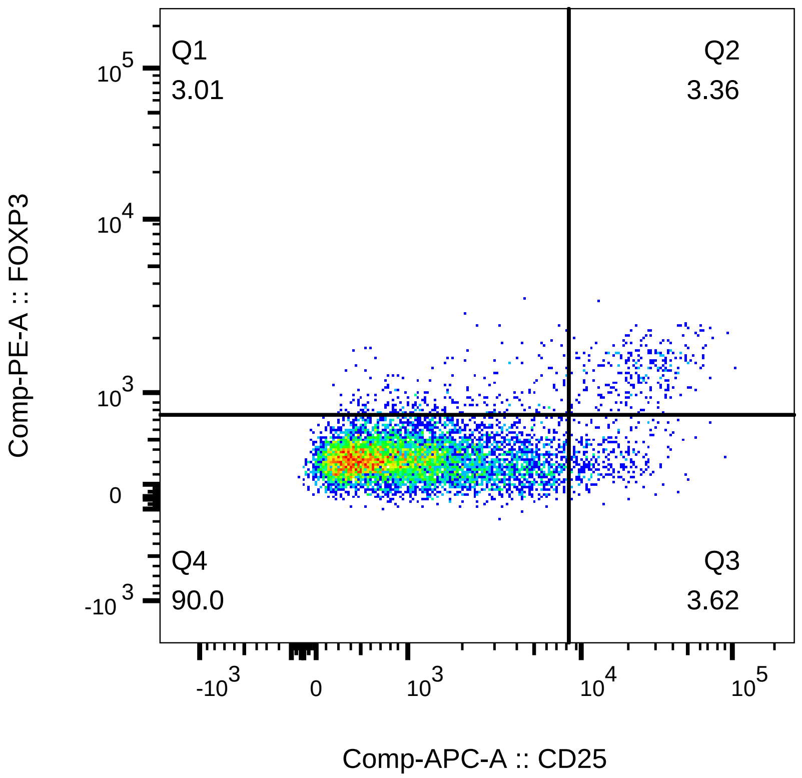

Specimen_003_ESA-3_011.fcs
CD4, SSC-A subset
8015

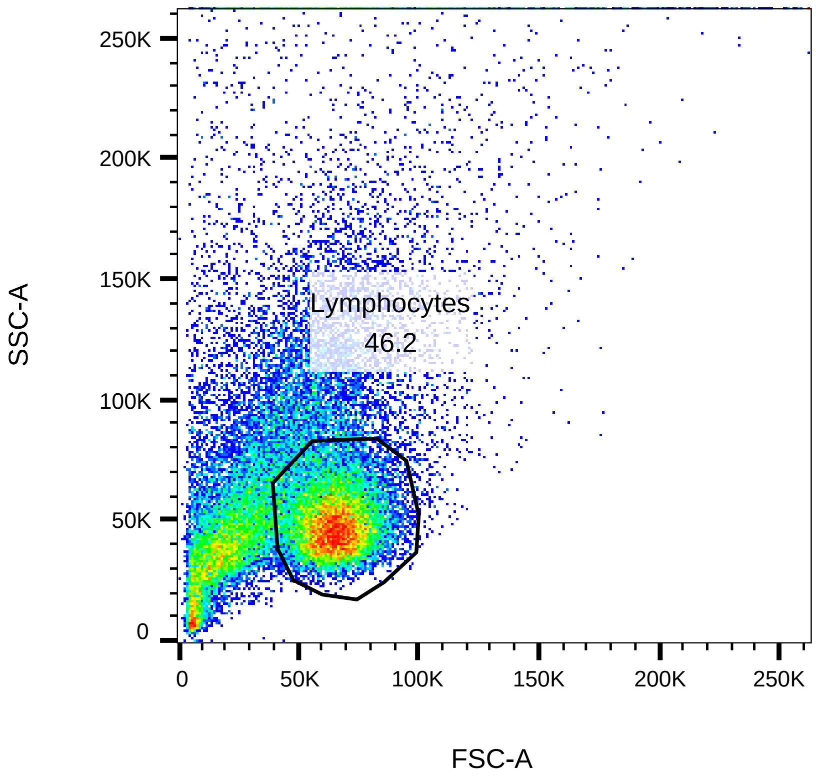

Specimen_003_LPS-1_018.fcs
Ungated
38431

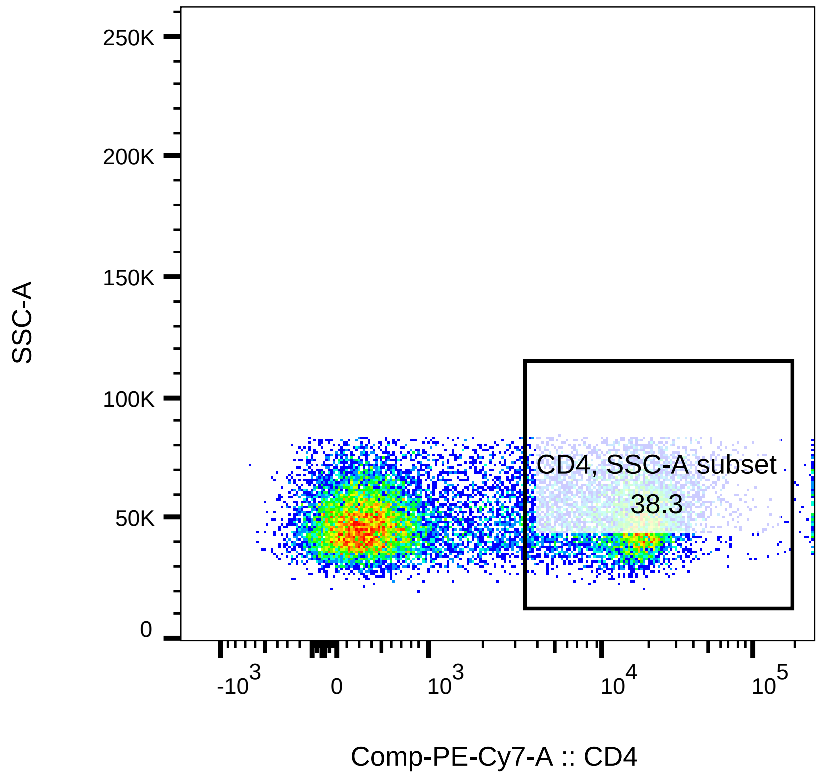

Specimen_003_LPS-1_018.fcs
Lymphocytes
17754

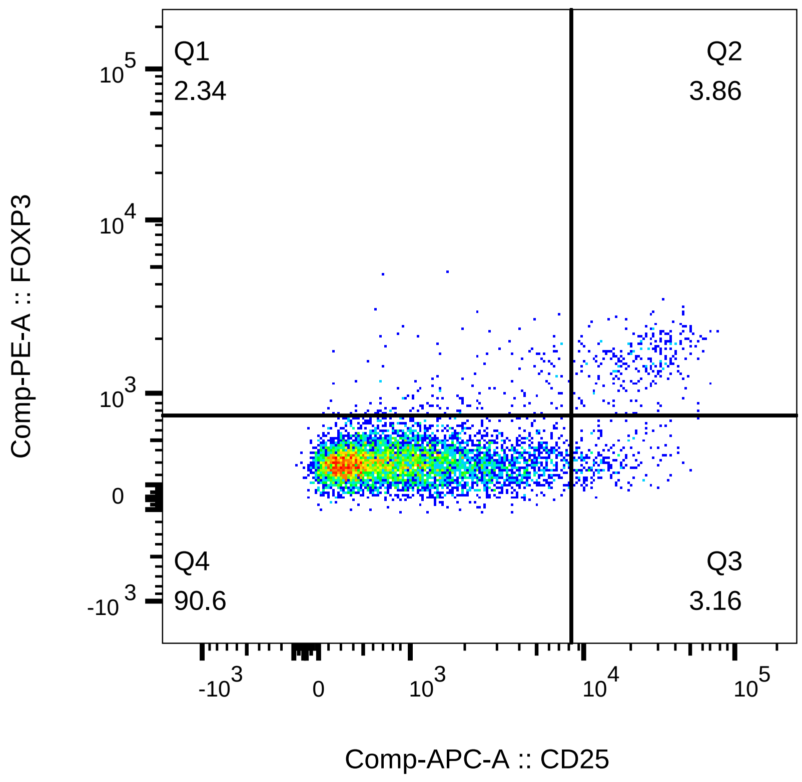

Specimen_003_LPS-1_018.fcs
CD4, SSC-A subset
6808

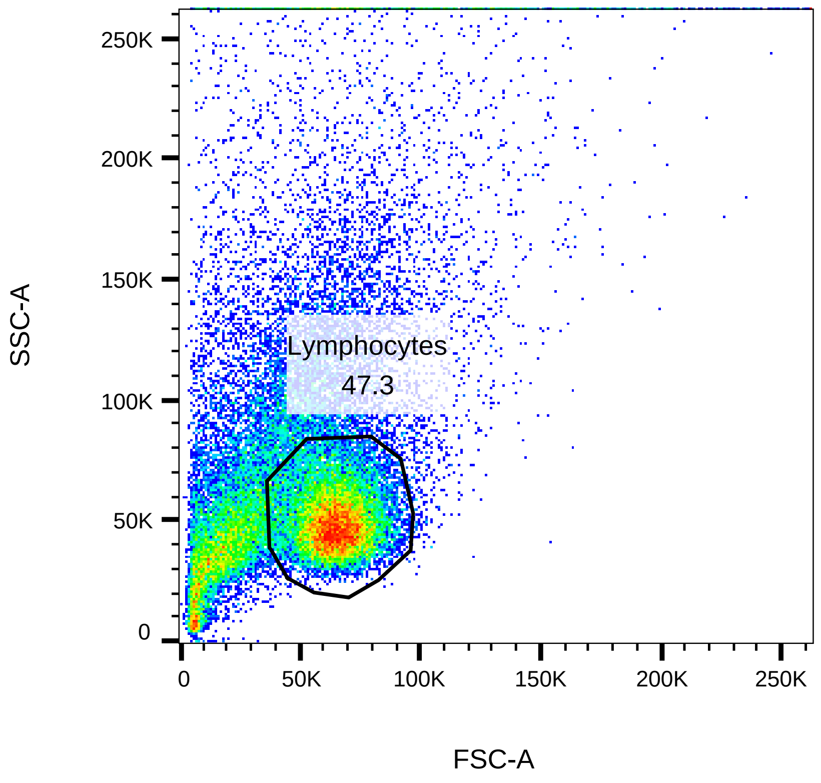

Specimen_003_LPS-2_019.fcs
Ungated
39479

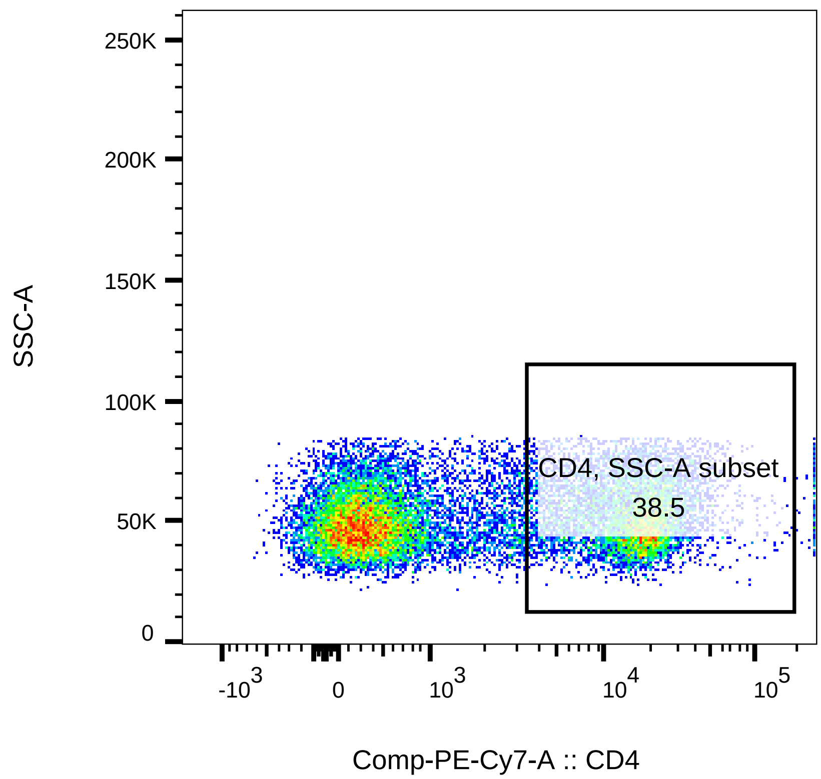

Specimen_003_LPS-2_019.fcs
Lymphocytes
18658

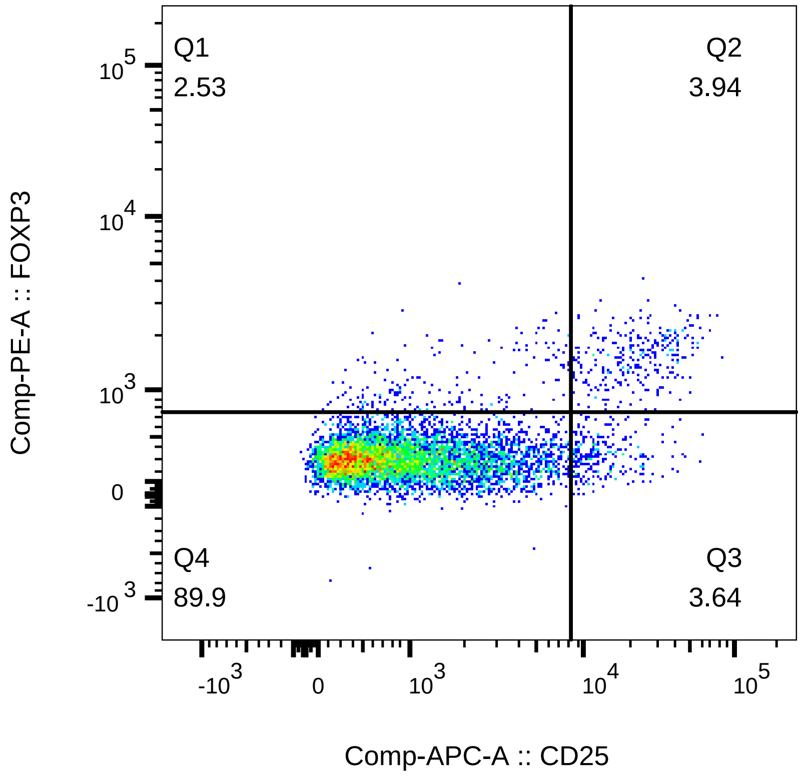

Specimen_003_LPS-2_019.fcs
CD4, SSC-A subset
7189

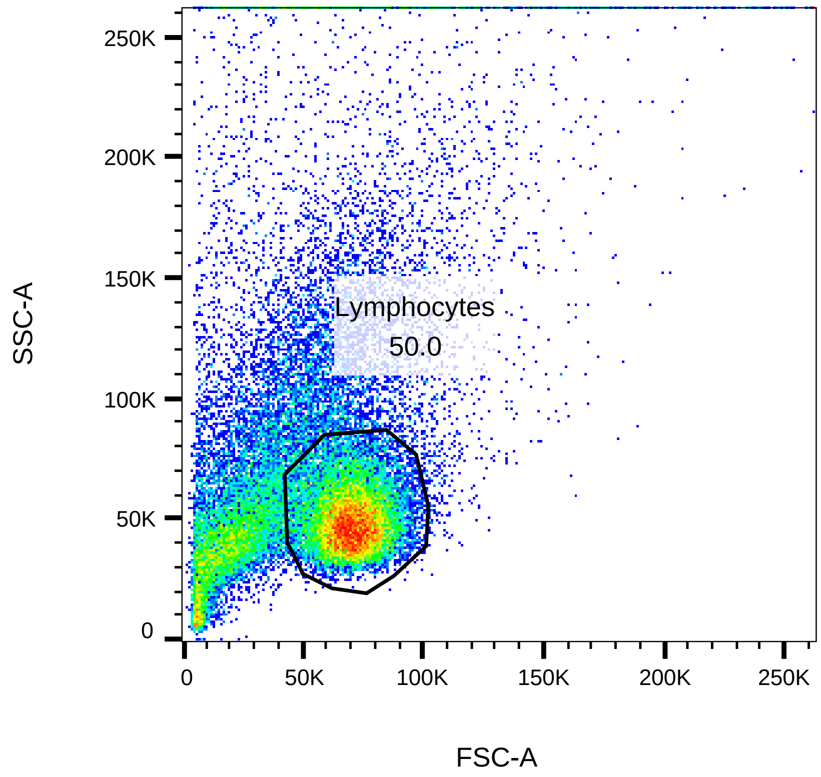

Specimen_003_LPS-3_020.fcs
Ungated
35657

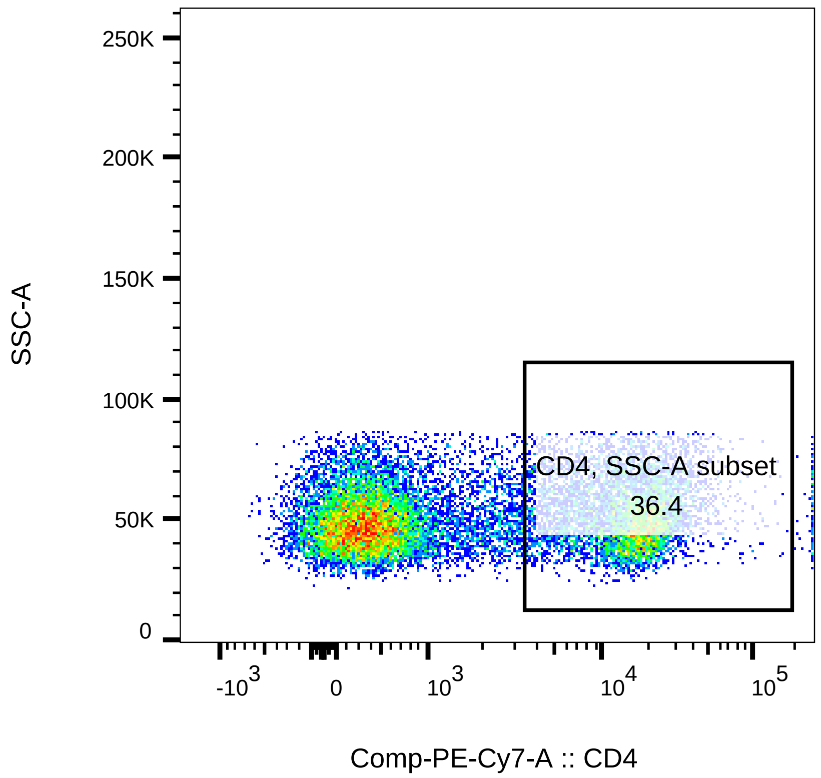

Specimen_003_LPS-3_020.fcs
Lymphocytes
17818

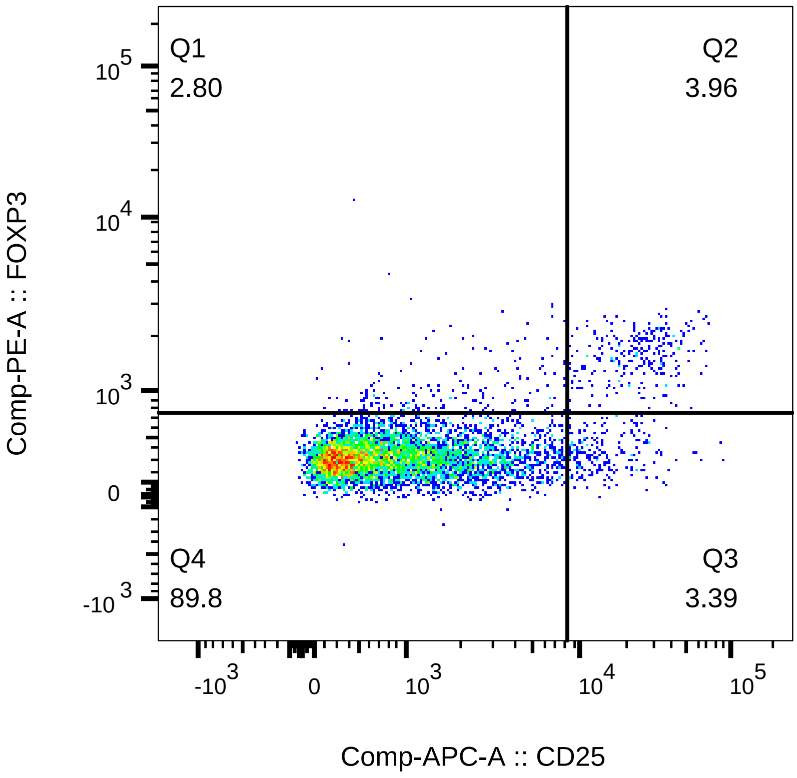

Specimen_003_LPS-3_020.fcs
CD4, SSC-A subset
6489

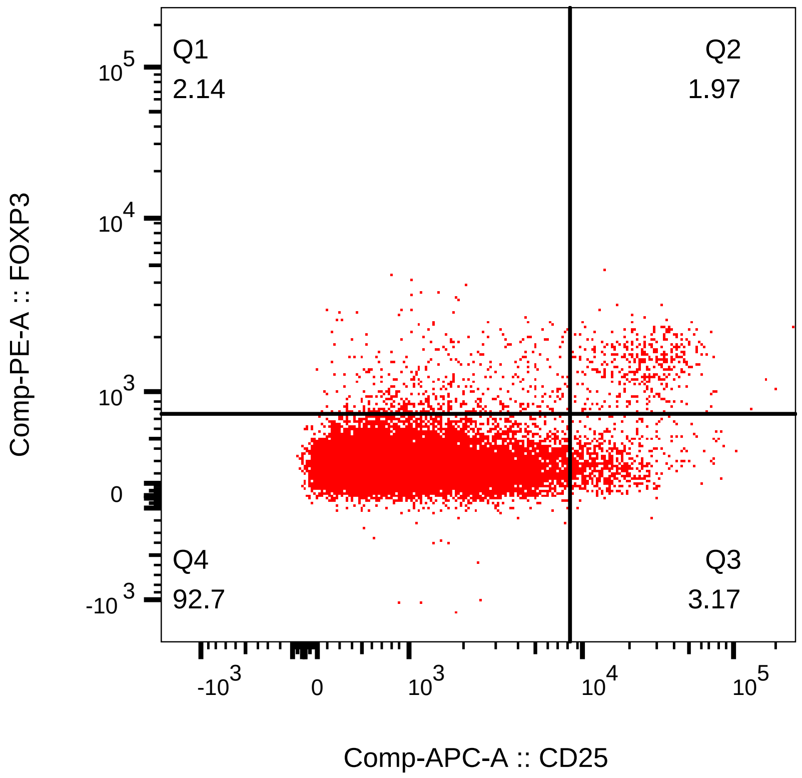

	Sample Name	Subset Name	Count
	Specimen_003_1640-3_008.fcs	CD4, SSC-A subset	8628
	Specimen_003_1640-2_007.fcs	CD4, SSC-A subset	7955
	Specimen_003_1640-1 10000_005.fcs	CD4, SSC-A subset	4254

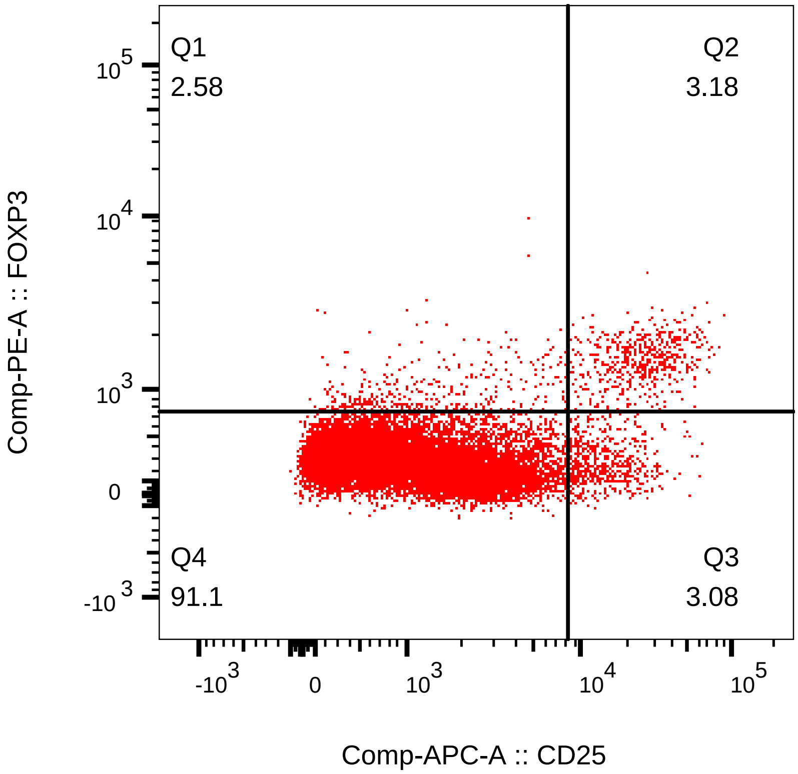

	Sample Name	Subset Name	Count
	Specimen_003_TPX-3_017.fcs	CD4, SSC-A subset	5744
	Specimen_003_TPX-2_016.fcs	CD4, SSC-A subset	6213
	Specimen_003_TPX-1_015.fcs	CD4, SSC-A subset	4993

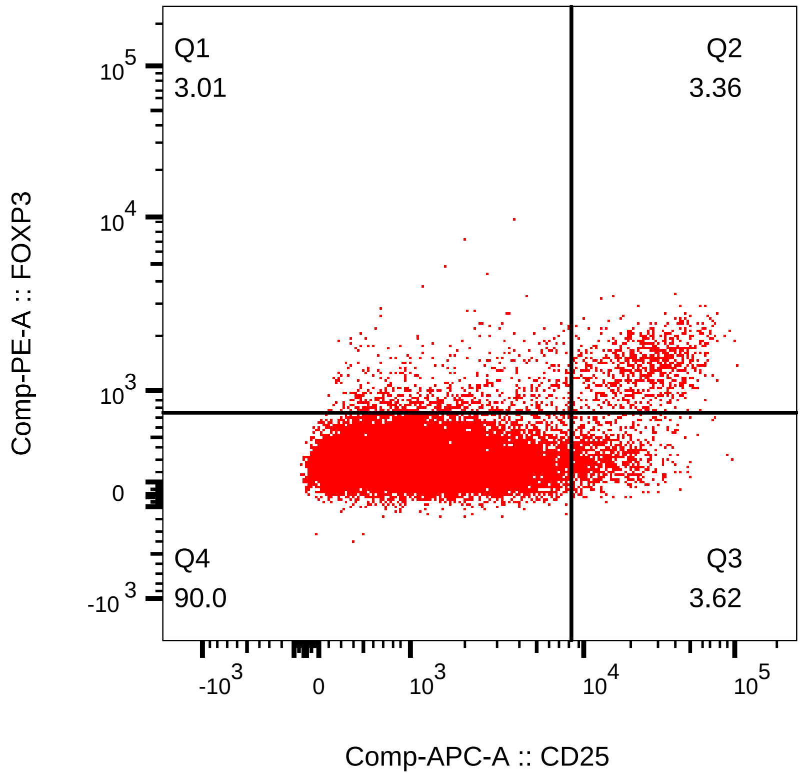

	Sample Name	Subset Name	Count
	Specimen_003_ESA-2_010.fcs	CD4, SSC-A subset	7592
	Specimen_003_ESA-1_009.fcs	CD4, SSC-A subset	10598
	Specimen_003_ESA-3_011.fcs	CD4, SSC-A subset	8015

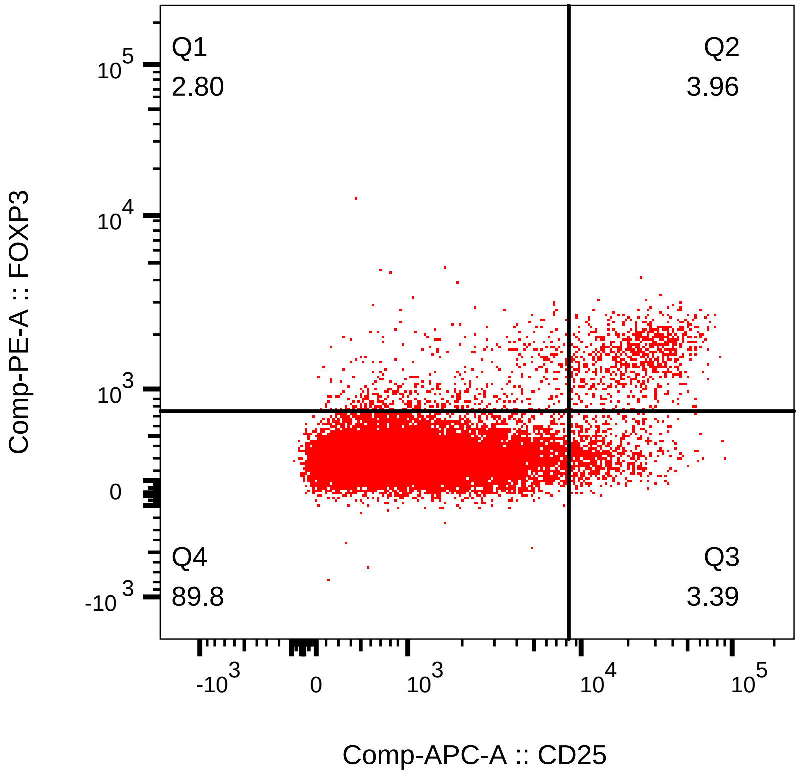

	Sample Name	Subset Name	Count
	Specimen_003_LPS-2_019.fcs	CD4, SSC-A subset	7189
	Specimen_003_LPS-1_018.fcs	CD4, SSC-A subset	6808
	Specimen_003_LPS-3_020.fcs	CD4, SSC-A subset	6489
